# The Early Stage of Bacterial Genome-Reductive Evolution in the Host

**DOI:** 10.1371/journal.ppat.1000922

**Published:** 2010-05-27

**Authors:** Han Song, Junghyun Hwang, Hyojeong Yi, Ricky L. Ulrich, Yan Yu, William C. Nierman, Heenam Stanley Kim

**Affiliations:** 1 Department of Medicine, College of Medicine, Korea University, Anam-Dong, Seongbuk-Gu, Seoul, Korea; 2 US Army Medical Research Institute of Infectious Diseases (USAMRIID), Fort Detrick, Maryland, United States of America; 3 J. Craig Venter Institute, Rockville, Maryland, United States of America; 4 The George Washington University School of Medicine, Department of Biochemistry and Molecular Biology, Washington, D.C., United States of America; University of Arizona, United States of America

## Abstract

The equine-associated obligate pathogen *Burkholderia mallei* was developed by reductive evolution involving a substantial portion of the genome from *Burkholderia pseudomallei*, a free-living opportunistic pathogen. With its short history of divergence (∼3.5 myr), *B. mallei* provides an excellent resource to study the early steps in bacterial genome reductive evolution in the host. By examining 20 genomes of *B. mallei* and *B. pseudomallei*, we found that stepwise massive expansion of IS (insertion sequence) elements IS*Bma*1, IS*Bma*2, and IS*407*A occurred during the evolution of *B. mallei*. Each element proliferated through the sites where its target selection preference was met. Then, IS*Bma*1 and IS*Bma*2 contributed to the further spread of IS*407*A by providing secondary insertion sites. This spread increased genomic deletions and rearrangements, which were predominantly mediated by IS*407*A. There were also nucleotide-level disruptions in a large number of genes. However, no significant signs of erosion were yet noted in these genes. Intriguingly, all these genomic modifications did not seriously alter the gene expression patterns inherited from *B. pseudomallei*. This efficient and elaborate genomic transition was enabled largely through the formation of the highly flexible IS-blended genome and the guidance by selective forces in the host. The detailed IS intervention, unveiled for the first time in this study, may represent the key component of a general mechanism for early bacterial evolution in the host.

## Author Summary

It has been known for some time that bacteria undergo genome-reduction when they transition from a free-living state to a constantly host-restricted state. High levels of IS element expansion were also found in these bacteria, and the IS elements were suggested to play a role in genome reductive evolution. Here we provide evidence for stepwise IS actions as the exclusive mechanism that mediates bacterial genomic changes during the early stage of constant host-bacterial association, by unveiling the processes that resulted in the development of *B. mallei* genome. We show the details of the multi-level interplay of IS elements, which facilitate the wide spread of the IS copies, and the overall mechanics in genome reduction and rearrangement. These processes appeared to operate as chain reactions mediating elaborate genomic transition, without seriously affecting the original gene expression patterns. The absence of differential gene expression in the resulting genome suggests that changes in transcriptional regulation that are often observed in other old bacterial genomes may take place subsequent to the IS-mediated steps, along with gradual nucleotide-level changes.

## Introduction

The genomes of host-adapted bacteria, including endosymbionts and obligatory intracellular pathogens, go through reductive evolution [1], [2], [3]. Such changes are partly due to a reduced pressure to maintain genes that are not essential for survival in the host. Similarly, decreased efficiency of purifying selection, resulting from the reduced population size from a restricted life, results in inactivated genes, including beneficial genes, through genetic drift [3]. During the early stage of the genome reduction process, the majority of genes are lost as large chromosomal fragments spanning multiple genes. Such genome reduction has been documented in diverse bacterial groups, including Firmicutes, Chlamydiae, Spirochetes, and γ-Proteobacteria [1], [3], [4], [5], [6], [7]. Most of these bacteria have large expansion of IS elements (insertion sequences), and thus it has been suggested that the IS elements may play an essential role during the genome reduction process [1], [3], [8], [9], [10].

*Burkholderia pseudomallei* and *Burkholderia mallei* belong to the ß-Proteobacteria family, and are the causative agents of melioidosis and glanders, respectively [11], [12], [13], [14], [15], [16], [17]. *B. mallei* has very recently (∼3.5 myr) evolved from a clone of *B. pseudomallei* through extensive genome reduction [18], [19], accounting for as much as 1.41 Mb or 20% of the genome, as estimated by the size difference between the genomes of *B. mallei* ATCC 23344 and *B. pseudomallei* K96243 [18], [20], [21]. Concomitant with this process, *B. mallei* became constantly associated with mammalian hosts, specifically equines [22], [23], while *B. pseudomallei* maintains an opportunistic pathogenic lifestyle [17]. Preliminary analyses of the two type strains, *B. mallei* ATCC 23344 and *B. pseudomallei* K96243, have suggested that genome reduction and rearrangement in *B. mallei* were mediated by IS elements that are widely spread throughout the genome [20], [21]. Genes that have been deleted from the *B. mallei* genome but are maintained in *B. pseudomallei* include genes that are required for environmental survival. Many of these genes encode metabolic functions for the synthesis of metabolites or the utilization of various sugars and amino acids, without which bacterial propagation in the environment could be significantly hindered [20].

While the genomic reduction during bacterial restriction to their hosts has been well documented [1], [8], [10], most of the stepwise processes have not yet been elucidated. The *B. mallei* genome has unique significance, as it is much younger than the other genomes in which the genome-reductive evolutionary processes have been most studied to date, including *Buchnera* (>150 million years) and other much older groups [1], [3], [4], [5], [6], [7]. The studies with these older genomes have been challenging due to the subsequent genomic- and nucleotide-level mutations that accumulated over a long evolutionary history. In this study, we dissected 10 genomes each of *B. pseudomallei* and *B. mallei* to understand the early-stage processes that drive genome-reductive evolution in host-associated bacteria.

## Results/Discussion

### Multiple IS elements with massive proliferation

**Figure 1:**
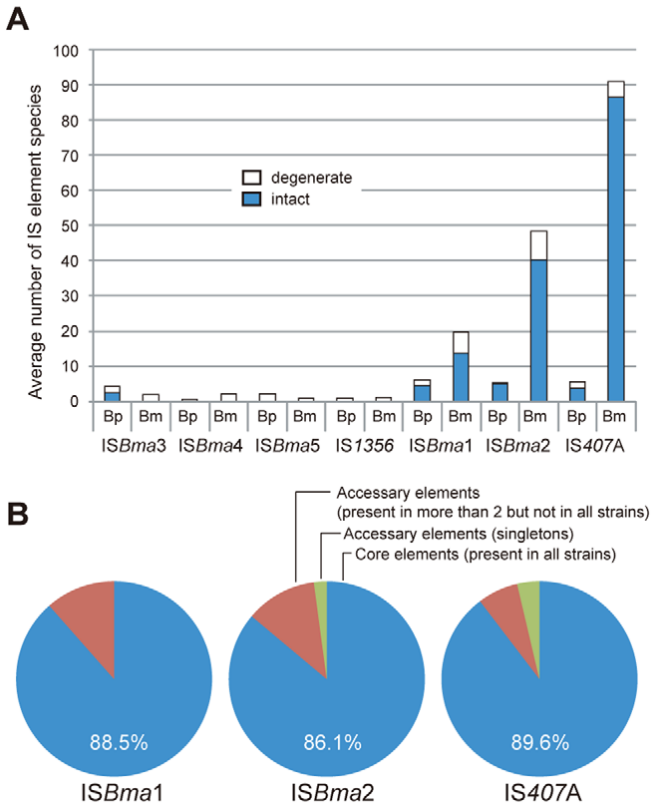
Expansion of a set of IS elements in the *B. mallei* genome. A. Average copy number of IS elements commonly present in *B. mallei* and *B. pseudomallei*. Three species of IS elements showed significant proliferation in *B. mallei* compared with their levels in *B. pseudomallei*. The number of representative IS elements, both as intact and degenerate (i.e., partly deleted) forms, are shown in graph. B. Distribution of the three types of IS element in the strains of *B. mallei*. The IS elements IS*Bma*1, IS*Bma*2, and IS*407*A can be divided into three groups depending on their distribution patterns in the *B. mallei* strains: 1) “Core” IS elements that are present in all the strains; 2) those IS elements present in more than two strains but not in all; and 3) those elements in only one strain. Groups 2 and 3 are collectively called “accessory IS elements” (for a scaled map with the IS insertion sites in all *B. mallei* and *B. pseudomallei* strains, see Fig. S1; for the patterns of genomic rearrangements in the strains of each species, see Fig. S2; for the actual comparative blast data, see Tables S1 and S2).

It is well known that bacteria specialized to a (host) niche, often have a large number of IS elements compared to their free-living relatives [1], [3], [8], [9], [10]. Likewise, by comparing genome sequences, we found that three types of IS elements, IS*Bma*1, IS*Bma*2, and IS*407*A, were significantly increased in *B. mallei* compared to *B. pseudomallei* (Fig. 1A). By contrast, other types of IS, including IS*1356*, IS*Bma*3, IS*Bma*4, and IS*Bma*5 were found in low copy number in both species of bacteria. These elements appeared to be mostly degenerate evolutionary remnants (i.e., part of the IS disrupted or deleted) of the *Burkholderia* lineage. IS*Bma*1, IS*Bma*2, and IS*407*A also had degenerate elements in each species; the IS*Bma*1 elements had the highest levels of degeneration (44%), followed by IS*Bma*2 (20%), and by IS*407*A (5%) (Fig. 1A).

Intriguingly, up to almost 90% of IS*Bma*1, IS*Bma*2, and IS*407*A (88.5%, 86.1%, and 89.6%, respectively) were found to be present at the corresponding loci in all 10 *B. mallei* strains, when examined after the rearranged genomic fragments in each strain were aligned against a reference genome of *B. pseudomallei* K96243 (Fig. 1B; for a scaled map with the IS insertion sites in all *B. mallei* and *B. pseudomallei* strains, see Fig. S1; for the patterns of genomic rearrangements in the strains of each species, see Fig. S2; for the actual comparative blast data, see Tables S1 and S2). In contrast to these “core” elements, those elements that were not present in all (singletons and those found in a few strains), collectively referred to as “accessory” elements, were much less common. That the core elements, expected to be associated with the speciation of *B. mallei* from *B. pseudomallei*, accounted for most of the elements clearly reflects the common origin of *B. mallei* strains from a clone of *B. pseudomallei* [18], [20]. More importantly, it also suggests that further transpositions were significantly slowed after subsequent geographical segregation of the bacteria. There are 13 core elements in *B. mallei* that have matching IS elements located at the same sites in *B. pseudomallei* strains (Table 1). These elements were found to be composed of elements of IS*Bma*1 and IS*Bma*2 but not of IS*407*A. This finding suggests that IS*Bma*1 and IS*Bma*2 have a longer history of association with *B. pseudomallei* than IS*407*A does.

**Table 1:** IS elements matched across strains of *B. mallei* and *B. pseudomallei*.

*B. pseudomallei*											*B. mallei*
IS name^b^	1^a^	2	3	4	5	6	7	8	9	10	IS name^c^
Bp_chr1_3_ISBma2							X^d^				Chr1_13ab_ISBma2_A
Bp_chr1_15_ISBma2									X		Chr1_52_ISBma2_A
Bp_chr1_27_ISBma2							X				Chr1_90_ISBma2_A
Bp_chr1_36_ISBma2	X	X	X	X	X	X	X	X		X	Chr1_118_ISBma2_A
Bp_chr1_38_ISBma2						X					Chr1_120_ISBma2_A
Bp_chr1_45_ISBma2		X	X								Chr1_147_ISBma2_A
Bp_chr1_48_ISBma2										X	Chr1_151ab_ISBma2_A
Bp_chr1_53_ISBma2						X					Chr1_159ab_ISBma2_A
Bp_chr2_12_ISBma2								X			Chr2_71ab_ISBma2_A
Bp_chr1_6_ISBma1									X		Chr1_26_ISBma1_A
Bp_chr1_49_ISBma1		X	X	X	X			X			Chr1_153_ISBma1_A
Bp_chr1_51_ISBma1						X			X		Chr1_156_ISBma1_A
Bp_chr2_11_ISBma1									X		Chr2_69_ISBma1_A

^*a*^Strains of *B. pseudomallei* are denoted by numbers (1: K96243; 2: 1106a; 3: 1106b; 4: 1710a; 5: 1710b; 6: 406e; 7: 668; 8: Pasteur; 9: S13; 10: 1655).

^*b*^Names of the IS elements present in *B. pseudomallei* can be found in Table S1.

^*c*^Names of the IS elements present in *B. mallei* can be found in Table S2. These elements are present in all strains of *B. mallei*.

^*d*^Presence of the IS element in the strain.

### IS-mediated large genomic deletions and rearrangements

**Figure 2:**
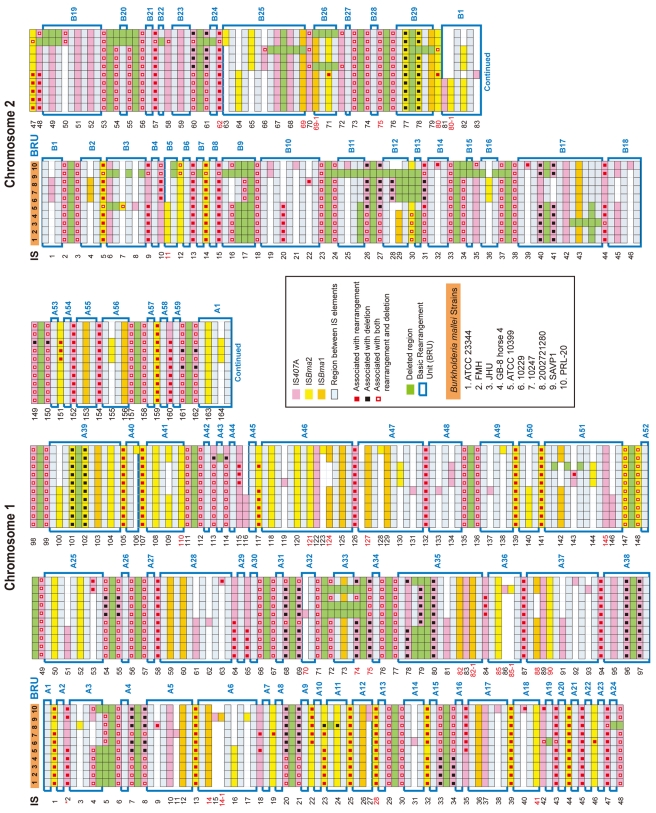
IS-blended *B. mallei* genomes. Locations of IS*Bma*1, IS*Bma*2, and IS*407*A and large deletions in each *B. mallei* strain were mapped back to the reference genome of *B. pseudomallei* K96243 to show their relative positions. For simplicity, these features are not displayed to scale, but are shown in boxes of equal sizes (for a scaled view of the same data, see Fig. S1). The IS elements were numbered in the order in which they appear in the reference chromosomes. The 2^nd^ element in chromosome 1 is denoted by an *, and is the IS*407*A insertion that disrupted *fliP*, which encodes a key factor for flagella formation. The numbers in red indicate the IS elements that were disrupted by a neighboring IS*407*A element. Each BRU is denoted as an open box and by sequential numbers, which are preceded by A or B for chromosome 1 or chromosome 2, respectively. For additional information, see Figure S1 and Tables S1 and S2, in which the IS elements are listed with their names, composed of the sequential numbers, the IS species to which they belong (i.e. IS*Bma*1, IS*Bma*2, and IS*407*A), and their distribution patterns among the *B. mallei* strains (i.e., _A: present in all the strains; _B: present in only some strains; and _C: present in a single strain).

**Figure 3:**
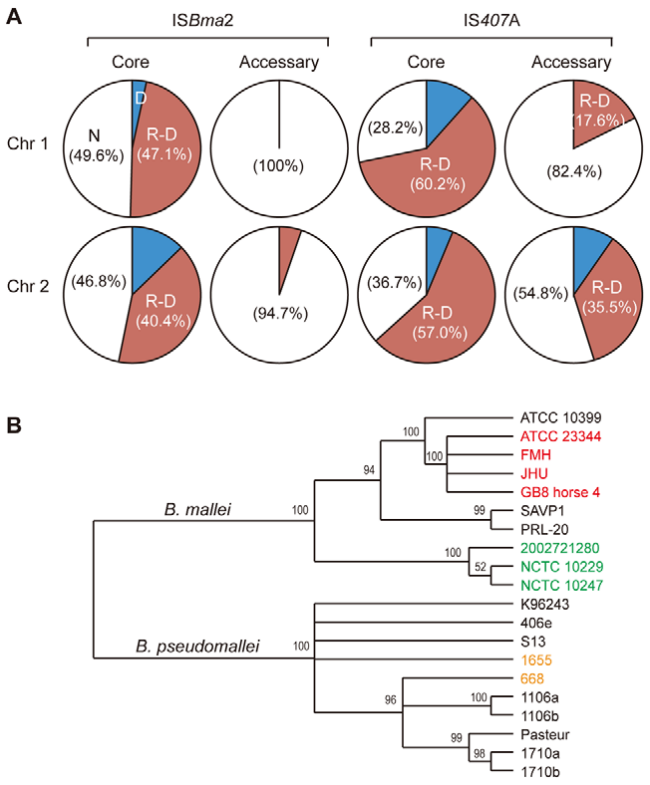
Patterns of IS-mediation in genomic deletions and rearrangements. A. Association of IS*407*A and IS*Bma*2 with genomic deletions and rearrangements. Core elements played a major role in such genomic modifications compared with the accessory elements. The latter elements had a more active role in chromosome 2 than in chromosome 1. B. A phylogenetic relationship of the *B. mallei* and *B. pseudomallei* strains based on the presence of the IS insertions and their association with the genomic deletions and rearrangements. Two strains of *B. pseudomallei* from Australia, 1655 and 668, are labeled in orange. The *B. mallei* strains of European origins, NCTC 10247, NCTC 10229, and 2002721280, are labeled in green, while ATCC 23344 and its direct derivatives are shown in red.

Among the three largely expanded elements, we found that IS*407*A and IS*Bma*2 were associated with almost all of the large genomic deletions and rearrangements in the *B. mallei* strains (Fig. 2; Figs. S1 and S2; Tables S1 and S2). The only exception to this was a large deletion found in the strain ATCC 23344 and its direct derivatives, FMH, JHU, and GB8 horse 4 [24], between the 43^rd^ and the 44^th^ elements in chromosome 2 (Fig. 2; Table S1). No genomic rearrangement was mediated by features other than the two IS elements. IS*Bma*1, which was significantly increased in *B. mallei*, was not directly involved in any of the genomic deletions or rearrangements, however as many as 35% of it served as secondary entry points for IS*407*A. The majority of the core elements of IS*407*A, 71.8% and 63.3% in chromosomes 1 and 2, respectively, mediated rearrangements, deletions, or both (Fig. 3A). By contrast, accessory elements of IS*407*A contributed less, but were more active in chromosome 2 than in chromosome 1. By contrast, 50.4% and 53.2% of the core elements of IS*Bma*2 in chromosomes 1 and 2, respectively, contributed to rearrangements and/or deletions, and the accessory elements in both chromosomes were very rarely involved (Figs. 2 and 3). We identified 59 and 28 genomic fragments in chromosomes 1 and 2, respectively, which were encompassed by core elements of IS*407*A or IS*Bma*2; these core elements mediated genomic rearrangements in at least one strain (Figs. 2; Table S1). We referred to these genomic fragments as BRUs (basic rearrangement units), a set of basic units for genomic reduction and rearrangement in *B. mallei*. The BRUs formed various rearrangement patterns in the *B. mallei* strains (Fig. S2A). By contrast, *B. pseudomallei* strains had little variation in genome arrangement among one another due to low levels of IS elements- a few rearrangements were found but were around non-IS repeat sequences (Fig. S2B).

When the pattern of the IS insertions and their involvement in genome-reductive and rearrangement processes in strains were used to construct a phylogenetic tree, strains sharing a recent common ancestry (e.g., ATCC 23344 and its immediate derivative isolates, FMH, JHU, and GB8 horse 4) or common recent geographical origins (e.g., strains NCTC 10257, NCTC 10229, and 2002721280 from European countries) were grouped together (Fig. 3B). This phylogenic relationship supports the hypothesis that the accessory IS elements, which provided the major determinants for the tree rather than the common core elements, occurred following the speciation and geographical segregation of the *B. mallei* strains. By contrast, such patterns were not obvious among the *B. pseudomallei* strains which did not go through IS element expansions; Australian strains 1655 and 668 did not branch separately from the South Asian strains.

The deletions and rearrangements that were mediated by accessory elements were most frequently noted in strains SAVP1 and 2002721280, which lost virulence after successive passages in laboratory cultures [25] (Figs. 2 and S1). Most of the extra deletions in these strains were more prominent in chromosome 2 than in chromosome 1. In SAVP1, an IS*407*A-mediated deletion removed a major group of virulence genes encoding the animal-type type III secretion system in the BRU B22 (Figs. 2 and S1); this deletion may be a major cause of the avirulence of that strain. By contrast, there is no obvious deletion that may be responsible for the loss of virulence in strain 2002721280. That the strains SAVP1 and 2002721280 obtained deleterious mutations from *in vitro* culturing suggests that maintenance of the genomic contents in *B. mallei* requires selective pressure for survival in the host environment. By contrast, the fully virulent strain PRL-20 showed more frequent deletions and rearrangements mediated by accessory elements than other virulent strains. This strain may represent one of the more evolved (more genome-reduced) strains of *B. mallei*.

Although extra deletions and rearrangements were noted, the actual number of the accessory IS elements was not significantly increased in PRL-20, SAVP1 or 2002721280. Furthermore, none of the direct derivatives of the strain ATCC 23344 (i.e. FMH, JHU, and GB8 horse 4) had new IS insertions (Fig. 2 and Table S1). These ATCC 23344 derivatives also did not have genomic rearrangements; the only change found was a single IS*407*A-mediated deletion located within the BRU B17 in the strain JHU (Fig. 2 and Table S1). These lines of evidence suggest that *B. mallei* genomes are structurally flexible with regard to deletions, however perhaps not as much anymore for additional IS transpositions or genomic rearrangements.

### Different insertion target preferences

**Figure 4:**
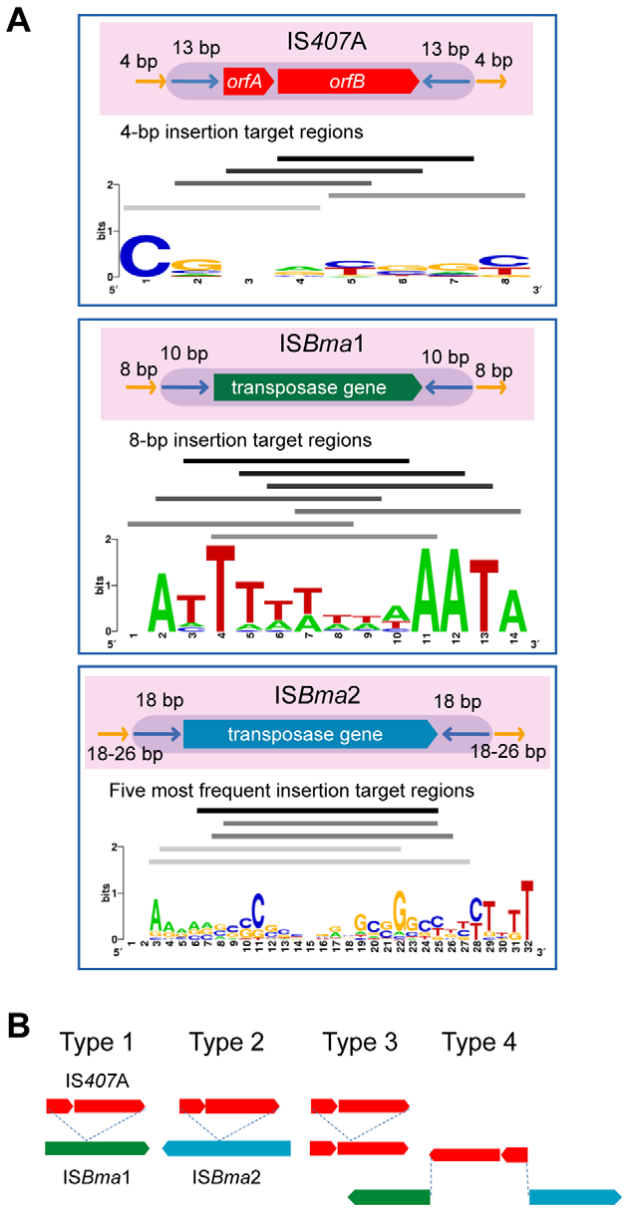
The main IS elements and their insertion target sequences. A. The structures of IS*Bma*1, IS*Bma*2, and IS*407*A and their duplicated target sequences in the *B. mallei* genomes. IS*407*A has a pair of two transposase genes, *orfA* and *orfB*, while IS*Bma*1 and IS*Bma*2 each contain a single transposase gene. These genes in each type of IS element are flanked by inverted repeats, which are denoted by blue arrows, and then by duplicated insertion target sequences, denoted by yellow arrows. Sequence logo displays [42] of the duplicated insertion target sequences are shown below the corresponding IS element. Solid lines above the Sequence logo display represent the specific regions to which actual target sequences matched, with the most abundant groups at the top. B. Patterns of a disruption of one type of core IS elements by another in *B. mallei*. Colored solid arrows represent transposases in each IS element. Four types of pattern were found (see Table S1 for details).

IS*407*A elements are known to generate 4-bp target region duplications as direct repeats around them when they transpose [26]. We found that IS*Bma*1 generates 8-bp target region duplications, and that IS*Bma*2 generates longer repeats of various lengths (18–26 bp) (Table 2; for the entire data, see Table S3). In addition to the various lengths of duplications, these target regions of the three types of IS had different nucleotide compositions and patterns. Most notably, the sequences of IS*Bma*1 contained homopolymers of A and/or T in up to 8-bp stretches of nucleotides (Fig. 4A). The target sequences of IS*Bma*2 had a loose pattern in which the GC-rich central region was encompassed by strands of As and Ts on either side. Target sequences of IS*407*A had the least characteristic composition. It is intriguing to note that each IS element showed different levels of copy number expansion, IS*Bma*1 with the lowest (3.3×), IS*Bma*2 with an increased level (9.5×), and IS*407*A with the highest (16.7×) (Fig. 1A). Perhaps this difference, at least in part, resulted from the availability of genomic sites suited for insertion targets.

**Table 2:** Repeat sequence pairs associated with the major IS elements in *B. mallei*.

	5′-end	3′-end
	IS*407*A	
Inverted Repeats^a^	TGACCTGCCCCCT b	AGGGGGCAGGTCA
Example direct target repeats^c^	TGTC	TGTC
	GTAG	GTAG
	TTCG	TTCG
	GGGC	GGGC
	ATCA	ATCA
	IS*Bma*1	
Inverted Repeats	GGTTCATCGC	GCGATGAACC d
Example direct target repeats	AATTTTTT	AATTTTTT
	AAAAAAAT	AAAAAAAT
	AAAAAATA	AAAAAATA
	CTTTTTTT	CTTTTTTT
	TTTTTCTT	TTTTTCTT
	IS*Bma*2	
Inverted Repeats	CAGATTGCTGACAAACCC	GGGTTCGTCAGCAGTCTG
Example direct target repeats	AGCCAGCTTGCGCTGGCT	AGCCAGCTTGCGCTGGCT
	ACCGCAGCGAAGGCTGCGGT	ACCGCAGCGAAGGCTGCGGT
	AATGGGCCGGAAACGGCCCATT	AATGGGCCGGAAACGGCCCATT
	AGGCCCGCCGAAGCGGGCTT	GGGCCGTTTCAAACGGCCCG
	GGGGCGCTTCGGCGCCCC	GGGGCGCTTCGGCGCCCC

^*a*^Inverted repeats are part of the IS elements, that are present on either side of the ends.

^*b*^Consensus sequences of the inverted repeats.

^*c*^Five examples of the direct repeat target duplicates, which encompass the IS elements. The entire data can be found in Table S3.

^*d*^GCGATGA overlaps with the 3′-terminus of the transposase gene in the upstream.

There were concordant patterns of disruption of the core elements of one type by another, in that IS*Bma*1 and IS*Bma*2 were intersected by transposed IS*407*A (Fig. 4B), while the reverse (IS*407*A disrupted by IS*Bma*1 or IS*Bma*2) was not found. A possible explanation for these insertion patterns may be that IS*Bma*1 and IS*Bma*2 could not transpose into IS*407*A due to the lack of sites suited for their rather uncommon target preferences, while IS*407*A did not have this problem. Consistent with this hypothesis, IS*Bma*1 and IS*Bma*2 also did not have self-disrupted elements, while there were several self-disrupted IS*407*A elements. The involvement of the three IS elements with different target sites increased the total number of IS insertions in the genome. Furthermore, this increase led to further spread of IS*407*A, because IS*Bma*1 and IS*Bma*2 provided neutral insertion points for the element. This in turn directly improved the efficiency of IS*407*A-mediated recombination in the genome, resulting in more sophisticated deletions and rearrangements.

We estimated that 83.7% of IS*407*A and 65.6% of IS*Bma*2 elements in the *B. mallei* genomes lost their matching target duplicates, while all of the elements from intact IS*Bma*1 elements were maintained (Table S3). Almost all of the IS*407*A (see Table S3 for details) and all of the IS*Bma*2 elements that contained matching repeats were not involved in genomic rearrangements in *B. mallei*. This indicates that recombination among the elements were the major cause of the loss of the matching target duplicates.

### Nucleotide-level mutations in *B. mallei*

*B. mallei* still has a high nucleotide-level identity (99%) to *B. pseudomallei*. Consistent with this, there was no AT-biased genome deviation in *B. mallei*, unlike that seen in many old symbionts or obligatory host-associated pathogens [1], [3]. Although the overall identity is still very high, significant nucleotide-level divergence exists, especially at the SSRs (simple sequence repeats), where there are intrinsically high mutation rates [27]. These SSRs were abundant in both *B. mallei* and *B. pseudomallei* at corresponding sites in the genomes. However, there were more genes that were disrupted by frameshift mutations in *B. mallei* compared to *B. pseudomallei* (Table S4). Most of these disrupted genes were commonly present in all *B. mallei* strains, reflecting the clonal origin of the strains. Some of these gene disruptions may have contributed to better adaptation of the bacteria (increased persistence) in the host environment or simply became obsolete [28]. One of the most characteristic loss of function or of surface structure in *B. mallei* is the loss of flagella [20]. A gene essential for flagellum biogenesis, *fliP*, [29] in the strain ATCC23344 was disrupted by a 65-kb fragment flanked by IS*407*A elements, and this mutation completely turned *B. mallei* flagella-less. This disruption in *fliP* is present in all *B. mallei* strains (Table S1; Fig. 2, between BRUs A2 and A3), implying the significance of losing flagella in the evolution of host-restricted *B. mallei*. The loss of flagella has been noted in other bacteria, including *Bordetella pertussis* and *Bordetella parapertussis* during their host specialization, derived from the strains of *Bordetella bronchiseptica*, [9] and *Yersinia pestis* during its conversion from a gut to a systemic pathogen [30]. Additional disrupted genes not present in all strains were found at approximately the same levels as in *B. pseudomallei*, suggesting that there were no significant increases in mutation rates in *B. mallei* after geographical segregation. There also was no significant level of erosion of these, so called, pseudogenes by purifying selection at levels high enough to contribute to the actual genome size reduction (data not shown).

### Genomic potential for gene expression divergence

The extensiveness of the genome-wide reduction and rearrangements as well as additional nucleotide-level mutations may suggest that there is a potential for altered gene expression patterns in *B. mallei*. A total of 341 potential regulatory genes survived the general IS-mediated genomic reduction in *B. mallei* (not taking into account the diverse strain-specific deletions that occurred after speciation). Among these genes, only a small fraction (about 10) in each strain had deleterious (e.g. frameshift, null, or IS-insertion) mutations (for the list of the genes, see Table S5). In addition, none of the predicted operons in *B. mallei*, which correspond to the putative operons previously found in *B. pseudomallei* K96243 [31], were disrupted by IS elements (data not shown). We also estimated the potential for changes in promoters. There were 2,473 upstream sequences of genes, many of which may overlap or contain promoters, in the reference genome of *B. pseudomallei* K96243 that have homologous sequences (with at least 95% identity over at least 95% of their lengths) in all other strains of *B. pseudomallei*. We found that up to 99% of these sequences also matched the corresponding regions in *B. mallei* ATTC23344 at the same homology levels (see Table S6 for the list of the 2,473 upstream sequences, associated gene information, and the blast data). Together, all these data from the analyses of the conserved genomic regions suggest that there is only a low potential for the genes in *B. mallei* to have significantly divergent gene expression patterns from *B. pseudomallei*.

By contrast, there were 56 genes with putative regulatory functions that were lost along with the commonly deleted genomic fragments of the *B. mallei* genome. These genes include potential global regulatory genes, such as those encoding a quorum-sensing system (genes BPSS1176 and BPSS1180 in the reference genome of *B. pseudomallei* K96243), a two-component regulatory system (the pair BPSS1994 and BpSS1995 in *B. pseudomallei* K96243), and a number of regulators of various families (Table S7). Whether the loss of any of these 56 regulatory genes affects the expression of the remaining genes in the *B. mallei* genome was yet to be examined.

### Similar gene expression profiles in *B. mallei* and *B. pseudomallei*

**Figure 5:**
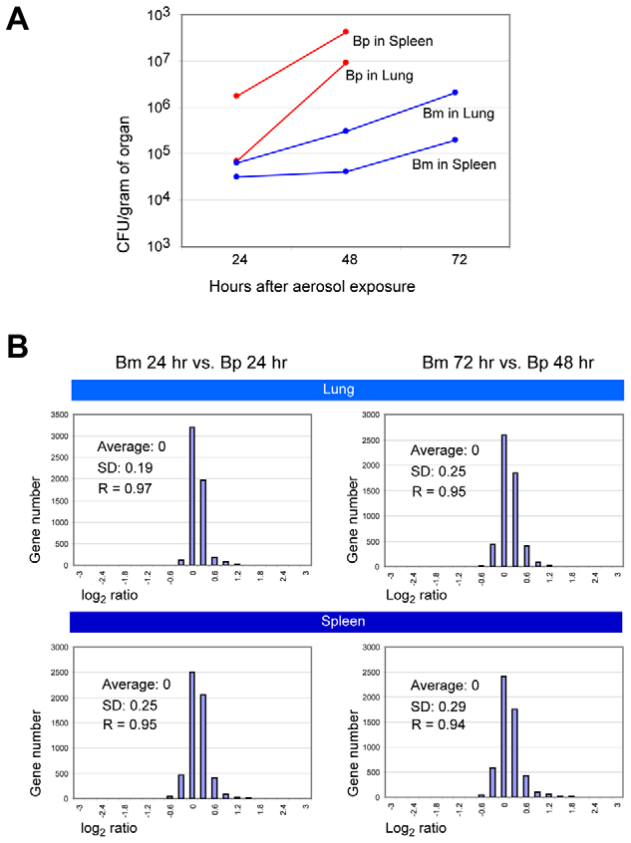
Comparison of *in vivo* gene expression patterns between *B. mallei* and *B. pseudomallei*. A. Bacterial loads within the lung and the spleen of *B. mallei* and *B. pseudomallei* aerosol-challenged mice. Bacterial loads are reported as the average cfu/g of tissue from two animals challenged with either *B. mallei* or *B. pseudomallei*. Due to animal mortality in the *B. pseudomallei* experimental group, only organ loads from 24 hr and 48 hr are reported. B. Histograms depicting the relative gene expression of *B. mallei* and *B. pseudomallei* in infected mouse organs. Comparison data from middle- and late stages of infection in two tissue types, spleen and lung, are displayed. Average ratios in the log_2_ scale, standard deviations of the ratios (SD), and the Pearson correlation coefficients (R's) between the *B. mallei* and *B. pseudomallei* samples are shown.

To experimentally estimate the possible transcriptomic divergence between *B. pseudomallei* and *B. mallei*, we infected female BALB/c mice with *B. mallei* ATCC 23344 or *B. pseudomallei* K96243, employing the previously established aerosol models of acute glanders and melioidosis [32]. Gene expression was compared in the bacteria that colonized the lungs and the spleens of the mice. Both *B. mallei*- and *B. pseudomallei*-challenged animals showed increases in the bacterial loads within these organs over time, with *B. pseudomallei* having slightly faster growth rates (Fig. 5). In our experience, *B. pseudomallei* also grew faster than *B. mallei in vitro* (data not shown). Unlike the mice infected by *B. mallei*, sampling the *B. pseudomallei*-challenged animals after 72 hr was not possible due to animal mortality from the more rapid disease progression. When gene expression profiles in the spleens and lungs were compared between *B. mallei* and *B. pseudomallei* at middle- (i.e., 24 hr for both bacteria) and late stages (i.e., 48 hr for *B. pseudomallei* and 72 hr for *B. mallei*) of infection (a total of four comparison pairs), conserved *B. mallei* and *B. pseudomallei* orthologs showed nearly identical patterns with high Pearson correlation coefficient (R) values ranging from 0.94 to 0.97, regardless of the host tissue type (Fig. 5). Therefore, there was no indication of significant modifications of the expression schemes in the genes required by *B. mallei* to thrive in BALB/c mice compared to those in *B. pseudomallei*. This is consistent with the findings of our previous gene expression studies in culture and *in vivo*, which also showed similar gene expression patterns in *B. mallei* and *B. pseudomallei* [20], [33], [34], [35]. These data suggest that, during the early stage, genomic reduction proceeds conservatively, not seriously affecting the indigenous gene expression patterns. In contrast to *B. mallei*, most of the transcription units in the insect symbiont *Buchnera* were altered, most likely due to complex genomic alterations accumulated over a long period of time [2].

### Conclusions

**Figure 6:**
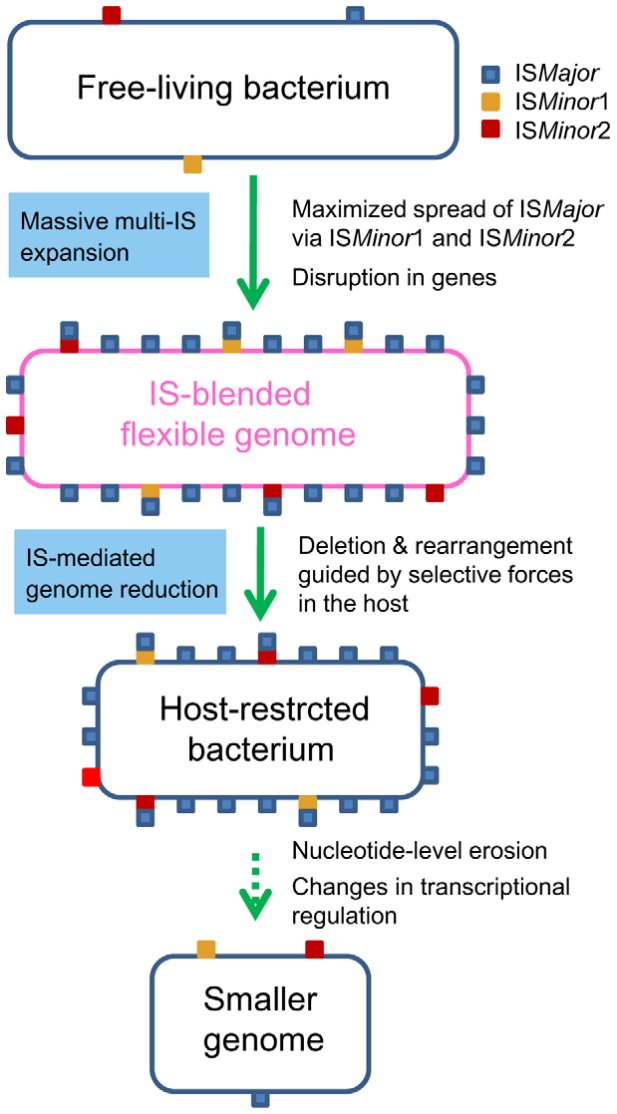
A proposed general model for the bacterial genome-reductive evolution in a specialized niche. Massive expansion of (multiple types of) IS elements may set the stage for extensive genome-reductive evolution in bacteria. When multiple elements are involved, expansion of some elements (e.g. IS*Minor*1 and IS*Minor*2) may lead to further spread of a major element (e.g. IS*Major*), by providing additional insertion sites in the regions, where the major element itself may rarely target. Gene deactivations by intersecting IS insertions can take place and extensive genomic deletions and rearrangements can occur through recombination reactions among the homologous IS copies. These processes can result in highly efficient deletions of dispensable genomic regions via the intrinsic high flexibility of the compactly IS-blended genome, guided by selective forces in the host. Slow and steady nucleotide-level mutations can accumulate after the IS-mediated genomic changes, eventually also contributing to the genome reduction and divergence in transcriptional regulatory patterns over time.

In this study, we unveiled the mechanics of genomic deletions and rearrangements that occur in the early stage of bacterial specialization in the host, by conducting comparative analyses of *B. mallei* and its parental species, *B. pseudomallei*. It became clear that stepwise IS intervention was the main driving force mediating a large genomic reduction in *B. mallei*. Expansion of IS*Bma*1 and IS*Bma*2 in a clone of *B. pseudomallei* set the stage for the wide spread of IS*407*A, allowing its proliferation to sites, to which the element itself may rarely target. Actual genomic deletions and rearrangements occurred through recombination reactions mainly among IS*407*A and also among IS*Bma*2 (Fig. 2). These processes achieved highly efficient deletions of dispensable genomic regions, causing only small disruptions to the portions of the genome that were maintained. This was possible due to the guidance by selective forces in the host and via the intrinsic flexibility of the compactly IS-blended genome. The *B. mallei* genome currently appears to still be structurally flexible with regard to deletions but is now less flexible with regard to genomic rearrangements and additional transpositions. This may indicate that the genomic evolution in *B. mallei* has been moving into a second stage, in which large-scale genomic alterations are reduced and nucleotide-level erosion has become more important. On the other hand, a large number of genes disrupted by frameshift mutations in SSRs were found in the *B. mallei* genome. The loss of function encoded by these genes and of flagella via disruption in *fliP* by IS*407*A (Table S1), could be part of the adaptive evolution for survival in the host environment, which will eventually lead to genome size reduction by erosion over time. Widespread relics of IS elements found in diverse symbionts and obligate pathogens [1], [3], [8] clearly suggest that a similar sequential IS intervention, modeled in Figure 6, may illustrate a general mechanism, by which elaborate genome transition occurs during early bacterial evolution after establishing constant association with the host.

## Materials and Methods

### Ethics statement

All research involving live animals was conducted in compliance with the Animal Welfare Act and other federal statutes and regulations relating to animals and experiments involving animals and adhered to the principles stated in the *Guide for the Care and Use of Laboratory Animals*, National Research Council, 1996. All mouse experiments conducted in the USAMRIID (US Army Medical Research Institute of Infectious Diseases) were approved by the Association for Assessment and Accreditation of Laboratory Animal Care International.

### Sequencing and annotation

The type strains for *B. mallei* (ATCC23344) [20] and *B. pseudomallei* (K96243) [21] were previously sequenced. Strains FMH, JHU, and GB8 horse 4 were direct derivatives of strain ATCC 23344 after passages in the human or horse, and these strains were also sequenced previously [24]. *B. mallei* strains NCTC10229, NCTC10247, and SAVP1 were sequenced with full closure and manually annotated as previously described [20]. The remaining three strains (2002721280, ATCC10399, and PRL-20) were sequenced to 8× Sanger sequence coverage by the whole genome shotgun method [36] without closure, and assembled using the Celera Assembler [37], and contigs were oriented by alignment to the reference strain ATCC23344 using PROMER [38]. ORFs were predicted and annotated automatically using GLIMMER [39], [40]. Pseudo-chromosomes were constructed from the ordered scaffolds, using manual examination where necessary. Similarly, *B. pseudomallei* strains 1106a, 1710b, and 668 were sequenced with full closure and manual annotation, while 1655, 406e, S13, and Pasteur 6068 were sequenced without closure and annotated automatically.

### Comparative genomic analyses with *B. mallei* and *B. pseudomallei*

For the analyses of genomic deletions and rearrangements in *B. mallei* and *B. pseudomallei*, 5,799 predicted protein sequences from the *B. pseudomallei* type strain K96243 were compared with the nucleotide sequences of the genomes of *B. mallei* (ATCC 23344, 2002721280, ATCC 10399, FMH, JHU, GB8 horse 4, PRL-20, NCTC 10229, NCTC 10247, SAVP1) and the other strains of *B. pseudomallei* (1106a, 1106b, 1655, 1710a, 1710b, 406e, 668, Pasteur, S13) using tblastn (http://blast.wustl.edu). For the mapping of the insertions of IS*Bma*1, IS*Bma*2, and IS*407*A in the genomes of *B. mallei* and *B. pseudomallei*, the entire sequences of the IS elements were searched against the 20 genomes using blastn (http://blast.wustl.edu). For the analysis of association of the IS elements with genomic deletions and rearrangements in *B. mallei* and of the target sequences in the genomes, strain ATCC 23344 represented all of its immediate derivatives, FMH, JHU, and GB8 horse 4, to avoid redundancy in the data, because the three strains showed identical patterns. To compare the patterns of genome rearrangements in the *B. mallei* strains, the positions of the BRUs in each strain of *B. mallei* relative to *B. pseudomallei* K96243 were visualized using a genome-comparative software tool ACT ([41]; http://www.sanger.ac.uk/Software/ACT), and the displays were compared in parallel among the strains.

We also examined *B. mallei* and *B. pseudomallei* for intergenic regions that potentially containing promoters, putative regulatory genes, and disruptions of putative operons to estimate the possibility of causing gene expression divergence. For intergenic region comparisons, up to 100 bp upstream of the start codon, or up to as much as available if the neighboring gene was closer, of the genes that contain at least 50 bp of an untranslated upstream region were retrieved from the genome of *B. pseudomallei* K96243. Then, these sequences (2,268 and 1,566 from chromosomes 1 and 2, respectively) were searched against the genomes of *B. mallei* and *B. pseudomallei* using blastn (http://blast.wustl.edu), and the length-match as well as the identity values of the orthologous regions were calculated. Putative operons reported by Rodrigues *et al.* from the genome of *B. pseudomallei* K6243 [31] were used to match the orthologous gene clusters in the genome of *B. mallei* ATCC 23344, and these gene clusters were examined for any disruptions caused by IS elements. All the genome sequences of *B. mallei* and *B. pseudomallei* used in this study are available through the Pathema web site (http://pathema.jcvi.org/cgi-bin/Burkholderia/PathemaHomePage.cgi) at the J. Craig Venter Institute (http://www.jcvi.org/).

### Construction of the phylogenetic tree

A phylogenetic tree was constructed with the strains of *B. mallei* and *B. pseudomallei* based on the insertion patterns of and the role played in the genomic deletions and rearrangements by the three major IS elements, IS*Bma*1, IS*Bma*2, and IS*407*A. All the data used are shown in Tables S1 and S2 and Figure 2. Bootstrapped maximum parsimony trees were calculated using the PAUP package with default parameters, and a consensus tree was produced from the bootstrap replicates. Branches with bootstrap scores of less than 50 were collapsed in the tree.

### Determination of the target sequence patterns

Among the duplicated target regions encompassing the IS elements IS*Bma*1, IS*Bma*2, and IS*407*A, those regions that had perfectly matching sequences were first collected. Then, among the sequences from unmatched pairs, those that occurred in more than two strains were assumed to be un-mutated valid sequences and, therefore, were added to the data pool for the analysis. Strain ATCC 23344 represented all its direct derivatives (FMH, JHU, and GB8 horse 4) in this analysis to avoid redundancy in the data. The collected sequences were aligned with Clustal X, and the alignments were graphically visualized using Sequence logos [42].

### Mouse infection, bacterial load estimation, and RNA preparation

Exposure of mice to bacterial aerosol was performed as described by Roy *et al.* [43]. Fresh overnight cultures of *B. pseudomallei* DD503 [44] and *B. mallei* ATCC 23344 were prepared in LB or in LBG (LB supplemented with 4% glycerol), respectively, at 37°C with aeration (250 rpm). Thirty female BALB/c mice six to eight weeks old (National Cancer Institute, Frederick, MD, USA) were infected with these bacteria: nine mice each with *B. pseudomallei* and *B. mallei* for the gene expression studies, and six mice each for the bacterial load assays. The mice infected with *B. mallei* received an inhaled dose of 7.2×10^3^ cfu (7.2×LD_50_), and those mice infected with *B. pseudomallei* received 1.8×10^4^ cfu (18×LD_50_), as estimated by colony counting on agar plates. The infected mice were provided with rodent feed and water ad libitum and maintained on a 12-hr light cycle. After 24 and 48 hr (for both *B. mallei* and *B. pseudomallei*) or 72 hr (for *B. mallei*) of infection, five mice from each point in time were euthanized in a CO2 chamber, and their spleens and lungs were removed. Due to animal mortality, a 72 hr point in time was not possible for *B. pseudomallei*. The organs from two randomly picked mice were saved for bacterial load estimations, and the rest were homogenized in 1 ml of Trizol (Invitrogen Corp., Carlsbad, CA, USA) using a Tissue-Tearor (BioSpec Products, Bartlesville, OK, USA). Total RNA was purified according to the manufacturer's recommendations (Invitrogen Corp., Carlsbad, CA, USA). The bacterial load in the mouse organs was estimated as described by Ulrich and DeShazer [32].

### RNA labeling and microarray analysis

Total RNA, both bacterial and mouse, from the same organ types from three mice was pooled to compensate for potential individual variation. These pooled RNA samples were used for the experiments without further purification of the bacterial RNA because RNA from mice does not cross-hybridize to the *B. mallei* microarray at a level affecting the legitimate interactions between the *B. mallei* array and the *Burkholderia* transcriptome [35]. The *B. mallei* whole genome array used in this study for both *B. mallei* and the closely related *B. pseudomallei* (average gene identity at the nucleotide level of 99%) was described in detail previously [33]. The *B. mallei-* and *B. pseudomallei*-infected organ samples were paired for the hybridization reactions based on early and late pathological states. A total of eight hybridization reactions or four different comparisons were performed, each of which was replicated in flip-dye pairs and the final ratios were calculated as log_2_ (*B. pseudomallei* gene expression intensity/*B. mallei* gene expression intensity). Labeling of the probes, slide hybridization, and slide scanning were carried out as previously described [35]. The independent TIFF slide images from each channel were analyzed using TIGR Spotfinder to assess the relative expression levels, and the data were normalized using a local regression technique LOWESS (LOcally WEighted Scatterplot Smoothing) with the MIDAS software (<http://www.jcvi.org/cms/research/software>, The J. Craig Venter Institute, Rockville, MD, USA). The resulting data were averaged from triplicate genes on each microarray and from duplicate flip-dye arrays for each experiment.

## Supporting Information

Figure S1 Graphical alignments of the genomes of *B. mallei* and *B. pseudomallei*. Genomes from strains of *B. mallei* and *B. pseudomallei* are aligned for close comparisons of the relative positions of IS elements and large deletions. Based on the genome of *B. pseudomallei* K96243 (displayed in the middle of the alignments), corresponding regions in *B. mallei* genomes (denoted by the upper green blocks) and those of the other *B. pseudomallei* genomes (denoted by the lower blue blocks) are displayed. Locations of IS*Bma*1, IS*Bma*2, and IS*407*A are denoted by red, yellow, and pink lines, respectively, in each strain.
(1.87 MB TIF)

Figure S2 The patterns of genomic rearrangements in *B. mallei*. A. Syntenic relationship among the *B. mallei* strains. Re-localization of BRUs in *B. mallei* strains (relative to *B. pseudomallei* strain K96243 as the reference) is shown with the comparative genomics display tool ACT (Wellcome Trust Sanger Institute) using blastn data. Blue and red connecting lines between the genomes indicate the same and opposite directions, respectively, of the corresponding BRUs relative to each other. B. Comparisons among the *B. pseudomallei* strains. Each strain was compared to the reference genome of strain K96243.
(2.42 MB TIF)

Table S1 Comparison of *B. mallei* genomes with that of *B. pseudomallei* strain K96243.
(8.84 MB PDF)

Table S2 Comparison of *B. pseudomallei* genomes with that of strain K96243.
(9.59 MB PDF)

Table S3 Specifics of the IS elements IS*407*A, IS*Bma*1, and IS*Bma*2 in the *B. mallei* strains.
(1.56 MB PDF)

Table S4 Disrupted genes that resulted from SSR-mediated frameshift mutations in *B. mallei* and *B. pseudomallei*.
(0.34 MB PDF)

Table S5 Potential regulatory genes that survived the genomic reduction in *B. mallei*.
(0.18 MB PDF)

Table S6 Comparison of the upstream regions of genes containing potential promoters.
(2.92 MB PDF)

Table S7 Putative regulatory genes deleted from *B. mallei* as IS-bounded genomic fragments.
(0.04 MB PDF)

## References

[1] Moran NA (2003). Tracing the evolution of gene loss in obligate bacterial symbionts.. Current Opinion in Microbiology.

[2] Moran NA, Mira A (2001). The process of genome shrinkage in the obligate symbiont *Buchnera aphidicola*.. Genome Biology.

[3] Moran NA, Plague GR (2004). Genomic changes following host restriction in bacteria.. Current Opinion in Genetics & Development.

[4] Nilsson AI, Koskiniemi S, Eriksson S, Kugelberg E, Hinton JCD (2005). Bacterial genome size reduction by experimental evolution.. Proc Natl Acad Sci U S A.

[5] Sallstrom B, Andersson SGE (2005). Genome reduction in the [alpha]-Proteobacteria.. Current Opinion in Microbiology.

[6] Batut J, Andersson SGE, O'Callaghan D (2004). The evolution of chronic infection strategies in the [alpha]-proteobacteria.. Nat Rev Micro.

[7] Moran NA, McLaughlin HJ, Sorek R (2009). The Dynamics and Time Scale of Ongoing Genomic Erosion in Symbiotic Bacteria.. Science.

[8] Mira A, Pushker R, Rodríguez-Valera F (2006). The Neolithic revolution of bacterial genomes.. Trends in Microbiology.

[9] Parkhill J, Sebaihia M, Preston A, Murphy LD, Thomson N (2003). Comparative analysis of the genome sequences of Bordetella pertussis, Bordetella parapertussis and Bordetella bronchiseptica.. Nat Genet.

[10] Treangen TJ, Abraham A-L, Touchon M, Rocha EPC (2009). Genesis, effects and fates of repeats in prokaryotic genomes.. FEMS Microbiology Reviews.

[11] Dance D (2000). Ecology of *Burkholderia pseudomallei* and the interactions between environmental *Burkholderia* spp. and human-animal hosts.. Acta Trop.

[12] Dharakul T, Songsivilai S (1999). The many facets of melioidosis.. Trends Microbiol.

[13] McGilvray C (1944). The transmission of glanders from horse to man.. Can J Public Health.

[14] Benenson A (1995). Control of Communicable Diseases Manual.

[15] DeShazer D, Waag D, Lindler L, Lebeda F, Korch GW (2004). Glanders: New Insights into an Old Disease.. Biological Weapons Defense: Infectious Diseases and Counterbioterrorism The Humana Press Inc.

[16] Cheng AC, Currie BJ (2005). Melioidosis: epidemiology, pathophysiology, and management.. Clin Microbiol Rev.

[17] Inglis TJJ, Sagripanti JL (2006). Environmental factors that affect the survival and persistence of *Burkholderia pseudomallei*.. Appl Environ Microbiol.

[18] Godoy D, Randle G, Simpson A, Aanensen D, Pitt T (2003). Multilocus sequence typing and evolutionary relationships among the causative agents of Melioidosis and Glanders, *Burkholderia pseudomallei* and *Burkholderia mallei*.. J Clin Microbiol.

[19] Lin CH, Bourque G, Tan P (2008). A Comparative Synteny Map of *Burkholderia* Species Links Large-Scale Genome Rearrangements to Fine-Scale Nucleotide Variation in Prokaryotes.. Mol Biol Evol.

[20] Nierman WC, DeShazer D, Kim HS, Tettelin H, Nelson KE (2004). Structural flexibility in the *Burkholderia* genome.. Proc Natl Acad Sci USA.

[21] Holden MTG, Titball RW, Peacock SJ, Cerdeño-Tárraga AM, Atkins T (2004). Genomic plasticity of the causative agent of melioidosis, *Burkholderia pseudomallei*.. Proc Natl Acad Sci USA.

[22] Wilkinson L (1981). Glanders: medicine and veterinary medicine in common pursuit of a contagious disease.. Med Hist.

[23] Whitlock GC, Estes DM, Torres AG (2007). Glanders: off to the races with *Burkholderia mallei*.. FEMS Microbiol Lett.

[24] Romero C, DeShazer D, Feldblyum T, Ravel J, Woods D (2006). Genome sequence alterations detected upon passage of *Burkholderia malle*i ATCC 23344 in culture and in mammalian hosts.. BMC Genomics.

[25] Schutzer SE, Schlater LR, Ronning CM, DeShazer D, Luft BJ (2008). Characterization of clinically-attenuated *Burkholderia mallei* by whole genome sequencing: candidate strain for exclusion from Select Agent lists.. PLoS ONE.

[26] DeShazer D, Waag DM, Fritz DL, Woods DE (2001). Identification of a *Burkholderia mallei* polysaccharide gene cluster by subtractive hybridization and demonstration that the encoded capsule is an essential virulence determinant.. Microbial Pathogenesis.

[27] Levinson G, Gutman GA (1987). Slipped-strand mispairing: a major mechanism for DNA sequence evolution.. Mol Biol Evol.

[28] Deitsch KW, Lukehart SA, Stringer JR (2009). Common strategies for antigenic variation by bacterial, fungal and protozoan pathogens.. Nat Rev Micro.

[29] Malakooti J, Ely B, Matsumura P (1994). Molecular characterization, nucleotide sequence, and expression of the *fliO*, *fliP*, *fliQ*, and *fliR* genes of *Esherichia coli*.. J Bacteriol.

[30] Parkhill J, Wren BW, Thomson NR, Titball RW, Holden MTG (2001). Genome sequence of Yersinia pestis, the causative agent of plague.. Nature.

[31] Rodrigues F, Sarkar-Tyson M, Harding SV, Sim S-H, Chua H-H (2006). Global map of growth-regulated gene expression in Burkholderia pseudomallei, the causative agent of melioidosis.. J Bacteriol.

[32] Ulrich RL, DeShazer D (2004). Type III secretion: a virulence factor delivery system essential for the pathogenicity of *Burkholderia mallei*.. Infect Immun.

[33] Tuanyok A, Kim HS, Nierman WC, Yu Y, Dunbar J (2005). Genome-wide expression analysis of iron regulation in *Burkholderia pseudomallei* and *Burkholderia mallei* using DNA microarrays.. FEMS Microbiol Lett.

[34] Moore RA, Reckseidler-Zenteno S, Kim H, Nierman B, Yu Y (2004). Contribution of gene loss to the pathogenic evolution of *Burkholderia pseudomallei* and *Burkholderia mallei*.. Infect Immun.

[35] Kim H, Schell MA, Yu Y, Ulrich RL, Sarria SH (2005). Bacterial genome adaptation to niches: Divergence of the potential virulence genes in three *Burkholderia* species of different survival strategies.. BMC Genomics.

[36] Fleischmann RD, Adams MD, White O, Clayton RA, Kirkness EF (1995). Whole-genome random sequencing and assembly of Haemophilus influenzae Rd.. Science.

[37] Myers EW, Sutton GG, Delcher AL, Dew IM, Fasulo DP (2000). A whole-genome assembly of Drosophila.. Science.

[38] Delcher AL, Phillippy A, Carlton J, Salzberg SL (2002). Fast algorithms for large-scale genome alignment and comparison.. Nucleic Acids Res.

[39] Delcher AL, Harmon D, Kasif S, White O, Salzberg SL (1999). Improved microbial gene identification with GLIMMER.. Nucleic Acids Res.

[40] Salzberg SL, Delcher AL, Kasif S, White O (1998). Microbial gene identification using interpolated Markov models.. Nucleic Acids Res.

[41] Carver TJ, Rutherford KM, Berriman M, Rajandream M-A, Barrell BG (2005). ACT: the Artemis comparison tool.. Bioinformatics.

[42] Schneider TD, Stephens RM (1990). Sequence logos: a new way to display consensus sequences.. Nucl Acids Res.

[43] Roy CJ, Hale M, Hartings JM, Pitt L, Duniho S (2003). Impact of inhalation exposure modality and particle size on the respiratory deposition of ricin in BALB/c mice.. Inhal Toxicol.

[44] Moore RA, DeShazer D, Reckseidler S, Weissman A, Woods DE (1999). Efflux-mediated aminoglycoside and macrolide resistance in *Burkholderia pseudomallei*.. Antimicrob Agents Chemother.

