# Supplementary figures and images for: The Early Stage of Bacterial Genome-Reductive Evolution in the Host
Source: PLoS Pathog. 2010 May 27;6(5):e1000922. doi: 10.1371/journal.ppat.1000922 (PMC2877748; doi:10.1371/journal.ppat.1000922)

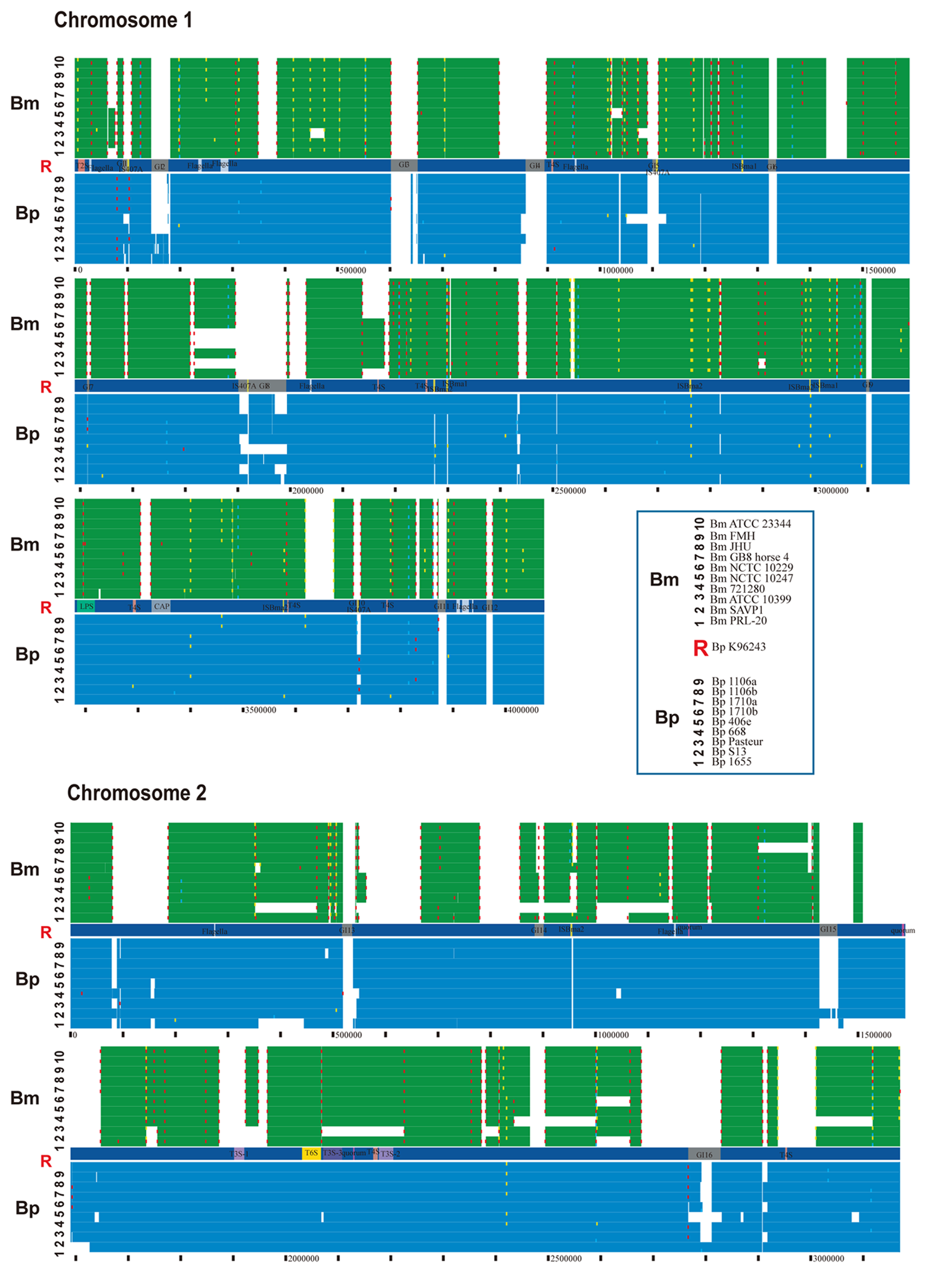

Supplement: Figure S1 — Graphical alignments of the genomes of B. mallei and B. pseudomallei. Genomes from strains of B. mallei and B. pseudomallei are aligned for close comparisons of the relative positions of IS elements and large deletions. Based on the genome of B. pseudomallei K96243 (displayed in the middle of the alignments), corresponding regions in B. mallei genomes (denoted by the upper green blocks) and those of the other B. pseudomallei genomes (denoted by the lower blue blocks) are displayed. Locations of ISBma1, ISBma2, and IS407A are denoted by red, yellow, and pink lines, respectively, in each strain. (1.87 MB TIF) [file ppat.1000922.s001.tif]

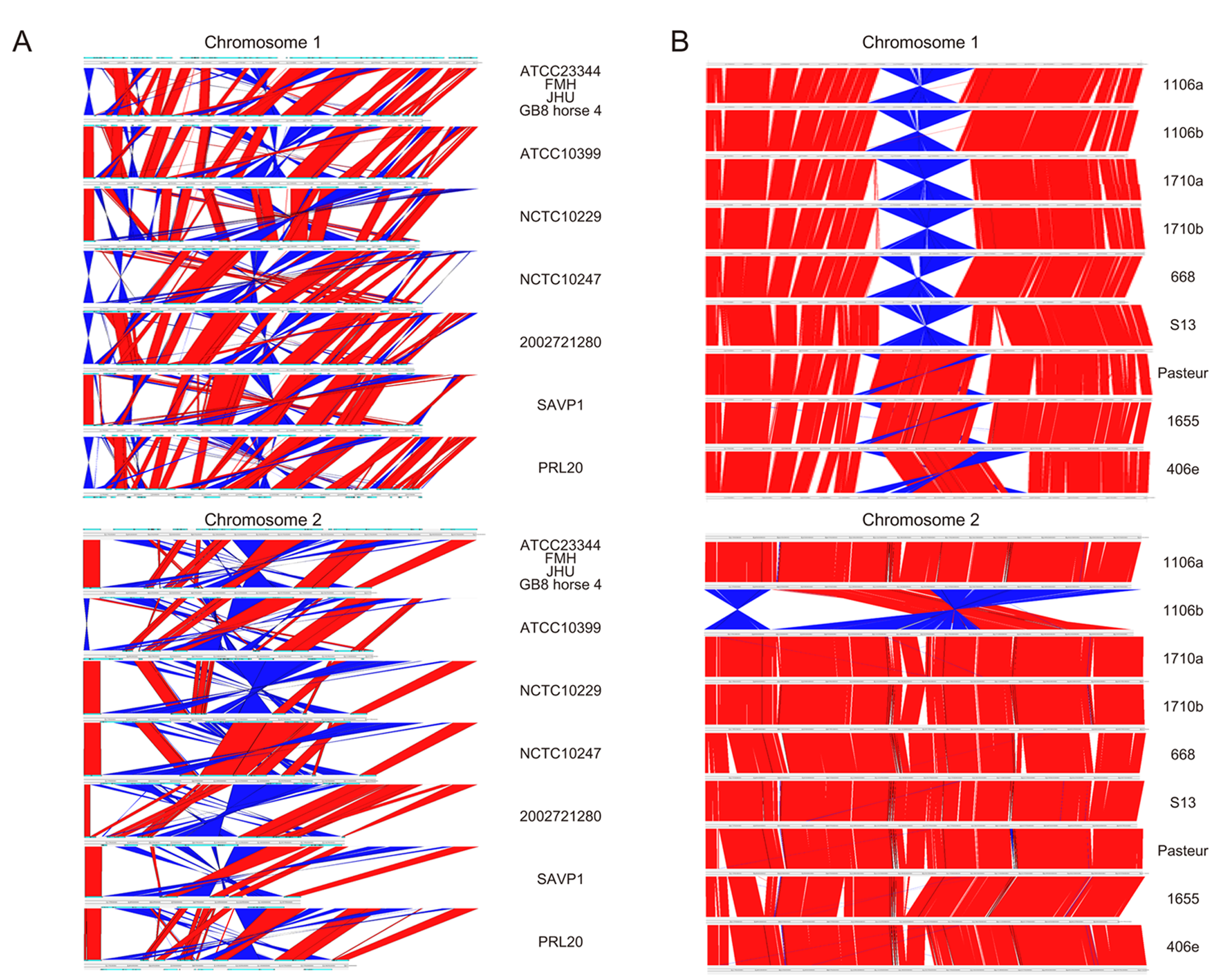

Supplement: Figure S2 — The patterns of genomic rearrangements in B. mallei. A. Syntenic relationship among the B. mallei strains. Re-localization of BRUs in B. mallei strains (relative to B. pseudomallei strain K96243 as the reference) is shown with the comparative genomics display tool ACT (Wellcome Trust Sanger Institute) using blastn data. Blue and red connecting lines between the genomes indicate the same and opposite directions, respectively, of the corresponding BRUs relative to each other. B. Comparisons among the B. pseudomallei strains. Each strain was compared to the reference genome of strain K96243. (2.42 MB TIF) [file ppat.1000922.s002.tif]
