# Supplementary material for: The Early Stage of Bacterial Genome-Reductive Evolution in the Host
Source: PLoS Pathog. 2010 May 27;6(5):e1000922. doi: 10.1371/journal.ppat.1000922 (PMC2877748; doi:10.1371/journal.ppat.1000922)
Supplement: Table S1 — Comparison of B. mallei genomes with that of B. pseudomallei strain K96243. (8.84 MB PDF) [file ppat.1000922.s003.pdf]

Table S1. Comparison of *B. mallei* genomes with that of *B. pseudomallei* strain K96243

IS elements are highligted in yellow	
Genomic deletions are highlighted in green	

				ATCC23344		FMH		JHU		GB-8 horse 4		ATCC 10399		NCTC 10229		NCTC 10247		2002721280		SAVP1		PRL-20	
w num	tags in Bp K	IS element	BRF	5'-end	3'-end	5'-end	3'-end	5'-end	3'-end	5'-end	3'-end	5'-end	3'-end	5'-end	3'-end	5'-end	3'-end	5'-end	3'-end	5'-end	3'-end	5'-end	3'-end
**Chromosome 1**																							
1	BPSL0001		1	3008497	3009609	2906416	2907528	2922158	2923270	2994742	2995854	3244191	3243079	1669406	1668294	2934607	2935719	2830401	2831513	3358492	3357380	3045976	3047088
2	BPSL0002		1	3007238	3008449	2905157	2906368	2920899	2922110	2993483	2994694	3245450	3244239	1670665	1669454	2933348	2934559	2829142	2830353	3359751	3358540	3044717	3045928
3	BPSL0003		1	3004445	3006496	2902364	2904415	2918106	2920157	2990690	2992741	3248243	3246192	1673458	1671407	2930555	2932606	2826349	2828400	3362535	3360493	3041933	3043975
4	BPSL0004		1	3004125	3003850	2902044	2901769	2917786	2917511	2990370	2990095	3248563	3248838	1673778	1674053	2930235	2929960	2826029	2825754	3362855	3363130	3041613	3041338
5		Chr1_1a_ISBma2_A		3002230	3003802	2900149	2901721	2915891	2917463	2988475	2990047	3248886	3250457	1674101	1675677	2928340	2929912	2824135	2825706	3363178	3364749	3039719	3041290
6		Chr1_1b_ISBma2_A		2879914	2881486	2776259	2777831	2791386	2792958	2866443	2868015	3029164	3030736	1930938	1932510	2622004	2623575			3479600	3481131	2896600	2898131
7	BPSL0005		2	2878493	2879830	2774838	2776175	2789965	2791302	2865022	2866359	3027743	3029080	1929517	1930854	2624996	2623659	2822714	2824051	3478179	3479516	2895179	2896516
8	BPSL0006		2	2877707	2878171	2774052	2774516	2789179	2789643	2864236	2864700	3026957	3027421	1928731	1929195	2625782	2625318	2821928	2822392	3477393	3477857	2894393	2894857
9	BPSL0007		2	2877309	2875039	2773654	2772416	2788781	2786511	2863838	2861568	3026559	3024289	1928333	1926063	2626180	2628450	2821530	2819260	3476995	3474725	2893995	2892760
10	BPSL0008		2	2875039	2873549	2770886	2769396	2786511	2785021	2861568	2860078	3024289	3022799	1926063	1924573	2628450	2629940	2819260	2817770	3474725	3473235	3030694	3029204
11	BPSL0009		2	2873541	2872327	2769388	2768174	2785013	2783799	2860070	2858856	3022791	3021577	1924565	1923351	2629948	2631162	2817762	2816548	3473227	3472013	3029196	3027982
12	BPSL0010		2	2871842	2872249	2767689	2768096	2783314	2783721	2858371	2858778	3021092	3021499	1922866	1923273	2631647	2631240	2816063	2816470	3471528	3471935	3027497	3027904
13	BPSL0011		2	2871694	2871245	2767541	2767092	2783166	2782717	2858223	2857774	3020944	3020495	1922718	1922269	2631795	2632244	2815915	2815466	3471380	3470931	3027349	3026900
14	BPSL0012		2	2871084	2870611	2766931	2766458	2782556	2782083	2857613	2857140	3020334	3019861	1922108	1921635	2632405	2632878	2815305	2814832	3470770	3470297	3026739	3026266
15	BPSL0013		2	2870605	2870204	2766452	2766051	2782077	2781676	2857134	2856733	3019855	3019454	1921629	1921228	2632884	2633285	2814826	2814425	3470291	3469890	3026260	3025859
16	BPSL0014		2	2870223	2869498	2766070	2765345	2781695	2780970	2856752	2856027	3019473	3018748	1921247	1920522	2633266	2633991	2814444	2813719	3469909	3469184	3025878	3025153
17	BPSL0015		2	2869313	2868246	2765120	2764053	2780785	2779718	2855842	2854775	3018563	3017496	1920337	1919270	2634176	2635243	2813534	2812467	3468999	3467932	3024968	3023901
18	BPSL0016		2	2868218	2866791	2764025	2763504	2779690	2778263	2854747	2853320	3017468	3016041	1919242	1917815	2635271	2636698	2812439	2811012	3467904	3466477	3023873	3022446
19	BPSL0017		2	2866791	2866288	2762160	2761657	2778263	2777760	2853320	2852817	3016041	3015538	1917815	1917312	2636698	2637201	2811012	2810509	3466477	3465974	3022446	3021943
20	BPSL0018		2	2866246	2865458	2761615	2760827	2777718	2776930	2852775	2851987	3015496	3014708	1917270	1916482	2637243	2638031	2810467	2809679	3465932	3465144	3021901	3021113
21	BPSL0019		2	2863468	2865078	2758837	2760447	2774940	2776550	2849969	2851579	3012795	3014405	1914618	1916228	2639881	2638271	2807822	2809432	3463294	3464904	3019270	3020880
22	BPSL0020		2	2862965	2863243	2758334	2758612	2774437	2774715	2849466	2849744	3012292	3012570	1914115	1914393	2640384	2640106	2807319	2807597	3462791	3463069	3018767	3019045
23	BPSL0021		2	2862766	2862278	2758135	2757647	2774238	2773750	2849267	2848779	3012093	3011605	1913916	1913428	2640583	2641071	2807120	2806632	3462592	3462104	3018568	3018080
24	BPSL0022		2	2862127	2860568	2757496	2755937	2773599	2772040	2848628	2847069	3011454	3009895	1913277	1911718	2641222	2642781	2806481	2804922	3461953	3460394	3017929	3016370
25	BPSL0023		2	2859582	2860502	2754951	2755871	2771054	2771974	2846083	2847003	3008909	3009829	1910732	1911652	2643767	2642847	2803936	2804856	3459408	3460328	3015384	3016304
26	BPSL0024		2	2859370	2858975	2754739	2754344	2770842	2770447	2845871	2845476	3008697	3008302	1910520	1910125	2643979	2644374	2803724	2803329	3459196	3458801	3015172	3014777
27	BPSL0025		2	2858903	2858184	2754272	2753553	2770375	2769656	2845404	2844685	3008230	3007511	1910053	1909334	2644446	2645165	2803257	2802538	3458729	3458010	3014705	3013986
28	BPSL0026		2	2857344	2856850	2752713	2752219	2768816	2768322	2843845	2843351	3006671	3006177	1908494	1908000	2646005	2646499	2801698	2801204	3457179	3456685	3013155	3012661
29	BPSL0027		2	2856824	2855829	2752193	2751198	2768296	2767301	2843325	2842330	3006151	3005156	1907974	1906979	2646525	2647520	2801178	2800183	3456659	3455664	3012635	3011640
30	BPSL0028		2	2855833	2855339	2751202	2750708	2767305	2766811	2842334	2841840	3005160	3004666	1906983	1906489	2647516	2648010	2800187	2799693	3455668	3455174	3011644	3011150
31	BPSL0029		2	2855168	2854677	2750537	2750046	2766640	2766149	2841669	2841178	3004495	3004004	1906348	1905857	2648151	2648642	2799552	2799061	3455003	3454512	3010979	3010488
32	BPSL0030_fl	partial	2	2854603	2854391	2749972	2749760	2766075	2765863	2841104	2840892	3003930	3003718	1905783	1905571	2648716	2648928	2798987	2798775	3454438	3454226	3010414	3010202
33		Chr1_2a_IS407A_A		2853155	2854390	2748524	2749759	2764627	2765862	2839656	2840891	3002482	3003717	1904335	1905570	2648929	2650164	2797539	2798774	3452990	3454225	3008966	3010201
34		Chr1_2b_IS407A_A		2786407	2787642	2681776	2683011	2697886	2699121	2773025	2774260	2968933	2970168							3221731	3222966	2830709	2831944
35	BPSL0030_fl	partial	3a	2786406	2785858	2681775	2681227	2697885	2697337	2773024	2772476	2970169	2970717	1904334	1903786	2650165	2650713	2797538	2796990	3222967	3223515	2830708	2830160
36	BPSL0031		3a	2785829	2785560	2681198	2680929	2697308	2697039	2772447	2772178	2970746	2971015	1903757	1903488	2650742	2651011	2796961	2796692	3223544	3223813	2830131	2829862
37	BPSL0032		3a	2785438	2784659	2680807	2680028	2696917	2696138	2772056	2771277	2971137	2971916	1903366	1902587	2651133	2651912	2796570	2795791	3223935	3224714	2829740	2828961

38 BPSL0033		3a	2783908	2784333	2678830	2679255	2695387	2695812	2770526	2770951	2972667	2972242	1901836	1902261	2652663	2652238	2795040	2795465	3225465	3225040	2828210	2828635
39 BPSL0034		3a	2783826	2782954	2678748	2677876	2695305	2694433	2770444	2769572	2972749	2973621	1901754	1900882	2652745	2653617	2794958	2794086	3225547	3226419	2828128	2827256
40 BPSL0035		3a	2782958	2782440	2677880	2677362	2694437	2693919	2769576	2769058	2973617	2974135	1900886	1900368	2653613	2654131	2794090	2793572	3226415	3226933	2827260	2826742
41 BPSL0036		3a	2782279	2781269	2677201	2676191	2693758	2692748	2768897	2767887	2974296	2975306	1900207	1899197	2654292	2655302	2793411	2792401	3227094	3228104	2826581	2825571
42 BPSL0037		3a	2781182	2780388	2676104	2675310	2692661	2691867	2767800	2767006	2975393	2976187	1899110	1898316	2655389	2656183	2792314	2791520	3228191	3228985	2825484	2824690
43 BPSL0038		3a	2780366	2779512	2675288	2674434	2691845	2690991	2766984	2766130	2976209	2977063	1898294	1897440	2656205	2657059	2791498	2790644	3229007	3229861	2824668	2823814
44 BPSL0039		3a	2779431	2778055	2674353	2672977	2690910	2689534	2766049	2764673	2977144	2978520	1897359	1895983	2657140	2658516	2790563	2789187	3229942	3231318	2823733	2822357
45 BPSL0040		3a	2778041	2777367	2672963	2672289	2689520	2688846	2764659	2763985	2978534	2979208	1895969	1895295	2658530	2659204	2789173	2788499	3231332	3232006	2822343	2821669
46 BPSL0041		3a	2777031	2775907	2671953	2670829	2688510	2687386	2763649	2762525	2979539	2980663	1894969	1893845	2659530	2660654	2788173	2787049	3232332	3233456	2821343	2820219
47	Chr1_3_ISBma2_C	3a															2785321	2786893				
48 BPSL0042		3a	2775428	2773419	2670350	2668341	2686907	2684898	2762046	2760037	2981143	2983152	1893366	1891357	2661133	2663142	2784977	2782968	3233935	3235944	2819740	2817731
49 BPSL0043		3a	2773375	2770349	2668297	2665271	2684854	2681828	2759993	2756967	2983196	2986222	1891313	1888287	2663186	2666212	2782924	2779898	3235988	3239014	2817687	2814661
50 BPSL0044		3a	2769808	2768711	2664730	2663633	2681287	2680190	2756426	2755329	2986763	2987860	1887746	1886649	2666753	2667850	2779357	2778260	3239555	3240652	2814120	2813023
51 BPSL0045		3a	2768308	2767355	2663230	2662277	2679787	2678834	2754926	2753973	2988263	2989216	1886246	1885293	2668253	2669206	2777857	2776904	3241055	3242008	2812620	2811667
52 BPSL0046		3a	2766495	2767223	2661417	2662145	2677974	2678702	2753113	2753841	2990076	2989348	1884433	1885161	2670066	2669338	2776044	2776772	3242868	3242140	2810807	2811535
53 BPSL0047		3a	2765400	2766119	2660322	2661041	2676879	2677598	2752018	2752737	2991171	2990452	1883338	1884057	2671161	2670442	2774949	2775668	3243963	3243244	2809712	2810431
54 BPSL0048		3a	2764354	2765400	2659276	2660322	2675833	2676879	2750972	2752018	2992217	2991171	1882292	1883338	2672207	2671161	2773903	2774949	3245009	3243963	2808666	2809712
55 BPSL0049		3a	2763479	2764354	2658401	2659276	2674958	2675833	2750097	2750972	2993092	2992217	1881417	1882292	2673082	2672207	2773028	2773903	3245884	3245009	2807791	2808666
56 BPSL0050		3a	2762236	2763471	2657158	2658393	2673715	2674950	2748854	2750089	2994335	2993100	1880174	1881409	2674325	2673090	2772025	2773020	3247127	3245892	2806548	2807783
57 BPSL0051		3a	2760493	2761995	2654915	2656417	2671718	2673220	2747110	2748612	2996078	2994576	1878431	1879933	2676068	2674566	2770227	2771729	3248870	3247368	3155697	3157199
58 BPSL0052		3a	2760045	2760434	2654467	2654856	2671270	2671659	2746662	2747051	2996526	2996137	1877983	1878372	2676516	2676127	2769779	2770168	3249318	3248929	3155249	3155638
59 BPSL0053		3a	2759225	2760031	2653647	2654453	2670450	2671256	2745842	2746648	2997346	2996540	1877163	1877969	2677336	2676530	2768959	2769765	3250138	3249332	3154429	3155235
60 BPSL0054		3a	2758382	2759176	2652804	2653598	2669607	2670401	2744999	2745793	2998189	2997395	1876320	1877114	2678179	2677385	2768116	2768910	3250981	3250187	3153586	3154380
61 BPSL0055		3a	2756648	2758294	2651070	2652716	2667873	2669519	2743265	2744911	2999923	2998277	1874586	1876232	2679913	2678267	2766382	2768028	3252715	3251069	3151852	3153498
62 BPSL0056		3a	2755920	2755582	2650342	2650004	2667145	2666807	2742537	2742199	3000651	3000989	1873858	1873520	2680641	2680979	2765654	2765316	3253443	3253781	3151124	3150786
63	Chr1_4_IS407A_B		2752854	2754089	2647276	2648511	2664079	2665314	2739471	2740706	3002482	3003717							3255150	3256385	3148182	3149417
64 BPSL0057-putative membrane protein-disrupted																						
65 BPSL0058		3b											1870725	1871708	2683774	2682791	2762521	2763504	3441355	3440372		
66 BPSL0059		3b											1869859	1870692	2684640	2683807	2761655	2762488	3442221	3441388		
67 BPSL0060		3b											1868327	1869403	2686172	2685096	2760123	2761199	3443753	3442677		
68 BPSL0061		3b											1868214	1867081	2686285	2687418	2760010	2758877	3443866	3444999		
69 BPSL0062		3b											1866471	1865269	2688028	2689230	2758267	2757065	3445609	3446007		
70 BPSL0063		3b											1865253	1863133	2689246	2691366	2757049	2754929	3446826	3448946		
71 BPSL0064		3b											1862766	1861636	2691733	2692863	2754562	2753432	3449313	3450443		
72 BPSL0065		3b											1860990	1861553	2693509	2692946	2752786	2753349	3451089	3450526		
73 BPSL0066		3b											1860190	1860651	2694309	2693848	2751986	2752447	3451889	3451428		
74 BPSL0067		3b											1859721	1859197	2694778	2695302	2751517	2750993	3452358	3452882		
75 BPSL0068		3b											1859207	1859073	2695292	2695426	2751003	2750869	3452872	3453006		
76	Chr1_5_IS407A_A												1857854	1859089	2695410	2696645	2749650	2750885	3452990	3454225		
77 BPSL0069																						
78 BPSL0070																						
79 BPSL0071																						
80 BPSL0072																						
81	Chr1_6_IS407A_A		6007	7242	3419381	3420616	3439108	3440343	5679	6914	3533677	3534912	2261519	2262754	6007	7242	3166175	3167410	2832213	2833448	34218	35453
82 BPSL0073		4	3081	5546	3416455	3418920	3436182	3438647	2753	5218	3537838	3535373	2265680	2263215	3081	5546	3163249	3165714	2829287	2831752	31292	33757
83 BPSL0074		4	1789	2889	3415163	3416263	3434890	3435990	1461	2561	3539130	3538030	2266972	2265872	1789	2889	3161957	3163057	2827995	2829095	30000	31100
84 BPSL0075		4	25	1623	3413459	3414997	3433174	3434724	3	1295	3540894	3539296	2268736	2267138	25	1623	3160193	3161791	2826231	2827829	28236	29834
85 BPSL0075a		4	3509727	3509596	3412532	3412401	3431898	3431767	3495522	3495391	3541242	3541373	2269182	2269313	3495266	3495135	3159747	3159616	2825785	2825654	27790	27659
86 BPSL0076		4	3509519	3509112	3412324	3411917	3431690	3431283	3495314	3494907	3541450	3541857	2269390	2269797	3495058	3494651	3159539	3159132	2825577	2825170	27582	27175
87 BPSL0077		4	3509029	3508763	3411834	3411568	3431200	3430934	3494824	3494558	3541940	3542206	2269880	2270146	3494568	3494302	3159049	3158783	2825087	2824821	27092	26826
88 BPSL0078		4	3508751	3507078	3411556	3409883	3430922	3429249	3494546	3492873	3542218	3543891	2270158	2271831	3494290	3492617	3158771	3157098	2824809	2823136	26814	25141

89	BPSL0079		4	3506553	3506915	3409358	3409720	3428724	3429086	3492348	3492710	3544416	3544054	2272356	2271994	3492092	3492454	3156573	3156935	2822611	2822973	24616	24978
90	BPSL0080		4	3506161	3504761	3408966	3407566	3428332	3426932	3491956	3490556	3544808	3546208	2272748	2274148	3491700	3490300	3156181	3154781	2822219	2820819	24224	22824
91		Chr1_7_IS407A_A		3503487	3504722	3406292	3407527	3425658	3426893	3489282	3490517	3546247	3547482	2274369	2275604	3488844	3490079	3153325	3154560	2819545	2820780	21550	22785
92	BPSL0081	GI1																					
93	BPSL0082	GI1																					
94	BPSL0083	GI1																					
95	BPSL0084	GI1																					
96	BPSL0085	GI1																					
97	BPSL0086	GI1																					
98	BPSL0087	GI1																					
99	BPSL0088	GI1																					
100	BPSL0089	GI1																					
101	BPSL0090	GI1_IS407A																					
102	BPSL0091	GI1_IS407A																					
103	BPSL0092	GI1																					
104	BPSL0093																						
105	BPSL0094																						
106	BPSL0095																						
107	BPSL0096																						
108		Chr1_8_IS407A_A		123747	124982	3538554	3539789	3559432	3560667	123374	124609	3546247	3547482	2274369	2275604	2264036	2265271	3153325	3154560	2819545	2820780	21550	22785
109	BPSL0097		5a	124984	125073	3539791	3539880	3560669	3560758	124611	124700	3547484	3547573	2275606	2275695	2265273	2265362	3153323	3153234	2819543	2819454	21548	21459
110	BPSL0098		5a	125292	125843	3540099	3540650	3560977	3561528	124919	125470	3547792	3548343	2275914	2276465	2265581	2266132	3153015	3152464	2819235	2818684	21240	20689
111	BPSL0099		5a	126462	126049	3541269	3540856	3562147	3561734	126089	125676	3548948	3548535	2277064	2276651	2266731	2266318	3151858	3152271	2818049	2818462	20069	20482
112		Chr1_9a_IS407A_C																?	?				
113		Chr1_9b_IS407A_C																3404613	3405848				
114	BPSL0100		5b	126864	127955	112	1203	1	780	126279	127058	282	1373	2277466	2278557	2267133	2268224	2	1027	2817647	2816556	19667	18576
115	BPSL0101		5b	127955	128893	1203	2141	780	1718	127058	127996	1373	2311	2278557	2279495	2268224	2269162	1027	1338	2816556	2815618	18576	17713
116	BPSL0102		5b	129068	130666	2316	3914	1893	3491	128171	129769	2486	4084	2279670	2281268	2269337	2270935	2141	3739	2815443	2813845	17462	15864
117	BPSL0103		5b	132136	130691	5384	3939	4961	3516	131239	129794	5554	4109	2282738	2281293	2272405	2270960	5209	3764	2812375	2813820	14394	15839
118	BPSL0104		5b	132948	132403	6196	5651	5773	5228	132051	131506	6366	5821	2283550	2283005	2273217	2272672	6021	5476	2811563	2812108	13582	14127
119	BPSL0105		5b	133405	133449	6653	6697	6230	6274	132508	132552	6817	6861	2284007	2284051	2273674	2273718	6478	6522	2811106	2811062	13125	13081
120	BPSL0106		5b	134110	133544	7358	6792	6935	6369	133213	132647	7522	6956	2284712	2284146	2274379	2273813	7183	6617	2810401	2810967	12420	12986
121	BPSL0107		5b	135181	134216	8429	7464	8006	7041	134284	133319	8593	7628	2285783	2284818	2275450	2274485	8254	7289	2809330	2810295	11349	12314
122	BPSL0108		5b	135324	136415	8572	9663	8149	9240	134427	135518	8736	9827	2285926	2287017	2275593	2276684	8397	9488	2809187	2808096	11206	10115
123	BPSL0109		5b	136447	137556	9695	10804	9272	10381	135550	136659	9859	10968	2287049	2288158	2276716	2277825	9520	10629	2808064	2806955	10083	8974
124	BPSL0110		5b	137570	138442	10818	11690	10395	11267	136673	137545	10982	11854	2288172	2289044	2277839	2278711	10643	11515	2806941	2806069	8960	8088
125	BPSL0111		5b	139379	140479	12627	13727	12204	13304	138482	139582	12791	13891	2289981	2291081	2280620	2281720	12452	13552	2805132	2804032	7151	6051
126	BPSL0112		5b	140839	140558	14087	13806	13664	13383	139942	139661	14251	13970	2291441	2291160	2282080	2281799	13912	13631	2803672	2803953	5691	5972
127	BPSL0113		5b	141318	140839	14566	14087	14143	13664	140421	139942	14730	14251	2291920	2291441	2282559	2282080	14391	13912	2803193	2803672	5212	5691
128		Chr1_10_IS407A_A	5b	141323	142558	14571	15806	14148	15383	140426	141661	14735	15970	2291924	2293159	2282563	2283798	14396	15631	2801953	2803188	3972	5207
129		Chr1_11_IS407A_C	5b													2300118	2301353						
130		Chr1_12_ISBma1_A	5b	142668	143972	15916	17220	15493	16797	141771	143075	17216	18520	2294405	2295709	2302599	2303903	16877	18181	2799403	2800707	1423	2727
131	BPSL0114		5b	144282	144683	17530	17931	17107	17508	143385	143786	18830	19231	2296019	2296420	2304213	2304614	18491	18892	2799093	2798692	1113	712
132	BPSL0115		5b	144724	145590	17972	18838	17549	18415	143827	144693	19272	20138	2296461	2297327	2304655	2305521	18933	19799	2798651	2797785	671	3
133	BPSL0116		5b	146730	145717	19978	18965	19555	18542	145833	144820	21278	20265	2298467	2297454	2306661	2305648	20939	19926	2796645	2797658	188096	187083
134	BPSL0117		5b	147180	148232	20428	21480	20005	21057	146283	147335	21728	22780	2298917	2299969	2307111	2308163	21389	22441	2796195	2795143	188546	189598
135	BPSL0118		5b	151066	148460	24314	21708	23891	21285	150169	147563	25614	23008	2302803	2300197	2310997	2308391	25275	22669	2792309	2794915	192432	189826
136	BPSL0119		5b	151648	151280	24896	24528	24473	24105	150751	150383	26196	25828	2303385	2303017	2311579	2311211	25857	25489	2791727	2792095	193014	192646
137	BPSL0120		5b	152832	151711	26080	24959	25657	24536	151935	150814	27380	26259	2304569	2303448	2312763	2311642	27041	25920	2790543	2791664	194198	193077
138	BPSL0121		5b	153127	153627	26375	26875	25952	26452	152230	152730	27675	28175	2304864	2305364	2313058	2313558	27336	27836	2790248	2789748	194493	194993
139	BPSL0122		5b	153662	154642	26910	27890	26487	27467	152765	153745	28210	29190	2305399	2306379	2313593	2314573	27871	28851	2789713	2788733	195028	196008

140	BPSL0123		5b	154766	155398	28014	28646	27591	28223	153869	154501	29314	29946	2306503	2307135	2314697	2315329	28975	29607	2788609	2787977	196132	196764
141	BPSL0124		5b	155511	156365	28759	29613	28336	29190	154614	155468	30059	30913	2307248	2308102	2315442	2316296	29720	30574	2787864	2787010	196877	197731
142	BPSL0125		5b	156746	158152	29994	31400	29571	30977	155849	157255	31294	32700	2308475	2309881	2316669	2318075	30947	32353	2786629	2785223	198112	199518
143	BPSL0126		5b	158152	158739	31400	31987	30977	31564	157255	157842	32700	33287	2309881	2310468	2318075	2318662	32353	32940	2785223	2784636	199518	200105
144	BPSL0127		5b	158744	161149	31992	34397	31569	33974	157847	160252	33292	35697	2310473	2312878	2318667	2321072	32945	35350	2784631	2782226	200110	202515
145	BPSL0128		5b	161153	161833	34401	35081	33978	34658	160256	160936	35701	36381	2312882	2313562	2321076	2321756	35354	36034	2782222	2781542	202519	203199
146	BPSL0129	GI2																					
147	BPSL0130	GI2																					
148	BPSL0130a	GI2																					
149	BPSL0131	GI2																					
150	BPSL0132	GI2																					
151	BPSL0133	GI2																					
152	BPSL0134	GI2																					
153	BPSL0135	GI2																					
154	BPSL0136	GI2																					
155	BPSL0137	GI2																					
156	BPSL0138	GI2																					
157	BPSL0139	GI2																					
158	BPSL0140	GI2																					
159	BPSL0141	GI2																					
160	BPSL0142	GI2																					
161	BPSL0143	GI2																					
162	BPSL0144	GI2																					
163	BPSL0145	GI2																					
164	BPSL0146	GI2																					
165	BPSL0147	GI2																					
166	BPSL0148	GI2																					
167	BPSL0149	GI2																					
168	BPSL0150	GI2																					
169	BPSL0151	GI2																					
170	BPSL0152	GI2																					
171	BPSL0153	GI2																					
172	BPSL0154	GI2																					
173	BPSL0155	GI2																					
174	BPSL0156	GI2																					
175	BPSL0157	GI2																					
176	BPSL0158	GI2																					
177	BPSL0159	GI2																					
178	BPSL0160	GI2																					
179	BPSL0161	GI2																					
180	BPSL0162	GI2																					
181	BPSL0163	GI2																					
182	BPSL0164	GI2																					
183	BPSL0165	GI2																					
184	BPSL0166	GI2																					
185	BPSL0167	GI2																					
186	BPSL0168	GI2																					
187	BPSL0169	GI2																					
188	BPSL0170	GI2																					
189	BPSL0171	GI2																					
190	BPSL0172	GI2_duplicate																					

191	BPSL0173	GI2_duplicate																					
192	BPSL0174	GI2_duplicate																					
193	BPSL0175	GI2_duplicate																					
194	BPSL0176	GI2																					
195	BPSL0177		5b	162304	163035	35552	36283	35129	35860	161407	162138	36852	37583	2314033	2314764	2322227	2322958	36505	37236	2781071	2780340	203670	204401
196	BPSL0178		5b	163132	163761	36380	37009	35957	36586	162235	162864	37680	38309	2314861	2315490	2323055	2323684	37333	37962	2780243	2779614	204498	205127
197	BPSL0179		5b	163784	164233	37032	37481	36609	37058	162887	163336	38332	38781	2315513	2315962	2323707	2324156	37985	38434	2779591	2779142	205150	205599
198	BPSL0180		5b	164658	165440	37906	38688	37483	38265	163761	164543	39206	39988	2316387	2317169	2324581	2325363	38859	39641	2778717	2777935	206024	206806
199	BPSL0181		5b	165440	166213	38688	39461	38265	39038	164543	165316	39988	40761	2317169	2317942	2325363	2326136	39641	40414	2777935	2777162	206806	207579
200	BPSL0182		5b	167778	166633	41012	39867	40589	39444	166881	165736	42207	41062	2319521	2318376	2327568	2326423	41916	40771	2775632	2776777	208990	207845
201	BPSL0183		5b	170198	167793	43432	41027	43009	40604	169301	166896	44627	42222	2321941	2319536	2329988	2327583	44336	41931	2773212	2775617	211272	209005
202	BPSL0184		5b	170894	170385	44128	43619	43705	43196	169997	169488	45323	44814	2322637	2322128	2330684	2330175	45032	44523	2772516	2773025	211929	211420
203	BPSL0185		5b	171964	170894	45198	44128	44775	43705	171067	169997	46393	45323	2323707	2322637	2331754	2330684	46102	45032	2771446	2772516	212999	211929
204	BPSL0186		5b	173127	172087	46361	45321	45938	44898	172230	171190	47556	46516	2324870	2323830	2332917	2331877	47265	46225	2770283	2771323	214162	213122
205	BPSL0187		5b	173499	173789	46733	47023	46310	46600	172602	172892	47928	48218	2325242	2325532	2333289	2333579	47637	47927	2769911	2769621	214534	214824
206	BPSL0188		5b	173905	175392	47139	48626	46716	48203	173008	174495	48334	49821	2325648	2327135	2333695	2335182	48043	49530	2769505	2768018	214940	216427
207	BPSL0189		5b	175398	176867	48632	50101	48209	49678	174501	175970	49827	51296	2327141	2328610	2335188	2336657	49536	51005	2768012	2766543	216433	217902
208	BPSL0190		5b	176997	177833	50231	51067	49808	50644	176100	176936	51426	52262	2328740	2329576	2336787	2337623	51135	51971	2766413	2765577	218032	218868
209	BPSL0191		5b	177868	178635	51102	51869	50679	51446	176971	177738	52297	53064	2329611	2330378	2337658	2338425	52006	52773	2765542	2764775	218903	219670
210		Chr1_13a_ISBma2_A		178751	180323	51985	53557	51562	53134	177854	179426	53180	54752	2330494	2332086	2338546	2340123	52889	54461	2763087	2764659	219786	221358
211		Chr1_13b_ISBma2_A		3346749	3348321	3247169	3248741	3265431	3267003	3333036	3334608	3421617	3423189	2207258	2208830	3400063	3401610	3218859	3220433	2884897	2886468	86972	88544
212	BPSL0192		6	3349662	3348601	3250082	3249021	3268344	3267283	3335949	3334888	3424467	3423406	2206022	2207083	3398827	3399888	3221669	3220608	2887711	2886650	89787	88726
213	Partial	Chr1_14_ISBma1_A	6	3349816	3351120	3250236	3251540	3268498	3269802	3336103	3337407	3424621	3425925	2204564	2205868	3397369	3398673	3221823	3223127	2887865	2888041	89941	91245
214		Chr1_15_IS407A_C	6																	2888034	2889269		
215	Partial	Chr1_14-1_ISBma1_A	6																	2889266	2890409		
216	BPSL0193		6	3351185	3352126	3251605	3252546	3269867	3270808	3337472	3338413	3425990	3426931	2204499	2203558	3397304	3396363	3223192	3224133	2890474	2891415	91310	92251
217	BPSL0194		6	3353441	3352506	3253861	3252926	3272123	3271188	3339728	3338793	3428246	3427311	2202234	2203178	3395039	3395983	3225457	3224513	2892730	2891795	93566	92631
218	BPSL0195		6	3354840	3353530	3255260	3253950	3273522	3272212	3341127	3339817	3429645	3428335	2200835	2202145	3393640	3394950	3226856	3225546	2894129	2892819	94965	93655
219	BPSL0196		6	3355466	3354855	3255886	3255275	3274148	3273537	3341753	3341142	3430271	3429660	2200209	2200820	3393014	3393625	3227482	3226871	2894755	2894144	95591	94980
220	BPSL0197		6	3356608	3355466	3257028	3255886	3275290	3274148	3342895	3341753	3431413	3430271	2199067	2200209	3391872	3393014	3228624	3227482	2895897	2894755	96733	95591
221	BPSL0198		6	3357650	3356964	3258070	3257384	3276332	3275646	3343937	3343251	3432455	3431769	2198025	2198711	3390830	3391516	3229666	3228980	2896939	2896253	97775	97089
222	BPSL0199		6	3358420	3357620	3258840	3258040	3277102	3276302	3344707	3343907	3433225	3432425	2197255	2198055	3390060	3390860	3230436	3229636	2897709	2896909	98545	97745
223	BPSL0200		6	3359316	3358420	3259736	3258840	3277998	3277102	3345603	3344707	3434121	3433225	2196359	2197255	3389164	3390060	3231332	3230436	2898605	2897709	99441	98545
224	BPSL0201		6	3359910	3361187	3260330	3261607	3278592	3279869	3346197	3347474	3434715	3435992	2195765	2194488	3388570	3387293	3231926	3233203	2899199	2900476	100035	101312
225	BPSL0202		6	3361201	3361749	3261621	3262169	3279883	3280431	3347488	3348036	3436006	3436554	2194474	2193926	3387279	3386731	3233217	3233765	2900490	2901038	101326	101874
226	BPSL0203		6	3363522	3362182	3263942	3262602	3282204	3280864	3349809	3348469	3438327	3436987	2192153	2193493	3384958	3386298	3235538	3234198	2902811	2901471	103647	102307
227	BPSL0204		6	3364068	3363535	3264488	3263955	3282750	3282217	3350355	3349822	3438873	3438340	2191607	2192140	3384412	3384945	3236084	3235551	2903357	2902824	104193	103660
228	BPSL0205		6	3364739	3364326	3265159	3264746	3283421	3283008	3351026	3350613	3439544	3439131	2190936	2191349	3383741	3384154	3236755	3236342	2904028	2903615	104864	104451
229	BPSL0206		6	3366404	3365295	3267236	3266127	3285758	3284649	3352688	3351579	3441300	3440191	2189271	2190380	3382076	3383185	3238496	3237387	2905693	2904584	3370746	3369637
230	BPSL0207		6	3367823	3366549	3268655	3267381	3287177	3285903	3354107	3352833	3442719	3441445	2187852	2189126	3380657	3381931	3239915	3238641	2907112	2905838	3372165	3370891
231	BPSL0208		6	3368844	3367915	3269676	3268747	3288198	3287269	3355128	3354199	3443740	3442811	2186831	2187760	3379636	3380565	3240936	3240007	2908133	2907204	3373186	3372257
232	BPSL0209		6	3369626	3368892	3270458	3269724	3288980	3288246	3355910	3355176	3444522	3443788	2186049	2186783	3378854	3379588	3241718	3240984	2908915	2908181	3373968	3373234
233	BPSL0210		6	3370519	3369653	3271351	3270485	3289873	3289007	3356803	3355937	3445415	3444549	2185156	2186022	3377961	3378827	3242611	3241745	2909808	2908942	3374861	3373995
234	BPSL0211		6	3371444	3370563	3272276	3271395	3290798	3289917	3357728	3356847	3446340	3445459	2184231	2185112	3377036	3377917	3243536	3242655	2910733	2909852	3375786	3374905
235	BPSL0212		6	3371710	3372894	3272542	3273726	3291064	3292248	3357994	3359178	3446606	3447790	2183965	2182781	3376770	3375586	3243802	3244986	2910999	2912183	3376052	3377236
236	BPSL0213		6	3373677	3374567	3274509	3275399	3293031	3293921	3359961	3360851	3448557	3449447	2182014	2181124	3374819	3373929	3245753	3246643	2912950	2913840	3378003	3378893
237	BPSL0214		6	3374846	3375097	3275678	3275929	3294200	3294451	3361085	3361288	3449726	3449977	2180845	2180594	3373650	3373399	3246922	3247173	2914119	2914370	3379172	3379423
238	BPSL0215		6	3375232	3376272	3276064	3277104	3294586	3295626	3361423	3362463	3450112	3451152	2180459	2179419	3373264	3372236	3247308	3248348	2914505	2915545	3379558	3380598
239	BPSL0216		6	3376503	3376321	3277335	3277153	3295857	3295675	3362694	3362512	3451383	3451201	2179188	2179370	3372005	3372187	3248579	3248397	2915776	2915594	3380829	3380647
240	BPSL0217		6	3377634	3376774	3278466	3277606	3296988	3296128	3363825	3362965	3452514	3451654	2178057	2178917	3370874	3371734	3249710	3248850	2916907	2916047	3381960	3381100
241	BPSL0218		6	3379260	3377974	3280092	3278806	3298614	3297328	3365451	3364165	3454140	3452854	2176431	2177717	3369248	3370534	3251336	3250050	2918533	2917247	3383586	3382300

242	BPSL0219		6	3379614	3379823	3280446	3280655	3298968	3299177	3365805	3366014	3454494	3454703	2176077	2175868	3368894	3368685	3251690	3251899	2918887	2919096	3383940	3384149
243	BPSL0220		6	3379867	3380895	3280699	3281727	3299221	3300249	3366058	3367086	3454747	3455775	2175824	2174796	3368641	3367613	3251943	3252971	2919140	2920168	3384193	3385221
244	BPSL0221		6	3381462	3381172	3282294	3282004	3301275	3301003	3367653	3367363	3456342	3456052							2920735	2920445	3385788	3385498
245	BPSL0222		6	3383001	3381586	3283833	3282418	3302814	3301399	3369192	3367777	3457881	3456466	2172679	2174094	3365496	3366911	3255088	3253673	2922274	2920859	3387327	3385912
246	BPSL0223		6	3384705	3384076	3285537	3284908	3304518	3303889	3370896	3370267	3459585	3458956	2170975	2171604	3363792	3364421	3256792	3256163	2923978	2923349	3389031	3388402
247	BPSL0224		6	3384939	3386579	3285771	3287411	3304752	3306392	3371130	3372770	3459819	3461459	2170741	2169101	3363558	3361918	3257026	3258666	2924212	2925852	3389265	3390905
248	BPSL0225		6	3388022	3386625	3289344	3288940	3308357	3308007	3373574	3372816	3462902	3461505	2167658	2169055	3360475	3361872	3260041	3259487	2927290	2925911	3392562	3392146
249	BPSL0226		6	3388507	3388058	3289829	3289380	3308842	3308393	3374501	3374052	3463387	3462938	2167173	2167622	3359990	3360439	3260526	3260077	2927775	2927326	3393047	3392598
250	BPSL0227		6	3390084	3388516	3291406	3289838	3310419	3308851	3376078	3374510	3464964	3463396	2165596	2167164	3358413	3359981	3262103	3260535	2929352	2927784	3394624	3393056
251	BPSL0228		6	3390758	3390081	3292080	3291403	3311093	3310416	3376752	3376075	3465638	3464961	2164922	2165599	3357739	3358416	3262777	3262100	2930026	2929349	3395298	3394621
252	BPSL0229		6	3391746	3390754	3293068	3292076	3312081	3311089	3377740	3376748	3466626	3465634	2163934	2164926	3356751	3357743	3263765	3262773	2931014	2930022	3396286	3395294
253	BPSL0230		6	3393532	3391739	3294854	3293061	3313867	3312074	3379526	3377733	3468412	3466619	2162148	2163941	3354965	3356758	3265551	3263758	2932800	2931007	3398072	3396279
254	BPSL0231		6	3393781	3394119	3295103	3295441	3314116	3314454	3379775	3380113	3468678	3469016	2161899	2161561	3354716	3354378	3265800	3266138	2933049	2933387	3398321	3398659
255	BPSL0232		6	3394255	3394686	3295577	3296008	3314590	3315021	3380249	3380680	3469152	3469583	2161425	2160994	3354242	3353811	3266274	3266705	2933523	2933954	3398795	3399226
256	BPSL0233		6	3394686	3395009	3296008	3296331	3315021	3315344	3380680	3381003	3469583	3469906	2160994	2160671	3353811	3353488	3266705	3267028	2933954	2934277	3399226	3399549
257	BPSL0234		6	3395122	3396624	3296444	3296584	3315457	3316959	3381116	3382618	3470019	3471521	2160558	2159056	3353375	3351873	3267141	3268643	2934390	2935892	3399662	3399748
258	BPSL0235		6	3396608	3396922	3298742	3299056	3316943	3317257	3382602	3382916	3471505	3471819	2159072	2158758	3351889	3351575	3268627	3268941	2935876	2936190	3400552	3400866
259	BPSL0236		6	3397118	3397861	3299252	3299995	3317453	3318196	3383112	3383855	3472015	3472758	2158562	2157819	3351379	3350636	3269137	3269880	2936386	2937129	3401062	3401805
260	BPSL0237		6	3398980	3397964	3301114	3300098	3319315	3318299	3384974	3383958	3473877	3472861	2156688	2157716	3349505	3350533	3271011	3269983	2938248	2937232	3402924	3401908
261	BPSL0238		6	3400582	3399182	3302716	3301316	3320917	3319517	3386576	3385176	3475479	3474079	2155086	2156486	3347903	3349303	3272613	3271213	2939850	2938450	3404526	3403126
262	BPSL0239		6	3401480	3400827	3303614	3302961	3321815	3321162	3387474	3386821	3476377	3475724	2154188	2154841	3347005	3347658	3273511	3272858	2940748	2940095	3405424	3404771
263		Chr1_16_ISBma2_B	6	3402607	3404179	3304741	3306313	3322942	3324514	3388601	3390173	3477498	3479070										
264	BPSL0240		6			3304353	3304243	3322554	3322444	3388213	3388103	3477110	3477000	2153449	2153559	3346266	3346376	3274250	3274140	2941487	2941377	3406163	3406053
265	BPSL0241		6	3404997	3404230	3307131	3306364	3325332	3324565	3390991	3390224	3479888	3479121	2152265	2153032	3345082	3345849	3275434	3274667	2942671	2941904	3407347	3406580
266	BPSL0242		6	3405863	3405213	3307997	3307347	3326198	3325548	3391857	3391207	3480754	3480104	2151399	2152049	3344216	3344866	3276300	3275650	2943537	2942887	3408213	3407563
267	BPSL0243		6	3406741	3405932	3308875	3308066	3327076	3326267	3392735	3391926	3481632	3480823	2150521	2151330	3343338	3344147	3277178	3276369	2944415	2943606	3409091	3408282
268	BPSL0244		6	3407311	3406751	3309445	3308885	3327646	3327086	3393305	3392745	3482202	3481642	2149951	2150511	3342768	3343328	3277748	3277188	2944985	2944425	3409661	3409101
269	BPSL0245		6	3407645	3408649	3309779	3310783	3327980	3328984	3393639	3394643	3482536	3483540	2149617	2148613	3342434	3341430	3278082	3279086	2945319	2946323	3409995	3410999
270	BPSL0246		6	3408724	3409842	3310858	3311976	3329059	3330177	3394718	3395836	3483615	3484733	2148538	2147420	3341355	3340237	3279161	3280279	2946398	2947516	3411074	3412192
271	BPSL0247		6	3409980	3410291	3312114	3312425	3330315	3330626	3395974	3396285	3484871	3485182	2147282	2146971	3340099	3339788	3280417	3280728	2947654	2947965	3412330	3412641
272	BPSL0248		6	3410526	3411815	3312660	3313949	3330861	3332150	3396520	3397809	3485417	3486706	2146736	2145447	3339553	3338264	3280963	3282252	2948200	2949489	3412876	3414165
273	BPSL0249		6	3412225	3413850	3314359	3315984	3332560	3334185	3398219	3399844	3487116	3488741	2145037	2143412	3337854	3336229	3282662	3284281	2949899	2951524	3414575	3416200
274	BPSL0250		6	3413965	3414978	3316099	3317112	3334300	3335313	3399959	3400972	3488856	3489869	2143297	2142284	3336114	3335101	3284396	3285409	2951639	2952652	3416315	3417328
275	BPSL0251		6	3415670	3415855	3317214	3317810	3335415	3336011	3401074	3401670	3489971	3490567	2142182	2141586	3334999	3334403	3285511	3286107	2952754	2953350	3417430	3418026
276	BPSL0252		6	3415860	3416849	3317994	3318983	3336195	3337184	3401854	3402843	3490751	3491740	2141402	2140413	3334219	3333230	3286291	3287280	2953534	2954523	3418210	3419199
277	BPSL0253		6	3416849	3417862	3318983	3319996	3337184	3338197	3402843	3403856	3491740	3492753	2140413	2139400	3333230	3332217	3287280	3288293	2954523	2955536	3419199	3420212
278	BPSL0254		6	3417978	3419003	3320523	3321473	3338313	3338399	3403972	3404997	3492869	3493894	2139284	2138259	3332101	3331076	3288409	3289434	2955652	2956677	3420328	3421353
279		Chr1_17_ISBma2_C	6																	2957135	2958707		
280	BPSL0255		6	3420436	3419483	3322906	3321953	3341338	3340385	3406430	3405477	3495327	3494374	2136826	2137779	3329643	3330596	3290867	3289914	2959701	2958748	3422786	3421833
281	BPSL0256		6	3421185	3420436	3323655	3322906	3342087	3341338	3407179	3406430	3496076	3495327	2136077	2136826	3328894	3329643	3291616	3290867	2960450	2959701	3423535	3422786
282	BPSL0257		6	3422569	3421808	3325039	3324278	3343471	3342710	3408563	3407802	3497451	3496690	2134693	2135454	3327510	3328271	3293000	3292239	2961834	2961073	3424919	3424158
283	BPSL0258		6	3423705	3422641	3326175	3325111	3344607	3343543	3409699	3408635	3498587	3497523	2133557	2134621	3326374	3327438	3294136	3293072	2962970	2961906	3426055	3424991
284	BPSL0259		6	3424358	3423705	3326828	3326175	3345260	3344607	3410352	3409699	3499240	3498587	2132904	2133557	3325721	3326374	3294789	3294136	2963623	2962970	3426708	3426055
285	BPSL0260		6	3424508	3425017	3326978	3327487	3345410	3345919	3410502	3411011	3499390	3499899	2132754	2132245	3325571	3325062	3294939	3295448	2963773	2964282	3426858	3427367
286	BPSL0261		6	3425848	3425255	3328318	3327725	3346750	3346157	3411842	3411249	3500730	3500137	2131414	2132007	3324231	3324824	3296279	3295686	2965113	2964520	3428198	3427605
287	BPSL0262		6	3425870	3427822	3328340	3330292	3346772	3348724	3411864	3413816	3500752	3502704	2131392	2129440	3324209	3322257	3296301	3298253	2965135	2967087	3428220	3430172
288	BPSL0263		6	3427947	3428903	3330417	3331373	3348849	3349805	3413941	3414897	3502829	3503785	2129315	2128359	3322132	3321176	3298378	3299334	2967212	2968168	3430297	3431253
289	BPSL0264		6	3428961	3429614	3331431	3332084	3349863	3350516	3414955	3415608	3503843	3504496	2128301	2127648	3321118	3320465	3299392	3300045	2968226	2968879	3431311	3431964
290	BPSL0265		6	3429614	3430852	3332084	3333322	3350516	3351754	3415608	3416846	3504496	3505734	2127648	2126410	3320465	3319227	3300045	3301283	2968879	2970117	3431964	3433202
291	BPSL0266		6	3431718	3431500	3334188	3333970	3352620	3352402	3417712	3417494	3506600	3506382	2125544	2125762	3318361	3318579	3302149	3301931	2970983	2970765	3434068	3433850
292	BPSL0267		6	3432637	3432203	3335107	3334673	3353539	3353105	3418631	3418197	3507519	3507085	2124625	2125059	3317442	3317876	3303068	3302634	2971902	2971468	3434987	3434553

293	BPSL0268		6	3433061	3432720	3335531	3335190	3353963	3353622	3419055	3418714	3507943	3507602	2124201	2124542	3317018	3317359	3303492	3303151	2972326	2971985	3435411	3435070
294	BPSL0269		6	3434001	3433168	3336471	3335638	3354903	3354070	3419995	3419162	3508883	3508050	2123261	2124094	3316078	3316911	3304432	3303599	2973266	2972433	3436351	3435518
295	BPSL0270		6	3434938	3435426	3337408	3337896	3355840	3356328	3420932	3421420	3509820	3510308	2122312	2121824	3315129	3314641	3305381	3305869	2974203	2974691	3437288	3437776
296	BPSL0271		6	3435513	3435935	3337983	3338405	3356415	3356837	3421507	3421929	3510395	3510817	2121737	2121315	3314554	3314132	3305956	3306378	2974778	2975200	3437863	3438285
297	BPSL0272		6	3436058	3436822	3338528	3339292	3356960	3357724	3422052	3422816	3510940	3511704	2121192	2120428	3314009	3313245	3306501	3307265	2975323	2976087	3438408	3439172
298	BPSL0273		6	3436853	3438091	3339323	3340561	3357755	3358993	3422847	3424085	3511735	3512973	2120397	2119159	3313214	3311976	3307296	3308534	2976118	2977356	3439203	3440441
299	BPSL0274		6	3438121	3438879	3340591	3341349	3359023	3359781	3424115	3424873	3513003	3513761	2119129	2118371	3311946	3311188	3308564	3309322	2977386	2978144	3440471	3441229
300	BPSL0275		6	3438916	3439701	3341386	3342171	3359818	3360603	3424910	3425695	3513798	3514583	2118334	2117549	3311151	3310366	3309359	3310144	2978181	2978966	3441266	3442051
301	BPSL0276		6	3439732	3440442	3342202	3342912	3360634	3361344	3425726	3426436	3514607	3515326	2117525	2116812	3310342	3309629	3310168	3310887	2978990	2979709	3442075	3442794
302	BPSL0277		6	3440460	3441641	3342930	3344111	3361362	3362543	3426454	3427635	3515344	3516525	2116794	2115613	3309611	3308430	3310905	3312086	2979727	2980908	3442812	3443993
303	BPSL0278		6	3441645	3442577	3344115	3345047	3362547	3363479	3427639	3428571	3516529	3517461	2115609	2114677	3308426	3307494	3312090	3313022	2980912	2981844	3443997	3444929
304	BPSL0279		6	3442796	3443551	3345266	3346021	3363698	3364453	3428790	3429545	3517680	3518435	2114458	2113703	3307275	3306520	3313241	3313996	2982063	2982818	3445148	3445903
305	BPSL0280		6	3443760	3445760	3346230	3348230	3364662	3366662	3429754	3431754	3518644	3520644	2113494	2111494	3306311	3304311	3314205	3316205	2983027	2985027	3446112	3448112
306	BPSL0281		6	3445779	3447008	3348249	3349478	3366681	3367910	3431773	3432963	3520663	3521892	2111475	2110246	3304292	3303063	3316224	3317453	2985046	2986275	3448131	3449282
307	BPSL0282		6	3447379	3448683	3349849	3351153	3368281	3369585	3433381	3434685	3522263	3523567	2109875	2108571	3302692	3301388	3317824	3319128	2986646	2987950	3450071	3451375
308	BPSL0283		6	3449757	3449074	3352227	3351544	3370659	3369976	3435759	3435076	3524641	3523958	2107491	2108174	3300308	3300991	3320208	3319525	2989030	2988347	3452455	3451772
309	BPSL0284		6	3449855	3450451	3352325	3352921	3370757	3371353	3435857	3436453	3524739	3525335	2107393	2106797	3300210	3299614	3320306	3320902	2989128	2989724	3452553	3453149
310	BPSL0285		6	3450451	3450978	3352921	3353448	3371353	3371880	3436453	3436980	3525335	3525862	2106797	2106270	3299614	3299087	3320902	3321429	2989724	2990251	3453149	3453676
311	BPSL0286		6	3451669	3451334	3354139	3353804	3372571	3372236	3437671	3437336	3526553	3526218	2105579	2105914	3298396	3298731	3322120	3321785	2990942	2990607	3454367	3454032
312	BPSL0287		6	3451783	3452124	3354253	3354594	3372685	3373026	3437785	3438126	3526667	3527008	2105465	2105124	3298282	3297941	3322234	3322575	2991056	2991397	3454481	3454822
313	BPSL0288		6	3452145	3452852	3354615	3355322	3373047	3373754	3438147	3438854	3527029	3527736	2105103	2104396	3297920	3297213	3322596	3323303	2991418	2992125	3454843	3455550
314	BPSL0289		6	3453399	3454529	3355869	3356999	3374301	3375431	3439401	3440531	3528283	3529413	2103849	2102719	3296666	3295536	3323850	3324980	2992672	2993802	3456097	3457227
315	BPSL0290		6	3455523	3454693	3357993	3357163	3376425	3375595	3441525	3440695	3530407	3529577	2101725	2102555	3294542	3295372	3325974	3325144	2994796	2993966	3458221	3457391
316	BPSL0291		6	3456134	3455574	3358604	3358044	3377036	3376476	3442136	3441576	3531018	3530458	2101114	2101674	3293931	3294491	3326585	3326025	2995407	2994847	3458832	3458272
317	BPSL0292		6	3457572	3456505	3360042	3358975	3378474	3377407	3443574	3442507	3532456	3531389	2099676	2100743	3292493	3293560	3328023	3326956	2996845	2995778	3460270	3459203
318	BPSL0293_1		6	3458791	3457568	3361261	3360038	3379693	3378470	3444793	3443570	3533675	3532452	2098457	2099680	3291274	3292497	3329242	3328019	2998064	2996841	3461489	3460266
319		Chr1_18a_IS407A_A		3458793	3460028	3361263	3362498	3379695	3380930	3444795	3446030	3533677	3534912	2097220	2098455	3290037	3291272	3329244	3330479	2998066	2999301	3461491	3462726
320		Chr1_18b_IS407A_A										163047	164282			2614655	2615890						
321	BPSL0293_4		7	3460073	3460029	3362543	3362499	3380975	3380931	3446075	3446031			2097175	2097219			3330524	3330480	2999346	2999302		
322	BPSL0294		7	3461984	3460458	3364454	3362928	3382886	3381360	3447986	3446460	161091	162617	2095264	2096790	2617846	2616320	3332435	3330909	3001257	2999731	3464682	3463156
323	BPSL0295		7	3463616	3465286	3366086	3367756	3384518	3386188	3449618	3451288	159459	157789	2093632	2091962	2619478	2621148	3334067	3335737	3002889	3004559	3466314	3467984
324	BPSL0296		7	3465848	3466069	3368318	3368539	3386750	3386971	3451850	3452071	157227	157006	2091400	2091179	2621710	2621931	3336299	3336520	3005121	3005342	3468546	3468767
325		Chr1_19a_ISBma2_A		3466142	3467714	3368613	3370185	3387044	3388616	3452144	3453716	155357	156927	2089532	2091106	2622004	2623575	3336593	3338164	3005415	3006987	3468840	3470412
326		Chr1_19b_ISBma2_A														2193940	2195512						
327	BPSL0297		8	3467866	3469224	3370337	3371695	3388768	3390126	3453868	3455226	155205	153847	2089380	2088022	2195664	2197022	3338316	3339674	3007139	3008497	3470564	3471922
328	BPSL0298		8	3469820	3471157	3372291	3373628	3390722	3392059	3455822	3457159	153251	151914	2087426	2086089	2197618	2198955	3340270	3341607	3009093	3010430	3472518	3473855
329	BPSL0299		8	3471322	3471954	3373793	3374425	3392224	3392856	3457324	3457956	151749	151117	2085924	2085292	2199120	2199752	3341772	3342404	3010595	3011227	3474020	3474652
330	BPSL0300		8	3472047	3472769	3374518	3375240	3392949	3393671	3458049	3458771	151024	150302	2085199	2084477	2199845	2200567	3342497	3343219	3011320	3012042	3474745	3475467
331	BPSL0301		8	3473094	3473339	3375565	3375810	3393996	3394241	3459096	3459341	149977	149732	2084152	2083907	2200892	2201137	3343544	3343789	3012367	3012612	3475792	3476037
332	BPSL0302		8	3476336	3473916	3378807	3376387	3397238	3394818	3462338	3459918	146735	149155	2080897	2083317	2204147	2201727	3346799	3344379	3015648	3013228	3479073	3476653
333	BPSL0303		8	3476981	3477652	3379452	3380123	3397883	3398554	3462983	3463654	146090	145419	2080252	2079581	2204792	2205463	3347444	3348115	3016293	3016964	3479718	3480389
334	BPSL0304		8	3477652	3478497	3380123	3380968	3398554	3399399	3463654	3464499	145419	144574	2079581	2078736	2205463	2206308	3348115	3348960	3016964	3017809	3480389	3481234
335	BPSL0305		8	3478497	3478994	3380968	3381465	3399399	3399896	3464499	3464996	144574	144077	2078736	2078239	2206308	2206805	3348960	3349457	3017809	3018306	3481234	3481731
336	BPSL0306		8	3479072	3479497	3381543	3381968	3399974	3400399	3465074	3465499	143999	143574	2078161	2077736	2206883	2207308	3349535	3349960	3018384	3018809	3481809	3482234
337	BPSL0307		8	3479599	3480897	3382070	3383368	3400501	3401799	3465601	3466899	143472	142174	2077634	2076336	2207410	2208708	3350062	3351360	3018911	3020209	3482336	3483634
338	BPSL0308		8	3480911	3482473	3383382	3384944	3401813	3403375	3466913	3468190	142160	140598	2076322	2074760	2208722	2210284	3351374	3352936	3020223	3021785	3483648	3485210
339	BPSL0309		8	3482473	3485682	3384944	3388153	3403375	3406584	3468477	3471686	140598	137389	2074760	2071551	2210284	2213493	3352936	3356145	3021785	3024994	3485210	3488419
340	BPSL0310		8	3485682	3486020	3388153	3388491	3406584	3406922	3471686	3472024	137389	137051	2071551	2071213	2213493	2213831	3356145	3356483	3024994	3025332	3488419	3488757
341	BPSL0311		8	3486224	3486667	3388695	3389138	3407126	3407569	3472228	3472671	136847	136404	2071009	2070566	2214035	2214478	3356687	3357130	3025536	3025979	3488961	3489404
342	BPSL0312		8	3488577	3486748	3391048	3389219	3409479	3407650	3474581	3472752	134494	136323	2068656	2070485	2216388	2214559	3359040	3357211	3027889	3026060	3491314	3489485
343	BPSL0313		8	3490002	3488644	3392473	3391115	3410904	3409546	3476006	3474648	133069	134427	2067231	2068589	2217813	2216455	3359565	3359107	3029314	3027956	3492739	3491381

344	BPSL0314		8	3491557	3490565	3394028	3393036	3413220	3412228	3477505	3476513	131514	132506	2065676	2066668	2219368	2218376	3360909	3359917	3030869	3029877	3494256	3493264
345	BPSL0315		8	3491958	3491557	3394429	3394028	3413621	3413220	3477906	3477505	131113	131514	2065275	2065676	2219769	2219368	3361310	3360909	3031270	3030869	3494657	3494256
346	BPSL0316		8	3492936	3492043	3395741	3394848	3415107	3414871			130083	130973	2064312	2065190	2220732	2219854			3032248	3031355	3495293	3494802
347	BPSL0317		8	3493004	3494191	3395809	3396996	3415175	3416362	3478799	3479986	130015	128828	2064244	2063057	2220800	2221987	3361454	3362551	3032316	3033503	186480	185293
348	BPSL0318		8	3494212	3494403	3397017	3397208	3416383	3416574	3480007	3480198	128807	128616	2063036	2062845	2222008	2222199	3362572	3362763	3033524	3033715	185272	185081
349	BPSL0319		8	3495234	3494701	3398039	3397506	3417405	3416872	3481029	3480496	127785	128318	2062014	2062547	2223030	2222497	3363594	3363061	3034546	3034013	184250	184783
350	BPSL0320		8	3496139	3495234	3398944	3398039	3418310	3417405	3481934	3481029	126880	127785	2061109	2062014	2223935	2223030	3364499	3363594	3035451	3034546	183345	184250
351	BPSL0321		8	3497587	3496139	3400392	3398944	3419758	3418310	3483382	3481934	125432	126880	2059661	2061109	2225383	2223935			3036899	3035451	181897	183345
352	BPSL0322		8	3498603	3497587	3401408	3400392	3420774	3419758	3484398	3483382	124416	125432	2058645	2059661	2226399	2225383	3366967	3365951	3037915	3036899	180881	181897
353	BPSL0323		8	3500333	3498777	3403138	3401582	3422504	3420948	3486128	3484572	122686	124242	2056915	2058471	2228129	2226573	3368697	3367141	3039645	3038089	179151	180707
354	BPSL0324		8	3501869	3500832	3404674	3403637	3424040	3423003	3487664	3486627	121150	122187	2055379	2056416	2229665	2228628	3370233	3369196	3041181	3040144	177615	178652
355	BPSL0325		8	3502298	3502723	3405103	3405528	3424469	3424894	3488093	3488518	120721	120296	2054950	2054525	2230094	2230519	3370662	3371087	3041610	3042035	177186	176761
356	BPSL0326		8	3503116	3503493	3405921	3406298	3425287	3425664	3488911	3489288	119903	119526	2054132	2053755	2230912	2231289	3371480	3371857	3042428	3042805	176368	175991
357		Chr1_20_IS407A_A		3503487	3504722	3406292	3407527	3425658	3426893	3489282	3490517	118297	119532	2052526	2053761	2231283	2232518	3371851	3373086	3042799	3044034	174762	175997
358	BPSL0327																						
359	BPSL0328																						
360	BPSL0329																						
361	BPSL0330																						
362	BPSL0331																						
363	BPSL0332																						
364	BPSL0333																						
365	BPSL0334																						
366	BPSL0335																						
367	BPSL0336																						
368	BPSL0337																						
369	BPSL0338																						
370	BPSL0339																						
371	BPSL0340																						
372	BPSL0341																						
373	BPSL0342																						
374	BPSL0343																						
375	BPSL0344																						
376	BPSL0345																						
377	BPSL0346																						
378	BPSL0347																						
379	BPSL0348																						
380	BPSL0349																						
381	BPSL0350																						
382	BPSL0352																						
383	BPSL0353																						
384	BPSL0354																						
385	BPSL0355																						
386	BPSL0356																						
387	BPSL0357																						
388		Chr1_21_IS407A_A		123747	124982	3538554	3539789	3559432	3560667	123374	124609	118297	119532	2052526	2053761	2231283	2232518	3371851	3373086	3042799	3044034	174762	175997
389	BPSL0358		9	123787	123260	3538594	3538067	3559472	3558945	123414	122887	118337	117810	2052566	2052039	2232478	2233005	3373079	3373573	3043994	3044521	174802	174275
390	BPSL0359		9	122800	122423	3537607	3537230	3558485	3558108	122427	122050	117350	116973	2051579	2051202	2233465	2233842	3374033	3374410	3044981	3045358	173815	173438
391	BPSL0360		9	122129	120693	3536936	3535500	3557814	3556378	121756	120320	116679	115243	2050903	2049467	2234141	2235577	3374709	3376145	3045652	3047088	173144	171708
392	BPSL0361		9	120657	119257	3535464	3534064	3556342	3554942	120284	118884	115207	113807	2049431	2048031	2235613	2237013	3376181	3377581	3047124	3048524	171672	170272
393	BPSL0362		9	119224	118415	3534031	3533222	3554909	3554100	118851	118042	113774	112965	2047998	2047189	2237046	2237855	3377614	3378423	3048557	3049366	170239	169430
394	BPSL0363		9	118033	117323	3532840	3532130	3553718	3553008	117660	116950	112583	111873	2046807	2046097	2238237	2238947	3378805	3379515	3049748	3050458	169048	168338

395	BPSL0364		9	116174	117190	3530981	3531997	3551859	3552875	115801	116817	110724	111740	2044948	2045964	2240096	2239080	3380664	3379648	3051607	3050591	167189	168205
396	BPSL0365		9	115388	116107	3530195	3530914	3551073	3551792	115015	115734	109938	110657	2044162	2044881	2240882	2240163	3381450	3380731	3052393	3051674	166403	167122
397	BPSL0366		9	114207	115388	3529014	3530195	3549892	3551073	113834	115015	108757	109938	2042981	2044162	2242063	2240882	3382631	3381450	3053574	3052393	165222	166403
398	BPSL0367		9	112864	114207	3527671	3529014	3548549	3549892	112491	113834	107414	108757	2041638	2042981	2243406	2242063	3383974	3382631	3054917	3053574	163879	165222
399	BPSL0368		9	111024	112214	3525831	3527021	3546709	3547899	110651	111841	105567	106847	2039792	2041072	2245252	2243972	3385820	3384540	3056764	3055484	162032	163312
400	BPSL0369		9	110343	110792	3525150	3525599	3546028	3546477	109970	110419	104886	105335	2039111	2039560	2245933	2245484	3386501	3386052	3057445	3056996	161351	161800
401	BPSL0370		9	109628	110308	3524435	3525115	3545313	3545993	109255	109935	104171	104851	2038396	2039076	2246648	2245968	3387216	3386536	3058160	3057480	160636	161316
402	BPSL0371		9	108404	109594	3523211	3524401	3544089	3545279	108031	109221	102947	104137	2037172	2038362	2247872	2246682	3388440	3387250	3059382	3058246	159414	160550
403	BPSL0372		9	107647	108414	3522454	3523221	3543332	3544099	107274	108041	102190	102957	2036415	2037182	2248629	2247862	3389197	3388430	3060139	3059372	158657	159424
404	BPSL0373		9	105778	107586	3520585	3522393	3541463	3543271	105405	107213	100321	102129	2034546	2036354	2250498	2248690	3391066	3389258	3062008	3060200	156788	158596
405	BPSL0374		9	105488	104412	3520295	3519219	3541173	3540097	105115	104039	100031	98955	2034256	2033180	2250788	2251864	3391356	3392432	3062298	3063374	156498	155422
406	BPSL0375		9	102468	104111	3517275	3518918	3538153	3539796	102095	103738	97011	98654	2031236	2032879	2253808	2252165	3394376	3392733	3065318	3063675	153478	155121
407	BPSL0376		9	100648	102231	3515455	3517038	3536333	3537916	100275	101858	95191	96774	2029416	2030999	2255628	2254045	3396196	3394613	3067138	3065555	151658	153241
408	BPSL0377		9																				
409	BPSL0378		9	99670	100134	3514477	3514941	3535355	3535819	99297	99761	94213	94677	2028438	2028902	2256606	2256142	3397174	3396710	3068116	3067652	150680	151144
410	BPSL0379		9	99430	98720	3514237	3513527	3535115	3534405	99057	98347	93973	93263	2028198	2027488	2256846	2257547	3397414	3398124	3068356	3069066	150440	149730
411	BPSL0380		9	97785	98561	3512592	3513368	3533470	3534246	97412	98188	92328	93104	2026553	2027329	2258482	2257706	3399059	3398283	3070001	3069225	148795	149571
412	BPSL0381		9	97130	97765	3511937	3512572	3532815	3533450	96757	97392	91673	92308	2025898	2026533	2259137	2258502	3399714	3399079	3070656	3070021	148140	148775
413	BPSL0382		9	96230	97075	3511037	3511882	3531915	3532760	95857	96702	90773	91618	2024998	2025843	2260037	2259192	3400614	3399769	3071556	3070711	147240	148085
414	BPSL0383		9	94367	96148	3509174	3510955	3530052	3531833	93994	95775	88910	90691	2023135	2024916	2261900	2260119	3402477	3400696	3073419	3071638	145377	147158
415	BPSL0384		9	94164	93844	3508971	3508651	3529849	3529529	93791	93471	88707	88387	2022932	2022612	2262103	2262423	3402680	3403000	3073622	3073942	145174	144854
416		Chr1_22a_ISBma2_A		92218	93790	3507025	3508597	3527903	3529475	91845	93417	86761	88333	2020996	2022558	2262477	2264039	3403054	3404616	3073996	3075559	143964	144800
417		Chr1_22b_ISBma2_A												1369886	1371461	3182889	3184461	3100401	3101948	254941	256513	2490911	2492483
418	BPSL0385		10	89303	92002	3504110	3506809	3524988	3527687	88930	91629	83846	86545	1374376	1371677	3179974	3182673	3097442	3100141	252026	254725	2487996	2490695
419	BPSL0386		10	88163	89239	3502970	3504046	3523848	3524924	87790	88866	82706	83782	1375516	1374440	3178834	3179910	3096302	3097378	250886	251962	2486856	2487932
420	BPSL0387		10	87718	87972	3502525	3502779	3523403	3523657	87345	87599	82261	82515	1375961	1375707	3178389	3178643	3095857	3096111	250441	250695	2486411	2486665
421	BPSL0388		10	86267	87532	3501074	3502339	3521952	3523217	85894	87159	80810	82075	1377412	1376147	3176938	3178203	3094406	3095671	248990	250255	2484960	2486225
422	BPSL0389		10	85274	86134	3499558	3500121	3520959	3521819	84901	85761	79817	80677	1378405	1377545	3175945	3176805	3093413	3094273	247997	248857	2483967	2484827
423	BPSL0390		10	85164	84175	3499448	3498459	3520849	3519860	84791	83802	79707	78718	1378515	1379504	3175835	3174846	3093303	3092314	247887	246898	2483857	2482868
424	BPSL0391		10	83975	83151	3498259	3497435	3519660	3518836	83602	82778	78518	77694	1379704	1380528	3174646	3173822	3092114	3091290	246698	245874	2482668	2481844
425	BPSL0392		10	83058	82261	3497342	3496710	3518743	3518006			77601	76804	1380621	1381418	3173729	3172932	3091197	3090400	245781	244984	2481751	2481137
426	BPSL0393		10	82221	80980	3496139	3494898	3517123	3515882	81894	80653	76764	75523	1381458	1382699	3172892	3171651	3090360	3089119	244944	243703	143786	142545
427	BPSL0394		10	80404	79532	3494322	3493450	3515306	3514434	80077	79205	74932	74060	1383281	1384153	3171064	3170192	3088538	3087666	243127	242255	141969	141097
428	BPSL0395		10	79388	78906	3493306	3492824	3514290	3513808	79061	78579	73916	73434	1384297	1384779	3170048	3169566	3087522	3087040	242111	241629	140953	140471
429	BPSL0396		10	78423	78842	3492341	3492760	3513325	3513744	78096	78515	72951	73370	1385262	1384843	3169083	3169502	3086557	3086976	241146	241565	139988	140407
430	BPSL0397		10	77534	78310	3491452	3492228	3512436	3513212	77207	77983	72062	72838	1386151	1385375	3168194	3168970	3085668	3086444	240257	241033	139099	139875
431	BPSL0398		10	76578	77534	3490496	3491452	3511480	3512436	76251	77207	71106	72062	1387107	1386151	3167238	3168194	3084712	3085668	239301	240257	138143	139099
432	BPSL0399		10	75305	76189	3489223	3490107	3510207	3511091	74978	75862	69833	70717	1388380	1387496	3165965	3166849	3083439	3084323	238028	238912	136888	137754
433	BPSL0400		10	73171	75228	3486815	3488872	3507407	3509464	72844	74901	67641	69698	1390514	1388457	3163831	3165888	3081251	3083308	235894	237951	134716	136773
434	BPSL0401		10	72901	71780	3486545	3485424	3507137	3506016	72574	71453	67371	66250	1390788	1391909	3163557	3162436	3080977	3079856	235624	234503	134446	133325
435	BPSL0402		10	71780	70941	3485424	3484585	3506016	3505177	71453	70614	66250	65411	1391909	1392748	3162436	3161597	3079856	3079017	234503	233664	133325	132486
436	BPSL0403		10	70914	69988	3484558	3483632	3505150	3504224	70587	69661	65384	64458	1392775	1393701	3161570	3160644	3078990	3078064	233637	232711	132459	131533
437	BPSL0404		10	69951	69331	3483595	3482975	3504187	3503567	69624	69004	64421	63801	1393738	1394358	3160607	3159987	3078027	3077407	232674	232054	131496	130876
438	BPSL0405		10	69296	68100	3482940	3481744	3503532	3502936	68969	67773	63766	62570	1394393	1394662	3159952	3159683	3077372	3077103	232019	230823	130841	130572
439	BPSL0406		10	67992	67669	3481636	3481313	3501753	3501430	67665	67342	62462	62139	1395557	1395880	3158788	3158465	3076208	3075885	230715	230392	129465	129142
440	BPSL0407		10	67619	66978	3481263	3480622	3501380	3500739	67292	66651	62089	61448	1395930	1396571	3158415	3157774	3075835	3075194	230342	229701	129092	128451
441	BPSL0408		10	66107	64899	3479751	3478543	3499868	3498660	65779	64571	60596	59388	1397461	1398669	3156915	3155707	3074304	3073096	228854	227646	127616	126408
442	BPSL0409		10	64806	63862	3478450	3477506	3498567	3497623	64478	63534	59295	58351	1398762	1399706	3155614	3154670	3073003	3072059	227553	226609	126315	125371
443	BPSL0410		10	63862	63557	3477506	3477201	3497623	3497318	63534	63229	58351	58046	1399706	1400011	3154670	3154365	3072059	3071754	226609	226304	125371	125066
444	BPSL0411		10	62578	63540	3476222	3477184	3496339	3497301	62250	63212	57067	58029	1400990	1400028	3153386	3154348	3070775	3071737	225325	226287	124087	125049
445	BPSL0412		10	62477	62154	3476121	3475798	3496238	3495915	62149	61826	56966	56643	1401091	1401414	3153285	3152962	3070674	3070351	225224	224901	123986	123663

446 BPSL0413		10	61986	61312	3475630	3474956	3495747	3495073	61658	60984	56475	55801	1401582	1402256	3152794	3152120	3070183	3069509	224733	224059	123495	122821
447 BPSL0414		10	61316	60330	3474960	3473974	3495077	3494091	60988	60002	55805	54819	1402252	1403238	3152124	3151138	3069513	3068527	224063	223077	122825	121839
448	Chr1_23a_ISBma2_A		58691	60263	3472335	3473907	3492452	3494024	58363	59935	53180	54752	1403305	1404881	3149499	3151071	3066888	3068460	221439	223010	120201	121772
449	Chr1_23b_ISBma2_A		3346749	3348321	3247169	3248741	3265431	3267003	3333036	3334608	3421617	3423189			2756502	2758099						
450 BPSL0415		11	3346239	3346616	3246659	3247036	3264921	3265298	3332526	3332903	3421107	3421484	1405391	1405014	2758609	2758232			220929	221306	119691	120068
451 BPSL0416		11	3344336	3346216	3244756	3246636	3263018	3264898	3330623	3332503	3419204	3421084	1407294	1405414	2760512	2758632			219026	220906	117788	119668
452 BPSL0417		11	3343492	3343154	3243912	3243574	3262174	3261836	3329779	3329441	3418368	3418030	1408130	1408468	2761340	2761678			218190	217852	116952	116614
453 BPSL0418		11	3342140	3343039	3242560	3243459	3260822	3261721	3328427	3329326	3417017	3417916	1409480	1408581	2762690	2761791			216839	217738	115601	116500
454 BPSL0419		11	3341985	3339862	3242405	3240282	3260667	3258544	3328272	3326149	3416862	3414739	1409635	1411758	2762845	2764968			216684	214561	115446	113323
455 BPSL0420		11	3339804	3338599	3240224	3239019	3258486	3257281	3326091	3324886	3414681	3413476	1411816	1413021	2765026	2766231			214503	213298	113265	112060
456 BPSL0421		11	3338586	3337459	3239006	3237879	3257268	3256141	3324873	3323746	3413463	3412336	1413034	1414161	2766244	2767371			213285	212158	112047	110920
457 BPSL0422		11	3337430	3336228	3237850	3236648	3256112	3254910	3323717	3322515	3412307	3411105	1414190	1415392	2767400	2768602			212129	210927	110891	109689
458 BPSL0423		11	3335938	3335636	3236358	3236056	3254620	3254318	3322225	3321923	3410815	3410513	1415682	1415984	2768892	2769194			210637	210335	109399	109097
459 BPSL0424		11	3335621	3333909	3236041	3234329	3254303	3252591	3321908	3320196	3410498	3408786	1415999	1417711	2769209	2770921			210320	208608	109082	107370
460 BPSL0425		11	3333538	3332255	3233958	3232675	3252220	3250937	3319825	3318542	3408415	3407132	1418082	1419365	2771292	2772575			208237	206954	106999	105716
461 BPSL0426		11	3332249	3331320	3232669	3232619	3250931	3250875	3318536	3318486	3407126	3407052	1419371	1420300	2772581	2773510			206948	206019	3369480	3368641
462 BPSL0427		11	3330237	3328885	3230390	3229038	3248281	3246929	3316514	3315162	3404276	3402924	1421383	1422735	2774593	2775945			204936	203584	3367558	3366206
463 BPSL0428		11	3328230	3328763	3228383	3228916	3246274	3246807	3314507	3315040	3402269	3402802	1423390	1422857	2776600	2776067			202929	203462	3365551	3366084
464 BPSL0429		11	3327937	3327350	3228090	3227503	3245981	3245394	3314214	3313627	3401976	3401389	1423683	1424270	2776893	2777480			202636	202049	3365258	3364671
465 BPSL0430		11	3325903	3326679	3226056	3226832	3243947	3244723	3312180	3312956	3399942	3400718	1425717	1424941	2778927	2778151			200602	201378	3363224	3364000
466 BPSL0431		11	3325404	3324943	3225557	3225096	3243448	3242987	3311681	3311220	3399443	3398982	1426224	1426685	2779442	2779903			200103	199642	3362725	3362264
467 BPSL0432		11	3322939	3324522	3223092	3224675	3240983	3242566	3309216	3310799	3396978	3398561	1428689	1427106	2781907	2780324			197638	199221	3360260	3361843
468 BPSL0433		11	3322550	3322798	3222703	3222951	3240594	3240842	3308827	3309075	3396589	3396837	1429078	1428830	2782296	2782048			197249	197497	3359871	3360119
469 BPSL0434		11	3322181	3321846	3222334	3221999	3240225	3239890	3308458	3308123	3396220	3395885	1429447	1429782	2782665	2783000			196880	196545	3359502	3359167
470 BPSL0435		11	3321780	3320413	3221933	3220566	3239824	3238457	3308057	3306690	3395819	3394452	1429848	1431215	2783066	2784433			196479	195112	3359101	3357734
471	Chr1_24_ISBma2_A		3318742	3320313	3218895	3220466	3236786	3238357	3305019	3306590	3392781	3394352	1432886	1432917	2784533	2786105	3066888	3068460	193441	195012	3356063	3357634
472 BPSL0436		11	3318485	3317160	3218638	3217313	3236529	3235204	3304762	3303437	3392524	3391199	1433174	1434499	2786362	2787687	3066631	3065306	193184	191859	3355806	3354481
473 BPSL0437		11	3317129	3316176	3217282	3216329	3235173	3234220	3303406	3302453	3391168	3390215	1434530	1435483	2787718	2788671	3065275	3064322	191828	190875	3354450	3353497
474 BPSL0438		11	3316000	3315530	3216153	3215683	3234044	3233574	3302277	3301807	3390039	3389569	1435659	1436129	2788847	2789317	3064146	3063676	190699	190229	3353321	3352851
475 BPSL0439		11	3315450	3315184	3215603	3215337	3233494	3233228	3301727	3301461	3389489	3389223	1436209	1436475	2789397	2789663	3063596	3063330	190149	189883	3352771	3352505
476 BPSL0440		11	3314985	3313216	3215138	3213369	3233029	3231260	3301262	3299493	3389024	3387255	1436674	1438443	2789862	2791631	3063131	3061362	189684	187915	3352306	3350537
477 BPSL0441		11	3312233	3312988	3212386	3213141	3230277	3231032	3298510	3299265	3386272	3387027	1439432	1438677	2792614	2791859	3060379	3061134	186932	187687	3349554	3350309
478 BPSL0442		11	3310554	3312125	3210707	3212278	3228598	3230169	3296831	3298402	3384593	3386164	1441111	1439540	2794293	2792722	3058700	3060271	185253	186824	3347875	3349446
479 BPSL0443		11	3309584	3310330	3209737	3210483	3227628	3228374	3295861	3296607	3383623	3384369	1442081	1441335	2795263	2794517	3057730	3058476	184283	185029	3346905	3347651
480 BPSL0444		11	3309450	3309046	3209603	3209199	3227494	3227090	3295727	3295323	3383489	3383085	1442215	1442619	2795397	2795801	3057596	3057192	184149	183745	3346771	3346367
481 BPSL0445		11	3309031	3308774	3209184	3208927	3227075	3226818	3295308	3295051	3383070	3382813	1442634	1442891	2795816	2796073	3057177	3056920	183730	183473	3346352	3346095
482 BPSL0446		11	3308654	3308178	3208807	3208331	3226698	3226222	3294931	3294455	3382693	3382217	1443011	1443487	2796193	2796669	3056800	3056324	183353	182877	3345975	3345499
483 BPSL0447		11	3308149	3307154	3208302	3207307	3226193	3225393	3294426	3293431	3382188	3381193	1443516	1444511	2796698	2797693	3056295	3055300	182848	181853	3345470	3344475
484 BPSL0448		11	3306998	3306468	3207151	3206621	3224192	3223662	3293275	3292745	3381037	3380507	1444667	1445197	2797849	2798379	3055144	3054614	181697	181167	3344319	3343789
485 BPSL0449		11	3305575	3306042	3205728	3206195	3222769	3223236	3291852	3292319	3379614	3380081	1446090	1445623	2799272	2798805	3053721	3054188	180274	180741	3342896	3343363
486 BPSL0450		11	3304791	3305516	3204944	3205669	3221985	3222710	3291068	3291793	3378830	3379555	1446874	1446149	2800056	2799331	3052937	3053662	179490	180215	3342112	3342837
487 BPSL0451		11	3304668	3303706	3204821	3203859	3221862	3220900	3290945	3289983	3378707	3377745	1446997	1447959	2800179	2801141	3052814	3051852	179367	178405	3341989	3341027
488 BPSL0452		11	3303505	3302993	3203658	3203146	3220699	3220187	3289782	3289270	3377544	3377032	1448160	1448672	2801342	2801854	3051651	3051139	178204	177692	3340826	3340314
489 BPSL0453		11	3302940	3301366	3203093	3201519	3220134	3218560	3289217	3287643	3376979	3375405	1448725	1450299	2801907	2803481	3051086	3049512	177639	176065	3340261	3338687
490 BPSL0454		11	3301319	3299715	3201472	3199868	3218513	3216909	3287596	3285992	3375358	3373754	1450346	1451950	2803528	2805132	3049465	3047861	176018	174414	3338640	3337036
491 BPSL0455		11	3299643	3299506	3199796	3199659	3216837	3216700	3285920	3285783	3373682	3373545	1452022	1452159	2805204	2805341	3047789	3047652	174342	174205	3336964	3336827
492 BPSL0456		11	3299481	3298879	3199634	3199032	3216675	3216073	3285758	3285156	3373520	3372918	1452184	1452786	2805366	2805968	3047627	3047025	174180	173578	3336802	3336200
493 BPSL0457		11	3298856	3298659	3199009	3198812	3216050	3215853	3285133	3284936	3372895	3372698	1452809	1453006	2805991	2806188	3047002	3046805	173555	173358	3336177	3335980
494 BPSL0458		11	3298521	3297667	3198674	3197820	3215715	3214861	3284798	3283944	3372560	3371706	1453144	1453998	2806326	2807180	3046667	3045813	173220	172366	3335842	3334988
495 BPSL0458a		11	3297295	3297501	3197448	3197654	3214489	3214695	3283572	3283778	3371334	3371540	1454370	1454164	2807552	2807346	3045441	3045647	171994	172200	3334616	3334822
496 BPSL0459		11	3297207	3296497	3197360	3196650	3214401	3213691	3283484	3282774	3371246	3370536	1454458	1455168	2807640	2808350	3045353	3044643	171906	171196	3334528	3333818

497 BPSL0460		11	3296432	3295791	3196585	3195944	3213626	3212985	3282709	3282068	3370471	3369830	1455233	1455874	2808415	2809056	3044578	3043937	171131	170490	3333753	3333112
498 BPSL0461		11	3295684	3294638	3195837	3194791	3212878	3211832	3281961	3280915	3369723	3368677	1455981	1457027	2809163	2810209	3043830	3042784	170383	169337	3333005	3331959
499 BPSL0462		11	3294622	3293723	3194775	3193876	3211816	3210917	3280899	3280000	3368661	3367762	1457043	1457942	2810225	2811124	3042768	3041869	169321	168422	3331943	3331044
500 BPSL0463		11	3293700	3293086	3193853	3193239	3210894	3210280	3279977	3279363	3367739	3367125	1457965	1458579	2811147	2811761	3041846	3041232	168399	167785	3331021	3330407
501 BPSL0464		11	3292710	3293000	3192410	3192700	3209303	3209593	3278962	3279252	3366678	3366968	1458955	1458665	2812137	2811847	3040856	3041146	167409	167699	3329553	3329843
502 BPSL0465		11	3292523	3290886	3192223	3190586	3209116	3207479	3278775	3277138	3366491	3364854	1459142	1460779	2812324	2813961	3040669	3039032	167222	165585	3329366	3327729
503 BPSL0466		11	3290733	3289918	3190433	3189618	3207326	3206511	3276985	3276170	3364701	3363886	1460932	1461747	2814114	2814929	3038879	3038064	165432	164617	3327576	3326761
504	Chr1_25a_ISBma2_A		3288285	3289857	3187985	3189557	3204878	3206450	3274537	3276109	3362254	3363825	1461808	1463379	2814990	2816561	3036431	3038003	163290	164556	3325434	3326700
505	Chr1_25b_ISBma2_A		3235804	3237379	3135195	3136770	3152397	3153972	3222056	3223631	3309804	3311376	1514301	1515875	2867420	2868994	2983988	2985560	109336	110910	3271661	3273235
506 BPSL0467		12	3237596	3238564	3136987	3137955	3154189	3155157	3223848	3224816	3311593	3312561	1514084	1513116	2867203	2866235	2985777	2986745	111127	112095	3273452	3274420
507 BPSL0468		12	3238916	3239809	3138307	3139200	3155509	3156402	3225168	3226061	3312903	3313796	1512784	1511891	2865903	2865010	2987077	2987970	112427	113320	3274752	3275645
508 BPSL0469		12	3239813	3240604	3139204	3139995	3156406	3157197	3226065	3226856	3313800	3314591	1511887	1511096	2865006	2864215	2987974	2988765	113324	114115	3275649	3276440
509 BPSL0470		12	3240814	3241521	3140205	3140912	3157407	3158114	3227066	3227773	3314801	3315508	1510886	1510179	2864005	2863298	2988975	2989682	114325	115032	3276650	3277171
510 BPSL0471		12	3241457	3242929	3140848	3142320	3158050	3159522	3227709	3229181	3315444	3316916	1510243	1508771	2863362	2861890	2989618	2991090	114968	116440	3277274	3278548
511 BPSL0472		12	3242938	3246126	3142329	3145517	3159531	3162719	3229190	3232378	3316925	3320113	1508762	1505574	2861881	2858693	2991099	2994287	116449	119637	3278557	3281745
512 BPSL0473		12	3247279	3246461	3146670	3145852	3163872	3163054	3233531	3232713	3321266	3320448	1504421	1505239	2857540	2858358	2995431	2994613	120790	119972	3282898	3282080
513 BPSL0474		12	3248516	3247326	3147907	3146717	3165109	3163919	3234768	3233578	3322503	3321313	1503184	1504374	2856303	2857493	2996668	2995478	122027	120837	3284135	3282945
514 BPSL0475		12	3249749	3248721	3149140	3148112	3166342	3165314	3236001	3234973	3323736	3322708	1501951	1502979	2855070	2856098	2997901	2996873	123260	122232	3285368	3284340
515 BPSL0476		12	3251147	3249798	3150538	3149189	3167740	3166391	3237399	3236050	3325134	3323785	1500553	1501902	2853672	2855021	2999299	2997950	124658	123309	3286766	3285417
516 BPSL0477		12	3251725	3251195	3151116	3150586	3168318	3167788	3237977	3237447	3325712	3325182	1499975	1500505	2853094	2853624	2999877	2999347	125236	124706	3287344	3286814
517 BPSL0478		12	3252891	3251803	3152282	3151194	3169484	3168396	3239143	3238055	3326878	3325790	1498809	1499897	2851928	2853016	3001043	2999955	126402	125314	3288510	3287422
518 BPSL0479		12	3253840	3252887	3153231	3152278	3170433	3169480	3240092	3239139	3327827	3326874	1497860	1498813	2850979	2851932	3001992	3001039	127351	126398	3289459	3288506
519 BPSL0480		12	3255027	3253963	3154418	3153354	3171620	3170556	3241279	3240215	3329014	3327950	1496673	1497737	2849792	2850856	3003179	3002115	128538	127474	3290646	3289582
520 BPSL0481		12	3255689	3255027	3155080	3154418	3172282	3171620	3241941	3241279	3329676	3329014	1496011	1496673	2849130	2849792	3003841	3003179	129200	128538	3291308	3290646
521 BPSL0482		12	3256880	3255732	3156271	3155123	3173473	3172325	3243132	3241984	3330867	3329719	1494820	1495968	2847939	2849087	3005032	3003884	130391	129243	3292499	3291351
522 BPSL0483		12	3258753	3256885	3158144	3156276	3175346	3173478	3245005	3243137	3332740	3330872	1492947	1494815	2846066	2847934	3006905	3005037	132264	130396	3294372	3292504
523 BPSL0484		12	3259790	3258753	3159181	3158144	3176383	3175346	3246042	3245005	3333777	3332740	1491910	1492947	2845029	2846066	3007942	3006905	133301	132264	3295409	3294372
524 BPSL0485		12	3261376	3259811	3160767	3159202	3177969	3176404	3247628	3246063	3335363	3333798	1490324	1491889	2843443	2845008	3009528	3007963	134887	133322	3296995	3295430
525 BPSL0486		12	3262580	3261387	3161971	3160778	3179173	3177980	3248832	3247639	3336567	3335374	1489120	1490313	2842239	2843432	3010732	3009539	136091	134898	3298199	3297006
526 BPSL0487		12	3263568	3262573	3162959	3161964	3180161	3179166	3249820	3248825	3337555	3336560	1488132	1489127	2841251	2842246	3011720	3010725	137079	136084	3299187	3298192
527 BPSL0488		12	3264514	3263576	3163905	3162967	3181107	3180169	3250766	3249828	3338501	3337563	1487186	1488124	2840305	2841243	3012666	3011728	138025	137087	3300133	3299195
528 BPSL0489		12	3265141	3264578	3164532	3163969	3181734	3181171	3251393	3250830	3339128	3338565	1486559	1487122	2839678	2840241	3013293	3012730	138652	138089	3300760	3300197
529 BPSL0490		12	3266025	3265141	3165416	3164532	3182618	3181734	3252277	3251393	3340012	3339128	1485675	1486559	2838794	2839678	3014177	3013293	139536	138652	3301644	3300760
530 BPSL0491		12	3266306	3266061	3165697	3165452	3182899	3182654	3252558	3252313	3340293	3340048	1485394	1485639	2838513	2838758	3014458	3014213	139817	139572	3301925	3301680
531 BPSL0492		12	3267546	3266365	3166937	3165756	3184139	3182958	3253798	3252617	3341533	3340352	1484154	1485335	2837273	2838454	3015698	3014517	141057	139876	3303165	3301984
532 BPSL0493		12	3269309	3267546	3168700	3166937	3185902	3184139	3255561	3253798	3343296	3341533	1482391	1484154	2835510	2837273	3017461	3015698	142820	141057	3304928	3303165
533 BPSL0494		12	3269743	3270663	3169134	3170054	3186336	3187256	3255995	3256915	3343730	3344650	1481957	1481037	2835076	2834156	3017895	3018815	143254	144174	3305362	3306282
534 BPSL0495		12	3271372	3272133	3170763	3171524	3187965	3188726	3257624	3258385	3345359	3346120	1480328	1479567	2833447	2832686	3019524	3020285	144883	145644	3306991	3307752
535 BPSL0496		12	3272235	3273335	3171626	3172726	3188828	3189928	3258487	3259587	3346222	3347322	1479465	1478365	2832584	2831484	3020387	3021487	145746	146846	3307854	3308954
536 BPSL0497		12	3273322	3274335	3172713	3173726	3189915	3190928	3259574	3260587	3347309	3348322	1478378	1477365	2831497	2830484	3021474	3022487	146833	147846	3308941	3309954
537 BPSL0498		12	3274372	3276987	3173763	3173945	3190965	3193580	3260624	3263239	3348359	3350974	1477328	1474713	2830447	2827832	3022524	3025139	147883	150498	3309991	3312606
538 BPSL0499		12	3277091	3278866	3176791	3178566	3193684	3195459	3263343	3265118	3351078	3352853	1474609	1472834	2827728	2825953	3025243	3027018	150602	152377	3312710	3314485
539 BPSL0500		12	3281972	3279474	3181672	3179174	3198565	3196067	3268224	3265726	3355959	3353461	1469665	1472163	2822847	2825345	3030145	3027647	155504	153006	3317612	3315114
540 BPSL0501		12	3284624	3283491	3184324	3183191	3201217	3200084	3270876	3269743	3358593	3357460	1467040	1468173	2820222	2821355	3032770	3031637	158156	157023	3320300	3319167
541 BPSL0502		12	3286257	3284665	3185957	3184365	3202850	3201258	3272509	3270917	3360226	3358634	1465407	1466999	2818589	2820181	3034403	3032811	159789	158197	3321933	3320341
542 BPSL0503		12	3286531	3286250	3186231	3185950	3203124	3202843	3272783	3272502	3360500	3360219	1465133	1465414	2818315	2818596	3034677	3034396	160063	159782	3322207	3321926
543	Chr1_26_ISBma1_A	12	3286945	3288251	3186645	3187951	3203538	3204844	3273197	3274503	3360914	3362220	1463413	1464719	2816595	2817901	3035091	3036397	160477	161783	3322621	3323927
544	Chr1_27_IS407A_B	12																	162059	163294	3324203	3325438
545	Chr1_28a_ISBma2_A		3288285	3289857	3187985	3189557	3204878	3206450	3274537	3276109	3362254	3363825	1461808	1463379	2814990	2816561	3036431	3038003	163290	164556	3325434	3326700
546	Chr1_28b_ISBma2_A		3235804	3237379	3135195	3136770	3152397	3153972	3222056	3223631	3309804	3311376	1514301	1515875	2867420	2868994	2983988	2985560	109336	110910	3271661	3273235
547 BPSL0504		13	3234800	3235732	3134191	3135123	3151393	3152325	3221052	3221984	3308800	3309732	1516879	1515947	2869998	2869066	2982984	2983916	108332	109264	3270657	3271589

548	BPSL0505		13	3234435	3233869	3133826	3133260	3151028	3150462	3220687	3220121	3308435	3307869	1517244	1517810	2870363	2870929	2982619	2982053	107967	107401	3270292	3269726
549	BPSL0506		13	3233845	3233441	3133236	3132832	3150438	3150034	3220097	3219693	3307845	3307441	1517834	1518238	2870953	2871357	2982029	2981625	107377	106973	3269702	3269298
550	BPSL0507		13	3231471	3233108	3130862	3132499	3148064	3149701	3217723	3219360	3305471	3307108	1520208	1518571	2873327	2871690	2979655	2981292	105003	106640	3267328	3268965
551	BPSL0508		13	3230187	3231032	3129578	3130423	3146780	3147625	3216439	3217284	3304187	3305032	1521492	1520647	2874611	2873766	2978371	2979216	103719	104564	3266044	3266889
552	BPSL0509		13	3228593	3229570	3127984	3128961	3145186	3146163	3214845	3215822	3302593	3303570	1523086	1522109	2876205	2875228	2976777	2977754	102125	103102	3264450	3265427
553	BPSL0510		13	3225741	3228593	3125132	3127984	3141614	3144319	3211993	3214845	3299741	3302593	1525938	1523086	2879057	2876205	2973925	2976777	99273	102125	3261598	3264450
554	BPSL0511		13	3225349	3225741	3124740	3125132	3141222	3141614	3211601	3211993	3299349	3299741	1526330	1525938	2879449	2879057	2973533	2973925	98881	99273	3261206	3261598
555	BPSL0512		13	3222836	3225277	3122227	3124668	3138709	3141150	3209088	3211529	3296836	3299277	1528843	1526402	2881962	2879521	2971020	2973461	96368	98809	3258693	3261134
556	BPSL0513		13	3222451	3221810	3121842	3121201	3138324	3137683	3208703	3208062	3296451	3295810	1529228	1529869	2882347	2882988	2970635	2969994	95983	95342	3258308	3257667
557	BPSL0514		13	3220331	3221647	3119722	3121038	3136204	3137520	3206583	3207899	3294331	3295647	1531348	1530032	2884467	2883151	2968515	2969831	93863	95179	3256188	3257504
558	BPSL0515		13	3220079	3219456	3119470	3118847	3135952	3135329	3206331	3205708	3294079	3293456	1531600	1532223	2884719	2885342	2968263	2967640	93611	92988	3255936	3255313
559	BPSL0516		13	3219342	3218845	3118733	3118236	3135215	3134718	3205594	3205097	3293342	3292845	1532337	1532834	2885456	2885953	2967526	2967029	92874	92377	3255199	3254702
560	BPSL0517		13	3218779	3218516	3118170	3117907	3134652	3134389	3205031	3204768	3292779	3292516	1532900	1533163	2886019	2886282	2966963	2966700	92311	92048	3254636	3254373
561	BPSL0518		13	3217264	3218331	3116655	3117722	3133137	3134204	3203516	3204583	3291264	3292331	1534415	1533348	2887534	2886467	2965448	2966515	90796	91863	3253121	3254188
562	BPSL0519		13	3216570	3217172	3115961	3116563	3132443	3133045	3202822	3203424	3290570	3291172	1535109	1534507	2888228	2887626	2964754	2965356	90102	90704	3252427	3253029
563	BPSL0520		13	3215830	3216453	3115221	3115844	3131703	3132326	3202082	3202705	3289830	3290453	1535849	1535226	2888968	2888345	2964014	2964637	89362	89985	3251687	3252310
564	BPSL0521		13	3214722	3215675	3114113	3115066	3130595	3131548	3200974	3201927	3288722	3289675	1536957	1536004	2890076	2889123	2962906	2963859	88254	89207	3250579	3251532
565	BPSL0522		13	3214577	3214732	3113968	3114123	3130450	3130605	3200829	3200984	3288577	3288732	1537102	1536947	2890221	2890066	2962761	2962916	88109	88264	3250434	3250589
566	BPSL0523		13	3213663	3214541	3113054	3113932	3129536	3130414	3199915	3200793	3287663	3288541	1538016	1537138	2891135	2890257	2961847	2962725	87195	88073	3249520	3250398
567	BPSL0524		13	3212982	3213626	3112373	3113017	3128855	3129499	3199234	3199878	3286982	3287626	1538709	1538053	2891828	2891172	2961154	2961810	86526	87158	3248851	3249483
568	BPSL0525		13	3211165	3212982	3110178	3111755	3127038	3128855	3197417	3199234	3285165	3286982	1540526	1538709	2893645	2891828	2959337	2961154	84709	86526	3247034	3248851
569	BPSL0526		13	3211076	3210261	3110089	3109274	3126949	3126134	3197328	3196513	3285076	3284261	1540615	1541430	2893734	2894549	2959248	2958433	84620	83805	3246945	3246130
570	BPSL0527		13	3210253	3209150	3109266	3108163	3126126	3125023	3196505	3195402	3284253	3283150	1541438	1542541	2894557	2895660	2958425	2957322	83797	82694	3246122	3245019
571	BPSL0528		13	3208127	3208756	3107140	3107769	3124000	3124629	3194379	3195008	3282127	3282756	1543564	1542935	2896683	2896054	2956299	2956928	81671	82300	3243996	3244625
572	BPSL0529		13	3207207	3208097	3106220	3107110	3123080	3123970	3193459	3194349	3281207	3282097	1544484	1543594	2897603	2896713	2955379	2956269	80751	81641	3243076	3243966
573	BPSL0530		13	3206153	3207118	3105166	3106131	3122026	3122991	3192405	3193370	3280153	3281118	1545538	1544573	2898657	2897692	2954325	2955290	79697	80662	3242022	3242987
574	BPSL0531		13	3205569	3206021	3104582	3105034	3121442	3121894	3191821	3192273	3279569	3280021	1546122	1545670	2899241	2898789	2953741	2954193	79113	79565	3241438	3241890
575	BPSL0532		13	3204923	3205279	3103936	3104292	3120796	3121152	3191175	3191531	3278923	3279279	1546768	1546412	2899887	2899531	2953095	2953451	78467	78823	3240792	3241148
576	BPSL0533		13	3203265	3204770	3102278	3103783	3119138	3120643	3189517	3191022	3277265	3278770	1548426	1546921	2901545	2900040	2951437	2952942	76809	78314	3239134	3240639
577	BPSL0534		13	3202320	3203093	3101333	3102106	3118193	3118966	3188572	3189345	3276320	3277093	1549371	1548598	2902490	2901717	2950492	2951265	75864	76637	3238189	3238962
578	BPSL0535		13	3201658	3202320	3100671	3101333	3117531	3118193	3187910	3188572	3275658	3276320	1550033	1549371	2903152	2902490	2949830	2950492	75202	75864	3237527	3238189
579	BPSL0536		13	3201025	3201636	3100038	3100649	3116898	3117509	3187277	3187888	3275025	3275636	1550666	1550055	2903785	2903174	2949197	2949808	74569	75180	3236894	3237505
580	BPSL0537		13	3200480	3201016	3099493	3100029	3116353	3116889	3186732	3187268	3274480	3275016	1551211	1550675	2904330	2903794	2948652	2949188	74024	74560	3236349	3236885
581	BPSL0538		13	3199497	3200477	3098510	3099490	3115370	3116350	3185749	3186729	3273497	3274477	1552194	1551214	2905313	2904333	2947669	2948649	73041	74021	3235366	3236346
582	BPSL0539		13	3199369	3197363	3098382	3096376	3115242	3113236	3185621	3183615	3273369	3271363	1552322	1554328	2905441	2907447	2947541	2945535	72913	70907	3235238	3233232
583	BPSL0540		13	3197276	3197166	3096289	3096179	3113149	3113039	3183528	3183418	3271276	3271166	1554415	1554525	2907534	2907644	2945448	2945338	70820	70710	3233145	3233035
584	BPSL0541		13	3196620	3196039	3095633	3095052	3112493	3111912	3182872	3182291	3270620	3270039	1555071	1555652	2908190	2908771	2944792	2944211	70164	69583	3232489	3231908
585	BPSL0542		13	3196023	3195169	3095036	3094182	3111896	3111042	3182275	3181421	3270023	3269169	1555668	1556522	2908787	2909641	2944195	2943341	69567	68713	3231892	3231038
586	BPSL0543		13	3195114	3194236	3094127	3093249	3110987	3110109	3181366	3180488	3269114	3268236	1556577	1557455	2909696	2910574	2943286	2942408	68658	67780	3230983	3230105
587	BPSL0544		13	3192903	3193934	3091916	3092947	3108776	3109807	3179155	3180186	3266903	3267934	1558788	1557757	2911907	2910876	2941075	2942106	66447	67478	3228772	3229803
588	BPSL0545		13	3189766	3192630	3088779	3091643	3105639	3108503	3176018	3178882	3263766	3266630	1561925	1559061	2915044	2912180	2937938	2940802	63310	66174	3225635	3228499
589	BPSL0546		13	3189552	3188368	3088565	3087381	3105425	3104241	3175804	3174620	3263552	3262368	1562139	1563323	2915258	2916442	2937724	2936540	63096	61912	3225421	3224237
590	BPSL0547		13	3188251	3187655	3087264	3086869	3104124	3103528	3174503	3173907	3262251	3261664	1563440	1564015	2916559	2917134	2936423	2935848	61795	61208	3224120	3223533
591		Chr1_29_IS407A_A		3186007	3187242	3084797	3086032	3101880	3103115	3172259	3173494	3260016	3261251	1564428	1565663	2917547	2918782	2934200	2935435	59560	60795	3221885	3223120
592	BPSL0548	GI3																					
593	BPSL0549	GI3																					
594	BPSL0550	GI3																					
595	BPSL0551	GI3																					
596	BPSL0552	GI3																					
597	BPSL0553	GI3																					
598	BPSL0554	GI3																					

599	BPSL0555	GI3																							
600	BPSL0556	GI3																							
601	BPSL0557	GI3																							
602	BPSL0558	GI3																							
603	BPSL0559	GI3																							
604	BPSL0560	GI3																							
605	BPSL0561	GI3																							
606	BPSL0562	GI3																							
607	BPSL0563	GI3																							
608	BPSL0564	GI3																							
609	BPSL0565	GI3																							
610	BPSL0566	GI3																							
611	BPSL0567	GI3																							
612	BPSL0568	GI3																							
613	BPSL0569	GI3																							
614	BPSL0570	GI3																							
615	BPSL0571	GI3																							
616	BPSL0572	GI3																							
617	BPSL0573	GI3																							
618	BPSL0574	GI3																							
619	BPSL0575	GI3																							
620	BPSL0576	GI3																							
621	BPSL0577	GI3																							
622	BPSL0578	GI3																							
623	BPSL0579	GI3																							
624	BPSL0580	GI3																							
625	BPSL0581	GI3																							
626	BPSL0582	GI3																							
627	BPSL0583	GI3																							
628	BPSL0584	GI3																							
629	BPSL0585	GI3																							
630	BPSL0586	GI3																							
631	BPSL0586a	GI3																							
632	BPSL0587	GI3																							
633	BPSL0588	GI3																							
634		Chr1_30_IS407A_A		6007	7242	3419381	3420616	3439108	3440343	5679	6914	163047	164282	2261519	2262754	2443334	2444569	3166175	3167410	2832213	2833448	34218	35453		
635	BPSL0589	GI3	14	7554	7243	3420928	3420617	3440655	3440344	7226	6915	164594	164283	2261207	2261518	2444881	2444570	3167722	3167411	2833760	2833449	35765	35454		
636	BPSL0590		14	13364	8292	3426738	3421666	3446465	3441393	13036	7964	170398	165332	2255403	2260469	2450685	2445619	3173526	3168460	2839564	2834498	41569	36503		
637		Chr1_31_IS407A_C	14											2252754	2253989										
638	BPSL0591		14	17169	13888	3430813	3430337	3450270	3446989	16841	13560	174203	170922	2250358	2252769	2454490	2451209	3177331	3174050	2843369	2840088	45374	42093		
639	BPSL0592		14	18737	18357	3432381	3432001	3451838	3451458	18409	18029	175771	175391	2248790	2249170	2456058	2455678	3178899	3178519	2844937	2844557	46942	46562		
640	BPSL0593		14	19192	18758	3432836	3432402	3452293	3451859	18864	18430	176226	175792	2248335	2248769	2456513	2456079	3179354	3178920	2845392	2844958	47397	46963		
641	BPSL0594		14	19763	19422	3433407	3433066	3452864	3452523	19435	19094	176797	176456	2247764	2248105	2457084	2456743	3179925	3179584	2845963	2845622	47968	47627		
642	BPSL0595		14	20641	20279	3434285	3433923	3453742	3453380	20313	19951	177675	177313	2246886	2247248	2457962	2457600	3180803	3180441	2846841	2846479	48846	48484		
643	BPSL0596		14	20889	21893	3434533	3435537	3453990	3454994	20561	21565	177923	178927	2246638	2245634	2458210	2459214	3181051	3182055	2847089	2848093	49094	50098		
644	BPSL0597		14	21907	25983	3435551	3439627	3455008	3459084	21579	25655	178941	183017	2245620	2241544	2459228	2463304	3182069	3186145	2848107	2852183	50112	54188		
645	BPSL0598		14	25983	26342	3439627	3439986	3459084	3459443	25655	26014	183017	183376	2241544	2241185	2463304	2463663	3186145	3186504	2852183	2852542	54188	54547		
646	BPSL0599		14	26347	26706	3439991	3440350	3459448	3459807	26019	26378	183381	183740	2241180	2240821	2463668	2464027	3186509	3186868	2852547	2852906	54552	54911		
647	BPSL0600		14	26801	29089	3440445	3442733	3459902	3462190	26473	28761	183835	186123	2240726	2238438	2464122	2466410	3186963	3189251	2853001	2855289	55076	57364		
648	BPSL0601		14	29162	29935	3442806	3443579	3462263	3463036	28834	29607	186196	186969	2238365	2237592	2466483	2467256	3189324	3190097	2855362	2856135	57437	58210		
649	BPSL0602		14	30421	32781	3444065	3446425	3463522	3465882	30093	32453	187455	189815	2237106	2234746	2467742	2470102	3190583	3192943	2856621	2858981	58696	61056		

650	BPSL0603		14	34230	32965	3447874	3446609	3467331	3466066	33902	32637	191273	190008	2233297	2234562	2471551	2470286	3194392	3193127	2860430	2859165	62505	61240
651	BPSL0604		14	35503	34310	3449147	3447954	3468604	3467411	35175	33982	192546	191353	2232024	2233217	2472824	2471631	3195665	3194472	2861703	2860510	63778	62585
652	BPSL0605		14	37130	35541	3450774	3449185	3470231	3468642	36802	35213	194173	192584	2230397	2231986	2474451	2472862	3197292	3195703	2863330	2861741	65405	63816
653	BPSL0606		14	37777	37130	3451421	3450774	3470878	3470231	37449	36802	194820	194173	2229750	2230397	2475098	2474451	3197939	3197292	2863977	2863330	66052	65405
654	BPSL0607		14	38155	39393	3451799	3453037	3471256	3472494	37827	39065	195198	196436	2229372	2228134	2475476	2476714	3198317	3199555	2864355	2865593	66430	67668
655	BPSL0608		14	39424	40155	3453068	3453799	3472525	3473256	39096	39827	196467	197198	2228103	2227366	2476745	2477482	3199586	3200323	2865624	2866361	67699	68436
656	BPSL0609		14	40903	42288	3454547	3455932	3474004	3475389	40575	41960	197946	199331	2226618	2225233	2478230	2479615	3201071	3202456	2867109	2868494	69184	70569
657	BPSL0610		14	44322	42349	3457966	3455993	3478083	3476377	43994	42021	201365	199392	2223199	2225172	2481649	2479676	3204490	3202517	2870528	2868555	72603	70630
658	BPSL0611		14	46838	44322	3460482	3457966	3480599	3478083	46510	43994	203881	201365	2220683	2223199	2484165	2481649	3207006	3204490	2873044	2870528	75119	72603
659	BPSL0612		14	47923	46838	3461567	3460482	3481684	3480599	47595	46510	204966	203881	2219598	2220683	2485250	2484165	3208091	3207006	2874129	2873044	76204	75119
660	BPSL0613		14	48894	47923	3462538	3461567	3482655	3481684	48566	47595	205937	204966	2218627	2219598	2486221	2485250	3209062	3208091	2875100	2874129	77175	76204
661	BPSL0614		14	50114	48894	3463758	3462538	3483875	3482655	49786	48566	207157	205937	2217407	2218627	2487441	2486221	3210282	3209062	2876320	2875100	78395	77175
662	BPSL0615		14	50413	50114	3464057	3463758	3484174	3483875	50085	49786	207456	207157	2217108	2217407	2487740	2487441	3210581	3210282	2876619	2876320	78694	78395
663	BPSL0616		14	51401	50610	3465045	3464254	3485162	3484371	51073	50282	208444	207653	2216120	2216911	2488728	2487937	3211569	3210778	2877607	2876816	79682	78891
664	BPSL0617		14	52268	53131	3465912	3466775	3486029	3486892	51940	52803	209311	210174	2215253	2214390	2489595	2490458	3212436	3213299	2878474	2879337	80549	81412
665	BPSL0618		14	53131	54546	3466775	3468190	3486892	3488307	52803	54218	210174	211589	2214390	2212975	2490458	2491873	3213299	3214714	2879337	2880752	81412	82827
666	BPSL0619		14	54621	55907	3468265	3469551	3488382	3489668	54293	55579	211664	212950	2212900	2211614	2491948	2493234	3214789	3216075	2880827	2882113	82902	84188
667	BPSL0620		14	56193	57332	3469837	3470976	3489954	3491093	55865	57004	213236	214375	2211328	2210189	2493520	2494659	3216361	3217500	2882399	2883538	84474	85613
668	BPSL0621		14	58263	57367	3471907	3471011	3492024	3491128	57935	57039	215306	214410	2209258	2210154	2495590	2494694	3218431	3217535	2884469	2883573	86544	85648
669		Chr1_32a_ISBma2_A		58691	60263	3472335	3473907	3492452	3494024	58363	59935	215734	217306	2207258	2208830	2496018	2497590	3218859	3220433	2884897	2886468	86972	88544
670		Chr1_32b_ISBma2_A		178751	180323	51985	53557	51562	53134	177854	179426			2330494	2332086	2338546	2340123	52889	54461	2763087	2764659	219786	221358
671	BPSL0622		15	180633	181064	53867	54298	53444	53875	179736	180167	217616	218047	2332396	2332827	2340433	2340864	54771	55202	2762777	2762346	221668	222099
672	BPSL0623		15	181159	182121	54393	55355	53970	54932	180262	181224	218142	219104	2332922	2333884	2340959	2341921	55297	56259	2762251	2761289	222194	223156
673	BPSL0624		15	183008	182097	56242	55331	55819	54908	182111	181200	219991	219080	2334771	2333860	2342808	2341897	57146	56235	2760402	2761313	224043	223132
674	BPSL0625		15	183115	184707	56349	57941	55926	57518	182218	183810	220098	221690	2334878	2336470	2342915	2344507	57253	58845	2760295	2758703	224150	225742
675	BPSL0626		15	184759	185190	57993	58424	57570	58001	183862	184293	221742	222173	2336522	2336953	2344559	2344990	58897	59328	2758651	2758220	225794	226225
676	BPSL0627		15	185892	185314	59126	58548	58703	58125	184995	184417	222875	222297	2337655	2337077	2345692	2345114	60030	59452	2757518	2758096	226927	226349
677	BPSL0628		15	187307	186045	60541	59279	60118	58856	186410	185148	224290	223028	2339070	2337808	2347107	2345845	61445	60183	2756103	2757365	228342	227080
678	BPSL0629		15	187445	188335	60679	61569	60256	61146	186548	187438	224428	225318	2339208	2340098	2347245	2348135	61583	62473	2755965	2755075	228480	229370
679	BPSL0630		15	188676	189764	61910	62998	61487	62575	187779	188867	225659	226747	2340439	2341527	2348476	2349564	62814	63902	2754734	2753646	229711	230799
680	BPSL0631		15	189894	190277	63128	63511	62705	63088	188997	189380	226877	227260	2341657	2342040	2349694	2350077	64032	64415	2753516	2753133	230929	231312
681	BPSL0632		15	191448	190627	64682	63861	64259	63438	190551	189730	228431	227610	2343211	2342390	2351248	2350427	65586	64765	2751962	2752783	232483	231662
682	BPSL0633		15	192991	191471	66225	64705	65802	64282	192094	190574	229974	228454	2344754	2343234	2352791	2351271	67129	65609	2750419	2751939	234026	232506
683	BPSL0634		15	193447	197523	66681	70757	66258	70334	192550	196626	230430	234506	2345210	2349286	2353247	2357323	67585	71661	2749963	2745887	234482	238558
684	BPSL0635		15	197561	198001	70795	71235	70372	70812	196664	197104	234544	234984	2349324	2349764	2357361	2357801	71699	72139	2745849	2745409	238596	239036
685	BPSL0636		15	198001	198411	71235	71645	70812	71222	197104	197514	234984	235394	2349764	2350174	2357801	2358211	72139	72549	2745409	2744999	239036	239446
686	BPSL0637		15	198462	199190	71696	72424	71273	72001	197565	198293	235445	236173	2350225	2350953	2358262	2358990	72600	73328	2744948	2744220	239497	240225
687	BPSL0638		15	199283	200251	72517	73485	72094	73062	198386	199354	236266	237234	2351046	2352014	2359083	2360051	73421	74389	2744127	2743159	240318	241286
688	BPSL0639		15	200335	201006	73569	74240	73146	73817	199438	200109	237318	237989	2352098	2352769	2360135	2360806	74473	75144	2743075	2742404	241370	242041
689	BPSL0640		15	201022	202596	74256	75830	73833	75407	200125	201699	238005	239579	2352785	2354359	2360822	2362396	75160	76734	2742388	2740814	242057	243631
690	BPSL0641		15	202923	203552	76157	76786	75734	76363	202026	202655	239906	240535	2354686	2355315	2362723	2363352	77061	77690	2740487	2739858	243958	244587
691	BPSL0642		15	203671	204009	76905	77243	76482	76820	202774	203112	240654	240992	2355434	2355772	2363471	2363809	77809	78147	2739739	2739401	244706	245044
692	BPSL0643		15	204083	204727	77317	77961	76894	77538	203186	203830	241066	241710	2355846	2356490	2363883	2364527	78221	78865	2739327	2738683	245118	245762
693	BPSL0644		15	204787	206586	78021	79820	77598	79397	203890	205689	241770	243569	2356550	2358349	2364587	2366386	78925	80724	2738623	2736824	245822	247621
694	BPSL0645		15	206798	207271	80032	80505	79609	80082	205901	206374	243781	244254	2358561	2359034	2366598	2367071	80936	81409	2736612	2736139	247833	248306
695	BPSL0646		15	207271	208542	80505	81776	80082	81353	206374	207645	244254	245525	2359034	2360305	2367071	2368342	81409	82680	2736139	2734868	248306	249577
696	BPSL0647		15	208735	209331	81969	82565	81546	82142	207838	208434	245718	246314	2360498	2361094	2368535	2369131	82873	83469	2734675	2734079	249770	250366
697	BPSL0648		15	209392	211176	82626	84410	82203	83987	208495	210279	246375	248159	2361155	2362939	2369192	2370976	83530	85314	2734018	2732234	250427	252211
698	BPSL0649		15	211337	213769	84571	87003	84148	86580	210454	212886	248320	250752	2363100	2365532	2371137	2373569	85573	88005	2732073	2729641	252546	254978
699	BPSL0650		15	213952	215148	87186	88382	86763	87959	213069	214265	250935	252131	2365715	2366911	2373752	2374948	88188	89384	2729458	2728262	255161	256357
700	BPSL0651		15	215246	216007	88480	89241	88057	88818	214363	215124	252229	252990	2367009	2367770	2375046	2375807	89482	90243	2728164	2727403	256455	257216

701	BPSL0652	15	216948	216127	90182	89361	89759	88938	216065	215244	253931	253110	2368711	2367890	2376748	2375927	91184	90363	2726462	2727283	258157	257336
702	BPSL0653	15	217082	217558	90316	90792	89893	90369	216199	216675	254065	254541	2368845	2369321	2376882	2377358	91318	91794	2726328	2725852	258291	258767
703	BPSL0654	15	218019	217609	91253	90843	90830	90420	217136	216726	255002	254592	2369782	2369372	2377819	2377409	92255	91845	2725391	2725801	259228	258818
704	BPSL0655	15	218326	220194	91560	93428	91137	93005	217443	219311	255309	257177	2370089	2371957	2378126	2379994	92562	94430	2725084	2723216	259535	261403
705	BPSL0656	15	221409	220693	94643	93927	94220	93504	220526	219810	258392	257676	2373172	2372456	2381209	2380493	95645	94929	2722001	2722717	262618	261902
706	BPSL0657	15	222447	221416	95681	94650	95258	94227	221564	220533	259430	258399	2374210	2373179	2382247	2381216	96683	95652	2720963	2721994	263656	262625
707	BPSL0658	15	222673	225033	95907	98267	95484	97844	221790	224150	259656	262016	2374436	2376796	2382473	2384833	96909	99269	2720737	2718377	263882	266242
708	BPSL0659	15	225097	226446	98331	99680	97908	99257	224214	225563	262080	263429	2376860	2378209	2384897	2386246	99333	100682	2718313	2716964	266306	267655
709	BPSL0660	15	226471	227502	99705	100736	99282	100313	225588	226619	263454	264485	2378234	2379265	2386271	2387302	100707	101738	2716939	2715908	267680	268711
710	BPSL0661	15	227537	228361	100771	101595	100348	101172	226654	227478	264520	265344	2379300	2380124	2387337	2388161	101773	102597	2715873	2715049	268746	269570
711	BPSL0662	15	229360	228617	102781	102584	102358	102161	228664	228467	266535	266338	2381355	2381158	2389367	2389170	103813	103616	2713783	2713980	270836	270639
712	BPSL0663	15	229925	230311	103159	103545	102736	103122	229042	229428	266913	267299	2381733	2382119	2389745	2390131	104191	104577	2713405	2713019	271214	271600
713	BPSL0664	15	231372	230530	104606	103764	104183	103341	230489	229647	268360	267518	2383180	2382338	2391192	2390350	105638	104796	2711958	2712800	272661	271819
714	BPSL0665	15	232232	231456	105466	104690	105043	104267	231349	230573	269220	268444	2384040	2383264	2392052	2391276	106498	105722	2711098	2711874	273521	272745
715	BPSL0666	15	232838	232278	106072	105512	105649	105089	231955	231395	269826	269266	2384646	2384086	2392658	2392098	107104	106544	2710492	2711052	274127	273567
716	BPSL0667	15	234955	232859	108189	106093	107766	105670	234072	231976	271943	269847	2386763	2384667	2394775	2392679	109221	107125	2708375	2710471	276244	274148
717	BPSL0668	15	235979	234975	109213	108209	108790	107786	235096	234092	272967	271963	2387787	2386783	2395799	2394795	110245	109241	2707351	2708355	277268	276264
718	BPSL0669	15	237941	236238	111817	111101	110752	109049	237058	235355	274929	273226	2389749	2388046	2397761	2396058	112270	111533	2705389	2707092	279230	277527
719	BPSL0670	15	238871	237987	112747	111863	111682	110798	237988	237104	275859	274975	2390679	2389795	2398691	2397807	113200	112316	2704459	2705343	280160	279276
720	BPSL0671	15	240005	239376	113881	113252	112816	112187	239122	238493	276993	276364	2391813	2391184	2399825	2399196	114334	113705	2703325	2703954	281294	280665
721	BPSL0672	15	240841	240029	114717	113905	113652	112840	239958	239146	277829	277017	2392649	2391837	2400661	2399849	115170	114358	2702489	2703301	282130	281318
722	BPSL0673	15	242577	241522	116453	115398	115388	114333	241694	240639	279629	278574	2394409	2393354	2402405	2401350	116890	115835	2700721	2701776	283570	282515
723	BPSL0674	15	243974	242604	117850	116480	116785	115415	243091	241721	281026	279656	2395806	2394436	2403802	2402432	118287	116917	2699324	2700694	284700	283597
724	BPSL0675	15	245392	244463	119268	118339	118203	117274	244509	243580	282444	281515	2397224	2396295	2405220	2404291	119705	118776	2697906	2698835	285992	285063
725	BPSL0676	15	245538	246707	119414	120583	118349	119518	244655	245824	282590	283759	2397370	2398539	2405366	2406535	119851	121020	2697760	2696591	286138	287307
726	BPSL0677	15	246844	247509	120720	121385	119655	120320	245961	246626	283896	284561	2398676	2399341	2406672	2407337	121157	121822	2696454	2695789	287444	288109
727	BPSL0678	15	247931	248650	121807	122526	120742	121461	247048	247767	284983	285702	2399763	2400482	2407759	2408478	122244	122963	2695367	2694648	288531	289250
728	BPSL0679	15	249407	248724	123283	122600	122218	121535	248524	247841	286459	285776	2401239	2400556	2409235	2408552	123720	123037	2693891	2694574	290007	289324
729	BPSL0680	15	250391	249540	124267	123416	123202	122351	249508	248657	287443	286592	2402223	2401372	2410219	2409368	124704	123853	2692907	2693758	290991	290140
730	BPSL0681	15	251701	251279	125577	125155	124512	124090	250818	250396	288753	288331	2403533	2403111	2411529	2411107	126014	125592	2691597	2692019	292301	291879
731	BPSL0682	15	253271	251742	127147	125618	126082	124553	252388	250859	290323	288794	2405103	2403574	2413099	2411570	127584	126055	2690027	2691556	293958	292429
732	BPSL0683	15	254670	253318	128546	127194	127481	126129	253787	252435	291722	290370	2406502	2405150	2414498	2413146	128983	127631	2688628	2689980	295357	294005
733	BPSL0684	15	256016	254712	129892	128588	128827	127523	255133	253829	293068	291764	2407848	2406544	2415844	2414540	130329	129025	2687282	2688586	296703	295399
734	BPSL0685	15	256460	256041	130336	129917	129271	128852	255577	255158	293512	293093	2408292	2407873	2416288	2415869	130773	130354	2686838	2687257	297147	296728
735	BPSL0686	15	258093	257380	131969	131256	130904	130191	257210	256497	295145	294432	2409925	2409212	2417921	2417208	132406	131693	2685205	2685918	298780	298067
736	BPSL0687	15	259708	258209	133584	132085	132519	131020	258825	257326	296760	295261	2411540	2410041	2419536	2418037	134021	132522	2683590	2685089	300395	298896
737	BPSL0688	15	261346	259817	135222	133693	134157	132628	260463	258934	298398	296869	2413178	2411649	2421174	2419645	135659	134130	2681952	2683481	302033	300504
738	BPSL0689	15	261514	261882	135390	135758	134325	134693	260631	260999	298566	298934	2413346	2413714	2421342	2421710	135827	136195	2681784	2681416	302201	302569
739	BPSL0690	15	262161	261955	136037	135831	134972	134766	261278	261072	299213	299007	2413993	2413787	2421989	2421783	136474	136268	2681137	2681343	302848	302642
740	BPSL0691	15	263306	262473	137182	136349	136117	135284	262423	261590	300358	299525	2415138	2414305	2423134	2422301	137619	136786	2679992	2680825	303993	303160
741	BPSL0692	15	263477	265147	137353	139023	136288	137958	262594	264264	300529	302199	2415309	2416979	2423305	2424975	137790	139460	2679821	2678151	304164	305834
742	BPSL0693	15	265453	266283	139329	140159	138264	139094	264570	265400	302505	303335	2417285	2418115	2425281	2426111	139766	140596	2677845	2677015	306140	306970
743	BPSL0694	15	266356	266853	140232	140729	139167	139664	265473	265970	303408	303905	2418188	2418685	2426184	2426681	140669	141166	2676942	2676445	307043	307510
744	BPSL0695	15	268451	266997	142327	140873	141262	139808	267568	266114	305503	304049	2420292	2418829	2428279	2426825	142764	141310	2674847	2676301	308851	307616
745	BPSL0696	15	269877	268582	143845	142550	143377	142082	269025	267730	307002	305707	2421718	2420423	2429705	2428410	144296	143001	2673421	2674716	310354	309059
746	BPSL0697	15	270658	269900	144626	143868	144158	143400	269806	269048	307783	307025	2422499	2421741	2430486	2429728	145077	144319	2672640	2673398	311135	310377
747	BPSL0698	15	271080	272003	145048	145971	144580	145503	270228	270587	308205	309128	2422921	2423844	2430908	2431831	145499	146422	2672218	2671295	311557	312480
748	BPSL0699	15	273589	272444	147557	146412	147089	145944	272281	271136	310724	309579	2425404	2424259	2433375	2432230	148014	146869	2669755	2670900	314010	312865
749	BPSL0700	15	274032	274376	148000	148344	147532	147876	272724	273068	311167	311511	2425847	2426191	2433818	2434162	148457	148801	2669312	2668968	314453	314797
750	BPSL0701	15	274997	274536	148965	148504	148497	148036	273689	273228	312141	311671	2426812	2426351	2434783	2434322	149440	148961	2668338	2668808	315418	314957
751	BPSL0702	15	275293	276972	149261	150940	148793	150472	273985	275664	312437	314116	2427108	2428787	2435079	2436758	149736	151415	2668042	2666363	315714	317393

752	BPSL0703		15	278836	276956	152804	150924	152336	150456	277528	275648	315980	314100	2430651	2428771	2438622	2436742	153279	151399	2664499	2666379	319257	317377
753	BPSL0704		15	280205	278889	154173	152857	153705	152389	278897	277581	317349	316033	2432020	2430704	2439991	2438675	154648	153332	2663130	2664446	320626	319310
754	BPSL0705		15	280994	281572	155183	155761	155181	155759	279669	280247	318214	318792	2432809	2433387	2440780	2441358	155488	156066	2662341	2661763	2341217	2340639
755	BPSL0706		15	283485	281746	157674	155935	157672	155933	282160	280421	320705	318966	2435300	2433561	2443271	2441532	157979	156240	2659850	2661589	2338726	2340465
756		Chr1_33_IS407A_A		283548	284783	157737	158972	157735	158970	282223	283458	320768	322003	2435363	2436598	2443334	2444569	158042	159277	2658552	2659787	2337504	2338663
757	BPSL0707																						
758	BPSL0708																						
759	BPSL0709																						
760	BPSL0710																						
761	BPSL0711																						
762	BPSL0712																						
763	BPSL0713																						
764	BPSL0714																						
765	BPSL0716																						
766	BPSL0717																						
767	BPSL0718																						
768	BPSL0719																						
769	BPSL0720																						
770	BPSL0721																						
771	BPSL0722																						
772	BPSL0723																						
773	BPSL0724																						
774	BPSL0725																						
775	BPSL0726																						
776	BPSL0727																						
777	BPSL0728																						
778	BPSL0729																						
779	BPSL0730																						
780	BPSL0731																						
781	BPSL0732																						
782	BPSL0733																						
783	BPSL0734																						
784	BPSL0735																						
785	BPSL0736																						
786	BPSL0737																						
787	BPSL0738																						
788	BPSL0739																						
789	BPSL0740																						
790	BPSL0741																						
791	BPSL0742																						
792	BPSL0743																						
793	BPSL0744																						
794	BPSL0745	GI4																					
795	BPSL0746	GI4																					
796	BPSL0747	GI4																					
797	BPSL0747a	GI4																					
798	BPSL0748	GI4																					
799	BPSL0749	GI4																					
800	BPSL0750	GI4																					
801	BPSL0751	GI4																					
802	BPSL0752	GI4																					

803	BPSL0753	GI4																					
804	BPSL0754	GI4																					
805	BPSL0756	GI4																					
806	BPSL0757	GI4																					
807	BPSL0758	GI4																					
808	BPSL0759	GI4																					
809	BPSL0760	GI4																					
810	BPSL0761	GI4																					
811	BPSL0762	GI4																					
812	BPSL0763	GI4																					
813	BPSL0764	GI4																					
814	BPSL0765	GI4																					
815	BPSL0766	GI4																					
816	BPSL0767	GI4																					
817	BPSL0768	GI4																					
818	BPSL0769	GI4																					
819	BPSL0770	GI4																					
820	BPSL0771	GI4																					
821	BPSL0772	GI4																					
822		Chr1_34_IS407A_A		283548	284783	157737	158972	157735	158970	282223	283458	338128	339363	2453363	2454598	24007	25242	176042	177277	2640788	2642023	2399992	2401227
823	BPSL0773		16	287428	285761	161617	159950	161615	159948	286103	284436	335483	337150	2450314	2451981	20958	22625	172993	174660	2645072	2643405	2404276	2402609
824	BPSL0774		16	289156	287642	163345	161831	163343	161829	287831	286317	333755	335269	2448586	2450100	19230	20744	171265	172779	2646800	2645286	2406004	2404490
825	BPSL0775		16	289923	289183	164112	163372	164110	163370	288598	287858	332988	333728	2447819	2448559	18463	19203	170498	171238	2647567	2646827	2406771	2406031
826	BPSL0776		16	290127	291194	164316	165383	164314	165381	288802	289869	332784	331717	2447615	2446548	18259	17192	170294	169227	2647771	2648838	2406975	2408042
827	BPSL0777		16	291563	292177	165752	166366	165750	166364	290238	290852	331348	330734	2446179	2445565	16823	16209	168858	168244	2649207	2649821	2408411	2409025
828	BPSL0778		16	292472	293080	166661	167269	166659	167267	291147	291755	330439	329831	2445270	2444662	15914	15306	167949	167341	2650116	2650724	2409320	2409928
829	BPSL0779		16	293163	294326	167352	168515	167350	168513	291838	293001	329748	328585	2444579	2443416	15223	14060	167258	166095	2650807	2651970	2410011	2411174
830	BPSL0780		16	294474	295352	168663	169541	168661	169539	293149	294027	328437	327559	2443268	2442390	13912	13034	165947	165069	2652118	2652996	2411322	2412200
831	BPSL0781		16	295407	296216	169596	170405	169594	170403	294082	294891	327504	326695	2442335	2441526	12979	12170	165014	164205	2653051	2653860	2412255	2413064
832	BPSL0782		16	296438	296953	170627	171142	170625	171140	295113	295628	326473	325958	2441304	2440789	11948	11433	163983	163468	2654082	2654597	2413286	2413801
833	BPSL0783		16	297164	298978	171353	173167	171351	173165	295839	297653	325747	323933	2440578	2438764	11222	9408	163257	161443	2654808	2656622	2414012	2415826
834	BPSL0784		16	299520	299990	173709	174179	173707	174177	298195	298665	323391	322921	2438222	2437752	8866	8396	160901	160431	2657164	2657634	2416368	2416838
835	BPSL0785		16	300230	300592	174419	174781	174417	174779	298905	299267	322681	322319	2437512	2437150	8156	7794	160191	159829	2657874	2658236	2417078	2417440
836		Chr1_35a_IS407A_A		300908	302143	175097	176332	175095	176330	299583	300818	320768	322003	2435363	2436598	6007	7242	158042	159277	2658552	2659787	2417756	2418991
837		Chr1_35b_IS407A_A										338128	339363	2453363	2454598	24007	25242	176042	177277	588962	590197	2350565	2351800
838	BPSL0786		17	302612	302145	176801	176334	176799	176332	301236	300820	339832	339365	2455067	2454600	25711	25244	177746	177279	590666	590199	2352269	2351802
839	BPSL0787		17	302759	302905	176948	177094	176946	177068	301373	301519	339979	341700	2455214	2456935	25858	27579	177893	179614	590813	592534	2352416	2354137
840	BPSL0788		17	305424	304804	179613	178993	180282	179662	304038	303418	342648	342028	2457883	2457263	28527	27907	180562	179942	593482	592862	2355085	2354465
841	BPSL0789		17	306451	305420	180640	179609	181309	180278	305065	304034	343675	342644	2458910	2457879	29554	28523	181589	180558	594509	593478	2356112	2355081
842	BPSL0790		17	306917	306474	181106	180663	181775	181332	305531	305088	344141	343698	2459376	2458933	30020	29577	182055	181612	594975	594532	2356578	2356135
843	BPSL0791		17	308089	307052	182278	181241	182947	181910	306703	305666	345313	344276	2460548	2459511	31192	30155	183227	182190	596147	595110	2357750	2356713
844	BPSL0792		17	308414	308220	183230	183036	184074	183880	307028	306834	345642	345448	2460873	2460679	31518	31324	183639	183445	596472	596278	321481	321287
845	BPSL0793		17	309403	308483	184219	183299	185063	184143	308017	307097	346631	345711	2461862	2460942	32507	31587	184628	183708	597461	596541	322470	321550
846	BPSL0794		17	309625	310371	184441	185187	185285	186031	308239	308985	346853	347599	2462084	2462830	32729	33475	184850	185596	597683	598429	322692	323438
847	BPSL0795		17	310371	310691	185187	185507	186031	186351	308985	309305	347599	347919	2462830	2463150	33475	33795	185596	185916	598429	598749	323438	323758
848	BPSL0796		17	310961	312151	185777	186967	186621	187811	309575	310765	348189	349379	2463420	2464610	34065	35255	186186	187376	599019	600209	324028	325218
849	BPSL0797		17	312489	313922	187305	188738	188149	189582	311103	312536	349717	351150	2464948	2466381	35593	37026	187714	189147	600547	601980	325518	326951
850	BPSL0798		17	314206	315267	189022	190083	189866	190927	312820	313881	351434	352495	2466665	2467726	37310	38371	189431	190492	602264	603325	327235	328296
851	BPSL0799		17	315460	316347	190276	191163	191120	192007	314074	314961	352688	353575	2467919	2468806	38564	39451	190685	191572	603518	604405	328489	329376
852	BPSL0800		17	316386	316901	191202	191717	192046	192561	315000	315515	353614	354129	2468845	2469360	39490	40005	191611	192126	604444	604959	329415	329930
853	BPSL0801		17	316984	318177	191800	192993	192644	193837	315598	316791	354212	355405	2469443	2470636	40088	41281	192209	193402	605042	606235	330013	331206

854 BPSL0802		17	318192	319214	193008	194030	193852	194874	316806	317828	355420	356442	2470651	2471673	41296	42318	193417	194439	606250	607272	331221	332243
855 BPSL0803		17	320610	319474	195426	194290	196270	195134	319224	318088	357850	356714	2473083	2471947	43748	42612	195861	194725	608662	607526	333639	332503
856 BPSL0804		17	321632	320718	196448	195534	197292	196378	320246	319332	358872	357958	2474105	2473191	44770	43856	196883	195969	609684	608770	334661	333747
857 BPSL0805		17	323471	322023	198287	196839	199131	197683	322085	320637	360735	359287	2475968	2474520	46573	45125	198782	197334	611475	610027	336464	335016
858 BPSL0806		17	323739	324398	198555	199214	199399	200058	322353	323012	361003	361662	2476236	2476895	46841	47500	199050	199709	611743	612402	336732	337391
859 BPSL0807		17	324405	325718	199221	200534	200065	201378	323019	324332	361669	362982	2476902	2478215	47507	48820	199716	201029	612409	613722	337398	338711
860 BPSL0808		17	326252	327667	201068	202483	201912	203327	324866	326281	363516	364931	2478749	2480164	49354	50769	201563	202978	614256	615671	339245	340660
861 BPSL0809		17	327830	328249	202646	203065	203490	203909	326444	326863	365094	365513	2480327	2480746	50932	51351	203141	203560	615834	616253	340823	341242
862 BPSL0810		17	328380	328742	203196	203558	204040	204402	326994	327356	365644	366006	2480877	2481239	51482	51844	203691	204053	616384	616746	341373	341735
863 BPSL0811		17	328773	329498	203589	204314	204433	205158	327387	328112	366037	366762	2481270	2481995	51875	52600	204084	204809	616777	617502	341766	342491
864 BPSL0812		17	330463	329831	205279	204647	206123	205491	329077	328445	367727	367095	2482960	2482328	53565	52933	205774	205142	618467	617835	343456	342824
865 BPSL0814		17	330873	332132	205689	206948	206533	207792	329487	330746	368137	368412	2483370	2484629	53975	55234	206184	207443	618877	620136	343866	345125
866 BPSL0815		17	332151	335348	206967	210164	207811	211008	330765	333962	369101	372298	2484648	2487845	55253	58450	207462	210659	620155	623352	345144	348341
867 BPSL0816		17	335355	336896	210171	211712	211015	212556	333969	335510	372305	373846	2487852	2489393	58457	59998	210666	212207	623359	624900	348348	349889
868	Chr1_36_ISBma1_A	17	337256	338560	212072	213376	212916	214220	335870	337174	374206	375510	2489753	2491057	60358	61662	212567	213871	625260	626564	350249	351553
869 BPSL0817_p	partial	17	339665	338796	214481	213612	215325	214456	338279	337410	376615	375746	2492051	2491293	62656	61898	214865	214107	627669	626800	352658	351789
870	Chr1_37_IS407A_A	17	339666	340901	214482	215717	215326	216561	338280	339515	376616	377851	2492041	2493276	62646	63881	214855	216090	627670	628905	352659	353894
871 BPSL0817_p	partial	17											2493853	2493278	64458	63883	216667	216092	629482	628907	354471	353896
872 BPSL0818	partial	17	340902	341138	215718	215954	216562	216798	339516	339752	377852	378088	2494354	2495310	64959	65915	217168	218124	629983	630939	354972	355658
873 BPSL0819		17	342462	341431	217278	216247	218122	217091	341076	340045	379412	378381	2496857	2495826	67462	66431	219662	218631	632468	631437	357108	356077
874 BPSL0820		17	342667	343770	217483	218586	218327	219430	341281	342384	379617	380720	2497062	2498165	67667	68770	219867	220970	632673	633776	357313	358416
875 BPSL0821		17	343787	344644	218603	219460	219447	220304	342401	343258	380737	381594	2498182	2499039	68787	69644	220987	221844	633793	634650	358433	359290
876 BPSL0822		17	345583	344795	220399	219611	221243	220455	344197	343409	382533	381745	2499978	2499190	70583	69795	222783	221995	635589	634801	360229	359441
877 BPSL0823		17	346448	345579	221264	220395	222108	221239	345062	344193	383398	382529	2500843	2499974	71448	70579	223648	222779	636454	635585	361094	360225
878 BPSL0824		17	347346	346474	222162	221290	223006	222134	345960	345088	384290	383424	2501741	2500869	72346	71474	224546	223674	637352	636480	361992	361120
879 BPSL0825		17	348086	347625	222902	222441	223746	223285	346700	346239	385009	384548	2502481	2502020	73086	72625	225286	224825	638079	637618	362719	362258
880 BPSL0826		17	348380	349153	223196	223969	224040	224813	346994	347767	385303	386076	2502775	2503548	73380	74153	225580	226353	638373	639146	363013	363786
881 BPSL0827		17	349161	350114	223977	224930	224821	225774	347775	348728	386084	387037	2503556	2504509	74161	75114	226361	227314	639154	640107	363794	364747
882 BPSL0828		17	350105	351442	224921	226258	225765	227102	348719	350056	387028	388365	2504500	2505837	75105	76442	227305	228642	640098	641435	364738	366096
883 BPSL0829		17	351645	352970	226461	227786	227305	228630	350259	351584	388568	389893	2506040	2507365	76645	77970	228845	230170	641638	642963	366299	367624
884 BPSL0830		17	353081	354022	228312	229253	229317	230258	351709	352650	390053	390994	2507476	2508417	78081	79022	230348	231289	643074	644015	367995	368936
885 BPSL0831		17	354022	354885	229253	230116	230258	231121	352650	353513	390994	391857	2508417	2509280	79022	79885	231289	232152	644015	644878	368936	369799
886 BPSL0832		17	354885	355580	230116	230811	231121	231816	353513	354208	391857	392552	2509280	2509975	79885	80580	232152	232847	644878	645573	369799	370494
887 BPSL0833		17	355590	356753	230821	231984	231826	232989	354218	355381	392562	393725	2509985	2511148	80590	81753	232857	234020	645583	646746	370504	371667
888 BPSL0834		17	358262	356964	233493	232195	234498	233200	356890	355592	395234	393936	2512657	2511359	83262	81964	235529	234231	648255	646957	373176	371878
889 BPSL0835		17	359517	358636	234748	233867	235753	234872	358145	357264	396489	395608	2513912	2513031	84517	83636	236784	235903	649510	648629	374460	373609
890 BPSL0836		17	359655	360848	234886	236079	235891	237084	358283	359476	396627	397820	2514050	2515243	84655	85848	236922	238115	649648	650841	374598	375791
891 BPSL0837		17	360848	362056	236079	237287	237084	237737	359476	360684	397820	399028	2515243	2516451	85848	87056	238115	239323	650841	652049	375791	376999
892 BPSL0838		17	363168	362224	238399	237455	240038	239094	361796	360852	400140	399196	2517563	2516619	88168	87224	240435	239491	653161	652217	378111	377167
893 BPSL0839		17	364727	363258	239958	238489	241597	240128	363161	361962	401699	400230	2519122	2517653	89727	88258	241994	240525	654720	653251	379571	378201
894 BPSL0840		17	366280	364841	241511	240072	243150	241711	364714	363275			2520675	2519236	91280	89841	243547	242108	656273	654834	381039	379675
895 BPSL0841		17	367336	366455	242567	241686	244206	243325	365770	364889	404304	403423	2521731	2520850	92336	91455	244603	243722	657329	656448	382095	381214
896 BPSL0842		17	367449	369065	242680	243360	244319	245935	365883	367499	404417	406033	2521844	2523460	92449	94065	244716	246332	657442	659058	382208	383824
897 BPSL0843		17	369190	370638	244734	246182	246060	247508	367624	369072	406158	407606	2523578	2525026	94183	95631	246450	247898	659183	660631	383949	385397
898 BPSL0844		17	370670	371599	246214	247143	247540	248469	369104	370033	407638	408567	2525058	2525987	95663	96592	247930	248859	660663	661592	385429	386358
899 BPSL0844a		17	372112	372342	247656	247886	248982	249212	370546	370776	409080	409310	2526500	2526730	97105	97335	249372	249602	662105	662335	386871	387101
900 BPSL0845		17	372342	373721	247886	249265	249212	250591	370776	372155	409310	410701	2526730	2528121	97335	98726	249602	250993	662335	663726	387101	388492
901 BPSL0846		17	374670	373753	250214	249297	251540	250623	373104	372187	411650	410733	2529070	2528153	99675	98758	251942	251025	664675	663758	389441	388524
902 BPSL0847		17	376019	374772	251563	250316	252889	251642	374453	373206	412999	411752	2530419	2529172	101024	99777	253291	252044	666024	664777	390790	389543
903 BPSL0848		17	376702	376064	252246	251608	253572	252934	375136	374498	413682	413044	2531102	2530464	101707	101069	253974	253336	666707	666069	391473	390835
904 BPSL0849		17	376832	377332	252376	252876	253702	254202	375266	375766	413812	414312	2531232	2531732	101837	102337	254104	254604	666837	667337	391603	392103

905 BPSL0850		17	377837	377325	253381	252869	254707	254195	376271	375759	414817	414305	2532237	2531725	102842	102330	255109	254597	667842	667330	392608	392096
906 BPSL0851		17	378212	378766	254087	254641	255082	255636	376525	377079	415192	415746	2532612	2533166	103217	103771	255484	256038	668217	668771	392983	393537
907 BPSL0852		17	380298	379129	256173	255004	257168	255999	378611	377442	417278	416109	2534698	2533529	105303	104134	257570	256401	670303	669134	395069	393900
908 BPSL0853		17	381941	380724	257816	256599	258811	257594	380254	379037	418921	417704	2536341	2535124	106946	105729	259213	257996	671946	670729	396712	395495
909	Chr1_38_IS407A_C	17																			2311172	2312193
910 BPSL0854		17	382735	382076	258610	257951	259605	258946	381048	380389	419715	419056	2537135	2536476	107740	107081	260007	259348	672740	672081	2310355	2311014
911 BPSL0855		17	382851	383714	258726	259589	259721	260584	381164	382027	419831	420694	2537251	2538114	107856	108719	260123	260986	672856	673719	2310239	2309376
912 BPSL0856		17	384130	383993	260769	260005	261764	261000	383207	382443	421874	421110	2539295	2538393	109900	108998	262167	261265	674900	673998	2308195	2309097
913 BPSL0857		17	385772	384894	261647	260769	262642	261764	384085	383207	422752	421874	2540173	2539295	110778	109900	263045	262167	675778	674900	2307317	2308195
914 BPSL0858		17	385957	386838	261832	262713	262827	263708	384270	385151	422937	423818	2540358	2541239	110963	111844	263230	264111	675963	676844	2307132	2306251
915 BPSL0859		17	388903	387362	264778	263237	265773	264232	387216	385675	425883	424342	2543304	2541763	113834	112368	266176	264635	678909	677368	2304186	2305727
916 BPSL0860		17	389451	388900	265326	264775	266321	265770	387764	387213	426431	425880	2543852	2543301	114449	113898	266724	266173	679457	678906	2303637	2304137
917 BPSL0861		17	389469	390695	265344	266570	266339	267565	387782	389008	426449	427675	2543870	2545096	114467	115693	266742	267968	679475	680701	2303619	2302393
918 BPSL0862		17	390711	391232	266586	267107	267581	268102	389024	389545	427691	428212	2545112	2545633	115709	116230	267984	268505	680717	681238	2302377	2301856
919 BPSL0863		17	391235	392233	267110	268108	268105	269103	389548	390546	428215	429213	2545636	2546634	116233	117231	268508	269506	681241	682239	2301853	2300855
920 BPSL0864		17	394073	392511	269948	268386	270943	269381	392386	390824	431053	429491	2548474	2546912	119071	117509	271346	269784	684079	682517	2299015	2300577
921 BPSL0865		17	394203	394691	270078	270566	271073	271561	392516	393004	431183	431671	2548604	2549092	119201	119689	271476	271964	684209	684697	2298885	2298397
922 BPSL0866		17	394803	395408	270678	271283	271673	272278	393116	393721	431783	432388	2549204	2549809	119801	120406	272076	272681	684809	685414	2298285	2297680
923 BPSL0867		17	396129	395647	272004	271522	272999	272517	394442	393960	433109	432627	2550530	2550048	121127	120645	273402	272920	686135	685653	2296959	2297441
924 BPSL0868		17	396266	397312	272141	273187	273136	274182	394579	395625	433246	434292	2550667	2551713	121264	122310	273539	274585	686272	687318	2296822	2295776
925 BPSL0869		17	398437	397511	274312	273386	275307	274381	396750	395824	435417	434491	2552838	2551912	123435	122509	275710	274784	688443	687517	2294651	2295577
926 BPSL0870		17	398851	399183	274726	275058	275721	276053	397164	397496	435831	436163	2553252	2553584	123849	124181	276124	276456	688857	689189	2294237	2293905
927 BPSL0871		17	399693	399418	275568	275293	276563	276288	398006	397731	436673	436398	2554094	2553819	124691	124416	276966	276691	689699	689424	2293395	2293670
928 BPSL0872		17	400098	401645	275973	277520	276968	278515	398411	399958	437078	438625	2554499	2556046	125096	126643	277371	278918	690102	691649	2292992	2291445
929 BPSL0873		17	401658	402368	277533	278243	278528	279238	399971	400681	438638	439477	2556059	2556898	126656	127495	278931	279770	691662	692501	2291432	2290593
930 BPSL0874		17	403516	402761	279391	278636	280386	279631	401829	401074	440497	439742	2557918	2557163	128515	127760	280790	280035	693521	692766	2289573	2290328
931	Chr1_39a_ISBma2_A		403674	405246	279549	281121	280544	282116	401987	403559	440655	442227	2558075	2559647	128672	130244	280947	282519	693678	695250	2287844	2289416
932	Chr1_39b_ISBma2_A		2362064	2363635	2254925	2256496	2270282	2271853	2350107	2351678	2511431	2513003	1061840	1063411	2113105	2114677	2318443	2320015	583222	584794	2344825	2346397
933 BPSL0875		18	2361340	2361999	2254201	2254860	2269558	2270217	2349383	2350042	2510707	2511366	1061116	1061775	2112381	2113040	2317719	2318378	582498	583157	2344101	2344760
934 BPSL0876		18	2360295	2361128	2253156	2253989	2268513	2269346	2348338	2349171	2509662	2510495	1060071	1060904	2111336	2112169	2316674	2317507	581453	582286	2343056	2343889
935 BPSL0877		18	2359960	2360163	2252821	2253024	2268178	2268381	2348003	2348206	2509327	2509530	1059736	1059939	2111001	2111204	2316339	2316542	581118	581321	2342721	2342924
936 BPSL0878		18	2359050	2359976	2251812	2252837	2267169	2268194	2346994	2348019	2508318	2509343	1058727	1059752	2109992	2111017	2315330	2316355	580109	581134	2341715	2342737
937	Chr1_40a_IS407A_C	18																			2337504	2338663
938	Chr1_40b_IS407A_C	18																			2312315	2313344
939 BPSL0879		18	2358492	2357113	2251354	2249975	2266711	2265332	2346587	2345208	2507983	2506604	1058269	1056890	2109534	2108155	2314872	2313493	579651	578272	2313587	2314966
940 BPSL0880		18	2356913	2356338	2249775	2249200	2265132	2264557	2345008	2344433	2506404	2505829	1056690	1056115	2107955	2107380	2313293	2312718	578072	577497	2315166	2315741
941	Chr1_41_ISBma2_A		2355339	2356228	2248201	2249090	2263558	2264447	2343434	2344323	2504830	2505719	1055116	1056005	2106381	2107270	2311719	2312608	576498	577387	2315851	2316740
942	Chr1_42a_IS407A_A																				2316739	2317974
943	Chr1_42b_IS407A_A		2354105	2355340	2246967	2248202	2262324	2263559	2342200	2343435	2503596	2504831	1053882	1055117	2105147	2106382	2310485	2311720	575340	576499	2337504	2338663
944 BPSL0882		19	2352858	2354060	2245849	2246922	2261077	2262279	2340953	2342155	2502349	2503551			2103900	2105102	2309238	2310440	574240	575403	2336404	2337567
945 BPSL0883		19	2351869	2352621	2244730	2245482	2260088	2260840	2339964	2340716	2501360	2502112			2102911	2103663	2308249	2309001	573251	574003	2335415	2336167
946 BPSL0884		19	2351784	2351260	2244645	2244121	2260003	2259479	2339879	2339355	2501275	2500751			2102826	2102302	2308164	2307640	573166	572642	2335330	2334806
947 BPSL0885		19	2351241	2350222	2244102	2243083	2259460	2258441	2339336	2338317	2500732	2499713			2102283	2101264	2307621	2306602	572623	571604	2334787	2333768
948 BPSL0886		19	2349695	2349093	2242556	2241954	2257914	2257312	2337790	2337188	2499186	2498584			2100721	2100119	2306059	2305457	571061	570459	2333225	2332623
949 BPSL0887		19	2347684	2348955	2240545	2241816	2255903	2257174	2335779	2337050	2497175	2498446			2098710	2099981	2304048	2305319	569050	570321	2331214	2332485
950 BPSL0888		19	2345602	2347038	2238463	2239899	2253821	2255257	2333697	2335133	2495045	2496481			2096588	2098024	2301950	2303386	566984	568420	2329068	2330504
951 BPSL0889		19	2345305	2345084	2238166	2237945	2253524	2253303	2333400	2333179	2494748	2494527			2096291	2095959	2301653	2301321	566687	566466	2328771	2328550
952 BPSL0890		19	2343701	2345059	2236562	2237920	2251920	2253278	2331796	2333154	2493144	2494502			2094622	2095980	2299984	2301342	565083	566441	2327102	2328424
953 BPSL0891		19	2342239	2342982	2235100	2235843	2250458	2251201	2330334	2331077	2491682	2492425			2093160	2093903	2298522	2299265	563621	564364	2325640	2326383
954 BPSL0892		19	2342070	2341228	2234931	2234089	2250289	2249447	2330165	2329323	2491513	2490671			2092991	2092149	2298353	2297511	563452	562610	2325471	2324629
955 BPSL0892a		19																				

956	BPSL0893		19	2335855	2337966	2228614	2230725	2244074	2246185	2323950	2326061	2485343	2487454			2086836	2088947	2292213	2294324	557301	559412	2319320	2321431
957	BPSL0894		19	2335045	2335551	2227804	2228310	2243264	2243770	2323140	2323646	2484533	2485039			2086026	2086532	2291403	2291909	556491	556997	2318510	2319016
958		Chr1_43a_IS407A_A		2333274	2334509	2226033	2227268	2241493	2242728	2321369	2322604	2482748	2483983	1053882	1055117	2084226	2085461	2289894	2291129	554720	555955	2316739	2317974
959		Chr1_43b_IS407A_A		511643	512878	387963	389198	389122	390357	509988	511223	551212	552447	1026593	1027828	134379	135614	403287	404522	699385	700620	2282474	2283709
960	BPSL0895		20	513603	513028	389923	389348	391082	390507	511948	511373	553172	552597	1028553	1027978	133654	134229	401322	401897	698660	699235	2284434	2283859
961	BPSL0896		20	513871	515127	390191	391447	391350	392606	512216	513472	553440	554696	1028821	1030077	133386	132130	401054	399798	698392	697136	2284702	2285958
962	BPSL0897		20	515298	516905	391618	393225	392777	394384	513643	515250	554867	556474	1030248	1031855	131959	130352	399627	398020	696965	695358	2286129	2287736
963		Chr1_44a_ISBma2_A		517013	518585	393333	394905	394492	396064	515358	516930	556580	558152	1031963	1033543	128672	130244	396339	397912	693678	695250	2287844	2289416
964		Chr1_44b_ISBma2_A		2362064	2363635	2254925	2256496	2270282	2271853	2350107	2351678	2511431	2513003	1061840	1063411	2113105	2114677	2318443	2320015	583222	584794	2344825	2346397
965	BPSL0898		21	2363887	2363687	2256748	2256548	2272105	2271905	2351930	2351730	2513174	2513055	1063663	1063463	2114929	2114729			585046	584846	2346649	2346449
966	BPSL0898a		21	2364420	2364731	2257281	2257592	2272638	2272949	2352463	2352774	2513328	2513639	1064196	1064507	2115462	2115773	2320085	2320141	585579	585890	2347182	2347493
967	BPSL0899		21	2364731	2367028	2257592	2259889	2272949	2275246	2352774	2355071	2513639	2515936	1064507	1066804	2115773	2118070	2320141	2321685	585890	588187	2347493	2349790
968	BPSL0900		21	2367131	2367802	2259992	2260663	2275349	2276020	2355174	2355845	2516039	2516710	1066907	1067578	2118173	2118844	2322627	2323298	588290	588961	2349893	2350564
969		Chr1_45a_IS407A_A		2367803	2369038	2260664	2261899	2276021	2277256	2355846	2357081	2516711	2517946	1067579	1068814	2118845	2120080	2323299	2324534	588962	590197	2350565	2351800
970		Chr1_45b_IS407A_A		2333274	2334509	2226033	2227268	2241493	2242728	2321369	2322604	2482748	2483983	1053882	1055117	2084226	2085461	2289894	2291129	2640788	2642023	2399992	2401227
971	BPSL0901		22	2333081	2332338	2225840	2225097	2241300	2240557	2321176	2320433	2482555	2481812	1053689	1052949	2084033	2083293	2289701	2288958	2640595	2639852	2399799	2399056
972	BPSL0902		22	2332170	2331391	2224929	2224150	2240389	2239610	2320265	2319486	2481644	2480865	1052781	1052002	2083125	2082346	2288790	2288011	2639684	2638905	2398888	2398109
973	BPSL0903		22	2330853	2331296	2223612	2224055	2239072	2239515	2318948	2319391	2480327	2480770	1051464	1051907	2081808	2082251	2287473	2287916	2638367	2638810	2397571	2398014
974	BPSL0904		22	2329578	2330786	2222337	2223545	2237797	2239005	2317673	2318881	2479052	2480260	1050189	1051397	2080533	2081741	2286198	2287406	2637092	2638300	2396296	2397504
975	BPSL0905		22	2329016	2329513	2221775	2222272	2237235	2237732	2317111	2317608	2478490	2478987	1049627	1050124	2079971	2080468	2285636	2286133	2636530	2637027	2395734	2396231
976	BPSL0906		22	2326179	2329013	2218938	2221772	2234398	2237232	2314274	2317108	2475653	2478487	1046790	1049624	2077134	2079968	2282799	2285633	2633693	2636527	2392897	2395731
977	BPSL0907		22	2325075	2326064	2217834	2218823	2233294	2234283	2313170	2314159	2474549	2475538	1045686	1046675	2076030	2077019	2281695	2282684	2632589	2633578	2391793	2392782
978	BPSL0908		22	2324950	2324291	2217709	2217050	2233169	2232510	2313045	2312386	2474424	2473765	1045561	1044902	2075905	2075246	2281570	2280911	2632464	2631805	2391668	2391009
979	BPSL0909		22	2324291	2323032	2217050	2215791	2232510	2231251	2312386	2311127	2473765	2472506	1044902	1043643	2075246	2073987	2280911	2279652	2631805	2630546	2391009	2389750
980	BPSL0910		22	2323017	2321668	2215776	2214427	2231236	2229887	2311112	2309763	2472491	2471142	1043628	1042279	2073972	2072623	2279637	2278288	2630531	2629182	2389735	2388386
981	BPSL0911		22	2321468	2320275	2214227	2213034	2229687	2228494	2309563	2308370	2470942	2469749	1042079	1040886	2072423	2071230	2278088	2276895	2628982	2627789	2388186	2386993
982	BPSL0912		22	2320103	2318970	2212862	2211729	2228322	2227189	2308198	2307065	2469577	2468444	1040714	1039581	2071058	2069925	2276723	2275590	2627617	2626484	2386821	2385688
983	BPSL0913		22	2318970	2318089	2211729	2210848	2227189	2226308	2307065	2306184	2468444	2467563	1039581	1038700	2069925	2069044	2275590	2274709	2626484	2625603	2385688	2384807
984	BPSL0914		22	2316248	2317891	2209007	2210650	2224467	2226110	2304343	2305986	2465722	2467365	1036859	1038502	2067203	2068846	2272868	2274511	2623762	2625405	2382966	2384609
985	BPSL0915		22	2315958	2316122	2208717	2208881	2224177	2224341	2304053	2304217	2465432	2465596	1036569	1036733	2066913	2067077	2272578	2272742	2623472	2623636	2382676	2382840
986	BPSL0916		22	2315707	2315937	2208466	2208696	2223926	2224156	2303802	2304032	2465181	2465411	1036318	1036548	2066662	2066892	2272327	2272557	2623221	2623451	2382425	2382655
987	BPSL0917		22	2314703	2315410	2207462	2208169	2222922	2223629	2302798	2303505	2464177	2464884	1035314	1036021	2065658	2066365	2271323	2272030	2622217	2622924	2381421	2382128
988	BPSL0918		22	2314438	2313986	2207197	2206745	2222657	2222205	2302533	2302081	2463912	2463460	1035049	1034597	2065393	2064941	2271058	2270606	2621952	2621500	2381156	2380704
989	BPSL0919		22	2313980	2313003	2206739	2205762	2222199	2221222	2302075	2301098	2463454	2462477	1034591	1033614	2064935	2063958	2270600	2269623	2621494	2620517	2380698	2379721
990		Chr1_46a_ISBma2_A		2311360	2312932	2204119	2205691	2219579	2221151	2299455	2301027	2460834	2462406	1031963	1033543	2062315	2063887	2267980	2269552	2618874	2620446	2378078	2379650
991		Chr1_46b_ISBma2_A												2558075	2559647								
992	BPSL0919A		23	2311145	2310627	2203904	2203386	2219364	2218846	2299240	2298722	2460619	2460101	2559859	2560377	2062103	2061585	2267768	2267250	2618659	2618141	2377863	2377345
993		Chr1_47a_IS407A_A		2309174	2310409	2201933	2203168	2217393	2218628	2297269	2298504	2458648	2459883	2560595	2561830	2060132	2061367	2265797	2267032	2616688	2617923	2375892	2377127
994		Chr1_47b_IS407A_A		2070441	2071676	1963199	1964434	1978651	1979886	2058486	2059721	2219825	2221060	907313	908548	252371	253606			935361	936596	2040335	2041570
995	BPSL0920		24	2070428	2069481	1963186	1962239	1978638	1977691	2058473	2057526	2219812	2218865	907316	906999	253603	253920			936609	937556	2040322	2039375
996	BPSL0921		24	2069467	2068301	1962225	1961059	1977677	1976511	2057512	2056346	2218851	2217685	906985	905819	253934	255100			937570	938736	2039361	2038195
997	BPSL0922		24	2068235	2067465	1960993	1960223	1976445	1975675	2056280	2055510	2217619	2216849	905753	904983	255166	255936			938802	939572	2038055	2037432
998	BPSL0923		24	2067462	2066749	1960220	1959507	1975672	1974959	2055507	2054794	2216846	2216133	904980	904267	255939	256652			939575	940288	2037429	2036716
999	BPSL0924		24	2066449	2065784	1959207	1958542	1974659	1973994	2054494	2053829	2215833	2215168	903967	903302	256952	257617			940588	941253	2036416	2035751
1000	BPSL0925		24	2065150	2065605	1957908	1958363	1973360	1973815	2053195	2053650	2214534	2214989	902668	903123	258251	257796			941887	941432	2035117	2035572
1001	BPSL0926		24	2063949	2065130	1956707	1957888	1972159	1973340	2051994	2053175	2213333	2214514	901467	902648	259452	258271			943088	941907	2033916	2035097
1002	BPSL0927		24	2062641	2063669	1955399	1956427	1970851	1971879	2050686	2051714	2212025	2213053	900159	901187	260760	259732			944396	943368	2032608	2033636
1003	BPSL0928		24	2062558	2061536	1955316	1954294	1970768	1969746	2050603	2049581	2211942	2210920	900076	899054	260843	261865			944479	945501	2032525	2031503
1004	BPSL0929		24	2061268	2061537	1954026	1954295	1969478	1969747	2049313	2049582	2210652	2210921	898786	899055	262133	261864			945769	945500	2031235	2031504
1005	BPSL0930		24	2060513	2061232	1953271	1953990	1968723	1969442	2048558	2049277	2209897	2210616	898031	898750	262888	262169			946524	945805	2030480	2031199
1006	BPSL0931		24	2060365	2060003	1953123	1952761	1968575	1968213	2048410	2048048	2209749	2209387	897883	897521	263036	263398			946672	947034	2030332	2029970

1007	BPSL0932		24	2059035	2059985	1951793	1952743	1967245	1968195	2047080	2048030	2208419	2209369	896544	897503	264375	263416			948002	947052	2029002	2029952
1008	BPSL0933		24	2057937	2058830	1950695	1951588	1966147	1967040	2045982	2046875	2207321	2208214	895446	896339	265473	264580			949100	948207	2027904	2028797
1009	BPSL0934		24	2057815	2056922	1950573	1949680	1966025	1965132	2045860	2044967	2207199	2206306	895324	894431	265595	266488			949222	950115	2027782	2026889
1010	BPSL0935		24	2056027	2056710	1948785	1949468	1964237	1964920	2044072	2044755	2205411	2206094	893536	894219	267383	266700			951010	950327	2025994	2026677
1011	BPSL0936		24	2055116	2056009	1947874	1948767	1963326	1964219	2043161	2044054	2204500	2205393	892625	893518	268294	267401			951921	951028	2025083	2025976
1012	BPSL0937		24	2054252	2055031	1947010	1947789	1962462	1963241	2042297	2043076	2203636	2204415	891761	892540	269158	268379			952785	952006	2024219	2024998
1013	BPSL0938		24	2054120	2053560	1946878	1946318	1962330	1961770	2042165	2041605	2203504	2202944	891629	891069	269290	269850			952917	953477	2024087	2023527
1014	BPSL0938A		24	2053517	2053062	1946275	1945820	1961727	1961272	2041562	2041107	2202901	2202446	891026	890571	269893	270348			953520	953975	2023484	2023029
1015		Chr1_48_IS407A_A		2051541	2052776	1944299	1945534	1959751	1960986	2039586	2040821	2200925	2202160	889050	890285	270634	271869			954151	955386	2021618	2022853
1016	BPSL0939																						
1017	BPSL0940																						
1018	BPSL0941																						
1019	BPSL0942																						
1020	BPSL0943																						
1021	BPSL0944	GI5																					
1022	BPSL0945	Gi5																					
1023	BPSL0946	GI5																					
1024	BPSL0947	GI5																					
1025	BPSL0948	GI5																					
1026	BPSL0949	GI5																					
1027	BPSL0950	GI5_IS407A																					
1028	BPSL0951	GI5_IS407A																					
1029	BPSL0952	GI5																					
1030		Chr1_49_IS407A_A		683232	684467	560092	561327	563350	564585	678191	679426	719456	720691	2955982	2957217	1661173	1662408	1867070	1868305	2356417	2357652	652265	653500
1031	BPSL0953	GI5_variation	25a	684553	684470	561413	561330	564671	564588	679512	679429	720777	720694	2957303	2957220	1661087	1661170	1866984	1867067	2356331	2356414	653586	653503
1032	BPSL0954		25a	685383	685048	562243	561908	565501	565166	680342	680007	721607	721272	2958133	2957798	1660257	1660592	1866154	1866489	2355501	2355836	654416	654081
1033	BPSL0955		25a	686321	685383	563181	562243	566439	565501	681280	680342	722545	721607	2959071	2958133	1659319	1660257	1865216	1866154	2354563	2355501	655354	654416
1034	BPSL0956		25a	686541	688217	563401	565077	566659	568335	681500	683176	722765	724441	2959291	2960967	1659099	1657423	1864996	1863320	2354343	2352667	655574	657250
1035	BPSL0957		25a	688245	688784	565105	565644	568363	568902	683204	683743	724469	725008	2960995	2961534	1657395	1656856	1863292	1862753	2352639	2352100	657278	657817
1036	BPSL0958		25a	688784	689530	565644	566390	568902	569648	683743	684489	725008	725754	2961534	2962280	1656856	1656110	1862753	1862007	2352100	2351354	657817	658563
1037	BPSL0959		25a	689614	690576	566474	567436	569732	570694	684573	685535	725838	726800	2962364	2963326	1656026	1655064	1861923	1860961	2351270	2350308	658647	659609
1038	BPSL0960		25a	690602	691915	567462	568775	570720	572033	685561	686874	726826	728139	2963352	2964665	1655038	1653725	1860935	1859622	2350282	2348969	659635	660948
1039	BPSL0961		25a	691915	692748	568775	569608	572033	572866	686874	687707	728139	728972	2964665	2965498	1653725	1652892	1859622	1858789	2348969	2348136	660948	661781
1040	BPSL0962		25a	693348	692965	570208	569825	573466	573083	688307	687924	729572	729189	2966098	2965715	1652292	1652675	1858189	1858572	2347536	2347919	662381	661998
1041	BPSL0963		25a	694531	693383	571391	570243	574649	573501	689490	688342	730755	729607	2967281	2966133	1651109	1652257	1857006	1858154	2346353	2347501	663564	662416
1042	BPSL0964		25a	695632	694538	572492	571398	575750	574656	690591	689497	731856	730762	2968382	2967288	1650008	1651102	1855905	1856999	2345252	2346346	664665	663571
1043	BPSL0965		25a	695753	697261	572613	574121	575871	577379	690712	692220	731977	733485	2968503	2970011	1649887	1648379	1855784	1854276	2345131	2343623	664786	666294
1044	BPSL0966		25a	697308	697721	574168	574581	577426	577839	692267	692680	733532	733945	2970058	2970471	1648332	1647919	1854229	1853816	2343576	2343163	666341	666754
1045	BPSL0967		25a	697746	698453	574606	575313	577864	578571	692705	693412	733970	734677	2970496	2971203	1647894	1647187	1853791	1853084	2343138	2342431	666779	667486
1046	BPSL0968		25a	698937	698617	575797	575477	579055	578735	693896	693576	735161	734841	2971687	2971367	1646703	1647023	1852600	1852920	2341947	2342267	667970	667650
1047		Chr1_50_ISBma2_A	25a	699018	700590	575878	577450	579136	580708	693979	695542	735242	736814	2971768	2973340	1645048	1646622	1850947	1852519	2340294	2341866	668438	670010
1048	BPSL0969		25a	702420	700750	579280	577610	582538	580868	697372	695702	738644	736974	2975170	2973500	1643218	1644888	1849117	1850787	2338465	2340135	671839	670169
1049	BPSL0970		25a	703490	703594	579458	580426	582716	583684	697550	698518	738822	739790	2975348	2976316	1643040	1642072	1848939	1847971	2338287	2337319	672017	672985
1050	BPSL0971		25a	703704	704591	580564	581451	583822	584709	698656	699543	739928	740815	2976454	2977341	1641934	1641047	1847833	1846946	2337190	2336303	673114	674001
1051	BPSL0972		25a	705684	704950	582544	581810	585802	585068	700636	699902	741908	741174	2978434	2977700	1639947	1640681	1845874	1846608	2335231	2335965	675073	674339
1052	BPSL0973		25a	706344	705886	583204	582746	586462	586004	701296	700838	742568	742110	2979094	2978636	1639287	1639745	1845214	1845672	2334571	2335029	675733	675275
1053	BPSL0974		25a	706998	706687	583858	583547	587116	586805	701950	701639	743222	742911	2979748	2979437	1638633	1638944	1844560	1844871	2333917	2334228	676387	676076
1054	BPSL0975		25a	707345	706998	584205	583858	587463	587116	702297	701950	743569	743222	2980095	2979748	1638286	1638633	1844213	1844560	2333570	2333917	676734	676387
1055	BPSL0976		25a	707885	709939	584745	586799	588003	590057	702837	704891	744109	746163	2980634	2982688	1637747	1635693	1843674	1841620	2333030	2330976	677274	679328
1056	BPSL0977		25a	710021	711019	586881	587879	590139	591137	704973	705971	746245	747243	2982770	2983768	1635611	1634613	1841538	1840540	2330894	2329896	679410	680408
1057	BPSL0978		25a	711019	711846	587879	588706	591137	591964	705971	706798	747243	748070	2983768	2984595	1634613	1633786	1840540	1839713	2329896	2329069	680408	681235

1058	BPSL0979		25a	711893	712948	588753	589808	592011	593066	706845	707900	748117	749172	2984642	2985697	1633739	1632684	1839666	1838611	2329022	2327967	681282	682337
1059	BPSL0980		25a	712958	713713	589818	590573	593076	593831	707910	708665	749182	749937	2985707	2986462	1632674	1631919	1838601	1837846	2327957	2327202	682347	682622
1060	BPSL0981		25a	713701	714297	590561	591157	593819	594415	708653	709249	749925	750521	2986450	2987046	1631931	1631335	1837858	1837262	2327214	2326618	683136	683732
1061	BPSL0982		25a	715279	714446	592139	591306	595397	594564	710231	709398	751503	750670	2988028	2987195	1630353	1631186	1836280	1837113	2325636	2326469	684714	683881
1062	BPSL0983		25a	716377	715448	593237	592308	596495	595566	711329	710400	752601	751672	2989126	2988197	1629255	1630184	1835182	1836111	2324538	2325467	685812	684883
1063	BPSL0984		25a	717435	716386	594295	593246	597821	597042	712387	711338	753659	752610	2990184	2989135	1628180	1628905	1834124	1835173	2323480	2324529	686870	685821
1064	BPSL0985		25a	718360	717431	595220	594291	598746	597817	713312	712383	754584	753655	2991109	2990180	1627255	1628184	1833199	1834128	2322555	2323484	687533	687411
1065	BPSL0986		25a	718911	718357	595771	595217	599297	598743	713863	713309	755135	754581	2991660	2991106	1626704	1627258	1832648	1833202	2322004	2322558	688084	687530
1066	BPSL0987		25a	719068	720525	595928	597385	599454	600911	714020	715477	755304	756761	2991799	2993256	1626547	1625090	1832485	1831028	2321841	2320384	688253	689710
1067	BPSL0988		25a	720809	721222	597669	598082	601195	601608	715761	716174	757045	757458	2993540	2993953	1624806	1624393	1830744	1830331	2320100	2319687	689994	690365
1068	BPSL0989		25a	721594	722214	598672	599322	602196	602816	716577	717197	757901	758521	2994325	2994945	1624021	1623401	1829959	1829339	2319315	2318695	690808	691428
1069	BPSL0990		25a	722723	722340	599831	599448	603325	602942	717706	717323	759030	758647	2995454	2995071	1622892	1623275	1828830	1829213	2318186	2318569	691937	691554
1070	BPSL0991		25a	723615	722779	600723	599887	604217	603381	718598	717762	759922	759086	2996346	2995510	1622000	1622836	1827938	1828774	2317294	2318130	692829	691993
1071	BPSL0992		25a	725246	724374	602354	601482	605848	604976	720229	719357	761553	760681	2997969	2997097	1620377	1621249	1826307	1827179	2315666	2316538	694457	693585
1072	BPSL0993		25a	725439	725254	602547	602362	606041	605856	720422	720237	761746	761561	2998162	2997977	1620184	1620369	1826114	1826299	2315473	2315658	694650	694465
1073	BPSL0994		25a	725648	727462	602756	604570	606250	608064	720631	722445	761955	763769	2998371	3000185	1619975	1618161	1825905	1824091	2315264	2313450	694859	696673
1074	BPSL0995		25a	727479	731594	604587	608702	608081	612196	722462	726577	763786	767901	3000202	3004317	1618144	1614029	1824074	1819959	2313433	2309318	696690	700805
1075	BPSL0996		25a	731948	732655	609056	609763	612550	613257	726931	727638	768255	768962	3004671	3005378	1613675	1612968	1819605	1818898	2308964	2308257	701159	701866
1076	BPSL0997		25a	732655	733665	609763	610773	613257	614267	727638	728648	768962	769972	3005378	3006388	1612968	1611958	1818898	1817888	2308257	2307247	701866	702876
1077	BPSL0998		25a	736194	734020	613302	611128	616796	614622	731177	729003	772501	770327	3008917	3006743	1609429	1611603	1815359	1817533	2304718	2306892	705405	703231
1078	BPSL0999		25a	737324	736680	614432	613788	617926	617282	732307	731663	773631	772987	3010047	3009403	1608299	1608943	1814229	1814873	2303588	2304232	706535	705891
1079	BPSL1000		25a	737706	738791	614814	615899	618308	619393	732689	733774	774013	775098	3010429	3011514	1607917	1606832	1813847	1812762	2303206	2302121	706917	708002
1080	BPSL1001		25a	738927	739463	616035	616571	619529	620065	733910	734446	775234	775770	3011650	3012186	1606696	1606160	1812626	1812090	2301985	2301449	708138	708674
1081	BPSL1002		25a	739570	740136	616678	617244	620172	620738	734553	735119	775877	776443	3012293	3012859	1606053	1605487	1811983	1811417	2301342	2300776	708781	709347
1082	BPSL1003		25a	740242	742518	617350	619626	620844	623120	735225	737501	776549	778825	3012965	3015241	1605381	1603105	1811311	1809035	2300670	2298394	709453	711729
1083	BPSL1004		25a	742524	743243	619632	620351	623126	623845	737507	738214	778831	779550	3015247	3015966	1603099	1602380	1809029	1808310	2298388	2297669	711735	712454
1084	BPSL1005		25a	744054	743383	621162	620491	624656	623985	739025	738354	780361	779690	3016777	3016106	1601569	1602240	1807499	1808170	2296858	2297529	713265	712594
1085	BPSL1006		25a	745496	744090	622604	621198	626098	624692	740467	739061	781803	780397	3018219	3016813	1600127	1601533	1806057	1807463	2295416	2296822	714707	713301
1086	BPSL1007		25a	747965	745668	625073	622776	628567	626270	742936	740639	784272	781975	3020688	3018391	1597658	1599955	1803588	1805885	2292947	2295244	717176	714879
1087		Chr1_51_IS407A_B	25a	748097	749332	625205	626440	628699	629934	743068	744303												
1088	BPSL1008		25a	750619	749912	627727	627020	631221	630514	745590	744883	785686	784979	3022093	3021386	1596253	1596960	1802183	1802890	2291628	2292335	718597	717890
1089	BPSL1009		25a	751145	750630	628253	627738	631747	631232	746116	745601	786212	785697	3022619	3022104	1595727	1596242	1801657	1802172	2291102	2291617	719123	718608
1090	BPSL1010		25a	751540	752526	628648	629634	632142	633128	746511	747497	786607	787593	3023014	3024000	1595332	1594346	1801262	1800276	2290707	2289721	719518	720504
1091	BPSL1011		25a	753517	752705	630625	629813	634119	633307	748488	747676	788584	787772	3024991	3024179	1593355	1594167	1799285	1800097	2288730	2289542	721495	720683
1092	BPSL1012		25a	753682	754452	630790	631560	634284	635054	748653	749423	788749	789519	3025156	3025926	1593190	1592420	1799120	1798350	2288565	2287795	721660	722430
1093		Chr1_52_ISBma2_A	25a	754776	756348	631884	633456	635378	636950	749747	751319	789822	791394	3026236	3027808	1590531	1592103	1796447	1798019	2285906	2287478	722740	724312
1094	BPSL1013		25a	759424	756443	636532	633551	640026	637045	754395	751414	794470	791489	3030884	3027903	1587455	1590436	1793371	1796352	2282830	2285811	727388	724407
1095	BPSL1014		25a	759537	759713	636645	636821	640139	640315	754508	754684	794583	794759	3030997	3031173	1587342	1587166	1793258	1793082	2282717	2282541	727501	727677
1096	BPSL1015		25a	759751	760737	636859	637845	640353	641339	754722	755708	794797	795783	3031211	3032197	1587128	1586142	1793044	1792058	2282503	2281517	727715	728701
1097	BPSL1016		25a	760737	762716	637845	639824	641339	643318	755708	757687	795783	797762	3032197	3034176	1586142	1584163	1792058	1790079	2281517	2279538	728701	730680
1098	BPSL1017		25a	762724	763911	639832	641019	643326	644513	757695	758882	797770	798957	3034184	3035371	1584155	1582968	1790071	1788884	2279530	2278343	730688	731875
1099	BPSL1018		25a	765402	764098	642510	641206	646004	644700	760373	759069	800448	799144	3036862	3035558	1581477	1582781	1787393	1788697	2276852	2278156	733366	732062
1100	BPSL1019		25a	766199	765444	643307	642552	646801	646046	761170	760415	801245	800490	3037659	3036904	1580680	1581435	1786596	1787351	2276055	2276810	734163	733408
1101	BPSL1020		25a	767727	766291	644835	643399	648329	646893	762698	761262	802773	801337	3039187	3037751	1579152	1580588	1785068	1786504	2274527	2275963	735691	734255
1102	BPSL1021		25a	767888	768412	644996	645520	648490	649014	762859	763383	802934	803458	3039348	3039872	1578991	1578467	1784907	1784383	2274366	2273842	735852	736376
1103	BPSL1022		25a									804479	803838	3040938	3040297	1577437	1578078						
1104	BPSL1023		25a					650269	650595									1783128	1782799			737640	737963
1105	BPSL1025		25a	771548	770373	648691	647516	652899	651724	766533	765358	806600	805425	3043105	3041930	1575259	1576434	1781168	1782343	2270699	2271874	1804039	1805214
1106	BPSL1026		25a	771639	773366	648782	650509	652990	654717	766624	768351	806691	808418	3043196	3044923	1575168	1573441	1781077	1779350	2270608	2268881	1803948	1802221
1107	BPSL1027		25a	773430	773765	650573	650908	654781	655116	768415	768750	808482	808817	3044987	3045322	1573377	1573042	1779286	1778951	2268817	2268482	1802157	1801822
1108	BPSL1028	transposase	25a																				

1109	BPSL1029		25a	775337	774240	652480	651383	656688	655591	770322	769225	810389	809292	3046894	3045797	1571470	1572567	1777379	1778476	2266910	2268007	1800250	1801347
1110		Chr1_53a_IS407A_B																		2265201	2266436	1798541	1799776
1111		Chr1_53b_IS407A_B																		967733	968968	2008080	2009315
1112	BPSL1030		25b	776999	776223	654142	653366	658350	657574	771984	771208	812051	811275	3048556	3047780	1569808	1570584	1775717	1776493	970161	969385	2006887	2007663
1113	BPSL1031		25b	777735	777025	654878	654168	659086	658376	772720	772010	812787	812077	3049292	3048582	1569072	1569782	1774981	1775691	970897	970187	2006151	2006861
1114	BPSL1032		25b	778421	777735	655564	654878	659772	659086	773406	772720	813473	812787	3049978	3049292	1568386	1569072	1774295	1774981	971583	970897	2005465	2006151
1115	BPSL1033		25b	779408	778635	656551	655778	660759	659986	774393	773620	814460	813687	3050965	3050192	1567399	1568172	1773308	1774081	972570	971797	2004478	2005251
1116	BPSL1034		25b	779801	780094	656944	657237	661152	661445	774786	775079	814853	815146	3051358	3051651	1567006	1566713	1772915	1772622	972963	973256	2004085	2003792
1117	BPSL1035		25b	781534	780839	658677	657982	662885	662190	776519	775824	816586	815891	3053091	3052396	1565273	1565968	1771182	1771877	974696	974001	2002352	2003047
1118	BPSL1036		25b	781623	782318	658766	659461	662974	663669	776608	777303	816675	817370	3053180	3053875	1565184	1564489	1771093	1770398	974785	975480	2002263	2001568
1119	BPSL1037		25b	782305	783615	659448	660758	663656	664966	777290	778600	817357	818667	3053862	3055172	1564502	1563192	1770411	1769101	975467	976777	2001581	2000271
1120	BPSL1038		25b	783958	783695	661101	660838	665309	665046	778943	778680	819010	818747	3055515	3055252	1562849	1563112	1768758	1769021	977120	976857	1999928	2000191
1121	BPSL1038a		25b	784360	784088	661503	661231	665711	665439	779345	779073	819412	819140	3055917	3055645	1562447	1562719	1768356	1768628	977522	977250	1999526	1999798
1122	BPSL1039		25b	784455	786209	661598	663352	665806	667560	779440	781194	819507	821261	3056012	3057766	1562352	1560598	1768261	1766507	977617	979371	1999431	1997677
1123	BPSL1040		25b	786266	787603	663409	664746	667617	668954	781251	782588	821318	822655	3057823	3059160	1560541	1559204	1766450	1765113	979428	980765	1997620	1996283
1124		Chr1_54_IS407A_A		788024	789259	665167	666402	669375	670610	783009	784244	823076	824311	3059581	3060816	1557548	1558783	1763457	1764692	980886	982121	1994927	1996162
1125	BPSL1041																						
1126	BPSL1042																						
1127		Chr1_55_IS407A_A		3110701	3111936	3008620	3009855	3024998	3026233	3096934	3098169	836600	837835	3059581	3060816	1557548	1558783	1763457	1764692	954151	955386	2021618	2022853
1128	BPSL1043		26	3112298	3111939	3010217	3009858	3026595	3026236	3098531	3098172	836238	836597	3061178	3060816	1557186	1557548	1763095	1763457	955748	955389	2021256	2021615
1129	BPSL1044		26	3112918	3114333	3010837	3012252	3027215	3028630	3099151	3100566	835618	834203	3061798	3063213	1556566	1555151	1762475	1761060	956368	957783	2020636	2019221
1130	BPSL1045		26	3114329	3114928	3012248	3012847	3028626	3029225	3100562	3101161	834207	833608	3063209	3063808	1555155	1554556	1761064	1760465	957779	958378	2019225	2018626
1131	BPSL1046		26	3115715	3115107	3013634	3013026	3030012	3029404	3101948	3101340	832821	833429	3064595	3063987	1553769	1554377	1759678	1760286	959165	958557	2017839	2018447
1132	BPSL1047		26																				
1133	BPSL1048		26	3117892	3116468	3015811	3014387	3032189	3030765	3104125	3102701	830664	832088	3066752	3065328	1551612	1553036	1757521	1758945	961372	959948	2015692	2017116
1134	BPSL1049		26	3118474	3117965	3016393	3015884	3032771	3032262	3104707	3104198	830082	830591	3067334	3066825	1551030	1551539	1756939	1757448	961954	961445	2015110	2015619
1135	BPSL1050		26	3119429	3119052	3017348	3016971	3033726	3033349	3105662	3105285	829127	829504	3068289	3067912	1550075	1550452	1755984	1756361	962909	962532	2014155	2014532
1136	BPSL1051		26	3119587	3119853	3017506	3017772	3033884	3034150	3105820	3106086	828969	828703	3068447	3068713	1549917	1549651	1755826	1755560	963067	963333	2013997	2013731
1137	BPSL1053		26	3120843	3120466	3018762	3018385	3035140	3034763	3107076	3106699	827713	828090	3069703	3069326	1548661	1549038	1754570	1754947	964323	963946	2012741	2013118
1138	BPSL1054		26	3121016	3121552	3018935	3019471	3035313	3035849	3107249	3107785	827540	827004	3069876	3070412	1548488	1547952	1754397	1753861	964496	965032	2012568	2012032
1139	BPSL1055		26	3122789	3121656	3020708	3019575	3037086	3035953	3109022	3107889	825767	826900	3071649	3070516	1546715	1547848	1752624	1753757	966269	965136	2010795	2011928
1140	BPSL1056		26	3123270	3123049	3021189	3020968	3037567	3037346	3109503	3109282	825286	825507	3072130	3071909	1546234	1546455	1752143	1752364	966758	966537	2010290	2010511
1141	BPSL1057		26	3123727	3124200	3021646	3022119	3038024	3038497	3109960	3110433	824829	824356	3072587	3073060	1545777	1545304	1751686	1751213	967215	967688	2009833	2009360
1142		Chr1_56_IS407A_A		3124245	3125480	3022164	3023399	3038542	3039777	3110478	3111713	823076	824311	3073105	3074340	1544024	1545259	1749933	1751168	967733	968968	2008080	2009315
1143	BPSL1058																						
1144	BPSL1059-conserved hypothetical protein																						
1145		Chr1_57_IS407A_A		2281870	2283105	2174629	2175864	2190089	2191324	2269939	2271174	2458648	2459883	2560595	2561830	2060132	2061367	2265797	2267032	699385	700620	2282474	2283709
1146	BPSL1060		27	2283108	2283338	2175867	2176097	2191327	2191557	2271177	2271407	2458645	2458415	2561833	2562063	2060129	2059899	2265794	2265564	700623	700853	2282471	2282241
1147	BPSL1061		27	2283338	2283553	2176097	2176312	2191557	2191772	2271407	2271622	2458415	2458200	2562063	2562278	2059899	2059684	2265564	2265349	700853	701068	2282241	2282026
1148	BPSL1062		27	2284070	2284333	2176829	2177092	2192289	2192552	2272139	2272402	2457683	2457420	2562795	2563058	2059167	2058904	2264832	2264569	701585	701848	2281509	2281246
1149	BPSL1063		27	2284356	2284697	2177115	2177456	2192575	2192916	2272425	2272766	2457397	2457056	2563081	2563422	2058881	2058540	2264546	2264205	701871	702212	2281223	2280882
1150	BPSL1064		27	2285727	2284804	2178486	2177563	2193946	2193023	2273796	2272873	2456026	2456949	2564452	2563529	2057510	2058433	2263175	2264098	703242	702319	2279852	2280775
1151	BPSL1065		27	2287334	2285751	2180093	2178510	2195553	2193970	2275403	2273820	2454419	2456002	2566059	2564476	2055903	2057486	2261568	2263151	704849	703266	2278245	2279828
1152	BPSL1066		27	2287962	2287783	2180721	2180542	2196181	2196002	2276031	2275852	2453791	2453970	2566687	2566508	2055275	2055454	2260940	2261119	705477	705298	2277617	2277796
1153	BPSL1067		27	2288336	2288788	2181095	2181547	2196555	2197007	2276405	2276857	2453424	2452972	2567061	2567513	2054901	2054449	2260566	2260114	705914	706366	2277159	2276707
1154	BPSL1068		27	2288959	2290902	2181718	2183661	2197178	2199121	2277028	2278971	2452801	2450858	2567684	2569627	2054278	2052335	2259943	2258000	706537	708480	2276536	2274593
1155	BPSL1069		27	2291111	2293090	2183870	2185849	2199330	2201309	2279180	2281159	2450649	2448670	2569836	2571815	2052126	2050147	2257791	2255812	708689	710668	2274384	2272405
1156	BPSL1070		27	2293152	2295467	2185911	2188226	2201371	2203686	2281221	2283536	2448608	2446293	2571877	2574192	2050085	2047770	2255750	2253435	710730	713045	2272343	2270028
1157	BPSL1071		27	2296163	2296618	2188922	2189377	2204382	2204837	2284232	2284687	2445597	2445142	2574888	2575343	2047074	2046619	2252739	2252284	713741	714196	2269332	2268877
1158	BPSL1072		27	2297628	2296747	2190387	2189506	2205847	2204966	2285697	2284816	2444132	2445013	2576353	2575472	2045609	2046490	2251274	2252155	715206	714325	2267867	2268748
1159	BPSL1073		27	2297722	2298186	2190481	2190945	2205941	2206405	2285791	2286255	2444038	2443574	2576447	2576911	2045515	2045051	2251180	2250716	715300	715764	2267773	2267309

1160	BPSL1074		27	2299375	2298416	2192134	2191175	2207594	2206635	2287444	2286485	2442385	2443344	2578100	2577141	2043862	2044821	2249527	2250486	716953	715994	2266120	2267079
1161	BPSL1075		27	2299591	2300901	2192350	2193660	2207810	2209120	2287660	2288970	2442169	2440859	2578316	2579626	2043646	2042336	2249311	2248001	717169	718479	2265904	2264594
1162	BPSL1076		27	2300906	2302165	2193665	2194924	2209125	2210384	2288975	2290234	2440854	2439595	2579631	2580890	2042331	2041072	2247996	2246737	718484	719743	2264589	2263330
1163	BPSL1078		27	2302479	2303498	2195238	2196257	2210698	2211717	2290548	2291567	2439295	2438276	2581204	2582223	2040758	2039739	2246423	2245404	720057	721076	2263016	2261997
1164	BPSL1079		27	2305018	2303828	2197777	2196587	2213237	2212047	2293087	2291897	2436764	2437954	2583871	2582681	2038075	2039265	2243724	2244914	722580	721390	2260493	2261683
1165	BPSL1080		27	2307525	2305168	2200284	2197927	2215744	2213387	2295620	2295012	2434257	2436614	2586378	2584021	2035568	2037925	2241217	2243574	725087	722730	2257986	2260343
1166	BPSL1081		27	2308653	2307757	2201412	2200516	2216872	2215976	2296748	2295852	2433129	2434025	2587506	2586610	2034440	2035336	2240089	2240985	726215	725319	2256858	2257754
1167	BPSL1082		27	2309125	2308661	2201884	2201420	2217344	2216880	2297220	2296756	2432657	2433121	2587978	2587514	2033968	2034432	2239617	2240081	726687	726223	2256386	2256850
1168		Chr1_58a_IS407A_A		2309174	2310409	2201933	2203168	2217393	2218628	2297269	2298504	2431373	2432608	2588026	2589261	2032685	2033920	2238334	2239569	726736	727971	2255102	2256337
1169		Chr1_58b_IS407A_A		2051541	2052776	1944299	1945534	1959751	1960986	2039586	2040821	2200925	2202160	889050	890285	270634	271869	403287	404522	954151	955386	1994927	1996162
1170	BPSL1083		28	2050338	2051537	1943096	1944295	1958548	1959747	2038383	2039582	2199710	2200909	887847	889046	273072	271873	405725	404526	983324	982125	1993724	1994923
1171	BPSL1084		28	2049390	2050295	1942148	1943053	1957600	1958505	2037435	2038340	2198762	2199667	886899	887804	274020	273115	406673	405768	984272	983367	1992776	1993681
1172	BPSL1085		28	2048606	2049286	1941364	1942044	1956816	1957496	2036651	2037331	2197978	2198658	886115	886795	274804	274124	407457	406777	985056	984376	1991992	1992672
1173	BPSL1086		28	2048470	2047004	1941228	1939762	1956680	1955214	2036515	2035049	2197842	2196376	885979	884513	274940	276406	407593	409059	985192	986658	1991856	1990390
1174	BPSL1087		28	2046865	2044970	1939623	1937728	1955075	1953180	2034910	2033015	2196237	2194342	884374	882479	276545	278440	409198	411093	986797	988692	1990251	1988356
1175	BPSL1088		28	2044958	2044368	1937716	1937126	1953168	1952578	2033003	2032413	2194330	2193740	882467	881877	278452	279042	411105	411695	988704	989294	1988344	1987754
1176	BPSL1089		28	2044368	2043892	1937126	1936650	1952578	1952102	2032413	2031937	2193740	2193264	881877	881401	279042	279518	411695	412171	989294	989770	1987754	1987278
1177	BPSL1091		28	2043638	2042712	1936396	1935470	1951848	1950922	2031683	2030757	2193010	2192084	881147	880221	279772	280698	412425	413351	990024	990950	1987024	1986098
1178	BPSL1092		28	2042517	2042281	1935275	1935039	1950727	1950491	2030562	2030326	2191889	2191653	880026	879790	280893	281129	413546	413782	991145	991381	1985903	1985667
1179	BPSL1093		28	2042281	2040734	1935039	1933492	1950491	1948944	2030326	2028779	2191653	2190106	879790	878243	281129	282676	413782	415329	991381	992928	1985667	1984120
1180	BPSL1094		28	2040171	2040602	1932929	1933360	1948381	1948812	2028216	2028647	2189543	2189974	877680	878111	283239	282808	415892	415461	993491	993060	1983557	1983988
1181	BPSL1095		28	2038852	2039802	1931610	1932560	1947062	1948012	2026897	2027847	2188224	2189174	876361	877311	284558	283608	417211	416261	994810	993860	1982238	1983188
1182	BPSL1096		28	2037233	2038447	1929991	1931205	1945443	1946657	2025278	2026492	2186605	2187819	874742	875956	286177	284963	418830	417616	996429	995215	1980619	1981833
1183	BPSL1097		28	2035974	2037146	1928732	1929904	1944184	1945356	2024019	2025191	2185346	2186518	873483	874655	287436	286264	420089	418917	997688	996516	1979360	1980532
1184	BPSL1098		28	2034919	2035758	1927677	1928516	1943129	1943968	2022964	2023803	2184291	2185130	872428	873267	288491	287652	421144	420305	998743	997904	1978305	1979144
1185	BPSL1099		28	2034685	2034329	1927443	1927087	1942895	1942539	2022730	2022374	2184057	2183701	872194	871838	288725	289081	421378	421734	998977	999333	1978071	1977715
1186	BPSL1100		28	2034285	2033920	1927043	1926678	1942495	1942130	2022330	2021965	2183657	2183292	871794	871429	289125	289490	421778	422143	999377	999742	1977671	1977306
1187		Chr1_59_ISBma1_A	28	2032534	2033838	1925292	1926596	1940744	1942048	2020579	2021883	2181906	2183210	870043	871347	289572	290876	422225	423530	999824	1001128	1975920	1977224
1188	BPSL1101		28	2031561	2032109	1924319	1924867	1939771	1940319	2019606	2020154	2180933	2181481	869069	869617	291850	291302	424504	423956	1002101	1001553	1974947	1975495
1189	BPSL1102		28	2031123	2030056	1923881	1922814	1939333	1938266	2019168	2018101	2180471	2179404	868631	867564	292296	293363	424934	426001	1002555	1003622	1974453	1973386
1190	BPSL1103		28	2029276	2029917	1922034	1922675	1937486	1938127	2017321	2017962	2178624	2179265	866784	867425	294143	293502	426781	426140	1004402	1003761	1972606	1973247
1191	BPSL1104		28	2028399	2029268	1920413	1920901	1936609	1937478	2016444	2017313	2177747	2178616	865907	866776	295020	294151	427658	426789	1005279	1004410	1971729	1972598
1192	BPSL1105		28	2028141	2027512	1920155	1919526	1936351	1935722	2016186	2015557	2177489	2176860	865649	865020	295278	295907	427916	428545	1005537	1006166	1971471	1970842
1193	BPSL1106		28	2025900	2027363	1917914	1919377	1934110	1935573	2013945	2015408	2175248	2176711	863408	864871	297519	296056	430157	428694	1007778	1006315	1969230	1970693
1194	BPSL1107		28	2023848	2025677	1915862	1917691	1932058	1933887	2011893	2013722	2173196	2175025	861356	863185	299571	297742	432209	430380	1009830	1008001	1967178	1969007
1195	BPSL1108		28	2023671	2022439	1915685	1914453	1931881	1930649	2011716	2010484	2173019	2171787	861179	859947	299748	300980	432386	433618	1010007	1011239	1967001	1965769
1196	BPSL1109		28			1908431	1909048	1924435	1925031													1960513	1961052
1197	BPSL1110		28	2016209	2016727	1907738	1908256	1923742	1924260	2004254	2004772	2165575	2166093	853693	854211	307198	306680	439836	439318	1017481	1016963	1959820	1960338
1198	BPSL1111		28	2015440	2014229	1906969	1905758	1922973	1921762	2003485	2002274	2164806	2163595	852924	851713	307967	309178	440605	441816	1018250	1019461	1959051	1957840
1199	BPSL1112		28	2014133	2013915	1905662	1905444	1921666	1921448	2002178	2001960	2163499	2163281	851617	851399	309274	309492	441912	442130	1019557	1019775	1957744	1957526
1200	BPSL1113		28	2013853	2013470	1905382	1904999	1921386	1921003	2001898	2001515	2163219	2162836	851337	850954	309554	309937	442192	442575	1019837	1020220	1957464	1957081
1201	BPSL1114		28	2012841	2011396	1904370	1902925	1920374	1918929	2000886	1999441	2162207	2160762	850325	848880	310566	312011	443204	444649	1020859	1022304	1956432	1955017
1202	BPSL1115		28	2009970	2010860	1901305	1902195	1916745	1917635	1998000	1998890	2159248	2160138	847462	848352	313437	312547	446150	445260	1023722	1022832	1953584	1954474
1203	BPSL1116		28	2009027	2009923	1900362	1901258	1915802	1916698	1997057	1997953	2158305	2159201	846519	847415	314380	313484	447093	446197	1024665	1023769	1952641	1953537
1204	BPSL1117		28	2008405	2004884	1899740	1896219	1915180	1911659	1996435	1992914	2157683	2154162	845897	842376	315002	318523	447715	451236	1025287	1028808	1952019	1948498
1205	BPSL1118		28	2004841	2003054	1896176	1894389	1911616	1909829	1992871	1991084	2154119	2152332	842333	840546	318566	320353	451279	453066	1028851	1030638	1948455	1946668
1206	BPSL1119		28	2002997	2001918	1894332	1893253	1909772	1908693	1991027	1989948	2152275	2151196	840489	839410	320410	321489	453123	454202	1030695	1031774	1946611	1945532
1207	BPSL1120		28	2001633	2000491	1892968	1891826	1908408	1907266	1989663	1988521	2150911	2149769	839125	837983	321774	322916	454487	455629	1032059	1033201	1945247	1944105
1208	BPSL1121		28	2000491	1999196	1891826	1890531	1907266	1905971	1988521	1987226	2149769	2148474	837983	836688	322916	324211	455629	456924	1033201	1034496	1944105	1942810
1209	BPSL1122		28	1999196	1997937	1890531	1889272	1905971	1904712	1987226	1985967	2148474	2147215	836688	835429	324211	325470	456924	458183	1034496	1035755	1942810	1941551
1210	BPSL1123		28	1997859	1996678	1889194	1888013	1904634	1903453	1985889	1984708	2147137	2145956	835351	834170	325548	326729	458261	459442	1035833	1037014	1941473	1940292

1211	BPSL1124		28	1995644	1996405	1886979	1887740	1902419	1903180	1983674	1984435	2144922	2145683	833136	833897	327763	327002	460476	459715	1038048	1037287	1939258	1940019
1212	BPSL1125		28	1995186	1994809	1886521	1886144	1901961	1901584	1983216	1982839	2144464	2144087	832678	832301	328221	328598	460934	461311	1038506	1038883	1938800	1938423
1213	BPSL1126		28	1993932	1994522	1885267	1885857	1900707	1901297	1981962	1982552	2143210	2143800	831424	832014	329475	328885	462188	461598	1039760	1039170	1937546	1938136
1214	BPSL1127		28	1992426	1993670	1883761	1885005	1899201	1900445	1980456	1981700	2141704	2142948	829918	831162	330981	329737	463694	462450	1041266	1040022	1936040	1937284
1215	BPSL1128		28	1991446	1992426	1882563	1882727	1898500	1899201	1979476	1980456	2140724	2141704	828938	829918	331961	330981	464674	463694	1042246	1041266	1935060	1936040
1216	BPSL1129		28	1990314	1990763	1881431	1881880	1896585	1897034	1978344	1978793	2139592	2140041	827806	828255	333093	332644	465806	465357	1043378	1042929	1933928	1934377
1217	BPSL1130		28	1988927	1989817	1880044	1880934	1895198	1896088	1976957	1977847	2138205	2139095	826419	827309	334480	333590	467193	466303	1044765	1043875	1932541	1933431
1218	BPSL1131		28	1988316	1988747	1879433	1879864	1894587	1895018	1976346	1976777	2137594	2138025	825808	826239	335091	334660	467804	467373	1045376	1044945	1931930	1932361
1219	BPSL1132		28	1987887	1988177	1879004	1879294	1894158	1894448	1975917	1976207	2137165	2137455	825379	825669	335520	335230	468233	467943	1045805	1045515	1931501	1931791
1220	BPSL1133		28	1987199	1986849	1878316	1877966	1893470	1893120	1975229	1974879	2136477	2136127	824691	824341	336208	336558	468921	469271	1046493	1046843	1930813	1930463
1221	BPSL1134		28	1985667	1984990	1876784	1876107	1891938	1891261	1973697	1973020	2134945	2134268	823159	822482	337740	338417	470453	471130	1048025	1048702	1929281	1928604
1222	BPSL1135		28	1984925	1983531	1876042	1874648	1891196	1889802	1972955	1971561	2134203	2132809	822417	821023	338482	339876	471195	472589	1048767	1050161	1928539	1927145
1223	BPSL1136		28	1982112	1983497	1873229	1874614	1888383	1889768	1970142	1971527	2131390	2132775	819604	820989	341295	339910	474008	472623	1051580	1050195	1925726	1927111
1224	BPSL1137	GI6																					
1225	BPSL1138	GI6																					
1226	BPSL1139	GI6																					
1227	BPSL1140	GI6																					
1228	BPSL1141	GI6																					
1229	BPSL1142	GI6																					
1230	BPSL1143	GI6																					
1231	BPSL1144	GI6																					
1232	BPSL1145	GI6																					
1233	BPSL1146	GI6																					
1234	BPSL1147	GI6																					
1235	BPSL1148	GI6																					
1236	BPSL1149	GI6																					
1237	BPSL1150	GI6																					
1238	BPSL1151	GI6																					
1239	BPSL1152	GI6																					
1240	BPSL1153	GI6																					
1241	BPSL1153A	GI6																					
1242	BPSL1154	GI6																					
1243	BPSL1155	GI6																					
1244	BPSL1156	GI6																					
1245	BPSL1157	GI6																					
1246	BPSL1158		28	1980328	1981791	1871445	1872908	1886599	1888062	1968358	1969821	2129606	2131069	817820	819283	343079	341616	475792	474329	1053364	1051901	1923942	1925405
1247	BPSL1159		28	1979690	1980316	1870807	1871433	1885961	1886587	1967720	1968346	2128968	2129594	817182	817808	343717	343091	476430	475804	1054002	1053376	1923304	1923930
1248	BPSL1160		28	1979167	1979634	1870284	1870751	1885438	1885905	1967197	1967664	2128445	2128912	816659	817126	344240	343773	476953	476486	1054525	1054058	1922781	1923248
1249	BPSL1161		28	1978646	1979107	1869763	1870224	1884917	1885378	1966676	1967137	2127924	2128385	816138	816599	344761	344300	477474	477013	1055046	1054585	1922260	1922721
1250	BPSL1162		28	1977889	1978638	1869006	1869755	1884160	1884909	1965919	1966668	2127167	2127916	815381	816130	345518	344769	478231	477482	1055803	1055054	1921503	1922252
1251	BPSL1163		28	1976969	1977889	1868086	1869006	1883240	1884160	1964999	1965919	2126247	2127167	814461	815381	346438	345518	479151	478231	1056723	1055803	1920583	1921503
1252	BPSL1164		28	1975561	1976835	1866678	1867952	1881832	1883106	1963591	1964865	2124839	2126113	813053	814327	347846	346572	480559	479285	1058131	1056857	1919175	1920449
1253	BPSL1165		28	1974722	1975447	1865839	1866564	1881182	1881718	1962752	1963477	2124000	2124725	812214	812939	348685	347960	481398	480673	1058970	1058245	1918336	1919061
1254	BPSL1166		28	1973577	1974224	1864358	1865005	1879089	1879736	1961626	1962273	2122855	2123502	811097	811744	349921	349274	482543	481896	1060122	1059475	1917233	1917880
1255	BPSL1167		28	1973319	1972543	1864100	1863324	1878831	1878055	1961368	1960592	2122597	2121821	810839	810063	350179	350955	482801	483577	1060380	1061156	1916975	1916199
1256	BPSL1168		28	1972302	1971913	1863083	1862694	1877814	1877425	1960351	1959962	2121580	2121191	809822	809433	351196	351585	483818	484207	1061397	1061786	1915958	1915569
1257	BPSL1169		28	1971087	1970116	1861868	1860897	1876599	1875628	1959136	1958165	2120357	2119386	808599	807628	352419	353390	485049	486020	1062620	1063591	1914735	1913764
1258	BPSL1170		28	1969648	1969559	1860429	1860340	1875160	1875071	1957697	1957608	2118918	2118829	807160	807071	353858	353947	486488	486577	1064059	1064148	1913296	1913207
1259	BPSL1171		28	1969559	1967754	1860340	1858535	1875071	1873266	1957608	1955803	2118829	2117024	807071	805266	353947	355752	486577	488382	1064148	1065953	1913207	1911402
1260	BPSL1172		28	1967655	1965598	1858436	1856379	1873167	1871110	1955704	1953647	2116925	2114868	805167	803110	355851	357899	488481	490538	1066052	1068109	1911303	1909246
1261	BPSL1173		28	1965582	1965004	1856363	1855785	1871094	1870516	1953631	1953053	2114852	2114274	803094	802516	357915	358493	490554	491132	1068125	1068703	1909230	1908652

1262	BPSL1174		28	1964715	1964572	1855496	1855353	1870227	1870084	1952764	1952621	2113985	2113842	802227	802084	358782	358925	491421	491564	1068992	1069135	1908363	1908220
1263	BPSL1175		28	1961743	1961048	1852523	1851828	1867255	1866560	1949781	1949086	2111013	2110318	799255	798560	361754	362449	494393	495088	1071964	1072659	1905391	1904696
1264	BPSL1176		28	1960663	1960370	1851443	1851150	1866175	1865882	1948701	1948408	2109933	2109640	798175	797882	362834	363127	495473	495766	1073044	1073337	1904311	1904018
1265	BPSL1177		28	1960151	1959834	1850931	1850614	1865663	1865346	1948189	1947872	2109421	2109104	797663	797346	363346	363663	495985	496302	1073556	1073873	1903799	1903482
1266	BPSL1178		28	1959639	1959025	1850419	1849805	1865151	1864537	1947677	1947063	2108909	2108295	797151	796537	363858	364472	496497	497111	1074068	1074682	1903287	1902673
1267	BPSL1179		28	1958761	1958282	1849541	1849062	1864273	1863794	1946799	1946320	2108031	2107552	796273	795794	364736	365215	497375	497854	1074946	1075425	1902409	1901930
1268	BPSL1180		28	1956392	1955949	1847172	1846729	1861904	1861461	1944430	1943987	2105662	2105219	793904	793461	367105	367548	499744	500187	1077315	1077758	1900040	1899597
1269	BPSL1181		28	1955835	1955107	1846615	1845887	1861347	1860619	1943873	1943145	2105105	2104377	793347	792619	367662	368390	500301	501029	1077872	1078600	1899483	1898755
1270	BPSL1182		28	1955107	1954469	1845887	1845249	1860619	1859981	1943145	1942507	2104377	2103739	792619	791981	368390	369028	501029	501667	1078600	1079238	1898755	1898228
1271	BPSL1183		28	1954122	1952731	1844902	1843511	1859634	1858243	1942160	1940769	2103392	2102001	791634	790243	369375	370766			1079585	1080976	1898105	1896714
1272		Chr1_60_ISBma1_A	28	1951374	1952678	1842154	1843458	1856886	1858190	1939412	1940716	2100644	2101948	788886	790190	370819	372123	502009	502817	1081029	1082333	1895357	1896661
1273	BPSL1184		28	1951158	1949845	1841938	1840625	1856670	1855357	1939196	1937883	2100443	2099133	788679	787366	372330	373643	503024	504337	1082540	1083853	1895150	1893837
1274	BPSL1185		28	1947612	1949516	1838392	1840296	1853124	1855028	1935650	1937554	2096900	2098804	785133	787037	375876	373972	506570	504666	1086086	1084182	1891604	1893508
1275	BPSL1186		28	1946282	1947094	1837062	1837874	1851794	1852606	1934320	1935132	2095577	2096389	783754	784566	377220	376408	507914	507102	1087423	1086611	1890225	1891037
1276	BPSL1187		28	1945334	1945918	1836114	1836698	1850846	1851430	1933372	1933956	2094629	2095213	782806	783390	378168	377584	508862	508278	1088371	1087787	1889277	1889861
1277	BPSL1188		28	1944618	1945334	1835398	1836114	1850130	1850846	1932656	1933372	2093913	2094629	782090	782806	378884	378168	509578	508862	1089087	1088371	1888561	1889277
1278	BPSL1189		28	1943911	1944378	1834691	1835158	1849423	1849890	1931949	1932416	2093206	2093673	781383	781850	379591	379124	510285	509818	1089794	1089327	1887854	1888321
1279	BPSL1190		28	1942793	1943824	1833573	1834604	1848305	1849336	1930831	1931862	2092088	2093119	780265	781296	380709	379678	511403	510372	1090912	1089881	1886736	1887767
1280	BPSL1191		28	1941833	1942768	1832613	1833548	1847345	1848280	1929871	1930806	2089813	2090313	779305	780240	381669	380734	512363	511428	1091872	1090937	1885776	1886711
1281	BPSL1192		28	1941275	1941739	1832055	1832519	1846787	1847251	1929313	1929777	2089255	2089719	778747	779211	382227	381763	512921	512457	1092430	1091966	1885218	1885682
1282	BPSL1193		28	1940471	1941223	1831251	1832003	1845983	1846735	1928509	1929261	2088451	2089203	777943	778695	383031	382279	513725	512973	1093234	1092482	1884414	1885166
1283	BPSL1194		28	1940018	1940437	1830798	1831217	1845620	1845949	1928056	1928475	2087998	2088417	777490	777909	383484	383065	514178	513759	1093687	1093268	1883961	1884380
1284	BPSL1195		28	1939461	1940018	1830241	1830798	1844347	1844895	1927499	1928056	2087441	2087998	776933	777490	384041	383484	514735	514178	1094244	1093687	1883404	1883961
1285	BPSL1196		28	1939065	1937305	1829845	1828085	1843951	1842191	1927103	1925343	2087045	2085285	776537	774777	384437	386197	515131	516891	1094640	1096400	1883008	1881248
1286	BPSL1197		28	1937190	1936702	1827970	1827482	1842076	1841588	1925228	1924740	2085170	2084682	774662	774174	386312	386800	517006	517494	1096515	1097003	1881133	1880645
1287	BPSL1198		28	1936616	1935603	1827396	1826383	1841502	1840489	1924654	1923641	2084596	2083583	774088	773075	386886	387899	517580	518593	1097089	1098102	1880559	1879546
1288	BPSL1199		28	1935478	1934831	1826258	1825611	1840364	1839717	1923516	1922869	2083458	2082811	772950	772303	388024	388671	518718	519365	1098227	1098874	1879421	1878774
1289	BPSL1200		28	1934811	1933942	1825591	1824722	1839697	1838828	1922849	1921980	2082791	2081922	772283	771414	388691	389560	519385	520254	1098894	1099763	1878754	1877885
1290	BPSL1201		28	1933363	1932032	1824143	1822812	1838249	1836918	1921401	1920070	2081343	2080012	770835	769504	390139	391470	520833	522164	1100342	1101673	1877306	1875975
1291		Chr1_61_IS407A_B	28	1930663	1931898	1821443	1822678	1835549	1836784	1918701	1919936	2078643	2079878										
1292	BPSL1202		28	1929107	1930651	1819887	1821431	1833993	1835537	1917145	1918689	2077087	2078631	767819	769363	393155	391611	523849	522305	1103358	1101814	1874289	1874840
1293	BPSL1203		28	1928415	1929047	1819195	1819827	1833301	1833933	1916453	1917085	2076395	2077027	767127	767759	393847	393215	524541	523909	1104050	1103418	1873597	1874229
1294	BPSL1204		28	1927470	1927967	1818250	1818747	1832356	1832853	1915508	1916005	2075450	2075947	766182	766679	394792	394295	525486	524989	1104995	1104498	1872652	1873149
1295	BPSL1205		28	1927095	1925821	1817875	1816601	1831981	1830707	1915133	1913859	2075075	2073801	765807	764533	395167	396441	525861	527135	1105370	1106644	1872277	1871003
1296	BPSL1206		28	1925712	1925446	1816492	1816226	1830598	1830332	1913750	1913484	2073692	2073426	764424	764158	396550	396816	527244	527510	1106753	1107019	1870894	1870628
1297	BPSL1207		28	1925139	1923001	1815919	1813781	1830025	1827887	1913177	1911039	2073119	2070981	763851	761713	397123	399261	527817	529955	1107326	1109464	1870321	1868183
1298	BPSL1208		28	1922882	1921866	1813662	1812646	1827768	1826752	1910920	1909904	2070862	2069846	761594	760578	399380	400396	530074	531090	1109583	1110599	1868064	1867048
1299	BPSL1209		28	1921758	1921006	1812538	1811786	1826644	1825892	1909796	1909044	2069738	2068986	760470	759718	400504	401256	531198	531950	1110707	1111459	1866940	1866188
1300	BPSL1210		28	1920923	1920549	1811703	1811329	1825809	1825435	1908961	1908587	2068903	2068529	759635	759261	401339	401713	532033	532407	1111542	1111916	1866105	1865731
1301	BPSL1211		28	1920197	1919841	1810977	1810621	1825083	1824727	1908235	1907879	2068177	2067821	758909	758553	402065	402421	532759	533115	1112268	1112624	1865379	1865023
1302	BPSL1212		28	1919817	1919299	1810597	1810079	1824703	1824185	1907855	1907337	2067797	2067279	758529	758011	402445	402963	533139	533657	1112648	1113166	1864999	1864481
1303	BPSL1213		28	1919275	1918676	1810055	1809456	1824161	1823562	1907313	1906714	2067255	2066656	757987	757388	402987	403586	533681	534280	1113190	1113789	1864457	1863858
1304	BPSL1214		28	1918662	1917412	1809442	1808192	1823548	1822298	1906700	1905450	2066642	2065392	757374	756124	403600	404850	534294	535544	1113803	1115053	1863844	1862594
1305	BPSL1215		28	1917240	1916758	1808020	1807538	1822126	1821644	1905278	1904796	2065220	2064738	755952	755470	405022	405504	535716	536198	1115225	1115707	1862422	1861940
1306	BPSL1216		28	1916758	1915451	1807538	1806231	1821644	1820337	1904796	1903489	2064738	2063431	755470	754163	405504	406811	536198	537505	1115707	1117014	1861940	1860633
1307	BPSL1217		28	1915365	1913038	1806145	1803818	1820251	1817924	1903403	1901076	2063345	2061018	754077	751750	406897	409224	537591	539918	1117100	1119427	1860547	1858220
1308	BPSL1218		28	1913034	1911973	1803814	1802753	1817920	1816859	1901072	1900011	2061014	2059953	751746	750685	409228	410289	539922	540983	1119431	1120492	1858216	1857155
1309	BPSL1219		28	1911954	1911469	1802734	1802249	1816840	1816355	1899992	1899507	2059934	2059449	750666	750181	410308	410793	541002	541487	1120511	1120996	1857136	1856651
1310	BPSL1220		28	1911356	1910703	1802136	1801483	1816242	1815589	1899394	1898741	2059336	2058683	750068	749415	410906	411559	541600	542253	1121109	1121762	1856538	1855885
1311	BPSL1221		28	1910681	1910379	1801461	1801159	1815567	1815265	1898719	1898417	2058661	2058359	749393	749091	411581	411883	542275	542577	1121784	1122086	1855863	1855561
1312	BPSL1222		28	1910358	1908322	1801138	1799102	1815244	1813208	1898396	1896360	2058338	2056302	749070	747034	411904	413940	542598	544634	1122107	1124143	1855540	1853504

1313	BPSL1223		28	1908303	1906816	1799083	1797596	1813189	1811702	1896341	1894854	2056283	2054796	747015	745528	413959	415446	544653	546140	1124162	1125649	1853485	1851998
1314	BPSL1224		28	1906788	1905319	1797568	1796099	1811674	1810205	1894826	1893357	2054768	2053299	745500	744031	415474	416943	546168	547637	1125677	1127146	1851970	1850501
1315	BPSL1225		28	1905316	1905014	1796096	1795794	1810202	1809900	1893354	1893052	2053296	2052994	744028	743726	416946	417248	547640	547942	1127149	1127451	1850498	1850196
1316	BPSL1226		28	1904974	1904387	1795754	1795167	1809860	1809273	1893012	1892425	2052954	2052367	743686	743099	417288	417875	547982	548569	1127491	1128078	1850156	1849569
1317	BPSL1227		28	1904232	1903813	1795012	1794593	1809118	1808699	1892270	1891851	2052212	2051793	742944	742525	418030	418449	548724	549143	1128233	1128652	1849414	1848995
1318	BPSL1228		28	1903749	1902556	1794529	1793336	1808635	1807442	1891787	1890594	2051729	2050536	742461	741268	418513	419706	549207	550400	1128716	1129909	1848931	1847738
1319	BPSL1229		28	1902541	1901411	1793321	1792191	1807427	1806297	1890579	1889449	2050521	2049391	741253	740123	419721	420851	550415	551545	1129924	1131054	1847723	1846593
1320	BPSL1230		28	1901367	1900912	1792147	1791692	1806253	1805798	1889405	1888950	2049347	2048892	740079	739624	420895	421350	551589	552044	1131098	1131553	1846549	1846094
1321	BPSL1231		28	1900912	1900433	1791692	1791213	1805798	1805319	1888950	1888471	2048892	2048413	739624	739145	421350	421829	552044	552523	1131553	1132032	1846094	1845615
1322	BPSL1233		28	1900337	1899075	1791117	1789855	1805223	1803961	1888375	1887113	2048317	2047055	739049	737787	421925	423187	552619	553881	1132128	1133390	1845519	1844257
1323	BPSL1234		28	1897568	1898791	1788348	1789571	1802454	1803677	1885606	1886829	2045548	2046771	736280	737503	424694	423471	555388	554165	1134897	1133674	1842750	1843973
1324	BPSL1235		28	1896418	1897521	1787198	1788301	1801304	1802407	1884456	1885559	2044398	2045501	735130	736233	425844	424741	556538	555435	1136047	1134944	1841600	1842703
1325	BPSL1236		28	1895677	1896387	1786457	1787167	1800563	1801273	1883715	1884425	2043657	2044367	734389	735099	426585	425875	557279	556569	1136788	1136078	1840859	1841569
1326	BPSL1237		28	1894804	1895664	1785584	1786444	1799690	1800550	1882842	1883702	2042784	2043644	733516	734376	427458	426598	558152	557292	1137661	1136801	1839986	1840846
1327	BPSL1238		28	1894654	1893983	1785434	1784763	1799540	1798869	1882692	1882021	2042634	2041963	733366	732695	427608	428279	558302	558973	1137811	1138482	1839836	1839165
1328	BPSL1239		28	1892595	1893746	1783375	1784526	1796964	1798115	1880633	1881784	2040575	2041726	731307	732458	429667	428516	560361	559210	1139870	1138719	1837777	1838928
1329	BPSL1240		28	1891860	1892525	1782640	1783305	1796229	1796894	1879898	1880563	2039840	2040505	730572	731237	430402	429737	561096	560431	1140605	1139940	1837042	1837707
1330	BPSL1241		28	1891802	1891023	1782582	1781803	1796171	1795392	1879840	1879061	2039782	2039003	730514	729735	430460	431239	561154	561933	1140663	1141442	1836984	1836205
1331	BPSL1242		28	1890961	1889810	1781741	1780590	1795330	1794179	1878999	1877848	2038941	2037790	729673	728522	431301	432452	561995	563146	1141504	1142655	1836143	1834992
1332	BPSL1243		28	1888937	1889593	1779717	1780373	1793306	1793962	1876975	1877631	2036917	2037573	727649	728305	433325	432669	564019	563363	1143528	1142872	1834119	1834775
1333	BPSL1244		28	1888142	1888813	1778922	1779593	1792511	1793182	1876339	1876851	2036122	2036793	726854	727525	434120	433449	564814	564143	1144323	1143652	1833324	1833995
1334	BPSL1245		28	1887167	1888072	1777947	1778852	1791536	1792441			2035147	2036052	725879	726784	435095	434190	565789	564884	1145298	1144393	1832349	1833254
1335	BPSL1246		28	1885816	1887030	1776596	1777810	1790185	1791399	1874386	1875600	2033793	2035010	724528	725742	436446	435232	567140	565926	1146649	1145435	1830998	1832212
1336	BPSL1247																						
1337	BPSL1248																						
1338	BPSL1249																						
1339	BPSL1250																						
1340	BPSL1251																						
1341	BPSL1252																						
1342	BPSL1253																						
1343	BPSL1254																						
1344	BPSL1255																						
1345	BPSL1256																						
1346	BPSL1257																						
1347	BPSL1258																						
1348	BPSL1259																						
1349	BPSL1260																						
1350	BPSL1261																						
1351	BPSL1262																						
1352	BPSL1263																						
1353	BPSL1264																						
1354	BPSL1265																						
1355	BPSL1266																						
1356	BPSL1267																						
1357	BPSL1268																						
1358		Chr1_62_IS407A_C	28									2032228	2033345										
1359	BPSL1269		28	1881146	1881643	1772175	1772672	1785584	1786081	1869927	1870424	2032085	2032192	719858	720355	441116	440619			1151319	1150822	1829563	1830060
1360	BPSL1270		28	1878639	1880597	1769668	1771626	1783077	1785035	1867525	1869378	2030244	2031536	717352	719310	443622	441664	569719	567761	1153826	1151868	1827239	1829014
1361	BPSL1271		28	1878382	1877870	1769411	1768899	1782820	1782308	1867309	1866797	2030010	2029498	717095	716583	443879	444391	569992	570441	1154083	1154595	1826960	1826448
1362	BPSL1272		28	1877777	1877319	1768806	1768549	1782215	1781757	1866704	1866246	2029405	2028947	716490	716032	444484	444942	570534	570992	1154688	1155146	1826355	1825897
1363	BPSL1273		28	1877049	1876426	1767979	1767356	1781487	1780864	1866025	1865402	2028625	2028035	715762	715139	445212	445835	571262	571885	1155416	1156039	1825627	1825004

1364	BPSL1274		28	1874974	1875357	1765904	1766287	1779412	1779795	1863950	1864333	2026583	2026966	713687	714070	447287	446904	573337	572954	1157491	1157108	1823552	1823935
1365	BPSL1275		28	1874827	1874363	1765757	1765293	1779265	1778801	1863803	1863339	2026436	2025972	713540	713076	447434	447898	573484	573948	1157638	1158102	1823405	1822941
1366	BPSL1276		28	1873439	1874215	1764369	1765145	1777877	1778653	1862513	1863250	2025048	2025824	712165	712941	448809	448033	574859	574083	1159013	1158237	1822030	1822806
1367	BPSL1277		28	1871731	1873434	1762661	1764364	1776169	1777872	1860805	1862508	2023340	2025043	710451	712160	450523	448814	576573	574864	1160727	1159018	1820316	1822025
1368	BPSL1278		28	1870686	1871738	1761616	1762668	1775124	1776176	1859760	1860812	2022295	2023347	709406	710458	451568	450516	577618	576566	1161772	1160720	1819271	1820323
1369	BPSL1279		28	1870446	1870270	1761376	1761200	1774884	1774708	1859520	1859344	2022055	2021879	709166	708990	451808	451984	577858	578034	1162012	1162188	1819031	1818855
1370	BPSL1280		28	1868761	1870122	1759691	1761052	1773199	1774560	1857835	1859196	2020370	2021731	707481	708842	453493	452132	579543	578182	1163697	1162336	1817346	1818707
1371	BPSL1281		28	1866300	1868573	1757230	1759503	1770738	1773011	1855374	1857647	2017909	2020182	705020	707293	455954	453681	582004	579731	1166158	1163885	1814885	1817158
1372	BPSL1282		28	1865311	1861970	1756255	1752914	1768860	1765519	1854392	1851051	2017025	2013684	704311	700970	456838	460179	582811	586152	1166923	1170264	1814204	1812441
1373	BPSL1283		28	1861970	1858161	1752914	1749105	1765519	1762031	1851051	1849885	2013684	2009875	700970	697161	460179	463988	586152	589961	1170264	1174073	1811002	1807193
1374	BPSL1284		28	1858161	1855468	1749105	1746412	1761159	1758517	1847592	1844899	2009875	2007182	697161	694468	463988	466681	589961	592654	1174073	1176766	1807193	1805655
1375		Chr1_63_IS407A_C	28																			3221885	3223120
1376	BPSL1285		28	1855468	1855067	1746412	1746011	1758517	1758116	1844899	1844498	2007182	2006781	694468	694067	466681	467082	592654	593055	1176766	1177167	3220614	3220213
1377	BPSL1286		28	1853388	1854164	1744332	1745108	1756437	1757213	1842819	1843595	2005142	2005918	692380	693156	468753	467977	594734	593958	1178806	1178030	3218566	3219342
1378	BPSL1287		28	1851421	1852350	1742365	1743294	1754470	1755399	1840852	1841781	2003175	2004104	690413	691342	470720	469791	596701	595772	1180764	1179844	3216608	3217528
1379	BPSL1288		28	1850926	1851147	1741870	1742091	1753975	1754196	1840357	1840578	2002680	2002901	689918	690139	471215	470994	597196	596975				
1380	BPSL1289		28	1850462	1850671	1741406	1741615	1753511	1753720	1839893	1840102	2002216	2002425	689454	689663	471679	471470	597660	597451				
1381		Chr1_64a_IS407A_A		1849090	1850325	1740034	1741269	1752139	1753374	1838521	1839756	2000844	2002079	688082	689317	471816	473051	597797	599032	1180766	1182001	3215347	3216582
1382		Chr1_64b_IS407A_A		3186007	3187242	3084797	3086032	3101880	3103115	3172259	3173494	836600	837835										
1383	BPSL1290		29	3183984	3185912	3082774	3084702	3099857	3101785	3170236	3172164	839858	837930	686059	687987	475074	473146	601055	599127	1184024	1182096	3213325	3215253
1384	BPSL1290a		29	3183353	3183709	3082143	3082499	3099226	3099582	3169605	3169961	840489	840133	685428	685784	475705	475349	601686	601330	1184655	1184299	3212694	3213050
1385	BPSL1291		29	3183334	3183032	3082124	3081822	3099207	3098905	3169586	3169284	840508	840810	685409	685107	475724	476026	601705	602007	1184674	1184976	3212675	3212373
1386	BPSL1292		29	3182868	3182047	3081658	3080837	3098741	3097920	3169120	3168299	840974	841795	684943	684122	476190	477011	602171	602992	1185140	1185961	3212209	3211388
1387	BPSL1293		29	3181633	3180920	3080423	3079710	3097506	3096793	3167885	3167172	842209	842922	683744	683031	477389	478102	603373	604086	1186375	1187088	3210974	3210261
1388	BPSL1294		29	3180901	3180113	3079691	3078903	3096774	3095986	3167153	3166365	842941	843729	683012	682224	478121	478909	604105	604893	1187107	1187895	3210242	3209454
1389	BPSL1295		29	3179656	3178724	3078446	3077514	3095529	3094597	3165908	3164976	844186	845118	681767	680835	479366	480298	605350	606282	1188352	1189284	3208997	3208065
1390	BPSL1296		29	3177177	3178370	3075967	3077160	3093050	3094243	3163429	3164622	846665	845472	679281	680474	481859	480666	607836	606643	1190859	1189666	3206511	3207704
1391	BPSL1297		29	3176898	3176113	3075688	3074903	3092771	3091986	3163150	3162365	846944	847729	679002	678217	482138	482923	608115	608900	1191138	1191923	3206232	3205447
1392	BPSL1298		29	3175978	3175571	3074768	3074361	3091851	3091444	3162230	3161823	847864	848271	678076	677669	483070	483477	609035	609442	1192100	1192507	3205300	3204893
1393	BPSL1299		29	3174385	3175209	3073175	3073999	3090258	3091082	3160637	3161461	849457	848633	676483	677307	484663	483839	610628	609804	1193693	1192869	3203707	3204531
1394	BPSL1300		29	3173120	3174145	3071910	3072935	3088993	3090018	3159372	3160397	850701	849676	675225	676250	485914	484889	611886	610861	1194986	1193961	3202435	3203460
1395	BPSL1301		29	3172064	3172963	3070854	3071753	3087937	3088836	3158316	3159215	851757	850858	674169	675068	486970	486071	612942	612043	1196042	1195143	3201379	3202278
1396	BPSL1302		29	3170022	3170843	3068812	3069633	3085895	3086716	3156274	3157095	853799	852978	672127	672948	489012	488191	614984	614163	1198075	1197254	3199346	3200167
1397	BPSL1303		29	3168873	3169931	3067663	3068721	3084746	3085804	3155125	3156183	854948	853890	670978	672036	490161	489103	616133	615075	1199224	1198166	3198197	3199255
1398	BPSL1304		29	3167800	3168813	3066590	3067603	3083673	3084686	3154052	3155065	856021	855008	669905	670918	491234	490221	617206	616193	1200297	1199284	3197124	3198137
1399	BPSL1305		29	3164301	3165452	3063091	3064242	3080174	3081325	3150553	3151704	859520	858369	666399	667550	494726	493575	620691	619540	1203810	1202659	3193618	3194769
1400	BPSL1306		29	3162571	3163932	3061361	3062722	3078444	3079805	3148823	3150184	861250	859889	664669	666030	496456	495095	622421	621060	1205556	1204195	3191864	3193225
1401	BPSL1307		29	3161884	3161285	3060674	3060075	3077757	3077158	3148136	3147537	861937	862536	663982	663383	497143	497742	623108	623707	1206243	1206842	3191181	3190582
1402	BPSL1308		29	3161229	3160435	3060019	3059225	3077102	3076308	3147481	3146687	862592	863386	663327	662533	497798	498592	623763	624557	1206898	1207692	3190526	3189732
1403	BPSL1309		29	3159401	3160036	3058172	3058807	3075274	3075909	3145653	3146288	864420	863785	661499	662134	499626	498991	625546	624956	1208726	1208091	3188698	3189333
1404	BPSL1310		29	3159279	3157741	3058050	3056512	3075152	3074937	3145531	3143993	864542	866080	661377	659839	499748	501286	625649	626959	1208848	1210386	3188576	3187239
1405	BPSL1311		29	3157038	3157529	3055809	3056300	3072140	3072631	3143290	3143781	866792	866301	659133	659624	502022	501531	627683	627192	1211086	1210595	3186540	3187031
1406	BPSL1312		29	3156879	3154966	3055650	3053737	3071981	3070068	3143131	3141218	866951	868864	658974	657061	502181	504094	627842	629755	1211245	1213158	3186381	3184468
1407	BPSL1313		29	3153418	3154746	3052189	3053517	3068520	3069848	3139670	3140998	870412	869084	655508	656836	505647	504319	631308	629980	1214706	1213378	3182920	3184248
1408	BPSL1314		29	3152692	3153393	3051463	3052164	3067275	3067568	3138944	3139645	871138	870437	654782	655483	506373	505672	632034	631333	1215432	1214731	3182194	3182895
1409	BPSL1315		29	3151629	3152525	3050400	3051296	3066212	3067108	3137881	3138777	872201	871305	653719	654615	507436	506540	633097	632201	1216495	1215599	3181131	3182027
1410	BPSL1316		29	3151378	3150608	3050149	3049379	3065961	3065191	3137630	3136860	872452	873222	653468	652698	507687	508457	633348	634118	1216746	1217516	3180880	3180110
1411	BPSL1317		29	3149021	3149209	3047792	3047980	3063604	3063792	3135273	3135461	874809	874621	651104	652474	510051	508681	635712	634342	1219103	1218915	3178523	3178711
1412	BPSL1318		29	3148171	3148575	3046942	3047346	3062754	3063158	3134423	3134827	875659	875255	650254	650658	510901	510497	636562	636158	1219953	1219549	3177673	3178077
1413	BPSL1319		29	3146357	3148171	3045128	3046942	3060940	3062754	3132609	3134423	877473	875659	648440	650254	512715	510901	638376	636562	1221767	1219953	3175859	3177673
1414	BPSL1320		29	3145839	3145648	3044610	3044419	3060422	3060231	3132091	3131900	877991	878182	647928	647737	513227	513418	638888	639079	1222285	1222476	3175341	3175150

1415	BPSL1321		29	3144093	3145565	3042864	3044336	3058676	3060148	3130345	3131817	879737	878265	646182	647654	514973	513501	640634	639162	1224031	1222559	3173595	3175067
1416	BPSL1322		29	3142987	3143385	3041758	3042156	3057570	3057968	3129239	3129637	880843	880445	645076	645474	516079	515681	641740	641342	1225137	1224739	3172489	3172887
1417	BPSL1323		29	3142518	3142952	3041289	3041723	3057101	3057535	3128770	3129204	881312	880878	644607	645041	516548	516114	642209	641775	1225606	1225172	3172020	3172454
1418	BPSL1323a		29	3142128	3141808	3040899	3040579	3056711	3056391	3128380	3128060	881702	882022	644217	643897	516938	517258	642599	642919	1225996	1226316	3171630	3171310
1419	BPSL1324		29	3140878	3139016	3039649	3037787	3055461	3053599	3127130	3125268	882946	884808	642988	641126	518185	520047	643837	645699	1227258	1229120	3170368	3168506
1420	BPSL1325		29	3138733	3137804	3037504	3036575	3053316	3052387	3124985	3124056	885091	886020	640843	639914	520330	521259	645982	646911	1229403	1230332	3168223	3167294
1421	BPSL1326		29	3136382	3137227	3035153	3035998	3050965	3051810	3122634	3123479	887442	886597	638492	639337	522681	521836	648333	647488	1231754	1230909	3165872	3166717
1422	BPSL1327		29	3136111	3135719	3034492	3034100	3050415	3050023	3122344	3121952	887784	888176	638221	637829	522952	523344	648667	649059	1232025	1232417	3165545	3165153
1423	BPSL1328		29	3135693	3134938	3034074	3033319	3049997	3049242	3121926	3121171	888202	888957	637803	637048	523370	524125	649085	649840	1232443	1233198	3165127	3164372
1424	BPSL1329		29	3134615	3132978	3032996	3031359	3048919	3047282	3120848	3119211	889370	891007	636728	635091	524454	526091	650160	651797	1233593	1235230	3163968	3162331
1425	BPSL1330		29	3132961	3132647	3031342	3031028	3047265	3046951	3119194	3118880	891024	891338	635074	634760	526108	526422	651814	652128	1235247	1235561	3162314	3162000
1426	BPSL1331		29	3132647	3131703	3031028	3030084	3046951	3046007	3118880	3117936	891338	892282	634760	633816	526422	527366	652128	653072	1235561	1236505	3162000	3161056
1427	BPSL1332		29	3131713	3130991	3030094	3029372	3046017	3045295	3117946	3117224	892272	892994	633826	633104	527356	528078	653062	653784	1236495	1237217	3161066	3160344
1428	BPSL1333		29	3130977	3130090	3029358	3028972	3045281	3044394	3117210	3116323	893008	893895	633090	632203	528092	528979	653798	654685	1237231	1238118	3160330	3159443
1429	BPSL1334		29	3129565	3128798	3027491	3026724	3043869	3043102	3115798	3115031	894423	895190	631708	630941	529468	530235	655382	656035	1238640	1239407	3158945	3158610
1430	BPSL1335		29	3128798	3127863	3026724	3025789	3043102	3042167	3115031	3114096	895190	896125	630941	630006	530235	531170	656035	656970	1239407	1240342	3158180	3157470
1431	BPSL1336		29	3127766	3127224	3025692	3025150	3042070	3041528	3113999	3113457	896222	896764	629909	629367	531267	531809	657067	657609	1240439	1240981	738196	738738
1432	BPSL1337		29	3126986	3126075	3024912	3024001	3041290	3040379	3113219	3112308	897164	898075	629048	628137	532173	533084	657973	658884	1241381	1242292	739165	740076
1433	BPSL1338_p	partial	29	3125680	3125477	3023599	3023396	3039977	3039774	3111913	3111710	898463	898666	627700	627497	533493	533696	659279	659482	1242673	1242876	740450	740653
1434		Chr1_65a_IS407A_A		3124245	3125480	3022164	3023399	3038542	3039777	3110478	3111713	898663	899898	626265	627500	533693	534928	659479	660714	1242873	1244108	740650	741885
1435		Chr1_65b_IS407A_A		788024	789259	665167	666402	669375	670610	783009	784244												
1436	BPSL1338_p	partial	30	789256	789702	666399	666845	670607	671053	784241	784687	899895	900341	626268	625822	534925	535371	660711	661157	1244105	1244551	741882	742328
1437	BPSL1339		30	791676	790192	668819	667335	673027	671543	786661	785177	902308	900824	623862	625346	537324	535840	663110	661626	1246511	1245027	744281	742797
1438	BPSL1340		30	792211	792540	669354	669683	673562	673891	787196	787525	902843	903172	623327	622998	537859	538188	663645	663974	1247046	1247375	744816	745145
1439	BPSL1341		30	793698	792979	670841	670122	675614	674895	788672	787953	904234	903515	621840	622559	539282	538563	665076	664357	1248477	1247758	746263	745544
1440	BPSL1342		30	794202	793759	671345	670902	676118	675675	789176	788733	904738	904295	621336	621779	539786	539343	665580	665137	1248981	1248538	746767	746324
1441	BPSL1343		30	795011	794202	672154	671345	676927	676118	789985	789176	905547	904738	620527	621336	540595	539786	666389	665580	1249790	1248981	747576	746767
1442	BPSL1344		30	795032	795835	672175	672978	676948	677751	790006	790809	905568	906371	620506	619703	540616	541419	666410	667213	1249811	1250614	747597	748400
1443	BPSL1345		30	795922	797580	673065	674723	677838	679496	790896	792554	906458	908116	619616	617958	541506	543164	667300	668958	1250701	1252359	748487	750145
1444	BPSL1346		30	797664	798953	674807	676096	679580	680869	792638	793927	908200	909489	617874	616585	543248	544537	669042	670331	1252443	1253732	750229	751518
1445	BPSL1347		30	800205	799069	677348	676212	682121	680985	795179	794043	910741	909605	615333	616469	545789	544653	671583	670447	1254984	1253848	752770	751634
1446	BPSL1348		30	800433	801452	677576	678595	682349	683368	795407	796426	910969	911988	615105	614086	546017	547036	671811	672830	1255212	1256231	752998	754017
1447	BPSL1349		30	801872	803014	679015	680157	683788	684930	796846	797988	912408	913550	613686	612544	547436	548578	673230	674372	1256651	1257793	754437	755579
1448	BPSL1350		30	803049	803708	680192	680851	684965	685624	798023	798682	913585	914244	612509	611850	548613	549272	674407	675066	1257828	1258487	755614	756273
1449	BPSL1351		30	803763	807014	680906	684157	685679	688930	798737	801988	914299	917550	611795	608544	549327	552578	675121	678372	1258542	1261793	756328	759579
1450	BPSL1352		30	807136	807609	684279	684752	689052	689525	802110	802583	917672	918145	608422	607949	552700	553173	678494	678967	1261915	1262388	759701	760174
1451	BPSL1353		30	807612	808112	684755	685255	689528	689860	802758	803108	918148	918648	607946	607446	553176	553676	678970	679470	1262391	1262891	760177	760677
1452	BPSL1354		30	808740	808189	685883	685332	691237	690740	803736	803185	919276	918725	606818	607369	554304	553753	680098	679547	1263519	1262968	761305	760754
1453	BPSL1355		30	808934	809593	686077	686736	691431	692090	803930	804589	919470	920129	606624	605965	554498	555157	680292	680951	1263713	1264372	761499	762158
1454	BPSL1356		30	809764	811647	686907	688790	692261	694144	804760	806643	920300	922183	605794	603911	555328	557211	681122	683005	1264543	1266426	762329	764212
1455	BPSL1357		30	811763	812650	688906	689793	694260	695147	806759	807646	922299	923186	603795	602908	557327	558214	683121	684008	1266542	1267429	764328	765215
1456	BPSL1358		30	812679	814034	689822	691177	695176	696531	807675	809030	923215	924570	602879	601524	558243	559598	684037	685392	1267458	1268813	765244	766599
1457	BPSL1359		30	814473	815498	691898	692923	697765	698790	809484	810509	925093	926118	601085	600060	560037	561062	685909	686934	1269252	1270277	767108	768133
1458	BPSL1360		30	815608	816591	693506	694489	698900	699883	810629	811141	926228	927211	599950	598967	561172	562155	687044	688027	1270387	1271370	768243	769226
1459	BPSL1361		30	816612	817481	694510	695379	699904	700773	811162	812031	927232	928101	598946	598077	562176	563045	688048	688917	1271391	1272260	769247	770116
1460	BPSL1362		30	817500	818345	695398	696243	700792	701637	812050	812895	928120	928965	598058	597213	563064	563909	688936	689781	1272279	1273124	770135	770980
1461	BPSL1363		30	818368	819069	696266	696967	701660	702361	812918	813619	928988	929689	597190	596489	563932	564633	689804	690505	1273147	1273848	771003	771704
1462	BPSL1364		30	819101	819799	696999	697697	702393	703091	813651	814349	929721	930419	596457	595759	564665	565363	690537	691235	1273880	1274578	771736	772434
1463	BPSL1365		30	819872	821179	697770	699077	703164	704471	814422	815729	930492	931799	595686	594379	565436	566743	691308	692615	1274651	1275958	772507	773814
1464	BPSL1366		30	823443	821386	701341	699284	706735	704678	817993	815936	934063	932006	592115	594172	569007	566950	694879	692822	1278222	1276165	776078	774021
1465	BPSL1367		30	823674	825230	701572	703128	706966	708522	818224	819780	934294	935850	591884	590328	569238	570794	695110	696666	1278453	1280009	776309	777865

1466	BPSL1368		30	826439	825549	704337	703447	709731	708841	820989	820099	937059	936169	589119	590009	572003	571113	697890	696985	1281218	1280328	779074	778184
1467	BPSL1369		30	826504	827574	704402	705472	709796	710866	821054	822124	937124	938194	589054	587984	572068	573138	697955	699025	1281283	1282353	779139	780209
1468	BPSL1370		30	827603	828265	705501	706163	710895	711557	822153	822815	938223	938885	587955	587293	573167	573829	699054	699716	1282382	1283044	780238	780900
1469	BPSL1371		30	828948	828694	706846	706592	712240	711986	823498	823244	939568	939314	586610	586864	574512	574258	700399	700145	1283727	1283473	781583	781329
1470	BPSL1372		30	829884	829627	707782	707525	713176	712919	824434	824177	940504	940247	585674	585931	575448	575191	701335	701078	1284663	1284406	782519	782262
1471	BPSL1373		30	830291	829884	708189	707782	713583	713176	824841	824434	940911	940504	585267	585674	575855	575448	701742	701335	1285070	1284663	782926	782519
1472	BPSL1374		30	830853	831311	708751	709209	714145	714603	825403	825861	941473	941931	584705	584247	576417	576875	702304	702762	1285632	1286090	783487	783945
1473	BPSL1375		30	831466	832281	709364	710179	714758	715573	826016	826831	942086	942901	584092	583277	577030	577845	702917	703732	1286245	1287060	784100	784915
1474	BPSL1376		30	832898	832638	710796	710536	716190	715930	827448	827188	943518	943258	582660	582920	578462	578202	704349	704089	1287677	1287417	785532	785272
1475	BPSL1377		30	832973	833161	710871	711059	716265	716453	827523	827711	943593	943781	582585	582397	578537	578725	704424	704612	1287752	1287940	785607	785795
1476	BPSL1378		30	834810	833422	712708	711320	718102	716714	829360	827972	945430	944042	580748	582136	580374	578986	706261	704873	1289589	1288201	787444	786056
1477	BPSL1379		30	835168	834821	713066	712719	718460	718113	829718	829371	945788	945441	580390	580737	580732	580385	706619	706272	1289947	1289600	787802	787455
1478	BPSL1380		30	837067	835406	714965	713304	720359	718698	831617	829956	947687	946026	578491	580152	582631	580970	708518	706857	1291846	1290185	789701	788040
1479	BPSL1381		30	838094	837384	715992	715282	721386	720676	832644	831934	948714	948004	577464	578174	583658	582948	709545	708835	1292873	1292163	790728	790018
1480	BPSL1382		30	839212	838382	717110	716280	722504	721674	833762	832932	949832	949002	576346	577176	584776	583946	710663	709833	1293991	1293161	791846	791016
1481		Chr1_66_IS407A_A		839215	840450	717113	718348	722507	723742	833765	835000	949835	951070	575108	576343	584779	586014	710666	711901	1293994	1295229	791849	793084
1482	BPSL1383																						
1483	BPSL1384	GI7																					
1484	BPSL1384a	GI7																					
1485	BPSL1385	GI7																					
1486	BPSL1386	GI7																					
1487	BPSL1387	GI7																					
1488	BPSL1388	GI7																					
1489	BPSL1389	GI7																					
1490	BPSL1390	GI7																					
1491	BPSL1391	GI7																					
1492	BPSL1392	GI7																					
1493	BPSL1393	GI7																					
1494		Chr1_67_IS407A_A		1533068	1534303	1420660	1421895	1430062	1431297	1523634	1524869	1686724	1687959	3386673	3387908	1230358	1231593	1436504	1437739	1950881	1952116	1479698	1480933
1495	BPSL1394		31	1531285	1531905	1418877	1419497	1428279	1428899	1521851	1522471	1684941	1685561	3389691	3389071	1228575	1229195	1434932	1435552	1949098	1949718	1477915	1478535
1496	BPSL1395		31	1529939	1531087	1417531	1418679	1426933	1428081	1520505	1521653	1683595	1684743	3391031	3389889	1227235	1228377	1433592	1434734	1947752	1948900	1476563	1477717
1497	BPSL1396		31	1528731	1529921	1416323	1417513	1425725	1426915	1519297	1520487	1682387	1683577	3392239	3391049	1226026	1226730	1432384	1433574	1946544	1947734	1475355	1476545
1498	BPSL1397		31	1527640	1528704	1415232	1416296	1424634	1425698	1518206	1519270	1681296	1682360	3393330	3392266	1224935	1225999	1431293	1432357	1945453	1946517	1474264	1475328
1499	BPSL1398		31	1527309	1526164	1414901	1413756	1424303	1423158	1517875	1516730	1680965	1679820	3393661	3394806	1224604	1223459	1430962	1429817	1945123	1943978	1473934	1472789
1500	BPSL1399		31	1526062	1525091	1413654	1412683	1423056	1422085	1516628	1515657	1679718	1678747	3394908	3395879	1223357	1222386	1429715	1428744	1943876	1942905	1472687	1471716
1501	BPSL1400		31	1524251	1524697	1411843	1412289	1421245	1421691	1514817	1515263	1677907	1678353	3396719	3396273	1221546	1221992	1427904	1428350	1942065	1942511	1470876	1471322
1502	BPSL1401		31	1522985	1524145	1410577	1411737	1419979	1421139	1513551	1514711	1676641	1677801	3397985	3396825	1220280	1221440	1426638	1427798	1940799	1941959	1469610	1470770
1503	BPSL1402		31	1522532	1521186	1410124	1408778	1419526	1418180	1513098	1511752	1676177	1674831	3398449	3399795	1219805	1218459	1426185	1424839	1940346	1939000	1469157	1467811
1504	BPSL1403		31	1521048	1520398	1408640	1407990	1418042	1417392	1511614	1510964	1674693	1674043	3399933	3400583	1218321	1217671	1424701	1424051	1938862	1938212	1467673	1467023
1505	BPSL1404		31	1520255	1518966	1407847	1406558	1417249	1415960	1510821	1509532	1673900	1672611	3400726	3402015	1217528	1216239	1423908	1422619	1938069	1936780	1466880	1465591
1506	BPSL1405		31	1518767	1516353	1406359	1403945	1415761	1413347	1509333	1506919	1672412	1669998	3402214	3404628	1216040	1213626	1422420	1420006	1936581	1934167	1465392	1462978
1507	BPSL1406		31	1515054	1515380	1402646	1402972	1412048	1412374	1505620	1505946	1668699	1669025	3405927	3405601	1212327	1212653	1418707	1419033	1932868	1933194	1461679	1462005
1508	BPSL1406A		31	1514871	1514656	1402463	1402248	1411865	1411650	1505437	1505222	1668516	1668301	3406110	3406325	1212144	1211929	1418524	1418309	1932685	1932470	1461496	1461281
1509	BPSL1407		31	1514578	1514165	1402170	1401757	1411572	1411159	1505144	1504731	1668223	1667810	3406403	3406816	1211851	1211438	1418231	1417818	1932392	1931979	1461203	1460790
1510	BPSL1408		31	1513897	1513757	1401489	1401349	1410891	1410751	1504463	1504323	1667542	1667402	3407084	3407224	1211170	1211030	1417550	1417410	1931711	1931571	1460522	1460382
1511	BPSL1409_putative deoxyribo nuclease		31																				
1512	BPSL1410		31	1512104	1510173	1399696	1397765	1409098	1407167	1502670	1500739	1665757	1663826	3408853	3410784	1209385	1207454	1415733	1413802	1929894	1927963	1458753	1456822
1513	BPSL1411		31	1509076	1509747	1396668	1397339	1406070	1406741	1499642	1500313	1662729	1663400	3411849	3411178	1206381	1207052	1412729	1413400	1926882	1927553	1455733	1456404
1514	BPSL1412		31	1509086	1508364	1396678	1395956	1406080	1405358	1499652	1498930	1662739	1662017	3411839	3412561	1206391	1205669	1412739	1412017	1926892	1926170	1455743	1455021
1515	BPSL1413		31	1506391	1508010	1393983	1395602	1403385	1405004	1496957	1498576	1660028	1661647	3414534	3412915	1203728	1205347	1410068	1411687	1924189	1925808	1453040	1454659
1516	BPSL1414		31	1504693	1506285	1392285	1393877	1401687	1403279	1495259	1496851	1658330	1659922	3416232	3414640	1202030	1203622	1408370	1409962	1922491	1924083	1451342	1452934

1517	BPSL1415		31	1503341	1504642	1390933	1392234	1400335	1401636	1493907	1495208	1656978	1658279	3417584	3416283	1200678	1201979	1407018	1408319	1921139	1922440	1449990	1451291
1518	BPSL1416		31	1503085	1499027	1390677	1386619	1400079	1396021	1493651	1489593	1656722	1652664	3417840	3421907	1200422	1196355	1406762	1402695	1920883	1916825	1449734	1445676
1519	BPSL1417		31	1499019	1498489	1386611	1386081	1396013	1395483	1489585	1489055	1652656	1652126	3421915	3422445	1196347	1195817	1402687	1402157	1916817	1916287	1445668	1445138
1520	BPSL1418		31	1497609	1498385	1385201	1385977	1394603	1395379	1488175	1488951	1651246	1652022	3423325	3422549	1194937	1195713	1401277	1402053	1915407	1916183	1444258	1445034
1521	BPSL1419		31	1497270	1497581	1384862	1385173	1394264	1394575	1487836	1488147	1650907	1651218	3423664	3423353	1194598	1194909	1400938	1401249	1915068	1915379	1443919	1444230
1522	BPSL1420		31	1496737	1497264	1384329	1384856	1393731	1394258	1487303	1487830	1650374	1650901	3424197	3423670	1194065	1194592	1400405	1400932	1914535	1915062	1443386	1443913
1523	BPSL1421		31	1496213	1496641	1383805	1384233	1393207	1393635	1486779	1487207	1649850	1650278	3424721	3424293	1193541	1193969	1399881	1400309	1914011	1914439	1442862	1443290
1524	BPSL1422		31	1494585	1496159	1382177	1383751	1391579	1393153	1485151	1486725	1648222	1649796	3426349	3424775	1191913	1193487	1398253	1399827	1912383	1913957	1441234	1442808
1525	BPSL1423		31	1494469	1492838	1382061	1380430	1391463	1389832	1485035	1483404	1648106	1646475	3426465	3428096	1191797	1190166	1398137	1396506	1912267	1910636	1441118	1439487
1526	BPSL1424		31	1492583	1490502	1380175	1378094	1389577	1387496	1483149	1481068	1646220	1644139	3428351	3430432	1189911	1187830	1396251	1394170	1910381	1908300	1439232	1437151
1527	BPSL1425		31	1490457	1489822	1378049	1377414	1387451	1386816	1481023	1480388	1644094	1643459	3430477	3431112	1187785	1187150	1394125	1393490	1908255	1907620	1437106	1436471
1528	BPSL1426		31	1489760	1488585	1377352	1376177	1386754	1385579	1480326	1479151	1643397	1642222	3431174	3432349	1187088	1185913	1393428	1392253	1907558	1906383	1436409	1435234
1529	BPSL1427		31	1487684	1488205	1375276	1375797	1384678	1385199	1478250	1478771	1641321	1641842	3433250	3432729	1185003	1185524	1391352	1391873	1905482	1906003	1434333	1434854
1530	BPSL1428		31	1486996	1486685	1374588	1374277	1383990	1383679	1477577	1477266	1640633	1640322	3433938	3434249	1184315	1184004	1390664	1390353	1904794	1904483	1433645	1433334
1531	BPSL1429		31	1486685	1486206	1374277	1373798	1383679	1383200	1477266	1476787	1640322	1639843	3434249	3434728	1184004	1183525	1390353	1389874	1904483	1904004	1433334	1432855
1532	BPSL1430		31	1486111	1485116	1373703	1372708	1383105	1382110	1476692	1475697	1639748	1638753	3434823	3435818	1183430	1182435	1389779	1388784	1903909	1902914	1432760	1431765
1533	BPSL1431		31	1485043	1484051	1372635	1371643	1382037	1381045	1475624	1474632	1638680	1637688	3435891	3436883	1182362	1181370	1388711	1387719	1902841	1901849	1431692	1430700
1534	BPSL1432		31	1483676	1483239	1371268	1370831	1380670	1380233	1474257	1473820	1637313	1636876	3437258	3437695	1180995	1180558	1387344	1386907	1901474	1901037	1430325	1429888
1535	BPSL1433		31	1482349	1483197	1369941	1370789	1379343	1380191	1472930	1473778	1635986	1636834	3438585	3437737	1179668	1180516	1386017	1386865	1900147	1900995	1428998	1429846
1536	BPSL1434		31	1481325	1482275	1368917	1369867	1378319	1379269	1471906	1472856	1634962	1635912	3439609	3438659	1178644	1179594	1384993	1385943	1899123	1900073	1427974	1428924
1537	BPSL1435		31	1481153	1480137	1368745	1367729	1378147	1377131	1471734	1470718	1634790	1633774	3439781	3440797	1178472	1177456	1384821	1383805	1898951	1897935	1427802	1426786
1538	BPSL1436		31	1480133	1479516	1367725	1367108	1377127	1376510	1470714	1470097	1633770	1633153	3440801	3441418	1177452	1176835	1383801	1383184	1897931	1897314	1426782	1426165
1539	BPSL1437		31	1479493	1478462	1367085	1366054	1376487	1375456	1470074	1469043	1633130	1632099	3441441	3442472	1176812	1175781	1383161	1382130	1897291	1896260	1426142	1425111
1540	BPSL1438		31	1478416	1477913	1366008	1365505	1375410	1374907	1468997	1468494	1632053	1631550	3442518	3443021	1175735	1175232	1382084	1381581	1896214	1895711	1425065	1424562
1541	BPSL1439		31	1477834	1477028	1365426	1364620	1374828	1374022	1468415	1467609	1631471	1630665	3443100	3443906	1175153	1174347	1381502	1380696	1895632	1894826	1424483	1423677
1542	BPSL1440		31	1477014	1476310	1364606	1363902	1374008	1373304	1467595	1466891	1630651	1629947	3443920	3444624	1174333	1173629	1380682	1379978	1894812	1894108	1423663	1422959
1543	BPSL1441		31	1476230	1475883	1363822	1363475	1373224	1372877	1466811	1466464	1629867	1629520	3444704	3445051	1173549	1173202	1379898	1379551	1894028	1893681	1422879	1422532
1544	BPSL1442		31	1475813	1474629	1363405	1362221	1372807	1371623	1466394	1465210	1629450	1628266	3445121	3446305	1173132	1171948	1379481	1378297	1893611	1892427	1422462	1421278
1545	BPSL1443		31	1474502	1473195	1362094	1360787	1371496	1370189	1465083	1463776	1628139	1626832	3446432	3447739	1171821	1170514	1378170	1376863	1892300	1890993	1421151	1419844
1546	BPSL1444		31	1472815	1471754	1360407	1359346	1369809	1368748	1463396	1462335	1626452	1625391	3448119	3449180	1170134	1169073	1376483	1375422	1890613	1889552	1419464	1418403
1547	BPSL1445		31	1470957	1471541	1358549	1359133	1367951	1368535	1461538	1462122	1624594	1625178	3449977	3449393	1168276	1168860	1374625	1375209	1888755	1889339	1417606	1418190
1548	BPSL1446		31	1470219	1470716	1357811	1358308	1367213	1367710	1460800	1461297	1623856	1624353	3450715	3450218	1167538	1168035	1373887	1374384	1888017	1888514	1416868	1417365
1549	BPSL1447		31	1469743	1470030	1357335	1357622	1366737	1367024	1460324	1460611	1623380	1623667	3451191	3450904	1167062	1167349	1373411	1373698	1887541	1887828	1416392	1416679
1550	BPSL1448		31	1469245	1468205	1356837	1355797	1366239	1365199	1459826	1458786	1622882	1621842	3451689	3452729	1166564	1165524	1372913	1371873	1887043	1886003	1415894	1414854
1551		Chr1_68_IS407A_A		1466275	1467510	1353867	1355102	1363269	1364504	1456856	1458091	1619910	1621145	3453424	3454659	1163594	1164829	1369943	1371178	1884073	1885308	1412924	1414159
1552	BPSL1449																						
1553	BPSL1450																						
1554	BPSL1451																						
1555	BPSL1452																						
1556	BPSL1453																						
1557		Chr1_69_IS407A_A		1466275	1467510	1353867	1355102	1363269	1364504	1456856	1458091	1109938	1111173	3453424	3454659	1163594	1164829	1369943	1371178	1884073	1885308	952619	953854
1558		Chr1_70_IS407A_C												3454610	3455717								
1559	BPSL1454		32	1465683	1466105	1353275	1353697	1362677	1363099	1456264	1456686	1111749	1111327	3456309	3455887	1163003	1163425	1369343	1369765	1883578	1884000	954469	954047
1560	BPSL1455		32	1464752	1465669	1352344	1353261	1361746	1362663	1455333	1456250	1112680	1111763	3457240	3456323	1162072	1162989	1368412	1369329	1882647	1883564	955400	954483
1561	BPSL1456		32	1464547	1463843	1352139	1351435	1361541	1360837	1455128	1454424	1112885	1113589	3457445	3458149	1161867	1161163	1368207	1367503	1882442	1881738	955605	956309
1562	BPSL1457		32	1463643	1462828	1351235	1350420	1360637	1359822	1454224	1453409	1113789	1114604	3458142	3458207	1160963	1160148	1367303	1366488	1881538	1880723	956509	957324
1563	BPSL1458		32	1462590	1462219	1350182	1349811	1359584	1359213	1453171	1452800	1114842	1115213	1194	1565	1159910	1159539	1366250	1365879	1880485	1880114	957562	957933
1564	BPSL1459		32	1462179	1461883	1349771	1349475	1359173	1358877	1452760	1452464	1115253	1115549	1605	1901	1159499	1159203	1365839	1365543	1880074	1879778	957973	958269
1565	BPSL1460		32	1461877	1461605	1349469	1349197	1358871	1358599	1452458	1452186	1115555	1115827	1907	2179	1159197	1158925	1365537	1365265	1879772	1879500	958275	958547
1566	BPSL1461		32	1461574	1461125	1349166	1348717	1358568	1358119	1452155	1451706	1115858	1116307	2210	2659	1158894	1158445	1365234	1364785	1879469	1879020	958578	959027
1567	BPSL1462		32	1460978	1459599	1348570	1347191	1357972	1356593	1451559	1450180	1116454	1117833	2806	4185	1158298	1156919	1364638	1363259	1878873	1877494	959174	960553

1568	BPSL1463	32	1459457	1458834	1347049	1346426	1356451	1355828	1450038	1449415	1117975	1118598	4327	4950	1156777	1156154	1363117	1362494	1877352	1876729	960695	961318
1569	BPSL1464	32	1458820	1457813	1346412	1345405	1355814	1354807	1449401	1448394	1118612	1119619	4964	5971	1156140	1155133	1362480	1361473	1876715	1875708	961332	962339
1570	BPSL1465	32	1456351	1457571	1343374	1344594	1352908	1353546	1447895	1448152	1121081	1119861	7433	6213	1153671	1154891	1360011	1361231	1874246	1875466	1358703	1359923
1571	BPSL1466	32	1454720	1456108	1341743	1343131	1350385	1351224	1445587	1446975	1122712	1121324	9064	7676	1152040	1153428	1358380	1359768	1872615	1874003	1357072	1358460
1572	BPSL1467	32	1453525	1453983	1340548	1341006	1349190	1349648	1444392	1444850	1123907	1123449	10259	9801	1150845	1151303	1357185	1357643	1871420	1871878	1355877	1356335
1573	BPSL1468	32	1452539	1453432	1339562	1340455	1348204	1349097	1443406	1444299	1124893	1124000	11245	10352	1149859	1150752	1356199	1357092	1870434	1871327	1354891	1355784
1574	BPSL1469	32	1450524	1452521	1337547	1339544	1346189	1348186	1441391	1443388	1126908	1124911	13260	11263	1147844	1149841	1354184	1356181	1868419	1870416	1352876	1354873
1575	BPSL1471	32	1449520	1450524	1336543	1337547	1345185	1346189	1440387	1441391	1127912	1126908	14264	13260	1146840	1147844	1353180	1354184	1867415	1868419	1351872	1352876
1576	BPSL1472	32	1448362	1449510	1335385	1336533	1344027	1345175	1439229	1440377	1129070	1127922	15422	14274	1145682	1146830	1352022	1353170	1866257	1867405	1350714	1351862
1577	BPSL1473	32	1447864	1448232	1334887	1335255	1343529	1343897	1438731	1439099	1129568	1129200	15920	15552	1145184	1145552	1351524	1351892	1865759	1866127	1350216	1350584
1578	BPSL1474	32	1446137	1447840	1333160	1334863	1341802	1343505	1437004	1438707	1131295	1129592	17647	15944	1143457	1145160	1349797	1351500	1864032	1865735	1348489	1350192
1579	BPSL1475	32	1445594	1445965	1332617	1332988	1341259	1341630	1436461	1436832	1131838	1131467	18190	17819	1142914	1143285	1349254	1349625	1863489	1863860	1347946	1348317
1580	BPSL1476	32	1445255	1445064	1332278	1332087	1340920	1340729	1436122	1435931	1132177	1132368	18529	18720	1142575	1142384	1348915	1348724	1863150	1862959	1347607	1347416
1581	BPSL1477	32	1443968	1442643	1330991	1329666	1339633	1338308	1434835	1433510	1133464	1134789	19816	21141	1141288	1139963	1347628	1346303	1861863	1860538	1346320	1344995
1582	BPSL1478	32	1442632	1441184	1329655	1328207	1338297	1336849	1433499	1432051	1134800	1136248	21152	22600	1139952	1138504	1346292	1344844	1860527	1859079	1344984	1343536
1583	BPSL1479	32	1440441	1439116	1327137	1325812	1335397	1334072	1431284	1429959	1137061	1138386	23343	24668	1137761	1136436	1343667	1342342	1858336	1857011	1342682	1341357
1584	BPSL1480	32	1439091	1438837	1325787	1325533	1334047	1333793	1429934	1429680	1138411	1138665	24693	24947	1136411	1136157	1342317	1342063	1856986	1856732	1341332	1341078
1585	BPSL1481	32	1438823	1438338	1325519	1325034	1333779	1333294	1429666	1429181	1138679	1139164	24961	25446	1136143	1135658	1342049	1341564	1856718	1856233	1341064	1340579
1586	BPSL1482	32	1437471	1438010	1324167	1324706	1332427	1332966	1428314	1428853	1140031	1139492	26313	25774	1134791	1135330	1340697	1341236	1855366	1855905	1339712	1340251
1587	BPSL1483	32	1437287	1436793	1323983	1323489	1332243	1331749	1428130	1427636	1140215	1140709	26497	26991	1134607	1134113	1340513	1340019	1855182	1854688	1339528	1339034
1588	BPSL1484	32	1436619	1433992	1323315	1322542	1331575	1328948	1427462	1424835	1140883	1143510	27165	29792	1133939	1131312	1339845	1337218	1854514	1851887	1338860	1336233
1589	BPSL1485	32	1433328	1433747	1319811	1320230	1328284	1328703	1424171	1424590	1144174	1143755	30456	30037	1130648	1131067	1336554	1336973	1851223	1851642	1335569	1335988
1590	BPSL1486	32	1432554	1433246	1319037	1319729	1327510	1328202	1423397	1424089	1144948	1144256	31230	30538	1129874	1130566	1335796	1335924	1850449	1851141	1334795	1335487
1591	BPSL1487	32	1432285	1431794	1318768	1318277	1327241	1326750	1423128	1422637	1145217	1145708	31499	31990	1129605	1129114	1335527	1335036	1850180	1849689	1334516	1334025
1592	BPSL1488	32	1431842	1431447	1318325	1317930	1326798	1326403	1422685	1422290	1145660	1146055	31942	32337	1129162	1128767	1335084	1334689	1849737	1849342	1334073	1333696
1593	BPSL1489	32	1429696	1431048	1316181	1317533	1324654	1326006	1420541	1421893	1147804	1146452	34086	32734	1127018	1128370	1332940	1334292	1847593	1848945	1331947	1333299
1594	BPSL1490	32	1427854	1429689	1314339	1316174	1322812	1324647	1418699	1420534	1149646	1147811	35928	34093	1125176	1127011	1331098	1332933	1845751	1847586	1330105	1331940
1595	BPSL1491	32	1427457	1427717	1313942	1314202	1322415	1322675	1418302	1418562	1150043	1149783	36325	36065	1124779	1125039	1330701	1330961	1845354	1845614	1329708	1329968
1596	BPSL1492	32	1426397	1427239	1312882	1313724	1321355	1322197	1417242	1418084	1151103	1150261	37385	36543	1123719	1124561	1329641	1330483	1844294	1845136	1328648	1329490
1597	BPSL1493	32	1424798	1426351	1311283	1312836	1319756	1321309	1415643	1417196	1152702	1151149	38984	37431	1122120	1123673	1328042	1329595	1842695	1844248	1327049	1328602
1598	BPSL1494	32	1424301	1424798	1310786	1311283	1319259	1319756	1415146	1415643	1153199	1152702	39481	38984	1121623	1122120	1327545	1328042	1842198	1842695	1326552	1327049
1599	BPSL1495	32	1422902	1424161	1309387	1310646	1317860	1319119	1413747	1415006	1154598	1153339	40880	39621	1120224	1121483	1326146	1327405	1840799	1842058	1325153	1326412
1600	BPSL1496	32	1422713	1422835	1309198	1309320	1317671	1317793	1413558	1413680	1154787	1154665	41069	40947	1120035	1120157	1325957	1326079	1840610	1840732	1324964	1325086
1601	BPSL1497	32	1422380	1422703	1308865	1309188	1317338	1317661	1413225	1413548	1155120	1154797	41402	41079	1119702	1120025	1325624	1325947	1840277	1840600	1324631	1324954
1602	BPSL1498	32	1421716	1419242	1308201	1306906	1316674	1315157	1411179	1410487	1155784	1158258	42066	44540	1119038	1116564	1323466	1322777	1839613	1837139	1323967	1322717
1603	BPSL1499	32	1419142	1418819	1305423	1305100	1313355	1313032	1410387	1410064	1158358	1158681	44640	44963	1116464	1116141	1322677	1322354	1837039	1836716	1321231	1320908
1604	BPSL1500	32	1418790	1418185	1305071	1304466	1313003	1312398	1410035	1409430	1158710	1159315	44992	45597	1116112	1115507	1322325	1321720	1836687	1836082	1320879	1320457
1605	BPSL1501	32	1418112	1416910	1304393	1303191	1312325	1311123	1409357	1408155	1159388	1160590	45670	46872	1115434	1114232	1321647	1320445	1836009	1834807	1320200	1318998
1606	BPSL1502	32	1416802	1416044	1303083	1302325	1311015	1310257	1408047	1407289	1160698	1161471	46980	47738	1114124	1113366	1320337	1319579	1834699	1833941	1318890	1318132
1607	BPSL1503	32	1416044	1415079	1302325	1301360	1310257	1309292	1407289	1406324	1161471	1162436	47738	48703	1113366	1112401	1319579	1318614	1833941	1832976	1318132	1317167
1608	BPSL1504	32	1415070	1414177	1301351	1300458	1309283	1308390	1406315	1405422	1162445	1163338	48712	49605	1112392	1111499	1318605	1317712	1832967	1832074	1317158	1316265
1609	BPSL1505	32	1414162	1413086	1300443	1299367	1308375	1307299	1405407	1404331	1163353	1164429	49620	50696	1111484	1110408	1317697	1316621	1832059	1830983	1316250	1315174
1610	BPSL1506	32	1413075	1412302	1299356	1298583	1307288	1306515	1404320	1403547	1164440	1165213	50707	51480	1110397	1109624	1316610	1315837	1830972	1830199	1315163	1314390
1611	BPSL1507	32	1411265	1412122	1297546	1298403	1305478	1306335	1402510	1403367	1166250	1165393	52517	51660	1108587	1109444	1314800	1315657	1829162	1830019	1313353	1314210
1612	BPSL1508	32	1411187	1409793	1297468	1296074	1305400	1304006	1402432	1401038	1166328	1167722	52595	53989	1108509	1107115	1314722	1313328	1829084	1827690	1313275	1311881
1613	BPSL1509	32	1409003	1409713	1295284	1295994	1303216	1303926	1400248	1400958	1168512	1167802	54779	54069	1106325	1107035	1312538	1313248	1826900	1827610	1311091	1311801
1614	BPSL1510	32	1408769	1408347	1295050	1294628	1302982	1302560	1400014	1399592	1168746	1169168	55013	55435	1106091	1105669	1312304	1311882	1826666	1826244	1310857	1310435
1615	BPSL1511	32	1408302	1407169	1294583	1293450	1302515	1301382	1399547	1398414	1169213	1170346	55480	56613	1105624	1104491	1311837	1310704	1826199	1825066	1310390	1309257
1616	BPSL1512	32	1407024	1405939	1293305	1292220	1301237	1300152	1398269	1397184	1170491	1171576	56758	57843	1104346	1103261	1310559	1309474	1824921	1823836	1309112	1308027
1617	BPSL1513	32	1405792	1404545	1292073	1290826	1300005	1298758	1397037	1395790	1171723	1172970	57990	59237	1103114	1101867	1309327	1308080	1823689	1822442	1307880	1306633
1618	BPSL1514	32	1404523	1403186	1290804	1289467	1298736	1297399	1395768	1394431	1172992	1174329	59259	60596	1101845	1100508	1308058	1306721	1822420	1821083	1306611	1305274

1619	BPSL1515		32	1403130	1402504	1289411	1288785	1297343	1296717	1394375	1393749	1174385	1175011	60652	61278	1100452	1099826	1306665	1306039	1821027	1820401	1305218	1304592
1620	BPSL1516		32	1402456	1401314	1288737	1287595	1296669	1295527	1393701	1392559	1175059	1176201	61326	62468	1099778	1098636	1305991	1304849	1820353	1819211	1304544	1303402
1621	BPSL1517		32	1401036	1399702	1287317	1285983	1295249	1293915	1392281	1390947	1176479	1177813	62746	64080	1098358	1097024	1304571	1303237	1818933	1817599	1303124	1301790
1622	BPSL1518		32	1399564	1399328	1285845	1285609	1293777	1293541	1390809	1390573	1177951	1178187	64218	64454	1096886	1096650	1303099	1302863	1817461	1817225	1301652	1301416
1623	BPSL1519		32	1399268	1398093	1285549	1284374	1293481	1292306	1390513	1389338	1178247	1179422	64514	65689	1096590	1095415	1302803	1301628	1817165	1815990	1301356	1300181
1624	BPSL1520		32	1398044	1396698	1284325	1282979	1292257	1290911	1389289	1387943	1179471	1180817	65738	67084	1095366	1094020	1301579	1300233	1815941	1814595	1300132	1298792
1625	BPSL1521		32	1396678	1395782	1282959	1282063	1290891	1289995			1180837	1181733	67104	68000	1094000	1093104	1300213	1299317	1814575	1813679	1298753	1297866
1626	BPSL1522		32	1395754	1395566	1282035	1281847	1289967	1289779	1387140	1386952	1181761	1181949	68028	68216	1093076	1092888	1299289	1299101	1813651	1813463	1297838	1297650
1627	BPSL1523		32	1395451	1394306	1281732	1280587	1289664	1288519	1386837	1385692	1182064	1183209	68331	69476	1092773	1091628	1298986	1297841	1813348	1812203	1297535	1296390
1628	BPSL1524		32	1394178	1392835	1280459	1279116	1288391	1287048	1385564	1384221	1183337	1184680	69604	70947	1091500	1090157	1297713	1296370	1812075	1810732	1296262	1294919
1629	BPSL1525		32	1392795	1392235	1279076	1278516	1287008	1286448	1384181	1383621	1184720	1185280	70987	71547	1090117	1089557	1296330	1295770	1810692	1810132	1294879	1294319
1630	BPSL1526		32	1391941	1390052	1278222	1276333	1286154	1284265	1383327	1381438	1185574	1187463	71841	73730	1089263	1087374	1295476	1293587	1809838	1807949	1294025	1292136
1631	BPSL1527		32	1387490	1389811	1273771	1276092	1281703	1284024	1378876	1381197	1190025	1187704	76292	73971	1084812	1087133	1291025	1293346	1805387	1807708	1289574	1291895
1632	BPSL1528		32	1387152	1386604	1273433	1272885	1281365	1280817	1378538	1377990	1190363	1190911	76630	77178	1084474	1083926	1290687	1290139	1805049	1804501	1289236	1288688
1633	BPSL1529		32	1386507	1386280	1272788	1272561	1280720	1280493	1377893	1377666	1191008	1191235	77275	77502	1083829	1083602	1290042	1289815	1804404	1804177	1288591	1288364
1634	BPSL1530		32	1386016	1383959	1272297	1270240	1280229	1278172	1377402	1375345	1191499	1193556	77766	79823	1083338	1081281	1289551	1287494	1803913	1801856	1288100	1286043
1635	BPSL1531		32	1382956	1383777	1269237	1270058	1277169	1277990	1374342	1375163	1194559	1193738	80826	80005	1080278	1081099	1286491	1287312	1800853	1801674	1285040	1285861
1636	BPSL1532		32	1382722	1381718	1269003	1267999	1276935	1275931	1374108	1373104	1194793	1195797	81060	82064	1080044	1079040	1286257	1285253	1800619	1799615	1284820	1283996
1637	BPSL1533		32	1381702	1380875	1267983	1267156	1275915	1275088	1373088	1372261	1195813	1196640	82080	82907	1079024	1078197	1285237	1284410	1799599	1798772	1283980	1283153
1638	BPSL1534		32	1380355	1378544	1266636	1264825	1274568	1272757	1371741	1369930	1197160	1198971	83427	85238	1077677	1075866	1283890	1282079	1798252	1796441	1282633	1282256
1639	BPSL1535		32	1378444	1377266	1264725	1263547	1272657	1271479	1369830	1368652	1199071	1200249	85338	86516	1075766	1074588	1281979	1280801	1796341	1795163	1280720	1279542
1640	BPSL1536		32	1377137	1376400	1263418	1262681	1271350	1270613	1368523	1367786	1200378	1201115	86645	87382	1074459	1073722	1280672	1279935	1795034	1794297	1279413	1278676
1641	BPSL1537		32	1376260	1375691	1262541	1261972	1270473	1269904	1367646	1367077	1201255	1201824	87522	88091	1073582	1073013	1279795	1279226	1794157	1793588	1278536	1277967
1642	BPSL1538		32	1375105	1373729	1261386	1260010	1269318	1267942	1366491	1365115	1202410	1203786	88677	90053	1072427	1071051	1278640	1277264	1793002	1791626	1277381	1276005
1643	BPSL1539		32	1373723	1372800	1260004	1259081	1267936	1267013	1365109	1364186	1203792	1204715	90059	90982	1071045	1070122	1277258	1276335	1791620	1790697	1275999	1275076
1644	BPSL1540		32	1372701	1371520	1258982	1257801	1266914	1265733	1364087	1362906	1204814	1205995	91081	92262	1070023	1068842	1276236	1275055	1790598	1789417	1274977	1273946
1645	BPSL1541		32	1371500	1370922	1257781	1257203	1265713	1265135	1362886	1362308	1206015	1206593	92282	92860	1068822	1068244	1275035	1274457	1789397	1788819	1273843	1273421
1646	BPSL1542		32	1370618	1369437	1256899	1255718	1264831	1263650	1362004	1360823	1206897	1208078	93164	94345	1067940	1066759	1274153	1272972	1788551	1787370	1273153	1271972
1647	BPSL1543		32	1368325	1369167	1254606	1255448	1262538	1263380	1359711	1360553	1209190	1208348	95457	94615	1065647	1066489	1271860	1272702	1786258	1787100	1270860	1271702
1648	BPSL1544		32	1367472	1366930	1253753	1253211	1261685	1261143	1358858	1358316	1210035	1210577	96308	96850	1064787	1064245	1270970	1270431	1785367	1784825	1269982	1269440
1649	BPSL1545		32	1365525	1366340	1251806	1252621	1259738	1260553	1356911	1357726	1212080	1211265	98339	97524	1062721	1063536	1268942	1269757	1783392	1784207	1267965	1268780
1650	BPSL1546		32	1364833	1365525	1251114	1251806	1259046	1259738	1356219	1356911	1212772	1212080	99031	98339	1062029	1062721	1268250	1268942	1782700	1783392	1267273	1267965
1651	BPSL1547		32	1364699	1362210	1250980	1248491	1258912	1256423	1356085	1353596	1212906	1215395	99165	101654	1061895	1059406	1268116	1267460	1782566	1780077	1267139	1264650
1652	BPSL1548		32	1361749	1360160	1248030	1246441	1255962	1254373	1353135	1351546	1215856	1217445	102115	103704	1058945	1057356	1265248	1263659	1779616	1778027	1264189	1262600
1653	BPSL1549		32	1359376	1360008	1245657	1246289	1253589	1254221	1350762	1351394	1218229	1217597	104488	103856	1056572	1057204	1262875	1263507	1777243	1777875	1261816	1262448
1654	BPSL1550		32	1359060	1357627	1245341	1243908	1253273	1251840	1350446	1349013	1218545	1219978	104804	106237	1056256	1054823	1262559	1261126	1776927	1775494	1261500	1260067
1655	BPSL1551		32	1357425	1356238	1243706	1242519	1251638	1250451	1348811	1347624	1220180	1221367	106439	107626	1054621	1053434	1260924	1260802	1775292	1774105	1259865	1258678
1656	BPSL1552		32	1356162	1355521	1242443	1241802	1250375	1249734	1347548	1346907	1221443	1222084	107702	108343	1053358	1052717	1259660	1259019	1774029	1773388	1258602	1257961
1657	BPSL1553		32	1355189	1355389	1241470	1241670	1249402	1249602	1346575	1346775	1222416	1222216	108675	108475	1052385	1052585	1258687	1258887	1773056	1773256	1257629	1257829
1658	BPSL1554		32	1353471	1354775	1239752	1241056	1247684	1248988	1344857	1346161	1224127	1222823	110393	109089	1050667	1051971	1256969	1258273	1771338	1772642	1255911	1257215
1659	BPSL1555		32	1353084	1351993	1239365	1238274	1247297	1246206	1344470	1343379	1224514	1225605	110780	111871	1050280	1049189	1256582	1255491	1770951	1769860	1255524	1254433
1660	BPSL1556		32	1351875	1350742	1238156	1237023	1246088	1244955	1343261	1342128	1225723	1226856	111989	113122	1049071	1047938	1255373	1254240	1769742	1768609	1254315	1253182
1661	BPSL1557		32	1350742	1349816	1237023	1236097	1244955	1244029	1342128	1341202	1226856	1227782	113122	114048	1047938	1047012	1254240	1253314	1768609	1767683	1253182	1252256
1662	BPSL1558		32	1349816	1349001	1236097	1235282	1244029	1243214	1341202	1340387	1227782	1228597	114048	114863	1047012	1046197	1253314	1252499	1767683	1766868	1252256	1251441
1663	BPSL1559		32	1348585	1348743	1234866	1235024	1242798	1242956	1339971	1340129	1229013	1228855	115279	115121	1045781	1045939	1252083	1252241	1766452	1766610	1251025	1251183
1664		Chr1_71_IS407A_A		1347348	1348583	1233629	1234864	1241561	1242796	1338734	1339969	1229015	1230250	115281	116516	1044544	1045779	1250846	1252081	1765215	1766450	1249788	1251023
1665	BPSL1560																						
1666	BPSL1561																						
1667	BPSL1562																						
1668	BPSL1563																						
1669	BPSL1564																						

1670	BPSL1565																						
1671		Chr1_72_IS407A_A		999459	1000694	878580	879815	885069	886304	992740	993975	1619910	1621145					871391	872626			1412924	1414159
1672	BPSL1566		33	1003263	1001362	882384	880483	888873	886972	996544	994643	1617341	1619242					875195	873294			1410355	1412256
1673	BPSL1567		33	1006436	1003269	885557	882390	892046	888879	999717	996550	1614168	1617335					878368	875201			1407182	1410349
1674	BPSL1568		33	1007548	1006436	886669	885557	893158	892046	1000829	999717	1613056	1614168					879480	878368			1406070	1407182
1675	BPSL1569		33	1007739	1008374	886860	887495	893349	893984	1001020	1001655	1612865	1612230					879671	880306			1405879	1405244
1676	BPSL1570		33	1010505	1008673	889626	887794	896115	894283	1003786	1001954	1610099	1611931					882437	880605			1403113	1404945
1677	BPSL1571		33	1011589	1010522	890710	889643	897199	896132	1004870	1003803	1609015	1610082					883521	882454			1402029	1403096
1678	BPSL1572		33	1011700	1012590	890821	891711	897310	898200	1004981	1005871	1608904	1608014					883632	884483			1401918	1401028
1679	BPSL1573		33	1013101	1012658	892222	891779	898711	898268	1006382	1005939	1607503	1607946					884994	884551			1400517	1400960
1680	BPSL1574		33	1013325	1014086	892446	893207	898935	899696	1006606	1007367	1607279	1606518					885218	885979			1400293	1399532
1681	BPSL1575		33	1014173	1015159	893294	894280	899783	900769	1007454	1008440	1606431	1605445					886066	887052			1399445	1398459
1682	BPSL1576		33	1015247	1016521	894368	895642	900857	902131	1008528	1009802	1605357	1604083					887140	888414			1398371	1397097
1683	BPSL1577		33	1016529	1017503	895650	896624	902139	903113	1009810	1010784	1604075	1603101					888422	889396			1397089	1396115
1684	BPSL1578		33	1018104	1019144	897225	898265	903714	904754	1011385	1012425	1602500	1601460					889997	891037			1395514	1394474
1685	BPSL1579		33	1019205	1019990	898326	899111	904815	905600	1012486	1013271	1601399	1600614					891098	891883			1394413	1393628
1686	BPSL1580		33	1020008	1020367	899129	899488	905618	905977	1013289	1013648	1600596	1600237					891901	892260			1393610	1393251
1687	BPSL1581		33	1021170	1020739	900291	899860	906780	906349	1014451	1014020	1599434	1599865					893008	892577			1392481	1392912
1688	BPSL1582		33	1021502	1022383	900623	901504	907112	907993	1014783	1015664	1599102	1598221					893340	894221			1392149	1391268
1689	BPSL1583		33	1023339	1022827	902460	901948	908949	908437	1016620	1016108	1597265	1597777					895177	894665			1390319	1390831
1690	BPSL1584		33	1023997	1024134	903118	903255	909607	909744	1017278	1017415	1596607	1596470					895835	895972			1389661	1389524
1691	BPSL1586		33	1024154	1024939	903275	904060	909764	910549	1017435	1018220	1596450	1595665					895992	896777			1389504	1388719
1692	BPSL1588		33	1025263	1025721	904660	905118	912106	912360	1018544	1019002	1595341	1594883					897101	897559			1388395	1387937
1693	BPSL1589		33	1025779	1026858	905176	906255	912418	913497	1019060	1020139	1594825	1593746					897617	898696			1387879	1386800
1694	BPSL1590		33	1027373	1026894	906770	906291	914012	913533	1020654	1020175	1593231	1593710					899211	898732			1386285	1386764
1695	BPSL1591		33	1027674	1028771	907071	908168	914313	915410	1020955	1022052	1592930	1591833					899512	900609			1385984	1384887
1696	BPSL1592		33	1029597	1028980	908994	908377	916236	915619	1022878	1022261	1591007	1591624					901435	900818			1384061	1384678
1697	BPSL1592A		33	1029755	1029639	909152	909036	916394	916278	1023036	1022920	1590849	1590965					901593	901477			1383903	1384019
1698	BPSL1593		33	1029927	1030934	909324	910331	916566	917573	1023208	1024215	1590677	1589670					901765	902772			1383731	1383198
1699	BPSL1594		33	1031298	1030951	910695	910348	917937	917590	1024579	1024232	1589306	1589653					903136	902789			1382361	1382708
1700	BPSL1595		33	1031787	1032224	911184	911621	918426	918863	1025068	1025505	1588817	1588380					903625	904062			1381872	1381435
1701	BPSL1596		33	1033139	1032879	912536	912276	919778	919518	1026427	1026167	1587430	1587690					905068	904808			1380506	1380766
1702	BPSL1597		33	1033232	1033600	912629	912997	919871	920239	1026520	1026888	1587337	1586969					905161	905529			1380413	1380045
1703	BPSL1598		33	1036373	1033950	915770	913347	923012	920589	1029661	1027238	1584196	1586619					908305	905879			1377272	1379695
1704	BPSL1599		33	1038878	1036740	918275	916137	925517	923379	1032166	1030028	1581698	1583836					910817	908679			1374767	1376905
1705	BPSL1600		33	1039422	1038931	918819	918328	926061	925570	1032710	1032219	1581154	1581645					911361	910870			1374223	1374714
1706	BPSL1601		33	1039848	1039447	919245	918844	926487	926086	1033136	1032735	1580728	1581129					911787	911386			1373797	1374198
1707	BPSL1602		33	1040120	1039854	919517	919251	926759	926493	1033408	1033142	1580456	1580722					912059	911793			1373525	1373791
1708	BPSL1603		33	1040661	1040134	920058	919531	927300	926773	1033949	1033422	1579915	1580442					912600	912073			1372984	1373511
1709	BPSL1604		33	1041494	1040661	920891	920058	928133	927300	1034782	1033949	1579082	1579915					913433	912600			1372151	1372984
1710	BPSL1605		33	1042405	1041494	921802	920891	929044	928133	1035693	1034782	1578171	1579082					914344	913433			1371240	1372151
1711	BPSL1606		33	1043652	1042405	923049	921802	930291	929044	1036940	1035693	1576924	1578171					915591	914344			1369993	1371240
1712	BPSL1607		33	1045792	1043828	925189	923225	932281	930467	1039080	1037116	1574784	1576748					917731	915767			1367853	1369817
1713	BPSL1608		33	1046443	1047498	925840	926895	933881	934936	1039731	1040786	1574133	1573078					918382	919437			1367202	1366147
1714	BPSL1609		33	1047523	1048524	926920	927921	934961	935962	1040811	1041812	1573053	1572052					919462	920463			1366122	1365121
1715	BPSL1610		33	1048524	1049261	927921	928658	935962	936699	1041812	1042549	1572052	1571315					920463	921200			1365121	1364384
1716	BPSL1611		33	1049439	1051025	928836	930422	936877	938463	1042727	1044313	1571137	1569551					921378	922964			1364206	1362620
1717	BPSL1612		33	1051205	1051630	930602	931027	938643	939068	1044493	1044918	1569371	1568946					923144	923569			1362440	1362015
1718	BPSL1613		33	1051849	1053558	931246	932742	939287	940996	1046371	1046871	1568727	1567018					923788	925497			1361796	1360147
1719	BPSL1614		33	1054985	1053939	934849	933803	942423	941377	1048298	1047252	1565591	1566637					926924	925878			963839	962793
1720	BPSL1615		33	1055983	1055021	935847	934885	943421	942459	1049296	1048334	1564593	1565555					927922	926960			964837	963875

1721	BPSL1616		33	1057369	1055993	937233	935857	944807	943431	1050682	1049306	1563207	1564583			929308	927932		966223	964847
1722	BPSL1617		33	1058852	1057413	938716	937277	946290	944851	1052165	1050726	1561724	1563163			930791	929352		967706	966267
1723	BPSL1618		33	1059687	1059652	939551	939516	947125	947090	1053000	1052965	1560889	1560924			931626	931591		968541	968506
1724	BPSL1619		33	1061496	1061813	940508	940996	948082	948570	1053957	1054445	1559937	1559449			932583	933071		969498	969986
1725	BPSL1621		33	1061952	1062299	941816	942163	949390	949737	1055265	1055612	1558629	1558282			933891	934238		970806	971153
1726	BPSL1622		33	1062435	1063193	942299	943057	949873	950631	1055748	1056506	1558146	1557388			934374	935132		971289	972047
1727	BPSL1623		33	1064587	1063502	944451	943366	952025	950940	1057900	1056815	1555994	1557079			936526	935441		973441	972356
1728	BPSL1624		33	1065070	1064933	944934	944797	952508	952371	1058383	1058246	1555511	1555648			937009	936872		973924	973787
1729	BPSL1625		33	1065172	1065636	945036	945500	952610	953074	1058485	1058949	1555409	1554945			937111	937575		974026	974490
1730		Chr1_73_ISBma1_A	33	1065697	1067001	945561	946865	953135	954439	1059010	1060314	1553580	1554884			968620	969496		974551	975855
1731	BPSL1626		33	1068520	1068633	947971	948381	955545	955955	1061420	1061830	1552474	1552064			940046	940456		976961	977371
1732	BPSL1627		33	1068735	1069502	948599	949366	956173	956940	1062048	1062815	1551846	1551079			940674	941441		977589	978356
1733	BPSL1628		33	1069512	1072061	949376	951925	956950	959499	1062825	1065374	1551069	1548520			941451	944000		978366	980915
1734	BPSL1629		33	1072061	1073023	951925	952887	959499	960461	1065374	1066336	1548520	1547558			944000	944962		980915	981877
1735	BPSL1630		33	1073803	1074051	953667	953915	961241	961489	1067116	1067364	1546778	1546530			945742	945990		982657	982905
1736	BPSL1631		33																	
1737	BPSL1632		33	1078592	1079074	958456	958938	966030	966512	1071905	1072387	1541989	1541507			950699	951181		987194	987676
1738	BPSL1633		33	1079375	1080037	959239	959901	966813	967475	1072688	1073350	1541206	1540544			951482	952144		987977	988639
1739		Chr1_74_IS407A_A		1080184	1081105	960048	960969	967622	968543	1073497	1074418	1539476	1540397			952291	953526		988786	989707
1740	BPSL1634																			
1741	BPSL1635																			
1742	BPSL1636																			
1743	BPSL1637	GI8																		
1744	BPSL1638	GI8																		
1745	BPSL1639	GI8																		
1746	BPSL1642	GI8																		
1747	BPSL1643	GI8																		
1748	BPSL1644	GI8																		
1749	BPSL1645	GI8																		
1750	BPSL1646	GI8																		
1751	BPSL1647	GI8																		
1752	BPSL1648	GI8																		
1753	BPSL1649	GI8																		
1754	BPSL1650	GI8																		
1755	BPSL1651	GI8																		
1756	BPSL1652	GI8																		
1757	BPSL1653	GI8																		
1758	BPSL1654	GI8																		
1759	BPSL1655	GI8																		
1760	BPSL1656	GI8																		
1761	BPSL1657	IS407A																		
1762	BPSL1658	IS407A																		
1763	BPSL1658a	GI8																		
1764	BPSL1659	GI8																		
1765	BPSL1660	GI8																		
1766	BPSL1661	GI8																		
1767	BPSL1662	GI8																		
1768	BPSL1663	GI8																		
1769	BPSL1664	GI8																		
1770	BPSL1665	GI8																		
1771	BPSL1666	GI8																		

1772	BPSL1667	GI8																					
1773	BPSL1668	GI8																					
1774	BPSL1669	GI8																					
1775	BPSL1670	GI8																					
1776	BPSL1671	GI8																					
1777	BPSL1672	GI8																					
1778	BPSL1673	GI8																					
1779	BPSL1674	GI8																					
1780	BPSL1675	GI8																					
1781	BPSL1676	GI8																					
1782	BPSL1677	GI8																					
1783	BPSL1678	GI8																					
1784	BPSL1679	GI8																					
1785	BPSL1680	GI8																					
1786	BPSL1681	GI8																					
1787	BPSL1682	GI8																					
1788	BPSL1683	GI8																					
1789	BPSL1684	GI8																					
1790	BPSL1685	GI8																					
1791	BPSL1686	GI8																					
1792	BPSL1687	GI8																					
1793	BPSL1688	GI8																					
1794	BPSL1689	GI8																					
1795	BPSL1690	GI8																					
1796	BPSL1691	GI8																					
1797	BPSL1692	GI8																					
1798	BPSL1693	GI8																					
1799	BPSL1694	GI8																					
1800	BPSL1695	GI8																					
1801	BPSL1696	GI8																					
1802	BPSL1697	GI8																					
1803	BPSL1699	GI8																					
1804	BPSL1700	GI8																					
1805	BPSL1702	GI8																					
1806	BPSL1703	GI8																					
1807	BPSL1704	GI8																					
1808	BPSL1704a	GI8																					
1809	BPSL1705	GI8																					
1810	BPSL1706	GI8																					
1811	BPSL1707	GI8																					
1812	BPSL1708	GI8																					
1813	BPSL1708A	GI8																					
1814	BPSL1709	GI8																					
1815		Chr1_75_IS407A_A		1080184	1081105	960048	960969	967622	968543	1073497	1074418	1539476	1540397	156544	157465	998173	999094	1205181	1206102	1454166	1455087	988786	989707
1816	BPSL1710		34	1084003	1081634	963867	961498	971441	969072	1077316	1074947	1536579	1538948	160365	157996	1001991	999622	1209001	1206632	1457986	1455617	992605	990236
1817	BPSL1711		34	1085327	1084131	965191	963995	972765	971569	1078640	1077444	1535255	1536451	161689	160493	1003315	1002119	1210325	1209129	1459310	1458114	993929	992733
1818		Chr1_76_IS407A_A		1085323	1086558	965187	966422	972761	973996	1078636	1079871	1534024	1535259	161685	162920	1003311	1004546	1210321	1211556	1459306	1460541	993925	995160
1819	BPSL1712																						
1820	BPSL1713																						
1821	BPSL1714																						
1822	BPSL1715																						

1823	BPSL1716																						
1824	BPSL1717																						
1825	BPSL1718																						
1826	BPSL1719																						
1827	BPSL1720																						
1828	BPSL1721																						
1829	BPSL1722																						
1830	BPSL1723																						
1831		Chr1_77_IS407A_A		1160172	1161407	1040287	1041522	1049331	1050566	1153315	1154550	1459172	1460407	234555	235790	925312	926547	1132308	1133543	1534145	1535380	1068513	1069748
1832	BPSL1724		35	1162381	1161410	1042496	1041525	1051540	1050569	1155524	1154553	1458198	1459169	236764	235793	924338	925309	1131334	1132305	1536354	1535383	1070722	1069751
1833	BPSL1725		35	1163327	1162446	1043442	1042561	1052486	1051605	1156470	1155589	1457252	1458133	237710	236829	923392	924273	1130388	1131269	1537300	1536419	1071668	1070787
1834	BPSL1726		35	1163538	1163317	1043653	1043432	1052697	1052476	1156681	1156460	1457041	1457262	237921	237700	923181	923402			1537511	1537290	1071879	1071658
1835	BPSL1727		35	1164582	1164965	1044697	1045080	1053741	1054124	1157725	1158108	1455997	1455614	238965	239348	922137	921754	1129132	1128749	1538556	1538939	1072925	1073308
1836	BPSL1728		35	1168085	1166865	1048200	1046980	1057244	1056024	1161092	1159872	1452494	1453714	242468	241248	918634	919854	1125629	1126849	1542059	1540839	1076428	1075208
1837	BPSL1729		35	1168433	1169230	1048548	1049345	1057592	1058389	1161440	1162237	1452146	1451349	242816	243613	918286	917489	1125281	1124484	1542407	1543204	1076776	1077498
1838	BPSL1730		35	1169398	1170333	1049513	1050448	1058557	1059492	1162405	1163340	1451181	1450246	243781	244716	917321	916386	1124316	1123381	1543372	1544307	1077712	1078647
1839	BPSL1731		35	1171039	1170530	1051154	1050645	1060198	1059689	1164046	1163537	1449540	1450049	245422	244913	915680	916189	1122675	1123184	1545013	1544504	1079353	1078844
1840	BPSL1732		35	1172819	1171080	1052934	1051195	1061978	1060239	1165826	1164087	1447760	1449499	247202	245463	913900	915639	1120895	1122634	1546793	1545054	1081133	1079394
1841	BPSL1733		35	1173720	1173535	1053835	1053650	1062879	1062694	1166727	1166542	1446859	1447044	248103	247918	912999	913184	1119994	1120179	1547694	1547509	1082034	1081849
1842	BPSL1734		35	1172072	1172104	1054175	1056205	1063219	1065249	1167067	1169097	1446519	1444489	248443	250473	912659	910629	1119654	1118254	1548034	1550064	1082374	1084404
1843	BPSL1735		35	1176164	1177564	1056279	1057679	1065323	1065391	1169171	1170571	1444415	1443015	250547	251947	910555	909155	1117498	1116098	1550138	1551538	1084478	1085878
1844	BPSL1736		35	1178822	1178055	1058937	1058170	1068651	1067884	1171845	1171078	1441854	1442621	253117	252350	908089	908856	1114936	1115703	1552812	1552045	1087384	1086617
1845	BPSL1737		35	1179239	1180174	1059354	1060289	1069068	1070003	1172262	1173197	1441459	1440524	253490	254425	907716	906781	1114563	1113628	1553196	1554131	1087768	1088703
1846	BPSL1738		35	1180234	1181391	1060349	1061506	1070063	1071220	1173257	1174414	1440464	1439307	254485	255642	906721	905564	1113568	1112411	1554191	1555348	1088763	1089920
1847	BPSL1739		35	1181391	1182326	1061506	1062441	1071220	1072155	1174414	1175349	1439307	1438372	255642	256577	905564	904629	1112411	1111476	1555348	1556283	1089920	1090849
1848	BPSL1740		35	1182326	1183141	1062441	1063256	1072155	1072970	1175349	1176164	1438372	1437557	256577	257392	904629	903814	1111476	1110661	1556283	1557098	1090856	1091671
1849	BPSL1741		35	1184367	1184696	1064482	1064811	1074196	1074525	1177390	1177719	1436331	1436002	258618	258947	902588	902259	1109435	1109106	1558324	1558653	1092897	1093226
1850	BPSL1742		35	1184860	1186317	1064975	1066432	1074689	1076146	1177883	1179340	1435838	1434381	259111	260568	902095	900638	1108942	1107485	1558817	1560274	1093390	1094847
1851	BPSL1743		35	1186338	1187591	1066453	1067706	1076167	1077420	1179361	1180614	1434360	1433107	260589	261842	900617	899364	1107464	1106211	1560295	1561548	1094868	1096121
1852	BPSL1744		35	1187659	1188666	1067774	1068781	1077488	1078495	1180682	1181689	1433039	1432032	261910	262917	899296	898289	1106143	1105136	1561616	1562623	1096189	1097196
1853	BPSL1745		35	1188758	1189693	1068873	1069808	1078587	1079522	1181781	1182716	1431940	1431005	263009	263944	898197	897262	1105044	1104109	1562715	1563650	1097288	1098223
1854	BPSL1746		35	1190733	1189942	1070848	1070057	1080562	1079771	1183756	1182965	1429965	1430756	264984	264193	896222	897013	1103069	1103860	1564690	1563899	1099263	1098472
1855	BPSL1747		35	1191179	1190733	1071294	1070848	1081008	1080562	1184202	1183756	1429519	1429965	265430	264984	895776	896222	1102623	1103069	1565136	1564690	1099709	1099263
1856	BPSL1748		35	1191277	1192194	1071392	1072309	1081106	1082023	1184300	1185217	1429421	1428504	265528	266445	895678	894761	1102525	1101608	1565234	1566151	1099807	1100724
1857	BPSL1749		35	1192482	1193183	1072597	1073298	1082311	1083012	1185505	1186206	1428216	1427515	266733	267434	894473	893772	1101320	1100619	1566439	1567140	1101012	1101713
1858	BPSL1750		35	1193739	1194272	1073854	1074387	1083568	1084101	1186762	1187295	1426959	1426426	267990	268523	893216	892683	1100063	1099530	1567696	1568229	1102269	1102802
1859	BPSL1751		35	1194498	1195610	1074613	1075725	1084327	1085439	1187521	1188633	1426200	1425088	268749	269861	892457	891345	1099304	1098192	1568455	1569567	1103028	1104140
1860	BPSL1752		35	1196402	1195911	1076517	1076026	1086231	1085740	1189425	1188934	1424296	1424787	270653	270162	890553	891044	1097400	1097891	1570359	1569868	1104932	1104441
1861	BPSL1753		35	1196526	1197707	1076641	1076961	1086355	1086834	1189549	1190730	1424172	1422991	270777	271958	890429	889248	1097276	1096095	1570483	1571664	1105056	1106237
1862	BPSL1754		35	1197824	1199392	1078561	1080129	1088514	1090082	1190847	1192415	1422874	1421306	272075	273643	889131	887563	1095978	1094410	1571781	1573349	1106354	1107922
1863	BPSL1755		35	1200199	1199477	1080936	1080214	1090889	1090167	1193222	1192500	1420499	1421221	274450	273728	886756	887478	1093603	1094325	1574156	1573434	1108729	1108007
1864	BPSL1756		35	1201266	1200541	1082003	1081278	1091956	1091231			1419432	1420157	275517	274792	885689	886414	1092536	1093261	1575223	1574498	1109738	1109511
1865	BPSL1757		35	1202360	1201272	1083097	1082009	1093050	1091962	1195219	1194131	1418338	1419426	276609	276511	884631	885683	1091444	1091542	1576317	1575229	1110832	1109744
1866	BPSL1758		35	1203586	1202363	1084323	1083100	1094276	1093053	1196445	1195222	1417112	1418335	277835	276612	883371	884594	1090218	1091441	1577543	1576320	1112058	1110835
1867	BPSL1759		35	1203984	1205429	1084721	1086166	1094674	1095645	1196843	1198288	1416714	1415269	278233	279678	882973	881528	1089820	1088375	1577941	1579386	1112316	1113254
1868	BPSL1760		35	1205425	1206048	1086162	1086785	1096767	1097390	1198284	1198907	1415273	1414650	279674	280297	881532	880909	1088379	1087756	1579382	1580005	1113362	1113985
1869	BPSL1761		35	1206048	1206779	1086785	1087516	1097390	1098121	1198907	1199638	1414650	1413919	280297	281028	880909	880178	1087756	1087025	1580005	1580736	1113985	1114716
1870	BPSL1762		35	1206779	1208620	1087516	1089357	1098121	1099962	1200456	1201157	1413919	1412072	281028	282875	880178	878331	1087025	1085178	1580736	1582583	1114716	1115315
1871	BPSL1763		35	1208955	1210325	1089692	1091062	1100297	1101667	1201492	1202862	1411737	1410367	283210	284580	877996	876626	1084843	1083473	1582918	1584288	1115662	1117032
1872	BPSL1764		35	1210691	1211014	1091428	1091751	1102035	1102358	1203228	1203551	1410001	1409678	284946	285269	876260	875937	1083107	1082784	1584654	1584977	1117392	1117715
1873	BPSL1765		35	1212047	1211364	1092784	1092101	1103391	1102708	1204584	1203901	1408645	1409328	286302	285619	874904	875587	1081751	1082434	1586010	1585327	1118748	1118065

1874	BPSL1766		35	1212989	1212378	1093726	1093115	1104333	1103722	1205530	1204943	1407703	1408314	287244	286633	873962	874573	1080809	1081420	1586952	1586341	1119690	1119079
1875	BPSL1767		35	1214254	1213073	1094991	1093810	1105598	1104417	1206652	1205759	1406396	1407619	288551	287328	872655	873878	1079502	1080725	1588259	1587036	1120997	1119774
1876	BPSL1768		35	1218578	1218045	1099315	1098782	1109922	1109389	1210976	1210443	1402072	1402605	292875	292342	868331	868864	1075178	1075711	1592583	1592050	1125321	1124788
1877	BPSL1769		35	1219718	1218648	1100455	1099385	1111062	1109992	1212116	1211046	1400932	1402002	294015	292945	867191	868261	1074038	1075108	1593723	1592653	1126461	1125391
1878	BPSL1770		35	1220901	1219843	1101638	1100580	1112245	1111187	1213299	1212241	1399749	1400807	295196	294279	866010	866927	1072857	1073774	1594906	1593848	1127644	1126586
1879	BPSL1771		35	1221990	1222439	1103220	1103669	1113334	1113783	1214388	1214837	1398660	1398211	296285	296734	864921	864547	1071768	1071319	1595995	1596444	1129214	1129543
1880	BPSL1772		35	1222501	1223100	1103731	1104330	1113845	1114444	1214899	1215498	1398149	1397550	296796	297395	864412	863813	1071257	1070658	1596506	1597105	1129649	1130248
1881	BPSL1773		35	1223108	1224721	1104338	1105951	1114452	1116065	1215506	1217119	1397542	1395929	297403	299016	863805	862192	1070650	1069037	1597113	1598726	1130256	1131740
1882	BPSL1774		35	1225870	1225034	1107100	1106264	1117214	1116378	1218213	1217377	1394780	1395616	300165	299329	861043	861879	1067888	1068724	1599875	1599039	1132561	1131842
1883	BPSL1775		35	1228196	1225938	1109426	1107168	1119540	1117282	1220539	1218281	1392454	1394712	302491	300233	858717	860975	1065562	1067820	1602201	1599943	1134887	1132629
1884	BPSL1776		35	1229599	1228196	1110829	1109426	1120943	1119540	1221942	1220539	1391051	1392454	303894	302491	857314	858717	1064159	1065562	1603604	1602201	1136290	1134887
1885	BPSL1777		35	1234968	1229746	1116833	1113138	1127152	1123376	1225471	1222379	1385682	1390904	309263	304041	851945	857167	1058790	1064012	1608973	1603751	1140437	1138800
1886	BPSL1778		35	1244883	1234993	1126748	1116858	1137561	1130896	1235663	1227066	1375776	1385657	319169	309288	842039	851920	1048884	1058765	1618888	1608998	1149991	1143674
1887	BPSL1779		35	1245455	1247197	1127320	1129062	1138133	1139875	1237375	1239117	1375204	1373462	319741	321483	841467	839725	1048312	1046570	1619460	1621202	1150563	1152305
1888	BPSL1780		35	1247687	1248259	1129552	1130124	1140365	1140937	1239607	1240179	1372972	1372400	321973	322545	839235	838663	1046080	1045508	1621692	1622264	1152795	1153367
1889	BPSL1781		35	1249475	1248450	1131340	1130315	1142153	1141128	1241395	1240370	1371184	1372209	323852	322827	837335	838360	1044208	1045233	1623480	1622455	1154583	1153558
1890	BPSL1782		35	1250446	1249643	1132311	1131508	1143124	1142321	1242366	1241563	1370213	1371016	324823	324020	836364	837167	1043237	1044040	1624451	1623648	1155554	1154751
1891	BPSL1783		35	1252563	1250446	1134562	1134497	1145241	1143124	1244429	1242537	1368096	1370213	326940	324823	834247	836364	1041120	1043237	1626568	1624451	1157614	1155554
1892	BPSL1784		35	1253450	1252563	1135449	1134562	1146128	1145241	1245316	1244429	1367209	1368096	327827	326940	833360	834247	1040233	1041120	1627455	1626568	1158642	1157755
1893	BPSL1785		35	1254504	1253491	1136503	1135490	1147182	1146169	1246370	1245357	1366155	1367168	328881	327868	832306	833319	1039179	1040192	1628509	1627496	1159696	1158683
1894	BPSL1786		35	1254805	1254566	1136804	1136565	1147483	1147244	1246671	1246432	1365854	1366093	329182	328943	832005	832244	1038878	1039117	1628810	1628571	1159997	1159758
1895	BPSL1787		35	1255484	1254864	1137483	1136863	1148162	1147542	1247350	1246730	1365175	1365795	329861	329241	831326	831946	1038199	1038819	1629489	1628869	1160676	1160056
1896	BPSL1788		35	1257385	1256642	1139294	1138641	1150710	1150573	1249264	1249112	1363286	1364029	331750	331007	829437	830180	1036405	1036542	1631371	1630628	1162558	1161815
1897	BPSL1789		35	1257551	1257387	1141752	1140397	1152248	1150863	1250802	1249417	1361748	1363133	333288	331903	827899	829284	1034867	1036252	1632909	1631524	1164096	1162711
1898	BPSL1790		35	1260045	1258957	1142874	1141786	1153370	1152282	1251924	1250836	1360626	1361714	334410	333322	826777	827865	1033745	1034833	1634031	1632943	1165218	1164130
1899	BPSL1791		35	1261276	1260275	1144105	1143104	1154601	1153600	1253155	1252154	1359395	1360396	335641	334640	825546	826547	1032514	1033515	1635262	1634261	1166449	1165448
1900	BPSL1792		35	1262808	1261282	1145637	1144111	1156133	1154607	1254687	1253161	1357863	1359389	337173	335647	824014	825540	1030982	1032508	1636794	1635268	1167981	1166455
1901	BPSL1793		35	1263814	1262873	1146643	1145702	1157139	1156198	1255693	1254752	1356857	1357798	338179	337238	823008	823949	1029976	1030917	1637800	1636859	1168987	1168046
1902	BPSL1794		35	1264935	1263922	1147764	1146751	1158260	1157247	1256814	1255801	1355736	1356749	339300	338287	821887	822900	1028855	1029868	1638921	1637908	1170108	1169095
1903	BPSL1795		35	1265654	1266367	1148483	1149196	1158979	1159692	1257533	1258246	1355017	1354304	340060	340773	821127	820414	1028095	1027382	1639681	1640394	1170868	1171581
1904	BPSL1796		35	1266447	1267739	1149276	1150568	1159772	1161064	1258326	1259618	1354224	1352932	340853	342145	820334	819042	1027302	1026010	1640474	1641766	1171661	1172953
1905		Chr1_78_IS407A_A	35	1267713	1268948	1150542	1151777	1161038	1162273	1259592	1260734	1351723	1352958	342119	343354	817833	819068	1024801	1026036	1641740	1642975	1172927	1174162
1906	BPSL1797		35									1351249	1349492	343828	345585	817359	815602	1024327	1022570	1643449	1645206		
1907	BPSL1798		35									1348413	1349435	346664	345642	814523	815545	1021491	1022513	1646285	1645263		
1908	BPSL1799		35									1347667	1348413	347410	346664	813777	814523	1020745	1021491	1647031	1646285		
1909	BPSL1800		35									1345119	1347584							1649571	1647106		
1910	BPSL1801		35									1344484	1344993							1650206	1649697		
1911	BPSL1802		35									1341137	1342684							1653549	1652002		
1912	BPSL1803		35									1338015	1341137	348452	347670	812735	813517	1019703	1020485	1656671	1653549		
1913	BPSL1804		35									1336799	1337995	349668	348472	811519	812715	1018502	1019683	1657887	1656691		
1914	BPSL1805		35									1336655	1335987	349812	350480	811375	810707	1018358	1017690	1658031	1658699		
1915	BPSL1806		35									1335701	1335102	351117	352217	810070	808970	1017408	1016602	1659335	1660435		
1916	BPSL1807		35									1334314	1335054	353005	352265	808182	808922	1015814	1016554	1661223	1660483		
1917	BPSL1808		35									1333586	1334308	353733	353011	807454	808176	1015086	1015808	1661951	1661229		
1918	BPSL1809		35									1332372	1333184	354947	354135	806240	807052	1013872	1014684	1663180	1662368		
1919	BPSL1810		35									1331112	1332089	356207	355230	804980	805957	1012612	1013589	1664440	1663463		
1920	BPSL1811		35									1330640	1331065	356679	356254	804508	804933	1012140	1012565	1664912	1664487		
1921	BPSL1812		35									1329653	1330483	357666	356836	803521	804351	1011153	1011983	1665899	1665069		
1922	BPSL1813		35									1328613	1329554	358706	357765	802481	803422	1010113	1011054	1666939	1665998		
1923	BPSL1814		35									1327621	1328613	359698	358706	801489	802481	1008532	1009077	1667931	1666939		
1924	BPSL1815		35									1326059	1327621	361260	359698	799927	801489	1006970	1008532	1669493	1667931		

1925	BPSL1816		35									1324827	1326059	362492	361260	798695	799927	1005648	1006715	1670725	1669493		
1926	BPSL1817		35									1324403	1324813	362916	362506	798271	798681	1005224	1005634	1671149	1670739		
1927	BPSL1818		35									1323050	1324366	364269	362953	796918	798234	1003871	1005187	1672502	1671186		
1928	BPSL1819		35									1321834	1322883	365485	364436	795702	796751	1002655	1003704	1673718	1672669		
1929	BPSL1820		35									1321333	1321818	366016	365501	795171	795686	1002124	1002639	1674249	1673734		
1930	BPSL1821		35									1321116	1321304	366233	366045	794954	795142	1001907	1002095	1674466	1674278		
1931	BPSL1822		35									1319575	1320543	367780	366812	793407	794375	1000366	1001334	1676007	1675039		
1932	BPSL1823		35									1318739	1319575	368616	367780	792571	793407	999530	1000366	1676843	1676007		
1933	BPSL1824		35									1317142	1318185	370123	369080	790983	792026	997951	998994	1678431	1677388		
1934	BPSL1825		35									1315585	1316886	371680	370379	789426	790727	996394	997695	1679988	1678687		
1935	BPSL1826		35									1313589	1315112	373676	372153	787430	788953	994398	995921	1681984	1680461		
1936	BPSL1827		35									1312168	1313430	375097	373835	786009	787271	992977	994239	1683405	1682143		
1937	BPSL1828		35									1310122	1312041	377143	375224	783963	785882	990931	992850	1685451	1683532		
1938		Chr1_79_IS407A_A										1308729	1309964	377301	378536	782570	783805	989538	990773	1685609	1686844		
1939	BPSL1829																						
1940	BPSL1830																						
1941	BPSL1831																						
1942	BPSL1832																						
1943	BPSL1833																						
1944	BPSL1834																						
1945		Chr1_80_IS407A_A		1267713	1268948	1150542	1151777	1161038	1162273	1259592	1260734	1308729	1309964	377301	378536	782570	783805	989538	990773	1685609	1686844	1172927	1174162
1946	BPSL1835		35	1270243	1269320	1153072	1152149	1163568	1162645	1262029	1261106	1307434	1308357	379831	378908	781275	782198	988243	989166	1688139	1687216		
1947	BPSL1836		35	1271368	1270316	1154197	1153145	1164693	1163641	1263154	1262102	1306309	1307361	380956	379904	780150	781202	987118	988170	1689264	1688212		
1948	BPSL1837		35	1272260	1271385	1155089	1154214	1165585	1164710	1264046	1263171	1305417	1306292	381848	380973	779258	780133	986226	987101	1690156	1689281		
1949	BPSL1838		35	1273318	1272428	1156147	1155257	1166643	1165753	1265104	1264214	1304334	1304405	382906	382016	778188	779078	985144	986034	1691226	1690336		
1950	BPSL1839		35	1274434	1273454	1157680	1156742	1168647	1167667	1266209	1265229	1303167	1304147	384022	383042	777072	778052	983951	984931	1692342	1691362	1176709	1175729
1951	BPSL1840		35	1274791	1275435	1158037	1158681	1169004	1169648	1266566	1267210	1302810	1302166	384379	385023	776715	776071	983594	982950	1692699	1693343	1177066	1177710
1952	BPSL1841		35	1275501	1275824	1158747	1159070	1169714	1170037	1267276	1267599	1302100	1301777	385089	385412	776005	775682	982884	982561	1693409	1693732	1177776	1178099
1953	BPSL1842		35	1275861	1276592	1159107	1159838	1170074	1170805	1267636	1268367	1301740	1301009	385449	386180	775645	774914	982524	981793	1693769	1694500	1178136	1178867
1954	BPSL1843		35	1277504	1277016	1160750	1160262	1171717	1171229	1269279	1268791	1300097	1300585	387092	386604	774002	774490	980881	981369	1695412	1694924	1179779	1179291
1955		Chr1_81_IS407A_B	35	1277696	1278931	1160942	1162177	1171909	1173144	1269471	1270706	1298670	1299905										
1956	BPSL1844		35	1278968	1279885	1162214	1162750	1173181	1174098	1270743	1271660	1298633	1297716	387316	388233	773778	772861	980657	979740	1695636	1696553	1180003	1180920
1957	BPSL1845		35	1279893	1280726	1162918	1163751	1174106	1174939	1271668	1272501	1297708	1296875	388241	389074	772853	772020	979732	978899	1696561	1697394	1180928	1181761
1958	BPSL1846		35	1281305	1280811	1164330	1163836	1175518	1175024	1273080	1272586	1296296	1296790	389653	389159	771441	771935	978320	978814	1697973	1697479	1182340	1181846
1959	BPSL1847		35	1282606	1281548	1165631	1164573	1176819	1175761	1274381	1273323	1294995	1296053	390954	389896	770140	771198	977019	978077	1699274	1698216	1183641	1182583
1960	BPSL1848		35	1284014	1282965	1167039	1165990	1178227	1177178	1275789	1274740	1293587	1294636	392362	391313	768732	769781	975611	976660	1700682	1699633	1185049	1184000
1961	BPSL1849		35	1284736	1284293	1167761	1167318	1178949	1178506	1276511	1276068	1292865	1293308	393084	392641	768010	768453	974889	975332	1701404	1700961	1185771	1185328
1962	BPSL1850		35	1284909	1285382	1167934	1168407	1179122	1179595	1276684	1277157	1292692	1292219	393257	393730	767837	767364	974716	974243	1701577	1702050	1185944	1186417
1963	BPSL1851		35	1286577	1287881	1169602	1170906	1180790	1182094	1278352	1279656	1291033	1289729	394916	396220	766178	764874	973057	971753	1703236	1704540	1187603	1188907
1964	BPSL1852		35	1288473	1289489	1171498	1172514	1182686	1183702	1280248	1281264	1289137	1288121	396812	397828	764282	763266	971161	970145	1705123	1706139	1189490	1190506
1965		Chr1_82_ISBma1_A	35	1290138	1291176	1173163	1174201	1184351	1185389	1281913	1282951	1286434	1287472	398477	399353	761741	762617	968620	969496	1706788	1707664	1191155	1192031
1966		Chr1_83_IS407A_A	35	1290984	1292219	1174009	1175244	1185197	1186432	1282759	1283994	1285391	1286626	399323	400558	760536	761771	967415	968650	1707634	1708869	1192001	1193236
1967		Chr1_82-1_ISBma1_A	35	1292202	1292680	1175227	1175705	1186415	1186893	1283977	1284455	1284930	1285408	399914	401019	760075	761180	966954	968059	1708804	1709330	1193171	1193697
1968	BPSL1853		35	1293259	1293008	1176284	1176033	1187472	1187221	1285034	1284783	1284351	1284602	401598	401347	759496	759747	966375	966626	1709909	1709658	1195269	1195018
1969	BPSL1854		35	1293578	1293336	1176603	1176361	1187791	1187549	1285353	1285111	1284032	1284274	401917	401675	759177	759419	966056	966298	1710228	1709986	1195588	1195346
1970	BPSL1855		35	1293875	1293663	1176900	1176688	1188088	1187876	1285650	1285438	1283735	1283947	402214	402002	758880	759092	965759	965971	1710525	1710313	1195885	1195673
1971	BPSL1856		35	1294971	1293967	1177996	1176992	1189184	1188180	1286746	1285742	1282639	1283643	403310	402306	757784	758788	964663	965667	1711621	1710617	1196981	1195977
1972	BPSL1857		35	1295789	1294971	1178814	1177996	1190002	1189184	1287564	1286746	1281821	1282639	404128	403310	756966	757784	963845	964663	1712439	1711621	1197799	1196981
1973	BPSL1858		35	1296964	1295810	1179989	1178835	1191177	1190023	1288739	1287585	1280646	1281800	405303	404149	755791	756945	962670	963824	1713614	1712460	1198974	1197820
1974	BPSL1859		35	1297300	1297761	1180325	1180786	1191513	1191974	1289075	1289536	1280310	1279849	405639	406100	755455	754994	962334	961873	1713950	1714411	1199310	1199771
1975	BPSL1860		35	1299363	1298224	1182388	1181249	1193576	1192437	1291077	1290859	1278247	1279386	407702	406563	753392	754531	960265	961404	1716013	1714874	1201373	1200234

1976	BPSL1861		35	1299731	1299396	1182756	1182421	1193944	1193609	1291445	1291110	1277879	1278214	408070	407735	753024	753359	959897	960232	1716381	1716046	1201741	1201406
1977	BPSL1862		35	1300233	1299739	1183258	1182764	1194446	1193952	1291947	1291453	1277377	1277871	408572	408078	752522	753016	959395	959889	1716883	1716389	1202243	1201749
1978	BPSL1863		35	1300952	1301494	1183977	1184519	1195165	1195707	1292666	1293208	1276658	1276116	409291	409833	751803	751261	958676	958134	1717602	1718144	1202962	1203504
1979	BPSL1864		35	1301506	1302270	1184531	1185295	1195719	1196483	1293220	1293984	1276104	1275340	409845	410609	751249	750485	958122	957358	1718156	1718920	1203516	1204280
1980	BPSL1865		35	1302307	1303134	1185332	1186159	1196520	1197347	1294021	1294848	1275303	1274476	410646	411473	750448	749621	957321	956494	1718957	1719784	1204317	1205144
1981	BPSL1866		35	1303242	1304267	1186267	1187292	1197455	1198480	1294956	1295981	1274368	1273343	411581	412606	749513	748488	956386	955361	1719892	1720917	1205252	1206277
1982	BPSL1867		35	1304390	1305181	1187415	1188206	1198603	1199394	1296104	1296895	1273220	1272429	412729	413520	748365	747574	955238	954447	1721040	1721831	1206400	1207191
1983	BPSL1868		35	1305314	1305970	1188339	1188995	1199527	1200183	1297028	1297684	1272296	1271640	413653	414309	747441	746785	954314	953658	1721964	1722620	1207324	1207980
1984		Chr1_84a_IS407A_A		1306102	1307337	1189127	1190362	1200315	1201550	1297816	1299051	1270273	1271508	414441	415676	745418	746653	952291	953526	1722752	1723987	1209165	1210140
1985		Chr1_84b_IS407A_A												156544	157465	1003311	1004546	1210321	1211556				
1986	BPSL1869		36	1308468	1307488	1191493	1190513	1202681	1201701	1300182	1299202	1269142	1270122	155413	156393	1005677	1004697	1212687	1211707	1725118	1724138	1211271	1210291
1987	BPSL1870		36	1309281	1308571	1192306	1191596	1203494	1202784	1300995	1300285	1268329	1269039	154600	155310	1006490	1005780	1213500	1212790	1725931	1725221	1212084	1211374
1988	BPSL1871		36	1310314	1309622	1193339	1192647	1204527	1203835	1302028	1301336	1267296	1267988	153567	154259	1007523	1006831	1214533	1213841	1726964	1726272	1213117	1212425
1989	BPSL1872		36	1311482	1310586	1194507	1193611	1205695	1204799	1303196	1302300	1266128	1267024	152399	153295	1008691	1007795	1215701	1214805	1728132	1727236	1214285	1213389
1990	BPSL1873		36	1312599	1311859	1195624	1194884	1206812	1206072	1304313	1303573	1265011	1265751	151282	152022	1009808	1009068	1216818	1216078	1729244	1728504	1215397	1214657
1991	BPSL1874		36	1315265	1312734	1198290	1195759	1209478	1206947	1306979	1304448	1262345	1264876	148616	151147	1012474	1009943	1219484	1216953	1731910	1729379	1218063	1215532
1992		Chr1_85_ISBma2_A	36	1315507	1317079	1198532	1200104	1209720	1211292	1307221	1308793	1260531	1262103	146802	148374	1012716	1014290	1219726	1221298	1732152	1733022	1218305	1219877
1993		Chr1_86_IS407A_C	36																	1733018	1734253		
1994		Chr1_85-1_ISBma2_A																		1733431	1734964		
1995	BPSL1875		36	1319208	1317499	1202233	1200524	1213421	1211712	1310922	1309213	1258402	1260111	144673	146382	1016419	1014710	1223427	1221718	1737093	1735384	1222006	1220297
1996	BPSL1876		36	1319710	1321227	1202735	1203745	1213923	1215440	1311424	1312941	1257900	1256383	144171	142654	1016921	1018438	1223929	1225446	1737595	1739112	1222508	1224025
1997	BPSL1877		36	1321517	1323040	1205111	1206634	1215730	1217253	1313231	1314754	1256093	1254570	142364	140841	1018728	1020251	1225736	1227259	1739402	1740925	1224315	1225838
1998	BPSL1878		36	1325110	1323401	1208704	1206995	1219323	1217614	1316824	1315115	1252521	1254230	138820	140529	1022272	1020563	1229273	1227564	1742953	1741244	1227852	1226143
1999	BPSL1879		36	1325783	1325160	1209377	1208754	1219996	1219373	1317497	1316874	1251848	1252471	138147	138770	1022945	1022322	1229946	1229323	1743626	1743003	1228525	1227902
2000	BPSL1880		36	1326451	1325930	1210045	1209524	1220664	1220143	1318165	1317644	1251180	1251701	137479	138000	1023613	1023092	1230614	1230093	1744294	1743773	1229193	1228672
2001	BPSL1881		36	1326898	1328115	1210492	1211709	1221111	1222328	1318612	1319829	1250733	1249516	137032	135815	1024060	1025277	1231061	1232278	1744741	1745958	1229640	1230857
2002	BPSL1882		36	1328335	1329288	1211929	1212882	1222548	1223501	1320049	1321002	1249296	1248343	135595	134642	1025497	1026450	1232498	1233451	1746178	1747131	1231077	1232030
2003	BPSL1883		36	1329476	1331245	1213070	1214839	1223689	1225458	1321140	1322903	1248155	1246386	134454	132685	1026638	1028407	1233639	1235408	1747319	1749088	1232218	1233987
2004	BPSL1884		36	1331308	1332276	1214902	1215870	1225521	1226489	1322966	1323934	1246323	1245355	132622	131654	1028470	1029228	1235471	1236439	1749151	1750119	1234050	1235018
2005	BPSL1885		36	1332727	1332458	1216321	1216052	1226940	1226671	1324296	1324249	1244904	1245173	131203	131472	1029890	1029621	1236890	1236621	1750570	1750301	1235469	1235200
2006	BPSL1885A		36	1333032	1333640	1216626	1217234	1227245	1227853	1324601	1325209	1244599	1243958	130898	130224	1030195	1030836	1237195	1237836	1750875	1751516	1235774	1236415
2007	BPSL1886		36	1334525	1333797	1218119	1217391	1228738	1228010	1326094	1325366	1243073	1243801	129339	130067	1031721	1030993	1238721	1237993	1752401	1751673	1237300	1236572
2008	BPSL1887		36	1335989	1334601	1219583	1218195	1230202	1228814	1327558	1326170	1241609	1242997	127875	129263	1033185	1031797	1240185	1238797	1753865	1752477	1238764	1237376
2009	BPSL1888		36	1337712	1336009	1225163	1224543	1231925	1230222	1329281	1327578	1239886	1241589	126152	127855	1034908	1033205	1241908	1240205	1755588	1753885	1240487	1238784
2010	BPSL1889		36	1338208	1337825	1225659	1225276	1232421	1232038	1329594	1329394	1239390	1239773	125656	126039	1035404	1035021	1242337	1242017	1756075	1755701	1240827	1240600
2011	BPSL1890		36	1339195	1338233	1223226	1222507	1233408	1232446	1330683	1329721	1238403	1239365	124669	125631	1036391	1035429	1243267	1242440	1757062	1756100	1241635	1240931
2012	BPSL1891		36	1340221	1339205	1224252	1223236	1234434	1233418	1331502	1330693	1237377	1238393	123643	124659	1037417	1036401	1243633	1243277	1758088	1757072	1242661	1241645
2013	BPSL1892		36	1341003	1340221	1227284	1226904	1235216	1234434	1332389	1331607	1236595	1237377	122861	123643	1038199	1037417	1244501	1243737	1758870	1758088	1243443	1242661
2014	BPSL1893		36	1342472	1342179	1228753	1228460	1236685	1236392	1333858	1333565	1235126	1235419	121392	121685	1039668	1039375	1245970	1245677	1760339	1760046	1244912	1244619
2015	BPSL1894		36	1343714	1342479	1229995	1228760	1237927	1236692	1335100	1333865	1233884	1235119	120150	121385	1040910	1039675	1247212	1245977	1761581	1760346	1246154	1244919
2016	BPSL1895		36	1345106	1343784	1231387	1230065	1239319	1237997	1336492	1335170	1232492	1233814	118758	120080	1042302	1040980	1248604	1247282	1762973	1761651	1247546	1246224
2017	BPSL1896		36	1346069	1345173	1232350	1231454	1240282	1239386	1337455	1336559	1231529	1232425	117795	118691	1043265	1042369	1249567	1248671	1763936	1763040	1248509	1247613
2018	BPSL1897		36	1346614	1346150	1232895	1232431	1240827	1240363	1338000	1337536	1230984	1231448	117250	117714	1043810	1043346	1250112	1249648	1764481	1764017	1249054	1248590
2019	BPSL1898		36	1347111	1346614	1233392	1232895	1241324	1240827	1338497	1338000	1230487	1230984	116753	117250	1044307	1043810	1250609	1250112	1764978	1764481	1249551	1249054
2020	BPSL1899		36	1347349	1347197	1233630	1233478	1241562	1241410	1338735	1338583	1230249	1230401	116515	116667	1044545	1044393	1250847	1250695	1765216	1765064	1249789	1249637
2021		Chr1_87a_IS407A_A		1347348	1348583	1233629	1234864	1241561	1242796	1338734	1339969	1229015	1230250	115281	116516	1044544	1045779	1250846	1252081	1765215	1766450	1249788	1251023
2022		Chr1_87b_IS407A_A		1085323	1086558	965187	966422	972761	973996	1078636	1079871	1534024	1535259	161685	162920	998173	999094	1210321	1211556	1459306	1460541	993925	995160
2023	BPSL1900		37	1086597	1086559	966461	966423	974035	973997	1079910	1079872									1460580	1460542	995199	995161
2024	BPSL1901		37	1087017	1088567	966881	968431	974455	976005	1080330	1081880	1533565	1532015	162922	164451	998171	996651	1205179	1203641	1461000	1462550	995619	997169
2025	BPSL1902		37	1088620	1089882	968484	969746	976058	977320	1081933	1083195	1531962	1530700	164504	165766	996598	995336	1203588	1202326	1462603	1463865	997222	998484
2026	BPSL1903		37	1090036	1091712	969900	971576	977474	979150	1083349	1085025	1530546	1528870	165920	167596	995182	993506	1202172	1200496	1464019	1465695	998638	1000314

2027	BPSL1904		37	1091938	1092417	971802	972281	979376	979855	1085251	1085730	1528644	1528165	167822	168301	993280	992801	1200270	1199791	1465921	1466400	1000540	1001019
2028	BPSL1905		37	1093067	1092660	972931	972524	980505	980098	1086380	1085973	1527515	1527922	168951	168544	992151	992558	1199141	1199548	1467050	1466643	1001669	1001262
2029	BPSL1906		37	1094359	1093265	974223	973129	981797	980703	1087672	1086578	1526223	1527317	170243	169149	990859	991953	1197849	1198943	1468342	1467248	1002961	1001867
2030	BPSL1907		37	1095896	1094469	975760	974333	983334	981907	1089209	1087782	1524686	1526113	171780	170353	989322	990749	1196312	1197739	1469879	1468452	1004498	1003071
2031	BPSL1908		37	1097270	1095999	977134	975863	984708	983437	1090480	1090175	1523312	1524583	173154	171883	987948	989219	1194938	1196209	1471253	1469982	1005872	1004601
2032	BPSL1909		37	1100262	1097401	980126	977265	987700	984839	1093472	1090611	1520320	1523181	176146	173285	984956	987817	1191946	1194807	1474245	1471384	1008864	1006003
2033	BPSL1911		37	1102427	1100604	982291	980468	989865	988042	1095637	1093814	1518155	1519978	178311	176488	982791	984614	1189781	1191604	1476410	1474587	1011029	1009206
2034	BPSL1912		37	1102720	1103214	982584	983078	990158	990652	1095930	1096424	1517862	1517368	178604	179098	982498	982004	1189488	1188994	1476703	1477197	1011322	1011816
2035	BPSL1913		37	1103317	1104810	983181	984674	990755	992248	1096527	1098020	1517265	1515772	179201	180694	981901	980408	1188891	1187398	1477300	1478793	1011919	1013412
2036	BPSL1914		37	1104881	1106113	984745	985977	992319	993551	1098091	1098678	1515701	1514469	180765	181997	980337	979105	1187327	1186095	1478864	1480096	1013483	1014715
2037	BPSL1915		37	1106139	1107695	986003	987559	993577	995133	1099271	1100827	1514443	1512887	182023	183579	979079	977523	1186069	1184513	1480122	1481678	1014741	1016297
2038	BPSL1916		37	1108904	1107972	988768	987836	996342	995410	1102036	1101104	1511678	1512610	184788	183856	976314	977246	1183304	1184236	1482887	1481955	1017506	1016574
2039	BPSL1917		37	1109299	1108934	989163	988798	996737	996372	1102431	1102066	1511283	1511648	185183	184818	975919	976284	1182909	1183274	1483282	1482917	1017901	1017536
2040	BPSL1918		37	1112332	1109408	992196	989272	1000508	997584	1105475	1102551	1508248	1511172	188216	185292	972886	975810	1179876	1182800	1486315	1483391	1021018	1018094
2041	BPSL1919		37	1113899	1112427	993680	992739	1002075	1000603	1107042	1105570	1506681	1508153	189783	188311	971319	972791	1178309	1179781	1487882	1486410	1022585	1021113
2042	BPSL1920		37	1114357	1113899	994138	993680	1002533	1002075	1107500	1107042	1506223	1506681	190241	189783	970861	971319	1177851	1178309	1488340	1487882	1023043	1022585
2043	BPSL1921		37	1116326	1114665	996107	994446	1004916	1004602	1109469	1107808	1504254	1505915	192210	190549	968892	970553	1175881	1177542	1490309	1488648	1025012	1023351
2044	BPSL1922		37	1117718	1116393	997499	996174	1006308	1004983	1110861	1109536	1502862	1504187	193602	192277	967500	968825	1174489	1175814	1491701	1490376	1026177	1025335
2045	BPSL1923		37	1119039	1118140	998820	997921	1007629	1006730	1112182	1111283	1501541	1502440	194923	194024	966179	967078	1173168	1174067	1493022	1492123	1027498	1026599
2046	BPSL1924		37	1119461	1119856	999242	999637	1008051	1008446	1112604	1112999	1501119	1500724	195345	195740	965757	965362	1172746	1172351	1493444	1493839	1027920	1028315
2047	BPSL1925		37	1120098	1120319	999879	1000100	1008688	1008909	1113241	1113462	1500482	1500261	195982	196203	965120	964899	1172109	1171888	1494081	1494302	1028557	1028778
2048	BPSL1926		37	1120798	1120484	1000579	1000265	1009388	1009074	1113941	1113627	1499782	1500096	196682	196368	964420	964734	1171409	1171723	1494781	1494467	1029257	1028943
2049	BPSL1927		37	1120999	1121718	1000780	1001499	1009589	1010308	1114142	1114861	1499581	1498862	196883	197602	964219	963500	1171208	1170489	1494982	1495701	1029458	1030177
2050	BPSL1929		37	1121960	1122235	1001741	1002016	1010550	1010825	1115103	1115378	1498620	1498345	197844	198119	963258	962983	1170247	1169972	1495943	1496218	1030419	1030694
2051	BPSL1930		37	1122387	1122641	1002168	1002422	1010977	1011231	1115530	1115784	1498193	1497939	198271	198525	962831	962577	1169820	1169566	1496370	1496624	1030846	1031100
2052	BPSL1931		37	1122984	1122697	1002765	1002478	1011574	1011287	1116127	1115840	1497596	1497883	198868	198581	962234	962521	1169223	1169510	1496967	1496680	1031443	1031156
2053	partial	Chr1_88_ISBma1_B	37	1123080	1123337	1002861	1003118	1011670	1011927	1116223	1116480	1497242	1497500							1497063	1497359	1031539	1031835
2054		Chr1_89_IS407A_A	37	1123337	1124572	1003118	1004353	1011927	1013162	1116480	1117715	1496007	1497242	198950	200185	960917	962152	1167906	1169141	1497321	1498556	1031797	1033032
2055	partial	Chr1_90_ISBma2_A	37	1124052	1125266	1003833	1005047	1012642	1013856	1117195	1118409	1495313	1496527	199692	200879	960223	961410	1167212	1168399	1498063	1499250	1032539	1033726
2056	BPSL1933		37	1126550	1125327	1006331	1005108	1015140	1013917	1119693	1118470	1494029	1495252	202163	200940	958939	960162	1165928	1167151	1500534	1499311	1035010	1033787
2057	BPSL1934		37	1126879	1127076	1006660	1006857	1015469	1015666	1120022	1120219	1493700	1493503	202492	202980	958610	958122	1165599	1165111	1500863	1501351	1035339	1035827
2058		Chr1_91_IS407A_B	37	1127077	1128312	1006858	1008093	1015667	1016902	1120220	1121455	1492267	1493502										
2059	BPSL1935		37	1128756	1129820	1008537	1009601	1017346	1018410	1121899	1122963	1491823	1490759	203129	204193	957973	956909	1164962	1163898	1501500	1502564	1035976	1037040
2060	BPSL1936_putative membrane protein		37																				
2061		Chr1_92_IS407A_C	37																			1037784	1039019
2062	BPSL1937		37	1130448	1132565	1010229	1012346	1019038	1021155	1123591	1125708	1490131	1488014	204821	206938	956281	954164	1163270	1161153	1503192	1503305	1039019	1041025
2063		Chr1_93_IS407A_C	37																	1503308	1504543		
2064	BPSL1938		37	1133094	1132693	1012875	1012474	1021684	1021283	1126237	1125836	1487485	1487886	207467	207066	953635	954036	1160624	1161025	1507078	1506677	1041554	1041153
2065	BPSL1939		37	1133500	1133156	1013281	1012937	1022090	1021746	1126643	1126299	1487079	1487423	207873	207529	953229	953573	1160218	1160562	1507484	1507140	1041960	1041616
2066	BPSL1940		37	1136062	1133633	1015843	1013414	1024652	1022223	1129205	1126776	1484517	1486946	210435	208006	950667	953096	1157656	1160085	1510046	1507617	1044522	1042093
2067	BPSL1941		37	1137163	1136153	1016944	1015934	1025753	1024743	1130306	1129296	1483416	1484426	211536	210526	949566	950576	1156555	1157565	1511147	1510137	1045623	1044613
2068	BPSL1942		37	1137671	1137315	1017452	1017096	1026261	1025905	1130814	1130458	1482908	1483264	212044	211688	949058	949414	1156047	1156403	1511655	1511299	1046131	1045775
2069	BPSL1943		37	1137899	1137705	1017680	1017486	1026489	1026295	1131042	1130848	1482680	1482874	212272	212078	948830	949024	1155819	1156013	1511883	1511689	1046359	1046165
2070	BPSL1944		37	1138628	1138095	1018409	1017876	1027218	1026685	1131771	1131238	1481951	1482484	213001	212468	948101	948634	1155090	1155623	1512612	1512079	1047088	1046555
2071	BPSL1945		37	1140573	1138669	1020354	1018450	1029163	1027259	1133716	1131812	1480006	1481910	214946	213042	946156	948060	1153145	1155049	1514557	1512653	1049033	1047129
2072	BPSL1946		37	1143269	1141035	1023050	1020816	1031859	1029625	1136412	1134178	1477310	1479544	217642	215408	943460	945694	1150449	1152683	1517253	1515019	1051729	1049495
2073	BPSL1947		37	1143667	1143317	1023448	1023098	1032257	1031907	1136810	1136460	1476912	1477262	218040	217690	943062	943412	1150051	1150401	1517651	1517301	1051977	1051777
2074	BPSL1948		37	1144646	1143744	1024761	1023859	1033805	1032903	1137789	1136887	1475933	1476835	219019	218117	942083	942985	1149072	1149974	1518630	1517728	1052998	1052096
2075	BPSL1949		37	1145199	1145960	1025285	1025317	1034329	1034361	1138313	1138345	1475409	1475377	219539	219571	941563	941531	1148552	1148520	1519150	1519182	1053518	1053550
2076	BPSL1950		37	1146882	1145980	1026997	1026095	1036041	1035139	1140025	1139123	1473697	1474599	221251	220349	939851	940753	1146840	1147742	1520862	1519960	1055230	1054328
2077	BPSL1951		37	1147070	1147624	1027185	1027739	1036229	1036783	1140213	1140767	1473509	1472955	221439	221993	939663	939109	1146652	1146098	1521050	1521604	1055418	1055972

2078	BPSL1952		37	1147642	1148739	1027757	1028854	1036801	1037898	1140785	1141882	1472937	1471840	222011	223108	939091	937994	1146080	1144983	1521622	1522719	1055990	1057087
2079	BPSL1953		37	1148786	1149505	1028901	1029620	1037945	1038664	1141929	1142648	1471793	1471074	223155	223874	937947	937228	1144936	1144217	1522766	1523485	1057134	1057853
2080	BPSL1954		37	1150539	1149901	1030654	1030016	1039698	1039060	1143682	1143044	1470040	1470678	224922	224284	936180	936818	1143176	1143814	1524512	1523874	1058880	1058242
2081	BPSL1955		37	1151245	1150544	1031360	1030659	1040404	1039703	1144388	1143687	1469334	1470035	225628	224927	935474	936175	1142470	1143171	1525218	1524517	1059586	1058885
2082	BPSL1956		37	1151498	1152040	1031613	1032155	1040657	1041199	1144641	1145183	1469081	1468539	225881	226423	935221	934679	1142217	1141675	1525471	1526013	1059839	1060381
2083	BPSL1957		37	1152614	1154143	1032729	1034258	1041773	1043302	1145757	1147286	1467965	1466436	226997	228526	934105	932576	1141101	1139572	1526587	1528116	1060955	1062484
2084	BPSL1958		37	1155075	1156145	1035190	1036260	1044234	1045304	1148218	1149288	1465504	1464434	229458	230528	931644	930574	1138640	1137570	1529048	1530118	1063416	1064486
2085	BPSL1959		37	1156955	1156527	1037070	1036642	1046114	1045686	1150098	1149670	1463624	1464052	231338	230910	929764	930192	1136760	1137188	1530928	1530500	1065296	1064868
2086	BPSL1960		37	1157779	1157003	1037894	1037118	1046938	1046162	1150922	1150146	1462800	1463576	232162	231386	928940	929716	1135936	1136712	1531752	1530976	1066120	1065344
2087	BPSL1961		37	1157960	1159630	1038075	1039745	1047119	1048789	1151103	1152773	1462619	1460949	232343	234013	928759	927089	1135755	1134085	1531933	1533603	1066301	1067971
2088		Chr1_94a_IS407A_A		1160172	1161407	1040287	1041522	1049331	1050566	1153315	1154550	1459172	1460407	234555	235790	925312	926547	1132308	1133543	1534145	1535380	1068513	1069748
2089		Chr1_94b_IS407A_A		999459	1000694	878580	879815	885069	886304	992740	993975	1109938	1111173	414441	415676	745418	746653	871391	872626	1454166	1455087	952619	953854
2090	BPSL1962		38	997675	998781	876796	877902	883285	884391	990956	992062	1108154	1109260	417895	416789	743199	744305	869172	870278	1452373	1453479	949824	950930
2091	BPSL1963		38	997104	997307	874102	875835	880990	882723	988661	990394	1105859	1107592	420190	418457	740904	742637	866878	867636	1450078	1451811	947529	949262
2092	BPSL1964		38	994816	995277	873538	873999	880426	880887	988097	988558	1105295	1105756	420754	420293	740340	740801	866314	866775	1449514	1449975	946965	947426
2093	BPSL1965		38	994599	994282	873321	873004	880209	879892	987880	987563	1105078	1104761	420971	421288	740123	739806	866097	865780	1449297	1448980	946748	946431
2094	BPSL1966		38	992803	994104	871525	872826	878413	879714	986084	987385	1103282	1104583	422764	421466	738330	739628	864304	865602	1447501	1448802	944952	946253
2095	BPSL1967		38	992688	992083	871410	870805	878298	877693	985969	985364	1103167	1102562	422879	423484	738215	737610	864189	863584	1447386	1446781	944837	944232
2096	BPSL1968		38	992083	991637	870805	870359	877693	877247	985364	984918	1102562	1102116	423484	423930	737610	737164	863584	863138	1446781	1446335	944232	943786
2097	BPSL1969		38	991599	990181	870321	868903	877209	875791	984880	983462	1102078	1100660	423968	425386	737126	735708	863100	861682	1446297	1444879	943748	942330
2098	BPSL1970		38	990146	989166	868868	867888	875756	874776	983427	982447	1100625	1099645	425421	426401	735673	734693	861647	860667	1444844	1443864	942295	941315
2099	BPSL1971		38	987878	988816	866600	867538	873488	874426	981159	982097	1098357	1099295	427689	426751	733405	734343	859379	860317	1442576	1443514	940027	940965
2100	BPSL1972		38	987720	985957	866442	864679	873330	871567	981001	979238	1098199	1096451	427847	429604	733247	731490	859221	857464	1442418	1440679	939869	938130
2101	BPSL1973		38	985922	983724	864644	862446	871532	869334	979203	977005	1096428	1094230	429627	431825	731467	729269	857441	855243	1440656	1438458	938107	935909
2102	BPSL1974		38	983724	983524	862446	862246	869334	869134	977005	976805	1094230	1094030	431825	432025	729269	729069	855243	855043	1438458	1438258	935909	935709
2103	BPSL1975		38	983519	982629	862241	861351	869129	868239	976800	975910	1094025	1093135	432030	432920	729064	728174	855038	854148	1438253	1437363	935704	934814
2104	BPSL1976		38	980610	982283	859332	861005	866220	867893	973891	975564	1091125	1092789	434947	433274	726147	727820	852121	853794	1435336	1437009	932497	934170
2105	BPSL1977		38	980258	979011	858980	857733	865868	864621	973539	972292	1090773	1089526	435299	436546	725795	724548	851769	850522	1434984	1433737	932145	930898
2106	BPSL1978		38	977882	978667	856604	857389	863492	864277	971163	971948	1088373	1089158	437667	436882	723427	724212	849409	850194	1432624	1433409	929793	930578
2107	BPSL1979		38	975522	977882	854244	856604	861132	863492	968803	971163	1086013	1088373	440027	437667	721067	723427	847049	849409	1430264	1432624	927433	929793
2108	BPSL1980		38	975382	972518	854104	851240	860992	858128	968663	965799	1085873	1083009	440167	443031	720927	718063	846909	844045	1430124	1427260	927293	924429
2109	BPSL1981		38	972428	971550	851150	850272	858038	857160	965709	964831	1082919	1082041	443121	443999	717973	717095	843955	843077	1427170	1426292	924339	923461
2110	BPSL1982		38	970869	971483	849591	850205	856479	857093	964150	964764	1081360	1081974	444680	444066	716414	717028	842396	843010	1425611	1426225	922780	923394
2111	BPSL1983		38	968387	970777	847109	849499	853997	856387	961668	964058	1078878	1081268	447162	444772	713932	716322	839914	842304	1423129	1425519	920298	922688
2112	BPSL1984		38	967030	968373	845752	847095	852640	853983	960311	961654	1077521	1078864	448519	447176	712575	713918	838557	839900	1421772	1423115	918941	920284
2113	BPSL1985		38	966287	967012	845009	845734	851897	852622	959568	960293	1076778	1077503	449262	448537	711832	712557	837814	838539	1421029	1421754	918198	918923
2114	BPSL1986		38	965597	965980	844319	844702	851207	851590	958878	959261	1076088	1076471	449952	449569	711142	711525	837124	837507	1420339	1420722	917508	917891
2115	BPSL1987		38	965091	965318	843813	844040	850701	850928	958372	958599	1075582	1075809	450458	450231	710636	710863	836618	836845	1419833	1420060	917002	917229
2116	BPSL1988		38	963889	964923	842611	843645	849499	850533	957170	958204	1074380	1075414	451660	450626	709434	710468	835416	836450	1419021	1419665	915800	916834
2117	BPSL1989		38	962857	963873	841579	842595	848467	849483	956138	957154	1073348	1074364	452692	451676	708402	709418	834384	835400	1417600	1418616	914768	915784
2118	BPSL1990		38	961983	962861	840705	841583	847593	848471	955264	956142	1072474	1073352	453566	452688	707528	708406	833510	834388	1416726	1417604	913894	914772
2119	BPSL1991		38	961738	960692	840460	839414	847348	846302	955019	953973	1072229	1071183	453811	454857	707283	706237	833265	832219	1416481	1415435	913649	912603
2120	BPSL1992		38	960588	959446	839310	838168	846198	845056	953869	952727	1071079	1069937	454961	456103	706133	704991	832115	830973	1415331	1414189	912499	911357
2121	BPSL1993		38	959430	958633	838152	837355	845040	844243	952711	951914	1069921	1069124	456119	456916	704975	704178	830957	830160	1414173	1413376	911341	910544
2122	BPSL1994		38	958616	956619	837338	835341	844226	842229	951897	949900	1069107	1067110	456933	458930	704161	702164	830143	828146	1413359	1411362	910527	908530
2123	BPSL1995		38	956605	954623	835327	833345	842215	840233	949886	947904	1067096	1065114	458944	460926	702150	700168	828132	826150	1411348	1409366	908516	906534
2124	BPSL1996		38	954623	953706	833345	832428	840233	839316	947904	946987	1065114	1064197	460926	461843	700168	699251	826150	825233	1409366	1408449	906534	905617
2125	BPSL1997		38	953703	952903	832425	831625	839313	838513	946984	946184	1064194	1063394	461846	462646	699248	698448	825230	824430	1408446	1407646	905614	904814
2126	BPSL1998		38	952079	952780	830801	831502	837689	838390	945360	946061	1062570	1063271	463470	462769	697624	698325	823606	824307	1406822	1407523	903990	904691
2127	BPSL1999		38	949797	952079	828519	830801	835407	837689	943078	945360	1060279	1062570	465752	463470	695351	696781	821324	823606	1404522	1406822	901699	903990
2128	BPSL2000		38	948869	949801	827591	828523	834479	835411	942150	943082	1059351	1060283	466680	465748	694423	695355	820396	821328	1403603	1404526	900780	901703

2129	BPSL2001		38	948415	948861	827137	827583	834025	834471	941696	942142	1058897	1059343	467134	466688	693969	694415	819942	820388	1403149	1403595	900326	900772
2130	BPSL2002		38	947025	948170	825747	826892	832635	833780	940306	941451	1057498	1058643	468524	467379	692579	693724	818552	819697	1401759	1402904	898936	900081
2131	BPSL2003		38	946809	946477	825531	825199	832419	832087	940090	939758	1057282	1056950	468740	469072	692363	692031	818336	818004	1401543	1401211	898720	898388
2132	BPSL2004		38	945745	946371	824467	825093	831355	831981	939026	939652	1056218	1056844	469804	469178	691299	691925	817272	817898	1400479	1401105	897656	898282
2133	BPSL2005		38	945531	944857	824253	823579	831141	830467	938812	938138	1056004	1055330	470018	470692	691085	690411	817058	816384	1400265	1399591	897442	896768
2134	BPSL2006		38	944180	944632	821809	822915	828697	829803	936368	937474	1053560	1054666	472462	471356	688641	689747	814614	815720	1397821	1398927	894998	896104
2135	BPSL2007_p	partial	38	942193	942774	820915	821496	827803	828384	935495	936055	1052666	1053247	473356	472775	687747	688328	813720	814301	1396927	1397508	894104	894685
2136		Chr1_95_IS407A_A	38	940960	942195	819682	820917	826570	827805	934492	935458	1051433	1052668	473354	474589	686514	687749	812487	813722	1395694	1396929	892871	894106
2137	BPSL2007_p	partial	38	940281	940958	819003	819680	825891	826568	933813	934490	1050754	1051431	475268	474591	685835	686512	811808	812485	1395015	1395692	892192	892869
2138	BPSL2008		38	940134	939172	818856	817894	825744	824782	933666	932704	1050607	1049645	475415	476377	685688	684726	811661	810699	1394868	1393918	892045	891095
2139	BPSL2009		38	936041	938662	814763	817384	821651	824272	929573	932194	1046514	1049135	479508	476887	681595	684216	807568	810189	1390787	1393408	887964	890585
2140	BPSL2010		38	935373	934156	814095	812878	820983	819766	928905	928129	1045882	1044665	480176	481393	680936	679719	806918	805701	1390146	1388929	887323	886106
2141	BPSL2011		38	933623	933826	811856	812059	818475	818678	927779	927982	1044319	1044450	481926	481723	679186	679389	805081	805284	1388396	1388599		
2142	BPSL2012		38	933342	931636	811575	809869	818194	816488	927498	925792	1044038	1042332	482207	483913	678905	677199	804800	803094	1388115	1386409	885650	883944
2143	BPSL2013		38	930828	931298	809061	809531	815680	816150	924984	925454	1041524	1041994	484721	484251	676391	676861	802286	802756	1385601	1386071	883136	883606
2144	BPSL2014		38	930414	930797	808647	809030	815266	815649	924570	924953	1041110	1041493	485135	484752	675977	676360	801872	802255	1385187	1385570	882722	883105
2145	BPSL2015		38	928089	928712	806322	806945	812941	813564	922245	922868	1038785	1039408	487460	486837	673652	674275	799547	800170	1382862	1383485	880397	881020
2146	BPSL2016		38	927786	927079	806019	805312	812638	811931	921942	921235	1038482	1037775	487763	488470	673349	672642	799244	798537	1382559	1381852	880094	879387
2147	BPSL2017		38	925621	927021	803854	805254	810473	811873	919777	921177	1036325	1037725	489920	488520	671192	672592	797087	798487	1380402	1381802	877937	879337
2148	BPSL2018		38	925465	923795	803698	802028	810317	808647	919621	917951	1036169	1034499	490076	491746	671036	669366	796931	795261	1380246	1378576	877781	876111
2149	BPSL2019		38	923600	922410	801833	800643	808452	807262	917756	916566	1034298	1033108	491947	493137	669165	667975	795060	793870	1378375	1377185	875910	874720
2150	BPSL2020		38	921788	920604	800021	798837	806640	805630	915944	914760	1032486	1031302	493759	494943	667353	666169	793248	792064	1376563	1375379	874098	872914
2151	BPSL2021		38	920428	918812	798661	797045	804803	803187	914584	912968	1031126	1029510	495119	496735	665993	664377	791888	790272	1375203	1373587	872738	871122
2152	BPSL2022		38	918678	917623	796911	795856	803053	801998	912834	911779	1029376	1028321	496869	497924	664243	663188	790138	789083	1373453	1372398	870988	869933
2153	BPSL2023		38	916924	917133	795157	795366	801299	801508	911080	911289	1027622	1027831	498623	498414	662489	662698	788384	788593	1371699	1371908	869234	869443
2154	BPSL2024		38	916199	916816	794432	795049	800574	801191	910355	910972	1026897	1027514	499348	498731	661764	662381	787659	788276	1370974	1371591	868509	869126
2155	BPSL2025		38	914105	916183	792338	794416	798480	800558	908261	910339	1024803	1026881	501442	499364	659670	661748	785565	787643	1368880	1370958	866415	868493
2156	BPSL2026		38	912729	913691	790962	791924	797104	798066	906885	907847	1023427	1024389	502818	501856	658294	659256	784189	785151	1367504	1368466	865039	866001
2157	BPSL2027		38	910364	911047	788597	789280	794739	795422	904520	905203	1021062	1021745	505182	502837	655929	656612	781824	782507	1365140	1367485	862674	863357
2158	BPSL2028		38	909417	910253	787650	788486	793792	794628	903573	904409	1020115	1020951	506129	505293	654982	655818	780877	781713	1364193	1365029	861727	862563
2159	BPSL2029		38	908875	909396	787108	787629	793250	793771	903031	903552	1019573	1020094	506671	506150	654440	654961	780335	780856	1363651	1364172	861185	861706
2160	BPSL2030		38	908238	908795	786471	787028	792613	793170	902394	902951	1018936	1019493	507308	506751	653803	654360	779698	780255	1363014	1363571	860548	861105
2161	BPSL2031		38	907640	908182	785873	786415	792015	792557	901796	902338	1018338	1018880	507906	507364	653205	653747	779100	779642	1362416	1362958	859950	860492
2162	BPSL2032		38	905930	906820	784163	785053	790305	791195	900086	900976	1016628	1017518	509616	508726	651495	652385	777390	778280	1360706	1361596	858240	859130
2163	BPSL2033		38	905513	904230	783746	782463	789888	788605	899669	898386	1016211	1014928	510033	511316	651078	649795	776973	775690	1360289	1359006	857823	856540
2164	BPSL2034		38	904182	903193	782415	781426	788557	787568	898338	897349	1014880	1013891	511364	512353	649747	648758	775642	774653	1358958	1357969	856492	855503
2165	BPSL2035		38	902722	902444	780955	780677	787097	786819	896878	896600	1013420	1013142	512824	513102	648287	648009	774182	773904	1357498	1357220	855032	854754
2166	BPSL2036		38	901919	902185	780152	780418	786294	786560	896075	896341	1012617	1012883	513627	513361	647484	647750	773379	773645	1356695	1356961	854229	854495
2167	BPSL2037		38																				
2168	BPSL2038		38	899032	899448	777265	777681	783407	783823	893188	893604	1009730	1010146	516514	516098	644597	645013	770492	770908	1353808	1354224	851342	851758
2169		Chr1_96_IS407A_A		897797	899032	776030	777265	782172	783407	891953	893188	1008495	1009730	516514	517749	643362	644597	769257	770492	1352573	1353808	850107	851342
2170	BPSL2039																						
2171	BPSL2040																						
2172	BPSL2041																						
2173	BPSL2042																						
2174	BPSL2042A																						
2175	BPSL2043																						
2176	BPSL2044																						
2177	BPSL2045																						
2178	BPSL2046																						
2179	BPSL2047																						

2180	BPSL2048																						
2181	BPSL2048A																						
2182		Chr1_97_IS407A_A		897797	899032	776030	777265	782172	783407	891953	893188	1008495	1009730	516514	517749	643362	644597	769257	770492	1352573	1353808	850107	851342
2183	BPSL2049		38	896777	897607	775010	775840	781152	781982	890933	891763	1007475	1008305	518769	517939	642342	643172	768237	769067	1351553	1352383	849087	849917
2184	BPSL2050		38	893967	896771	772200	775004	778342	781146	888123	890927	1004665	1007469	521579	518775	639532	642336	765427	768231	1348743	1351547	846277	849081
2185	BPSL2051		38	893446	893183	771679	771416	777821	777558	887602	887339	1004144	1003881	522100	522363	639011	638748	764906	764643	1348222	1347959	845756	845493
2186	BPSL2052		38	893183	891744	771416	769977	777558	776119	887339	885900	1003881	1002442	522363	523802	638748	637309	764643	763204	1347959	1346520	845493	844282
2187	BPSL2053		38	889863	891710	768096	769943	774238	776085	884019	885866	1000561	1002408	525683	523836	635430	635624	761323	763170	1344639	1346486	842360	844180
2188	BPSL2054		38	889033	889449	767266	767682	773408	773824	883189	883605	999731	1000147	526513	526097	634600	635016	760493	760909	1343809	1344225	841530	841946
2189	BPSL2055		38	887739	887972	765972	766205	772114	772347	881895	882128	998427	999260	527811	526978	633302	634135	759195	760028	1342511	1343344	840232	841065
2190	BPSL2056		38	887367	884809	765600	763042	771742	769184	881523	878965	998055	995497	528183	530741	632930	630372	758823	756265	1342139	1339581	839860	837302
2191	BPSL2057		38	884779	884294	763012	762527	769154	768669	878935	878450	995467	994982	530771	531256	630342	629857	756235	755750	1339551	1339066	837272	836787
2192	BPSL2058		38	884256	883699	762489	761932	768631	768074	878412	877855	994944	994387	531294	531851	629819	629262	755712	755155	1339028	1338471	836749	836192
2193	BPSL2059		38	883650	883411	761883	761644	768025	767786	877806	877567	994338	994099	531900	532139	629213	628974	755106	754867	1338422	1338183	836143	835904
2194	BPSL2060		38	882641	882982	760874	761215	767016	767357	876797	877138	993329	993670	532909	532568	628204	628545	754097	754438	1337413	1337754	835134	835475
2195	BPSL2061		38	881095	882621	759328	760854	765470	766996	875251	876777	991783	993309	534455	532929	626658	628184	752551	754077	1335867	1337393	833588	835114
2196	BPSL2062		38	880383	881090	758616	759323	764758	765465	874539	875246	991071	991778	535167	534460	625946	626653	751839	752546	1335155	1335862	832876	833583
2197	BPSL2063		38	877015	880299	755248	758532	761390	764674	871171	874455	987703	990987	538535	535251	622578	625862	748471	751755	1331787	1335071	829508	832792
2198	BPSL2064		38	876831	876941	755064	755174	761206	761316	870987	871097	987519	987629	538731	538609	622382	622504	748275	748397	1331603	1331713	829324	829434
2199	BPSL2065		38	875944	876651	754177	754884	760319	761026	870100	870807	986632	987339	539618	538911	621495	622202	747388	748095	1330716	1331423	828437	829144
2200	BPSL2066		38	874594	874962	752827	753195	758969	759337	868750	869118	985282	985650	540968	540600	620145	620513	746038	746406	1329366	1329734	827087	827455
2201	BPSL2067		38	874368	874580	752601	752813	758743	758955	868524	868736	985056	985268	541194	540982	619919	620131	745812	746024	1329140	1329352	826861	827073
2202	BPSL2068		38	873402	874181	751635	752414	757777	758556	867558	868337	984090	984869	542160	541381	618953	619732	744846	745625	1328174	1328953	825895	826674
2203	BPSL2069		38	872056	872985	750289	751218	756431	757360	866212	867141	982744	983673	543506	542577	617607	618536	743500	744429	1326828	1327757	824549	825478
2204	BPSL2070		38	870353	871741	748586	749974	754728	756116	864509	865897	981041	982429	545209	543821	615904	617292	741797	743185	1325125	1326513	822846	824234
2205	BPSL2071		38	869414	869602	747647	747835	753789	753977	863570	863758	980102	980290										
2206	BPSL2072		38	868734	868519	746967	746752	753109	752894	862890	862675	979410	979195	546815	547030	614298	614083	740185	739970	1323513	1323298	821234	821019
2207	BPSL2073		38	867808	866333	746041	744566	752183	750708	861964	860489	978484	977009	547741	549216	613372	611897	739259	737784	1322587	1321112	820308	818833
2208	BPSL2074		38	865875	862432	744108	740665	750250	749783	860031	859465	976551	973108	549674	553117	611439	607996	737326	733883	1320654	1317211	818375	814932
2209	BPSL2075		38	862432	859040	740665	737273	746207	742815	856982	853590	973108	969716	553117	556509	607996	604604	733883	730491	1317211	1313819	814932	811540
2210	BPSL2076		38	859040	858300	737273	736533	742815	742075	853590	852850	969716	968976	556509	557249	604604	603864	730491	729751	1313819	1313079	811540	810800
2211	BPSL2077		38	856775	854670	735008	732903	740550	738445	851325	849220	967451	965346	558774	560879	602339	600234	728226	726121	1311554	1309449	809275	807170
2212	BPSL2078		38	853191	852901	732873	731410	738415	736952	849190	847727	965316	963853	560909	562372	600204	598741	726091	724628	1309419	1307956	807140	805677
2213	BPSL2079		38	852901	850682	731134	730619	736676	736176	847451	845232	963577	961358	562648	564867	598465	596246	724352	722133	1307680	1305461	805401	804982
2214	BPSL2080		38	850682	847893	728580	725791	733974	731185	845232	842443	961358	958569	564867	567656	596246	593457	722133	719344	1305461	1302672	803316	800527
2215	BPSL2081		38	847187	847672	725085	725570	730479	730964	841737	842222	957863	958348	568371	567886	592751	593236	718638	719123	1301966	1302451	799821	800306
2216	BPSL2082		38	846696	847067	724594	724965	729988	730359	841246	841617	957316	957687	568862	568491	592260	592631	718147	718518	1301475	1301846	799330	799701
2217	BPSL2083		38	846473	845016	724371	722914	729765	728308	841023	839566	957093	955636	569085	570542	592037	590580	717924	716467	1301252	1299795	799107	797650
2218	BPSL2084		38	842967	844943	720865	722841	726259	728235	837517	839493	953587	955563	572591	570615	588531	590507	714418	716394	1297746	1299722	795601	797577
2219	BPSL2085		38	842308	842030	720206	719928	725600	725322	836858	836580	952928	952650	573250	573528	587872	587594	713759	713481	1297087	1296809	794942	794664
2220	BPSL2086		38	841883	841383	719781	719281	725175	724675	836433	835933	952503	952003	573675	574175	587447	586947	713334	712834	1296662	1296162	794517	794017
2221	BPSL2087		38	841387	841064	719285	718962	724679	724356	835937	835614	952007	951684	574171	574494	586951	586628	712838	712515	1296166	1295843	794021	793698
2222		Chr1_98_IS407A_A		839215	840450	717113	718348	722507	723742	833765	835000	949835	951070	575108	576343	584779	586014	710666	711901	1293994	1295229	791849	793084
2223	BPSL2087A																						
2224	BPSL2088																						
2225		Chr1_99_IS407A_A		1533068	1534303	1420660	1421895	1430062	1431297	1523634	1524869	1686724	1687959	3386673	3387908	1230358	1231593	1436504	1437739	1950881	1952116	1479698	1480933
2226	BPSL2089		39	1534644	1534303	1422236	1421895	1431638	1431297	1525210	1524869	1688300	1687959	3386332	3386673	1231934	1231593	1438080	1437739	1952457	1952116	1481274	1480933
2227	BPSL2090		39	1535215	1536048	1422807	1423640	1432209	1433042	1525781	1526614	1688871	1689704	3385761	3384928	1232505	1233338	1438651	1439484	1953028	1953861	1481845	1482678
2228	BPSL2091		39	1537336	1536095	1424928	1423687	1434330	1433089	1527902	1526661	1690992	1689751	3383640	3384881	1234626	1233385	1440772	1439531	1955149	1953908	1483966	1482725
2229	BPSL2092		39	1538834	1537422	1426426	1425014	1435828	1434416	1529400	1527988	1692490	1691078	3382142	3383554	1236124	1234712	1442270	1440858	1956647	1955235	1485464	1484052
2230	BPSL2093		39	1539416	1539081	1427008	1426673	1436410	1436075	1529982	1529647	1693072	1692737	3381560	3381895	1236706	1236371	1442852	1442517	1957229	1956894	1486046	1485711

2231	BPSL2094		39	1539638	1540360	1427230	1427952	1436632	1437354	1530204	1530926	1693294	1694016	3381338	3380616	1236928	1237650	1443074	1443796	1957451	1958173	1486268	1486990
2232	BPSL2095		39	1540430	1541785	1428022	1429377	1437424	1438779	1530996	1532351	1694086	1695441	3380546	3379191	1237720	1239075	1443866	1445221	1958243	1959598	1487060	1488415
2233	BPSL2096		39	1541987	1542532	1429579	1430124	1438981	1439526	1532553	1533098	1695643	1696188	3378989	3378444	1239277	1239822	1445423	1445968	1959800	1960345	1488617	1489162
2234	BPSL2097		39	1542613	1543137	1430205	1430729	1439607	1440131	1533179	1533703	1696269	1696793	3378363	3377839	1239903	1240427	1446049	1446573	1960426	1960950	1489243	1489767
2235	BPSL2098		39	1543997	1543512	1431589	1431104	1440991	1440506	1534563	1534078	1697653	1697168	3376989	3377474	1241277	1240792	1447423	1446938	1961810	1961325	1490627	1490142
2236	BPSL2099		39	1544748	1544041	1432340	1431633	1441742	1441035	1535314	1534607	1698404	1697697	3376238	3376945	1242028	1241321	1448174	1447467	1962561	1961854	1491378	1490671
2237	BPSL2100		39	1544769	1548335	1432361	1435927	1441763	1445329	1535335	1538901	1698425	1701991	3376217	3372651	1242049	1245615	1448195	1451761	1962582	1966148	1491399	1494965
2238	BPSL2101		39	1549588	1550754	1437180	1438346	1446582	1447748	1540154	1541320	1703258	1704424	3371342	3370176	1247015	1248181	1453077	1454243	1967415	1968581	1496225	1497391
2239	BPSL2102		39	1550819	1551847	1438411	1439439	1447813	1448841	1541385	1542413	1704489	1705517	3370111	3369083	1248246	1249274	1454308	1455336	1968646	1969674	1497456	1498484
2240	BPSL2103		39	1552358	1552762	1439950	1440354	1449352	1449756	1542924	1543328	1706028	1706432	3368572	3368168	1249785	1250189	1455847	1456251	1970185	1970589	1498995	1499399
2241	BPSL2104		39	1555878	1553323	1443470	1440915	1452872	1450317	1546444	1543889	1709548	1706993	3365052	3367607	1253305	1250750	1459367	1456812	1973705	1971150	1502515	1499960
2242	BPSL2105		39	1556357	1557034	1443949	1444626	1453351	1454028	1546923	1547600	1710027	1710704	3364573	3363896	1253784	1254461	1459846	1460523	1974184	1974861	1502994	1503671
2243	BPSL2106		39	1557152	1558363	1444827	1446038	1454146	1455357	1547732	1548943	1710822	1712033	3363778	3362567	1254579	1255790	1460683	1461894	1974979	1976190	1503863	1505074
2244		Chr1_100_ISBma2_B	39	1558411	1559983	1446086	1447658	1455405	1456977	1548991	1550563	1712081	1713653										
2245	BPSL2107		39	1560145	1560573	1447820	1448248	1457139	1457567	1550725	1551153	1713815	1714243	3362380	3361952	1255977	1256405	1462081	1462509	1976377	1976805	1505261	1505689
2246	BPSL2108		39	1560889	1561383	1448564	1449058	1457883	1458377	1551469	1551963	1714559	1715053	3361636	3361142	1256721	1257215	1462825	1463319	1977121	1977615	1506005	1506499
2247		Chr1_101_ISBma2_A		1561424	1562996	1449099	1450671	1458418	1459990	1552004	1553576	1715094	1716666	3359529	3361101	1257256	1258828	1463360	1464932	1977656	1979228	1506540	1508112
2248	BPSL2109																						
2249	BPSL2110																						
2250	BPSL2111																						
2251	BPSL2112																						
2252	BPSL2113																						
2253	BPSL2114																						
2254		Chr1_102_ISBma2_A		1561424	1562996	1449099	1450671	1458418	1459990	1552004	1553576	1715094	1716666	3359529	3361101	1257256	1258828	1463360	1464932	1977656	1979228	1506540	1508112
2255	BPSL2115		39	1564372	1563863	1452047	1451538	1461366	1460857	1554952	1554443	1718064	1717555	3358164	3358673	1260193	1259684	1466308	1465799	1980582	1980073		
2256	BPSL2116		39	1565382	1564372	1453057	1452047	1462376	1461366	1555962	1554952	1719074	1718064	3357154	3358164	1261203	1260193	1467318	1466308	1981592	1980582	1510476	1509466
2257	BPSL2117		39	1566135	1565614	1453810	1453289	1463129	1462608	1556715	1556194	1719827	1719306	3356401	3356922	1261956	1261435	1468071	1467550	1982345	1981824	1511229	1510708
2258	BPSL2118		39	1567085	1566135	1454760	1453810	1464079	1463129	1557665	1556715	1720777	1719827	3355451	3356401	1262906	1261956	1469021	1468071	1983295	1982345	1512179	1511229
2259	BPSL2119		39	1568169	1567234	1455844	1454909	1465163	1464228	1558749	1557814	1721861	1720926	3354367	3355302	1263990	1263055	1470105	1469170	1984379	1983444	1513263	1512328
2260	BPSL2120		39	1569712	1568183	1457387	1455858	1466706	1465177	1560292	1558763	1723404	1721875	3352824	3354353	1265533	1264004	1471648	1470119	1985922	1984393	1514806	1513277
2261	BPSL2121		39	1570529	1569828	1458204	1457503	1467523	1466822	1561109	1560408	1724221	1723520	3352007	3352708	1266350	1265649	1472465	1471764	1986739	1986038	1515623	1514922
2262		Chr1_103_ISBma1_A	39	1570802	1572106	1458477	1459781	1467796	1469100	1561382	1562686	1724494	1725798	3350430	3351734	1266623	1267927	1472738	1474042	1987012	1988316	1515896	1517200
2263	BPSL2122		39	1573098	1572160	1460773	1459835	1470092	1469154	1563678	1562740	1726790	1725852	3349438	3350376	1268919	1267981	1475034	1474096	1989308	1988370	1518192	1517254
2264	BPSL2123		39	1573678	1573157	1461353	1460832	1470672	1470151	1564258	1563737	1727370	1726849	3348858	3349379	1269499	1268978	1475614	1475093	1989888	1989367	1518772	1518251
2265	BPSL2124		39	1574148	1573750	1461823	1461425	1471142	1470744	1564728	1564330	1727840	1727442	3348388	3348786	1269969	1269571	1476084	1475686	1990358	1989960	1519242	1518844
2266	BPSL2125		39	1575454	1574951	1463129	1462626	1472448	1471945	1566034	1565531	1729146	1728643	3347082	3347585	1271275	1270772	1477390	1476887	1991664	1991161	1520548	1520045
2267	BPSL2126		39	1575822	1577039	1463497	1464714	1472816	1474033	1566402	1567619	1729514	1730731	3346714	3345497	1271643	1272860	1477758	1478975	1992032	1993249	1520916	1522133
2268	BPSL2127		39	1580018	1578378	1467693	1466053	1477012	1475372	1570598	1568958	1733710	1732070	3342518	3344158	1275839	1274199	1481954	1480314	1996228	1994588	1525112	1523472
2269	BPSL2128		39	1580775	1580125	1468450	1467800	1478504	1478100	1571355	1570705	1734467	1733817	3341761	3342411	1276596	1275946	1482711	1482061	1996985	1996335	1525869	1525219
2270	BPSL2129		39	1582242	1580785	1469917	1468460	1479971	1478514	1572822	1571365	1735934	1734477	3340294	3341751	1278063	1276606	1484178	1482721	1998452	1996995	1527336	1525879
2271	BPSL2130		39	1583424	1582663	1471099	1470338	1481153	1480392	1574004	1573243	1737116	1736355	3339112	3339873	1279245	1278484	1485360	1484599	1999634	1998873	1528518	1527757
2272	BPSL2131		39	1583629	1584330	1471236	1471340	1481290	1481394	1574141	1574245	1737253	1737357	3338975	3338871	1279382	1279486	1485497	1485601	1999771	1999875	1528655	1528759
2273	BPSL2133		39	1584812	1585492	1472487	1473167	1482541	1483221	1575392	1576072	1738504	1739184	3337724	3337044	1280633	1281313	1486748	1487428	2001022	2001702	1529906	1530586
2274	BPSL2134		39	1585830	1586795	1473505	1474470	1483559	1484524	1576410	1577375	1739522	1740487	3336706	3335741	1281651	1282616	1487766	1488731	2002040	2003005	1530924	1531889
2275	BPSL2135		39	1587266	1586946	1474941	1474621	1484995	1484675	1577846	1577526	1740958	1740638	3335270	3335590	1283087	1282767	1489202	1488882	2003476	2003156	1532360	1532040
2276	BPSL2136		39	1587709	1587275	1475384	1474950	1485438	1485004	1578289	1577855	1741401	1740967	3334827	3335261	1283530	1283096	1489645	1489211	2003919	2003485	1532803	1532369
2277	BPSL2137		39	1587807	1588250	1475482	1475925	1485536	1485979	1578387	1578830	1741499	1741942	3334729	3334286	1283628	1284071	1489743	1490186	2004017	2004460	1532901	1533344
2278	BPSL2138		39	1589369	1588425	1477044	1476100	1487098	1486154	1579949	1579005	1743061	1742117	3333167	3334111	1285190	1284246	1491305	1490361	2005579	2004635	1534463	1533519
2279	BPSL2139		39	1589883	1589452	1477558	1477127	1487612	1487181	1580463	1580032	1743575	1743144	3332653	3333084	1285704	1285273	1491819	1491388	2006093	2005662	1534977	1534546
2280	BPSL2140		39	1592410	1590014	1480085	1477689	1490139	1487743	1582990	1580594	1746102	1743706	3330126	3332522	1288231	1285835	1494346	1491950	2008620	2006224	1537504	1535108
2281	BPSL2141		39	1593988	1593158	1481663	1480833	1491717	1490887	1584568	1583738	1747680	1746850	3328548	3329378	1289809	1288979	1495924	1495094	2010198	2009368	1539082	1538252

2282	BPSL2142		39	1596120	1594222	1483795	1481897	1493849	1491951	1586700	1584802	1749806	1747908	3326410	3328308	1291947	1290049	1498062	1496164	2012342	2010444	1541220	1539322
2283	BPSL2143		39	1596655	1597467	1484330	1485142	1494384	1495196	1587235	1588047	1750341	1751153	3325875	3325063	1292482	1293294	1498597	1499409	2012877	2013689	1541755	1542567
2284	BPSL2144		39	1598460	1597675	1486135	1485350	1496189	1495404	1589040	1588255	1752146	1751361	3324070	3324855	1294287	1293502	1500402	1499617	2014682	2013897	1543560	1542775
2285	BPSL2145		39	1599101	1598460	1486776	1486135	1496830	1496189	1589681	1589040	1752787	1752146	3323429	3324070	1294928	1294287	1501043	1500402	2015323	2014682	1544201	1543560
2286	BPSL2146		39	1600267	1599104	1487942	1486779	1497996	1496833	1590847	1589684	1753953	1752790	3322263	3323426	1296094	1294931	1502209	1501046	2016489	2015326	1545367	1544204
2287	BPSL2147		39	1601059	1600274	1488734	1487949	1498788	1498003	1591639	1590854	1754745	1753960	3321471	3322256	1296886	1296101	1503001	1502216	2017281	2016496	1546159	1545374
2288	BPSL2148		39	1601624	1601118	1489299	1488793	1499353	1498847	1592204	1591698	1755310	1754804	3320906	3321412	1297451	1296945	1503566	1503060	2017846	2017340	1546724	1546218
2289	BPSL2149		39	1602813	1601731	1490488	1489406	1500542	1499460	1593393	1592311	1756499	1755417	3319717	3320799	1298640	1297558	1504755	1503673	2019035	2017953	1547913	1546831
2290	BPSL2150		39	1603363	1602833	1491038	1490508	1501092	1500562	1593943	1593413	1757049	1756519	3319167	3319697	1299190	1298660	1505305	1504775	2019585	2019055	1548463	1547933
2291	BPSL2151		39	1605710	1603404	1493385	1491079	1503439	1501133	1596290	1593984	1759396	1757090	3316820	3319126	1301537	1299231	1507652	1505346	2021932	2019626	1550810	1548504
2292	BPSL2152		39	1607166	1605778	1494841	1493453	1504895	1503507	1597746	1596358	1760852	1759464	3315364	3316752	1302993	1301605	1509108	1507720	2023388	2022000	1552266	1550878
2293	BPSL2153		39	1608380	1607187	1496055	1494862	1506109	1504916	1598960	1597767	1762066	1760873	3314150	3315343	1304207	1303014	1510322	1509129	2024602	2023409	1553480	1552287
2294	BPSL2154		39	1609216	1608398	1496891	1496073	1506945	1506127	1599796	1598978	1762902	1762084	3313314	3314132	1305043	1304225	1511158	1510340	2025438	2024620	1554316	1553498
2295	BPSL2155		39	1609992	1609213	1497667	1496888	1507721	1506942	1600572	1599793	1763678	1762899	3312538	3313317	1305819	1305040	1511934	1511155	2026214	2025435	1555092	1554313
2296	BPSL2156		39	1610600	1610043	1498275	1497718	1508329	1507772	1601180	1600623	1764286	1763729	3311930	3312487	1306427	1305870	1512542	1511985	2026822	2026265	1555700	1555143
2297	BPSL2157		39	1611404	1610694	1499079	1498369	1509133	1508423	1601984	1601274	1765090	1764380	3311126	3311836	1307231	1306521	1513346	1512636	2027626	2026916	1556504	1555794
2298	BPSL2158		39	1612464	1611586	1500139	1499261	1510193	1509315	1603044	1602166	1766150	1765272	3310066	3310944	1308291	1307413	1514406	1513528	2028686	2027808	1557564	1556686
2299	BPSL2159		39	1613387	1612650	1501062	1500325	1511116	1510379	1603967	1603230	1767073	1766336	3309143	3309880	1309214	1308477	1515329	1514592	2029609	2028872	1558487	1557750
2300	BPSL2160		39	1613869	1614681	1501544	1502356	1511598	1512410	1604449	1605261	1767555	1768367	3308661	3307849	1309696	1310508	1515811	1516623	2030091	2030903	1558969	1559781
2301	BPSL2161		39	1614850	1617423	1502525	1505098	1512579	1515152	1605430	1608003	1768536	1771109	3307680	3305107	1310677	1313250	1516792	1519365	2031072	2033645	1559950	1562523
2302	BPSL2162		39	1618383	1619129	1506262	1507008	1516980	1517726	1608856	1609602	1772069	1772815	3304147	3303401	1314210	1314956	1520325	1521071	2034605	2035351	1563279	1564025
2303	BPSL2163		39	1619860	1619330	1507739	1507209	1518457	1517927	1610333	1609803	1773546	1773016	3302670	3303200	1315687	1315157	1521802	1521272	2036082	2035552	1564756	1564226
2304	BPSL2164		39	1621932	1619860	1509808	1507739	1520529	1518457	1612405	1610333	1775618	1773546	3300598	3302670	1317759	1315687	1523874	1521802	2038154	2036082	1566828	1564756
2305	BPSL2165		39	1623641	1622373	1511835	1510567	1522238	1520970	1614114	1612846	1777327	1776059	3298889	3300157	1319468	1318200	1525583	1524315	2039863	2038595	1568537	1567269
2306	BPSL2166		39	1627220	1623711	1515414	1511905	1525817	1522308	1617693	1614184	1780906	1777397	3295310	3298819	1323047	1319538	1529162	1525653	2043442	2039933	1572116	1568607
2307	BPSL2167		39	1628318	1627374	1516512	1515568	1526915	1525971	1618791	1617847	1782004	1781060	3294212	3295156	1324145	1323201	1530260	1529316	2044540	2043596	1573214	1572270
2308	BPSL2168		39	1628583	1629812	1516777	1518006	1527180	1528409	1619056	1620285	1782269	1782661	3293947	3292718	1324410	1325639	1530525	1531754	2044805	2046034	1573469	1574698
2309	BPSL2169		39	1629872	1630696	1518066	1518890	1528469	1529293	1620345	1621169	1783560	1784384	3292658	3291834	1325699	1326523	1531814	1532638	2046094	2046918	1574758	1575582
2310	BPSL2170		39	1630699	1631064	1518893	1519258	1529296	1529661	1621172	1621537	1784387	1784752	3291831	3291460	1326526	1326897	1532641	1533012	2046921	2047292	1575585	1575956
2311	BPSL2171		39	1631129	1632277	1519323	1520471	1529726	1530226	1621602	1622738	1784817	1785965	3291395	3290247	1326962	1328110	1533077	1534225	2047357	2048505	1576021	1576482
2312	BPSL2172		39	1632294	1633214	1520488	1521408	1531289	1532209	1622755	1623675	1785982	1786902	3290230	3289310	1328127	1329047	1534242	1535162	2048522	2049442	1577322	1578242
2313	BPSL2173		39																				
2314	BPSL2174		39	1633400	1634836	1521594	1523030	1532395	1533831	1623861	1625297	1787088	1788524	3289124	3287688	1329233	1330669	1535348	1536784	2049628	2051064	1578428	1579864
2315	BPSL2175		39	1635431	1634955	1523625	1523149	1534426	1533950	1625892	1625416	1789119	1788643	3287093	3287569	1331264	1330788	1537379	1536903	2051659	2051183	1580459	1579983
2316	BPSL2176		39	1635577	1637514	1523771	1525708	1534572	1536509	1626038	1627975	1789265	1791202	3286947	3285010	1331410	1333347	1537525	1539462	2051805	2053742	1580605	1582542
2317	BPSL2177		39	1637937	1637608	1526131	1525802	1536932	1536603	1628398	1628069	1791625	1791296	3284587	3284916	1333770	1333441	1539885	1539556	2054165	2053836	1582965	1582636
2318	BPSL2178		39	1639411	1638038	1527605	1526232	1538406	1537033	1629872	1628499	1793099	1791726	3283113	3284486	1335244	1333871	1541359	1539986	2055639	2054266	1584439	1583066
2319	BPSL2179		39	1640492	1639425	1528686	1527619	1539487	1538420	1630953	1629886	1794180	1793113	3282032	3283099	1336325	1335258	1542440	1541373	2056720	2055653	1585520	1584453
2320	BPSL2180		39	1640704	1642008	1528898	1530202	1539699	1541003	1631165	1632469	1794392	1795696	3281820	3280516	1336537	1337841	1542652	1543956	2056932	2058236	1585732	1587036
2321	BPSL2181		39	1642122	1642928	1530316	1531122	1541117	1541923	1632583	1633389	1795810	1796616	3280402	3279596	1337955	1338761	1544070	1544876	2058350	2059156	1587150	1587956
2322	BPSL2182		39	1644295	1643318	1532489	1531512	1543290	1542313	1634756	1633779	1798001	1797024	3278235	3279212	1340116	1339139	1546273	1545296	2060577	2059600	1589359	1588382
2323	BPSL2183		39	1645146	1644295	1533313	1532489	1544141	1543290	1635574	1634756	1798852	1798001	3277384	3278235	1340967	1340116	1547124	1546273	2061428	2060577	1590210	1589359
2324	BPSL2184		39	1646110	1645631	1534469	1533990	1545660	1545181	1636387	1635908	1799825	1799346	3276402	3276881	1341949	1341470	1548115	1547636	2062410	2061931	1591210	1590731
2325	BPSL2185		39	1646883	1646125	1535242	1534484	1546433	1545675	1637160	1636402	1800598	1799840	3275629	3276387	1342722	1341964	1548888	1548130	2063183	2062425	1591983	1591225
2326	BPSL2186		39	1647053	1647319	1535412	1535678	1546603	1546869	1637330	1637596	1800768	1801034	3275459	3275193	1342892	1343158	1549058	1549324	2063353	2063619	1592153	1592419
2327	BPSL2187		39	1647957	1649315	1536316	1537674	1547507	1548865	1638234	1639592	1801672	1803030	3274555	3273197	1343796	1345154	1549962	1551320	2064257	2065615	1593057	1594415
2328		Chr1_104_ISBma2_A	39	1649393	1650965	1537752	1539324	1548943	1550515	1639670	1641242	1803108	1804680	3271547	3273119	1345232	1346804	1551398	1552969	2065693	2067265	1594486	1596058
2329	BPSL2188		39	1651302	1652606	1539661	1540965	1550852	1552156	1641579	1642883	1805018	1806322	3271209	3269905	1347142	1348446	1553307	1554611	2067603	2068907	1596396	1597700
2330	BPSL2189		39	1653303	1652872	1541662	1541231	1552853	1552422	1643580	1643149	1807019	1806588	3269208	3269639	1349143	1348712	1555308	1554877	2069604	2069173	1598397	1597966
2331	BPSL2190		39	1654485	1653586	1542844	1541945	1554035	1553136	1644228	1643863	1808201	1807302	3268026	3268925	1350325	1349426	1556490	1555591	2070786	2069887	1599579	1598680
2332	BPSL2191		39	1654645	1655424	1543004	1543783	1554195	1554974	1644574	1645353	1808361	1809140	3267866	3267087	1350485	1351264	1556650	1557429	2070946	2071725	1599739	1600518

2333	BPSL2192	39	1655480	1657069	1543839	1545428	1555030	1556619	1645409	1645549	1809196	1810785	3267031	3265442	1351320	1352909	1557485	1559074	2071781	2073370	1600574	1602163
2334	BPSL2193	39	1658355	1657951	1546721	1546317	1557905	1557501	1648049	1647645	1811861	1811457	3264352	3264756	1354013	1353609	1560178	1559774	2074460	2074056	1603316	1602912
2335	BPSL2194	39	1658900	1658406	1547266	1546772	1558450	1557956	1648594	1648100	1812406	1811912	3263807	3264301	1354558	1354064	1560723	1560229	2075005	2074511	1603861	1603367
2336	BPSL2195	39	1659030	1659890	1547396	1547839	1558580	1559440	1648724	1649584	1812536	1813396	3263677	3262817	1354688	1355548	1560853	1561713	2075135	2075995	1603991	1604851
2337	BPSL2196	39	1660978	1660514	1549748	1549284	1560528	1560064	1650586	1650122	1814484	1814020	3261747	3262211	1356618	1356154	1562801	1562337	2077074	2076610	1605921	1605457
2338	BPSL2197	39	1663179	1661773	1551949	1550543	1562451	1561045	1652787	1651381	1816685	1815279	3259546	3260952	1358819	1357413	1565002	1563596	2079275	2077869	1608122	1606716
2339	BPSL2198	39	1663746	1664678	1552516	1553448	1563018	1563596	1653354	1654286	1817252	1818184	3258973	3258041	1359392	1360324	1565575	1566507	2079842	2080774	1608689	1609621
2340	BPSL2199	39	1665551	1664850	1554321	1553620	1565399	1564698	1655159	1654458	1819057	1818356	3257168	3257869	1361197	1360496	1567380	1566679	2081647	2080946	1610494	1609793
2341	BPSL2200	39	1667336	1665690	1556106	1554460	1567184	1565538	1656944	1655298	1820842	1819196	3255383	3257029	1362982	1361336	1569165	1567519	2083432	2081786	1612279	1610633
2342	BPSL2201	39	1668430	1667336	1557200	1556106	1568278	1567184	1658038	1656944	1821936	1820842	3254289	3255383	1364076	1362982	1570259	1569165	2084526	2083432	1613373	1612279
2343	BPSL2202	39	1669472	1668435	1558242	1557205	1569320	1568283	1659080	1658043	1822978	1821941	3253247	3254284	1365118	1364081	1571301	1570264	2085568	2084531	1614415	1613378
2344	BPSL2203	39	1671613	1669664	1560383	1558434	1571461	1569512	1661221	1659272	1825119	1823170	3251106	3253055	1367259	1365310	1573442	1571493	2087709	2085760	1616556	1614607
2345	BPSL2204	39	1671734	1672522	1560504	1561292	1571582	1572370	1661342	1662130	1825240	1826028	3250985	3250197	1367380	1368168	1573563	1574351	2087830	2088618	1616677	1617465
2346	BPSL2205	39	1673130	1672780	1561900	1561550	1572978	1572628	1662738	1662388	1826629	1826279	3249589	3249939	1368776	1368426	1574959	1574609	2089226	2088876	1618073	1617723
2347	BPSL2206	39	1673384	1673602	1562154	1562372	1573232	1573450	1662992	1663210	1826883	1827101	3249335	3249117	1369030	1369248	1575213	1575431	2089480	2089698	1618327	1618545
2348	BPSL2207	39	1676144	1673826	1564914	1562596	1575992	1573674	1665752	1663434	1829643	1827325	3246575	3248893	1371790	1369472	1577973	1575655	2092240	2089922	1621087	1618769
2349	BPSL2208	39	1677422	1676652	1566192	1565422	1577270	1576500	1667030	1666260	1830921	1830151	3245297	3246067	1373068	1372298	1579251	1578481	2093518	2092748	1622365	1621595
2350	BPSL2209	39	1678419	1677433	1567189	1566203	1578267	1577281	1668027	1667041	1831918	1830932	3244738	3245286	1373627	1373079	1579810	1579262	2094515	2093529	1623362	1622376
2351	BPSL2210	39	1678841	1678506	1567611	1567276	1578689	1578354	1668449	1668114	1832340	1832005	3243880	3244215	1374485	1374150	1580668	1580333	2094937	2094602	1623784	1623449
2352	BPSL2211	39	1679322	1679885	1568092	1568583	1579651	1579734	1668930	1669493	1832821	1833384	3243397	3242834	1374968	1375531	1581150	1581701	2095418	2095981	1624265	1624828
2353	BPSL2212	39	1680780	1679965	1569709	1568924	1580629	1579814	1670388	1669573	1834279	1833464	3241939	3242754	1376426	1375611	1582609	1581794	2096876	2096061	1625723	1624908
2354	BPSL2213	39	1681004	1680783	1569933	1569712	1580853	1580632	1670612	1670391	1834503	1834282	3241715	3241936	1376650	1376429	1582833	1582612	2097100	2096879	1625947	1625726
2355	BPSL2214	39	1683978	1681291	1572907	1570220	1583827	1581140	1673586	1670899	1837471	1834790	3238735	3241428	1379636	1376937	1585813	1583120	2100068	2097387	1628915	1626234
2356	BPSL2215	39	1685122	1684061	1574051	1572990	1584971	1583910	1674730	1673669	1838615	1837554	3237591	3238652	1380780	1379719	1586957	1585896	2101212	2100151	1630059	1628998
2357	BPSL2216	39	1686326	1685130	1575255	1574059	1586175	1584979	1675934	1674738	1839819	1838623	3236387	3237583	1381984	1380788	1588161	1586965	2102416	2101220	1631263	1630067
2358	BPSL2217	39	1687314	1686352	1576243	1575281	1587163	1586201	1676922	1675960	1840807	1839845	3235399	3236361	1382972	1382010	1589149	1588187	2103404	2102442	1632251	1631289
2359	BPSL2218	39	1688288	1687473	1577217	1576402	1588137	1587322	1677896	1677081	1841781	1840966	3234425	3235240	1383946	1383131	1590123	1589308	2104378	2103563	1633225	1632410
2360	BPSL2219	39	1689465	1688374	1578394	1577303	1589314	1588223	1679073	1677982	1842958	1841867	3233248	3234339	1385123	1384032	1591300	1590209	2105555	2104464	1634402	1633311
2361	BPSL2220	39	1690633	1689509	1579562	1578438	1590482	1589358	1680241	1679117	1844126	1843002	3232080	3233204	1386291	1385167	1592468	1591344	2106723	2105599	1635570	1634446
2362	BPSL2221	39	1691803	1690688	1580732	1579617	1591652	1590537	1681411	1680296	1845296	1844181	3230910	3232025	1387461	1386346	1593638	1592523	2107893	2106778	1636740	1635625
2363	BPSL2222	39	1692717	1691809	1581646	1580738	1592566	1591658	1682325	1681417	1846210	1845302	3229996	3230904	1388375	1387467	1594552	1593644	2108807	2107899	1637654	1636746
2364	BPSL2223	39	1693553	1692717	1582482	1581646	1593402	1592566	1683161	1682325	1847046	1846210	3229160	3229996	1389211	1388375	1595388	1594552	2109643	2108807	1638490	1637654
2365	BPSL2224	39	1694592	1693561	1583521	1582490	1594441	1593410	1684200	1683169	1848085	1847054	3228121	3229152	1390250	1389219	1596427	1595396	2110682	2109651	1639529	1638498
2366	BPSL2225	39	1695419	1694643	1584348	1583572	1595268	1594492	1685027	1684251	1848912	1848136	3227294	3228070	1391077	1390301	1597254	1596478	2111509	2110733	1640356	1639580
2367	BPSL2226	39	1696287	1695442	1585216	1584371	1596136	1595291	1685895	1685050	1849780	1848935	3226426	3227271	1391945	1391100	1598122	1597277	2112377	2111532	1641224	1640379
2368	BPSL2227	39	1697235	1696360	1586164	1585289	1597084	1596209	1686843	1685968	1850728	1849853	3225478	3226353	1392893	1392018	1599070	1598195	2113325	2112450	1642172	1641297
2369	BPSL2228	39	1698037	1697243	1586966	1586172	1597886	1597092	1687645	1686851	1851530	1850736	3224676	3225470	1393695	1392901	1599872	1599078	2114127	2113333	1642974	1642180
2370	BPSL2229	39	1703877	1698037	1596002	1595937	1606902	1603396	1696661	1693155	1860546	1857040	3215660	3219166	1402711	1399205	1608888	1605382	2123143	2119637	1651990	1648484
2371	BPSL2230	39	1708453	1707056	1597402	1596005	1608302	1606905	1698061	1696664	1861946	1860549	3214260	3215657	1404111	1402714	1610288	1608891	2124538	2123465	1653385	1652312
2372	BPSL2231	39	1709971	1708673	1598920	1597622	1609820	1608522	1699579	1698281	1863464	1862166	3212742	3214040	1405629	1404331	1611806	1610508	2126056	2124758	1654903	1653605
2373	BPSL2232	39	1711931	1710042	1600880	1598991	1611780	1609891	1701539	1699650	1865424	1863535	3210782	3212671	1407589	1405700	1613766	1611877	2128016	2126127	1656863	1654974
2374	BPSL2233	39	1713418	1711931	1602367	1600880	1613267	1611780	1703026	1701539	1866911	1865424	3209295	3210782	1409076	1407589	1615253	1613766	2129503	2128016	1658350	1656863
2375	BPSL2234	39	1714821	1716095	1603770	1605044	1614670	1615944	1704429	1705703	1868314	1869588	3207892	3206618	1410479	1411753	1616656	1617930	2130906	2132180	1659753	1661027
2376	BPSL2235	39	1716095	1719238	1605044	1608187	1615944	1619087	1705703	1708846	1869588	1872731	3206618	3203475	1411753	1414896	1617930	1621073	2132180	2135323	1661027	1664170
2377	BPSL2236	39	1719319	1720536	1608268	1609461	1619168	1620361	1708927	1710120	1872812	1874005	3203394	3202177	1414977	1416194	1621154	1622347	2135404	2136603	1664251	1665444
2378	BPSL2237	39	1722410	1720581	1611633	1609804	1622917	1621088	1712034	1710205	1875955	1874126	3200303	3202132	1418068	1416239	1624325	1622496	2138490	2136661	1667385	1665556
2379	BPSL2238	39	1723648	1722605	1612871	1611828	1624155	1623112	1713272	1712229	1877193	1876150	3199065	3200108	1419306	1418263	1625563	1624520	2139728	2138685	1668623	1667580
2380	BPSL2239	39	1726177	1724930	1615400	1614153	1626684	1625437	1715801	1714554	1879722	1878475	3196529	3197776	1421835	1420588	1628092	1626845	2142257	2141010	1671152	1669905
2381	BPSL2240	39	1727987	1726521	1617210	1615744	1628494	1627028	1717611	1716145	1881532	1880066	3194719	3196185	1423645	1422179	1629902	1628436	2144067	2142601	1672962	1671496
2382	BPSL2241	39	1729002	1728034	1618225	1617257	1629509	1628541	1718626	1717658	1882547	1881579	3193704	3194672	1424660	1423692	1630917	1629949	2145082	2144114	1673977	1673009
2383	BPSL2242	39	1730044	1729109	1619267	1618332	1630551	1629616	1719668	1718733	1883589	1882654	3192662	3193597	1425702	1424767	1631959	1631024	2146124	2145189	1675019	1674084

2384	BPSL2243	39	1731489	1730095	1620712	1619318	1631996	1630602	1721113	1719719	1885034	1883640	3191217	3192611	1427147	1425753	1633404	1632010	2147569	2146175	1676464	1675070
2385	BPSL2244	39	1731778	1732440	1621001	1621663	1632285	1632947	1721402	1722064	1885323	1885985	3190928	3190266	1427436	1428098	1633693	1634355	2147858	2148520	1676753	1677415
2386	BPSL2245	39	1732500	1733072	1621723	1622295	1633007	1633579	1722124	1722696	1886045	1886617	3190206	3189634	1428158	1428730	1634415	1634987	2148580	2149152	1677475	1678047
2387	BPSL2246	39	1733156	1733644	1622379	1622867	1633663	1634151	1722780	1723268	1886701	1887189	3189550	3189062	1428814	1429302	1635071	1635559	2149236	2149724	1678131	1678619
2388	BPSL2247	39	1733711	1734469	1622934	1623692	1634218	1634976	1723335	1724093	1887256	1888014	3188995	3188237	1429369	1430127	1635626	1636384	2149791	2150549	1678686	1679444
2389	BPSL2248	39	1735593	1734811	1624816	1624034	1636100	1635318	1725217	1724435	1889138	1888356	3187113	3187895	1431251	1430469	1637508	1636726	2151673	2150891	1680568	1679786
2390	BPSL2249	39	1736695	1735787	1625918	1625010	1637202	1636294	1726319	1725411	1890240	1889332	3186011	3186919	1432353	1431445	1638610	1637702	2152775	2151867	1681670	1680762
2391	BPSL2250	39	1736996	1737796	1626219	1627019	1637503	1638303	1726620	1727420	1890541	1891341	3185710	3184910	1432654	1433454	1638911	1639711	2153076	2153876	1681971	1682771
2392	BPSL2251	39	1738351	1738082	1627574	1627305	1638858	1638589	1727975	1727706	1891896	1891627	3184355	3184624	1434009	1433740	1640266	1639997	2154431	2154162	1683326	1683057
2393	BPSL2252	39	1739693	1742365	1628916	1631588	1640200	1642872	1729317	1731989	1893238	1895910	3183013	3180341	1435351	1438023	1641608	1644280	2155773	2158445	1684668	1687340
2394	BPSL2253	39	1742355	1743650	1631578	1632873	1642862	1644157	1731979	1733274	1895900	1897195	3180351	3179056	1438013	1439308	1644270	1645565	2158435	2159730	1687330	1688625
2395	BPSL2254	39	1744055	1743636	1633278	1632859	1644562	1644143	1733679	1733260	1897600	1897181	3178651	3179070	1439713	1439294	1645970	1645551	2160135	2159716	1689030	1688611
2396	BPSL2255	39	1744270	1745430	1633493	1634653	1644777	1645937	1733894	1735054	1897815	1898975	3178436	3177276	1439928	1441088	1646185	1647345	2160350	2161510	1689245	1690405
2397	BPSL2256	39	1746969	1746196	1636192	1635419	1647476	1646703	1736593	1735820	1900514	1899741	3175737	3176510	1442627	1441854	1648884	1648111	2163049	2162276	1691944	1691171
2398	BPSL2257	39	1748116	1746977	1637339	1636200	1648623	1647484	1737740	1736601	1901661	1900522	3174590	3175729	1443774	1442635	1650031	1648892	2164196	2163057	1693091	1691952
2399	BPSL2258	39	1749129	1748230	1638352	1637453	1649636	1648737	1738753	1737854	1902674	1901775	3173577	3174476	1444787	1443888	1651044	1650145	2165209	2164310	1694104	1693205
2400	BPSL2259	39	1749791	1749177	1639014	1638400	1650298	1649684	1739415	1738801	1903336	1902722	3172915	3173529	1445449	1444835	1651706	1651092	2165871	2165257	1694766	1694152
2401	BPSL2260	39	1750990	1749791	1640213	1639014	1651497	1650298	1740614	1739415	1904535	1903336	3171716	3172915	1446648	1445449	1652905	1651706	2167070	2165871	1695965	1694766
2402	BPSL2261	39	1751664	1751002	1640887	1640225	1652171	1651509	1741288	1740626	1905209	1904547	3171042	3171704	1447322	1446660	1653579	1652917	2167744	2167082	1696639	1695977
2403	BPSL2262	39	1752392	1751766	1641615	1640989	1652899	1652273	1742016	1741390	1905937	1905311	3170314	3170940	1448050	1447424	1654307	1653681	2168472	2167846	1697367	1696741
2404	BPSL2263	39	1753296	1752460	1642519	1641683	1653803	1652967	1742920	1742084	1906841	1906005	3169410	3170246	1448954	1448118	1655211	1654375	2169376	2168540	1698271	1697435
2405	BPSL2264	39	1754411	1753524	1643634	1642747	1654918	1654031	1744035	1743148	1907956	1907069	3168295	3169182	1450069	1449182	1656326	1655439	2170491	2169604	1699386	1698499
2406	BPSL2265	39	1755418	1754570	1644641	1643793	1655925	1655077	1745042	1744194	1908963	1908115	3167288	3168136	1451076	1450228	1657333	1656485	2171498	2170650	1700393	1699545
2407	BPSL2266	39	1755595	1756116	1644818	1645339	1656102	1656623	1745219	1745740	1909140	1909661	3167111	3166590	1451253	1451774	1657510	1658031	2171675	2172196	1700570	1701091
2408	BPSL2267	39	1756158	1757105	1645381	1646328	1656665	1657612	1745782	1746729	1909703	1910650	3166548	3165601	1451816	1452763	1658073	1659020	2172238	2173185	1701133	1702080
2409	BPSL2268	39	1757192	1757524	1646415	1646747	1657699	1658031	1746816	1747148	1910737	1911069	3165514	3165182	1452850	1453182	1659107	1659439	2173272	2173604	1702167	1702499
2410	BPSL2269	39	1758184	1757756	1647407	1646979	1658691	1658263	1747808	1747380	1911729	1911301	3164522	3164950	1453842	1453414	1660099	1659671	2174264	2173836	1703159	1702731
2411	BPSL2270	39	1759559	1758279	1648782	1647502	1660066	1658786	1749183	1747903	1913104	1911824	3163147	3164427	1455217	1453937	1661474	1660194	2175639	2174359	1704534	1703254
2412	BPSL2271	39	1760517	1759666	1649740	1648889	1661024	1660173	1750141	1749290	1914062	1913211	3162189	3163040	1456175	1455324	1662432	1661581	2176597	2175746	1705492	1704641
2413	BPSL2272	39	1762175	1760517	1651398	1649740	1662682	1661024	1751799	1750141	1915720	1914062	3160531	3162189	1457833	1456175	1664090	1662432	2178255	2176597	1707150	1705492
2414	BPSL2273	39	1763259	1762435	1652482	1651658	1663766	1662942	1752883	1752059	1916804	1915980	3159447	3160271	1458917	1458093	1665174	1664350	2179339	2178515	1708234	1707410
2415	BPSL2274	39	1765866	1763299	1655877	1654108	1667003	1665735	1755490	1752923	1919411	1916844	3156840	3159407	1461524	1458957	1667781	1665214	2181946	2179379	1710287	1709466
2416	BPSL2275	39	1766665	1765880	1656676	1655891	1667802	1667017	1756289	1755504	1920210	1919425	3156041	3156826	1462323	1461538	1668580	1667795	2182745	2181960	1711086	1710301
2417	BPSL2276	39	1767392	1766673	1657403	1656684	1668529	1667810	1757016	1756297	1920937	1920218	3155314	3156033	1463050	1462331	1669307	1668588	2183472	2182753	1711813	1711094
2418	BPSL2277	39	1768665	1767415	1658676	1657426	1669802	1668552	1758289	1757039	1922210	1920960	3154041	3155291	1464323	1463073	1670580	1669330	2184745	2183495	1713086	1711836
2419	BPSL2278	39	1769237	1770310	1659248	1660321	1670374	1671033	1758861	1759934	1922782	1923855	3153469	3152396	1464895	1465968	1671152	1672225	2185317	2186390	1713658	1714731
2420	BPSL2279	39	1770329	1772020	1660340	1662031	1672178	1673869	1759953	1761644	1923874	1925565	3152377	3150686	1465987	1467678	1672244	1673935	2186409	2188100	1714750	1716441
2421	BPSL2280	39	1772386	1773489	1662397	1663500	1674235	1675338	1762010	1763113	1925931	1927034	3150320	3149217	1468044	1469147	1674301	1675404	2188466	2189569	1716807	1717910
2422	BPSL2281	39	1773585	1775108	1663596	1665119	1675434	1676957	1763209	1764732	1927130	1928653	3149121	3147598	1469243	1470766	1675500	1677023	2189665	2191188	1718006	1719529
2423	BPSL2282	39	1775471	1776193	1665482	1666204	1677320	1678042	1765095	1765817	1929016	1929738	3147227	3146505	1471137	1471859	1677394	1678116	2191551	2192273	1719892	1720614
2424	BPSL2283	39	1776819	1776613	1666830	1666624	1678668	1678462	1766443	1766237	1930364	1930158	3145879	3146085	1472485	1472279	1678742	1678536	2192899	2192693	1721240	1721034
2425	BPSL2284	39	1777179	1776841	1667190	1666852	1679028	1678690	1766803	1766465	1930724	1930386	3145519	3145857	1472845	1472507	1679102	1678764	2193259	2192921	1721600	1721262
2426	BPSL2285	39	1779140	1777275	1669151	1667286	1680989	1679124	1768764	1766899	1932685	1930820	3143558	3145423	1474806	1472941	1681063	1679198	2195220	2193355	1723561	1721696
2427	BPSL2286	39	1779733	1779209	1669744	1669220	1681582	1681058	1769357	1768833	1933278	1932754	3142965	3143489	1475399	1474875	1681656	1681132	2195813	2195289	1724154	1723630
2428	BPSL2287	39	1780169	1779849	1670180	1669860	1682018	1681698	1769793	1769473	1933714	1933394	3142529	3142849	1475835	1475515	1682092	1681772	2196249	2195929	1724590	1724270
2429	BPSL2288	39	1780659	1780255	1670670	1670266	1682508	1682104	1770283	1769879	1934204	1933800	3142039	3142443	1476325	1475921	1682582	1682178	2196739	2196335	1725080	1724676
2430	BPSL2289	39	1781948	1780728	1671959	1670739	1683797	1682577	1771572	1770352	1935493	1934273	3140750	3141970	1477614	1476394	1683871	1682651	2198028	2196808	1726369	1725149
2431	BPSL2290	39	1782556	1782020	1672567	1672031	1684405	1683869	1772180	1771644	1935846	1935565	3140142	3140678	1478222	1477686	1684479	1683943	2198636	2198100	1726977	1726441
2432	BPSL2291	39	1783137	1782661	1673148	1672672	1684986	1684510	1772761	1772285	1936501	1936025	3139561	3140037	1478803	1478327	1685060	1684584	2199217	2198741	1727558	1727082
2433	BPSL2292	39	1783489	1783232	1673500	1673243	1685338	1685081	1773113	1772856	1936853	1936596	3139209	3139466	1479155	1478898	1685412	1685155	2199569	2199312	1727910	1727653
2434	BPSL2293	39	1785051	1783603	1675062	1673614	1686900	1685452	1774675	1773227	1938415	1936967	3137647	3139095	1480717	1479269	1686974	1685526	2201131	2199683	1729472	1728024

2435	BPSL2294		39	1785922	1785203	1675933	1675214	1687771	1687052	1775546	1774827	1939286	1938567	3136769	3137488	1481595	1480876	1687852	1687133	2202002	2201283	1730343	1729624
2436	BPSL2295		39	1786105	1786914	1676116	1676925	1687954	1688763	1775729	1776538	1939469	1940278	3136586	3135777	1481778	1482587	1688035	1688844	2202185	2202994	1730526	1731335
2437	BPSL2296_IS	Chr1_105a_ISBma2_A		1787046	1788506	1677057	1678517	1688895	1690355	1776670	1778130	1940410	1941870	3134127	3135699	1482719	1484179	1688976	1690436	2203126	2204586	1731467	1732927
2438		Chr1_105b_ISBma2_A		1791043	1792615	1681054	1682626	1692892	1694464	1780667	1782239	1944407	1945979	3130076	3131648	1486716	1488288	1692973	1694545	2207123	2208695	1740573	1742147
2439	BPSL2297		40	1789899	1790975	1679910	1680986	1691748	1692824	1779523	1780599	1943263	1944339	3132792	3131716	1485572	1486648	1691829	1692905	2205979	2207055	1739429	1740505
2440	BPSL2298		40	1789187	1788621	1679198	1678632	1691036	1690470	1778811	1778245	1942551	1941985	3133504	3134070	1484860	1484294	1691117	1690551	2205267	2204701	1738717	1738151
2441		Chr1_106_ISBma2_C																				1736522	1738094
2442		Chr1_107a_ISBma2_A		1787046	1788506	1677057	1678517	1688895	1690355	1776670	1778130	1940410	1941870	3134127	3135699	1482719	1484179	1688976	1690436	2203126	2204586	1731467	1732927
2443		Chr1_107b_ISBma2_A		1791043	1792615	1681054	1682626	1692892	1694464	1780667	1782239	1944407	1945979	3130076	3131648	1486716	1488288	1692973	1694545	2207123	2208695	1740573	1742147
2444	BPSL2299		41	1794587	1792821	1684598	1682832	1696436	1694670	1784211	1782445	1947951	1946185	3128104	3129870	1490260	1488494	1696517	1694751	2210667	2208901	1744119	1742353
2445	BPSL2300		41	1796484	1795825	1686708	1686358	1699193	1698999	1786108	1785449	1949848	1949189	3126207	3126866	1492157	1491498	1698414	1697755	2212564	2211905		
2446	BPSL2301		41	1799313	1796620	1689537	1686844	1702022	1699329	1788937	1786244	1952677	1949984	3123378	3126071	1494986	1492293	1701243	1698550	2215393	2212700	1748506	1745813
2447	BPSL2302		41	1799556	1802093	1689780	1692317	1702265	1704802	1789180	1791717	1952920	1955457	3123135	3120598	1495229	1497766	1701486	1704023	2215636	2218173	1748749	1751286
2448	BPSL2303		41	1802093	1802728	1692317	1692952	1704802	1705437	1791717	1792352	1955457	1956092	3120598	3119963	1497766	1498401	1704023	1704658	2218173	2218808	1751286	1751921
2449	BPSL2304		41	1803048	1803902	1693272	1694126	1705757	1706611	1792672	1793526	1956412	1957266	3119643	3118789	1498721	1499575	1704978	1705832	2219128	2219982	1752241	1753095
2450	BPSL2305		41	1804583	1806679	1694807	1696903	1707292	1709388	1794207	1796303	1957939	1960035	3118108	3116012	1500256	1502352	1706521	1708617	2220679	2222775	1753792	1755888
2451	BPSL2306		41	1808114	1806915	1698338	1697139	1710823	1709624	1797738	1796539	1961470	1960271	3114577	3115776	1503787	1502588	1710052	1708853	2224210	2223011	1757323	1756124
2452	BPSL2307		41	1808837	1810114	1699061	1700338	1711546	1712823	1798461	1799738	1962193	1963470	3113854	3112577	1504510	1505787	1710775	1712052	2224940	2226217	1758044	1759321
2453	BPSL2308		41	1810161	1811543	1700385	1701767	1712870	1714252	1799785	1801167	1963517	1964899	3112530	3111148	1505834	1507216	1712099	1713481	2226264	2227646	1759368	1760750
2454	BPSL2309		41	1811797	1815519	1702021	1705743	1714506	1718228	1801421	1805143	1965153	1968875	3110894	3107172	1507470	1511192	1713735	1717457	2227900	2231622	1761004	1764726
2455	BPSL2310		41	1815519	1817069	1705743	1707293	1718228	1719778	1805143	1806693	1968875	1970425	3107172	3105622	1511192	1512742	1717457	1719007	2231622	2233172	1764726	1766276
2456	BPSL2311		41	1817096	1817770	1707320	1707994	1719805	1720479	1806720	1807394	1970452	1971126	3105595	3104921	1512769	1513443	1719034	1719708	2233199	2233873	1766303	1766977
2457	BPSL2312		41	1817810	1818493	1708034	1708717	1720519	1721202	1807434	1808117	1971166	1971849	3104881	3104198	1513483	1514166	1719748	1720431	2233913	2234596	1767017	1767700
2458	BPSL2313		41	1818638	1820569	1709297	1711228	1721687	1723618	1808262	1810193	1971995	1973926	3104053	3102122	1514311	1516242	1720576	1722507	2234741	2236672	1768056	1769987
2459	BPSL2314		41	1820596	1821294	1711255	1711953	1723645	1724343	1810220	1810918	1973953	1974651	3102095	3101397	1516269	1516967	1722534	1723232	2236699	2237397	1770014	1770712
2460	BPSL2315		41	1821444	1822217	1712103	1712876	1724493	1725266	1811068	1811841	1974801	1975574	3101247	3100474	1517117	1517890	1723382	1724155	2237547	2238320	1770862	1771635
2461		Chr1_108_ISBma2_A	41	1822448	1824020	1713107	1714679	1725497	1727069	1812072	1813644	1975805	1977377	3098671	3100243	1518121	1519693	1724386	1725958	2238551	2240122	1771866	1773437
2462	BPSL2316		41	1825634	1824102	1716293	1714761	1728683	1727151	1815258	1813726	1978992	1977460	3097056	3098588	1521308	1519776	1727573	1726041	2241737	2240205	1775052	1773520
2463	BPSL2317		41	1826766	1825675	1717425	1716334	1729815	1728724	1816390	1815299	1980124	1979033	3095924	3097015	1522440	1521349	1728705	1727614	2242869	2241778	1776184	1775093
2464		Chr1_109_ISBma2_B	41	1826885	1828457	1717544	1719116	1729934	1731506	1816509	1818081									2242987	2244559	1776302	1777874
2465	BPSL2318		41	1829990	1828578	1720649	1719237	1733039	1731627	1819614	1818202	1981754	1980342	3094294	3095706	1524070	1522658	1730335	1728923	2246092	2244680	1779407	1777995
2466	BPSL2319		41	1830371	1830856	1721030	1721515	1733420	1733905	1819995	1820480	1982135	1982620	3093913	3093428	1524451	1524936	1730716	1731201	2246473	2246958	1779788	1780273
2467	BPSL2320		41	1831930	1831115	1722589	1721774	1734979	1734164	1821554	1820739	1983694	1982879	3092354	3093169	1526010	1525195	1732275	1731460	2248032	2247217	1781347	1780532
2468	BPSL2321		41	1832760	1831930	1723419	1722589	1735809	1734979	1822384	1821554	1984524	1983694	3091524	3092354	1526840	1526010	1733105	1732275	2248862	2248032	1782177	1781347
2469	BPSL2322		41	1834293	1833307	1724952	1723966	1737342	1736356	1823917	1822931	1986057	1985071	3089991	3090977	1528373	1527387	1734638	1733652	2250395	2249409	1783710	1782724
2470	BPSL2323		41	1835302	1834313	1725961	1724972	1738351	1737362	1824926	1823937	1987066	1986077	3088982	3089971	1529382	1528393	1735647	1734658	2251404	2250415	1784719	1783730
2471	BPSL2324		41	1839297	1835407	1730241	1727311	1742346	1738456	1828951	1825031	1991060	1990725	3084987	3088877	1533377	1529487	1739249	1735752	2255399	2251509	1788714	1784824
2472	BPSL2325		41	1839555	1840928	1730499	1731872	1742604	1743977	1829209	1830582	1991318	1992691	3084729	3083356	1533635	1535008	1739544	1740917	2255657	2257030	1788997	1790370
2473	BPSL2326		41	1840984	1841256	1731928	1732200	1744033	1744305	1830638	1830910	1992747	1993019	3083300	3083028	1535064	1535336	1740973	1741245	2257086	2257358	1790426	1790698
2474	BPSL2327		41	1842775	1841435	1733719	1732379	1745824	1744484	1832429	1831089	1994538	1993198	3081509	3082849	1536855	1535515	1742764	1741424	2258877	2257537	1792217	1790877
2475	BPSL2328		41	1844072	1842975	1735016	1733919	1747121	1746024	1833726	1832629	1995835	1994738	3080212	3081309	1538152	1537055	1744061	1742964	2260174	2259077	1793514	1792417
2476	BPSL2329		41	1846288	1844498	1737232	1735442	1749337	1747547	1835942	1834152	1998042	1996252	3077996	3079786	1540368	1538578	1746277	1744487	2262399	2260609	1795739	1793949
2477	BPSL2330		41	1846397	1847296	1737341	1738240	1749446	1750345	1836051	1836347	1998151	1999050	3077887	3076988	1540477	1541376	1746386	1747285	2262508	2263407	1795848	1796747
2478		Chr1_110_ISBma2_A		1847646	1849097	1738590	1740041	1750695	1752146	1837077	1838528	1999400	2000851	3075066	3076638	1541726	1543298	1747635	1749207	2263757	2265208	1797097	1798548
2479		Chr1_111_IS407A_A		1849090	1850325	1740034	1741269	1752139	1753374	1838521	1839756	2000844	2002079	3073105	3074340	1544024	1545259	1749933	1751168	2265201	2266436	1798541	1799776
2480	BPSL2331																						
2481	BPSL2332																						
2482	BPSL2333																						
2483	BPSL2334																						
2484		Chr1_112_IS407A_A		683232	684467	560092	561327	563350	564585	678191	679426	719456	720691	2955982	2957217	1661173	1662408	1867070	1868305	2356417	2357652	652265	653500
2485	BPSL2335		42	682620	683231	559480	560091	562738	563349	677579	678190	718844	719455	2955370	2955981	1663020	1662409	1868917	1868306	2358264	2357653	651653	652264

2486	BPSL2336	42	681251	682582	558111	559442	561369	562700	676210	677541	717475	718806	2954001	2955332	1664389	1663058	1870286	1868955	2359633	2358302	650284	651615
2487	BPSL2337	42	680427	681215	557287	558075	560545	561333	675386	676174	716651	717439	2953177	2953965	1665213	1664425	1871110	1870322	2360457	2359669		
2488	BPSL2338	42	678605	679405	555465	556265	558723	559523	673564	674364	714829	715629	2951355	2952155	1667035	1666235	1872932	1872132	2362279	2361479	647626	648426
2489	BPSL2339	42	676979	678358	553839	555218	557097	558476	671938	673317	713203	714582	2949729	2951108	1668661	1667282	1874558	1873179	2363905	2362526	646000	647379
2490	BPSL2340	42	675742	676962	552602	553822	555860	557080	670701	671921	711966	713186	2948492	2949712	1669898	1668678	1875795	1874575	2365142	2363922	644763	645983
2491	BPSL2341	42	675086	675742	551946	552602	555204	555860	670045	670701	711310	711966	2947836	2948492	1670554	1669898	1876451	1875795	2365798	2365142	644107	644763
2492	BPSL2342	42	673398	675083	550258	551943	553516	555201	668357	670042	709622	711307	2946148	2947833	1672242	1670557	1878139	1876454	2367486	2365801	642419	644104
2493	BPSL2343	42	672660	673352	549520	550212	552778	553470	667619	668311	708884	709576	2945410	2946102	1672980	1672288	1878877	1878185	2368224	2367532	641681	642373
2494	BPSL2344	42	671132	672652	547992	549512	551250	552770	666091	667611	707356	708876	2943882	2945402	1674508	1672988	1880405	1878885	2369752	2368232	640153	641673
2495	BPSL2345	42	669672	670460	546532	547320	549790	550578	664631	665419	705896	706684	2942422	2943210	1675968	1675180	1881865	1881077	2371212	2370424	638693	639481
2496	BPSL2346	42	668557	669564	545417	546424	548675	549682	663516	664523	704781	705788	2941307	2942314	1677083	1676076	1882980	1881973	2372327	2371320	637578	638585
2497	BPSL2347	42	666614	667336	543474	544196	546143	546865	661573	662295	702832	703554	2939370	2940092	1679026	1678304	1884923	1884201	2374276	2373554	635635	636357
2498	BPSL2348	42	665092	665760	541952	542620	544621	545289	660051	660719	701310	701978	2937848	2938516	1680548	1679880	1886445	1885777	2375798	2375130	634113	634781
2499	BPSL2349	42	663550	665007	540410	541867	543079	544536	658509	659966	699768	701225	2936306	2937763	1682090	1680633	1887987	1886530	2377340	2375883	632602	632991
2500	BPSL2350	42	663131	661968	539991	538828	542660	541497	658090	656927	699349	698186	2935887	2934724	1682509	1683672	1888406	1889569	2377759	2378922	632183	631020
2501	BPSL2351	42	661243	658958	538103	535818	540772	538487	656202	653917	697461	695176	2933999	2931714	1684397	1686682	1890294	1892447	2379647	2381932	630295	628010
2502	BPSL2352	42	658670	658533	535722	535525	537718	537521	653819	653622	695017	694820	2931631	2931434	1686765	1686962	1892740	1892937	2382015	2382212	627730	627533
2503	BPSL2354	42	658477	657347	535324	534194	537320	536190	653421	652291	694619	693489	2931233	2930103	1687163	1688293	1893138	1894268	2382413	2383543	627332	626202
2504	BPSL2355	42	657207	655987	534054	532834	536050	534830	652151	650931	693349	692129	2929963	2928743	1688433	1689653	1894408	1895628	2383683	2384903	626062	624842
2505	BPSL2356	42	655386	653626	532233	530473	534229	532469	650330	648570	691528	689768	2928142	2926382	1690254	1692014	1896229	1897989	2385504	2387264	624241	622481
2506	BPSL2357	42	653620	653411	530467	530258	532463	532254	648564	648355	689762	689553	2926376	2926167	1692020	1692229	1897995	1898204	2387270	2387479	622475	622266
2507	BPSL2358	42	653389	652673	530236	529520	532232	531516	648333	647617	689531	688815	2926145	2925429	1692251	1692967	1898226	1898507	2387501	2388217	622244	621528
2508	BPSL2359	42	651969	652412	528816	529259	530812	531255	646913	647356	688111	688554	2924725	2925168	1693671	1693228	1899647	1899204	2388921	2388478	620824	621267
2509	BPSL2360	42	651450	651926	528297	528773	530293	530769	646394	646870	687592	688068	2924206	2924682	1694190	1693714	1900166	1899690	2389440	2388964	620305	620781
2510	BPSL2361	42	650509	651393	527356	528240	529352	530236	645453	646337	686649	687533	2923263	2924147	1695133	1694249	1901109	1900225	2390383	2389499	619362	620246
2511	BPSL2362	42	649434	650465	526281	527312	528277	529308	644378	645409	685574	686605	2922188	2923219	1696208	1695177	1902184	1901153	2391458	2390427	618287	619318
2512	BPSL2363	42	648128	649342	524975	526189	526971	528185	643072	644286	684268	685482	2920882	2922096	1697494	1696280	1903490	1902276	2392764	2391550	616981	618195
2513	BPSL2364	42	647908	646595	524755	523442	526751	525438	642852	641539	684048	682735	2920662	2919349	1697714	1699027	1903710	1905023	2392984	2394297	616761	615448
2514	BPSL2365	42	645923	646612	522770	523459	524766	525455	640867	641556	682063	682752	2918677	2919366	1699699	1699010	1905695	1905006	2394969	2394280	614776	615465
2515	BPSL2366	42	644469	645911	521316	522758	523312	524754	639413	640855	680609	682051	2917223	2918665	1701153	1699711	1907149	1905707	2396423	2394981	613322	614764
2516	BPSL2367	42	642392	644311	519239	521158	521235	523154	637336	639255	678532	680451	2915146	2917065	1703230	1701311	1909227	1908220	2398500	2396581	611245	613164
2517	BPSL2368	42	641858	640665	518705	517512	520701	519508	636802	635609	677998	676805	2914612	2913419	1703764	1704957	1909761	1910954	2399034	2400227	610711	609518
2518	BPSL2369	42	640173	639022	517020	515869	519016	517865	635117	633966	676313	675162	2912927	2911776	1705449	1706600	1911446	1912597	2400719	2401870	609026	607875
2519	BPSL2370	42	638468	638124	515315	514971	517311	516967	633412	633068	674608	674264	2911222	2910878	1707154	1707498	1913151	1913495	2402424	2402768	607321	606977
2520	BPSL2371	42	638116	636884	514963	513731	516959	515727	633060	631828	674256	673024	2910870	2909638	1707506	1708738	1913503	1914735	2402776	2404008	606969	605737
2521	BPSL2372	42	636879	635650	513726	512497	515722	514493	631823	630594	673019	671790	2909633	2908404	1708743	1709972	1914740	1915969	2404013	2405242	605732	604503
2522	BPSL2373	42	635650	635162	512497	512009	514493	514005	630594	630106	671790	671302	2908404	2907916	1709972	1710460	1915969	1916457	2405242	2405730	604503	604015
2523	BPSL2374	42	635162	634617	512009	511464	514005	513460	630106	629561	671302	670757	2907916	2907371	1710460	1711005	1916457	1917002	2405730	2406275	604015	603470
2524	BPSL2375	42	634020	634316	510867	511163	512863	513159	628964	629260	670160	670456	2906774	2907070	1711602	1711306	1917599	1917303	2406872	2406576	602873	603169
2525	BPSL2376	42	633829	633344	510676	510191	512672	512187	628773	628288	669969	669484	2906583	2906098	1711793	1712278	1917790	1918275	2407063	2407548	602682	602197
2526	BPSL2377	42	632678	633124	509525	509971	511521	511967	627622	628068	668810	669256	2905432	2905878	1712932	1712498	1918949	1918503	2408214	2407768	601531	601977
2527	BPSL2378	42	632299	631412	509146	508259	511142	510255	627243	626356	668431	667544	2905053	2904166	1713311	1714198	1919328	1920215	2408593	2409480	601152	600265
2528	BPSL2379	42	631405	631121	508252	507968	510248	509964	626349	626065	667537	667253	2904159	2903875	1714205	1714489	1920222	1920506	2409487	2409771	600258	599974
2529	BPSL2380	42	629124	628810	506260	505976	508256	507972	624357	624073	665545	665261	2902167	2901883	1716197	1716481	1922214	1922498	2411479	2411763	598266	597982
2530	BPSL2381	42	628806	628477	505653	505324	507649	507320	623750	623421	664938	664609	2901560	2901231	1716804	1717133	1922821	1923150	2412086	2412415	597659	597330
2531	BPSL2382	42	628320	627091	505167	503938	507163	505934	623264	622035	664452	663223	2901074	2899845	1717290	1718519	1923307	1924536	2412572	2413801	597173	595944
2532	BPSL2383	42	626022	626813	502869	503660	504865	505656	620966	621757	662147	662938	2898783	2899574	1719581	1718790	1925598	1924807	2414877	2414086	594854	595645
2533	BPSL2384	42	625701	625261	502548	502108	504544	504104	620645	620205	661826	661386	2898462	2898022	1719902	1720342	1925919	1926359	2415198	2415638	594533	594093
2534	BPSL2385	42	624162	625208	501009	502055	503005	504051	619106	620152	660287	661333	2896923	2897969	1721441	1720395	1927458	1926412	2416737	2415691	592994	594040
2535	BPSL2386	42	622838	624175	499685	501022	501681	503018	617782	619119	658963	660300	2895599	2896936	1722765	1721428	1928782	1927445	2418061	2416724	591670	593007
2536	BPSL2387	42	621361	622821	498208	499668	500204	501664	616305	617765	657486	658946	2894245	2895582	1724119	1722782	1930136	1928799	2419538	2418078	590193	591653

2537	BPSL2388		42	620325	621353	497172	498200	499168	500196	615269	616297	656450	657478	2893100	2894128	1725264	1724236	1931281	1930253	2420574	2419546	589157	590185
2538	BPSL2389		42	619288	620325	496135	497172	498131	499168	614232	615269	655413	656450	2892063	2893100	1726301	1725264	1932318	1931281	2421611	2420574	588120	589157
2539	BPSL2390		42	618010	619239	494857	496086	496853	498082	612954	614183	654135	655364	2890785	2892014	1727579	1726350	1933596	1932367	2422889	2421660	586842	588071
2540	BPSL2391		42	616223	617266	493070	494113	495066	496109	611167	612210	652348	653391	2888998	2890041	1729367	1728324	1935383	1934340	2424676	2423633	585055	586098
2541	BPSL2392		42	615410	616201	492257	493048	494253	495044	610354	611145	651535	652326	2888185	2888976	1730180	1729389	1936196	1935405	2425489	2424698	584242	585033
2542	BPSL2393		42	614675	615385	491522	492232	493518	494228	609619	610329	650800	651510	2887450	2888160	1730915	1730205	1936931	1936221	2426224	2425514	583507	584217
2543	BPSL2394		42	614589	614675	491436	491522	493432	493518	609533	609619	650714	650800	2887364	2887450	1731001	1730915	1937017	1936931	2426310	2426224	583421	583507
2544	BPSL2395_putative lipoprotein		42																				
2545		Chr1_113_IS407A_A		613104	614339	489951	491186	491947	493182	608048	609283	649478	650713	2886128	2887363	1731002	1732237	1937018	1938253	2426311	2427546	582185	583420
2546	BPSL2396		43	601822	600779	478669	477626	480665	479622	596766	595723	638215	637172	2874879	2873836	1743480	1744523	1949502	1950545			581060	582103
2547	BPSL2397		43	602706	601822	479553	478669	481549	480665	597650	596766	639099	638215	2875763	2874879	1742596	1743480	1948618	1949502			580176	581060
2548	BPSL2398		43	604131	602713	480978	479560	482974	481556	599075	597657	640524	639106	2877188	2875770	1741171	1742589	1947193	1948611			578751	580169
2549	BPSL2399		43	605421	604246	482268	481093	484264	483089	600365	599190	641814	640639	2878478	2877303	1739881	1741056	1945903	1947078			577461	578636
2550	BPSL2400		43	606048	607103	482895	483950	484891	485946	600992	602047	642441	643496	2879105	2880160	1739254	1738199	1945276	1944221			576834	575779
2551	BPSL2401		43	608570	607545	485417	484392	487413	486388	603514	602489	644956	643931	2881606	2880581	1736753	1737778	1942775	1943800			574305	575330
2552	BPSL2402		43	609093	609953	485940	486800	487936	488796	604037	604897	645467	646327	2882117	2882977	1736248	1735388	1942264	1941404			573794	572934
2553	BPSL2403		43	610626	612725	487473	489572	489469	491568	605570	607669	647000	649099	2883650	2885749	1734715	1732616	1940731	1938632			572235	571408
2554		Chr1_114_IS407A_A		599462	600697	476309	477544	478305	479540	594406	595641	635855	637090	2872519	2873754	1744605	1745840	1950627	1951862	2426311	2427546	570173	571408
2555	BPSL2404		44	598801	597656	475648	474503	477644	476499	593745	592600	635187	634042	2871858	2870713	1746508	1747653	1952523	1953668	2428207	2429352	569512	568367
2556	BPSL2405		44	597554	596385	474401	473232	476397	475228	592498	591329	633940	632771	2870611	2869442	1747755	1748924	1953770	1954939	2429454	2430623	568265	567096
2557	BPSL2406		44	595817	596269	472664	473116	474660	475112	590761	591213	632203	632655	2868828	2869286	1749538	1749080	1955553	1955095	2431237	2430779	566482	566940
2558	BPSL2407		44	594829	595758	471676	472605	473672	474601	590481	590702	631217	631654	2867813	2868769	1750553	1749597	1956568	1955612	2432252	2431296	565467	566423
2559	BPSL2408		44	593498	594559	470345	471406	472341	473402	588520	589581	629886	630947	2866482	2867543	1751884	1750823	1957899	1956838	2433583	2432522	564136	565197
2560	BPSL2409		44	592933	591071	469780	467918	471776	469914	587955	586093	629321	627459	2865917	2864055	1752449	1754311	1958464	1960326	2434148	2436010	563571	561709
2561	BPSL2410		44	589630	591036	466477	467883	468473	469879	584652	586058	626018	627424	2862614	2864020	1755752	1754346	1961767	1960361	2437451	2436045	560268	561674
2562	BPSL2411		44	588824	589630	465671	466477	467667	468473	583846	584652	625212	626018	2861808	2862614	1756558	1755752	1962573	1961767	2438257	2437451	559462	560268
2563	BPSL2412		44	588464	587808	465311	464655	467307	466651	583486	582830	624852	624196	2861448	2860792	1756918	1757574	1962933	1963589	2438617	2439273	559102	558446
2564	BPSL2413		44	587778	587335	464625	464182	466621	466178	582800	582357	624166	623723	2860762	2860319	1757604	1758047	1963619	1964062	2439303	2439746	558416	557973
2565	BPSL2414		44	587321	586452	464168	463299	466164	465295	582343	581474	623709	622840	2860305	2859436	1758061	1758930	1964076	1964945	2439760	2440629	557959	557090
2566	BPSL2415		44	585812	586312	462659	463159	464655	465155	580834	581334	622200	622700	2858796	2859296	1759570	1759070	1965585	1965085	2441269	2440769	556450	556950
2567	BPSL2416		44	584712	585575	461559	462422	463555	464418	579734	580597	621100	621963	2857696	2858559	1760670	1759807	1966685	1965822	2442369	2441506	555350	556213
2568	BPSL2417		44	584585	583680	461432	460527	463428	462523	579607	578702	620973	620068	2857569	2856664	1760797	1761702	1966812	1967717	2442496	2443401	555223	554318
2569	BPSL2418		44	583510	583001	460357	459848	462353	461844	578532	578023	619898	619389	2856494	2855985	1761872	1762381	1967887	1968396	2443571	2444080	554148	553639
2570	BPSL2419		44	580694	581284	457541	458131	459537	460127	575716	576306	617082	617672	2853678	2854268	1764553	1763963	1970703	1970113	2446252	2445662	551467	552057
2571	BPSL2420		44	578317	580563	455164	457410	457160	459406	573339	575585	614705	616951	2851301	2853547	1766930	1764684	1973080	1970834	2448629	2446383	549090	551336
2572	BPSL2421		44	578099	576882	454946	453729	456942	455725	573121	571904	614487	613270	2851083	2849866	1767148	1768365	1973298	1974515	2448847	2450064	548872	547655
2573	BPSL2422		44	576689	576135	453536	452982	455532	454978	571711	571157	613077	612523	2849673	2849119	1768558	1769112	1974708	1975262	2450257	2450811	547462	546908
2574	BPSL2423		44	575861	574467	452708	451314	454704	453310	570883	569489	612249	610855	2848845	2847451	1769386	1770780	1975536	1976930	2451085	2452479	546634	545240
2575	BPSL2424		44	573322	574347	450169	451194	452165	453190	568344	569369	609710	610735	2846306	2847331	1771925	1770900	1978075	1977056	2453624	2452599	544095	545120
2576	BPSL2425		44	572863	573291	449710	450138	451706	452134	567885	568313	609251	609679	2845847	2846275	1772384	1771956	1978534	1978106	2454083	2453655	543636	544064
2577	BPSL2426		44	572084	572854	448931	449701	450927	451697	567106	567876	608472	609242	2845068	2845838	1773163	1772393	1979313	1978543	2454862	2454092	542857	543627
2578	BPSL2427		44	571260	572084	448107	448931	450103	450927	566282	567106	607648	608472	2844244	2845068	1773987	1773163	1980137	1979313	2455686	2454862	542033	542857
2579	BPSL2428		44	570341	571237	447188	448084	449184	450080	565363	566259	606729	607625	2843325	2844221	1774906	1774010	1981056	1980160	2456605	2455709	541114	542010
2580	BPSL2429		44	568868	570268	445715	447115	447711	449111	563890	565290	605256	606656	2841852	2843252	1776379	1774979	1982529	1981129	2458078	2456678	539641	541041
2581	BPSL2430		44	567824	568714	444671	445561	446667	447557	562846	563736	604212	605102	2840808	2841698	1777423	1776533	1983573	1982683	2459122	2458232	538597	539487
2582	BPSL2431		44	566015	567805	442862	444652	444858	446648	561037	562827	602403	604193	2838999	2840789	1779232	1777442	1985382	1983592	2460931	2459141	536788	538578
2583	BPSL2432		44	565520	565771	442367	442618	444363	444614	560542	560793	601908	602159	2838504	2838755	1779727	1779476	1985877	1985626	2461426	2461175	536293	536544
2584	BPSL2433		44	564066	565520	440913	442367	442909	444363	559088	560542	600454	601908	2837050	2838504	1781181	1779727	1987331	1985877	2462880	2461426	534839	536293
2585	BPSL2434		44	562946	563995	439793	440842	441789	442838	557968	559017	599334	600383	2835930	2836979	1782301	1781252	1988451	1987402	2464000	2462951	533719	534768
2586	BPSL2435		44	562338	562937	439185	439784	441181	441780	557360	557959	598726	599325	2835322	2835921	1782909	1782310	1989059	1988460	2464608	2464009	533111	533710
2587	BPSL2436		44	561655	562251	438502	439098	440498	441094	556677	557273	598043	598639	2834639	2835235	1783592	1782996	1989742	1989146	2465291	2464695	532428	533024

2588	BPSL2437		44	561076	561546	437923	438393	439919	440389	556098	556568	597464	597934	2834060	2834530	1784171	1783701	1990321	1989851	2465870	2465400	531849	532319
2589	BPSL2438		44	559826	561061	436673	437908	438669	439904	554848	556083	596214	597449	2832810	2834045	1785421	1784186	1991571	1990336	2467120	2465885	530599	531834
2590	BPSL2439		44	559430	559666	436277	436513	438273	438509	554452	554688	595818	596054	2832414	2832650	1785817	1785581	1991967	1991731	2467516	2467280	530203	530439
2591	BPSL2440		44	558537	559283	435384	436130	437380	438126	553559	554305	594925	595671	2831521	2832267	1786710	1785964	1992860	1992114	2468409	2467663	529310	530056
2592	BPSL2441		44	557554	558483	434401	435330	436397	437326	552576	553505	593942	594871	2830538	2831467	1787693	1786764	1993843	1992914	2469392	2468463	528327	529256
2593	BPSL2442		44	556494	557480	433341	434327	435337	436323	551516	552502	592882	593868	2829478	2830464	1788753	1787767	1994903	1993917	2470452	2469466	527267	528253
2594	BPSL2443		44	555388	556491	432235	433338	434231	435334	550410	551513	591776	592879	2828372	2829475	1789859	1788756	1996009	1994906	2471558	2470455	526161	527264
2595	BPSL2444		44	555070	555246	431917	432093	433913	434089	550092	550268	591458	591634	2828054	2828230	1790177	1790001	1996327	1996151	2471876	2471700	525843	526019
2596	BPSL2445		44	554341	554970	431188	431817	433184	433813	549363	549992	590729	591358	2827325	2827954	1790906	1790277	1997056	1996427	2472605	2471976	525114	525743
2597	BPSL2446		44	554097	553453	430944	430300	432940	432296	549119	548475	590485	589841	2827081	2826437	1791150	1791794	1997300	1997944	2472849	2473493	524870	524226
2598	BPSL2447		44	553453	552734	430300	429581	432296	431577	548475	547756	589841	589122	2826437	2825718	1791794	1792513	1997944	1998663	2473493	2474212	524226	523507
2599	BPSL2448		44	551691	552689	428538	429536	430534	431532	546713	547711	588079	589077	2824675	2825673	1793556	1792558	1999706	1998708	2475255	2474257	522464	523462
2600	BPSL2449		44	551286	551678	428133	428525	430129	430521	546308	546700	587674	588066	2824270	2824662	1793961	1793569	2000111	1999719	2475660	2475268	522059	522451
2601	BPSL2450		44	551035	551286	427882	428133	429878	430129	546057	546308	587423	587674	2824019	2824270	1794212	1793961	2000362	2000111	2475911	2475660	521808	522059
2602	BPSL2451		44	549981	550985	426828	427832	428824	429828	545003	546007	586369	587373	2822965	2823969	1795266	1794262	2001416	2000412	2476965	2475961	520754	521758
2603	BPSL2452		44	549217	545948	426064	424205	428060	426309			585605	582336	2822201	2818941	1796030	1799290	2002180	2005440	2477729	2480998	519990	516721
2604	BPSL2453		44	545775	544696	422220	421141	423678	422599	540809	539997	582163	581084	2818768	2817689	1799463	1800542	2005613	2006692	2481171	2482250	516548	515469
2605	BPSL2454		44	544677	544039	421122	420484	422580	421942	539978	539766	581065	580427	2817670	2817032	1800561	1801199	2006711	2007349	2482269	2482907	515450	515220
2606	BPSL2455		44	543924	542617	420369	419062	421827	420520	539328	538828	580312	579005	2816917	2815622	1801314	1802609	2007464	2008771	2483022	2484329	514134	512827
2607	BPSL2456		44	542577	542293	419022	418738	420480	420196			578965	578681	2815582	2815298	1802649	1802933	2008811	2009095	2484369	2484653	512787	512503
2608	BPSL2457		44	542293	541793	418738	418238	420196	419696			578681	578181	2815298	2814798	1802933	1803433	2009095	2009595	2484653	2485153	512503	512003
2609	BPSL2458		44	540072	541544	416517	417989	417975	419447	538216	538788	576460	577932	2813077	2814549	1805154	1803682	2011316	2009844	2486874	2485402	510282	511754
2610	BPSL2459		44	539086	540072	415531	416517	416989	417975	537230	538216	575474	576460	2812091	2813077	1806140	1805154	2012302	2011316	2487860	2486874	509296	510282
2611	BPSL2460		44	537578	539017	414023	415462	415481	416920	535722	537161	573966	575405	2810583	2812022	1807648	1806209	2013810	2012371	2489368	2487929	507788	509227
2612	BPSL2461		44	536800	537519	413245	413964	414703	415422	534944	535663	573188	573907	2809805	2810524	1808426	1807707	2014588	2013869	2490146	2489427	507010	507729
2613	BPSL2462		44	535516	536712	411961	413157	413419	414615	533660	534856	571904	573100	2808521	2809717	1809710	1808514	2015872	2014676	2491430	2490234	505726	506922
2614	BPSL2463		44	535247	534927	411692	411372	413150	413091	533391	533164	571635	571315	2808252	2807932	1809979	1810299	2016141	2016353	2491699	2492019	505457	505137
2615	BPSL2464		44	534436	533960	410971	410495	412504	412028	532781	532305	571277	570963	2807441	2806965	1810790	1811266	2016708	2017184	2492510	2492986	504646	504170
2616	BPSL2465		44	533473	532574	410008	409109	411541	410642	531818	530919	570490	569591	2806506	2805607	1811725	1812624	2017657	2018556	2493466	2494365	503676	502777
2617	BPSL2466		44	531862	532383	408493	408918	409930	410451	530207	530728	568879	569400	2804895	2805416	1813336	1812815	2019268	2018747	2495077	2494556	502065	502586
2618	BPSL2467		44	531542	531324	407862	407644	409610	409392	529887	529669	568559	568341	2804575	2804357	1813656	1813874	2019588	2019806	2495397	2495615	501745	501527
2619	BPSL2468		44	531196	529793	407516	406113	409264	407861	529541	528138	568213	566810	2804229	2802826	1814002	1815405	2019934	2021337	2495743	2497146	501399	499996
2620		Chr1_115a_IS407A_A		528284	529519	404604	405839	406352	407587	526629	527864	565315	566550	2801289	2802524	1815700	1816935	2021618	2022853	2497406	2498641	498494	499729
2621		Chr1_115b_IS407A_A												907313	908548	252371	253606	387955	389190				
2622		Chr1_116_IS407A_B		525748	526983	402068	403303	403816	405051	524093	525328												
2623	BPSL2469		45	525411	524020	401731	400340	403479	402088	523756	522365	564978	563587	908885	910276	252034	250643	389527	390918	2498978	2500369	498157	496766
2624	BPSL2470		45	523032	523535	399352	399855	401100	401603	521377	521880	562599	563102	911264	910761	249655	250158	391906	391403	2501357	2500854	495778	496281
2625	BPSL2471		45	522079	521372	398399	397692	400147	399440	520424	519717	561646	560939	912202	912909	248709	248002	392845	393552	2502303	2503010	494839	494132
2626	BPSL2472		45	521213	518643	397533	394963	399281	397494	519558	516988	560780	558210	913068	915638	247843	245273	393711	396281	2503169	2505694	493973	491448
2627		Chr1_117a_ISBma2_A		517013	518585	393333	394905	394492	396064	515358	516930	556580	558152	915696	917293	243623	245215	396339	397912	2505752	2507325	489817	491390
2628		Chr1_117b_ISBma2_A		403674	405246	279549	281121	280544	282116	401987	403559	440655	442227					280947	282519				
2629	BPSL2473		46	406273	405305	282148	281180	283143	282175	404586	403618	443254	442286	918322	917354	242594	243562	283548	282580	2508352	2507384	488790	489758
2630	BPSL2474		46	407715	406333	283590	282208	284585	283203	406028	404646	444696	443314	919764	918382	241152	242534	284990	283608	2509794	2508412	487348	488730
2631	BPSL2474a		46	408513	409877	284388	285752	285383	285943	406826	407554	445494	446858	920562	921926	240354	238990	285788	287152	2510592	2511956	396874	397539
2632	BPSL2475		46	410475	411932	286350	287807	287954	289411	408789	410246	447456	448913	922542	923999	238383	236926	287777	289234	2512599	2514056	398173	399630
2633	BPSL2476		46	412143	412643	288018	288518	289622	290122	410457	410957	449124	449624	924210	924710	236715	236215	289445	289945	2514267	2514767	399841	400341
2634	BPSL2477_IS	Chr1_118_ISBma2_A	46	414238	412778	290113	288653	291717	290257	412552	411092	451219	449759	926305	924845	234620	236080	291540	290080	2516362	2514902	400418	401990
2635	BPSL2478		46	415745	414378	291620	290253	293224	291857	414059	412692	452726	451359	927812	926445	233113	234480	293047	291680	2517869	2516502	403443	402076
2636	BPSL2479		46	415891	416496	291766	292371	293370	293975	414205	414810	452872	453477	927958	928563	232967	232362	293193	293798	2518015	2518620	403589	404194
2637	BPSL2480		46	416480	417097	292355	292972	293959	294576	414794	415411	453461	454078	928547	929164	232378	231761	293782	294399	2518604	2519221	404178	404795
2638	BPSL2481		46	417953	417351	293828	293226	295432	294830	416267	415665	454934	454332	930020	929418	230905	231507	295255	294653	2520077	2519475	405651	405049

2639	BPSL2482		46	418072	419334	293947	295209	295551	296813	416386	417648	455053	456315	930139	931401	230786	229524	295374	296636	2520196	2521458	405770	407032
2640	BPSL2483		46	419334	420275	295209	296150	296813	297754	417648	418589	456315	457256	931401	932342	229524	228583	296636	297577	2521458	2522399	407032	407973
2641	BPSL2484		46	420347	420661	296222	296536	297826	298140	418661	418975	457328	457642	932414	932728	228511	228197	297649	297963	2522471	2522785	408045	408359
2642	BPSL2485		46	421820	420834	297695	296709	299299	298313	420134	419148	458801	457815	933887	932901	227038	228024	299122	298136	2523944	2522958	409518	408532
2643	BPSL2486		46	422553	421873	298428	297748	300032	299352	420867	420187	459534	458854	934620	933940	226305	226985	299855	299175	2524677	2523997	410251	409571
2644	BPSL2487		46	423465	424052	299340	299927	300944	301531	421779	422366	460476	461063	935550	936137	225381	224794	300785	301372	2525601	2526188	411175	411762
2645	BPSL2488_ISBma1		46																				
2646	BPSL2489		46	424765	424379	300640	300254	302244	301858	423079	422693	461776	461390	936850	936464	224081	224467	302085	301699	2526901	2526515	412475	412089
2647	BPSL2490		46	425697	424906	301572	300781	303176	302385	424011	423220	462708	461917	937782	936991	223149	223940	303017	302226	2527833	2527042	413407	412616
2648	BPSL2491		46	426334	425702	302209	301577	303813	303181	424648	424016	463345	462713	938419	937787	222512	223144	303654	303022	2528470	2527838	414044	413412
2649	BPSL2492		46	426687	426436	302562	302311	304166	303915	425001	424750	463698	463447	938772	938521	222159	222410	304007	303756	2528823	2528572	414397	414146
2650	BPSL2493		46	426903	428018	302778	303893	304382	305497	425217	426332	463914	465029	938988	940103	221943	220828	304223	305338	2529039	2530154	414613	415728
2651	BPSL2494		46	428128	428592	304003	304467	305607	306071	426442	426906	465139	465603	940213	940677	220718	220254	305448	305912	2530264	2530728	415838	416302
2652	BPSL2495		46	429787	428633	305662	304508	307266	306112	428101	426947	466798	465644	941872	940718	219059	220213	307107	305953	2531923	2530769	417497	416343
2653	BPSL2496		46	430384	429899	306259	305774	307863	307378	428698	428213	467395	466910	942469	941984	218462	218947	307704	307219	2532520	2532035	418094	417609
2654		Chr1_119_IS407A_C	46											942572	943807								
2655	BPSL2497		46	430532	431815	306407	307690	308011	309294	428846	430129	467543	468826	943857	945140	218314	217031	307852	309135	2532668	2533951	418242	419525
2656	BPSL2498		46	432652	431936	309596	308514	311200	310118	432035	430953	470732	469650	947046	945964	215125	216207	311041	309959	2535857	2534775	421431	420349
2657	BPSL2499		46	434769	433837	310644	309712	312248	311316	433083	432151	471780	470848	948094	947162	214077	215009	312089	311157	2536905	2535973	422479	421547
2658	BPSL2500		46	435534	434788	311409	310663	313013	312267	433848	433102	472545	471799	948859	948113	213312	214058	312854	312108	2537670	2536924	423244	422498
2659	BPSL2501		46	436555	435764	312430	311639	314034	313243	434869	434078	473566	472775	949880	949089	212291	213082	313875	313084	2538691	2537900	424265	423474
2660	BPSL2502		46	437282	436632	313157	312507	314761	314111	435596	434946	474293	473643	950607	949957	211564	212214	314602	313952	2539418	2538768	424992	424342
2661	BPSL2503		46	438306	437275	314181	313150	315785	314754	436620	435589	475317	474286	951631	950600	210540	211571	315626	314595	2540442	2539411	426016	424985
2662	BPSL2504		46	438615	439454	314490	315329	316094	316933	436929	437768	475626	476465	951940	952779	210231	209392	315935	316774	2540751	2541590	426325	427164
2663	BPSL2505		46	440723	439803	316598	315678	318202	317282	439037	438117	477734	476814	954048	953128	208123	209043	318043	317123	2542859	2541939	428433	427513
2664	BPSL2506		46	440851	442194	316726	318069	318330	319673	439165	440508	477862	479205	954176	955519	207995	206646	318171	319514	2542987	2544330	428561	429904
2665	BPSL2507		46	443325	442426	319200	318301	320804	319905	441639	440740	480336	479437	956650	955751	205515	206414	320645	319746	2545461	2544562	431035	430136
2666	BPSL2508		46	443966	443652	319841	319527	321445	321131	442280	441966	480977	480663	957291	956977	204874	205188	321286	320972	2546102	2545788	431676	431362
2667	BPSL2509		46	445030	444041	320905	319916	322509	321520	443344	442355	482041	481052	958355	957366	203810	204799	322350	321361	2547166	2546177	432740	431751
2668	BPSL2510		46	446012	445092	321887	320967	323491	322571	444326	443406	483023	482103	959337	958417	202828	203748	323332	322412	2548148	2547228	433722	432802
2669	BPSL2511		46	447444	446047	323319	321922	324923	323526	445758	444361	484455	483058	960769	959372	201396	202793	324764	323367	2549580	2548183	435154	433757
2670	BPSL2512		46	448734	447568	324609	323443	326213	325047	447048	445882	485745	484579	962059	960893	200106	201272	326054	324888	2550870	2549704	436444	435278
2671	BPSL2513		46	449260	448790	325135	324665	326739	326269	447574	447104	486271	485801	962585	962115	199580	200050	326580	326110	2551396	2550926	436970	436500
2672		Chr1_120_ISBma2_A	46	449261	450833	325136	326708	326740	328312	447575	449147	486272	487844	962586	964158	198007	199579	326581	328153	2551397	2552969	436971	438543
2673	BPSL2514		46	451221	450901	327096	326776	328700	328380	449535	449215	488232	487912	964546	964226	197619	197939	328541	328221	2553357	2553037	438931	438611
2674	BPSL2515		46	452956	451247	328831	327122	330435	328726	451270	449561	489967	488258	966281	964572	195884	197593	330276	328567	2555092	2553383	440666	438957
2675	BPSL2516		46	453811	453104	329686	328979	331290	330583	452125	451418	490822	490115	967136	966429	195029	195736	331131	330424	2555947	2555240	441521	440814
2676	BPSL2517		46	456069	453811	331944	329686	333548	331290	454383	452125	493080	490822	969394	967136	192771	195029	333389	331131	2558205	2555947	443779	441521
2677	BPSL2518		46	457316	456237	333191	332112	334795	333716	455630	454551	494327	493248	970641	969562	191524	192603	334636	333557	2559452	2558373	445026	443947
2678	BPSL2519		46	458433	457354	334308	333229	335912	334833	456747	455668	495444	494365	971758	970679	190407	191486	335753	334674	2560569	2559490	446143	445064
2679	BPSL2520		46	459203	458610	335078	334485	336682	336089	457517	456924	496214	495621	972528	971935	189637	190230	336523	335930	2561339	2560746	446913	446320
2680	BPSL2521		46	461902	459305	337777	335180	339381	336784	460216	457619	498913	496316	975227	972630	186938	189535	339222	336625	2564038	2561441	449612	447015
2681	BPSL2522		46	462413	463084	338288	338959	339892	340563	460727	461398	499424	500095	975738	976409	186427	185756	339733	340404	2564549	2565220	450123	450794
2682	BPSL2523		46	463256	463951	339131	339826	340735	341430	461570	462265	500267	500962	976581	977276	185584	184889	340576	341271	2565392	2566087	450966	451661
2683	BPSL2524		46	463980	464702	339855	340577	341459	342181	462294	463016	500991	501713	977305	978027	184860	184138	341300	342022	2566116	2566838	451690	452412
2684		Chr1_121_ISBma2_A	46	465119	465259	340994	341134	342598	342738	463433	463573	502130	502270	978444	978584	183581	183721	342439	342579	2567255	2567395	452829	452969
2685		Chr1_122_IS407A_A	46	465256	466491	341131	342366	342735	343970	463570	464805	502267	503502	978581	979816	182349	183584	342576	343811	2567392	2568627	452966	454201
2686		Chr1_123_ISBma2_C	46																			454308	455880
2687		Chr1_124_ISBma1_B	46	466488	467560	342363	343435	343967	345039	464802	465874	503499	504571							2568741	2570045	455910	457214
2688	BPSL2525		46	467798	468481	343673	344356	345277	345960	466112	466795	504809	505492	980159	980842	182006	181323	344154	344837	2570283	2570966	457452	458135
2689	BPSL2526		46	468928	468668	344803	344543	346407	346147	467242	466982	505939	505679	981289	981029	180876	181136	345284	345024	2571413	2571153	458582	458322

2690	BPSL2527		46	470136	469033	346011	344908	347615	346512	468450	467347	507147	506044	982497	981394	179668	180771	346492	345389	2572621	2571518	459790	458687
2691	BPSL2528		46	470225	470737	346100	346612	347704	348216	468539	469051	507236	507748	982586	983098	179579	179067	346581	347093	2572710	2573222	459879	460391
2692	BPSL2529		46	470737	472299	346612	348174	348216	349778	469051	470613	507748	509310	983098	984660	179067	177505	347093	348655	2573222	2574784	460391	461953
2693	BPSL2530		46	472317	475268	348192	351143	349796	352747	470631	473582	509328	512279	984678	987629	177487	174536	348673	351624	2574802	2577753	461971	464922
2694	BPSL2531		46	475279	475536	351154	351411	352758	353015	473593	473850	512290	512547	987640	987897	174525	174268	351635	351892	2577764	2578021	464933	465190
2695	BPSL2532		46	477582	475918	353457	351793	355061	353397	475896	474232	514614	512950	989978	988314	172229	173893	353882	352218	2580081	2578417	467264	465600
2696	BPSL2533		46	478249	479556	354124	355383	355728	357035	476563	477870	515281	516588	990645	991952	171562	170255	354549	355856	2580748	2582055	467931	469238
2697	BPSL2534		46	481243	479951	357565	356273	358722	357430	479557	478265	518275	516983	993639	992347	168568	169860	357543	356251	2583742	2582450	470925	469633
2698	BPSL2535		46	482802	481735	359124	358057	360281	359214	481116	480049	519834	518767	995198	994131	167009	168076	359105	358035	2585301	2584234	472484	471417
2699	BPSL2536		46	483669	485042	359991	361364	361148	362521	481983	483356	520701	522074	996065	997438	166142	164769	359972	361345	2586168	2587541	473351	474724
2700	BPSL2537		46	485154	485660	361476	361982	362633	363139	483468	483974	522186	522692	997550	998056	164657	164151	361457	361963	2587653	2588159	474836	475342
2701	BPSL2538		46	486172	487191	362494	363513	363651	364670	484486	485505	523204	524223	998568	999587	163639	162620	362475	363494	2588671	2589690	475854	476873
2702	BPSL2539		46	487236	488258	363558	364580	364715	365737	485550	486572	524268	525290	999632	1000654	162575	161553	363539	364561	2589735	2590757	476918	477940
2703	BPSL2540		46	488313	489722	364635	366044	365792	367201	486627	488036	525355	526764	1000719	1002128	161488	160079	364626	366035	2590822	2592231	478005	479414
2704	BPSL2541		46	489751	491058	366073	367380	367230	368537	488065	489372	526793	528100	1002157	1003464	160050	158743	366064	367371	2592260	2593567	479443	480750
2705	BPSL2542		46	491549	492118	367871	368440	369028	369597	489894	490463	528591	529160	1003955	1004524	158252	157683	367862	368431	2594058	2594627	481146	481715
2706	BPSL2543		46	493168	492458	369488	368778	370647	369937	491513	490803	530210	529500	1005574	1004864	156633	157343	369481	368771	2595677	2594967	482765	482055
2707	BPSL2544		46	493389	496088	369709	372408	370868	373567	491734	494433	530431	533130	1005795	1008494	156412	153713	369702	372401	2595898	2598597	482986	485685
2708	BPSL2545		46	498733	496442	375053	372762	376212	373921	497078	494787	535766	533475	1011148	1008857	151059	153350	375046	372755	2601242	2598951	2360446	2358155
2709	BPSL2546		46	498850	499779	375170	376099	376329	377258	497195	498124	535883	536812	1011265	1012194	150942	150013	375163	376092	2601359	2602288	2360563	2361492
2710	BPSL2547		46	499912	500925	376232	377245	377391	378404	498257	499270	536945	537958	1012327	1013340	149880	148867	376225	377238	2602421	2603434	2361625	2362638
2711		Chr1_125_ISBma1_A	46	501195	502499	377515	378819	378674	379978	499540	500844	538228	539532	1013610	1014914	147293	148597	377508	378812	2603704	2605008	2362908	2364212
2712	BPSL2548		46	503121	503594	379441	379914	380600	381073	501466	501939	540154	540627	1015536	1016009	146671	146198	379434	379907	2605630	2606103	2364834	2365307
2713	BPSL2549		46	503766	504011	380086	380331	381245	381490	502111	502356	540799	541044	1016181	1016426	146026	145781	380079	380324	2606275	2606520	2365479	2365724
2714	BPSL2550		46	504672	505016	380992	381336	382151	382495	503017	503361	541705	542049	1017087	1017431	145120	144776	380985	381329	2607181	2607525	2366385	2366729
2715	BPSL2551		46	505794	505309	382114	381629	383273	382788	504139	503654	542827	542342	1018209	1017724	143998	144483	382107	381622	2608303	2607818	2367507	2367022
2716	BPSL2552		46	507062	505794	383382	382114	384541	383273	505407	504139	544095	542827	1019477	1018209	142730	143998	383375	382107	2609571	2608303	2368775	2367507
2717	BPSL2553		46	509386	507053	385706	383373	386865	384532	507731	505398	546419	544086	1021801	1019468	140406	142739	385699	383366	2611895	2609562	2371099	2368766
2718	BPSL2554		46	510009	509536	386329	385856	387488	387015	508354	507881	547042	546569	1022424	1021951	139783	140256	386322	385849	2612518	2612045	2371722	2371249
2719	BPSL2555		46	510535	511125	386855	387445	388014	388604	508880	509470	547568	548158	1022950	1023540	139257	138667	386848	387438	2613044	2613634	2372248	2372838
2720	BPSL2556		46											1024055	1023855	138152	138352	387953	387753				
2721		Chr1_126a_IS407A_A		511643	512878	387963	389198	389122	390357	509988	511223	548676	549911	1024057	1025292	136915	138150	387955	389190	2614152	2615387	2373356	2374591
2722		Chr1_126b_IS407A_A		2179445	2180680	2072203	2073438	2087663	2088898	2167514	2168749	2330125	2331360	2690743	2691978	1927000	1928235	2132568	2133803	826175	827410	2149515	2150750
2723	BPSL2558		47											2690437	2690177	1928541	1928801	2134109	2134369				
2724		Chr1_127_ISBma1_A	47	2180681	2181011	2073439	2073769	2088899	2089229	2168750	2169080	2331091	2332356	2688853	2690159	1928819	1930125	2134387	2135693	825845	826242	2150683	2151080
2725	BPSL2559		47	2181522	2182658	2074280	2075416	2089740	2090876	2169591	2170727					1930636	1931772	2136204	2137340			2151591	2152727
2726		Chr1_128_ISBma2_C	47																			2152775	2154347
2727		Chr1_129_ISBma1_A	47	2182712	2184016	2075470	2076774	2090930	2092234	2170781	2172085	2331091	2332356	2688853	2690159	1931826	1933130	2137833	2138804	825845	826242	2155991	2157225
2728	BPSL2560		47	2184765	2184205	2077523	2076963	2092983	2092423	2172834	2172274	2333105	2332545	2688104	2688664	1933879	1933319	2139553	2138993	825096	825656	2157974	2157414
2729	BPSL2561		47	2187672	2185306	2080430	2078064	2095890	2093524	2175741	2173375	2336012	2333646	2685197	2687563	1936786	1934420	2142460	2140094	822189	824555	2160881	2158515
2730	BPSL2562		47	2187942	2187742	2080700	2080500	2096160	2095960	2176011	2175811	2336282	2336082	2684927	2685127	1937056	1936856	2142730	2142530	821919	822119	2161151	2160951
2731	BPSL2563		47	2188686	2188006	2081444	2080764	2096904	2096224	2176755	2176075	2337026	2336346	2684183	2684863	1937800	1937120	2143474	2142794	821175	821855	2161895	2161215
2732	BPSL2564		47	2189616	2188696	2082374	2081454	2097834	2096914	2177685	2176765	2337956	2337036	2683253	2684173	1938730	1937810	2144404	2143484	820245	821165	2162825	2161905
2733	BPSL2565		47	2189788	2190516	2082546	2083274	2098006	2098734	2177857	2178585	2338128	2338856	2683081	2682353	1938902	1939630	2144576	2145304	820073	819345	2162997	2163725
2734	BPSL2566		47	2190522	2191145	2083280	2083903	2098740	2099363	2178591	2179214	2338862	2339485	2682347	2681724	1939636	1940259	2145310	2145933	819339	818716	2163731	2164354
2735	BPSL2567		47	2191145	2192359	2083903	2085117	2099363	2100577	2179214	2180428	2339485	2340699	2681724	2680510	1940259	1941473	2145933	2147147	818716	817502	2164354	2165568
2736	BPSL2568	GI9																					
2737	BPSL2569	GI9																					
2738	BPSL2570	GI9																					
2739	BPSL2571	GI9																					
2740	BPSL2572	GI9																					

2741	BPSL2573	GI9																					
2742	BPSL2574	GI9																					
2743	BPSL2575	GI9																					
2744	BPSL2576	GI9																					
2745	BPSL2577	GI9																					
2746	BPSL2578	GI9																					
2747	BPSL2579	GI9																					
2748	BPSL2580	GI9																					
2749	BPSL2581	GI9																					
2750	BPSL2582	GI9																					
2751	BPSL2583	GI9																					
2752	BPSL2584	GI9																					
2753	BPSL2585	GI9																					
2754	BPSL2586	GI9																					
2755	BPSL2588		47	2194172	2193597	2086930	2086355	2102390	2101815	2182241	2181666	2342524	2341949	2678709	2679284	1943274	1942699	2148948	2148373	815653	816228	2167393	2166818
2756	BPSL2589		47	2194346	2194837	2087104	2087595	2102564	2103055	2182415	2182906	2342698	2343189	2678535	2678044	1943448	1943939	2149122	2149478	815479	814988	2167567	2168058
2757	BPSL2590		47	2195289	2196599	2088047	2089357	2103507	2104817	2183358	2184668	2343641	2344951	2677592	2676282	1944411	1945721	2150060	2151370	814546	813236	2168500	2169810
2758	BPSL2591		47	2196783	2196962	2089541	2089720	2105001	2105180	2184852	2185031	2345135	2345314	2676098	2675919	1945905	1946084	2151554	2151733	813052	812873	2169994	2170173
2759	BPSL2592		47	2198022	2197003	2090780	2089761	2106240	2105221	2186091	2185072	2346374	2345355	2674859	2675878	1947144	1946125	2152793	2151774	811813	812832	2171233	2170214
2760	BPSL2593		47	2198184	2199836	2090942	2092594	2106402	2108054	2186253	2187905	2346536	2348188	2674697	2673045	1947306	1948958	2152955	2154607	811651	809999	2171395	2173047
2761	BPSL2594		47	2200375	2199959	2093133	2092717	2108593	2108177	2188444	2188028	2348727	2348311	2672506	2672922	1949497	1949081	2155146	2154730	809460	809876	2173586	2173170
2762	BPSL2595		47	2200751	2200500	2093509	2093258	2108969	2108718	2188820	2188569	2349103	2348852	2672130	2672381	1949873	1949622	2155522	2155271	809084	809335	2173962	2173711
2763	BPSL2596		47	2201579	2200767	2094337	2093525	2109797	2108985	2189648	2188836	2349931	2349119	2671302	2672114	1950701	1949889	2156350	2155538	808256	809068	2174790	2173978
2764	BPSL2597		47	2202450	2201641	2095208	2094399	2110668	2109859	2190519	2189710	2350802	2349993	2670431	2671240	1951572	1950763	2157221	2156412	807385	808194	2175661	2174852
2765	BPSL2598		47	2203128	2202607	2095886	2095365	2111346	2110825	2191197	2190676	2351480	2350959	2669753	2670274	1952250	1951729	2157899	2157378	806707	807228	2176339	2175818
2766	BPSL2599		47	2204054	2204323	2096812	2097081	2112272	2112541	2192123	2192392	2352406	2352675	2668827	2668558	1953176	1953445	2158825	2159094	805781	805512	2177265	2177534
2767	BPSL2600		47	2206057	2204759	2098815	2097517	2114275	2112977	2194126	2192828	2354409	2353111	2666824	2668122	1955179	1953881	2160828	2159530	803778	805076	2179268	2177970
2768	BPSL2601		47	2206170	2206478	2098928	2099236	2114388	2114696	2194239	2194547	2354522	2354830	2666711	2666403	1955292	1955600	2160941	2161249	803665	803357	2179381	2179689
2769	BPSL2602		47	2207888	2206524	2100646	2099282	2116106	2114742	2195957	2194593	2356240	2354876	2664993	2666357	1957010	1955646	2162659	2161295	801947	803311	2181099	2179735
2770	BPSL2603		47	2208745	2208032	2101503	2100790	2116963	2116250	2196814	2196101	2357097	2356384	2664136	2664849	1957867	1957154	2163516	2162803	801090	801803	2181956	2181243
2771	BPSL2604		47	2211271	2208806	2104029	2101564	2119489	2117024	2199340	2196875	2359623	2357158	2661610	2664075	1960393	1957928	2166042	2163577	798564	801029	2184482	2182017
2772	BPSL2605		47	2211437	2212396	2104195	2105154	2119655	2120614	2199506	2200465	2359789	2360748	2661444	2660485	1960559	1961518	2166208	2167167	798398	797439	2184648	2185607
2773	BPSL2606		47	2212567	2213301	2105332	2106060	2120837	2121520	2200636	2201370	2360919	2361653	2660314	2659580	1961689	1962423	2167338	2168072	797268	796534	2185778	2186512
2774	BPSL2607		47	2213330	2213947	2106089	2106706	2121549	2122166	2201399	2202016	2361682	2362299	2659551	2658934	1962452	1963069	2168101	2168718	796505	795888	2186541	2187158
2775	BPSL2608		47	2215301	2214186	2108060	2106945	2123520	2122405	2203370	2202255	2363653	2362538	2657580	2658695	1964423	1963308	2170072	2168957	794534	795649	2188512	2187397
2776	BPSL2609		47	2216259	2215405	2109018	2108164	2124478	2123624	2204328	2203474	2364611	2363757	2656622	2657476	1965381	1964527	2171030	2170176	793576	794430	2189470	2188616
2777	BPSL2610		47	2217187	2216252	2109946	2109011	2125406	2124471	2205256	2204321	2365539	2364604	2655694	2656629	1966309	1965374	2171958	2171023	792648	793583	2190398	2189463
2778	BPSL2611		47	2218591	2217347	2111350	2110106	2126810	2125566	2206660	2205416	2366943	2365699	2654290	2655534	1967713	1966469	2173362	2172118	791244	792488	2191802	2190558
2779	BPSL2612		47	2219050	2220516	2111809	2113275	2127269	2128735	2207119	2208585	2367402	2368868	2653831	2652365	1968172	1969638	2173821	2175287	790785	789319	2192261	2193727
2780	BPSL2613		47	2220607	2221284	2113366	2114043	2128826	2129503	2208676	2209353	2368959	2369636	2652274	2651597	1969729	1970406	2175378	2176055	789228	788551	2193818	2194495
2781	BPSL2614		47	2221265	2223187	2114024	2115946	2129484	2131406	2209334	2211256	2369617	2371539	2651616	2649694	1970387	1972309	2176036	2177958	788570	786648	2194476	2196398
2782	BPSL2615		47	2223661	2224452	2116420	2117211	2131880	2132671	2211730	2212521	2372013	2372804	2649220	2648429	1972783	1973574	2178432	2179223	786174	785383	2196872	2197663
2783	BPSL2616		47	2224541	2225197	2117300	2117956	2132760	2133416	2212610	2213266	2372893	2373549	2648340	2647684	1973663	1974319	2179312	2179968	785294	784638	2197752	2198408
2784	BPSL2617		47	2225247	2225969	2118006	2118728	2133466	2134188	2213316	2214038	2373599	2374321	2647634	2646912	1974369	1975091	2180018	2180740	784588	783866	2198458	2199180
2785	BPSL2618		47	2226043	2227059	2118802	2119818	2134262	2135278	2214112	2215128	2374395	2375411	2646838	2645822	1975165	1976181	2180814	2181830	783792	782776	2199254	2200270
2786	BPSL2619		47	2227059	2227523	2119818	2120282	2135278	2135742	2215128	2215592	2375411	2375875	2645822	2645358	1976181	1976645	2181830	2182294	782776	782312	2200270	2200734
2787	BPSL2620		47	2228838	2227639	2121597	2120398	2137057	2135858	2216907	2215708	2377190	2375991	2644043	2645242	1977960	1976761	2183609	2182410	780997	782196	2202049	2200850
2788	BPSL2621		47	2228925	2229542	2121684	2122301	2137144	2137761	2216994	2217611	2377277	2377894	2643956	2643339	1978047	1978664	2183696	2184313	780910	780293	2202136	2202753
2789	BPSL2622		47	2229645	2230100	2122404	2122859	2137864	2138319	2217714	2218169	2377997	2378452	2643236	2642781	1978767	1979222	2184416	2184871	780190	779735	2202856	2203311
2790	BPSL2623		47	2230537	2231817	2123296	2124576	2138756	2140036	2218606	2219886	2378898	2380178	2642317	2641037	1979677	1980957	2185326	2186606	779316	778036	2203721	2205001
2791	BPSL2624		47	2231849	2232988	2124608	2125747	2140068	2141207	2219918	2221057	2380210	2381349	2641005	2639866	1980989	1982128	2186638	2187777	778004	776865	2205033	2206172

2792	BPSL2625		47	2233006	2233617	2125765	2126376	2141225	2141836	2221075	2221686	2381367	2381978	2639848	2639237	1982146	1982757	2187795	2188406	776847	776236	2206190	2206801
2793	BPSL2626		47	2234083	2235216	2126842	2127975	2142302	2143435	2222152	2223285	2382444	2383577	2638771	2637638	1983223	1984356	2188872	2190005	775770	774637	2207267	2208400
2794	BPSL2627		47	2235299	2235817	2128058	2128576	2143518	2144036	2223368	2223886	2383660	2384178	2637555	2637037	1984439	1984957	2190088	2190606	774554	774036	2208483	2209001
2795	BPSL2628		47	2235817	2236251	2128576	2129010	2144036	2144470	2223886	2224320	2384178	2384612	2637037	2636603	1984957	1985391	2190606	2191040	774036	773602	2209001	2209435
2796	BPSL2629		47	2236262	2237509	2129021	2130268	2144481	2145728	2224331	2225578	2384623	2385870	2636592	2635345	1985402	1986649	2191051	2192298	773591	772344	2209446	2210693
2797	BPSL2630		47	2239236	2238172	2131995	2130931	2147455	2146391	2227305	2226241	2387501	2386437	2633588	2634652	1988370	1987306	2193995	2192931	770671	771735	2212414	2211350
2798	BPSL2631		47	2239861	2241417	2132620	2134176	2148080	2149636	2227930	2229486	2388126	2389682	2632963	2631407	1988995	1990551	2194620	2196176	770046	768490	2213039	2214595
2799	BPSL2632		47	2241461	2242132	2134220	2134891	2149680	2150351	2229530	2230201	2389726	2390397	2631363	2630692	1990595	1991266	2196220	2196891	768446	767775	2214639	2215310
2800	BPSL2633		47	2243572	2242592	2136331	2135351	2151791	2150811	2231641	2230661	2391837	2390857	2629252	2630232	1992706	1991726	2198331	2197351	766335	767315	2216750	2215770
2801	BPSL2634		47	2244027	2243737	2136786	2136496	2152246	2151956	2232096	2231806	2392292	2392002	2628797	2629087	1993161	1992871	2198786	2198496	765880	766170	2217205	2216915
2802	BPSL2635		47	2244453	2245586	2137212	2138345	2152672	2153805	2232522	2233655	2392718	2393851	2628371	2627238	1993587	1994720	2199212	2200345	765454	764321	2217631	2218764
2803	BPSL2636		47	2245672	2246232	2138431	2138991	2153891	2154451	2233741	2234301	2393938	2394498	2627152	2626592	1994806	1995366	2200431	2200991	764235	763675	2218850	2219410
2804	BPSL2637		47	2246615	2246262	2139374	2139021	2154834	2154481	2234684	2234331	2394881	2394528	2626209	2626562	1995749	1995396	2201374	2201021	763292	763645	2219793	2219440
2805	BPSL2638		47	2247025	2246642	2139784	2139401	2155244	2154861	2235094	2234711	2395291	2394908	2625799	2626182	1996159	1995776	2201784	2201401	762882	763265	2220203	2219820
2806	BPSL2639		47	2247635	2248852	2140394	2141611	2155854	2157071	2235704	2236921	2395901	2397118	2625189	2623972	1996769	1997986	2202394	2203611	762272	761055	2220813	2222030
2807		Chr1_130_ISBma2_B	47											2622284	2623857	1998101	1999674	2203726	2205299				
2808	BPSL2640		47	2249851	2249126	2142610	2141885	2158070	2157345	2237920	2237195	2398117	2397392	2621376	2622101	2000582	1999857	2206207	2205482	760056	760781	2223029	2222304
2809	BPSL2641		47	2251333	2250089	2144092	2142848	2159552	2158308	2239402	2238158	2399599	2398355	2619894	2621138	2002064	2000820	2207689	2206445	758574	759818	2224511	2223267
2810	BPSL2642		47	2251728	2252519	2144487	2145278	2159947	2160738	2239797	2240588	2399994	2400785	2619499	2618708	2002459	2003250	2208084	2208875	758179	757388	2224906	2225697
2811	BPSL2643		47	2253512	2252685	2146271	2145444	2161731	2160904	2241581	2240754	2401778	2400951	2617715	2618542	2004243	2003416	2209868	2209041	756395	757222	2226690	2225863
2812	BPSL2644		47	2254170	2253592	2146929	2146351	2162389	2161811	2242239	2241661	2402436	2401858	2617057	2617635	2004901	2004323	2210526	2209948	755737	756315	2227348	2226770
2813	BPSL2645		47	2254478	2254170	2147237	2146929	2162697	2162389	2242547	2242239	2402744	2402436	2616749	2617057	2005209	2004901	2210834	2210526	755429	755737	2227656	2227348
2814	BPSL2646		47	2254666	2256057	2147425	2148816	2162885	2164276	2242735	2244126	2402932	2404323	2616561	2615170	2005397	2006788	2211022	2212413	755241	753850	2227844	2229235
2815	BPSL2647		47	2256094	2256396	2148853	2149155	2164313	2164615	2244163	2244465	2404360	2404662	2615133	2614831	2006825	2007127	2212450	2212752	753813	753511	2229272	2229574
2816	BPSL2648		47	2256506	2257156	2149265	2149915	2164725	2165375	2244575	2245225	2404772	2405422	2614721	2614071	2007237	2007887	2212862	2213512	753401	752751	2229684	2230334
2817	BPSL2649		47	2257174	2257494	2149933	2150253	2165393	2165713	2245243	2245563	2405440	2405760	2614053	2613733	2007905	2008225	2213530	2213850	752733	752413	2230352	2230672
2818	BPSL2650		47	2258909	2257584	2151668	2150343	2167128	2165803	2246978	2245653	2407184	2405859	2612309	2613634	2009649	2008324	2215274	2213949	750989	752314	2232096	2230771
2819	BPSL2651		47	2259264	2260568	2152023	2153327	2167483	2168787	2247333	2248637	2407539	2408843	2611954	2610650	2010004	2011308	2215629	2216933	750634	749330	2232451	2233755
2820	BPSL2652		47	2260666	2262285	2153425	2155044	2168885	2170504	2248735	2250354	2408941	2410560	2610552	2608933	2011406	2013025	2217031	2218650	749232	747613	2233853	2235472
2821	BPSL2653		47	2262290	2262391	2155049	2155150	2170509	2170610	2250359	2250460	2410565	2410666	2608928	2608827	2013030	2013131	2218655	2218756	747608	747507	2235477	2235578
2822		Chr1_131_IS407A_C	47									2411631	2412866										
2823	BPSL2654		47	2263442	2264302	2156201	2157061	2171661	2172521	2251511	2252371	2412957	2413817	2607776	2606916	2014182	2015042	2219807	2220667	746456	745596	2236629	2237489
2824	BPSL2655		47	2264310	2264999	2157069	2157758	2172529	2173218	2252379	2253068	2413825	2414514	2606908	2606219	2015050	2015739	2220675	2221364	745588	744899	2237497	2238186
2825	BPSL2656		47	2265124	2266125	2157883	2158884	2173343	2174344	2253193	2254194	2414639	2415640	2606094	2605093	2015864	2016865	2221489	2222490	744774	743773	2238311	2239312
2826	BPSL2657		47	2266350	2266649	2159109	2159408	2174569	2174868	2254419	2254718	2415865	2416164	2604868	2604569	2017090	2017389	2222715	2223014	743548	743249	2239537	2239836
2827	BPSL2658		47	2266742	2267044	2159501	2159803	2174961	2175263	2254811	2255113	2416257	2416559	2604476	2604174	2017482	2017784	2223107	2223409	743156	742854	2239929	2240231
2828	BPSL2659		47	2267112	2268815	2159871	2161574	2175331	2177034	2255181	2256884	2416627	2418330	2604106	2602403	2017852	2019555	2223477	2225180	742786	741083	2240299	2242002
2829	BPSL2660		47	2268872	2269387	2161631	2162146	2177091	2177606	2256941	2257456	2418387	2418890	2602382	2601744	2019576	2020202	2225201	2225851	741062	740454	2242023	2242619
2830	BPSL2661		47	2269377	2270054	2162136	2162813	2177596	2178273	2257446	2258123	2418880	2419557	2601754	2601077	2020192	2020869	2225841	2226518	740464	739787	2242609	2243286
2831	BPSL2662		47	2270088	2270735	2162847	2163494	2178307	2178954	2258157	2258804	2419591	2420238	2601043	2600396	2020903	2021550	2226552	2227199	739753	739106	2243320	2243967
2832	BPSL2663		47	2272349	2270967	2165108	2163726	2180568	2179186	2260418	2259036	2421852	2420470	2598782	2600164	2023164	2021782	2228813	2227431	737492	738874	2245581	2244199
2833	BPSL2664		47	2273271	2272390	2166030	2165149	2181490	2180609	2261340	2260459	2422774	2421893	2597860	2598741	2024086	2023205	2229735	2228854	736570	737451	2246503	2245622
2834	BPSL2665		47	2274282	2273290	2167041	2166049	2182501	2181509	2262351	2261359	2423785	2422793	2596849	2597841	2025097	2024105	2230746	2229754	735559	736551	2247514	2246522
2835	BPSL2666		47	2275857	2274466	2168616	2167225	2184076	2182685	2263926	2262535	2425360	2423969	2595274	2596665	2026672	2025281	2232321	2230930	733984	735375	2249089	2247698
2836	BPSL2667		47	2276116	2277372	2168875	2170131	2184335	2185591	2264185	2265441	2425619	2426875	2595015	2593759	2026931	2028187	2232580	2233836	733725	732469	2249348	2250604
2837	BPSL2668		47	2277372	2278205	2170131	2170964	2185591	2186424	2265441	2266274	2426875	2427708	2593759	2592926	2028187	2029020	2233836	2234669	732469	731636	2250604	2251437
2838	BPSL2669		47	2278237	2279391	2170996	2172150	2186456	2187610	2266306	2267460	2427740	2428894	2592894	2591740	2029052	2030206	2234701	2235855	731604	730450	2251469	2252623
2839	BPSL2670		47	2279409	2280428	2172168	2173187	2187628	2188647	2267478	2268497	2428912	2429931	2591722	2590703	2030224	2031243	2235873	2236892	730432	729413	2252641	2253660
2840	BPSL2671		47	2280459	2281562	2173218	2174321	2188678	2189781	2268528	2269631	2429962	2431065	2590672	2589569	2031274	2032377	2236923	2238026	729382	728279	2253691	2254794
2841		Chr1_132a_IS407A_A		2281870	2283105	2174629	2175864	2190089	2191324	2269939	2271174	2431373	2432608	2588026	2589261	2032685	2033920	2238334	2239569	726736	727971	2255102	2256337
2842		Chr1_132b_IS407A_A		2070441	2071676	1963199	1964434	1978651	1979886	2058486	2059721	2219825	2221060	2801289	2802524	1815700	1816935	2021618	2022853	935361	936596	2040335	2041570

2843	BPSL2672		48	2073662	2071752	1966420	1964510	1981872	1979962	2061707	2059797	2223046	2221136	2799303	2801213	1818921	1817011	2024839	2022929	933375	935285	2043556	2041646
2844	BPSL2673		48	2074679	2073672	1967437	1966430	1982889	1981882	2062724	2061717	2224063	2223056	2798286	2799293	1819938	1818931	2025856	2024849	932358	933365	2044573	2043566
2845		Chr1_133_IS407A_C	48									2224765	2226000										
2846	BPSL2674		48	2075662	2074700	1968420	1967458	1983872	1982910	2063707	2062745	2226286	2225996	2797303	2798265	1820921	1819959	2026839	2025877	931375	932337	2045556	2044594
2847	BPSL2675		48	2076489	2075662	1969247	1968420	1984699	1983872	2064534	2063707	2227113	2226286	2796476	2797303	1821748	1820921	2027666	2026839	930548	931375	2046383	2045556
2848	BPSL2676		48	2078405	2076567	1971163	1969325	1986615	1984777	2066450	2064612	2229029	2227191	2794560	2796398	1823664	1821826	2029582	2027744	928632	930470	2048299	2046461
2849	BPSL2677		48	2080181	2078460	1972939	1971218	1988391	1986670	2068226	2066505	2230805	2229084	2792784	2794505	1825440	1823719	2031358	2029637	926856	928577	2050075	2048354
2850	BPSL2678		48	2081108	2080188	1973866	1972946	1989318	1988398	2069153	2068233	2231732	2230812	2791857	2792777	1826367	1825447	2032285	2031365	925929	926849	2051002	2050082
2851	BPSL2679		48	2082220	2081132	1974978	1973890	1990430	1989342	2070265	2069177	2232844	2231756	2790745	2791833	1827479	1826391	2033397	2032309	924817	925905	2052114	2051026
2852	BPSL2680		48	2083458	2082223	1976216	1974981	1991668	1990433	2071503	2070268	2234082	2232847	2789507	2790742	1828717	1827482	2034635	2033400	923579	924814	2053352	2052117
2853	BPSL2681		48	2084864	2083458	1977622	1976216	1993074	1991668	2072909	2071503	2235488	2234082	2788101	2789507	1830123	1828717	2036041	2034635	922173	923579	2054758	2053352
2854	BPSL2682		48	2085687	2084857	1978445	1977615	1993897	1993067	2073732	2072902	2236311	2235481	2787278	2788108	1830946	1830116	2036864	2036034	921350	922180	2055581	2054751
2855	BPSL2683		48	2086610	2085717	1979368	1978475	1994820	1993927	2074655	2073762	2237234	2236341	2786355	2787248	1831869	1830976	2037787	2036894	920427	921320	2056504	2055611
2856	BPSL2684		48	2087158	2086610	1979916	1979368	1995368	1994820	2075203	2074655	2237782	2237234	2785807	2786355	1832417	1831869	2038335	2037787	919879	920427	2057052	2056504
2857	BPSL2685		48	2088036	2087146	1980794	1979904	1996246	1995356	2076081	2075191	2238660	2237770	2784929	2785819	1833295	1832405	2039213	2038323	919001	919891	2057930	2057040
2858	BPSL2686		48	2089109	2088051	1981867	1980809	1997319	1996261	2077154	2076096	2239733	2238675	2783856	2784914	1834368	1833310	2040286	2039228	917928	918986	2059003	2057945
2859	BPSL2687		48	2089487	2090332	1982245	1983090	1997697	1998542	2077532	2078377	2240111	2240956	2783478	2782633	1834746	1835591	2040664	2041509	917550	916705	2059381	2060226
2860	BPSL2688		48	2091334	2090468	1984092	1983226	1999544	1998678	2079379	2078513	2241958	2241092	2781631	2782497	1836593	1835727	2042511	2041645	915703	916569	2061228	2060362
2861	BPSL2689		48	2092626	2091352	1985384	1984110	2000836	1999562	2080671	2079397	2243250	2241976	2780339	2781613	1837885	1836611	2043803	2042529	914411	915685	2062520	2061246
2862	BPSL2690		48	2093749	2092721	1986507	1985479	2001959	2000931	2081794	2080766	2244373	2243345	2779216	2780244	1839008	1837980	2044926	2043898	913288	914316	2063643	2062615
2863	BPSL2691		48	2094348	2093833	1987106	1986591	2002558	2002043	2082393	2081878	2244972	2244457	2778617	2779132	1839607	1839092	2045525	2045010	912689	913204	2064242	2063727
2864	BPSL2692		48	2094775	2094338	1987533	1987096	2002985	2002548	2082820	2082383	2245399	2244962	2778190	2778627	1840034	1839597	2045952	2045515	912262	912699	2064669	2064232
2865	BPSL2693		48	2095350	2094775	1988108	1987533	2003560	2002985	2083395	2082820	2245974	2245399	2777615	2778190	1840609	1840034	2046527	2045952	911687	912262	2065244	2064669
2866	BPSL2694		48	2096887	2095559	1989645	1988317	2005097	2003769	2084932	2083604	2247511	2246183	2776078	2777406	1842146	1840818	2048064	2046736	910150	911478	2066781	2065453
2867	BPSL2695		48	2097156	2096989	1989914	1989747	2005366	2005199	2085201	2085034	2247780	2247613	2775809	2775976	1842415	1842248	2048333	2048166	909881	910048	2067050	2066883
2868	BPSL2696		48	2097376	2098221	1990134	1990979	2005586	2006431	2085421	2086266	2248000	2248845	2775589	2774744	1842635	1843480	2048553	2049398	909661	908816	2067270	2068115
2869	BPSL2697		48	2100307	2098670	1993065	1991428	2008517	2006880	2088360	2086723	2250839	2249202	2772722	2774359	1845478	1843841	2051444	2049807	906714	908351	2070185	2068548
2870	BPSL2698		48	2100648	2100358	1993406	1993116	2008858	2008568	2088701	2088411	2251180	2250890	2772381	2772671	1845819	1845529	2051785	2051495	906373	906663	2070526	2070236
2871	BPSL2699_putative exported protein		48											2769632	2771788	1848568	1846412	2054534	2052378				
2872	BPSL2700		48	2103929	2103498	1996687	1996256	2012139	2011708	2091982	2091551	2254461	2254030	2768140	2768571	1850060	1849629	2056026	2055595	903092	903523	2073807	2073376
2873	BPSL2701		48	2104178	2105182	1996936	1997940	2012388	2013392	2092231	2093235	2254710	2255714	2767891	2766887	1850309	1851313	2056275	2057279	902843	901839	2074056	2075060
2874	BPSL2702		48	2106084	2105212	1998842	1997970	2014294	2013422	2094137	2093265	2256616	2255744	2765985	2766857	1852215	1851343	2058181	2057309	900937	901809	2075962	2075090
2875	BPSL2703		48	2107183	2106671	1999941	1999429	2015393	2014881	2095236	2094724	2257715	2257203	2764886	2765398	1853314	1852802	2059280	2058768	899838	900350	2077061	2076549
2876	BPSL2704		48	2108204	2107302	2000962	2000060	2016414	2015512	2096257	2095355	2258736	2257834	2763865	2764767	1854335	1853433	2060301	2059399	898817	899719	2078082	2077180
2877	BPSL2705		48	2109454	2108204	2002212	2000962	2017664	2016414	2097507	2096257	2259986	2258736	2762615	2763865	1855585	1854335	2061551	2060301	897567	898817	2079332	2078082
2878	BPSL2706		48	2111112	2109517	2003870	2002275	2019322	2017727	2099165	2097570	2261644	2260049	2760957	2762552	1857243	1855648	2063209	2061614	895909	897504	2080990	2079395
2879	BPSL2707		48	2112296	2111856	2005054	2004614	2020506	2020066	2100349	2099909	2262828	2262388	2759773	2760213	1858427	1857987	2064393	2063953	894725	895165	2082174	2081734
2880	BPSL2708		48	2112577	2113806	2005335	2006564	2020787	2022016	2100630	2101859	2263109	2264338	2759492	2758263	1858708	1859937	2064674	2065903	894444	893215	2082455	2083684
2881	BPSL2709		48	2113991	2114575	2006749	2007333	2022201	2022785	2102044	2102628	2264523	2265107	2758078	2757494	1860122	1860706	2066088	2066672	893030	892446	2083869	2084453
2882	BPSL2710		48	2114575	2115099	2007333	2007857	2022785	2023309	2102628	2103152	2265107	2265631	2757494	2756970	1860706	1861230	2066672	2067196	892446	891922	2084453	2084977
2883	BPSL2711		48	2115452	2116072	2008210	2008830	2023662	2024282	2103505	2104125	2265984	2266604	2756617	2755997	1861583	1862203	2067549	2068169	891569	890949	2085330	2085950
2884	BPSL2712		48	2116549	2117682	2009307	2010440	2024759	2025892	2104602	2105735	2267072	2268205	2755502	2754369	1862698	1863831	2068664	2069797	890481	889348	2086418	2087551
2885	BPSL2713		48	2117815	2119254	2010573	2012012	2026025	2027464	2105868	2107307	2268338	2269777	2754236	2752797	1863964	1865403	2069930	2071369	889215	887776	2087684	2089123
2886	BPSL2714		48	2119754	2120539	2012512	2013297	2027964	2028749	2107807	2108592	2270277	2271062	2752297	2751512	1865903	1866688	2071869	2072654	887276	886491	2089623	2090408
2887	BPSL2715		48	2122722	2120878	2015480	2013636	2030932	2029088	2110775	2108931	2273245	2271401	2749329	2751173	1868871	1867027	2074837	2072993	884308	886152	2092591	2090747
2888	BPSL2716		48	2125427	2123331	2018185	2016089	2033637	2031541	2113480	2111384	2275950	2273854	2746624	2748720	1871576	1869480	2077542	2075446	881603	883699	2095278	2093200
2889	BPSL2717		48	2125915	2125550	2018673	2018308	2034125	2033760	2113968	2113603	2276438	2276073	2746136	2746501	1872064	1871699	2078030	2077665	881115	881480	2095726	2095382
2890	BPSL2718		48	2126309	2127817	2019067	2020575	2034519	2036027	2114362	2115870	2276841	2278349	2745742	2744234	1872458	1873966	2078424	2079932	880712	879204	2096129	2097637
2891	BPSL2719		48	2127918	2128553	2020676	2021311	2036128	2036763	2115971	2116606	2278450	2279085	2744133	2743498	1874067	1874702	2080033	2080668	879103	878468	2097738	2098373
2892	BPSL2720		48	2128630	2129574	2021388	2022332	2036840	2037784	2116683	2117627	2279162	2280106	2743421	2742477	1874779	1875723	2080745	2081689	878391	877447	2098450	2099394
2893	BPSL2721		48	2130775	2129663	2023533	2022421	2038985	2037873	2118828	2117716	2281307	2280195	2741276	2742388	1876924	1875812	2082890	2081778	876246	877358	2100637	2100203

2894	BPSL2722		48	2131799	2131008	2024557	2023766	2040009	2039218	2119852	2119061	2282331	2281540	2740252	2741043	1877948	1877157	2083914	2083123	875222	876013	2101661	2100870
2895	BPSL2723		48	2132590	2131796	2025348	2024554	2040800	2040006	2120643	2119849	2283122	2282328	2739461	2740255	1878739	1877945	2084705	2083911	874431	875225	2102452	2101658
2896	BPSL2724		48	2134902	2132635	2027660	2025393	2043112	2040845	2122955	2120688	2285434	2283167	2737149	2739416	1881051	1878784	2087017	2084750	872119	874386	2104764	2102497
2897	BPSL2725		48	2135863	2135396	2028621	2028154	2044073	2043606	2123916	2123449	2286395	2285928	2736188	2736655	1882012	1881545	2087978	2087511	871158	871625	2105725	2105258
2898	BPSL2726		48	2136764	2135985	2029522	2028743	2044974	2044195	2124817	2124038	2287296	2286517	2735287	2736066	1882913	1882134	2088879	2088100	870257	871036	2106626	2105847
2899	BPSL2727		48	2139510	2137150	2032268	2029908	2047720	2045360	2127563	2125203	2290042	2287682	2732541	2734901	1885659	1883299	2091625	2089265	867511	869871	2109372	2107012
2900	BPSL2728		48	2141056	2139542	2033814	2032300	2049266	2047752	2129109	2127595	2291588	2290074	2730995	2732509	1887205	1885691	2093171	2091657	865965	867479	2110918	2109404
2901	BPSL2729		48	2141336	2142556	2034094	2035314	2049546	2050766	2129389	2130609	2291868	2293088	2730715	2729495	1887485	1888705	2093451	2094671	865685	864465	2111198	2112418
2902	BPSL2730		48	2143962	2143015	2036720	2035773	2052172	2051225	2132015	2131068	2294458	2293511	2728107	2729054	1890093	1889146	2096086	2095139	863068	864015	2113797	2112850
2903	BPSL2731		48	2143993	2144988	2036751	2037746	2052203	2053198	2132046	2133041	2294489	2295484	2728076	2727081	1890124	1891119	2096117	2097112	863037	862042	2113828	2114823
2904	BPSL2732		48	2146230	2145019	2038988	2037777	2054440	2053229	2134283	2133072	2296726	2295515	2725839	2727050	1892361	1891150	2098354	2097143	860800	862011	2116065	2114854
2905		Chr1_134_IS407A_B	48											2724302	2725537	1892663	1893898	2098656	2099891				
2906	BPSL2733		48	2147128	2148105	2039886	2040863	2055338	2056315	2135181	2136158	2297624	2298601	2724305	2723358	1894499	1895476	2100492	2101469	859902	858925	2116963	2117940
2907	BPSL2734		48	2148155	2149156	2040913	2041914	2056365	2057366	2136208	2137209	2298651	2299652	2723308	2722307	1895526	1896527	2101519	2102520	858875	857874	2117990	2118991
2908	BPSL2735		48	2149587	2150243	2042345	2043001	2057797	2058453	2137640	2138296	2300091	2300747	2721868	2721212	1896982	1897638	2102959	2103615	857443	856787	2119430	2120086
2909	BPSL2736		48	2150362	2151528	2043120	2044286	2058572	2059738	2138415	2139581	2300866	2302032	2721093	2719927	1897757	1898923	2103734	2104900	856668	855502	2120205	2121371
2910	BPSL2737		48	2151799	2152992	2044557	2045750	2060009	2061202	2139852	2141045	2302303	2303496	2719663	2718470	1899187	1900380	2105164	2106357	855231	854038	2121642	2122835
2911	BPSL2738		48	2154723	2153419	2047481	2046177	2062933	2061629	2142776	2141472	2305227	2303923	2716739	2718043	1902111	1900807	2108088	2106784	852307	853611	2124566	2123262
2912	BPSL2739		48	2156072	2154723	2048830	2047481	2064282	2062933	2144125	2142776	2306576	2305227	2715390	2716739	1903460	1902111	2109140	2108967	850958	852307	2125915	2124566
2913	BPSL2740		48	2157428	2156076	2050186	2048834	2065638	2064286	2145481	2144129	2307932	2306580	2714034	2715386	1904816	1903464	2110496	2109144	849602	850954	2127271	2125919
2914	BPSL2741		48	2157646	2157437	2050404	2050195	2065856	2065647	2145699	2145490	2308150	2307941	2713816	2714025	1905034	1904825	2110714	2110505	849384	849593	2127489	2127280
2915	BPSL2742		48	2159350	2157692	2052108	2050450	2067560	2065902	2147403	2145745	2309854	2308196	2712112	2713770	1906738	1905080	2112418	2110760	847680	849338	2129193	2127535
2916	BPSL2743		48	2160326	2159373	2053084	2052131	2068536	2067583	2148379	2147426	2310830	2309877	2711136	2712089	1907714	1906761	2113394	2112441	846704	847657	2130169	2129216
2917	BPSL2744		48	2160583	2161386	2053341	2054144	2068793	2069596	2148636	2149439	2311087	2311890	2710879	2710076	1907971	1908774	2113651	2114454	846447	845644	2130426	2131229
2918	BPSL2745		48	2162204	2161752	2054962	2054510	2070414	2069962	2150257	2149805	2312708	2312256	2709258	2709710	1909592	1909140	2115272	2114820	844826	845278	2132067	2131615
2919	BPSL2746		48	2163481	2162660	2056239	2055418	2071699	2070878	2151550	2150729	2314161	2313340	2707941	2708762	1911037	1910216	2116605	2115784	843373	844194	2133552	2132731
2920	BPSL2747		48	2163471	2164709	2056229	2057467	2071689	2072927	2151540	2152778	2314151	2315389	2707951	2706713	1911027	1912265	2116595	2117833	843383	842145	2133542	2134780
2921	BPSL2748		48	2165601	2164966	2058359	2057724	2073819	2073184	2153670	2153035	2316281	2315646	2705821	2706456	1913157	1912522	2118725	2118090	841253	841888	2135672	2135037
2922	BPSL2749		48	2165929	2166228	2058687	2058986	2074147	2074446	2153998	2154297	2316609	2316908	2705493	2705194	1913485	1913784	2119053	2119352	840925	840626	2136000	2136299
2923	BPSL2750		48	2166355	2166741	2059113	2059499	2074573	2074959	2154424	2154810	2317035	2317421	2705067	2704681	1913911	1914297	2119479	2119865	840499	840113	2136426	2136812
2924	BPSL2751		48	2166794	2167249	2059552	2060007	2075012	2075467	2154863	2155318	2317474	2317929	2704628	2704173	1914350	1914805	2119918	2120373	840060	839605	2136865	2137320
2925	BPSL2752		48	2167809	2167360	2060567	2060118	2076027	2075578	2155878	2155429	2318489	2318040	2703613	2704062	1915365	1914916	2120933	2120484	839045	839494	2137880	2137431
2926	BPSL2753		48	2168425	2167832	2061183	2060590	2076643	2076050	2156494	2155901	2319105	2318512	2702997	2703590	1915981	1915388	2121549	2120956	838429	839022	2138496	2137903
2927	BPSL2754		48	2169210	2168428	2061968	2061186	2077428	2076646	2157279	2156497	2319890	2319108	2702212	2702994	1916766	1915984	2122334	2121552	837644	838426	2139281	2138499
2928	BPSL2755		48	2169632	2169210	2062390	2061968	2077850	2077428	2157701	2157279	2320312	2319890	2701790	2702212	1917188	1916766	2122756	2122334	837222	837644	2139703	2139281
2929	BPSL2756		48	2170174	2169617	2062932	2062375	2078392	2077835	2158243	2157686	2320854	2320297	2701248	2701805	1917730	1917173	2123298	2122741	836680	837237	2140245	2139688
2930	BPSL2757		48	2170839	2170363	2063597	2063121	2079057	2078581	2158908	2158432	2321519	2321043	2700583	2701059	1918395	1917919	2123963	2123487	836015	836491	2140910	2140434
2931	BPSL2758		48	2172093	2170849	2064851	2063607	2080311	2079067	2160162	2158918	2322773	2321529	2699329	2700573	1919649	1918405	2125217	2123973	834761	836005	2142164	2140920
2932	BPSL2759		48	2172545	2173288	2065303	2066046	2080763	2081506	2160614	2161357	2323225	2323968	2698877	2698134	1920101	1920844	2125669	2126412	834309	833566	2142616	2143359
2933	BPSL2760		48	2173388	2173879	2066146	2066637	2081606	2082097	2161457	2161948	2324068	2324559	2698034	2697543	1920944	1921435	2126512	2127003	833466	832975	2143459	2143950
2934	BPSL2761		48	2173948	2174733	2066706	2067491	2082166	2082951	2162017	2162802	2324628	2325413	2697474	2696689	1921504	1922289	2127072	2127857	832906	832121	2144019	2144804
2935	BPSL2762		48	2174747	2175193	2067505	2067951	2082965	2083411	2162816	2163262	2325427	2325873	2696675	2696229	1922303	1922749	2127871	2128317	832107	831661	2144818	2145264
2936	BPSL2763		48	2175193	2176215	2067951	2068973	2083411	2084433	2163262	2164284	2325873	2326895	2696229	2695207	1922749	1923771	2128317	2129339	831661	830639	2145264	2146286
2937	BPSL2764		48	2176315	2177613	2069073	2070371	2084533	2085831	2164384	2165682	2326995	2328293	2695107	2693809	1923871	1925169	2129439	2130737	830539	829241	2146386	2147684
2938	BPSL2765		48	2177634	2178143	2070392	2070901	2085852	2086361	2165703	2166212	2328314	2328823	2693788	2693279	1925190	1925699	2130758	2131267	829220	828711	2147705	2148214
2939	BPSL2766		48	2178166	2178912	2070924	2071670	2086384	2087130	2166235	2166981	2328846	2329592	2693256	2692510	1925722	1926468	2131290	2132036	828688	827942	2148237	2148983
2940		Chr1_135_IS407A_A		2179445	2180680	2072203	2073438	2087663	2088898	2167514	2168749	2330125	2331360	2690743	2691978	1927000	1928235	2132568	2133803	826175	827410	2149515	2150750
2941	BPSL2767																						
2942	BPSL2768																						
2943	BPSL2769																						
2944	BPSL2770																						

2945	BPSL2771																						
2946	BPSL2772																						
2947	BPSL2773																						
2948	BPSL2774																						
2949	BPSL2775																						
2950	BPSL2776																						
2951	BPSL2777																						
2952	BPSL2778																						
2953	BPSL2779																						
2954	BPSL2780																						
2955	BPSL2781																						
2956	BPSL2782																						
2957	BPSL2783																						
2958	BPSL2784																						
2959		Chr1_136_IS407A_A		2367803	2369038	2260664	2261899	2276021	2277256	2355846	2357081	2516711	2517946	1067579	1068814	2118845	2120080	2323299	2324534	554720	555955	2417756	2418991
2960	BPSL2785		49	2369039	2369482	2261900	2262343	2277257	2277700	2357082	2357525	2517947	2518390	1068815	1069258	2120081	2120524	2324535	2324978	554719	554276	2418992	2419435
2961	BPSL2786		49	2370641	2369949	2263502	2262810	2278859	2278167	2358684	2357992	2519549	2518857	1070417	1069725	2121683	2120991	2326137	2325445	553117	553809	2420594	2419902
2962	BPSL2787		49	2371966	2370650	2264827	2263511	2280184	2278868	2360009	2358693	2520874	2519558	1071742	1070426	2123008	2121692	2327462	2326146	551792	553108	2421919	2420603
2963	BPSL2788		49	2372948	2372034	2265809	2264895	2281166	2280252	2360991	2360077	2521856	2520942	1072724	1071810	2123990	2123076	2328444	2327530	550810	551724	2422901	2421987
2964	BPSL2789		49	2380585	2372948	2273446	2265809	2288803	2281166	2368628	2360991	2529493	2521856	1080361	1072724	2131627	2123990	2336081	2328444	543173	550810	2430538	2422901
2965	BPSL2790		49	2382120	2380591	2274981	2273452	2290338	2288809	2370163	2368634	2531028	2529499	1081896	1080367	2133162	2131633	2337616	2336087	541638	543167	2432073	2430544
2966	BPSL2791		49	2382910	2382125	2275771	2274986	2291128	2290343	2370953	2370168	2531818	2531033	1082686	1081901	2133952	2133167	2338406	2337621	540848	541633	2432863	2432078
2967	BPSL2792		49	2384109	2382910	2276970	2275771	2292327	2291128	2372152	2370953	2533017	2531818	1083885	1082686	2135151	2133952	2339605	2338406	539649	540848	2434062	2432863
2968	BPSL2793		49	2384945	2384379	2277806	2277240	2293163	2292597	2372988	2372422	2533853	2533287	1084721	1084155	2135987	2135421	2340441	2339875	538813	539379	2434898	2434332
2969	BPSL2794		49	2385634	2384945	2278495	2277806	2293852	2293163	2373677	2372988	2534542	2533853	1085410	1084721	2136676	2135987	2341130	2340441	538124	538813	2435587	2434898
2970	BPSL2795		49	2386229	2385639	2279090	2278500	2294447	2293857	2374272	2373682	2535137	2534547	1086005	1085415	2137271	2136681	2341725	2341135	537529	538119	2436182	2435592
2971	BPSL2796		49	2387251	2386214	2280112	2279075	2295469	2294432	2375294	2374257	2536159	2535122	1087027	1085990	2138293	2137256	2342747	2341710	536507	537544	2437204	2436167
2972	BPSL2797		49	2388280	2387270	2281141	2280131	2296498	2295488	2376323	2375313	2537188	2536178	1088056	1087046	2139322	2138312	2343776	2342766	535478	536488	2438233	2437223
2973	BPSL2798		49	2389132	2388293	2281993	2281154	2297350	2296511	2377175	2376336	2538040	2537201	1088908	1088069	2140174	2139335	2344628	2343789	534626	535465	2439085	2438246
2974		Chr1_137_IS407A_C	49									2538164	2539399										
2975	BPSL2799		49	2390067	2389132	2282928	2281993	2298285	2297350	2378110	2377175	2540215	2539400	1089843	1088908	2141109	2140174	2345563	2344628	533691	534626	2440020	2439085
2976	BPSL2800		49	2391878	2390085	2284739	2282946	2300096	2298303	2379921	2378128	2542026	2540233	1091654	1089861	2142920	2141127	2347374	2345581	531880	533673	2441831	2440038
2977	BPSL2801		49	2392816	2391878	2285677	2284739	2301034	2300096	2380859	2379921	2542964	2542026	1092592	1091654	2143858	2142920	2348312	2347374	530942	531880	2442769	2441831
2978	BPSL2802		49	2394148	2392829	2287009	2285690	2302366	2301047	2382191	2380872	2544296	2542977	1093924	1092605	2145190	2143871	2349644	2348325	529610	530929	2444101	2442782
2979	BPSL2803		49	2395694	2394174	2288555	2287035	2303912	2302392	2383737	2382217	2545842	2544322	1095470	1093950	2146736	2145216	2351190	2349670	528064	529584	2445647	2444127
2980	BPSL2804		49	2396383	2395730	2289244	2288591	2304601	2303948	2384426	2383773	2546531	2545878	1096159	1095506	2147425	2146772	2351879	2351226	527375	528028	2446336	2445683
2981	BPSL2805		49	2397162	2396383	2290023	2289244	2305380	2304601	2385205	2384426	2547310	2546531	1096938	1096159	2148204	2147425	2352658	2351879	526596	527375	2447115	2446336
2982	BPSL2806		49	2398317	2397172	2291178	2290033	2306535	2305390	2386360	2385215	2548465	2547320	1098093	1096948	2149359	2148214	2353813	2352668	525441	526586	2448270	2447125
2983	BPSL2807		49	2399961	2398801	2292822	2291662	2308179	2307019	2388004	2386844	2550109	2548949	1099737	1098577	2151003	2149843	2355457	2354297	523797	524957	2449914	2448754
2984	BPSL2808		49	2400142	2401290	2293003	2294151	2308360	2309508	2388185	2389333	2550290	2551438	1099918	1101066	2151184	2152332	2355638	2356786	523616	522468	2450095	2451243
2985	BPSL2809		49	2401294	2403306	2294155	2296167	2309512	2311524	2389337	2391349	2551442	2553454	1101070	1103082	2152336	2154348	2356790	2358802	522464	520452	2451247	2453259
2986	BPSL2810		49	2403707	2405131	2296568	2297992	2311925	2313349	2391750	2393174	2553855	2555279	1103483	1104907	2154749	2156173	2359203	2360627	520051	518627	2453660	2455084
2987	BPSL2811		49	2405396	2406238	2298257	2299099	2313614	2314456	2393439	2394281	2555544	2556386	1105172	1106014	2156438	2157280	2360892	2361734	518362	517520	2455349	2456191
2988	BPSL2812		49	2406307	2407176	2299168	2300037	2314525	2315394	2394350	2395219	2556455	2557324	1106083	1106952	2157349	2158218	2361803	2362672	517451	516582	2456260	2457129
2989	BPSL2813		49	2408243	2407503	2301104	2300364	2316461	2315721	2396286	2395546	2558391	2557651	1108019	1107279	2159285	2158545	2363739	2362999	515515	516255	2458196	2457456
2990	BPSL2814		49	2408365	2410416	2301226	2303277	2316583	2318634	2396408	2398459	2558513	2560564	1108141	1110192	2159407	2161458	2363861	2365912	515393	513342	2458318	2460369
2991	BPSL2815		49	2410416	2411387	2303277	2304248	2318634	2319605	2398459	2399430	2560564	2561535	1110192	1111163	2161458	2162429	2365912	2366883	513342	512371	2460369	2461340
2992	BPSL2816		49	2411809	2411411	2304670	2304272	2320027	2319629	2399852	2399454	2561957	2561559	1111585	1111187	2162851	2162453	2367305	2366907	511949	512347	2461762	2461364
2993	BPSL2817	transposase	49																				
2994	BPSL2818		49	2413152	2412100	2306013	2304961	2321370	2320318	2401195	2400143	2563300	2562248	1112928	1111876	2164194	2163142	2368648	2367596	510606	511658	2463105	2462053
2995	BPSL2819		49	2413451	2414173	2306312	2307034	2321669	2322391	2401494	2402216	2563599	2564321	1113227	1113949	2164493	2165215	2368947	2369669	510307	509585	2463404	2464126

2996	BPSL2820		49	2414198	2414881	2307059	2307742	2322416	2323099	2402241	2402924	2564346	2565029	1113974	1114657	2165240	2165923	2369694	2370377	509560	508877	2464151	2464834
2997	BPSL2821		49	2414881	2416425	2307742	2309286	2323099	2324643	2402924	2404468	2565029	2566573	1114657	1116201	2165923	2167467	2370377	2371921	508877	507333	2464834	2466378
2998	BPSL2822		49	2416465	2416989	2309326	2309850	2324683	2325207	2404508	2405032	2566613	2567137	1116241	1116765	2167507	2168031	2371961	2372485	507293	506769	2466418	2466945
2999	BPSL2823		49	2416989	2417669	2309850	2310530	2325207	2325887	2405032	2405712	2567137	2567817	1116765	1117445	2168031	2168711	2372485	2373165	506769	506089	2466945	2467625
3000	BPSL2824		49	2417737	2418549	2310598	2311410	2325955	2326767	2405780	2406592	2567885	2568697	1117513	1118325	2168779	2169591	2373233	2374045	506021	505209	2467693	2468505
3001	BPSL2825		49	2420746	2418731	2313607	2311592	2328964	2326949	2408789	2406774	2570894	2568879	1120522	1118507	2171788	2169773	2376242	2374227	503012	505027	2470702	2468687
3002	BPSL2826		49	2421898	2420771	2314759	2313632	2330116	2328989	2409941	2408814	2572046	2570919	1121674	1120547	2172940	2171813	2377394	2376267	501860	502987	2471854	2470727
3003	BPSL2827		49	2424117	2422168	2316978	2315029	2332335	2330386	2412160	2410211	2574265	2572316	1123893	1121944	2175159	2173210	2379614	2377722	499641	501590	2474073	2472124
3004	BPSL2828		49	2424694	2424266	2317555	2317127	2332912	2332484	2412737	2412309	2574842	2574414	1124470	1124042	2175736	2175308	2380191	2379763	499064	499492	2474650	2474222
3005	BPSL2829		49	2425267	2424713	2318128	2317574	2333485	2332931	2413310	2412756	2575415	2574861	1125043	1124489	2176309	2175755	2380764	2380210	498491	499045	2475223	2474669
3006	BPSL2830		49	2426171	2425767	2319032	2318628	2334389	2333985	2414214	2413810	2576319	2575915	1125947	1125543	2177213	2176809	2381668	2381264	497587	497991	2476127	2475723
3007	BPSL2831		49	2427280	2426180	2320141	2319041	2335498	2334398	2415323	2414223	2577428	2576328	1127056	1125956	2178322	2177222	2382777	2381677	496478	497578	2477236	2476136
3008	BPSL2832		49	2428475	2427456	2321336	2320317	2336693	2335674	2416518	2415499	2578623	2577604	1128251	1127232	2179517	2178498	2383972	2382953	495283	496302	2478431	2477412
3009	BPSL2833		49	2428609	2429508	2321470	2322369	2336827	2337726	2416652	2417551	2578757	2579656	1128385	1129284	2179651	2180550	2384106	2385005	495149	494250	2478565	2479464
3010	BPSL2834		49	2429541	2431187	2322402	2324048	2337759	2339405	2417584	2419230	2579689	2581335	1129317	1130963	2180583	2182229	2385038	2386684	494217	492571	2479497	2481035
3011		Chr1_138_ISBma2_C	49																			2727692	2728537
3012	BPSL2835		49	2434372	2431592	2327233	2324453	2342590	2339810	2422371	2419591	2584520	2581740	1134148	1131368	2185414	2182634	2389869	2387089	489386	492166	2724729	2727509
3013	BPSL2836		49	2434533	2438723	2327394	2331584	2342751	2346941	2422532	2426722	2584681	2588871	1134309	1138499	2185575	2189765	2390030	2394220	489225	485035	2724568	2720378
3014	BPSL2837		49	2438778	2439602	2331639	2332463	2346996	2347820	2426777	2427601	2588926	2589750	1138554	1139378	2189820	2190644	2394275	2395099	484980	484156	2720323	2719499
3015	BPSL2838		49	2439685	2441178	2332546	2334039	2347903	2349396	2427684	2429177	2589833	2591326	1139461	1140954	2190727	2192220	2395182	2396675	484073	482580	2719416	2717923
3016	BPSL2839		49	2441591	2442661	2334452	2335522	2349809	2350879	2429590	2430660	2591739	2592809	1141367	1142437	2192633	2193703	2397088	2398158	482167	481097	2717510	2716440
3017		Chr1_139a_ISBma2_A		2442898	2444470	2335759	2337331	2351116	2352688	2430897	2432469	2593046	2594618	1142674	1144265	2193940	2195512	2398395	2399967	479288	480860	2714631	2716203
3018		Chr1_139b_ISBma2_A		2525029	2526601	2418316	2419888	2434455	2436027	2513056	2514628	2675065	2676637	1224724	1226315	2576418	2577992	2478896	2480470	335753	337325	2571166	2572738
3019	BPSL2840		50	2523752	2524957	2417039	2418244	2433178	2434383	2511779	2512984	2673788	2674993	1223447	1224652	2575141	2576346	2477619	2478824	334476	335681	2569889	2571094
3020	BPSL2841		50	2523068	2523637	2416355	2416924	2432494	2433063	2511095	2511664	2673104	2673673	1222763	1223332	2574457	2575026	2476935	2477504	333792	334361	2569205	2569774
3021	BPSL2842		50	2522904	2521498	2416191	2414785	2432330	2430924	2510931	2509525	2672940	2671534	1222599	1221193	2574293	2572887	2476771	2475365	333628	332222	2569041	2567635
3022	BPSL2843		50	2521430	2519940	2414717	2413227	2430856	2429366	2509457	2507967	2671466	2669976	1221125	1219635	2572819	2571329	2475297	2473807	332154	330664	2567567	2566077
3023	BPSL2844		50	2519835	2518750	2413122	2412037	2429261	2428176	2507862	2506777	2669871	2668786	1219530	1218445	2571224	2570139	2473702	2472617	330559	329474	2565972	2564887
3024	BPSL2845		50	2518740	2517517	2412027	2410804	2428166	2426943	2506767	2505544	2668776	2667553	1218435	1217212	2570129	2568906	2472607	2471384	329464	328241	2564877	2563654
3025	BPSL2846		50	2517295	2516600	2410582	2409887	2426721	2426026	2505322	2504627	2667331	2666636	1216990	1216295	2568684	2567989	2471162	2470467	328019	327324	2563432	2562737
3026	BPSL2847		50	2516561	2515752	2409848	2409039	2425987	2425178	2504588	2503779	2666597	2665788	1216256	1215447	2567950	2567141	2470428	2469619	327285	326476	2562698	2561889
3027	BPSL2848		50	2514497	2515309	2407784	2408596	2423923	2424735	2502524	2503336	2664533	2665345	1214192	1215004	2565886	2566698	2468364	2469176	325221	326033	2560634	2561446
3028	BPSL2849		50	2513505	2514497	2406792	2407784	2422931	2423923	2501532	2502524	2663541	2664533	1213200	1214192	2564894	2565886	2467372	2468364	324229	325221	2559642	2560634
3029	BPSL2850		50	2512451	2513419	2405738	2406706	2421877	2422845	2500443	2501381	2662483	2663310	1212146	1213114	2563840	2564808	2466226	2467173	323175	324143	2558588	2559556
3030	BPSL2851		50	2511135	2512262	2404422	2405549	2420561	2421688	2499127	2500254	2661167	2662294	1210830	1211957	2562524	2563651	2464910	2466037	321859	322986	2557272	2558399
3031	BPSL2852		50	2510323	2511117	2403610	2404404	2419749	2420543	2498315	2499109	2660355	2661149	1210018	1210812	2561712	2562506	2464098	2464892	321047	321841	2556460	2557254
3032	BPSL2853		50	2509544	2510311	2402831	2403598	2418970	2419737	2497536	2498303	2659576	2660343	1209239	1210006	2560933	2561700	2463319	2464086	320268	321035	2555681	2556448
3033	BPSL2854		50	2508573	2509544	2401860	2402831	2417999	2418970	2496565	2497536	2658605	2659576	1208268	1209239	2559962	2560933	2462348	2463319	319297	320268	2554710	2555681
3034	BPSL2855		50	2507359	2508573	2400646	2401860	2416785	2417999	2495351	2496565	2657391	2658605	1207054	1208268	2558748	2559962	2461134	2462348	318083	319297	2553496	2554710
3035	BPSL2856		50	2506730	2507353	2400017	2400640	2416156	2416779	2494722	2495345	2656762	2657385	1206425	1207048	2558119	2558742	2460505	2461128	317454	318077	2552867	2553490
3036	BPSL2857		50	2506185	2506730	2399472	2400017	2415611	2416156	2494177	2494722	2656217	2656762	1205880	1206425	2557574	2558119	2459960	2460505	316909	317454	2552322	2552867
3037	BPSL2858		50	2506052	2505237	2399339	2398524	2415478	2414663	2494044	2493229	2656084	2655269	1205747	1204932	2557441	2556626	2459827	2459012	316776	315961	2552189	2551374
3038	BPSL2859		50	2505244	2504432	2398531	2397719	2414670	2413858	2493236	2492424	2655276	2654464	1204939	1204127	2556633	2555821	2459019	2458207	315968	315156	2551381	2551169
3039	BPSL2860		50	2504432	2503881	2397719	2397168	2413858	2413307	2492424	2491873	2654464	2653913	1204127	1203576	2555821	2555270	2458207	2457656	315156	314605	2550652	2550101
3040	BPSL2861		50	2502546	2503493	2395833	2396780	2411972	2412919	2490538	2491485	2652573	2653520	1202241	1203188	2553935	2554882	2456321	2457268	313265	314212	2548751	2549698
3041	BPSL2862		50	2501688	2502218	2394975	2395505	2411114	2411644	2489680	2490210	2651706	2652236	1201374	1201904	2553068	2553598	2455454	2455984	312380	312910	2547875	2548405
3042	BPSL2863		50	2500898	2501383	2394185	2394670	2410324	2410809	2488890	2489375	2650914	2651327	1200575	1201060	2552269	2552754	2454655	2455140	311581	312066	2547076	2547561
3043	BPSL2864		50	2500525	2500695	2393812	2393982	2409951	2410121	2488517	2488687	2650541	2650711	1200202	1200372	2551896	2552066	2454282	2454452	311208	311378	2546703	2546873
3044	BPSL2865		50	2498181	2500424	2391468	2393711	2407607	2409850	2486173	2488416	2648197	2650440	1197858	1200101	2549552	2551795	2451938	2454181	308864	311107	2544359	2546602
3045	BPSL2866		50	2496796	2497752	2390083	2391039	2406222	2407178	2484788	2485744	2646812	2647768	1196473	1197429	2548167	2549123	2450553	2451509	307479	308435	2542974	2543930
3046	BPSL2867		50	2494414	2496657	2387713	2389944	2403840	2406083	2482406	2484649	2644430	2646673	1194091	1196334	2545785	2548028	2448171	2450414	305097	307340	2540793	2542835

3047	BPSL2868		50	2493797	2492718	2386693	2385614	2402553	2401474	2481789	2480710	2643813	2642734	1193474	1192395	2545168	2544089	2447671	2446592	304480	303401	2540400	2539321
3048	BPSL2869		50	2492454	2491264	2385350	2384160	2401210	2400020	2480446	2479256	2642486	2641296	1192147	1190957	2543841	2542651	2446336	2445146	303137	301947	2539041	2537851
3049	BPSL2870		50	2490876	2490553	2383772	2383449	2399632	2399309	2478868	2478545	2640908	2640585	1190569	1190246	2542263	2541940	2444758	2444435	301559	301236	2537463	2537140
3050	BPSL2871		50	2490442	2488418	2383338	2381314	2399198	2397174	2478434	2476410	2640474	2638450	1190135	1188111	2541829	2539805	2444324	2442300	301125	299101	2537029	2535005
3051	BPSL2872		50	2488399	2487461	2381295	2380357	2397155	2396217	2476391	2475453	2638431	2637493	1188092	1187154	2539786	2538848	2442281	2441343	299082	298144	2534986	2534048
3052	BPSL2873		50	2486590	2487210	2379486	2380106	2395346	2395966	2474582	2475202	2636622	2637242	1186283	1186903	2537977	2538597	2440472	2441092	297273	297893	2533177	2533797
3053	BPSL2874		50	2485911	2486486	2378807	2379382	2394667	2395242	2473903	2474478	2635943	2636518	1185604	1186179	2537298	2537873	2439793	2440368	296594	297169	2532498	2533073
3054	BPSL2875		50	2485284	2485841	2378180	2378737	2394040	2394597	2473276	2473833	2635316	2635873	1184977	1185534	2536671	2537228	2439166	2439723	295967	296524	2531871	2532428
3055	BPSL2876		50	2484709	2485269	2377605	2378165	2393465	2394025	2472701	2473261	2634741	2635301	1184402	1184962	2536096	2536656	2438591	2439151	295392	295952	2531296	2531856
3056	BPSL2877		50	2484444	2483791	2377340	2376687	2393200	2392547	2472436	2471783	2634476	2633823	1184137	1183484	2535831	2535178	2438326	2437673	295127	294474	2531031	2530378
3057	BPSL2878		50	2483791	2483132	2376687	2376028	2392547	2391888	2471783	2471124	2633823	2633164	1183484	1182825	2535178	2534519	2437673	2437014	294474	293815	2530378	2529719
3058	BPSL2879		50	2483142	2481484	2376038	2374380	2391898	2390240	2471134	2469476	2633174	2631516	1182835	1181177	2534529	2532871	2437024	2435366	293825	292167	2529729	2528071
3059	BPSL2880		50	2481484	2480717	2374380	2373613	2390240	2389473	2469476	2468709	2631516	2630749	1181177	1180410	2532871	2532104	2435366	2434599	292167	291400	2528071	2527304
3060	BPSL2881		50	2479936	2479724	2372832	2372620	2388154	2387942	2467928	2467716	2629968	2627854	1179629	1177515	2531323	2529209	2433818	2431704	290610	288496	2526514	2524400
3061	BPSL2882		50	2477651	2476362	2370547	2369258	2385869	2384580	2465643	2464354	2627666	2626377	1177327	1176038	2529021	2527732	2431516	2430227	288308	287019	2524212	2522923
3062	BPSL2883		50	2475036	2474629	2367897	2367490	2383254	2382847	2463035	2462628	2625163	2624756	1174810	1174403	2526518	2526111	2428908	2428501	285602	285195	2521499	2521092
3063	BPSL2884		50	2474177	2473494	2367038	2366355	2382395	2381712	2462176	2461493	2624304	2623621	1173969	1173286	2525677	2524994	2428061	2427378	284629	283946	2520592	2519909
3064	BPSL2885		50	2471622	2473073	2364483	2365934	2379840	2381291	2459621	2461072	2621770	2623221	1171449	1172900	2523164	2524615	2425541	2426992	282074	283525	2518044	2519495
3065	BPSL2886		50	2471296	2471622	2364157	2364483	2379514	2379840	2459295	2459621	2621444	2621770	1171123	1171449	2522838	2523164	2425215	2425541	281748	282074	2517718	2518044
3066	BPSL2887		50	2470144	2471280	2363005	2364141	2378362	2379498	2458143	2459279	2620292	2621428	1169971	1171107	2521686	2522822	2424063	2425199	280596	281732	2516566	2517702
3067	BPSL2888		50	2469900	2469394	2362761	2362255	2378118	2377612	2457899	2457393	2620048	2619542	1169727	1169221	2521442	2520936	2423819	2423313	280352	279846	2516322	2515816
3068	BPSL2889		50	2469394	2468246	2362255	2361107	2377612	2376464	2457393	2456245	2619542	2618394	1169221	1168073	2520936	2519788	2423313	2422165	279846	278698	2515816	2514668
3069	BPSL2890		50	2468186	2466582	2361047	2359443	2376404	2374800	2456185	2454581	2618334	2616730	1168013	1166409	2519728	2518124	2422105	2420501	278638	277034	2514608	2513004
3070		Chr1_140_ISBma2_B	50	2464767	2466339	2357628	2359200	2372985	2374557	2452766	2454338	2614915	2616487										
3071	BPSL2891		50	2464109	2464714	2356970	2357575	2372327	2372932	2452108	2452713	2614257	2614862	1163917	1164522	2517242	2517847	2419619	2420224	276152	276757	2512122	2512727
3072	BPSL2892		50	2463645	2462239	2356506	2355100	2371863	2370457	2451644	2450238	2613793	2612387	1163440	1162034	2516765	2515359	2419142	2417736	275688	274282	2511658	2510252
3073	BPSL2893		50	2462191	2461001	2355052	2353862	2370409	2369219	2450190	2449000	2612339	2611149	1161986	1160796	2515311	2514121	2417688	2416498	274234	273044	2510204	2509014
3074	BPSL2894		50	2460790	2459729	2353651	2352590	2369008	2367947	2448789	2447728	2610938	2609877	1160585	1159524	2513910	2512849	2416287	2415226	272833	271772	2508803	2507742
3075	BPSL2895		50	2459729	2459499	2352590	2352360	2367947	2367717	2447728	2447498	2609877	2609647	1159524	1159294	2512849	2512619	2415226	2414996	271772	271542	2507742	2507512
3076	BPSL2896		50	2459451	2457889	2352312	2350750	2367669	2366107	2447450	2445888	2609599	2608037	1159246	1157684	2512571	2511009	2414948	2413386	271494	269932	2507464	2505902
3077	BPSL2897		50	2457519	2456980	2350380	2349841	2365737	2365198	2445518	2444979	2607667	2607128	1157314	1156775	2510639	2510100	2413016	2412477	269562	269023	2505532	2504993
3078	BPSL2898		50	2456903	2456325	2349764	2349186	2365121	2364543	2444902	2444324	2607051	2606473	1156698	1156120	2510023	2509445	2412400	2411822	268946	268368	2504916	2504338
3079	BPSL2899		50	2456154	2455087	2349015	2347948	2364372	2363305	2444153	2443086	2606302	2605235	1155949	1154882	2509274	2508207	2411651	2410584	268197	267130	2504167	2503100
3080	BPSL2900		50	2455058	2454105	2347919	2346966	2363276	2362323	2443057	2442104	2605206	2604253	1154853	1153900	2508178	2507225	2410555	2409602	267101	266148	2503071	2502118
3081	BPSL2901		50	2453970	2452831	2346831	2345692	2362188	2361049	2441969	2440830	2604118	2602979	1153765	1152626	2507090	2505951	2409467	2408328	266013	264874	2501983	2500844
3082	BPSL2902		50	2451983	2452669	2344844	2345530	2360201	2360887	2439982	2440668	2602131	2602817	1151778	1152464	2505103	2505789	2407480	2408166	264026	264712	2499996	2500682
3083	BPSL2903		50	2451496	2451972	2344357	2344833	2359714	2360190	2439495	2439971	2601644	2602120	1151291	1151767	2504616	2505092	2406993	2407469	263539	264015	2499509	2499985
3084	BPSL2904		50	2450184	2451422	2343045	2344283	2358402	2359640	2438183	2439421	2600332	2601570	1149979	1151217	2503304	2504542	2405681	2406919	262227	263465	2498197	2499435
3085	BPSL2905		50	2450053	2448845	2342914	2341706	2358271	2357063	2438052	2436844	2600201	2598993	1149848	1148640	2503173	2501965	2405550	2404342	262096	260888	2498066	2496858
3086	BPSL2906		50	2448033	2448728	2340894	2341589	2356251	2356946	2436032	2436727	2598181	2598876	1147828	1148523	2501153	2501848	2403530	2404225	260076	260771	2496046	2496741
3087	BPSL2907		50	2447324	2448025	2340185	2340886	2355542	2356243	2435323	2436024	2597472	2598173	1147119	1147820	2500444	2501145	2402821	2403522	259367	260068	2495337	2496038
3088	BPSL2908		50	2447231	2446332	2340092	2339193	2355449	2354550	2435230	2434331	2597379	2596480	1147026	1146127	2500351	2499452	2402728	2401829	259274	258375	2495244	2494345
3089	BPSL2909		50	2445793	2446158	2338654	2339019	2354011	2354376	2433792	2434157	2595941	2596306	1145588	1145953	2498913	2499278	2401290	2401655	257836	258201	2493806	2494171
3090	BPSL2910		50	2445194	2445583	2338055	2338444	2353412	2353801	2433193	2433582	2595342	2595731	1144989	1145378	2498314	2498703	2400691	2401080	257237	257626	2493207	2493596
3091	BPSL2911		50	2444754	2445179	2337615	2338040	2352972	2353397	2432753	2433178	2594902	2595327	1144549	1144974	2497874	2498299	2400251	2400676	256797	257222	2492767	2493192
3092		Chr1_141a_ISBma2_A		2442898	2444470	2335759	2337331	2351116	2352688	2430897	2432469	2593046	2594618	1142674	1144265	2496018	2497590	2398395	2399967	254941	256513	2490911	2492483
3093		Chr1_141b_ISBma2_A		2525029	2526601	2418316	2419888	2434455	2436027	2513056	2514628	2675065	2676637	1224724	1226315	2576418	2577992	2478896	2480470	335753	337325	2571166	2572738
3094	BPSL2912		51a	2527011	2527430	2420298	2420717	2436437	2436856	2515038	2515457	2677047	2677466	1226725	1227144	2578402	2578821	2480880	2481299	337735	338154	2573148	2573567
3095	BPSL2913		51a	2528927	2528100	2422214	2421387	2438353	2437526	2516954	2516127	2678963	2678136	1228641	1227814	2580318	2579491	2482796	2481969	339651	338824	2575064	2574237
3096	BPSL2914		51a	2530335	2529280	2423622	2422567	2439761	2438706	2518362	2517307	2680371	2679316	1230049	1228994	2581726	2580671	2484195	2483140	341059	340004	2576472	2575417
3097	BPSL2915		51a	2530545	2531456	2423832	2424743	2439971	2440882	2518572	2519483	2680581	2681492	1230259	1231170	2581936	2582847	2484405	2485115	341269	342180	2576682	2577593

3098	BPSL2916		51a	2532244	2531909	2425531	2425196	2441670	2441335	2520271	2519936	2682280	2681945	1231958	1231623	2583635	2583300	342968	342633	2578381	2578046
3099	BPSL2917		51a	2533049	2532633	2426336	2425920	2442475	2442059	2521076	2520660	2683105	2682689	1232763	1232347	2584440	2584024	343773	343357	2579186	2578770
3100	BPSL2918		51a	2533555	2533118	2426842	2426405	2442981	2442544	2521582	2521145	2683611	2683174	1233269	1232832	2584946	2584509	344279	343842	2579692	2579255
3101	BPSL2919		51a	2534018	2534332	2427305	2427619	2443444	2443758	2522045	2522359	2684074	2684388	1233732	1234046	2585409	2585723	344742	345056	2580155	2580469
3102	BPSL2920		51a	2534630	2534881	2427917	2428168	2444056	2444307	2522657	2522908	2684686	2684937	1234344	1234595	2586021	2586272	345354	345605	2580767	2581018
3103	BPSL2921		51a	2535719	2534997	2429006	2428284	2445145	2444423	2523746	2523024	2685775	2685053	1235433	1234711	2587110	2586388	346443	345721	2581856	2581134
3104	BPSL2922		51a	2536393	2535719	2429680	2429006	2445819	2445145	2524420	2523746	2686449	2685775	1236107	1235433	2587784	2587110	347117	346443	2582530	2581856
3105	BPSL2923		51a	2537133	2536396	2430420	2429683	2446559	2445822	2525160	2524423	2687189	2686452	1236847	1236110	2588524	2587787	347857	347120	2583270	2582533
3106		Chr1_142_ISBma1_B	51a	2538026	2539332	2431313	2432619	2447452	2448758	2526053	2527359	2688082	2689388					348750	350056	2584163	2585469
3107	BPSL2924		51a	2539433	2539332	2432720	2432619	2448859	2448758	2526052	2525246	2688081	2687275	1237832	1236933	2589509	2588610	350157	350056	2584162	2583356
3108	BPSL2925		51a	2540961	2539660	2434248	2432947	2450387	2449086	2528988	2527687	2691017	2689716	1239360	1238059	2591037	2589736	351685	350384	2587098	2585797
3109	BPSL2926		51a	2542292	2541345	2435579	2434632	2451718	2450771	2530319	2529372	2692348	2691401	1240691	1239744	2592368	2591421	353016	352069	2588429	2587482
3110	BPSL2927		51a	2542710	2544161	2435997	2437448	2452136	2453587	2530737	2532188	2692766	2694217	1241109	1242560	2592786	2594237	353434	354885	2588847	2590298
3111	BPSL2928		51a	2544209	2545657	2437496	2438944	2453635	2455083	2532236	2533684	2694265	2695713	1242608	1244056	2594285	2595733	354933	356381	2590346	2591794
3112	BPSL2929		51a	2546272	2545757	2439559	2439044	2455698	2455183	2534299	2533784	2696328	2695813	1244671	1244156	2596348	2595833	356996	356481	2592409	2591894
3113	BPSL2930		51a	2547647	2546289	2440934	2439576	2457073	2455715	2535674	2534316	2697703	2696345	1246046	1244688	2597723	2596365	358371	357013	2593784	2592426
3114	BPSL2931		51a	2548496	2547870	2441783	2441157	2457922	2457296	2536523	2535897	2698552	2697926	1246895	1246269	2598572	2597946	359220	358594	2594633	2594007
3115	BPSL2932		51a	2550368	2548518	2443655	2441805	2459794	2457944	2538395	2536545	2700424	2698574	1248767	1246917	2600444	2598594	361092	359242	2596505	2594655
3116	BPSL2933		51a	2550666	2551661	2443953	2444948	2460092	2461087	2538693	2539688	2700722	2701717	1249065	1250060	2600742	2601737	361390	362385	2596803	2597798
3117	BPSL2934		51a	2552534	2552115	2445821	2445402	2461960	2461541	2540561	2540142	2702590	2702171	1250933	1250514	2602610	2602191	363264	362845	2598677	2598258
3118	BPSL2935		51a	2553928	2552555	2447215	2445842	2463354	2461981	2541955	2540582	2703984	2702611	1252327	1250954	2604004	2602631	364658	363285	2600071	2598698
3119	BPSL2936		51a	2555048	2553963	2448335	2447250	2464474	2463389	2543075	2541990	2705104	2704019	1253447	1252362	2605124	2604039	365778	364693	2601191	2600106
3120	BPSL2937		51a	2555645	2555097	2448932	2448384	2465071	2464523	2543672	2543124	2705701	2705153	1254044	1253496	2605721	2605173	366375	365827	2601788	2601240
3121	BPSL2938		51a	2558281	2555690	2451568	2448977	2467707	2465116	2546308	2543717	2708337	2705746	1256680	1254089	2608357	2605766	369011	366420	2604424	2601833
3122	BPSL2939		51a	2558986	2558459	2452273	2451746	2468412	2467885	2547013	2546486	2709042	2708515	1257385	1256858	2609062	2608535	369716	369189	2605129	2604602
3123	BPSL2940		51a	2559734	2559030	2453021	2452317	2469160	2468456	2547761	2547057	2709790	2709086	1258133	1257429	2609810	2609106	370464	369760	2605877	2605173
3124	BPSL2941		51a	2560569	2559766	2453856	2453053	2469995	2469192	2548596	2547793	2710625	2709822	1258968	1258165	2610645	2609842	371299	370496	2606712	2605909
3125	BPSL2942		51a	2561470	2560643	2454947	2454432	2471595	2471128	2549497	2548670	2711526	2710699	1259869	1259042	2611546	2610719				
3126	BPSL2943		51a	2561585	2562010	2455062	2455487	2471710	2472135	2549612	2550037	2711641	2712066	1259984	1260409	2611661	2612086	372204	372629	2607617	2608042
3127	BPSL2944		51a	2562533	2562153	2456010	2455630	2472658	2472278	2550560	2550180	2712589	2712209	1260932	1260552	2612609	2612229	373152	372772	2608565	2608185
3128	BPSL2945		51a	2563694	2562687	2457171	2456164	2473819	2472812	2551721	2550714	2713750	2712743	1262093	1261086	2613770	2612763	374313	373306	2609726	2608719
3129		Chr1_143a_IS407A_B												1262978	1264213	2614655	2615890				
3130		Chr1_143b_IS407A_B														3290037	3291272				
3131	BPSL2946		51b	2565154	2563835	2458631	2457312	2475279	2473960	2553181	2551862	2715210	2713891	1264793	1264200	3289457	3290050	375773	374454	2611186	2609867
3132	BPSL2947		51b	2565400	2566086	2458877	2459563	2475525	2476211	2553427	2554113	2715456	2716142	1265039	1265725	3289211	3288525	376019	376705	2611432	2612118
3133	BPSL2948		51b	2567661	2566729	2461138	2460206	2477786	2476854	2555681	2554749	2717696	2716764	1267293	1266361	3286957	3287889	378385	377453	2613728	2612796
3134	BPSL2949		51b	2567776	2568432	2461253	2461909	2477901	2478557	2555796	2556452	2717811	2718467	1267408	1268064	3286842	3286186	378500	379156	2613843	2614499
3135	BPSL2950		51b	2568522	2568989	2461999	2462466	2478647	2479114	2556542	2557009	2718557	2719024	1268154	1268621	3286096	3285629	379246	379713	2614589	2615056
3136	BPSL2951		51b	2569173	2570369	2462650	2463846	2479298	2480494	2557193	2558389	2719208	2720404	1268805	1270001	3285445	3284249	379897	381093	2615240	2616436
3137	BPSL2952		51b	2571791	2570784	2465268	2464261	2481916	2480909	2559811	2558804	2721826	2720819	1271423	1270416	3282827	3283834	382515	381508	2617858	2616851
3138	BPSL2953		51b	2573909	2571885	2467386	2465362	2484034	2482010	2561929	2559905	2723944	2721920	1273541	1271517	3280709	3282733	384633	382609	2619976	2617952
3139	BPSL2954		51b	2574172	2575026	2467649	2468503	2484297	2485151	2562192	2563046	2724207	2725061	1273804	1274658	3280446	3279592	384896	385750	2620239	2621093
3140	BPSL2955		51b	2575069	2575482	2468546	2468959	2485194	2485607	2563089	2563502	2725104	2725517	1274701	1275114	3279549	3279136	385793	386206	2621136	2621549
3141	BPSL2956		51b	2575509	2576246	2468986	2469723	2485634	2486371	2563529	2564266	2725544	2726281	1275141	1275878	3279109	3278372	386233	386970	2621576	2622313
3142	BPSL2957		51b	2577049	2576654	2470526	2470131	2487174	2486779	2565069	2564674	2727084	2726689	1276681	1276286	3277569	3277964	387773	387378	2623116	2622721
3143	BPSL2958		51b	2577662	2577234	2471139	2470711	2487787	2487359	2565682	2565254	2727697	2727269	1277294	1276866	3276956	3277384	388386	387958	2623729	2623301
3144	BPSL2959		51b	2580299	2577993	2473776	2471470	2490424	2488118	2568319	2566013	2730334	2728028	1279931	1277625	3274319	3276625	391023	388717	2626366	2624060
3145	BPSL2960		51b	2580501	2581499	2473978	2474976	2490626	2491624	2568521	2569519	2730536	2731534	1280133	1281131	3274117	3273119	391225	392223	2626568	2627566
3146	BPSL2961		51b	2581636	2582106	2475113	2475583	2491761	2492231	2569656	2570126	2731671	2732141	1281268	1281738	3272982	3272512	392360	392830	2627703	2628173
3147	BPSL2962		51b	2582153	2582650	2475630	2476127	2492278	2492775	2570173	2570670	2732188	2732685	1281785	1282282	3272465	3271968	392877	393374	2628220	2628717
3148	BPSL2963		51b	2583763	2582939	2477240	2476416	2493888	2493064	2571783	2570959	2733798	2732974	1283395	1282571	3270855	3271679	394487	393663	2629830	2629006

3149	BPSL2964		51b	2584843	2583863	2478320	2477340	2494968	2493988	2572863	2571883	2734878	2733898	1284475	1283495	3269775	3270755			395567	394587	2630910	2629930
3150	BPSL2965		51b	2585786	2584983	2479263	2478460	2495911	2495108	2573806	2573003	2735821	2735018	1285418	1284615	3268832	3269635			396510	395707	2631853	2631050
3151	BPSL2966		51b	2586829	2585828	2480306	2479305	2496954	2495953	2574849	2573848	2736864	2735863	1286461	1285460	3267789	3268790			397553	396552	2632896	2631895
3152	BPSL2967		51b	2588476	2586947	2481953	2480424	2498601	2497072	2576496	2574967	2738511	2736982	1288108	1286579	3266142	3267671			399200	397671	2634543	2633014
3153	BPSL2968		51b	2589539	2588547	2483016	2482024	2499664	2498672	2577559	2576567	2739574	2738582	1289171	1288179	3265079	3266071			400263	399271	2635606	2634614
3154	BPSL2969		51b	2590448	2589672	2483925	2483149	2500573	2499797	2578468	2577692	2740483	2739707	1290080	1289304	3264170	3264946			401172	400396	2636515	2635739
3155	BPSL2970		51b	2591074	2590448	2484551	2483925	2501199	2500573	2579094	2578468	2741109	2740483	1290706	1290080	3263544	3264170			401798	401172	2637141	2636515
3156	BPSL2971		51b	2592185	2591175	2485662	2484652	2502310	2501300	2580205	2579195	2742220	2741210	1291817	1290807	3262433	3263443			402909	401899	2638252	2637242
3157	BPSL2972		51b	2593099	2592185	2486576	2485662	2503224	2502310	2581119	2580205	2743134	2742220	1292731	1291817	3261519	3262433			403823	402909	2639166	2638252
3158	BPSL2973		51b	2593795	2593367	2487272	2486844	2503920	2503492	2581815	2581387	2743830	2743402	1293427	1292999	3260823	3261251			404519	404091	2639862	2639434
3159	BPSL2974		51b	2595490	2593859	2488967	2487336	2505615	2503984	2583510	2581879	2745525	2743894	1295122	1293491	3259128	3260759			406214	404583	2641557	2639926
3160	BPSL2975		51b	2596720	2595953	2490197	2489430	2506845	2506078	2584740	2583973	2746755	2745988	1296352	1295585	3257898	3258665			407444	406677	2642787	2642020
3161	BPSL2976		51b	2597619	2596744	2491096	2490221	2507744	2506869	2585639	2584764	2747654	2746779	1297251	1296376	3256999	3257874			408343	407468	2643686	2642811
3162	BPSL2977		51b	2599694	2597619	2493171	2491096	2509819	2507744	2587714	2585639	2749729	2747654	1299326	1297251	3254924	3256999			410418	408343	2645761	2643686
3163	BPSL2978		51b	2600387	2599821	2493864	2493298	2510512	2509946	2588407	2587841	2750422	2749856	1300019	1299453	3254231	3254797			411111	410545	2646454	2645888
3164	BPSL2979		51b	2601868	2600387	2495345	2493864	2511993	2510512	2589888	2588407	2751903	2750422	1301500	1300019	3252750	3254231			412592	411111	2647935	2646454
3165	BPSL2980		51b	2601990	2602643	2495467	2496120	2512115	2512768	2590010	2590663	2752025	2752678	1301622	1302275	3252628	3251975			412714	413367	2648057	2648710
3166	BPSL2981		51b	2602717	2603196	2496194	2496673	2512842	2513321	2590737	2591216	2752752	2753231	1302349	1302828	3251901	3251422			413441	413920	2648784	2649263
3167	BPSL2982		51b	2603395	2603844	2496872	2497321	2513520	2513969	2591415	2591864	2753430	2753879	1303027	1303476	3251223	3250774			414119	414568	2649462	2649911
3168	BPSL2983		51b	2603915	2604385	2497392	2497862	2514040	2514510	2591935	2592405	2753950	2754420	1303547	1304017	3250703	3250233			414639	415109	2649982	2650452
3169	BPSL2984		51b	2604473	2605837	2497950	2499314	2514598	2515962	2592493	2593857	2754508	2755872	1304105	1305469	3250145	3248781			415197	416561	2650540	2651904
3170	BPSL2985		51b	2605843	2606742	2499320	2500219	2515968	2516867	2593863	2594762	2755878	2756777	1305475	1306374	3248775	3247876			416567	417466	2651910	2652809
3171	BPSL2986		51b	2606793	2608217	2500270	2501694	2516918	2518342	2594813	2596237	2756828	2758252	1306425	1307849	3247825	3246401			417517	418941	2652860	2654284
3172	BPSL2987		51b	2608288	2608788	2501765	2502265	2518413	2518913	2596308	2596808	2758323	2758823	1307920	1308420	3246330	3245830			419012	419512	2654355	2654855
3173	BPSL2988		51b	2608892	2609827	2502369	2503304	2519017	2519952	2596912	2597847	2758927	2759862	1308524	1309459	3245726	3244791			419616	420551	2654959	2655894
3174	BPSL2989		51b	2609854	2610324	2503331	2503801	2519979	2520449	2597874	2598344	2759889	2760359	1309486	1309956	3244764	3244294			420578	421048	2655921	2656391
3175	BPSL2990_Histone H1-like protein		51b																				
3176	BPSL2991		51b	2612511	2611462	2505988	2504939	2522636	2521587	2600531	2599482	2762591	2761542	1312173	1311124	3242077	3243126			423265	422216	2658608	2657559
3177	BPSL2992		51b	2616025	2613041	2509502	2506518	2526150	2523166	2604045	2601061	2766105	2763121	1315687	1312703	3238563	3241547			426779	423795	2662122	2659138
3178	BPSL2993		51b	2617086	2616499	2510563	2509976	2527211	2526624	2605106	2604519	2767166	2766579	1316748	1316161	3237502	3238089			427840	427253	2663183	2662596
3179	BPSL2993a		51b	2617403	2617086	2510880	2510563	2527528	2527211	2605423	2605106	2767483	2767166	1317065	1316748	3237185	3237502			428157	427840	2663500	2663183
3180	BPSL2994		51b	2618316	2617420	2511793	2510897	2528441	2527545	2606336	2605440	2768396	2767500	1317978	1317082	3236272	3237168			429070	428174	2664413	2663517
3181	BPSL2995		51b	2618404	2619768	2511881	2513245	2528529	2529893	2606424	2607788	2768484	2769848	1318066	1319430	3236184	3234820			429158	430522	2664501	2665865
3182	BPSL2996		51b	2619869	2620417	2513346	2513894	2529994	2530542	2607889	2608437	2769949	2770497	1319531	1320079	3234719	3234171			430623	431171	2665966	2666514
3183	BPSL2997		51b	2621213	2620614	2514690	2514091	2531338	2530739	2609233	2608634	2771293	2770694	1320875	1320276	3233375	3233974			431967	431368	2667310	2666711
3184	BPSL2998		51b	2622981	2621248	2516458	2514725	2533106	2531373	2611001	2609268	2773061	2771328	1322643	1320910	3231607	3233340	3148933	3150372	433735	432002	2669078	2667345
3185	BPSL2999		51b	2623199	2623846	2516676	2517323	2533324	2533971	2611219	2611866	2773279	2773926	1322861	1323508	3231389	3230742	3148715	3148068	433953	434600	2669296	2669943
3186	BPSL3000		51b	2623905	2624489	2517382	2517966	2534030	2534614	2611925	2612509	2773985	2774569	1323567	1324151	3230683	3230099	3148009	3147425	434659	435243	2670002	2670586
3187	BPSL3001_IS	Chr1_144_ISBma2_B	51b											1324314	1325889	3228362	3229936	3145687	3147259				
3188	BPSL3002		51b	2625819	2624704	2519296	2518181	2535944	2534829	2613839	2612724	2775899	2774784	1327075	1325960	3227176	3228291	3144501	3145616	436573	435458	2671916	2670801
3189	BPSL3003		51b	2627022	2625907	2520499	2519384	2537147	2536032	2615042	2613927	2777102	2775987	1328278	1327163	3225973	3227088	3143298	3144413	437776	436661	2673119	2672004
3190	BPSL3004		51b	2627470	2627210	2520947	2520687	2537595	2537335	2615490	2615230	2777550	2777290	1328726	1328466	3225525	3225785	3142850	3143110	438224	437964	2673567	2673307
3191	BPSL3005		51b	2627810	2627502	2521287	2520979	2537935	2537627	2615830	2615522	2777890	2777582	1329066	1328758	3225185	3225493	3142510	3142818	438564	438256	2673907	2673599
3192	BPSL3006		51b	2628026	2629021	2521503	2522498	2538151	2539146	2616046	2617041	2778106	2779101	1329282	1330277	3224969	3223974	3142294	3141299	438780	439775	2674123	2675118
3193		Chr1_145_IS407A_A	51b	2630161	2631063	2523638	2524540	2540286	2541188	2618181	2619083	2780241	2781476	1331417	1332652	3221599	3222834	3139134	3140369	440915	442150	2676258	2677493
3194		Chr1_146_IS407A_B	51b	2631064	2632299	2524541	2525776	2541189	2542424	2619084	2620319												
3195	BPSL3007		51b	2633929	2635221	2527406	2528698	2544054	2545346	2621949	2623241	2783106	2784398	1334282	1335574	3219969	3218677	3137504	3136212	443780	445072	2679123	2680415
3196	BPSL3008		51b	2635324	2636580	2528801	2530057	2545449	2546705	2623344	2624600	2784501	2785757	1335677	1336933	3218574	3217318	3136109	3134853	445175	446431	2680518	2681774
3197	BPSL3009		51b	2636580	2637794	2530057	2531271	2546705	2547919	2624600	2625814	2785757	2786971	1336933	1338147	3217318	3216104	3134853	3133639	446431	447645	2681774	2682988
3198	BPSL3010		51b	2637827	2638753	2531304	2532230	2547952	2548878	2625847	2626773	2787004	2787930	1338180	1339106	3216071	3215145	3133606	3132680	447678	448604	2683021	2683947
3199	BPSL3011		51b	2638760	2639368	2532237	2532845	2548885	2549493	2626780	2627388	2787937	2788545	1339113	1339721	3215138	3214530	3132673	3132065	448611	449219	2683954	2684562

3200	BPSL3012		51b	2639520	2640272	2532997	2533749	2549645	2550397	2627540	2628292	2788697	2789449	1339873	1340625	3214378	3213626	3131913	3131161	449371	450123	2684714	2685466
3201	BPSL3012a		51b	2640311	2640511	2533788	2533988	2550436	2550636	2628331	2628531	2789488	2789688	1340664	1340864	3213587	3213387	3131122	3130922	450162	450362	2685505	2685705
3202	BPSL3013		51b	2641099	2640653	2534576	2534130	2551224	2550778	2629119	2628673	2790276	2789830	1341452	1341006	3212799	3213245	3130334	3130780	450950	450504	2686293	2685847
3203	BPSL3014		51b	2641965	2641099	2535442	2534576	2552090	2551224	2629985	2629119	2791142	2790276	1342318	1341452	3211933	3212799	3129468	3130334	451816	450950	2687159	2686293
3204	BPSL3015		51b	2643227	2641989	2536704	2535466	2553352	2552114	2631247	2630009	2792404	2791166	1343580	1342342	3210671	3211909	3128206	3129444	453078	451840	2688421	2687183
3205	BPSL3016		51b	2646177	2643385	2539654	2536862	2556302	2553510	2634197	2631405	2795354	2792562	1346530	1343738	3207721	3210513	3125256	3128048	456028	453236	2691371	2688579
3206	BPSL3017		51b	2646895	2647371	2540372	2540848	2557020	2557496	2634915	2635391	2796042	2796518	1347248	1347724	3207099	3206623	3124586	3124110	456650	457126	2691993	2692469
3207	BPSL3018		51b	2648682	2647768	2542159	2541245	2558807	2557893	2636702	2635788	2797829	2796915	1349035	1348121	3205312	3206226	3122799	3123713	458437	457523	2693780	2692866
3208	BPSL3019		51b	2649430	2648927	2542907	2542404	2559555	2559052	2637450	2636947	2798577	2798074	1349783	1349280	3204564	3205067	3122051	3122554	459185	458682	2694528	2694025
3209	BPSL3020		51b	2650812	2649619	2544289	2543096	2560937	2559744	2638832	2637639	2799959	2798766	1351165	1349972	3203182	3204375	3120669	3121862	460567	459374	2695910	2694717
3210	BPSL3021		51b	2652153	2650924	2545630	2544401	2562278	2561049	2640173	2638944	2801300	2800071	1352506	1351277	3201841	3203070	3119328	3120557	461908	460679	2697251	2696022
3211	BPSL3022		51b	2652932	2652183	2546409	2545660	2563057	2562308	2640952	2640203	2802079	2801330	1353285	1352536	3201062	3201811	3118549	3119298	462687	461938	2698030	2697281
3212	BPSL3023		51b	2653891	2652956	2547368	2546433	2564016	2563081	2641911	2640976	2803038	2802103	1354244	1353309	3200103	3201038	3117590	3118525	463646	462711	2698989	2698054
3213	BPSL3024		51b	2655290	2653896	2548767	2547373	2565415	2564021	2643310	2641916	2804437	2803043	1355643	1354249	3198704	3200098	3116191	3117585	465045	463651	2700388	2698994
3214	BPSL3025		51b	2656390	2655290	2549867	2548767	2566515	2565415	2644410	2643310	2805537	2804437	1356743	1355643	3197604	3198704	3115091	3116191	466145	465045	2701488	2700388
3215	BPSL3026		51b	2657804	2656515	2551281	2549992	2567929	2566640	2645824	2644535	2806951	2805662	1358157	1356868	3196190	3197479	3113677	3114966	467559	466270	2702902	2701613
3216	BPSL3027		51b	2659291	2657804	2552768	2551281	2569416	2567929	2647311	2645824	2808438	2806951	1359644	1358157	3194703	3196190	3112190	3113677	469046	467559	2704389	2702902
3217	BPSL3028		51b	2660510	2659344	2553987	2552821	2570635	2569469	2648530	2647364	2809657	2808491	1360863	1359697	3193484	3194650	3110971	3112137	470265	469099	2705608	2704442
3218	BPSL3029		51b	2661931	2660528	2555408	2554005	2572056	2570653	2649951	2648548	2811078	2809675	1362284	1360881	3192063	3193466	3109550	3110953	471686	470283	2707029	2705626
3219	BPSL3030		51b	2663472	2661931	2556949	2555408	2573597	2572056	2651492	2649951	2812619	2811078	1363825	1362284	3190522	3192063	3108009	3109550	473227	471686	2708570	2707029
3220	BPSL3031		51b	2665313	2663472	2558790	2556949	2575438	2573597	2653333	2651492	2814460	2812619	1365666	1363825	3188681	3190522	3106168	3108009	475068	473227	2710411	2708570
3221	BPSL3032		51b	2665639	2665313	2559116	2558790	2575764	2575438	2653659	2653333	2814786	2814460	1365992	1365666	3188355	3188681	3105842	3106168	475394	475068	2710737	2710411
3222	BPSL3033		51b	2666577	2665639	2560054	2559116	2576702	2575764	2654597	2653659	2815724	2814786	1366930	1365992	3187417	3188355	3104904	3105842	476332	475394	2711675	2710737
3223	BPSL3034		51b	2667029	2666604	2560506	2560081	2577154	2576729	2655049	2654624	2816176	2815751	1367382	1366957	3186965	3187390	3104452	3104877	476784	476359	2712127	2711702
3224	BPSL3035		51b	2668043	2667420	2561520	2560897	2578168	2577545	2656063	2655440	2817190	2816567	1368396	1367773	3185951	3186574	3103438	3104061	477798	477175	2713141	2712518
3225	BPSL3036		51b	2668324	2669481	2561801	2562958	2578449	2579606	2656344	2657501	2817471	2818628	1368677	1369834	3185670	3184513	3103157	3102000	478079	479236	2713422	2714579
3226		Chr1_147_ISBma2_A		2669533	2671096	2563010	2564573	2579658	2581221	2657553	2659116	2818680	2820243	1369886	1371461	3182889	3184461	3100401	3101948	479288	480860	2714631	2716203
3227	BPSL3037																						
3228	BPSL3038																						
3229	BPSL3040	Chr2																					
3230	BPSL3041	Chr2																					
3231	BPSL3042	Chr2																					
3232	BPSL3043	Chr2																					
3233	BPSL3044	Chr2																					
3234	BPSL3045	Chr2																					
3235	BPSL3046	Chr2																					
3236	BPSL3047	Chr2																					
3237	BPSL3048	Chr2																					
3238	BPSL3049	Chr2																					
3239	BPSL3050	Chr2																					
3240	BPSL3051	Chr2																					
3241	BPSL3052	Chr2																					
3242	BPSL3053	Chr2																					
3243	BPSL3054	Chr2																					
3244	BPSL3055	Chr2																					
3245	BPSL3056	Chr2																					
3246	BPSL3057	Chr2																					
3247	BPSL3058	Chr2																					
3248	BPSL3059	Chr2																					
3249	BPSL3060	Chr2																					
3250	BPSL3061	Chr2																					

3251	BPSL3062	Chr2																					
3252	BPSL3063	Chr2																					
3253	BPSL3064	Chr2																					
3254	BPSL3065	Chr2																					
3255	BPSL3066	Chr2																					
3256	BPSL3067	Chr2																					
3257	BPSL3068	Chr2																					
3258	BPSL3069	Chr2																					
3259	BPSL3070	Chr2																					
3260	BPSL3071	Chr2																					
3261	BPSL3072	Chr2																					
3262	BPSL3073	Chr2																					
3263	BPSL3074	Chr2																					
3264	BPSL3075	Chr2																					
3265	BPSL3076	Chr2																					
3266	BPSL3077	Chr2																					
3267	BPSL3078	Chr2																					
3268		Chr1_148_ISBma2_A		2888639	2890211	2784984	2786556	2800111	2801683	2875168	2876717	3037889	3039461	1787702	1789301	3140775	3142322	2648477	2650049	21273	22845	2904760	2906332
3269	BPSL3080		52	2891628	2890369	2787973	2786714	2803100	2801841	2878134	2876875	3040878	3039619	1786285	1787544	3139358	3140617	2651466	2650207	24262	23003	2907749	2906490
3270	BPSL3081		52	2892843	2892229	2789188	2788574	2804315	2803701	2879349	2878735	3042093	3041479	1785070	1785684	3138143	3138757	2652681	2652067	25477	24863	2908964	2908350
3271	BPSL3082		52	2894611	2893331	2790956	2789676	2806083	2804803	2881117	2879837	3043861	3042581	1783302	1784582	3136375	3137655	2654449	2653169	27245	25965	2910732	2909452
3272	BPSL3083		52	2894770	2895411	2791115	2791756	2806242	2806883	2881276	2881917	3044020	3044661	1783143	1782502	3136216	3135575	2654608	2655249	27404	28045	2910891	2911532
3273	BPSL3084		52	2895752	2896372	2792097	2792717	2807224	2807844	2882258	2882878	3045002	3045622	1782161	1781541	3135234	3134614	2655590	2656210	28386	29006	2911873	2912493
3274	BPSL3085		52	2897356	2896463	2793701	2792808	2808828	2807935	2883862	2882969	3046606	3045713	1780557	1781450	3133630	3134523	2657194	2656301	29990	29097	2913477	2912584
3275	BPSL3086		52	2898674	2897487	2795019	2793832	2810146	2808959	2885180	2883993	3047924	3046737	1779239	1780426	3132312	3133499	2658512	2657325	31308	30121	2914795	2913608
3276	BPSL3087		52	2900179	2898980	2796524	2795325	2811651	2810452	2886685	2885486	3049429	3048230	1777734	1778933	3130807	3132006	2660017	2658818	32813	31614	2916300	2915101
3277	BPSL3088		52	2900944	2900501	2797289	2796846	2812416	2811973	2887450	2887007	3050194	3049751	1776934	1777377	3130007	3130450	2660817	2660374	33578	33135	2917065	2916622
3278	BPSL3089		52	2901606	2903768	2797951	2800113	2813078	2815240	2888112	2890274	3050856	3053018	1776272	1774110	3129345	3127183	2661479	2663641	34240	36402	2917727	2919889
3279	BPSL3090		52	2904794	2905060	2801139	2801405	2816266	2816532	2891300	2891566	3054044	3054310	1773084	1772818	3126157	3125891	2664667	2664933	37428	37694	2920915	2921181
3280	BPSL3091		52	2905166	2905393	2801511	2801738	2816638	2816865	2891672	2891899	3054416	3054643	1772712	1772485	3125785	3125558	2665039	2665266	37800	38027	2921287	2921514
3281	BPSL3092		52	2906012	2907277	2802357	2803622	2817484	2818749	2892518	2893783	3055262	3056527	1771866	1770601	3124939	3123674	2665885	2667150	38646	39911	2922133	2923398
3282	BPSL3093		52	2907295	2909553	2803640	2805898	2818767	2821025	2893801	2896059	3056545	3058803	1770583	1768325	3123656	3121398	2667168	2669426	39929	42187	2923416	2925674
3283	BPSL3094		52	2909553	2910998	2805898	2807343	2821025	2822470	2896059	2897504	3058803	3060254	1768325	1766880	3121398	3119953	2669426	2670871	42187	43632	2925674	2927119
3284	BPSL3095		52	2911955	2911215	2808300	2807560	2823427	2822687	2898461	2897721	3061211	3060471	1765923	1766663	3118996	3119736	2671828	2671088	44589	43849	2928076	2927336
3285	BPSL3096		52	2912567	2913142	2808912	2809487	2824039	2824614	2899073	2899648	3061823	3062398	1765311	1764736	3118384	3117809	2672440	2673015	45201	45776	2928688	2929263
3286	BPSL3097		52	2913417	2917307	2809762	2813652	2824889	2828779	2899923	2903813	3062673	3066563	1764461	1760571	3117534	3113644	2673290	2677180	46051	49941	2929538	2933428
3287	BPSL3098		52	2917307	2918293	2813652	2814638	2828779	2829765	2903813	2904799	3066563	3067549	1760571	1759585	3113644	3112658	2677180	2678166	49941	50927	2933428	2934414
3288	BPSL3099		52	2918301	2919230	2814646	2815575	2829773	2830702	2904807	2905736	3067557	3068486	1759577	1758648	3112650	3111721	2678174	2679103	50935	51864	2934422	2935351
3289	BPSL3100		52	2920553	2919435	2816898	2815780	2832025	2830907	2907059	2905941	3069809	3068691	1757325	1758443	3110398	3111516	2680426	2679308	53187	52069	2936674	2935556
3290	BPSL3101		52	2923336	2920646	2819681	2816991	2834808	2832118	2909842	2907152	3072592	3069902	1754566	1757232	3107639	3110305	2683185	2680519	55970	53280	2939457	2936767
3291	BPSL3102		52	2924470	2923373	2820815	2819718	2835942	2834845	2910976	2909879	3073726	3072629	1753432	1754529	3106505	3107602	2684319	2683222	57104	56007	2940591	2939494
3292	BPSL3103		52	2926260	2924437	2822605	2820782	2837732	2835909	2912766	2910943	3075516	3073693	1751642	1753465	3104722	3104802	2686109	2684286	58894	57071	2942381	2940558
3293	BPSL3104	GI10	52	2926864	2926355	2823209	2822700	2838333	2837827	2913370	2912861	3076117	3075611	1751038	1751547	3104118	3104627	2686713	2686204	59498	58989	2942976	2942476
3294		Chr1_149_IS407A_A		2926926	2928161	2823968	2825203	2839030	2840265	2913457	2914692	3076183	3077418	1749741	1750976	3102821	3104056	2686821	2687644	59560	60795	2944646	2945881
3295	BPSL3105																						
3296	BPSL3106																						
3297	BPSL3107																						
3298	BPSL3108																						
3299	BPSL3109																						
3300	BPSL3110																						
3301	BPSL3111																						

3302	BPSL3113																						
3303	BPSL3114	GI10																					
3304	BPSL3115	GI10																					
3305	BPSL3116	IS407A																					
3306	BPSL3117	IS407A																					
3307	BPSL3118	GI10																					
3308		Chr1_150_IS407A_A		2786407	2787642	2681776	2683011	2697886	2699121	2773025	2774260	2968933	2970168	1857854	1859089	2695410	2696645	2686821	2687644	3221731	3222966	2830709	2831944
3309	BPSL3119		53	2791409	2790855	2686778	2686224	2702888	2702334	2778027	2777473	2965186	2965740	1854087	1854641	2700412	2699858	2691411	2690857	3220161	3220715	2833514	2832960
3310	BPSL3120		53	2792054	2791446	2687423	2686815	2703533	2702925	2778672	2778064	2964541	2965149	1853442	1854050	2701057	2700449	2692056	2691448	3219516	3220124	2834159	2833551
3311	BPSL3121		53	2792905	2792150	2688274	2687519	2704384	2703629	2779523	2778768	2963690	2964445	1852591	1853346	2701908	2701153	2692907	2692152	3218665	3219420	2835010	2834255
3312	BPSL3122		53	2794310	2792931	2689679	2688300	2705789	2704410	2780928	2779549	2962285	2963664	1851186	1852565	2703313	2701934	2694312	2692933	3217260	3218639	2836415	2835036
3313	BPSL3123		53	2794934	2794314	2690303	2689683	2706413	2705793	2781552	2780932	2961661	2962281	1850562	1851182	2703937	2703317	2694936	2694316	3216636	3217256	2837039	2836419
3314	BPSL3124		53	2795898	2795155	2691267	2690524	2707377	2706634	2782516	2781773	2960697	2961440	1849598	1850341	2704901	2704158	2695900	2695157	3215672	3216415	2838003	2837260
3315	BPSL3125		53	2795920	2797125	2691289	2692494	2707399	2708604	2782538	2783743	2960675	2959470	1849576	1848371	2704923	2706128	2696062	2697126	3215650	3214445	2838025	2839230
3316	BPSL3126		53	2798241	2797462	2693610	2692831	2709720	2708941	2784859	2784080	2958354	2959133	1847255	1848034	2707244	2706465	2698242	2697463	3213329	3214108	2840346	2839567
3317	BPSL3127		53	2798807	2798283	2694176	2693652	2710286	2709762	2785425	2784901	2957788	2958312	1846689	1847213	2707810	2707286	2698808	2698284	3212763	3213287	2840912	2840388
3318	BPSL3128		53	2799067	2798837	2694436	2694206	2710546	2710316	2785685	2785455	2957528	2957758	1846429	1846659	2708070	2707840	2699068	2698838	3212503	3212733	2841172	2840942
3319	BPSL3129		53	2799521	2799159	2694890	2694528	2711000	2710638	2786139	2785777	2957074	2957436	1845975	1846337	2708524	2708162	2699522	2699160	3212049	3212411	2841626	2841264
3320	BPSL3130		53	2799939	2799553	2695308	2694922	2711418	2711032	2786557	2786171	2956656	2957042	1845557	1845943	2708942	2708556	2699940	2699554	3211631	3212017	2842044	2841658
3321	BPSL3131		53	2800360	2799995	2695729	2695364	2711839	2711474	2786978	2786613	2956235	2956600	1845136	1845501	2709363	2708998	2700361	2699996	3211210	3211575	2842465	2842100
3322	BPSL3132		53	2800773	2800360	2696142	2695729	2712252	2711839	2787391	2786978	2955822	2956235	1844723	1845136	2709776	2709363	2700774	2700361	3210797	3211210	2842878	2842465
3323	BPSL3133		53	2801558	2800773	2696927	2696142	2713037	2712252	2788176	2787391	2955037	2955822	1843938	1844723	2710561	2709776	2701559	2700774	3210012	3210797	2843663	2842878
3324	BPSL3134		53	2802381	2801629	2697750	2696998	2713860	2713108	2788999	2788247	2954214	2954966	1843115	1843867	2711384	2710632	2702382	2701630	3209189	3209941	2844486	2843734
3325	BPSL3135		53	2803190	2802552	2698559	2697921	2714669	2714031	2789808	2789170	2953405	2954043	1842306	1842944	2712193	2711555	2703191	2702553	3208380	3209018	2845295	2844657
3326	BPSL3136		53	2803807	2803190	2699176	2698559	2715286	2714669	2790425	2789808	2952788	2953405	1841689	1842306	2712810	2712193	2703808	2703191	3207763	3208380	2845912	2845295
3327	BPSL3137		53	2804450	2803866	2699819	2699235	2715929	2715345	2791068	2790484	2952145	2952729	1841046	1841630	2713453	2712869	2704451	2703867	3207120	3207704	2846555	2845971
3328	BPSL3138		53	2805584	2804517	2700953	2699886	2717063	2715996	2792202	2791135	2951011	2952078	1839912	1840979	2714587	2713520	2705585	2704518	3205986	3207053	2847689	2846622
3329	BPSL3139		53	2807076	2805742	2702445	2701111	2718555	2717221	2793694	2792360	2949519	2950853	1838420	1839754	2716079	2714745	2707077	2705743	3204494	3205828	2849181	2847847
3330	BPSL3140		53	2807774	2807121	2703143	2702490	2719253	2718600	2794392	2793739	2948821	2949474	1837722	1838375	2716777	2716124	2707775	2707122	3203796	3204449	2849879	2849226
3331	BPSL3141		53	2809120	2807774	2704489	2703143	2720599	2719253	2795738	2794392	2947475	2948821	1836376	1837722	2718123	2716777	2709121	2707775	3202450	3203796	2851225	2849879
3332	BPSL3142		53	2809373	2809137	2704742	2704506	2720852	2720616	2795991	2795755	2947222	2947458	1836123	1836359	2718376	2718140	2709374	2709138	3202197	3202433	2851478	2851242
3333	BPSL3143		53	2810214	2809462	2705583	2704831	2721693	2720941	2796832	2796080	2946381	2947133	1835282	1836034	2719217	2718465	2710215	2709463	3201356	3202108	2852319	2851567
3334	BPSL3144		53	2811152	2810214	2706521	2705583	2722631	2721693	2797770	2796832	2945443	2946381	1834344	1835282	2720155	2719217	2711153	2710215	3200418	3201356	2853257	2852319
3335	BPSL3145		53	2811702	2811436	2707071	2706805	2723181	2722915	2798320	2798054	2944893	2945159	1833794	1834060	2720705	2720439	2711703	2711437	3199868	3200134	2853694	2853428
3336	BPSL3146		53	2812331	2811702	2707700	2707071	2723810	2723181	2798949	2798320	2944264	2944893	1833165	1833794	2721334	2720705	2712332	2711703	3199239	3199868	2854238	2853903
3337	BPSL3147		53	2813456	2812449	2708825	2707818	2724935	2723928	2800074	2799067	2943139	2944146	1832040	1833047	2722459	2721452	2713457	2712450	3198114	3199121	2855363	2854356
3338	BPSL3148		53	2814013	2813456	2709382	2708825	2725492	2724935	2800631	2800074	2942582	2943139	1831483	1832040	2723016	2722459	2714014	2713457	3197557	3198114	2855920	2855363
3339	BPSL3149		53	2814793	2814029	2710162	2709398	2726272	2725508	2801411	2800647	2941802	2942566	1830703	1831467	2723796	2723032	2714794	2714030	3196777	3197541	2856700	2855936
3340	BPSL3150		53	2815608	2814793	2710977	2710162	2727087	2726272	2802226	2801411	2940987	2941802	1829888	1830703	2724611	2723796	2715609	2714794	3195962	3196777	2857515	2856700
3341	BPSL3151		53	2816811	2815711	2712180	2711080	2728290	2727190	2803429	2802329	2939784	2940884	1828685	1829785	2725814	2724714	2716812	2715712	3194759	3195859	2858718	2857618
3342	BPSL3152		53	2817623	2816811	2712992	2712180	2729102	2728290	2804241	2803429	2938972	2939784	1827873	1828685	2726626	2725814	2717624	2716812	3193947	3194759	2859530	2858718
3343	BPSL3153		53	2817873	2817679	2713242	2713048	2729352	2729158	2804491	2804297	2938722	2938916	1827623	1827817	2726876	2726682	2717874	2717680	3193697	3193891	2859780	2859586
3344	BPSL3154		53	2819049	2817925	2714418	2713294	2730528	2729404	2805667	2804543	2937546	2938670	1826447	1827571	2728052	2726928	2719050	2717926	3192521	3193645	2860956	2859832
3345	BPSL3155		53	2819604	2821421	2714973	2716790	2731083	2732900	2806222	2808039	2936991	2935174	1825892	1824075	2728607	2730424	2719605	2721422	3191966	3190149	2861511	2863328
3346	BPSL3156		53	2822238	2821687	2717607	2717056	2733710	2733159	2808673	2808305	2934350	2934901	1823286	1823837	2731212	2730661	2722218	2721667	3189332	3189883	2864145	2863594
3347	BPSL3157		53	2823752	2822244	2719121	2717613	2735224	2733716	2810253	2808745	2932836	2934344	1821772	1823280	2732726	2731218	2723732	2722224	3187818	3189326	2865659	2864151
3348	BPSL3158		53	2825554	2824037	2720923	2719406	2737026	2735509	2812055	2810538	2931034	2932551	1819970	1821487	2734528	2733011	2725534	2724017	3186016	3187533	2867461	2865944
3349	BPSL3159		53	2830297	2825597	2725666	2720966	2741769	2737069	2816798	2812098	2926291	2930991	1815227	1819927	2739271	2734571	2730277	2725577	3181273	3185973	2872204	2867504
3350	BPSL3160		53	2831301	2830591	2726670	2725960	2742773	2742063	2817802	2817092	2925287	2925997	1814223	1814933	2740275	2739565	2731281	2730571	3180269	3180979	2873208	2872498
3351	BPSL3161		53	2831494	2832222	2726863	2727591	2742966	2743694	2817995	2818723	2925094	2924366	1814030	1813302	2740468	2741196	2731474	2732202	3180076	3179348	2873401	2874129
3352	BPSL3162		53	2833132	2832374	2728501	2727743	2744604	2743846	2819633	2818875	2923456	2924214	1812392	1813150	2742106	2741348	2733112	2732354	3178438	3179196	2875039	2874281

3353	BPSL3163		53	2834259	2833180	2729628	2728549	2745731	2744652	2820760	2819681	2922329	2923408	1811265	1812344	2743233	2742154	2734239	2733160	3177311	3178390	2876166	2875087
3354	BPSL3164		53	2835130	2834288	2730499	2729657	2746602	2745760	2821631	2820789	2921458	2922300	1810394	1811236	2744104	2743262	2735110	2734268	3176440	3177282	2877037	2876195
3355	BPSL3165		53	2836011	2835130	2731380	2730499	2747483	2746602	2822512	2821631	2920577	2921458	1809513	1810394	2744985	2744104	2735991	2735110	3175559	3176440	2877918	2877037
3356	BPSL3166		53	2837467	2836148	2732836	2731517	2748939	2747620	2823968	2822649	2919121	2920440	1808057	1809376	2746441	2745122	2737447	2736128	3174103	3175422	2879374	2878055
3357	BPSL3167		53	2838784	2837555	2734153	2732924	2750256	2749027	2825285	2824056	2917804	2919033	1806740	1807969	2747758	2746529	2738764	2737535	3172786	3174015	2880691	2879462
3358	BPSL3168		53	2839912	2838836	2735281	2734205	2751384	2750308	2826413	2825337	2916676	2917752	1805612	1806688	2748886	2747810	2739892	2738816	3171658	3172734	2881819	2880743
3359	BPSL3169		53	2840463	2839912	2735832	2735281	2751935	2751384	2826964	2826413	2916125	2916676	1805061	1805612	2749437	2748886	2740443	2739892	3171107	3171658	2882370	2881819
3360	BPSL3170		53	2841881	2840547	2737250	2735916	2753353	2752019	2828382	2827048	2914707	2916041	1803643	1804977	2750855	2749521	2741861	2740527	3169689	3171023	2883788	2882454
3361	BPSL3171		53	2843227	2842271	2738596	2737640	2754699	2753743	2829728	2828772	2913361	2914317	1802297	1803253	2752201	2751245	2743207	2742251	3168343	3169299	2884546	2884178
3362	BPSL3172		53	2843904	2843227	2739273	2738596	2755376	2754699	2830405	2829728	2912684	2913361	1801620	1802297	2752878	2752201	2743884	2743207	3167666	3168343	2885359	2884682
3363	BPSL3173		53	2844839	2843904	2740208	2739273	2756311	2755376	2831340	2830405	2911740	2912684	1800676	1801620	2753822	2752878	2744828	2743884	3166722	3167666	2886329	2886102
3364	BPSL3174		53	2845152	2847470	2740521	2742839	2756624	2758942	2831653	2833971	2911427	2909109	1800363	1798045	2754135	2756453	2745141	2746325	3166409	3164091	2886642	2888960
3365		Chr1_151a_ISBma2_A		2847519	2849091	2742888	2744460	2758991	2760563	2834020	2835592	2907488	2909060	1796424	1797996	2756502	2758049	2747570	2749142	3162470	3164042	2889009	2890581
3366		Chr1_151b_ISBma2_A												1930938	1932510	3401631	3401662	2565718	2567265				
3367	BPSL3175		54	2849510	2849187	2744879	2744556	2760982	2760659	2836011	2835688	2907069	2907392	1932929	1932606	3402081	3401758	2565249	2565572	3162051	3162374	2891000	2890677
3368	BPSL3175A		54	2849547	2849828	2744916	2745197	2761019	2761300	2836048	2836329	2907032	2906751	1932966	1933247	3402118	3402399	2565212	2564928	3162014	3161733	2891037	2891318
3369	BPSL3176		54	2849842	2851110	2745211	2746479	2761314	2762582	2836343	2837611	2906737	2905469	1933261	1934529	3402413	3403681	2564914	2563646	3161719	3160451	2891332	2892600
3370	BPSL3177		54	2852085	2851375	2747454	2746744	2763557	2762847	2838586	2837876	2904500	2905210	1935504	1934794	3404656	3403946	2562671	2563381	3159476	3160186	2805629	2806339
3371	BPSL3178		54	2853102	2852110	2748471	2747479	2764574	2763582	2839603	2838611	2903483	2904475	1936521	1935529	3405673	3404681	2561654	2562646	3158459	3159451	2804612	2805604
3372		Chr1_152a_IS407A_A		2853155	2854390	2748524	2749759	2764627	2765862	2839656	2840891	2902195	2903430	1936574	1937809	3405726	3406961	2560366	2561601	3157171	3158406	2803324	2804559
3373		Chr1_152b_IS407A_A		2705320	2706555	2598865	2600100	2615744	2616979	2692936	2694171	2855128	2856363	1984096	1985331	3453248	3454483	2512937	2514172	3109637	3110872	2756293	2757528
3374	BPSL3179		55	2707875	2706688	2601420	2600233	2618299	2617112	2695491	2694304	2857683	2856496	1982776	1983963	3451928	3453115	2515492	2514305	3112192	3111005	2758848	2757661
3375	BPSL3180		55	2709979	2707883	2603524	2601428	2620403	2618307	2697595	2695499	2859787	2857691	1980672	1982768	3449824	3451920	2517596	2515500	3114296	3112200	2760952	2758856
3376	BPSL3181		55	2710835	2710191	2604380	2603736	2621259	2620615	2698451	2697807	2860643	2859999	1979816	1980460	3448968	3449612	2518452	2517808	3115152	3114508	2761808	2761164
3377	BPSL3182		55	2711052	2711708	2604597	2605253	2621476	2622132	2698668	2699324	2860860	2861516	1979599	1978943	3448751	3448095	2518669	2519325	3115369	3116025	2762025	2762681
3378	BPSL3183		55	2711929	2712990	2605474	2606535	2622353	2623414	2699545	2700606	2861737	2862798	1978722	1977661	3447874	3446813	2519546	2520607	3116246	3117307	2762902	2763963
3379	BPSL3184		55	2715092	2713212	2608637	2606757	2625516	2623636	2702708	2700828	2864900	2863020	1975559	1977439	3444711	3446591	2522709	2520829	3119409	3117529	2766065	2764185
3380	BPSL3185		55	2715435	2715088	2608980	2608633	2625859	2625512	2703051	2702704	2865243	2864896	1975216	1975563	3444368	3444715	2523052	2522705	3119752	3119405	2766408	2766061
3381	BPSL3186		55	2715965	2715573	2609510	2609118	2626389	2625997	2703581	2703189	2865773	2865381	1974686	1975078	3443838	3444230	2523582	2523190	3120282	3119890	2766938	2766546
3382	BPSL3187		55	2717083	2716109	2610628	2609654	2627507	2626533	2704699	2703725	2866891	2865917	1973568	1974542	3442720	3443694	2524700	2523726	3121400	3120426	2768056	2767082
3383	BPSL3188		55	2717834	2717214	2611379	2610759	2628258	2627638	2705450	2704830	2867642	2867022	1972817	1973437	3441969	3442589	2525539	2525006	3122151	3121531	2768807	2768187
3384	BPSL3189		55	2718386	2717988	2611931	2611533	2628810	2628412	2706002	2705604	2868194	2867796	1972265	1972663	3441417	3441815	2526091	2525693	3122703	3122305	2769359	2768961
3385	BPSL3190		55	2718775	2718413	2612320	2611958	2629199	2628837	2706391	2706029	2868583	2868221	1971876	1972238	3441028	3441390	2526480	2526118	3123092	3122730	2769748	2769386
3386	BPSL3191		55	2718930	2718817	2612475	2612362	2629354	2629241	2706546	2706433	2868738	2868625	1971721	1971834	3440873	3440986	2526635	2526522	3123247	3123134	2769903	2769790
3387	BPSL3192		55	2719169	2718954	2612714	2612499	2629593	2629378	2706785	2706570	2868977	2868762	1971482	1971697	3440634	3440849	2526874	2526659	3123486	3123271	2770142	2769927
3388	BPSL3193		55	2720543	2719182	2614088	2612727	2630967	2629606	2708159	2706798	2870351	2868990	1970108	1971469	3439260	3440621	2528248	2526887	3124860	3123499	2771516	2770155
3389	BPSL3194		55	2721004	2720573	2614549	2614118	2631428	2630997	2708620	2708189	2870812	2870381	1969647	1970078	3438799	3439230	2528709	2528278	3125321	3124890	2771977	2771546
3390	BPSL3195		55	2721215	2721036	2614760	2614581	2631639	2631460	2708831	2708652	2871023	2870844	1969436	1969615	3438588	3438767	2528920	2528741	3125532	3125353	2772188	2772009
3391	BPSL3196		55	2721749	2721234	2615294	2614779	2632173	2631658	2709365	2708850	2871557	2871042	1968902	1969417	3438054	3438569	2529454	2528939	3126066	3125551	2772722	2772207
3392	BPSL3197		55	2722128	2721766	2615673	2615311	2632552	2632190	2709744	2709382	2871936	2871574	1968523	1968885	3437675	3438037	2529833	2529471	3126445	3126083	2773101	2772739
3393	BPSL3198		55	2722671	2722144	2616216	2615689	2633095	2632568	2710287	2709760	2872479	2871952	1967980	1968507	3437132	3437659	2530376	2529849	3126988	3126461	2773644	2773117
3394	BPSL3199		55	2723087	2722695	2616632	2616240	2633511	2633119	2710703	2710311	2872895	2872503	1967564	1967956	3436716	3437108	2530792	2530400	3127404	3127012	2774060	2773668
3395	BPSL3200		55	2723407	2723105	2616952	2616650	2633831	2633529	2711023	2710721	2873215	2872913	1967244	1967546	3436396	3436698	2531112	2530810	3127724	3127422	2774380	2774078
3396	BPSL3201		55	2723954	2723418	2617499	2616963	2634378	2633842	2711570	2711034	2873762	2873226	1966697	1967233	3435849	3436385	2531659	2531123	3128271	3127735	2774927	2774391
3397	BPSL3202		55	2724280	2723975	2617825	2617520	2634704	2634399	2711896	2711591	2874088	2873783	1966371	1966676	3435523	3435828	2531985	2531680	3128597	3128292	2775253	2774948
3398	BPSL3203		55	2724658	2724293	2618203	2617838	2635082	2634717	2712274	2711909	2874466	2874101	1965993	1966358	3435145	3435510	2532363	2531998	3128975	3128610	2775631	2775266
3399	BPSL3204	30S ribosomal protein	55	2725240	2724971	2618785	2618516	2635664	2635395			2875048	2874779	1965411	1965680	3434563	3434832	2532945	2532676	3129557	3129288	2776213	2775944
3400	BPSL3205	50S ribosomal protein	55	2725431	2725240	2618976	2618785	2635855	2635664			2875240	2875079	1965220	1965411	3434372	3434563	2533136	2532945	3129748	3129557	2776404	2776213
3401	BPSL3206	50S ribosomal protein	55	2725857	2725444	2619402	2618989	2636281	2635868			2875666	2875253	1964794	1965207	3433946	3434359	2533562	2533149	3130174	3129761	2776830	2776417
3402	BPSL3207	30S ribosomal protein	55	2726660	2725863	2620205	2619408	2637084	2636287			2876469	2875672	1963991	1964788	3433143	3433940	2534365	2533568	3130977	3130180	2777633	2776836
3403	BPSL3208		55	2727001	2726675	2620546	2620220	2637425	2637099	2713637	2713311	2876810	2876484	1963650	1963976	3432802	3433128	2534706	2534380	3131318	3130992	2777974	2777648

3404	BPSL3209		55	2727289	2727017	2620834	2620562	2637713	2637441	2713925	2713653	2877098	2876826	1963362	1963634	3432514	3432786	2534994	2534722	3131606	3131334	2778262	2777990
3405	BPSL3210		55	2728127	2727303	2621672	2620848	2638551	2637727	2714763	2713939	2877936	2877112	1962524	1963348	3431676	3432500	2535832	2535008	3132444	3131620	2779100	2778276
3406	BPSL3211		55	2728444	2728133	2621989	2621678	2638868	2638557	2715080	2714769	2878253	2877942	1962207	1962518	3431359	3431670	2536149	2535838	3132761	3132450	2779417	2779106
3407	BPSL3212		55	2729061	2728444	2622606	2621989	2639485	2638868	2715697	2715080	2878870	2878253	1961590	1962207	3430742	3431359	2536766	2536149	3133378	3132761	2780034	2779417
3408	BPSL3213		55	2729720	2729064	2623265	2622609	2640144	2639488	2716356	2715700	2879529	2878873	1960931	1961587	3430083	3430739	2537425	2536769	3134037	3133381	2780693	2780037
3409	BPSL3214		55	2730218	2729910	2623763	2623455	2640642	2640334	2716854	2716546	2880027	2879719	1960433	1960741	3429585	3429893	2537923	2537615	3134535	3134227	2781191	2780883
3410	BPSL3215		55	2731561	2730374	2625942	2624755	2663196	2662009	2718178	2716991	2901312	2900125	1959090	1960277	3428242	3429429	2559483	2558296	3135878	3134691	2802740	2801553
3411	BPSL3216		55	2733705	2731606	2628086	2625987	2644477	2642378	2720322	2718223	2883046	2880947	1956946	1959045	3426098	3428197	2541217	2539118	3138022	3135923	2784410	2782311
3412	BPSL3217		55	2734297	2733830	2628678	2628211	2645069	2644602	2720914	2720447	2883638	2883171	1956354	1956821	3425506	3425973	2541809	2541342	3138614	3138147	2785002	2784535
3413	BPSL3218		55	2734848	2734471	2629229	2628852	2645620	2645243	2721465	2721088	2884189	2883812	1955803	1956180	3424955	3425332	2542360	2541983	3139165	3138788	2785553	2785176
3414	BPSL3219		55	2736998	2735067	2631379	2629448	2647770	2645839	2723615	2721684	2886339	2884408	1953653	1955584	3422805	3424736	2544510	2542579	3141315	3139384	2787703	2785772
3415		Chr1_153_ISBma1_A	55	2737048	2738352	2631429	2632733	2614893	2615748	2723665	2724969	2886389	2887693	1952299	1953603	3421451	3422755	2544560	2545864	3141365	3142669	2787753	2789057
3416	BPSL3220		55	2742663	2738428	2637044	2632809	2653435	2649200	2729280	2725045	2892004	2887769	1947988	1952223	3417140	3421375	2550175	2545940	3146980	3142745	2793368	2789133
3417	BPSL3221		55	2746791	2742688	2641172	2637069	2657563	2653460	2733408	2729305	2896132	2892029	1943860	1947963	3413012	3417115	2554303	2550200	3151108	3147005	2797496	2793393
3418	BPSL3222		55	2747487	2747158	2641868	2641539	2658259	2657930	2734104	2733775	2896828	2896499	1943164	1943493	3412316	3412645	2554999	2554670	3151804	3151475	2798192	2797863
3419	BPSL3223		55	2748090	2747596	2642471	2641977	2658862	2658368	2734707	2734213	2897431	2896937	1942561	1943055	3411713	3412207	2555602	2555108	3152407	3151913	2798795	2798301
3420	BPSL3224		55	2749086	2748391	2643467	2642772	2659858	2659163	2735703	2735008	2898427	2897732	1941565	1942260	3410717	3411412	2556598	2555903	3153403	3152708	2799791	2799096
3421	BPSL3225		55	2749518	2749090	2643899	2643471	2660290	2659862	2736135	2735707	2898859	2898431	1941133	1941561	3410285	3410713	2557030	2556602	3153835	3153407	2800223	2799795
3422	BPSL3226		55	2750221	2749667	2644643	2644089	2661446	2660892	2736838	2736284	2899562	2899008	1940430	1940984	3409582	3410136	2557733	2557179	3154538	3153984	2800990	2800436
3423	BPSL3227		55	2750603	2750226	2645025	2644648	2661828	2661451	2737220	2736843	2899944	2899567	1940048	1940425	3409200	3409577	2558115	2557738	3154920	3154543	2801372	2800995
3424	BPSL3228		55	2751971	2750784	2646393	2645206	2663196	2662009	2738588	2737401	2901312	2900125	1938680	1939867	3407832	3409019	2559483	2558296	3156288	3155101	2802740	2801553
3425		Chr1_154a_IS407A_A		2752854	2754089	2647276	2648511	2664079	2665314	2739471	2740706	2902195	2903430	1936574	1937809	3405726	3406961	2560366	2561601	3157171	3158406	2803324	2804559
3426		Chr1_154b_IS407A_A		2671093	2672328	2564570	2565805	2581218	2582453	2659248	2660362	2820240	2821475	2019764	2020999	3488844	3490079	?	?	3075556	3076791	2728534	2729133
3427	BPSL3229		56	2679640	2678366	2573034	2571760	2589560	2588286	2667258	2665984	2828856	2827582	2012453	2013727	3481533	3482807	2485703	2485143	3084102	3082828	2730686	2729412
3428	BPSL3230		56	2680827	2681822	2574213	2575208	2590739	2591734	2668445	2669440	2829884	2830879	2011848	2010853	3480928	3479933	2486420	2487415	3084753	3085748	2731337	2732332
3429	BPSL3231		56	2681888	2682169	2575274	2575555	2591800	2592081	2669506	2669787	2830945	2831226	2010787	2010506	3479867	3479586	2487481	2487762	3085814	3086095	2732398	2732679
3430	BPSL3232		56	2682183	2682983	2575569	2576369	2592095	2592895	2669801	2670601	2831240	2832040	2010492	2009692	3479572	3478772	2487776	2488576	3086109	3086909	2732693	2733493
3431	BPSL3233		56	2683022	2683639	2576408	2577025	2592934	2593551	2670640	2671257	2832079	2832696	2009379	2009035	3478459	3478115	2488889	2489233	3086948	3087565	2733532	2734149
3432	BPSL3234		56	2683644	2684729	2577030	2578115	2593556	2594641	2671262	2672347	2832701	2833786	2009030	2007945	3478110	3477025	2489238	2490323	3087570	3088655	2734154	2735239
3433	BPSL3235		56	2684854	2685681	2578399	2579226	2595278	2596105	2672470	2673297	2834641	2835468	2007820	2006993	3476900	3476073	2490512	2491339	3088780	3089607	2735436	2736263
3434	BPSL3236		56	2685711	2686307	2579256	2579852	2596135	2596731	2673327	2673923	2835498	2836094	2006963	2006367	3476043	3475447	2491369	2491965	3089637	3090233	2736293	2736889
3435	BPSL3237		56	2686537	2687196	2580082	2580741	2596961	2597620	2674153	2674812	2836324	2836983	2006137	2005478	3475217	3474558	2492195	2492854	3090463	3091122	2737119	2737778
3436	BPSL3238		56	2687833	2687321	2581378	2580866	2598257	2597745	2675449	2674937	2837620	2837108	2004841	2005353	3473921	3474433	2493491	2492979	3091759	3091247	2738415	2737903
3437	BPSL3239		56	2687985	2689079	2581530	2582624	2598409	2599503	2675601	2676695	2837772	2838866	2004689	2003595	3473769	3472675	2493643	2494002	3091911	3093005	2738567	2739661
3438		Chr1_155_ISBma2_B	56											2001853	2003425	3470928	3472505	2494952	2496499				
3439	BPSL3240		56	2689515	2689321	2583060	2582866	2599939	2599745	2677131	2676937	2839302	2839108	2001562	2001756	3470637	3470831	2496790	2496596	3093441	3093247	2740097	2739903
3440	BPSL3241		56	2689835	2693413	2583380	2586958	2600259	2603837	2677451	2681029	2839622	2843200	2001242	1997664	3470317	3466739	2497110	2500688	3093761	3097339	2740417	2743995
3441	BPSL3242		56	2696171	2693904	2589716	2587449	2606595	2604328	2683787	2681520	2845979	2843712	1994829	1997096	3463981	3466248	2503439	2501172	3100139	3097872	2746795	2744528
3442	BPSL3243		56	2696700	2697383	2590245	2590928	2607124	2607807	2684316	2684999	2846508	2847191	1994300	1993617	3463452	3462769	2503968	2504651	3100668	3101351	2747324	2748007
3443	BPSL3244		56	2697688	2698281	2591233	2591826	2608112	2608705	2685304	2685897	2847496	2848089	1993312	1992719	3462464	3461871	2504956	2505549	3101656	3102249	2748312	2748905
3444	BPSL3245		56	2698667	2699239	2592212	2592784	2609091	2609663	2686283	2686855	2848475	2849047	1992333	1991761	3461485	3460913	2505935	2506507	3102635	3103207	2749291	2749863
3445	BPSL3246		56	2699395	2700336	2592940	2593881	2609819	2610760	2687011	2687952	2849203	2850144	1991605	1990664	3460757	3459816	2506663	2507604	3103363	3104304	2750019	2750960
3446	BPSL3247		56	2700784	2702184	2594329	2595729	2611208	2612608	2688400	2689800	2850592	2851992	1990216	1988816	3459368	3457968	2508052	2509452	3104752	3106152	2751408	2752808
3447	BPSL3248		56	2702314	2703561	2595859	2597106	2612738	2613985	2689930	2691177	2852122	2853369	1988686	1987439	3457838	3456591	2509582	2510829	3106282	3107529	2752938	2754185
3448	BPSL3249		56	2703584	2704411	2597129	2597956	2614008	2614835	2691200	2692027	2853392	2854219	1987416	1986589	3456568	3455741	2510852	2511679	3107552	3108379	2754208	2755035
3449		Chr1_156_ISBma1_A	56	2704469	2705324	2598014	2598869	2614893	2615748	2692085	2692940	2854277	2855132	1985332	1986531	3454484	3455683	2511737	2512936	3108437	3109636	2755093	2756292
3450		Chr1_157_IS407A_A		2705320	2706555	2598865	2600100	2615744	2616979	2692936	2694171	2855128	2856363	1984096	1985331	3453248	3454483	2512937	2514172	3109637	3110872	2756293	2757528
3451	BPSL3250																						
3452	BPSL3251																						
3453	BPSL3252																						
3454	BPSL3253																						

3455	BPSL3254																										
3456	BPSL3254A																										
3457	BPSL3254B																										
3458	BPSL3255																										
3459		Chr1_158_IS407A_A		2881994	2883229	2778339	2779574	2793466	2794701	2868523	2869758	3031244	3032479	1794681	1795916	3147756	3148991	2749650	2750885	14627	15862	2898114	2899349
3460	BPSL3256		57	2883628	2885031	2779973	2781376	2795100	2796503	2870157	2871560	3032878	3034281	1794282	1792879	3147357	3145954	2643506	2644909	16262	17665	2899749	2901152
3461	BPSL3257	GI11																					
3462	BPSL3258	GI11																					
3463	BPSL3259	GI11																					
3464	BPSL3260	GI11																					
3465	BPSL3261	GI11																					
3466	BPSL3262	GI11																					
3467	BPSL3263	GI11																					
3468	BPSL3264	GI11																					
3469	BPSL3265	GI11																					
3470	BPSL3266	GI11																					
3471	BPSL3267	GI11																					
3472	BPSL3268	GI11																					
3473	BPSL3269	GI11																					
3474	BPSL3270	GI11																					
3475	BPSL3271		57	2885695	2885351	2782040	2781696	2797167	2796823	2872224	2871880	3034945	3034601	1792215	1792559	3145290	3145634	2645573	2645229	18329	17985	2901816	2901472
3476	BPSL3272		57	2886510	2885713	2782855	2782058	2797982	2797185	2873039	2872242	3035760	3034963	1791430	1792197	3144505	3145272	2646238	2645591	19144	18347	2902631	2901834
3477	BPSL3273		57	2887107	2886532	2783452	2782877	2798579	2798004	2873636	2873061	3036357	3035782	1790833	1791408	3143908	3144483	2646945	2646370	19741	19166	2903228	2902653
3478	BPSL3274		57	2887664	2887233	2784009	2783578	2799136	2798705	2874193	2873762	3036914	3036483	1790276	1790707	3143351	3143782	2647502	2647071	20298	19867	2903785	2903354
3479	BPSL3275		57	2887711	2888595	2784056	2784940	2799183	2800067	2874240	2875124	3036961	3037845	1790229	1789345	3143304	3142420	2647549	2648433	20345	21229	2903832	2904716
3480		Chr1_159a_ISBma2_A		2888639	2890211	2784984	2786556	2800111	2801683	2875168	2876003	3037889	3039461	1789270	1789301	3142345	3142376	2648477	2650049	21273	22845	2904760	2906332
3481		Chr1_159b_ISBma2_A		3002230	3003802	2900149	2901721	2915891	2917463	2988475	2990047	3248886	3250457	1674101	1675677	2928340	2929912	2565718	2567265	3363178	3364749	3039719	3041290
3482	BPSL3276		58	3001521	3002153	2899440	2900072	2915182	2915814	2987766	2988398	3251166	3250534	1676386	1675754	2927631	2928263	2567974	2567342	3365458	3364826	3039010	3039642
3483	BPSL3277		58	3000920	3000279	2898839	2898198	2914581	2913940	2987165	2986524	3251767	3252408	1676987	1677628	2927030	2926389	2568575	2569216	3366059	3366700	3038409	3037768
3484	BPSL3278		58	3000279	2999626	2898198	2897545	2913940	2913287	2986524	2985871	3252408	3253061	1677628	1678281	2926389	2925736	2569216	2569869	3366700	3367353	3037768	3037115
3485	BPSL3279		58	2999565	2999077	2897484	2896996	2913226	2912738	2985810	2985322	3253122	3253610	1678342	1678830	2925675	2925187	2569930	2570418	3367414	3367902	3037054	3036566
3486	BPSL3280		58	2997705	2998892	2895624	2896811	2911366	2912553	2983950	2985137	3254982	3253795	1680202	1679015	2923815	2925002	2571790	2570603	3369274	3368087	3035194	3036381
3487	BPSL3281		58	2997172	2997462	2895091	2895381	2910833	2911123	2983417	2983707	3255515	3255225	1680735	1680445	2923282	2923572	2572323	2572033	3369807	3369517	3034661	3034951
3488	BPSL3282		58	2995981	2996784	2893900	2894703	2909642	2910445	2982226	2983029	3256706	3255903	1681926	1681123	2922091	2922894	2573514	2572711	3370998	3370195	3033470	3034273
3489	BPSL3283		58	2995704	2994589	2893623	2892508	2909365	2908250	2981949	2980834	3256983	3258098	1682203	1683318	2921814	2920699	2573791	2574588	3371275	3372390	3033193	3032078
3490	BPSL3284		58	2993253	2994392	2890971	2892110	2906480	2907619	2979498	2980637	3259435	3258440	1684654	1683515	2919363	2920502	2576125	2574986	3373726	3372587	2944065	2943082
3491	BPSL3285_putative oxidoreductase		58	2992674	2992793	2890392	2890511	2905901	2906020	2978919	2979038	3260014	3259895	1685233	1685114	2918784	2918903	2576704	2576585	3374305	3374186	2944644	2944525
3492		Chr1_160a_IS407A_A		2991437	2992672	2889155	2890390	2904664	2905899	2977682	2978917	3260016	3261251	1685235	1686470	2917547	2918782	2576706	2577325	3374307	3375542	2944646	2945881
3493		Chr1_160b_IS407A_A		2926926	2928161	2823968	2825203	2839030	2840265	2913457	2914692	3076183	3077418	1749741	1750976	3102821	3104056	2641872	2643107				
3494	BPSL3285_putative oxidoreductase		59	2929142	2928162	2826184	2825204	2841246	2840266	2915673	2914693	3078399	3077419	1748760	1749740	3101840	3102820	2640891	2641871	3376523	3375543	2946862	2945882
3495	BPSL3286		59	2929469	2930158	2826511	2827200	2841573	2842262	2916000	2916689	3078726	3079415	1748433	1747744	3101513	3100824	2640564	2639875	3376850	3377539	2947189	2947878
3496	BPSL3287		59	2930258	2931745	2827300	2828787	2842362	2843849	2916789	2918276	3079515	3081002	1747644	1746157	3100724	3099237	2639775	2638288	3377639	3379126	2947978	2949465
3497	BPSL3288		59	2932762	2931935	2829804	2828977	2844866	2844039	2919293	2918466	3082019	3081192	1745140	1745967	3098220	3099047	2637271	2638098	3380143	3379316	2950482	2949655
3498	BPSL3289		59	2933133	2932783	2830175	2829825	2845237	2844887	2919664	2919314	3082390	3082040	1744769	1745119	3097849	3098199	2636900	2637250	3380514	3380164	2950853	2950503
3499	BPSL3290		59	2934718	2933300	2831760	2830342	2846822	2845404	2921249	2919831	3083975	3082557	1743184	1744602	3096264	3097682	2635315	2636733	3382099	3380681	2952438	2951020
3500	BPSL3291		59	2935803	2935075	2832845	2832117	2847907	2847179	2922334	2921606	3085060	3084332	1742099	1742827	3095179	3095907	2634230	2634958	3383184	3382456	2953523	2952795
3501	BPSL3292		59	2936640	2935831	2833682	2832873	2848744	2847935	2923171	2922362	3085897	3085088	1741262	1742071	3094342	3095151	2633393	2634202	3384021	3383212	2954360	2953551
3502	BPSL3293		59	2938376	2937735	2835418	2834777	2850480	2849839	2924907	2924266	3087633	3086992	1739526	1740167	3092606	3093247	2631657	2632298	3385757	3385116	2956096	2955455
3503	BPSL3294		59	2940475	2938376	2837517	2835418	2852579	2850480	2927006	2924907	3089732	3087633	1737427	1739526	3090507	3092606	2629558	2631657	3387856	3385757	2958195	2956096
3504	BPSL3295		59	2941689	2940475	2838731	2837517	2853793	2852579	2928220	2927006	3090946	3089732	1736213	1737427	3089293	3090507	2628344	2629558	3389070	3387856	2959409	2958195
3505	BPSL3296		59	2942885	2942388	2839927	2839430	2854989	2854492	2929416	2928919	3092142	3091645	1735017	1735514	3088097	3088594	2627148	2627645	3390266	3389769	2960605	2960108

3506	BPSL3297		59	2944238	2943075	2841280	2840117	2856342	2855179	2930769	2929606	3093495	3092332	1733664	1734827	3086744	3087907	2625795	2626958	3391619	3390456	2961958	2960795
3507	BPSL3298		59	2944848	2944348	2841890	2841390	2856952	2856452	2931379	2930879	3094105	3093605	1733054	1733554	3086134	3086634	2625185	2625685	3392229	3391729	2962568	2962068
3508	BPSL3299		59	2945774	2945049	2842816	2842091	2857878	2857153	2932305	2931580	3095031	3094306	1732128	1732853	3085208	3085933	2624259	2624984	3393155	3392430	2963494	2962769
3509	BPSL3300		59	2946204	2945779	2843246	2842821	2858308	2857883	2932735	2932310	3095461	3095036	1731698	1732123	3084778	3085203	2623829	2624254	3393585	3393160	2963924	2963499
3510	BPSL3301		59	2947623	2946523	2844665	2843565	2859727	2858627	2934154	2933054	3096880	3095780	1730279	1731379	3083755	3084459	2622410	2623510	3395004	3393904	2965343	2964243
3511	BPSL3302		59	2948324	2947623	2845366	2844665	2860428	2859727	2934855	2934154	3097581	3096880	1729578	1730279	3082660	3083361	2621709	2622410	3395705	3395004	2966044	2965343
3512	BPSL3303		59	2949268	2948324	2846494	2846069	2861972	2861544	2935807	2935385	3098574	3098164	1728634	1729578	3081716	3082660	2621233	2621709	3396639	3396178	2966921	2966502
3513	BPSL3304		59	2951293	2949275	2848519	2846501	2863997	2861979	2937832	2935814	3100599	3098581	1726609	1728627	3079691	3081709	2617958	2619976	3398664	3396646	2968946	2966928
3514	BPSL3305		59	2951861	2951337	2849087	2848563	2864565	2864041	2938400	2937876	3101167	3100643	1726041	1726565	3079123	3079647	2617390	2617914	3399232	3398708	2969514	2968990
3515	BPSL3306		59	2953832	2951895	2850416	2849121	2865900	2864599	2939921	2938434	3102529	3101201	1724070	1726007	3077152	3079089	2615154	2616158	3401203	3399266	2970891	2969548
3516	BPSL3307		59	2954607	2954230	2852325	2851948	2867833	2867456	2940849	2940472	3103983	3103606	1723295	1723672	3076377	3076754	2614742	2615119	3401978	3401601	2972131	2971754
3517	BPSL3308		59	2955658	2955275	2853376	2852993	2868884	2868501	2941900	2941517	3105034	3104651	1722244	1722627	3075326	3075709	2613691	2614074	3403029	3402646	2973182	2972799
3518	BPSL3309		59	2956535	2955678	2854253	2853396	2869761	2868904	2942777	2941920	3105911	3105054	1721367	1722224	3074449	3075306	2612814	2613671	3403906	3403049	2974059	2973202
3519	BPSL3310		59	2957280	2956732	2854998	2854450	2870506	2869958	2943522	2942974	3106656	3106108	1720622	1721170	3073704	3074252	2612069	2612617	3404651	3404103	2974804	2974256
3520	BPSL3311		59	2957694	2957377	2855412	2855095	2870920	2870603	2943936	2943619	3107070	3106753	1720208	1720525	3073290	3073607	2611655	2611972	3405065	3404748	2975218	2974901
3521	BPSL3312		59	2958288	2959349	2856006	2857067	2871514	2872575	2944530	2945591	3107664	3108725	1719614	1718553	3072696	3071635	2611061	2610000	3405659	3406720	2975812	2976873
3522	BPSL3313		59	2959752	2959459	2857470	2857177	2872978	2872685	2945994	2945701	3109128	3108835	1718150	1718443	3071232	3071525	2609597	2609890	3407123	3406830	2977276	2976983
3523	BPSL3314		59	2960058	2960759	2857776	2858477	2873284	2873985	2946300	2947001	3109434	3110135	1717844	1717143	3070926	3070225	2609291	2608590	3407429	3408130	2977582	2978283
3524	BPSL3315		59	2961793	2960954	2859511	2858672	2875019	2874180	2948035	2947196	3111169	3110330	1716109	1716948	3069191	3070030	2607556	2608395	3409164	3408325	2979317	2978478
3525	BPSL3316		59	2962782	2961895	2860500	2859613	2876008	2875121	2949024	2948137	3112158	3111271	1715120	1716007	3068202	3069089	2606567	2607454	3410153	3409266	2980306	2979419
3526	BPSL3317		59	2962933	2963538	2860651	2861256	2876159	2876764	2949175	2949780	3112309	3112914	1714969	1714364	3068051	3067446	2606416	2605811	3410304	3410909	2980457	2981062
3527	BPSL3318		59	2964140	2963931	2861858	2861649	2877366	2877157	2950382	2950173	3113516	3113307	1713762	1713971	3066844	3067053	2605209	2605418	3411511	3411302	2981664	2981455
3528	BPSL3319		59	2964698	2965861	2862416	2863579	2877924	2879087	2950940	2952103	3114074	3115237	1713204	1712041	3066286	3065123	2604651	2603488	3412069	3413232	2982222	2983385
3529	BPSL3320		59	2966014	2967531	2863732	2865249	2879240	2880757	2952256	2953773	3115390	3116907	1711888	1710371	3064970	3063453	2603335	2601818	3413385	3414902	2983538	2985055
3530	BPSL3321		59	2967548	2967859	2865266	2865577	2880774	2881085	2953790	2954101	3116924	3117235	1710354	1710043	3063436	3063125	2601801	2601490	3414919	3415227	2985072	2985380
3531	BPSL3322		59	2968085	2970412	2865803	2868130	2881311	2883638	2954327	2956654	3117461	3119788	1709817	1707490	3062899	3060572	2601264	2598937	3415456	3417783	2985609	2987936
3532	BPSL3323		59	2970412	2971557	2868130	2869275	2883638	2884783	2956654	2957799	3119788	3120933	1707490	1706345	3060572	3059427	2598937	2597792	3417783	3418928	2987936	2989081
3533	BPSL3324		59	2971557	2972588	2869275	2870306	2884783	2885814	2957799	2958830	3120933	3121964	1706345	1705314	3059427	3058396	2597792	2596761	3418928	3419959	2989081	2990112
3534	BPSL3325		59	2972603	2972821	2870321	2870539	2885829	2886047	2958845	2959063	3121979	3122197	1705299	1705081	3058381	3058163	2596746	2596528	3419974	3420192	2990127	2990345
3535	BPSL3326		59	2972827	2973888	2870545	2871606	2886053	2887114	2959069	2960130	3122203	3123264	1705075	1704014	3058157	3057096	2596522	2595461	3420198	3421259	2990351	2991412
3536	BPSL3327		59	2973888	2974649	2871606	2872367	2887114	2887875	2960130	2960891	3123264	3124025	1704014	1703253	3057096	3056335	2595461	2594700	3421259	3422020	2991412	2992173
3537	BPSL3328		59	2974652	2975281	2872370	2872999	2887878	2888507	2960894	2961523	3124028	3124657	1703250	1702621	3056332	3055703	2594697	2594068	3422023	3422652	2992176	2992805
3538	BPSL3329		59	2975286	2976347	2873004	2874065	2888512	2889573	2961528	2962589	3124662	3125723	1702616	1701555	3055698	3054637	2594063	2593002	3422657	3423718	2992810	2993871
3539	BPSL3330		59	2977167	2976433	2874885	2874151	2890393	2889659	2963409	2962675	3126543	3125809	1700735	1701469	3053817	3054551	2592182	2592916	3424538	3423804	2994691	2993957
3540	BPSL3331		59	2977893	2977495	2875611	2875213	2891119	2890721	2964135	2963737	3127269	3126871	1700009	1700407	3053091	3053489	2591456	2591854	3425264	3424866	2995417	2995019
3541	BPSL3332		59	2978460	2979080	2876178	2876798	2891686	2892246	2964702	2965322	3127836	3128456	1699442	1698822	3052524	3051904	2590889	2590269	3425831	3426451	2995984	2996604
3542	BPSL3333		59	2981117	2979387	2878835	2877105	2894344	2892614	2967359	2965629	3130493	3128763	1696785	1698515	3049867	3051597	2588232	2589962	3428488	3426758	2998641	2996911
3543	BPSL3334		59	2982263	2981580	2879981	2879298	2895490	2894807	2968505	2967822	3131639	3130956	1695639	1696322	3048721	3049404	2587086	2587769	3429634	3428951	2999787	2999104
3544	BPSL3335		59	2982688	2982263	2880406	2879981	2895915	2895490	2968930	2968505	3132064	3131639	1695214	1695639	3048296	3048721	2586661	2587086	3430059	3429634	3000212	2999787
3545	BPSL3336		59	2983422	2982751	2881140	2880469	2896649	2895978	2969664	2968993	3132798	3132127	1694480	1695151	3047562	3048233	2585927	2586598	3430793	3430122	3000946	3000275
3546	BPSL3337		59	2984460	2985278	2882178	2882996	2897687	2898505	2970702	2971520	3133836	3134654	1693442	1692624	3046524	3045706	2584889	2584071	3431831	3432649	3001984	3002802
3547	BPSL3338		59	2987151	2985337	2884869	2883055	2900378	2898564	2973393	2971579	3136527	3134713	1690756	1692246	3043838	3045328	2582198	2584012	3434522	3432708	3004675	3002861
3548	BPSL3339		59	2989081	2987423	2886799	2885141	2902308	2900650	2975326	2973668	3138457	3136799	1688826	1690484	3041908	3043566	2580268	2581926	3436452	3434794	3006605	3004947
3549	BPSL3340		59	2989965	2989267	2887683	2886985	2903192	2902494	2976210	2975512	3139341	3138643	1687942	1688640	3041024	3041722	2579384	2580082	3437336	3436638	3007489	3006791
3550	BPSL3341		59	2990731	2990417	2888449	2888135	2903958	2903644	2976976	2976662	3140107	3139793	1687176	1687490	3040258	3040572	2578618	2578932	3438102	3437788	3008255	3007941
3551		Chr1_161_IS407A_A		2991437	2992672	2889155	2890390	2904664	2905899	2977682	2978917	3140813	3142048	1685235	1686470	3038317	3039552	2577427	2577912	3438813	3440048	3008966	3010201
3552	BPSL3342	GI12																					
3553	BPSL3343	GI12																					
3554	BPSL3344	GI12																					
3555	BPSL3345	GI12																					
3556	BPSL3346	GI12																					

3557	BPSL3347	GI12																					
3558	BPSL3348	GI12																					
3559	BPSL3349	GI12																					
3560	BPSL3350	GI12																					
3561	BPSL3351	GI12																					
3562	BPSL3352	GI12																					
3563	BPSL3353	GI12																					
3564		Chr1_162_IS407A_A		3110701	3111936	3008620	3009855	3024998	3026233	3096934	3098169	3140813	3142048	1564428	1565663	3038317	3039552	2934200	2935435	3255150	3256385	3148182	3149417
3565	BPSL3354		1	3109151	3108255	3007070	3006174	3023448	3022552	3095384	3094806	3143598	3144494	1567213	1568109	3036767	3035871	2932650	2931754	3257935	3258831	3146632	3145736
3566	BPSL3355		1	3105829	3107928	3003748	3005847	3020126	3022225	3092081	3094180	3146920	3144821	1570535	1568436	3033445	3035544	2929328	2931427	3261257	3259158	3143310	3145409
3567	BPSL3356		1	3105579	3104740	3003498	3002659	3019876	3019037	3091831	3090992	3147170	3148009	1570785	1571624	3033195	3032356	2929078	2928239	3261507	3262346	3143060	3142221
3568	BPSL3357		1	3104740	3103940	3002659	3001859	3019037	3018237	3090992	3090192	3148009	3148809	1571624	1572424	3032356	3031556	2928239	2927439	3262346	3263146	3142221	3141421
3569	BPSL3358		1	3103474	3102374	3001393	3000293	3017771	3016671	3089726	3088626	3149275	3150375	1572890	1573990	3031090	3029990	2926973	2925873	3263612	3264712	3140955	3139855
3570	BPSL3359		1	3102199	3101843	3000118	2999762	3016496	3016140	3088451	3088095	3150550	3150906	1574165	1574521	3029815	3029459	2925698	2925342	3264887	3265243	3139680	3139324
3571	BPSL3360		1	3101033	3099918	2998952	2997837	3015323	3014208	3087278	3086163	3151681	3152796	1575303	1576418	3028740	3027625	2924602	2923487	3266018	3267133	3138535	3137420
3572	BPSL3361		1	3099821	3099444	2997740	2997363	3014111	3013734	3086066	3085689	3152893	3153270	1576515	1576892	3027528	3027151	2923390	2923013	3267230	3267607	3137323	3136946
3573	BPSL3362		1	3099136	3096212	2997055	2994131	3013426	3010502	3085381	3082457	3153578	3156502	1577200	1580124	3026843	3023919	2922705	2919781	3267915	3270839	3136638	3133714
3574	BPSL3363		1	3096057	3094942	2993976	2992861	3010347	3009232	3082302	3081187	3156657	3157772	1580279	1581394	3023764	3022649	2919626	2918511	3270994	3272109	3133559	3132444
3575	BPSL3364		1	3094905	3093520	2992824	2991439	3009195	3007810	3081150	3079765	3157809	3159194	1581431	1582816	3022612	3021227	2918474	2917089	3272146	3273531	3132407	3131022
3576	BPSL3365		1	3092301	3093479	2990220	2991398	3006591	3007769	3078546	3079724	3160413	3159235	1584035	1582857	3020008	3021186	2915870	2917048	3274750	3273572	3129803	3130981
3577	BPSL3366		1	3092073	3090373	2989992	2988292	3006363	3004663	3078318	3076618	3160641	3162341	1584263	1585963	3019780	3018080	2915642	2913942	3274978	3276678	3129575	3127875
3578	BPSL3367		1	3090341	3089277	2988260	2987196	3004631	3003567	3076586	3075522	3162373	3163437	1585995	1587059	3018048	3016984	2913910	2912846	3276710	3277774	3127843	3126779
3579	BPSL3368		1	3088076	3089152	2985995	2987071	3002366	3003442	3074321	3075397	3164638	3163562	1588260	1587184	3015783	3016859	2911645	2912721	3278975	3277899	3125578	3126654
3580	BPSL3369		1	3087831	3086314	2985750	2984233	3002121	3000604	3074076	3072559	3164883	3166400	1588505	1590022	3015538	3014021	2911400	2909883	3279220	3280737	3125333	3123816
3581	BPSL3370		1	3085799	3086041	2983718	2983960	3000089	3000331	3072044	3072286	3166914	3166672	1590537	1590295	3013506	3013748	2909368	2909610	3281252	3281010	3123301	3123543
3582	BPSL3371		1	3084930	3085730	2982849	2983649	2999220	3000020	3071175	3071975	3167783	3166983	1591406	1590606	3012637	3013437	2908499	2909299	3282121	3281321	3122432	3123232
3583	BPSL3372_ethanolamine ammonia-lyase		1	3083587	3084930	2981506	2982849	2997877	2999220	3069832	3071175	3169126	3167783	1592749	1591406	3011294	3012637	2907156	2908499	3283464	3282121	3121089	3122432
3584	BPSL3372_2	Chr1_163_ISBma2_A	1	3082021	3083593	2979940	2981512	2996311	2997883	3068266	3069838	3169120	3170692	1592743	1594343	3009753	3011300	2905615	2907162	3283458	3285029	3119525	3121095
3585	BPSL3372_ethanolamine ammonia-lyase		1	3081945	3082022	2979864	2979941	2996235	2996312	3068190	3068267	3170768	3170691	1594419	1594342	3009621	3009698	2905383	2905460	3285105	3285028	3119449	3119526
3586	BPSL3373		1	3080479	3081885	2978398	2979804	2994769	2996175	3066724	3068130	3172234	3170828	1595885	1594479	3008155	3009561	2903917	2905323	3286571	3285165	3117983	3119389
3587	BPSL3374		1	3079862	3078687	2977781	2976606	2994152	2992977	3066107	3064932	3172851	3174026	1596502	1597677	3007538	3006363	2903300	2902125	3287188	3288363	3117366	3116191
3588	BPSL3375		1	3078644	3078465	2976563	2976384	2992934	2992755	3064889	3064710	3174069	3174248	1597720	1597899	3006320	3006141	2902082	2901903	3288406	3288585	3116148	3115969
3589	BPSL3376		1	3076800	3078308	2974719	2976227	2991090	2992598	3063045	3064553	3175913	3174405	1599564	1598056	3004476	3005984	2900238	2901746	3290250	3288742	3114304	3115812
3590	BPSL3378		1	3073008	3075503	2970927	2973422	2987298	2989793	3059253	3061748	3179711	3177210	1603356	1600861	3000678	3003179	2896445	2898079	3294048	3291547	3110506	3113007
3591	BPSL3379		1	3072925	3072497	2970844	2970416	2987215	2986787	3059170	3058742	3179794	3180222	1603439	1603867	3000595	3000167	2896362	2895934	3294131	3294559	3110423	3109995
3592	BPSL3380		1	3072394	3071756	2970313	2969675	2986684	2986046	3058639	3058001	3180325	3180963	1603970	1604608	3000064	2999426	2895831	2895193	3294662	3295300	3109892	3109254
3593	BPSL3381		1	3070941	3071693	2968860	2969612	2985231	2985983	3057186	3057938	3181778	3181026	1605423	1604671	2998611	2999363	2894378	2895130	3296115	3295363	3108439	3109191
3594	BPSL3383		1	3069951	3070742	2967870	2968661	2984241	2985032	3056196	3056987	3182768	3181977	1606413	1605622	2997621	2998412	2893388	2894179	3297105	3296314	3107449	3108240
3595	BPSL3384		1	3068707	3069894	2966626	2967813	2982997	2984184	3054952	3056139	3184012	3182825	1607657	1606470	2996377	2997564	2892144	2893331	3298349	3297162	3106205	3107392
3596	BPSL3385		1	3067215	3067946	2965134	2965865	2981505	2982236	3053460	3054191	3185472	3184741	1609099	1608368	2994943	2995674	2890710	2891441	3299777	3299046	3104761	3105492
3597	BPSL3386		1	3067216	3065579	2965135	2963498	2981506	2979869	3053461	3051824	3185471	3187108	1609098	1610735	2994944	2993307	2890711	2889074	3299776	3301413	3104762	3103125
3598	BPSL3387		1	3065460	3063916	2963379	2961835	2979750	2978206	3051705	3050161	3187227	3188771	1610854	1612398	2993188	2991644	2888955	2887411	3301532	3303076	3103006	3101462
3599	BPSL3388		1	3062534	3063670	2960453	2961589	2976824	2977960	3048779	3049915	3190153	3189017	1613780	1612644	2990262	2991398	2886029	2887165	3304458	3303322	3100080	3101216
3600	BPSL3389		1	3058520	3062446	2956439	2960365	2972810	2976736	3044765	3048691	3194167	3190241	1617794	1613868	2986248	2990174	2882015	2885941	3308472	3304546	3096066	3099992
3601	BPSL3390		1	3055788	3058052	2953707	2955971	2970078	2972342	3042033	3044297	3196899	3194635	1620526	1618262	2983516	2985780	2879283	2881547	3311204	3308940	3093334	3095598
3602	BPSL3391		1	3054253	3055344	2952172	2953263	2968543	2969634	3040498	3041589	3198434	3197343	1622061	1620970	2981981	2983072	2877748	2878839	3312739	3311648	3091799	3092890
3603	BPSL3392		1	3054113	3053331	2952032	2951250	2968403	2967621	3040358	3039576	3198574	3199356	1622201	1622983	2981841	2981059	2877608	2876826	3312879	3313661	3091659	3090877
3604	BPSL3393		1	3052814	3053125	2950733	2951044	2967104	2967415	3039059	3039370	3199873	3199562	1623500	1623189	2980542	2980853	2876309	2876620	3314178	3313867	3090360	3090671
3605	BPSL3394		1	3050497	3052224	2948416	2950143	2964787	2966514	3036742	3038469	3202190	3200463	1625817	1624090	2978225	2979952	2873992	2875719	3316495	3314768	3088043	3089770
3606		Chr1_164_ISBma2_B	1											1625978	1627550	2976463	2978010	2872259	2873831				
3607	BPSL3395		1	3049839	3050261	2947758	2948180	2964129	2964551	3036084	3036506	3202848	3202426	1628069	1627647	2975944	2976366	2871740	2872162	3317153	3316731	3087385	3087807

3608	BPSL3396	1	3048015	3049406	2945934	2947325	2962305	2963696	3034260	3035651	3204672	3203281	1629893	1628502	2974120	2975511	2869916	2871307	3318977	3317586	3085561	3086952
3609	BPSL3397	1	3047060	3047932	2944979	2945851	2961350	2962222	3033305	3034177	3205627	3204755	1630848	1629976	2973165	2974037	2868961	2869833	3319932	3319060	3084606	3085478
3610	BPSL3398	1	3045447	3046985	2943366	2944904	2959108	2960646	3031692	3033230	3207241	3205703	1632461	1630923	2971552	2973090	2867347	2868885	3321545	3320007	3082923	3084461
3611	BPSL3399	1	3044861	3045397	2942780	2943316	2958522	2959058	3031106	3031642	3207827	3207291	1633047	1632511	2970966	2971502	2866761	2867297	3322131	3321595	3082337	3082873
3612	BPSL3400	1	3044418	3044855	2942337	2942774	2958079	2958516	3030663	3031100	3208270	3207833	1633490	1633053	2970523	2970960	2866318	2866755	3322574	3322137	3081894	3082331
3613	BPSL3401	1	3043985	3044257	2941904	2942176	2957646	2957918	3030230	3030502	3208703	3208431	1633923	1633651	2970090	2970362	2865885	2866157	3323007	3322735	3081461	3081733
3614	BPSL3402	1	3043045	3043911	2940964	2941830	2956706	2957572	3029290	3030156	3209643	3208777	1634863	1633997	2969150	2970016	2864945	2865811	3323947	3323081	3080521	3081387
3615	BPSL3403	1	3042420	3042947	2940339	2940866	2956081	2956608	3028665	3029192	3210268	3209741	1635488	1634961	2968525	2969052	2864320	2864847	3324572	3324045	3079896	3080423
3616	BPSL3404	1	3040998	3042146	2938917	2940065	2954659	2955807	3027243	3028391	3211690	3210542	1636910	1635762	2967103	2968251	2862898	2864046	3325994	3324846	3078474	3079622
3617	BPSL3405	1	3040121	3041005	2938040	2938924	2953782	2954666	3026366	3027250	3212567	3211683	1637787	1636903	2966226	2967110	2862021	2862905	3326871	3325987	3077597	3078481
3618	BPSL3406	1	3039291	3040082	2937210	2938001	2952952	2953743	3025536	3026327	3213397	3212606	1638617	1637826	2965396	2966187	2861191	2861982	3327701	3326910	3076767	3077558
3619	BPSL3407	1	3038570	3039259	2936489	2937178	2952231	2952920	3024815	3025504	3214118	3213429	1639338	1638649	2964675	2965364	2860470	2861159	3328422	3327733	3076046	3076735
3620	BPSL3408	1	3036594	3038564	2934513	2936483	2950255	2952225	3022839	3024809	3216094	3214124	1641314	1639344	2962699	2964669	2858494	2860464	3330398	3328428	3074070	3076040
3621	BPSL3409	1	3035677	3036429	2933596	2934348	2949338	2950090	3021922	3022674	3217011	3216259	1642231	1641479	2961782	2962534	2857577	2858329	3331315	3330563	3073153	3073905
3622	BPSL3410	1	3033890	3035671	2931809	2933590	2947551	2949332	3020135	3021916	3218798	3217017	1644018	1642237	2959995	2961776	2855789	2857465	3333102	3331321	3071366	3073147
3623	BPSL3411	1	3032830	3033882	2930749	2931801	2946491	2947543	3019075	3020127	3219858	3218806	1645078	1644026	2958935	2959987	2854729	2855781	3334162	3333110	3070306	3071358
3624	BPSL3412	1	3031360	3032514	2929279	2930433	2945021	2946175	3017605	3018759	3221328	3220174	1646548	1645394	2957465	2958619	2853259	2854413	3335632	3334478	3068836	3069990
3625	BPSL3413	1	3029804	3031036	2927723	2928955	2943465	2944697	3016049	3017281	3222884	3221652	1648104	1646872	2955909	2957141	2851703	2852935	3337188	3335956	3067280	3068512
3626	BPSL3414	1	3028417	3029391	2926336	2927310	2942078	2943052	3014662	3015636	3224271	3223297	1649491	1648517	2954522	2955496	2850316	2851290	3338575	3337601	3065893	3066867
3627	BPSL3415	1	3027536	3028417	2925455	2926336	2941197	2942078	3013781	3014662	3225152	3224271	1650372	1649491	2953641	2954522	2849435	2850316	3339456	3338575	3065012	3065893
3628	BPSL3416	1	3026200	3027393	2924119	2925312	2939861	2941054	3012445	3013638	3226488	3225295	1651706	1651671	2952307	2952342	2848101	2848136	3340792	3339599	3063676	3064869
3629	BPSL3417	1	3025377	3026129	2923296	2924048	2939038	2939790	3011622	3012374	3227311	3226559	1652529	1651777	2951484	2952236	2847278	2848030	3341615	3340863	3062853	3063605
3630	BPSL3418	1	3024601	3025377	2922520	2923296	2938262	2939038	3010846	3011622	3228087	3227311	1653305	1652529	2950708	2951484	2846502	2847278	3342391	3341615	3062077	3062853
3631	BPSL3419	1	3022416	3024098	2920335	2922017	2936077	2937759	3008661	3010343	3230272	3228590	1655490	1653808	2948523	2950205	2844317	2845999	3344576	3342894	3059892	3061574
3632	BPSL3420	1	3020715	3022238	2918634	2920157	2934376	2935899	3006960	3008483	3231973	3230450	1657191	1655668	2946822	2948345	2842616	2844139	3346277	3344754	3058191	3059714
3633	BPSL3421	1	3020574	3019678	2918493	2917597	2934235	2933339	3006819	3005923	3232114	3233010	1657332	1658228	2946681	2945785	2842475	2841579	3346418	3347314	3058050	3057154
3634	BPSL3422	1	3019284	3018757	2917203	2916676	2932945	2932418	3005529	3005002	3233404	3233931	1658622	1659149	2945391	2944864	2841185	2840658	3347708	3348235	3056760	3056233
3635	BPSL3423	1	3018102	3018650	2916021	2916569	2931763	2932311	3004347	3004895	3234586	3234038	1659804	1659256	2944209	2944757	2840003	2840551	3348890	3348342	3055578	3056126
3636	BPSL3424	1	3018018	3017128	2915937	2915047	2931679	2930789	3004263	3003373	3234670	3235560	1659888	1660778	2944125	2943235	2839919	2839029	3348974	3349864	3055494	3054604
3637	BPSL3425	1	3017041	3016739	2914960	2914658	2930702	2930400	3003286	3002984	3235647	3235949	1660865	1661167	2943148	2942846	2838942	2838640	3349951	3350253	3054517	3054215
3638	BPSL3426	1	3016301	3015612	2914220	2913531	2929962	2929273	3002546	3001857	3236387	3237076	1661605	1662294	2942408	2941719	2838202	2837513	3350691	3351380	3053777	3053088
3639	BPSL3427	1	3015502	3014018	2913421	2911937	2929163	2927679	3001747	3000263	3237186	3238670	1662404	1663885	2941609	2940128	2837403	2835922	3351490	3352971	3052978	3051497
3640	BPSL3428	1	3013061	3013570	2910980	2911489	2926722	2927231	2999306	2999815	3239627	3239118	1664842	1664333	2939171	2939680	2834965	2835474	3353928	3353419	3050540	3051049
3641	BPSL3429	1	3011966	3012748	2909663	2909890	2925405	2925632	2997989	2998216	3240944	3240717	1666159	1665932	2937854	2938081	2833648	2833875	3355245	3355018	3049223	3049450
3642	BPSL3430	1	3010807	3011514	2908726	2909433	2924468	2925175	2997052	2997759	3241881	3241174	1667096	1666389	2936917	2937624	2832711	2833418	3356182	3355475	3048286	3048993
3643	BPSL3431	1	3010126	3010764	2908045	2908683	2923787	2924425	2996371	2997009	3242562	3241924	1667777	1667139	2936236	2936874	2832030	2832668	3356863	3356225	3047605	3048243
**Chromosome 2**																						
1	BPSS0001	1	1139	2323	10653	11837	10601	11785	1092	2276	9524	10708	1496454	1497638	1139	2323	1780428	1779244	1165330	1166514	32271	33455
2	BPSS0002	1	3463	2423	12977	11937	12925	11885	3416	2376	11848	10808	1498778	1497738	3463	2423	1778104	1779144	1167654	1166614	34595	33555
3	BPSS0003	1	4057	3641	13571	13155	13519	13103	4010	3594	12442	12026	1499372	1498956	4057	3641	1777510	1777926	1168248	1167832	35189	34773
4	BPSS0004	1	4694	4089	14208	13603	14156	13551	4647	4042	13079	12474	1500009	1499404	4694	4089	1776873	1777478	1168885	1168280	35826	35221
5	BPSS0005	1	4862	6058	14376	15572	14324	15520	4815	6011	13247	14443	1500177	1501373	4862	6058	1776705	1775509	1169053	1170249	35994	37190
6	BPSS0006	1	6074	7102	15588	16616	15536	16564	6027	7055	14459	15487	1501389	1502417	6074	7102	1775493	1774465	1170265	1171293	37206	38234
7	BPSS0007	1	8093	7443	17607	16957	17555	16905	8046	7396	16478	15828	1503408	1502758	8093	7443	1773474	1774124	1172284	1171634	39225	38575
8	BPSS0008	1	8183	8797	17697	18311	17645	18259	8136	8750	16568	17182	1503498	1504112	8183	8797	1773384	1772770	1172374	1172988	39315	39929
9	BPSS0009	1	8901	9377	18415	18891	18363	18839	8854	9330	17286	17762	1504216	1504692	8901	9377	1772666	1772190	1173092	1173568	40033	40509
10	BPSS0010	1	10618	9605	20132	19119	20080	19067	10571	9558	19003	17990	1505933	1504920	10618	9605	1770949	1771962	1174809	1173796	41750	40737
11	BPSS0011	1	12006	10924	21520	20438	21468	20386	11959	10877	20391	19309	1507321	1506239	12006	10924	1769561	1770643	1176218	1175136	43159	42077
12	BPSS0012	1	12175	13089	21689	22603	21637	22551	12128	13042	20560	21474	1507490	1508404	12175	13089	1769392	1768478	1176387	1177301	43328	44242

13 BPSS0013		1	13578	14252	23092	23766	23040	23714	13531	14205	22012	22686	1508935	1509609	13599	14273	1767982	1767335	1177790	1178464	44738	45412
14 BPSS0014		1	14470	15090	23984	24604	23932	24552	14423	15043	22894	23514	1509807	1510427	14471	15091	1767230	1766610	1178662	1179282	45610	46230
15 BPSS0015		1	15992	15531	25506	25045	25454	24993	15945	15484	24416	23955	1511329	1510868	15993	15532	1765708	1766169	1180184	1179723	47132	46671
16 BPSS0016		1	17244	16075	26758	25589	26706	25537	17197	16028	25668	24499	1512581	1511412	17245	16076	1764456	1765625	1181436	1180267	48384	47215
17 BPSS0017		1	18092	17310	27606	26824	27554	26772	18045	17263	26516	25734	1513429	1512647	18093	17311	1763608	1764390	1182284	1181502	49232	48450
18 BPSS0018		1	18879	18142	28393	27656	28341	27604	18832	18095	27303	26566	1514216	1513479	18880	18143	1762821	1763558	1183071	1182334	50019	49282
19 BPSS0019		1	19338	19931	28852	29445	28800	29393	19291	19884	27762	28355	1514675	1515268	19339	19932	1762362	1761769	1183530	1184123	50478	51071
20 BPSS0020		1	20250	19960	29764	29474	29712	29422	20203	19913	28674	28384	1515587	1515297	20251	19961	1761450	1761740	1184442	1184152	51390	51100
21 BPSS0021		1	21786	20962	31300	30476	31248	30424	21739	20915	30210	29386	1517123	1516299	21787	20963	1759914	1760738	1185978	1185154	52926	52102
22 BPSS0022		1	22533	21829	32047	31343	31995	31291	22486	21782	30957	30253	1517870	1517166	22534	21830	1759167	1759871	1186725	1186021	53673	52969
23 BPSS0023		1	22941	24344	32455	33858	32403	33806	22894	24297	31365	32768	1518278	1519681	22942	24345	1758759	1757356	1187133	1188536	54081	55484
24 BPSS0024		1	24497	24694	34011	34208	33959	34156	24450	24647	32921	33118	1519834	1520031	24498	24695	1757203	1757006	1188689	1188886	55637	55834
25 BPSS0025		1	24871	25719	34385	35233	34333	35181	24824	25672	33295	34143	1520208	1521056	24872	25720	1756829	1755981	1189063	1189911	56011	56859
26 BPSS0026		1	25719	26549	35233	36063	35181	36011	25672	26502	34143	34973	1521056	1521886	25720	26550	1755981	1755151	1189911	1190741	56859	57689
27 BPSS0027		1	26549	28489	36063	38003	36011	37951	26502	28442	34973	36913	1521886	1523826	26550	28490	1755151	1753211	1190741	1192681	57689	59629
28 BPSS0028		1	28506	29492	38020	39006	37968	38954	28459	29445	36930	37916	1523843	1524829	28507	29493	1753194	1752208	1192698	1193684	59646	60632
29 BPSS0029		1	30287	29670	39801	39184	39749	39132	30240	29623	38711	38094	1525624	1525007	30288	29671	1751413	1752030	1194479	1193862	61427	60810
30 BPSS0030		1	30395	31285	39909	40799	39857	40747	30348	31238	38819	39709	1525732	1526622	30396	31286	1751305	1750877	1194587	1195477	61535	62425
31 BPSS0031		1	32090	31338	41604	40852	41552	40800	32043	31291	40514	39762	1527427	1526675	32091	31339	1749522	1750274	1196282	1195530	63230	62478
32 BPSS0032		1	32707	32243	42221	41757	42169	41705	32660	32196	41131	40667	1528044	1527580	32708	32244	1748905	1749369	1196899	1196435	63847	63383
33 BPSS0033		1	33095	33358	42609	42872	42557	42820	33048	33311	41519	41782	1528432	1528695	33096	33359	1748517	1748254	1197287	1197550	64235	64498
34 BPSS0034		1	34127	35704	43641	45218	43589	45166	34080	35657	42551	44128	1529464	1531041	34128	35705	1747485	1745908	1198319	1199896	65267	66844
35 BPSS0035		1	35704	36651	45218	46165	45166	46113	35657	36604	44128	45075	1531041	1531433	35705	36097	1744278	1743721	1199896	1200843	66844	67791
36	Chr2_1_IS407A_B	1											1531434	1532669	36098	37333	1744280	1745515				
37 BPSS0036		1	37848	37435	47362	46949	47310	46897	37801	37388	46272	45859	1534417	1534004	39137	38724	1742532	1742945	1202048	1201635	68988	68575
38 BPSS0037		1	38103	38348	47617	47862	47565	47810	38056	38301	46527	46772	1534672	1534917	39392	39637	1742277	1742032	1202303	1202548	69243	69488
39 BPSS0038		1	38475	39395	47989	48909	47937	48857	38428	39348	46899	47819	1535044	1535964	39764	40684	1741905	1740985	1202675	1203595	69615	70535
40 BPSS0039		1	40390	39590	49911	49111	49859	49059	40343	39543	48877	48077	1537001	1536201	41770	40970	1740004	1740804	1204625	1203825	71516	70716
41 BPSS0040		1	40945	40394	50466	49915	50414	49863	40898	40347	49432	48881	1537556	1537005	42325	41774	1739449	1740000	1205180	1204629	72071	71520
42 BPSS0041		1	42183	41260	51704	50781	51652	50729	42136	41213	50670	49747	1538794	1537871	43563	42640	1738211	1739134	1206418	1205495	73309	72386
43 BPSS0042		1	42297	43517	51818	53038	51766	52986	42250	43470	50784	52004	1538908	1540128	43677	44897	1738097	1736877	1206532	1207752	73423	74643
44 BPSS0043		1	43728	44438	53249	53959	53197	53907	43681	44391	52215	52925	1540339	1541049	45108	45818	1736666	1735956	1207963	1208673	74854	75564
45 BPSS0044		1	44460	45113	53981	54634	53929	54582	44413	45066	52947	53600	1541071	1541724	45840	46493	1735934	1735281	1208695	1209348	75586	76239
46 BPSS0045		1	45213	46607	54734	56128	54682	56076	45166	46560	53700	55094	1541824	1543218	46593	47987	1735181	1733787	1209448	1210842	76339	77733
47 BPSS0046		1	46623	47405	56144	56926	56092	56874	46576	47358	55110	55892	1543234	1544016	48003	48785	1733771	1732989	1210858	1211640	77749	78531
48 BPSS0047		1	47424	47807	56945	57328	56893	57276	47377	47760	55911	56294	1544035	1544418	48804	49187	1732970	1732587	1211659	1212042	78550	78933
49 BPSS0048		1	47989	49341	57510	58862	57458	58810	47942	49294	56487	57839	1544589	1545941	49358	50710	1732416	1731064	1212224	1213576	79115	80467
50 BPSS0049		1	49660	49415	59181	58936	59129	58884	49613	49368	58158	57913	1546260	1546015	51029	50784	1730745	1730990	1213895	1213650	80786	80541
51 BPSS0050		1	50378	49764	59899	59285	59847	59233	50331	49717	58876	58262	1546978	1546364	51747	51133	1730027	1730641	1214613	1213999	81504	80890
52 BPSS0051		1	51829	50576	61350	60097	61298	60045	51782	50529	60327	59074	1548429	1547176	53198	51945	1728576	1729829	1216064	1214811	82955	81702
53 BPSS0052		1	52244	51819	61765	61340	61713	61288	52197	51772	60742	60317	1548844	1548419	53613	53188	1728161	1728586	1216479	1216054	83370	82945
54 BPSS0053		1	53836	52649	63357	62170	63305	62118	53789	52602	62334	61147	1550436	1549249	55205	54018	1726569	1727756	1218071	1216884	84962	83775
55 BPSS0054		1	54828	53836	64349	63357	64297	63305	54781	53789	63326	62334	1551428	1550436	56197	55205	1725577	1726569	1219063	1218071	85954	84962
56 BPSS0055		1	55803	56348	65324	65869	65272	65817	55756	56301	64289	64834	1552391	1552936	57184	57729	1724602	1724057	1220002	1220547	86893	87438
57 BPSS0056		1	56404	56703	65925	66224	65873	66172	56357	56656	64890	65189	1552992	1553291	57785	58084	1724001	1723702	1220603	1220902	87494	87793
58 BPSS0057		1	56742	57407	66263	66928	66211	66876	56695	57360	65228	65893	1553330	1553995	58123	58788	1723663	1722998	1220941	1221606	87832	88497
59 BPSS0058		1	57887	63796	67408	73317	67356	73265	57840	63749	66373	72282	1554475	1560384	59268	65177	1722518	1716609	1222086	1227995	88977	94886
60 BPSS0059		1	65093	64836	74614	74357	74562	74305	65046	64789	73579	73322	1561681	1561424	66474	66217	1715312	1715569	1229292	1229035	96183	95926
61 BPSS0060		1	65736	66005	75257	75526	75205	75474	65689	65958	74222	74491	1562324	1562593	67117	67386	1714669	1714400	1229935	1230204	96826	97095
62 BPSS0060A		1	66195	66635	75675	75860	75664	75846	66148	66588	74681	75046	1562783	1563223	67576	68016	1714210	1713770	1230394	1230834	97285	97725
63 BPSS0061		1	67140	68489	76365	77714	76698	77453	67093	68442	2330556	2329207	1563716	1565065	68509	69858	1713277	1712648	1231339	1231626	98230	98517

64	BPSS0062		1	68545	69426	77770	78651	78937	79818	68498	69379	2329151	2328270	1565121	1566002	69914	70795	1711803	1710922	1232743	1233624	99634	100515
65	BPSS0063		1	70449	71792	79674	81017	80841	82184	70402	71598	2327247	2325904	1567025	1568368	71818	73161	1709899	1708556	1234647	1235990	101538	102881
66	BPSS0064		1	73576	73259	82801	82484	83968	83651	73555	73238	2324120	2324437	1570152	1569835	74945	74628	1706772	1707089	1237774	1237457	104665	104348
67	BPSS0065		1	74036	75985	83261	85210	84428	86377	74015	75964	2323653	2321704	1570612	1572561	75405	77354	1706312	1704363	1238234	1240183	105125	107074
68	BPSS0066		1	76239	76742	85464	85967	86631	87134	76218	76721	2321450	2320947	1572815	1573318	77608	78111	1704109	1703606	1240437	1240940	107328	107831
69	BPSS0067		1	77117	79309	86342	88534	87509	89701	77096	79288	2320572	2318380	1573693	1575885	78486	80678	1703231	1701039	1241315	1243507	108206	110398
70	BPSS0068		1	79816	80325	89041	89550	90208	90717	79795	80304	2317873	2317364	1576392	1576901	81185	81694	1700532	1700023	1244014	1244523	110905	111414
71		Chr2_2_IS407A_A		80328	81563	89553	90788	90720	91955	80307	81542	2316126	2317361	1576904	1578139	81697	82932	1698785	1700020	1244526	1245761	111417	112652
72	BPSS0069																						
73	BPSS0070																						
74	BPSS0072																						
75	BPSS0072A																						
76	BPSS0073																						
77	BPSS0074																						
78	BPSS0075																						
79	BPSS0076																						
80	BPSS0077																						
81	BPSS0077A																						
82	BPSS0078																						
83	BPSS0079																						
84	BPSS0080																						
85	BPSS0080a																						
86	BPSS0081																						
87	BPSS0082																						
88	BPSS0083																						
89	BPSS0084																						
90	BPSS0085																						
91	BPSS0086																						
92	BPSS0087																						
93	BPSS0088																						
94	BPSS0089																						
95	BPSS0090																						
96	BPSS0091																						
97	BPSS0092																						
98	BPSS0093																						
99	BPSS0094																						
100	BPSS0095																						
101	BPSS0096																						
102	BPSS0097																						
103	BPSS0098																						
104	BPSS0099																						
105	BPSS0100																						
106	BPSS0101																						
107	BPSS0102																						
108	BPSS0103																						
109	BPSS0104																						
110	BPSS0105																						
111	BPSS0106																						
112	BPSS0108																						
113	BPSS0109																						
114	BPSS0110																						

115	BPSS0111																						
116	BPSS0112																						
117	BPSS0113																						
118	BPSS0114																						
119	BPSS0115																						
120	BPSS0116																						
121	BPSS0117																						
122	BPSS0118																						
123	BPSS0119																						
124	BPSS0120																						
125	BPSS0121																						
126	BPSS0122																						
127	BPSS0123																						
128	BPSS0124																						
129	BPSS0125																						
130	BPSS0126																						
131	BPSS0127																						
132	BPSS0128																						
133	BPSS0129																						
134	BPSS0130																						
135	BPSS0131																						
136	BPSS0132																						
137	BPSS0133																						
138	BPSS0134																						
139	BPSS0135																						
140	BPSS0136																						
141	BPSS0137																						
142	BPSS0138																						
143	BPSS0139																						
144	BPSS0140																						
145	BPSS0141																						
146	BPSS0142																						
147	BPSS0143																						
148		Chr2_3_IS407A_A		2135943	2137178	2173035	2174270	2146619	2147854	2128166	2129401	2097872	2099107	1307203	1308438	2164773	2166008	2030650	2031885	977425	978660	1972722	1973957
149	BPSS0144		2			2172880	2173035	2146464	2146619	2128011	2128166	2097717	2097872							977270	977425	1972567	1972722
150	BPSS0145		2	2133495	2134889	2170587	2171981	2144171	2145565	2125718	2127112	2095410	2096804	1305814	1307202	2163384	2164772	2029261	2030649	974956	976350	1970260	1971654
151	BPSS0146		2	2132672	2133298	2169764	2170390	2143348	2143974			2094587	2095213	1304991	1305617	2162561	2163187	2028438	2029064	974133	974759	1969437	1970063
152	BPSS0147		2	2131325	2131837	2168417	2168929	2142001	2142513	2123587	2124099	2093240	2093752	1303644	1304156	2161214	2161726	2027091	2027603	972786	973298	1968090	1968602
153	BPSS0148		2	2130094	2131281	2167186	2168373	2140770	2141957	2122356	2123543	2092009	2093196	1302413	1303600	2159983	2161170	2025860	2027047	971555	972742	1966859	1968046
154	BPSS0149		2	2129914	2129015	2167006	2166107	2140590	2139691	2122176	2121277	2091829	2090930	1302233	1301334	2159803	2158904	2025680	2024781	971375	970476	1966679	1965780
155	BPSS0150		2	2127861	2128667	2164953	2165759	2138537	2139343	2120123	2120929	2089776	2090582	1300180	1300986	2157750	2158556	2023627	2024433	969322	970128	1964626	1965432
156	BPSS0151		2	2127666	2126503	2164758	2163595	2138342	2137179	2119928	2118765	2089572	2088409	1299976	1298813	2157537	2156374	2023432	2022269	969127	967964	1964431	1963268
157	BPSS0152		2	2125998	2125561	2163090	2162653	2136674	2136237	2118260	2117823			1298308	1297871	2155869	2155432	2021764	2021327	967469	967032	1962773	1962336
158	BPSS0153		2	2124053	2124949	2161145	2162041	2134729	2135625	2116315	2117211	2086049	2086945	1296350	1297246	2153937	2154833	2019812	2020708	965505	966401	1960784	1961680
159	BPSS0154		2	2122642	2123886	2159734	2160978	2133318	2134562	2114904	2116148	2084638	2085882	1294939	1296183	2152526	2153770	2018401	2019645	964094	965338	1959373	1960617
160	BPSS0155		2	2121557	2122486	2158649	2159578	2132233	2133162	2113819	2114748	2083553	2084482	1293854	1294783	2151441	2152370	2017316	2018245	963009	963938	1958288	1959217
161	BPSS0156		2	2120251	2121009	2157343	2158101	2130927	2131685	2112513	2113271	2082247	2083005	1292548	1293306	2150135	2150893	2016010	2016768	961703	962461	1956982	1957740
162	BPSS0157		2	2119882	2119466	2156974	2156558	2130558	2130142	2112144	2111728	2081878	2081462	1292179	1291763	2149766	2149350	2015641	2015225	961334	960918	1956613	1956197
163	BPSS0158		2	2119021	2117108	2156113	2154200	2129697	2127784	2111283	2109370	2081017	2079104	1291318	1289405	2148905	2146992	2014780	2012867	960473	958560	1955752	1953839
164	BPSS0159		2	2116830	2113936	2153922	2151028	2127506	2124612	2109092	2106198	2078826	2075932	1289127	1286233	2146714	2143820	2012589	2009695	958282	955388	1953561	1950667
165		Chr2_4_ISBma1_B	2											1284442	1285746	2142029	2143333	2007904	2009208				

166 BPSS0160	2	2113331	2112276	2150423	2149368	2124007	2122952	2105593	2104538	2075327	2074272	1284315	1283260	2141902	2140847	2007777	2006722	954783	953728	1950062	1949007
167 BPSS0161	2	2112276	2111383	2149368	2148475	2122952	2122059	2104538	2103645	2074272	2073379	1283260	1282367	2140847	2139954	2006722	2005829	953728	952835	1949007	1948114
168 BPSS0162	2	2111372	2109870	2148464	2146962	2122048	2120546	2103634	2102132	2073368	2071866	1282356	1280854	2139943	2138441	2005818	2004316	952824	951322	1948103	1946601
169 BPSS0163	2	2109821	2107956	2146913	2145048	2120497	2118632	2102083	2100218	2071817	2069952	1280805	1278940	2138392	2136527	2004267	2002402	951273	949408	1946552	1944687
170 BPSS0164	2	2107924	2106956	2145016	2144048	2118600	2117632	2100186	2099218	2069920	2068952	1278908	1277940	2136495	2135527	2002370	2001402	949376	948408	1944655	1943687
171 BPSS0165	2	2106898	2105975	2143990	2143067	2117574	2116651	2099160	2098237	2068894	2067971	1277882	1276959	2135469	2134546	2001344	2000421	948350	947427	1943629	1942706
172 BPSS0166	2	2104767	2105726	2141859	2142818	2115443	2116402	2097029	2097988	2066763	2067722	1275751	1276710	2133338	2134297	1999213	2000172	946219	947178	1941498	1942457
173 BPSS0167	2	2101988	2103544	2136564	2139191	2109594	2110364	2091734	2094361	2061468	2064095	1270456	1273083	2128043	2130670	1993918	1996545	940924	943551	1936203	1938830
174 BPSS0168	2	2098178	2099440	2135270	2136532	2108300	2109562	2090440	2091702	2060174	2061436	1269162	1270424	2126749	2128011	1992624	1993886	939630	940892	1934909	1936171
175 BPSS0169	2	2096743	2098086	2133835	2135178	2106865	2108208	2089005	2090348	2058739	2060082	1267727	1269070	2125314	2126657	1991189	1992532	938195	939538	1933474	1934817
176 BPSS0170	2	2096209	2096718	2133301	2133810	2106331	2106840	2088471	2088980	2058205	2058714	1267193	1267702	2124780	2125289	1990655	1991164	937661	938170	1932940	1933449
177 BPSS0171	2	2095626	2096108	2132718	2133200	2105748	2106230	2087888	2088370	2057622	2058104	1266610	1267092	2124197	2124679	1990072	1990554	937078	937560	1932357	1932839
178 BPSS0172	2	2094010	2095506	2131102	2132598	2104132	2105628	2086272	2087768	2056006	2057502	1264994	1266490	2122581	2124077	1988456	1989952	935462	936958	1930741	1932237
179 BPSS0173	2	2093394	2093972	2130486	2131064	2103516	2104094	2085656	2086234	2055390	2055968	1264378	1264956	2121965	2122543	1987840	1988418	934846	935424	1930125	1930703
180 BPSS0174	2	2090435	2091040	2127527	2128132	2100557	2101162	2082697	2083302	2052431	2053036	1261419	1262024	2119006	2119611	1984881	1985486	931887	932492	1927166	1927771
181 BPSS0175	2	2089902	2089186	2126994	2126278	2100024	2099308	2082164	2081448	2051898	2051182	1260886	1260170	2118473	2117757	1984348	1983632	931354	930638	1926633	1925917
182 BPSS0176	2	2089186	2088224	2126278	2125316	2099308	2098346	2081448	2080486	2051182	2050220	1260170	1259208	2117757	2116795	1983632	1982670	930638	929676	1925917	1924955
183 BPSS0177	2	2088180	2087656	2125272	2124748	2098302	2097778	2080442	2079918	2050176	2049652	1259164	1258640	2116751	2116227	1982626	1982102	929632	929108	1924911	1924387
184 BPSS0178	2	2087622	2085736	2124714	2122828	2097744	2095858	2079884	2077998	2049618	2047732	1258606	1256720	2116193	2114307	1982068	1980182	929074	927188	1924353	1922467
185 BPSS0179	2	2085686	2084652	2122813	2121743	2095843	2094773	2077983	2076913	2047717	2046647	1256705	1255635	2114292	2113222	1980167	1979097	927173	926103	1922452	1921382
186 BPSS0180	2	2084652	2083552	2121743	2120643	2094773	2093673	2076913	2075813	2046647	2045547	1255635	1254535	2113222	2112122	1979097	1977997	926103	925003	1921382	1920282
187 BPSS0181	2	2083477	2081153	2120568	2118244	2093598	2091274	2075738	2073414	2045472	2043148	1254460	1252136	2112047	2109723	1977922	1975598	924928	922604	1920207	1917883
188 BPSS0182	2	2081118	2078644	2118209	2115735	2091239	2088765	2073379	2070905	2043113	2040639	1252101	1249627	2109688	2107214	1975563	1973089	922569	920095	1917848	1915374
189 BPSS0183	2	2078644	2077565	2115735	2114656	2088765	2087686	2070905	2069826	2040639	2039560	1249627	1248548	2107214	2106135	1973089	1972010	920095	919016	1915374	1914295
190 BPSS0184	2	2077380	2076724	2114471	2113815	2087501	2086845	2069641	2068985	2039375	2038719	1248363	1247707	2105950	2105294	1971825	1971169	918831	918175	1914110	1913454
191 BPSS0185	2	2076674	2076303	2113765	2113394	2086795	2086424	2068935	2068564	2038669	2038298	1247657	1247286	2105244	2104873	1971119	1970748	918125	917754	1913404	1913033
192 BPSS0186	2	2073664	2075577	2110755	2112668	2083785	2085698	2065925	2067838	2035671	2037584	1244641	1246554	2102246	2104159	1968121	1970034	915133	917046	1910412	1912325
193 BPSS0187	2	2072431	2073321	2109522	2110412	2082552	2083442	2064692	2065582	2034438	2035328	1243408	1244298	2101013	2101903	1966888	1967778	913900	914790	1909179	1910069
194 BPSS0188	2	2072335	2071562	2109426	2108653	2082456	2081683	2064596	2063823	2034342	2033569	1243312	1242539	2100917	2100144	1966792	1966019	913804	913031	1909083	1908310
195 BPSS0189	2	2071533	2071096	2108624	2108187	2081654	2081217	2063794	2063357	2033540	2033103	1242510	1242073	2100115	2099678	1965990	1965553	913002	912565	1908281	1907844
196 BPSS0190	2	2069264	2070556	2106355	2107647	2079385	2080677	2061525	2062817	2031271	2032563	1240241	1241533	2097846	2099138	1963721	1965013	910733	912025	1906012	1907304
197 BPSS0191	2	2068590	2068979	2105681	2106070	2078711	2079100	2060851	2061240	2030597	2030986	1239567	1239956	2097172	2097561	1963047	1963436	910059	910448	1905338	1905727
198 BPSS0192	2	2067409	2068251	2104500	2105342	2077530	2078372	2059670	2060512	2029416	2030258	1238386	1239228	2095991	2096833	1961866	1962708	908878	909720	1904157	1904999
199 BPSS0193	2	2065360	2066712	2102451	2103803	2075481	2076833	2057621	2058973	2027367	2028719	1236344	1237696	2093942	2095294	1959817	1961169	906829	908181	1902108	1903460
200 BPSS0194	2	2064169	2065170	2101260	2102261	2074290	2075291	2056430	2057431	2026176	2027177	1235153	1236154	2092751	2093752	1958626	1959627	905638	906639	1900917	1901918
201 BPSS0195	2	2063304	2064176	2100395	2101267	2073425	2074297	2055565	2056437	2025311	2026183	1234288	1235160	2091886	2092758	1957761	1958633	904773	905645	1900052	1900924
202 BPSS0196	2	2061862	2063304	2098953	2100395	2071983	2073425	2054123	2055565	2023869	2025311	1232846	1234288	2090444	2091886	1956319	1957761	903331	904773	1898610	1900052
203 BPSS0197	2	2061590	2061853	2098681	2098944	2071711	2071974	2053851	2054114	2023597	2023860	1232574	1232837	2090172	2090435	1956047	1956310	903059	903322	1898338	1898601
204 BPSS0198	2	2060800	2061597	2097891	2098688	2070921	2071718	2053061	2053858	2022807	2023604	1231784	1232581	2089382	2090179	1955257	1956054	902269	903066	1897548	1898345
205 BPSS0199	2	2060532	2059621	2097623	2096712	2070653	2069742	2052793	2051882	2022539	2021628	1231516	1230605	2089114	2088203	1954989	1954078	902001	901090	1897280	1896369
206 BPSS0200	2	2058812	2056302	2095903	2093393	2068933	2066423	2051073	2048563	2020839	2018329	1229852	1227342	2087492	2084982	1953188	1950678	900343	897833	1895469	1892959
207 BPSS0201	2	2055912	2054977	2093003	2092068	2066033	2065098	2048173	2047238	2017939	2017004	1226952	1226017	2084592	2083657	1950288	1949353	897443	896508	1892569	1891634
208 BPSS0202	2	2054813	2053923	2091904	2091014	2064934	2064044	2047074	2046184	2016840	2015950	1225853	1224963	2083493	2082603	1949189	1948299	896344	895454	1891470	1890580
209 BPSS0203	2	2051815	2053824	2088906	2090915	2061936	2063945	2044076	2046085	2013842	2015851	1222855	1224864	2080495	2082504	1946191	1948200	893346	895355	1888472	1890481
210 BPSS0204	2	2049207	2050832	2086298	2087923	2059328	2060953	2041475	2043100	2011227	2012852	1220163	1221788	2077894	2079519	1943527	1945152	890745	892370	1885934	1887559
211 BPSS0205	2	2046773	2048854	2083864	2085945	2056894	2058975	2039041	2041122	2008793	2010874	1217729	1219810	2075460	2077541	1941093	1943174	888311	890392	1883500	1885581
212 BPSS0206	2	2046556	2045666	2083647	2082757	2056677	2055787	2038795	2037905	2008576	2007686	1217512	1216622	2075243	2074353	1940876	1939986	888094	887204	1883283	1882393
213 BPSS0207	2	2045614	2044445	2082705	2081536	2055735	2054566	2037853	2036684	2007634	2006465	1216570	1215401	2074301	2073132	1939934	1938765	887152	885983	1882341	1881172
214 BPSS0208	2	2044368	2041777	2081459	2078868	2054489	2051898	2036607	2034016	2006388	2003797	1215324	1212733	2073055	2070464	1938688	1936097	885906	883315	1881095	1878507
215 BPSS0209	2	2041615	2040428	2078707	2078474	2051737	2050550	2033855	2032668	2003636	2002449	1212572	1211385	2070303	2069116	1935936	1934749	883154	881967	1878346	1877159
216 BPSS0210	2	2039929	2040369	2076700	2077140	2050051	2050491	2032169	2032609	2001950	2002390	1210886	1211326	2068617	2069057	1934250	1934690	881468	881908	1876660	1877100

217	BPSS0211		2	2038255	2038067	2075040	2074852	2048377	2048189	2030509	2030321	2000528	2000340	1209625	1209437	2067237	2067049	1933234	1933046	880193	880005	1875315	1875127
218	BPSS0212		2	2037971	2037336	2074756	2074121	2048093	2047458	2030225	2029590	2000244	1999609	1209341	1208706	2066953	2066318	1932950	1932315	879909	879274	1875031	1874396
219	BPSS0213		2	2037280	2036693	2074065	2073478	2047402	2046815	2029534	2028947	1999553	1998966	1208650	1208063	2066262	2065675	1932259	1931672	879218	878631	1874340	1873753
220	BPSS0214		2	2036611	2035139	2073396	2071924	2046733	2045261	2028865	2027393	1998884	1997412	1207981	1206509	2065593	2064121	1931590	1930118	878549	877077	1873671	1872199
221	BPSS0215		2	2034964	2033354	2071749	2070142	2045086	2043476	2027218	2025608	1997237	1995627	1206334	1204724	2063946	2062336	1929943	1928333	876902	875292	1872024	1870414
222	BPSS0216		2	2031526	2032062	2068309	2068845	2041648	2042184	2023780	2024316	1993799	1994335	1202896	1203432	2060508	2061044	1926505	1927041	873464	874000	1868586	1869122
223	BPSS0217		2	2031397	2030534	2068180	2067317	2041519	2040656	2023651	2022788	1993670	1992807	1202767	1201904	2060379	2059516	1926376	1925513	873335	872472	1868457	1867594
224	BPSS0218		2	2029345	2030445	2066128	2067228	2039467	2040567	2021599	2022699	1991618	1992718	1200715	1201815	2058327	2059427	1924324	1925424	871283	872383	1866714	1867364
225	BPSS0219		2	2029233	2028541	2066016	2065324	2039355	2038663	2021487	2020795	1991506	1990814	1200603	1199911	2058215	2057523	1924212	1923520	871171	870479	1866602	1865910
226	BPSS0220		2	2028218	2026971	2064658	2063780	2038340	2037093	2020472	2019225	1990491	1989244	1199588	1198341	2057200	2055947	1923197	1921938	870156	868897	1865587	1865351
227	BPSS0221		2	2026771	2026619	2063580	2063428	2036893	2036741	2019025	2018873	1989044	1988892	1198141	1197989	2055747	2055595	1921738	1921586	868697	868545	1864328	1864176
228	BPSS0222		2	2025611	2025144	2062420	2061953	2035733	2035266	2017865	2017398	1987884	1987417	1196981	1196514	2054587	2054120	1920578	1920111	867537	867070	1863168	1862701
229	BPSS0223		2	2024495	2024935	2061304	2061744	2034617	2035057	2016749	2017189	1986768	1987208	1195865	1196305	2053471	2053911	1919462	1919902	866421	866861	1862052	1862492
230	BPSS0224		2	2020864	2021793	2057673	2058602	2030986	2031915	2013118	2014047	1983151	1984080	1192227	1193156	2049835	2050764	1915866	1916795	862804	863733	1858428	1859357
231	BPSS0225		2	2020260	2019436	2057069	2056245	2030382	2029558	2012514	2011690	1982517	1981693	1191611	1190787	2049255	2048431	1915250	1914426	862158	861334	1857758	1857531
232	BPSS0226		2	2018659	2017241	2055317	2053899	2027978	2026560	2010882	2009464	1980801	1979383	1190010	1188592	2047654	2046236	1913622	1912204	860557	859139	1856156	1854738
233	BPSS0227		2	2017083	2013163	2053741	2049821	2026402	2025149	2009306	2007690	1979225	1975305	1188434	1184514	2046078	2042158	1912046	1908126	858981	855061	1854580	1851992
234	BPSS0228		2	2012771	2013031	2049429	2049689	2021811	2022071	2005088	2005348	1974913	1975173	1184122	1184382	2041766	2042026	1907734	1907994	854669	854929		
235	BPSS0229		2	2010784	2012442	2047442	2049100	2019824	2021482	2003101	2004759	1972926	1974584	1182135	1183793	2039779	2041437	1905747	1907405	852682	854340	1848532	1850190
236	BPSS0230		2	2009811	2010200	2046252	2046641	2018851	2019240	2002128	2002517	1971953	1972342	1181162	1181551	2038806	2039195	1904774	1905163	851709	852098	1847559	1847948
237	BPSS0231		2	2009402	2009683	2045843	2046124	2018442	2018723	2001719	2002000	1971544	1971825	1180753	1181034	2038397	2038678	1904365	1904646	851300	851581	1847150	1847431
238	BPSS0232		2	2009233	2008166	2045674	2044607	2018273	2017206	2001550	2000483	1971375	1970308	1180584	1179517	2038228	2037161	1904196	1903129	851131	850064	1846981	1845914
239	BPSS0233_LysR-family regulatory protein		2																				
240	BPSS0233a		2	2007310	2007119	2043751	2043560	2016350	2016159	1999627	1999436	1969452	1969261	1178661	1178470	2036305	2036114	1902273	1902082	849208	849017	1845058	1844867
241	BPSS0234		2	2005626	2006630	2042067	2043071	2014666	2015670	1997943	1998947	1967768	1968772	1176977	1177981	2034621	2035625	1900589	1901593	847524	848528	1843374	1844378
242	BPSS0235		2	2004178	2005587	2040619	2042028	2013218	2014627	1996495	1997904	1966320	1967729	1175529	1176938	2033173	2034582	1899141	1900550	846076	847485	1841926	1843335
243	BPSS0236		2	2003582	2002572	2039938	2038928	2011789	2010779	1995899	1994889	1965723	1964713	1174933	1173923	2032578	2031568	1898545	1897535	845480	844470	1841322	1840312
244	BPSS0237		2	2001623	2002597	2037979	2038953	2009830	2010804	1993940	1994914	1963780	1964688	1172974	1173948	2030619	2031593	1896586	1897560	843539	844495	1839381	1840337
245	BPSS0238		2	2001467	1999308	2037823	2035871	2009674	2007515	1993784	1991625	1963624	1961465	1172818	1170659	2030463	2028613	1896430	1894580	843383	841224	1839225	1837066
246	BPSS0239		2	1999292	1998687	2035515	2034910	2007499	2006894	1991609	1991004	1961449	1960844	1170643	1170038	2028263	2027658	1894230	1893625	841208	840603	1837050	1836445
247	BPSS0240		2	1997817	1998632	2034040	2034855	2006024	2006839	1990134	1990949	1959974	1960789	1169168	1169983	2026788	2027603	1892755	1893570	839733	840548	1835575	1836390
248	BPSS0241		2	1996289	1997407	2032512	2033630	2004496	2005614	1988606	1989724	1958410	1959528	1167526	1168644			1891239	1892357	838202	839320	1834026	1835144
249	BPSS0242		2	1995094	1996017	2031317	2032240	2003301	2004224	1987411	1988334	1957224	1958147	1166322	1167245	2024080	2025003	1889990	1890913	837016	837939	1832840	1833763
250	BPSS0243		2	1993946	1995094	2030169	2031317	2002153	2003301	1986263	1987411	1956076	1957224	1165174	1166322	2022932	2024080	1888842	1889990	835868	837016	1831692	1832840
251	BPSS0244		2	1991586	1993886	2027809	2030109	1999793	2002093	1983903	1986203	1953716	1956016	1162814	1165114	2020572	2022872	1886482	1888782	833508	835808	1829332	1831632
252	BPSS0245		2	1990883	1991107	2027106	2027330	1999090	1999314	1983200	1983424	1953013	1953237	1162111	1162335	2019869	2020093	1885779	1886003	832805	833029	1828629	1828853
253	BPSS0246		2	1989825	1990718	2026048	2026941	1998032	1998925	1982142	1983035	1951955	1952848	1161053	1161946	2018811	2019704	1884721	1885614	831747	832640	1827571	1828464
254	BPSS0247		2	1989658	1988813	2025881	2025036	1997865	1997020	1981975	1981130	1951788	1950943	1160886	1160041	2018644	2017799	1884554	1883709	831580	830735	1827404	1826559
255	BPSS0248		2	1988805	1987765	2025028	2023988	1997012	1995972	1981122	1980082	1950935	1949895	1160033	1158993	2017791	2016751	1883701	1882661	830727	829687	1826551	1825511
256	BPSS0249		2	1987735	1986650	2023958	2022873	1995942	1994857	1980052	1978967	1949865	1948780	1158963	1157878	2016721	2015636	1882631	1881546	829657	828572	1825481	1824396
257	BPSS0250		2	1986657	1985812	2022880	2022035	1994864	1994019	1978974	1978129	1948787	1947942	1157885	1157040	2015643	2014798	1881553	1880708	828579	827734	1824403	1823558
258	BPSS0251		2	1985795	1985286	2022018	2021509	1994002	1993493	1978112	1977603	1947925	1947416	1157023	1156514	2014781	2014272	1880691	1880182	827717	827208	1823541	1823032
259	BPSS0252		2	1985216	1984020	2021439	2020243	1993423	1992227	1977533	1976337	1947346	1946150	1156444	1155248	2014202	2013006	1880112	1878916	827138	825942	1822962	1821766
260	BPSS0253		2	1982999	1983958	2019222	2020181	1991206	1992165	1975316	1976275	1945129	1946088	1154227	1155186	2011985	2012944	1877895	1878854	824921	825880	1820745	1821704
261	BPSS0254		2	1982470	1982961	2018693	2019184	1990677	1991168	1974787	1975278	1944600	1945091	1153698	1154189	2011456	2011947	1877366	1877857	824392	824883	1820216	1820707
262	BPSS0255		2	1982231	1980717	2018454	2016940	1990438	1988924	1974548	1973034	1944361	1942847	1153459	1151945	2011217	2009703	1877127	1875613	824153	822639	1819977	1818463
263	BPSS0256		2	1980705	1980004	2016928	2016227	1988912	1988211	1973022	1972321	1942835	1942134	1151933	1151232	2009691	2008990	1875601	1874900	822627	821926	1818451	1817750
264	BPSS0257		2	1979789	1979745	2016012	2015968	1987996	1987952	1972106	1972062	1941919	1941875	1151017	1150973	2008775	2008731	1874685	1874641	821711	821667	1817535	1817491
265	BPSS0258		2	1978738	1977971	2014961	2014194	1986945	1986178	1971055	1970288	1940868	1940101	1149966	1149199	2007724	2006957	1873623	1872856	820660	819893	1816484	1815717
266	BPSS0259		2	1977902	1976769	2014125	2012992	1986109	1984976	1970219	1969086	1940032	1938899	1149130	1147997	2006888	2005755	1872787	1871654	819824	818691	1815648	1814515
267	BPSS0260		2	1976761	1975760	2012984	2011983	1984968	1983967	1969078	1968077	1938891	1937890	1147989	1146988	2005747	2004746	1871646	1870645	818683	817682	1814507	1813506

268	BPSS0261	2	1975760	1975104	2011983	2011327	1983967	1983311	1968077	1967421	1937890	1937234	1146988	1146332	2004746	2004090	1870645	1869989	817682	817026	1813506	1812850
269	BPSS0262	2	1974594	1975064	2010817	2011287	1982801	1983271	1966911	1967381	1936724	1937194	1145822	1146292	2003580	2004050	1869479	1869949	816516	816986	1812340	1812810
270	BPSS0263	2	1974142	1974594	2010365	2010817	1982349	1982801	1966459	1966911	1936272	1936724	1145370	1145822	2003128	2003580	1869027	1869479	816064	816516	1811888	1812340
271	BPSS0264	2	1973461	1973718	2009684	2009941	1981668	1981925	1965778	1966035	1935591	1935848	1144689	1144946	2002447	2002704	1868346	1868603	815383	815640	1811207	1811464
272	Chr2_5a_ISBma2_A		1971717	1973289	2007940	2009512	1979924	1981496	1964034	1965606	1933847	1935419	1142945	1144517	2000703	2002275	1866602	1868174	813639	815211	1809463	1811035
273	Chr2_5b_ISBma2_A		1638049	1639625	1668531	1670107	1632858	1634434	1631568	1633144			2146943	2148516	719172	720746	1189961	1191534			1506314	1507887
274	BPSS0265_putative porin-related protein	3	1637546	1637986	1668028	1668468	1632355	1632795	1631065	1631505			2149019	2148579	721249	720809	1189458	1189898			1505811	1506251
275	Chr2_6_IS407A_A	3	1636309	1637544	1666791	1668026	1631118	1632353	1629828	1631063			2149021	2150256	721251	722486	1188221	1189456			1504574	1505809
276	BPSS0265_putative porin-related protein	3	1635601	1636308	1666083	1666790	1630410	1631117	1629120	1629827			2150964	2150257	723194	722487	1187513	1188220			1503866	1504573
277	BPSS0266	3	1635288	1634491	1665770	1664973	1630097	1629300	1628807	1628010			2151277	2152074	723507	724304	1187200	1186403			1503553	1502756
278	BPSS0267	3	1632900	1634012	1663382	1664494	1627709	1628821	1626419	1627531			2153665	2152553	725895	724783	1184812	1185924			1501165	1502277
279	BPSS0268	3	1632628	1630517	1663110	1660999	1627437	1625326	1626147	1624036			2153937	2156048	726167	728278	1184540	1182429			1500893	1498782
280	BPSS0269	3	1629416	1630195	1659898	1660677	1624225	1625004	1622935	1623714			2157149	2156370	729379	728600	1181328	1182107			1497699	1498478
281	BPSS0270	3	1628283	1629179	1658765	1659661	1623092	1623988	1621802	1622698			2158282	2157386	730512	729616	1180195	1181091			1496566	1497462
282	BPSS0271	3	1628147	1626963	1658629	1657445	1622956	1621772	1621666	1620482			2158418	2159602	730648	731832	1180059	1178875			1496430	1495246
283	Chr2_7_ISBma2_C	3									1600353	1601926										
284	BPSS0272	3	1626686	1625373	1657168	1655855	1621495	1620182	1620205	1618892	1600164	1598851	2159879	2161192	732109	733422	1178598	1177285			1494969	1493656
285	BPSS0273	3	1624102	1624818	1654584	1655300	1618911	1619627	1617621	1618337	1597580	1598296	2162463	2161747	734693	733977	1176014	1176730			1492385	1493101
286	BPSS0274	3	1622884	1623738	1653366	1654220	1617693	1618547	1616403	1617257	1596362	1597216	2163681	2162827	735911	735057	1174796	1175650			1491167	1492021
287	BPSS0275	3	1622719	1621520	1653201	1652002	1617528	1616329	1616238	1615039	1596197	1595004	2163846	2165045	736076	737275	1174631	1173432			1491002	1489803
288	BPSS0276	3	1620732	1619191	1651214	1649673	1615541	1614000	1614251	1612710	1594216	1592675	2165833	2167374	738063	739604	1172644	1171103			1489015	1487474
289	BPSS0277	3	1618084	1619097	1648566	1649579	1612893	1613906	1611603	1612616	1591568	1592581	2168481	2167468	740711	739698	1169996	1171009			1486367	1487380
290	BPSS0278	3	1616626	1617663	1647108	1648145	1611435	1612472	1610145	1611182	1590110	1591147	2169939	2168902	742169	741132	1168538	1169575			1484909	1485946
291	BPSS0279	3	1615682	1616626	1646164	1647108	1610491	1611435	1609201	1610145	1589166	1590110	2170883	2169939	743113	742169	1167594	1168538			1483965	1484909
292	BPSS0280	3	1612461	1613927	1642943	1644409	1607270	1608736	1605980	1607446	1585945	1587411	2174104	2172638	746334	744868	1164373	1165839			1480744	1482210
293	BPSS0281	3	1611120	1612400	1641602	1642882	1605929	1607209	1604639	1605919	1584604	1585884	2175445	2174165	747675	746395	1163032	1164312			1479403	1480683
294	BPSS0282	3	1610978	1609290	1641460	1639772	1605787	1604150	1604497	1604225	1584462	1582771	2175587	2177278	747817	749508	1162890	1161199			1479261	1477570
295	BPSS0283	3	1607876	1609318	1638358	1639800	1602685	1604127	1601460	1602902	1581306	1582748	2178743	2177301	750973	749531	1159734	1161176			1476105	1477547
296	BPSS0284	3	1607058	1607822	1637540	1638304	1601867	1602631	1600642	1601406	1580488	1581252	2179570	2178806	751791	751027	1158916	1159680			1475287	1476051
297	BPSS0285	3	1606153	1607058	1636635	1637540	1600962	1601867	1599737	1600642	1579583	1580488	2180475	2179570	752696	751791	1158011	1158916			1474382	1475287
298	BPSS0285A_putative outer membrane pc	3																				
299	BPSS0286	3	1604404	1605234	1634886	1635716	1599213	1600043	1597988	1598818	1577834	1578664	2182224	2181394	754445	753615	1156262	1157092			1472633	1473463
300	BPSS0287	3	1604186	1603125	1634668	1633607	1598995	1597934	1597770	1596709	1577616	1576555	2182442	2183503	754663	755724	1156044	1154983			1472415	1471354
301	BPSS0288	3	1601763	1600705	1632245	1631187	1596572	1595514	1595347	1594289	1575193	1574135	2184865	2185923	757086	758144	1153621	1152563			1469992	1468934
302	BPSS0289	3	1600169	1600459	1630651	1630941	1594978	1595268	1593753	1594043	1573599	1573889	2186459	2186169	758680	758390	1152027	1152317			1468398	1468688
303	BPSS0290	3	1598240	1599241	1628722	1629723	1593049	1594050	1591824	1592825	1571678	1572679	2188436	2187435	760617	759616	1150090	1151091			1466461	1467462
304	BPSS0291	3	1598147	1597200	1628629	1627682	1592956	1592009	1591731	1590784	1571585	1570638	2188529	2189476	760710	761657	1149997	1149050			1466368	1465421
305	BPSS0292	3	1597147	1595921	1627629	1626403	1591956	1590730	1590731	1589505	1570585	1569359	2189529	2190755	761710	762936	1148997	1147771			1465368	1464142
306	BPSS0293	3	1595856	1592674	1626338	1623156	1590665	1587483	1589440	1586258	1569294	1566112	2190820	2194002	763001	766183	1147706	1144524			1464077	1460895
307	BPSS0294	3	1592660	1591131	1623142	1621613	1587469	1585940	1586244	1584715	1566098	1564569	2194016	2195545	766197	767726	1144510	1142981			1460881	1459352
308	BPSS0295	3	1589001	1590239	1619483	1620721	1583810	1585048	1582585	1583823	1562439	1563677	2197739	2196501	769920	768682	1140819	1142057			1457190	1458428
309	BPSS0296	3	1588358	1589014	1618840	1619496	1583167	1583823	1581942	1582598	1561796	1562452	2198382	2197726	770563	769907	1140176	1140832			1456547	1457203
310	BPSS0297	3	1588202	1587735	1618684	1618217	1583011	1582544	1581786	1581319	1561640	1561173	2198538	2199005	770719	771186	1140020	1139553			1456391	1455924
311	BPSS0298	3	1586645	1587541	1617127	1618023	1581454	1582350	1580229	1581125	1560083	1560979	2200095	2199199	772276	771380	1138463	1139359			1454834	1455730
312	BPSS0299	3	1584327	1586189	1614809	1616671	1579136	1580998	1577905	1579773	1557747	1559609	2202431	2200569	774612	772750	1136121	1137983			1452528	1454390
313	BPSS0300	3	1583411	1584307	1613893	1614789	1578220	1579116	1576989	1577885	1556831	1557727	2203347	2202451	775528	774632	1135205	1136101			1451612	1452508
314	BPSS0301	3	1582979	1583407	1613461	1613889	1577788	1578216	1576557	1576985	1556399	1556827	2203779	2203351	775960	775532	1134773	1135201			1451180	1451608
315	BPSS0302	3	1581052	1582908	1611534	1613390	1575861	1577717	1574630	1576486	1554472	1556328	2205706	2203850	777887	776031	1132846	1134702			1449253	1451109
316	BPSS0303	3	1579763	1581019	1610245	1611501	1574572	1575828	1573341	1574597	1553183	1554439	2206995	2205739	779176	777920	1131557	1132813			1447964	1449220
317	BPSS0304	3	1578285	1579763	1608767	1610245	1572309	1573010	1571863	1573341	1551705	1553183	2208473	2206995	780654	779176	1130346	1130882			1446521	1446796
318	BPSS0305	3	1577389	1577850	1607871	1608332	1571413	1571874	1570967	1571428	1550809	1551270	2209369	2208908	781550	781089	1129450	1129911			1445625	1446086

319	BPSS0306		3	1565126	1570378	1595608	1600860	1557961	1558176	1558704	1563956	1538546	1543798	2221623	2216380	793804	788561	1117196	1122439			1434049	1435902
320	BPSS0307		3	1563669	1565078	1594151	1595560	1556504	1557913	1557237	1557509	1537089	1538498	2223080	2221671	795261	793852	1115739	1117148			1432592	1434001
321	BPSS0308		3	1562254	1563672	1592736	1594154	1554201	1554956	1555822	1557240	1535674	1537092	2224495	2223077	796676	795258	1114324	1115742			1431177	1432595
322	BPSS0309		3	1561166	1562242	1591648	1592724	1553113	1554189	1554734	1555810	1534586	1535662	2225583	2224507	797764	796688	1113236	1114312			1430089	1431165
323	BPSS0310		3	1559676	1560191	1590158	1590673	1551623	1552138	1553244	1553759	1533064	1533579	2227050	2226535	799244	798729	1111762	1112277			1428608	1429123
324	BPSS0311		3	1551048	1559573	1580630	1582630	1541942	1546162	1544752	1551855	1524436	1532961	2235678	2227153	807873	807355	1103320	1110051			1420193	1424377
325		Chr2_8_IS407A_C	3									1523165	1524400										
326	BPSS0312		3	1549854	1549153	1579436	1578735	1540748	1540047	1543558	1542857	1522002	1521301	2236872	2237573	809067	809768	1102126	1101425			1418999	1418298
327	BPSS0313		3	1548820	1547984	1578402	1577566	1539714	1538878	1542524	1541688	1520968	1520132	2237906	2238742	810101	810937	1101092	1100256			1417964	1417128
328	BPSS0314		3	1547963	1546572	1577545	1576154	1538857	1537466	1541667	1540276	1520111	1518720	2238763	2240154	810958	812349	1100235	1098844			1417107	1415716
329	BPSS0315		3	1546500	1545361	1576082	1575351	1537394	1536255	1540204	1539065	1518648	1517509	2240226	2241365	812421	813560	1098772	1097633			1415644	1414505
330	BPSS0316		3	1545365	1544712	1574414	1573761	1536259	1535606	1539069	1538416	1517513	1516860	2241361	2242014	813556	814209	1097637	1096984			1414509	1413856
331	BPSS0317		3	1543953	1542775	1573002	1571824	1534847	1533669	1537657	1536479	1516101	1514923	2242785	2243963	814980	816158	1096195	1095017			1413097	1411919
332	BPSS0318		3	1541843	1542667	1570892	1571716	1532737	1533561	1535547	1536371	1513991	1514815	2244895	2244071	817090	816266	1094085	1094909			1410987	1411811
333	BPSS0319		3	1540948	1541544	1569997	1570593	1531842	1532438	1534652	1535248	1513096	1513692	2245790	2245194	817985	817389	1093190	1093786			1410092	1410688
334	BPSS0320		3	1540208	1540924	1569257	1569973	1531102	1531818	1533912	1534628	1512356	1513072	2246530	2245814	818725	818009	1092448	1093113			1409352	1410068
335	BPSS0321		3	1539806	1539003	1568855	1568052	1530700	1529897	1533510	1532707	1511954	1511151	2246922	2247725	819117	819920	1092056	1091253			1408940	1408137
336	BPSS0322		3	1539003	1537558	1568052	1566607	1529897	1528452	1532707	1531262	1511151	1509706	2247725	2249170	819920	821365	1091253	1089808			1408137	1406692
337	BPSS0323		3	1537558	1536836	1566607	1565885	1528452	1527730	1531262	1530540	1509706	1508984	2249170	2249892	821365	822087	1089808	1089086			1406692	1405970
338	BPSS0324		3	1536465	1534750	1565514	1564699	1527359	1526547	1530169	1528454	1508613	1506898	2250263	2251978	822458	824173	1088715	1087000			1405529	1404753
339	BPSS0325		3	1534418	1533984	1563018	1562584	1524778	1524344	1528122	1527688	1506566	1506132	2252310	2252744	824505	824939	1086668	1086234			1404421	1403987
340	BPSS0326		3	1532250	1533782	1560850	1562382	1522610	1524142	1525954	1527486	1504398	1505930	2254478	2252946	826673	825141	1084500	1086032			1402253	1403785
341	BPSS0327		3	1530690	1531979	1558865	1560154	1521050	1522339	1524394	1525683	1502838	1504127	2256038	2254749	828233	826944	1082940	1084229			1400767	1401762
342	BPSS0328		3	1529314	1530345	1557489	1558520	1519674	1520705	1523018	1524049	1501462	1502493	2257414	2256383	829609	828578	1081564	1082595			1399391	1400422
343	BPSS0329		3	1527729	1529318	1555904	1557493	1518089	1519678	1521433	1523022	1499877	1501466	2258999	2257410	831194	829605	1079979	1081568			1397806	1399395
344	BPSS0330		3	1526937	1527650	1554269	1555126	1516454	1517311	1519798	1520655	1498242	1499099	2260634	2259777	832829	831972	1078344	1079201			1396171	1397028
345	BPSS0331		3	1524713	1525630	1552503	1553420	1514817	1515734	1518416	1519333	1496861	1497778	2262015	2261098	834210	833293	1076963	1077880			1394790	1395707
346	BPSS0332		3	1524462	1523533	1552252	1551323	1514566	1513637	1518165	1517236	1496610	1495681	2262266	2263195	834461	835390	1076712	1075783			1394539	1393610
347	BPSS0333		3	1523533	1522394	1551323	1550709	1513637	1512498	1517236	1516097	1495681	1494542	2263195	2264334	835390	836538	1075783	1074644			1393610	1392471
348	BPSS0334		3	1522394	1522164	1550198	1549968	1512498	1512268	1516097	1515867	1494542	1494312	2264334	2264564	836538	836768	1074644	1074414			1392471	1392241
349	BPSS0335		3	1522164	1520875	1549968	1549291	1512268	1511996	1515867	1514578	1494312	1493023	2264564	2265853	836768	838057	1074414	1073125			1392241	1391330
350	BPSS0336		3	1520680	1519967	1547983	1547270	1509972	1509259	1514383	1513670	1492828	1492115	2266048	2266761	838252	838965	1072930	1072217			1390901	1390344
351	BPSS0337		3	1519497	1519763	1546800	1547066	1508789	1509055	1513200	1513466	1491645	1491911	2267231	2266965	839435	839169	1071747	1072013			748509	748243
352		Chr2_9a_IS407A_A		1517607	1518842	1544910	1546145	1506899	1508134	1511310	1512545	1489755	1490990	2267886	2269121	840090	841325	1069857	1071092			749164	750399
353		Chr2_9b_IS407A_A		869730	870965	883719	884954	944285	945520	865037	866272	1236549	1237784							1718700	1719935	845139	846374
354	BPSS0338		4	871763	870966	885752	884955	946318	945521	867070	866273	1235751	1236548	2269919	2269122	842123	841326	1069059	1069856	1720733	1719936	847172	846375
355	BPSS0339		4	873973	871922	887962	885911	948528	946477	869280	867229	1233541	1235592	2272129	2270078	844333	842282	1066849	1068900	1722943	1720892	849382	847331
356	BPSS0340		4	874233	874685	888222	888674	948788	949240	869540	869992	1233281	1232829	2272389	2272841	844593	845045	1066589	1066137	1723203	1723655	849642	850094
357	BPSS0341		4	874685	875536	888674	889525	949240	950091	869992	870843	1232829	1231978	2272841	2273692	845045	845896	1066137	1065286	1723655	1724506	850094	850945
358	BPSS0342		4	875587	876930	889576	890919	950142	951485	870894	872237	1231927	1230584	2273743	2275086	845947	847290	1065235	1064114	1724557	1725900	850996	852339
359	BPSS0343		4	878296	877190	892285	891179	952851	951745	873603	872497	1229218	1230324	2276600	2275494	848804	847698	1062683	1063789	1727266	1726160	853705	852599
360	BPSS0344		4	878534	879613	892523	893602	953089	954168	873878	874957	1228980	1227901	2276838	2277917	849042	850121	1062445	1061366	1727504	1728583	853943	855022
361	BPSS0345		4	879673	880767	893662	894756	954228	955322	875017	876111	1227841	1226747	2277977	2279071	850181	851275	1061306	1060212	1728643	1729737	855082	856176
362	BPSS0346		4	880748	881692	894737	895681	955303	956079	876092	877036	1226766	1225822	2279052	2279996	851256	852200	1060231	1059287	1729718	1730662	856157	857101
363	BPSS0347		4	881685	882542	895674	896531	956835	957692	877029	877886	1225829	1224972	2279989	2280846	852193	853050	1059294	1058437	1730655	1731512	857094	857951
364	BPSS0348		4	882589	883857	896578	897846	957739	959007	877933	879201	1224925	1223657	2280893	2282161	853097	854365	1058390	1057122	1731559	1732827	857998	859266
365	BPSS0349		4	883866	885308	897855	899297	959016	960458	879210	880652	1223648	1222206	2282170	2283612	854374	855816	1057113	1055671	1732836	1734278	859275	860717
366	BPSS0350		4	885316	885864	899305	899853	960466	961014	880660	881208	1222198	1221650	2283620	2284093	855824	856372	1055663	1055115	1734286	1734834	860725	861273
367	BPSS0351_putative copper-related MerR		4																				
368		Chr2_10a_IS407A_A		891974	893209	905698	906933	967328	968563	887110	888345	1214324	1215557	6183	7418	862482	863717	1052451	1053615	6022	7257	867383	868618
369		Chr2_10b_IS407A_A												20142	21377	876441	877676	106411	107646				

370		Chr2_11_ISBma2_B		893528	895100	907252	909260	968882	970454	888664	890236	1214269	1214325										
371	BPSS0352		5	896391	895549	910551	909709	971745	970903	891527	890685	1212978	1213820	18914	19756	875213	876055	105183	106025				
372	BPSS0353		5	897511	896543	911671	910703	972865	971897	892647	891679	1211858	1212826	17794	18762	874093	875061	104063	105031				
373	BPSS0354		5	897699	898484	911859	912644	973053	973838	892835	893620	1211670	1210885	17606	16821	873905	873120	103875	103090				
374		Chr2_12_ISBma2_A	5	898637	900210	912797	914370	973991	975564	893773	895346	1209160	1210732	15095	16668	871394	872967	101363	102937	1707496	1709043	876295	877868
375	BPSS0355		6	901498	900302	915658	914462	976852	975656	896634	895438	1207872	1209068	13807	15003	870106	871302	100075	101271	1710371	1709175	875007	876203
376	BPSS0356		6	901786	903873	915946	918033	977140	979227	896922	899009	1207584	1205497	13519	11432	869818	867731	99787	97700	1710659	1712746	874719	872632
377	BPSS0357		6	904100	904648	918260	918808	979454	980002	899236	899784	1205270	1204722	11205	10657	867504	866956	97473	96925	1712973	1713521	872405	871857
378	BPSS0358		6	904711	905037	918871	919197	980065	980391	899847	900173	1204659	1204333	10594	10268	866893	866567	96862	96536	1713584	1713910	871794	871468
379	BPSS0359		6	905100	905939	919260	920099	980454	981293	900236	901075	1204270	1203431	10205	9366	866504	865665	96473	96255	1713973	1714812	871405	870566
380	BPSS0360		6	905939	907336	920099	921496	981293	982690	901075	902472	1203431	1202034	9366	7969	865665	864268	95633	94236	1714812	1716209	870566	869169
381	BPSS0361		6	907399	907806	921559	921966	982753	983160	902535	902942	1201971	1201564	7906	7499	864205	863798	94173	93766	1716272	1716679	869106	868699
382		Chr2_13a_IS407A_A		907887	909122	922047	923283	983241	984476	903023	904258	1200293	1201483	6183	7418	862482	863717	92450	93685	1716760	1717995	867383	868618
383		Chr2_13b_IS407A_A		1643058	1644293	1673540	1674775	1637867	1639102	1636577	1637812	1605359	1606594	2142275	2143510	714504	715739	1194967	1196202			1511320	1512555
384	BPSS0362		7	1642654	1642914	1673136	1673396	1637463	1637723	1636173	1636433	1604955	1605215	2143914	2143654	716143	715883	1194563	1194823			1510916	1511176
385	BPSS0363		7	1642025	1642507	1672507	1672989	1636834	1637316	1635544	1636026	1604326	1604808	2144543	2144061	716772	716290	1193934	1194416			1510287	1510769
386	BPSS0364		7	1641034	1641903	1671516	1672385	1635843	1636712	1634553	1635422	1603335	1604204	2145534	2144665	717763	716894	1192943	1193812			1509296	1510165
387	BPSS0365		7	1640911	1639943	1671393	1670425	1635720	1634752	1634430	1633462	1603212	1602244	2145657	2146625	717886	718854	1192820	1191852			1509173	1508205
388		Chr2_14a_ISBma2_A		1638049	1639625	1668531	1670107	1632858	1634434	1631568	1633144	1600353	1601926	2146943	2148516	719172	720746	1189961	1191534			1506314	1507887
389		Chr2_14b_ISBma2_A		1971717	1973289	2007940	2009512	1979924	1981496	1964034	1965606	1933847	1935419	1142945	1144517	2000703	2002275	1866602	1868174	813639	815211	1809463	1811035
390	BPSS0366		8	1971202	1971621	2007425	2007844	1979409	1979828	1963519	1963938	1933332	1933751	1142430	1142849	2000188	2000607	1866087	1866506	813124	813543	1808948	1809367
391	BPSS0367		8	1970454	1971179	2006677	2007402	1978661	1979386	1962771	1963496	1932584	1933309	1141682	1142407	1999440	2000165	1865339	1866064	812376	813101	1808200	1808925
392	BPSS0368		8	1969728	1970414	2005951	2006637	1977935	1978621	1962045	1962731	1931858	1932544	1140956	1141642	1998714	1999400	1864613	1865299	811650	812336	1807474	1808160
393	BPSS0369		8	1969213	1969449	2005436	2005672	1977420	1977656	1961530	1961766	1931343	1931579	1140441	1140677	1998199	1998435	1864098	1864334	811135	811371	1806959	1807195
394	BPSS0370		8	1968209	1969078	2004432	2005301	1976416	1977285	1960526	1961395	1930339	1931208	1139437	1140306	1997195	1998064	1863094	1863963	810131	811000	1805955	1806824
395	BPSS0371		8	1967686	1968159	2003909	2004382	1975893	1976366	1960003	1960476	1929816	1930289	1138914	1139387	1996672	1997145	1862571	1863044	809608	810081	1805432	1805905
396	BPSS0372		8	1965996	1967528	2002219	2003751	1974203	1975735	1958313	1959845	1928126	1929658	1137224	1138756	1994982	1996514	1860881	1862413	807918	809450	1803742	1805274
397	BPSS0373		8	1965348	1965953	2001571	2002176	1973045	1973098	1957665	1958270	1927478	1928083	1136576	1137181	1994334	1994939	1860233	1860838	807270	807875	1803094	1803699
398	BPSS0374		8	1964220	1964675	2000443	2000898	1971917	1972372	1956537	1956992	1926350	1926805	1135448	1135903	1993206	1993661	1859105	1859560	806142	806597	1802403	1802858
399	BPSS0375		8	1963930	1961951	2000153	1998174	1971627	1969648	1956247	1954268	1926060	1924081	1135158	1133179	1992916	1990937	1858815	1856836	805852	803873	1800359	1798380
400	BPSS0376		8	1961850	1961797	1998073	1998020	1969547	1969494	1954167	1954114	1923980	1923927	1133078	1133025	1990836	1990783	1856735	1856682	803772	803719	1798279	1798226
401	BPSS0377		8	1961477	1959465	1997700	1995688	1969174	1967162	1953794	1951782	1923607	1921595	1132705	1130693	1990463	1988451	1856362	1854350	803399	801387	1797906	1795894
402	BPSS0378	GI13																					
403	BPSS0379	GI13																					
404	BPSS0380	GI13																					
405	BPSS0380A	GI13																					
406	BPSS0380B	GI13																					
407	BPSS0381	GI13																					
408	BPSS0382	GI13																					
409	BPSS0383	GI13																					
410	BPSS0384	GI13																					
411	BPSS0384A	GI13																					
412	BPSS0385	GI13																					
413	BPSS0386	GI13																					
414	BPSS0387	GI13																					
415	BPSS0388	GI13																					
416	BPSS0389	GI13																					
417	BPSS0390	GI13																					
418	BPSS0391	GI13																					
419	BPSS0391A	GI13																					
420	BPSS0392	GI13																					

421	BPSS0393	GI13																					
422	BPSS0394	GI13																					
423	BPSS0395	GI13																					
424	BPSS0396	GI13																					
425	BPSS0397	GI13																					
426	BPSS0397A	GI13																					
427	BPSS0398	GI13																					
428	BPSS0399	GI13																					
429	BPSS0400	GI13																					
430	BPSS0401	GI13																					
431	BPSS0402		8	1958858	1959106	1995081	1995329	1966555	1966803	1951175	1951423	1920988	1921236	1130086	1130334	1987844	1988092	1853743	1853991	800780	801028	1795287	1795535
432		Chr2_15a_IS407A_A		1957621	1958856	1993844	1995079	1965318	1966553	1949938	1951173	1919751	1920986	1128849	1130084	1986607	1987842	1852506	1853741	799543	800778	1794050	1795285
433		Chr2_15b_IS407A_A		560460	561695	571425	572660	600352	601587	559127	560362	515849	517084	966292	967527	1824067	1825302	1052451	1053615			541189	542423
434	BPSS0403		9	561889	563346	572854	574311	601781	603238	560556	562013	517278	518735	966098	964641	1823873	1822416	1052257	1051055			542617	544074
435	BPSS0404		9	564121	564768	574365	575087	603292	604014	562067	562789	518789	519511	964587	963865	1822362	1821640	1050744	1050022			544128	544850
436	BPSS0405		9	564928	565146	575893	576111	604820	605038	563595	563813	520317	520535	963059	962130	1820834	1819905	1049216	1048287				
437		Chr2_16_IS407A_B	9	565147	566382	576112	577347	605039	606274	563814	565049	520536	521771									545708	546943
438	BPSS0406		9											961929	961729	1819704	1819504	1048086	1047886				
439	BPSS0407		9											961701	961003	1819476	1818778	1047858	1047160				
440	BPSS0408		9											960228	960959	1818003	1818734	1046385	1047116				
441	BPSS0409		9											951691	960234	1809466	1818009	1037848	1046391				
442		Chr2_17_IS407A_A												950458	951693	1808233	1809468	1036615	1037850				
443	BPSS0410																						
444	BPSS0411																						
445	BPSS0412																						
446	BPSS0413																						
447	BPSS0414																						
448	BPSS0415																						
449	BPSS0416																						
450	BPSS0416A																						
451	BPSS0417																						
452	BPSS0418																						
453	BPSS0419																						
454	BPSS0420																						
455	BPSS0421																						
456	BPSS0422																						
457	BPSS0423																						
458	BPSS0424																						
459	BPSS0425																						
460	BPSS0426																						
461	BPSS0427																						
462	BPSS0428																						
463	BPSS0429																						
464	BPSS0430																						
465	BPSS0431																						
466	BPSS0432																						
467	BPSS0433																						
468	BPSS0434																						
469	BPSS0435																						
470	BPSS0436																						
471	BPSS0437																						

472	BPSS0438											
473	BPSS0439											
474	BPSS0440											
475	BPSS0441											
476	BPSS0442											
477	BPSS0443											
478	BPSS0444											
479	BPSS0445											
480	BPSS0446											
481	BPSS0447											
482	BPSS0448											
483	BPSS0449											
484	BPSS0450											
485	BPSS0451											
486	BPSS0452											
487	BPSS0453											
488	BPSS0454											
489	BPSS0455											
490	BPSS0456											
491	BPSS0457											
492	BPSS0458											
493	BPSS0459											
494	BPSS0460											
495	BPSS0461											
496	BPSS0462											
497	BPSS0463											
498	BPSS0464											
499	BPSS0465											
500	BPSS0466											
501	BPSS0467											
502	BPSS0468											
503	BPSS0469											
504	BPSS0470											
505	BPSS0471											
506	BPSS0472											
507	BPSS0473											
508	BPSS0474											
509	BPSS0475											
510	BPSS0476											
511	BPSS0477											
512	BPSS0478											
513	BPSS0479											
514	BPSS0480											
515	BPSS0481											
516	BPSS0482											
517	BPSS0483											
518	BPSS0484											
519	BPSS0485											
520	BPSS0486											
521	BPSS0487											
522	BPSS0488											

523	BPSS0488A																						
524	BPSS0489																						
525	BPSS0490																						
526		Chr2_18_IS407A_A		1957621	1958856	1993844	1995079	1965318	1966553	1949938	1951173	1919751	1920986	1128849	1130084	1986607	1987842	1852506	1853741	799543	800778	1794050	1795285
527	BPSS0491		10a	1956647	1957630	1992870	1993853	1964344	1965327	1948964	1949947	1918777	1919760	1127875	1128858	1985633	1986616	1851532	1852515	798569	799552	1793076	1794059
528	BPSS0492		10a	1955880	1956440	1992103	1992663	1963577	1964137	1948197	1948757	1918010	1918570	1127108	1127668	1984866	1985426	1850765	1851325	797802	798362	1792309	1792869
529	BPSS0493		10a	1954523	1955617	1990746	1991840	1962220	1963314	1946840	1947934	1916653	1917747	1125751	1126845	1983509	1984603	1849408	1850502	796445	797539	1790952	1792046
530	BPSS0494		10a	1953462	1953962	1989685	1990185	1961159	1961659	1945779	1946279	1915592	1916092	1124690	1125190	1982448	1982948	1848347	1848847	795384	795884	1789891	1790391
531	BPSS0495		10a	1952764	1953384	1988987	1989607	1960461	1961081	1945081	1945701	1914894	1915514	1123992	1124612	1981750	1982370	1847649	1848269	794686	795306	1789193	1789813
532	BPSS0496		10a	1952051	1950618	1988274	1986841	1959748	1958315	1944368	1942935	1914181	1912748	1123279	1121846	1981037	1979604	1846936	1845503	793973	792540	1788480	1787047
533	BPSS0497		10a	1950520	1949582	1986743	1985805	1958217	1957279	1942837	1941899	1912650	1911712	1121748	1120810	1979506	1978568	1845405	1844467	792442	791504	1786949	1786011
534	BPSS0498		10a	1949570	1948617	1985793	1984840	1957267	1956314	1941887	1940934	1911700	1910747	1120798	1119845	1978556	1977603	1844455	1843502	791492	790539	1785999	1785046
535	BPSS0499		10a	1948477	1945058	1984700	1981281	1956174	1952755	1940794	1937375	1910607	1907188	1119705	1116286	1977463	1974044	1843362	1839943	790399	786980	1784906	1781487
536	BPSS0500		10a	1944867	1942222	1981090	1978445	1952564	1949919	1937184	1934539	1906997	1904352	1116095	1113450	1973853	1971208	1839752	1837107	786789	784144	1781296	1778651
537	BPSS0501		10a	1942228	1941209	1978451	1977432	1949925	1948906	1934545	1933526	1904358	1903339	1113456	1112437	1971214	1970195	1837113	1836094	784150	783131	1778657	1777638
538	BPSS0502		10a	1939516	1941015	1975739	1977238	1947213	1948712	1931833	1933332	1901646	1903145	1110744	1112243	1968502	1970001	1834401	1835900	781438	782937	1776166	1776234
539	BPSS0503		10a	1939388	1938204	1975611	1974427	1947085	1945901	1931705	1930521	1901518	1900334	1110616	1109432	1968374	1967190	1834273	1833089	781310	780126	1776038	1774854
540	BPSS0504		10a	1936814	1937884	1973037	1974107	1944511	1945581	1929131	1930201	1898944	1900014	1108042	1109112	1965800	1966870	1831699	1832769	778736	779806	1773464	1774534
541	BPSS0505		10a	1935610	1936572	1971833	1972795	1943307	1944269	1927927	1928889	1897740	1898702	1106838	1107800	1964596	1965558	1830495	1831457	777532	778494	1772258	1773220
542	BPSS0506		10a	1935468	1934773	1971691	1970996	1943165	1942470	1927785	1927090	1897598	1896903	1106696	1106001	1964454	1963759	1830353	1829658	777390	776695	1772116	1771421
543	BPSS0507		10a	1933557	1934621	1969780	1970844	1941254	1942318	1925874	1926938	1895687	1896751	1104785	1105849	1962543	1963607	1828442	1829506	775479	776543	1770205	1771269
544	BPSS0508		10a	1932365	1932403	1968588	1968626	1940062	1940100	1924682	1924720	1894495	1894533	1103593	1103631	1961351	1961389	1827250	1827288	774287	774325	1769013	1769051
545	BPSS0509		10a	1930734	1932365	1966957	1968588	1938431	1940062	1923051	1924682	1892864	1894495	1101962	1103593	1959720	1961351	1825619	1827250	772656	774287	1767382	1769013
546	BPSS0510		10a	1929901	1930680	1966124	1966903	1937598	1938377	1922218	1922997	1892031	1892810	1101129	1101908	1958887	1959666	1824790	1825518	771823	772602	1766549	1767328
547	BPSS0511		10a	1928876	1929874	1965099	1966097	1936573	1937571	1921193	1922191	1891006	1892004	1100104	1101102	1957862	1958860	1823765	1824763	770798	771796	1765524	1766522
548	BPSS0512		10a	1928113	1928874	1964336	1965097	1935810	1936571	1920430	1921191	1890243	1891004	1099341	1100102	1957099	1957860	1823002	1823763	770035	770796	1764761	1765522
549	BPSS0513		10a	1927427	1928113	1963650	1964336	1935124	1935810	1919744	1920430	1889557	1890243	1098655	1099341	1956413	1957099	1822316	1823002	769349	770035	1764075	1764761
550	BPSS0514		10a	1927152	1925551	1963375	1961774	1934849	1933248	1919469	1917868	1889282	1887681	1098380	1096779	1956138	1954537	1822041	1820440	769074	767473	1763800	1762199
551		Chr2_19_IS407A_B	10a																	764597	765832	1759323	1760558
552	BPSS0515		10a	1923839	1922904	1960062	1959127	1931536	1930601	1916156	1915221	1885969	1885034	1095067	1094132	1952825	1951890	1818728	1817793	764521	763586	1759247	1758312
553	BPSS0516_conserved hypothetical protei		10a	1922867	1922784	1959090	1959007	1930564	1930481	1915184	1915101	1884997	1884914	1094095	1093550	1951853	1951308	1817756	1817211	763549	763004	1758275	1757730
554		Chr2_20a_IS407A_B		1921548	1922783	1957771	1959006	1929245	1930480	1913865	1915100	1883678	1884913										
555		Chr2_20b_IS407A_B		432859	434094	442944	444179	472071	473306	431867	433102	387487	388722										
556	BPSS0516_conserved hypothetical protei		10b	434096	434560	444181	444645	473308	473772	433104	433568	388724	389188										
557	BPSS0517		10b	434560	436068	444645	446153	473772	475280	433568	435076	389188	390696	1093550	1092042	1951308	1949800	1817211	1815703	763004	761496	1757730	1756222
558	BPSS0518		10b	436215	436739	446300	446824	475427	475951	435223	435747	390843	391367	1091895	1091371	1949653	1949129	1815556	1815032	761349	760825	1756075	1755551
559	BPSS0519		10b	436783	437250	446868	447335	475995	476462	435791	436258	391411	391878	1091327	1090860	1949085	1948618	1814988	1814521	760781	760314	1755507	1755040
560	BPSS0520		10b	437267	439126	447352	449211	476479	478338	436275	438134	391895	393754	1090843	1088984	1948601	1946742	1814504	1812645	760297	758438	1755023	1753164
561	BPSS0521		10b	439126	440112	449211	450197	478338	479324	438134	439120	393754	394740	1088984	1087998	1946742	1945756	1812645	1811659	758438	757452	1753164	1752178
562	BPSS0522		10b	440118	442985	450203	453070	479330	482197	439126	441993	394746	397613	1087992	1085125	1945750	1942883	1811653	1808786	757446	754579	431730	433688
563	BPSS0523		10b	442979	445222	453064	455307	482191	484434	441987	444230	399977	402262	1085131	1082888	1942889	1940646	1808792	1806549	754585	752342	433682	435925
564	BPSS0524		10b	445391	447676	455476	457761	484603	486888	444399	446684	399977	402262	1082719	1080434	1940477	1938192	1806380	1804095	752173	749888	436094	438379
565	BPSS0525		10b	447683	449896	457768	459981	486895	489108	446691	448904	402269	404482	1080427	1078214	1938185	1935972	1804088	1801875	749881	747668	438386	440599
566	BPSS0526		10b	449896	450966	459981	461051	489108	490178	448904	449974	404482	405552	1078214	1077144	1935972	1934902	1801875	1800805	747668	746598	440599	441669
567	BPSS0527		10b	450972	451685	461057	461770	490184	490897	449980	450693	405558	406271	1077138	1076425	1934896	1934183	1800799	1800086	746592	745879	441675	442388
568	BPSS0528		10b	451731	452117	461816	462202	490943	491329	450739	451125	406317	406703	1076379	1075993	1934137	1933751	1800040	1799654	745833	745447	442434	442820
569	BPSS0529		10b	452126	452716	462211	462801	491338	491928	451134	451724	406712	407302	1075984	1075394	1933742	1933152	1799645	1799055	745438	744848	442829	443419
570	BPSS0530		10b	452716	454074	462801	464159	491928	493286	451724	453109	407302	408660	1075394	1074036	1933152	1931794	1799055	1797697	744848	743490	443419	444777
571	BPSS0531		10b	454160	455815	464245	465900	493372	495027	453195	454850	408746	410401	1073950	1072271	1931708	1930053	1797611	1795932	743404	741749	444863	446518
572	BPSS0532		10b	455815	459315	465900	469400	495027	498527	454850	458350	410401	413901	1072271	1068771	1930053	1926553	1795932	1792432	741749	740439	446518	450018
573	BPSS0533		10b	459377	459733	469462	469818	498589	498945	458412	458768	413963	414319	1068709	1068353	1926491	1926135	1792370	1792014	738191	737835	450080	450436

574	BPSS0534		10b	459759	460181	469844	469930	498971	499393	458794	459216	414345	414767	1068327	1067905	1926109	1925687	1791988	1791566	737809	737387	450462	450575
575	BPSS0535		10b	460409	460777	471144	471512	499621	499989	459444	459812	414995	415363	1067677	1067309	1925459	1925091	1791338	1790970	737159	736791	450809	451177
576		Chr2_21_IS407A_C	10b																			920084	921319
577	BPSS0536		10b	461331	462227	472066	472962	500543	501439	460366	461262	416791	417687	1066755	1065859	1924537	1923641	1790416	1789703	736237	735341	921440	922336
578		Chr2_22_IS407A_C																529400	530635				
579	BPSS0537		10c	462452	463768	951670	952986	1014307	1015623	461487	462794	417912	419228	1065634	1064318	1923416	1922100	531876	533192	735116	733800	922561	923877
580	BPSS0538		10c	463768	465351	474681	476201	503353	504936	462832	464346	419228	420811	1064318	1062735	1922100	1920517	533192	534775	733800	732217	923877	925460
581	BPSS0539		10c	465619	466611	476469	477461	505204	506196	935693	936685	421091	422083	1062425	1061433	1920225	1919233	535055	536047	731949	730957	925722	926714
582	BPSS0540		10c	466919	468511	477769	479361	506504	508096	465681	467273	422420	423997	1061125	1059545	1918925	1917333	536355	537947	730649	729057	927022	928614
583	BPSS0541		10c	469082	470245	479932	481095	508667	509830	467844	469007	424568	425731	1058974	1057811	1916762	1915599	744	1907	728486	727323	929185	930348
584	BPSS0542		10c	472514	470853	483364	481703	512099	510438	471276	469615	427992	426331	1055558	1057219	1913338	1914999	4152	2491	725086	726747	455415	453754
585	BPSS0543		10c	474212	472650	485062	483500	513797	512235	472974	471412	429690	428128	1053860	1055422	1911640	1913202	5850	4288	723388	724950	457113	455551
586	BPSS0544		10c	474401	475414	485251	486264	513986	514999	473163	474176	429879	430892	1053693	1052680	1911473	1910460	6039	7052	723199	722186	457302	458315
587	BPSS0545		10c	475857	477053	486707	487903	515442	516638	474619	475815	431356	432552	1052216	1051020	1909982	1908786	7523	8719	721743	720547	458758	459954
588	BPSS0546		10c	478259	477237	489109	488087	517844	516822	477021	475999	433758	432736	1049814	1050836	1907580	1908602	9925	8903	719341	720363	461160	460138
589	BPSS0547		10c	478678	479964	489528	490814	518263	519549	477440	478726	434177	435463	1049395	1048109	1907161	1905875	10344	11630	718922	717636	461579	462865
590	BPSS0548		10c	480040	481008	490890	491858	519625	520593	478802	479770	435539	436507	1048033	1047065	1905799	1904831	11706	12674	717560	716592	462941	463909
591	BPSS0549		10c	481162	481692	492012	492542	520747	521277	479924	480454	436661	437191	1046911	1046381	1904677	1904147	12828	13358	716438	715908	464063	464593
592	BPSS0550		10c	481756	483816	492606	494666	521341	523401	480518	482578	437255	439315	1046317	1044257	1904083	1902023	13422	15482	715844	713784	464657	466717
593	BPSS0551		10c	483822	485744	494672	496594	523407	525329	482584	484506	439321	441243	1044251	1042329	1902017	1900095	15488	17410	713778	711856	466723	468645
594	BPSS0552		10c	485752	486921	496602	497771	525337	526506	484514	485683	441251	442420	1042321	1041152	1900087	1898918	17418	18587	711848	710679	468653	469822
595	BPSS0553		10c	486921	487703	497771	498553	526506	527288	485683	486465	442420	443202	1041152	1040370	1898918	1898136	18587	19369	710679	709897	469822	470604
596	BPSS0554		10c	487731	488996	498581	499846	527316	528581	486493	487758	443230	444495	1040342	1039077	1898108	1896843	19397	20662	709869	708604	470632	471897
597	BPSS0555		10c	489020	490162	499870	501012	528605	529747	487782	488924	444519	445661	1039053	1037911	1896819	1895677	20686	21828	708580	707438	471921	473063
598	BPSS0556		10c	490275	491135	501125	501985	529860	530720	489037	489897	445774	446634	1037798	1036938	1895564	1894704	21941	22801	707325	706465	473176	474036
599	BPSS0557		10c	491319	492971	502169	503821	530904	532556	490081	491733	446818	448470	1036754	1035102	1894520	1892868	22985	24637	706281	704629	474220	475872
600	BPSS0558		10c	493936	493064	504786	503914	533521	532649	492698	491826	449435	448563	1034137	1035009	1891903	1892775	25602	24730	703664	704536	476837	475965
601	BPSS0559		10c	494979	494083	505829	504933	534564	533668	493741	492845	450478	449582	1033094	1033990	1890860	1891756	26645	25749	702621	703517	477880	476984
602	BPSS0560		10c	495115	496665	505965	507515	534700	536250	493877	495427	450614	452164	1032958	1031408	1890724	1889174	26863	28335	702485	700935	478016	479566
603	BPSS0561		10c	497310	497555	507554	508171	536289	536906	495466	496083	452203	452820	1031369	1030752	1889135	1888518	28374	28991	700896	700279	479605	480222
604	BPSS0562		10c	497786	498925	508636	509775	537371	538510	496548	497687	453285	454424	1030287	1029148	1888053	1886914	29456	30595	699814	698675	480687	481826
605	BPSS0563		10c	500209	498986	511059	509836	539794	538571	498887	497748	455708	454485	1027864	1029087	1885630	1886853	31879	30656	697391	698614	483110	481887
606	BPSS0564		10c	502030	500330	512880	511180	541615	539915	500819	499119	457630	455930	1026043	1027743	1883809	1885509	33844	32144	695570	697270	484982	483282
607	BPSS0565		10c	503446	502505	514296	513355	543031	542090	502235	501294	459046	458105	1024627	1025568	1882393	1883334	35260	34319	694154	695095	486398	485457
608	BPSS0566		10c	503714	504661	514564	515511	543299	544246	502503	503450	459314	460261	1024359	1023412	1882125	1881178	35528	36475	693886	692939	486666	487613
609	BPSS0567		10c	504747	505670	515597	516520	544332	545255	503536	504459	460347	461270	1023326	1022403	1881092	1880169	36561	37484	692853	691930	487699	488622
610	BPSS0568		10c	505791	506753	516641	517603	545376	546338	504580	505542	461391	462353	1022282	1021320	1880048	1879086	37606	38568	691809	690847	488743	489705
611	BPSS0569		10c	506774	507253	517624	518103	546359	546838	505563	506042	462374	462853	1021299	1020820	1879065	1878586	38589	39068	690826	690347	489726	490205
612	BPSS0570		10c	507292	508248	518142	519098	546877	547833	506081	507037	462892	463848	1020781	1019825	1878547	1877591	39107	40063	690308	689352	490244	491200
613	BPSS0571		10c	508906	508601	519756	519451	548491	548186	507695	507390	464506	464201	1019167	1019472	1876933	1877238	40721	40416	688694	688999	491858	491553
614	BPSS0572_hypothetical protein		10c									465012	464743			1876427	1876696	41227	40958				
615		Chr2_23_IS407A_A		509411	510646	520261	521496	548996	550231	508200	509435	465011	466246	1017427	1018662	1875193	1876428	41226	42461	686954	688189	492363	493598
616	BPSS0573																						
617	BPSS0574																						
618	BPSS0575																						
619	BPSS0576																						
620	BPSS0577																						
621	BPSS0578																						
622	BPSS0579																						
623	BPSS0580																						
624	BPSS0581																						

625	BPSS0582																					
626	BPSS0583																					
627	BPSS0584																					
628	BPSS0585																					
629	BPSS0586																					
630	BPSS0587																					
631	BPSS0588																					
632	BPSS0589																					
633	BPSS0590																					
634	BPSS0591																					
635	BPSS0592																					
636	BPSS0593																					
637	BPSS0594																					
638	BPSS0595																					
639	BPSS0596																					
640	BPSS0597																					
641	BPSS0598																					
642	BPSS0599																					
643	BPSS0600																					
644	BPSS0601																					
645	BPSS0602																					
646	BPSS0603																					
647	BPSS0604																					
648	BPSS0605																					
649	BPSS0606																					
650	BPSS0607																					
651	BPSS0608																					
652	BPSS0609																					
653	BPSS0610																					
654	BPSS0611																					
655	BPSS0612																					
656	BPSS0613																					
657	BPSS0614																					
658	BPSS0615																					
659	BPSS0616																					
660	BPSS0617																					
661	BPSS0618																					
662	BPSS0619																					
663	BPSS0620																					
664	BPSS0621																					
665	BPSS0622																					
666	BPSS0623																					
667	BPSS0624																					
668	BPSS0625																					
669	BPSS0626																					
670		Chr2_24_IS407A_A	838658	839893	852556	853791	881257	882492	836729	837964	1438414	1439649	22152	23387	1533196	1534431	382829	384064			749164	750399
671	BPSS0627	11	839895	840005	853793	853903	882494	882604	837966	838076	1439651	1439761	23389	23499	1533194	1533084	384066	384176			750401	750511
672	BPSS0628	11	841443	840532	855341	854430	884042	883131	839514	838603	1441181	1440270	24910	23999	1531673	1532584	385587	384676			751922	751011
673	BPSS0629	11									1441226	1441333			1531628	1531521	385632	385964				
674	BPSS0630	11	841982	842689	855880	856587	884581	885288	840053	840760	1441765	1442472	25431	26138	1531116	1530409	386090	386797			752434	753141
675	BPSS0631	11	843080	844192	856978	858090	885679	886791	841151	842263	1442864	1443976	26529	27641	1530018	1528906	387188	388300			753532	754644

676	BPSS0632		11	846844	844535	860742	858433	889443	887134	844915	842606	1446628	1444319	30293	27984	1526254	1528563	390952	388643			757149	754987
677	BPSS0633		11	847630	848691	861528	862589	890229	891290	845701	846762	1447486	1448547	31115	32176	1525444	1524383	391750	392811			758090	759151
678	BPSS0634		11	849191	849778	863089	863676	891790	892377	847262	847849	1449047	1449634	32676	33263	1523883	1523296	393311	393898			759651	760238
679	BPSS0635		11	850074	852200	863972	866098	892673	894019	848145	848621	1449930	1451363	33568	35694	1522991	1520865	394203	395543			760534	760887
680	BPSS0636		11	852299	852559	866197	866457	926854	927114	850259	850519	1452356	1452589	35793	36053	1520766	1520506	396302	396562			761724	761984
681	BPSS0637		11	853785	852799	867683	866697	928340	927354			1453588	1452692	37279	36293	1519280	1520266	397788	396802			763210	762224
682	BPSS0639		11	854150	854623	868048	868521	928705	929178	851160	851321	1453953	1454426	37644	38117	1518915	1518442					763575	764048
683	BPSS0640		11	854925	855581	869559	870215	929480	930136	851623	852279			38419	39075	1518140	1517484	398271	398774			764350	765006
684	BPSS0641		11	855625	856332	870259	870696	930180	930887	852323	852940	1454565	1455272	39119	39826	1517440	1516733	398818	399525			765050	765757
685	BPSS0642		11	859594	858218	873583	872207	934149	932773	854901	853525	1310016	1311392	43116	41740	1513457	1514833	402613	401237			769012	767636
686	BPSS0643		11	860253	859594	874242	873583	934808	934149	855560	854901	1309357	1310016	43775	43116	1512798	1513457	403272	402613			769671	769012
687	BPSS0644		11	860414	861271	874403	875260	934969	935826	855721	856578	1309196	1308339	43936	44793	1512637	1511780	403433	404290			769832	770689
688	BPSS0645		11	861660	861926	875649	875915	936215	936481	856967	857233	1307950	1307684	45182	45448	1511391	1511125	404679	404945			771078	771344
689	BPSS0646		11	862484	862149	876473	876138	937039	936704	857791	857456	1307126	1307461	46006	45671	1510567	1510902	405503	405168			771902	771567
690	BPSS0647		11	863041	862577	877030	876566	937596	937132	858348	857884	1306569	1307033	46563	46099	1510010	1510474	406060	405596			772459	771995
691	BPSS0648		11	863148	864101	877137	878090	937703	938656	858455	859408	1306462	1305509	46670	47623	1509903	1508950	406167	407120			772566	773519
692	BPSS0649		11	864692	865153	878681	879142	939247	939708	859999	860460	1304928	1304467	48194	48655	1508379	1507918	407691	408152			774090	774551
693	BPSS0650		11	865220	866668	879209	880657	939775	941223	860527	861975	1304400	1302952	48722	50170	1507851	1506403	408219	409667			774618	776066
694	BPSS0651		11	866781	867146	880770	881135	941336	941701	862088	862453	1302839	1302474	50283	50648	1506290	1505925	409780	410145			776179	776544
695	BPSS0652		11	867676	868251	881665	882240	942231	942806	862983	863558	1301944	1301369	51178	51753	1505395	1504820	410675	411250			777074	777649
696		Chr2_25_IS407A_C	11																			778318	779553
697	BPSS0652a		11	869058	869363									52611	52850	1503962	1503723	412108	412347				
698	BPSS0653_transposase (partial_IS407A or		11	869345	869563	883334	883552	943900	944118	864652	864870	1300275	1300057	52835	53053	1503738	1503520	412332	412550			779983	780201
699	BPSS0654		11											53470	55203	1503103	1501370	412967	414700			780618	782351
700	BPSS0655		11											55460	56518	1501113	1499452	414957	416618			782608	783042
701	BPSS0656		11													1499417	1499043	416653	417027				
702	BPSS0657_transposase (partial_IS407A or		11	869345	869563	883334	883552	943900	944118	864652	864870	1300275	1300057	52835	53053	1503738	1503520	412332	412550			779983	780201
703		Chr2_26_IS407A_A		869730	870965	883719	884954	944285	945520	865037	866272	1298656	1299890	56520	57755	1497556	1498791	418100	419335			783043	784278
704	BPSS0658	GI14																					
705	BPSS0659	GI14																					
706	BPSS0660	GI14																					
707	BPSS0661	GI14																					
708	BPSS0663	GI14																					
709	BPSS0664	GI14																					
710	BPSS0664a	GI14																					
711	BPSS0665	GI14																					
712		Chr2_27_IS407A_A		1225576	1226811	1246300	1247535	1310457	1311650	1219713	1220948	1298656	1299890	56520	57755	1497556	1498791	418100	419335			783043	784278
713	BPSS0666		12	1224944	1225576	1245668	1246300	1309825	1310457	1219081	1219713	1298024	1298656	58387	57755	1496924	1497556	419967	419335			784910	784278
714	BPSS0667_acetyltransferase (GNAT) famil		12																				
715	BPSS0668		12	1224200	1223985	1244924	1244709	1309081	1308866	1218337	1218122	1297280	1297065	59131	59346	1496180	1495965	420711	420926			785654	785869
716	BPSS0669		12	1222561	1223787	1243285	1244511	1307442	1308668	1216698	1217924	1295641	1296867	60770	59544	1494541	1495767	422350	421124			787293	786067
717	BPSS0670		12	1220747	1222141	1241471	1242865	1305628	1307022	1214884	1216278	1293827	1295221	62584	61190	1492727	1494121	424164	422770			789107	787713
718	BPSS0671		12	1220069	1218378	1240050	1238575	1304634	1302943	1214206	1212515	1293149	1291458	63262	64953	1492049	1490358	424842	426533			789785	791476
719	BPSS0672		12	1217974	1217165	1238171	1237362	1302539	1301730	1212111	1211302	1291054	1290245	65357	66166	1489954	1489145	426937	427746			791880	792689
720	BPSS0673		12	1217097	1216507	1237294	1236704	1301662	1301072	1211234	1210644	1290177	1289587	66234	66824	1489077	1488487	427814	428404			792757	793347
721	BPSS0674		12	1216482	1216183	1236679	1236380	1301047	1300748	1210619	1210320	1289562	1289263	66849	67148	1488462	1488163	428429	428728			793372	793671
722	BPSS0675		12	1215198	1216085	1235395	1236282	1299763	1300650	1209335	1210222	1288278	1289165	68133	67246	1487178	1488065	429713	428826			794656	793769
723	BPSS0676		12	1214674	1213853	1234871	1234050	1299239	1298418	1208811	1207990	1287754	1286933	68657	69478	1486654	1485833	430237	431058			795180	796001
724	BPSS0677		12	1213632	1211665	1233829	1231862	1298197	1296230	1207769	1205802	1286712	1284745	69699	71666	1485612	1483645	431279	433246			796222	798189
725	BPSS0678		12	1211356	1211607	1231553	1231804	1295921	1296172	1205493	1205744	1284436	1284687	71975	71724	1483336	1483587	433555	433304			798498	798247
726	BPSS0679		12	1208290	1210518	1228155	1230383	1292170	1292589	1202680	1204458	1281370	1283598	75041	72813	1480270	1482498	436621	434393			801564	799336

727	BPSS0680		12	1207826	1208284	1227691	1228149	1291706	1292164	1202216	1202674	1280906	1281364	75505	75047	1479806	1480264	437085	436627			802028	801570
728	BPSS0681		12	1207625	1206645	1227490	1226597	1291505	1290525	1202015	1201035	1280705	1279725	75706	76686	1479605	1478625	437286	438266			802229	803209
729	BPSS0682		12	1206421	1206026	1226318	1225923	1290301	1289906	1200811	1200416	1279501	1279106	76910	77305	1478401	1478006	438490	438885			803433	803828
730	BPSS0683		12	1205235	1205588	1225132	1225485	1289115	1289468	1199625	1199978	1278315	1278668	78096	77743	1477215	1477568	439676	439323			804619	804266
731	BPSS0684		12	1204830	1203595	1224727	1223492	1288710	1287475	1199220	1197985	1277910	1276675	78501	79736	1476810	1475575	440081	441316			805024	806259
732	BPSS0685		12	1203587	1202376	1223484	1222273	1287467	1286256	1197977	1196766	1276667	1275456	79744	80955	1475567	1474356	441324	442535			806267	807478
733	BPSS0686		12	1202285	1200885	1222182	1220782	1286165	1284765	1196675	1195275	1275365	1273965	81046	82446	1474265	1472865	442626	444026			807569	808969
734	BPSS0686a		12	1200885	1200799	1220767	1220696	1284765	1284679	1195275	1195189	1273965	1273879	82446	82532	1472865	1472779	444026	444112			808969	809055
735	BPSS0687		12	1199278	1200603	1218706	1219728	1283158	1284483	1193668	1194993	1272358	1273683	84053	82728	1471258	1472583	445633	444308			810576	809251
736	BPSS0688		12	1198555	1199274	1217983	1218702	1282435	1283154	1192945	1193664	1271635	1272354	84776	84057	1470535	1471254	446356	445637			811299	810580
737	BPSS0689		12	1198347	1197571	1217775	1216999	1282227	1281451	1192737	1191961	1271427	1270651	84984	85760	1470327	1469551	446564	447340			811507	812283
738	BPSS0690		12	1197544	1196360	1216972	1215788	1281424	1280240	1191934	1190750	1270624	1269440	85787	86971	1469524	1468340	447367	448551			812310	813494
739	BPSS0691		12	1195876	1196310	1215304	1215738	1279756	1280190	1190266	1190700	1268956	1269390	87455	87021	1467856	1468290	449035	448601			813978	813544
740	BPSS0692		12	1195710	1195024	1215138	1214452	1279590	1278904	1190100	1189414	1268790	1268104	87621	88307	1467690	1467004	449201	449887			814144	814830
741	BPSS0693		12	1195024	1194263	1214452	1213691	1278904	1278143	1189414	1188653	1268104	1267343	88307	89068	1467004	1466243	449887	450648			814830	815591
742	BPSS0694		12	1194236	1192776	1213664	1212204	1278116	1276656	1188626	1187166	1267316	1265856	89095	90555	1466216	1464756	450675	452135			815618	817078
743	BPSS0695		12	1192703	1191858	1212131	1211286	1276583	1275738	1187093	1186248	1265783	1264938	90628	91473	1464683	1463838	452208	453053			817151	817996
744	BPSS0696		12	1191797	1191408	1211225	1210836	1275677	1275288	1186187	1185798	1264877	1264488	91534	91923	1463777	1463388	453114	453503			818057	818446
745	BPSS0697		12	1191382	1190582	1210810	1210010	1275262	1274462	1185744	1185061	1264462	1263662	91949	92749	1463362	1462562	453529	454329			818472	819272
746	BPSS0698		12	1190205	1189825	1209997	1209611	1274449	1274063	1185048	1184662	1263649	1263263	92762	93148	1462549	1462163	454342	454728			819285	819671
747	BPSS0699		12	1188985	1189824	1208413	1209252	1272865	1273704	1183464	1184303	1262065	1262904	94346	93507	1460965	1461804	455926	455087			820869	820030
748	BPSS0700		12	1188708	1187746	1208136	1207174	1272588	1271626	1183187	1182225	1261788	1260826	94623	95585	1460688	1459726	456203	457165			821146	822108
749	BPSS0701		12	1186559	1187647	1205987	1207075	1270439	1271527	1181038	1182126	1259639	1260727	96772	95684	1458539	1459627	458352	457264			823295	822207
750	BPSS0702		12	1185014	1186078	1204442	1205506	1268894	1269958	1179493	1180557	1258094	1259158	98324	97260	1456980	1458044	459918	458854			824833	823769
751	BPSS0703		12	1184737	1185018	1202682	1204196	1267134	1268648	1177733	1179247	1256334	1257848	100084	98570	1455220	1456734	461678	460164			826593	825079
752	BPSS0704		12	1182065	1183150	1201494	1202579	1265946	1267031	1176545	1177630	1255146	1256231	101272	100187	1454032	1455117	462866	461781			827781	826696
753	BPSS0705		12	1181936	1181268	1201365	1200697	1265817	1265149	1176416	1175748	1255017	1254349	101401	102069	1453903	1453235	462995	463663			827910	828578
754	BPSS0706		12	1181268	1179766	1200697	1199195	1265149	1263647	1175748	1174246	1254349	1252847	102069	103571	1453235	1451733	463663	465165			828578	830080
755	BPSS0707		12	1178265	1179131	1197694	1198560	1262146	1263012	1172745	1173611	1251346	1252212	104988	104206	1450316	1451098	466582	465800			831581	830715
756		Chr2_28_IS407A_B	12											104943	106178	1449126	1450361	466537	467772				
757		Chr2_29_ISBma1_B	12	1175066	1176372	1194495	1195801	1258947	1260253	1169546	1170852												
758	BPSS0708		12	1175037	1173838	1194466	1193267	1258918	1257719	1169517	1168318	1249424	1248225									833503	834702
759	BPSS0709_I	Chr2_30_ISBma2_A		1172245	1173705	1191674	1193134	1256126	1257586	1166725	1168185	1246632	1248092									836295	834835
760	BPSS0710		13									1245161	1244379									837778	838560
761	BPSS0711		13									1242929	1244050									839985	838864
762	BPSS0712		13									1242400	1242023									840514	840891
763	BPSS0713		13									1241855	1240623									841059	842291
764	BPSS0714		13									1240615	1239644									842299	843270
765	BPSS0715		13									1238889	1239419									844025	843495
766	BPSS0716_hypothetical protein		13									1237808	1238641									845115	844282
767		Chr2_31a_IS407A_A										1236549	1237784									845139	846374
768		Chr2_31b_IS407A_A		1517607	1518842	1544910	1546145	1506899	1508134	1511310	1512545	1198234	1199469	104943	106178	1449126	1450361	466537	467772	535045	536280	726908	728143
769	BPSS0717		14a	1516341	1517606	1543644	1544909	1505633	1506898	1510044	1511309	1196968	1198233	107444	106179	1447860	1449125	469038	467773	533779	535044	729409	728144
770	BPSS0718		14a	1515355	1514714	1542658	1542458	1504647	1504006	1509058	1508417	1195954	1195313	108421	109062	1446874	1446233	470068	470709	532766	532125	730431	731072
771	BPSS0719		14a	1514166	1513489	1540945	1540268	1503458	1502781	1507869	1507192	1194765	1194088	109610	110287	1445685	1445008	471257	471934	531577	530900	731620	732297
772	BPSS0720		14a	1513197	1512103	1539976	1538882	1502489	1501395	1506900	1505806	1193796	1192702	110579	111673	1444716	1443622	472226	473320	530608	529514	732589	733683
773	BPSS0721		14a	1511128	1510370	1537907	1537149	1500420	1499662	1504831	1504073	1191727	1190969	112648	113406	1442647	1441889	474295	475053	528539	527781	734658	735416
774	BPSS0721a		14a	1510124	1509636	1536903	1536415	1499416	1498928	1503827	1503339	1190743	1190255	113643	114131	1441652	1441164	475290	475778	527511	527023	735664	736152
775	BPSS0723		14a	1509486	1508419	1536265	1535198	1498778	1497711	1503189	1502122	1190105	1189038	114281	115348	1441014	1439947	475928	476995	526873	525806	736302	737369
776	BPSS0724		14a	1508290	1507898	1535069	1534677	1497582	1497190	1501993	1501601	1188909	1188517	115477	115869	1439818	1439426	477124	477516	525677	525285	737498	737890
777	BPSS0725		14a	1506823	1507614	1533602	1534393	1496115	1496906	1500526	1501317	1187424	1188215	116953	116162	1438342	1439133	478600	477809	524201	524992	738965	738174

778	BPSS0726		14a	1504972	1506477	1531751	1533256	1494264	1495769	1498675	1500180	1185573	1187078	118804	117299	1436491	1437996	480451	478946	522359	523864	740807	739302
779	BPSS0727		14a	1504868	1504152	1531647	1530931	1494160	1493444	1498571	1497855	1185469	1184753	118908	119624	1436387	1435671	480555	481271	522255	521539	740911	741627
780	BPSS0728		14a	1502975	1503664	1529754	1530443	1492267	1492956	1496678	1497367	1183576	1184265	120793	120104	1434494	1435183	482448	481759	520362	521051	742812	742123
781	BPSS0730		14a	1502156	1502857	1528935	1529636	1491448	1492149	1495859	1496560	1182757	1183458	121612	120911	1433675	1434376	483267	482566	519543	520244	743631	742930
782	BPSS0731		14a	1501854	1500196	1528633	1526975	1491146	1489488	1495557	1493899	1182455	1180797	121914	123572	1433373	1431715	483569	485227	519241	517583	743942	745600
783	BPSS0732_hypothetical protein		14a																				
784	BPSS0733		14a	1499450	1499872	1524547	1526301	1487060	1488814	1491471	1493225	1178369	1180123	125998	123896	1429287	1431041	487655	485901	515155	516909	747962	746274
785		Chr2_32a_IS407A_C																				749164	750399
786		Chr2_32b_IS407A_C																				878187	878956
787	BPSS0734		14b	1495567	1497423	1522346	1524202	1484859	1486715	1489270	1491126	1176168	1178024	128199	126343	1427086	1428942	489856	488000	512954	514810	880932	879076
788	BPSS0735		14b	1493542	1495506	1520321	1522285	1482834	1484798	1487245	1489209	1174142	1176007	130224	128260	1425061	1427025	491881	489917	510929	512893	882957	880993
789	BPSS0736		14b	1492214	1493536	1518993	1520315	1481506	1482828	1485917	1487239	1172814	1174136	131552	130230	1423733	1425055	493209	491887	509601	510923	884285	882963
790	BPSS0737		14b	1490660	1490752	1517439	1517531	1479952	1480044	1484363	1484455	1171260	1171352	133106	133014	1422179	1422271	494763	494671	508047	508139	885839	885747
791	BPSS0738		14b	1489622	1489296	1516401	1516075	1478914	1478588	1483325	1482999	1170222	1169896	134144	134470	1421141	1420815	495801	496127	507009	506683	886877	887203
792	BPSS0739		14b	1487914	1488405	1514693	1515184	1476329	1476820	1481617	1482108	1168505	1168996	135870	135379	1419415	1419906	497554	497063	505283	505774	888603	888112
793	BPSS0740		14b	1486978	1487760	1513757	1514539	1475393	1476175	1480681	1481463	1167569	1168351	136806	136024	1418479	1419261	498490	497708	504347	505129	889539	888757
794	BPSS0741		14b	1485074	1486021	1511853	1512800	1473489	1474436	1478777	1479724	1165686	1166633	138703	137756	1416589	1417536	500394	499447	502457	503404	891428	890481
795	BPSS0742		14b	1484970	1483618	1511749	1510397	1473385	1472033	1478673	1477321	1165582	1164230	138807	140159	1416485	1415133	500498	501850	502353	501001	891532	892884
796	BPSS0743		14b	1483404	1481890	1510183	1508669	1471819	1470305	1477107	1475593	1164016	1162502	140373	141887	1414919	1413405	502064	503578	500787	499273	893098	894612
797		Chr2_33_IS407A_A		1480359	1481594	1507138	1508373	1468774	1470009	1474062	1475297	1160971	1162206	142183	143418	1411874	1413109	503874	505109	497742	498977	894908	896143
798	BPSS0744																						
799	BPSS0745																						
800		Chr2_34_IS407A_A		592085	593320	603189	604424	631977	633212	590631	591866	547403	548638	924390	925625	1782102	1783337	134525	135760				
801	BPSS0746		15	594435	593305	605539	604409	634327	633197	592981	591851	549753	548623	923275	924405	1780987	1782117	136875	135745				
802	BPSS0747		15	594954	596048	606058	607152	634846	635940	593500	594594	550272	551366	922756	921662	1780468	1779374	137394	138488				
803	BPSS0748		15	597041	596673	608145	607777	636933	636565	595587	595219	552359	551991	920669	921037	1778381	1778749	139481	139113				
804	BPSS0749		15	598008	597211	609112	608315	637900	637103	596554	595757	553326	552529	919702	920499	1777414	1778211	140448	139651				
805	BPSS0750		15	598130	599536	609234	610640	638022	639428	596676	598082	553448	554854	919580	918174	1777292	1775886	140570	141976				
806	BPSS0751		15	599581	600708	610685	611812	639473	640600	598127	599254	554899	556026	918129	917002	1775841	1774714	142021	143148				
807	BPSS0752		15	602348	600705	613452	611809	642240	640597	600894	599251	557666	556023	915362	917005	1773074	1774717	144788	143145				
808	BPSS0753		15	602843	602526	613947	613630	642735	642418	601389	601072	558161	557844	914867	915184	1772579	1772896	145283	144966				
809	BPSS0754		15	605105	602871	616209	613975	644997	642763	603651	601417	560423	558189	912605	914839	1770317	1772551	147545	145311				
810	BPSS0755		15	605316	606323	616420	617427	645208	646215	603862	604869	560634	561641	912394	911387	1770106	1769099	147756	148763				
811	BPSS0756		15	607131	606832	618235	617936	647023	646724	605677	605378	562449	562150	910579	910878	1768291	1768590	149571	149272				
812	BPSS0757		15	607450	608601	618554	619705	647342	648493	605996	607147	562768	563919	910260	909109	1767972	1766821	149890	151041				
813	BPSS0758		15	608718	609179	619822	620283	648610	649071	607264	607725	564036	564497	908992	908531	1766704	1766243	151158	151619				
814	BPSS0759		15	609732	610991	620836	622095	649624	650883	608278	609537	565050	566309	907978	906719	1765690	1764431	152172	153431				
815	BPSS0760		15	610999	612228	622103	623332	650891	652120	609545	610774	566317	567546	906711	905482	1764423	1763194	153439	154668				
816	BPSS0761		15	612231	613484	623335	624588	652123	653376	610777	612030	567549	568802	905479	904226	1763191	1761938	154671	155924				
817	BPSS0762		15	614891	613557	625995	624661	654783	653449	613437	612103	570209	568875	902805	904139	1760524	1761858	157338	156004				
818	BPSS0763		15	616019	615090	627123	626194	655911	654982	614565	613636	571337	570408	901677	902606	1759396	1760325	158466	157537				
819	BPSS0764		15	616858	616064	627962	627168	656750	655956	615404	614610	572176	571382	900838	901632	1758557	1759351	159305	158511				
820	BPSS0765		15	617689	616976	628793	628080	657581	656868	616235	615522	573007	572294	900007	900720	1757726	1758439	160136	159423				
821	BPSS0766		15	618036	619379	629140	630483	657928	659271	616582	617925	573354	574697	899660	898317	1757379	1756036	160483	161826				
822	BPSS0767		15	620029	619709	631133	630813	659921	659601	618575	618255	575347	575027	897667	897987	1755386	1755706	162476	162156				
823	BPSS0768		15	624166	624128	635270	635232	664058	664020	620698	620501	577470	577273	893488	893526	1751228	1751266	164599	164402				
824	BPSS0769		15	625168	624191	636272	635295	665060	664083	623721	622744	580493	579516	892486	893463	1750226	1751203	167601	166624				
825	BPSS0770		15	626342	625707	637446	636811	666234	665599	624895	624260	581667	581032	891312	891947	1749052	1749687	168775	168140				
826	BPSS0771		15	627517	627735	638621	638839	667409	667627	626070	626288	582842	583060	890137	889919	1747877	1747659	169950	170168				
827	BPSS0772		15	628340	628819	639444	639923	668232	668711	626893	627372	583665	584144	889314	888835	1747054	1746575	170773	171252				
828	BPSS0773		15	629647	629123	640751	640227	669539	669015	628200	627676	584972	584448	888007	888531	1745747	1746271	172080	171556				

829	BPSS0774		15	629655	630395	640759	641499	669547	670287	628208	628948	584980	585720	887999	887259	1745739	1744999	172088	172828				
830	BPSS0776		15	630663	631595	641767	642699	670555	671487	629216	630148	585988	586920	886991	886059	1744731	1743799	173096	174028				
831	BPSS0777		15	632286	632699	643390	643803	672178	672591	630845	631258	587584	587997	885356	884943	1743131	1742718	174678	175091				
832	BPSS0778		15	633228	632791	644332	643895	673120	672683	631787	631350	588526	588089	884414	884851	1742189	1742626	175620	175183				
833	BPSS0779		15	635023	635847	646127	646951	674915	675739	633582	634406	590321	591145	882619	881795	1740394	1739570	177415	178239				
834	BPSS0780		15	636017	637462	647121	647528	675909	677354	634576	636021	591315	592760	881625	880180	1739400	1737955	178409	179854				
835	BPSS0781		15	638073	638459	648787	649392	677375	677980	636042	636647	592781	593386	880159	879554	1737934	1737329	179875	180480				
836	BPSS0782		15	639559	640761	650863	652065	679451	680653	638118	639320	594857	596059	878083	876881	1735858	1734656	181951	183153				
837	BPSS0783		15	641298	642434	652602	653738	681190	682326	639857	640993	596684	597820	876176	875040	1733839	1732703	183674	184810				
838	BPSS0784		15	642510	644231	653814	655535	682402	684123	641069	642790	597896	599617	874964	873243	1732627	1730906	184886	186607				
839	BPSS0785		15	645323	644502	656627	655806	685215	684394	643882	643061	600709	599888	872151	872972	1729814	1730635	187699	186878				
840	BPSS0786		15	645512	646522	656816	657826	685404	686414	644071	645081	600898	601908	871962	870952	1729625	1728615	187888	188898				
841	BPSS0787		15	646522	648075	657826	659379	686414	687967	645081	646634	601908	603461	870952	869399	1728615	1727062	188898	190451				
842	BPSS0788		15	648068	649090	659372	660394	687960	688982	646627	647649	603454	604476	869406	868384	1727069	1726047	190444	191268				
843	BPSS0789		15	649090	649407	660394	660711	688982	689299	647649	647966	604476	604793	868384	868067	1726047	1725730	191468	191785				
844	BPSS0790		15	649445	650764	660749	662068	689337	690656	648004	649323	604831	606150	868029	866710	1725692	1724373	191823	193142				
845	BPSS0791		15	651039	651875	662591	663388	690931	691767	649598	650434	606425	607261	866435	865599	1724098	1723262	193417	194253				
846	BPSS0792		15	652018	652758	663531	664271	691910	692650	650577	651317	607404	608144	865456	864716	1723119	1722379	194396	195136				
847	BPSS0793		15	652837	653676	664350	665189	692729	693568	651396	652235	608223	609062	864637	863798	1722300	1721461	195215	196054				
848	BPSS0794		15	653831	654604	665344	666117	693723	694496	652390	653163	609217	609990	863643	862870	1721306	1720533	196209	196982				
849	BPSS0795		15	654643	654978	666156	666491	694535	694870	653202	653537	610029	610364	862831	862496	1720494	1720159	197021	197356				
850		Chr2_35_IS407A_A		656707	657942	668220	669455	696599	697834	655266	656501	612093	613328	859532	860767	1717195	1718430	199085	200320			545708	546943
851	BPSS0796_putative surface-exposed prot		16																				
852	BPSS0797		16	665118	664381	676631	675894	705010	704273	663677	662940	620504	619767	851840	852577	1709503	1710240	208012	207275			554119	553382
853	BPSS0798		16	665697	665236	677210	676749	705589	705128	664256	663795	621083	620622	851261	851722	1708924	1709385	208591	208130			554698	554237
854	BPSS0799		16	666933	665716	678446	677229	706825	705608	665492	664275	622319	621102	850025	851242	1707688	1708905	209827	208610			555934	554717
855	BPSS0800		16	668424	667813	679937	679326	708316	707705	666983	666372	623810	623199	848534	849145	1706197	1706808	211318	210707			557425	556814
856	BPSS0801		16	668539	668973	680052	680486	708431	708865	667098	667532	623925	624359	848419	847985	1706082	1705648	211433	211867			557540	557974
857	BPSS0802		16	669125	670264	680638	681777	709017	710156	667684	668823	624511	625650	847833	846694	1705496	1704357	212019	213158			558126	559265
858	BPSS0803		16	670729	671361	682242	682874	710621	711253	669288	669920	626115	626747	846229	845597	1703892	1703260	213623	214255			559734	560366
859	BPSS0804		16	671799	671425	683312	682938	711691	711317	670358	669984	627185	626811	845159	845533	1702822	1703196	214693	214319			560804	560430
860	BPSS0805		16	674501	672438	686014	683951	714393	712330	673060	670997	629887	627824	842457	844520	1700120	1702183	217395	215332			563506	561443
861	BPSS0806		16	675295	674831	686808	686344	715187	714723	673854	673390	630681	630217	841663	842127	1699326	1699790	218189	217725			564300	563836
862	BPSS0807		16	675628	677010	687141	688523	715520	716902	674187	675569	631014	632396	841330	839948	1698993	1697611	218522	219904			564633	566015
863	BPSS0808		16	677045	678244	688558	689757	716937	718136	675604	676803	632431	633630	839913	838714	1697576	1696377	219939	221138			566050	567249
864	BPSS0809		16	678579	678965	690092	690478	718471	718857	677138	677524	633965	634351	838379	837993	1696042	1695656	221473	221859			567601	567987
865	BPSS0810		16	680112	680723	691625	692236	720004	720615	678671	679282	635498	636109	836846	836235	1694509	1693898	223006	223617			569134	569745
866	BPSS0811		16	681625	680894	693138	692407	721517	720786	680184	679453	637011	636280	835333	836064	1692996	1693727	224519	223788			570647	569916
867	BPSS0812		16	683085	682966	694598	694479	722977	722858	681644	681525	638471	638352	833873	833992	1691536	1691655	225979	225860			572107	571988
868	BPSS0813		16	684661	686073	696174	697586	724553	725965	683220	684632	640047	641459	832273	830861	1689941	1688529	227573	228985			573695	575107
869	BPSS0814		16	686087	686452	697600	697965	725979	726344	684646	685011	641473	641838	830847	830482	1688515	1688150	228999	229364			575121	575486
870	BPSS0815		16	686439	687056	697952	698569	726331	726948	684998	685615	641825	642442	830495	829878	1688163	1687546	229351	229968			575473	576090
871	BPSS0816		16	689640	687097	701714	701652	729532	726989	688199	685656	645026	642483	827296	829449	1684964	1687117	232547	230397			578656	576131
872	BPSS0817		16	696097	689933	708171	702007	735989	729825	694656	688492	651525	645367	820827	826991	1678489	1684659	239031	232867			585113	578949
873	BPSS0818		16	696650	697108	708724	709182	736542	737000	695209	695667	652094	652552	820282	819824	1677944	1677486	239576	240034			585682	586140
874	BPSS0819		16	698271	698738	710345	710812	738163	738630	696830	697297	653724	654191	818661	818194	1676323	1675856	241197	241664			587312	587779
875	BPSS0820		16	700242	700937	710812	711879	738630	739787	697297	698817	654191	655711	818194	816674	1675856	1674336	241664	243184			587779	589170
876	BPSS0821		16	700937	701854	713540	714457	740830	741747	699496	700413	656390	657307	815995	815078	1673657	1672740	243863	244780			589945	590862
877	BPSS0822		16	701860	702759	714463	715362	741753	742652	700419	701318	657313	658212	815072	814173	1672734	1671835	244786	245685			590868	591767
878	BPSS0823		16	702759	704369	715362	716972	742652	744262	701318	702928	658212	659822	814173	812563	1671835	1670225	245685	247295			591767	593377
879	BPSS0824		16	704365	705255	716968	717858	744258	745148	702924	703814	659818	660708	812567	811677	1670229	1669339	247291	248181			593373	594263

880	BPSS0825		16	705929	705225	718532	717828	745822	745118	704488	703784	661382	660678	811003	811707	1668665	1669369	248855	248151			594937	594233
881	BPSS0826		16	705941	707095	718544	719698	745834	746988	704500	705654	661394	662548	810991	809837	1668653	1667499	248867	250021			594949	596103
882	BPSS0827		16	708108	710042	720711	722645	748001	749935	706667	708601	663561	665495	808838	806904	1666500	1664566	251020	252954			597102	599036
883	BPSS0828		16	710672	710337	723008	722940	750565	750230	709231	708896	666125	665790	806274	806609	1663936	1664271	253584	253249			599666	599331
884	BPSS0829		16	712484	711321	725310	724147	752377	751214	711043	709880	667937	666774	804462	805625	1662124	1663287	255396	254233			601478	600315
885	BPSS0830		16	713825	713085	726651	725911	753718	752978	712384	711644	669278	668538	803121	803861	1660783	1661523	256737	255997			602819	602079
886	BPSS0831		16	714197	714382	727023	727208	754090	754275	712756	712941	669650	669835	802749	802564	1660411	1660226	257109	257294			603162	603347
887	BPSS0832		16	715131	714646	727957	727472	755024	754539	713690	713205	670584	670099	801815	802300	1659477	1659962	258043	257558			604096	603611
888	BPSS0833		16	715321	715608	728147	728434	755214	755501	713880	714167	670774	671061	801625	801338	1659287	1659000	258233	258520			604286	604573
889	BPSS0834		16	715673	715906	728499	728732	755566	755799	714232	714465	671126	671359	801273	801040	1658935	1658702	258585	258818			604638	604871
890	BPSS0835		16	716635	715991	729461	728817	756528	755884	715194	714550	672088	671444	800311	800955	1657973	1658617	259547	258903			605600	604956
891	BPSS0836		16	717197	717652	730023	730478	757090	757545	715756	716211	672650	673105	799749	799294	1657411	1656956	260109	260564			606162	606617
892		Chr2_36_ISBma2_B	16											797475	799047	1655136	1656709	260811	262383				
893	BPSS0837		16	718861	718025	731687	730851	758754	757918	717420	716584	674314	673478	796487	797095	1654148	1654756	263370	262534			607826	606990
894	BPSS0838		16	719352	718861	732178	731687	759245	758754	717911	717420	674805	674314	795996	796487	1653657	1654148	263861	263370			608317	607826
895	BPSS0839		16	720373	719537	733199	732363	760266	759430	718932	718096	675826	674990	794975	795811	1652636	1653472	264882	264046			609338	608502
896	BPSS0840		16	720623	721660	733449	734486	760516	761553	719182	720219	676076	677113	794725	793688	1652386	1651349	265132	266169			609588	610625
897	BPSS0841		16	722144	723187	734970	736013	762037	763080	720703	721746	677583	678626	793218	792175	1650879	1649836	266639	267682			611095	612138
898	BPSS0842		16	723379	724584	736205	737410	763272	764477	721938	723143	678818	680023	791983	790778	1649644	1648439	267874	269079			612330	613535
899	BPSS0843		16	725007	725765	737833	738591	764900	765658	723566	724324	680446	681204	790355	789597	1648016	1647258	269502	270260			613958	614716
900	BPSS0844		16	725784	726650	738610	739476	765677	766543	724343	725209	681223	682089	789578	788712	1647239	1646373	270279	271145			614735	615601
901	BPSS0845		16	727563	728945	740389	741771	767456	768838	726122	727504	683002	684384	787793	786411	1645460	1644078	272058	273440			616514	617896
902	BPSS0845a		16	729744	729505	742570	742331	769637	769398	728303	728064	685183	684944	785612	785851	1643279	1643518	274239	274000			618695	618456
903	BPSS0846		16	729889	730182	742715	743008	769782	770075	728448	728741	685328	685621	785467	785174	1643134	1642841	274384	274677			618840	619133
904	BPSS0847		16	731895	730582	744721	743408	771788	770475	730454	729141	687334	686021	783461	784774	1641128	1642441	276391	275465			620846	619533
905	BPSS0848		16	732814	732320	745640	745146	772707	772213	731373	730879	688253	687759	782542	783036	1640209	1640703	277310	276816			621765	621271
906	BPSS0849		16	733852	732935	746678	745761	773745	772828	732411	731494	689291	688374	781504	782421	1639171	1640088	278348	277431			622803	621886
907		Chr2_37_IS407A_A		733880	735115	746706	747941	773773	775008	732439	733674	689319	690554	780241	781476	1637908	1639143	278376	279611			622831	624066
908	BPSS0850																						
909	BPSS0851																						
910	BPSS0852																						
911	BPSS0853																						
912	BPSS0854																						
913	BPSS0855																						
914		Chr2_38_IS407A_A		1480359	1481594	1507138	1508373	1468774	1470009	1474062	1475297	1160971	1162206	675587	676822	878410	879645	1036615	1037850	379739	380974	1013932	1015167
915	BPSS0856		17	1480357	1478750	1507136	1505529	1468772	1467165	1474060	1472453	1160969	1159362	675585	673978	879647	881254	1036613	1035006	379737	378130	1015169	1016776
916	BPSS0857		17	1478712	1478089	1505491	1504868	1467127	1466504	1472415	1471792	1159324	1158044	673940	672660	881292	882572	1034968	1033688	378092	376812	1016814	1018094
917	BPSS0858_N-carbamoyl-L-amino acid hy		17																				
918	BPSS0860		17	1476659	1475139	1503438	1501918	1465074	1463554	1470362	1468842	1157266	1155746	671882	670362	883350	884870	1032910	1031390	376034	374514	1018872	1020392
919	BPSS0861		17	1473840	1474736	1500619	1501515	1462255	1463151	1467543	1468439	1154447	1155343	669063	669959	886169	885273	1030091	1030987	373215	374111	1021691	1020795
920	BPSS0862		17	1473750	1473055	1500529	1499834	1462165	1461470	1467453	1466758	1154357	1153662	668973	668278	886259	886954	1030001	1029306	373125	372430	1021781	1022476
921	BPSS0863		17	1473076	1472756	1499855	1499535	1461491	1461171	1466779	1466459	1153683	1153363	668299	667979	886933	887253	1029327	1029007	372451	372131	1022455	1022742
922		Chr2_39_IS407A_C	17																			1023389	1024624
923	BPSS0864		17	1472422	1471478	1499201	1498257	1460837	1459893	1466125	1465181	1153029	1152085	667645	666701	887587	888531	1028673	1027729	371797	370853	1390218	1389274
924	BPSS0865		17	1470993	1470130	1497773	1496910	1459408	1458545	1464696	1463833	1151591	1150728	666189	665326	888998	889861	1027226	1026363	370269	369406	1388834	1387971
925	BPSS0866		17	1470052	1469300	1496832	1496080	1458467	1457715	1463755	1463003	1150650	1149898	665248	664496	889939	890691	1026285	1025533	369328	368576	1387893	1387141
926	BPSS0867		17	1468854	1468150	1495634	1494930	1457269	1456565	1462557	1461853	1149452	1148748	664050	663346	891116	891820	1025108	1024404	368123	367419	1386688	1385984
927	BPSS0868		17	1467890	1466391	1494670	1493171	1456305	1454806	1461593	1460094	1148488	1146989	663086	661587	892080	893579	1024144	1022645	367159	365660	1385724	1385248
928	BPSS0869		17	1466350	1465004	1493130	1491784	1454765	1453419	1460053	1458707	1146948	1145602	661546	660200	893620	894966	1022604	1021258	365619	364273	1384481	1383135
929	BPSS0870		17	1464792	1463305	1491572	1490085	1453207	1451720	1458495	1457008	1145390	1143954	659988	658501	895178	896665	1021046	1019559	364061	362574	1382923	1381436
930	BPSS0871		17	1463305	1462688	1490085	1489468	1451720	1451103	1457008	1456391	1143851	1143408	658501	657884	896665	897282	1019559	1018942	362574	361957	1381436	1380819

931	BPSS0872		17	1462688	1461672	1489468	1488452	1451103	1450087	1456391	1455375	1143408	1142392	657884	656868	897282	898298	1018942	1017926	361957	360941	1380819	1379803
932	BPSS0873		17	1461439	1460240	1488219	1487020	1449854	1448655	1455142	1453943	1142159	1140960	656635	655436	898531	899730	1017693	1016494	360708	359509	1379570	1378371
933	BPSS0874		17	1459973	1459773	1486753	1486553	1448388	1448188	1453676	1453476	1140693	1140493	655169	654969	899997	900197	1016227	1016027	359242	359042	1378104	1377904
934	BPSS0875		17	1458946	1459554	1485726	1486334	1447361	1447969	1452649	1453257	1139666	1140274	654142	654750	901024	900416	1015200	1015808	358215	358823	1377077	1377685
935	BPSS0876		17	1458774	1458298	1485554	1485078	1447189	1446713	1452477	1452001	1139494	1139018	653970	653494	901196	901672	1015028	1014552	358043	357567	1376905	1376429
936	BPSS0877		17	1457093	1458151	1483873	1484931	1445508	1446566	1450796	1451854	1137813	1138871	652289	653347	902877	901819	1013347	1014405	356362	357420	1375224	1376282
937	BPSS0878		17	1456738	1457040	1483518	1483820	1445153	1445455	1450441	1450743	1137458	1137760	651934	652236	903232	902930	1012992	1013294	356007	356309	1374869	1375171
938	BPSS0879		17	1455408	1454281	1482188	1481061	1443823	1443551	1449111	1447984	1136128	1135001	650604	649477	904562	905689	1011662	1010535	354677	353550	1373539	1372412
939	BPSS0880		17	1453650	1454057	1480430	1480837	1442066	1442473	1447353	1447760	1134370	1134777	648846	649253	906320	905913	1009904	1010311	352919	353326	1371781	1372188
940	BPSS0881		17	1453142	1452804	1479922	1479584	1441558	1441220	1446845	1446507	1133862	1133524	648338	648000	906828	907166	1009396	1009058	352411	352073	1371273	1370935
941	BPSS0882		17	1452132	1452593	1478912	1479373	1440548	1441009	1445835	1446296	1132852	1133313	647328	647789	907838	907377	1008386	1008847	351401	351862	1370263	1370724
942	BPSS0883		17	1451955	1451305	1478735	1478085	1440371	1439721	1445658	1445008	1132675	1132025	647151	646501	908015	908665	1008209	1007559	351224	350574	1370086	1369436
943	BPSS0884		17	1450491	1451336	1477271	1478116	1438907	1439752	1444194	1445039	1131211	1132056	645687	646532	909479	908634	1006745	1007590	349760	350605	1368622	1369467
944	BPSS0885		17	1449818	1450426	1476598	1477206	1438234	1438842	1443521	1444129	1130538	1131146	645014	645622	910152	909544	1006072	1006680	349087	349695	1367949	1368557
945	BPSS0886		17	1449150	1449590	1475930	1476370	1437566	1438006	1442853	1443293	1129870	1130310	644346	644786	910820	910380	1005404	1005844	348419	348859	1367281	1367721
946	BPSS0887		17	1449076	1448360	1475856	1475140	1437492	1436776	1442779	1442063	1129796	1129080	644272	643556	910894	911610	1005330	1004614	348345	347629	1367207	1366491
947	BPSS0888		17	1447605	1448318	1474385	1475098	1436021	1436734	1441308	1442021	1128325	1129038	642801	643514	912365	911652	1003859	1004572	346874	347587	1365736	1366449
948	BPSS0889		17	1445706	1446908	1472486	1473688	1434122	1435324	1439409	1440611	1126118	1127320	640916	642118	914313	913111	1001897	1003099	344856	346058	1363648	1364850
949	BPSS0890		17	1445060	1445641	1471840	1472421	1433476	1434057	1438763	1439344	1125472	1126053	640270	640851	914959	914378	1001251	1001832	344210	344791	1363002	1363583
950	BPSS0891		17	1444927	1444304	1471707	1471084	1433343	1432720	1438630	1438007	1125339	1124716	640137	639514	915092	915715	1001118	1000495	344077	343454	1362869	1362246
951	BPSS0892		17	1444214	1443267	1470994	1470047	1432630	1431683	1437917	1436970	1124626	1123679	639424	638477	915805	916752	1000405	999458	343364	342417	1362156	1361209
952	BPSS0893		17	1441868	1442953	1468648	1469733	1430284	1431369	1435571	1436656	1122280	1123365	637078	638163	918151	917066	998059	999144	341018	342103	1359810	1360895
953	BPSS0894		17	1441433	1441119	1468213	1467899	1429849	1429535	1435136	1434822	1121845	1121531	636643	636329	918586	918900	997624	997310	340583	340269	1359375	1359061
954	BPSS0895		17	1441114	1440806	1467894	1467586	1429530	1429222	1434817	1434509	1121526	1121218	636324	636016	918905	919213	997305	996997	340264	339956	1359056	1358748
955	BPSS0896		17	1440768	1439704	1467548	1466484	1429184	1428120	1434471	1433407	1121180	1120116	635978	634914	919251	920315	996959	995895	339918	338854	1358710	1357646
956	BPSS0897		17	1439692	1438937	1466472	1465717	1428108	1427353	1433395	1432640	1120104	1119349	634902	634147	920327	921082	995883	995128	338842	338087	1357634	1356879
957	BPSS0898		17	1438910	1438371	1465690	1465151	1427326	1426787	1432613	1432074	1119322	1118783	634120	633581	921109	921648	995101	994562	338060	337521	1356852	1356313
958	BPSS0899		17	1438351	1437449	1465131	1464229	1426767	1425865	1432054	1431152	1118763	1117861	633561	632659	921668	922570	994542	993640	337501	336599	1356293	1355391
959	BPSS0900		17	1437449	1436598	1464229	1463378	1425865	1425014	1431152	1430301	1117861	1117010	632659	631808	922570	923421	993640	992789	336599	335748	1355391	1354540
960	BPSS0901		17	1436598	1435804	1463378	1462584	1425014	1424220	1430301	1429507	1117010	1116216	631808	631014	923421	924215	992789	991995	335748	334954	1354540	1353746
961	BPSS0902		17	1435808	1434126	1462588	1460906	1424224	1422542	1429511	1427829	1116220	1114526	631018	629348	924211	925881	991999	990329	334958	333276	1353299	1352067
962	BPSS0903		17	1434130	1433318	1460910	1460098	1422546	1421734	1427833	1427021	1114530	1113718	629352	628540	925877	926689	990333	989521	333280	332468	1352071	1351259
963	BPSS0904		17	1433289	1431802	1460069	1458582	1421705	1420218	1426992	1425505	1113689	1112202	628511	627024	926718	928205	989492	988005	332439	330952	1351230	1349743
964	BPSS0905		17	1431782	1430820	1458562	1457600	1420198	1419236	1425485	1424523	1112182	1111220	627004	626042	928225	929187	987985	987023	330932	329970	1349723	1348761
965	BPSS0906		17	1430791	1429580	1457571	1456360	1419207	1417996	1424494	1423283	1111191	1109980	626013	624802	929216	930427	986994	985783	329941	328730	1348732	1347521
966	BPSS0907		17	1429542	1428139	1456322	1454919	1417958	1416555	1423245	1421842	1109942	1108539	624764	623361	930465	931868	985745	984342	328692	327289	1347483	1346080
967	BPSS0908		17	1427431	1425119	1454211	1451899	1415847	1413535	1421134	1418822	1107831	1105519	622653	620341	932576	934888	983634	981322	326581	324269	1345372	1343060
968	BPSS0909		17	1425057	1424230	1451837	1451010	1413473	1412646	1418760	1417933	1105457	1104630	620279	619452	934950	935777	981260	980433	324207	323380	1342998	1342171
969	BPSS0910		17	1423632	1424063	1450412	1450843	1412048	1412479	1417335	1417766	1104032	1104463	618854	619285	936375	935944	979835	980266	322782	323213	1341573	1342004
970	BPSS0911		17	1423098	1421608	1449878	1448388	1411514	1410024	1416801	1415311	1103498	1102008	618320	616830	936909	938399	979301	977811	322257	320767	1341039	1339549
971	BPSS0912		17	1420958	1421437	1447738	1448217	1409374	1409853	1414661	1415140	1101358	1101837	616180	616659	939049	938570	977161	977640	320117	320596	1338899	1339378
972	BPSS0913		17	1420770	1419472	1447550	1446252	1409186	1407888	1414473	1413175	1101170	1099872	615992	614694	939237	940535	976973	975675	319929	318631	1338711	1337413
973	BPSS0914		17	1419077	1419298	1445857	1446078	1407493	1407714	1412780	1413001	1099477	1099698	614299	614520	940930	940709	975280	975501	318236	318457	1337018	1337239
974	BPSS0915		17	1418640	1417942	1445420	1444722	1407056	1406358	1412343	1411645	1099040	1098342	613862	613164	941367	942065	974843	974145	317799	317101	1336581	1335883
975	BPSS0916		17	1417822	1416953	1444602	1443733	1406238	1405369	1411525	1410656	1098222	1097353	613044	612175	942185	943054	974025	973156	316981	316112	1335763	1334894
976	BPSS0917		17	1416654	1416100	1443434	1442880	1405070	1404516	1410357	1409803	1097054	1096500	611876	611322	943353	943907	972857	972303	315813	315259	1334595	1334041
977	BPSS0918		17	1415516	1415974	1442296	1442754	1403932	1404390	1409219	1409677	1095916	1096374	610738	611196	944491	944033	971719	972177	314675	315133	1333457	1333915
978	BPSS0919		17	1415386	1414583	1442166	1441363	1403802	1402999	1409089	1408286	1095786	1094983	610608	609805	944621	945424	971589	970786	314545	313742	1333327	1332524
979		Chr2_40_IS407A_A	17	1412717	1413952	1439497	1440732	1401133	1402368	1406420	1407655	1093117	1094352	607939	609174	946055	947290	968920	970155	311876	313111	1330658	1331893
980	BPSS0920_LysR family regulatory protein		17																				
981	BPSS0921_putative peptidase		17																				

982	BPSS0922		17											607937	606687	947292	948542	968918	967668				
983	BPSS0923		17											605150	606100	950079	949129	966131	967081				
984	BPSS0924		17											604224	605135	951005	950094	965205	966116				
985	BPSS0925		17											603301	604224	951928	951005	964282	965205				
986		Chr2_41_IS407A_B	17	1412717	1413952	1439497	1440732	1401133	1402368	1406420	1407655	1093117	1094352							311876	313111	1330658	1331893
987	BPSS0926		17	1412128	1412715	1438908	1439495	1400544	1401131	1405831	1406418	1092528	1093115	601684	603270	953545	951959	962665	964251	311287	311874	1330069	1330656
988	BPSS0927		17	1411232	1411879	1438012	1438659	1399648	1400295	1404935	1405582	1091632	1092279	600788	601435	954441	953794	961769	962416	310391	311038	1329173	1329820
989	BPSS0928		17	1410062	1411042	1436842	1437822	1398478	1399458	1403765	1404745	1090462	1091442	599618	600598	955611	954631	960599	961579	309221	310201	1328003	1328983
990	BPSS0929		17	1409131	1409934	1435911	1436714	1397547	1398350	1402834	1403637	1089531	1090334	598687	599490	956542	955739	959668	960471	308290	309093	1327072	1327875
991	BPSS0930		17	1408486	1409049	1435266	1435829	1396902	1397465	1402189	1402752	1088886	1089449	598042	598605	957187	956624	959023	959586	307645	308208	1326427	1326990
992	BPSS0931		17	1407765	1406971	1434143	1433349	1396181	1395387	1401468	1400674	1088165	1087371	597321	596527	957908	958702	958302	957508	306924	306130	1325706	1324912
993	BPSS0932		17	1405419	1406930	1431797	1433308	1393835	1395346	1399122	1400633	1085819	1087330	594975	596486	960254	958743	955956	957467	304578	306089	1323360	1324871
994	BPSS0933		17	1405169	1403628	1431547	1430006	1393585	1392044	1398872	1397331	1085569	1084028	594725	593184	960504	962045	955706	954165	304328	302787	1323110	1321569
995	BPSS0934		17	1401728	1403389	1428106	1429767	1390144	1391805	1395403	1397064	1082128	1083789	591284	592945	963945	962284	952265	953926	300887	302548	1319669	1321330
996	BPSS0935		17	1400177	1401082	1426555	1427460	1388593	1389498	1393852	1394757	1080577	1081482	589733	590638	965496	964591	950714	951619	299336	300241	1318118	1319023
997	BPSS0936		17	1399998	1398673	1426376	1425051	1388414	1388217	1393673	1392348	1080398	1079073	589554	588229	965675	967000	950535	949210	299157	297832	1317939	1316614
998	BPSS0937		17	1398402	1397296	1424780	1423674	1386817	1385711	1392077	1390971	1078802	1077696	587958	586852	967271	968377	948939	947833	297561	296455	1316343	1315237
999	BPSS0938		17	1396349	1397236	1422727	1423614	1384764	1385651	1390024	1390911	1076749	1077636	585905	586792	969324	968437	946886	947773	295508	296395	1314431	1314787
1000	BPSS0939		17	1395966	1395571	1422344	1422087	1384381	1383986	1389641	1389246	1076366	1075971	585522	585127	969707	970102	946503	946108	295125	294730	1314048	1313689
1001	BPSS0940		17	1394952	1394008	1420470	1419526	1383345	1382413	1388627	1387683	1075424	1074966	584524	583580	970705	971649	945465	944521	294055	293111	1313202	1312258
1002	BPSS0941		17	1393161	1394030	1418679	1419548	1381566	1382435	1386836	1387705	1073668	1074537	582733	583602	972496	971627	943674	944543	292264	293133	1311411	1312280
1003	BPSS0942		17	1391969	1393120	1417487	1418638	1380374	1381525	1385644	1386795	1072476	1073627	581541	582692	973688	972537	942482	943633	291072	292223	1310219	1311370
1004	BPSS0943		17	1391548	1390397	1417066	1415915	1379953	1378802	1385223	1384072	1072055	1070904	581120	579969	974109	975260	942061	940910	290651	289500	1309798	1308647
1005	BPSS0944		17	1388981	1389889	1414499	1415407	1377386	1378294	1382656	1383564	1069488	1070396	578553	579461	976676	975768	939635	940543	288084	288992	1307231	1308139
1006	BPSS0945		17	1388363	1387419	1413881	1413366	1376768	1375824	1382038	1381094	1068870	1067926	577935	576991	977294	978238	939017	938073	287466	286522	1306613	1305669
1007	BPSS0946		17	1387241	1386357	1412636	1411743	1375646	1374762	1380916	1380032	1067748	1066864	576813	575929	978416	979300	937895	937011	286344	285460	1305490	1304606
1008	BPSS0948		17	1385623	1384754	1411017	1410148	1373303	1372434	1379298	1378429	1066130	1065261	575195	574326	980034	980903	936277	935408	284726	283857	1303872	1303003
1009	BPSS0949		17	1384306	1384638	1409700	1410032	1371986	1372318	1377981	1378313	1064813	1065145	573878	574210	981351	981019	934960	935292	283409	283741	1302555	1302887
1010	BPSS0950		17	1382676	1383461	1408070	1408855	1370356	1371141	1376351	1377136	1063183	1063968	572248	573033	982981	982196	933330	934115	281779	282564	1300925	1301710
1011	BPSS0951		17	1381819	1382685	1407213	1408079	1369499	1370365	1375494	1376360	1062326	1063192	571391	572257	983838	982972	932473	933339	280922	281788	1300068	1300934
1012	BPSS0952		17	1380876	1381814	1406270	1407208	1368556	1369494	1374551	1375489	1061383	1062321	570448	571386	984781	983843	931530	932468	279979	280917	1299125	1300063
1013	BPSS0953		17	1379763	1380830	1405157	1406224	1367443	1368510	1373438	1374505	1060270	1061337	569335	570402	985894	984827	930417	931484	278866	279933	1298012	1299079
1014	BPSS0954		17	1378141	1379739	1403535	1405133	1365821	1367419	1371816	1373414	1058648	1060246	567713	569311	987516	985918	928795	930393	277244	278842	1296390	1297988
1015	BPSS0955		17	1377423	1377989	1402817	1403383	1365103	1365669	1371098	1371664	1057930	1058496	566995	567561	988234	987668	928077	928643	276526	277092	1295672	1296238
1016	BPSS0956		17	1376513	1377400	1401907	1402794	1364193	1365080	1370188	1371075	1057020	1057907	566085	566972	989144	988257	927167	928054	275616	276503	1294762	1295649
1017	BPSS0957		17	1374311	1375519	1399705	1400913	1361991	1363199	1368108	1369316	1054818	1056026	563883	565091	991346	990138	924965	926173	273414	274622	1292571	1293779
1018	BPSS0958		17	1374021	1372039	1399415	1397433	1361701	1359719	1367818	1365836	1054528	1052546	563593	561611	991636	993618	924675	922693	273124	271142	1292281	1290299
1019	BPSS0959		17	1371944	1371513	1397338	1396907	1359624	1359193	1365741	1365310	1052451	1052020	561516	561085	993713	994144	922598	922167	271047	270616	1290204	1289773
1020	BPSS0960		17	1371458	1366836	1396852	1392230	1359138	1354516	1365255	1360633	1051965	1047343	561030	556408	994199	998821	922112	917490	270561	265939	1289718	1285096
1021	BPSS0961		17	1366826	1366350	1392220	1391744	1354506	1354030	1360623	1360147	1047333	1046857	556398	555922	998831	999307	917480	917004	265929	265453	1285086	1284610
1022	BPSS0962		17	1365304	1361912	1390698	1387306	1352984	1349592	1359101	1355709	1045811	1042419	554876	551484	1000353	1003745	915958	912566	264407	261015	1283564	1280172
1023	BPSS0963		17	1361863	1361186	1387257	1386580	1349543	1348866	1355660	1354983	1042370	1041693	551435	550758	1003794	1004471	912517	911840	260966	260289	1280123	1279446
1024	BPSS0964		17	1359395	1360984	1384789	1386378	1347075	1348664	1353192	1354781	1039902	1041491	548967	550556	1006262	1004673	910049	911638	258498	260087	1277655	1279244
1025	BPSS0965		17	1359109	1357850	1384503	1383244	1346789	1345530	1352906	1351647	1039616	1038357	548681	547422	1006548	1007807	909763	908504	258212	256953	1277369	1276110
1026	BPSS0966		17	1357730	1356438	1383124	1381832	1345410	1344118	1351527	1350235	1038237	1036945	547302	546010	1007927	1009219	908384	907092	256833	255541	1275990	1274698
1027	BPSS0967		17	1355547	1355996	1380941	1381390	1343227	1343676	1349344	1349793	1036054	1036503	545119	545568	1010110	1009661	906201	906650	254650	255099	1273807	1274256
1028	BPSS0968		17	1354390	1355295	1379784	1380689	1342070	1342975	1348187	1349092	1034897	1035802	543962	544867	1011267	1010362	905044	905949	253493	254398	1272650	1273555
1029	BPSS0969		17	1352053	1354374	1377447	1379768	1339733	1342054	1345850	1348171	1032560	1034881	541625	543946	1013604	1011283	902707	905028	251156	253477	1270313	1272634
1030	BPSS0970		17	1351874	1350459	1377268	1375853	1339554	1338139	1345671	1344256	1032381	1030966	541446	540031	1013783	1015198	902528	901113	250977	249562	1270134	1268719
1031	BPSS0971		17	1350385	1350029	1375779	1375423	1338065	1337709	1344135	1343896	1030892	1030536	539957	539601	1015272	1015628	901039	900683	249488	249132	1268645	1268289
1032	BPSS0972		17	1349124	1349864	1374518	1375258	1336104	1336844	1342991	1343731	1029631	1030371	538696	539436	1016533	1015793	899778	900518	248227	248967	1267290	1268030

1033	BPSS0973		17	1346262	1349045	1371656	1374439	1333242	1336025	1340129	1342912	1026769	1029552	535834	538617	1019395	1016612	896916	899699	245365	248148	1264428	1267211
1034	BPSS0974		17	1343903	1345519	1369297	1370913	1330883	1332499	1337770	1339386	1024410	1026026	533475	535091	1021754	1020138	894557	896173	243006	244622	1262069	1263685
1035	BPSS0975		17	1342362	1341850	1367756	1367244	1329342	1328830	1336229	1335717	1022869	1022357	531934	531422	1023295	1023807	893016	892504	241465	240953	1260528	1260016
1036	BPSS0976		17	1341847	1340237	1367241	1365631	1328827	1327217	1335714	1334104	1022354	1020744	531419	529809	1023810	1025420	892501	890891	240950	239340	1260013	1258403
1037	BPSS0977		17	1340189	1338879	1365583	1364273	1327169	1325859	1334056	1332746	1020696	1019386	529761	528451	1025468	1026778	890843	889533	239292	237982	1258355	1257045
1038	BPSS0978		17	1338684	1338274	1364078	1363668	1325664	1325254	1332551	1332141	1019191	1018781	528256	527846	1026973	1027383	889338	888928	237787	237377	1256850	1256440
1039	BPSS0979		17	1338218	1337106	1363612	1362500	1325198	1324086	1332085	1330973	1018725	1017613	527790	526678	1027439	1028551	888872	887760	237321	236209	1256384	1255272
1040	BPSS0980		17	1336101	1336901	1361495	1362295	1323081	1323881	1329968	1330768	1016608	1017408	525673	526473	1029556	1028756	886755	887555	235204	236004	1254267	1255067
1041	BPSS0981		17	1335634	1334948	1361028	1360342	1322614	1321928	1329501	1328815	1016141	1015455	525206	524520	1030023	1030709	886288	885602	234737	234051	1253800	1253114
1042	BPSS0982		17	1334948	1334238	1360342	1359632	1321928	1321218	1328815	1328105	1015455	1014745	524520	523810	1030709	1031419	885602	884892	234051	233341	1253114	1252404
1043	BPSS0983		17	1334166	1333402	1359560	1358796	1321146	1320382	1328033	1327269	1014673	1013909	523738	522974	1031491	1032255	884820	884056	233269	232505	1252332	1251568
1044	BPSS0984		17	1332034	1333332	1357192	1358490	1319009	1320307	1325900	1327198	1012539	1013837	521605	522903	1033624	1032326	882687	883985	231136	232434	1250198	1251496
1045	BPSS0985		17	1331793	1331302	1356951	1356460	1318768	1318277	1325659	1325168	1012298	1011807	521364	520873	1033865	1034356	882446	881958	230895	230404	1249957	1249466
1046	BPSS0986		17	1331045	1330299	1356203	1355457	1318020	1317274	1324911	1324165	1011550	1010804	520616	519870	1034613	1035359	881701	880955	230147	229401	1249209	1248463
1047	BPSS0987		17	1329834	1330175	1354992	1355333	1316809	1317150	1323700	1324041	1010339	1010680	519405	519746	1035824	1035483	880490	880831	228936	229277	1247998	1248339
1048	BPSS0988		17	1329199	1327943	1354357	1353101	1316174	1314918	1323065	1322385	1009704	1008448	518770	517514	1036459	1037715	879855	878599	228301	227045	1247363	1246107
1049	BPSS0989		17	1327353	1327892	1352511	1353050	1314328	1314867	1321196	1321735	1007858	1008397	516924	517463	1038305	1037766	878009	878548	226455	226994	1245517	1246056
1050	BPSS0990		17	1326758	1325742	1351916	1350900	1313733	1312717	1320601	1319585	1007263	1006247	516336	515320	1038893	1039909	877421	876405	225867	224851	1244929	1243913
1051		Chr2_42_IS407A_A	17	1324421	1325656	1349579	1350814	1311687	1312631	1318264	1319499	1004926	1006161	513999	515234	1039995	1041230	875084	876319	223530	224765	1242592	1243827
1052	BPSS0991		17	1324109	1323768	1349267	1348926			1317952	1317611	1004614	1004273	513687	513346	1041542	1041883	874772	874431	223218	222877	1242280	1241939
1053	BPSS0993		17	1323452	1321938	1348610	1347096			1317295	1315781	1003957	1002443	513030	511744	1042199	1043485	874115	872829	222561	221047	1241623	1240109
1054	BPSS0994		17	1320007	1321467	1344800	1346248			1313876	1315315	1000520	1001980	509622	511082	1045599	1044139	870699	872159	219140	220600	1238307	1239605
1055	BPSS0995		17	1318264	1319820	1343057	1344613			1312133	1313689	998777	1000333	507879	509435	1047342	1045786	868956	870512	217397	218953	1236564	1238120
1056	BPSS0996		17	1317093	1318094	1341886	1342887			1310962	1311963	997606	998607	506708	507709	1048513	1047512	867785	868786	216226	217227	1235393	1236394
1057	BPSS0997		17	1316505	1316924	1340107	1341411			1309183	1310487	995827	997131	504929	506233	1050292	1048988	866006	867310	214447	215751	1233614	1234918
1058	BPSS0998		17	1314028	1315269	1338821	1340062			1307897	1309138	994541	995782	503643	504884	1051578	1050337	864720	865961	213161	214402	1232328	1233569
1059	BPSS0999		17	1313802	1314038	1338595	1338831			1307671	1307907	994315	994551	503417	503653	1051804	1051568	864494	864730	212935	213171	1232102	1232338
1060	BPSS1000		17	1313002	1313745	1337795	1338538			1306871	1307614	993515	994258	502617	503360	1052604	1051861	863694	864437	212135	212878	1231302	1232045
1061	BPSS1001		17	1312195	1312962	1336988	1337755			1306064	1306831	992708	993475	501810	502577	1053411	1052644	862887	863654	211328	212095	1230495	1231262
1062		Chr2_43_ISBma1_A	17	1310749	1312053	1335542	1336846			1304618	1305922	991262	992566	500364	501668	1053553	1054857	861441	862745	209882	211186	1229130	1230350
1063	BPSS1002_pfragmentation by ISBma1		17	1309629	1310747	1334422	1335540			1303498	1304616	990142	991260	499244	500362	1055977	1054859	860321	861439	208762	209880	1227929	1229047
1064	BPSS1003		17	1309179	1309514	1333972	1334307			1303048	1303383	989692	990027	498794	499129	1056427	1056092	859871	860206	208312	208647	1227479	1227814
1065	BPSS1004		17	1307052	1308233	1331845	1333026			1300921	1302102	987565	988746	496667	497848	1058554	1057373	857744	858925	206185	207366	1225512	1226414
1066	BPSS1005		17	1305438	1306667	1330231	1331460			1299307	1300536	985951	987180	495053	496282	1060168	1058939	856130	857359	204571	205800	1223898	1225127
1067	BPSS1006		17	1304815	1300589	1329608	1325382			1298684	1294458	985328	981102	494430	490204	1060791	1065017	855507	853555	203948	199722	1223275	1219436
1068	BPSS1007		17	1278214	1277519	1314533	1311342			1275196	1272002	971661	962575	467772	467077	1087982	1091938	835815	832633	190272	181165	1209292	1200653
1069	BPSS1008		17	1273752	1270264	1297363	1293875			1267845	1264357	954275	952482	463286	459798	1091938	1095426	824445	822781	172951	169463	1192535	1189047
1070	BPSS1009		17	1259134	1254494	1280241	1275601			1253227	1248587	939631	934991	448614	443947	1106574	1111241	810182	805524	158315	153666	1178380	1173731
1071	BPSS1010		17	1254471	1252753	1275578	1273860			1248564	1246846	934968	933250	443924	442206	1111264	1112982	805501	803783	153643	151925	1173708	1171990
1072	BPSS1011		17	1252667	1251234	1273774	1272341			1246760	1245327	933164	931731	442120	440687	1113068	1114501	803697	802264	151839	150406	1171904	1170471
1073	BPSS1012		17	1248692	1250593	1269799	1271700			1242785	1244686	929189	931090	438145	440046	1117054	1115153	799711	801612	147864	149765	1167929	1169830
1074	BPSS1013		17	1248244	1248654	1269351	1269761			1242337	1242747	928741	929151	437697	438107	1117502	1117092	799263	799673	147416	147826	1167481	1167891
1075	BPSS1014		17	1246910	1248100	1268017	1269207			1241003	1242193	927407	928597	436363	437553	1118836	1117646	797929	799119	146082	147272	1166147	1167337
1076	BPSS1015		17	1246557	1245589	1267664	1266696			1240650	1239682	927054	926086	436010	435042	1119189	1120157	797576	796608	145729	144761	1165794	1164826
1077	BPSS1016		17	1243922	1245061	1265029	1266168			1238015	1239154	924419	925558	433375	434514	1121824	1120685	794941	796080	143094	144233	1163159	1164298
1078	BPSS1017		17	1243258	1242497	1264365	1263604			1237351	1236590	923755	922994	432711	431950	1122488	1123249	794277	793516	142430	141669	1162495	1161734
1079	BPSS1018		17	1241239	1242144	1262346	1263251			1235308	1236213	921736	922641	430692	431597	1124507	1123602	792258	793163	140411	141316	1160476	1161381
1080	BPSS1019		17	1240359	1241060	1261466	1262167			1234428	1235129	920856	921557	429812	430513	1125387	1124686	791378	792079	139531	140232	1159596	1160297
1081	BPSS1020		17	1239149	1240174	1260256	1261281			1233225	1234250	919646	920671	428602	429627	1126597	1125572	790168	791193	138321	139346	1158400	1159395
1082	BPSS1021		17	1238455	1238300	1259562	1259407			1232531	1232376	918994	918839	427950	427795	1127249	1127404	789502	789347	137683	137528	1157755	1157600
1083	BPSS1022		17	1238281	1237763	1259388	1258870			1232357	1231839	918820	918302	427776	427258	1127423	1127941	789328	788810	137509	136991	1157581	1157063

1084	BPSS1023		17	1237763	1236942	1258870	1258049			1231839	1231018	918302	917481	427258	426437	1127941	1128762	788810	787989	136991	136170	1157063	1156242
1085	BPSS1024		17	1236696	1235887	1257803	1257711			1230833	1230024	917235	916426	426191	425382	1129008	1129817	787743	786934	135924	135115	1155996	1155187
1086	BPSS1025		17	1235844	1235488	1256568	1256212			1229981	1229625	916383	916027	425339	424983	1129860	1130216	786891	786535	135072	134716	1155144	1154788
1087	BPSS1026		17	1235488	1235264	1256212	1255988			1229625	1229401	916027	915803	424983	424759	1130216	1130440	786535	786311	134716	134492	1154788	1154564
1088	BPSS1027		17	1234987	1234481	1255711	1255205			1229124	1228618	915526	915020	424482	423976	1130717	1131223	786034	785528	134215	133709	1154287	1154198
1089	BPSS1028		17	1234399	1233482	1255123	1254206			1228536	1227619	914938	914021	423894	422977	1131305	1132222	785446	784529	133627	132710	1153780	1153655
1090	BPSS1029		17	1233160	1230710	1253884	1251434			1227297	1224847	913699	911249	422655	420205	1132544	1134994	784207	781757	132388	129938	1152558	1150108
1091	BPSS1030		17	1229515	1230492	1250239	1251216			1223652	1224629	910054	911031	419010	419987	1136189	1135212	780562	781539	128743	129720	1149204	1149890
1092	BPSS1031		17															779840	780301				
1093	BPSS1032		17											416472	418286	1138727	1136913	778026	779840	126205	128019	1144003	1145817
1094	BPSS1033		17											415426	416472	1139773	1138727	776980	778026	125159	126205	1142957	1144003
1095	BPSS1034		17											414805	414068	1140394	1141131	776359	775622	124538	123801	1142336	1141599
1096	BPSS1035		17											413491	413739	1141708	1141460	775045	775293	123224	123472	1141022	1141270
1097	BPSS1036		17											412069	413325	1143130	1141874	773623	774879	121802	123058	1139600	1140856
1098	BPSS1037		17	1227833	1228897	1248557	1249621			1221970	1223034	908372	909436	410380	411846	1144819	1143353	771934	773400	120113	121579	1137911	1139377
1099		Chr2_44a_IS407A_A		1225576	1226811	1246300	1247535			1219713	1220948	906115	907350	408123	409358	1145841	1147076	769677	770912	117856	119091	1135654	1136889
1100		Chr2_44b_IS407A_A		1061754	1062989	1080393	1081628	1143190	1144425	1056512	1057747	793863	795098							6022	7257	1023389	1024624
1101	BPSS1038		18	1063331	1063600	1081970	1082239	1144767	1145036	1058089	1058358	795440	795709	407781	407512	1147418	1147687	769335	769066	7599	7868	1024966	1025235
1102	BPSS1039		18	1065110	1063719	1083749	1082358	1146546	1145155	1059868	1058477	797219	795828	406011	407402	1149188	1147797	767565	768956	9378	7987	1026745	1025354
1103	BPSS1040		18	1065796	1065110	1084435	1083749	1147232	1146546	1060554	1059868	797905	797219	405325	406011	1149874	1149188	766879	767565	10064	9378	1027431	1026745
1104	BPSS1041		18	1069036	1065806	1087675	1084445	1150472	1147242	1063794	1060564	801145	797915	402085	405315	1153114	1149884	763639	766869	13304	10074	1030671	1027441
1105	BPSS1042		18	1070551	1069085	1089190	1087724	1151987	1150521	1065309	1063843	802660	801194	400570	402036	1154629	1153163	762124	763590	14819	13353	1032186	1030720
1106	BPSS1043		18	1071920	1070565	1090559	1089204	1153356	1152001	1066678	1065323	804029	802674	399201	400556	1155998	1154643	760755	762110	16188	14833	1033555	1032200
1107	BPSS1044		18	1073005	1072868	1091644	1091507	1154441	1154304	1067763	1067626	805114	804977	398116	398253	1157083	1156946	759670	759807	17273	17136	1034640	1034503
1108	BPSS1045		18	1074146	1073661	1092785	1092300	1155582	1155097	1068904	1068419	806261	805776	396958	397443	1158241	1157756	758511	758996	18432	17947	1035799	1035314
1109	BPSS1046		18	1074473	1074955	1093112	1093594	1155909	1156391	1069231	1069713	806588	807070	396631	396149	1158568	1159050	758184	757702	18759	19241	1036126	1036608
1110	BPSS1047	GI15																					
1111	BPSS1048	GI15																					
1112	BPSS1049	GI15																					
1113	BPSS1050	GI15																					
1114	BPSS1051	GI15																					
1115	BPSS1052	GI15																					
1116	BPSS1053	GI15																					
1117	BPSS1054	GI15																					
1118	BPSS1055	GI15																					
1119	BPSS1056	GI15																					
1120	BPSS1057	GI15																					
1121	BPSS1058	GI15																					
1122	BPSS1059	GI15																					
1123	BPSS1060	GI15																					
1124	BPSS1061	GI15																					
1125	BPSS1062	GI15																					
1126	BPSS1063	GI15																					
1127	BPSS1064	GI15																					
1128	BPSS1065	GI15																					
1129	BPSS1066	GI15																					
1130	BPSS1067	GI15																					
1131	BPSS1068	GI15																					
1132	BPSS1069	GI15																					
1133	BPSS1070	GI15																					
1134	BPSS1071	GI15																					

1135	BPSS1072	GI15																					
1136	BPSS1073	GI15																					
1137	BPSS1074	GI15																					
1138	BPSS1075	GI15																					
1139	BPSS1076	GI15																					
1140	BPSS1077	GI15																					
1141	BPSS1078	GI15																					
1142	BPSS1079	GI15																					
1143	BPSS1080	GI15																					
1144	BPSS1081	GI15																					
1145	BPSS1082	GI15																					
1146	BPSS1083	GI15																					
1147	BPSS1084	GI15																					
1148	BPSS1085	GI15																					
1149	BPSS1086	GI15																					
1150	BPSS1087	GI15																					
1151	BPSS1088	GI15																					
1152	BPSS1089	GI15																					
1153	BPSS1090																						
1154	BPSS1091																						
1155	BPSS1092																						
1156	BPSS1093																						
1157	BPSS1094																						
1158	BPSS1095																						
1159	BPSS1096																						
1160	BPSS1097																						
1161	BPSS1098																						
1162	BPSS1099																						
1163	BPSS1100																						
1164	BPSS1101																						
1165	BPSS1102																						
1166	BPSS1103																						
1167	BPSS1104																						
1168	BPSS1105																						
1169	BPSS1106																						
1170	BPSS1107																						
1171	BPSS1108																						
1172	BPSS1109																						
1173	BPSS1110																						
1174	BPSS1111																						
1175	BPSS1112		18	1075226	1075681	1093865	1094320	1156662	1157117	1069984	1070439	807341	807796	395878	395423	1159321	1159776	757431	756976	19512	19967	1036879	1037334
1176	BPSS1113		18	1076800	1077486	1095439	1096125	1158236	1158922	1071558	1072244	808915	809601	394304	393618	1160895	1161581	755857	755171	21086	21772	1038453	1039139
1177	BPSS1114		18	1079614	1077617	1098253	1096256	1161050	1159053	1074372	1072375	811743	809746	391476	393473	1163723	1161726	753036	755033	23914	21917	1041281	1039284
1178	BPSS1115		18	1080172	1079939	1098811	1098578	1161608	1161375	1074930	1074697	812301	812068	390910	391419	1164289	1163780	752470	752979	24472	24239	1041839	1041606
1179	BPSS1116		18	1080945	1082750	1099584	1101389	1162381	1164084	1075703	1076101	813074	814879	390137	388332	1165062	1166867	751697	749892	25245	26510	1042612	1043877
1180	BPSS1117		18	1083591	1082887	1102230	1101526	1165028	1164324	1078324	1077620	815720	815016	387491	388195	1167708	1167004	749051	749755	27878	27174	1045245	1044541
1181	BPSS1118		18	1083706	1084800	1102345	1103439	1165143	1166237	1078439	1079533	815835	816929	387376	386282	1167823	1168917	748936	747842	27993	29087	1045360	1046454
1182	BPSS1119		18	1084854	1087898	1103493	1106537	1166291	1169335	1079587	1082631	816983	820027	386228	383184	1168971	1172015	747788	744744	29141	32185	1046508	1049552
1183	BPSS1120		18	1088036	1089571	1106675	1108210	1169473	1171008	1082769	1083197	820165	821700	383046	381511	1172153	1173688	744606	743071	32323	33858	1049690	1050190
1184	BPSS1121		18	1089904	1090545	1108543	1109184	1171341	1171982	1084633	1085274	822033	822674	381178	380537	1174021	1174662	742738	742097	34191	34832	1050981	1051622
1185	BPSS1122		18	1090545	1092239	1109184	1110878	1171982	1173676	1085274	1086968	822674	824368	380537	378843	1174662	1176356	742097	740403	34832	36526	1051622	1053316

1186	BPSS1123	18	1092239	1092898	1110878	1111537	1173676	1174335	1086968	1087627	824368	825027	378843	378184	1176356	1177015	740403	739744	36526	37185	1053316	1053975
1187	BPSS1124																					
1188	BPSS1125																					
1189	BPSS1126																					
1190	BPSS1127																					
1191	BPSS1128																					
1192	BPSS1129																					
1193	BPSS1130																					
1194	BPSS1131																					
1195	BPSS1132																					
1196	BPSS1133																					
1197	BPSS1134																					
1198	BPSS1135																					
1199	BPSS1136																					
1200	BPSS1137																					
1201	BPSS1138																					
1202	BPSS1139																					
1203	BPSS1140																					
1204	BPSS1141																					
1205	BPSS1142																					
1206	BPSS1143																					
1207	BPSS1144																					
1208	BPSS1145																					
1209	BPSS1146																					
1210	BPSS1147																					
1211	BPSS1148																					
1212	BPSS1149																					
1213	BPSS1150																					
1214	BPSS1151																					
1215	BPSS1152																					
1216	BPSS1153																					
1217	BPSS1154																					
1218	BPSS1155																					
1219	BPSS1156																					
1220	BPSS1157																					
1221	BPSS1158																					
1222	BPSS1159																					
1223	BPSS1160																					
1224	BPSS1161																					
1225	BPSS1162																					
1226	BPSS1163																					
1227	BPSS1164																					
1228	BPSS1165																					
1229	BPSS1166																					
1230	BPSS1167																					
1231	BPSS1168																					
1232	BPSS1169																					
1233	BPSS1170																					
1234	BPSS1171																					
1235	BPSS1172																					
1236	BPSS1173																					

1237	BPSS1174																						
1238	BPSS1175																						
1239	BPSS1176																						
1240	BPSS1177																						
1241	BPSS1178																						
1242	BPSS1179																						
1243	BPSS1180																						
1244	BPSS1181																						
1245	BPSS1182																						
1246	BPSS1183																						
1247	BPSS1184																						
1248	BPSS1185																						
1249	BPSS1185a																						
1250	BPSS1187																						
1251	BPSS1188																						
1252	BPSS1190																						
1253	BPSS1191																						
1254	BPSS1192																						
1255	BPSS1193																						
1256	BPSS1194																						
1257	BPSS1195																						
1258	BPSS1196																						
1259	BPSS1197																						
1260	BPSS1198																						
1261	BPSS1199																						
1262	BPSS1200																						
1263	BPSS1201																						
1264	BPSS1202																						
1265	BPSS1203																						
1266	BPSS1204																						
1267	BPSS1205																						
1268	BPSS1206																						
1269	BPSS1207																						
1270	BPSS1208																						
1271	BPSS1209																						
1272	BPSS1210																						
1273	BPSS1211																						
1274	BPSS1212																						
1275	BPSS1213																						
1276	BPSS1214																						
1277	BPSS1215																						
1278	BPSS1216																						
1279	BPSS1217																						
1280	BPSS1218																						
1281		Chr2_45_IS407A_B	18	1096730	1097965	1115369	1116604	1178167	1179402	1091459	1092694	828859	830094							41016	42251	1057806	1059041
1282	BPSS1219		18	1098143	1099960	1116782	1118599	1179580	1181397	1092872	1094689	830272	832089	374178	372361	1181021	1182838	735738	733921	42429	44246	1059219	1061036
1283	BPSS1220		18	1100359	1101357	1118998	1119996	1181796	1182794	1095088	1096086	832481	833479	371955	370957	1183237	1184235	733536	732538	44638	45636	1061428	1062426
1284	BPSS1221		18	1101419	1102189	1120058	1120828	1182856	1183626	1096148	1096918	833541	834311	370895	370125	1184297	1185067	732476	731706	45698	46468	1062488	1063258
1285	BPSS1222		18	1103960	1102230	1122599	1120869	1185397	1183667	1098689	1096959	836082	834352	368354	370084	1186838	1185108	729935	731665	48239	46509	1065029	1063299
1286	BPSS1223		18	1105197	1106096	1123836	1124735	1186634	1187533	1099926	1100825	837319	838218	367117	366218	1188075	1188974	728698	727799	49476	50375	1066266	1067165
1287	BPSS1224		18	1106200	1108170	1124839	1126782	1187637	1189607	1100929	1102899	838322	840292	366114	364144	1189078	1191048	727695	725725	50479	52449	1067269	1069239

1288	BPSS1225		18	1108187	1108540	1126827	1126955	1189624	1189977	1102916	1103269	840309	840662	364127	363774	1191065	1191418	725708	725355	52466	52819	1069256	1069609
1289	BPSS1226		18	1108780	1109055	1127573	1127848	1190217	1190492	1103509	1103784	840902	841177	363534	363259	1191650	1191925	725115	724840	53059	53334	1069849	1070124
1290	BPSS1227		18	1109689	1109219	1128482	1128012	1191126	1190656	1104418	1103948	841811	841341	362625	363095	1192559	1192089	724206	724676	53968	53498	1070758	1070288
1291	BPSS1228		18	1110867	1109848	1129660	1128641	1192304	1191285	1105596	1104577	842989	841970	361447	362466	1193737	1192718	723028	724047	55146	54127	1071936	1070917
1292	BPSS1229		18	1111380	1112159	1130173	1130952	1192817	1193596	1106109	1106888	843502	844281	360934	360155	1194250	1195029	722515	721736	55659	56438	1072449	1073228
1293	BPSS1230		18	1112175	1113329	1130968	1132122	1193612	1194766	1106904	1108058	844297	845451	360139	358985	1195045	1196199	721720	720566	56454	57608	1073244	1074398
1294	BPSS1231		18	1114211	1113744	1133004	1132537	1195648	1195181	1108940	1108473	846277	845810	358117	358584	1197060	1196593	719670	720137	58469	58002	1075238	1074771
1295	BPSS1232		18	1114988	1116271	1133781	1135064	1196425	1197708	1109717	1111000	847054	848337	357340	356063	1197837	1199114	718893	717616	59246	60523	1076015	1077292
1296	BPSS1233		18	1116470	1117345	1135263	1136138	1197907	1198782	1111199	1112074	848536	849411	355891	355016	1199286	1200161	717453	716578	60704	61579	1077482	1078357
1297	BPSS1234		18	1117336	1118145	1136129	1136938	1198773	1199582	1112065	1112874	849402	850211	355025	354216	1200152	1200961	716587	715778	61570	62379	1078348	1079157
1298	BPSS1235		18	1118170	1119201	1136963	1137994	1199607	1200638	1112899	1113930	850236	851267	354191	353160	1200986	1202017	715753	714722	62404	63435	1079182	1080213
1299	BPSS1236		18	1119232	1120398	1138025	1139191	1200669	1201835	1113961	1115127	851298	852464	353129	351963	1202048	1203214	714691	713525	63466	64632	1080244	1081410
1300	BPSS1237		18	1120398	1121393	1139191	1140186	1201835	1202830	1115127	1116122	852464	853459	351963	350968	1203214	1204209	713525	712530	64632	65627	1081410	1082405
1301	BPSS1238		18	1121393	1122817	1140186	1141610	1202830	1204254	1116122	1117546	853459	854883	350968	349544	1204209	1205633	712530	711106	65627	67051	1082405	1083829
1302	BPSS1239		18	1123437	1124435	1142230	1143228	1204874	1205872	1118166	1119164	855503	856501	348923	347925	1206254	1207252	710486	709488	67671	68654	1084449	1085432
1303	BPSS1240		18	1124760	1126514	1143553	1145307	1206197	1206391	1119489	1121243	856826	858580	347600	345846	1207577	1209331	709163	707409	68762	70516	1085540	1087234
1304	BPSS1241		18	1131331	1127093	1150124	1145886	1213317	1209079	1126060	1121822	863389	859151	341037	345275	1214140	1209902	702592	706548	75325	71087	1091239	1087679
1305		Chr2_46_IS407A_C	18																			1091241	1092476
1306	BPSS1242		18	1131740	1131393	1150533	1150186	1213726	1213379	1126469	1126122	863798	863451	340628	340975	1214549	1214202	702183	702530	75734	75387	1093566	1093219
1307	BPSS1243		18	1134360	1131781	1153153	1150574	1216346	1213767	1129089	1126510	866418	863839	338008	340587	1217169	1214590	699563	702142	78354	75775	1096186	1093607
1308	BPSS1244		18	1135702	1134380	1154495	1153173	1217688	1216366	1130431	1129109	867760	866438	336666	337988	1218511	1217189	698221	699543	79696	78374	1097528	1096206
1309	BPSS1245		18	1136577	1137518	1155541	1156482	1219085	1220026	1131058	1131999	868643	869584	335791	334850	1219386	1220327	697346	696405	80579	81520	1098109	1098861
1310	BPSS1246		18	1137623	1138216	1156587	1157180	1220131	1220724	1132104	1132697	869689	870282	334745	334152	1220432	1221025	696300	695707	81625	82218	1098966	1099559
1311	BPSS1247		18	1138216	1139295	1157180	1158259	1220724	1221803	1132697	1133776	870282	871361	334152	333073	1221025	1222104	695707	694628	82218	83297	1099559	1100638
1312	BPSS1248		18	1140200	1139571	1159164	1158535	1222708	1222079	1134681	1134052	872266	871637	332168	332797	1223009	1222380	693723	694352	84202	83573	1101543	1100914
1313	BPSS1249		18	1141765	1143954	1160729	1162918	1224273	1226462	1136246	1138435	873831	876020	330603	328414	1224574	1226763	692174	689985	85767	87956	1103108	1105297
1314	BPSS1250		18	1145523	1144504	1164487	1163468	1228031	1227012	1140004	1138985	877589	876570	326845	327864	1228332	1227313	688416	689435	89525	88506	1106866	1105847
1315	BPSS1251		18	1146933	1145557	1165897	1164521	1229441	1228065	1141414	1140038	878999	877623	325435	326811	1229742	1228366	687006	688382	90935	89559	1108276	1106900
1316	BPSS1252		18	1148292	1146961	1167256	1165925	1230800	1229469	1142773	1141442	880358	879027	324076	325407	1231101	1229770	685647	686978	92294	90963	1109635	1108304
1317	BPSS1253		18	1148481	1148561	1167445	1167525	1230989	1231069	1142962	1143042	880547	880627	323887	323807	1231290	1231370	685458	685378	92483	92563	1109824	1109904
1318	BPSS1254		18	1149978	1149559	1168942	1168523	1232486	1232067	1144459	1144040	882044	881625	322390	322809	1232787	1232368	683961	684380	93980	93561	1111321	1110902
1319	BPSS1255		18	1150140	1150027	1169994	1169101	1233538	1232645	1145511	1144618	883096	882203	321338	322231	1233839	1232946	682909	683802	95032	94139	1112373	1111480
1320	BPSS1256		18	1151154	1151894	1170118	1170858	1233662	1234402	1145635	1146375	883220	883960	321214	320474	1233963	1234703	682785	682045	95156	95896	1112497	1113237
1321	BPSS1257		18	1153625	1152210	1172589	1171174	1236133	1234718	1148106	1146691	885691	884276	318743	320158	1236434	1235019	680314	681729	97627	96212	1114968	1113553
1322	BPSS1258		18	1155694	1154660	1175122	1174088	1238926	1237892	1150174	1149140	887687	886653	316667	317701	1238629	1237595	678088	679122	99668	98634	1117402	1116368
1323	BPSS1259		18	1156916	1155762	1176344	1175190	1240148	1238994	1151396	1150242	888909	887755	315445	316599	1239851	1238697	676866	678020	100890	99736	1118624	1117470
1324	BPSS1260		18	1158741	1160552	1178169	1179980	1241973	1243784	1153221	1155032	890734	892545	313620	311809	1241676	1243487	675041	673230	102715	104526	1120449	1122260
1325	BPSS1261		18	1161129	1160800	1180557	1180228	1244361	1244032	1155609	1155280	893122	892793	311232	311561	1244064	1243735	672653	672982	105103	104774	1122837	1122508
1326	BPSS1262		18	1161393	1162172	1180821	1181600	1244625	1245404	1155873	1156652	893386	894165	310968	310189	1244328	1245107	672389	671610	105367	106146	1123101	1123880
1327	BPSS1263		18	1162186	1164999	1181614	1184427	1245418	1248228	1156666	1159479									106160	108973	1123894	1126707
1328	BPSS1264		18	1164999	1165643	1184427	1185071			1159479	1160123	897232	897876	307122	306478	1248174	1248818	668543	667899	108973	109617	1126707	1127351
1329	BPSS1265_conserved hypothetical protei		18																				
1330	BPSS1266		18	1169952	1166707	1189381	1187321	1253833	1250588	1164432	1161187	902185	898940	302169	305414	1253127	1249882	663590	666835	113926	110681	1131724	1129664
1331	BPSS1267		18	1170205	1170002	1189634	1189431	1254086	1253883	1164685	1164482	902438	902235	301916	302119	1253380	1253177	663337	663540	114179	113976	1131977	1131774
1332	BPSS1268		18	1171513	1170227	1190942	1189656	1255394	1254108	1165993	1164707	903746	902460	300608	301894	1254688	1253402	662029	663315	115487	114201	1133285	1131999
1333	BPSS1269_partial		18	1172237	1171521	1191666	1190950	1256118	1255402	1166717	1166001	904470	903754	299884	300600	1255430	1254696	661305	662021	116211	115495	1134009	1133293
1334		Chr2_47_ISBma2_A		1172191	1173763	1191620	1193192	1256072	1257644	1166671	1168243	904424	905996	298358	299930	1255366	1256940	659779	661351	116165	117737	1133963	1135535
1335		Chr2_48_IS407A_A		1061754	1062989	1080393	1081628	1143190	1144425	1056512	1057747	1438414	1439649	297003	298238	1257060	1258295	658424	659659			1135654	1136889
1336	BPSS1270		19a	1048735	1049730	1067374	1068369	1130171	1131166	1043493	1044488	1425407	1426402	284002	284997	1271296	1270301	645422	646417				
1337	BPSS1271		19a	1048163	1048492	1066802	1067131	1129599	1129928	1042921	1043250	1424835	1425164	283430	283759	1271868	1271539	644850	645179				
1338	BPSS1272		19a	1046401	1048191	1065040	1066830	1127837	1129627	1041159	1042949	1423073	1424863	281668	283458	1273630	1271840	643088	644878				

1339	BPSS1273		19a	1044644	1046401	1063283	1065040	1126080	1127837	1039402	1041159	1421316	1423073	279911	281668	1275387	1273630	641331	643088				
1340	BPSS1274		19a	1042815	1044644	1061454	1063283	1124251	1126080	1037573	1039402	1419487	1421316	278082	279911	1277216	1275387	639502	641331				
1341	BPSS1275		19a	1042219	1041698	1060858	1060337	1123655	1123134	1036977	1036456	1418891	1418370	277486	276965	1277812	1278333	638906	638385				
1342	BPSS1276		19a	1040917	1040330	1059556	1058969	1122353	1121766	1035675	1035088	1417573	1416986	276191	275604	1279107	1279694	637603	637016				
1343	BPSS1277		19a	1040197	1039124	1058836	1058570	1121633	1121373	1034955	1034689	1416853	1416587	275471	274398	1279827	1280900	636883	636617				
1344	BPSS1278		19a	1036329	1038401	1054552	1056624	1117211	1119283	1031103	1033175	1412896	1414968	271597	273669	1283701	1281629	632966	635038				
1345	BPSS1279	variation_fragmentation	19a	1035175	1036329	1053398	1054552	1116057	1117211	1029949	1031103	1411742	1412896	268753	269223	1286545	1286075	631812	632966				
1346		Chr2_49_IS407A_A	19a	1033957	1035192	1052180	1053415	1114839	1116074	1028731	1029966	1410524	1411759	269225	270460	1284838	1286073	630594	631829				
1347	BPSS1280		19a											268342	268088	1286956	1287210	629711	629457				
1348	BPSS1281		19a											267072	267995	1288226	1287303	628441	629364				
1349	BPSS1282		19a	1032146	1032685	1049839	1050297	1112102	1112284	1026930	1027388	1408709	1410202	265176	266669	1290122	1288629	626545	628038				
1350	BPSS1283		19a	1030837	1032129	1048530	1049822	1110793	1112085	1025621	1026913	1407400	1408692	263867	265159	1291431	1290139	625236	626528				
1351	BPSS1284		19a	1029802	1030821	1047495	1048514	1109758	1110777	1024586	1025605	1406365	1407384	262832	263851	1292466	1291447	624201	625220				
1352		Chr2_50_IS407A_B																		379739	380974	1013932	1015167
1353	BPSS1285		19b	1027661	1029802	1045354	1047495	1107617	1109758	1022445	1024586	1404224	1406365	260691	262832	1294607	1292466	622060	624201	381061	380975	1013845	1013931
1354	BPSS1286		19b	1027327	1027542	1045020	1045235	1107283	1107498	1022111	1022326	1403890	1404105	260357	260572	1294941	1294726	621726	621941	381395	381180	1013511	1013726
1355	BPSS1286a		19b	1026940	1027110	1044633	1044803	1106896	1107066	1021724	1021894	1403503	1403673	259970	260140	1295328	1295158	621339	621509	381782	381612	1013124	1013294
1356	BPSS1287		19b	1026571	1025234	1044264	1042927	1106527	1105190	1021355	1020018	1403134	1401797	259601	258264	1295697	1297034	620970	619633	382151	383488	1012755	1011418
1357	BPSS1288		19b	1023313	1025031	1041006	1042724	1103269	1104987	1018097	1019815	1399876	1401594	256343	258061	1298955	1297237	617712	619430	385409	383691	1009497	1011215
1358	BPSS1289		19b	1022445	1021930	1040138	1039983	1102401	1101886	1017229	1016714	1399008	1398493	255486	254971	1299812	1300327	616855	616340	386301	386816	1008611	1008096
1359	BPSS1290		19b	1021597	1021082	1038934	1038419	1101553	1101038	1016381	1015866	1398160	1397645	254638	254123	1300660	1301175	616007	615492	387149	387664	1007763	1007410
1360	BPSS1291		19b	1020554	1015761	1037891	1036071	1098223	1096799	1015572	1013605	1396448	1391670	253499	248706	1301931	1306724	614880	610087	388192	392985	1006892	1005126
1361		Chr2_51_IS407A_A	19b	1014356	1015591	1031221	1032456	1092993	1094228	1009373	1010608	1390265	1391500	247301	248536	1306894	1308129	608682	609917	393155	394390	1000862	1002097
1362	BPSS1292		19b	1013889	1012597	1030754	1029462	1092526	1091441	1008906	1007614	1389798	1388506	246834	245542	1308596	1309888	608215	606923	394857	396149	1000395	999103
1363	BPSS1293		19b	1011723	1011598	1028588	1028463	1090361	1090236	1006740	1006615	1387448	1387323	244572	244447	1310866	1310991	605921	605796	397119	397244	998141	998016
1364	BPSS1294		19b	1010956	1011468	1027821	1028333	1089594	1090106	1005973	1006485	1386681	1387193	243805	244317	1311633	1311121	605154	605666	397886	397374	997374	997886
1365	BPSS1295		19b	1010741	1009626	1027606	1026491	1089379	1088264	1005758	1004643	1386466	1385351	243590	242475	1311848	1312963	604939	603824	398101	399216	997159	996044
1366	BPSS1296		19b	1008457	1009122	1025322	1025987	1087095	1087760	1003474	1004139	1384182	1384847	241306	241971	1314132	1313467	602655	603320	400385	399720	994875	995540
1367	BPSS1297		19b	1006277	1007794	1023142	1024659	1084915	1086432	1001294	1002811	1382058	1383575	239038	240555	1316392	1314875	600419	601936	402597	401080	992655	994172
1368	BPSS1298		19b	1005607	1004939	1022472	1021804	1084245	1083577	1000624	999956	1381388	1380720	238368	237700	1317062	1317730	599749	599081	403267	403935	991985	991317
1369	BPSS1299		19b	1003189	1004190	1020054	1021055	1081219	1082220	998206	999207	1378970	1379971	235950	236951	1319480	1318479	597331	598332	405683	404691	989569	990561
1370	BPSS1300		19b	1003081	1002380	1019946	1019245	1081111	1080410	998098	997397	1378862	1378161	235842	235141	1319588	1320289	597223	596522	405791	406492	989461	988760
1371	BPSS1301		19b	1002374	1001784	1019239	1018649	1080404	1079910	997391	996801	1378155	1377565	235135	234545	1320295	1320885	596516	595926	406498	407088	988754	988164
1372	BPSS1302		19b	1000699	1001523	1017564	1018388	1078134	1078958	995716	996540	1376474	1377298	233446	234270	1321984	1321160	594827	595651	408185	407361	987067	987891
1373	BPSS1303		19b	999353	1000696	1016218	1017561	1076788	1078131	994370	995713	1375119	1376471	232100	233443	1323330	1321987	593472	594824	409531	408188	985721	987064
1374	BPSS1304		19b	998445	999350	1015310	1016215	1075880	1076785	993462	994367	1374211	1375116	231192	232097	1324238	1323333	592564	593469	410439	409534	984813	985718
1375	BPSS1305		19b	997517	998434	1014382	1015299	1074952	1075869	992534	993451	1373283	1374200	230264	231181	1325166	1324249	591636	592553	411367	410450	983885	984802
1376	BPSS1306		19b	995818	997377	1012683	1014242	1073253	1074812	990835	992394	1371584	1373143	228565	230124	1326865	1325306	589937	591496	413066	411507	982186	983745
1377	BPSS1307		19b	993600	995627	1010465	1012492	1071035	1073062	988617	990644	1369366	1371393	226340	228358	1329074	1327056	587719	589746	415293	413257	979905	981995
1378	BPSS1308		19b	992489	993556	1009354	1010421	1069924	1070991	987506	988573	1368267	1369322	225253	226296	1330161	1329118	586632	587675	416380	415337	978846	979472
1379	BPSS1309		19b	991530	992489	1008395	1009354	1068965	1069924	986547	987506	1367308	1368267	224294	225253	1331120	1330161	586333	586632	417339	416380	977887	978846
1380	BPSS1310		19b	991196	989169	1008061	1006034	1068631	1066604	986213	984186	1366974	1364947	223960	221933	1331454	1333481	585356	583329	417673	419700	977553	975526
1381	BPSS1311		19b	987556	988767	1004320	1005531	1064355	1065566	982573	983784	1363364	1364575	220250	221461	1335014	1333803	581656	582867	421283	420072	972681	973892
1382	BPSS1312		19b	986962	985505	1003726	1002269	1063761	1062304	981979	980522	1362770	1361313	219656	218199	1335608	1337065	581062	579605	421877	423334	972087	970630
1383	BPSS1313		19b	985014	984376	1001778	1001140	1061813	1061175	980031	979393	1360822	1360184	217708	217070	1337564	1338202	579114	578476	423825	424463	970139	969501
1384	BPSS1314_hypothetical protein		19b																				
1385	BPSS1315		19b	980548	983448	997312	1000212	1057347	1060247	975565	978465	1356308	1359208	213226	216126	1342046	1339146	574632	577532	428318	425439	965625	968525
1386	BPSS1316		19b	979526	980461	996290	997225	1056325	1057260	974543	975478	1355295	1356221	212204	213139	1343068	1342133	573610	574545	429340	428405	964603	965538
1387	BPSS1317		19b	978810	979526	995574	996290	1055609	1056325	973827	974543	1354579	1355295	211488	212204	1343784	1343068	572894	573610	430056	429340	963887	964603
1388	BPSS1318		19b	976686	978233	993450	994997	1053485	1055032	971703	973250	1352455	1354002	209350	210897	1345908	1344361	570770	572317	432180	430633	961763	963310
1389	BPSS1319		19b	976313	976600	993077	993364	1053112	1053399	971330	971617	1352082	1352369	208977	209264	1346281	1345994	570397	570684	432553	432266	961390	961677

1390	BPSS1320		19b	975395	976249	992159	993013	1052194	1053048	970412	971266	1351164	1352018	208059	208913	1347199	1346345	569479	570333	433471	432617	960472	961326
1391	BPSS1321		19b	974289	975368	991053	992132	1051088	1052167	969306	970385	1350059	1351156	206954	208051	1348304	1347207	568374	569471	434576	433479	959367	960464
1392	BPSS1322		19b	973553	972684	990317	989448	1050352	1049483	968570	967701	1349323	1348454	206218	205349	1349040	1349909	567638	566769	435312	436181	958631	957762
1393	BPSS1323		19b	971528	972484	988292	989248	1048327	1049283	966545	967501	1347298	1348254	204193	205149	1351065	1350109	565613	566569	437337	436381	956606	957562
1394	BPSS1324		19b	970271	971284	987035	988048	1047070	1048083	965288	966301	1346041	1347054	202936	203949	1352322	1351309	564356	565369	438594	437581	955349	956362
1395	BPSS1325		19b	970078	969572	986842	986336	1046877	1046371	965095	964589	1345848	1345342	202743	202237	1352515	1353021	564163	563657	438787	439293	955156	954650
1396	BPSS1326		19b	969139	967682	985903	984446	1045938	1044481	964156	962699	1344909	1343452	201804	200347	1353454	1354911	563224	561767	439726	441183	954217	952760
1397	BPSS1327		19b	726717	726746	982888	984228	1042923	1044263	961141	962481	1341894	1343234	198789	200129	1356469	1355129	560209	561549	442741	441401	951202	952542
1398	BPSS1328		19b	966016	965294	982780	982058	1042815	1042093	961033	960311	1341786	1341064	198681	197959	1356577	1357299	560101	559379	442849	443571	951094	950372
1399	BPSS1329		19b	965231	963810	981995	980574	1042030	1040609	960248	958827	1341001	1339580	197896	196475	1357362	1358783	559316	557895	443634	445055	950309	948888
1400	BPSS1330		19b	963755	962868	980519	979632	1040554	1039667	958772	957885	1339525	1338638	196420	195533	1358838	1359725	557840	556953	445110	445997	948833	947946
1401	BPSS1331		19b	962519	960975	979283	977739	1039318	1037774	957536	955992	1338289	1336745	195184	193640	1360074	1361618	556604	555060	446346	447890	947597	946053
1402	BPSS1332		19b	960975	959356	977739	976120	1037774	1037664	955992	954373	1336745	1335126	193640	192021	1361618	1363237	555060	553441	447890	449509	946053	944434
1403	BPSS1333		19b	959174	958317	975938	975081	1035972	1035115	954191	953334	1334944	1334087	191839	190982	1363419	1364276	553259	552402	449691	450548	944252	943395
1404	BPSS1334		19b	957637	957356	974401	974120	1034435	1034154	952654	952373	1333407	1333126	190302	190021	1364956	1365237	551722	551441	451228	451509	942715	942434
1405	BPSS1335		19b	956998	956789	973762	973553	1033796	1033587	952015	951806	1332768	1332559	189663	189454	1365595	1365804	551083	550874	451867	452076	942076	941867
1406	BPSS1336		19b	955892	956452	972656	973216	1032690	1033250	950909	951469	1331662	1332222	188557	189117	1366701	1366141	549977	550537	452973	452413	940970	941530
1407	BPSS1337		19b	955438	955848	972202	972612	1032236	1032646	950455	950865	1331208	1331618	188103	188513	1367155	1366745	549523	549933	453427	453017	940516	940926
1408	BPSS1338		19b	954993	955424	971757	972188	1031791	1032222	950010	950441	1330763	1331194	187658	188089	1367600	1367169	549078	549509	453872	453441	940071	940502
1409	BPSS1339		19b	954683	955000	971447	971764	1031481	1031798	949700	950017	1330453	1330770	187348	187665	1367910	1367593	548768	549085	454182	453865	939761	940078
1410	BPSS1340		19b	954142	953612	970906	970376	1030940	1030410	949159	948629	1329912	1329382	186807	186277	1368451	1368981	548227	547697	454723	455253	939220	938690
1411	BPSS1341		19b	951344	953323	968108	970087	1028142	1030121	946361	948340	1327114	1329093	184009	185988	1371249	1369270	545429	547408	457521	455542	936422	938401
1412	BPSS1342		19b	950320	951333	967084	968097	1027118	1028131	945337	946350	1326090	1327103	182985	183998	1372273	1371260	544405	545418	458545	457532	935398	936411
1413	BPSS1343		19b	949999	950280	966763	967044	1026797	1027078	945016	945297	1325769	1326050	182664	182945	1372594	1372313	544084	544365	458866	458585	935077	935358
1414	BPSS1344		19b	949013	949999	965777	966763	1025811	1026797	944030	945016	1324783	1325769	181678	182664	1373580	1372594	543098	544084	459852	458866	934091	935077
1415	BPSS1345		19b	948431	948895	965195	965659	1025229	1025693	943448	943912	1324201	1324665	181096	181560	1374162	1373698	542516	542980	460434	459970	933509	933973
1416	BPSS1346		19b	948082	945968	964846	962732	1024880	1022766	943099	940985	1323852	1321738	180747	178633	1374511	1376625	542167	540053	460783	462897	933160	931046
1417	BPSS1347		19b	944052	945302	960816	962066	1020850	1022100	939069	940319	1457289	1456039	176757	178007	1378541	1377291	538431	539403	464781	463531	929098	930348
1418	BPSS1348		19b	941976	943568	477769	479361	1018774	1020366	936993	938585	1459350	1457773	174693	176273	1380617	1379025	536355	537947	466857	465265	927022	928614
1419	BPSS1349		19b	940676	941668	955734	956726	1017474	1018466	935693	936685	1460679	1459687	173393	174385	1381917	1380925	535055	536047	468157	467165	925722	926714
1420	BPSS1350		19b	938825	940408	474681	476201	1015623	1017206	933961	934008	1462542	1460959	171500	173083	1383792	1382209	533192	534775	470008	468425	923877	925460
1421	BPSS1351		19b	937434	938825	951595	952986	1014232	1015623	932570	933961	1463933	1462542	170109	171500	1385183	1383792	531801	533192	471399	470008	922486	923877
1422	BPSS1352		19b	936388	937284	950549	951445	1013186	1014082	931524	932420	1464979	1464083	169063	169959	1386229	1385333	530755	531651	472445	471549	921440	922336
1423		Chr2_52_IS407A_A	19b	935032	936267	949193	950428	1011830	1013065	930168	931403	1465100	1466335	167708	168943	1386349	1387584	529400	530635	472566	473801	920084	921319
1424	BPSS1353		19b	934742	934158	948903	948319	1011540	1010956	929878	929294	1466625	1467209	167418	166834	1387874	1388458	529110	528526	474091	474675	919794	919210
1425	BPSS1354		19b	934112	932646	948273	946807	1010910	1009444	929248	927782	1467255	1468721	166788	165322	1388504	1389970	528479	527013	474721	476187	919164	917698
1426	BPSS1355		19b	932623	930929	946784	945090	1009421	1007727	927759	926065	1468744	1470438	165299	163605	1389993	1391687	526990	525296	476210	477904	917675	915981
1427	BPSS1356		19b	929846	926472	944007	940633	1006644	1003270	924982	921608	1471521	1474895	162522	159148	1392770	1396144	524213	520839	478987	482361	914898	911524
1428	BPSS1357		19b	926244	924811	940405	938972	1002481	1001048	921380	919947	1475123	1476556	158920	157487	1396372	1397805	520611	519178	482589	484022	911296	909863
1429	BPSS1358		19b	923249	924724	937410	938885	999486	1000961	918385	919860	1478118	1476643	155925	157400	1399367	1397892	517616	519091	485584	484109	908301	909776
1430	BPSS1359		19b	922800	923249	936961	937410	999037	999486	917936	918385	1478567	1478118	155476	155925	1399816	1399367	517167	517616	486033	485584	907852	908301
1431	BPSS1360		19b	920583	922793	934744	936954	996820	999030	915719	917929	1480784	1478574	153259	155469	1402033	1399823	514950	517160	488250	486040	905635	907845
1432	BPSS1361		19b	919552	920034	933713	934195	995789	996271	914688	915170	1481815	1481333	152228	152710	1403064	1402582	513919	514401	489281	488799	904604	905086
1433	BPSS1362		19b	918530	919510	932691	933671	994767	995747	913666	914646	1482837	1481857	151206	152186	1404086	1403106	512897	513877	490303	489323	903582	904562
1434	BPSS1363		19b	917821	917393	931982	931554	994058	993630	912957	912529	1483546	1483974	150497	150069	1404795	1405223	512188	511760	491012	491440	902873	902445
1435	BPSS1364		19b	916409	916609	930570	930770	992646	992846	911545	911745	1484958	1484758	149085	149285	1406207	1406007	510776	510976	492424	492224	901461	901661
1436	BPSS1364a		19b	915756	915547	929917	929708	991993	991784	910892	910683	1485611	1485820	148432	148223	1406860	1407069	510123	509914	493077	493286	900808	900599
1437	BPSS1365		19b	914445	914903	928606	929064	990682	991140	909581	910039	1486922	1486464	147121	147579	1408171	1407713	508812	509270	494388	493930	899497	899955
1438	BPSS1366		19b	914239	914442	927975	928409	990051	990485	908950	909384	1487553	1487119	146490	146924	1408802	1408368	508181	508615	495019	494585	898866	899300
1439	BPSS1367		19b	911092	913821	925253	927982	987329	990058	906228	908957	1489753	1487546	143492	146497	1411800	1408795	505183	508188	497741	495012	896144	898873
1440	BPSS1368		19b											143418	143492	1411874	1411800	505109	505183				

1441		Chr2_53_IS407A_A		909856	911091	924017	925252	986093	987328	904992	906227	1489755	1490990	142183	143418	1411874	1413109	503874	505109	497742	498977	894908	896143
1442	BPSS1369																						
1443	BPSS1370																						
1444	BPSS1371																						
1445	BPSS1372																						
1446	BPSS1373																						
1447	BPSS1374																						
1448	BPSS1375																						
1449	BPSS1376																						
1450	BPSS1377																						
1451	BPSS1378																						
1452	BPSS1379																						
1453	BPSS1380																						
1454	BPSS1381																						
1455	BPSS1382																						
1456	BPSS1383																						
1457	BPSS1383a																						
1458	BPSS1384																						
1459	BPSS1384a																						
1460	BPSS1385																						
1461	BPSS1386																						
1462	BPSS1387																						
1463	BPSS1388																						
1464	BPSS1389																						
1465	BPSS1390																						
1466	BPSS1391																						
1467	BPSS1392																						
1468	BPSS1393																						
1469	BPSS1394																						
1470	BPSS1395																						
1471	BPSS1396																						
1472	BPSS1397																						
1473	BPSS1398																						
1474	BPSS1399																						
1475	BPSS1400																						
1476	BPSS1401																						
1477	BPSS1402																						
1478	BPSS1403																						
1479	BPSS1404																						
1480	BPSS1405																						
1481	BPSS1406																						
1482	BPSS1407																						
1483	BPSS1408																						
1484	BPSS1409																						
1485	BPSS1410																						
1486		Chr2_54_IS407A_A		592085	593320	603189	604424	631977	633212	590631	591866	547403	548638	924390	925625	1782102	1783337	134525	135760				
1487	BPSS1411		20	591873	590761	602977	601865	631765	630653	590419	589307	547191	546079	925837	926949	1783549	1784661	134313	133201				
1488	BPSS1412		20	589849	590328	600953	601432	629741	630220	588395	588874	545167	545646	927861	927382	1785573	1785094	132289	132768				
1489	BPSS1413		20	589101	589751	600205	600855	628993	629643	587647	588297	544419	545069	928609	927959	1786321	1785671	131541	132191				
1490	BPSS1414		20	588949	588248	600053	599352	628841	628140	587495	586794	544267	543566	928761	929462	1786473	1787174	131389	130688				
1491	BPSS1415		20	588166	587279	599270	598383	628058	627171	586712	585825	543484	542597	929544	930431	1787256	1788143	130606	129719				

1492	BPSS1416		20	587265	585889	598369	596993	627157	625781	585811	584435	542583	541207	930445	931797	1788157	1789557	129705	128353				
1493	BPSS1417		20	585889	585251	596993	596355	625781	625143	584435	583797	541207	540569	931797	932435	1789557	1790195	128353	127715				
1494	BPSS1418		20	585159	583825	596263	594929	625051	623717	583705	582371	540477	539143	932527	933861	1790287	1791621	127623	126289				
1495	BPSS1419		20	583740	582967	594844	594071	623632	622859	582286	581513	539058	538285	933946	934719	1791706	1792479	126204	125431				
1496	BPSS1420		20	582936	581968	594040	593072	622828	621860	581482	580514	538254	537286	934750	935718	1792510	1793478	125400	124432				
1497	BPSS1421		20	581076	580288	592180	591392	620968	620180	579622	578834	536459	535671	936584	937372	1794331	1795119	123553	122765				
1498	BPSS1422		20	578496	579401	589600	590505	618388	619293	577042	577947	533879	534784	939164	938259	1796911	1796006	120973	121878				
1499	BPSS1423		20	577172	578119	588137	589084	617064	618011	575747	576511	532555	533502	940495	939548	1798242	1797295	119642	120589				
1500	BPSS1424		20	576073	577068	587038	588033	615965	616960	574648	575643	531456	532451	941594	940599	1799341	1798346	118543	119538				
1501	BPSS1425		20	575048	575947	586013	586912	614940	615839	573715	574146	530431	531330	942619	941720	1800366	1799467	117518	118417				
1502	BPSS1426		20	573886	575052	584851	586017	613778	614944	572553	573719	529269	530435	943781	942615	1801528	1800362	116356	117522				
1503	BPSS1427		20	573085	572438	584050	583403	612977	612330	571752	571105	528468	527821	944591	945238	1802329	1802976	115555	114908				
1504	BPSS1428		20	571841	571629	582806	582594	611733	611521	570508	570296	527230	527018	945809	946021	1803573	1803785	114305	114093				
1505	BPSS1429		20	571527	570631	582492	581596	611419	610523	570194	569298	526916	526020	946123	947019	1803887	1804783	113991	113095				
1506	BPSS1430		20	570156	569824	581121	580789	610048	609716	568823	568491	525545	525213	947494	947826	1805258	1805590	112620	112288				
1507	BPSS1431		20	569851	569348	580816	580313	609743	609240	568518	568015	525240	524737	947799	948302	1805563	1806066	112315	111812				
1508	BPSS1432		20	569310	568819	580275	579784	609202	608711	567977	567486	524699	524208	948340	948831	1806104	1806595	111774	111283				
1509	BPSS1433		20	568753	567686	579718	578651	608645	607578	567420	566353	524142	523075	948897	949964	1806661	1807728	111217	110150				
1510		Chr2_55_IS407A_A		565147	566382	576112	577347	605039	606274	563814	565049	520536	521771	950458	951693	1808233	1809468	108421	109656				
1511	BPSS1434																						
1512	BPSS1437																						
1513	BPSS1438																						
1514	BPSS1439																						
1515		Chr2_56_IS407A_A		838658	839893	852556	853791	881257	882492	836729	837964	793863	795098	675587	676822	1533196	1534431	382829	384064	535045	536280	726908	728143
1516	BPSS1442		21	837310	836156	851208	850054	879909	878755	835381	834227	792515	791361	678170	679324	1535779	1536933	381481	380327	537628	538782	725560	724406
1517	BPSS1443		21	836107	834986	850005	848884	878706	877585	834178	833057	791312	790191	679373	680494	1536982	1538103	380278	379157	538831	539952	724357	723236
1518	BPSS1444		21	833141	834319	847039	848217	875740	876918	831212	832390	788346	789524	682339	681161	1539948	1538770	377312	378490	541792	540614	721396	722574
1519	BPSS1445		21	831045	833036	844943	846934	873644	875635	829416	830855	786237	786464	684435	682444	1542044	1540053	375216	377207	543888	541897	719300	721291
1520	BPSS1446		21	830080	830862	843978	844760	872679	873461	828451	829233	785272	786054	685400	684618	1543009	1542227	374251	375033	544853	544071	718335	719117
1521	BPSS1447		21	828452	830056	842350	843954	871051	872655	826823	828427	783644	785248	687028	685424	1544637	1543033	372617	374227	546481	544877	716707	718311
1522	BPSS1448		21	827248	828426	841146	842324	869847	871025	825619	826797	782440	783618	688232	687054	1545841	1544663	371413	372591	547685	546507	715503	716681
1523	BPSS1449		21	826968	826279	840866	840177	869567	868878	825339	824650	782160	781471	688512	689201	1546121	1546810	371133	370444	547965	548654	715223	714534
1524	BPSS1450		21	825320	824715	839106	838519	867919	867314	823691	823086	780528	779923	690144	690749	1547753	1548358	369501	368896	549589	550194	713583	712978
1525	BPSS1452		21	822861	821401	836665	835205	865460	864000	821232	819772	778069	776609	692603	694063	1550212	1551672	367042	365582	552048	553508	711124	709664
1526	BPSS1453		21	820862	817614	834666	831418	863461	860213	819233	815985	776070	772804	694602	697859	1552211	1555468	365043	361786	554047	557313	709125	705841
1527	BPSS1454		21	817462	816263	831266	830067	860061	858862	815833	814634	772652	771453	698011	699210	1555620	1556819	361634	360435	557465	558664	705689	704490
1528	BPSS1455		21	816258	815470	830062	829274	858857	858069	814629	813841	771448	770660	699215	700003	1556824	1557612	360430	359642	558669	559457	704485	703697
1529	BPSS1456		21	815079	814462	828608	827991	857677	857060	813450	812833	770269	769652	700396	701013	1558005	1558622	359251	358634	559848	560465	703306	702689
1530	BPSS1457		21	814318	813308	827847	826837	856916	856593	812689	811679	769508	768498	701157	702167	1558766	1559776	358490	357480	560609	561619	702545	701535
1531	BPSS1458		21	811307	812689	824850	826232	853311	854693	809678	811060	766644	768026	704090	702900	1561824	1560634	355677	356867	563459	562077	699646	701028
1532	BPSS1459		21	810389	811168	823932	824711	852393	853172	808760	809539	765726	766505	705008	704229	1562742	1561963	354759	355538	564377	563598	698728	699507
1533	BPSS1460		21	808414	809772	821957	823315	849907	851265	806785	808143	763751	765109	706983	705625	1564717	1563359	352784	354142	566352	564994	696719	697753
1534	BPSS1461		21	807752	808408	821295	821951	849245	849901	806123	806779	763089	763745	707645	706989	1565379	1564723	352122	352778	567014	566358	696057	696713
1535	BPSS1462		21	807553	807167	821096	820710	849046	848660	805924	805538	762890	762504	707844	708230	1565578	1565964	351923	351537	567213	567599	695858	695472
1536	BPSS1463		21	806279	805815	819822	819358	847772	847308	804641	804177	761608	761144	709206	709670	1566860	1567324	350673	350209	568439	568903	694648	694184
1537	BPSS1464		21	805790	805485	819333	819028	847283	846978	804152	803847	761119	760814	709695	710000	1567349	1567654	350184	349879	568928	569233	694159	693854
1538	BPSS1465		21	803408	802266	816951	815809	844901	843759	801770	800628	758737	757595	712077	713219	1569731	1570873	347802	346660	571310	572452	691777	690635
1539	BPSS1466		21	800737	802245	814280	815788	842230	843738	799099	800607	756066	757574	714748	713240	1572402	1570894	345131	346639	573981	572473	689106	690614
1540	BPSS1467		21	800405	800575	812976	813986	840926	841936	797795	798805	754762	755772	716052	715042	1573706	1572696	343827	344837	575285	574275	687802	688812
1541	BPSS1468		21	798389	799405	811932	812948	839882	840898	796751	797767	753718	754734	717096	716080	1574738	1573734	342783	343799	576329	575313	686758	687774
1542	BPSS1469		21	798214	797309	811757	810852	839707	838802	796576	795671	753543	752638	717271	718176	1574913	1575818	342608	341703	576504	577409	686583	685678

1543	BPSS1470		21	796530	795232	810073	808775	838023	836725	794892	793594	751859	750561	718955	720253	1576597	1577895	340924	339626	578188	579486	684899	683601
1544	BPSS1471		21	795176	794481	808719	808024	836669	835974	793538	792843	750505	749810	720309	721004	1577951	1578646	339570	338875	579542	580237	683545	682850
1545	BPSS1472_transposase		21																				
1546	BPSS1473		21	792218	794008	805761	807551	833711	835501	790580	792370	747547	749337	723266	721476	1580908	1579118	336613	338403	582500	580710	680587	682377
1547	BPSS1474		21	790733	791488	804276	805031	832226	832981	789095	789850	746062	746817	724751	723996	1582393	1581638	335128	335883	583985	583230	679102	679857
1548	BPSS1475		21	788759	790111	801571	802140	829389	829763	787121	788473	744088	745440	726725	725373	1584367	1583015	333154	334506	585959	584607	677128	678480
1549	BPSS1476		21	786977	788443	799789	801255	827607	829073	785339	786805	742306	743772	728507	727041	1586149	1584683	331372	332838	587741	586275	675346	676812
1550	BPSS1477		21	786805	786080	799617	798892	827435	826710	785167	784442	742134	741409	728679	729404	1586321	1587046	331200	330475	587913	588638	675174	674449
1551	BPSS1478		21	786055	784850	798867	797662	826685	825480	784417	783212	741384	740179	729429	730634	1587071	1588276	330450	329245	588663	589868	674424	673219
1552	BPSS1479		21	784798	783770	797610	796582	825428	824400	783160	782132	740127	739099	730686	731714	1588328	1589356	329193	328165	589920	590948	673167	672139
1553	BPSS1480		21	782814	782632	795626	795444	823444	823262	781176	780994	738158	737976	732657	732839	1590299	1590481	327217	327035	591905	592087	671182	671000
1554	BPSS1481		21	782516	781641	795328	794453	823146	822271	780878	780018	737860	736985	732955	733830	1590597	1591472	326919	326044	592203	593078	670884	670009
1555	BPSS1482		21	781193	780342	794005	793154	821823	820972	779779	778928	736544	735693	734278	735129	1591934	1592785	325624	324773	593554	594405	669582	668731
1556	BPSS1483		21	779059	780081	791871	792893	819689	820711	777645	778667	734410	735432	736403	735390	1594059	1593046	323394	324221	595688	594666	667401	668417
1557	BPSS1484		21	778947	777733	791759	790545	819577	818363	777533	776319	734298	733084	736515	737729	1594171	1595385	323282	322068	595800	597014	667289	666075
1558	BPSS1485		21	777733	776888	790545	789700	818363	817518	776319	775474	733084	732239	737729	738574	1595385	1596230	322068	321223	597014	597859	666075	665230
1559	BPSS1486		21	776839	776402	789651	789214	817469	817032	775425	774988	732190	731753	738623	739060	1596279	1596716	321174	320737	597908	598345	665181	664744
1560	BPSS1487		21	775991	774432	788803	787244	816621	815062	774577	773018	731342	729783	739471	741030	1597127	1598686	320322	318763	598756	600315	664333	662774
1561	BPSS1488		21	774404	773571	787216	786383	815034	814201	772990	772157	729755	728922	741058	741891	1598714	1599547	318735	317902	600343	601176	662646	661972
1562	BPSS1489		21	773571	772960	786383	785772	814201	813590	772157	771546	728922	728311	741891	742502	1599547	1600158	317902	317291	601176	601787	661972	661361
1563	BPSS1490		21	771918	770896	784730	783708	812548	811526	770504	769482	727269	726247	743544	744566	1601200	1602222	316249	315227	602829	603851	660319	659297
1564	BPSS1491		21	770605	769391	783417	782203	811235	810021	769191	767977	725956	724742	744857	746071	1602513	1603727	314936	313722	604142	605356	659006	657792
1565	BPSS1492_hypothetical protein		21																				
1566	BPSS1493		21	765507	767429	778333	780255	806137	808059	764093	766015	721472	722749	749941	748037	1607624	1605675	309856	311760	608655	607366	654479	655768
1567	BPSS1494		21	764773	765507	777599	778333	805403	806137	763359	764093	720138	720872	750675	749941	1608358	1607624	309122	309856	609986	609252	653148	653882
1568	BPSS1495		21	762932	764773	775758	777599	803562	805403	761518	763359	718297	720138	752519	750675	1610199	1608358	307281	309122	611827	609986	651307	653148
1569	BPSS1496		21	762672	762181	775498	775007	803302	802811	761258	760767	718037	717546	752779	753270	1610459	1610950	307021	306530	612087	612578	651047	650556
1570	BPSS1497		21	762154	760658	774980	773484	802784	801288	760740	759244	717519	716023	753297	754793	1610977	1612473	306503	305007	612605	614101	650529	649033
1571	BPSS1498		21	760435	759929	773261	772755	801065	800559	759021	758515	715800	715294	755016	755522	1612696	1613202	304784	304278	614324	614830	648810	648304
1572	BPSS1499		21	759933	759475	772759	772301	800563	800105	758519	758061	715298	714840	755518	755976	1613198	1613656	304282	303824	614826	615284	648308	647850
1573	BPSS1500		21	759435	757696	772261	770522	800065	798326	758021	756282	714800	713061	756016	757755	1613696	1615435	303784	302045	615324	617063	647810	646071
1574	BPSS1501		21	757705	756686	770531	769512	798335	797505	756291	755272	713070	712051	757746	758765	1615426	1616445	302054	301035	617054	618073	646080	645061
1575	BPSS1502		21	756696	753655	769522	766481	797183	794142	755282	752214	712061	709020	758755	761805	1616435	1619479	301045	298004	618063	621104	645071	645006
1576	BPSS1503		21	753586	750566	766412	763392	794073	791053	752145	749125	708888	705868	761865	764885	1619509	1622529	297962	294942	621182	624202	642061	639041
1577	BPSS1504		21	750537	747898	763363	760724	791024	788385	749096	746457	705839	703200	764914	767553	1622558	1625197	294913	292274	624231	626870	639012	636373
1578	BPSS1505		21	747877	746816	760703	759642	788364	787303	746436	745375	703179	702118	767574	768635	1625218	1626279	292253	291192	626891	627952	636352	635291
1579	BPSS1506		21	746816	746058	759642	758884	787303	786545	745375	744617	702118	701360	768635	769393	1626279	1627037	291192	290434	627952	628710	635291	634533
1580	BPSS1507		21	746026	745637	758852	758463	786513	786124	744585	744196	701328	700939	769425	769814	1627069	1627458	290402	290013	628742	629131	634501	634112
1581	BPSS1508		21	745504	744830	758330	757656	785991	785317	744063	743389	700806	700132	769947	770621	1627591	1628265	289880	289206	629264	629938	633979	633305
1582	BPSS1509		21	744830	743400	757656	756226	785317	783887	743389	741959	700132	698702	770621	772051	1628265	1629695	289206	287776	629938	631368	633305	631875
1583	BPSS1510		21	743382	742738	756208	755564	783869	783225	741941	741297	698684	698040	772069	772713	1629713	1630357	287758	287114	631386	632030	631857	631213
1584	BPSS1511		21	740992	738695	753818	751521	783210	781831	739551	737254	698025	694078	772728	776693	1630372	1634328	287099	283143	633875	636109	631198	630812
1585	BPSS1512		21	737589	736738	750415	749564	777482	776631	736148	735297	693028	692177	777767	778618	1635434	1636285	282085	281234	637199	638050	626540	625689
1586	BPSS1513		21	736571	736281	749397	749107	776464	776174	735130	734840	692010	691720	778785	779075	1636452	1636742	281067	280777	638217	638507	625522	625232
1587	BPSS1514		21	736273	735644	749099	748470	776166	775537	734832	734203	691712	691083	779083	779712	1636750	1637379	280769	280140	638515	639144	625224	624595
1588	BPSS1515		21	735527	735225	748353	748051	775420	775118	734086	733784	690966	690664	779829	780131	1637496	1637798	280023	279721	639261	639563	624478	624176
1589		Chr2_57a_IS407A_A		733880	735115	746706	747941	773773	775008	732439	733674	689319	690554	780241	781476	1637908	1639143	278376	279611	639673	640908	622831	624066
1590		Chr2_57b_IS407A_A		1643058	1644293	1673540	1674775	1637867	1639102	1636577	1637812	1605359	1606594	2142275	2143510	714504	715739	1194967	1196202			1511320	1512555
1591	BPSS1516		22									1607916	1606546	2140953	2142275	713182	714504	1197524	1196202			1513877	1512555
1592	BPSS1517		22	1645619	1645179	1676101	1675661	1640428	1639988	1639138	1638698	1608511	1608071	2140358	2140798	712587	713027	1198119	1197679			1514472	1514032
1593	BPSS1518		22	1645979	1645701	1676461	1676183	1640788	1640510	1639498	1639220	1608871	1608593	2139998	2140276	712227	712505	1198479	1198201			1514832	1514554

1594	BPSS1519	22	1645897	1646445	1676379	1676927	1640706	1641254	1639416	1639964	1608789	1609337	2140080	2139532	712309	711761	1198397	1198945	1514750	1515298
1595	BPSS1520	22	1647593	1646535	1678075	1677017	1642402	1641344	1641112	1640054	1610485	1609427	2138384	2139442	710613	711671	1200093	1199035	1516446	1515388
1596	BPSS1521	22	1648419	1647979	1678901	1678461	1644000	1643560	1641938	1641498	1611332	1610892	2137544	2137984	709779	710219	1200926	1200486	1517287	1516847
1597	BPSS1522	22	1649346	1648678	1679828	1679160	1644927	1644259	1642865	1642197	1612259	1611591	2136617	2137285	708852	709520	1201853	1201185	1518214	1517546
1598	BPSS1523	22	1650045	1649593	1680527	1680075	1645626	1645174	1643564	1643112	1612958	1612506	2135918	2136370	708153	708605	1202552	1202100	1518913	1518461
1599	BPSS1524	22	1651735	1650200	1682217	1680682	1647316	1645781	1645254	1643719	1614648	1613113	2134228	2135763	706463	707998	1204242	1202707	1520603	1519068
1600	BPSS1525	22	1652361	1653143	1682843	1683625	1647942	1648724	1645880	1646662	1615274	1616056	2133602	2132820	705837	705055	1204799	1205581	1521229	1522011
1601	BPSS1526	22	1654160	1653600	1684642	1684082	1649741	1649181	1647679	1647119	1617064	1616504	2131749	2132309	703948	704508	1206661	1206101	1523172	1522612
1602	BPSS1527	22	1654438	1654160	1684920	1684642	1650019	1649741	1647957	1647679	1617342	1617064	2131471	2131749	703670	703948	1206939	1206661	1523450	1523172
1603	BPSS1528	22	1657128	1654555	1687815	1687132	1652673	1650136	1650647	1648074	1620041	1617522	2128787	2131471	700995	703670	1209623	1206939	1526140	1525229
1604	BPSS1529	22	1658162	1657233	1688849	1687920	1654156	1653227	1651681	1650752	1621075	1620146	2127753	2128682	699961	700890	1210657	1209728	1527174	1526245
1605	BPSS1530	22	1658526	1658227	1689213	1688914	1654520	1654221	1652045	1651746	1621439	1621140	2127389	2127688	699597	699896	1211021	1210722	1527538	1527239
1606	BPSS1531	22	1659907	1658651	1690826	1690296	1655901	1654645	1653426	1652170	1622820	1621564	2126008	2127264	698216	699472	1212402	1211146	1528919	1527663
1607	BPSS1532	22	1661811	1659952	1692730	1690871	1657805	1655946	1655330	1653471	1624724	1622865	2124104	2125963	696312	698171	1214306	1212447	1530823	1528964
1608	BPSS1533	22	1662345	1661833	1693264	1692752	1658339	1657827	1655864	1655352	1625258	1624746	2123570	2124082	695778	696290	1214840	1214328	1531357	1530845
1609	BPSS1534	22	1663706	1662474	1694625	1693393	1659700	1658468	1657225	1655993	1626619	1625387	2122209	2123441	694417	695649	1216201	1214969	1532718	1531486
1610	BPSS1535	22	1664465	1663713	1695384	1694632	1660459	1659707	1657984	1657232	1627378	1626626	2121450	2122202	693658	694410	1216960	1216208	1533477	1532725
1611	BPSS1536	22	1664753	1664502	1695672	1695421	1660747	1660496	1658272	1658021	1627666	1627415	2121162	2121413	693370	693621	1217248	1216997	1533765	1533514
1612	BPSS1537	22	1665469	1664792	1696388	1695711	1661463	1660786	1658988	1658311	1628382	1627705	2120446	2121123	692654	693331	1217964	1217287	1534481	1533804
1613	BPSS1538	22	1666442	1665462	1697361	1696381	1662436	1661456	1659961	1658981	1629355	1628375	2119449	2120453	691657	692661	1218961	1217957	1535466	1534474
1614	BPSS1539	22	1667686	1666442	1698605	1697361	1662849	1662436			1630599	1629355	2118205	2119449	690413	691657			1535819	1535466
1615	BPSS1540	22	1668128	1667667	1699047	1698586	1664899	1664438	1661601	1661140	1631041	1630580	2117763	2118224	689971	690432	1220647	1220186	1536790	1536329
1616	BPSS1541	22	1669435	1668128	1700354	1699047	1666206	1664899	1662908	1661601	1632348	1631041	2116456	2117763	688664	689971	1221954	1220647	1538097	1536790
1617	BPSS1542	22	1669839	1669435	1700758	1700354	1666610	1666206	1663312	1662908	1632752	1632348	2116052	2116456	688260	688664	1222358	1221954	1538501	1538097
1618	BPSS1543	22	1671923	1669854	1702842	1700773	1668694	1666625	1665396	1663327	1634836	1632767	2113968	2116037	686176	688245	1224442	1222373	1540585	1538516
1619	BPSS1544	22	1673080	1671962	1703999	1702881	1669851	1668733	1666553	1665435	1635993	1634875	2112811	2113929	685019	686137	1225599	1224481	1541742	1540624
1620	BPSS1545	22	1674954	1673080	1706387	1706145	1672352	1671861	1668427	1666553	1637867	1635993	2110937	2112811	683145	685019	1227473	1225599	1543489	1543424
1621	BPSS1546	22	1675609	1674971	1707042	1706404	1673007	1672369	1669082	1668444	1638522	1637884	2110282	2110920	682490	683128	1228128	1227490	1544144	1543506
1622	BPSS1547	22	1676120	1677403	1707553	1708176	1673518	1674801	1669593	1670876	1639033	1640316	2109771	2108488	681979	680696	1228639	1229922	1544655	1545938
1623	BPSS1548	22	1677403	1677669	1709078	1709344	1674801	1675067	1670876	1671142	1640316	1640582	2108488	2108222	680696	680430	1229922	1230188	1545938	1546204
1624	BPSS1549	22	1677727	1678026	1709402	1709701	1675125	1675424	1671200	1671499	1640640	1640939	2108164	2107865	680372	680073	1230246	1230545	1546262	1546561
1625	BPSS1550	22	1678034	1678981	1709709	1710434	1675432	1676022	1671507	1672379	1640947	1641903	2107857	2106910	680065	679118	1230553	1231434	1546569	1547195
1626	BPSS1551	22	1678981	1679565	2357503	2357745	1677391	1677975	1672397	1672981	1641895	1642479	2106910	2106326	679118	678534	1231575	1232159	1547298	1547825
1627	BPSS1552	22	1679537	1680328	2357717	2358301	1677947	1678561	1672953	1673744	1642451	1643242	2106354	2105563	678562	677771	1232131	1232922	1547797	1548588
1628	BPSS1553	22	1681325	1682293	1712424	1713392	1679727	1680656	1674741	1675709	1644239	1645207	2104571	2103603	676779	675811	1233914	1234882	1549585	1550553
1629	BPSS1554	22	1682308	1682745	1713407	1713844	1680985	1681323	1675724	1676161	1645222	1645659	2103588	2103151	675796	675359	1234897	1235334	1550568	1551005
1630	BPSS1555	22	1683758	1685452	1714857	1716551	1682336	1684030	1677174	1678868	1646672	1648366	2102138	2100444	674346	672652	1236347	1238077	1552018	1553712
1631	BPSS1556	22	1687049	1686564	1718148	1717663	1685627	1685142	1680465	1679980	1649963	1649478	2098847	2099332	671055	671540	1239674	1239189	1555309	1554824
1632	BPSS1557	22	1687376	1687831	1718475	1718930	1685954	1686409	1680792	1681247	1650290	1650745	2098520	2098065	670728	670273	1240001	1240456	1555636	1556091
1633	BPSS1558	22	1688398	1689714	1719497	1720813	1686976	1688292	1681814	1683130	1651312	1652628	2097498	2096182	669706	668390	1241023	1242339	1556658	1557974
1634	BPSS1559	22	1690965	1690081	1722064	1721180	1689543	1688659	1684381	1683497	1653879	1652995	2094931	2095815	667139	668023	1243590	1242706	1559225	1558341
1635	BPSS1560	22	1691066	1692286	1722165	1723385	1689644	1690864	1684482	1685702	1653980	1655200	2094830	2093610	667038	665818	1243691	1244911	1559326	1560546
1636	BPSS1561	22	1694077	1692416	1725176	1723515	1692655	1690994	1687493	1685832	1656991	1655330	2091819	2093480	664027	665688	1246702	1245041	1562337	1560676
1637	BPSS1562	22	1696408	1694837	1727507	1725936	1694986	1693415	1689824	1688253	1659295	1657724	2089518	2091104	661663	663249	1249076	1247490	1564650	1563079
1638	BPSS1563	22	1697453	1696842	1728552	1727941	1696031	1695420	1690869	1690258	1660340	1659729	2088473	2089084	660618	661229	1250121	1249510	1565695	1565084
1639	BPSS1564	22	1697587	1698048	1728686	1729147	1696165	1696626	1691003	1691464	1660474	1660935	2088339	2087878	660484	660023	1250255	1250716	1565829	1566290
1640	BPSS1565	22	1698378	1698776	1729477	1729875	1696956	1697354	1691794	1692192	1661265	1661663	2087516	2087118	659653	659255	1251118	1251516	1566620	1567018
1641	BPSS1566	22	1699173	1700756	1730272	1731855	1697751	1699334	1692589	1694172	1662060	1663643	2086721	2085138	658858	657275	1251913	1253496	1567415	1568998
1642	BPSS1567	22	1700909	1701688	1732008	1732787	1699487	1700266	1694325	1695104	1663796	1664575	2084985	2084206	657122	656343	1253649	1254428	1569151	1569930
1643	BPSS1568	22	1702271	1703875	1733370	1734974	1700849	1702453	1695687	1697291	1665158	1666762	2083623	2082019	655760	654156	1255011	1256615	1570513	1572117
1644	BPSS1569	22	1704646	1705335	1735745	1736434	1703224	1703913	1698062	1698751	1667533	1668222	2081248	2080559	653385	652696	1257386	1258075	1572888	1573577

1645	BPSS1570	22	1705664	1706269	1736763	1737368	1704242	1704847	1699080	1699685	1668551	1669156	2080230	2079625	652367	651762	1258404	1259009	1573906	1574511
1646	BPSS1571	22	1706566	1707927	1737665	1739026	1705144	1706379	1699982	1701343	1669453	1670814	2079328	2077967	651465	650104	1259306	1260667	1574808	1576169
1647	BPSS1572	22	1709133	1708279	1740232	1739378	1708638	1707784	1702549	1701695	1672020	1671166	2076761	2077615	648898	649752	1261873	1261019	1577375	1576521
1648	BPSS1573	22	1709912	1709133	1741011	1740232	1709417	1708638	1703328	1702549	1672799	1672020	2075982	2076761	648119	648898	1262652	1261873	1578154	1577375
1649	BPSS1574	22	1710682	1709936	1741781	1741035	1710187	1709441	1704098	1703352	1673569	1672823	2075212	2075958	647349	648095	1263422	1262676	1578924	1578178
1650	BPSS1575	22	1710630	1710932	1741729	1742031	1710135	1710437	1704046	1704348	1673517	1673819	2075264	2074962	647401	647099	1263370	1263672	1578872	1579174
1651	BPSS1576	22	1712924	1711371	1744023	1742470	1712429	1710876	1706340	1704787	1675811	1674258	2072970	2074523	645107	646660	1265664	1264111	1581166	1579613
1652	BPSS1577	22	1715504	1712961	1746603	1744060	1715009	1712466	1708910	1708137	1678379	1675848	2070390	2072933	642527	645070	1268244	1265701	1583733	1583443
1653	BPSS1578	22	1716364	1715504	1747463	1746603	1715869	1715009	1709770	1708910	1679239	1678379	2069530	2070390	641667	642527	1269104	1268244	1584593	1583733
1654	BPSS1579	22	1718819	1716573	1749918	1747672	1719108	1717303	1712225	1709979	1681694	1679448	2067075	2069321	639212	641458	1271559	1269313	1586992	1586927
1655	BPSS1580	22	1723287	1718848	1752646	1749947	1723576	1719137	1716693	1712254	1686157	1683704	2062607	2067046	634744	639183	1276027	1271588		
1656	BPSS1581	22	1724861	1723569	1756220	1756149	1725927	1725853	1718066	1716975	1687734	1686439	2061033	2062325	633170	634462	1277451	1276309	1591456	1590323
1657	BPSS1582	22	1727268	1725118	1758627	1756477	1728334	1726184	1720607	1718457	1690141	1687991	2058644	2060794	630781	632931	1280187	1278037	1594164	1592014
1658	BPSS1582a	22	1727858	1728418	1759237	1759797	1729663	1730223	1721197	1721757	1690731	1691291	2058054	2057494	630191	629631	1280777	1281337	1594754	1595314
1659	BPSS1583	22	1728577	1728978	1759956	1760357	1730382	1730783	1721916	1722317	1691450	1691851	2057335	2056934	629472	629071	1281496	1281897	1595473	1595874
1660	BPSS1584	22	1728978	1730327	1760357	1761706	1730783	1732132	1722317	1723666	1691851	1693200	2056934	2055585	629071	627722	1281897	1283246	1595874	1597223
1661	BPSS1585	22	1730939	1730490	1762318	1761869	1732744	1732295	1724278	1723829	1693845	1693396	2054962	2055411	627099	627548	1283869	1283420	1597846	1597397
1662	BPSS1586	22	1732263	1731352	1763642	1762731	1734068	1733157	1725602	1724691	1695169	1694258	2053617	2054528	625754	626665	1285200	1284289	1599163	1598252
1663	BPSS1587	22	1732430	1733380	1763809	1764759	1734235	1735185	1725769	1726719	1695336	1696286	2053450	2052500	625587	624637	1285367	1286317	1599330	1600280
1664	BPSS1588	22	1733994	1735391	1765373	1766770	1736632	1738029	1727333	1728730	1696900	1698297	2051886	2050489	624023	622626	1286931	1288328	1600894	1602291
1665	BPSS1589	22	1736740	1737537	1768119	1768916	1739378	1740175	1730079	1730876	1699645	1700442	2049149	2048352	621295	620498	1289675	1290472	1603646	1604443
1666	BPSS1590	22	1737795	1738013	1769174	1769392	1740433	1740651	1731134	1731352	1700700	1700918	2048094	2047876	620240	620022	1290730	1290948	1604701	1604919
1667	BPSS1591	22	1738167	1739048	1769546	1770427	1740805	1741686	1731506	1732387	1701072	1701953	2047722	2046841	619868	618987	1291102	1291983	1605073	1605954
1668	BPSS1592	22	1740918	1739122	1772297	1770501	1743556	1741760	1734277	1733639	1703823	1702027	2044971	2046767	617117	618913	1293853	1292057	1607824	1606028
1669	BPSS1593	22	1742706	1741048	1774085	1772427	1745344	1743686	1736065	1734407	1705611	1703953	2043183	2044841	615329	616987	1295641	1293983	1609612	1607954
1670	BPSS1594	22	1743194	1742718	1774573	1774097	1745832	1745356	1736553	1736077	1706099	1705623	2042695	2043171	614841	615317	1296129	1295653	1610100	1609624
1671	BPSS1595	22	1743788	1743237	1775167	1774616	1746426	1745875	1737147	1736596	1706693	1706142	2042101	2042652	614247	614798	1296723	1296172	1610694	1610143
1672	BPSS1596	22	1744897	1743824	1776276	1775203	1747535	1746462	1738256	1737183	1707802	1706729	2040992	2042065	613138	614211	1297832	1296759	1611803	1610730
1673	BPSS1597	22	1746497	1744890	1777876	1776269	1749135	1747528	1739856	1738249	1709402	1707795	2039392	2040999	611538	613145	1299432	1297825	1613349	1611796
1674	BPSS1598	22	1746997	1746497	1778376	1777876	1749635	1749135	1740356	1739856	1709902	1709402	2038892	2039392	611038	611538	1299932	1299432	1613713	1613453
1675	BPSS1599	22	1748306	1747011	1779685	1778390	1750944	1749649	1741665	1740370	1711211	1709916	2037583	2038878	609729	611024	1301241	1299946	1614945	1614625
1676	BPSS1600	22	1750090	1748321	1782134	1781664	1752728	1750959	1743302	1743039	1712997	1712965	2035799	2037568	607945	609714	1303025	1301256	1616729	1614960
1677	BPSS1601	22	1750659	1750093	1782564	1782334	1754001	1753735	1743871	1743305	1713566	1713000	2035230	2035796	607376	607942	1303594	1303028	1617298	1616732
1678	BPSS1602	22	1750976	1752079	1782881	1783984	1754318	1755421	1744188	1745045	1713883	1714986	2034913	2033810	607059	605956	1303911	1305014	1617615	1618718
1679	BPSS1603	22	1754093	1752306	1786200	1784863	1758263	1757406	1747251	1745464	1716257	1715475	2031796	2033583	603942	605729	1307028	1305241	1620732	1618945
1680	BPSS1604	22	1754839	1754093	1786946	1786200	1759009	1758263	1747997	1747251	1717003	1716257	2031050	2031796	603196	603942	1307774	1307028	1621478	1620732
1681	BPSS1605	22	1755975	1754893	1788082	1787000	1760145	1759063	1749133	1748051	1718139	1717057	2029914	2030996	602060	603142	1308910	1307828	1622614	1621532
1682	BPSS1606	22	1756747	1756106	1788854	1788213	1760917	1760276	1749905	1749264	1718911	1718270	2029142	2029783	601288	601929	1309682	1309041	1623386	1622745
1683	BPSS1607	22	1756956	1757441	1789063	1789548	1761126	1761611	1750114	1750599	1719120	1719605	2028933	2028448	601079	600594	1309891	1310376	1623595	1624080
1684	BPSS1608	22	1758630	1757971	1790737	1790078	1762800	1762141	1751788	1751129	1720794	1720135	2027259	2027918	599405	600064	1311565	1310906	1625262	1624603
1685	BPSS1609	22	1758839	1759075	1790946	1791182	1763009	1763245	1751997	1752233	1721003	1721239	2027050	2026814	599196	598960	1311774	1312010	1625471	1625707
1686	BPSS1610	22	1761465	1760035	1793572	1792142	1765635	1764205	1754623	1753193	1723629	1722199	2024424	2025854	596570	598000	1314400	1312970	1628097	1626667
1687	BPSS1611	22	1762132	1761680	1794239	1793787	1766302	1765850	1755290	1754838	1724296	1723844	2023757	2024209	595903	596355	1315067	1314615	1628764	1628312
1688	BPSS1612	22	1762413	1762228	1794520	1794335	1766583	1766398	1755571	1755386	1724577	1724392	2023476	2023661	595622	595807	1315348	1315163	1629045	1628860
1689	BPSS1613	22	1762828	1762580	1795493	1795245	1772558	1772310	1755984	1755736	1724993	1724745	2023061	2023309	595207	595455	1315763	1315515	1629494	1629246
1690	BPSS1614	22	1763834	1763436	1796499	1796101	1768526	1768128	1756990	1756592	1725999	1725601	2022055	2022453	594201	594599	1316769	1316371		
1691	BPSS1615	22	1764899	1764033	1797564	1796698	1769591	1768725	1758055	1757189	1727064	1726198	2020990	2021856	593136	594002	1317834	1316968	1631576	1630710
1692	BPSS1616	22	1765159	1764899	1797824	1797564	1769851	1769591	1758315	1758055	1727324	1727064	2020730	2020990	592876	593136	1318094	1317834	1631836	1631576
1693	BPSS1617	22	1765850	1765203	1798515	1797868	1770542	1769895	1759006	1758359	1728015	1727368	2020039	2020686	592185	592832	1318785	1318138	1632527	1631880
1694	BPSS1618	22	1767135	1765840	1800052	1799891	1771827	1770532	1760056	1759514	1729300	1728005	2018754	2020049	590900	592195	1320070	1318775	1633704	1632952
1695	BPSS1619	22	1767380	1767168	1800691	1800287	1772072	1771860	1760695	1760291	1729939	1729535	2018115	2018519	590261	590665	1320709	1320305	1634343	1633939

1696	BPSS1620		22	1769843	1767774	1802760	1800691	1775337	1773544	1762764	1760695	1732008	1729939	2016046	2018115	588192	590261	1322778	1320709			1636326	1634551
1697	BPSS1621		22	1770922	1769843	1803839	1802760	1776416	1775337	1763843	1762764	1733087	1732008	2014967	2016046	587113	588192	1323857	1322778			1637315	1636326
1698	BPSS1622		22	1771158	1771730	1804075	1804647	1776652	1777224	1764079	1764651	1733323	1733895	2014731	2014159	586877	586305	1324093	1324530			1637535	1638107
1699	BPSS1623		22	1771747	1772160	1804664	1805077	1777241	1777654	1764668	1765081	1733912	1734325	2014142	2013729	586288	585875	1324684	1325097			1638124	1638537
1700	BPSS1624		22	1772160	1772432	1805077	1805349	1777654	1777926	1765081	1765353	1734325	1734597	2013729	2013457	585875	585603	1325097	1325369			1638537	1638809
1701	BPSS1625		22	1773009	1773683	1805926	1806114	1779375	1780019	1765928	1766569	1735174	1735848	2012880	2012206	585026	584352	1325946	1326620			1639386	1640060
1702	BPSS1626		22	1773671	1774384	1807266	1807979	1780007	1780720	1766557	1767270	1735836	1736549	2012218	2011505	584364	583651	1326608	1327321			1640048	1640761
1703	BPSS1627		22	1774384	1775709	1807979	1809238	1780720	1782045	1767270	1768595	1736549	1737874	2011505	2010180	583651	582326	1327321	1327353			1640761	1642086
1704	BPSS1628		22	1775709	1776233	1809520	1809924	1782045	1782569	1768595	1769119	1737874	1738398	2010180	2009656	582326	581802	1328647	1329171			1642086	1642610
1705	BPSS1629		22	1776233	1777234	1809924	1810925	1782569	1783570	1769119	1770120	1738398	1739417	2009656	2008673	581802	580783	1329171	1330181			1642610	1643638
1706	BPSS1630		22	1777605	1779368	1811296	1813059	1783941	1785704	1770491	1772254	1739788	1741551	2008302	2006539	580340	578577	1330552	1332315			1644009	1645784
1707	BPSS1631		22	1780581	1779619	1814272	1813310	1786917	1785955	1773467	1772505	1742764	1741802	2005326	2006288	577364	578326	1333528	1332566			1646997	1646035
1708	BPSS1632		22	1790632	1780640	1824323	1814331	1796968	1786976	1783518	1773526	1752815	1742823	1995275	2005267	567313	577305	1343579	1333587			1657048	1647056
1709		Chr2_58_IS407A_A		1790656	1791891	1824347	1825582	1796992	1798227	1783542	1784777	1752839	1754074	1994016	1995251	566054	567289	1343603	1344838	1705942	1707177	1657072	1658307
1710	BPSS1633		23	1793073	1791892	1826764	1825583	1799409	1798228	1785959	1784778	1755256	1754075	1992834	1994015	564872	566053	1346020	1344839	1704760	1705941	1659489	1658308
1711	BPSS1634		23	1796451	1791892	1830142	1825583	1802787	1798228	1789337	1784778	1758634	1754075	1989456	1994015	561494	566053	1349398	1344839	1701382	1705941	1662867	1658308
1712	BPSS1635		23	1799525	1796649	1833216	1830340	1805861	1802985	1792411	1789535	1761708	1758832	1986382	1989258	558420	561296	1352472	1349596	1698308	1701184	1665941	1663065
1713	BPSS1636		23	1802899	1801172	1836590	1834863	1809235	1807508	1795785	1794058	1765082	1763355	1983008	1984735	555046	556773	1355846	1354119	1694934	1696661	1669315	1667588
1714	BPSS1637		23	1804237	1802903	1837928	1836594	1810573	1809239	1797123	1795789	1766420	1765086	1981670	1983004	553708	555042	1357184	1355850	1693596	1694930	1670653	1669319
1715	BPSS1638		23	1804783	1804259	1838474	1837950	1811119	1810595	1797669	1797145	1766966	1766442	1981124	1981648	553162	553686	1357730	1357206	1693050	1693574	1671199	1670675
1716	BPSS1639		23	1805701	1804823	1839392	1838514	1812037	1811159	1798587	1797709	1767884	1767006	1980206	1981084	552244	553122	1358648	1357770	1692132	1693010	1672117	1671239
1717	BPSS1640		23	1806717	1805809	1840408	1839500	1813053	1812145	1799603	1798695	1768900	1767992	1979190	1980098	551228	552136	1359664	1358756	1691116	1692024	1673133	1672225
1718	BPSS1641		23	1806869	1807180	1840560	1840871	1813205	1813516	1799755	1800066	1769052	1769363	1979038	1978727	551076	550765	1359816	1360127	1690964	1690653	1673285	1673596
1719	BPSS1643		23	1808190	1807282	1841881	1840973	1814526	1813618	1801068	1800217	1770373	1769465	1977717	1978625	549755	550663	1361137	1360229	1689643	1690551	1674606	1673698
1720	BPSS1644		23	1808308	1809702	1841999	1843393	1814644	1816038	1801186	1802580	1770491	1771885	1977599	1976205	549637	548243	1361255	1362649	1689525	1688131	1674724	1676118
1721	BPSS1645		23	1809702	1810097	1843393	1843788	1816038	1816433	1802580	1802975	1771885	1772280	1976205	1975810	548243	547848	1362649	1363044	1688131	1687736	1676118	1676513
1722	BPSS1646		23	1811432	1810266	1845123	1843957	1817768	1816602	1804310	1803144	1773615	1772449	1974475	1975641	546513	547679	1364379	1363213	1686401	1687567	1677848	1676682
1723	BPSS1647		23	1812603	1811428	1846294	1845119	1818939	1817764	1805481	1804306	1774786	1773611	1973304	1974479	545342	546517	1365550	1364375	1685230	1686405	1679019	1677844
1724	BPSS1648		23	1814103	1812679	1847794	1846370	1820439	1819015	1806981	1805557	1776286	1774862	1971804	1973228	543842	545266	1367050	1365626	1683730	1685154	1680519	1679095
1725	BPSS1649		23	1814489	1816906	1848180	1850597	1820825	1823242	1807367	1809784	1776672	1779089	1971418	1969001	543456	541039	1367436	1369853	1683344	1680927	1680905	1683322
1726	BPSS1650		23	1816906	1817394	1850597	1851085	1823242	1823730	1809784	1810272	1779089	1779577	1969001	1968513	541039	540551	1369853	1370341	1680927	1680439	1683322	1683810
1727	BPSS1651		23	1817384	1817995	1851075	1851686	1823720	1824331	1810262	1810873	1779567	1780178	1968523	1967912	540561	539950	1370331	1370942	1680449	1679838	1683800	1684411
1728	BPSS1652		23	1819097	1818102	1852788	1851793	1825433	1824438	1811975	1810980	1781280	1780285	1966810	1967805	538848	539843	1372044	1371049	1678736	1679731	1685513	1684518
1729	BPSS1653		23	1820287	1819721	1853978	1853412	1826623	1826057	1813165	1812599	1782470	1781904	1965620	1966186	537658	538224	1373234	1372668	1677546	1678112	1686703	1686137
1730	BPSS1654		23	1822709	1820358	1856400	1854049	1829045	1826694	1815587	1813236	1784892	1782541	1963198	1965549	535236	537587	1375656	1373305	1675124	1677475	1689125	1686774
1731	BPSS1655		23	1822988	1823125	1856679	1856816	1829324	1829461	1815866	1816003	1785171	1785308	1962919	1962782	534957	534820	1375935	1376072	1674845	1674708	1689404	1689541
1732	BPSS1656		23	1823200	1824453	1856891	1858144	1829536	1830789	1816078	1817331	1785383	1786636	1962707	1961454	534745	533492	1376147	1377400	1674633	1673380	1689616	1690869
1733	BPSS1657		23	1825195	1827387	1858886	1861078	1831531	1833723	1818073	1820265	1787384	1789576	1960712	1958520	532750	530558	1378148	1380340	1672626	1670434	1691623	1693815
1734	BPSS1658		23	1827875	1828363	1861566	1862054	1834211	1834699	1820753	1821241	1790064	1790552	1958032	1957544	530070	529582	1380828	1381316	1669946	1669458	1694303	1694791
1735	BPSS1659		23	1828507	1828791	1862198	1862482	1834843	1835127	1821385	1821669	1790696	1790980	1957400	1957116	529438	529154	1381460	1381744	1669314	1669030	1694935	1695219
1736	BPSS1660		23	1828874	1829353	1862565	1863044	1835210	1835689	1821752	1822231	1791063	1791542	1957033	1956554	529071	528592	1381827	1382306	1668947	1668468	1695302	1695781
1737	BPSS1661		23	1829425	1829637	1863116	1863328	1835761	1835973	1822303	1822515	1791614	1791826	1956482	1956270	528520	528308	1382378	1382590	1668396	1668184	1695853	1696065
1738	BPSS1662		23	1832146	1830224	1865837	1863915	1838482	1836560	1825024	1823102	1794335	1792413	1953761	1955683	525799	527721	1385099	1383177	1665675	1667597	1698574	1696652
1739	BPSS1663		23	1833585	1832146	1867736	1867077	1839921	1838482	1826463	1825024	1795774	1794335	1952322	1953761	524360	525799	1386538	1385099	1664236	1665675	1700013	1698574
1740	BPSS1664		23	1834550	1833585	1868701	1867736	1840886	1839921	1827428	1826463	1796739	1795774	1951357	1952322	523395	524360	1387503	1386538	1663271	1664236	1700978	1700013
1741	BPSS1665		23	1835351	1834728	1869502	1868879	1841687	1841064	1828229	1827606	1797540	1796917	1950556	1951179	522594	523217	1388304	1387681	1662470	1663093	1701779	1701156
1742	BPSS1666		23	1836262	1835351	1870413	1869502	1842598	1841687	1829140	1828229	1798451	1797540	1949645	1950556	521683	522594	1389215	1388304	1661559	1662470	1702690	1701779
1743	BPSS1667		23	1839344	1836276	1873495	1870427	1845680	1842612	1832222	1829154	1801533	1798465	1946563	1949631	518601	521669	1392297	1389229	1658477	1661545	1705772	1702704
1744	BPSS1669		23	1841052	1840288	1875203	1874439	1847388	1846624	1833930	1833166	1803269	1802505	1944883	1945647	516914	517678	1393977	1393213	1656762	1657526	1707487	1706723
1745	BPSS1670		23	1842240	1841308	1876391	1875459	1848576	1847644	1835118	1834186	1804457	1803525	1943695	1944627	515726	516658	1395165	1394233	1655574	1656506	1708675	1707743
1746	BPSS1671		23	1844668	1842761	1878819	1876912	1851004	1849097	1837546	1835639	1806894	1804987	1941231	1943138	513271	515178	1397647	1395740	1653128	1655035	1711121	1709214

1747	BPSS1672		23	1846106	1844787	1880257	1878938	1852442	1851123	1838984	1837665	1808332	1807013	1939793	1941112	511833	513152	1399085	1397766	1651690	1653009	1712559	1711240
1748	BPSS1673		23	1847163	1846165	1881314	1880316	1853499	1852501	1840041	1839043	1809389	1808391	1938736	1939734	510776	511774	1400142	1399144	1650633	1651631	1713616	1712618
1749	BPSS1674		23	1848419	1847469	1882570	1881620	1854755	1853805	1841268	1840318	1810662	1809712	1937463	1938413	509503	510453	1401415	1400465	1649360	1650310	1714889	1713939
1750	BPSS1675		23	1849056	1848508	1883207	1882659	1855392	1854844	1841905	1841357	1811299	1810751	1936826	1937374	508866	509414	1402052	1401504	1648723	1649271	1715526	1714978
1751	BPSS1676		23	1851112	1850084	1885263	1884235	1857448	1856420	1843961	1842933	1813235	1812207	1934777	1935805	506804	507832	1404056	1403028	1646947	1647975	1717494	1716466
1752	BPSS1677		23	1851793	1852023	1885944	1885973	1858129	1858359	1844635	1844865	1813948	1814178	1934094	1933864	506130	505900	1404732	1404962	1646294	1646064	1718180	1718410
1753	BPSS1678		23	1852286	1853206	1886954	1887874	1858622	1859542	1845128	1846048	1814441	1815361	1933601	1932681	505637	504717	1405225	1406145	1645801	1644881	1718673	1719593
1754	BPSS1679		23	1853667	1854818	1888335	1889486	1860003	1861154	1846509	1847660	1815822	1816973	1932220	1931069	504256	503105	1406606	1407757	1644420	1643269	1720054	1721205
1755	BPSS1680		23	1857021	1856602	1891689	1891270	1863357	1862938	1849863	1849444	1819176	1818757	1928851	1929270	500894	501313	1409968	1409549	1641066	1641485	1723408	1722989
1756	BPSS1681		23	1857317	1858441	1891985	1893109	1863653	1864777	1850159	1851283	1819472	1820596	1928555	1927431	500598	499474	1410264	1411388	1640770	1639646	1723704	1724828
1757	BPSS1682		23	1859862	1858978	1894530	1893646	1866198	1865314	1852704	1851820	1822017	1821133	1926010	1926894	498053	498937	1412809	1411925	1638225	1639109	1726249	1725365
1758	BPSS1683		23	1861299	1861670	1895183	1895965	1866851	1867633	1853357	1854139	1822670	1823452	1925357	1924575	497400	496618	1413462	1414244	1637572	1636790	1726902	1727684
1759	BPSS1684		23	1864111	1861790	1898779	1896458	1870821	1868803	1856953	1854632	1826266	1823945	1921761	1924082	493804	496125	1417058	1414737	1633976	1636297	1730498	1728177
1760	BPSS1685		23	1865712	1864249	1900380	1898917	1872422	1870959	1858554	1857091	1827867	1826404	1920160	1921623	492203	493666	1418659	1417196	1632375	1633838	1732099	1730636
1761	BPSS1686		23	1867197	1865764	1901865	1900432	1873907	1872474	1860039	1858606	1829352	1827919	1918675	1920108	490718	492151	1420144	1418711	1630890	1632323	1733584	1732151
1762	BPSS1687		23	1868342	1869397	1903010	1904065	1875052	1876107	1861184	1862239	1830470	1831525	1917530	1916475	489573	488518	1421289	1422344	1629745	1628690	1734729	1735784
1763	BPSS1688		23	1869436	1870476	1904104	1905144	1876146	1877186	1862278	1863318	1831564	1832604	1916436	1915396	488479	487439	1422383	1423423	1628651	1627611	1735823	1736863
1764	BPSS1689		23	1871317	1871424	1905134	1906021	1877176	1878063	1863308	1864195	1832594	1833481	1915406	1914519	487449	486562	1423413	1424300	1627621	1626734	1736853	1737740
1765	BPSS1690		23	1871792	1871415	1906460	1906083	1878502	1878125	1864634	1864257	1833920	1833543	1914080	1914457	486123	486500	1424739	1424362	1626295	1626672	1738179	1737802
1766	BPSS1691		23	1873311	1872097	1907979	1906765	1880021	1878807	1866153	1864939	1835439	1834225	1912481	1913695	484524	485738	1426328	1425114	1624706	1625920	1739768	1738554
1767	BPSS1692		23	1875075	1873543	1909743	1908211	1881785	1880253	1867917	1866385	1837203	1835671	1910717	1912249	482760	484292	1428092	1426560	1622942	1624474	1741532	1740000
1768	BPSS1693		23	1875622	1875131	1910290	1909799	1882332	1881841	1868464	1867973	1837750	1837259	1910170	1910661	482213	482704	1428639	1428148	1622395	1622886	1742079	1741588
1769	BPSS1694		23	1876369	1875632	1911037	1910300	1883079	1882342	1869211	1868474	1838497	1837760	1909423	1910160	481466	482203	1429386	1428649	1621648	1622385	1742826	1742089
1770	BPSS1695		23	1877787	1876477	1912455	1911145	1884497	1883187	1870629	1869319	1839915	1838605	1908005	1909315	480048	481358	1430804	1429494	1620230	1621540	1744244	1742934
1771	BPSS1696		23	1878771	1877902	1913439	1912570	1885481	1884612	1871613	1870744	1840899	1840030	1907021	1907890	479064	479933	1431788	1430919	1619246	1620115	1745228	1744359
1772	BPSS1697		23	1879666	1878854	1914334	1913522	1886376	1885564	1872508	1871696	1841794	1840982	1906126	1906938	478169	478981	1432683	1431871	1618351	1619163	1746123	1745311
1773	BPSS1698		23	1880558	1879713	1915226	1914381	1887268	1886423	1873400	1872555	1842686	1841841	1905234	1906079	477277	478122	1433575	1432730	1617459	1618304	1747015	1746170
1774	BPSS1699		23	1881761	1880571	1916429	1915239	1888471	1887281	1874603	1873413	1843889	1842699	1904031	1905221	476074	477264	1434778	1433588	1616256	1617446	1748218	1747028
1775	BPSS1700		23	1882597	1881836	1917265	1916504	1889307	1888546	1875439	1874678	1844725	1843964	1903195	1903956	475238	475999	1435614	1434853	1615420	1616181	1749054	1748293
1776	BPSS1701		23	1883409	1882597	1918077	1917265	1890119	1889307	1876251	1875439	1845537	1844725	1902383	1903195	474426	475238	1436426	1435614	1614608	1615420	1749866	1749054
1777	BPSS1702		23	1884538	1884032	1919207	1918077	1891033	1890119	1877381	1876251	1846667	1845537	1901253	1902383	473296	474426	1437556	1436426	1613478	1614608	1750761	1750300
1778	BPSS1703		23			1920089	1919367			1877657	1877541											1751259	1750921
1779	BPSS1704		23	1887289	1886171	1922562	1921444	1894986	1893868	1879845	1878727	1849418	1848300	1898502	1899620	470545	471663	1440307	1439189	1610727	1611845	430270	431388
1780	BPSS1705		23	1888698	1887634	1923971	1922907	1896395	1895331	1881254	1880190	1850827	1849763	1897093	1898157	469136	470200	1441716	1440652	1609318	1610382	428861	429925
1781	BPSS1706		23	1889397	1888750	1924670	1924023	1897094	1896447	1881953	1881306	1851526	1850879	1896394	1897041	468437	469084	1442415	1441768	1608619	1609266	428162	428809
1782	BPSS1706a		23	1889618	1889478	1924891	1924751	1897315	1897175	1882174	1882034	1851747	1851607	1896173	1896313	468216	468356	1442636	1442496	1608398	1608538	427941	428081
1783	BPSS1707		23	1891030	1889624	1926303	1924897	1898727	1897321	1883586	1882180	1853159	1851753	1894761	1896167	466804	468210	1444048	1442642	1606986	1608392	426529	427935
1784	BPSS1708		23	1892717	1891425	1927990	1926698	1900414	1899122	1885273	1883981	1854846	1853554	1893074	1894366	465117	466409	1445735	1444443	1605299	1606591	424842	426134
1785	BPSS1709		23	1893387	1892713	1928660	1927986	1901084	1900410	1885943	1885269	1855516	1854842	1892404	1893078	464447	465121	1446405	1445731	1604629	1605303	424172	424846
1786	BPSS1710		23	1894904	1894380	1930177	1929653	1902601	1902077	1887460	1886936	1857033	1856509	1890887	1891411	462930	463454	1447922	1447398	1603112	1603636	422655	423179
1787	BPSS1711		23	1895065	1897785	1930338	1930742	1902762	1905482	1887621	1890341	1857194	1859914	1890726	1888006	462769	460049	1448083	1450803	1602951	1600231	422494	419774
1788	BPSS1712		23	1899284	1898043	1935507	1934413	1906981	1905740	1891840	1890599	1861413	1860172	1886507	1887748	458550	459791	1452302	1451061	1598732	1599973	418275	419516
1789	BPSS1713_conserved hypothetical protei		23	1901770	1901666	1937993	1937889	1909467	1909363	1892982	1892701	1862555	1862274	1884021	1884125	456064	456168	1453444	1453163	1596246	1596350	417133	417414
1790		Chr2_59_IS407A_A	23	1900428	1901663	1936651	1937886	1908125	1909360	1892984	1894219	1862557	1863793	1884128	1885363	456171	457406	1453446	1454681	1596353	1597588	415896	417131
1791	BPSS1714		23	1901846	1902817	1938069	1939040	1909543	1910514	1894402	1894776	1863976	1864947	1883945	1882974	455988	455017	1454864	1455835	1596170	1595199	415713	414742
1792	BPSS1715		23	1904218	1902920	1940441	1939143	1911915	1910617	1896535	1895924	1866348	1865050	1881573	1882871	453616	454914	1457236	1455938	1593798	1595096	413341	414639
1793	BPSS1716		23	1904543	1904274	1940766	1940497	1912240	1911971	1896860	1896591	1866673	1866404	1881248	1881517	453291	453560	1457561	1457292	1593473	1593742	413016	413285
1794	BPSS1717		23	1905246	1904548	1941469	1940771	1912943	1912245	1897563	1896865	1867376	1866678	1880545	1881243	452588	453286	1458264	1457566	1592770	1593468	412313	413011
1795	BPSS1718		23	1907043	1905271	1943266	1941494	1914740	1912968	1899360	1897588	1869173	1867401	1878748	1880520	450791	452563	1460061	1458289	1590973	1592745	410516	412288
1796	BPSS1719		23	1907416	1907051	1943639	1943274	1915113	1914748	1899733	1899368	1869546	1869181	1878375	1878740	450418	450783	1460434	1460069	1590600	1590965	410143	410508
1797	BPSS1720		23	1907837	1907424	1944060	1943647	1915534	1915121	1900154	1899741	1869967	1869554	1877954	1878367	449997	450410	1460855	1460442	1590179	1590592	409722	410135

1798	BPSS1721		23	1908837	1908040	1945060	1944263	1916534	1915737	1901154	1900357	1870967	1870170	1876954	1877751	448997	449794	1461855	1461058	1589179	1589976	408722	409519
1799	BPSS1722		23	1909136	1910116	1945359	1946339	1916833	1917813	1901453	1902433	1871266	1872246	1876655	1875675	448698	447718	1462154	1463134	1588880	1587900	408423	407443
1800	BPSS1723		23	1910341	1911333	1946564	1947556	1918038	1919030	1902658	1903650	1872471	1873463	1875450	1874458	447493	446501	1463359	1464351	1587675	1586683	407218	406226
1801	BPSS1724		23	1911493	1911999	1947716	1948222	1919190	1919696	1903810	1904316	1873623	1874129	1874298	1873792	446341	445835	1464511	1465017	1586523	1586017	406066	405560
1802	BPSS1725		23	1912079	1913527	1948302	1949750	1919776	1921224	1904396	1905844	1874209	1875657	1873712	1872264	445755	444307	1465097	1466545	1585937	1584489	405480	404032
1803	BPSS1726		23	1913578	1916292	1949801	1952515	1921275	1923989	1905895	1908609	1875708	1878422	1872213	1869499	444256	441542	1466596	1469310	1584438	1581724	403981	401267
1804	BPSS1727		23	1919277	1916545	1955500	1952768	1926974	1924242	1911594	1908862	1881407	1878675	1866514	1869246	438557	441289	1472295	1469563	1578739	1581471	398282	401014
1805	BPSS1728		23	1921051	1919363	1957274	1955586	1928748	1927060	1913368	1911680	1883181	1881493	1864740	1866428	436783	438471	1474069	1472381	1576965	1578653	396508	398196
1806		Chr2_60_IS407A_A		1921548	1922783	1957771	1959006	1929245	1930480	1913865	1915100	1883678	1884913	1863008	1864243	435051	436286	1474566	1475801	1575233	1576468	394776	396011
1807	BPSS1729																						
1808	BPSS1730																						
1809	BPSS1731																						
1810	BPSS1732																						
1811	BPSS1733																						
1812		Chr2_61_IS407A_A		432859	434094	442944	444179	472071	473306	431867	433102	387487	388722	1863008	1864243	435051	436286	1474566	1475801			394776	396011
1813	BPSS1734		24	432863	432429	442948	442514	472075	471641	431871	431437	387491	387057	1863012	1862578	435055	434621	1475797	1476231			394780	394346
1814	BPSS1735		24	431969	432328	441590	442054	470717	471181	430513	430977	386133	386597	1861654	1862118	433697	434161	1477155	1476691			393422	393886
1815	BPSS1736		24	430206	431471	440291	441556	469418	470683	429214	430479	384834	386099	1860355	1861620	432398	433663	1478454	1477189			392123	393388
1816	BPSS1737		24	429068	430111	439153	440196	468280	469323	428076	429119	383696	384739	1859217	1860260	431260	432303	1479592	1478549			390985	392028
1817	BPSS1738		24	427893	428990	437978	439075	467105	468202	426901	427998	382521	383618	1858042	1859139	430085	431182	1480767	1479670			389810	390907
1818	BPSS1739		24	427784	426258	437869	436343	466996	465470	426792	425266	382412	380886	1857933	1856407	429976	428450	1480876	1482402			389701	388175
1819	BPSS1740		24	425119	426150	435204	436235	464331	465362	424127	425158	379747	380778	1855268	1856299	427311	428342	1483541	1482510			387036	388067
1820	BPSS1741		24	425005	425112	434107	435090	463234	464217	423030	424013	378650	379633	1854171	1855154	426214	427197	1484638	1483655			385939	386922
1821	BPSS1742		24	421605	423695	431690	433780	460817	462907	420613	422703	376233	378323	1851754	1853844	423797	425887	1487055	1484965			383522	385612
1822	BPSS1743		24	420978	421370	431063	431455	460190	460582	419986	420378	375606	375998	1851127	1851519	423170	423562	1487682	1487290			382895	383287
1823	BPSS1744		24	420281	420814	430366	430899	459493	460026	419289	419822	374909	375442	1850430	1850963	422473	423006	1488379	1487846			382198	382731
1824	BPSS1745		24	420012	419380	430097	429465	459224	458592	419020	418388	374640	374008	1850161	1849529	422204	421572	1488648	1489280			381929	381297
1825	BPSS1746		24	418482	419243	428637	429104	457694	458455	417490	418251	373110	373871	1848631	1849392	420674	421435	1490178	1489417			380399	381160
1826	BPSS1747		24	417886	418200	428041	428355	457098	457412	416894	417208	372514	372828	1848035	1848349	420078	420392	1490774	1490460			379803	380117
1827	BPSS1748		24	417109	417444	427264	427599	456321	456656	416117	416452	371737	372072	1847258	1847593	419301	419636	1491551	1491216			379026	379361
1828	BPSS1749		24	415501	416913	425656	427068	454713	456125	414509	415921	370129	371541	1845650	1847062	417693	419105	1493159	1491747			377418	378830
1829	BPSS1750		24	414431	415042	424586	425197	453643	454254	413439	414050	369059	369670	1844580	1845191	416623	417234	1494229	1493618			376348	376959
1830	BPSS1751		24	413467	414402	423622	424557	452679	453614	412475	413410	368095	369030	1843616	1844551	415659	416594	1495193	1494258			375384	376319
1831	BPSS1752		24	412046	413467	422201	423622	451258	452679	411054	412475	366674	368095	1842195	1843616	414238	415659	1496614	1495193			373963	375384
1832	BPSS1753		24	411409	410552	421564	420707	450621	449764	410417	409560	366037	365180	1841558	1840701	413601	412744	1497251	1498108			373326	372469
1833	BPSS1754		24	410485	409943	420640	420098	449697	449155	409493	408951	365113	364571	1840634	1840092	412677	412135	1498175	1498717			372402	371860
1834		Chr2_62a_IS407A_A		408708	409943	418863	420098	447920	449155	407716	408951	363336	364571	1838857	1840092	410900	412135	1498717	1499952			370625	371860
1835		Chr2_62b_IS407A_A		329696	330729	339193	340226	367812	368845	328902	329935	284510	285543	1759985	1761018	332069	333102	1577349	1578382	1496137	1497372	292289	293524
1836		Chr2_63_ISBma2_B																		1493768	1495340	289920	291492
1837	BPSS1755		25	327125	329164	336622	338661	365241	367280	326331	328370	281939	283978	1757414	1759453	329498	331537	1580953	1578914	1491340	1493379	287492	289531
1838	BPSS1756		25	324807	326678	334304	336175	362923	364794	324013	325884	279621	281492	1755096	1756967	327180	329051	1583271	1581400	1489022	1490893	285174	287045
1839	BPSS1757		25	324266	324709	333763	334206	362382	362825	323472	323915	279080	279523	1754555	1754998	326639	327082	1583812	1583369	1488481	1488924	284633	285076
1840	BPSS1758		25	323887	324096	333384	333593	362003	362212	323093	323302	278701	278910	1754178	1754294	326260	326469	1584191	1583982	1488102	1488311	284254	284463
1841	BPSS1759		25	322584	323804	332081	333301	360700	361920	321790	323010	277398	278618	1752875	1754095	324957	326177	1585494	1584274	1486799	1488019	282951	284171
1842	BPSS1760		25	322444	321407	331941	330904	360560	359523	321650	320613	277258	276221	1752735	1751698	324817	323780	1585634	1586671	1486659	1485622	282811	281774
1843		Chr2_64_ISBma2_B	25	319482	321054	328979	330551	357598	359170	318688	320260	274296	275868										
1844	BPSS1761		25	318608	319414	328105	328911	356724	357530	317814	318620	273422	274228	1750495	1751301	322577	323383	1587874	1587068	1484419	1485225	280571	281377
1845	BPSS1762		25	316553	318454	326050	327951	354669	356570	315759	317660	271367	273268	1748440	1750341	320522	322423	1589929	1588028	1482364	1484265	278516	280417
1846	BPSS1763		25	315585	316466	325082	325963	353701	354582	314791	315672	270399	271280	1747472	1748353	319554	320435	1590897	1590016	1481396	1482277	277548	278429
1847	BPSS1764		25	315295	315585	324792	325082	353411	353701	314501	314791	270109	270399	1747182	1747472	319264	319554	1591187	1590897	1481106	1481396	277258	277548
1848	BPSS1765		25	315031	313922	324528	323419	353147	352038	314237	313128	269845	268736	1746918	1745809	319000	317891	1591451	1592560	1480842	1479733	276994	275885

1849		Chr2_65_ISBma2_B	25											1744134	1745709	316219	317791	1592660	1594232	1478060	1479632	274212	275784
1850	BPSS1766		25	313771	312905	323268	322402	351887	351021	312977	312111	268585	267719	1744064	1743198	316149	315283	1594302	1595168	1477989	1477123	274141	273275
1851	BPSS1767		25	311968	312840	321465	322337	350084	350956	311174	312046	266782	267654	1742261	1743133	314346	315218	1596105	1595233	1476186	1477058	272338	273210
1852	BPSS1768		25	311254	309464	320751	318961	349370	347580	310460	308670	266068	264278	1741547	1739757	313632	311842	1596819	1598609	1475472	1473682	271624	269834
1853	BPSS1769		25	307710	309089	317207	318586	345826	347205	306916	308295	262566	263945	1738003	1739382	310116	311495	1600363	1598984	1471935	1473314	268101	269480
1854	BPSS1770		25	304611	307388	314108	316885	342727	345504	303817	306594	259467	262244	1734904	1737681	307017	309794	1603462	1600685	1468836	1471613	265002	267779
1855	BPSS1771		25	304594	303863	314091	313360	342710	341979	303800	303069	259450	258719	1734887	1734156	307000	306269	1603479	1604210	1468819	1468088	264985	264254
1856	BPSS1772		25	303442	303161	312939	312658	341558	341277	302648	302367	258298	258017	1733735	1733454	305848	305567	1604631	1604912	1467667	1467386	263833	263552
1857	BPSS1773		25	302498	302010	311995	311507	340614	340126	301704	301216	257354	256866	1732791	1732303	304904	304416	1605575	1606063	1466723	1466235	262889	262401
1858	BPSS1774		25	299903	301126	309400	310623	338019	339242	299109	300332	254759	255982	1730196	1731419	302309	303532	1608170	1606947	1464128	1465351	260294	261517
1859		Chr2_66_IS407A_B	25											1728529	1729764	300642	301877	1608602	1609837				
1860	BPSS1774A		25	299113	299169	308610	308666	337229	337285	298319	298375	253969	254025	1728166	1728222	300279	300335			1463338	1463394	259504	259560
1861	BPSS1775		25	298966	298700	308463	308197	337082	336816	298172	297906	253822	253556	1728019	1727753	300132	299866			1463191	1462925	259357	259091
1862	BPSS1776		25	298682	298191	308179	307688	336798	336307	297888	297397	253538	253047	1727735	1727244	299848	299357			1462907	1462416	259073	258582
1863	BPSS1777		25	296386	297909	305883	307406	334502	336025	295592	297115	251242	252765	1725439	1726962	297552	299075			1460611	1462134	256777	258300
1864	BPSS1778		25	296230	295835	305727	305332	334346	333951	295436	295041	251086	250691	1725283	1724888	297396	297001			1460455	1460060	256621	256226
1865	BPSS1779		25	295738	294746	305235	304243	333854	332862	294944	293952	250594	249602	1724791	1723799	296904	295912			1459963	1458971	256129	255137
1866	BPSS1780		25	294752	293943	304249	303440	332868	332059	293958	293149	249608	248799	1723805	1722996	295918	295109			1458977	1458168	255143	254334
1867	BPSS1781		25	293437	292988	302934	302485	331553	331104	292643	292194	248293	247844	1722490	1722041	294603	294154			1457662	1457213	253828	253379
1868	BPSS1782		25	292847	292431	302344	301928	330963	330547	292053	291637	247703	247287	1721900	1721484	294013	293597			1457072	1456656	253238	252822
1869	BPSS1783		25	292157	292312	301654	301809	330273	330428	291363	291518	247013	247168	1721210	1721365	293323	293478			1456382	1456537	252548	252703
1870	BPSS1784		25	291014	292057	300511	301554	329130	330173	290220	291263	245870	246913	1720067	1721110	292180	293223			1455239	1456282	251405	252448
1871	BPSS1785		25	290652	289966	300149	299463	328768	328082	289858	289172	245508	244822	1719705	1719019	291818	291132			1454877	1454191	251043	250357
1872	BPSS1786		25	289523	288726	299020	298223	327639	326842	288729	287932	244378	243581	1718575	1717778	290688	289891			1453747	1452950	249913	249116
1873	BPSS1787		25	288710	288039	298207	297536	326826	326155	287916	287245	243565	242894	1717762	1717091	289875	289204			1452934	1452263	249100	248429
1874	BPSS1788		25	288034	287336	297531	296833	326150	325452	287240	286542	242889	242191	1717086	1716388	289199	288501			1452258	1451560	248424	247726
1875	BPSS1789		25	286001	286786	295498	296283	324117	324902	285207	285992	240856	241641	1715053	1715838	287166	287951			1450225	1451010	246391	247176
1876	BPSS1790		25	283849	285903	293346	295400	321965	324019	283055	285109	238704	240758	1712901	1714955	285014	287068			1448073	1450127	244239	246293
1877	BPSS1791		25	283403	283783	292900	293280	321519	321899	282609	282989	238258	238638	1712455	1712835	284568	284948			1447627	1448007	243793	244173
1878	BPSS1792		25	283305	282433	292802	291930	321421	320987	282511	281639	238160	237288	1712357	1711485	284470	283598			1447529	1446657	243695	243207
1879	BPSS1793		25	282363	281446	291860	290943	320212	319295	281569	280652	237218	236301	1711415	1710498	283528	282611			1446587	1445670	242893	241976
1880	BPSS1794		25	281419	280574	290916	290071	319268	318423	280625	279780	236274	235429	1710471	1709626	282584	281739			1445643	1444798	241949	241104
1881	BPSS1795		25	280485	279148	289982	288645	318334	316997	279691	278354	235340	234003	1709537	1708200	281650	280313			1444709	1443372	241015	239678
1882	BPSS1796		25	279148	278522	288645	288019	316997	316371	278354	277728	234003	233377	1708200	1707574	280313	279687			1443372	1442746	239678	239052
1883	BPSS1797		25	276717	278357	286214	287854	314566	316206	275923	277563	231572	233212	1705769	1707409	277882	279522			1440941	1442581	237247	238887
1884	BPSS1798		25	276374	275784	285871	285281	314223	313633	275580	274990	231229	230639	1705426	1704836	277539	276949			1440598	1440008	236904	236314
1885	BPSS1799		25	275432	274980	284929	284477	313281	312829	274638	274186	230287	229835	1704484	1704032	276597	276145			1439656	1439204	235962	235510
1886	BPSS1800		25	273674	274909	283171	284406	311523	312758	272880	274115	228529	229764	1702726	1703961	274839	276074			1437898	1439133	234204	235439
1887	BPSS1801		25	273448	273014	282945	282511	311297	310863	272654	272220	228303	227869	1702500	1702066	274613	274179			1437672	1437238	233978	233544
1888	BPSS1802		25	272143	272766	281640	282263	309992	310615	271349	271972	226998	227621	1701195	1701818	273308	273931			1436367	1436990	232673	233296
1889	BPSS1803		25	272080	271235	281577	280732	309929	309084	271286	270441	226935	226090	1701132	1700281	273245	272394			1436304	1435459	232610	231765
1890	BPSS1804		25	270549	269626	280046	279123	308398	307475	269755	268832	225404	224481	1699595	1698672	271708	270785			1434773	1433850	231079	230156
1891		Chr2_67_IS407A_A		268389	269624	277886	279121	306238	307473	267595	268830	223244	224479	1697435	1698670	269548	270783			1432613	1433848	228919	230154
1892	BPSS1805																						
1893	BPSS1806																						
1894	BPSS1807																						
1895	BPSS1808																						
1896	BPSS1809																						
1897	BPSS1810																						
1898	BPSS1811																						
1899	BPSS1812																						

1900	BPSS1813																						
1901	BPSS1814																						
1902	BPSS1815																						
1903	BPSS1816																						
1904	BPSS1817																						
1905	BPSS1818																						
1906	BPSS1819																						
1907	BPSS1820																						
1908	BPSS1821																						
1909	BPSS1822																						
1910	BPSS1823																						
1911	BPSS1824																						
1912	BPSS1825																						
1913	BPSS1826																						
1914	BPSS1827																						
1915	BPSS1828																						
1916	BPSS1829																						
1917	BPSS1830																						
1918	BPSS1831																						
1919	BPSS1832																						
1920	BPSS1833																						
1921		Chr2_68_IS407A_A		268389	269624	277886	279121	306238	307473	267595	268830	223244	224479	1697435	1698670	269548	270783			1432613	1433848	228919	230154
1922	BPSS1834		25	267312	268388	276809	277885	305161	306237	266518	267594	222167	223243	1696358	1697434	268471	269547			1431536	1432612	227842	228918
1923	BPSS1835		25	266856	265390	276353	274887	304705	303239	266062	264596	221711	220245	1695902	1694436	268015	266549			1431080	1429614	227386	225920
1924	BPSS1836		25	264310	265311	273807	274808	302159	303160	263516	264517	219165	220166	1693356	1694357	265469	266470			1428534	1429535	224840	225841
1925	BPSS1837		25	263176	263730	272673	273227	301025	301579	262382	262936	218031	218585	1692222	1692776	264335	264889			1427400	1427954	223706	224260
1926	BPSS1838		25	261789	263123	271286	272620	299638	300972	260995	262329	216644	217978	1690835	1692169	262948	264282			1426013	1427347	222319	223653
1927	BPSS1839		25	261255	261674	270751	271170	299104	299523	260461	260880	216110	216529	1690301	1690720	262414	262833			1425479	1425898	221785	222204
1928	BPSS1840		25	259944	260981	269440	270477	297793	298830	259150	260187	214799	215836	1688990	1690027	261103	262140			1424168	1425205	220474	221511
1929	BPSS1841		25	258131	259396	267627	268892	295980	297245	257337	258602	212986	214251	1687177	1688442	259290	260555			1422355	1423620	218661	219926
1930	BPSS1842		25	257595	258131	267091	267627	295444	295980	256801	257337	212450	212986	1686641	1687177	258754	259290			1421819	1422355	218125	218661
1931	BPSS1843		25	257160	257570	266656	267066	295009	295419	256366	256776	212015	212425	1686206	1686616	258319	258729			1421384	1421794	217690	218100
1932	BPSS1844		25	256640	256990	266136	266486	294489	294839	255846	256196	211495	211845	1685686	1686036	257799	258149			1420864	1421214	217171	217326
1933	BPSS1845		25	256279	254408	265775	263904	294128	292257	255485	253614	211134	209263	1685325	1683454	257438	255567			1420503	1418632	216810	215146
1934	BPSS1846		25	253627	253250	263123	262746	291476	291099	252833	252456	208482	208105	1682673	1682296	254786	254409			1417851	1417474	214666	214289
1935	BPSS1847		25	253198	252617	262694	262113	291047	290466	252404	251823	208053	207472	1682244	1681663	254357	253776			1417422	1416841	214237	213656
1936	BPSS1848		25	252422	251331	261918	260827	290271	289180	251628	250537	207277	206186	1681468	1680377	253581	252490			1416646	1415555	213461	212370
1937	BPSS1849		25	250636	251109	260132	260605	288485	288958	249842	250315	205491	205964	1679682	1680155	251795	252268			1414860	1415333	211675	212148
1938	BPSS1850		25	248417	250510	257913	260006	286266	288359	247623	249716	203272	205365	1677463	1679556	249576	251669			1412641	1414734	209456	211549
1939	BPSS1851		25	247950	246748	257446	256244	285799	284597	247156	245954	202805	201603	1676996	1675794	249109	247907			1412174	1410972	208989	207787
1940	BPSS1852		25	245081	246094	254577	255590	282930	283943	244287	245300	199936	200949	1674136	1675149	246240	247253			1409296	1410309	206111	207124
1941	BPSS1853		25	243894	244688	253390	254184	281743	282537	243100	243894	198791	199585	1672879	1673673	245032	245826			1408165	1408959	204924	205718
1942	BPSS1854		25	242902	243669	252398	253165	280751	281518	242108	242875	197799	198566	1671887	1672654	244040	244807			1407173	1407940	203932	204699
1943	BPSS1855		25	242179	242865	251675	252361	280028	280714	241385	242071	197076	197762	1671164	1671850	243317	244003			1406450	1407136	203209	203895
1944	BPSS1856		25	241106	242179	250602	251675	278955	280028	240312	241385	196003	197076	1670091	1671164	242244	243317			1405377	1406450	202136	203209
1945	BPSS1857		25	239877	241106	249373	250602	277726	278955	239083	240312	194774	196003	1668862	1670091	241015	242244			1404148	1405377	200907	202136
1946	BPSS1858		25	239479	238556	248968	248045	277328	276405	238685	237762	194306	193383	1668513	1667590	240624	239701			1403666	1402743	200530	199607
1947	BPSS1859		25	238461	238063	247950	247552	276310	275912	237667	237269	193288	192890	1667495	1667109	239606	239220			1402648	1402250	199512	199114
1948	BPSS1860		25	236733	237917	246222	247406	274582	275766	235939	237123	191560	192744	1665779	1666963	237890	239074			1400920	1402104	197784	198968
1949	BPSS1861		25	234959	235837	244448	245326	272808	273686	234165	235043	189786	190664	1664005	1664883	236116	236994			1399146	1400024	196010	196888
1950	BPSS1862		25	234268	232262	243757	241751	272117	270111	233474	231468	189095	187089	1663314	1661308	235425	233419			1398455	1396449	195319	193313

1951	BPSS1863		25	231192	231917	240681	241406	269041	269766	230398	231123	186019	186744	1660238	1660963	232349	233074			1395379	1396104	192243	192968
1952	BPSS1864		25	231113	230076	240602	239565	268962	267925	230319	229282	185940	184903	1660159	1659122	232270	231233			1395300	1394263	192164	191127
1953	BPSS1865		25	229619	228582	239108	238071	267468	266431	228825	227788	184446	183409	1658677	1657640	230788	229751			1393812	1392775	190670	189633
1954	BPSS1866		25	228095	227442	237584	236931	265944	265291	227301	226648	182908	182255	1657153	1656500	229257	228604			1392281	1391628	189132	188479
1955	BPSS1867		25	226589	225648	236078	235137	264438	263497	225795	224854	181402	180461	1655647	1654706	227751	226810			1390775	1389834	187626	186685
1956	BPSS1868		25	224159	225439	233648	234928	262008	263288	223546	224277	178972	180252	1653217	1654497	225321	226601			1388345	1389625	185196	186476
1957	BPSS1869		25	222748	223806	232237	233295	260597	261655	222135	223193	177561	178619	1651806	1652864	223910	224968			1386934	1387992	183785	184843
1958	BPSS1870		25	221668	222693	231157	232182	259517	260542	221055	222080	176481	177506	1650726	1651751	222830	223855			1385854	1386879	182705	183730
1959	BPSS1871		25	219067	221658	228556	231147	256662	257168	218454	221045	173880	176471	1648125	1650716	220229	222820			1383253	1385844	180104	182695
1960	BPSS1872		25	218336	219067	227825	228556	255931	256662	217723	218454	173149	173880	1647394	1648125	219498	220229			1382522	1383253	179373	180104
1961	BPSS1873		25	216301	218352	225790	227841	253373	254386			171114	173165	1645359	1647410	217463	219514			1380487	1382538	177868	178764
1962	BPSS1874		25	215762	216301	225251	225790	252834	253373	215455	215994	170575	171114	1644820	1645359	216924	217463			1379948	1380487	177329	177868
1963	BPSS1875		25	214075	215748	223564	225237	251147	252820	213768	215441	168888	170561	1643133	1644806	215237	216910			1378261	1379934	175642	177315
1964	BPSS1876		25	212807	213901	222296	223390	249879	250973	212500	213594	167620	168714	1641865	1642959	213969	215063			1376993	1378087	174374	175468
1965	BPSS1877		25	211991	211659	221480	221148	249063	248731	211684	211352	166804	166472	1641049	1640717	213153	212821			1376177	1375845	173558	173226
1966	BPSS1878		25	209943	211373	219432	220862	247015	248445	209636	211066	164756	166186	1639001	1640431	211105	212535			1374129	1375559	171510	172940
1967	BPSS1879		25	208211	209848	217700	219337	245283	246920	207904	209541	163024	164661	1637269	1638906	209373	211010			1372397	1374034	169778	171415
1968	BPSS1880		25	206284	207900	215773	217389	243356	244972	205977	207593	161097	162713	1635342	1636958	207446	209062			1370470	1372086	167851	169467
1969	BPSS1881		25	205521	206120	214325	215047	241908	242630	204529	205251	159649	160371	1633894	1634616	205998	206720			1369022	1369744	166403	167125
1970	BPSS1882		25	204371	204532	213860	214021	241443	241604	204064	204225	159184	159345	1633429	1633590	205533	205694			1368557	1368718	165938	166099
1971	BPSS1883		25	204095	202446	213584	212919	241167	239518	203788	202139	158908	157259	1633153	1631504	205257	203608			1368281	1366632	165662	164013
1972	BPSS1884		25	201957	202373	211457	211873	239029	239445	201650	202066	156770	157186	1631015	1631431	203119	203535			1366143	1366559	163524	163940
1973	BPSS1885		25	200514	201752	210014	211252	237586	238824	200207	201445	155327	156565	1629572	1630810	201676	202914			1364700	1365938	162081	163319
1974	BPSS1886		25	200198	200524	209698	210024	237270	237596	199891	200217	155011	155337	1629256	1629582	201360	201686			1364384	1364710	161765	162091
1975	BPSS1887		25	199661	200173	209161	209673	236733	237245	199354	199866	154474	154986	1628719	1629231	200823	201335			1363847	1364359	161228	161740
1976	BPSS1888		25	198408	199661	207908	209161	235480	236733	198101	199354	153221	154474	1627466	1628719	199570	200823			1362594	1363847	159975	161228
1977	BPSS1889		25	198208	197171	207708	206671	235280	234243	197901	196864	153021	151984	1627266	1626229	199370	198333			1362394	1361357	159775	158738
1978	BPSS1890		25	196231	197115	205731	206615	233303	234187	195924	196808	151044	151928	1625289	1626173	197393	198277			1360417	1361301	157798	158682
1979	BPSS1891		25	196141	195011	205641	204511	233213	232083	195834	194704	150954	149824	1625199	1624069	197303	196173			1360327	1359197	157708	156578
1980	BPSS1892		25	194973	194074	204473	203574	232045	231146	194666	193767	149786	148887	1624031	1623132	196135	195236			1359159	1358260	156540	155641
1981	BPSS1893		25	194033	193746	203533	203246	231105	230818	193726	193439	148846	148559	1623091	1622804	195195	194908			1358219	1357932	155600	155313
1982	BPSS1894		25	193291	193620	202791	203120	230363	230692	192984	193313	148104	148433	1622349	1622678	194453	194782			1357477	1357806	154858	155187
1983	BPSS1895		25	192697	193287	202197	202787	229769	230359	192390	192980	147510	148100	1621755	1622345	193859	194449			1356883	1357473	154264	154854
1984	BPSS1896		25	190676	192679	200176	202179	227748	229751	190369	192372	145489	147492	1619734	1621737	191838	193841			1354862	1356865	152243	154246
1985	BPSS1897		25	189786	190670	199286	200170	226858	227742	189479	190363	144599	145483	1618844	1619728	190948	191832			1353972	1354856	151353	152237
1986	BPSS1898		25	187028	189058	196528	198558	224100	226130	186721	188751	141841	143871	1616086	1618116	188190	190220			1351214	1353244	148595	150625
1987	BPSS1899		25	186878	185829	196378	195329	223950	222901	186571	185522	141691	140642	1615936	1614887	188040	186991			1351064	1350015	148445	147396
1988	BPSS1900		25	184925	185869	194425	195369	221997	222941	184618	185562	139738	140682	1613983	1614927	186087	187031			1349111	1350055	146492	147436
1989	BPSS1901		25	184830	183835	194330	193335	221902	220907	184523	183528	139643	138648	1613888	1612893	185992	184997			1349016	1348021	146397	145402
1990	BPSS1902		25	182740	183651	192240	193151	219812	220723	182433	183344	137553	138464	1611798	1612709	183902	184813			1346926	1347837	144307	145218
1991	BPSS1903		25	182516	181152	192016	190652	219588	218224	182209	180845	137329	135965	1611574	1610210	183678	182314			1346702	1345338	144083	142719
1992	BPSS1904		25	181152	180664	190652	190164	218224	217736	180845	180357	135965	135477	1610210	1609722	182314	181826			1345338	1344850	142719	142231
1993	BPSS1905		25	180617	179601	190117	189101	217689	216673	180310	179294	135430	134414	1609675	1608659	181779	180763			1344803	1343787	142184	141168
1994	BPSS1906		25	179601	178810	189101	188310	216673	215882	179294	178503	134414	133623	1608659	1607868	180763	179972			1343787	1342996	141168	140377
1995	BPSS1907		25	177885	178760	187385	188260	214957	215832	177578	178453	132698	133573	1606943	1607818	179047	179922			1342071	1342946	139452	140327
1996	BPSS1908		25	177482	177000	186982	186500	214554	214072	177175	176693	132295	131813	1606540	1606058	178644	178162			1341668	1341186	139049	138567
1997	BPSS1909		25	177000	176431	186500	185931	214072	213503	176693	176124	131813	131244	1606058	1605489	178162	177593			1341186	1340617	138567	137998
1998	BPSS1910		25	176405	175356	185905	184856	213477	212428	176098	175049	131218	130169	1605463	1604414	177567	176518			1340591	1339542	137972	137451
1999	BPSS1911		25	174726	173245	184226	182745	211798	210317	174419	172938	129539	128058	1603784	1602303	175888	174407			1338912	1337431	136297	134816
2000	BPSS1912_I	Chr2_69_ISBma1_A	25	171828	173071	181328	182571	208900	210143	171521	172764	126655	127898	1600893	1602136	172990	174233			1336007	1337250	133392	134635
2001		Chr2_70_IS407A_A		170594	171829	180094	181329	207666	208901	170287	171522	125421	126656	1599659	1600894	171756	172991	1608602	1609837	1334773	1336008	132158	133393

2002	BPSS1912_I	Chr2_69-1_ISBma1_A	26	170527	170594	180027	180094	207599	207666	170220	170747	125354	125421	171689	171756	1609838	1609906	1334706	1334773
2003	BPSS1913		26	168879	170438	178379	179938	205951	207510	168572	170131	123706	125265	170041	171600	1611552	1609993	1333058	1334617
2004		Chr2_71a_ISBma2_A	26									121539	123111						
2005		Chr2_71b_ISBma2_A	26	166712	168284	176212	177784	203784	205356	166405	167977	2229185	2230757	167874	169446	1612147	1613720	1330891	1332463
2006	BPSS1914		26	165754	166641	175254	176141	202826	203713	165447	166334	2231715	2230828	166916	167803	1614678	1613791	1329933	1330820
2007	BPSS1915		26	165040	163664	174540	173164	202112	200736	164733	163357	2232429	2233805	166202	164826	1615392	1616768	1329219	1327843
2008	BPSS1916		26	162321	163064	171821	172564	199393	200136	162014	162757	2235148	2234405	163483	164226	1618111	1617368	1326500	1327243
2009	BPSS1917		26	161656	160877	171156	170377	198728	197949	161349	160570	2235813	2236592	162818	162039	1618776	1619555	1325835	1325056
2010	BPSS1918		26	159682	160665	169182	170165	196754	197737	159375	160358	2237787	2236804	160844	161827	1620750	1619767	1323861	1324844
2011	BPSS1919		26	159455	159679	168955	169179	196527	196751	159148	159372	2238014	2237790	160617	160841	1620977	1620753	1323634	1323858
2012	BPSS1920		26	158696	158271	168196	167771	195768	195343	158389	157964	2238766	2239191	159851	159426	1621743	1622168	1322875	1322450
2013	BPSS1921		26	157559	157879	167059	167379	194631	194951	157252	157572	2239903	2239583	158714	159034	1622880	1622560	1321738	1322058
2014	BPSS1922		26	157198	156323	166698	165823	194270	193395	156891	156016	2240264	2241139	158353	157478	1623241	1624116	1321377	1320502
2015	BPSS1923		26	155993	155607	165493	165107	193065	192679	155686	155300	2241469	2241855	157148	156762	1624446	1624832	1320172	1319786
2016	BPSS1924		26	154352	154999	163852	164499	165407	166054	154237	154863	2243110	2242463	155507	156154	1626087	1625440	1318531	1319178
2017	BPSS1925		26	152859	154010	162359	163510	163914	165065	152744	153895	2244631	2243480	154007	155158	1627594	1626443	1316996	1318147
2018	BPSS1926		26	151917	152558	161417	162058	162972	163613	151802	152443	2245573	2244932	153065	153706	1628536	1627895	1316054	1316695
2019	BPSS1927		26	149976	151673	159476	161173	161031	162728	149861	151558	2247514	2245817	151124	152821	1630477	1628780	1314113	1315810
2020	BPSS1928		26	149085	148147	158585	157647	160140	159202	148970	148032	2248405	2249343	150233	149295	1631368	1632306	1313222	1312284
2021	BPSS1929		26	146641	147951	156141	157451	157696	159006	146526	147836	2250849	2249539	147789	149099	1633812	1632502	1310778	1312088
2022	BPSS1930		26	145578	146660	155078	156160	156633	157715	145463	146545	2251912	2250830	146726	147808	1634875	1633793	1309715	1310797
2023	BPSS1931		26	144883	145578	154383	155078	155938	156633	144768	145463	2252607	2251912	146031	146726	1635570	1634875	1309020	1309715
2024	BPSS1932		26	143744	144877	153244	154377	154799	155932	143629	144762	2253746	2252613	144892	146025	1636709	1635576	1307881	1309014
2025	BPSS1933		26	143496	143744	152996	153244	154551	154799	143381	143629	2253994	2253746	144644	144892	1636957	1636709	1307633	1307881
2026	BPSS1934		26	142993	143460	152493	152960	154048	154515	142878	143345	2254497	2254030	144141	144608	1637460	1636993	1307130	1307597
2027	BPSS1935		26	141911	142774	151411	152274	152966	153829	141796	142659	2255579	2254716	143059	143922	1638542	1637679	1306048	1306911
2028	BPSS1936		26	141644	140121	151144	149621	152699	151176	141529	140006	2255846	2257369	142792	141269	1638809	1640290	1305781	1304258
2029	BPSS1937		26	140121	139636	149621	149136	151176	150691	140006	139521	2257369	2257854	141269	140784	1640327	1640812	1304258	1303773
2030	BPSS1938		26	139055	135843	148555	145343	150110	146898	138940	135728	2258435	2261647	140203	136967	1641393	1644629	1303192	1299980
2031	BPSS1939		26	135837	134716	145337	144216	146892	145771	135722	134601	2261653	2262774	136961	135840	1644635	1645756	1299974	1298853
2032	BPSS1940		26	134563	132731	144063	142231	145618	143786	134448	132616	2262927	2264759	135687	133855	1645909	1647717	1298700	1296868
2033	BPSS1941		26	130822	132381	140322	141881	141877	143436	130707	132266	2266668	2265109	131946	133505	1649675	1648116	1294953	1296512
2034	BPSS1942		26	130278	129772	139778	139272	141333	140827	130163	129657	2267212	2267718	131402	130896	1650219	1650725	1294409	1293903
2035	BPSS1943		26	129208	129708	138708	139208	140263	140763	129093	129593	2268282	2267782	130332	130832	1651289	1650789	1293339	1293839
2036	BPSS1944		26	128106	129128	137606	138628	139161	140183	127991	129013	2269384	2268362	129230	130252	1652391	1651369	1292237	1293259
2037	BPSS1945		26	127043	127888	136543	137388	138098	138943	126928	127773	2270447	2269602	128167	129012	1653454	1652609	1291174	1292019
2038	BPSS1946		26	125034	127043	134534	136543	135412	135978	124919	126928	2272456	2270447	126158	128167	1655463	1653454	1289165	1291174
2039	BPSS1947		26	124301	125047	133801	134547			124186	124932	2273189	2272443	125425	126171	1656196	1655450	1288432	1289178
2040	BPSS1948		26	124039	124284	133539	133784	134431	134676	123924	124169	2273451	2273206	125163	125408	1656458	1656213	1288167	1288415
2041	BPSS1949		26	123341	124039	132841	133539	133733	134431	123226	123924	2274149	2273451	124465	125163	1657156	1656458	1287469	1288167
2042	BPSS1950		26	123045	123341	132545	132841	133437	133733	122930	123226	2274445	2274149	124169	124465	1657452	1657156	1287173	1287469
2043	BPSS1951		26	122582	123040	132082	132540	132974	133432	122467	122925	2274908	2274450	123706	124164	1657915	1657457	1286710	1287168
2044	BPSS1952		26	122100	122552	131600	132052	132492	132944	122079	122357	2275390	2274938	123224	123676	1658397	1657945	1286228	1286680
2045	BPSS1953		26	120587	122107	130087	131607	130979	132499	120566	122086	2276903	2275383	121711	123231	1659910	1658390	1284715	1286235
2046	BPSS1954		26	120145	118355	129645	127855	130537	128747	120124	118334	2277345	2279135	121269	119479	1660352	1662142	1284273	1282483
2047	BPSS1955		26	118341	116941	127841	126441	128733	127333	118320	116920	2279149	2280549	119465	118065	1662156	1663556	1282469	1281069
2048	BPSS1956		26	116941	115766	126441	125266	127333	126158	116920	115745	2280549	2281724	118065	116890	1663556	1664731	1281069	1279894
2049	BPSS1957		26	114189	115127	123689	124627	124581	125519	114168	115106	2283301	2282363	115313	116251	1666308	1665370	1278317	1279255
2050	BPSS1958		26	112951	113595	122465	123109	123343	123987	112930	113574	2284679	2284035	114243	114887	1667441	1666797	1277086	1277730
2051	BPSS1959		26	112033	112422	121547	121936	122425	122814	112012	112401	2285624	2285235	113334	113723	1668359	1667970	1276177	1276566
2052	BPSS1960		26	110433	111761	119658	120542	120825	122153	110412	111740	2287232	2285904	111734	113062	1669959	1668631	1274577	1275905

2053	BPSS1961		26	109754	109578	118979	118803	120146	119970	109733	109557	2287911	2288087			111055	110879	1670638	1670814	1273898	1273722		
2054	BPSS1962		26	108493	109272	117718	118497	118885	119664	108472	109251	2289172	2288393			109794	110573	1671899	1671120	1272637	1273416		
2055	BPSS1963		26	107205	108476	116430	117701	117597	118868	107184	108455	2290460	2289189			108506	109777	1673187	1671916	1271349	1272620		
2056	BPSS1964		26	105430	106659	114655	115884	115822	117051	105409	106638	2292238	2291000			106740	107978	1674950	1673721	1269565	1270821		
2057	BPSS1965		26	104657	105298	113882	114523	115049	115690	104636	105277	2293011	2292370			105967	106608	1675723	1675082	1268792	1269433		
2058		Chr2_72_IS407A_A		103191	104426	112416	113651	113583	114818	103170	104405	2293242	2294477	1599659	1600894	104501	105736	1675954	1677189	1267326	1268561	1972722	1973957
2059	BPSS1966		27	103143	102241	112368	111466	113535	112633	103122	102220	2294525	2295427	1599611	1598709	104453	103551	1677237	1678139	1267278	1266376	1974005	1974907
2060	BPSS1967		27	101374	102021	110599	111246	111766	112413	101353	102000	2296294	2295647	1597842	1598489	102684	103331	1679006	1678359	1265509	1266156	1975774	1975127
2061	BPSS1968		27	101271	100366	110496	109591	111663	110758	101250	100345	2296397	2297302	1597739	1596834	102581	101676	1679109	1680014	1265406	1264501	1975877	1976782
2062	BPSS1969		27	100366	98771	109591	107996	110758	109163	100345	98750	2297302	2298897	1596834	1595239	101676	100081	1680014	1681609	1264501	1262906	1976782	1978377
2063	BPSS1970		27	98771	97791	107996	107016	109163	108183	98750	97770	2298897	2299877	1595239	1594253	100081	99095	1681609	1682595	1262906	1261926	1978377	1979357
2064	BPSS1971		27	97108	95525	106333	104750	107500	105917	97087	95504	2300560	2302143	1593570	1591987	98412	96829	1683278	1684861	1261243	1259660	1980040	1981623
2065	BPSS1972		27	95362	94916	104587	104141	105754	105308	95341	94895	2302306	2302752	1591824	1591378	96666	96220	1685024	1685470	1259497	1259051	1981786	1982232
2066	BPSS1973		27	93977	92046	103202	101271	104369	102438	93956	92025	2303691	2305622	1590439	1588508	95281	93350	1686409	1688340	1258112	1256181	1983171	1985102
2067	BPSS1974		27	91158	89407	100383	98632	101550	99799	91137	89386	2306517	2308259	1587683	1585950	92469	90736	1689221	1690918	1255314	1253572	1986077	1987759
2068	BPSS1975		27	89313	88033	98538	97258	99705	98425	89292	88012	2308353	2309633	1585856	1584576	90642	89362	1691012	1692292	1253478	1252198	120404	119124
2069	BPSS1976		27	87213	85846	96438	95071	97605	96238	87192	85825	2310476	2311843	1583789	1582422	88582	87215	1693135	1694502	1251411	1250044	118302	116935
2070	BPSS1977		27	85811	84486	95036	93711	96203	94878	85790	84465	2311878	2313203	1582387	1581062	87180	85855	1694537	1695862	1250009	1248684	116900	115575
2071	BPSS1978		27	84358	81998	93583	91223	94750	92390	84337	81977	2313331	2315691	1580934	1578574	85727	83367	1695990	1698350	1248556	1246196	115447	113087
2072		Chr2_73_IS407A_A		80328	81563	89553	90788	90720	91955	80307	81542	2316126	2317361	1576904	1578139	81697	82932	1698785	1700020	1244526	1245761	111417	112652
2073	BPSS1979																						
2074	BPSS1980																						
2075	BPSS1981																						
2076	BPSS1982																						
2077	BPSS1983																						
2078	BPSS1984																						
2079	BPSS1985																						
2080	BPSS1986																						
2081	BPSS1987																						
2082	BPSS1988																						
2083	BPSS1989																						
2084	BPSS1990																						
2085	BPSS1991																						
2086	BPSS1992																						
2087	BPSS1993																						
2088	BPSS1994																						
2089	BPSS1995																						
2090	BPSS1996																						
2091	BPSS1997																						
2092	BPSS1998																						
2093	BPSS1999																						
2094	BPSS2000																						
2095	BPSS2001																						
2096	BPSS2002																						
2097	BPSS2003																						
2098	BPSS2004																						
2099	BPSS2005																						
2100	BPSS2006																						
2101	BPSS2007																						
2102	BPSS2008																						
2103	BPSS2009																						

2104	BPSS2010										
2105	BPSS2011										
2106	BPSS2012										
2107	BPSS2013										
2108	BPSS2014										
2109	BPSS2015										
2110	BPSS2016										
2111	BPSS2017										
2112	BPSS2018										
2113	BPSS2019										
2114	BPSS2020										
2115	BPSS2021										
2116	BPSS2022										
2117	BPSS2023										
2118	BPSS2024										
2119	BPSS2025										
2120	BPSS2026										
2121	BPSS2027										
2122	BPSS2028										
2123	BPSS2029										
2124	BPSS2030										
2125	BPSS2031										
2126	BPSS2032										
2127	BPSS2033										
2128	BPSS2034										
2129	BPSS2035										
2130	BPSS2036										
2131	BPSS2037										
2132	BPSS2038										
2133	BPSS2039										
2134	BPSS2040										
2135	BPSS2041										
2136	BPSS2042										
2137	BPSS2043										
2138	BPSS2044										
2139	BPSS2045										
2140	BPSS2046										
2141	BPSS2047										
2142	BPSS2048										
2143	BPSS2049										
2144	BPSS2050	gene remnant									
2145	BPSS2051	GI16									
2146	BPSS2052	GI16									
2147	BPSS2053	GI16									
2148	BPSS2054	GI16									
2149	BPSS2055	GI16									
2150	BPSS2056	GI16									
2151	BPSS2057	GI16_transposase									
2152	BPSS2058	GI16									
2153	BPSS2059	GI16									
2154	BPSS2060	GI16									

2155	BPSS2061	GI16																					
2156	BPSS2061A	GI16																					
2157	BPSS2062	GI16																					
2158	BPSS2063	GI16																					
2159	BPSS2064	GI16																					
2160	BPSS2065	GI16																					
2161	BPSS2066	GI16																					
2162	BPSS2067	GI16																					
2163	BPSS2068	GI16																					
2164	BPSS2069	GI16																					
2165	BPSS2070	GI16																					
2166	BPSS2071	GI16																					
2167	BPSS2072	GI16																					
2168	BPSS2073	GI16																					
2169	BPSS2074	GI16																					
2170	BPSS2074a	GI16																					
2171	BPSS2075	GI16																					
2172	BPSS2076	GI16																					
2173	BPSS2078	GI16																					
2174	BPSS2079	GI16																					
2175	BPSS2080	GI16																					
2176	BPSS2081	GI16																					
2177	BPSS2082	GI16																					
2178	BPSS2083	GI16																					
2179	BPSS2084	GI16																					
2180	BPSS2085	GI16																					
2181	BPSS2086	GI16																					
2182	BPSS2087	GI16																					
2183	BPSS2088	GI16																					
2184	BPSS2089	GI16																					
2185	BPSS2090	GI16																					
2186		Chr2_74_IS407A_A		408708	409943	418863	420098	447920	449155	407716	408951	363336	364571	1838857	1840092	410900	412135	1498717	1499952	1575233	1576468	370625	371860
2187	BPSS2091		28	407753	408310	417908	418465	446965	447522	406761	407318	362381	362938	1837902	1838459	409945	410502	1500907	1500350	1574278	1574835	369670	370227
2188	BPSS2092		28	407027	407119	417182	417274	446239	446331	406035	406127	361655	361747	1837176	1837268	409219	409311	1501633	1501541	1573552	1573644	368944	369036
2189	BPSS2093		28	404807	407008	414962	417163	444019	446220	403815	406016	359435	361636	1834956	1837157	406999	409200	1503853	1501652	1571332	1573533	366724	368925
2190	BPSS2094		28	402174	404807	412329	414962	441386	444019	401182	403815	356802	359435	1832323	1834956	404366	406999	1506486	1503853	1568699	1571332	364091	366724
2191	BPSS2095		28	400687	402174	410842	412329	439899	441386	399695	401182	355327	356802	1830848	1832323	402891	404366	1507961	1506486	1567224	1568699	362616	364091
2192	BPSS2096		28	398819	400687	408974	410842	438031	439899	397827	399695	353459	355327	1828980	1830848	401023	402891	1509829	1507961	1565356	1567224	360751	362616
2193	BPSS2097		28	398287	398811	408442	408966	437499	438023	397295	397819	352927	353451	1828448	1828972	400491	401015	1510361	1509837	1564824	1565348	360219	360743
2194	BPSS2098		28	397744	398232	407899	408387	436956	437444	396752	397240	352384	352872	1827905	1828393	399948	400436	1510904	1510416	1564281	1564769	359676	360164
2195	BPSS2099		28	396176	397681	406331	407836	435388	436893	395184	396689	350816	352321	1826337	1827842	398380	399885	1512472	1510967	1562713	1564218	358108	359613
2196	BPSS2100		28	395605	396180	405760	406335	434817	435392	394613	395188	350245	350820	1825766	1826341	397809	398384	1513043	1512468	1562142	1562717	357537	358112
2197	BPSS2101		28	394463	395539	404618	405694	433675	434751	393471	394547	349103	350179	1824624	1825700	396667	397743	1514185	1513109	1561000	1562076	356395	357471
2198	BPSS2102		28	391813	394407	402038	403321	431025	433619	390821	393415	346453	349047	1821974	1824568	394017	396611	1516835	1514241	1558359	1560944	353930	355639
2199	BPSS2103		28											1820858	1821760	392901	393803	1517951	1517049				
2200	BPSS2104		28	387061	390687	397286	400912	426273	429899	386069	389695	341701	345327	1817232	1820858	389275	392901	1521577	1517951	1553607	1557233	349178	352804
2201	BPSS2105		28	385742	387055	395967	397280	424954	426267	384750	386063	340382	341695	1815913	1817226	387956	389269	1522896	1521583	1552288	1553601	347859	349172
2202	BPSS2106		28	384338	385723	394563	395948	423550	424935	383346	384731	338978	340363	1814509	1815894	386552	387937	1524300	1522915	1550884	1552269	346455	347840
2203	BPSS2107		28	383808	384323	394033	394548	423020	423535	382816	383331	338448	338963	1813979	1814494	386022	386537	1524830	1524315	1550354	1550869	345925	346440
2204	BPSS2108		28	383117	382770	393342	392995	422329	421982	382125	381778	337757	337410	1813288	1812941	385331	384984	1525521	1525868	1549663	1549316	345234	344887
2205	BPSS2109		28	382712	381279	392937	391504	421924	420491	381720	380287	337352	335919	1812883	1811450	384926	383493	1525926	1527359	1549258	1547825	344829	343396

2206	BPSS2110		28	379335	381230	389560	391455	418547	420442	378343	380238	333975	335870	1809506	1811401	381549	383444	1529303	1527408	1545881	1547776	341452	343347
2207	BPSS2111		28	378907	378857	389132	389082	418119	418069	377915	377865	333547	333497	1809078	1809028	381121	381071	1529731	1529781	1545453	1545403	341024	340974
2208	BPSS2112		28	377312	376998	387537	387223	416524	416210	376320	376006	331952	331638	1807483	1807169	379526	379212	1531326	1531640	1543858	1543544	339429	339115
2209	BPSS2113		28	376946	376773	387171	386998	416158	415985	375954	375781	331586	331413	1807117	1806944	379160	378987	1531692	1531865	1543492	1543319	339063	338890
2210	BPSS2114		28	375504	376205	385729	386430	414716	415417	374512	375213	330174	330875	1805745	1806446	377798	378499	1533084	1532383	1542090	1542791	337701	338402
2211	BPSS2115		28	375337	374411	385562	384636	414549	413623	374345	373419	330007	329081	1805578	1804652	377631	376705	1533251	1534177	1541923	1540997	337534	336608
2212	BPSS2116		28	372922	373377	383147	383602	412134	412589	371930	372385	327592	328047	1803175	1803630	375228	375683	1535654	1535199	1539508	1539963	335119	335574
2213	BPSS2117		28	372825	371887	383050	382112	412037	411099	371833	370895	327495	326557	1803078	1802140	375131	374193	1535751	1536689	1539411	1538473	335022	334084
2214	BPSS2118		28	370196	370885	380421	381110	409408	410097	369204	369893	324866	325555	1800449	1801138	372502	373191	1538395	1537706	1536782	1537471	332393	333082
2215	BPSS2119		28	370141	369404	380366	379629	409353	408616	369149	368412	324811	324074	1800394	1799657	372447	371710	1538450	1539187	1536727	1535990	332338	331601
2216	BPSS2120		28	369396	368158	379621	378383	408608	407370	368404	367166	324066	322828	1799649	1798411	371702	370464	1539195	1540433	1535982	1534744	331593	330355
2217	BPSS2121		28	368108	366990	378333	377215	407320	406202	367116	365998	322778	321660	1798361	1797243	370414	369296	1540483	1541601	1534694	1533576	330305	329187
2218	BPSS2122		28	366098	366493	376323	376718	405310	405705	365106	365501	320768	321163	1796351	1796746	368404	368799	1542493	1542098	1532684	1533079	328295	328690
2219	BPSS2123		28	365594	364314	375819	374539	404806	403526	364602	363322	320264	318984	1795847	1794567	367900	366620	1542997	1544277	1532180	1530900	327791	326511
2220	BPSS2124		28	363545	362625	373762	372842	402749	401829	362561	361641	318262	317342	1793846	1792926	365930	365010	1544952	1545872	1530185	1529265	325763	324843
2221	BPSS2125		28	362557	361418	372774	371635	401761	400622	361573	360434	317274	316135	1792858	1791719	364942	363803	1545940	1547079	1529197	1528058	324775	323636
2222	BPSS2126		28	360383	361228	370600	371445	399587	400432	359399	360244	315100	315945	1790684	1791529	362768	363613	1548114	1547269	1527023	1527868	322601	323446
2223	BPSS2127		28	359872	359051	370089	369268	399076	398255	358888	358067	314589	313768	1790173	1789352	362257	361436			1526512	1525691	322090	321269
2224	BPSS2128		28	358757	357357	368716	367316	397684	396284	357858	356458	313474	312074	1789058	1787658	361142	359742	1549076	1550476	1525397	1523997	320907	319507
2225	BPSS2129		28	357168	356620	367127	366579	396095	395547	356269	355721	311885	311337	1787469	1786921	359553	359005	1550794	1551219	1523808	1523260	319318	318770
2226	BPSS2130		28	353701	356109	363660	366068	392628	395036	352951	354423	308418	310826	1784002	1786410	356086	358494	1554146	1554057	1520341	1522749	315851	318259
2227	BPSS2131		28	351917	352204	361414	361701	390033	390320	351123	351410	306634	306921	1782218	1782505	354302	354589	1556133	1555846	1518557	1518844	314239	314526
2228	BPSS2131a		28	350902	351456	360399	360953	389018	389572	350108	350662	305619	306173	1781203	1781757	353287	353841	1557148	1556594	1517542	1518096	313224	313778
2229	BPSS2132		28	349087	350604	358584	360101	387203	388720	348293	349810	303901	305418	1779388	1780905	351472	352989	1558963	1557446	1515727	1517244	311879	312583
2230	BPSS2133		28	348743	347382	358240	356879	386859	385498	347949	346588	303557	302196	1779044	1777683	351128	349767	1559308	1560669	1515383	1514022	311535	310174
2231	BPSS2134		28	347021	346779	356518	356276	385137	384895	346227	345985	301835	301593	1777322	1777080	349406	349164	1561030	1561272	1513661	1513419	309813	309571
2232	BPSS2135		28	346621	345632	356118	355129	384737	383748	345827	344838	301435	300446	1776922	1775933	349006	348017	1561430	1562422	1513261	1512272	309413	308424
2233	BPSS2136		28	343855	345294	353352	354791	381971	383410	343061	344500	298669	300108	1774156	1775595	346240	347679	1564210	1562771	1510495	1511934	306647	308086
2234	BPSS2137		28	342546	343676	352043	353173	380662	381792	341752	342882	297360	298490	1772847	1773977	344931	346061	1565520	1564519	1509186	1510316	305338	306468
2235	BPSS2138		28	341560	342546	351057	352043	379676	380662	340766	341752	296374	297360	1771861	1772847	343945	344931	1566506	1565520	1508200	1509186	304352	305338
2236	BPSS2139		28	340656	341555	350153	351052	378772	379671	339862	340761	295470	296369	1770957	1771856	343041	343940	1567410	1566511	1507296	1508195	303448	304347
2237	BPSS2140		28	339706	340641	349203	350138	377822	378757	338912	339847	294520	295455	1770007	1770942	342091	343026	1568360	1567425	1506346	1507281	302498	303433
2238	BPSS2141		28	338024	339685	347521	349182	376140	377801	337230	338891	292838	294499	1768325	1769986	340409	342070	1570042	1568381	1504664	1506325	300816	302477
2239	BPSS2142		28	337307	337885	346804	347382	375423	376001	336513	337091	292121	292699	1767608	1768186	339692	340270	1570759	1570181	1503947	1504525	300099	300677
2240	BPSS2143		28	336671	335172	346168	344669	374787	373288	335877	334378	291485	289986	1766972	1765473	339056	337557	1571395	1572894	1503311	1501812	299463	297964
2241	BPSS2144		28	334730	334530	344227	344027	372846	372646	333936	333736	289544	289344	1765031	1764831	337115	336915	1573336	1573536	1501370	1501170	297522	297322
2242	BPSS2145		28	332777	334453	342274	343950	370893	372569	331983	333659	287591	289267	1763066	1764754	335150	336838	1575301	1573613	1499417	1501093	295569	297245
2243	BPSS2146		28	331485	332495	340982	341992	369601	370611	330691	331701	286299	287309	1761774	1762784	333858	334868	1576593	1575583	1498125	1499135	294277	295287
2244	BPSS2147		28	331030	331281	340527	340778	369146	369397	330236	330487	285844	286095	1761319	1761570	333403	333654	1577048	1576797	1497670	1497921	293822	294073
2245	BPSS2148		28	330730	330876	340227	340373	368846	368992	329936	330082	285544	285690	1761019	1761165	333103	333249	1577348	1577202	1497373	1497516	1795287	1795592
2246		Chr2_75_IS407A_A		329696	330729	339193	340226	367812	368845	328902	329935	284510	285543	1759985	1761018	332069	333102	1577349	1578382	1496137	1497372	291658	292532
2247	BPSS2148a																						
2248	BPSS2149																						
2249	BPSS2150																						
2250	BPSS2151																						
2251	BPSS2152																						
2252	BPSS2153																						
2253	BPSS2154																						
2254	BPSS2155																						
2255		Chr2_76_IS407A_A		2135943	2137178	2173035	2174270	2146619	2147854	2128166	2129401	2097872	2099107	1307203	1308438	2164773	2166008	2030650	2031885	977425	978660	132158	133393
2256	BPSS2156		29	2137922	2137281	2175014	2174373	2148598	2147957	2130145	2129504	2099851	2099210	1309182	1308541	2166752	2166111	2032630	2032088	979404	978763	131414	132055

2257	BPSS2157		29	2139235	2138579	2176327	2175671	2149911	2149255	2131458	2130802	2101164	2100508	1310495	1309839	2168065	2167409	2033943	2033287	980717	980061	130101	130757
2258	BPSS2158		29	2140058	2139312	2177150	2176404	2150734	2149988	2132281	2131535	2101987	2101241	1311318	1310572	2168888	2168142	2034766	2034020	981540	980794	129278	130024
2259	BPSS2159		29	2140302	2141204	2177394	2178296	2150978	2151880	2132525	2133427	2102231	2103133	1311562	1312464	2169132	2170034	2035010	2035912	981784	982686	129034	128132
2260	BPSS2160		29	2141523	2142599	2178615	2179691	2152199	2153275	2133746	2134822	2103452	2104528	1312783	1313859	2170353	2171429	2036231	2037307	983005	984081	127813	126737
2261	BPSS2161		29	2142644	2144119	2179736	2181211	2153320	2154795	2134867	2136342	2104573	2106048	1313904	1315379	2171474	2172949	2037352	2038827	984126	985601	126692	125217
2262	BPSS2162		29	2145327	2144203	2182419	2181295	2156003	2154879	2137550	2136426	2107256	2106132	1316587	1315463	2174157	2173033	2040035	2038911	986808	985684	124010	125134
2263	BPSS2163		29	2146620	2145730	2183712	2182822	2157296	2156406	2138843	2137953	2108549	2107659	1317880	1316990	2175450	2174560	2041328	2040438	988101	987211	122717	123607
2264	BPSS2164		29	2146913	2146620	2184005	2183712	2157589	2157296	2139136	2138843	2108842	2108549	1318173	1317880	2175743	2175450	2041621	2041328	988394	988101	122424	122717
2265	BPSS2165		29	2147074	2148078	2184166	2185170	2157750	2158754	2139297	2140301	2109003	2110007	1318334	1319338	2175904	2176908	2041782	2042786	988555	989559	122263	121259
2266	BPSS2166		29			2185428	2185694			2140559	2140720											1987889	1988464
2267	BPSS2167		29	2151200	2150019	2188587	2187406	2161858	2160695	2143248	2142067	2113129	2111948	1322448	1321267	2180018	2178837	2045908	2044727	992681	991500	1989796	1988696
2268	BPSS2168		29	2151594	2152532	2188981	2189919	2162765	2163703	2143642	2144580	2113523	2114461	1322842	1323780	2180412	2181350	2046302	2047240	993075	994013	1990270	1991208
2269	BPSS2169		29	2152548	2153705	2189935	2191092	2163719	2164876	2144596	2145753	2114477	2115634	1323796	1324953	2181366	2182523	2047256	2048413	994029	995186	1991224	1992381
2270	BPSS2170		29	2154053	2154403	2191440	2191790	2165224	2165574	2146101	2146451	2115982	2116332	1325301	1325651	2182871	2183221	2048761	2049111	995534	995884	1992729	1993079
2271	BPSS2171		29	2154955	2156310	2192342	2193697	2166126	2167481	2147003	2148358	2116884	2118239	1326203	1327558	2183773	2185128	2049663	2051018	996436	997791	1993631	1994986
2272		Chr2_77_ISBma2_A		2157201	2158773	2194588	2196160	2168372	2169944	2149249	2150821	2119130	2120702	1328449	1330024	2186019	2187594	2051909	2053481	998682	1000254	1995877	1997449
2273	BPSS2172																						
2274	BPSS2173																						
2275	BPSS2174																						
2276	BPSS2175																						
2277	BPSS2176																						
2278	BPSS2177																						
2279	BPSS2178																						
2280	BPSS2179																						
2281	BPSS2180																						
2282	BPSS2181																						
2283	BPSS2182																						
2284	BPSS2183																						
2285	BPSS2184																						
2286	BPSS2185																						
2287	BPSS2186																						
2288	BPSS2187																						
2289	BPSS2188																						
2290	BPSS2189																						
2291	BPSS2190																						
2292	BPSS2191																						
2293	BPSS2192																						
2294	BPSS2193																						
2295	BPSS2194																						
2296	BPSS2195																						
2297	BPSS2196																						
2298	BPSS2197																						
2299	BPSS2198																						
2300	BPSS2199																						
2301	BPSS2200																						
2302	BPSS2201																						
2303	BPSS2202																						
2304	BPSS2203																						
2305	BPSS2204																						
2306	BPSS2205																						
2307	BPSS2206																						

2308	BPSS2207																					
2309	BPSS2208																					
2310	BPSS2209																					
2311	BPSS2210																					
2312	BPSS2211																					
2313	BPSS2212																					
2314	BPSS2213																					
2315	BPSS2214																					
2316	BPSS2215																					
2317	BPSS2216																					
2318	BPSS2217																					
2319	BPSS2218																					
2320	BPSS2219																					
2321	BPSS2220																					
2322	BPSS2221																					
2323	BPSS2222																					
2324	BPSS2223																					
2325	BPSS2224																					
2326	BPSS2225																					
2327	BPSS2226																					
2328	BPSS2227																					
2329	BPSS2227A																					
2330	BPSS2228																					
2331	BPSS2229																					
2332	BPSS2230																					
2333	BPSS2231																					
2334	BPSS2232																					
2335	BPSS2233																					
2336	BPSS2234																					
2337	BPSS2235																					
2338		Chr2_78_ISBma2_A	2157201	2158773	2194588	2196160	2168372	2169944	2149249	2150821	2119130	2120702	1328449	1330024	2186019	2187594			998682	1000254	1995877	1997449
2339	BPSS2236	29	2159481	2158843	2196868	2196230	2170652	2170014	2151529	2150891	2121410	2120772	1330732	1330094	2188302	2187664			1000962	1000324	1998157	1997519
2340	BPSS2237	29	2160174	2159572	2197561	2196959	2171345	2170743	2152222	2151620	2122103	2121501	1331425	1330823	2188995	2188393			1001655	1001053	1998850	1998248
2341	BPSS2238	29	2161069	2160215	2198456	2197602	2172240	2171386	2153117	2152263	2122998	2122144	1332320	1331466	2189890	2189036			1002550	1001696	1999745	1998891
2342	BPSS2239	29	2161846	2161283	2199233	2198670	2173017	2172454	2153894	2153331	2123775	2123212	1333097	1332534	2190667	2190104			1003327	1002764	2000522	1999959
2343	BPSS2240	29	2162356	2162099	2199975	2199757	2174180	2173950	2154352	2154095	2124285	2124028	1333607	1333350	2191177	2190920			1003837	1003580	2000927	2000670
2344	BPSS2241	29	2162769	2162563	2200388	2200182	2174593	2174387	2154765	2154559	2124698	2124492	1334020	1333814	2191590	2191384			1004250	1004044	2001340	2001134
2345	BPSS2242	29	2163591	2162779	2201210	2200398	2175415	2174603	2155587	2154775	2125520	2124708	1334842	1334030	2192412	2191600			1005072	1004260	2002162	2001350
2346	BPSS2243	29	2163747	2163622	2201366	2201241	2175571	2175446	2155743	2155618	2125676	2125551	1334998	1334873	2192568	2192443			1005228	1005103	2002318	2002193
2347	BPSS2244	29	2164336	2165721	2201955	2203340	2176160	2177545	2156332	2157717	2126265	2127650	1335578	1336963	2193148	2194533			1005817	1007202	2002906	2004291
2348	BPSS2245	29	2165721	2166764	2203340	2204383	2177545	2178588	2157717	2158760	2127650	2128693	1336963	1338006	2194533	2195576			1007202	1008245	2004291	2005334
2349	BPSS2246	29	2169214	2166899	2206833	2204518	2181038	2178723	2161210	2158895	2131143	2128828	1340456	1338141	2198026	2195711			1010695	1008380	2007784	2005469
2350	BPSS2247	29	2169346	2170035	2206965	2207654	2181170	2181859	2161342	2162031	2131275	2131964	1340588	1341277	2198158	2198847			1010827	1011516	2007916	2008605
2351	BPSS2248	29	2170056	2170961	2207675	2208580	2181880	2182368	2162052	2162957	2131985	2132890	1341298	1342203	2198868	2199773			1011537	1012442	2008626	2009531
2352	BPSS2249	29	2172358	2170991	2209977	2208610	2184984	2183617	2164354	2162987	2134287	2132920	1343600	1342233	2201170	2199803			1013839	1012472	2010928	2009561
2353	BPSS2250	29	2172747	2173712	2210366	2211331	2185373	2186338	2164743	2165708	2134676	2135641	1343989	1344954	2201559	2202524			1014228	1015193	2011317	2012282
2354	BPSS2251	29	2174217	2173951	2212510	2211827	2187517	2186834	2166887	2166204	2136820	2136137	1346142	1345459	2203712	2203029			1016372	1015689	2013460	2012777
2355	BPSS2252	29	2175996	2174884	2213615	2212503	2188622	2187510	2167992	2166880	2137925	2136813	1347247	1346135	2204817	2203705			1017477	1016365	2014565	2013453
2356	BPSS2253	29	2176994	2175996	2214613	2213615	2189620	2188622	2168990	2167992	2138923	2137925	1348245	1347247	2205815	2204817			1018475	1017477	2015563	2014565
2357	BPSS2254	29	2178745	2176994	2216364	2214613	2191371	2189620	2170741	2168990	2140674	2138923	1349996	1348245	2207566	2205815			1020226	1018475	2017314	2015563
2358	BPSS2255	29	2180626	2178752	2218245	2216371	2193252	2191378	2172622	2170748	2142546	2140681	1351877	1350003	2209438	2207573			1022116	1020233	2019195	2017321

2359	BPSS2256	29	2181246	2180626	2218865	2218245	2193872	2193252	2173242	2172622	2143166	2142546	1352497	1351877	2210058	2209438	1022736	1022116	2019815	2019195
2360	BPSS2257	29	2182000	2181248	2219619	2218867	2194626	2193874	2173996	2173244	2143920	2143168	1353251	1352499	2210812	2210060	1023490	1022738	2020569	2019817
2361	BPSS2258	29	2182707	2182216	2220326	2219835	2195333	2194842	2174703	2174212	2144627	2144136	1353958	1353467	2211519	2211028	1024197	1023706	2021276	2020785
2362	BPSS2259	29	2185637	2183133	2223256	2220752	2198263	2195759	2177633	2175129	2147568	2144605	1356899	1353936	2214460	2211497	1027138	1024175	2024217	2021254
2363	BPSS2260	29	2186802	2185738	2224421	2223357	2200272	2200048	2178798	2177734	2148733	2147669	1358064	1357000	2215625	2214561	1028303	1027239	2025382	2024318
2364	BPSS2261	29	2188793	2187495	2226750	2225452	2202263	2200965	2180797	2179499	2150839	2149541	1360068	1358770	2217629	2216331	1030307	1029009	2027852	2026554
2365	BPSS2262	29	2189662	2188958	2227619	2226915	2203132	2202428	2181666	2180962	2151708	2151004	1360937	1360233	2218498	2217794	1031176	1030472	2028721	2028017
2366	BPSS2263	29	2189990	2189748	2227947	2227705	2203460	2203218	2181994	2181752	2152036	2151794	1361265	1361023	2218826	2218584	1031504	1031262	2029049	2028807
2367	BPSS2264	29	2191197	2190313	2229154	2228270	2204667	2203783	2183201	2182317	2153259	2152375	1362464	1361580	2220025	2219141	1032703	1031819	2030248	2029364
2368	BPSS2265	29	2192687	2191341	2230644	2229298	2206157	2204811	2184691	2183345	2154749	2153403	1363954	1362608	2221515	2220169	1034193	1032847	2031738	2030392
2369	BPSS2266	29	2193783	2192971	2231740	2230928	2207253	2206441	2185787	2184975	2155845	2155033	1365050	1364238	2222611	2221799	1035289	1034477	2032834	2032022
2370	BPSS2267	29	2195254	2194004	2233211	2231961	2208724	2207474	2187258	2186008	2157316	2156066	1366521	1365271	2224082	2222832	1036760	1035510	2034305	2033055
2371	BPSS2268	29	2196090	2195620	2234047	2233577	2209560	2209090	2188094	2187624	2158152	2157682	1367357	1366887	2224918	2224448	1037596	1037126	2035141	2034671
2372	BPSS2269	29	2198139	2196985	2236096	2234942	2211609	2210455	2190143	2188989	2160201	2159047	1369406	1368252	2226967	2225813	1039645	1038491	2037190	2036036
2373	BPSS2270	29	2200192	2198795	2238149	2236752	2213662	2212265	2192196	2190799	2162254	2160857	1371459	1370062	2229020	2227623	1041698	1040301	2039243	2037846
2374	BPSS2271	29	2201648	2200200	2239605	2238157	2215118	2213670	2193652	2192204	2163710	2162262	1372915	1371467	2230476	2229028	1043154	1041706	2040699	2039251
2375	BPSS2272	29	2202693	2201653	2240650	2239610	2216163	2215123	2194697	2193657	2164755	2163715	1373960	1372920	2231521	2230481	1044199	1043159	2041744	2040704
2376	BPSS2273	29	2203931	2202702	2241888	2240659	2217401	2216172	2195935	2194706	2165993	2164764	1375198	1373969	2232759	2231530	1045437	1044208	2042982	2041753
2377	BPSS2274	29	2205184	2205621	2243141	2243578	2218654	2219013	2197188	2197625	2167246	2167683	1376451	1376888	2234012	2234449	1046690	1047127	2044235	2044672
2378	BPSS2275	29	2207074	2205962	2245031	2243919	2221033	2219921	2199078	2197966	2169136	2168024	1378341	1377229	2235902	2234790	1048580	1047468	2046125	2045013
2379	BPSS2276	29	2207372	2208304	2245329	2246261	2221331	2222263	2199376	2200308	2169434	2170366	1378639	1379571	2236200	2237132	1048878	1049810	2046423	2047355
2380	BPSS2277	29	2208489	2208917	2246446	2246874	2222448	2222876	2200493	2200921	2170551	2170979	1379756	1380184	2237317	2237745	1049995	1050423	2047540	2047968
2381	BPSS2278	29	2209211	2210875	2247168	2248832	2223170	2224834	2201215	2202879	2171273	2172937	1380478	1382142	2238039	2239703	1050717	1052381	2048262	2049926
2382	BPSS2279	29	2213462	2211192	2251419	2249149	2227421	2225151	2205466	2203196	2175524	2173254	1384729	1382459	2242290	2240020	1054968	1052698	2052513	2050243
2383	BPSS2280	29	2213990	2213598	2251947	2251555	2227949	2227557	2205994	2205602	2176052	2175660	1385257	1384865	2242818	2242426	1055496	1055104	2053041	2052649
2384	BPSS2281	29	2215357	2216619	2253314	2254576	2229316	2230578	2207361	2208623	2177439	2178701	1386634	1387857	2244200	2245423	1056883	1058145	2054418	2055680
2385	BPSS2282	29	2216871	2218709	2254828	2256666	2230830	2232668	2208875	2210713	2178953	2180791	1388145	1389983	2245711	2247549	1058397	1060235	2055932	2057770
2386	BPSS2283	29	2218889	2220586	2256846	2256920	2232848	2234545	2210893	2212590	2180971	2182668	1390163	1391860	2247729	2249426	1060415	1062112	2057950	2059647
2387	BPSS2284	29	2221117	2222145	2260414	2261442	2235076	2236104	2213121	2214149	2183200	2184228	1392386	1393414	2249952	2250980	1062644	1063672	2060179	2061207
2388	BPSS2285	29	2222145	2223974	2261442	2263271	2236104	2237933	2214149	2215978	2184228	2186057	1393414	1395243	2250980	2252809	1063672	1065501	2061207	2063036
2389	BPSS2286	29	2224875	2224360	2264172	2263657	2238834	2238319	2216879	2216364	2186958	2186443	1396144	1395629	2253710	2253195	1066402	1065887	2063730	2063422
2390	BPSS2287	29	2225260	2225910	2264557	2265207	2239219	2239869	2217264	2217914	2187343	2187993	1396529	1397179	2254095	2254745	1066787	1067437	2064226	2064876
2391	BPSS2288	29	2226415	2225984	2265712	2265281	2240374	2239943	2218419	2217988	2188498	2188067	1397684	1397253	2255250	2254819	1067942	1067511	2065381	2064950
2392	BPSS2289	29	2227948	2226824	2267245	2266121	2241907	2240783	2219952	2218828	2190031	2188907	1399217	1398093	2256783	2255659	1069475	1068351	2066914	2065790
2393	BPSS2290	29	2229833	2228031	2269130	2267328	2243792	2241990	2221837	2220035	2191916	2190114	1401102	1399300	2258668	2256866	1071360	1069558	2068799	2066997
2394	BPSS2291	29	2232899	2230149	2272196	2269446	2246858	2244108	2224903	2222153	2194982	2192232	1404168	1401418	2261734	2258984	1074426	1071676	2071865	2069115
2395	BPSS2292	29	2233063	2234121	2272360	2272713	2247022	2247234	2225067	2226125	2195146	2196204	1404332	1405390	2261898	2262956	1074590	1075648	2072029	2072208
2396	BPSS2293	29	2234227	2235207	2274169	2275149	2248805	2249785	2226231	2227211	2196310	2197290	1405496	1406476	2263062	2264042	1075754	1076734	2073100	2074080
2397	BPSS2294	29	2235212	2236057	2275154	2275999	2249790	2250635	2227216	2228061	2197295	2198140	1406481	1407326	2264047	2264892	1076739	1077584	2074085	2074930
2398	BPSS2295	29	2236159	2237175	2276101	2277117	2250737	2251753	2228163	2229179	2198242	2199258	1407428	1408444	2264994	2266010	1077686	1078702	2075032	2076048
2399	BPSS2296	29	2237345	2238667	2277287	2278609	2251923	2253245	2229349	2230671	2199428	2200750	1408614	1409936	2266180	2267502	1078872	1080194	2076218	2077540
2400	BPSS2297	29	2238693	2239034	2278635	2278976	2253271	2253612	2230697	2231038	2200776	2201369	1409962	1410555	2267528	2268121	1080220	1080813	2077566	2078159
2401	BPSS2298	29											1410555	1410590			1080813	1080848		
2402	BPSS2299	29	2239991	2242864	2279933	2282806	2254569	2257442	2231995	2234868	2202073	2204946	1411259	1414132	2268832	2271705	1081517	1084390	2078863	2081736
2403	BPSS2300	29	2242881	2243675	2282823	2283617	2257459	2258253	2234885	2235679	2204963	2205757	1414149	1414943	2271722	2272516	1084407	1085201	2081753	2082547
2404	BPSS2301	29	2243736	2244683	2283678	2284625	2258314	2259261	2235740	2236687	2205818	2206765	1415004	1415951	2272577	2273524	1085262	1086209	2082608	2083555
2405	BPSS2302	29	2245817	2246701	2285759	2286643	2260395	2261279	2237821	2238705	2207913	2208797	1417076	1417960	2274595	2275479	1087424	1088308	2084785	2085669
2406	BPSS2303	29	2247180	2248568	2287122	2288510	2261758	2263146	2239184	2240572	2209276	2210664	1418439	1419827	2275958	2277346	1088787	1090175	2086148	2087536
2407	BPSS2304	29	2251273	2248757	2291215	2288699	2265851	2263335	2243277	2240761	2213369	2210853	1422532	1420016	2280051	2277535	1092880	1090364	2090241	2087725
2408	BPSS2305	29	2253539	2251974	2293952	2292387	2268716	2267151	2245504	2243939	2215765	2214326	1424856	1423291	2282303	2280738	1095170	1093605	2092508	2091525
2409	BPSS2306	29	2255406	2254237	2295819	2294650	2270583	2269414	2247371	2246202	2217632	2216463	1426723	1425554	2284170	2283001	1097037	1095868	2094375	2093206

2410	BPSS2307		29	2257317	2255734	2297730	2296147	2272494	2270911	2249282	2247699	2219543	2217960	1428634	1427051	2286081	2284498			1098948	1097365	2096342	2094759
2411	BPSS2308		29	2257832	2258152	2298245	2298565	2273009	2273329	2249787	2250107	2220008	2220328	1429149	1429469	2286536	2286856			1099603	1099923	2096877	2097197
2412	BPSS2309		29	2258922	2258350	2299335	2298763	2274099	2273527	2250877	2250305	2221098	2220526	1430239	1429667	2287626	2287054			1100693	1100121	2097967	2097395
2413	BPSS2310		29	2259476	2258922	2299889	2299335	2274653	2274099	2251431	2250877	2221652	2221098	1430793	1430239	2288180	2287626			1101247	1100693	2098521	2097967
2414	BPSS2311		29	2260842	2259484	2301255	2299897	2276019	2274661	2252797	2251439	2223018	2221660	1432159	1430801	2289546	2288188			1102613	1101255	2099887	2098529
2415	BPSS2312		29	2262275	2260845	2302688	2301258	2277452	2276022	2254230	2252800	2224451	2223021	1433592	1432162	2290979	2289549			1104046	1102616	2101320	2099890
2416	BPSS2313		29	2262618	2262271	2303031	2302684	2277795	2277448	2254573	2254226	2224794	2224447	1433935	1433588	2291322	2290975			1104389	1104042	2101663	2101316
2417	BPSS2314		29	2263491	2264888	2303904	2305301	2278668	2280065	2255446	2256843	2225667	2227064	1434808	1436205	2292195	2293592			1105262	1106659	2102536	2103933
2418	BPSS2315		29	2264888	2265532	2305301	2305945	2280065	2280709	2256843	2257487	2227064	2227708	1436205	1436849	2293592	2294236			1106659	1107303	2103933	2104577
2419		Chr2_79_ISBma1_A	29	2265680	2266984	2306093	2307397	2280857	2282161	2257635	2258939	2227856	2229160	1436997	1438301	2294384	2295688			1107451	1108755	2104725	2106029
2420		Chr2_80a_ISBma2_A		2267009	2267068	2307422	2307481	2282186	2282245	2258964	2259023	2229185	2230757	1438326	1439898	2295713	2297285	2051909	2053481	1108780	1110352	2106054	2107626
2421		Chr2_81_IS407A_B		2267062	2268297	2307475	2308710	2282239	2283474	2259017	2260252												
2422		Chr2_80a-1_ISBma2_A		2268298	2269821	2308711	2310234	2283475	2284998	2260253	2261776												
2423		Chr2_80b_ISBma2_A										121539	123111										
2424	BPSS2316		1	2270469	2269876	2310882	2310289	2285646	2285053	2262424	2261831	120891	121484	1440546	1439953	2297933	2297340	2054129	2053536	1111000	1110407	2108274	2107681
2425	BPSS2317		1	2271526	2270765	2311939	2311178	2286703	2285942	2263481	2262720	119834	120595	1441603	1440842	2298990	2298229	2055186	2054425	1112057	1111296	2109331	2108570
2426	BPSS2318		1	2273977	2271539	2314390	2311952	2289154	2286716	2265932	2263494	117383	119821	1444054	1441616	2301441	2299003	2057637	2055199	1114508	1112070	2111782	2109344
2427	BPSS2319		1	2275364	2274297	2315777	2314710	2290541	2289474	2267319	2266252	115996	117063	1445441	1444374	2302828	2301761	2059024	2057957	1115895	1114828	2112884	2112102
2428	BPSS2320		1	2276186	2275494	2316599	2315907	2291363	2290671	2268141	2267449	115174	115866	1446263	1445571	2303650	2302958	2059846	2059154	1116717	1116025	2113472	2112987
2429	BPSS2321		1	2276717	2276322	2317130	2316735	2291894	2291499	2268672	2268277	114643	115038	1446794	1446399	2304181	2303786	2060377	2059982	1117248	1116853	2114003	2113608
2430	BPSS2322		1	2278055	2276760	2318468	2317173	2293232	2291937	2270010	2268715	113305	114600	1448132	1446837	2305519	2304224	2061715	2060420	1118586	1117291	2115341	2114046
2431	BPSS2323		1	2278834	2278055	2319247	2318468	2294011	2293232	2270789	2270010	112526	113305	1448911	1448132	2306298	2305519	2062494	2061715	1119365	1118586	2116120	2115341
2432	BPSS2324		1	2280318	2278894	2320731	2319307	2295495	2294071	2272273	2270849	111042	112466	1450395	1448971	2307782	2306358	2063978	2062554	1120849	1119425	2117604	2116180
2433	BPSS2325		1	2281019	2280321	2321432	2320734	2296196	2295498	2272974	2272276	110341	111039	1451096	1450398	2308483	2307785	2064679	2063981	1121550	1120852	2118305	2117607
2434	BPSS2326		1	2282500	2281022	2322913	2321435	2297677	2296199	2274455	2272977	108857	110338	1452580	1451099	2309967	2308486	2066163	2064682	1123034	1121553	2119789	2118308
2435	BPSS2327		1	2286645	2282527	2327494	2325938	2302590	2301028	2278610	2276979	104712	108830	1456725	1452607	2314112	2309994	2070308	2066190	1127179	1123061	2123553	2122474
2436	BPSS2328		1	2293658	2292318	2334807	2333872	2309603	2308263	2281657	2278610	97741	99081	1463609	1459494	2320972	2314112	2077216	2073077	1134078	1132738	1023	1
2437	BPSS2329		1	2295408	2293765	2336557	2334914	2311353	2309710	2287299	2285656	95991	97634	1465359	1463716	2322722	2321079	2078966	2077323	1135828	1134185	2773	1130
2438	BPSS2330		1	2296004	2298634	2337153	2339783	2311949	2314579	2287895	2290525	95395	92765	1465955	1468585	2323318	2325948	2079562	2082192	1136424	1139054	3369	5999
2439	BPSS2331		1	2298630	2299619	2339779	2340768	2314575	2315564	2290521	2291510	92769	91780	1468581	1469570	2325944	2326942	2082188	2083177	1139050	1140039	5995	6984
2440	BPSS2332		1	2300409	2299864	2341558	2341013	2316354	2315809	2292300	2291755	90990	91535	1470360	1469815	2327732	2327187	2083967	2083422	1140829	1140284	7774	7229
2441	BPSS2333		1	2301799	2300456	2342948	2341605	2317744	2316401	2293690	2292347	89600	90943	1471750	1470407	2329122	2327779	2085357	2084014	1142219	1140876	9164	7821
2442	BPSS2334		1	2302866	2301799	2344015	2342948	2318811	2317744	2294757	2293690	88533	89600	1472817	1471750	2330189	2329122	2086424	2085357	1143286	1142219	10231	9164
2443	BPSS2335		1	2303607	2302882	2344756	2344031	2319552	2318827	2295498	2294773	87792	88517	1473558	1472833	2330930	2330205	2087165	2086440	1144027	1143302	10972	10247
2444	BPSS2336		1	2304260	2303607	2345409	2344756	2320205	2319552	2296151	2295498	87139	87792	1474211	1473558	2331583	2330930	2087818	2087165	1144680	1144027	11625	10972
2445	BPSS2337		1	2305077	2304328	2346226	2345477	2321022	2320273	2296968	2296219	86322	87071	1475028	1474279	2332400	2331651	2088635	2087886	1145497	1144748	12442	11693
2446	BPSS2338		1	2306232	2305528	2347381	2346677	2322177	2321473	2298123	2297419	85167	85871	1476183	1475479	2333555	2332851	2089790	2089086	1146652	1145948	13597	12893
2447	BPSS2339		1	2308212	2306242	2349361	2347391	2324157	2322187	2300103	2298133	83187	85157	1478163	1476193	2335535	2333565	2091770	2089800	1148632	1146662	15577	13607
2448	BPSS2340		1	2309388	2308543	2350537	2349692	2325333	2324488	2301279	2300434	82011	82856	1479339	1478494	2336711	2335866	2092946	2092101	1149808	1148963	16753	15908
2449	BPSS2341		1	2309933	2310835	2351082	2351984	2325878	2326780	2302170	2302676	81466	80564	1479884	1480786	2337256	2338158	2093491	2094393	1150353	1151255	17298	18200
2450	BPSS2342		1	2311820	2310888	2352969	2352037	2327765	2326833	2303661	2302729	79579	80511	1481771	1480839	2339143	2338211	2095378	2094446	1152240	1151308	19185	18253
2451	BPSS2343		1	2312428	2311820	2353577	2352969	2328373	2327765	2304269	2303661	78971	79579	1482379	1481771	2339751	2339143	2095986	2095378	1152848	1152240	19793	19185
2452	BPSS2344		1	2312801	2312436	2353950	2353585	2328746	2328381	2304642	2304277	78598	78963	1482752	1482387	2340124	2339759	2096359	2095994	1153221	1152856	20166	19801
2453	BPSS2345		1	2313307	2315586	2354456	2356735	2329252	2331531	2305148	2306680	78092	75813	1483258	1485537	2340630	2342909	2096865	2099144	1153727	1156006	20672	22951
2454	BPSS2346		1	2316411	2315857			2332356	2331802			75084	75542	1486362	1485808	2343734	2343180	2099820	2099416	1156824	1156270	23769	23215
2455	BPSS2347		1	2316775	2317377	678	1280	77	679	2306842	2307444	148	750	1486726	1487328	2344098	2344700	1789589	1788987	1157188	1157790	24133	24735
2456	BPSS2347a		1	2317361	2317555	1264	1458	663	857	2307428	2307622	734	928	1487312	1487506	2344684	2344878	1789003	1788809	1157774	1157968	24719	24913
2457	BPSS2348		1	2318245	2317892	2148	1795	1547	1194	2308312	2307959	1618	1265	1488202	1487849	2345580	2345227	1788113	1788466	1158664	1158311	25603	25250
2458	BPSS2349		1	2318674	2319333	2577	3236	1976	2635	2308741	2309400	2040	2699	1488624	1489283	2346002	2346661	1787691	1787032	1159086	1159745	26025	26684
2459	BPSS2350		1	2319361	2320425	3264	4328	2663	3727	2309428	2310492	2727	3791	1489311	1490375	2346689	2347753	1787004	1785940	1159773	1160837	26712	27776
2460		Chr2_82_ISBma2_B	1	2320471	2322043	4374	5946	3773	5345	2310538	2312110			1490409	1491981	2347787	2349359	1784937	1785906				

2461	BPSS2351		1	2323568	2322120	7471	6023	6870	5422	2313635	2312187	5336	3888	1493505	1492057	2350883	2349435	1783378	1784826	1162382	1160934	29321	27873
2462		Chr2_83_IS407A_C	1									6354	7589										
