# Supplementary material for: The Early Stage of Bacterial Genome-Reductive Evolution in the Host
Source: PLoS Pathog. 2010 May 27;6(5):e1000922. doi: 10.1371/journal.ppat.1000922 (PMC2877748; doi:10.1371/journal.ppat.1000922)
Supplement: Table S2 — Comparison of B. pseudomallei genomes with that of strain K96243. (9.59 MB PDF) [file ppat.1000922.s004.pdf]

Table S2. Comparison of *B. pseudomallei* genomes with that of strain K96243

				1106a		1106b		1710a		1710b		406e		668		Pasteur		S13		1655	
	**locus_tags in Bp K962**	**Additional genes**	**IS element**	**5'-end**	**3'-end**	**5'-end**	**3'-end**	**5'-end**	**3'-end**	**5'-end**	**3'-end**	**5'-end**	**3'-end**	**5'-end**	**3'-end**	**5'-end**	**3'-end**	**5'-end**	**3'-end**	**5'-end**	**3'-end**
**Chromosome 1**																					
1	BPSL0001			1116	4	3250495	3249383	244698	243586	223896	222784	3960220	3959108	1116	4	4016601	4015489	4094729	4093617	3888983	3887871
2	BPSL0002			2375	1164	3251754	3250543	245957	244746	225155	223944	3961479	3960268	2375	1164	4017860	4016649	4095988	4094777	3890242	3889031
3	BPSL0003			5168	3117	3254547	3252496	248750	246699	227948	225897	3964272	3962221	5159	3117	4020653	4018602	4098781	4096730	3893035	3890984
4	BPSL0004			5488	5763	3254867	3255142	249070	249345	228268	228543	3964592	3964867	5479	5754	4020973	4021248	4099101	4099376	3893355	3893630
5	BPSL0005			7209	5872	3256588	3255251	250791	249454	229989	228652	3966313	3964976	7200	5863	4022694	4021357	4100822	4099485	3895076	3893739
6	BPSL0006			8013	7531	3257392	3256910	251595	251113	230793	230311	3967117	3966635	8004	7522	4023498	4023016	4101626	4101144	3895880	3895398
7	BPSL0007			8395	10665	3257774	3258997	251977	254247	231175	233445	3967499	3968734	8386	10656	4023880	4025109	4102008	4104278	3896262	3898514
8	BPSL0008			10665	12155	3260045	3261535	254247	255737	233445	234935	3969815	3971305	10656	12146	4026151	4027641	4104278	4105768	3898514	3900004
9	BPSL0009			12163	13377	3261543	3262757	255745	256959	234943	236157	3971313	3972527	12154	13368	4027649	4028863	4105776	4106990	3900012	3901226
10	BPSL0010			13862	13455	3263242	3262835	257444	257037	236642	236235	3973012	3972605	13853	13446	4029348	4028941	4107475	4107068	3901711	3901304
11	BPSL0011			14010	14459	3263390	3263839	257592	258041	236790	237239	3973160	3973609	14001	14450	4029496	4029945	4107623	4108072	3901859	3902308
12	BPSL0012			14617	15093	3263997	3264473	258202	258675	237400	237873	3973767	3974243	14611	15084	4030106	4030579	4108233	4108706	3902469	3902942
13	BPSL0013			15099	15500	3264479	3264880	258681	259082	237879	238280	3974249	3974650	15090	15491	4030585	4030986	4108712	4109113	3902948	3903349
14	BPSL0014			15481	16206	3264861	3265586	259063	259788	238261	238986	3974631	3975356	15472	16197	4030967	4031692	4109094	4109819	3903330	3904055
15	BPSL0015			16391	17458	3265771	3266838	259973	261040	239171	240238	3975541	3976608	16382	17449	4031877	4032944	4110004	4111071	3904240	3905307
16	BPSL0016			17486	18913	3266866	3268293	261068	262495	240266	241693	3976636	3978063	17477	18904	4032972	4034399	4111099	4112526	3905335	3906762
17	BPSL0017			18913	19416	3268293	3268796	262495	262998	241693	242196	3978063	3978566	18904	19407	4034399	4034902	4112526	4113029	3906762	3907265
18	BPSL0018			19458	20246	3268838	3269626	263040	263828	242238	243026	3978608	3979396	19449	20237	4034944	4035732	4113071	4113859	3907307	3908095
19	BPSL0019			22110	20500	3271490	3269880	265643	264033	244841	243231	3981380	3979692	22045	20438	4037638	4036028	4115767	4114145	3909925	3908324
20	BPSL0020			22604	22335	3271984	3271715	266137	265868	245335	245066	3981874	3981605	22551	22270	4038132	4037863	4116270	4115992	3910428	3910150
21	BPSL0021			22803	23291	3272183	3272671	266336	266824	245534	246022	3982073	3982561	22750	23238	4038331	4038819	4116469	4116957	3910627	3911115
22	BPSL0022			23442	25001	3272822	3274381	266975	268534	246173	247732	3982712	3984271	23389	24948	4038970	4040529	4117108	4118667	3911265	3912824
23	BPSL0023			25987	25067	3275367	3274447	269520	268600	248718	247798	3985257	3984337	25934	25014	4041515	4040595	4119653	4118733	3913810	3912890
24	BPSL0024			26202	26594	3275582	3275974	269735	270127	248933	249325	3985472	3985864	26146	26541	4041730	4042122	4119868	4120260	3914025	3914417
25	BPSL0025			26666	27385	3276046	3276765	270199	270918	249397	250116	3985936	3986655	26613	27332	4042194	4042913	4120332	4121051	3914489	3915208
26		BURPS1106A_0027		27877	28116																
27		BURPS1106B_A3312				3277496	3277257														
28		BURPS406E_A0911										3987376	3987086								
29		BURPSPAST_Y0262														4043634	4043344				
30	BPSL0026			28217	28711	3277597	3278091	271740	272234	250938	251432	3987477	3987971	28150	28644	4043735	4044229	4121873	4122367	3916030	3916524
31	BPSL0027			28737	29732	3278117	3279112	272260	273255	251458	252453	3987997	3988992	28670	29665	4044255	4045250	4122393	4123388	3916550	3917545
32	BPSL0028			29728	30222	3279108	3279602	273251	273745	252449	252943	3988988	3989482	29661	30155	4045246	4045740	4123384	4123878	3917541	3918035
33	BPSL0029			30393	30884	3279773	3280264	273916	274407	253114	253605	3989653	3990144	30326	30817	4045911	4046402	4124049	4124540	3918206	3918697
34	BPSL0030			30958	31716	3280338	3281096	274481	275239	253679	254437	3990218	3990976	30891	31649	4046476	4047234	4124614	4125372	3918771	3919529
35	BPSL0031			31745	32014	3281125	3281394	275268	275537	254466	254735	3991005	3991274	31678	31947	4047263	4047532	4125401	4125670	3919558	3919827
36	BPSL0032			32136	32915	3281516	3282295	275659	276438	254857	255636	3991396	3992175	32069	32848	4047654	4048442	4125792	4126571	3919949	3920728
37	BPSL0033			33666	33241	3283046	3282621	277189	276764	256387	255962	3992926	3992501	33599	33174	4049295	4048870	4127322	4126897	3921479	3921054
38	BPSL0034			33748	34620	3283128	3284000	277271	278143	256469	257341	3993008	3993880	33681	34553	4049377	4050249	4127404	4128276	3921561	3922433
39	BPSL0035			34616	35134	3283996	3284514	278139	278657	257337	257855	3993876	3994394	34549	35067	4050245	4050418	4128272	4128790	3922429	3922947
40	BPSL0036			35295	36305	3284675	3285685	278818	279828	258016	259026	3994555	3995565	35228	36238	4050922	4051932	4128951	4129961	3923108	3924118
41	BPSL0037			36392	37186	3285772	3286566	279915	280709	259113	259907	3995652	3996446	36325	37119	4052019	4052813	4130048	4130842	3924205	3924999
42	BPSL0038			37208	38062	3286588	3287442	280731	281585	259929	260783	3996468	3997322	37141	37995	4052835	4053689	4130864	4131718	3925021	3925875
43	BPSL0039			38143	39519	3287523	3288899	281666	283042	260864	262240	3997403	3998779	38076	39452	4053770	4055146	4131799	4133175	3925956	3927332
44	BPSL0040			39533	40207	3288913	3289587	283056	283730	262254	262928	3998793	3999467	39466	40140	4055160	4055834	4133189	4133863	3927346	3928020
45	BPSL0041			40533	41657	3289913	3291037	284056	285180	263254	264378	3999788	4000912	40461	41585	4056160	4057284	4134189	4135313	3928341	3929465
46	BPSL0042			42136	44145	3291516	3293525	285659	287668	264857	266866	4001370	4003379	42064	44073	4057763	4059772	4135792	4137801	3929939	3931948
47	BPSL0043			44189	47215	3293569	3296595	287712	290738	266910	269936	4003423	4006449	44117	47143	4059816	4062842	4137845	4140871	3931992	3935018
48	BPSL0044			47756	48853	3297136	3298233	291279	292376	270477	271574	4006990	4008087	47677	48774	4063383	4064480	4141412	4142509	3935552	3936649
49	BPSL0045			49256	50212	3298636	3299592	292779	293735	271977	272933	4008490	4009446	49177	50130	4064884	4065840	4142912	4143865	3937052	3938005
50	BPSL0046			51069	50341	3300449	3299721	294592	293864	273790	273062	4010303	4009575	50990	50262	4066697	4065969	4144725	4143997	3938865	3938137
51	BPSL0047			52164	51445	3301544	3300825	295687	294968	274885	274166	4011398	4010679	52085	51366	4067792	4067073	4145820	4145101	3939960	3939241

52	BPSL0048			53210	52164	3302590	3301544	296733	295687	275931	274885	4012444	4011398	53131	52085	4068838	4067792	4146866	4145820	3941006	3939960
53	BPSL0049			54085	53210	3303465	3302590	297608	296733	276806	275931	4013319	4012444	54006	53131	4069713	4068838	4147741	4146866	3941881	3941006
54	BPSL0050			55328	54093	3304708	3303473	298851	297616	278049	276814	4014562	4013327	55249	54014	4070956	4069721	4148984	4147749	3943124	3941889
55	BPSL0051			57071	55569	3306524	3305022	300594	299092	279792	278290	4016377	4014875	56992	55490	4072789	4071287	4150788	4149286	3944948	3943446
56	BPSL0052			57518	57129	3306971	3306582	301042	300653	280240	279851	4016825	4016436	57440	57051	4073237	4072848	4151236	4150847	3945396	3945007
57	BPSL0053			58338	57532	3307791	3306985	301862	301056	281060	280254	4017645	4016839	58260	57454	4074057	4073251	4152056	4151250	3946216	3945410
58	BPSL0054			59181	58387	3308634	3307840	302705	301911	281903	281109	4018488	4017694	59103	58309	4074900	4074106	4152899	4152105	3947059	3946265
59	BPSL0055			60936	59269	3310389	3308722	304460	302793	283658	281991	4020243	4018576	60858	59191	4076655	4074988	4154654	4152987	3948814	3947147
60	BPSL0056			61663	62001	3311116	3311454	305187	305525	284385	284723	4020970	4021308	61583	61921	4077382	4077720	4155379	4155717	3949539	3949877
61	BPSL0057			62001	63185	3311454	3312638	305525	306709	284723	285907	4021308	4022492	61921	63105	4077720	4078904	4155717	4156901	3949877	3951043
62	BPSL0058			64847	63864	3314300	3313317	308371	307388	287569	286586	4024154	4023171	64767	63784	4080566	4079583	4158563	4157580	3952705	3951722
63	BPSL0059			65713	64880	3315166	3314333	309237	308404	288435	287602	4025020	4024187	65633	64800	4081432	4080599	4159429	4158596	3953571	3952738
64	BPSL0060			67268	66156	3316721	3315609	310792	309680	289990	288878	4026564	4025476	67180	66077	4082966	4081875	4160975	4159872	3955103	3954027
65	BPSL0061			67381	68514	3316834	3317967	310905	312038	290103	291236	4026677	4027810	67293	68426	4083079	4084212	4161088	4162221	3955216	3956349
66		BURPS668_0072												68477	68809						
67	BPSL0062			69172	70374	3318625	3319827	312707	313909	291905	293107	4028479	4029681	69070	70272	4084866	4086068	4162890	4164092	3957002	3958204
68	BPSL0063			70390	72510	3319843	3321963	313925	316045	293123	295243	4029697	4031817	70288	72408	4086084	4088204	4164108	4166228	3958220	3960340
69	BPSL0064			72919	74049	3322372	3323502	316444	317574	295642	296772	4032208	4033338	72845	73975	4088611	4089741	4166621	4167751	3960755	3961885
70	BPSL0065			74695	74132	3324148	3323585	318220	317657	297418	296855	4033984	4033421	74621	74058	4090387	4089824	4168397	4167834	3962531	3961968
71	BPSL0066			75507	75046	3324960	3324499	319020	318559	298218	297757	4034784	4034323	75421	74960	4091187	4090726	4169209	4168748	3963337	3962876
72	BPSL0067			75976	76500	3325429	3325953	319489	320013	298687	299211	4035253	4035777	75890	76414	4091656	4092180	4169678	4170202	3963805	3964329
73	BPSL0068			76490	76855	3325943	3326308	320003	320368	299201	299566	4035767	4036132	76404	76769	4092170	4092535	4170192	4170557	3964319	3964684
74	BPSL0069			78302	77547	3327755	3327000	321797	321042	300995	300240	4037561	4036806	78207	77452	4093964	4093209	4171983	4171237	3966113	3965358
75	BPSL0070			78814	78302	3328267	3327755	322309	321797	301507	300995	4038073	4037561	78719	78207	4094476	4093964	4172495	4171983	3966625	3966113
76	BPSL0071			78975	80057	3328428	3329510	322470	323552	301668	302750	4038234	4039316	78880	79962	4094637	4095719	4172656	4173738	3966786	3967868
77	BPSL0072			80057	80581	3329510	3330034	323552	324076	302750	303274	4039316	4039840	79962	80486	4095719	4096243	4173738	4174262	3967868	3968392
78			Bp_chr1_1_IS407	82576	83054	3332029	3332507	326357	327498	305269	306505					4098238	4099474				
79		BURPS1106A_0087		81137	82036																
80		BURPS1106A_0088		82233	82385																
81		BURPS1106A_0089		82644	82907																
82		BURPS1106A_0090		82931	83092																
83		BURPS1106A_0091		83097	83387																
84		BURPS1106A_0092		83381	84235																
85		BURPS1106A_0093		84389	85225																
86		BURPS1106A_0094		85693	86940																
87		BURPS1106A_0095		87362	87685																
88		BURPS1106A_0096		87678	88547																
89		BURPS1106A_0097		88777	91245																
90		BURPS1106A_0098		91595	92542																
91		BURPS1106A_0099		92566	95835																
92		BURPS1106A_0100		95976	96527																
93		BURPS1106B_A3372				3331686	3331838														
94		BURPS1106B_A3375				3332550	3332840														
95		BURPS1106B_A3376				3332834	3333688														
96		BURPS1106B_A3377				3334678	3333842														
97		BURPS1106B_A3378				3336393	3335146														
98		BURPS1106B_A3379				3336815	3337138														
99		BURPS1106B_A3380				3337131	3338000														
100		BURPS1106B_A3381				3338230	3340698														
101		BURPS1106B_A3382				3341048	3341995														
102		BURPS1106B_A3383				3342019	3345288														
103		BURPS1106B_A3384				3345285	3345980														
104		BURPS1710A_0346						324632	325531												
105		BURPS1710A_0347						325728	325880												
106		BURPSPAST_Y0322														4096799	4097698				
107		BURPSPAST_Y0323														4097895	4098047				
108	BPSL0073			99739	97274	3349192	3346727	330177	327712	309184	306719	4043177	4040712	83828	81363	4102153	4099688	4177601	4175136	3971732	3969267
109	BPSL0074			101031	99931	3350484	3349384	331468	330368	310475	309375	4044468	4043368	85120	84020	4103444	4102344	4178892	4177792	3973024	3971924

110	BPSL0075			102795	101197	3352248	3350650	333232	331634	312239	310641	4046232	4044634	86884	85286	4105208	4103610	4180656	4179058	3974788	3973190
111	BPSL0075a			103241	103372	3352694	3352825	333678	333809	312685	312816	4046678	4046809	87330	87461	4105654	4105785	4181103	4181234	3975234	3975365
112	BPSL0076			103449	103856	3352902	3353309	333886	334293	312893	313300	4046886	4047293	87538	87945	4105862	4106269	4181311	4181718	3975442	3975849
113	BPSL0077			103939	104205	3353392	3353658	334376	334642	313383	313649	4047376	4047642	88028	88294	4106352	4106618	4181801	4182067	3975932	3976198
114	BPSL0078			104217	105890	3353670	3355343	334654	336327	313661	315334	4047654	4049327	88306	89979	4106630	4108303	4182079	4183752	3976210	3977883
115	BPSL0079			106415	106053	3355868	3355506	336851	336489	315858	315496	4049851	4049489	90504	90142	4108827	4108465	4184279	4183917	3978408	3978046
116	BPSL0080			106807	108207	3356260	3357660	337243	338643	316250	317650	4050243	4051643	90896	92296	4109219	4110619	4184671	4186071	3978800	3980200
117	BPSL0081			108364	109605	3357817	3359058	338800	340041	317807	319048			92453	93694	4110776	4112017			3980357	3981598
118	BPSL0082			113123	110259	3362576	3359712	343559	340695	322566	319702			97213	94349	4115263	4113362	1094800	1097664		
119	BPSL0083			113589	113251	3363042	3362704	344025	343687	323032	322694			97679	97341	4115729	4115391	1094334	1094672	3985580	3985242
120	BPSL0084			113843	113589	3363296	3363042	344279	344025	323286	323032			97933	97679	4115983	4115729	1094080	1094334	3985834	3985580
121	BPSL0085			114012	113839	3363465	3363292	344448	344275	323455	323282			98102	97929	4116152	4115979	1093911	1094084	3986003	3985830
122	BPSL0086			114859	114383	3364312	3363836	345295	344819	324302	323826			98949	98473	4116999	4116523	1093064	1093540	3986850	3986374
123	BPSL0087			115773	115255	3365226	3364708	346209	345691	325216	324698			99863	99345	4117913	4117395	1092150	1092668	3987764	3987246
124	BPSL0088			117098	116184	3366551	3365637	347534	346620	326541	325627			101188	100274	4119238	4118324	1090825	1091739	3989089	3988175
125	BPSL0089			118092	117094	3367545	3366547	348528	347530	327535	326537			102182	101184	4120232	4119234	1089831	1090829	3990083	3989085
126		BURPS1655_F0356																		3990358	3990209
127		BURPS1655_F0357																		3991602	3991414
128		BURPS1655_F0358																		3993952	3992252
129		BURPS668_0105												102912	103490						
130		BURPS668_0106												103512	105677						
131	BPSL0090		Bp_chr1_2_IS407	119285	118455	3368738	3367908	349721	348891	328728	327898					4121425	4120595				
132	BPSL0091		Bp_chr1_2_IS407	119572	119312	3369025	3368765	350008	349748	329015	328755					4121712	4121452				
133	BPSL0092			120803	119874	3370256	3369327	351194	350310	330201	329317	4052770	4051841	107573	106644	4122898	4122014	4187198	4186269	3995873	3994944
134	BPSL0093			121171	122325	3370624	3371778	351562	352716	330569	331723	4053138	4054292	107940	109094	4123266	4124420	4187566	4188720	3996241	3997395
135	BPSL0094			122391	122543	3371844	3371996	352782	352934	331789	331941	4054358	4054510	109159	109311	4124486	4124638	4188786	4188938	3997461	3997613
136	BPSL0095			122903	123205	3372356	3372658	353285	353593	332292	332600	4054861	4055169	109661	109969	4124989	4125297	4189287	4189595	3997964	3998272
137	BPSL0096			123226	123783	3372679	3373236	353614	354171	332621	333178	4055190	4055747	109990	110547	4125318	4125875	4189616	4190173	3998293	3998850
138	BPSL0097			124275	124616	3373728	3374069	354664	355005	333671	334012	4056239	4056580	111039	111380	4126368	4126709	4190665	4191006	3999342	3999683
139	BPSL0098			124839	125390	3374292	3374387	355224	355775	334231	334782	4056803	4057354	111594	112145	4126928	4127479	4191224	4191775	3999902	4000453
140	BPSL0099			126006	125593			356398	355985	335405	334992	4057978	4057565	112719	112306	4128038	4127625	4192414	4192001	4001079	4000666
141	BPSL0100			126408	127499	3374596	3375687	356741	357847	335807	336898	1117	2208	113120	114211	3	302	64	1155	262	1353
142	BPSL0101			127499	128437	3375687	3376625	357847	358785	336898	337836	2208	3146	114211	115149	302	1240	1155	2093	1353	2291
143	BPSL0102			128611	130209	3376799	3378397	358959	360557	338010	339608	3320	4918	115323	116921	1414	3012	2267	3865	2465	4063
144	BPSL0103			131674	130229	3379862	3378417	362022	360577	341073	339628	6383	4938	118391	116946	4482	3037	5335	3890	5533	4088
145	BPSL0104			132485	131940	3380673	3380128	362833	362288	341884	341339	7194	6649	119202	118657	5293	4748	6146	5601	6344	5799
146		BURPS1655_K1229																		6736	6852
147	BPSL0105			132917	132961	3381105	3381149	363271	363315	342322	342366	7585	7698	119641	119682	5731	5775				
148	BPSL0106			133622	133056	3381810	3381244	363976	363410	343027	342461	8359	7793	120346	119780	6436	5870	7330	6764	7510	6944
149	BPSL0107			134693	133728	3382881	3381916	365047	364082	344098	343133	9430	8465	121417	120452	7507	6542	8401	7436	8581	7616
150	BPSL0108			134836	135927	3383024	3384115	365190	366281	344241	345332	9573	10664	121560	122651	7650	8741	8544	9635	8724	9815
151	BPSL0109			135959	137068	3384147	3385256	366313	367422	345364	346473	10696	11805	122683	123792	8773	9882	9667	10776	9847	10956
152	BPSL0110			137082	137954	3385270	3386142	367436	368308	346487	347359	11819	12691	123806	124678	9896	10768	10790	11662	10970	11842
153		BURPS1106A_0143		138094	138558																
154		BURPS1106B_A3427				3386746	3386282														
155		BURPS668_0128												125584	125703						
156		BURPS668_0129												125700	126725						
157		BURPS668_0130												126722	127603						
158		BURPS668_0131												127600	128502						
159		BURPS668_0132												128639	129652						
160		BURPSPAST_Z0832														11372	10908				
161	BPSL0111			138945	140045	3387133	3388233	369298	370398	348349	349449	13924	15033	130480	131580	11759	12856	12653	13750	12832	13932
162		BURPS1106A_0146		140092	140448																
163		BURPS1106A_0147		141073	141897																
164		BURPSS13_G0104																12763	12326		
165	BPSL0112							370759	370478	349810	349529	15394	15113	131941	131660	13220	12939	14114	13833	14293	14012
166	BPSL0113							371238	370759	350289	349810	15873	15394	132420	131941	13699	13220	14593	14114	14772	14293
167		BURPS1655_K1217																		16996	15443

168	BURPS1655_K1218																	15459	15256
169	BURPS1710A_0404					374671	374246												
170	BURPS668_0138											132905	133108						
171	BURPS668_0139											133092	134867						
172	BURPSS13_G0099															16078	17424		
173	BURPSS13_G0098															17529	18290		
174	BURPSS13_G0097															19577	18336		
175	BURPSS13_G0096															20479	21066		
176	BURPSS13_G0095															22087	21356		
177	BURPSS13_G0094															22411	24240		
178	BURPSS13_G0093															24308	24868		
179	BURPSS13_G0092															24904	25953		
180	BURPSS13_G0091															26270	26881		
181	BURPSS13_G0090															27198	27947		
182	BURPSS13_G0089															28140	29420		
183	BURPSS13_G0088															30226	32343		
184	BURPSS13_G0087															34921	34640		
185	BURPSS13_G0086															32347	34680		
186	BURPSS13_G0085															35084	38227		
187	BURPSS13_G0084															38230	40134		
188	BURPSS13_G0083															41568	40585		
189	BURPSS13_G0082															41542	41676		
190	BURPSS13_G0081															43369	41930		
191	BURPSS13_G0080															44646	43537		
192	BURPSS13_G0079															46026	44713		
193	BURPSS13_G0078															46289	46609		
194	BURPSS13_G0077															46922	46623		
195	BURPSS13_G0076															47857	47051		
196	BURPSS13_G0075															48949	47921		
197	BURPSS13_G0074															49857	48946		
198	BURPSS13_G0073															50891	49899		
199	BURPSS13_G0072															51935	50967		
200	BURPSS13_G0071															52069	52233		
201	BURPSS13_G0070															53331	52639		
202	BURPSS13_G0069															53780	55021		
203	BURPSS13_G0068															55497	57446		
204	BURPSS13_G0067															59033	57819		
205	BURPSS13_G0066															58981	60177		
206 BPSL0114		142499	142900	3390687	3391088	375260	375661	354311	354712	18294	18695	135259	135660	17721	18122	60936	61337	17609	18010
207 BPSL0115		142941	143807	3391129	3391995	375702	376568	354753	355619	18736	19602	135701	136567	18163	19029	61378	62244	18051	18917
208 BPSL0116		144947	143934	3393135	3392122	377708	376695	356759	355746	20742	19729	137707	136694	20169	19156	63384	62371	20058	19045
209 BPSL0117		145386	146438	3393574	3394626	378147	379199	357198	358250	21181	22233	138146	139198	20608	21660	63823	64875	20497	21549
210 BPSL0118		149275	146669	3397463	3394857	382033	379427	361084	358478	25070	22464	142034	139428	24494	21888	67709	65103	24383	21777
211 BPSL0119		149857	149489	3398045	3397677	382615	382247	361666	361298	25652	25284	142616	142248	25076	24708	68291	67923	24965	24597
212 BPSL0120		151041	149920	3399229	3398108	383799	382678	362850	361729	26836	25715	143800	142679	26260	25139	69475	68354	26149	25028
213 BPSL0121		151336	151836	3399524	3400024	384094	384594	363145	363645	27131	27631	144095	144595	26555	27055	69770	70270	26444	26944
214 BPSL0122		151871	152851	3400059	3401039	384629	385609	363680	364660	27666	28646	144630	145610	27090	28070	70305	71285	26979	27959
215 BPSL0123		152975	153607	3401163	3401795	385733	386365	364784	365416	28770	29402	145734	146366	28194	28826	71409	72041	28083	28715
216 BPSL0124		153720	154574	3401908	3402762	386478	387332	365529	366383	29515	30369	146479	147333	28939	29793	72154	73008	28828	29682
217	BURPSS13_G0050															73176	73340		
218 BPSL0125		155171	156577	3403359	3404765	387823	389229	366874	368280	30862	32268	147778	149184	30276	31682	73453	74859	30287	31693
219 BPSL0126		156577	157164	3404765	3405352	389229	389816	368280	368867	32268	32855	149184	149771	31682	32269	74859	75446	31693	32280
220 BPSL0127		157169	159574	3405357	3407762	389821	392226	368872	371277	32860	35265	149776	152181	32274	34679	75451	77856	32285	34690
221 BPSL0128		159578	160258	3407766	3408446	392230	392910	371281	371961	35269	35949	152185	152865	34683	35363	77860	78540	34694	35374
222 BPSL0129														36568	35441	79735	78725		
223 BPSL0130														40228	37439	83395	80606	3784473	3787262
224	BURPS1655_F0150																	3788155	3789264
225 BPSL0130a														40494	40243	83661	83410	3784207	3784458

226	BPSL0131									40859	40494	84026	83661	3783842	3784207
227		BURPSS13_G0039										84226	84020		
228	BPSL0132									41059	40856			3783642	3783845
229	BPSL0133									41467	41231	84634	84398	3783234	3783470
230	BPSL0134									41680	41474	84847	84641	3783021	3783227
231	BPSL0135									41906	41712	85073	84879	3782795	3782989
232	BPSL0136									42130	41915			3782571	3782786
233	BPSL0137									42691	42149			3782010	3782552
234	BPSL0138									43025	42780	85642	85397	3781676	3781921
235	BPSL0139									43309	43016	85926	85633	3781392	3781685
236		BURPSS13_G0031										86249	86680		
237		BURPSS13_G0030										86730	87473		
238		BURPSS13_G0029										87555	88412		
239	BPSL0140									43507	43316			3781194	3781385
240	BPSL0141													3781113	3780613
241	BPSL0142													3780557	3779811
242	BPSL0143													3779765	3779268
243	BPSL0144									46846	45761	89517	88423	3778160	3779254
244	BPSL0145									47271	46849	89942	89520	3777735	3778157
245	BPSL0146									50255	47292			3774751	3777714
246	BPSL0147									50365	50255	93036	92926	3774641	3774751
247	BPSL0148									50718	50377	93389	93048	3774288	3774629
248	BPSL0149									51285	50779	93956	93450	3773721	3774227
249	BPSL0150									52473	51304	95135	93975	3772533	3773699
250	BPSL0151									53200	52532	95870	95202	3771806	3772474
251	BPSL0152									55589	53220	98259	95890	3769417	3771786
252	BPSL0153									56145	55594	98815	98264	3768861	3769412
253	BPSL0154									57043	56141	99713	98811	3767963	3768865
254	BPSL0155									57402	57043	100072	99713	3767604	3767963
255	BPSL0156									58079	57402	100503	100303	3766927	3767604
256	BPSL0157									58253	58993	100677	101417	3766753	3766013
257		BURPSS13_G0015										102122	101619		
258	BPSL0158														
259	BPSL0159									60319	59855	102743	102279	3764508	3764966
260		BURPS1655_F0120												3765090	3765848
261	BPSL0160									60732	60319			3764095	3764508
262	BPSL0161									61000	60728	103422	103150	3763827	3764099
263	BPSL0162									61277	60840	103699	103262	3763550	3763987
264	BPSL0163									62086	61277	104508	103699	3762741	3763550
265	BPSL0164									62355	62086	104777	104508	3762472	3762741
266	BPSL0165									62701	62360	105123	104782	3762126	3762467
267	BPSL0166									62922	62719	105344	105141	3761905	3762108
268	BPSL0167									63170	62922	105592	105344	3761657	3761905
269	BPSL0168									63649	63173	106071	105595	3761178	3761654
270	BPSL0169									64438	63755	106860	106174	3760389	3761075
271	BPSL0170									65448	64438	107870	106860	3759379	3760389
272	BPSL0171									66291	65485	108713	107907	3758536	3759342
273	BPSL0172									66435	68198	108857	110620	3758392	3756629
274	BPSL0173									68201	69253	119957	121009	3756626	3755574
275		BURPSPAST_Z0765								69595	69867				
276		BURPSPAST_Z0764								71819	71118				
277		BURPSS13_I0968										121119	121391		
278		BURPSS13_I0967										121357	121623		
279		BURPSS13_I0966										123908	123678		
280		BURPSS13_I0965										124690	124040		
281	BPSL0174													3532435	3532788
282		BURPS1106B_A3431		3390085	3389261										
283	BPSL0175													3532136	3532429

284	BPSL0176																				
285	BPSL0177			160730	161461	3408918	3409649	393382	394113	372433	373164	36421	37152	153337	154068	73472	74203	126319	127050	35845	36576
286	BPSL0178			161558	162187	3409746	3410375	394210	394839	373261	373890	37249	37878	154166	154795	74300	74929	127147	127776	36673	37302
287	BPSL0179			162210	162659	3410398	3410847	394862	395311	373913	374362	37901	38350	154818	155267	74952	75401	127799	128248	37325	37774
288	BPSL0180			163087	163869	3411275	3412057	395735	396517	374786	375568	38775	39557	155691	156473	75825	76607	128673	129455	38199	38981
289	BPSL0181			163869	164642	3412057	3412830	396517	397290	375568	376341	39557	40330	156473	157246	76607	77380	129455	130228	38981	39754
290	BPSL0182			166170	165025	3414358	3413213	398715	397570	377766	376621	41880	40735	158711	157566	78915	77770	131708	130563	41215	40070
291	BPSL0183			168611	166185	3416799	3414373	401138	398730	380189	377781	44303	41895	161134	158726	81338	78930	134131	131723	43647	41230
292	BPSL0184			169307	168798	3417495	3416986	401834	401325	380885	380376	44999	44490	161830	161321			134827	134318	44343	43834
293	BPSL0185			170377	169307	3418565	3417495	402904	401834	381955	380885	46069	44999	162900	161830	82657	81587	135897	134827	45413	44343
294	BPSL0186			171540	170500	3419728	3418688	404067	403027	383118	382078	47232	46192	164063	163023	83820	82780	137060	136020	46576	45536
295	BPSL0187			171912	172202	3420100	3420390	404438	404728	383489	383779	47604	47894	164432	164722	84191	84481	137432	137722	46948	47238
296	BPSL0188			172318	173805	3420506	3421993	404844	406331	383895	385382	48010	49497	164838	166325	84597	86084	137838	139325	47354	48841
297	BPSL0189			173811	175280	3421999	3423468	406337	407806	385388	386857	49503	50972	166331	167800	86090	87559	139331	140800	48847	50316
298	BPSL0190			175410	176246	3423598	3424434	407937	408773	386987	387823	51102	51938	167930	168766	87694	88530	140930	141766	50446	51282
299	BPSL0191			176281	177048	3424469	3425236	408809	409576	387859	388626	51973	52740	168801	169568	88565	89332	141801	142568	51318	52085
300		BURPSPAST_Z0746														89429	89731				
301			Bp_chr1_3_ISBma2											169674	171246						
302	BPSL0192			178410	177349	3426591	3425530	410947	409886	389997	388936	54153	53092	172587	171526	90773	89712	143949	142888	53461	52400
303	BPSL0193			178620	179561	3426801	3427742	411157	412098	390207	391148	54363	55304	172797	173738	90985	91926	144161	145102	53673	54614
304	BPSL0194			180882	179941	3429063	3428122	413419	412478	392469	391528	56625	55684	175062	174118	93247	92306	146423	145482	55918	54977
305	BPSL0195			182293	180983	3430474	3429164	414830	413520	393880	392570	58036	56726	176473	175163	94658	93348	147834	146524	57329	56019
306	BPSL0196			182919	182308	3431100	3430489	415456	414845	394506	393895	58662	58051	177099	176488	95284	94673	148460	147849	57955	57344
307	BPSL0197			184061	182919	3432242	3431100	416598	415456	395648	394506	59804	58662	178241	177099	96426	95284	149602	148460	59097	57955
308	BPSL0198			185103	184417	3433284	3432598	417639	416950	396689	396000	60842	60156	179280	178594	97467	96778	150647	149958	60136	59450
309	BPSL0199			185873	185073	3434054	3433254	418409	417609	397459	396659	61612	60812	180050	179250	98237	97437	151417	150617	60906	60106
310	BPSL0200			186769	185873	3434950	3434054	419305	418409	398355	397459	62508	61612	180946	180050	99133	98237	152313	151417	61802	60906
311	BPSL0201			187363	188640	3435544	3436821	419899	421176	398949	400226	63102	64379	181540	182817	99727	101004	152907	154184	62396	63673
312	BPSL0202			188654	189202	3436835	3437383	421190	421738	400240	400788	64393	64941	182831	183379	101018	101566	154198	154746	63687	64235
313		BURPS406E_B0509										66191	65355								
314	BPSL0203			190975	189635	3439156	3437816	423502	422162	402552	401212	68165	66825	185152	183812	103330	101990	156519	155179	66008	64668
315	BPSL0204			191521	190988	3439702	3439169	424048	423515	403098	402565	68711	68178	185698	185165	103876	103343	157065	156532	66554	66021
316	BPSL0205			192199	191786	3440380	3439967	424719	424306	403769	403356	69382	68969	186362	185949	104540	104127	157736	157323	67225	66812
317	BPSL0206			193872	192763	3442053	3440944	426381	425272	405431	404322	71062	69974	188025	186916	106273	105164	159459	158350	68865	67756
318	BPSL0207			195291	194017	3443472	3442198	427800	426526	406850	405576	72481	71207	189435	188170	107692	106418	160878	159604	70284	69010
319	BPSL0208			196312	195383	3444493	3443564	428821	427892	407871	406942	73502	72573	190456	189527	108713	107784	161899	160970	71305	70376
320	BPSL0209			197094	196360	3445275	3444541	429596	428862	408646	407912	74270	73536	191231	190497	109481	108747	162681	161947	72080	71346
321	BPSL0210			197987	197121	3446168	3445302	430489	429623	409539	408673	75163	74297	192124	191258	110374	109508	163574	162708	72973	72107
322	BPSL0211			198912	198031	3447093	3446212	431414	430533	410464	409583	76088	75207	193049	192168	111299	110418	164499	163618	73898	73017
323	BPSL0212			199179	200363	3447360	3448544	431680	432864	410730	411914	76354	77538	193315	194499	111565	112749	164766	165950	74164	75348
324	BPSL0213			201131	202021	3449312	3450202	433631	434521	412681	413571	78305	79195	195266	196156	113516	114406	166718	167608	76116	77006
325	BPSL0214			202302	202553	3450483	3450734	434800	435051	413850	414101	79474	79725	196426	196677	114685	114936	167878	168129	77285	77536
326	BPSL0215			202688	203728	3450869	3451909	435186	436226	414236	415276	79860	80900	196812	197852	115071	116111	168264	169304	77671	78711
327	BPSL0216			204003	203821	3452184	3452002	436501	436319	415551	415369	81176	80994	198127	197945	116386	116204	169579	169397	78986	78804
328	BPSL0217			205134	204274	3453315	3452455	437632	436772	416682	415822	82307	81447	199259	198399	117517	116657	170710	169850	80117	79257
329	BPSL0218			206778	205492	3454959	3453673	439258	437972	418308	417022	83933	82647	200900	199617	119161	117875	172354	171068	81761	80475
330	BPSL0219			207132	207341	3455313	3455522	439612	439821	418662	418871	84287	84496	201254	201463	119515	119724	172708	172917	82115	82324
331	BPSL0220			207385	208413	3455566	3456594	439865	440893	418915	419943	84540	85568	201507	202535	119768	120796	172961	173989	82368	83396
332		BURPS1106A_0225		208693	209028																
333		BURPS1106B_A3508				3457209	3456874														
334		BURPS1710b_0407								419858	421999										
335		BURPSS13_I0912																174604	174269		
336	BPSL0221													203120	202818					83975	83673
337	BPSL0222			210503	209088	3458684	3457269	442915	441500	421965	420550	87590	86175	204659	203244	122821	121406	176079	174664	85504	84089
338	BPSL0223			212217	210541	3460398	3458722	444629	442953	423679	422003	89304	87628	206373	204697	124535	122859	177793	176117	87218	85542
339	BPSL0224			212451	214091	3460632	3462272	444864	446504	423914	425554	89538	91178	206607	208247	124770	126410	178028	179668	87452	89092
340	BPSL0225			215534	214137	3463715	3462318	447956	446550	427006	425600	92359	92087	209681	208293	127868	126456	181129	180734	90535	89138
341	BPSL0226			216019	215570	3464200	3463751	448441	447992	427491	427042	92844	92395	210166	209717	128353	127904	181614	181165	91020	90571

342	BPSL0227		217596	216028	3465777	3464209	450027	448450	429077	427500	94421	92853	211743	210175	129930	128362	183191	181623	92597	91029
343	BPSL0228		218270	217593	3466451	3465774	450701	450024	429751	429074	95095	94418	212417	211740	130604	129927	183865	183188	93271	92594
344	BPSL0229		219258	218266	3467439	3466447	451689	450697	430739	429747	96083	95091	213405	212413	131592	130600	184853	183861	94259	93267
345	BPSL0230		221044	219251	3469225	3467432	453475	451682	432525	430732	97869	96076	215191	213398	133378	131585	186639	184846	96045	94252
346	BPSL0231		221293	221631	3469474	3469812	453724	454062	432774	433112	98118	98456	215440	215778	133627	133965	186887	187225	96294	96632
347	BPSL0232		221756	222187	3469937	3470368	454198	454629	433248	433679	98581	99012	215914	216345	134101	134532	187350	187781	96768	97199
348	BPSL0233		222187	222510	3470368	3470691	454629	454952	433679	434002	99012	99335	216345	216668	134532	134855	187781	188104	97199	97522
349	BPSL0234		222623	224122	3470804	3472303			434115	435614	99745	101088	216781	218268	134968	136467	188217	189731	97635	97874
350	BPSL0235		224122	224436	3472303	3472617	456565	456879	435614	435928	101088	101402	218268	218582	136467	136781	189731	190045	98757	99071
351	BPSL0236		224632	225375	3472813	3473556	457075	457818	436124	436867	101598	102341	218776	219519	136977	137720	190239	190982	99265	100008
352	BPSL0237		226491	225469	3474672	3473650	458943	457921	437992	436970	103466	102444	220651	219629	138845	137823	192098	191076	101134	100112
353	BPSL0238		228093	226693	3476274	3474874	460545	459145	439594	438194	105068	103668	222253	220853	140446	139046	193700	192300	102736	101336
354	BPSL0239		228991	228338	3477172	3476519	461443	460790	440492	439839	105966	105313	223159	222506	141344	140691	194598	193945	103634	102981
355	BPSL0240		229848	228991	3478029	3477172	462294	461443	441343	440492	106802	105966	224004	223159	142213	141344	195467	194598	104479	103634
356	BPSL0241		231032	230265	3479213	3478446	463478	462711	442527	441760	107986	107219	225188	224421	143397	142630	196651	195884	105663	104896
357	BPSL0242		231898	231248	3480079	3479429	464344	463694	443393	442743	108852	108202	225922	225734	144263	143613	197517	196867	106529	105879
358	BPSL0243		232776	231967	3480957	3480148	465222	464413	444271	443462	109730	108921	226807	225998	145141	144332	198395	197586	107407	106598
359	BPSL0244		233346	232786	3481527	3480967	465792	465232	444841	444281	110300	109740	227377	226817	145711	145151	198965	198405	107977	107417
360		BURPS1655_K1121																	108001	108147
361	BPSL0245		233680	234684	3481861	3482865	466126	467130	445175	446179	110634	111638	227711	228715	146045	147049	199299	200303	108266	109270
362	BPSL0246		234759	235877	3482940	3484058	467205	468323	446254	447372	111713	112831	228790	229908	147124	148242	200378	201496	109345	110463
363	BPSL0247		236015	236326	3484196	3484507	468461	468772	447510	447821	112969	113280	230046	230357	148380	148691	201634	201945	110601	110912
364	BPSL0248		236562	237851	3484743	3486032	469008	470297	448057	449346	113514	114803	230592	231881	148927	150216	202180	203469	111147	112436
365	BPSL0249		238285	239910	3486466	3488091	470725	472350	449774	451399	115231	116856	232309	233934	150644	152269	203903	205528	112864	114489
366	BPSL0250		240025	241032	3488206	3489213	472465	473472	451514	452521	116971	117978	234049	235056	152384	153391	205643	206650	114604	115611
367	BPSL0251		241134	242048	3489315	3490229	473574	474488	452623	453537	118080	118994	235158	236072	153493	154407	206752	207666	115713	116627
368	BPSL0252		242053	243042	3490234	3491223	474493	475482	453542	454531	118999	119988	236077	237066	154412	155401	207671	208660	116632	117621
369	BPSL0253		243042	244055	3491223	3492236	475482	476495	454531	455544	119988	121001	237066	238079	155401	156414	208660	209673	117621	118634
370	BPSL0254		244171	245196	3492352	3493377	476730	477755	455660	456685	121117	122142	238195	239220	156530	157555	209789	210814	118750	119775
371	BPSL0255		246633	245680	3494814	3493861	479193	478240	458123	457170	123579	122626	240658	239705	158992	158039	212251	211298	121210	120257
372	BPSL0256		247382	246633	3495563	3494814	479942	479193	458872	458123	124328	123579	241407	240658	159741	158992	213000	212251	121959	121210
373		BURPS1106B_A3547			3495584	3496156														
374	BPSL0257		248756	247995	3496937	3496176	481316	480555	460246	459485	125702	124941	242792	242031	161115	160354	214387	213626	123346	122585
375	BPSL0258		249894	248818	3498075	3496999	482454	481378	461384	460308	126840	125764	243930	242854	162253	161177	215525	214449	124484	123408
376	BPSL0259		250547	249894	3498728	3498075	483107	482454	462037	461384	127493	126840	244583	243930	162906	162253	216178	215525	125137	124484
377	BPSL0260		250697	251206	3498878	3499387	483257	483766	462187	462696	127643	128152	244733	245242	163056	163565	216328	216837	125288	125797
378	BPSL0261		252077	251484	3500258	3499665	484613	484020	463543	462950	129015	128422	246121	245528	164420	163827	217716	217123	126636	126043
379	BPSL0262		252099	254051	3500280	3502232	484635	486587	463565	465517	129037	130989	246143	248095	164442	166394	217738	219690	126658	128610
380	BPSL0263		254176	255132	3502357	3503313	486712	487668	465642	466598	131114	132070	248220	249176	166519	167475	219815	220771	128735	129691
381	BPSL0264		255190	255843	3503371	3504024	487726	488379	466656	467309	132128	132781	249237	249890	167533	168186	220829	221482	129749	130402
382	BPSL0265		255843	257081	3504024	3505262	488379	489617	467309	468547	132781	134019	249890	251128	168186	169424	221482	222720	130402	131640
383	BPSL0266		257954	257736	3506135	3505917	490490	490272	469420	469202	134892	134674	252014	251796	170297	170079	223606	223388	132519	132349
384	BPSL0267		258889	258455	3507070	3506636	491409	490975	470339	469905	135811	135377	252949	252515	171216	170782	224525	224091	133454	133020
385	BPSL0268		259313	258972	3507494	3507153	491833	491492	470763	470422	136235	135894	253373	253032	171640	171299	224949	224608	133878	133537
386	BPSL0269		260340	259420	3508521	3507601	492860	491940	471790	470870	137262	136342	254400	253480	172667	171747	225976	225056	134905	133985
387	BPSL0270		261200	261688	3509381	3509869	493726	494214	472656	473144	138128	138616	255278	255766	173557	174045	226842	227330	135771	136259
388	BPSL0271		261775	262197	3509956	3510378	494301	494723	473231	473653	138703	139125	255853	256275	174132	174554	227417	227839	136346	136768
389	BPSL0272		262320	263093	3510501	3511274	494846	495619	473776	474549	139248	140021	256398	257171	174677	175450	227962	228735	136891	137664
390	BPSL0273		263124	264362	3511305	3512543	495650	496888	474580	475818	140052	141290	257202	258440	175481	176719	228766	230004	137695	138933
391	BPSL0274		264392	265150	3512573	3513331	496918	497676	475848	476606	141320	142078	258470	259228	176749	177507	230034	230792	138963	139721
392	BPSL0275		265187	265972	3513368	3514153	497713	498498	476643	477428	142115	142900	259265	260050	177544	178329	230829	231614	139758	140543
393	BPSL0276		265996	266715	3514177	3514896	498522	499241	477452	478171	142924	143643	260074	260793	178353	179072	231638	232357	140567	141286
394	BPSL0277		266733	267914	3514914	3516095	499259	500440	478189	479370	143661	144842	260814	261992	179090	180271	232375	233556	141304	142485
395	BPSL0278		267918	268850	3516099	3517031	500444	501376	479374	480306	144846	145778	261996	262928	180275	181207	233560	234492	142489	143421
396	BPSL0279		269069	269824	3517250	3518005	501595	502350	480525	481280	145997	146752	263147	263902	181426	182181	234711	235466	143640	144395
397	BPSL0280		270022	272022	3518203	3520203	502548	503801	481478	483478	146961	148961	264111	266111	182390	184390	235664	237664	144604	146604
398	BPSL0281		272041	273270	3520222	3521451	504679	505908	483497	484726	148980	150209	266130	267359	184409	185638	237683	238912	146623	147852
399	BPSL0282		273641	274945	3521822	3523126	506279	507583	485097	486401	150580	151884	267708	269012	186009	187313	239283	240587	148222	149526

400 BPSL0283			276044	275166	3524225	3523347	508694	507816	487512	486634	152983	152105	270106	269228	188412	187534	241674	240796	150620	149742
401 BPSL0284			276142	276738	3524323	3524919	508792	509388	487610	488206	153081	153677	270204	270800	188510	189106	241772	242368	150718	151314
402 BPSL0285			276738	277265	3524919	3525446	509388	509915	488206	488733	153677	154204	270800	271327	189106	189633	242368	242895	151314	151841
403 BPSL0286			277933	277598	3526114	3525779	510587	510252	489405	489070	154872	154537	271995	271660	190305	189970	243563	243228	152503	152168
404 BPSL0287			278047	278388	3526228	3526569	510701	511042	489519	489860	154986	155327	272109	272450	190419	190760	243677	244018	152617	152958
405 BPSL0288			278409	279146	3526590	3527327	511063	511800	489881	490618	155348	156085	272471	273178	190781	191518	244039	244746	152979	153716
406 BPSL0289			279690	280820	3527871	3529001	512344	513474	491162	492292	156629	157759	273725	274855	192062	193192	245293	246423	154260	155390
407 BPSL0290			281814	280984	3529995	3529165	514456	513626	493274	492444	158753	157923	275849	275019	194186	193356	247417	246587	156372	155542
408 BPSL0291			282425	281865	3530606	3530046	515067	514507	493885	493325	159364	158804	276460	275900	194797	194237	248028	247468	156983	156423
409 BPSL0292			283863	282796	3532044	3530977	516505	515438	495323	494256	160802	159735	277899	276832	196235	195168	249466	248399	158421	157354
410 BPSL0293			285124	283859	3533305	3532040	517766	516501	496584	495319	162063	160798	279160	277895	197496	196231	250727	249462	159682	158417
411 BPSL0294			287035	285509	3535216	3533690	519677	518151	498495	496969	163974	162448	281071	279545	199407	197881	252638	251112	161593	160067
412 BPSL0295			288677	290350	3536858	3538531	521320	522975	500138	501793	165617	167308	282707	284380	201046	202719	254280	255953	163236	164909
413 BPSL0296			290912	291133	3539093	3539314	523548	523769	502366	502587	167881	168102	284953	285174	203281	203502	256515	256736	165482	165703
414		Bp_chr1_4_ISBma1					523848	525152	502666	503970					203581	204885				
415 BPSL0297			291333	292691	3539514	3540872	525282	526640	504100	505458	168302	169660	285373	286731	205015	206373	256936	258294	165903	167261
416	BURPS1655_K1056																		167841	167578
417 BPSL0298			293287	294624	3541468	3542805	527236	528573	506054	507391	170256	171593	287327	288664	206969	208306	258890	260227	167911	169248
418 BPSL0299			294789	295421	3542970	3543602	528738	529370	507556	508188	171758	172390	288832	289452	208471	209103	260402	261034	169413	170045
419 BPSL0300			295514	296236	3543695	3544417	529463	530185	508281	509003	172483	173205	289543	290265	209196	209918	261127	261849	170138	170860
420 BPSL0301			296561	296806	3544742	3544987	530510	530755	509328	509573	173530	173775	290590	290835	210243	210488	262174	262419	171185	171430
421	BURPS668_0308												290814	291104						
422 BPSL0302			299838	297418	3548019	3545599	533733	531313	512551	510131	176833	174413	293819	291399	213533	211113	265436	263016	174427	172007
423 BPSL0303			300492	301163	3548673	3549344	534398	535069	513216	513887	177503	178174	294493	295170	214203	214874	266090	266761	175072	175743
424 BPSL0304			301163	302008	3549344	3550189	535069	535914	513887	514732	178174	179019	295170	296015	214874	215719	266761	267606	175743	176588
425 BPSL0305			302008	302505	3550189	3550686	535914	536411	514732	515229	179019	179516	296015	296512	215719	216216	267606	268103	176588	177085
426 BPSL0306			302542	303006	3550723	3551187	536448	536912	515266	515730	179594	180019	296549	297013	216294	216719	268140	268604	177163	177588
427 BPSL0307			303108	304406	3551289	3552587	537014	538312	515832	517130	180121	181419	297115	298413	216821	218119	268706	270004	177690	178988
428 BPSL0308			304420	305988	3552601	3554169	538326	539894	517144	518712	181433	183001	298427	299995	218133	219710	270018	271586	179002	180570
429 BPSL0309			305988	309197	3554169	3557378	539894	543103	518712	521921	183001	186210	299995	303204	219710	222919	271586	274795	180570	183779
430 BPSL0310			309197	309535	3557378	3557716	543103	543441	521921	522259	186210	186548	303204	303542	222919	223257	274795	275133	183779	184117
431 BPSL0311			309748	310191	3557929	3558372	543645	544088	522463	522906	186761	187204	303746	304189	223461	223904	275337	275780	184321	184764
432 BPSL0312			312101	310272	3560282	3558453	545998	544169	524816	522987	189114	187285	306099	304270	225814	223985	277690	275861	186674	184845
433 BPSL0313			313526	312168	3561707	3560349	547423	546065	526241	524883	190539	189181	307524	306166	227239	225881	279115	277757	188099	186741
434 BPSL0314			315099	314107	3563280	3562288	548996	548004	527814	526822	192092	191100	309088	308096			280673	279681	189749	188757
435 BPSL0315			315500	315099	3563681	3563280	549397	548996	528215	527814	192493	192092	309489	309088			281074	280673	190150	189749
436 BPSL0316			316463	315585	3564711	3563833	550417	549539	529178	528300	192799	192635	310452	309574	227792	227718	281999	281214	191140	190955
437 BPSL0317			316531	317718	3564779	3565966	550485	551672	529246	530433	192867	194054	310520	311707	227860	229047	282067	283254	191208	192395
438 BPSL0318			317739	317930	3565987	3566178	551693	551884	530454	530645	194075	194266	311728	311919	229068	229259	283275	283466	192416	192607
439 BPSL0319			318761	318225	3567009	3566473	552715	552179	531476	530940	195097	194561	312750	312214	230090	229554	284297	283761	193438	192902
440 BPSL0320			319666	318761	3567914	3567009	553620	552715	532381	531476	196002	195097	313655	312750	230997	230536	285202	284297	194343	193438
441 BPSL0321			321132	319666	3569380	3567914	555074	553620	533835	532381	197456	196002	315154	313655	232460	230997	286677	285202	195818	194343
442 BPSL0322			322148	321132	3570396	3569380	556090	555074	534851	533835	198472	197456	316170	315154	233476	232460	287693	286677	196834	195818
443 BPSL0323			323878	322322	3572126	3570570	557820	556264	536581	535025	200202	198646	317901	316345	235206	233650	289423	287867	198564	197008
444	BURPS406E_B0650										200782	201084								
445	BURPS406E_B0651										201081	201941								
446 BPSL0324			325529	324492	3573793	3572756	559163	558534	538213	537176	203123	202086	319536	318499	236774	235737	291033	289996	200198	199161
447 BPSL0325			325960	326385	3574224	3574649	559594	560019	538644	539069	203554	203979	319967	320392	237205	237630	291464	291889	200629	201054
448 BPSL0326			326796	327869	3575060	3576133	560430	561503	539480	540553	204390	205463	320803	321876	238041	239114	292300	293373	201447	202520
449 BPSL0327			328032	328991	3576296	3577255	561666	562625	540716	541675	205626	206585	322038	322997	239277	240236	293536	294495	202683	203642
450 BPSL0328			330399	329443	3578663	3577707	564033	563077	543083	542127	207993	207037	324415	323459	241644	240688	295886	294930	205034	204078
451 BPSL0329			330638	331585	3578902	3579849	564272	565219	543322	544269	208232	209179	324654	325601	241883	242830	296125	297072	205273	206220
452 BPSL0330			332158	331673	3580422	3579937	565792	565307	544842	544357	209752	209267	326174	325689	243403	242918	297645	297160	206793	206308
453 BPSL0331			332270	333181	3580534	3581445	565904	566815	544954	545865	209864	210775	326286	327197	243515	244426	297757	298668	206905	207816
454		Bp_chr1_5_ISBm	333431	334735	3581695	3582999					211065	212369								
455 BPSL0332			335618	335115	3583882	3583379	567969	567466	547019	546516	213252	212749	328294	327791	245562	245059	299860	299357	208940	208437
456 BPSL0333			336609	335677	3584873	3583941	568960	568028	548010	547078	214243	213311	329285	328353	246553	245621	300851	299919	209932	209000
457 BPSL0334			337636	336698	3585900	3584962	569987	569049	549037	548099	215270	214332	330312	329374	247580	246642	301878	300940	210959	210021

458	BPSL0335		338054	337644	3586318	3585908	570405	569995	549455	549045	215688	215278	330730	330320	247998	247588	302296	301886	211377	210967
459	BPSL0336		338207	338854	3586471	3587118	570558	571205	549608	550255	215841	216488	330883	331530	248151	248798	302449	303096	211530	212177
460	BPSL0337		339296	338889	3587560	3587153	571647	571240	550697	550290	216930	216523	331972	331565	249240	248833	303538	303131	212619	212212
461	BPSL0338		341551	339437	3589815	3587701	573902	571788	552952	550838	219185	217071	334233	332119	251495	249381	305793	303679	214877	212763
462	BPSL0339		341894	341601	3590158	3589865	574245	573952	553295	553002	219528	219235	334576	334283	251838	251545	306135	305842	215220	214927
463	BPSL0340		342590	342135	3590854	3590399	574941	574486	553991	553536	220224	219769	335272	334817	252534	252079	306831	306376	215916	215461
464	BPSL0341		342715	343440	3590979	3591704	575066	575791	554116	554841	220349	221074	335397	336122	252659	253384	306956	307681	216041	216766
465	BPSL0342		343440	343739	3591704	3592003	575791	576090	554841	555140	221074	221373	336122	336421	253384	253683	307681	307980	216766	217065
466	BPSL0343		343739	344569	3592003	3592833	576090	576920	555140	555970	221373	222203	336421	337251	253683	254513	307980	308810	217065	217895
467	BPSL0344		345529	344939	3593793	3593203	577880	577290	556930	556340	223163	222573	337765	337640	255473	254883	309770	309180	218855	218265
468	BPSL0345		348286	345560	3596550	3593824	580637	577911	559687	556961	225920	223194			258230	255504	312527	309801	221612	218886
469	BPSL0346		350257	349358	3598521	3597622	582608	581709	561658	560759	227894	226995	339320	338424	260204	259305	314422	313523	223583	222684
470	BPSL0347		350682	350524	3599295	3598927	583381	582875	562431	561925	228668	228300			260977	260471	315196	314828	224356	223850
471	BPSL0348		352199	351429	3600463	3599693	584549	583779	563599	562829	229836	229066			262145	261375	316364	315594	225524	224754
472	BPSL0349		352345	352899	3600609	3601163	584695	585249	563745	564299	229982	230536			262291	262845	316510	317064	225670	226224
473	BPSL0350		353373	354026	3601637	3602290	585722	586375	564772	565425	231009	231662			263318	263971	317538	318191	226697	227350
474	BPSL0352		354498	355508	3602762	3603772	586847	587857	565897	566907	232134	233144	340058	341068	264443	265453	318663	319673	227822	228832
475	BPSL0353		356014	357702	3604278	3605966	588363	590051	567413	569101	233650	235338	341575	343263	265959	267647	320179	321867	229338	231026
476	BPSL0354		357777	358178	3606041	3606442	590126	590527	569176	569577	235413	235814	343339	343740	267722	268123	321943	322344	231101	231502
477	BPSL0355		358295	359293	3606559	3607557	590644	591642	569694	570692	235901	236899	343857	344855	268240	269238	322461	323459	231619	232617
478	BPSL0356		359513	363085	3607777	3611349	591862	595434	570912	574484	237119	240691	345075	348647	269458	273030	323679	327251	232837	236409
479	BPSL0357		365015	363351	3613277	3611613	597372	595708	576422	574758	242590	240926	350594	348930	274920	273256	329213	327549	238356	236692
480	BPSL0358		365327	366613	3613589	3614875	597684	598970	576734	578020	242902	244188	350906	352192	275232	276518	329525	330811	238668	239954
481	BPSL0359		367071	367448	3615333	3615710	599428	599805	578478	578855	244728	245105	352649	353026	276976	277353	331344	331721	240364	240741
482	BPSL0360		367747	369183	3616009	3617445	600104	601540	579154	580590	245404	246840	353324	354760	277652	279088	332020	333456	241035	242471
483	BPSL0361		369219	370619	3617481	3618881	601576	602976	580626	582026	246876	248276	354796	356196	279124	280524	333492	334892	242507	243907
484	BPSL0362		370652	371482	3618914	3619744	603009	603818	582059	582868	248309	249130	356229	357050	280557	281360	334925	335728	243940	244752
485		BURPS1106A_0407	371585	371803																
486		BURPS1106B_A3690			3619847	3620065														
487	BPSL0363		371864	372574	3620126	3620836	604207	604917	583257	583967	249512	250222	357425	358135	281736	282446	336103	336813	245127	245837
488	BPSL0364		373723	372707	3621985	3620969	606066	605050	585116	584100	251371	250355	359284	358268	283595	282579	337962	336946	246986	245970
489	BPSL0365		374509	373790	3622771	3622052	606852	606133	585902	585183	252157	251438	360070	359351	284381	283662	338748	338029	247772	247053
490	BPSL0366		375690	374509	3623952	3622771	608033	606852	587083	585902	253338	252157	361251	360070	285562	284381	339929	338748	248953	247772
491	BPSL0367		377033	375690	3625295	3623952	609376	608033	588426	587083	254681	253338	362594	361251	286905	285562	341272	339929	250296	248953
492	BPSL0368		378880	377600	3627142	3625862	611222	609942	590272	588992	256528	255248	364441	363161	288752	287472	343119	341839	252143	250863
493	BPSL0369		379561	379112	3627823	3627374	611903	611454	590953	590504	257209	256760	365096	364653	289433	288984	343800	343351	252824	252375
494	BPSL0370		380270	379596	3628532	3627858	612612	611938	591662	590988	257918	257244	365805	365131	290142	289468	344509	343835	253533	252859
495	BPSL0371		381494	380304	3629756	3628566	613836	612646	592886	591696	259142	257952	367029	365839	291366	290176	345733	344543	254757	253567
496	BPSL0372		382251	381484	3630513	3629746	614593	613826	593643	592876	259899	259132	367786	367019	292123	291356	346490	345723	255514	254747
497	BPSL0373		384120	382312	3632382	3630574	616462	614654	595512	593704	261768	259960	369655	367847	293992	292184	348359	346551	257383	255575
498	BPSL0374		384410	385486	3632672	3633748	616751	617824	595801	596874	262057	263133	369943	371019	294281	295357	348648	349724	257672	258748
499	BPSL0375		387422	385779	3635684	3634041	619803	618160	598853	597210	265101	263458	372970	371327	297325	295682	351692	350049	260676	259033
500	BPSL0376		389242	387659	3637504	3635921	621623	620040	600673	599090	266921	265338	374790	373207	299145	297562	353512	351929	262496	260913
501	BPSL0377		390023	389343	3638285	3637605	622260	621724	601310	600774	267702	267022	375571	374891	299926	299246	354149	353613	263265	262597
502	BPSL0378		390686	390222	3638948	3638484	623048	622584	602098	601634	268365	267901	376234	375770	300589	300125	354937	354473	263928	263464
503	BPSL0379		390925	391635	3639187	3639897	623287	623997	602337	603047	268604	269314	376473	377183	300828	301538	355176	355886	264167	264877
504	BPSL0380		392562	391786	3640824	3640048	624932	624156	603982	603206	270249	269473	378118	377342	302465	301689	356821	356045	265804	265028
505	BPSL0381		393217	392582	3641479	3640844	625587	624952	604637	604002	270904	270269	378773	378138	303120	302485	357476	356841	266459	265824
506	BPSL0382		394117	393272	3642379	3641534	626487	625642	605537	604692	271804	270959	379673	378828	304020	303175	358376	357531	267362	266514
507	BPSL0383		395980	394199	3644242	3642461	628350	626569	607400	605619	273667	271886	381536	379755	305883	304102	360239	358458	269225	267444
508	BPSL0384		396183	396503	3644445	3644765	628553	628873	607603	607923	273870	274190	381739	382059	306086	306406	360442	360762	269428	269748
509	BPSL0385		399452	396753	3647714	3645015	631822	629123	610872	608173	277139	274440	385008	382309	309355	306656	363711	361012	272697	269998
510	BPSL0386		400592	399516	3648854	3647778	632962	631886	612012	610936	278279	277203	386148	385072	310495	309419	364851	363775	273837	272761
511	BPSL0387		401037	400783	3649299	3649045	633407	633153	612457	612203	278725	278471	386593	386339	310941	310687	365296	365042	274282	274028
512	BPSL0388		402488	401223	3650750	3649485	634858	633593	613908	612643	279876	278911	388044	386779	312392	311127	366747	365482	275733	274468
513	BPSL0389		403481	402621	3651743	3650883	635851	634991	614901	614041	280677	279979	389037	388177	313385	312525	367740	366880	276822	276037
514	BPSL0390		403591	404580	3651853	3652842	635961	636950	615011	616000	280787	281776	389147	390136	313495	314484	367850	368839	276932	277921
515		BURPS1655_K0943																	277921	278154

516	BPSL0391		404802	405626	3653064	3653888	637161	637985	616211	617035	281987	282811	390351	391175	314695	315519	369050	369874	278151	278975
517	BPSL0392		405719	406507	3653981	3654769	638078	638884	617128	617934	282904	283545	391268	392062	315612	316400	369967	370584	279068	279721
518	BPSL0393		406547	407788	3654809	3656050	638924	640165	617974	619215	283817	285058	392102	393346	316440	317681	370800	372041	279966	281207
519	BPSL0394		408399	409271	3656651	3657523	640761	641633	619811	620683	285619	286491	393902	394774	318287	319159	372637	373509	281888	282760
520	BPSL0395		409415	409897	3657667	3658149	641777	642259	620827	621309	286635	287117	394918	395400	319303	319785	373653	374135	282904	283386
521	BPSL0396		410368	409949	3658620	3658201	642736	642317	621786	621367	287594	287175	395883	395464	320256	319837	374606	374187	283857	283438
522	BPSL0397		411257	410481	3659509	3658733	643625	642849	622675	621899	288483	287707	396772	395996	321145	320369	375495	374719	284746	283970
523		BURPS1710A_0709					644612	644316												
524	BPSL0398		412213	411257	3660465	3659509	645186	644626	623631	622675	289439	288483	397728	396772	322127	321831	376451	375495	285702	284746
525	BPSL0399		413481	412597	3661733	3660849	646236	646087	624884	624000	290575	289823	399006	398122	323097	322516	377704	376820	286980	286096
526	BPSL0400		415615	413558	3663867	3661810	648370	646313	627018	624961	292753	290696	401140	399083	325276	323219	379838	377781	289114	287057
527	BPSL0401		415881	417002	3664133	3665254	648636	649757	627284	628405	293019	294140	401406	402527	325542	326663	380104	381225	289380	290501
528	BPSL0402		417002	417841	3665254	3666093	649757	650596	628405	629244	294140	294979	402527	403366	326663	327502	381225	382064	290501	291340
529	BPSL0403		417868	418794	3666120	3667046	650623	651549	629271	630197	295006	295932	403393	404319	327529	328455	382091	383017	291367	292293
530	BPSL0404		418831	419451	3667083	3667703	651586	652206	630234	630854	295969	296589	404356	404976	328492	329112	383054	383674	292330	292950
531	BPSL0405		419486	420694	3667738	3668946	652241	653407	630889	632097	296624	297832	405011	406219	329147	330355	383709	384917	292985	294193
532	BPSL0406		420803	421129	3669055	3669381	653559	653885	632206	632532	297941	298267	406328	406654	330464	330790	385026	385352	294302	294628
533	BPSL0407		421180	421821	3669432	3670073	653935	654576	632582	633223	298317	298958	406694	407335	330841	331482	385402	386043	294680	295321
534		BURPS1106B_A3741			3670067	3670189														
535	BPSL0408		422673	423881	3670925	3672133	655423	656631	634070	635278	299798	301006	408264	409472	332353	333561	386847	388055	296130	297338
536	BPSL0409		423974	424918	3672226	3673170	656724	657668	635371	636315	301099	302043	409565	410509	333654	334598	388148	389092	297431	298375
537	BPSL0410		424918	425223	3673170	3673475	657668	657973	636315	636620	302043	302348	410509	410814	334598	334903	389092	389397	298375	298680
538	BPSL0411		426202	425240	3674454	3673492	658952	657990	637599	636637	303327	302365	411793	410831	335882	334920	390376	389414	299659	298697
539	BPSL0412		426303	426626	3674555	3674878	659053	659376	637700	638023	303428	303751	411894	412217	335983	336306	390477	390800	299760	300083
540	BPSL0413		426785	427459	3675037	3675711	659535	660209	638182	638856	303910	304584	412394	413068	336465	337139	390959	391633	300269	300943
541	BPSL0414		427455	428441	3675707	3676693	660205	661191	638852	639838	304580	305566	413064	414050	337135	338121	391629	392615	300939	301925
542	BPSL0415		429009	428632	3677261	3676884	661753	661382	640400	640029	306140	305769			338695	338324	393189	392818	302493	302116
543	BPSL0416		430912	429032	3679164	3677284	663653	661773	642300	640420	308040	306160	416488	414611	340595	338715	395089	393209	304396	302516
544		BURPS1655_K0913																	304721	304992
545		BURPS1710A_0734					664061	663948												
546	BPSL0417		431818	432156	3680070	3680408	664522	664860	643169	643507	308971	309309	417354	417692	341517	341855	396003	396341	305365	305703
547	BPSL0418		433306	432407	3681558	3680659	665986	665087	644633	643734	310467	309568	418882	417983	342965	342066	397483	396584	306837	305938
548	BPSL0419		433461	435584	3681713	3683836	666141	668264	644788	646911	310622	312745	419037	421160	343120	345243	397638	399761	306992	309115
549	BPSL0420		435642	436847	3683894	3685099	668322	669527	646969	648174	312803	314008	421218	422423	345301	346506	399819	401024	309173	310378
550	BPSL0421		436860	437987	3685112	3686239	669540	670667	648187	649314	314021	315148	422436	423563	346519	347646	401037	402164	310391	311518
551	BPSL0422		438016	439218	3686268	3687470	670696	671898	649343	650545	315177	316379	423592	424794	347675	348877	402193	403395	311547	312749
552	BPSL0423		439662	439964	3687914	3688216	672350	672652	650997	651299	316831	317133	425182	425484	349241	349543	403703	404005	313201	313503
553	BPSL0424		439979	441691	3688231	3689943	672667	674379	651314	653026	317148	318860	425499	427211	349558	351270	404020	405732	313518	315230
554	BPSL0425		442062	443345	3690314	3691597	674750	676033	653397	654680	319231	320514	427581	428864	351641	352924	406103	407386	315601	316884
555	BPSL0426		443351	445381	3691603	3691659	676039	676086	654686	656716	320520	320576	428870	430900	352930	352977	407392	407439	316890	316961
556	BPSL0427		445433	446785	3693776	3695128	678204	679556	656768	658120	322681	324033	430952	432304	354918	356270	409259	410611	319062	320414
557	BPSL0428		447440	446907	3695783	3695250	680211	679678	658775	658242	324688	324155	432959	432426	356925	356392	411266	410733	321069	320536
558	BPSL0429		447733	448320	3696076	3696663	680504	681091	659068	659655	324981	325568	433252	433839	357218	357805	411559	412146	321362	321949
559		BURPS668_0461											434058	434285						
560	BPSL0430		449789	449013	3698132	3697356	682552	681776	661116	660340	327037	326261	435309	434533	359047	358271	413615	412839	323411	322635
561	BPSL0431		450286	450747	3698629	3699090	683041	683502	661605	662066	327534	327995	435814	436275	359562	360023	414112	414573	323900	324361
562	BPSL0432		452751	451168	3701094	3699511	685526	683943	664090	662507	330029	328446	438305	436722	362081	360498	416589	415006	326371	324788
563	BPSL0433		453140	452892	3701484	3701236	685915	685667	664479	664231	330418	330170	438694	438446	362470	362222	416978	416730	326760	326512
564	BPSL0434		453509	453844	3701853	3702188	686284	686619	664848	665183	330787	331122	439063	439398	362839	363174	417347	417682	327129	327464
565	BPSL0435		453895	455379	3702239	3703723	686670	688154	665234	666718	331173	332690	439449	440933	363225	364628	417733	419217	327515	328999
566	BPSL0436		455757	457082	3704101	3705426	688511	689836	667075	668400	333057	334382	441176	442501	364963	366288	419551	420876	329323	330648
567	BPSL0437		457113	458066	3705457	3706410	689867	690820	668431	669384	334413	335366	442532	443485	366319	367272	420907	421860	330679	331632
568	BPSL0438		458242	458712	3706586	3707056	690996	691466	669560	670030	335542	336012	443661	444131	367448	367918	422036	422506	331808	332275
569	BPSL0439		458792	459058	3707136	3707402	691546	691812	670110	670376	336092	336358	444211	444477	367998	368264	422586	422852	332355	332621
570	BPSL0440		459257	461026	3707601	3709370	692011	693780	670575	672344	336557	338326	444676	446445	368463	370232	423051	424820	332820	334589
571		BURPS1655_K0886																	334619	334801
572		BURPS668_0475											446445	446654						
573	BPSL0441		462075	461320	3710419	3709664	694811	694056	673375	672620	339393	338638	447437	446682	371269	370514	425885	425121	335584	334829

574 BPSL0442	463772	462204	3712116	3710548	696508	694940	675072	673504	341090	339522	449116	447545	372966	371398	427582	426014	337263	335692
575 BPSL0443	464742	463996	3713086	3712340	697478	696732	676042	675296	342060	341314	450086	449340	373936	373190	428552	427806	338233	337487
576 BPSL0444	464876	465280	3713220	3713624	697612	698016	676176	676580	342194	342598	450220	450624	374070	374474	428686	429090	338367	338771
577 BPSL0445	465295	465552	3713639	3713896	698031	698288	676595	676852	342613	342870	450639	450896	374489	374746	429105	429362	338786	339043
578 BPSL0446	465672	466148	3714016	3714492	698408	698884	676972	677448	342990	343466	451016	451492	374866	375342	429482	429958	339163	339639
579 BPSL0447	466177	467172	3714521	3715516	698913	699908	677477	678472	343495	344490	451521	452516	375371	376366	429987	430982	339668	340663
580 BPSL0448	467328	467858	3715672	3716202	700064	700594	678628	679158	344646	345176	452672	453202	376522	377052	431138	431668	340819	341349
581 BPSL0449	468751	468284	3717095	3716628	701487	701020	680051	679584	346069	345602	454099	453632	377945	377478	432550	432083	342242	341775
582 BPSL0450	469545	468820	3717889	3717164	702271	701546	680835	680110	346853	346128	454893	454168	378729	378004	433344	432619	343036	342311
583 BPSL0451	469668	470630	3718012	3718974	702394	703356	680958	681920	346976	347938	455016	455978	378852	379814	433467	434429	343159	344121
584 BPSL0452	470831	471343	3719175	3719687	703557	704069	682121	682633	348139	348651	456179	456691	380015	380527	434630	435142	344322	344834
585 BPSL0453	471396	472970	3719740	3721314	704122	705696	682686	684260	348704	350278	456744	458318	380580	382154	435195	436769	344887	346461
586 BPSL0454	473017	474621	3721361	3722965	705743	707347	684307	685911	350325	351929	458365	459969	382201	383805	436816	438420	346508	348112
587 BPSL0455	474693	474830	3723037	3723174	707419	707556	685983	686120	352001	352138	460041	460178	383877	384014	438492	438629	348184	348321
588 BPSL0456	474855	475457	3723199	3723801	707581	708183	686145	686747	352163	352765	460203	460805	384039	384641	438654	439256	348346	348948
589 BPSL0457	475480	475677	3723824	3724021	708206	708403	686770	686967	352788	352985	460828	461025	384664	384861	439279	439476	348971	349168
590 BPSL0458	475815	476669	3724159	3725013	708541	709395	687105	687959	353123	353977	461162	462016	384999	385853	439614	440468	349305	350159
591 BPSL0458a	477041	476835	3725385	3725179	709767	709561	688331	688125	354349	354143	462388	462182	386225	386019	440840	440634	350531	350325
592 BPSL0459	477129	477839	3725473	3726183	709855	710565	688419	689129	354437	355147	462476	463186	386313	387023	440928	441638	350619	351329
593 BPSL0460	477904	478545	3726248	3726889	710630	711271	689194	689835	355212	355853	463251	463892	387088	387729	441703	442344	351394	352035
594 BPSL0461	478652	479698	3726996	3728042	711378	712424	689942	690988	355960	357006	463999	465045	387836	388882	442451	443497	352142	353188
595 BPSL0462	479714	480613	3728058	3728957	712440	713339	691004	691903	357022	357921	465061	465960	388898	389797	443513	444412	353204	354103
596 BPSL0463	480636	481250	3728980	3729594	713362	713976	691926	692540	357944	358558	465983	466597	389820	390434	444435	445049	354126	354740
597 BPSL0464	481626	481336	3729970	3729680	714352	714062	692916	692626	359003	358713	466973	466683	390901	390611	445494	445204	355174	354959
598 BPSL0465	481813	483450	3730157	3731794	714539	716176	693103	694740	359190	360827	467160	468797	391088	392725	445681	447318	355361	356998
599 BPSL0466	483604	484419	3731948	3732763	716329	717144	694893	695708	360980	361795	468950	469765	392878	393693	447472	448287	357152	357967
600 BPSL0467	484673	485641	3733017	3733985	717398	718366	695962	696930	362049	363017	470019	470987	393948	394916	448541	449509	358221	359189
601 BPSL0468	485963	486856	3734307	3735200	718688	719581	697252	698145	363329	364222	471309	472202	395208	396101	449831	450724	359511	360404
602 BPSL0469	486860	487651	3735204	3735995	719585	720376	698149	698940	364226	365017	472206	472997	396105	396896	450728	451519	360408	361199
603 BPSL0470	487861	488568	3736205	3736912	720586	721293	699150	699857	365227	365934	473207	473914	397106	397813	451729	452436	361409	362116
604 BPSL0471	488504	489976	3736848	3738320	721229	722701	699793	701265	365870	366223	473850	475322	397749	399221	452372	452812	362052	363524
605 BPSL0472	489985	493200	3738329	3741544	722710	725925	701274	704489	367311	370526	475331	478546	399230	402445	453900	457115	363533	366748
606 BPSL0473	494496	493678	3742840	3742022	727069	726251	705633	704815	371670	370852	479753	478935	403589	402771	458384	457566	367910	367092
607 BPSL0474	495733	494543	3744077	3742887	728306	727116	706870	705680	372907	371717	480990	479800	404826	403636	459621	458431	369147	367957
608 BPSL0475	496966	495938	3745310	3744282	729539	728511	708103	707075	374140	373112	482223	481195	406059	405031	460854	459826	370380	369352
609 BPSL0476	498364	497015	3746708	3745359	730937	729588	709501	708152	375538	374189	483621	482272	407457	406108	462252	460903	371778	370429
610 BPSL0477	498942	498412	3747286	3746756	731515	730985	710079	709549	376116	375586	484199	483669	408035	407505	462830	462300	372356	371826
611 BPSL0478	500108	499020	3748452	3747364	732681	731593	711245	710157	377282	376194	485365	484277	409201	408113	463996	462908	373522	372434
612 BPSL0479	501057	500104	3749401	3748448	733630	732677	712194	711241	378231	377278	486314	485361	410150	409197	464945	463992	374471	373518
613 BPSL0480	502217	501153	3750561	3749497	734790	733726	713354	712290	379391	378327	487474	486410	411310	410246	466105	465041	375622	374558
614 BPSL0481	502879	502217	3751223	3750561	735452	734790	714016	713354	380053	379391	488136	487474	411972	411310	466767	466105	376284	375622
615 BPSL0482	504070	502922	3752414	3751266	736643	735495	715207	714059	381244	380096	489327	488179	413163	412015	467958	466810	377475	376327
616 BPSL0483	505943	504075	3754287	3752419	738516	736648	717080	715212	383117	381249	491200	489332	415036	413168	469831	467963	379348	377480
617 BPSL0484	506980	505943	3755324	3754287	739553	738516	718117	717080	384154	383117	492237	491200	416073	415036	470868	469831	380385	379348
618 BPSL0485	508566	507001	3756910	3755345	741139	739574	719703	718138	385740	384175	493823	492258	417659	416094	472454	470889	381971	380406
619 BPSL0486	509770	508577	3758114	3756921	742343	741150	720907	719714	386944	385751	495027	493834	418863	417670	473658	472465	383175	381982
620 BPSL0487	510758	509763	3759102	3758107	743331	742336	721895	720900	387932	386937	496015	495020	419851	418856	474646	473651	384163	383168
621 BPSL0488	511704	510766	3760048	3759110	744277	743339	722841	721903	388878	387940	496961	496023	420797	419859	475592	474654	385109	384171
622 BPSL0489	512331	511768	3760675	3760112	744904	744341	723468	722905	389505	388942	497588	497025	421424	420861	476219	475656	385736	385173
623 BPSL0490	513215	512331	3761559	3760675	745788	744904	724352	723468	390389	389505	498472	497588	422308	421424	477103	476219	386620	385736
624 BPSL0491	513496	513251	3761840	3761595	746069	745824	724633	724388	390670	390425	498753	498508	422589	422344	477384	477139	386901	386656
625 BPSL0492	514736	513555	3763080	3761899	747318	746128	725882	724692	391910	390729	500002	498812	423838	422648	478624	477443	388168	386960
626 BPSL0493	516507	514744	3764851	3763088	749089	747326	727653	725890	393681	391918	501765	500002	425609	423846	480395	478632	389939	388176
627 BPSL0494	516941	517861	3765285	3766205	749523	750443	728087	729007	394115	395035	502200	503120	426043	426963	480829	481749	390373	391293
628 BPSL0495	518570	519331	3766914	3767675	751159	751920	729723	730484	395744	396505	503826	504587	427679	428440	482458	483219	392002	392763
629 BPSL0496	519433	520533	3767777	3768877	752022	753122	730586	731686	396607	397707	504689	505789	428542	429642	483321	484421	392865	393965
630 BPSL0497	520520	521533	3768864	3769877	753109	754122	731673	732686	397694	398707	505776	506789	429629	430642	484408	485421	393952	394965
631 BPSL0498	521570	524203	3769914	3772547	754159	756789	732723	735353	398744	401353	506826	509450	430679	433288	485458	488079	395002	397611

632	BPSL0499			524307	526076	3772651	3774420	756893	758668	735457	737232	401457	403226	509554	511323	433392	435161	488183	489958	397715	399484
633	BPSL0500			529214	526716	3777565	3775067	761809	759311	740373	737875	406399	403901	514468	511970	438348	435850	493183	490685	402657	400159
634	BPSL0501			531869	530736	3780220	3779087	764475	763342	743037	741904	409071	407938	517129	515996	440996	439863	495820	494687	405320	404187
635	BPSL0502			533502	531910	3781853	3780261	766108	764516	744670	743078	410704	409112	518762	517170	442629	441037	497453	495861	406953	405361
636	BPSL0503			533776	533495	3782127	3781846	766382	766101	744944	744663	410978	410697	519036	518755	442903	442622	497727	497446	407227	406946
637			Bp_chr1_6_ISBma1															498141	499445		
638	BPSL0504			535192	534260	3783543	3782611	767798	766866	746360	745428	412394	411462	520453	519521	444319	443387	500456	499524	408641	407709
639	BPSL0505			535557	536123	3783908	3784474	768163	768729	746725	747291	412759	413325	520818	521384	444684	445250	500821	501387	409006	409572
640	BPSL0506			536147	536551	3784498	3784902	768753	769157	747315	747719	413349	413753	521408	521812	445274	445678	501411	501815	409596	410000
641	BPSL0507			538509	536872	3786860	3785223	771117	769480	749679	748042	415711	414074	523762	522125	447638	446001	503785	502148	411970	410333
642	BPSL0508			539792	538947	3788143	3787298	772400	771555	750962	750117	416994	416149	525045	524200	448921	448076	505068	504223	413253	412408
643	BPSL0509			541393	540416	3789744	3788767	773994	773017	752556	751579	418588	417611	526646	525669	450515	449538	506624	505647	414848	413871
644	BPSL0510			544245	541393	3792596	3789744	776846	773994	755408	752556	421440	418588	529498	526646	453367	450515	509476	506624	417700	414848
645	BPSL0511			544637	544245	3792988	3792596	777238	776846	755800	755408	421832	421440	529890	529498	453759	453367	509868	509476	418092	417700
646	BPSL0512			547157	544716	3795508	3793067	779758	777317	758320	755879	424352	421911	532410	529969	456279	453838	512388	509947	420612	418171
647	BPSL0513			547542	548183	3795893	3796534	780143	780784	758705	759346	424737	425378	532795	533436	456664	457305	512773	513414	420996	421637
648	BPSL0514			549662	548346	3798041	3797949	782263	780947	760825	759509	426857	425541	534915	533599	458784	457468	514893	513577	423116	421800
649	BPSL0515			549914	550537	3798293	3798916	782515	783138	761077	761700	427109	427732	535167	535790	459036	459659	515144	515767	423368	423991
650	BPSL0516			550651	551148	3799030	3799527	783252	783749	761814	762311	427846	428343	535904	536401	459773	460270	515881	516378	424105	424602
651	BPSL0517			551214	551477	3799593	3799856	783815	784078	762377	762640	428409	428672	536467	536730	460336	460599	516444	516707	424668	424931
652	BPSL0518			552729	551662	3801108	3800041	785330	784263	763892	762825	429924	428857	537973	536906	461851	460784	517959	516892	426183	425116
653	BPSL0519			553423	552821	3801802	3801200	786024	785422	764586	763984	430618	430016	538667	538065	462545	461943	518653	518051	426877	426275
654	BPSL0520			554163	553540	3802542	3801919	786764	786141	765326	764703	431358	430735	539407	538784	463285	462662	519393	518770	427617	426994
655	BPSL0521			555271	554318	3803650	3802697	787872	786919	766434	765481	432466	431513	540515	539562	464393	463440	520501	519548	428725	427772
656	BPSL0522			555416	555261	3803795	3803640	788017	787862	766579	766424	432611	432456	540660	540505	464538	464383	520646	520491	428870	428715
657	BPSL0523			556330	555452	3804709	3803831	788931	788053	767493	766615	433525	432647	541574	540696	465452	464574	521560	520682	429859	429140
658	BPSL0524			556987	556367	3805378	3804746	789588	788968	768150	767530	434182	433562	542231	541611	466109	465489	522217	521597	430516	429896
659	BPSL0525			558804	556987	3807195	3805378	791405	789588	769967	768150	435999	434182	544048	542231	467926	466109	524034	522217	432333	430516
660	BPSL0526			558893	559720	3807284	3808111	791494	792321	770056	770883	436088	436915	544137	544964	468015	468842	524123	524950	432422	433249
661	BPSL0527			559728	560831	3808119	3809222	792329	793432	770891	771994	436923	438026	544972	546075	468850	469953	524958	526061	433257	434360
662	BPSL0528			561854	561225	3810245	3809616	794461	793832	773023	772394	439055	438426	547098	546469	470982	470353	527084	526455	435389	434760
663	BPSL0529			562774	561884	3811165	3810275	795381	794491	773943	773053	439975	439085	548018	547128	471902	471012	528004	527114	436309	435419
664	BPSL0530			563828	562863	3812219	3811254	796435	795470	774997	774032	441029	440064	549063	548098	472956	471991	529058	528093	437363	436398
665	BPSL0531			564412	563960	3812803	3812351	797019	796567	775581	775129	441613	441161	549647	549195	473540	473088	529642	529190	437947	437495
666	BPSL0532			565058	564702	3813449	3813093	797665	797309	776227	775871	442259	441903	550293	549937	474186	473830	530288	529932	438593	438237
667	BPSL0533			566716	565211	3815107	3813602	799323	797818	777885	776380	443917	442412	551951	550446	475844	474339	531946	530441	440251	438746
668	BPSL0534			567661	566888	3816052	3815279	800268	799495	778830	778057	444862	444089	552896	552123	476789	476016	532891	532118	441196	440423
669	BPSL0535			568323	567661	3816714	3816052	800930	800268	779492	778830	445524	444862	553558	552896	477451	476789	533553	532891	441858	441196
670	BPSL0536			568956	568345	3817347	3816736	801563	800952	780125	779514	446157	445546	554191	553580	478084	477473	534186	533575	442491	441880
671	BPSL0537			569501	568965	3817892	3817356	802108	801572	780670	780134	446702	446166	554736	554200	478629	478093	534731	534195	443036	442500
672	BPSL0538			570484	569504	3818875	3817895	803091	802111	781653	780673	447685	446705	555719	554739	479612	478632	535714	534734	444019	443039
673	BPSL0539			570612	572618	3819003	3821009	803219	805225	781781	783787	447813	449819	555847	557853	479740	481746	535842	537848	444147	446153
674	BPSL0540			572706	573266	3821097	3821657	805312	805872	783874	784434	449906	450466	557940	558500	481833	482393	537936	538496	446240	446800
675	BPSL0541			573363	573974	3821754	3822365	805969	806580	784531	785142	450563	451174	558597	559208	482490	483101	538593	539204	446897	447508
676	BPSL0542			573990	574844	3822381	3823235	806596	807450	785158	786012	451190	452044	559224	560078	483117	483971	539220	540074	447524	448378
677	BPSL0543			574899	575777	3823290	3824168	807505	808383	786067	786945	452099	452977	560133	561011	484026	484904	540129	541007	448433	449311
678	BPSL0544			577113	576082	3825504	3824473	809720	808689	788282	787251	454313	453282	562348	561317	486240	485209	542343	541312	450647	449616
679	BPSL0545			580232	577368	3828623	3825759	812839	809975	791401	788537	457450	454586	565485	562621	489359	486495	545480	542616	453766	450902
680	BPSL0546			580446	581630	3828837	3830021	813053	814237	791615	792799	457664	458848	565699	566883	489573	490757	545694	546878	453980	455164
681	BPSL0547			581747	582298	3830138	3830689	814354	814908	792916	793470	458965	459519	567000	567536	490874	491428	546995	547546	455281	455832
682		BURPS1710A_0887						817396	816359												
683		BURPS1710A_0888						818258	817653												
684			Bp_chr1_7_IS407A					818262	819497	796824	798059										
685		BURPS1710A_0891						822438	820927												
686		BURPS1710A_0893						824050	823931												
687		BURPS1710A_0895						824006	824914												
688		BURPS1710A_0896						825114	825404												
689		BURPS1710A_0897						825398	826252												

690		BURPS1710b_0784								799489	801000										
691		BURPS1710b_0785								803661	803966										
692		BURPS1710b_0786								803960	804814										
693		BURPS406E_B0907										462007	460970								
694		BURPS406E_B0908										463229	462264								
695		BURPS406E_B0909										464744	464298								
696		BURPS406E_B0911										467377	468285								
697	BPSL0548		GI3																		
698	BPSL0549		GI3																		
699	BPSL0550		GI3																		
700	BPSL0551		GI3																		
701	BPSL0552		GI3																		
702	BPSL0553		GI3																		
703	BPSL0554		GI3																		
704	BPSL0555		GI3																		
705	BPSL0556		GI3																		
706	BPSL0557		GI3																		
707	BPSL0558		GI3																		
708	BPSL0559		GI3																		
709	BPSL0560		GI3																		
710	BPSL0561		GI3																		
711	BPSL0562		GI3																		
712	BPSL0563		GI3																		
713	BPSL0564		GI3																		
714	BPSL0565		GI3																		
715	BPSL0566		GI3																		
716	BPSL0567		GI3																		
717	BPSL0568		GI3																		
718	BPSL0569		GI3																		
719	BPSL0570		GI3																		
720	BPSL0571		GI3																		
721	BPSL0572		GI3																		
722	BPSL0573		GI3																		
723	BPSL0574		GI3																		
724	BPSL0575		GI3																		
725	BPSL0576		GI3																		
726	BPSL0577		GI3																		
727	BPSL0578			583144	582416	3831535	3830807	827696	826968	806258	805530	469855	469127	568382	567654	492274	491546	548392	547664	456678	456040
728	BPSL0579			583488	585065	3831879	3833456	828039	829616	806601	808178	470199	471776	568730	570307	492618	494195	548735	550312	457021	458598
729	BPSL0580			585517	585269	3833908	3833660	830050	829802	808612	808364	472192	471944	570750	570502	494638	494390	550728	550480	459032	458784
730	BPSL0581			586007	585693	3834398	3834084	830540	830226	809102	808788	472682	472368	571240	570926	495128	494814	551218	550904	459522	459208
731		BURPS1106A_0618		586923	587057																
732		BURPS1106B_A3903				3835314	3835448														
733		BURPS1655_K0761																		460785	460483
734		BURPS1655_K0762																		460486	459626
735		BURPS1710A_0904						830702	830869												
736		BURPS1710A_0905						831286	831155												
737		BURPS1710A_0906						832285	831362												
738		BURPS668_0603												571579	573417						
739		BURPSS13_I0528																553925	555187		
740		BURPSS13_I0529																553439	551673		
741	BPSL0582		GI3																		
742	BPSL0583		GI3																		
743	BPSL0584		GI3																		
744	BPSL0585		GI3																		
745	BPSL0586		GI3																		
746	BPSL0586a		GI3	587689	588387	3836080	3836778					474364	475062			496810	497508				
747	BPSL0587		GI3																		

748	BPSL0588		GI3																		
749	BPSL0589		GI3	588847	588500	3837238	3836891	832778	832434	811340	810996	475521	475177	573846	573499	497967	497623	555491	555171	463938	463591
750	BPSL0590			595212	589120	3843603	3837511	839143	833051	817705	811613	481886	475794	580211	574119	504333	501526	561856	555764	470303	464211
751	BPSL0591			598518	595237	3846909	3843628	842449	839168	821011	817730	485192	481911	583517	580236	507639	504358	565162	561881	473609	470328
752		BURPS1655_K0755																		474260	476134
753		BURPS1655_K0754																		476144	477430
754		BURPS1655_K0753																		477460	478512
755		BURPS1655_K0752																		478541	482647
756		BURPS1655_K0751																		482644	483735
757			Bp_chr1_8_ISBma1									486097	487400								
758	BPSL0592			600086	599706	3848477	3848097	844017	843637	822579	822199	488072	487692	585094	584714	509207	508827	566724	566344		
759	BPSL0593			600541	600107	3848932	3848498	844472	844038	823034	822600	488527	488093	585549	585115	509662	509228	567179	566745		
760	BPSL0594			601115	600771	3849506	3849162	845046	844702	823608	823264	489101	488757	586120	585779	510236	509892	567753	567409		
761	BPSL0595			601994	601632	3850385	3850023	845925	845563	824487	824125	489980	489618	586998	586636	511115	510753	568631	568269	484823	484461
762	BPSL0596			602242	603246	3850633	3851637	846173	847177	824735	825739	490228	491232	587246	588250	511363	512367	568879	569883	485071	486075
763	BPSL0597			603260	607336	3851651	3855727	847191	851267	825753	829829	491246	495319	588264	592340	512381	516457	569897	573973	486089	490165
764	BPSL0598			607336	607695	3855727	3856086	851267	851626	829829	830188	495319	495678	592340	592699	516457	516816	573973	574332	490165	490524
765	BPSL0599			607700	608059	3856091	3856450	851631	851990	830193	830552	495683	496042	592704	593063	516821	517180	574337	574696	490529	490888
766	BPSL0600			608154	610451	3856545	3858842	852085	854373	830647	832935	496137	496451	593158	595464	517275	519572	574791	577079	490984	493281
767	BPSL0601			610524	611297	3858915	3859688	854446	855219	833008	833781	498262	499035	595537	596310	519645	520418	577152	577925	493354	494127
768	BPSL0602			611783	614143	3860174	3862534	855704	858064	834266	836626	499521	501881	596796	599156	520904	523264	578411	580771	494613	496973
769	BPSL0603			615592	614327	3863983	3862718	859513	858248	838075	836810	503382	502117	600605	599340	524713	523448	582233	580968	498461	497196
770	BPSL0604			616865	615672	3865256	3864063	860786	859593	839348	838155	504655	503462	601878	600685	525986	524793	583506	582313	499734	498541
771	BPSL0605			618492	616903	3866883	3865294	862413	860824	840975	839386	506282	504693	603505	601916	527613	526024	585133	583544	501361	499772
772	BPSL0606			619139	618492	3867530	3866883	863060	862413	841622	840975	506929	506282	604152	603505	528260	527613	585780	585133	502008	501361
773	BPSL0607			619517	620755	3867908	3869146	863438	864676	842000	843238	507307	508545	604530	605768	528638	529876	586158	587396	502386	503624
774	BPSL0608			620786	621517	3869177	3869908	864707	865438	843269	844000	508576	509307	605799	606530	529907	530638	587427	588158	503655	504383
775	BPSL0609			622265	623650	3870656	3872041	866186	867571	844748	846133	510055	511440	607278	608663	531386	532771	588906	590291	505131	506303
776	BPSL0610			625696	623711	3874087	3872102	869605	867632	848167	846194	513474	511501	610697	608724	534817	532832	592337	590352	508218	506407
777	BPSL0611			628212	625696	3876602	3876300	872121	869605	850683	848167	515990	513474	613213	610697	537333	534817	594853	592337	510734	508218
778	BPSL0612			629297	628212	3877687	3876602	873206	872121	851768	850683	517075	515990	614298	613213	538418	537333	595938	594853	511819	510734
779	BPSL0613			630268	629297	3878658	3877687	874177	873206	852739	851768	518046	517075	615269	614298	539389	538418	596909	595938	512790	511819
780	BPSL0614			631488	630268	3879878	3878658	875397	874177	853959	852739	519266	518046	616489	615269	540609	539389	598129	596909	514010	512790
781	BPSL0615			631787	631488	3880177	3879878	875696	875397	854258	853959	519565	519266	616782	616489	540908	540609	598428	598129	514309	514010
782	BPSL0616			632775	631984	3881165	3880374	876684	875893	855246	854455	520553	519762	617769	616978	541896	541105	599416	598625	515297	514506
783	BPSL0617			633642	634505	3882032	3882895	877551	878414	856113	856976	521420	522283	618636	619499	542763	543626	600283	601146	516164	517027
784	BPSL0618			634505	635920	3882895	3884310	878414	879829	856976	858391	522283	523698	619499	620914	543626	545041	601146	602561	517027	518442
785	BPSL0619			635995	637281	3884385	3885671	879904	881190	858466	859752	523773	525059	620989	622275	545116	546402	602636	603922	518517	519803
786	BPSL0620			637564	638706	3885954	3887096	881473	882615	860035	861177	525345	526484	622561	623700	546685	547827	604208	605347	520089	521228
787	BPSL0621			639637	638741	3888027	3887131	883546	882650	862108	861212	527415	526519	624631	623735	548758	547862	606278	605382	522159	521263
788			Bp_chr1_9_ISBma2																	522588	524160
789	BPSL0622			640354	640785	3888744	3889175	884303	884734	862865	863296	528133	528564	625363	625794	549476	549907	607025	607456	524470	524901
790	BPSL0623			640880	641842	3889270	3890232	884829	885791	863391	864353	528659	529621	625889	626851	550002	550964	607551	608513	524996	525958
791	BPSL0624			642729	641818	3891119	3890208	886678	885767	865240	864329	530508	529597	627738	626827	551851	550940	609400	608489	526845	525934
792	BPSL0625			642836	644428	3891226	3892818	886785	888377	865347	866939	530615	532207	627845	629437	551958	553550	609507	611099	526952	528544
793	BPSL0626			644480	644911	3892870	3893301	888429	888860	866991	867422	532259	532690	629489	629920	553602	554033	611151	611582	528596	529027
794	BPSL0627			645613	645035	3894003	3893425	889551	888973	868113	867535	533392	532814	630584	630006	554735	554157	612284	611706	529729	529151
795	BPSL0628			647043	645766	3895433	3894156	890981	889704	869543	868266	534822	533545	632014	630737	556165	554888	613714	612437	531159	529882
796	BPSL0629			647181	648071	3895571	3896461	891119	892009	869681	870571	534960	535850	632152	633042	556303	557193	613852	614742	531297	532187
797	BPSL0630			648076	649554	3896466	3897944	892014	893492	870576	872054	535855	537333	633047	634525	557198	558676	614747	616225	532192	533670
798		BURPS668_0659												634539	634658						
799	BPSL0631			649682	650065	3898072	3898455	893620	894003	872182	872565	537463	537846	634655	635038	558804	559187	616353	616736	533792	534175
800	BPSL0632			651232	650411	3899622	3898801	895171	894350	873733	872912	539012	538191	636227	635406	560355	559534	617905	617084	535356	534535
801	BPSL0633			652775	651255	3901165	3899645	896714	895194	875276	873756	540555	539035	637770	636250	561898	560378	619398	618730	536899	535379
802	BPSL0634			653231	657307	3901621	3905697	897170	901246	875732	879808	541011	545087	638226	642302	562354	566430	619854	623930	537355	541431
803	BPSL0635			657345	657785	3905735	3906175	901284	901724	879846	880286	545125	545565	642340	642780	566468	566908	623968	624408	541469	541909
804	BPSL0636			657785	658195	3906175	3906585	901724	902134	880286	880696	545565	545975	642780	643190	566908	567318	624408	624818	541909	542319
805	BPSL0637			658246	658974	3906636	3907364	902185	902913	880747	881475	546026	546754	643241	643969	567369	568097	624869	625597	542370	543098

806	BPSL0638		659067	660035	3907457	3908425	903006	903974	881568	882536	546847	547164	644062	645033	568190	569158	625690	626658	543191	544159
807	BPSL0639		660119	660790	3908509	3909180	904058	904729	882620	883291	547976	548647	645117	645788	569242	569913	626742	627413	544243	544914
808	BPSL0640		660806	662380	3909196	3910770	904745	906319	883307	884881	548663	550237	645804	647378	569929	571503	627429	629003	544930	546504
809	BPSL0641		662707	663345	3911097	3911735	906655	907293	885217	885855	550564	551202	647714	648352	571839	572477	629330	629968	546831	547469
810	BPSL0642		663449	663787	3911839	3912177	907397	907735	885959	886297	551306	551644	648456	648794	572581	572919	630072	630410	547573	547911
811	BPSL0643		663861	664505	3912251	3912895	907809	908453	886371	887015	551718	552362	648868	649512	572993	573637	630484	631128	547985	548629
812	BPSL0644		664565	666364	3912955	3914754	908513	910312	887075	888874	552422	554221	649572	651371	573697	575496	631188	632987	548689	550488
813		BURPS1106B_A3979			3914791	3914970														
814		BURPS668_0676											651408	651587						
815	BPSL0645		666577	667050	3914967	3915440	910525	910998	889087	889560	554433	554906	651584	652057	575709	576182	633199	633672	550700	551173
816	BPSL0646		667050	668321	3915440	3916711	910998	912269	889560	890831	554906	556177	652057	653328	576182	577453	633672	634943	551173	552444
817	BPSL0647		668514	669110	3916904	3917500	912462	913058	891024	891620	556370	556966	653521	654117	577646	578242	635136	635732	552637	553233
818	BPSL0648		669171	670955	3917561	3919345	913119	914903	891681	893465	557027	558811	654178	655962	578303	580087	635793	637577	553294	555078
819	BPSL0649		671116	673548	3919506	3921938	915064	917496	893626	896058	558972	561404	656123	658555	580248	582680	637738	640170	555644	558076
820	BPSL0650		673731	674927	3922121	3923317	917679	918875	896241	897437	561691	562887	658686	659882	582863	584059	640353	641549	558207	559403
821	BPSL0651		675025	675786	3923415	3924176	918973	919734	897535	898296	562985	563746	659980	660741	584157	584918	641647	642408	559501	560262
822	BPSL0652		676727	675906	3925117	3924296	920675	919854	899237	898416	564687	563866	661682	660861	585859	585038	643349	642528	561203	560382
823	BPSL0653		676862	677338	3925252	3925728	920810	921286	899372	899848	564822	565298	661817	662293	585994	586470	643484	643960	561338	561814
824	BPSL0654		677799	677389	3926189	3925779	921747	921337	900309	899899	565759	565349	662764	662354	586931	586521	644421	644011	562261	561851
825	BPSL0655		678106	679974	3926496	3928364	922054	923922	900616	902484	566066	567934	663070	664938	587238	589106	644728	646596	562568	564436
826	BPSL0656		681189	680473	3929579	3928863	925137	924421	903699	902983	569149	568433	666153	665437	590321	589605	647811	647095	565651	564935
827	BPSL0657		682227	681196	3930617	3929586	926175	925144	904737	903706	570187	569156	667191	666160	591359	590328	648849	647818	566689	565658
828	BPSL0658		682440	684800	3930830	3933190	926388	928748	904950	907310	570400	572760	667404	669764	591572	593932	649062	651422	566902	569262
829	BPSL0659		684864	686213	3933254	3934603	928812	930161	907374	908723	572824	574173	669828	671177	593996	595345	651486	652835	569326	570675
830	BPSL0660		686238	687284	3934628	3935674	930186	931217	908748	909779	574198	575229	671202	672263	595370	596416	652860	653906	570700	571731
831	BPSL0661		687319	688143	3935709	3936533	931252	932076	909814	910638	575264	576088	672298	673122	596451	597275	653941	654765	571766	572590
832	BPSL0662		689386	688451	3937776	3936841	933279	932344	911841	910906	577316	576381	674340	673408	598523	597588	656003	655068	573793	572861
833	BPSL0663		689764	690150	3938154	3938540	933657	934043	912219	912605	577694	578080	674718	675104	598901	599287	656381	656767	574171	574557
834	BPSL0664		691216	690374	3939606	3938764	935104	934262	913666	912824	579141	578299	676170	675328	600348	599506	657828	656986	575618	574776
835	BPSL0665		692076	691300	3940466	3939690	935964	935188	914526	913750	580001	579225	677030	676254	601208	600432	658688	657912	576478	575702
836	BPSL0666		692682	692122	3941072	3940512	936570	936010	915132	914572	580607	580047	677636	677076	601814	601254	659294	658734	577084	576524
837	BPSL0667		694799	692703	3943189	3941093	938687	936591	917249	915153	582724	580628	679753	677657	603931	601835	661411	659315	579201	577105
838	BPSL0668		695823	694819	3944213	3943209	939711	938707	918273	917269	583748	582744	680777	679773	604955	603951	662435	661431	580225	579221
839	BPSL0669		697785	696082	3946175	3944472	941673	939970	920235	918532	585761	585069	682739	681036	606987	606235	664397	662694	582187	580484
840	BPSL0670		698715	697831	3947105	3946221	942603	941719	921165	920281	586691	585807	683669	682785	607917	607033	665327	664443	583117	582233
841	BPSL0671		699867	699238	3948257	3947628	943748	943119	922310	921681	587843	587214	684821	684192	609052	608423	666479	665850	584289	583660
842	BPSL0672		700703	699891	3949093	3948281	944584	943772	923146	922334	588679	587867	685666	684845	609888	609076	667315	666503	585125	584313
843	BPSL0673		702476	701415	3950874	3949813	946279	945218	924841	923780	590405	589344	687410	686349	611583	610522	669050	667989	586860	585799
844	BPSL0674		703873	702503	3952271	3950901	947676	946306	926238	924868	591802	590432	688807	687437	612980	611610	670447	669077	588257	586887
845		BURPS1106A_0725	703914	704351																
846		BURPS1106B_A4013			3952749	3952312														
847		BURPS1655_K0659																	588757	588309
848		BURPS668_0711											688804	689298						
849	BPSL0675		705280	704351	3953678	3952749	949094	948165	927656	926727	593220	592291	690227	689298	614410	613481	671876	670947	589686	588757
850	BPSL0676		705426	706595	3953824	3954993	949240	950409	927802	928971	593366	594535	690373	691542	614556	615725	672022	673191	589832	591001
851	BPSL0677		706732	707397	3955130	3955795	950546	951211	929108	929773	594672	595337	691679	692344	615862	616527	673328	673993	591138	591803
852	BPSL0678		707824	708543	3956222	3956941	951633	952352	930195	930914	595758	596477	692766	693485	616949	617668	674420	675139	592225	592944
853	BPSL0679		709300	708617	3957698	3957015	953109	952426	931671	930988	597234	596551	694242	693559	618425	617742	675896	675213	593701	593018
854	BPSL0680		710284	709433	3958682	3957831	954093	953242	932655	931804	598218	597367	695226	694375	619409	618558	676880	676029	594685	593834
855	BPSL0681		711599	710376	3959997	3958774	955408	954185	933970	932747	599533	598310	696541	695318	620724	619501	678195	676972	596000	594777
856	BPSL0682		713169	711640	3961567	3960038	956978	955449	935540	934011	601103	599574	698111	696582	622294	620765	679765	678236	597570	596041
857	BPSL0683		714568	713216	3962966	3961614	958377	957025	936939	935587	602502	601150	699510	698158	623693	622341	681164	679812	598969	597617
858	BPSL0684		715914	714610	3964312	3963008	959723	958419	938285	936981	603848	602544	700856	699552	625039	623735	682510	681206	600315	599011
859	BPSL0685		716358	715939	3964756	3964337	960167	959748	938729	938310	604292	603873	701300	700881	625483	625064	682954	682535	600759	600340
860	BPSL0686		717990	717277	3966388	3965675	961799	961086	940362	939649	605933	605220	702933	702220	627115	626402	684587	683874	602393	601680
861	BPSL0687		719605	718106	3968003	3966504	963414	961915	941977	940478	607548	606049	704548	703049	628730	627231	686202	684703	604008	602509
862	BPSL0688		721243	719714	3969641	3968112	965052	963523	943615	942086	609186	607657	706186	704657	630368	628839	687840	686311	605646	604117
863	BPSL0689		721411	721779	3969809	3970177	965220	965588	943783	944151	609354	609722	706354	706722	630536	630904	688008	688376	605814	606182

864	BPSL0690		722058	721852	3970456	3970250	965867	965661	944430	944224	610001	609795	707001	706795	631183	630977	688655	688449	606461	606255
865	BPSL0691		723201	722368	3971599	3970766	967010	966177	945573	944740	611146	610313	708146	707313	632328	631495	689798	688965	607606	606773
866	BPSL0692		723372	725042	3971770	3973440	967181	968851	945744	947414	611317	612987	708317	709987	632499	634169	689969	691639	607777	609447
867	BPSL0693		725348	726178	3973746	3974576	969157	969987	947720	948550	613293	614123	710293	711123	634475	635305	691945	692775	609753	610583
868	BPSL0694		726251	726748	3974649	3975146	970060	970557	948623	949120	614196	614693	711196	711693	635378	635875	692848	693345	610654	611151
869	BPSL0695		728344	726890	3976742	3975288	972153	970699	950716	949262	616243	614837	713291	711837	637473	636019	694941	693487	612749	611295
870		BURPSS13_I0403															694983	695171		
871	BPSL0696		729770	728475	1352	57	973579	972284	952142	950847	617719	616424	714717	713422	638974	637679	696844	695549	613944	612928
872	BPSL0697		730551	729793	2133	1375	974360	973602	952923	952165	618500	617742	715498	714740	639755	638997	697625	696867	614725	613967
873	BPSL0698		730976	731899	2558	3481	974781	975704	953344	954267	618925	619848	715920	716843	640176	641099	698050	698973	615150	616073
874	BPSL0699		733582	732437	5172	4027	977318	976173	955881	954736	621508	620363	718398	717253	642795	641650	700579	699434	617653	616508
875		BURPSS13_I0398															702023	700761		
876	BPSL0700		734039	734383	5629	5973	977760	978104	956323	956667	621950	622294	718842	719186	643237	643581	702505	702849	618095	618439
877	BPSL0701		734964	734521	6554	6111	978716	978264	957279	956827	622866	622432	719789	719346	644193	643741	703439	702987	619039	618596
878	BPSL0702		735260	736939	6850	8529	979012	980691	957575	959254	623074	624753	720085	721764	644489	646168	703728	705407	619335	621014
879	BPSL0703		738803	736923	10393	8513	982555	980675	961118	959238	626626	624737	723628	721748	648032	646152	707278	705779	622878	620998
880	BPSL0704		740172	738856	11762	10446	983924	982608	962487	961171	627995	626679	724997	723681	649401	648085	708647	707331	624247	622931
881	BPSL0705		740960	741538	12550	13128	984712	985290	963275	963853	628783	629361	725784	726362	650189	650767	709435	710013	625035	625613
882	BPSL0706		743460	741712	15050	13302	987212	985464	965775	964027	631283	629535	728284	726536	652689	650941	711935	710187	627535	625787
883	BPSL0707		746112	744355	17702	15945	989864	988107	968427	966670	633935	632178	730934	729177	655341	653584	714587	712830	630187	628430
884		BURPS1106A_0763	746287	746883																
885		BURPS1710b_0930							968585	969010										
886	BPSL0708		746883	746488	18473	18078	990635	990240	969198	968803	634706	634311	731354	731310	656112	655717	715358	714963	630958	630563
887		BURPS1106B_A0015			18473	17877														
888		BURPS1710A_1050					990635	990039												
889		BURPS406E_B1058									634706	634110								
890		BURPSPAST_Z0192													656112	655516				
891		BURPSS13_I0386															715358	714762		
892	BPSL0709		746914	747735	18504	19325	990666	991487	969229	970050	634737	635558	731741	732568	656143	656964	715389	716210	630989	631804
893	BPSL0710		747788	748864	19378	20454	991540	992616	970103	971179	635611	636687	732621	733697	657017	658093	716263	717339	631857	632933
894	BPSL0711		749592	749257	21182	20847	993332	992997	971895	971560	637415	637080	734425	734090	658809	658474	718067	717732	633661	633326
895	BPSL0712		751184	749814	22774	21404	994924	993554	973487	972117	638996	637626	736006	734636	660401	659031	719659	718289	635253	633883
896	BPSL0713		752926	751196	24516	22786	996666	994936	975229	973499	640786	639008	737748	736018	662143	660413	721401	719671	636995	635265
897	BPSL0714		753213	754616	24803	26206	996953	998356	975516	976919	641073	642476	738035	739438	662430	663833	721688	723091	637298	638662
898	BPSL0716		754631	755317	26221	26907	998371	999057	976934	977620	642491	643177	739453	740139	663848	664534	723106	723792	638677	639363
899	BPSL0717		755317	756669	26907	28259	999057	1000409	977620	978972	643177	644529	740139	741491	664534	665886	723792	725144	639363	640715
900	BPSL0718		756823	757527	28413	29117	1000563	1001267	979126	979830	644683	645396	741645	742349	666040	666744	725298	726002	640869	641573
901	BPSL0719		760697	757581	32287	29171	1004434	1001321	982997	979884	648474	645817	745519	742403	669911	666798	729169	726056	644740	641627
902	BPSL0720		762633	761197	34223	32787	1006370	1004934	984933	983497	650247	648811	747464	746010	671667	670411	731105	729669	646733	645384
903	BPSL0721		763265	762618	34855	34208	1006993	1006355	985556	984918	650870	650232	748087	747449	672181	671771	731728	731090	646993	646718
904	BPSL0722		764119	763265	37668	34855	1009842	1006993	988405	985556	653700	651991	748920	748087	675021	672181	734577	731728	650157	647917
905	BPSL0723		767306	766206	38788	37688	1010962	1009862	989525	988425	654838	653720	752119	750992	676141	675041	735697	734597	651295	650177
906	BPSL0724		769185	767362	40667	38844	1012841	1011018	991404	989581	656732	654909	754013	752190	678020	676197	737576	735753	653187	651364
907	BPSL0725		769583	770680	41065	42162	1013224	1014321	991787	992884	657121	658218	754412	755509	678414	679511	737974	739071	653584	654681
908	BPSL0726		770693	771661	42175	43143	1014339	1015304	992902	993867	658231	658758	755522	756490	679529	680494	739084	740052	654699	655664
909	BPSL0727		771661	772500	43143	43982	1015304	1016143	993867	994706	659308	660153	756490	757329	680494	681333	740052	740915	655664	656521
910	BPSL0728		772586	774181	44068	45663	1016229	1017824	994792	996387	660239	661834	757415	759010	681419	683014	741001	742596	656607	658202
911	BPSL0729		776168	774234	47650	45716	1019811	1017877	998374	996440	663821	661887	760997	759063	685001	683067	744583	742649	660189	658255
912	BPSL0730		776876	779374	48358	50856	1020519	1023017	999082	1001580	664529	667027	761705	764203	685709	688207	745291	747789	660897	663395
913	BPSL0731		779374	780651	50856	52139	1023017	1024234	1001580	1002797	667027	668250	764377	765540	688369	689532	748083	749246	663395	664648
914	BPSL0732		782649	780844	54137	52332	1026232	1024427	1004795	1002990	670248	668443	767538	765733	691530	689725	751244	749439	666646	664841
915	BPSL0733		783161	782649	54649	54137	1026744	1026232	1005307	1004795	670760	670248	768050	767538	692042	691530	751756	751244	667158	666646
916	BPSL0734		784133	783174	55621	54662	1027715	1026756	1006278	1005319	671731	670772	769021	768062	693013	692054	752728	751769	668130	667171
917	BPSL0735		784430	784987	55918	56475	1028012	1028569	1006575	1007132	672028	672588	769319	769357	693310	693867	753025	753582	668427	668873
918		BURPS1655_K0550																	710937	709618
919		BURPS1655_K0551																	709216	709497
920		BURPS1655_K0552																	707732	706713
921		BURPS1655_K0553																	706302	705970

922	BURPS1655_K0554																	704661	704969
923	BURPS1655_K0555																	705937	704942
924	BURPS1655_K0556																	703186	704559
925	BURPS1655_K0557																	702967	702815
926	BURPS1655_K0558																	700843	702456
927	BURPS1655_K0559																	699746	700846
928	BURPS1655_K0560																	698475	699749
929	BURPS1655_K0561																	695516	698485
930	BURPS1655_K0562																	695542	695417
931	BURPS1655_K0563																	694689	694826
932	BURPS1655_K0564																	693697	694278
933	BURPS1655_K0565																	694589	694275
934	BURPS1655_K0568																	693428	693285
935	BURPS1655_K0569																	692534	692418
936	BURPS1655_K0570																	692409	691591
937	BURPS1655_K0571																	691125	689941
938	BURPS1655_K0572																	689944	688157
939	BURPS1655_K0573																	686903	687862
940	BURPS1655_K0574																	683503	683631
941	BURPS1655_K0575																	683077	682328
942	BURPS1655_K0576																	682165	680819
943	BURPS1655_K0577																	680753	680361
944	BURPS1655_K0578																	679012	680097
945	BURPS1655_K0579																	677980	678936
946	BURPS1655_K0580																	677343	677864
947	BURPS1655_K0581																	677189	677311
948	BURPS1655_K0582																	677190	676315
949	BURPS1655_K0583																	675080	676291
950	BURPS1655_K0584																	673763	675076
951	BURPS1655_K0585																	672669	673766
952	BURPS1655_K0586																	671396	672631
953	BURPS1655_K0587																	670483	671385
954	BURPS1655_K0590																	668427	668983
955	BURPS1710A_1090					1032306	1031452												
956	BURPS1710A_1091					1032590	1032300												
957	BURPS1710b_0960							1010015	1010869										
958	BURPS1710b_0961							1010863	1011168										
959	BURPS406E_B1088									674353	676287								
960	BURPS406E_B1089									676287	676955								
961	BURPS406E_B1090									676955	680587								
962	BURPS406E_B1091									682087	682917								
963	BURPS406E_B1092									684301	683564								
964	BURPS668_0790											771423	772325						
965	BURPS668_0791											772336	773571						
966	BURPS668_0792											773609	774706						
967	BURPS668_0793											774703	776016						
968	BURPS668_0794											776020	777231						
969	BURPS668_0795											777255	778130						
970	BURPS668_0796											778129	778251						
971	BURPS668_0797											778283	778804						
972	BURPS668_0798											778920	779876						
973	BURPS668_0799											779952	781037						
974	BURPS668_0800											781301	781693						
975	BURPS668_0801											781759	783105						
976	BURPS668_0802											783129	783263						
977	BURPS668_0803											783268	784017						
978	BURPS668_0804											784443	784571						
979	BURPSPAST_Z0156													697888	697598				

980		BURPSPAST_Z0157														697604	696750				
981		BURPSS13_I0336																768098	768226		
982		BURPSS13_I0337																767672	766923		
983		BURPSS13_I0338																766823	765414		
984		BURPSS13_I0339																765348	764956		
985		BURPSS13_I0340																763607	764692		
986		BURPSS13_I0341																762575	763531		
987		BURPSS13_I0342																761944	762459		
988		BURPSS13_I0343																761784	761906		
989		BURPSS13_I0344																761785	760910		
990		BURPSS13_I0345																759675	760886		
991		BURPSS13_I0346																758358	759671		
992		BURPSS13_I0347																757264	758361		
993		BURPSS13_I0348																755991	757226		
994		BURPSS13_I0349																755078	755980		
995	BPSL0736			788030	787113	59518	58601	1032826	1032632	1011389	1011195					698124	697930				
996	BPSL0737			788189	788503	59677	59991	1032985	1033299	1011548	1011862					698283	698597				
997	BPSL0738			788503	789357	59991	60845	1033299	1034153	1011862	1012716					698597	699451				
998	BPSL0739			789784	789473	61272	60961	1034580	1034269	1013143	1012832					699878	699567				
999	BPSL0740			789981	789820	61469	61308	1034777	1034616	1013340	1013179					700075	699914				
1000	BPSL0741			790976	790422	62464	61910	1035772	1035218	1014335	1013781					701070	700516				
1001	BPSL0742			791820	790981	63308	62469	1036616	1035777	1015179	1014340					701914	701075				
1002	BPSL0743			792765	791920	64253	63408	1037561	1036716	1016124	1015279					702859	702014				
1003	BPSL0744			794157	792802	65645	64290	1038953	1037598	1017516	1016161					704251	702896				
1004	BPSL0745		GI4																		
1005	BPSL0746		GI4																		
1006	BPSL0747		GI4																		
1007	BPSL0747a		GI4																		
1008	BPSL0748		GI4																		
1009	BPSL0749		GI4																		
1010	BPSL0750		GI4																		
1011	BPSL0751		GI4																		
1012	BPSL0752		GI4																		
1013	BPSL0753		GI4																		
1014	BPSL0754		GI4																		
1015	BPSL0756		GI4																		
1016	BPSL0757		GI4																		
1017	BPSL0758		GI4																		
1018	BPSL0759		GI4																		
1019	BPSL0760		GI4																		
1020	BPSL0761		GI4																		
1021	BPSL0762		GI4																		
1022	BPSL0763		GI4																		
1023	BPSL0764		GI4																		
1024	BPSL0765		GI4																		
1025	BPSL0766		GI4																		
1026	BPSL0767		GI4																		
1027	BPSL0768		GI4																		
1028	BPSL0769		GI4																		
1029	BPSL0770		GI4																		
1030	BPSL0771		GI4																		
1031	BPSL0772		GI4																		
1032	BPSL0773			796230	794563	67718	66051	1041025	1039358	1019588	1017921	686434	684767	788188	786521	706323	704656	771843	770176	713007	711340
1033	BPSL0774			797997	796444	69485	67932	1042792	1041239	1021355	1019802	688201	686648	789955	788402	708090	706537	773610	772057	714774	713221
1034	BPSL0775			798764	798024	70252	69512	1043559	1042819	1022122	1021382	688968	688228	790722	789982	708857	708117	774377	773637	715541	714801
1035	BPSL0776			798968	800035	70456	71523	1043763	1044830	1022326	1023393	689172	690239	790926	791993	709061	710128	774581	775648	715745	716812
1036	BPSL0777			800404	801018	71892	72506	1045199	1045813	1023762	1024376	690608	691222	792362	792976	710497	711111	776017	776631	717181	717795
1037	BPSL0778			801313	801921	72801	73409	1046108	1046716	1024671	1025279	691517	692125	793271	793879	711406	712014	776926	777534	718090	718698

1038	BPSL0779			802004	803167	73492	74655	1046799	1047962	1025362	1026525	692208	693371	793962	795125	712097	713260	777617	778780	718781	719944
1039	BPSL0780			803314	804192	74802	75680	1048110	1048988	1026673	1027551	693518	694396	795273	796151	713408	714286	778928	779806	720091	720969
1040	BPSL0781			804247	805056	75735	76544	1049043	1049852	1027606	1028415	694451	695260	796206	797015	714341	715150	779861	780670	721024	721833
1041	BPSL0782			805278	805793	76766	77281	1050074	1050589	1028637	1029152	695482	695997	797237	797752	715372	715887	780892	781407	722055	722570
1042	BPSL0783			806004	807818	77492	79306	1050800	1052614	1029363	1031177	696208	698022	797963	799777	716098	717912	781618	783432	722781	724595
1043		BURPS1655_K0536																		724595	725168
1044	BPSL0784			808382	808852	79870	80340	1053213	1053683	1031776	1032246	698621	699091	800313	800783	718497	718967	783982	784452	725165	725635
1045	BPSL0785			809092	809454	80580	80942	1053923	1054285	1032486	1032848	699332	699694	801022	801384	719207	719569	784694	785056	725875	726237
1046		BURPSS13_I0316																786142	786483		
1047		BURPSS13_I0315																786523	787866		
1048			Bp_chr1_10_IS407A															788732	789966		
1049		BURPSS13_I0312																790328	790696		
1050		BURPSS13_I0311																791147	791296		
1051		BURPSS13_I0310																791496	792260		
1052		BURPSS13_I0309																792836	793021		
1053	BPSL0786			810148	809657	81636	81145	1054979	1054491	1033542	1033054	700385	699894	802078	801590	720263	719772	794701	794225	726928	726437
1054	BPSL0787			810291	812012	81779	83500	1055122	1056843	1033685	1035406	700534	702255	802222	803943	720412	722133	794846	796567	727077	728798
1055	BPSL0788			812956	812336	84444	83824	1057791	1057171	1036354	1035734	703203	702583	804877	804257	723081	722461	797515	796895	729746	729126
1056	BPSL0789			813983	812952	85471	84440	1058818	1057787	1037381	1036350	704230	703199	805904	804873	724108	723077	798542	797511	730773	729742
1057	BPSL0790			814449	814006	85937	85494	1059284	1058841	1037847	1037404	704696	704253	806370	805927	724574	724131	799008	798565	731239	730796
1058	BPSL0791			815621	814584	87109	86072	1060456	1059419	1039019	1037982	705868	704831	807542	806505	725746	724709	800180	799143	732411	731374
1059	BPSL0792			815946	815752	87435	87241	1060781	1060587	1039344	1039150	706275	706081	807867	807673	726071	725877	800505	800311	732736	732542
1060	BPSL0793			816935	816015	88424	87504	1061770	1060850	1040333	1039413	707264	706344	808856	807936	727060	726140	801494	800574	733725	732805
1061	BPSL0794			817157	817903	88646	89392	1061992	1062738	1040555	1041301	707486	708232	809078	809824	727282	728028	801716	802462	733947	734693
1062	BPSL0795			817903	818223	89392	89712	1062738	1063058	1041301	1041621	708232	708552	809824	810144	728028	728348	802462	802782	734693	735013
1063	BPSL0796			818493	819683	89982	91172	1063328	1064518	1041891	1043081	708822	710012	810414	811604	728618	729787	803052	804182	735283	736473
1064	BPSL0797			820021	821454	91510	92943	1064856	1066289	1043419	1044852	710371	711804	811942	813375	730042	731475	804466	805899	736811	738244
1065			Bp_chr1_11_ISBma1									711902	713208								
1066	BPSL0798			821747	822808	93236	94297	1066573	1067634	1045136	1046197	713403	714464	813660	814721	731759	732820	806183	807244	738528	739589
1067	BPSL0799			823001	823888	94490	95377	1067827	1068714	1046390	1047277	714657	715544	814914	815801	733013	733900	807437	808324	739782	740669
1068	BPSL0800			823927	824442	95416	95931	1068753	1069268	1047316	1047831	715583	716098	815840	816355	733939	734454	808363	808878	740708	741223
1069	BPSL0801			824525	825718	96014	97207	1069351	1070544	1047914	1049107	716181	717374	816438	817631	734537	735730	808961	810154	741306	742499
1070	BPSL0802			825733	826755	97222	98244	1070565	1071584	1049128	1050147	717389	718411	817652	818671	735751	736770	810169	811191	742514	743536
1071		BURPS1106B_A0097				98302	98487														
1072		BURPS1655_K0513																		743594	743743
1073		BURPS1710b_1004								1050205	1050396										
1074		BURPSPAST_Z0113														736828	737037				
1075	BPSL0803			828233	827097	99722	98586	1073068	1071932	1051631	1050495	719873	718737	820123	818987	738272	737136	812685	811549	744978	743842
1076	BPSL0804			829255	828341	100744	99830	1074090	1073176	1052653	1051739	720895	719981	821145	820231	739294	738380	813707	812793	746001	745087
1077	BPSL0805			831142	829694	102631	101183	1075929	1074481	1054492	1053044	722710	721262	822971	821523	741085	739637	815522	814074	747792	746344
1078	BPSL0806			831410	832069	102899	103558	1076197	1076856	1054760	1055419	722976	723635	823239	823898	741353	742012	815790	816449	748058	748717
1079	BPSL0807			832076	833389	103565	104878	1076863	1078176	1055426	1056739	723642	724955	823905	825218	742019	743332	816456	817769	748724	750037
1080	BPSL0808			833923	835338	105412	106827	1078710	1080125	1057273	1058688	725487	726902	825752	827167	743864	745279	818303	819718	750569	751984
1081	BPSL0809			835501	835920	106990	107409	1080288	1080707	1058851	1059270	727065	727484	827330	827749	745442	745861	819881	820300	752147	752566
1082	BPSL0810			836051	836413	107540	107902	1080838	1081200	1059401	1059763	727615	727977	827880	828242	745992	746354	820431	820793	752697	753059
1083	BPSL0811			836444	837169	107933	108658	1081231	1081956	1059794	1060519	728008	728733	828273	828998	746385	747110	820824	821549	753090	753815
1084	BPSL0812			838145	837513	109634	109002	1082921	1082289	1061484	1060852	729692	729060	829969	829337	748075	747443	822536	821904	754780	754148
1085	BPSL0814			838555	839814	110044	111303	1083331	1084590	1061894	1063153	730102	731361	830380	831639	748485	749744	822946	824205	755190	756449
1086	BPSL0815			839833	843030	111322	114519	1084609	1087806	1063172	1066369	731380	734577	831658	834855	749763	752960	824224	827421	756468	759665
1087	BPSL0816			843037	844578	114526	116067	1087813	1089354	1066376	1067917	734584	735387	834862	836403	752967	754508	827428	828969	759672	761213
1088		BURPS1106B_A0114				116669	116349														
1089		BURPS668_0857												836557	837003						
1090	BPSL0817			846622	845180	118111	116669	1091385	1089943	1069948	1068506	738075	736633	838445	837003	756539	755097	831013	829571	763233	761791
1091	BPSL0818			846951	848372	118440	119861	1091714	1093135	1070277	1071698	738404	739825	838774	840195	756868	758289	831342	832763	763562	764983
1092	BPSL0819			849714	848683	121203	120172	1094444	1093410	1073007	1071973	741134	740100	841817	840786	759598	758564	834069	833038	766283	765249
1093	BPSL0820			849919	851022	121408	122511	1094649	1095752	1073212	1074315	741339	742442	842022	843125	759803	760906	834274	835377	766488	767591
1094	BPSL0821			851039	851896	122528	123385	1095769	1096626	1074332	1075189	742459	743316	843142	843999	760923	761780	835394	836251	767608	768465
1095	BPSL0822			852835	852047	124324	123536	1097565	1096777	1076128	1075340	744255	743467	844938	844150	762719	761931	837190	836402	769404	768616

1096	BPSL0823			853700	852831	125189	124320	1098430	1097561	1076993	1076124	745120	744251	845803	844934	763584	762715	838055	837186	770269	769400
1097	BPSL0824			854598	853726	126087	125215	1099328	1098456	1077891	1077019	746018	745146	846701	845829	764482	763610	838953	838081	771167	770295
1098	BPSL0825			855338	854877	126827	126366	1100068	1099607	1078631	1078170	746758	746297	847429	846968	765204	764743	839693	839232	771907	771446
1099	BPSL0826			855632	856405	127121	127894	1100362	1101135	1078925	1079698	747052	747825	847723	848496	765498	766271	839987	840760	772201	772974
1100	BPSL0827			856413	857366	127902	128855	1101143	1102096	1079706	1080659	747833	748786	848504	849457	766279	767232	840768	841721	772982	773935
1101	BPSL0828			857357	858850	128846	130339	1102087	1103580	1080650	1082143	748777	750270	849448	850941	767223	768716	841712	843205	773926	775419
1102	BPSL0829			858904	860229	130393	131718	1103634	1104959	1082197	1083522	750324	751649	850995	852320	768770	770095	843259	844584	775473	776798
1103	BPSL0830			860340	861281	131829	132770	1105139	1106080	1083633	1084574	751834	752775	852431	853372	770280	771221	844780	845721	776986	777927
1104	BPSL0831			861281	862144	132770	133633	1106080	1106943	1084574	1085437	752775	753638	853372	854235	771221	772084	845721	846584	777927	778790
1105	BPSL0832			862144	862839	133633	134328	1106943	1107638	1085437	1086132	753638	754333	854235	854930	772084	772779	846584	847279	778790	779485
1106	BPSL0833			862849	864012	134338	135501	1107648	1108811	1086142	1087305	754343	755506	854940	856103	772789	773952	847289	848452	779495	780658
1107	BPSL0834			865535	864237	137024	135726	1110326	1109028	1088820	1087522	757015	755717	857561	856263	775461	774163	849967	848669	782157	780859
1108	BPSL0835			866875	865994	138364	137483	1111656	1110775	1090149	1089268	758334	757453	858898	858017	776851	775970	851192	850311	783539	782658
1109	BPSL0836			867013	868206	138502	139695	1111794	1112987	1090287	1091480	758472	759665	859036	860229	776989	778182	851330	852523	783677	784870
1110	BPSL0837			868206	869414	139695	140903	1112987	1114195	1091480	1092688	759665	760837	860229	861437	778182	779390	852523	853731	784870	786078
1111	BPSL0838			870526	869582	142015	141071	1115318	1114374	1093811	1092867	762077	761133	862560	861616	780491	779547	854843	853899	787201	786257
1112	BPSL0839			872094	870616	143583	142105	1116886	1115408	1095379	1093901	763645	762167	864128	862650	782059	780581	856411	854933	788769	787291
1113	BPSL0840			873647	872208	145136	143697	1118439	1117000	1096932	1095493	765198	763759	865681	864242	783612	782173	857964	856525	790322	788883
1114	BPSL0841			874721	873822	146210	145311	1119513	1118614	1098006	1097107	766272	765373	866744	865845	784686	783787	859038	858139	791396	790497
1115	BPSL0842			874834	876450	146323	147939	1119626	1121242	1098119	1099735	766385	768001	866857	868473	784799	785482	859151	859885	791509	792168
1116	BPSL0843			876589	878037	148078	149526	1121367	1122815	1099860	1101308	768126	768956	868598	870046	786542	787990	860984	862432	793274	794722
1117	BPSL0844			878069	878998	149558	150487	1122847	1123776	1101340	1102269	769528	769713	870078	871007	788022	788951	862464	863393	794754	795683
1118	BPSL0844a			879510	879740	150999	151229	1124288	1124518	1102781	1103011	770975	771205	871514	871744	789464	789694	863906	864136	796199	796429
1119	BPSL0845			879740	881131	151229	152620	1124518	1125909	1103011	1104402	771205	772596	871744	873135	789694	791085	864136	865527	796429	797820
1120	BPSL0846			882080	881163	153569	152652	1126858	1125941	1105351	1104434	773545	772628	874084	873167	792034	791117	866476	865559	798769	797852
1121	BPSL0847			883429	882182	154918	153671	1128207	1126960	1106700	1105453	774894	773647	875433	874186	793383	792136	867825	866578	800118	798871
1122	BPSL0848			884112	883474	155601	154963	1128890	1128252	1107383	1106745	775577	774939	876116	875478	794066	793428	868508	867870	800801	800163
1123	BPSL0849			884242	884742	155731	156231	1129020	1129520	1107513	1108013	775707	776207	876246	876746	794196	794696	868638	869138	800931	801431
1124	BPSL0850			885247	884735	156736	156224	1130025	1129513	1108518	1108006	776712	776200	877251	876739	795201	794689	869643	869131	801936	801424
1125	BPSL0851			885622	886176	157111	157665	1130418	1130972	1108911	1109465	777078	777632	877626	878180	795577	796131	870018	870572	802311	802865
1126	BPSL0852			887717	886539	159206	158028	1132495	1131335	1110988	1109828	779155	777995	879721	878543	797672	796494	872131	870935	804397	803228
1127	BPSL0853			889360	888143	160849	159632	1134138	1132921	1112631	1111414	780798	779581	881364	880147	799315	798098	873774	872557	806040	804823
1128	BPSL0854			890154	889495	161643	160984	1134933	1134274	1113425	1112766	781592	780933	882158	881499	800109	799450	874568	873909	806834	806175
1129	BPSL0855			890270	891133	161759	162622	1135049	1135912	1113541	1114404	781708	782571	882274	883137	800225	801088	874684	875547	806950	807813
1130		BURPS1655_K0455																		808049	807813
1131	BPSL0856			892314	891412	163803	162901	1137097	1136195	1115589	1114687	783752	782850	884317	883415	802273	801371	876732	875830	808998	808096
1132	BPSL0857			893192	892314	164681	163803	1137975	1137097	1116467	1115589	784630	783752	885195	884317	803151	802273	877610	876732	809876	808998
1133	BPSL0858			893377	894258	164866	165747	1138160	1139041	1116652	1117533	784811	785692	885376	886257	803336	804217	877791	878672	810057	810938
1134	BPSL0859			896323	894782	167812	166271	1141106	1139565	1119598	1118057	787758	786217	888323	886782	806282	804741	880737	879196	813004	811463
1135	BPSL0860			896871	896320	168360	167809	1141654	1141103	1120146	1119595	788306	787755	888871	888320	806830	806279	881285	880734	813552	813001
1136	BPSL0861			896889	898115	168378	169604	1141672	1142898	1120164	1121390	788324	789550	888889	890115	806848	808074	881303	882529	813570	814796
1137	BPSL0862			898171	898653	169660	170142	1142954	1143436	1121446	1121928	789566	790087	890171	890653	808130	808612	882585	883067	814812	815333
1138	BPSL0863			898656	899654	170145	171143	1143439	1144437	1121931	1122929	790090	791088	890656	891654	808615	809613	883070	884068	815336	816334
1139	BPSL0864			901507	899945	172996	171434	1146290	1144728	1124782	1123220	792941	791379	893501	891939	811466	809904	885908	884346	818174	816612
1140	BPSL0865			901637	902125	173126	173614	1146420	1146908	1124912	1125400	793071	793559	893631	894119	811596	812084	886038	886526	818304	818792
1141	BPSL0866			902237	902842	173726	174331	1147020	1147628	1125512	1126120	793671	794276	894231	894839	812196	812801	886638	887243	818904	819512
1142	BPSL0867			903563	903081	175052	174570	1148346	1147864	1126838	1126356	794997	794515	895557	895075	813522	813040	887964	887482	820230	819748
1143	BPSL0868			903700	904746	175189	176235	1148483	1149529	1126975	1128021	795134	796180	895694	896740	813659	814705	888101	889147	820367	821413
1144	BPSL0869			905864	904938	177353	176427	1150653	1149727	1129145	1128219	797298	796372	897852	896926	815823	814897	890265	889339	822607	821681
1145	BPSL0870			906278	906610	177767	178099	1151067	1151399	1129559	1129891	797712	798044	898266	898598	816237	816566	890679	891011	823021	823353
1146	BPSL0871			907120	906845	178609	178334	1151924	1151649	1130416	1130141	798554	798279	899108	898833	817094	816819	891536	891261	823862	823587
1147	BPSL0872			907525	909072	179014	180561	1152329	1153876	1130821	1132368	798951	800498	899513	901060	817499	819046	891941	893488	824249	825796
1148	BPSL0873			909085	909924	180574	181413	1153889	1154728	1132381	1133220	800511	801350	901073	901912	819059	819898	893501	894340	825809	826648
1149	BPSL0874			910954	910199	182443	181688	1155758	1155003	1134250	1133495	802378	801623	902940	902185	820928	820173	895370	894615	827678	826923
1150			Bp_chr1_12_ISBma2									802538	804110								
1151	BPSL0875			911814	911155	183303	182644	1156618	1155959	1135110	1134451	804834	804175	903800	903141	821788	821129	896230	895571	828538	827879
1152	BPSL0876			912859	912026	184348	183515	1157663	1156830	1136155	1135322	805879	805046	904845	904012	822833	822000	897275	896442	829583	828750
1153	BPSL0877			913194	912991	184683	184480	1157998	1157795	1136490	1136287	806214	806011	905180	904977	823168	822965	897610	897407	829918	829715

1154	BPSL0878		914203	913178	185692	184667	1159007	1157982	1137499	1136474	807223	806198	906189	905164	824177	823152	898619	897594	830927	829902
1155	BPSL0879		914661	916040	186150	187529	1159458	1160837	1137950	1139329	807681	809060	906640	908019	824635	826014	899070	900449	831378	832757
1156	BPSL0880		916240	916815	187729	188304	1161037	1161612	1139529	1140104	809260	809835	908219	908794	826214	826789	900649	901224	832957	833532
1157	BPSL0881		917253	918518	188742	190007	1162050	1163315	1140543	1141808	810273	811538	909232	910497	827227	828492	901662	902927	833970	835235
1158	BPSL0882		920468	919266	191957	190755	1165300	1164098	1143793	1142591	813533	812331	912429	911227	830507	829305	904877	903675	837195	835993
1159	BPSL0883		921457	920705	192946	192194	1166289	1165537	1144782	1144030	814522	813770	913407	912655	831496	830744	905866	905114	838184	837432
1160	BPSL0884		921542	922066	193031	193555	1166374	1166898	1144867	1145391	814607	815131	913492	914016	831581	832105	905951	906499	838269	838793
1161	BPSL0885		922085	923104	193574	194593	1166917	1167936	1145410	1146429	815150	816169	914035	915054	832124	833143	906518	907537	838812	839831
1162	BPSL0886		923647	924249	195136	195738	1168463	1169065	1146956	1147558	816704	817306	915644	916246	833678	834280	908104	908706	840333	840935
1163	BPSL0887		925637	924366	197126	195855	1170523	1169252	1149016	1147745	818694	817423	917725	916454	835724	834453	910094	908823	842393	841122
1164	BPSL0888		927728	926292	199217	197781	1172618	1171182	1151111	1149675	820782	819346	919900	918464	837796	836360	912161	910725	844536	843100
1165	BPSL0889		928025	928357	199514	199846	1172915	1173247	1151408	1151740	821080	821301	920197	920529	838093	838425	912458	912790	844833	845165
1166	BPSL0890		929685	928336	201174	199825	1174065	1173226	1152558	1151719	822666	821326	921857	920508	839744	838404	914118	912769	846511	845144
1167	BPSL0891		931148	930405	202637	201894	1175160	1174417	1153653	1152910	824129	823386	923320	922577	841185	840442	915581	914838	847952	847209
1168	BPSL0892		931317	932159	202806	203648	1175329	1176171	1153822	1154664	824298	825140	923489	924331	841354	842196	915750	916592	848121	848963
1169		BURPS1106B_A0203			205930	204611														
1170		BURPS1655_K0408																	851365	849926
1171		BURPS1710A_1245					1179188	1177128												
1172		BURPS1710b_1103							1155187	1157238										
1173		BURPS1710b_1104							1155621	1157681										
1174		BURPSPAST_Z0007													845228	843153				
1175		BURPSS13_I0188															917115	919004		
1176	BPSL0892a																			
1177	BPSL0893		937805	935694	209294	207183	1181915	1179804	1160408	1158297	830665	828554	930091	927980	847955	845844	922175	920064	854728	852617
1178	BPSL0894		938612	938106	210101	209595	1182734	1182228	1161227	1160721	831481	830975	930901	930395	848774	848268	922985	922479	855538	855032
1179	BPSL0895		939863	939288	211352	210777	1183985	1183410	1162478	1161903	832732	832157	932152	931577	850025	849450	924236	923661	856789	856214
1180	BPSL0896		940131	941387	211620	212876	1184253	1185509	1162746	1164002	833000	834256	932419	933675	850293	851549	924504	925760	857057	858313
1181	BPSL0897		941558	943165	213047	214654	1185680	1187302	1164173	1165795	834427	836034	933846	935453	851720	853327	925931	927538	858484	860112
1182	BPSL0898		943501	943301	214990	214790	1187638	1187438	1166131	1165931	836370	836170	935789	935589	853655	853620	927874	927674	860448	860248
1183		BURPSPAST_A1259													853655	853620				
1184	BPSL0898a		944034	944345	215533	215844	1188171	1188482	1166664	1166975	836903	837214	936321	936632	854188	854499	928407	928718	860981	861292
1185	BPSL0899		944345	946642	215844	218141	1188482	1190779	1166975	1169272	837214	838212	936632	938929	854499	854597	928718	931015	861292	863589
1186		Bp_chr1_13_ISBma2									879847	881419								
1187	BPSL0900		946745	948118	218244	219617	1190882	1192255	1169375	1170748			939032	940405	856900	858273	931118	932491	863701	865068
1188	BPSL0901		948194	948934	219693	220433	1192283	1193026	1170776	1171519			940481	941221	858301	859044	932567	933307	865144	865884
1189	BPSL0902		949102	949881	220601	221380	1193194	1193973	1171687	1172466			941389	942168	859212	859991	933475	934254	866052	866831
1190	BPSL0903		950419	949976	221918	221475	1194511	1194068	1173004	1172561			942706	942263	860522	860079	934785	934342	867369	866926
1191	BPSL0904		951694	950486	223193	221985	1195786	1194578	1174279	1173071			943981	942773	861797	860589	936060	934852	868644	867436
1192	BPSL0905		952256	951759	223755	223258	1196348	1195851	1174841	1174344			944543	944046	862359	861862	936622	936125	869206	868709
1193	BPSL0906		955093	952259	226592	223758	1199185	1196351	1177678	1174844			947380	944546	865196	862362	939459	936625	872043	869209
1194	BPSL0907		956197	955208	227696	226707	1200289	1199300	1178782	1177793			948485	947496	866300	865311	940563	939574	873147	872158
1195	BPSL0908		956322	956981	227821	228480	1200414	1201073	1178907	1179566			948610	949269	866425	867084	940688	941347	873272	873931
1196	BPSL0909		956981	958240	228480	229739	1201073	1202332	1179566	1180825			949269	950528	867084	868343	941347	942606	873931	875190
1197	BPSL0910		958255	959604	229754	231103	1202347	1203696	1180840	1182189			950543	951892	868358	869707	942621	943970	875205	876554
1198	BPSL0911		959804	960997	231303	232496	1203896	1205089	1182389	1183582			952092	953285	869907	871100	944170	945363	876754	877947
1199	BPSL0912		961169	962302	232668	233801	1205261	1206394	1183754	1184887			953457	954590	871272	872405	945535	946668	878119	879252
1200	BPSL0913		962302	963183	233801	234682	1206394	1207275	1184887	1185768			954590	955471	872405	873286	946668	947549	879252	880133
1201		BURPS1106A_0981	963188	963406																
1202		BURPS1106B_A0232			234687	234905														
1203		BURPS1655_K0379																	880138	880356
1204	BPSL0914		965024	963381	236523	234880	1209117	1207474	1187610	1185967			957313	955670	875128	873485	949391	947748	881974	880331
1205	BPSL0915		965314	965150	236813	236649	1209407	1209243	1187900	1187736			957603	957439	875418	875254	949681	949517	882264	882100
1206	BPSL0916		965565	965335	237064	236834	1209658	1209428	1188151	1187921			957854	957624	875669	875439	949932	949702	882515	882285
1207	BPSL0917		966569	965862	238068	237361	1210663	1209968	1189155	1188448			958858	958151	876673	875966	950936	950229	883519	882812
1208	BPSL0918		966834	967286	238333	238785	1210928	1211380	1189420	1189872			959123	959575	876938	877390	951201	951653	883790	884242
1209	BPSL0919		967292	968266	238791	239765	1211386	1212363	1189878	1190855			959581	960558	877396	878373	951659	952636	884248	885225
1210		BURPS1106B_A0239			239917	241020														
1211	BPSL0919A		969885	970403	241384	241902	1212623	1213141	1191115	1191633			960818	961336	878633	879151	952896	953411	885484	886002

1212	BPSL0920			970629	971576	242128	243075	1213367	1214314	1191859	1192806			961562	962509	879377	880324	953637	954584	886228	887175
1213	BPSL0921			971590	972756	243089	244255	1214328	1215494	1192820	1193986			962523	963689	880338	881504	954598	955764	887189	888355
1214	BPSL0922			972822	973592	244321	245091	1215560	1216330	1194052	1194822			963755	964525	881570	882340	955830	956600	888421	889191
1215	BPSL0923			973595	974308	245094	245807	1216333	1217046	1194825	1195538			964528	965241	882343	883056	956603	957316	889194	889907
1216	BPSL0924			974625	975290	246124	246789	1217352	1218017	1195844	1196509			965541	966206	883362	884027	957622	958287	890213	890878
1217	BPSL0925			975924	975469	247423	246968	1218628	1218173	1197120	1196665			966840	966385	884638	884183	958921	958466	891496	891041
1218	BPSL0926			977125	975944	248624	247443	1219829	1218648	1198321	1197140			968041	966860	885839	884658	960122	958941	892697	891516
1219	BPSL0927			978433	977405	249932	248904	1221137	1220109	1199629	1198601			969349	968321	887147	886119	961430	960402	894005	892977
1220	BPSL0928			978516	979538	250015	251037	1221220	1222242	1199712	1200734			969432	970454	887230	888252	961513	962535	894088	895110
1221	BPSL0929			979806	979537	251305	251036	1222510	1222241	1201002	1200733			970722	970453	888520	888251	962803	962534	895378	895109
1222	BPSL0930			980561	979842	252060	251341	1223265	1222546	1201757	1201038			971477	970758	889275	888556	963558	962839	896133	895414
1223	BPSL0931			980709	981071	252208	252570	1223413	1223775	1201905	1202267			971625	971987	889423	889785	963706	964068	896280	896642
1224	BPSL0932			982048	981089	253547	252588	1224752	1223793	1203244	1202285			972964	972005	890762	889803	965045	964086	897619	896660
1225	BPSL0933			983146	982253	254645	253752	1225850	1224957	1204342	1203449			974062	973169	891860	890967	966143	965250	898717	897824
1226	BPSL0934			983268	984161	254767	255660	1225972	1226865	1204464	1205357			974184	975077	891982	892875	966265	967158	898839	899732
1227	BPSL0935			985107	984424	256606	255923	1227768	1227082	1206260	1205574			976012	975329	893778	893092	968077	967394	900643	899960
1228	BPSL0936			986018	985125	257517	256624	1228679	1227786	1207171	1206278			976923	976030	894689	893796	968988	968095	901554	900661
1229	BPSL0937			986882	986103	258381	257602	1229543	1228764	1208035	1207256			977787	977008	895553	894774	969852	969073	902418	901639
1230	BPSL0938			987014	987574	258513	259073	1229675	1230235	1208167	1208727			977919	978479	895685	896245	969984	970544	902550	903110
1231	BPSL0938A			987617	988090	259116	259589	1230278	1230751	1208770	1209243			978522	978977	896288	896761	970587	971042	903159	903608
1232		BURPS1655_K0350																		904788	904213
1233		BURPS1655_K0349																		907162	906251
1234		BURPS1655_K0348																		908648	908529
1235		BURPS1655_K0347																		909045	909167
1236		BURPS1655_K0346																		909591	909767
1237		BURPS1655_K0345																		909760	910062
1238		BURPS1655_K0344																		910055	910267
1239		BURPS1655_K0343																		910260	910607
1240		BURPS1655_K0342																		910617	910943
1241		BURPS1655_K0340																		915760	914546
1242	BPSL0939																				
1243	BPSL0940																				
1244	BPSL0941																				
1245	BPSL0942																				
1246	BPSL0943																				
1247	BPSL0944		GI5																		
1248	BPSL0945		GI5																		
1249	BPSL0946		GI5																		
1250	BPSL0947		GI5																		
1251	BPSL0948		GI5																		
1252	BPSL0949		GI5																		
1253	BPSL0950		Bp_chr1_14_IS407A																		
1254	BPSL0951		Bp_chr1_14_IS407A																		
1255	BPSL0952		GI5																		
1256	BPSL0953		GI5																		
1257	BPSL0954			988701	988366	260200	259865	1231293	1231027	1209785	1209519			979605	979270	897303	897037	971670	971335	916402	916067
1258	BPSL0955			989639	988701	261138	260200	1232299	1231361	1210791	1209853			980543	979605	898309	897371	972608	971670	917340	916402
1259	BPSL0956			989859	991535	261358	263034	1232519	1234195	1211011	1212687			980763	982439	898529	900205	972828	974504	917560	919236
1260	BPSL0957			991563	992102	263062	263601	1234223	1234762	1212715	1213254			982467	983006	900233	900772	974532	975071	919264	919803
1261	BPSL0958			992102	992848	263601	264347	1234762	1235508	1213254	1214000			983006	983752	900772	901518	975071	975817	919803	920549
1262	BPSL0959			992932	993894	264431	265393	1235592	1236554	1214084	1215046			983836	984798	901602	902564	975901	976863	920633	921595
1263	BPSL0960			993920	995233	265419	266732	1236580	1237893	1215072	1216385			984824	986137	902590	903903	976889	978202	921621	922934
1264	BPSL0961			995233	996066	266732	267565	1237893	1238726	1216385	1217218			986137	986970	903903	904736	978202	979035	922934	923767
1265	BPSL0962			996666	996283	268165	267782	1239326	1238943	1217818	1217435			987570	987187	905336	904953	979635	979252	924367	923984
1266	BPSL0963			997849	996701	269348	268200	1240509	1239361	1219001	1217853			988753	987605	906519	905371	980818	979670	925550	924402
1267	BPSL0964			998950	997856	270449	269355	1241610	1240516	1220102	1219008			989854	988760	907620	906526	981919	980825	926651	925557
1268	BPSL0965			999071	1000579	270570	272078	1241731	1243239	1220223	1221731			989975	991483	907741	909249	982040	983548	926772	928280
1269	BPSL0966			1000626	1001039	272125	272538	1243286	1243699	1221778	1222191			991530	991943	909296	909709	983595	984008	928327	928740

1270	BPSL0967			1001064	1001351	272563	273279	1243724	1244428	1222216	1222920			991968	992675	909734	910438	984033	984749	928765	929472
1271	BPSL0968			1002265	1001945	273763	273443	1244915	1244595	1223407	1223087			993159	992839	910925	910605	985233	984913	929956	929636
1272	BPSL0969			1004152	1002482	275650	273980	1246802	1245132	1225294	1223624	917424	915754	995046	993376	912812	911142	987481	985811	932343	930673
1273	BPSL0970			1004330	1005322	275828	276820	1246980	1247972	1225472	1226464	917602	918585	995224	996234	912990	913982	987659	988696	932521	933522
1274	BPSL0971			1005432	1006319	276930	277817	1248082	1248969	1226574	1227461	918695	919582	996344	997231	914092	914979	988806	989693	933632	934519
1275	BPSL0972			1007419	1006685	278917	278183	1250063	1249329	1228562	1227828	920710	919976	998347	997613	916087	915353	990773	990039	935749	935015
1276	BPSL0973			1008079	1007621	279577	279119	1250723	1250265	1229222	1228764	921370	920912	999007	998549	916747	916289	991433	990975	936409	935951
1277	BPSL0974			1008733	1008422	280231	279920	1251377	1251066	1229876	1229565	922024	921713	999661	999350	917401	917090	992087	991776	937063	936752
1278	BPSL0975			1009080	1008733	280578	280231	1251724	1251377	1230223	1229876	922371	922024	1000008	999661	917748	917401	992434	992087	937410	937063
1279	BPSL0976			1009619	1011673	281117	281602	1252263	1254317	1230762	1232816	922910	924964	1000547	1002601	918287	920341	992973	995027	937949	940003
1280	BPSL0977			1011755	1012753	283252	284250	1254411	1255409	1232910	1233908	925046	925855	1002683	1003681	920435	921433	995109	996107	940085	941083
1281	BPSL0978			1012753	1013562	284250	285059	1255409	1256227	1233908	1234726	926046	926873	1003681	1004508	921433	922251	996107	996916	941083	941892
1282	BPSL0979			1013617	1014672	285114	286169	1256275	1257330	1234774	1235829	926920	927975	1004555	1005610	922299	923354	996971	998026	941947	943002
1283	BPSL0980			1014682	1015437	286179	286934	1257340	1258095	1235839	1236594	927985	928740	1005620	1006375	923364	924119	998036	998791	943012	943767
1284	BPSL0981			1015425	1016021	286922	287518	1258083	1258679	1236582	1237178	928728	929324	1006363	1006959	924107	924703	998779	999375	943755	944351
1285	BPSL0982			1017003	1016170	288500	287667	1259661	1258828	1238160	1237327	930306	929476	1007941	1007108	925685	924852	1000357	999524	945335	944502
1286	BPSL0983			1018101	1017172	289598	288669	1260759	1259830	1239258	1238329	931404	930475	1009039	1008110	926783	925854	1001455	1000526	946441	945512
1287	BPSL0984			1019159	1018110	290656	289607	1261817	1260768	1240316	1239267	932462	931413	1010097	1009048	927841	926792	1002513	1001464	947499	946450
1288	BPSL0985			1020096	1019155	291593	290652	1262754	1261813	1241253	1240312	933399	932458	1011034	1010093	928778	927837	1003150	1002509	948436	947495
1289	BPSL0986			1020647	1020093	292144	291590	1263305	1262751	1241804	1241250	933950	933396	1011585	1011031	929329	928775	1003788	1003252	948987	948433
1290	BPSL0987			1020822	1022279	292319	293776	1263450	1264907	1241949	1243406	934137	935594	1011790	1013247	929510	930967	1003957	1005414	949132	950589
1291	BPSL0988			1022563	1022976	294060	294473	1265191	1265604	1243690	1244103	935878	936291	1013536	1013949	931251	931664	1005698	1006111	950873	951286
1292	BPSL0989			1023338	1023958	294835	295455	1265976	1266596	1244475	1245095	936742	937362	1014321	1014941	932119	932739	1006473	1007093	951655	952275
1293	BPSL0990			1024462	1024079	295959	295576	1267100	1266717	1245599	1245216	937866	937483	1015445	1015062	933243	932860	1007597	1007214	952784	952401
1294	BPSL0991			1025354	1024518	296851	296015	1267992	1267156	1246491	1245655	938758	937922	1016337	1015501	934135	933299	1008489	1007653	953676	952840
1295		BURPSS13_I0095																1009181	1008486		
1296	BPSL0992			1026918	1026046	298415	297543	1269655	1268783	1248154	1247282	940341	939469	1017917	1017045	935782	934910	1010053	1009181	955251	954379
1297	BPSL0993			1027111	1026926	298608	298423	1269848	1269663	1248347	1248162	940534	940349	1018110	1017925	935975	935790	1010246	1010061	955444	955259
1298	BPSL0994			1027320	1029134	298817	300631	1270055	1271869	1248554	1250368	940741	942555	1018317	1020131	936184	937998	1010455	1012269	955651	957465
1299	BPSL0995			1029151	1033266	300648	304763	1271886	1276001	1250385	1254500	942572	946687	1020148	1024263	938015	939511	1012286	1016401	957482	961606
1300	BPSL0996			1033620	1034327	305117	305824	1276353	1277060	1254852	1255559	947038	947745	1024614	1025321	942109	942816	1016755	1017462	961959	962666
1301	BPSL0997			1034327	1035337	305824	306834	1277060	1278070	1255559	1256569	947745	948755	1025321	1026331	942816	943826	1017462	1018472	962666	963676
1302	BPSL0998			1037866	1035692	309363	307189	1280606	1278432	1259105	1256931	951291	949117	1028869	1026695	946362	944188	1021001	1018827	966214	964040
1303	BPSL0999			1038995	1038351	310492	309848	1281735	1281091	1260234	1259590	952420	951776	1029998	1029354	947491	946847	1022130	1021486	967343	966699
1304	BPSL1000			1039376	1040461	310873	311958	1282117	1283202	1260616	1261701	952799	953884	1030377	1031462	947873	948958	1022512	1023597	967726	968811
1305	BPSL1001			1040597	1041133	312094	312630	1283338	1283874	1261837	1262373	954020	954556	1031598	1032134	949094	949630	1023733	1024269	968947	969483
1306	BPSL1002			1041240	1041806	312737	313303	1283981	1284547	1262480	1263046	954663	955229	1032241	1032807	949737	950303	1024376	1024942	969590	970156
1307	BPSL1003			1041912	1044188	313409	315685	1284653	1286929	1263152	1265428	955335	957611	1032913	1035189	950409	952685	1025048	1027324	970263	972539
1308	BPSL1004			1044194	1044913	315691	316410	1286935	1287654	1265434	1266153	957617	958336	1035195	1035902	952691	953407	1027330	1028049	972545	973252
1309	BPSL1005			1045724	1045053	317221	316550	1288465	1287794	1266964	1266293	959147	958476	1036713	1036042	954218	953547	1028860	1028189	974063	973392
1310	BPSL1006			1047166	1045760	318663	317257	1289907	1288501	1268406	1267000	960589	959183	1038155	1036749	955660	954254	1030302	1028896	975505	974099
1311	BPSL1007			1049636	1047339	321132	320860	1292376	1290079	1270875	1268578	963058	960761	1040624	1038327	958129	955832	1032771	1030474	977974	975677
1312	BPSL1008			1051143	1050436	322639	321932	1293877	1293170	1272376	1271669	964529	963822	1042197	1041490	959546	958839	1034287	1033580	979484	978777
1313	BPSL1009			1051669	1051154	323165	322650	1294403	1293888	1272902	1272387	965055	964540	1042723	1042208	960072	959557	1034813	1034298	980010	979495
1314	BPSL1010			1052063	1053049	323559	324545	1294798	1295784	1273297	1274283	965450	966436	1043119	1044105	960467	961453	1035207	1036193	980403	981389
1315	BPSL1011			1054040	1053228	325536	324724	1296775	1295963	1275274	1274462	967427	966615	1045096	1044284	962444	961632	1037184	1036372	982379	981567
1316	BPSL1012			1054205	1054975	325701	326471	1296940	1297710	1275439	1276209	967592	968362	1045261	1046031	962609	963379	1037349	1038119	982544	983314
1317			Bp_chr1_15_ISBma2															1038443	1040015		
1318	BPSL1013			1058331	1055350	329827	326846	1301080	1298099	1279579	1276598	971704	968723	1049371	1046390	966754	963773	1043091	1040110	986675	983694
1319	BPSL1014			1058444	1058620	329940	330116	1301193	1301369	1279692	1279868	971817	971972	1049484	1049660	966867	967043	1043204	1043380	986788	986964
1320	BPSL1015			1058658	1059644	330154	331140	1301407	1302393	1279906	1280892	972027	973013	1049698	1050684	967081	968067	1043418	1044404	987002	987988
1321	BPSL1016			1059644	1061623	331140	333119	1302393	1304372	1280892	1282871	973013	974992	1050684	1052663	968067	970046	1044404	1046383	987988	989967
1322	BPSL1017			1061631	1062818	333127	334314	1304380	1305567	1282879	1284066	975000	976187	1052671	1053858	970054	971241	1046391	1047578	989975	991162
1323	BPSL1018			1064297	1062993	335793	334489	1307046	1305742	1285545	1284241	977666	976362	1055337	1054033	972720	971416	1049057	1047753	992641	991337
1324	BPSL1019			1065094	1064339	336590	335835	1307843	1307088	1286342	1285587	978463	977708	1056134	1055379	973517	972762	1049854	1049099	993438	992683
1325	BPSL1020			1066622	1065186	338118	336682	1309369	1307933	1287868	1286432	979991	978555	1057660	1056224	975045	973609	1051380	1049944	994964	993528
1326	BPSL1021			1066783	1067307	338279	338803	1309530	1310054	1288029	1288553	980152	980676	1057821	1058345	975206	975730	1051541	1052065	995125	995649
1327		BURPS1655_K0263																		996634	995711

1328		BURPS1710A_1374					1310970	1310116												
1329		BURPS1710b_1238							1288615	1289469										
1330		BURPS406E_R0141									981574	980738								
1331		BURPSPAST_A1138													976628	975792				
1332		BURPSS13_I0062															1053005	1052127		
1333	BPSL1022		1068313	1067672	339800	339159														
1334	BPSL1023		1068610	1069086	340097	340573	1311267	1311743	1289766	1290242	981871	982347	1059501	1059974	976925	977401	1053303	1053779	996931	997407
1335	BPSL1025		1070491	1069316	341978	340803	1313148	1311973	1291647	1290472	983751	982576	1061379	1060204	978806	977631	1055183	1054008	998812	997637
1336	BPSL1026		1070582	1072309	342069	343796	1313239	1314966	1291738	1293465	983842	985569	1061470	1063197	978897	980624	1055274	1057001	998903	1000630
1337	BPSL1027		1072373	1072708	343860	344195	1315030	1315365	1293529	1293864	985633	985968	1063261	1063596	980688	981023	1057065	1057400	1000694	1001029
1338	BPSL1028	Bp_chr1_16_ISBma1																		
1339	BPSL1029		1074280	1073183	345767	344670	1316927	1315830	1295426	1294329	987542	986445	1065156	1064059	982585	981488	1058950	1057853	1002589	1001492
1340	BPSL1030		1075942	1075166	347429	346653	1318589	1317813	1297088	1296312	989204	988428	1066818	1066042	984247	983471	1060612	1059836	1004252	1003476
1341	BPSL1031		1076678	1075968	348165	347455	1319325	1318615	1297824	1297114	989940	989230	1067554	1066844	984983	984273	1061348	1060638	1004988	1004278
1342	BPSL1032		1077364	1076678	348851	348165	1320011	1319325	1298510	1297824	990626	989940	1068240	1067554	985669	984983	1062034	1061348	1005674	1004988
1343	BPSL1033		1078351	1077578	349838	349065	1320998	1320225	1299497	1298724	991613	990840	1069227	1068454	986656	985883	1063021	1062248	1006661	1005888
1344	BPSL1034		1078746	1079441	350233	350928	1321393	1322088	1299892	1300587	992008	992703	1069620	1070315	987049	987744	1063416	1064111	1007054	1007749
1345	BPSL1035		1080490	1079795	351977	351282	1323139	1322444	1301638	1300943	993753	993058	1071363	1070668	988792	988097	1065159	1064464	1008797	1008102
1346	BPSL1036		1080579	1081301	352066	352788	1323228	1323950	1301727	1302449	993842	994564	1071452	1072174	988881	989603	1065248	1065970	1008886	1009608
1347	BPSL1037		1081288	1082598	352775	354085	1323937	1325247	1302436	1303746	994551	995861	1072161	1073471	989590	990900	1065957	1067267	1009595	1010905
1348	BPSL1038		1082941	1082678	354428	354165	1325590	1325327	1304089	1303826	996204	995941	1073814	1073551	991243	990980	1067578	1067315	1011232	1010969
1349	BPSL1038a		1083343	1083071	354830	354558	1325992	1325720	1304491	1304219	996606	996334	1074204	1073944	991645	991373	1067980	1067708	1011634	1011362
1350	BPSL1039		1083438	1085192	354925	356679	1326087	1327841	1304586	1306340	996701	998455	1074312	1076066	991740	993494	1068075	1069829	1011729	1013483
1351	BPSL1040		1085249	1086586	356736	358073	1327898	1329235	1306397	1307734	998512	999849	1076123	1077460	993551	994888	1069886	1071223	1013540	1014877
1352	BPSL1041		1086917	1086759	358404	358246	1329507	1329409	1308006	1307908	1000180	1000022	1077792	1077634	995160	995062	1071555	1071397	1015209	1015051
1353		BURPS1710A_1393					1329560	1329390												
1354		BURPSPAST_A1118													995213	995049				
1355	BPSL1042		1087480	1086977	358967	358464	1330123	1329620	1308622	1308119	1000743	1000240	1078355	1077852	995776	995273	1072118	1071615	1015772	1015269
1356	BPSL1043		1088028	1087480	359515	358967	1330671	1330123	1309170	1308622	1001291	1000743	1078903	1078355	996324	995776	1072666	1072118	1016320	1015772
1357	BPSL1044		1088647	1090062	360134	361549	1331290	1332705	1309789	1311204	1001910	1003325	1079522	1080937	996943	998358	1073285	1074700	1016939	1017175
1358	BPSL1045		1090058	1090657	361545	362144	1332701	1333300	1311200	1311799	1003321	1003920	1080933	1081532	998354	998953	1074696	1075295	1018231	1018830
1359	BPSL1046		1091444	1090836	362931	362323	1334087	1333479	1312586	1311978	1004707	1004099	1082319	1081711	999740	999132	1076082	1075474	1019617	1019009
1360	BPSL1047		1091803	1092057	363290	363544					1005066	1005320					1076441	1076695	1019976	1020230
1361		BURPS1106A_1112	1092277	1093380																
1362		BURPS1106B_A0366			364867	363764														
1363	BPSL1048		1094957	1093533	366444	365020	1336234	1334810	1314733	1313309	1006872	1005448	1084613	1083189	1001887	1000463	1078228	1076804	1021786	1020362
1364	BPSL1049		1095539	1095030	367026	366517	1336816	1336307	1315315	1314806	1007454	1006945	1085195	1084686	1002469	1001960	1078810	1078301	1022368	1021859
1365	BPSL1050		1096494	1096117	367981	367604	1337768	1337391	1316267	1315890	1008409	1008032	1086150	1085773	1003421	1003044	1079765	1079388	1023323	1022946
1366	BPSL1051		1096652	1096918	368139	368405	1337926	1338192	1316425	1316691	1008567	1008833	1086308	1086574	1003579	1003845	1079923	1080189	1023481	1023747
1367	BPSL1053		1097908	1097531	369395	369018	1339182	1338805	1317681	1317304	1009821	1009444	1087564	1087187	1004835	1004458	1081177	1080800	1024737	1024360
1368	BPSL1054		1098081	1098617	369568	370104	1339355	1339891	1317854	1318390	1009994	1010530	1087737	1088273	1005008	1005544	1081350	1081886	1024910	1025446
1369	BPSL1055		1099854	1098721	371341	370208	1341128	1339995	1319627	1318494	1011767	1010634	1089510	1088377	1006781	1005648	1083123	1081990	1026683	1025550
1370	BPSL1056		1100319	1100098	371806	371585	1341665	1341444	1320164	1319943	1012216	1011995	1089999	1089778	1007278	1007057	1083628	1083407	1027196	1026975
1371	BPSL1057		1100776	1101249	372263	372736	1342122	1342595	1320621	1321094	1012673	1013146	1090455	1090928	1007735	1008208	1084085	1084558	1027652	1028125
1372		BURPS1655_K0217																	1029823	1039122
1373		BURPS1655_K0216																	1039538	1039690
1374		BURPS1655_K0215																	1040003	1041715
1375		BURPS1655_K0214																	1042147	1042704
1376		BURPS1655_K0213																	1045075	1043996
1377		BURPS1655_K0212																	1045028	1045228
1378		BURPS1655_K0209																	1049944	1050945
1379		BURPS1655_K0208																	1051229	1051957
1380		BURPS1655_K0207																	1052336	1052614
1381		BURPS1655_K0206																	1053139	1053441
1382		BURPS1655_K0205																	1053434	1053646
1383		BURPS1655_K0204																	1053639	1053986
1384		BURPS1655_K0203																	1053996	1054340
1385		BURPS1655_K0201																	1060445	1059231

1386	BURPS1106A_1129	1102888	1112313																
1387	BURPS1106A_1130	1112436	1112675																
1388	BURPS1106A_1131	1112960	1113301																
1389	BURPS1106A_1132	1112987	1114693																
1390	BURPS1106B_A0384			374375	383800														
1391	BURPS1106B_A0385			383923	384162														
1392	BURPS1106B_A0386			384468	386180														
1393	BURPS1710A_1415					1343288	1343425												
1394	BURPS1710A_1416					1344283	1344152												
1395	BURPS1710A_1417					1344269	1353664												
1396	BURPS1710A_1418					1353787	1354026												
1397	BURPS1710A_1419					1354038	1354262												
1398	BURPS1710A_1420					1354332	1356044												
1399	BURPS1710A_1421					1356077	1356274												
1400	BURPS1710A_1422					1356399	1357031												
1401	BURPS1710A_1423					1357114	1357263												
1402	BURPS1710A_1424					1358532	1357561												
1403	BURPS1710A_1425					1358416	1358685												
1404	BURPS1710A_1426					1358724	1358978												
1405	BURPS1710A_1427					1359008	1359202												
1406	BURPS1710A_1430					1360013	1359900												
1407	BURPS1710A_1431					1360542	1361585												
1408	BURPS1710A_1432					1361921	1361805												
1409	BURPS1710A_1433					1361997	1362368												
1410	BURPS1710A_1434					1363111	1362536												
1411	BURPS1710A_1435					1363402	1364403												
1412	BURPS1710A_1436					1364558	1365415												
1413	BURPS1710A_1437					1365534	1365797												
1414	BURPS1710A_1438					1365794	1366072												
1415	BURPS1710A_1439					1366428	1366604												
1416	BURPS1710A_1440					1366597	1366899												
1417	BURPS1710A_1441					1366925	1367104												
1418	BURPS1710A_1442					1367097	1367444												
1419	BURPS1710A_1443					1367454	1367798												
1420	BURPS1710A_1445					1372385	1371123												
1421	BURPS1710A_1446					1373016	1372888												
1422	BURPS1710A_1447					1373167	1373934												
1423	BURPS1710A_1448					1375352	1374138												
1424	BURPS1710b_1277							1322768	1332163										
1425	BURPS1710b_1278							1332786	1334543										
1426	BURPS1710b_1279							1334880	1335530										
1427	BURPS1710b_1280							1336060	1336971										
1428	BURPS1710b_1284							1341035	1341610										
1429	BURPS1710b_1285							1342952	1343914										
1430	BURPS1710b_1286							1344296	1344571										
1431	BURPS1710b_1287							1345953	1346297										
1432	BURPS1710b_1289							1349622	1350938										
1433	BURPS1710b_1290							1352637	1353851										
1434	BURPS406E_R0179									1014844	1024143								
1435	BURPS406E_R0180									1025024	1026736								
1436	BURPS406E_R0182									1027529	1028629								
1437	BURPS406E_R0183									1028811	1028662								
1438	BURPS406E_R0184									1029665	1028889								
1439	BURPS406E_R0185									1030003	1029674								
1440	BURPS406E_R0186									1031436	1030117								
1441	BURPS406E_R0187									1031885	1031436								
1442	BURPS406E_R0188									1032385	1031882								
1443	BURPS406E_R0189									1032819	1032484								

1444	BURPS406E_R0190					1032993	1032874						
1445	BURPS406E_R0191					1034028	1034213						
1446	BURPS406E_R0192					1034385	1034816						
1447	BURPS406E_R0193					1034818	1035162						
1448	BURPS406E_R0194					1035625	1035170						
1449	BURPS406E_R0195					1035627	1036085						
1450	BURPS406E_R0196					1036459	1036929						
1451	BURPS406E_R0197					1037049	1039541						
1452	BURPS406E_R0198					1039767	1040354						
1453	BURPS406E_R0199					1040735	1041583						
1454	BURPS406E_R0200					1042336	1042905						
1455	BURPS406E_R0201					1042742	1044856						
1456	BURPS406E_R0202					1044867	1045073						
1457	BURPS406E_R0203					1045070	1046563						
1458	BURPS406E_R0204					1046560	1047660						
1459	BURPS406E_R0205					1047687	1048031						
1460	BURPS406E_R0206					1048097	1049092						
1461	BURPS406E_R0207					1049096	1049386						
1462	BURPS406E_R0208					1049388	1049918						
1463	BURPS406E_R0209					1049908	1050441						
1464	BURPS406E_R0210					1050444	1051124						
1465	BURPS406E_R0211					1051187	1051384						
1466	BURPS406E_R0212					1051381	1051725						
1467	BURPS406E_R0213					1051722	1052615						
1468	BURPS406E_R0214					1052608	1053183						
1469	BURPS406E_R0215					1053171	1054976						
1470	BURPS406E_R0216					1054989	1055675						
1471	BURPS406E_R0217					1055739	1056908						
1472	BURPS406E_R0218					1056919	1057422						
1473	BURPS406E_R0219					1058296	1058093						
1474	BURPS406E_R0220					1059278	1059580						
1475	BURPS406E_R0221					1059534	1059650						
1476	BURPS406E_R0222					1059668	1062076						
1477	BURPS406E_R0223					1062085	1062966						
1478	BURPS406E_R0224					1062941	1063147						
1479	BURPS406E_R0225					1063157	1064209						
1480	BURPS406E_R0226					1064285	1064479						
1481	BURPS406E_R0227					1064481	1064969						
1482	BURPS406E_R0228					1064969	1065514						
1483	BURPS406E_R0230					1066954	1066775						
1484	BURPS406E_R0231					1067598	1067446						
1485	BURPS406E_R0232					1068079	1068585						
1486	BURPS406E_R0234					1069951	1069361						
1487	BURPS406E_R0238					1071568	1071293						
1488	BURPS406E_R0239					1071822	1071571						
1489	BURPS406E_R0240					1072118	1072789						
1490	BURPS406E_R0241					1073472	1074293						
1491	BURPS406E_R0242					1074366	1075961						
1492	BURPS668_1121							1092478	1092609				
1493	BURPS668_1122							1092565	1101999				
1494	BURPS668_1123							1102095	1102334				
1495	BURPS668_1124							1102649	1103050				
1496	BURPS668_1125							1102796	1104382				
1497	BURPSPAST_A1053									1057036	1058067		
1498	BURPSPAST_A1054									1056040	1056849		
1499	BURPSPAST_A1055									1054334	1055857		
1500	BURPSPAST_A1056									1052995	1053723		
1501	BURPSPAST_A1057									1050212	1052845		

1502	BURPSPAST_A1059							1048783	1048944			
1503	BURPSPAST_A1060							1048640	1047960			
1504	BURPSPAST_A1061							1047954	1047514			
1505	BURPSPAST_A1062							1047514	1046198			
1506	BURPSPAST_A1063							1046084	1045755			
1507	BURPSPAST_A1064							1045746	1044967			
1508	BURPSPAST_A1065							1044922	1044740			
1509	BURPSPAST_A1066							1044585	1043734			
1510	BURPSPAST_A1067							1042576	1043715			
1511	BURPSPAST_A1069							1042178	1040964			
1512	BURPSPAST_A1070							1039211	1037949			
1513	BURPSPAST_A1072							1034279	1034623			
1514	BURPSPAST_A1073							1033922	1034269			
1515	BURPSPAST_A1074							1033750	1033929			
1516	BURPSPAST_A1075							1033422	1033724			
1517	BURPSPAST_A1076							1032622	1032897			
1518	BURPSPAST_A1077							1031383	1032240			
1519	BURPSPAST_A1078							1030227	1031228			
1520	BURPSPAST_A1079							1029936	1029361			
1521	BURPSPAST_A1080							1028746	1028630			
1522	BURPSPAST_A1081							1027367	1028410			
1523	BURPSPAST_A1086							1025310	1025510			
1524	BURPSPAST_A1087							1025297	1024437			
1525	BURPSPAST_A1088							1023558	1024412			
1526	BURPSPAST_A1089							1023301	1023564			
1527	BURPSPAST_A1090							1022727	1022876			
1528	BURPSPAST_A1091							1022012	1022644			
1529	BURPSPAST_A1092							1021690	1021887			
1530	BURPSPAST_A1093							1019951	1021657			
1531	BURPSPAST_A1094							1020043	1019924			
1532	BURPSPAST_A1095							1019409	1019639			
1533	BURPSPAST_A1096							1009882	1019277			
1534	BURPSS13_I0022									1085580	1087376	
1535	BURPSS13_I0021									1088808	1088236	
1536	BURPSS13_I0011									1100400	1099111	
1537	BURPSS13_I0009									1100738	1101877	
1538	BURPSS13_I0008									1102882	1101896	
1539	BURPSS13_I0007									1103084	1102902	
1540	BURPSS13_I0006									1103914	1103129	
1541	BURPSS13_I0005									1104141	1103917	
1542	BURPSS13_I0004									1105867	1105418	
1543	BURPSS13_I0003									1106469	1105864	
1544	BURPSS13_I0002									1106801	1106466	
1545	BURPSS13_I0001									1107041	1106853	
1546	BURPSS13_H0001									1108263	1110755	
1547	BURPSS13_H0002									1110973	1111560	
1548	BURPSS13_H0003									1112281	1112162	
1549	BURPSS13_H0004									1113098	1112547	
1550	BURPSS13_H0005									1113103	1115214	
1551	BURPSS13_H0006									1115225	1115431	
1552	BURPSS13_H0007									1115428	1116921	
1553	BURPSS13_H0008									1116918	1118018	
1554	BURPSS13_H0009									1118045	1118389	
1555	BURPSS13_H0010									1118424	1119449	
1556	BURPSS13_H0011									1119453	1119743	
1557	BURPSS13_H0012									1119745	1120272	
1558	BURPSS13_H0013									1120265	1120798	
1559	BURPSS13_H0014									1120801	1121481	

1560	BURPSS13_H0015															1121546	1121752		
1561	BURPSS13_H0016															1121749	1122093		
1562	BURPSS13_H0017															1122090	1122983		
1563	BURPSS13_H0018															1122976	1123551		
1564	BURPSS13_H0019															1123539	1125002		
1565	BURPSS13_H0020															1125018	1125470		
1566	BURPSS13_H0021															1125536	1126705		
1567	BURPSS13_H0022															1126716	1127219		
1568	BURPSS13_H0023															1127289	1127591		
1569	BURPSS13_H0024															1127545	1127661		
1570	BURPSS13_H0025															1127679	1130093		
1571	BURPSS13_H0026															1130102	1130983		
1572	BURPSS13_H0027															1130949	1131164		
1573	BURPSS13_H0028															1131174	1132226		
1574	BURPSS13_H0029															1132302	1132496		
1575	BURPSS13_H0030															1132498	1132986		
1576	BURPSS13_H0031															1132983	1133531		
1577	BURPSS13_H0032															1133675	1134463		
1578	BURPSS13_H0033															1134807	1137434		
1579	BURPSS13_H0034															1137434	1138312		
1580	BURPSS13_H0035															1138803	1140446		
1581	BURPSS13_H0036															1143775	1140719		
1582	BURPSS13_H0037															1145513	1143966		
1583 BPSL1058		1115979	1115680	387466	387167	1376285	1375986	1354784	1354485	1076990	1076691	1105665	1105366	1059091	1058792	1147040	1146741	1061378	1061079
1584 BPSL1059		1116424	1116705	387911	388192	1376730	1377011	1355229	1355510	1077435	1077716	1106118	1106399	1059536	1059817	1147485	1147766	1061823	1062104
1585 BPSL1060		1116719	1117018	388206	388505	1377025	1377324	1355524	1355823	1077730	1078029	1106413	1106712	1059831	1060130	1147780	1148079	1062118	1062417
1586 BPSL1061		1117018	1117233	388505	388720	1377324	1377539	1355823	1356038	1078029	1078244	1106712	1106927	1060130	1060345	1148079	1148294	1062417	1062632
1587 BPSL1062		1117750	1118013	389237	389500	1378056	1378319	1356555	1356818	1078761	1079024	1107444	1107707	1060862	1061125	1148811	1149074	1063149	1063412
1588 BPSL1063		1118036	1118173	389523	389660	1378342	1378479	1356841	1356978	1079047	1079184	1107730	1107867	1061148	1061489	1149097	1149234	1063435	1063572
1589 BPSL1064		1119403	1118480	390890	389967	1379709	1378786	1358208	1357285	1080414	1079491	1109097	1108174	1062519	1061596	1150464	1149541	1064802	1063879
1590 BPSL1065		1121010	1119427	392497	390914	1381316	1379733	1359815	1358232	1082021	1080438	1110704	1109121	1064126	1062543	1152071	1150488	1066409	1064826
1591 BPSL1066		1121638	1121459	393125	392946	1381944	1381765	1360443	1360264	1082649	1082470	1111332	1111153	1064754	1064575	1152699	1152520	1067037	1066858
1592 BPSL1067		1121984	1122436	393471	393923	1382269	1382721	1360768	1361220	1082995	1083447	1111769	1112221	1065079	1065531	1153052	1153504	1067355	1067807
1593 BPSL1068		1122607	1124550	394094	396037	1382892	1384835	1361391	1363334	1083618	1085561	1112392	1114335	1065702	1067645	1153675	1155618	1067978	1069921
1594 BPSL1069		1124759	1126738	396246	398225	1385044	1387023	1363543	1365522	1085770	1087749	1114544	1116523	1067854	1069833	1155827	1157806	1070130	1072109
1595 BPSL1070		1126800	1129115	398287	400602	1387085	1389400	1365584	1367899	1087811	1090126	1116585	1118900	1069895	1072210	1157868	1160183	1072171	1074486
1596	BURPS668_1139											1119176	1119571						
1597 BPSL1071		1129811	1130266	401298	401753	1390096	1390551	1368595	1369050	1090823	1091278	1119605	1120060	1072906	1073361	1160880	1161335	1075181	1075636
1598 BPSL1072		1131315	1130434	402802	401921	1391575	1390694	1370074	1369193	1092356	1091475	1121070	1120189	1074385	1073504	1162377	1161496	1076646	1075765
1599 BPSL1073		1131409	1131873	402896	403360	1391669	1392133	1370168	1370632	1092450	1092914	1121170	1121634	1074479	1074943	1162471	1162935	1076740	1077204
1600 BPSL1074		1133047	1132088	404534	403575	1393322	1392363	1371821	1370862	1094103	1093144	1122808	1121849	1076132	1075173	1164124	1163165	1078378	1077419
1601 BPSL1075		1133263	1134573	404750	406060	1393538	1394848	1372037	1373347	1094319	1095629	1123024	1124334	1076348	1077658	1164340	1165650	1078594	1079904
1602 BPSL1076		1134578	1135837	406065	407324	1394853	1396112	1373352	1374611	1095634	1096893	1124339	1125598	1077663	1078922	1165655	1166914	1079909	1081168
1603 BPSL1078		1136172	1137194	407659	408681	1396398	1397420	1374897	1375919	1097235	1098257	1125940	1126962	1079215	1080237	1167291	1168313	1081510	1082532
1604	BURPS668_1149											1126962	1127159						
1605 BPSL1079		1138778	1137588	410265	409075	1398988	1397798	1377487	1376297	1099889	1098699	1128474	1127284	1081781	1080591	1169889	1168699	1084036	1082846
1606 BPSL1080		1141285	1138928	412772	410415	1401495	1399138	1379994	1377637	1102396	1100039	1130981	1128624	1084288	1081931	1172396	1170039	1086543	1084186
1607 BPSL1081		1142404	1141508	413891	412995	1402614	1401718	1381113	1380217	1103515	1102619	1132100	1131204	1085407	1084511	1173515	1172619	1087662	1086766
1608 BPSL1082		1142876	1142412	414363	413899	1403086	1402622	1381585	1381121	1103987	1103523	1132572	1132108	1085879	1085415	1173987	1173523	1088134	1087670
1609 BPSL1083		1144134	1142935	415621	414422	1404344	1403145	1382843	1381644	1105245	1104046	1133830	1132631	1087137	1085938	1175245	1174046	1089392	1088193
1610 BPSL1084		1145082	1144177	416569	415664	1405292	1404387	1383791	1382886	1106193	1105288	1134778	1133873	1088085	1087180	1176193	1175288	1090340	1089435
1611 BPSL1085		1145859	1145179	417346	416666	1406076	1405396	1384575	1383895	1106977	1106297	1135562	1134882	1088862	1088182	1176970	1176290	1091124	1090444
1612 BPSL1086		1145995	1147461	417482	418948	1406212	1407678	1384711	1386177	1107113	1108579	1135698	1137164	1088998	1090464	1177106	1178572	1091260	1092726
1613 BPSL1087		1147600	1149495	419087	420982	1407817	1409712	1386316	1388211	1108718	1110613	1137303	1139198	1090603	1092498	1178711	1180606	1092865	1094760
1614 BPSL1088		1149507	1150097	420994	421584	1409724	1410314	1388223	1388813	1110625	1111215	1139210	1139800	1092510	1093100	1180618	1181208	1094772	1095362
1615 BPSL1089		1150097	1150573	421584	422060	1410314	1410790	1388813	1389289	1111215	1111691	1139800	1140276	1093100	1093576	1181208	1181684	1095362	1095838
1616 BPSL1091		1150840	1151766	422327	423253	1411044	1411970	1389543	1390469	1111958	1112884	1140530	1141456	1093830	1094756	1181951	1182877	1096105	1097031
1617 BPSL1092		1151961	1152197	423448	423684	1412165	1412401	1390664	1390900	1113081	1113317	1141652	1141888	1094951	1095187	1183072	1183308	1097226	1097462

1618	BPSL1093			1152197	1153744	423684	425231	1412401	1413948	1390900	1392447	1113317	1114864	1141888	1143435	1095187	1096734	1183308	1184855	1097462	1099009
1619	BPSL1094			1154309	1153878	425796	425365	1414513	1414082	1393012	1392581	1115429	1114998	1144000	1143569	1097299	1096868	1185420	1184989	1099574	1099143
1620	BPSL1095			1155628	1154678	427115	426165	1415831	1414881	1394330	1393380	1116747	1115797	1145320	1144370	1098615	1097665	1186738	1185788	1100890	1099940
1621	BPSL1096			1157247	1156033	428734	427520	1417450	1416236	1395949	1394735	1118366	1117152	1146939	1145725	1100234	1099020	1188357	1187143	1102509	1101295
1622	BPSL1097			1158506	1157334	429993	428821	1418718	1417546	1397217	1396045	1119625	1118453	1148198	1147026	1101493	1100321	1189616	1188444	1103777	1102605
1623	BPSL1098			1159533	1158694	431020	430181	1419773	1418934	1398272	1397433	1120680	1119841	1149253	1148414	1102548	1101709	1190671	1189832	1104832	1103993
1624	BPSL1099			1159767	1160123	431254	431610	1420007	1420363	1398506	1398862	1120914	1121270	1149487	1149843	1102782	1103138	1190905	1191261	1105066	1105422
1625	BPSL1100			1160167	1160532	431654	432019	1420407	1420772	1398906	1399271	1121314	1121679	1149887	1150252	1103182	1103547	1191305	1191670	1105466	1105831
1626	BPSL1101			1161575	1161027	433062	432514	1421815	1421267	1400314	1399766	1122722	1122174	1151284	1150736	1104590	1104042	1192713	1192165	1106863	1106315
1627	BPSL1102			1161997	1163064	433484	434551	1422269	1423336	1400768	1401835	1123224	1124291	1151754	1152821	1104980	1106047	1193183	1194250	1107301	1108368
1628	BPSL1103			1163844	1163203	435331	434690	1424116	1423475	1402615	1401974	1125071	1124430	1153601	1152960	1106827	1106186	1195030	1194389	1109147	1108506
1629	BPSL1104			1164721	1163852	436208	435339	1424993	1424124	1403492	1402623	1125921	1125079	1154478	1153609	1107766	1107203	1195319	1195038	1110119	1109538
1630	BPSL1105			1164979	1165608	436466	437095	1425251	1425880	1403750	1404379	1126179	1126808	1154736	1155365	1108024	1108653	1196259	1196888	1110377	1111006
1631	BPSL1106			1167220	1165757	438707	437244	1427492	1426029	1405991	1404528	1128420	1126957	1156977	1155514	1110265	1108802	1198500	1197037	1112618	1111155
1632	BPSL1107			1169272	1167443	440759	438930	1429544	1427715	1408043	1406214	1130472	1128643	1159029	1157200	1112317	1110488	1200552	1198723	1114670	1112841
1633	BPSL1108			1169449	1170681	440936	442168	1429721	1430953	1408220	1409452	1130649	1131881	1159206	1160438	1112494	1113726	1200729	1201961	1114847	1116079
1634		BURPS1106A_1192		1170707	1170841																
1635		BURPS1106B_A0446				442194	442328														
1636		BURPS1710A_1507						1430979	1431107												
1637		BURPS1710b_1342								1409478	1409606										
1638		BURPS668_1184												1160464	1160586						
1639		BURPSPAST_A0994														1113752	1113880				
1640		BURPSS13_H0095																1201987	1202121		
1641		BURPSS13_H0100																1205859	1206548		
1642	BPSL1109			1176542	1170990	448029	442477	1436808	1431256	1415307	1409755	1137557	1136901	1166236	1160735	1119646	1118807				
1643	BPSL1110			1177235	1176717	448722	448204	1437501	1436983	1416000	1415482	1138250	1137732	1166929	1166411	1120339	1119821	1208640	1208122	1122740	1122222
1644	BPSL1111			1177996	1179207	449483	450694	1438262	1439473	1416761	1417972	1139000	1140211	1167699	1168910	1121100	1122311	1209398	1210609	1123510	1124721
1645	BPSL1112			1179313	1179531	450800	451018	1439579	1439797	1418078	1418296	1140317	1140535	1169016	1169234	1122427	1122645	1210725	1210943	1124837	1125055
1646	BPSL1113			1179593	1179976	451080	451463	1439859	1440242	1418358	1418741	1140597	1140980	1169296	1169679	1122707	1123090	1211005	1211388	1125117	1125500
1647	BPSL1114			1180625	1182070	452112	453557	1440871	1442214	1419370	1420815	1141589	1143034	1170318	1171760	1123729	1125174	1212027	1213472	1126139	1127581
1648		BURPS1655_K0135																		1128124	1127975
1649	BPSL1115			1183466	1182576	454953	454063	1443736	1442846	1422234	1421344	1144551	1143661	1173125	1172235	1127080	1126190	1215119	1214229	1129041	1128151
1650	BPSL1116			1184409	1183513	455896	455000	1444679	1443783	1423177	1422281	1145494	1144598	1174068	1173172	1128023	1127127	1216062	1215166	1129984	1129088
1651		BURPS406E_R0311										1145663	1146136								
1652	BPSL1117			1185049	1188570	456536	460057	1445301	1448822	1426021	1427319	1146142	1149663	1174682	1178203	1128645	1132166	1216684	1220205	1130606	1134127
1653	BPSL1118			1188613	1190400	460100	461887	1448865	1450652	1427362	1429149	1149706	1151493	1178246	1180033	1132209	1133996	1220248	1222035	1134170	1135957
1654	BPSL1119			1190457	1191536	461944	463023	1450709	1451788	1429206	1430285	1151550	1152629	1180090	1181169	1134053	1135132	1222092	1223171	1136014	1137093
1655	BPSL1120			1191821	1192963	463308	464450	1452073	1453215	1430570	1431712	1152914	1154056	1181454	1182596	1135417	1136559	1223456	1224598	1137378	1138529
1656	BPSL1121			1192963	1194258	464450	465745	1453215	1454510	1431712	1433007	1154056	1155351	1182596	1183897	1136559	1137854	1224598	1225893	1138529	1139824
1657	BPSL1122			1194258	1195517	465745	467004	1454510	1455769	1433007	1434266	1155351	1156610	1183897	1185156	1137854	1139113	1225893	1227152	1139824	1141083
1658	BPSL1123			1195595	1196776	467082	468263	1455847	1457028	1434344	1435525	1156688	1157869	1185234	1186415	1139191	1140372	1227230	1228411	1141161	1142342
1659	BPSL1124			1197810	1197049	469297	468536	1458062	1457301	1436559	1435798	1158903	1158142	1187449	1186688	1141406	1140645	1229445	1228684	1143376	1142615
1660	BPSL1125			1198268	1198645	469755	470132	1458520	1458897	1437017	1437394	1159361	1159738	1187907	1188284	1141864	1142241	1229903	1230280	1143834	1144211
1661	BPSL1126			1199522	1198932	471009	470419	1459774	1459184	1438271	1437681	1160615	1160025	1189161	1188571	1143118	1142528	1231157	1230567	1145088	1144498
1662	BPSL1127			1201034	1199775	472521	471262	1461304	1460045	1439801	1438542	1162124	1160868	1190733	1189468	1144630	1143371	1232711	1231446	1146648	1145377
1663	BPSL1128			1202014	1201034	473501	472521	1462284	1461304	1440781	1439801	1163083	1162124	1191713	1190733	1145610	1144630	1233691	1232711		
1664	BPSL1129			1203167	1202718	474654	474205	1463430	1462981	1441927	1441478	1164255	1163806	1192866	1192417	1146770	1146321	1234837	1234388	1148817	1148368
1665	BPSL1130			1204549	1203659	476036	475146	1464819	1463929	1443316	1442426	1165651	1164761	1194262	1193372	1148166	1147276	1236224	1235334	1150206	1149316
1666	BPSL1131			1205161	1204976	476648	476463	1465431	1465246	1443928	1443743	1166263	1166078	1194873	1194442	1148778	1148593	1236836	1236651	1150817	1150386
1667	BPSL1132			1205590	1205300	477077	476787	1465860	1465570	1444357	1444067	1166692	1166402	1195302	1195012	1149207	1148917	1237265	1236975	1151246	1150956
1668	BPSL1133			1206287	1206637	477774	478124	1466555	1466905	1445052	1445402	1167380	1167730	1195991	1196335	1149895	1150245	1237962	1238312	1151941	1152285
1669		BURPS1710A_1541						1466938	1467474												
1670	BPSL1134			1207825	1208502	479312	479989	1468073	1468750	1446570	1447247	1168910	1169587	1197504	1198181	1151435	1152112	1239480	1240157	1153486	1154163
1671	BPSL1135			1208567	1209961	480054	481448	1468815	1470209	1447312	1448706	1169652	1171046	1198246	1199640	1152177	1153571	1240222	1241616	1154228	1155622
1672	BPSL1136			1211380	1209995	482867	481482	1471628	1470243	1450125	1448740	1172465	1171080	1201059	1199674	1154990	1153605	1243035	1241650	1157041	1155656
1673	BPSL1137		GI6																		
1674	BPSL1138		GI6																		
1675	BPSL1139		GI6																		

1676	BPSL1140		GI6																		
1677	BPSL1141		GI6																		
1678	BPSL1142		GI6																		
1679	BPSL1143		GI6																		
1680	BPSL1144		GI6																		
1681	BPSL1145		GI6																		
1682	BPSL1146		GI6																		
1683	BPSL1147		GI6																		
1684	BPSL1148		GI6																		
1685	BPSL1149		GI6																		
1686	BPSL1150		GI6																		
1687	BPSL1151		GI6																		
1688	BPSL1152		GI6																		
1689	BPSL1153		GI6																		
1690	BPSL1153A		GI6																		
1691	BPSL1154		GI6																		
1692	BPSL1155		GI6																		
1693	BPSL1156		GI6																		
1694	BPSL1157		GI6																		
1695	BPSL1158			1213164	1211701	484651	483188	1473412	1471949	1451909	1450446	1174249	1172786	1202843	1201380	1156774	1155311	1244819	1243356	1158825	1157362
1696	BPSL1159			1213802	1213176	485289	484663	1474050	1473424	1452547	1451921	1174887	1174261	1203481	1202855	1157412	1156786	1245457	1244831	1159463	1158837
1697	BPSL1160			1214325	1213858	485812	485345	1474573	1474106	1453070	1452603	1175410	1174943	1204004	1203537	1157935	1157468	1245980	1245513	1159986	1159519
1698	BPSL1161			1214846	1214385	486333	485872	1475094	1474633	1453591	1453130	1175931	1175470	1204525	1204064	1158456	1157995	1246501	1246040	1160507	1160046
1699	BPSL1162			1215603	1214854	487090	486341	1475851	1475102	1454348	1453599	1176688	1175939	1205282	1204533	1159213	1158464	1247258	1246509	1161264	1160515
1700	BPSL1163			1216523	1215603	488010	487090	1476771	1475851	1455268	1454348	1177608	1176688	1206202	1205282	1160133	1159213	1248178	1247258	1162184	1161264
1701	BPSL1164			1217931	1216657	489418	488144	1478179	1476905	1456676	1455402	1179016	1177742	1207610	1206336	1161541	1160267	1249586	1248312	1163592	1162318
1702	BPSL1165			1218770	1218045	490257	489532	1479192	1479094	1457515	1456790	1179855	1179130	1208449	1207724	1162380	1161655	1250425	1249700	1164431	1163706
1703	BPSL1166			1219958	1219311	491439	490792	1481224	1480577	1458682	1458035	1180966	1180319	1209575	1208928	1163606	1162959	1251557	1250910	1165514	1164867
1704	BPSL1167			1220216	1220992	491697	492473	1481482	1482081	1458940	1459716	1181224	1182000	1209833	1210609	1163864	1164640	1251815	1252591	1165772	1166548
1705	BPSL1168			1221234	1221623	492715	493104	1482393	1482782	1459976	1460365	1182233	1182622	1210830	1211219	1164873	1165262	1252830	1253219	1166769	1167158
1706	BPSL1169			1222592	1223563	494073	495044	1483704	1484675	1461287	1462258	1183544	1184515	1212085	1213056	1166168	1167139	1254093	1255064	1168061	1169032
1707		BURPS668_1239												1213065	1213379						
1708	BPSL1170			1224031	1224120	495512	495601	1485143	1485232	1462726	1462815	1184983	1185072	1213522	1213611	1167607	1167696	1255532	1255621	1169500	1169589
1709	BPSL1171			1224120	1225925	495601	497406	1485232	1487037	1462815	1464620	1185072	1186877	1213611	1215416	1167696	1169501	1255621	1257426	1169589	1171394
1710	BPSL1172			1226024	1228081	497505	499562	1487136	1489193	1464719	1466776	1186976	1189033	1215515	1217596	1169600	1171657	1257525	1259582	1171493	1173550
1711	BPSL1173			1228097	1228675	499578	500156	1489209	1489787	1466792	1467370	1189049	1189627	1217612	1218190	1171673	1172251	1259598	1260176	1173566	1174144
1712	BPSL1174			1228963	1231941	500444	503422	1490075	1493053	1467658	1470636	1189915	1192803	1218478	1221456	1172539	1175427	1260447	1263425	1174415	1177408
1713	BPSL1175			1231937	1232632	503418	504113	1493049	1493744	1470632	1471327	1193007	1193702	1221452	1222147	1175532	1176182	1263421	1264116	1177404	1178099
1714	BPSL1176			1233025	1233318	504506	504799	1494130	1494423	1471713	1472006	1194102	1194395	1222532	1222825	1176568	1176861	1264502	1264795	1178470	1178763
1715	BPSL1177			1233537	1233854	505018	505335	1494642	1494959	1472225	1472542	1194614	1194931	1223044	1223361	1177080	1177397	1265014	1265331	1178982	1179299
1716	BPSL1178			1234049	1234663	505530	506144	1495154	1495768	1472737	1473351	1195126	1195740	1223556	1224170	1177592	1178206	1265526	1266140	1179494	1180108
1717	BPSL1179			1234663	1237302	506144	508783	1495768	1498413	1473351	1475990	1195740	1198379	1224170	1226818	1178206	1180845	1266140	1268779	1180108	1182726
1718	BPSL1180			1237369	1237812	508850	509293	1498480	1498923	1476057	1476500	1198446	1198889	1226885	1227328	1180912	1181355	1268846	1269289	1182793	1182834
1719	BPSL1181			1237926	1238654	509407	510135	1499037	1499765	1476614	1477342	1199003	1199731	1227442	1228170	1181469	1182197	1269403	1270131	1183407	1184135
1720	BPSL1182			1238654	1239292	510135	510773	1499765	1500403	1477342	1477980	1199731	1200369	1228170	1228808	1182197	1182835	1270131	1270769	1184135	1184773
1721	BPSL1183			1239639	1241030	511120	512511	1500750	1502141	1478327	1479718	1200716	1202107	1229155	1230546	1183182	1184573	1271116	1272507	1185120	1186511
1722	BPSL1184			1241275	1242576	512756	514057	1502385	1503695	1479962	1481272	1202358	1203671	1230797	1232110	1184817	1186127	1272758	1274071	1186750	1188060
1723	BPSL1185			1244797	1242893	516278	514374	1505916	1504012	1483493	1481589	1205892	1203988	1234343	1232439	1188348	1186444	1276292	1274388	1190293	1188389
1724	BPSL1186			1246148	1245336	517629	516817	1507281	1506469	1484858	1484046	1207194	1206382	1235680	1234868	1189699	1188887	1277608	1276796	1191630	1190818
1725	BPSL1187			1247096	1246512	518577	517993	1508229	1507645	1485806	1485222	1208143	1207559	1236641	1236057	1190647	1190063	1278556	1277972	1192578	1191994
1726	BPSL1188			1247812	1247096	519293	518577	1508945	1508229	1486522	1485806	1208859	1208143	1237357	1236641	1191363	1190647	1279272	1278556	1193294	1192578
1727	BPSL1189			1248519	1248052	520000	519533	1509652	1509185	1487229	1486762	1209566	1209099	1238064	1237597	1192070	1191603	1279979	1279512	1194001	1193534
1728	BPSL1190			1249637	1248606	521118	520087	1510770	1509739	1488347	1487316	1210684	1209653	1239182	1238151	1193188	1192157	1281097	1280066	1195119	1194088
1729	BPSL1191			1250597	1249662	522078	521143	1511730	1510795	1489307	1488372	1211644	1210709	1240142	1239207	1194148	1193213	1282057	1281122	1196079	1195144
1730	BPSL1192			1251155	1250691	522636	522172	1512288	1511824	1489865	1489401	1212202	1211738	1240700	1240236	1194706	1194242	1282615	1282151	1196637	1196173
1731	BPSL1193			1251959	1251207	523440	522688	1513092	1512340	1490669	1489917	1213006	1212254	1241504	1240752	1195510	1194758	1283419	1282667	1197441	1196689
1732	BPSL1194			1252412	1251993	523893	523474	1513545	1513126	1491122	1490703	1213459	1213040	1241957	1241538	1195963	1195544	1283873	1283580	1197894	1197475
1733	BPSL1195			1252969	1252412	524450	523893	1514102	1513545	1491679	1491122	1214016	1213459	1242514	1241957	1196520	1195963	1284430	1283873	1198451	1197894

1734 BPSL1196			1253365	1255125	524846	526606	1514498	1516258	1492075	1493835	1214412	1216172	1242910	1244670	1196916	1198676	1284826	1286586	1198847	1200607
1735 BPSL1197			1255240	1255728	526721	527209	1516373	1516861	1493950	1494438	1216287	1216775	1244785	1245273	1198791	1199279	1286701	1287189	1200722	1201210
1736 BPSL1198			1255814	1256827	527295	528308	1516947	1517960	1494524	1495537	1216861	1217874	1245359	1246372	1199365	1200378	1287275	1288288	1201296	1202309
1737 BPSL1199			1256952	1257599	528433	529080	1518085	1518732	1495662	1496309	1217999	1218646	1246497	1247144	1200503	1201150	1288413	1289060	1202434	1203081
1738 BPSL1200			1257619	1258488	529100	529969	1518752	1519621	1496329	1497198	1218666	1219535	1247164	1248033	1201170	1202039	1289080	1289949	1203101	1203970
1739 BPSL1201			1258850	1260394	530331	531875	1519984	1521528	1497561	1499105	1219897	1221441	1248395	1249939	1202402	1203946	1290312	1291856	1204333	1205877
1740 BPSL1202			1262079	1260535	533560	532016	1523213	1521669	1500790	1499246	1223126	1221582	1251624	1250080	1205631	1204087	1293541	1291997	1207562	1206018
1741 BPSL1203			1262771	1262139	534252	533620	1523905	1523273	1501482	1500850	1223819	1223187	1252317	1251685	1206323	1205691	1294233	1293601	1208254	1207622
1742 BPSL1204			1263715	1263218	535196	534699	1524849	1524352	1502426	1501929	1224763	1224266	1253261	1252764	1207267	1206770	1295177	1294680	1209198	1208701
1743 BPSL1205			1264094	1265368	535575	536849	1525228	1526502	1502805	1504079	1225142	1226416	1253645	1254919	1207646	1208920	1295556	1296830	1209577	1210851
1744 BPSL1206			1265477	1265743	536958	537224	1526611	1526877	1504188	1504454	1226525	1226791	1255028	1255294	1209029	1209295	1296939	1297205	1210960	1211226
1745 BPSL1207			1266050	1268188	537531	539669	1527184	1529322	1504761	1506899	1227098	1229236	1255601	1257739	1209602	1211740	1297512	1299650	1211533	1213671
1746 BPSL1208			1268307	1269323	539788	540804	1529441	1530457	1507018	1508034	1229355	1230371	1257858	1258874	1211859	1212875	1299769	1300785	1213790	1214806
1747 BPSL1209			1269431	1270183	540912	541664	1530565	1531317	1508142	1508894	1230479	1231231	1258982	1259734	1212983	1213735	1300893	1301645	1214914	1215666
1748 BPSL1210			1270266	1270640	541747	542121	1531400	1531774	1508977	1509351	1231314	1231688	1259817	1260191	1213818	1214192	1301728	1302102	1215749	1216123
1749 BPSL1211			1270992	1271348	542473	542829	1532126	1532482	1509703	1510059	1232040	1232396	1260543	1260899	1214544	1214900	1302454	1302810	1216475	1216831
1750 BPSL1212			1271372	1271890	542853	543371	1532506	1533024	1510083	1510601	1232420	1232938	1260923	1261441	1214924	1215442	1302834	1303352	1216855	1217373
1751 BPSL1213			1271914	1272513	543395	543994	1533048	1533647	1510625	1511224	1232962	1233561	1261465	1262064	1215466	1216065	1303376	1303975	1217397	1217996
1752 BPSL1214			1272527	1273777	544008	545258	1533661	1534911	1511238	1512488	1233574	1234824	1262078	1263328	1216079	1217329	1303989	1305239	1218010	1219260
1753 BPSL1215			1273949	1274431	545430	545912	1535083	1535565	1512660	1513142	1234996	1235478	1263500	1263982	1217501	1217983	1305411	1305893	1219432	1219914
1754 BPSL1216			1274431	1275738	545912	547219	1535565	1536872	1513142	1514449	1235478	1236785	1263982	1265289	1217983	1219290	1305893	1307200	1219914	1221221
1755 BPSL1217			1275824	1278151	547305	549632	1536958	1539285	1514535	1516862	1236871	1239198	1265375	1267702	1219376	1221703	1307286	1309613	1221307	1223634
1756 BPSL1218			1278155	1279216	549636	550697	1539289	1540350	1516866	1517927	1239202	1240263	1267706	1268767	1221707	1222768	1309617	1310678	1223638	1224699
1757 BPSL1219			1279235	1279720	550716	551201	1540369	1540854	1517946	1518431	1240282	1240767	1268786	1269271	1222787	1223272	1310697	1311182	1224718	1225203
1758 BPSL1220			1279833	1280486	551314	551967	1540967	1541620	1518544	1519197	1240880	1241533	1269384	1270037	1223385	1224038	1311295	1311948	1225316	1225969
1759 BPSL1221			1280508	1280810	551989	552291	1541642	1541944	1519219	1519521	1241555	1241857	1270059	1270361	1224060	1224362	1311970	1312272	1225991	1226293
1760 BPSL1222			1280831	1282867	552312	554348	1541965	1544001	1519542	1521578	1241878	1243914	1270382	1272418	1224383	1226419	1312293	1314329	1226314	1228350
1761 BPSL1223			1282886	1284373	554367	555854	1544020	1545507	1521597	1523084	1243933	1245420	1272437	1273924	1226438	1227925	1314348	1315835	1228369	1229856
1762 BPSL1224			1284401	1285870	555882	557351	1545535	1547004	1523112	1524581	1245448	1246917	1273952	1275421	1227953	1229422	1315863	1317332	1229884	1231353
1763 BPSL1225			1285873	1286175	557354	557656	1547007	1547309	1524584	1524886	1246920	1247222	1275424	1275726	1229425	1229727	1317335	1317637	1231356	1231658
1764 BPSL1226			1286215	1286802	557696	558283	1547349	1547936	1524926	1525513	1247262	1247849	1275766	1276353	1229767	1230354	1317677	1318264	1231698	1232285
1765 BPSL1227			1286957	1287376	558438	558857	1548091	1548510	1525668	1526087	1248004	1248423	1276508	1276927	1230509	1230928	1318419	1318838	1232440	1232859
1766 BPSL1228			1287440	1288633	558921	560114	1548574	1549767	1526151	1527344	1248487	1249680	1276991	1278184	1230992	1232185	1318902	1320095	1232923	1234116
1767 BPSL1229			1288648	1289778	560129	561259	1549782	1550912	1527359	1528489	1249695	1250825	1278199	1279329	1232200	1233330	1320110	1321240	1234131	1235261
1768 BPSL1230			1289822	1290277	561303	561758	1550956	1551411	1528533	1528988	1250869	1251324	1279373	1279828	1233374	1233829	1321284	1321739	1235305	1235760
1769 BPSL1231			1290277	1290756	561758	562237	1551411	1551890	1528988	1529467	1251324	1251803	1279828	1280307	1233829	1234308	1321739	1322218	1235760	1236239
1770 BPSL1233			1290852	1292111	562333	563592	1551986	1553245	1529563	1530822	1251899	1253158	1280403	1281662	1234404	1235663	1322314	1323573	1236335	1237594
1771 BPSL1234			1293624	1292401	565105	563882	1554751	1553528	1532328	1531105	1254664	1253441	1283173	1281950	1237169	1235946	1325086	1323863	1239100	1237877
1772 BPSL1235			1294774	1293671	566255	565152	1555901	1554798	1533478	1532375	1255814	1254711	1284323	1283220	1238319	1237216	1326236	1325133	1240250	1239147
1773 BPSL1236			1295515	1294805	566996	566286	1556645	1555932	1534219	1533509	1256555	1255845	1285064	1284354	1239060	1238350	1326977	1326267	1240991	1240281
1774 BPSL1237			1296388	1295528	567869	567009	1557518	1556658	1535092	1534232	1257428	1256568	1285937	1285077	1239933	1239073	1327850	1326990	1241864	1241004
1775 BPSL1238			1296538	1297209	568019	568690	1557668	1558339	1535242	1535913	1257578	1258249	1286090	1286761	1240084	1240755	1328000	1328671	1242014	1242685
1776 BPSL1239			1298597	1297446	570078	568927	1559716	1558565	1537290	1536139	1259626	1258475	1288160	1287009	1242104	1240953	1330048	1328897	1244063	1243053
1777 BPSL1240			1299332	1298667	570813	570148	1560451	1559786	1538025	1537360	1260361	1259696	1288895	1288230	1242839	1242174	1330783	1330118	1244798	1244133
1778 BPSL1241			1299390	1300169	570871	571650	1560509	1561288	1538083	1538862	1260419	1261198	1288953	1289732	1242897	1243676	1330841	1331620	1244856	1245635
1779 BPSL1242			1300231	1301382	571712	572863	1561350	1562501	1538924	1540075	1261260	1262411	1289794	1290945	1243738	1244889	1331682	1332833	1245697	1246848
1780 BPSL1243			1302255	1301599	573736	573080	1563374	1562718	1540948	1540292	1263284	1262628	1291818	1291162	1245762	1245106	1333706	1333050	1247721	1247065
1781 BPSL1244			1303050	1302379	574531	573860	1564169	1563498	1541743	1541072	1264079	1263408	1292613	1291942	1246557	1245886	1334501	1333830	1248516	1247845
1782 BPSL1245			1304025	1303120	575506	574601	1565144	1564239	1542718	1541813	1265054	1264149	1293552	1292683	1247532	1246627	1335476	1334571	1249491	1248586
1783 BPSL1246			1305376	1304162	576857	575643	1565526	1565281	1544069	1542855	1266405	1265191	1294940	1293726	1248883	1247669	1336827	1335613	1250842	1249628
1784		BURPSS13_R0001															1337268	1337543		
1785		BURPSS13_R0002															1337908	1338459		
1786 BPSL1247			1312058	1313944	580865	582751	1567068	1568954	1550753	1552639	2852882	2850996	1301703	1303589	2476877	2474991	1339718	1341604	2459453	2457567
1787 BPSL1248			1315166	1314096	583973	582903	1570176	1569106	1553861	1552791	2849774	2850844	1304870	1303800	2473769	2474839	1342831	1341761	2456340	2457410
1788 BPSL1249			1315353	1316393	584160	585200	1570363	1571403	1554048	1555088	2849587	2848547	1305057	1306097	2473582	2472542	1343018	1344058	2456153	2455113
1789 BPSL1250			1316716	1317987	585523	586794	1571726	1572997	1555411	1556682	2848224	2846953	1306417	1307688	2472219	2470948	1344380	1345651	2454794	2453523
1790 BPSL1251			1318550	1319011	587357	587818	1573560	1574021	1557245	1557706	2846390	2845929	1308251	1308712	2470385	2469924	1346215	1346676	2452960	2452499
1791 BPSL1252			1320459	1319437	589266	588244	1575469	1574447	1559154	1558132	2844481	2845503	1310160	1309138	2468483	2469505	1348117	1347095	2451058	2452080

1792	BPSL1253		1321348	1323072	590155	591879	1576358	1578082	1560043	1561767	2843592	2841868	1311047	1312771	2467594	2465870	1349006	1350730	2450169	2448445
1793	BPSL1254		1323593	1324666	592400	593473	1578603	1579676	1562288	1563361	2841347	2840274	1313282	1314355	2465342	2464269	1351251	1352324	2447924	2446851
1794	BPSL1255		1324709	1324852	593516	593659	1579719	1579862	1563404	1563547	2840231	2840088	1314398	1314541	2464226	2464083	1352367	1352510	2446808	2446665
1795	BPSL1256		1325083	1325796	593890	594603	1580093	1580806	1563778	1564491	2839857	2839144	1314772	1315485	2463852	2463139	1352741	1353454	2446434	2445721
1796	BPSL1257		1325799	1327088	594606	595895	1580809	1582098	1564494	1565783	2839141	2837852	1315488	1316777	2463136	2461847	1353457	1354746	2445718	2444429
1797	BPSL1258		1327099	1327278	595906	596085	1582109	1582288	1565794	1565973	2837841	2837662	1316788	1316967	2461836	2461657	1354757	1354936	2444418	2444239
1798	BPSL1259		1327294	1327863	596101	596670	1582304	1582873	1565989	1566558	2837646	2837077	1316983	1317552	2461641	2461072	1354952	1355521	2444223	2443654
1799	BPSL1260		1327853	1329475	596660	598282	1582863	1584485	1566548	1568170	2837087	2835465	1317542	1319164	2461082	2459460	1355511	1357133	2443664	2442042
1800	BPSL1261		1329475	1330260	598282	599067	1584485	1585270	1568170	1568955	2835465	2834680	1319164	1319949	2459460	2458675	1357133	1357918	2442042	2441257
1801	BPSL1262		1332313	1330862	601127	599676	1587288	1585837	1570973	1569522	2832669	2834120	1321959	1320508	2456622	2458073	1359957	1358506	2439261	2440712
1802	BPSL1263		1334090	1332351	602904	601165	1589065	1587326	1572750	1571011	2830892	2832631	1323736	1321997	2454845	2456584	1361734	1359995	2437484	2439223
1803	BPSL1264		1334383	1334928	603197	603742	1589358	1589903	1573043	1573588	2830599	2830054	1324029	1324574	2454552	2454007	1362027	1362572	2437191	2436646
1804	BPSL1265		1336078	1335458	604892	604272	1591013	1590393	1574698	1574078	2828899	2829519	1325714	1325094	2452857	2453477	1363727	1363107	2435496	2436116
1805	BPSL1266		1340002	1336703	608816	605517	1594937	1591638	1578622	1575323	2824975	2828274	1329638	1326339	2448933	2452232	1367651	1364352	2431569	2434868
1806	BPSL1267		1343113	1340006	611927	608820	1598048	1594941	1581733	1578626	2821864	2824971	1332749	1329642	2445822	2448929	1370762	1367655	2428458	2431565
1807	BPSL1268		1344464	1343130	613278	611944	1599399	1598065	1583084	1581750	2820513	2821847	1334091	1332766	2444471	2445805	1372113	1370779	2427116	2428441
1808	BPSL1269		1345774	1344950	614588	613764	1600710	1599886	1584395	1583571	2819139	2819963	1335402	1334578	2443160	2443318	1373424	1372600	2425832	2426656
1809	BPSL1270		1348282	1346324	617096	615138	1603219	1601261	1586904	1584946	2816630	2818588	1337913	1335955	2440651	2442609	1375934	1373976	2423247	2423342
1810	BPSL1271		1348533	1349051	617347	617865	1603470	1603988	1587155	1587673	2816379	2815861	1338164	1338682	2440400	2439882	1376185	1376703	2422996	2422478
1811	BPSL1272		1349144	1349602	617958	618416	1604081	1604539	1587766	1588224	2815768	2815310	1338775	1339233	2439789	2439331	1376796	1377254	2422385	2421927
1812	BPSL1273		1349872	1350495	618686	619309	1604809	1605432	1588494	1589117	2815040	2814417	1339507	1340130	2439061	2438438	1377524	1378147	2421657	2421034
1813	BPSL1274		1351948	1351295	620762	620109	1606886	1606233	1590571	1589918	2812964	2813617	1341583	1340930	2436984	2437637	1379600	1378947	2419589	2420242
1814	BPSL1275		1352095	1352559	620909	621373	1607033	1607497	1590718	1591182	2812817	2812353	1341730	1342194	2436837	2436373	1379747	1380211	2419442	2418978
1815	BPSL1276		1353470	1352694	622284	621508	1608411	1607635	1592096	1591320	2811442	2812218	1343092	1342316	2435459	2436235	1381119	1380343	2418070	2418846
1816	BPSL1277		1355184	1353475	623998	622289	1610125	1608416	1593810	1592101	2809728	2811437	1344806	1343097	2433745	2435454	1382833	1381124	2416356	2418065
1817	BPSL1278		1356229	1355177	625043	623991	1611170	1610118	1594855	1593803	2808683	2809735	1345851	1344799	2432700	2433752	1383878	1382826	2415311	2416363
1818	BPSL1279		1356469	1356645	625283	625459	1611410	1611586	1595095	1595271	2808443	2808267	1346091	1346267	2432460	2432284	1384118	1384294	2415071	2414895
1819	BPSL1280		1358164	1356803	626978	625617	1613105	1611744	1596790	1595429	2806738	2808099	1347766	1346405	2430765	2432126	1385813	1384452	2413386	2414747
1820	BPSL1281		1360625	1358352	629439	627166	1615566	1613293	1599251	1596978	2804277	2806550	1350227	1347954	2428304	2430577	1388274	1386001	2410925	2413198
1821	BPSL1282		1361271	1364612	630092	633433	1616191	1619532	1599876	1603217	2803729	2800388	1350852	1354193	2427700	2424359	1388885	1392226	2410314	2406973
1822	BPSL1283		1364612	1368421	633433	637242	1619532	1623341	1603217	1607026	2800388	2796579	1354193	1357981	2424359	2420550	1392226	1396035	2406973	2403164
1823	BPSL1284		1368421	1371090	637242	639911	1623341	1626034	1607026	1609719	2796579	2795386	1357981	1360674	2420550	2417857	1396035	1398704	2403164	2402031
1824	BPSL1285		1371090	1371491	639911	640312	1626034	1626435	1609719	1610120	2793959	2793558	1360674	1361075	2417857	2417456	1398704	1399105	2400608	2400207
1825		BURPS1106A_1393	1371628	1371765																
1826		BURPS1106B_A0641			640449	640586														
1827		BURPS1710A_1716					1626572	1626709												
1828	BPSL1286		1373158	1371950	641979	640771	1628095	1626887	1611780	1610572	2791930	2793138	1362678	1361470	2415804	2417012	1400765	1399557	2398583	2399791
1829	BPSL1287		1375030	1374101	643851	642922	1630053	1629124	1613738	1612809	2789951	2790880	1364844	1363915	2413846	2414775	1402637	1401708	2396610	2397539
1830		BURPS1655_A2372																	2397650	2398411
1831	BPSL1288		1375525	1375304	644346	644125	1630548	1630327	1614233	1614012	2789477	2789698	1365328	1365107	2413351	2413572	1403132	1402911	2396115	2396336
1832	BPSL1289		1375989	1375780	644810	644601	1631012	1630803	1614697	1614488	2789013	2789222	1365792	1365583	2412887	2413096	1403596	1403387	2395651	2395860
1833	BPSL1290		1378144	1376216	646965	645037	1633167	1631239	1616852	1614924	2786858	2788786	1367947	1366019	2410732	2412660	1405751	1403823	2393496	2395424
1834	BPSL1290a		1378765	1378472	647586	647293	1633798	1633442	1617483	1617127	2786227	2786583			2410101	2410457	1406382	1406026	2392865	2393221
1835	BPSL1291		1378784	1379086	647605	647907	1633817	1634119	1617502	1617804	2786208	2785906	1368591	1368893	2410082	2409780	1406401	1406703	2392846	2392544
1836	BPSL1292		1379250	1380071	648071	648892	1634283	1635104	1617968	1618789	2785742	2784921	1369057	1369878	2409616	2408795	1406867	1407688	2392380	2391559
1837	BPSL1293		1380485	1381198	649306	650019	1635524	1636267	1619209	1619952	2784510	2783782	1370304	1370993	2408363	2407620	1408120	1408863	2391145	2390444
1838	BPSL1294		1381217	1382005	650038	650826	1636286	1637074	1619971	1620759	2783763	2782975	1371012	1371800	2407601	2406813	1408882	1409670	2390425	2389637
1839	BPSL1295		1382441	1383373	651262	652194	1637510	1638442	1621195	1622127	2782539	2781607	1372257	1373189	2406377	2405445	1410127	1411059	2389180	2388248
1840	BPSL1296		1384955	1383762	653762	652569	1640052	1638859	1623737	1622544	2780004	2781197	1374770	1373577	2403744	2404937	1412652	1411459	2386718	2387911
1841	BPSL1297		1385234	1386019	654041	654826	1640331	1641116	1624016	1624801	2779725	2778940			2403465	2402680	1412931	1413716	2386439	2385654
1842	BPSL1298		1386163	1386570	654970	655377	1641260	1641667	1624945	1625352	2778796	2778389			2402530	2402123	1413863	1414270	2385522	2385115
1843	BPSL1299		1387756	1386932	656563	655739	1642853	1642029	1626538	1625714	2777203	2778027	1375857	1375033	2400937	2401761	1415456	1414632	2383929	2384753
1844	BPSL1300		1389014	1387989	657821	656796	1644111	1643086	1627796	1626771	2775966	2776991	1377108	1376083	2399686	2400711	1416707	1415682	2382692	2383717
1845	BPSL1301		1390104	1389193	658911	658000	1645201	1644290	1628886	1627975	2774876	2775787	1378198	1377287	2398596	2399507	1417797	1416886	2381602	2382513
1846	BPSL1302		1392129	1391308	660936	660115	1647235	1646414	1630920	1630099	2772824	2773645	1380231	1379410	2396562	2397383	1419849	1419028	2379552	2380373
1847	BPSL1303		1393299	1392241	662106	661048	1648391	1647333	1632076	1631018	2771654	2772712	1381387	1380329	2395406	2396464	1421026	1419968	2378375	2379433
1848	BPSL1304		1394372	1393359	663179	662166	1649482	1648451	1633167	1632136	2770554	2771585	1382469	1381447	2394315	2395346	1422099	1421086	2377284	2378315
1849	BPSL1305		1408204	1407053	677011	675860	1652998	1651847	1636683	1635532	2767029	2768180	1386045	1384894	2390729	2391880	1425694	1424543	2373757	2374908

1850		BURPS1106A_1448		1408241	1408570																
1851		BURPS1106B_A0694				677048	677377														
1852		BURPS1655_A2344																		2376149	2376649
1853	BPSL1306			1409942	1408581	678749	677388	1654728	1653367	1638413	1637052	2765290	2766651	1387815	1386454	2388991	2390352	1427400	1426039	2372003	2373364
1854	BPSL1307			1410663	1411262	679470	680069	1655441	1656040	1639126	1639725	2764589	2763990	1388528	1389127	2388282	2387683	1428113	1428712	2371302	2370703
1855	BPSL1308			1411318	1412112	680125	680919	1656096	1656890	1639781	1640575	2763934	2763140	1389183	1389977	2387627	2386833	1428768	1429562	2370647	2369853
1856	BPSL1309			1413156	1412521	681963	681328	1657927	1657292	1641612	1640977	2761997	2762632	1391003	1390368	2385789	2386424	1430620	1429985	2368816	2369451
1857	BPSL1310			1413278	1414816	682085	683623	1658049	1659587	1641734	1643272	2761875	2760337	1391125	1392663	2385667	2384129	1430742	1432280	2368694	2367156
1858	BPSL1311			1415576	1415085	684383	683892	1660392	1659901	1644077	1643586	2759538	2760029	1393450	1392959	2383318	2383809	1433025	1432534	2366398	2366889
1859	BPSL1312			1415735	1417663	684542	686470	1660551	1662449	1644236	1646134	2759379	2757481	1393609	1395522	2383159	2381261	1433184	1435097	2366237	2364339
1860	BPSL1313			1419207	1417879	688014	686686	1663993	1662665	1647678	1646350	2755937	2757265	1397106	1395778	2379717	2381045	1436641	1435313	2362760	2364088
1861		BURPS1655_A2326																		2364339	2364061
1862	BPSL1314			1419939	1419232	688746	688039	1664719	1664018	1648404	1647703	2755211	2755912	1397829	1397131	2378991	2379692	1437364	1436666	2362034	2362735
1863	BPSL1315			1421002	1420106	689809	688913	1665783	1664887	1649468	1648572	2754148	2755044	1398895	1397999	2377927	2378823	1438431	1437535	2360971	2361867
1864	BPSL1316			1421253	1422023	690060	690830	1666034	1666804	1649719	1650489	2753897	2753127	1399146	1399916	2377676	2376906	1438682	1439452	2360720	2359950
1865		BURPS1106A_1464		1423655	1423975																
1866		BURPS668_1433												1401516	1401938						
1867	BPSL1317			1423662	1422247	692469	691054	1668461	1667028	1652146	1650713	2752267	2752746	1401519	1400140			1441091	1439676	2358335	2359726
1868		BURPS1710A_1768						1668858	1668454												
1869		BURPSS13_P0105																1441488	1441084		
1870	BPSL1318			1424502	1424098	693309	692905	1669293	1668889	1652978	1652574	2751427	2751831	1402373	1401969	2374411	2374815	1441923	1441519	2357485	2357889
1871		BURPSPAST_AA0104														2374846	2375250				
1872	BPSL1319			1426316	1424502	695123	693309	1671107	1669293	1654792	1652978	2749613	2751427	1404187	1402373	2372597	2374411	1443737	1441923	2355671	2357485
1873	BPSL1320			1426915	1427106	695722	695913	1671635	1671826	1655320	1655511	2749049	2748858	1404760	1404951	2372074	2371883	1444277	1444468	2355103	2354912
1874	BPSL1321			1428661	1427189	697468	695996	1673381	1671909	1657066	1655594	2747303	2748775	1406515	1405034	2370328	2371800	1446023	1444551	2353357	2354829
1875	BPSL1322			1429778	1429380	698585	698187	1674489	1674091	1658174	1657776	2746214	2746612	1407620	1407222	2369224	2369622	1447131	1446733	2352262	2352660
1876	BPSL1323			1430247	1429813	699054	698620	1674958	1674524	1658643	1658209	2745745	2746179	1408089	1407655	2368755	2369189	1447600	1447166	2351793	2352227
1877	BPSL1323a			1430640	1430960	699447	699767	1675351	1675671	1659036	1659356	2745352	2745032	1408482	1408802	2368362	2368042	1447993	1448313	2351400	2351080
1878		BURPS1710A_1778	BURPS1710A_1778					1676206	1676006												
1879		BURPS668_1443	BURPS668_1443											1408896	1409372						
1880	BPSL1324			1431841	1433703	700648	702510	1676574	1678436	1660259	1662121	2744066	2742204	1409769	1411631	2367119	2365257	1449246	1451108	2350190	2348328
1881		BURPS406E_H1501	BURPS406E_H1501									2744618	2744463								
1882	BPSL1325			1433987	1434916	702794	703723	1678720	1679649	1662405	1663334	2741920	2740991	1411914	1412843	2364973	2364044	1451392	1452321	2348044	2347115
1883	BPSL1326			1436338	1435493	705145	704300	1681059	1680214	1664744	1663899	2739569	2740414	1414264	1413419	2362634	2363479	1453757	1452912	2345712	2346557
1884	BPSL1327			1436609	1437001	705416	705808	1681334	1681726	1665019	1665411	2739231	2738839	1414535	1414927	2362258	2361866	1454093	1454485	2345431	2345039
1885	BPSL1328			1437027	1437782	705834	706589	1681752	1682507	1665437	1666192	2738813	2738058	1414953	1415708	2361840	2361085	1454511	1455266	2345013	2344258
1886	BPSL1329			1438086	1439729	706893	708536	1682802	1684445	1666487	1668130	2737753	2736110	1416004	1417647	2360790	2359147	1455561	1457204	2343954	2342311
1887	BPSL1330			1439746	1440060	708553	708867	1684462	1684776	1668147	1668461	2736093	2735779	1417664	1417978	2359130	2358816	1457221	1457535	2342294	2341980
1888	BPSL1331			1440060	1441004	708867	709811	1684776	1685720	1668461	1669405	2735779	2734835	1417978	1418922	2358816	2357872	1457535	1458479	2341980	2341036
1889	BPSL1332			1440994	1441716	709801	710523	1685710	1686432	1669395	1670117	2734845	2734123	1418912	1419634	2357882	2357160	1458469	1459191	2341046	2340324
1890	BPSL1333			1441730	1442617	710537	711424	1686446	1687330	1670131	1671015	2734109	2733222	1419648	1420535	2357146	2356262	1459205	1460092		
1891	BPSL1334			1443142	1443909	711949	712716	1687840	1688607	1671525	1672292	2732649	2732278	1421123	1421890	2355680	2355303	1460587	1460919	2338802	2338035
1892	BPSL1335			1443909	1444844	712716	713651	1688607	1689542	1672292	1673227			1421890	1422825	2354968	2354033	1461395	1462330	2338035	2337100
1893	BPSL1336			1444941	1445483	713748	714290	1689639	1690181	1673324	1673866	2732174	2731710	1422922	1423464	2353936	2353394	1462427	1462969	2337003	2336461
1894	BPSL1337			1445991	1446902	714753	715664	1690797	1691708	1674482	1675393	2731382	2730471	1423810	1424721	2352958	2352047	1463504	1464415	2335818	2334907
1895	BPSL1338			1447339	1447965	716101	716727	1692103	1692729	1675788	1676414	2730083	2729457	1425144	1425770	2351561	2350935	1464859	1465485	2334456	2333830
1896	BPSL1339			1450009	1448525	718771	717287	1694752	1693268	1678437	1676953	2727447	2728931	1427716	1426232	2349072	2350412	1467608	1466124	2331812	2333296
1897	BPSL1340			1450544	1450873	719306	719635	1695287	1695616	1678972	1679301	2726912	2726583	1428252	1428581	2348537	2348208	1468143	1468472	2331278	2330949
1898	BPSL1341			1451972	1451253	720734	720015	1696734	1696015	1680419	1679700	2725492	2726211	1429605	1428886	2347106	2347825	1469542	1468823	2329866	2330585
1899	BPSL1342			1452476	1452033	721238	720795	1697238	1696795	1680923	1680480	2724988	2725431	1430109	1429666	2346602	2347045	1470046	1469603	2329362	2329805
1900	BPSL1343			1453285	1452476	722047	721238	1698047	1697238	1681732	1680923	2724179	2724988	1430918	1430109	2345793	2346602	1470855	1470046	2328554	2328625
1901	BPSL1344			1453306	1454109	722068	722871	1698068	1698871	1681753	1682556	2724158	2723355	1430939	1431742	2345772	2344969	1470876	1471679	2328533	2327730
1902	BPSL1345			1454196	1455854	722958	724616	1698958	1700616	1682643	1684301	2723268	2721610	1431829	1433487	2344882	2343224	1471766	1473424	2327643	2325985
1903	BPSL1346			1455940	1457229	724702	725991	1700700	1701989	1684385	1685674	2721526	2720237	1433571	1434860	2343140	2341851	1473510	1474787	2325901	2324612
1904	BPSL1347			1458481	1457342	727243	726104	1703241	1702102	1686926	1685787	2718985	2720124	1436112	1434973	2340599	2341738	1476039	1474900		
1905	BPSL1348			1458709	1459728	727471	728490	1703469	1704488	1687154	1688173	2718757	2717738	1436326	1437345	2340371	2339352	1476267	1477286	2323126	2322107
1906	BPSL1349			1460146	1461288	728908	730050	1704906	1706048	1688591	1689733	2717320	2716178	1437771	1438913	2338934	2337792	1477704	1478846	2321687	2320545
1907	BPSL1350			1461323	1461982	730085	730744	1706083	1706742	1689768	1690427	2716143	2715484	1438948	1439607	2337757	2337098	1478881	1479540	2320510	2319851

1908	BPSL1351		1462037	1465288	730799	734050	1706797	1710048	1690482	1693733	2715429	2712178	1439662	1442913	2337043	2333792	1479595	1482846	2319796	2316545
1909	BPSL1352		1465410	1465883	734172	734645	1710170	1710643	1693855	1694328	2712056	2711583	1443035	1443508	2333670	2333197	1482968	1483441	2316423	2315950
1910	BPSL1353		1465886	1466386	734648	735148	1710646	1711146	1694331	1694831	2711580	2711080	1443511	1444011	2333194	2332694	1483444	1483944	2315947	2315447
1911	BPSL1354		1467014	1466463	735776	735225	1711774	1711223	1695459	1694908	2710452	2711003	1444639	1444088	2332066	2332617	1484572	1484021	2314819	2315370
1912	BPSL1355		1467208	1467867	735970	736629	1711968	1712627	1695653	1696312	2710258	2709599	1444833	1445492	2331872	2331213	1484766	1485425	2314625	2313966
1913	BPSL1356		1468038	1469921	736800	738683	1712798	1714681	1696483	1698366	2709428	2707545	1445663	1447546	2331042	2329159	1485596	1487479	2313795	2311912
1914	BPSL1357		1470037	1470924	738799	739686	1714797	1715684	1698482	1699369	2707429	2706542	1447662	1448546	2329043	2328156	1487595	1488482	2311796	2310909
1915	BPSL1358		1470953	1472308	739715	741070	1715713	1717068	1699398	1700753	2706513	2705158	1448578	1449933	2328127	2326772	1488511	1489866	2310880	2309525
1916	BPSL1359		1472747	1473772	741509	742534	1717507	1718532	1701192	1702217	2704642	2703617	1450372	1451397	2326271	2325246			2308999	2307974
1917	BPSL1360		1473882	1474865	742644	743627	1718642	1719625	1702327	1703310	2703507	2702524	1451507	1452490	2325136	2324153	1490119	1491102	2307864	2306881
1918	BPSL1361		1474886	1475755	743648	744517	1719646	1720515	1703331	1704200	2702503	2701634	1452511	1453380	2324132	2323263	1491123	1491992	2306860	2305991
1919	BPSL1362		1475774	1476619	744536	745381	1720534	1721379	1704219	1705064	2701615	2700770	1453399	1454244	2323244	2322399	1492011	1492856	2305972	2305127
1920	BPSL1363		1476642	1477343	745404	746105	1721402	1722103	1705087	1705788	2700747	2700046	1454267	1454968	2322376	2321675	1492879	1493580	2305104	2304403
1921	BPSL1364		1477375	1478073	746137	746835	1722135	1722833	1705820	1706518	2700014	2699316	1455000	1455698	2321643	2320945	1493612	1494310	2304371	2303673
1922	BPSL1365		1478146	1479453	746908	748215	1722906	1724213	1706591	1707898	2699243	2697936	1455770	1457077	2320872	2319565	1494383	1495690	2303600	2302293
1923	BPSL1366		1481715	1479658	750477	748420	1726475	1724418	1710160	1708103	2695674	2697731	1459341	1457284	2317301	2319358	1497952	1495895	2300029	2302086
1924	BPSL1367		1481946	1483502	750708	752264	1726706	1728262	1710391	1711947	2695443	2693887	1459572	1461128	2317067	2315514	1498183	1499739	2299798	2298242
1925	BPSL1368		1484711	1483821	753473	752583	1729469	1728669	1713156	1712266	2692663	2693568	1462322	1461447	2314305	2315195	1500948	1500058	2297048	2297923
1926	BPSL1369		1484777	1485847	753539	754609	1729535	1730605	1713222	1714292	2692597	2691527	1462388	1463458	2314239	2313169	1501014	1502075	2296982	2295912
1927	BPSL1370		1485876	1486538	754638	755300	1730634	1731296	1714321	1714983	2691498	2690836	1463487	1464149	2313140	2312478	1502104	1502766	2295883	2295221
1928	BPSL1371		1487219	1486965	755981	755727	1731977	1731723	1715664	1715410	2690151	2690405	1464831	1464577	2311797	2312051	1503449	1503195	2294538	2294792
1929	BPSL1372		1488155	1487898	756917	756660	1732912	1732655	1716599	1716342	2689216	2689473			2310862	2311119	1504385	1504128	2293602	2293859
1930	BPSL1373		1488562	1488155	757324	756917	1733319	1732912	1717006	1716599	2688809	2689216	1466175	1465768	2310455	2310862	1504792	1504385	2293195	2293602
1931		BURPS1710A_1838					1739046	1735645												
1932		BURPS1710A_1839					1740355	1739615												
1933		BURPS1710A_1840					1742438	1741023												
1934		BURPS1710A_1841					1742786	1742442												
1935		BURPS1710A_1842					1745593	1742783												
1936		BURPS1710A_1843					1746489	1746361												
1937		BURPS1710A_1844					1747282	1746845												
1938		BURPS1710A_1845					1749622	1748285												
1939		BURPS1710A_1846					1751183	1749849												
1940		BURPS1710A_1847					1751786	1751193												
1941		BURPS1710A_1848					1753395	1751806												
1942		BURPS1710A_1849					1756472	1755828												
1943		BURPS1710A_1850					1758060	1758383												
1944		BURPS1710A_1851					1758729	1759343												
1945		BURPS1710A_1852					1759571	1759768												
1946		BURPS1710A_1853					1760020	1760391												
1947		BURPS1710A_1854					1760391	1760642												
1948		BURPS1710A_1855					1760899	1761186												
1949		BURPS1710A_1856					1761702	1762220												
1950		BURPS1710A_1857					1762253	1762384												
1951		BURPS1710A_1858					1762675	1763676												
1952		BURPS1710A_1859					1763673	1763819												
1953		BURPS1710A_1860					1763954	1764112												
1954		BURPS1710A_1861					1764403	1765323												
1955		BURPS1710A_1862					1765493	1765654												
1956		BURPS1710A_1863					1765651	1766214												
1957		BURPS1710A_1864					1766261	1768663												
1958		BURPS1710A_1865					1768684	1769514												
1959		BURPS1710A_1866					1770092	1770868												
1960		BURPS1710A_1867					1771014	1771229												
1961		BURPS1710A_1868					1771343	1772014												
1962		BURPS1710A_1869					1772024	1772614												
1963		BURPS1710A_1870					1772852	1772965												
1964		BURPS1710A_1871					1773258	1773455												
1965		BURPS1710A_1872					1773726	1773917												

1966	BURPS1710A_1873			1774417	1776726
1967	BURPS1710A_1874			1776767	1777585
1968	BURPS1710A_1875			1777646	1777776
1969	BURPS1710A_1876			1777737	1777916
1970	BURPS1710A_1877			1777999	1778205
1971	BURPS1710A_1878			1780466	1779000
1972	BURPS1710A_1880			1781828	1780728
1973	BURPS1710A_1881			1782052	1781828
1974	BURPS1710A_1882			1782098	1782322
1975	BURPS1710A_1883			1782335	1782613
1976	BURPS1710A_1884			1782622	1782867
1977	BURPS1710A_1885			1783911	1782940
1978	BURPS1710A_1886			1784246	1783908
1979	BURPS1710A_1887			1784400	1784236
1980	BURPS1710A_1888			1784728	1784471
1981	BURPS1710A_1889			1786653	1784725
1982	BURPS1710A_1890			1786946	1786668
1983	BURPS1710A_1891			1787341	1786946
1984	BURPS1710A_1892			1788009	1787344
1985	BURPS1710A_1893			1788730	1788023
1986	BURPS1710A_1894			1789561	1789004
1987	BURPS1710A_1895			1790107	1789832
1988	BURPS1710A_1896			1789620	1789858
1989	BURPS1710A_1897			1790609	1791502
1990	BURPS1710A_1898			1791657	1792946
1991	BURPS1710A_1899			1794733	1794515
1992	BURPS1710A_1900			1794402	1794518
1993	BURPS1710A_1901			1794909	1795388
1994	BURPS1710A_1902			1795952	1796620
1995	BURPS1710A_1903			1796625	1797761
1996	BURPS1710A_1904			1797758	1798048
1997	BURPS1710A_1905			1798083	1798904
1998	BURPS1710A_1906			1798901	1799161
1999	BURPS1710A_1907			1799330	1800307
2000	BURPS1710A_1908			1800991	1800833
2001	BURPS1710A_1909			1800304	1800855
2002	BURPS1710A_1910			1801237	1801662
2003	BURPS1710A_1911			1801665	1801997
2004	BURPS1710A_1912			1802048	1802233
2005	BURPS1710A_1913			1802242	1802889
2006	BURPS1710A_1914			1805082	1805306
2007	BURPS1710A_1915			1805645	1809106
2008	BURPS1710A_1916			1810287	1809901
2009	BURPS1710A_1917			1810541	1810284
2010	BURPS1710A_1918			1810602	1810958
2011	BURPS1710A_1920			1811106	1811591
2012	BURPS1710A_1921			1811601	1813316
2013	BURPS1710A_1922			1813313	1814626
2014	BURPS1710A_1923			1814607	1815293
2015	BURPS1710A_1924			1815296	1816552
2016	BURPS1710A_1925			1816844	1817206
2017	BURPS1710A_1926			1817394	1817537
2018	BURPS1710A_1927			1817530	1817952
2019	BURPS1710A_1928			1817949	1818296
2020	BURPS1710A_1929			1818358	1818816
2021	BURPS1710A_1930			1818844	1819308
2022	BURPS1710A_1931			1819308	1819592
2023	BURPS1710A_1932			1819606	1823670

2024	BURPS1710A_1933			1823640	1824005							
2025	BURPS1710A_1934			1824014	1825402							
2026	BURPS1710A_1935			1825399	1826082							
2027	BURPS1710A_1936			1826132	1826884							
2028	BURPS1710A_1937			1826881	1827465							
2029	BURPS1710A_1938			1827462	1830767							
2030	BURPS1710A_1939			1830872	1831078							
2031	BURPS1710A_1940			1831078	1831812							
2032	BURPS1710A_1941			1832114	1832551							
2033	BURPS1710A_1942			1832593	1833096							
2034	BURPS1710A_1943			1833239	1834027							
2035	BURPS1710A_1944			1834280	1835014							
2036	BURPS1710A_1945			1836515	1837606							
2037	BURPS1710A_1946			1838200	1837739							
2038	BURPS1710A_1947			1838592	1839137							
2039	BURPS1710b_1634					1719332	1722733					
2040	BURPS1710b_1635					1723302	1724042					
2041	BURPS1710b_1636					1724710	1726125					
2042	BURPS1710b_1637					1726129	1726473					
2043	BURPS1710b_1638					1726470	1729280					
2044	BURPS1710b_1639					1729277	1729717					
2045	BURPS1710b_1640					1733536	1734831					
2046	BURPS1710b_1641					1734880	1735473					
2047	BURPS1710b_1642					1735493	1737082					
2048	BURPS1710b_1643					1739515	1740384					
2049	BURPS1710b_1644					1740980	1741699					
2050	BURPS1710b_1645					1743707	1744078					
2051	BURPS1710b_1646					1744078	1744329					
2052	BURPS1710b_1647					1746068	1747363					
2053	BURPS1710b_1648					1748090	1749010					
2054	BURPS1710b_1649					1750026	1752350					
2055	BURPS1710b_1650					1752347	1753201					
2056	BURPS1710b_1651					1754913	1755701					
2057	BURPS1710b_1652					1758105	1760414					
2058	BURPS1710b_1653					1762688	1764136					
2059	BURPS1710b_1655					1764416	1765516					
2060	BURPS1710b_1656					1766023	1766301					
2061	BURPS1710b_1657					1766280	1766555					
2062	BURPS1710b_1658					1766628	1767638					
2063	BURPS1710b_1659					1768413	1770386					
2064	BURPS1710b_1660					1770634	1771029					
2065	BURPS1710b_1661					1771032	1771697					
2066	BURPS1710b_1662					1771711	1772505					
2067	BURPS1710b_1663					1772692	1773717					
2068	BURPS1710b_1664					1774932	1775462					
2069	BURPS1710b_1665					1775514	1775621					
2070	BURPS1710b_1666					1776280	1777434					
2071	BURPS1710b_1667					1777544	1778878					
2072	BURPS1710b_1668					1780841	1781320					
2073	BURPS1710b_1669					1781335	1782552					
2074	BURPS1710b_1670					1782557	1783693					
2075	BURPS1710b_1671					1784015	1784836					
2076	BURPS1710b_1672					1785304	1786239					
2077	BURPS1710b_1673					1786236	1786787					
2078	BURPS1710b_1674					1788174	1788821					
2079	BURPS1710b_1675					1791577	1795038					
2080	BURPS1710b_1676					1795833	1796300					
2081	BURPS1710b_1677					1796534	1796890					

2082		BURPS1710b_1678								1797038	1797523										
2083		BURPS1710b_1679								1797524	1799248										
2084		BURPS1710b_1680								1799308	1800558										
2085		BURPS1710b_1681								1800539	1801225										
2086		BURPS1710b_1682								1801228	1802484										
2087		BURPS1710b_1683								1802776	1803138										
2088		BURPS1710b_1684								1803462	1803884										
2089		BURPS1710b_1685								1803881	1804228										
2090		BURPS1710b_1686								1804290	1804748										
2091		BURPS1710b_1687								1804776	1805240										
2092		BURPS1710b_1688								1805538	1809602										
2093		BURPS1710b_1689								1809599	1809937										
2094		BURPS1710b_1690								1809946	1811334										
2095		BURPS1710b_1691								1811331	1812014										
2096		BURPS1710b_1692								1812064	1812816										
2097		BURPS1710b_1693								1812831	1813397										
2098		BURPS1710b_1694								1813421	1816699										
2099		BURPS1710b_1695								1817992	1818483										
2100		BURPS1710b_1696								1818483	1819028										
2101		BURPS1710b_1697								1819171	1819959										
2102		BURPS1710b_1698								1820212	1820946										
2103		BURPS1710b_1699								1822420	1823538										
2104		BURPS1710b_1700								1823671	1824312										
2105		BURPS1710b_1701								1824368	1825069										
2106	BPSL1374			1489124	1489582	757886	758344	1839573	1840037	1825505	1825969	2688248	2687790	1466737	1467195	2309892	2309434	1505353	1505811	2292633	2292175
2107	BPSL1375			1489737	1490552	758499	759314	1840192	1841007	1826124	1826939	2687635	2686820	1467350	1468165	2309279	2308464	1505966	1506781	2292020	2291205
2108	BPSL1376			1491152	1490892	759914	759654	1841625	1841365	1827557	1827297	2686220	2686480	1468764	1468504	2307847	2308107	1507380	1507120	2290606	2290866
2109	BPSL1377			1491227	1491415	759989	760177	1841700	1841888	1827632	1827820	2686145	2685957	1468839	1469027	2307772	2307584	1507455	1507643	2290531	2290343
2110	BPSL1378			1493063	1491675	761825	760437	1843551	1842163	1829483	1828095	2684309	2685697	1470681	1469293	2305928	2307316	1509292	1507904	2288680	2290068
2111	BPSL1379			1493421	1493074	762183	761836	1843909	1843562	1829841	1829494	2683951	2684298	1471039	1470692	2305570	2305917	1509650	1509303	2288322	2288669
2112	BPSL1380			1495320	1493656	764082	762418	1845841	1844177	1831773	1830109	2682052	2683716	1472971	1471307	2303638	2305302	1511582	1509918	2286390	2288051
2113	BPSL1381			1496347	1495637	765109	764399	1846868	1846158	1832800	1832090	2681025	2681735	1474003	1473284	2302611	2303321	1512609	1511899	2285363	2286073
2114	BPSL1382			1497540	1496635	766302	765397	1848061	1847156	1833993	1833088	2679832	2680737	1475195	1474290	2301418	2302323	1513802	1512897	2284170	2285075
2115	BPSL1383			1498088	1498915	766850	767677	1848609	1849436	1834541	1835368	2679284	2678457	1475743	1476570	2300870	2300043	1514350	1515177	2283622	2282795
2117		BURPS1710A_1962						1850635	1850925												
2118		BURPS1710A_1963						1852962	1855736												
2119		BURPS1710A_1964						1855822	1857399												
2120		BURPS1710A_1965						1857783	1858013												
2121		BURPS1710A_1966						1858866	1859102												
2122		BURPS1710A_1967						1859600	1860001												
2123		BURPS1710A_1968						1861599	1860778												
2124		BURPS1710A_1969						1862046	1861786												
2125			Bp_chr1_17_IS407A					1862107	1863342	1848039	1849274										
2126		BURPS1710A_1972						1863798	1864904												
2127		BURPS1710A_1973						1866663	1866544												
2128		BURPS1710b_1715								1836567	1836857										
2129		BURPS1710b_1716								1838894	1841668										
2130		BURPS1710b_1717								1841754	1843331										
2131		BURPS1710b_1718								1845532	1845933										
2132		BURPS1710b_1721								1851765	1852124										
2133		BURPS1710b_1722								1853156	1854175										
2134		BURPS668_1515												1478230	1478352						
2135		BURPS668_1516												1478529	1478657						
2136		BURPS668_1517												1480168	1482942						
2137		BURPS668_1518												1483430	1484104						
2138		BURPS668_1519												1484471	1484611						
2139		BURPS668_1520												1484811	1484960						
2140		BURPS668_1521												1485258	1485413						

2141		BURPS668_1522												1487372	1487830						
2142		BURPS668_1523												1486968	1487375						
2143			Bp_chr1_18_ISBma2											1488589	1490161						
2144	BPSL1384		GI7									2677774	2678142	1490529	1490212						
2145	BPSL1384a		GI7	1499606	1499809	768368	768571	1868796	1868999	1854728	1854931	2677766	2677563	1490537	1490740	2299352	2299149	1515868	1516071	2282104	2281901
2146	BPSL1385		GI7	1500546	1499836	769308	768598	1869736	1869026	1855668	1854958	2676826	2677536	1491477	1490767	2298412	2299122	1516808	1516098	2281164	2281874
2147		BURPS1106B_A0794				770256	769402														
2148		BURPS1106B_A0795				770540	770250														
2149		BURPS1710A_1977						1870684	1869830												
2150		BURPS1710A_1978						1870968	1870678												
2151	BPSL1386		GI7	1500614	1500558	771035	770583	1871463	1871011	1855736	1855680	2676311	2676814	1491992	1491489	2296685	2297137	1517323	1516820	2280649	2281152
2152		BURPS1106A_1549		1500640	1501494																
2153		BURPS1106A_1550		1501488	1501778																
2154		BURPS1710b_1724								1855762	1856616										
2155		BURPS1710b_1725								1856610	1856915										
2156		BURPSPAST_AA0188														2297467	2298318				
2157		BURPSPAST_AA0189														2297207	2297470				
2158	BPSL1387		GI7	1502694	1502248	771456	771010	1871884	1871438	1857816	1857370	2675890	2676336	1492413	1491967	2296264	2296710	1517744	1517298	2280228	2280674
2159	BPSL1388		GI7	1503214	1502714	771976	771476	1872404	1871904	1858336	1857836	2675370	2675870	1492933	1492433	2295744	2296244	1518264	1517764	1392320	1391820
2160	BPSL1389		GI7	1503639	1503220	772401	771982	1872829	1872410	2765812	2766234	2674945	2675364	1493358	1492936	2295322	2295741	1518686	1518267	1392745	1392323
2161	BPSL1390		GI7	2351529	2351921	1619466	1619858	2781245	2781637	2765136	2765528	1790548	1790156	2317827	2318219	1391535	1391143	2365170	2365562	1393421	1393029
2162		BURPS1106A_2384		2353643	2354077																
2163		BURPS1106B_A1615				1621868	1622014														
2164		BURPS1710A_2887						2783134	2783793												
2165		BURPS668_2343												2319932	2320375						
2166	BPSL1391		GI7	2351275	2351051	1619212	1618988	2780991	2780767	2764882	2764658	1790802	1791026	2317576	2317352	1391789	1392013	2364916	2364692	1393671	1393895
2167	BPSL1392		GI7	2350779	2351009	1618716	1618946	2780495	2780725	2764386	2764616	1791298	1791068	2317074	2317304	1392285	1392055	2364420	2364650	1394167	1393937
2168	BPSL1393		GI7	2350197	2350700	1618134	1618637	2779913	2780416	2763804	2764307	1791850	1791377	2316492	2316995	1392867	1392364	2363868	2364341	1394749	1394246
2169	BPSL1394			2348372	2348992	1616309	1616929	2778087	2778707	2761978	2762598	1793707	1793087	2314738	2315358	1394692	1394072	2362009	2362629	1396575	1395955
2170	BPSL1395			2347026	2348174	1614963	1616111	2776741	2777889	2760632	2761780	1795053	1793905	2313392	2314540	1396038	1394890	2360663	2361811	1397921	1396773
2171	BPSL1396			2345818	2347008	1613755	1614945	2775533	2776723	2759424	2760614	1796261	1795071	2312184	2313374	1397246	1396056	2359455	2360645	1399129	1397939
2172	BPSL1397			2344727	2345791	1612664	1613728	2774442	2775506	2758333	2759397	1797352	1796288	2311093	2312157	1398337	1397273	2358364	2359428	1400220	1399156
2173	BPSL1398			2344396	2343251	1612333	1611188	2774111	2772966	2758002	2756857	1797683	1798828	2310761	2309616	1398668	1399813	2358033	2356888	1400551	1401696
2174	BPSL1399			2343149	2342166	1611086	1610103	2772864	2771893	2756755	2755784	1798930	1799913	2309514	2308519	1399915	1400886	2356786	2355815	1401798	1402781
2175	BPSL1400			2341324	2341770	1609261	1609707	2771053	2771499	2754944	2755390	1800753	1800307	2307679	2308125	1401726	1401280	2354975	2355421	1403621	1403175
2176	BPSL1401			2340058	2341218	1607995	1609155	2769787	2770947	2753678	2754838	1802019	1800859	2306407	2307573	1402992	1401832	2353709	2354869	1404886	1403726
2177	BPSL1402			2339616	2338270	1607553	1606207	2769338	2767992	2753229	2751883	1802468	1803814	2305957	2304611	1403441	1404787	2353267	2351921	1405339	1406685
2178	BPSL1403			2338132	2337482	1606069	1605419	2767854	2767204	2751745	2751095	1803952	1804602	2304473	2303823	1404925	1405575	2351783	2351133	1406823	1407473
2179	BPSL1404			2337339	2336050	1605276	1603987	2767061	2765772	2750952	2749663	1804745	1806034	2303680	2302391	1405718	1407007	2350990	2349701	1407616	1408905
2180	BPSL1405			2335851	2333437	1603788	1601374	2765573	2763159	2749464	2747050	1806233	1808647	2302192	2299778	1407206	1409620	2349502	2347088	1409104	1411518
2181	BPSL1406			2332073	2332399	1600010	1600336	2761797	2762123	2745688	2746014	1810009	1809683	2298417	2298743	1410983	1410657			1412880	1412554
2182	BPSL1406A			2331890	2331675	1599827	1599612	2761614	2761399	2745505	2745290					1411166	1411381			1413063	1413278
2183	BPSL1407			2331597	2331184	1599534	1599121	2761321	2760908	2745212	2744799	1810470	1810883	2297943	2297530	1411459	1411872			1413356	1413769
2184	BPSL1408			2330916	2330776	1598853	1598713	2760640	2760500	2744531	2744391	1811151	1811291	2297259	2297119	1412140	1412280			1414038	1414178
2185	BPSL1409			2330040	2329831	1597977	1597768	2759785	2759576	2743676	2743467	1812014	1812223	2296405	2296211	1412995	1413204	2344078	2343884	1414901	1415095
2186		BURPSS13_P0973																2344834	2346096		
2187	BPSL1410			2329002	2327071	1596939	1595008	2758842	2756911	2742733	2740802	1812965	1814896	2295437	2293506	1413946	1415877	2343136	2341205	1415821	1417752
2188			Bp_chr1_19_ISBma2																	1417823	1419395
2189	BPSL1411			2325974	2326645	1593911	1594582	2755726	2756397	2739617	2740288	1815955	1815284	2292393	2293064	1417022	1416351	2340146	2340817	1420404	1419733
2190		BURPS1655_A1415																		1421534	1421262
2191	BPSL1412			2325984	2325262	1593921	1593199	2755736	2755014	2739627	2738905	1815945	1816667	2292403	2291681	1417012	1417734	2340156	2339434	1420394	1421116
2192	BPSL1413			2323241	2324860	1591178	1592797	2753033	2754652	2736924	2738543	1818664	1817045	2289571	2291190	1419691	1418072	2337413	2339032	1423153	1421534
2193	BPSL1414			2321543	2323135	1589480	1591072	2751335	2752927	2735226	2736818	1820362	1818770	2287873	2289465	1421389	1419797	2335715	2337307	1424851	1423259
2194	BPSL1415			2320191	2321492	1588128	1589429	2749983	2751284	2733874	2735175	1821714	1820413	2286521	2287822	1422741	1421440	2334363	2335664	1426203	1424902
2195	BPSL1416			2319935	2315859	1587872	1583796	2749727	2745660	2733618	2729551	1821970	1826037	2286265	2282198	1422997	1427055	2334107	2330040	1426459	1430526
2196	BPSL1417			2315851	2315321	1583788	1583258	2745652	2745122	2729543	2729013	1826045	1826575	2282190	2281660	1427063	1427593	2330032	2329502	1430534	1431064
2197	BPSL1418			2314441	2315217	1582378	1583154	2744242	2745018	2728133	2728909	1827455	1826679	2280780	2281556	1428473	1427697	2328622	2329398	1431944	1431168
2198	BPSL1419			2314102	2314413	1582039	1582350	2743903	2744214	2727794	2728105	1827794	1827483	2280441	2280752	1428812	1428501	2328283	2328594	1432283	1431972

2199	BPSL1420		2313569	2314096	1581506	1582033	2743370	2743897	2727261	2727788	1828327	1827800	2279908	2280435	1429345	1428818	2327750	2328277	1432816	1432289
2200	BPSL1421		2313045	2313473	1580982	1581410	2742846	2743274	2726737	2727165	1828851	1828423	2279384	2279812	1429869	1429441	2327226	2327654	1433340	1432912
2201	BPSL1422		2311417	2312991	1579354	1580928	2741218	2742792	2725109	2726683	1830479	1828905	2277756	2279330	1431497	1429923	2325598	2327172	1434968	1433394
2202	BPSL1423		2311301	2309664	1579238	1577601	2741102	2739459	2724993	2723350	1830595	1832232	2277640	2276003	1431613	1433256	2325482	2323845	1435084	1436721
2203	BPSL1424		2309417	2307336	1577354	1575273	2739204	2737123	2723095	2721014	1832479	1834560	2275744	2273663	1433511	1435592	2323598	2321517	1436968	1439049
2204	BPSL1425		2307291	2306656	1575228	1574593	2737078	2736443	2720969	2720334	1834605	1835240	2273618	2272983	1435637	1436272	2321472	2320837	1439094	1439729
2205	BPSL1426		2306594	2305419	1574531	1573356	2736381	2735206	2720272	2719097	1835302	1836477	2272921	2271746	1436334	1437509	2320775	2319600	1439791	1440966
2206	BPSL1427		2304503	2305039	1572440	1572976	2734314	2734835	2718205	2718726	1837393	1836857	2270830	2271366	1438342	1437806	2318684	2319220	1441882	1441346
2207	BPSL1428		2303826	2303515	1571763	1571452	2733620	2733309	2717511	2717200	1838070	1838381	2270153	2269842	1438892	1439203	2318007	2317696	1442562	1442873
2208	BPSL1429		2303515	2303036	1571452	1570973	2733309	2732830	2717200	2716721	1838381	1838860	2269842	2269363	1439203	1439682	2317696	2317217	1442873	1443352
2209	BPSL1430		2302941	2301946	1570878	1569883	2732735	2731740	2716626	2715631	1838955	1839950	2269268	2268273	1439777	1440772	2317122	2316127	1443447	1444442
2210	BPSL1431		2301873	2300881	1569810	1568818	2731667	2730675	2715558	2714566	1840023	1841015	2268200	2267208	1440845	1441837	2316054	2315062	1444515	1445507
2211	BPSL1432		2300506	2300069	1568443	1568006	2730308	2729871	2714199	2713762	1841382	1841819	2266832	2266395	1442204	1442641	2314695	2314258	1445881	1446318
2212	BPSL1433		2299179	2300027	1567116	1567964	2728981	2729829	2712872	2713720	1842709	1841861	2265505	2266353	1443531	1442683	2313368	2314216	1447208	1446360
2213	BPSL1434		2298155	2299105	1566092	1567042	2727957	2728907	2711848	2712798	1843733	1842783	2264481	2265431	1444555	1443605	2312344	2313294	1448232	1447282
2214	BPSL1435		2297983	2296967	1565920	1564904	2727785	2726769	2711676	2710660	1843905	1844921	2264309	2263293	1444727	1445743	2312172	2311156	1448404	1449420
2215	BPSL1436		2296963	2296346	1564900	1564283	2726765	2726148	2710656	2710039	1844925	1845542	2263289	2262672	1445747	1446364	2311152	2310535	1449424	1450041
2216	BPSL1437		2296323	2295292	1564260	1563229	2726125	2725094	2710016	2708985	1845565	1846596	2262649	2261618	1446387	1447418	2310512	2309481	1450064	1451095
2217	BPSL1438		2295246	2294743	1563183	1562680	2725048	2724545	2708939	2708436	1846642	1847145	2261572	2261069	1447464	1447967	2309435	2308932	1451141	1451644
2218	BPSL1439		2294664	2293858	1562601	1561795	2724466	2723660	2708357	2707551	1847224	1848030	2260990	2260184	1448046	1448852	2308853	2308047	1451723	1452529
2219	BPSL1440		2293844	2293119	1561781	1561056	2723646	2722921	2707537	2706812	1848044	1848763	2260170	2259451	1448866	1449591	2308033	2307314	1452543	1453262
2220	BPSL1441		2293039	2292692	1560976	1560629	2722841	2722494	2706732	2706385	1848843	1849190	2259371	2259024	1449671	1450018	2307234	2306887	1453342	1453689
2221	BPSL1442		2292622	2291438	1560559	1559375	2722424	2721240	2706315	2705131	1849260	1850444	2258954	2257770	1450088	1451272	2306817	2305633	1453759	1454943
2222	BPSL1443		2291233	2289980	1559170	1557917	2721053	2719800	2704944	2703691	1850571	1851878	2257583	2256330	1451459	1452712	2305464	2304211	1455070	1456401
2223	BPSL1444		2289601	2288540	1557538	1556477	2719420	2718359	2703311	2702250	1852258	1853319	2255951	2254890	1453092	1454153	2303831	2302770	1456793	1457854
2224	BPSL1445		2287742	2288326	1555679	1556263	2717561	2718145	2701452	2702036	1854117	1853533	2254092	2254676	1454951	1454367	2301972	2302556	1458652	1458068
2225	BPSL1446		2287004	2287501	1554941	1555438	2716823	2717320	2700714	2701211	1854867	1854358	2253342	2253851	1455689	1455192	2301234	2301731	1459390	1458893
2226	BPSL1447		2286528	2286815	1554465	1554752	2716347	2716634	2700238	2700525	1855333	1855046	2252876	2253163	1456165	1455878	2300759	2301046	1459866	1459579
2227	BPSL1448		2286030	2284990	1553967	1552927	2715849	2714809	2699740	2698700	1855831	1856871	2252378	2251338	1456663	1457703	2300261	2299221	1460364	1461404
2228	BPSL1449		2284341	2284808	1552278	1552745	2714160	2714627	2698051	2698518	1857520	1857053	2250689	2251156	1458352	1457885	2298572	2299039	1462053	1461586
2229	BPSL1450		2282664	2283566	1550601	1551503	2712496	2713398	2696387	2697289	1859184	1858282	2249024	2249926	1460016	1459114	2296908	2297810	1463691	1462789
2230	BPSL1451		2281778	2282563	1549715	1550500	2711610	2712395	2695501	2696286	1860070	1859285	2248138	2248923	1460902	1460117	2296022	2296807	1464577	1463792
2231	BPSL1452		2279886	2281658	1547823	1549595	2709718	2711490	2693609	2695381	1861962	1860190	2246246	2248018	1462794	1461022	2294130	2295902	1466439	1464697
2232	BPSL1453		2279723	2278815	1547660	1546752	2709555	2708647	2693446	2692538	1862125	1863033	2246083	2245175	1462957	1463865	2293968	2293060	1466636	1467544
2233	BPSL1454		2278087	2278509	1546016	1546438	2708055	2708477	2691946	2692368	1863606	1863184	2244577	2244999	1464457	1464035	2292416	2292838	1468083	1467688
2234	BPSL1455		2277156	2278073	1545085	1546002	2706435	2707352	2691015	2691932	1864537	1863620	2243646	2244563	1465388	1464471	2291485	2292402	1469042	1468125
2235		BURPS1710A_2794					2707540	2707361												
2236	BPSL1456		2276950	2276246	1544879	1544175	2706229	2705525	2690809	2690105	1864743	1865447	2243440	2242736	1465594	1466298	2291279	2290575	1469248	1469952
2237	BPSL1457		2276046	2275117	1543975	1543046	2705325	2704396	2689905	2688976			2242743	2241595	1466498	1467427	2290375	2289566	1470152	1470982
2238	BPSL1458		2274879	2274508	1542808	1542437	2704158	2703787	2688738	2688367	1866805	1867176	2241357	2240986	1467665	1468036	2289328	2288957	1471220	1471591
2239	BPSL1459		2274468	2274172	1542397	1542101	2703747	2703451	2688327	2688031	1867216	1867512	2240946	2240650	1468076	1468372	2288917	2288621	1471631	1471927
2240	BPSL1460		2274166	2273894	1542095	1541823	2703445	2703173	2688025	2687753	1867518	1867790	2240644	2240372	1468378	1468650	2288615	2288343	1471933	1472205
2241	BPSL1461		2273863	2273414	1541792	1541343	2703142	2702693	2687722	2687273	1867821	1868270	2240341	2239892	1468681	1469130	2288312	2287863	1472236	1472685
2242	BPSL1462		2273267	2271888	1541196	1539817	2702546	2701167	2687126	2685747	1868417	1869796	2239745	2238366	1469277	1470656	2287716	2286337	1472832	1474211
2243	BPSL1463		2271746	2271123	1539675	1539052	2701025	2700402	2685605	2684982	1869938	1870561	2238224	2237601	1470798	1471421	2286195	2285572	1474353	1474976
2244	BPSL1464		2271109	2270102	1539038	1538031	2700388	2699381	2684968	2683961	1870575	1871582	2237587	2236580	1471435	1472442	2285558	2284551	1474990	1475997
2245	BPSL1465		2268649	2269860	1536578	1537789	2697919	2699139	2682499	2683719	1873145	1872183	2235127	2236338	1473895	1472684	2283099	2284310	1477458	1476238
2246	BPSL1466		2267018	2268406	1534947	1536335	2696288	2697676	2680868	2682256	1874776	1873388	2233496	2234884	1475526	1474138	2281468	2282856	1479089	1477701
2247	BPSL1467		2265823	2266281	1533752	1534210	2695093	2695551	2679673	2680131	1875971	1875513	2232301	2232759	1476721	1476263	2280273	2280731	1480284	1479826
2248	BPSL1468		2264837	2265730	1532766	1533659	2694107	2695000	2678687	2679580	1876957	1876064	2231315	2232208	1477707	1476814	2279287	2280180	1481270	1480377
2249	BPSL1469		2262823	2264820	1530752	1532749	2692092	2694089	2676672	2678669	1878971	1876974	2229300	2231297	1479721	1477724	2277273	2279270	1483285	1481288
2250	BPSL1471		2261819	2262823	1529748	1530752	2691088	2692092	2675668	2676672	1879975	1878971	2228296	2229300	1480725	1479721	2276269	2277273	1484289	1483285
2251	BPSL1472		2260661	2261809	1528590	1529738	2689930	2691078	2674510	2675658	1881133	1879985	2227138	2228286	1481883	1480735	2275111	2276259	1485447	1484299
2252	BPSL1473		2260163	2260531	1528092	1528460	2689432	2689800	2674012	2674380	1881631	1881263	2226640	2227008	1482381	1482013	2274613	2274981	1485945	1485577
2253	BPSL1474		2258436	2260139	1526365	1528068	2687705	2689408	2672285	2673988	1883358	1881655	2224913	2226616	1484108	1482405	2272886	2274589	1487672	1485969
2254	BPSL1475		2257893	2258264	1525822	1526193	2687162	2687533	2671742	2672113	1883901	1883530	2224370	2224741	1484651	1484280	2272343	2272714	1488215	1487844
2255	BPSL1476		2257554	2256319	1525483	1524248	2686823	2685588	2671403	2670168	1884240	1885475	2224031	2222796	1484990	1486225	2272004	2270769	1488554	1489789
2256	BPSL1477		2256271	2254946	1524200	1522875	2685540	2684215	2670120	2668795	1885523	1886848	2222748	2221423	1486273	1487598	2270721	2269396	1489837	1491162

2257	BPSL1478		2254935	2253487	1522864	1521416	2684204	2682756	2668784	2667336	1886859	1888307	2221412	2219964	1487609	1489057	2269385	2267937	1491173	1492621
2258	BPSL1479		2252722	2251397	1520651	1519326	2681997	2680672	2666578	2665253	1889623	1890948	2219189	2217864	1489867	1491192	2267154	2265829	1493473	1494798
2259	BPSL1480		2251372	2251118	1519301	1519047	2680647	2680393	2665228	2664974	1890973	1891227	2217839	2217585	1491217	1491471	2265804	2265550	1494823	1495077
2260	BPSL1481		2251104	2250619	1519033	1518548	2680379	2679894	2664960	2664475	1891241	1891726	2217571	2217086	1491485	1491970	2265536	2265051	1495091	1495576
2261	BPSL1482		2249746	2250285	1517675	1518214	2679021	2679560	2663602	2664141	1892599	1892060	2216214	2216753	1492843	1492304	2264178	2264717	1496449	1495910
2262	BPSL1483		2249562	2249068	1517491	1516997	2678837	2678343	2663418	2662924	1892783	1893277	2216030	2215536	1493027	1493521	2263994	2263500	1496633	1497127
2263	BPSL1484		2248894	2246267	1516823	1514196	2678169	2675542	2662750	2660123	1893451	1896078	2215362	2212735	1493695	1496322	2263326	2260699	1497301	1499928
2264	BPSL1485		2245593	2246012	1513522	1513941	2674873	2675292	2659454	2659873	1896747	1896328	2212061	2212480	1496996	1496577	2260025	2260444	1500602	1500183
2265	BPSL1486		2244819	2245511	1512748	1513440	2674099	2674791	2658680	2659372	1897521	1896829	2211287	2211979	1497770	1497078	2259251	2259943	1501376	1500684
2266	BPSL1487		2244550	2244059	1512479	1511988	2673814	2673323	2658395	2657904	1897806	1898297	2211010	2210519	1498039	1498530	2258982	2258491	1501653	1502144
2267	BPSL1488		2244107	2243712	1512036	1511641	2673371	2672976	2657952	2657557	1898249	1898644	2210567	2210172	1498482	1498877	2258539	2258144	1502096	1502491
2268	BPSL1489		2241963	2243315	1509892	1511244	2671227	2672579	2655808	2657160	1900393	1899041	2208423	2209775	1500626	1499274	2256395	2257747	1504241	1502889
2269	BPSL1490		2240121	2241956	1508050	1509885	2669385	2671220	2653966	2655801	1902235	1900400	2206581	2208416	1502468	1500633	2254553	2256388	1506083	1504248
2270	BPSL1491		2239724	2239984	1507653	1507913	2668988	2669248	2653569	2653829	1902632	1902372	2206184	2206444	1502865	1502605	2254156	2254416	1506481	1506221
2271	BPSL1492		2238664	2239506	1506593	1507435	2667928	2668770	2652509	2653351	1903692	1902850	2205124	2205966	1503925	1503083	2253096	2253938	1507541	1506699
2272	BPSL1493		2237065	2238618	1504994	1506547	2666329	2667882	2650910	2652463	1905291	1903738	2203525	2205078	1505524	1503971	2251497	2253050	1509140	1507587
2273	BPSL1494		2236568	2237065	1504497	1504994	2665832	2666329	2650413	2650910	1905788	1905291	2203028	2203525	1506021	1505524	2251000	2251497	1509637	1509140
2274	BPSL1495		2235169	2236428	1503098	1504357	2664433	2665692	2649014	2650273	1907187	1905928	2201630	2202889	1507420	1506161	2249601	2250860	1511036	1509777
2275	BPSL1496		2234980	2235102	1502909	1503031	2664244	2664366	2648825	2648947	1907376	1907254	2201441	2201563	1507609	1507487	2249412	2249534	1511225	1511103
2276	BPSL1497		2234647	2234970	1502576	1502899	2663911	2664234	2648492	2648815	1907709	1907386	2201108	2201431	1507942	1507619	2249079	2249402	1511558	1511235
2277	BPSL1498		2233983	2231509	1501912	1499438	2663243	2660778	2647824	2645359	1908376	1909971	2200444	2197967	1509962	1510939				
2278	BPSL1499		2231409	2231086	1499338	1499015	2660678	2660355	2645259	2644936	1910921	1911244	2197867	2197544	1511039	1511362	2245982	2245659	1514430	1514753
2279	BPSL1500		2231057	2230452	1498986	1498381	2660326	2659721	2644907	2644302	1911273	1911878	2197515	2196910	1511391	1511996	2245630	2245025	1514782	1515387
2280	BPSL1501		2230379	2229177	1498308	1497106	2659648	2658446	2644229	2643027	1911951	1913153	2196837	2195635	1512069	1513271	2244952	2243750	1515460	1516662
2281	BPSL1502		2229069	2228311	1496998	1496240	2658338	2657580	2642919	2642161	1913261	1914019	2195527	2194769	1513379	1514137	2243642	2242884	1516770	1517528
2282	BPSL1503		2228311	2227346	1496240	1495275	2657580	2656615	2642161	2641196	1914019	1914984	2194769	2193804	1514137	1515102	2242884	2241919	1517528	1518493
2283	BPSL1504		2227337	2226444	1495266	1494373	2656606	2655713	2641187	2640294	1914993	1915886	2193795	2192902	1515111	1516004	2241910	2241017	1518502	1519395
2284	BPSL1505		2226429	2225353	1494358	1493282	2655698	2654622	2640279	2639203	1915901	1916977	2192887	2191811	1516019	1517095	2241002	2239926	1519410	1520486
2285	BPSL1506		2225342	2224569	1493271	1492498	2654611	2653838	2639192	2638419	1916988	1917761	2191800	2191027	1517106	1517879	2239915	2239142	1520497	1521270
2286	BPSL1507		2223517	2224374	1491446	1492303	2652801	2653658	2637382	2638239	1918823	1917966	2189980	2190837	1518916	1518059	2238105	2238962	1522317	1521460
2287	BPSL1508		2223439	2222045	1491368	1489974	2652723	2651329	2637304	2635910	1918901	1920295	2189902	2188508	1518994	1520388	2238027	2236633	1522395	1523789
2288	BPSL1509		2221255	2221965	1489184	1489894	2650539	2651249	2635120	2635830	1921085	1920375	2187698	2188408	1521178	1520468	2235843	2236553	1524600	1523890
2289	BPSL1510		2221021	2220599	1488950	1488528	2650305	2649883	2634886	2634464	1921319	1921741	2187464	2187042	1521412	1521834	2235609	2235187	1524834	1525256
2290	BPSL1511		2220554	2219421	1488483	1487350	2649838	2648705	2634419	2633286	1921786	1922919	2186997	2185864	1521879	1523012	2235142	2234009	1525301	1526434
2291	BPSL1512		2219276	2218191	1487205	1486120	2648560	2647475	2633141	2632056	1923064	1924149	2185719	2184634	1523857	1524243	2233864	2232779	1526579	1527664
2292	BPSL1513		2218044	2216797	1485973	1484726	2647328	2646081	2631909	2630662	1924296	1925543	2184487	2183240	1524390	1525637	2232632	2231385	1527811	1529058
2293	BPSL1514		2216775	2215438	1484704	1483367	2646059	2644722	2630640	2629303	1925565	1926902	2183218	2181881	1525659	1526996	2231363	2230026	1529080	1530417
2294	BPSL1515		2215382	2214756	1483311	1482685	2644666	2644040	2629247	2628621	1926958	1927584	2181825	2181199	1527052	1527678	2229970	2229344	1530473	1531099
2295	BPSL1516		2214708	2213566	1482637	1481495	2643992	2642850	2628573	2627431	1927632	1928774	2181151	2180009	1527726	1528868	2229296	2228154	1531147	1532289
2296	BPSL1517		2213287	2211953	1481216	1479882	2642571	2641237	2627152	2625818	1929053	1930387	2179730	2178396	1529147	1530481	2227875	2226541	1532567	1533901
2297	BPSL1518		2211815	2211579	1479744	1479508	2641099	2640863	2625680	2625444	1930525	1930761	2178258	2178022	1530619	1530855	2226403	2226167	1534039	1534275
2298	BPSL1519		2211519	2210344	1479448	1478273	2640803	2639628	2625384	2624209	1930821	1931996	2177962	2176787	1530915	1532090	2226107	2224932	1534335	1535510
2299	BPSL1520		2210295	2208934	1478224	1476863	2639579	2638218	2624160	2622799	1932045	1933406	2176738	2175377	1532139	1533500	2224883	2223522	1535559	1536920
2300	BPSL1521		2208914	2208018	1476843	1475947	2638198	2637302	2622779	2621883	1933426	1934322	2175357	2174461	1533520	1534416	2223502	2222606	1536940	1537836
2301	BPSL1522		2207990	2207802	1475919	1475731	2637274	2637086	2621855	2621667	1934350	1934538	2174433	2174245	1534444	1534632	2222578	2222390	1537864	1538052
2302	BPSL1523		2207687	2206542	1475616	1474471	2636971	2635826	2621552	2620407	1934653	1935798	2174130	2172985	1534747	1535892	2222275	2221130	1538167	1539312
2303	BPSL1524		2206414	2205071	1474343	1473000	2635698	2634355	2620279	2618936	1935926	1937269	2172857	2171514	1536020	1537363	2221002	2219659	1539440	1540783
2304	BPSL1525		2205031	2204471	1472960	1472400	2634315	2633755	2618896	2618336	1937309	1937869	2171474	2170914	1537403	1537963	2219619	2219059	1540823	1541383
2305		Bp_chr1_20_ISBma1					2632381	2633685	2616962	2618266	1937939	1939243			1538033	1539337				
2306	BPSL1526		2204175	2202286	1472104	1470215	2632147	2630258	2616728	2614839	1939477	1941366	2170619	2168730	1539571	1541460	2218764	2216875	1541679	1543568
2307		Bp_chr1_21_ISBma1															2215373	2216677		
2308	BPSL1527		2199723	2202044	1467652	1469973	2627697	2630018	2612278	2614599	1943929	1941608	2166170	2168491	1544023	1541702	2212998	2215319	1546133	1543812
2309	BPSL1528		2199385	2198837	1467314	1466766	2627359	2626811	2611940	2611392	1944267	1944815	2165832	2165287	1544361	1544909	2212660	2212112	1546471	1547019
2310	BPSL1529		2198740	2198513	1466669	1466442	2626714	2626487	2611295	2611068	1944912	1945139	2165190	2164963	1545006	1545233	2212015	2211788	1547116	1547343
2311	BPSL1530		2198249	2196201	1466178	1464130	2626223	2624166	2610804	2608747	1945403	1947460	2164699	2162642	1545497	1547554	2211524	2209467	1547607	1549664
2312	BPSL1531		2195198	2196019	1463127	1463948	2623163	2623984	2607744	2608565	1948463	1947642	2161645	2162466	1548557	1547736	2208464	2209285	1550667	1549846
2313	BPSL1532		2194964	2193960	1462893	1461889	2622929	2621925	2607510	2606506	1948697	1949701	2161411	2160407	1548791	1549795	2208230	2207202	1550901	1551905
2314	BPSL1533		2193944	2193117	1461873	1461046	2621909	2621082	2606490	2605663	1949717	1950544	2160391	2159564	1549811	1550638	2207186	2206359	1551921	1552748

No.	BPSL	Locus	IS	G1 start	G1 end	G2 start	G2 end	G3 start	G3 end	G4 start	G4 end	G5 start	G5 end	G6 start	G6 end	G7 start	G7 end	G8 start	G8 end	G9 start	G9 end
2315	BPSL1534			2192597	2190786	1460526	1458715	2620562	2618760	2605143	2603341	1951076	1952887	2159032	2157236	1551170	1552981	2205839	2204028	1553280	1555076
2316	BPSL1535			2190686	2189508	1458615	1457437	2618660	2617482	2603241	2602063	1952987	1954165	2157136	2155958	1553081	1554259	2203928	2202750	1555176	1556354
2317	BPSL1536			2189379	2188642	1457308	1456571	2617353	2616616	2601934	2601197	1954294	1955031	2155829	2155092	1554388	1555125	2202621	2201884	1556483	1557220
2318	BPSL1537			2188502	2187933	1456431	1455862	2616476	2615907	2601057	2600488	1955171	1955740	2154952	2154383	1555265	1555834	2201744	2201175	1557360	1557929
2319	BPSL1538			2187338	2185962	1455267	1453891	2615254	2613878	2599835	2598459	1956335	1957711	2153771	2152395	1556455	1557831	2200632	2199256	1558517	1559893
2320	BPSL1539			2185956	2185033	1453885	1452962	2613872	2612949	2598453	2597530	1957717	1958640	2152389	2151466	1557837	1558760	2199250	2198327	1559899	1560822
2321	BPSL1540			2184934	2183753	1452863	1451682	2612850	2611669	2597431	2596250	1958739	1959920	2151367	2150186	1558859	1560040	2198228	2197047	1560921	1562102
2322	BPSL1541			2183733	2183155	1451662	1451084	2611649	2611071	2596230	2595652	1959940	1960038	2150166	2149588	1560060	1560638	2197027	2196452	1562122	1562700
2323	BPSL1542			2182851	2181670	1450780	1449599	2610767	2609586	2595348	2594167	1960889	1962070	2149284	2148103	1560942	1562123	2196148	2194967	1563004	1564185
2324	BPSL1543			2180558	2181400	1448487	1449329	2608474	2609316	2593055	2593897	1963182	1962340	2146991	2147833	1563235	1562393	2193855	2194697	1565297	1564455
2325		BURPS406E_H0785										1963195	1963965								
2326	BPSL1544			2179672	2179130	1447601	1447059	2607567	2607025	2592148	2591606	1964036	1964578	2146205	2145663	1564041	1564583	2192904	2192362	1566163	1566705
2327		BURPSS13_P0812																2193842	2193264		
2328	BPSL1545			2177586	2178401	1445508	1446323	2605648	2606463	2590229	2591044	1966045	1965230	2144196	2145011	1566057	1565242	2190895	2191710	1568172	1567357
2329	BPSL1546			2176894	2177586	1444816	1445508	2604956	2605648	2589537	2590229	1966737	1966045	2143504	2144196	1566749	1566057	2190203	2190895	1568864	1568172
2330	BPSL1547			2176760	2174289	1444682	1442211	2604822	2602333	2589403	2586914	1966871	1968454	2143370	2140881	1566883	1569372	2190069	2187580	1568998	1571469
2331	BPSL1548			2173828	2172239	1441750	1440161	2601871	2600282	2586452	2584863	1969804	1971393	2140420	2138837	1569834	1571423	2187119	2185530	1571930	1573519
2332		BURPS668_2149												2137632	2138588						
2333			Bp_chr1_22_IS407A											2136715	2137571						
2334		BURPS668_2145												2136358	2136639						
2335		BURPS668_2144												2135718	2136344						
2336		BURPS668_2143												2135095	2135271						
2337		BURPS668_2142												2132026	2134911						
2338		BURPS668_2141												2130739	2132022						
2339		BURPS668_2140												2129364	2130680						
2340		BURPS668_2139												2128199	2129026						
2341		BURPS668_2138												2127351	2128046						
2342		BURPS668_2137												2126251	2126394						
2343		BURPS668_2136												2125143	2126012						
2344		BURPS668_2135												2124855	2125094						
2345		BURPS1106A_2187		2173872	2174252																
2346		BURPS1106B_A1422				1442174	1441794														
2347			Bp_chr1_23_IS407A											2123570	2124805						
2348	BPSL1549			2171453	2172085	1439375	1440007	2599498	2600130	2584079	2584711	1972178	1971546	2122044	2122676	1572208	1571576	2184745	2185377	1574305	1573673
2349		BURPS668_2132												2123479	2123595						
2350	BPSL1550			2171137	2169704	1439059	1437626	2599182	2597749	2583763	2582330	1972494	1973927	2121728	2120295	1572524	1573957	2184429	2182996	1574621	1576054
2351	BPSL1551			2169502	2168315	1437424	1436237	2597547	2596360	2582128	2580941	1974129	1975316	2120094	2118907	1574159	1575346	2182794	2181607	1576256	1577443
2352	BPSL1552			2168239	2167598	1436161	1435520	2596284	2595643	2580865	2580224	1975392	1976033	2118831	2118190	1575422	1576063	2181531	2180890	1577519	1578160
2353	BPSL1553			2167267	2167467	1435189	1435389	2595311	2595511	2579892	2580092	1976365	1976165	2117858	2118058	1576395	1576195	2180558	2180758	1578492	1578292
2354	BPSL1554			2165549	2166853	1433471	1434775	2593578	2594882	2578159	2579463	1978089	1976785	2116175	2117479	1578120	1576816	2178863	2180167	1580215	1578911
2355		BURPS668_2125												2117502	2117696						
2356	BPSL1555			2165162	2164071	1433084	1431993	2593192	2592101	2577773	2576682	1978476	1979567	2115788	2114697	1578507	1579598	2178476	2177385	1580602	1581693
2357	BPSL1556			2163953	2162820	1431875	1430742	2591983	2590850	2576564	2575431	1979685	1980818	2114579	2113446	1579716	1580849	2177267	2176134	1581811	1582944
2358	BPSL1557			2162820	2161894	1430742	1429816	2590850	2589924	2575431	2574505	1980818	1981744	2113446	2112520	1580849	1581775	2176134	2175208	1582944	1583870
2359	BPSL1558			2161894	2161079	1429816	1429001	2589924	2589109	2574505	2573690	1981744	1982559	2112520	2111705	1581775	1582590	2175208	2174393	1583870	1584685
2360	BPSL1559			2160015	2160818	1427937	1428740	2588045	2588848	2572626	2573429	1983617	1982814	2110642	2111445	1583651	1582848	2173329	2174132	1585746	1584943
2361	BPSL1560			2158305	2159972	1426227	1427894	2586335	2588002	2570916	2572583	1985327	1983660	2108932	2110599	1585361	1583694	2171619	2173286	1587456	1585789
2362	BPSL1561			2157331	2158251	1425253	1426173	2585361	2586281	2569942	2570862	1986301	1985381	2107958	2108878	1586335	1585415	2170645	2171565	1588430	1587510
2363	BPSL1562			2157164	2155758	1425086	1423680	2585194	2583794	2569775	2568375	1986468	1987868	2107791	2106391	1586502	1587902	2170478	2169072	1588597	1589997
2364	BPSL1563			2155442	2154882	1423364	1422804	2583478	2582918	2568059	2567499	1988184	1988744			1588218	1588778	2168756	2168196	1590315	1590875
2365	BPSL1564			2154868	2154647	1422790	1422569	2582904	2582683	2567485	2567264	1988868	1989047			1588792	1589013	2168182	2167961	1590889	1591110
2366	BPSL1565			2152232	2153278	1420154	1421200	2580280	2581326	2564861	2565907	1991438	1990392	2097869	2098894	1591406	1590360	2165546	2166592	1593503	1592457
2367		BURPS668_2108												2098979	2099182						
2368		BURPS668_2109												2099209	2099940						
2369		BURPS668_2110												2099500	2101335						
2370		BURPS668_2111												2101332	2102390						
2371		BURPS668_2112												2102559	2103776						
2372		BURPS668_2113												2106164	2106391						

2373		BURPS668_2114											2103862	2106186						
2374	BPSL1566		2149873	2151774	1417795	1419696	2577923	2579824	2562504	2564405	1993797	1991896	2095555	2097216	1593765	1591864	2163187	2165088	1595862	1593961
2375		BURPS668_2106											2097497	2097679						
2376	BPSL1567		2146700	2149867	1414622	1417789	2574750	2577917	2559331	2562498	1996970	1993803	2092382	2095549	1596938	1593771	2160014	2163181	1599035	1595868
2377	BPSL1568		2145588	2146700	1413510	1414622	2573638	2574750	2558219	2559331	1998082	1996970	2091270	2092382	1598050	1596938	2158902	2160014	1600147	1599035
2378	BPSL1569		2145397	2144762	1413319	1412684	2573447	2572812	2558028	2557393	1998273	1998908	2091079	2090444	1598241	1598876	2158711	2158076	1600338	1600973
2379	BPSL1570		2142634	2144466	1410556	1412388	2570681	2572513	2555262	2557094	2001041	1999209	2088316	2090148	1601009	1599177	2155948	2157780	1603106	1601274
2380	BPSL1571		2141550	2142617	1409472	1410539	2569597	2570664	2554178	2555245	2002125	2001058	2087232	2088299	1602093	1601026	2154864	2155931	1604190	1603123
2381	BPSL1572		2141439	2140540	1409361	1408462	2569486	2568587	2554067	2553168	2002236	2003135	2087121	2086222	1602204	1603103	2154753	2153854	1604301	1605200
2382	BPSL1573		2140029	2140472	1407951	1408394	2568076	2568519	2552657	2553100	2003646	2003203	2085711	2086154	1603614	1603171	2153343	2153786	1605711	1605268
2383	BPSL1574		2139805	2139044	1407727	1406966	2567852	2567091	2552433	2551672	2003870	2004631	2085487	2084726	1603838	1604599	2153119	2152358	1605935	1606696
2384	BPSL1575		2138957	2137971	1406879	1405893	2567004	2566018	2551585	2550599	2004718	2005704	2084639	2083653	1604686	1605672	2152271	2151285	1606783	1607769
2385	BPSL1576		2137883	2136609	1405805	1404531	2565930	2564656	2550511	2549237	2005792	2007066	2083565	2082291	1605760	1607034	2151197	2149923	1607857	1609131
2386	BPSL1577		2136601	2135627	1404523	1403549	2564648	2563674	2549229	2548255	2007074	2008048	2082283	2081309	1607042	1608016	2149915	2148941	1609139	1610113
2387	BPSL1578		2135016	2133976	1402938	1401898	2563063	2562023	2547644	2546604	2008649	2009689	2080708	2079668	1608627	1609667	2148320	2147280	1610714	1611754
2388	BPSL1579		2133915	2133130	1401837	1401052	2561962	2561177	2546543	2545758	2009750	2010535	2079607	2078822	1609728	1610513	2147219	2146434	1611815	1612600
2389	BPSL1580		2133112	2132753	1401034	1400675	2561159	2560800	2545740	2545381	2010553	2010912	2078804	2078445	1610531	1610890	2146416	2146057	1612618	1612977
2390	BPSL1581		2131949	2132380	1399871	1400302	2559952	2560383	2544533	2544964	2011661	2011230	2077620	2078051	1611727	1611296	2145120	2145551	1613714	1613283
2391	BPSL1582		2131617	2130736	1399539	1398658	2559620	2558739	2544201	2543320	2011993	2012874	2077291	2076410	1612059	1612940	2144788	2143907	1614043	1614924
2392	BPSL1583		2129663	2130175	1397585	1398097	2557802	2558314	2542383	2542895	2013810	2013298	2075459	2075971	1613877	1613365	2142953	2143483	1615903	1615391
2393		BURPS406E_H0832									2014503	2014273								
2394	BPSL1584		2129453	2128863	1397375	1396785	2557588	2556983	2542169	2541564					1614091	1614684	2142294	2142157	1616117	1616704
2395	BPSL1586		2128843	2128058	1396765	1395980	2556963	2556616	2541544	2541197	2016715	2017062	2072538	2072191	1614704	1615021	2142137	2141355	1616724	1617509
2396		BURPS668_2087											2074750	2074980						
2397	BPSL1588		2127734	2127276	1395656	1395198	2555844	2555386	2540425	2539967	2017836	2018294	2071419	2070961	1615317	1615775	2141031	2140573	1617873	1618331
2398	BPSL1589		2127218	2126139	1395140	1394061	2555328	2554249	2539909	2538830	2018352	2019431	2070903	2069824	1615833	1616912	2140515	2139436	1618389	1619468
2399	BPSL1590		2125624	2126103	1393546	1394025	2553734	2554213	2538315	2538794	2019946	2019467	2069309	2069788	1617427	1616948	2138921	2139400	1619983	1619504
2400	BPSL1591		2125323	2124226	1393245	1392148	2553433	2552438	2538014	2536917	2020247	2021344	2069008	2067911	1617728	1618825	2138620	2137523	1620284	1621381
2401	BPSL1592		2123400	2124017	1391322	1391939	2551514	2552131	2536094	2536711	2022170	2021553	2067085	2067702	1619648	1619031	2136697	2137314	1622207	1621590
2402	BPSL1592A		2123242	2123358	1391164	1391280	2551356	2551472	2535936	2536052	2022328	2022212	2066927	2067043	1619806	1619690	2136539	2136655	1622365	1622249
2403	BPSL1593		2123070	2122063	1390992	1389985	2551184	2550177	2535764	2534757	2022500	2023507	2066755	2065748	1619978	1620985	2136367	2135963	1622537	1623544
2404	BPSL1594		2121699	2122049	1389621	1389971	2549813	2550163	2534393	2534743	2023871	2023521	2065384	2065734	1621349	1620999	2135007	2135357	1623908	1623558
2405	BPSL1595		2121210	2120773	1389132	1388695	2549324	2548887	2533904	2533467	2024360	2024797	2064919	2064482	1621838	1622275	2134518	2134081	1624406	1624843
2406	BPSL1596		2119886	2120146	1387808	1388068	2548079	2548339	2532659	2532919	2025758	2025498	2063535	2063795	1623120	1622860	2133071	2133331	1625747	1625487
2407	BPSL1597		2119793	2119716	1387715	1387638	2547986	2547909			2025851	2025928	2063442	2063368	1623213	1623290	2132978	2132901	1625840	1625917
2408	BPSL1598		2116659	2119085	1384581	1387007	2544839	2547265	2529419	2531845	2028973	2026547	2060349	2062775	1626349	1625120	2129831	2132257	1628995	1626569
2409	BPSL1599		2114093	2116231	1382015	1384153	2542340	2544472	2526920	2529052	2031450	2029318	2057773	2059911	1628861	1626723	2127221	2129359	1631473	1629335
2410		BURPS1106A_2124	2116231	2116587																
2411		BURPS1106B_A1359			1384153	1384509														
2412		BURPS668_2069											2059911	2060339						
2413	BPSL1600		2113549	2114040	1381471	1381962	2541789	2542280	2526369	2526860	2032001	2031510	2057229	2057720	1629405	1628914	2126677	2127168	1632017	1631526
2414	BPSL1601		2113123	2113524	1381045	1381446	2541363	2541764	2525943	2526344	2032427	2032026	2056803	2057204	1629831	1629430	2126251	2126652	1632443	1632042
2415	BPSL1602		2112851	2113117	1380773	1381039	2541091	2541357	2525671	2525937	2032699	2032433	2056531	2056797	1630103	1629837	2125979	2126245	1632715	1632449
2416	BPSL1603		2112310	2112837	1380232	1380759	2540550	2541077	2525130	2525657	2033240	2032713	2055990	2056517	1630644	1630117	2125438	2125965	1633256	1632729
2417	BPSL1604		2111477	2112310	1379399	1380232	2539717	2540550	2524297	2525130	2034073	2033240	2055157	2055990	1631477	1630644	2124605	2125438	1634089	1633256
2418	BPSL1605		2110566	2111477	1378488	1379399	2538806	2539717	2523386	2524297	2034984	2034073	2054246	2055157	1632388	1631477	2123694	2124605	1635000	1634089
2419	BPSL1606		2109319	2110566	1377241	1378488	2537559	2538806	2522139	2523386	2036231	2034984	2052999	2054246	1633635	1632388	2122447	2123694	1636247	1635000
2420	BPSL1607		2107171	2109135	1375093	1377057	2535419	2537383	2519999	2521963	2038371	2036407	2050830	2052800	1635775	1633811	2120307	2122271	1638395	1636431
2421		BURPS406E_H0861									2039030	2038647								
2422		BURPSPAST_AA0803													1636358	1636113				
2423	BPSL1608		2106489	2105434	1374411	1373356	2534737	2533682	2519317	2518262	2039052	2040107	2050149	2049094	1636456	1637511	2119637	2118582	1639065	1640120
2424		BURPS668_2059											2050171	2050554						
2425	BPSL1609		2105409	2104408	1373331	1372330	2533657	2532656	2518237	2517236	2040132	2041133	2049070	2048051	1637535	1638554	2118557	2117556	1640145	1641146
2426	BPSL1610		2104408	2103671	1372330	1371593	2532656	2531919	2517236	2516499	2041133	2041870	2048051	2047314	1638554	1639291	2117556	2116819	1641146	1641883
2427	BPSL1611		2103493	2101907	1371415	1369829	2531741	2530155	2516321	2514735	2042048	2043634	2047136	2045550	1639469	1641055	2116641	2115055	1642061	1643647
2428	BPSL1612		2101727	2101302	1369649	1369224	2529975	2529550	2514555	2514130	2043758	2044240	2045109	2044945	1641179	1641661	2114615	2114451	1643827	1644252
2429	BPSL1613		2101084	2099375	1369006	1367297	2529332	2527623	2513912	2512203	2044458	2046167	2044727	2043018	1641879	1643588	2114233	2112524	1644470	1646044
2430	BPSL1614		2097954	2099000	1365876	1366922	2526211	2527248	2510791	2511828	2047536	2046499	2041600	2042637	1645024	1643987	2111110	2112156	1647596	1646541

2431	BPSL1615			2096956	2097918	1364878	1365840	2525213	2526175	2509793	2510755	2048534	2047572	2040602	2041564	1646022	1645060	2110112	2111074	1648594	1647632
2432	BPSL1616			2095570	2096946	1363492	1364868	2523827	2525203	2508407	2509783	2049920	2048544	2039216	2040592	1647408	1646032	2108726	2110102	1649980	1648604
2433	BPSL1617			2094087	2095526	1362009	1363448	2522344	2523783	2506924	2508363	2051403	2049964	2037733	2039172	1648891	1647452	2107243	2108682	1651463	1650024
2434	BPSL1618			2093251	2093946	1361173	1361868	2521508	2522203	2506088	2506783	2052239	2051544	2036896	2037591	1649727	1649032	2106407	2107102	1652299	1651604
2435		BURPS1655_A1666																		1653101	1652736
2436		BURPSPAST_AA0788														1650610	1650437				
2437	BPSL1619			2091456	2091139	1360231	1359377	2520557	2519703	2504284	2503967	2053201	2054370	2035064	2034747	1650673	1651842	2105390	2104602	1654103	1654420
2438		BURPS1710A_2594						2520620	2520793												
2439	BPSL1621			2091001	2090789	1358923	1358711	2519249	2519037	2503829	2503617	2054509	2054856	2034608	2034396	1651981	1652193			1654583	1654891
2440	BPSL1622			2090501	2089734	1358423	1357656	2518750	2517983	2503330	2502563	2054992	2055750	2034109	2033351	1652480	1653247	2103663	2102905	1655027	1655794
2441		BURPSS13_P0720																2104200	2103796		
2442	BPSL1623			2088340	2089425	1356262	1357347	2516589	2517674	2501169	2502254	2057144	2056059	2031957	2033042	1654641	1653556	2101511	2102596	1657188	1656103
2443	BPSL1624			2022808	2022837	1355778	1355915	2516106	2516243	2500686	2500823	2057628	2057491	2031475	2031663	1655124	1654987	2101028	2101165	1657670	1657482
2444	BPSL1625			2087754	2087290	1355676	1355212	2516004	2515540	2500584	2500120	2057730	2058194	2031373	2030909	1655226	1655690	2100926	2100462	1657772	1658236
2445	BPSL1626			2086131	2085604	1354053	1353526	2514382	2513855	2498962	2498435	2059353	2059880	2029732	2029205	1656821	1657348	2099304	2098777	1659393	1659920
2446		BURPS668_2034												2030155	2030418						
2447		BURPS668_2035												2030426	2030584						
2448	BPSL1627			2085502	2084735	1353424	1352657	2513753	2512986	2498333	2497566	2059982	2060749	2029103	2028336	1657450	1658217	2098675	2097908	1660020	1660787
2449	BPSL1628			2084725	2082176	1352647	1350098	2512976	2510427	2497556	2495007	2060759	2063308	2028326	2025777	1658227	1658601	2097898	2095349	1660797	1663346
2450		BURPSPAST_AA0776														1659736	1659473				
2451		BURPSPAST_AA0777														1659476	1658625				
2452	BPSL1629			2082176	2081214	1350098	1349136	2510427	2509465	2495007	2494045	2063308	2064270	2025777	2024815	1661988	1662950	2095349	2094387	1663346	1664308
2453	BPSL1630			2080425	2080177	1348347	1348099	2508685	2508437	2493265	2493017	2065050	2065298	2024024	2023776	1663730	1663978	2093607	2093359	1665097	1665345
2454	BPSL1631																				
2455	BPSL1632			2075068	2074586	1342990	1342508	2503737	2503255	2488317	2487835	2069994	2070476	2019328	2018846	1668939	1669421	2089167	2088685	1670306	1670788
2456		BURPS668_2027												2020672	2023560						
2457	BPSL1633			2074296	2073634	1342218	1341556	2502965	2502303	2487545	2486883	2070777	2071439	2018556	2017894	1669711	1670373	2088395	2087733	1671078	1671740
2458	BPSL1634			2073631	2070386	1341553	1338308	2502300	2499055	2486880	2483635	2071442	2074687	2017891	2014646	1670376	1673621	2087730	2084485	1671743	1674988
2459	BPSL1635			2070155	2069265	1338077	1337187	2498824	2497934	2483404	2482514	2074918	2075808	2014415	2013525	1673852	1674742	2084254	2083364	1675219	1676109
2460	BPSL1636			2068895	2068590	1336817	1336512	2496608	2496303	2481188	2480883	2077152	2077457	2012182	2011877	1676078	1676383	2082046	2081741	1677453	1677758
2461		BURPSS13_P0699																2082958	2083170		
2462	BPSL1637		GI8	2067260	2068327	1335182	1336249	2494973	2496040	2479553	2480620	2078787	2077720	2010547	2011614	1677713	1676646	2080411	2081478	1679088	1678021
2463		BURPS1655_A1697																		1679527	1679375
2464		BURPS1655_A1698																		1679658	1679825
2465		BURPS406E_H0907										2079715	2079488								
2466		BURPS406E_H0908										2080165	2079749								
2467		BURPS406E_H0909										2080442	2080858								
2468		BURPS406E_H0910										2081204	2080998								
2469		BURPSPAST_AA0750														1678286	1678453				
2470		BURPSPAST_AA0751														1678155	1678003				
2471	BPSL1638		GI8					2494133	2493837	2478713	2478417			2009709	2009413	1678553	1678849			1679925	1680221
2472		BURPS1710A_2564						2494400	2494233												
2473		BURPS1710A_2565						2494531	2494683												
2474		BURPS668_2012												2009738	2009974						
2475		BURPS668_2013												2010105	2010257						
2476	BPSL1639		GI8					2493059	2493823	2477639	2478403			2008635	2009399	1679627	1678863			1680999	1680235
2477	BPSL1642		GI8					2492817	2492050	2477397	2476630			2008393	2008040	1679869	1680636			1681241	1682008
2478	BPSL1643		GI8					2491751	2491167	2476331	2475747					1680937	1681521			1682307	1682891
2479	BPSL1644		GI8					2491174	2490293	2475754	2474873					1681514	1682395			1682884	1683765
2480	BPSL1645		GI8					2490297	2489344	2474877	2473924					1682391	1683344			1683761	1684714
2481	BPSL1646		GI8					2489232	2488192	2473812	2472772					1683456	1684496			1684826	1685866
2482	BPSL1647		GI8					2488145	2486676	2472725	2471256					1684543	1686012			1685913	1687382
2483	BPSL1648		GI8					2486603	2486292	2471183	2470872					1686085	1686396			1687451	1687762
2484	BPSL1649		GI8					2486219	2485191	2470799	2469771					1686469	1687497			1687835	1688863
2485	BPSL1650		GI8					2485109	2484228	2469689	2468808					1687579	1688460			1688945	1689823
2486	BPSL1651		GI8					2484223	2483432	2468803	2468012					1688465	1689256			1689831	1690622
2487	BPSL1652		GI8					2483412	2482324	2467992	2466904					1689276	1690364			1690642	1691730
2488	BPSL1653		GI8					2482340	2481579	2466920	2466159					1690348	1691109			1691714	1692475

2489	BPSL1654		GI8					2481554	2480097	2466134	2464677					1691134	1692591			1692500	1693957
2490	BPSL1655		GI8					2479950	2478835	2464530	2463415					1692738	1693853			1694104	1695219
2491	BPSL1656		GI8					2478707	2478420	2463287	2463000					1693981	1694268			1695347	1695634
2492	BPSL1657		Bp_chr1_24_IS407A																		
2493	BPSL1658		Bp_chr1_24_IS407A																		
2494	BPSL1658a		GI8	2064121	2063738	1332043	1331660	2477687	2477304	2462267	2461884	2081926	2082309			1695000	1695383	2077273	2076890	1696367	1696750
2495		BURPS1106A_2067		2064843	2065049																
2496		BURPS1106A_2068		2065189	2065605																
2497		BURPS1106A_2069		2065882	2066298																
2498		BURPS1106A_2070		2066332	2066559																
2499		BURPS1106A_2071		2066581	2066700																
2500		BURPS1106A_2072		2066827	2066970																
2501		BURPS1106B_A1304				1333527	1333111														
2502		BURPS1106B_A1305				1334035	1334220														
2503		BURPS1106B_A1306				1334329	1334481														
2504		BURPS1106B_A1307				1334622	1334503														
2505		BURPS1106B_A1308				1334749	1334892														
2506		BURPSS13_P0689																2077994	2078200		
2507		BURPSS13_P0690																2078756	2078340		
2508		BURPSS13_P0691																2079033	2079449		
2509		BURPSS13_P0692																2079543	2079710		
2510		BURPSS13_P0693																2079978	2080121		
2511	BPSL1659		GI8	2063248	2062352	1331170	1330274	2476814	2475918	2461394	2460498	2082799	2083695			1695873	1696769	2076398	2075502	1697240	1698136
2512	BPSL1660		GI8	2062220	2060424	1330142	1328346	2475786	2473990	2460366	2458570	2083827	2085623			1696901	1698697	2075370	2073574	1698268	1700064
2513	BPSL1661		GI8	2060310	2050624	1327635	1319500	2473876	2464190	2457859	2449724	2085737	2095423			1698811	1708497	2072863	2064728	1700775	1708910
2514	BPSL1662		GI8	2050525	2050019	1319401	1318895	2464091	2463585	2449625	2449119	2095522	2096028			1708596	1709102	2064629	2064123	1709009	1709515
2515	BPSL1663		GI8	2050019	2049147	1318895	1318023	2463585	2462713	2449119	2448247	2096028	2096900			1709102	1709974	2064123	2063251	1709515	1710387
2516	BPSL1664		GI8	2049118	2046821	1317994	1315697	2462684	2460387	2448218	2445921	2096929	2099226			1710003	1712300	2063222	2060925	1710416	1712713
2517	BPSL1665		GI8	2046827	2045415	1315703	1314291	2460393	2458981	2445927	2444515	2099220	2100632			1712294	1713706	2060931	2059519	1712707	1714119
2518	BPSL1666		GI8	2045301	2040940	1314177	1309816	2458867	2454506	2444401	2440040	2100746	2105107			1713820	1718181	2059405	2055044	1714233	1718594
2519	BPSL1667		GI8	2040940	2039273	1309816	1308149	2454506	2452839	2440040	2438373	2105107	2106774			1718181	1719848	2055044	2053377	1718594	1720261
2520	BPSL1668		GI8	2039238	2039128	1308114	1308004	2452804	2451956	2438338	2437490	2106809	2107657			1719883	1720731	2053342	2053232	1720296	1721144
2521	BPSL1669		GI8	2037376	2036666	1306252	1305542	2450939	2450229	2436473	2435763	2108674	2109384			1721748	1722458	2051479	2050769	1722161	1722871
2522	BPSL1670		GI8	2035582	2036262	1304458	1305138	2449131	2449823	2434665	2435357	2110480	2109788					2049672	2050121	1723968	1723276
2523	BPSL1671		GI8	2035128	2034652	1304004	1303528	2448677	2448201	2434211	2433735	2110934	2111410			1724009	1724485	2049218	2048742	1724422	1724898
2524	BPSL1672		GI8	2034448	2033405	1303324	1302281	2447997	2446954	2433531	2432488	2111614	2112657			1724689	1725732	2048538	2047495	1725102	1726145
2525	BPSL1673		GI8	2033077	2032808	1301953	1301684	2446626	2446357	2432160	2431891	2112985	2113254			1726056	1726325	2047171	2046902	1726473	1726742
2526	BPSL1674		GI8	2031294	2032391	1300170	1301267	2444843	2445940	2430377	2431474	2114768	2113671			1727839	1726742	2045388	2046485	1728256	1727159
2527	BPSL1675		GI8	2030536	2031000	1299412	1299876	2444085	2444549	2429619	2430083	2115526	2115062			1728615	1728133	2044630	2045094	1729014	1728550
2528	BPSL1676		GI8	2028818	2030449	1297694	1299325	2442367	2443998	2427901	2429532	2117244	2115613			1730333	1728702	2042912	2044543	1730732	1729101
2529	BPSL1677		GI8	2027517	2028797	1296393	1297673	2441066	2442346	2426600	2427880	2118545	2117265			1731634	1730354	2041611	2042891	1732033	1730753
2530	BPSL1678		GI8	2025801	2027438	1294677	1296314	2439350	2440987	2424884	2426521	2120261	2118624			1733350	1731713	2039895	2041532	1733749	1732112
2531	BPSL1679		GI8	2024691	2025779	1293567	1294655	2438240	2439328	2423774	2424862	2121370	2120405			1734460	1733372	2039307	2039873	1734859	1733771
2532	BPSL1680		GI8	2023620	2024507	1292496	1293383	2437169	2438056	2422703	2423590	2122441	2121554			1735531	1734644	2037706	2038593	1735930	1735043
2533	BPSL1681		GI8	2023520	2022138	1292396	1291014	2437069	2435687	2422603	2421221	2122541	2123923			1735631	1735717	2037606	2036224	1736030	1737412
2534	BPSL1682		GI8	2022138	2020861	1291014	1289737	2435687	2434410	2421221	2419944	2123923	2125200			1736922	1738199	2036224	2034947	1737412	1738689
2535	BPSL1683		GI8	2020473	2020802	1289349	1289678	2434022	2434351	2419556	2419885	2125588	2125259			1738587	1738258	2034559	2034888	1739077	1738748
2536	BPSL1684		GI8	2019810	2020256	1288686	1289132	2433359	2433805	2418893	2419339	2126251	2125805			1739250	1738804	2033896	2034342	1739740	1739294
2537	BPSL1685		GI8									2126539	2126294			1739538	1739293	2033608	2033853	1740033	1739749
2538	BPSL1686		GI8	2019095	2018562	1287971	1287438	2432644	2432111	2418178	2417645	2126968	2127501			1739967	1740500	2033178	2032645	1740462	1740995
2539	BPSL1687		GI8	2018562	2017924	1287438	1286800	2432111	2431473	2417645	2417007	2127501	2128139			1740500	1741138	2032645	2032007	1740995	1741261
2540	BPSL1688		GI8	2017192	2017899	1286068	1286775	2430741	2431448	2416275	2416982	2128871	2128164			1741870	1741163	2031275	2031982	1742355	1741648
2541	BPSL1689		GI8	2016692	2017192	1285568	1286068	2430241	2430741	2415775	2416275	2129371	2128871			1742370	1741870	2030775	2031275	1742855	1742355
2542	BPSL1690		GI8	2015907	2016683	1284783	1285559	2429456	2430232	2414990	2415766	2130156	2129380			1743155	1742379	2029990	2030766	1743640	1742864
2543	BPSL1691		GI8	2015006	2015914	1283882	1284790	2428555	2429463	2414089	2414997	2131057	2130149			1744056	1743148	2029089	2029997	1744541	1743633
2544	BPSL1692		GI8	2014661	2014969	1283537	1283845	2428210	2428518	2413744	2414052	2131402	2131094			1744401	1744093	2028744	2029052	1744886	1744578
2545	BPSL1693		GI8					2427636	2427265	2413170	2412799	2131976	2132347			1744975	1745346			1745460	1745831
2546	BPSL1694		GI8					2426654	2427070	2412188	2412604	2132958	2132542			1745958	1745575			1746442	1746026

2547	BPSL1695		GI8					2425783	2426448	2411317	2411982	2133829	2133164			1746829	1746164			1747313	1746648
2548	BPSL1696		GI8					2423744	2423103	2409278	2408637	2135868	2136509			1748868	1749509			1749352	1749993
2549	BPSL1697		GI8					2422070	2422831	2407604	2408365	2137542	2136781			1750542	1749781			1751026	1750265
2550	BPSL1699		GI8					2421677	2421967	2407211	2407501	2137935	2137645			1750935	1750645			1751418	1751128
2551	BPSL1700		GI8					2421431	2420664	2406965	2406198	2138181	2138948			1751181	1751948			1751664	1752431
2552	BPSL1702		GI8					2419697	2420287	2405231	2405821	2139915	2139325			1752915	1752325			1753398	1752808
2553	BPSL1703		GI8					2418971	2419402	2404505	2404936	2140641	2140210			1753641	1753210			1754124	1753693
2554	BPSL1704		GI8					2418567	2418929	2404101	2404463	2141045	2140683			1754045	1753683			1754528	1754166
2555	BPSL1704a		GI8					2417267	2416788	2402801	2402322	2142345	2142824			1755345	1755824			1755827	1756306
2556		BURPS1655_A1767																		1756557	1761071
2557	BPSL1705		GI8					2416394	2411577	2401928	2397111	2143218	2148035			1756218	1760447				
2558	BPSL1706		GI8					2409875	2409561	2395409	2395095	2149737	2150051			1762738	1763052			1762770	1763084
2559	BPSL1707		GI8					2409108	2406796	2394642	2392330	2150504	2152816			1763505	1764260			1763537	1765849
2560	BPSL1708		GI8					2406726	2406301	2392260	2391835	2152886	2153311			1765888	1766313			1765919	1766344
2561	BPSL1708A		GI8					2406119	2405865	2391653	2391399	2153493	2153747			1766495	1766749			1766526	1766780
2562	BPSL1709		GI8					2405755	2405351	2391289	2390885	2153857	2154261			1766859	1767263	2028062	2027919	1766890	1767294
2563	BPSL1710			2010835	2013204	1279711	1282080	2402348	2404717	2387882	2390251	2157264	2154895	2005370	2007739	1770264	1767895	2025044	2027413	1770295	1767926
2564	BPSL1711			2008989	2010707	1277865	1279583	2400502	2402220	2386036	2387754	2159110	2157392	2003531	2005249	1772110	1770392	2023204	2024922	1772141	1770423
2565	BPSL1712			1990080	2008895	1258956	1277771	2381587	2400408	2367121	2385942	2178031	2159204	1984625	2003437	1791013	1772204	2004301	2023110	1791056	1772235
2566	BPSL1713			1989511	1990080	1258387	1258956	2381018	2381587	2366552	2367121	2178600	2178031	1984056	1984622	1791582	1791013	2003732	2004301	1791625	1791056
2567	BPSL1714			1988294	1989451	1257170	1258327	2379801	2380958	2365335	2366492	2179766	2178660	1982839	1983996	1792799	1791642	2002515	2003672	1792839	1791685
2568	BPSL1715			1985567	1988248	1254443	1257124	2377074	2379755	2362608	2365289	2182545	2179870	1980112	1982793	1795569	1792942	1999788	2002469	1795294	1792943
2569	BPSL1716			1984399	1985511	1253275	1254387	2375906	2377018	2361440	2362552	2183713	2182601	1978944	1980056	1796737	1795625	1998620	1999732	1796462	1795350
2570	BPSL1717			1983749	1984387	1252625	1253263	2375256	2375894	2360790	2361428	2184363	2183725	1978294	1978932	1797387	1796749	1997970	1998608	1797112	1796474
2571	BPSL1718			1982520	1983749	1251396	1252625	2374027	2375256	2359561	2360790	2185592	2184363	1977065	1978294	1798616	1797387	1996741	1997970	1798341	1797112
2572	BPSL1719			1981549	1982463	1250425	1251339	2373056	2373970	2358590	2359504	2186563	2185649	1976094	1977008	1799587	1798673	1995770	1996684	1799312	1798398
2573	BPSL1720			1980152	1981549	1249028	1250425	2371659	2373056	2357193	2358590	2187960	2186563	1974697	1976094	1800984	1799587	1994373	1995770	1800709	1799312
2574	BPSL1721			1978941	1980152	1247817	1249028	2370448	2371659	2355982	2357193	2189171	2187960	1973486	1974697	1802195	1800984	1993162	1994373	1801920	1800709
2575	BPSL1722			1978082	1978897	1246958	1247773	2369589	2370404	2355123	2355938	2190030	2189215	1972627	1973442	1803054	1802239	1992303	1993118	1802779	1801964
2576	BPSL1723			1976926	1978023	1245802	1246899	2368433	2369530	2353967	2355064	2191186	2190089	1971471	1972568	1804210	1803113	1991147	1992244	1803935	1802838
2577	BPSL1724			1975906	1976913	1244782	1245789	2367413	2368420	2352947	2353954	2192206	2191199	1970451	1971458	1805230	1804223	1990127	1991134	1804955	1803948
2578	BPSL1725			1974960	1975841	1243836	1244717	2366467	2367348	2352001	2352882	2193152	2192271	1969505	1970386	1806176	1805295	1989181	1990062	1805901	1805020
2579	BPSL1726			1974749	1974970	1243625	1243846	2366256	2366477	2351790	2352011	2193363	2193142	1969294	1969515	1806387	1806166	1988970	1989191	1806112	1805891
2580	BPSL1727			1973741	1971927	1242617	1240803	2365220	2363400	2350754	2348934	2194399	2196225	1968258	1966444	1807424	1809244	1987962	1986148	1807120	1808934
2581	BPSL1728			1970243	1971463	1239119	1240339	2361759	2362979	2347293	2348513	2197863	2196646	1964761	1965981	1810931	1809711	1984467	1985684	1810618	1809401
2582	BPSL1729			1969895	1969098	1238771	1237974	2361404	2360607	2346938	2346141	2198211	2199008	1964427	1963630	1811279	1812076	1984112	1983315	1810973	1811770
2583	BPSL1730			1968930	1967995	1237806	1236871	2360439	2359504	2345973	2345038	2199176	2200111	1963462	1962527	1812244	1813179	1983147	1982212	1811938	1812873
2584	BPSL1731			1967289	1967798	1236165	1236674	2358798	2359307	2344332	2344841	2200817	2200308	1961821	1962330	1813885	1813376	1981506	1982015	1813579	1813070
2585	BPSL1732			1965509	1967248	1234385	1236124	2357018	2358757	2342552	2344291	2202597	2200858	1960041	1961780	1815665	1813926	1979726	1981465	1815359	1813620
2586	BPSL1733			1964607	1964792	1233483	1233668	2356117	2356302	2341651	2341836	2203498	2203313	1959121	1959306	1816566	1816381	1978825	1979010	1816261	1816076
2587	BPSL1734			1964264	1962234	1233140	1231110	2355787	2353757	2341321	2339291	2203828	2205858	1958797	1956761	1816896	1818926	1978475	1976448	1816587	1818620
2588	BPSL1735			1962160	1960760	1231036	1229636	2353683	2352283	2339217	2337817	2205932	2207332	1956687	1955287	1819006	1820406	1976374	1974974	1818699	1820096
2589	BPSL1736			1959630	1960397	1228506	1229273	2351209	2351976	2336743	2337510	2208390	2207623	1954234	1955001	1821432	1820665	1973868	1974635	1821178	1820411
2590	BPSL1737			1959268	1958333	1228144	1227209	2350847	2349912	2336381	2335446	2208752	2209687	1953872	1952937	1821794	1822729	1973473	1972538	1821529	1822464
2591	BPSL1738			1958273	1957116	1227149	1225992	2349852	2348695	2335386	2334229	2209747	2210904	1952877	1951720	1822789	1822812	1972478	1971321	1822524	1823681
2592	BPSL1739			1957116	1956181	1225992	1225057	2348695	2347760	2334229	2333294	2210904	2211839	1951720	1950785	1823449	1824384	1971321	1970386	1823681	1824616
2593	BPSL1740			1956181	1955366	1225057	1224242	2347760	2346945	2333294	2332479	2211839	2212654	1950785	1949970	1824384	1825199	1970386	1969571	1824616	1825431
2594		BURPSPAST_AA0633														1825238	1825774				
2595	BPSL1741			1954521	1954192	1223397	1223068	2346188	2345859	2331722	2331393	2213880	2214209	1948747	1948418	1826044	1826373	1968726	1968397	1826667	1826996
2596		BURPS1106A_1991		1954791	1955327																
2597		BURPS1106B_A1228				1224203	1223667														
2598		BURPSS13_P0618																1969532	1968996		
2599	BPSL1742			1954029	1952572	1222905	1221448	2345696	2344239	2331230	2329773	2214372	2215829	1948255	1946798	1826536	1827993	1968233	1966776	1827159	1828616
2600	BPSL1743			1952551	1951298	1221427	1220174	2344218	2342965	2329752	2328499	2215850	2217103	1946777	1945524	1828014	1829267	1966755	1965502	1828637	1829890
2601	BPSL1744			1951230	1950223	1220106	1219099	2342897	2341890	2328431	2327424	2217171	2218178	1945456	1944449	1829335	1830342	1965434	1964427	1829958	1830965
2602	BPSL1745			1950131	1949196	1219007	1218072	2341798	2340863	2327332	2326397	2218270	2219205	1944357	1943422	1830434	1831369	1964335	1963400	1831057	1831992
2603	BPSL1746			1948156	1948947	1217032	1217823	2339823	2340614	2325357	2326148	2220137	2219346	1942393	1943184	1832398	1831607	1962371	1963162	1833032	1832241
2604	BPSL1747			1947710	1948156	1216586	1217032	2339377	2339823	2324911	2325357	2220583	2220137	1941947	1942393	1832844	1832398	1961925	1962371	1833478	1833032

2605 BPSL1748		1947612	1946695	1216488	1215571	2339279	2338362	2324813	2323896	2220681	2221598	1941849	1940932	1832942	1833859	1961827	1960910	1833576	1834493
2606 BPSL1749		1946407	1945706	1215283	1214582	2338075	2337374	2323609	2322908	2221886	2222587	1940645	1939944	1834147	1834848	1960623	1959922	1834781	1835482
2607 BPSL1750		1945160	1944792	1214036	1213668	2336826	2336290	2322360	2321824	2223135	2223467	1939396	1938860	1835396	1835932	1959376	1959008	1836030	1836566
2608 BPSL1751		1944401	1943289	1213277	1212165	2336066	2334954	2321600	2320488	2223883	2224995	1938637	1937525	1836155	1837267	1958617	1957505	1836789	1837901
2609 BPSL1752		1942484	1942975	1211360	1211851	2334149	2334640	2319683	2320174	2225800	2225309	1936707	1937198	1838072	1837581	1956713	1957204	1838706	1838215
2610 BPSL1753		1942360	1941179	1211236	1210055	2334025	2332844	2319559	2318378	2225924	2227105	1936583	1935402	1838196	1839377	1956589	1955408	1838830	1839996
2611 BPSL1754		1941052	1939484	1209928	1208360	2332716	2331148	2318250	2316682	2227233	2228801	1935274	1933706	1839505	1841073	1955281	1953713	1840127	1841695
2612 BPSL1755		1938677	1939399	1207553	1208275	2330341	2331063	2315875	2316597	2229608	2228886	1932899	1933621	1841880	1841158	1952906	1953628	1842502	1841780
2613	BURPS1655_A1830																	1842698	1842516
2614 BPSL1756		1937610	1938335	1206486	1207211	2329274	2329999	2314808	2315533	2230658	2229933	1931832	1932557	1842947	1842222	1951839	1952564	1843485	1842760
2615 BPSL1757		1936516	1937604	1205392	1206480	2328180	2329268	2313714	2314802	2231752	2230664	1930738	1931826	1844041	1842953	1950745	1951833	1844579	1843491
2616 BPSL1758		1935290	1936513	1204166	1205389	2326954	2328177	2312488	2313711	2232978	2231755	1929512	1930735	1845267	1844044	1949519	1950742	1845805	1844582
2617 BPSL1759		1934892	1933447	1203768	1202323	2326556	2325111	2312090	2310645	2233376	2233987	1929114	1927669	1845665	1847110	1949121	1947676	1846203	1847648
2618 BPSL1760		1933451	1932828	1202327	1201704	2325115	2324492	2310649	2310026	2234723	2235346	1927673	1927050	1847106	1847729	1947680	1947057	1847644	1848267
2619 BPSL1761		1932828	1932097	1201704	1200973	2324492	2323761	2310026	2309295	2235346	2236077	1927050	1926319	1847729	1848460	1947057	1946326	1848267	1848998
2620 BPSL1762		1932097	1930271	1200973	1199147	2323761	2321914	2309295	2307448	2236077	2237903	1926319	1924472	1848460	1849338	1946326	1944479	1848998	1850821
2621 BPSL1763		1929935	1928565	1198811	1197441	2321578	2320208	2307112	2305742	2238239	2239609	1924136	1922766	1850235	1851605	1944144	1942774	1851157	1852527
2622 BPSL1764		1928199	1927876	1197075	1196752	2319842	2319519	2305376	2305053	2239975	2240298	1922400	1922077	1851971	1852294	1942408	1942085	1852893	1853216
2623 BPSL1765		1926843	1927526	1195719	1196402	2318486	2319169	2304020	2304703	2241331	2240648	1921044	1921727	1853334	1852651	1941052	1941735	1854249	1853566
2624	BURPSPAST_AA0597													1857051	1861874				
2625	BURPSPAST_AA0598													1856508	1856873				
2626	BURPSPAST_AA0599													1853515	1856511				
2627 BPSL1766		1925889	1926500	1194765	1195376	2307092	2307703	2292626	2293237	2242273	2241662	1920102	1920713	1864771	1864160	1940104	1940715	1855215	1854604
2628	BURPS1710A_2404					2309815	2308118												
2629	BURPS1710A_2405					2314768	2309987												
2630	BURPS1710A_2406					2315311	2314946												
2631	BURPS1710A_2407					2318304	2315308												
2632	BURPS1710b_2104							2295521	2300302										
2633	BURPS1710b_2105							2300480	2300845										
2634	BURPS1710b_2106							2300842	2304021										
2635 BPSL1767		1924582	1925805	1193458	1194681	2305785	2307008	2291319	2292542	2243414	2242605	1918795	1920018	1865391	1864855	1938797	1940020	1856522	1855299
2636	BURPSPAST_AA0591													1866716	1866219				
2637 BPSL1768		1920103	1923945	1188979	1192821	2301278	2305120	2286812	2290654	2247893	2244051	1914243	1918085	1870558	1866716	1934290	1938132	1861029	1857187
2638	BURPS1106A_1955	1923945	1924442																
2639	BURPS1106B_A1192			1192821	1193318														
2640 BPSL1769		1918963	1920033	1187839	1188909	2300138	2301208	2285672	2286742	2249033	2247963	1913103	1914173	1871698	1870628	1933150	1934220	1862169	1861099
2641 BPSL1770		1917780	1918838	1186656	1187714	2298955	2300013	2284489	2285547	2250216	2249158	1911920	1912978	1872881	1871823	1931967	1933025	1863352	1862294
2642 BPSL1771		1916677	1916240	1185553	1185116	2297850	2297401	2283384	2282935	2251319	2251762	1910812	1910375	1873718	1874167	1930864	1930427	1864455	1864898
2643 BPSL1772		1916178	1915579	1185054	1184455	2297339	2296740	2282873	2282274	2251824	2252423	1910313	1909714	1874229	1874828	1930365	1929766	1864960	1865559
2644 BPSL1773		1915571	1913976	1184447	1182852	2296732	2295137	2282266	2280671	2252431	2254053	1909706	1908102	1874836	1876431	1929758	1928145	1865567	1867189
2645 BPSL1774		1912827	1913663	1181703	1182539	2293988	2294824	2279522	2280358	2255202	2254366	1906988	1907824	1877580	1876744	1926978	1927814	1868329	1867493
2646 BPSL1775		1910498	1912759	1179374	1181635	2291659	2293920	2277193	2279454	2257531	2255270	1904659	1906920	1879909	1877648	1924649	1926910	1870658	1868397
2647 BPSL1776		1909095	1910498	1177971	1179374	2290256	2291659	2275790	2277193	2258934	2257531	1903256	1904659	1881312	1879909	1923246	1924649	1872061	1870658
2648 BPSL1777		1903726	1908948	1172602	1177824	2284866	2290109	2270400	2275643	2264381	2259267	1897875	1903109	1885622	1881411	1917856	1923099	1877451	1872208
2649 BPSL1778		1893832	1903701	1162708	1172577	2274578	2278969	2260515	2270375	2274331	2265515	1887981	1897850	1895507	1885647	1907815	1910505	1887360	1877476
2650 BPSL1779		1893248	1891506	1162124	1160382	2274006	2272264	2259943	2258201	2274915	2276657	1887407	1885665	1896079	1897821	1907229	1905487	1887934	1889676
2651 BPSL1780		1891015	1890443	1159891	1159319	2271773	2271201	2257710	2257138	2277148	2277720	1885168	1884596	1898322	1898894	1904996	1904424	1890167	1890739
2652	BURPS1106B_A1176			1159908	1160279														
2653 BPSL1781		1889234	1890259	1158110	1159135	2269978	2271003	2255915	2256940	2278929	2277904	1883324	1884349	1900131	1899106	1903215	1904240	1891985	1890960
2654 BPSL1782		1888263	1889066	1157139	1157942	2268980	2269783	2254917	2255720	2279900	2279097	1882353	1883156	1901102	1900299	1902244	1903047	1892956	1892153
2655 BPSL1783		1886146	1888263	1155022	1157139	2266863	2268980	2252800	2254917	2282017	2279900	1880236	1882353	1903219	1901102	1900127	1902244	1895073	1892956
2656 BPSL1784		1885259	1886146	1154135	1155022	2265976	2266863	2251913	2252800	2282904	2282017	1879349	1880236	1904106	1903219	1899240	1900127	1895960	1895073
2657 BPSL1785		1884205	1885218	1153081	1154094	2264922	2265935	2250859	2251872	2283958	2282945	1878295	1879308	1905160	1904147	1898186	1899199	1897014	1896001
2658 BPSL1786		1883904	1884143	1152780	1153019	2264615	2264860	2250552	2250797	2284259	2284020	1877994	1878233	1905461	1905222	1897885	1898124	1897315	1897076
2659 BPSL1787		1883225	1883845	1152101	1152721	2263936	2264556	2249873	2250493	2284938	2284318	1877315	1877935	1906140	1905520	1897206	1897826	1897994	1897374
2660	BURPS1655_A1868																	1898607	1898847
2661	BURPSPAST_AA0569													1906468	1907001				
2662 BPSL1788		1881329	1882072	1150205	1150948	2262033	2262776	2247970	2248713	2286076	2285936	1875393	1876136	1908034	1907291	1895538	1896281	1899856	1899689

2663	BPSL1789		1881163	1881327	1148667	1150052	2260495	2261880	2247804	2247968	2287614	2286229	1875227	1875391	1909573	1908188	1894000	1895385		
2664	BPSL1790		1878669	1879757	1147545	1148633	2259373	2260461	2245310	2246398	2288736	2287648	1872733	1873821	1910695	1909607	1892878	1893966	1902518	1901430
2665	BPSL1791		1877447	1878463	1146323	1147339	2258151	2259167	2244088	2245104	2289958	2288942	1871496	1872512	1911917	1910901	1891656	1892672	1903764	1902748
2666	BPSL1792		1875915	1877441	1144791	1146317	2256619	2258145	2242556	2244082	2291490	2289964	1869964	1871490	1913449	1911923	1890124	1891650	1905296	1903770
2667	BPSL1793		1874909	1875850	1143785	1144726	2255613	2256554	2241550	2242491	2292493	2291555	1868958	1869899	1914455	1913514	1889121	1890059	1906302	1905361
2668	BPSL1794		1873788	1874801	1142664	1143677	2254492	2255505	2240429	2241442	2293614	2292601	1867837	1868850	1915576	1914563	1888000	1889013	1907423	1906410
2669	BPSL1795		1873028	1872315	1141904	1141191	2253732	2253019	2239669	2238956	2294374	2295087	1867077	1866364	1916336	1917049	1887240	1886527	1908183	1908896
2670	BPSL1796		1872235	1870637	1141111	1139513	2252939	2251341	2238876	2237278	2295167	2296765	1866284	1864692	1917129	1918727	1886447	1884849	1908976	1910574
2671	BPSL1797		1870500	1868743	1139376	1137619	2251204	2249447	2237141	2235384	2296902	2298659	1864555	1862798	1918864	1920621	1884712	1883780	1910711	1911799
2672	BPSL1798		1867664	1868686	1136540	1137562	2248368	2249390	2234305	2235327	2299738	2298716	1861719	1862741	1921700	1920678	1881810	1882832	1913548	1912526
2673	BPSL1799		1866918	1867664	1135794	1136540	2247622	2248368	2233559	2234305	2300484	2299738	1860973	1861719	1922446	1921700	1881064	1881810	1914294	1913548
2674	BPSL1800		1864271	1866736	1133147	1135612	2245047	2247512	2230984	2233449	2303131	2300666	1858424	1860889	1925033	1922568	1878471	1880936	1916843	1914378
2675	BPSL1801		1863636	1864145	1132512	1133021	2244412	2244921	2230349	2230858	2303766	2303257	1857789	1858298	1925668	1925159	1877836	1878345	1917478	1916969
2676		BURPS1655_A1886																	1919211	1918945
2677		BURPS1655_A1887																	1919398	1919267
2678		BURPS406E_H1079									2304337	2305104								
2679	BPSL1802		1860132	1861679	1129008	1130555	2240932	2242479	2226868	2228415	2307335	2307171	1854334	1855881	1929220	1929119	1874395	1875942	1920953	1919406
2680	BPSL1803		1857004	1860132	1125880	1129008	2237804	2240932	2223740	2226868	2310463	2307335	1851206	1854334	1932348	1929220	1871267	1874395	1924081	1920953
2681	BPSL1804		1855788	1856984	1124664	1125860	2236588	2237784	2222524	2223720	2311679	2310483	1849990	1851186	1933564	1932368	1870051	1871247	1925297	1924101
2682	BPSL1805		1855644	1854976	1124520	1123852	2236444	2235776	2222380	2221712	2311823	2312491	1849846	1849178	1933708	1934376	1869907	1869239	1925441	1926091
2683	BPSL1806		1854457	1853219	1123229	1121991	2235257	2234019	2221193	2219955	2313299	2314537	1848656	1847415	1934970	1936208	1868643	1867405	1926018	1926293
2684	BPSL1807		1852431	1853171	1121203	1121943	2233231	2233971	2219167	2219907	2315325	2314585	1846627	1847367	1936996	1936256	1866617	1867357	1927078	1926341
2685	BPSL1808		1851703	1852425	1120475	1121197	2232503	2233225	2218439	2219161	2316053	2315331	1845899	1846621	1937724	1937002	1865889	1866611	1927806	1927084
2686	BPSL1809		1850489	1851301	1119261	1120073	2231289	2232101	2217225	2218037	2317267	2316455	1844738	1845550	1938938	1938126	1864675	1865487	1929005	1928193
2687	BPSL1810		1849229	1850206	1118001	1118978	2230024	2231001	2215960	2216937	2318527	2317550	1843489	1844466	1940198	1939221	1863415	1864392	1930264	1929287
2688		BURPS668_1881											1844433	1844615						
2689	BPSL1811		1848757	1849182	1117529	1117954	2229552	2229977	2215488	2215913	2318999	2318574	1843017	1843442	1940670	1940245	1862943	1863368	1930736	1930311
2690	BPSL1812		1847711	1848757	1116483	1117529	2228497	2229552	2214433	2215488	2320009	2318999	1841998	1843017	1941716	1940670	1861870	1862943	1931737	1930736
2691	BPSL1813		1846671	1847612	1115443	1116384	2227457	2228398	2213393	2214334	2321049	2320108	1840967	1841908	1942756	1941815	1860830	1861771	1932231	1931836
2692	BPSL1814		1845679	1846671	1114451	1115443	2226465	2227457	2212401	2213393	2321956	2321822	1839975	1840967	1943741	1943598	1859838	1860830	1932825	1932334
2693	BPSL1815		1844108	1845679	1112880	1114451	2224999	2226465	2210836	2212401	2323419	2321956	1838404	1839975	1944976	1943741	1858414	1859838	1934216	1932825
2694	BPSL1816		1842876	1844108	1111648	1112880	2223666	2224898	2209604	2210836	2324687	2323524	1837172	1838404	1946202	1945105	1857144	1858310	1935568	1934336
2695	BPSL1817		1842452	1842862	1111224	1111634	2223242	2223652	2209180	2209590	2325111	2324701	1836748	1837158	1946626	1946216	1856720	1857130	1935992	1935582
2696	BPSL1818		1841099	1842415	1109871	1111187	2221889	2223205	2207827	2209143	2326464	2325148	1835395	1836711	1947979	1946663	1855367	1856683	1937345	1936029
2697	BPSL1819		1839883	1840932	1108654	1109574	2220673	2221722	2206611	2207660	2327680	2326631	1834179	1835228	1949195	1948146	1854151	1855200	1938561	1937512
2698	BPSL1820		1839352	1839867	1108123	1108638	2220142	2220657	2206080	2206595	2328211	2327696	1833648	1834163	1949726	1949211	1853620	1854135	1939092	1938577
2699	BPSL1821		1839135	1839323	1107906	1108094	2219925	2220113	2205863	2206051	2328428	2328240	1833431	1833619	1949943	1949755	1853403	1853591	1939309	1939121
2700	BPSL1822		1837607	1838575	1106378	1107346	2218379	2219347	2204317	2205285	2329950	2328982	1831915	1832883	1951459	1950491	1851863	1852831	1940837	1939869
2701	BPSL1823		1836771	1837607	1105542	1106378	2217543	2218379	2203481	2204317	2330786	2329950	1831079	1831915	1952295	1951459	1851027	1851863	1941676	1940837
2702	BPSL1824		1835273	1836316	1104017	1105060	2215991	2217034	2201929	2202972	2332321	2331278	1829599	1830642	1953766	1952723	1849610	1850653	1943183	1942140
2703	BPSL1825		1833716	1835017	1102460	1103761	2214434	2215735	2200372	2201673	2333878	2332577	1828042	1829343	1955323	1954022	1848053	1849354	1944740	1943439
2704	BPSL1826		1831763	1833286	1100507	1102030	2212438	2213961	2198376	2199899	2335889	2334366	1826046	1827569	1957319	1955796	1846047	1847570	1946736	1945213
2705		BURPS1106B_A1114			1102000	1102290														
2706	BPSL1827		1830342	1831604	1099086	1100348	2211017	2212279	2196955	2198217	2337310	2336048	1824625	1825887	1958740	1957478	1844626	1845888	1948157	1946895
2707	BPSL1828		1828296	1830215	1097040	1098959	2208971	2210890	2194909	2196828	2339356	2337437	1822579	1824498	1960786	1958867	1842580	1844499	1950203	1948284
2708	BPSL1829		1825541	1827442	1094285	1096186	2206216	2208117	2192154	2194055	2342139	2340481	1819808	1821694	1963544	1961640	1839811	1841712	1952973	1951072
2709	BPSL1830		1824378	1825349	1093122	1094093	2205053	2206024	2190991	2191962			1818645	1819616	1964707	1963736	1838648	1839619	1954136	1953165
2710	BPSL1831		1823350	1824378	1092094	1093122	2204025	2205053	2189963	2190991	2344354	2343326	1817617	1818645	1965735	1964707	1837620	1838648	1955164	1954136
2711	BPSL1832		1822307	1823317	1091051	1092061	2202982	2203992	2188920	2189930	2345397	2344387	1816574	1817584	1966778	1965768	1836577	1837587	1956207	1955197
2712	BPSL1833		1820665	1822233	1089409	1090977	2201340	2202908	2187278	2188846	2347037	2346684	1814932	1816500	1968420	1966852	1834935	1836503	1957849	1956281
2713	BPSL1834		1819563	1820522	1088307	1089266	2200235	2201194	2186173	2187132	2348142	2347183	1813830	1814789	1969525	1968566	1833833	1834792	1958954	1957995
2714	BPSL1835		1818278	1819201	1087022	1087945	2198950	2199873	2184888	2185811	2349428	2348505	1812546	1813469	1970810	1969887	1832549	1833472	1960239	1959316
2715	BPSL1836		1817153	1818205	1085897	1086949	2197825	2198877	2183763	2184815	2350553	2349501	1811421	1812473	1971935	1970883	1831424	1832476	1961364	1960312
2716	BPSL1837		1816261	1817136	1085005	1085880	2196933	2197808	2182871	2183746	2351445	2350570	1810529	1811404	1972827	1971952	1830532	1831407	1962256	1961381
2717	BPSL1838		1815215	1816105	1083959	1084849	2195887	2196777	2181825	2182715	2352479	2351589	1809471	1810361	1973861	1972971	1829486	1830376	1962954	1962412
2718	BPSL1839		1814099	1815079	1082843	1083823	2194771	2195751	2180709	2181689	2353677	2352697	1808355	1809335	1975046	1974066	1828288	1829268	1964108	1963128
2719	BPSL1840		1813742	1813098	1082486	1081842	2194414	2193770	2180352	2179708	2354034	2354678	1807998	1807354	1975403	1976047	1827931	1827287	1964465	1965109
2720	BPSL1841		1813032	1812709	1081776	1081453	2193704	2193381	2179642	2179319	2354744	2355067	1807288	1806965	1976113	1976436	1827221	1826898	1965175	1965498

2721	BPSL1842		1812672	1811941	1081416	1080685	2193344	2192613	2179282	2178551	2355104	2355835	1806928	1806197	1976473	1977204	1826861	1826130	1965535	1966266
2722	BPSL1843		1811036	1811524	1079780	1080268	2191680	2192168	2177618	2178106	2356754	2356266	1805285	1805773	1978158	1977670	1825224	1825712	1967178	1966690
2723	BPSL1844		1810812	1809895	1079556	1078639	2191456	2190539	2177394	2176477	2356978	2357895	1805061	1804144	1978382	1979299	1825000	1824083	1967402	1968319
2724	BPSL1845		1809887	1809054	1078631	1077798	2190531	2189698	2176469	2175636	2357903	2358736	1804136	1803303	1979307	1980140	1824075	1823242	1968327	1969160
2725	BPSL1846		1808448	1808942	1077192	1077686	2189092	2189586	2175030	2175524	2359342	2358848	1802697	1803191	1980746	1980252	1822636	1823130	1969766	1969272
2726	BPSL1847		1807151	1808209	1075895	1076953	2187795	2188853	2173733	2174791	2360643	2359585	1801396	1802454	1982043	1980985	1821339	1822397	1971067	1970009
2727	BPSL1848		1805743	1806792	1074487	1075536	2186387	2187436	2172325	2173374	2362072	2361023	1799988	1801037	1983451	1982402	1819931	1820980	1972475	1971426
2728	BPSL1849		1805010	1805453	1073754	1074197	2185665	2186108	2171603	2172046	2362805	2362362	1799264	1799707	1984184	1983741	1819209	1819652	1973208	1972765
2729	BPSL1850		1804837	1804364	1073581	1073108	2185492	2185019	2171430	2170957	2362978	2363451	1799091	1798618	1984357	1984830	1819036	1818563	1973381	1973854
2730	BPSL1851		1803180	1801876	1071924	1070620	2183836	2182532	2169774	2168470	2364633	2365937	1797428	1796124	1986012	1987316	1817379	1816075	1974921	1976225
2731		BURPS1710A_2289					2185013	2184015												
2732		BURPS1710b_1999							2169972	2170433										
2733		BURPSS13_P0480															1818557	1817577		
2734	BPSL1852		1801295	1800279	1070039	1069023	2181942	2180926	2167880	2166864	2366527	2367543	1795533	1794517	1987897	1988169	1815484	1814468	1976817	1977833
2735	BPSL1853		1799046	1799297	1067790	1068041	2179693	2179944	2165631	2165882	2368776	2368525	1793284	1793535	1990058	1989807	1813258	1813509	1979052	1978801
2736	BPSL1854		1798727	1798969	1067471	1067713	2179374	2179616	2165312	2165554	2369095	2368853	1792965	1793207	1990377	1990135	1812939	1813181	1979371	1979129
2737	BPSL1855		1798431	1798643	1067175	1067387	2179078	2179290	2165016	2165228	2369391	2369179	1792669	1792881	1990673	1990461	1812643	1812855	1979667	1979455
2738	BPSL1856		1797334	1798338	1066078	1067082	2177982	2178986	2163920	2164924	2370487	2369483	1791597	1792577	1991769	1990765	1811546	1812550	1980763	1979759
2739	BPSL1857		1796528	1797334	1065272	1066078	2177164	2177982	2163102	2163920	2371305	2370487	1790791	1791597	1992575	1991769	1810740	1811546	1981581	1980763
2740	BPSL1858		1795353	1796507	1064097	1065251	2175989	2177143	2161927	2163081	2372480	2371326	1789616	1790770	1993750	1992596	1809565	1810719	1982756	1981602
2741	BPSL1859		1795017	1794556	1063761	1063300	2175652	2175191	2161590	2161129	2372816	2373277	1789280	1788819	1994076	1994537	1809229	1808768	1983085	1983546
2742	BPSL1860		1792954	1794093	1061698	1062837	2173589	2174728	2159527	2160666	2374879	2373740	1787219	1788358	1996139	1995000	1807165	1808292	1985148	1984009
2743	BPSL1861		1792586	1792921	1061330	1061665	2173221	2173556	2159159	2159494	2375247	2374912	1786851	1787186	1996507	1996172	1806797	1807132	1985516	1985181
2744	BPSL1862		1792084	1792578	1060828	1061322	2172719	2173213	2158657	2159151	2375749	2375255	1786349	1786843	1997009	1996515	1806295	1806789	1986018	1985524
2745	BPSL1863		1791364	1790822	1060108	1059566	2171999	2171457	2157937	2157395	2376469	2377011	1785630	1785088	1997729	1998271	1805575	1805033	1986738	1987280
2746	BPSL1864		1790810	1790046	1059554	1058790	2171445	2170681	2157383	2156619	2377023	2377787	1785076	1784312	1999283	1999047	1805021	1804257	1987292	1988056
2747	BPSL1865		1790009	1789182	1058753	1057926	2170644	2169817	2156582	2155755	2377824	2378651	1784275	1783448	1999084	1999911	1804220	1803393	1988093	1988920
2748	BPSL1866		1789074	1788049	1057818	1056793	2169709	2168684	2155647	2154622	2378759	2379784	1783340	1782315	2000019	2001044	1803285	1802260	1989028	1990053
2749	BPSL1867		1787926	1787135	1056670	1055879	2168561	2167770	2154499	2153708	2379907	2380698	1782192	1781401	2001166	2001957	1802137	1801346	1990176	1990967
2750	BPSL1868		1787002	1786346	1055746	1055090	2167637	2166981	2153575	2152919	2380831	2381487	1781268	1780612	2002090	2002746	1801213	1800557	1991100	1991756
2751	BPSL1869		1784958	1785938	1053702	1054682	2165593	2166573	2151531	2152511	2382874	2381894	1779226	1780206	2004134	2003154	1799175	1800155	1993142	1992162
2752	BPSL1870		1784145	1784855	1052889	1053599	2164780	2165490	2150718	2151428	2383687	2382977	1778413	1779123	2004947	2004237	1798362	1799072	1993955	1993245
2753	BPSL1871		1783112	1783804	1051856	1052548	2163747	2164439	2149685	2150377	2384720	2384028	1777381	1778073	2005980	2005288	1797329	1798021	1994988	1994296
2754	BPSL1872		1781944	1782840	1050688	1051584	2162579	2163475	2148517	2149413	2385888	2384992	1776213	1777109	2007148	2006252	1796161	1797057	1996156	1995260
2755	BPSL1873		1780827	1781567	1049571	1050311	2161462	2162202	2147400	2148140	2387005	2386265	1775096	1775836	2008265	2007525	1795044	1795784	1997273	1996533
2756	BPSL1874		1778161	1780692	1046905	1049436	2158796	2161327	2144734	2147265	2389671	2387140	1772430	1774961	2010931	2008400	1792378	1794909	1999939	1997408
2757	BPSL1875		1775809	1777518	1044553	1046262	2156444	2158153	2142382	2144091	2392023	2390314	1770078	1771787	2013283	2011574	1790026	1791735	2002291	2000582
2758	BPSL1876		1775307	1773790	1044051	1042534	2155942	2154425	2141880	2140363	2392525	2394042	1769576	1768059	2013785	2015302	1789525	1788008	2002793	2004310
2759		BURPS406E_H1174									2394365	2395627								
2760	BPSL1877		1773491	1771968	1042235	1040712	2152684	2151161	2138622	2137099	2395763	2397286	1767761	1766238	2015596	2017119	1787708	1786185	2004609	2006132
2761		BURPS1710A_2253					2154082	2152820												
2762		BURPS1710b_1964							2138758	2140074										
2763		BURPS1710b_1965							2139424	2139582										
2764	BPSL1878		1769785	1771494	1038529	1040238	2149007	2150716	2134938	2136647	2399421	2397712	1764119	1765828	2019301	2017592	1784106	1785815	2008182	2006473
2765	BPSL1879		1769112	1769735	1037856	1038479	2148334	2148957	2134265	2134888	2400094	2399471	1763446	1764069	2019974	2019351	1783433	1784056	2008717	2008232
2766	BPSL1880		1768444	1768965	1037188	1037709	2147666	2148187	2133597	2134118	2400762	2400241	1762778	1763299	2020642	2020121	1782765	1783286		
2767	BPSL1881		1767998	1766781	1036742	1035525	2147220	2146003	2133151	2131934	2401208	2402425	1762384	1761167	2021088	2022305	1782319	1781102	2009157	2010095
2768		BURPS668_1786											1762541	1762744						
2769	BPSL1882		1766561	1765614	1035305	1034358	2145783	2144836	2131714	2130767	2402645	2403598	1760947	1760000	2022525	2023478	1780882	1779935	2011298	2011444
2770	BPSL1883		1765426	1763657	1034170	1032401	2144648	2142879	2130579	2128810	2403786	2405555	1759812	1758043	2023666	2025435	1779747	1777978	2011632	2013401
2771	BPSL1884		1763594	1762638	1032338	1031382	2142816	2141848	2128747	2127779	2405618	2406586	1757980	1757024	2025498	2026466	1777915	1776947	2013464	2014420
2772	BPSL1885		1762175	1762444	1030919	1031188	2141410	2141679	2127341	2127610	2407024	2406755	1756573	1756842	2026904	2026635	1776509	1776778	2014859	2014590
2773	BPSL1885A		1761870	1761223	1030614	1029967	2141105	2140458	2127036	2126389	2407329	2407976	1756268	1755696	2027209	2027856			2015164	2015781
2774	BPSL1886		1760338	1761066	1029082	1029810	2139573	2140301	2125504	2126232	2408861	2408133	1754670	1755398	2028741	2028013	1774573	1775301	2016666	2015938
2775	BPSL1887		1758874	1760262	1027618	1029006	2138109	2139497	2124040	2125428	2410325	2408937	1753206	1754594	2030205	2028817	1773109	1774497	2018130	2016742
2776	BPSL1888		1757151	1758854	1025895	1027598	2136386	2138089	2122317	2124020	2412048	2410345	1751483	1753186	2031928	2030225	1771385	1772029	2019853	2018150
2777	BPSL1889		1756661	1757038	1025405	1025782	2135896	2136273	2121827	2122204	2412538	2412161	1751005	1751370	2032340	2032041	1771063	1771272	2020256	2019966
2778	BPSL1890		1755674	1756636	1024418	1025380	2134909	2135871	2120840	2121802	2413526	2412678	1750018	1750980	2033249	2032443	1770719	1770949	2020987	2020436

2779 BPSL1891			1754648	1755664	1023392	1024408	2133883	2134899	2119814	2120830	2414552	2413536	1748980	1750008	2034275	2033259	1769681	1770709	2022025	2020997
2780 BPSL1892			1753644	1754648	1022388	1023392	2132879	2133883	2118810	2119814	2415556	2414552	1747976	1748980	2035279	2034275	1768677	1769681	2022789	2022616
2781 BPSL1893			1752308	1753648	1021052	1022392	2131543	2132883	2117474	2118814	2416892	2415552	1746640	1747980	2036615	2035275	1767341	1768681	2024125	2022785
2782 BPSL1894			1751066	1752301	1019810	1021045	2130301	2131536	2116232	2117467	2418134	2416899	1745398	1746633	2037857	2036622	1766099	1767334	2025367	2024132
2783 BPSL1895			1749674	1750996	1018418	1019740	2128909	2130231	2114840	2116162	2419526	2418204	1744006	1745328	2039249	2037927	1764707	1766029	2026759	2025437
2784 BPSL1896			1748711	1749607	1017455	1018351	2127946	2128842	2113877	2114773	2420489	2419593	1743043	1743939	2040212	2039316	1763744	1764640	2027722	2026826
2785 BPSL1897			1748164	1748628	1016908	1017372	2127399	2127863	2113330	2113794	2421036	2420572	1742496	1742960	2040759	2040295	1763197	1763661	2028269	2027805
2786 BPSL1898			1747667	1748164	1016411	1016908	2126902	2127399	2112833	2113330	2421533	2421036	1741999	1742496	2041256	2040759	1762700	1763197	2028766	2028269
2787 BPSL1899			1747414	1747581	1016158	1016325	2126649	2126816	2112580	2112747	2421786	2421619	1741746	1741913	2041509	2041342	1762447	1762614	2029019	2028852
2788 BPSL1900			1747169	1747303	1015913	1016047	2126404	2126538	2112335	2112469	2422031	2421897	1741501	1741635	2041754	2041620	1762202	1762336	2029264	2029130
2789 BPSL1901			1746749	1745151	1015493	1013895	2125984	2124374	2111915	2110305	2422451	2424049	1741081	1739480	2042174	2043784	1761782	1760184	2029684	2031270
2790 BPSL1902			1745098	1743836	1013842	1012580	2124321	2123059	2110252	2108990	2424102	2425364	1739427	1738165	2043837	2045099	1760131	1758869	2031323	2032585
2791 BPSL1903			1743682	1742006	1012426	1010750	2122905	2121229	2108836	2107160	2425518	2427194	1738011	1736335	2045253	2046929	1758715	1757039	2032739	2034415
2792 BPSL1904			1741783	1741304	1010527	1010048	2121002	2120523	2106933	2106454	2427421	2427900	1736109	1735630	2047156	2047635	1756816	1756337	2034638	2035117
2793 BPSL1905			1740654	1741061	1009398	1009805	2119873	2120280	2105804	2106211	2428539	2428132	1734980	1735387	2048285	2047878	1755687	1756094	2035767	2035360
2794 BPSL1906			1739362	1740456	1008106	1009200	2118581	2119675	2104512	2105606	2429831	2428737	1733688	1734782	2049577	2048483	1754395	1755489	2037059	2035965
2795 BPSL1907			1737825	1739252	1006569	1007996	2117044	2118471	2102975	2104402	2431368	2429941	1732151	1733578	2051114	2049687	1752858	1754285	2038596	2037169
2796 BPSL1908			1736460	1737722	1005204	1006466	2115667	2116941	2101598	2102872	2432733	2431471	1730774	1732048	2052491	2051217	1751481	1752755	2039973	2038699
2797 BPSL1909			1733461	1736322	1002205	1005066	2112668	2115529	2098599	2101460	2435732	2432871	1727782	1730643	2055490	2052629	1748482	1751343	2042972	2040111
2798 BPSL1910		Bp_chr1_25_ISBma2											1725910	1727482	2055790	2057362				
2799 BPSL1911			1731296	1733119	1000040	1001863	2110503	2112326	2096434	2098257	2437897	2436074	1724026	1725849	2059246	2057423	1746317	1748140	2045137	2043314
2800 BPSL1912			1731003	1730509	999747	999253	2110210	2109716	2096141	2095647	2438190	2438684	1723733	1723239	2059539	2060033	1746022	1745528	2045430	2045924
2801 BPSL1913			1730406	1728913	999150	997657	2109613	2108120	2095544	2094051	2438787	2440280	1723136	1721643	2060136	2061629	1745425	1743932	2046027	2047520
2802 BPSL1914			1728842	1727610	997586	996354	2108049	2106817	2093980	2092748	2440351	2441583	1721572	1720340	2061700	2062932	1743861	1742629	2047591	2048823
2803 BPSL1915			1727584	1726028	996328	994772	2106791	2105235	2092722	2091166	2441609	2443165	1720314	1718758	2062958	2064514	1742603	1741047	2048849	2050405
2804 BPSL1916			1724820	1725752	993564	994496	2104027	2104959	2089958	2090890	2444374	2443442	1717549	1718481	2065722	2064790	1739838	1740770	2051613	2050681
2805 BPSL1917			1724425	1724790	993169	993534	2103632	2103997	2089563	2089928	2444769	2444404	1717154	1717519	2066117	2065752	1739443	1739808	2052008	2051643
2806 BPSL1918			1721392	1724316	990136	993060	2100599	2103523	2086530	2089454	2447889	2444965	1714121	1717045	2069464	2066540	1736332	1739256	2054912	2052201
2807 BPSL1919			1719825	1721297	988569	990041	2099032	2100504	2084963	2086435	2449456	2447984	1712554	1714026	2071031	2069559	1734765	1736237	2056479	2055007
2808 BPSL1920			1719367	1719825	988111	988569	2098574	2099032	2084505	2084963	2449914	2449456	1712096	1712554	2071489	2071031	1734307	1734765	2056937	2056479
2809 BPSL1921			1717398	1719059	986142	987803	2096605	2098266	2082536	2084197	2451883	2450222	1710127	1711788	2073301	2071913	1732338	1733999	2058906	2057245
2810 BPSL1922			1715964	1717331	984708	986075	2095171	2096538	2081102	2082469	2453278	2451950	1708696	1710060	2074845	2073478	1730904	1732271	2060337	2058973
2811 BPSL1923			1714643	1715542	983387	984286	2093850	2094749	2079781	2080680	2454599	2453700	1707375	1708274	2076166	2075267	1729583	1730482	2061658	2060759
2812 BPSL1924			1714221	1713826	982965	982570	2093428	2093033	2079359	2078964	2455021	2455416	1706942	1706547	2076588	2076983	1729161	1728766	2062080	2062475
2813 BPSL1925			1713584	1713363	982328	982107	2092791	2092570	2078722	2078501	2455658	2455879	1706305	1706084	2077225	2077446	1728524	1728303	2062717	2062938
2814 BPSL1926			1712884	1713198	981628	981942	2092089	2092403	2078020	2078334	2456358	2456044	1705605	1705919	2077925	2077611	1727824	1728138	2063419	2063105
2815 BPSL1927			1712683	1711964	981427	980708	2091888	2091169	2077819	2077100	2456559	2457278	1705404	1704685	2078126	2078845	1727623	1726904	2063620	2064339
2816 BPSL1929			1711722	1711447	980466	980191	2090927	2090652	2076858	2076583	2457520	2457795	1704443	1704168	2079087	2079362	1726662	1726387	2064581	2064856
2817 BPSL1930			1711295	1711041	980039	979785	2090500	2090246	2076431	2076177	2457947	2458201	1704016	1703762	2079514	2079768	1726235	1725981	2065008	2065262
2818 BPSL1931			1710698	1710985	979442	979729	2089903	2090190	2075834	2076121	2458544	2458257	1703419	1703706	2080111	2079824	1725638	1725925	2065605	2065318
2819	BURPS406E_H1233										2460316	2460666								
2820 BPSL1932		Bp_chr1_26_ISBma1																		
2821		Bp_chr1_27_ISBma2											1701319	1702891						
2822 BPSL1933			1708910	1710133	977654	978877	2088113	2089336	2074044	2075267	2460333	2459110	1700037	1701260	2081901	2080678	1723850	1725073	2067148	2066684
2823	BURPSS13_P0378																1723867	1723517		
2824 BPSL1934			1708581	1708093	977325	976837	2087784	2087296	2073715	2073227	2460663	2461151	1699706	1699218	2082230	2082718	1723520	1723032	2067476	2067964
2825 BPSL1935			1707950	1706886	976694	975630	2087141	2086077	2073072	2072008	2461306	2462370	1699051	1697987	2082873	2083937	1722889	1721825	2068107	2069171
2826 BPSL1936			1706171	1704990	974915	973734	2085362	2084181	2071293	2070112	2463085	2464266	1697271	1696090	2084652	2085833	1721116	1719935	2069883	2070179
2827 BPSL1937			1704487	1702370	973231	971114	2083678	2081561	2069609	2067492	2464769	2466886	1695587	1693470	2086336	2088453	1719432	1717315	2071569	2073686
2828 BPSL1938			1701841	1702242	970585	970986	2081032	2081433	2066963	2067364	2467415	2467014	1692946	1693347	2088982	2088581	1716786	1717187	2074215	2073814
2829 BPSL1939			1701435	1701779	970179	970523	2080626	2080970	2066557	2066901	2467821	2467477	1692540	1692884	2089388	2089044	1716380	1716724	2074621	2074277
2830 BPSL1940			1698873	1701302	967617	970046	2078064	2080493	2063995	2066424	2470383	2467954	1689978	1692407	2091950	2089521	1713818	1716247	2077183	2074754
2831 BPSL1941			1697772	1698782	966516	967526	2076963	2077973	2062894	2063904	2471484	2470474	1688877	1689887	2093051	2092041	1712717	1713727	2078284	2077274
2832 BPSL1942			1697264	1697620	966008	966364	2076455	2076811	2062386	2062742	2471992	2471636	1688369	1688725	2093559	2093203	1712209	1712565	2078792	2078436
2833 BPSL1943			1697036	1697230	965780	965974	2076227	2076421	2062158	2062352	2472220	2472026	1688141	1688335	2093787	2093593	1711981	1712175	2079020	2078826
2834 BPSL1944			1696307	1696840	965051	965584	2075498	2076031	2061429	2061962	2472949	2472416	1687412	1687945	2094516	2093983	1711252	1711785	2079749	2079216
2835 BPSL1945			1694362	1696266	963106	965010	2073553	2075457	2059484	2061388	2474894	2472990	1685467	1687371	2096461	2094557	1709307	1711211	2081694	2079790
2836 BPSL1946			1691666	1693900	960410	962644	2070856	2073090	2056787	2059021	2477590	2475356	1682770	1685004	2099157	2096923	1706611	1708845	2084390	2082156

2837 BPSL1947		1691268	1691618	960012	960362	2070457	2070807	2056389	2056739	2477988	2477638	1682372	1682722	2099555	2099205	1706213	1706563	2084788	2084438
2838 BPSL1948		1690289	1691191	959033	959935	2069478	2070380	2055410	2056312	2478967	2478065	1681393	1682295	2100620	2099718	1705143	1706045	2086186	2085284
2839 BPSL1949		1689769	1689737	958513	958481	2068958	2068926	2054890	2054858	2479487	2479519			2101140	2101172	1704623	1704591	2086706	2086738
2840 BPSL1950		1688057	1688959	956801	957703	2067246	2068148	2053178	2054080	2481199	2480297	1678261	1679163	2102852	2101950	1702911	1703813	2088418	2087516
2841	BURPS668_1705											1679212	1679655						
2842 BPSL1951		1687869	1687315	956613	956059	2067058	2066504	2052990	2052436	2481387	2481941	1678073	1677519	2103040	2103531	1702723	1702169	2088606	2089160
2843 BPSL1952		1687297	1686200	956041	954944	2066486	2065389	2052418	2051321	2481959	2483056	1677501	1676404	2103633	2104682	1702151	1701054	2089178	2090275
2844 BPSL1953		1686153	1685434	954897	954178	2065342	2064623	2051274	2050555	2483103	2483822	1676357	1675638	2104729	2105448	1701007	1700288	2090322	2091041
2845 BPSL1954		1684389	1685027	953133	953771	2063554	2064192	2049486	2050124	2484905	2484267	1674486	1675124	2106531	2105893	1699184	1699822	2092068	2091430
2846 BPSL1955		1683683	1684384	952427	953128	2062848	2063549	2048780	2049481	2485611	2484910	1673780	1674481	2107237	2106536	1698478	1699179	2092774	2092073
2847 BPSL1956		1683430	1682888	952174	951632	2062595	2062053	2048527	2047985	2485864	2486406	1673527	1672985	2107490	2108032	1698225	1697683	2093027	2093569
2848 BPSL1957		1682293	1680764	951037	949508	2061458	2059929	2047390	2045861	2486980	2488509	1672410	1670881	2108627	2110156	1697088	1695559	2094142	2095671
2849 BPSL1958		1679832	1678762	948576	947506	2058997	2058395	2044929	2044327	2489441	2490511	1669949	1668879	2111088	2112158	1694627	1693557	2096602	2097672
2850 BPSL1959		1677950	1678378	946694	947122	2057583	2058011	2043515	2043943	2491323	2490895	1668067	1668495	2112970	2112542	1692745	1693173	2098484	2098056
2851 BPSL1960		1677126	1677902	945870	946646	2056759	2057535	2042691	2043467	2492147	2491371	1667243	1668019	2113794	2113018	1691921	1692697	2099308	2098532
2852 BPSL1961		1676945	1675275	945689	944019	2056578	2054908	2042510	2040840	2492328	2493998	1667062	1665392	2113975	2115645	1691740	1690070	2099489	2101159
2853 BPSL1962		1672514	1673620	941258	942364	2053609	2054715	2039541	2040647	2495297	2494191	1664048	1665154	2116944	2115838	1688771	1689877	2102458	2101352
2854	BURPS1106A_1714	1673775	1674611																
2855	BURPS1106B_A0958			943355	942519														
2856 BPSL1963		1670215	1672146	938959	940890	2051310	2053241	2037242	2039173	2497596	2495665	1661749	1663680	2119125	2117323	1686472	1688403	2104758	2102827
2857 BPSL1964		1669651	1670112	938395	938856	2050746	2051207	2036678	2037139	2498160	2497699	1661185	1661646	2119689	2119228	1685908	1686369	2105322	2104861
2858 BPSL1965		1669434	1669117	938178	937861	2050529	2050212	2036461	2036144	2498377	2498694	1660968	1660651	2119906	2120223	1685691	1685374	2105539	2105856
2859 BPSL1966		1667557	1668858	936328	937629	2048724	2050025	2034656	2035957	2500182	2498881	1659145	1660446	2121756	2120455	1683823	1685124	2107371	2106070
2860 BPSL1967		1667442	1666837	936213	935608	2048609	2048004	2034541	2033936	2500297	2500902	1659030	1658425	2121871	2122476	1683708	1683103	2107486	2108091
2861 BPSL1968		1666837	1666391	935608	935162	2048004	2047558	2033936	2033490	2500902	2501348	1658425	1657979	2122476	2122922	1683103	1682657	2108091	2108537
2862 BPSL1969		1666353	1664935	935124	933706	2047520	2046102	2033452	2032034	2501386	2502804	1657941	1656523	2122960	2124378	1682619	1681201	2108575	2109993
2863 BPSL1970		1664900	1663932	933671	932703	2046067	2045075	2031999	2031007	2502839	2503807	1656488	1655508	2124413	2125405	1681166	1680186	2110028	2111008
2864 BPSL1971		1662639	1663577	931410	932348	2043782	2044720	2029714	2030652	2505100	2504162	1654215	1655153	2126698	2125760	1678893	1679831	2112301	2111363
2865 BPSL1972		1662481	1660715	931252	929486							1654057	1652264	2126856	2128811	1678735	1676978	2112459	2114207
2866 BPSL1973		1660692	1658494	929463	927265	2041745	2039547	2027677	2025479	2507056	2509254	1652241	1650043	2128762	2130960	1676955	1674757	2114230	2116428
2867 BPSL1974		1658494	1658294	927265	927065	2039547	2039347	2025479	2025279	2509254	2509454	1650043	1649843	2130960	2131160	1674757	1674557	2116428	2116628
2868 BPSL1975		1658289	1657399	927060	926170	2039342	2038452	2025274	2024384	2509459	2510349	1649838	1648948	2131165	2132055	1674552	1673662	2116633	2117523
2869	BURPS1655_A2087																	2117613	2117903
2870 BPSL1976		1655369	1657042	924140	925813	2036441	2038114	2022373	2024046	2512360	2510687	1646936	1648609	2134067	2132394	1671645	1673318	2119552	2117879
2871	BURPS1106A_1695	1657018	1657308																
2872	BURPS1106B_A0939			926079	925789														
2873	BURPS1710A_2139					2038361	2038068												
2874 BPSL1977		1655017	1653767	923788	922538	2036089	2034839	2022021	2020771	2512712	2513959	1646584	1645334	2134419	2135669	1671293	1670043	2119904	2121154
2875 BPSL1978		1652639	1653424	921410	922195	2033751	2034536	2019683	2020468	2515079	2514294	1644109	1644894	2136797	2136012	1668843	1669628	2122307	2121522
2876 BPSL1979		1650279	1652639	919050	921410	2031391	2033751	2017323	2019683	2517439	2515079	1641749	1644109	2139157	2136797	1666483	1668843	2124667	2122307
2877 BPSL1980		1650139	1647275	918910	916046	2031251	2028387	2017183	2014319	2517579	2520443	1641609	1638745	2139297	2142161	1666343	1663479	2124807	2127671
2878 BPSL1981		1647185	1646307	915956	915078	2028297	2027419	2014229	2013351	2520533	2521411	1638655	1637777	2142251	2143129	1663389	1662511	2127761	2128639
2879 BPSL1982		1645626	1646240	914397	915011	2026738	2027352	2012670	2013284	2522092	2521478	1637096	1637710	2143810	2143196	1661830	1662444	2129320	2128706
2880 BPSL1983		1643144	1645534	911915	914305	2024256	2026646	2010188	2012578	2524574	2522184	1634614	1637004	2146292	2143902	1659348	1661738	2131802	2129412
2881 BPSL1984		1641787	1643130	910558	911901	2022899	2024242	2008831	2010174	2525931	2524588	1633257	1634600	2147649	2146306	1657991	1659334	2133159	2131816
2882 BPSL1985		1641044	1641769	909815	910540	2022156	2022881	2008088	2008813	2526674	2525949	1632514	1633239	2148392	2147667	1657248	1657973	2133902	2133177
2883 BPSL1986		1640354	1640737	909125	909508	2021466	2021849	2007398	2007781	2527364	2526981	1631824	1632207	2149082	2148699	1656558	1656941	2134592	2134209
2884 BPSL1987		1639848	1640075	908619	908846	2020960	2021187	2006892	2007119	2527870	2527643	1631318	1631545	2149588	2149361	1656052	1656279	2135098	2134871
2885 BPSL1988		1638646	1639680	907417	908451	2019758	2020792	2005690	2006724	2529072	2528038	1630166	1631200	2150790	2149756	1654850	1655884	2136301	2135267
2886 BPSL1989		1637614	1638630	906385	907401	2018726	2019742	2004658	2005674	2530104	2529088	1629134	1630150	2151822	2150806	1653818	1654834	2137333	2136317
2887 BPSL1990		1636740	1637618	905511	906389	2017852	2018730	2003784	2004662	2530978	2530100	1628260	1629138	2152696	2151818	1652944	1653822	2138207	2137329
2888 BPSL1991		1636495	1635449	905266	904220	2017607	2016561	2003539	2002493	2531223	2532269	1628015	1626969	2152941	2153987	1652699	1651653	2138452	2139498
2889 BPSL1992		1635345	1634203	904116	902974	2016457	2015315	2002389	2001247	2532373	2533515	1626865	1625723	2154091	2155233	1651549	1650407	2139602	2140744
2890 BPSL1993		1634187	1633390	902958	902161	2015299	2014502	2001231	2000434	2533531	2534328	1625707	1624910	2155249	2156046	1650391	1649594	2140760	2141557
2891 BPSL1994		1633373	1631376	902144	900147	2014485	2012488	2000417	1998420	2534345	2536342	1624893	1622896	2156063	2158060	1649577	1647580	2141574	2143571
2892 BPSL1995		1631362	1629380	900133	898151	2012474	2010492	1998406	1996424	2536356	2538338	1622882	1620900	2158074	2160056	1647566	1645584	2143585	2145567
2893 BPSL1996		1629380	1628463	898151	897234	2010492	2009575	1996424	1995507	2538338	2539255	1620900	1619983	2160056	2160973	1645584	1644667	2145567	2146484
2894 BPSL1997		1628460	1627660	897231	896431	2009572	2008772	1995504	1994704	2539258	2540058	1619980	1619180	2160976	2161776	1644664	1643864	2146487	2147287

2895	BPSL1998			1626836	1627537	895607	896308	2007948	2008649	1993880	1994581	2540882	2540181	1618356	1619057	2162600	2161899	1643040	1643741	2148111	2147410
2896	BPSL1999			1624563	1626005	893334	894776	2005675	2007105	1991607	1993037	2543191	2540882	1616065	1618356	2164873	2163443	1640695	1643040		
2897	BPSL2000			1623635	1624567	892406	893338	2004747	2005679	1990679	1991611	2544119	2543187	1615137	1616069	2165801	2164869	1639767	1640699	2151465	2150533
2898	BPSL2001			1623181	1623627	891952	892398	2004293	2004739	1990225	1990671	2544573	2544127	1614683	1615129	2166255	2165809	1639313	1639759	2151919	2151473
2899	BPSL2002			1621791	1622936	890562	891707	2002903	2004048	1988835	1989980	2545972	2544827	1613302	1614447	2167645	2166500	1637932	1639077	2153327	2152182
2900	BPSL2003			1621574	1621242	890345	890013	2002687	2002355	1988619	1988287	2546188	2546520	1613086	1612754	2167861	2168193	1637716	1637384	2153544	2153876
2901	BPSL2004			1620510	1621136	889281	889907	2001623	2002249	1987555	1988181	2547252	2546626	1612022	1612648	2168925	2168299	1636652	1637278	2154608	2153982
2902	BPSL2005			1620296	1619622	889067	888393	2001409	2000735	1987341	1986667	2547466	2548140	1611809	1611135	2169139	2169813	1636439	1635765	2154821	2155495
2903	BPSL2006			1617844	1619397	886615	888168	1998957	2000510	1984889	1986442	2549926	2548373	1609357	1610910	2171600	2170047	1633987	1635540	2157273	2155720
2904		BURPS406E_H1314										2551866	2550247								
2905	BPSL2007					884634	886280													2159281	2157608
2906	BPSL2008			1615725	1614763	884487	883525	1996912	1995950	1982844	1981882	2551989	2552951	1607265	1606303	2173638	2174600	1631904	1630942	2159428	2160390
2907		BURPS1106A_1662		1615872	1617512																
2908		BURPS668_1639												1607412	1609025						
2909		BURPSS13_P0297																1632027	1633655		
2910	BPSL2009			1611604	1614225	880366	882987	1992780	1995401	1978712	1981333	2556110	2553489	1603179	1605800	2177776	2175155	1627777	1630398	2163520	2160899
2911	BPSL2010			1611052	1609835	879814	878597	1992147	1990930	1978079	1976862	2556866	2558083	1602535	1601318	2178454	2179671	1627189	1625972	2164056	2165273
2912	BPSL2011			1609297	1609500	878059	878262	1990392	1990595	1976324	1976527	2558392	2558285	1600780	1600983	2180130	2179927	1625325	1625528	2165937	2165734
2913	BPSL2012			1609014	1607308	877776	876070	1990109	1988403	1976041	1974335	2558675	2560381	1600497	1598791	2180413	2182119	1625042	1623336	2166220	2166579
2914	BPSL2013			1606488	1606958	875250	875720	1987555	1988025	1973487	1973957	2561215	2560745	1597964	1598434	2182953	2182483	1622516	1622986	2168786	2168316
2915	BPSL2014			1606074	1606457	874836	875219	1987141	1987524	1973073	1973456	2561629	2561246	1597550	1597933	2183367	2182984	1622102	1622485	2169200	2168817
2916	BPSL2015			1603738	1605783	872500	874545	1984805	1986850	1970737	1972782	2563965	2561920	1595214	1597259	2185703	2183658	1619766	1621811	2171536	2169491
2917	BPSL2016			1603435	1602728	872197	871490	1984502	1983795	1970434	1969727	2564268	2564975	1594911	1594204	2186006	2186713	1619463	1618756	2171839	2172546
2918	BPSL2017			1601278	1602678	870040	871440	1982328	1983719	1968260	1969651	2566416	2565025	1592754	1594145	2188145	2186763	1617306	1618706	2174023	2172605
2919	BPSL2018			1601122	1599452	869884	868214	1982172	1980502	1968104	1966434	2566572	2568242	1592598	1590928	2188301	2189971	1617150	1615480	2174179	2175849
2920			Bp_chr1_29_ISBma2									2568302	2569874								
2921	BPSL2019			1599251	1598061	868013	866823	1980301	1979111	1966233	1965043	2570035	2571225	1590732	1589542	2190172	2191362	1615279	1614089	2176056	2177246
2922	BPSL2020			1597434	1596250	866196	865012	1978489	1977305	1964421	1963237	2571852	2573036	1588915	1587731	2191989	2193173	1613462	1612278	2177873	2179057
2923	BPSL2021			1596065	1594449	864827	863211	1977120	1975504	1963052	1961436	2573212	2574828	1587546	1585930	2193358	2194974	1612093	1610477	2179242	2180858
2924	BPSL2022			1594315	1592738	863077	861500	1975370	1973793	1961302	1959725	2574962	2576539	1585793	1584231	2195108	2196685	1610343	1608775	2181001	2182569
2925	BPSL2023			1592536	1592745	861298	861507	1973591	1973800	1959523	1959732	2576741	2576532			2196887	2196678	1608573	1608782	2182771	2182562
2926	BPSL2024			1591811	1592428	860573	861190	1972866	1973483	1958798	1959415	2577466	2576849	1582117	1582734	2197612	2196995	1607848	1608465	2183496	2182879
2927		BURPS668_1619												1582899	1583222						
2928	BPSL2025			1589717	1591795	858479	860557	1970772	1972850	1956704	1958782	2579560	2577482	1580008	1582101	2199706	2197628	1605754	1607832	2185590	2183512
2929	BPSL2026			1588341	1589303	857103	858065	1969396	1970358	1955328	1956290	2580936	2579974	1578632	1579594	2201082	2200120	1604378	1605340	2186966	2186004
2930	BPSL2027			1585977	1588322	854739	857084	1967032	1969377	1952964	1955309	2583300	2580955	1576268	1578613	2203446	2201101	1602014	1604359	2189330	2186985
2931	BPSL2028			1585030	1585866	853792	854628	1966085	1966921	1952017	1952853	2584247	2583411	1575321	1576157	2204393	2203557	1601067	1601903	2190277	2189441
2932	BPSL2029			1584488	1585009	853250	853771	1965543	1966064	1951475	1951996	2584789	2584268	1574779	1575300	2204935	2204414	1600525	1601046	2190819	2190298
2933	BPSL2030			1583851	1584408	852613	853170	1964906	1965463	1950838	1951395	2585426	2584869	1574142	1574699	2205572	2205015	1599888	1600445	2191456	2190899
2934	BPSL2031			1583253	1583795	852015	852557	1964308	1964850	1950240	1950782	2586024	2585482	1573544	1574086	2206170	2205628	1599290	1599832	2192054	2191512
2935	BPSL2032			1581543	1582433	850305	851195	1962598	1963488	1948530	1949420	2587737	2586847	1571834	1572724	2207883	2206993	1597577	1598467	2193741	2192851
2936	BPSL2033			1581126	1579843	849888	848605	1962181	1960898	1948113	1946830	2588154	2589437	1571417	1570134	2208300	2209583	1597160	1595877	2194157	2195440
2937	BPSL2034			1579795	1578815	848557	847577	1960850	1959870	1946782	1945802	2589485	2590465	1570086	1569115	2209631	2210611	1595829	1594849	2195488	2196459
2938	BPSL2035			1578344	1578066	847106	846828	1959399	1959121	1945331	1945053	2590936	2591214	1568644	1568366	2211082	2211360	1594379	1594101	2196930	2197208
2939			Bp_chr1_30_ISBma1					1957654	1958958	1943586	1944890										
2940	BPSL2036			1577530	1577796	846292	846558	1957272	1957538	1943204	1943470	2591739	2591473	1567828	1568097	2211895	2211629	1593576	1593842	2197743	2197477
2941		BURPS1655_A2160																		2198332	2198192
2942		BURPS1655_A2161																		2198574	2198413
2943		BURPS1655_A2162																		2199950	2198637
2944		BURPS1655_A2163																		2199975	2200151
2945		BURPS406E_H1353										2592328	2592188								
2946		BURPS406E_H1354										2593737	2592634								
2947		BURPS406E_H1355										2593947	2593744								
2948		BURPS406E_H1356										2593972	2594148								
2949		BURPS406E_H1358										2596593	2596480								
2950		BURPS406E_H1359										2596895	2596695								
2951		BURPS406E_H1362										2596958	2597926								
2952		BURPS406E_H1363										2598755	2598291								

2953		BURPS406E_H1364									2599351	2598905								
2954		BURPS406E_H1365									2599825	2600061								
2955		BURPS406E_H1366									2600132	2600275								
2956		BURPS406E_H1367									2606238	2603215								
2957		BURPS406E_H1368									2607257	2606241								
2958	BPSL2037		1577161	1576985	845923	845747							1567460	1567284	2212264	2212440				
2959	BPSL2038						1953939	1954802	1939871	1940734					2214233	2213370			2201076	2200213
2960		BURPS1710A_2059					1955040	1954864												
2961		BURPS1710A_2060					1955065	1956378												
2962		BURPS1710A_2061					1956441	1956602												
2963		BURPS1710A_2062					1956683	1956823												
2964		BURPS1710A_2063					1956915	1957028												
2965		BURPS1710b_1781							1940997	1942310										
2966	BPSL2039						1953545	1953766	1939477	1939698					2214918	2214406			2201470	2201249
2967		BURPS1655_A2167																	2203669	2203358
2968		BURPS1655_A2168																	2204520	2204086
2969		BURPS1655_A2169																	2204561	2204728
2970		BURPS1655_A2170																	2205183	2205061
2971		BURPS1655_A2171																	2210688	2207665
2972		BURPS1655_A2172																	2211707	2210691
2973		BURPSPAST_AA0262													2223184	2222264				
2974		BURPSPAST_AA0263													2222133	2221882				
2975		BURPSPAST_AA0264													2221885	2221016				
2976		BURPSPAST_AA0266													2219875	2219618				
2977		BURPSPAST_AA0267													2219151	2218459				
2978		BURPSPAST_AA0268													2217719	2217886				
2979		BURPSPAST_AA0269													2217699	2217244				
2980		BURPSPAST_AA0270													2217157	2216516				
2981	BPSL2040		1571305	1572177	840067	840939	1941155	1942027	1927087	1927959			1560272	1561144	2227017	2226145	1586355	1587227		
2982		BURPS1710A_2047					1946039	1946290												
2983		BURPS1710A_2048					1946287	1947156												
2984		BURPS1710A_2052					1949798	1950100												
2985		BURPS1710A_2053					1950431	1950288												
2986		BURPS1710A_2054					1950738	1950502												
2987		BURPS1710A_2055					1951212	1951658												
2988		BURPS1710b_1774							1930809	1931840										
2989		BURPS1710b_1775							1931956	1932222										
2990		BURPS1710b_1776							1932267	1933088										
2991		BURPS668_1594											1565156	1565407						
2992		BURPS668_1595											1565404	1566273						
2993		BURPSS13_P0255															1591343	1591167		
2994		BURPSS13_P0256															1593018	1592662		
2995		BURPSS13_P0257															1591368	1592681		
2996	BPSL2041		1568463	1571300	837225	840062	1938313	1941150	1924245	1927082	2610095	2607315	1557430	1560267	2229859	2227022	1583513	1586350	2214545	2211765
2997	BPSL2042		1568145	1568447	836907	837209	1937995	1938297	1923927	1924229	2610413	2610111	1557112	1557414	2230177	2229875	1583195	1583497	2214863	2214561
2998	BPSL2042A		1567864	1568124	836626	836886	1937713	1937973	1923645	1923905	2610694	2610434	1556831	1557091	2230459	2230199	1582914	1583174	2215144	2214884
2999	BPSL2043		1567113	1567649	835875	836411	1936962	1937498	1922894	1923430	2611446	2610913	1556084	1556248	2231210	2230674	1582163	1582699	2215895	2215359
3000	BPSL2044		1566801	1567019	835563	835781	1936650	1936868	1922582	1922800	2611758	2611540	1555772	1555990	2231522	2231304	1581851	1582069	2216207	2215989
3001	BPSL2045		1566305	1566793	835067	835555	1936154	1936642	1922086	1922574	2612254	2611766	1555276	1555764	2232018	2231530	1581355	1581843	2216703	2216215
3002	BPSL2046		1562992	1566324	831754	835086	1932841	1936173	1918773	1922105	2615567	2612235	1551963	1555295	2235331	2231999	1578042	1581374	2220016	2216684
3003	BPSL2047		1560475	1562880	829237	831642	1930324	1932729	1916256	1918661	2618084	2615679	1549446	1551851	2237848	2235443	1575526	1576890		
3004	BPSL2048		1559885	1560475	828647	829237	1929734	1930324	1915666	1916256	2618674	2618084	1548856	1549446	2238438	2237848	1574936	1575526		
3005		BURPSPAST_AA0248													2239160	2238561				
3006	BPSL2048A		1559340	1559885	828102	828647	1928451	1928915	1914383	1914847	2619234	2618674	1548302	1548856	2239721	2239257	1574382	1574936	2223677	2223123
3007	BPSL2049		1558361	1559191	827123	827953	1927481	1928311	1913413	1914243	2620204	2619374	1547332	1548162	2240691	2239861	1573412	1574242	2224647	2223817
3008	BPSL2050		1555551	1558355	824313	827117	1924671	1927475	1910603	1913407	2623014	2620210	1544522	1547326	2243501	2240697	1570611	1573406	2227457	2224653
3009	BPSL2051		1555031	1554768	823793	823530	1924150	1923887	1910082	1909819	2623535	2623798	1544001	1543738	2244022	2244285	1570090	1569827	2227978	2228241
3010	BPSL2052		1554768	1553329	823530	822091	1923887	1922448	1909819	1908380	2623798	2625237	1543738	1542299	2244285	2245724	1569827	1568388	2228241	2229680

3011 BPSL2053		1551448	1553295	820210	822057	1920567	1922414	1906499	1908346	2627118	2625271	1540418	1542265	2247605	2245758	1566507	1568354	2231561	2229714
3012 BPSL2054		1550553	1550621	819315	819383	1919680	1920153	1905612	1906085	2628005	2627532			2248492	2248019	1565605	1565673	2232455	2232387
3013 BPSL2055		1549309	1550142	818071	818904	1918465	1919298	1904397	1905230	2629220	2628387	1538999	1539832	2249707	2248874	1564390	1565223	2233669	2232836
3014 BPSL2056		1548937	1546379	817699	815141	1918093	1915529	1904025	1901461	2629592	2632156	1538627	1536069	2250079	2251671	1564018	1561460	2234041	2236599
3015 BPSL2057		1546349	1545864	815111	814626	1915499	1915014	1901431	1900946	2632186	2632671	1536039	1535554	2252668	2253153	1561430	1560945	2236629	2237114
3016 BPSL2058		1545826	1545269	814588	814031	1914976	1914419	1900908	1900351	2632709	2633266	1535516	1534959	2253191	2253748	1560907	1560350	2237152	2237709
3017 BPSL2059		1545220	1544990	813982	813752	1914370	1914140	1900302	1900072	2633315	2633545	1534910	1534680	2253797	2254036	1560301	1560062	2237758	2237988
3018 BPSL2060		1544184	1544525	812946	813287	1913334	1913675	1899266	1899607	2634351	2634010	1533874	1534215	2254842	2254501	1559256	1559597	2238794	2238453
3019 BPSL2061		1542638	1544164	811400	812926	1911788	1913314	1897720	1899246	2635897	2634371	1532328	1533854	2256388	2254862	1557710	1559236	2240340	2238814
3020 BPSL2062		1541926	1542633	810688	811395	1911076	1911783	1897008	1897715	2636609	2635902	1531616	1532323	2257100	2256393	1556998	1557705	2241052	2240345
3021 BPSL2063		1538558	1541842	807320	810604	1907708	1910992	1893640	1896924	2639977	2636693	1528248	1531532	2260468	2257184	1553630	1556914	2244420	2241136
3022	BURPSPAST_AA0228													2260716	2260585				
3023 BPSL2064										2640182	2640051								
3024 BPSL2065		1537475	1538182	806237	806944	1906649	1907356	1892581	1893288	2641072	2640365	1527165	1527872	2261527	2260820	1552523	1553230	2245504	2244797
3025	BURPS1710A_2016					1907460	1907591												
3026	BURPS1710b_1749							1893392	1893523										
3027 BPSL2066		1536128	1536496	804890	805258	1905301	1905669	1891233	1891601	2642419	2642051	1525818	1526186	2262875	2262507	1551176	1551544	2246853	2246485
3028 BPSL2067		1535902	1536114	804664	804876	1905075	1905287	1891007	1891219	2642645	2642433	1525595	1525804	2263101	2262889	1550950	1551162	2247079	2246867
3029 BPSL2068		1534937	1535716	803699	804478	1904114	1904893	1890046	1890825	2643611	2642832	1524634	1525413	2264062	2263283	1549984	1550763	2248040	2247261
3030 BPSL2069		1533591	1534520	802353	803282	1902768	1903697	1888700	1889629	2644991	2644062	1523258	1524187	2265408	2264479	1548606	1549535	2249410	2248481
3031	BURPS668_1556											1524175	1524321						
3032 BPSL2070		1531888	1533276	800650	802038	1901065	1902453	1886997	1888385	2646694	2645306	1521533	1522921	2267111	2265723	1546903	1548291	2251113	2249725
3033	BURPS406E_H1406									2647615	2647442								
3034 BPSL2071		1530934	1531137	799696	799899									2268053	2267862				
3035 BPSL2072		1530260	1530045	799022	798807	1899421	1899206	1885353	1885138	2648312	2648527	1519979	1519764	2268751	2268966	1545293	1545078	2252713	2252928
3036 BPSL2073		1529320	1527845	798082	796607	1898486	1897011	1884418	1882943	2649247	2650722	1519042	1517567	2269686	2271161	1544333	1542858	2253648	2255123
3037 BPSL2074		1527360	1523920	796122	792682	1896553	1893110	1882485	1879042	2651188	2654649	1517100	1513660	2271619	2275062	1542406	1538966	2255581	2259024
3038 BPSL2075		1523920	1520528	792682	789290	1893110	1889718	1879042	1875650	2654649	2658041	1513660	1510268	2275062	2278454	1538966	1535574	2259024	2262416
3039 BPSL2076		1520528	1518315	789290	787077	1889718	1887505	1875650	1873437	2658041	2660254	1510268	1508055	2278454	2280667	1535574	1533361	2262416	2264629
3040 BPSL2077		1518259	1516154	787021	784916	1887449	1885344	1873381	1871276	2660310	2662415	1507999	1505894	2280723	2282828	1533305	1531200	2264685	2266790
3041 BPSL2078		1516124	1514223	784886	782985	1885314	1883413	1871246	1869345	2662445	2664346	1505864	1503963	2282858	2284759	1531170	1529269	2266820	2268721
3042 BPSL2079		1514223	1512004	782985	780766	1883413	1881194	1869345	1867126	2664346	2666565	1503963	1501738	2284759	2286978	1529269	1527050	2268721	2270940
3043 BPSL2080		1512004	1509215	780766	777977	1881194	1878405	1867126	1864337	2666565	2669354	1501738	1498949	2286978	2289767	1527050	1524261	2270940	2273729
3044 BPSL2081		1508518	1509003	777280	777765	1877708	1878193	1863640	1864125	2670066	2669581	1498237	1498722	2290443	2289958	1523565	1524050	2274405	2273920
3045 BPSL2082		1508027	1508401	776789	777163	1877217	1877591	1863149	1863523	2670557	2670183	1497746	1498120	2290934	2290560	1523074	1523448	2274896	2274522
3046 BPSL2083		1507804	1506347	776566	775109	1876994	1875537	1862926	1861469	2670780	2672237	1497523	1496066	2291157	2292614	1522851	1521394	2275119	2276576
3047 BPSL2084		1504298	1506274	773060	775036	1873488	1875464	1859420	1861396	2674286	2672310	1494017	1495993	2294663	2292687	1519345	1521321	2278625	2276649
3048 BPSL2085		1503639	1503361	772401	772123	1872829	1872551	1858761	1858483	2674945	2675223	1493358	1493080	2295322	2295600	2365846	2366124	2279284	2279562
3049 BPSL2086		1503220	1502714	771982	771476	1872410	1871904	1858342	1857836	2675364	2675870	1492933	1492433	2295744	2296244	2366268	2366774	1392320	1391820
3050 BPSL2087		1502718	1502395	771480	771157	1871908	1871585	1857840	1857517	2675866	2676189	1492437	1492114	2296240	2296563	2366770	2367093	1391824	1391501
3051	BURPSS13_P0997															2367575	2367721		
3052 BPSL2087A										1788602	1788003			1389589	1389392				
3053 BPSL2088										1787993	1787274								
3054	Bp_chr1_31_ISBma1													1388085	1389389				
3055 BPSL2089		2354887	2354831	1623204	1622830	2784983	2784609	2768494	2768438	1786813	1787187	2321560	2321129	1386484	1386858	2368911	2368537	1389683	1390057
3056	BURPSPAST_AA1068													1387820	1387674				
3057 BPSL2090		2355825	2356664	1623762	1624601	2785553	2786386	2769444	2770277	1786255	1785416	2322130	2322969	1385914	1385081	2369482	2370315	1389112	1388279
3058 BPSL2091		2357964	2356711	1625901	1624648	2787686	2786433	2771577	2770324	1784116	1785369	2324254	2323016	1383781	1385034	2371612	2370362	1386982	1388232
3059 BPSL2092		2359462	2358050	1627399	1625987	2789184	2787772	2773075	2771663	1782618	1784030	2325752	2324340	1382283	1383695	2373110	2371698	1385484	1386896
3060 BPSL2093		2360033	2359698	1627970	1627635	2789766	2789431	2773657	2773322	1782036	1782371	2326334	2325999	1381701	1382036	2373692	2373357		
3061 BPSL2094		2360255	2360977	1628192	1628914	2789988	2790710	2773879	2774601	1781814	1781092	2326556	2327278	1381479	1380757	2373914	2374636	1384681	1383959
3062 BPSL2095		2361047	2362402	1628984	1630339	2790780	2792135	2774671	2776026	1781022	1779667	2327348	2328703	1380687	1379332	2374706	2376061	1383889	1382534
3063 BPSL2096		2362604	2363149	1630541	1631086	2792337	2792882	2776228	2776773	1779465	1778920	2328905	2329450	1379130	1378585	2376263	2376808	1382333	1381788
3064 BPSL2097		2363230	2363754	1631167	1631691	2792963	2793487	2776854	2777378	1778839	1778315	2329531	2330055	1378504	1377980	2376889	2377413	1381707	1381183
3065	BURPS1106A_2399	2363977	2364129																
3066	BURPS1106B_A1630			1632066	1631914														
3067	BURPS668_2356											2330269	2330421						
3068	BURPSS13_P1011															2377788	2377636		

3069	BPSL2098		2364614	2364129	1632551	1632066	2794348	2793863	2778239	2777754	1777455	1777940	2330906	2330421	1377119	1377604	2378273	2377788	1380323	1380808
3070		BURPS1655_A1364																	1380808	1380960
3071		BURPS406E_H0588									1777940	1778092								
3072	BPSL2099		2365365	2364658	1633302	1632595	2795099	2794392	2778990	2778283	1776704	1777411	2331657	2330950	1376368	1377075	2379024	2378317	1379572	1380279
3073	BPSL2100		2365386	2368952	1633323	1636889	2795120	2798686	2779011	2782577	1776683	1773117	2331678	2335244	1376347	1372781	2379045	2382611	1379551	1375985
3074	BPSL2101		2370147	2371313	1638084	1639250	2800028	2801194	2783919	2785085	1771880	1770714	2336392	2337558	1371593	1370427	2383736	2384902	1374797	1373631
3075	BPSL2102		2371378	2372406	1639315	1640343	2801259	2802287	2785150	2786178	1770649	1769621	2337623	2338651	1370362	1369334	2384967	2385995	1373566	1372538
3076	BPSL2103		2372917	2373321	1640854	1641258	2802798	2803202	2786689	2787093	1769110	1768706	2339162	2339566	1368823	1368419	2386506	2386910	1372027	1371623
3077		BURPS1106B_A1638			1641536	1641213														
3078		BURPSS13_P1020															2387188	2386865		
3079	BPSL2104		2376434	2373879	1644371	1641816	2806315	2803760	2790206	2787651	1765593	1768148	2342680	2340125	1365306	1367861	2390023	2387468	1368508	1371063
3080		BURPS406E_H0579									1768428	1768751								
3081		BURPSPAST_AA1092													1368141	1368464				
3082	BPSL2105		2376914	2377591	1644851	1645528	2806795	2807472	2790686	2791363	1765113	1764436	2343150	2343827	1364826	1364149	2390503	2391180	1368028	1367351
3083	BPSL2106		2377709	2378920	1645646	1646857	2807590	2807877	2791481	2792692	1764318	1763107	2343945	2345156	1363945	1362719	2391298	2392509	1367236	1366025
3084		BURPSS13_P1027															2393420	2392584		
3085	BPSL2107		2379107	2379535	1647044	1647472	2809101	2809529	2792879	2793307	1762920	1762492	2345343	2345771	1362532	1362104	2394157	2394585	1364377	1363949
3086		BURPS1655_A1348																	1364581	1365114
3087		BURPS1655_A1349																	1365114	1365950
3088	BPSL2108		2379850	2380344	1647787	1648281	2809844	2810338	2793622	2794116	1762177	1761683	2346086	2346580	1361789	1361295	2394900	2395394	1363634	1363140
3089	BPSL2109		2381415	2380531	1649352	1648468	2811409	2810525	2795187	2794303	1760611	1761495	2347651	2346767	1360224	1361108	2396465	2395581	1362069	1362953
3090	BPSL2110		2381559	2381765	1649496	1649702	2811553	2811759	2795331	2795537	1760467	1760261	2347795	2348001	1360080	1359874	2396609	2396815	1361925	1361719
3091	BPSL2111		2382508	2383434	1650445	1651371	2812504	2813430	2796282	2797208	1759515	1758589	2348745	2349671	1359129	1358203	2397560	2398486	1360976	1360050
3092	BPSL2112		2385119	2383692	1653056	1651629	2815115	2813688	2798893	2797466	1756908	1758335	2351380	2349953	1356530	1357957	2400237	2398810	1358333	1359760
3093	BPSL2113		2385679	2386035	1653616	1653972	2815675	2816031	2799453	2799809	1756348	1755992	2351940	2352296	1355970	1355614	2400797	2401153	1357773	1357417
3094	BPSL2114		2386088	2387287	1654025	1655224	2816084	2817283	2799862	2801061	1755939	1754740	2352349	2353548	1355561	1354362	2401206	2402405	1357364	1356165
3095	BPSL2115		2388667	2388158	1656604	1656095	2818663	2818154	2802441	2801932	1753351	1753860	2354926	2354417	1352982	1353491	2403786	2403277	1354798	1355307
3096	BPSL2116		2389677	2388667	1657614	1656604	2819673	2818663	2803451	2802441	1752341	1753351	2355936	2354926	1351972	1352982	2404796	2403786	1353788	1354798
3097	BPSL2117		2390430	2389909	1658367	1657846	2820426	2819905	2804204	2803683	1751588	1752109	2356689	2356168	1351219	1351740	2405549	2405028	1353035	1353556
3098	BPSL2118		2391380	2390430	1659317	1658367	2821376	2820426	2805154	2804204	1750638	1751588	2357639	2356689	1350269	1351219	2406499	2405549	1352085	1353035
3099	BPSL2119		2392482	2391529	1660419	1659466	2822505	2821525	2806283	2805303	1749527	1750489	2358723	2357788	1349140	1350120	2407610	2406648	1350983	1351936
3100	BPSL2120		2394025	2392496	1661962	1660433	2824048	2822519	2807826	2806297	1747984	1749513	2360266	2358737	1347597	1349126	2409153	2407624	1349440	1350969
3101	BPSL2121		2394842	2394141	1662779	1662078	2824865	2824164	2808643	2807942	1747167	1747868	2361083	2360382	1346780	1347481	2409970	2409269	1348623	1349324
3102	BPSL2122		2396098	2395160	1664035	1663097	2826121	2825183	2809899	2808961	1745911	1746849	2362339	2361401	1345524	1346462	2411225	2410287	1347367	1348305
3103	BPSL2123		2396678	2396157	1664615	1664094	2826701	2826180	2810479	2809958	1745331	1745852	2362919	2362398	1344944	1345465	2411805	2411284	1346787	1347308
3104	BPSL2124		2397148	2396750	1665085	1664687	2827171	2826773	2810949	2810551	1744861	1745259	2363389	2362991	1344474	1344872	2412275	2411877	1346317	1346715
3105		BURPSS13_P1052															2412957	2412757		
3106	BPSL2125		2398456	2397953	1666393	1665890	2828479	2827976	2812257	2811754	1743553	1744056	2364674	2364171	1343166	1343669	2413581	2413078	1345009	1345512
3107	BPSL2126		2398824	2400041	1666761	1667978	2828847	2830064	2812625	2813842	1743185	1741968	2365042	2366259	1342798	1341581	2413949	2415166	1344641	1343424
3108		BURPS1106A_2445	2401716	2402951																
3109		BURPS1106A_2446	2402954	2405290																
3110		BURPS1106A_2447	2405764	2406165																
3111		BURPS1106A_2448	2406277	2406402																
3112		BURPS1106A_2449	2407165	2407290																
3113		BURPS1106A_2450	2407877	2408038																
3114		BURPS1106A_2451	2408883	2409323																
3115		BURPS1106A_2452	2409301	2410227																
3116		BURPS1106A_2453	2410243	2410422																
3117		BURPS1106B_A1674			1670888	1669653														
3118		BURPS1106B_A1675			1673161	1670891														
3119		BURPS1106B_A1676			1674102	1673701														
3120		BURPS1106B_A1677			1674214	1674339														
3121		BURPS1106B_A1678			1675690	1674368														
3122		BURPS1106B_A1679			1676820	1677260														
3123		BURPS1106B_A1680			1677238	1678164														
3124		BURPS1106B_A1681			1678180	1678359														
3125		BURPS1710A_2947					2832974	2831739												
3126		BURPS1710A_2948					2835787	2832977												

3127	BURPS1710A_2949					2836470	2835787												
3128	BURPS1710A_2950					2836300	2836425												
3129	BURPS1710A_2951					2837776	2836454												
3130	BURPS1710A_2952					2838061	2837900												
3131	BURPS1710A_2953					2838906	2839346												
3132	BURPS1710A_2954					2839324	2840250												
3133	BURPS1710A_2955					2840266	2840445												
3134	BURPS1710b_2542							2815517	2816752										
3135	BURPS1710b_2543							2816755	2819565										
3136	BURPS1710b_2544							2823072	2824028										
3137	BURPS1710b_2545							2824044	2824223										
3138	BURPSS13_P1058															2418067	2416832		
3139	BURPSS13_P1059															2420880	2418070		
3140	BURPSS13_P1060															2422869	2421547		
3141	BURPSS13_P1061															2424094	2423957		
3142	BURPSS13_P1062															2424387	2425343		
3143	BURPSS13_P1063															2425359	2425538		
3144 BPSL2127	BPSL2127	2412777	2411137	1680714	1679074	2842800	2841160	2826578	2824938	1733686	1735326	2369247	2367607	1328845	1330485	2427893	2426253	1330688	1332328
3145	BURPS1655_A1315																	1333222	1333043
3146	BURPS1655_A1316																	1334164	1333238
3147	BURPS1655_A1317																	1337018	1337701
3148	BURPS1655_A1318																	1337701	1340511
3149	BURPS1655_A1319																	1340514	1341749
3150	BURPS406E_H0541									1735640	1736371								
3151	BURPS406E_H0542									1736973	1738142								
3152	BURPS406E_H0543									1738130	1739002								
3153	BURPS406E_H0544									1739394	1740380								
3154	BURPSPAST_AA1126													1338671	1339906				
3155	BURPSPAST_AA1127													1335858	1338668				
3156	BURPSPAST_AA1128													1335175	1335858				
3157	BURPSPAST_AA1129													1332351	1331395				
3158	BURPSPAST_AA1130													1331379	1331200				
3159 BPSL2128		2413534	2412884	1681471	1680821	2843557	2842907	2827335	2826685	1732929	1733579	2370004	2369354	1328065	1328532	2428650	2428000	1329931	1330581
3160 BPSL2129		2415001	2413544	1682938	1681481	2845024	2843567	2828802	2827345	1731462	1732919	2371471	2370014	1326598	1328055	2430117	2428660	1328464	1329921
3161 BPSL2130		2416182	2415421	1684119	1683358	2846205	2845444	2829983	2829222	1730280	1731041	2372649	2371888	1325417	1326178	2431298	2430537	1327282	1328043
3162 BPSL2131		2416319	2417089	1684256	1685026	2846342	2847112	2830120	2830890	1730143	1729373	2372786	2372824	1325280	1324510	2431435	2432205	1327145	1326375
3163	BURPS1710b_2554							2830436	2832346										
3164 BPSL2133		2417571	2418251	1685508	1686188	2847594	2848274	2831372	2832052	1728891	1728211			1324028	1323348	2432687	2433367	1325893	1325213
3165 BPSL2134		2418589	2419554	1686526	1687491	2848612	2849577	2832390	2833355	1727873	1726908	2373601	2374566	1323010	1322045	2433705	2434670	1324875	1323910
3166 BPSL2135		2420025	2419705	1687962	1687642	2850048	2849728	2833826	2833506	1726437	1726757	2375030	2374710	1321574	1321894	2435134	2434814	1323425	1323745
3167 BPSL2136		2420468	2420034	1688405	1687971	2850491	2850057	2834269	2833835	1725994	1726428	2375473	2375039	1321131	1321565	2435577	2435143	1322982	1323416
3168 BPSL2137		2420566	2421009	1688503	1688946	2850589	2851032	2834367	2834810	1725896	1725453	2375571	2376014	1321033	1320590	2435675	2436118	1322884	1322441
3169 BPSL2138		2422136	2421192	1690073	1689129	2852159	2851215	2835937	2834993	1724326	1725270	2377142	2376198	1319463	1320407	2437231	2436311	1321313	1322257
3170 BPSL2139		2422650	2422219	1690587	1690156	2852674	2852597	2836451	2836020	1723812	1724243	2377656	2377225	1318949	1319380	2437745	2437314	1320799	1321230
3171 BPSL2140		2425177	2422781	1693114	1690718	2855201	2852805	2838978	2836582	1721285	1723681	2380183	2377787	1316422	1318818	2440272	2437876	1318272	1320668
3172 BPSL2141		2426732	2425902	1694669	1693839	2856779	2855949	2840556	2839726	1719703	1720542	2381744	2380914	1314844	1315674	2441850	2441020	1316694	1317524
3173 BPSL2142		2428885	2426987	1696822	1694924	2858983	2857085	2842760	2840862	1717520	1719418	2383909	2382011	1312646	1314544	2444036	2442135	1314523	1316421
3174 BPSL2143		2429420	2430232	1697357	1698169	2859518	2860330	2843295	2844107	1716985	1716173	2384444	2385256	1312111	1311299	2444571	2445383	1313988	1313176
3175 BPSL2144		2431225	2430440	1699162	1698377	2861323	2860538	2845100	2844315	1715180	1715965	2386256	2385471	1310306	1311091	2446376	2445591	1312183	1312968
3176 BPSL2145		2431866	2431225	1699803	1699162	2861964	2861323	2845741	2845100	1714539	1715180	2386897	2386256	1309665	1310306	2447017	2446376	1311542	1312183
3177 BPSL2146		2433032	2431869	1700969	1699806	2863130	2861967	2846907	2845744	1713373	1714536	2388063	2386900	1308499	1309662	2448183	2447020	1310376	1311539
3178 BPSL2147		2433824	2433039	1701761	1700976	2863922	2863137	2847699	2846914	1712581	1713366	2388855	2388070	1307707	1308492	2448975	2448190	1309584	1310369
3179 BPSL2148		2434389	2433883	1702326	1701820	2864487	2863981	2848264	2847758	1712016	1712522	2389420	2388914	1307142	1307648	2449540	2449034	1309019	1309525
3180 BPSL2149		2435578	2434496	1703515	1702433	2865676	2864594	2849453	2848371	1710827	1711909	2390609	2389527	1305953	1307035	2450729	2449647	1307830	1308912
3181 BPSL2150		2436128	2435598	1704065	1703535	2866226	2865696	2850003	2849473	1710277	1710807	2391159	2390629	1305403	1305933	2451279	2450749	1307280	1307810
3182 BPSL2151		2438475	2436169	1706412	1704106	2868573	2866267	2852350	2850044	1707930	1710236	2393506	2391200	1303056	1305362	2453626	2451320	1304933	1307239
3183 BPSL2152		2439931	2438543	1707868	1706480	2870029	2868641	2853806	2852418	1706474	1707862	2394962	2393574	1301600	1302988	2455082	2453694	1303477	1304865
3184 BPSL2153		2441145	2439952	1709082	1707889	2871243	2870050	2855020	2853827	1705260	1706453	2396176	2394983	1300386	1301579	2456296	2455103	1302263	1303456

3185	BPSL2154		2441981	2441163	1709918	1709100	2872079	2871261	2855856	2855038	1704424	1705242	2397012	2396194	1299550	1300368	2457132	2456314	1301427	1302245
3186	BPSL2155		2442757	2441978	1710694	1709915	2872855	2872076	2856632	2855853	1703648	1704427	2397788	2397009	1298774	1299553	2457908	2457129	1300651	1301430
3187	BPSL2156		2443365	2442808	1711302	1710745	2873463	2872906	2857240	2856683	1703040	1703597	2398396	2397839	1298166	1298723	2458516	2457959	1300043	1300600
3188	BPSL2157		2444169	2443459	1712106	1711396	2874267	2873557	2858044	2857334	1702236	1702946	2399200	2398490	1297362	1298072	2459320	2458610	1299239	1299949
3189	BPSL2158		2445229	2444351	1713166	1712288	2875327	2874449	2859104	2858226	1701176	1702054	2400260	2399382	1296302	1297180	2460380	2459502	1298179	1299057
3190	BPSL2159		2446152	2445415	1714089	1713352	2876250	2875513	2860027	2859290	1700253	1700990	2401183	2400446	1295379	1296116	2461303	2460566	1297256	1297993
3191	BPSL2160		2446632	2447444	1714569	1715381	2876733	2877545	2860510	2861322	1699770	1698958	2401666	2402478	1294896	1294084	2461786	2462598	1296776	1295964
3192	BPSL2161		2447618	2450191	1715555	1718128	2877719	2880292	2861496	2864069	1698784	1696211	2402645	2405218	1293915	1293760	2462772	2465345	1295793	1293220
3193	BPSL2162		2451175	2451921	1719112	1719858	2881300	2882046	2865077	2865823	1695130	1694384	2406211	2406957	1290601	1289855	2466338	2467084	1292236	1291490
3194	BPSL2163		2452652	2452122	1720589	1720059	2882777	2882247	2866554	2866024	1693653	1694183	2407688	2407158	1289124	1289654	2467815	2467285	1290759	1291289
3195	BPSL2164		2454724	2452652	1722661	1720589	2884849	2882777	2868626	2866554	1691581	1693653	2409760	2407688	1287052	1289124	2469887	2467815	1288687	1290759
3196		BURPS1106A_2500	2454763	2455092																
3197		BURPS1106B_A1727			1723035	1722700														
3198		BURPS1710b_2589							2868654	2869016										
3199		BURPSS13_P1108															2469975	2470334		
3200	BPSL2165		2456447	2455164	1724384	1723101	2886574	2885291	2870351	2869068	1689858	1691141	2411481	2410201	1285326	1286609	2471610	2470327	1286963	1288246
3201		BURPS406E_H0499									1691213	1691542								
3202	BPSL2166		2460026	2456517	1727963	1724454	2890153	2886644	2873930	2870421	1686279	1689788	2415063	2411554	1281747	1285256	2475189	2471680	1283384	1286893
3203	BPSL2167		2461122	2460178	1729059	1728115	2891249	2890305	2875026	2874082	1685183	1686127	2416159	2415215	1280651	1281595	2476285	2475341	1282288	1283232
3204	BPSL2168		2461387	2462616	1729324	1730553	2891514	2892743	2875291	2876520	1684918	1683689	2416424	2417653	1280386	1279157	2476550	2477779	1282023	1280794
3205	BPSL2169		2462676	2463500	1730613	1731437	2892803	2893627	2876580	2877404	1683629	1682805	2417713	2418537	1279097	1278273	2477839	2478663	1280734	1279910
3206	BPSL2170		2463503	2463874	1731440	1731811	2893630	2894001	2877407	2877778	1682802	1682431	2418540	2418911	1278270	1277899	2478666	2479037	1279907	1279536
3207	BPSL2171		2463939	2465087	1731876	1733024	2894066	2895214	2877843	2878991	1682366	1681902	2418976	2420124	1277834	1276686	2479102	2480250	1279471	1278323
3208	BPSL2172		2465104	2466024	1733041	1733961	2895231	2896151	2879008	2879928	1681099	1680179	2420141	2421061	1276669	1275749	2480267	2481187	1278306	1277386
3209	BPSL2173		2466031	2466270	1733968	1734207					1680172	1679963	2421068	2421292	1275742	1275518	2481194	2481433	1277379	1277170
3210	BPSL2174		2466270	2467706	1734207	1735643	2896412	2897848	2880189	2881625	1679963	1678527	2421292	2422728	1275518	1274082	2481433	2482869	1277170	1275734
3211	BPSL2175		2468292	2467816	1736229	1735753	2898434	2897958	2882211	2881735	1677941	1678417	2423323	2422847	1273486	1273962	2483455	2482979	1275139	1275615
3212	BPSL2176		2468438	2470375	1736375	1738312	2898580	2900517	2882357	2884294	1677795	1675858	2423469	2425406	1273340	1271403	2483601	2485538	1274993	1273056
3213	BPSL2177		2470798	2470469	1738735	1738406	2900940	2900611	2884717	2884388	1675435	1675764	2425829	2425500	1270980	1271309	2485961	2485632	1272633	1272962
3214	BPSL2178		2472272	2470899	1740209	1738836	2902414	2901041	2886191	2884818	1673961	1675334	2427303	2425930	1269506	1270879	2487435	2486062	1271159	1272532
3215	BPSL2179		2473353	2472286	1741290	1740223	2903495	2902428	2887272	2886205	1672880	1673947	2428384	2427317	1268425	1269492	2488516	2487449	1270078	1271145
3216	BPSL2180		2473565	2474869	1741502	1742806	2903707	2905011	2887484	2888788	1672668	1671364	2428596	2429900	1268213	1266909	2488728	2490032	1269866	1269225
3217	BPSL2181		2474983	2475789	1742920	1743726	2905125	2905931	2888902	2889708	1671250	1670444	2430014	2430820	1266795	1265989	2490146	2490952	1268380	1267574
3218	BPSL2182		2477198	2476221	1745135	1744158	2907382	2906405	2891159	2890182	1669017	1669994	2432217	2431240	1264568	1265545	2492319	2491342	1266183	1267160
3219	BPSL2183		2478049	2477198	1745986	1745135	2908233	2907382	2892010	2891159	1668166	1669017	2433068	2432217	1263717	1264568	2493170	2492319	1265332	1266183
3220	BPSL2184		2479031	2478552	1746968	1746489	2909254	2908775	2893031	2892552	1667157	1667636	2434050	2433571	1262672	1263151	2494152	2493673	1264326	1264805
3221	BPSL2185		2479804	2479046	1747741	1746983	2910027	2909269	2893804	2893046	1666384	1667142	2434823	2434065	1261899	1262657	2494925	2494167	1263553	1264311
3222	BPSL2186		2479974	2480240	1747911	1748177	2910197	2910463	2893974	2894240	1666198	1665932	2434993	2435259	1261729	1261463	2495095	2495361	1263383	1263117
3223	BPSL2187		2480869	2482227	1748806	1750164	2911101	2912459	2894878	2896236	1665308	1663950	2435880	2437235	1260834	1259476	2495990	2497348	1262477	1261119
3224	BPSL2188		2482622	2483926	1750559	1751863	2912854	2914158	2896631	2897935	1663555	1662251	2437629	2438933	1259081	1257777	2497743	2499047	1260724	1259420
3225	BPSL2189		2484623	2484192	1752560	1752129	2914855	2914424	2898632	2898201	1661554	1661985	2439630	2439199	1257080	1257511	2499744	2499313	1258724	1259155
3226	BPSL2190		2485805	2484906	1753742	1752843	2916037	2915138	2899814	2898915	1660372	1661271	2440812	2439913	1255898	1256797	2500926	2500027	1257542	1258441
3227	BPSL2191		2485965	2486744	1753902	1754681	2916197	2916976	2899974	2900753	1660212	1659433	2440972	2441721	1255738	1254959	2501086	2501865	1257382	1256603
3228	BPSL2192		2486800	2488389	1754737	1756326	2917032	2918621	2900809	2902398	1659377	1657788	2441806	2443395	1254903	1253314	2501921	2503510	1256547	1254958
3229	BPSL2193		2489444	2489040	1757388	1756984	2919577	2919173	2903354	2902950	1656837	1657241	2444374	2443970	1252335	1252739	2504488	2504084	1253966	1254370
3230	BPSL2194		2489989	2489495	1757933	1757439	2920122	2919628	2903899	2903405	1656292	1656786	2444919	2444425	1251790	1252284	2505033	2504539	1253421	1253915
3231	BPSL2195		2490119	2490979	1758063	1758923	2920252	2921112	2904029	2904889	1656162	1655302	2445049	2445909	1251660	1250800	2505163	2506023	1253291	1252431
3232		BURPS668_2479											2445909	2446121						
3233	BPSL2196		2492053	2491589	1759997	1759533	2922182	2921718	2905959	2905495	1654228	1654692	2447025	2446561	1249748	1250212	2507067	2506603	1251379	1251843
3234	BPSL2197		2494548	2493142	1762267	1760861	2924521	2923211	2908453	2907047	1651733	1653139	2449520	2448114	2479933	2478527	2509561	2508155	2463520	2462114
3235	BPSL2198		2495113	2496045	1762832	1763764	2925295	2926227	2909112	2910044	1651120	1650188	2450097	2451029	2480586	2481329	2510140	2511072	2464139	2465071
3236	BPSL2199		2496925	2496224	1764644	1763943	2927100	2926399	2910917	2910216	1649315	1650016	2451902	2451201	2482392	2481691	2511945	2511244	2465944	2465243
3237	BPSL2200		2498710	2497064	1766429	1764783	2928885	2927239	2912702	2911056	1647530	1649176	2453687	2452041	2484177	2482531	2513730	2512084	2467711	2466083
3238	BPSL2201		2499804	2498710	1767523	1766429	2929979	2928885	2913796	2912702	1646436	1647530	2454781	2453687	2485271	2484177	2514824	2513730	2468805	2467711
3239	BPSL2202		2500846	2499809	1768565	1767528	2931021	2929984	2914838	2913801	1645394	1646431	2455823	2454786	2486313	2485276	2515866	2514829	2469847	2468810
3240	BPSL2203		2502987	2501038	1770706	1768757	2933162	2931213	2916979	2915030	1643253	1645202	2457964	2456015	2488454	2486505	2518007	2516058	2471988	2470039
3241	BPSL2204		2503108	2503896	1770827	1771615	2933283	2934071	2917100	2917888	1643132	1642344	2458085	2458873	2488575	2489363	2518128	2518916	2472109	2472897
3242	BPSL2205		2504505	2504155	1772224	1771874	2934680	2934330	2918497	2918147	1641735	1642085	2459481	2459131	2489972	2489622	2519525	2519175	2473504	2473154

3243	BPSL2206		2504759	2504977	1772478	1772696	2934934	2935152	2918751	2918969	1641481	1641263	2459735	2459953	2490226	2490444	2519779	2519997	2473758	2473976
3244	BPSL2207		2507535	2505217	1775254	1772936	2937726	2935408	2921543	2919225	1638673	1640991	2462495	2460177	2493002	2490684	2522539	2520221	2476518	2474200
3245		BURPS1655_H0306																	2476588	2476896
3246	BPSL2208		2508813	2508043	1776532	1775762	2939004	2938234	2922821	2922051	1637395	1638165	2463773	2463003	2494280	2493510	2523817	2523047	2477797	2477027
3247	BPSL2209		2509807	2508824	1777526	1776543	2939999	2939457	2923284	2922832	1636400	1636942	2464236	2463784	2495275	2494733	2524811	2523828	2478792	2478250
3248	BPSL2210		2510253	2509918	1777972	1777637	2940421	2940086	2924238	2923903	1635978	1636313	2465211	2464876	2495697	2495362	2525257	2524922	2479214	2478879
3249	BPSL2211		2510732	2511295	1778451	1779014	2940900	2941463	2924717	2925280	1635499	1634936	2465690	2466253	2496175	2496738	2525736	2526299	2479703	2480266
3250	BPSL2212		2512190	2511375	1779909	1779094	2942358	2941543	2926175	2925360	1634041	1634856	2467148	2466333	2497633	2496818	2527194	2526379	2481161	2480346
3251	BPSL2213		2512414	2512193	1780133	1779912	2942582	2942361	2926399	2926178	1633817	1634038	2467372	2467151	2497857	2497636	2527418	2527197	2481385	2481164
3252		BURPSPAST_R0311													2498144	2497848				
3253	BPSL2214		2515412	2512701	1783131	1780420	2945580	2942869	2929397	2926686	1630789	1633530	2470394	2467659	2500861	2498144	2530352	2527647	2484383	2481672
3254		BURPS406E_H0440									1633530	1633826								
3255	BPSL2215		2516556	2515495	1784275	1783214	2946724	2945663	2930541	2929480	1629645	1630706	2471538	2470477	2502005	2500944	2531496	2530435	2485534	2484473
3256	BPSL2216		2517760	2516564	1785479	1784283	2947928	2946732	2931745	2930549	1628441	1629637	2472742	2471546	2503209	2502013	2532700	2531504	2486738	2485542
3257	BPSL2217		2518748	2517786	1786467	1785505	2948916	2947954	2932733	2931771	1627453	1628415	2473730	2472768	2504197	2503235	2533688	2532726	2487726	2486764
3258	BPSL2218		2519722	2518907	1787441	1786626	2949890	2949075	2933707	2932892	1626479	1627294	2474699	2473884	2505171	2504356	2534662	2533847	2488700	2487885
3259	BPSL2219		2520907	2519816	1788626	1787535	2951075	2949984	2934892	2933801	1625294	1626385	2475876	2474785	2506356	2505265	2535847	2534756	2489877	2488786
3260	BPSL2220		2522075	2520951	1789794	1788670	2952243	2951119	2936060	2934936	1624126	1625250	2477044	2475920	2507524	2506400	2537015	2535891	2491045	2489921
3261	BPSL2221		2523272	2522130	1790991	1789849	2953431	2952298	2937248	2936115	1622938	1624071	2478214	2477099	2508721	2507579	2538194	2537070	2492233	2491100
3262	BPSL2222		2524186	2523278	1791905	1790997	2954345	2953437	2938162	2937254	1622024	1622932	2479128	2478220	2509635	2508727	2539108	2538200	2493147	2492239
3263	BPSL2223		2525022	2524186	1792741	1791905	2955181	2954345	2938998	2938162	1621188	1622024	2479964	2479128	2510471	2509635	2539944	2539108	2493983	2493147
3264	BPSL2224		2526061	2525030	1793780	1792749	2956220	2955189	2940037	2939006	1620149	1621180	2481003	2479972	2511510	2510479	2540983	2539952	2495022	2493991
3265	BPSL2225		2526888	2526112	1794607	1793831	2957047	2956271	2940864	2940088	1619322	1620098	2481830	2481054	2512337	2511561	2541810	2541034	2495849	2495073
3266	BPSL2226		2527756	2526911	1795475	1794630	2957915	2957070	2941732	2940887	1618454	1619299	2482698	2481853	2513205	2512360	2542678	2541833	2496717	2495872
3267	BPSL2227		2528704	2527829	1796423	1795548	2958863	2957988	2942680	2941805	1617506	1618381	2483646	2482771	2514153	2513278	2543626	2542751	2497665	2496790
3268	BPSL2228		2529506	2528712	1797225	1796431	2959665	2958871	2943482	2942688	1616704	1617498	2484448	2483654	2514955	2514161	2544428	2543634	2498467	2497673
3269	BPSL2229		2538526	2529506	1806245	1797225	2968685	2959665	2952502	2943482	1607684	1616704	2493468	2484448	2523975	2514955	2553448	2544428	2507408	2503248
3270	BPSL2230		2539926	2538529	1807645	1806248	2970085	2968688	2953902	2952505	1606284	1607681	2494868	2493471	2525375	2523978	2554848	2553451	2508808	2507411
3271	BPSL2231		2541586	2540288	1809305	1808007	2971625	2970327	2955442	2954144	1604744	1606042	2496408	2495110	2526915	2525617	2556468	2555170	2510381	2509083
3272	BPSL2232		2543546	2541657	1811265	1809376	2973585	2971696	2957402	2955513	1602784	1604673	2498368	2496479	2528875	2526986	2558428	2556539	2512341	2510452
3273	BPSL2233		2545042	2543546	1812761	1811265	2975081	2973585	2958898	2957402	1601288	1602784	2499864	2498368	2530371	2528875	2559924	2558428	2513837	2512341
3274	BPSL2234		2546445	2547716	1814164	1815435	2976484	2977761	2960301	2961578	1599858	1598581	2501267	2502541	2531774	2533051	2561327	2562598	2515240	2516511
3275	BPSL2235		2547716	2550859	1815435	1818578	2977761	2980904	2961578	2964721	1598581	1595438	2502541	2505684	2533051	2536194	2562598	2565741	2516511	2519654
3276	BPSL2236		2550940	2552157	1818659	1819876	2980985	2982202	2964802	2966019	1595357	1594320	2505765	2506982	2536275	2537495	2565822	2567018	2519735	2520904
3277	BPSL2237		2554031	2552202	1821750	1819921	2984079	2982250	2967893	2966064	1592380	1594209	2508884	2507055	2539369	2537540	2568990	2567161	2522849	2521020
3278	BPSL2238		2555271	2554228	1822990	1821947	2985319	2984276	2969133	2968090	1591131	1592174	2510133	2509090	2540609	2539566	2570230	2569187	2524097	2523054
3279		BURPS406E_H0408									1589888	1588650								
3280		BURPS406E_H0407									1587487	1588569								
3281		BURPS406E_H0406									1586774	1587481								
3282		BURPS406E_H0405									1586232	1586753								
3283		BURPS406E_H0404									1585249	1585022								
3284		BURPS406E_H0403									1584998	1584126								
3285		BURPS406E_H0402									1584120	1582426								
3286		BURPS406E_H0401									1582426	1581866								
3287		BURPS406E_H0400									1581859	1580708								
3288		BURPS406E_H0399									1580322	1576918								
3289		BURPS406E_H0398									1576743	1575943								
3290		BURPS406E_H0397									1575937	1575407								
3291		BURPS406E_H0396									1575410	1575006								
3292		BURPS406E_H0395									1573894	1574754								
3293		BURPS406E_H0394									1573595	1573897								
3294		BURPS406E_H0393									1572696	1573514								
3295		BURPS406E_H0392									1572377	1570347								
3296		BURPS406E_H0391									1570202	1568409								
3297		BURPS406E_H0390									1568113	1567868								
3298		BURPS406E_H0389									1567848	1567459								
3299		BURPS406E_H0388									1567320	1566604								
3300		BURPS406E_H0387									1566487	1565693								

3301	BURPS406E_H0386					1565580	1565221						
3302	BURPS406E_H0385					1564628	1563813						
3303	BURPS406E_H0384					1563418	1562900						
3304	BURPS406E_H0383					1562805	1562203						
3305	BURPS406E_H0382					1562108	1561458						
3306	BURPS406E_H0381					1561396	1560287						
3307	BURPS406E_H0380					1560188	1559892						
3308	BURPS406E_H0379					1559806	1557539						
3309	BURPS406E_H0378					1556882	1556580						
3310	BURPS406E_H0377					1556583	1555723						
3311	BURPS406E_H0376					1555605	1554382						
3312	Bp_chr1_32_ISBma1					1553380	1554229						
3313	BURPS406E_H0375					1553726	1554226						
3314	BURPS406E_H0374					1552802	1553653						
3315	BURPS406E_H0372					1552582	1551509						
3316	BURPS406E_H0373					1552515	1552805						
3317	BURPS406E_H0371					1548802	1550754						
3318	BURPS406E_H0370					1543926	1548821						
3319	BURPS406E_H0369					1541134	1543929						
3320	BURPS406E_H0368					1535855	1541122						
3321	BURPS668_2539							2510793	2511260				
3322	BURPSS13_P1199										2571490	2572728	
3323	BURPSS13_P1200										2573931	2572804	
3324	BURPSS13_P1201										2574623	2573892	
3325	BURPSS13_P1202										2575141	2574620	
3326	BURPSS13_P1203										2576125	2576352	
3327	BURPSS13_P1204										2576376	2577248	
3328	BURPSS13_P1205										2577254	2578948	
3329	BURPSS13_P1206										2578948	2579508	
3330	BURPSS13_P1207										2579512	2580666	
3331	BURPSS13_P1208										2581055	2584456	
3332	BURPSS13_P1209										2584633	2585433	
3333	BURPSS13_P1210										2585430	2585969	
3334	BURPSS13_P1211										2585966	2586379	
3335	BURPSS13_P1212										2586572	2588602	
3336	BURPSS13_P1213										2590280	2589039	
3337	BURPSS13_P1214										2590616	2590921	
3338	BURPSS13_P1215										2590941	2591330	
3339	BURPSS13_P1216										2591468	2592181	
3340	BURPSS13_P1217										2592300	2593094	
3341	BURPSS13_P1218										2593208	2593567	
3342	BURPSS13_P1219										2593914	2593666	
3343	BURPSS13_P1220										2594370	2594888	
3344	BURPSS13_P1221										2594982	2595584	
3345	BURPSS13_P1222										2595623	2596336	
3346	BURPSS13_P1223										2596398	2597507	
3347	BURPSS13_P1224										2597603	2597896	
3348	BURPSS13_P1225										2597981	2600248	
3349	BURPSS13_P1226										2602266	2602150	
3350	BURPSS13_P1227										2600376	2602169	
3351	BURPSS13_P1228										2602447	2602686	
3352	BURPSS13_P1229										2602812	2603414	
3353	BURPSS13_P1230										2604053	2604775	
3354	BURPSS13_P1231										2604772	2605329	
3355	BURPSS13_P1232										2605343	2605864	
3356	BURPSS13_P1233										2605866	2608025	
3357	BURPSS13_P1234										2608045	2608791	
3358	BURPSS13_P1235										2610595	2609420	

3359		BURPSS13_P1236															2610869	2610570		
3360		BURPSS13_P1237															2612979	2613329		
3361		BURPSS13_P1238															2613326	2613565		
3362		BURPSS13_P1239															2613582	2613953		
3363		BURPSS13_P1240															2613961	2614383		
3364		BURPSS13_P1241															2614380	2615057		
3365		BURPSS13_P1242															2615066	2615941		
3366		BURPSS13_P1243															2615931	2617397		
3367		BURPSS13_P1244															2617402	2617809		
3368		BURPSS13_P1245															2617809	2620697		
3369		BURPSS13_P1246															2620694	2621440		
3370		BURPSS13_P1247															2621642	2622139		
3371		BURPSS13_P1248															2622535	2624652		
3372		BURPSS13_P1249															2624885	2625070		
3373		BURPSS13_P1250															2625100	2625441		
3374		BURPSS13_P1251															2631960	2626711		
3375		BURPSS13_P1252															2634531	2632063		
3376		BURPSS13_P1253															2634769	2636031		
3377		BURPSS13_P1254															2636522	2636722		
3378		BURPSS13_P1255															2636752	2637795		
3379		BURPSS13_P1256															2638368	2637820		
3380		BURPSS13_P1257															2639458	2638358		
3381		BURPSS13_P1258															2639831	2640931		
3382		BURPSS13_P1259															2642154	2640964		
3383		BURPSS13_P1260															2643474	2642296		
3384		BURPSS13_P1261															2644889	2643471		
3385		BURPSS13_P1262															2645145	2644843		
3386		BURPSS13_P1263															2645936	2645142		
3387		BURPSS13_P1264															2646829	2645933		
3388		BURPSS13_P1265															2647928	2646822		
3389		BURPSS13_P1266															2649041	2647968		
3390		BURPSS13_P1267															2649150	2650094		
3391		BURPSS13_P1268															2650192	2651802		
3392		BURPSS13_P1269															2651772	2652803		
3393		BURPSS13_P1270															2652891	2653358		
3394		BURPSS13_P1271															2653355	2654296		
3395		BURPSS13_P1272															2654248	2655717		
3396		BURPSS13_P1273															2655714	2656061		
3397		BURPSS13_P1274															2656074	2657579		
3398		BURPSS13_P1275															2657816	2657589		
3399		BURPSS13_P1276															2658160	2658918		
3400		BURPSS13_P1277															2658911	2659831		
3401		BURPSS13_P1278															2661979	2660480		
3402		BURPSS13_P1279															2662418	2664337		
3403		BURPSS13_P1280															2664536	2667418		
3404		BURPSS13_P1281															2667415	2669211		
3405		BURPSS13_P1282															2671663	2674392		
3406		BURPSS13_P1283															2674513	2676282		
3407	BPSL2239		2557807	2556560	1825526	1824279	2987848	2986601	2971662	2970415	1533501	1534748	2512659	2511412	2543131	2541884	2678109	2676862	2526610	2525363
3408	BPSL2240		2559626	2558151	1827345	1825870	2989667	2988192	2973481	2972006	1531691	1533157	2514469	2513003	2544950	2543475	2679928	2678453	2528420	2526954
3409	BPSL2241		2560641	2559673	1828360	1827392	2990682	2989714	2974496	2973528	1530676	1531644	2515484	2514516	2545965	2544997	2680943	2679975	2529435	2528467
3410	BPSL2242		2561683	2560748	1829402	1828467	2991724	2990789	2975538	2974603	1529634	1530569	2516526	2515591	2547007	2546072	2681985	2681050	2530477	2529542
3411	BPSL2243		2563128	2561734	1830847	1829453	2993169	2991775	2976983	2975589	1528189	1529583	2517971	2516577	2548452	2547058	2683430	2682036	2531922	2530528
3412	BPSL2244		2563417	2564079	1831136	1831798	2993458	2994120	2977272	2977934	1527900	1527238	2518260	2518922	2548741	2549403	2683719	2684381	2532211	2532873
3413	BPSL2245		2564139	2564711	1831858	1832430	2994180	2994752	2977994	2978566	1527178	1526606	2518982	2519554	2549463	2550035	2684441	2685013	2532933	2533505
3414	BPSL2246		2564795	2565283	1832514	1833002	2994836	2995324	2978650	2979138	1526522	1526034	2519638	2520126	2550119	2550193	2685097	2685585	2533589	2534077
3415	BPSL2247		2565350	2566108	1833069	1833827	2995391	2996149	2979205	2979963	1525967	1525209	2520193	2520951	2550790	2551548	2685652	2686410	2534144	2534902
3416	BPSL2248		2567232	2566450	1834951	1834169	2997273	2996491	2981087	2980305	1524085	1524867	2522074	2521292	2552672	2551890	2687534	2686752	2536026	2535244

3417	BPSL2249			2568358	2567426	1836077	1835145	2998398	2997466	2982212	2981280	1522989	1523891	2523176	2522268	2553791	2552865	2688654	2687728	2537122	2536220
3418	BPSL2250			2568659	2569459	1836378	1837178	2998699	2999499	2982513	2983313	1522688	1521888	2523477	2524277	2554092	2554892	2688955	2689755	2537423	2538223
3419	BPSL2251			2570014	2569745	1837733	1837464	3000042	2999773	2983856	2983587	1521333	1521602	2524821	2524552	2555447	2555178	2690310	2690041	2538778	2538509
3420			Bp_chr1_33_ISBn	2570546	2571850	1838265	1839569											2690842	2692146		
3421		BURPS1106A_2608		2570687	2571847																
3422		BURPSS13_P1305																2694094	2693261		
3423		BURPSS13_P1306																2695230	2694091		
3424		BURPSS13_P1307																2695694	2695855		
3425		BURPSS13_P1308																2697835	2695883		
3426		BURPSS13_P1309																2698629	2698180		
3427		BURPSS13_P1310																2698619	2698879		
3428		BURPSS13_P1311																2700143	2699187		
3429		BURPSS13_P1312																2700435	2700118		
3430		BURPSS13_P1316																2702862	2703518		
3431		BURPSS13_P1317																2703515	2704258		
3432		BURPSS13_P1318																2705082	2704294		
3433		BURPSS13_P1319																2705774	2705226		
3434		BURPSS13_P1320																2706268	2705771		
3435		BURPSS13_P1321																2706455	2706261		
3436		BURPSS13_P1322																2707583	2706531		
3437		BURPSS13_P1323																2707808	2707593		
3438		BURPSS13_P1324																2708655	2707774		
3439		BURPSS13_P1325																2711078	2708664		
3440		BURPSS13_P1326																2711212	2711096		
3441		BURPSS13_P1327																2711468	2711166		
3442		BURPSS13_P1328																2712041	2711538		
3443		BURPSS13_P1329																2713221	2712052		
3444		BURPSS13_P1330																2713739	2713287		
3445		BURPSS13_P1331																2715218	2713755		
3446		BURPSS13_P1332																2715781	2715206		
3447		BURPSS13_P1333																2716667	2715774		
3448		BURPSS13_P1334																2717008	2716664		
3449		BURPSS13_P1335																2717211	2717005		
3450		BURPSS13_P1336																2717956	2717276		
3451		BURPSS13_P1337																2718492	2717959		
3452		BURPSS13_P1338																2719012	2718485		
3453		BURPSS13_P1339																2719304	2719014		
3454		BURPSS13_P1340																2720333	2719308		
3455		BURPSS13_P1341																2720712	2720368		
3456		BURPSS13_P1342																2721839	2720739		
3457		BURPSS13_P1343																2723329	2721836		
3458		BURPSS13_P1344																2723532	2723326		
3459		BURPSS13_P1345																2725654	2723543		
3460		BURPSS13_P1346																2725659	2726210		
3461		BURPSS13_P1347																2727784	2727197		
3462		BURPSS13_P1348																2730494	2728002		
3463		BURPSS13_P1349																2731084	2730614		
3464		BURPSS13_V0265																2767260	2765959		
3465		BURPSS13_V0266																2765284	2765757		
3466		BURPSS13_V0267																2765051	2765275		
3467		BURPSS13_V0268																2763516	2764832		
3468		BURPSS13_V0269																2763067	2763516		
3469		BURPSS13_V0270																2762567	2763070		
3470		BURPSS13_V0271																2762133	2762468		
3471		BURPSS13_V0272																2761962	2762081		
3472		BURPSS13_V0273																2760749	2761312		
3473		BURPSS13_V0274																2760373	2759846		
3474		BURPSS13_V0275																2759832	2759500		

3475	BURPSS13_V0276															2758793	2758335		
3476	BURPSS13_V0277															2757961	2757491		
3477	BURPSS13_V0278															2757371	2754879		
3478	BURPSS13_V0279															2754661	2754074		
3479	BURPSS13_V0280															2753353	2753472		
3480	BURPSS13_V0281															2752536	2753087		
3481	BURPSS13_V0282															2752531	2750420		
3482	BURPSS13_V0283															2750409	2750203		
3483	BURPSS13_V0284															2750206	2748713		
3484	BURPSS13_V0285															2748716	2747616		
3485	BURPSS13_V0286															2747589	2747245		
3486	BURPSS13_V0287															2747210	2746185		
3487	BURPSS13_V0288															2746181	2745891		
3488	BURPSS13_V0289															2745889	2745362		
3489	BURPSS13_V0290															2745369	2744836		
3490	BURPSS13_V0291															2744839	2744153		
3491	BURPSS13_V0292															2744088	2743882		
3492	BURPSS13_V0293															2743885	2743541		
3493	BURPSS13_V0294															2743544	2742651		
3494	BURPSS13_V0295															2742658	2742083		
3495	BURPSS13_V0296															2742095	2740632		
3496	BURPSS13_V0297															2740616	2740164		
3497	BURPSS13_V0298															2740098	2738929		
3498	BURPSS13_V0299															2738918	2738415		
3499	BURPSS13_V0300															2738345	2738043		
3500	BURPSS13_V0301															2737955	2735541		
3501	BURPSS13_V0302															2735532	2734651		
3502	BURPSS13_V0303															2734685	2734470		
3503	BURPSS13_V0304															2734460	2733408		
3504	BURPSS13_V0305															2733332	2733138		
3505	BURPSS13_V0306															2733136	2732648		
3506	BURPSS13_V0307															2732648	2732103		
3507	BURPSS13_V0308															2731959	2731321		
3508 BPSL2252		2572670	2575342	1840389	1843061	3001384	3004053	2985198	2987867	1519989	1517320	2526163	2528835	2556787	2559456	2767438	2770101	2540120	2542792
3509 BPSL2253		2575332	2576627	1843051	1844346	3004043	3005338	2987857	2989152	1517330	1516035	2528825	2530120	2559446	2560741	2770091	2771386	2542782	2544077
3510 BPSL2254		2577155	2576613	1844874	1844332	3005866	3005324	2989680	2989138	1515507	1516049	2530648	2530106	2561269	2560727	2771914	2771372	2544605	2544063
3511 BPSL2255		2577248	2578408	1844967	1846127	3005959	3007119	2989773	2990933	1515414	1514254	2530741	2531901	2561362	2562522	2772007	2773167	2544698	2545858
3512 BPSL2256		2579914	2579141	1847633	1846860	3008626	3007853	2992440	2991667	1512748	1513521	2533407	2532634	2564028	2563255	2774673	2773900	2547364	2546591
3513 BPSL2257		2581061	2579922	1848780	1847641	3009773	3008634	2993587	2992448	1511601	1512740	2534554	2533415	2565175	2564036	2775820	2774681	2548511	2547372
3514 BPSL2258		2582074	2581175	1849793	1848894	3010786	3009887	2994600	2993701	1510588	1511487	2535567	2534668	2566188	2565289	2776833	2775934	2549524	2548625
3515 BPSL2259		2582736	2582122	1850455	1849841	3011448	3010834	2995262	2994648	1509929	1510540	2536229	2535615	2566847	2566236	2777495	2776881	2550183	2549572
3516 BPSL2260		2583935	2582736	1851654	1850455	3012647	3011448	2996461	2995262	1508730	1509929	2537428	2536229	2568046	2566847	2778694	2777495	2551382	2550183
3517 BPSL2261		2584609	2583947	1852328	1851666	3013321	3012659	2997135	2996473	1508056	1508718	2538102	2537440	2568720	2568058	2779368	2778706	2552056	2551394
3518 BPSL2262		2585337	2584711	1853056	1852430	3014049	3013423	2997863	2997237	1507328	1507954	2538830	2538204	2569448	2568822	2780096	2779470	2552784	2552158
3519 BPSL2263		2586241	2585405	1853960	1853124	3014953	3014117	2998767	2997931	1506424	1507260	2539734	2538898	2570352	2569516	2781000	2780164	2553688	2552852
3520 BPSL2264		2587362	2586475	1855081	1854194	3016074	3015187	2999888	2999001	1505296	1506183	2540870	2539983	2571464	2570577	2782112	2781225	2554816	2553929
3521 BPSL2265		2588368	2587520	1856087	1855239	3017082	3016234	3000896	3000048	1504290	1505138	2541877	2541029	2572470	2571622	2783120	2782272	2555823	2554975
3522 BPSL2266		2588545	2589066	1856264	1856785	3017259	3017780	3001073	3001594	1504113	1503592	2542054	2542575	2572647	2573168	2783297	2783818	2556000	2556521
3523 BPSL2267		2589108	2590055	1856827	1857774	3017822	3018769	3001636	3002583	1503550	1502603	2542617	2543564	2573210	2574157	2783860	2784807	2556563	2557510
3524 BPSL2268		2590142	2590486	1857861	1858205	3018856	3019200	3002670	3003014	1502516	1502172	2543651	2543995	2574244	2574588	2784894	2785238	2557597	2557941
3525 BPSL2269		2591143	2590715	1858862	1858434	3019857	3019429	3003671	3003243	1501512	1501940	2544652	2544224	2575245	2574817	2785898	2785470	2558598	2558170
3526 BPSL2270		2592518	2591238	1860237	1858957	3021232	3019952	3005046	3003766	1500137	1501417	2546027	2544747	2576620	2575340	2787273	2785993	2559973	2558693
3527 BPSL2271		2593476	2592625	1861195	1860344	3022190	3021339	3006004	3005153	1499179	1500030	2546985	2546134	2577578	2576727	2788231	2787380	2560931	2560080
3528 BPSL2272		2595134	2593476	1862853	1861195	3023848	3022190	3007662	3006004	1497521	1499179	2548643	2546985	2579236	2577578	2789889	2788231	2562589	2560931
3529 BPSL2273		2596218	2595394	1863937	1863113	3024931	3024107	3008745	3007921	1496437	1497261	2549726	2548902	2580319	2579495	2790973	2790149	2563673	2562849
3530 BPSL2274		2598825	2596258	1866544	1863977	3027402	3025810	3011352	3008785	1493830	1496397	2552333	2549766	2582926	2580359	2792110	2791013	2566280	2563713
3531 BPSL2275		2599624	2598839	1867343	1866558	3028201	3027416	3012151	3011366	1493031	1493816	2553132	2552347	2583725	2582940	2794383	2793598	2567079	2566294
3532 BPSL2276		2600351	2599632	1868070	1867351	3028928	3028209	3012878	3012159	1492304	1493023	2553859	2553140	2584452	2583733	2795110	2794391	2567806	2567087

3533	BPSL2277			2601624	2600374	1869343	1868093	3030201	3028951	3014151	3012901	1491031	1492281	2555132	2553882	2585725	2584475	2796383	2795133	2569079	2567829
3534		BURPS668_2585												2555279	2555533						
3535	BPSL2278			2602192	2603265	1869911	1870984	3030769	3031842	3014719	3015792	1490464	1489391	2555703	2556776	2586292	2587365	2796951	2798024	2569652	2570725
3536	BPSL2279			2603284	2604975	1871003	1872694	3031861	3033552	3015811	3017502	1489372	1487681	2556795	2558486	2587384	2589075	2798043	2799734	2570744	2572435
3537	BPSL2280			2605339	2606442	1873058	1874161	3033916	3035019	3017866	3018969	1487317	1486214	2558857	2559960	2589439	2590542	2800098	2801201	2572801	2573904
3538	BPSL2281			2606537	2608060	1874256	1875779	3035115	3036638	3019065	3020588	1486119	1484596	2560056	2561579	2590636	2592159	2801296	2802819	2573999	2575522
3539	BPSL2282			2608495	2609217	1876214	1876936	3037040	3037762	3020990	3021712	1484241	1483519	2561933	2562655	2592513	2593235	2803174	2803896	2575893	2576615
3540	BPSL2283			2609856	2609650	1877575	1877369	3038401	3038195	3022351	3022145	1482880	1483086	2563279	2563073	2593883	2593677	2804535	2804329	2577263	2577057
3541	BPSL2284			2610216	2609878	1877935	1877597	3038761	3038423	3022711	3022373	1482520	1482858	2563639	2563301	2594243	2593905	2804895	2804557	2577623	2577285
3542	BPSL2285			2612177	2610312	1879896	1878031	3040722	3038857	3024672	3022807	1480559	1482424	2565600	2563735	2596205	2596143	2806856	2804991	2579584	2577719
3543	BPSL2286			2612762	2612238	1880481	1879957	3041307	3040783	3025257	3024733	1479974	1480498	2566185	2565661	2596790	2596266	2807441	2806917	2580169	2579645
3544	BPSL2287			2613198	2612878	1880917	1880597	3041743	3041423	3025693	3025373	1479538	1479858	2566621	2566301	2597226	2596906	2807877	2807557	2580605	2580285
3545	BPSL2288			2613688	2613284	1881407	1881003	3042233	3041829	3026183	3025779	1479048	1479452	2567111	2566707	2597716	2597312	2808367	2807963	2581095	2580691
3546	BPSL2289			2614977	2613757	1882696	1881476	3043522	3042302	3027472	3026252	1477759	1478979	2568400	2567180	2599005	2597785	2809656	2808436	2582384	2581164
3547	BPSL2290			2615585	2615049	1883304	1882768	3044130	3043594	3028080	3027544	1477151	1477687	2569008	2568472	2599613	2599077	2810264	2809728	2582992	2582456
3548	BPSL2291			2616166	2615690	1883885	1883409	3044711	3044235	3028661	3028185	1476570	1477046	2569589	2569113	2600194	2599718	2810845	2810369	2583573	2583097
3549	BPSL2292			2616518	2616261	1884237	1883980	3045063	3044806	3029013	3028756	1476218	1476475	2569941	2569684	2600546	2600289	2811197	2810940	2583925	2583668
3550	BPSL2293			2618080	2616632	1885799	1884351	3046625	3045177	3030575	3029127	1474656	1476104	2571503	2570055	2602108	2600660	2812759	2811311	2585487	2584039
3551	BPSL2294			2618944	2618225	1886663	1885944	3047489	3046770	3031439	3030720	1473792	1474511	2572367	2571648	2602972	2602253	2813623	2812904	2586351	2585632
3552	BPSL2295			2619127	2619936	1886846	1887655	3047672	3048481	3031622	3032431	1473609	1472800	2572550	2573359	2603155	2603964	2813806	2814615	2586534	2587343
3553	BPSL2296		Bp_chr1_34_ISBr	2620013	2621584	1887732	1889303	3048559	3050131	3032509	3034081			2573437	2575009	2604042	2605614				
3554	BPSL2297			2622726	2621650	1890445	1889369	3051275	3050199	3035225	3034149	1471601	1472677	2576153	2575077	2606758	2605682	2815814	2814738	2588541	2587465
3555	BPSL2298			2623438	2624004	1891157	1891723	3051987	3052553	3035937	3036503	1470889	1470323	2576865	2577431	2607470	2608036	2816526	2817092	2589253	2589819
3556	BPSL2299			2626034	2624268	1893753	1891987	3054572	3052806	3038522	3036756	1468304	1470070	2579443	2577677	2610048	2608282	2819160	2817394	2591894	2590128
3557	BPSL2300			2627988	2626345	1895707	1894064	3056523	3054883	3040473	3038833	1466380	1467993	2581382	2579754	2611881	2611423	2821111	2819471	2593752	2593246
3558	BPSL2301			2630817	2628124	1898536	1895843	3059352	3056659	3043302	3040609	1463523	1466216	2584211	2581518	2614710	2612017	2823940	2821247	2596581	2593888
3559	BPSL2302			2631060	2633597	1898779	1901316	3059595	3062132	3043545	3046082	1463280	1460743	2584454	2586991	2614953	2617490	2824183	2826720	2596824	2599361
3560	BPSL2303			2633597	2634232	1901316	1901951	3062132	3062767	3046082	3046717	1460743	1460108	2586991	2587626	2617490	2618125	2826720	2827355	2599361	2599996
3561	BPSL2304			2634551	2635405	1902270	1903124	3063087	3063941	3047037	3047891	1459788	1458934	2587945	2588799	2618445	2619299	2827674	2828528	2600316	2601170
3562		BURPS1106B_A1895				1903553	1903440														
3563	BPSL2305			2636114	2638210	1903833	1905929	3064661	3066757	3048611	3050707	1458198	1456102	2589511	2591607	2620019	2622115	2829240	2831336	2601906	2604002
3564	BPSL2306			2639665	2638448	1907384	1906167	3068192	3066993	3052142	3050943	1454665	1455864	2593051	2591843	2623550	2622351	2832791	2831574	2605338	2604139
3565	BPSL2307			2640444	2641721	1908163	1909440	3068964	3070241	3052907	3054184	1453944	1452667	2593843	2595120	2624329	2625606	2833542	2834819	2606109	2607386
3566	BPSL2308			2641768	2643150	1909487	1910869	3070288	3071670	3054231	3055613	1452620	1451238	2595167	2596549	2625653	2627035	2834866	2836248	2607433	2608815
3567	BPSL2309			2643404	2647126	1911123	1914845	3071924	3075646	3055867	3059589	1450984	1447262	2596803	2600525	2627289	2631011	2836502	2840224	2609069	2612791
3568	BPSL2310			2647126	2648676	1914845	1916395	3075646	3077196	3059589	3061139	1447262	1445712	2600525	2602075	2631011	2632561	2840224	2841774	2612791	2614341
3569	BPSL2311			2648703	2649377	1916422	1917096	3077223	3077909	3061166	3061852	1445685	1445011	2602102	2602776	2632588	2633274	2841801	2842475	2614368	2615042
3570	BPSL2312			2649417	2650100	1917136	1917819	3077949	3078632	3061892	3062575	1444971	1444288	2602816	2603499	2633314	2633997	2842515	2843198	2615082	2615765
3571	BPSL2313			2650245	2652176	1917967	1919898	3078777	3080708	3062720	3064651	1444030	1442099	2603644	2605575	2634143	2636074	2843343	2845274	2615980	2617911
3572	BPSL2314			2652203	2652901	1919925	1920623	3080735	3081433	3064678	3065376	1442072	1441398	2605602	2606300	2636101	2636799	2845301	2845999	2617938	2618636
3573	BPSL2315			2653050	2653823	1920772	1921545	3081582	3082355	3065525	3066298	1441249	1440476	2606449	2607222	2636948	2637721	2846148	2846921	2618785	2619558
3574	BPSL2316			2655649	2654117	1923371	1921839	3084181	3082649	3068124	3066592	1438649	1440181	2609049	2607517	2639548	2638016	2848747	2847215	2621384	2619852
3575	BPSL2317			2656781	2655690	1924503	1923412	3085313	3084222	3069256	3068165	1437517	1438608	2610181	2609090	2640680	2639589	2849879	2848788	2622516	2621425
3576	BPSL2318			2658411	2656999	1926133	1924721	3086943	3085531	3070886	3069474	1435887	1437299	2611811	2610399	2642310	2640898	2851509	2850097	2624146	2622734
3577	BPSL2319			2658792	2659277	1926514	1926999	3087324	3087809	3071267	3071752	1435506	1435021	2612192	2612677	2642691	2643176	2851890	2852375	2624527	2625012
3578	BPSL2320			2660331	2659516	1928053	1927238	3088856	3088041	3072799	3071984	1433947	1434762	2613751	2612936	2644223	2643408	2853447	2852632	2626086	2625271
3579	BPSL2321			2661161	2660331	1928883	1928053	3089686	3088856	3073629	3072799	1433117	1433947	2614581	2613751	2645053	2644223	2854277	2853447	2626916	2626086
3580	BPSL2322			2662694	2661708	1930416	1929430	3091233	3090247	3075176	3074190	1431584	1432570	2616140	2615154	2646586	2645600	2855775	2854789	2628443	2627457
3581	BPSL2323			2663703	2662714	1931425	1930436	3092242	3091253	3076185	3075196	1430575	1431564	2617149	2616160	2647595	2646606	2856784	2855795	2629452	2628463
3582	BPSL2324			2667698	2663808	1935420	1931530	3096237	3092347	3080180	3076290	1426519	1429500	2621144	2617254	2651590	2647700	2860779	2856889	2633447	2629557
3583	BPSL2325			2667956	2669329	1935678	1937051	3096495	3097868	3080438	3081811	1426261	1424888	2621402	2622775	2651967	2653340	2861037	2862410	2633714	2635087
3584	BPSL2326			2669386	2669658	1937108	1937380	3097924	3098196	3081867	3082139	1424832	1424560	2622831	2623103	2653396	2653668	2862466	2862738	2635143	2635415
3585	BPSL2327			2671177	2669837	1938899	1937559	3099715	3098375	3083658	3082318	1423042	1424382	2624622	2623282	2655187	2653847	2864256	2862916	2636934	2635594
3586	BPSL2328			2672474	2671377	1940196	1939099	3101012	3099915	3084955	3083858	1421745	1422842	2625919	2624822	2656484	2655387	2865553	2864456	2638231	2637134
3587	BPSL2329			2674792	2673008	1942514	1940730	3103228	3101438	3087171	3085381	1419529	1421319	2628123	2626327	2658691	2656901	2867895	2866105	2640438	2638648
3588	BPSL2330			2674901	2675800	1942623	1943522	3103337	3104236	3087280	3088179	1419420	1418521	2628232	2629131	2658800	2659699	2868004	2868903	2640547	2641446
3589		BURPS1106A_2706		2676250	2676603																
3590		BURPS1106A_2707		2676906	2677760																

3591		BURPS1106A_2708	2677754	2678044																
3592		BURPS1106B_A1927			1944325	1943972														
3593		BURPS1106B_A1928			1945482	1944628														
3594		BURPS1106B_A1929			1945766	1945476														
3595		BURPS668_2651											2629579	2629932						
3596	BPSL2331						3104945	3104661	3088888	3088604					2660408	2660124	2869612	2869328		
3597	BPSL2332						3106081	3105212	3090024	3089155					2661544	2660675	2870748	2869879		
3598	BPSL2333						3106366	3106118	3090309	3090061					2661829	2661581	2871033	2870785		
3599	BPSL2334		2678970	2678515	1946692	1946237	3106835	3106380	3090778	3090323	1416608	1417063	2631087	2630632	2662298	2661843	2871502	2871047	2643404	2642949
3600		BURPS406E_H0256									1417796	1418134								
3601	BPSL2335		2680641	2679217	1948363	1946939	3108506	3107082	3092449	3091025	1414937	1416361	2632749	2631334	2663969	2662545	2873173	2871749	2645075	2643651
3602	BPSL2336		2682010	2680679	1949732	1948401	3109875	3108544	3093818	3092487	1413568	1414899	2634119	2632788	2665338	2664007	2874542	2873211	2646444	2645113
3603	BPSL2337		2682834	2682046	1950556	1949768	3110699	3109911	3094642	3093854	1412744	1413532	2634943	2634155	2666162	2665374	2875366	2874578	2647268	2646480
3604	BPSL2338		2684645	2683845	1952367	1951567	3112510	3111710	3096453	3095653	1410933	1411733	2636765	2635965	2667973	2667173	2877177	2876377	2649079	2648279
3605	BPSL2339		2686271	2684892	1953993	1952614	3114136	3112757	3098079	3096700	1409307	1410686	2638391	2637012	2669599	2668220	2878803	2877424	2650705	2649326
3606	BPSL2340		2687508	2686288	1955230	1954010	3115373	3114153	3099316	3098096	1408070	1409290	2639628	2638408	2670836	2669616	2880040	2878820	2651942	2650722
3607	BPSL2341		2688164	2687508	1955886	1955230	3116029	3115373	3099972	3099316	1407414	1408070	2640284	2639628	2671492	2670836	2880696	2880040	2652598	2651942
3608	BPSL2342		2689852	2688167	1957574	1955889	3117717	3116032	3101660	3099975	1405726	1407411	2641972	2640287	2673180	2671495	2882384	2880699	2654286	2652601
3609	BPSL2343		2690590	2689898	1958312	1957620	3118455	3117763	3102398	3101706	1404988	1405680	2642710	2642018	2673918	2673226	2883122	2882430	2655024	2654332
3610	BPSL2344		2692118	2690598	1959840	1958320	3119983	3118463	3103926	3102406	1403460	1404980	2644238	2642718	2675446	2673926	2884650	2883130	2656552	2655032
3611		BURPS1106B_A1947			1961254	1960673														
3612		BURPS1106B_A1948			1961777	1961205														
3613	BPSL2345		2694935	2694147	1962658	1961870	3121443	3120655	3105386	3104598	1402000	1402788	2645698	2644910	2676906	2676118	2886110	2885322	2658012	2657224
3614	BPSL2346		2696050	2695043	1963773	1962766	3122558	3121551	3106501	3105494	1400885	1401892	2646813	2645806	2678021	2677014	2887225	2886218	2659133	2658120
3615		BURPS1655_H0141																	2659147	2659449
3616	BPSL2347		2697987	2697265	1965710	1964988	3124483	3123761	3108426	3107704	1398960	1399682	2648741	2648019	2679952	2679230	2889143	2888421	2661059	2660337
3617	BPSL2348		2699510	2698842	1967233	1966565	3126005	3125337	3109948	3109280	1397462	1398130	2650273	2649605	2681450	2680782	2890666	2889998	2662601	2661933
3618	BPSL2349		2701052	2699595	1968775	1967318	3127547	3126090	3111490	3110033	1395920	1397377	2651815	2650358	2682992	2681535	2892208	2890751	2664143	2662686
3619	BPSL2350		2701472	2702635	1969195	1970358	3127967	3129130	3111910	3113073	1395500	1394337	2652235	2653398	2683411	2684574	2892628	2893791	2664562	2665725
3620	BPSL2351		2703456	2705741	1971187	1973472	3129879	3132164	3113822	3116107	1393596	1391311	2654203	2656488	2685363	2687426	2894540	2896825	2666482	2668764
3621	BPSL2352		2706029	2706166	1973555	1973752	3132247	3132444	3116395	3116532	1391149	1390952	2656776	2656913			2896987	2897184	2668849	2669046
3622	BPSL2354		2706223	2707353	1973954	1975084	3132646	3133776	3116589	3117719	1390750	1389620	2656970	2658100	2687528	2688583	2897385	2898515	2669248	2670378
3623	BPSL2355		2707502	2708722	1975233	1976453	3133925	3135145	3117868	3119088	1389480	1388260	2658228	2659448	2688723	2689943	2898664	2899884	2670527	2671747
3624	BPSL2356		2709323	2711083	1977054	1978814	3135746	3137506	3119689	3121449	1387646	1385886	2660049	2661809	2690543	2692303	2900485	2902245	2672348	2674108
3625	BPSL2357		2711089	2711298	1978820	1979029	3137512	3137721	3121455	3121664	1385880	1385671	2661815	2662024	2692309	2692518	2902251	2902460	2674114	2674323
3626	BPSL2358		2711320	2712036	1979051	1979767	3137743	3138459	3121686	3122402	1385649	1384933	2662046	2662762	2692540	2693256	2902482	2903198	2674345	2675061
3627	BPSL2359		2712740	2712297	1980471	1980028	3139163	3138720	3123106	3122663	1384229	1384672	2663478	2663035	2693953	2693510	2903907	2903464	2675765	2675322
3628	BPSL2360		2713259	2712783	1980990	1980514	3139682	3139206	3123625	3123149	1383710	1384186	2663997	2663521	2694472	2693996	2904426	2903950	2676284	2675808
3629	BPSL2361		2714202	2713318	1981933	1981049	3140625	3139741	3124568	3123684	1382767	1383651	2664940	2664056	2695415	2694531	2905369	2904485	2677227	2676343
3630	BPSL2362		2715277	2714246	1983008	1981977	3141700	3140669	3125643	3124612	1381692	1382723	2666015	2664984	2696490	2695459	2906444	2905413	2678302	2677271
3631	BPSL2363		2716583	2715369	1984314	1983100	3143006	3141792	3126949	3125735	1380386	1381600	2667321	2666107	2697796	2696582	2907750	2906536	2679608	2678394
3632	BPSL2364		2716803	2718116	1984534	1985847	3143226	3144539	3127169	3128482	1380166	1378853	2667541	2668854	2698016	2699329	2907970	2909283	2679828	2681141
3633	BPSL2365		2718788	2718099	1986519	1985830	3145211	3144522	3129154	3128465	1378181	1378870	2669526	2668837	2700001	2699312	2909955	2909266	2681813	2681124
3634	BPSL2366		2720242	2718800	1987973	1986531	3146665	3145223	3130608	3129166	1376727	1378169	2670980	2669538	2701455	2700013	2911409	2909967	2683267	2681825
3635	BPSL2367		2722319	2720400	1990050	1988131	3148742	3146823	3132685	3130766	1374650	1376569	2673057	2671138	2703532	2701613	2913486	2911567	2685344	2683425
3636	BPSL2368		2722853	2724046	1990584	1991777	3149290	3150483	3133233	3134426	1374102	1372909	2673591	2674784	2704066	2705259	2914020	2915213	2685878	2687071
3637	BPSL2369		2724538	2725989	1992269	1993720	3150975	3152426	3134918	3136369	1372418	1370967	2675276	2676727	2705751	2707202	2915705	2917156	2687563	2689014
3638	BPSL2370		2726001	2726789	1993732	1994520	3152439	3153227	3136382	3137170	1370954	1370166	2676739	2677527	2707214	2708002	2917169	2917957	2689026	2689814
3639	BPSL2371		2726797	2728029	1994528	1995760	3153235	3154467	3137178	3138410	1370158	1368926	2677535	2678767	2708010	2709242	2917965	2919197	2689822	2691054
3640	BPSL2372		2728034	2729263	1995765	1996994	3154472	3155701	3138415	3139644	1368921	1367692	2678772	2680001	2709247	2710476	2919202	2920431	2691059	2692288
3641	BPSL2373		2729263	2729751	1996994	1997482	3155701	3156189	3139644	3140132	1367692	1367204	2680001	2680489	2710476	2710964	2920431	2920919	2692288	2692776
3642	BPSL2374		2729751	2730296	1997482	1998027	3156189	3156734	3140132	3140677	1367204	1366659	2680489	2681034	2710964	2711509	2920919	2921464	2692776	2693321
3643	BPSL2375		2730893	2730597	1998624	1998328	3157331	3157035	3141274	3140978	1366059	1366355	2681634	2681338	2712106	2711810	2922064	2921768	2693918	2693622
3644	BPSL2376		2731084	2731569	1998815	1999300	3157522	3158007	3141465	3141950	1365868	1365383	2681825	2682310	2712297	2712782	2922255	2922740	2694109	2694594
3645	BPSL2377		2732223	2731789	1999954	1999520	3158641	3158219	3142584	3142162	1364729	1365163	2682952	2682518	2713436	2713002	2923374	2922952	2695240	2694806
3646	BPSL2378		2732602	2733489	2000333	2001220	3159020	3159907	3142963	3143850	1364350	1363463	2683331	2684218	2713815	2714702	2923753	2924640	2695619	2696506
3647	BPSL2379		2733496	2735499	2001227	2003230	3159914	3161917	3143857	3145860	1363456	1361453	2684225	2686228	2714709	2716712	2924647	2926650	2696513	2698516
3648	BPSL2380		2735502	2736104	2003233	2003835	3161920	3162522	3145863	3146465	1361450	1360848	2686231	2686833	2716715	2717317	2926653	2927255	2698519	2699121

3649	BPSL2381		2736108	2736437	2003839	2004168	3162526	3162855	3146469	3146798	1360844	1360515	2686837	2687166	2717321	2717650	2927259	2927588	2699125	2699454
3650	BPSL2382		2736594	2737823	2004325	2005554	3163001	3164230	3146944	3148173	1360369	1359140	2687323	2688558	2717807	2719036	2927745	2928974	2699617	2700852
3651	BPSL2383		2738997	2738206	2006721	2005930	3165467	3164676	3149396	3148605	1358057	1358848	2689641	2688850	2720167	2719376	2930081	2929290	2701921	2701130
3652	BPSL2384		2739318	2739758	2007042	2007482	3165788	3166228	3149717	3150157	1357736	1357296	2689962	2690402	2720488	2720928	2930402	2930842	2702242	2702682
3653	BPSL2385		2740843	2739797	2008567	2007521	3167313	3166267	3151242	3150196	1356211	1357257	2691501	2690455	2722027	2720981	2931927	2930881	2703767	2702721
3654	BPSL2386		2742167	2740830	2009891	2008554	3168637	3167300	3152566	3151229	1354887	1356224	2692825	2691488	2723351	2722014	2933251	2931914	2705091	2703754
3655	BPSL2387		2743644	2742184	2011368	2009908	3170114	3168654	3154043	3152583	1353410	1354870	2694302	2692842	2724828	2723368	2934728	2933268	2706568	2705108
3656	BPSL2388		2744680	2743652	2012404	2011376	3171150	3170122	3155079	3154051	1352374	1353402	2695338	2694310	2725864	2724836	2935764	2934736	2707604	2706576
3657	BPSL2389		2745735	2744680	2013459	2012404	3172205	3171150	3156134	3155079	1351319	1352374	2696393	2695338	2726919	2725864	2936819	2935764	2708659	2707604
3658	BPSL2390		2747013	2745784	2014737	2013508	3173483	3172254	3157412	3156183	1350041	1351270	2697671	2696442	2728196	2726967	2938097	2936868	2709936	2708707
3659	BPSL2391		2748798	2747755	2016522	2015479	3175268	3174225	3159197	3158154	1348256	1349299	2699458	2698415	2729942	2728899	2939941	2938898	2711722	2710679
3660	BPSL2392		2749611	2748820	2017335	2016544	3176081	3175290	3160010	3159219	1347443	1348234	2700271	2699480	2730755	2729964	2940754	2939963	2712535	2711744
3661	BPSL2393		2750346	2749636	2018070	2017360	3176816	3176106	3160745	3160035	1346708	1347418	2701006	2700296	2731490	2730780	2941489	2940779	2713270	2712560
3662	BPSL2394		2751032	2750346	2018756	2018070	3177502	3176816	3161431	3160745	1346022	1346708	2701692	2701006	2732176	2731490	2942175	2941489	2713956	2713270
3663	BPSL2395		2751908	2751351	2019632	2019075	3178378	3177821	3162307	3161750	1345146	1345703	2702568	2702011	2733052	2732495	2943049	2942492	2714830	2714273
3664		BURPSS13_V0082															2943938	2943270		
3665	BPSL2396		2753804	2752761	2021521	2020478	3180108	3179065	3164037	3162994	1343239	1344282	2704387	2703344	2735008	2733965	2945022	2943979	2716618	2715575
3666	BPSL2397		2754688	2753804	2022405	2021521	3180974	3180108	3164903	3164037	1342355	1343239	2705271	2704387	2735892	2735008	2945906	2945022	2717502	2716618
3667	BPSL2398		2756113	2754695	2023830	2022412	3182399	3180981	3166328	3164910	1340930	1342348	2706696	2705278	2737317	2735899	2947331	2945913	2718927	2717509
3668	BPSL2399		2757403	2756228	2025120	2023945	3183692	3182514	3167621	3166443	1339640	1340815	2707986	2706811	2738607	2737432	2948621	2947446	2720217	2719042
3669	BPSL2400		2758030	2759085	2025747	2026802	3184319	3185374	3168248	3169303	1339013	1337958	2708613	2709668	2739234	2740289	2949248	2950303	2720844	2721899
3670		BURPS668_2741											2709668	2710207						
3671	BPSL2401		2760594	2759569	2028311	2027286	3186908	3185883	3170837	3169812	1336452	1337477	2711212	2710187	2741805	2740780	2951763	2950738	2723412	2722387
3672	BPSL2402		2761117	2761977	2028834	2029694	3187419	3188279	3171348	3172208	1335953	1335093	2711735	2712595	2742346	2743206	2952286	2953146	2723929	2724789
3673	BPSL2403		2762626	2764725	2030343	2032442	3188964	3191063	3172893	3174992	1334408	1332309	2713250	2715349	2743927	2746026	2953795	2955894	2725489	2727588
3674	BPSL2404		2765569	2766714	2033286	2034431	3191983	3193128	3175912	3177057	1331500	1330355	2716144	2717289	2746908	2748053	2956759	2957904	2728446	2729591
3675	BPSL2405		2766816	2767985	2034533	2035702	3193230	3194399	3177159	3178328	1330253	1329084	2717391	2718560	2748155	2749324	2958006	2959175	2729693	2730862
3676	BPSL2406		2768605	2768147	2036322	2035864	3195019	3194561	3178948	3178490	1328464	1328922	2719179	2718721	2749944	2749486	2959795	2959337	2731482	2731024
3677	BPSL2407		2769632	2768664	2037349	2036381	3196134	3195478	3179975	3179007	1327366	1328106	2720206	2719238	2750971	2750003	2960096	2959854	2732484	2731834
3678	BPSL2408		2770963	2769902	2038680	2037619	3197465	3196404	3181306	3180245	1326035	1327096	2721537	2720476	2752302	2751241	2962179	2961118	2733815	2732754
3679	BPSL2409		2771530	2773392	2039247	2041109	3198032	3199894	3181873	3183735	1325468	1323606	2722104	2723966	2752867	2754729	2962746	2964608	2734382	2736244
3680	BPSL2410		2774833	2773427	2042550	2041144	3201335	3199929	3185176	3183770	1322165	1323571	2725407	2724001	2756170	2754764	2966049	2964643	2737685	2736279
3681	BPSL2411		2775639	2774833	2043356	2042550	3202141	3201335	3185982	3185176	1321359	1322165	2726213	2725407	2756976	2756170	2966855	2966049	2738491	2737685
3682	BPSL2412		2775999	2776655	2043716	2044372	3202501	3203157	3186342	3186998	1320999	1320343	2726560	2727216	2757336	2757992	2967209	2967865	2738851	2739507
3683	BPSL2413		2776685	2777128	2044402	2044845	3203187	3203630	3187028	3187471	1320313	1319870	2727246	2727689	2758022	2758465	2967895	2968338	2739537	2739980
3684	BPSL2414		2777142	2778011	2044859	2045728	3203644	3204513	3187485	3188354	1319856	1318987	2727703	2728572	2758479	2759348	2968352	2969221	2739994	2740863
3685	BPSL2415		2778652	2778152	2046369	2045869	3205154	3204654	3188995	3188495	1318346	1318846	2729213	2728713	2759989	2759489	2969862	2969362	2741504	2741004
3686	BPSL2416		2779732	2778869	2047449	2046586	3206234	3205371	3190075	3189212	1317266	1318129	2730293	2729430	2761069	2760206	2970942	2970079	2742584	2741721
3687	BPSL2417		2779859	2780764	2047576	2048481	3206361	3207266	3190202	3191107	1317139	1316234	2730420	2731325	2761196	2762101	2971069	2971974	2742711	2743616
3688	BPSL2418		2780932	2781441	2048649	2049158	3207436	3207945	3191277	3191786	1316064	1315555	2731495	2732004	2762271	2762780	2972142	2972651	2743786	2744295
3689	BPSL2419		2783740	2783150	2051457	2050867	3210244	3209654	3194085	3193495	1313256	1313846	2734311	2733721	2765079	2764489	2974950	2974360	2746601	2746011
3690	BPSL2420		2786117	2783871	2053834	2051588	3212621	3210375	3196462	3194216	1310879	1313125	2736688	2734442	2767456	2765210	2977327	2975081	2748966	2746732
3691	BPSL2421		2786326	2787543	2054043	2055260	3212830	3214047	3196671	3197888	1310661	1309444	2736908	2738113	2767674	2768891	2977536	2978753	2749184	2750401
3692	BPSL2422		2787731	2788285	2055448	2056002	3214240	3214794	3198081	3198635	1309251	1308697	2738306	2738860	2769084	2769638	2978946	2979500	2750594	2751148
3693	BPSL2423		2788559	2789953	2056276	2057670	3215068	3216462	3198909	3200303	1308423	1307029	2739134	2740528	2769912	2771306	2979774	2981168	2751422	2752816
3694	BPSL2424		2791098	2790073	2058815	2057790	3217607	3216582	3201448	3200423	1305881	1306906	2741673	2740648	2772454	2771429	2982313	2981288	2753961	2752936
3695	BPSL2425		2791557	2791129	2059274	2058846	3218066	3217638	3201907	3201479	1305422	1305850	2742132	2741704	2772913	2772485	2982772	2982344	2754420	2753992
3696	BPSL2426		2792336	2791566	2060053	2059283	3218845	3218075	3202686	3201916	1304643	1305413	2742911	2742141	2773692	2772922	2983551	2982781	2755199	2754429
3697	BPSL2427		2793160	2792336	2060877	2060053	3219669	3218845	3203510	3202686	1303819	1304643	2743735	2742911	2774516	2773692	2984375	2983551	2756023	2755199
3698	BPSL2428		2794079	2793183	2061796	2060900	3220588	3219692	3204429	3203533	1302900	1303796	2744654	2743758	2775435	2774539	2985294	2984398	2756942	2756046
3699	BPSL2429		2795552	2794152	2063269	2061869	3222061	3220661	3205902	3204502	1301427	1302827	2746127	2744727	2776908	2775508	2986767	2985367	2758415	2757015
3700	BPSL2430		2796596	2795706	2064313	2063423	3223105	3222215	3206946	3206056	1300383	1301273	2747171	2746281	2777952	2777062	2987811	2986921	2759459	2758569
3701	BPSL2431		2798405	2796615	2066122	2064332	3224914	3223124	3208755	3206965	1298574	1300364	2748980	2747190	2779761	2777971	2989620	2987830	2761268	2759478
3702	BPSL2432		2798901	2798650	2066618	2066367	3225410	3225159	3209251	3209000	1298079	1298330	2749475	2749224	2780257	2780006	2990116	2989865	2761763	2761512
3703	BPSL2433		2800355	2798901	2068072	2066618	3226864	3225410	3210705	3209251	1296625	1298079	2750929	2749475	2781711	2780257	2991570	2990116	2763217	2761763
3704	BPSL2434		2801475	2800426	2069192	2068143	3227984	3226935	3211825	3210776	1295505	1296554	2752049	2751000	2782831	2781782	2992690	2991641	2764337	2763288
3705	BPSL2435		2802083	2801484	2069800	2069201	3228592	3227993	3212433	3211834	1294897	1295496	2752657	2752058	2783439	2782840	2993298	2992699	2764945	2764346
3706	BPSL2436		2802766	2802170	2070483	2069887	3229275	3228679	3213116	3212520	1294214	1294810	2753340	2752744	2784122	2783526	2993981	2993385	2765628	2765032

3707	BPSL2437			2803345	2802875	2071062	2070592	3229854	3229384	3213695	3213225	1293635	1294105	2753919	2753449	2784701	2784231	2994560	2994090	2766207	2765737
3708	BPSL2438			2804595	2803360	2072312	2071077	3231104	3229869	3214945	3213710	1292385	1293620	2755169	2753934	2785951	2784716	2995810	2994575	2767457	2766222
3709	BPSL2439			2804991	2804755	2072708	2072472	3231500	3231264	3215341	3215105	1291989	1292225	2755565	2755329	2786347	2786111	2996206	2995970	2767853	2767617
3710	BPSL2440			2805884	2805138	2073601	2072855	3232393	3231647	3216234	3215488	1291096	1291842	2756458	2755712	2787240	2786494	2997099	2996353	2768746	2768000
3711	BPSL2441			2806867	2805938	2074584	2073655	3233376	3232447	3217217	3216288	1290113	1291042	2757441	2756512	2788223	2787294	2998082	2997153	2769729	2768800
3712	BPSL2442			2807927	2806941	2075644	2074658	3234436	3233450	3218277	3217291	1289053	1290039	2758501	2757515	2789283	2788297	2999142	2998156	2770789	2769803
3713	BPSL2443			2809033	2807930	2076750	2075647	3235542	3234439	3219383	3218280	1287947	1289050	2759607	2758504	2790389	2789286	3000248	2999145	2771895	2770792
3714	BPSL2444			2809352	2809176	2077069	2076893	3235861	3235685	3219702	3219526	1287628	1287804	2759926	2759750	2790708	2790532	3000567	3000391	2772214	2772038
3715	BPSL2445			2810081	2809452	2077798	2077169	3236590	3235961	3220431	3219802	1286899	1287528	2760655	2760026	2791437	2790808	3001296	3000667	2772943	2772314
3716	BPSL2446			2810325	2810969	2078042	2078686	3236834	3237478	3220675	3221319	1286655	1286011	2760899	2761543	2791681	2792325	3001540	3002184	2773187	2773831
3717	BPSL2447			2810969	2811688	2078686	2079405	3237478	3238197	3221319	3222038	1286011	1285292	2761543	2762262	2792325	2793044	3002184	3002903	2773831	2774550
3718	BPSL2448			2812731	2811733	2080448	2079450	3239240	3238242	3223081	3222083	1284249	1285247	2763305	2762307	2794087	2793089	3003946	3002948	2775593	2774595
3719	BPSL2449			2813136	2812744	2080853	2080461	3239645	3239253	3223486	3223094	1283844	1284236	2763710	2763318	2794492	2794100	3004351	3003959	2775998	2775606
3720	BPSL2450			2813792	2813136	2081509	2080853	3240301	3239645	3224142	3223486	1283188	1283844	2764366	2763710	2795148	2794492	3005007	3004351	2776654	2775998
3721	BPSL2451			2814805	2813801	2082522	2081518	3241314	3240310	3225155	3224151	1282175	1283179	2765379	2764375	2796161	2795157	3006020	3005016	2777667	2776663
3722	BPSL2452			2815528	2818791	2083245	2086508	3242037	3245300	3225878	3229141	1281452	1278375	2766102	2769365	2796884	2799796	3006743	3009700		
3723	BPSL2453			2818964	2820043	2086681	2087760	3245473	3246552	3229314	3230393	1277972	1276893	2769538	2770617	2800259	2801338	3010247	3011326	2780320	2781141
3724	BPSL2454			2820062	2820700	2087779	2088417	3246571	3247209	3230412	3231050	1276874	1276236	2770636	2771274	2801357	2801995	3011345	3011983	2781159	2781797
3725	BPSL2455			2820815	2822110	2088532	2089827	3247324	3248619	3231165	3232460	1276121	1274826	2771389	2772684	2802110	2803405	3012098	3013393	2782156	2783208
3726	BPSL2456			2822150	2822434	2089867	2090151	3248659	3248943	3232500	3232784	1274786	1274502	2772724	2773008	2803445	2803729	3013433	3013717	2783248	2783532
3727	BPSL2457			2822434	2822934	2090151	2090651	3248943	3249443	3232784	3233284	1274502	1274002	2773008	2773508	2803729	2804229	3013717	3014217	2783532	2784032
3728	BPSL2458			2824647	2823175	2092364	2090892	3251156	3249684	3234997	3233525	1272289	1273761	2775221	2773749	2805942	2804470	3015930	3014458	2785745	2784273
3729	BPSL2459			2825633	2824647	2093350	2092364	3252142	3251156	3235983	3234997	1271303	1272289	2776195	2775221	2806928	2805942	3016916	3015930	2786731	2785745
3730	BPSL2460			2827141	2825702	2094858	2093419	3253650	3252211	3237491	3236052	1269795	1271234	2777703	2776264	2808412	2806997	3018424	3016985	2788239	2786800
3731	BPSL2461			2827919	2827200	2095636	2094917	3254428	3253709	3238269	3237550	1269017	1269736	2778481	2777762	2809213	2808494	3019202	3018483	2789017	2788298
3732	BPSL2462			2829203	2828007	2096920	2095724	3255712	3254516	3239553	3238357	1267805	1268929	2779764	2778568	2810497	2809301	3020486	3019290	2790301	2789105
3733	BPSL2463			2829472	2829792	2097189	2097509	3255981	3256301	3239822	3240142			2780033	2780353	2810766	2811086	3020755	3021075	2790570	2790779
3734			Bp_chr1_35_ISBma2											2780737	2782168						
3735	BPSL2464			2830283	2830759	2097681	2097986	3256789	3257265	3240633	3241109	2859611	2860087	2782909	2783385	2811612	2812088	3021538	3022014	2791130	2791606
3736	BPSL2465			2831223	2832122	2098450	2099349	3257761	3258660	3241605	3242504	2860539	2861438	2783906	2784805	2812542	2813441	3022475	3023374	2792060	2792959
3737	BPSL2466			2832834	2832313	2100061	2099540	3259372	3258851	3243216	3242695	2862150	2861629	2785516	2784995	2814153	2813632	3024086	3023565	2793671	2793150
3738	BPSL2467			2833154	2833372	2100381	2100599	3259692	3259910	3243536	3243754	2862470	2862688	2785836	2786051	2814473	2814691	3024406	3024624	2793991	2794206
3739	BPSL2468			2833500	2834903	2100727	2102130	3260038	3261441	3243882	3245285	2862816	2864219	2786182	2787585	2814819	2816222	3024752	3026155	2794337	2795740
3740	BPSL2469			2835502	2836893	2102729	2104120	3262033	3263424	3245884	3247275	2864798	2866189	2788210	2789601	2816800	2818191	3026804	3028195	2796375	2797766
3741	BPSL2470			2837881	2837378	2105108	2104605	3264426	3263923	3248277	3247774	2867175	2866672	2790599	2790096	2819171	2818668	3029183	3028680	2798754	2798251
3742		BURPS406E_E0690										2868039	2867815								
3743	BPSL2471			2838822	2839529	2106049	2106756	3265415	3266122	3249266	3249973	2868124	2868831	2791556	2792263	2820143	2820850	3030111	3030818	2799705	2800412
3744	BPSL2472			2839688	2842264	2106915	2109491	3266281	3268842	3250132	3252693	2868990	2871011	2792422	2794998	2821009	2823570	3030977	3033538	2800571	2803132
3745	BPSL2473			2843328	2842360	2110555	2109587	3269906	3268938	3253757	3252789	2872500	2871532	2796062	2795094	2824634	2823666	3034602	3033634	2804196	2803228
3746	BPSL2474			2844770	2843388	2111997	2110615	3271348	3269966	3255199	3253817	2873942	2872560	2797504	2796122	2826076	2824694	3036044	3034662	2805638	2804256
3747	BPSL2474a			2845568	2846932	2112795	2114159	3272146	3273510	3255997	3257361	2874740	2876104	2798302	2799657	2826874	2828238	3036842	3037504	2806436	2807791
3748	BPSL2475			2847590	2849047	2114817	2116274	3274159	3275616	3258010	3259467	2876744	2878201	2800324	2801781	2828896	2830353	3038930	3040387	2808458	2809915
3749	BPSL2476			2849258	2849758	2116485	2116985	3275832	3276332	3259683	3260183	2878412	2878912	2801992	2802492	2830569	2831069	3040598	3041098	2810126	2810626
3750	BPSL2477		Bp_chr1_36_ISBm	2849834	2851406	2117061	2118570	3276408	3277977	3260259	3261831	2878988	2880561	2802568	2804140	2831145	2832717			2810702	2812274
3751	BPSL2478			2852857	2851490	2120021	2118654	3279429	3278062	3263283	3261916	2882013	2880646	2805592	2804225	2834169	2832802	3042602	3041235	2813726	2812359
3752	BPSL2479			2853003	2853608	2120167	2120772	3279575	3280177	3263429	3264031	2882159	2882764	2805738	2806352	2834315	2834917	3042748	3043353	2813872	2814477
3753	BPSL2480			2853592	2854209	2120756	2121373	3280173	3280790	3264027	3264644	2882748	2883365	2806336	2806953	2834913	2835530	3043337	3043954	2814461	2815078
3754	BPSL2481			2855098	2854496	2122262	2121660	3281673	3281071	3265527	3264925	2884254	2883652	2807843	2807241	2836413	2835811	3044810	3044208	2815968	2815366
3755	BPSL2482			2855217	2856473	2122381	2123637	3281792	3283048	3265646	3266902	2884373	2885629	2807962	2809218	2836532	2837788	3044929	3046185	2816087	2817343
3756	BPSL2483			2856473	2857414	2123637	2124578	3283048	3283989	3266902	3267843	2885629	2886570	2809218	2810159	2837788	2838729	3046185	3047126	2817343	2818284
3757	BPSL2484			2857486	2857800	2124650	2124964	3284061	3284375	3267915	3268229	2886642	2886956	2810229	2810543	2838801	2839115	3047198	3047512	2818356	2818670
3758	BPSL2485			2858959	2857973	2126123	2125137	3285534	3284548	3269388	3268402	2888115	2887129	2811702	2810716	2840274	2839288	3048671	3047685	2819829	2818843
3759	BPSL2486			2859692	2859012	2126856	2126176	3286267	3285587	3270121	3269441	2888848	2888168	2812435	2811755	2841007	2840327	3049404	3048724	2820562	2819882
3760	BPSL2487			2860589	2861176	2127753	2128340	3287176	3287763	3271030	3271617	2889785	2890372	2813338	2813925	2841916	2842503	3050294	3050881	2821501	2822088
3761	BPSL2488		Bp_chr1_37_ISBma1																		
3762	BPSL2489			2861882	2861496	2129046	2128660	3288477	3288091	3272331	3271945	2891078	2890692	2814631	2814245	2843217	2842831	3051587	3051201	2822789	2822403
3763	BPSL2490			2862814	2862023	2129978	2129187	3289409	3288618	3273263	3272472	2892010	2891219	2815563	2814772	2844149	2843358	3052519	3051728	2823721	2822930
3764	BPSL2491			2863451	2862819	2130615	2129983	3290046	3289414	3273900	3273268	2892647	2892015	2816200	2815568	2844786	2844154	3053156	3052524	2824358	2823726

3765	BPSL2492			2863804	2863553	2130968	2130717	3290399	3290148	3274253	3274002	2893000	2892749	2816553	2816302	2845139	2844888	3053509	3053258	2824711	2824460
3766	BPSL2493			2864020	2865135	2131184	2132299	3290615	3291730	3274469	3275584	2893216	2894331	2816769	2817884	2845355	2846470	3053725	3054840	2824927	2826042
3767	BPSL2494			2865245	2865709	2132409	2132873	3291840	3292304	3275694	3276158	2894441	2894905	2817994	2818458	2846580	2847044	3054950	3055414	2826152	2826616
3768	BPSL2495			2866904	2865750	2134068	2132914	3293499	3292345	3277353	3276199	2896100	2894946	2819647	2818499	2848239	2847085	3056609	3055455	2827811	2826657
3769	BPSL2496			2867501	2867016	2134665	2134180	3294096	3293611	3277950	3277465	2896697	2896212	2820244	2819759	2848836	2848351	3057206	3056721	2828408	2827923
3770	BPSL2497			2867649	2868932	2134813	2136096	3294244	3295527	3278098	3279381	2896845	2898128	2820392	2821675	2848984	2850267	3057354	3058637	2828556	2829839
3771	BPSL2498			2870840	2869053	2138004	2136217	3297434	3295647	3281288	3279501	2900036	2898249	2823582	2821795	2852174	2850387	3060545	3058758	2831746	2829959
3772	BPSL2499			2871889	2870957	2139053	2138121	3298483	3297551	3282337	3281405	2901085	2900153	2824631	2823699	2853223	2852291	3061594	3060662	2832795	2831863
3773	BPSL2500			2872654	2871908	2139818	2139072	3299248	3298502	3283102	3282356	2901850	2901104	2825396	2824650	2853988	2853242	3062359	3061613	2833560	2832814
3774	BPSL2501			2873681	2872884	2140845	2140048	3300275	3299478	3284129	3283332	2902883	2902080	2826423	2825626	2855015	2854218	3063380	3062589	2834587	2833790
3775	BPSL2502			2874408	2873758	2141572	2140922	3301002	3300352	3284856	3284206	2903610	2902960	2827150	2826500	2855742	2855092	3064107	3063457	2835314	2834664
3776	BPSL2503			2875432	2874401	2142596	2141565	3302026	3300995	3285880	3284849	2904634	2903603	2828174	2827143	2856766	2855735	3065131	3064100	2836338	2835307
3777	BPSL2504			2875740	2876579	2142904	2143743	3302336	3303175	3286190	3287029	2904943	2905782	2828483	2829322	2857075	2857914	3065440	3066279	2836645	2837484
3778	BPSL2505			2877844	2876924	2145008	2144088	3304444	3303524	3288298	3287378	2907051	2906131	2830583	2829663	2859183	2858263	3067548	3066628	2838753	2837833
3779	BPSL2506			2877972	2879327	2145136	2146491	3304572	3305945	3288426	3289799	2907179	2908516	2830711	2832054	2859311	2860660	3067676	3069013	2838881	2840224
3780	BPSL2507			2880459	2879560	2147623	2146724	3307077	3306178	3290931	3290032	2909648	2908749	2833186	2832287	2861792	2860893	3070145	3069246	2841356	2840457
3781	BPSL2508			2881118	2880804	2148282	2147968	3307745	3307431	3291599	3291285	2910318	2910004	2833854	2833540	2862451	2862137	3070840	3070526	2842006	2841692
3782	BPSL2509			2882182	2881193	2149346	2148357	3308809	3307820	3292663	3291674	2911382	2910393	2834918	2833929	2863515	2862526	3071904	3070915	2843070	2842081
3783	BPSL2510			2883164	2882244	2150328	2149408	3309791	3308871	3293645	3292725	2912364	2911444	2835900	2834980	2864497	2863577	3072886	3071966	2844052	2843132
3784	BPSL2511			2884596	2883199	2151760	2150363	3311223	3309826	3295077	3293680	2913796	2912399	2837332	2835935	2865929	2864532	3074318	3072921	2845484	2844087
3785	BPSL2512			2885886	2884720	2153050	2151884	3312513	3311347	3296367	3295201	2915086	2913920	2838622	2837456	2867219	2866053	3075608	3074442	2846774	2845608
3786	BPSL2513			2886454	2885942	2153618	2153106	3313081	3312569	3296935	3296423	2915612	2915142	2839190	2838678	2867787	2867275	3076176	3075664	2847342	2846830
3787			Bp_chr1_38_ISBma2									2915613	2917185								
3788	BPSL2514			2886780	2886460	2153944	2153624	3313407	3313087	3297261	3296941	2917573	2917253	2839516	2839196	2868113	2867793	3076502	3076182	2847668	2847348
3789	BPSL2515			2888515	2886806	2155679	2153970	3315142	3313433	3298996	3297287	2919308	2917599	2841251	2839542	2869848	2868139	3078237	3076528	2849403	2847694
3790	BPSL2516			2889369	2888662	2156533	2155826	3315997	3315290	3299851	3299144	2920163	2919456	2842105	2841398	2870703	2869996	3079092	3078385	2850258	2849551
3791	BPSL2517			2891627	2889369	2158791	2156533	3318255	3315997	3302109	3299851	2922421	2920163	2844363	2842105	2872961	2870703	3081350	3079092	2852516	2850258
3792	BPSL2518			2892936	2891857	2160100	2159021	3319411	3318332	3303265	3302186	2923730	2922651	2845686	2844607	2874270	2873191	3082659	3081580	2853825	2852746
3793	BPSL2519			2894053	2892974	2161217	2160138	3320528	3319449	3304382	3303303	2924847	2923768	2846803	2845724	2875387	2874308	3083776	3082697	2854942	2853863
3794	BPSL2520			2894823	2894230	2161987	2161394	3321298	3320705	3305152	3304559	2925617	2925024	2847573	2846980	2876157	2875564	3084546	3083953	2855712	2855119
3795	BPSL2521			2897524	2894927	2164688	2162091	3323997	3321400	3307851	3305254	2928316	2925719	2850272	2847675	2878858	2876261	3087247	3084650	2858413	2855816
3796	BPSL2522			2898033	2898704	2165197	2165868	3324506	3325177	3308360	3309031	2928825	2929496	2850783	2851454	2879367	2880038	3087756	3088427	2858924	2859595
3797	BPSL2523			2898876	2899571	2166040	2166735	3325349	3326044	3309203	3309898	2929668	2930363	2851626	2852321	2880210	2880905	3088599	3089294	2859767	2860462
3798	BPSL2524			2899600	2900322	2166764	2167486	3326073	3326795	3309927	3310649	2930392	2931114	2852350	2853072	2880934	2881656	3089323	3090045	2860491	2861213
3799		BURPSPAST_C1310														2882387	2882773				
3800		BURPSPAST_C1311														2882997	2883518				
3801		BURPSPAST_C1312														2883569	2883853				
3802		BURPSPAST_C1313														2884371	2883856				
3803		BURPSPAST_C1314														2885003	2884443				
3804		BURPSPAST_C1315														2885506	2885000				
3805		BURPSPAST_C1316														2885717	2885535				
3806		BURPSPAST_C1317														2885891	2886175				
3807		BURPSPAST_C1318														2886271	2886903				
3808		BURPSPAST_C1319														2886967	2888037				
3809		BURPSPAST_C1320														2890261	2888174				
3810		BURPSPAST_C1321														2891000	2890335				
3811		BURPSPAST_C1322														2892135	2891008				
3812		BURPSPAST_C1323														2892603	2892199				
3813		BURPSPAST_C1324														2893265	2892603				
3814		BURPSPAST_C1325														2894392	2893262				
3815		BURPSPAST_C1326														2895356	2894397				
3816		BURPSPAST_C1327														2897356	2895353				
3817		BURPSPAST_C1328														2898129	2897614				
3818		BURPSPAST_C1329														2897355	2897624				
3819		BURPSPAST_C1330														2898251	2898126				
3820		BURPSPAST_C1331														2898709	2898296				
3821		BURPSPAST_C1332														2899218	2898787				
3822		BURPSPAST_C1333														2900791	2899277				

3823	BURPSPAST_C1334							2901048	2900866		
3824	BURPSPAST_C1335							2901908	2901060		
3825	BURPSPAST_C1336							2902101	2901901		
3826	BURPSPAST_C1337							2902445	2902101		
3827	BURPSPAST_C1338							2903353	2902442		
3828	BURPSPAST_C1339							2903653	2903357		
3829	BURPSPAST_C1340							2903877	2903650		
3830	BURPSPAST_C1341							2904993	2904001		
3831	BURPSPAST_C1342							2906547	2905087		
3832	BURPSPAST_C1343							2907030	2906557		
3833	BURPSPAST_C1344							2907253	2907062		
3834	BURPSPAST_C1345							2907568	2907263		
3835	BURPSPAST_C1346							2907903	2907631		
3836	BURPSPAST_C1347							2908061	2907900		
3837	BURPSPAST_C1348							2908311	2908087		
3838	BURPSPAST_C1349							2908508	2908311		
3839	BURPSPAST_C1350							2908515	2908646		
3840	BURPSPAST_C1351							2908831	2908709		
3841	BURPSPAST_C1352							2909202	2908918		
3842	BURPSPAST_C1353							2909342	2909199		
3843	BURPSPAST_C1354							2910704	2909367		
3844	BURPSPAST_C1355							2912140	2910713		
3845	BURPSPAST_C1356							2912615	2912130		
3846	BURPSPAST_C1357							2913323	2912658		
3847	BURPSPAST_C1358							2913408	2913563		
3848	BURPSPAST_C1359							2913590	2913901		
3849	BURPSPAST_C1360							2913931	2914086		
3850	BURPSPAST_C1361							2914115	2914294		
3851	BURPSPAST_C1362							2914325	2914957		
3852	BURPSPAST_C1363							2915005	2915271		
3853	BURPSPAST_C1364							2915362	2915565		
3854	BURPSPAST_C1365							2915604	2915858		
3855	BURPSPAST_C1366							2917090	2916362		
3856	BURPSPAST_C1367							2915920	2916405		
3857	BURPSPAST_C1368							2917241	2917068		
3858	BURPSPAST_C1369							2917333	2917587		
3859	BURPSPAST_C1370							2917571	2917906		
3860	BURPSPAST_C1375							2919835	2919581		
3861	BURPSPAST_C1376							2920241	2919786		
3862	BURPSPAST_C1377							2920351	2920593		
3863	BURPSPAST_C1378							2920900	2921853		
3864	BURPSPAST_C1379							2921846	2922511		
3865	BURPSPAST_C1380							2922553	2924538		
3866	BURPSPAST_C1381							2924535	2925707		
3867	BURPSPAST_C1382							2925988	2926146		
3868	BURPSPAST_C1383							2926155	2926484		
3869	BURPSPAST_C1384							2926563	2926730		
3870	BURPSPAST_C1385							2926717	2927067		
3871	BURPSPAST_C1386							2927064	2927564		
3872	BURPSPAST_C1387							2927566	2927919		
3873	BURPSPAST_C1388							2927921	2928121		
3874	BURPSPAST_C1389							2928121	2928648		
3875	BURPSPAST_C1390							2928685	2928894		
3876	BURPSPAST_C1391							2928891	2929526		
3877	BURPSPAST_C1392							2929749	2931371		
3878	BURPSPAST_C1393							2931361	2931717		
3879	BURPSPAST_C1394							2931729	2932016		
3880	BURPSPAST_C1395							2932112	2932285		

3881		BURPSPAST_C1396													2932385	2932684				
3882		BURPSPAST_C1397													2932705	2933703				
3883		BURPSPAST_C1398													2933700	2934299				
3884		BURPSPAST_M0053													2941488	2942723				
3885		BURPSPAST_M0054													2941050	2941262				
3886		BURPSPAST_M0055													2940826	2941053				
3887		BURPSPAST_M0056													2940635	2940829				
3888		BURPSPAST_M0057													2940201	2940470				
3889		BURPSPAST_M0058													2939912	2940208				
3890		BURPSPAST_M0059													2939341	2939742				
3891		BURPSPAST_M0060													2938277	2939344				
3892		BURPSPAST_M0061													2938149	2938277				
3893		BURPSPAST_M0062													2937202	2938152				
3894		BURPSPAST_M0063													2936708	2937199				
3895		BURPSPAST_M0064													2935527	2936708				
3896		BURPSPAST_M0065													2934805	2935416				
3897	BPSL2525		2900975	2901658	2168139	2168822	3327448	3328131	3311302	3311985	2931767	2932450	2853724	2854407	2943141	2943824	3090697	3091380	2861865	2862548
3898	BPSL2526		2902105	2901845	2169269	2169009	3328578	3328318	3312432	3312172	2932897	2932637	2854854	2854594	2944271	2944011	3091827	3091567	2862995	2862735
3899	BPSL2527		2903313	2902210	2170477	2169374	3329786	3328683	3313640	3312537	2934105	2933002	2856062	2854959	2945479	2944376	3093035	3091932	2864203	2863100
3900	BPSL2528		2903402	2903914	2170566	2171078	3329875	3330387	3313729	3314241	2934194	2934706	2856151	2856663	2945568	2946080	3093124	3093636	2864292	2864804
3901	BPSL2529		2903914	2905476	2171078	2172640	3330387	3331949	3314241	3315803	2934706	2936268	2856663	2858225	2946080	2947642	3093636	3095198	2864804	2866366
3902	BPSL2530		2905494	2908445	2172658	2175609	3331967	3334918	3315821	3318772	2936286	2939237	2858243	2861194	2947660	2950611	3095216	3098167	2866384	2869335
3903	BPSL2531		2908456	2908713	2175620	2175877	3334929	3335186	3318783	3319040	2939248	2939505	2861205	2861462	2950622	2950879	3098178	3098435	2869346	2869603
3904	BPSL2532		2910738	2909074	2176807	2176238	3337204	3335540	3321058	3319394	2941530	2939866	2863508	2861844	2952904	2951240	3100439	3098775	2871623	2869959
3905	BPSL2533		2911405	2912712	2178570	2179877	3337871	3339178	3321725	3323032	2942197	2943504	2864175	2865482	2953571	2954878	3101106	3102413	2872290	2873597
3906	BPSL2534		2914399	2913107	2181564	2180272	3340865	3339573	3324719	3323427	2945191	2943899	2867169	2865877	2956565	2955273	3104100	3102808	2875284	2873992
3907	BPSL2535		2915952	2914891	2183117	2182056	3342394	3341357	3326272	3325211	2946668	2945697	2868725	2867670	2958118	2957057	3105554	3104592	2876846	2875785
3908		BURPS668_2911											2869043	2869528						
3909		BURPSPAST_M0037													2958915	2958430				
3910	BPSL2536		2916819	2918192	2183984	2185357	3343288	3344661	3327138	3328511	2947641	2949014	2869597	2870970	2958984	2960357	3106225	3107598	2877713	2879086
3911	BPSL2537		2918304	2918810	2185469	2185975	3344772	3345278	3328622	3329128	2949126	2949632	2871082	2871588	2960468	2960974	3107710	3108216	2879198	2879704
3912	BPSL2538		2919355	2920374	2186520	2187539	3345823	3346842	3329673	3330692	2950177	2951196	2872133	2873152	2961519	2962538	3108761	3109780	2880248	2881267
3913	BPSL2539		2920419	2921441	2187584	2188606	3346887	3347909	3330737	3331759	2951241	2952263	2873197	2874219	2962583	2963605	3109825	3110847	2881312	2882334
3914	BPSL2540		2921506	2922915	2188671	2190080	3347974	3349383	3331824	3333233	2952328	2953737	2874284	2875693	2963670	2965079	3110912	3112321	2882399	2883808
3915	BPSL2541		2922944	2924251	2190109	2191416	3349412	3350719	3333262	3334569	2953766	2955073	2875722	2877029	2965108	2966415	3112350	3113657	2883837	2885144
3916	BPSL2542		2924742	2925311	2191907	2192476	3351210	3351779	3335060	3335629	2955564	2956133	2877517	2878086	2966906	2967475	3114148	3114717	2885635	2886204
3917	BPSL2543		2926361	2925651	2193526	2192816	3352829	3352119	3336679	3335969	2957183	2956473	2879136	2878426	2968525	2967815	3115767	3115057	2887254	2886544
3918	BPSL2544		2926582	2929281	2193747	2196446	3353050	3355749	3336900	3339599	2957404	2960019	2879357	2882056	2968746	2971445	3115988	3118687	2887475	2890174
3919	BPSL2545		2931926	2929635	2199091	2196800	3358393	3356102	3342243	3339952	2962648	2960357	2884691	2882400	2974090	2971799	3121332	3119041	2892808	2890517
3920	BPSL2546		2932043	2932972	2199208	2200137	3358510	3359439	3342360	3343289	2962765	2963694	2884808	2885737	2974207	2975136	3121449	3122378	2892925	2893854
3921	BPSL2547		2933105	2934118	2200270	2201283	3359571	3360584	3343421	3344434	2963827	2964840	2885870	2886883	2975268	2976281	3122511	3123524	2893987	2895000
3922	BPSL2548		2935001	2935474	2202166	2202639	3361466	3361939	3345316	3345789	2965723	2966196	2888064	2888237	2977163	2977636	3124407	3124880	2895882	2896355
3923	BPSL2549		2935646	2935891	2202811	2203056	3362111	3362356	3345961	3346206	2966368	2966613	2888409	2888654	2977808	2978053	3125052	3125297	2896527	2896772
3924	BPSL2550		2936550	2936894	2203715	2204059	3363018	3363362	3346868	3347212	2967272	2967616	2889311	2889655	2978712	2979056	3125956	3126300	2897433	2897777
3925	BPSL2551		2937681	2937187	2204846	2204352	3364149	3363655	3347999	3347505	2968390	2967896	2890468	2889974	2979830	2979336	3127100	3126606	2898551	2898057
3926	BPSL2552		2938949	2937681	2206114	2204846	3365417	3364149	3349267	3347999	2969658	2968390	2891736	2890468	2981098	2979830	3128368	3127100	2899819	2898551
3927	BPSL2553		2941273	2938940	2208438	2206105	3367741	3365408	3351591	3349258	2971982	2969649	2894060	2891727	2983422	2981089	3130692	3128359	2902143	2899810
3928	BPSL2554		2941896	2941423	2209061	2208588	3368364	3367891	3352214	3351741	2972605	2972132	2894683	2894210	2984045	2983572	3131315	3130842	2902766	2902293
3929	BPSL2555		2942422	2943012	2209587	2210177	3368890	3369480	3352740	3353330	2973131	2973721	2895209	2895799	2984571	2985161	3131841	3132431	2903292	2903882
3930	BPSL2556		2944469	2943327	2211634	2210492	3370937	3369795	3354787	3353645	2975178	2974036	2897254	2896112	2986546	2985476	3133886	3132744	2905339	2904197
3931		BURPSPAST_S0045													2987550	2988243				
3932		BURPSPAST_S0046													2986593	2987267				
3933		BURPSS13_O0004															3137685	3137386		
3934	BPSL2557		2944818	2944618	2211983	2211783	3371161	3371096	3355136	3354936	2975486	2975340	2897603	2897403			3138948	3138778	2905688	2905488
3935		BURPS1106B_A2225			2212178	2212468														
3936		BURPS1106B_A2226			2212462	2213316														
3937	BPSL2558		2945221	2945481	2213598	2213858	3371564	3371824	3355539	3355799	2975889	2976149	2898006	2898266			3139351	3139611	2906091	2906351
3938	BPSL2559		2946000	2947136	2214377	2215513	3372343	3373479	3356318	3357454	2976668	2977804	2898785	2899921	2988886	2990022	3140130	3141266	2906870	2907319

3939			Bp_chr1_39_ISBma2															3141314	3142886		
3940	BPSL2560			2947930	2947370	2216307	2215747	3374273	3373713	3358248	3357688	2978598	2978038	2900715	2900155	2990816	2990256	3143655	3143095	2908520	2907963
3941	BPSL2561			2950838	2948472	2219215	2216849	3377182	3376403	3361156	3358790	2981506	2979140	2903622	2901256	2993724	2991358	3146563	3144197	2911427	2909061
3942	BPSL2562			2951108	2950908	2219485	2219285	3377452	3377252	3361426	3361226	2981776	2981576	2903892	2903692	2993994	2993794	3146833	3146633	2911697	2911497
3943	BPSL2563			2951852	2951172	2220229	2219549	3378196	3377516	3362170	3361490	2982520	2981840	2904636	2903956	2994738	2994058	3147577	3146897	2912441	2911761
3944	BPSL2564			2952782	2951862	2221159	2220239	3379126	3378206	3363100	3362180	2983450	2982530	2905566	2904646	2995668	2994748	3148507	3147587	2913371	2912451
3945	BPSL2565			2952954	2953682	2221331	2222059	3379298	3380026	3363272	3364000	2983622	2984350	2905738	2906466	2995840	2996568	3148679	3149407	2913543	2914271
3946	BPSL2566			2953688	2954311	2222065	2222688	3380032	3380655	3364006	3364629	2984356	2984979	2906472	2907095	2996574	2997197	3149413	3150036	2914277	2914900
3947	BPSL2567			2954311	2955525	2222688	2223902	3380655	3381869	3364629	3365843	2984979	2986193	2907095	2908309	2997197	2998411	3150036	3151250	2914900	2916114
3948	BPSL2568		GI9	1394925	1395440	663732	664247														
3949	BPSL2569		GI9	1395440	1395862	664247	664669														
3950	BPSL2570		GI9	1396781	1396227	665588	665034														
3951	BPSL2571		GI9	1397086	1396781	665893	665588														
3952		BURPS1106A_1428		1397687	1397848																
3953		BURPS1106B_A0675				666655	666494														
3954	BPSL2572		GI9																		
3955	BPSL2573		GI9																		
3956	BPSL2574		GI9																		
3957	BPSL2575		GI9																		
3958	BPSL2576		GI9	1399365	1398733	668172	667540														
3959		BURPS1106A_1432		1399693	1399860																
3960	BPSL2577		GI9	1399696	1399397	668503	668204														
3961		BURPS1106B_A0679				668667	668500														
3962	BPSL2578		GI9	1400054	1399860	668861	668667														
3963		BURPS1106A_1434		1400057	1400836																
3964		BURPS1106A_1435		1400820	1401590																
3965		BURPS1106A_1436		1401587	1401925																
3966		BURPS1106A_1437		1401936	1402145																
3967		BURPS1106A_1438		1402256	1402381																
3968		BURPS1106A_1439		1402420	1402890																
3969		BURPS1106B_A0681				669643	668864														
3970		BURPS1106B_A0682				670397	669627														
3971		BURPS1106B_A0683				670732	670394														
3972		BURPS1106B_A0684				670952	670743														
3973		BURPS1106B_A0685				671063	671188														
3974		BURPS1106B_A0686				671697	671227														
3975	BPSL2579		GI9																		
3976	BPSL2580		GI9																		
3977	BPSL2581		GI9																		
3978	BPSL2582		GI9																		
3979	BPSL2583		GI9																		
3980	BPSL2584		GI9	1403291	1403082	672098	671889														
3981	BPSL2585		GI9	1403571	1403287	672378	672094														
3982		BURPS1106A_1442		1403655	1404869																
3983		BURPS1106B_A0689				673676	672462														
3984	BPSL2586		GI9																		
3985	BPSL2588			2957314	2956739	2225691	2225116	3383658	3383083	3367632	3367057	2987982	2987407	2909941	2909570	3000200	2999625	3153039	3152464	2917903	2917328
3986	BPSL2589			2957488	2957979	2225865	2226356	3383832	3384323	3367806	3368297	2988156	2988647	2910272	2910763	3000374	3000865	3153213	3153704	2918077	2918568
3987	BPSL2590			2958401	2959711	2226778	2228088	3384745	3386055	3368719	3370029	2989099	2990409	2911185	2912495	3001287	3002597	3154126	3155436	2919000	2920310
3988	BPSL2591			2959895	2960074	2228272	2228451	3386239	3386418	3370213	3370392	2990593	2990772	2912679	2912858	3002781	3002960	3155620	3155799	2920494	2920673
3989	BPSL2592			2961134	2960115	2229511	2228492	3387478	3386459	3371452	3370433	2991832	2990813	2913918	2912899	3004020	3003001	3156859	3155840	2921733	2920714
3990	BPSL2593			2961296	2962954	2229673	2231331	3387640	3389292	3371614	3373266	2991994	2993646	2914080	2915738	3004182	3005840	3157021	3158679	2921895	2923538
3991	BPSL2594			2963493	2963077	2231870	2231454	3389831	3389415	3373805	3373389	2994185	2993769	2916277	2915861	3006379	3005963	3159218	3158802	2924077	2923661
3992	BPSL2595			2963869	2963618	2232246	2231995	3390207	3389956	3374181	3373930	2994561	2994310	2916653	2916402	3006755	3006504	3159594	3159343	2924453	2924202
3993	BPSL2596			2964697	2963885	2233074	2232262	3391035	3390223	3375009	3374197	2995389	2994577	2917481	2916669	3007583	3006771	3160422	3159610	2925281	2924469
3994	BPSL2597			2965568	2964759	2233945	2233136	3391906	3391097	3375880	3375071	2996260	2995451	2918352	2917543	3008454	3007645	3161293	3160484	2926152	2925343
3995		BURPSS13_N0033																3162011	3162703		
3996	BPSL2598			2966294	2965725	2234671	2234102	3392632	3392063	3376606	3376037	2996986	2996417	2919078	2918509	3009180	3008611	3162019	3161450	2926878	2926309

3997	BURPS1710A_3528					3392849	3393316												
3998	BURPS406E_K0031									2997203	2997670								
3999	BURPSPAST_AB0369													3009397	3009864				
4000 BPSL2599		2967211	2967480	2235588	2235857	3393549	3393818	3377523	3377792	2997907	2998176	2919990	2920259	3010097	3010366	3162936	3163205	2927792	2928067
4001	BURPS668_2972											2921263	2921484						
4002	BURPS668_2973											2921676	2922791						
4003	BURPS668_2974											2922770	2923075						
4004	BURPS668_2975											2923078	2923272						
4005	BURPS668_2976											2923400	2923582						
4006	BURPS668_2977											2923683	2923862						
4007	BURPS668_2978											2923970	2925748						
4008	BURPS668_2979											2925758	2926051						
4009	BURPS668_2980											2926319	2927143						
4010	BURPS668_2981											2927309	2928493						
4011	BURPS668_2982											2928543	2928689						
4012	BURPS668_2983											2928809	2929228						
4013	BURPS668_2984											2929833	2930444						
4014 BPSL2600		2969214	2967916	2237591	2236293	3395552	3394254	3379526	3378228	2999910	2998612	2932013	2930715	3012100	3010802	3164939	3163641	2929801	2928503
4015 BPSL2601		2969327	2969635	2237704	2238012	3395665	3395973	3379639	3379947	3000023	3000331	2932126	2932434	3012213	3012521	3165052	3165360	2929914	2930222
4016 BPSL2602		2971045	2969681	2239422	2238058	3397383	3396019	3381357	3379993	3001741	3000377	2933844	2932480	3013931	3012567	3166770	3165406	2931632	2930268
4017 BPSL2603		2971910	2971197	2240287	2239574	3398229	3397516	3382203	3381490	3002587	3001874	2934699	2933980	3014777	3014064	3167619	3166906	2932483	2931776
4018 BPSL2604		2974436	2971971	2242813	2240348	3400755	3398290	3384729	3382264	3005113	3002648	2937225	2934760	3017303	3014838	3170145	3167680	2935009	2932544
4019 BPSL2605		2974602	2975561	2242979	2243938	3400921	3401880	3384895	3385854	3005279	3006238	2937391	2938350	3017469	3018428	3170311	3171270	2935175	2936134
4020 BPSL2606		2975730	2976464	2244107	2244841			3386023	3386757	3006468	3007202	2938521	2939255	3018597	3019331	3171439	3172173	2936303	2937037
4021 BPSL2607		2976493	2977110	2244870	2245487	3403091	3403708	3386786	3387403	3007231	3007848	2939284	2939901	3019360	3019977	3172202	3172819	2937066	2937683
4022 BPSL2608		2978464	2977349	2246841	2245726	3405062	3403947	3388757	3387642	3009202	3008087	2941255	2940140	3021331	3020216	3174173	3173058	2939037	2937922
4023 BPSL2609		2979422	2978568	2247799	2246945	3406020	3405166	3389715	3388861	3010160	3009306	2942213	2941359	3022289	3021435	3175131	3174277	2939995	2939141
4024 BPSL2610		2980350	2979415	2248727	2247792	3406948	3406013	3390643	3389708	3011088	3010153	2943141	2942206	3023217	3022282	3176059	3175124	2940923	2939988
4025 BPSL2611		2981754	2980510	2250131	2248887	3408352	3407108	3392047	3390803	3012492	3011248	2944545	2943301	3024621	3023377	3177463	3176219	2942327	2941083
4026 BPSL2612		2982213	2983679	2250590	2252056	3408809	3410275	3392504	3393970	3012951	3014417	2945004	2946470	3025080	3026546	3177922	3179388	2942786	2944252
4027 BPSL2613		2983770	2984447	2252147	2252824	3410366	3411043	3394061	3394738	3014508	3015185	2946561	2947238	3026637	3027314	3179479	3180156	2944343	2945020
4028 BPSL2614		2984428	2986350	2252805	2254727	3411024	3412946	3394719	3396641	3015166	3017088	2947219	2949141	3027295	3029217	3180137	3182059	2945001	2946923
4029 BPSL2615		2986812	2987609	2255189	2255986	3413420	3414217	3397115	3397912	3017594	3018391	2949650	2950447	3029691	3030488	3182538	3183335	2947402	2948199
4030 BPSL2616		2987698	2988354	2256075	2256731	3414306	3414962	3398001	3398657	3018480	3019136	2950536	2951192	3030577	3031233	3183424	3184080	2948288	2948944
4031 BPSL2617		2988404	2989126	2256781	2257503	3415012	3415734	3398707	3399429	3019186	3019638	2951242	2951964	3031283	3032005	3184130	3184852	2948994	2949716
4032	BURPS1710b_3093							3398748	3400466										
4033 BPSL2618		2989200	2990216	2257577	2258593	3415809	3416825	3399504	3400520	3019980	3020996	2952039	2953055	3032079	3033095	3184927	3185943	2949790	2950806
4034 BPSL2619		2990216	2990680	2258593	2259057	3416825	3417289	3400520	3400984	3020996	3021460	2953055	2953519	3033095	3033559	3185943	3186407	2950806	2951270
4035 BPSL2620		2992028	2990796	2260405	2259173	3418637	3417405	3402332	3401100	3022808	3021576	2954863	2953631	3034907	3033675	3187755	3186523	2952618	2951386
4036 BPSL2621		2992115	2992165	2260492	2260542	3418724	3419341	3402419	3403036	3022895	3023512	2954950	2955567	3034994	3035611	3187842	3188459	2952705	2952755
4037 BPSL2622		2992837	2993292	2261214	2261669	3419450	3419905	3403145	3403600	3023621	3024076	2955670	2956125	3035714	3036169	3188568	3189023	2953421	2953876
4038 BPSL2623		2993799	2995079	2262176	2263456	3420353	3421633	3404048	3405328	3024515	3025795	2956525	2957805	3036587	3037867	3189471	3190751	2954333	2955613
4039 BPSL2624		2995111	2996244	2263488	2264621	3421665	3422798	3405360	3406493	3025827	3026960	2957837	2958976	3037899	3039038	3190783	3191916	2955645	2956784
4040 BPSL2625		2996262	2996873	2264639	2265250	3422816	3423427	3406511	3407122	3026978	3027589	2958994	2959605	3039056	3039667	3191934	3192545	2956802	2957413
4041 BPSL2626		2997339	2998472	2265716	2266849	3423893	3425026	3407588	3408721	3028056	3029189	2960071	2961204	3040134	3041267	3193011	3194144	2957879	2959012
4042 BPSL2627		2998555	2999073	2266932	2267450	3425109	3425627	3408804	3409322	3029272	3029790	2961287	2961805	3041350	3041868	3194227	3194745	2959095	2959613
4043 BPSL2628		2999073	2999507	2267450	2267884	3425627	3426061	3409322	3409756	3029790	3030224	2961805	2962239	3041868	3042302	3194745	3195179	2959613	2960047
4044 BPSL2629		2999518	3000765	2267895	2269142	3426072	3427319	3409767	3411014	3030235	3031482	2962250	2963497	3042313	3043560	3195190	3196437	2960058	2961305
4045 BPSL2630		3002462	3001398	2270839	2269775	3429034	3427970	3412729	3411665	3033185	3032121	2965230	2964166	3045209	3044145	3198224	3197160	2963002	2961938
4046 BPSL2631		3003087	3004643	2271464	2273020	3429659	3431215	3413354	3414910	3033810	3035366	2965855	2967411	3045834	3047390	3198849	3200405	2963627	2965183
4047 BPSL2632		3004687	3005358	2273064	2273735	3431259	3431930	3414954	3415625	3035410	3036081	2967455	2968126	3047434	3048105	3200449	3201120	2965227	2965898
4048 BPSL2633		3006791	3005811	2275168	2274188	3433373	3432393	3417068	3416088	3037521	3036541	2969556	2968576	3049546	3048566	3202553	3201573	2967333	2966353
4049 BPSL2634		3007246	3006956	2275623	2275333	3433828	3433538	3417523	3417233	3037976	3037686	2970010	2969720	3050001	3049711	3203008	3202718	2967788	2967498
4050 BPSL2635		3007670	3008803	2276047	2277180	3434253	3435386	3417948	3419081	3038402	3039535	2970435	2971568	3050426	3051559	3203517	3204650	2968213	2969346
4051 BPSL2636		3008889	3009449	2277266	2277826	3435472	3436032	3419167	3419727	3039621	3040181	2971654	2972214	3051645	3052205	3204736	3205296	2969432	2969992
4052 BPSL2637		3009832	3009479	2278209	2277856	3436415	3436062	3420110	3419757	3040564	3040211	2972597	2972244	3052588	3052235	3205679	3205326	2970375	2970022
4053 BPSL2638		3010242	3009859	2278619	2278236	3436825	3436442	3420520	3420137	3040974	3040591	2973007	2972624	3052998	3052615	3206089	3205706	2970785	2970402
4054 BPSL2639		3010852	3012069	2279229	2280446	3437435	3438652	3421130	3422347	3041584	3042801	2973617	2974834	3053608	3054825	3206699	3207916	2971394	2972611

4055	BPSL2640		3013068	3012343	2281445	2280720	3439651	3438926	3423346	3422621	3043800	3043075	2975833	2975108	3055824	3055099	3208915	3208190	2973610	2972885
4056		BURPS668_3037											2977540	2979024						
4057		BURPS668_3038											2979105	2979401						
4058		BURPS668_3039											2979497	2981050						
4059		BURPS668_3040											2981351	2981779						
4060		BURPS668_3041											2983935	2984228						
4061		BURPS668_3042											2985643	2985861						
4062		BURPS668_3043											2986345	2986665						
4063		BURPS668_3044											2987057	2987314						
4064		BURPS668_3045											2987935	2989479						
4065		BURPS668_3046											2989547	2991061						
4066		BURPS668_3047											2991895	2993040						
4067		BURPS668_3048											2993112	2993600						
4068		BURPS668_3049											2994085	2994453						
4069		BURPS668_3050											2994508	2994867						
4070		BURPS668_3051											2994867	2995217						
4071		BURPS668_3052											2995271	2995651						
4072		BURPS668_3053											2997463	2997693						
4073		BURPS668_3054											2997692	3000337						
4074	BPSL2641		3014562	3013315	2282939	2281692	3440992	3439889	3424687	3423584	3045315	3044047			3057324	3056077	3210409	3209162		
4075		BURPS406E_K0088									3047513	3046251								
4076		BURPS406E_K0089									3048716	3048552								
4077		BURPS406E_K0090									3049720	3048866								
4078		BURPS406E_K0091									3049977	3049714								
4079		BURPS406E_K0092									3051721	3050168								
4080		BURPS406E_K0093									3052547	3051993								
4081		BURPS406E_K0094									3052735	3053997								
4082	BPSL2642		3014957	3015751	2283334	2284128	3441520	3442314	3425215	3426009	3054396	3055187	3000886	3001680	3057719	3058513	3210804	3211598	2975491	2976282
4083	BPSL2643		3016742	3015915	2285119	2284292	3443307	3442480	3427002	3426175	3056180	3055353	3002673	3001846	3059503	3058676	3212589	3211762	2977273	2976446
4084	BPSL2644		3017400	3016822	2285777	2285199	3443965	3443387	3427660	3427082	3056838	3056260	3003331	3002753	3060161	3059583	3213247	3212669	2977931	2977353
4085	BPSL2645		3017708	3017400	2286085	2285777	3444273	3443965	3427968	3427660	3057146	3056838	3003639	3003331	3060469	3060161	3213555	3213247	2978239	2977931
4086	BPSL2646		3017896	3019287	2286273	2287664	3444461	3445852	3428156	3429547	3057334	3058725	3003827	3005218	3060657	3062048	3213743	3215134	2978427	2979818
4087	BPSL2647		3019326	3019628	2287703	2288005	3445889	3446191	3429584	3429886	3058762	3059064	3005255	3005557	3062085	3062387	3215171	3215473	2979855	2980157
4088		BURPS1106B_A2313			2289272	2288169														
4089	BPSL2648		3021095	3021745	2289472	2290122	3446301	3446951	3429996	3430646	3059174	3059824	3005667	3006317	3062497	3063147	3215583	3216233	2980267	2980917
4090	BPSL2649		3021763	3022083	2290140	2290460	3446969	3447289	3430664	3430984	3059842	3060162	3006335	3006655	3063165	3063485	3216251	3216571	2980935	2981255
4091	BPSL2650		3023507	3022182	2291884	2290559	3448713	3447388	3432408	3431083	3061586	3060261	3008079	3006754	3064909	3063584	3217995	3216670	2982679	2981354
4092	BPSL2651		3023861	3025165	2292238	2293542	3449067	3450371	3432762	3434066	3061940	3063244	3008433	3009737	3065263	3066567	3218348	3219652	2983033	2984337
4093	BPSL2652		3025263	3026882	2293640	2295259	3450469	3452088	3434164	3435783	3063342	3064961	3009836	3011455	3066665	3068284	3219750	3221369	2984436	2986055
4094	BPSL2653		3026887	3028086	2295264	2296463	3452093	3453292	3435788	3436987	3064966	3066165	3011460	3012659	3068289	3069488	3221374	3222573	2986060	2987259
4095	BPSL2654		3028086	3028946	2296463	2297323	3453292	3454152	3436987	3437847	3066165	3067025	3012659	3013519	3069488	3070348	3222573	3223433	2987259	2988119
4096	BPSL2655		3028954	3029643	2297331	2298020	3454160	3454849	3437855	3438544	3067033	3067722	3013527	3014216	3070356	3071045	3223441	3224130	2988127	2988816
4097	BPSL2656		3029746	3030738	2298123	2299115	3454952	3455944	3438647	3439639	3067847	3068848	3014319	3015311	3071170	3072171	3224266	3225258	2988941	2989933
4098	BPSL2657		3030963	3031262	2299340	2299639	3456169	3456468	3439864	3440163	3069073	3069372	3015536	3015835	3072396	3072695	3225483	3225782	2990158	2990457
4099	BPSL2658		3031355	3031657	2299732	2300034	3456561	3456863	3440256	3440558	3069465	3069767	3015928	3016230	3072788	3073090	3225875	3226177	2990550	2990852
4100	BPSL2659		3031725	3033428	2300102	2301805	3456931	3458634	3440626	3442329	3069835	3071538	3016298	3018001	3073158	3074861	3226245	3227948	2990920	2992623
4101	BPSL2660		3033449	3034069	2301826	2302440	3458655	3459263	3442350	3442958	3071559	3072167	3018022	3018636	3074882	3075508	3227969	3228589	2992644	2993264
4102	BPSL2661		3034059	3034736	2302430	2303107	3459253	3459930	3442948	3443625	3072157	3072834	3018626	3019303	3075498	3076175	3228579	3229256	2993254	2993931
4103	BPSL2662		3034770	3035417	2303141	2303788	3459964	3460611	3443659	3444306	3072868	3073515	3019337	3019984	3076209	3076856	3229290	3229937	2993965	2994612
4104	BPSL2663		3037030	3035648	2305401	2304019	3462237	3460855	3445932	3444550	3075141	3073759	3021607	3020240	3078481	3077099	3231562	3230180	2996226	2994844
4105	BPSL2664		3037952	3037071	2306323	2305442	3463159	3462278	3446854	3445973	3076063	3075182	3022529	3021648	3079403	3078522	3232484	3231603	2997148	2996267
4106	BPSL2665		3038963	3037971	2307334	2306342	3464170	3463178	3447865	3446873	3077074	3076082	3023540	3022548	3080414	3079422	3233495	3232503	2998159	2997167
4107	BPSL2666		3040538	3039147	2308909	2307518	3465745	3464354	3449440	3448049	3078648	3077257	3025114	3023723	3081989	3080598	3235070	3233679	2999734	2998343
4108	BPSL2667		3040796	3042052	2309167	2310423	3466003	3467259	3449698	3450954	3078907	3080163	3025373	3026629	3082248	3083504	3235328	3236584	2999993	3001249
4109	BPSL2668		3042052	3042885	2310423	2311256	3467259	3468092	3450954	3451787	3080163	3080996	3026629	3027462	3083504	3084337	3236584	3237417	3001249	3002082
4110	BPSL2669		3042917	3044071	2311288	2312442	3468124	3469278	3451819	3452973	3081028	3082182	3027494	3028648	3084369	3085523	3237449	3238603	3002114	3003268
4111	BPSL2670		3044089	3045108	2312460	2313479	3469296	3470315	3452991	3454010	3082200	3083219	3028666	3029685	3085541	3086560	3238621	3239640	3003286	3004305
4112	BPSL2671		3045139	3046242	2313510	2314613	3470346	3471449	3454041	3455144	3083250	3084353	3029716	3030819	3086591	3087694	3239671	3240774	3004336	3005439

4113	BPSL2672		3048532	3046622	2316903	2314993	3473739	3471829	3457434	3455524	3086643	3084733	3033108	3031198	3089984	3088074	3243063	3241153	3007730	3006888
4114	BPSL2673		3049549	3048542	2317920	2316913	3474756	3473749	3458451	3457444	3087660	3086653	3034125	3033118	3091001	3089994	3244080	3243073	3008747	3007740
4115	BPSL2674		3050532	3049570	2318903	2317941	3475739	3474777	3459434	3458472	3088643	3087681	3035108	3034146	3091984	3091022	3245063	3244101	3009730	3008768
4116	BPSL2675		3051359	3050532	2319730	2318903	3476566	3475739	3460261	3459434	3089470	3088643	3035935	3035108	3092811	3091984	3245890	3245063	3010551	3009730
4117	BPSL2676		3053275	3051437	2321646	2319808	3478482	3476644	3462177	3460339	3091386	3089548	3037851	3036013	3094727	3092889	3247806	3245968	3012473	3010635
4118	BPSL2677		3055051	3053330	2323422	2321701	3480258	3478537	3463953	3462232	3093162	3091441	3039627	3037906	3096503	3094782	3249582	3247861	3014249	3012528
4119	BPSL2678		3055978	3055058	2324349	2323429	3481185	3480265	3464880	3463960	3094089	3093169	3040554	3039634	3097430	3096510	3250509	3249589	3015176	3014256
4120	BPSL2679		3057090	3056002	2325461	2324373	3482297	3481209	3465992	3464904	3095201	3094113	3041666	3040578	3098542	3097454	3251621	3250533	3016288	3015200
4121	BPSL2680		3058328	3057093	2326699	2325464	3483535	3482300	3467230	3465995	3096439	3095204	3042904	3041669	3099780	3098545	3252859	3251624	3017526	3016291
4122	BPSL2681		3059734	3058328	2328105	2326699	3484941	3483535	3468636	3467230	3097845	3096439	3044310	3042904	3101186	3099780	3254265	3252859	3018932	3017526
4123	BPSL2682		3060557	3059727	2328928	2328098	3485764	3484934	3469459	3468629	3098668	3097838	3045133	3044303	3102009	3101179	3255088	3254258	3019755	3018925
4124	BPSL2683		3061480	3060587	2329851	2328958	3486687	3485794	3470382	3469489	3099591	3098698	3046057	3045167	3102932	3102039	3256011	3255118	3020678	3019785
4125	BPSL2684		3062028	3061480	2330399	2329851	3487235	3486687	3470930	3470382	3100139	3099591	3046605	3046057	3103480	3102932	3256559	3256011	3021226	3020678
4126	BPSL2685		3062906	3062016	2331277	2330387	3488113	3487223	3471808	3470918	3101017	3100127	3047483	3046593	3104358	3103468	3257437	3256547	3022104	3021214
4127	BPSL2686		3063979	3062921	2332350	2331292	3489186	3488128	3472881	3471823	3102090	3101032	3048556	3047498	3105431	3104373	3258510	3257452	3023177	3022119
4128	BPSL2687		3064357	3065202	2332728	2333573	3489564	3490409	3473259	3474104	3102468	3103313	3048934	3049779	3105809	3106654	3258888	3259733	3023555	3024400
4129	BPSL2688		3066204	3065338	2334575	2333709	3491411	3490545	3475106	3474240	3104315	3103449	3050781	3049915	3107656	3106790	3260735	3259869	3025402	3024536
4130	BPSL2689		3067496	3066222	2335867	2334593	3492703	3491429	3476398	3475124	3105607	3104333	3052073	3050799	3108948	3107674	3262027	3260753	3026694	3025420
4131	BPSL2690		3068619	3067591	2336990	2335962	3493826	3492798	3477521	3476493	3106730	3105702	3053196	3052168	3110071	3109043	3263150	3262122	3027817	3026789
4132	BPSL2691		3069218	3068703	2337589	2337074	3494425	3493910	3478120	3477605	3107329	3106814	3053795	3053280	3110670	3110155	3263749	3263234	3028416	3027901
4133	BPSL2692		3069645	3069208	2338016	2337579	3494852	3494415	3478547	3478110	3107756	3107319	3054222	3053785	3111097	3110660	3264176	3263739	3028843	3028406
4134	BPSL2693		3070220	3069645	2338591	2338016	3495427	3494852	3479122	3478547	3108331	3107756	3054797	3054222	3111672	3111097	3264751	3264176	3029418	3028843
4135	BPSL2694		3071757	3070429	2340128	2338800	3496964	3495636	3480659	3479331	3109868	3108540	3056334	3055006	3113209	3111881	3266288	3264960	3030955	3029627
4136	BPSL2695		3072026	3071859	2340397	2340230	3497233	3497066	3480928	3480761	3110137	3109970	3056603	3056436	3113478	3113311	3266557	3266390	3031221	3031054
4137	BPSL2696		3072246	3073091	2340617	2341462	3497453	3498298	3481148	3481993	3110357	3111202	3056823	3057668	3113698	3114543	3266777	3267622	3031441	3032286
4138	BPSL2697		3075165	3073528	2343544	2341907	3500316	3498679	3484011	3482374	3113316	3111679	3059691	3058054	3116585	3114948	3269608	3267971	3034456	3032819
4139	BPSL2698		3075506	3075216	2343885	2343595	3500657	3500367	3484352	3484062	3113657	3113367	3060032	3059742	3116926	3116636	3269949	3269659	3034797	3034507
4140	BPSL2699		3078255	3076099	2346634	2344478														
4141	BPSL2700		3079747	3079316	2348126	2347695	3504778	3504347	3488473	3488042	3117058	3116627	3063760	3063329	3121047	3120616	3273950	3273519	3038918	3038487
4142	BPSL2701		3079996	3081000	2348375	2349379	3505027	3506031	3488722	3489726	3117307	3118311	3064009	3065013	3121296	3122300	3274199	3275203	3039166	3040170
4143	BPSL2702		3081995	3081030	2350374	2349409	3507026	3506061	3490721	3489756	3119306	3118341	3066008	3065043	3123295	3122330	3276198	3275233	3041165	3040200
4144		BURPS1655_E0642																	3041240	3041506
4145	BPSL2703		3083055	3082543	2351434	2350922	3508086	3507574	3491781	3491269	3120366	3119854	3067068	3066556	3124355	3123843	3277258	3276746	3042224	3041712
4146	BPSL2704		3084076	3083174	2352455	2351553	3509107	3508205	3492802	3491900	3121387	3120485	3068089	3067187	3125376	3124474	3278279	3277377	3043245	3042343
4147	BPSL2705		3085326	3084076	2353705	2352455	3510357	3509107	3494052	3492802	3122637	3121387	3069339	3068089	3126626	3125376	3279529	3278279	3044495	3043245
4148	BPSL2706		3086984	3085389	2355363	2353768	3512015	3510420	3495710	3494115	3124295	3122700	3070997	3069402	3128284	3126689	3281187	3279592	3046153	3044558
4149	BPSL2707		3088168	3087728	2356547	2356107	3513197	3512763	3496892	3496458	3125478	3125035	3072179	3071736	3129466	3129032	3282370	3281927	3047336	3046893
4150	BPSL2708		3088449	3089678	2356828	2358057	3513477	3514706	3497172	3498401	3125758	3126987	3072459	3073688	3129746	3130975	3282651	3283880	3047616	3048845
4151	BPSL2709		3089863	3090447	2358242	2358826	3514891	3515475	3498586	3499170	3127172	3127756	3073871	3074455	3131160	3131744	3284065	3284649	3049030	3049614
4152	BPSL2710		3090447	3090971	2358826	2359350	3515475	3515999	3499170	3499694	3127756	3128280	3074455	3074991	3131744	3132268	3284649	3285173	3049614	3050138
4153	BPSL2711		3091324	3091944	2359703	2360323	3516352	3516972	3500047	3500667	3128642	3129262	3075344	3075964	3132621	3133241	3285526	3286146	3050491	3051111
4154	BPSL2712		3092432	3093565	2360811	2361944	3517437	3518570	3501132	3502265	3129750	3130883	3076410	3077543	3133706	3134839	3286640	3287773	3051569	3052702
4155	BPSL2713		3093698	3095137	2362077	2363516	3518703	3520142	3502398	3503837	3131016	3132455	3077676	3079115	3134972	3136411	3287906	3289345	3052835	3054274
4156	BPSL2714		3095637	3096422	2364016	2364801	3520642	3521427	3504337	3505122	3132954	3133739	3079642	3080427	3136911	3137696	3289844	3290629	3054774	3055559
4157	BPSL2715		3098605	3096761	2366984	2365140	3523610	3521766	3507305	3505461	3135922	3134078	3082622	3080766	3139879	3138035	3292812	3290968	3057730	3055886
4158	BPSL2716		3101310	3099214	2369689	2367593	3526315	3524219	3510010	3507914	3138627	3136531	3085327	3083231	3142584	3140488	3295517	3293421	3060435	3058339
4159	BPSL2717		3101798	3101433	2370177	2369812	3526803	3526438	3510498	3510133	3139115	3138750	3085815	3085450	3143072	3142707	3296005	3295640	3060923	3060558
4160		BURPSPAST_AB0229													3143122	3143400				
4161	BPSL2718		3102208	3103698	2370587	2372077	3527197	3528687	3510892	3512382	3139509	3140999	3086222	3087700	3143474	3144982	3296408	3297898	3061313	3062827
4162		BURPS406E_K0185									3139597	3139112								
4163	BPSL2719		3103799	3104434	2372178	2372813	3528788	3529423	3512483	3513118	3141100	3141735	3087827	3088462	3145083	3145718	3297999	3298634	3062928	3063563
4164	BPSL2720		3104511	3105455	2372890	2373834	3529500	3530444	3513195	3514139	3141812	3142756	3088539	3089483	3145795	3146739	3298711	3299655	3063640	3064584
4165	BPSL2721		3106656	3105544	2375035	2373923	3531645	3530533	3515340	3514228	3143942	3142845	3090684	3089572	3147940	3146828	3300871	3299744	3065785	3064673
4166	BPSL2722		3107681	3106656	2376060	2375035	3532685	3531645	3516380	3515340	3144982	3143942	3091709	3090684	3148980	3147940	3301911	3300871	3066561	3065785
4167	BPSL2723		3108472	3107678	2376851	2376057	3533476	3532682	3517171	3516377	3145773	3144979	3092500	3091706	3149771	3148977	3302702	3301908	3067596	3066802
4168	BPSL2724		3110784	3108517	2379163	2376896	3535782	3533515	3519477	3517210	3148088	3145812	3094806	3092539	3152077	3149810	3305014	3302747	3069902	3067635
4169	BPSL2725		3111745	3111278	2380124	2379657	3536743	3536276	3520438	3519971	3149049	3148582	3095769	3095302	3153038	3152571	3305975	3305508	3070863	3070396
4170	BPSL2726		3112646	3111867	2381025	2380246	3537644	3536865	3521339	3520560	3149950	3149171	3096677	3095898	3153939	3153160	3306876	3306097	3071764	3070985

4171	BPSL2727			3115407	3113047	2383786	2381426	3540391	3538031	3524086	3521726	3152697	3150337	3099432	3097072	3156686	3154326	3309623	3307263	3074518	3072158
4172	BPSL2728			3116953	3115439	2385332	2383818	3541937	3540423	3525632	3524118	3154243	3152729	3100978	3099464	3158232	3156718	3311169	3309655	3076064	3074550
4173	BPSL2729			3117233	3118453	2385612	2386832	3542217	3543437	3525912	3527132	3154523	3155743	3101258	3102478	3158512	3159732	3311449	3312669	3076344	3077564
4174	BPSL2730			3119903	3118956	2388282	2387335	3544896	3543949	3528591	3527644	3157148	3156201	3103848	3102901	3161164	3160217	3314074	3313127	3078925	3077978
4175	BPSL2731			3119934	3120929	2388313	2389308	3544927	3545922	3528622	3529617	3157179	3158174	3103879	3104874	3161195	3162190	3314105	3315100	3078956	3079951
4176	BPSL2732			3122171	3120960	2390550	2389339	3547164	3545953	3530859	3529648	3159416	3158205	3106116	3104905	3163432	3162221	3316342	3315131	3081193	3079982
4177	BPSL2733			3123069	3124046	2391448	2392425	3548062	3549039	3531757	3532734	3160315	3161292	3107013	3107990	3164330	3165307	3317254	3318231	3082091	3083068
4178	BPSL2734			3124096	3125097	2392475	2393476	3549089	3550090	3532784	3533785	3161342	3162343	3108040	3109041	3165357	3166358	3318281	3319282	3083118	3084119
4179	BPSL2735			3125665	3126321	2394052	2394708	3550618	3551274	3534313	3534969	3162903	3163559	3109466	3110122	3166910	3167566	3319915	3320571	3084679	3085335
4180	BPSL2736			3126440	3127606	2394827	2395993	3551393	3552559	3535088	3536254	3163678	3164844	3110241	3111407	3167685	3168851	3320690	3321856	3085454	3086620
4181	BPSL2737			3127854	3128111	2396241	2396498	3552817	3554010	3536512	3537705	3165115	3166308	3111658	3112851	3169109	3170302	3322127	3323320	3086868	3088061
4182		BURPSS13_A0203																3323436	3323696		
4183	BPSL2738			3130788	3129484	2399175	2397871	3555734	3554430	3539429	3538125	3168032	3166728	3114583	3113282	3172026	3170722	3325068	3323764	3089796	3088492
4184	BPSL2739			3132137	3130788	2400524	2399175	3557083	3555734	3540778	3539429	3169381	3168032	3115932	3114583	3173375	3172026	3326417	3325068	3091145	3089796
4185	BPSL2740			3133493	3132141	2401880	2400528	3558439	3557087	3542134	3540782	3170737	3169385	3117288	3115936	3174731	3173379	3327773	3326421	3092501	3091149
4186	BPSL2741			3133720	3133493	2402107	2401880	3558666	3558439	3542361	3542134	3170964	3170737	3117515	3117288	3174958	3174731	3328000	3327773	3092728	3092501
4187	BPSL2742			3135424	3133766	2403811	2402153	3560370	3558712	3544065	3542407	3172668	3171010	3119219	3117561	3176662	3175004	3329704	3328046	3094432	3092774
4188	BPSL2743			3136400	3135447	2404787	2403834	3561346	3560393	3545041	3544088	3173644	3172691	3120195	3119242	3177638	3176685	3330680	3329727	3095408	3094455
4189	BPSL2744			3136657	3137460	2405044	2405847	3561603	3562406	3545298	3546101	3173901	3174704	3120452	3121255	3177895	3178698	3330937	3331740	3095665	3096468
4190		BURPS1710b_3236								3546371	3549595										
4191	BPSL2745			3138373	3137921	2406760	2406308	3563328	3562876	3547023	3546571	3175634	3175182	3122184	3121732	3179620	3179168	3332654	3332202	3097385	3096933
4192	BPSL2746			3139743	3138922	2408138	2407317	3564453	3563632	3548148	3547327	3176863	3176042	3123421	3122600	3180721	3179900	3333843	3333022	3098529	3097708
4193		BURPS1106A_3224		3140995	3141276																
4194		BURPS1106B_A2439				2409390	2409671														
4195		BURPS406E_K0224										3178115	3178396								
4196	BPSL2747			3139733	3140971	2408128	2409366	3564443	3565681	3548138	3549376	3176853	3178091	3123411	3124649	3180711	3181949	3333833	3335071	3098519	3099757
4197			Bp_chr1_40_ISBma2															3335277	3336849		
4198	BPSL2748			3141865	3141230	2410260	2409625	3566577	3565942	3550272	3549637	3178985	3178350	3125541	3124906	3182841	3182206	3337561	3336926	3100653	3100018
4199	BPSL2749			3142193	3142492	2410588	2410887	3566905	3567204	3550600	3550899	3179313	3179612	3125869	3126168	3183169	3183468	3337889	3338188	3100981	3101280
4200	BPSL2750			3142619	3143005	2411014	2411400	3567331	3567717	3551026	3551412	3179739	3180125	3126295	3126681	3183595	3183981	3338315	3338701	3101407	3101793
4201	BPSL2751			3143058	3143513	2411453	2411908	3567770	3568225	3551465	3551920	3180178	3180633	3126734	3127189	3184034	3184489	3338754	3339209	3101846	3102301
4202	BPSL2752			3144067	3143618	2412462	2412013	3568785	3568336	3552480	3552031	3181184	3180735	3127758	3127309	3185049	3184600	3339769	3339320	3102864	3102415
4203	BPSL2753			3144683	3144090	2413078	2412485	3569401	3568808	3553096	3552503	3181800	3181207	3128374	3127781	3185665	3185072	3340385	3339792	3103480	3102887
4204	BPSL2754			3145468	3144686	2413863	2413081	3570186	3569404	3553881	3553099	3182585	3181803	3129159	3128377	3186450	3185668	3341170	3340388	3104265	3103483
4205	BPSL2755			3145890	3145468	2414285	2413863	3570608	3570186	3554303	3553881	3183007	3182585	3129581	3129159	3186872	3186450	3341592	3341170	3104687	3104265
4206	BPSL2756			3146432	3145875	2414827	2414270	3571150	3570593	3554845	3554288	3183549	3182992	3130123	3129566	3187414	3186857	3342134	3341577	3105229	3104672
4207	BPSL2757			3147097	3146621	2415492	2415016	3571815	3571339	3555510	3555034	3184214	3183738	3130788	3130312	3188079	3187603	3342799	3342323	3105894	3105418
4208	BPSL2758			3148351	3147107	2416746	2415502	3573069	3571825	3556764	3555520	3185468	3184224	3132042	3130798	3189333	3188089	3344053	3342809	3107148	3105904
4209	BPSL2759			3148803	3149546	2417198	2417941	3573519	3574262	3557214	3557957	3185918	3186661	3132494	3133237	3189783	3190526	3344505	3345248	3107600	3108343
4210	BPSL2760			3149646	3150137	2418041	2418532	3574362	3574853	3558057	3558548	3186761	3187252	3133337	3133828	3190626	3191117	3345348	3345839	3108443	3108934
4211	BPSL2761			3150206	3150991	2418601	2419386	3574922	3575707	3558617	3559402	3187321	3188106	3133897	3134682	3191186	3191971	3345908	3346693	3109003	3109788
4212	BPSL2762			3151005	3151451	2419400	2419846	3575721	3576167	3559416	3559862	3188120	3188566	3134696	3135142	3191985	3192431	3346707	3347153	3109802	3110248
4213	BPSL2763			3151451	3152473	2419846	2420868	3576167	3577189	3559862	3560884	3188566	3189588	3135142	3136164	3192431	3193453	3347153	3348175	3110248	3111270
4214	BPSL2764			3152573	3153871	2420968	2422266	3577289	3578587	3560984	3562282	3189688	3190986	3136264	3137562	3193553	3194851	3348275	3349573	3111370	3112668
4215	BPSL2765			3153892	3154401	2422287	2422796	3578608	3579117	3562303	3562812	3191007	3191516	3137583	3138092	3194872	3195381	3349594	3350103	3112689	3113198
4216	BPSL2766			3154424	3155170	2422819	2423565	3579140	3579886	3562835	3563581	3191539	3192285	3138115	3138861	3195404	3196150	3350126	3350872	3113221	3113967
4217		BURPS406E_K0245										3193799	3193017								
4218		BURPS406E_K0246										3195212	3194052								
4219		BURPS406E_K0247										3195776	3195901								
4220		BURPS406E_K0248										3195916	3196452								
4221		BURPS406E_K0249										3196705	3196523								
4222		BURPS406E_K0250										3197121	3198425								
4223		BURPS406E_K0251										3198516	3198638								
4224		BURPS406E_K0252										3199113	3199841								
4225		BURPS406E_K0253										3200215	3200553								
4226		BURPS406E_K0254										3201028	3200828								
4227		BURPS406E_K0255										3201815	3201354								
4228		BURPS406E_K0256										3202241	3202053								

4229	BURPS406E_K0257									3203085	3202399								
4230	BURPS406E_K0258									3203791	3203420								
4231	BURPS406E_K0259									3204219	3203866								
4232	BURPS406E_K0260									3205773	3204382								
4233	BURPS406E_K0261									3206690	3205770								
4234	BURPS406E_K0262									3206984	3206784								
4235	BURPS406E_K0263									3208417	3207584								
4236	BURPS406E_K0264									3210067	3208667								
4237 BPSL2767		3156179	3155949	2424574	2424344	3580895	3580665	3564590	3564360	3211182	3210952	3139859	3139629	3197158	3196928	3351880	3351650	3114966	3114736
4238 BPSL2768		3156668	3156444	2425063	2424839	3581379	3581320	3565074	3565015	3211666	3211607	3140348	3140124	3197647	3197423	3352369	3352145	3115450	3115391
4239 BPSL2769		3156894	3157775	2425289	2426170	3581605	3582486	3565300	3566181	3211892	3212773	3140574	3141455	3197873	3198754	3352595	3353476	3115676	3116557
4240 BPSL2770		3159028	3158036	2427423	2426431	3583739	3582747	3567434	3566442	3214026	3213034	3142708	3141716	3200010	3199015	3354729	3353737	3117783	3116818
4241 BPSL2771		3159623	3159075	2428018	2427470	3584334	3583786	3568029	3567481	3214621	3214073	3143303	3142755	3200605	3200057	3355324	3354776	3118404	3117856
4242 BPSL2772		3160465	3159623	2428860	2428018	3585176	3584334	3568871	3568029	3215463	3214621	3144145	3143303	3201447	3200605	3356166	3355324	3119246	3118404
4243 BPSL2773		3161712	3161635	2430107	2430030	3586421	3585264	3570116	3568959	3216708	3215551	3145393	3144233	3202695	3201535	3357411	3356254	3120494	3119334
4244 BPSL2774		3162653	3161712	2431048	2430107	3587362	3586421	3571057	3570116	3217649	3216708	3146334	3145393	3203636	3202695	3358352	3357411	3121435	3120494
4245 BPSL2775		3163186	3162653	2431581	2431048	3587895	3587362	3571590	3571057	3218182	3217649	3146867	3146334	3204169	3203636	3358885	3358352	3121968	3121435
4246 BPSL2776		3164384	3163191	2432779	2431586	3589093	3587900	3572788	3571595	3219380	3218187	3148065	3146872	3205367	3204174	3360083	3358890	3123166	3121973
4247 BPSL2777		3165691	3164420	2434086	2432815	3590400	3589129	3574095	3572824	3220687	3219416	3149372	3148101	3206674	3205403	3361387	3360119	3124473	3123202
4248 BPSL2778		3167992	3165755	2436387	2434150	3592713	3590476	3576408	3574171	3222988	3220751	3151685	3149448	3208975	3206738	3363695	3361458	3126774	3124537
4249 BPSL2779		3168468	3168037	2436863	2436432	3593189	3592758	3576884	3576453	3223464	3223033	3152161	3151730	3209451	3209020	3364171	3363740	3127250	3126819
4250 BPSL2780		3169667	3168468	2438062	2436863	3594388	3593189	3578083	3576884	3224663	3223464	3153360	3152161	3210650	3209451	3365370	3364171	3128449	3127250
4251 BPSL2781		3170851	3169733	2439246	2438128	3595572	3594454	3579267	3578149	3225847	3224729	3154544	3153426	3211834	3210716	3366554	3365436	3129633	3128515
4252 BPSL2782		3172078	3171035	2440473	2439430	3596799	3595756	3580494	3579451	3227074	3226031	3155771	3154728	3213061	3212018	3367781	3366738	3130860	3129817
4253 BPSL2783		3172864	3173433	2441259	2441828	3597588	3598157	3581283	3581852	3227863	3228432	3156560	3157129	3213850	3214419	3368567	3369136	3131649	3132218
4254 BPSL2784		3173633	3174985	2442028	2443380	3598357	3599709	3582052	3583404	3228632	3229987	3157363	3158685	3214619	3215971	3369336	3370691	3132418	3133770
4255 BPSL2785		3175030	3176199	2443425	2444594	3599754	3600923	3583449	3584618	3230032	3231201	3158730	3159899	3216016	3217185	3370736	3371905	3133815	3134984
4256 BPSL2786		3177358	3176666	2445753	2445061	3602082	3601390	3585777	3585085	3232363	3231668	3161058	3160366	3218344	3217652	3373067	3372372	3136146	3135451
4257 BPSL2787		3178683	3177367	2447078	2445762	3603407	3602091	3587102	3585786	3233691	3232375	3162383	3161067	3219669	3218353	3374395	3373079	3137480	3136164
4258 BPSL2788		3179665	3178751	2448060	2447146	3604389	3603475	3588084	3587170	3234673	3233759	3163365	3162451	3220651	3219737	3375377	3374463	3138462	3137548
4259 BPSL2789		3187302	3179665	2455697	2448060	3612026	3604389	3595721	3588084	3242310	3234673	3171002	3163365	3228288	3220651	3383014	3375377	3146098	3140417
4260 BPSL2790		3188837	3187308	2457232	2455703	3613561	3612032	3597256	3595727	3243845	3242316	3172537	3171008	3229823	3228294	3384549	3383020	3147633	3146104
4261 BPSL2791		3189627	3188842	2458022	2457237	3614351	3613566	3598046	3597261	3244635	3243850	3173327	3172542	3230613	3229828	3385339	3384554	3148423	3147638
4262 BPSL2792		3190826	3189627	2459221	2458022	3615550	3614351	3599245	3598046	3245834	3244635	3174526	3173327	3231812	3230613	3386538	3385339	3149622	3148423
4263 BPSL2793		3191662	3191096	2460057	2459491	3616386	3615820	3600081	3599515	3246670	3246104	3175362	3174796	3232648	3232082	3387374	3386808	3150458	3149892
4264 BPSL2794		3192351	3191662	2460746	2460057	3617075	3616386	3600770	3600081	3247359	3246670	3176051	3175362	3233337	3232648	3388063	3387374	3151147	3150458
4265 BPSL2795		3192946	3192356	2461341	2460751	3617670	3617080	3601365	3600775	3247954	3247364	3176646	3176056	3233932	3233342	3388658	3388068	3151742	3151152
4266 BPSL2796		3193968	3192931	2462363	2461326	3618692	3617655	3602387	3601350	3248976	3247939	3177668	3176631	3234954	3233917	3389680	3388643	3152764	3151727
4267 BPSL2797		3194997	3193987	2463392	2462382	3619721	3618711	3603416	3602406	3250005	3248995	3178697	3177687	3235983	3234973	3390709	3389699	3153793	3152783
4268 BPSL2798		3195849	3195010	2464244	2463405	3620573	3619734	3604268	3603429	3250857	3250018	3179549	3178710	3236835	3235996	3391561	3390722	3154645	3153806
4269 BPSL2799		3196784	3195849	2465179	2464244	3621508	3620573	3605203	3604268	3251792	3250857	3180484	3179549	3237770	3236835	3392496	3391561	3155580	3154645
4270 BPSL2800		3198595	3196802	2466990	2465197	3623319	3621526	3607014	3605221	3253603	3251810	3182295	3180502	3239581	3237788	3394307	3392514	3157391	3155598
4271 BPSL2801		3199533	3198595	2467928	2466990	3624257	3623319	3607952	3607014	3254541	3253603	3183233	3182295	3240519	3239581	3395245	3394307	3158329	3157391
4272 BPSL2802		3200865	3199546	2469260	2467941	3625589	3624270	3609284	3607965	3255873	3254554	3184565	3183246	3241851	3240532	3396577	3395258	3159661	3158342
4273 BPSL2803		3202411	3200891	2470806	2469286	3627135	3625615	3610830	3609310	3257419	3255899	3186111	3184591	3243397	3241877	3398123	3396603	3161207	3159687
4274 BPSL2804		3203100	3202447	2471495	2470842	3627824	3627171	3611519	3610866	3258108	3257455	3186800	3186147	3244086	3243433	3398812	3398159	3161896	3161243
4275 BPSL2805		3203879	3203100	2472274	2471495	3628603	3627824	3612298	3611519	3258887	3258108	3187579	3186800	3244865	3244086	3399591	3398812	3162675	3161896
4276 BPSL2806		3205034	3203889	2473429	2472284	3629758	3628613	3613453	3612308	3260042	3258897	3188734	3187589	3246020	3244875	3400746	3399601	3163830	3162685
4277 BPSL2807		3206678	3205518	2475073	2473913	3631402	3630242	3615097	3613937	3261686	3260526	3190378	3189218	3247664	3246504	3402390	3401230	3165474	3164314
4278 BPSL2808		3206858	3208003	2475253	2476398	3631583	3632728	3615278	3616423	3261866	3263011	3190559	3191704	3247845	3248990	3402571	3403716	3165654	3166799
4279 BPSL2809		3208010	3210022	2476405	2478417	3632735	3634747	3616430	3618442	3263018	3265030	3191711	3193723	3248997	3251009	3403723	3405735	3166806	3168818
4280 BPSL2810		3210424	3211848	2478819	2480243	3635149	3636573	3618844	3620268	3265432	3266856	3194125	3195549	3251411	3252835	3406137	3407561	3169220	3170644
4281 BPSL2811		3212113	3212955	2480508	2481350	3636838	3637680	3620533	3621375	3267121	3267963	3195814	3196656	3253100	3253942	3407826	3408668	3170897	3171739
4282 BPSL2812		3213024	3213893	2481419	2482288	3637749	3638618	3621444	3622313	3268032	3268901	3196726	3197595	3254011	3254880	3408737	3409606	3171808	3172677
4283 BPSL2813		3214960	3214220	2483355	2482615	3639685	3638945	3623380	3622640	3269968	3269228	3198662	3197922	3255944	3255204	3410673	3409933	3173744	3173004
4284 BPSL2814		3215082	3217133	2483477	2485528	3639817	3641859	3623512	3625554	3270090	3272141	3198794	3200836	3256066	3258108	3410795	3412837	3173866	3175908
4285 BPSL2815		3217133	3218104	2485528	2486499	3641859	3642830	3625554	3626525	3272141	3273112	3200836	3201807	3258108	3259079	3412837	3413808	3175908	3176879
4286 BPSL2816		3218526	3218128	2486921	2486523	3643252	3642854	3626947	3626549	3273534	3273136	3202229	3201831	3259501	3259103	3414230	3413832	3177301	3176903

4287	BPSL2817		Bp_chr1_41_ISBma1															3414365	3415669		
4288	BPSL2818			3219869	3218817	2488264	2487212	3644595	3643543	3628290	3627238	3274925	3273873	3203669	3202617	3260844	3259792	3416788	3415736	3178545	3177493
4289	BPSL2819			3220168	3220890	2488563	2489285	3644894	3645616	3628589	3629311	3275224	3275946	3203968	3204690	3261143	3261865	3417087	3417809	3178844	3179566
4290	BPSL2820			3220915	3221598	2489310	2489993	3645641	3646324	3629336	3630019	3275971	3276654	3204715	3205398	3261890	3262573	3417834	3418517	3179591	3180274
4291	BPSL2821			3221598	3223142	2489993	2491537	3646324	3647868	3630019	3631563	3276654	3278198	3205398	3206942	3262573	3264117	3418517	3420061	3180274	3181818
4292	BPSL2822			3223182	3223706	2491577	2492101	3647908	3648432	3631603	3632127	3278238	3278762	3206982	3207506	3264157	3264681	3420101	3420625	3181858	3182382
4293	BPSL2823			3223706	3224386	2492101	2492781	3648432	3649112	3632127	3632807	3278762	3279442	3207506	3208186	3264681	3265361	3420625	3421305	3182382	3183062
4294	BPSL2824			3224454	3225266	2492849	2493661	3649180	3649992	3632875	3633687	3279510	3280322	3208254	3209066	3265429	3266241	3421373	3422185	3183130	3183942
4295	BPSL2825			3227474	3225456	2495869	2493851	3652197	3650182	3635892	3633877	3282527	3280512	3211263	3209248	3268446	3266431	3424393	3422375	3186148	3184133
4296	BPSL2826			3228626	3227499	2497021	2495894	3653349	3652222	3637044	3635917	3283679	3282552	3212415	3211288	3269598	3268471	3425545	3424418	3187300	3186173
4297	BPSL2827			3230845	3228896	2499240	2497291	3655568	3653619	3639263	3637314	3285898	3283949	3214634	3212685	3271817	3269868	3427764	3425815	3189519	3187570
4298	BPSL2828			3231422	3230994	2499817	2499389	3656145	3655717	3639840	3639412	3286475	3286047	3215210	3214782	3272394	3271966	3428341	3427913	3190096	3189668
4299	BPSL2829			3231995	3231441	2500390	2499836	3656718	3656164	3640413	3639859	3287048	3286494	3215782	3215228	3272967	3272413	3428914	3428360	3190669	3190115
4300	BPSL2830			3232899	3232495	2501294	2500890	3657622	3657218	3641317	3640913	3287952	3287548	3216686	3216282	3273871	3273467	3429818	3429414	3191572	3191168
4301	BPSL2831			3234008	3232908	2502403	2501303	3658731	3657631	3642426	3641326	3289061	3287961	3217795	3216695	3274980	3273880	3430927	3429827	3192681	3191581
4302	BPSL2832			3235203	3234184	2503598	2502579	3659926	3658907	3643621	3642602	3290256	3289237	3218990	3217971	3276175	3275156	3432122	3431103	3193876	3192857
4303	BPSL2833			3235337	3236236	2503732	2504631	3660060	3660959	3643755	3644654	3290390	3291289	3219124	3220023	3276309	3277208	3432256	3433155	3194010	3194909
4304	BPSL2834			3236269	3237915	2504664	2506310	3660992	3662638	3644687	3646333	3291322	3292965	3220056	3221702	3277241	3278887	3433188	3434834	3194942	3196588
4305	BPSL2835			3241100	3238320	2509495	2506715	3665823	3663043	3649518	3646738	3295936	3293156	3224890	3222110	3281679	3279334	3438079	3435299	3199773	3196993
4306	BPSL2836			3241261	3245451	2509656	2513846	3665984	3670174	3649679	3653869	3296097	3300287	3225051	3229241	3281901	3286091	3438240	3442430	3199930	3204120
4307	BPSL2837			3245506	3246330	2513901	2514725	3670229	3671053	3653924	3654748	3300342	3301166	3229296	3230120	3286146	3286970	3442485	3443309	3204175	3204999
4308	BPSL2838			3246413	3247906	2514808	2516301	3671136	3672629	3654831	3656324	3301249	3302742	3230203	3231696	3287053	3288546	3443392	3444885	3205082	3206575
4309	BPSL2839			3248319	3249389	2516714	2517784	3673042	3674112	3656737	3657807	3303155	3304225	3232109	3233179	3288959	3290029	3445298	3446368	3206988	3208058
4310			Bp_chr1_42_ISBma2					3674349	3675921	3658044	3659616					3290266	3291837				
4311	BPSL2840			3250879	3249674	2519274	2518069	3677199	3675994	3660894	3659689	3305632	3304427	3234669	3233464	3293115	3291910	3447858	3446653	3209548	3208343
4312	BPSL2841			3251563	3250994	2519958	2519389	3677883	3677314	3661578	3661009	3306316	3305747	3235341	3234787	3293799	3293230	3448542	3447973	3210232	3209663
4313	BPSL2842			3251727	3253133	2520122	2521528	3678047	3679453	3661742	3663148	3306480	3307886	3235505	3236911	3293963	3295369	3448706	3450112	3210396	3211802
4314	BPSL2843			3253201	3254691	2521596	2523086	3679521	3681011	3663216	3664706	3307954	3309444	3236979	3238469	3295437	3296927	3450180	3451670	3211870	3213360
4315	BPSL2844			3254796	3255881	2523191	2524276	3681116	3682201	3664811	3665896	3309549	3310634	3238574	3239659	3297032	3298117	3451775	3452860	3213465	3214550
4316	BPSL2845			3255891	3257114	2524286	2525509	3682211	3683434	3665906	3667129	3310644	3311867	3239669	3240892	3298127	3299350	3452870	3454093	3214560	3215783
4317	BPSL2846			3257350	3258045	2525745	2526440	3683670	3684365	3667365	3668060	3312116	3312811	3241155	3241850	3299586	3300281	3454329	3455024	3216046	3216741
4318	BPSL2847			3258082	3258891	2526477	2527286	3684403	3685212	3668098	3668907	3312849	3313658	3241888	3242697	3300319	3301128	3455062	3455871	3216779	3217588
4319		BURPS1106A_3335		3259118	3259375																
4320		BURPS1106B_A2551				2527770	2527513														
4321		BURPS1710A_3831						3685696	3685439												
4322	BPSL2848			3260166	3259354	2528561	2527749	3686487	3685675	3670182	3669370	3314933	3314121	3243959	3243147	3302403	3301591	3457112	3456300	3218863	3218051
4323	BPSL2849			3261158	3260166	2529553	2528561	3687479	3686487	3671174	3670182	3315925	3314933	3244951	3243959	3303395	3302403	3458104	3457112	3219855	3218863
4324	BPSL2850			3262212	3261244	2530607	2529639	3688533	3687565	3672228	3671260	3316979	3316011	3246005	3245037	3304273	3303557	3459121	3458285	3220853	3220041
4325	BPSL2851			3263528	3262401	2531923	2530796	3689849	3688722	3673544	3672417	3318295	3317168	3247321	3246194	3305589	3304462	3460437	3459310	3222169	3221042
4326	BPSL2852			3264340	3263546	2532735	2531941	3690661	3689867	3674356	3673562	3319107	3318313	3248133	3247339	3306401	3305607	3461249	3460455	3222981	3222187
4327	BPSL2853			3265119	3264352	2533514	2532747	3691440	3690673	3675135	3674368	3319886	3319119	3248912	3248145	3307180	3306413	3462028	3461261	3223760	3222993
4328	BPSL2854			3266090	3265119	2534485	2533514	3692411	3691440	3676106	3675135	3320905	3319886	3249883	3248912	3308151	3307180	3463011	3462028	3224731	3223760
4329	BPSL2855			3267295	3266090	2535690	2534485	3693622	3692411	3677317	3676106	3322116	3320905	3251076	3249883	3309362	3308151	3464222	3463011	3225936	3224731
4330	BPSL2856			3267924	3267298	2536319	2535693	3694251	3693628	3677946	3677323	3322745	3322122	3251705	3251082	3309991	3309368	3464851	3464228	3226565	3225942
4331	BPSL2857			3268469	3267924	2536864	2536319	3694805	3694251	3678500	3677946	3323299	3322745	3252286	3251705	3310545	3309991	3465396	3464851	3227119	3226565
4332	BPSL2858			3268602	3269417	2536997	2537812	3694938	3695753	3678633	3679448	3323432	3324247	3252419	3253234	3310678	3311493	3465529	3466344	3227252	3228067
4333	BPSL2859			3269410	3270204	2537805	2538599	3695746	3696540	3679441	3680235	3324240	3325034	3253227	3254003	3311486	3312280			3228060	3228281
4334	BPSL2860			3270204	3270755	2538599	2539150	3696540	3697091	3680235	3680786	3325034	3325585	3254003	3254554	3312280	3312831	3467176	3467727	3228714	3229265
4335	BPSL2861			3272073	3271126	2540468	2539521	3698470	3697523	3682165	3681218	3326949	3326002	3255926	3254979	3314190	3313243	3469092	3468145	3230624	3229677
4336	BPSL2862			3272987	3272925	2541382	2541320	3699337	3698804	3683032	3682499	3327807	3327274	3256862	3256329	3315057	3314527	3469968	3469438	3231491	3230961
4337	BPSL2863			3273734	3273249	2542129	2541644	3700099	3699614	3683794	3683309	3328569	3328084	3257691	3257206	3315918	3315433	3470822	3470337	3232270	3231785
4338	BPSL2864			3274107	3273937	2542502	2542332	3700472	3700302	3684167	3683997	3328942	3328772	3258070	3257894	3316291	3316121	3471195	3471025	3232643	3232473
4339	BPSL2865			3276452	3274209	2544847	2542604	3702817	3700574	3686512	3684269	3331287	3329044	3260423	3258180	3318636	3316393	3473540	3471297	3234988	3232745
4340	BPSL2866			3277837	3276881	2546232	2545276	3704202	3703246	3687897	3686941	3332672	3331716	3261807	3260851	3320021	3319065	3474925	3473969	3236373	3235417
4341	BPSL2867			3280219	3277976	2548614	2546371	3706584	3704341	3690279	3688036	3335036	3332811	3264189	3261946	3322352	3320160	3477298	3475064	3238647	3236512
4342	BPSL2868			3280836	3281915	2549231	2550310	3707201	3708280	3690896	3691975	3335429	3336508	3264806	3265885	3322859	3323938	3477744	3478823	3238972	3240051
4343	BPSL2869			3282163	3283353	2550558	2551748	3708528	3709718	3692223	3693413	3336796	3337986	3266293	3267483	3324226	3325416	3479163	3480353	3240379	3241560
4344	BPSL2870			3283781	3284104	2552176	2552499	3710151	3710474	3693846	3694169	3338420	3338743	3267793	3268116	3325849	3326172	3480792	3481115	3241959	3242282

4345	BPSL2871			3284215	3286236	2552610	2554631	3710585	3712606	3694280	3696301	3338854	3340728	3268226	3270250	3326283	3328304	3481225	3483243	3242392	3244413
4346	BPSL2872			3286255	3287202	2554650	2555597	3712625	3713572	3696320	3697267	3340954	3341901	3270269	3271216	3328323	3329270	3483262	3484209	3244432	3245379
4347	BPSL2873			3288074	3287454	2556469	2555849	3714426	3713824	3698121	3697519	3342772	3342152	3272088	3271468	3330124	3329522	3485081	3484461	3246253	3245633
4348	BPSL2874			3288753	3288178	2557148	2556573	3716008	3715433	3699703	3699128	3343451	3342876	3272767	3272192	3331706	3331131	3485760	3485185	3246931	3246356
4349	BPSL2875			3289380	3288823	2557775	2557218	3716635	3716078	3700330	3699773	3344078	3343521	3273394	3272837	3332333	3331776	3486387	3485830	3247558	3247001
4350	BPSL2876			3289955	3289395	2558350	2557790	3717210	3716650	3700905	3700345	3344653	3344093	3273969	3273409	3332908	3332348	3486962	3486402	3248133	3247573
4351	BPSL2877			3290220	3290873	2558615	2559268	3717475	3718128	3701170	3701823	3344918	3345571	3274234	3274887	3333173	3333826	3487227	3487880	3248398	3249051
4352	BPSL2878			3290873	3291532	2559268	2559927	3718128	3718787	3701823	3702482	3345571	3346230	3274887	3275546	3333826	3334485	3487880	3488539	3249051	3249710
4353	BPSL2879			3291522	3293180	2559917	2561575	3718777	3720435	3702472	3704130	3346220	3347878	3275536	3277194	3334475	3336133	3488529	3490187	3249700	3251358
4354	BPSL2880			3293180	3293947	2561575	2562342	3720435	3721202	3704130	3704897	3347878	3348606	3277194	3277967	3336133	3336900	3490187	3490954	3251358	3252134
4355	BPSL2881			3294826	3296940	2563221	2565335	3722133	3724247	3705828	3707942	3349626	3351740	3278899	3281013	3337783	3339897	3491845	3493959	3252992	3255106
4356	BPSL2882			3297128	3298417	2565523	2566812	3724419	3725708	3708114	3709403	3351912	3353201	3281185	3282474	3340069	3341358	3494131	3495420	3255278	3256567
4357	BPSL2883			3299877	3300284	2568300	2568707	3727147	3727554	3710835	3711242	3354556	3354963	3283816	3284223	3342648	3343055	3496796	3497203	3257922	3258329
4358	BPSL2884			3300868	3301551	2569303	2569986	3728048	3728731	3711736	3712419	3355589	3356272	3284723	3285406	3343561	3344244	3497775	3498458	3258775	3259458
4359		BURPS668_3349												3285591	3285833						
4360		BURPSS13_A0033																3498521	3498940		
4361	BPSL2885			3303534	3302083	2571969	2570518	3730700	3729249	3714388	3712937	3358185	3356734	3287284	3285833	3346186	3344735	3500363	3498912	3261428	3259977
4362	BPSL2886			3303860	3303534	2572295	2571969	3731026	3730700	3714714	3714388	3358511	3358185	3287610	3287284	3346512	3346186	3500689	3500363	3261754	3261428
4363	BPSL2887			3305012	3303876	2573447	2572311	3732178	3731042	3715866	3714730	3359663	3358527	3288762	3287626	3347664	3346528	3501841	3500705	3262906	3261770
4364	BPSL2888			3305256	3305762	2573691	2574197	3732422	3732928	3716110	3716616	3359907	3360413	3289006	3289512	3347908	3348414	3502085	3502591	3263150	3263656
4365	BPSL2889			3305762	3306910	2574197	2575345	3732928	3734076	3716616	3717764	3360413	3361561	3289512	3290660	3348414	3349562	3502591	3503739	3263656	3264804
4366	BPSL2890			3307035	3308672	2575470	2577107	3734201	3735838	3717889	3719526	3361686	3363323	3290785	3292422	3349687	3351324	3503864	3505501	3264929	3266566
4367			Bp_chr1_43_ISBr	3308915	3310487	2577350	2578922														
4368	BPSL2891			3311145	3310540	2579580	2578975	3736718	3736113	3720406	3719801	3364202	3363597	3293302	3292697	3352203	3351598	3506380	3505775	3267446	3266841
4369		BURPS1655_E0421																		3267948	3267496
4370	BPSL2892			3311703	3313109	2580138	2581544	3737276	3738682	3720964	3722370	3364760	3366166	3293860	3295266	3352761	3354167	3506944	3508350	3268000	3269406
4371	BPSL2893			3313157	3314347	2581592	2582782	3738730	3739920	3722418	3723608	3366214	3367404	3295314	3296504	3354215	3355405	3508398	3509588	3269454	3270644
4372	BPSL2894			3314558	3315619	2582993	2584054	3740131	3741192	3723819	3724880	3367615	3368676	3296715	3297776	3355616	3356677	3509799	3510860	3270855	3271916
4373	BPSL2895			3315619	3315849	2584054	2584284	3741192	3741422	3724880	3725110	3368676	3368906	3297776	3298006	3356677	3356907	3510860	3511090	3271916	3272146
4374	BPSL2896			3315897	3317459	2584332	2585894	3741470	3743032	3725158	3726720	3368954	3370516	3298054	3299616	3356955	3358517	3511138	3512700	3272194	3273756
4375	BPSL2897			3317834	3318373	2586269	2586808	3743402	3743941	3727090	3727629	3370886	3371425	3299988	3300527	3358888	3359427	3513070	3513609	3274126	3274665
4376	BPSL2898			3318450	3319028	2586885	2587463	3744018	3744596	3727706	3728284	3371502	3372080	3300602	3301180	3359504	3360082	3513686	3514264	3274742	3275320
4377	BPSL2899			3319199	3320266	2587634	2588701	3744767	3745834	3728455	3729522	3372265	3373332	3301365	3302432	3360267	3361334	3514435	3515502	3275491	3276558
4378	BPSL2900			3320295	3321248	2588730	2589683	3745863	3746816	3729551	3730504	3373361	3374314	3302461	3303414	3361363	3362316	3515531	3516484	3276587	3277540
4379	BPSL2901			3321383	3322522	2589818	2590957	3746951	3748090	3730639	3731778	3374449	3375588	3303549	3304688	3362451	3363590	3516619	3517758	3277675	3278577
4380	BPSL2902			3323370	3322684	2591805	2591119	3748938	3748252	3732626	3731940	3376436	3375750	3305545	3304859	3364438	3363752	3518606	3517920	3279664	3278978
4381	BPSL2903			3323857	3323381	2592292	2591816	3749425	3748949	3733113	3732637	3376923	3376447	3306032	3305556	3364925	3364449	3519093	3518617	3280151	3279675
4382	BPSL2904			3325169	3323931	2593604	2592366	3750737	3749499	3734425	3733187	3378235	3376997	3307344	3306106	3366237	3364999	3520405	3519167	3281463	3280225
4383	BPSL2905			3325300	3326508	2593735	2594943	3750868	3752076	3734556	3735764	3378366	3379574	3307474	3308682	3366368	3367576	3520536	3521744	3281599	3282801
4384	BPSL2906			3327315	3326620	2595750	2595055	3752883	3752188	3736571	3735876	3380386	3379691	3309489	3308794	3368383	3367688	3522551	3521856	3283613	3282918
4385	BPSL2907			3328024	3327323	2596459	2595758	3753592	3752891	3737280	3736579	3381095	3380394	3310198	3309497	3369092	3368391	3523260	3522559	3284322	3283621
4386	BPSL2908			3328117	3329016	2596552	2597451	3753685	3754584	3737373	3738272	3381188	3382087	3310291	3311190	3369185	3370084	3523353	3524252	3284415	3285314
4387	BPSL2909			3329555	3329190	2597990	2597625	3755124	3754759	3738812	3738447	3382627	3382262	3311736	3311371	3370624	3370259	3524791	3524426	3285853	3285488
4388	BPSL2910			3330154	3329765	2598589	2598200	3755723	3755334	3739411	3739022	3383226	3382837	3312335	3311946	3371223	3370834	3525390	3525001	3286452	3286063
4389	BPSL2911			3330594	3330169	2598735	2598604	3756163	3755738	3739851	3739426	3383666	3383241	3312775	3312350	3371663	3371238	3525785	3525405	3286892	3286467
4390		BURPSPAST_Q0001														3375503	3375204				
4391		BURPSPAST_Q0002														3375661	3375500				
4392		BURPSPAST_Q0003														3376019	3375705				
4393		BURPSPAST_Q0004														3376761	3376051				
4394		BURPSPAST_Q0005														3377719	3376802				
4395		BURPSPAST_Q0006														3378246	3377770				
4396		BURPSPAST_Q0007														3378269	3378409				
4397		BURPSPAST_Q0008														3378498	3378880				
4398		BURPSPAST_U0001														3373521	3374970				
4399	BPSL2912			3331264	3331683	2598882	2599301	3756793	3757212	3740521	3740940	3384336	3384755	3313445	3313864	3379093	3379512			3287562	3287981
4400	BPSL2913			3333195	3332356	2600813	2599974	3758724	3757885	3742452	3741613	3386267	3385428	3315376	3314537	3381024	3380185	3526984	3526145	3289493	3288654
4401	BPSL2914			3334603	3333548	2602221	2601166	3760132	3759077	3743860	3742805	3387675	3386620	3316775	3315720	3382441	3381386	3528392	3527337	3290901	3289846
4402	BPSL2915			3334813	3335724	2602431	2602826	3760342	3761253	3744070	3744981	3387885	3388796	3316985	3317896	3382651	3383562	3528602	3529513	3291111	3292022

4403	BPSL2916		3336508	3336173	2604127	2603792	3762041	3761706	3745769	3745434	3389584	3389249	3318684	3318349	3384350	3384015	3530297	3529962	3292810	3292475
4404	BPSL2917		3337294	3336878	2604913	2604497	3762820	3762404	3746548	3746132	3390399	3389983	3319453	3319037	3385136	3384720	3531083	3530667	3293589	3293173
4405	BPSL2918		3337800	3337363	2605419	2604982	3763326	3762889	3747054	3746617	3390905	3390468	3319959	3319522	3385642	3385205	3531589	3531152	3294095	3293658
4406	BPSL2919		3338263	3338577	2605882	2606196	3763789	3764103	3747517	3747831	3391368	3391682	3320419	3320733	3386105	3386419	3532052	3532366	3294558	3294872
4407	BPSL2920		3338875	3339126	2606494	2606745	3764401	3764652	3748129	3748380	3391980	3392231	3321031	3321282	3386694	3386945	3532664	3532915	3295165	3295416
4408	BPSL2921		3339965	3339243	2607584	2606862	3765491	3764769	3749219	3748497	3393069	3392347	3322120	3321398	3387784	3387062	3533754	3533032	3296254	3295532
4409	BPSL2922		3340639	3339965	2608258	2607584	3766165	3765491	3749893	3749219	3393743	3393069	3322794	3322120	3388458	3387784	3534428	3533754	3296928	3296254
4410	BPSL2923		3341379	3340642	2608998	2608261	3766905	3766168	3750633	3749896	3394483	3393746	3323534	3322797	3389198	3388461	3535168	3534431	3297668	3296931
4411	BPSL2924		3342364	3341465	2609983	2609084	3767890	3766991	3751618	3750719	3395468	3394569	3324519	3323620	3390183	3389284	3536153	3535254	3298653	3297754
4412	BPSL2925		3343892	3342591	2611511	2610210	3769418	3768117	3753146	3751845	3396996	3395695	3326047	3324746	3391711	3390410	3537681	3536380	3300181	3298880
4413	BPSL2926		3345223	3344276	2612842	2611895	3770749	3769802	3754477	3753530	3398327	3397380	3327378	3326431	3393042	3392095	3539012	3538065	3301512	3300565
4414	BPSL2927		3345641	3347095	2613260	2614714	3771167	3772621	3754895	3756349	3398745	3400199	3327796	3329250	3393460	3394914	3539430	3540884	3301930	3303384
4415	BPSL2928		3347143	3348591	2614762	2616210	3772669	3774117	3756397	3757845	3400247	3401695	3329298	3330746	3394962	3396410	3540932	3542380	3303432	3304880
4416	BPSL2929		3349233	3348718	2616852	2616337	3774756	3774244	3758484	3757972	3402337	3401822	3331388	3330873	3397052	3396537	3543022	3542507	3305522	3305007
4417	BPSL2930		3350608	3349250	2618227	2616869	3776131	3774773	3759859	3758501	3403712	3402354	3332763	3331405	3398427	3397069	3544397	3543039	3306897	3305539
4418	BPSL2931		3351458	3350832	2619077	2618451	3776980	3776354	3760708	3760082	3404563	3403937	3333612	3332986	3399276	3398650	3545248	3544622	3307746	3307120
4419	BPSL2932		3353330	3351480	2620949	2619099	3778852	3777002	3762580	3760730	3406435	3404585	3335484	3333634	3401148	3399298	3547120	3545270	3309618	3307768
4420	BPSL2933		3353628	3354623	2621247	2622242	3779150	3780145	3762878	3763873	3406733	3407686	3335782	3336777	3401446	3402441	3547418	3548413	3309916	3310911
4421		BURPS1106A_3445	3354764	3354952																
4422		BURPS1106B_A2662			2622571	2622383														
4423		BURPS1710A_3939					3780471	3780289												
4424		BURPS668_3410											3336918	3337121						
4425	BPSL2934		3355551	3355132	2623170	2622751	3781072	3780653	3764800	3764381	3408694	3408275	3337711	3337292	3403385	3402966	3549294	3548875	3311856	3311437
4426	BPSL2935		3356945	3355572	2624564	2623191	3782466	3781093	3766194	3764821	3410088	3408715	3339105	3337732	3404779	3403406	3550688	3549315	3313250	3311877
4427	BPSL2936		3358065	3356980	2625684	2624599	3783592	3782507	3767320	3766235	3411208	3410123	3340225	3339140	3405905	3404820	3551808	3550723	3314370	3313285
4428	BPSL2937		3358662	3358114	2626281	2625733	3784189	3783641	3767917	3767369	3411805	3411257	3340822	3340274	3406502	3405954	3552405	3551857	3314967	3314419
4429	BPSL2938		3361298	3358707	2628917	2626326	3786825	3784234	3770553	3767962	3414441	3411850	3343458	3340867	3409138	3406547	3555041	3552450	3317603	3315012
4430	BPSL2939		3362003	3361476	2629622	2629095	3787530	3787003	3771258	3770731	3415146	3414619	3344151	3343636	3409843	3409316	3555746	3555219	3318308	3317781
4431	BPSL2940		3362751	3362047	2630370	2629666	3788278	3787574	3772006	3771302	3415894	3415190	3344899	3344195	3410591	3409887	3556494	3555790	3319056	3318352
4432	BPSL2941		3363586	3362783	2631205	2630402	3789113	3788310	3772841	3772038	3416729	3415926	3345734	3344931	3411426	3410623	3557329	3556526	3319891	3319088
4433	BPSL2942		3364487	3363660	2632106	2631279	3790008	3789187	3773736	3772915	3417624	3416803	3346635	3345808	3412310	3411588	3558230	3557403	3320786	3319965
4434	BPSL2943		3364602	3365027	2632221	2632646	3790123	3790548	3773851	3774276	3417739	3418164	3346750	3347175	3412425	3412850	3558345	3558770	3320901	3321326
4435	BPSL2944		3365677	3365156	2633296	2632775	3791212	3790691	3774940	3774419	3418828	3418307	3347839	3347318	3413514	3412993	3559420	3558899	3321990	3321469
4436	BPSL2945		3366701	3365694	2634320	2633313	3792236	3791229	3775964	3774957	3419852	3418845	3348863	3347856	3414538	3413531	3560444	3559437	3323014	3322007
4437	BPSL2946		3368161	3366842	2635780	2634461	3793695	3792376	3777423	3776104	3421312	3419993	3350323	3349004	3415997	3414678	3561904	3560585	3324474	3323155
4438	BPSL2947		3368407	3369093	2636026	2636712	3793941	3794627	3777669	3778355	3421558	3422244	3350569	3351255	3416243	3416929	3562150	3562836	3324720	3325406
4439	BPSL2948		3370646	3369714	2638272	2637340	3796199	3795267	3779927	3778995	3423830	3422898	3352867	3351935	3418508	3417576	3564399	3563467	3327006	3326074
4440	BPSL2949		3370761	3371417	2638387	2639043	3796314	3796970	3780042	3780698	3423945	3424601	3352982	3353638	3418623	3419279	3564514	3565170	3327121	3327777
4441	BPSL2950		3371507	3371974	2639133	2639600	3797060	3797527	3780788	3781255	3424691	3425158	3353728	3354195	3419369	3419836	3565260	3565727	3327867	3328334
4442	BPSL2951		3372159	3373355	2639785	2640981	3797712	3798908	3781440	3782636	3425334	3426530	3354380	3355576	3420021	3421217	3565911	3567107	3328519	3329715
4443	BPSL2952		3374776	3373769	2642402	2641395	3800330	3799323	3784058	3783051	3427952	3426945	3356998	3355991	3422639	3421632	3568528	3567521	3331137	3330130
4444	BPSL2953		3376894	3374870	2644520	2642496	3802448	3800424	3786176	3784152	3430070	3428046	3359116	3357092	3424757	3422733	3570646	3568622	3333255	3331231
4445	BPSL2954		3377157	3378011	2644783	2645637	3802711	3803565	3786439	3787293	3430333	3431187	3359379	3360233	3425020	3425874	3570909	3571763	3333518	3334372
4446	BPSL2955		3378054	3378467	2645680	2646093	3803608	3804021	3787336	3787749	3431230	3431643	3360276	3360689	3425917	3426330	3571806	3572219	3334415	3334828
4447	BPSL2956		3378494	3379231	2646120	2646857	3804048	3804785	3787776	3788513	3431670	3432407	3360716	3361453	3426357	3427094	3572246	3572983	3334855	3335592
4448	BPSL2957		3380036	3379641	2647662	2647267	3805525	3805130	3789253	3788858	3433212	3432817	3362257	3361862	3427834	3427439	3573787	3573392	3336396	3336001
4449	BPSL2958		3380649	3380221	2648275	2647847	3806138	3805710	3789866	3789438	3433825	3433397	3362870	3362442	3428447	3428019	3574400	3573972	3337009	3336581
4450	BPSL2959		3383287	3380981	2650913	2648607	3808776	3806470	3792504	3790198	3436463	3434157	3365505	3363202	3431085	3428779	3577038	3574732	3339647	3337341
4451	BPSL2960		3383489	3384487	2651115	2652113	3808978	3809976	3792706	3793704	3436665	3437663	3365716	3366714	3431287	3432285	3577240	3578238	3339849	3340847
4452	BPSL2961		3384624	3385094	2652250	2652720	3810113	3810583	3793841	3794311	3437800	3438270	3366851	3367321	3432422	3432892	3578375	3578845	3340984	3341454
4453	BPSL2962		3385141	3385638	2652767	2653264	3810630	3811127	3794358	3794855	3438317	3438814	3367368	3367865	3432939	3433436	3578892	3579389	3341501	3341998
4454	BPSL2963		3386751	3385927	2654377	2653553	3812240	3811416	3795968	3795144	3439927	3439103	3368987	3368163	3434549	3433725	3580502	3579678	3343133	3342309
4455	BPSL2964		3387831	3386851	2655457	2654477	3813320	3812340	3797048	3796068	3441007	3440027	3370067	3369087	3435629	3434649	3581582	3580602	3344213	3343233
4456	BPSL2965		3388774	3387971	2656400	2655597	3814263	3813460	3797991	3797188	3441950	3441147	3371010	3370207	3436572	3435769	3582525	3581722	3345156	3344353
4457	BPSL2966		3389817	3388816	2657443	2656442	3815306	3814305	3799034	3798033	3442993	3441992	3372053	3371052	3437615	3436614	3583568	3582567	3346199	3345198
4458	BPSL2967		3391464	3389935	2659090	2657561	3816953	3815424	3800681	3799152	3444640	3443111	3373704	3372175	3439262	3437733	3585215	3583686	3347846	3346317
4459	BPSL2968		3392527	3391535	2660153	2659161	3818016	3817024	3801744	3800752	3445703	3444711	3374767	3373775	3440325	3439333	3586278	3585286	3348909	3347917
4460	BPSL2969		3393436	3392660	2661062	2660286	3818925	3818149	3802653	3801877	3446612	3445836	3375676	3374900	3441234	3440458	3587187	3586411	3349818	3349042

4461	BPSL2970			3394062	3393436	2661688	2661062	3819551	3818925	3803279	3802653	3447238	3446612	3376302	3375676	3441860	3441234	3587813	3587187	3350444	3349818
4462	BPSL2971			3395173	3394163	2662799	2661789	3820662	3819652	3804390	3803380	3448349	3447339	3377413	3376403	3442971	3441961	3588924	3587914	3351555	3350545
4463	BPSL2972			3396087	3395173	2663713	2662799	3821576	3820662	3805304	3804390	3449263	3448349	3378327	3377413	3443885	3442971	3589838	3588924	3352469	3351555
4464	BPSL2973			3396783	3396355	2664409	2663981	3822272	3821844	3806000	3805572	3449959	3449531	3379023	3378595	3444581	3444153	3590534	3590106	3353165	3352737
4465	BPSL2974			3398480	3396849	2666106	2664475	3823967	3822336	3807695	3806064	3451656	3450025	3380718	3379087	3446276	3444645	3592231	3590600	3354860	3353229
4466	BPSL2975			3399711	3398944	2667337	2666570	3825198	3824431	3808926	3808159	3452887	3452120	3381949	3381182	3447507	3446740	3593462	3592695	3356090	3355323
4467	BPSL2976			3400610	3399735	2668236	2667361	3826097	3825222	3809825	3808950	3453786	3452911	3382848	3381973	3448406	3447531	3594361	3593486	3356989	3356114
4468	BPSL2977			3402685	3400610	2670311	2668236	3828172	3826097	3811900	3809825	3455861	3453786	3384923	3382848	3450481	3448406	3596436	3594361	3359064	3356989
4469	BPSL2978			3403378	3402812	2671004	2670438	3828865	3828299	3812593	3812027	3456554	3455988	3385616	3385050	3451174	3450608	3597129	3596563	3359757	3359191
4470	BPSL2979			3404859	3403378	2672485	2671004	3830346	3828865	3814074	3812593	3458035	3456554	3387097	3385616	3452655	3451174	3598610	3597129	3361238	3359757
4471	BPSL2980			3404981	3405634	2672607	2673260	3830468	3831121	3814196	3814849	3458157	3458810	3387219	3387872	3452777	3453430	3598732	3599385	3361360	3362013
4472	BPSL2981			3405708	3406187	2673334	2673813	3831195	3831674	3814923	3815402	3458884	3459363	3387946	3388425	3453504	3453983	3599459	3599938	3362087	3362566
4473	BPSL2982			3406386	3406835	2674012	2674461	3831873	3832322	3815601	3816050	3459562	3460011	3388624	3389073	3454182	3454631	3600137	3600586	3362765	3363214
4474	BPSL2983			3406906	3407376	2674532	2675002	3832393	3832863	3816121	3816591	3460082	3460552	3389144	3389614	3454702	3455172	3600657	3601127	3363285	3363755
4475	BPSL2984			3407464	3408828	2675090	2676454	3832951	3834315	3816679	3818043	3460640	3462004	3389702	3391066	3455260	3456624	3601215	3602579	3363843	3365207
4476	BPSL2985			3408834	3409733	2676460	2677359	3834321	3835220	3818049	3818948	3462010	3462909	3391072	3391971	3456630	3457529	3602585	3603484	3365213	3366112
4477	BPSL2986			3409784	3411208	2677410	2678834	3835271	3836695	3818999	3820423	3462960	3464396	3392022	3393446	3457580	3459004	3603535	3604959	3366163	3367596
4478	BPSL2987			3411279	3411779	2678905	2679405	3836766	3837266	3820494	3820994	3464467	3464967	3393517	3394017	3459075	3459575	3605030	3605530	3367667	3368167
4479	BPSL2988			3411883	3412818	2679509	2680444	3837370	3838305	3821098	3822033	3465071	3466006	3394120	3395055	3459679	3460614	3605634	3606569	3368271	3369206
4480	BPSL2989			3412845	3413315	2680471	2680941	3838332	3838802	3822060	3822530	3466033	3466503	3395082	3395552	3460641	3461111	3606596	3607066	3369233	3369703
4481	BPSL2990			3414147	3413569	2681773	2681195	3839634	3839056	3823362	3822784	3467335	3466757	3396384	3395806	3461943	3461365	3607898	3607320	3370519	3369956
4482	BPSL2991			3415634	3414585	2683260	2682211	3841121	3840072	3824849	3823800	3468822	3467773	3397871	3396822	3463430	3462381	3609385	3608336	3372006	3370957
4483	BPSL2992			3419148	3416164	2686774	2683790	3844635	3841651	3828363	3825379	3472336	3469352	3401385	3398401	3466944	3463960	3612899	3609915	3375520	3372536
4484	BPSL2993			3420209	3419622	2687835	2687248	3845696	3845109	3829424	3828837	3473397	3472810	3402446	3401859	3468005	3467418	3613960	3613373	3376581	3375994
4485	BPSL2993a			3420526	3420209	2688152	2687835	3846013	3845696	3829741	3829424	3473714	3473397	3402763	3402446	3468322	3468005	3614277	3613960	3376898	3376581
4486	BPSL2994			3421439	3420543	2689065	2688169	3846926	3846030	3830654	3829758	3474627	3473731	3403676	3402780	3469235	3468339	3615190	3614294	3377811	3376915
4487	BPSL2995			3421527	3422891	2689153	2690517	3847014	3848378	3830742	3832106	3474715	3476079	3403764	3405128	3469323	3470687	3615278	3616642	3377899	3379263
4488	BPSL2996			3422992	3423540	2690618	2691166	3848479	3849027	3832207	3832755	3476180	3476728	3405229	3405777	3470788	3471129	3616743	3617291	3379364	3379912
4489	BPSL2997			3424336	3423737	2691962	2691363	3849823	3849224	3833551	3832952	3477524	3476925	3406585	3405986	3472134	3471535	3618087	3617488	3380708	3380109
4490	BPSL2998			3426104	3424371	2693730	2691997	3851591	3849858	3835319	3833586	3479292	3477559	3408353	3406620	3473902	3472169	3619855	3618122	3382476	3380743
4491	BPSL2999			3426322	3426969	2693948	2694595	3851809	3852456	3835537	3836184	3479511	3480158	3408572	3409219	3474120	3474767	3620073	3620720	3382694	3383341
4492	BPSL3000			3427028	3427612	2694654	2695238	3852515	3853099	3836243	3836827	3480217	3480801	3409278	3409862	3474826	3475410	3620779	3621363	3383400	3383984
4493	BPSL3001		Bp_chr1_44_ISBma2																	3384147	3385719
4494	BPSL3002			3428943	3427828	2696569	2695454	3854429	3853314	3838157	3837042	3482131	3481016	3411190	3410075	3476740	3475625	3622694	3621579	3386905	3385790
4495	BPSL3003			3430146	3429031	2697772	2696657	3855632	3854517	3839360	3838245	3483334	3482219	3412393	3411278	3477943	3476828	3623897	3622782	3388108	3386993
4496	BPSL3004			3430594	3430334	2698220	2697960	3856080	3855820	3839808	3839548	3483782	3483522	3412841	3412581	3478391	3478131	3624345	3624085	3388556	3388296
4497	BPSL3005			3430934	3430626	2698560	2698252	3856420	3856112	3840148	3839840	3484122	3483814	3413181	3412873	3478731	3478423	3624685	3624377	3388896	3388588
4498	BPSL3006			3431150	3432145	2698776	2699771	3856636	3857631	3840364	3841359	3484338	3485333	3413397	3414392	3478947	3479942	3624901	3625896	3389112	3390107
4499		BURPS668_3493												3414673	3416388						
4500		BURPS668_3494												3416395	3416706						
4501		BURPS668_3495												3416923	3417498						
4502		BURPS668_3496												3417875	3417991						
4503		BURPS668_3497												3417975	3418229						
4504		BURPS668_3498												3419897	3420337						
4505		BURPS668_3499												3420505	3422643						
4506		BURPS668_3500												3422771	3422893						
4507		BURPS668_3501												3422998	3423993						
4508		BURPS668_3502												3425700	3426017						
4509		BURPS668_3503												3427890	3429890						
4510		BURPS668_3504												3429924	3430703						
4511	BPSL3007			3432429	3433721	2700055	2701347	3857916	3859208	3841644	3842936	3485617	3486909	3432623	3433912	3480226	3481518	3626180	3627472	3390392	3391684
4512	BPSL3008			3433824	3435080	2701450	2702706	3859311	3860567	3843039	3844295	3487012	3488268	3434018	3435274	3481621	3482877	3627575	3628831	3391787	3393043
4513	BPSL3009			3435080	3436294	2702706	2703920	3860567	3861781	3844295	3845509	3488268	3489482	3435274	3436488	3482877	3484091	3628831	3630045	3393043	3394257
4514	BPSL3010			3436327	3437253	2703953	2704879	3861814	3862740	3845542	3846468	3489515	3490441	3436521	3437447	3484124	3485050	3630078	3631004	3394290	3395216
4515	BPSL3011			3437260	3437868	2704886	2705494	3862747	3863355	3846475	3847083	3490448	3491056	3437454	3438062	3485057	3485665	3631011	3631619	3395223	3395831
4516	BPSL3012			3438020	3438772	2705646	2706398	3863507	3864259	3847235	3847987	3491208	3491960	3438214	3438966	3485817	3486569	3631771	3632523	3395983	3396735
4517	BPSL3012a			3438811	3439011	2706437	2706637	3864298	3864498	3848026	3848226	3491999	3492199	3439005	3439205	3486608	3486808	3632562	3632762	3396774	3396974
4518	BPSL3013			3439599	3439153	2707225	2706779	3865086	3864640	3848814	3848368	3492787	3492341	3439795	3439349	3487396	3486950	3633350	3632904	3397562	3397116

4519	BPSL3014			3440465	3439599	2708091	2707225	3865952	3865086	3849680	3848814	3493653	3492787	3440661	3439795	3488262	3487396	3634216	3633350	3398428	3397562
4520	BPSL3015			3441727	3440489	2709353	2708115	3867214	3865976	3850942	3849704	3494915	3493677	3441923	3440685	3489524	3488286	3635478	3634240	3399690	3398452
4521	BPSL3016			3444677	3441885	2712303	2709511	3870164	3867372	3853892	3851100	3497853	3495073	3444813	3442021	3492474	3489682	3638428	3635636	3402640	3399848
4522	BPSL3017			3445389	3445865	2713015	2713491	3870858	3871334	3854586	3855062	3498565	3499041	3445477	3445953	3493174	3493650	3639140	3639616	3403364	3403840
4523	BPSL3018			3447176	3446262	2714802	2713888	3872645	3871731	3856373	3855459	3500352	3499438	3447264	3446350	3494961	3494047	3640927	3640013	3405151	3404237
4524	BPSL3019			3447924	3447421	2715550	2715047	3873393	3872890	3857121	3856618	3501100	3500597	3448012	3447509	3495709	3495206	3641675	3641172	3405899	3405396
4525	BPSL3020			3449306	3448113	2716932	2715739	3874775	3873582	3858503	3857310	3502482	3501289	3449394	3448201	3497091	3495898	3643057	3641864	3407281	3406088
4526	BPSL3021			3450647	3449418	2718273	2717044	3876116	3874887	3859844	3858615	3503823	3502594	3450735	3449506	3498432	3497203	3644398	3643169	3408622	3407393
4527	BPSL3022			3451426	3450677	2719052	2718303	3876895	3876146	3860623	3859874	3504602	3503853	3451514	3450765	3499211	3498462	3645177	3644428	3409401	3408652
4528	BPSL3023			3452385	3451450	2720011	2719076	3877854	3876919	3861582	3860647	3505561	3504626	3452473	3451538	3500170	3499235	3646136	3645201	3410360	3409425
4529	BPSL3024			3453784	3452390	2721410	2720016	3879253	3877859	3862981	3861587	3506960	3505566	3453872	3452478	3501569	3500175	3647535	3646141	3411759	3410365
4530	BPSL3025			3454884	3453784	2722510	2721410	3880353	3879253	3864081	3862981	3508060	3506960	3454972	3453872	3502669	3501569	3648635	3647535	3412859	3411759
4531	BPSL3026			3456298	3455009	2723924	2722635	3881767	3880478	3865495	3864206	3509474	3508185	3456386	3455097	3504083	3502794	3650049	3648760	3414273	3412984
4532	BPSL3027			3457785	3456298	2725411	2723924	3883254	3881767	3866982	3865495	3510961	3509474	3457873	3456386	3505570	3504083	3651536	3650049	3415760	3414273
4533	BPSL3028			3459004	3457838	2726630	2725464	3884473	3883307	3868201	3867035	3512180	3511014	3459092	3457926	3506789	3505623	3652755	3651589	3416979	3415813
4534	BPSL3029			3460425	3459022	2728053	2727700	3885894	3884491	3869622	3868219	3513601	3512198	3460513	3459110	3508210	3506807	3654176	3652773	3418400	3416997
4535	BPSL3030			3461966	3460425	2729594	2728053	3887435	3885894	3871163	3869622	3515142	3513601	3462054	3460513	3509751	3508210	3655717	3654176	3419941	3418400
4536	BPSL3031			3463807	3461966	2731435	2729594	3889276	3887435	3873004	3871163	3516983	3515142	3463895	3462054	3511592	3509751	3657558	3655717	3421782	3419941
4537	BPSL3032			3464133	3463807	2731761	2731435	3889602	3889276	3873330	3873004	3517309	3516983	3464221	3463895	3511918	3511592	3657884	3657558	3422108	3421782
4538	BPSL3033			3465071	3464133	2732699	2731761	3890540	3889602	3874268	3873330	3518247	3517309	3465159	3464221	3512856	3511918	3658822	3657884	3423046	3422108
4539	BPSL3034			3465523	3465098	2733151	2732726	3890992	3890567	3874720	3874295	3518699	3518274	3465611	3465186	3513308	3512883	3659274	3658849	3423498	3423073
4540	BPSL3035			3466537	3465914	2734165	2733542	3892006	3891383	3875734	3875111	3519713	3519090	3466625	3466002	3514323	3513700	3660288	3659665	3424512	3423889
4541	BPSL3036			3466818	3467975	2734446	2735603	3892287	3893444	3876015	3877172	3519994	3521151	3466906	3468063	3514604	3515761	3660569	3661726	3424793	3425950
4542			Bp_chr1_45_ISBn	3468028	3469600	2735656	2737228														
4543	BPSL3037			3471480	3469810	2739108	2737438	3895356	3893686	3879084	3877414	3523063	3521393	3469975	3468305	3517673	3516003	3663638	3661968	3427862	3426192
4544	BPSL3038			3473695	3471623	2741323	2739251	3897571	3895499	3881299	3879227	3525278	3523206	3472190	3470118	3519888	3517816	3665853	3663781	3430077	3428005
4545		BURPS406E_K0572										3525686	3525988								
4546		BURPS406E_K0573										3526483	3526746								
4547		BURPS406E_K0574										3526740	3527594								
4548		BURPS406E_K0575										3527596	3528057								
4549	BPSL3040			3480813	3480022	2746554	2745763	3904522	3903731	3888417	3887626	3534929	3534138	3479308	3478517	3532702	3532166	3672913	3672122	3438956	3438165
4550	BPSL3041			3480926	3482629	2746667	2748370	3904635	3906338	3888530	3890233	3535042	3536745	3479421	3481124	3532815	3534518	3673026	3674729	3439069	3440772
4551	BPSL3042			3482706	3483920	2748447	2749661	3906415	3907629	3890310	3891524	3536822	3538036	3481201	3482415	3534595	3535809	3674806	3676020	3440849	3442063
4552	BPSL3043			3484019	3484807	2749760	2750548	3907728	3908516	3891623	3892411	3538098	3538886	3482477	3483265	3535908	3536696	3676119	3676907	3442162	3442950
4553	BPSL3044			3484896	3485285	2750637	2751026	3908605	3908994	3892500	3892889	3538975	3539364	3483354	3483743	3536785	3537174	3676996	3677385	3443039	3443428
4554	BPSL3045			3485326	3486621	2751067	2752362	3909035	3910330	3892930	3894225	3539405	3540700	3483784	3485079	3537215	3538510	3677426	3678721	3443469	3444764
4555	BPSL3046			3488070	3486955	2753811	2752696	3911779	3910664	3895674	3894559	3542149	3541034	3486531	3485416	3539959	3538844	3680170	3679055	3446213	3445098
4556	BPSL3047			3488661	3488290	2754402	2754031	3912370	3911999	3896265	3895894	3542740	3542369	3487122	3486751	3540550	3540179	3680761	3680390	3446804	3446433
4557	BPSL3048			3488798	3489526	2754539	2755267	3912507	3913235	3896402	3897130	3542877	3543605	3487259	3487987	3540687	3541415	3680898	3681626	3446941	3447669
4558	BPSL3049			3489526	3490260	2755267	2756001	3913235	3913969	3897130	3897864	3543605	3544339	3487987	3488721	3541415	3542149	3681626	3682360	3447669	3448403
4559	BPSL3050			3490626	3492116	2756367	2757857	3914335	3915825	3898230	3899720	3544705	3546195	3489087	3490577	3542515	3544005	3682726	3684216	3448769	3450259
4560	BPSL3051			3492133	3492720	2757874	2758461	3915842	3916429	3899737	3900324	3546212	3546799	3490594	3491181	3544022	3544609	3684233	3684820	3450276	3450863
4561	BPSL3052			3492741	3493769	2758482	2759510	3916450	3917478	3900345	3901373	3546820	3547848	3491202	3492230	3544630	3545658	3684841	3685869	3450884	3451912
4562	BPSL3053			3493835	3494617	2759576	2760358	3917544	3918326	3901439	3902221	3547914	3548696	3492296	3493078	3545724	3546506	3685935	3686717	3451978	3452760
4563	BPSL3054			3494631	3495275	2760372	2761016	3918340	3918984	3902235	3902879	3548710	3549348	3493092	3493730	3546520	3547158	3686731	3687369	3452774	3453412
4564	BPSL3055			3495371	3496105	2761112	2761846	3919080	3919814	3902975	3903709	3549444	3550178	3493826	3494560	3547254	3547988	3687465	3688199	3453508	3454242
4565	BPSL3056			3496923	3496330	2762664	2762071	3920632	3920039	3904527	3903934	3550996	3550403	3495378	3494785	3548806	3548213	3689016	3688423	3455060	3454467
4566	BPSL3057			3498891	3497065	2764632	2762806	3922609	3920774	3906504	3904669	3552973	3551138	3497346	3495520	3550783	3548948	3690984	3689158	3457028	3455202
4567	BPSL3058			3499728	3498994	2765469	2764735	3923446	3922712	3907341	3906607	3553810	3553076	3498183	3497449	3551620	3550886	3691821	3691087	3457865	3457131
4568	BPSL3059			3501002	3499824	2766743	2765565	3924720	3923542	3908615	3907437	3555084	3553906	3499457	3498279	3552894	3551716	3693095	3691917	3459139	3457961
4569	BPSL3060			3501317	3501442	2767058	2767183	3925035	3925160	3908930	3909055	3555399	3555524	3499772	3499897	3553209	3553334	3693410	3693535	3459454	3459579
4570	BPSL3061			3501876	3502238	2767617	2767979	3925620	3925982	3909515	3909877	3556000	3556362	3500357	3500719	3553794	3554156	3694004	3694366	3460039	3460401
4571	BPSL3062			3502751	3503842	2768492	2769583	3926463	3927554	3910358	3911449	3556883	3557974	3501280	3502371	3554637	3555728	3694871	3695962	3460934	3462025
4572	BPSL3063			3504452	3504952	2770193	2770693	3928164	3928664	3912059	3912559	3558584	3559084	3503004	3503504	3556338	3556838	3696587	3697087	3462646	3463146
4573	BPSL3064			3505059	3505799	2770800	2771540	3928771	3929511	3912666	3913406	3559191	3559931	3503611	3504351	3556945	3557685	3697194	3697934	3463253	3463993
4574	BPSL3065			3507277	3505877	2773018	2771618	3930989	3929589	3914884	3913484	3561409	3560009	3505820	3504420	3559163	3557763	3699412	3698012	3465471	3464071
4575	BPSL3066			3509518	3508541	2775259	2774282	3933227	3932253	3917122	3916148	3563650	3562673	3508059	3507082	3561401	3560427	3701653	3700676	3467712	3466735
4576	BPSL3067			3510265	3511929	2776006	2777670	3933965	3935629	3917860	3919524	3564379	3566043	3508788	3510452	3562139	3563803	3702391	3704055	3468450	3470114

4577	BPSL3068			3512892	3512272	2778633	2778013	3936591	3935971	3920486	3919866	3567094	3566474	3511391	3510771	3564774	3564154	3704881	3704321	3471099	3470479
4578	BPSL3069			3513416	3512892	2779157	2778633	3937115	3936591	3921010	3920486	3567618	3567094	3511915	3511391	3565298	3564774	3705405	3704881	3471623	3471099
4579		BURPS1655_E0202																		3471814	3471671
4580	BPSL3070			3515386	3513575	2781127	2779316	3939085	3937274	3922980	3921169	3569593	3567782	3513890	3512079	3567273	3565462	3707380	3705569	3473598	3471787
4581	BPSL3071			3515659	3515408	2781400	2781149	3939358	3939107	3923253	3923002	3569866	3569615	3514163	3513912	3567546	3567295	3707653	3707402	3473871	3473620
4582	BPSL3072			3516232	3517554	2781973	2783295	3939931	3941253	3923826	3925148	3570439	3571761	3514736	3516058	3568119	3569441	3708226	3709548	3474444	3475766
4583	BPSL3073			3517624	3518703	2783365	2784444	3941323	3942402	3925218	3926297	3571831	3572910	3516128	3517207	3569511	3570590	3709618	3710697	3475836	3476915
4584	BPSL3074			3518750	3519604	2784491	2785345	3942449	3943303	3926344	3927198	3572957	3573811	3517254	3518108	3570637	3571491	3710744	3711598	3476962	3477816
4585	BPSL3075			3519687	3519992	2785428	2785733	3943386	3943691	3927281	3927586	3573894	3574199	3518191	3518496	3571574	3571879	3711681	3711986	3477899	3478204
4586	BPSL3076			3520010	3520603	2785751	2786344	3943709	3944302	3927604	3928197	3574217	3574810	3518514	3519107	3571897	3572490	3712004	3712597	3478222	3478815
4587	BPSL3077			3520890	3521675	2786631	2787416	3944589	3945374	3928484	3929269	3575097	3575882	3519394	3520179	3572777	3573562	3712884	3713669	3479102	3479887
4588		BURPS406E_I0636										3576181	3575861								
4589		BURPSPAST_P0314														3573847	3573656				
4590		BURPSS13_S0174																3713947	3713648		
4591	BPSL3078			3523652	3522054	2789393	2787795	3947344	3945746	3931239	3929641	3577887	3576289	3522318	3520720	3575532	3573934	3715653	3714055	3481888	3480290
4592	BPSL3079			3524086	3524286	2789827	2790027	3947778	3947978	3931673	3931873	3578321	3578521	3522752	3522952	3575966	3576166	3716087	3716287	3482322	3482522
4593	BPSL3080			3525738	3524479	2791479	2790220	3949430	3948171	3933325	3932066	3579973	3578714	3524404	3523145	3577618	3576359	3717739	3716480	3483974	3482715
4594		BURPS1106B_A2838				2791603	2791800														
4595		BURPS1655_E0183																		3485233	3485808
4596		BURPS1655_E0186																		3484098	3484265
4597		BURPS1710A_4121						3950706	3951281												
4598		BURPS406E_I0626										3581240	3581815								
4599		BURPSPAST_P0305														3578911	3579486				
4600		BURPSS13_S0165																3718920	3719495		
4601	BPSL3081			3527077	3526463	2792818	2792204	3950756	3950142	3934651	3934037	3581290	3580676	3525681	3525067	3578961	3578347	3718970	3718356	3485283	3484669
4602	BPSL3082			3528845	3527565	2794586	2793306	3952524	3951244	3936419	3935139	3583058	3581778	3527449	3526169	3580729	3579449	3720738	3719458	3487051	3485771
4603	BPSL3083			3529004	3529645	2794745	2795386	3952683	3953336	3936578	3937231	3583217	3583858	3527608	3528249	3580888	3581541	3720897	3721538	3487210	3487851
4604	BPSL3084			3530007	3530627	2795748	2796368	3953677	3954297	3937572	3938192	3584199	3584819	3528597	3529217	3581882	3582502	3721879	3722499	3488192	3488812
4605	BPSL3085			3531624	3530725	2797365	2796466	3955287	3954388	3939182	3938283	3585810	3584917	3530214	3529315	3583492	3582593	3723496	3722597	3489803	3488910
4606	BPSL3086			3532942	3531755	2798683	2797496	3956605	3955418	3940500	3939313	3587128	3585941	3531532	3530345	3584810	3583623	3724814	3723627	3491121	3489934
4607		BURPS668_3610												3532952	3533395						
4608	BPSL3087			3534447	3533248	2800188	2798989	3958110	3956911	3942005	3940806	3588633	3587434	3533037	3531838	3586315	3585116	3726319	3725120	3492626	3491427
4609	BPSL3088			3535247	3534804	2800988	2800545	3958910	3958467	3942805	3942362	3589433	3588990	3533835	3533392	3587115	3586672	3727119	3726676	3493426	3492983
4610	BPSL3089			3535908	3538070	2801649	2803811	3959571	3961733	3943466	3945628	3590094	3592247	3534496	3536658	3587776	3589929	3727780	3729942	3494087	3496249
4611	BPSL3090			3539096	3539362	2804837	2805103	3962759	3963025	3946654	3946920	3593273	3593539	3537684	3537950	3590955	3591221	3730968	3731234	3497264	3497530
4612	BPSL3091			3539468	3539695	2805209	2805436	3963131	3963358	3947026	3947253	3593645	3593872	3538056	3538283	3591327	3591554	3731340	3731567	3497636	3497863
4613	BPSL3092			3540314	3541579	2806055	2807320	3963977	3965242	3947872	3949137	3594491	3595756	3538902	3540167	3592173	3593438	3732186	3733454	3498482	3499747
4614	BPSL3093			3541597	3543855	2807338	2809596	3965260	3967518	3949155	3951413	3595774	3598032	3540185	3542443	3593456	3595714	3733472	3735730	3499765	3502023
4615	BPSL3094			3543855	3545300	2809596	2811041	3967518	3968963	3951413	3952858	3598032	3599477	3542443	3543888	3595714	3597159	3735730	3737175	3502023	3503468
4616	BPSL3095			3546268	3545516	2812009	2811257	3969931	3969179	3953826	3953074	3600445	3599693	3544838	3544104	3598145	3597375	3738143	3737391	3504436	3503684
4617	BPSL3096			3546880	3547455	2812621	2813196	3970543	3971118	3954438	3955013	3601057	3601632	3545450	3546025	3598757	3599332	3738755	3739330	3505048	3505623
4618	BPSL3097			3547730	3551620	2813471	2817361	3971393	3975283	3955288	3959178	3601907	3605797	3546300	3550190	3599607	3603497	3739619	3743509	3505898	3509788
4619	BPSL3098			3551620	3552606	2817361	2818347	3975283	3976269	3959178	3960164	3605797	3606783	3550190	3551176	3603497	3604483	3743509	3744495	3509788	3510774
4620	BPSL3099			3552614	3553543	2818355	2819284	3976277	3977206	3960172	3961101	3606791	3607720	3551184	3552113	3604491	3605420	3744503	3745432	3510782	3510943
4621	BPSL3100			3554866	3553748	2820607	2819489	3978529	3977411	3962424	3961306	3609050	3607932	3553436	3552318	3606743	3605625	3746755	3745637	3513094	3511976
4622	BPSL3101			3557625	3554959	2823366	2820700	3981288	3978622	3965183	3962517	3611809	3609143	3556195	3553529	3609502	3606836	3749514	3746848	3515853	3513187
4623	BPSL3102			3558759	3557662	2824500	2823403	3982422	3981325	3966317	3965220	3612943	3611846	3557329	3556232	3610636	3609539	3750648	3749551	3516987	3515890
4624	BPSL3103			3560549	3558726	2826290	2824467	3984212	3982389	3968107	3966284	3614733	3612910	3559119	3557296	3612426	3610603	3752438	3750615	3518777	3516954
4625	BPSL3104			3561153	3560644	2826894	2826385	3984816	3984307	3968711	3968202	3615331	3614828	3559723	3559214	3613024	3612521	3753036	3752533	3519375	3518872
4626	BPSL3105			3561684	3561184	2827425	2826925	3985347	3984847	3969242	3968742	3615934	3615434	3560254	3559754	3614169	3613669	3753659	3753159	3519999	3519499
4627	BPSL3106			3563247	3561760	2828988	2827501	3986910	3985423	3970805	3969318	3617497	3616010	3561817	3560330	3615732	3614245	3755222	3753735	3521562	3520075
4628	BPSL3107			3563782	3563267	2829523	2829008	3987445	3986930	3971340	3970825	3618032	3617517	3562352	3561837	3616267	3615752	3755757	3755242	3522097	3521582
4629	BPSL3108			3564490	3563822	2830231	2829563	3988153	3987485	3972048	3971380	3618740	3618072	3563060	3562392	3616975	3616307	3756465	3755797	3522805	3522137
4630	BPSL3109			3564864	3565475	2830605	2831216	3988527	3989138	3972422	3973033	3619115	3619726	3563435	3564046	3617349	3617960	3756839	3757450	3523180	3523791
4631	BPSL3110			3565587	3566930	2831328	2832671	3989250	3990593	3973145	3974488	3619838	3621181	3564158	3565501	3618072	3619415	3757562	3758905	3523903	3525246
4632	BPSL3111			3566930	3567712	2832671	2833453	3990593	3991375	3974488	3975270	3621181	3621963	3565501	3566283	3619415	3620197	3758905	3759687	3525246	3526028
4633			Bp_chr1_46_ISBm	3570786	3572090	2836527	2837831														
4634		BURPS1106A_3670		3570789	3571949																

4635	BURPS1106A_3671	3572859	3573389		
4636	BURPS1106A_3672	3574186	3575289		
4637	BURPS1106A_3673	3575381	3577201		
4638	BURPS1106A_3674	3577817	3579688		
4639	BURPS1106A_3675	3579691	3583689		
4640	BURPS1106A_3676	3583686	3585311		
4641	BURPS1106A_3677	3585308	3588439		
4642	BURPS1106A_3678	3588455	3592840		
4643	BURPS1106A_3679	3593047	3593241		
4644	BURPS1106A_3680	3593469	3596606		
4645	BURPS1106A_3681	3597115	3598149		
4646	BURPS1106A_3682	3598216	3598467		
4647	BURPS1106A_3683	3599423	3600391		
4648	BURPS1106A_3684	3600561	3601463		
4649	BURPS1106A_3685	3601519	3601752		
4650	BURPS1106A_3686	3601781	3602713		
4651	BURPS1106A_3687	3603971	3605074		
4652	BURPS1106A_3688	3605210	3605329		
4653	BURPS1106A_3689	3605219	3606025		
4654	BURPS1106A_3690	3606153	3606452		
4655	BURPS1106A_3691	3606462	3606719		
4656	BURPS1106A_3692	3607235	3609004		
4657	BURPS1106A_3693	3609075	3609731		
4658	BURPS1106A_3694	3610808	3611203		
4659	BURPS1106A_3695	3613308	3614393		
4660	BURPS1106A_3696	3614512	3615657		
4661	BURPS1106A_3697	3615733	3617271		
4662	BURPS1106A_3698	3617281	3617493		
4663	BURPS1106A_3699	3618449	3618799		
4664	BURPS1106A_3700	3618922	3620025		
4665	BURPS1106A_3701	3620175	3621005		
4666	BURPS1106B_A2885			2838600	2839130
4667	BURPS1106B_A2886			2839717	2841030
4668	BURPS1106B_A2887			2842942	2841122
4669	BURPS1106B_A2888			2845429	2843558
4670	BURPS1106B_A2889			2849430	2845432
4671	BURPS1106B_A2890			2851052	2849427
4672	BURPS1106B_A2891			2854180	2851049
4673	BURPS1106B_A2892			2858581	2854196
4674	BURPS1106B_A2893			2858788	2858982
4675	BURPS1106B_A2894			2862404	2859210
4676	BURPS1106B_A2895			2863905	2862856
4677	BURPS1106B_A2896			2864208	2863957
4678	BURPS1106B_A2897			2865026	2866132
4679	BURPS1106B_A2898			2866122	2867204
4680	BURPS1106B_A2899			2867260	2867493
4681	BURPS1106B_A2900			2867411	2868454
4682	BURPS1106B_A2901			2870815	2869712
4683	BURPS1106B_A2902			2871070	2870951
4684	BURPS1106B_A2903			2870960	2871766
4685	BURPS1106B_A2904			2871894	2872193
4686	BURPS1106B_A2905			2872460	2872203
4687	BURPS1106B_A2906			2872976	2874745
4688	BURPS1106B_A2907			2874816	2875472
4689	BURPS1106B_A2908			2876668	2876549
4690	BURPS1106B_A2909			2880134	2879049
4691	BURPS1106B_A2910			2881398	2880253
4692	BURPS1106B_A2911			2883012	2881474

4693	BURPS1106B_A2912			2883234	2883022														
4694	BURPS1106B_A2913			2884540	2884190														
4695	BURPS1106B_A2914			2884663	2885766														
4696	BURPS1106B_A2915			2886746	2885916														
4697	BURPS1655_E0141																	3530656	3530793
4698	BURPS1655_E0144																	3527262	3526603
4699	BURPS1710A_4163					3992314	3991949												
4700	BURPS1710A_4167					3995119	3995643												
4701	BURPS1710A_4168					3996423	3997637												
4702	BURPS1710A_4169					3997801	3997682												
4703	BURPS1710A_4170					4000684	3997949												
4704	BURPS1710A_4171					4002387	4000684												
4705	BURPS1710A_4172					4004149	4002398												
4706	BURPS1710A_4173					4007037	4004149												
4707	BURPS1710A_4174					4007106	4007234												
4708	BURPS1710A_4175					4007386	4007270												
4709	BURPS1710A_4176					4008361	4007585												
4710	BURPS1710A_4177					4009095	4008508												
4711	BURPS1710A_4178					4009633	4009761												
4712	BURPS1710A_4179					4009988	4010215												
4713	BURPS1710A_4180					4010308	4011273												
4714	BURPS1710A_4181					4012172	4012008												
4715	BURPS1710A_4182					4012376	4012239												
4716	BURPS1710A_4183					4012377	4013300												
4717	BURPS1710A_4184					4014292	4014179												
4718	BURPS1710A_4185					4014645	4015451												
4719	BURPS1710A_4186					4016415	4016158												
4720	BURPS1710A_4187					4016088	4016204												
4721	BURPS1710A_4188					4016482	4016835												
4722	BURPS1710A_4189					4017065	4018906												
4723	BURPS1710A_4190					4019070	4019768												
4724	BURPS1710A_4191					4019974	4022790												
4725	BURPS1710A_4192					4023063	4023212												
4726	BURPS1710A_4193					4023197	4023601												
4727	BURPS1710A_4194					4023601	4023783												
4728	BURPS1710A_4195					4026183	4024438												
4729	BURPS1710A_4196					4027868	4026780												
4730	BURPS1710A_4197					4027947	4028063												
4731	BURPS1710A_4198					4028097	4028321												
4732	BURPS1710A_4199					4030120	4028564												
4733	BURPS1710A_4200					4030785	4030120												
4734	BURPS1710A_4201					4032857	4031460												
4735	BURPS1710A_4202					4034183	4032966												
4736	BURPS1710b_3654							3980318	3981532										
4737	BURPS1710b_3655							3981844	3984579										
4738	BURPS1710b_3656							3984579	3986282										
4739	BURPS1710b_3657							3986293	3988044										
4740	BURPS1710b_3658							3988044	3990926										
4741	BURPS1710b_3659							3994203	3995168										
4742	BURPS1710b_3660							3995903	3996067										
4743	BURPS1710b_3661							3998540	3999346										
4744	BURPS1710b_3662							4000822	4002801										
4745	BURPS1710b_3663							4002848	4003663										
4746	BURPS1710b_3664							4003887	4006685										
4747	BURPS1710b_3665							4008333	4010123										
4748	BURPS1710b_3666							4010675	4011916										
4749	BURPS1710b_3667							4012459	4014015										
4750	BURPS1710b_3668							4014015	4015226										

4751		BURPS1710b_3669								4016861	4018249										
4752		BURPSPAST_P0245														3653327	3652107				
4753		BURPSPAST_P0246														3648003	3646444				
4754		BURPSPAST_P0247														3645738	3645595				
4755		BURPSPAST_P0248														3643988	3645160				
4756		BURPSPAST_P0249														3641947	3643788				
4757		BURPSPAST_P0250														3641948	3641784				
4758		BURPSPAST_P0251														3640638	3641276				
4759		BURPSPAST_P0252														3641583	3641254				
4760		BURPSPAST_P0253														3640337	3640636				
4761		BURPSPAST_P0254														3639260	3640231				
4762		BURPSPAST_P0255														3638346	3639263				
4763		BURPSPAST_P0256														3637322	3638317				
4764		BURPSPAST_P0257														3636283	3637248				
4765		BURPSPAST_P0258														3635303	3633801				
4766		BURPSPAST_P0259														3633804	3632173				
4767		BURPSPAST_P0260														3632180	3631017				
4768		BURPSPAST_P0261														3631020	3628027				
4769		BURPSPAST_P0262														3628017	3625156				
4770		BURPSPAST_P0263														3625154	3623886				
4771	BPSL3113											3622799	3622464	3567110	3566775			3760513	3760178		
4772	BPSL3114		GI10									3623234	3624031	3567544	3568341			3760948	3761745		
4773	BPSL3115		GI10									3624041	3625108	3568351	3569418			3761755	3762822		
4774	BPSL3116		Bp_chr1_47_IS407A									3625327	3625655	3569636	3569964			3763040	3763368		
4775	BPSL3117		Bp_chr1_47_IS407A									3625655	3626562	3569964	3570871			3763368	3764275		
4776	BPSL3118		GI10									3629517	3628765	3573827	3571230			3767231	3764634		
4777	BPSL3119			3621855	3621301	2887596	2887042	4035009	4034455	4018904	4018350	3630314	3629760	3574609	3574070	3654172	3653618	3768028	3767474	3535169	3534609
4778	BPSL3120			3622500	3621892	2888241	2887633	4035654	4035046	4019549	4018941	3630959	3630351	3575270	3574662	3654817	3654209	3768673	3768065	3535814	3535206
4779	BPSL3121			3623351	3622596	2889092	2888337	4036505	4035750	4020400	4019645	3631810	3631055	3576121	3575366	3655668	3654913	3769524	3768769	3536665	3535910
4780	BPSL3122			3624756	3623377	2890497	2889118	4037910	4036531	4021805	4020426	3633215	3631836	3577526	3576147	3657073	3655694	3770929	3769550	3538070	3536691
4781	BPSL3123			3625380	3624760	2891121	2890501	4038534	4037914	4022429	4021809	3633839	3633219	3578150	3577530	3657697	3657077	3771553	3770933	3538694	3538074
4782	BPSL3124			3626344	3625601	2892085	2891342	4039498	4038755	4023393	4022650	3634803	3634060	3579114	3578371	3658661	3657918	3772517	3771774	3539658	3538915
4783	BPSL3125			3626366	3627571	2892107	2893312	4039520	4040725	4023415	4024620	3634825	3636030	3579136	3580341	3658683	3659888	3772539	3773744	3539680	3540885
4784		BURPSPAST_P0236														3660153	3659929				
4785	BPSL3126			3628697	3627918	2894438	2893659	4041855	4041076	4025750	4024971	3637198	3636419	3581471	3580692	3661004	3660225	3774873	3774094	3542015	3541236
4786	BPSL3127			3629263	3628739	2895004	2894480	4042421	4041897	4026316	4025792	3637764	3637240	3582037	3581513	3661570	3661046	3775439	3774915	3542578	3542057
4787	BPSL3128			3629523	3629293	2895264	2895034	4042681	4042451	4026576	4026346	3638024	3637794	3582297	3582067	3661830	3661600	3775699	3775469	3542838	3542608
4788	BPSL3129			3629977	3629615	2895718	2895356	4043135	4042773	4027030	4026668	3638478	3638116	3582751	3582389	3662284	3661922	3776153	3775791	3543292	3542930
4789	BPSL3130			3630395	3630009	2896136	2895750	4043553	4043167	4027448	4027062	3638896	3638510	3583169	3582783	3662702	3662316	3776571	3776185	3543710	3543324
4790	BPSL3131			3630816	3630451	2896557	2896192	4043974	4043609	4027869	4027504	3639317	3638952	3583590	3583225	3663123	3662758	3776992	3776627	3544131	3543766
4791	BPSL3132			3631229	3630816	2896970	2896557	4044387	4043974	4028282	4027869	3639730	3639317	3584003	3583590	3663536	3663123	3777405	3776992	3544544	3544131
4792	BPSL3133			3632014	3631229	2897755	2896970	4045172	4044387	4029067	4028282	3640515	3639730	3584788	3584003	3664321	3663536	3778190	3777405	3545329	3544544
4793	BPSL3134			3632837	3632085	2898578	2897826	4045995	4045243	4029890	4029138	3641338	3640586	3585611	3584859	3665144	3664392	3779013	3778261	3546152	3545400
4794	BPSL3135			3633646	3633008	2899387	2898749	4046804	4046166	4030699	4030061	3642147	3641509	3586420	3585782	3665953	3665315	3779822	3779184	3546961	3546323
4795	BPSL3136			3634263	3633646	2900004	2899387	4047421	4046804	4031316	4030699	3642764	3642147	3587037	3586420	3666570	3665953	3780439	3779822	3547578	3546961
4796	BPSL3137			3634906	3634322	2900647	2900063	4048064	4047480	4031959	4031375	3643407	3642823	3587680	3587096	3667213	3666629	3781082	3780498	3548221	3547637
4797	BPSL3138			3636040	3634973	2901781	2900714	4049198	4048131	4033093	4032026	3644541	3643474	3588814	3587747	3668347	3667280	3782216	3781149	3549355	3548288
4798	BPSL3139			3637533	3636199	2903274	2901940	4050691	4049357	4034586	4033252	3646034	3644700	3590307	3588973	3669840	3668506	3783709	3782375	3550848	3549514
4799	BPSL3140			3638231	3637578	2903972	2903319	4051389	4050736	4035284	4034631	3646732	3646079	3591005	3590352	3670538	3669885	3784407	3783754	3551546	3550893
4800	BPSL3141			3639577	3638231	2905318	2903972	4052735	4051389	4036630	4035284	3648078	3646732	3592351	3591005	3671884	3670538	3785753	3784407	3552892	3551546
4801	BPSL3142			3639830	3639594	2905571	2905335	4052988	4052752	4036883	4036647	3648331	3648095	3592604	3592368	3672137	3671901	3786006	3785770	3553145	3552909
4802	BPSL3143			3640671	3639919	2906412	2905660	4053829	4053077	4037724	4036972	3649172	3648420	3593445	3592693	3672978	3672226	3786847	3786095	3553986	3553234
4803	BPSL3144			3641609	3640671	2907350	2906412	4054767	4053829	4038662	4037724	3650110	3649172	3594383	3593445	3673916	3672978	3787785	3786847	3554924	3553986
4804	BPSL3145			3642159	3641893	2907900	2907634	4055317	4055051	4039212	4038946	3650660	3650394	3594933	3594667	3674466	3674200	3788335	3788069	3555474	3555208
4805	BPSL3146			3642788	3642159	2908529	2907900	4055946	4055317	4039841	4039212	3651289	3650660	3595562	3594933	3675095	3674466	3788964	3788335	3556103	3555474
4806	BPSL3147			3643913	3642906	2909654	2908647	4057071	4056064	4040966	4039959	3652414	3651407	3596687	3595680	3676220	3675213	3790089	3789082	3557228	3556221
4807	BPSL3148			3644470	3643913	2910211	2909654	4057634	4057071	4041529	4040966	3652971	3652414	3597253	3596687	3676783	3676220	3790646	3790089	3557800	3557228
4808	BPSL3149			3645250	3644486	2910991	2910227	4058414	4057650	4042309	4041545	3653751	3652987	3598033	3597269	3677563	3676799	3791426	3790662	3558580	3557816

4809	BPSL3150			3646065	3645250	2911806	2910991	4059229	4058414	4043124	4042309	3654566	3653751	3598848	3598033	3678378	3677563	3792241	3791426	3559395	3558580
4810	BPSL3151			3647268	3646168	2913009	2911909	4060432	4059332	4044327	4043227	3655769	3654669	3600051	3598951	3679581	3678481	3793444	3792344	3560598	3559498
4811	BPSL3152			3648080	3647268	2913821	2913009	4061244	4060432	4045139	4044327	3656581	3655769	3600863	3600051	3680393	3679581	3794256	3793444	3561410	3560598
4812	BPSL3153			3648330	3648136	2914071	2913877	4061494	4061300	4045389	4045195	3656831	3656637	3601113	3600919	3680643	3680449	3794506	3794312	3561660	3561466
4813	BPSL3154			3649506	3648382	2915247	2914123	4062670	4061546	4046565	4045441	3658007	3656883	3602289	3601165	3681819	3680695	3795682	3794558	3562836	3561712
4814	BPSL3155			3650061	3651878	2915802	2917619	4063225	4065042	4047120	4048937	3658562	3660379	3602844	3604661	3682374	3684191	3796237	3798054	3563391	3565208
4815	BPSL3156			3652633	3652082	2918374	2917823	4065829	4065278	4049724	4049173	3661145	3660594	3605440	3604889	3684963	3684412	3798832	3798281	3565963	3565412
4816	BPSL3157			3654147	3652639	2919888	2918380	4067343	4065835	4051238	4049730	3662659	3661151	3606954	3605446	3686477	3684969	3800346	3798838	3567477	3565969
4817	BPSL3158			3655947	3654430	2921688	2920171	4069143	4067626	4053038	4051521	3664459	3662942	3608755	3607238	3688278	3686761	3802146	3800629	3569278	3567761
4818	BPSL3159			3660690	3655990	2926431	2921731	4073886	4069186	4057781	4053081	3669202	3664502	3613498	3608798	3693021	3688321	3806889	3802189	3574021	3569321
4819	BPSL3160			3661695	3660985	2927436	2926726	4074891	4074181	4058786	4058076	3670206	3669496	3614502	3613792	3694026	3693316	3807893	3807183	3575025	3574315
4820	BPSL3161			3661888	3662616	2927629	2928357	4075084	4075812	4058979	4059707	3670399	3671127	3614695	3615423	3694219	3694947	3808086	3808814	3575218	3575946
4821	BPSL3162			3663526	3662768	2929267	2928509	4076722	4075964	4060617	4059859	3672037	3671279	3616333	3615575	3695857	3695099	3809724	3808966	3576856	3576098
4822	BPSL3163			3664653	3663574	2930394	2929315	4077849	4076770	4061744	4060665	3673164	3672085	3617460	3616381	3696984	3695905	3810851	3809772	3577982	3576903
4823	BPSL3164			3665524	3664682	2931265	2930423	4078720	4077878	4062615	4061773	3674035	3673193	3618331	3617489	3697855	3697013	3811722	3810880	3578853	3578011
4824	BPSL3165			3666405	3665524	2932146	2931265	4079601	4078720	4063496	4062615	3674916	3674035	3619212	3618331	3698736	3697855	3812603	3811722	3579734	3578853
4825	BPSL3166			3667861	3666542	2933602	2932283	4081057	4079738	4064952	4063633	3676372	3675053	3620668	3619349	3700192	3698873	3814059	3812740	3581190	3579871
4826	BPSL3167			3669178	3667949	2934919	2933690	4082374	4081145	4066269	4065040	3677689	3676460	3621985	3620756	3701509	3700280	3815376	3814147	3582507	3581278
4827	BPSL3168			3670306	3669230	2936047	2934971	4083502	4082426	4067397	4066321	3678817	3677741	3623113	3622037	3702637	3701561	3816504	3815428	3583635	3582559
4828	BPSL3169			3670857	3670306	2936598	2936047	4084053	4083502	4067948	4067397	3679368	3678817	3623664	3623113	3703188	3702637	3817055	3816504	3584186	3583635
4829	BPSL3170			3672284	3670941	2938025	2936682	4085471	4084137	4069366	4068032	3680780	3679452	3625082	3623748	3704606	3703272	3818473	3817139	3585604	3584270
4830	BPSL3171			3673630	3672674	2939371	2938415	4086817	4085861	4070712	4069756	3682126	3681170	3626428	3625472	3705952	3704996	3819819	3818863	3586950	3585994
4831	BPSL3172			3674307	3673630	2940048	2939371	4087494	4086817	4071389	4070712	3682803	3682126	3627105	3626428	3706629	3705952	3820496	3819819	3587627	3586950
4832	BPSL3173			3675251	3674307	2940992	2940048	4088438	4087494	4072333	4071389	3683747	3682803	3628049	3627105	3707369	3706629	3821440	3820496	3588571	3587627
4833	BPSL3174			3675565	3677883	2941306	2943624	4088751	4091069	4072646	4074964	3684060	3686378	3628362	3630680	3707388	3709706	3821753	3824071	3588884	3591202
4834			Bp_chr1_48_ISBma2																	3591253	3592825
4835	BPSL3175			3678328	3678005	2944069	2943746	4091514	4091191	4075409	4075086	3686823	3686500	3631125	3630802	3710151	3709828	3824516	3824193	3593248	3592925
4836	BPSL3175A			3678365	3678646	2944106	2944387	4091551	4091832	4075446	4075727	3686860	3687141	3631162	3631443	3710188	3710469	3824553	3824834	3593285	3593566
4837	BPSL3176			3678660	3679928	2944401	2945669	4091846	4093114	4075741	4077009	3687155	3688423	3631457	3632725	3710483	3711751	3824848	3826116	3593580	3594848
4838	BPSL3177			3680903	3680196	2946644	2945937	4094083	4093373	4077978	4077268	3689402	3688692	3633694	3632984	3712720	3712010	3827084	3826374	3595817	3595107
4839	BPSL3178			3681920	3680928	2947661	2946669	4095100	4094108	4078995	4078003	3690419	3689427	3634711	3633719	3713737	3712745	3828101	3827109	3596834	3595842
4840	BPSL3179			3683288	3682101	2949029	2947842	4096468	4095281	4080363	4079176	3691787	3690600	3636079	3634892	3715105	3713918	3829469	3828282	3598202	3597015
4841	BPSL3180			3685392	3683296	2951133	2949037	4098572	4096476	4082467	4080371	3693891	3691795	3638183	3636087	3717209	3715113	3831573	3829477	3600306	3598210
4842	BPSL3181			3686248	3685604	2951989	2951345	4099428	4098784	4083323	4082679	3694747	3694103	3639039	3638395	3718065	3717421	3832429	3831785	3601162	3600518
4843	BPSL3182			3686465	3687121	2952206	2952862	4099645	4100301	4083540	4084196	3694964	3695620	3639256	3639912	3718282	3718938	3832646	3833302	3601379	3602035
4844	BPSL3183			3687342	3688403	2953083	2954144	4100522	4101583	4084417	4085478	3695841	3696902	3640133	3641194	3719159	3720220	3833523	3834584	3602256	3603317
4845	BPSL3184			3690505	3688625	2956246	2954366	4103685	4101805	4087580	4085700	3699004	3697124	3643293	3641413	3722322	3720442	3836686	3834806	3605419	3603539
4846	BPSL3185			3690848	3690501	2956589	2956242	4104028	4103681	4087923	4087576	3699347	3699000	3643636	3643289	3722665	3722318	3837029	3836682	3605762	3605415
4847	BPSL3186			3691378	3690986	2957119	2956727	4104558	4104166	4088453	4088061	3699877	3699485	3644166	3643774	3723195	3722803	3837559	3837167	3606292	3605900
4848	BPSL3187			3692496	3691522	2958237	2957263	4105676	4104702	4089571	4088597	3700995	3700021	3645284	3644310	3724313	3723339	3838677	3837703	3607410	3606436
4849	BPSL3188			3693247	3692627	2958988	2958368	4106427	4105807	4090322	4089702	3701746	3701126	3646035	3645415	3725064	3724444	3839428	3838808	3608161	3607541
4850	BPSL3189			3693799	3693401	2959540	2959142	4106979	4106581	4090874	4090476	3702298	3701900	3646587	3646189	3725616	3725218	3839980	3839582	3608713	3608315
4851	BPSL3190			3694188	3693826	2959929	2959567	4107368	4107006	4091263	4090901	3702687	3702325	3646976	3646614	3726005	3725643	3840369	3840007	3609102	3608740
4852	BPSL3191			3694343	3694230	2960084	2959971	4107523	4107410	4091418	4091305	3702842	3702729	3647131	3647018	3726160	3726047	3840524	3840411	3609257	3609144
4853	BPSL3192			3694582	3694367	2960323	2960108	4107762	4107547	4091657	4091442	3703081	3702866	3647370	3647155	3726399	3726184	3840763	3840548	3609496	3609281
4854	BPSL3193			3695956	3694595	2961697	2960336	4109136	4107775	4093031	4091670	3704455	3703094	3648744	3647383	3727773	3726412	3842137	3840776	3610870	3609509
4855	BPSL3194			3696417	3695986	2962158	2961727	4109597	4109166	4093492	4093061	3704916	3704485	3649205	3648774	3728234	3727803	3842598	3842167	3611331	3610900
4856	BPSL3195			3696628	3696449	2962369	2962190	4109808	4109629	4093703	4093524	3705127	3704948	3649416	3649237	3728445	3728266	3842809	3842630	3611542	3611363
4857	BPSL3196			3697162	3696647	2962903	2962388	4110342	4109827	4094237	4093722	3705661	3705146	3649950	3649435	3728979	3728464	3843343	3842828	3612076	3611561
4858	BPSL3197			3697541	3697179	2963282	2962920	4110721	4110359	4094616	4094254	3706040	3705678	3650329	3649967	3729358	3728996	3843722	3843360	3612455	3612093
4859	BPSL3198			3698084	3697557	2963825	2963298	4111264	4110737	4095159	4094632	3706583	3706056	3650872	3650345	3729901	3729374	3844265	3843738	3612998	3612471
4860	BPSL3199			3698500	3698108	2964241	2963849	4111680	4111288	4095575	4095183	3706999	3706607	3651288	3650896	3730317	3729925	3844681	3844289	3613414	3613022
4861	BPSL3200			3698820	3698518	2964561	2964259	4112000	4111698	4095895	4095593	3707319	3707017	3651608	3651306	3730637	3730335	3845001	3844699	3613734	3613432
4862	BPSL3201			3699367	3698831	2965108	2964572	4112547	4112011	4096442	4095906	3707866	3707330	3652155	3651619	3731184	3730648	3845548	3845012	3614281	3613745
4863	BPSL3202			3699693	3699388	2965434	2965129	4112873	4112568	4096768	4096463	3708192	3707887	3652481	3652176	3731510	3731205	3845874	3845569	3614607	3614302
4864	BPSL3203			3700071	3699706	2965812	2965447	4113251	4112886	4097146	4096781	3708570	3708205	3652859	3652494	3731888	3731523	3846252	3845887	3614985	3614620
4865	BPSL3204			3700653	3700384	2966394	2966125	4113833	4113564	4097728	4097459	3709152	3708883	3653441	3653172	3732470	3732201	3846834	3846565	3615567	3615298
4866	BPSL3205			3700844	3700653	2966585	2966394	4114024	4113833	4097919	4097728	3709343	3709152	3653632	3653441	3732661	3732470	3847025	3846834	3615758	3615567

4867	BPSL3206			3701270	3700857	2967011	2966598	4114450	4114037	4098345	4097932	3709769	3709356	3654058	3653645	3733087	3732674	3847451	3847038	3616184	3615771
4868	BPSL3207			3702073	3701276	2967814	2967017	4115253	4114456	4099148	4098351	3710572	3709775	3654861	3654064	3733890	3733093	3848254	3847457	3616987	3616190
4869	BPSL3208			3702414	3702088	2968155	2967829	4115594	4115268	4099489	4099163	3710913	3710587	3655202	3654876	3734231	3733905	3848595	3848269	3617328	3617002
4870	BPSL3209			3702702	3702430	2968443	2968171	4115882	4115610	4099777	4099505	3711201	3710929	3655490	3655218	3734519	3734247	3848883	3848611	3617616	3617344
4871	BPSL3210			3703540	3702716	2969281	2968457	4116720	4115896	4100615	4099791	3712100	3712020	3656328	3655504	3735357	3734533	3849721	3848897	3618454	3617630
4872	BPSL3211			3703857	3703546	2969598	2969287	4117037	4116726	4100932	4100621	3712417	3712106	3656645	3656334	3735674	3735363	3850038	3849727	3618771	3618460
4873	BPSL3212			3704474	3703857	2970215	2969598	4117654	4117037	4101549	4100932	3713034	3712417	3657262	3656645	3736291	3735674	3850655	3850038	3619388	3618771
4874	BPSL3213			3705133	3704477	2970874	2970218	4118313	4117657	4102208	4101552	3713693	3713037	3657921	3657265	3736950	3736294	3851314	3850658	3620047	3619391
4875	BPSL3214			3705632	3705324	2971373	2971065	4118812	4118504	4102707	4102399	3714192	3713884	3658420	3658112	3737449	3737141	3851813	3851505	3620546	3620238
4876	BPSL3215			3728597	3727410	2972716	2971529	1262	75	4104050	4102863	3734139	3732952	3659763	3658576	3738859	3737672	3871939	3870752	3640793	3639606
4877	BPSL3216			3709119	3707020	2974860	2972761	3406	1307	4106194	4104095	3717098	3714999	3661907	3659808	3741003	3738904	3854985	3852886	3623755	3621656
4878	BPSL3217			3709711	3709244	2975452	2974985	3998	3531	4106786	4106319	3717690	3717223	3662499	3662032	3741595	3741128	3855577	3855110	3624347	3623880
4879	BPSL3218			3710262	3709885	2976003	2975626	4549	4172	4107337	4106960	3718241	3717864	3663050	3662673	3742146	3741769	3856128	3855751	3624898	3624521
4880	BPSL3219			3712412	3710481	2978153	2976222	6699	4768	4109487	4107556	3720391	3718460	3665200	3663269	3744296	3742365	3858278	3856347	3627048	3625117
4881			Bp_chr1_49_ISBr	3712462	3712752	2978203	2978493	6749	8053	4109537	4110841					3744346	3745650				
4882		BURPS1106A_3811		3712465	3712821																
4883		BURPS1106A_3813		3712705	3712995																
4884		BURPS1106A_3814		3712989	3713843																
4885		BURPS1106B_A3027				2978446	2978736														
4886		BURPS1106B_A3028				2978730	2979584														
4887			Bp_chr1_49-1_IS	3713869	3714978	2979610	2980719														
4888	BPSL3220			3719289	3715054	2985030	2980795	12364	8129	4115152	4110917	3724743	3720508	3669552	3665317	3749961	3745726	3862630	3858395	3631400	3627165
4889	BPSL3221			3723417	3719314	2989158	2985055	16492	12389	4119280	4115177	3728871	3724768	3673680	3669577	3754089	3749986	3866758	3862655	3635528	3631425
4890	BPSL3222			3724113	3723784	2989854	2989525	17188	16859	4119976	4119647	3729567	3729238	3674376	3674047	3754785	3754456	3867454	3867125	3636224	3635895
4891	BPSL3223			3724716	3724222	2990457	2989963	17791	17297	4120579	4120085	3730170	3729676	3674979	3674485	3755388	3754894	3868057	3867563	3636827	3636333
4892	BPSL3224			3725712	3725017	2991453	2990758	18787	18092	4121575	4120880	3731166	3730471	3675975	3675280	3756384	3755689	3869053	3868358	3637823	3637128
4893	BPSL3225			3726144	3725716	2991885	2991457	19220	19113	4122007	4121579	3731598	3731170	3676407	3675979	3756816	3756388	3869485	3869057	3638255	3637827
4894	BPSL3226			3726847	3726293	2992588	2992034	19923	19369	4122710	4122156	3732389	3731835	3677110	3676556	3757592	3757038	3870189	3869635	3639043	3638489
4895	BPSL3227			3727229	3726852	2992970	2992593	20305	19928	4123092	4122715	3732771	3732394	3677492	3677115	3757974	3757597	3870571	3870194	3639425	3639048
4896	BPSL3228			3728597	3727410	2994338	2993151	21673	20486	4124460	4123273	3734139	3732952	3678860	3677673	3759342	3758155	3871939	3870752	3640793	3639606
4897			Bp_chr1_50_IS407A					22518	23753	4125305	248					3760187	3761423				
4898	BPSL3229			3736873	3735599	3000733	2999459	28754	27480	7647	6373	3739758	3738484	3686097	3685870	3527461	3526187	3875172	3873901	3642157	3640886
4899	BPSL3230			3737470	3738465	3001330	3002325	29453	30448	8346	9341	3740433	3741428	3687893	3688888	3528160	3529155	3875921	3876916	3642776	3643771
4900	BPSL3231			3738531	3738812	3002391	3002672	30514	30795	9407	9688	3741494	3741775	3688954	3689235	3529221	3529502	3876982	3877263	3643837	3644118
4901	BPSL3232			3738826	3739626	3002686	3003486	30809	31609	9702	10502	3741789	3742589	3689249	3690049	3529516	3530316	3877277	3878077	3644132	3644932
4902	BPSL3233			3739665	3740282	3003525	3004142	31648	32265	10541	11158	3742628	3743245	3690088	3690705	3530355	3530972	3878116	3878733	3644971	3645588
4903	BPSL3234			3740287	3741372	3004147	3005232	32270	33355	11163	12248	3743250	3744335	3690710	3691795	3530977	3532062	3878738	3879823	3645593	3646678
4904	BPSL3235			3741497	3742324	3005357	3006184	33566	34393	12373	13200	3745039	3745866	3691920	3692747	3762337	3763164	3880550	3881377	3646847	3647494
4905	BPSL3236			3742354	3742950	3006214	3006810	34423	35019	13230	13826	3745896	3746492	3692777	3693373	3763194	3763790	3881407	3882003	3647524	3648120
4906	BPSL3237			3743180	3743839	3007040	3007699	35231	35908	14038	14715	3746722	3747381	3693580	3694263	3763997	3764680	3882210	3882893	3648327	3649010
4907	BPSL3238			3744475	3743963	3008335	3007823	36545	36033	15352	14840	3748017	3747505	3694900	3694388	3765317	3764805	3883530	3883018	3649647	3649135
4908	BPSL3239			3744627	3745721	3008487	3009581	36697	37791	15504	16598	3748169	3749263	3695052	3696146	3765469	3766563	3883682	3884776	3649799	3650893
4909	BPSL3240			3746154	3745963	3010014	3009823	38227	38033	17034	16840	3749696	3749505	3696582	3696388	3766999	3766805	3885212	3885018	3651329	3651135
4910	BPSL3241			3746474	3750052	3010334	3013912	38547	42125	17354	20932	3750016	3753594	3696902	3700480	3767319	3770897	3885532	3889110	3651649	3655227
4911		BURPS1106B_A3053				3013968	3014345														
4912		BURPS668_3787												3700536	3700934						
4913	BPSL3242			3752852	3750585	3016719	3014452	44960	42693	23774	21507	3756408	3754141	3703308	3701041	3773691	3771424	3891945	3889678	3658109	3655842
4914	BPSL3243			3753382	3754065	3017249	3017932	45489	46172	24303	24986	3756938	3757621	3703838	3704521	3774220	3774903	3892475	3893158	3658638	3659321
4915	BPSL3244			3754370	3754963	3018237	3018830	46477	47070	25291	25884	3757926	3758519	3704827	3705420	3775208	3775801	3893463	3894056	3659626	3660219
4916	BPSL3245			3755352	3755924	3019219	3019791	47460	48032	26274	26846	3758908	3759480	3705810	3706382	3776186	3776758	3894446	3895018	3660605	3661177
4917	BPSL3246			3756080	3757021	3019947	3020888	48188	49129	27002	27943	3759636	3760577	3706537	3707478	3776914	3777855	3895174	3896115	3661333	3662274
4918	BPSL3247			3757469	3758869	3021336	3022736	49577	50977	28391	29791	3761025	3762425	3707926	3709326	3778303	3779703	3896563	3897963	3662722	3664122
4919	BPSL3248			3758998	3760245	3022865	3024112	51107	52354	29921	31168	3762554	3763801	3709455	3710702	3779833	3781080	3898093	3899340	3664251	3665498
4920	BPSL3249			3760268	3761095	3024135	3024659	52377	52895	31191	32018	3763824	3764651	3710725	3711552	3781103	3781930	3899363	3900190	3665521	3666216
4921			Bp_chr1_51_ISBma1									3764711	3766015					3900248	3901028		
4922		BURPSS13_D0002																3901053	3901499		
4923		BURPSS13_D0001																3901808	3902369		
4924	BPSL3250			3762223	3761288	3025729	3024794	53955	53020	33146	32211	3766983	3766126	3712680	3711745	3783108	3782173	3903404	3902469	3667063	3666251

4925	BPSL3251			3762406	3763584	3025912	3027090	54138	55316	33329	34507	3767166	3768344	3712863	3714041	3783291	3784469	3903587	3904765	3667246	3668424
4926	BPSL3252			3764436	3763681	3027942	3027187	56168	55413	35359	34604	3769196	3768441	3714890	3714138	3785321	3784566	3905617	3904862	3669275	3668520
4927	BPSL3253			3765314	3764448	3028820	3027954	57046	56180	36237	35371	3770074	3769208	3715771	3714905	3786199	3785333	3906495	3905629	3670153	3669287
4928	BPSL3254			3765441	3766193	3028947	3029699	57173	57925	36364	37116	3770201	3770953	3715898	3716650	3786326	3787078	3906622	3907374	3670280	3671032
4929	BPSL3254A			3766377	3766601	3029883	3030107	58087	58311	37278	37502	3771115	3771339	3716801	3717025	3787240	3787464	3907536	3907760	3671194	3671418
4930	BPSL3254B			3767644	3768048	3031150	3031554	59354	59758	38545	38949	3772380	3772784	3718066	3718470	3788507	3788911	3908801	3909205	3672468	3672866
4931	BPSL3255			3769577	3769263	3033083	3032769	61287	60973	40478	40164	3774313	3773999	3719999	3719685	3790440	3790126	3910734	3910420	3674395	3674081
4932	BPSL3256			3770092	3771495	3033598	3035001	61802	63205	40993	42396	3774828	3776231	3720514	3721917	3790955	3792358	3911249	3912652	3674910	3676313
4933			Bp_chr1_52_IS40	3772960	3774195	3036466	3037701														
4934		BURPS1106A_3875		3771636	3771752																
4935		BURPS1106A_3876		3771946	3772233																
4936		BURPS1106A_3877		3773007	3773840																
4937		BURPS1106A_3878		3773864	3774127																
4938		BURPS1106A_3879		3774386	3776071																
4939		BURPS1106A_3880		3776738	3786217																
4940		BURPS1106A_3881		3787047	3787445																
4941		BURPS1106A_3882		3787780	3788070																
4942		BURPS1106A_3883		3788064	3788918																
4943		BURPS1106A_3884		3788943	3789419																
4944		BURPS1106A_3885		3790351	3790641																
4945		BURPS1106A_3886		3790635	3791489																
4946		BURPS1106A_3887		3791874	3793514																
4947		BURPS1106A_3888		3793511	3795406																
4948		BURPS1106A_3889		3795730	3796833																
4949		BURPS1106A_3890		3797130	3798020																
4950		BURPS1106A_3891		3798020	3801670																
4951		BURPS1106A_3892		3801915	3802847																
4952		BURPS1106A_3893		3802876	3803259																
4953		BURPS1106A_3894		3803256	3803636																
4954		BURPS1106A_3895		3804128	3805138																
4955		BURPS1106B_A3081				3035258	3035142														
4956		BURPS1106B_A3082				3035452	3035739														
4957		BURPS1106B_A3085				3039577	3037892														
4958		BURPS1106B_A3086				3049723	3040244														
4959		BURPS1106B_A3087				3050553	3050951														
4960		BURPS1106B_A3088				3051286	3051576														
4961		BURPS1106B_A3089				3051570	3052424														
4962		BURPS1106B_A3090				3052764	3054449														
4963		BURPS1106B_A3091				3054446	3056341														
4964		BURPS1106B_A3092				3056665	3057768														
4965		BURPS1106B_A3093				3058041	3058955														
4966		BURPS1106B_A3094				3058955	3062605														
4967		BURPS1106B_A3095				3063782	3062850														
4968		BURPS1106B_A3096				3064182	3063811														
4969		BURPS1106B_A3097				3064571	3064191														
4970		BURPS1106B_A3098				3066073	3065063														
4971	BPSL3257		GI11																		
4972	BPSL3258		GI11																		
4973	BPSL3259		GI11																		
4974	BPSL3260		GI11																		
4975	BPSL3261		GI11																		
4976	BPSL3262		GI11																		
4977	BPSL3263		GI11																		
4978	BPSL3264		GI11																		
4979	BPSL3265		GI11																		
4980	BPSL3266		GI11																		
4981	BPSL3267		GI11																		
4982	BPSL3268		GI11																		

4983	BPSL3269	GI11																		
4984	BPSL3270	GI11																		
4985	BPSL3271		3807042	3806698	3067977	3067633	63869	63525	43060	42716	3776895	3776551	3722589	3722245	3793022	3792678	3913316	3912972	3676977	3676633
4986	BPSL3272		3807857	3807060	3068792	3067995	64684	63887	43875	43078	3777710	3776913	3723404	3722607	3793837	3793040	3914131	3913334	3677792	3676995
4987	BPSL3273		3808454	3807879	3069389	3068814	65281	64706	44472	43897	3778307	3777732	3724001	3723426	3794434	3793859	3914728	3914153	3678389	3677814
4988	BPSL3274		3809011	3808580	3069946	3069515	65838	65407	45029	44598	3778864	3778433	3724558	3724127	3794991	3794560	3915285	3914854	3678946	3678515
4989	BPSL3275		3809058	3809942	3069993	3070877	65885	66769	45076	45960	3778911	3779795	3724605	3725489	3795038	3795922	3915332	3916216	3678993	3679877
4990		Bp_chr1_53_ISBma2									3779839	3781411								
4991	BPSL3276		3810675	3810043	3071610	3070978	67502	66870	46693	46061	3782120	3781488	3726222	3725590	3796655	3796023	3916949	3916317	3680610	3679978
4992	BPSL3277		3811276	3811917	3072211	3072852	68103	68744	47294	47935	3782721	3783362	3726823	3727464	3797256	3797897	3917550	3918191	3681211	3681852
4993	BPSL3278		3811917	3812570	3072852	3073505	68744	69397	47935	48588	3783362	3784015	3727464	3728117	3797897	3798550	3918191	3918844	3681852	3682505
4994	BPSL3279		3812631	3813119	3073566	3074054	69458	69946	48649	49137	3784076	3784564	3728178	3728666	3798611	3799099	3918905	3919393	3682566	3683054
4995	BPSL3280		3814491	3813304	3075426	3074239	71318	70131	50509	49322	3785936	3784749	3730038	3728851	3800471	3799284	3920765	3919578	3684426	3683239
4996	BPSL3281		3815024	3814734	3075959	3075669	71851	71561	51042	50752	3786470	3786180	3730571	3730281	3801004	3800714	3921298	3921008	3684960	3684670
4997	BPSL3282		3816215	3815412	3077150	3076347	73042	72239	52233	51430	3787661	3786858	3731762	3730959	3802195	3801392	3922489	3921686	3686151	3685348
4998	BPSL3283		3816492	3817607	3077427	3078542	73319	74434	52510	53625	3787938	3789053	3732039	3733154	3802472	3803587	3922766	3923881	3686428	3687543
4999	BPSL3284		3818941	3817802	3079877	3078738	75770	74631	54961	53822	3790472	3789333	3734488	3733349	3804922	3803783	3925217	3924078	3688879	3687740
5000	BPSL3285		3820497	3819400	3081433	3080336	77326	76229	56517	55420	3792028	3790931	3736045	3734948	3806478	3805381	3926773	3925676	3690435	3689338
5001	BPSL3286		3820824	3821513	3081760	3082449	77653	78342	56844	57533	3792355	3793044	3736372	3737061	3806805	3807494	3927100	3927789	3690762	3691451
5002	BPSL3287		3821611	3823098	3082547	3084034	78440	79927	57631	59118	3793143	3794630	3737160	3738647	3807592	3809079	3927888	3929375	3691550	3693037
5003	BPSL3288		3824115	3823288	3085051	3084224	80944	80117	60135	59308	3795647	3794820	3739664	3738837	3810096	3809269	3930392	3929565	3694066	3693239
5004	BPSL3289		3824486	3824136	3085422	3085072	81315	80965	60506	60156	3796018	3795668	3740035	3739685	3810467	3810117	3930763	3930413	3694437	3694087
5005	BPSL3290		3826071	3824653	3087007	3085589	82900	81482	62091	60673	3797603	3796185	3741620	3740202	3812052	3810634	3932348	3930930	3696022	3694604
5006	BPSL3291		3827156	3826428	3088092	3087364	83985	83257	63176	62448	3798688	3797960	3742705	3741977	3813137	3812409	3933433	3932705	3697107	3696379
5007	BPSL3292		3827993	3827184	3088929	3088120	84822	84013	64013	63204	3799525	3798716	3743542	3742733	3813974	3813165	3934270	3933461	3697944	3697135
5008	BPSL3293		3829737	3827989	3090673	3088925	86566	84818	65757	64009	3801269	3799521	3745286	3743538	3815718	3813970	3936014	3934266	3699688	3697940
5009	BPSL3294		3831836	3829737	3092772	3090673	88665	86566	67856	65757	3803368	3801269	3747385	3745286	3817817	3815718	3938113	3936014	3701787	3699688
5010	BPSL3295		3833050	3831836	3093986	3092772	89879	88665	69070	67856	3804582	3803368	3748599	3747385	3819031	3817817	3939327	3938113	3703001	3701787
5011	BPSL3296		3834246	3833749	3095182	3094685	91075	90578	70266	69769	3805778	3805281	3749795	3749298	3820227	3819730	3940523	3940026	3704182	3703685
5012	BPSL3297		3835737	3834436	3096673	3095372	92566	91265	71757	70456	3807269	3805968	3751288	3749987	3821718	3820417	3942014	3940713	3705673	3704372
5013	BPSL3298		3836263	3835763	3097199	3096699	93092	92592	72283	71783	3807795	3807295	3751814	3751314	3822244	3821744	3942540	3942040	3706199	3705699
5014	BPSL3299		3837189	3836464	3098125	3097400	94018	93293	73209	72484	3808721	3807996	3752740	3752015	3823170	3822445	3943466	3942741	3707125	3706400
5015	BPSL3300		3837619	3837194	3098555	3098130	94448	94023	73639	73214	3809151	3808726	3753170	3752745	3823600	3823175	3943896	3943471	3707555	3707130
5016	BPSL3301		3839038	3837938	3099974	3098874	95867	94767	75058	73958	3810570	3809470	3754589	3753489	3825019	3823919	3945315	3944215	3708974	3707874
5017	BPSL3302		3839739	3839038	3100675	3099974	96568	95867	75759	75058	3811271	3810570	3755290	3754589	3825720	3825019	3946016	3945315	3709675	3708974
5018	BPSL3303		3840683	3839739	3101696	3101301	97512	96568	76703	75759	3811774	3811271	3756234	3755290	3826664	3825720	3946960	3946016	3710443	3710258
5019	BPSL3304		3842708	3840690	3103721	3101703	99537	97519	78728	76710	3813697	3811880	3758253	3756241			3948985	3946967	3712468	3710450
5020	BPSL3305		3843276	3842752	3104289	3103765	100105	99581	79296	78772	3814265	3813741	3758821	3758297	3829383	3828859	3949553	3949029	3713036	3712512
5021	BPSL3306		3845241	3843310	3106254	3104323	102070	100139	81261	79330	3815624	3814299	3761140	3758855	3831348	3829417	3950819	3949587	3715013	3713070
5022	BPSL3307		3846016	3845639	3107029	3106652	102845	102468	82036	81659	3816789	3816412	3761552	3761175	3832123	3831746	3952202	3951825	3715788	3715411
5023	BPSL3308		3847069	3846050	3108082	3107063	103898	102879	83089	82070	3817842	3816823	3762605	3761586	3833176	3832157	3953255	3952236	3716841	3715822
5024	BPSL3309		3847946	3847089	3108959	3108102	104775	103918	83966	83109	3818719	3817862	3763482	3762625	3834053	3833196	3954132	3953275	3717718	3716861
5025	BPSL3310		3848691	3848143	3109704	3109156	105520	104972	84711	84163	3819464	3818916	3764227	3763679	3834800	3834252	3954877	3954329	3718465	3717917
5026	BPSL3311		3849105	3848788	3110118	3109801	105934	105617	85125	84808	3819878	3819561	3764641	3764324	3835214	3834897	3955291	3954974	3718879	3718562
5027	BPSL3312		3849699	3850760	3110712	3111773	106528	107589	85719	86780	3820471	3821532	3765234	3766295	3835808	3836869	3955884	3956945	3719472	3720533
5028	BPSL3313		3851163	3850870	3112176	3111883	107992	107699	87183	86890	3821935	3821642	3766698	3766405	3837272	3836979	3957348	3957055	3720936	3720643
5029	BPSL3314		3851469	3852170	3112482	3113183	108298	108999	87489	88190	3822241	3822942	3767004	3767705	3837578	3838279	3957654	3958355	3721242	3721943
5030	BPSL3315		3853204	3852365	3114217	3113378	110033	109194	89224	88385	3823976	3823137	3768739	3767900	3839313	3838474	3959389	3958550	3722979	3722140
5031	BPSL3316		3854193	3853306	3115206	3114319	111020	110133	90211	89324	3824965	3824078	3769726	3768839	3840300	3839413	3960376	3959489	3723966	3723079
5032	BPSL3317		3854344	3854949	3115357	3115962	111171	111776	90362	90967	3825116	3825721	3769877	3770482	3840451	3841056	3960527	3961132	3724117	3724722
5033	BPSL3318		3855551	3855342	3116564	3116355	112378	112169	91569	91360	3826323	3826114	3771084	3770875	3841658	3841449	3961734	3961525	3725324	3725115
5034	BPSL3319		3856109	3857272	3117122	3118285	112936	114099	92127	93290	3826881	3828044	3771642	3772805	3842216	3843379	3962292	3963455	3725882	3727045
5035	BPSL3320		3857425	3858942	3118438	3119955	114252	115769	93443	94960	3828197	3829714	3772958	3774475	3843532	3845049	3963608	3965125	3727198	3728715
5036	BPSL3321		3858959	3859270	3119972	3120283	115786	116097	94977	95288	3829731	3830042	3774492	3774803	3845066	3845377	3965142	3965453	3728732	3729043
5037	BPSL3322		3859496	3861823	3120509	3122836	116323	118650	95514	97841	3830268	3832595	3775029	3777356	3845603	3847927	3965679	3968006	3729269	3731596
5038	BPSL3323		3861823	3862968	3122836	3123981	118650	119795	97841	98986	3832595	3833740	3777356	3778501	3847927	3849072	3968006	3969151	3731596	3732741
5039	BPSL3324		3862968	3863999	3123981	3125012	119795	120826	98986	100017	3833740	3834771	3778501	3779532	3849072	3850103	3969151	3970182	3732741	3733619
5040	BPSL3325		3864014	3864232	3125027	3125245	120841	121059	100032	100250	3834786	3835004	3779547	3779765	3850118	3850336	3970197	3970415	3733788	3734006

5041	BPSL3326		3864238	3865299	3125251	3126312	121065	122126	100256	101317	3835010	3836071	3779771	3780832	3850342	3851403	3970421	3971482	3734012	3735073
5042	BPSL3327		3865299	3866060	3126312	3127073	122126	122887	101317	102078	3836071	3836832	3780832	3781593	3851403	3852164	3971482	3972243	3735073	3735834
5043	BPSL3328		3866063	3866692	3127076	3127705	122890	123519	102081	102710	3836835	3837464	3781596	3782225	3852167	3852796	3972246	3972875	3735837	3736466
5044	BPSL3329		3866697	3867758	3127710	3128771	123524	124585	102715	103776	3837469	3838530	3782230	3783291	3852801	3853862	3972880	3973941	3736471	3737532
5045	BPSL3330		3868578	3867844	3129591	3128857	125405	124671	104596	103862	3839350	3838616	3784111	3783377	3854682	3853948	3974761	3974027	3738352	3737618
5046	BPSL3331		3869301	3868906	3130314	3129919	126131	125733	105322	104924	3840073	3839678	3784834	3784439	3855408	3855010	3975484	3975089	3739078	3738680
5047	BPSL3332		3869861	3870481	3130874	3131494	126698	127318	105889	106509	3840633	3841253	3785394	3786014	3855975	3856595	3976053	3976673	3739644	3740264
5048	BPSL3333		3872518	3870788	3133531	3131801	129355	127625	108546	106816	3843290	3841560	3788051	3786321	3858632	3856902	3978710	3976980	3742301	3740571
5049		BURPS1655_F0088																	3742760	3742517
5050	BPSL3334		3873664	3872981	3134677	3133994	130514	129831	109705	109022	3844436	3843753	3789205	3788522	3859778	3859095	3979869	3979186	3743455	3742772
5051	BPSL3335		3874089	3873664	3135102	3134677	130939	130514	110130	109705	3844861	3844436	3789624	3789208	3860203	3859778	3980294	3979869	3743886	3743464
5052	BPSL3336		3874826	3874152	3135839	3135165	131676	131002	110867	110193	3845598	3844924	3790361	3789687	3860940	3860266	3981031	3980357	3744623	3743949
5053	BPSL3337		3875859	3876677	3136872	3137672	132713	133531	111904	112722	3846635	3847453	3791397	3792215	3861977	3862795	3982068	3982886	3745660	3746478
5054	BPSL3338		3878550	3876736	3139564	3137750	135404	133590	114595	112781	3849326	3847512	3794088	3792274	3864668	3862854	3984759	3982945	3748351	3746537
5055		BURPSS13_F0204															3986639	3986863		
5056	BPSL3339		3880480	3878822	3141494	3139836	137334	135676	116525	114867	3851309	3849651	3796018	3794360	3866598	3864940	3986689	3985031	3750281	3748623
5057	BPSL3340		3881370	3880672	3142384	3141686	138218	137520	117409	116711	3852199	3851501	3796908	3796210	3867487	3866789	3987574	3986876	3751165	3750467
5058	BPSL3341		3882068	3881829	3143082	3142843	138917	138678	118108	117869	3852898	3852659	3797608	3797369	3868183	3867947	3988273	3988034	3751933	3751619
5059		BURPS1106A_3981	3882213	3882461																
5060		BURPS1106A_3982	3882618	3883697																
5061		BURPS1106A_3983	3884286	3884402																
5062		BURPS1106A_3984	3884750	3885853																
5063		BURPS1106A_3985	3886560	3887168																
5064		BURPS1106A_3987	3888582	3888713																
5065		BURPS1106B_A3183			3143475	3143227														
5066		BURPS1106B_A3184			3144711	3143632														
5067		BURPS1106B_A3185			3145300	3145416														
5068		BURPS1106B_A3186			3145764	3146867														
5069		BURPS1106B_A3187			3148182	3147574														
5070		BURPS1106B_A3189			3149596	3149727														
5071		BURPS1655_F0102																	3754148	3754351
5072		BURPS1655_F0103																	3755232	3754960
5073		BURPS1655_F0104																	3755464	3755192
5074		BURPS1710A_0158					139310	139062												
5075		BURPS1710A_0159					140546	139467												
5076		BURPS1710A_0160					141259	140546												
5077		BURPS1710A_0161					142969	142052												
5078		BURPS1710A_0162					144409	143666												
5079		BURPS1710b_0115							118253	118501										
5080		BURPS1710b_0116							118658	119878										
5081		BURPS406E_A1033									3856641	3856033								
5082		BURPS406E_A1034									3854668	3853448								
5083		BURPS406E_A1035									3853291	3853043								
5084		BURPS668_3899											3797729	3798001						
5085		BURPS668_3900											3798158	3799237						
5086		BURPS668_3901											3799826	3799942						
5087		BURPS668_3902											3800744	3801694						
5088		BURPS668_3904											3802555	3802923						
5089		BURPS668_3905											3803199	3803399						
5090		BURPS668_3906											3803668	3804816						
5091		BURPSPAST_Y0099													3868578	3868330				
5092		BURPSPAST_Y0100													3869814	3868735				
5093		BURPSPAST_Y0101													3871171	3870524				
5094		BURPSPAST_Y0102													3872237	3871320				
5095		BURPSPAST_Y0104													3873342	3873473				
5096		BURPSPAST_Y0105													3873997	3874428				
5097		BURPSPAST_Y0106													3874533	3875672				
5098		BURPSPAST_Y0107													3877031	3878557				

5099		BURPSPAST_Y0108														3878976	3879500				
5100		BURPSPAST_Y0109														3879497	3880258				
5101		BURPSPAST_Y0110														3880271	3881488				
5102		BURPSPAST_Y0111														3884542	3881966				
5103		BURPSPAST_Y0112														3886123	3884552				
5104		BURPSPAST_Y0113														3887325	3886123				
5105		BURPSPAST_Y0114														3892685	3887322				
5106		BURPSPAST_Y0115														3894507	3894674				
5107		BURPSPAST_Y0116														3894946	3895128				
5108		BURPSPAST_Y0117														3895223	3896188				
5109		BURPSPAST_Y0118														3896262	3897257				
5110		BURPSPAST_Y0119														3897292	3898224				
5111		BURPSPAST_Y0120														3899572	3900378				
5112		BURPSPAST_Y0121														3900476	3900670				
5113		BURPSPAST_Y0122														3901341	3901084				
5114		BURPSPAST_Y0123														3901519	3901761				
5115		BURPSPAST_Y0124														3902223	3903833				
5116		BURPSPAST_Y0125														3903979	3904692				
5117		BURPSPAST_Y0126														3904912	3905376				
5118		BURPSPAST_Y0127														3907972	3909252				
5119		BURPSPAST_Y0128														3910663	3909365				
5120		BURPSPAST_Y0129														3910832	3910668				
5121		BURPSPAST_Y0130														3911077	3911442				
5122		BURPSPAST_Y0131														3912773	3911502				
5123		BURPSPAST_Y0132														3914464	3912854				
5124		BURPSPAST_Y0133														3914850	3914500				
5125		BURPSPAST_Y0134														3915427	3914861				
5126		BURPSPAST_Y0135														3916459	3915512				
5127		BURPSS13_F0198																3993765	3993022		
5128		BURPSS13_F0199																3992016	3991408		
5129		BURPSS13_F0200																3990043	3988823		
5130		BURPSS13_F0201																3988666	3988418		
5131	BPSL3342		GI12																		
5132	BPSL3343		GI12																		
5133	BPSL3344		GI12																		
5134	BPSL3345		GI12											3806606	3805833						
5135		BURPS668_3908												3807378	3808724						
5136		BURPS668_3909												3808721	3809647						
5137		BURPS668_3911												3809922	3811898						
5138		BURPS668_3912												3811906	3812217						
5139		BURPS668_3913												3812296	3812505						
5140	BPSL3346		GI12																		
5141	BPSL3347		GI12																		
5142	BPSL3348		GI12																		
5143	BPSL3349		GI12																		
5144	BPSL3350		GI12																		
5145	BPSL3351		GI12																		
5146	BPSL3352		GI12											3812890	3812603						
5147		BURPS668_3915												3813495	3813710						
5148	BPSL3353		GI12																		
5149	BPSL3354			3889944	3890840	3150958	3151854	145435	146331	124626	125522	3859417	3860313	3814542	3815438	3917043	3917939	3994792	3995688	3789682	3790578
5150		BURPS1106A_3989		3890827	3890943																
5151	BPSL3355			3893253	3891169	3154267	3152183	148741	146657	127932	125848	3862726	3860642	3817841	3815757	3920352	3918268	3998101	3996017	3792991	3790907
5152	BPSL3356			3893503	3894342	3154517	3155356	148991	149830	128182	129021	3862976	3863815	3818091	3818930	3920602	3921441	3998351	3999190	3793241	3794080
5153	BPSL3357			3894342	3895142	3155356	3156156	149830	150630	129021	129821	3863815	3864615	3818930	3819730	3921441	3922241	3999190	3999990	3794080	3794880
5154	BPSL3358			3895616	3896716	3156630	3157730	151104	152204	130295	131395	3865089	3866189	3820196	3821296	3922715	3923815	4000464	4001564	3795346	3796446
5155	BPSL3359			3896891	3897247	3157905	3158261	152379	152735	131570	131926	3866364	3866720	3821471	3821827	3923990	3924346	4001739	4002095	3796621	3796977
5156	BPSL3360			3898169	3899284	3159092	3160207	153608	154723	132806	133921	3867502	3868617	3822875	3823990	3925135	3926250	4002905	4004020	3797745	3798860

5157	BPSL3361		3899381	3899758	3160304	3160681	154820	155197	134018	134395	3868714	3869091	3824087	3824464	3926348	3926725	4004117	4004494	3798957	3799334
5158	BPSL3362		3900507	3903416	3161430	3164339	155750	158674	134948	137872	3869644	3872568	3825066	3827990	3927278	3930202	4005047	4007971	3799937	3802861
5159	BPSL3363		3903571	3904686	3164494	3165609	158829	159944	138027	139142	3872723	3873838	3828145	3829260	3930357	3931472	4008126	4009241	3803016	3804131
5160	BPSL3364		3904723	3906108	3165646	3167031	159981	161366	139179	140564	3873875	3875260	3829297	3830682	3931509	3932894	4009278	4010663	3804168	3805553
5161	BPSL3365		3907327	3906149	3168250	3167072	162585	161407	141783	140605	3876479	3875301	3831901	3830723	3934113	3932935	4011882	4010704	3806772	3805594
5162	BPSL3366		3907551	3909251	3168474	3170174	162813	164513	142011	143711	3876707	3878407	3832129	3833829	3934337	3936037	4012110	4013810	3807000	3808700
5163	BPSL3367		3909283	3910347	3170206	3171270	164545	165609	143743	144807	3878439	3879503	3833861	3834925	3936069	3937133	4013842	4014906	3808732	3809796
5164	BPSL3368		3911548	3910472	3172471	3171395	166810	165734	146008	144932	3880704	3879628	3836126	3835050	3938334	3937258	4016107	4015031	3810997	3809921
5165	BPSL3369		3911793	3913310	3172716	3174233	167055	168572	146253	147770	3880949	3882466	3836371	3837888	3938579	3940096	4016352	4017869	3811242	3812759
5166		BURPS406E_A1010									3882809	3884071								
5167	BPSL3370		3913823	3913581	3174746	3174504	169085	168843	148283	148041	3884434	3884192	3838403	3838161	3940609	3940367	4018381	4018139	3813257	3813015
5168	BPSL3371		3914692	3913892	3175615	3174815	169954	169154	149152	148352	3885303	3884503	3839272	3838472	3941478	3940678	4019250	4018450	3814126	3813326
5169	BPSL3372		3916086	3914692	3177009	3175615	171348	169954	150546	149152	3886697	3885303	3840666	3839272	3942872	3941478	4020644	4019250	3815520	3814126
5170	BPSL3373		3917552	3916146	3178475	3177069	172814	171408	152012	150606	3888163	3886757	3842132	3840726	3944338	3942932	4022110	4020704	3816986	3815580
5171	BPSL3374		3918170	3919345	3179093	3180268	173432	174607	152630	153805	3888781	3889956	3842749	3843924	3944966	3946141	4022728	4023903	3817604	3818779
5172	BPSL3375		3919388	3919567	3180311	3180490	174650	174829	153848	154027	3889999	3890178	3843967	3844143	3946227	3946364	4023946	4024125	3818822	3819001
5173	BPSL3376		3921232	3919724	3182155	3180647	176494	174986	155692	154184	3891843	3890335	3845811	3844303	3948029	3946521	4025790	4024282	3820666	3819158
5174	BPSL3378		3925045	3922550	3185968	3183473	180268	177791	159466	156989	3895642	3893147	3849583	3847100	3951855	3949354	4029619	4027136	3824406	3821911
5175	BPSL3379		3925128	3925556	3186051	3186479	180351	180779	159549	159977	3895725	3896153	3849666	3850094	3951938	3952366	4029702	4030130	3824489	3824917
5176	BPSL3380		3925659	3926297	3186582	3187220	180882	181520	160080	160718	3896256	3896894	3850197	3850835	3952469	3953107	4030233	4030871	3825020	3825658
5177	BPSL3381		3927112	3926360	3188035	3187283	182335	181583	161533	160781	3897709	3896957	3851650	3850898	3953922	3953170	4031686	4030934	3826473	3825721
5178	BPSL3383		3928102	3927311	3189025	3188234	183325	182534	162523	161732	3898699	3897908	3852640	3851849	3954912	3954121	4032676	4031885	3827463	3826672
5179	BPSL3384		3929346	3928159	3190269	3189082	184569	183382	163767	162580	3899943	3898756	3853884	3852697	3956156	3954969	4033920	4032733	3828707	3827520
5180	BPSL3385		3930836	3930105	3191759	3191028	185961	185215	165159	164413	3901441	3900710	3855324	3854578	3957664	3956918	4035700	4034954	3830141	3829410
5181	BPSL3386		3930835	3932472	3191758	3193395	185960	187597	165158	166795	3901440	3903077	3855323	3856960	3957663	3959300	4035699	4037336	3830140	3831777
5182	BPSL3387		3932592	3934136	3193515	3195059	187716	189260	166914	168458	3903196	3904740	3857079	3858623	3959421	3960965	4037455	4038999	3831897	3833441
5183	BPSL3388		3935518	3934382	3196441	3195305	190642	189506	169840	168704	3906122	3904986	3860005	3858869	3962355	3961219	4040373	4039237	3834823	3833687
5184	BPSL3389		3939532	3935606	3200455	3196529	194656	190730	173854	169928	3910136	3906210	3864019	3860093	3966369	3962443	4044387	4040461	3838837	3834911
5185	BPSL3390		3942264	3940000	3203187	3200923	197388	195124	176586	174322	3912868	3910604	3866751	3864487	3969101	3966837	4047119	4044855	3841569	3839305
5186	BPSL3391		3943799	3942708	3204722	3203631	198923	197832	178121	177030	3914403	3913312	3868286	3867195	3970636	3969545	4048654	4047563	3843104	3842013
5187	BPSL3392		3943939	3944721	3204862	3205644	199063	199845	178261	179043	3914543	3915325	3868426	3869208	3970776	3971558	4048794	4049576	3843244	3844026
5188	BPSL3393		3945238	3944927	3206161	3205850	200362	200051	179560	179249	3915842	3915531	3869725	3869414	3972075	3971764	4050093	4049782	3844543	3844232
5189	BPSL3394		3947555	3945828	3208478	3206751	202679	200952	181877	180150	3918159	3916432	3872042	3870315	3974392	3972665	4052410	4050683	3846860	3845133
5190	BPSL3395		3948213	3947791	3209136	3208714	203337	202915	182535	182113	3918791	3918369	3872700	3872278	3975050	3974628	4053068	4052646	3847518	3847096
5191	BPSL3396		3949943	3948552	3210866	3209475	205067	203676	184265	182874	3920615	3919224	3874430	3873039	3976967	3975576	4054892	4053501	3849341	3847953
5192	BPSL3397		3950898	3950026	3211821	3210949	206022	205150	185220	184348	3921570	3920698	3875385	3874513	3977922	3977050	4055664	4054975	3850296	3849424
5193	BPSL3398		3952511	3950973	3213435	3211897	207635	206097	186833	185295	3923182	3921644	3877000	3875462	3979535	3977997	4057690	4056152	3851909	3850371
5194	BPSL3399		3953097	3952561	3214021	3213485	208221	207685	187419	186883	3923768	3923232	3877586	3877050	3980121	3979585	4058276	4057740	3852495	3851959
5195	BPSL3400		3953540	3953103	3214464	3214027	208664	208227	187862	187425	3924211	3923774	3878029	3877592	3980564	3980127	4058719	4058282	3852938	3852501
5196	BPSL3401		3953973	3953701	3214897	3214625	209097	208825	188295	188023	3924644	3924372	3878462	3878190	3980997	3980725	4059152	4058880	3853371	3853099
5197	BPSL3402		3954913	3954047	3215837	3214971	210037	209171	189235	188369	3925584	3924718	3879402	3878536	3981937	3981071	4060092	4059226	3854311	3853445
5198	BPSL3403		3955538	3955011	3216462	3215935	210662	210135	189860	189333	3926209	3925682	3880027	3879500	3982562	3982035	4060717	4060190	3854936	3854409
5199	BPSL3404		3956960	3955812	3217884	3216736	212084	210936	191282	190134	3927631	3926483	3881449	3880301	3983984	3982836	4062139	4060991	3856358	3855210
5200	BPSL3405		3957837	3956953	3218761	3217877	212961	212077	192159	191275	3928508	3927624	3882326	3881442	3984861	3983977	4063016	4062132	3857235	3856351
5201	BPSL3406		3958667	3957876	3219591	3218800	213791	213000	192989	192198	3929338	3928547	3883156	3882365	3985691	3984900	4063846	4063055	3858065	3857274
5202	BPSL3407		3959388	3958699	3220312	3219623	214512	213823	193710	193021	3930059	3929370	3883877	3883188	3986413	3985724	4064567	4063878	3858786	3858097
5203	BPSL3408		3961364	3959394	3222288	3220318	216488	214518	195686	193716	3932035	3930065	3885853	3883883	3988389	3986419	4066543	4064573	3860762	3858792
5204	BPSL3409		3962281	3961529	3223205	3222453	217405	216653	196603	195851	3932952	3932200	3886770	3886018	3989306	3988554	4067460	4066708	3861679	3860927
5205	BPSL3410		3964068	3962287	3224992	3223211	219192	217411	198390	196609	3934739	3932958	3888557	3886776	3991093	3989312	4069247	4067466	3863466	3861685
5206	BPSL3411		3965128	3964076	3226052	3225000	220252	219200			3935799	3934747	3889617	3888565	3992153	3991101	4070307	4069255	3864526	3863474
5207	BPSL3412		3966598	3965444	3227522	3226368	221722	220568	200920	199766	3937269	3936115	3891087	3889933	3993623	3992469	4071777	4070623	3865996	3864842
5208	BPSL3413		3968154	3966922	3229078	3227846	223278	222049	202476	201247	3938825	3937593	3892643	3891411	3995179	3993947	4073333	4072101	3867563	3866484
5209	BPSL3414		3969541	3968567	3230465	3229491	224665	223691	203863	202889	3940212	3939238	3894030	3893056	3996566	3995592	4074720	4073746	3868950	3867976
5210	BPSL3415		3970422	3969541	3231346	3230465	225546	224665	204744	203863	3941093	3940212	3894911	3894030	3997447	3996566	4075601	4074720	3869831	3868950
5211	BPSL3416		3971758	3970565	3232682	3231489	226882	225689	206080	204887	3942429	3941236	3896247	3895054	3998783	3997590	4076937	4075744	3871167	3869974
5212	BPSL3417		3972581	3971829	3233505	3232753	227705	226953	206903	206151	3943252	3942500	3897070	3896318	3999606	3998854	4077760	4077008	3871990	3871238
5213	BPSL3418		3973357	3972581	3234281	3233505	228481	227705	207679	206903	3944028	3943252	3897846	3897070	4000382	3999606	4078536	4077760	3872766	3871990
5214	BPSL3419		3975542	3973860	3236466	3234784	230666	228984	209864	208182	3946213	3944531	3900031	3898349	4002566	4000884	4080721	4079039	3874951	3873269

5215 BPSL3420		3977243	3975720	3238167	3236644	232367	230844	211565	210042	3947914	3946391	3901732	3900209	4004270	4002747	4082422	4080899	3876652	3875129
5216 BPSL3421		3977384	3978280	3238308	3239204	232508	233404	211706	212602	3948055	3948951	3901873	3902769	4004411	4005307	4082563	4083459	3876793	3877689
5217	BURPSPAST_Y0221													4005320	4005643				
5218 BPSL3422		3978675	3979202	3239599	3240126	233798	234325	212996	213523	3949345	3949872	3903163	3903690	4005701	4006228	4083854	4084381	3878083	3878610
5219 BPSL3423		3979857	3979309	3240781	3240233	234980	234432	214178	213630	3950527	3949979	3904345	3903797	4006883	4006335	4085036	4084488	3879265	3878717
5220 BPSL3424		3979941	3980831	3240865	3241755	235064	235954	214262	215152	3950611	3951501	3904429	3905319	4006967	4007857	4085120	4086010	3879349	3880239
5221 BPSL3425		3980918	3981220	3241842	3242144	236041	236343	215239	215541	3951588	3951890	3905406	3905708	4007944	4008246	4086097	4086399	3880326	3880628
5222 BPSL3426		3981658	3982347	3242582	3243271	236781	237470	215979	216668	3952328	3953017	3906146	3906835	4008684	4009373	4086837	4087526	3881066	3881755
5223 BPSL3427		3982457	3983938	3243381	3244862	237580	239061	216778	218259	3953127	3954608	3906945	3908426	4009483	4010964	4087636	4089117	3881865	3883346
5224 BPSL3428		3984902	3984393	3245826	3245317	240018	239509	219216	218707	3955551	3955042	3909383	3908874	4011921	4011412	4090060	4089551	3884303	3883794
5225 BPSL3429		3986324	3985215	3247248	3246139	241440	240331	220638	219529	3956973	3955864	3910805	3909696	4013343	4012234	4091482	4090373	3885725	3884616
5226 BPSL3430		3987261	3986554	3248185	3247478	242388	241681	221586	220879	3957910	3957203	3911753	3911046	4014291	4013584	4092419	4091712	3886673	3885966
5227 BPSL3431		3987942	3987304	3248866	3248228	243069	242431	222267	221629	3958591	3957953	3912434	3911796	4014972	4014334	4093100	4092462	3887354	3886716
**Chromosome 2**																			
1 BPSS0001		1	1185	1962563	1961379	2325201	2326385	1839078	1840262	9969	11153	1	1185	11661	12845	8469	9653	8075	9259
2 BPSS0002		2325	1285	1960239	1961279	2327525	2326485	1841402	1840362	12293	11253	2325	1285	13985	12945	10793	9753	10399	9359
3 BPSS0003		2919	2503	1959645	1960061	2328119	2327703	1841996	1841580	12887	12471	2919	2503	14579	14163	11387	10971	10993	10577
4 BPSS0004		3556	2951	1959008	1959613	2328756	2328151	1842633	1842028	13524	12919	3556	2951	15216	14611	12024	11419	11630	11025
5 BPSS0005		3734	4930	1958830	1957634	2328924	2330120	1842801	1843997	13692	14888	3724	4920	15384	16580	12202	13398	11798	12994
6 BPSS0006		4946	5974	1957618	1956590	2330136	2331164	1844013	1845041	14904	15932	4936	5964	16596	17624	13414	14442	13010	14038
7 BPSS0007		6972	6322	1955592	1956242	2332155	2331505	1846032	1845382	16958	16308	7053	6403	18615	17965	15440	14790	15036	14386
8 BPSS0008		7062	7676	1955502	1954888	2332245	2332859	1846122	1846736	17048	17662	7143	7757	18705	19319	15530	16144	15126	15740
9 BPSS0009		7780	8256	1954784	1954308	2332963	2333439	1846840	1847316	17766	18242	7861	8337	19423	19899	16248	16724	15844	16320
10	BURPS1106A_A0011	8229	8480																
11	BURPS1655_B0099																	16544	16293
12	BURPSS13_B0075															16948	16697		
13 BPSS0010		9497	8484	1953067	1954080	2334680	2333667	1848557	1847544	19483	18470	9578	8565	21140	20127	17965	16952	17561	16548
14	BURPS1106B_1980			1954084	1954335														
15 BPSS0011		10885	9803	1951679	1952761	2336068	2334986	1849945	1848863	20871	19789	10948	9866	22528	21446	19353	18271	18949	17867
16 BPSS0012		11054	11968	1951510	1950596	2336237	2337151	1850114	1851028	21040	21954	11117	12031	22697	23611	19522	20436	19118	20032
17 BPSS0013		12520	13194	1950044	1949370	2337703	2338377	1851580	1852254	22478	23152	12562	13236	24170	24844	20946	21620	20521	21195
18	BURPS1710A_A2500					2338417	2338539												
19 BPSS0014		13422	14042	1949142	1948522	2338586	2339206	1852463	1853083	23390	24010	13424	14044	25082	25702	21858	22478	21403	22023
20 BPSS0015		14944	14483	1947620	1948081	2340108	2339647	1853985	1853524	24912	24451	14937	14476	26604	26143	23380	22919	22925	22464
21 BPSS0016		16196	15027	1946368	1947537	2341360	2340191	1855237	1854068	26164	24995	16189	15020	27856	26687	24647	23478	24177	23008
22 BPSS0017		17044	16262	1945520	1946302	2342208	2341426	1856085	1855303	27012	26230	17037	16255	28704	27922	25495	24713	25025	24243
23 BPSS0018		17831	17094	1944733	1945470	2342995	2342258	1856872	1856135	27799	27062	17824	17087	29491	28754	26282	25545	25812	25075
24 BPSS0019		18290	18883	1944274	1943681	2343454	2344047	1857331	1857924	28258	28851	18283	18876	29950	30543	26741	27334	26271	26864
25 BPSS0020		19202	18912	1943362	1943652	2344366	2344076	1858243	1857953	29170	28880	19195	18905	30862	30572	27653	27363	27183	26893
26 BPSS0021		20732	19908	1941832	1942656	2345902	2345078	1859779	1858955	30706	29882	20731	19907	32398	31574	29183	28359	28719	27895
27 BPSS0022		21476	20775	1941088	1941789	2346646	2345945	1860523	1859822	31450	30749	21475	20774	33142	32441	29948	29226	29466	28762
28	BURPS668_A0031											21983	22258						
29	BURPS668_A0032											22331	23008						
30	BURPS668_A0033											23433	24437						
31	Bp_chr2_1_IS407A											26971	28206						
32	BURPS668_A0037											28300	28458						
33	BURPS668_A0038											28825	30609						
34	BURPS668_A0039											30596	32395						
35	BURPS668_A0040											32701	32865						
36	BURPS668_A0041											33011	34027						
37	BURPS668_A0042											34039	34188						
38 BPSS0023		21879	23282	1940685	1939282	2347049	2348452	1860926	1862329	31853	33256	35648	37051	33545	34948	30351	31754	29874	31277
39 BPSS0024		23435	23632	1939129	1938932	2348605	2348802	1862482	1862679	33409	33606	37204	37401	35101	35298	31907	32104	31430	31627
40 BPSS0025		23809	24657	1938755	1937907	2348979	2349827	1862856	1863704	33783	34631	37578	38426	35475	36323	32281	33129	31804	32652
41 BPSS0026		24657	25487	1937907	1937077	2349827	2350645	1863704	1864522	34631	35461	38426	39256	36323	37153	33129	33959	32652	33482
42 BPSS0027		25487	27427	1937077	1935137	2350645	2352585	1864522	1866462	35461	37401	39256	41196	37153	39093	33959	35899	33482	35422
43 BPSS0028		27444	28430	1935120	1934134	2352602	2353588	1866479	1867465	37418	38404	41213	42199	39110	40096	35916	36902	35439	36425

44 BPSS0029		29225	28608	1933339	1933956	2354383	2353766	1868260	1867643	39199	38582	42994	42377	40891	40274	37697	37080	37220	36603
45 BPSS0030		29333	30223	1933231	1932341	2354491	2355381	1868368	1869258	39307	40197	43102	43992	40999	41889	37805	38695	37328	38218
46 BPSS0031		31048	30296	1931516	1932268	2356214	2355462	1870091	1869339	41040	40288	44842	44090	42732	41980	39518	38766	39032	38280
47 BPSS0032		31665	31201	1930899	1931363	2356831	2356367	1870708	1870244	41657	41193	45459	44995	43349	42885	40135	39671	39649	39185
48 BPSS0033		32053	32316	1930511	1930248	2357215	2357478	1871092	1871355	42045	42308	45847	46110	43733	43996	40523	40786	40037	40300
49 BPSS0034		33094	34671	1929470	1927893	2358256	2359833	1872133	1873710	43087	44664	46891	48468	44775	46352	41574	43151	41079	42656
50 BPSS0035		34671	35618	1927893	1926946	2359833	2360780	1873710	1874657	44664	45611	48468	49415	46352	47299	43151	44098	42656	43603
51	BURPS1710b_A1545							1875153	1875353										
52	BURPS668_A0059											49475	50236						
53	BURPSPAST_X0058													47999	47787				
54 BPSS0036		36806	36393	1925758	1926171	2361932	2361519	1875809	1875396	46743	46330	50704	50291	48455	48042	45227	44814	44780	44367
55 BPSS0037		37061	37306	1925503	1925258	2362187	2362432	1876064	1876309	46999	47244	50960	51205	48710	48955	45483	45683	45036	45281
56 BPSS0038		37433	38353	1925131	1924211	2362559	2363479	1876436	1877356	47370	48290	51332	52252	49082	50002	45788	46453	45408	46328
57	BURPSS13_B0037															46477	46788		
58 BPSS0039		39391	38591	1923153	1923953	2364607	2363807	1878449	1877649	49320	48520	53359	52559	51067	50267	47546	46746	47365	46565
59 BPSS0040		39946	39395	1922598	1923149	2365162	2364611	1879004	1878453	49875	49324	53914	53363	51622	51071	48101	47550	47920	47369
60 BPSS0041		41184	40261	1921360	1922283	2366400	2365477	1880242	1879319	51113	50190	55152	54229	52860	51937	49339	48416	49158	48235
61 BPSS0042		41298	42518	1921246	1920026	2366514	2367734	1880356	1881576	51227	52447	55266	56486	52974	54194	49453	50673	49272	50492
62 BPSS0043		42729	43439	1919815	1919105	2367945	2368655	1881787	1882497	52658	53368	56697	57407	54405	55115	50884	51594	50703	51413
63 BPSS0044		43461	44114	1919083	1918430	2368677	2369330	1882519	1883172	53390	54043	57429	58082	55137	55790	51616	52269	51435	52088
64 BPSS0045		44214	45608	1918330	1916936	2369430	2370824	1883272	1884666	54143	55537	58182	59576	55890	57284	52369	53763	52188	53582
65 BPSS0046		45624	46406	1916920	1916138	2370840	2371622	1884682	1885464	55553	56335	59592	60374	57300	58082	53779	54561	53598	54380
66 BPSS0047		46425	46808	1916119	1915736	2371641	2372024	1885483	1885866	56354	56737	60393	60776	58101	58484	54580	54963	54399	54782
67 BPSS0048		46990	48342	1915554	1914202	2372206	2373558	1886048	1887400	56908	58260	60947	62299	58666	60018	55145	56497	54964	56316
68 BPSS0049		48661	48416	1913883	1914128	2373877	2373632	1887719	1887474	58579	58334	62618	62373	60337	60092	56816	56571	56635	56390
69	BURPS1710b_A1559							1887820	1889742										
70 BPSS0050		49379	48765	1913165	1913779	2374595	2373981	1888437	1887823	59297	58683	63336	62722	61055	60441	57534	56920	57353	56739
71	BURPS1106A_A0063	50726	51649																
72	BURPS1655_B0039																	59624	58701
73	BURPS1655_B0040																	58643	57606
74	BURPS1710A_A2547					2375884	2374847												
75	BURPS1710A_A2548					2376865	2375942												
76	BURPS1710b_A1560							1889784	1890707										
77	BURPS406E_Q0081									60587	59550								
78	BURPS406E_Q0082									61568	60645								
79	BURPS668_A0075											64096	64212						
80	BURPS668_A0076											64368	65393						
81	BURPS668_A0078											66266	66745						
82	BURPS668_A0079											66772	67809						
83	BURPS668_A0080											67867	68790						
84	BURPSPAST_X0040													63325	62402				
85	BURPSPAST_X0041													62344	61307				
86	BURPSS13_B0019															64549	63626		
87	BURPSS13_B0020															63568	62531		
88	BURPSS13_B0022															62492	62280		
89	BURPSS13_B0023															61940	61821		
90	BURPSS13_B0024															61435	58817		
91	BURPSS13_B0025															58820	58023		
92 BPSS0051		53021	51768	1909523	1910776	2378237	2376984	1892079	1890826	62940	61687	70162	68909	64697	63444	65921	64668	60996	59743
93	BURPS1106B_1928			1910895	1911818														
94 BPSS0052		53436	53011	1909108	1909533	2378652	2378227	1892494	1892069	63355	62930	70577	70152	65112	64687	66336	65911	61411	60986
95 BPSS0053		55028	53841	1907516	1908703	2380244	2379057	1894086	1892899	64947	63760	72169	70982	66704	65517	67928	66741	63003	61816
96 BPSS0054		56020	55028	1906524	1907516	2381236	2380244	1895078	1894086	65939	64947	73161	72169	67696	66704	68920	67928	63995	63003
97 BPSS0055		56938	57483	1905606	1905061	2382202	2382747	1896044	1896589	66881	67426	74103	74648	68650	69195	69838	70383	64973	65518
98 BPSS0056		57539	57835	1905005	1904709	2382803	2383102	1896645	1896944	67482	67781	74704	75003	69251	69550	70439	70732	65574	65873
99 BPSS0057		57874	58539	1904670	1904005	2383141	2383806	1896983	1897648	67820	68485	75042	75707	69589	70254	70774	71439	65912	66577
100 BPSS0058		59019	64928	1903525	1897616	2384286	2390198	1898128	1904040	68965	74877	76187	82099	70736	76648	71919	77828	67057	72969
101	BURPSS13_B0007															77927	78070		

102	BPSS0059		66154	65897	1896390	1896647	2391495	2391238	1905336	1905079	76173	75916	83395	83138	77953	77696	79045	78788	74267	74010
103		BURPS1655_B0003																	89107	88664
104		BURPS1655_B0004																	88634	88290
105		BURPS1655_B0005																	79953	88280
106		BURPS1655_B0006																	79936	79676
107		BURPS1655_B0007																	79548	79432
108		BURPS1655_B0008																	79338	79183
109		BURPS1655_B0009																	79178	78720
110		BURPS1655_B0010																	78638	78522
111		BURPS1655_B0011																	78525	78358
112		BURPS1655_B0012																	77523	77636
113		BURPS1655_B0013																	77379	77251
114		BURPS1655_B0014																	77278	76895
115		BURPS1655_B0015																	76914	76702
116		BURPS1655_B0016																	76480	76626
117		BURPS1655_B0017																	76242	75913
118		BURPS1655_B0018																	75559	75903
119		BURPS1655_B0019																	75086	75529
120	BPSS0060		66805	67074	1895739	1895470	2392154	2392423	1905995	1906264	76832	77101	84054	84323	78604	78873	79704	79973	89194	89394
121	BPSS0060A		67264	67704	1895280	1894840	2392613	2392744	1906454	1906894	77291	77521	84513	84953	79063	79503	80163	80498		
122	BPSS0061		68210	69559	1894334	1892985	2393610	2394959	1907400	1908749	78256	79605	85459	86808	80009	81358	81022	82371	90115	91464
123	BPSS0062		69615	71543	1892929	1891001	2395015	2396943	1908805	1910733	79661	81589	86864	88792	81414	83342	82427	84355	91520	93448
124	BPSS0063		71530	72873	1891014	1889671	2396930	2398273	1910720	1912063	81576	82919	88779	90122	83329	84672	84342	85685	93435	94778
125	BPSS0064		74757	74341	1887787	1888203	2400156	2399740	1913946	1913530	84802	84386	92013	91597	86555	86139	87569	87153	96660	96244
126	BPSS0065		75164	77104	1887380	1885440	2400585	2402534	1914375	1916324	85224	87173	92420	92671	86984	88933	87976	89916	97066	99006
127	BPSS0066		77359	77868	1885185	1884676	2402789	2403298	1916579	1917088	87427	87936	94615	95124	89188	89697	90171	90680	99260	99769
128	BPSS0067		78243	80435	1884301	1882109	2403673	2405865	1917463	1919655	88311	90503	95478	97670	90072	92264	91055	93247	100144	102336
129		BURPS1106A_A0093	81301	81666																
130		BURPS1106A_A0094	82340	82975																
131		BURPS1106A_A0095	83684	83809																
132		BURPS1106A_A0096	84481	85209																
133		BURPS1106A_A0097	85469	85618																
134		BURPS1655_C0693																	107517	107368
135		BURPS1655_C0694																	106191	107108
136		BURPS1655_C0695																	105586	105711
137		BURPS1655_C0696																	103568	103000
138		BURPS1710A_A2581					2407096	2406839												
139		BURPS1710A_A2582					2408786	2407770												
140		BURPS1710A_A2583					2409105	2409239												
141		BURPS1710A_A2584					2409314	2409427												
142		BURPS1710A_A2585					2409455	2409571												
143		BURPS1710A_A2586					2409722	2410639												
144		BURPS1710A_A2587					2411048	2410899												
145		BURPS1710b_A1578							1922595	1923014										
146		BURPS1710b_A1579							1923512	1924429										
147		BURPS1710b_A1580							1924416	1925270										
148		BURPS1710b_A1581							1924940	1926298										
149		BURPS406E_P0442									95686	95537								
150		BURPS406E_P0443									94360	95277								
151		BURPS406E_P0444									93743	93877								
152		BURPSPAST_X0004													100851	100588				
153		BURPSPAST_X0005													100594	99740				
154		BURPSPAST_X0007													97549	98907				
155		BURPSPAST_X0008													97447	97298				
156		BURPSPAST_X0009													96193	97038				
157		BURPSPAST_X0010													95504	95638				
158		BURPSPAST_X0011													93495	93238				
159	BPSS0068												98177	98908			93777	94262		

160	BPSS0069																	96876	94939		
161	BPSS0070											99387	100631			94986	96230				
162	BPSS0072											101599	101276			97198	96875				
163	BPSS0072A											102084	102248			97683	97847				
164	BPSS0073											103267	102251			98866	97850				
165	BPSS0074											103359	103718			98958	99317				
166	BPSS0075											104224	105141			99822	100739				
167	BPSS0076											105128	105979			100726	101577				
168	BPSS0077											105979	107007			101577	102605				
169	BPSS0077A			87721	88023	1874823	1874521	2413151	2413453	1926941	1927243	97789	98091	107598	107900	1666773	1666471	1628788	1628486	109620	109922
170		BURPS1106B_1891				1876926	1877075														
171		BURPS1106B_1893				1878860	1878735														
172		BURPS1106B_1894				1880070	1880204														
173		BURPS1106B_1895				1880878	1881243														
174	BPSS0078			88356	91124	1873971	1873546	2413786	2416164	1929954	1930205	98424	101177	108232	111006	101398	104172	1628153	1625946	1456473	1453699
175	BPSS0079			91141	93378	1873529	1871292	2416432	2418669	1930222	1932459	101194	103431	111023	113260	104189	104791	1625361	1623124	113045	115282
176		BURPSPAST_I0001														105489	106634				
177		BURPSPAST_N0037														107712	109193				
178		BURPSPAST_N0036														110253	111668				
179		BURPSPAST_N0034														111939	113018				
180		BURPSPAST_N0035														120399	112987				
181		BURPSPAST_N0033														120593	120396				
182		BURPSPAST_N0032														125221	120635				
183		BURPSPAST_N0031														125346	125224				
184		BURPSPAST_N0030														125379	125594				
185		BURPSPAST_N0029														126255	126127				
186		BURPSPAST_N0028														126713	126426				
187		BURPSPAST_N0027														127276	126902				
188		BURPSPAST_N0026														127468	127277				
189		BURPSPAST_N0025														127866	127681				
190		BURPSPAST_N0024														128420	128238				
191		BURPSPAST_N0023														128481	128885				
192		BURPSPAST_N0022														129029	129457				
193		BURPSPAST_N0021														129650	130405				
194		BURPSPAST_N0020														130755	131108				
195		BURPSPAST_N0019														131779	131474				
196		BURPSPAST_N0018														132049	132180				
197		BURPSPAST_N0017														132375	132500				
198		BURPSPAST_N0016														132551	132745				
199		BURPSPAST_N0015														132767	132982				
200		BURPSPAST_N0014														132993	133259				
201		BURPSPAST_N0013														133412	134299				
202		BURPSPAST_N0012														134415	134789				
203			Bp_chr2_2_IS407A													135207	136442				
204		BURPSPAST_N0011														136087	135254				
205		BURPSPAST_N0010														136374	136111				
206		BURPSPAST_N0009														136533	137428				
207		BURPSPAST_N0008														138209	138349				
208		BURPSPAST_N0007														139129	138821				
209		BURPSPAST_N0006														139413	139526				
210		BURPSPAST_N0005														139928	147481				
211		BURPSPAST_N0004														147505	150474				
212		BURPSPAST_N0003														150604	155019				
213		BURPSPAST_N0002														155075	157933				
214		BURPSPAST_N0001														158163	164438				
215	BPSS0080							2418991	2420343	1932781	1934133	103753	105105					105394	106746		
216	BPSS0080a			95068	95328	1869602	1869342	2420356	2420616	1934146	1934406	105118	105378	114950	115210	165563	165823	106759	107019	116972	117232
217	BPSS0081			96165	95749	1868505	1868921	2421453	2421037	1935243	1934827	106215	105799	116047	115631	166660	166244	107856	107440	118069	117653

218	BPSS0082		96511	96188	1868159	1868482	2421799	2421476	1935589	1935266	106561	106238	116393	116070	167006	166683	108202	107879	118415	118092
219		BURPSS13_J0027															109868	108765		
220	BPSS0083		96983	97672	1867687	1866998	2422271	2422960	1936061	1936750	107033	107722	116864	117553	167478	168167	110031	110720	118887	119576
221	BPSS0084		98307	98170	1866363	1866500	2423595	2423458	1937385	1937248	108357	108220	118188	118051	168802	168665	111355	111218	120211	120074
222	BPSS0085		98884	98264	1865786	1866406	2424172	2423552	1937962	1937342	108934	108314	118765	118145	169379	168759	111932	111312	120788	120168
223		BURPSS13_J0032															112066	111935		
224	BPSS0086		100596	99004	1864074	1865666	2425878	2424286	1939668	1938076	110706	109114	120495	118903	171079	169487	113698	112106	122470	120878
225		BURPS1710A_A2603					2426397	2426143												
226	BPSS0087		103203	101749	1861467	1862921	2428485	2427031	1942275	1940821	113315	111861	123102	121648	173686	172232	116307	114853	125079	123625
227		BURPS1106A_A0121	104775	107015																
228		BURPS668_A0145											124674	126872						
229	BPSS0088		105341	107194	1859329	1857476					115201	117054	125198	127051	175488	177341	117866	119710	127174	129027
230		BURPS1106B_1868			1859895	1857655														
231	BPSS0089		108751	107267	1855919	1857403	2433445	2431961	1947235	1945751	118611	117127	128608	127124	178898	177414	121267	119783	130584	129100
232	BPSS0090		109567	109016	1855103	1855654	2434261	2433710	1948051	1947500	119427	118876	129425	128874	179714	179163	122083	121532	131400	130849
233	BPSS0091		110152	110694	1854518	1853976	2434846	2435388	1948636	1949178	120012	120554	130010	130552	180299	180841	122668	123210	131986	132528
234	BPSS0092		110762	111496	1853908	1853174	2435456	2436190	1949246	1949980	120622	121356	130620	131354	180909	181643	123278	124012	132596	133330
235	BPSS0093		111723	114353	1852947	1850317	2436417	2439047	1950207	1952837	121583	124213	131581	134211	181870	184500	124239	126869	133557	136187
236	BPSS0094		114349	114918	1850321	1849752	2439043	2439612	1952833	1953402	124209	124778	134207	134776	184496	185065	126865	127434	136183	136752
237	BPSS0095		114986	115639	1849684	1849031	2439680	2440333	1953470	1954123	124846	125499	134844	135497	185133	185786	127502	128155	136820	137473
238	BPSS0096		115645	117318	1849025	1847352	2440339	2442012	1954129	1955802	125505	127178	135503	137176	185792	187465	128161	129834	137479	139152
239	BPSS0097		117711	118247	1846959	1846423	2442405	2442941	1956195	1956731	127571	128107	137568	138104	187858	188394	130191	130727	139544	140080
240	BPSS0098		118284	119780	1846386	1844890	2442978	2444474	1956768	1958264	128144	129640	138141	139637	188431	189927	130764	132260	140117	141613
241	BPSS0099		119983	120462	1844687	1844208	2444677	2445156	1958467	1958946	129843	130322	139840	140319	190130	190609	132463	132942	141816	142295
242	BPSS0100		120653	121132	1844017	1843538	2445347	2445826	1959137	1959616	130513	130992	140510	140989	190886	191365	133133	133612	142569	143048
243	BPSS0101		121141	122487	1843529	1842183	2445835	2447181	1959625	1960971	131001	132347	140998	142344	191374	192720	133621	134967	143057	144403
244	BPSS0102		122487	123785	1842183	1840885	2447181	2448479	1960971	1962269	132347	133645	142344	143642	192720	194018	134967	136265	144403	145701
245	BPSS0103		123803	127708	1840867	1836962	2448497	2452402	1962287	1966192	133663	137568	143660	147565	194036	197941	136283	140188	145719	149624
246	BPSS0104		127901	128467	1836769	1836203	2452595	2453161	1966385	1966951	137761	138327	147758	148324	198134	198700	140381	140947	149817	150383
247	BPSS0105		128535	131219	1836135	1833451	2453229	2455349	1967019	1969613	138395	140617	148392	151076	198768	200969	141015	143669	150451	153120
248		BURPS1710A_A2633					2455410	2455850												
249	BPSS0106		131327	135064	1833343	1829606	2455932	2459669	1969697	1973434	140868	144605	151286	155023	201439	205176	143753	147490	153204	156941
250	BPSS0108		136318	134963	1828352	1829707	2460923	2459568	1974688	1973333	145859	144504	156277	154922	206430	205075	148744	147389	158195	156840
251	BPSS0109		137379	136354	1827291	1828316	2461984	2460959	1975749	1974724	146920	145895	157338	156313	207491	206466	149805	148780	159256	158231
252	BPSS0110		138504	137437	1826166	1827233	2463109	2462042	1976874	1975807	148045	146978	158463	157396	208616	207549	150930	149863	160381	159314
253	BPSS0111		139572	138526	1825098	1826144	2464177	2463131	1977942	1976896	149113	148067	159531	158485	209684	208638	151998	150952	161449	160403
254	BPSS0112		141449	139572	1823221	1825098	2466054	2464177	1979819	1977942	150990	149113	161408	159531	211561	209684	153875	151998	163326	161449
255	BPSS0113		141969	141454	1822701	1823216	2466574	2466059	1980339	1979824	151510	150995	161928	161413	212081	211566	154395	153880	163846	163331
256	BPSS0114		142810	141959	1821860	1822711	2467415	2466564	1981180	1980329	152351	151500	162769	161918	212922	212071	155236	154385	164687	163836
257	BPSS0115		143849	142797	1820821	1821873	2468454	2467402	1982219	1981167	153390	152338	163808	162756	213961	212909	156275	155223	165726	164674
258	BPSS0116		144279	146921	1820391	1817749	2468884	2471526	1982649	1985291	153820	156462	164238	166880	214391	217033	156705	159347	166156	168798
259	BPSS0117		150359	147318	1814311	1817352	2474964	2471923	1988729	1985688	159900	156859	170318	167277	220471	217430	162785	159744	172236	169195
260		BURPS1655_C0638																	173585	173752
261		BURPS1655_C0639																	173415	173242
262		BURPS406E_P0387									161248	161415								
263		BURPS406E_P0388									161111	160905								
264		BURPS668_A0181											170361	170492						
265		BURPS668_A0182											170520	170633						
266		BURPS668_A0183											170677	170823						
267		BURPS668_A0184											171102	171296						
268		BURPS668_A0185											171324	171530						
269		BURPS668_A0186											171667	171834						
270	BPSS0118		151030	151287	1813640	1813383	2475636	2475893	1989401	1989658					221143	221400	163457	163714		
271	BPSS0119		152623	151964	1812047	1812706	2477229	2476570	1990994	1990335	162164	161505	172583	171924	222736	222077	165050	164391	174501	173842
272		BURPS1655_C0574																	229369	229494
273		BURPS1655_C0575																	228720	229082
274		BURPS1655_C0576																	228039	227272
275		BURPS1655_C0577																	227092	227229

276	BURPS1655_C0578									226710	227051
277	BURPS1655_C0579									225646	226713
278	BURPS1655_C0580									224716	225630
279	BURPS1655_C0581									223340	224698
280	BURPS1655_C0582									222862	223215
281	BURPS1655_C0583									221688	222851
282	BURPS1655_C0584									221032	221688
283	BURPS1655_C0585									220247	221035
284	BURPS1655_C0586									219490	220215
285	BURPS1655_C0587									218299	219486
286	BURPS1655_C0588									217163	218302
287	BURPS1655_C0589									216731	217126
288	BURPS1655_C0590									215135	215929
289	BURPS1655_C0591									214723	215148
290	BURPS1655_C0592									214448	214726
291	BURPS1655_C0593									213683	214393
292	BURPS1655_C0594									213213	213677
293	BURPS1655_C0595									212037	213188
294	BURPS1655_C0596									211735	212040
295	BURPS1655_C0597									211338	211724
296	BURPS1655_C0598									209979	211304
297	BURPS1655_C0599									209458	209895
298	BURPS1655_C0600									208154	209461
299	BURPS1655_C0601									207429	208157
300	BURPS1655_C0602									206416	207432
301	BURPS1655_C0603									204840	206438
302	BURPS1655_C0604									204415	204747
303	BURPS1655_C0605									203580	202732
304	BURPS1655_C0606									202728	202381
305	BURPS1655_C0607									202384	201617
306	BURPS1655_C0608									201617	201345
307	BURPS1655_C0609									201340	200564
308	BURPS1655_C0610									200571	199432
309	BURPS1655_C0611									197169	197375
310	BURPS1655_C0612									199435	197345
311	BURPS1655_C0613									196123	197172
312	BURPS1655_C0614									195001	196065
313	BURPS1655_C0615									194331	194933
314	BURPS1655_C0616									193529	194341
315	BURPS1655_C0617									191027	193408
316	BURPS1655_C0618									190533	191006
317	BURPS1655_C0619									188247	190523
318	BURPS1655_C0620									187879	188250
319	BURPS1655_C0621									186936	187871
320	BURPS1655_C0622									186066	186917
321	BURPS1655_C0623									185939	186058
322	BURPS1655_C0624									185769	185918
323	BURPS1655_C0625									184764	185654
324	BURPS1655_C0626									183496	184650
325	BURPS1655_C0627									182856	183515
326	BURPS1655_C0628									182272	182880
327	BURPS1655_C0629									181487	182275
328	BURPS1655_C0630									180559	181494
329	BURPS1655_C0631									178976	180562
330	BURPS1655_C0632									178977	178861
331	BURPS1655_C0633									178819	178670
332	BURPS1655_C0634									178673	176181
333	BURPS1655_C0635									175411	176115

334	BURPS1655_C0636										175360	175235
335	BURPS406E_P0322					217011	217136					
336	BURPS406E_P0323					216362	216724					
337	BURPS406E_P0324					215682	214915					
338	BURPS406E_P0325					214681	214872					
339	BURPS406E_P0326					214353	214694					
340	BURPS406E_P0327					213289	214356					
341	BURPS406E_P0328					212359	213273					
342	BURPS406E_P0329					210983	212341					
343	BURPS406E_P0330					210505	210858					
344	BURPS406E_P0331					209316	210494					
345	BURPS406E_P0332					208675	209331					
346	BURPS406E_P0333					207890	208678					
347	BURPS406E_P0334					207133	207858					
348	BURPS406E_P0335					205942	207129					
349	BURPS406E_P0336					205220	205945					
350	BURPS406E_P0337					204807	205223					
351	BURPS406E_P0338					204375	204770					
352	BURPS406E_P0339					204216	203515					
353	BURPS406E_P0340					203132	202890					
354	BURPS406E_P0341					202367	202792					
355	BURPS406E_P0342					202092	202370					
356	BURPS406E_P0343					201327	202037					
357	BURPS406E_P0344					200857	201321					
358	BURPS406E_P0345					199690	200832					
359	BURPS406E_P0346					199388	199693					
360	BURPS406E_P0347					198991	199377					
361	BURPS406E_P0348					197632	198957					
362	BURPS406E_P0349					197111	197548					
363	BURPS406E_P0350					195807	197114					
364	BURPS406E_P0351					195082	195810					
365	BURPS406E_P0352					194069	195085					
366	BURPS406E_P0353					192493	194091					
367	BURPS406E_P0354					192068	192400					
368	BURPS406E_P0355					191231	190383					
369	BURPS406E_P0356					190379	190044					
370	BURPS406E_P0357					190047	189280					
371	BURPS406E_P0358					189280	189008					
372	BURPS406E_P0359					189003	188227					
373	BURPS406E_P0360					188234	187095					
374	BURPS406E_P0361					184832	185038					
375	BURPS406E_P0362					187098	185008					
376	BURPS406E_P0363					183786	184835					
377	BURPS406E_P0364					182664	183728					
378	BURPS406E_P0365					181994	182596					
379	BURPS406E_P0366					181087	182004					
380	BURPS406E_P0367					178690	181071					
381	BURPS406E_P0368					178196	178669					
382	BURPS406E_P0369					175910	178186					
383	BURPS406E_P0370					175542	175913					
384	BURPS406E_P0371					174599	175534					
385	BURPS406E_P0372					173729	174580					
386	BURPS406E_P0373					173602	173721					
387	BURPS406E_P0374					173432	173581					
388	BURPS406E_P0375					172427	173317					
389	BURPS406E_P0376					171159	172313					
390	BURPS406E_P0377					170519	171178					
391	BURPS406E_P0378					169935	170543					

392	BURPS406E_P0379					169150	169938					
393	BURPS406E_P0380					168222	169157					
394	BURPS406E_P0381					166639	168225					
395	BURPS406E_P0382					166640	166524					
396	BURPS406E_P0383					166456	163844					
397	BURPS406E_P0384					163032	163778					
398	BURPS406E_P0385					163023	162898					
399	BURPS668_A0188							172666	172866			
400	BURPS668_A0189							173181	173396			
401	BURPS668_A0190							173494	174198			
402	BURPS668_A0191							174264	176756			
403	BURPS668_A0192							176944	177060			
404	BURPS668_A0193							177059	178645			
405	BURPS668_A0194							178642	179577			
406	BURPS668_A0195							179570	180358			
407	BURPS668_A0196							180355	180963			
408	BURPS668_A0197							180939	181598			
409	BURPS668_A0198							181579	182733			
410	BURPS668_A0199							182847	183737			
411	BURPS668_A0200							184022	184141			
412	BURPS668_A0201							184149	185000			
413	BURPS668_A0202							185019	185954			
414	BURPS668_A0203							185962	186333			
415	BURPS668_A0204							186330	188606			
416	BURPS668_A0205							188616	189089			
417	BURPS668_A0206							189110	191491			
418	BURPS668_A0207							191612	192424			
419	BURPS668_A0208							192414	193016			
420	BURPS668_A0209							193084	194148			
421	BURPS668_A0210							194206	195255			
422	BURPS668_A0211							195341	195457			
423	BURPS668_A0212							195427	197517			
424	BURPS668_A0213							197514	198653			
425	BURPS668_A0214							198646	199422			
426	BURPS668_A0215							199427	199699			
427	BURPS668_A0216							199699	200466			
428	BURPS668_A0217							200463	200810			
429	BURPS668_A0218							200814	201746			
430	BURPS668_A0219							201752	201880			
431	BURPS668_A0220							201922	202071			
432	BURPS668_A0221							202228	202356			
433	BURPS668_A0222							202498	202830			
434	BURPS668_A0223							202923	204521			
435	BURPS668_A0224							204502	205515			
436	BURPS668_A0225							205512	206240			
437	BURPS668_A0226							206237	207544			
438	BURPS668_A0227							207541	207978			
439	BURPS668_A0228							208054	209379			
440	BURPS668_A0229							209413	209799			
441	BURPS668_A0230							209810	210115			
442	BURPS668_A0231							210112	211254			
443	BURPS668_A0232							211279	211743			
444	BURPS668_A0233							211749	212459			
445	BURPS668_A0234							212514	212792			
446	BURPS668_A0235							212789	213214			
447	BURPS668_A0236							213201	213995			
448	BURPS668_A0237							214818	215192			
449	BURPS668_A0238							215229	215645			

450		BURPS668_A0239											215642	216367						
451		BURPS668_A0240											216364	217551						
452		BURPS668_A0241											217555	218280						
453		BURPS668_A0242											218312	219100						
454		BURPS668_A0243											219097	219753						
455		BURPS668_A0244											219753	220916						
456		BURPS668_A0245											220927	221280						
457		BURPS668_A0246											221405	222763						
458		BURPS668_A0247											222781	223695						
459		BURPS668_A0248											223711	224778						
460		BURPS668_A0249											224784	225116						
461		BURPS668_A0250											225157	225294						
462		BURPS668_A0251											225337	226104						
463		BURPS668_A0252											226060	226245						
464		BURPS668_A0253											226373	226693						
465		BURPS668_A0254											226975	227145						
466		BURPS668_A0255											227374	227607						
467		BURPS668_A0256											227604	227765						
468		BURPS668_A0257											227969	228103						
469	BPSS0120		155556	153175	1809114	1811495	2480162	2477781	1993927	1991546					225669	223288	167983	165602		
470	BPSS0121		156627	155920	1808043	1808750	2481233	2480526	1994998	1994291					226740	226033	169054	168347		
471	BPSS0122		157750	157106	1806920	1807564	2482356	2481712	1996121	1995477					227863	227219	170177	169533		
472	BPSS0123		158642	157989	1806028	1806681	2483248	2482595	1997013	1996360					228755	228102	171069	170416		
473	BPSS0124		159650	160339	1805020	1804331	2484253	2484942	1998018	1998707			228044	228733	229760	230449	172080	172769		
474	BPSS0125		160944	160654	1803726	1804016	2485708	2485418	1999473	1999183	218917	218627	229499	229209	231054	230764	173374	173084	231275	230985
475	BPSS0126		161435	162757	1803235	1801913	2486199	2487521	1999964	2001286	219408	220730	229991	231313	231545	232867	173865	175187	231766	233088
476	BPSS0127		162834	163997	1801836	1800673	2487598	2488761	2001363	2002526	220807	221970	231390	232553	232944	234107	175264	176427	233165	234328
477	BPSS0128		164163	165614	1800507	1799056	2488957	2490408	2002722	2004173	222146	223597	232739	234190	234313	235299	176603	178054	234504	235955
478	BPSS0129		165876	167093	1798794	1797577	2490670	2491887	2004435	2005652	223859	225076	234440	235654	236019	237233	178316	179533	236217	237431
479	BPSS0130		168157	170961	1796513	1793709	2492951	2495755	2006716	2009520	226140	228944	236719	239523	238307	241111	180597	183401	238505	241309
480	BPSS0131		170998	171981	1793672	1792689	2495792	2496775	2009557	2010540	228981	229964	239560	240543	241148	242131	183438	184421	241346	242329
481	BPSS0132		172008	172400	1792662	1792270	2496802	2497194	2010567	2010959	229991	230383	240570	240962	242158	242550	184448	184840	242356	242748
482	BPSS0133		172646	173668	1792024	1791002	2497440	2498462	2011205	2012227	230639	231661	241228	242250	242796	243818	185086	186108	242994	244016
483	BPSS0134		174653	173715	1790017	1790955	2499447	2498509	2013212	2012274	232646	231708	243235	242297	244803	243865	187093	186155	245001	244063
484	BPSS0135		174720	175136	1789950	1789534	2499514	2499930	2013279	2013695	232713	233129	243302	243718	244870	245286	187160	187576	245068	245484
485	BPSS0136		176557	175172	1788113	1789498	2501365	2499980	2015130	2013745	234590	233205	245147	243762	246707	245322	188997	187612	246912	245527
486	BPSS0137		177844	178128	1786826	1786542	2502652	2502936	2016417	2016701	235876	236160	246433	246717	247994	248278	190284	190568	248199	248483
487	BPSS0138		178307	178609	1786363	1786061	2503115	2503417	2016880	2017182	236339	236641	246896	247198	248457	248759	190747	191049	248662	248964
488	BPSS0139		179324	180700	1785346	1783970	2504130	2505506	2017895	2019271	237356	238732	247911	249287	249472	250848	191762	193138	249679	251055
489	BPSS0140		181783	180839	1782887	1783831	2506589	2505645	2020354	2019410	239822	238878	250377	249433	251931	250987	194221	193277	252145	251201
490	BPSS0141		182894	181863	1781776	1782807	2507700	2506669	2021465	2020434	240933	239902	251488	250457	253042	252011	195332	194301	253256	252225
491	BPSS0142		184490	182940	1780180	1781730	2509296	2507746	2023061	2021511	242529	240979	253084	251534	254638	253088	196928	195378	254852	253302
492	BPSS0143		185727	184480	1778943	1780190	2510533	2509286	2024298	2023051	243766	242519	254321	253074	255875	254628	198165	196918	256089	254842
493	BPSS0144		189246	186634	1775424	1778036	2514013	2511404	2027778	2025169	247246	244637	257843	255231	259394	256785	201773	199161	259389	256780
494		BURPS1106A_A0201	189540	189953																
495		BURPS1655_C0539																	260102	259533
496		BURPS1710A_A2692					2514748	2514158												
497		BURPS406E_P0287									247987	247391								
498		BURPS668_A0290											257942	258742						
499		BURPSPAST_E0271													260106	259552				
500		BURPSS13_J0110															202624	201872		
501	BPSS0145		191347	189953	1773323	1774717	2516142	2514748	2029907	2028513	249381	247987	260136	258742	261500	260106	204018	202624	261496	260102
502		BURPS1106B_1790			1774717	1775130														
503	BPSS0146		192039	191515	1772631	1773155	2516936	2516310	2030701	2030075	250175	249549	260959	260333	262294	261668	204841	204215	262290	261664
504	BPSS0147		193358	192048	1771312	1772622	2518284	2516974	2032049	2030739	251523	250213	262307	260997	263642	262332	206196	205105	263638	262328
505	BPSS0148		194589	193402	1770081	1771268	2519515	2518328	2033280	2032093	252754	251567	263538	262351	264873	263686	207427	206240	264869	263682
506		BURPSS13_J0116															208808	207705		
507	BPSS0149		194768	195667	1769902	1769003	2519694	2520593	2033459	2034358	252934	253833	263718	264617	265053	265952	208964	209863	265049	265948

508	BPSS0150			196819	196013	1767851	1768657	2521761	2520955	2035526	2034720	254987	254181	265757	264951	267099	266293	211015	210209	267116	266310
509	BPSS0151			197016	198179	1767654	1766491	2521938	2523101	2035703	2036866	255175	256338	265934	267097	267276	268439	211212	212375	267293	268456
510	BPSS0152			198674	199111	1765996	1765559	2523606	2524043	2037371	2037808	256833	257114	267592	268029	268934	269371	212870	213307	268951	269388
511		BURPS1710A_A2703						2524579	2524034												
512		BURPS1710b_A1668								2037799	2038344										
513			Bp_chr2_3_ISBma2																	269938	271510
514	BPSS0153			200632	199736	1764038	1764934	2525568	2524672	2039333	2038437	259009	258113	269591	268695	270958	270062	214816	213920	272491	271595
515	BPSS0154			202042	200798	1762628	1763872	2526979	2525735	2040744	2039500	260419	259175	271001	269757	272369	271125	216226	214982	273901	272657
516	BPSS0155			203127	202198	1761543	1762472	2528064	2527135	2041829	2040900	261504	260575	272086	271157	273454	272525	217311	216382	274986	274057
517		BURPS668_A0308												272126	272497						
518	BPSS0156			204434	203676	1760236	1760994	2529370	2528612	2043135	2042377	262811	262053	273386	272628	274761	274003	218618	217860	276286	275528
519	BPSS0157			204803	205219	1759867	1759451	2529739	2530155	2043504	2043920	263180	263596	273755	274171	275083	275313	218987	219403	276655	277071
520		BURPS1655_C0515																		277290	277439
521	BPSS0158			205679	207592	1758991	1757078	2530600	2532513	2044365	2046278	264041	265954	274616	276529	275927	277840	219863	221776	277515	279428
522	BPSS0159			207869	210763	1756801	1753907	2532792	2535686	2046557	2049451	266232	269126	276807	279701	278118	281012	222053	224947	279705	282599
523	BPSS0160			211366	212421	1753304	1752249	2536289	2537344	2050054	2051109	269731	270786	280303	281358	281615	282670	225552	226607	283202	284257
524	BPSS0161			212421	213332	1752249	1751338	2537344	2538255	2051109	2052020	270786	271697	281358	282269	282670	283581	226607	227518	284257	285168
525	BPSS0162			213340	214842	1751330	1749828	2538263	2539765	2052028	2053530	271705	273207	282277	283779	283589	285091	227526	229028	285176	286678
526	BPSS0163			214891	216756	1749779	1747914	2539814	2541679	2053579	2055444	273256	275121	283828	285693	285140	287005	229077	230942	286727	288592
527	BPSS0164			216788	217756	1747882	1746914	2541711	2542679	2055476	2056444	275153	276121	285725	286693	287037	288005	230974	231942	288624	289592
528	BPSS0165			217814	218737	1746856	1745933	2542737	2543660	2056502	2057425	276179	277102	286751	287674	288063	288986	232000	232923	289650	290573
529	BPSS0166			219946	218987	1744724	1745683	2544869	2543910	2058634	2057675	278311	277352	288882	287923	290195	289236	234132	233173	291782	290823
530	BPSS0167			225244	221171	1739426	1743499	2550167	2546094	2063932	2059859	283609	279536	294180	290107	295493	291420	239430	235357	297083	293007
531	BPSS0168			226538	225276	1738132	1739394	2551461	2550199	2065226	2063964	284903	283641	295474	294212	296787	295525	240724	239462	298377	297115
532	BPSS0169			227973	226630	1736697	1738040	2552896	2551553	2066661	2065318	286338	284995	296909	295566	298222	296879	242159	240816	299812	298469
533	BPSS0170			228507	227998	1736163	1736672	2553430	2552921	2067195	2066686	286872	286363	297443	296934	298756	298247	242693	242184	300346	299837
534	BPSS0171			229090	228608	1735580	1736062	2554013	2553534	2067778	2067299	287455	286973	298026	297544	299339	298857	243276	242794	300929	300447
535	BPSS0172			230706	229210	1733964	1735460	2555629	2554133	2069394	2067898	289071	287575	299642	298146	300955	299459	244892	243396	302545	301049
536	BPSS0173			231322	230744	1733348	1733926	2556245	2555667	2070010	2069432	289687	289109	300258	299680	301571	300993	245508	244930	303161	302583
537	BPSS0174			234297	231361	1730373	1733309	2559208	2556284	2072973	2070049	292662	289726	303221	300297	304558	301610	248483	245547	306148	303200
538	BPSS0175			234829	235548	1729841	1729122	2559741	2560457	2073506	2074222	293195	293911	303754	304470	305091	305807	249015	249734	306681	307397
539	BPSS0176			235548	236510	1729122	1728160	2560457	2561419	2074222	2075184	293911	294873	304470	305432	305807	306769	249734	250696	307397	308359
540	BPSS0177			236554	237078	1728116	1727592	2561463	2561987	2075228	2075752	294917	295441	305476	306000	306813	307337	250740	251264	308403	308927
541	BPSS0178			237112	238998	1727558	1725672	2562021	2563907	2075786	2077672	295475	297361	306034	307920	307371	309257	251298	253184	308961	310847
542	BPSS0179			239013	240083	1725657	1724587	2563922	2564992	2077687	2078757	297376	298446	307935	309005	309272	310342	253199	254269	310862	311932
543	BPSS0180			240083	241192	1724587	1723478	2564992	2566092	2078757	2079857	298446	299546	309005	310105	310342	311442	254269	255378	311932	313044
544	BPSS0181			241270	243594	1723400	1721076	2566167	2568491	2079932	2082256	299621	301945	310180	312504	311517	313841	255456	257780	313119	315443
545	BPSS0182			243629	246103	1721041	1718567	2568526	2571000	2082291	2084765	301980	304454	312539	315013	313876	316350	257815	260289	315478	317949
546	BPSS0183			246103	247182	1718567	1717488	2571000	2572079	2084765	2085844	304454	305533	315013	316092	316350	317429	260289	261368	317952	319031
547	BPSS0184			247367	248023	1717303	1716647	2572264	2572920	2086029	2086685	305718	306374	316277	316933	317614	318270	261553	262209	319216	319872
548	BPSS0185			248073	248444	1716597	1716226	2572970	2573341	2086735	2087106	306424	306795	316983	317354	318320	318691	262259	262630	319922	320293
549	BPSS0186			251103	249190	1713567	1715480	2575962	2574049	2089727	2087814	309410	307497	319998	318085	321348	319435	265300	263387	322932	321019
550	BPSS0187			252336	251446	1712334	1713224	2577195	2576305	2090960	2090070	310644	309754	321232	320342	322582	321692	266533	265643	324166	323276
551	BPSS0188			252432	253205	1712238	1711465	2577291	2578064	2091056	2091829	310740	311513	321328	322101	322678	323451	266629	267402	324262	325035
552	BPSS0189			253234	253671	1711436	1710999	2578093	2578530	2091858	2092295	311542	311979	3044630	3044659	323480	323917	267431	267868	325064	325501
553	BPSS0190			255503	254211	1709167	1710459	2580362	2579070	2094127	2092835	313811	312519	323332	323135	325749	324457	269700	268408	327341	326049
554	BPSS0191			256179	255790	1708491	1708880	2581038	2580649	2094803	2094414	314487	314098	325089	324700	326425	326036	270374	269985	327942	327553
555	BPSS0192			257396	256554	1707274	1708116	2582237	2581395	2096002	2095160	315713	314871	326297	325455	327606	326764	271555	270713	329123	328281
556	BPSS0193			259538	258186	1705132	1706484	2584379	2583027	2098144	2096792	317883	316531	328458	327106	329760	328408	273697	272345	331291	329939
557	BPSS0194			260729	259728	1703941	1704942	2585570	2584569	2099335	2098334	319074	318073	329649	328648	330951	329950	274888	273887	332482	331481
558	BPSS0195			261594	260722	1703076	1703948	2586435	2585563	2100200	2099328	319939	319067	330514	329642	331816	330944	275753	274881	333347	332475
559	BPSS0196			263036	261594	1701634	1703076	2587877	2586435	2101642	2100200	321381	319939	331956	330514	333258	331816	277195	275753	334789	333347
560	BPSS0197			263308	263045	1701362	1701625	2588149	2587886	2101914	2101651	321653	321390	332228	331965	333530	333267	277467	277204	335061	334798
561	BPSS0198			264098	263301	1700572	1701369	2588939	2588142	2102704	2101907	322443	321646	333018	332221	334320	333523	278257	277460	335851	335054
562	BPSS0199			264366	265277	1700304	1699393	2589207	2590118	2102972	2103883	322711	323622	333286	334197	334588	335499	278525	279436	336119	337030
563	BPSS0200			266076	268586	1698587	1696077	2590855	2593365	2104641	2107151	324671	327181	334890	337400	336225	338735	280178	282688	337749	340259
564	BPSS0201			268976	269911	1695687	1694752	2593754	2594689	2107540	2108475	327568	328503	337787	338722	339124	340059	283078	284013	340649	341584
565	BPSS0202			270132	270959	1694595	1694530	2594846	2594911	2108632	2108697	328660	329550	338943	339770	340216	340281	284170	284235	341819	342646

566	BPSS0203		273085	271076	1691578	1693587	2597845	2595836	2111631	2109622	331658	329649	341896	339887	343233	341224	287187	285178	344754	342745
567	BPSS0204		275602	273977	1689054	1690679	2600271	2598646	2114057	2112432	334175	332550	344413	342788	345722	344097	289739	288114	347299	345674
568	BPSS0205		278036	275955	1686620	1688701	2602714	2600624	2116500	2114410	336615	334528	346874	344766	348162	346075	292173	290092	349733	347652
569	BPSS0206		278253	279143	1686403	1685513	2602931	2603821	2116717	2117607	336832	337722	347091	347981	348379	349269	292390	293280	349950	350840
570	BPSS0207		279195	280364	1685461	1684292	2603873	2605042	2117659	2118828	337774	338943	348033	349202	349321	350490	293332	294501	350892	352061
571	BPSS0208		280441	283032	1684215	1681624	2605119	2607710	2118905	2121496	339020	341611	349279	351870	350567	353158	294578	297169	352138	354729
572	BPSS0209		283193	284380	1681463	1680276	2607871	2609058	2121657	2122844	341772	342959	352031	353218	353319	354506	297330	298517	354890	356077
573	BPSS0210		284879	284439	1679777	1680217	2609557	2609117	2123343	2122903	343458	343018	353717	353277	355005	354565	299016	298576	356576	356136
574		BURPS1710A_A2786					2610056	2609829												
575		BURPSPAST_E0181													355209	355817				
576	BPSS0211		285947	286135	1678730	1678542	2610679	2610867	2124465	2124653	344419	344607	354807	354995	356141	356329	300168	300356	357728	357916
577	BPSS0212		286231	286866	1678446	1677811	2610963	2611598	2124749	2125384	344703	345338	355091	355726	356425	357060	300452	301087	358012	358647
578	BPSS0213		286922	287509	1677755	1677168	2611654	2612241	2125440	2126027	345394	345981	355782	356369	357116	357703	301143	301730	358703	359290
579	BPSS0214		287591	289063	1677086	1675614	2612323	2613795	2126109	2127581	346063	347535	356451	357923	357785	359257	301812	303284	359372	360844
580		BURPS1106A_A0299	289075	289215																
581		BURPS406E_P0190									347535	347756								
582		BURPS668_A0391											357935	358099						
583		BURPSS13_J0206															303284	303547		
584	BPSS0215		289266	290876	1675411	1673801	2614007	2615617	2127793	2129403	347796	349400	358139	359731	359525	361135	303587	305197	361069	362679
585		BURPS1106B_1694			1675602	1675462														
586	BPSS0216		292695	292159	1671982	1672518	2617440	2615806	2131226	2129592	351143	349509	361518	361411	362958	361324	307065	305431	364700	363066
587	BPSS0217		292824	293687	1671853	1670990	2617569	2618432	2131355	2132218	351272	352135	361647	362510	363087	363950	307194	308057	364829	365692
588	BPSS0218		294876	293776	1669801	1670901	2619621	2618521	2133407	2132307	353324	352224	363699	362599	365139	364039	309246	308146	366881	365781
589	BPSS0219		294987	295679	1669690	1668998	2619732	2620424	2133518	2134210	353435	354127	363810	364502	365250	365942	309357	310049	366992	367684
590	BPSS0220		296080	297207	1668597	1667470	2620876	2622096	2134662	2135882	354540	355796	364825	366075	366394	367614	310372	311625	368007	369296
591		BURPS1710b_A1752							2136037	2137875										
592	BPSS0221		297407	297559	1667270	1667118	2622296	2622448	2136082	2136234	355996	356148	366275	366427	367814	367966	311825	311977	369496	369648
593		BURPS406E_P0183									357125	356415								
594		BURPSS13_J0216															312643	312906		
595	BPSS0222		298611	299078	1666066	1665599	2623496	2623963	2137282	2137749	357190	357657	367439	367906	369008	369475	313009	313476	370722	371189
596	BPSS0223		299727	299287	1664950	1665390	2624612	2624172	2138398	2137958	358306	357866	368555	368115	370124	369684	314125	313685	371857	371417
597		BURPS1655_C0420																	372100	372324
598	BPSS0224		303396	302467	1661281	1662210	2628281	2627352	2142067	2141138	361982	361053	372171	371242	373807	372878	317770	316841	375512	374583
599	BPSS0225		304016	304840	1660673	1659849	2628921	2629745	2142707	2143531	362616	363440	372878	373702	374477	375301	318392	319216	376086	376910
600		BURPS668_A0408											373950	374309						
601		BURPSPAST_E0161													375544	375873				
602	BPSS0226		305617	307035	1659072	1657654	2630621	2632039	2144329	2145747	364341	365759	374531	375949	376099	377517	320019	321437	377705	379123
603	BPSS0227		307193	311146	1657496	1653543	2632197	2636147	2145905	2149855	365917	369867	376108	380070	377675	379819	321595	325548	379281	383192
604	BPSS0228		311537	311277	1653152	1653412	2636538	2636278	2150246	2149986	370258	369998	380462	380202	381759	381499	325939	325679		
605	BPSS0229		313524	311866	1651165	1652823	2638525	2636867	2152233	2150575	372245	370587	382449	380791	383739	382081	327926	326268	385089	383431
606		BURPS1106A_A0325	313788	314165																
607		BURPSS13_J0231															328190	328567		
608	BPSS0230		314521	314132	1650168	1650557	2639498	2639109	2153206	2152817	373218	372829	383422	383036	384712	384323	328923	328534	386072	385683
609		BURPS1106B_1670			1650901	1650524														
610	BPSS0231		314930	314649	1649759	1650040	2639907	2639626	2153615	2153334	373627	373346	383831	383550	385121	384840	329332	329051	386481	386200
611	BPSS0232		315099	316166	1649590	1648523	2640076	2641143	2153784	2154851	373796	374863	384000	385067	385290	386357	329501	330568	386650	387717
612		BURPS668_A0419											385070	385489						
613		BURPS668_A0420											385532	386563						
614		BURPS668_A0421											386562	387764						
615		BURPS668_A0422											387791	388597						
616		BURPS668_A0423											388673	389788						
617	BPSS0233						2641762	2641175	2155470	2154883	375482	374895			386976	386389	331188	330718	387865	387749
618	BPSS0233a		317022	317213	1647667	1647476	2641996	2642187	2155704	2155895	375716	375907	390162	390353	387210	387401	331422	331613	388572	388763
619	BPSS0234		318706	317702	1645983	1646987	2643680	2642676	2157388	2156384	377400	376396	391846	390842	388894	387890	333106	332102	390256	389252
620	BPSS0235		320154	318745	1644535	1645944	2645128	2643719	2158836	2157427	378848	377439	393294	391885	390342	388933	334554	333145	391704	390295
621	BPSS0236		320756	321766	1643777	1642767	2647083	2648093	2159437	2160447	379501	380511	393890	394900	390938	391948	335355	336365	392303	393313
622	BPSS0237		322715	321741	1641818	1642792	2649042	2648068	2161396	2160422	381460	380486	395849	394875	392897	391923	337314	336340	394262	393288
623	BPSS0238		322871	325030	1641662	1639503	2649198	2651357	2161552	2163711	381616	383775	396005	398164	393053	395212	337470	339629	394418	396577

624	BPSS0239		325046	325651	1639487	1638882	2651373	2651978	2163727	2164332	383791	384396	398180	398785	395228	395833	339645	340250	396593	397198
625	BPSS0240		326521	325706	1638012	1638827	2652848	2652033	2165202	2164387	385266	384451	399655	398840	396703	395888	341120	340305	398068	397253
626		BURPS1106A_A0344	328031	328324																
627	BPSS0241		328034	326916	1636499	1637617	2654424	2653306	2166778	2165660	386584	385661	401177	400059	398260	397142	342624	341506	399596	398478
628		BURPS1710A_A2840					2654723	2654421												
629	BPSS0242		329247	328324	1635286	1636209	2655646	2654723	2168000	2167077	387902	386979	402489	401566	399464	398541	343837	342914	400743	399820
630		BURPS1106B_1651			1636209	1636502														
631	BPSS0243		330395	329247	1634138	1635286	2656794	2655646	2169148	2168000	389050	387902	403637	402489	400612	399464	344985	343837	401882	400743
632	BPSS0244		332731	330455	1631802	1634078	2659145	2656854	2171499	2169208	391386	389110	405988	403697	402963	400672	347321	345045	404218	401942
633	BPSS0245		333432	333208	1631101	1631325	2659848	2659624	2172202	2171978	392089	391865	406691	406467	403666	403442	348024	347800	404921	404697
634	BPSS0246		334490	333597	1630043	1630936	2660906	2660013	2173260	2172367	393147	392254	407746	406853	404724	403831	349082	348189	405979	405086
635	BPSS0247		334657	335502	1629876	1629031	2661073	2661918	2173427	2174272	393314	394159	407913	408758	404891	405736	349248	350093	406146	406991
636	BPSS0248		335510	336550	1629023	1627983	2661926	2662966	2174280	2175320	394167	395207	408766	409806	405744	406784	350101	351141	406999	408039
637	BPSS0249		336580	337665	1627953	1626868	2662996	2664081	2175350	2176435	395237	396322	409836	410921	406814	407899	351171	352256	408069	409154
638	BPSS0250		337658	338503	1626875	1626030	2664074	2664919	2176428	2177273	396315	397160	410914	411759	407892	408737	352249	353094	409147	409992
639	BPSS0251		338520	339029	1626013	1625504	2664936	2665445	2177290	2177799	397177	397686	411776	412285	408754	409263	353111	353620	410009	410518
640	BPSS0252		339099	340295	1625434	1624238	2665515	2666711	2177869	2179065	397756	398952	412355	412600	409333	410529	353690	354886	410588	411784
641	BPSS0253		341316	340357	1623217	1624176	2667732	2666773	2180086	2179127	399973	399014	414573	413614	411554	410595	355907	354948	412805	411846
642	BPSS0254		341845	341354	1622688	1623179	2668261	2667770	2180615	2180124	400502	400011	415102	414611	412083	411592	356436	355945	413334	412843
643	BPSS0255		342084	343598	1622449	1620935	2668500	2670014	2180854	2182368	400741	402255	415340	416854	412322	413836	356675	358189	413573	415087
644	BPSS0256		343610	344584	1620923	1619949	2670026	2671000	2182380	2183354	402267	403241	416866	417840	413848	414822	358201	359175	415099	416073
645	BPSS0257		344619	345644	1619914	1618889	2671035	2672060	2183389	2184414	403276	404301	417875	418900	414857	415882	359210	360235	416108	417145
646	BPSS0258		345659	346426	1618874	1618107	2672075	2672842	2184429	2185196	404316	405083	418915	419682	415897	416664	360250	361017	417160	417927
647	BPSS0259		346495	347628	1618038	1616905	2672911	2674044	2185265	2186398	405152	406285	419751	420884	416733	417866	361086	362219	417996	419129
648	BPSS0260		347636	348637	1616897	1615896	2674052	2675053	2186406	2187407	406293	407294	420892	421893	417874	418875	362227	363228	419137	420138
649	BPSS0261		348637	349293	1615896	1615240	2675053	2675709	2187407	2188063	407294	407950	421893	422549	418875	419531	363228	363884	420138	420794
650	BPSS0262		349512	349333	1614732	1615049	2676217	2675900	2188282	2188103	408446	407976	423059	422589	420039	419722	364394	363924	421304	420834
651	BPSS0263		350253	349801	1614280	1614732	2676669	2676217	2189023	2188571	408898	408446	423511	423059	420491	420039	364846	364394	421756	421304
652	BPSS0264		350924	350667	1613609	1613866	2677340	2677083	2189694	2189437	409578	409321	424189	423932	421162	420905	365527	365270	422436	422179
653	BPSS0265		352280	351135	1612253	1613398	2678696	2677551	2191050	2189905	410934	409789	425546	424401	422518	421373	366882	365737	423792	422647
654	BPSS0266		352593	353390	1611940	1611143	2679009	2679806	2191363	2192160	411247	412044	425859	426656	422831	423628	367195	367992	424105	424902
655	BPSS0267		354981	353869	1609552	1610664	2681400	2680288	2193754	2192642	413638	412526	428247	427135	425233	424121	369583	368471	426504	425392
656	BPSS0268		355254	357365	1609279	1607168	2681673	2683784	2194027	2196138	413910	416021	428518	430629	425505	427616	369856	371967	426777	428714
657	BPSS0269		358538	357759	1605995	1606774	2684894	2684115	2197248	2196469	417113	416334	431676	430897	428717	427938	373140	372361		
658	BPSS0270		359671	358775	1604862	1605758	2686027	2685131	2198381	2197485	418246	417350	432809	431913	429850	428954	374273	373377		
659	BPSS0271		359807	360991	1604726	1603542	2686163	2687347	2198517	2199701	418382	419566	432945	434129	429986	431170	374409	375593		
660	BPSS0272		361268	362581	1603265	1601952	2687638	2688951	2199992	2201305	419843	421156	434420	435733	431461	432774	375870	377183		
661	BPSS0273		363886	363170	1600647	1601363	2690263	2689547	2202617	2201901	422497	421781	437007	436291	434094	433378	378558	377842		
662	BPSS0274		365104	364250	1599429	1600283	2691481	2690627	2203835	2202981	423715	422861	438225	437371	435312	434458	379776	378922		
663	BPSS0275		365269	366468	1599264	1598065	2691646	2692845	2204000	2205199	423880	425079	438390	439589	435477	436676	379941	381140		
664		BURPSS13_J0290															381362	381246		
665	BPSS0276		367256	368797	1597277	1595736	2693633	2695174	2205987	2207528	425867	427408	440377	441918	437464	439005	381928	383469		
666	BPSS0277		369914	368904	1594619	1595629	2696291	2695281	2208645	2207635	428508	427495	443035	442025	440122	439112	384576	383563		
667	BPSS0278		371372	370335	1593161	1594198	2697749	2696712	2210103	2209066	429966	428929	444492	443455	441580	440543	386034	384997		
668	BPSS0279		372316	371372	1592217	1593161	2698693	2697749	2211047	2210103	430910	429966	445436	444492	442524	441580	386978	386034		
669		BURPS1106A_A0393	372646	373038																
670		BURPS1710A_A2889					2699023	2699415												
671		BURPS406E_P0101									431790	431248								
672		BURPS668_A0491											445766	446158						
673	BPSS0280		375531	374065	1589002	1590468	2701896	2700430	2214250	2212784	434122	432656	448648	447182	445727	444261	389883	388417		
674		BURPS1106B_1600			1591887	1591495														
675	BPSS0281		376872	375592	1587661	1588941	2703237	2701957	2215591	2214311	435463	434183	449989	448709	447068	445788	391224	389944		
676	BPSS0282		377014	378705	1587519	1585828	2703379	2705070	2215733	2217424	435605	437281	450131	451813	447210	448901	391366	393057		
677	BPSS0283		380209	378728	1584324	1585805	2706574	2705093	2218928	2217447	438779	437304	453314	451836	450405	448924	394561	393080	951746	951291
678	BPSS0284		381042	380278	1583491	1584255	2707416	2706652	2219770	2219006	439591	438827	454132	453368	451238	450474	395394	394630	952576	951812
679	BPSS0285		381947	381042	1582586	1583491	2708321	2707416	2220675	2219770	440496	439591	455037	454132	452143	451238	396299	395394		
680		BURPS406E_P0092									441126	441278								
681	BPSS0285A												455380	455922						

682	BPSS0286			383696	382866	1580837	1581667	2710071	2709439	2221854	2221603	442245	441415	456789	455959	453893	453261	398048	397218		
683	BPSS0287			383916	384977	1580617	1579556	2710291	2711352	2222645	2223706	442418	443479	457009	458070	454113	455174	398268	399329		
684		BURPSS13_J0312																400123	400659		
685	BPSS0288			386307	387365	1578226	1577168	2712686	2713744	2225040	2226098	444813	445871	459367	460425	456508	457566	400672	401730		
686	BPSS0289			387901	387611	1576632	1576922	2714280	2713990	2226634	2226344	446407	446117	460952	460662	458102	457812	402266	401976		
687			Bp_chr2_4_ISBma1															402947	404251		
688	BPSS0290			389814	388813	1574719	1575720	2716193	2715192	2228547	2227546	448320	447319	462873	461872	460015	459014	405508	404507		
689	BPSS0291			389907	390854	1574626	1573679	2716286	2717233	2228640	2229587	448413	449360	462966	463913	460108	461055	405601	406548		
690	BPSS0292			390907	392133	1573626	1572400	2717286	2718512	2229640	2230866	449413	450639	463967	465193	461108	462334	406601	407827		
691	BPSS0293			392198	395380	1572335	1569153	2718577	2721759	2230931	2234113	450704	453886	465258	468440	462399	465581	407892	411074		
692	BPSS0294			395394	396923	1569139	1567610	2721773	2723302	2234127	2235656	453900	455429	468454	469983	465595	467124	411088	412617		
693	BPSS0295			399076	397838	1565457	1566695	2725431	2724193	2237785	2236547	457574	456336	472225	470987	469359	468121	414786	413548		
694	BPSS0296			399719	399063	1564814	1565470	2726074	2725418	2238428	2237772	458217	457561	472868	472212	470002	469346	415429	414773		
695	BPSS0297			399875	400342	1564658	1564191	2726230	2726697	2238584	2239051	458373	458840	473024	473491	470158	470625	415585	416052		
696	BPSS0298			401432	400536	1563101	1563997	2727787	2726891	2240141	2239245	459930	459034	474574	473678	471715	470819	417142	416246		
697	BPSS0299			403714	401852	1560819	1562681	2730069	2728207	2242423	2240561	462218	460356	476875	475013	474039	472177	419424	417562		
698	BPSS0300			404630	403734	1559903	1560799	2730985	2730089	2243339	2242443	463134	462238	477791	476895	474955	474059	420340	419444		
699	BPSS0301			405062	404634	1559471	1559899	2731417	2730989	2243771	2243343	463566	463138	478223	477795	475387	474959	420772	420344		
700	BPSS0302			406989	405133	1557544	1559400	2733344	2731488	2245698	2243842	465493	463637	480150	478294	477314	475458	422699	420843		
701	BPSS0303			408278	407022	1556255	1557511	2734633	2733377	2246987	2245731	466782	465526	481439	480183	478603	477347	423988	422732		
702	BPSS0304			409756	408278	1554777	1556255	2736111	2734633	2248465	2246987	468260	466782	482917	481439	480150	479389	425466	423988		
703	BPSS0305			410808	409756	1553725	1554777	2737163	2736111	2249517	2248465	469312	468260	483969	482917	481202	480150	426518	425466		
704	BPSS0306			423230	410817	1541303	1553716	2749540	2737172	2261894	2249526	481689	469321	496343	483978	493621	481211	438927	432358		
705	BPSS0307			424687	423278	1539846	1541255	2751011	2749602	2263365	2261956	483160	481751	497814	496405	495092	493683	440398	438989		
706	BPSS0308			426102	424684	1538431	1539849	2752426	2751008	2264780	2263362	484575	483157	499229	497811	496507	495089	441813	440395		
707	BPSS0309			427190	426114	1537343	1538419	2753514	2752438	2265868	2264792	485663	484587	500317	499241	497595	496519	442901	441825		
708	BPSS0310			428622	428107	1535911	1536426	2754934	2754419	2267366	2266851	487146	486631	501821	501306	499051	498536	444342	443827		
709	BPSS0311			437229	428725	1527304	1535808	2763554	2756526	2275973	2267469	495022	488024	510437	501924	507580	500522	452991	444445		
710	BPSS0312			438447	439148	1526086	1525385	2764772	2765473	2277191	2277892	496240	496941	511686	512387	508798	509499	454209	454910		
711	BPSS0313			439481	440317	1525052	1524216	2765806	2766642	2278225	2279061	497274	498110	512730	513566	509832	510668	455243	456079		
712	BPSS0314			440338	441729	1524195	1522804	2766663	2768054	2279082	2280473	498131	499522	513587	514978	510689	512080	456100	457491		
713	BPSS0315			441801	442940	1522732	1521593	2768126	2769265	2280545	2281684	499594	500733	515050	516192	512152	513291	457563	458702		
714	BPSS0316			442936	443589	1521597	1520944	2769261	2769914	2281680	2282333	500729	501382	516188	516841	513287	513940	458698	459351		
715	BPSS0317			444338	445537	1520195	1518996	2770687	2771886	2283106	2284305	502131	503330	517601	518800	514707	515906	460118	461317	428627	429757
716	BPSS0318			446469	445645	1518064	1518888	2772818	2771994	2285237	2284413	504262	503438	519732	518908	516838	516014	462249	461425	430689	429865
717	BPSS0319			447364	446768	1517169	1517765	2773713	2773117	2286132	2285536	505157	504561	520627	520031	517728	517132	463144	462548	431584	430988
718	BPSS0320			448104	447388	1516429	1517145	2774465	2773749	2286884	2286168	505913	505197	521383	520667	518484	517768	463900	463184	432340	431624
719	BPSS0321			448487	449290	1516046	1515243	2774878	2775681	2287297	2288100	506296	507096	521776	522579	518976	519521	464283	465086	432723	433526
720	BPSS0322			449290	450735	1515243	1513798	2775681	2777126	2288100	2289545	507099	508544	522579	524024	519521	520966	465086	466531	433526	434971
721	BPSS0323			450735	451457	1513798	1513076	2777126	2777848	2289545	2290267	508544	509266	524024	524746	520966	521688	466531	467253	434971	435693
722	BPSS0324			451828	453543	1512705	1510990	2778219	2779934	2290638	2292353	509637	511352	525123	526838	522059	522871	467624	469339	436064	437017
723	BPSS0325			453848	454282	1510685	1510251	2780239	2780673	2292658	2293092	511657	512091	527152	527586	523718	524152	469644	470078	438085	438519
724	BPSS0326			456016	454484	1508517	1510049	2782407	2780875	2294826	2293294	513825	512293	529329	527788	525886	524354	471812	470280	440262	438721
725	BPSS0327			457576	456287	1506957	1508246	2783967	2782678	2296386	2295097	515403	514114	530896	529607	527482	526193	473390	472101	441840	440551
726	BPSS0328			458946	457915	1505587	1506618	2785333	2784296	2297752	2296715	516773	515742	532256	531225	528850	527819	474766	473735	443210	442179
727	BPSS0329			460531	458942	1504002	1505591	2786918	2785329	2299337	2297748	518358	516769	533841	532252	530435	528846	476351	474762	444795	443206
728	BPSS0330			462232	460610	1502301	1503923	2788619	2786997	2301038	2299416	520059	518437	535542	533920	532136	530514	478052	476430	446496	444874
729		BURPS1106A_A0470		462349	462606																
730	BPSS0331			463655	462738	1500874	1501791	2790000	2789083	2302419	2301502	521556	520639	536973	536056	533451	532534	479502	478585	447936	447019
731		BURPS1106B_1524				1501923	1502180														
732	BPSS0332			463906	464835	1500623	1499694	2790251	2791180	2302670	2303599	521807	522736	537224	538153	533702	534631	479753	480682	448187	449116
733	BPSS0333			464835	465965	1499694	1498564	2791180	2792310	2303599	2304729	522736	523866	538153	539283	534631	535761	480682	481812	449116	450246
734	BPSS0334			465965	466195	1498564	1498334	2792310	2792540	2304729	2304959	523866	524096	539283	539513	535761	535991	481812	482042	450246	450476
735	BPSS0335			466195	467484	1498334	1497045	2792540	2793829	2304959	2306248	524096	525385	539513	540802	535991	537280	482042	483331	450476	450967
736	BPSS0336			467679	468392	1496850	1496137	2794036	2794749	2306455	2307168	525580	526293	540997	541710	537487	538200	483526	484239	452031	452744
737	BPSS0337			468862	468596	1495667	1495933	2795219	2794953	2307638	2307372	526763	526497	542179	541913	538670	538404	484709	484443	453214	452948
738	BPSS0338			470300	469080	1494229	1495449	2796658	2795438	2309077	2307857	528206	527091	543630	542398	540109	538889	486152	485037	454640	453543
739	BPSS0339			472503	470452	1492026	1494077	2798854	2796803	2311273	2309222	530402	528351	545905	543845	542305	540254	488348	486297	456843	454792

740	BPSS0340			472763	473215	1491766	1491314	2799114	2799566	2311533	2311985	530662	531114	546163	546615	542565	543017	488608	489060	457103	457555
741	BPSS0341			473215	474066	1491314	1490463	2799566	2800417	2311985	2312836	531114	531965	546615	547466	543017	543868	489060	489911	457555	458406
742	BPSS0342			474118	475461	1490411	1489068	2800468	2801775	2312887	2314230	532017	533360	547517	548860	543919	545262	489962	491305	458457	459800
743	BPSS0343			476975	475869	1487554	1488660	2803097	2802003	2315745	2314639	534939	533833	550226	549120			492861	491755	461291	460185
744	BPSS0344			477213	478292	1487316	1486237	2803335	2804414	2315983	2317062	535177	536256	550464	551543	545531	546556	493099	494178	461529	462608
745	BPSS0345			478352	479446	1486177	1485083	2804474	2805568	2317122	2318216	536316	537410	551603	552697	546616	547710	494238	495332	462668	463762
746	BPSS0346			479427	480371	1485102	1484158	2805549	2806493	2318197	2319141	537391	538335	552678	553622	547691	548635	495313	496257	463743	464687
747	BPSS0347			480364	481215	1483783	1483313	2806486	2807337	2319134	2319985	538328	539179	553615	554466	548628	549479	496250	497101	464680	465531
748	BPSS0348			481262	482530	1483266	1481998	2807384	2808652	2320032	2321300	539226	540494	554513	555781	549526	550794	497148	498416	465578	466846
749	BPSS0349			482539	483981	1481989	1480547	2808661	2810091	2321309	2322751	540503	541357	555790	557232	550803	552245	498425	499867	466855	468297
750	BPSS0350			483989	484537	1480539	1479991			2322759	2323307	541954	542502	557240	557788	552253	552801	499875	500423	468305	468778
751		BURPSS13_W0001																500825	500604		
752		BURPSS13_W0002																501180	501061		
753		BURPSS13_W0003																501354	501217		
754		BURPSS13_W0004																501134	501262		
755	BPSS0351			490819	491220	1242939	1243340	2811307	2811708	2329654	2330055	548725	549126	564126	564527	554300	554701	503590	503991	469655	470056
756	BPSS0352			492927	492085	1245047	1244205	2813415	2812573	2331762	2330920	550833	549991	566234	565392	556408	555566	505698	504856	471763	470921
757	BPSS0353			494047	493079	1246167	1245199	2814535	2813567	2332882	2331914	551953	550985	567354	566386	557528	556560	506818	505850	472883	471915
758	BPSS0354			494235	495020	1246355	1247140	2814723	2815508	2333070	2333855	552141	552926	567542	568327	557716	558501	507006	507791	473071	473856
759	BPSS0355			496452	495256	1248572	1247376	2816936	2815740	2335283	2334087	554354	553158	569754	568558	559929	558733	509223	508027	475288	474092
760	BPSS0356			496740	498827	1248860	1250947	2817224	2819311	2335571	2337658	554642	556729	570041	572128	560217	562304	509511	511598	475576	477663
761	BPSS0357			499054	499602	1251174	1251722	2819538	2820086	2337885	2338433	556956	557504	572355	572903	562531	563079	511825	512373	477890	478438
762	BPSS0358			499665	499991	1251785	1252111	2820146	2820472	2338493	2338819	557564	557890	572963	573289	563139	563465	512436	512762	478501	478827
763	BPSS0359			500054	500893	1252174	1253013	2820535	2821374	2338882	2339721	557953	558792	573352	574191	563528	564367	512825	513664	478890	479729
764	BPSS0360			500893	502290	1253013	1254410	2821374	2822771	2339721	2341118	558792	560189	574191	575588	564367	565764	513664	515061	479729	481126
765	BPSS0361			502353	502760	1254473	1254880	2822834	2823241	2341181	2341588	560252	560659	575651	576058	565827	566234	515124	515531	481189	481596
766	BPSS0362			503329	503069	1255449	1255189	2823810	2823550	2342157	2341897	561228	560968	576627	576367	566802	566542	516100	515840	482165	481905
767	BPSS0363			503958	503476	1256078	1255596	2824441	2823959	2342788	2342306	561858	561376	577257	576775	567432	566950	516730	516248	482795	482313
768	BPSS0364			504949	504080	1257069	1256200	2825432	2824563	2343779	2342910	562849	561980	578248	577379	568423	567554	517721	516852	483786	482917
769	BPSS0365			505072	506040	1257192	1258160	2825555	2826523	2343902	2344870	562972	563940	578371	579339	568546	569514	517844	518812	483909	484877
770			Bp_chr2_5_ISBma2															519131	520703		
771	BPSS0366			506848	506429	1258968	1258549	2827331	2826912	2345678	2345259	564748	564329	580148	579729	570322	569903	521219	520800	485685	485266
772	BPSS0367			507596	506871	1259716	1258991	2828079	2827354	2346426	2345701	565496	564771	580896	580171	571070	570345	521967	521242	486433	485708
773	BPSS0368			508328	507636	1260448	1259756	2828805	2828119	2347152	2346466	566228	565536	581628	580936	571802	571110	522693	522007	487165	486473
774	BPSS0369			508843	508607	1260963	1260727	2829320	2829084	2347667	2347431	566743	566507	582154	581918	572317	572081	523208	522972	487680	487444
775	BPSS0370			509847	508978	1261967	1261098	2830324	2829455	2348671	2347802	567747	566878	583158	582289	573321	572452	524212	523343	488683	487814
776	BPSS0371			510370	509897	1262490	1262017	2830847	2830374	2349194	2348721	568270	567797	583681	583208	573844	573371	524735	524262	489206	488733
777	BPSS0372			512060	510528	1264180	1262648	2832537	2831005	2350884	2349352	569960	568428	585371	583839	575534	574002	526425	524893	490896	489364
778	BPSS0373			512708	512103	1264828	1264223	2833185	2832580	2351532	2350927	570608	570003	586019	585414	576182	575577	527073	526468	491544	490939
779	BPSS0374			513840	512944	1265960	1265064	2834317	2833421	2352664	2351768	571740	570844	587151	586255	577314	576418	528205	527309	492676	491780
780	BPSS0375			514130	516109	1266250	1268229	2834607	2836586	2352954	2354933	572030	574009	587441	589420	577604	579394	528495	530474	492966	494945
781	BPSS0376			516210	516587	1268330	1268707	2836687	2837064	2355034	2355411	574110	574487	589521	589898	579581	579958	530575	530952	495046	495423
782	BPSS0377			516584	518596	1268704	1270716	2837061	2839073	2355408	2357420	574484	576496	589895	591907	579955	581967	530949	532961	495420	497432
783		BURPSPAST_D0350														582668	583033				
784		BURPSPAST_D0351														585986	585387				
785		BURPSPAST_D0352														586460	587908				
786		BURPSPAST_D0353														588264	589061				
787		BURPSS13_L0034																533144	533548		
788		BURPSS13_L0035																533773	534027		
789		BURPSS13_L0036																536980	536381		
790		BURPSS13_L0037																537454	538902		
791		BURPSS13_L0038																539258	540055		
792		BURPSS13_L0039																541282	541458		
793			Bp_chr2_6_IS407A											592154	593390						
794		BURPS668_A0633												592222	592485						
795		BURPS668_A0634												592509	593342						
796		BURPS668_A0635												593459	593974						
797		BURPS668_A0636												594096	596834						

798		BURPS668_A0638												599284	599838						
799		BURPS668_A0639												599850	600137						
800		BURPS668_A0640												600509	601333						
801			Bp_chr2_7_IS407A											600590	601688						
802		BURPS668_A0641												601357	601620						
803		BURPS668_A0642												601686	601844						
804		BURPS668_A0643												601716	602684						
805		BURPS668_A0644												602875	603024						
806		BURPS668_A0645												603057	603806						
807		BURPS668_A0646												604424	604630						
808	BPSS0378		GI13																		
809	BPSS0379		GI13																		
810	BPSS0380		GI13																		
811	BPSS0380A		GI13																		
812	BPSS0380B		GI13																		
813	BPSS0381		GI13																		
814	BPSS0382		GI13																		
815	BPSS0383		GI13																		
816	BPSS0384		GI13																		
817	BPSS0384A		GI13																		
818	BPSS0385		GI13																		
819	BPSS0386		GI13																		
820	BPSS0387		GI13																		
821	BPSS0388		GI13																		
822	BPSS0389		GI13																		
823	BPSS0390		GI13																		
824	BPSS0391		GI13																		
825	BPSS0391A		GI13																		
826	BPSS0392		GI13	519272	518898	1271392	1271018	2839749	2839375	2358096	2357722	577172	576798							498108	497734
827	BPSS0393		GI13	524155	524388	1276275	1276508	2840008	2839772	2358355	2358119	581754	581990			592423	592509	543417	543503	502691	502927
828		BURPSPAST_D0354														592457	591570				
829		BURPSS13_L0040																543451	542564		
830	BPSS0394		GI13	523453	523734	1275573	1275854	2840709	2840428	2359056	2358775	581052	581333							501989	502270
831	BPSS0395		GI13	523248	523466	1275368	1275586	2840914	2840696	2359261	2359043	580848	580889							501785	501826
832	BPSS0396		GI13	522628	522356	1274748	1274476	2841534	2841806	2359881	2360153	580247	579975							501183	500911
833	BPSS0397		GI13	522343	522215	1274463	1274335	2841819	2841947	2360166	2360294	579962	579834							500898	500770
834	BPSS0397A		GI13	522210	522106	1274330	1274226	2841952	2842056	2360299	2360403	579829	579725							500765	500661
835		BURPS1710A_A3036						2842198	2842641												
836	BPSS0398		GI13									579695	579477							500631	500416
837	BPSS0399		GI13	521270	521533	1273390	1273653	2842892	2842629	2361239	2360976	579223	579486							500159	500422
838		BURPS1106A_A0539		521521	521964																
839		BURPS1106B_1459				1274084	1273641														
840	BPSS0400		GI13	520382	521077	1272502	1273197	2843781	2843692	2362127	2361432										
841	BPSS0401		GI13	520240	520094	1272360	1272214	2843923	2844069	2362269	2362415	578137	577994							499076	498930
842	BPSS0402			520094	518955	1272214	1271075	2844069	2845241	2362415	2363587	577994	576855							498930	497791
843	BPSS0403			525767	527224	1277887	1279344	2846244	2847701	2364590	2366047	583347	584804			592951	594408	543945	545402	504284	505741
844	BPSS0404			527278	528645	1279398	1280765	2847755	2849122	2366101	2367468	584858	586225			594462	595829	545456	546823	505795	507162
845	BPSS0405			528805	529734	1280925	1281854	2849282	2850211	2367628	2368557	586385	587314			595989	596918	546983	547912	507322	508251
846	BPSS0406			529935	529994	1282055	1282114	2850412	2850612	2368758	2368958	587515	587715			597119	597319	548113	548172	508452	508652
847	BPSS0407			530162	530860	1282282	1282980	2850640	2851338	2368986	2369684	587743	588453			597347	597805	548340	549038	508680	509378
848	BPSS0408			531635	530904	1283755	1283024	2852113	2851382	2370459	2369728	589217	588486	605826	605095	598819	598088	549813	549082	510153	509422
849	BPSS0409			544021	531629	1296141	1283749	2864501	2861856	2382845	2370453	601603	589211	618212	605820	611205	598813	562199	549807	522539	510147
850	BPSS0410			547583	544008	1299703	1296128	2868063	2864488	2386407	2382832	605165	601590	621774	618199	614767	611192	565761	562186	526101	522526
851	BPSS0411			548477	547704	1300597	1299824	2868957	2868184	2387301	2386528	606059	605286	622668	621895	615661	614888	566655	565882	526995	526222
852	BPSS0412			548934	548890	1301054	1301010	2869415	2868993	2387759	2387337	606517	606095	623126	622704	616118	616074	567113	566691	527453	527031
853	BPSS0413			549470	549874	1301590	1301994	2869951	2870355	2388295	2388699	607053	607457	623662	624066	616654	617058	567649	568053	527989	528393
854	BPSS0414			550116	551825	1302236	1303945	2870597	2872306	2388941	2390650	607699	609408	624308	626017	617300	619009	568295	570004	528635	530344
855	BPSS0415			554975	551991	1307095	1304111	2875456	2872472	2393800	2390816	612564	609574	629167	626183	622159	619175	573154	570170	533494	530510

856	BPSS0416		555621	554980	1307741	1307100	2876102	2875461	2394446	2393805	613210	612569	629813	629172	622805	622164	573800	573159	534140	533499
857	BPSS0416A		555714	556067	1307834	1308187	2876195	2876548	2394539	2394892	613303	613656	629906	630250	622898	623251	573893	574246	534233	534586
858	BPSS0417		557036	559489	1309156	1311609	2877517	2879970	2395861	2398314	614628	617084	631222	633678	624220	626673	575215	577668	535555	538005
859	BPSS0418		559497	560711	1311617	1312831	2879978	2881192	2398322	2399536	617092	618306	633686	634900	626681	627895	577676	578890	538013	539227
860	BPSS0419		560711	561484	1312831	1313604	2881192	2881965	2399536	2400309	618306	619079	634900	635673	627895	628668	578890	579663	539227	540000
861	BPSS0420		561490	562560	1313610	1314680	2881971	2883041	2400315	2401385	619085	620155	635679	636749	628674	629744	579669	580739	540006	541076
862	BPSS0421		562571	563920	1314691	1316040	2883052	2884401	2401396	2402745	620166	621515	636760	638109	629755	631104	580750	582099	541087	542436
863	BPSS0422		563949	565706	1316069	1317826	2884430	2886187	2402774	2404531	621544	623301	638138	639895	631133	632890	582128	583885	542465	544222
864	BPSS0423		565958	567310	1318078	1319430	2886439	2887791	2404783	2406135	623553	624905	640147	641499	633142	634494	584137	585489	544474	545826
865	BPSS0424		567310	568368	1319430	1320488	2887791	2888849	2406135	2407193	624905	625963	641499	642557	634494	635552	585489	586547	545826	546884
866	BPSS0425		568544	569797	1320664	1321917	2889025	2890278	2407369	2408622	626139	627392	642733	643986	635728	636981	586723	587976	547060	548313
867	BPSS0426		569706	570758	1321826	1322878	2890187	2891239	2408531	2409583	627301	628353	643895	644947	636890	637942	587885	588937	548222	549274
868	BPSS0427		570754	571320	1322874	1323440	2891235	2891801	2409579	2410145	628349	628915	644943	645509	637938	638504	588933	589499	549270	549836
869	BPSS0428		571335	572378	1323455	1324498	2891816	2892859	2410160	2411203	628930	629973	645524	646567	638519	639562	589514	590557	549851	550894
870	BPSS0429		572385	573431	1324505	1325551	2892866	2893912	2411210	2412256	629980	631026	646574	647620	639569	640615	590564	591610	550901	551947
871	BPSS0430						2894792	2894280	2413136	2412624					641635	641123	592199	591978	552867	552355
872	BPSS0431		575266	574370	1327386	1326490	2895778	2894882	2414122	2413226	632881	631985	649540	648644	642621	641725	593399	592503	553853	552957
873	BPSS0432		575396	576118	1327516	1328238	2895908	2896630	2414252	2414974	633011	633733	649670	650392	642751	643473	593529	594251	553983	554705
874		BURPSS13_L0077															594438	594235		
875	BPSS0433		576396	577073	1328516	1329193	2896914	2897591	2415258	2415935	634017	634694	650730	651407	643743	644420	594538	595215	555002	555679
876	BPSS0434		578155	577109	1330275	1329229	2898673	2897627	2417017	2415971	635776	634730	652489	651443	645502	644456	596297	595251	556761	555715
877	BPSS0435		578494	579417	1330614	1331537	2899012	2899935	2417356	2418279	636115	637044	652828	653745	645841	646764	596636	597559	557100	558029
878	BPSS0436		580381	579482	1332501	1331602	2900899	2900000	2419243	2418344	638008	637109	654709	653810	647734	646829	598523	597624	558993	558094
879	BPSS0437		581933	580734	1334053	1332854	2902451	2901252	2420795	2419596	639560	638361	656261	655062	649286	648087	600075	598876	560545	559346
880	BPSS0438		582339	583247	1334459	1335367	2902857	2903765	2421201	2422109	639966	640874	656667	657575	649692	650600	600481	601389	560951	561859
881	BPSS0439		584211	583528	1336331	1335648	2904733	2904050	2423077	2422394	641964	641281	658537	657854	651568	650885	602356	601673	562814	562131
882	BPSS0440		584743	585054	1336863	1337174	2905260	2905571	2423604	2423915	642491	642802	659064	659375	652095	652406	602883	603194	563341	563652
883	BPSS0441		585050	586945	1337170	1339065	2905567	2907462	2423911	2425806	642798	644693	659371	661266	652402	654297	603190	605085	563648	565543
884	BPSS0442		587169	588398	1339289	1340518	2907686	2908915	2426030	2427259	644917	646146	661469	662701	654521	655750	605293	606522	565765	566994
885	BPSS0443		588734	590080	1340854	1342200	2909239	2910585	2427583	2428929	646503	647849	663013	664359	656074	657420	606870	608216	567333	568679
886	BPSS0444		590649	591467	1342769	1343587	2911154	2911972	2429498	2430316	648418	649236	664928	665746	657989	658807	608798	609616	569248	570066
887	BPSS0445		591892	592026	1343617	1344027	2912002	2912544	2430346	2430888	649266	649712	665776	666186	658837	659247	609646	610056	570096	570506
888	BPSS0446		592347	592973	1344467	1345093	2912865	2913491	2431209	2431835	650130	650756	666626	667252	659680	660306	610489	611115	570942	571568
889		BURPS1710A_A3100					2913536	2914141												
890	BPSS0447		593646	594248	1345773	1346375	2914138	2914740	2432482	2433084	651442	652044	667875	668477	660967	661569	611788	612390	572234	572836
891	BPSS0448		594840	594391	1346967	1346518	2915332	2914883	2433676	2433227	652636	652187	669069	668620	662161	661712	612982	612533	573428	572979
892	BPSS0449		596420	595089	1348547	1347216	2916915	2915584	2435259	2433928	654204	652873	670663	669332	663758	662427	614577	613246	575002	573671
893	BPSS0450		596509	597123	1348636	1349250	2917004	2917618	2435348	2435962	654293	654907	670752	671366	663847	664461	614666	615280	575091	575705
894		BURPS668_A0703											671544	671738						
895		BURPSPAST_D0416													664823	664560				
896	BPSS0451		598570	597485	1350697	1349612	2919065	2917980	2437409	2436324	656356	655271	672813	671728	665908	664823	616727	615642	577153	576068
897		BURPS1710A_A3109					2919511	2919158												
898	BPSS0452		599682	601328	1351809	1353455	2920236	2922185	2438580	2440529	657485	659404	673928	675853	667061	669022	617862	619805	578274	580223
899	BPSS0453		601916	602320	1354043	1354447	2922457	2922861	2440801	2441205	659674	660078	676102	676506	669294	669698	620077	620481	580493	580897
900	BPSS0454		602421	602714	1354548	1354841	2922962	2923255	2441306	2441599	660174	660467	676602	676892	669799	670092	620582	620875	580993	581286
901	BPSS0455		602714	604375	1354841	1356502	2923255	2924916	2441599	2443260	660467	662140	676892	678553	670092	671753	620875	622536	581286	582947
902	BPSS0456		604381	605673	1356508	1357800	2924922	2926214	2443266	2444558	662146	663438	678559	679851	671759	673051	622542	623834	582953	584245
903	BPSS0457		605795	606148	1357922	1358275	2926336	2926689	2444680	2445033	663560	663913	679973	680335	673173	673526	623956	624327	584367	584720
904	BPSS0458		606201	606578	1358328	1358705	2926742	2927119	2445086	2445463	663966	664343	680388	680765	673579	673956	624380	624757	584773	585150
905	BPSS0459		606581	607522	1358708	1359649	2927122	2928063	2445466	2446407	664346	665287	680768	681709	673959	674900	624760	625701	585153	586094
906	BPSS0460		607826	609673	1359953	1361800	2928367	2930211	2446711	2448555	665591	667435	682013	683860	675204	677048	626005	627849	586398	588233
907	BPSS0461		611276	609987	1363403	1362114	2931814	2930525	2450158	2448869	669038	667749	685463	684174	678651	677362	629452	628163	589836	588547
908	BPSS0462		612678	611350	1364805	1363477	2933216	2931888	2451560	2450232	670440	669112	686865	685537	680053	678725	630854	629526	591238	589910
909	BPSS0463		614356	612785	1366483	1364912	2934894	2933323	2453238	2451667	672118	670547	688543	686972	681731	680160	632532	630961	592916	591345
910	BPSS0464		615354	614428	1367481	1366555	2935892	2934966	2454236	2453310	673116	672190	689541	688615	682729	681803	633530	632604	593914	592988
911	BPSS0465		616331	615354	1368458	1367481	2936869	2935892	2455213	2454236	674093	673116	690518	689541	683706	682729	634507	633530	594891	593914
912	BPSS0466		617482	616331	1369609	1368458	2938020	2936869	2456364	2455213	675244	674093	691669	690518	684857	683706	635658	634507	596042	594891
913	BPSS0467		618793	617675	1370920	1369802	2939331	2938213	2457675	2456557	676555	675437	692980	691862	686168	685050	636969	635851	597353	596235

914	BPSS0468	620279	618864	1372406	1370991	2940501	2939863	2459161	2457746	678041	676626	694466	693051	687654	686239	638455	637040	598839	597424
915	BPSS0469	621649	620318	1373776	1372445	2941871	2940540	2460531	2459200	679411	678080	695836	694505	689024	687693	639825	638494	600209	598878
916	BPSS0470	622476	621679	1374603	1373806	2942698	2941901	2461358	2460561	680238	679441	696663	695866	689851	689054	640652	639855	601036	600239
917	BPSS0471	622711	623115	1374838	1375242	2942933	2943337	2461593	2461997	680473	680877	696898	697302	690086	690490	640887	641291	601271	601675
918	BPSS0472	623418	623966	1375545	1376093	2943640	2944188	2462300	2462848	681180	681728	697605	698153	690793	691341	641594	642142	601978	602526
919	BPSS0473	624079	625566	1376206	1377693	2944313	2945800	2462973	2464460	681857	683344	698282	699769	691466	692953	642267	643754	602635	604122
920	BPSS0474	625700	626659	1377827	1378786	2945934	2946893	2464594	2465553	683478	684437	699903	700862	693087	694046	643888	644847	604256	605215
921	BPSS0475	626781	627191	1378908	1379318	2947015	2947425	2465675	2466085	684559	684969	700984	701391	694168	694578	644969	645379	605337	605747
922	BPSS0476	627332	627619	1379459	1379746	2947622	2947909	2466282	2466569	685110	685397	701577	701864	694775	695062	645576	645863	605944	606231
923	BPSS0477	627644	629287	1379771	1381414	2947934	2949571	2466594	2468231	685422	687059	701889	703532	695087	696724	645888	647531	606256	607893
924	BPSS0478	629797	629354	1381924	1381481	2950082	2949639	2468742	2468299	687570	687127	704042	703599	697235	696792	648041	647598	608404	607961
925	BPSS0479	630404	632935	1382531	1385062	2950690	2953221	2469350	2471881	688178	690709	704649	707180	697843	700374	648648	651179	609012	611543
926	BPSS0480	633499	633618	1385626	1385745	2953792	2953893	2472452	2472553	691290	691391	707744	707863	700945	701046	651743	651862	612125	612226
927	BPSS0481	633972	635594	1386099	1387721	2954247	2955869	2472907	2474529	691745	693367	708218	709840	701400	703022	652217	653839	612580	614202
928	BPSS0482	635684	636571	1387811	1388698	2955959	2956846	2474619	2475506	693457	694344	709930	710817	703112	703999	653929	654816	614292	615155
929	BPSS0483	636574	637677	1388701	1389804	2956849	2957952	2475509	2476612	694347	695450	710820	711323	704002	705105	654819	655922	615158	616261
930	BPSS0484	637670	638665	1389797	1390792	2957945	2958940	2476605	2477600	695443	696438	711876	712871	705098	706093	655915	656910	616254	617249
931	BPSS0485	638683	639570	1390810	1391697	2958958	2959845	2477618	2478505	696456	697343	712889	713776	706111	706998	656928	657815	617267	618154
932	BPSS0486	639701	643549	1391828	1395676	2959976	2963848	2478636	2482508	697474	701346	713907	717779	707129	711007	657946	661830	618285	622151
933	BPSS0487	643566	645698	1395693	1397825	2963865	2965997	2482525	2484657	701363	703495	717796	719928	711024	713156	661847	663979	622168	624300
934	BPSS0488	646068	646436	1398195	1398563	2966367	2966735	2485027	2485395	703904	704239	720298	720666	713526	713894	664349	664717	624670	625038
935	BPSS0488A	646780	647220	1398907	1399347	2967066	2967503	2485726	2486163	704680	705024			714225	714662	665158	665502	625485	625829
936	BPSS0489	647272	647724	1399399	1399851	2967558	2968010	2486218	2486670	705076	705528	721510	721962	714717	715169	665554	666006	625881	626333
937	BPSS0490	648220	648426	1400347	1400553	2968506	2968712	2487166	2487372	706024	706230	722506	722712	715665	715871	666502	666708	626877	627083
938	BPSS0491	650290	648686	1402417	1400813	2970576	2968972	2489236	2487632	708094	706490	724576	722972	717735	716131	668572	666968	628947	627343
939	BPSS0492	651057	650497	1403184	1402624	2971343	2970783	2490003	2489443	708906	708346	725342	724782	718502	717942	669339	668779	629714	629154
940	BPSS0493	652414	651320	1404541	1403447	2972700	2971606	2491360	2490266	710269	709169	726699	725605	719859	718765	670696	669602	631071	629977
941	BPSS0494	653477	652977	1405604	1405104	2973763	2973263	2492423	2491923	711332	710832	727762	727262	720922	720422	671757	671257	632134	631634
942	BPSS0495	654175	653555	1406302	1405682	2974461	2973841	2493121	2492501	712030	711410	728460	727840	721620	721000	672455	671835	632832	632212
943	BPSS0496	654889	656322	1407016	1408449	2975174	2976607	2493834	2495267	712743	714176	729174	730607	722333	723766	673169	674602	633546	634979
944	BPSS0497	656420	657358	1408547	1409485	2976705	2977643	2495365	2496303	714274	715212	730705	731643	723864	724802	674700	675638	635077	636015
945	BPSS0498	657370	658323	1409497	1410450	2977655	2978608	2496315	2497268	715224	716177	731655	732608	724814	725767	675650	676603	636027	636980
946	BPSS0499	658463	661882	1410590	1414009	2978748	2982167	2497408	2500827	716317	719730	732748	736167	725907	729326	676743	680162	637120	640539
947	BPSS0500	662073	664718	1414200	1416845	2982358	2985003	2501018	2503663	719921	722566	736358	739003	729517	732162	680353	682998	640730	643375
948	BPSS0501	664712	665761	1416839	1417888	2984997	2986016	2503657	2504676	722560	723579	738997	740016	732156	733190	682992	684011	643369	644388
949	BPSS0502	667454	665955	1419581	1418082	2987709	2986210	2506369	2504870	725272	723773	741817	740318	734883	733384	685746	684247	646081	644582
950	BPSS0503	667582	668766	1419709	1420893	2987837	2989021	2506497	2507681	725400	726584	741945	743129	735011	736195	685874	687058	646209	647393
951	BPSS0504	670164	669094	1422291	1421221	2990419	2989349	2509079	2508009	727982	726912	744511	743441	737593	736523	688456	687386	648799	647729
952	BPSS0505	671368	670406	1423495	1422533	2991623	2990661	2510283	2509321	729186	728224	745722	744760	738797	737835	689660	688698	650003	649041
953	BPSS0506	671510	672205	1423637	1424332	2991765	2992460	2510425	2511120	729328	730023	745864	746559	738939	739634	689802	690497	650145	650840
954	BPSS0507	673421	672357	1425548	1424484	2993676	2992612	2512336	2511272	731239	730175	747775	746711	740850	739786	691713	690649	652056	650992
955	BPSS0508	674617	673439	1426744	1425566	2994872	2993694	2513532	2512354	732435	731257	748971	747793	742046	740868	692909	691731	653252	652074
956	BPSS0509	676248	674617	1428375	1426744	2996503	2994872	2515163	2513532	734066	732435	750602	748971	743677	742046	694540	692909	654883	653252
957	BPSS0510	677081	676302	1429208	1428429	2997336	2996557	2515996	2515217	734899	734120	751435	750656	744510	743731	695373	694594	655716	654937
958	BPSS0511	678106	677108	1430233	1429235	2998361	2997363	2517021	2516023	735924	734926	752460	751462	745535	744537	696398	695400	656741	655743
959	BPSS0512	678876	678106	1431003	1430233	2999131	2998361	2517791	2517021	736694	735924	753230	752460	746305	745535	697168	696398	657511	656741
960	BPSS0513	679562	678876	1431689	1431003	2999811	2999131	2518471	2517791	737380	736694	753916	753230	746991	746305	697854	697168	658197	657511
961	BPSS0514	679837	681438	1431964	1433565	3000086	3001687	2518746	2520347	737655	739256	754205	755791	747266	748867	698129	699730	658472	660073
962	BPSS0515	683162	684097	1435289	1436224	3003411	3004346	2522071	2523006	740969	741904	757515	758450	750580	751515	701454	702389	661796	662731
963	BPSS0516	684134	684679	1436261	1436806	3004383	3004928	2523043	2523588	741941	742486	758487	759032	751552	752097	702426	702971	662768	663313
964	BPSS0517	684679	686187	1436806	1438314	3004928	3006436	2523588	2525096	742486	743994	759032	760540	752097	753605	702971	704479	663313	664821
965	BPSS0518	686334	686858	1438461	1438985	3006583	3007107	2525243	2525767	744141	744665	760687	761211	753752	754276	704626	705150	664968	665492
966	BPSS0519	686902	687369	1439029	1439496	3007151	3007618	2525811	2526278	744709	745176	761255	761722	754320	754787	705194	705661	665536	666003
967	BPSS0520	687386	689245	1439513	1441372	3007635	3009494	2526295	2528154	745193	747052	761739	763598	754804	756663	705678	707537	666020	667879
968	BPSS0521	689245	690231	1441372	1442358	3009494	3010480	2528154	2529140	747052	748038	763598	764584	756663	757649	707537	708523	667879	668865
969	BPSS0522	690237	693104	1442364	1445231	3010486	3013353	2529146	2532013	748044	750911	764590	767457	757655	760522	708529	711387	668871	671738
970	BPSS0523	693098	695386	1445225	1447513	3013347	3015635	2532007	2534295	750905	753193	767451	769739	760516	762804	711381	713669	671732	674020
971	BPSS0524	695555	697840	1445225	1447513	3013347	3015635	2534464	2536749	750905	753193	769908	772193	760516	762804	711381	713669	674189	676474

972	BPSS0525		697847	700060	1449974	1452187	3018096	3020309	2536756	2538969	755654	757867	772200	774413	765265	767478	716130	718343	676481	678694
973	BPSS0526		700060	701130	1452187	1453257	3020309	3021379	2538969	2540039	757867	758937	774413	775483	767478	768548	718343	719413	678694	679764
974	BPSS0527		701136	701849	1453263	1453976	3021385	3022098	2540045	2540758	758943	759656	775489	776202	768554	769267	719419	720132	679770	680483
975	BPSS0528		701895	702281	1454022	1454408	3022144	3022530	2540804	2541190	759702	760088	776248	776634	769313	769699	720178	720564	680529	680915
976	BPSS0529		702290	702880	1454417	1455007	3022539	3023129	2541199	2541789	760097	760687	776643	777233	769708	770298	720573	721163	680924	681514
977	BPSS0530		702880	704238	1455007	1456365	3023129	3024487	2541789	2543147	760687	762045	777233	778591	770298	771656	721163	722521	681514	682872
978	BPSS0531		704324	705979	1456451	1458106	3024573	3026228	2543233	2544888	762131	763786	778677	780332	771742	773397	722607	724262	682958	684613
979	BPSS0532		705979	709479	1458106	1461606	3026228	3029728	2544888	2548388	763786	767286	780332	783832	773397	776897	724262	727762	684613	688113
980	BPSS0533		709541	709897	1461668	1462024	3029790	3030146	2548450	2548806	767348	767704	783894	784250	776959	777315	727824	728180	688175	688531
981	BPSS0534		709923	710345	1462050	1462472	3030172	3030594	2548832	2549254	767730	768152	784276	784704	777341	777760	728206	728628	688557	688979
982	BPSS0535		710573	710941	1462700	1463068	3030822	3031190	2549482	2549850	768380	768748	784932	785300	777988	778356	728856	729224	689207	689575
983		BURPS1655_L0002																	691987	691556
984	BPSS0536		711495	712391	369717	370613	1014559	1013663	2550404	2551300	769302	770198	785854	786750	1877791	1876895	730785	731678	1665182	1664286
985	BPSS0537		712628	713944	370850	372166	3032271	3033587	2551537	2552853	1925238	1923922	786987	788303	1876670	1875354	731903	733219	1664036	1662720
986	BPSS0538		713944	715524	372166	373749	3033587	3035170	2552853	2554436	1923922	1922339	788303	789886	1875354	1873771	733219	734802	1662720	1661137
987	BPSS0539		715780	716772	374005	374997	3035414	3036406	2554680	2555672	771115	772044	790106	791110	779330	780322	735082	736074	1660881	1659889
988	BPSS0540		717080	718660	375305	376885	3036714	3038294	2555980	2557560	772352	773932	791418	792998	780630	782210	736382	737962	1659581	1658001
989		BURPS1106A_A0731	718979	719167																
990	BPSS0541		719489	720652	377714	378877	3039160	3040023	2558131	2559294	774761	775924	793815	794978	782781	783944	738791	739954	692084	693247
991	BPSS0542		722842	721181	381067	379406	3042204	3040543	2561475	2559814	778114	776453	797181	795520	786124	784463	742144	740483	695446	693785
992	BPSS0543		724540	722978	382765	381203	3043902	3042340	2563173	2561611	779812	778250	798879	797317	787822	786260	743842	742280	697144	695582
993	BPSS0544		724729	725742	382954	383967	3044091	3045104	2563362	2564375	780001	781014	799068	800081	788011	789024	744031	745044	697333	698346
994	BPSS0545		726185	727381	384410	385606	3045617	3046813	2564888	2566084	781464	782660	800560	801756	789495	790691	745487	746683	698796	699992
995	BPSS0546		728587	727565	386812	385790	3048019	3046997	2567290	2566268	783866	782844	802962	801940	791897	790875	747889	746867	701198	700176
996	BPSS0547		729005	730291	387230	388516	3048438	3049724	2567709	2568995	784285	785571	803380	804666	792316	793602	748307	749593	701619	702905
997	BPSS0548		730367	731335	388592	389560	3049800	3050768	2569071	2570039	785647	786615	804742	805710	793678	794646	749669	750637	702981	703949
998	BPSS0549		731489	732019	389714	390244	3050922	3051452	2570193	2570723	786769	787299	805864	806394	794800	795330	750791	751321	704103	704633
999	BPSS0550		732083	734143	390308	392368	3051516	3053576	2570787	2572847	787363	789423	806458	808518	795394	797454	751385	753445	704697	706757
1000	BPSS0551		734149	736071	392374	394296	3053582	3055504	2572853	2574775	789429	791351	808524	810446	797460	799382	753451	755373	706763	708685
1001	BPSS0552		736079	737248	394304	395473	3055512	3056681	2574783	2575952	791359	792528	810454	811623	799390	800559	755381	756550	708693	709862
1002	BPSS0553		737248	738030	395473	396255	3056681	3057463	2575952	2576734	792528	793310	811623	812405	800559	801341	756550	757332	709862	710644
1003	BPSS0554		738058	739323	396283	397548	3057491	3058756	2576762	2578027	793338	794603	812433	813698	801369	802634	757360	758625	710672	711937
1004	BPSS0555		739347	740489	397572	398714	3058780	3059922	2578051	2579193	794627	795769	813722	814864	802658	803800	758649	759791	711961	713103
1005	BPSS0556		740602	741462	398827	399687	3060035	3060895	2579306	2580166	795882	796742	814977	815837	803913	804773	759904	760764	713216	714076
1006	BPSS0557		741646	743298	399871	401523	3061079	3062731	2580350	2582002	796926	798578	816021	817673	804957	806609	760948	762600	714260	715912
1007	BPSS0558		744263	743391	402488	401616	3063696	3062824	2582967	2582095	799543	798671	818638	817766	807574	806702	763565	762693	716877	716005
1008	BPSS0559		745312	744416	403537	402641	3064739	3063843	2584010	2583114	800592	799696	819681	818785	808617	807721	764608	763712	717920	717024
1009	BPSS0560		745448	746998	403673	405223	3064875	3066425	2584146	2585696	800728	802278	819817	821367	808753	810303	764744	766294	718056	719606
1010	BPSS0561		747037	748032	405262	406257	3066464	3067459	2585735	2586730	802317	803312	821406	822401	810342	811337	766333	767328	719645	720640
1011	BPSS0562		748263	749402	406488	407627	3067690	3068829	2586961	2588100	803543	804682	822632	823771	811568	812707	767559	768698	720871	722010
1012	BPSS0563		750686	749463	408911	407688	3070113	3068890	2589384	2588161	805966	804743	825055	823832	813991	812768	769982	768759	723294	722071
1013		BURPSS13_C0153															770226	770038		
1014	BPSS0564		752507	750807	410732	409032	3071934	3070234	2591205	2589505	807853	806153	826875	825175	815812	814112	772092	770392	725115	723415
1015	BPSS0565		753923	752982	412148	411207	3073350	3072409	2592621	2591680	809269	808328	828291	827350	817228	816287	773508	772567	726531	725590
1016	BPSS0566		754191	755138	412416	413363	3073618	3074565	2592889	2593836	809537	810484	828559	829506	817496	818443	773776	774723	726799	727746
1017	BPSS0567		755224	756150	413449	414375	3074651	3075574	2593922	2594845	810570	811496	829592	830515	818529	819455	774809	775735	727832	728758
1018	BPSS0568		756268	757230	414493	415455	3075695	3076657	2594966	2595928	811614	812576	830636	831598	819573	820535	775853	776815	728876	729838
1019	BPSS0569		757251	757730	415476	415955	3076678	3077157	2595949	2596428	812597	813076	831619	832098	820556	821035	776836	777315	729859	730338
1020	BPSS0570		757769	758725	415994	416950	3077196	3078152	2596467	2597423	813115	814071	832137	833093	821074	822030	777354	778310	730377	731333
1021	BPSS0571		759383	759078	417608	417303	3078810	3078505			814729	814424	833750	833445	822688	822383	778968	778663	731991	731686
1022	BPSS0572		760605	759619	418830	417844	3080033	3079047			815951	814965	834972	833986	823911	822925	780191	779205	733214	732228
1023	BPSS0573		761114	760827	419339	419052	3080542	3080255	2599813	2599526	816460	816173	835481	835194	824420	824133	780700	780413	733723	733436
1024	BPSS0574		763128	761887	421353	420112	3082556	3081315	2601827	2600586	818474	817233	837494	836253	826434	825193	782714	781473	735737	734496
1025	BPSS0575		764090	765202	422315	423427	3083446	3084558	2602717	2603829	819364	820476	838401	839513	827324	828436	783620	784732	736635	737747
1026	BPSS0576		765399	766322	423624	424547	3084755	3085678	2604026	2604949	820673	821596	839709	840632	828633	829556	784929	785852	737944	738867
1027	BPSS0577		766373	767602	424598	425827	3085729	3086958	2605000	2606229	821647	822876	840683	841912	829607	830836	785903	787132	738918	740147
1028	BPSS0578		767602	768372	425827	426597	3086958	3087728	2606229	2606999	822876	823646	841912	842682	830836	831606	787132	787902	740147	740917
1029	BPSS0579		768375	769073	426600	427298	3087731	3088429	2607002	2607700	823649	824347	842685	843383	831609	832307	787905	788603	740920	741618

1030 BPSS0580		769811	771043	428036	429268	3089167	3090399	2608438	2609670	825085	826317	844121	845353	833045	834277	789341	790573	742356	743588
1031 BPSS0581		772813	771362	431038	429587	3092178	3090727	2611449	2609998	828089	828039	847132	845681	836047	834596	792341	790893	745358	743907
1032 BPSS0582		773115	772813	431340	431038	3092480	3092178	2611751	2611449	828391	828089	847434	847132	836349	836047	792643	792341	745660	745358
1033 BPSS0583		773885	773115	432110	431340	3093250	3092480	2612521	2611751	829161	828391	848204	847434	837119	836349	793413	792643	746430	745660
1034 BPSS0584		775525	773885	433750	432110	3094890	3093250	2614161	2612521	830801	829161	849844	848204	838759	837119	795053	793413	748070	746430
1035 BPSS0585		776177	777115	434402	435340	3095542	3096480	2614813	2615751	831405	832343	850484	851422	839411	840349	795706	796644	748722	749660
1036 BPSS0586		777245	781711	435470	439936	3096610	3101142	2615881	2620413	832473	836720	851552	856054	840479	845017	797783	801142	749790	754310
1037 BPSS0587		781711	787779	439936	446004	3101142	3107144	2620413	2626415	836980	840738	861205	862323	845017	851082	801142	804657	754310	760354
1038 BPSS0588		787779	788831	446004	447056	3107144	3108196	2626415	2627467	842916	843968	862323	863375	851082	852134	806943	807995	760354	761406
1039 BPSS0589		788831	790555	447056	448780	3108196	3109920	2627467	2629191	843968	845692	863375	865099	852134	853858	807995	809719	761406	763130
1040 BPSS0590		790555	792336	448780	450561	3109920	3111701	2629191	2630972	845692	847473	865099	866880	853858	855639	809719	811500	763130	764911
1041 BPSS0591		792416	794671	450641	452896	3111781	3114036	2631052	2633307	847553	849808	866960	869215	855719	857974	811580	813835	764991	767246
1042 BPSS0592		794674	794937	452899	453162	3114039	3114302	2633310	2633573	849811	850074	869218	869481	857977	858240	813842	814084	767249	767509
1043 BPSS0593		794942	796453	453167	454678	3114307	3115818	2633578	2635089	850079	851590	869486	870997	858245	859756	814089	815600	767514	769025
1044 BPSS0594		796473	797741	454698	455966	3115838	3117106	2635109	2636377	851610	852878	871017	872285	859776	861044	815620	816888	769045	770313
1045 BPSS0595		798409	798122	456634	456347	3117774	3117487	2637045	2636758	853546	853259	873157	872870	861712	861425	817556	817269	770980	770693
1046 BPSS0596		800724	798787	458949	457012	3120138	3118201	2639409	2637472	855861	853924	875479	873542	864083	862146	819885	817948	773337	771400
1047 BPSS0597		800994	801185	459219	459410	3120408	3120599	2639679	2639870	856131	856322	875749	875940	864353	864544	820155	820346	773607	773798
1048 BPSS0598		801871	802155	460096	460380	3121285	3121569	2640556	2640840	857008	857292	876627	876911	865228	865512	821032	821316	774485	774769
1049 BPSS0599		802223	802378	460448	460603	3121637	3121792	2640908	2641063	857360	857515	876979	877134	865580	865735	821384	821539	774837	774971
1050 BPSS0600		802378	802659	460603	460884	3121792	3122064	2641063	2641335	857515	857796	877134	877421	865735	866016	821539	821832		
1051 BPSS0601		804382	803084	462607	461309	3123855	3122557	2643126	2641828	859608	858310	879276	877978	867793	866495	823694	822396	776459	775161
1052 BPSS0602		804625	805248	462850	463473	3124088	3124711	2643359	2643982	859852	860475	879509	880132	868036	868659	823937	824560	776703	777326
1053 BPSS0603		806069	806368	464294	464593	3125524	3125823	2644795	2645094					869464	869763	825389	825688		
1054 BPSS0604		807565	807182	465790	465407	3127026	3126643	2646297	2645914	862867	862484	882484	882101	870966	870583	826877	826494	779668	779285
1055	BURPS1655_L0083																	779840	780496
1056	BURPS668_A0904											882586	883254						
1057	BURPSS13_C0057															827127	827363		
1058 BPSS0605		809044	808322	467269	466547	3128494	3127772	2647765	2647043	864336	863614	883961	883239	872437	871715	828367	827645	781201	780479
1059 BPSS0606		809161	810057	467386	468282	3128611	3129507	2647882	2648778	864453	865349	884078	884974	872554	873450	828484	829380	781318	782214
1060 BPSS0607		811562	810093	469787	468318	3131012	3129543	2650283	2648814	866854	865385	886479	885010	874955	873486	830885	829416	783719	782250
1061 BPSS0608		812479	811604	470704	469829	3131929	3131054	2651200	2650325	867771	866896	887396	886521	875872	874997	831802	830927	784636	783761
1062 BPSS0609		813647	812418	471872	470643	3133097	3131868	2652368	2651139	868939	867710	888576	887335	877040	875811	832970	831741	785804	784575
1063 BPSS0610		814555	813647	472780	471872	3134005	3133097	2653276	2652368	869847	868939	889484	888576	877948	877040	833878	832970	786712	785804
1064 BPSS0611		815931	815026	474156	473251	3135381	3134476	2654652	2653747	871223	870318	890860	889955	879324	878419	835254	834349	788088	787183
1065 BPSS0612		817759	815972	475984	474197	3137209	3135422	2656480	2654693	873051	871264	892688	890901	881152	879365	837082	835295	789916	788129
1066 BPSS0613		818700	817816	476925	476041	3138150	3137266	2657421	2656537	873992	873108	893629	892745	882093	881209	838023	837139	790857	789973
1067 BPSS0614		820004	819237	478229	477462	3139428	3138661	2658699	2657932	875270	874503	894924	894157	883371	882604	839335	838568	792177	791410
1068 BPSS0615		821509	820007	479734	478232	3140933	3139431	2660204	2658702	876775	875273	896435	894927	884876	883374	840840	839338	793682	792180
1069 BPSS0616		822720	821596	480945	479821	3142144	3141020	2661415	2660291	877986	876862	897646	896522	886087	884963	842051	840927	794893	793769
1070 BPSS0617		822820	823950	481045	482175	3142244	3143374	2661515	2662645	878086	879216	897746	898876	886187	887317	842151	843281	794993	796123
1071 BPSS0618		824308	826008	482533	484233	3143634	3145334	2662905	2664605	879574	881274	899430	901130	887577	889277	843688	845388	796432	798132
1072 BPSS0619		826080	827606	484305	485831	3145406	3146932	2664677	2666203	881346	882872	901202	902728	889349	890875	845460	846986	798204	799730
1073 BPSS0620		827721	828614	485946	486839	3147047	3147940	2666318	2667211	882987	883880	902843	903736	890990	891883	847101	847994	799845	800738
1074 BPSS0621		828617	829408	486842	487633	3147943	3148734	2667214	2668005	883883	884674	903739	904530	891886	892677	847997	848788	800741	801532
1075 BPSS0622		829408	830553	487633	488778	3148734	3149879	2668005	2669150	884674	885819	904530	905675	892677	893822	848788	849933	801532	802677
1076 BPSS0623		832391	831012	490616	489237	3151717	3150338	2670988	2669609	887675	886296	907519	906140	895735	894356	851789	850410	804511	803132
1077 BPSS0624		834362	832404	492587	490629	3153688	3151730	2672959	2671001	889646	887688	909490	907532	897706	895748	853760	851802	806482	804524
1078 BPSS0625		835567	834368	493792	492593	3154893	3153694	2674164	2672965	890851	889652	910692	909496	898911	897712	854965	853766	807687	806488
1079 BPSS0626		835896	836987	494121	495212	3155222	3156313	2674493	2675584	891180	892271	911020	912111	899240	900331	855294	856385	808016	809107
1080 BPSS0627		837363	838082	495588	496307	3156686	3157405	2675957	2676676	892653	893372	912471	913190	900713	901432	856767	857486	809489	810208
1081 BPSS0628		839474	838563	497699	496788	3158797	3157886	2678068	2677157	894764	893853	914583	913672	902834	901923	858945	858034	811631	810720
1082 BPSS0629		839519	839824	497744	498049	3158842	3159147	2678113	2678418	894809	895114			902879	903184	858990	859295	811676	811990
1083 BPSS0630		839950	840657	498175	498882	3159273	3159980	2678544	2679251	895240	895947	915095	915802	903310	904017	859421	860128	812116	812823
1084 BPSS0631		841048	842160	499273	500385	3160371	3161483	2679642	2680754	896338	897450	916193	917305	904408	905520	860519	861631	813214	814326
1085	BURPSS13_C0012															861747	861917		
1086 BPSS0632		844848	842512	503073	500737	3164165	3161835	2683436	2681106	900141	897802	919957	917648	908211	905872	864322	861983	816978	814669
1087 BPSS0633		845599	846660	503824	504885	3164894	3165955	2684162	2685223	900936	901997	920731	921792	908994	910055	865069	866130	817752	818813

1088	BPSS0634			847214	847801	505439	506026	3166473	3167060	2685741	2686328	902506	903093	922310	922897	910564	911151	866640	867227	819358	819945
1089	BPSS0635			848169	850295	506394	508520	3167347	3168867	2686615	2688741	903380	905506	923184	925310	911447	912778	867541	868848	820241	820987
1090	BPSS0636			850394	850654	508619	508879	3169574	3169834	2688840	2689100	905605	905865	925409	925669	913673	913933	869801	869998		
1091	BPSS0637			851928	850894	510153	509119	3171207	3171139	2690392	2689340	907155	907087			914894	914172	1829412	1828783		
1092	BPSS0639			852266	852739	510491	510964	3171545	3172018	2690728	2691201	907493	907966	927259	927732					822363	822836
1093	BPSS0640			853051	853707	511276	511932	3172314	3172970	2691497	2692153	908278	908934	928050	928706					823130	823786
1094	BPSS0641			853751	854458	511976	512683	3173014	3173721	2692197	2692904	908978	909685	928750	929457			1830005	1830712	823830	824537
1095		BURPS1655_L0122																		824756	824990
1096	BPSS0642			857724	856348	515949	514573	1800	424	2696160	2694784	912801	911425	932720	931344	916879	915503	872053	870677	826731	825355
1097	BPSS0643			858383	857724	516608	515949	2459	1800	2696819	2696160	913460	912801	933379	932720	917538	916879	872712	872053	827390	826731
1098	BPSS0644			858544	859401	516769	517626	2620	3477	2696980	2697837	913621	914478	933540	934397	917699	918556	872873	873730	827551	828408
1099	BPSS0645			859790	860056	518015	518281	3866	4132	2698226	2698492	914867	915133	934785	935051	918945	919211	874119	874385	828797	829063
1100	BPSS0646			860602	860279	518827	518504	4688	4365	2699048	2698725	915689	915366	935598	935275	919767	919444	874931	874608	829609	829286
1101	BPSS0647			861159	860695	519384	518920	5245	4781	2699605	2699141	916246	915782	936155	935691	920324	919860	875488	875024	830166	829702
1102	BPSS0648			861266	862219	519491	520444	5352	6305	2699712	2700665	916353	917306	936262	937215	920431	921384	875595	876548	830273	831226
1103		BURPS1106A_A0873		862481	862711																
1104		BURPS1106B_1112				520706	520936														
1105		BURPS1655_I0130																		831430	831780
1106		BURPS1710A_A0010						6576	6797												
1107		BURPS406E_G0033										917568	917798								
1108		BURPSPAST_J0249														921675	921896				
1109		BURPSS13_T0010																876834	877049		
1110	BPSS0649			862796	863257	521021	521482	6882	7358	2701242	2701718	917883	918344	937782	938243	921981	922457	877134	877595	831824	832300
1111	BPSS0650			863324	864772	521549	522997	7425	8873	2701785	2703233	918411	919859	938310	939758	922524	923972	877662	879110	832367	833815
1112	BPSS0651			864885	865250	523110	523475	8986	9351	2703346	2703711	919972	920337	939871	940236	924085	924450	879223	879588	833928	834287
1113	BPSS0652			865780	866355	524005	524580	9886	10461	2704246	2704821	920867	921442	940765	941340	924980	925555	880118	880693	834817	835392
1114		BURPS668_A0971												941386	941628						
1115	BPSS0652a		GI14	867238	867477	525463	525702	11344	11583	2705704	2705943	922325	922564	942224	942463	926438	926677	881576	881815	836275	836526
1116	BPSS0653		GI14	867462	867680	525687	525905	11568	11786	2705928	2706146	922549	922767	942448	942666	926662	926880	881800	882018	836511	836729
1117	BPSS0654		GI14	868097	869830	526322	528055	12203	13936	2706563	2708296	923184	924917	943083	944816	927297	929030	882435	884168	837146	838879
1118	BPSS0655		GI14	870087	871748	528312	529973	14193	14987	2708553	2710214	925174	926835	945073	946734	929287	930948	884425	886086	839136	840797
1119	BPSS0656		GI14	871783	872157	530008	530382	15888	16262	2710249	2710623	926870	927244	946769	947143	930983	931357	886121	886495	840832	841206
1120	BPSS0657		GI14	872892	872449	531117	530674	16997	16554	2711358	2710915	927979	927536	947880	947695	932092	931649	887230	886787	841941	841498
1121	BPSS0658		GI14	873713	875740	531938	533965	17817	19844	2712178	2714205	928800	930827	948701	950728	932912	934939	888051	890078	842762	844789
1122	BPSS0659		GI14	876102	876533	534327	534758	20206	20637	2714567	2714998	931189	931620	951090	951521	935301	935732	890440	890871	845149	845580
1123	BPSS0660		GI14	877007	877285	535232	535510	21000	21278	2715361	2715639	931983	932258			936095	936373	891234	891509	846054	846332
1124	BPSS0661		GI14	877333	878142	535558	536367	21326	22135	2715687	2716496	932306	932878	952213	952275	936421	937230	891557	892129	846380	847189
1125	BPSS0663		GI14	878897	879289	537122	537514	22890	23282	2717251	2717643	933871	934263	953031	953423	937985	938377	893122	893514	847944	848336
1126	BPSS0664		GI14	880081	880788	538306	539013	24075	24782	2718436	2719143	935056	935763	954585	954923	939170	939877	894307	895014	849129	849836
1127	BPSS0664a		GI14	881558	881932	539783	540157	25552	25926	2719913	2720287	936533	936907	955691	956065	940647	941021	895783	896157	850603	850977
1128	BPSS0665		GI14	882238	883131	540463	541356	26232	27125	2720593	2721486	937213	938106	956371	957264	941327	942220	896463	897356	851283	852176
1129	BPSS0666			885432	883498	543657	541723	29426	27492	2723787	2721853	940407	938473	959557	957623	944521	942587	899657	897723	854477	852543
1130		BURPS1106A_A0901		885668	886306																
1131	BPSS0667							29629	30120	2723990	2724481					944724	945209			854680	855165
1132	BPSS0668			886176	886391	544401	544616	30173	30388	2724534	2724749	941150	941365	960294	960509	945247	945462	900401	900616	855203	855418
1133	BPSS0669			887832	886606	546057	544831	31829	30603	2726190	2724964	942806	941580	961930	960704	946903	945677	902057	900831	856859	855633
1134	BPSS0670			889645	888251	547870	546476	33643	32249	2728004	2726610	944620	943226	963744	962350	948717	947323	903870	902476	858673	857279
1135	BPSS0671			890291	891985	548516	550210	34321	36018	2728682	2730379	945303	946994	964422	966116	949268	950959	904548	906239	859349	861046
1136	BPSS0672			892389	893198	550614	551423	36422	37231	2730783	2731592	947398	948207	966520	967329	951363	952172	906643	907452	861450	862259
1137	BPSS0673			893266	893856	551491	552081	37299	37889	2731660	2732250	948275	948865	967397	967987	952240	952830	907520	908110	862327	862917
1138	BPSS0674			893878	894177	552103	552402	37914	38213	2732275	2732574	948890	949189	968012	968311	952855	953154	908135	908434	862942	863241
1139	BPSS0675			895163	894276	553388	552501	39199	38312	2733560	2732673	950175	949288	969297	968410	954140	953253	909420	908533	864227	863340
1140	BPSS0676			895693	896514	553918	554739	39723	40544	2734084	2734905	950705	951526	969833	970654	954670	955491	909944	910765	864736	865557
1141	BPSS0677			896735	898702	554960	556927	40765	42732	2735126	2737093	951747	953714	970875	972842	955712	957679	910986	912953	865778	867745
1142	BPSS0678			899233	898760	557458	556985	43263	42790	2737624	2737151	954245	953772	973371	972898	958210	957737	913484	913011	868276	867803
1143	BPSS0679			902089	899861	560314	558086	46119	43891	2740480	2738252	957115	954887	976190	973962	961080	958852	916340	914112	871132	868904
1144	BPSS0680			902553	902095	560778	560320	46583	46125	2740944	2740486	957579	957121	976654	976196	961544	961086	916804	916346	871596	871138
1145	BPSS0681			902754	903734	560979	561959	46784	47764	2741145	2742125	957780	958760	976856	977836	961745	962725	917005	917985	871797	872777

1146	BPSS0682			903940	904335	562165	562560	47986	48381	2742347	2742742	958964	959359	978042	978437	962931	963326	918189	918584	872999	873394
1147	BPSS0683			905126	904773	563351	562998	49172	48819	2743533	2743180	960142	959789	979221	978868	964117	963764	919367	919014	874177	873824
1148	BPSS0684			905531	906766	563756	564991	49577	50812	2743938	2745173	960547	961782	979626	980861	964522	965757	919772	921007	874582	875817
1149	BPSS0685			906774	907985	564999	566210	50820	52031	2745181	2746392	961790	963001	980869	982080	965765	966976	921015	922226	875825	877036
1150	BPSS0686			908081	909481	566306	567706	52129	53529	2746490	2747890	963092	964492	982160	983560	967064	968464	922317	923717	877126	878526
1151	BPSS0686a			909481	909567	567706	567792	53529	53732	2747890	2748093	964492	964578	983560	983763	968464	968550	923717	923803	878526	878612
1152	BPSS0687			911086	909761	569311	567986	55134	53809	2749495	2748170	966097	964772	985165	983840	970069	968744	925322	923997	880133	878808
1153	BPSS0688			911809	911090	570034	569315	55857	55138	2750218	2749499	966820	966101	985888	985169	970792	970073	926045	925326	880856	880137
1154	BPSS0689			912017	912793	570242	571018	56065	56841	2750426	2751202	967028	967804	986096	986872	971000	971776	926253	927029	881064	881840
1155	BPSS0690			912820	914004	571045	572229	56868	58052	2751229	2752413	967829	969013	986899	988083	971803	972987	927056	928240	881867	883051
1156	BPSS0691			914488	914054	572713	572279	58536	58102	2752897	2752463	969497	969063	988567	988133	973471	973037	928724	928290	883535	883101
1157	BPSS0692			914654	915340	572879	573565	58702	59388	2753063	2753749	969663	970349	988742	989428	973637	974323	928890	929576	883701	884387
1158	BPSS0693			915340	916101	573565	574326	59388	60149	2753749	2754510	970349	971110	989428	990189	974323	975084	929576	930337	884387	885148
1159	BPSS0694			916129	917589	574354	575814	60176	61636	2754537	2755997	971137	972597	990216	991676	975111	976571	930365	931825	885175	886635
1160	BPSS0695			917662	918507	575887	576732	61709	62554	2756070	2756915	972670	973515	991749	992594	976644	977489	931898	932743	886708	887553
1161	BPSS0696			918568	918957	576793	577182	62615	63004	2756976	2757365	973576	973965	992655	993044	977550	977939	932804	933193	887614	888003
1162	BPSS0697			918983	919783	577208	578008	63030	63830	2757391	2758191	973991	974791	993070	993870	977965	978765	933219	934019	888029	888829
1163	BPSS0698			919796	920599	578021	578824	63843	64646	2758204	2759007	974804	975607	993883	994686	978778	979581	934032	934835	888842	889645
1164	BPSS0699			921437	920610	579662	578835	65474	64641	2759835	2759002	976446	975607	995525	994686	980420	979581	935674	934835	890484	889645
1165	BPSS0700			921714	922676	579939	580901	65751	66713	2760112	2761074	976723	977685	995802	996764	980697	981659	935951	936913	890761	891723
1166	BPSS0701			923863	922775	582088	581000	67900	66812	2762261	2761173	978872	977784	997951	996863	982846	981758	938100	937012	892910	891822
1167		BURPS668_A1033												997976	998299						
1168	BPSS0702			925430	924366	583655	582591	69438	68374	2763799	2762735	980431	979367	999539	998475	984391	983327	939652	938588	894469	893405
1169	BPSS0703			927192	925426	585417	583651	71200	69434	2765561	2763795	982190	980427	1001301	999535	986153	984387	941411	939648	896228	894465
1170	BPSS0704			928380	927298	586605	585523	72388	71303	2766749	2765664	983378	982293	1002489	1001404	987341	986256	942599	941514	897416	896331
1171	BPSS0705			928509	929177	586734	587402	72517	73185	2766878	2767546	983507	984175	1002618	1003286	987470	988138	942728	943396	897545	898213
1172	BPSS0706			929177	930679	587402	588904	73185	74687	2767546	2769048	984175	985677	1003286	1004788	988138	989640	943396	944898	898213	899715
1173	BPSS0707			932165	931299	590390	589524	76172	75306	2770533	2769667	987327	986461	1006421	1005555	991126	990260	946400	945534	901364	900498
1174		BURPS1655_I0207																		902284	901833
1175	BPSS0708			934075	935274	592300	593499	78078	79277	2772439	2773638	989237	990436	1008346	1009545	993036	994235	948310	949509	903263	904462
1176		BURPS1710A_A0099						79376	79489												
1177		BURPSPAST_J0332														994334	994645				
1178	BPSS0709		BP_Chr2_8_ISBma2																		
1179	BPSS0710			936784	937566	595009	595791	80751	81533	2775112	2775894	991939	992721	1011078	1011860	995896	996678	951013	951795	905942	906724
1180	BPSS0711			938993	937872	597218	596097	82958	81837	2777319	2776198	994146	993025	1013296	1012175	998104	996983	953220	952099	908243	907122
1181	BPSS0712			939522	939899	597747	598124	83487	83864	2777848	2778225	994675	995052	1013825	1014202	998633	999010	953749	954126	908772	909149
1182	BPSS0713			940067	941299	598292	599524	84032	85264	2778393	2779625	995220	996452	1014370	1015602	999178	1000410	954294	955526	909317	910549
1183	BPSS0714			941307	942278	599532	600503	85272	86243	2779633	2780604	996460	997431	1015610	1016581	1000418	1001389	955534	956505	910557	911528
1184	BPSS0715			943033	942503	601258	600728	86988	86467	2781349	2780828	998177	997656	1017327	1016806	1002134	1001613	957260	956730	912274	911753
1185	BPSS0716			944105	943272	602330	601497	88060	87227	2782421	2781588	999249	998416	1018399	1017566	1003206	1002373	958341	957508	913346	912513
1186	BPSS0717			945390	944101	603615	602326	89345	88056	2783706	2782417	1000534	999245	1019684	1018395	1004491	1003202	959626	958337	914631	913342
1187		BURPS406E_G0130										1001116	1001238								
1188		BURPSS13_T0108																960244	960366		
1189	BPSS0718			946466	947107	604691	605332	90341	90982	2784702	2785343	1001617	1002258	1020671	1021312	1005601	1006242	960745	961386	915637	916278
1190	BPSS0719			947655	948332	605880	606557	91530	92207	2785891	2786568	1002806	1003483	1021860	1022537	1006790	1007467	961934	962611	916826	917503
1191	BPSS0720			948615	949709	606840	607934	92500	93594	2786861	2787955	1003775	1004869	1022866	1023960	1007759	1008853	962903	963997	917786	918880
1192	BPSS0721			950686	951444	608911	609669	94560	95318	2788921	2789679	1005822	1006580	1024938	1025696	1009840	1010598	964974	965732	919870	920628
1193		BURPS1710A_A0119						95342	95467												
1194	BPSS0721a			951705	952193	609930	610418	95633	96121	2789994	2790482	1006832	1007320	1025929	1026417	1010849	1011337	966011	966499	920835	921323
1195	BPSS0723			952343	953410	610568	611635	96271	97338	2790632	2791699	1007470	1008537	1026567	1027634	1011487	1012554	966649	967716	921473	922540
1196	BPSS0724			953544	953936	611769	612161	97467	97859	2791828	2792220	1008666	1009058	1027768	1028160	1012683	1013075	967850	968242	922674	923066
1197	BPSS0725			954993	954202	613218	612427	98934	98143	2793295	2792504	1010133	1009342	1029208	1028417	1014141	1013350	969308	968517	924168	923377
1198	BPSS0726			956818	955313	615043	613538	100830	99325	2795191	2793686	1012020	1010515	1031069	1029564	1016001	1014496	971133	969628	926037	924532
1199	BPSS0727			956922	957638	615147	615863	100934	101650	2795295	2796011	1012124	1012840	1031173	1031889	1016105	1016821	971237	971953	926141	926857
1200		BURPS668_A1074												1032268	1033089						
1201		BURPSS13_T0125																973143	972421		
1202	BPSS0728											1014002	1013619								
1203	BPSS0730			959582	958875	617807	617100	103627	102920	2797988	2797281	1014827	1014120	1033842	1033135	1018798	1018091	973897	973190	928795	928088

1204	BPSS0731		959893	961551	618118	619776	103938	105596	2798299	2799957	1015126	1016784	1034129	1035787	1019097	1020755	974208	975866	929106	930764
1205	BPSS0732						105938	106633	2800299	2800994			1036129	1036824	1021097	1021792				
1206	BPSS0733		963957	961873	622182	620098	108854	106752	2803215	2801113	1019190	1017106	1039021	1036943	1024013	1021911	978290	976188	933172	931088
1207	BPSS0734		966165	964309	624390	622534	111055	109199	2805416	2803560	1021389	1019533	1041254	1039398	1026221	1024365	980498	978642	935380	933524
1208	BPSS0735		968190	966226	626415	624451	113080	111116	2807441	2805477	1023414	1021450	1043279	1041315	1028246	1026282	982523	980559	937405	935441
1209	BPSS0736		969518	968196	627743	626421	114408	113086	2808769	2807447	1024742	1023420	1044607	1043285	1029574	1028252	983851	982529	938733	937411
1210	BPSS0737		971083	969536	629308	627761	115973	114426	2810334	2808787	1026307	1024760	1046172	1044625	1031139	1029592	985416	983869	940298	938751
1211	BPSS0738		972119	972445	630344	630670	117009	117335	2811370	2811696	1027343	1027669	1047209	1047535	1032175	1032501	986452	986778	941334	941660
1212		BURPSPAST_J0374													1033140	1033370				
1213	BPSS0739		973854	973363	632079	631588	118744	118253	2813105	2812614	1029045	1028554	1048952	1048461	1033890	1033399	988178	987687	943071	942580
1214	BPSS0740		974790	974008	633015	632233	119677	118895	2814038	2813256	1029981	1029199	1049888	1049106	1034826	1034044	989114	988332	944007	943225
1215		BURPSPAST_J0378													1035339	1035578				
1216	BPSS0741		976683	975736	634908	633961	121573	120626	2815934	2814987	1031876	1030929	1051794	1050847	1036706	1035759	991007	990060	945897	944950
1217	BPSS0742		976787	978139	635012	636364	121677	123029	2816038	2817390	1031980	1033332	1051898	1053250	1036810	1038162	991111	992463	946001	947353
1218	BPSS0743		978353	979867	636578	638092	123243	124757	2817604	2819118	1033546	1035060	1053464	1054978	1038376	1039890	992677	994191	947567	949081
1219	BPSS0744		980397	980158	638622	638383	125287	125048	2819648	2819409	1035590	1035351	1055508	1055269	1040420	1040181	994721	994482		
1220		BURPS1655_I0264																	953169	952573
1221		BURPS1655_I0265																	953285	953503
1222		BURPS1655_I0266																	953771	953908
1223		BURPS1655_I0267																	956022	954889
1224		BURPS1655_I0268																	956261	956848
1225		BURPS1655_I0269																	957372	958139
1226		BURPS1655_I0270																	959477	961039
1227		BURPS1655_I0271																	961039	962226
1228		BURPS1655_I0272																	962366	963151
1229		BURPS1655_I0273																	963241	964326
1230		BURPS1655_I0274																	965067	969119
1231		BURPS1655_I0275																	969166	970293
1232		BURPS1655_I0276																	970303	970647
1233		BURPS1655_I0277																	970668	971783
1234		BURPS1655_I0278																	971892	972674
1235		BURPS1655_I0279																	974071	975693
1236		BURPS1655_I0280																	975797	976042
1237		BURPS1655_I0281																	976367	978655
1238		BURPS1655_I0282																	979314	979427
1239		BURPS1655_I0283																	980387	979509
1240		BURPS1655_I0284																	980371	980604
1241		BURPS1655_I0285																	980674	981807
1242		BURPS1655_I0286																	981809	982738
1243		BURPS1655_I0287																	982743	984131
1244		BURPS1655_I0288																	985335	984382
1245		BURPS1655_I0289																	985529	986926
1246		BURPS1655_I0290																	988399	989673
1247		BURPS1655_I0291																	990698	989892
1248		BURPS1655_I0292																	990897	991961
1249		BURPS1655_I0293																	992033	993382
1250		BURPS1655_I0294																	993385	994350
1251		BURPS1655_I0295																	994355	995248
1252		BURPS1655_I0296																	995276	996394
1253		BURPS1655_I0297																	996408	996749
1254		BURPS1655_I0298																	996980	997099
1255		BURPS1655_I0299																	997142	998479
1256		BURPS1655_I0300																	998469	999509
1257		BURPS1655_I0301																	999511	1000356
1258		BURPS1655_I0302																	1000387	1001196
1259		BURPS1655_I0303																	1001236	1002078
1260		BURPS1655_I0304																	1002492	1003535
1261		BURPS1655_I0305																	1003887	1003633

1262	BPSS0745		981034	980549	639259	638774	125924	125439	2820285	2819800	1036227	1035742	1056145	1055660	1041057	1040572	995358	994873	1004538	1004053
1263	BPSS0746		982816	981587	641041	639812	127706	126477	2822067	2820838	1038009	1036780	1057926	1056697	1042839	1041610	997140	995911	1006320	1005091
1264	BPSS0747		983336	984430	641561	642655	128226	129320	2822587	2823681	1038529	1039623	1058446	1059540	1043359	1044453	997660	998754	1006840	1007934
1265	BPSS0748		985423	985055	643648	643280	130313	129945	2824674	2824306	1040615	1040247	1060533	1060165	1045446	1045078	999747	999379	1008927	1008559
1266	BPSS0749		986390	985593	644615	643818	131280	130483	2825641	2824844	1041582	1040785	1061500	1060703	1046413	1045616	1000714	999917	1009894	1009097
1267	BPSS0750		986512	987921	644737	646146	131402	132808	2825763	2827169	1041704	1043110	1061622	1063028	1046535	1047941	1000836	1002242	1010016	1011422
1268	BPSS0751		987966	989093	646191	647318	132853	133980	2827214	2828341	1043155	1044282	1063073	1064200	1047986	1049104	1002287	1003414	1011467	1012594
1269	BPSS0752		990733	989090	648958	647315	135620	133977	2829981	2828338	1045922	1044279	1065840	1064197	1050744	1049101	1005054	1003411	1014234	1012591
1270	BPSS0753		991228	990911	649453	649136	136114	135797	2830475	2830158	1046417	1046100	1066335	1066018	1051238	1050921	1005549	1005232	1014729	1014412
1271	BPSS0754		993490	991256	651715	649481	138376	136142	2832737	2830503	1048673	1046445	1068597	1066363	1053506	1051266	1007823	1005577	1016979	1014757
1272	BPSS0755		993701	994708	651926	652933	138587	139594	2832948	2833955	1048884	1049891	1068808	1069815	1053717	1054724	1008034	1009041	1017190	1018197
1273	BPSS0756		995546	995247	653771	653472	140450	140151	2834811	2834512	1050718	1050419	1070624	1070325	1055562	1055263	1009899	1009600	1019007	1018708
1274	BPSS0757		995865	997016	654090	655241	140769	141920	2835130	2836281	1051037	1052188	1070943	1072094	1055881	1057032	1010218	1011369	1019326	1020477
1275	BPSS0758		997133	997594	655358	655819	142037	142498	2836398	2836859	1052305	1052766	1072211	1072672	1057149	1057610	1011486	1011947	1020594	1021055
1276	BPSS0759		998147	999406	656372	657631	143052	144311	2837413	2838672	1053319	1054578	1073226	1074485	1058163	1059422	1012500	1013759	1021609	1022868
1277	BPSS0760		999414	1000643	657639	658868	144319	145548	2838680	2839909	1054586	1055815	1074493	1075722	1059430	1060659	1013767	1014996	1022876	1024105
1278	BPSS0761		1000646	1001899	658871	660124	145551	146804	2839912	2841165	1055818	1057071	1075725	1076978	1060662	1061915	1014999	1016252	1024108	1025361
1279	BPSS0762		1003299	1001965	661524	660190	148204	146870	2842565	2841231	1058471	1057137	1078413	1077079	1063329	1061995	1017694	1016360	1026775	1025441
1280	BPSS0763		1004427	1003498	662652	661723	149332	148403	2843693	2842764	1059599	1058670	1079541	1078612	1064457	1063528	1018822	1017893	1027903	1026974
1281	BPSS0764		1005266	1004472	663491	662697	150171	149377	2844532	2843738	1060438	1059644	1080380	1079586	1065296	1064502	1019661	1018867	1028742	1027948
1282	BPSS0765		1006097	1005384	664322	663609	151002	150289	2845363	2844650	1061269	1060556	1081206	1080493	1066127	1065414	1020492	1019779	1029573	1028860
1283	BPSS0766		1006451	1007794	664676	666019	151356	152699	2845717	2847060	1061623	1062966	1081558	1082901	1066481	1067824	1020846	1022189	1029927	1031270
1284	BPSS0767		1008444	1008124	666669	666349	153349	153029	2847710	2847390	1063616	1063296	1083551	1083231	1068474	1068154	1022839	1022519	1031920	1031600
1285		BURPS1106A_A1042	1008763	1010373																
1286		BURPS1106B_0947			668598	666988														
1287		BURPS1655_I0336																	1033577	1032234
1288		BURPS1655_I0337																	1033588	1033749
1289		BURPS1710A_A0184					155290	153668												
1290		BURPS406E_G0197									1065557	1063935								
1291		BURPSPAST_J0410													1070403	1068793				
1292		BURPSS13_T0175															1024763	1023153		
1293	BPSS0768		1012578	1012540	670810	670772	155494	155297	2851841	2851803	1065761	1065564	1084039	1083809	1072621	1072583	1025009	1024770	1034048	1033851
1294	BPSS0769		1013580	1012603	671812	670835	158482	157505	2852843	2851866	1068728	1067751	1085054	1084074	1073623	1072646	1026011	1025034	1037077	1036100
1295		BURPS1106A_A1046	1013596	1013961																
1296		BURPS1710A_A0188					158498	158863												
1297	BPSS0770		1014760	1014125	672992	672357	159655	159020	2854016	2853381	1069902	1069267	1086250	1085615	1074797	1074162	1027184	1026549	1038251	1037616
1298	BPSS0771		1015950	1016168	674182	674400	160845	161063	2855206	2855424	1071077	1071295	1087425	1087643	1075972	1076190	1028359	1028577	1039441	1039659
1299		BURPS1655_I0350																	1039738	1040022
1300	BPSS0772		1016761	1017240	674993	675472	161656	162135	2856017	2856496	1071900	1072379	1088245	1088724	1076795	1077274	1029182	1029661	1040247	1040726
1301	BPSS0773		1018092	1017484	676324	675716	162977	162369	2857338	2856730	1073217	1072693	1089401	1088958	1078116	1077508	1030499	1029975	1041568	1040960
1302	BPSS0774		1018100	1018840	676332	677072	162985	163725	2857346	2858086	1073225	1073965	1089576	1090316	1078124	1078864	1030507	1031247	1041576	1042316
1303	BPSS0776		1019082	1020014	677314	678246	164007	164939	2858368	2859300	1074233	1075165	1090570	1091502	1079146	1080078	1031528	1032460	1042583	1043515
1304	BPSS0777		1020667	1021080	678899	679312	165638	166051	2859999	2860412	1075785	1076198	1092040	1092453	1080761	1081174	1033193	1033606	1044134	1044547
1305	BPSS0778		1021609	1021172	679841	679404	166580	166143	2860941	2860504	1076727	1076290	1092982	1092545	1081703	1081266	1034135	1033698	1045076	1044639
1306	BPSS0779		1023404	1024228	681636	682460	168375	169199	2862736	2863560	1078522	1079346			1083497	1084321	1035930	1036754	1046860	1047684
1307	BPSS0780		1024398	1025843	682630	684075	169369	170814	2863730	2865175	1079516	1080961			1084491	1085936	1036924	1038369	1047854	1049299
1308	BPSS0781		1025864	1027717	684096	685949	170835	172688	2865196	2867049	1080982	1082835			1085957	1087810	1038390	1040243	1049320	1051173
1309	BPSS0782		1027982	1029184	686214	687416	173001	174203	2867362	2868564	1083132	1084334			1088086	1089288	1040508	1041710	1051433	1052635
1310	BPSS0783		1029673	1030809	687905	689041	174620	175756	2868981	2870117	1084855	1085991			1089665	1090801	1042167	1043303	1053068	1054204
1311	BPSS0784		1030885	1032606	689117	690838	175832	177553	2870193	2871914	1086067	1087788	1094661	1095146	1090877	1092598	1043379	1045100	1054280	1056001
1312	BPSS0785		1033698	1032877	691930	691109	178644	177823	2873005	2872184	1088879	1088058	1096205	1095384	1093689	1092868	1046192	1045371	1057093	1056272
1313	BPSS0786		1033887	1034897	692119	693129	178833	179843	2873194	2874204	1089068	1090078	1096394	1097404	1093878	1094888	1046381	1047367	1057282	1058292
1314	BPSS0787		1034897	1036450	693129	694682	179843	181396	2874204	2875757	1090078	1091631	1097404	1098957	1094888	1096441	1047367	1048920	1058292	1059845
1315	BPSS0788		1036443	1037465	694675	695697	181389	182411	2875750	2876772	1091624	1092646	1098950	1099972	1096434	1097456	1048913	1049935	1059838	1060860
1316	BPSS0789		1037465	1037782	695697	696014	182411	182728	2876772	2877089	1092646	1092963	1099972	1100289	1097456	1097773	1049935	1050252	1060860	1061177
1317	BPSS0790		1037820	1039139	696052	697371	182766	184085	2877127	2878446	1093001	1094320	1100327	1101646	1097811	1099130	1050290	1051609	1061215	1062534
1318	BPSS0791		1039414	1040250	697646	698482	184360	185196	2878721	2879557	1094595	1095431	1101915	1102751	1099405	1100241	1051884	1052720	1062884	1063720
1319	BPSS0792		1040393	1041133	698625	699365	185339	186079	2879700	2880440	1095574	1096314	1102894	1103634	1100384	1101124	1052863	1053603	1063863	1064603

1320	BPSS0793		1041212	1042051	699444	700283	186158	186997	2880519	2881358	1096393	1097232	1103713	1104552	1101203	1102042	1053682	1054521	1064682	1065521
1321	BPSS0794		1042206	1042979	700438	701211	187152	187925	2881513	2882286	1097381	1098154	1104707	1105480	1102197	1102970	1054676	1055449	1065676	1066449
1322	BPSS0795		1043018	1043353	701250	701585	187964	188299	2882325	2882660	1098193	1098528	1105519	1105854	1103009	1103344	1055488	1055823	1066488	1066823
1323		BURPS1106A_A1090	1045325	1050334																
1324		BURPS1106B_0900			703557	708566														
1325		BURPS1655_I0382																	1068796	1074384
1326		BURPS1710A_A0235					190271	195352												
1327		BURPS406E_G0244									1100500	1105059								
1328		BURPSPAST_J0459													1105316	1110397				
1329		BURPSS13_T0219															1058003	1062202		
1330	BPSS0796												1108004	1112941						
1331	BPSS0797		1052499	1051762	710731	709994	197505	196768	2891866	2891129	1107202	1106465	1115098	1114361	1112540	1111803	1064367	1063630	1076533	1075796
1332	BPSS0798		1053078	1052617	711310	710849	198084	197623	2892445	2891984	1107781	1107320	1115677	1115216	1113119	1112658	1064946	1064485	1077113	1076652
1333	BPSS0799		1054314	1053097	712546	711329	199320	198103	2893681	2892464	1109017	1107800	1116913	1115696	1114355	1113138	1066182	1064965	1078349	1077132
1334	BPSS0800		1055805	1055194	714037	713426	200810	200199	2895171	2894560	1110507	1109896	1118404	1117793	1115846	1115235	1067672	1067061	1079840	1079229
1335	BPSS0801		1055920	1056354	714152	714586	200925	201359	2895286	2895720	1110622	1111056	1118519	1118953	1115961	1116395	1067787	1068221	1079955	1080389
1336	BPSS0802		1056506	1057645	714738	715877	201511	202650	2895872	2897011	1111214	1112353	1119102	1120241	1116547	1117686	1068373	1069512	1080541	1081680
1337	BPSS0803		1058122	1058754	716354	716986	203127	203759	2897488	2898120	1112818	1113450	1120714	1121346	1118175	1118807	1069989	1070621	1082157	1082789
1338	BPSS0804		1059192	1058818	717424	717050	204197	203823	2898558	2898184	1113888	1113514	1121784	1121410	1119245	1118871	1071059	1070685	1083227	1082853
1339	BPSS0805		1061904	1059841	720136	718073	206909	204846	2901270	2899207	1116600	1114537	1124496	1122433	1120707	1119751	1073761	1071698	1085939	1083876
1340	BPSS0806		1062698	1062234	720930	720466	207703	207239	2902064	2901600	1117394	1116930	1125290	1124826	1121487	1121023	1074541	1074077	1086709	1086245
1341	BPSS0807		1063031	1064413	721263	722645	208036	209418	2902397	2903779	1117727	1119109	1125623	1127005	1121820	1123202	1074874	1076256	1087042	1088424
1342	BPSS0808		1064448	1065647	722680	723879	209453	210652	2903814	2905013	1119144	1120343	1127039	1128238	1123237	1124436	1076291	1077490	1088459	1089658
1343	BPSS0809		1065982	1066368	724214	724600	210987	211373	2905348	2905734	1120677	1121063	1128573	1128959	1124770	1125156	1077825	1078211	1089986	1090372
1344	BPSS0810		1067488	1068108	725720	726340	212521	213141	2906882	2907502	1122210	1122821	1130105	1130725	1126303	1126914	1079358	1079969	1091519	1092130
1345	BPSS0811		1069010	1068279	727242	726511	214043	213312	2908404	2907673	1123723	1122992	1131627	1130896	1127816	1127085	1080871	1080140	1093032	1092301
1346	BPSS0812		1070471	1069041	728703	727273	215503	215384	2909864	2909745	1125183	1125064	1133087	1132968	1129276	1129157	1082331	1082212	1094492	1094373
1347	BPSS0813		1072095	1073507	730327	731739	217154	218566	2911515	2912927	1126819	1128231	1134705	1136117	1130871	1132283	1083866	1085278	1096192	1097604
1348	BPSS0814		1073521	1073886	731753	732118	218580	218945	2912941	2913306	1128245	1128610	1136131	1136496	1132297	1132662	1085292	1085657	1097618	1097983
1349	BPSS0815		1073873	1074490	732105	732722	218932	219549	2913293	2913910	1128597	1129214	1136483	1137100	1132649	1133266	1085644	1086261	1097970	1098587
1350	BPSS0816		1077065	1074531	735297	732763	222160	219590	2916521	2913951	1131792	1129255	1139714	1137141	1135721	1133307	1088869	1086302	1101012	1098628
1351		BURPS1106A_A1128	1083435	1083722																
1352	BPSS0817		1083438	1077289	741670	735521	228611	222453	2922972	2916814	1138225	1132067	1146087	1139929	1142303	1136145	1095188	1089030	1107508	1101344
1353		BURPS1106B_0861			741954	741667														
1354	BPSS0818		1083960	1084511	742192	742743	229141	229692	2923502	2924053	1138755	1139306	1146599	1147150	1142833	1143384	1095710	1096261	1108020	1108571
1355	BPSS0819		1085670	1086137	743902	744369	230739	231206	2925100	2925567	1140353	1140820	1148226	1148693	1144431	1144898	1097337	1097804	1109667	1110134
1356	BPSS0820		1086137	1088338	744369	746570	231206	233407	2925567	2927768	1140820	1143021	1148693	1150894	1144898	1147099	1097804	1100005	1110134	1112335
1357	BPSS0821		1088338	1089255	746570	747487	233407	234324	2927768	2928685	1143021	1143938	1150894	1151811	1147099	1148016	1100005	1100922	1112335	1113252
1358	BPSS0822		1089261	1090160	747493	748392	234330	235229	2928691	2929590	1143944	1144843	1151817	1152716	1148022	1148921	1100928	1101827	1113258	1114157
1359	BPSS0823		1090160	1091770	748392	750002	235229	236839	2929590	2931200	1144843	1146453	1152716	1154326	1148921	1150531	1101827	1103437	1114157	1115767
1360	BPSS0824		1091766	1092656	749998	750888	236835	237725	2931196	2932086	1146449	1147339	1154322	1155212	1150527	1151417	1103433	1104323	1115763	1116653
1361	BPSS0825		1093330	1092626	751562	750858	238399	237695	2932760	2932056	1148013	1147309	1155886	1155182	1152091	1151387	1104997	1104293	1117327	1116623
1362	BPSS0826		1093342	1094496	751574	752728	238411	239565	2932772	2933926	1148025	1149179	1155898	1157052	1152103	1153257	1105009	1106163	1117339	1118493
1363	BPSS0827		1095481	1097415	753713	755647	240550	242484	2934911	2936845	1150164	1152098	1158037	1159971	1154242	1156176	1107148	1109082	1119478	1121412
1364		BURPS406E_G0290									1152098	1152307								
1365		BURPS668_A1217											1159971	1160180						
1366	BPSS0828		1098048	1097713	756280	755945	243113	242778	2937475	2937140	1152729	1152394	1160602	1160267	1156805	1156470	1109715	1109380	1122057	1121722
1367	BPSS0829		1099867	1098704	758099	756936	244932	243769	2939294	2938131	1154550	1153387	1162401	1161238	1158624	1157461	1111534	1110371	1123869	1122706
1368	BPSS0830		1101208	1100468	759440	758700	246273	245533	2940635	2939895	1155891	1155151	1163744	1163004	1159965	1159225	1112875	1112135	1125212	1124472
1369	BPSS0831		1101580	1101765	759812	759997	246645	246830	2941007	2941192	1156263	1156448	1164116	1164301	1160337	1160522	1113247	1113432	1125584	1125769
1370	BPSS0832		1102523	1102038	760755	760270	247580	247095	2941942	2941457	1157206	1156721	1165053	1164568	1161272	1160787	1114222	1113737	1126531	1126046
1371	BPSS0833		1102713	1103000	760945	761232	247770	248057	2942132	2942419	1157396	1157683	1165243	1165530	1161462	1161749	1114412	1114699	1126721	1127008
1372	BPSS0834		1103072	1103305	761304	761537	248129	248362	2942491	2942724	1157748	1157981	1165602	1165835	1161821	1162054	1114764	1114997	1127073	1127306
1373	BPSS0835		1104034	1103390	762266	761622	249091	248447	2943453	2942809	1158710	1158066	1166564	1165920	1162783	1162139	1115726	1115082	1128035	1127391
1374	BPSS0836		1104602	1105057	762834	763289	249653	250108	2944015	2944470	1159278	1159733	1167138	1167593	1163345	1163800	1116294	1116749	1128597	1129052
1375	BPSS0837		1106278	1105442	764510	763674	251320	250484	2945682	2944846	1160954	1160118	1168802	1167966	1165012	1164176	1117970	1117134	1130270	1129434
1376	BPSS0838		1106769	1106278	765001	764510	251811	251320	2946173	2945682	1161545	1161513	1169293	1168802	1165503	1165012	1118461	1117970	1130761	1130270
1377	BPSS0839		1107780	1106944	766012	765176	252832	251996	2947194	2946358	1162556	1161720	1170304	1169468	1166524	1165688	1119444	1118608	1131750	1130914

1378	BPSS0840		1108030	1109067	766262	767299	253082	254119	2947444	2948481	1162806	1163843	1170554	1171591	1166774	1167811	1119694	1120731	1132000	1133037
1379	BPSS0841		1109482	1110525	767714	768757	254567	255610	2948929	2949972	1164301	1165344	1172034	1173077	1168259	1169302	1121177	1122220	1133479	1134522
1380	BPSS0842		1110715	1111920	768947	770152	255800	257005	2950162	2951367	1165536	1166741	1173269	1174474	1169494	1170699	1122412	1123617	1134714	1135919
1381	BPSS0843		1112434	1113192	770666	771424	257447	258205	2951809	2952567	1167247	1168005	1174980	1175738	1171141	1171899	1124131	1124889	1136441	1137199
1382	BPSS0844		1113211	1114077	771443	772309	258224	259090	2952586	2953452	1168024	1168890	1175757	1176623	1171918	1172784	1124908	1125774	1137218	1138084
1383		BURPSS13_T0290															1126755	1126279		
1384	BPSS0845		1115043	1116425	773281	774663	260092	261474	2954454	2955836	1169815	1171197	1177591	1178973	1173774	1175156	1126783	1128165	1139057	1140439
1385	BPSS0845a		1117224	1116985	775462	775223	262273	262034	2956635	2956396	1171996	1171757	1179774	1179535	1175955	1175716	1128964	1128725	1141238	1140999
1386	BPSS0846		1117369	1117662	775607	775900	262418	262711	2956780	2957073	1172141	1172434	1179919	1180212	1176100	1176393	1129109	1129402	1141383	1141676
1387	BPSS0847		1119375	1118062	777613	776300	264424	263111	2958786	2957473	1174147	1172834	1181925	1180612	1178106	1176793	1131116	1129803	1143389	1142076
1388	BPSS0848		1120294	1119800	778532	778038	265343	264849	2959705	2959211	1175066	1174572	1182844	1182350	1179025	1178531	1132035	1131541	1144308	1143814
1389	BPSS0849		1121332	1120415	779570	778653	266381	265464	2960743	2959826	1176104	1175187	1183882	1182965	1180063	1179146	1133073	1132156	1145346	1144429
1390	BPSS0850		1121943	1122284	780181	780522	266992	267333	2961354	2961695	1176715	1177056	1184498	1184839	1180674	1181015	1133684	1134025	1145957	1146298
1391	BPSS0851		1122452	1123795	780690	782033	267501	268844	2961863	2963206	1177224	1178567	1185007	1186350	1181183	1182526	1134193	1135536	1146456	1147799
1392	BPSS0852		1124294	1125262	782532	783500	269343	270311	2963705	2964673	1179078	1180046	1186892	1187860	1183025	1183993	1136035	1137003	1148322	1149290
1393	BPSS0853		1126221	1125292	784459	783530	271270	270341	2965632	2964703	1181005	1180076	1188819	1187890	1184952	1184023	1137962	1137033	1150249	1149320
1394	BPSS0854		1126516	1127004	784754	785242	271565	272053	2965927	2966415	1181300	1181788	1189114	1189602	1185247	1185735	1138257	1138745	1150544	1151032
1395	BPSS0855		1127226	1128917	785464	787155	272275	273966	2966637	2968328	1182009	1183700	1189823	1191514	1185957	1187648	1138967	1140658	1151254	1152945
1396	BPSS0856		1128917	1131181	787155	789419	273966	276230	2968328	2970592	1183700	1185964	1191514	1193778	1187648	1189912	1140658	1142922	1152945	1155209
1397	BPSS0857		1131219	1132508	789457	790746	276268	277557	2970630	2971919	1186002	1187273	1193816	1195096	1189950	1191239	1142960	1143316	1155247	1156518
1398	BPSS0858		1132498	1133088	790736	791326	277547	278137	2971909	2972499	1187263	1187853	1195086	1195676	1191229	1191819	1144232	1144822		
1399	BPSS0860		1133286	1134806	791524	793044	278335	279855	2972697	2974217	1188051	1189571	1195874	1197394	1192017	1193537	1145020	1146540	1157252	1158772
1400	BPSS0861		1136105	1135209	794343	793447	281154	280258	2975516	2974620	1190870	1189974	1198693	1197797	1194836	1193940	1147839	1146943	1160071	1159175
1401	BPSS0862		1136199	1136894	794437	795132	281248	281943	2975610	2976305	1190960	1191655	1198783	1199478	1194930	1195625	1147933	1148628	1160161	1160856
1402	BPSS0863																		1161059	1161154
1403	BPSS0864		1137285	1138229	795523	796467	282429	283373	2976791	2977735	1192140	1193084	1199963	1200907	1196111	1197055	1149113	1150057	1161488	1162432
1404	BPSS0865		1138633	1139496	796871	797734	283768	284631	2978130	2978993	1193470	1194333	1201311	1202174	1197432	1198295	1150479	1151342	1162773	1163636
1405	BPSS0866		1139574	1140326	797812	798564	284709	285461	2979071	2979823	1194411	1195163	1202252	1203004	1198373	1199125	1151420	1152172	1163714	1164466
1406	BPSS0867		1140793	1141497	799031	799735	285970	286674	2980332	2981036	1195672	1196376	1203471	1204175	1199606	1200310	1152652	1153356	1164940	1165644
1407	BPSS0868		1141757	1143256	799995	801494	286934	288433	2981296	2982795	1196636	1198135	1204435	1205934	1200570	1202069	1153616	1155115	1165904	1167403
1408	BPSS0869		1143297	1144643	801535	802881	288474	289820	2982836	2984182	1198176	1199522	1205975	1207321	1202110	1203456	1155156	1156502	1167444	1168790
1409	BPSS0870		1144855	1146342	803093	804580	290032	291519	2984394	2985881	1199734	1201221	1207533	1209020	1203668	1205155	1156714	1158201	1169002	1170489
1410	BPSS0871		1146342	1146959	804580	805197	291519	292136	2985881	2986498	1201221	1201838	1209020	1209637	1205155	1205772	1158201	1158818	1170489	1171106
1411	BPSS0872		1146959	1147975	805197	806213	292136	293152	2986498	2987514	1201838	1202854	1209637	1210653	1205772	1206788	1158818	1159834	1171120	1172136
1412	BPSS0873		1148208	1149407	806446	807645	293385	294584	2987747	2988946	1203087	1204286	1210886	1212085	1207021	1208220	1160067	1161266	1172369	1173568
1413	BPSS0874		1149674	1149988	807912	808226	294851	295165	2989213	2989527	1204553	1204867	1212393	1212665	1208487	1208801	1161533	1161847	1173835	1174035
1414	BPSS0875		1150711	1150103	808949	808341	295888	295280	2990250	2989642	1205590	1204982	1213388	1212780	1209524	1208916	1162570	1161962	1174860	1174348
1415	BPSS0876		1150883	1151359	809121	809597	296060	296536	2990422	2990898	1205762	1206238	1213560	1214036	1209696	1210172	1162742	1163218	1175032	1175508
1416	BPSS0877		1152557	1151499	810795	809737	297741	296683	2992103	2991045	1207443	1206385	1215234	1214176	1211377	1210319	1164416	1163358	1176713	1175655
1417	BPSS0878		1152912	1152610	811150	810848	298096	297794	2992458	2992156	1207798	1207496	1215589	1215287	1211732	1211430	1164771	1164469	1177068	1176766
1418	BPSS0879		1154244	1155365	812482	813603	299425	300546	2993787	2994908	1209128	1210249	1216926	1218047	1213061	1214182	1166101	1167222	1178398	1179519
1419	BPSS0880		1156000	1155593	814238	813831	301178	300771	2995540	2995133	1210884	1210477	1218682	1218275	1214814	1214407	1167854	1167447	1180152	1179745
1420	BPSS0881		1156508	1156846	814746	815084	301685	302023	2996047	2996385	1211392	1211730	1219190	1219528	1215322	1215660	1168361	1168699	1180660	1180998
1421	BPSS0882		1157518	1157057	815756	815295	302695	302234	2997057	2996596	1212402	1211941	1220200	1219739	1216332	1215871	1169371	1168910	1181670	1181209
1422	BPSS0883		1157695	1158345	815933	816583	302872	303522	2997234	2997884	1212579	1213229	1220377	1221027	1216509	1217159	1169548	1170198	1181847	1182497
1423	BPSS0884		1159159	1158314	817397	816552	304336	303491	2998698	2997853	1214043	1213198	1221841	1220996	1217973	1217128	1171012	1170167	1183311	1182466
1424	BPSS0885		1159832	1159224	818070	817462	305009	304401	2999371	2998763	1214717	1214361	1222514	1221906	1218646	1218038	1171685	1171077	1183984	1183376
1425	BPSS0886		1160500	1160060	818738	818298	305677	305237	3000039	2999599	1215385	1214945	1223182	1222742	1219314	1218874	1172353	1171913	1184652	1184212
1426	BPSS0887		1160574	1161290	818812	819528	305751	306467	3000113	3000829	1215459	1216175	1223256	1223972	1219388	1219759	1172427	1173143	1184726	1185442
1427	BPSS0888		1162045	1161332	820283	819570	307222	306509	3001585	3000872	1216930	1216217	1224727	1224014	1220852	1220139	1173898	1173185	1186197	1185484
1428	BPSS0889		1163916	1162714	822168	820966	309037	307835	3003400	3002198	1218808	1217606	1226682	1225480	1222681	1221479	1175783	1174581	1187998	1186796
1429	BPSS0890		1164563	1163982	822815	822234	309684	309103	3004047	3003466	1219455	1218874	1227329	1226748	1223328	1222747	1176430	1175849	1188645	1188064
1430	BPSS0891		1164696	1165319	822948	823571	309817	310440	3004180	3004803	1219588	1220211	1227462	1228085	1223461	1224084	1176563	1177186	1188778	1189401
1431	BPSS0892		1165409	1166353	823661	824605	310530	311474	3004893	3005837	1220301	1221245	1228175	1229122	1224404	1225348	1177276	1178223	1189491	1190438
1432	BPSS0893		1167764	1166679	826016	824931	312885	311800	3007248	3006163	1222656	1221571	1230530	1229445	1226759	1225674	1179631	1178546	1191846	1190761
1433	BPSS0894		1168198	1168512	826450	826764	313320	313634	3007683	3007997	1223091	1223405	1230965	1231279	1227193	1227507	1180066	1180380	1192281	1192595
1434	BPSS0895		1168517	1168825	826769	827077	313639	313947	3008002	3008310	1223410	1223718	1231284	1231592	1227512	1227820	1180385	1180693	1192600	1192908
1435	BPSS0896		1168863	1169927	827115	828179	313985	315049	3008348	3009412	1223756	1224820	1231630	1232694	1227858	1228922	1180731	1181795	1192946	1194010

1436	BPSS0897		1169939	1170694	828191	828946	315061	315816	3009424	3010179	1224832	1225587	1232706	1233461	1228934	1229689	1181807	1182562	1194022	1194777
1437	BPSS0898		1170721	1171260	828973	829512	315843	316382	3010206	3010745	1225614	1226153	1233488	1234027	1229716	1230255	1182589	1183128	1194804	1195343
1438	BPSS0899		1171280	1172182	829532	830434	316402	317304	3010765	3011667	1226173	1226439	1234047	1234955	1230275	1231183	1183148	1184050	1195363	1196265
1439	BPSS0900		1172182	1173033	830434	831285	317304	318155	3011667	3012518	1227077	1227928	1234955	1235806	1231183	1232034	1184050	1184901	1196265	1197116
1440	BPSS0901		1173033	1173827	831285	832079	318155	318949	3012518	3013312	1227928	1228722	1235806	1236600	1232034	1232828	1184901	1185695	1197116	1197910
1441	BPSS0902		1173823	1175493	832075	833745	318945	320615	3013308	3014978	1228718	1230388	1236596	1238266	1232824	1234494	1185691	1187361	1197906	1199576
1442	BPSS0903		1175489	1176301	833741	834553	320611	321423	3014974	3015786	1230384	1231196	1238262	1239074	1234490	1235302	1187357	1188169	1199572	1200384
1443	BPSS0904		1176330	1177817	834582	836069	321452	322939	3015815	3017302	1231225	1232712	1239103	1240590	1235331	1236818	1188198	1189685	1200413	1201900
1444	BPSS0905		1177837	1178799	836089	837051	322959	323921	3017322	3018284	1232732	1233694	1240610	1241572	1236838	1237800	1189705	1190667	1201920	1202882
1445	BPSS0906		1178828	1180039	837080	838291	323950	325161	3018313	3019524	1233723	1234934	1241601	1242812	1237829	1239040	1190696	1191907	1202911	1204122
1446	BPSS0907		1180077	1181480	838329	839732	325199	326602	3019562	3020965	1234972	1236375	1242850	1244253	1239078	1240481	1191945	1193348	1204160	1205563
1447	BPSS0908		1182188	1184500	840440	842752	327310	329622	3021673	3023985	1237083	1239395	1244961	1247273	1241189	1243501	1194056	1196368	1206271	1208583
1448	BPSS0909		1184562	1185389	842814	843641	329684	330511	3024047	3024874	1239457	1240284	1247335	1248162	1243563	1244390	1196430	1197257	1208645	1209472
1449	BPSS0910		1185987	1185556	844239	843808	331109	330678	3025472	3025041	1240882	1240451	1248760	1248329	1244988	1244557	1197855	1197424	1210070	1209639
1450	BPSS0911		1186512	1188002	844764	846254	331649	333139	3026012	3027502	1241422	1242912	1249294	1250784	1245513	1247003	1198380	1199870	1210613	1212103
1451	BPSS0912		1188652	1188173	846904	846425	333789	333310	3028152	3027673	1243562	1243083	1251434	1250955	1247653	1247174	1200520	1200041	1212753	1212274
1452	BPSS0913		1188840	1190138	847092	848390	333977	335275	3028340	3029638	1243750	1245048	1251622	1252917	1247841	1249139	1200708	1202006	1212941	1214239
1453	BPSS0914		1190533	1190312	848785	848564	335670	335449	3030033	3029812	1245443	1245222	1253315	1253094	1249534	1249313	1202401	1202180	1214634	1214413
1454	BPSS0915		1190970	1191668	849222	849920	336107	336805	3030470	3031168	1245880	1246578	1253752	1254450	1249971	1250669	1202838	1203536	1215071	1215769
1455	BPSS0916		1191788	1192657	850040	850909	336925	337794	3031288	3032157	1246698	1247567	1254570	1255439	1250789	1251658	1203656	1204525	1215889	1216758
1456	BPSS0917		1192946	1193500	851198	851752	338097	338651	3032460	3033014	1247870	1248424	1255742	1256296	1251961	1252515	1204804	1205358	1217061	1217615
1457	BPSS0918		1194084	1193626	852336	851878	339235	338777	3033598	3033140	1249008	1248550	1256880	1256422	1253099	1252641	1205942	1205484	1218207	1217749
1458	BPSS0919		1194214	1195122	852466	853374	339365	340273	3033728	3034636	1249138	1250046	1257009	1257917	1253229	1254137	1206072	1206980	1218336	1219244
1459		BURPS668_A1338											1258417	1260393						
1460		BURPS668_A1339											1260889	1261008						
1461	BPSS0920		1196852	1195920	855104	854172	342003	341071	3036366	3035434	1251776	1250844	1262476	1261544	1255856	1254924	1208710	1207778	1220961	1220029
1462	BPSS0921		1196970	1198385	855222	856637	342121	343536	3036484	3037899	1251894	1253309	1262594	1264009	1255974	1257389	1208828	1210243	1221079	1222494
1463	BPSS0922		1198415	1199689	856667	857941	343566	344840	3037929	3039203	1253339	1254613	1264039	1265313	1257419	1258693	1210273	1211547	1222524	1223798
1464	BPSS0923		1201209	1200259	859461	858511	346360	345410	3040723	3039773	1256133	1255183	1266832	1265882	1260213	1259263	1213067	1212117	1225325	1224375
1465	BPSS0924		1202135	1201224	860387	859476	347286	346375	3041649	3040738	1257059	1256148	1267758	1266847	1261139	1260228	1213993	1213082	1226251	1225340
1466	BPSS0925		1203058	1202135	861310	860387	348209	347286	3042572	3041649	1257982	1257059	1268681	1267758	1262062	1261139	1214916	1213993	1227174	1226251
1467	BPSS0926		1204675	1203089	862927	861341	349826	348240	3044189	3042603	1259599	1258013	1270298	1268712	1263679	1262093	1216533	1214947	1228791	1227205
1468	BPSS0927		1205571	1204924	863823	863176	350722	350075	3045085	3044438	1260495	1259848	1271194	1270547	1264575	1263928	1217429	1216782	1229687	1229040
1469	BPSS0928		1206755	1205775	865007	864027	351927	350947	3046290	3045310	1261679	1260699	1272378	1271398	1265780	1264800	1218627	1217647	1230878	1229898
1470	BPSS0929		1207686	1206883	865938	865135	352871	352068	3047234	3046431	1262610	1261807	1273309	1272509	1266724	1265921	1219558	1218755	1231809	1231006
1471	BPSS0930		1208331	1207768	866583	866020	353516	352953	3047879	3047316	1263255	1262692	1273954	1273391	1267369	1266806	1220203	1219640	1232454	1231891
1472	BPSS0931		1209061	1209855	867313	868107	354237	355031	3048600	3049394	1263984	1264778	1274674	1275468	1268090	1268884	1220932	1221726	1233174	1233968
1473	BPSS0932		1211407	1209896	869659	868148	356583	355072	3050946	3049435	1266330	1264819	1277020	1275509	1270436	1268925	1223278	1221767	1235520	1234009
1474	BPSS0933		1211646	1213199	869898	871451	356822	358375	3051185	3052738	1266569	1268122	1277259	1278812	1270675	1270761	1223517	1225070	1235759	1237312
1475	BPSS0934		1215189	1213528	873441	871780	360302	358641	3054665	3053004	1270022	1268361	1280740	1279079	1274138	1272477	1226970	1225309	1239212	1237551
1476	BPSS0935		1216740	1215835	874992	874087	361852	360947	3056215	3055310	1271580	1270675	1282289	1281384	1275689	1274784	1228521	1227616	1240763	1239858
1477	BPSS0936		1216919	1218244	875171	876496	362031	363356	3056394	3057719	1271759	1273084	1282468	1283793	1275868	1277193	1228700	1230025	1240942	1242267
1478	BPSS0937		1218524	1219630	876776	877882	363636	364742	3057999	3059105	1273364	1274470	1284064	1285170	1277473	1278579	1230305	1231411	1242538	1243644
1479	BPSS0938		1220584	1219697	878836	877949	365703	364816	3060066	3059179	1275431	1274544	1286124	1285237	1279540	1278653	1232368	1231481	1244601	1243714
1480	BPSS0939		1220966	1221361	879218	879613	366078	366473	3060441	3060836	1275821	1276216	1286514	1286909	1279930	1280325	1232736	1233131	1244984	1245379
1481	BPSS0940		1221972	1222922	880224	881174	367236	368186	3061599	3062549	1276795	1277622	1287495	1288445	1280912	1281862	1233910	1234707	1246111	1247061
1482	BPSS0941		1223769	1222900	882021	881152	369033	368164	3063396	3062527	1278593	1277724	1289292	1288423	1282718	1281840	1235778	1234909	1247908	1247039
1483	BPSS0942		1224973	1223810	883225	882062	370237	369074	3064600	3063437	1279797	1278634	1290496	1289333	1283922	1282759	1236982	1235819	1249100	1247949
1484	BPSS0943		1225394	1226545	883646	884797	370658	371809	3065021	3066172	1280218	1281369	1290917	1292068	1284343	1285494	1237403	1238554	1249521	1250672
1485	BPSS0944		1227961	1227053	886213	885305	373225	372317	3067588	3066680	1282785	1281877	1293484	1292576	1286910	1286002	1239970	1239062	1252088	1251180
1486	BPSS0945		1228579	1229523	886831	887775	373841	374785	3068204	3069148	1283403	1284347	1294099	1295043	1287528	1288472	1240586	1241530	1252706	1253650
1487	BPSS0946		1229701	1230585	887953	888837	374963	375847	3069326	3070210	1284525	1285409	1295228	1296112	1288650	1289534	1241708	1242592	1253828	1254712
1488	BPSS0948		1231307	1232176	889559	890428	376581	377450	3070944	3071813	1286143	1287012	1296834	1297703	1290259	1291128	1243326	1244195	1255446	1256315
1489	BPSS0949		1232625	1232293	890877	890545	377898	377566	3072261	3071929	1287460	1287128	1298151	1297819	1291577	1291245	1244643	1244311	1256763	1256431
1490		BURPS1106B_0681			891462	891749														
1491		BURPS1655_I0595																	1257593	1256745
1492		BURPS668_A1386											1298321	1298785						
1493	BPSS0950		1234267	1233482	892519	891734	379540	378755	3073903	3073118	1289107	1288322	1299786	1299001	1293219	1292434	1246291	1245506	1258405	1257620

1494	BPSS0951		1235124	1234258	893376	892510	380397	379531	3074760	3073894	1289964	1289098	1300643	1299777	1294076	1293210	1247148	1246282	1259262	1258396
1495	BPSS0952		1236067	1235129	894319	893381	381340	380402	3075703	3074765	1290907	1289969	1301586	1300648	1295019	1294081	1248091	1247153	1260205	1259267
1496	BPSS0953		1237180	1236113	895432	894365	382453	381386	3076816	3075749	1292020	1290953	1302699	1301632	1296132	1295065	1249204	1248137	1261318	1260251
1497	BPSS0954		1238802	1237204	897054	895456	384075	382477	3078438	3076840	1293642	1292044	1304321	1302723	1297754	1296156	1250826	1249228	1262940	1261342
1498	BPSS0955		1239520	1238954	897772	897206	384793	384227	3079156	3078590	1294360	1293794	1305030	1304464	1298472	1297906	1251544	1250978	1263667	1263101
1499	BPSS0956		1240430	1239543	898682	897795	385703	384816	3080066	3079179	1295270	1294383	1305940	1305053	1299391	1298495	1252454	1251567	1264577	1263690
1500		BURPSS13_T0442															1253218	1252901		
1501		BURPSS13_T0443															1252567	1252914		
1502	BPSS0957		1242632	1241424	900884	899676	387907	386699	3082270	3081062	1297472	1296264	1308142	1306934	1301589	1300381	1254558	1253350	1266773	1265565
1503	BPSS0958		1242922	1244904	901174	903156	388197	390179	3082560	3084542	1297762	1299744	1308432	1310414	1301879	1303861	1254848	1256830	1267063	1269045
1504	BPSS0959		1244999	1245436	903251	903688	390274	390711	3084637	3085074	1299839	1300276	1310509	1310946	1303956	1304393	1256925	1257362	1269140	1269577
1505	BPSS0960		1245491	1250113	903743	908365	390766	395388	3085129	3089751	1300331	1304953	1311001	1315623	1304448	1309070	1257417	1262039	1269632	1274254
1506	BPSS0961		1250123	1250599	908375	908851	395398	395874	3089761	3090237	1304963	1305439	1315633	1316109	1309080	1309556	1262049	1262525	1274264	1274740
1507	BPSS0962		1251645	1255037	909897	913289	396920	400312	3091283	3094675	1306485	1309877	1317154	1320546	1310602	1313994	1263571	1266963	1275783	1279175
1508	BPSS0963		1255086	1255763	913338	914015	400361	401038	3094724	3095401	1309920	1310603	1320589	1321272	1314043	1314720	1267006	1267689	1279218	1279901
1509		BURPS1106A_A1330	1255754	1255999																
1510		BURPS1106B_0658			914006	914251														
1511		BURPS1710A_A0483					401029	401274												
1512		BURPS406E_G0480									1310594	1310839								
1513		BURPS668_A1410											1321263	1321508						
1514		BURPSPAST_J0685													1314711	1314956				
1515		BURPSS13_T0454															1267680	1267925		
1516	BPSS0964		1257554	1255965	915806	914217	402829	401240	3097192	3095603	1312394	1310805	1323063	1321474	1316511	1314922	1269480	1267891	1281693	1280104
1517	BPSS0965		1257840	1259099	916092	917351	403115	404374	3097478	3098737	1312680	1313939	1323349	1324608	1316797	1318056	1269766	1271025	1281979	1283247
1518	BPSS0966		1259235	1260527	917487	918779	404510	405802	3098873	3100165	1314075	1315367	1324744	1326036	1318192	1319484	1271145	1272437	1283383	1284675
1519	BPSS0967		1261428	1260979	919680	919231	406703	406254	3101066	3100617	1316268	1315819	1326937	1326488	1320395	1319946	1273338	1272889	1285566	1285117
1520	BPSS0968		1262585	1261680	920837	919932	407860	406955	3102223	3101318	1317425	1316520	1328094	1327189	1321552	1320647	1274495	1273590	1286723	1285818
1521	BPSS0969		1264922	1262601	923174	920853	410197	407876	3104560	3102239	1319762	1317441	1330431	1328110	1323889	1321568	1276832	1274511	1289060	1286739
1522	BPSS0970		1265101	1266516	923353	924768	410376	411791	3104739	3106154	1319941	1321356	1330610	1332025	1324068	1325483	1277011	1278426	1289239	1290654
1523	BPSS0971		1266590	1266946	924842	925198	411865	412221	3106228	3106584	1321430	1321786	1332099	1332455	1325557	1325907	1278500	1278856	1290728	1291084
1524	BPSS0972		1267851	1267111	926103	925363	413126	412386	3107489	3106749	1322691	1321951	1333360	1332620	1326897	1326157	1279761	1279021	1291937	1291197
1525	BPSS0973		1270713	1267930	928965	926182	415988	413205	3110351	3107568	1325553	1322770	1336222	1333439	1329759	1326976	1282623	1279840	1294799	1292016
1526	BPSS0974		1273072	1271456	931324	929708	418347	416731	3112710	3111094	1327912	1326296	1338581	1336965	1332116	1330500	1284982	1283366	1297158	1295542
1527		BURPS1710A_A0498					418861	418607												
1528		BURPS668_A1427											1338841	1339095						
1529		BURPS668_A1430											1339357	1339497						
1530		BURPSS13_T0469															1285388	1285822		
1531	BPSS0975		1274640	1275152	932892	933404	419894	420406	3114257	3114769	1329483	1329995	1340153	1340665	1333693	1334205	1286580	1287092	1298785	1299297
1532	BPSS0976		1275155	1276765	933407	935017	420409	422019	3114772	3116382	1329998	1331608	1340668	1342278	1334208	1335818	1287095	1288705	1299300	1300910
1533	BPSS0977		1276813	1278123	935065	936375	422067	423377	3116430	3117740	1331656	1332966	1342326	1343636	1335866	1337176	1288753	1290063	1300958	1302268
1534	BPSS0978		1278318	1278728	936570	936980	423572	423982	3117935	3118345	1333047	1333457	1343832	1344242	1337371	1337781	1290259	1290669	1302463	1302873
1535	BPSS0979		1278784	1279896	937036	938148	424038	425150	3118401	3119513	1333513	1334625	1344298	1345410	1337837	1338781	1290725	1291837	1302929	1304041
1536	BPSS0980		1280901	1280101	939153	938353	426155	425355	3120518	3119718	1335629	1334829	1346415	1345615	1339854	1339054	1292842	1292042	1305045	1304245
1537	BPSS0981		1281368	1282054	939620	940306	426623	427309	3120986	3121672	1336096	1336782	1346882	1347568	1340321	1341007	1293310	1293996	1305513	1306199
1538	BPSS0982		1282054	1282764	940306	941016	427309	428019	3121672	3122382	1336782	1337492	1347568	1348278	1341007	1341717	1293996	1294706	1306199	1306909
1539	BPSS0983		1282836	1283600	941088	941852	428091	428855	3122454	3123218	1337564	1338328	1348350	1349114	1341789	1342553	1294778	1295542	1306981	1307745
1540	BPSS0984		1284969	1283671	943221	941923	430224	428926	3124587	3123289	1339697	1338399	1350483	1349185	1343922	1342624	1296911	1295613	1309114	1307816
1541	BPSS0985		1285225	1285716	943477	943968	430475	430966	3124838	3125329	1339948	1340439	1350732	1351223	1344134	1344625	1297185	1297676	1309366	1309857
1542	BPSS0986		1285975	1286721	944227	944973	431223	431969	3125586	3126332	1340698	1341444	1351473	1352219	1344882	1345628	1297933	1298679	1310116	1310862
1543	BPSS0987		1287186	1286845	945438	945097	432434	432093	3126797	3126456	1341909	1341568	1352684	1352343	1346093	1345752	1299144	1298803	1311327	1310986
1544	BPSS0988		1287812	1289086	946064	947338	433069	434343	3127432	3128706	1342544	1343818	1353319	1354593	1346728	1348002	1299779	1301053	1311962	1313236
1545	BPSS0989		1289676	1289137	947928	947389	434933	434394	3129296	3128757	1344408	1343869	1355183	1354644	1348592	1348053	1301643	1301104	1313826	1313287
1546	BPSS0990		1290325	1291341	948577	949593	435864	436529	3130227	3130892	1344962	1345978	1355795	1356811	1349523	1350188			1314424	1315440
1547	BPSS0991		1291346	1292044	949598	950296	436534	436893	3130897	3131256	1345983	1346342	1357204	1357545	1350193	1350552	1303062	1303421	1315445	1315804
1548	BPSS0993		1292360	1293874	950612	952126	437580	439094	3131943	3133457	1347029	1348543	1357861	1359375	1351239	1352753	1304108	1305622	1316491	1318005
1549	BPSS0994		1295771	1294305	954023	952557	440983	439517	3135346	3133880	1350528	1349062	1361336	1359870	1354682	1353216	1307551	1306085	1319862	1318396
1550	BPSS0995		1297508	1295952	955760	954204	442720	441164	3137083	3135527	1352386	1350830	1363073	1361517	1356423	1355119	1309288	1307732	1321599	1320043
1551	BPSS0996		1298679	1297678	956931	955930	443891	442890	3138254	3137253	1353557	1352556	1364244	1363243	1357594	1356593	1310459	1309458	1322770	1321769

1552 BPSS0997		1300524	1298848	958776	957100	445736	444060	3140099	3138423	1355402	1353726	1366089	1364413	1359439	1357763	1312304	1310628	1324615	1322939
1553 BPSS0998		1301810	1300569	960062	958821	447022	445781	3141385	3140144	1356688	1355447	1367375	1366134	1360725	1359484	1313590	1312349	1325901	1324660
1554 BPSS0999		1302036	1301800	960288	960052	447248	447012	3141611	3141375	1356914	1356678	1367601	1367365	1360951	1360715	1313816	1313580	1326127	1325891
1555 BPSS1000		1302836	1302093	961088	960345	448048	447305	3142411	3141668	1357714	1356971	1368401	1367658	1361751	1361008	1314616	1313873	1326927	1326184
1556 BPSS1001		1303643	1302876	961895	961128	448855	448088	3143218	3142451	1358521	1357754	1369208	1368441	1362558	1361791	1315423	1314656	1327734	1326967
1557 BPSS1002		1304896	1303640	963148	961892	450108	448852	3144471	3143215	1359774	1358518	1370461	1369205	1363811	1362555	1316676	1315420	1328987	1327731
1558 BPSS1003		1305346	1305011	963598	963263	450558	450223	3144921	3144586	1360224	1359889	1370911	1370576	1364261	1363926	1317126	1316791	1329437	1329102
1559	BURPS1106A_A1387	1306173	1307378																
1560 BPSS1004		1308924	1307755	967176	966007	454135	452966	3148498	3147329	1362347	1361172	1373030	1371855	1366378	1365209	1319279	1318086	1331586	1330393
1561 BPSS1005		1310542	1309313	968794	967565	455753	454524	3150116	3148887	1363965	1362736	1374648	1373419	1367996	1366767	1320897	1319668	1333204	1331975
1562 BPSS1006		1311165	1323101	969417	981353	456376	468273	3150739	3162636	1364587	1366644	1382087	1389043	1368618	1370591	1321519	1328673	1333827	1345826
1563 BPSS1007		1335004	1342713	993247	1000956	470060	474907	3164423	3169159	1378429	1388700	1399032	1406780	1386307	1392078	1335416	1344700	1352112	1356287
1564 BPSS1008		1342713	1350323	1000956	1008566	487631	489562	223	8571	1396227	1399604	1406780	1414357	1399312	1402689	1353034	1361367	1364422	1367844
1565 BPSS1009		1357762	1362450	1015996	1020684	502770	507440	15359	20029	1411428	1416098	1421835	1426508	1415805	1419323	1368155	1372804	1379140	1383813
1566 BPSS1010		1362473	1364191	1020707	1022425	507463	509181	20052	21770	1416121	1417839	1426531	1428249	1419346	1421064	1372827	1374545	1383836	1385554
1567 BPSS1011		1364283	1365722	1022517	1023956	509273	510712	21862	23301	1417925	1419364	1428335	1429774	1421156	1422595	1374631	1376070	1385640	1387079
1568	BURPS1106B_0589			1024022	1024231														
1569 BPSS1012		1368190	1366322	1026424	1024556	513130	511277	25719	23866	1421873	1420005	1432281	1430413	1425043	1423160	1378516	1376648	1389612	1387696
1570 BPSS1013		1368638	1368228	1026872	1026462	513578	513168	26167	25757	1422321	1421911	1432729	1432319	1425491	1425081	1378964	1378554	1390060	1389650
1571 BPSS1014		1369972	1368782	1028206	1027016	514912	513722	27501	26311	1423655	1422465	1434063	1432873	1426825	1425635	1380298	1379108	1391394	1390204
1572 BPSS1015		1370318	1371286	1028552	1029520	515265	516233	27854	28822	1424008	1424976	1434409	1435377	1427178	1428146	1380644	1381612	1391740	1392708
1573	BURPS1655_I0688																	1392767	1393273
1574	BURPS668_A1490											1435426	1435968						
1575	BURPSS13_T0525															1381671	1382195		
1576 BPSS1016		1372999	1371860	1031233	1030094	517905	516766	30494	29355	1426648	1425509	1437083	1435944	1429818	1428679	1383310	1382171	1394405	1393266
1577	BURPS1655_I0689																	1394626	1395072
1578	BURPSS13_T0526															1383521	1383988		
1579 BPSS1017		1373670	1374455	1031904	1032689	518576	519361	31165	31950	1427319	1428104	1437754	1438539	1430489	1431274	1383985	1384770	1395069	1395854
1580 BPSS1018		1375713	1374808	1033947	1033042	520619	519714	33208	32303	1429362	1428457	1439788	1438883	1432532	1431627	1386028	1385123	1397112	1396207
1581 BPSS1019		1376593	1375892	1034827	1034126	521499	520798	34088	33387	1429948	1429541	1440668	1439967	1433412	1432711	1386908	1386207	1397992	1397291
1582 BPSS1020		1377815	1376772	1036050	1035007	522721	521678	35310	34267	1430956	1430054	1441884	1440841	1434634	1433591	1388130	1387087	1399190	1398165
1583	BURPS406E_G0560									1431214	1431450								
1584	BURPS668_A1498											1441894	1442388						
1585 BPSS1021		1378461	1378616	1036696	1036851	523430	523585	36019	36174	1431602	1431757	1442540	1442695	1435343	1435498	1388811	1388966	1399846	1400001
1586 BPSS1022		1378635	1379153	1036870	1037388	523604	524122	36193	36711	1431776	1432294	1442714	1443232	1435517	1436035	1388985	1389503	1400020	1400538
1587 BPSS1023		1379153	1379974	1037388	1038209	524122	524943	36711	37532			1443232	1444053	1436035	1436856	1389503	1389868	1400538	1401359
1588 BPSS1024		1380209	1381018	1038444	1039253	525184	525993	37773	38582	1433395	1434204	1444281	1445090	1437097	1437906	1390174	1390983	1401605	1402414
1589 BPSS1025		1381061	1381417	1039296	1039652	526036	526392	38625	38981	1434247	1434603	1445133	1445489	1437949	1438305	1391026	1391382	1402457	1402813
1590 BPSS1026		1381417	1381641	1039652	1039876	526392	526616	38981	39205	1434603	1434827	1445604	1445693	1438420	1438509	1391382	1391606	1402813	1403037
1591 BPSS1027		1381918	1382424	1040153	1040659	526893	527399	39482	39988	1435104	1435274	1445970	1446476	1438786	1439148	1391883	1392389	1403314	1403820
1592 BPSS1028		1382424	1383425	1040659	1041660	527399	528400	39988	40989	1435611	1436612	1446476	1447477			1392389	1393390	1403820	1404821
1593 BPSS1029		1383747	1386197	1041982	1044432	528722	531172	41311	43761	1436934	1439384	1447790	1450252	1439506	1441968	1393721	1396183	1405179	1405571
1594 BPSS1030		1387399	1386422	1045634	1044657	532374	531397	44963	43986	1440579	1439602	1451447	1450470	1443170	1442193	1397378	1396401	1408894	1407917
1595 BPSS1031		1388477	1387395	1046712	1045630	533452	532370	46041	44959	1441657	1440575	1452525	1451443	1444068	1443166	1398456	1397374	1409972	1408890
1596 BPSS1032		1390279	1388477	1048514	1046712	535209	533452	47798	46041	1443501	1441657	1454339	1452525	1445934	1444171	1400279	1398456	1411771	1409972
1597 BPSS1033		1391325	1390279	1049560	1048514					1444547	1443501	1455385	1454339	1446980	1445934	1401325	1400279	1412817	1411771
1598 BPSS1034		1391957	1392694	1050192	1050929	536588	537325	49177	49914	1445178	1445915	1456016	1456753	1447612	1448349	1401957	1402694	1413449	1414186
1599 BPSS1035		1393304	1393056	1051539	1051291	537913	537665	50502	50254	1446525	1446277	1457292	1457044	1448937	1448689	1403293	1403045	1414774	1414526
1600 BPSS1036		1394726	1393470	1052961	1051705	539335	538079	51924	50668	1447947	1446691	1458714	1457458	1450359	1449103	1404715	1403459	1416196	1414940
1601	BURPS1655_I0715																	1416478	1416212
1602 BPSS1037		1396415	1394949	1054650	1053184	541008	539542	53597	52131	1449636	1448170	1460403	1458937	1452032	1450566	1406388	1404922	1417901	1416441
1603	BURPS1106A_A1433	1396505	1396684																
1604	BURPS1106B_0557			1054919	1054740														
1605	BURPS1655_I0717																	1417993	1418196
1606	BURPS406E_G0580									1449728	1449931								
1607 BPSS1038		1397724	1397993	1055959	1056228	542303	542572	54892	55161	1450998	1451267	1461683	1461952	1453327	1453596	1407681	1407950	1419222	1419491
1608 BPSS1039		1399494	1398103	1057729	1056338	544064	542673	56653	55262	1452768	1451377	1463474	1462083	1455088	1453697	1409442	1408051	1420992	1419601
1609 BPSS1040		1400180	1399494	1058415	1057729	544750	544064	57339	56653	1453454	1452768	1464160	1463474	1455774	1455088	1410128	1409442	1421678	1420992

1610 BPSS1041		1403420	1400190	1061655	1058425	547990	544760	60579	57349	1456694	1453464	1467400	1464170	1459014	1455784	1413368	1410138	1424918	1421688
1611 BPSS1042		1404935	1403469	1063170	1061704	549505	548039	62094	60628	1458209	1456743	1468915	1467449	1460529	1459063	1414883	1413417	1426466	1424967
1612 BPSS1043		1406304	1404949	1064539	1063184	550874	549519	63463	62108	1459578	1458223	1470284	1468929	1461898	1460543	1416252	1414897	1427835	1426480
1613 BPSS1044		1407390	1407253	1065625	1065488	551962	551825	64551	64414	1460664	1460527	1471368	1471231	1462985	1462848	1417338	1417201	1428920	1428783
1614	BURPS1106A_A1447	1407856	1408056																
1615	BURPS1106B_0543			1066091	1066291														
1616 BPSS1045		1408532	1408047	1066767	1066282	553093	552608	65682	65197	1461819	1461334	1472543	1472058	1464116	1463631	1418575	1418090	1430092	1429607
1617	BURPS1655_I0730																	1430321	1431298
1618	BURPS668_A1534											1472771	1473748						
1619 BPSS1046		1408864	1409346	1067099	1067581	553425	553907	66014	66496	1462142	1462624	1473853	1474335	1464448	1464930	1418907	1419389	1431403	1431885
1620	BURPS1655_I0732																	1433277	1432516
1621	BURPS1655_I0733																	1434157	1433867
1622	BURPS1655_I0734																	1434304	1434564
1623 BPSS1047																1419936	1419601		
1624 BPSS1048																1420317	1421096		
1625 BPSS1049																1422162	1422371		
1626 BPSS1050																1422576	1423814		
1627 BPSS1051																1423817	1424020		
1628 BPSS1052																1424023	1425813		
1629 BPSS1053																1425832	1426095		
1630 BPSS1054																1426095	1426298		
1631 BPSS1055																1426388	1427041		
1632 BPSS1056																1427245	1427397		
1633 BPSS1057																1427503	1428033		
1634 BPSS1058																1428033	1428767		
1635 BPSS1059																1428779	1429897		
1636 BPSS1060																1430426	1430785	1434634	1434825
1637 BPSS1061																1430785	1431144	1434825	1435184
1638 BPSS1062																1432246	1431194		
1639 BPSS1063																1434048	1432246		
1640 BPSS1064																1434156	1434962		
1641 BPSS1065																1434999	1436009		
1642 BPSS1066																1436009	1436692		
1643 BPSS1067																1436798	1437274		
1644 BPSS1068																1437277	1437525		
1645 BPSS1069																1437525	1437728		
1646 BPSS1070																1437746	1438087		
1647 BPSS1071																1438092	1438361		
1648 BPSS1072																1438361	1439170		
1649 BPSS1073																1439170	1439607		
1650 BPSS1074																1439715	1440128		
1651 BPSS1075																1440128	1440592		
1652 BPSS1076																			
1653 BPSS1077																			
1654 BPSS1078																			
1655 BPSS1079																			
1656 BPSS1080																1440629	1440808		
1657 BPSS1081																1440804	1441355		
1658 BPSS1082																1441369	1443729		
1659 BPSS1083																1443749	1444417		
1660 BPSS1084																1444484	1445644		
1661 BPSS1085																1445663	1446169		
1662 BPSS1086																1446230	1446571		
1663 BPSS1087																			
1664 BPSS1088																			
1665 BPSS1089																1449418	1450230		
1666 BPSS1090		1410819	1409707	1069054	1067942	555380	554268	67969	66857	1464097	1462985	1475828	1474716	1466403	1465291	1452163	1451051	1436975	1435863
1667	BURPS668_A1538											1476107	1476310						

1668 BPSS1091		1411873	1411547	1070108	1069782	556433	556107	69022	68696	1465150	1464824	1476881	1476555	1467456	1467130	1453217	1452891	1438029	1437703
1669 BPSS1092		1412489	1411890	1070724	1070125	557049	556450	69638	69039	1465766	1465167	1477509	1476898	1468072	1467473	1453833	1453234	1438645	1438046
1670 BPSS1093		1412873	1413706	1071108	1071941	557433	558266	70022	70855	1466150	1466983	1477904	1478737	1468456	1469289	1454217	1455050	1439029	1439862
1671 BPSS1094		1414077	1414649	1072312	1072884	558637	559209	71226	71798	1467354	1467569	1479109	1479681	1469660	1470232	1455421	1455993	1440227	1440799
1672 BPSS1095		1414649	1416490	1072884	1074725	559209	561053	71798	73642	1467927	1469771	1479681	1481525	1470232	1472076	1455993	1457834	1440799	1442643
1673 BPSS1096		1416500	1419304	1074735	1077539	561060	563864	73649	76453	1469778	1472582	1481532	1484336	1472083	1474887	1457844	1460648	1442650	1444086
1674 BPSS1097		1420826	1419600	1079061	1077835	565386	564160	77975	76749	1474108	1472882	1485862	1484636	1476409	1475183	1462169	1460943		
1675 BPSS1098		1421567	1421310	1079802	1079545	566127	565870	78716	78459	1474863	1474606	1486573	1486316	1477150	1476893	1462902	1462645		
1676	BURPS1106A_A1463	1421957	1422424																
1677	BURPS1106B_0526			1080659	1080192														
1678	BURPSPAST_J0802													1478007	1477540				
1679 BPSS1099		1423625	1423005	1081860	1081240	568185	567565	80774	80154	1476913	1476293	1488632	1488012	1479208	1478588	1464954	1464334		
1680 BPSS1100		1426190	1423680	1084425	1081915	570750	568240	83339	80829	1479478	1476968	1491197	1488687	1481773	1479263	1467519	1465009		
1681 BPSS1101		1426767	1426291	1085002	1084526	571327	570851	83916	83440	1480055	1479579	1491774	1491298	1482350	1481874	1468096	1467620		
1682	BURPS1710A_A0629					571399	572061												
1683	BURPS668_A1557											1491933	1492508						
1684 BPSS1102		1427757	1428599	1085992	1086834	572321	573163	84910	85752	1481045	1481887	1492768	1493610	1483416	1484258	1469090	1469932		
1685	BURPS1710b_A0058							85732	87330										
1686 BPSS1103		1430287	1428860	1088522	1087095	574850	573423	86275	86012	1483574	1482147	1495298	1493871	1485945	1484518	1471619	1470192		
1687 BPSS1104		1431249	1430380	1089484	1088615	575806	574943	88396	87533	1484530	1483667	1496254	1495391	1486904	1486038	1472581	1471712		
1688 BPSS1105		1432145	1431408	1090380	1089643	576722	575985	89312	88575	1485439	1484702	1497170	1496433	1487804	1487067	1473477	1472740		
1689 BPSS1106		1433150	1432164	1091385	1090399	577727	576741	90317	89331	1486444	1485458	1498175	1497189	1488809	1487823	1474482	1473496		
1690 BPSS1107		1433607	1433155	1091842	1091390	578184	577732	90774	90322	1486901	1486449	1498632	1498180	1489266	1488814	1474939	1474487		
1691 BPSS1108		1434167	1433607	1092402	1091842	578744	578184	91334	90774	1487461	1486901	1499192	1498632	1489826	1489266	1475499	1474939		
1692 BPSS1109		1434666	1435457	1092901	1093692	579243	580034	91833	92624	1487960	1488751	1499691	1500482	1490325	1491116	1475998	1476789		
1693 BPSS1110		1435517	1436662	1093752	1094897	580094	581239	92684	93829	1488811	1489956	1500542	1501687	1491176	1492321	1476859	1478004		
1694 BPSS1111		1437698	1436865	1095933	1095100	582274	581441	94864	94031	1490991	1490158	1502730	1501897	1493356	1492523	1479040	1478207		
1695	BURPSPAST_J0826													1493526	1493993				
1696 BPSS1112		1438418	1439905	1096653	1098140	582969	584456	95559	97046	1491686	1493173	1503447	1504934	1494062	1495549	1479735	1481222		
1697 BPSS1113		1440136	1440822	1098371	1099057	584687	585373	97277	97963	1493404	1494090	1505165	1505851	1495780	1496466	1481445	1482131		
1698 BPSS1114		1443006	1441009	1101241	1099244	587515	585518	100105	98108	1496239	1494242	1507993	1505996	1498608	1496611	1484280	1482283		
1699 BPSS1115		1443572	1443063	1101807	1101298	588081	587572	100671	100162	1496805	1496296	1508559	1508050	1499174	1498665	1484846	1484337		
1700	BURPS1106A_A1491	1444277	1444390																
1701	BURPS1106B_0498			1102512	1102625														
1702 BPSS1116		1444345	1446138	1102580	1104373	588854	590647	101444	103237	1497582	1499375	1509333	1511126	1499947	1501740	1485619	1487412		
1703 BPSS1117		1446979	1446275	1105214	1104510	591488	590784	104078	103374	1500216	1499512	1511967	1511263	1502581	1501877	1488253	1487549		
1704 BPSS1118		1447094	1448188	1105329	1106423	591603	592697	104193	105287	1500331	1501425	1512082	1513176	1502696	1503790	1488368	1489462		
1705 BPSS1119		1448242	1451295	1106477	1109530	592751	595804	105341	108394	1501479	1504532	1513230	1516283	1503844	1506897	1489516	1492569		
1706 BPSS1120		1451433	1452968	1109668	1111203	595942	596382	108532	110064	1504670	1506205	1516409	1517944	1507035	1507907	1492772	1493515		
1707 BPSS1121		1453301	1453942	1111536	1112177	597592	598233	110397	111038	1506538	1507179	1518277	1518918	1508904	1509545	1493848	1494489		
1708 BPSS1122		1453942	1455636	1112177	1113871	598233	599927	111038	112732	1507179	1508873	1518918	1520612	1509545	1511239	1494489	1496183		
1709 BPSS1123		1455636	1456343	1113871	1114578	599927	600634	112732	113439	1508873	1509580	1520612	1521319	1511239	1511946	1496183	1496890		
1710 BPSS1124		1456343	1457323	1114578	1115558	600634	601614	113439	114419	1509580	1510560	1521319	1522299	1511946	1512926	1496890	1497870		
1711 BPSS1125		1457465	1458649	1115700	1116884	601736	602920	114541	115725	1510673	1511857	1522412	1523614	1513048	1514232	1497992	1499185		
1712 BPSS1126		1458712	1459761	1116947	1117996	602983	604032	115788	116837	1511920	1512969	1523677	1524726	1514295	1515344	1499248	1500297		
1713 BPSS1127		1459767	1460627	1118002	1118862	604038	604898	116843	117703	1512975	1513835	1524732	1525592	1515350	1516210	1500303	1501163		
1714 BPSS1128		1460662	1462062	1118897	1120297	604933	606333	117738	119138	1513870	1515270	1525627	1527027	1516245	1517645	1501198	1502598		
1715 BPSS1129		1462130	1462627	1120365	1120862	606401	606898	119206	119703	1515338	1515835	1527095	1527592	1517713	1518210	1502666	1503163		
1716 BPSS1130		1462649	1464055	1120884	1122290	606920	608326	119725	121131	1515857	1517263	1527614	1529020	1518232	1519638	1503185	1504591		
1717 BPSS1131		1465076	1464276	1123311	1122511	609346	608546	122151	121351	1518284	1517484	1530030	1529230	1520658	1519858	1505612	1504812		
1718 BPSS1132		1465877	1465461	1124112	1123696	610146	609730	122951	122535	1519085	1518669	1530831	1530415	1521458	1521042	1506413	1505997		
1719 BPSS1133		1468049	1465998	1126284	1124233	612318	610267	125123	123072	1521257	1519206	1533003	1530952	1523630	1521579	1508585	1506534		
1720 BPSS1134		1468165	1468719	1126400	1126954	612434	612988	125239	125793	1521373	1521927	1533119	1533673	1523746	1524300	1508701	1509255		
1721 BPSS1135		1468978	1469805	1127213	1128040	613247	614074	126052	126879	1522186	1523013	1533932	1534759	1524559	1525386	1509514	1510341		
1722 BPSS1136		1470327	1471187	1128562	1129422	614596	615456	127401	128261	1523535	1524395	1535281	1536141	1525908	1526768	1510863	1511723		
1723 BPSS1137		1471305	1472189	1129540	1130424	615574	616458	128379	129263	1524513	1525397	1536259	1537143	1526886	1527770	1511841	1512725		
1724 BPSS1138		1473081	1472455	1131316	1130690	617350	616724	130155	129529	1526289	1525663	1538035	1537409	1528662	1528036	1513617	1512991		
1725 BPSS1139		1473486	1473232	1131721	1131467	617755	617501	130560	130306	1526694	1526440	1538440	1538186	1529067	1528813	1514022	1513768		

1726	BPSS1140		1473869	1474828	1132104	1133063	618089	619048	130894	131853	1527077	1528036	1538823	1539782	1529401	1530360	1514405	1515364
1727	BPSS1141		1475466	1475053	1133701	1133288	619686	619273	132491	132078	1528674	1528261	1540420	1540007	1530998	1530585	1516002	1515589
1728	BPSS1142		1476415	1475900	1134650	1134135	620635	620120	133440	132925	1529614	1529099	1541369	1540854	1531947	1531432	1516951	1516436
1729	BPSS1143		1478070	1476418	1136305	1134653	622290	620638	135095	133443	1531242	1529617	1543024	1541372	1533602	1531950	1518606	1516954
1730	BPSS1144		1479603	1478146	1137838	1136381	623823	622366	136628	135171	1532775	1531318	1544557	1543100	1535135	1533678	1520139	1518682
1731	BPSS1145		1480255	1479599	1138490	1137834	624475	623819	137280	136624	1533427	1532771	1545209	1544553	1535787	1535131	1520791	1520135
1732	BPSS1146		1481206	1480259	1139441	1138494	625426	624479	138231	137284	1534378	1533431	1546160	1545213	1536738	1535791	1521742	1520795
1733	BPSS1147		1483206	1481203	1141441	1139438	627426	625423	140231	138228	1536378	1534375	1548160	1546157	1538738	1536735	1523742	1521739
1734	BPSS1148		1483565	1484335	1141800	1142570	627785	628555	140590	141360	1536737	1537507	1548519	1549289	1539097	1539867	1524101	1524871
1735	BPSS1149		1484565	1485059	1142800	1143294	628785	629279	141590	142084	1537737	1538231	1549519	1550013	1540097	1540591	1525101	1525595
1736	BPSS1150		1485120	1485377	1143355	1143612	629340	629597	142145	142402	1538292	1538549	1550074	1550331	1540652	1540909	1525656	1525913
1737	BPSS1151		1485377	1486228	1143612	1144463	629597	630448	142402	143253	1538549	1539400	1550331	1551182	1540909	1541760	1525913	1526764
1738	BPSS1152		1486338	1486553	1144573	1144788	630558	630773	143363	143578	1539510	1539725	1551292	1551507	1541870	1542085	1526874	1527089
1739	BPSS1153		1486597	1486920	1144832	1145155	630817	631140	143622	143945	1539769	1540092	1551551	1551874	1542129	1542452	1527133	1527456
1740	BPSS1154		1488202	1486931	1146437	1145166	632422	631151	145227	143956	1541374	1540103	1553156	1551885	1543734	1542463	1528738	1527467
1741	BPSS1155		1489024	1488230	1147259	1146465	633244	632450	146049	145255	1542196	1541402	1553978	1553184	1544556	1543762	1529560	1528766
1742	BPSS1156		1489727	1489047	1147962	1147282	633947	633267	146752	146072	1542899	1542219	1554681	1554001	1545259	1544579	1530263	1529583
1743	BPSS1157		1490624	1489938	1148859	1148173	634815	634129	147620	146934	1543767	1543081	1555539	1554853	1546127	1545441	1531131	1530445
1744	BPSS1158		1492126	1490624	1150361	1148859	636317	634815	149122	147620	1545269	1543767	1557041	1555539	1547629	1546127	1532633	1531131
1745	BPSS1159		1495950	1492150	1154185	1150385	640141	636341	152946	149146	1549093	1545293	1560865	1557065	1551453	1547653	1536457	1532657
1746	BPSS1160		1496725	1498689	1154960	1156924	640911	642875	153716	155680	1549863	1551827	1561635	1563599	1552223	1554187	1537227	1539191
1747	BPSS1161		1498689	1499333	1156924	1157568	642875	643519	155680	156324	1551827	1552471	1563599	1564243	1554187	1554831	1539191	1539835
1748	BPSS1162		1499898	1500305	1158133	1158540	644129	644536	156935	157342	1553036	1553443	1564815	1565222	1555434	1555841	1540428	1540835
1749	BPSS1163		1500321	1501097	1158556	1159332	644552	645328	157358	158134	1553459	1554235	1565238	1566014	1555857	1556633	1540851	1541627
1750	BPSS1164		1503137	1501488	1161372	1159723	647397	645748	160203	158554	1556293	1554644	1568054	1566405	1558691	1557042	1543696	1542047
1751	BPSS1165		1503835	1504677	1162070	1162912	648095	648937	160901	161743	1556991	1557833	1568752	1569594	1559389	1560231	1544394	1545236
1752	BPSS1166		1506181	1504934	1164416	1163169	650453	649194	163259	162000	1559337	1558090	1571097	1569850	1561735	1560488	1546740	1545493
1753	BPSS1167		1506944	1506240	1165179	1164475	651216	650512	164022	163318	1560100	1559396	1571860	1571156	1562498	1561794	1547503	1546799
1754	BPSS1168		1507938	1507096	1166173	1165331	652210	651368	165016	164174	1561103	1560261	1572890	1572048	1563492	1562650	1548497	1547655
1755	BPSS1169		1509535	1507946	1167770	1166181	653807	652218	166613	165024	1562700	1561111	1574487	1572898	1565089	1563500	1550094	1548505
1756	BPSS1170		1512016	1509551	1170251	1167786	656288	653823	169094	166629	1565181	1562716	1576968	1574503	1567570	1565105	1552575	1550110
1757	BPSS1171		1515473	1512000	1173708	1170235	659745	656272	172551	169078	1568638	1565165	1580425	1576952	1571027	1567554	1556032	1552559
1758	BPSS1172		1519502	1515516	1177737	1173751	663774	659788	176580	172594	1572667	1568681	1584454	1580468	1575056	1571070	1560061	1556075
1759	BPSS1173		1524087	1519498	1182322	1177733	668359	663770	181165	176576	1577252	1572663	1589033	1584450	1579641	1575052	1564643	1560057
1760	BPSS1174		1528687	1524098	1186922	1182333	672971	668370	185777	181176	1581851	1580916	1593645	1589044	1584253	1579652	1569249	1564654
1761	BPSS1175		1528882	1530948	1187117	1189183	673166	675232	185972	188038	1582046	1584112	1593840	1595906	1584448	1586514	1569444	1571510
1762	BPSS1176		1531512	1532213	1189747	1190448	675796	676497	188602	189303	1584676	1585377	1596469	1597170	1587078	1587779	1572075	1572776
1763	BPSS1177		1533190	1532336	1191425	1190571	677474	676620	190280	189426	1586354	1585500	1598147	1597293	1588756	1587902	1573753	1572899
1764	BPSS1178		1534590	1533244	1192825	1191479	678874	677528	191680	190334	1587754	1586408	1599547	1598201	1590156	1588810	1575153	1573807
1765	BPSS1179		1535070	1535510	1193305	1193745	679354	679794	192160	192600	1588234	1588674	1600027	1600467	1590639	1591073	1575633	1576073
1766	BPSS1180		1535596	1536213	1193831	1194448	679880	680497	192686	193303	1588760	1589377	1600553	1601170	1591159	1591776	1576159	1576776
1767	BPSS1181		1536285	1537799	1194520	1196034	680569	682083	193375	194889	1589449	1590963	1601242	1602756	1591848	1593362	1576848	1578362
1768	BPSS1182		1537799	1538035	1196034	1196270	682083	682319	194889	195125	1590963	1591199	1602756	1602992	1593362	1593598	1578362	1578598
1769	BPSS1183		1538176	1539096	1196411	1197331	682460	683380	195266	196186	1591340	1592260	1603127	1604047	1593739	1594659	1578747	1579667
1770	BPSS1184		1539190	1539483	1197425	1197718	683474	683767	196280	196573	1592354	1592647	1604141	1604434	1594753	1595046	1579761	1580054
1771	BPSS1185		1539686	1539913	1197921	1198148	683971	684063	196777	196869	1592850	1592942	1604638	1605276	1595250	1595342		
1772		BURPS1710A_A0745					684912	684361										
1773		BURPS1710A_A0747					685979	685572										
1774		BURPS1710A_A0748					687560	686187										
1775		BURPS1710A_A0749					687730	687557										
1776		BURPS1710A_A0751					688934	689299										
1777		BURPS1710A_A0752					690026	689676										
1778		BURPS1710A_A0753					690115	690002										
1779		BURPS1710A_A0754					691306	690452										
1780		BURPS1710A_A0755					691563	691300										
1781		BURPS1710A_A0756					691898	691764										
1782		BURPS1710A_A0757					699231	691978										
1783		BURPS1710A_A0758					702319	699461										

1784	BURPS1710A_A0759			706832	702375				
1785	BURPS1710A_A0760			709889	706920				
1786	BURPS1710A_A0761			717568	709913				
1787	BURPS1710A_A0762			717981	717868				
1788	BURPS1710A_A0763			719535	719666				
1789	BURPS1710A_A0764			720661	719966				
1790	Bp_chr2_9_IS407A			720952	722187	233758	234993	1628139	1629374
1791	BURPS1710A_A0765			721020	721283				
1792	BURPS1710A_A0766			721307	722140				
1793	BURPS1710A_A0767			722979	722605				
1794	BURPS1710A_A0768			724199	724086				
1795	BURPS1710A_A0769			724573	724412				
1796	BURPS1710A_A0770			724843	724649				
1797	BURPS1710A_A0771			725019	724894				
1798	BURPS1710A_A0772			725624	725511				
1799	BURPS1710A_A0773			725660	725920				
1800	BURPS1710A_A0774			726047	726196				
1801	BURPS1710A_A0775			726639	726286				
1802	BURPS1710A_A0776			727744	726989				
1803	BURPS1710A_A0777			728365	727937				
1804	BURPS1710A_A0778			729144	728509				
1805	BURPS1710A_A0779			729528	729713				
1806	BURPS1710A_A0780			729926	730117				
1807	BURPS1710A_A0781			730118	730492				
1808	BURPS1710A_A0782			731182	731066				
1809	BURPS1710A_A0783			731462	731196				
1810	BURPS1710A_A0784			732015	731800				
1811	BURPS1710A_A0785			732173	736759				
1812	BURPS1710A_A0786			737130	744407				
1813	BURPS1710A_A0787			744448	745064				
1814	BURPS1710A_A0788			748037	746943				
1815	BURPS1710A_A0789			745121	746946				
1816	BURPS1710A_A0790			748340	748119				
1817	BURPS1710A_A0791			748390	748743				
1818	BURPS1710b_A0150					198378	198785		
1819	BURPS1710b_A0151					198993	200363		
1820	BURPS1710b_A0152					200357	200557		
1821	BURPS1710b_A0154					201740	202105		
1822	BURPS1710b_A0155					202482	202832		
1823	BURPS1710b_A0156					203258	204109		
1824	BURPS1710b_A0157					204106	204369		
1825	BURPS1710b_A0158					204570	204737		
1826	BURPS1710b_A0159					204784	212037		
1827	BURPS1710b_A0160					212267	215125		
1828	BURPS1710b_A0161					215181	219638		
1829	BURPS1710b_A0162					219726	222695		
1830	BURPS1710b_A0163					222719	230272		
1831	BURPS1710b_A0164					232157	232612		
1832	BURPS1710b_A0165					232772	233410		
1833	BURPS1710b_A0166					233826	234089		
1834	BURPS1710b_A0167					234113	234946		
1835	BURPS1710b_A0168					235411	235722		
1836	BURPS1710b_A0169					238891	239532		
1837	BURPS1710b_A0170					239795	240478		
1838	BURPS1710b_A0171					241315	242073		
1839	BURPS1710b_A0172					243007	243171		
1840	BURPS1710b_A0173					242924	243298		
1841	BURPS1710b_A0174					244606	244830		

1842		BURPS1710b_A0175							244979	249565										
1843		BURPS1710b_A0176							249936	257213										
1844		BURPS1710b_A0177							257254	264630										
1845		BURPS1710b_A0178							264627	265709										
1846		BURPS406E_G0730									1593890	1593240								
1847		BURPS406E_G0731									1594858	1594451								
1848		BURPS406E_G0732									1596439	1595066								
1849		BURPS406E_G0733									1596591	1596436								
1850		BURPS406E_G0735									1597813	1598178								
1851		BURPS406E_G0736									1598905	1598555								
1852		BURPS406E_G0737									1598994	1598881								
1853		BURPS406E_G0738									1599821	1599693								
1854		BURPS406E_G0739									1607160	1599907								
1855		BURPS406E_G0740									1610248	1607390								
1856		BURPS406E_G0741									1614719	1610304								
1857		BURPS406E_G0742									1617818	1614849								
1858		BURPS406E_G0743									1625500	1617842								
1859		BURPS406E_G0744									1625913	1625800								
1860		BURPS406E_G0745									1628102	1627899								
1861		BURPS406E_C0001									1666604	1665522								
1862		BURPS406E_C0002									1658149	1665525								
1863		BURPS406E_C0003									1666919	1666698								
1864		BURPS406E_G0746									1628207	1628470								
1865		BURPS406E_G0747									1628494	1629327								
1866		BURPS406E_G0748									1630166	1629792								
1867		BURPS406E_G0749									1631386	1631273								
1868		BURPS406E_G0750									1631772	1631599								
1869		BURPS406E_G0751									1632030	1631836								
1870		BURPS406E_G0752									1632961	1633104								
1871		BURPS406E_G0753									1633213	1633914								
1872		BURPS406E_G0754									1634931	1634176								
1873		BURPS406E_G0755									1635552	1635124								
1874		BURPS406E_G0756									1636610	1635696								
1875		BURPS406E_G0757									1637305	1637679								
1876		BURPS406E_G0758									1638649	1638383								
1877		BURPS406E_G0759									1639202	1638987								
1878		BURPS406E_G0760									1639360	1643946								
1879		BURPS406E_G0761									1644182	1651594								
1880		BURPS406E_G0762									1651635	1658039								
1881		BURPSPAST_AD0041													1619497	1619850				
1882		BURPSPAST_AD0042													1619426	1619226				
1883		BURPSPAST_AD0043													1610677	1618053				
1884		BURPSPAST_AD0044													1619144	1618050				
1885		BURPSPAST_J0922													1596290	1595640				
1886		BURPSPAST_J0923													1598839	1597466				
1887		BURPSPAST_J0924													1598917	1599030				
1888		BURPSPAST_J0926													1600213	1600578				
1889		BURPSPAST_J0927													1601305	1600955				
1890		BURPSPAST_J0928													1601394	1601281				
1891		BURPSPAST_J0929													1602585	1601731				
1892		BURPSPAST_J0930													1602842	1602579				
1893		BURPSPAST_J0931													1603177	1603043				
1894		BURPSPAST_J0932													1610510	1603257				
1895	BPSS1185a										1668018	1668140	1605275	1605397						
1896	BPSS1187		1540472	1541275	1198707	1199510	750059	750862	267743	268546	1668638	1669441	1605891	1606694	1621166	1621969	1581512	1582315		
1897	BPSS1188		1544173	1542089	1202408	1200324	753760	751676	271444	269360	1672446	1672288	1609592	1607508	1624867	1622783	1585213	1583129		
1898	BPSS1190		1545117	1544173	1203352	1202408	754704	753760	272388	271444	1673390	1672446	1610536	1609592	1625811	1624867	1586157	1585213		
1899	BPSS1191		1546193	1545132	1204428	1203367	755780	754719	273464	272403	1674466	1673405	1611612	1610551	1626887	1625826	1587233	1586172		

1900	BPSS1192		1547509	1546193	1205744	1204428	757090	755780	274774	273464	1675782	1674466	1612922	1611612	1628203	1626887	1588549	1587233		
1901	BPSS1193		1552209	1547509	1210444	1205744	761791	760181	279474	274774	1680482	1675782	1617622	1612922	1632903	1628203	1593249	1588549		
1902	BPSS1194		1554899	1552230	1213134	1210465	764499	761812	282182	279495	1682176	1680503	1620342	1617643	1635611	1632924	1595939	1593270		
1903	BPSS1195		1556267	1554930	1214502	1213165	765846	764530	283529	282213	1683104	1682280	1621701	1620373	1636961	1635642	1597307	1595970		
1904	BPSS1196		1557325	1556267	1215560	1214502	766904	765846	284587	283529	1684162	1683104	1622759	1621701	1638019	1636961	1598365	1597307		
1905	BPSS1197		1566812	1557354	1225047	1215589	776394	766933	294077	284616	1693727	1692015	1632273	1622788	1647545	1638048	1607955	1606444		
1906	BPSS1198		1567571	1566816	1225806	1225051	777153	776398	294836	294081	1694486	1693731	1633032	1632277	1648304	1647549	1608714	1607959		
1907	BPSS1199		1567871	1567611	1226106	1225846	777453	777193	295136	294876	1694786	1694526	1633332	1633072	1648604	1648344	1609014	1608754		
1908	BPSS1200		1568391	1569935	1226626	1228170	777973	779517	295656	297200	1695343	1696887	1633890	1635434	1649123	1650667	1609534	1611078		
1909		BURPS1710A_A0811					778035	777766												
1910	BPSS1201		1570170	1570616	1228405	1228851	779752	780198	297435	297881	1697122	1697568	1635670	1636116	1650902	1651348	1611313	1611759		
1911	BPSS1202		1571032	1571073	1228851	1229264	780198	780650	297881	298333	1697568	1698020	1636116	1636568	1651348	1651800	1611759	1612166		
1912	BPSS1203		1571611	1572903	1229846	1231138	781188	782480	298871	300163	1698545	1699837	1637106	1638398	1652326	1653618	1612748	1614040		
1913	BPSS1204		1573331	1575553	1231566	1233788	782904	785126	300587	302809	1700261	1702483	1638810	1641032	1654046	1656268	1614463	1616685	1444136	1446358
1914	BPSS1205		1575558	1576301	1233793	1234536	785131	785874	302814	303557	1702488	1703231	1641037	1641780	1656273	1657016	1616690	1617433	1446363	1447106
1915	BPSS1206		1576301	1576981	1234536	1235216	785874	786554	303557	304237	1703231	1703911	1641780	1642460	1657016	1657696	1617433	1618113	1447106	1447786
1916	BPSS1207		1577617	1577414	1235852	1235649	787190	786987	304873	304670	1704547	1704344	1643101	1642898	1658332	1658129	1618750	1618547	1448428	1448225
1917	BPSS1208		1578099	1577701	1236334	1235936	787672	787274	305355	304957	1705029	1704631	1643583	1643185	1658814	1658416	1619232	1618834	1448910	1448512
1918	BPSS1209		1578492	1578136	1236727	1236371	788071	787709	305754	305392	1705422	1705066	1643982	1643620	1659207	1658851	1619631	1619269	1449309	1448947
1919	BPSS1210		1578929	1578678	1237164	1236913	788508	788257	306191	305940	1705859	1705608	1644419	1644168	1659644	1659393	1620068	1619817	1449746	1449498
1920		BURPSS13_K0162															1621487	1622590		
1921	BPSS1211		1580495	1578951	1238730	1237186					1707425	1705881								
1922	BPSS1212		1582817	1580628	1241052	1238863	2416480	2418666	310079	307893	1709747	1707561	1647398	1646121	1663298	1661919	1625313	1623127	1453634	1451448
1923	BPSS1213		1585656	1582882	1241596	1241117	2413786	2416164	312897	310144	98424	101177			101398	104172	1628153	1626003	1456473	1453699
1924	BPSS1214		1587168	1587860	367899	367207	795968	796660	314401	315093	1714030	1714722			1667642	1668334	1629657	1629893	1457985	1458677
1925	BPSS1215		1587836	1589113	367231	365954	796636	797913	315069	316346	1714698	1715975			1668310	1669587			1458653	1459930
1926	BPSS1216		1589152	1589748	365915	365319	798924	799520	316385	316981	1716014	1716610			1669626	1670222	1630336	1630932	1459969	1460565
1927	BPSS1217		1591025	1591621	364042	363446	801813	802409	318253	318849	1717887	1718483	1657713	1658309	1671494	1672090	1632189	1632785	1461842	1462438
1928		BURPS1106A_A1622	1592155	1592277																
1929		BURPS668_A1709											1658799	1658921						
1930	BPSS1218		1592561	1594111	362506	360956	803349	804899	319789	321339	1719434	1720984	1659206	1660756	1673019	1674569	1633748	1635298	1463367	1464917
1931		BURPS1106B_0368			362912	362790														
1932	BPSS1219		1594677	1596494	360390	358573	805483	807300	321923	323740	1721549	1723366	1661354	1663171	1675153	1676970	1635903	1637720	1465485	1467299
1933	BPSS1220		1596916	1597914	358151	357153	807698	808696	324138	325136	1723855	1724853	1663660	1664658	1677368	1678366	1638121	1639119	1467742	1468740
1934		BURPS1655_I0766																	1467762	1467475
1935	BPSS1221		1597976	1598746	357091	356321	808758	809528	325198	325968	1724915	1725685	1664720	1665490	1678428	1679198	1639181	1639951	1468802	1469572
1936		BURPS1106A_A1629	1599158	1599340																
1937		BURPS1106A_A1630	1599550	1599729																
1938		BURPS1106A_A1631	1600478	1601254																
1939		BURPS1106A_A1632	1601379	1602722																
1940		BURPS1106A_A1633	1602921	1603889																
1941		BURPS1106A_A1634	1603999	1605111																
1942		BURPS1710A_A0851					810122	809940												
1943		BURPS1710A_A0852					810332	810511												
1944		BURPS1710A_A0853					810752	812029												
1945		BURPS1710A_A0854					812154	813049												
1946		BURPS1710A_A0855					813106	814453												
1947		BURPS1710A_A0856					814652	815620												
1948		BURPS1710A_A0859					816479	817663												
1949		BURPS1710b_A0216							327192	328469										
1950		BURPS1710b_A0217							328507	329937										
1951		BURPS1710b_A0218							330136	331104										
1952		BURPS1710b_A0219							331142	332326										
1953		BURPS406E_C1589									1726288	1726097								
1954		BURPS406E_C1590									1726923	1728200								
1955		BURPS406E_C1591									1728325	1729668								
1956		BURPS406E_C1592									1729747	1730835								
1957		BURPS406E_C1593									1730873	1732057								

1958		BURPS668_A1716											1665902	1666084						
1959		BURPS668_A1717											1666342	1666473						
1960		BURPS668_A1718											1667222	1667998						
1961		BURPS668_A1719											1668123	1669466						
1962		BURPS668_A1720											1669665	1670633						
1963		BURPS668_A1721											1670743	1671855						
1964		BURPS668_A1722											1671975	1672220						
1965		BURPSPAST_V0016													1679801	1679610				
1966		BURPSPAST_V0017													1680429	1681706				
1967		BURPSPAST_V0018													1681831	1683174				
1968		BURPSPAST_V0019													1683253	1684341				
1969		BURPSPAST_V0020													1684379	1685563				
1970	BPSS1222		1607225	1605477	347842	349590	819861	818113	334440	332692	1734237	1732489	1673959	1672220	1687585	1685840	1641740	1639992	1471361	1469613
1971		BURPS1106B_0356			351140	349956														
1972		BURPS1106B_0357			352146	351178														
1973		BURPS1106B_0358			353688	352345														
1974		BURPS1106B_0359			355051	353813														
1975		BURPS1106B_0360			355517	355338														
1976		BURPS1106B_0361			355727	355909														
1977	BPSS1223		1608455	1609354	346612	345713	821091	821990	335670	336569	1735464	1736363	1675186	1676085	1688812	1689711	1642970	1643869	1472588	1473487
1978	BPSS1224		1609458	1611428	345609	343639	822094	824064	336673	338643	1736467	1738437	1676189	1678159	1689815	1691785	1643973	1645943	1473591	1475546
1979	BPSS1225		1611445	1611798	343622	343269	824081	824434	338660	339013	1738454	1738807	1678176	1678529	1691802	1692155	1645960	1646313	1475563	1475916
1980	BPSS1226		1612030	1612305	343037	342762	824682	824957	339261	339536	1739071	1739346	1678801	1679076	1692403	1692678	1646553	1646828	1476164	1476439
1981	BPSS1227		1612939	1612469	342128	342598	825591	825121	340170	339700	1739981	1739511	1679710	1679240	1693312	1692842	1647545	1647075	1477076	1476606
1982	BPSS1228		1614117	1613098	340950	341969	826769	825750	341348	340329	1741159	1740140	1680890	1679871	1694490	1693471	1648723	1647704	1478254	1477235
1983	BPSS1229		1614631	1615410	340436	339657	827289	828068	341868	342647	1741679	1742458	1681404	1682183	1695005	1695784	1649236	1650015	1478767	1479546
1984	BPSS1230		1615426	1616580	339641	338487	828084	829238	342663	343817	1742474	1743628	1682199	1683353	1695800	1696954	1650031	1651185	1479562	1480716
1985		BURPS668_A1736											1683368	1683532						
1986	BPSS1231		1617462	1616995	337619	338086	830092	829625	344671	344204	1744510	1744043	1684263	1683796	1697815	1697348	1652046	1651579	1481601	1481134
1987		BURPS1710A_A0877					830610	830278												
1988	BPSS1232		1618254	1619531	336827	335550	830863	832140	345442	346719	1745291	1746568	1685024	1686301	1698596	1699873	1652838	1654115	1482376	1483653
1989	BPSS1233		1619677	1620552	335404	334529	832278	833153	346857	347732	1746706	1747581	1686483	1687358	1700011	1700886	1654253	1655128	1483852	1484727
1990	BPSS1234		1620543	1621352	334538	333729	833144	833953	347723	348532	1747572	1748381	1687349	1688158	1700877	1701686	1655119	1655928	1484718	1485527
1991	BPSS1235		1621377	1622408	333704	332673	833978	835009	348557	349588	1748406	1749437	1688183	1689214	1701711	1702742	1655953	1656984	1485552	1486583
1992	BPSS1236		1622439	1623605	332642	331476	835040	836206	349619	350785	1749468	1750634	1689245	1690411	1702773	1703939	1657015	1658181	1486614	1487780
1993	BPSS1237		1623605	1624570	331476	330511	836206	837264	350785	351843	1750634	1751692	1690411	1691376	1703939	1704997	1658181	1659146	1487780	1488775
1994	BPSS1238		1624654	1626111	330427	328970	837264	838721	351843	353300	1751692	1753149	1691460	1692917	1704997	1706454	1659230	1660687	1488775	1490232
1995	BPSS1239		1626731	1627729	328350	327352	839341	840339	353920	354918	1753769	1754767	1693537	1694535	1707074	1708072	1661307	1662305	1490852	1491850
1996	BPSS1240		1628042	1629796	327039	325285	840652	842406	355231	356985	1755103	1756842	1694877	1696616	1708409	1710163	1662641	1664380	1492192	1493931
1997		BURPSS13_K0203															1664699	1664583		
1998	BPSS1241		1634633	1630395	320448	324686	847175	844638	359441	357576	1761663	1757425	1701426	1697188	1715004	1710766	1669181	1667223	1498429	1494185
1999	BPSS1242		1635042	1634695	320039	320386	847584	847237	362163	361816	1762072	1761725	1701835	1701488	1715413	1715066	1669590	1669243	1498838	1498491
2000	BPSS1243		1637662	1635083	317419	319998	850204	847625	364783	362204	1764693	1764574	1704455	1701876	1718033	1715454	1672210	1669631	1501458	1498879
2001	BPSS1244		1639004	1637682	316077	317399	851546	850224	366125	364803	1766035	1764713	1705797	1704475	1719375	1718053	1673552	1672230	1502800	1501478
2002	BPSS1245		1639837	1640778	315244	314303	852406	853347	366985	367926	1766926	1767867	1706630	1707571	1720235	1721176	1674399	1675340	1503626	1504567
2003	BPSS1246		1640883	1641476	314198	313605	853452	854045	368031	368624	1767972	1768565	1707676	1708269	1721281	1721874	1675445	1676038	1504672	1505265
2004	BPSS1247		1641476	1642555	313605	312526	854045	855124	368624	369703	1768565	1769644	1708269	1709348	1721874	1722953	1676038	1677117	1505265	1506344
2005		BURPS668_A1761											1709362	1709652						
2006	BPSS1248		1643460	1642831	311621	312250	856043	855414	370622	369993	1770563	1769934	1710281	1709652	1723858	1723229	1678050	1677421	1507249	1506620
2007		BURPS1710A_A0904					856280	856161												
2008	BPSS1249		1645057	1647246	310024	307835	857656	859845	372235	374424	1772144	1774333	1711871	1714060	1725463	1727652	1679671	1681860	1508832	1511021
2009	BPSS1250		1648806	1647787	306275	307294	861414	860395	375993	374974	1775902	1774883	1715620	1714601	1729221	1728202	1683423	1682404	1512601	1511582
2010	BPSS1251		1650216	1648840	304865	306241	862824	861448	377403	376027	1777312	1775936	1717023	1715683	1730631	1729255	1684833	1683457	1514011	1512635
2011	BPSS1252		1651575	1650244	303506	304837	864183	862852	378762	377431	1778671	1777340	1718388	1717057	1731990	1730659	1686192	1684861	1515370	1514039
2012	BPSS1253		1651764	1652699	303317	302382	864372	865307	378951	379886	1778860	1779795	1718577	1719512	1732179	1733114	1686381	1687316	1515559	1516494
2013	BPSS1254		1653300	1652875	301781	302206	865908	865483	380487	380062	1780396	1779971	1720085	1719663	1733684	1733265	1687917	1687492	1517069	1516644
2014	BPSS1255		1654353	1653349	300728	301732	866961	865957	381540	380536	1781449	1780445	1721138	1720134	1734737	1733733	1688970	1687966	1518122	1517118
2015	BPSS1256		1654477	1655217	300604	299864	867085	867825	381664	382404	1781573	1781953	1721262	1722002	1734861	1735601	1689094	1689834	1518246	1518986

2016	BURPS1655_I0823																	1519368	1518949
2017 BPSS1257		1657028	1655613	298003	299418	869556	868141	384135	382720	1784128	1782713	1723743	1722328			1691589	1690174	1520777	1519362
2018	BURPS1655_I0826																	1521801	1521052
2019 BPSS1258		1659176	1658142	295848	296882	871834	870800	386203	385169	1786238	1785204	1725873	1724839	1737112	1736078	1693790	1692756	1523020	1521986
2020	BURPS1106B_0299			297068	297583														
2021 BPSS1259		1660398	1659244	294626	295780	873056	871902	387425	386271	1787460	1786306	1727095	1725941	1738334	1737180	1695012	1693858	1524242	1523088
2022	BURPS1106A_A1697	1661565	1661843																
2023	BURPS1655_I0833																	1525309	1525596
2024 BPSS1260		1662316	1664127	292708	290897	874940	876751	389309	391120	1789382	1791193	1729037	1730848	1740193	1742004	1696875	1698674	1526101	1527912
2025	BURPS1106B_0293			293459	293181														
2026 BPSS1261		1664725	1664396	290299	290628	877321	876992	391690	391361	1791791	1791462	1731446	1731117	1742602	1742273	1699272	1698943	1528510	1528181
2027 BPSS1262		1664995	1665774	290029	289250	877591	878370	391960	392739	1792063	1792842	1731713	1732468	1742874	1743653	1699542	1700321	1528791	1529570
2028	BURPS1710A_A0933					878384	880960												
2029	BURPS1710b_A0266							392753	395329										
2030	BURPSPAST_V0091													1743667	1746003				
2031 BPSS1263		1665788	1668601	289236	286423					1792856	1795669	1732482	1735295			1700335	1703148	1529584	1532397
2032 BPSS1264		1668601	1669245	286423	285779	880957	881601	395326	395970	1795669	1796313	1735295	1735939	1746000	1746644	1703148	1703792	1532397	1533041
2033	BURPS668_A1789											1735935	1736711						
2034 BPSS1265		1669232	1670053	285792	284971	881588	882442	395957	396811	1796300	1797118					1703779	1704594	1533028	1533918
2035	BURPS1106A_A1705	1670150	1670374																
2036 BPSS1266		1673589	1670344	281435	284680	885979	882734	400348	397103	1800655	1797410	1740246	1737001	1750965	1747720	1708130	1704885	1537454	1534209
2037	BURPS1106B_0285			284874	284650														
2038 BPSS1267		1673842	1673639	281182	281385	886232	886029	400601	400398	1800908	1800705	1740499	1740296	1751218	1751015	1708383	1708180	1537707	1537504
2039 BPSS1268		1675150	1673864	279874	281160	887540	886254	401909	400623	1802216	1800930	1741807	1740521	1752526	1751240	1709691	1708405	1539015	1537729
2040 BPSS1269		1687952	1683201	267072	271823	900264	887548	414633	401917	1815021	1810258	1754609	1749663	1765205	1752534	1722484	1717721	1551682	1539023
2041 BPSS1270		1689000	1688005	266024	267019	901297	900317	415666	414686	1816069	1815074	1755669	1754662	1766253	1765258	1723532	1722537	1552736	1551735
2042 BPSS1271		1689639	1689310	265385	265714	901979	901650	416348	416019	1816687	1816358	1756271	1755942	1766843	1766514	1724150	1723821	1553333	1553004
2043 BPSS1272		1691401	1689611	263623	265413	903741	901951	418110	416320	1818449	1816659	1758033	1756243	1768605	1766815	1725912	1724122	1555095	1553305
2044 BPSS1273		1693158	1691401	261866	263623	905498	903741	419867	418110	1820206	1818449	1759790	1758033	1770362	1768605	1727669	1725912	1556852	1555095
2045 BPSS1274		1694987	1693158	260037	261866	907327	905498	421696	419867	1822035	1820206	1761619	1759790	1772190	1771678	1729498	1727669	1558681	1556852
2046 BPSS1275		1695584	1696105	259440	258919	907924	908445	422293	422814	1822632	1823153	1762220	1762741	1772787	1773308	1730095	1730616	1559278	1559799
2047 BPSS1276		1696884	1697471	258140	257553	909211	909798	423580	424167	1823909	1824496	1763528	1764115	1774090	1774677	1731374	1731961	1560581	1561168
2048 BPSS1277		1697604	1698677	257420	256347	909931	911004	424300	425373	1824629	1824895	1764248	1765321	1774810	1775076	1732094	1732174	1561301	1561444
2049 BPSS1278		1701517	1699445	253507	255579	913828	911756	428197	426125	1828566	1826494	1768146	1766074	1778767	1776695	1735858	1733786	1565150	1563078
2050 BPSS1279		1703121	1701517	251903	253507	915432	913828	429801	428197	1830170	1828566	1769750	1768146	1780371	1778767	1737462	1735858	1566754	1565150
2051 BPSS1280		1703531	1703785	251493	251239	915842	916096	430211	430465	1830580	1830834	1770160	1770414	1780781	1781035	1737872	1738126	1567164	1567418
2052 BPSS1281		1704801	1703878	250223	251146	917112	916189	431481	430558	1831850	1830927	1771430	1770507	1782051	1781128	1739142	1738219	1568433	1567510
2053	BURPS1710A_A0961					917116	917598												
2054	BURPSPAST_V0118													1782644	1782096				
2055 BPSS1282		1706751	1705258	248273	249766	919271	917778	433640	432147	1833789	1832296	1773325	1771832	1784188	1782695	1741092	1739599	1570243	1569944
2056 BPSS1283		1708060	1706768	246964	248256	920580	919288	434949	433657	1835098	1833806	1774634	1773342	1785497	1784205	1742401	1741109	1571552	1570260
2057 BPSS1284		1709095	1708076	245929	246948	921615	920596	435984	434965	1836133	1835114	1775669	1774650	1786532	1785513	1743436	1742417	1572587	1571568
2058 BPSS1285		1711236	1709095	243788	245929	923756	921615	438125	435984	1838274	1836133	1777810	1775669	1788673	1786532	1745577	1743436	1574728	1572587
2059 BPSS1286		1711570	1711355	243454	243669	924090	923875	438459	438244	1838608	1838393	1778144	1777929	1789007	1788792	1745911	1745696	1575062	1574847
2060 BPSS1286a		1711957	1711787	243067	243237	924477	924307	438846	438676	1838995	1838825	1778531	1778361	1789394	1789224	1746298	1746128	1575449	1575279
2061 BPSS1287		1712326	1713663	242698	241361	924846	926183	439215	440552	1839364	1840701	1778900	1780237	1789763	1791100	1746667	1748004	1575818	1577155
2062 BPSS1288		1715590	1713872	239434	241152	928148	926430	442517	440799	1842628	1840910	1782164	1780446	1793043	1791325	1749931	1748213	1579098	1577380
2063	BURPS668_A1824											1782224	1782475						
2064 BPSS1289		1716529	1717044	238489	237974	929061	929576	443430	443945	1843516	1844031	1783053	1783568	1793932	1794447	1750805	1751320	1579938	1580453
2065 BPSS1290		1717376	1717891	237642	237127	929922	930437	444291	444806	1844377	1844892	1783915	1784430	1794793	1795308	1751652	1752167	1580786	1581301
2066 BPSS1291		1718430	1723226	236588	231792	931081	935874	445450	450243	1845454	1850286	1784977	1789770	1796000	1800793	1752819	1755128	1581972	1584008
2067 BPSS1292		1723847	1725139	231171	229879	936495	937787	450864	452156	1850907	1851113	1790384	1791676	1801414	1802016	1758149	1759441	1587651	1588943
2068	BURPS1710b_A0311							452537	455173										
2069	BURPS668_A1833											1791637	1791816						
2070	BURPSS13_K0284															1759441	1759617		
2071 BPSS1293		1726045	1726170	228973	228848														
2072 BPSS1294		1726790	1726278	228228	228740	939411	938899	453780	453268	1853800	1853288	1793358	1792846	1803794	1803282	1761074	1760562	1590393	1589881
2073 BPSS1295		1727005	1728120	228013	226898	939626	940741	453995	455110	1854015	1855130	1793571	1794686	1804009	1805124	1761289	1762404	1590608	1591723

2074 BPSS1296		1729289	1728624	225729	226394	941914	941249	456283	455618	1856303	1855638	1795858	1795193	1806297	1805632	1763577	1762912	1592895	1592230
2075	BURPS406E_C1714									1856663	1856541								
2076	BURPSS13_K0291															1763965	1763843		
2077 BPSS1297		1731470	1729953	223548	225065	944051	942534	458420	456903	1858384	1856867	1798026	1796509	1808466	1806949	1765686	1764169	1595056	1593539
2078 BPSS1298		1732132	1732800	222886	222218	944719	945387	459088	459756	1859052	1859720	1798693	1799361	1809134	1809802	1766356	1767024	1595724	1596392
2079	BURPS668_A1843											1799393	1800184						
2080 BPSS1299		1734538	1733537	220480	221481	947130	946129	461499	460498	1861473	1860472	1801185	1800184	1811547	1810546	1768767	1767766	1598094	1597093
2081 BPSS1300		1734646	1735347	220372	219671	947238	947939	461607	462308	1861581	1862282	1801293	1801994	1811655	1812356	1768875	1769576	1598202	1598903
2082 BPSS1301		1735353	1735943	219665	219075	947945	948535	462314	462904	1862288	1862878	1802000	1802590	1812362	1812952	1769582	1770172	1598909	1599499
2083	BURPS1655_I0897																	1599649	1599807
2084	BURPS668_A1847											1802744	1802929						
2085 BPSS1302		1737223	1736399	217802	218626	949727	948903	464096	463272	1864099	1863275	1803837	1803013	1813961	1813770	1771515	1770760	1600703	1599867
2086 BPSS1303		1738578	1737226	216447	217799	951055	949730	465424	464099	1865445	1864102	1805165	1803840	1815289	1813964	1772852	1771518	1602067	1600706
2087 BPSS1304		1739486	1738581	215539	216444	951963	951058	466332	465427	1866353	1865448	1806073	1805168	1816197	1815292	1773760	1772855	1602975	1602070
2088 BPSS1305		1740414	1739497	214611	215528	952891	951974	467260	466343	1867281	1866364	1807001	1806084	1817125	1816208	1774688	1773771	1603903	1602986
2089 BPSS1306		1742113	1740554	212912	214471	954590	953031	468959	467400	1868980	1867421	1808700	1807141	1818824	1817265	1776387	1774828	1605602	1604043
2090 BPSS1307		1744347	1742320	210678	212705	956851	954797	471220	469166	1871173	1869155	1810898	1808907	1821074	1819047	1778573	1776546	1607866	1605857
2091 BPSS1308		1745422	1744391	209603	210634	957938	956895	472307	471264	1872260	1871217	1811985	1810942	1822161	1821118	1779660	1778617	1608953	1607910
2092 BPSS1309		1746381	1745422	208644	209603	958897	957938	473266	472307	1873219	1872260	1812944	1811985	1823120	1822161	1780619	1779660	1609912	1608953
2093 BPSS1310		1746715	1748742	208310	206283	959231	961258	473600	475627	1873553	1875580	1813278	1815305	1823454	1825481	1780953	1782980	1610246	1612273
2094 BPSS1311		1750494	1749283	204531	205742	962730	961519	477099	475888	1877013	1875802	1816667	1815456	1826874	1825663	1784413	1783202	1613636	1612425
2095 BPSS1312		1751088	1752545	203937	202480	963324	964781	477693	479150	1877632	1879089	1817286	1818743	1827493	1828950	1785032	1786489	1614255	1615703
2096 BPSS1313		1753028	1753666	201997	201359	965264	965902	479633	480271	1879596	1880234	1819265	1819903	1829441	1830079	1786988	1787626	1616233	1616871
2097 BPSS1314		1754066	1753719	200959	201306	966309	965962	480678	480331	1880641	1880294	1820315	1819968	1830479	1830132	1788033	1787686	1617285	1616938
2098 BPSS1315		1757517	1754599	197508	200426	969760	966842	484129	481211	1884076	1881158	1823701	1820801	1833912	1831012	1791466	1788566	1620670	1617770
2099 BPSS1316		1758539	1757604	196486	197421	970782	969847	485151	484216	1885098	1884163	1824723	1823788	1834934	1833999	1792488	1791553	1621692	1620757
2100 BPSS1317		1759255	1758539	195770	196486	971498	970782	485867	485151	1885814	1885098	1825439	1824723	1835650	1834934	1793204	1792488	1622408	1621692
2101	BURPS1106A_A1786	1759304	1759732																
2102	BURPS1655_I0918																	1622981	1622457
2103	BURPS668_A1872											1825456	1825857						
2104	BURPSPAST_V0171													1835917	1835699				
2105 BPSS1318		1761374	1759827	193651	195198	973596	972049	487967	486420	1887738	1886440	1827570	1826023	1837532	1836168	1795255	1793708	1624552	1623335
2106	BURPS1106B_0202			195293	195721														
2107 BPSS1319		1761753	1761460	193272	193565	973975	973682	488346	488053	1888117	1887824	1827949	1827656	1838095	1837802	1795634	1795341	1624937	1624644
2108 BPSS1320		1762671	1761817	192354	193208	974893	974039	489264	488410	1889035	1888181	1828867	1828013	1839013	1838159	1796552	1795698	1625855	1625001
2109 BPSS1321		1763776	1762679	191249	192346	975998	974901	490369	489272	1890140	1889043	1829972	1828875	1840118	1839021	1797657	1796560	1626960	1625863
2110 BPSS1322		1764512	1765381	190513	189644	976734	977603	491105	491974	1890876	1891745	1830709	1831578	1840854	1841723	1798393	1799262	1627703	1628572
2111 BPSS1323		1766546	1765590	188479	189435	978751	977795	493122	492166	1892902	1891946	1832734	1831778	1842871	1841915	1800410	1799454	1629728	1628772
2112 BPSS1324		1767814	1766801	187211	188224	980019	979006	494390	493377	1894181	1893168	1833980	1832967	1844139	1843126	1801678	1800665	1630985	1629972
2113 BPSS1325		1768007	1768513	187018	186512	980212	980718	494583	495089	1894374	1894880	1834173	1834679	1844332	1844838	1801871	1802377	1631178	1631684
2114	BURPS1106A_A1796	1768479	1768700																
2115	BURPS1710A_A1032					981052	980684												
2116	BURPS1710b_A0346							495055	495423										
2117	BURPS406E_C1749									1895019	1894846								
2118	BURPSPAST_V0180													1845067	1844804				
2119	BURPSS13_K0324															1802516	1802343		
2120 BPSS1326		1768950	1770398	186075	184627	981151	982599	495522	496970	1895315	1896772	1835138	1836595	1845271	1846719	1802810	1804258	1632102	1633550
2121	BURPS1106B_0193			186325	186546														
2122 BPSS1327		1771960	1770608	183065	184417	984161	982809	498532	497180	1898334	1896982	1838157	1836805	1848281	1846929	1805820	1804468	1635112	1633760
2123 BPSS1328		1772068	1772790	182957	182235	984269	984991	498640	499362	1898442	1899164	1838265	1838987	1848389	1849111	1805928	1806650	1635220	1635942
2124 BPSS1329		1772853	1774274	182172	180751	985054	986475	499425	500846	1899227	1900648	1839050	1840471	1849174	1850595	1806713	1808134	1636005	1637426
2125 BPSS1330		1774329	1775213	180696	179812	986530	987417	500901	501788	1900703	1901590	1840526	1841422	1850650	1851537	1808189	1809076	1637481	1638368
2126 BPSS1331		1775562	1777106	179463	177919	987766	989310	502137	503681	1901939	1903483	1841772	1843316	1851886	1853430	1809425	1810969	1638715	1640259
2127	BURPS1106B_0186			179467	179604														
2128 BPSS1332		1777106	1778725	177919	176300	989310	990929	503681	505300	1903483	1905102	1843316	1844935	1853430	1855049	1810969	1812588	1640259	1641878
2129 BPSS1333		1778907	1779764	176118	175261	991111	991968	505482	506339	1905284	1906141	1845117	1845974	1855231	1856088	1812770	1813627	1642060	1642917
2130 BPSS1334		1780444	1780725	174581	174300	992647	992928	507018	507299	1906820	1907101	1846655	1846936	1856767	1857048	1814308	1814589	1643597	1643878
2131 BPSS1335		1781068	1781277	173957	173748	993286	993495	507657	507866	1907460	1907669	1847299	1847508	1857407	1857616	1814952	1815161	1644242	1644451

2132	BPSS1336		1782174	1781614	172851	173411	994392	993832	508763	508203	1908573	1908013	1848412	1847852	1858520	1857960	1816058	1815498	1645348	1644788
2133	BPSS1337		1782628	1782218	172397	172807	994846	994436	509217	508807	1909027	1908617	1848866	1848456	1858974	1858564	1816512	1816102	1645802	1645392
2134	BPSS1338		1783073	1782642	171952	172383	995291	994860	509662	509231	1909472	1909041	1849311	1848880	1859419	1858988	1816957	1816526	1646247	1645816
2135	BPSS1339		1783383	1783066	171642	171959	995601	995284	509972	509655	1909782	1909465	1849621	1849304	1859729	1859412	1817267	1816950	1646557	1646240
2136	BPSS1340		1783924	1784454	171101	170571	996142	996672	510513	511043	1910323	1910853	1850162	1850692	1860270	1860800	1817808	1818338	1647098	1647628
2137	BPSS1341		1786730	1784751	168295	170274	998949	998683	513319	511340	1913129	1911150	1852960	1850981	1862854	1861097	1820614	1818635	1649904	1647925
2138	BPSS1342		1787754	1786741	167271	168284	999973	998960	514343	513330	1914153	1913140	1853984	1852971	1863876	1862956	1821638	1820625	1650928	1649915
2139	BPSS1343		1788075	1787794	166950	167231	1000294	1000013	514664	514383	1914456	1914196	1854305	1854024	1864197	1863916	1821959	1821678	1651249	1650968
2140	BPSS1344		1789061	1788075	165964	166950	1001280	1000294	515650	514664	1915442	1914456	1855291	1854305	1865183	1864197	1822945	1821959	1652235	1651249
2141	BPSS1345		1789643	1789179	165382	165846	1001862	1001398	516232	515768	1916024	1915560	1855873	1855409	1865765	1865301	1823527	1823063	1652817	1652353
2142	BPSS1346		1790010	1792124	165015	162901	1002229	1004343	516599	518713	1916373	1918487	1856222	1858336	1866132	1868246	1823894	1826008	1653166	1655280
2143	BPSS1347		1794107	1792857	151276	152526	1006219	1005797	520590	519340	1920450	1919200	1860197	1858947	1870131	1868881	738704	739954	691997	693247
2144		BURPS1106A_A1826	1794342	1794530																
2145		BURPS668_A1915											1860207	1860620						
2146	BPSS1348		1796429	1794849	148954	150534	1008947	1007367	522666	521086	772352	773932	1862519	1860939	1872195	1870615	736382	737962	1659581	1658001
2147	BPSS1349		1797729	1796737	147654	148646	1010247	1009255	523966	522974	771115	772044	1863831	1862827	1873495	1872503	735082	736074	1660881	1659889
2148	BPSS1350		1799565	1797985	145815	147398	1012074	1010491	525793	524210	1923922	1922339	1865634	1864051	1875354	1873771	733219	734802	1662720	1661137
2149		BURPSS13_C0199															734896	735015		
2150	BPSS1351		1800956	1799565	144424	145815	1013465	1012074	527184	525793	1925313	1923922	1867025	1865634	1876745	1875354	731828	733219	1664111	1662720
2151	BPSS1352		1802014	1801118	143366	144262	1014559	1013663	528278	527382	1927541	1926645	1868083	1867187	1877791	1876895	730785	731678	1665182	1664286
2152	BPSS1353		1802996	1803580	142384	141800	1015541	1016125	529260	529844	1928523	1929107	1869065	1869649	1878773	1879357	1836181	1836765	1666164	1666748
2153	BPSS1354		1803626	1805092	141754	140288	1016171	1017637	529890	531356	1929153	1930619	1869695	1871161	1879403	1880869	1836811	1838277	1666794	1668260
2154	BPSS1355		1805115	1806809	140265	138571	1017660	1019354	531379	533073	1930642	1932336	1871184	1872878	1880892	1882586	1838300	1839994	1668283	1669977
2155	BPSS1356		1807937	1811311	137443	134069	1020437	1023811	534156	537530	1933464	1936838	1873999	1877373	1883669	1887043	1841078	1844452	1671104	1674478
2156	BPSS1357		1811508	1812941	133872	132439	1024008	1025441	537727	539160	1937033	1938466	1877582	1879015	1887240	1888673	1844661	1846085	1674687	1676120
2157	BPSS1358		1814503	1813028	130877	132352	1027003	1025528	540722	539247	1940028	1938553	1880577	1879102	1890235	1888760	1847647	1846172	1677682	1676207
2158	BPSS1359		1814952	1814503	130428	130877	1027452	1027003	541171	540722	1940477	1940028	1881026	1880577	1890684	1890235	1848096	1847647	1678131	1677682
2159	BPSS1360		1817169	1814959	128211	130421	1029636	1027459	543355	541178	1942694	1940484	1883210	1881033	1892868	1890691	1850280	1848103	1680348	1678138
2160	BPSS1361		1818198	1817716	127182	127664	1030701	1030219	544420	543938	1943725	1943243	1884296	1883814	1893933	1893451	1851345	1850863	1681393	1680911
2161	BPSS1362		1819220	1818240	126160	127140	1031723	1030743	545442	544462	1944747	1943767	1885318	1884338	1894955	1893975	1852367	1851387	1682415	1681435
2162	BPSS1363		1819929	1820399	125451	124981	1032432	1032902	546151	546621	1945456	1945926	1886027	1886497	1895664	1896134	1853076	1853528	1683124	1683594
2163	BPSS1364		1821382	1821182	123998	124198	1033885	1033685	547604	547404	1946909	1946709	1887480	1887280	1897117	1896917	1854511	1854311	1684578	1684378
2164	BPSS1364a		1822034	1822243	123346	123137	1034537	1034746	548256	548465	1947561	1947770	1888132	1888341	1897770	1897979	1855163	1855372	1685231	1685440
2165	BPSS1365		1823335	1822877	122045	122503	1035845	1035387	549564	549106	1948884	1948426	1889451	1888993	1899093	1898635	1856464	1856006	1686543	1686085
2166	BPSS1366		1823970	1823338	121410	122042	1036480	1035848	550199	549567	1949519	1948887	1890086	1889454	1899728	1899096	1857099	1856467	1687178	1686546
2167	BPSS1367		1826968	1823963	118412	121417	1039478	1036473	553197	550192	1952517	1949512	1893084	1890079	1902726	1899721	1860097	1857092	1690176	1687171
2168	BPSS1368		1827255	1826968	118125	118412	1039765	1039478	553484	553197	1952804	1952517	1893371	1893084	1903013	1902726	1860384	1860097	1690463	1690176
2169	BPSS1369		1828876	1827635	116504	117745	1041288	1040047	555007	553766	1954474	1953233	1894796	1893555	1904732	1903491	1861907	1860666	1692035	1690794
2170	BPSS1370		1830278	1828896	115102	116484	1042690	1041308	556409	555027	1955876	1954494	1896198	1894816	1906134	1904752	1863309	1861927	1693437	1692055
2171	BPSS1371		1830566	1831795	114814	113585	1042978	1044165	556697	557884	1956164	1957345	1896486	1897673	1906422	1907627	1863597	1864760	1693725	1694918
2172	BPSS1372		1832497	1832174	112883	113206	1044867	1044544	558586	558263	1958133	1957810	1898375	1898052	1908329	1908006	1865409	1865086	1695637	1695314
2173	BPSS1373		1833319	1832618	112061	112762	1045689	1044988	559408	558707	1958955	1958254	1899197	1898496	1909151	1908450	1866231	1865530	1696459	1695758
2174	BPSS1374		1834334	1833558	111046	111822	1046704	1045928	560423	559647	1959970	1959194	1900212	1899436	1910166	1909390	1867246	1866470	1697474	1696698
2175		BURPS1710A_A1113					1047772	1047209												
2176		BURPS1710b_A0401							560541	562817										
2177		BURPSPAST_T0247													1911227	1910664				
2178	BPSS1375		1835955	1835407	109425	109973	1048339	1047791	562058	561510	1961582	1961034	1901832	1901284	1911794	1911246	1868851	1868303	1699081	1698533
2179		BURPS1106A_A1871	1835968	1836366																
2180		BURPS1655_I1000																	1699220	1699510
2181		BURPS668_A1964											1901845	1902261						
2182		BURPSPAST_T0249													1911807	1912226				
2183		BURPSS13_X0040															1868864	1869283		
2184	BPSS1376		1837611	1836604	107769	108776	1050017	1049010	563736	562729	1962675	1962253	1903505	1902498	1913471	1912464	1870528	1869521	1700754	1699747
2185		BURPS1106B_0108			109412	109014														
2186	BPSS1377		1839046	1837604	106334	107776	1051001	1050432	564154	563729	1964070	1963501	1904940	1903498	1914455	1913886	1871963	1870521	1702189	1700747
2187	BPSS1378		1839706	1839284	105674	106096	1051661	1051239	565380	564958	1964730	1964308	1905599	1905177	1915115	1914693	1872623	1872201	1702849	1702427
2188	BPSS1379		1839885	1840619	105495	104761	1051856	1052575	565575	566294	1964909	1965643	1905792	1906508	1915294	1916028	1872818	1873537	1703028	1703762
2189	BPSS1380		1840661	1840948	104719	104432	1052617	1052904	566336	566623	1965685	1965972	1906553	1906840	1916070	1916357	1873579	1873866	1703804	1704091

2190	BPSS1381		1841023	1843206	104357	102174	1053292	1055163	566698	568881	1966047	1968230	1906915	1909098	1916432	1918615	1873941	1876124	1704166	1706349
2191	BPSS1382		1843206	1844219	102174	101161	1055163	1056176	568881	569894	1968230	1968457	1909098	1910111	1918615	1919628	1876124	1877137	1706349	1707362
2192	BPSS1383		1844219	1844590	101161	100790	1056176	1056547	569894	570265	1969244	1969615	1910111	1910482	1919628	1919999	1877137	1877508	1707362	1707733
2193	BPSS1383a		1845602	1844871	99778	100509	1057559	1056828	571277	570546	1970627	1969896	1911496	1910765	1921011	1920280	1878520	1877789	1708745	1708014
2194	BPSS1384		1847292	1845766	98088	99614	1059249	1057723	572967	571441	1972317	1970791	1913192	1911660	1922701	1921175	1880210	1878684	1710435	1708909
2195	BPSS1384a						1060153	1059788	573871	573506					1923605	1923240	1881114	1880749	1711338	1710973
2196	BPSS1385						1061334	1060351	575052	574069					1924786	1923803	1882295	1881312	1712519	1711536
2197		BURPSS13_X0055															1882842	1883945		
2198	BPSS1386		1852204	1848392	93176	96988	1065776	1061940	579494	575658	1977259	1973417	1918093	1914290	1929228	1925392	1888094	1884258	1716961	1713125
2199	BPSS1387		1852761	1852279	92619	93101	1066333	1065851	580051	579569	1977816	1977334	1918653	1918171	1929785	1929303	1888651	1888169	1717518	1717036
2200	BPSS1388		1854594	1852789	90786	92591	1068166	1066361	581884	580079	1979649	1977844	1920486	1918681	1931618	1929813	1890484	1888679	1719351	1717546
2201	BPSS1389		1856278	1854875	89102	90505	1069850	1068447	583568	582165	1981333	1979930	1922170	1920767	1933302	1931899	1892168	1890765	1721035	1719632
2202	BPSS1390		1858438	1856675	86942	88705	1072010	1070247	585728	583965	1983493	1981730	1924330	1922567	1935462	1933699	1894328	1892565	1723195	1721432
2203	BPSS1391		1859931	1858483	85449	86897	1073503	1072055	587221	585773	1984986	1983538	1925823	1924375	1936955	1935507	1895821	1894373	1724688	1723240
2204	BPSS1392		1860924	1860109	84456	85271	1074496	1073681	588214	587399	1985979	1985164	1926816	1926001	1937948	1937133	1896814	1895999	1725681	1724866
2205	BPSS1393		1861427	1860924	83953	84456	1074999	1074496	588717	588214	1986482	1985979	1927319	1926816	1938451	1937948	1897317	1896814	1726184	1725681
2206	BPSS1394		1862797	1861451	82583	83929	1076369	1075023	590087	588741	1987852	1986506	1928689	1927343	1939821	1938475	1898687	1897341	1727554	1726208
2207	BPSS1395		1863642	1862806	81738	82574	1077235	1076369	590953	590087	1988718	1987852	1929555	1928689	1940687	1939821	1899553	1898687	1728420	1727554
2208	BPSS1396		1864394	1863630	80986	81750	1077987	1077223	591705	590941	1989470	1988706	1930307	1929543	1941439	1940675	1900305	1899541	1729172	1728408
2209	BPSS1397		1865233	1864412	80147	80968	1078826	1078005	592544	591723	1990309	1989488	1931146	1930325	1942278	1941457	1901144	1900323	1730011	1729190
2210	BPSS1398		1865657	1865274	79723	80106	1079250	1078867	592968	592585	1990733	1990350	1931570	1931187	1942702	1942319	1901568	1901185	1730435	1730052
2211	BPSS1399		1866263	1865697	79117	79683	1079856	1079290	593574	593008	1991339	1990773	1932177	1931611	1943308	1942742	1902174	1901608	1731041	1730475
2212	BPSS1400		1866550	1867605	78830	77775	1080143	1081198	593861	594916	1991626	1992681	1932464	1933519	1943595	1944650	1902461	1903516	1731328	1732383
2213	BPSS1401		1867624	1869738	77756	75642	1081217	1083331	594935	597049	1992700	1994814	1933538	1935652	1944669	1946783	1903535	1905649	1732402	1734516
2214	BPSS1402		1869738	1870421	75642	74959	1083331	1084014	597049	597732	1994814	1995497	1935652	1936335	1946783	1947466	1905649	1906332	1734516	1735199
2215	BPSS1403		1870793	1871722	74587	73658	1084386	1085315	598104	599033	1995869	1996798	1936707	1937636	1947838	1948767	1906704	1907633	1735571	1736500
2216	BPSS1404		1871709	1872356	73671	73024	1085302	1085949	599020	599667	1996785	1997432	1937623	1938270	1948754	1949401	1907620	1908267	1736487	1737134
2217	BPSS1405		1872382	1872642	72998	72738	1085975	1086235	599693	599953	1997458	1997718	1938296	1938556	1949427	1949687	1908293	1908553	1737160	1737420
2218	BPSS1406		1872650	1873600	72730	71780	1086243	1087193	599961	600911	1997726	1998676	1938564	1939514	1949695	1950645	1908561	1909511	1737428	1738378
2219	BPSS1407		1873746	1874792	71634	70588	1087339	1088385	601057	602103	1998822	1999868	1939660	1940706	1950791	1951837	1909657	1910703	1738524	1739570
2220	BPSS1408		1874850	1875095	70530	70285	1088443	1088688	602161	602406	1999926	2000171	1940764	1941009	1951895	1952140	1910761	1911006	1739628	1739873
2221	BPSS1409		1875228	1875512	70152	69868	1088821	1089105	602539	602823	2000304	2000588	1941142	1941426	1952273	1952557	1911139	1911423	1740006	1740290
2222	BPSS1410		1875628	1876080	69752	69300	1089221	1089673	602939	603391	2000704	2001156	1941542	1941994	1952673	1953125	1911539	1911991	1740406	1740858
2223	BPSS1411		1876374	1877486	69006	67894	1090013	1091125	603731	604843	2001496	2002608	1942334	1943446	1953465	1954577	1912331	1913443	1741198	1742310
2224	BPSS1412		1878398	1877919	66982	67461	1092037	1091558	605755	605276	2003520	2003041	1944358	1943879	1955489	1955010	1914355	1913876	1743222	1742743
2225	BPSS1413		1879146	1878496	66234	66884	1092785	1092135	606503	605853	2004268	2003618	1945100	1944450	1956237	1955587	1915103	1914453	1743970	1743320
2226	BPSS1414		1879298	1879999	66082	65381	1092937	1093638	606655	607356	2004420	2005121	1945252	1945953	1956389	1957090	1915255	1915956	1744122	1744823
2227	BPSS1415		1880081	1880968	65299	64412	1093720	1094607	607438	608325	2005203	2006090	1946035	1946922	1957172	1958059	1916038	1916925	1744905	1745792
2228	BPSS1416		1880982	1882358	64398	63022	1094621	1095997	608339	609715	2006104	2007456	1946936	1948312	1958073	1959449	1916939	1918339	1745806	1747206
2229	BPSS1417		1882358	1882996	63022	62384	1095997	1096635	609715	610353	2007456	2008094	1948312	1948950	1959449	1960087	1918339	1918977	1747206	1747844
2230	BPSS1418		1883088	1884422	62292	60958	1096727	1098061	610445	611779	2008186	2009520	1949042	1950376	1960179	1961513	1919069	1920403	1747936	1749270
2231	BPSS1419		1884507	1885280	60873	60100	1098146	1098919	611864	612637	2009605	2010378	1950461	1951234	1961598	1962371	1920488	1921261	1749355	1750128
2232	BPSS1420		1885311	1886279	60069	59101	1098950	1099918	612668	613636	2010409	2011377	1951265	1952233	1962402	1963370	1921292	1922260	1750159	1751127
2233		BURPS1655_I1055																	1751208	1751927
2234		BURPS1710A_A1172					1100038	1100760												
2235		BURPSPAST_T0306													1963451	1964173				
2236	BPSS1421		1887053	1887841	58327	57539	1100772	1101560	614490	615278	2012179	2012967	1952955	1953743	1964185	1964973	1923101	1923889	1752039	1752818
2237	BPSS1422		1889669	1888725	55711	56655	1103404	1102460	617122	616178	2014818	2013874	1955600	1954656	1966817	1965873	1925701	1924796	1754705	1753761
2238	BPSS1423		1890953	1890006	54427	55374	1104688	1103741	618406	617459	2016095	2015148	1956891	1955944	1968101	1967154	1927018	1926071	1755982	1755035
2239	BPSS1424		1892052	1891057	53328	54323	1105787	1104792	619505	618510	2017194	2016199	1957990	1956995	1969200	1968205	1928117	1927122	1757081	1756086
2240	BPSS1425		1893077	1892178	52303	53202	1106811	1105912	620529	619630	2018219	2017320	1959015	1958116	1970225	1969326	1929141	1928242	1758106	1757207
2241	BPSS1426		1894239	1893073	51141	52307	1107973	1106807	621691	620525	2019270	2018428	1960177	1959011	1971387	1970221	1930303	1929137	1759268	1758102
2242		BURPS668_A2028											1960941	1961291						
2243		BURPSS13_X0102															1930871	1930350		
2244	BPSS1427		1895031	1895678	50349	49702	1108765	1109412	622483	623130	2020062	2020709	1961384	1962031	1972179	1972826	1931094	1931741	1760468	1761115
2245	BPSS1428		1896262	1896474	49118	48906	1110008	1110220	623726	623938	2021311	2021523	1962621	1962833	1973410	1973622	1932331	1932543	1761700	1761912
2246	BPSS1429		1896576	1897472	48804	47908	1110322	1111218	624040	624936	2021625	2022521	1962935	1963831	1973724	1974620	1932645	1933541	1762014	1762208
2247		BURPS1106A_A1940	1897590	1898273																

2248	BPSS1430		1897938	1898270	47442	47110	1111684	1112016	625402	625734	2022987	2023319	1964308	1964640	1975086	1975418	1934007	1934339	1762262	1762594
2249		BURPS1106B_0040			47790	47107														
2250	BPSS1431		1898243	1898746	47137	46634	1111989	1112492	625707	626210	2023292	2023795	1964613	1965116	1975391	1975894	1934312	1934815	1762567	1762914
2251	BPSS1432		1898784	1899275	46596	46105	1112530	1113021	626248	626739	2023833	2024324	1965154	1965645	1975932	1976423	1934853	1935344	1763106	1763597
2252	BPSS1433		1899341	1900408	46039	44972	1113087	1114154	626805	627872	2024390	2025457	1965711	1966778	1976489	1977556	1935410	1936477	1763663	1764730
2253		BURPS1106A_A1945	1902108	1904618																
2254		BURPS1106A_A1946	1904035	1909785																
2255		BURPS1710A_A1194					1118673	1115854												
2256		BURPS1710A_A1195					1122348	1118686												
2257		BURPS1710b_A0458							629572	632391										
2258		BURPS1710b_A0459							628976	637249										
2259		BURPS1710b_A0460							632404	636066										
2260		BURPS668_A2041											1970755	1974285						
2261		BURPSPAST_T0326													1983157	1979255				
2262	BPSS1434																			
2263	BPSS1437		1909938	1910258	35442	35122	1123684	1124004	637402	637722	2033634	2033954	1975900	1976220	1984493	1984813	1944633	1944953	1771851	1772171
2264		BURPS1106B_0032			40625	35595														
2265		BURPS1106B_0033			40657	43272														
2266	BPSS1438		1910791	1912584	34589	32796	1124529	1126322	638247	640040	2034473	2036266	1976758	1978551	1985338	1987131	1945507	1947300	1772704	1774497
2267		BURPS1106A_A1951	1913443	1918269																
2268		BURPS1655_D1805																	1778974	1775912
2269		BURPS1710A_A1201					1131027	1127737												
2270		BURPS1710b_A0463							640898	645748										
2271		BURPS1710b_A0464							641455	645360										
2272		BURPS406E_D1007									2041564	2037695								
2273		BURPS668_A2047											1979952	1983551						
2274		BURPSPAST_T0331													1991776	1988546				
2275		BURPSS13_X0118															1951181	1948716		
2276	BPSS1439																			
2277	BPSS1442		1918263	1919417	27118	25964	1132024	1133178	645742	646896	2041907	2043061	1984065	1985219	1992704	1993858	1951983	1953137	1779941	1781095
2278	BPSS1443		1919466	1920587	25915	24794	1133227	1134348	646945	648066	2043110	2044231	1985268	1986389	1993907	1995028	1953186	1954307	1781144	1782265
2279	BPSS1444		1922117	1920939	23264	24442	1135878	1134700	649596	648418	2045761	2044583	1987919	1986741	1996558	1995380	1955843	1954665	1783795	1782617
2280	BPSS1445		1924213	1922222	21168	23159	1137980	1135983	651698	649701	2047848	2045857	1990021	1988024	1998660	1996663	1957939	1955948	1785897	1783900
2281	BPSS1446		1925169	1924387	20212	20994	1138926	1138144	652644	651862	2048804	2048022	1990976	1990194	1999606	1998824	1958885	1958103	1786843	1786061
2282	BPSS1447		1926797	1925193	18584	20188	1140554	1138950	654272	652668	2050432	2048828	1992604	1991000	2001234	1999630	1960513	1958909	1788471	1786867
2283	BPSS1448		1928001	1926823	17380	18558	1141758	1140580	655476	654298	2051636	2050458	1993808	1992630	2002438	2001260	1961717	1960539	1789675	1788497
2284	BPSS1449		1928281	1928970	17100	16411	1142038	1142727	655756	656445	2051916	2052605	1994088	1994777	2002718	2003407	1961997	1962686	1789955	1790644
2285	BPSS1450		1929913	1930518	15468	14863	1143670	1144275	657388	657993	2053548	2054153	1995706	1996311	2004350	2004955	1963629	1964234	1791563	1793881
2286	BPSS1452		1932372	1933832	13009	11549	1146133	1147593	659851	661311	2056010	2057470	1998168	1999628	2006812	2008272	1966088	1967548	1794022	1795482
2287	BPSS1453		1934371	1937700	11010	7681	1148132	1151443	661850	665161	2058009	2061311	2000167	2003424	2008811	2012086	1968087	1971380	1796021	1798213
2288	BPSS1454		1937852	1939051	7529	6330	1151595	1152794	665313	666512	2061463	2062662	2003576	2004769	2012238	2013437	1971532	1972731	1799511	1800710
2289	BPSS1455		1939056	1939844	6325	5537	1152799	1153587	666517	667305	2062667	2063455	2004774	2005562	2013442	2014230	1972736	1973524	1800715	1801503
2290	BPSS1456		1940235	1940852	5146	4529	1153978	1154595	667696	668313	2063850	2064467	2005953	2006570	2014621	2015238	1973915	1974532	1801894	1802511
2291	BPSS1457		1940996	1942006	4385	3375	1154739	1155749	668457	669467	2064611	2065621	2006714	2007724	2015382	2016392	1974676	1975686	1802655	1803665
2292	BPSS1458		1943918	1942536	1449	2831	1157619	1156237	671344	669962	2067433	2066051	2009438	2008059	2018057	2016675	1977612	1976230	1805549	1804167
2293	BPSS1459		1944836	1944057	531	1310	1158537	1157758	672262	671483	2068351	2067572	2010359	2009580	2018975	2018196	1978530	1977751	1806467	1805688
2294		BURPS668_A2072											2010492	2010956						
2295	BPSS1460		1946802	1945444	3116451	3117767	1160512	1159154	674237	672879	2070433	2069075	2012323	2010965	2020929	2019571	1980484	1979126	1808430	1807051
2296	BPSS1461		1947464	1946808	3115789	3116445	1161174	1160518	674899	674243	2071095	2070439	2012985	2012329	2021591	2020935	1981146	1980490	1809092	1808436
2297	BPSS1462		1947663	1948049	3115590	3115204	1161373	1161759	675098	675484	2071294	2071680	2013184	2013570	2021790	2022176	1981345	1981731	1809291	1809677
2298	BPSS1463		1948814	1949278	3114439	3113975	1162556	1163020	676281	676745	2072344	2072808	2014539	2015003	2022965	2023429	1982555	1983019	1810574	1811038
2299	BPSS1464		1949303	1951639	3113950	3111614	1163045	1165381	676770	679106	2072833	2075169	2015028	2017373	2023454	2025790	1983044	1985380	1811063	1813399
2300	BPSS1465		1951692	1952804	3111561	3110449	1165434	1166546	679159	680271	2075222	2076334	2017426	2018538	2025843	2026955	1985433	1986545	1813452	1814564
2301	BPSS1466		1954333	1952825	3108920	3110428	1168075	1166567	681800	680292	2077863	2076355	2020067	2018559	2028484	2026976	1988074	1986566	1816093	1814585
2302	BPSS1467		1955757	1954504	3107496	3108749	1169499	1168246	683224	681971	2079287	2078034	2021491	2020238	2029745	2029518	1989498	1988245	1817508	1816255
2303	BPSS1468		1956801	1955785	3106452	3107468	1170543	1169527	684268	683252	2080331	2079315	2022535	2021519	2030789	2029773	1990542	1989526	1818522	1817536
2304	BPSS1469		1956976	1957881	3106277	3105372	1170718	1171623	684443	685348	2080506	2081411	2022710	2023615	2030964	2031869	1990717	1991622	1818797	1819702
2305	BPSS1470		1958687	1959985	3104566	3103268	1172417	1173715	686142	687440	2082202	2083500	2024409	2025707	2032678	2033976	1992428	1993726	1820481	1821779

2306	BPSS1471		1960041	1960736	3103212	3102517	1173771	1174466	687496	688191	2083556	2084251	2025763	2026458	2034032	2034727	1993782	1994477	1821835	1822530
2307		BURPSPAST_T0367													2035148	2034696				
2308	BPSS1472		1307378	1306176	965630	964428	452590	451388											454640	453624
2309	BPSS1473		1962994	1961204	3100259	3102049	1176724	1174934	690449	688659	2086512	2084722	2028720	2026930	2037010	2035220	1996735	1994945	1824802	1823012
2310		BURPS1106A_A1996	1964395	1965045																
2311		BURPS668_A2089											2028749	2029192						
2312	BPSS1474		1964473	1963718	3098780	3099535	1178216	1177461	691941	691186	2087991	2087236	2030225	2029470	2038504	2037962	1998214	1997459	1826281	1825526
2313	BPSS1475		1966433	1965081	3096820	3098172	1180190	1178838	693915	692563	2089951	2088599	2032199	2030847	2040480	2039761	2000174	1998822	1828269	1826917
2314		BURPSPAST_T0372													2040708	2038966				
2315	BPSS1476		1968206	1966740	3095047	3096513	1181963	1180497	695688	694222	2091721	2090255	2033969	2032503	2042253	2040787	2001947	2000481	1830039	1828573
2316	BPSS1477		1968379	1969104	3094874	3094149	1182135	1182860	695860	696585	2091895	2092620	2034140	2034865	2042425	2043150	2002120	2002845	1830211	1830936
2317	BPSS1478		1969129	1970334	3094124	3092919	1182885	1184090	696610	697815	2092645	2093850	2034890	2036095	2043175	2044380	2002870	2004075	1830961	1832166
2318	BPSS1479		1970386	1971414	3092867	3091839	1184142	1185170	697867	698895	2093902	2094930	2036147	2037175	2044432	2045460	2004127	2005155	1832218	1833246
2319	BPSS1480		1972367	1972549	3090886	3090704	1186109	1186291	699834	700016	2095866	2096048	2038123	2038305	2046378	2046560	2006050	2006232	1834163	1834345
2320	BPSS1481		1972665	1973540	3090588	3089713	1186407	1187282	700132	701007	2096164	2097039	2038421	2039296	2046676	2047551	2006348	2007223	1834461	1835336
2321		BURPSPAST_T0380													2048319	2047756				
2322	BPSS1482		1974135	1974986	3089118	3088267	1187878	1188729	701603	702454	2097572	2098423	2039766	2040617	2048462	2049313	2007719	2008570	1835724	1836575
2323	BPSS1483		1976269	1975247	3086984	3088006	1190012	1188990	703737	702715	2099706	2098684	2041876	2040878	2050672	2049887	2009853	2008831	1837849	1836836
2324	BPSS1484		1976381	1977595	3086872	3085658	1190124	1191338	703849	705063	2099818	2101032	2041988	2043202	2050784	2051998	2009965	2011179	1837961	1839175
2325	BPSS1485		1977595	1978440	3085658	3084813	1191338	1192183	705063	705908	2101032	2101877	2043202	2044047	2051998	2052843	2011179	2012024	1839175	1840020
2326	BPSS1486		1978489	1978926	3084764	3084327	1192232	1192669	705957	706394	2101926	2102363	2044096	2044533	2052892	2053329	2012073	2012510	1840069	1840506
2327	BPSS1487		1979336	1980883	3083917	3082370	1193079	1194638	706804	708363	2102846	2104393	2044943	2046502	2053739	2055298	2013070	2014617	1840916	1842475
2328	BPSS1488		1980911	1981744	3082342	3081509	1194666	1195499	708391	709224	2104421	2105254	2046540	2047349	2055326	2056159	2014645	2015478	1842503	1843336
2329	BPSS1489		1981744	1982355	3081509	3080898	1195499	1196110	709224	709835	2105254	2105865	2047349	2047960	2056159	2056770	2015478	2016089	1843336	1843947
2330		BURPS406E_D0946									2106238	2106074								
2331		BURPS668_A2110											2048169	2048333						
2332		BURPSPAST_T0388													2057143	2056979				
2333		BURPSS13_X0182															2016462	2016298		
2334	BPSS1490		1983396	1984418	3079857	3078835	1197175	1198197	710900	711922	2106930	2107952	2049023	2050057	2057835	2058857	2017153	2018175	1844988	1846010
2335	BPSS1491		1984690	1985946	3078563	3077307	1198469	1199725	712194	713450	2108224	2109480	2050329	2051543	2059148	2060404	2018447	2019703	1846282	1847538
2336		BURPS668_A2118											2051578	2052714						
2337	BPSS1492		1985972	1987519	3077281	3075734	1199751	1201298	713476	715023	2109506	2111053			2060430	2061977	2019729	2021276	1847564	1849126
2338	BPSS1493		1989556	1988225	3073697	3075028	1203287	1202004	717012	715729	2113039	2111759	2055317	2053419	2063949	2062678	2023292	2021982		
2339	BPSS1494		1990821	1990087	3072432	3073166	1204618	1203884	718343	717609	2114373	2113639	2056051	2055317	2065283	2064549	2024560	2023826	1852395	1851850
2340	BPSS1495		1992662	1990821	3070591	3072432	1206444	1204618	720169	718343	2116214	2114373	2057880	2056051	2067124	2065283	2026401	2024560	1854236	1852395
2341	BPSS1496		1992922	1993413	3070331	3069840	1206704	1207195	720429	720920	2116474	2116965	2058140	2058631	2067384	2067875	2026661	2027152	1854496	1854987
2342	BPSS1497		1993440	1994936	3069813	3068317	1207222	1208718	720947	722443	2116992	2118488	2058658	2060154	2067902	2069398	2027179	2028675	1855014	1856510
2343	BPSS1498		1995159	1995665	3068094	3067588	1208941	1209447	722666	723172	2118711	2119217	2060377	2060883	2069621	2070127	2028898	2029404	1856733	1857239
2344	BPSS1499		1995661	1996119	3067592	3067134	1209443	1209901	723168	723626	2119213	2119671	2060879	2061337	2070123	2070581	2029400	2029858	1857235	1857693
2345	BPSS1500		1996159	1997898	3067094	3065355	1209941	1211680	723666	725405	2119711	2121450	2061377	2063116	2070621	2072360	2029898	2031637	1857733	1859472
2346	BPSS1501		1997889	1998908	3065364	3064345	1211671	1212690	725396	726415	2121441	2122460	2063107	2064126	2072351	2073370	2031628	2032647	1859463	1860482
2347	BPSS1502		1998898	2001951	3064355	3061302	1212680	1215757	726405	729482	2122450	2125521	2064116	2067130	2073360	2076377	2032637	2035681	1860472	1863513
2348	BPSS1503		2001981	2005001	3061272	3058252	1215835	1218855	729560	732580	2125551	2128571	2067163	2070183	2076407	2079427	2035711	2038731	1863573	1866593
2349	BPSS1504		2005030	2007660	3058223	3055593	1218884	1221523	732609	735248	2128600	2131239	2070212	2072851	2079456	2082086	2038760	2041387	1866622	1869261
2350	BPSS1505		2007681	2008742	3055572	3054511	1221544	1222605	735269	736330	2131260	2132321	2072872	2073933	2082107	2083168	2041408	2042469	1869282	1870343
2351	BPSS1506		2008742	2009494	3054511	3053759	1222605	1223354	736330	737079	2132321	2133070	2073933	2074682	2083168	2083917	2042469	2043221	1870343	1871095
2352	BPSS1507		2009526	2009915	3053727	3053338	1223386	1223775	737111	737500	2133102	2133491	2074714	2075103	2083949	2084338	2043253	2043642	1871127	1871516
2353	BPSS1508		2010048	2010722	3053205	3052531	1223908	1224582	737633	738307	2133624	2134298	2075236	2075910	2084471	2085145	2043775	2044449	1871649	1872323
2354	BPSS1509		2010722	2012152	3052531	3051101	1224582	1226012	738307	739737	2134298	2135728	2075910	2077340	2085145	2086575	2044449	2045879	1872323	1873753
2355	BPSS1510		2012170	2012814	3051083	3050439	1226030	1226674	739755	740399	2135746	2136390	2077358	2078002	2086593	2087237	2045897	2046541	1873771	1874415
2356	BPSS1511		2012829	2016830	3050424	3048994	1226689	1230681	740437	744405	2136405	2137019	2078017	2082000	2089100	2091334	2046556	2050521	1876242	1878476
2357		BURPS1106A_A2041	2016849	2016998																
2358		BURPS668_A2139											2082000	2082155						
2359	BPSS1512		2017927	2018778	3045168	3044317	1231786	1232637	745510	746361	2141669	2142520	2083060	2083908	2092479	2093330	2051697	2052548	1879602	1880450
2360		BURPS1106B_3141			3046246	3046097														
2361	BPSS1513		2018945	2019235	3044150	3043860	1232804	1233094	746528	746818	2142687	2142977	2084075	2084365	2093571	2093792	2052789	2053010	1880613	1880903
2362	BPSS1514		2019243	2019872	3043852	3043223	1233102	1233731	746826	747455	2142985	2143614	2084373	2085002	2093800	2094429	2053018	2053647	1880911	1881540
2363		BURPS668_A2147											2085578	2085754						

2364	BURPS668_A2149											2086855	2087313						
2365 BPSS1515		2019989	2020291	3043106	3042804	1233848	1234150	747572	747874	2143731	2144033			2094546	2094848	2053764	2054066	1881657	1881959
2366 BPSS1516		2022345	2020939	3040750	3042156	1236204	1234798	749928	748522	2146087	2144681			2096902	2095496	2056120	2054714	1884013	1882607
2367 BPSS1517		2022940	2022500	3040155	3040595	1236799	1236359	750523	750083	2146682	2146242			2097497	2097057	2056715	2056275	1884608	1884168
2368 BPSS1518		2023312	2023028	3039783	3040067	1237171	1236887	750895	750611	2147054	2146770			2097869	2097585	2057087	2056803	1884980	1884696
2369	BURPSS13_X0222															2057383	2057535		
2370 BPSS1519		2023614	2024210	3039481	3038885	1237517	1238068	751241	751792	2147356	2147952			2098215	2098766	2057430	2057984	1885326	1885877
2371	BURPS406E_D0903									2149099	2149545								
2372 BPSS1520		2025358	2024300	3037737	3038795	1239216	1238158	752940	751882	2149100	2148042	2086856	2085798	2099914	2098856	2059132	2058074	1887025	1885967
2373 BPSS1521		2026328	2025888	3036791	3037231	1240053	1239613	753777	753337	2149960	2149520	2087728	2087288	2100835	2100395	2060077	2059637	1887998	1887558
2374 BPSS1522		2027237	2026569	3035882	3036550	1240980	1240312	754704	754036	2150869	2150201	2088637	2087969	2101744	2101076	2060986	2060318	1888907	1888239
2375 BPSS1523		2027928	2027476	3035191	3035643	1241679	1241227	755403	754951	2151560	2151108	2089336	2088884	2102435	2101983	2061677	2061225	1889598	1889146
2376 BPSS1524		2029618	2028083	3033501	3035036	1243358	1241823	757082	755547	2153250	2151715	2091037	2089502	2104114	2102579	2063356	2061821	1891277	1889742
2377 BPSS1525		2030244	2031026	3032875	3032093	1243991	1244773	757715	758497	2153883	2154665	2091663	2092445	2104747	2105529	2063989	2064771	1891903	1892685
2378 BPSS1526		2032107	2031547	3031012	3031572	1245764	1245204	759488	758928	2155673	2155113	2093514	2092954	2106542	2105982	2065807	2065247	1893649	1893089
2379 BPSS1527		2032385	2032107	3030734	3031012	1246042	1245764	759766	759488	2155951	2155673	2093792	2093514	2106820	2106542	2066085	2065807	1893927	1893649
2380 BPSS1528		2035036	2032385	3028083	3030734	1248738	1246159	762462	759883	2158542	2155951	2096422	2093792	2109504	2106820	2068754	2066085	1896548	1893927
2381 BPSS1529		2036068	2035139	3027051	3027980	1249770	1248841	763494	762565	2159574	2158645	2097454	2096525	2110536	2109607	2069786	2068857	1897580	1896651
2382 BPSS1530		2036432	2036133	3026687	3026986	1250134	1249835	763858	763559	2159938	2159639	2097818	2097519	2110900	2110601	2070150	2069851	1897944	1897645
2383 BPSS1531		2037813	2036557	3025306	3026562	1251515	1250259	765239	763983	2161319	2160063	2099199	2097943	2112281	2111025	2071531	2070275	1899325	1898069
2384 BPSS1532		2039717	2037858	3023402	3025261	1253419	1251560	767143	765284	2163223	2161364	2101103	2099244	2114185	2112326	2073435	2071576	1901229	1899370
2385 BPSS1533		2040251	2039739	3022868	3023380	1253953	1253441	767677	767165	2163757	2163245	2101637	2101125	2114719	2114207	2073969	2073457	1901763	1901251
2386 BPSS1534		2041612	2040380	3021507	3022739	1255314	1254082	769038	767806	2165118	2163886	2102983	2101766	2116080	2114848	2075330	2074098	1903124	1901892
2387 BPSS1535		2042371	2041619	3020748	3021500	1256073	1255321	769797	769045	2165877	2165125	2103742	2102990	2116839	2116087	2076089	2075337	1903883	1903131
2388 BPSS1536		2042659	2042408	3020460	3020711	1256361	1256110	770085	769834	2166165	2165914	2104030	2103779	2117127	2116876	2076377	2076126	1904171	1903920
2389 BPSS1537		2043375	2042698	3019744	3020421	1257079	1256402	770803	770126	2166881	2166204	2104746	2104069	2117844	2117167	2077093	2076416	1904888	1904211
2390 BPSS1538		2044336	2043368	3018783	3019751	1258028	1257072	771752	770796	2167830	2166874	2105707	2104739	2118805	2117837	2078066	2077086	1905885	1904881
2391 BPSS1539		2045598	2044336	3017521	3018783	1259314	1258028	773038	771752			2107008	2105707	2120006	2119260	2079328	2078066		
2392 BPSS1540		2046040	2045579	3017079	3017540	1259756	1259295	773480	773019	2169606	2169145	2107450	2106989	2120448	2119987	2079770	2079309	1907624	1907163
2393 BPSS1541		2047344	2046040	3015775	3017079	1261060	1259756	774784	773480	2170898	2169606	2108754	2107450	2121752	2120448	2081074	2079770	1908928	1907624
2394 BPSS1542		2047748	2047344	3015371	3015775	1261464	1261060	775188	774784	2171315	2170911	2109158	2108754	2122156	2121752	2081478	2081074	1909332	1908928
2395 BPSS1543		2049832	2047763	3013287	3015356	1263548	1261479	777272	775203	2173399	2171330	2111242	2109173	2124240	2122171	2083562	2081493	1911416	1909347
2396 BPSS1544		2050989	2049871	3012130	3013248	1264705	1263587	778429	777311	2174556	2173438	2112399	2111281	2125397	2124279	2084719	2083601	1912573	1911455
2397 BPSS1545		2052863	2050989	3010256	3012130	1266564	1264705	779505	778429	2176430	2174556	2114249	2112399	2127271	2125397	2086594	2086202	1914447	1912573
2398 BPSS1546		2053516	2052878	3009603	3010241	1267219	1266581	780944	780306	2177085	2176447	2114904	2114266	2127926	2127288	2087247	2086609	1915102	1914464
2399 BPSS1547		2054027	2055310	3009092	3007809	1267730	1269013	781455	782738	2177596	2178879	2115415	2116698	2128437	2129720	2087758	2089041	1915613	1916896
2400 BPSS1548		2055310	2055576	3007809	3007543	1269013	1269279	782738	783004	2178879	2179145	2116698	2116964	2129720	2129986	2089041	2089307	1916896	1917162
2401 BPSS1549		2055639	2055938	3007480	3007181	1269342	1269641	783067	783366	2179208	2179507	2117027	2117326	2130049	2130348	2089370	2089669	1917225	1917524
2402 BPSS1550		2055946	2056890	3007173	3006280	1269649	1270593	783374	784318	2179515	2180459	2117334	2118278	2130356	2131300	2089677	2090621	1917532	1918476
2403 BPSS1551		2056890	2057474	3006177	3005758	1270593	1271177	784318	784902	2180459	2181043	2118278	2118862	2131300	2131884	2090621	2091205	1918476	1919060
2404 BPSS1552		2057446	2058225	3005786	3005007	1271149	1271940	784874	785665	2181015	2181806	2118834	2119616	2131856	2132647	2091177	2091956	1919032	1919814
2405 BPSS1553		2059229	2060197	3004003	3003035	1272944	1273912	786669	787637	2182803	2183780	2120620	2121588	2133651	2134619	2092960	2093928	1920811	1921779
2406 BPSS1554		2060212	2060649	3003020	3002583	1273927	1274364	787652	788089	2183795	2184232	2121603	2122040	2134634	2135071	2093943	2094380	1921794	1922231
2407 BPSS1555		2061696	2063390	3001536	2999842	1275411	1277105	789136	790830	2185279	2186973	2123054	2124748	2136118	2137812	2095427	2097121	1923277	1924971
2408 BPSS1556		2065003	2064518	2998229	2998714	1278718	1278233	792443	791958	2188586	2188101	2126349	2125864	2139425	2138940	2098734	2098249	1926544	1926059
2409 BPSS1557		2065330	2065785	2997902	2997447	1279045	1279500	792770	793225	2188912	2189367	2126675	2127130	2139751	2140206	2099060	2099515	1926870	1927325
2410 BPSS1558		2066355	2067671	2996877	2995561	1280070	1281386	793795	795111	2189937	2191253	2127697	2129013	2140776	2142092	2100085	2101401	1927894	1929210
2411 BPSS1559		2068923	2068039	2994309	2995193	1282772	1281888	796379	795495	2192529	2191645	2130273	2129389	2143416	2142532	2102669	2101785	1930251	1929367
2412 BPSS1560		2069024	2070244	2994208	2992988	1282873	1284093	796480	797700	2192630	2193850	2130374	2131594	2143517	2144737	2102770	2103990	1930352	1931572
2413 BPSS1561		2072035	2070374	2991197	2992858	1285884	1284223	799491	797830	2195641	2193980	2133385	2131724	2146528	2144867	2105781	2104120	1933363	1931702
2414	BURPS668_A2201											2133551	2134009						
2415 BPSS1562		2074372	2072786	2988878	2990464	1288167	1286581	801774	800188	2197924	2196338	2135621	2134035	2148811	2147225	2108043	2106457	1935628	1934042
2416 BPSS1563		2075429	2074806	2987821	2988444	1289224	1288601	802831	802208	2198981	2198358	2136673	2136050	2149868	2149245	2109111	2108488	1936680	1936057
2417 BPSS1564		2075563	2076024	2987687	2987226	1289358	1289819	802965	803426	2199115	2199576	2136807	2137268	2150002	2150463	2109245	2109706	1936814	1937275
2418 BPSS1565		2076362	2076763	2986888	2986487	1290139	1290540	803746	804147	2199895	2200296	2137649	2138050	2150790	2151191	2110092	2110493	1937585	1937986
2419 BPSS1566		2077142	2078725	2986108	2984525	1290921	1292504	804528	806111	2200652	2202235	2138431	2140014	2151572	2153155	2110887	2112470	1938253	1939836
2420 BPSS1567		2078878	2079657	2984372	2983593	1292657	1293436	806264	807043	2202388	2203167	2140167	2140946	2153308	2154087	2112623	2113402	1939989	1940768
2421 BPSS1568		2080243	2081847	2983007	2981403	1294022	1295626	807629	809233	2203766	2205370	2141545	2143149	2154673	2156277	2113999	2115603	1941354	1942958

2422 BPSS1569		2082618	2083307	2980632	2979943	1296397	1297086	810004	810693	2206141	2206830	2143921	2144610	2157048	2157737	2116374	2117063	1943730	1944419
2423	BURPS1655_D1634																	1944684	1944460
2424	BURPSPAST_T0488													2157837	2158091				
2425 BPSS1570		2083633	2084238	2979617	2979012	1297413	1298018	811020	811625	2207157	2207762	2144929	2145534	2158064	2158669	2117380	2117985	1944753	1945358
2426 BPSS1571		2084544	2085905	2978706	2977345	1298324	1299685	811931	813292	2208068	2209429	2145840	2147201	2158975	2160336	2118291	2119652	1945664	1947025
2427 BPSS1572		2087111	2086257	2976139	2976993	1300891	1300037	814498	813644	2210635	2209781	2148407	2147553	2161542	2160688	2120858	2120004	1948231	1947377
2428 BPSS1573		2087890	2087111	2975360	2976139	1301670	1300891	815277	814498	2211414	2210635	2149186	2148407	2162321	2161542	2121637	2120858	1949010	1948231
2429 BPSS1574		2088921	2087914	2974329	2975336	1302701	1301694	816308	815301	2212445	2211438	2150217	2149210	2163352	2162345	2122668	2121661	1950041	1949034
2430 BPSS1575		2089224	2090054	2974026	2973196	1302994	1303824	816601	817431	2212718	2213548	2150490	2151320	2163655	2164485	2122941	2123771	1950304	1951134
2431 BPSS1576		2092039	2090486	2971211	2972764	1305828	1304275	819435	817882	2215552	2213999	2153324	2151771	2166470	2164917	2125775	2124222	1953126	1951573
2432 BPSS1577		2094619	2092076	2968631	2971174	1308402	1305865	822009	819472	2218243	2217776	2155898	2153361	2169044	2166507	2128349	2125812	1955700	1953163
2433 BPSS1578		2095479	2094619	2967771	2968631	1309262	1308402	822869	822009	2219103	2218243	2156758	2155898	2169904	2169044	2129221	2128349	1956548	1955700
2434 BPSS1579		2097928	2095688	2965322	2967562	1311699	1309471	825306	823078	2221558	2219312	2159213	2156967	2172314	2170113	2131658	2129430	1958997	1956757
2435 BPSS1580		2102378	2097957	2961703	2965293	1316170	1311728	829777	825335	2225635	2223794	2163684	2159242	2176807	2172365	2136189	2131687	1963450	1959026
2436 BPSS1581		2103952	2102660	2959297	2960589	1317648	1316341	831240	829948	2227209	2225917	2165147	2163855	2178253	2178182	2137493	2136189	1964913	1963621
2437	BURPS1106B_3038			2960571	2961872														
2438 BPSS1582		2106377	2104227	2956872	2959022	1320031	1317881	833623	831473	2229614	2227464	2167530	2165380	2180636	2178486	2139885	2137735	1967311	1965161
2439 BPSS1582a		2106941	2107528	2956308	2955721	1320595	1321182	834187	834774	2230204	2230764	2168120	2168680	2181200	2181787	2140449	2141036	1967901	1968461
2440 BPSS1583		2107687	2108094	2955562	2955155	1321341	1321748	834933	835340	2230923	2231324	2168839	2169246	2181980	2182384	2141195	2141608	1968620	1969027
2441 BPSS1584		2108094	2109443	2955155	2953806	1321748	1323097	835340	836689	2231324	2232673	2169246	2170595	2182384	2183733	2141608	2142957	1969027	1970376
2442 BPSS1585		2110055	2109606	2953194	2953643	1323687	1323238	837279	836830	2233296	2232847	2171196	2170747	2184323	2183874	2143569	2143120	1970999	1970550
2443 BPSS1586		2111393	2110482	2951856	2952767	1325004	1324093	838596	837685	2234641	2233730	2172515	2171604	2185640	2184729	2144900	2143989	1972348	1971437
2444 BPSS1587		2111560	2112510	2951689	2950739	1325171	1326121	838763	839713	2234808	2235758	2172682	2173632	2185807	2186757	2145067	2146017	1972515	1973465
2445 BPSS1588		2113119	2114516	2950130	2948733	1326735	1328132	840327	841724	2236376	2237773	2174249	2175646	2187371	2188768	2146629	2148026	1974080	1975477
2446	BURPS1106A_A2160	2114535	2114834																
2447	BURPS1710b_A0645							844479	846026										
2448 BPSS1589		2116001	2116798	2947248	2946451	1330963	1331760	844555	845352	2239176	2239973	2177026	2177823	2190187	2190984	2149445	2150242	1976816	1977613
2449	BURPS1106B_3022			2948714	2948415														
2450 BPSS1590		2117056	2117283	2946193	2945966	1332016	1332243	845608	845835	2240229	2240456	2178081	2178308	2191240	2191467	2150500	2150727	1977871	1978098
2451 BPSS1591		2117437	2118318	2945812	2944931	1332397	1333278	845989	846870	2240610	2241491	2178463	2179344	2191621	2192502	2150881	2151762	1978252	1979133
2452 BPSS1592		2120188	2118392	2943061	2944857	1335154	1333352	848746	846944	2243367	2241565	2181220	2179418	2194372	2192576	2153638	2151836	1981009	1979207
2453 BPSS1593		2121978	2120308	2941271	2942941	1336944	1335274	850536	848866	2245157	2243487	2183010	2181340	2196162	2194492	2155428	2153758	1982799	1981129
2454 BPSS1594		2122466	2121990	2940783	2941259	1337432	1336956	851024	850548	2245645	2245169	2183498	2183022	2196650	2196174	2155916	2155440	1983287	1982811
2455 BPSS1595		2123060	2122509	2940189	2940740	1338026	1337475	851618	851067	2246239	2245688	2184092	2183541	2197244	2196693	2156510	2155959	1983881	1983330
2456 BPSS1596		2124169	2123096	2939080	2940153	1339135	1338062	852727	851654	2247348	2246275	2185201	2184128	2198353	2197280	2157619	2156546	1984990	1983917
2457 BPSS1597		2125769	2124162	2937480	2939087	1340735	1339128	854327	852720	2248948	2247341	2186801	2185194	2199953	2198346	2159219	2157612	1986590	1984983
2458 BPSS1598		2126269	2125769	2936980	2937480	1341235	1340735	854827	854327	2249448	2248948	2187301	2186801	2200453	2199953	2159719	2159219	1987090	1986590
2459 BPSS1599		2127578	2126283	2935671	2936966	1342544	1341249	856136	854841	2250757	2249462	2188610	2187315	2201762	2200467	2161028	2159733	1988399	1987104
2460 BPSS1600		2129362	2127593	2933887	2935656	1344325	1342559	857917	856151	2252541	2250772	2190394	2188625	2203546	2201777	2162812	2161043	1989974	1989717
2461 BPSS1601		2129931	2129365	2933318	2933884	1344894	1344328	858486	857920	2253110	2252544	2190993	2190397	2204115	2203549	2163381	2162815	1990558	1989977
2462 BPSS1602		2130248	2131351	2933001	2931898	1345211	1346176	858803	859906	2253427	2254530	2191310	2192413	2204432	2205535	2163698	2164801	1990875	1991978
2463 BPSS1603		2133387	2131600	2929862	2931649	1348330	1346543	861920	860133	2256156	2254969	2194422	2192629	2206524	2205841	2166916	2166365	1993437	1992388
2464 BPSS1604		2134133	2133387	2929116	2929862	1349076	1348330	862666	861920	2256902	2256156	2195168	2194422	2207270	2206524	2167662	2166916	1994183	1993437
2465 BPSS1605		2135269	2134187	2927980	2929062	1350212	1349130	863802	862720	2258038	2256956	2196304	2195222	2208407	2207325	2168798	2167716	1995320	1994238
2466 BPSS1606		2136041	2135400	2927208	2927849	1350984	1350343	864574	863933	2258810	2258169	2197076	2196435	2209179	2208538	2169570	2168929	1996089	1995451
2467 BPSS1607		2136250	2136735	2926999	2926514	1351199	1351684	864789	865274	2259025	2259510	2197285	2197770	2209388	2209873	2169785	2170270	1996298	1996783
2468	BURPS1655_D1586																	1997329	1997078
2469 BPSS1608		2137924	2137265	2925325	2925984	1352950	1352291	866540	865881	2260720	2260061	2198952	2198293	2211097	2210438	2171515	2170856	1997988	1997329
2470 BPSS1609		2138133	2138777	2925116	2924472	1353159	1353803	866749	867393	2260929	2261573	2199161	2199805	2211306	2211950	2171724	2172368	1998197	1998841
2471	BURPS1710A_A1447					1354358	1354077												
2472 BPSS1610		2140781	2139339	2922468	2923910	1355800	1354358	869390	867948	2263577	2262135	2201795	2200356	2213947	2212505	2174372	2172930	2000866	1999424
2473 BPSS1611		2141448	2140996	2921801	2922253	1356467	1356015	870057	869605	2264244	2263792	2202462	2202010	2214614	2214162	2175039	2174587	2001533	2001081
2474 BPSS1612		2141729	2141544	2921520	2921705	1356748	1356563	870338	870153	2264525	2264340	2202743	2202558	2214895	2214710	2175320	2175135	2001814	2001629
2475 BPSS1613		2142144	2141896	2921105	2921353	1357163	1356915	870753	870505	2264990	2264742	2203158	2202910	2215149	2215090	2175735	2175487		
2476 BPSS1614		2143155	2142175	2920094	2921074	1358174	1357194	871764	870784	2266001	2265021	2204169	2203189	2216160	2215180	2176746	2175766	2002483	2002076
2477 BPSS1615		2144220	2143354	2919029	2919895	1359239	1358373	872829	871963	2267066	2266200	2205234	2204368	2217225	2216359	2177811	2176945	2003548	2002682
2478 BPSS1616		2144480	2144220	2918769	2919029	1359499	1359239	873089	872829	2267326	2267066	2205494	2205234	2217485	2217225	2178071	2177811	2003808	2003548
2479 BPSS1617		2145171	2144524	2918078	2918725	1360190	1359543	873780	873133	2268017	2267370	2206185	2205538	2218176	2217529	2178762	2178115	2004499	2003852

2480	BPSS1618		2146468	2145161	2916781	2918088	1361463	1360180	875053	873770	2269302	2268007	2207446	2206175	2219461	2218166	2180035	2178752	2005896	2005627
2481	BPSS1619		2147109	2146501	2916140	2916748	1362104	1361496	875694	875086	2269943	2269335	2208087	2207479	2220102	2219494	2180676	2180068	2006537	2005929
2482	BPSS1620		2149178	2147109	2914071	2916140	1364173	1362104	877763	875694	2272012	2269943	2210156	2208087	2222171	2220102	2182745	2180676	2008606	2006537
2483	BPSS1621		2150257	2149178	2912992	2914071	1365252	1364173	878842	877763	2273091	2272012	2211235	2210156	2223250	2222171	2183824	2182745	2009685	2008606
2484	BPSS1622		2150493	2151071	2912756	2912178	1365488	1366066	879078	879656	2273327	2273905	2211471	2212049	2223486	2224064	2184060	2184638	2009921	2010499
2485	BPSS1623		2151088	2151501	2912161	2911748	1366083	1366496	879673	880086	2273922	2274335	2212066	2212479	2224081	2224494	2184655	2185068	2010516	2010929
2486	BPSS1624		2151501	2152352	2911748	2910897	1366496	1367347	880086	880937	2274335	2275132	2212479	2213330	2224494	2225303	2185068	2185919	2010929	2011630
2487	BPSS1625		2152352	2153026	2910897	2910223	1367347	1368021	880937	881611	2275236	2275877	2213330	2214004			2185919	2186593	2011731	2012396
2488	BPSS1626		2153014	2153727	2910235	2909522	1368009	1368722	881599	882312	2275865	2276578	2213992	2214705	2225974	2226687	2186581	2187294	2012384	2013097
2489	BPSS1627		2153727	2155052	2909522	2908197	1368722	1370047	882312	883637	2276578	2277903	2214705	2216030	2226687	2228012	2187294	2188619	2013097	2014422
2490	BPSS1628		2155052	2155576	2908197	2907673	1370047	1370571	883637	884161	2277903	2278427	2216030	2216554	2228012	2228536	2188619	2189143	2014422	2014946
2491	BPSS1629		2155576	2156532	2907673	2906717	1370571	1371554	884161	885153	2278427	2279383	2216554	2217510	2229301	2229414	2189143	2190216	2014946	2015965
2492	BPSS1630		2156912	2158675	2906337	2904574	1371925	1373688	885524	887287	2279763	2281526	2217889	2219652	2229786	2231549	2190587	2192350	2016426	2018189
2493		BURPS1106A_A2211	2158675	2158926																
2494		BURPS1655_D1562																	2018189	2018440
2495		BURPS1710A_A1472					1373703	1373939												
2496		BURPS406E_D0768									2281541	2281777								
2497	BPSS1631		2159888	2158926	2903361	2904323	1374901	1373939	888500	887538	2282739	2281777	2220865	2219903	2232748	2231786	2193547	2192585	2019402	2018440
2498		BURPS1106B_2971			2904574	2904323														
2499	BPSS1632		2178219	2174245	2885027	2889001	1393205	1386741	906804	900340	2297785	2293811	2233097	2220924	2245887	2249615	2208493	2205359	2034384	2030329
2500	BPSS1633		2191592	2187765	2871655	2875482	1405888	1393175	920093	906774	2311086	2297755			2263738	2260211	2219948	2219028	2046481	2042951
2501	BPSS1634		2201530	2191592	2861717	2871655	1416475	1406495	930073	920093	2321064	2313556			2275764	2270167	2229889	2219948		
2502	BPSS1635		2204603	2201727	2858644	2861520	1419548	1416672	933146	930270	2324137	2321261	2242015	2239139	2278837	2275961	2232963	2230087	2052933	2050057
2503	BPSS1636		2207977	2206250	2855270	2856997	1422922	1421195	936520	934793	2327499	2325772	2245389	2243662	2282211	2280484	2236337	2234610	2056308	2054581
2504	BPSS1637		2209315	2207981	2853932	2855266	1424260	1422926	937858	936524	2328837	2327503	2246727	2245393	2283549	2282215	2237675	2236341	2057646	2056312
2505	BPSS1638		2209861	2209337	2853386	2853910	1424806	1424282	938404	937880	2329383	2328859	2247273	2246749	2284095	2283571	2238221	2237697	2058192	2057668
2506	BPSS1639		2210779	2209901	2852468	2853346	1425724	1424846	939322	938444	2330301	2329423	2248191	2247313	2285013	2284135	2239139	2238261	2059110	2058232
2507	BPSS1640		2211795	2210887	2851452	2852360	1426740	1425832	940338	939430	2331317	2330409	2249207	2248299	2286029	2285121	2240155	2239247	2060126	2059218
2508	BPSS1641		2211947	2212258	2851300	2850989	1426892	1427203	940490	940801	2331469	2331780	2249359	2249670	2286181	2286492	2240307	2240618	2060278	2060589
2509	BPSS1643		2213268	2212360	2849979	2850887	1428213	1427305	941811	940903	2332790	2331882	2250680	2249772	2287502	2286594	2241628	2240720	2061599	2060691
2510	BPSS1644		2213386	2214780	2849861	2848467	1428331	1429725	941929	943323	2332908	2334302	2250798	2252192	2287620	2289014	2241746	2243140	2061717	2063111
2511	BPSS1645		2214780	2215175	2848467	2848072	1429725	1430120	943323	943718	2334302	2334697	2252192	2252587	2289014	2289409	2243140	2243535	2063111	2063506
2512	BPSS1646		2216510	2215344	2846737	2847903	1431455	1430289	945053	943887	2336032	2334866	2253922	2252756	2290744	2289578	2244870	2243704	2064841	2063675
2513	BPSS1647		2217681	2216506	2845566	2846741	1432626	1431451	946224	945049	2337203	2336028	2255093	2253918	2291915	2290740	2246041	2244866	2066012	2064837
2514	BPSS1648		2219181	2217757	2844066	2845490	1434126	1432702	947724	946300	2338703	2337279	2256593	2255169	2293415	2291991	2247541	2246117	2067512	2066088
2515	BPSS1649		2219567	2221984	2843680	2841263	1434513	1436930	948111	950528	2339089	2341506	2256979	2259396	2293825	2296242	2247927	2250344	2067911	2070328
2516	BPSS1650		2221984	2222472	2841263	2840775	1436930	1437418	950528	951016	2341506	2341994	2259396	2259884	2296242	2296730	2250344	2250832	2070328	2070816
2517	BPSS1651		2222462	2223073	2840785	2840174	1437408	1438019	951006	951617	2341984	2342595	2259874	2260485	2296720	2297331	2250822	2251433	2070806	2071417
2518	BPSS1652		2224173	2223178	2839074	2840069	1439119	1438124	952717	951722	2343695	2342700	2261587	2260592	2298431	2297436	2252535	2251540	2072519	2071524
2519	BPSS1653		2224967	2224797	2837885	2838280	1440308	1439913	953511	953341	2344884	2344489	2262758	2262192	2299620	2299225	2253724	2253329	2073709	2073143
2520	BPSS1654		2227784	2225433	2835463	2837814	1442730	1440379	956328	953977	2347306	2344955	2265180	2262829	2302042	2299691	2256146	2253795	2076131	2073780
2521	BPSS1655		2228009	2228143	2835238	2835104	1443008	1443145	956606	956743	2347584	2347721	2265471	2265608	2302321	2302458	2256425	2256562	2076409	2076546
2522	BPSS1656		2228218	2229471	2835029	2833776	1443220	1444473	956818	958071	2347796	2349049	2265686	2266939	2302533	2303786	2256637	2257890	2076621	2077874
2523	BPSS1657		2230201	2232393	2833046	2830854	1445227	1447419	958825	961017	2349815	2352007	2267651	2269843	2304546	2306738	2258632	2260824	2078610	2080802
2524	BPSS1658		2232881	2233369	2830366	2829878	1447907	1448395	961505	961993	2352495	2352983	2270331	2270819	2307226	2307714	2261312	2261800	2081290	2081778
2525		BURPS1655_D1527																	2081992	2083809
2526	BPSS1659		2233513	2233797	2829734	2829450	1448539	1448823	962137	962421	2353127	2353411			2307858	2308142	2261944	2262228		
2527	BPSS1660		2233880	2234359	2829367	2828888	1448906	1449385	962504	962983	2353494	2353973			2308225	2308704	2262311	2262790		
2528	BPSS1661		2234431	2234643	2828816	2828604	1449457	1449669	963055	963267	2354045	2354257			2308776	2308988	2262862	2263074		
2529	BPSS1662		2237151	2235229	2826096	2828018	1452177	1450255	965775	963853	2356765	2354843	2314366	2312444	2311496	2309574	2265591	2263669	2086069	2084147
2530	BPSS1663		2238590	2237151	2824657	2826096	1453616	1452177	967214	965775	2358204	2356765	2315805	2314366	2312935	2311496	2267030	2265591	2087508	2086069
2531	BPSS1664		2239555	2238590	2823692	2824657	1454581	1453616	968179	967214	2359169	2358204	2316770	2315805	2313888	2312935	2267995	2267030	2087621	2087508
2532	BPSS1665		2240356	2239733	2822891	2823514	1455382	1454759	968980	968357	2359978	2359355	2317579	2316956	2314656	2314033	2268796	2268173	2088425	2087802
2533	BPSS1666		2241267	2240356	2821980	2822891	1456293	1455382	969891	968980	2360889	2359978	2318490	2317579	2315567	2314656	2269707	2268796	2089336	2088425
2534	BPSS1667		2244349	2241281	2818898	2821966	1459375	1456307	972973	969905	2363971	2360903	2321572	2318504	2318649	2315581	2272789	2269721	2092418	2089350
2535		BURPS1710A_A1527					1459422	1459688												
2536	BPSS1669		2246170	2245406	2817098	2817862	1461075	1460311	974673	973909	2365692	2364928	2323343	2322579	2320377	2319613	2274491	2273727	2094111	2093347
2537	BPSS1670		2247358	2246426	2815910	2816842	1462262	1461330	975860	974928	2366880	2365948	2324530	2323598	2321564	2320632	2275679	2274747	2095298	2094366

2538	BPSS1671		2249814	2247907	2813454	2815361	1466206	1464299	979803	977896	2369381	2367474	2327030	2325123	2324056	2322149	2278161	2276254	2097789	2095882
2539	BPSS1672		2251252	2249933	2812016	2813335	1467644	1466325	981241	979922	2370819	2369500	2328468	2327149	2325494	2324175	2279599	2278280	2099227	2097908
2540	BPSS1673		2252309	2251311	2810959	2811957	1468701	1467703	982298	981300	2371876	2370878	2329525	2328527	2326551	2325553	2280656	2279658	2100284	2099286
2541	BPSS1674		2253466	2252516	2809802	2810752	1469858	1468908	983455	982505	2373033	2372083	2330798	2329848	2327708	2326758	2281813	2280863	2101561	2100611
2542	BPSS1675		2254103	2253555	2809165	2809713	1470495	1469947	984092	983544	2373670	2373122	2331435	2330887	2328345	2327797	2282450	2281902	2102198	2101650
2543	BPSS1676		2256086	2255058	2807182	2808210	1472416	1471388	986020	984992	2375939	2374911	2333392	2332364	2330492	2329464	2284386	2283358	2103946	2102918
2544	BPSS1677		2256873	2257103	2806416	2806186	1473131	1473361	986728	986958	2376776	2377006	2334059	2334289	2331179	2331409	2285138	2285368	2104702	2104932
2545	BPSS1678		2257366	2258286	2805923	2805003	1473624	1474544	987221	988141	2377269	2378189	2334552	2335472	2331672	2332592	2285631	2286551	2105197	2106117
2546	BPSS1679		2258748	2259899	2804541	2803390	1475005	1476156	988602	989753	2378650	2379801	2335933	2337084	2333053	2334204	2287012	2288163	2106578	2107729
2547	BPSS1680		2262150	2261731	2801139	2801558	1478397	1477978	991994	991575	2382038	2381619	2339311	2338892	2336440	2336021	2290400	2289981	2109930	2109511
2548	BPSS1681		2262446	2263570	2800843	2799719	1478693	1479817	992290	993414	2382334	2383458	2339607	2340731	2336736	2337860	2290696	2291820	2110226	2111350
2549	BPSS1682		2265012	2264128	2798277	2799161	1481245	1480361	994842	993958	2384963	2384079	2342193	2341309	2339302	2338418	2293241	2292357	2112806	2111922
2550	BPSS1683		2265665	2266819	2797624	2796470	1481898	1483052	995495	996649	2385616	2386770	2342846	2344000	2339955	2341109	2293894	2295048	2113459	2114613
2551	BPSS1684		2269260	2266939	2794029	2796350	1485493	1483172	999090	996769	2389211	2386890	2346441	2344120	2343550	2341229	2297489	2295168	2117054	2114733
2552	BPSS1685		2270861	2269398	2792428	2793891	1487094	1485631	1000691	999228	2390812	2389349	2348042	2346579	2345151	2343688	2299090	2297627	2118655	2117192
2553	BPSS1686		2273096	2270913	2790193	2792376	1489329	1487146	1002926	1000743	2393047	2390864	2350277	2348094	2347380	2345203	2301325	2299142	2120884	2118707
2554		BURPS1655_D1490																	2121053	2121376
2555	BPSS1687		2273651	2274706	2789638	2788583	1489884	1490939	1003481	1004536	2393602	2394657	2350832	2351887	2347934	2348989	2301880	2302935	2121434	2122489
2556	BPSS1688		2274745	2275785	2788544	2787504	1490978	1492018	1004575	1005615	2394696	2395736	2351926	2352966	2349028	2350068	2302974	2304014	2122528	2123568
2557	BPSS1689		2275775	2276740	2787514	2786549	1492008	1492973	1005605	1006570	2395726	2396691	2352956	2353921	2350058	2351023	2304004	2304969	2123558	2124523
2558	BPSS1690		2277108	2276731	2786181	2786558	1493341	1492964	1006938	1006561	2397059	2396682	2354289	2353912	2351391	2351014	2305337	2304960	2124891	2124514
2559	BPSS1691		2278678	2277464	2784611	2785825	1494911	1493697	1008508	1007294	2398642	2397428	2355880	2354666	2352974	2351760	2306907	2305693	2126484	2125270
2560	BPSS1692		2280442	2278910	2782847	2784379	1496675	1495143	1010272	1008740	2400406	2398874	2357644	2356112	2354738	2353206	2308671	2307139	2128248	2126716
2561	BPSS1693		2280989	2280498	2782300	2782791	1497222	1496731	1010819	1010328	2400953	2400462	2358191	2357700	2355285	2354794	2309218	2308727	2128795	2128304
2562	BPSS1694		2281736	2280999	2781553	2782290	1497969	1497232	1011566	1010829	2401700	2400963	2358938	2358201	2356032	2355295	2309965	2309228	2129542	2128805
2563	BPSS1695		2283154	2281844	2780135	2781445	1499387	1498077	1012984	1011674	2403118	2401808	2360356	2359046	2357450	2356140	2311383	2310073	2130960	2129650
2564	BPSS1696		2284138	2283269	2779151	2780020	1500371	1499502	1013968	1013099	2404102	2403233	2361340	2360471	2358434	2357565	2312367	2311498	2131944	2131075
2565	BPSS1697		2285033	2284221	2778256	2779068	1501266	1500454	1014863	1014051	2404997	2404185	2362235	2361423	2359329	2358517	2313262	2312450	2132839	2132027
2566	BPSS1698		2285926	2285081	2777363	2778208	1502159	1501314	1015756	1014911	2405890	2405045	2363127	2362282	2360222	2359377	2314155	2313310	2133731	2132886
2567	BPSS1699		2287129	2285939	2776160	2777350	1503362	1502172	1016959	1015769	2407093	2405903	2364330	2363140	2361425	2360235	2315358	2314168	2134934	2133744
2568	BPSS1700		2287965	2287204	2775324	2776085	1504198	1503437	1017795	1017034	2407929	2407168	2365166	2364405	2362261	2361500	2316194	2315433	2135770	2135009
2569	BPSS1701		2288777	2287965	2774512	2775324	1505010	1504198	1018607	1017795	2408741	2407929	2365978	2365166	2363073	2362261	2317006	2316194	2136582	2135770
2570	BPSS1702		2289907	2288777	2773382	2774512	1506140	1505010	1019737	1018607	2409349	2408741	2367123	2365978	2364203	2363073	2318136	2317006	2137712	2136582
2571	BPSS1703		2291344	2290067	2771945	2773222	1507579	1506665	1021174	1019897	2410302	2409922	2368548	2367283	2365640	2364363				
2572	BPSS1704		2292676	2291558	2770613	2771731	1508911	1507793	1022506	1021388	2411634	2410516	2369880	2368762	2366972	2365854	2320887	2319769	2139650	2138532
2573	BPSS1705		2294085	2293021	2769204	2770268	1510320	1509256	1023915	1022851	2413046	2411982	2371290	2370226	2368384	2367320	2322299	2321235	2141062	2139998
2574	BPSS1706		2294784	2294137	2768505	2769152	1511019	1510372	1024614	1023967	2413745	2413098	2371989	2371342	2369083	2368436	2322998	2322351	2141761	2141114
2575	BPSS1706a		2295005	2294865	2768284	2768424	1511240	1511100	1024835	1024695	2413966	2413826	2372210	2372070	2369304	2369164	2323219	2323079	2141982	2141842
2576	BPSS1707		2296417	2295011	2766872	2768278	1512652	1511246	1026247	1024841	2415378	2413972	2373622	2372216	2370716	2369310	2324631	2323225	2143394	2141988
2577	BPSS1708		2298104	2296812	2765185	2766477	1514339	1513047	1027934	1026642	2417065	2415773	2375309	2374017	2372403	2371111	2326318	2325026	2145081	2143789
2578	BPSS1709		2298774	2298100	2764515	2765189	1515009	1514335	1028604	1027930	2417735	2417061	2375979	2375305	2373073	2372399	2326988	2326314	2145751	2145077
2579	BPSS1710		2300292	2299768	2762997	2763521	1516526	1516002	1030121	1029597	2419253	2418729	2377496	2376972	2374590	2374066	2328506	2327982	2147268	2146744
2580	BPSS1711		2300453	2303173	2762836	2760116	1516687	1519407	1030282	1033002	2419414	2422134	2377657	2380377	2374751	2377471	2328667	2331387	2147429	2150149
2581	BPSS1712		2304672	2303431	2758617	2759858	1520908	1519667	1034503	1033262	2423635	2422394	2381878	2380637	2378972	2377731	2332886	2331645	2151649	2150408
2582	BPSS1713		2305919	2305533	2757370	2757756	1522155	1521769	1035750	1035364	2424882	2424496	2383125	2382739	2380219	2379833	2334133	2333747	2152896	2152510
2583	BPSS1714		2305995	2306966	2757294	2756323	1522231	1523202	1035826	1036797	2424958	2425404	2383201	2384172	2380295	2381266	2334209	2335180	2152972	2153943
2584	BPSS1715		2308367	2307069	2754922	2756220	1524603	1523305	1038198	1036900	2427440	2426142	2385573	2384275	2382667	2381369	2336581	2335283	2155344	2154046
2585	BPSS1716		2308692	2308423	2754597	2754866	1524928	1524659	1038523	1038254	2427765	2427496	2385898	2385629	2382992	2382723	2336906	2336637	2155669	2155400
2586	BPSS1717		2309395	2308697	2753894	2754592	1525631	1524933	1039226	1038528	2428468	2427770	2386601	2385903	2383695	2382997	2337609	2336911	2156372	2155674
2587	BPSS1718		2311192	2309420	2752097	2753869	1527428	1525656	1041023	1039251	2430265	2428493	2388398	2386626	2385492	2383720	2339406	2337634	2158169	2156397
2588	BPSS1719		2311565	2311200	2751724	2752089	1527801	1527436	1041396	1041031	2430638	2430273	2388771	2388406	2385865	2385500	2339779	2339414	2158542	2158177
2589	BPSS1720		2311986	2311573	2751303	2751716	1528222	1527809	1041817	1041404	2431059	2430646	2389192	2388779	2386286	2385873	2340200	2339787	2158963	2158550
2590	BPSS1721		2312986	2312189	2750303	2751100	1529222	1528425	1042817	1042020	2432059	2431262	2390192	2389395	2387286	2386489	2341200	2340403	2159963	2159166
2591	BPSS1722		2313285	2314265	2750004	2749024	1529521	1530501	1043116	1044096	2432358	2433338	2390491	2391471	2387585	2388565	2341499	2342479	2160262	2161242
2592	BPSS1723		2314490	2315482	2748799	2747807	1530726	1531718	1044321	1045313	2433563	2434555	2391696	2392688	2388790	2389782	2342704	2343696	2161467	2162459
2593	BPSS1724		2315642	2316148	2747647	2747141	1531878	1532384	1045473	1045979	2434715	2435221	2392848	2393354	2389942	2390448	2343856	2344362	2162619	2163125
2594	BPSS1725		2316228	2317676	2747061	2745613	1532464	1533912	1046059	1047507	2435301	2436749	2393434	2394882	2390528	2391976	2344442	2345890	2163205	2164653
2595	BPSS1726		2317727	2320441	2745562	2742848	1533963	1536677	1047558	1050272	2436800	2439514	2394933	2397647	2392027	2394741	2345941	2348655	2164704	2167418

2596	BPSS1727		2323435	2320694	2739854	2742595	1539672	1536931	1053267	1050526	2442500	2439768	2400641	2397900	2397736	2394995	2351649	2348908	2170403	2167671
2597	BPSS1728		2325209	2323521	2738080	2739768	1541446	1539758	1055041	1053353	2444274	2442586	2402415	2400727	2399510	2397822	2353423	2351735	2172177	2170489
2598	BPSS1729		2328570	2326489	2734719	2736800	1544807	1542726	1058402	1056321	2447635	2445554	2405778	2403697	2402872	2400791	2356784	2354703	2175538	2173457
2599	BPSS1730		2329419	2329051	2733870	2734238	1545665	1545297	1059260	1058892	2448493	2448125	2406637	2406269	2403721	2403353	2357633	2357265	2176450	2176082
2600	BPSS1731		2331346	2329736	2731943	2733553	1547592	1545982	1061187	1059577	2450420	2448810	2408564	2406954	2405648	2404038	2359560	2357950	2178377	2176767
2601	BPSS1732		2332279	2331653	2731010	2731636	1548528	1547899	1062123	1061494	2451356	2450727	2409485	2408859	2406581	2405955	2360493	2359867	2179298	2178672
2602	BPSS1733		2333274	2333405	2730015	2729884	1549523	1549654	1063118	1063249	2452351	2452482	2410480	2410611	2407576	2407707	2361488	2361619	2180293	2180424
2603	BPSS1734		2333755	2335500	2729534	2727789	1550004	1551749	1063599	1065344	2452832	2454577	2410961	2412706	2408057	2409802	2361969	2363714	2180774	2182519
2604	BPSS1735		2336437	2335601	2726852	2727688	1552686	1551850	1066281	1065445	2455514	2454678	2413643	2412807	2410739	2409903	2364651	2363815	2183456	2182620
2605	BPSS1736		2337736	2336471	2725553	2726818	1553985	1552720	1067580	1066315	2456813	2455548	2414942	2413677	2412038	2410773	2365950	2364685	2184755	2183490
2606	BPSS1737		2338874	2337831	2724415	2725458	1555123	1554080	1068718	1067675	2457951	2456908	2416080	2415037	2413176	2412133	2367088	2366045	2185893	2184850
2607	BPSS1738		2340049	2338952	2723240	2724337	1556298	1555201	1069893	1068796	2459126	2458029	2417255	2416158	2414351	2413254	2368263	2367166	2187068	2185971
2608	BPSS1739		2340158	2341684	2723131	2721605	1556407	1557933	1070002	1071528	2459235	2460761	2417364	2418890	2414460	2415986	2368372	2369898	2187177	2188703
2609	BPSS1740		2342828	2341797	2720461	2721492	1559077	1558046	1072672	1071641	2461905	2460874	2420034	2419003	2417130	2416099	2371042	2370011	2189847	2188816
2610	BPSS1741		2343926	2342835	2719363	2720454	1560175	1559084	1073770	1072679	2463003	2461912	2421132	2420041	2418228	2417137	2372140	2371049	2190945	2189854
2611	BPSS1742		2346355	2344253	2716934	2719036	1562604	1560502	1076199	1074097	2465432	2463330	2423549	2421459	2420657	2418555	2374569	2372467	2193374	2191272
2612	BPSS1743		2346982	2346590	2716307	2716699	1563231	1562839	1076826	1076434	2466059	2465667	2424176	2423784	2421284	2420892	2375196	2374804	2194001	2193609
2613	BPSS1744		2347679	2347146	2715610	2716143	1563928	1563395	1077523	1076990	2466756	2466223	2424873	2424340	2421981	2421448	2375893	2375360	2194698	2194165
2614	BPSS1745		2347948	2348580	2715341	2714709	1564197	1564829	1077792	1078424	2467025	2467657	2425142	2425774	2422250	2422882	2376162	2376794	2194967	2195599
2615	BPSS1746		2349479	2348718	2713810	2714571	1565727	1564966	1079322	1078561	2468558	2467797	2426678	2425917	2423783	2423022	2377693	2376932	2196497	2195736
2616	BPSS1747		2350076	2349762	2713213	2713527	1566324	1566010	1079919	1079605	2469155	2468841	2427274	2426960	2424380	2424066	2378290	2377976	2197094	2196780
2617	BPSS1748		2350853	2350518	2712436	2712771	1567101	1566766	1080696	1080361	2469932	2469597	2428055	2427720	2425148	2424813	2379058	2378723	2197874	2197539
2618	BPSS1749		2352461	2351049	2710828	2712240	1568709	1567297	1082304	1080892	2471540	2470128	2429663	2428251	2426756	2425344	2380666	2379254	2199482	2198070
2619	BPSS1750		2353531	2352920	2709758	2710369	1569807	1569196	1083402	1082791	2472609	2471998	2430733	2430122	2427825	2427214	2381799	2381188	2200551	2199940
2620	BPSS1751		2354489	2353560	2708800	2709729	1570765	1569836	1084360	1083431	2473573	2472638	2431697	2430762	2428789	2427854	2382757	2381828	2201515	2200580
2621	BPSS1752		2355910	2354489	2707379	2708800	1572186	1570765	1085781	1084360	2474994	2473573	2433118	2431697	2430210	2428789	2384178	2382757	2202936	2201515
2622	BPSS1753		2356547	2357404	2706742	2705885	1572823	1573680	1086418	1087275	2475632	2476489	2433756	2434613	2430848	2431705	2384815	2385672	2203573	2204430
2623	BPSS1754		2357471	2358634	2705818	2704655	1573747	1574910	1087342	1088505	2476556	2477719	2434680	2435843	2431772	2432923	2385739	2386902	2204497	2205660
2624	BPSS1755		2361564	2359525	2701725	2703764	1577840	1575801	1091435	1089396	2480651	2478612	2438771	2436732	2435666	2433627	2389830	2387791	2208425	2206386
2625	BPSS1756		2363882	2362011	2699407	2701278	1580158	1578287	1093753	1091882	2482969	2481098	2441089	2439218	2437984	2436113	2392148	2390277	2210743	2208872
2626	BPSS1757		2364423	2363980	2698866	2699309	1580699	1580256	1094294	1093851	2483510	2483067	2441630	2441187	2438525	2438082	2392689	2392246	2211284	2210841
2627	BPSS1758		2364802	2364593	2698487	2698696	1581078	1580869	1094673	1094464	2483889	2483680	2442009	2441800	2438904	2438695	2393068	2392859	2211663	2211454
2628	BPSS1759		2366105	2364885	2697184	2698404	1582381	1581161	1095976	1094756	2485192	2483972	2443312	2442092	2440207	2438987	2394371	2393151	2212966	2211746
2629	BPSS1760		2366245	2367282	2697044	2696007	1582521	1583558	1096116	1097153	2485332	2486369	2443452	2444489	2440347	2441384	2394511	2395548	2213106	2214143
2630	BPSS1761		2368486	2367680	2694803	2695609	1584792	1583986	1098387	1097581	2487603	2486797	2445730	2444924	2442618	2441812	2396752	2395946	2215353	2214547
2631	BPSS1762		2370541	2368640	2692748	2694649	1586847	1584946	1100442	1098541	2489658	2487757	2447785	2445884	2444673	2442772	2398807	2396906	2217408	2215507
2632	BPSS1763		2371509	2370628	2691780	2692661	1587815	1586934	1101410	1100529	2490626	2489745	2448753	2447872	2445641	2444760	2399775	2398894	2218376	2217495
2633	BPSS1764		2371799	2371509	2691490	2691780	1588105	1587815	1101700	1101410	2490916	2490626	2449043	2448753	2445931	2445641	2400065	2399775	2218666	2218376
2634	BPSS1765		2372063	2373172	2691226	2690117	1588369	1589478	1101964	1103073	2491180	2492289	2449307	2450416	2446195	2447304	2400329	2401438	2218930	2220039
2635		Bp_chr2_10_ISBn	2373272	2374844	2688445	2690017	1589578	1591150	1103173	1104745			2450515	2452087	2447405	2448977				
2636	BPSS1766		2374914	2375780	2688375	2687509	1591220	1592086	1104815	1105681	2492440	2493306	2452156	2453022	2449047	2449913	2401589	2402455	2220190	2221056
2637	BPSS1767		2376717	2375845	2686572	2687444	1593023	1592151	1106618	1105746	2494243	2493371	2453959	2453087	2450850	2449978	2403392	2402520	2221993	2221121
2638	BPSS1768		2377436	2379226	2685853	2684063	1593743	1595533	1107338	1109128	2494963	2496753	2454679	2456469	2451570	2453360	2404111	2405901	2222713	2224503
2639		BURPS1106B_2779			2683893	2684066														
2640		BURPS1655_D1389																	2224673	2224500
2641		BURPS1710A_A1673					1595703	1595530												
2642		BURPSPAST_AC0520													2453530	2453357				
2643	BPSS1769		2380917	2379538	2682372	2683751	1597140	1595761	1110735	1109356	2498458	2497079	2458125	2456746	2454967	2453588	2407578	2406199	2226173	2224794
2644	BPSS1770		2384007	2381239	2679282	2682050	1600230	1597462	1113825	1111057	2501548	2498780	2461215	2458447	2458057	2455289	2410668	2407900	2229263	2226495
2645	BPSS1771		2384024	2384755	2679265	2678534	1600247	1600978	1113842	1114573	2501565	2502296	2461232	2461963	2458074	2458805	2410685	2411416	2229280	2230011
2646	BPSS1772		2385176	2385457	2678113	2677832	1601399	1601680	1114994	1115275	2502717	2502998	2462384	2462665	2459226	2459507	2411837	2412118	2230432	2230713
2647	BPSS1773		2386120	2386608	2677169	2676681	1602343	1602831	1115938	1116426	2503651	2504139	2463322	2463810	2460170	2460658	2412781	2413269	2231377	2231865
2648	BPSS1774		2388716	2387493	2674573	2675796	1604939	1603716	1118534	1117311	2506247	2505024			2462766	2461543	2415377	2414154	2233973	2232750
2649	BPSS1774A		2389651	2389451	2673638	2673838	1605874	1605674	1119469	1119269	2507182	2506982	1265340	1265369	2463557	2463501	2416312	2416112	2234909	2234763
2650	BPSS1775		2389652	2389918	2673637	2673371	1605875	1606141	1119470	1119736	2507183	2507449			2463704	2463970	2416313	2416579	2234910	2235176
2651	BPSS1776		2389936	2390427	2673353	2672862	1606159	1606650	1119754	1120245	2507467	2507958			2463988	2464479	2416597	2417088	2235194	2235685
2652	BPSS1777		2392232	2390709	2671057	2672580	1608454	1606931	1122049	1120526	2509762	2508239	2465490	2463967	2466284	2464761	2418893	2417370	2237490	2235967
2653	BPSS1778		2392388	2392783	2670901	2670506	1608610	1609005	1122205	1122600	2509918	2510313	2465646	2466041	2466440	2466835	2419049	2419444	2237646	2238041

2654	BPSS1779		2392880	2393872	2670409	2669417	1609102	1610094	1122697	1123689	2510410	2511402	2466138	2467130	2466932	2467924	2419541	2420533	2238138	2239130
2655	BPSS1780		2393866	2394675	2669423	2668614	1610088	1610897	1123683	1124492	2511396	2512205	2467124	2467933	2467918	2468727	2420527	2421336	2239124	2239933
2656	BPSS1781		2395174	2395623	2668115	2667666	1611403	1611852	1124998	1125447	2512711	2513160	2468439	2468888	2469226	2469675	2421842	2422291	2240437	2240886
2657	BPSS1782		2395764	2396180	2667525	2667109	1611993	1612409	1125588	1126004	2513301	2513717	2469029	2469445	2469816	2470232	2422432	2422848	2241027	2241443
2658	BPSS1783		2396454	2396299	2666835	2666990	1612684	1612529	1126278	1126123	2513991	2513836	2469719	2469564	2470819	2470664	2423124	2422969	2242161	2242006
2659	BPSS1784		2397597	2396554	2665692	2666735	1613827	1612784	1127421	1126378	2515134	2514091	2470862	2469819	2471962	2470919	2424267	2423224	2243304	2242261
2660	BPSS1785		2397959	2398645	2665330	2664644	1614189	1614875	1127783	1128469	2515496	2516182	2471224	2471910	2472324	2473010	2424629	2425315	2243666	2244352
2661	BPSS1786		2399129	2399926	2664160	2663363	1615430	1616227	1129024	1129821	2516737	2517534	2472490	2473287	2473517	2474314	2425815	2426612	2244933	2245730
2662	BPSS1787		2399942	2400613	2663347	2662676	1616243	1616914	1129837	1130508	2517550	2518221	2473303	2473974	2474330	2475001	2426628	2427299	2245746	2246417
2663	BPSS1788		2400618	2401316	2662671	2661973	1616919	1617617	1130513	1131211	2518226	2518924	2473979	2474677	2475006	2475704	2427304	2428002	2246422	2247120
2664	BPSS1789		2402650	2401865	2660639	2661424	1618951	1618166	1132545	1131760	2520258	2519473	2476010	2475225	2477038	2476253	2429336	2428551	2248454	2247669
2665	BPSS1790		2404829	2402748	2658460	2660541	1621112	1619049	1134706	1132643	2522446	2520356	2478171	2476108	2479208	2477136	2431533	2429434	2250615	2248552
2666	BPSS1791		2405275	2404895	2658014	2658394	1621558	1621178	1135152	1134772	2522892	2522512	2478617	2478237	2479654	2479274	2431979	2431599	2251061	2250681
2667	BPSS1792		2405373	2406245	2657916	2657044	1622041	1622529	1135250	1136122	2522990	2523862	2478715	2479587	2479752	2480624	2432077	2432949	2251159	2252031
2668	BPSS1793		2406315	2407232	2656974	2656057	1622599	1623516	1136192	1137109	2523932	2524849	2479657	2480574	2480694	2481611	2433019	2433936	2252101	2253018
2669	BPSS1794		2407265	2408104	2656024	2655185	1623543	1624388	1137136	1137981	2524882	2525721	2480601	2481446	2481644	2482483	2433969	2434808	2253045	2253890
2670	BPSS1795		2408193	2409530	2655096	2653759	1624477	1625814	1138070	1139407	2525810	2527147	2481535	2482872	2482572	2483909	2434897	2436234	2253979	2255316
2671	BPSS1796		2409530	2410156	2653759	2653133	1625814	1626440	1139407	1140033	2527147	2527773	2482872	2483498	2483909	2484535	2436234	2436860	2255316	2255942
2672	BPSS1797		2411961	2410321	2651328	2652968	1628245	1626605	1141838	1140198	2529578	2527938	2485303	2483663	2486340	2484700	2438665	2437025	2257747	2256107
2673	BPSS1798		2412237	2412947	2651052	2650342	1628521	1629231	1142114	1142824	2529854	2530564	2485579	2486289	2486616	2487326	2438941	2439651	2258023	2258733
2674		BURPS1710A_A1719					1629241	1629468												
2675	BPSS1799		2413299	2413751	2649990	2649538	1629582	1630034	1143175	1143627	2530906	2531358	2486641	2487093	2487677	2488129	2440003	2440455	2259085	2259537
2676	BPSS1800		2415057	2413822	2648232	2649467	1631340	1630105	1144933	1143698	2532664	2531429	2488399	2487164	2489435	2488200	2441761	2440526	2260843	2259608
2677	BPSS1801		2415283	2415717	2648006	2647572	1631566	1632000	1145159	1145593	2532890	2533324	2488625	2489059	2489661	2490095	2441987	2442421	2261069	2261503
2678	BPSS1802		2416588	2415965	2646701	2647324	1632871	1632248	1146464	1145841	2534195	2533572	2489930	2489307	2490966	2490343	2443292	2442669	2262374	2261751
2679	BPSS1803		2416651	2417502	2646638	2645787	1632934	1633785	1146527	1147378	2534258	2535109	2489993	2490841	2491029	2491880	2443355	2444206	2262437	2263288
2680	BPSS1804		2418190	2419275	2645099	2644014	1634471	1635556	1148064	1149149	2535796	2536881	2491532	2492617	2492566	2493651	2444894	2445979	2263976	2265061
2681	BPSS1805		2419929	2419348	2643360	2643941	1636210	1635629	1149803	1149222	2537535	2536954	2493271	2492690	2494305	2493724	2446633	2446052	2265715	2265134
2682	BPSS1806		2420407	2419988	2642882	2643301	1636688	1636269	1150281	1149862	2538013	2537594	2493749	2493330	2494783	2494364	2447111	2446692	2266193	2265774
2683	BPSS1807		2421488	2420448	2641801	2642841	1637769	1636729	1151362	1150322	2539094	2538054	2494830	2493790	2495864	2494824	2448192	2447152	2267274	2266234
2684	BPSS1808		2422378	2421488	2640911	2641801	1638659	1637769	1152252	1151362	2539984	2539094	2495720	2494830	2496754	2495864	2449082	2448192	2268164	2267274
2685	BPSS1809		2423217	2422378	2640072	2640911	1639498	1638659	1153091	1152252	2540823	2539984	2496559	2495720	2497593	2496754	2449921	2449082	2269009	2268164
2686	BPSS1810		2424158	2423217	2639131	2640072	1640439	1639498	1154032	1153091	2541764	2540823	2497500	2496559	2498534	2497593	2450862	2449921	2269950	2269009
2687	BPSS1811		2424856	2424176	2638433	2639113	1641137	1640457	1154730	1154050	2542462	2541782	2498198	2497518	2499232	2498552	2451560	2450880	2270648	2269968
2688	BPSS1812		2425661	2424867	2637628	2638422	1641942	1641148	1155535	1154741	2543267	2542473	2499003	2498209	2500037	2499243	2452365	2451571	2271453	2270659
2689	BPSS1813		2426570	2425668	2636719	2637621	1642851	1641949	1156444	1155542	2544176	2543274	2499912	2499010	2500946	2500044	2453274	2452372	2272362	2271460
2690	BPSS1814		2426927	2426670	2636362	2636619	1643208	1642951	1156801	1156544	2544533	2544276	2500269	2500012	2501303	2501046	2453631	2453374	2272719	2272462
2691	BPSS1815		2428513	2426930	2634776	2636359	1644794	1643208	1158387	1156801	2546119	2544533	2501855	2500269	2502889	2501303	2455217	2453631	2274305	2272719
2692	BPSS1816		2430042	2429752	2633247	2633537	1646322	1646032	1159915	1159625	2547648	2547358	2503377	2503087	2504417	2504127	2456696	2456406	2275838	2275548
2693	BPSS1817		2430559	2430101	2632730	2633188	1646839	1646381	1160432	1159974	2548165	2547707	2503894	2503436	2504934	2504476	2457213	2456755	2276355	2275897
2694	BPSS1818		2431359	2430709	2631930	2632580	1647609	1646959	1161202	1160552	2548965	2548315	2504694	2504044	2505704	2505054	2458013	2457363	2277155	2276505
2695	BPSS1819		2431481	2432212	2631808	2631077	1647731	1648462	1161324	1162055	2549087	2549818	2504816	2505547	2505826	2506557	2458135	2458866	2277277	2278008
2696	BPSS1820		2433010	2432312	2630279	2630977	1649267	1648569	1162860	1162162	2550623	2549925	2506345	2505647	2507362	2506664	2459664	2458966	2278813	2278115
2697	BPSS1821		2433426	2433010	2629863	2630279	1649683	1649267	1163276	1162860	2551039	2550623	2506761	2506345	2507778	2507362	2460080	2459664	2279229	2278813
2698	BPSS1822		2435065	2434160	2628224	2629129	1651322	1650417	1164915	1164010	2552678	2551773	2508400	2507495	2509417	2508512	2461719	2460814	2280868	2279963
2699	BPSS1823		2435569	2435907	2627720	2627382	1651826	1652164	1165419	1165757	2553182	2553520	2508904	2509242	2509921	2510259	2462223	2462561	2281372	2281710
2700	BPSS1824		2435973	2436737	2627316	2626552	1652230	1652994	1165823	1166587	2553586	2554350	2509308	2510072	2510325	2511089	2462627	2463391	2281776	2282540
2701	BPSS1825		2438195	2437032	2625094	2626257	1654452	1653289	1168045	1166882	2555808	2554645	2511530	2510367	2512547	2511384	2464849	2463686	2283979	2282816
2702	BPSS1826		2439379	2438198	2623910	2625091	1655636	1654455	1169229	1168048	2556992	2555811	2512714	2511533	2513731	2512550	2466033	2464852	2285163	2283982
2703	BPSS1827		2440851	2439379	2622438	2623910	1657108	1655636	1170701	1169229	2558464	2556992	2514186	2512714	2515203	2513731	2467505	2466033	2286635	2285163
2704	BPSS1828		2442002	2440866	2621287	2622423	1658259	1657123	1171852	1170716	2559615	2558479	2515337	2514201	2516354	2515218	2468656	2467520	2287786	2286650
2705	BPSS1829		2442993	2442049	2620296	2621240	1659250	1658306	1172843	1171899	2560606	2559662	2516328	2515384	2517345	2516401	2469647	2468703	2288777	2287833
2706	BPSS1830		2445242	2443026	2618047	2620263	1661499	1659283	1175092	1172876	2562855	2560639	2518577	2516361	2519594	2517378	2471896	2469680	2291026	2288810
2707	BPSS1831		2446468	2445296	2616821	2617993	1662725	1661553	1176318	1175146	2564081	2562909	2519803	2518631	2520820	2519648	2473122	2471950	2292252	2291080
2708	BPSS1832		2446896	2446465	2616393	2616824	1663153	1662722	1176746	1176315	2564509	2564078	2520231	2519800	2521248	2520817	2473550	2473119	2292680	2292249
2709	BPSS1833		2448340	2446922	2614949	2616367	1664597	1663179	1178190	1176772	2565953	2564535	2521675	2520257	2522692	2521274	2474994	2473576	2294124	2292706
2710	BPSS1834		2449801	2448389	2613488	2614900	1666058	1664646	1179651	1178239	2567414	2566002	2523136	2521724	2524153	2522741	2476455	2475043	2295585	2294173
2711	BPSS1835		2450258	2451715	2613031	2611574	1666514	1667971	1180107	1181564	2567870	2569330	2523592	2525049	2524609	2526066	2476911	2478377	2296041	2297510

2712 BPSS1836		2452795	2451794	2610494	2611495	1669051	1668050	1182644	1181643	2570410	2569409	2526129	2525128	2527146	2526145	2479457	2478456	2298590	2297589
2713 BPSS1837		2453929	2453375	2609360	2609914	1670185	1669631	1183778	1183224	2571544	2570990	2527262	2526708	2528280	2527726	2480591	2480037	2299724	2299170
2714 BPSS1838		2455316	2453982	2607973	2609307	1671572	1670238	1185165	1183831	2572931	2571597	2528649	2527315	2529667	2528333	2481978	2480644	2301111	2299777
2715 BPSS1839		2455850	2455431	2607439	2607858	1672106	1671687	1185699	1185280	2573463	2573044	2529181	2528762	2530201	2529782	2482512	2482093	2301641	2301222
2716 BPSS1840		2457155	2456124	2606134	2607165	1673411	1672380	1187004	1185973	2574768	2573737	2530486	2529455	2531512	2530475	2483817	2482786	2302946	2301915
2717 BPSS1841		2458967	2457702	2604322	2605587	1675252	1673987	1188845	1187580	2576609	2575344	2532313	2531048	2533360	2532095	2485643	2484378	2304759	2303494
2718 BPSS1842		2459503	2458967	2603786	2604322	1675788	1675252	1189381	1188845	2577145	2576609	2532849	2532313	2533896	2533360	2486179	2485643	2305295	2304759
2719 BPSS1843		2459938	2459528	2603351	2603761	1676223	1675813	1189816	1189406	2577580	2577170	2533284	2532874	2534331	2533921	2486614	2486204	2305730	2305320
2720 BPSS1844		2460458	2460108	2602831	2603181	1676742	1676392	1190335	1189985	2578100	2577750	2533804	2533454	2534850	2534500	2487133	2486783	2306249	2305899
2721 BPSS1845		2460820	2462691	2602469	2600598	1677112	1678983	1190705	1192576	2578461	2580332	2534166	2536037	2535220	2537091	2487558	2489429	2306635	2308506
2722 BPSS1846		2463484	2463861	2599805	2599428	1679775	1680152	1193368	1193745	2581125	2581502	2536827	2537204	2537883	2538260	2490221	2490598	2309299	2309676
2723 BPSS1847		2463913	2464494	2599376	2598795	1680204	1680785	1193797	1194378	2581554	2582135	2537256	2537837	2538312	2538893	2490650	2491231	2309728	2310309
2724 BPSS1848		2464689	2465780	2598600	2597509	1680980	1682071	1194573	1195664	2582330	2583421	2538032	2539123	2539088	2540179	2491426	2492517	2310504	2311595
2725 BPSS1849		2466475	2466002	2596814	2597287	1682766	1682293	1196359	1195886	2584104	2583631	2539818	2539345	2540874	2540401	2493199	2492726	2312289	2311816
2726 BPSS1850		2468696	2466603	2594593	2596686	1684987	1682894	1198580	1196487	2586325	2584232	2542034	2539941	2543095	2541002	2495420	2493327	2314511	2312418
2727 BPSS1851		2469163	2470365	2594126	2592924	1685454	1686656	1199047	1200249	2586792	2587994	2542501	2543703	2543562	2544764	2495887	2497089	2314978	2316180
2728	BURPSPAST_AC0412													2544789	2545112				
2729 BPSS1852		2472070	2471057	2591219	2592232	1688352	1687339	1201945	1200932	2589679	2588666	2545417	2544404	2546504	2545491	2498741	2497728	2317866	2316853
2730 BPSS1853		2473215	2472421	2590074	2590868	1689420	1688626	1203013	1202219	2590782	2589988	2546537	2545743	2547593	2546799	2499966	2499172	2318962	2318168
2731 BPSS1854		2474225	2473458	2589064	2589831	1690414	1689647	1204007	1203240	2591776	2591009	2547559	2546792	2548608	2547841	2500981	2500214	2319979	2319212
2732 BPSS1855		2474948	2474262	2588341	2589027	1691137	1690451	1204730	1204044	2592499	2591813	2548282	2547596	2549331	2548645	2501704	2501018	2320702	2320016
2733 BPSS1856		2476021	2474948	2587268	2588341	1692210	1691137	1205803	1204730	2593572	2592499	2549355	2548282	2550404	2549331	2502777	2501704	2321775	2320702
2734 BPSS1857		2477250	2476021	2586039	2587268	1693439	1692210	1207032	1205803	2594801	2593572	2550584	2549355	2551633	2550404	2504006	2502777	2323004	2321775
2735 BPSS1858		2477656	2478579	2585619	2584696	1693775	1694698	1207368	1208291	2595080	2596003	2550799	2551722	2551849	2552772	2504375	2505298	2323369	2324292
2736 BPSS1859		2478674	2479072	2584601	2584203	1694793	1695191	1208386	1208784	2596098	2596496	2551817	2552215	2552867	2553265	2505393	2505791	2324387	2324785
2737 BPSS1860		2480400	2479216	2582875	2584059	1696521	1695337	1210114	1208930	2597817	2596642	2553544	2552360	2554595	2553411	2507119	2505935	2326113	2324929
2738	BURPS1655_D1275																	2326339	2326851
2739	BURPS1710A_A1802					1696747	1697226												
2740	BURPS1710b_A0951							1210340	1210819										
2741 BPSS1861		2482206	2481328	2581069	2581947	1698294	1697416	1211887	1211009	2599591	2598713	2555325	2554447	2556379	2555501	2508915	2508037	2327919	2327041
2742	BURPS1106B_2654			2582649	2582137														
2743 BPSS1862		2482888	2484894	2580387	2578381	1698976	1700982	1212569	1214575	2600273	2602279	2556016	2558022	2557079	2559085	2509620	2511626	2328619	2330625
2744 BPSS1863		2485960	2485235	2577315	2578040	1702048	1701323	1215641	1214916	2603349	2602624	2559068	2558343	2560151	2559426	2512671	2511946	2331692	2330967
2745 BPSS1864		2486039	2487076	2577236	2576199	1702127	1703164	1215720	1216757	2603428	2604465	2559147	2560199	2560230	2561267	2512750	2513787	2331771	2332823
2746 BPSS1865		2487659	2488696	2575616	2574579	1703663	1704700	1217256	1218293	2604970	2606007	2560642	2561679	2561736	2562773	2514398	2515435	2333346	2334383
2747 BPSS1866		2489162	2489815	2574113	2573460	1705167	1705820	1218760	1219413	2606488	2607141	2562129	2562782	2563254	2563907	2515951	2516604	2334849	2335472
2748 BPSS1867		2490672	2491613	2572603	2571662	1706678	1707619	1220271	1221212	2607999	2608940	2563640	2564584	2564765	2565706	2517462	2518403	2336338	2337279
2749 BPSS1868		2493102	2491822	2570173	2571453	1709118	1707838	1222711	1221431	2610439	2609159	2566073	2564793	2567205	2565925	2519892	2518612	2338768	2337488
2750	BURPSS13_X0677															2520054	2520203		
2751 BPSS1869		2494498	2493467	2568777	2569808	1710586	1709519	1224179	1223112	2611847	2610780	2566896	2566402	2568625	2567558	2521312	2520245	2340167	2339109
2752	BURPS1106B_2640			2570020	2569850														
2753 BPSS1870		2495573	2494548	2567702	2568727	1711641	1710616	1225234	1224209	2612899	2611877	2568437	2567412	2569632	2568655	2522367	2521342	2341223	2340198
2754 BPSS1871		2498174	2495583	2565101	2567692	1714242	1711651	1227835	1225244	2615500	2612909	2571038	2568447	2571663	2569642	2524968	2522377	2343824	2341233
2755 BPSS1872		2498905	2498174	2564370	2565101	1714973	1714242	1228566	1227835	2616231	2615500	2571775	2571038	2572920	2572189	2525699	2524968	2344555	2343824
2756 BPSS1873		2500940	2498889	2562335	2564386	1717008	1714957	1230601	1228550	2617698	2616796	2573810	2571759	2574953	2573550	2527716	2525683	2346429	2345431
2757 BPSS1874		2501479	2500940	2561796	2562335	1717547	1717008	1231140	1230601	2618237	2617698	2574349	2573810	2575492	2574953	2528255	2527716	2346968	2346429
2758 BPSS1875		2503166	2501493	2560109	2561782	1719234	1717561	1232827	1231154	2619924	2618251	2576036	2574363	2577179	2575506	2529942	2528269	2348655	2346982
2759 BPSS1876		2504434	2503340	2558841	2559935	1720502	1719408	1234095	1233001	2621193	2620099	2577304	2576210	2578447	2577353	2531210	2530116	2349923	2348829
2760 BPSS1877		2505250	2505588	2558025	2557687	1721318	1721668	1234911	1235261	2622009	2622347	2578109	2578447	2579263	2579613	2532026	2532376	2350739	2351077
2761 BPSS1878		2507304	2505874	2555971	2557401	1723384	1721954	1236977	1235547	2624063	2622633	2580138	2578708	2581329	2579899	2534092	2532662	2352793	2351363
2762 BPSS1879		2509025	2507388	2554250	2555887	1725116	1723479	1238709	1237072	2625795	2624158	2581870	2580233	2583061	2581424	2535825	2535505	2354525	2352888
2763 BPSS1880		2510952	2509336	2552323	2553939	1727042	1725426	1240635	1239019	2627722	2626106	2583797	2582181	2584987	2583371	2537752	2536136	2356452	2354836
2764	BURPS1655_D1246																	2356637	2356473
2765	BURPS406E_D0445									2627907	2627743								
2766	BURPSPAST_AC0368													2585157	2585008				
2767 BPSS1881		2512399	2511116	2550876	2552159	1728449	1727166	1242042	1240759	2629169	2627886	2585204	2583921	2586419	2585136	2539159	2537876	2357899	2356616
2768	BURPS1106B_2622			2552138	2552302														
2769 BPSS1882		2512864	2512703	2550411	2550572	1728914	1728753	1242507	1242346	2629634	2629473	2585670	2585248	2586884	2586723	2539625	2539203	2358364	2358203

2770	BPSS1883			2513143	2514792	2550132	2548483	1729193	1730842	1242786	1244435	2629910	2631559	2585949	2587598	2587160	2588809	2539904	2541568	2358644	2360293
2771	BPSS1884			2515281	2514865	2547994	2548410	1731331	1730915	1244924	1244508	2632048	2631632	2588087	2587671	2589298	2588882	2542057	2541641	2360782	2360366
2772	BPSS1885			2516724	2515486	2546551	2547789	1732772	1731534	1246365	1245127	2633491	2632253	2589528	2588290	2590741	2589503	2543500	2542262	2362225	2360987
2773	BPSS1886			2517040	2516714	2546235	2546561	1733088	1732762	1246681	1246355	2633807	2633481	2589844	2589518	2591057	2590731	2543816	2543490	2362541	2362215
2774	BPSS1887			2517577	2517065	2545698	2546210	1733625	1733113	1247218	1246706	2634344	2633832	2590381	2589869	2591594	2591082	2544353	2543841	2363078	2362566
2775	BPSS1888			2518830	2517577	2544445	2545698	1734878	1733625	1248471	1247218	2635597	2634344	2591634	2590381	2592844	2591594	2545606	2544353	2364331	2363078
2776	BPSS1889			2519030	2520067	2544245	2543208	1735078	1736115	1248671	1249708	2635797	2636834	2591856	2592872	2593047	2594084	2545806	2546843	2364531	2364554
2777	BPSS1890			2521028	2520144	2542247	2543131	1737076	1736192	1250669	1249785	2637795	2636911	2593833	2592949	2595024	2594140	2547804	2546920	2366530	2365646
2778	BPSS1891			2521118	2522248	2542157	2541027	1737166	1738296	1250759	1251889	2637885	2639015	2593923	2595053	2595114	2596244	2547894	2549024	2366620	2367750
2779	BPSS1892			2522286	2523185	2540989	2540090	1738334	1739233	1251927	1252826	2639053	2639952	2595091	2595990	2596282	2597181	2549062	2549961	2367788	2368687
2780	BPSS1893			2523226	2523513	2540049	2539762	1739274	1739561	1252867	1253154	2639993	2640280	2596031	2596318	2597222	2597509	2550002	2550289	2368728	2369015
2781	BPSS1894			2523968	2523639	2539307	2539636	1740016	1739687	1253609	1253280	2640735	2640406	2596773	2596444	2597964	2597635	2550744	2550415	2369470	2369141
2782	BPSS1895			2524562	2523972	2538713	2539303	1740610	1740020	1254203	1253613	2641329	2640739	2597367	2596777	2598558	2597968	2551338	2550748	2370064	2369474
2783	BPSS1896			2526589	2524580	2536686	2538695	1742631	1740628	1256224	1254221	2643350	2641347	2599388	2597385	2600579	2598576	2553365	2551356	2372085	2370082
2784	BPSS1897			2527480	2526596	2535795	2536679	1743522	1742638	1257115	1256231	2644241	2643357	2600279	2599395	2601470	2600586	2554255	2553371	2372976	2372092
2785		BURPS1710A_A1855						1744124	1744294												
2786	BPSS1898			2530255	2528225	2533020	2535050	1746297	1744267	1259890	1257860	2647016	2644986	2603081	2601051	2604245	2602215	2557035	2555005	2375745	2373715
2787	BPSS1899			2530405	2531454	2532870	2531821	1746447	1747496	1260040	1261089	2647166	2648215	2603231	2604280	2604395	2605444	2557185	2558234	2375895	2376944
2788	BPSS1900			2532358	2531414	2530917	2531861	1748400	1747456	1261993	1261049	2649119	2648175	2605184	2604240	2606348	2605404	2559138	2558194	2377848	2376904
2789	BPSS1901			2532453	2533448	2530822	2529827	1748495	1749493	1262088	1263086	2649214	2650209	2605279	2606274	2606443	2607441	2559233	2560228	2377943	2378941
2790	BPSS1902			2534544	2533633	2528731	2529642	1750589	1749678	1264182	1263271	2651305	2650394	2607369	2606458	2608537	2607626	2561324	2560413	2380037	2379126
2791	BPSS1903			2534768	2536132	2528507	2527143	1750813	1752177	1264406	1265770	2651529	2652893	2607593	2608957	2608761	2610125	2561548	2562912	2380261	2381625
2792	BPSS1904			2536132	2536620	2527143	2526655	1752177	1752665	1265770	1266258	2652893	2653381	2608957	2609445	2610125	2610613	2562912	2563400	2381625	2382113
2793	BPSS1905			2536667	2537683	2526608	2525592	1752712	1753728	1266305	1267321	2653428	2654444	2609492	2610508	2610660	2611676	2563447	2564463	2382160	2383176
2794	BPSS1906			2537683	2538474	2525592	2524801	1753728	1754519	1267321	1268112	2654444	2655235	2610508	2611299	2611676	2612467	2564463	2565254	2383176	2383967
2795	BPSS1907			2539399	2538524	2523876	2524751	1755444	1754569	1269037	1268162	2656160	2655285	2612224	2611349	2613392	2612517	2566179	2565304	2384892	2384017
2796	BPSS1908			2539802	2540284	2523473	2522991	1755857	1756339	1269450	1269932	2656573	2657055	2612637	2613119	2613805	2614287	2566592	2567074	2385305	2385787
2797	BPSS1909			2540284	2540853	2522991	2522422	1756339	1756908	1269932	1270501	2657055	2657624	2613119	2613688	2614287	2614856	2567074	2567643	2385787	2386356
2798	BPSS1910			2540879	2541928	2522396	2521347	1756934	1757983	1270527	1271576	2657650	2658117	2613714	2614763	2614882	2615931	2567669	2568718	2386382	2387431
2799	BPSS1911			2542558	2544039	2520717	2519236	1758602	1760083	1272195	1273676	2659384	2660865	2615382	2616863	2616550	2618031	2569348	2570829	2388042	2389523
2800	BPSS1912		Bp_chr2_11_ISBma1															2571003	2572307		
2801	BPSS1913			2545852	2544293	2517416	2518975	1761903	1760344	1275496	1273937	2662699	2661140	2618648	2617089	2619851	2618292	2573955	2572396	2391336	2389777
2802			Bp_chr2_12_ISBma2													2620446	2622018				
2803	BPSS1914			2547424	2546537	2515844	2516731	1763433	1762546	1277026	1276139	2664229	2663342	2620178	2619291	2622977	2622090	2575485	2574598	2392880	2391993
2804	BPSS1915			2548138	2549514	2515130	2513754	1764147	1765523	1277740	1279116	2664943	2666319	2620892	2622268	2623691	2625067	2576199	2577575	2393592	2394968
2805	BPSS1916			2550857	2550114	2512411	2513154	1766865	1766122	1280458	1279715	2667661	2666918	2623618	2622875	2626410	2625667	2578926	2578183	2396318	2395575
2806	BPSS1917			2551522	2552301	2511746	2510967	1767530	1768309	1281123	1281902	2668326	2669105	2624283	2625062	2627075	2627854	2579591	2580370	2396983	2397762
2807	BPSS1918			2553496	2552513	2509772	2510755	1769504	1768521	1283097	1282114	2670300	2669317	2626257	2625274	2629049	2628066	2581565	2580582	2398957	2397974
2808	BPSS1919			2553723	2553499	2509545	2509769	1769731	1769507	1283324	1283100	2670527	2670303	2626484	2626260	2629276	2629052	2581792	2581568	2399184	2398960
2809	BPSS1920			2554468	2554893	2508800	2508375	1770483	1770908	1284076	1284501	2671279	2671704	2627243	2627668	2630021	2630446	2582530	2582955	2399915	2400340
2810	BPSS1921			2555629	2555309	2507639	2507959	1771608	1771288	1285201	1284881	2672424	2672104	2628375	2628055	2631182	2630862	2583663	2583343	2401060	2400740
2811	BPSS1922			2555978	2556853	2507290	2506415	1771957	1772832	1285550	1286425	2672773	2673648	2628724	2629599	2631531	2632406	2584012	2584887	2401415	2402290
2812	BPSS1923			2557183	2557569	2506085	2505699	1773161	1773547	1286754	1287140	2673989	2674375	2629950	2630336	2632736	2633122	2585228	2585614	2402630	2403016
2813	BPSS1924			2558813	2558166	2504455	2505102	1774802	1774155	1288395	1287748	2675610	2674963	2631590	2630943	2634366	2633719	2586849	2586202	2404271	2403624
2814	BPSS1925			2560443	2559268	2502825	2504000	1776339	1775164	1289932	1288757	2677117	2675966	2633175	2631988	2636003	2634828	2588349	2587198	2405790	2404615
2815	BPSS1926			2561385	2560744	2501883	2502524	1777281	1776640	1290874	1290233	2678059	2677418	2634117	2633476	2636945	2636304	2589291	2588650	2406732	2406091
2816		BURPS1710b_A1030								1292787	1294640										
2817	BPSS1927			2563317	2561629	2499951	2501639	1779213	1777525	1292806	1291118	2679991	2678303	2636049	2634361	2638877	2637189	2591223	2589535	2408664	2406976
2818	BPSS1928			2564208	2565146	2499060	2498122	1780106	1781044	1293699	1294637	2680883	2681821	2636940	2637878	2639767	2640705	2592114	2593052	2409554	2410492
2819	BPSS1929			2566668	2565187	2496600	2498081	1782566	1781085	1296159	1294678	2683343	2681862	2639400	2637919	2642227	2640746	2594574	2593093	2412014	2410533
2820	BPSS1930			2567731	2566649	2495537	2496619	1783629	1782547	1297222	1296140	2684406	2683324	2640463	2639381	2643296	2642208	2595637	2594555	2413077	2411995
2821	BPSS1931			2568426	2567731	2494842	2495537	1784324	1783629	1297917	1297222	2685101	2684406	2641158	2640463	2643991	2643296	2596332	2595637	2413772	2413077
2822	BPSS1932			2569565	2568432	2493703	2494836	1785463	1784330	1299056	1297923	2686240	2685107	2642297	2641164	2645130	2643997	2597471	2596338	2414911	2413778
2823	BPSS1933			2569813	2569565	2493455	2493703	1785711	1785463	1299304	1299056	2686488	2686240	2642545	2642297	2645378	2645130	2597719	2597471	2415159	2414911
2824	BPSS1934			2570316	2569849	2492952	2493419	1786214	1785747	1299807	1299340	2686991	2686524	2643048	2642581	2645881	2645414	2598222	2597755	2415662	2415195
2825	BPSS1935			2571398	2570535	2491870	2492733	1787296	1786433	1300889	1300026	2688073	2687210	2644130	2643267	2646949	2646695	2599304	2598441	2416744	2415881
2826	BPSS1936			2571692	2573215	2491576	2490053	1787583	1789106	1301176	1302699	2688367	2689890	2644424	2645947	2647236	2648759	2599598	2601121	2417038	2418561
2827	BPSS1937			2573215	2574285	2490053	2488983	1789106	1790176	1302699	1303769	2689890	2690960	2645947	2647017	2648759	2649829	2601121	2602191	2418561	2419631

2828	BPSS1938		2574285	2577482	2488983	2485786	1790176	1791957	1303769	1307014	2690960	2694205	2647017	2650283	2649829	2653086	2602191	2605436	2419631	2422885
2829	BPSS1939		2577488	2578609	2485780	2484659	1793428	1794549	1307020	1308141	2694211	2695332	2650289	2651410	2653092	2654213	2605442	2606563	2422891	2424012
2830	BPSS1940		2578762	2580594	2484506	2482674	1794702	1796534	1308294	1310126	2695485	2697317	2651563	2653395	2654366	2656198	2606716	2608548	2424165	2425997
2831	BPSS1941		2582500	2580938	2480768	2482330	1798434	1796872	1312026	1310464	2699253	2697691	2655331	2653769	2658128	2656566	2610490	2608928	2427939	2426377
2832	BPSS1942		2583041	2583547	2480227	2479721	1798975	1799481	1312567	1313073	2699794	2700300	2655872	2656378	2658669	2659175	2611031	2611537	2428480	2428986
2833	BPSS1943		2584111	2583611	2479157	2479657	1800045	1799545	1313637	1313137	2700864	2700364	2656942	2656442	2659739	2659239	2612101	2611601	2429550	2429050
2834	BPSS1944		2585213	2584191	2478055	2479077	1801130	1800108	1314722	1313700	2701966	2700944	2658044	2657022	2660841	2659819	2613203	2612181	2430652	2429630
2835		BURPS406E_D0353									2702200	2702036								
2836	BPSS1945		2586320	2585475	2476948	2477793	1802213	1801368	1315805	1314960	2702928	2702200	2659095	2658250	2661916	2661071	2614246	2613401	2431715	2430870
2837		BURPS1106B_2532			2477793	2478014														
2838	BPSS1946		2588317	2586320	2474951	2476948	1804204	1802213	1317796	1315805	2705045	2703054	2661104	2659095	2663913	2661916	2616231	2614246	2433712	2431718
2839	BPSS1947		2589050	2588304	2474218	2474964	1804937	1804191	1318529	1317783	2705778	2705032	2661837	2661091	2664646	2663900	2616964	2616218	2434445	2433699
2840	BPSS1948		2589312	2589067	2473956	2474201	1805199	1804954	1318791	1318546	2706040	2705795	2662099	2661854	2664908	2664663	2617226	2616981	2434707	2434462
2841	BPSS1949		2590010	2589312	2473258	2473956	1805897	1805199	1319489	1318791	2706738	2706040	2662797	2662099	2665606	2664908	2617924	2617226	2435405	2434707
2842	BPSS1950		2590306	2590010	2472962	2473258	1806193	1805897	1319785	1319489	2707034	2706738	2663093	2662797	2665902	2665606	2618220	2617924	2435701	2435405
2843	BPSS1951		2590784	2590311	2472484	2472957	1806656	1806198	1320248	1319790	2707497	2707039	2663571	2663098	2666380	2665907	2618799	2618752	2436164	2435706
2844	BPSS1952		2591260	2590808	2472008	2472460	1807138	1806686	1320730	1320278	2707979	2707527	2664047	2663595	2666856	2666404	2619275	2618823	2436646	2436194
2845	BPSS1953		2592773	2591253	2470495	2472015	1808651	1807131	1322243	1320723	2709540	2707972	2665560	2664040	2668369	2666849	2620788	2619268	2438171	2436639
2846	BPSS1954		2593197	2594987	2470071	2468281	1809075	1810865	1322667	1324457	2709964	2711754	2666002	2667792	2668793	2670583	2621212	2623002	2438613	2440403
2847	BPSS1955		2595001	2596401	2468267	2466867	1810879	1812279	1324471	1325871	2711768	2713168	2667806	2669206	2670597	2671997	2623016	2624416	2440417	2441817
2848	BPSS1956		2596401	2597576	2466867	2465692	1812279	1813454	1325871	1327046	2713168	2714343	2669206	2670381	2671997	2673172	2624416	2625591	2441817	2442992
2849	BPSS1957		2599248	2598310	2464020	2464958	1815128	1814190	1328720	1327782	2715997	2715059	2672128	2671190	2674653	2673715	2627265	2626327	2444473	2443535
2850		BURPS1106B_2517			2464958	2465383														
2851		BURPS406E_D0336									2716996	2717655								
2852	BPSS1958		2600297	2599653	2462971	2463615	1816275	1815631	1329860	1329216	2717018	2716374	2673177	2672533	2675632	2674988	2628300	2627656	2445564	2444920
2853	BPSS1959		2601259	2600870	2461991	2462380	1817247	1816858	1330832	1330443	2718035	2717646	2674221	2673832	2676621	2676232	2629289	2628900	2446571	2446182
2854		BURPS406E_D0335									2718316	2718032								
2855		BURPSS13_X0801															2629609	2629286		
2856	BPSS1960		2602890	2601562	2460360	2461688	1818887	1817559	1332472	1331144	2719641	2718316	2675899	2674571	2678253	2676925	2630937	2629609	2448227	2446899
2857		BURPS1655_D1133																	2448717	2448457
2858	BPSS1961		2603569	2603745	2459681	2459505	1819566	1819742	1333151	1333327	2720320	2720496	2676682	2676858	2678932	2679108	2631616	2631792	2449010	2449186
2859	BPSS1962		2604830	2604051	2458420	2459199	1820827	1820048	1334412	1333633	2721581	2720802	2677943	2677164	2680193	2679414	2632877	2632098	2450259	2449480
2860	BPSS1963		2606118	2604847	2457132	2458403	1822115	1820844	1335700	1334429	2722869	2721598	2679231	2677960	2681481	2680210	2634165	2632894	2451547	2450276
2861	BPSS1964		2607877	2606681	2455508	2456242	1823856	1822660	1337441	1336245	2724593	2723520	2681047	2679806	2683222	2682026	2635912	2634716	2453318	2452068
2862	BPSS1965		2608650	2608009	2454735	2455376	1824629	1823988	1338214	1337573	2725366	2724725	2681820	2681179	2683995	2683354	2636685	2636044	2454091	2453450
2863	BPSS1966		2608924	2609826	2454461	2453559	1824903	1825805	1338488	1339390	2725640	2726548	2682103	2683005	2684269	2685171	2636959	2637861	2454365	2455267
2864	BPSS1967		2610693	2610043	2452692	2453342	1826672	1826022	1340257	1339607	2727415	2726765	2683872	2683222	2686038	2685388	2638728	2638078	2456134	2455484
2865	BPSS1968		2610796	2611701	2452589	2451684	1826775	1827680	1340360	1341265	2727518	2728423	2683975	2684880	2686141	2687046	2638831	2639736	2456237	2457142
2866	BPSS1969		2611701	2613296	2451684	2450089	1827680	1829275	1341265	1342860	2728423	2730018	2684880	2686475	2687046	2688641	2639736	2641331	2457142	2458737
2867	BPSS1970		2613296	2614276	2450089	2449109	1829275	1830255	1342860	1343840	2730018	2730998	2686475	2687455	2688641	2689621	2641331	2642311	2458737	2459717
2868	BPSS1971		2614941	2616524	2448444	2446861	1830929	1832512	1344514	1346097	2731689	2733272	2688118	2689701	2690304	2691887	2643002	2644585	2460382	2461965
2869	BPSS1972		2616687	2617133	2446698	2446252	1832675	1833121	1346260	1346706	2733435	2733881	2689864	2690310	2692050	2692496	2644748	2645194	2462128	2462574
2870	BPSS1973		2618072	2620003	2445313	2443382	1834060	1835991	1347645	1349576	2734821	2736752	2691249	2693180	2693435	2695111	2646125	2648056	2463506	2465437
2871	BPSS1974		2620970	2622664	2442415	2440721	1836795	1838408	1350380	1352140	2737563	2739368			2696171	2697931	2648954	2650681	2466290	2468077
2872	BPSS1975		2622758	2624038	2440627	2439347	1838650	1839930	1352234	1353514	2739462	2740742	2696058	2697338	2698025	2699305	2650775	2652055	2468172	2469452
2873		BURPS1106B_2492			2441823	2442641														
2874	BPSS1976		2624771	2626138	2438614	2437247	1840755	1842122	1354339	1355706	2741513	2742880	2698085	2699452	2700104	2701471	2652826	2654193	2470218	2471585
2875	BPSS1977		2626173	2627498	2437212	2435887	1842157	1843482	1355741	1357066	2742915	2744240	2699487	2700812	2701506	2702831	2654228	2655553	2471620	2472945
2876	BPSS1978		2627626	2629986	2435759	2433399	1843610	1845970	1357194	1359554	2744368	2746728	2700940	2703300	2702959	2705319	2655681	2658041	2473073	2475433
2877	BPSS1979		2630543	2631649	2432842	2431736	1846527	1847633	1360111	1361217	2747285	2748391	2703857	2704969	2705876	2706982	2658598	2659704	2475990	2477096
2878	BPSS1980		2631775	2632275	2431610	2431110	1847759	1848259	1361343	1361843	2748517	2749017	2705095	2705583	2707108	2707608	2659830	2660330	2477222	2477722
2879	BPSS1981		2632561	2632833	2430824	2430552	1848545	1848817	1362129	1362401	2749303	2749575	2705871	2706143	2707894	2708166	2660616	2660888	2478008	2478280
2880	BPSS1982		2632849	2634114	2430536	2429271	1848833	1850098	1362417	1363682	2749591	2750856	2706159	2707424	2708182	2709447	2660904	2662169	2478296	2479561
2881	BPSS1983		2634897	2635946	2428488	2427439	1850881	1851930	1364465	1365514	2751639	2752688	2708207	2709256	2710230	2711279	2662952	2664001	2480344	2481393
2882	BPSS1984		2636021	2638924	2427364	2424461	1852005	1854908	1365589	1368492	2752763	2755666	2709331	2712234	2711354	2714257	2664076	2666979	2481468	2484371
2883	BPSS1985		2638939	2639703	2424446	2423682	1854923	1855687	1368507	1369271	2755681	2756445	2712249	2713013	2714272	2715036	2666994	2667758	2484386	2485150
2884	BPSS1986		2639751	2640503	2423634	2422882	1855735	1856487	1369319	1370071	2756493	2757245	2713061	2713813	2715084	2715836	2667806	2668558	2485198	2485950
2885	BPSS1987		2640507	2642126	2422878	2421259	1856491	1858110	1370075	1371694	2757249	2758868	2713817	2715436	2715840	2717459	2668562	2670181	2485954	2487573

2886	BPSS1988		2642126	2643139	2421259	2420246	1858110	1859123	1371694	1372707	2758868	2759881	2715436	2716449	2717459	2718472	2670181	2671194	2487573	2488586
2887	BPSS1989		2643165	2644316	2420220	2419069	1859149	1860300	1372733	1373884	2759907	2761058	2716475	2717626	2718498	2719649	2671220	2672371	2488612	2489763
2888	BPSS1990		2645175	2644426	2418210	2418959	1861159	1860410	1374743	1373994	2761917	2761168	2718485	2717736	2720508	2719759	2673230	2672481	2490622	2489873
2889	BPSS1991		2645446	2646117	2417939	2417268	1861430	1862101	1375014	1375685	2762188	2762859	2718756	2719427	2720779	2721450	2673501	2674172	2490893	2491564
2890	BPSS1992		2648147	2646258	2415238	2417127	1864131	1862242	1377715	1375826	2764889	2763000	2721466	2719577	2723480	2721591	2676211	2674322	2493603	2491714
2891	BPSS1993		2649991	2648492	2413394	2414893	1865933	1864434	1379517	1378018	2766733	2765234	2723310	2721811	2725324	2723825	2678055	2676556	2495447	2493948
2892	BPSS1994		2651692	2652381	2411693	2411004	1867634	1868323	1381218	1381907	2768425	2769114	2725011	2725700	2727034	2727723	2679756	2680445	2497175	2497864
2893	BPSS1995		2652381	2653832	2411004	2409553	1868323	1869774	1381907	1383358	2769114	2770565	2725700	2727151	2727723	2729174	2680445	2681896	2497864	2499315
2894	BPSS1996		2653935	2654324	2409450	2409061	1869877	1870266	1383461	1383850	2770668	2771057	2727254	2727643	2729277	2729666	2681999	2682388	2499418	2499807
2895	BPSS1997		2655289	2654483	2408096	2408902	1871231	1870425	1384815	1384009	2772022	2771216	2728608	2727802	2730631	2729825	2683353	2682547	2500772	2499966
2896	BPSS1998		2658800	2655939	2404585	2407446	1874717	1871856	1388301	1385440	2775533	2772672	2732119	2729258	2734117	2731256	2686864	2684003	2504283	2501422
2897	BPSS1999		2660041	2659208	2403344	2404177	1875958	1875125	1389542	1388709	2776774	2775941	2733360	2732527	2735364	2734531	2688105	2687272	2505524	2504691
2898	BPSS2000		2660396	2660058	2402989	2403327	1876313	1875975	1389897	1389559	2777129	2776791	2733715	2733377	2735719	2735381	2688460	2688122	2505879	2505541
2899	BPSS2001		2661605	2661360	2401780	2402025	1877522	1877277	1391106	1390861	2778338	2778093	2734924	2734679	2736928	2736683	2689669	2689424	2507088	2506843
2900	BPSS2002		2662671	2661937	2400714	2401448	1878588	1877854	1392172	1391438	2779404	2778670	2735990	2735256	2737994	2737260	2690735	2690001	2508154	2507420
2901	BPSS2003		2663139	2663624	2400246	2399761	1879056	1879541	1392640	1393125	2779872	2780357	2736458	2736943	2738462	2738947	2691203	2691688	2508622	2509107
2902	BPSS2004		2664274	2664002	2399111	2399383	1880191	1879919	1393775	1393503	2781007	2780735	2737593	2737396	2739597	2739325	2692338	2692066	2509756	2509499
2903	BPSS2005		2664590	2664282	2398795	2399103	1880507	1880199	1394091	1393783	2781323	2781015	2737897	2737601	2739913	2739605	2692654	2692346	2510072	2509764
2904		BURPS1106B_2438			2393442	2392330														
2905		BURPS1106B_2439			2395167	2393467														
2906		BURPS1106B_2440			2396252	2395218														
2907		BURPS1106B_2441			2397919	2396303														
2908		BURPS1106B_2443			2398733	2398539														
2909		BURPS1655_D1063																	2515425	2516537
2910		BURPS1655_D1064																	2513700	2515400
2911		BURPS1655_D1065																	2512576	2513649
2912		BURPS1655_D1066																	2510948	2512564
2913		BURPS1710A_A2015					1880569	1880763												
2914		BURPS1710A_A2017					1881383	1882999												
2915		BURPS1710A_A2018					1883050	1884084												
2916		BURPS1710A_A2019					1884135	1885835												
2917		BURPS1710A_A2020					1885860	1886972												
2918		BURPS1710b_A1115							1394967	1396583										
2919		BURPS1710b_A1116							1396595	1397668										
2920		BURPS1710b_A1117							1397719	1399419										
2921		BURPS1710b_A1118							1399516	1400556										
2922		BURPS406E_D0263									2786676	2787788								
2923		BURPS406E_D0264									2784951	2786651								
2924		BURPS406E_D0265									2783827	2784900								
2925		BURPS406E_D0266									2782199	2783815								
2926		BURPSS13_X0871															2693530	2695146		
2927		BURPSS13_X0872															2695158	2696231		
2928		BURPSS13_X0873															2696282	2697982		
2929		BURPSS13_X0874															2698007	2699119		
2930	BPSS2006		2671297	2671160	2392088	2392225	1887214	1887077	1400798	1400661	2788030	2787893	2744603	2744466	2740113	2739976	2699361	2699224	2516779	2516642
2931	BPSS2007		2672475	2671312	2390910	2392073	1888392	1887229	1401976	1400813	2789208	2788045	2745781	2744618	2741291	2740128	2700539	2699376	2517951	2516794
2932	BPSS2008		2672846	2673463	2390539	2389922	1888763	1889380	1402347	1402964	2789579	2790196	2746146	2746769	2741656	2742279	2700910	2701527	2518322	2518939
2933	BPSS2009		2675511	2673691	2387874	2389694	1891422	1889608	1405006	1403192	2792238	2790424	2748805	2746991	2744321	2742507	2703575	2701755	2520981	2519167
2934	BPSS2010		2676637	2675543	2386748	2387842	1892550	1891432	1406134	1405016	2793366	2792248	2749933	2748815	2745449	2744331	2704703	2703585	2522109	2520991
2935	BPSS2011		2678842	2676731	2384543	2386654	1894763	1892646	1408347	1406230	2795573	2793462	2752129	2750027	2747656	2745545	2706910	2704799	2524316	2522205
2936	BPSS2012		2679987	2679175	2383398	2384210	1895908	1895096	1409492	1408680	2796718	2795906	2753274	2752462	2748801	2747989	2708055	2707243	2525461	2524649
2937	BPSS2013		2680983	2680003	2382402	2383382	1896937	1895924	1410521	1409508	2797759	2796734	2754351	2753290	2749815	2748817	2709066	2708071	2526493	2525477
2938	BPSS2014		2682313	2681339	2381072	2382046	1898168	1897194	1411752	1410778	2798990	2798016	2755582	2754608	2751046	2750072	2710423	2709449	2527724	2526750
2939	BPSS2015		2683708	2682335	2379677	2381050	1899563	1898190	1413147	1411774	2800385	2799012	2756977	2755604	2752441	2751068	2711815	2710445	2529119	2527746
2940	BPSS2016		2685159	2686370	2378226	2377015	1901005	1902216	1414589	1415800	2801839	2803050	2758381	2759592	2753883	2755094	2713263	2714474	2530561	2531772
2941		BURPS1710A_A2039					1903051	1902812												
2942	BPSS2017		2688521	2687646	2374864	2375739	1904366	1903491	1417950	1417075	2805211	2804336	2761746	2760871	2757251	2756376	2716623	2715748	2533963	2533088
2943	BPSS2018		2688657	2689412	2374728	2373973	1904502	1905257	1418086	1418841	2805347	2806102	2761882	2762637	2757387	2758142	2716759	2717514	2534099	2534854

2944	BPSS2019			2689559	2689888	2373826	2373497	1905404	1905733	1418988	1419317	2806249	2806560	2762784	2763113	2758289	2758618	2717661	2717990	2535001	2535321
2945	BPSS2020			2690129	2690554	2373256	2372831	1905974	1906399	1419558	1419983	2806801	2807226	2763342	2763767	2758859	2759284	2718231	2718656	2535559	2535984
2946	BPSS2021			2692258	2690780	2371127	2372605	1908103	1906625	1421687	1420209	2808942	2807464	2765471	2763993	2760988	2759510	2720372	2718894	2537700	2536222
2947	BPSS2022			2692428	2693300	2370957	2370085	1908273	1909145	1421857	1422729	2809112	2809984	2765641	2766513	2761158	2762030	2720542	2721414	2537870	2538742
2948	BPSS2023			2693667	2693927	2369718	2369458	1909512	1909772	1423096	1423356	2810365	2810625	2766867	2767127	2762396	2762656	2721795	2722055	2539109	2539369
2949	BPSS2024			2695150	2693945	2368235	2369440	1910995	1909790	1424579	1423374	2811848	2810643	2768350	2767145	2763879	2762674	2723278	2722073	2540592	2539387
2950		BURPS1655_D1033																		2540896	2541054
2951	BPSS2025			2697127	2695607	2366258	2367778	1913051	1911531	1426635	1425115	2813872	2812352	2770378	2768858	2765895	2764375	2725263	2723743	2542601	2541081
2952	BPSS2026			2699016	2697406	2364369	2365979	1914940	1913330	1428524	1426914	2815761	2814151	2772267	2770657	2767784	2766174	2727152	2725542	2544490	2542880
2953	BPSS2027			2699984	2699613	2363401	2363772	1915928	1915557	1429512	1429141	2816744	2816373	2773265	2772894	2768772	2768401	2728120	2727749	2545458	2545087
2954	BPSS2028			2700835	2700206	2362550	2363179	1916779	1916150	1430363	1429734	2817595	2816966	2774116	2773490	2769623	2768994	2728971	2728342	2546309	2545680
2955	BPSS2029			2703602	2702433	2359783	2360952	1918189	1917020	1431773	1430604	2819005	2817836	2775526	2774357	2771033	2769864	2730381	2729212	2547719	2546550
2956		BURPS1106B_2404				2362219	2361116														
2957	BPSS2030			2703919	2704788	2359466	2358597	1918504	1919373	1432088	1432957	2819320	2820189	2775840	2776712	2771348	2772217	2730693	2731565	2548033	2548905
2958	BPSS2031			2704793	2706406	2358592	2356979	1919378	1920991	1432962	1434575	2820194	2821807	2776717	2778330	2772222	2773835	2731570	2733183	2548910	2550523
2959	BPSS2032			2706422	2707570	2356963	2355815	1921007	1922155	1434591	1435739	2821823	2822971	2778346	2779494	2773851	2774999	2733199	2734347	2550539	2551687
2960	BPSS2033			2707580	2708419	2355805	2354966	1922165	1923004	1435749	1436588	2822981	2823820	2779504	2780343	2775376	2775849	2734357	2735196	2551697	2552536
2961	BPSS2034			2708419	2710422	2354966	2352963	1923004	1925013	1436588	1438597	2823820	2825829	2780343	2782352	2775849	2777858	2735196	2737205	2552536	2554545
2962	BPSS2035			2710460	2711341	2352925	2352044	1925051	1925932	1438635	1439516	2825867	2826748	2782390	2783271	2777896	2778777	2737243	2738124	2554584	2555465
2963	BPSS2036			2711707	2713365	2351678	2350020	1926298	1927956	1439882	1441540	2827114	2828772	2783637	2785295	2779143	2780801	2738490	2740148	2555831	2557489
2964		BURPS1655_D1018																		2557471	2558283
2965	BPSS2037			2714472	2715380	2348913	2348005	1929063	1929971	1442647	1443555	2829878	2830786	2786401	2787309	2781907	2782815	2741255	2742163	2558594	2559502
2966	BPSS2038			2715409	2715690	2347976	2347695	1930000	1930281	1443584	1443865	2830815	2831096	2787338	2787619	2782844	2783125	2742192	2742473	2559531	2559812
2967	BPSS2039			2715706	2716638	2347679	2346747	1930297	1931229	1443881	1444813	2831112	2832044	2787635	2788567	2783141	2784073	2742489	2743421	2559828	2560760
2968	BPSS2040			2716638	2717675	2346747	2345710	1931229	1932266	1444813	1445850	2832044	2833081	2788567	2789604	2784073	2785110	2743421	2744458	2560760	2561794
2969	BPSS2041			2717719	2718741	2345666	2344644	1932310	1933332	1445894	1446916	2833125	2834147	2789648	2790670	2785154	2786176	2744502	2745524	2561841	2562863
2970	BPSS2042			2718741	2720591	2344644	2342794	1933332	1935182	1446916	1448766	2834147	2835997	2790670	2792520	2786176	2788026	2745524	2747374	2562863	2564713
2971	BPSS2043			2720608	2721024	2342777	2342361	1935199	1935615	1448783	1449199	2836014	2836430	2792537	2792953	2788043	2788459	2747391	2747807	2564730	2565146
2972	BPSS2044			2721196	2722002	2342189	2341383	1935787	1936593	1449371	1450177	2836602	2837408	2793125	2793931	2788631	2789437	2747979	2748785	2565318	2566124
2973	BPSS2045			2722475	2723068	2340910	2340317	1937066	1937659	1450650	1451243			2794403	2794996	2789910	2790503	2749258	2749851	2566598	2567191
2974		BURPS1655_D1007																		2567402	2568067
2975	BPSS2046			2723308	2724033	2340077	2339352	1937899	1938624	1451483	1452208	2837835	2838560	1647435	1648160	2790743	2791468	2750091	2750816	2571181	2570456
2976	BPSS2047											2840856	2839426	1649346	1650776						
2977	BPSS2048											2839400	2838747	1650802	1651455					2569616	2570269
2978		BURPS668_A1700												1651544	1652983						
2979		BURPS668_A1701												1653717	1653869						
2980		BURPS668_A1702												1653839	1654795						
2981	BPSS2049			2723308	2724033	2340077	2339352	1937899	1938624	1451483	1452208	2841804	2841079	1655622	1654897	2790743	2791468	2750091	2750816	2571181	2570456
2982		BURPS1710A_A2084						1939271	1938831												
2983			Bp_chr2_13_IS40	2725193	2726428	2336957	2338192	1939784	1941019	1453368	1454603					2792628	2793863	2751976	2753211		
2984		BURPS1106B_2379				2338705	2339145														
2985	BPSS2050																			2571394	2571224
2986	BPSS2051			2726984	2726430	2336401	2336955	1941575	1941024	1455159	1454608					2794419	2793868	2753767	2753213	2572952	2571816
2987	BPSS2052			2728711	2726987	2334674	2336398	1943302	1941578	1456886	1455162	2843957	2842233			2796146	2794422	2755494	2753770	2574679	2572955
2988		BURPS1106B_2373				2334817	2334668														
2989		BURPS406E_D0204										2844182	2844379								
2990	BPSS2053			2738612	2729382	2324773	2334003	1953203	1943973	1466787	1457557	2853978	2844928			2806047	2796817	2765395	2756165	2584580	2575350
2991		BURPS1655_D0995																		2590419	2585824
2992		BURPS1655_D0996																		2585732	2585550
2993	BPSS2054			2744448	2739820	2318937	2323565	1959039	1954411	1472623	1467995	2859814	2855186			2811883	2807255	2771231	2766603		
2994	BPSS2055			2745730	2744468	2317655	2318917	1960321	1959059	1473905	1472643	2861096	2859834			2813165	2811903	2772513	2771251	2591674	2590412
2995	BPSS2056			2747950	2745749	2315435	2317636	1962541	1960340	1476125	1473924	2863314	2861944			2815385	2813184	2774733	2772532	2593894	2591693
2996		BURPS1655_D0990																		2596358	2595351
2997		BURPS406E_D0196										2865667	2864771								
2998	BPSS2057		Bp_chr2_14_ISBma1									2866427	2867102							2596662	2597337
2999		BURPS1655_D0988																		2597977	2598546
3000		BURPS1655_D0989																		2597354	2597821
3001		BURPS406E_D0195										2867119	2867586								

3002	BPSS2058										2869930	2869334							2600166	2599744
3003	BPSS2059										2870365	2869958							2600601	2600194
3004	BPSS2060																		2601771	2600719
3005	BPSS2061																		2601994	2602635
3006		BURPS1655_D0983																	2603706	2603137
3007	BPSS2061A																		2604200	2603994
3008	BPSS2062																		2604852	2604418
3009	BPSS2063																		2605414	2604815
3010	BPSS2064																		2607168	2606092
3011	BPSS2065																		2609019	2607583
3012	BPSS2066																		2610405	2609224
3013	BPSS2067																		2611312	2610419
3014	BPSS2068																		2612076	2611309
3015	BPSS2069																		2612954	2612094
3016	BPSS2070																		2613919	2612954
3017	BPSS2071																		2614938	2613943
3018	BPSS2072																		2616178	2614979
3019	BPSS2073																		2616902	2616192
3020	BPSS2074																		2617034	2617921
3021	BPSS2074a																		2618159	2617986
3022	BPSS2075																		2618995	2618369
3023	BPSS2076		2749895	2749506	2313490	2313879	1964486	1964097	1478070	1477681	2871765	2871157			2817330	2816941	2776678	2776289	2620565	2619276
3024	BPSS2078		2750272	2749928	2313113	2313457	1964863	1964519	1478447	1478103	2872142	2871798			2817707	2817363	2777055	2776711	2620942	2620598
3025		BURPSPAST_AC0115													2818390	2819241				
3026		BURPSPAST_AC0116													2818130	2818393				
3027	BPSS2079		2750676	2750272	2312709	2313113	1965267	1964863	1478851	1478447	2872546	2872142			2819323	2819270	2777459	2777055	2621346	2620942
3028	BPSS2080		2753142	2751112	2310243	2312273	1967733	1965703	1481317	1479287	2875012	2872982			2821789	2819759	2779925	2777895	2623812	2621782
3029	BPSS2081		2755554	2753386	2307831	2309999	1970145	1967977	1483729	1481561	2877424	2875256			2824201	2822033	2782337	2780169	2626224	2624056
3030	BPSS2082		2756410	2755562	2306975	2307823	1971001	1970153	1484585	1483737	2878280	2877432			2825057	2824209	2783193	2782345	2627080	2626232
3031	BPSS2083		2757364	2756414	2306021	2306971	1971955	1971005	1485539	1484589	2879234	2878284			2826011	2825061	2784147	2783197	2628034	2627084
3032	BPSS2084		2758638	2757400	2304747	2305985	1973229	1971991	1486813	1485575	2880508	2879270			2827285	2826047	2785421	2784183	2629308	2628070
3033	BPSS2085		2759918	2758677	2303467	2304708	1974509	1973268	1488093	1486852	2881788	2880547			2828565	2827324	2786701	2785460	2630588	2629347
3034	BPSS2086		2761931	2759964	2301454	2303421	1976521	1974938	1488528	1488139	2883801	2881834			2830577	2828994	2788714	2786747	2632601	2630634
3035	BPSS2087		2763430	2762375	2299955	2301010	1978020	1976965	1491604	1490549	2885300	2884245			2832076	2831021	2790213	2789158	2634100	2633045
3036	BPSS2088		2763723	2763983	2299662	2299402	1978314	1978574	1491898	1492158	2885594	2885851			2832370	2832630	2790507	2790767	2634394	2634654
3037	BPSS2089		2764823	2765605	2298562	2297780	1979414	1980196	1492998	1493780	2887113	2887895			2833470	2834252	2791607	2792389	2635495	2636277
3038	BPSS2090		2766768	2766343	2296617	2297042	1981359	1980934	1494943	1494518	2889058	2888633			2835415	2834990	2793552	2793127	2637440	2637015
3039		BURPS1655_D0942																	2647566	2646751
3040		BURPS1655_D0943																	2644915	2644766
3041		BURPS1655_D0944																	2644495	2644202
3042		BURPS1655_D0945																	2644205	2643315
3043		BURPS1655_D0947																	2641757	2640938
3044		BURPS1655_D0948																	2639474	2639569
3045		BURPS1655_D0950																	2638257	2637809
3046	BPSS2091		2767545	2766889	2295840	2296496	1982136	1981480	1495720	1495064	2889833	2889099			2836192	2835536	2794329	2793673	2649512	2648955
3047		BURPS1655_D0941																	2649584	2647719
3048	BPSS2092		2768269	2767562	2295116	2295823	1982860	1982153	1496444	1495737	2890557	2889850	2802572	2801919	2836916	2836209	2795053	2794346	2650236	2649529
3049	BPSS2093		2770489	2768288	2292896	2295097	1985080	1982879	1498664	1496463	2892777	2890576	2804792	2802591	2839136	2836935	2797273	2795072	2652456	2650255
3050	BPSS2094		2773134	2770489	2290251	2292896	1987725	1985080	1501309	1498664	2895422	2892777	2807437	2804792	2841781	2839136	2799918	2797273	2655101	2652456
3051	BPSS2095		2774610	2773186	2288775	2290199	1989200	1987725	1502784	1501309	2896898	2895474	2808900	2807437	2843256	2841781	2801394	2799970	2656577	2655153
3052	BPSS2096		2776478	2774610	2286907	2288775	1991068	1989200	1504652	1502784	2898766	2896898	2810768	2808900	2845124	2843256	2803262	2801394	2658445	2656577
3053	BPSS2097		2777019	2776486	2286366	2286899	1991600	1991076	1505184	1504660	2899298	2898774	2811300	2810776	2845656	2845132	2803803	2803270	2658989	2658453
3054	BPSS2098		2777541	2777053	2285844	2286332	1992143	1991655	1505727	1505239	2899841	2899353	2811843	2811355	2846199	2845711	2804325	2803837	2659532	2659044
3055	BPSS2099		2779109	2777604	2284276	2285781	1993711	1992206	1507295	1505790	2901409	2899904	2813411	2811906	2847767	2846262	2805893	2804388	2661100	2659595
3056	BPSS2100		2779680	2779105	2283705	2284280	1994282	1993707	1507866	1507291	2901980	2901405	2813982	2813407	2848338	2847763	2806464	2805889	2661671	2661096
3057	BPSS2101		2780822	2779746	2282563	2283639	1995424	1994348	1509008	1507932	2903122	2902046	2815124	2814048	2849480	2848404	2807606	2806530	2662813	2661737
3058	BPSS2102		2783460	2780878	2279925	2282507	1998128	1995480	1511712	1509064	2905783	2903756	2817774	2815180	2852166	2849536	2810183	2808186	2665475	2662869
3059	BPSS2103		2784639	2783674	2278746	2279711	1999253	1998342	1512837	1511926	2906944	2905997	2818980	2818249	2853291	2852380	2811344	2810397	2666652	2665687

3060 BPSS2104		2788265	2784639	2275120	2278746	2002879	1999253	1516463	1512837	2910570	2906944	2822606	2818980	2856917	2853291	2814970	2811344	2670278	2666652
3061 BPSS2105		2789584	2788271	2273801	2275114	2004198	2002885	1517782	1516469	2911889	2910576	2823925	2822612	2858236	2856923	2816289	2814976	2671597	2670284
3062 BPSS2106		2790988	2789603	2272397	2273782	2005602	2004217	1519186	1517801	2913293	2911908	2825329	2823944	2859640	2858255	2817693	2816308	2673001	2671616
3063 BPSS2107		2791518	2791003	2271867	2272382	2006132	2005617	1519716	1519201	2913823	2913308	2825859	2825344	2860170	2859655	2818223	2817708	2673531	2673016
3064 BPSS2108		2792209	2792556	2271176	2270829	2006823	2007170	1520407	1520754	2914514	2914861	2826550	2826897	2860861	2861208	2818914	2819261	2674222	2674569
3065 BPSS2109		2792614	2794047	2270771	2269338	2007228	2008661	1520812	1522245	2914919	2916352	2826955	2828385	2861266	2862699	2819319	2820752	2674627	2676060
3066 BPSS2110		2795998	2794097	2267387	2269288	2010611	2008710	1524195	1522294	2918296	2916401	2830329	2828434	2864649	2862748	2822709	2820802	2678004	2676109
3067 BPSS2111		2796429	2797709	2266956	2265676	2011038	2012321	1524622	1525905	2918726	2920003	2830758	2832038	2865076	2866359	2823140	2824420	2678432	2679715
3068 BPSS2112		2798022	2798336	2265363	2265049	2012646	2012948	1526230	1526532	2920318	2920632	2832353	2832667	2866684	2866986	2824733	2825047	2680028	2680342
3069 BPSS2113		2798388	2798561	2264997	2264824	2013000	2013173	1526584	1526757	2920684	2920857	2832837	2832899	2867038	2867211	2825099	2825272	2680394	2680567
3070 BPSS2114		2799770	2799069	2263616	2264317	2014372	2013671	1527996	1527295	2922046	2921345	2834128	2833427	2868400	2867699	2826411	2825710	2681706	2681005
3071 BPSS2115		2799937	2800863	2263449	2262523	2014539	2015465	1528163	1529089	2922213	2923139	2834295	2835221	2868567	2869121	2826578	2827504	2681872	2682798
3072 BPSS2116		2802359	2801025	2261027	2262361	2016959	2015625	1530583	1529249	2924669	2923335	2836788	2835454	2870988	2869654	2828988	2827654	2684377	2683043
3073 BPSS2117		2802456	2803394	2260930	2259992	2017056	2017994	1530680	1531618	2924766	2925704	2836885	2837823	2871085	2872023	2829085	2830023	2684474	2685412
3074 BPSS2118		2805098	2804409	2258288	2258977	2019685	2018996	1533309	1532620	2927408	2926719			2873714	2873025	2831727	2831038	2687103	2686414
3075 BPSS2119		2805153	2805890	2258233	2257496	2019740	2020477	1533364	1534101	2927463	2928200			2873769	2874506	2831782	2832519	2687158	2687895
3076 BPSS2120		2805898	2807136	2257488	2256250	2020485	2021723	1534109	1535347	2928208	2929446			2874514	2875752	2832527	2833765	2687903	2689141
3077 BPSS2121		2807186	2808304	2256200	2255082	2021773	2022891	1535397	1536515	2929496	2930614			2875802	2876920	2833815	2834933	2689191	2690309
3078 BPSS2122		2809196	2808888	2254190	2254498	2023782	2023387	1537406	1537011	2931506	2931300	2840714	2840619	2877811	2877416	2835824	2835618	2691199	2690804
3079 BPSS2123		2809719	2810999	2253667	2252387	2024286	2025566	1537910	1539190	2932010	2933290	2841218	2842498	2878315	2879595	2836328	2837608	2691703	2692983
3080	BURPS1655_D0657																	2693358	2693098
3081	BURPS1710A_A2157					2025932	2025723												
3082	BURPS406E_D0135									2933290	2933940								
3083 BPSS2124		2811630	2812550	2251740	2250820	2026226	2027146	1539850	1540770	2933950	2934870	2843167	2844087	2880179	2881099	2838223	2839143	2693632	2694552
3084	BURPS1106B_2307			2252018	2252251														
3085 BPSS2125		2812618	2813757	2250752	2249613	2027214	2028353	1540838	1541977	2934938	2936077	2844155	2845294	2881167	2882306	2839211	2840350	2694620	2695759
3086 BPSS2126		2814792	2813947	2248578	2249423	2029381	2028536	1543005	1542160	2937105	2936260	2846322	2845477	2883334	2882489	2841385	2840540	2696794	2695949
3087 BPSS2127		2815303	2816124	2248067	2247246	2029892	2030713	1543516	1544337	2937599	2938420	2846861	2847682	2883845	2884666	2841896	2842717	2697305	2698126
3088 BPSS2128		2816427	2817827	2246905	2245505	2031135	2032535	1544646	1546046	2938729	2940129	2847985	2849385	2884975	2886375	2843026	2844426	2698429	2699829
3089 BPSS2129		2818016	2818564	2245316	2244768	2032724	2033272	1546235	1546783	2940318	2940863	2849574	2850122	2886564	2887112	2844615	2845160	2700018	2700566
3090 BPSS2130		2821497	2819089	2241834	2243699	2036212	2033804	1549723	1547315	2943799	2941391	2853055	2850647	2890052	2887644	2848110	2845702	2703534	2701126
3091 BPSS2131		2823281	2822994	2240050	2240337			1551507	1551220			2854839	2854552	2891883	2891596	2849673	2849455		
3092 BPSS2131a		2824296	2823742	2239035	2239589	2038676	2038122	1552522	1551968	2946002	2945451	2855854	2855300	2892898	2892344	2850694	2850134	2705531	2704980
3093 BPSS2132		2826120	2824603	2237212	2238297	2040500	2038983	1554346	1552829	2947895	2946378	2857678	2856179	2894722	2893205	2852512	2850995	2707421	2705904
3094 BPSS2133		2826464	2827825	2236868	2235507	2040843	2042204	1554689	1556050	2948239	2949600	2858023	2859384	2895065	2896426	2852856	2854217	2707766	2709127
3095 BPSS2134		2828186	2828428	2235146	2234904	2042565	2042807	1556411	1556653	2949961	2950203	2859745	2859987	2896759	2897001	2854673	2854915	2709488	2709730
3096 BPSS2135		2828586	2829575	2234746	2233757	2042965	2043954	1556811	1557800	2950361	2951350	2860145	2861134	2897159	2898148	2855073	2856062	2709888	2710877
3097 BPSS2136		2831352	2829913	2231980	2233419	2045731	2044292	1559577	1558138	2953127	2951688	2862911	2861472	2899925	2898486	2857850	2856411	2712654	2711215
3098 BPSS2137		2832661	2831531	2230671	2231801	2047040	2045910	1560886	1559756	2954436	2953306	2864220	2863090	2901234	2900104	2859159	2858029	2713963	2712833
3099 BPSS2138		2833647	2832661	2229685	2230671	2048026	2047040	1561872	1560886	2955422	2954436	2865206	2864220	2902220	2901234	2860145	2859159	2714949	2713963
3100 BPSS2139		2834551	2833652	2228781	2229680	2048930	2048031	1562776	1561877	2956326	2955427	2866110	2865211	2903124	2902225	2861049	2860150	2715853	2714954
3101 BPSS2140		2835501	2834566	2227831	2228766	2049880	2048945	1563726	1562791	2957276	2956341	2867060	2866125	2904074	2903139	2861999	2861064	2716803	2715868
3102 BPSS2141		2837183	2835522	2226149	2227810	2051562	2049901	1565408	1563747	2958958	2957297	2868742	2867081	2905756	2904095	2863681	2862020	2718485	2716824
3103 BPSS2142		2837900	2837322	2225432	2226010	2052279	2051701	1566125	1565547	2959675	2959097	2869459	2868881	2906473	2905895	2864398	2863820	2719202	2718624
3104 BPSS2143		2838536	2840035	2224796	2223297	2052915	2054414	1566761	1568260	2960311	2961810	2870095	2871594	2907109	2908608	2865034	2866533	2719838	2721337
3105 BPSS2144		2840477	2840677	2222855	2222655	2054857	2055057	1568703	1568903	2962252	2962452	2872037	2872237	2909051	2909251	2866975	2867175	2721780	2721980
3106 BPSS2145		2842442	2840754	2220890	2222578	2056822	2055134	1570668	1568980	2964217	2962529	2874002	2872314	2911016	2909328	2868940	2867252	2723745	2722057
3107 BPSS2146		2843762	2842752	2219570	2220580	2058114	2057104	1571960	1570950	2965518	2964508	2875343	2874333	2912308	2911298	2870232	2869222	2725044	2724034
3108 BPSS2147		2844217	2843966	2219115	2219366	2058569	2058318	1572415	1572164	2965973	2965722	2875798	2875547	2912763	2912512	2870687	2870436	2725499	2725248
3109 BPSS2148																			
3110 BPSS2148a																			
3111 BPSS2149						2060829	2059321	1574675	1573167			2878058	2876550	2915023	2913515			2727759	2726251
3112 BPSS2150						2061698	2060829	1575544	1574675			2878927	2878058	2915892	2915023			2728628	2727759
3113 BPSS2151						2062993	2061698	1576839	1575544			2880225	2878927	2917187	2915892			2729923	2728628
3114 BPSS2152						2064220	2063057	1578066	1576903			2881450	2880287	2918414	2917251			2731137	2729974
3115 BPSS2153						2064861	2064277	1578707	1578123			2882091	2881507	2919055	2918471			2731778	2731194
3116 BPSS2154						2066324	2064897	1580170	1578743			2883554	2882127	2920518	2919091			2733241	2731814
3117 BPSS2155						2068321	2066861	1582167	1580707			2885551	2884091	2922515	2921055			2735238	2733778

3118	BPSS2156		2844997	2843966	2218335	2219366	2069446	2068415	1583292	1582261	2966753	2965722	2886676	2885645	2923640	2922609	2871467	2870436	2736363	2735332
3119	BPSS2157		2846312	2845038	2217020	2218294	2070761	2069487	1584607	1583333	2968068	2966794	2887991	2886717	2924955	2923681	2872787	2871513	2737678	2736404
3120	BPSS2158		2847135	2846389	2216197	2216943	2071584	2070838	1585430	1584684	2968891	2968145	2888814	2888068	2925778	2925032	2873610	2872864	2738501	2737755
3121	BPSS2159		2847379	2848281	2215953	2215051	2071829	2072731	1585675	1586577	2969136	2970038	2889059	2889961	2926022	2926924	2873854	2874756	2738746	2739648
3122	BPSS2160		2848609	2849685	2214723	2213647	2073050	2074126	1586896	1587972	2970357	2971433	2890280	2891356	2927243	2928319	2875075	2876151	2739959	2741035
3123	BPSS2161		2849730	2851202	2213602	2212130	2074171	2075643	1588017	1589489	2971478	2972950	2891401	2892873	2928364	2929836	2876196	2877668	2741080	2742549
3124	BPSS2162		2852408	2851284	2210924	2212048	2076849	2075725	1590695	1589571	2974156	2973032	2894080	2892956	2931042	2929918	2878869	2877754	2743756	2742632
3125	BPSS2163		2853700	2852810	2209632	2210522	2078141	2077251	1591987	1591097	2975448	2974558	2895372	2894482	2932334	2931444	2880161	2879271	2745048	2744158
3126	BPSS2164		2853993	2853700	2209339	2209632	2078434	2078141	1592280	1591987	2975741	2975448	2895665	2895372	2932627	2932334	2880454	2880161	2745341	2745048
3127	BPSS2165		2854154	2855158	2209178	2208174	2078595	2079599	1592441	1593445	2975902	2976906	2895826	2896830	2932788	2933792	2880615	2881619	2745502	2746506
3128	BPSS2166		2855416	2856981	2207916	2206351	2080725	2081540	1593703	1595268	2977164	2977601	2897088	2898653	2934050	2934388	2881877	2882527	2747713	2748246
3129	BPSS2167		2858422	2857235	2204910	2206097	2082803	2081772	1596687	1595500	2979425	2978265	2900077	2898890	2935988	2935944	2884409	2883222	2749677	2748490
3130	BPSS2168		2858816	2859754	2204516	2203578	2083303	2084241	1597081	1598019	2979948	2980886	2900471	2901409	2936382	2937320	2884803	2885741	2750071	2751009
3131	BPSS2169		2859770	2860927	2203562	2202405	2084257	2085414	1598035	1599192	2980902	2982059	2901425	2902582	2937336	2938493	2885757	2886914	2751025	2752182
3132	BPSS2170		2861274	2861624	2202058	2201708	2085761	2086111	1599539	1599889	2982406	2982756	2902932	2903282	2938840	2939190	2887262	2887612	2752532	2752882
3133	BPSS2171		2862156	2863511	2201176	2199821	2086643	2087998	1600421	1601776	2983318	2984673	2903834	2905189	2939722	2941077	2888194	2889549	2753444	2754799
3134	BPSS2172		2864320	2864784	2199012	2198548	2088803	2089267	1602581	1603045	2985482	2985946	2905998	2906462	2941886	2942350	2890353	2890817	2755609	2756073
3135	BPSS2173		2867338	2865263	2195994	2198069	2091845	2089770	1605623	1603548	2988621	2986546	2909043	2906968	2944898	2942823	2893395	2891320	2758675	2756600
3136	BPSS2174		2868090	2867584	2195242	2195748	2092597	2092091	1606375	1605869	2989373	2988867	2909806	2909300	2945650	2945144	2894158	2893652	2759438	2758932
3137	BPSS2175		2869160	2868111	2194172	2195221	2093667	2092618	1607445	1606396	2990443	2989394	2910876	2909827	2946720	2945671	2895228	2894179	2760508	2759459
3138	BPSS2176		2871021	2869516	2192311	2193816	2095528	2094023	1609306	1607801	2992304	2990799	2912746	2911241	2948581	2947076	2897089	2895584	2762365	2760860
3139	BPSS2177		2871182	2872135	2192150	2191197	2095689	2096642	1609467	1610420	2992465	2993418	2912907	2913860	2948742	2949695	2897250	2898203	2762526	2763479
3140	BPSS2178		2872993	2872607	2190339	2190725	2097482	2097096	1611260	1610874	2994253	2993867	2914688	2914302	2950553	2950167	2899026	2898640	2764296	2763910
3141	BPSS2179		2873252	2873701	2190080	2189631	2097741	2098190	1611519	1611968	2994512	2994961	2914930	2915379	2950812	2951261	2899285	2899734	2764558	2764827
3142	BPSS2180		2874138	2873755	2189194	2189577	2098627	2098244	1612405	1612022	2995406	2995023	2915816	2915433	2951698	2951315	2900179	2899796	2765451	2765068
3143	BPSS2181		2874881	2874138	2188451	2189194	2099370	2098627	1613148	1612405	2996149	2995406	2916559	2915816	2952441	2951698	2900922	2900179	2766194	2765451
3144	BPSS2182		2875970	2874912	2187362	2188420	2100459	2099401	1614237	1613179	2997238	2996180	2917648	2916590	2953530	2952472	2902011	2900953	2767283	2766225
3145	BPSS2183		2877379	2875958	2185953	2187374	2101868	2100447	1615646	1614225	2998647	2997226	2919057	2917636	2954941	2953523	2903420	2901999	2768692	2767271
3146	BPSS2184		2878617	2877379	2184715	2185953	2103106	2101868	1616884	1615646	2999885	2998647	2920295	2919057	2956179	2954941	2904658	2903420	2769930	2768692
3147	BPSS2185		2879741	2879908	2183591	2183424	2104230	2104397	1618008	1618175	3001010	3001177	2921419	2921586	2957303	2957470	2905782	2905949	2771054	2771221
3148	BPSS2186		2880018	2880233	2183314	2183099	2104507	2104722	1618285	1618500	3001287	3001502	2921696	2921911	2957580	2957795	2906059	2906274	2771331	2771546
3149	BPSS2187		2880328	2880864	2183004	2182468	2104817	2105353	1618595	1619131	3001597	3002133	2922006	2922542	2957890	2958426	2906369	2906905	2771641	2772177
3150	BPSS2188		2880907	2882286	2182425	2181046	2105396	2106775	1619174	1620553	3002176	3003555	2922585	2923964	2958469	2959848	2906948	2908327	2772220	2773599
3151	BPSS2189		2882335	2883177	2180997	2180155	2106824	2107666	1620602	1621444	3003604	3004446	2924013	2924855	2959897	2960739	2908376	2909218	2773648	2774490
3152	BPSS2190		2883294	2885363	2180038	2177969	2107783	2109852	1621561	1623630	3004563	3006266	2924972	2927041			2909335	2911404	2774607	2776676
3153	BPSS2191		2885388	2885690	2177944	2177642	2109877	2110179	1623655	1623957	3006370	3006546	2927066	2927368	2962936	2963238	2911429	2911731	2776701	2777003
3154	BPSS2192		2885784	2886971	2177548	2176361	2110273	2111460	1624051	1625238	3006640	3007827	2927462	2928649	2963332	2964519	2911825	2913012	2777097	2778284
3155	BPSS2193		2886998	2887537	2176334	2175795	2111487	2112026	1625265	1625804	3007854	3008393	2928676	2929215	2964546	2965085	2913039	2913578	2778311	2778850
3156	BPSS2194		2887537	2887995	2175795	2175337	2112026	2112484	1625804	1626262	3008393	3008851	2929215	2929673	2965085	2965543	2913578	2914036	2778850	2779308
3157	BPSS2195		2888021	2889226	2175311	2174106	2112510	2113715	1626288	1627493	3008877	3010082	2929699	2930904	2965569	2966774	2914062	2915267	2779334	2780539
3158	BPSS2196		2889251	2890615	2174081	2172717	2113740	2115104	1627518	1628882	3010107	3011471	2930929	2932293	2966799	2968163	2915292	2916656	2780564	2781928
3159	BPSS2197		2890630	2891619	2172702	2171713	2115119	2116108	1628897	1629886	3011486	3012475	2932308	2933297	2968178	2969167	2916671	2917660	2781943	2782932
3160	BPSS2198		2891677	2892633	2171655	2170699	2116166	2117122	1629944	1630900	3012533	3013489	2933355	2934311	2969225	2970181	2917718	2918674	2782990	2783946
3161	BPSS2199		2893513	2893040	2169819	2170292	2118049	2117576	1631827	1631354	3014391	3013918	2935211	2934738	2971092	2970619	2919559	2919086	2784875	2784402
3162	BPSS2200		2893748	2894962	2169584	2168370	2118284	2119498	1632062	1633276	3014626	3015840	2935446	2936660	2971327	2972541	2919794	2920066	2785110	2786324
3163	BPSS2201		2895189	2895578	2168143	2167754	2119725	2120114	1633503	1633892	3016120	3016509	2936887	2937276	2972855	2973244	2921242	2921631	2786551	2786940
3164	BPSS2202		2895749	2896819	2167583	2166513	2120285	2121355	1634063	1635133	3016680	3017750	2937447	2938517	2973415	2974485	2921802	2922872	2787111	2788181
3165	BPSS2203		2896902	2898329	2166430	2165003	2121438	2122865	1635216	1636643	3017833	3019260	2938600	2940027	2974568	2975995	2922955	2924382	2788264	2789691
3166	BPSS2204		2899176	2898412	2164156	2164920	2123712	2122948	1637490	1636726	3020107	3019343	2940839	2940075	2976842	2976078	2925229	2924465	2790538	2789774
3167	BPSS2205		2901208	2899673	2162124	2163659	2125744	2124209	1639522	1637987	3022139	3020604	2942871	2941336	2978874	2977339	2927261	2925726	2792570	2791035
3168	BPSS2206		2902512	2901208	2160820	2162124	2127048	2125744	1640826	1639522	3023443	3022139	2944175	2942871	2980178	2978874	2928565	2927261	2793874	2792570
3169	BPSS2207		2902641	2903552	2160691	2159780	2127177	2128088	1640955	1641866	3023572	3024483	2944304	2945215	2980307	2981218	2928694	2929605	2794003	2794914
3170	BPSS2208		2905973	2903850	2157359	2159482	2130509	2128386	1644287	1642164	3026904	3024781	2947636	2945513	2983639	2981516	2932026	2929903	2797335	2795212
3171	BPSS2209		2906908	2907282	2156424	2156050	2131444	2131818	1645222	1645596	3027838	3028212	2948571	2948945	2984574	2984948	2932960	2933334	2798268	2798642
3172	BPSS2210		2907332	2908648	2156000	2154684	2131868	2133184	1645646	1646962	3028262	3029578	2948995	2950311	2984998	2986314	2933384	2934700	2798692	2800008
3173	BPSS2211		2912802	2909314	2150530	2154018	2137376	2133906	1651181	1647684	3033647	3030150	2954358	2950888	2990409	2986921	2938775	2935290	2804007	2800528
3174		BURPS1106B_2184			2154654	2154268														
3175	BPSS2212		2913978	2912875	2149354	2150457	2138577	2137480	1652354	1651254	3034812	3033718	2955534	2954431	2991601	2990480	2939933	2938848	2805174	2804080

3176	BPSS2213		2914563	2914063	2148769	2149269	2139162	2138662	1652939	1652439	3035397	3034897	2956119	2955619	2992186	2991686	2940518	2940018	2805759	2805259
3177	BPSS2214		2916712	2914589	2146620	2148743	2141311	2139188	1655088	1652965	3037546	3035423	2958268	2956145	2994335	2992212	2942667	2940544	2807908	2805785
3178	BPSS2215		2917279	2916851	2146053	2146481	2141878	2141450	1655655	1655227	3038113	3037685	2958835	2958407	2994975	2994547	2943234	2942806		
3179	BPSS2216		2917475	2917317	2145857	2146015	2142074	2141916	1655851	1655693	3038309	3038151	2959031	2958873	2995171	2995013	2943430	2943272	2808537	2808379
3180	BPSS2217		2917708	2917523	2145624	2145809	2142307	2142122	1656084	1655899	3038542	3038357	2959264	2959079	2995404	2995219	2943663	2943478	2808770	2808585
3181	BPSS2218		2919251	2917779	2144081	2145553	2143852	2142380	1657625	1656153	3040085	3038613	2960807	2959335	2996945	2995473	2945204	2943732	2810313	2808841
3182	BPSS2219		2919933	2920688	2143399	2142644	2144556	2145311	1658329	1659084	3040762	3041517	2961647	2962402	2997642	2998397	2945881	2946636	2810988	2811743
3183	BPSS2220		2920993	2921643	2142339	2141689	2145616	2146266	1659389	1660039	3041822	3042472	2962707	2963357	2998702	2999352	2946941	2947591	2812048	2812698
3184	BPSS2221		2922525	2922019	2140794	2141300	2147272	2146766	1661045	1660539	3043393	3042887	2964161	2963655	3000182	2999676	2948408	2947902	2813572	2813066
3185	BPSS2222		2923668	2922994	2139651	2140325	2148324	2147650	1662097	1661423	3044445	3043771	2965217	2964540	3001324	3000650	2949551	2948877	2814627	2814040
3186	BPSS2223		2924837	2923983	2138482	2139336	2149493	2148639	1663266	1662412	3045614	3044760	2966386	2965532	3002493	3001639	2950720	2949866	2817499	2816645
3187	BPSS2224		2925551	2925237	2137768	2138082	2150194	2149880	1663967	1663653	3046345	3046031	2967085	2966792	3003194	3002880	2951421	2951107	2818213	2817899
3188	BPSS2225		2925923	2925570	2137396	2137749	2150566	2150213	1664339	1663986	3046717	3046364	2967448	2967104	3003566	3003213	2951793	2951440	2818585	2818232
3189		BURPS1710A_A2303					2152229	2151768												
3190		BURPS1710b_A1352							1665537	1665998										
3191	BPSS2226		2928024	2927728	2135295	2135591	2152619	2152323	1666388	1666092			2969538	2969242	3005615	3005319	2953833	2953537	2820670	2820374
3192	BPSS2227		2928317	2928562	2135002	2134757	2152912	2153157	1666681	1666926	3048373	3048618	2969831	2970076	3005908	3006153	2954126	2954371	2821051	2821296
3193	BPSS2227A		2928670	2928801	2134649	2134518	2153282	2153413	1667051	1667182	3048726	3048857	2970197	2970328	3006278	3006409	2954479	2954610	2821404	2821535
3194	BPSS2228		2929927	2929286	2133392	2134033	2154539	2153898	1668308	1667667	3049993	3049352	2971454	2970813	3007535	3006894	2955736	2955095	2822661	2822020
3195		BURPS1710A_A2310					2154918	2154730												
3196		BURPS1710b_A1359							1668499	1668687										
3197	BPSS2229		2930515	2930943	2132804	2132376	2155127	2155555	1668896	1669324	3050630	3051058	2972062	2972490	3008118	3008546	2956324	2956752	2823233	2823661
3198	BPSS2230		2931092	2931580	2132227	2131739	2155704	2156192	1669473	1669961	3051207	3051695	2972639	2973127	3008695	3009183	2956901	2957389	2823810	2824298
3199	BPSS2231		2932249	2931803	2131070	2131516	2156860	2156414	1670629	1670183	3052364	3051918	2973797	2973351	3009852	3009406	2958058	2957612	2824967	2824521
3200	BPSS2232		2932358	2932711	2130961	2130608	2156969	2157322	1670738	1671091	3052473	3052826	2973906	2974259	3009961	3010314	2958167	2958520	2825076	2825429
3201	BPSS2233		2932711	2933379	2130608	2129940	2157322	2157990	1671091	1671759	3052826	3053494	2974259	2974927	3010314	3010982	2958520	2959188	2825431	2826096
3202	BPSS2234		2933461	2934912	2129858	2128407	2158072	2159541	1671841	1673310	3053576	3055027	2974998	2976458	3011064	3012524	2959270	2960730	2826181	2827641
3203	BPSS2235		2936200	2935028	2127119	2128291	2160829	2159657	1674598	1673426	3056315	3055143	2977746	2976574	3013752	3012640	2962018	2960846	2828929	2827757
3204	BPSS2236		2937254	2936616	2126065	2126703	2162039	2161401	1675697	1675059	3057370	3056732	2978800	2978162	3014537	3013899	2963073	2962435	2829991	2829353
3205	BPSS2237		2937947	2937345	2125372	2125974	2162732	2162130	1676390	1675788	3058063	3057461	2979493	2978891	3015230	3014628	2963766	2963164	2830673	2830071
3206	BPSS2238		2938842	2937988	2124477	2125331	2163627	2162773	1677285	1676431	3058958	3058104	2980388	2979534	3016059	3015271	2964661	2963807	2831568	2830714
3207	BPSS2239		2939619	2939056	2123700	2124263	2164404	2163841	1678062	1677499	3059735	3059172	2981165	2980602			2965438	2964875	2832345	2831782
3208	BPSS2240		2940174	2939866	2123145	2123453	2164959	2164651	1678617	1678309	3060233	3059925	2981736	2981428	3016641	3016333	2966002	2965694	2832906	2832598
3209		BURPS1106B_2148			2123536	2123709														
3210	BPSS2241		2940538	2940332	2122781	2122987	2165323	2165117	1678981	1678775	3060597	3060391	2982100	2981894	3017005	3016799	2966366	2966160	2833270	2833064
3211	BPSS2242		2941360	2940548	2121959	2122771	2166145	2165333	1679803	1678991	3061419	3060607	2982922	2982110	3017827	3017015	2967188	2966376	2834092	2833280
3212	BPSS2243		2941879	2941391	2121440	2121928	2166676	2166176	1680334	1679834	3061938	3061450	2983441	2982953	3018358	3017858	2967719	2967219	2834611	2834123
3213	BPSS2244		2942110	2943495	2121209	2119824	2166907	2168292	1680565	1681950	3062169	3063554	2983672	2985057	3018589	3019974	2967950	2969335	2834842	2836227
3214	BPSS2245		2943495	2944538	2119824	2118781	2168292	2169335	1681950	1682993	3063554	3064597	2985057	2986100	3019974	3021017	2969335	2970378	2836227	2837270
3215	BPSS2246		2946988	2944673	2116331	2118646	2171779	2169470	1685437	1683128	3067041	3064732	2988550	2986235	3023461	3021152	2972822	2970513	2839720	2837405
3216	BPSS2247		2947120	2947809	2116199	2115510	2171911	2172600	1685569	1686258	3067173	3067862	2988682	2989371	3023593	3024282	2972954	2973643	2839852	2840541
3217	BPSS2248		2947830	2948735	2115489	2114584	2172621	2173526	1686279	1687184	3067883	3068788	2989392	2990297	3024303	3024821	2973664	2974569	2840562	2841467
3218	BPSS2249		2950132	2948765	2113187	2114554	2174923	2173556	1688581	1687214	3070185	3068818	2991694	2990327	3026607	3025240	2975966	2974599	2842864	2841497
3219	BPSS2250		2950521	2951486	2112798	2111833	2175312	2176277	1688970	1689935	3070574	3071536	2992083	2993048	3026996	3027961	2976355	2977320	2843253	2844218
3220	BPSS2251		2952691	2951744	2110628	2111575	2177465	2176518	1691122	1690175	3072726	3071779	2994256	2993309	3029148	3028201	2978525	2977578	2845405	2844458
3221	BPSS2252		2953796	2952684	2109523	2110635	2178570	2177458	1692227	1691115	3073831	3072719	2995361	2994249	3030253	3029141	2979630	2978518	2846510	2845398
3222	BPSS2253		2954794	2953796	2108525	2109523	2179568	2178570	1693225	1692227	3074829	3073831	2996359	2995361	3031251	3030253	2980628	2979630	2847508	2846510
3223	BPSS2254		2956545	2954794	2106774	2108525	2181319	2179568	1694976	1693225	3076580	3074829	2998110	2996359	3033002	3031251	2982379	2980628	2849259	2847508
3224	BPSS2255		2958417	2956552	2104902	2106767	2183254	2182298	1696911	1695955	3078452	3076587	2999982	2998117	3034865	3033009	2984296	2983337	2851131	2849266
3225	BPSS2256		2959037	2958417	2104282	2104902	2183874	2183254	1697531	1696911	3079072	3078452	3000614	2999982	3035485	3034865	2984916	2984296	2851763	2851131
3226	BPSS2257		2959783	2959031	2103536	2104288	2184620	2183868	1698277	1697525	3079818	3079066	3001360	3000608	3036231	3035479	2985662	2984910	2852509	2851757
3227		Bp_chr2_15_ISBn	2959935	2961239	2102080	2103384														
3228	BPSS2258		2961803	2961312	2101516	2102007	2185327	2184836	1698984	1698493	3080525	3080034	3002067	3001576	3036938	3036447	2986369	2985878	2853216	2852725
3229	BPSS2259		2964744	2961781	2098575	2101538	2188268	2185305	1701925	1698962	3083466	3080503	3005008	3002045	3039879	3036916	2989310	2986347	2856142	2853194
3230	BPSS2260		2965889	2964825	2097430	2098494	2189433	2188369	1703090	1702026	3084631	3083567	3006173	3005109	3041023	3039980	2990475	2989411	2857307	2856243
3231	BPSS2261		2967924	2966626	2095395	2096693	2191644	2190346	1705301	1704003	3086883	3085585	3008209	3006920	3043271	3041973	2992844	2991555	2859341	2858043
3232		BURPS1106B_2124			2096743	2098497														
3233	BPSS2262		2968793	2968089	2094526	2095230	2192513	2191809	1706170	1705466	3087752	3087048	3009078	3008374	3044140	3043436	2993713	2993009	2860210	2859506

3234	BPSS2263			2969121	2968879	2094198	2094440	2192841	2192599	1706498	1706256	3088080	3087838	3009406	3009164	3044468	3044226	2994041	2993799	2860538	2860296
3235		BURPS1710A_A2351						2192941	2193159												
3236	BPSS2264			2970328	2969444	2092991	2093875	2194031	2193147	1707688	1706804	3089238	3088354	3010564	3009680	3045674	3044790	2995224	2994340	2861712	2860828
3237	BPSS2265			2971818	2970472	2091501	2092847	2195521	2194175	1709178	1707832	3090728	3089382	3012054	3010708	3047164	3045818	2996714	2995368	2863202	2861856
3238	BPSS2266			2972914	2972102	2090405	2091217	2196617	2195805	1710274	1709462	3091824	3091012	3013150	3012338	3048260	3047448	2997810	2996998	2864298	2863486
3239	BPSS2267			2974385	2973135	2088934	2090184	2198088	2196838	1711745	1710495	3093295	3092045	3014621	3013371	3049731	3048481	2999281	2998031	2865769	2864519
3240	BPSS2268			2975221	2974751	2088098	2088568	2198924	2198454	1712581	1712111	3094131	3093661	3015457	3014987	3050567	3050097	3000117	2999647	2866605	2866135
3241	BPSS2269			2977285	2976131	2086034	2087188	2200988	2199834	1714645	1713491	3096195	3095041	3017515	3016361	3052631	3051477	3002181	3001027	2868663	2867509
3242	BPSS2270			2979338	2977941	2083981	2085378	2203041	2201644	1716698	1715301	3098248	3096851	3019568	3018171	3054684	3053287	3004234	3002837	2870716	2869319
3243	BPSS2271			2980800	2979346	2082519	2083973	2204497	2203049	1718154	1716706	3099704	3098256	3021030	3019576	3056140	3054692	3005690	3004242	2872172	2870724
3244	BPSS2272			2981845	2980805	2081474	2082514	2205542	2204502	1719199	1718159	3100749	3099709	3022075	3021035	3057185	3056145	3006735	3005695	2873217	2872177
3245	BPSS2273			2983083	2981854	2080236	2081465	2206780	2205551	1720437	1719208	3101987	3100758	3023313	3022084	3058423	3057194	3007973	3006744	2874455	2873226
3246	BPSS2274			2984336	2984773	2078983	2078546	2208033	2208470	1721690	1722127	3103240	3103677	3024566	3025003	3059676	3060113	3009226	3009663	2875706	2876143
3247		BURPS1106B_2101				2079902	2078955														
3248	BPSS2275			2986226	2985114	2077093	2078205	2209890	2208778	1723547	1722435	3105130	3104018	3026456	3025344	3061533	3060421	3011116	3010004	2877547	2876435
3249	BPSS2276			2986524	2987456	2076795	2075863	2210188	2211120	1723845	1724777	3105428	3106360	3026754	3027686	3061831	3062763	3011414	3012346	2877845	2878777
3250	BPSS2277			2987634	2988062	2075685	2075257	2211298	2211726	1724955	1725383	3106538	3106966	3027864	3028292	3062942	3063370	3012524	3012952	2878955	2879383
3251	BPSS2278			2988356	2990020	2074963	2073299	2212020	2213684	1725677	1727341	3107260	3108924	3028586	3030250	3063664	3065328	3013246	3014910	2879677	2881341
3252	BPSS2279			2992580	2990310	2070739	2073009	2216250	2213980	1729907	1727637	3111484	3109214	3032825	3030555	3067894	3065624	3017470	3015200	2883911	2881641
3253		BURPS406E_0485										3111992	3112444								
3254		BURPSS13_0197																3017978	3018430		
3255	BPSS2280			2993108	2992716	2070211	2070603	2216778	2216386	1730435	1730043	3112012	3111620	3033353	3032961	3068422	3068030	3017998	3017606	2884439	2884047
3256	BPSS2281			2994463	2995731	2068856	2067588	2218133	2219401	1731790	1733058	3113428	3114690	3034737	3035999	3069777	3071045	3019414	3020676	2885821	2887083
3257	BPSS2282			2995950	2997788	2067369	2065531	2219620	2221458	1733277	1735115	3114909	3116747	3036254	3038092	3071263	3073101	3020883	3022721	2887314	2889152
3258	BPSS2283			2997968	2999674	2065351	2063645	2221638	2223344	1735295	1737001	3116927	3118633	3038270	3039976	3073281	3074672	3022901	3024607	2889332	2891038
3259		BURPS1655_J0079																		2891038	2891466
3260	BPSS2284			3000194	3001222	2063125	2062097	2223858	2224886	1737515	1738543	3119161	3120189	3040446	3041474	3075352	3076380	3025135	3026163	2891604	2892632
3261	BPSS2285			3001222	3003051	2062097	2060268	2224886	2226715	1738543	1740372	3120189	3122048	3041474	3043303	3076380	3078209	3026163	3027992	2892632	2894458
3262	BPSS2286			3004028	3003522	2059286	2059792	2227691	2227176	1741348	1740833	3123024	3122509	3044286	3043771	3079185	3078670	3028965	3028450	2895435	2894929
3263	BPSS2287			3004411	3005061	2058903	2058253	2228081	2228731	1741738	1742388	3123409	3124059	3044671	3045321	3079560	3080210	3029350	3030000	2895828	2896478
3264	BPSS2288			3005566	3005135	2057748	2058179	2229238	2228807	1742895	1742464	3124564	3124133	3045826	3045395	3080715	3080284	3030505	3030074	2896983	2896552
3265	BPSS2289			3007079	3005955	2056235	2057359	2230771	2229647	1744428	1743304	3126097	3124973			3082249	3081125	3032038	3030914	2898517	2897393
3266		BURPS1106B_2078				2057443	2057751														
3267	BPSS2290			3008964	3007162	2054350	2056152	2232656	2230854	1746313	1744511	3127982	3126180			3084134	3082332	3033922	3033299	2900402	2898600
3268	BPSS2291			3012031	3009269	2051283	2054045	2235723	2232961	1749380	1746618	3131154	3128908			3087190	3084428	3037000	3034238	2903480	2900718
3269	BPSS2292			3012195	3013253	2051119	2050061	2235887	2236945	1749544	1750602							3037164	3038222	2903644	2904702
3270	BPSS2293			3013359	3014339	2049955	2048975	2237051	2238031	1750708	1751688	3132537	3133517			3088578	3089558	3038328	3039308	2904808	2905788
3271	BPSS2294			3014344	3015189	2048970	2048125	2238036	2238881	1751693	1752538	3133522	3134367			3089563	3090408	3039313	3040158	2905793	2906638
3272	BPSS2295			3015291	3016307	2048023	2047007	2238983	2239999	1752640	1753656	3134469	3135485			3090510	3091526	3040260	3041276	2906740	2907756
3273	BPSS2296			3016477	3017799	2046837	2045515	2240169	2241491	1753826	1755148	3135655	3136977			3091696	3093018	3041446	3042768	2907926	2909248
3274	BPSS2297			3017825	3018418	2045489	2044896	2241517	2242110	1755174	1755767	3137003	3137596			3093044	3093637	3042794	3043387	2909274	2909867
3275	BPSS2298			3018418	3019137	2044896	2044177	2242110	2242829	1755767	1756486	3137596	3138315			3093637	3094356	3043387	3044106	2909867	2910586
3276	BPSS2299			3019723	3022596	2043591	2040718	2243401	2246274	1757058	1759931	3138894	3141767	3046834	3049707	3094928	3097801	3044706	3047579	2911201	2914074
3277	BPSS2300			3022613	3023407	2040701	2039907	2246291	2247085	1759948	1760742	3141784	3142578	3049724	3050518	3097818	3098612	3047596	3048390	2914091	2914885
3278	BPSS2301			3023468	3024415	2039846	2038899	2247146	2248093	1760803	1761750	3142639	3143586	3050579	3051526	3098673	3099620	3048451	3049398	2914946	2915455
3279	BPSS2302			3025428	3026315	2037886	2036999	2249157	2250044	1762814	1763701	3144843	3145730	3052700	3053587	3100708	3101595	3050725	3051612	2916822	2917709
3280	BPSS2303			3026783	3028171	2036531	2035143	2250512	2251900	1764169	1765557	3146210	3147598	3054090	3055478	3102063	3103451	3052092	3053480	2918176	2919564
3281	BPSS2304			3030886	3028370	2032428	2034944	2254615	2252099	1768272	1765756	3150303	3147787	3058193	3055677	3106166	3103650	3056071	3053663	2922266	2919750
3282	BPSS2305			3033218	3031653	2030096	2031661	2256931	2255366	1770588	1769023	3152516	3150951	3060363	3058798	3108496	3106931	3058277	3056712	2924292	2922727
3283	BPSS2306			3035090	3033921	2028224	2029393	2258803	2257634	1772460	1771291	3154392	3153223	3062225	3061056	3110368	3109199	3060153	3058984	2926168	2924999
3284	BPSS2307			3037001	3035418	2026313	2027896	2260715	2259132	1774372	1772789	3156304	3154721	3064141	3062558	3112280	3110697	3062041	3060482	2928168	2927899
3285	BPSS2308			3037576	3037896	2025698	2025378	2261150	2261470	1774807	1775127	3156871	3157191	3064629	3064949	3112835	3113155	3062696	3063016	2928743	2929063
3286	BPSS2309			3038666	3038094	2024608	2025180	2262240	2261668	1775897	1775325	3157961	3157389	3065719	3065147	3113925	3113353	3063786	3063214	2929833	2929261
3287	BPSS2310			3039220	3038666	2024054	2024608	2262794	2262240	1776451	1775897	3158515	3157961	3066273	3065719	3114479	3113925	3064340	3063786	2930387	2929833
3288	BPSS2311			3040586	3039228	2022688	2024046	2264160	2262802	1777817	1776459	3159881	3158523	3067639	3066281	3115845	3114487	3065706	3064348	2931753	2930395
3289	BPSS2312			3042019	3040589	2021255	2022685	2265593	2264163	1779250	1777820	3161314	3159884	3069072	3067642	3117278	3115848	3067139	3065709	2933186	2931756
3290	BPSS2313			3042365	3042015	2020909	2021259	2265936	2265589	1779593	1779246	3161657	3161310	3069166	3069068	3117621	3117274	3067482	3067135	2933529	2933182
3291			Bp_chr2_16_ISBma1					2266184	2267488	1779841	1781144					3117869	3119173				

3292 BPSS2314		3043238	3044635	2020036	2018639	2268122	2269519	1781778	1783175	3162530	3163927	3070292	3071689	3119807	3121204	3068355	3069752	2934402	2935799
3293 BPSS2315		3044635	3045279	2018639	2017995	2269519	2270163	1783175	1783819	3163927	3164571	3071689	3072333	3121204	3121848	3069752	3070396	2935799	2936443
3294 BPSS2316		3046072	3045479	2017202	2017795	2270956	2270363	1784612	1784019	3165364	3164771	3073126	3072533	3122641	3122048	3071189	3070596	2937236	2936643
3295 BPSS2317		3047129	3046368	2016145	2016906	2272013	2271252	1785669	1784908	3166421	3165660	3074183	3073422	3123698	3122937	3072246	3071485	2938293	2937532
3296 BPSS2318		3049580	3047142	2013694	2016132	2274464	2272026	1788120	1785682	3168872	3166434	3076634	3074196	3126149	3123711	3074697	3072259	2940744	2938306
3297 BPSS2319		3050967	3049900	2012307	2013374	2275851	2274784	1789507	1788440	3170259	3169192	3078021	3076954	3127536	3126469	3076084	3075017	2942131	2941064
3298 BPSS2320		3051858	3051097	2011416	2012177	2276742	2275981	1790398	1789637	3171150	3170389	3078913	3078236	3128427	3127666	3076975	3076214	2943022	2942261
3299 BPSS2321		3052319	3051924	2010955	2011350	2277203	2276808	1790859	1790464	3171611	3171216	3079374	3078979	3128888	3128493	3077436	3077041	2943483	2943088
3300 BPSS2322		3053657	3052362	2009617	2010912	2278541	2277246	1792197	1790902	3172949	3171654	3080712	3079417	3130226	3128931	3078774	3077479	2944821	2943526
3301 BPSS2323		3054436	3053657	2008838	2009617	2279320	2278541	1792976	1792197	3173728	3172949	3081491	3080712	3131005	3130226	3079553	3078774	2945600	2944821
3302 BPSS2324		3055920	3054496	2007354	2008778	2280804	2279380	1794460	1793036	3175203	3173788	3082975	3081551	3132480	3131065	3081037	3079613	2947084	2945660
3303 BPSS2325		3056621	3055923	2006653	2007351	2281505	2280807	1795161	1794463	3175904	3175206	3083676	3082978	3133181	3132483	3081738	3081040	2947785	2947087
3304 BPSS2326		3058102	3056624	2005172	2006650	2282986	2281508	1796642	1795164	3177391	3175907	3085157	3083679	3134668	3133184	3083219	3081741	2949272	2947788
3305 BPSS2327		3062274	3058156	2001000	2005118	2287143	2283025	1800799	1796681	3181475	3180066	3089314	3085196	3138825	3137749	3087403	3083285	2953426	2949308
3306 BPSS2328		3065297	3062274	1994227	2000028	2293961	2287143	1807617	1800799	3188332	3181475	3096129	3089314	3145652	3138825	3094239	3087403	2960394	2959051
3307 BPSS2329		3070797	3069154	1992477	1994120	2295699	2294068	1809355	1807724	3190079	3188439	3097879	3096236	3147390	3145759	3095977	3094346	2962141	2960501
3308 BPSS2330		3071393	3074023	1991881	1989251	2296211	2298841	1809941	1812571	3190723	3193353	3098476	3101106	3147993	3150623	3096573	3099203	2962737	2965367
3309 BPSS2331		3074019	3074972	1989255	1988302	2298837	2299817	1812567	1813547	3193349	3194218	3101102	3102127	3150619	3151617	3099199	3100176	2965363	2966379
3310 BPSS2332		3075735	3075190	1987539	1988084	2300604	2300059	1814334	1813789	3195071	3194526	3102918	3102373	3152404	3151859	3101041	3100493	2967159	2966614
3311 BPSS2333		3077125	3075782	1986149	1987492	2301994	2300651	1815724	1814381	3196461	3195118	3104308	3102965	3153794	3152451	3102431	3101088	2968549	2967206
3312 BPSS2334		3078192	3077125	1985082	1986149	2303061	2301994	1816791	1815724	3197528	3196461	3105375	3104308	3154861	3153794	3103498	3102431	2969616	2968549
3313 BPSS2335		3078933	3078208	1984341	1985066	2303802	2303077	1817532	1816807	3198269	3197544	3106116	3105391	3155602	3154877	3104239	3103514	2970357	2969632
3314 BPSS2336		3079586	3078933	1983688	1984341	2304455	2303802	1818185	1817532	3198922	3198269	3106769	3106116	3156255	3155602	3104892	3104239	2971010	2970357
3315 BPSS2337		3080403	3079654	1982871	1983620	2305272	2304523	1819002	1818253	3199739	3198990	3107586	3106837	3157072	3156323	3105709	3104960	2971827	2971078
3316 BPSS2338		3081558	3080854	1981716	1982420	2306426	2305722	1820156	1819452	3200894	3200190	3108740	3108036	3158227	3157523	3106864	3106160	2972981	2972277
3317 BPSS2339		3083538	3081568	1979736	1981706	2308406	2306436	1822136	1820166	3202874	3200904	3110720	3108750	3160207	3158237	3108844	3106874	2974961	2972991
3318 BPSS2340		3084714	3083869	1978560	1979405	2309582	2308737	1823312	1822467	3204050	3203205	3111896	3111051	3161383	3160538	3110020	3109175		
3319 BPSS2341		3085259	3086161	1978015	1977113	2310127	2311029	1823857	1824759	3204595	3205497	3112441	3113343	3161928	3162830	3110565	3111467	2975889	2976791
3320 BPSS2342		3087146	3086214	1976128	1977060	2312014	2311082	1825744	1824812	3206482	3205550	3114328	3113396	3163815	3162883	3112452	3111520	2977776	2976844
3321 BPSS2343		3087754	3087146	1975520	1976128	2312622	2312014	1826352	1825744	3207090	3206482	3114936	3114328	3164423	3163815	3113060	3112452	2978384	2977776
3322 BPSS2344		3088127	3087762	1975147	1975512	2312995	2312630	1826725	1826360	3207463	3207098	3115309	3114944	3164796	3164431	3113433	3113068	2978757	2978392
3323 BPSS2345		3088633	3090912	1974641	1972362	2313501	2315780	1827231	1829510	3207969	3210248	3115815	3118094	3165302	3167581	3113939	3116218	2979263	2980354
3324 BPSS2346		3091892	3091338	1971382	1971936	2316773	2316219	1830504	1829950	3211139	3210681	3118937	3118383	3168505	3167951	3117247	3116693	2982321	2981767
3325 BPSS2347		3092256	3092858	1971102	1970500	2316967	2317569	1830868	1831470	187	789	3119301	3119903	3401	4003	139	741	1	447
3326 BPSS2347a		3092842	3093036	1970516	1970322	2317553	2317747	1831454	1831648	773	967	3119887	3120081	3987	4181	725	919	431	625
3327 BPSS2348		3093711	3093358	1969647	1970000	2318422	2318069	1832323	1831970	1642	1289	3120762	3120409	4856	4503	1594	1241	1303	950
3328 BPSS2349		3094549	3095208	1968809	1968150	2318965	2319624	1832842	1833501	2273	2932	3121211	3121870	5425	6084	2231	2890	1829	2488
3329 BPSS2350		3095236	3096300	1968122	1967058	2319652	2320716	1833529	1834593	2960	4024	3121898	3122962	6112	7176	2918	3982	2516	3580
3330	BURPS406E_Q0007									4643	5479								
3331 BPSS2351		3097845	3096397	1965513	1966961	2322261	2320813	1836138	1834690	7029	5581	3124507	3123059	8721	7273	5527	4079	5125	3677
3332	BURPS1655_B0114																	6135	7124
