# Supplementary material for: The Early Stage of Bacterial Genome-Reductive Evolution in the Host
Source: PLoS Pathog. 2010 May 27;6(5):e1000922. doi: 10.1371/journal.ppat.1000922 (PMC2877748; doi:10.1371/journal.ppat.1000922)
Supplement: Table S3 — Specifics of the IS elements IS407A, ISBma1, and ISBma2 in the B. mallei strains. (1.56 MB PDF) [file ppat.1000922.s005.pdf]

Table S3. Specifics of the IS elements IS*407* A, IS*Bma* 1, and IS*Bma* 2 in the *B. mallei* strains.

IS element	Role	Strain	Strain	position	romoso	IS type	5'-ext	5'-IR	*orfA*	intergenic region	*orfB*	3'-IR	3'-ext	5'-DR	3'-DR	et duplication
**Chromosome 1**																
**IS *407* A**																
								TGACCTGCCCCCT				AGGGGGCAGGTCA				
2*Chr1_89_IS407A_A	N	fmh	2	1003118-1004353	Chr1	IS407A	GCCCC	TGACCTGCCCCCTACA	ATGAAGAAGCGCTTTACG	GCCCGCAAGCCAAACGCG	TGTTGGC	AGGGACTTTGCTAGAA	GCGCGGA	CCAG	GCGC	N
7*Chr1_76_IS407A 7*Chr1_84b_IS407A_A	D-R	NCTC_10247	7	1003311-1004546	Chr1	IS407A	CGGGA	TGACCTGCCCCCTACA	ATGAAGAAGCGCTTTACG	GCCCGCAAGCCAAACGCG	TGTTGGC	AGGGACTTTGCTAGAA	GTCGGGC	GCCG	GTCG	N
2*Chr1_91_IS407A_B	N	fmh	2	1006858-1008093	Chr1	IS407A	GACGC	TGACCTGCCCCCTACA	ATGAAGAAGCGCTTTACG	GCCCGCAAGCCAAACGCG	TGTTGGC	AGGGACTTTGCTAGAA	TGTCGAC	TGTC	TGTC	Y
5*Chr1_96_IS407A 5*Chr1_97_IS407A_A	D	ATCC_10399	5	1008495-1009730	Chr1	IS407A	GGCAC	TGACCTGCCCCCTACA	ATGAAGAAGCGCTTTACG	GCCCGCAAGCCAAACGCG	TGTTGGC	AGGGACTTTGCTAGAA	ACCGCCC	CCCC	ACCG	N
3*Chr1_89_IS407A_A	N	JHU	3	1011927-1013162	Chr1	IS407A	GCCCC	TGACCTGCCCCCTACA	ATGAAGAAGCGCTTTACG	GCCCGCAAGCCAAACGCG	TGTTGGC	AGGGACTTTGCTAGAA	GCGCGGA	CCAG	GCGC	N
3*Chr1_91_IS407A_B	N	JHU	3	1015667-1016902	Chr1	IS407A	GACGC	TGACCTGCCCCCTACA	ATGAAGAAGCGCTTTACG	GCCCGCAAGCCAAACGCG	TGTTGGC	AGGGACTTTGCTAGAA	TGTCGAC	TGTC	TGTC	Y
6*Chr1_126a_IS407A_A	R	NCTC_10229	6	1024057-1025292	Chr1	IS407A	TGCCG	TGACCTGCCCCCTACA	ATGAAGAAGCGCTTTACG	GCCCGCAAGCCAAACGCG	TGTTGGC	AGGGACTTTGCTAGAA	CCTGCGA	AAAA	CCTG	N
8*Chr1_78_IS407A_A	N	2002721280	8	1024801-1026036	Chr1	IS407A	CAGCA	TGACCTGCCCCCTACA	ATGAAGAAGCGCTTTACG	GCCCGCAAGCCAAACGCG	TGTTGGC	AGGGACTTTGCTAGAA	GTAGCTG	GTAG	GTAG	Y
6*Chr1_43b_IS407A_A	D-R	NCTC_10229	6	1026593-1027828	Chr1	IS407A	AAAAA	TGACCTGCCCCCTACA	ATGAAGAAGCGCTTTACG	GCCCGCAAGCCAAACGCG	TGTTGGC	AGGGACTTTGCTAGAA	CGGGCGG	AAGC	CGGG	N
10*Chr1_89_IS407A_A	N	PRL20	10	1031797-1033032	Chr1	IS407A	GAGCC	TGACCTGCCCCCTACA	ATGAAGAAGCGCTTTACG	GCCCGCAAGCCAAACGCG	TGTTGGC	AGGGACTTTGCTAGAA	CTGGATG	CGCA	CTGG	N
10*Chr1_92_IS407A_C	N	PRL20	10	1037784-1039019	Chr1	IS407A	CGCGC	TGACCTGCCCCCTACA	ATGAAGAAGCGCTTTACG	GCCCGCAAGCCAAACGCG	TGTTGGC	AGGGACTTTGCTAGAA	TTCGTGC	TTCG	TTCG	Y
2*Chr1_77_IS407A 2*Chr1_94a_IS407A_A	D-R	fmh	2	1040287-1041522	Chr1	IS407A	ATGAT	TGACCTGCCCCCTACA	ATGAAGAAGCGCTTTACG	GCCCGCAAGCCAAACGCG	TGTTGGC	AGGGACTTTGCTAGAA	AACCGAT	AGGT	AACC	N
7*Chr1_71_IS407A 7*Chr1_87a_IS407A_A	D-R	NCTC_10247	7	1044544-1045779	Chr1	IS407A	GAGCG	TGACCTGCCCCCTACA	ATGAAGAAGCGCTTTACG	GCCCGCAAGCCAAACGCG	TGTTGGC	AGGGACTTTGCTAGAA	TCGCTTCT	GAGC	TCGC	N
3*Chr1_77_IS407A 3*Chr1_94a_IS407A_A	D-R	JHU	3	1049331-1050566	Chr1	IS407A	ATGAT	TGACCTGCCCCCTACA	ATGAAGAAGCGCTTTACG	GCCCGCAAGCCAAACGCG	TGTTGGC	AGGGACTTTGCTAGAA	AACCGAT	AGGT	AACC	N
5*Chr1_95_IS407A_A	N	ATCC_10399	5	1051433-1052668	Chr1	IS407A	GGCGC	TGACCTGCCCCCTACA	ATGAAGAAGCGCTTTACG	GCCCGCAAGCCAAACGCG	TGTTGGC	AGGGACTTTGCTAGAA	GCCCGCC	GATG	GCCC	N
6*Chr1_42b_IS407 6*Chr1_43a_IS40 6*Chr1_45b_I	D-R	NCTC_10229	6	1053882-1055117	Chr1	IS407A	GCGCT	TGACCTGCCCCCTACA	ATGAAGAAGCGCTTTACG	GCCCGCAAGCCAAACGCG	TGTTGGC	AGGGACTTTGCTAGAA	GCGGTGC	TGCG	GCGG	N
6*Chr1_136_IS407 6*Chr1_45a_IS407A_A	D-R	NCTC_10229	6	1067579-1068814	Chr1	IS407A	GATCG	TGACCTGCCCCCTACA	ATGAAGAAGCGCTTTACG	GCCCGCAAGCCAAACGCG	TGTTGGC	AGGGACTTTGCTAGAA	GATGCTC	GAAC	GATG	N
10*Chr1_77_IS407 10*Chr1_94a_IS407A_A	D-R	PRL20	10	1068513-1069748	Chr1	IS407A	TCAGA	TGACCTGCCCCCTACA	ATGAAGAAGCGCTTTACG	GCCCGCAAGCCAAACGCG	TGTTGGC	AGGGACTTTGCTAGAA	ACCTGTC	GGTT	ACCT	N
4*Chr1_74_IS407A 4*Chr1_75_IS407A_A	D	gb8_horse_4	4	1073497-1074418	Chr1	IS407A	#ISBma	~~~~~~~~~~~~~~	~~~~~~~~~~~~~~~~~	GCCCATGCCCACGCAGCA	CGAGATG	AGGGACTTTGCTAGAA	GGTCATG	#	GGTC	N.D.
4*Chr1_76_IS407A 4*Chr1_87b_IS407A_A	D-R	gb8_horse_4	4	1078636-1079871	Chr1	IS407A	CGGGA	TGACCTGCCCCCTACA	ATGAAGAAGCGCTTTACG	GCCCGCAAGCCAAACGCG	TGTTGGC	AGGGACTTTGCTAGAA	CGAGCGC	GCCG	CGAG	N
1*Chr1_74_IS407A 1*Chr1_75_IS407A_A	D	ATCC_23344	1	1080184-1081105	Chr1	IS407A	#ISBma	~~~~~~~~~~~~~~	~~~~~~~~~~~~~~~~~	GCCCATGCCCACGCAGCA	CGAGATG	AGGGACTTTGCTAGAA	GGTCATG	#	GGTC	N.D.
1*Chr1_76_IS407A 1*Chr1_87b_IS407A_A	D-R	ATCC_23344	1	1085323-1086558	Chr1	IS407A	CGGGA	TGACCTGCCCCCTACA	ATGAAGAAGCGCTTTACG	GCCCGCAAGCCAAACGCG	TGTTGGC	AGGGACTTTGCTAGAA	CGAGCGC	GCCG	CGAG	N
5*Chr1_69_IS407A 5*Chr1_94b_IS407A_A	D-R	ATCC_10399	5	1109938-1111173	Chr1	IS407A	GGCGA	TGACCTGCCCCCTACA	ATGAAGAAGCGCTTTACG	GCCCGCAAGCCAAACGCG	TGTTGGC	AGGGACTTTGCTAGAA	TGACGTTG	CTGG	TGAC	N
4*Chr1_89_IS407A_A	N	gb8_horse_4	4	1116480-1117715	Chr1	IS407A	GCCCC	TGACCTGCCCCCTACA	ATGAAGAAGCGCTTTACG	GCCCGCAAGCCAAACGCG	TGTTGGC	AGGGACTTTGCTAGAA	GCGCGGA	CCAG	GCGC	N
4*Chr1_91_IS407A_B	N	gb8_horse_4	4	1120220-1121455	Chr1	IS407A	GACGC	TGACCTGCCCCCTACA	ATGAAGAAGCGCTTTACG	GCCCGCAAGCCAAACGCG	TGTTGGC	AGGGACTTTGCTAGAA	TGTCGAC	TGTC	TGTC	Y
1*Chr1_89_IS407A_A	N	ATCC_23344	1	1123337-1124572	Chr1	IS407A	GCCCC	TGACCTGCCCCCTACA	ATGAAGAAGCGCTTTACG	GCCCGCAAGCCAAACGCG	TGTTGGC	AGGGACTTTGCTAGAA	GCGCGGA	CCAG	GCGC	N
1*Chr1_91_IS407A_B	N	ATCC_23344	1	1127077-1128312	Chr1	IS407A	GACGC	TGACCTGCCCCCTACA	ATGAAGAAGCGCTTTACG	GCCCGCAAGCCAAACGCG	TGTTGGC	AGGGACTTTGCTAGAA	TGTCGAC	TGTC	TGTC	Y
8*Chr1_77_IS407A 8*Chr1_94a_IS407A_A	D-R	2002721280	8	1132308-1133543	Chr1	IS407A	ATGAT	TGACCTGCCCCCTACA	ATGAAGAAGCGCTTTACG	GCCCGCAAGCCAAACGCG	TGTTGGC	AGGGACTTTGCTAGAA	AACCGAT	AGGT	AACC	N
2*Chr1_78_IS407A 2*Chr1_80_IS407A_A	D	fmh	2	1150542-1151777	Chr1	IS407A	GGGAC	TGACCTGCCCCCTACA	ATGAAGAAGCGCTTTACG	GCCCGCAAGCCAAACGCG	TGTTGGC	AGGGACTTTGCTAGAA	GTAGCTG	GTCA	GTAG	N
6*Chr1_71_IS407A 6*Chr1_87a_IS407A_A	D-R	NCTC_10229	6	115281-116516	Chr1	IS407A	GAGCG	TGACCTGCCCCCTACA	ATGAAGAAGCGCTTTACG	GCCCGCAAGCCAAACGCG	TGTTGGC	AGGGACTTTGCTAGAA	TCGCTTCT	GAGC	TCGC	N
4*Chr1_77_IS407A 4*Chr1_94a_IS407A_A	D-R	gb8_horse_4	4	1153315-1154550	Chr1	IS407A	ATGAT	TGACCTGCCCCCTACA	ATGAAGAAGCGCTTTACG	GCCCGCAAGCCAAACGCG	TGTTGGC	AGGGACTTTGCTAGAA	AACCGAT	AGGT	AACC	N
1*Chr1_77_IS407A 1*Chr1_94a_IS407A_A	D-R	ATCC_23344	1	1160172-1161407	Chr1	IS407A	ATGAT	TGACCTGCCCCCTACA	ATGAAGAAGCGCTTTACG	GCCCGCAAGCCAAACGCG	TGTTGGC	AGGGACTTTGCTAGAA	AACCGAT	AGGT	AACC	N
2*Chr1_81_IS407A_B	N	fmh	2	1160942-1162177	Chr1	IS407A	GCAAG	TGACCTGCCCCCTACA	ATGAAGAAGCGCTTTACG	GCCCGCAAGCCAAACGCG	TGTTGGC	AGGGACTTTGCTAGAA	GGGCCAT	GGGC	GGGC	Y
3*Chr1_78_IS407A 3*Chr1_80_IS407A_A	D	JHU	3	1161038-1162273	Chr1	IS407A	GGGAC	TGACCTGCCCCCTACA	ATGAAGAAGCGCTTTACG	GCCCGCAAGCCAAACGCG	TGTTGGC	AGGGACTTTGCTAGAA	GTAGCTG	GTCA	GTAG	N
7*Chr1_68_IS407A 7*Chr1_69_IS407A_A	D	NCTC_10247	7	1163594-1164829	Chr1	IS407A	CCTAA	TGACCTGCCCCCTACA	ATGAAGAAGCGCTTTACG	GCCCGCAAGCCAAACGCG	TGTTGGC	AGGGACTTTGCTAGAA	CAGTTTGT	ACGT	CAGT	N
8*Chr1_89_IS407A_A	N	2002721280	8	1167906-1169141	Chr1	IS407A	GCGAA	TGACCTGCCCCCTACA	ATGAAGAAGCGCTTTACG	GCCCGCAAGCCAAACGCG	TGTTGGC	AGGGACTTTGCTAGAA	CTGGATG	CCGC	CTGG	N
3*Chr1_81_IS407A_B	N	JHU	3	1171909-1173144	Chr1	IS407A	GCAAG	TGACCTGCCCCCTACA	ATGAAGAAGCGCTTTACG	GCCCGCAAGCCAAACGCG	TGTTGGC	AGGGACTTTGCTAGAA	GGGCCAT	GGGC	GGGC	Y
10*Chr1_78_IS407 10*Chr1_80_IS407A_A	D	PRL20	10	1172927-1174162	Chr1	IS407A	CAGCA	TGACCTGCCCCCTACA	ATGAAGAAGCGCTTTACG	GCCCGCAAGCCAAACGCG	TGTTGGC	AGGGACTTTGCTAGAA	GTAGCTG	GTAG	GTAG	Y
2*Chr1_83_IS407A_A	N	fmh	2	1174009-1175244	Chr1	IS407A	CAAGT	TGACCTGCCCCCTACA	ATGAAGAAGCGCTTTACG	GCCCGCAAGCCAAACGCG	TGTTGGC	AGGGACTTTGCTAGAA	ATCAGGC	GGAT	ATCA	N
9*Chr1_64a_IS407A_A	N	SAVP1	9	1180766-1182001	Chr1	IS407A	CCAGA	TGACCTGCCCCCTACA	ATGAAGAAGCGCTTTACG	GCCCGCAAGCCAAACGCG	TGTTGGC	AGGGACTTTGCTAGAA	TCGCTTCT	CTTG	TCGC	N
5*Chr1_20_IS407A 5*Chr1_21_IS407A_A	D	ATCC_10399	5	118297-119532	Chr1	IS407A	ATGTC	TGACCTGCCCCCTACA	ATGAAGAAGCGCTTTACG	GCCCGCAAGCCAAACGCG	TGTTGGC	AGGGACTTTGCTAGAA	GTCCACG	CCGG	GTCC	N
3*Chr1_83_IS407A_A	N	JHU	3	1185197-1186432	Chr1	IS407A	CAAGT	TGACCTGCCCCCTACA	ATGAAGAAGCGCTTTACG	GCCCGCAAGCCAAACGCG	TGTTGGC	AGGGACTTTGCTAGAA	ATCAGGC	GGAT	ATCA	N
2*Chr1_84a_IS407A_A	N	fmh	2	1189127-1190362	Chr1	IS407A	GTGCC	TGACCTGCCCCCTACA	ATGAAGAAGCGCTTTACG	GCCCGCAAGCCAAACGCG	TGTTGGC	AGGGACTTTGCTAGAA	GTCGGGC	AAAG	GTCG	N
10*Chr1_83_IS407A_A	N	PRL20	10	1192001-1193236	Chr1	IS407A	TTACG	TGACCTGCCCCCTACA	ATGAAGAAGCGCTTTACG	GCCCGCAAGCCAAACGCG	TGTTGGC	AGGGACTTTGCTAGAA	ATCCACC	TGAT	ATCC	N
3*Chr1_84a_IS407A_A	N	JHU	3	1200315-1201550	Chr1	IS407A	GTGCC	TGACCTGCCCCCTACA	ATGAAGAAGCGCTTTACG	GCCCGCAAGCCAAACGCG	TGTTGGC	AGGGACTTTGCTAGAA	GTCGGGC	AAAG	GTCG	N
8*Chr1_75_IS407A_A	D-R	2002721280	8	1205181-1206102	Chr1	IS407A	#ISBma	~~~~~~~~~~~~~~	~~~~~~~~~~~~~~~~~	GCCCATGCCCACGCAGCA	CGAGATG	AGGGACTTTGCTAGAA	CCATGGC	#	CCAT	N.D.
10*Chr1_84a_IS407A_A	N	PRL20	10	1209165-1210140	Chr1	IS407A	CGCGT	TGACCTGCCCCCTACA	ATGAAGAAGCGCTTTACG	GCCCGCAAGCCAAACGCG	TGTTGGC	#	#contig_br	CGAC	#con	N
8*Chr1_76_IS407A 8*Chr1_84b_IS40 8*Chr1_87b_I	D-R	2002721280	8	1210321-1211556	Chr1	IS407A	CGGGA	TGACCTGCCCCCTACA	ATGAAGAAGCGCTTTACG	GCCCGCAAGCCAAACGCG	TGTTGGC	AGGGACTTTGCTAGAA	GTCGGGC	GCCG	GTCG	N
5*Chr1_71_IS407A 5*Chr1_87a_IS407A_A	D-R	ATCC_10399	5	1229015-1230250	Chr1	IS407A	TTCGA	TGACCTGCCCCCTACA	ATGAAGAAGCGCTTTACG	GCCCGCAAGCCAAACGCG	TGTTGGC	AGGGACTTTGCTAGAA	GCTCGAC	GCGA	GCTC	N

7*Chr1_99_IS407A	7*Chr1_67_IS407A_A	D-R	NCTC_10247	7	1230358-1231593	Chr1	IS407A	TTCAT	TGACCTGCCCCCTACA	ATGAAGAAGCGCTTTACG	GCCCGCAAGCCAAACGCG	TGTTGGC	AGGGACTTTGCTAGAA	GATCGAG	GTGG	GATC	N
2*Chr1_71_IS407A	2*Chr1_87a_IS407A_A	D-R	fmh	2	1233629-1234864	Chr1	IS407A	TTCGA	TGACCTGCCCCCTACA	ATGAAGAAGCGCTTTACG	GCCCGCAAGCCAAACGCG	TGTTGGC	AGGGACTTTGCTAGAA	GCTCGAC	GCGA	GCTC	N
4*Chr1_21_IS407A	4*Chr1_8_IS407A_A	D-R	gb8_horse_4	4	123374-124609	Chr1	IS407A	GGCGC	TGACCTGCCCCCTACA	ATGAAGAAGCGCTTTACG	GCCCGCAAGCCAAACGCG	TGTTGGC	AGGGACTTTGCTAGAA	GTCCACG	GACG	GTCC	N
1*Chr1_21_IS407A	1*Chr1_8_IS407A_A	D-R	ATCC_23344	1	123747-124982	Chr1	IS407A	GGCGC	TGACCTGCCCCCTACA	ATGAAGAAGCGCTTTACG	GCCCGCAAGCCAAACGCG	TGTTGGC	AGGGACTTTGCTAGAA	GTCCACG	GACG	GTCC	N
3*Chr1_71_IS407A	3*Chr1_87a_IS407A_A	D-R	JHU	3	1241561-1242796	Chr1	IS407A	TTCGA	TGACCTGCCCCCTACA	ATGAAGAAGCGCTTTACG	GCCCGCAAGCCAAACGCG	TGTTGGC	AGGGACTTTGCTAGAA	GCTCGAC	GCGA	GCTC	N
9*Chr1_65a_IS407A_A		N	SAVP1	9	1242873-1244108	Chr1	IS407A	GCGAC	TGACCTGCCCCCTACA	ATGAAGAAGCGCTTTACG	GCCCGCAAGCCAAACGCG	TGTTGGC	AGGGACTTTGCTAGAA	GGGGGCA	GAAT	GGGG	N
10*Chr1_71_IS407	10*Chr1_87a_IS407A_A	D-R	PRL20	10	1249788-1251023	Chr1	IS407A	TTCGA	TGACCTGCCCCCTACA	ATGAAGAAGCGCTTTACG	GCCCGCAAGCCAAACGCG	TGTTGGC	AGGGACTTTGCTAGAA	GCTCGAC	GCGA	GCTC	N
8*Chr1_71_IS407A	8*Chr1_87a_IS407A_A	D-R	2002721280	8	1250846-1252081	Chr1	IS407A	GAGCG	TGACCTGCCCCCTACA	ATGAAGAAGCGCTTTACG	GCCCGCAAGCCAAACGCG	TGTTGGC	AGGGACTTTGCTAGAA	TCGCTTCT	GAGC	TCGC	N
4*Chr1_78_IS407A	4*Chr1_80_IS407A_A	D	gb8_horse_4	4	1259592-1260734	Chr1	IS407A	GGGAC	TGACCTGCCCCCTACA	ATGAAGAAGCGCTTTACG	GCCCGCAAGCCAAACGCG	TGTTGGC	AGGGACTTTGCTAGAA	GTAGCTG	GTCA	GTAG	N
6*Chr1_143a_IS407A_B		N	NCTC_10229	6	1262978-1264213	Chr1	IS407A	TTCGC	TGACCTGCCCCCTACA	ATGAAGAAGCGCTTTACG	GCCCGCAAGCCAAACGCG	TGTTGGC	AGGGACTTTGCTAGAA	ATCAAGC	ATCA	ATCA	Y
1*Chr1_78_IS407A	1*Chr1_80_IS407A_A	D	ATCC_23344	1	1267713-1268948	Chr1	IS407A	GGGAC	TGACCTGCCCCCTACA	ATGAAGAAGCGCTTTACG	GCCCGCAAGCCAAACGCG	TGTTGGC	AGGGACTTTGCTAGAA	GTAGCTG	GTCA	GTAG	N
4*Chr1_81_IS407A_B		N	gb8_horse_4	4	1269471-1270706	Chr1	IS407A	GCAAG	TGACCTGCCCCCTACA	ATGAAGAAGCGCTTTACG	GCCCGCAAGCCAAACGCG	TGTTGGC	AGGGACTTTGCTAGAA	GGGCCAT	GGGC	GGGC	Y
5*Chr1_84a_IS407A_A		N	ATCC_10399	5	1270273-1271508	Chr1	IS407A	GTGCC	TGACCTGCCCCCTACA	ATGAAGAAGCGCTTTACG	GCCCGCAAGCCAAACGCG	TGTTGGC	AGGGACTTTGCTAGAA	GTCGGGC	AAAG	GTCG	N
1*Chr1_81_IS407A_B		N	ATCC_23344	1	1277696-1278931	Chr1	IS407A	GCAAG	TGACCTGCCCCCTACA	ATGAAGAAGCGCTTTACG	GCCCGCAAGCCAAACGCG	TGTTGGC	AGGGACTTTGCTAGAA	GGGCCAT	GGGC	GGGC	Y
4*Chr1_83_IS407A_A		N	gb8_horse_4	4	1282759-1283994	Chr1	IS407A	CAAGT	TGACCTGCCCCCTACA	ATGAAGAAGCGCTTTACG	GCCCGCAAGCCAAACGCG	TGTTGGC	AGGGACTTTGCTAGAA	ATCAGGC	GGAT	ATCA	N
5*Chr1_83_IS407A_A		N	ATCC_10399	5	1285391-1286626	Chr1	IS407A	CAAGT	TGACCTGCCCCCTACA	ATGAAGAAGCGCTTTACG	GCCCGCAAGCCAAACGCG	TGTTGGC	AGGGACTTTGCTAGAA	ATCAGGC	GGAT	ATCA	N
1*Chr1_83_IS407A_A		N	ATCC_23344	1	1290984-1292219	Chr1	IS407A	CAAGT	TGACCTGCCCCCTACA	ATGAAGAAGCGCTTTACG	GCCCGCAAGCCAAACGCG	TGTTGGC	AGGGACTTTGCTAGAA	ATCAGGC	GGAT	ATCA	N
9*Chr1_98_IS407A	9*Chr1_66_IS407A_A	D-R	SAVP1	9	1293994-1295229	Chr1	IS407A	CTCGT	TGACCTGCCCCCTACA	ATGAAGAAGCGCTTTACG	GCCCGCAAGCCAAACGCG	TGTTGGC	AGGGACTTTGCTAGAA	GTAGGCA	CCCG	GTAG	N
4*Chr1_84a_IS407A_A		N	gb8_horse_4	4	1297816-1299051	Chr1	IS407A	GTGCC	TGACCTGCCCCCTACA	ATGAAGAAGCGCTTTACG	GCCCGCAAGCCAAACGCG	TGTTGGC	AGGGACTTTGCTAGAA	GTCGGGC	AAAG	GTCG	N
5*Chr1_81_IS407A_B		N	ATCC_10399	5	1298670-1299905	Chr1	IS407A	GCAAG	TGACCTGCCCCCTACA	ATGAAGAAGCGCTTTACG	GCCCGCAAGCCAAACGCG	TGTTGGC	AGGGACTTTGCTAGAA	GGGCCAT	GGGC	GGGC	Y
1*Chr1_84a_IS407A_A		N	ATCC_23344	1	1306102-1307337	Chr1	IS407A	GTGCC	TGACCTGCCCCCTACA	ATGAAGAAGCGCTTTACG	GCCCGCAAGCCAAACGCG	TGTTGGC	AGGGACTTTGCTAGAA	GTCGGGC	AAAG	GTCG	N
5*Chr1_79_IS407A	5*Chr1_80_IS407A_A	D	ATCC_10399	5	1308729-1309964	Chr1	IS407A	GGGAC	TGACCTGCCCCCTACA	ATGAAGAAGCGCTTTACG	GCCCGCAAGCCAAACGCG	TGTTGGC	AGGGACTTTGCTAGAA	CAAGCAG	GTCA	CAAG	N
6*Chr1_145_IS407A_A		N	NCTC_10229	6	1331417-1332652	Chr1	IS407A	AAGCC	TGACCTGCCCCCTACA	ATGAAGAAGCGCTTTACG	GCCCGCAAGCCAAACGCG	TGTTGGC	AGGGACTTTGCTAGAA	GGCTTCG	ATAG	GGCT	N
4*Chr1_71_IS407A	4*Chr1_87a_IS407A_A	D-R	gb8_horse_4	4	1338734-1339969	Chr1	IS407A	TTCGA	TGACCTGCCCCCTACA	ATGAAGAAGCGCTTTACG	GCCCGCAAGCCAAACGCG	TGTTGGC	AGGGACTTTGCTAGAA	GCTCGAC	GCGA	GCTC	N
7*Chr1_43b_IS407A_A		R	NCTC_10247	7	134379-135614	Chr1	IS407A	AAAAA	TGACCTGCCCCCTACA	ATGAAGAAGCGCTTTACG	GCCCGCAAGCCAAACGCG	TGTTGGC	AGGGACTTTGCTAGAA	CGGGCGG	AAGC	CGGG	N
1*Chr1_71_IS407A	1*Chr1_87a_IS407A_A	D-R	ATCC_23344	1	1347348-1348583	Chr1	IS407A	TTCGA	TGACCTGCCCCCTACA	ATGAAGAAGCGCTTTACG	GCCCGCAAGCCAAACGCG	TGTTGGC	AGGGACTTTGCTAGAA	GCTCGAC	GCGA	GCTC	N
5*Chr1_78_IS407A_A		N	ATCC_10399	5	1351723-1352958	Chr1	IS407A	CAGCA	TGACCTGCCCCCTACA	ATGAAGAAGCGCTTTACG	GCCCGCAAGCCAAACGCG	TGTTGGC	AGGGACTTTGCTAGAA	GTAGCTG	GTAG	GTAG	Y
9*Chr1_96_IS407A	9*Chr1_97_IS407A_A	D	SAVP1	9	1352573-1353808	Chr1	IS407A	GGCAC	TGACCTGCCCCCTACA	ATGAAGAAGCGCTTTACG	GCCCGCAAGCCAAACGCG	TGTTGGC	AGGGACTTTGCTAGAA	ACCGCCC	CCCC	ACCG	N
2*Chr1_68_IS407A	2*Chr1_69_IS407A_A	D	fmh	2	1353867-1355102	Chr1	IS407A	CCTAA	TGACCTGCCCCCTACA	ATGAAGAAGCGCTTTACG	GCCCGCAAGCCAAACGCG	TGTTGGC	AGGGACTTTGCTAGAA	CCAGTTTG	ACGT	CCAG	N
3*Chr1_68_IS407A	3*Chr1_69_IS407A_A	D	JHU	3	1363269-1364504	Chr1	IS407A	CCTAA	TGACCTGCCCCCTACA	ATGAAGAAGCGCTTTACG	GCCCGCAAGCCAAACGCG	TGTTGGC	AGGGACTTTGCTAGAA	CCAGTTTG	ACGT	CCAG	N
7*Chr1_126a_IS407A_A		R	NCTC_10247	7	136915-138150	Chr1	IS407A	TGCCG	TGACCTGCCCCCTACA	ATGAAGAAGCGCTTTACG	GCCCGCAAGCCAAACGCG	TGTTGGC	AGGGACTTTGCTAGAA	CCTGCGA	AAAA	CCTG	N
8*Chr1_68_IS407A	8*Chr1_69_IS407A_A	D	2002721280	8	1369943-1371178	Chr1	IS407A	CCTAA	TGACCTGCCCCCTACA	ATGAAGAAGCGCTTTACG	GCCCGCAAGCCAAACGCG	TGTTGGC	AGGGACTTTGCTAGAA	CCAGTTTG	ACGT	CCAG	N
9*Chr1_95_IS407A_A		N	SAVP1	9	1395694-1396929	Chr1	IS407A	GGCGC	TGACCTGCCCCCTACA	ATGAAGAAGCGCTTTACG	GCCCGCAAGCCAAACGCG	TGTTGGC	AGGGACTTTGCTAGAA	GCCCGCC	GATG	GCCC	N
4*Chr1_10_IS407A_A		N	gb8_horse_4	4	140426-141661	Chr1	IS407A	TCCGT	TGACCTGCCCCCTACA	ATGAAGAAGCGCTTTACG	GCCCGCAAGCCAAACGCG	TGTTGGC	AGGGACTTTGCTAGAA	CCCTATGC	ACAT	CCCT	N
10*Chr1_68_IS407	10*Chr1_72_IS407A_A	D-R	PRL20	10	1412924-1414159	Chr1	IS407A	CCAGA	TGACCTGCCCCCTACA	ATGAAGAAGCGCTTTACG	GCCCGCAAGCCAAACGCG	TGTTGGC	AGGGACTTTGCTAGAA	ACGTTGCT	CGCG	ACGT	N
1*Chr1_10_IS407A_A		N	ATCC_23344	1	141323-142558	Chr1	IS407A	TCCGT	TGACCTGCCCCCTACA	ATGAAGAAGCGCTTTACG	GCCCGCAAGCCAAACGCG	TGTTGGC	AGGGACTTTGCTAGAA	CCCTATGC	ACAT	CCCT	N
3*Chr1_10_IS407A_A		N	JHU	3	14148-15383	Chr1	IS407A	TCCGT	TGACCTGCCCCCTACA	ATGAAGAAGCGCTTTACG	GCCCGCAAGCCAAACGCG	TGTTGGC	AGGGACTTTGCTAGAA	CCCTATGC	ACAT	CCCT	N
2*Chr1_99_IS407A	2*Chr1_67_IS407A_A	D-R	fmh	2	1420660-1421895	Chr1	IS407A	TTCAT	TGACCTGCCCCCTACA	ATGAAGAAGCGCTTTACG	GCCCGCAAGCCAAACGCG	TGTTGGC	AGGGACTTTGCTAGAA	GATCGAG	GTGG	GATC	N
3*Chr1_99_IS407A	3*Chr1_67_IS407A_A	D-R	JHU	3	1430062-1431297	Chr1	IS407A	TTCAT	TGACCTGCCCCCTACA	ATGAAGAAGCGCTTTACG	GCCCGCAAGCCAAACGCG	TGTTGGC	AGGGACTTTGCTAGAA	GATCGAG	GTGG	GATC	N
8*Chr1_99_IS407A	8*Chr1_67_IS407A_A	D-R	2002721280	8	1436504-1437739	Chr1	IS407A	TTCAT	TGACCTGCCCCCTACA	ATGAAGAAGCGCTTTACG	GCCCGCAAGCCAAACGCG	TGTTGGC	AGGGACTTTGCTAGAA	ACTTGATT	GTGG	ACTT	N
8*Chr1_10_IS407A_A		N	2002721280	8	14396-15631	Chr1	IS407A	CGCCA	TGACCTGCCCCCTACA	ATGAAGAAGCGCTTTACG	GCCCGCAAGCCAAACGCG	TGTTGGC	AGGGACTTTGCTAGAA	CCCTATGC	CCCT	CCCT	Y
9*Chr1_75_IS407A	9*Chr1_94b_IS407A_A	D-R	SAVP1	9	1454166-1455087	Chr1	IS407A	#ISBma	~~~~~~~~~~~~~~	~~~~~~~~~~~~~~~~~	GCCCATGCCCACGCAGCA	CGAGATG	AGGGACTTTGCTAGAA	ACGTTGCT	#	ACGT	N.D.
4*Chr1_68_IS407A	4*Chr1_69_IS407A_A	D	gb8_horse_4	4	1456856-1458091	Chr1	IS407A	CCTAA	TGACCTGCCCCCTACA	ATGAAGAAGCGCTTTACG	GCCCGCAAGCCAAACGCG	TGTTGGC	AGGGACTTTGCTAGAA	CCAGTTTG	ACGT	CCAG	N
2*Chr1_10_IS407A_A		N	fmh	2	14571-15806	Chr1	IS407A	TCCGT	TGACCTGCCCCCTACA	ATGAAGAAGCGCTTTACG	GCCCGCAAGCCAAACGCG	TGTTGGC	AGGGACTTTGCTAGAA	CCCTATGC	ACAT	CCCT	N
5*Chr1_77_IS407A	5*Chr1_94a_IS407A_A	D-R	ATCC_10399	5	1459172-1460407	Chr1	IS407A	ATGAT	TGACCTGCCCCCTACA	ATGAAGAAGCGCTTTACG	GCCCGCAAGCCAAACGCG	TGTTGGC	AGGGACTTTGCTAGAA	AACCGAT	AGGT	AACC	N
9*Chr1_76_IS407A	9*Chr1_87b_IS407A_A	D-R	SAVP1	9	1459306-1460541	Chr1	IS407A	AATGT	TGACCTGCCCCCTACA	ATGAAGAAGCGCTTTACG	GCCCGCAAGCCAAACGCG	TGTTGGC	AGGGACTTTGCTAGAA	CGGCACG	CTCG	CGGC	N
9*Chr1_158_IS407A_A		D-R	SAVP1	9	14627-15862	Chr1	IS407A	AGCGA	TGACCTGCCCCCTACA	ATGAAGAAGCGCTTTACG	GCCCGCAAGCCAAACGCG	TGTTGGC	AGGGACTTTGCTAGAA	AATCCACT	TGTT	AATC	N
1*Chr1_68_IS407A	1*Chr1_69_IS407A_A	D	ATCC_23344	1	1466275-1467510	Chr1	IS407A	CCTAA	TGACCTGCCCCCTACA	ATGAAGAAGCGCTTTACG	GCCCGCAAGCCAAACGCG	TGTTGGC	AGGGACTTTGCTAGAA	CCAGTTTG	ACGT	CCAG	N
5*Chr1_10_IS407A_A		N	ATCC_10399	5	14735-15970	Chr1	IS407A	CGCCA	TGACCTGCCCCCTACA	ATGAAGAAGCGCTTTACG	GCCCGCAAGCCAAACGCG	TGTTGGC	AGGGACTTTGCTAGAA	CCCTATGC	CCCT	CCCT	Y
10*Chr1_99_IS407	10*Chr1_67_IS407A_A	D-R	PRL20	10	1479698-1480933	Chr1	IS407A	TTCAT	TGACCTGCCCCCTACA	ATGAAGAAGCGCTTTACG	GCCCGCAAGCCAAACGCG	TGTTGGC	AGGGACTTTGCTAGAA	GATCGAG	GTGG	GATC	N
5*Chr1_91_IS407A_B		N	ATCC_10399	5	1492267-1493502	Chr1	IS407A	GACGC	TGACCTGCCCCCTACA	ATGAAGAAGCGCTTTACG	GCCCGCAAGCCAAACGCG	TGTTGGC	AGGGACTTTGCTAGAA	TGTCGAC	TGTC	TGTC	Y
5*Chr1_89_IS407A_A		N	ATCC_10399	5	1496007-1497242	Chr1	IS407A	GCCCC	TGACCTGCCCCCTACA	ATGAAGAAGCGCTTTACG	GCCCGCAAGCCAAACGCG	TGTTGGC	AGGGACTTTGCTAGAA	GGCGCGG	CCAG	GGCG	N
9*Chr1_89_IS407A_A		N	SAVP1	9	1497321-1498556	Chr1	IS407A	GAGCC	TGACCTGCCCCCTACA	ATGAAGAAGCGCTTTACG	GCCCGCAAGCCAAACGCG	TGTTGGC	AGGGACTTTGCTAGAA	CTGGATG	CGCA	CTGG	N
9*Chr1_93_IS407A_C		N	SAVP1	9	1503308-1504543	Chr1	IS407A	CGCGC	TGACCTGCCCCCTACA	ATGAAGAAGCGCTTTACG	GCCCGCAAGCCAAACGCG	TGTTGGC	AGGGACTTTGCTAGAA	TTCGTGCG	TTCG	TTCG	Y
4*Chr1_99_IS407A	4*Chr1_67_IS407A_A	D-R	gb8_horse_4	4	1523634-1524869	Chr1	IS407A	TTCAT	TGACCTGCCCCCTACA	ATGAAGAAGCGCTTTACG	GCCCGCAAGCCAAACGCG	TGTTGGC	AGGGACTTTGCTAGAA	GATCGAG	GTGG	GATC	N
1*Chr1_99_IS407A	1*Chr1_67_IS407A_A	D-R	ATCC_23344	1	1533068-1534303	Chr1	IS407A	TTCAT	TGACCTGCCCCCTACA	ATGAAGAAGCGCTTTACG	GCCCGCAAGCCAAACGCG	TGTTGGC	AGGGACTTTGCTAGAA	GATCGAG	GTGG	GATC	N
5*Chr1_76_IS407A	5*Chr1_87b_IS407A_A	D-R	ATCC_10399	5	1534024-1535259	Chr1	IS407A	CGGGA	TGACCTGCCCCCTACA	ATGAAGAAGCGCTTTACG	GCCCGCAAGCCAAACGCG	TGTTGGC	AGGGACTTTGCTAGAA	CGAGCGC	GCCG	CGAG	N
9*Chr1_77_IS407A	9*Chr1_94a_IS407A_A	D-R	SAVP1	9	1534145-1535380	Chr1	IS407A	ATGAT	TGACCTGCCCCCTACA	ATGAAGAAGCGCTTTACG	GCCCGCAAGCCAAACGCG	TGTTGGC	AGGGACTTTGCTAGAA	AACCGAT	AGGT	AACC	N

5*Chr1_74_IS407A	5*Chr1_75_IS407A_A	D	ATCC_10399	5	1539476-1540397	Chr1	IS407A	#ISBma	~~~~~~~~~~~~~~~	~~~~~~~~~~~~~~~~~	GCCCATGCCCACGCAGCA	CGAGATG	AGGGACTTTGCTAGAA	GGTCATG	#	GGTC	N.D.
7*Chr1_111_IS407	7*Chr1_56_IS407A_A	D-R	NCTC_10247	7	1544024-1545259	Chr1	IS407A	TGGAA	TGACCTGCCCCCTACA	ATGAAGAAGCGCTTTACG	GCCCGCAAGCCAAACGCG	TGTTGGC	AGGGACTTTGCTAGAA	CAGGCGC	GCGG	CAGG	N
7*Chr1_54_IS407A	7*Chr1_55_IS407A_A	D	NCTC_10247	7	1557548-1558783	Chr1	IS407A	ATCAC	TGACCTGCCCCCTACA	ATGAAGAAGCGCTTTACG	GCCCGCAAGCCAAACGCG	TGTTGGC	AGGGACTTTGCTAGAA	GCGGGTC	GCGT	GCGG	N
6*Chr1_162_IS407	6*Chr1_29_IS407A_A	D-R	NCTC_10229	6	1564428-1565663	Chr1	IS407A	ATTTAT	TGACCTGCCCCCTACA	ATGAAGAAGCGCTTTACG	GCCCGCAAGCCAAACGCG	TGTTGGC	AGGGACTTTGCTAGAA	ATCAGCA	CATT	ATCA	N
6*Chr1_75_IS407A	6*Chr1_84b_IS407A_A	D-R	NCTC_10229	6	156544-157465	Chr1	IS407A	#ISBma	~~~~~~~~~~~~~~~	~~~~~~~~~~~~~~~~~	GCCCATGCCCACGCAGCA	CGAGATG	AGGGACTTTGCTAGAA	GTCGGGC	#	GTCG	N.D.
3*Chr1_33_IS407A	3*Chr1_34_IS407A_A	D	JHU	3	157735-158970	Chr1	IS407A	CCGCA	TGACCTGCCCCCTACA	ATGAAGAAGCGCTTTACG	GCCCGCAAGCCAAACGCG	TGTTGGC	AGGGACTTTGCTAGAA	GCAACGG	CAAT	GCAA	N
2*Chr1_33_IS407A	2*Chr1_34_IS407A_A	D	fmh	2	157737-158972	Chr1	IS407A	CCGCA	TGACCTGCCCCCTACA	ATGAAGAAGCGCTTTACG	GCCCGCAAGCCAAACGCG	TGTTGGC	AGGGACTTTGCTAGAA	GCAACGG	CAAT	GCAA	N
8*Chr1_33_IS407A	8*Chr1_35a_IS407A_A	D-R	2002721280	8	158042-159277	Chr1	IS407A	CCGCA	TGACCTGCCCCCTACA	ATGAAGAAGCGCTTTACG	GCCCGCAAGCCAAACGCG	TGTTGGC	AGGGACTTTGCTAGAA	GGGGCAG	CAAT	GGGG	N
6*Chr1_76_IS407A	6*Chr1_87b_IS407A_A	D-R	NCTC_10229	6	161685-162920	Chr1	IS407A	CGGGA	TGACCTGCCCCCTACA	ATGAAGAAGCGCTTTACG	GCCCGCAAGCCAAACGCG	TGTTGGC	AGGGACTTTGCTAGAA	CCATGGC	GCCG	CCAT	N
5*Chr1_68_IS407A	5*Chr1_72_IS407A_A	D-R	ATCC_10399	5	1619910-1621145	Chr1	IS407A	CCAGA	TGACCTGCCCCCTACA	ATGAAGAAGCGCTTTACG	GCCCGCAAGCCAAACGCG	TGTTGGC	AGGGACTTTGCTAGAA	TGACGTT	CGCG	TGAC	N
9*Chr1_27_IS407A_B		N	SAVP1	9	162059-163294	Chr1	IS407A	ATGGG	TGACCTGCCCCCTACA	ATGAAGAAGCGCTTTACG	GCCCGCAAGCCAAACGCG	TGTTGGC	AGGGACTTTGCTAGAA	GGTGGCC	GGTG	GGTG	Y
5*Chr1_18b_IS407	5*Chr1_30_IS407A_A	D-R	ATCC_10399	5	163047-164282	Chr1	IS407A	CGTCG	TGACCTGCCCCCTACA	ATGAAGAAGCGCTTTACG	GCCCGCAAGCCAAACGCG	TGTTGGC	AGGGACTTTGCTAGAA	CGCCGCA	CACC	CGCC	N
9*Chr1_78_IS407A_A		N	SAVP1	9	1641740-1642975	Chr1	IS407A	CAGCA	TGACCTGCCCCCTACA	ATGAAGAAGCGCTTTACG	GCCCGCAAGCCAAACGCG	TGTTGGC	AGGGACTTTGCTAGAA	GTAGCTG	GTAG	GTAG	Y
7*Chr1_112_IS407	7*Chr1_49_IS407A_A	D-R	NCTC_10247	7	1661173-1662408	Chr1	IS407A	GCCCT	TGACCTGCCCCCTACA	ATGAAGAAGCGCTTTACG	GCCCGCAAGCCAAACGCG	TGTTGGC	AGGGACTTTGCTAGAA	TTGACGC	GCCC	TTGA	N
6*Chr1_160a_IS40	6*Chr1_161_IS407A_A	D-R	NCTC_10229	6	1685235-1686470	Chr1	IS407A	TACGA	TGACCTGCCCCCTACA	ATGAAGAAGCGCTTTACG	GCCCGCAAGCCAAACGCG	TGTTGGC	AGGGACTTTGCTAGAA	GTCCGGG	TTTT	GTCC	N
9*Chr1_79_IS407A	9*Chr1_80_IS407A_A	D	SAVP1	9	1685609-1686844	Chr1	IS407A	GGGAC	TGACCTGCCCCCTACA	ATGAAGAAGCGCTTTACG	GCCCGCAAGCCAAACGCG	TGTTGGC	AGGGACTTTGCTAGAA	CAAGCAG	GTCA	CAAG	N
5*Chr1_99_IS407A	5*Chr1_67_IS407A_A	D-R	ATCC_10399	5	1686724-1687959	Chr1	IS407A	TTCAT	TGACCTGCCCCCTACA	ATGAAGAAGCGCTTTACG	GCCCGCAAGCCAAACGCG	TGTTGGC	AGGGACTTTGCTAGAA	GATCGAG	GTGG	GATC	N
9*Chr1_83_IS407A_A		N	SAVP1	9	1707634-1708869	Chr1	IS407A	TTACG	TGACCTGCCCCCTACA	ATGAAGAAGCGCTTTACG	GCCCGCAAGCCAAACGCG	TGTTGGC	AGGGACTTTGCTAGAA	ATCCACC	TGAT	ATCC	N
9*Chr1_84a_IS407A_A		N	SAVP1	9	1722752-1723987	Chr1	IS407A	GTGCC	TGACCTGCCCCCTACA	ATGAAGAAGCGCTTTACG	GCCCGCAAGCCAAACGCG	TGTTGGC	AGGGACTTTGCTAGAA	GTCGGGC	AAAG	GTCG	N
7*Chr1_113_IS407A_A		N	NCTC_10247	7	1731002-1732237	Chr1	IS407A	TTTGCT	TGACCTGCCCCCTACA	ATGAAGAAGCGCTTTACG	GCCCGCAAGCCAAACGCG	TGTTGGC	AGGGACTTTGCTAGAA	ATCTACCT	CCTC	ATCT	N
9*Chr1_86_IS407A_C		N	SAVP1	9	1733018-1734253	Chr1	IS407A	GCGGT	TGACCTGCCCCCTACA	ATGAAGAAGCGCTTTACG	GCCCGCAAGCCAAACGCG	TGTTGGC	AGGGACTTTGCTAGAA	GTCCCGC	GTCC	GTCC	Y
2*Chr1_111_IS407	2*Chr1_64a_IS407A_A	D-R	fmh	2	1740034-1741269	Chr1	IS407A	CTTGTT	TGACCTGCCCCCTACA	ATGAAGAAGCGCTTTACG	GCCCGCAAGCCAAACGCG	TGTTGGC	AGGGACTTTGCTAGAA	CTGCTGC	TCGC	CTGC	N
7*Chr1_114_IS407A_A		N	NCTC_10247	7	1744605-1745840	Chr1	IS407A	TGATT	TGACCTGCCCCCTACA	ATGAAGAAGCGCTTTACG	GCCCGCAAGCCAAACGCG	TGTTGGC	AGGGACTTTGCTAGAA	GATCCGG	GAAG	GATC	N
10*Chr1_20_IS407	10*Chr1_21_IS407A_A	D	PRL20	10	174762-175997	Chr1	IS407A	ATGTC	TGACCTGCCCCCTACA	ATGAAGAAGCGCTTTACG	GCCCGCAAGCCAAACGCG	TGTTGGC	AGGGACTTTGCTAGAA	GTCCACGT	CCGG	GTCC	N
6*Chr1_149_IS407	6*Chr1_160b_IS407A_A	D-R	NCTC_10229	6	1749741-1750976	Chr1	IS407A	CGCGC	TGACCTGCCCCCTACA	ATGAAGAAGCGCTTTACG	GCCCGCAAGCCAAACGCG	TGTTGGC	AGGGACTTTGCTAGAA	CCTCACG	GTCC	CCTC	N
8*Chr1_111_IS407	8*Chr1_56_IS407A_A	D-R	2002721280	8	1749933-1751168	Chr1	IS407A	TGGAA	TGACCTGCCCCCTACA	ATGAAGAAGCGCTTTACG	GCCCGCAAGCCAAACGCG	TGTTGGC	AGGGACTTTGCTAGAA	CAGGCGC	GCGG	CAGG	N
3*Chr1_35a_IS407A_A		N	JHU	3	175095-176330	Chr1	IS407A	GTGAA	TGACCTGCCCCCTACA	ATGAAGAAGCGCTTTACG	GCCCGCAAGCCAAACGCG	TGTTGGC	AGGGACTTTGCTAGAA	CCGGAAC	GATT	CCGG	N
2*Chr1_35a_IS407A_A		N	fmh	2	175097-176332	Chr1	IS407A	GTGAA	TGACCTGCCCCCTACA	ATGAAGAAGCGCTTTACG	GCCCGCAAGCCAAACGCG	TGTTGGC	AGGGACTTTGCTAGAA	CCGGAAC	GATT	CCGG	N
3*Chr1_111_IS407	3*Chr1_64a_IS407A_A	D-R	JHU	3	1752139-1753374	Chr1	IS407A	CTTGTT	TGACCTGCCCCCTACA	ATGAAGAAGCGCTTTACG	GCCCGCAAGCCAAACGCG	TGTTGGC	AGGGACTTTGCTAGAA	CTGCTGC	TCGC	CTGC	N
8*Chr1_34_IS407A	8*Chr1_35b_IS407A_A	D-R	2002721280	8	176042-177277	Chr1	IS407A	GTGAA	TGACCTGCCCCCTACA	ATGAAGAAGCGCTTTACG	GCCCGCAAGCCAAACGCG	TGTTGGC	AGGGACTTTGCTAGAA	GGGGGAT	GATT	GGGG	N
8*Chr1_54_IS407A	8*Chr1_55_IS407A_A	D	2002721280	8	1763457-1764692	Chr1	IS407A	ATCAC	TGACCTGCCCCCTACA	ATGAAGAAGCGCTTTACG	GCCCGCAAGCCAAACGCG	TGTTGGC	AGGGACTTTGCTAGAA	GCGGGTC	GCGT	GCGG	N
9*Chr1_71_IS407A	9*Chr1_87a_IS407A_A	D-R	SAVP1	9	1765215-1766450	Chr1	IS407A	TTCGA	TGACCTGCCCCCTACA	ATGAAGAAGCGCTTTACG	GCCCGCAAGCCAAACGCG	TGTTGGC	AGGGACTTTGCTAGAA	GCTCGAC	GCGA	GCTC	N
6*Chr1_158_IS407A_A		D-R	NCTC_10229	6	1794681-1795916	Chr1	IS407A	AACCC	TGACCTGCCCCCTACA	ATGAAGAAGCGCTTTACG	GCCCGCAAGCCAAACGCG	TGTTGGC	AGGGACTTTGCTAGAA	ATCCACTC	ATGG	ATCC	N
10*Chr1_111_IS40	10*Chr1_53a_IS407A_B	D-R	PRL20	10	1798541-1799776	Chr1	IS407A	GAAGG	TGACCTGCCCCCTACA	ATGAAGAAGCGCTTTACG	GCCCGCAAGCCAAACGCG	TGTTGGC	AGGGACTTTGCTAGAA	CTGCTGC	ACGG	CTGC	N
7*Chr1_115a_IS40	7*Chr1_132b_IS407A_A	R	NCTC_10247	7	1815700-1816935	Chr1	IS407A	TTCGC	TGACCTGCCCCCTACA	ATGAAGAAGCGCTTTACG	GCCCGCAAGCCAAACGCG	TGTTGGC	AGGGACTTTGCTAGAA	GGACGGG	GATG	GGAC	N
2*Chr1_61_IS407A_B		N	fmh	2	1821443-1822678	Chr1	IS407A	TTTGCT	TGACCTGCCCCCTACA	ATGAAGAAGCGCTTTACG	GCCCGCAAGCCAAACGCG	TGTTGGC	AGGGACTTTGCTAGAA	CCCGCAC	CCCG	CCCG	Y
7*Chr1_122_IS407A_A		N	NCTC_10247	7	182349-183584	Chr1	IS407A	GCGCA	TGACCTGCCCCCTACA	ATGAAGAAGCGCTTTACG	GCCCGCAAGCCAAACGCG	TGTTGGC	AGGGACTTTGCTAGAA	GTGCTGC	GAAG	GTGC	N
3*Chr1_61_IS407A_B		N	JHU	3	1835549-1836784	Chr1	IS407A	TTTGCT	TGACCTGCCCCCTACA	ATGAAGAAGCGCTTTACG	GCCCGCAAGCCAAACGCG	TGTTGGC	AGGGACTTTGCTAGAA	CCCGCAC	CCCG	CCCG	Y
4*Chr1_111_IS407	4*Chr1_64a_IS407A_A	D-R	gb8_horse_4	4	1838521-1839756	Chr1	IS407A	CTTGTT	TGACCTGCCCCCTACA	ATGAAGAAGCGCTTTACG	GCCCGCAAGCCAAACGCG	TGTTGGC	AGGGACTTTGCTAGAA	CTGCTGC	TCGC	CTGC	N
1*Chr1_111_IS407	1*Chr1_64a_IS407A_A	D-R	ATCC_23344	1	1849090-1850325	Chr1	IS407A	CTTGTT	TGACCTGCCCCCTACA	ATGAAGAAGCGCTTTACG	GCCCGCAAGCCAAACGCG	TGTTGGC	AGGGACTTTGCTAGAA	CTGCTGC	TCGC	CTGC	N
6*Chr1_150_IS407	6*Chr1_5_IS407A_A	D-R	NCTC_10229	6	1857854-1859089	Chr1	IS407A	ATCGC	TGACCTGCCCCCTACA	ATGAAGAAGCGCTTTACG	GCCCGCAAGCCAAACGCG	TGTTGGC	AGGGACTTTGCTAGAA	GCGCCCG	CAAG	GCGC	N
8*Chr1_112_IS407	8*Chr1_49_IS407A_A	D-R	2002721280	8	1867070-1868305	Chr1	IS407A	GCCCT	TGACCTGCCCCCTACA	ATGAAGAAGCGCTTTACG	GCCCGCAAGCCAAACGCG	TGTTGGC	AGGGACTTTGCTAGAA	TTGACGC	GCCC	TTGA	N
9*Chr1_68_IS407A	9*Chr1_69_IS407A_A	D	SAVP1	9	1884073-1885308	Chr1	IS407A	CCTAA	TGACCTGCCCCCTACA	ATGAAGAAGCGCTTTACG	GCCCGCAAGCCAAACGCG	TGTTGGC	AGGGACTTTGCTAGAA	ATCAGCC	ACGT	ATCA	N
7*Chr1_134_IS407A_B		N	NCTC_10247	7	1892663-1893898	Chr1	IS407A	CCTAT	TGACCTGCCCCCTACA	ATGAAGAAGCGCTTTACG	GCCCGCAAGCCAAACGCG	TGTTGGC	AGGGACTTTGCTAGAA	GCACGGC	GCAC	GCAC	Y
6*Chr1_2a_IS407A_A		null	NCTC_10229	6	1904335-1905570	Chr1	IS407A	GATGC	TGACCTGCCCCCTACA	ATGAAGAAGCGCTTTACG	GCCCGCAAGCCAAACGCG	TGTTGGC	AGGGACTTTGCTAGAA	ACGAGCT	GACG	ACGA	N
4*Chr1_61_IS407A_B		N	gb8_horse_4	4	1918701-1919936	Chr1	IS407A	TTTGCT	TGACCTGCCCCCTACA	ATGAAGAAGCGCTTTACG	GCCCGCAAGCCAAACGCG	TGTTGGC	AGGGACTTTGCTAGAA	CCCGCAC	CCCG	CCCG	Y
7*Chr1_126b_IS40	7*Chr1_135_IS407A_A	D-R	NCTC_10247	7	1927000-1928235	Chr1	IS407A	CTGGA	TGACCTGCCCCCTACA	ATGAAGAAGCGCTTTACG	GCCCGCAAGCCAAACGCG	TGTTGGC	AGGGACTTTGCTAGAA	CCGGAAG	ATCG	CCGG	N
1*Chr1_61_IS407A_B		N	ATCC_23344	1	1930663-1931898	Chr1	IS407A	TTTGCT	TGACCTGCCCCCTACA	ATGAAGAAGCGCTTTACG	GCCCGCAAGCCAAACGCG	TGTTGGC	AGGGACTTTGCTAGAA	CCCGCAC	CCCG	CCCG	Y
6*Chr1_152a_IS40	6*Chr1_154a_IS407A_A	R	NCTC_10229	6	1936574-1937809	Chr1	IS407A	CGCAG	TGACCTGCCCCCTACA	ATGAAGAAGCGCTTTACG	GCCCGCAAGCCAAACGCG	TGTTGGC	AGGGACTTTGCTAGAA	GGGACGC	CTGT	GGGA	N
8*Chr1_113_IS407A_A		N	2002721280	8	1937018-1938253	Chr1	IS407A	TTTGCT	TGACCTGCCCCCTACA	ATGAAGAAGCGCTTTACG	GCCCGCAAGCCAAACGCG	TGTTGGC	AGGGACTTTGCTAGAA	ATCTACCT	CCTC	ATCT	N
2*Chr1_48_IS407A	2*Chr1_58b_IS407A_A	D-R	fmh	2	1944299-1945534	Chr1	IS407A	CGTCG	TGACCTGCCCCCTACA	ATGAAGAAGCGCTTTACG	GCCCGCAAGCCAAACGCG	TGTTGGC	AGGGACTTTGCTAGAA	GAAGTGG	GTGA	GAAG	N
8*Chr1_114_IS407A_A		N	2002721280	8	1950627-1951862	Chr1	IS407A	TGATT	TGACCTGCCCCCTACA	ATGAAGAAGCGCTTTACG	GCCCGCAAGCCAAACGCG	TGTTGGC	AGGGACTTTGCTAGAA	GATCCGG	GAAG	GATC	N
9*Chr1_99_IS407A	9*Chr1_67_IS407A_A	D-R	SAVP1	9	1950881-1952116	Chr1	IS407A	TTCAT	TGACCTGCCCCCTACA	ATGAAGAAGCGCTTTACG	GCCCGCAAGCCAAACGCG	TGTTGGC	AGGGACTTTGCTAGAA	GATCGAG	GTGG	GATC	N
3*Chr1_48_IS407A	3*Chr1_58b_IS407A_A	D-R	JHU	3	1959751-1960986	Chr1	IS407A	CGTCG	TGACCTGCCCCCTACA	ATGAAGAAGCGCTTTACG	GCCCGCAAGCCAAACGCG	TGTTGGC	AGGGACTTTGCTAGAA	GAAGTGG	GTGA	GAAG	N
2*Chr1_132b_IS40	2*Chr1_47b_IS407A_A	R	fmh	2	1963199-1964434	Chr1	IS407A	TGAGG	TGACCTGCCCCCTACA	ATGAAGAAGCGCTTTACG	GCCCGCAAGCCAAACGCG	TGTTGGC	AGGGACTTTGCTAGAA	GGACGGG	CCTT	GGAC	N
3*Chr1_132b_IS40	3*Chr1_47b_IS407A_A	R	JHU	3	1978651-1979886	Chr1	IS407A	TGAGG	TGACCTGCCCCCTACA	ATGAAGAAGCGCTTTACG	GCCCGCAAGCCAAACGCG	TGTTGGC	AGGGACTTTGCTAGAA	GGACGGG	CCTT	GGAC	N
6*Chr1_152b_IS40	6*Chr1_157_IS407A_A	D-R	NCTC_10229	6	1984096-1985331	Chr1	IS407A	GAAAC	TGACCTGCCCCCTACA	ATGAAGAAGCGCTTTACG	GCCCGCAAGCCAAACGCG	TGTTGGC	AGGGACTTTGCTAGAA	GATGCCTT	GGTC	GATG	N
6*Chr1_89_IS407A_A		N	NCTC_10229	6	198950-200185	Chr1	IS407A	GCGAA	TGACCTGCCCCCTACA	ATGAAGAAGCGCTTTACG	GCCCGCAAGCCAAACGCG	TGTTGGC	AGGGACTTTGCTAGAA	CTGGATG	CCGC	CTGG	N

10*Chr1_54_IS407	10*Chr1_58b_IS407A_A	D-R	PRL20	10	1994927-1996162	Chr1	IS407A	CGTCG	TGACCTGCCCCCTACA	ATGAAGAAGCGCTTTACGC	GCCCGCAAGCCAAACGCG	TGTTGGC	AGGGACTTTGCTAGAA	CGAACAC	GTGA	CGAA	N
5*Chr1_111_IS407	5*Chr1_64a_IS407A_A	D-R	ATCC_10399	5	2000844-2002079	Chr1	IS407A	CTTGTT	TGACCTGCCCCCTACA	ATGAAGAAGCGCTTTACGC	GCCCGCAAGCCAAACGCG	TGTTGGC	AGGGACTTTGCTAGAA	CTGCTGC	TCGC	CTGC	N
10*Chr1_53b_IS40	10*Chr1_56_IS407A_A	D-R	PRL20	10	2008080-2009315	Chr1	IS407A	TGGAA	TGACCTGCCCCCTACA	ATGAAGAAGCGCTTTACGC	GCCCGCAAGCCAAACGCG	TGTTGGC	AGGGACTTTGCTAGAA	ACGGCGA	GCGG	ACGG	N
6*Chr1_154b_IS407A_A		R	NCTC_10229	6	2019764-2020999	Chr1	IS407A	CGCGC	TGACCTGCCCCCTACA	ATGAAGAAGCGCTTTACGC	GCCCGCAAGCCAAACGCG	TGTTGGC	AGGGACTTTGCTAGAA	AACCCTC	GCTT	AACC	N
8*Chr1_115a_IS40	8*Chr1_132b_IS407A_A	R	2002721280	8	2021618-2022853	Chr1	IS407A	TTCGC	TGACCTGCCCCCTACA	ATGAAGAAGCGCTTTACGC	GCCCGCAAGCCAAACGCG	TGTTGGC	AGGGACTTTGCTAGAA	GGACGGG	GATG	GGAC	N
10*Chr1_48_IS407	10*Chr1_55_IS407A_A	D-R	PRL20	10	2021618-2022853	Chr1	IS407A	ATCAC	TGACCTGCCCCCTACA	ATGAAGAAGCGCTTTACGC	GCCCGCAAGCCAAACGCG	TGTTGGC	AGGGACTTTGCTAGAA	TTCCGGC	GCGG	TTCC	N
5*Chr1_62_IS407A_C		N	ATCC_10399	5	2032228-2033345	Chr1	IS407A	#contig	~~~~~~~~~~~~~~~	ACTTAATTAATTAAGTGTG	GCCCGCAAGCCAAACGCG	TGTTGGC	AGGGACTTTGCTAGAA	TGTGTGN	#	#contig_break	N.D.
7*Chr1_132a_IS40	7*Chr1_58a_IS407A_A	R	NCTC_10247	7	2032685-2033920	Chr1	IS407A	ACGCC	TGACCTGCCCCCTACA	ATGAAGAAGCGCTTTACGC	GCCCGCAAGCCAAACGCG	TGTTGGC	AGGGACTTTGCTAGAA	GTTCGCTC	GGAC	GTTC	N
4*Chr1_48_IS407A	4*Chr1_58b_IS407A_A	D-R	gb8_horse_4	4	2039586-2040821	Chr1	IS407A	CGTCG	TGACCTGCCCCCTACA	ATGAAGAAGCGCTTTACGC	GCCCGCAAGCCAAACGCG	TGTTGGC	AGGGACTTTGCTAGAA	GAAGTGG	GTGA	GAAG	N
10*Chr1_132b_IS4	10*Chr1_47b_IS407A_A	R	PRL20	10	2040335-2041570	Chr1	IS407A	TGAGG	TGACCTGCCCCCTACA	ATGAAGAAGCGCTTTACGC	GCCCGCAAGCCAAACGCG	TGTTGGC	AGGGACTTTGCTAGAA	GGACGGG	CCTT	GGAC	N
1*Chr1_48_IS407A	1*Chr1_58b_IS407A_A	D-R	ATCC_23344	1	2051541-2052776	Chr1	IS407A	CGTCG	TGACCTGCCCCCTACA	ATGAAGAAGCGCTTTACGC	GCCCGCAAGCCAAACGCG	TGTTGGC	AGGGACTTTGCTAGAA	GAAGTGG	GTGA	GAAG	N
6*Chr1_20_IS407A	6*Chr1_21_IS407A_A	D	NCTC_10229	6	2052526-2053761	Chr1	IS407A	ATGTC	TGACCTGCCCCCTACA	ATGAAGAAGCGCTTTACGC	GCCCGCAAGCCAAACGCG	TGTTGGC	AGGGACTTTGCTAGAA	GTCCACG	CCGG	GTCC	N
4*Chr1_132b_IS40	4*Chr1_47b_IS407A_A	R	gb8_horse_4	4	2058486-2059721	Chr1	IS407A	TGAGG	TGACCTGCCCCCTACA	ATGAAGAAGCGCTTTACGC	GCCCGCAAGCCAAACGCG	TGTTGGC	AGGGACTTTGCTAGAA	GGACGGG	CCTT	GGAC	N
7*Chr1_47a_IS407	7*Chr1_57_IS407A_A	D-R	NCTC_10247	7	2060132-2061367	Chr1	IS407A	TCCGC	TGACCTGCCCCCTACA	ATGAAGAAGCGCTTTACGC	GCCCGCAAGCCAAACGCG	TGTTGGC	AGGGACTTTGCTAGAA	CGTGGAC	AAGG	CGTG	N
1*Chr1_132b_IS40	1*Chr1_47b_IS407A_A	R	ATCC_23344	1	2070441-2071676	Chr1	IS407A	TGAGG	TGACCTGCCCCCTACA	ATGAAGAAGCGCTTTACGC	GCCCGCAAGCCAAACGCG	TGTTGGC	AGGGACTTTGCTAGAA	GGACGGG	CCTT	GGAC	N
2*Chr1_126b_IS40	2*Chr1_135_IS407A_A	D-R	fmh	2	2072203-2073438	Chr1	IS407A	TGGAC	TGACCTGCCCCCTACA	ATGAAGAAGCGCTTTACGC	GCCCGCAAGCCAAACGCG	TGTTGGC	AGGGACTTTGCTAGAA	CAATCAG	TCGC	CAAT	N
5*Chr1_61_IS407A_B		N	ATCC_10399	5	2078643-2079878	Chr1	IS407A	TTTGCT	TGACCTGCCCCCTACA	ATGAAGAAGCGCTTTACGC	GCCCGCAAGCCAAACGCG	TGTTGGC	AGGGACTTTGCTAGAA	CCCGCAC	CCCG	CCCG	Y
7*Chr1_43a_IS407	7*Chr1_45b_IS407A_A	R	NCTC_10247	7	2084226-2085461	Chr1	IS407A	TTCTG	TGACCTGCCCCCTACA	ATGAAGAAGCGCTTTACGC	GCCCGCAAGCCAAACGCG	TGTTGGC	AGGGACTTTGCTAGAA	CATTGGC	CCGC	CATT	N
3*Chr1_126b_IS40	3*Chr1_135_IS407A_A	D-R	JHU	3	2087663-2088898	Chr1	IS407A	TGGAC	TGACCTGCCCCCTACA	ATGAAGAAGCGCTTTACGC	GCCCGCAAGCCAAACGCG	TGTTGGC	AGGGACTTTGCTAGAA	CAATCAG	TCGC	CAAT	N
6*Chr1_18a_IS407A_A		N	NCTC_10229	6	2097220-2098455	Chr1	IS407A	CGTCG	TGACCTGCCCCCTACA	ATGAAGAAGCGCTTTACGC	GCCCGCAAGCCAAACGCG	TGTTGGC	AGGGACTTTGCTAGAA	CACCGGC	CACC	CACC	Y
8*Chr1_134_IS407A_B		N	2002721280	8	2098656-2099891	Chr1	IS407A	CCTAT	TGACCTGCCCCCTACA	ATGAAGAAGCGCTTTACGC	GCCCGCAAGCCAAACGCG	TGTTGGC	AGGGACTTTGCTAGAA	GCACGGC	GCAC	GCAC	Y
7*Chr1_42b_IS407A_A		N	NCTC_10247	7	2105147-2106382	Chr1	IS407A	GCGCT	TGACCTGCCCCCTACA	ATGAAGAAGCGCTTTACGC	GCCCGCAAGCCAAACGCG	TGTTGGC	AGGGACTTTGCTAGAA	CCCAAAC	TGCG	CCCA	N
7*Chr1_136_IS407	7*Chr1_45a_IS407A_A	D-R	NCTC_10247	7	2118845-2120080	Chr1	IS407A	GATCG	TGACCTGCCCCCTACA	ATGAAGAAGCGCTTTACGC	GCCCGCAAGCCAAACGCG	TGTTGGC	AGGGACTTTGCTAGAA	GATGCTC	GAAC	GATG	N
8*Chr1_126b_IS40	8*Chr1_135_IS407A_A	D-R	2002721280	8	2132568-2133803	Chr1	IS407A	CTGGA	TGACCTGCCCCCTACA	ATGAAGAAGCGCTTTACGC	GCCCGCAAGCCAAACGCG	TGTTGGC	AGGGACTTTGCTAGAA	CCGGAAG	ATCG	CCGG	N
2*Chr1_37_IS407A_A		N	fmh	2	214482-215717	Chr1	IS407A	CGTCG	TGACCTGCCCCCTACA	ATGAAGAAGCGCTTTACGC	GCCCGCAAGCCAAACGCG	TGTTGGC	AGGGACTTTGCTAGAA	GACCCGG	CCAG	GACC	N
8*Chr1_37_IS407A_A		N	2002721280	8	214855-216090	Chr1	IS407A	CGCGA	TGACCTGCCCCCTACA	ATGAAGAAGCGCTTTACGC	GCCCGCAAGCCAAACGCG	TGTTGGC	AGGGACTTTGCTAGAA	GGTCGCC	CGCG	GGTC	N
10*Chr1_126b_IS4	10*Chr1_135_IS407A_A	D-R	PRL20	10	2149515-2150750	Chr1	IS407A	GCTCG	TGACCTGCCCCCTACA	ATGAAGAAGCGCTTTACGC	GCCCGCAAGCCAAACGCG	TGTTGGC	AGGGACTTTGCTAGAA	CGATGTG	GATT	CGAT	N
3*Chr1_37_IS407A_A		N	JHU	3	215326-216561	Chr1	IS407A	CGTCG	TGACCTGCCCCCTACA	ATGAAGAAGCGCTTTACGC	GCCCGCAAGCCAAACGCG	TGTTGGC	AGGGACTTTGCTAGAA	GACCCGG	CCAG	GACC	N
10*Chr1_7_IS407A	10*Chr1_8_IS407A_A	D	PRL20	10	21550-22785	Chr1	IS407A	GGCGC	TGACCTGCCCCCTACA	ATGAAGAAGCGCTTTACGC	GCCCGCAAGCCAAACGCG	TGTTGGC	AGGGACTTTGCTAGAA	TTTCCAGT	GACG	TTTC	N
4*Chr1_126b_IS40	4*Chr1_135_IS407A_A	D-R	gb8_horse_4	4	2167514-2168749	Chr1	IS407A	TGGAC	TGACCTGCCCCCTACA	ATGAAGAAGCGCTTTACGC	GCCCGCAAGCCAAACGCG	TGTTGGC	AGGGACTTTGCTAGAA	CAATCAG	TCGC	CAAT	N
2*Chr1_132a_IS40	2*Chr1_57_IS407A_A	D-R	fmh	2	2174629-2175864	Chr1	IS407A	ACGCC	TGACCTGCCCCCTACA	ATGAAGAAGCGCTTTACGC	GCCCGCAAGCCAAACGCG	TGTTGGC	AGGGACTTTGCTAGAA	CGTGGAC	GGAC	CGTG	N
1*Chr1_126b_IS40	1*Chr1_135_IS407A_A	D-R	ATCC_23344	1	2179445-2180680	Chr1	IS407A	TGGAC	TGACCTGCCCCCTACA	ATGAAGAAGCGCTTTACGC	GCCCGCAAGCCAAACGCG	TGTTGGC	AGGGACTTTGCTAGAA	CAATCAG	TCGC	CAAT	N
3*Chr1_132a_IS40	3*Chr1_57_IS407A_A	D-R	JHU	3	2190089-2191324	Chr1	IS407A	ACGCC	TGACCTGCCCCCTACA	ATGAAGAAGCGCTTTACGC	GCCCGCAAGCCAAACGCG	TGTTGGC	AGGGACTTTGCTAGAA	CGTGGAC	GGAC	CGTG	N
5*Chr1_48_IS407A	5*Chr1_58b_IS407A_A	D-R	ATCC_10399	5	2200925-2202160	Chr1	IS407A	CGGCA	TGACCTGCCCCCTACA	ATGAAGAAGCGCTTTACGC	GCCCGCAAGCCAAACGCG	TGTTGGC	AGGGACTTTGCTAGAA	GAAGTGG	CCCC	GAAG	N
2*Chr1_47a_IS407	2*Chr1_58a_IS407A_A	R	fmh	2	2201933-2203168	Chr1	IS407A	GAGTT	TGACCTGCCCCCTACA	ATGAAGAAGCGCTTTACGC	GCCCGCAAGCCAAACGCG	TGTTGGC	AGGGACTTTGCTAGAA	CCTTGGC	AACG	CCTT	N
3*Chr1_47a_IS407	3*Chr1_58a_IS407A_A	R	JHU	3	2217393-2218628	Chr1	IS407A	GAGTT	TGACCTGCCCCCTACA	ATGAAGAAGCGCTTTACGC	GCCCGCAAGCCAAACGCG	TGTTGGC	AGGGACTTTGCTAGAA	CCTTGGC	AACG	CCTT	N
5*Chr1_132b_IS40	5*Chr1_47b_IS407A_A	R	ATCC_10399	5	2219825-2221060	Chr1	IS407A	TGAGG	TGACCTGCCCCCTACA	ATGAAGAAGCGCTTTACGC	GCCCGCAAGCCAAACGCG	TGTTGGC	AGGGACTTTGCTAGAA	GGACGGG	CCTT	GGAC	N
5*Chr1_133_IS407A_C		N	ATCC_10399	5	2224765-2226000	Chr1	IS407A	CCCGA	TGACCTGCCCCCTACA	ATGAAGAAGCGCTTTACGC	GCCCGCAAGCCAAACGCG	TGTTGGC	AGGGACTTTGCTAGAA	GCGGCGA	GCGG	GCGG	Y
2*Chr1_43a_IS407	2*Chr1_45b_IS407A_A	R	fmh	2	2226033-2227268	Chr1	IS407A	GGTTG	TGACCTGCCCCCTACA	ATGAAGAAGCGCTTTACGC	GCCCGCAAGCCAAACGCG	TGTTGGC	AGGGACTTTGCTAGAA	GCGGTGC	GCGG	GCGG	Y
7*Chr1_20_IS407A	7*Chr1_21_IS407A_A	D	NCTC_10247	7	2231283-2232518	Chr1	IS407A	ATGTC	TGACCTGCCCCCTACA	ATGAAGAAGCGCTTTACGC	GCCCGCAAGCCAAACGCG	TGTTGGC	AGGGACTTTGCTAGAA	GTCCACG	CCGG	GTCC	N
8*Chr1_132a_IS40	8*Chr1_58a_IS407A_A	R	2002721280	8	2238334-2239569	Chr1	IS407A	ACGCC	TGACCTGCCCCCTACA	ATGAAGAAGCGCTTTACGC	GCCCGCAAGCCAAACGCG	TGTTGGC	AGGGACTTTGCTAGAA	GTTCGCTC	GGAC	GTTC	N
3*Chr1_43a_IS407	3*Chr1_45b_IS407A_A	R	JHU	3	2241493-2242728	Chr1	IS407A	GGTTG	TGACCTGCCCCCTACA	ATGAAGAAGCGCTTTACGC	GCCCGCAAGCCAAACGCG	TGTTGGC	AGGGACTTTGCTAGAA	GCGGTGC	GCGG	GCGG	Y
2*Chr1_42b_IS407A_A		N	fmh	2	2246967-2248202	Chr1	IS407A	GCGCT	TGACCTGCCCCCTACA	ATGAAGAAGCGCTTTACGC	GCCCGCAAGCCAAACGCG	TGTTGGC	AGGGACTTTGCTAGAA	CCCAAAC	TGCG	CCCA	N
6*Chr1_31_IS407A_C		N	NCTC_10229	6	2252754-2253989	Chr1	IS407A	GCTAT	TGACCTGCCCCCTACA	ATGAAGAAGCGCTTTACGC	GCCCGCAAGCCAAACGCG	TGTTGGC	AGGGACTTTGCTAGAA	CGATGAC	CGAT	CGAT	Y
10*Chr1_132a_IS4	10*Chr1_58a_IS407A_A	R	PRL20	10	2255102-2256337	Chr1	IS407A	ACGCC	TGACCTGCCCCCTACA	ATGAAGAAGCGCTTTACGC	GCCCGCAAGCCAAACGCG	TGTTGGC	AGGGACTTTGCTAGAA	CGTTCGCT	GGAC	CGTT	N
2*Chr1_136_IS407	2*Chr1_45a_IS407A_A	D-R	fmh	2	2260664-2261899	Chr1	IS407A	GATCG	TGACCTGCCCCCTACA	ATGAAGAAGCGCTTTACGC	GCCCGCAAGCCAAACGCG	TGTTGGC	AGGGACTTTGCTAGAA	GATGCTC	GAAC	GATG	N
6*Chr1_30_IS407A	6*Chr1_6_IS407A_A	D-R	NCTC_10229	6	2261519-2262754	Chr1	IS407A	TTGAG	TGACCTGCCCCCTACA	ATGAAGAAGCGCTTTACGC	GCCCGCAAGCCAAACGCG	TGTTGGC	AGGGACTTTGCTAGAA	CGCCGCA	TCGG	CGCC	N
3*Chr1_42b_IS407A_A		N	JHU	3	2262324-2263559	Chr1	IS407A	GCGCT	TGACCTGCCCCCTACA	ATGAAGAAGCGCTTTACGC	GCCCGCAAGCCAAACGCG	TGTTGGC	AGGGACTTTGCTAGAA	CCCAAAC	TGCG	CCCA	N
7*Chr1_8_IS407A_A		D-R	NCTC_10247	7	2264036-2265271	Chr1	IS407A	GGCGC	TGACCTGCCCCCTACA	ATGAAGAAGCGCTTTACGC	GCCCGCAAGCCAAACGCG	TGTTGGC	AGGGACTTTGCTAGAA	AACCCTC	GACG	AACC	N
9*Chr1_111_IS407	9*Chr1_53a_IS407A_B	D-R	SAVP1	9	2265201-2266436	Chr1	IS407A	GAAGG	TGACCTGCCCCCTACA	ATGAAGAAGCGCTTTACGC	GCCCGCAAGCCAAACGCG	TGTTGGC	AGGGACTTTGCTAGAA	CTGCTGC	ACGG	CTGC	N
8*Chr1_47a_IS407	8*Chr1_57_IS407A_A	D-R	2002721280	8	2265797-2267032	Chr1	IS407A	TCCGC	TGACCTGCCCCCTACA	ATGAAGAAGCGCTTTACGC	GCCCGCAAGCCAAACGCG	TGTTGGC	AGGGACTTTGCTAGAA	CGTGGAC	AAGG	CGTG	N
4*Chr1_132a_IS40	4*Chr1_57_IS407A_A	D-R	gb8_horse_4	4	2269939-2271174	Chr1	IS407A	ACGCC	TGACCTGCCCCCTACA	ATGAAGAAGCGCTTTACGC	GCCCGCAAGCCAAACGCG	TGTTGGC	AGGGACTTTGCTAGAA	CGTGGAC	GGAC	CGTG	N
6*Chr1_7_IS407A_	6*Chr1_8_IS407A_A	D	NCTC_10229	6	2274369-2275604	Chr1	IS407A	GGCGC	TGACCTGCCCCCTACA	ATGAAGAAGCGCTTTACGC	GCCCGCAAGCCAAACGCG	TGTTGGC	AGGGACTTTGCTAGAA	TGAGAGC	GACG	TGAG	N
3*Chr1_136_IS407	3*Chr1_45a_IS407A_A	D-R	JHU	3	2276021-2277256	Chr1	IS407A	GATCG	TGACCTGCCCCCTACA	ATGAAGAAGCGCTTTACGC	GCCCGCAAGCCAAACGCG	TGTTGGC	AGGGACTTTGCTAGAA	GATGCTC	GAAC	GATG	N
1*Chr1_132a_IS40	1*Chr1_57_IS407A_A	D-R	ATCC_23344	1	2281870-2283105	Chr1	IS407A	ACGCC	TGACCTGCCCCCTACA	ATGAAGAAGCGCTTTACGC	GCCCGCAAGCCAAACGCG	TGTTGGC	AGGGACTTTGCTAGAA	CGTGGAC	GGAC	CGTG	N
10*Chr1_43b_IS40	10*Chr1_57_IS407A_A	D-R	PRL20	10	2282474-2283709	Chr1	IS407A	CCCGT	TGACCTGCCCCCTACA	ATGAAGAAGCGCTTTACGC	GCCCGCAAGCCAAACGCG	TGTTGGC	AGGGACTTTGCTAGAA	CGTGGAC	CCCG	CGTG	N
7*Chr1_10_IS407A_A		N	NCTC_10247	7	2282563-2283798	Chr1	IS407A	GGCGC	TGACCTGCCCCCTACA	ATGAAGAAGCGCTTTACGC	GCCCGCAAGCCAAACGCG	TGTTGGC	AGGGACTTTGCTAGAA	CCTATGC	GACG	CCTA	N
8*Chr1_43a_IS407	8*Chr1_45b_IS407A_A	R	2002721280	8	2289894-2291129	Chr1	IS407A	GGCTT	TGACCTGCCCCCTACA	ATGAAGAAGCGCTTTACGC	GCCCGCAAGCCAAACGCG	TGTTGGC	AGGGACTTTGCTAGAA	GCGGTGC	GTGC	GCGG	N

6*Chr1_10_IS407A_A			N	NCTC_10229	6	2291924-2293159	Chr1	IS407A	CGCCA	TGACCTGCCCCCTACA	ATGAAGAAGCGCTTTACG	GCCCGCAAGCCAAACGCG	TGTTGGC	AGGGACTTTGCTAGAA	CCTATGC	CCCT	CCTA	N
4*Chr1_47a_IS407	4*Chr1_58a_IS407A_A		R	gb8_horse_4	4	2297269-2298504	Chr1	IS407A	GAGTT	TGACCTGCCCCCTACA	ATGAAGAAGCGCTTTACG	GCCCGCAAGCCAAACGCG	TGTTGGC	AGGGACTTTGCTAGAA	CCTTGGC	AACG	CCTT	N
7*Chr1_11_IS407A_C			N	NCTC_10247	7	2300118-2301353	Chr1	IS407A	CGCCA	TGACCTGCCCCCTACA	ATGAAGAAGCGCTTTACG	GCCCGCAAGCCAAACGCG	TGTTGGC	AGGGACTTTGCTAGAA	CCTATGC	CCCT	CCTA	N
1*Chr1_47a_IS407	1*Chr1_58a_IS407A_A		R	ATCC_23344	1	2309174-2310409	Chr1	IS407A	GAGTT	TGACCTGCCCCCTACA	ATGAAGAAGCGCTTTACG	GCCCGCAAGCCAAACGCG	TGTTGGC	AGGGACTTTGCTAGAA	CCTTGGC	AACG	CCTT	N
8*Chr1_42b_IS407A_A			N	2002721280	8	2310485-2311720	Chr1	IS407A	GCGCT	TGACCTGCCCCCTACA	ATGAAGAAGCGCTTTACG	GCCCGCAAGCCAAACGCG	TGTTGGC	AGGGACTTTGCTAGAA	CCCAAAC	TGCG	CCCA	N
10*Chr1_38_IS407A_C			N	PRL20	10	2311172-2312193	Chr1	IS407A	#contig	~~~~~~~~~~~~~~	~~~~~~~~~~~~~~~~~	GCCCGCAAGCCAAACGCG	TGTTGGC	AGGGACTTTGCTAGAA	CGTGAAC	#	CGTG	N.D.
10*Chr1_40b_IS407A_C			R	PRL20	10	2312315-2313344	Chr1	IS407A	#contig	~~~~~~~~~~~~~~	ATGAAGAAGCGCTTTACG	GCCCGCAAGCCAAACGCG	TGTTGGC	~~~~~~~~~	#contig_br	#	#con	N.D.
10*Chr1_42a_IS40	10*Chr1_43a_IS407A_A		R	PRL20	10	2316739-2317974	Chr1	IS407A	GCGCT	TGACCTGCCCCCTACA	ATGAAGAAGCGCTTTACG	GCCCGCAAGCCAAACGCG	TGTTGGC	AGGGACTTTGCTAGAA	CCGCAGC	TGCG	CCGC	N
4*Chr1_43a_IS407	4*Chr1_45b_IS407A_A		R	gb8_horse_4	4	2321369-2322604	Chr1	IS407A	GGTTG	TGACCTGCCCCCTACA	ATGAAGAAGCGCTTTACG	GCCCGCAAGCCAAACGCG	TGTTGGC	AGGGACTTTGCTAGAA	GCGGTGC	GCGG	GCGG	Y
8*Chr1_136_IS407	8*Chr1_45a_IS407A_A		D-R	2002721280	8	2323299-2324534	Chr1	IS407A	GATCG	TGACCTGCCCCCTACA	ATGAAGAAGCGCTTTACG	GCCCGCAAGCCAAACGCG	TGTTGGC	AGGGACTTTGCTAGAA	GATGCTC	GAAC	GATG	N
5*Chr1_126b_IS40	5*Chr1_135_IS407A_A		D-R	ATCC_10399	5	2330125-2331360	Chr1	IS407A	TGGAC	TGACCTGCCCCCTACA	ATGAAGAAGCGCTTTACG	GCCCGCAAGCCAAACGCG	TGTTGGC	AGGGACTTTGCTAGAA	CCTCGAC	TCGC	CCTC	N
1*Chr1_43a_IS407	1*Chr1_45b_IS407A_A		R	ATCC_23344	1	2333274-2334509	Chr1	IS407A	GGTTG	TGACCTGCCCCCTACA	ATGAAGAAGCGCTTTACG	GCCCGCAAGCCAAACGCG	TGTTGGC	AGGGACTTTGCTAGAA	GCGGTGC	GCGG	GCGG	Y
10*Chr1_33_IS407	10*Chr1_40a_IS4	10*Chr1_42b_	D-R	PRL20	10	2337504-2338663	Chr1	IS407A	CCGCA	TGACCTGCCCCCTACA	ATGAAGAAGCGCTTTACG	GCCCGCAAGCCAAACGCG	TGTTGGC	CCACACCGGCT	#can not f	CAAT	#can	N.D.
4*Chr1_42b_IS407A_A			N	gb8_horse_4	4	2342200-2343435	Chr1	IS407A	GCGCT	TGACCTGCCCCCTACA	ATGAAGAAGCGCTTTACG	GCCCGCAAGCCAAACGCG	TGTTGGC	AGGGACTTTGCTAGAA	CCCAAAC	TGCG	CCCA	N
6*Chr1_77_IS407A	6*Chr1_94a_IS407A_A		D-R	NCTC_10229	6	234555-235790	Chr1	IS407A	ATGAT	TGACCTGCCCCCTACA	ATGAAGAAGCGCTTTACG	GCCCGCAAGCCAAACGCG	TGTTGGC	AGGGACTTTGCTAGAA	AACCGAT	AGGT	AACC	N
10*Chr1_35b_IS40	10*Chr1_45a_IS407A_A		R	PRL20	10	2350565-2351800	Chr1	IS407A	GTGAA	TGACCTGCCCCCTACA	ATGAAGAAGCGCTTTACG	GCCCGCAAGCCAAACGCG	TGTTGGC	AGGGACTTTGCTAGAA	GATGCTC	GATT	GATG	N
1*Chr1_42b_IS407A_A			N	ATCC_23344	1	2354105-2355340	Chr1	IS407A	GCGCT	TGACCTGCCCCCTACA	ATGAAGAAGCGCTTTACG	GCCCGCAAGCCAAACGCG	TGTTGGC	AGGGACTTTGCTAGAA	CCCAAAC	TGCG	CCCA	N
4*Chr1_136_IS407	4*Chr1_45a_IS407A_A		D-R	gb8_horse_4	4	2355846-2357081	Chr1	IS407A	GATCG	TGACCTGCCCCCTACA	ATGAAGAAGCGCTTTACG	GCCCGCAAGCCAAACGCG	TGTTGGC	AGGGACTTTGCTAGAA	GATGCTC	GAAC	GATG	N
9*Chr1_112_IS407	9*Chr1_49_IS407A_A		D-R	SAVP1	9	2356417-2357652	Chr1	IS407A	TCATC	TGACCTGCCCCCTACA	ATGAAGAAGCGCTTTACG	GCCCGCAAGCCAAACGCG	TGTTGGC	AGGGACTTTGCTAGAA	GGGCTGA	TCAA	GGGC	N
1*Chr1_136_IS407	1*Chr1_45a_IS407A_A		D-R	ATCC_23344	1	2367803-2369038	Chr1	IS407A	GATCG	TGACCTGCCCCCTACA	ATGAAGAAGCGCTTTACG	GCCCGCAAGCCAAACGCG	TGTTGGC	AGGGACTTTGCTAGAA	GATGCTC	GAAC	GATG	N
10*Chr1_126a_IS407A_A			R	PRL20	10	2373356-2374591	Chr1	IS407A	AAAAA	TGACCTGCCCCCTACA	ATGAAGAAGCGCTTTACG	GCCCGCAAGCCAAACGCG	TGTTGGC	AGGGACTTTGCTAGAA	TTTTTCAT	AAGC	TTTT	N
10*Chr1_47a_IS407A_A			R	PRL20	10	2375892-2377127	Chr1	IS407A	TCCGC	TGACCTGCCCCCTACA	ATGAAGAAGCGCTTTACG	GCCCGCAAGCCAAACGCG	TGTTGGC	AGGGACTTTGCTAGAA	CCTGCGAT	AAGG	CCTG	N
10*Chr1_34_IS407	10*Chr1_45b_IS407A_A		D-R	PRL20	10	2399992-2401227	Chr1	IS407A	TTCTG	TGACCTGCCCCCTACA	ATGAAGAAGCGCTTTACG	GCCCGCAAGCCAAACGCG	TGTTGGC	AGGGACTTTGCTAGAA	GGGGGAT	CCGC	GGGG	N
7*Chr1_34_IS407A	7*Chr1_35b_IS407A_A		D-R	NCTC_10247	7	24007-25242	Chr1	IS407A	GTGAA	TGACCTGCCCCCTACA	ATGAAGAAGCGCTTTACG	GCCCGCAAGCCAAACGCG	TGTTGGC	AGGGACTTTGCTAGAA	GGGGGAT	GATT	GGGG	N
5*Chr1_131_IS407A_C			N	ATCC_10399	5	2411631-2412866	Chr1	IS407A	CGGCA	TGACCTGCCCCCTACA	ATGAAGAAGCGCTTTACG	GCCCGCAAGCCAAACGCG	TGTTGGC	AGGGACTTTGCTAGAA	GAGGAAG	GAGG	GAGG	Y
10*Chr1_136_IS40	10*Chr1_35a_IS407A_A		D-R	PRL20	10	2417756-2418991	Chr1	IS407A	GATCG	TGACCTGCCCCCTACA	ATGAAGAAGCGCTTTACG	GCCCGCAAGCCAAACGCG	TGTTGGC	AGGGACTTTGCTAGAA	CCGGAAC	GAAC	CCGG	N
9*Chr1_113_IS407	9*Chr1_114_IS407A_A		D	SAVP1	9	2426311-2427546	Chr1	IS407A	CGAGC	TGACCTGCCCCCTACA	ATGAAGAAGCGCTTTACG	GCCCGCAAGCCAAACGCG	TGTTGGC	AGGGACTTTGCTAGAA	GATCCGG	AGAT	GATC	N
5*Chr1_132a_IS40	5*Chr1_58a_IS407A_A		R	ATCC_10399	5	2431373-2432608	Chr1	IS407A	ACGCC	TGACCTGCCCCCTACA	ATGAAGAAGCGCTTTACG	GCCCGCAAGCCAAACGCG	TGTTGGC	AGGGACTTTGCTAGAA	CGTTCGCT	GGAC	CGTT	N
6*Chr1_33_IS407A	6*Chr1_35a_IS407A_A		D-R	NCTC_10229	6	2435363-2436598	Chr1	IS407A	CCGCA	TGACCTGCCCCCTACA	ATGAAGAAGCGCTTTACG	GCCCGCAAGCCAAACGCG	TGTTGGC	AGGGACTTTGCTAGAA	GGGGCAG	CAAT	GGGG	N
7*Chr1_30_IS407A	7*Chr1_33_IS407A_A		D-R	NCTC_10247	7	2443334-2444569	Chr1	IS407A	CCGCA	TGACCTGCCCCCTACA	ATGAAGAAGCGCTTTACG	GCCCGCAAGCCAAACGCG	TGTTGGC	AGGGACTTTGCTAGAA	CGCCGCA	CAAT	CGCC	N
6*Chr1_34_IS407A	6*Chr1_35b_IS407A_A		D-R	NCTC_10229	6	2453363-2454598	Chr1	IS407A	GTGAA	TGACCTGCCCCCTACA	ATGAAGAAGCGCTTTACG	GCCCGCAAGCCAAACGCG	TGTTGGC	AGGGACTTTGCTAGAA	GGGGGAT	GATT	GGGG	N
5*Chr1_47a_IS407	5*Chr1_57_IS407A_A		D-R	ATCC_10399	5	2458648-2459883	Chr1	IS407A	TCCGC	TGACCTGCCCCCTACA	ATGAAGAAGCGCTTTACG	GCCCGCAAGCCAAACGCG	TGTTGGC	AGGGACTTTGCTAGAA	CGTGGAC	AAGG	CGTG	N
5*Chr1_43a_IS407	5*Chr1_45b_IS407A_A		R	ATCC_10399	5	2482748-2483983	Chr1	IS407A	TTCTG	TGACCTGCCCCCTACA	ATGAAGAAGCGCTTTACG	GCCCGCAAGCCAAACGCG	TGTTGGC	AGGGACTTTGCTAGAA	AAGGGGG	CCGC	AAGG	N
6*Chr1_37_IS407A_A			N	NCTC_10229	6	2492041-2493276	Chr1	IS407A	CGCGA	TGACCTGCCCCCTACA	ATGAAGAAGCGCTTTACG	GCCCGCAAGCCAAACGCG	TGTTGGC	AGGGACTTTGCTAGAA	GGTCGCC	CGCG	GGTC	N
9*Chr1_115a_IS407A_A			N	SAVP1	9	2497406-2498641	Chr1	IS407A	TTCGC	TGACCTGCCCCCTACA	ATGAAGAAGCGCTTTACG	GCCCGCAAGCCAAACGCG	TGTTGGC	AGGGACTTTGCTAGAA	GATGACA	GATG	GATG	Y
5*Chr1_42b_IS407A_A			N	ATCC_10399	5	2503596-2504831	Chr1	IS407A	GCGCT	TGACCTGCCCCCTACA	ATGAAGAAGCGCTTTACG	GCCCGCAAGCCAAACGCG	TGTTGGC	AGGGACTTTGCTAGAA	CCCAAAC	TGCG	CCCA	N
8*Chr1_152b_IS40	8*Chr1_157_IS407A_A		D-R	2002721280	8	2512937-2514172	Chr1	IS407A	GAAAC	TGACCTGCCCCCTACA	ATGAAGAAGCGCTTTACG	GCCCGCAAGCCAAACGCG	TGTTGGC	AGGGACTTTGCTAGAA	GATGCCTT	GGTC	GATG	N
5*Chr1_136_IS407	5*Chr1_45a_IS407A_A		D-R	ATCC_10399	5	2516711-2517946	Chr1	IS407A	GATCG	TGACCTGCCCCCTACA	ATGAAGAAGCGCTTTACG	GCCCGCAAGCCAAACGCG	TGTTGGC	AGGGACTTTGCTAGAA	GATGCTC	GAAC	GATG	N
2*Chr1_145_IS407A_A			N	fmh	2	2523638-2524540	Chr1	IS407A	#IS407	~~~~~~~~~~~~~~	~~~~~~~~~~~~~~~~~	ACCCGCAAGCCAAACGCG	TGTTGGC	AGGGACTTTGCTAGAA	GGCTTCG	#	GGCT	N.D.
7*Chr1_115b_IS40	7*Chr1_47b_IS407A_A		R	NCTC_10247	7	252371-253606	Chr1	IS407A	GAAGC	TGACCTGCCCCCTACA	ATGAAGAAGCGCTTTACG	GCCCGCAAGCCAAACGCG	TGTTGGC	AGGGACTTTGCTAGAA	GATGACA	GAGC	GATG	N
2*Chr1_146_IS407A_B			N	fmh	2	2524541-2525776	Chr1	IS407A	AAGCC	TGACCTGCCCCCTACA	ATGAAGAAGCGCTTTACG	GCCCGCAAGCCAAACGCG	TGTTGGC	AGGGACTTTGCTAGAA	CCCGCAA	ATAG	CCCG	N
5*Chr1_137_IS407A_C			N	ATCC_10399	5	2538164-2539399	Chr1	IS407A	CGGCG	TGACCTGCCCCCTACA	ATGAAGAAGCGCTTTACG	GCCCGCAAGCCAAACGCG	TGTTGGC	AGGGACTTTGCTAGAA	ATACGGTT	ATAC	ATAC	Y
3*Chr1_145_IS407A_A			N	JHU	3	2540286-2541188	Chr1	IS407A	#IS407	~~~~~~~~~~~~~~	~~~~~~~~~~~~~~~~~	ACCCGCAAGCCAAACGCG	TGTTGGC	AGGGACTTTGCTAGAA	GGCTTCG	#	GGCT	N.D.
3*Chr1_146_IS407A_B			N	JHU	3	2541189-2542424	Chr1	IS407A	AAGCC	TGACCTGCCCCCTACA	ATGAAGAAGCGCTTTACG	GCCCGCAAGCCAAACGCG	TGTTGGC	AGGGACTTTGCTAGAA	CCCGCAA	ATAG	CCCG	N
8*Chr1_152a_IS40	8*Chr1_154a_IS407A_A		R	2002721280	8	2560366-2561601	Chr1	IS407A	GCAGC	TGACCTGCCCCCTACA	ATGAAGAAGCGCTTTACG	GCCCGCAAGCCAAACGCG	TGTTGGC	AGGGACTTTGCTAGAA	GGGACGC	CCCC	GGGA	N
6*Chr1_47a_IS407	6*Chr1_57_IS407A_A		D-R	NCTC_10229	6	2560595-2561830	Chr1	IS407A	TCCGC	TGACCTGCCCCCTACA	ATGAAGAAGCGCTTTACG	GCCCGCAAGCCAAACGCG	TGTTGGC	AGGGACTTTGCTAGAA	CGTGGAC	AAGG	CGTG	N
2*Chr1_154b_IS407A_A			R	fmh	2	2564570-2565805	Chr1	IS407A	CGCGC	TGACCTGCCCCCTACA	ATGAAGAAGCGCTTTACG	GCCCGCAAGCCAAACGCG	TGTTGGC	AGGGACTTTGCTAGAA	AACCCTC	GCTT	AACC	N
9*Chr1_122_IS407A_A			N	SAVP1	9	2567392-2568627	Chr1	IS407A	TAATG	TGACCTGCCCCCTACA	ATGAAGAAGCGCTTTACG	GCCCGCAAGCCAAACGCG	TGTTGGC	AGGGACTTTGCTAGAA	CTTCGTG	GCAC	CTTC	N
8*Chr1_160a_IS407A_A			R	2002721280	8	2576706-2577325	Chr1	IS407A	#contig	~~~~~~~~~~~~~~	~~~~~~~~~~~~~~~~~	~~~~~~~~~~~~~~~~~	~~~~~~~	AGGGACTTTGCTAGAA	GTCCGGG	#	GTCC	N.D.
8*Chr1_161_IS407A_A			D-R	2002721280	8	2577952-2576666	Chr1	IS407A	TACGA	TGACCTGCCCCCTACA	ATGAAGAAGCGCTTTACG	GCCCGCAAGCCAAACGCG	TGTTGGC	AGGGACTTTGCTAGAA	GTCCGGG	TTTT	GTCC	N
3*Chr1_154b_IS407A_A			R	JHU	3	2581218-2582453	Chr1	IS407A	CGCGC	TGACCTGCCCCCTACA	ATGAAGAAGCGCTTTACG	GCCCGCAAGCCAAACGCG	TGTTGGC	AGGGACTTTGCTAGAA	AACCCTC	GCTT	AACC	N
6*Chr1_132a_IS40	6*Chr1_58a_IS407A_A		R	NCTC_10229	6	2588026-2589261	Chr1	IS407A	ACGCC	TGACCTGCCCCCTACA	ATGAAGAAGCGCTTTACG	GCCCGCAAGCCAAACGCG	TGTTGGC	AGGGACTTTGCTAGAA	GTTCGCTC	GGAC	GTTC	N
2*Chr1_152b_IS40	2*Chr1_157_IS407A_A		D-R	fmh	2	2598865-2600100	Chr1	IS407A	GAAAC	TGACCTGCCCCCTACA	ATGAAGAAGCGCTTTACG	GCCCGCAAGCCAAACGCG	TGTTGGC	AGGGACTTTGCTAGAA	GCCTGAT	GGTC	GCCT	N
9*Chr1_126a_IS407A_A			R	SAVP1	9	2614152-2615387	Chr1	IS407A	AAAAA	TGACCTGCCCCCTACA	ATGAAGAAGCGCTTTACG	GCCCGCAAGCCAAACGCG	TGTTGGC	AGGGACTTTGCTAGAA	TTTTTCAT	AAGC	TTTT	N
7*Chr1_143a_IS40	7*Chr1_18b_IS407A_A		R	NCTC_10247	7	2614655-2615890	Chr1	IS407A	CGTCG	TGACCTGCCCCCTACA	ATGAAGAAGCGCTTTACG	GCCCGCAAGCCAAACGCG	TGTTGGC	AGGGACTTTGCTAGAA	ATCAAGCT	CACC	ATCA	N
3*Chr1_152b_IS40	3*Chr1_157_IS407A_A		D-R	JHU	3	2615744-2616979	Chr1	IS407A	GAAAC	TGACCTGCCCCCTACA	ATGAAGAAGCGCTTTACG	GCCCGCAAGCCAAACGCG	TGTTGGC	AGGGACTTTGCTAGAA	GCCTGAT	GGTC	GCCT	N
9*Chr1_47a_IS407A_A			R	SAVP1	9	2616688-2617923	Chr1	IS407A	TCCGC	TGACCTGCCCCCTACA	ATGAAGAAGCGCTTTACG	GCCCGCAAGCCAAACGCG	TGTTGGC	AGGGACTTTGCTAGAA	CCTGCGAT	AAGG	CCTG	N
4*Chr1_145_IS407A_A			N	gb8_horse_4	4	2618181-2619083	Chr1	IS407A	#IS407	~~~~~~~~~~~~~~	~~~~~~~~~~~~~~~~~	ACCCGCAAGCCAAACGCG	TGTTGGC	AGGGACTTTGCTAGAA	GGCTTCG	#	GGCT	N.D.

4*Chr1_146_IS407A_B		N	gb8_horse_4	4	2619084-2620319	Chr1	IS407A	AAGCC	TGACCTGCCCCCTACA	ATGAAGAAGCGCTTTACG	GCCCGCAAGCCAAACGCG	TGTTGGCC	AGGGACTTTGCTAGAA	CCCGCAA	ATAG	CCCG	N
1*Chr1_145_IS407A_A		N	ATCC_23344	1	2630161-2631063	Chr1	IS407A	#IS407	~~~~~~~~~~~~~~	~~~~~~~~~~~~~~~~~~	ACCCGCAAGCCAAACGCG	TGTTGGCC	AGGGACTTTGCTAGAA	GGCTTCGC	#	GGCT	N.D.
1*Chr1_146_IS407A_B		N	ATCC_23344	1	2631064-2632299	Chr1	IS407A	AAGCC	TGACCTGCCCCCTACA	ATGAAGAAGCGCTTTACG	GCCCGCAAGCCAAACGCG	TGTTGGCC	AGGGACTTTGCTAGAA	CCCGCAA	ATAG	CCCG	N
9*Chr1_34_IS407A	9*Chr1_45a_IS407A_A	D-R	SAVP1	9	2640788-2642023	Chr1	IS407A	CAGAA	TGACCTGCCCCCTACA	ATGAAGAAGCGCTTTACG	GCCCGCAAGCCAAACGCG	TGTTGGCC	AGGGACTTTGCTAGAA	GCGGTGC	CCCC	GCGG	N
8*Chr1_160b_IS407A_A		R	2002721280	8	2641872-2643107	Chr1	IS407A	CGCGC	TGACCTGCCCCCTACA	ATGAAGAAGCGCTTTACG	GCCCGCAAGCCAAACGCG	TGTTGGCC	AGGGACTTTGCTAGAA	ATCCACTC	GTCC	ATCC	N
2*Chr1_154a_IS407	2*Chr1_4_IS407A_B	D-R	fmh	2	2647276-2648511	Chr1	IS407A	GCAGC	TGACCTGCCCCCTACA	ATGAAGAAGCGCTTTACG	GCCCGCAAGCCAAACGCG	TGTTGGCC	AGGGACTTTGCTAGAA	GACAGGG	CCCC	GACA	N
7*Chr1_2a_IS407A_A		null	NCTC_10247	7	2648929-2650164	Chr1	IS407A	GATGC	TGACCTGCCCCCTACA	ATGAAGAAGCGCTTTACG	GCCCGCAAGCCAAACGCG	TGTTGGCC	AGGGACTTTGCTAGAA	ACGAGCT	GACG	ACGA	N
9*Chr1_33_IS407A	9*Chr1_35a_IS407A_A	D-R	SAVP1	9	2658552-2659787	Chr1	IS407A	CCGCA	TGACCTGCCCCCTACA	ATGAAGAAGCGCTTTACG	GCCCGCAAGCCAAACGCG	TGTTGGCC	AGGGACTTTGCTAGAA	CCGGAAC	CAAT	CCGG	N
4*Chr1_154b_IS407A_A		R	gb8_horse_4	4	2659248-2660362	Chr1	IS407A	CGCGC	TGACCTGCCCCCTACA	ATGAAGAAGCGCTTTACG	GCCCGCAAGCCAAACGCG	TGTTGGCC	#	#IS407A o	GCTT	#IS4	N.D.
3*Chr1_154a_IS407	3*Chr1_4_IS407A_B	D-R	JHU	3	2664079-2665314	Chr1	IS407A	GCAGC	TGACCTGCCCCCTACA	ATGAAGAAGCGCTTTACG	GCCCGCAAGCCAAACGCG	TGTTGGCC	AGGGACTTTGCTAGAA	GACAGGG	CCCC	GACA	N
1*Chr1_154b_IS407A_A		R	ATCC_23344	1	2671093-2672328	Chr1	IS407A	CGCGC	TGACCTGCCCCCTACA	ATGAAGAAGCGCTTTACG	GCCCGCAAGCCAAACGCG	TGTTGGCC	AGGGACTTTGCTAGAA	AACCCTCC	GCTT	AACC	N
10*Chr1_145_IS407A_A		N	PRL20	10	2676258-2677493	Chr1	IS407A	AAGCC	TGACCTGCCCCCTACA	ATGAAGAAGCGCTTTACG	GCCCGCAAGCCAAACGCG	TGTTGGCC	AGGGACTTTGCTAGAA	GGCTTCGC	ATAG	GGCT	N
2*Chr1_150_IS407	2*Chr1_2b_IS407A_A	D-R	fmh	2	2681776-2683011	Chr1	IS407A	ATCGC	TGACCTGCCCCCTACA	ATGAAGAAGCGCTTTACG	GCCCGCAAGCCAAACGCG	TGTTGGCC	AGGGACTTTGCTAGAA	ACGAGCT	CAAG	ACGA	N
8*Chr1_149_IS407	8*Chr1_150_IS407A_A	D	2002721280	8	2686821-2687644	Chr1	IS407A	ATCGC	TGACCTGCCCCCTACA	ATGAAGAAGCGCTTTACG	GCCCGCAAGCCAAACGCG	TGTTGGCC	#	#contig_br	CAAG	#con	N.D.
6*Chr1_126b_IS40	6*Chr1_135_IS407A_A	D-R	NCTC_10229	6	2690743-2691978	Chr1	IS407A	CTGGA	TGACCTGCCCCCTACA	ATGAAGAAGCGCTTTACG	GCCCGCAAGCCAAACGCG	TGTTGGCC	AGGGACTTTGCTAGAA	CCGGAAG	ATCG	CCGG	N
4*Chr1_152b_IS40	4*Chr1_157_IS407A_A	D-R	gb8_horse_4	4	2692936-2694171	Chr1	IS407A	GAAAC	TGACCTGCCCCCTACA	ATGAAGAAGCGCTTTACG	GCCCGCAAGCCAAACGCG	TGTTGGCC	AGGGACTTTGCTAGAA	GCCTGATC	GGTC	GCCT	N
7*Chr1_150_IS407	7*Chr1_5_IS407A_A	D-R	NCTC_10247	7	2695410-2696645	Chr1	IS407A	ATCGC	TGACCTGCCCCCTACA	ATGAAGAAGCGCTTTACG	GCCCGCAAGCCAAACGCG	TGTTGGCC	AGGGACTTTGCTAGAA	GCGCCCG	CAAG	GCGC	N
3*Chr1_150_IS407	3*Chr1_2b_IS407A_A	D-R	JHU	3	2697886-2699121	Chr1	IS407A	ATCGC	TGACCTGCCCCCTACA	ATGAAGAAGCGCTTTACG	GCCCGCAAGCCAAACGCG	TGTTGGCC	AGGGACTTTGCTAGAA	ACGAGCT	CAAG	ACGA	N
1*Chr1_152b_IS40	1*Chr1_157_IS407A_A	D-R	ATCC_23344	1	2705320-2706555	Chr1	IS407A	GAAAC	TGACCTGCCCCCTACA	ATGAAGAAGCGCTTTACG	GCCCGCAAGCCAAACGCG	TGTTGGCC	AGGGACTTTGCTAGAA	GCCTGATC	GGTC	GCCT	N
7*Chr1_48_IS407A	7*Chr1_58b_IS407A_A	D-R	NCTC_10247	7	270634-271869	Chr1	IS407A	CGTCG	TGACCTGCCCCCTACA	ATGAAGAAGCGCTTTACG	GCCCGCAAGCCAAACGCG	TGTTGGCC	AGGGACTTTGCTAGAA	GAAGTGG	GTGA	GAAG	N
6*Chr1_134_IS407A_B		N	NCTC_10229	6	2724302-2725537	Chr1	IS407A	TTCCG	TGACCTGCCCCCTACA	ATGAAGAAGCGCTTTACG	GCCCGCAAGCCAAACGCG	TGTTGGCC	AGGGACTTTGCTAGAA	GCACGGC	ACAC	GCAC	N
10*Chr1_154b_IS407A_A		R	PRL20	10	2728534-2729133	Chr1	IS407A	#contig	~~~~~~~~~~~~~~	~~~~~~~~~~~~~~~~~~	~~~~~~~~~~~~~~~~~	~~~~~~~~	AGGGACTTTGCTAGAA	AACCCTCC	#	AACC	N.D.
4*Chr1_154a_IS407	4*Chr1_4_IS407A_B	D-R	gb8_horse_4	4	2739471-2740706	Chr1	IS407A	GCAGC	TGACCTGCCCCCTACA	ATGAAGAAGCGCTTTACG	GCCCGCAAGCCAAACGCG	TGTTGGCC	AGGGACTTTGCTAGAA	GACAGGG	CCCC	GACA	N
2*Chr1_152a_IS407	2*Chr1_2a_IS407A_A	R	fmh	2	2748524-2749759	Chr1	IS407A	GATGC	TGACCTGCCCCCTACA	ATGAAGAAGCGCTTTACG	GCCCGCAAGCCAAACGCG	TGTTGGCC	AGGGACTTTGCTAGAA	GGGACGC	GACG	GGGA	N
8*Chr1_158_IS407	8*Chr1_5_IS407A_A	D-R	2002721280	8	2749650-2750885	Chr1	IS407A	AACCC	TGACCTGCCCCCTACA	ATGAAGAAGCGCTTTACG	GCCCGCAAGCCAAACGCG	TGTTGGCC	AGGGACTTTGCTAGAA	GCGCCCG	ATGG	GCGC	N
1*Chr1_154a_IS407	1*Chr1_4_IS407A_B	D-R	ATCC_23344	1	2752854-2754089	Chr1	IS407A	GCAGC	TGACCTGCCCCCTACA	ATGAAGAAGCGCTTTACG	GCCCGCAAGCCAAACGCG	TGTTGGCC	AGGGACTTTGCTAGAA	GACAGGG	CCCC	GACA	N
10*Chr1_152b_IS4	10*Chr1_157_IS407A_A	D-R	PRL20	10	2756293-2757528	Chr1	IS407A	GAAAC	TGACCTGCCCCCTACA	ATGAAGAAGCGCTTTACG	GCCCGCAAGCCAAACGCG	TGTTGGCC	AGGGACTTTGCTAGAA	GATGCCTT	GGTC	GATG	N
3*Chr1_152a_IS407	3*Chr1_2a_IS407A_A	R	JHU	3	2764627-2765862	Chr1	IS407A	GATGC	TGACCTGCCCCCTACA	ATGAAGAAGCGCTTTACG	GCCCGCAAGCCAAACGCG	TGTTGGCC	AGGGACTTTGCTAGAA	GGGACGC	GACG	GGGA	N
4*Chr1_150_IS407	4*Chr1_2b_IS407A_A	D-R	gb8_horse_4	4	2773025-2774260	Chr1	IS407A	ATCGC	TGACCTGCCCCCTACA	ATGAAGAAGCGCTTTACG	GCCCGCAAGCCAAACGCG	TGTTGGCC	AGGGACTTTGCTAGAA	ACGAGCT	CAAG	ACGA	N
2*Chr1_158_IS407A_A		D-R	fmh	2	2778339-2779574	Chr1	IS407A	AACCC	TGACCTGCCCCCTACA	ATGAAGAAGCGCTTTACG	GCCCGCAAGCCAAACGCG	TGTTGGCC	AGGGACTTTGCTAGAA	ATCCACTC	ATGG	ATCC	N
5*Chr1_145_IS407A_A		N	ATCC_10399	5	2780241-2781476	Chr1	IS407A	AAGCC	TGACCTGCCCCCTACA	ATGAAGAAGCGCTTTACG	GCCCGCAAGCCAAACGCG	TGTTGGCC	AGGGACTTTGCTAGAA	GGCTTCGC	ATAG	GGCT	N
1*Chr1_150_IS407	1*Chr1_2b_IS407A_A	D-R	ATCC_23344	1	2786407-2787642	Chr1	IS407A	ATCGC	TGACCTGCCCCCTACA	ATGAAGAAGCGCTTTACG	GCCCGCAAGCCAAACGCG	TGTTGGCC	AGGGACTTTGCTAGAA	ACGAGCT	CAAG	ACGA	N
3*Chr1_158_IS407A_A		D-R	JHU	3	2793466-2794701	Chr1	IS407A	AACCC	TGACCTGCCCCCTACA	ATGAAGAAGCGCTTTACG	GCCCGCAAGCCAAACGCG	TGTTGGCC	AGGGACTTTGCTAGAA	ATCCACTC	ATGG	ATCC	N
8*Chr1_2a_IS407A_A		null	2002721280	8	2797539-2798774	Chr1	IS407A	GATGC	TGACCTGCCCCCTACA	ATGAAGAAGCGCTTTACG	GCCCGCAAGCCAAACGCG	TGTTGGCC	AGGGACTTTGCTAGAA	ACGAGCT	GACG	ACGA	N
6*Chr1_115a_IS407	6*Chr1_132b_IS407A_A	R	NCTC_10229	6	2801289-2802524	Chr1	IS407A	TTCGC	TGACCTGCCCCCTACA	ATGAAGAAGCGCTTTACG	GCCCGCAAGCCAAACGCG	TGTTGGCC	AGGGACTTTGCTAGAA	GGACGGG	GATG	GGAC	N
9*Chr1_10_IS407A_A		N	SAVP1	9	2801953-2803188	Chr1	IS407A	CGCCA	TGACCTGCCCCCTACA	ATGAAGAAGCGCTTTACG	GCCCGCAAGCCAAACGCG	TGTTGGCC	AGGGACTTTGCTAGAA	CCCTATGC	CCCT	CCCT	Y
10*Chr1_152a_IS4	10*Chr1_154a_IS407A_A	R	PRL20	10	2803324-2804559	Chr1	IS407A	CCTGG	TGACCTGCCCCCTACA	ATGAAGAAGCGCTTTACG	GCCCGCAAGCCAAACGCG	TGTTGGCC	AGGGACTTTGCTAGAA	GGGACGC	AAAA	GGGA	N
9*Chr1_7_IS407A_	9*Chr1_8_IS407A_A	D	SAVP1	9	2819545-2820780	Chr1	IS407A	GGCGC	TGACCTGCCCCCTACA	ATGAAGAAGCGCTTTACG	GCCCGCAAGCCAAACGCG	TGTTGGCC	AGGGACTTTGCTAGAA	TTTCCAGT	GACG	TTTC	N
5*Chr1_154b_IS407A_A		R	ATCC_10399	5	2820240-2821475	Chr1	IS407A	CGCGC	TGACCTGCCCCCTACA	ATGAAGAAGCGCTTTACG	GCCCGCAAGCCAAACGCG	TGTTGGCC	AGGGACTTTGCTAGAA	AACCCTCC	GCTT	AACC	N
4*Chr1_33_IS407A	4*Chr1_34_IS407A_A	D	gb8_horse_4	4	282223-283458	Chr1	IS407A	CCGCA	TGACCTGCCCCCTACA	ATGAAGAAGCGCTTTACG	GCCCGCAAGCCAAACGCG	TGTTGGCC	AGGGACTTTGCTAGAA	GCAACGG	CAAT	GCAA	N
2*Chr1_149_IS407	2*Chr1_160b_IS407A_A	D-R	fmh	2	2823968-2825203	Chr1	IS407A	CGCGC	TGACCTGCCCCCTACA	ATGAAGAAGCGCTTTACG	GCCCGCAAGCCAAACGCG	TGTTGGCC	AGGGACTTTGCTAGAA	CCTCACG	GTCC	CCTC	N
10*Chr1_150_IS40	10*Chr1_2b_IS407A_A	D-R	PRL20	10	2830709-2831944	Chr1	IS407A	CGTGC	TGACCTGCCCCCTACA	ATGAAGAAGCGCTTTACG	GCCCGCAAGCCAAACGCG	TGTTGGCC	AGGGACTTTGCTAGAA	ACGAGCT	GTCG	ACGA	N
9*Chr1_30_IS407A	9*Chr1_6_IS407A_A	D-R	SAVP1	9	2832213-2833448	Chr1	IS407A	TTGAG	TGACCTGCCCCCTACA	ATGAAGAAGCGCTTTACG	GCCCGCAAGCCAAACGCG	TGTTGGCC	AGGGACTTTGCTAGAA	CGCCGCA	TCGG	CGCC	N
1*Chr1_33_IS407A	1*Chr1_34_IS407A_A	D	ATCC_23344	1	283548-284783	Chr1	IS407A	CCGCA	TGACCTGCCCCCTACA	ATGAAGAAGCGCTTTACG	GCCCGCAAGCCAAACGCG	TGTTGGCC	AGGGACTTTGCTAGAA	GCAACGG	CAAT	GCAA	N
3*Chr1_149_IS407	3*Chr1_160b_IS407A_A	D-R	JHU	3	2839030-2840265	Chr1	IS407A	CGCGC	TGACCTGCCCCCTACA	ATGAAGAAGCGCTTTACG	GCCCGCAAGCCAAACGCG	TGTTGGCC	AGGGACTTTGCTAGAA	CCTCACG	GTCC	CCTC	N
4*Chr1_152a_IS407	4*Chr1_2a_IS407A_A	R	gb8_horse_4	4	2839656-2840891	Chr1	IS407A	GATGC	TGACCTGCCCCCTACA	ATGAAGAAGCGCTTTACG	GCCCGCAAGCCAAACGCG	TGTTGGCC	AGGGACTTTGCTAGAA	GGGACGC	GACG	GGGA	N
1*Chr1_152a_IS407	1*Chr1_2a_IS407A_A	R	ATCC_23344	1	2853155-2854390	Chr1	IS407A	GATGC	TGACCTGCCCCCTACA	ATGAAGAAGCGCTTTACG	GCCCGCAAGCCAAACGCG	TGTTGGCC	AGGGACTTTGCTAGAA	GGGACGC	GACG	GGGA	N
5*Chr1_152b_IS40	5*Chr1_157_IS407A_A	D-R	ATCC_10399	5	2855128-2856363	Chr1	IS407A	GAAAC	TGACCTGCCCCCTACA	ATGAAGAAGCGCTTTACG	GCCCGCAAGCCAAACGCG	TGTTGGCC	AGGGACTTTGCTAGAA	GCCTGATC	GGTC	GCCT	N
4*Chr1_158_IS407A_A		D-R	gb8_horse_4	4	2868523-2869758	Chr1	IS407A	AACCC	TGACCTGCCCCCTACA	ATGAAGAAGCGCTTTACG	GCCCGCAAGCCAAACGCG	TGTTGGCC	AGGGACTTTGCTAGAA	ATCCACTC	ATGG	ATCC	N
6*Chr1_114_IS407A_A		N	NCTC_10229	6	2872519-2873754	Chr1	IS407A	TGATTC	TGACCTGCCCCCTACA	ATGAAGAAGCGCTTTACG	GCCCGCAAGCCAAACGCG	TGTTGGCC	AGGGACTTTGCTAGAA	GATCCGG	GAAG	GATC	N
1*Chr1_158_IS407A_A		D-R	ATCC_23344	1	2881994-2883229	Chr1	IS407A	AACCC	TGACCTGCCCCCTACA	ATGAAGAAGCGCTTTACG	GCCCGCAAGCCAAACGCG	TGTTGGCC	AGGGACTTTGCTAGAA	ATCCACTC	ATGG	ATCC	N
6*Chr1_113_IS407A_A		N	NCTC_10229	6	2886128-2887363	Chr1	IS407A	TTTGCT	TGACCTGCCCCCTACA	ATGAAGAAGCGCTTTACG	GCCCGCAAGCCAAACGCG	TGTTGGCC	AGGGACTTTGCTAGAA	ATCTACCT	CCTC	ATCT	N
9*Chr1_15_IS407A_C		N	SAVP1	9	2888034-2889269	Chr1	IS407A	CGCCG	TGACCTGCCCCCTACA	ATGAAGAAGCGCTTTACG	GCCCGCAAGCCAAACGCG	TGTTGGCC	AGGGACTTTGCTAGAA	TTACTGGC	TTAC	TTAC	Y
2*Chr1_160a_IS407	2*Chr1_161_IS407A_A	D-R	fmh	2	2889155-2890390	Chr1	IS407A	TACGA	TGACCTGCCCCCTACA	ATGAAGAAGCGCTTTACG	GCCCGCAAGCCAAACGCG	TGTTGGCC	AGGGACTTTGCTAGAA	GTCCGGG	TTTT	GTCC	N
10*Chr1_158_IS407A_A		D-R	PRL20	10	2898114-2899349	Chr1	IS407A	CACCA	TGACCTGCCCCCTACA	ATGAAGAAGCGCTTTACG	GCCCGCAAGCCAAACGCG	TGTTGGCC	AGGGACTTTGCTAGAA	AATCCACT	CTTC	AATC	N
5*Chr1_152a_IS407	5*Chr1_154a_IS407A_A	R	ATCC_10399	5	2902195-2903430	Chr1	IS407A	GCAGC	TGACCTGCCCCCTACA	ATGAAGAAGCGCTTTACG	GCCCGCAAGCCAAACGCG	TGTTGGCC	AGGGACTTTGCTAGAA	GGGACGC	CCCC	GGGA	N
3*Chr1_160a_IS407	3*Chr1_161_IS407A_A	D-R	JHU	3	2904664-2905899	Chr1	IS407A	TACGA	TGACCTGCCCCCTACA	ATGAAGAAGCGCTTTACG	GCCCGCAAGCCAAACGCG	TGTTGGCC	AGGGACTTTGCTAGAA	GTCCGGG	TTTT	GTCC	N
4*Chr1_149_IS407	4*Chr1_160b_IS407A_A	D-R	gb8_horse_4	4	2913457-2914692	Chr1	IS407A	CGCGC	TGACCTGCCCCCTACA	ATGAAGAAGCGCTTTACG	GCCCGCAAGCCAAACGCG	TGTTGGCC	AGGGACTTTGCTAGAA	CCTCACG	GTCC	CCTC	N

7*Chr1_160a_IS40	7*Chr1_29_IS407A_A	D-R	NCTC_10247	7	2917547-2918782	Chr1	IS407A	ATTTAT	TGACCTGCCCCCTACA	ATGAAGAAGCGCTTTACG	GCCCGCAAGCCAAACGCG	TGTTGGC	AGGGACTTTGCTAGAA	GTCCGGG	CATT	GTCC	N
1*Chr1_149_IS407	1*Chr1_160b_IS407A_A	D-R	ATCC_23344	1	2926926-2928161	Chr1	IS407A	CGCGC	TGACCTGCCCCCTACA	ATGAAGAAGCGCTTTACG	GCCCGCAAGCCAAACGCG	TGTTGGC	AGGGACTTTGCTAGAA	CCTCACG	GTCC	CCTC	N
8*Chr1_162_IS407	8*Chr1_29_IS407A_A	D-R	2002721280	8	2934200-2935435	Chr1	IS407A	ATTTAT	TGACCTGCCCCCTACA	ATGAAGAAGCGCTTTACG	GCCCGCAAGCCAAACGCG	TGTTGGC	AGGGACTTTGCTAGAA	ATCAGCA	CATT	ATCA	N
10*Chr1_149_IS40	10*Chr1_160a_IS407A_A	D-R	PRL20	10	2944646-2945881	Chr1	IS407A	CGCGC	TGACCTGCCCCCTACA	ATGAAGAAGCGCTTTACG	GCCCGCAAGCCAAACGCG	TGTTGGC	AGGGACTTTGCTAGAA	GTCCGGG	GTCC	GTCC	Y
6*Chr1_112_IS407	6*Chr1_49_IS407A_A	D-R	NCTC_10229	6	2955982-2957217	Chr1	IS407A	GCCCT	TGACCTGCCCCCTACA	ATGAAGAAGCGCTTTACG	GCCCGCAAGCCAAACGCG	TGTTGGC	AGGGACTTTGCTAGAA	TTGACGC	GCCC	TTGA	N
5*Chr1_150_IS407	5*Chr1_2b_IS407A_A	D-R	ATCC_10399	5	2968933-2970168	Chr1	IS407A	ATCGC	TGACCTGCCCCCTACA	ATGAAGAAGCGCTTTACG	GCCCGCAAGCCAAACGCG	TGTTGGC	AGGGACTTTGCTAGAA	ACGAGCT	CAAG	ACGA	N
4*Chr1_160a_IS40	4*Chr1_161_IS407A_A	D-R	gb8_horse_4	4	2977682-2978917	Chr1	IS407A	TACGA	TGACCTGCCCCCTACA	ATGAAGAAGCGCTTTACG	GCCCGCAAGCCAAACGCG	TGTTGGC	AGGGACTTTGCTAGAA	GTCCGGG	TTTT	GTCC	N
1*Chr1_160a_IS40	1*Chr1_161_IS407A_A	D-R	ATCC_23344	1	2991437-2992672	Chr1	IS407A	TACGA	TGACCTGCCCCCTACA	ATGAAGAAGCGCTTTACG	GCCCGCAAGCCAAACGCG	TGTTGGC	AGGGACTTTGCTAGAA	GTCCGGG	TTTT	GTCC	N
4*Chr1_35a_IS407A_A		N	gb8_horse_4	4	299583-300818	Chr1	IS407A	GTGAA	TGACCTGCCCCCTACA	ATGAAGAAGCGCTTTACG	GCCCGCAAGCCAAACGCG	TGTTGGC	AGGGACTTTGCTAGAA	CCGGAAC	GATT	CCGG	N
9*Chr1_18a_IS407A_A		N	SAVP1	9	2998066-2999301	Chr1	IS407A	CGTCG	TGACCTGCCCCCTACA	ATGAAGAAGCGCTTTACG	GCCCGCAAGCCAAACGCG	TGTTGGC	AGGGACTTTGCTAGAA	CACCGGC	CACC	CACC	Y
5*Chr1_2a_IS407A	5*Chr1_4_IS407A_B	D-R	ATCC_10399	5	3002482-3003717	Chr1	IS407A	GATGC	TGACCTGCCCCCTACA	ATGAAGAAGCGCTTTACG	GCCCGCAAGCCAAACGCG	TGTTGGC	AGGGACTTTGCTAGAA	GACAGGG	GACG	GACA	N
2*Chr1_162_IS407	2*Chr1_55_IS407A_A	D-R	fmh	2	3008620-3009855	Chr1	IS407A	ATCAC	TGACCTGCCCCCTACA	ATGAAGAAGCGCTTTACG	GCCCGCAAGCCAAACGCG	TGTTGGC	AGGGACTTTGCTAGAA	ATCAGCA	GCGG	ATCA	N
10*Chr1_161_IS40	10*Chr1_2a_IS407A_A	D-R	PRL20	10	3008966-3010201	Chr1	IS407A	GATGC	TGACCTGCCCCCTACA	ATGAAGAAGCGCTTTACG	GCCCGCAAGCCAAACGCG	TGTTGGC	AGGGACTTTGCTAGAA	GGTCAAA	GACG	GGTC	N
1*Chr1_35a_IS407A_A		N	ATCC_23344	1	300908-302143	Chr1	IS407A	GTGAA	TGACCTGCCCCCTACA	ATGAAGAAGCGCTTTACG	GCCCGCAAGCCAAACGCG	TGTTGGC	AGGGACTTTGCTAGAA	CCGGAAC	GATT	CCGG	N
2*Chr1_56_IS407A	2*Chr1_65a_IS407A_A	D-R	fmh	2	3022164-3023399	Chr1	IS407A	TGGAA	TGACCTGCCCCCTACA	ATGAAGAAGCGCTTTACG	GCCCGCAAGCCAAACGCG	TGTTGGC	AGGGACTTTGCTAGAA	GGGGGCA	GCGG	GGGG	N
3*Chr1_162_IS407	3*Chr1_55_IS407A_A	D-R	JHU	3	3024998-3026233	Chr1	IS407A	ATCAC	TGACCTGCCCCCTACA	ATGAAGAAGCGCTTTACG	GCCCGCAAGCCAAACGCG	TGTTGGC	AGGGACTTTGCTAGAA	ATCAGCA	GCGG	ATCA	N
5*Chr1_158_IS407A_A		D-R	ATCC_10399	5	3031244-3032479	Chr1	IS407A	AACCC	TGACCTGCCCCCTACA	ATGAAGAAGCGCTTTACG	GCCCGCAAGCCAAACGCG	TGTTGGC	AGGGACTTTGCTAGAA	ATCCACT	ATGG	ATCC	N
7*Chr1_161_IS407	7*Chr1_162_IS407A_A	D	NCTC_10247	7	3038317-3039552	Chr1	IS407A	TACGA	TGACCTGCCCCCTACA	ATGAAGAAGCGCTTTACG	GCCCGCAAGCCAAACGCG	TGTTGGC	AGGGACTTTGCTAGAA	ATCAGCA	TTTT	ATCA	N
3*Chr1_56_IS407A	3*Chr1_65a_IS407A_A	D-R	JHU	3	3038542-3039777	Chr1	IS407A	TGGAA	TGACCTGCCCCCTACA	ATGAAGAAGCGCTTTACG	GCCCGCAAGCCAAACGCG	TGTTGGC	AGGGACTTTGCTAGAA	GGGGGCA	GCGG	GGGG	N
9*Chr1_20_IS407A	9*Chr1_21_IS407A_A	D	SAVP1	9	3042799-3044034	Chr1	IS407A	ATGTC	TGACCTGCCCCCTACA	ATGAAGAAGCGCTTTACG	GCCCGCAAGCCAAACGCG	TGTTGGC	AGGGACTTTGCTAGAA	GTCCACG	CCGG	GTCC	N
6*Chr1_54_IS407A	6*Chr1_55_IS407A_A	D	NCTC_10229	6	3059581-3060816	Chr1	IS407A	ATCAC	TGACCTGCCCCCTACA	ATGAAGAAGCGCTTTACG	GCCCGCAAGCCAAACGCG	TGTTGGC	AGGGACTTTGCTAGAA	GCGGGTC	GCGT	GCGG	N
6*Chr1_111_IS407	6*Chr1_56_IS407A_A	D-R	NCTC_10229	6	3073105-3074340	Chr1	IS407A	TGGAA	TGACCTGCCCCCTACA	ATGAAGAAGCGCTTTACG	GCCCGCAAGCCAAACGCG	TGTTGGC	AGGGACTTTGCTAGAA	CAGGCGC	GCGG	CAGG	N
9*Chr1_154b_IS407A_A		R	SAVP1	9	3075556-3076791	Chr1	IS407A	GGCTT	TGACCTGCCCCCTACA	ATGAAGAAGCGCTTTACG	GCCCGCAAGCCAAACGCG	TGTTGGC	GAAGGGGGCAGGTCA	AACCCTC	GCTT	AACC	N
5*Chr1_149_IS407	5*Chr1_160b_IS407A_A	D-R	ATCC_10399	5	3076183-3077418	Chr1	IS407A	TCGAA	TGACCTGCCCCCTACA	ATGAAGAAGCGCTTTACG	GCCCGCAAGCCAAACGCG	TGTTGGC	AGGGACTTTGCTAGAA	GGACCAC	GAGG	GGAC	N
2*Chr1_29_IS407A	2*Chr1_64b_IS407A_A	D-R	fmh	2	3084797-3086032	Chr1	IS407A	ATTTAT	TGACCTGCCCCCTACA	ATGAAGAAGCGCTTTACG	GCCCGCAAGCCAAACGCG	TGTTGGC	AGGGACTTTGCTAGAA	TCGCTTCT	CATT	TCGC	N
4*Chr1_162_IS407	4*Chr1_55_IS407A_A	D-R	gb8_horse_4	4	3096934-3098169	Chr1	IS407A	ATCAC	TGACCTGCCCCCTACA	ATGAAGAAGCGCTTTACG	GCCCGCAAGCCAAACGCG	TGTTGGC	AGGGACTTTGCTAGAA	ATCAGCA	GCGG	ATCA	N
3*Chr1_29_IS407A	3*Chr1_64b_IS407A_A	D-R	JHU	3	3101880-3103115	Chr1	IS407A	ATTTAT	TGACCTGCCCCCTACA	ATGAAGAAGCGCTTTACG	GCCCGCAAGCCAAACGCG	TGTTGGC	AGGGACTTTGCTAGAA	TCGCTTCT	CATT	TCGC	N
7*Chr1_149_IS407	7*Chr1_160b_IS407A_A	D-R	NCTC_10247	7	3102821-3104056	Chr1	IS407A	CGCGC	TGACCTGCCCCCTACA	ATGAAGAAGCGCTTTACG	GCCCGCAAGCCAAACGCG	TGTTGGC	AGGGACTTTGCTAGAA	CCTCACG	GTCC	CCTC	N
9*Chr1_152b_IS40	9*Chr1_157_IS407A_A	D-R	SAVP1	9	3109637-3110872	Chr1	IS407A	GAAAC	TGACCTGCCCCCTACA	ATGAAGAAGCGCTTTACG	GCCCGCAAGCCAAACGCG	TGTTGGC	AGGGACTTTGCTAGAA	GATGCCT	GGTC	GATG	N
4*Chr1_56_IS407A	4*Chr1_65a_IS407A_A	D-R	gb8_horse_4	4	3110478-3111713	Chr1	IS407A	TGGAA	TGACCTGCCCCCTACA	ATGAAGAAGCGCTTTACG	GCCCGCAAGCCAAACGCG	TGTTGGC	AGGGACTTTGCTAGAA	GGGGGCA	GCGG	GGGG	N
1*Chr1_162_IS407	1*Chr1_55_IS407A_A	D-R	ATCC_23344	1	3110701-3111936	Chr1	IS407A	ATCAC	TGACCTGCCCCCTACA	ATGAAGAAGCGCTTTACG	GCCCGCAAGCCAAACGCG	TGTTGGC	AGGGACTTTGCTAGAA	ATCAGCA	GCGG	ATCA	N
1*Chr1_56_IS407A	1*Chr1_65a_IS407A_A	D-R	ATCC_23344	1	3124245-3125480	Chr1	IS407A	TGGAA	TGACCTGCCCCCTACA	ATGAAGAAGCGCTTTACG	GCCCGCAAGCCAAACGCG	TGTTGGC	AGGGACTTTGCTAGAA	GGGGGCA	GCGG	GGGG	N
8*Chr1_145_IS407A_A		N	2002721280	8	3139134-3140369	Chr1	IS407A	CAAGC	TGACCTGCCCCCTACA	ATGAAGAAGCGCTTTACG	GCCCGCAAGCCAAACGCG	TGTTGGC	AGGGACTTTGCTAGAA	CTATCGC	GAAT	CTAT	N
5*Chr1_161_IS407	5*Chr1_162_IS407A_A	D	ATCC_10399	5	3140813-3142048	Chr1	IS407A	TACGA	TGACCTGCCCCCTACA	ATGAAGAAGCGCTTTACG	GCCCGCAAGCCAAACGCG	TGTTGGC	AGGGACTTTGCTAGAA	ATCAGCA	TTTT	ATCA	N
7*Chr1_158_IS407A_A		D-R	NCTC_10247	7	3147756-3148991	Chr1	IS407A	AACCC	TGACCTGCCCCCTACA	ATGAAGAAGCGCTTTACG	GCCCGCAAGCCAAACGCG	TGTTGGC	AGGGACTTTGCTAGAA	ATCCACT	ATGG	ATCC	N
10*Chr1_162_IS40	10*Chr1_4_IS407A_B	D-R	PRL20	10	3148182-3149417	Chr1	IS407A	ACGTT	TGACCTGCCCCCTACA	ATGAAGAAGCGCTTTACG	GCCCGCAAGCCAAACGCG	TGTTGGC	AGGGACTTTGCTAGAA	CGGCACA	TGAT	CGGC	N
8*Chr1_7_IS407A_	8*Chr1_8_IS407A_A	D	2002721280	8	3153325-3154560	Chr1	IS407A	GGCGC	TGACCTGCCCCCTACA	ATGAAGAAGCGCTTTACG	GCCCGCAAGCCAAACGCG	TGTTGGC	AGGGACTTTGCTAGAA	TGAGAGC	GACG	TGAG	N
9*Chr1_152a_IS40	9*Chr1_154a_IS407A_A	R	SAVP1	9	3157171-3158406	Chr1	IS407A	GCAGC	TGACCTGCCCCCTACA	ATGAAGAAGCGCTTTACG	GCCCGCAAGCCAAACGCG	TGTTGGC	AGGGACTTTGCTAGAA	GGGACGC	CCCC	GGGA	N
8*Chr1_30_IS407A	8*Chr1_6_IS407A_A	D-R	2002721280	8	3166175-3167410	Chr1	IS407A	TTGAG	TGACCTGCCCCCTACA	ATGAAGAAGCGCTTTACG	GCCCGCAAGCCAAACGCG	TGTTGGC	AGGGACTTTGCTAGAA	CGCCGCA	TCGG	CGCC	N
4*Chr1_29_IS407A	4*Chr1_64b_IS407A_A	D-R	gb8_horse_4	4	3172259-3173494	Chr1	IS407A	ATTTAT	TGACCTGCCCCCTACA	ATGAAGAAGCGCTTTACG	GCCCGCAAGCCAAACGCG	TGTTGGC	AGGGACTTTGCTAGAA	TCGCTTCT	CATT	TCGC	N
1*Chr1_29_IS407A	1*Chr1_64b_IS407A_A	D-R	ATCC_23344	1	3186007-3187242	Chr1	IS407A	ATTTAT	TGACCTGCCCCCTACA	ATGAAGAAGCGCTTTACG	GCCCGCAAGCCAAACGCG	TGTTGGC	AGGGACTTTGCTAGAA	TCGCTTCT	CATT	TCGC	N
5*Chr1_33_IS407A	5*Chr1_35a_IS407A_A	D-R	ATCC_10399	5	320768-322003	Chr1	IS407A	CCGCA	TGACCTGCCCCCTACA	ATGAAGAAGCGCTTTACG	GCCCGCAAGCCAAACGCG	TGTTGGC	AGGGACTTTGCTAGAA	CCGGAAC	CAAT	CCGG	N
10*Chr1_64a_IS407A_A		N	PRL20	10	3215347-3216582	Chr1	IS407A	AAGGA	TGACCTGCCCCCTACA	ATGAAGAAGCGCTTTACG	GCCCGCAAGCCAAACGCG	TGTTGGC	AGGGACTTTGCTAGAA	CGCTTCTC	GGGC	CGCT	N
7*Chr1_145_IS407A_A		N	NCTC_10247	7	3221599-3222834	Chr1	IS407A	AAGCC	TGACCTGCCCCCTACA	ATGAAGAAGCGCTTTACG	GCCCGCAAGCCAAACGCG	TGTTGGC	AGGGACTTTGCTAGAA	GGCTTCG	ATAG	GGCT	N
9*Chr1_150_IS407	9*Chr1_2b_IS407A_A	D-R	SAVP1	9	3221731-3222966	Chr1	IS407A	CGTGC	TGACCTGCCCCCTACA	ATGAAGAAGCGCTTTACG	GCCCGCAAGCCAAACGCG	TGTTGGC	AGGGACTTTGCTAGAA	ACGAGCT	GTCG	ACGA	N
10*Chr1_29_IS407A_A		D-R	PRL20	10	3221885-3223120	Chr1	IS407A	ATTTAT	TGACCTGCCCCCTACA	ATGAAGAAGCGCTTTACG	GCCCGCAAGCCAAACGCG	TGTTGGC	AGGGACTTTGCTAGAA	CCTCACG	CATT	CCTC	N
9*Chr1_162_IS407	9*Chr1_4_IS407A_B	D-R	SAVP1	9	3255150-3256385	Chr1	IS407A	ACGTT	TGACCTGCCCCCTACA	ATGAAGAAGCGCTTTACG	GCCCGCAAGCCAAACGCG	TGTTGGC	AGGGACTTTGCTAGAA	CGGCACA	TGAT	CGGC	N
5*Chr1_160a_IS40	5*Chr1_29_IS407A_A	D-R	ATCC_10399	5	3260016-3261251	Chr1	IS407A	ATTTAT	TGACCTGCCCCCTACA	ATGAAGAAGCGCTTTACG	GCCCGCAAGCCAAACGCG	TGTTGGC	AGGGACTTTGCTAGAA	GTCCGGG	CATT	GTCC	N
7*Chr1_143b_IS40	7*Chr1_18a_IS407A_A	R	NCTC_10247	7	3290037-3291272	Chr1	IS407A	TTCGC	TGACCTGCCCCCTACA	ATGAAGAAGCGCTTTACG	GCCCGCAAGCCAAACGCG	TGTTGGC	AGGGACTTTGCTAGAA	CACCGGC	ATCA	CACC	N
10*Chr1_27_IS407A_B		N	PRL20	10	3324203-3325438	Chr1	IS407A	ATGGG	TGACCTGCCCCCTACA	ATGAAGAAGCGCTTTACG	GCCCGCAAGCCAAACGCG	TGTTGGC	AGGGACTTTGCTAGAA	GGTGGCC	GGTG	GGTG	Y
8*Chr1_18a_IS407A_A		N	2002721280	8	3329244-3330479	Chr1	IS407A	CGTCG	TGACCTGCCCCCTACA	ATGAAGAAGCGCTTTACG	GCCCGCAAGCCAAACGCG	TGTTGGC	AGGGACTTTGCTAGAA	CACCGGC	CACC	CACC	Y
2*Chr1_18a_IS407A_A		N	fmh	2	3361263-3362498	Chr1	IS407A	CGTCG	TGACCTGCCCCCTACA	ATGAAGAAGCGCTTTACG	GCCCGCAAGCCAAACGCG	TGTTGGC	AGGGACTTTGCTAGAA	CACCGGC	CACC	CACC	Y
8*Chr1_20_IS407A	8*Chr1_21_IS407A_A	D	2002721280	8	3371851-3373086	Chr1	IS407A	CTGAC	TGACCTGCCCCCTACA	ATGAAGAAGCGCTTTACG	GCCCGCAAGCCAAACGCG	TGTTGGC	AGGGACTTTGCTAGAA	CCGGCGC	GGAC	CCGG	N
9*Chr1_160a_IS407A_A		N	SAVP1	9	3374307-3375542	Chr1	IS407A	CGCGC	TGACCTGCCCCCTACA	ATGAAGAAGCGCTTTACG	GCCCGCAAGCCAAACGCG	TGTTGGC	AGGGACTTTGCTAGAA	GTCCGGG	GTCC	GTCC	Y
3*Chr1_18a_IS407A_A		N	JHU	3	3379695-3380930	Chr1	IS407A	CGTCG	TGACCTGCCCCCTACA	ATGAAGAAGCGCTTTACG	GCCCGCAAGCCAAACGCG	TGTTGGC	AGGGACTTTGCTAGAA	CACCGGC	CACC	CACC	Y
5*Chr1_34_IS407A	5*Chr1_35b_IS407A_A	D-R	ATCC_10399	5	338128-339363	Chr1	IS407A	GTGAA	TGACCTGCCCCCTACA	ATGAAGAAGCGCTTTACG	GCCCGCAAGCCAAACGCG	TGTTGGC	AGGGACTTTGCTAGAA	GCAACGG	GATT	GCAA	N
4*Chr1_37_IS407A_A		N	gb8_horse_4	4	338280-339515	Chr1	IS407A	CGTCG	TGACCTGCCCCCTACA	ATGAAGAAGCGCTTTACG	GCCCGCAAGCCAAACGCG	TGTTGGC	AGGGACTTTGCTAGAA	GACCCGG	CCAG	GACC	N
6*Chr1_99_IS407A	6*Chr1_67_IS407A_A	D-R	NCTC_10229	6	3386673-3387908	Chr1	IS407A	TTCATA	TGACCTGCCCCCTACA	ATGAAGAAGCGCTTTACG	GCCCGCAAGCCAAACGCG	TGTTGGC	AGGGACTTTGCTAGAA	GATCGAG	GTGG	GATC	N

1*Chr1_37_IS407A_A		N	ATCC_23344	1	339666-340901	Chr1	IS407A	CGTCG	TGACCTGCCCCCTACA	ATGAAGAAGCGCTTTACG	GCCCGCAAGCCAAACGCG	TGTTGGC	AGGGACTTTGCTAGAA	GACCCGG	CCAG	GACC	N
8*Chr1_9b_IS407A_C		R	2002721280	8	3404613-3405848	Chr1	IS407A	CGCGC	TGACCTGCCCCCTACA	ATGAAGAAGCGCTTTACG	GCCCGCAAGCCAAACGCG	TGTTGGC	AGGGACTTTGCTAGAA	AACCCTC	GCTT	AACC	N
7*Chr1_152a_IS40	7*Chr1_154a_IS407A_A	R	NCTC_10247	7	3405726-3406961	Chr1	IS407A	CGCAG	TGACCTGCCCCCTACA	ATGAAGAAGCGCTTTACG	GCCCGCAAGCCAAACGCG	TGTTGGC	AGGGACTTTGCTAGAA	GGGACGC	CTGT	GGGA	N
2*Chr1_20_IS407A	2*Chr1_7_IS407A_A	D-R	fmh	2	3406292-3407527	Chr1	IS407A	ATGTC	TGACCTGCCCCCTACA	ATGAAGAAGCGCTTTACG	GCCCGCAAGCCAAACGCG	TGTTGGC	AGGGACTTTGCTAGAA	TTTCCAGT	CCGG	TTTC	N
2*Chr1_122_IS407A_A		N	fmh	2	341131-342366	Chr1	IS407A	CAGAT	TGACCTGCCCCCTACA	ATGAAGAAGCGCTTTACG	GCCCGCAAGCCAAACGCG	TGTTGGC	AGGGACTTTGCTAGAA	CTTCGTGC	CTGC	CTTC	N
2*Chr1_30_IS407A	2*Chr1_6_IS407A_A	D-R	fmh	2	3419381-3420616	Chr1	IS407A	TTGAG	TGACCTGCCCCCTACA	ATGAAGAAGCGCTTTACG	GCCCGCAAGCCAAACGCG	TGTTGGC	AGGGACTTTGCTAGAA	CGCCGCA	TCGG	CGCC	N
6*Chr1_78_IS407A_A		N	NCTC_10229	6	342119-343354	Chr1	IS407A	CAGCA	TGACCTGCCCCCTACA	ATGAAGAAGCGCTTTACG	GCCCGCAAGCCAAACGCG	TGTTGGC	AGGGACTTTGCTAGAA	GTAGCTG	GTAG	GTAG	Y
10*Chr1_30_IS407	10*Chr1_6_IS407A_A	D-R	PRL20	10	34218-35453	Chr1	IS407A	TTGAG	TGACCTGCCCCCTACA	ATGAAGAAGCGCTTTACG	GCCCGCAAGCCAAACGCG	TGTTGGC	AGGGACTTTGCTAGAA	CGCCGCA	TCGG	CGCC	N
3*Chr1_20_IS407A	3*Chr1_7_IS407A_A	D-R	JHU	3	3425658-3426893	Chr1	IS407A	ATGTC	TGACCTGCCCCCTACA	ATGAAGAAGCGCTTTACG	GCCCGCAAGCCAAACGCG	TGTTGGC	AGGGACTTTGCTAGAA	TTTCCAGT	CCGG	TTTC	N
8*Chr1_122_IS407A_A		N	2002721280	8	342576-343811	Chr1	IS407A	GCGCA	TGACCTGCCCCCTACA	ATGAAGAAGCGCTTTACG	GCCCGCAAGCCAAACGCG	TGTTGGC	AGGGACTTTGCTAGAA	GTGCTGC	GAAG	GTGC	N
3*Chr1_122_IS407A_A		N	JHU	3	342735-343970	Chr1	IS407A	CAGAT	TGACCTGCCCCCTACA	ATGAAGAAGCGCTTTACG	GCCCGCAAGCCAAACGCG	TGTTGGC	AGGGACTTTGCTAGAA	CTTCGTGC	CTGC	CTTC	N
9*Chr1_161_IS407A_A		D-R	SAVP1	9	3438813-3440048	Chr1	IS407A	CTTAG	TGACCTGCCCCCTACA	ATGAAGAAGCGCTTTACG	GCCCGCAAGCCAAACGCG	TGTTGGC	AGGGACTTTGCTAGAA	TGTCGATC	GACC	TGTC	N
3*Chr1_30_IS407A	3*Chr1_6_IS407A_A	D-R	JHU	3	3439108-3440343	Chr1	IS407A	TTGAG	TGACCTGCCCCCTACA	ATGAAGAAGCGCTTTACG	GCCCGCAAGCCAAACGCG	TGTTGGC	AGGGACTTTGCTAGAA	CGCCGCA	TCGG	CGCC	N
4*Chr1_18a_IS407A_A		N	gb8_horse_4	4	3444795-3446030	Chr1	IS407A	CGTCG	TGACCTGCCCCCTACA	ATGAAGAAGCGCTTTACG	GCCCGCAAGCCAAACGCG	TGTTGGC	AGGGACTTTGCTAGAA	CACCGGC	CACC	CACC	Y
9*Chr1_2a_IS407A	9*Chr1_5_IS407A_A	D-R	SAVP1	9	3452990-3454225	Chr1	IS407A	GATGC	TGACCTGCCCCCTACA	ATGAAGAAGCGCTTTACG	GCCCGCAAGCCAAACGCG	TGTTGGC	AGGGACTTTGCTAGAA	GCGCCCG	GACG	GCGC	N
7*Chr1_152b_IS40	7*Chr1_157_IS407A_A	D-R	NCTC_10247	7	3453248-3454483	Chr1	IS407A	GAAAC	TGACCTGCCCCCTACA	ATGAAGAAGCGCTTTACG	GCCCGCAAGCCAAACGCG	TGTTGGC	AGGGACTTTGCTAGAA	GATGCCTT	GGTC	GATG	N
6*Chr1_68_IS407A	6*Chr1_69_IS407A_A	D	NCTC_10229	6	3453424-3454659	Chr1	IS407A	CCTAA	TGACCTGCCCCCTACA	ATGAAGAAGCGCTTTACG	GCCCGCAAGCCAAACGCG	TGTTGGC	AGGGACTTTGCTAGAA	CTACACCT	ACGT	CTAC	N
6*Chr1_70_IS407A_C		N	NCTC_10229	6	3454610-3455717	Chr1	IS407A	#IS407	~~~~~~~~~~~~~~	~~~~~~~~~~~~~~~~~	GCCCGCAAGCCAAACGCG	TGTTGGC	AGGGACTTTGCTAGAA	CCAGTTTG	#	CCAG	N.D.
1*Chr1_18a_IS407A_A		N	ATCC_23344	1	3458793-3460028	Chr1	IS407A	CGTCG	TGACCTGCCCCCTACA	ATGAAGAAGCGCTTTACG	GCCCGCAAGCCAAACGCG	TGTTGGC	AGGGACTTTGCTAGAA	CACCGGC	CACC	CACC	Y
10*Chr1_18a_IS407A_A		N	PRL20	10	3461491-3462726	Chr1	IS407A	CGTCG	TGACCTGCCCCCTACA	ATGAAGAAGCGCTTTACG	GCCCGCAAGCCAAACGCG	TGTTGGC	AGGGACTTTGCTAGAA	CACCGGC	CACC	CACC	Y
7*Chr1_154b_IS40	7*Chr1_7_IS407A_A	D-R	NCTC_10247	7	3488844-3490079	Chr1	IS407A	CGCGC	TGACCTGCCCCCTACA	ATGAAGAAGCGCTTTACG	GCCCGCAAGCCAAACGCG	TGTTGGC	AGGGACTTTGCTAGAA	TGAGAGC	GCTT	TGAG	N
4*Chr1_20_IS407A	4*Chr1_7_IS407A_A	D-R	gb8_horse_4	4	3489282-3490517	Chr1	IS407A	ATGTC	TGACCTGCCCCCTACA	ATGAAGAAGCGCTTTACG	GCCCGCAAGCCAAACGCG	TGTTGGC	AGGGACTTTGCTAGAA	TTTCCAGT	CCGG	TTTC	N
1*Chr1_20_IS407A	1*Chr1_7_IS407A_A	D-R	ATCC_23344	1	3503487-3504722	Chr1	IS407A	ATGTC	TGACCTGCCCCCTACA	ATGAAGAAGCGCTTTACG	GCCCGCAAGCCAAACGCG	TGTTGGC	AGGGACTTTGCTAGAA	TTTCCAGT	CCGG	TTTC	N
10*Chr1_37_IS407A_A		N	PRL20	10	352659-353894	Chr1	IS407A	TGGGC	TGACCTGCCCCCTACA	ATGAAGAAGCGCTTTACG	GCCCGCAAGCCAAACGCG	TGTTGGC	AGGGACTTTGCTAGAA	GACCCGG	GACC	GACC	Y
5*Chr1_18a_IS407	5*Chr1_6_IS407A_A	D-R	ATCC_10399	5	3533677-3534912	Chr1	IS407A	TTGAG	TGACCTGCCCCCTACA	ATGAAGAAGCGCTTTACG	GCCCGCAAGCCAAACGCG	TGTTGGC	AGGGACTTTGCTAGAA	CACCGGC	TCGG	CACC	N
2*Chr1_21_IS407A	2*Chr1_8_IS407A_A	D-R	fmh	2	3538554-3539789	Chr1	IS407A	GGCGC	TGACCTGCCCCCTACA	ATGAAGAAGCGCTTTACG	GCCCGCAAGCCAAACGCG	TGTTGGC	AGGGACTTTGCTAGAA	GTCCACGT	GACG	GTCC	N
5*Chr1_7_IS407A_	5*Chr1_8_IS407A_A	D	ATCC_10399	5	3546247-3547482	Chr1	IS407A	GGCGC	TGACCTGCCCCCTACA	ATGAAGAAGCGCTTTACG	GCCCGCAAGCCAAACGCG	TGTTGGC	AGGGACTTTGCTAGAA	TTTCCAGT	GACG	TTTC	N
3*Chr1_21_IS407A	3*Chr1_8_IS407A_A	D-R	JHU	3	3559432-3560667	Chr1	IS407A	GGCGC	TGACCTGCCCCCTACA	ATGAAGAAGCGCTTTACG	GCCCGCAAGCCAAACGCG	TGTTGGC	AGGGACTTTGCTAGAA	GTCCACGT	GACG	GTCC	N
5*Chr1_37_IS407A_A		N	ATCC_10399	5	376616-377851	Chr1	IS407A	CGTCG	TGACCTGCCCCCTACA	ATGAAGAAGCGCTTTACG	GCCCGCAAGCCAAACGCG	TGTTGGC	AGGGACTTTGCTAGAA	GACCCGG	CCAG	GACC	N
6*Chr1_79_IS407A	6*Chr1_80_IS407A_A	D	NCTC_10229	6	377301-378536	Chr1	IS407A	GGGAC	TGACCTGCCCCCTACA	ATGAAGAAGCGCTTTACG	GCCCGCAAGCCAAACGCG	TGTTGGC	AGGGACTTTGCTAGAA	CAAGCAG	GTCA	CAAG	N
8*Chr1_115b_IS40	8*Chr1_126a_IS407A_A	R	2002721280	8	387955-389190	Chr1	IS407A	TGCCG	TGACCTGCCCCCTACA	ATGAAGAAGCGCTTTACG	GCCCGCAAGCCAAACGCG	TGTTGGC	AGGGACTTTGCTAGAA	GATGACA	AAAA	GATG	N
2*Chr1_126a_IS40	2*Chr1_43b_IS407A_A	R	fmh	2	387963-389198	Chr1	IS407A	CCCGT	TGACCTGCCCCCTACA	ATGAAGAAGCGCTTTACG	GCCCGCAAGCCAAACGCG	TGTTGGC	AGGGACTTTGCTAGAA	TTTTTCAT	CCCG	TTTT	N
3*Chr1_126a_IS40	3*Chr1_43b_IS407A_A	R	JHU	3	389122-390357	Chr1	IS407A	CCCGT	TGACCTGCCCCCTACA	ATGAAGAAGCGCTTTACG	GCCCGCAAGCCAAACGCG	TGTTGGC	AGGGACTTTGCTAGAA	TTTTTCAT	CCCG	TTTT	N
10*Chr1_10_IS407A_A		N	PRL20	10	3972-5207	Chr1	IS407A	CGCCA	TGACCTGCCCCCTACA	ATGAAGAAGCGCTTTACG	GCCCGCAAGCCAAACGCG	TGTTGGC	AGGGACTTTGCTAGAA	CCCTATGC	CCCT	CCCT	Y
6*Chr1_83_IS407A_A		N	NCTC_10229	6	399323-400558	Chr1	IS407A	TTACG	TGACCTGCCCCCTACA	ATGAAGAAGCGCTTTACG	GCCCGCAAGCCAAACGCG	TGTTGGC	AGGGACTTTGCTAGAA	ATCCACC	TGAT	ATCC	N
2*Chr1_116_IS407A_B		N	fmh	2	402068-403303	Chr1	IS407A	AAAAA	TGACCTGCCCCCTACA	ATGAAGAAGCGCTTTACG	GCCCGCAAGCCAAACGCG	TGTTGGC	AGGGACTTTGCTAGAA	GATGACA	AAGC	GATG	N
8*Chr1_43b_IS407	8*Chr1_58b_IS407A_A	R	2002721280	8	403287-404522	Chr1	IS407A	CGTCG	TGACCTGCCCCCTACA	ATGAAGAAGCGCTTTACG	GCCCGCAAGCCAAACGCG	TGTTGGC	AGGGACTTTGCTAGAA	GCTTCGG	GTGA	GCTT	N
3*Chr1_116_IS407A_B		N	JHU	3	403816-405051	Chr1	IS407A	AAAAA	TGACCTGCCCCCTACA	ATGAAGAAGCGCTTTACG	GCCCGCAAGCCAAACGCG	TGTTGGC	AGGGACTTTGCTAGAA	GATGACA	AAGC	GATG	N
2*Chr1_115a_IS407A_A		N	fmh	2	404604-405839	Chr1	IS407A	TTCGC	TGACCTGCCCCCTACA	ATGAAGAAGCGCTTTACG	GCCCGCAAGCCAAACGCG	TGTTGGC	AGGGACTTTGCTAGAA	CCTGCGAT	GATG	CCTG	N
3*Chr1_115a_IS407A_A		N	JHU	3	406352-407587	Chr1	IS407A	TTCGC	TGACCTGCCCCCTACA	ATGAAGAAGCGCTTTACG	GCCCGCAAGCCAAACGCG	TGTTGGC	AGGGACTTTGCTAGAA	CCTGCGAT	GATG	CCTG	N
6*Chr1_84a_IS407	6*Chr1_94b_IS407A_A	R	NCTC_10229	6	414441-415676	Chr1	IS407A	GTGCC	TGACCTGCCCCCTACA	ATGAAGAAGCGCTTTACG	GCCCGCAAGCCAAACGCG	TGTTGGC	AGGGACTTTGCTAGAA	GTAAAGC	AAAG	GTAA	N
9*Chr1_145_IS407A_A		N	SAVP1	9	440915-442150	Chr1	IS407A	AAGCC	TGACCTGCCCCCTACA	ATGAAGAAGCGCTTTACG	GCCCGCAAGCCAAACGCG	TGTTGGC	AGGGACTTTGCTAGAA	GGCTTCG	ATAG	GGCT	N
10*Chr1_122_IS407A_A		N	PRL20	10	452966-454201	Chr1	IS407A	TAATG	TGACCTGCCCCCTACA	ATGAAGAAGCGCTTTACG	GCCCGCAAGCCAAACGCG	TGTTGGC	AGGGACTTTGCTAGAA	CTTCGTGC	GCAC	CTTC	N
4*Chr1_122_IS407A_A		N	gb8_horse_4	4	463570-464805	Chr1	IS407A	CAGAT	TGACCTGCCCCCTACA	ATGAAGAAGCGCTTTACG	GCCCGCAAGCCAAACGCG	TGTTGGC	AGGGACTTTGCTAGAA	CTTCGTGC	CTGC	CTTC	N
1*Chr1_122_IS407A_A		N	ATCC_23344	1	465256-466491	Chr1	IS407A	CAGAT	TGACCTGCCCCCTACA	ATGAAGAAGCGCTTTACG	GCCCGCAAGCCAAACGCG	TGTTGGC	AGGGACTTTGCTAGAA	CTTCGTGC	CTGC	CTTC	N
7*Chr1_64a_IS407A_A		N	NCTC_10247	7	471816-473051	Chr1	IS407A	CTTGTT	TGACCTGCCCCCTACA	ATGAAGAAGCGCTTTACG	GCCCGCAAGCCAAACGCG	TGTTGGC	AGGGACTTTGCTAGAA	TCGCTTCT	TCGC	TCGC	Y
6*Chr1_95_IS407A_A		N	NCTC_10229	6	473354-474589	Chr1	IS407A	GGCGC	TGACCTGCCCCCTACA	ATGAAGAAGCGCTTTACG	GCCCGCAAGCCAAACGCG	TGTTGGC	AGGGACTTTGCTAGAA	GCCCGCC	GATG	GCCC	N
2*Chr1_114_IS407A_A		N	fmh	2	476309-477544	Chr1	IS407A	TGATT	TGACCTGCCCCCTACA	ATGAAGAAGCGCTTTACG	GCCCGCAAGCCAAACGCG	TGTTGGC	AGGGACTTTGCTAGAA	GATCCGG	GAAG	GATC	N
3*Chr1_114_IS407A_A		N	JHU	3	478305-479540	Chr1	IS407A	TGATT	TGACCTGCCCCCTACA	ATGAAGAAGCGCTTTACG	GCCCGCAAGCCAAACGCG	TGTTGGC	AGGGACTTTGCTAGAA	GATCCGG	GAAG	GATC	N
2*Chr1_113_IS407A_A		N	fmh	2	489951-491186	Chr1	IS407A	TTTGCT	TGACCTGCCCCCTACA	ATGAAGAAGCGCTTTACG	GCCCGCAAGCCAAACGCG	TGTTGGC	AGGGACTTTGCTAGAA	CAACGGC	CCTC	CAAC	N
3*Chr1_113_IS407A_A		N	JHU	3	491947-493182	Chr1	IS407A	TTTGCT	TGACCTGCCCCCTACA	ATGAAGAAGCGCTTTACG	GCCCGCAAGCCAAACGCG	TGTTGGC	AGGGACTTTGCTAGAA	CAACGGC	CCTC	CAAC	N
10*Chr1_115a_IS407A_A		N	PRL20	10	498494-499729	Chr1	IS407A	TTCGC	TGACCTGCCCCCTACA	ATGAAGAAGCGCTTTACG	GCCCGCAAGCCAAACGCG	TGTTGGC	AGGGACTTTGCTAGAA	GATGACA	GATG	GATG	Y
5*Chr1_122_IS407A_A		N	ATCC_10399	5	502267-503502	Chr1	IS407A	CAGAT	TGACCTGCCCCCTACA	ATGAAGAAGCGCTTTACG	GCCCGCAAGCCAAACGCG	TGTTGGC	AGGGACTTTGCTAGAA	CTTCGTGC	CTGC	CTTC	N
4*Chr1_126a_IS40	4*Chr1_43b_IS407A_A	R	gb8_horse_4	4	509988-511223	Chr1	IS407A	CCCGT	TGACCTGCCCCCTACA	ATGAAGAAGCGCTTTACG	GCCCGCAAGCCAAACGCG	TGTTGGC	AGGGACTTTGCTAGAA	TTTTTCAT	CCCG	TTTT	N
1*Chr1_126a_IS40	1*Chr1_43b_IS407A_A	R	ATCC_23344	1	511643-512878	Chr1	IS407A	CCCGT	TGACCTGCCCCCTACA	ATGAAGAAGCGCTTTACG	GCCCGCAAGCCAAACGCG	TGTTGGC	AGGGACTTTGCTAGAA	TTTTTCAT	CCCG	TTTT	N
6*Chr1_96_IS407A	6*Chr1_97_IS407A_A	D	NCTC_10229	6	516514-517749	Chr1	IS407A	GGCAC	TGACCTGCCCCCTACA	ATGAAGAAGCGCTTTACG	GCCCGCAAGCCAAACGCG	TGTTGGC	AGGGACTTTGCTAGAA	ACCGCCC	CCCC	ACCG	N
4*Chr1_116_IS407A_B		N	gb8_horse_4	4	524093-525328	Chr1	IS407A	AAAAA	TGACCTGCCCCCTACA	ATGAAGAAGCGCTTTACG	GCCCGCAAGCCAAACGCG	TGTTGGC	AGGGACTTTGCTAGAA	GATGACA	AAGC	GATG	N
1*Chr1_116_IS407A_B		N	ATCC_23344	1	525748-526983	Chr1	IS407A	AAAAA	TGACCTGCCCCCTACA	ATGAAGAAGCGCTTTACG	GCCCGCAAGCCAAACGCG	TGTTGGC	AGGGACTTTGCTAGAA	GATGACA	AAGC	GATG	N

4*Chr1_115a_IS407A_A		N	gb8_horse_4	4	526629-527864	Chr1	IS407A	TTCGC(	TGACCTGCCCCCTACA	ATGAAGAAGCGCTTTACG(	GCCCGCAAGCCAAACGCG/	TGTTGGC(	AGGGACTTTGCTAGAA(	CCTGCGAT	GATG	CCTG	N
1*Chr1_115a_IS407A_A		N	ATCC_23344	1	528284-529519	Chr1	IS407A	TTCGC(	TGACCTGCCCCCTACA	ATGAAGAAGCGCTTTACG(	GCCCGCAAGCCAAACGCG/	TGTTGGC(	AGGGACTTTGCTAGAA(	CCTGCGAT	GATG	CCTG	N
7*Chr1_65a_IS407A_A		N	NCTC_10247	7	533693-534928	Chr1	IS407A	GCGAC	TGACCTGCCCCCTACA	ATGAAGAAGCGCTTTACG(	GCCCGCAAGCCAAACGCG/	TGTTGGC(	AGGGACTTTGCTAGAA(	GGGGGCA	GAAT	GGGG	N
5*Chr1_126a_IS407A_A		R	ATCC_10399	5	548676-549911	Chr1	IS407A	AAAAA	TGACCTGCCCCCTACA	ATGAAGAAGCGCTTTACG(	GCCCGCAAGCCAAACGCG/	TGTTGGC(	AGGGACTTTGCTAGAA(	TTTTTCAT(	AAGC	TTTT	N
5*Chr1_43b_IS407A_A		R	ATCC_10399	5	551212-552447	Chr1	IS407A	CCCGT(	TGACCTGCCCCCTACA	ATGAAGAAGCGCTTTACG(	GCCCGCAAGCCAAACGCG/	TGTTGGC(	AGGGACTTTGCTAGAA(	CCTGCGAT	CCCG	CCTG	N
9*Chr1_136_IS407/	9*Chr1_43a_IS407A_A	D-R	SAVP1	9	554720-555955	Chr1	IS407A	GATCG.	TGACCTGCCCCCTACA	ATGAAGAAGCGCTTTACG(	GCCCGCAAGCCAAACGCG/	TGTTGGC(	AGGGACTTTGCTAGAA(	CCGCAGC(	GAAC	CCGC	N
2*Chr1_112_IS407/	2*Chr1_49_IS407A_A	D-R	fmh	2	560092-561327	Chr1	IS407A	TCATC(	TGACCTGCCCCCTACA	ATGAAGAAGCGCTTTACG(	GCCCGCAAGCCAAACGCG/	TGTTGGC(	AGGGACTTTGCTAGAA(	GGGCTGAT	TCAA	GGGC	N
3*Chr1_112_IS407/	3*Chr1_49_IS407A_A	D-R	JHU	3	563350-564585	Chr1	IS407A	TCATC(	TGACCTGCCCCCTACA	ATGAAGAAGCGCTTTACG(	GCCCGCAAGCCAAACGCG/	TGTTGGC(	AGGGACTTTGCTAGAA(	GGGCTGAT	TCAA	GGGC	N
5*Chr1_115a_IS407A_A		N	ATCC_10399	5	565315-566550	Chr1	IS407A	TTCGC(	TGACCTGCCCCCTACA	ATGAAGAAGCGCTTTACG(	GCCCGCAAGCCAAACGCG/	TGTTGGC(	AGGGACTTTGCTAGAA(	GATGACA(	GATG	GATG	Y
4*Chr1_30_IS407A	4*Chr1_6_IS407A_A	D-R	gb8_horse_4	4	5679-6914	Chr1	IS407A	TTGAG/	TGACCTGCCCCCTACA	ATGAAGAAGCGCTTTACG(	GCCCGCAAGCCAAACGCG/	TGTTGGC(	AGGGACTTTGCTAGAA(	CGCCGCAT	TCGG	CGCC	N
10*Chr1_114_IS407A_A		N	PRL20	10	570173-571408	Chr1	IS407A	ACGCG	TGACCTGCCCCCTACA	ATGAAGAAGCGCTTTACG(	GCCCGCAAGCCAAACGCG/	TGTTGGC(	AGGGACTTTGCTAGAA(	GATCCGG(	GCAC	GATC	N
6*Chr1_98_IS407A	6*Chr1_66_IS407A_A	D-R	NCTC_10229	6	575108-576343	Chr1	IS407A	CTCGT(	TGACCTGCCCCCTACA	ATGAAGAAGCGCTTTACG(	GCCCGCAAGCCAAACGCG/	TGTTGGC(	AGGGACTTTGCTAGAA(	GTAGGCA/	CCCG	GTAG	N
9*Chr1_42b_IS407A_A		N	SAVP1	9	575340-576499	Chr1	IS407A	GCGCT	TGACCTGCCCCCTACA	ATGAAGAAGCGCTTTACG(	GCCCGCAAGCCAAACGCG/	TGTTGGC(	CCACACCGGCT	#can not f	TGCG	#can	N.D.
10*Chr1_113_IS407A_A		N	PRL20	10	582185-583420	Chr1	IS407A	TGATT(	TGACCTGCCCCCTACA	ATGAAGAAGCGCTTTACG(	GCCCGCAAGCCAAACGCG/	TGTTGGC(	AGGGACTTTGCTAGAA(	ATCTACCT	GAAG	ATCT	N
7*Chr1_98_IS407A	7*Chr1_66_IS407A_A	D-R	NCTC_10247	7	584779-586014	Chr1	IS407A	CTCGT(	TGACCTGCCCCCTACA	ATGAAGAAGCGCTTTACG(	GCCCGCAAGCCAAACGCG/	TGTTGGC(	AGGGACTTTGCTAGAA(	GTAGGCA/	CCCG	GTAG	N
9*Chr1_35b_IS407/	9*Chr1_45b_IS407A_A	R	SAVP1	9	588962-590197	Chr1	IS407A	GTGAA	TGACCTGCCCCCTACA	ATGAAGAAGCGCTTTACG(	GCCCGCAAGCCAAACGCG/	TGTTGGC(	AGGGACTTTGCTAGAA(	GATGCTC/	GATT	GATG	N
4*Chr1_114_IS407A_A		N	gb8_horse_4	4	594406-595641	Chr1	IS407A	TGATT(	TGACCTGCCCCCTACA	ATGAAGAAGCGCTTTACG(	GCCCGCAAGCCAAACGCG/	TGTTGGC(	AGGGACTTTGCTAGAA(	GATCCGG(	GAAG	GATC	N
9*Chr1_149_IS407/	9*Chr1_29_IS407A_A	D-R	SAVP1	9	59560-60795	Chr1	IS407A	ATTTAT	TGACCTGCCCCCTACA	ATGAAGAAGCGCTTTACG(	GCCCGCAAGCCAAACGCG/	TGTTGGC(	AGGGACTTTGCTAGAA(	CCTCACG/	CATT	CCTC	N
8*Chr1_64a_IS407A_A		N	2002721280	8	597797-599032	Chr1	IS407A	CTTGTT	TGACCTGCCCCCTACA	ATGAAGAAGCGCTTTACG(	GCCCGCAAGCCAAACGCG/	TGTTGGC(	AGGGACTTTGCTAGAA(	TCGCTTCT	TCGC	TCGC	Y
1*Chr1_114_IS407A_A		N	ATCC_23344	1	599462-600697	Chr1	IS407A	TGATT(	TGACCTGCCCCCTACA	ATGAAGAAGCGCTTTACG(	GCCCGCAAGCCAAACGCG/	TGTTGGC(	AGGGACTTTGCTAGAA(	GATCCGG(	GAAG	GATC	N
1*Chr1_30_IS407A	1*Chr1_6_IS407A_A	D-R	ATCC_23344	1	6007-7242	Chr1	IS407A	TTGAG/	TGACCTGCCCCCTACA	ATGAAGAAGCGCTTTACG(	GCCCGCAAGCCAAACGCG/	TGTTGGC(	AGGGACTTTGCTAGAA(	CGCCGCAT	TCGG	CGCC	N
7*Chr1_35a_IS407/	7*Chr1_6_IS407A_A	R	NCTC_10247	7	6007-7242	Chr1	IS407A	TTGAG/	TGACCTGCCCCCTACA	ATGAAGAAGCGCTTTACG(	GCCCGCAAGCCAAACGCG/	TGTTGGC(	AGGGACTTTGCTAGAA(	GGGGCAG	TCGG	GGGG	N
4*Chr1_113_IS407A_A		N	gb8_horse_4	4	608048-609283	Chr1	IS407A	TTTGCT	TGACCTGCCCCCTACA	ATGAAGAAGCGCTTTACG(	GCCCGCAAGCCAAACGCG/	TGTTGGC(	AGGGACTTTGCTAGAA(	CAACGGC/	CCTC	CAAC	N
1*Chr1_113_IS407A_A		N	ATCC_23344	1	613104-614339	Chr1	IS407A	TTTGCT	TGACCTGCCCCCTACA	ATGAAGAAGCGCTTTACG(	GCCCGCAAGCCAAACGCG/	TGTTGGC(	AGGGACTTTGCTAGAA(	CAACGGC/	CCTC	CAAC	N
2*Chr1_51_IS407A_B		N	fmh	2	625205-626440	Chr1	IS407A	AGGCG	TGACCTGCCCCCTACA	ATGAAGAAGCGCTTTACG(	GCCCGCAAGCCAAACGCG/	TGTTGGC(	AGGGACTTTGCTAGAA(	GATGCGGT	GATG	GATG	Y
6*Chr1_65a_IS407A_A		N	NCTC_10229	6	626265-627500	Chr1	IS407A	GCGAC	TGACCTGCCCCCTACA	ATGAAGAAGCGCTTTACG(	GCCCGCAAGCCAAACGCG/	TGTTGGC(	AGGGACTTTGCTAGAA(	GGGGGCA	GAAT	GGGG	N
7*Chr1_37_IS407A_A		N	NCTC_10247	7	62646-63881	Chr1	IS407A	CGCGA	TGACCTGCCCCCTACA	ATGAAGAAGCGCTTTACG(	GCCCGCAAGCCAAACGCG/	TGTTGGC(	AGGGACTTTGCTAGAA(	GGTCGCC(	CGCG	GGTC	N
9*Chr1_37_IS407A_A		N	SAVP1	9	627670-628905	Chr1	IS407A	TGGGC	TGACCTGCCCCCTACA	ATGAAGAAGCGCTTTACG(	GCCCGCAAGCCAAACGCG/	TGTTGGC(	AGGGACTTTGCTAGAA(	GACCCGGT	GACC	GACC	Y
3*Chr1_51_IS407A_B		N	JHU	3	628699-629934	Chr1	IS407A	AGGCG	TGACCTGCCCCCTACA	ATGAAGAAGCGCTTTACG(	GCCCGCAAGCCAAACGCG/	TGTTGGC(	AGGGACTTTGCTAGAA(	GATGCGGT	GATG	GATG	Y
5*Chr1_114_IS407A_A		N	ATCC_10399	5	635855-637090	Chr1	IS407A	TGATT(	TGACCTGCCCCCTACA	ATGAAGAAGCGCTTTACG(	GCCCGCAAGCCAAACGCG/	TGTTGGC(	AGGGACTTTGCTAGAA(	GATCCGG(	GAAG	GATC	N
7*Chr1_96_IS407A	7*Chr1_97_IS407A_A	D	NCTC_10247	7	643362-644597	Chr1	IS407A	GGCAC	TGACCTGCCCCCTACA	ATGAAGAAGCGCTTTACG(	GCCCGCAAGCCAAACGCG/	TGTTGGC(	AGGGACTTTGCTAGAA(	ACCGCCC(	CCCC	ACCG	N
5*Chr1_113_IS407A_A		N	ATCC_10399	5	649478-650713	Chr1	IS407A	TTTGCT	TGACCTGCCCCCTACA	ATGAAGAAGCGCTTTACG(	GCCCGCAAGCCAAACGCG/	TGTTGGC(	AGGGACTTTGCTAGAA(	ATCTACCT	CCTC	ATCT	N
10*Chr1_112_IS40	10*Chr1_49_IS407A_A	D-R	PRL20	10	652265-653500	Chr1	IS407A	TCATC(	TGACCTGCCCCCTACA	ATGAAGAAGCGCTTTACG(	GCCCGCAAGCCAAACGCG/	TGTTGGC(	AGGGACTTTGCTAGAA(	GGGCTGAT	TCAA	GGGC	N
8*Chr1_65a_IS407A_A		N	2002721280	8	659479-660714	Chr1	IS407A	GCGAC	TGACCTGCCCCCTACA	ATGAAGAAGCGCTTTACG(	GCCCGCAAGCCAAACGCG/	TGTTGGC(	AGGGACTTTGCTAGAA(	GGGGGCA	GAAT	GGGG	N
2*Chr1_54_IS407A	2*Chr1_65b_IS407A_A	D-R	fmh	2	665167-666402	Chr1	IS407A	GCGAC	TGACCTGCCCCCTACA	ATGAAGAAGCGCTTTACG(	GCCCGCAAGCCAAACGCG/	TGTTGGC(	AGGGACTTTGCTAGAA(	GCGGGTCT	GAAT	GCGG	N
3*Chr1_54_IS407A	3*Chr1_65b_IS407A_A	D-R	JHU	3	669375-670610	Chr1	IS407A	GCGAC	TGACCTGCCCCCTACA	ATGAAGAAGCGCTTTACG(	GCCCGCAAGCCAAACGCG/	TGTTGGC(	AGGGACTTTGCTAGAA(	GCGGGTCT	GAAT	GCGG	N
4*Chr1_112_IS407/	4*Chr1_49_IS407A_A	D-R	gb8_horse_4	4	678191-679426	Chr1	IS407A	TCATC(	TGACCTGCCCCCTACA	ATGAAGAAGCGCTTTACG(	GCCCGCAAGCCAAACGCG/	TGTTGGC(	AGGGACTTTGCTAGAA(	GGGCTGAT	TCAA	GGGC	N
1*Chr1_112_IS407/	1*Chr1_49_IS407A_A	D-R	ATCC_23344	1	683232-684467	Chr1	IS407A	TCATC(	TGACCTGCCCCCTACA	ATGAAGAAGCGCTTTACG(	GCCCGCAAGCCAAACGCG/	TGTTGGC(	AGGGACTTTGCTAGAA(	GGGCTGAT	TCAA	GGGC	N
7*Chr1_95_IS407A_A		N	NCTC_10247	7	686514-687749	Chr1	IS407A	GGCGC	TGACCTGCCCCCTACA	ATGAAGAAGCGCTTTACG(	GCCCGCAAGCCAAACGCG/	TGTTGGC(	AGGGACTTTGCTAGAA(	GCCCGCC(	GATG	GCCC	N
6*Chr1_64a_IS407A_A		N	NCTC_10229	6	688082-689317	Chr1	IS407A	CTTGTT	TGACCTGCCCCCTACA	ATGAAGAAGCGCTTTACG(	GCCCGCAAGCCAAACGCG/	TGTTGGC(	AGGGACTTTGCTAGAA(	TCGCTTCT	TCGC	TCGC	Y
9*Chr1_43b_IS407/	9*Chr1_57_IS407A_A	D-R	SAVP1	9	699385-700620	Chr1	IS407A	CCCGT(	TGACCTGCCCCCTACA	ATGAAGAAGCGCTTTACG(	GCCCGCAAGCCAAACGCG/	TGTTGGC(	AGGGACTTTGCTAGAA(	CGTGGAC(	CCCG	CGTG	N
8*Chr1_98_IS407A	8*Chr1_66_IS407A_A	D-R	2002721280	8	710666-711901	Chr1	IS407A	CTCGT(	TGACCTGCCCCCTACA	ATGAAGAAGCGCTTTACG(	GCCCGCAAGCCAAACGCG/	TGTTGGC(	AGGGACTTTGCTAGAA(	GTAGGCA/	CCCG	GTAG	N
2*Chr1_98_IS407A	2*Chr1_66_IS407A_A	D-R	fmh	2	717113-718348	Chr1	IS407A	CTCGT(	TGACCTGCCCCCTACA	ATGAAGAAGCGCTTTACG(	GCCCGCAAGCCAAACGCG/	TGTTGGC(	AGGGACTTTGCTAGAA(	GTAGGCA/	CCCG	GTAG	N
5*Chr1_112_IS407/	5*Chr1_49_IS407A_A	D-R	ATCC_10399	5	719456-720691	Chr1	IS407A	TCATC(	TGACCTGCCCCCTACA	ATGAAGAAGCGCTTTACG(	GCCCGCAAGCCAAACGCG/	TGTTGGC(	AGGGACTTTGCTAGAA(	GGGCTGAT	TCAA	GGGC	N
3*Chr1_98_IS407A	3*Chr1_66_IS407A_A	D-R	JHU	3	722507-723742	Chr1	IS407A	CTCGT(	TGACCTGCCCCCTACA	ATGAAGAAGCGCTTTACG(	GCCCGCAAGCCAAACGCG/	TGTTGGC(	AGGGACTTTGCTAGAA(	GTAGGCA/	CCCG	GTAG	N
9*Chr1_132a_IS40	9*Chr1_58a_IS407A_A	R	SAVP1	9	726736-727971	Chr1	IS407A	ACGCC	TGACCTGCCCCCTACA	ATGAAGAAGCGCTTTACG(	GCCCGCAAGCCAAACGCG/	TGTTGGC(	AGGGACTTTGCTAGAA(	CGTTCGCT	GGAC	CGTT	N
10*Chr1_65a_IS407A_A		N	PRL20	10	740650-741885	Chr1	IS407A	GCGAC	TGACCTGCCCCCTACA	ATGAAGAAGCGCTTTACG(	GCCCGCAAGCCAAACGCG/	TGTTGGC(	AGGGACTTTGCTAGAA(	GGGGGCA	GAAT	GGGG	N
4*Chr1_51_IS407A_B		N	gb8_horse_4	4	743068-744303	Chr1	IS407A	AGGCG	TGACCTGCCCCCTACA	ATGAAGAAGCGCTTTACG(	GCCCGCAAGCCAAACGCG/	TGTTGGC(	AGGGACTTTGCTAGAA(	GATGCGGT	GATG	GATG	Y
7*Chr1_84a_IS407/	7*Chr1_94b_IS407A_A	R	NCTC_10247	7	745418-746653	Chr1	IS407A	GTGCC(	TGACCTGCCCCCTACA	ATGAAGAAGCGCTTTACG(	GCCCGCAAGCCAAACGCG/	TGTTGGC(	AGGGACTTTGCTAGAA(	GTAAAGCT	AAAG	GTAA	N
1*Chr1_51_IS407A_B		N	ATCC_23344	1	748097-749332	Chr1	IS407A	AGGCG	TGACCTGCCCCCTACA	ATGAAGAAGCGCTTTACG(	GCCCGCAAGCCAAACGCG/	TGTTGGC(	AGGGACTTTGCTAGAA(	GATGCGGT	GATG	GATG	Y
7*Chr1_83_IS407A_A		N	NCTC_10247	7	760536-761771	Chr1	IS407A	TTACG(	TGACCTGCCCCCTACA	ATGAAGAAGCGCTTTACG(	GCCCGCAAGCCAAACGCG/	TGTTGGC(	AGGGACTTTGCTAGAA(	ATCCACC(	TGAT	ATCC	N
8*Chr1_96_IS407A	8*Chr1_97_IS407A_A	D	2002721280	8	769257-770492	Chr1	IS407A	GGCAC	TGACCTGCCCCCTACA	ATGAAGAAGCGCTTTACG(	GCCCGCAAGCCAAACGCG/	TGTTGGC(	AGGGACTTTGCTAGAA(	ACCGCCC(	CCCC	ACCG	N
2*Chr1_96_IS407A	2*Chr1_97_IS407A_A	D	fmh	2	776030-777265	Chr1	IS407A	GGCAC	TGACCTGCCCCCTACA	ATGAAGAAGCGCTTTACG(	GCCCGCAAGCCAAACGCG/	TGTTGGC(	AGGGACTTTGCTAGAA(	ACCGCCC(	CCCC	ACCG	N
3*Chr1_96_IS407A	3*Chr1_97_IS407A_A	D	JHU	3	782172-783407	Chr1	IS407A	GGCAC	TGACCTGCCCCCTACA	ATGAAGAAGCGCTTTACG(	GCCCGCAAGCCAAACGCG/	TGTTGGC(	AGGGACTTTGCTAGAA(	ACCGCCC(	CCCC	ACCG	N
7*Chr1_79_IS407A	7*Chr1_80_IS407A_A	D	NCTC_10247	7	782570-783805	Chr1	IS407A	GGGAC	TGACCTGCCCCCTACA	ATGAAGAAGCGCTTTACG(	GCCCGCAAGCCAAACGCG/	TGTTGGC(	AGGGACTTTGCTAGAA(	CAAGCAG(	GTCA	CAAG	N
4*Chr1_54_IS407A	4*Chr1_65b_IS407A_A	D-R	gb8_horse_4	4	783009-784244	Chr1	IS407A	GCGAC	TGACCTGCCCCCTACA	ATGAAGAAGCGCTTTACG(	GCCCGCAAGCCAAACGCG/	TGTTGGC(	AGGGACTTTGCTAGAA(	GCGGGTCT	GAAT	GCGG	N
1*Chr1_54_IS407A	1*Chr1_65b_IS407A_A	D-R	ATCC_23344	1	788024-789259	Chr1	IS407A	GCGAC	TGACCTGCCCCCTACA	ATGAAGAAGCGCTTTACG(	GCCCGCAAGCCAAACGCG/	TGTTGGC(	AGGGACTTTGCTAGAA(	GCGGGTCT	GAAT	GCGG	N
10*Chr1_98_IS407/	10*Chr1_66_IS407A_A	D-R	PRL20	10	791849-793084	Chr1	IS407A	CTCGT(	TGACCTGCCCCCTACA	ATGAAGAAGCGCTTTACG(	GCCCGCAAGCCAAACGCG/	TGTTGGC(	AGGGACTTTGCTAGAA(	GTAGGCA/	CCCG	GTAG	N

8*Chr1_95_IS407A_A			N	2002721280	8	812487-813722	Chr1	IS407A	GGCGC	TGACCTGCCCCCTACA	ATGAAGAAGCGCTTTACG	GCCCGCAAGCCAAACGCG	TGTTGGC	AGGGACTTTGCTAGAA	GCCCGCC	GATG	GCCC	N
7*Chr1_78_IS407A_A			N	NCTC_10247	7	817833-819068	Chr1	IS407A	CAGCA	TGACCTGCCCCCTACA	ATGAAGAAGCGCTTTACG	GCCCGCAAGCCAAACGCG	TGTTGGC	AGGGACTTTGCTAGAA	GTAGCTG	GTAG	GTAG	Y
2*Chr1_95_IS407A_A			N	fmh	2	819682-820917	Chr1	IS407A	GGCGC	TGACCTGCCCCCTACA	ATGAAGAAGCGCTTTACG	GCCCGCAAGCCAAACGCG	TGTTGGC	AGGGACTTTGCTAGAA	GCCCGCC	GATG	GCCC	N
5*Chr1_54_IS407A	5*Chr1_56_IS407A_A		D-R	ATCC_10399	5	823076-824311	Chr1	IS407A	TGGAA	TGACCTGCCCCCTACA	ATGAAGAAGCGCTTTACG	GCCCGCAAGCCAAACGCG	TGTTGGC	AGGGACTTTGCTAGAA	GCGGGTC	GCGG	GCGG	Y
9*Chr1_126b_IS40	9*Chr1_135_IS407A_A		D-R	SAVP1	9	826175-827410	Chr1	IS407A	GCTCG	TGACCTGCCCCCTACA	ATGAAGAAGCGCTTTACG	GCCCGCAAGCCAAACGCG	TGTTGGC	AGGGACTTTGCTAGAA	CGATGTG	GATT	CGAT	N
3*Chr1_95_IS407A_A			N	JHU	3	826570-827805	Chr1	IS407A	GGCGC	TGACCTGCCCCCTACA	ATGAAGAAGCGCTTTACG	GCCCGCAAGCCAAACGCG	TGTTGGC	AGGGACTTTGCTAGAA	GCCCGCC	GATG	GCCC	N
4*Chr1_98_IS407A	4*Chr1_66_IS407A_A		D-R	gb8_horse_4	4	833765-835000	Chr1	IS407A	CTCGT	TGACCTGCCCCCTACA	ATGAAGAAGCGCTTTACG	GCCCGCAAGCCAAACGCG	TGTTGGC	AGGGACTTTGCTAGAA	GTAGGCA	CCCG	GTAG	N
5*Chr1_55_IS407A	5*Chr1_64b_IS407A_A		D-R	ATCC_10399	5	836600-837835	Chr1	IS407A	ATCAC	TGACCTGCCCCCTACA	ATGAAGAAGCGCTTTACG	GCCCGCAAGCCAAACGCG	TGTTGGC	AGGGACTTTGCTAGAA	GCGCTTCT	GCGG	GCGC	N
1*Chr1_98_IS407A	1*Chr1_66_IS407A_A		D-R	ATCC_23344	1	839215-840450	Chr1	IS407A	CTCGT	TGACCTGCCCCCTACA	ATGAAGAAGCGCTTTACG	GCCCGCAAGCCAAACGCG	TGTTGGC	AGGGACTTTGCTAGAA	GTAGGCA	CCCG	GTAG	N
10*Chr1_96_IS407	10*Chr1_97_IS407A_A		D	PRL20	10	850107-851342	Chr1	IS407A	GGCAC	TGACCTGCCCCCTACA	ATGAAGAAGCGCTTTACG	GCCCGCAAGCCAAACGCG	TGTTGGC	AGGGACTTTGCTAGAA	ACCGCCC	CCCC	ACCG	N
8*Chr1_72_IS407A	8*Chr1_94b_IS407A_A		D-R	2002721280	8	871391-872626	Chr1	IS407A	CCAGA	TGACCTGCCCCCTACA	ATGAAGAAGCGCTTTACG	GCCCGCAAGCCAAACGCG	TGTTGGC	AGGGACTTTGCTAGAA	GTAAAGC	CGCG	GTAA	N
2*Chr1_72_IS407A	2*Chr1_94b_IS407A_A		D-R	fmh	2	878580-879815	Chr1	IS407A	CCAGA	TGACCTGCCCCCTACA	ATGAAGAAGCGCTTTACG	GCCCGCAAGCCAAACGCG	TGTTGGC	AGGGACTTTGCTAGAA	TGACGTT	CGCG	TGAC	N
3*Chr1_72_IS407A	3*Chr1_94b_IS407A_A		D-R	JHU	3	885069-886304	Chr1	IS407A	CCAGA	TGACCTGCCCCCTACA	ATGAAGAAGCGCTTTACG	GCCCGCAAGCCAAACGCG	TGTTGGC	AGGGACTTTGCTAGAA	TGACGTT	CGCG	TGAC	N
6*Chr1_48_IS407A	6*Chr1_58b_IS407A_A		D-R	NCTC_10229	6	889050-890285	Chr1	IS407A	CGTCG	TGACCTGCCCCCTACA	ATGAAGAAGCGCTTTACG	GCCCGCAAGCCAAACGCG	TGTTGGC	AGGGACTTTGCTAGAA	GAAGTGG	GTGA	GAAG	N
4*Chr1_96_IS407A	4*Chr1_97_IS407A_A		D	gb8_horse_4	4	891953-893188	Chr1	IS407A	GGCAC	TGACCTGCCCCCTACA	ATGAAGAAGCGCTTTACG	GCCCGCAAGCCAAACGCG	TGTTGGC	AGGGACTTTGCTAGAA	ACCGCCC	CCCC	ACCG	N
10*Chr1_95_IS407A_A			N	PRL20	10	892871-894106	Chr1	IS407A	GGCGC	TGACCTGCCCCCTACA	ATGAAGAAGCGCTTTACG	GCCCGCAAGCCAAACGCG	TGTTGGC	AGGGACTTTGCTAGAA	GCCCGCC	GATG	GCCC	N
1*Chr1_96_IS407A	1*Chr1_97_IS407A_A		D	ATCC_23344	1	897797-899032	Chr1	IS407A	GGCAC	TGACCTGCCCCCTACA	ATGAAGAAGCGCTTTACG	GCCCGCAAGCCAAACGCG	TGTTGGC	AGGGACTTTGCTAGAA	ACCGCCC	CCCC	ACCG	N
5*Chr1_65a_IS407A_A			N	ATCC_10399	5	898663-899898	Chr1	IS407A	GCGAC	TGACCTGCCCCCTACA	ATGAAGAAGCGCTTTACG	GCCCGCAAGCCAAACGCG	TGTTGGC	AGGGACTTTGCTAGAA	GGGGGCA	GAAT	GGGG	N
6*Chr1_115b_IS40	6*Chr1_47b_IS407A_A		R	NCTC_10229	6	907313-908548	Chr1	IS407A	GAAGC	TGACCTGCCCCCTACA	ATGAAGAAGCGCTTTACG	GCCCGCAAGCCAAACGCG	TGTTGGC	AGGGACTTTGCTAGAA	GATGACA	GAGC	GATG	N
7*Chr1_77_IS407A	7*Chr1_94a_IS407A_A		D-R	NCTC_10247	7	925312-926547	Chr1	IS407A	ATGAT	TGACCTGCCCCCTACA	ATGAAGAAGCGCTTTACG	GCCCGCAAGCCAAACGCG	TGTTGGC	AGGGACTTTGCTAGAA	AACCGAT	AGGT	AACC	N
4*Chr1_95_IS407A_A			N	gb8_horse_4	4	934492-935458	Chr1	IS407A	GGCGC	TGACCTGCCCCCTACA	ATGAAGAAGCGCTTTACG	GCCCGCAAGCCAAACGCG	TGTTGGC	#	#contig_br	GATG	#con	N.D.
9*Chr1_132b_IS40	9*Chr1_47b_IS407A_A		R	SAVP1	9	935361-936596	Chr1	IS407A	TGAGG	TGACCTGCCCCCTACA	ATGAAGAAGCGCTTTACG	GCCCGCAAGCCAAACGCG	TGTTGGC	AGGGACTTTGCTAGAA	GGACGGG	CCTT	GGAC	N
1*Chr1_95_IS407A_A			N	ATCC_23344	1	940960-942195	Chr1	IS407A	GGCGC	TGACCTGCCCCCTACA	ATGAAGAAGCGCTTTACG	GCCCGCAAGCCAAACGCG	TGTTGGC	AGGGACTTTGCTAGAA	GCCCGCC	GATG	GCCC	N
6*Chr1_119_IS407A_C			N	NCTC_10229	6	942572-943807	Chr1	IS407A	GGATA	TGACCTGCCCCCTACA	ATGAAGAAGCGCTTTACG	GCCCGCAAGCCAAACGCG	TGTTGGC	AGGGACTTTGCTAGAA	GATGCGC	GATG	GATG	Y
5*Chr1_98_IS407A	5*Chr1_66_IS407A_A		D-R	ATCC_10399	5	949835-951070	Chr1	IS407A	CTCGT	TGACCTGCCCCCTACA	ATGAAGAAGCGCTTTACG	GCCCGCAAGCCAAACGCG	TGTTGGC	AGGGACTTTGCTAGAA	GTAGGCA	CCCG	GTAG	N
8*Chr1_74_IS407A	8*Chr1_84a_IS407A_A		D-R	2002721280	8	952291-953526	Chr1	IS407A	GTGCC	TGACCTGCCCCCTACA	ATGAAGAAGCGCTTTACG	GCCCGCAAGCCAAACGCG	TGTTGGC	AGGGACTTTGCTAGAA	GGTCATG	AAAG	GGTC	N
10*Chr1_69_IS407	10*Chr1_94b_IS407A_A		D-R	PRL20	10	952619-953854	Chr1	IS407A	TGGCG	TGACCTGCCCCCTACA	ATGAAGAAGCGCTTTACG	GCCCGCAAGCCAAACGCG	TGTTGGC	AGGGACTTTGCTAGAA	AAGGGCC	CCTG	AAGG	N
9*Chr1_48_IS407A	9*Chr1_55_IS407	9*Chr1_58b_I	D-R	SAVP1	9	954151-955386	Chr1	IS407A	ATCAC	TGACCTGCCCCCTACA	ATGAAGAAGCGCTTTACG	GCCCGCAAGCCAAACGCG	TGTTGGC	AGGGACTTTGCTAGAA	TTCCGGC	GCGG	TTCC	N
2*Chr1_74_IS407A	2*Chr1_75_IS407A_A		D	fmh	2	960048-960969	Chr1	IS407A	#ISBma	~~~~~~~~~~~~~~	~~~~~~~~~~~~~~~~~	GCCCATGCCCACGCAGCA	CGAGATG	AGGGACTTTGCTAGAA	GGTCATG	#	GGTC	N.D.
7*Chr1_89_IS407A_A			N	NCTC_10247	7	960917-962152	Chr1	IS407A	GCGAA	TGACCTGCCCCCTACA	ATGAAGAAGCGCTTTACG	GCCCGCAAGCCAAACGCG	TGTTGGC	AGGGACTTTGCTAGAA	CTGGATG	CCGC	CTGG	N
2*Chr1_76_IS407A	2*Chr1_87b_IS407A_A		D-R	fmh	2	965187-966422	Chr1	IS407A	CGGGA	TGACCTGCCCCCTACA	ATGAAGAAGCGCTTTACG	GCCCGCAAGCCAAACGCG	TGTTGGC	AGGGACTTTGCTAGAA	CGAGCGC	GCCG	CGAG	N
8*Chr1_83_IS407A_A			N	2002721280	8	967415-968650	Chr1	IS407A	TTACG	TGACCTGCCCCCTACA	ATGAAGAAGCGCTTTACG	GCCCGCAAGCCAAACGCG	TGTTGGC	AGGGACTTTGCTAGAA	ATCCACC	TGAT	ATCC	N
3*Chr1_74_IS407A	3*Chr1_75_IS407A_A		D	JHU	3	967622-968543	Chr1	IS407A	#ISBma	~~~~~~~~~~~~~~	~~~~~~~~~~~~~~~~~	GCCCATGCCCACGCAGCA	CGAGATG	AGGGACTTTGCTAGAA	GGTCATG	#	GGTC	N.D.
9*Chr1_53b_IS407	9*Chr1_56_IS407A_A		D-R	SAVP1	9	967733-968968	Chr1	IS407A	TGGAA	TGACCTGCCCCCTACA	ATGAAGAAGCGCTTTACG	GCCCGCAAGCCAAACGCG	TGTTGGC	AGGGACTTTGCTAGAA	ACGGCGA	GCGG	ACGG	N
3*Chr1_76_IS407A	3*Chr1_87b_IS407A_A		D-R	JHU	3	972761-973996	Chr1	IS407A	CGGGA	TGACCTGCCCCCTACA	ATGAAGAAGCGCTTTACG	GCCCGCAAGCCAAACGCG	TGTTGGC	AGGGACTTTGCTAGAA	CGAGCGC	GCCG	CGAG	N
6*Chr1_122_IS407A_A			N	NCTC_10229	6	978581-979816	Chr1	IS407A	GCGCA	TGACCTGCCCCCTACA	ATGAAGAAGCGCTTTACG	GCCCGCAAGCCAAACGCG	TGTTGGC	AGGGACTTTGCTAGAA	GTGCTGC	GAAG	GTGC	N
9*Chr1_54_IS407A_A			D-R	SAVP1	9	980886-982121	Chr1	IS407A	CGTCG	TGACCTGCCCCCTACA	ATGAAGAAGCGCTTTACG	GCCCGCAAGCCAAACGCG	TGTTGGC	AGGGACTTTGCTAGAA	CGAACAC	GTGA	CGAA	N
10*Chr1_74_IS407	10*Chr1_75_IS407A_A		D	PRL20	10	988786-989707	Chr1	IS407A	#ISBma	~~~~~~~~~~~~~~	~~~~~~~~~~~~~~~~~	GCCCATGCCCACGCAGCA	CGAGATG	AGGGACTTTGCTAGAA	GGTCATG	#	GGTC	N.D.
8*Chr1_79_IS407A	8*Chr1_80_IS407A_A		D	2002721280	8	989538-990773	Chr1	IS407A	GGGAC	TGACCTGCCCCCTACA	ATGAAGAAGCGCTTTACG	GCCCGCAAGCCAAACGCG	TGTTGGC	AGGGACTTTGCTAGAA	CAAGCAG	GTCA	CAAG	N
4*Chr1_72_IS407A	4*Chr1_94b_IS407A_A		D-R	gb8_horse_4	4	992740-993975	Chr1	IS407A	CCAGA	TGACCTGCCCCCTACA	ATGAAGAAGCGCTTTACG	GCCCGCAAGCCAAACGCG	TGTTGGC	AGGGACTTTGCTAGAA	TGACGTT	CGCG	TGAC	N
10*Chr1_76_IS407	10*Chr1_87b_IS407A_A		D-R	PRL20	10	993925-995160	Chr1	IS407A	AATGT	TGACCTGCCCCCTACA	ATGAAGAAGCGCTTTACG	GCCCGCAAGCCAAACGCG	TGTTGGC	AGGGACTTTGCTAGAA	CGGCACG	CTCG	CGGC	N
7*Chr1_75_IS407A	7*Chr1_87b_IS407A_A		D-R	NCTC_10247	7	998173-999094	Chr1	IS407A	#ISBma	~~~~~~~~~~~~~~	~~~~~~~~~~~~~~~~~	GCCCATGCCCACGCAGCA	CGAGATG	AGGGACTTTGCTAGAA	CCATGGC	#	CCAT	N.D.
1*Chr1_72_IS407A	1*Chr1_94b_IS407A_A		D-R	ATCC_23344	1	999459-1000694	Chr1	IS407A	CCAGA	TGACCTGCCCCCTACA	ATGAAGAAGCGCTTTACG	GCCCGCAAGCCAAACGCG	TGTTGGC	AGGGACTTTGCTAGAA	TGACGTT	CGCG	TGAC	N

IS *Bma* 1

								5'-ext	5'-IR	ORF	3'-IR	3'-ext	5'-DR	3'-DR	rget duplicates
									GGTTCATCGC		GCGATGA ACC				
2*Chr1_88_ISBma1_B	N	fmh	2	1002861-1003118	Chr1	ISBma1		#IS407	~~~~~~~~~~~~~~	~~~~~~~~~~~~~~~~~	ACC	ATTTTTTT	#IS407A	ATTTTTTT	N.D.
3*Chr1_88_ISBma1_B	N	JHU	3	1011670-1011927	Chr1	ISBma1		#IS407	~~~~~~~~~~~~~~	~~~~~~~~~~~~~~~~~	ACC	ATTTTTTT	#IS407A	ATTTTTTT	N.D.
6*Chr1_125_ISBma1_A	N	NCTC_10229	6	1013610-1014914	Chr1	ISBma1		AGGCA	GGTTCATCGCAGAAAA	TTGCTCGATCGCAAGGCAC	ACC	AATTTTTT	AATTTTTT	AATTTTTT	Y
10*Chr1_88_ISBma1_B	N	PRL20	10	1031539-1031835	Chr1	ISBma1		#IS407	~~~~~~~~~~~~~~	~~~~~~~~~~~~~~~~~	ACC	ATTTTTTT	#IS407A	ATTTTTTT	N.D.
4*Chr1_73_ISBma1_A	N	gb8_horse_4	4	1059010-1060314	Chr1	ISBma1		GAGAG	GGTTCATCGCAGAAAA	TTGCTCGATCGCAAGGCAC	ACC	AAAAAAA	AAAAAAAT	AAAAAAA	Y
1*Chr1_73_ISBma1_A	N	ATCC_23344	1	1065697-1067001	Chr1	ISBma1		GAGAG	GGTTCATCGCAGAAAA	TTGCTCGATCGCAAGGCAC	ACC	AAAAAAA	AAAAAAAT	AAAAAAA	Y
9*Chr1_60_ISBma1_A	N	SAVP1	9	1081029-1082333	Chr1	ISBma1		CGGTA	GGTTCATCGCAGAAAA	TTGCTCGATCGCAAGGCAC	ACC	AAAAAAT	AAAAAATA	AAAAAAT	Y
4*Chr1_88_ISBma1_B	N	gb8_horse_4	4	1116223-1116480	Chr1	ISBma1		#IS407	~~~~~~~~~~~~~~	~~~~~~~~~~~~~~~~~	ACC	ATTTTTTT	#IS407A	ATTTTTTT	N.D.
1*Chr1_88_ISBma1_B	N	ATCC_23344	1	1123080-1123337	Chr1	ISBma1		#IS407	~~~~~~~~~~~~~~	~~~~~~~~~~~~~~~~~	ACC	ATTTTTTT	#IS407A	ATTTTTTT	N.D.
2*Chr1_82_ISBma1_A	N	fmh	2	1173163-1174201	Chr1	ISBma1		CGAAA	GGTTCATCGCAGAAAA	TTGCTCGATCGCAAGGCAC	#	#IS407A	TTTCTTTT	#IS407A	N
2*Chr1_82-1_ISBma1_A	N	fmh	2	1175227-1175705	Chr1	ISBma1		#IS407	~~~~~~~~~~~~~~	~~~~~~~~~~~~~~~~~	ACC	TTTCTTTT	#IS407A	TTTCTTTT	N.D.

3*Chr1_82_ISBma1_A	N	JHU	3	1184351-1185389	Chr1	ISBma1	CGAAA	GGTTCATCGCAGAAAA	TTGCTCGATCGCAAGGCAC	#	#IS407A	TTTCTTTT	#IS407A	N
3*Chr1_82-1_ISBma1_A	N	JHU	3	1186415-1186893	Chr1	ISBma1	#IS407A	~~~~~~~~~~~~~~~	~~~~~~~~~~~~~~~~~~	ACC	TTTCTTTC	#IS407A	TTTCTTTT	N.D.
10*Chr1_82_ISBma1_A	N	PRL20	10	1191155-1192031	Chr1	ISBma1	CGAAA	GGTTCATCGCAGAAAA	TTGCTCGATCGCAAGGCAC	#	#IS407A	TTTCTTTT	#IS407A	N
10*Chr1_82-1_ISBma1_A	N	PRL20	10	1193171-1193697	Chr1	ISBma1	#IS407A	~~~~~~~~~~~~~~~	~~~~~~~~~~~~~~~~~~	ACC	TTTCTTTC	#IS407A	TTTCTTTT	N.D.
7*Chr1_103_ISBma1_A	N	NCTC_10247	7	1266623-1267927	Chr1	ISBma1	AGTCG	GGTTCATCGCAGAAAA	TTGCTCGATCGCAAGGCAC	ACC	CTTTTTTC	CTTTTTTT	CTTTTTTT	Y
4*Chr1_82_ISBma1_A	N	gb8_horse_4	4	1281913-1282951	Chr1	ISBma1	CGAAA	GGTTCATCGCAGAAAA	TTGCTCGATCGCAAGGCAC	#	#IS407A	TTTCTTTT	#IS407A	N
4*Chr1_82-1_ISBma1_A	N	gb8_horse_4	4	1283977-1284455	Chr1	ISBma1	#IS407A	~~~~~~~~~~~~~~~	~~~~~~~~~~~~~~~~~~	ACC	TTTCTTTC	#IS407A	TTTCTTTT	N.D.
5*Chr1_82-1_ISBma1_A	N	ATCC_10399	5	1284930-1285408	Chr1	ISBma1	#IS407A	~~~~~~~~~~~~~~~	~~~~~~~~~~~~~~~~~~	ACC	TTTCTTTC	#IS407A	TTTCTTTT	N.D.
5*Chr1_82_ISBma1_A	N	ATCC_10399	5	1286434-1287472	Chr1	ISBma1	CGAAA	GGTTCATCGCAGAAAA	TTGCTCGATCGCAAGGCAC	#	#IS407A	TTTCTTTT	#IS407A	N
1*Chr1_82_ISBma1_A	N	ATCC_23344	1	1290138-1291176	Chr1	ISBma1	CGAAA	GGTTCATCGCAGAAAA	TTGCTCGATCGCAAGGCAC	#	#IS407A	TTTCTTTT	#IS407A	N
1*Chr1_82-1_ISBma1_A	N	ATCC_23344	1	1292202-1292680	Chr1	ISBma1	#IS407A	~~~~~~~~~~~~~~~	~~~~~~~~~~~~~~~~~~	ACC	TTTCTTTC	#IS407A	TTTCTTTT	N.D.
4*Chr1_12_ISBma1_A	N	gb8_horse_4	4	141771-143075	Chr1	ISBma1	GAATT	GGTTCATCGCAGAAAA	TTGCTCGATCGCAAGGCAC	ACC	TTTTTCTTT	TTTTTCTT	TTTTTCTT	Y
10*Chr1_12_ISBma1_A	N	PRL20	10	1423-2727	Chr1	ISBma1	GAATT	GGTTCATCGCAGAAAA	TTGCTCGATCGCAAGGCAC	ACC	TTTTTCTTT	TTTTTCTT	TTTTTCTT	Y
1*Chr1_12_ISBma1_A	N	ATCC_23344	1	142668-143972	Chr1	ISBma1	GAATT	GGTTCATCGCAGAAAA	TTGCTCGATCGCAAGGCAC	ACC	TTTTTCTTT	TTTTTCTT	TTTTTCTT	Y
2*Chr1_103_ISBma1_A	N	fmh	2	1458477-1459781	Chr1	ISBma1	AGTCG	GGTTCATCGCAGAAAA	TTGCTCGATCGCAAGGCAC	ACC	CTTTTTTC	CTTTTTTT	CTTTTTTT	Y
6*Chr1_26_ISBma1_A	N	NCTC_10229	6	1463413-1464719	Chr1	ISBma1	GCGTG	GGTTCATCGCAGAAAA	TTGCTCGATCGCAAGGCAC	ACC	ATAAAAAA	ATAAAAAA	ATAAAAAA	Y
3*Chr1_103_ISBma1_A	N	JHU	3	1467796-1469100	Chr1	ISBma1	AGTCG	GGTTCATCGCAGAAAA	TTGCTCGATCGCAAGGCAC	ACC	CTTTTTTC	CTTTTTTT	CTTTTTTT	Y
8*Chr1_103_ISBma1_A	N	2002721280	8	1472738-1474042	Chr1	ISBma1	AGTCG	GGTTCATCGCAGAAAA	TTGCTCGATCGCAAGGCAC	ACC	CTTTTTTC	CTTTTTTT	CTTTTTTT	Y
7*Chr1_125_ISBma1_A	N	NCTC_10247	7	147293-148597	Chr1	ISBma1	AGGCA	GGTTCATCGCAGAAAA	TTGCTCGATCGCAAGGCAC	ACC	AATTTTTC	AATTTTTT	AATTTTTT	Y
9*Chr1_88_ISBma1_B	N	SAVP1	9	1497063-1497359	Chr1	ISBma1	#IS407A	~~~~~~~~~~~~~~~	~~~~~~~~~~~~~~~~~~	ACC	ATTTTTTC	#IS407A	ATTTTTTT	N.D.
5*Chr1_88_ISBma1_B	N	ATCC_10399	5	1497242-1497500	Chr1	ISBma1	#IS407A	~~~~~~~~~~~~~~~	~~~~~~~~~~~~~~~~~~	ACC	ATTTTTTC	#IS407A	ATTTTTTT	N.D.
10*Chr1_103_ISBma1_A	N	PRL20	10	1515896-1517200	Chr1	ISBma1	AGTCG	GGTTCATCGCAGAAAA	TTGCTCGATCGCAAGGCAC	ACC	CTTTTTTC	CTTTTTTT	CTTTTTTT	Y
3*Chr1_12_ISBma1_A	N	JHU	3	15493-16797	Chr1	ISBma1	GAATT	GGTTCATCGCAGAAAA	TTGCTCGATCGCAAGGCAC	ACC	TTTTTCTTT	TTTTTCTT	TTTTTCTT	Y
5*Chr1_73_ISBma1_A	N	ATCC_10399	5	1553580-1554884	Chr1	ISBma1	GAGAG	GGTTCATCGCAGAAAA	TTGCTCGATCGCAAGGCAC	ACC	AAAAAAAT	AAAAAAAT	AAAAAAAT	Y
4*Chr1_103_ISBma1_A	N	gb8_horse_4	4	1561382-1562686	Chr1	ISBma1	AGTCG	GGTTCATCGCAGAAAA	TTGCTCGATCGCAAGGCAC	ACC	CTTTTTTC	CTTTTTTT	CTTTTTTT	Y
1*Chr1_103_ISBma1_A	N	ATCC_23344	1	1570802-1572106	Chr1	ISBma1	AGTCG	GGTTCATCGCAGAAAA	TTGCTCGATCGCAAGGCAC	ACC	CTTTTTTC	CTTTTTTT	CTTTTTTT	Y
2*Chr1_12_ISBma1_A	N	fmh	2	15916-17220	Chr1	ISBma1	GAATT	GGTTCATCGCAGAAAA	TTGCTCGATCGCAAGGCAC	ACC	TTTTTCTTT	TTTTTCTT	TTTTTCTT	Y
9*Chr1_26_ISBma1_A	N	SAVP1	9	160477-161783	Chr1	ISBma1	GCGTG	GGTTCATCGCAGAAAA	TTGCTCGATCGCAAGGCAC	ACC	ATAAAAAA	ATAAAAAA	ATAAAAAA	Y
8*Chr1_12_ISBma1_A	N	2002721280	8	16877-18181	Chr1	ISBma1	GAATT	GGTTCATCGCAGAAAA	TTGCTCGATCGCAAGGCAC	ACC	TTTTTCTTT	TTTTTCTT	TTTTTCTT	Y
9*Chr1_82_ISBma1_A	N	SAVP1	9	1706788-1707664	Chr1	ISBma1	CGAAA	GGTTCATCGCAGAAAA	TTGCTCGATCGCAAGGCAC	#	#IS407A	TTTCTTTT	#IS407A	N.D.
9*Chr1_82-1_ISBma1_A	N	SAVP1	9	1708804-1709330	Chr1	ISBma1	#IS407A	~~~~~~~~~~~~~~~	~~~~~~~~~~~~~~~~~~	ACC	TTTCTTTC	#IS407A	TTTCTTTT	N.D.
5*Chr1_12_ISBma1_A	N	ATCC_10399	5	17216-18520	Chr1	ISBma1	GAATT	GGTTCATCGCAGAAAA	TTGCTCGATCGCAAGGCAC	ACC	TTTTTCTTT	TTTTTCTT	TTTTTCTT	Y
5*Chr1_103_ISBma1_A	N	ATCC_10399	5	1724494-1725798	Chr1	ISBma1	AGTCG	GGTTCATCGCAGAAAA	TTGCTCGATCGCAAGGCAC	ACC	CTTTTTTC	CTTTTTTT	CTTTTTTT	Y
2*Chr1_60_ISBma1_A	N	fmh	2	1842154-1843458	Chr1	ISBma1	CGGTA	GGTTCATCGCAGAAAA	TTGCTCGATCGCAAGGCAC	ACC	AAAAAATA	AAAAAATA	AAAAAATA	Y
3*Chr1_60_ISBma1_A	N	JHU	3	1856886-1858190	Chr1	ISBma1	CGGTA	GGTTCATCGCAGAAAA	TTGCTCGATCGCAAGGCAC	ACC	AAAAAATA	AAAAAATA	AAAAAATA	Y
10*Chr1_60_ISBma1_A	N	PRL20	10	1895357-1896661	Chr1	ISBma1	CGGTA	GGTTCATCGCAGAAAA	TTGCTCGATCGCAAGGCAC	ACC	AAAAAATA	AAAAAATA	AAAAAATA	Y
2*Chr1_59_ISBma1_A	N	fmh	2	1925292-1926596	Chr1	ISBma1	CACCG	GGTTCATCGCAGAAAA	TTGCTCGATCGCAAGGCAC	ACC	AAAAAAAT	AAAAAAAT	AAAAAAAT	Y
7*Chr1_127_ISBma1_A	N	NCTC_10247	7	1928819-1930125	Chr1	ISBma1	GCCGC	GGTTCATCGCAGAAAA	TTGCTCGATCGCAAGGCAC	ACC	AAAAAAAA	AAAAAAAA	AAAAAAAA	Y
7*Chr1_129_ISBma1_A	N	NCTC_10247	7	1931826-1933130	Chr1	ISBma1	CGCGC	GGTTCATCGCAGAAAA	TTGCTCGATCGCAAGGCAC	ACC	TTTTTTTAT	TTTTTTTA	TTTTTTTA	Y
4*Chr1_60_ISBma1_A	N	gb8_horse_4	4	1939412-1940716	Chr1	ISBma1	CGGTA	GGTTCATCGCAGAAAA	TTGCTCGATCGCAAGGCAC	ACC	AAAAAATA	AAAAAATA	AAAAAATA	Y
3*Chr1_59_ISBma1_A	N	JHU	3	1940744-1942048	Chr1	ISBma1	CACCG	GGTTCATCGCAGAAAA	TTGCTCGATCGCAAGGCAC	ACC	AAAAAAAT	AAAAAAAT	AAAAAAAT	Y
1*Chr1_60_ISBma1_A	N	ATCC_23344	1	1951374-1952678	Chr1	ISBma1	CGGTA	GGTTCATCGCAGAAAA	TTGCTCGATCGCAAGGCAC	ACC	AAAAAATA	AAAAAATA	AAAAAATA	Y
6*Chr1_153_ISBma1_A	N	NCTC_10229	6	1952299-1953603	Chr1	ISBma1	CCGAT	GGTTCATCGCAGAAAA	TTGCTCGATCGCAAGGCAC	ACC	TTTTTTTAT	TTTTTTTA	TTTTTTTA	Y
10*Chr1_59_ISBma1_A	N	PRL20	10	1975920-1977224	Chr1	ISBma1	CACCG	GGTTCATCGCAGAAAA	TTGCTCGATCGCAAGGCAC	ACC	AAAAAAAT	AAAAAAAT	AAAAAAAT	Y
6*Chr1_156_ISBma1_A	N	NCTC_10229	6	1985332-1986531	Chr1	ISBma1	CCGCG	GGTTCATCGCAGAAAA	TTGCTCGATCGCAAGGCAC	#	#IS407A	AAAAAAAA	#IS407A	N.D.
9*Chr1_103_ISBma1_A	N	SAVP1	9	1987012-1988316	Chr1	ISBma1	AGTCG	GGTTCATCGCAGAAAA	TTGCTCGATCGCAAGGCAC	ACC	CTTTTTTC	CTTTTTTT	CTTTTTTT	Y
4*Chr1_59_ISBma1_A	N	gb8_horse_4	4	2020579-2021883	Chr1	ISBma1	CACCG	GGTTCATCGCAGAAAA	TTGCTCGATCGCAAGGCAC	ACC	AAAAAAAT	AAAAAAAT	AAAAAAAT	Y
1*Chr1_59_ISBma1_A	N	ATCC_23344	1	2032534-2033838	Chr1	ISBma1	CACCG	GGTTCATCGCAGAAAA	TTGCTCGATCGCAAGGCAC	ACC	AAAAAAAT	AAAAAAAT	AAAAAAAT	Y
2*Chr1_127_ISBma1_A	N	fmh	2	2073439-2073769	Chr1	ISBma1	#IS407A	~~~~~~~~~~~~~~~	~~~~~~~~~~~~~~~~~~	ACC	AAAAAAAA	#IS407A	AAAAAAAA	N.D.
2*Chr1_129_ISBma1_A	N	fmh	2	2075470-2076774	Chr1	ISBma1	CGCGC	GGTTCATCGCAGAAAA	TTGCTCGATCGCAAGGCAC	ACC	TTTTTTTAT	TTTTTTTA	TTTTTTTA	Y
3*Chr1_127_ISBma1_A	N	JHU	3	2088899-2089229	Chr1	ISBma1	#IS407A	~~~~~~~~~~~~~~~	~~~~~~~~~~~~~~~~~~	ACC	AAAAAAAA	#IS407A	AAAAAAAA	N.D.
3*Chr1_129_ISBma1_A	N	JHU	3	2090930-2092234	Chr1	ISBma1	CGCGC	GGTTCATCGCAGAAAA	TTGCTCGATCGCAAGGCAC	ACC	TTTTTTTAT	TTTTTTTA	TTTTTTTA	Y
5*Chr1_60_ISBma1_A	N	ATCC_10399	5	2100644-2101948	Chr1	ISBma1	CGGTA	GGTTCATCGCAGAAAA	TTGCTCGATCGCAAGGCAC	ACC	AAAAAATA	AAAAAATA	AAAAAATA	Y
2*Chr1_36_ISBma1_A	N	fmh	2	212072-213376	Chr1	ISBma1	GCGGC	GGTTCATCGCAGAAAA	TTGCTCGATCGCAAGGCAC	ACC	TTTTTTCTT	TTTTTTCT	TTTTTTCT	Y
8*Chr1_36_ISBma1_A	N	2002721280	8	212567-213871	Chr1	ISBma1	GCGGC	GGTTCATCGCAGAAAA	TTGCTCGATCGCAAGGCAC	ACC	TTTTTTCTT	TTTTTTCT	TTTTTTCT	Y
3*Chr1_36_ISBma1_A	N	JHU	3	212916-214220	Chr1	ISBma1	GCGGC	GGTTCATCGCAGAAAA	TTGCTCGATCGCAAGGCAC	ACC	TTTTTTCTT	TTTTTTCT	TTTTTTCT	Y
8*Chr1_127_ISBma1_A	N	2002721280	8	2134387-2135693	Chr1	ISBma1	GCCGC	GGTTCATCGCAGAAAA	TTGCTCGATCGCAAGGCAC	ACC	AAAAAAAA	AAAAAAAA	AAAAAAAA	Y
8*Chr1_129_ISBma1_A	N	2002721280	8	2137833-2138804	Chr1	ISBma1	~~~~~	~~~~~~~~~~~~~~~	~~~~~~~~~~~~~~~~~~	ACC	TTTTTTTAT	~~~~~~~~	TTTTTTTA	N.D.

10*Chr1_127_ISBma1_A		N	PRL20	10	2150683-2151080	Chr1	ISBma1	#IS407/	~~~~~~~~~~~~~~~	~~~~~~~~~~~~~~~~~	ACC	AAAAAAA/	#IS407A	AAAAAAA/	N.D.
10*Chr1_129_ISBma1_A		N	PRL20	10	2155991-2157225	Chr1	ISBma1	#IS407/	~~~~~~~~~~~~~~~	CATCGAATCGTTCAACGGC	ACC	TTTTTTTAT	#IS407A	TTTTTTTA	N.D.
4*Chr1_127_ISBma1_A		N	gb8_horse_4	4	2168750-2169080	Chr1	ISBma1	#IS407/	~~~~~~~~~~~~~~~	~~~~~~~~~~~~~~~~~	ACC	AAAAAAA/	#IS407A	AAAAAAA/	N.D.
4*Chr1_129_ISBma1_A		N	gb8_horse_4	4	2170781-2172085	Chr1	ISBma1	CGCGC	GGTTCATCGCAGAAAA	TTGCTCGATCGCAAGGCAC	ACC	TTTTTTTAT	TTTTTTTA	TTTTTTTA	Y
1*Chr1_127_ISBma1_A		N	ATCC_23344	1	2180681-2181011	Chr1	ISBma1	#IS407/	~~~~~~~~~~~~~~~	~~~~~~~~~~~~~~~~~	ACC	AAAAAAA/	#IS407A	AAAAAAA/	N.D.
5*Chr1_59_ISBma1_A		N	ATCC_10399	5	2181906-2183210	Chr1	ISBma1	CACCG	GGTTCATCGCAGAAAA	TTGCTCGATCGCAAGGCAC	ACC	AAAAAAAT	AAAAAAAT	AAAAAAAT	Y
1*Chr1_129_ISBma1_A		N	ATCC_23344	1	2182712-2184016	Chr1	ISBma1	CGCGC	GGTTCATCGCAGAAAA	TTGCTCGATCGCAAGGCAC	ACC	TTTTTTTAT	TTTTTTTA	TTTTTTTA	Y
6*Chr1_14_ISBma1_A		N	NCTC_10229	6	2204564-2205868	Chr1	ISBma1	AAACA	GGTTCATCGCAGAAAA	TTGCTCGATCGCAAGGCAC	ACC	TTTTTTCC(	TTTTTTCC	TTTTTTCC	Y
6*Chr1_12_ISBma1_A		N	NCTC_10229	6	2294405-2295709	Chr1	ISBma1	GAATT(	GGTTCATCGCAGAAAA	TTGCTCGATCGCAAGGCAC	ACC	TTTTTCTTT	TTTTTCTT	TTTTTCTT	Y
7*Chr1_12_ISBma1_A		N	NCTC_10247	7	2302599-2303903	Chr1	ISBma1	GAATT(	GGTTCATCGCAGAAAA	TTGCTCGATCGCAAGGCAC	ACC	TTTTTCTTT	TTTTTCTT	TTTTTCTT	Y
5*Chr1_127_ISBma	5*Chr1_129_ISBma1_A	N	ATCC_10399	5	2331091-2332356	Chr1	ISBma1	#IS407/	CGTCACGCTGAAGTTG	ATGCCTTAACGAGCACTGG	ACC	TTTTTTTAT	#IS407A	TTTTTTTA	N.D.
10*Chr1_125_ISBma1_A		N	PRL20	10	2362908-2364212	Chr1	ISBma1	AGGCA	GGTTCATCGCAGAAAA	TTGCTCGATCGCAAGGCAC	ACC	AATTTTTT(	AATTTTTT	AATTTTTT	Y
2*Chr1_142_ISBma1_B		N	fmh	2	2431313-2432619	Chr1	ISBma1	GCGGC	GGTTCATCGCAGAAAA	TTGCTCGATCGCAAGGCAC	ACC	AAAAAAAT	AAAAAAAT	AAAAAAAT	Y
3*Chr1_142_ISBma1_B		N	JHU	3	2447452-2448758	Chr1	ISBma1	GCGGC	GGTTCATCGCAGAAAA	TTGCTCGATCGCAAGGCAC	ACC	AAAAAAAT	AAAAAAAT	AAAAAAAT	Y
6*Chr1_36_ISBma1_A		N	NCTC_10229	6	2489753-2491057	Chr1	ISBma1	GCGGC	GGTTCATCGCAGAAAA	TTGCTCGATCGCAAGGCAC	ACC	TTTTTTCTT	TTTTTTCT	TTTTTTCT	Y
8*Chr1_156_ISBma1_A		N	2002721280	8	2511737-2512936	Chr1	ISBma1	CCGCG	GGTTCATCGCAGAAAA	TTGCTCGATCGCAAGGCAC	#	#IS407A	AAAAAAAA	#IS407A	N.D.
4*Chr1_142_ISBma1_B		N	gb8_horse_4	4	2526053-2527359	Chr1	ISBma1	GCGGC	GGTTCATCGCAGAAAA	TTGCTCGATCGCAAGGCAC	ACC	AAAAAAAT	AAAAAAAT	AAAAAAAT	Y
1*Chr1_142_ISBma1_B		N	ATCC_23344	1	2538026-2539332	Chr1	ISBma1	GCGGC	GGTTCATCGCAGAAAA	TTGCTCGATCGCAAGGCAC	ACC	AAAAAAAT	AAAAAAAT	AAAAAAAT	Y
8*Chr1_153_ISBma1_A		N	2002721280	8	2544560-2545864	Chr1	ISBma1	CCGAT(	GGTTCATCGCAGAAAA	TTGCTCGATCGCAAGGCAC	ACC	TTTTTTTAT	TTTTTTTA	TTTTTTTA	Y
9*Chr1_124_ISBma1_B		N	SAVP1	9	2568741-2570045	Chr1	ISBma1	TCAGC/	GGTTCATCGCAGAAAA	TTGCTCGATCGCAAGGCAC	ACC	TTTTTCTTT	TTTTTCTT	TTTTTCTT	Y
10*Chr1_142_ISBma1_B		N	PRL20	10	2584163-2585469	Chr1	ISBma1	GCGGC	GGTTCATCGCAGAAAA	TTGCTCGATCGCAAGGCAC	ACC	AAAAAAAT	AAAAAAAT	AAAAAAAT	Y
2*Chr1_156_ISBma1_A		N	fmh	2	2598014-2598869	Chr1	ISBma1	CCGCG	GGTTCATCGCAGAAAA	TTGCTCGATCGCAAGGCAC	#	#IS407A	AAAAAAAA	#IS407A	N.D.
9*Chr1_125_ISBma1_A		N	SAVP1	9	2603704-2605008	Chr1	ISBma1	AGGCA	GGTTCATCGCAGAAAA	TTGCTCGATCGCAAGGCAC	ACC	AATTTTTT(	AATTTTTT	AATTTTTT	Y
3*Chr1_153_ISBma	3*Chr1_156_ISBma1_A	N	JHU	3	2614893-2615748	Chr1	ISBma1	CCGCG	GGTTCATCGCAGAAAA	TTGCTCGATCGCAAGGCAC	#	#IS406A	AAAAAAAA	#IS406A	N.D.
2*Chr1_153_ISBma1_A		N	fmh	2	2631429-2632733	Chr1	ISBma1	CCGAT(	GGTTCATCGCAGAAAA	TTGCTCGATCGCAAGGCAC	ACC	TTTTTTTAT	TTTTTTTA	TTTTTTTA	Y
5*Chr1_142_ISBma1_B		N	ATCC_10399	5	2688082-2689388	Chr1	ISBma1	GCGGC	GGTTCATCGCAGAAAA	TTGCTCGATCGCAAGGCAC	ACC	AAAAAAAT	AAAAAAAT	AAAAAAAT	Y
6*Chr1_127_ISBma	6*Chr1_129_ISBma1_A	N	NCTC_10229	6	2688853-2690159	Chr1	ISBma1	GCCGC	GGTTCATCGCAGAAAA	TTGCTCGATCGCAAGGCAC	ACC	TTTTTTTAT	AAAAAAAA	TTTTTTTA	N
4*Chr1_156_ISBma1_A		N	gb8_horse_4	4	2692085-2692940	Chr1	ISBma1	CCGCG	GGTTCATCGCAGAAAA	TTGCTCGATCGCAAGGCAC	#	#IS407A	AAAAAAAA	#IS407A	N.D.
1*Chr1_156_ISBma1_A		N	ATCC_23344	1	2704469-2705324	Chr1	ISBma1	CCGCG	GGTTCATCGCAGAAAA	TTGCTCGATCGCAAGGCAC	#	#IS407A	AAAAAAAA	#IS407A	N.D.
4*Chr1_153_ISBma1_A		N	gb8_horse_4	4	2723665-2724969	Chr1	ISBma1	CCGAT(	GGTTCATCGCAGAAAA	TTGCTCGATCGCAAGGCAC	ACC	TTTTTTTAT	TTTTTTTA	TTTTTTTA	Y
1*Chr1_153_ISBma1_A		N	ATCC_23344	1	2737048-2738352	Chr1	ISBma1	CCGAT(	GGTTCATCGCAGAAAA	TTGCTCGATCGCAAGGCAC	ACC	TTTTTTTAT	TTTTTTTA	TTTTTTTA	Y
10*Chr1_156_ISBma1_A		N	PRL20	10	2755093-2756292	Chr1	ISBma1	CCGCG	GGTTCATCGCAGAAAA	TTGCTCGATCGCAAGGCAC	#	#IS407A	AAAAAAAA	#IS407A	N.D.
10*Chr1_153_ISBma1_A		N	PRL20	10	2787753-2789057	Chr1	ISBma1	CCGAT(	GGTTCATCGCAGAAAA	TTGCTCGATCGCAAGGCAC	ACC	TTTTTTTAT	TTTTTTTA	TTTTTTTA	Y
9*Chr1_12_ISBma1_A		N	SAVP1	9	2799403-2800707	Chr1	ISBma1	GAATT(	GGTTCATCGCAGAAAA	TTGCTCGATCGCAAGGCAC	ACC	TTTTTCTTT	TTTTTCTT	TTTTTCTT	Y
7*Chr1_26_ISBma1_A		N	NCTC_10247	7	2816595-2817901	Chr1	ISBma1	GCGTG	GGTTCATCGCAGAAAA	TTGCTCGATCGCAAGGCAC	ACC	ATAAAAA/	ATAAAAAA	ATAAAAA/	Y
5*Chr1_156_ISBma1_A		N	ATCC_10399	5	2854277-2855132	Chr1	ISBma1	CCGCG	GGTTCATCGCAGAAAA	TTGCTCGATCGCAAGGCAC	#	#IS407A	AAAAAAAA	#IS407A	N.D.
5*Chr1_153_ISBma1_A		N	ATCC_10399	5	2886389-2887693	Chr1	ISBma1	CCGAT(	GGTTCATCGCAGAAAA	TTGCTCGATCGCAAGGCAC	ACC	TTTTTTTAT	TTTTTTTA	TTTTTTTA	Y
9*Chr1_14_ISBma1_A		N	SAVP1	9	2887865-2888041	Chr1	ISBma1	#IS407/	~~~~~~~~~~~~~~~	~~~~~~~~~~~~~~~~~	ACC	TTTTTTCC(	#IS407A	TTTTTTCC	N.D.
9*Chr1_14-1_ISBma1_A		N	SAVP1	9	2889266-2890409	Chr1	ISBma1	AAACA	GGTTCATCGCAGAAAA	TTGCTCGATCGCAAGGCAC	#	#IS407A	TTTTTTCC	#IS407A	N.D.
7*Chr1_59_ISBma1_A		N	NCTC_10247	7	289572-290876	Chr1	ISBma1	CACCG	GGTTCATCGCAGAAAA	TTGCTCGATCGCAAGGCAC	ACC	AAAAAAAT	AAAAAAAT	AAAAAAAT	Y
8*Chr1_26_ISBma1_A		N	2002721280	8	3035091-3036397	Chr1	ISBma1	GCGTG	GGTTCATCGCAGAAAA	TTGCTCGATCGCAAGGCAC	ACC	ATAAAAA/	ATAAAAAA	ATAAAAA/	Y
9*Chr1_156_ISBma1_A		N	SAVP1	9	3108437-3109636	Chr1	ISBma1	CCGCG	GGTTCATCGCAGAAAA	TTGCTCGATCGCAAGGCAC	#	#IS407A	AAAAAAAA	#IS407A	N.D.
9*Chr1_153_ISBma1_A		N	SAVP1	9	3141365-3142669	Chr1	ISBma1	CCGAT(	GGTTCATCGCAGAAAA	TTGCTCGATCGCAAGGCAC	ACC	TTTTTTTAT	TTTTTTTA	TTTTTTTA	Y
2*Chr1_26_ISBma1_A		N	fmh	2	3186645-3187951	Chr1	ISBma1	GCGTG	GGTTCATCGCAGAAAA	TTGCTCGATCGCAAGGCAC	ACC	ATAAAAA/	ATAAAAAA	ATAAAAA/	Y
3*Chr1_26_ISBma1_A		N	JHU	3	3203538-3204844	Chr1	ISBma1	GCGTG	GGTTCATCGCAGAAAA	TTGCTCGATCGCAAGGCAC	ACC	ATAAAAA/	ATAAAAAA	ATAAAAA/	Y
8*Chr1_14_ISBma1_A		N	2002721280	8	3221823-3223127	Chr1	ISBma1	AAACA	GGTTCATCGCAGAAAA	TTGCTCGATCGCAAGGCAC	ACC	TTTTTTCC(	TTTTTTCC	TTTTTTCC	Y
2*Chr1_14_ISBma1_A		N	fmh	2	3250236-3251540	Chr1	ISBma1	AAACA	GGTTCATCGCAGAAAA	TTGCTCGATCGCAAGGCAC	ACC	TTTTTTCC(	TTTTTTCC	TTTTTTCC	Y
3*Chr1_14_ISBma1_A		N	JHU	3	3268498-3269802	Chr1	ISBma1	AAACA	GGTTCATCGCAGAAAA	TTGCTCGATCGCAAGGCAC	ACC	TTTTTTCC(	TTTTTTCC	TTTTTTCC	Y
4*Chr1_26_ISBma1_A		N	gb8_horse_4	4	3273197-3274503	Chr1	ISBma1	GCGTG	GGTTCATCGCAGAAAA	TTGCTCGATCGCAAGGCAC	ACC	ATAAAAA/	ATAAAAAA	ATAAAAA/	Y
1*Chr1_26_ISBma1_A		N	ATCC_23344	1	3286945-3288251	Chr1	ISBma1	GCGTG	GGTTCATCGCAGAAAA	TTGCTCGATCGCAAGGCAC	ACC	ATAAAAA/	ATAAAAAA	ATAAAAA/	Y
10*Chr1_26_ISBma1_A		N	PRL20	10	3322621-3323927	Chr1	ISBma1	GCGTG	GGTTCATCGCAGAAAA	TTGCTCGATCGCAAGGCAC	ACC	ATAAAAA/	ATAAAAAA	ATAAAAA/	Y
4*Chr1_14_ISBma1_A		N	gb8_horse_4	4	3336103-3337407	Chr1	ISBma1	AAACA	GGTTCATCGCAGAAAA	TTGCTCGATCGCAAGGCAC	ACC	TTTTTTCC(	TTTTTTCC	TTTTTTCC	Y
1*Chr1_14_ISBma1_A		N	ATCC_23344	1	3349816-3351120	Chr1	ISBma1	AAACA	GGTTCATCGCAGAAAA	TTGCTCGATCGCAAGGCAC	ACC	TTTTTTCC(	TTTTTTCC	TTTTTTCC	Y
6*Chr1_103_ISBma1_A		N	NCTC_10229	6	3350430-3351734	Chr1	ISBma1	AGTCG	GGTTCATCGCAGAAAA	TTGCTCGATCGCAAGGCAC	ACC	CTTTTTTT(	CTTTTTTT	CTTTTTTT	Y
4*Chr1_36_ISBma1_A		N	gb8_horse_4	4	335870-337174	Chr1	ISBma1	GCGGC	GGTTCATCGCAGAAAA	TTGCTCGATCGCAAGGCAC	ACC	TTTTTTCTT	TTTTTTCT	TTTTTTCT	Y
5*Chr1_26_ISBma1_A		N	ATCC_10399	5	3360914-3362220	Chr1	ISBma1	GCGTG	GGTTCATCGCAGAAAA	TTGCTCGATCGCAAGGCAC	ACC	ATAAAAA/	ATAAAAAA	ATAAAAA/	Y
1*Chr1_36_ISBma1_A		N	ATCC_23344	1	337256-338560	Chr1	ISBma1	GCGGC	GGTTCATCGCAGAAAA	TTGCTCGATCGCAAGGCAC	ACC	TTTTTTCTT	TTTTTTCT	TTTTTTCT	Y
7*Chr1_14_ISBma1_A		N	NCTC_10247	7	3397369-3398673	Chr1	ISBma1	AAACA	GGTTCATCGCAGAAAA	TTGCTCGATCGCAAGGCAC	ACC	TTTTTTCC(	TTTTTTCC	TTTTTTCC	Y

7*Chr1_153_ISBma1_A		N	NCTC_10247	7	3421451-3422755	Chr1	ISBma1	CCGAT(	GGTTCATCGCAGAAAA	TTGCTCGATCGCAAGGCAC	ACC	TTTTTTTAT	TTTTTTTA	TTTTTTTA	Y
2*Chr1_124_ISBma1_B		N	fmh	2	342363-343435	Chr1	ISBma1	#IS407/	~~~~~~~~~~~~~~~	~~~~~~~~~~~~~~~~~	ACC	TTTTTCTTT	#IS407A	TTTTTCTT	N.D.
5*Chr1_14_ISBma1_A		N	ATCC_10399	5	3424621-3425925	Chr1	ISBma1	AAACA	GGTTCATCGCAGAAAA	TTGCTCGATCGCAAGGCAC	ACC	TTTTTTCC(	TTTTTTCC	TTTTTTCC	Y
3*Chr1_124_ISBma1_B		N	JHU	3	343967-345039	Chr1	ISBma1	#IS407/	~~~~~~~~~~~~~~~	~~~~~~~~~~~~~~~~~	ACC	TTTTTCTTT	#IS407A	TTTTTCTT	N
7*Chr1_156_ISBma1_A		N	NCTC_10247	7	3454484-3455683	Chr1	ISBma1	CCGCG	GGTTCATCGCAGAAAA	TTGCTCGATCGCAAGGCAC	#	#IS407A	AAAAAAAA	#IS407A	N.D.
9*Chr1_142_ISBma1_B		N	SAVP1	9	348750-350056	Chr1	ISBma1	GCGGC	GGTTCATCGCAGAAAA	TTGCTCGATCGCAAGGCAC	ACC	AAAAAAA	AAAAAAAT	AAAAAAA	Y
10*Chr1_36_ISBma1_A		N	PRL20	10	350249-351553	Chr1	ISBma1	GCGGC	GGTTCATCGCAGAAAA	TTGCTCGATCGCAAGGCAC	ACC	TTTTTTCTT	TTTTTTCT	TTTTTTCT	Y
7*Chr1_60_ISBma1_A		N	NCTC_10247	7	370819-372123	Chr1	ISBma1	CGGTA	GGTTCATCGCAGAAAA	TTGCTCGATCGCAAGGCAC	ACC	AAAAAAT/	AAAAAATA	AAAAAAT/	Y
5*Chr1_36_ISBma1_A		N	ATCC_10399	5	374206-375510	Chr1	ISBma1	GCGGC	GGTTCATCGCAGAAAA	TTGCTCGATCGCAAGGCAC	ACC	TTTTTTCTT	TTTTTTCT	TTTTTTCT	Y
8*Chr1_125_ISBma1_A		N	2002721280	8	377508-378812	Chr1	ISBma1	AGGCA	GGTTCATCGCAGAAAA	TTGCTCGATCGCAAGGCAC	ACC	AATTTTTT(	AATTTTTT	AATTTTTT	Y
2*Chr1_125_ISBma1_A		N	fmh	2	377515-378819	Chr1	ISBma1	AGGCA	GGTTCATCGCAGAAAA	TTGCTCGATCGCAAGGCAC	ACC	AATTTTTT(	AATTTTTT	AATTTTTT	Y
3*Chr1_125_ISBma1_A		N	JHU	3	378674-379978	Chr1	ISBma1	AGGCA	GGTTCATCGCAGAAAA	TTGCTCGATCGCAAGGCAC	ACC	AATTTTTT(	AATTTTTT	AATTTTTT	Y
6*Chr1_82_ISBma1_A		N	NCTC_10229	6	398477-399353	Chr1	ISBma1	CGAAA	GGTTCATCGCAGAAAA	TTGCTCGATCGCAAGGCAC	#	#IS407A	TTTCTTTT	#IS407A	N.D.
6*Chr1_82-1_ISBma1_A		N	NCTC_10229	6	399914-401019	Chr1	ISBma1	#IS407/	~~~~~~~~~~~~~~~	~~~~~~~~~~~~~~~~~	ACC	TTTCTTTT(	#IS407A	TTTCTTTT	N.D.
8*Chr1_59_ISBma1_A		N	2002721280	8	422225-423530	Chr1	ISBma1	CACCG	GGTTCATCGCAGAAAA	TTGCTCGATCGCAAGGCAC	ACC	AAAAAAA	AAAAAAAT	AAAAAAA	Y
10*Chr1_124_ISBma1_B		N	PRL20	10	455910-457214	Chr1	ISBma1	TCAGC/	GGTTCATCGCAGAAAA	TTGCTCGATCGCAAGGCAC	ACC	TTTTTCTTT	TTTTTCTT	TTTTTCTT	Y
4*Chr1_124_ISBma1_B		N	gb8_horse_4	4	464802-465874	Chr1	ISBma1	#IS407/	~~~~~~~~~~~~~~~	~~~~~~~~~~~~~~~~~	ACC	TTTTTCTTT	#IS407A	TTTTTCTT	N.D.
1*Chr1_124_ISBma1_B		N	ATCC_23344	1	466488-467560	Chr1	ISBma1	#IS407/	~~~~~~~~~~~~~~~	~~~~~~~~~~~~~~~~~	ACC	TTTTTCTTT	#IS407A	TTTTTCTT	N.D.
4*Chr1_125_ISBma1_A		N	gb8_horse_4	4	499540-500844	Chr1	ISBma1	AGGCA	GGTTCATCGCAGAAAA	TTGCTCGATCGCAAGGCAC	ACC	AATTTTTT(	AATTTTTT	AATTTTTT	Y
1*Chr1_125_ISBma1_A		N	ATCC_23344	1	501195-502499	Chr1	ISBma1	AGGCA	GGTTCATCGCAGAAAA	TTGCTCGATCGCAAGGCAC	ACC	AATTTTTT(	AATTTTTT	AATTTTTT	Y
8*Chr1_60_ISBma1_A		N	2002721280	8	502009-502817	Chr1	ISBma1	CGGTA	GGTTCATCGCAGAAAA	TTGCTCGATCGCAAGGCAC	#	#contig_br	AAAAAATA	#contig_	N.D.
5*Chr1_124_ISBma1_B		N	ATCC_10399	5	503499-504571	Chr1	ISBma1	#IS407/	~~~~~~~~~~~~~~~	~~~~~~~~~~~~~~~~~	ACC	TTTTTCTTT	#IS407A	TTTTTCTT	N.D.
5*Chr1_125_ISBma1_A		N	ATCC_10399	5	538228-539532	Chr1	ISBma1	AGGCA	GGTTCATCGCAGAAAA	TTGCTCGATCGCAAGGCAC	ACC	AATTTTTT(	AATTTTTT	AATTTTTT	Y
7*Chr1_36_ISBma1_A		N	NCTC_10247	7	60358-61662	Chr1	ISBma1	GCGGC	GGTTCATCGCAGAAAA	TTGCTCGATCGCAAGGCAC	ACC	TTTTTTCTT	TTTTTTCT	TTTTTTCT	Y
9*Chr1_36_ISBma1_A		N	SAVP1	9	625260-626564	Chr1	ISBma1	GCGGC	GGTTCATCGCAGAAAA	TTGCTCGATCGCAAGGCAC	ACC	TTTTTTCTT	TTTTTTCT	TTTTTTCT	Y
7*Chr1_82-1_ISBma1_A		N	NCTC_10247	7	760075-761180	Chr1	ISBma1	#IS407/	~~~~~~~~~~~~~~~	~~~~~~~~~~~~~~~~~	ACC	TTTCTTTT(	#IS407A	TTTCTTTT	N.D.
7*Chr1_82_ISBma1_A		N	NCTC_10247	7	761741-762617	Chr1	ISBma1	CGAAA	GGTTCATCGCAGAAAA	TTGCTCGATCGCAAGGCAC	#	#IS407A	TTTCTTTT	#IS407A	N.D.
6*Chr1_60_ISBma1_A		N	NCTC_10229	6	788886-790190	Chr1	ISBma1	CGGTA	GGTTCATCGCAGAAAA	TTGCTCGATCGCAAGGCAC	ACC	AAAAAAT/	AAAAAATA	AAAAAAT/	Y
9*Chr1_127_ISBma	9*Chr1_129_ISBma1_A	N	SAVP1	9	825845-826242	Chr1	ISBma1	#IS407/	~~~~~~~~~~~~~~~	~~~~~~~~~~~~~~~~~	ACC	TTTTTTTAT	#IS407A	TTTTTTTA	N.D.
6*Chr1_59_ISBma1_A		N	NCTC_10229	6	870043-871347	Chr1	ISBma1	CACCG	GGTTCATCGCAGAAAA	TTGCTCGATCGCAAGGCAC	ACC	AAAAAAA	AAAAAAAT	AAAAAAA	Y
10*Chr1_14_ISBma1_A		N	PRL20	10	89941-91245	Chr1	ISBma1	AAACA	GGTTCATCGCAGAAAA	TTGCTCGATCGCAAGGCAC	ACC	TTTTTTCC(	TTTTTTCC	TTTTTTCC	Y
2*Chr1_73_ISBma1_A		N	fmh	2	945561-946865	Chr1	ISBma1	GAGAG	GGTTCATCGCAGAAAA	TTGCTCGATCGCAAGGCAC	ACC	AAAAAAA	AAAAAAAT	AAAAAAA	Y
3*Chr1_73_ISBma1_A		N	JHU	3	953135-954439	Chr1	ISBma1	GAGAG	GGTTCATCGCAGAAAA	TTGCTCGATCGCAAGGCAC	ACC	AAAAAAA	AAAAAAAT	AAAAAAA	Y
8*Chr1_82-1_ISBma1_A		N	2002721280	8	966954-968059	Chr1	ISBma1	#IS407/	~~~~~~~~~~~~~~~	~~~~~~~~~~~~~~~~~	ACC	TTTCTTTT(	#IS407A	TTTCTTTT	N.D.
8*Chr1_82_ISBma1	8*Chr1_73_ISBma1_A	N	2002721280	8	968620-969496	Chr1	ISBma1	CGAAA	GGTTCATCGCAGAAAA	TTGCTCGATCGCAAGGCAC	#	#IS407A	TTTCTTTT	#IS407A	N.D.
10*Chr1_73_ISBma1_A		N	PRL20	10	974551-975855	Chr1	ISBma1	GAGAG	GGTTCATCGCAGAAAA	TTGCTCGATCGCAAGGCAC	ACC	AAAAAAA	AAAAAAAT	AAAAAAA	Y
9*Chr1_59_ISBma1_A		N	SAVP1	9	999824-1001128	Chr1	ISBma1	CACCG	GGTTCATCGCAGAAAA	TTGCTCGATCGCAAGGCAC	ACC	AAAAAAA	AAAAAAAT	AAAAAAA	Y

IS *Bma* 2

								5'-ext	5'-IR	ORF	3'-IR	3'-ext	5'-DR	3'-DR	rget duplicates
									CAGATTGCTGACAAACCC		GGGTTCGTCAGCAGTCTG				
2*Chr1_90_ISBma2_A		N	fmh	2	1003833-1005047	Chr1	ISBma2	#IS407/	~~~~~~~~~~~~~~~	~~~~~~~~~~~~~~~~~	TCCCAGAAAGACAAAACC(	AAAGCCC(	#IS407A	AAAGCCC(	N.D.
3*Chr1_90_ISBma2_A		N	JHU	3	1012642-1013856	Chr1	ISBma2	#IS407/	~~~~~~~~~~~~~~~	~~~~~~~~~~~~~~~~~	TCCCAGAAAGACAAAACC(	AAAGCCC(	#IS407A	AAAGCCC(	N.D.
7*Chr1_85_ISBma2_A		N	NCTC_10247	7	1012716-1014290	Chr1	ISBma2	GCCTC/	CAGATTGCTGACAAAC	ATGCTGAAGACGCCCATG(	TCCCAGAAAGACAAAACC(	AGCCAGC	AGCCAGCTTGCGCTGG(	AGCCAGC	N
6*Chr1_44a_ISBma	6*Chr1_46a_ISBma2_A	R	NCTC_10229	6	1031963-1033543	Chr1	ISBma2	ACGGC	CAGATTGCTGACAAAC	ATGCTGAAGACGCCCATG(	TCCCAGAAAGACAAAACC(	AAAAGCG	AACCCGGCTGGAAGCC	AAAAGCG	N
10*Chr1_90_ISBma2_A		N	PRL20	10	1032539-1033726	Chr1	ISBma2	#IS407/	~~~~~~~~~~~~~~~	~~~~~~~~~~~~~~~~~	TCCCAGAAAGACAAAACC(	AAAGCCC(	#IS407A	AAAGCCC(	N.D.
6*Chr1_41_ISBma2_A		N	NCTC_10229	6	1055116-1056005	Chr1	ISBma2	AATCA(	CAGATTGCTGACAAAC	ATGCTGAAGACGCCCATG(	#	#IS407A	CCCTCCACTGGAGGGC	#IS407A	N.D.
6*Chr1_39b_ISBma	6*Chr1_44b_ISBma2_A	R	NCTC_10229	6	1061840-1063411	Chr1	ISBma2	GCAGG	CAGATTGCTGACAAAC	ATGCTGAAGACGCCCATG(	TCCCAGAAAGACAAAACC(	AAAAACC(	CCGCCCGCTCCGGGCG	AAAAACC(	N
9*Chr1_25b_ISBma	9*Chr1_28b_ISBma2_A	R	SAVP1	9	109336-110910	Chr1	ISBma2	CGCCG	CAGATTGCTGACAAAC	ATGCTGAAGACGCCCATG(	TCCCAGAAAGACAAAACC(	AGGCCCG	CTGTCGCAGGACGGCG	AGGCCCG	N
4*Chr1_90_ISBma2_A		N	gb8_horse_4	4	1117195-1118409	Chr1	ISBma2	#IS407/	~~~~~~~~~~~~~~~	~~~~~~~~~~~~~~~~~	TCCCAGAAAGACAAAACC(	AAAGCCC(	#IS407A	AAAGCCC(	N.D.
1*Chr1_90_ISBma2_A		N	ATCC_23344	1	1124052-1125266	Chr1	ISBma2	#IS407/	~~~~~~~~~~~~~~~	~~~~~~~~~~~~~~~~~	TCCCAGAAAGACAAAACC(	AAAGCCC(	#IS407A	AAAGCCC(	N.D.
6*Chr1_139a_ISBm	6*Chr1_141a_ISBma2_A	R	NCTC_10229	6	1142674-1144265	Chr1	ISBma2	GCCCT(	CAGATTGCTGACAAAC	ATGCTGAAGACGCCCATG(	TCCCAGAAAGACAAAACC(	AGCCCGA/	CCGGCGCATGGCGCCG	AGCCCGA/	N
8*Chr1_90_ISBma2_A		N	2002721280	8	1167212-1168399	Chr1	ISBma2	#IS407/	~~~~~~~~~~~~~~~	~~~~~~~~~~~~~~~~~	TCCCAGAAAGACAAAACC(	AAAGCCC(	#IS407A	AAAGCCC(	N.D.
2*Chr1_85_ISBma2_A		N	fmh	2	1198532-1200104	Chr1	ISBma2	GCCTC/	CAGATTGCTGACAAAC	ATGCTGAAGACGCCCATG(	TCCCAGAAAGACAAAACC(	AGCCAGC	AGCCAGCTTGCGCTGG(	AGCCAGC	Y
10*Chr1_23a_ISBma2_A		N	PRL20	10	120201-121772	Chr1	ISBma2	CGCGC	CAGATTGCTGACAAAC	ATGCTGAAGACGCCCATG(	TCCCAGAAAGACAAAACC(	ACCGCAG(	ACCGCAGCGAAGGCTG	ACCGCAG(	Y
3*Chr1_85_ISBma2_A		N	JHU	3	1209720-1211292	Chr1	ISBma2	GCCTC/	CAGATTGCTGACAAAC	ATGCTGAAGACGCCCATG(	TCCCAGAAAGACAAAACC(	AGCCAGC	AGCCAGCTTGCGCTGG(	AGCCAGC	Y
10*Chr1_85_ISBma2_A		N	PRL20	10	1218305-1219877	Chr1	ISBma2	GCCTC/	CAGATTGCTGACAAAC	ATGCTGAAGACGCCCATG(	TCCCAGAAAGACAAAACC(	AGCCAGC	AGCCAGCTTGCGCTGG(	AGCCAGC	Y

8*Chr1_85_ISBma2_A		N	2002721280	8	1219726-1221298	Chr1	ISBma2	GCCTC	CAGATTGCTGACAAAC	ATGCTGAAGACGCCCATG	TCCCAGAAAGACAAAACC	AGCCAGC	AGCCAGCTTGCGCTGG	AGCCAGC	Y
6*Chr1_139b_ISBm	6*Chr1_141b_ISBma2_A	R	NCTC_10229	6	1224724-1226315	Chr1	ISBma2	CCTCC	CAGATTGCTGACAAAC	ATGCTGAAGACGCCCATG	TCCCAGAAAGACAAAACC	AGCCCGG	CCGAACCAGCGGTTCG	AGCCCGG	N
7*Chr1_101_ISBma	7*Chr1_102_ISBma2_A	D	NCTC_10247	7	1257256-1258828	Chr1	ISBma2	GCATC	CAGATTGCTGACAAAC	ATGCTGAAGACGCCCATG	TCCCAGAAAGACAAAACC	GGGCCGT	AGGCCCGCCGAAGCG	GGGCCGT	N
5*Chr1_85_ISBma2_A		N	ATCC_10399	5	1260531-1262103	Chr1	ISBma2	GCCTC	CAGATTGCTGACAAAC	ATGCTGAAGACGCCCATG	TCCCAGAAAGACAAAACC	AGCCAGC	AGCCAGCTTGCGCTGG	AGCCAGC	Y
7*Chr1_39a_ISBma	7*Chr1_44a_ISBma2_A	R	NCTC_10247	7	128672-130244	Chr1	ISBma2	ACGGC	CAGATTGCTGACAAAC	ATGCTGAAGACGCCCATG	TCCCAGAAAGACAAAACC	AGCCCTC	AACCCGGCTGGAAGCC	AGCCCTC	N
4*Chr1_85_ISBma2_A		N	gb8_horse_4	4	1307221-1308793	Chr1	ISBma2	GCCTC	CAGATTGCTGACAAAC	ATGCTGAAGACGCCCATG	TCCCAGAAAGACAAAACC	AGCCAGC	AGCCAGCTTGCGCTGG	AGCCAGC	Y
1*Chr1_85_ISBma2_A		N	ATCC_23344	1	1315507-1317079	Chr1	ISBma2	GCCTC	CAGATTGCTGACAAAC	ATGCTGAAGACGCCCATG	TCCCAGAAAGACAAAACC	AGCCAGC	AGCCAGCTTGCGCTGG	AGCCAGC	Y
6*Chr1_144_ISBma2_B		N	NCTC_10229	6	1324314-1325889	Chr1	ISBma2	GCGGC	CAGATTGCTGACAAAC	ATGCTGAAGACGCCCATG	TCCCAGAAAGACAAAACC	AGCCCGC	AGCCCGCGCATGCGGG	AGCCCGC	Y
7*Chr1_104_ISBma2_A		N	NCTC_10247	7	1345232-1346804	Chr1	ISBma2	TACCC	CAGATTGCTGACAAAC	ATGCTGAAGACGCCCATG	TCCCAGAAAGACAAAACC	ACCGGCG	ACCGGCGCATTCGCG	ACCGGCG	Y
6*Chr1_22b_ISBma	6*Chr1_147_ISBma2_A	D-R	NCTC_10229	6	1369886-1371461	Chr1	ISBma2	GCCGC	CAGATTGCTGACAAAC	ATGCTGAAGACGCCCATG	TCCCAGAAAGACAAAACC	AGGCGCC	AGCCCGCGAGACGCG	AGGCGCC	N
6*Chr1_23a_ISBma2_A		N	NCTC_10229	6	1403305-1404881	Chr1	ISBma2	CGCGC	CAGATTGCTGACAAAC	ATGCTGAAGACGCCCATG	TCCCAGAAAGACAAAACC	ACCGCAG	ACCGCAGCGAAGGCTG	ACCGCAG	Y
6*Chr1_24_ISBma2_A		N	NCTC_10229	6	1432886-1432917	Chr1	ISBma2	GGCAA	CAGATTGCTGACAAAC	ATGCTGAAGACGCCCATG	TCCCAGAAAGACAAAACC	AACCCGC	AACCCGCCTCGGAGGC	AACCCGC	Y
10*Chr1_22a_ISBma2_A		R	PRL20	10	143964-144800	Chr1	ISBma2	#contig	~~~~~~~~~~~~~~~	~~~~~~~~~~~~~~~~~	TCCCAGAAAGACAAAACC	AGCCCGC	#contig_break	AGCCCGC	N.D.
2*Chr1_100_ISBma2_B		N	fmh	2	1446086-1447658	Chr1	ISBma2	GCGCG	CAGATTGCTGACAAAC	ATGCTGAAGACGCCCATG	TCCCAGAAAGACAAAACC	AATGGGC	AATGGGCCGGAAACG	AATGGGC	Y
2*Chr1_101_ISBma	2*Chr1_102_ISBma2_A	D	fmh	2	1449099-1450671	Chr1	ISBma2	GCATC	CAGATTGCTGACAAAC	ATGCTGAAGACGCCCATG	TCCCAGAAAGACAAAACC	GGGCCGT	AGGCCCGCCGAAGCG	GGGCCGT	N
3*Chr1_100_ISBma2_B		N	JHU	3	1455405-1456977	Chr1	ISBma2	GCGCG	CAGATTGCTGACAAAC	ATGCTGAAGACGCCCATG	TCCCAGAAAGACAAAACC	AATGGGC	AATGGGCCGGAAACG	AATGGGC	Y
3*Chr1_101_ISBma	3*Chr1_102_ISBma2_A	D	JHU	3	1458418-1459990	Chr1	ISBma2	GCATC	CAGATTGCTGACAAAC	ATGCTGAAGACGCCCATG	TCCCAGAAAGACAAAACC	GGGCCGT	AGGCCCGCCGAAGCG	GGGCCGT	N
6*Chr1_25a_ISBma	6*Chr1_28a_ISBma2_A	R	NCTC_10229	6	1461808-1463379	Chr1	ISBma2	TCGAA	CAGATTGCTGACAAAC	ATGCTGAAGACGCCCATG	TCCCAGAAAGACAAAACC	AGCCCGC	CCCGCGCGTCGCGCGG	AGCCCGC	N
8*Chr1_101_ISBma	8*Chr1_102_ISBma2_A	D	2002721280	8	1463360-1464932	Chr1	ISBma2	GCATC	CAGATTGCTGACAAAC	ATGCTGAAGACGCCCATG	TCCCAGAAAGACAAAACC	GGGCCGT	AGGCCCGCCGAAGCG	GGGCCGT	N
6*Chr1_85_ISBma2_A		N	NCTC_10229	6	146802-148374	Chr1	ISBma2	GCCTC	CAGATTGCTGACAAAC	ATGCTGAAGACGCCCATG	TCCCAGAAAGACAAAACC	AGCCAGC	AGCCAGCTTGCGCTGG	AGCCAGC	N
7*Chr1_105a_ISBm	7*Chr1_107a_ISBma2_A	R	NCTC_10247	7	1482665-1484237	Chr1	ISBma2	TCGGG	CAGATTGCTGACAAAC	ATGCTGAAGACGCCCATG	TCCCAGAAAGACAAAACC	AAGCCCC	CCCGCTGGTTTCAGCG	AAGCCCC	N
7*Chr1_105b_ISBm	7*Chr1_107b_ISBma2_A	R	NCTC_10247	7	1486716-1488288	Chr1	ISBma2	AACGA	CAGATTGCTGACAAAC	ATGCTGAAGACGCCCATG	TCCCAGAAAGACAAAACC	AAGCCCC	CCCGCTGGTTTCAGCG	AAGCCCC	N
5*Chr1_90_ISBma2_A		N	ATCC_10399	5	1495313-1496527	Chr1	ISBma2	#IS407	~~~~~~~~~~~~~~~	~~~~~~~~~~~~~~~~~	TCCCAGAAAGACAAAACC	AAAGCCC	#IS407A	AAAGCCC	N.D.
9*Chr1_90_ISBma2_A		N	SAVP1	9	1498063-1499250	Chr1	ISBma2	#IS407	~~~~~~~~~~~~~~~	~~~~~~~~~~~~~~~~~	TCCCAGAAAGACAAAACC	AAAGCCC	#IS407A	AAAGCCC	N.D.
10*Chr1_101_ISBm	10*Chr1_102_ISBma2_A	D	PRL20	10	1506540-1508112	Chr1	ISBma2	GCATC	CAGATTGCTGACAAAC	ATGCTGAAGACGCCCATG	TCCCAGAAAGACAAAACC	GGGCCGT	AGGCCCGCCGAAGCG	GGGCCGT	N
6*Chr1_25b_ISBma	6*Chr1_28b_ISBma2_A	R	NCTC_10229	6	1514301-1515875	Chr1	ISBma2	CGCCG	CAGATTGCTGACAAAC	ATGCTGAAGACGCCCATG	TCCCAGAAAGACAAAACC	AGGCCCG	CTGTCGCAGGACGGCG	AGGCCCG	N
7*Chr1_108_ISBma2_A		N	NCTC_10247	7	1518121-1519693	Chr1	ISBma2	CGTGG	CAGATTGCTGACAAAC	ATGCTGAAGACGCCCATG	TCCCAGAAAGACAAAACC	GGGGCGC	GGGGCGCTTCGGCGCC	GGGGCGC	Y
2*Chr1_104_ISBma2_A		N	fmh	2	1537752-1539324	Chr1	ISBma2	TACCC	CAGATTGCTGACAAAC	ATGCTGAAGACGCCCATG	TCCCAGAAAGACAAAACC	ACCGGCG	ACCGGCGCATTCGCG	ACCGGCG	Y
7*Chr1_110_ISBma2_A		N	NCTC_10247	7	1541726-1543298	Chr1	ISBma2	TCCCG	CAGATTGCTGACAAAC	ATGCTGAAGACGCCCATG	TCCCAGAAAGACAAAACC	AAGCCCT	GCCCTGCGAGTGCAGG	AAGCCCT	N
3*Chr1_104_ISBma2_A		N	JHU	3	1548943-1550515	Chr1	ISBma2	TACCC	CAGATTGCTGACAAAC	ATGCTGAAGACGCCCATG	TCCCAGAAAGACAAAACC	ACCGGCG	ACCGGCGCATTCGCG	ACCGGCG	Y
4*Chr1_100_ISBma2_B		N	gb8_horse_4	4	1548991-1550563	Chr1	ISBma2	GCGCG	CAGATTGCTGACAAAC	ATGCTGAAGACGCCCATG	TCCCAGAAAGACAAAACC	AATGGGC	AATGGGCCGGAAACG	AATGGGC	Y
8*Chr1_104_ISBma2_A		N	2002721280	8	1551398-1552969	Chr1	ISBma2	TACCC	CAGATTGCTGACAAAC	ATGCTGAAGACGCCCATG	TCCCAGAAAGACAAAACC	ACCGGCG	ACCGGCGCATTCGCG	ACCGGCG	Y
4*Chr1_101_ISBma	4*Chr1_102_ISBma2_A	D	gb8_horse_4	4	1552004-1553576	Chr1	ISBma2	GCATC	CAGATTGCTGACAAAC	ATGCTGAAGACGCCCATG	TCCCAGAAAGACAAAACC	GGGCCGT	AGGCCCGCCGAAGCG	GGGCCGT	N
5*Chr1_19a_ISBma2_A		N	ATCC_10399	5	155357-156927	Chr1	ISBma2	CACCC	ACGATTGCTGACAAAC	ATGCTGAAGACGCCCATG	TCCCAGAAAGACAAAACC	AGCGGAG	GGGCGTCGCCCCTCCG	AGCGGAG	N
1*Chr1_100_ISBma2_B		N	ATCC_23344	1	1558411-1559983	Chr1	ISBma2	GCGCG	CAGATTGCTGACAAAC	ATGCTGAAGACGCCCATG	TCCCAGAAAGACAAAACC	AATGGGC	AATGGGCCGGAAACG	AATGGGC	Y
1*Chr1_101_ISBma	1*Chr1_102_ISBma2_A	D	ATCC_23344	1	1561424-1562996	Chr1	ISBma2	GCATC	CAGATTGCTGACAAAC	ATGCTGAAGACGCCCATG	TCCCAGAAAGACAAAACC	GGGCCGT	AGGCCCGCCGAAGCG	GGGCCGT	N
7*Chr1_52_ISBma2_A		N	NCTC_10247	7	1590531-1592103	Chr1	ISBma2	CCGGA	CAGATTGCTGACAAAC	ATGCTGAAGACGCCCATG	TCCCAGAAAGACAAAACC	ACGGCCG	ACGGCCGCGCTCACGC	ACGGCCG	Y
6*Chr1_163_ISBma2_A		N	NCTC_10229	6	1592743-1594343	Chr1	ISBma2	CGCTT	CAGATTGCTGACAAAC	ATGCTGAAGACGCCCATG	TCCCAGAAAGACAAAACC	TGCTCGC	TGCTCGCGAAGGCGAG	TGCTCGC	Y
10*Chr1_104_ISBma2_A		N	PRL20	10	1594486-1596058	Chr1	ISBma2	TACCC	CAGATTGCTGACAAAC	ATGCTGAAGACGCCCATG	TCCCAGAAAGACAAAACC	ACCGGCG	ACCGGCGCATTCGCG	ACCGGCG	Y
6*Chr1_164_ISBma2_B		N	NCTC_10229	6	1625978-1627550	Chr1	ISBma2	ATGCC	CAGATTGCTGACAAAC	ATGCTGAAGACGCCCATG	TCCCAGAAAGACAAAACC	AGGCAGC	AGGCAGCCCGAGGGG	AGGCAGC	Y
9*Chr1_25a_ISBma	9*Chr1_28a_ISBma2_A	R	SAVP1	9	163290-164556	Chr1	ISBma2	TCGAA	CAGATTGCTGACAAAC	ATGCTGAAGACGCCCATG	#	#IS407A	CCCGCGCGTCGCGCGG	#IS407A	N.D.
4*Chr1_104_ISBma2_A		N	gb8_horse_4	4	1639670-1641242	Chr1	ISBma2	TACCC	CAGATTGCTGACAAAC	ATGCTGAAGACGCCCATG	TCCCAGAAAGACAAAACC	ACCGGCG	ACCGGCGCATTCGCG	ACCGGCG	Y
7*Chr1_50_ISBma2_A		N	NCTC_10247	7	1645048-1646622	Chr1	ISBma2	GCGAC	CAGATTGCTGACAAAC	ATGCTGAAGACGCCCATG	TCCCAGAAAGACAAAACC	GGGGCGC	GGGGCGCGACGTACTC	GGGGCGC	Y
1*Chr1_104_ISBma2_A		N	ATCC_23344	1	1649393-1650965	Chr1	ISBma2	TACCC	CAGATTGCTGACAAAC	ATGCTGAAGACGCCCATG	TCCCAGAAAGACAAAACC	ACCGGCG	ACCGGCGCATTCGCG	ACCGGCG	Y
6*Chr1_1a_ISBma2	6*Chr1_159b_ISBma2_A	R	NCTC_10229	6	1674101-1675677	Chr1	ISBma2	AGCTG	CAGATTGCTGACAAAC	ATGCTGAAGACGCCCATG	TCCCAGAAAGACAAAACC	GGGCCGC	CCCGCTGGTTTCAGCG	GGGCCGC	N
2*Chr1_105a_ISBm	2*Chr1_107a_ISBma2_A	R	fmh	2	1677003-1678575	Chr1	ISBma2	TCGGG	CAGATTGCTGACAAAC	ATGCTGAAGACGCCCATG	TCCCAGAAAGACAAAACC	AAGCCCC	CCCGCTGGTTTCAGCG	AAGCCCC	N
2*Chr1_105b_ISBm	2*Chr1_107b_ISBma2_A	R	fmh	2	1681054-1682626	Chr1	ISBma2	AACGA	CAGATTGCTGACAAAC	ATGCTGAAGACGCCCATG	TCCCAGAAAGACAAAACC	AAGCCCC	CCCGCTGGTTTCAGCG	AAGCCCC	N
3*Chr1_105a_ISBm	3*Chr1_107a_ISBma2_A	R	JHU	3	1688841-1690413	Chr1	ISBma2	TCGGG	CAGATTGCTGACAAAC	ATGCTGAAGACGCCCATG	TCCCAGAAAGACAAAACC	AAGCCCC	CCCGCTGGTTTCAGCG	AAGCCCC	N
8*Chr1_105a_ISBm	8*Chr1_107a_ISBma2_A	R	2002721280	8	1688922-1690494	Chr1	ISBma2	TCGGG	CAGATTGCTGACAAAC	ATGCTGAAGACGCCCATG	TCCCAGAAAGACAAAACC	AAGCCCC	CCCGCTGGTTTCAGCG	AAGCCCC	N
3*Chr1_105b_ISBm	3*Chr1_107b_ISBma2_A	R	JHU	3	1692892-1694464	Chr1	ISBma2	AACGA	CAGATTGCTGACAAAC	ATGCTGAAGACGCCCATG	TCCCAGAAAGACAAAACC	AAGCCCC	CCCGCTGGTTTCAGCG	AAGCCCC	N
8*Chr1_105b_ISBm	8*Chr1_107b_ISBma2_A	R	2002721280	8	1692973-1694545	Chr1	ISBma2	AACGA	CAGATTGCTGACAAAC	ATGCTGAAGACGCCCATG	TCCCAGAAAGACAAAACC	AAGCCCC	CCCGCTGGTTTCAGCG	AAGCCCC	N
5*Chr1_100_ISBma2_B		N	ATCC_10399	5	1712081-1713653	Chr1	ISBma2	GCGCG	CAGATTGCTGACAAAC	ATGCTGAAGACGCCCATG	TCCCAGAAAGACAAAACC	AATGGGC	AATGGGCCGGAAACG	AATGGGC	Y
2*Chr1_108_ISBma2_A		N	fmh	2	1713107-1714679	Chr1	ISBma2	GCGTG	CAGATTGCTGACAAAC	ATGCTGAAGACGCCCATG	TCCCAGAAAGACAAAACC	GGGGCGC	GGGGCGCTTCGGCGCC	GGGGCGC	Y
5*Chr1_101_ISBma	5*Chr1_102_ISBma2_A	D	ATCC_10399	5	1715094-1716666	Chr1	ISBma2	GCATC	CAGATTGCTGACAAAC	ATGCTGAAGACGCCCATG	TCCCAGAAAGACAAAACC	GGGCCGT	AGGCCCGCCGAAGCG	GGGCCGT	N
2*Chr1_109_ISBma2_B		N	fmh	2	1717544-1719116	Chr1	ISBma2	AGGCA	CAGATTGCTGACAAAC	ATGCTGAAGACGCCCATG	TCCCAGAAAGACAAAACC	GGGACGG	GGGACGGCGGGACGC	GGGACGG	Y
8*Chr1_108_ISBma2_A		N	2002721280	8	1724386-1725958	Chr1	ISBma2	CGTGG	CAGATTGCTGACAAAC	ATGCTGAAGACGCCCATG	TCCCAGAAAGACAAAACC	GGGGCGC	GGGGCGCTTCGGCGCC	GGGGCGC	Y
3*Chr1_108_ISBma2_A		N	JHU	3	1725497-1727069	Chr1	ISBma2	GCGTG	CAGATTGCTGACAAAC	ATGCTGAAGACGCCCATG	TCCCAGAAAGACAAAACC	GGGGCGC	GGGGCGCTTCGGCGCC	GGGGCGC	Y
3*Chr1_109_ISBma2_B		N	JHU	3	1729934-1731506	Chr1	ISBma2	AGGCA	CAGATTGCTGACAAAC	ATGCTGAAGACGCCCATG	TCCCAGAAAGACAAAACC	GGGACGG	GGGACGGCGGGACGC	GGGACGG	Y

10*Chr1_105a_ISB	10*Chr1_107a_ISBma2_A	R	PRL20	10	1731413-1732985	Chr1	ISBma2	TCGGG	CAGATTGCTGACAAAC	ATGCTGAAGACGCCCATG	TCCCAGAAAGACAAAACC	GCGCTCC	CCCGCTGGTTTCAGCG	GCGCTCC	N
9*Chr1_85_ISBma2_A		N	SAVP1	9	1732152-1733022	Chr1	ISBma2	#IS407	~~~~~~~~~~~~~~~	~~~~~~~~~~~~~~~~~~	TCCCAGAAAGACAAAACC	AGCCAGC	#IS407A	AGCCAGC	N.D.
9*Chr1_85-1_ISBma2_A		N	SAVP1	9	1733431-1734964	Chr1	ISBma2	GCCTC	CAGATTGCTGACAAAC	ATGCTGAAGACGCCCATG	ACGCTGCGAGAAAGCCCG	#IS407A	AGCCAGCTTGCGCTGG	#IS407A	N.D.
10*Chr1_106_ISBma2_C		N	PRL20	10	1736522-1738094	Chr1	ISBma2	TCAGC	CAGATTGCTGACAAAC	ATGCTGAAGACGCCCATG	TCCCAGAAAGACAAAACC	AAGCCCC	GCCCCGTCAATACGGG	AAGCCCC	N
2*Chr1_110_ISBma2_A		N	fmh	2	1738590-1740041	Chr1	ISBma2	#IS407	~~~~~~~~~~~~~~~	~~~~~~~~~~~~~~~~~~	TCCCAGAAAGACAAAACC	AAGCCCT	#IS407A	AAGCCCT	N.D.
10*Chr1_105b_ISB	10*Chr1_107b_ISBma2_A	R	PRL20	10	1740573-1742147	Chr1	ISBma2	AACGA	CAGATTGCTGACAAAC	ATGCTGAAGACGCCCATG	TCCCAGAAAGACAAAACC	AAGCCCC	CCCGCTGGTTTCAGCG	AAGCCCC	N
8*Chr1_110_ISBma2_A		N	2002721280	8	1747635-1749207	Chr1	ISBma2	TCCCG	CAGATTGCTGACAAAC	ATGCTGAAGACGCCCATG	TCCCAGAAAGACAAAACC	AAGCCCT	GCCCTGCGAGTGCAGG	AAGCCCT	N
3*Chr1_110_ISBma2_A		N	JHU	3	1750695-1752146	Chr1	ISBma2	#IS407	~~~~~~~~~~~~~~~	~~~~~~~~~~~~~~~~~~	TCCCAGAAAGACAAAACC	AAGCCCT	#IS407A	AAGCCCT	N.D.
10*Chr1_108_ISBma2_A		N	PRL20	10	1771866-1773437	Chr1	ISBma2	CGTGG	CAGATTGCTGACAAAC	ATGCTGAAGACGCCCATG	TCCCAGAAAGACAAAACC	GGGGCGC	GGGGCGCTTCGGCGCC	GGGGCGC	Y
10*Chr1_109_ISBma2_B		N	PRL20	10	1776302-1777874	Chr1	ISBma2	AGGCA	CAGATTGCTGACAAAC	ATGCTGAAGACGCCCATG	TCCCAGAAAGACAAAACC	GGACGGC	GGACGGCGGGACGCC	GGACGGC	Y
4*Chr1_105a_ISBm	4*Chr1_107a_ISBma2_A	R	gb8_horse_4	4	1776616-1778188	Chr1	ISBma2	TCGGG	CAGATTGCTGACAAAC	ATGCTGAAGACGCCCATG	TCCCAGAAAGACAAAACC	AAGCCCC	CCCGCTGGTTTCAGCG	AAGCCCC	N
4*Chr1_13a_ISBma	4*Chr1_32b_ISBma2_A	R	gb8_horse_4	4	177854-179426	Chr1	ISBma2	GCGAC	CAGATTGCTGACAAAC	ATGCTGAAGACGCCCATG	TCCCAGAAAGACAAAACC	GAGCGGC	GACCGCGTGGGCGGTC	GAGCGGC	N
4*Chr1_105b_ISBm	4*Chr1_107b_ISBma2_A	R	gb8_horse_4	4	1780667-1782239	Chr1	ISBma2	AACGA	CAGATTGCTGACAAAC	ATGCTGAAGACGCCCATG	TCCCAGAAAGACAAAACC	AAGCCCC	CCCGCTGGTTTCAGCG	AAGCCCC	N
1*Chr1_105a_ISBm	1*Chr1_107a_ISBma2_A	R	ATCC_23344	1	1786992-1788564	Chr1	ISBma2	TCGGG	CAGATTGCTGACAAAC	ATGCTGAAGACGCCCATG	TCCCAGAAAGACAAAACC	AAGCCCC	CCCGCTGGTTTCAGCG	AAGCCCC	N
1*Chr1_13a_ISBma	1*Chr1_32b_ISBma2_A	R	ATCC_23344	1	178751-180323	Chr1	ISBma2	GCGAC	CAGATTGCTGACAAAC	ATGCTGAAGACGCCCATG	TCCCAGAAAGACAAAACC	GAGCGGC	GACCGCGTGGGCGGTC	GAGCGGC	N
6*Chr1_148_ISBma	6*Chr1_159a_ISBma2_A	D-R	NCTC_10229	6	1787702-1789301	Chr1	ISBma2	GGATT	CAGATTGCTGACAAAC	ATGCTGAAGACGCCCATG	TCCCAGAAAGACAAAACC	AGGCCCC	AGGGCCGCATGAAGCG	AGGCCCC	N
1*Chr1_105b_ISBm	1*Chr1_107b_ISBma2_A	R	ATCC_23344	1	1791043-1792615	Chr1	ISBma2	AACGA	CAGATTGCTGACAAAC	ATGCTGAAGACGCCCATG	TCCCAGAAAGACAAAACC	AAGCCCC	CCCGCTGGTTTCAGCG	AAGCCCC	N
6*Chr1_151a_ISBma2_A		R	NCTC_10229	6	1796424-1797996	Chr1	ISBma2	ATTTC	CAGATTGCTGACAAAC	ATGCTGAAGACGCCCATG	TCCCAGAAAGACAAAACC	AACCCGC	GCCCCGCTTCGGCGGG	AACCCGC	N
8*Chr1_52_ISBma2_A		N	2002721280	8	1796447-1798019	Chr1	ISBma2	CCGGA	CAGATTGCTGACAAAC	ATGCTGAAGACGCCCATG	TCCCAGAAAGACAAAACC	ACGGCG	ACGGCGCGCTCACGC	ACGGCG	Y
10*Chr1_110_ISBma2_A		N	PRL20	10	1797097-1798548	Chr1	ISBma2	#IS407	~~~~~~~~~~~~~~~	~~~~~~~~~~~~~~~~~~	TCCCAGAAAGACAAAACC	AAGCCCT	#IS407A	AAGCCCT	N.D.
5*Chr1_104_ISBma2_A		N	ATCC_10399	5	1803108-1804680	Chr1	ISBma2	TACCC	CAGATTGCTGACAAAC	ATGCTGAAGACGCCCATG	TCCCAGAAAGACAAAACC	ACCGGCG	ACCGGCGCATTCGCG	ACCGGCG	Y
4*Chr1_108_ISBma2_A		N	gb8_horse_4	4	1812072-1813644	Chr1	ISBma2	GCGTG	CAGATTGCTGACAAAC	ATGCTGAAGACGCCCATG	TCCCAGAAAGACAAAACC	GGGGCGC	GGGGCGCTTCGGCGCC	GGGGCGC	Y
4*Chr1_109_ISBma2_B		N	gb8_horse_4	4	1816509-1818081	Chr1	ISBma2	AGGCA	CAGATTGCTGACAAAC	ATGCTGAAGACGCCCATG	TCCCAGAAAGACAAAACC	GGGACGG	GGGACGGCGGGACGC	GGGACGG	Y
1*Chr1_108_ISBma2_A		N	ATCC_23344	1	1822448-1824020	Chr1	ISBma2	GCGTG	CAGATTGCTGACAAAC	ATGCTGAAGACGCCCATG	TCCCAGAAAGACAAAACC	GGGGCGC	GGGGCGCTTCGGCGCC	GGGGCGC	Y
1*Chr1_109_ISBma2_B		N	ATCC_23344	1	1826885-1828457	Chr1	ISBma2	AGGCA	CAGATTGCTGACAAAC	ATGCTGAAGACGCCCATG	TCCCAGAAAGACAAAACC	GGGACGG	GGGACGGCGGGACGC	GGGACGG	Y
7*Chr1_121_ISBma2_A		N	NCTC_10247	7	183581-183721	Chr1	ISBma2	#IS407	~~~~~~~~~~~~~~~	~~~~~~~~~~~~~~~~~~	TCCCAGAAAGACAAAACC	AAGCCCTT	#IS407A	AAGCCCTT	N.D.
4*Chr1_110_ISBma2_A		N	gb8_horse_4	4	1837077-1838528	Chr1	ISBma2	#IS407	~~~~~~~~~~~~~~~	~~~~~~~~~~~~~~~~~~	TCCCAGAAAGACAAAACC	AAGCCCT	#IS407A	AAGCCCT	N.D.
1*Chr1_110_ISBma2_A		N	ATCC_23344	1	1847646-1849097	Chr1	ISBma2	#IS407	~~~~~~~~~~~~~~~	~~~~~~~~~~~~~~~~~~	TCCCAGAAAGACAAAACC	AAGCCCT	#IS407A	AAGCCCT	N.D.
8*Chr1_50_ISBma2_A		N	2002721280	8	1850947-1852519	Chr1	ISBma2	GCGAC	CAGATTGCTGACAAAC	ATGCTGAAGACGCCCATG	TCCCAGAAAGACAAAACC	GGGGCGC	GGGGCGCGACGTACTC	GGGGCGC	Y
6*Chr1_1b_ISBma2	6*Chr1_151b_ISBma2_A	R	NCTC_10229	6	1930938-1932510	Chr1	ISBma2	AGCCG	CAGATTGCTGACAAAC	ATGCTGAAGACGCCCATG	TCCCAGAAAGACAAAACC	AACCCGC	AAGCGGCCTTTCGGCC	AACCCGC	N
9*Chr1_24_ISBma2_A		N	SAVP1	9	193441-195012	Chr1	ISBma2	GGCAA	CAGATTGCTGACAAAC	ATGCTGAAGACGCCCATG	TCCCAGAAAGACAAAACC	AGCCCGC	AACCCGCCTCGGAGGC	AGCCCGC	N
5*Chr1_105a_ISBm	5*Chr1_107a_ISBma2_A	R	ATCC_10399	5	1940356-1941928	Chr1	ISBma2	TCGGG	CAGATTGCTGACAAAC	ATGCTGAAGACGCCCATG	TCCCAGAAAGACAAAACC	AAGCCCC	CCCGCTGGTTTCAGCG	AAGCCCC	N
5*Chr1_105b_ISBm	5*Chr1_107b_ISBma2_A	R	ATCC_10399	5	1944407-1945979	Chr1	ISBma2	AACGA	CAGATTGCTGACAAAC	ATGCTGAAGACGCCCATG	TCCCAGAAAGACAAAACC	AAGCCCC	CCCGCTGGTTTCAGCG	AAGCCCC	N
5*Chr1_108_ISBma2_A		N	ATCC_10399	5	1975805-1977377	Chr1	ISBma2	CGTGG	CAGATTGCTGACAAAC	ATGCTGAAGACGCCCATG	TCCCAGAAAGACAAAACC	GGGGCGC	GGGGCGCTTCGGCGCC	GGGGCGC	Y
9*Chr1_101_ISBma	9*Chr1_102_ISBma2_A	D	SAVP1	9	1977656-1979228	Chr1	ISBma2	GCATC	CAGATTGCTGACAAAC	ATGCTGAAGACGCCCATG	TCCCAGAAAGACAAAACC	GGGCCGT	AGGCCCGCCGAAGCG	GGGCCGT	N
7*Chr1_120_ISBma2_A		N	NCTC_10247	7	198007-199579	Chr1	ISBma2	AGCCA	CAGATTGCTGACAAAC	ATGCTGAAGACGCCCATG	TCCCAGAAAGACAAAACC	GGCGCCC	GGCGCCCCGCAAGGG	GGCGCCC	Y
6*Chr1_90_ISBma2_A		N	NCTC_10229	6	199692-200879	Chr1	ISBma2	#IS407	~~~~~~~~~~~~~~~	~~~~~~~~~~~~~~~~~~	TCCCAGAAAGACAAAACC	AAAGCCC	#IS407A	AAAGCCC	N.D.
7*Chr1_130_ISBma2_B		N	NCTC_10247	7	1998101-1999674	Chr1	ISBma2	GTCCA	CAGATTGCTGACAAAC	ATGCTGAAGACGCCCATG	TCCCAGAAAGACAAAACC	AGGCGAT	AGGCGATGCGCGCAGC	AGGCGAT	Y
5*Chr1_110_ISBma2_A		N	ATCC_10399	5	1999400-2000851	Chr1	ISBma2	#IS407	~~~~~~~~~~~~~~~	~~~~~~~~~~~~~~~~~~	TCCCAGAAAGACAAAACC	AAGCCCT	#IS407A	AAGCCCT	N.D.
6*Chr1_155_ISBma2_B		N	NCTC_10229	6	2001853-2003425	Chr1	ISBma2	CCTTC	CAGATTGCTGACAAAC	ATGCTGAAGACGCCCATG	TCCCAGAAAGACAAAACC	GGCTGGA	GGCTGGAATCCCGGGA	GGCTGGA	Y
6*Chr1_22a_ISBma2_A		R	NCTC_10229	6	2020996-2022558	Chr1	ISBma2	~~~~~	AGGGGGCAGGTCAAA	ATGCTGAAGACGCCCATG	TCCCAGAAAGACAAAACC	AGCCCGC	AGCCCATTGGCCCTATC	AGCCCGC	N
7*Chr1_46a_ISBma2_A		N	NCTC_10247	7	2062315-2063887	Chr1	ISBma2	GGAAA	CAGATTGCTGACAAAC	ATGCTGAAGACGCCCATG	TCCCAGAAAGACAAAACC	AAAAGCG	AAAAGCGCGCCCTAGG	AAAAGCG	Y
9*Chr1_104_ISBma2_A		N	SAVP1	9	2065693-2067265	Chr1	ISBma2	TACCC	CAGATTGCTGACAAAC	ATGCTGAAGACGCCCATG	TCCCAGAAAGACAAAACC	ACCGGCG	ACCGGCGCATTCGCG	ACCGGCG	Y
6*Chr1_19a_ISBma2_A		N	NCTC_10229	6	2089532-2091106	Chr1	ISBma2	GCACG	CAGATTGCTGACAAAC	ATGCTGAAGACGCCCATG	TCCCAGAAAGACAAAACC	AGCGGAG	AGCGGAGGGCGTCGCC	AGCGGAG	Y
7*Chr1_41_ISBma2_A		N	NCTC_10247	7	2106381-2107270	Chr1	ISBma2	AATCA	CAGATTGCTGACAAAC	ATGCTGAAGACGCCCATG	#	#IS407A	CCCTCCACTGGAGGGC	#IS407A	N.D.
7*Chr1_39b_ISBma	7*Chr1_44b_ISBma2_A	R	NCTC_10247	7	2113105-2114677	Chr1	ISBma2	GCAGG	CAGATTGCTGACAAAC	ATGCTGAAGACGCCCATG	TCCCAGAAAGACAAAACC	AAAAACC	CCGCCCGCTCCGGGCG	AAAAACC	N
9*Chr1_148_ISBma	9*Chr1_159a_ISBma2_A	D-R	SAVP1	9	21273-22845	Chr1	ISBma2	GGATT	CAGATTGCTGACAAAC	ATGCTGAAGACGCCCATG	TCCCAGAAAGACAAAACC	AGGCCCC	AGGGCCGCATGAAGCG	AGGCCCC	N
10*Chr1_128_ISBma2_C		N	PRL20	10	2152775-2154347	Chr1	ISBma2	CCCCT	CAGATTGCTGACAAAC	ATGCTGAAGACGCCCATG	TCCCAGAAAGACAAAACC	AAGCGCC	AAGCGCCCCGCTCGGG	AAGCGCC	Y
5*Chr1_32a_ISBma2_A		N	ATCC_10399	5	215734-217306	Chr1	ISBma2	TGTGT	CAGATTGCTGACAAAC	ATGCTGAAGACGCCCATG	TCCCAGAAAGACAAAACC	GAGCGGC	AGAGCGGCCCGAAGG	GAGCGGC	N
7*Chr1_19b_ISBma	7*Chr1_139a_ISBma2_A	R	NCTC_10247	7	2193940-2195512	Chr1	ISBma2	GCCCT	CAGATTGCTGACAAAC	ATGCTGAAGACGCCCATG	TCCCAGAAAGACAAAACC	AGCGGAG	AAGCCCGGCGCATGGC	AGCGGAG	N
10*Chr1_13a_ISBm	10*Chr1_32b_ISBma2_A	R	PRL20	10	219786-221358	Chr1	ISBma2	GCGAC	CAGATTGCTGACAAAC	ATGCTGAAGACGCCCATG	TCCCAGAAAGACAAAACC	GAGCGGC	GACCGCGTGGGCGGTC	GAGCGGC	N
9*Chr1_105a_ISBm	9*Chr1_107a_ISBma2_A	R	SAVP1	9	2203072-2204644	Chr1	ISBma2	TCGGG	CAGATTGCTGACAAAC	ATGCTGAAGACGCCCATG	TCCCAGAAAGACAAAACC	AAGCCCC	CCCGCTGGTTTCAGCG	AAGCCCC	N
8*Chr1_130_ISBma2_B		N	2002721280	8	2203726-2205299	Chr1	ISBma2	GTCCA	CAGATTGCTGACAAAC	ATGCTGAAGACGCCCATG	TCCCAGAAAGACAAAACC	AGGCGAT	AGGCGATGCGCGCAGC	AGGCGAT	Y
2*Chr1_46a_ISBma2_A		N	fmh	2	2204119-2205691	Chr1	ISBma2	AATAC	CAGATTGCTGACAAAC	ATGCTGAAGACGCCCATG	TCCCAGAAAGACAAAACC	AAAAGCG	AAAAGCGCGCCCTAGG	AAAAGCG	Y
9*Chr1_105b_ISBm	9*Chr1_107b_ISBma2_A	R	SAVP1	9	2207123-2208695	Chr1	ISBma2	AACGA	CAGATTGCTGACAAAC	ATGCTGAAGACGCCCATG	TCCCAGAAAGACAAAACC	AAGCCCC	CCCGCTGGTTTCAGCG	AAGCCCC	N
6*Chr1_13b_ISBma	6*Chr1_32a_ISBma2_A	R	NCTC_10229	6	2207258-2208830	Chr1	ISBma2	TGTGT	CAGATTGCTGACAAAC	ATGCTGAAGACGCCCATG	TCCCAGAAAGACAAAACC	AACGGAC	AGAGCGGCCCGAAGG	AACGGAC	N
9*Chr1_23a_ISBma2_A		N	SAVP1	9	221439-223010	Chr1	ISBma2	CGCGC	CAGATTGCTGACAAAC	ATGCTGAAGACGCCCATG	TCCCAGAAAGACAAAACC	ACCGCAG	ACCGCAGCGAAGGCTG	ACCGCAG	Y
3*Chr1_46a_ISBma2_A		N	JHU	3	2219579-2221151	Chr1	ISBma2	AATAC	CAGATTGCTGACAAAC	ATGCTGAAGACGCCCATG	TCCCAGAAAGACAAAACC	AAAAGCG	AAAAGCGCGCCCTAGG	AAAAGCG	Y

9*Chr1_108_ISBma2_A		N	SAVP1	9	2238551-2240122	Chr1	ISBma2	CGTGG	CAGATTGCTGACAAAC	ATGCTGAAGACGCCCATG	TCCCAGAAAGACAAAACC	GGGGCGC	GGGGCGCTTCGGCGCC	GGGGCGC	Y
9*Chr1_109_ISBma2_B		N	SAVP1	9	2242987-2244559	Chr1	ISBma2	AGGCA	CAGATTGCTGACAAAC	ATGCTGAAGACGCCCATG	TCCCAGAAAGACAAAACC	GGACGGC	GGACGGCGGGACGCC	GGACGGC	Y
2*Chr1_41_ISBma2_A		N	fmh	2	2248201-2249090	Chr1	ISBma2	AATCA	CAGATTGCTGACAAAC	ATGCTGAAGACGCCCATG	#	#IS407A	CCCTCCACTGGAGGGC	#IS407A	N.D.
2*Chr1_39b_ISBma	2*Chr1_44b_ISBma2_A	R	fmh	2	2254925-2256496	Chr1	ISBma2	GCAGG	CAGATTGCTGACAAAC	ATGCTGAAGACGCCCATG	TCCCAGAAAGACAAAACC	AAAAACC	CCGCCCGCTCCGGGCG	AAAAACC	N
7*Chr1_22a_ISBma2_A		R	NCTC_10247	7	2262477-2264039	Chr1	ISBma2	~~~~~	AGGGGGCAGGTCAAA	ATGCTGAAGACGCCCATG	TCCCAGAAAGACAAAACC	AGCCCGC	AGCCCATTGGCCCTAT	AGCCCGC	N
3*Chr1_41_ISBma2_A		N	JHU	3	2263558-2264447	Chr1	ISBma2	AATCA	CAGATTGCTGACAAAC	ATGCTGAAGACGCCCATG	#	#IS407A	CCCTCCACTGGAGGGC	#IS407A	N.D.
9*Chr1_110_ISBma2_A		N	SAVP1	9	2263757-2265208	Chr1	ISBma2	#IS407	~~~~~~~~~~~~~~~	~~~~~~~~~~~~~~~~~~	TCCCAGAAAGACAAAACC	AAGCCCT	#IS407A	AAGCCCT	N
8*Chr1_46a_ISBma2_A		N	2002721280	8	2267980-2269552	Chr1	ISBma2	GGAAA	CAGATTGCTGACAAAC	ATGCTGAAGACGCCCATG	TCCCAGAAAGACAAAACC	AAAAGCG	AAAAGCGCGCCCTAGG	AAAAGCG	Y
3*Chr1_39b_ISBma	3*Chr1_44b_ISBma2_A	R	JHU	3	2270282-2271853	Chr1	ISBma2	GCAGG	CAGATTGCTGACAAAC	ATGCTGAAGACGCCCATG	TCCCAGAAAGACAAAACC	AAAAACC	CCGCCCGCTCCGGGCG	AAAAACC	N
9*Chr1_52_ISBma2_A		N	SAVP1	9	2285906-2287478	Chr1	ISBma2	CCGGA	CAGATTGCTGACAAAC	ATGCTGAAGACGCCCATG	TCCCAGAAAGACAAAACC	ACGGCCG	ACGGCCGCGCTCACGC	ACGGCCG	Y
10*Chr1_39a_ISBm	10*Chr1_44a_ISBma2_A	R	PRL20	10	2287844-2289416	Chr1	ISBma2	ACGGC	CAGATTGCTGACAAAC	ATGCTGAAGACGCCCATG	TCCCAGAAAGACAAAACC	AGCCCTC	AACCCGGCTGGAAGCC	AGCCCTC	N
4*Chr1_46a_ISBma2_A		N	gb8_horse_4	4	2299455-2301027	Chr1	ISBma2	AATAC	CAGATTGCTGACAAAC	ATGCTGAAGACGCCCATG	TCCCAGAAAGACAAAACC	AAAAGCG	AAAAGCGCGCCCTAGG	AAAAGCG	Y
1*Chr1_46a_ISBma2_A		N	ATCC_23344	1	2311360-2312932	Chr1	ISBma2	AATAC	CAGATTGCTGACAAAC	ATGCTGAAGACGCCCATG	TCCCAGAAAGACAAAACC	AAAAGCG	AAAAGCGCGCCCTAGG	AAAAGCG	Y
8*Chr1_41_ISBma2_A		N	2002721280	8	2311719-2312608	Chr1	ISBma2	AATCA	CAGATTGCTGACAAAC	ATGCTGAAGACGCCCATG	#	#IS407A	CCCTCCACTGGAGGGC	#IS407A	N.D.
10*Chr1_41_ISBma2_A		N	PRL20	10	2315851-2316740	Chr1	ISBma2	AATCA	CAGATTGCTGACAAAC	ATGCTGAAGACGCCCATG	#	#IS407A	CCCTCCACTGGAGGGC	#IS407A	N.D.
8*Chr1_39b_ISBma	8*Chr1_44b_ISBma2_A	R	2002721280	8	2318443-2320015	Chr1	ISBma2	GCAGG	CAGATTGCTGACAAAC	ATGCTGAAGACGCCCATG	TCCCAGAAAGACAAAACC	AAAAACC	CCGCCCGCTCCGGGCG	AAAAACC	N
6*Chr1_13a_ISBma	6*Chr1_32b_ISBma2_A	R	NCTC_10229	6	2330494-2332086	Chr1	ISBma2	GCGAC	CAGATTGCTGACAAAC	ATGCTGAAGACGCCCATG	TCCCAGAAAGACAAAACC	GAGCGGC	GACCGCGTGGGCGGTC	GAGCGGC	N
2*Chr1_139a_ISBm	2*Chr1_141a_ISBma2_A	R	fmh	2	2335759-2337331	Chr1	ISBma2	GCCCT	CAGATTGCTGACAAAC	ATGCTGAAGACGCCCATG	TCCCAGAAAGACAAAACC	AGCCCGA	CCGGCGCATGGCGCCG	AGCCCGA	N
7*Chr1_13a_ISBma	7*Chr1_32b_ISBma2_A	R	NCTC_10247	7	2338546-2340123	Chr1	ISBma2	GCGAC	CAGATTGCTGACAAAC	ATGCTGAAGACGCCCATG	TCCCAGAAAGACAAAACC	GAGCGGC	GACCGCGTGGGCGGTC	GAGCGGC	N
9*Chr1_50_ISBma2_A		N	SAVP1	9	2340294-2341866	Chr1	ISBma2	AGCGA	CAGATTGCTGACAAAC	ATGCTGAAGACGCCCATG	TCCCAGAAAGACAAAACC	GGGGCGC	GGGGCGCGACGTACTC	GGGGCGC	Y
4*Chr1_41_ISBma2_A		N	gb8_horse_4	4	2343434-2344323	Chr1	ISBma2	AATCA	CAGATTGCTGACAAAC	ATGCTGAAGACGCCCATG	#	#IS407A	CCCTCCACTGGAGGGC	#IS407A	N.D.
10*Chr1_39b_ISBm	10*Chr1_44b_ISBma2_A	R	PRL20	10	2344825-2346397	Chr1	ISBma2	GCAGG	CAGATTGCTGACAAAC	ATGCTGAAGACGCCCATG	TCCCAGAAAGACAAAACC	AAAAACC	CCGCCCGCTCCGGGCG	AAAAACC	N
7*Chr1_118_ISBma2_A		N	NCTC_10247	7	234566-236138	Chr1	ISBma2	CGATC	CAGATTGCTGACAAAC	ATGCTGAAGACGCCCATG	TCCCAGAAAGACAAAACC	AAGGCGC	AAGGCGCCGCGGCAAC	AAGGCGC	Y
4*Chr1_39b_ISBma	4*Chr1_44b_ISBma2_A	R	gb8_horse_4	4	2350107-2351678	Chr1	ISBma2	GCAGG	CAGATTGCTGACAAAC	ATGCTGAAGACGCCCATG	TCCCAGAAAGACAAAACC	AAAAACC	CCGCCCGCTCCGGGCG	AAAAACC	N
3*Chr1_139a_ISBm	3*Chr1_141a_ISBma2_A	R	JHU	3	2351116-2352688	Chr1	ISBma2	GCCCT	CAGATTGCTGACAAAC	ATGCTGAAGACGCCCATG	TCCCAGAAAGACAAAACC	AGCCCGA	CCGGCGCATGGCGCCG	AGCCCGA	N
1*Chr1_41_ISBma2_A		N	ATCC_23344	1	2355339-2356228	Chr1	ISBma2	AATCA	CAGATTGCTGACAAAC	ATGCTGAAGACGCCCATG	#	#IS407A	CCCTCCACTGGAGGGC	#IS407A	N.D.
2*Chr1_140_ISBma2_B		N	fmh	2	2357628-2359200	Chr1	ISBma2	GCGCA	CAGATTGCTGACAAAC	ATGCTGAAGACGCCCATG	TCCCAGAAAGACAAAACC	ACGCCCC	ACGCCCCGGCCGGGGC	ACGCCCC	Y
1*Chr1_39b_ISBma	1*Chr1_44b_ISBma2_A	R	ATCC_23344	1	2362064-2363635	Chr1	ISBma2	GCAGG	CAGATTGCTGACAAAC	ATGCTGAAGACGCCCATG	TCCCAGAAAGACAAAACC	AAAAACC	CCGCCCGCTCCGGGCG	AAAAACC	N
3*Chr1_140_ISBma2_B		N	JHU	3	2372985-2374557	Chr1	ISBma2	GCGCA	CAGATTGCTGACAAAC	ATGCTGAAGACGCCCATG	TCCCAGAAAGACAAAACC	ACGCCCC	ACGCCCCGGCCGGGGC	ACGCCCC	Y
10*Chr1_46a_ISBma2_A		N	PRL20	10	2378078-2379650	Chr1	ISBma2	AATAC	CAGATTGCTGACAAAC	ATGCTGAAGACGCCCATG	TCCCAGAAAGACAAAACC	AAAAGCG	AAAAGCGCGCCCTAGG	AAAAGCG	Y
8*Chr1_139a_ISBm	8*Chr1_141a_ISBma2_A	R	2002721280	8	2398395-2399967	Chr1	ISBma2	GCCCT	CAGATTGCTGACAAAC	ATGCTGAAGACGCCCATG	TCCCAGAAAGACAAAACC	AGCCCGA	CCGGCGCATGGCGCCG	AGCCCGA	N
2*Chr1_139b_ISBm	2*Chr1_141b_ISBma2_A	R	fmh	2	2418316-2419888	Chr1	ISBma2	CCTCC	CAGATTGCTGACAAAC	ATGCTGAAGACGCCCATG	TCCCAGAAAGACAAAACC	AGCCCGG	CCGAACCAGCGGTTCG	AGCCCGG	N
4*Chr1_139a_ISBm	4*Chr1_141a_ISBma2_A	R	gb8_horse_4	4	2430897-2432469	Chr1	ISBma2	GCCCT	CAGATTGCTGACAAAC	ATGCTGAAGACGCCCATG	TCCCAGAAAGACAAAACC	AGCCCGA	CCGGCGCATGGCGCCG	AGCCCGA	N
3*Chr1_139b_ISBm	3*Chr1_141b_ISBma2_A	R	JHU	3	2434455-2436027	Chr1	ISBma2	CCTCC	CAGATTGCTGACAAAC	ATGCTGAAGACGCCCATG	TCCCAGAAAGACAAAACC	AGCCCGG	CCGAACCAGCGGTTCG	AGCCCGG	N
7*Chr1_117a_ISBma2_A		N	NCTC_10247	7	243623-245215	Chr1	ISBma2	TCGAC	CAGATTGCTGACAAAC	ATGCTGAAGACGCCCATG	TCCCAGAAAGACAAAACC	AACCCGC	CCCGCTGGTTTCAGCG	AACCCGC	N
1*Chr1_139a_ISBm	1*Chr1_141a_ISBma2_A	R	ATCC_23344	1	2442898-2444470	Chr1	ISBma2	GCCCT	CAGATTGCTGACAAAC	ATGCTGAAGACGCCCATG	TCCCAGAAAGACAAAACC	AGCCCGA	CCGGCGCATGGCGCCG	AGCCCGA	N
4*Chr1_140_ISBma2_B		N	gb8_horse_4	4	2452766-2454338	Chr1	ISBma2	GCGCA	CAGATTGCTGACAAAC	ATGCTGAAGACGCCCATG	TCCCAGAAAGACAAAACC	ACGCCCC	ACGCCCCGGCCGGGGC	ACGCCCC	Y
5*Chr1_46a_ISBma2_A		N	ATCC_10399	5	2460834-2462406	Chr1	ISBma2	AATAC	CAGATTGCTGACAAAC	ATGCTGAAGACGCCCATG	TCCCAGAAAGACAAAACC	AAAAGCG	AAAAGCGCGCCCTAGG	AAAAGCG	Y
1*Chr1_140_ISBma2_B		N	ATCC_23344	1	2464767-2466339	Chr1	ISBma2	GCGCA	CAGATTGCTGACAAAC	ATGCTGAAGACGCCCATG	TCCCAGAAAGACAAAACC	ACGCCCC	ACGCCCCGGCCGGGGC	ACGCCCC	Y
8*Chr1_139b_ISBm	8*Chr1_141b_ISBma2_A	R	2002721280	8	2478896-2480470	Chr1	ISBma2	CCTCC	CAGATTGCTGACAAAC	ATGCTGAAGACGCCCATG	TCCCAGAAAGACAAAACC	AGCCCGG	CCGAACCAGCGGTTCG	AGCCCGG	N
10*Chr1_22b_ISBm	10*Chr1_141a_ISBma2_A	R	PRL20	10	2490911-2492483	Chr1	ISBma2	GCCGC	CAGATTGCTGACAAAC	ATGCTGAAGACGCCCATG	TCCCAGAAAGACAAAACC	AGCCCGA	AGCCCGCGAGACGCGC	AGCCCGA	N
8*Chr1_155_ISBma2_B		N	2002721280	8	2494952-2496499	Chr1	ISBma2	~CTTC	CAGATTGCTGACAAAC	ATGCTGAAGACGCCCATG	TCCCAGAAAGACAAAACC	GGCTGGA	GGCTGGAATCCCGGGA	GGCTGGA	Y
7*Chr1_32a_ISBma	7*Chr1_141a_ISBma2_A	R	NCTC_10247	7	2496018-2497590	Chr1	ISBma2	TGTGT	CAGATTGCTGACAAAC	ATGCTGAAGACGCCCATG	TCCCAGAAAGACAAAACC	AGCCCGA	AGAGCGGCCCGAAGGC	AGCCCGA	N
5*Chr1_41_ISBma2_A		N	ATCC_10399	5	2504830-2505719	Chr1	ISBma2	AATCA	CAGATTGCTGACAAAC	ATGCTGAAGACGCCCATG	#	#IS407A	CCCTCCACTGGAGGGC	#IS407A	N.D.
9*Chr1_117a_ISBma2_A		N	SAVP1	9	2505752-2507325	Chr1	ISBma2	TTTCGA	CAGATTGCTGACAAAC	ATGCTGAAGACGCCCATG	TTCCAGAAAGACAAAACC	AACCCGC	AACCCGGCTGGAAGCC	AACCCGC	N
5*Chr1_39b_ISBma	5*Chr1_44b_ISBma2_A	R	ATCC_10399	5	2511431-2513003	Chr1	ISBma2	GCAGG	CAGATTGCTGACAAAC	ATGCTGAAGACGCCCATG	TCCCAGAAAGACAAAACC	AAAAACC	CCGCCCGCTCCGGGCG	AAAAACC	N
4*Chr1_139b_ISBm	4*Chr1_141b_ISBma2_A	R	gb8_horse_4	4	2513056-2514628	Chr1	ISBma2	CCTCC	CAGATTGCTGACAAAC	ATGCTGAAGACGCCCATG	TCCCAGAAAGACAAAACC	AGCCCGG	CCGAACCAGCGGTTCG	AGCCCGG	N
9*Chr1_118_ISBma2_A		N	SAVP1	9	2514844-2516416	Chr1	ISBma2	CGATC	CAGATTGCTGACAAAC	ATGCTGAAGACGCCCATG	TCCCAGAAAGACAAAACC	AAGGCGC	AAGGCGCCGCGGCAAC	AAGGCGC	Y
1*Chr1_139b_ISBm	1*Chr1_141b_ISBma2_A	R	ATCC_23344	1	2525029-2526601	Chr1	ISBma2	CCTCC	CAGATTGCTGACAAAC	ATGCTGAAGACGCCCATG	TCCCAGAAAGACAAAACC	AGCCCGG	CCGAACCAGCGGTTCG	AGCCCGG	N
9*Chr1_22b_ISBma	9*Chr1_141a_ISBma2_A	R	SAVP1	9	254941-256513	Chr1	ISBma2	GCCGC	CAGATTGCTGACAAAC	ATGCTGAAGACGCCCATG	TCCCAGAAAGACAAAACC	AGCCCGA	AGCCCGCGAGACGCGC	AGCCCGA	N
9*Chr1_120_ISBma2_A		N	SAVP1	9	2551397-2552969	Chr1	ISBma2	AGCCA	CAGATTGCTGACAAAC	ATGCTGAAGACGCCCATG	TTCCAGAAAGACAAAACC	GGCGCCC	GGCGCCCGCAAGGGC	GGCGCCC	Y
6*Chr1_39a_ISBma	6*Chr1_46b_ISBma2_A	R	NCTC_10229	6	2558075-2559647	Chr1	ISBma2	GGAAA	CAGATTGCTGACAAAC	ATGCTGAAGACGCCCATG	TCCCAGAAAGACAAAACC	AGCCCTC	AGCGCGCCCTAGGGCG	AGCCCTC	N
2*Chr1_147_ISBma2_A		D-R	fmh	2	2563010-2564573	Chr1	ISBma2	~~~~~	AGGGGGCAGGTCAAA	ATGCTGAAGACGCCCATG	TCCCAGAAAGACAAAACC	AGGCGCC	AAGCCCATTGGCCCTA	AGGCGCC	N
8*Chr1_151b_ISBm	8*Chr1_159b_ISBma2_A	R	2002721280	8	2565718-2567265	Chr1	ISBma2	~~~~~	NNNNNNNNNNNNNNN	ATGCTGAAGACGCCCATG	TCCCAGAAAGACAAAACC	GGGCGCC	~~~~~~~~~~~~~~N	GGGCGCC	N.D.
9*Chr1_121_ISBma2_A		N	SAVP1	9	2567255-2567395	Chr1	ISBma2	#IS407	~~~~~~~~~~~~~~~	~~~~~~~~~~~~~~~~~~	TCCCAGAAAGACAAAACC	AAGCCCT	#IS407A	AAGCCCT	N.D.
10*Chr1_139b_ISB	10*Chr1_141b_ISBma2_A	R	PRL20	10	2571166-2572738	Chr1	ISBma2	CCTCC	CAGATTGCTGACAAAC	ATGCTGAAGACGCCCATG	TCCCAGAAAGACAAAACC	GGCCCGG	CCGAACCAGCGGTTCG	GGCCCGG	N
7*Chr1_139b_ISBm	7*Chr1_141b_ISBma2_A	R	NCTC_10247	7	2576418-2577992	Chr1	ISBma2	CCTCC	CAGATTGCTGACAAAC	ATGCTGAAGACGCCCATG	TCCCAGAAAGACAAAACC	AGCCCGG	CCGAACCAGCGGTTCG	AGCCCGG	N
3*Chr1_147_ISBma2_A		D-R	JHU	3	2579658-2581221	Chr1	ISBma2	~~~~~	AGGGGGCAGGTCAAA	ATGCTGAAGACGCCCATG	TCCCAGAAAGACAAAACC	AGGCGCC	AAGCCCATTGGCCCTA	AGGCGCC	N

5*Chr1_139a_ISBm	5*Chr1_141a_ISBma2_A	R	ATCC_10399	5	2593046-2594618	Chr1	ISBma2	GCCCT	CAGATTGCTGACAAAC	ATGCTGAAGACGCCCATG	TCCCAGAAAGACAAAACC	AGCCCGA	CCGGCGCATGGCGCCG	AGCCCGA	N
5*Chr1_140_ISBma2_B		N	ATCC_10399	5	2614915-2616487	Chr1	ISBma2	GCGCA	CAGATTGCTGACAAAC	ATGCTGAAGACGCCCATG	TCCCAGAAAGACAAAACC	ACGCCCC	ACGCCCCGGCCGGGGC	ACGCCCC	Y
9*Chr1_46a_ISBma2_A		N	SAVP1	9	2618874-2620446	Chr1	ISBma2	AATAC	CAGATTGCTGACAAAC	ATGCTGAAGACGCCCATG	TCCCAGAAAGACAAAACC	AAAAGCG	AAAAGCGCGCCCTAGG	AAAAGCG	Y
7*Chr1_1b_ISBma2	7*Chr1_19a_ISBma2_A	R	NCTC_10247	7	2622004-2623575	Chr1	ISBma2	GCACG	CAGATTGCTGACAAAC	ATGCTGAAGACGCCCATG	TCCCAGAAAGACAAAACC	AACCCGC	GAGGGCGTCGCCCTCC	AACCCGC	N
6*Chr1_130_ISBma2_B		N	NCTC_10229	6	2622284-2623857	Chr1	ISBma2	GTCCA	CAGATTGCTGACAAAC	ATGCTGAAGACGCCCATG	TCCCAGAAAGACAAAACC	AGGCGAT	AGGCGATGCGCGCAGC	AGGCGAT	Y
8*Chr1_148_ISBma	8*Chr1_159a_ISBma2_A	D-R	2002721280	8	2648477-2650049	Chr1	ISBma2	GGATT	CAGATTGCTGACAAAC	ATGCTGAAGACGCCCATG	TCCCAGAAAGACAAAACC	AGGCCCC	AGGGCCGCATGAAGCG	AGGCCCC	N
4*Chr1_147_ISBma2_A		D-R	gb8_horse_4	4	2657553-2659116	Chr1	ISBma2	~~~~~	AGGGGGCAGGTCAAA	ATGCTGAAGACGCCCATG	TCCCAGAAAGACAAAACC	AGGCGCC	AAGCCCATTGGCCCTA	AGGCGCC	N
1*Chr1_147_ISBma2_A		D-R	ATCC_23344	1	2669533-2671096	Chr1	ISBma2	~~~~~	AGGGGGCAGGTCAAA	ATGCTGAAGACGCCCATG	TCCCAGAAAGACAAAACC	AGGCGCC	AAGCCCATTGGCCCTA	AGGCGCC	N
5*Chr1_139b_ISBm	5*Chr1_141b_ISBma2_A	R	ATCC_10399	5	2675065-2676637	Chr1	ISBma2	CCTCC	CAGATTGCTGACAAAC	ATGCTGAAGACGCCCATG	TCCCAGAAAGACAAAACC	AGCCCGG	CCGAACCAGCGGTTCG	AGCCCGG	N
10*Chr1_139a_ISB	10*Chr1_147_ISBma2_A	D-R	PRL20	10	2714631-2716203	Chr1	ISBma2	GCCCT	CAGATTGCTGACAAAC	ATGCTGAAGACGCCCATG	TCCCAGAAAGACAAAACC	AGGCGCC	CCGGCGCATGGCGCCG	AGGCGCC	N
10*Chr1_138_ISBma2_C		N	PRL20	10	2727692-2728537	Chr1	ISBma2	~~~~~	AGGGGGCAGGTCAAA	ATGCTGAAGACGCCCATG	#	#contig_br	AAGCCCATTGGCCCTA	#contig_br	N.D.
2*Chr1_151a_ISBma2_A		N	fmh	2	2742888-2744460	Chr1	ISBma2	AGCCG	CAGATTGCTGACAAAC	ATGCTGAAGACGCCCATG	TCCCAGAAAGACAAAACC	AGCCCGC	AAGCGGCCTTTCGGCC	AGCCCGC	N
8*Chr1_151a_ISBma2_A		R	2002721280	8	2747570-2749142	Chr1	ISBma2	ATTTC	CAGATTGCTGACAAAC	ATGCTGAAGACGCCCATG	TCCCAGAAAGACAAAACC	AACCCGC	GCCCCGCTTCGGCGGG	AACCCGC	N
7*Chr1_23b_ISBma	7*Chr1_151a_ISBma2_A	R	NCTC_10247	7	2756502-2758099	Chr1	ISBma2	CGCGC	CAGATTGCTGACAAAC	ATGCTGAAGACGCCCATG	TCCCAGAAAGACAAAACC	AACCCGC	ACCGCAGCGAAGGCTG	AACCCGC	N
3*Chr1_151a_ISBma2_A		N	JHU	3	2758991-2760563	Chr1	ISBma2	AGCCG	CAGATTGCTGACAAAC	ATGCTGAAGACGCCCATG	TCCCAGAAAGACAAAACC	AGCCCGC	AAGCGGCCTTTCGGCC	AGCCCGC	N
9*Chr1_13a_ISBma	9*Chr1_32b_ISBma2_A	R	SAVP1	9	2763087-2764659	Chr1	ISBma2	GCGAC	CAGATTGCTGACAAAC	ATGCTGAAGACGCCCATG	TCCCAGAAAGACAAAACC	GAGCGGC	GACCGCGTGGGCGGTC	GAGCGGC	N
2*Chr1_1b_ISBma2_A		R	fmh	2	2776259-2777831	Chr1	ISBma2	ATTTC	CAGATTGCTGACAAAC	ATGCTGAAGACGCCCATG	TCCCAGAAAGACAAAACC	AACCCGC	GCCCCGCTTCGGCGGG	AACCCGC	N
7*Chr1_24_ISBma2_A		N	NCTC_10247	7	2784533-2786105	Chr1	ISBma2	GGCAA	CAGATTGCTGACAAAC	ATGCTGAAGACGCCCATG	TCCCAGAAAGACAAAACC	AACCCGC	AACCCGCCTCGGAGGC	AACCCGC	Y
2*Chr1_148_ISBma	2*Chr1_159a_ISBma2_A	D-R	fmh	2	2784984-2786556	Chr1	ISBma2	GGATT	CAGATTGCTGACAAAC	ATGCTGAAGACGCCCATG	TCCCAGAAAGACAAAACC	AGGCCCC	AGGGCCGCATGAAGCG	AGGCCCC	N
8*Chr1_3_ISBma2_C		N	2002721280	8	2785321-2786893	Chr1	ISBma2	AAAGG	CAGATTGCTGACAAAC	ATGCTGAAGACGCCCATG	TCCCAGAAAGACAAAACC	AGCCCGG	AGCCCGGCATGAGCCG	AGCCCGG	Y
3*Chr1_1b_ISBma2_A		R	JHU	3	2791386-2792958	Chr1	ISBma2	ATTTC	CAGATTGCTGACAAAC	ATGCTGAAGACGCCCATG	TCCCAGAAAGACAAAACC	AACCCGC	GCCCCGCTTCGGCGGG	AACCCGC	N
2*Chr1_39a_ISBma	2*Chr1_117b_ISBma2_A	R	fmh	2	279549-281121	Chr1	ISBma2	TTTCG	CAGATTGCTGACAAAC	ATGCTGAAGACGCCCATG	TCCCAGAAAGACAAAACC	AAGCCCT	AACCCGGCTGGAAGCC	AAGCCCT	N
3*Chr1_148_ISBma	3*Chr1_159a_ISBma2_A	D-R	JHU	3	2800111-2801683	Chr1	ISBma2	GGATT	CAGATTGCTGACAAAC	ATGCTGAAGACGCCCATG	TCCCAGAAAGACAAAACC	AGGCCCC	AGGGCCGCATGAAGCG	AGGCCCC	N
3*Chr1_39a_ISBma	3*Chr1_117b_ISBma2_A	R	JHU	3	280544-282116	Chr1	ISBma2	TTTCG	CAGATTGCTGACAAAC	ATGCTGAAGACGCCCATG	TCCCAGAAAGACAAAACC	AAGCCCT	AACCCGGCTGGAAGCC	AAGCCCT	N
8*Chr1_39a_ISBma	8*Chr1_117b_ISBma2_A	R	2002721280	8	280947-282519	Chr1	ISBma2	TCGAC	CAGATTGCTGACAAAC	ATGCTGAAGACGCCCATG	TCCCAGAAAGACAAAACC	AGCCCTC	CCCGCTGGTTTCAGCG	AGCCCTC	N
7*Chr1_25a_ISBma	7*Chr1_28a_ISBma2_A	R	NCTC_10247	7	2814990-2816561	Chr1	ISBma2	TCGAA	CAGATTGCTGACAAAC	ATGCTGAAGACGCCCATG	TCCCAGAAAGACAAAACC	AGCCCGC	CCCGCGCGTCGCGCGG	AGCCCGC	N
5*Chr1_147_ISBma2_A		D-R	ATCC_10399	5	2818680-2820243	Chr1	ISBma2	~~~~~	AGGGGGCAGGTCAAA	ATGCTGAAGACGCCCATG	TCCCAGAAAGACAAAACC	AGGCGCC	AAGCCCATTGGCCCTA	AGGCGCC	N
8*Chr1_1a_ISBma2_A		N	2002721280	8	2824135-2825706	Chr1	ISBma2	AGCTG	CAGATTGCTGACAAAC	ATGCTGAAGACGCCCATG	TCCCAGAAAGACAAAACC	AACCCGC	CCCGCTGGTTTCAGCG	AACCCGC	N
4*Chr1_151a_ISBma2_A		N	gb8_horse_4	4	2834020-2835592	Chr1	ISBma2	AGCCG	CAGATTGCTGACAAAC	ATGCTGAAGACGCCCATG	TCCCAGAAAGACAAAACC	AGCCCGC	AAGCGGCCTTTCGGCC	AGCCCGC	N
1*Chr1_151a_ISBma2_A		N	ATCC_23344	1	2847519-2849091	Chr1	ISBma2	AGCCG	CAGATTGCTGACAAAC	ATGCTGAAGACGCCCATG	TCCCAGAAAGACAAAACC	AGCCCGC	AAGCGGCCTTTCGGCC	AGCCCGC	N
4*Chr1_1b_ISBma2_A		R	gb8_horse_4	4	2866443-2868015	Chr1	ISBma2	ATTTC	CAGATTGCTGACAAAC	ATGCTGAAGACGCCCATG	TCCCAGAAAGACAAAACC	AACCCGC	GCCCCGCTTCGGCGGG	AACCCGC	N
7*Chr1_25b_ISBma	7*Chr1_28b_ISBma2_A	R	NCTC_10247	7	2867420-2868994	Chr1	ISBma2	CGCCG	CAGATTGCTGACAAAC	ATGCTGAAGACGCCCATG	TCCCAGAAAGACAAAACC	AGGCCCG	CTGTCGCAGGACGGCG	AGGCCCG	N
8*Chr1_164_ISBma2_B		N	2002721280	8	2872259-2873831	Chr1	ISBma2	ATGCC	CAGATTGCTGACAAAC	ATGCTGAAGACGCCCATG	TCCCAGAAAGACAAAACC	AGGCAGC	AGGCAGCCCGAGGGG	AGGCAGC	Y
4*Chr1_148_ISBma	4*Chr1_159a_ISBma2_A	D-R	gb8_horse_4	4	2875168-2876717	Chr1	ISBma2	GGATT	CAGATTGCTGACAAAC	ATGCTGAAGACGCCCATG	TCCCAGAAAGACAAAACC	AGGCCCC	AGGGCCGCATGAAGCG	AGGCCCC	N
1*Chr1_1b_ISBma2_A		R	ATCC_23344	1	2879914-2881486	Chr1	ISBma2	ATTTC	CAGATTGCTGACAAAC	ATGCTGAAGACGCCCATG	TCCCAGAAAGACAAAACC	AACCCGC	GCCCCGCTTCGGCGGG	AACCCGC	N
9*Chr1_13b_ISBma	9*Chr1_32a_ISBma2_A	R	SAVP1	9	2884897-2886468	Chr1	ISBma2	TGTGT	CAGATTGCTGACAAAC	ATGCTGAAGACGCCCATG	TCCCAGAAAGACAAAACC	AACGGAC	AGAGCGGCCCGAAGG	AACGGAC	N
2*Chr1_118_ISBma2_A		N	fmh	2	288595-290167	Chr1	ISBma2	CGATC	CAGATTGCTGACAAAC	ATGCTGAAGACGCCCATG	TCCCAGAAAGACAAAACC	AAGGCGC	AAGGCGCCGCGGCAA	AAGGCGC	Y
1*Chr1_148_ISBma	1*Chr1_159a_ISBma2_A	D-R	ATCC_23344	1	2888639-2890211	Chr1	ISBma2	GGATT	CAGATTGCTGACAAAC	ATGCTGAAGACGCCCATG	TCCCAGAAAGACAAAACC	AGGCCCC	AGGGCCGCATGAAGCG	AGGCCCC	N
10*Chr1_151a_ISBma2_A		N	PRL20	10	2889009-2890581	Chr1	ISBma2	AGCCG	CAGATTGCTGACAAAC	ATGCTGAAGACGCCCATG	TCCCAGAAAGACAAAACC	AACCCGC	AAGCGGCCTTTCGGCC	AACCCGC	N
10*Chr1_1b_ISBma2_A		R	PRL20	10	2896600-2898131	Chr1	ISBma2	#IS407	~~~~~~~~~~~~~~~	GGTCAGAAGACGCCCATG	TCCCAGAAAGACAAAACC	AACCCGC	#IS407A	AACCCGC	N.D.
2*Chr1_1a_ISBma2	2*Chr1_159b_ISBma2_A	R	fmh	2	2900149-2901721	Chr1	ISBma2	AGCTG	CAGATTGCTGACAAAC	ATGCTGAAGACGCCCATG	TCCCAGAAAGACAAAACC	GGGCCGC	CCCGCTGGTTTCAGCG	GGGCCGC	N
8*Chr1_118_ISBma2_A		N	2002721280	8	290022-291594	Chr1	ISBma2	CGATC	CAGATTGCTGACAAAC	ATGCTGAAGACGCCCATG	TCCCAGAAAGACAAAACC	AAGGCGC	AAGGCGCCGCGGCAA	AAGGCGC	Y
3*Chr1_118_ISBma2_A		N	JHU	3	290199-291771	Chr1	ISBma2	CGATC	CAGATTGCTGACAAAC	ATGCTGAAGACGCCCATG	TCCCAGAAAGACAAAACC	AAGGCGC	AAGGCGCCGCGGCAA	AAGGCGC	Y
10*Chr1_148_ISBm	10*Chr1_159a_ISBma2_A	D-R	PRL20	10	2904760-2906332	Chr1	ISBma2	GGATT	CAGATTGCTGACAAAC	ATGCTGAAGACGCCCATG	TCCCAGAAAGACAAAACC	AGGCCCC	AGGGCCGCATGAAGCG	AGGCCCC	N
8*Chr1_163_ISBma2_A		N	2002721280	8	2905615-2907162	Chr1	ISBma2	~~~~~	CAGATTGCTGACAAAC	ATGCTGAAGACGCCCATG	TCCCAGAAAGACAAAACC	TGCTCGC	TGCTCGCGAAGGCGAG	TGCTCGC	Y
5*Chr1_151a_ISBma2_A		N	ATCC_10399	5	2907488-2909060	Chr1	ISBma2	AGCCG	CAGATTGCTGACAAAC	ATGCTGAAGACGCCCATG	TCCCAGAAAGACAAAACC	AGCCCGC	AAGCGGCCTTTCGGCC	AGCCCGC	N
3*Chr1_1a_ISBma2	3*Chr1_159b_ISBma2_A	R	JHU	3	2915891-2917463	Chr1	ISBma2	AGCTG	CAGATTGCTGACAAAC	ATGCTGAAGACGCCCATG	TCCCAGAAAGACAAAACC	GGGCCGC	CCCGCTGGTTTCAGCG	GGGCCGC	N
7*Chr1_1a_ISBma2	7*Chr1_159b_ISBma2_A	R	NCTC_10247	7	2928340-2929912	Chr1	ISBma2	AGCTG	CAGATTGCTGACAAAC	ATGCTGAAGACGCCCATG	TCCCAGAAAGACAAAACC	GGGCCGC	CCCGCTGGTTTCAGCG	GGGCCGC	N
9*Chr1_17_ISBma2_C		N	SAVP1	9	2957135-2958707	Chr1	ISBma2	TGAGC	CAGATTGCTGACAAAC	ATGCTGAAGACGCCCATG	TCCCAGAAAGACAAAACC	AAGCGCC	AAGCGCCCTCGGGCGC	AAGCGCC	Y
6*Chr1_50_ISBma2_A		N	NCTC_10229	6	2971768-2973340	Chr1	ISBma2	GCGAC	CAGATTGCTGACAAAC	ATGCTGAAGACGCCCATG	TCCCAGAAAGACAAAACC	GGGGCGC	GGGGCGCGACGTACTC	GGGGCGC	Y
7*Chr1_164_ISBma2_B		N	NCTC_10247	7	2976463-2978010	Chr1	ISBma2	~~~~~	CAGATTGCTGACAAAC	ATGCTGAAGACGCCCATG	TCCCAGAAAGACAAAACC	AGGCAGC	AGGCAGCCCGAGGGG	AGGCAGC	Y
2*Chr1_163_ISBma2_A		N	fmh	2	2979940-2981512	Chr1	ISBma2	CGCTT	CAGATTGCTGACAAAC	ATGCTGAAGACGCCCATG	TCCCAGAAAGACAAAACC	TGCTCGC	TGCTCGCGAAGGCGAG	TGCTCGC	Y
8*Chr1_25b_ISBma	8*Chr1_28b_ISBma2_A	R	2002721280	8	2983988-2985560	Chr1	ISBma2	CGCCG	CAGATTGCTGACAAAC	ATGCTGAAGACGCCCATG	TCCCAGAAAGACAAAACC	AGGCCCG	CTGTCGCAGGACGGCG	AGGCCCG	N
4*Chr1_1a_ISBma2	4*Chr1_159b_ISBma2_A	R	gb8_horse_4	4	2988475-2990047	Chr1	ISBma2	AGCTG	CAGATTGCTGACAAAC	ATGCTGAAGACGCCCATG	TCCCAGAAAGACAAAACC	GGGCCGC	CCCGCTGGTTTCAGCG	GGGCCGC	N
3*Chr1_163_ISBma2_A		N	JHU	3	2996311-2997883	Chr1	ISBma2	CGCTT	CAGATTGCTGACAAAC	ATGCTGAAGACGCCCATG	TCCCAGAAAGACAAAACC	TGCTCGC	TGCTCGCGAAGGCGAG	TGCTCGC	Y
1*Chr1_1a_ISBma2	1*Chr1_159b_ISBma2_A	R	ATCC_23344	1	3002230-3003802	Chr1	ISBma2	AGCTG	CAGATTGCTGACAAAC	ATGCTGAAGACGCCCATG	TCCCAGAAAGACAAAACC	GGGCCGC	CCCGCTGGTTTCAGCG	GGGCCGC	N
9*Chr1_19a_ISBma2_A		N	SAVP1	9	3005415-3006987	Chr1	ISBma2	GCACG	CAGATTGCTGACAAAC	ATGCTGAAGACGCCCATG	TCCCAGAAAGACAAAACC	AGCGGAG	AGCGGAGGGCGTCGCC	AGCGGAG	Y
7*Chr1_163_ISBma2_A		N	NCTC_10247	7	3009753-3011300	Chr1	ISBma2	~~~~~	CAGATTGCTGACAAAC	ATGCTGAAGACGCCCATG	TCCCAGAAAGACAAAACC	TGCTCGC	TGCTCGCGAAGGCGAG	TGCTCGC	Y

6*Chr1_52_ISBma2_A		N	NCTC_10229	6	3026236-3027808	Chr1	ISBma2	CCGGA	CAGATTGCTGACAAAC	ATGCTGAAGACGCCCATG	TCCCAGAAAGACAAAACC	ACGGCCG	ACGGCCGCGCTCACGC	ACGGCCG	Y
5*Chr1_1b_ISBma2_A		R	ATCC_10399	5	3029164-3030736	Chr1	ISBma2	ATTTC	CAGATTGCTGACAAAC	ATGCTGAAGACGCCCATG	TCCCAGAAAGACAAAACC	AACCCGC	GCCCCGCTTCGGCGGG	AACCCGC	N
8*Chr1_25a_ISBma	8*Chr1_28a_ISBma2_A	R	2002721280	8	3036431-3038003	Chr1	ISBma2	TCGAA	CAGATTGCTGACAAAC	ATGCTGAAGACGCCCATG	TCCCAGAAAGACAAAACC	AGCCCGC	CCCGCGCGTCGCGCGG	AGCCCGC	N
5*Chr1_148_ISBma	5*Chr1_159a_ISBma2_A	D-R	ATCC_10399	5	3037889-3039461	Chr1	ISBma2	GGATT	CAGATTGCTGACAAAC	ATGCTGAAGACGCCCATG	TCCCAGAAAGACAAAACC	AGGCCCC	AGGGCCGCATGAAGCG	AGGCCCC	N
10*Chr1_1a_ISBma	10*Chr1_159b_ISBma2_A	R	PRL20	10	3039719-3041290	Chr1	ISBma2	AGCTG	CAGATTGCTGACAAAC	ATGCTGAAGACGCCCATG	TCCCAGAAAGACAAAACC	GGGCCGC	CCCGCTGGTTTCAGCG	GGGCCGC	N
8*Chr1_23a_ISBma	8*Chr1_24_ISBma2_A	D	2002721280	8	3066888-3068460	Chr1	ISBma2	GGCAA	CAGATTGCTGACAAAC	ATGCTGAAGACGCCCATG	TCCCAGAAAGACAAAACC	ACCGCAG	ACCCGCCTCGGAGGCG	ACCGCAG	N
4*Chr1_163_ISBma2_A		N	gb8_horse_4	4	3068266-3069838	Chr1	ISBma2	CGCTT	CAGATTGCTGACAAAC	ATGCTGAAGACGCCCATG	TCCCAGAAAGACAAAACC	TGCTCGC	TGCTCGCGAAGGCGAG	TGCTCGC	Y
9*Chr1_22a_ISBma2_A		R	SAVP1	9	3073996-3075559	Chr1	ISBma2	~~~~~	AGGGGGCAGGTCAAA	ATGCTGAAGACGCCCATG	TCCCAGAAAGACAAAACC	AGCCCGC	AGCCCATTGGCCCTAT	AGCCCGC	N
6*Chr1_110_ISBma2_A		N	NCTC_10229	6	3075066-3076638	Chr1	ISBma2	TCCCG	CAGATTGCTGACAAAC	ATGCTGAAGACGCCCATG	TCCCAGAAAGACAAAACC	AAGCCCT	GCCCTGCGAGTGCAGG	AAGCCCT	N
1*Chr1_163_ISBma2_A		N	ATCC_23344	1	3082021-3083593	Chr1	ISBma2	CGCTT	CAGATTGCTGACAAAC	ATGCTGAAGACGCCCATG	TCCCAGAAAGACAAAACC	TGCTCGC	TGCTCGCGAAGGCGAG	TGCTCGC	Y
6*Chr1_108_ISBma2_A		N	NCTC_10229	6	3098671-3100243	Chr1	ISBma2	CGTGG	CAGATTGCTGACAAAC	ATGCTGAAGACGCCCATG	TCCCAGAAAGACAAAACC	GGGGCGC	GGGGCGCTTCGGCGCC	GGGGCGC	Y
8*Chr1_22b_ISBma	8*Chr1_147_ISBma2_A	D-R	2002721280	8	3100401-3101948	Chr1	ISBma2	GCCGC	CAGATTGCTGACAAAC	ATGCTGAAGACGCCCATG	TCCCAGAAAGACAAAACC	AGGCGCC	AGCCCGCGAGACGCG	AGGCGCC	N
10*Chr1_163_ISBma2_A		N	PRL20	10	3119525-3121095	Chr1	ISBma2	CGCTT	CAGATTGCTGACAAAC	ATGCTGAAGACGCCCATG	TCCCAGAAAGACAAAACC	TGCTCGC	TGCTCGCGAAGGCGAG	TGCTCGC	Y
6*Chr1_105b_ISBm	6*Chr1_107b_ISBma2_A	R	NCTC_10229	6	3130076-3131648	Chr1	ISBma2	AACGA	CAGATTGCTGACAAAC	ATGCTGAAGACGCCCATG	TCCCAGAAAGACAAAACC	AAGCCCC	CCCGCTGGTTTCAGCG	AAGCCCC	N
6*Chr1_105a_ISBm	6*Chr1_107a_ISBma2_A	R	NCTC_10229	6	3134127-3135699	Chr1	ISBma2	TCGGG	CAGATTGCTGACAAAC	ATGCTGAAGACGCCCATG	TCCCAGAAAGACAAAACC	AAGCCCC	CCCGCTGGTTTCAGCG	AAGCCCC	N
2*Chr1_25b_ISBma	2*Chr1_28b_ISBma2_A	R	fmh	2	3135195-3136770	Chr1	ISBma2	CGCCG	CAGATTGCTGACAAAC	ATGCTGAAGACGCCCATG	TCCCAGAAAGACAAAACC	AGGCCCG	CTGTCGCAGGACGGCG	AGGCCCG	N
7*Chr1_148_ISBma	7*Chr1_159a_ISBma2_A	D-R	NCTC_10247	7	3140775-3142376	Chr1	ISBma2	~~~~~	CAGATTGCTGACAAAC	ATGCTGAAGACGCCCATG	TCCCAGAAAGACAAAACC	AGGCCCC	AGGGCCGCATGAAGCG	AGGCCCC	N
8*Chr1_144_ISBma2_B		N	2002721280	8	3145687-3147259	Chr1	ISBma2	GCGGC	CAGATTGCTGACAAAC	ATGCTGAAGACGCCCATG	TCCCAGAAAGACAAAACC	AGGCCCC	AGCCCGCGCATGCGGG	AGGCCCC	N
7*Chr1_23a_ISBma2_A		R	NCTC_10247	7	3149499-3151071	Chr1	ISBma2	ATTTC	CAGATTGCTGACAAAC	ATGCTGAAGACGCCCATG	TCCCAGAAAGACAAAACC	ACCGCAG	GCCCCGCTTCGGCGGG	ACCGCAG	N
3*Chr1_25b_ISBma	3*Chr1_28b_ISBma2_A	R	JHU	3	3152397-3153972	Chr1	ISBma2	CGCCG	CAGATTGCTGACAAAC	ATGCTGAAGACGCCCATG	TCCCAGAAAGACAAAACC	AGGCCCG	CTGTCGCAGGACGGCG	AGGCCCG	N
9*Chr1_151a_ISBma2_A		N	SAVP1	9	3162470-3164042	Chr1	ISBma2	AGCCG	CAGATTGCTGACAAAC	ATGCTGAAGACGCCCATG	TCCCAGAAAGACAAAACC	AACCCGC	AAGCGGCCTTTCGGCC	AACCCGC	N
5*Chr1_163_ISBma2_A		N	ATCC_10399	5	3169120-3170692	Chr1	ISBma2	CGCTT	CAGATTGCTGACAAAC	ATGCTGAAGACGCCCATG	TCCCAGAAAGACAAAACC	TGCTCGC	TGCTCGCGAAGGCGAG	TGCTCGC	Y
7*Chr1_22b_ISBma	7*Chr1_147_ISBma2_A	D-R	NCTC_10247	7	3182889-3184461	Chr1	ISBma2	GCCGC	CAGATTGCTGACAAAC	ATGCTGAAGACGCCCATG	TCCCAGAAAGACAAAACC	AGGCGCC	AGCCCGCGAGACGCG	AGGCGCC	N
2*Chr1_25a_ISBma	2*Chr1_28a_ISBma2_A	R	fmh	2	3187985-3189557	Chr1	ISBma2	TCGAA	CAGATTGCTGACAAAC	ATGCTGAAGACGCCCATG	TCCCAGAAAGACAAAACC	AGCCCGC	CCCGCGCGTCGCGCGG	AGCCCGC	N
3*Chr1_25a_ISBma	3*Chr1_28a_ISBma2_A	R	JHU	3	3204878-3206450	Chr1	ISBma2	TCGAA	CAGATTGCTGACAAAC	ATGCTGAAGACGCCCATG	TCCCAGAAAGACAAAACC	AGCCCGC	CCCGCGCGTCGCGCGG	AGCCCGC	N
8*Chr1_13b_ISBma	8*Chr1_32a_ISBma2_A	R	2002721280	8	3218859-3220433	Chr1	ISBma2	TGTGT	CAGATTGCTGACAAAC	ATGCTGAAGACGCCCATG	TCCCAGAAAGACAAAACC	AACGGAC	AGAGCGGCCCGAAGG	AACGGAC	N
2*Chr1_24_ISBma2_A		N	fmh	2	3218895-3220466	Chr1	ISBma2	GGCAA	CAGATTGCTGACAAAC	ATGCTGAAGACGCCCATG	TCCCAGAAAGACAAAACC	AACCCGC	AACCCGCCTCGGAGGC	AACCCGC	Y
4*Chr1_25b_ISBma	4*Chr1_28b_ISBma2_A	R	gb8_horse_4	4	3222056-3223631	Chr1	ISBma2	CGCCG	CAGATTGCTGACAAAC	ATGCTGAAGACGCCCATG	TCCCAGAAAGACAAAACC	AGGCCCG	CTGTCGCAGGACGGCG	AGGCCCG	N
7*Chr1_144_ISBma2_B		N	NCTC_10247	7	3228362-3229936	Chr1	ISBma2	GCGGC	CAGATTGCTGACAAAC	ATGCTGAAGACGCCCATG	TCCCAGAAAGACAAAACC	AGCCCGC	AGCCCGCGCATGCGGG	AGCCCGC	Y
1*Chr1_25b_ISBma	1*Chr1_28b_ISBma2_A	R	ATCC_23344	1	3235804-3237379	Chr1	ISBma2	CGCCG	CAGATTGCTGACAAAC	ATGCTGAAGACGCCCATG	TCCCAGAAAGACAAAACC	AGGCCCG	CTGTCGCAGGACGGCG	AGGCCCG	N
3*Chr1_24_ISBma2_A		N	JHU	3	3236786-3238357	Chr1	ISBma2	GGCAA	CAGATTGCTGACAAAC	ATGCTGAAGACGCCCATG	TCCCAGAAAGACAAAACC	AACCCGC	AACCCGCCTCGGAGGC	AACCCGC	Y
2*Chr1_13b_ISBma	2*Chr1_23b_ISBma2_A	R	fmh	2	3247169-3248741	Chr1	ISBma2	CGCGC	CAGATTGCTGACAAAC	ATGCTGAAGACGCCCATG	TCCCAGAAAGACAAAACC	AACGGAC	ACCGCAGCGAAGGCTG	AACGGAC	N
5*Chr1_1a_ISBma2	5*Chr1_159b_ISBma2_A	R	ATCC_10399	5	3248886-3250457	Chr1	ISBma2	AGCTG	CAGATTGCTGACAAAC	ATGCTGAAGACGCCCATG	TCCCAGAAAGACAAAACC	GGGCCGC	CCCGCTGGTTTCAGCG	GGGCCGC	N
2*Chr1_120_ISBma2_A		N	fmh	2	325136-326708	Chr1	ISBma2	AGCCA	CAGATTGCTGACAAAC	ATGCTGAAGACGCCCATG	TCCCAGAAAGACAAAACC	GGCGCCC	GGCGCCCCGCAAGGG	GGCGCCC	Y
3*Chr1_13b_ISBma	3*Chr1_23b_ISBma2_A	R	JHU	3	3265431-3267003	Chr1	ISBma2	CGCGC	CAGATTGCTGACAAAC	ATGCTGAAGACGCCCATG	TCCCAGAAAGACAAAACC	AACGGAC	ACCGCAGCGAAGGCTG	AACGGAC	N
8*Chr1_120_ISBma2_A		N	2002721280	8	326581-328153	Chr1	ISBma2	AGCCA	CAGATTGCTGACAAAC	ATGCTGAAGACGCCCATG	TCCCAGAAAGACAAAACC	GGCGCCC	GGCGCCCCGCAAGGG	GGCGCCC	Y
3*Chr1_120_ISBma2_A		N	JHU	3	326740-328312	Chr1	ISBma2	AGCCA	CAGATTGCTGACAAAC	ATGCTGAAGACGCCCATG	TCCCAGAAAGACAAAACC	GGCGCCC	GGCGCCCCGCAAGGG	GGCGCCC	Y
6*Chr1_104_ISBma2_A		N	NCTC_10229	6	3271547-3273119	Chr1	ISBma2	TACCC	CAGATTGCTGACAAAC	ATGCTGAAGACGCCCATG	TCCCAGAAAGACAAAACC	ACCGGCG	ACCGGCGCATTCGCGC	ACCGGCG	Y
10*Chr1_25b_ISBm	10*Chr1_28b_ISBma2_A	R	PRL20	10	3271661-3273235	Chr1	ISBma2	CGCCG	CAGATTGCTGACAAAC	ATGCTGAAGACGCCCATG	TCCCAGAAAGACAAAACC	AGGCCCG	CTGTCGCAGGACGGCG	AGGCCCG	N
4*Chr1_25a_ISBma	4*Chr1_28a_ISBma2_A	R	gb8_horse_4	4	3274537-3276109	Chr1	ISBma2	TCGAA	CAGATTGCTGACAAAC	ATGCTGAAGACGCCCATG	TCCCAGAAAGACAAAACC	AGCCCGC	CCCGCGCGTCGCGCGG	AGCCCGC	N
9*Chr1_163_ISBma2_A		N	SAVP1	9	3283458-3285029	Chr1	ISBma2	CGCTT	CAGATTGCTGACAAAC	ATGCTGAAGACGCCCATG	TCCCAGAAAGACAAAACC	TGCTCGC	TGCTCGCGAAGGCGAG	TGCTCGC	Y
1*Chr1_25a_ISBma	1*Chr1_28a_ISBma2_A	R	ATCC_23344	1	3288285-3289857	Chr1	ISBma2	TCGAA	CAGATTGCTGACAAAC	ATGCTGAAGACGCCCATG	TCCCAGAAAGACAAAACC	AGCCCGC	CCCGCGCGTCGCGCGG	AGCCCGC	N
2*Chr1_16_ISBma2_B		N	fmh	2	3304741-3306313	Chr1	ISBma2	CAAGA	CAGATTGCTGACAAAC	ATGCTGAAGACGCCCATG	TCCCAGAAAGACAAAACC	ACCGGAG	ACCGGAGCGCGAGGCT	ACCGGAG	Y
4*Chr1_24_ISBma2_A		N	gb8_horse_4	4	3305019-3306590	Chr1	ISBma2	GGCAA	CAGATTGCTGACAAAC	ATGCTGAAGACGCCCATG	TCCCAGAAAGACAAAACC	AACCCGC	AACCCGCCTCGGAGGC	AACCCGC	Y
5*Chr1_25b_ISBma	5*Chr1_28b_ISBma2_A	R	ATCC_10399	5	3309804-3311376	Chr1	ISBma2	CGCCG	CAGATTGCTGACAAAC	ATGCTGAAGACGCCCATG	TCCCAGAAAGACAAAACC	AGGCCCG	CTGTCGCAGGACGGCG	AGGCCCG	N
1*Chr1_24_ISBma2_A		N	ATCC_23344	1	3318742-3320313	Chr1	ISBma2	GGCAA	CAGATTGCTGACAAAC	ATGCTGAAGACGCCCATG	TCCCAGAAAGACAAAACC	AACCCGC	AACCCGCCTCGGAGGC	AACCCGC	Y
3*Chr1_16_ISBma2_B		N	JHU	3	3322942-3324514	Chr1	ISBma2	CAAGA	CAGATTGCTGACAAAC	ATGCTGAAGACGCCCATG	TCCCAGAAAGACAAAACC	ACCGGAG	ACCGGAGCGCGAGGCT	ACCGGAG	Y
10*Chr1_25a_ISBm	10*Chr1_28a_ISBma2_A	R	PRL20	10	3325434-3326700	Chr1	ISBma2	TCGAA	CAGATTGCTGACAAAC	ATGCTGAAGACGCCCATG	#	#IS407A	CCCGCGCGTCGCGCGG	#IS407A	N.D.
4*Chr1_13b_ISBma	4*Chr1_23b_ISBma2_A	R	gb8_horse_4	4	3333036-3334608	Chr1	ISBma2	CGCGC	CAGATTGCTGACAAAC	ATGCTGAAGACGCCCATG	TCCCAGAAAGACAAAACC	AACGGAC	ACCGCAGCGAAGGCTG	AACGGAC	N
8*Chr1_19a_ISBma2_A		N	2002721280	8	3336593-3338164	Chr1	ISBma2	GCACG	CAGATTGCTGACAAAC	ATGCTGAAGACGCCCATG	TCCCAGAAAGACAAAACC	ACCGGAG	AGCGGAGGGCGTCGCC	ACCGGAG	N
1*Chr1_13b_ISBma	1*Chr1_23b_ISBma2_A	R	ATCC_23344	1	3346749-3348321	Chr1	ISBma2	CGCGC	CAGATTGCTGACAAAC	ATGCTGAAGACGCCCATG	TCCCAGAAAGACAAAACC	AACGGAC	ACCGCAGCGAAGGCTG	AACGGAC	N
10*Chr1_24_ISBma2_A		N	PRL20	10	3356063-3357634	Chr1	ISBma2	GGCAA	CAGATTGCTGACAAAC	ATGCTGAAGACGCCCATG	TCCCAGAAAGACAAAACC	AGCCCGC	AACCCGCCTCGGAGGC	AGCCCGC	N
9*Chr1_139b_ISBm	9*Chr1_141b_ISBma2_A	R	SAVP1	9	335753-337325	Chr1	ISBma2	CCTCC	CAGATTGCTGACAAAC	ATGCTGAAGACGCCCATG	TCCCAGAAAGACAAAACC	GGCCCGG	CCGAACCAGCGGTTCG	GGCCCGG	N
6*Chr1_101_ISBma	6*Chr1_102_ISBma2_A	D	NCTC_10229	6	3359529-3361101	Chr1	ISBma2	GCATC	CAGATTGCTGACAAAC	ATGCTGAAGACGCCCATG	TCCCAGAAAGACAAAACC	GGGCCGT	AGGCCCGCCGAAGCG	GGGCCGT	N
5*Chr1_25a_ISBma	5*Chr1_28a_ISBma2_A	R	ATCC_10399	5	3362254-3363825	Chr1	ISBma2	TCGAA	CAGATTGCTGACAAAC	ATGCTGAAGACGCCCATG	TCCCAGAAAGACAAAACC	AGCCCGC	CCCGCGCGTCGCGCGG	AGCCCGC	N
9*Chr1_1a_ISBma2	9*Chr1_159b_ISBma2_A	R	SAVP1	9	3363178-3364749	Chr1	ISBma2	AGCTG	CAGATTGCTGACAAAC	ATGCTGAAGACGCCCATG	TCCCAGAAAGACAAAACC	GGGCCGC	CCCGCTGGTTTCAGCG	GGGCCGC	N
2*Chr1_19a_ISBma2_A		N	fmh	2	3368613-3370185	Chr1	ISBma2	CACGG	CAGATTGCTGACAAAC	ATGCTGAAGACGCCCATG	TCCCAGAAAGACAAAACC	AGCGGAG	AGCGGAGGGGCGTCGC	AGCGGAG	N
3*Chr1_19a_ISBma2_A		N	JHU	3	3387044-3388616	Chr1	ISBma2	GCACG	CAGATTGCTGACAAAC	ATGCTGAAGACGCCCATG	TCCCAGAAAGACAAAACC	AGCGGAG	AGCGGAGGGCGTCGCC	AGCGGAG	Y

4*Chr1_16_ISBma2_B		N	gb8_horse_4	4	3388601-3390173	Chr1	ISBma2	CAAGA	CAGATTGCTGACAAAC	ATGCTGAAGACGCCCATG(	TCCCAGAAAGACAAAACC(	ACCGGAG	ACCGGAGCGCGAGGCT	ACCGGAG	Y
5*Chr1_24_ISBma2_A		N	ATCC_10399	5	3392781-3394352	Chr1	ISBma2	GGCAA	CAGATTGCTGACAAAC	ATGCTGAAGACGCCCATG(	TCCCAGAAAGACAAAACC(	AACCCGC(	AACCCGCCTCGGAGGC	AACCCGC(	Y
7*Chr1_13b_ISBma	7*Chr1_151b_ISBma2_A	R	NCTC_10247	7	3400063-3401662	Chr1	ISBma2	~~~~~	CAGATTGCTGACAAAC	ATGCTGAAGACGCCCATG(	TCCCAGAAAGACAAAACC(	AACGGAC	AAGCGGCCTTTCGGCC(	AACGGAC	N
1*Chr1_16_ISBma2_B		N	ATCC_23344	1	3402607-3404179	Chr1	ISBma2	CAAGA	CAGATTGCTGACAAAC	ATGCTGAAGACGCCCATG(	TCCCAGAAAGACAAAACC(	ACCGGAG	ACCGGAGCGCGAGGCT	ACCGGAG	Y
8*Chr1_22a_ISBma2_A		R	2002721280	8	3403054-3404616	Chr1	ISBma2	~~~~~	AGGGGGCAGGTCAAA	ATGCTGAAGACGCCCATG(	TCCCAGAAAGACAAAACC(	AGCCCGC(	AGCCCATTGGCCCTAT(	AGCCCGC(	N
2*Chr1_121_ISBma2_A		N	fmh	2	340994-341134	Chr1	ISBma2	#IS407/	~~~~~~~~~~~~~~~	~~~~~~~~~~~~~~~~~	TCCCAGAAAGACAAAACC(	AAGCCCTT	#IS407A	AAGCCCTT	N.D.
5*Chr1_13b_ISBma	5*Chr1_23b_ISBma2_A	R	ATCC_10399	5	3421617-3423189	Chr1	ISBma2	CGCGC	CAGATTGCTGACAAAC	ATGCTGAAGACGCCCATG(	TCCCAGAAAGACAAAACC(	AACGGAC	ACCGCAGCGAAGGCTG	AACGGAC	N
8*Chr1_121_ISBma2_A		N	2002721280	8	342439-342579	Chr1	ISBma2	#IS407/	~~~~~~~~~~~~~~~	~~~~~~~~~~~~~~~~~	TCCCAGAAAGACAAAACC(	AAGCCCTT	#IS407A	AAGCCCTT	N.D.
3*Chr1_121_ISBma2_A		N	JHU	3	342598-342738	Chr1	ISBma2	#IS407/	~~~~~~~~~~~~~~~	~~~~~~~~~~~~~~~~~	TCCCAGAAAGACAAAACC(	AAGCCCTT	#IS407A	AAGCCCTT	N.D.
4*Chr1_19a_ISBma2_A		N	gb8_horse_4	4	3452144-3453716	Chr1	ISBma2	GCACG	CAGATTGCTGACAAAC	ATGCTGAAGACGCCCATG(	TCCCAGAAAGACAAAACC(	AGCGGAG	AGCGGAGGGCGTCGCC	AGCGGAG	Y
1*Chr1_19a_ISBma2_A		N	ATCC_23344	1	3466142-3467714	Chr1	ISBma2	GCACG	CAGATTGCTGACAAAC	ATGCTGAAGACGCCCATG(	TCCCAGAAAGACAAAACC(	AGCGGAG	AGCGGAGGGCGTCGCC	AGCGGAG	Y
10*Chr1_19a_ISBma2_A		N	PRL20	10	3468840-3470412	Chr1	ISBma2	GCACG	CAGATTGCTGACAAAC	ATGCTGAAGACGCCCATG(	TCCCAGAAAGACAAAACC(	AGCGGAG	AGCGGAGGGCGTCGCC	AGCGGAG	Y
7*Chr1_155_ISBma2_B		N	NCTC_10247	7	3470928-3472505	Chr1	ISBma2	CCTTCA	CAGATTGCTGACAAAC	ATGCTGAAGACGCCCATG(	TCCCAGAAAGACAAAACC(	GGCTGGA	GGCTGGAATCCCGGGA	GGCTGGA	Y
2*Chr1_23a_ISBma	2*Chr1_32a_ISBma2_A	R	fmh	2	3472335-3473907	Chr1	ISBma2	TGTGT(	CAGATTGCTGACAAAC	ATGCTGAAGACGCCCATG(	TCCCAGAAAGACAAAACC(	ACCGCAG	AGAGCGGCCCGAAGG(	ACCGCAG	N
5*Chr1_16_ISBma2_B		N	ATCC_10399	5	3477498-3479070	Chr1	ISBma2	CAAGA	CAGATTGCTGACAAAC	ATGCTGAAGACGCCCATG(	TCCCAGAAAGACAAAACC(	ACCGGAG	ACCGGAGCGCGAGGCT	ACCGGAG	N
9*Chr1_1b_ISBma2_A		R	SAVP1	9	3479600-3481131	Chr1	ISBma2	#IS407/	~~~~~~~~~~~~~~~	GGTCAGAAGACGCCCATG(	TCCCAGAAAGACAAAACC(	AACCCGCT	#IS407A	AACCCGCT	N.D.
3*Chr1_23a_ISBma	3*Chr1_32a_ISBma2_A	R	JHU	3	3492452-3494024	Chr1	ISBma2	TGTGT(	CAGATTGCTGACAAAC	ATGCTGAAGACGCCCATG(	TCCCAGAAAGACAAAACC(	ACCGCAG	AGAGCGGCCCGAAGG(	ACCGCAG	N
2*Chr1_22a_ISBma2_A		N	fmh	2	3507025-3508597	Chr1	ISBma2	GCCGC	CAGATTGCTGACAAAC	ATGCTGAAGACGCCCATG(	TCCCAGAAAGACAAAACC(	AGCCCGC(	AGCCCGCGAGACGCG(	AGCCCGC(	Y
3*Chr1_22a_ISBma2_A		N	JHU	3	3527903-3529475	Chr1	ISBma2	GCCGC	CAGATTGCTGACAAAC	ATGCTGAAGACGCCCATG(	TCCCAGAAAGACAAAACC(	AGCCCGC(	AGCCCGCGAGACGCG(	AGCCCGC(	Y
2*Chr1_44a_ISBma	2*Chr1_117a_ISBma2_A	R	fmh	2	393333-394905	Chr1	ISBma2	ACGGC	CAGATTGCTGACAAAC	ATGCTGAAGACGCCCATG(	TCCCAGAAAGACAAAACC(	AACCCGC(	AACCCGGCTGGAAGCC	AACCCGC(	N
3*Chr1_44a_ISBma	3*Chr1_117a_ISBma2_A	R	JHU	3	394492-396064	Chr1	ISBma2	ACGGC	CAGATTGCTGACAAAC	ATGCTGAAGACGCCCATG(	TCCCAGAAAGACAAAACC(	AACCCGC(	AACCCGGCTGGAAGCC	AACCCGC(	N
8*Chr1_44a_ISBma	8*Chr1_117a_ISBma2_A	R	2002721280	8	396339-397912	Chr1	ISBma2	ACGGC	CAGATTGCTGACAAAC	ATGCTGAAGACGCCCATG(	TCCCAGAAAGACAAAACC(	AACCCGC(	AACCCGGCTGGAAGCC	AACCCGC(	N
10*Chr1_118_ISBma2_A		N	PRL20	10	400418-401990	Chr1	ISBma2	CGATC(	CAGATTGCTGACAAAC	ATGCTGAAGACGCCCATG(	TCCCAGAAAGACAAAACC(	AAGGCGC	AAGGCGCCGCGGCAA(	AAGGCGC	Y
4*Chr1_39a_ISBma	4*Chr1_117b_ISBma2_A	R	gb8_horse_4	4	401987-403559	Chr1	ISBma2	TTTCGA	CAGATTGCTGACAAAC	ATGCTGAAGACGCCCATG(	TCCCAGAAAGACAAAACC(	AAGCCCT(	AACCCGGCTGGAAGCC	AAGCCCT(	N
1*Chr1_39a_ISBma	1*Chr1_117b_ISBma2_A	R	ATCC_23344	1	403674-405246	Chr1	ISBma2	TTTCGA	CAGATTGCTGACAAAC	ATGCTGAAGACGCCCATG(	TCCCAGAAAGACAAAACC(	AAGCCCT(	AACCCGGCTGGAAGCC	AAGCCCT(	N
4*Chr1_118_ISBma2_A		N	gb8_horse_4	4	411034-412606	Chr1	ISBma2	CGATC(	CAGATTGCTGACAAAC	ATGCTGAAGACGCCCATG(	TCCCAGAAAGACAAAACC(	AAGGCGC	AAGGCGCCGCGGCAA(	AAGGCGC	Y
1*Chr1_118_ISBma2_A		N	ATCC_23344	1	412720-414292	Chr1	ISBma2	CGATC(	CAGATTGCTGACAAAC	ATGCTGAAGACGCCCATG(	TCCCAGAAAGACAAAACC(	AAGGCGC	AAGGCGCCGCGGCAA(	AAGGCGC	Y
10*Chr1_120_ISBma2_A		N	PRL20	10	436971-438543	Chr1	ISBma2	AGCCA	CAGATTGCTGACAAAC	ATGCTGAAGACGCCCATG(	TTCCAGAAAGACAAAACCC	GGCGCCC	GGCGCCCCGCAAGGG(	GGCGCCC	Y
5*Chr1_39a_ISBma	5*Chr1_117b_ISBma2_A	R	ATCC_10399	5	440655-442227	Chr1	ISBma2	TTTCGA	CAGATTGCTGACAAAC	ATGCTGAAGACGCCCATG(	TCCCAGAAAGACAAAACC(	AAGCCCT(	AACCCGGCTGGAAGCC	AAGCCCT(	N
4*Chr1_120_ISBma2_A		N	gb8_horse_4	4	447575-449147	Chr1	ISBma2	AGCCA	CAGATTGCTGACAAAC	ATGCTGAAGACGCCCATG(	TCCCAGAAAGACAAAACC(	GGCGCCC	GGCGCCCCGCAAGGG(	GGCGCCC	Y
1*Chr1_120_ISBma2_A		N	ATCC_23344	1	449261-450833	Chr1	ISBma2	AGCCA	CAGATTGCTGACAAAC	ATGCTGAAGACGCCCATG(	TCCCAGAAAGACAAAACC(	GGCGCCC	GGCGCCCCGCAAGGG(	GGCGCCC	Y
5*Chr1_118_ISBma2_A		N	ATCC_10399	5	449701-451273	Chr1	ISBma2	CGATC(	CAGATTGCTGACAAAC	ATGCTGAAGACGCCCATG(	TCCCAGAAAGACAAAACC(	AAGGCGC	AAGGCGCCGCGGCAA(	AAGGCGC	Y
10*Chr1_121_ISBma2_A		N	PRL20	10	452829-452969	Chr1	ISBma2	#IS407/	~~~~~~~~~~~~~~~	~~~~~~~~~~~~~~~~~	TCCCAGAAAGACAAAACC(	AAGCCCTT	#IS407A	AAGCCCTT	N.D.
10*Chr1_123_ISBma2_C		N	PRL20	10	454308-455880	Chr1	ISBma2	TGCGA	CAGATTGCTGACAAAC	ATGCTGAAGACGCCCATG(	TCCCAGAAAGACAAAACC(	AGCCCGC(	AGCCCGCCTGAAGAGG	AGCCCGC(	Y
4*Chr1_121_ISBma2_A		N	gb8_horse_4	4	463433-463573	Chr1	ISBma2	#IS407/	~~~~~~~~~~~~~~~	~~~~~~~~~~~~~~~~~	TCCCAGAAAGACAAAACC(	AAGCCCTT	#IS407A	AAGCCCTT	N.D.
1*Chr1_121_ISBma2_A		N	ATCC_23344	1	465119-465259	Chr1	ISBma2	#IS407/	~~~~~~~~~~~~~~~	~~~~~~~~~~~~~~~~~	TCCCAGAAAGACAAAACC(	AAGCCCTT	#IS407A	AAGCCCTT	N.D.
9*Chr1_139a_ISBm	9*Chr1_147_ISBma2_A	D-R	SAVP1	9	479288-480860	Chr1	ISBma2	GCCCT(	CAGATTGCTGACAAAC	ATGCTGAAGACGCCCATG(	TCCCAGAAAGACAAAACC(	AGGCGCC	CCGGCGCATGGCGCCG	AGGCGCC	N
5*Chr1_120_ISBma2_A		N	ATCC_10399	5	486272-487844	Chr1	ISBma2	AGCCA	CAGATTGCTGACAAAC	ATGCTGAAGACGCCCATG(	TCCCAGAAAGACAAAACC(	GGCGCCC	GGCGCCCCGCAAGGG(	GGCGCCC	Y
10*Chr1_117a_ISBma2_A		N	PRL20	10	489817-491390	Chr1	ISBma2	TTTCGA	CAGATTGCTGACAAAC	ATGCTGAAGACGCCCATG(	TTCCAGAAAGACAAAACCC	AACCCGC(	AACCCGGCTGGAAGCC	AACCCGC(	N
5*Chr1_121_ISBma2_A		N	ATCC_10399	5	502130-502270	Chr1	ISBma2	#IS407/	~~~~~~~~~~~~~~~	~~~~~~~~~~~~~~~~~	TCCCAGAAAGACAAAACC(	AAGCCCTT	#IS407A	AAGCCCTT	N.D.
4*Chr1_44a_ISBma	4*Chr1_117a_ISBma2_A	R	gb8_horse_4	4	515358-516930	Chr1	ISBma2	ACGGC	CAGATTGCTGACAAAC	ATGCTGAAGACGCCCATG(	TCCCAGAAAGACAAAACC(	AACCCGC(	AACCCGGCTGGAAGCC	AACCCGC(	N
3*Chr1_13a_ISBma	3*Chr1_32b_ISBma2_A	R	JHU	3	51562-53134	Chr1	ISBma2	GCGAC	CAGATTGCTGACAAAC	ATGCTGAAGACGCCCATG(	TCCCAGAAAGACAAAACC(	GAGCGGC	GACCGCGTGGGCGGTC	GAGCGGC	N
1*Chr1_44a_ISBma	1*Chr1_117a_ISBma2_A	R	ATCC_23344	1	517013-518585	Chr1	ISBma2	ACGGC	CAGATTGCTGACAAAC	ATGCTGAAGACGCCCATG(	TCCCAGAAAGACAAAACC(	AACCCGC(	AACCCGGCTGGAAGCC	AACCCGC(	N
2*Chr1_13a_ISBma	2*Chr1_32b_ISBma2_A	R	fmh	2	51985-53557	Chr1	ISBma2	GCGAC	CAGATTGCTGACAAAC	ATGCTGAAGACGCCCATG(	TCCCAGAAAGACAAAACC(	GAGCGGC	GACCGCGTGGGCGGTC	GAGCGGC	N
8*Chr1_13a_ISBma	8*Chr1_32b_ISBma2_A	R	2002721280	8	52889-54461	Chr1	ISBma2	GCGAC	CAGATTGCTGACAAAC	ATGCTGAAGACGCCCATG(	TCCCAGAAAGACAAAACC(	GAGCGGC	GACCGCGTGGGCGGTC	GAGCGGC	N
5*Chr1_13a_ISBma	5*Chr1_23a_ISBma2_A	R	ATCC_10399	5	53180-54752	Chr1	ISBma2	GCGAC	CAGATTGCTGACAAAC	ATGCTGAAGACGCCCATG(	TCCCAGAAAGACAAAACC(	ACCGCAG	GACCGCGTGGGCGGTC	ACCGCAG	N
5*Chr1_44a_ISBma	5*Chr1_117a_ISBma2_A	R	ATCC_10399	5	556580-558152	Chr1	ISBma2	GCACG	CAGATTGCTGACAAAC	ATGCTGAAGACGCCCATG(	TCCCAGAAAGACAAAACC(	AACCCGC(	AAAACCCGGCTGGAAG	AACCCGC(	N
2*Chr1_50_ISBma2_A		N	fmh	2	575878-577450	Chr1	ISBma2	GCGAC	CAGATTGCTGACAAAC	ATGCTGAAGACGCCCATG(	TCCCAGAAAGACAAAACC(	GGGGCGC	GGGGCGCGACGTACTC	GGGGCGC	Y
9*Chr1_41_ISBma2_A		N	SAVP1	9	576498-577387	Chr1	ISBma2	AATCA(	CAGATTGCTGACAAAC	ATGCTGAAGACGCCCATG(	#	#IS407A	CCCTCCACTGGAGGGC	#IS407A	N.D.
3*Chr1_50_ISBma2_A		N	JHU	3	579136-580708	Chr1	ISBma2	GCGAC	CAGATTGCTGACAAAC	ATGCTGAAGACGCCCATG(	TCCCAGAAAGACAAAACC(	GGGGCGN	GGGGCGCGACGTACTC	GGGGCGN	Y
9*Chr1_39b_ISBma	9*Chr1_44b_ISBma2_A	R	SAVP1	9	583222-584794	Chr1	ISBma2	GCAGG	CAGATTGCTGACAAAC	ATGCTGAAGACGCCCATG(	TCCCAGAAAGACAAAACC(	AAAAACC(	CCGCCCGCTCCGGGCG	AAAAACC(	N
4*Chr1_23a_ISBma	4*Chr1_32a_ISBma2_A	R	gb8_horse_4	4	58363-59935	Chr1	ISBma2	TGTGT(	CAGATTGCTGACAAAC	ATGCTGAAGACGCCCATG(	TCCCAGAAAGACAAAACC(	ACCGCAG	AGAGCGGCCCGAAGG(	ACCGCAG	N
1*Chr1_23a_ISBma	1*Chr1_32a_ISBma2_A	R	ATCC_23344	1	58691-60263	Chr1	ISBma2	TGTGT(	CAGATTGCTGACAAAC	ATGCTGAAGACGCCCATG(	TCCCAGAAAGACAAAACC(	ACCGCAG	AGAGCGGCCCGAAGG(	ACCGCAG	N
2*Chr1_52_ISBma2_A		N	fmh	2	631884-633456	Chr1	ISBma2	CCGGA	CAGATTGCTGACAAAC	ATGCTGAAGACGCCCATG(	TCCCAGAAAGACAAAACC(	ACGGCCG	ACGGCCGCGCTCACGC	ACGGCCG	Y
3*Chr1_52_ISBma2_A		N	JHU	3	635378-636950	Chr1	ISBma2	CCGGA	CAGATTGCTGACAAAC	ATGCTGAAGACGCCCATG(	TCCCAGAAAGACAAAACC(	ACGGCCG	ACGGCCGCGCTCACGC	ACGGCCG	Y
10*Chr1_50_ISBma2_A		N	PRL20	10	668438-670010	Chr1	ISBma2	AGCGA	CAGATTGCTGACAAAC	ATGCTGAAGACGCCCATG(	TCCCAGAAAGACAAAACC(	#contig_br	GGGGGCGCGACGTACT	#contig_br	N
9*Chr1_39a_ISBma	9*Chr1_44a_ISBma2_A	R	SAVP1	9	693678-695250	Chr1	ISBma2	ACGGC	CAGATTGCTGACAAAC	ATGCTGAAGACGCCCATG(	TCCCAGAAAGACAAAACC(	AGCCCTC(	AACCCGGCTGGAAGCC	AGCCCTC(	N
4*Chr1_50_ISBma2_A		N	gb8_horse_4	4	693979-695542	Chr1	ISBma2	GCGAC	CAGATTGCTGACAAAC	ATGCTGAAGACGCCCATG(	TCCCAGAAAGACAAAACC(	#contig_br	GGGGCGCGACGTACTC	#contig_br	N.D.

1*Chr1_50_ISBma2_A		N	ATCC_23344	1	699018-700590	Chr1	ISBma2	GCGAC	CAGATTGCTGACAAAC	ATGCTGAAGACGCCCATG	TCCCAGAAAGACAAAACC	GGGGCGC	GGGGCGCGACGTACTC	GGGGCGC	Y		
10*Chr1_52_ISBma2_A		N	PRL20	10	722740-724312	Chr1	ISBma2	CCGGA	CAGATTGCTGACAAAC	ATGCTGAAGACGCCCATG	TCCCAGAAAGACAAAACC	ACGGCCG	ACGGCCGCGCTCACGC	ACGGCCG	Y		
5*Chr1_50_ISBma2_A		N	ATCC_10399	5	735242-736814	Chr1	ISBma2	GCGAC	CAGATTGCTGACAAAC	ATGCTGAAGACGCCCATG	TCCCAGAAAGACAAAACC	GGGGCGC	GGGGCGCGACGTACTC	GGGGCGC	Y		
4*Chr1_52_ISBma2_A		N	gb8_horse_4	4	749747-751319	Chr1	ISBma2	CCGGA	CAGATTGCTGACAAAC	ATGCTGAAGACGCCCATG	TCCCAGAAAGACAAAACC	ACGGCCG	ACGGCCGCGCTCACGC	ACGGCCG	Y		
1*Chr1_52_ISBma2_A		N	ATCC_23344	1	754776-756348	Chr1	ISBma2	CCGGA	CAGATTGCTGACAAAC	ATGCTGAAGACGCCCATG	TCCCAGAAAGACAAAACC	ACGGCCG	ACGGCCGCGCTCACGC	ACGGCCG	Y		
5*Chr1_52_ISBma2_A		N	ATCC_10399	5	789822-791394	Chr1	ISBma2	CCGGA	CAGATTGCTGACAAAC	ATGCTGAAGACGCCCATG	TCCCAGAAAGACAAAACC	ACGGCCG	ACGGCCGCGCTCACGC	ACGGCCG	Y		
5*Chr1_22a_ISBma2_A		N	ATCC_10399	5	86761-88333	Chr1	ISBma2	GCCGC	CAGATTGCTGACAAAC	ATGCTGAAGACGCCCATG	TCCCAGAAAGACAAAACC	AGCCCGC	AGCCCGCGAGACGCG	AGCCCGC	Y		
10*Chr1_13b_ISBm	10*Chr1_32a_ISBma2_A	R	PRL20	10	86972-88544	Chr1	ISBma2	TGTGT	CAGATTGCTGACAAAC	ATGCTGAAGACGCCCATG	TCCCAGAAAGACAAAACC	AACGGAC	AGAGCGGCCCGAAGG	AACGGAC	N		
6*Chr1_117a_ISBma2_A		N	NCTC_10229	6	915696-917293	Chr1	ISBma2	TCGAC	CAGATTGCTGACAAAC	ATGCTGAAGACGCCCATG	TCCCAGAAAGACAAAACC	AACCCGC	CCCGCTGGTTTCAGCG	AACCCGC	N		
4*Chr1_22a_ISBma2_A		N	gb8_horse_4	4	91845-93417	Chr1	ISBma2	GCCGC	CAGATTGCTGACAAAC	ATGCTGAAGACGCCCATG	TCCCAGAAAGACAAAACC	AGCCCGC	AGCCCGCGAGACGCG	AGCCCGC	Y		
1*Chr1_22a_ISBma2_A		N	ATCC_23344	1	92218-93790	Chr1	ISBma2	GCCGC	CAGATTGCTGACAAAC	ATGCTGAAGACGCCCATG	TCCCAGAAAGACAAAACC	AGCCCGC	AGCCCGCGAGACGCG	AGCCCGC	Y		
6*Chr1_118_ISBma2_A		N	NCTC_10229	6	924787-926359	Chr1	ISBma2	CGATC	CAGATTGCTGACAAAC	ATGCTGAAGACGCCCATG	TCCCAGAAAGACAAAACC	AAGGCGC	AAGGCGCCGCGGCAA	AAGGCGC	Y		
7*Chr1_90_ISBma2_A		N	NCTC_10247	7	960223-961410	Chr1	ISBma2	#IS407	~~~~~~~~~~~~~~~	~~~~~~~~~~~~~~~~~~	TCCCAGAAAGACAAAACC	AAAGCCC	#IS407A	AAAGCCC	N.D.		
6*Chr1_120_ISBma2_A		N	NCTC_10229	6	962586-964158	Chr1	ISBma2	AGCCA	CAGATTGCTGACAAAC	ATGCTGAAGACGCCCATG	TCCCAGAAAGACAAAACC	GGCGCCC	GGCGCCCCGCAAGGG	GGCGCCC	Y		
6*Chr1_121_ISBma2_A		N	NCTC_10229	6	978444-978584	Chr1	ISBma2	#IS407	~~~~~~~~~~~~~~~	~~~~~~~~~~~~~~~~~~	TCCCAGAAAGACAAAACC	AAGCCCT	#IS407A	AAGCCCT	N.D.		

Chromosome 2

IS *407* A

								5'-ext	5'-IR	*orfA*	intergenic region	*orfB*	3'-IR	3'-ext	5'-DR	3'-DR	et duplicates
									TGACCTGCCCCCT				AGGGGGCAGGTCA				
10*Chr2_51_IS407A_A		N	Bm_PRL20	10	1000862-1002097	Chr2	IS407A	CGGCC	TGACCTGCCCCC~TAC	ATGAAGAAGCGCTTTACG	GCCCGCAAGCCAAACGCG	TGTTGGC	AGGGACTTTGCTAGAA	GGCGGCC	GGCG	GGCG	Y
5*Chr2_42_IS407A_A		N	Bm_ATCC_1039	5	1004926-1006161	Chr2	IS407A	ATCGA	TGACCTGCCCCC~TAC	ATGAAGAAGCGCTTTACG	GCCCGCAAGCCAAACGCG	TGTTGGC	AGGGACTTTGCTAGAA	CTGCGGC	CTGC	CTGC	Y
4*Chr2_51_IS407A_A		N	Bm_gb8_horse_	4	1009373-1010608	Chr2	IS407A	CGGCC	TGACCTGCCCCC~TAC	ATGAAGAAGCGCTTTACG	GCCCGCAAGCCAAACGCG	TGTTGGC	AGGGACTTTGCTAGAA	GGCGGCC	GGCG	GGCG	Y
3*Chr2_52_IS407A_A		N	Bm_JHU	3	1011830-1013065	Chr2	IS407A	GAATT	TGACCTGCCCCC~TAC	ATGAAGAAGCGCTTTACG	GCCCGCAAGCCAAACGCG	TGTTGGC	AGGGACTTTGCTAGAA	CCGTCAC	GGAT	CCGT	N
10*Chr2_38_IS407	10*Chr2_50_IS407A_B	D-R	Bm_PRL20	10	1013932-1015167	Chr2	IS407A	GTCCA	TGACCTGCCCCC~TAC	ATGAAGAAGCGCTTTACG	GCCCGCAAGCCAAACGCG	TGTTGGC	AGGGACTTTGCTAGAA	CGCGAGA	GAAC	CGCG	N
1*Chr2_51_IS407A_A		N	Bm_ATCC_2334	1	1014356-1015591	Chr2	IS407A	CGGCC	TGACCTGCCCCC~TAC	ATGAAGAAGCGCTTTACG	GCCCGCAAGCCAAACGCG	TGTTGGC	AGGGACTTTGCTAGAA	GGCGGCC	GGCG	GGCG	Y
6*Chr2_23_IS407A_A		D-R	Bm_NCTC_102	6	1017427-1018662	Chr2	IS407A	CGCCG	TGACCTGCCCCC~TAC	ATGAAGAAGCGCTTTACG	GCCCGCAAGCCAAACGCG	TGTTGGC	AGGGACTTTGCTAGAA	ATGGATTT	CGAT	ATGG	N
10*Chr2_39_IS407	10*Chr2_44b_IS407A_A	R	Bm_PRL20	10	1023389-1024624	Chr2	IS407A	GACAG	TGACCTGCCCCC~TAC	ATGAAGAAGCGCTTTACG	GCCCGCAAGCCAAACGCG	TGTTGGC	AGGGACTTTGCTAGAA	CATGCGC	CGCC	CATG	N
4*Chr2_49_IS407A_A		N	Bm_gb8_horse_	4	1028731-1029966	Chr2	IS407A	CCCAA	TGACCTGCCCCC~TAC	ATGAAGAAGCGCTTTACG	GCCCGCAAGCCAAACGCG	TGTTGGC	AGGGACTTTGCTAGAA	AGCGACG	CCCA	AGCG	N
2*Chr2_51_IS407A_A		N	Bm_fmh	2	1031221-1032456	Chr2	IS407A	CGGCC	TGACCTGCCCCC~TAC	ATGAAGAAGCGCTTTACG	GCCCGCAAGCCAAACGCG	TGTTGGC	AGGGACTTTGCTAGAA	GGCGGCC	GGCG	GGCG	Y
4*Chr2_72_IS407A_A		N	Bm_gb8_horse_	4	103170-104405	Chr2	IS407A	CACAT	TGACCTGCCCCC~TAC	ATGAAGAAGCGCTTTACG	GCCCGCAAGCCAAACGCG	TGTTGGC	AGGGACTTTGCTAGAA	GATGGAT	GATG	GATG	Y
1*Chr2_72_IS407A_A		N	Bm_ATCC_2334	1	103191-104426	Chr2	IS407A	CACAT	TGACCTGCCCCC~TAC	ATGAAGAAGCGCTTTACG	GCCCGCAAGCCAAACGCG	TGTTGGC	AGGGACTTTGCTAGAA	GATGGAT	GATG	GATG	Y
1*Chr2_49_IS407A_A		N	Bm_ATCC_2334	1	1033957-1035192	Chr2	IS407A	CCCAA	TGACCTGCCCCC~TAC	ATGAAGAAGCGCTTTACG	GCCCGCAAGCCAAACGCG	TGTTGGC	AGGGACTTTGCTAGAA	AGCGACG	CCCA	AGCG	N
8*Chr2_17_IS407A	8*Chr2_38_IS407A_A	D-R	Bm_200272128	8	1036615-1037850	Chr2	IS407A	GTCCA	TGACCTGCCCCC~TAC	ATGAAGAAGCGCTTTACG	GCCCGCAAGCCAAACGCG	TGTTGGC	AGGGACTTTGCTAGAA	GTGCGCT	GAAC	GTGC	N
7*Chr2_42_IS407A_A		N	Bm_NCTC_102	7	1039995-1041230	Chr2	IS407A	ATCGA	TGACCTGCCCCC~TAC	ATGAAGAAGCGCTTTACG	GCCCGCAAGCCAAACGCG	TGTTGGC	AGGGACTTTGCTAGAA	CTGCGGC	CTGC	CTGC	Y
7*Chr2_72_IS407A_A		N	Bm_NCTC_102	7	104501-105736	Chr2	IS407A	CACAT	TGACCTGCCCCC~TAC	ATGAAGAAGCGCTTTACG	GCCCGCAAGCCAAACGCG	TGTTGGC	AGGGACTTTGCTAGAA	GATGGAT	GATG	GATG	Y
6*Chr2_28_IS407A	6*Chr2_31b_IS407A_A	D	Bm_NCTC_102	6	104943-106178	Chr2	IS407A	GAACG	TGACCTGCCCCC~TAC	ATGAAGAAGCGCTTTACG	GCCCGCAAGCCAAACGCG	TGTTGGC	AGGGACTTTGCTAGAA	CGATGCT	GATC	CGAT	N
2*Chr2_49_IS407A_A		N	Bm_fmh	2	1052180-1053415	Chr2	IS407A	CCCAA	TGACCTGCCCCC~TAC	ATGAAGAAGCGCTTTACG	GCCCGCAAGCCAAACGCG	TGTTGGC	AGGGACTTTGCTAGAA	AGCGACG	CCCA	AGCG	N
8*Chr2_10a_IS407	8*Chr2_15b_IS407A_A	R	Bm_200272128	8	1052451-1053615	Chr2	IS407A	#contig	~~~~~~~~~~~~~~	NNNAAGAAGCGCTTTACG	GCCCGCAAGCCAAACGCG	TGTTGGC	AGGGACTTTGCTAGAA	ACAGTTTT	reak	ACAG	N
4*Chr2_44b_IS407	4*Chr2_48_IS407A_A	R	Bm_gb8_horse_	4	1056512-1057747	Chr2	IS407A	GACAG	TGACCTGCCCCC~TAC	ATGAAGAAGCGCTTTACG	GCCCGCAAGCCAAACGCG	TGTTGGC	AGGGACTTTGCTAGAA	CGGCAGC	CGCC	CGGC	N
10*Chr2_45_IS407A_B		N	Bm_PRL20	10	1057806-1059041	Chr2	IS407A	CGGGC	TGACCTGCCCCC~TAC	ATGAAGAAGCGCTTTACG	GCCCGCAAGCCAAACGCG	TGTTGGC	AGGGACTTTGCTAGAA	GCCCGCC	CTTT	GCCC	N
1*Chr2_44b_IS407	1*Chr2_48_IS407A_A	R	Bm_ATCC_2334	1	1061754-1062989	Chr2	IS407A	GACAG	TGACCTGCCCCC~TAC	ATGAAGAAGCGCTTTACG	GCCCGCAAGCCAAACGCG	TGTTGGC	AGGGACTTTGCTAGAA	CGGCAGC	CGCC	CGGC	N
8*Chr2_10b_IS407A_A		R	Bm_200272128	8	106411-107646	Chr2	IS407A	GCCTC	TGACCTGCCCCC~TAC	ATGAAGAAGCGCTTTACG	GCCCGCAAGCCAAACGCG	TGTTGGC	AGGGACTTTGCTAGAA	TTTGCTAG	GCGG	TTTG	N
8*Chr2_9a_IS407A_A		N	Bm_200272128	8	1069857-1071092	Chr2	IS407A	CATGC	TGACCTGCCCCC~TAC	ATGAAGAAGCGCTTTACG	GCCCGCAAGCCAAACGCG	TGTTGGC	AGGGACTTTGCTAGAA	CAAGCAG	CAAG	CAAG	Y
2*Chr2_44b_IS407	2*Chr2_48_IS407A_A	R	Bm_fmh	2	1080393-1081628	Chr2	IS407A	GACAG	TGACCTGCCCCC~TAC	ATGAAGAAGCGCTTTACG	GCCCGCAAGCCAAACGCG	TGTTGGC	AGGGACTTTGCTAGAA	CGGCAGC	CGCC	CGGC	N
8*Chr2_55_IS407A_A		D-R	Bm_200272128	8	108421-109656	Chr2	IS407A	AGCGT	TGACCTGCCCCC~TAC	ATGAAGAAGCGCTTTACG	GCCCGCAAGCCAAACGCG	TGTTGGC	AGGGACTTTGCTAGAA	GGAGTTC	GCCC	GGAG	N
10*Chr2_46_IS407A_C		N	Bm_PRL20	10	1091241-1092476	Chr2	IS407A	GGCGA	TGACCTGCCCCC~TAC	ATGAAGAAGCGCTTTACG	GCCCGCAAGCCAAACGCG	TGTTGGC	AGGGACTTTGCTAGAA	CAGCAGA	CAGC	CAGC	Y
4*Chr2_45_IS407A_B		N	Bm_gb8_horse_	4	1091459-1092694	Chr2	IS407A	GCCGC	TGACCTGCCCCC~TAC	ATGAAGAAGCGCTTTACG	GCCCGCAAGCCAAACGCG	TGTTGGC	AGGGACTTTGCTAGAA	GAAAGGC	GGGC	GAAA	N
3*Chr2_51_IS407A_A		N	Bm_JHU	3	1092993-1094228	Chr2	IS407A	CGGCC	TGACCTGCCCCC~TAC	ATGAAGAAGCGCTTTACG	GCCCGCAAGCCAAACGCG	TGTTGGC	AGGGACTTTGCTAGAA	GGCGGCC	GGCG	GGCG	Y
5*Chr2_40_IS407A	5*Chr2_41_IS407A_B	D	Bm_ATCC_1039	5	1093117-1094352	Chr2	IS407A	GAAGG	TGACCTGCCCCC~TAC	ATGAAGAAGCGCTTTACG	GCCCGCAAGCCAAACGCG	TGTTGGC	AGGGACTTTGCTAGAA	CGAACTG	AGCG	CGAA	N
1*Chr2_45_IS407A_B		N	Bm_ATCC_2334	1	1096730-1097965	Chr2	IS407A	GCCGC	TGACCTGCCCCC~TAC	ATGAAGAAGCGCTTTACG	GCCCGCAAGCCAAACGCG	TGTTGGC	AGGGACTTTGCTAGAA	GAAAGGC	GGGC	GAAA	N
10*Chr2_2_IS407A	10*Chr2_73_IS407A_A	D-R	Bm_PRL20	10	111417-112652	Chr2	IS407A	CGCTG	TGACCTGCCCCC~TAC	ATGAAGAAGCGCTTTACG	GCCCGCAAGCCAAACGCG	TGTTGGC	AGGGACTTTGCTAGAA	CAACGCA	GTGT	CAAC	N
3*Chr2_49_IS407A_A		N	Bm_JHU	3	1114839-1116074	Chr2	IS407A	CCCAA	TGACCTGCCCCC~TAC	ATGAAGAAGCGCTTTACG	GCCCGCAAGCCAAACGCG	TGTTGGC	AGGGACTTTGCTAGAA	AGCGACG	CCCA	AGCG	N
2*Chr2_45_IS407A_B		N	Bm_fmh	2	1115369-1116604	Chr2	IS407A	GCCGC	TGACCTGCCCCC~TAC	ATGAAGAAGCGCTTTACG	GCCCGCAAGCCAAACGCG	TGTTGGC	AGGGACTTTGCTAGAA	GAAAGGC	GGGC	GAAA	N
2*Chr2_72_IS407A_A		N	Bm_fmh	2	112416-113651	Chr2	IS407A	CACAT	TGACCTGCCCCC~TAC	ATGAAGAAGCGCTTTACG	GCCCGCAAGCCAAACGCG	TGTTGGC	AGGGACTTTGCTAGAA	GATGGAT	GATG	GATG	Y
6*Chr2_15a_IS407	6*Chr2_18_IS407A_A	D-R	Bm_NCTC_102	6	1128849-1130084	Chr2	IS407A	AGACC	TGACCTGCCCCC~TAC	ATGAAGAAGCGCTTTACG	GCCCGCAAGCCAAACGCG	TGTTGGC	AGGGACTTTGCTAGAA	GGATCAG	AGAT	GGAT	N
10*Chr2_44a_IS40	10*Chr2_48_IS407A_A	D-R	Bm_PRL20	10	1135654-1136889	Chr2	IS407A	CGATC	TGACCTGCCCCC~TAC	ATGAAGAAGCGCTTTACG	GCCCGCAAGCCAAACGCG	TGTTGGC	AGGGACTTTGCTAGAA	AGGGGTA	GCGG	AGGG	N

3*Chr2_72_IS407A_A		N	Bm_JHU	3	113583-114818	Chr2	IS407A	CACAT	TGACCTGCCCCC~TAC	ATGAAGAAGCGCTTTACG	GCCCGCAAGCCAAACGCG	TGTTGGC	AGGGACTTTGCTAGAA	GATGGAT	GATG	GATG	Y
3*Chr2_44b_IS407	3*Chr2_48_IS407A_A	D-R	Bm_JHU	3	1143190-1144425	Chr2	IS407A	GACAG	TGACCTGCCCCC~TAC	ATGAAGAAGCGCTTTACG	GCCCGCAAGCCAAACGCG	TGTTGGC	AGGGACTTTGCTAGAA	CGGCAGC	CGCC	CGGC	N
7*Chr2_44a_IS407A_A		N	Bm_NCTC_102	7	1145841-1147076	Chr2	IS407A	GACAG	TGACCTGCCCCC~TAC	ATGAAGAAGCGCTTTACG	GCCCGCAAGCCAAACGCG	TGTTGGC	AGGGACTTTGCTAGAA	AGGGGTA	CGCC	AGGG	N
5*Chr2_33_IS407A	5*Chr2_38_IS407A_A	D-R	Bm_ATCC_103	5	1160971-1162206	Chr2	IS407A	GTCCA	TGACCTGCCCCC~TAC	ATGAAGAAGCGCTTTACG	GCCCGCAAGCCAAACGCG	TGTTGGC	AGGGACTTTGCTAGAA	GTCCAGC	GAAC	GTCC	N
3*Chr2_45_IS407A_B		N	Bm_JHU	3	1178167-1179402	Chr2	IS407A	GCCGC	TGACCTGCCCCC~TAC	ATGAAGAAGCGCTTTACG	GCCCGCAAGCCAAACGCG	TGTTGGC	AGGGACTTTGCTAGAA	GAAAGGC	GGGC	GAAA	N
9*Chr2_44a_IS407A_A		R	Bm_SAVP1	9	117856-119091	Chr2	IS407A	CGATC	TGACCTGCCCCC~TAC	ATGAAGAAGCGCTTTACG	GCCCGCAAGCCAAACGCG	TGTTGGC	AGGGACTTTGCTAGAA	AGGGGTA	GCGG	AGGG	N
8*Chr2_6_IS407A_A		N	Bm_200272128	8	1188221-1189456	Chr2	IS407A	GCGCA	TGACCTGCCCCC~TAC	ATGAAGAAGCGCTTTACG	GCCCGCAAGCCAAACGCG	TGTTGGC	AGGGACTTTGCTAGAA	CGTGAAC	CGTG	CGTG	Y
8*Chr2_13b_IS407	8*Chr2_57b_IS407A_A	R	Bm_200272128	8	1194967-1196202	Chr2	IS407A	CAAGC	TGACCTGCCCCC~TAC	ATGAAGAAGCGCTTTACG	GCCCGCAAGCCAAACGCG	TGTTGGC	AGGGACTTTGCTAGAA	ATCTTCCC	GGTT	ATCT	N
5*Chr2_31b_IS407A_A		R	Bm_ATCC_103	5	1198234-1199469	Chr2	IS407A	GAACG	TGACCTGCCCCC~TAC	ATGAAGAAGCGCTTTACG	GCCCGCAAGCCAAACGCG	TGTTGGC	AGGGACTTTGCTAGAA	GGAGTTC	GATC	GGAG	N
5*Chr2_13a_IS407A_A		R	Bm_ATCC_103	5	1200293-1201483	Chr2	IS407A	GAGCC	TGACCTGCCCCC~TAC	ATGAAGAAGCGCTTTACG	GCCCGCAAGCCAAACGCG	TGTTGGC	AGNNNNNNNNNNNN	#	CTCG	#	N.D.
5*Chr2_10a_IS407A_A		N	Bm_ATCC_103	5	1214324-1215557	Chr2	IS407A	CGCGC	TGACCTGCCCCC~TAC	ATGAAGAAGCGCTTTACG	GCCCGCAAGCCAAACGCG	TGTTGGC	AGGGACTTTGCTAGAA	TCACGCC	GCTT	TCAC	N
4*Chr2_27_IS407A	4*Chr2_44a_IS407A_A	D-R	Bm_gb8_horse	4	1219713-1220948	Chr2	IS407A	CTGGC	TGACCTGCCCCC~TAC	ATGAAGAAGCGCTTTACG	GCCCGCAAGCCAAACGCG	TGTTGGC	AGGGACTTTGCTAGAA	AGGGGTA	CGAG	AGGG	N
1*Chr2_27_IS407A	1*Chr2_44a_IS407A_A	D-R	Bm_ATCC_233	1	1225576-1226811	Chr2	IS407A	CTGGC	TGACCTGCCCCC~TAC	ATGAAGAAGCGCTTTACG	GCCCGCAAGCCAAACGCG	TGTTGGC	AGGGACTTTGCTAGAA	AGGGGTA	CGAG	AGGG	N
5*Chr2_9b_IS407A	5*Chr2_31a_IS407A_A	R	Bm_ATCC_103	5	1236549-1237784	Chr2	IS407A	CATGC	TGACCTGCCCCC~TAC	ATGAAGAAGCGCTTTACG	GCCCGCAAGCCAAACGCG	TGTTGGC	AGGGACTTTGCTAGAA	GATCGAG	CAAG	GATC	N
10*Chr2_42_IS407A_A		N	Bm_PRL20	10	1242592-1243827	Chr2	IS407A	ATCGA	TGACCTGCCCCC~TAC	ATGAAGAAGCGCTTTACG	GCCCGCAAGCCAAACGCG	TGTTGGC	AGGGACTTTGCTAGAA	CTGCGGC	CTGC	CTGC	Y
9*Chr2_2_IS407A_	9*Chr2_73_IS407A_A	D-R	Bm_SAVP1	9	1244526-1245761	Chr2	IS407A	CGCTG	TGACCTGCCCCC~TAC	ATGAAGAAGCGCTTTACG	GCCCGCAAGCCAAACGCG	TGTTGGC	AGGGACTTTGCTAGAA	CAACGCA	GTGT	CAAC	N
2*Chr2_27_IS407A	2*Chr2_44a_IS407A_A	D-R	Bm_fmh	2	1246300-1247535	Chr2	IS407A	CTGGC	TGACCTGCCCCC~TAC	ATGAAGAAGCGCTTTACG	GCCCGCAAGCCAAACGCG	TGTTGGC	AGGGACTTTGCTAGAA	AGGGGTA	CGAG	AGGG	N
5*Chr2_70_IS407A_A		N	Bm_ATCC_103	5	125421-126656	Chr2	IS407A	CAACA	TGACCTGCCCCC~TAC	ATGAAGAAGCGCTTTACG	GCCCGCAAGCCAAACGCG	TGTTGGC	AGGGACTTTGCTAGAA	GTACCGC	GTAC	GTAC	Y
7*Chr2_48_IS407A_A		N	Bm_NCTC_102	7	1257060-1258295	Chr2	IS407A	CATTC	TGACCTGCCCCC~TAC	ATGAAGAAGCGCTTTACG	GCCCGCAAGCCAAACGCG	TGTTGGC	AGGGACTTTGCTAGAA	GCCGCTC	GCCG	GCCG	Y
9*Chr2_72_IS407A_A		N	Bm_SAVP1	9	1267326-1268561	Chr2	IS407A	CACAT	TGACCTGCCCCC~TAC	ATGAAGAAGCGCTTTACG	GCCCGCAAGCCAAACGCG	TGTTGGC	AGGGACTTTGCTAGAA	GATGGAT	GATG	GATG	Y
7*Chr2_49_IS407A_A		N	Bm_NCTC_102	7	1284838-1286073	Chr2	IS407A	CCGAC	TGACCTGCCCCC~TAC	ATGAAGAAGCGCTTTACG	GCCCGCAAGCCAAACGCG	TGTTGGC	AGGGACTTTGCTAGAA	AGCGACG	AGCG	AGCG	Y
5*Chr2_26_IS407A	5*Chr2_27_IS407A_A	D	Bm_ATCC_103	5	1298656-1299890	Chr2	IS407A	CTGGC	TGACCTGCCCCC~TAC	ATGAAGAAGCGCTTTACG	GCCCGCAAGCCAAACGCG	TGTTGGC	AGGGACTTTGCTAGAA	CTGGATA	CGAG	CTGG	N
7*Chr2_51_IS407A_A		N	Bm_NCTC_102	7	1306894-1308129	Chr2	IS407A	CGGCC	TGACCTGCCCCC~TAC	ATGAAGAAGCGCTTTACG	GCCCGCAAGCCAAACGCG	TGTTGGC	AGGGACTTTGCTAGAA	GGCGGCC	GGCG	GGCG	Y
6*Chr2_3_IS407A_	6*Chr2_76_IS407A_A	D-R	Bm_NCTC_102	6	1307203-1308438	Chr2	IS407A	GGGCA	TGACCTGCCCCC~TAC	ATGAAGAAGCGCTTTACG	GCCCGCAAGCCAAACGCG	TGTTGGC	AGGGACTTTGCTAGAA	TCTTCCTT	GATG	TCTT	N
3*Chr2_27_IS407A_A		D-R	Bm_JHU	3	1310457-1311650	Chr2	IS407A	CTGGC	TGACCTGCCCCC~TAC	ATGAAGAAGCGCTTTACG	GCCCGCAAGCCAAACGCG	TGTTGGC	GGGACNNNNNCACAC	#contig_br	CGAG	#con	N
3*Chr2_42_IS407A_A		D-R	Bm_JHU	3	1311687-1312631	Chr2	IS407A	#contig	~~~~~~~~~~~~~~	~~~~~~~~~~~~~~~~~	GCCCGCAAGCCAAACGCG	TGTTGGC	AGGGACTTTGCTAGAA	CTGCGGC	reak	CTGC	N
4*Chr2_42_IS407A_A		N	Bm_gb8_horse	4	1318264-1319499	Chr2	IS407A	ATCGA	TGACCTGCCCCC~TAC	ATGAAGAAGCGCTTTACG	GCCCGCAAGCCAAACGCG	TGTTGGC	AGGGACTTTGCTAGAA	CTGCGGC	CTGC	CTGC	Y
10*Chr2_70_IS407	10*Chr2_76_IS407A_A	D-R	Bm_PRL20	10	132158-133393	Chr2	IS407A	CAACA	TGACCTGCCCCC~TAC	ATGAAGAAGCGCTTTACG	GCCCGCAAGCCAAACGCG	TGTTGGC	AGGGACTTTGCTAGAA	TCTTCCTT	GTAC	TCTT	N
1*Chr2_42_IS407A_A		N	Bm_ATCC_233	1	1324421-1325656	Chr2	IS407A	ATCGA	TGACCTGCCCCC~TAC	ATGAAGAAGCGCTTTACG	GCCCGCAAGCCAAACGCG	TGTTGGC	AGGGACTTTGCTAGAA	CTGCGGC	CTGC	CTGC	Y
10*Chr2_40_IS407	10*Chr2_41_IS407A_B	D	Bm_PRL20	10	1330658-1331893	Chr2	IS407A	GAAGG	TGACCTGCCCCC~TAC	ATGAAGAAGCGCTTTACG	GCCCGCAAGCCAAACGCG	TGTTGGC	AGGGACTTTGCTAGAA	CGAACTG	AGCG	CGAA	N
9*Chr2_70_IS407A_A		N	Bm_SAVP1	9	1334773-1336008	Chr2	IS407A	CAACA	TGACCTGCCCCC~TAC	ATGAAGAAGCGCTTTACG	GCCCGCAAGCCAAACGCG	TGTTGGC	AGGGACTTTGCTAGAA	GTACCGC	GTAC	GTAC	Y
8*Chr2_58_IS407A_A		N	Bm_200272128	8	1343603-1344838	Chr2	IS407A	CGCGG	TGACCTGCCCCC~TAC	ATGAAGAAGCGCTTTACG	GCCCGCAAGCCAAACGCG	TGTTGGC	AGGGACTTTGCTAGAA	ATCCGGG	CGAC	ATCC	N
8*Chr2_34_IS407A	8*Chr2_54_IS407A_A	D-R	Bm_200272128	8	134525-135760	Chr2	IS407A	CGCGG	TGACCTGCCCCC~TAC	ATGAAGAAGCGCTTTACG	GCCCGCAAGCCAAACGCG	TGTTGGC	AGGGACTTTGCTAGAA	GGATAGA	GAGG	GGAT	N
2*Chr2_42_IS407A_A		N	Bm_fmh	2	1349579-1350814	Chr2	IS407A	ATCGA	TGACCTGCCCCC~TAC	ATGAAGAAGCGCTTTACG	GCCCGCAAGCCAAACGCG	TGTTGGC	AGGGACTTTGCTAGAA	CTGCGGC	CTGC	CTGC	Y
7*Chr2_52_IS407A_A		N	Bm_NCTC_102	7	1386349-1387584	Chr2	IS407A	GAATT	TGACCTGCCCCC~TAC	ATGAAGAAGCGCTTTACG	GCCCGCAAGCCAAACGCG	TGTTGGC	AGGGACTTTGCTAGAA	CGTCACG	GGAT	CGTC	N
5*Chr2_51_IS407A_A		N	Bm_ATCC_103	5	1390265-1391500	Chr2	IS407A	CGGCC	TGACCTGCCCCT~TAC	ATGAAGAAGCGCTTTACG	GCCCGCAAGCCAAACGCG	TGTTGGC	AGGGACTTTGCTAGAA	GGCGGCC	GGCG	GGCG	Y
3*Chr2_40_IS407A	3*Chr2_41_IS407A_B	D	Bm_JHU	3	1401133-1402368	Chr2	IS407A	GAAGG	TGACCTGCCCCC~TAC	ATGAAGAAGCGCTTTACG	GCCCGCAAGCCAAACGCG	TGTTGGC	AGGGACTTTGCTAGAA	CGAACTG	AGCG	CGAA	N
4*Chr2_40_IS407A	4*Chr2_41_IS407A_B	D	Bm_gb8_horse	4	1406420-1407655	Chr2	IS407A	GAAGG	TGACCTGCCCCC~TAC	ATGAAGAAGCGCTTTACG	GCCCGCAAGCCAAACGCG	TGTTGGC	AGGGACTTTGCTAGAA	CGAACTG	AGCG	CGAA	N
5*Chr2_49_IS407A_A		N	Bm_ATCC_103	5	1410524-1411759	Chr2	IS407A	CCCAA	TGACCTGCCCCC~TAC	ATGAAGAAGCGCTTTACG	GCCCGCAAGCCAAACGCG	TGTTGGC	AGGGACTTTGCTAGAA	AGCGACG	CCCA	AGCG	N
7*Chr2_33_IS407A	7*Chr2_53_IS407A_A	D-R	Bm_NCTC_102	7	1411874-1413109	Chr2	IS407A	GTGCC	TGACCTGCCCCC~TAC	ATGAAGAAGCGCTTTACG	GCCCGCAAGCCAAACGCG	TGTTGGC	AGGGACTTTGCTAGAA	GTCCAGC	ACGG	GTCC	N
1*Chr2_40_IS407A	1*Chr2_41_IS407A_B	D	Bm_ATCC_233	1	1412717-1413952	Chr2	IS407A	GAAGG	TGACCTGCCCCC~TAC	ATGAAGAAGCGCTTTACG	GCCCGCAAGCCAAACGCG	TGTTGGC	AGGGACTTTGCTAGAA	CGAACTG	AGCG	CGAA	N
6*Chr2_33_IS407A	6*Chr2_53_IS407A_A	D-R	Bm_NCTC_102	6	142183-143418	Chr2	IS407A	GTGCC	TGACCTGCCCCC~TAC	ATGAAGAAGCGCTTTACG	GCCCGCAAGCCAAACGCG	TGTTGGC	AGGGACTTTGCTAGAA	GTCCAGC	ACGG	GTCC	N
9*Chr2_67_IS407A	9*Chr2_68_IS407A_A	N	Bm_SAVP1	9	1432613-1433848	Chr2	IS407A	CACGC	TGACCTGCCCCC~TAC	ATGAAGAAGCGCTTTACG	GCCCGCAAGCCAAACGCG	TGTTGGC	AGGGACTTTGCTAGAA	CCCTGCC	GGTC	CCCT	N
5*Chr2_24_IS407A	5*Chr2_48_IS407A_A	D-R	Bm_ATCC_103	5	1438414-1439649	Chr2	IS407A	CGGGA	TGACCTGCCCCC~TAC	ATGAAGAAGCGCTTTACG	GCCCGCAAGCCAAACGCG	TGTTGGC	AGGGACTTTGCTAGAA	CGGCAGC	CCAC	CGGC	N
2*Chr2_40_IS407A	2*Chr2_41_IS407A_B	D	Bm_fmh	2	1439497-1440732	Chr2	IS407A	GAAGG	TGACCTGCCCCC~TAC	ATGAAGAAGCGCTTTACG	GCCCGCAAGCCAAACGCG	TGTTGGC	AGGGACTTTGCTAGAA	CGAACTG	AGCG	CGAA	N
7*Chr2_28_IS407A	7*Chr2_31b_IS407A_A	D	Bm_NCTC_102	7	1449126-1450361	Chr2	IS407A	GAACG	TGACCTGCCCCC~TAC	ATGAAGAAGCGCTTTACG	GCCCGCAAGCCAAACGCG	TGTTGGC	AGGGACTTTGCTAGAA	CGATGCT	GATC	CGAT	N
8*Chr2_59_IS407A_A		N	Bm_200272128	8	1453446-1454681	Chr2	IS407A	CCGCG	TGACCTGCCCCC~TAC	ATGAAGAAGCGCTTTACG	GCCCGCAAGCCAAACGCG	TGTTGGC	AGGGACTTTGCTAGAA	GGCGGAT	GTGC	GGCG	N
5*Chr2_52_IS407A_A		N	Bm_ATCC_103	5	1465100-1466335	Chr2	IS407A	GAATT	TGACCTGCCCCC~TAC	ATGAAGAAGCGCTTTACG	GCCCGCAAGCCAAACGCG	TGTTGGC	AGGGACTTTGCTAGAA	CCGTCAC	GGAT	CCGT	N
3*Chr2_33_IS407A	3*Chr2_38_IS407A_A	D-R	Bm_JHU	3	1468774-1470009	Chr2	IS407A	GTCCA	TGACCTGCCCCC~TAC	ATGAAGAAGCGCTTTACG	GCCCGCAAGCCAAACGCG	TGTTGGC	AGGGACTTTGCTAGAA	GTCCAGC	GAAC	GTCC	N
4*Chr2_33_IS407A	4*Chr2_38_IS407A_A	D-R	Bm_gb8_horse	4	1474062-1475297	Chr2	IS407A	GTCCA	TGACCTGCCCCC~TAC	ATGAAGAAGCGCTTTACG	GCCCGCAAGCCAAACGCG	TGTTGGC	AGGGACTTTGCTAGAA	GTCCAGC	GAAC	GTCC	N
8*Chr2_60_IS407A	8*Chr2_61_IS407A_A	D	Bm_200272128	8	1474566-1475801	Chr2	IS407A	CGCCG	TGACCTGCCCCC~TAC	ATGAAGAAGCGCTTTACG	GCCCGCAAGCCAAACGCG	TGTTGGC	AGGGACTTTGCTAGAA	CGACGGC	CGTG	CGAC	N
1*Chr2_33_IS407A	1*Chr2_38_IS407A_A	D-R	Bm_ATCC_233	1	1480359-1481594	Chr2	IS407A	GTCCA	TGACCTGCCCCC~TAC	ATGAAGAAGCGCTTTACG	GCCCGCAAGCCAAACGCG	TGTTGGC	AGGGACTTTGCTAGAA	GTCCAGC	GAAC	GTCC	N
5*Chr2_9a_IS407A	5*Chr2_53_IS407A_A	D-R	Bm_ATCC_103	5	1489755-1490990	Chr2	IS407A	GCGCA	TGACCTGCCCCC~TAC	ATGAAGAAGCGCTTTACG	GCCCGCAAGCCAAACGCG	TGTTGGC	AGGGACTTTGCTAGAA	CAAGCAG	CATC	CAAG	N
9*Chr2_62b_IS407	9*Chr2_75_IS407A_A	D-R	Bm_SAVP1	9	1496137-1497372	Chr2	IS407A	CTAGA	TGACCTGCCCCC~TAC	ATGAAGAAGCGCTTTACG	GCCCGCAAGCCAAACGCG	TGTTGGC	AGGGACTTTGCTAGAA	CACGGTC	GATC	CACG	N
7*Chr2_26_IS407A	7*Chr2_27_IS407A_A	D	Bm_NCTC_102	7	1497556-1498791	Chr2	IS407A	GGTTT	TGACCTGCCCCC~TAC	ATGAAGAAGCGCTTTACG	GCCCGCAAGCCAAACGCG	TGTTGGC	AGGGACTTTGCTAGAA	CTCGGCG	GTCG	CTCG	N
8*Chr2_62a_IS407	8*Chr2_74_IS407A_A	D-R	Bm_200272128	8	1498717-1499952	Chr2	IS407A	CCATC	TGACCTGCCCCC~TAC	ATGAAGAAGCGCTTTACG	GCCCGCAAGCCAAACGCG	TGTTGGC	AGGGACTTTGCTAGAA	CGGCTTC	TGAT	CGGC	N
10*Chr2_6_IS407A_A		N	Bm_PRL20	10	1504574-1505809	Chr2	IS407A	GCGCA	TGACCTGCCCCC~TAC	ATGAAGAAGCGCTTTACG	GCCCGCAAGCCAAACGCG	TGTTGGC	AGGGACTTTGCTAGAA	CGTGAAC	CGTG	CGTG	Y
3*Chr2_9a_IS407A	3*Chr2_31b_IS407A_A	D-R	Bm_JHU	3	1506899-1508134	Chr2	IS407A	GAACG	TGACCTGCCCCC~TAC	ATGAAGAAGCGCTTTACG	GCCCGCAAGCCAAACGCG	TGTTGGC	AGGGACTTTGCTAGAA	CAAGCAG	GATC	CAAG	N

2*Chr2_33_IS407A	2*Chr2_38_IS407A_A	D-R	Bm_fmh	2	1507138-1508373	Chr2	IS407A	GTCCA	TGACCTGCCCCC~TAC	ATGAAGAAGCGCTTTACG	GCCCGCAAGCCAAACGCG	TGTTGGC	AGGGACTTTGCTAGAA	GTCCAGC	GAAC	GTCC	N
4*Chr2_9a_IS407A	4*Chr2_31b_IS407A_A	D-R	Bm_gb8_horse	4	1511310-1512545	Chr2	IS407A	GAACG	TGACCTGCCCCC~TAC	ATGAAGAAGCGCTTTACG	GCCCGCAAGCCAAACGCG	TGTTGGC	AGGGACTTTGCTAGAA	CAAGCAG	GATC	CAAG	N
10*Chr2_13b_IS40	10*Chr2_57b_IS407A_A	R	Bm_PRL20	10	1511320-1512555	Chr2	IS407A	CAAGC	TGACCTGCCCCC~TAC	ATGAAGAAGCGCTTTACG	GCCCGCAAGCCAAACGCG	TGTTGGC	AGGGACTTTGCTAGAA	ATCTTCCC	GGTT	ATCT	N
1*Chr2_9a_IS407A	1*Chr2_31b_IS407A_A	D-R	Bm_ATCC_233	1	1517607-1518842	Chr2	IS407A	GAACG	TGACCTGCCCCC~TAC	ATGAAGAAGCGCTTTACG	GCCCGCAAGCCAAACGCG	TGTTGGC	AGGGACTTTGCTAGAA	CAAGCAG	GATC	CAAG	N
5*Chr2_8_IS407A_C		N	Bm_ATCC_103	5	1523165-1524400	Chr2	IS407A	CGCAG	TGACCTGCCCCC~TAC	ATGAAGAAGCGCTTTACG	GCCCGCAAGCCAAACGCG	TGTTGGC	AGGGACTTTGCTAGAA	CATTGCCT	CATT	CATT	Y
6*Chr2_1_IS407A_B		N	Bm_NCTC_102	6	1531434-1532669	Chr2	IS407A	CCAGA	TGACCTGCCCCC~TAC	ATGAAGAAGCGCTTTACG	GCCCGCAAGCCAAACGCG	TGTTGGC	AGGGACTTTGCTAGAA	CGGCGAA	CGGC	CGGC	Y
7*Chr2_24_IS407A	7*Chr2_56_IS407A_A	D-R	Bm_NCTC_102	7	1533196-1534431	Chr2	IS407A	CGGGA	TGACCTGCCCCC~TAC	ATGAAGAAGCGCTTTACG	GCCCGCAAGCCAAACGCG	TGTTGGC	AGGGACTTTGCTAGAA	CATCGGC	CCAC	CATC	N
2*Chr2_9a_IS407A	2*Chr2_31b_IS407A_A	D-R	Bm_fmh	2	1544910-1546145	Chr2	IS407A	GAACG	TGACCTGCCCCC~TAC	ATGAAGAAGCGCTTTACG	GCCCGCAAGCCAAACGCG	TGTTGGC	AGGGACTTTGCTAGAA	CAAGCAG	GATC	CAAG	N
9*Chr2_60_IS407A	9*Chr2_74_IS407A_A	D-R	Bm_SAVP1	9	1575233-1576468	Chr2	IS407A	CGCCG	TGACCTGCCCCC~TAC	ATGAAGAAGCGCTTTACG	GCCCGCAAGCCAAACGCG	TGTTGGC	AGGGACTTTGCTAGAA	CGGCTTC	CGTG	CGGC	N
6*Chr2_2_IS407A_	6*Chr2_73_IS407A_A	D-R	Bm_NCTC_102	6	1576904-1578139	Chr2	IS407A	CGCTG	TGACCTGCCCCC~TAC	ATGAAGAAGCGCTTTACG	GCCCGCAAGCCAAACGCG	TGTTGGC	AGGGACTTTGCTAGAA	CAACGCA	GTGT	CAAC	N
8*Chr2_62b_IS407	8*Chr2_75_IS407A_A	D-R	Bm_200272128	8	1577349-1578382	Chr2	IS407A	#IS407	~~~~~~~~~~~~~~	~~~~~~~~~~~~~~~~~	GCCCGCAAGCCAAACGCG	TGTTGGC	AGGGACTTTGCTAGAA	GTCGATC	IS407A?	GTCG	N.D.
9*Chr2_59_IS407A_A		N	Bm_SAVP1	9	1596353-1597588	Chr2	IS407A	CCGCG	TGACCTGCCCCC~TAC	ATGAAGAAGCGCTTTACG	GCCCGCAAGCCAAACGCG	TGTTGGC	AGGGACTTTGCTAGAA	GGCGGAT	GTGC	GGCG	N
6*Chr2_70_IS407A	6*Chr2_72_IS407A_A	D-R	Bm_NCTC_102	6	1599659-1600894	Chr2	IS407A	CAACA	TGACCTGCCCCC~TAC	ATGAAGAAGCGCTTTACG	GCCCGCAAGCCAAACGCG	TGTTGGC	AGGGACTTTGCTAGAA	GATGGAT	GTAC	GATG	N
5*Chr2_13b_IS407	5*Chr2_57b_IS407A_A	R	Bm_ATCC_103	5	1605359-1606594	Chr2	IS407A	TATCG	TGACCTGCCCCC~TAC	ATGAAGAAGCGCTTTACG	GCCCGCAAGCCAAACGCG	TGTTGGC	AGGGACTTTGCTAGAA	AACCCAA	AGAT	AACC	N
8*Chr2_66_IS407A	8*Chr2_70_IS407A_A	D-R	Bm_200272128	8	1608602-1609837	Chr2	IS407A	TCAGT	TGACCTGCCCCC~TAC	ATGAAGAAGCGCTTTACG	GCCCGCAAGCCAAACGCG	TGTTGGC	AGGGACTTTGCTAGAA	GTACCGC	TGGC	GTAC	N
4*Chr2_6_IS407A_A		N	Bm_gb8_horse	4	1629828-1631063	Chr2	IS407A	GCGCA	TGACCTGCCCCC~TAC	ATGAAGAAGCGCTTTACG	GCCCGCAAGCCAAACGCG	TGTTGGC	AGGGACTTTGCTAGAA	CGTGAAC	CGTG	CGTG	Y
3*Chr2_6_IS407A_A		N	Bm_JHU	3	1631118-1632353	Chr2	IS407A	GCGCA	TGACCTGCCCCC~TAC	ATGAAGAAGCGCTTTACG	GCCCGCAAGCCAAACGCG	TGTTGGC	AGGGACTTTGCTAGAA	CGTGAAC	CGTG	CGTG	Y
1*Chr2_6_IS407A_A		N	Bm_ATCC_233	1	1636309-1637544	Chr2	IS407A	GCGCA	TGACCTGCCCCC~TAC	ATGAAGAAGCGCTTTACG	GCCCGCAAGCCAAACGCG	TGTTGGC	AGGGACTTTGCTAGAA	CGTGAAC	CGTG	CGTG	Y
4*Chr2_13b_IS407	4*Chr2_57b_IS407A_A	R	Bm_gb8_horse	4	1636577-1637812	Chr2	IS407A	CGCAG	TGACCTGCCCCC~TAC	ATGAAGAAGCGCTTTACG	GCCCGCAAGCCAAACGCG	TGTTGGC	AGGGACTTTGCTAGAA	AACCCAA	TCGG	AACC	N
3*Chr2_13b_IS407	3*Chr2_57b_IS407A_A	R	Bm_JHU	3	1637867-1639102	Chr2	IS407A	CGCAG	TGACCTGCCCCC~TAC	ATGAAGAAGCGCTTTACG	GCCCGCAAGCCAAACGCG	TGTTGGC	AGGGACTTTGCTAGAA	AACCCAA	TCGG	AACC	N
7*Chr2_37_IS407A	7*Chr2_57a_IS407A_A	D-R	Bm_NCTC_102	7	1637908-1639143	Chr2	IS407A	CCCTT	TGACCTGCCCCC~TAC	ATGAAGAAGCGCTTTACG	GCCCGCAAGCCAAACGCG	TGTTGGC	AGGGACTTTGCTAGAA	CGCCCGC	ACCT	CGCC	N
1*Chr2_13b_IS407	1*Chr2_57b_IS407A_A	R	Bm_ATCC_233	1	1643058-1644293	Chr2	IS407A	CGCAG	TGACCTGCCCCC~TAC	ATGAAGAAGCGCTTTACG	GCCCGCAAGCCAAACGCG	TGTTGGC	AGGGACTTTGCTAGAA	AACCCAA	TCGG	AACC	N
10*Chr2_58_IS407A_A		N	Bm_PRL20	10	1657072-1658307	Chr2	IS407A	GCCGG	TGACCTGCCCCC~TAC	ATGAAGAAGCGCTTTACG	GCCCGCAAGCCAAACGCG	TGTTGGC	AGGGACTTTGCTAGAA	GTCGAGC	GGAT	GTCG	N
2*Chr2_6_IS407A_A		N	Bm_fmh	2	1666791-1668026	Chr2	IS407A	GCGCA	TGACCTGCCCCC~TAC	ATGAAGAAGCGCTTTACG	GCCCGCAAGCCAAACGCG	TGTTGGC	AGGGACTTTGCTAGAA	CGTGAAC	CGTG	CGTG	Y
2*Chr2_13b_IS407	2*Chr2_57b_IS407A_A	R	Bm_fmh	2	1673540-1674775	Chr2	IS407A	CGCAG	TGACCTGCCCCC~TAC	ATGAAGAAGCGCTTTACG	GCCCGCAAGCCAAACGCG	TGTTGGC	AGGGACTTTGCTAGAA	AACCCAA	TCGG	AACC	N
8*Chr2_72_IS407A_A		N	Bm_200272128	8	1675954-1677189	Chr2	IS407A	CACAT	TGACCTGCCCCC~TAC	ATGAAGAAGCGCTTTACG	GCCCGCAAGCCAAACGCG	TGTTGGC	AGGGACTTTGCTAGAA	GATGGAT	GATG	GATG	Y
6*Chr2_52_IS407A_A		N	Bm_NCTC_102	6	167708-168943	Chr2	IS407A	GAATT	TGACCTGCCCCC~TAC	ATGAAGAAGCGCTTTACG	GCCCGCAAGCCAAACGCG	TGTTGGC	AGGGACTTTGCTAGAA	CGTCACG	GGAT	CGTC	N
6*Chr2_67_IS407A	6*Chr2_68_IS407A_A	N	Bm_NCTC_102	6	1697435-1698670	Chr2	IS407A	CACGC	TGACCTGCCCCC~TAC	ATGAAGAAGCGCTTTACG	GCCCGCAAGCCAAACGCG	TGTTGGC	AGGGACTTTGCTAGAA	CCCTGCC	GGTC	CCCT	N
8*Chr2_2_IS407A_	8*Chr2_73_IS407A_A	D-R	Bm_200272128	8	1698785-1700020	Chr2	IS407A	CGCTG	TGACCTGCCCCC~TAC	ATGAAGAAGCGCTTTACG	GCCCGCAAGCCAAACGCG	TGTTGGC	AGGGACTTTGCTAGAA	CAACGCA	GTGT	CAAC	N
4*Chr2_70_IS407A_A		N	Bm_gb8_horse	4	170287-171522	Chr2	IS407A	CAACA	TGACCTGCCCCC~TAC	ATGAAGAAGCGCTTTACG	GCCCGCAAGCCAAACGCG	TGTTGGC	AGGGACTTTGCTAGAA	GTACCGC	GTAC	GTAC	Y
1*Chr2_70_IS407A_A		N	Bm_ATCC_233	1	170594-171829	Chr2	IS407A	CAACA	TGACCTGCCCCC~TAC	ATGAAGAAGCGCTTTACG	GCCCGCAAGCCAAACGCG	TGTTGGC	AGGGACTTTGCTAGAA	GTACCGC	GTAC	GTAC	Y
9*Chr2_58_IS407A_A		D-R	Bm_SAVP1	9	1705942-1707177	Chr2	IS407A	GTTAA	TGACCTGCCCCC~TAC	ATGAAGAAGCGCTTTACG	GCCCGCAAGCCAAACGCG	TGTTGGC	AGGGACTTTGCTAGAA	GTCGAGC	AGTG	GTCG	N
9*Chr2_13a_IS407A_A		D-R	Bm_SAVP1	9	1716760-1717995	Chr2	IS407A	GAGCC	TGACCTGCCCCC~TAC	ATGAAGAAGCGCTTTACG	GCCCGCAAGCCAAACGCG	TGTTGGC	AGGGACTTTGCTAGAA	CATGCGC	CTCG	CATG	N
7*Chr2_35_IS407A_A		N	Bm_NCTC_102	7	1717195-1718430	Chr2	IS407A	CGATC	TGACCTGCCCCC~TAC	ATGAAGAAGCGCTTTACG	GCCCGCAAGCCAAACGCG	TGTTGGC	AGGGACTTTGCTAGAA	CATCAGC	CATC	CATC	Y
7*Chr2_70_IS407A_A		N	Bm_NCTC_102	7	171756-172991	Chr2	IS407A	CAACA	TGACCTGCCCCC~TAC	ATGAAGAAGCGCTTTACG	GCCCGCAAGCCAAACGCG	TGTTGGC	AGGGACTTTGCTAGAA	GTACCGC	GTAC	GTAC	Y
9*Chr2_9b_IS407A_A		D-R	Bm_SAVP1	9	1718700-1719935	Chr2	IS407A	CATGC	TGACCTGCCCCC~TAC	ATGAAGAAGCGCTTTACG	GCCCGCAAGCCAAACGCG	TGTTGGC	AGGGACTTTGCTAGAA	TTTGCTAG	CAAG	TTTG	N
6*Chr2_66_IS407A_B		N	Bm_NCTC_102	6	1728529-1729764	Chr2	IS407A	TCAGT	TGACCTGCCCCC~TAC	ATGAAGAAGCGCTTTACG	GCCCGCAAGCCAAACGCG	TGTTGGC	AGGGACTTTGCTAGAA	TGGCAGT	TGGC	TGGC	Y
8*Chr2_1_IS407A_B		N	Bm_200272128	8	1744280-1745515	Chr2	IS407A	CCAGA	TGACCTGCCCCC~TAC	ATGAAGAAGCGCTTTACG	GCCCGCAAGCCAAACGCG	TGTTGGC	AGGGACTTTGCTAGAA	CGGCGAA	CGGC	CGGC	Y
5*Chr2_58_IS407A_A		N	Bm_ATCC_103	5	1752839-1754074	Chr2	IS407A	CGCGG	TGACCTGCCCCC~TAC	ATGAAGAAGCGCTTTACG	GCCCGCAAGCCAAACGCG	TGTTGGC	AGGGACTTTGCTAGAA	ATCCGGG	CGAC	ATCC	N
10*Chr2_19_IS407A_B		N	Bm_PRL20	10	1759323-1760558	Chr2	IS407A	TTATG	TGACCTGCCCCC~TAC	ATGAAGAAGCGCTTTACG	GCCCGCAAGCCAAACGCG	TGTTGGC	AGGGACTTTGCTAGAA	GGGTATTT	GGGT	GGGT	Y
6*Chr2_62b_IS407	6*Chr2_75_IS407A_A	D-R	Bm_NCTC_102	6	1759985-1761018	Chr2	IS407A	#IS407	~~~~~~~~~~~~~~	~~~~~~~~~~~~~~~~~	GCCCGCAAGCCAAACGCG	TGTTGGC	AGGGACTTTGCTAGAA	GTCGATC	IS407A?	GTCG	N.D.
7*Chr2_34_IS407A	7*Chr2_54_IS407A_A	D-R	Bm_NCTC_102	7	1782102-1783337	Chr2	IS407A	CGCGG	TGACCTGCCCCC~TAC	ATGAAGAAGCGCTTTACG	GCCCGCAAGCCAAACGCG	TGTTGGC	AGGGACTTTGCTAGAA	GGATAGA	GAGG	GGAT	N
4*Chr2_58_IS407A_A		N	Bm_gb8_horse	4	1783542-1784777	Chr2	IS407A	CGCGG	TGACCTGCCCCC~TAC	ATGAAGAAGCGCTTTACG	GCCCGCAAGCCAAACGCG	TGTTGGC	AGGGACTTTGCTAGAA	ATCCGGG	CGAC	ATCC	N
1*Chr2_58_IS407A_A		N	Bm_ATCC_233	1	1790656-1791891	Chr2	IS407A	CGCGG	TGACCTGCCCCC~TAC	ATGAAGAAGCGCTTTACG	GCCCGCAAGCCAAACGCG	TGTTGGC	AGGGACTTTGCTAGAA	ATCCGGG	CGAC	ATCC	N
10*Chr2_15a_IS40	10*Chr2_18_IS407A_A	D-R	Bm_PRL20	10	1794050-1795285	Chr2	IS407A	AGACC	TGACCTGCCCCC~TAC	ATGAAGAAGCGCTTTACG	GCCCGCAAGCCAAACGCG	TGTTGGC	AGGGACTTTGCTAGAA	GGATCAG	AGAT	GGAT	N
3*Chr2_58_IS407A_A		N	Bm_JHU	3	1796992-1798227	Chr2	IS407A	CGCGG	TGACCTGCCCCC~TAC	ATGAAGAAGCGCTTTACG	GCCCGCAAGCCAAACGCG	TGTTGGC	AGGGACTTTGCTAGAA	ATCCGGG	CGAC	ATCC	N
2*Chr2_70_IS407A_A		N	Bm_fmh	2	180094-181329	Chr2	IS407A	CAACA	TGACCTGCCCCC~TAC	ATGAAGAAGCGCTTTACG	GCCCGCAAGCCAAACGCG	TGTTGGC	AGGGACTTTGCTAGAA	GTACCGC	GTAC	GTAC	Y
7*Chr2_17_IS407A	7*Chr2_55_IS407A_A	D-R	Bm_NCTC_102	7	1808233-1809468	Chr2	IS407A	GTAAA	TGACCTGCCCCC~TAC	ATGAAGAAGCGCTTTACG	GCCCGCAAGCCAAACGCG	TGTTGGC	AGGGACTTTGCTAGAA	GTGCGCT	CCCC	GTGC	N
7*Chr2_15b_IS407A_A		R	Bm_NCTC_102	7	1824067-1825302	Chr2	IS407A	CGCGC	TGACCTGCCCCC~TAC	ATGAAGAAGCGCTTTACG	GCCCGCAAGCCAAACGCG	TGTTGGC	AGGGACTTTGCTAGAA	ACAGTTTT	GCTT	ACAG	N
2*Chr2_58_IS407A_A		N	Bm_fmh	2	1824347-1825582	Chr2	IS407A	CGCGG	TGACCTGCCCCC~TAC	ATGAAGAAGCGCTTTACG	GCCCGCAAGCCAAACGCG	TGTTGGC	AGGGACTTTGCTAGAA	ATCCGGG	CGAC	ATCC	N
6*Chr2_62a_IS407	6*Chr2_74_IS407A_A	D-R	Bm_NCTC_102	6	1838857-1840092	Chr2	IS407A	CCATC	TGACCTGCCCCC~TAC	ATGAAGAAGCGCTTTACG	GCCCGCAAGCCAAACGCG	TGTTGGC	AGGGACTTTGCTAGAA	CGGCTTC	TGAT	CGGC	N
8*Chr2_15a_IS407	8*Chr2_18_IS407A_A	D-R	Bm_200272128	8	1852506-1853741	Chr2	IS407A	AGACC	TGACCTGCCCCC~TAC	ATGAAGAAGCGCTTTACG	GCCCGCAAGCCAAACGCG	TGTTGGC	AGGGACTTTGCTAGAA	GGATCAG	AGAT	GGAT	N
5*Chr2_59_IS407A_A		N	Bm_ATCC_103	5	1862557-1863793	Chr2	IS407A	CCGCG	TGACCTGCCCCCcTAC	ATGAAGAAGCGCTTTACG	GCCCGCAAGCCAAACGCG	TGTTGGC	AGGGACTTTGCTAGAA	GGCGGAT	GTGC	GGCG	N
6*Chr2_60_IS407A	6*Chr2_61_IS407A_A	D	Bm_NCTC_102	6	1863008-1864243	Chr2	IS407A	CGCCG	TGACCTGCCCCC~TAC	ATGAAGAAGCGCTTTACG	GCCCGCAAGCCAAACGCG	TGTTGGC	AGGGACTTTGCTAGAA	CGACGGC	CGTG	CGAC	N
7*Chr2_23_IS407A_A		D-R	Bm_NCTC_102	7	1875193-1876428	Chr2	IS407A	CGCCG	TGACCTGCCCCC~TAC	ATGAAGAAGCGCTTTACG	GCCCGCAAGCCAAACGCG	TGTTGGC	AGGGACTTTGCTAGAA	ATGGATTT	CGAT	ATGG	N
5*Chr2_20a_IS407	5*Chr2_60_IS407A_A	D-R	Bm_ATCC_103	5	1883678-1884913	Chr2	IS407A	CGCCG	TGACCTGCCCCC~TAC	ATGAAGAAGCGCTTTACG	GCCCGCAAGCCAAACGCG	TGTTGGC	AGGGACTTTGCTAGAA	GTACACC	CGTG	GTAC	N
6*Chr2_59_IS407A_A		N	Bm_NCTC_102	6	1884128-1885363	Chr2	IS407A	CCGCG	TGACCTGCCCCC~TAC	ATGAAGAAGCGCTTTACG	GCCCGCAAGCCAAACGCG	TGTTGGC	AGGGACTTTGCTAGAA	GGCGGAT	GTGC	GGCG	N
4*Chr2_59_IS407A_A		N	Bm_gb8_horse	4	1892984-1894219	Chr2	IS407A	CCGCG	TGACCTGCCCCC~TAC	ATGAAGAAGCGCTTTACG	GCCCGCAAGCCAAACGCG	TGTTGGC	AGGGACTTTGCTAGAA	GGCGGAT	GTGC	GGCG	N

1*Chr2_59_IS407A_A		N	Bm_ATCC_233	1	1900428-1901663	Chr2	IS407A	CCGCG	TGACCTGCCCCC~TAC	ATGAAGAAGCGCTTTACG	GCCCGCAAGCCAAACGCG	TGTTGGC	AGGGACTTTGCTAGAA	GGCGGAT	GTGC	GGCG	N
3*Chr2_59_IS407A_A		N	Bm_JHU	3	1908125-1909360	Chr2	IS407A	CCGCG	TGACCTGCCCCC~TAC	ATGAAGAAGCGCTTTACG	GCCCGCAAGCCAAACGCG	TGTTGGC	AGGGACTTTGCTAGAA	GGCGGAT	GTGC	GGCG	N
4*Chr2_20a_IS407	4*Chr2_60_IS407A_A	D-R	Bm_gb8_horse	4	1913865-1915100	Chr2	IS407A	CGCCG	TGACCTGCCCCC~TAC	ATGAAGAAGCGCTTTACG	GCCCGCAAGCCAAACGCG	TGTTGGC	AGGGACTTTGCTAGAA	GTACACC	CGTG	GTAC	N
5*Chr2_15a_IS407	5*Chr2_18_IS407A_A	D-R	Bm_ATCC_103	5	1919751-1920986	Chr2	IS407A	AGACC	TGACCTGCCCCC~TAC	ATGAAGAAGCGCTTTACG	GCCCGCAAGCCAAACGCG	TGTTGGC	AGGGACTTTGCTAGAA	GGATCAG	AGAT	GGAT	N
1*Chr2_20a_IS407	1*Chr2_60_IS407A_A	D-R	Bm_ATCC_233	1	1921548-1922783	Chr2	IS407A	CGCCG	TGACCTGCCCCC~TAC	ATGAAGAAGCGCTTTACG	GCCCGCAAGCCAAACGCG	TGTTGGC	AGGGACTTTGCTAGAA	GTACACC	CGTG	GTAC	N
3*Chr2_20a_IS407	3*Chr2_60_IS407A_A	D-R	Bm_JHU	3	1929245-1930480	Chr2	IS407A	CGCCG	TGACCTGCCCCC~TAC	ATGAAGAAGCGCTTTACG	GCCCGCAAGCCAAACGCG	TGTTGGC	AGGGACTTTGCTAGAA	GTACACC	CGTG	GTAC	N
2*Chr2_59_IS407A_A		N	Bm_fmh	2	1936651-1937886	Chr2	IS407A	CCGCG	TGACCTGCCCCC~TAC	ATGAAGAAGCGCTTTACG	GCCCGCAAGCCAAACGCG	TGTTGGC	AGGGACTTTGCTAGAA	GGCGGAT	GTGC	GGCG	N
4*Chr2_15a_IS407	4*Chr2_18_IS407A_A	D-R	Bm_gb8_horse	4	1949938-1951173	Chr2	IS407A	AGACC	TGACCTGCCCCC~TAC	ATGAAGAAGCGCTTTACG	GCCCGCAAGCCAAACGCG	TGTTGGC	AGGGACTTTGCTAGAA	GGATCAG	AGAT	GGAT	N
1*Chr2_15a_IS407	1*Chr2_18_IS407A_A	D-R	Bm_ATCC_233	1	1957621-1958856	Chr2	IS407A	AGACC	TGACCTGCCCCC~TAC	ATGAAGAAGCGCTTTACG	GCCCGCAAGCCAAACGCG	TGTTGGC	AGGGACTTTGCTAGAA	GGATCAG	AGAT	GGAT	N
2*Chr2_20a_IS407	2*Chr2_60_IS407A_A	D-R	Bm_fmh	2	1957771-1959006	Chr2	IS407A	CGCCG	TGACCTGCCCCC~TAC	ATGAAGAAGCGCTTTACG	GCCCGCAAGCCAAACGCG	TGTTGGC	AGGGACTTTGCTAGAA	GTACACC	CGTG	GTAC	N
3*Chr2_15a_IS407	3*Chr2_18_IS407A_A	D-R	Bm_JHU	3	1965318-1966553	Chr2	IS407A	AGACC	TGACCTGCCCCC~TAC	ATGAAGAAGCGCTTTACG	GCCCGCAAGCCAAACGCG	TGTTGGC	AGGGACTTTGCTAGAA	GGATCAG	AGAT	GGAT	N
10*Chr2_3_IS407A	10*Chr2_72_IS407A_A	D-R	Bm_PRL20	10	1972722-1973957	Chr2	IS407A	AGTCG	TGACCTGCCCCC~TAC	ATGAAGAAGCGCTTTACG	GCCCGCAAGCCAAACGCG	TGTTGGC	AGGGACTTTGCTAGAA	GATGGAT	GGGT	GATG	N
7*Chr2_15a_IS407	7*Chr2_18_IS407A_A	D-R	Bm_NCTC_102	7	1986607-1987842	Chr2	IS407A	AGACC	TGACCTGCCCCC~TAC	ATGAAGAAGCGCTTTACG	GCCCGCAAGCCAAACGCG	TGTTGGC	AGGGACTTTGCTAGAA	GGATCAG	AGAT	GGAT	N
8*Chr2_35_IS407A_A		N	Bm_200272128	8	199085-200320	Chr2	IS407A	CGATC	TGACCTGCCCCC~TAC	ATGAAGAAGCGCTTTACG	GCCCGCAAGCCAAACGCG	TGTTGGC	AGGGACTTTGCTAGAA	CATCAGC	CATC	CATC	Y
2*Chr2_15a_IS407	2*Chr2_18_IS407A_A	D-R	Bm_fmh	2	1993844-1995079	Chr2	IS407A	AGACC	TGACCTGCCCCC~TAC	ATGAAGAAGCGCTTTACG	GCCCGCAAGCCAAACGCG	TGTTGGC	AGGGACTTTGCTAGAA	GGATCAG	AGAT	GGAT	N
6*Chr2_58_IS407A_A		N	Bm_NCTC_102	6	1994016-1995251	Chr2	IS407A	CGCGG	TGACCTGCCCCC~TAC	ATGAAGAAGCGCTTTACG	GCCCGCAAGCCAAACGCG	TGTTGGC	AGGGACTTTGCTAGAA	ATCCGGG	CGAC	ATCC	N
6*Chr2_10b_IS407A_A		R	Bm_NCTC_102	6	20142-21377	Chr2	IS407A	GCCTC	TGACCTGCCCCC~TAC	ATGAAGAAGCGCTTTACG	GCCCGCAAGCCAAACGCG	TGTTGGC	AGGGACTTTGCTAGAA	TTTGCTAG	GCGG	TTTG	N
8*Chr2_3_IS407A_	8*Chr2_76_IS407A_A	D-R	Bm_200272128	8	2030650-2031885	Chr2	IS407A	GGGCA	TGACCTGCCCCC~TAC	ATGAAGAAGCGCTTTACG	GCCCGCAAGCCAAACGCG	TGTTGGC	AGGGACTTTGCTAGAA	TCTTCCTT	GATG	TCTT	N
3*Chr2_70_IS407A_A		N	Bm_JHU	3	207666-208901	Chr2	IS407A	CAACA	TGACCTGCCCCC~TAC	ATGAAGAAGCGCTTTACG	GCCCGCAAGCCAAACGCG	TGTTGGC	AGGGACTTTGCTAGAA	GTACCGC	GTAC	GTAC	Y
5*Chr2_3_IS407A_	5*Chr2_76_IS407A_A	D-R	Bm_ATCC_103	5	2097872-2099107	Chr2	IS407A	AGTCG	TGACCTGCCCCC~TAC	ATGAAGAAGCGCTTTACG	GCCCGCAAGCCAAACGCG	TGTTGGC	AGGGACTTTGCTAGAA	TCTTCCTT	GGGT	TCTT	N
4*Chr2_3_IS407A_	4*Chr2_76_IS407A_A	D-R	Bm_gb8_horse	4	2128166-2129401	Chr2	IS407A	AGTCG	TGACCTGCCCCC~TAC	ATGAAGAAGCGCTTTACG	GCCCGCAAGCCAAACGCG	TGTTGGC	AGGGACTTTGCTAGAA	TCTTCCTT	GGGT	TCTT	N
1*Chr2_3_IS407A_	1*Chr2_76_IS407A_A	D-R	Bm_ATCC_233	1	2135943-2137178	Chr2	IS407A	AGTCG	TGACCTGCCCCC~TAC	ATGAAGAAGCGCTTTACG	GCCCGCAAGCCAAACGCG	TGTTGGC	AGGGACTTTGCTAGAA	TCTTCCTT	GGGT	TCTT	N
6*Chr2_13b_IS407	6*Chr2_57b_IS407A_A	R	Bm_NCTC_102	6	2142275-2143510	Chr2	IS407A	CAAGC	TGACCTGCCCCC~TAC	ATGAAGAAGCGCTTTACG	GCCCGCAAGCCAAACGCG	TGTTGGC	AGGGACTTTGCTAGAA	ATCTTCCC	GGTT	ATCT	N
3*Chr2_3_IS407A_	3*Chr2_76_IS407A_A	D-R	Bm_JHU	3	2146619-2147854	Chr2	IS407A	AGTCG	TGACCTGCCCCC~TAC	ATGAAGAAGCGCTTTACG	GCCCGCAAGCCAAACGCG	TGTTGGC	AGGGACTTTGCTAGAA	TCTTCCTT	GGGT	TCTT	N
6*Chr2_6_IS407A_A		N	Bm_NCTC_102	6	2149021-2150256	Chr2	IS407A	GCGCA	TGACCTGCCCCC~TAC	ATGAAGAAGCGCTTTACG	GCCCGCAAGCCAAACGCG	TGTTGGC	AGGGACTTTGCTAGAA	CGTGAAC	CGTG	CGTG	Y
7*Chr2_3_IS407A_	7*Chr2_76_IS407A_A	D-R	Bm_NCTC_102	7	2164773-2166008	Chr2	IS407A	GGGCA	TGACCTGCCCCC~TAC	ATGAAGAAGCGCTTTACG	GCCCGCAAGCCAAACGCG	TGTTGGC	AGGGACTTTGCTAGAA	TCTTCCTT	GATG	TCTT	N
2*Chr2_3_IS407A_	2*Chr2_76_IS407A_A	D-R	Bm_fmh	2	2173035-2174270	Chr2	IS407A	AGTCG	TGACCTGCCCCC~TAC	ATGAAGAAGCGCTTTACG	GCCCGCAAGCCAAACGCG	TGTTGGC	AGGGACTTTGCTAGAA	TCTTCCTT	GGGT	TCTT	N
6*Chr2_24_IS407A_A		D-R	Bm_NCTC_102	6	22152-23387	Chr2	IS407A	CGGGA	TGACCTGCCCCC~TAC	ATGAAGAAGCGCTTTACG	GCCCGCAAGCCAAACGCG	TGTTGGC	AGGGACTTTGCTAGAA	GGAGTTC	CCAC	GGAG	N
5*Chr2_67_IS407A	5*Chr2_68_IS407A_A	N	Bm_ATCC_103	5	223244-224479	Chr2	IS407A	CACGC	TGACCTGCCCCC~TAC	ATGAAGAAGCGCTTTACG	GCCCGCAAGCCAAACGCG	TGTTGGC	AGGGACTTTGCTAGAA	CCCTGCC	GGTC	CCCT	N
9*Chr2_42_IS407A_A		N	Bm_SAVP1	9	223530-224765	Chr2	IS407A	ATCGA	TGACCTGCCCCC~TAC	ATGAAGAAGCGCTTTACG	GCCCGCAAGCCAAACGCG	TGTTGGC	AGGGACTTTGCTAGAA	CTGCGGC	CTGC	CTGC	Y
4*Chr2_81_IS407A_B		N	Bm_gb8_horse	4	2259017-2260252	Chr2	IS407A	AGTTC	TGACCTGCCCCC~TAC	ATGAAGAAGCGCTTTACG	GCCCGCAAGCCAAACGCG	TGTTGGC	AGGGACTTTGCTAGAA	ACGCCAT	ACGC	ACGC	Y
1*Chr2_81_IS407A_B		N	Bm_ATCC_233	1	2267062-2268297	Chr2	IS407A	AGTTC	TGACCTGCCCCC~TAC	ATGAAGAAGCGCTTTACG	GCCCGCAAGCCAAACGCG	TGTTGGC	AGGGACTTTGCTAGAA	ACGCCAT	ACGC	ACGC	Y
6*Chr2_9a_IS407A_A		N	Bm_NCTC_102	6	2267886-2269121	Chr2	IS407A	CATGC	TGACCTGCCCCC~TAC	ATGAAGAAGCGCTTTACG	GCCCGCAAGCCAAACGCG	TGTTGGC	AGGGACTTTGCTAGAA	CAAGCAG	CAAG	CAAG	Y
3*Chr2_81_IS407A_B		N	Bm_JHU	3	2282239-2283474	Chr2	IS407A	AGTTC	TGACCTGCCCCC~TAC	ATGAAGAAGCGCTTTACG	GCCCGCAAGCCAAACGCG	TGTTGGC	AGGGACTTTGCTAGAA	ACGCCAT	ACGC	ACGC	Y
10*Chr2_67_IS407	10*Chr2_68_IS407A_A	N	Bm_PRL20	10	228919-230154	Chr2	IS407A	CACGC	TGACCTGCCCCC~TAC	ATGAAGAAGCGCTTTACG	GCCCGCAAGCCAAACGCG	TGTTGGC	AGGGACTTTGCTAGAA	CCCTGCC	GGTC	CCCT	N
5*Chr2_72_IS407A_A		N	Bm_ATCC_103	5	2293242-2294477	Chr2	IS407A	CACAT	TGACCTGCCCCC~TAC	ATGAAGAAGCGCTTTACG	GCCCGCAAGCCAAACGCG	TGTTGGC	AGGGACTTTGCTAGAA	GATGGAT	GATG	GATG	Y
2*Chr2_81_IS407A_B		N	Bm_fmh	2	2307475-2308710	Chr2	IS407A	AGTTC	TGACCTGCCCCC~TAC	ATGAAGAAGCGCTTTACG	GCCCGCAAGCCAAACGCG	TGTTGGC	AGGGACTTTGCTAGAA	ACGCCAT	ACGC	ACGC	Y
5*Chr2_2_IS407A_	5*Chr2_73_IS407A_A	D-R	Bm_ATCC_103	5	2316126-2317361	Chr2	IS407A	CGCTG	TGACCTGCCCCC~TAC	ATGAAGAAGCGCTTTACG	GCCCGCAAGCCAAACGCG	TGTTGGC	AGGGACTTTGCTAGAA	CAACGCA	GTGT	CAAC	N
6*Chr2_51_IS407A_A		N	Bm_NCTC_102	6	247301-248536	Chr2	IS407A	CGGCC	TGACCTGCCCCC~TAC	ATGAAGAAGCGCTTTACG	GCCCGCAAGCCAAACGCG	TGTTGGC	AGGGACTTTGCTAGAA	GGCGGCC	GGCG	GGCG	Y
4*Chr2_67_IS407A	4*Chr2_68_IS407A_A	N	Bm_gb8_horse	4	267595-268830	Chr2	IS407A	CACGC	TGACCTGCCCCC~TAC	ATGAAGAAGCGCTTTACG	GCCCGCAAGCCAAACGCG	TGTTGGC	AGGGACTTTGCTAGAA	CCCTGCC	GGTC	CCCT	N
1*Chr2_67_IS407A	1*Chr2_68_IS407A_A	N	Bm_ATCC_233	1	268389-269624	Chr2	IS407A	CACGC	TGACCTGCCCCC~TAC	ATGAAGAAGCGCTTTACG	GCCCGCAAGCCAAACGCG	TGTTGGC	AGGGACTTTGCTAGAA	CCCTGCC	GGTC	CCCT	N
6*Chr2_49_IS407A_A		N	Bm_NCTC_102	6	269225-270460	Chr2	IS407A	CCGAC	TGACCTGCCCCC~TAC	ATGAAGAAGCGCTTTACG	GCCCGCAAGCCAAACGCG	TGTTGGC	AGGGACTTTGCTAGAA	AGCGACG	AGCG	AGCG	Y
7*Chr2_67_IS407A	7*Chr2_68_IS407A_A	N	Bm_NCTC_102	7	269548-270783	Chr2	IS407A	CACGC	TGACCTGCCCCC~TAC	ATGAAGAAGCGCTTTACG	GCCCGCAAGCCAAACGCG	TGTTGGC	AGGGACTTTGCTAGAA	CCCTGCC	GGTC	CCCT	N
2*Chr2_67_IS407A	2*Chr2_68_IS407A_A	N	Bm_fmh	2	277886-279121	Chr2	IS407A	CACGC	TGACCTGCCCCC~TAC	ATGAAGAAGCGCTTTACG	GCCCGCAAGCCAAACGCG	TGTTGGC	AGGGACTTTGCTAGAA	CCCTGCC	GGTC	CCCT	N
8*Chr2_37_IS407A	8*Chr2_57a_IS407A_A	D-R	Bm_200272128	8	278376-279611	Chr2	IS407A	CCCTT	TGACCTGCCCCC~TAC	ATGAAGAAGCGCTTTACG	GCCCGCAAGCCAAACGCG	TGTTGGC	AGGGACTTTGCTAGAA	CGCCCGC	ACCT	CGCC	N
5*Chr2_62b_IS407	5*Chr2_75_IS407A_A	D-R	Bm_ATCC_103	5	284510-285543	Chr2	IS407A	#IS407	~~~~~~~~~~~~~~	~~~~~~~~~~~~~~~~~	GCCCGCAAGCCAAACGCG	TGTTGGC	AGGGACTTTGCTAGAA	GTCGATC	IS607A?	GTCG	N.D.
10*Chr2_75_IS407A_A		D-R	Bm_PRL20	10	291658-292532	Chr2	IS407A	#IS407	~~~~~~~~~~~~~~	~~~~~~~~~~~~~~~~~	GCCCGCAAGCCAAACGCG	TGTTGGC	#	#IS407A	IS607A?	#IS4	N.D.
10*Chr2_62b_IS407A_A		R	Bm_PRL20	10	292289-293524	Chr2	IS407A	CTAGA	TGACCTGCCCCC~TAC	ATGAAGAAGCGCTTTACG	GCCCGCAAGCCAAACGCG	TGTTGGC	AGGGACTTTGCTAGAA	CACGGTC	GATC	CACG	N
6*Chr2_48_IS407A_A		N	Bm_NCTC_102	6	297003-298238	Chr2	IS407A	CATTC	TGACCTGCCCCC~TAC	ATGAAGAAGCGCTTTACG	GCCCGCAAGCCAAACGCG	TGTTGGC	AGGGACTTTGCTAGAA	GCCGCTC	GCCG	GCCG	Y
7*Chr2_66_IS407A_B		N	Bm_NCTC_102	7	300642-301877	Chr2	IS407A	TCAGT	TGACCTGCCCCC~TAC	ATGAAGAAGCGCTTTACG	GCCCGCAAGCCAAACGCG	TGTTGGC	AGGGACTTTGCTAGAA	TGGCAGT	TGGC	TGGC	Y
3*Chr2_67_IS407A	3*Chr2_68_IS407A_A	N	Bm_JHU	3	306238-307473	Chr2	IS407A	CACGC	TGACCTGCCCCC~TAC	ATGAAGAAGCGCTTTACG	GCCCGCAAGCCAAACGCG	TGTTGGC	AGGGACTTTGCTAGAA	CCCTGCC	GGTC	CCCT	N
9*Chr2_40_IS407A	9*Chr2_41_IS407A_B	D	Bm_SAVP1	9	311876-313111	Chr2	IS407A	GAAGG	TGACCTGCCCCC~TAC	ATGAAGAAGCGCTTTACG	GCCCGCAAGCCAAACGCG	TGTTGGC	AGGGACTTTGCTAGAA	CGAACTG	AGCG	CGAA	N
4*Chr2_62b_IS407	4*Chr2_75_IS407A_A	D-R	Bm_gb8_horse	4	328902-329935	Chr2	IS407A	#IS407	~~~~~~~~~~~~~~	~~~~~~~~~~~~~~~~~	GCCCGCAAGCCAAACGCG	TGTTGGC	AGGGACTTTGCTAGAA	GTCGATC	IS607A?	GTCG	N.D.
1*Chr2_62b_IS407	1*Chr2_75_IS407A_A	D-R	Bm_ATCC_233	1	329696-330729	Chr2	IS407A	#IS407	~~~~~~~~~~~~~~	~~~~~~~~~~~~~~~~~	GCCCGCAAGCCAAACGCG	TGTTGGC	AGGGACTTTGCTAGAA	GTCGATC	IS607A?	GTCG	N.D.
7*Chr2_62b_IS407	7*Chr2_75_IS407A_A	D-R	Bm_NCTC_102	7	332069-333102	Chr2	IS407A	#IS407	~~~~~~~~~~~~~~	~~~~~~~~~~~~~~~~~	GCCCGCAAGCCAAACGCG	TGTTGGC	AGGGACTTTGCTAGAA	GTCGATC	IS607A?	GTCG	N.D.
2*Chr2_62b_IS407	2*Chr2_75_IS407A_A	D-R	Bm_fmh	2	339193-340226	Chr2	IS407A	#IS407	~~~~~~~~~~~~~~	~~~~~~~~~~~~~~~~~	GCCCGCAAGCCAAACGCG	TGTTGGC	AGGGACTTTGCTAGAA	GTCGATC	IS607A?	GTCG	N.D.
7*Chr2_1_IS407A_B		N	Bm_NCTC_102	7	36098-37333	Chr2	IS407A	CCAGA	TGACCTGCCCCC~TAC	ATGAAGAAGCGCTTTACG	GCCCGCAAGCCAAACGCG	TGTTGGC	AGGGACTTTGCTAGAA	CGGCGAA	CGGC	CGGC	Y
5*Chr2_62a_IS407	5*Chr2_74_IS407A_A	D-R	Bm_ATCC_103	5	363336-364571	Chr2	IS407A	CCATC	TGACCTGCCCCC~TAC	ATGAAGAAGCGCTTTACG	GCCCGCAAGCCAAACGCG	TGTTGGC	AGGGACTTTGCTAGAA	CGGCTTC	TGAT	CGGC	N

3*Chr2_62b_IS407	3*Chr2_75_IS407A_A	D-R	Bm_JHU	3	367812-368845	Chr2	IS407A	#IS407	~~~~~~~~~~~~~~	~~~~~~~~~~~~~~~~~	GCCCGCAAGCCAAACGCG	TGTTGGC	AGGGACTTTGCTAGAA	GTCGATC	IS607A?	GTCG	N.D.
10*Chr2_62a_IS40	10*Chr2_74_IS407A_A	D-R	Bm_PRL20	10	370625-371860	Chr2	IS407A	CCATC	TGACCTGCCCCC~TAC	ATGAAGAAGCGCTTTACG	GCCCGCAAGCCAAACGCG	TGTTGGC	AGGGACTTTGCTAGAA	CGGCTTC	TGAT	CGGC	N
9*Chr2_38_IS407A	9*Chr2_50_IS407A_B	D-R	Bm_SAVP1	9	379739-380974	Chr2	IS407A	GTCCA	TGACCTGCCCCC~TAC	ATGAAGAAGCGCTTTACG	GCCCGCAAGCCAAACGCG	TGTTGGC	AGGGACTTTGCTAGAA	CGCGAGA	GAAC	CGCG	N
8*Chr2_24_IS407A	8*Chr2_56_IS407A_A	D-R	Bm_200272128	8	382829-384064	Chr2	IS407A	CGGGA	TGACCTGCCCCC~TAC	ATGAAGAAGCGCTTTACG	GCCCGCAAGCCAAACGCG	TGTTGGC	AGGGACTTTGCTAGAA	CATCGGC	CCAC	CATC	N
5*Chr2_20b_IS407	5*Chr2_61_IS407A_A	D-R	Bm_ATCC_103	5	387487-388722	Chr2	IS407A	CGCGA	TGACCTGCCCCC~TAC	ATGAAGAAGCGCTTTACG	GCCCGCAAGCCAAACGCG	TGTTGGC	AGGGACTTTGCTAGAA	CGACGGC	GTAC	CGAC	N
9*Chr2_51_IS407A_A		N	Bm_SAVP1	9	393155-394390	Chr2	IS407A	CGGCC	TGACCTGCCCCC~TAC	ATGAAGAAGCGCTTTACG	GCCCGCAAGCCAAACGCG	TGTTGGC	AGGGACTTTGCTAGAA	GGCGGCC	GGCG	GGCG	Y
10*Chr2_60_IS407	10*Chr2_61_IS407A_A	D	Bm_PRL20	10	394776-396011	Chr2	IS407A	CGCCG	TGACCTGCCCCC~TAC	ATGAAGAAGCGCTTTACG	GCCCGCAAGCCAAACGCG	TGTTGGC	AGGGACTTTGCTAGAA	CGACGGC	CGTG	CGAC	N
4*Chr2_62a_IS407	4*Chr2_74_IS407A_A	D-R	Bm_gb8_horse_	4	407716-408951	Chr2	IS407A	CCATC	TGACCTGCCCCC~TAC	ATGAAGAAGCGCTTTACG	GCCCGCAAGCCAAACGCG	TGTTGGC	AGGGACTTTGCTAGAA	CGGCTTC	TGAT	CGGC	N
6*Chr2_44a_IS407A_A		N	Bm_NCTC_102	6	408123-409358	Chr2	IS407A	GACAG	TGACCTGCCCCC~TAC	ATGAAGAAGCGCTTTACG	GCCCGCAAGCCAAACGCG	TGTTGGC	AGGGACTTTGCTAGAA	AGGGGTA	CGCC	AGGG	N
1*Chr2_62a_IS407	1*Chr2_74_IS407A_A	D-R	Bm_ATCC_233	1	408708-409943	Chr2	IS407A	CCATC	TGACCTGCCCCC~TAC	ATGAAGAAGCGCTTTACG	GCCCGCAAGCCAAACGCG	TGTTGGC	AGGGACTTTGCTAGAA	CGGCTTC	TGAT	CGGC	N
9*Chr2_45_IS407A_B		N	Bm_SAVP1	9	41016-42251	Chr2	IS407A	CGGGC	TGACCTGCCCCC~TAC	ATGAAGAAGCGCTTTACG	GCCCGCAAGCCAAACGCG	TGTTGGC	AGGGACTTTGCTAGAA	GCCCGCC	CTTT	GCCC	N
7*Chr2_62a_IS407	7*Chr2_74_IS407A_A	D-R	Bm_NCTC_102	7	410900-412135	Chr2	IS407A	CCATC	TGACCTGCCCCC~TAC	ATGAAGAAGCGCTTTACG	GCCCGCAAGCCAAACGCG	TGTTGGC	AGGGACTTTGCTAGAA	CGGCTTC	TGAT	CGGC	N
8*Chr2_23_IS407A_A		D-R	Bm_200272128	8	41226-42461	Chr2	IS407A	CGCCG	TGACCTGCCCCC~TAC	ATGAAGAAGCGCTTTACG	GCCCGCAAGCCAAACGCG	TGTTGGC	AGGGACTTTGCTAGAA	ATGGATTT	CGAT	ATGG	N
10*Chr2_59_IS407A_A		N	Bm_PRL20	10	415896-417131	Chr2	IS407A	CCGCG	TGACCTGCCCCC~TAC	ATGAAGAAGCGCTTTACG	GCCCGCAAGCCAAACGCG	TGTTGGC	AGGGACTTTGCTAGAA	GGCGGAT	GTGC	GGCG	N
8*Chr2_26_IS407A	8*Chr2_27_IS407A_A	D	Bm_200272128	8	418100-419335	Chr2	IS407A	CTGGC	TGACCTGCCCCC~TAC	ATGAAGAAGCGCTTTACG	GCCCGCAAGCCAAACGCG	TGTTGGC	AGGGACTTTGCTAGAA	CCCGAGC	CGAG	CCCG	N
2*Chr2_62a_IS407	2*Chr2_74_IS407A_A	D-R	Bm_fmh	2	418863-420098	Chr2	IS407A	CCATC	TGACCTGCCCCC~TAC	ATGAAGAAGCGCTTTACG	GCCCGCAAGCCAAACGCG	TGTTGGC	AGGGACTTTGCTAGAA	CGGCTTC	TGAT	CGGC	N
4*Chr2_20b_IS407	4*Chr2_61_IS407A_A	D-R	Bm_gb8_horse_	4	431867-433102	Chr2	IS407A	CGCGA	TGACCTGCCCCC~TAC	ATGAAGAAGCGCTTTACG	GCCCGCAAGCCAAACGCG	TGTTGGC	AGGGACTTTGCTAGAA	CGACGGC	GTAC	CGAC	N
1*Chr2_20b_IS407	1*Chr2_61_IS407A_A	D-R	Bm_ATCC_233	1	432859-434094	Chr2	IS407A	CGCGA	TGACCTGCCCCC~TAC	ATGAAGAAGCGCTTTACG	GCCCGCAAGCCAAACGCG	TGTTGGC	AGGGACTTTGCTAGAA	CGACGGC	GTAC	CGAC	N
7*Chr2_60_IS407A	7*Chr2_61_IS407A_A	D	Bm_NCTC_102	7	435051-436286	Chr2	IS407A	CGCCG	TGACCTGCCCCC~TAC	ATGAAGAAGCGCTTTACG	GCCCGCAAGCCAAACGCG	TGTTGGC	AGGGACTTTGCTAGAA	CGACGGC	CGTG	CGAC	N
2*Chr2_20b_IS407	2*Chr2_61_IS407A_A	D-R	Bm_fmh	2	442944-444179	Chr2	IS407A	CGCGA	TGACCTGCCCCC~TAC	ATGAAGAAGCGCTTTACG	GCCCGCAAGCCAAACGCG	TGTTGGC	AGGGACTTTGCTAGAA	CGACGGC	GTAC	CGAC	N
3*Chr2_62a_IS407	3*Chr2_74_IS407A_A	D-R	Bm_JHU	3	447920-449155	Chr2	IS407A	CCATC	TGACCTGCCCCC~TAC	ATGAAGAAGCGCTTTACG	GCCCGCAAGCCAAACGCG	TGTTGGC	AGGGACTTTGCTAGAA	CGGCTTC	TGAT	CGGC	N
7*Chr2_59_IS407A_A		N	Bm_NCTC_102	7	456171-457406	Chr2	IS407A	CCGCG	TGACCTGCCCCC~TAC	ATGAAGAAGCGCTTTACG	GCCCGCAAGCCAAACGCG	TGTTGGC	AGGGACTTTGCTAGAA	GGCGGAT	GTGC	GGCG	N
5*Chr2_23_IS407A_A		D-R	Bm_ATCC_103	5	465011-466246	Chr2	IS407A	CGCCG	TGACCTGCCCCC~TAC	ATGAAGAAGCGCTTTACG	GCCCGCAAGCCAAACGCG	TGTTGGC	AGGGACTTTGCTAGAA	ATGGATTT	CGAT	ATGG	N
8*Chr2_28_IS407A	8*Chr2_31b_IS407A_A	D	Bm_200272128	8	466537-467772	Chr2	IS407A	GAACG	TGACCTGCCCCC~TAC	ATGAAGAAGCGCTTTACG	GCCCGCAAGCCAAACGCG	TGTTGGC	AGGGACTTTGCTAGAA	CGATGCT	GATC	CGAT	N
3*Chr2_20b_IS407	3*Chr2_61_IS407A_A	D-R	Bm_JHU	3	472071-473306	Chr2	IS407A	CGCGA	TGACCTGCCCCC~TAC	ATGAAGAAGCGCTTTACG	GCCCGCAAGCCAAACGCG	TGTTGGC	AGGGACTTTGCTAGAA	CGACGGC	GTAC	CGAC	N
9*Chr2_52_IS407A_A		N	Bm_SAVP1	9	472566-473801	Chr2	IS407A	GAATT	TGACCTGCCCCC~TAC	ATGAAGAAGCGCTTTACG	GCCCGCAAGCCAAACGCG	TGTTGGC	AGGGACTTTGCTAGAA	CCGTCAC	GGAT	CCGT	N
10*Chr2_23_IS407A_A		D-R	Bm_PRL20	10	492363-493598	Chr2	IS407A	AATGG	TGACCTGCCCCC~TAC	ATGAAGAAGCGCTTTACG	GCCCGCAAGCCAAACGCG	TGTTGGC	AGGGACTTTGCTAGAA	ATCGGCC	CCAT	ATCG	N
9*Chr2_33_IS407A	9*Chr2_53_IS407A_A	D-R	Bm_SAVP1	9	497742-498977	Chr2	IS407A	GCGAC	TGACCTGCCCCC~TAC	ATGAAGAAGCGCTTTACG	GCCCGCAAGCCAAACGCG	TGTTGGC	AGGGACTTTGCTAGAA	GTCCAGC	CCTG	GTCC	N
8*Chr2_33_IS407A	8*Chr2_53_IS407A_A	D-R	Bm_200272128	8	503874-505109	Chr2	IS407A	GTGCC	TGACCTGCCCCC~TAC	ATGAAGAAGCGCTTTACG	GCCCGCAAGCCAAACGCG	TGTTGGC	AGGGACTTTGCTAGAA	GTCCAGC	ACGG	GTCC	N
4*Chr2_23_IS407A_A		D-R	Bm_gb8_horse_	4	508200-509435	Chr2	IS407A	AATGG	TGACCTGCCCCC~TAC	ATGAAGAAGCGCTTTACG	GCCCGCAAGCCAAACGCG	TGTTGGC	AGGGACTTTGCTAGAA	ATCGGCC	CCAT	ATCG	N
1*Chr2_23_IS407A_A		D-R	Bm_ATCC_233	1	509411-510646	Chr2	IS407A	AATGG	TGACCTGCCCCC~TAC	ATGAAGAAGCGCTTTACG	GCCCGCAAGCCAAACGCG	TGTTGGC	AGGGACTTTGCTAGAA	ATCGGCC	CCAT	ATCG	N
6*Chr2_42_IS407A_A		N	Bm_NCTC_102	6	513999-515234	Chr2	IS407A	ATCGA	TGACCTGCCCCC~TAC	ATGAAGAAGCGCTTTACG	GCCCGCAAGCCAAACGCG	TGTTGGC	AGGGACTTTGCTAGAA	CTGCGGC	CTGC	CTGC	Y
5*Chr2_15b_IS407A_A		R	Bm_ATCC_103	5	515849-517084	Chr2	IS407A	CGCGC	TGACCTGCCCCC~TAC	ATGAAGAAGCGCTTTACG	GCCCGCAAGCCAAACGCG	TGTTGGC	AGGGACTTTGCTAGAA	ACAGTTTT	GCTT	ACAG	N
2*Chr2_23_IS407A_A		D-R	Bm_fmh	2	520261-521496	Chr2	IS407A	AATGG	TGACCTGCCCCC~TAC	ATGAAGAAGCGCTTTACG	GCCCGCAAGCCAAACGCG	TGTTGGC	AGGGACTTTGCTAGAA	ATCGGCC	CCAT	ATCG	N
5*Chr2_16_IS407A	5*Chr2_55_IS407A_A	D-R	Bm_ATCC_103	5	520536-521771	Chr2	IS407A	CGATC	TGACCTGCCCCC~TAC	ATGAAGAAGCGCTTTACG	GCCCGCAAGCCAAACGCG	TGTTGGC	AGGGACTTTGCTAGAA	CGTAATCC	CCGC	CGTA	N
8*Chr2_22_IS407A	8*Chr2_52_IS407A_A	R	Bm_200272128	8	529400-530635	Chr2	IS407A	GAATT	TGACCTGCCCCC~TAC	ATGAAGAAGCGCTTTACG	GCCCGCAAGCCAAACGCG	TGTTGGC	AGGGACTTTGCTAGAA	CGTCACG	GGAT	CGTC	N
9*Chr2_31b_IS407	9*Chr2_56_IS407A_A	D-R	Bm_SAVP1	9	535045-536280	Chr2	IS407A	GAACG	TGACCTGCCCCC~TAC	ATGAAGAAGCGCTTTACG	GCCCGCAAGCCAAACGCG	TGTTGGC	AGGGACTTTGCTAGAA	CATCGGC	GATC	CATC	N
10*Chr2_15b_IS407A_A		R	Bm_PRL20	10	541189-542423	Chr2	IS407A	CGCGC	TGACCTGCCCCC~TAC	ATGAAGAAGCGCTTTACG	GCCCGCAAGCCAAACGCG	TGTTGGC	AGGGACTTTGCTAGAA	ACAGTTTT	GCTT	ACAG	N
10*Chr2_16_IS407	10*Chr2_35_IS407A_A	D-R	Bm_PRL20	10	545708-546943	Chr2	IS407A	CGATC	TGACCTGCCCCC~TAC	ATGAAGAAGCGCTTTACG	GCCCGCAAGCCAAACGCG	TGTTGGC	AGGGACTTTGCTAGAA	CCAGCAA	CATC	CCAG	N
5*Chr2_34_IS407A	5*Chr2_54_IS407A_A	D-R	Bm_ATCC_103	5	547403-548638	Chr2	IS407A	CGCGG	TGACCTGCCCCC~TAC	ATGAAGAAGCGCTTTACG	GCCCGCAAGCCAAACGCG	TGTTGGC	AGGGACTTTGCTAGAA	GGATAGA	GAGG	GGAT	N
3*Chr2_23_IS407A_A		D-R	Bm_JHU	3	548996-550231	Chr2	IS407A	AATGG	TGACCTGCCCCC~TAC	ATGAAGAAGCGCTTTACG	GCCCGCAAGCCAAACGCG	TGTTGGC	AGGGACTTTGCTAGAA	ATCGGCC	CCAT	ATCG	N
4*Chr2_15b_IS407A_A		R	Bm_gb8_horse_	4	559127-560362	Chr2	IS407A	CGCGC	TGACCTGCCCCC~TAC	ATGAAGAAGCGCTTTACG	GCCCGCAAGCCAAACGCG	TGTTGGC	AGGGACTTTGCTAGAA	ACAGTTTT	GCTT	ACAG	N
1*Chr2_15b_IS407A_A		R	Bm_ATCC_233	1	560460-561695	Chr2	IS407A	CGCGC	TGACCTGCCCCC~TAC	ATGAAGAAGCGCTTTACG	GCCCGCAAGCCAAACGCG	TGTTGGC	AGGGACTTTGCTAGAA	ACAGTTTT	GCTT	ACAG	N
4*Chr2_16_IS407A	4*Chr2_55_IS407A_A	D-R	Bm_gb8_horse_	4	563814-565049	Chr2	IS407A	CGATC	TGACCTGCCCCC~TAC	ATGAAGAAGCGCTTTACG	GCCCGCAAGCCAAACGCG	TGTTGGC	AGGGACTTTGCTAGAA	CGTAATCC	CCGC	CGTA	N
1*Chr2_16_IS407A	1*Chr2_55_IS407A_A	D-R	Bm_ATCC_233	1	565147-566382	Chr2	IS407A	CGATC	TGACCTGCCCCC~TAC	ATGAAGAAGCGCTTTACG	GCCCGCAAGCCAAACGCG	TGTTGGC	AGGGACTTTGCTAGAA	CGTAATCC	CCGC	CGTA	N
6*Chr2_26_IS407A	6*Chr2_27_IS407A_A	D	Bm_NCTC_102	6	56520-57755	Chr2	IS407A	TTCAC	TGACCTGCCCCC~TAC	ATGAAGAAGCGCTTTACG	GCCCGCAAGCCAAACGCG	TGTTGGC	AGGGACTTTGCTAGAA	CTCGGCG	TATG	CTCG	N
7*Chr2_58_IS407A_A		N	Bm_NCTC_102	7	566054-567289	Chr2	IS407A	CGCGG	TGACCTGCCCCC~TAC	ATGAAGAAGCGCTTTACG	GCCCGCAAGCCAAACGCG	TGTTGGC	AGGGACTTTGCTAGAA	ATCCGGG	CGAC	ATCC	N
2*Chr2_15b_IS407A_A		R	Bm_fmh	2	571425-572660	Chr2	IS407A	CGCGC	TGACCTGCCCCC~TAC	ATGAAGAAGCGCTTTACG	GCCCGCAAGCCAAACGCG	TGTTGGC	AGGGACTTTGCTAGAA	ACAGTTTT	GCTT	ACAG	N
2*Chr2_16_IS407A	2*Chr2_55_IS407A_A	D-R	Bm_fmh	2	576112-577347	Chr2	IS407A	CGATC	TGACCTGCCCCC~TAC	ATGAAGAAGCGCTTTACG	GCCCGCAAGCCAAACGCG	TGTTGGC	AGGGACTTTGCTAGAA	CGTAATCC	CCGC	CGTA	N
4*Chr2_34_IS407A	4*Chr2_54_IS407A_A	D-R	Bm_gb8_horse_	4	590631-591866	Chr2	IS407A	CGCGG	TGACCTGCCCCC~TAC	ATGAAGAAGCGCTTTACG	GCCCGCAAGCCAAACGCG	TGTTGGC	AGGGACTTTGCTAGAA	GGATAGA	GAGG	GGAT	N
1*Chr2_34_IS407A	1*Chr2_54_IS407A_A	D-R	Bm_ATCC_233	1	592085-593320	Chr2	IS407A	CGCGG	TGACCTGCCCCC~TAC	ATGAAGAAGCGCTTTACG	GCCCGCAAGCCAAACGCG	TGTTGGC	AGGGACTTTGCTAGAA	GGATAGA	GAGG	GGAT	N
3*Chr2_15b_IS407A_A		R	Bm_JHU	3	600352-601587	Chr2	IS407A	CGCGC	TGACCTGCCCCC~TAC	ATGAAGAAGCGCTTTACG	GCCCGCAAGCCAAACGCG	TGTTGGC	AGGGACTTTGCTAGAA	ACAGTTTT	GCTT	ACAG	N
9*Chr2_10a_IS407	9*Chr2_44b_IS407A_A	D-R	Bm_SAVP1	9	6022-7257	Chr2	IS407A	GACAG	TGACCTGCCCCC~TAC	ATGAAGAAGCGCTTTACG	GCCCGCAAGCCAAACGCG	TGTTGGC	AGGGACTTTGCTAGAA	AAGCCGT	CGCC	AAGC	N
2*Chr2_34_IS407A	2*Chr2_54_IS407A_A	D-R	Bm_fmh	2	603189-604424	Chr2	IS407A	CGCGG	TGACCTGCCCCC~TAC	ATGAAGAAGCGCTTTACG	GCCCGCAAGCCAAACGCG	TGTTGGC	AGGGACTTTGCTAGAA	GGATAGA	GAGG	GGAT	N
3*Chr2_16_IS407A	3*Chr2_55_IS407A_A	D-R	Bm_JHU	3	605039-606274	Chr2	IS407A	CGATC	TGACCTGCCCCC~TAC	ATGAAGAAGCGCTTTACG	GCCCGCAAGCCAAACGCG	TGTTGGC	AGGGACTTTGCTAGAA	CGTAATCC	CCGC	CGTA	N
6*Chr2_40_IS407A_A		N	Bm_NCTC_102	6	607939-609174	Chr2	IS407A	CGCGT	TGACCTGCCCCC~TAC	ATGAAGAAGCGCTTTACG	GCCCGCAAGCCAAACGCG	TGTTGGC	AGGGACTTTGCTAGAA	CGAACTG	CAGG	CGAA	N
8*Chr2_51_IS407A_A		N	Bm_200272128	8	608682-609917	Chr2	IS407A	CGGCC	TGACCTGCCCCC~TAC	ATGAAGAAGCGCTTTACG	GCCCGCAAGCCAAACGCG	TGTTGGC	AGGGACTTTGCTAGAA	GGCGGCC	GGCG	GGCG	Y
5*Chr2_35_IS407A_A		N	Bm_ATCC_103	5	612093-613328	Chr2	IS407A	CGATC	TGACCTGCCCCC~TAC	ATGAAGAAGCGCTTTACG	GCCCGCAAGCCAAACGCG	TGTTGGC	AGGGACTTTGCTAGAA	CATCAGC	CATC	CATC	Y

6*Chr2_10a_IS407/	6*Chr2_13a_IS407A_A		R	Bm_NCTC_102:	6	6183-7418	Chr2	IS407A	GAGCC	TGACCTGCCCCC~TAC	ATGAAGAAGCGCTTTACG(	GCCCGCAAGCCAAACGCG,	TGTTGGC(	AGGGACTTTGCTAGAA(	AAGCCGT1	CTCG	AAGC	N
10*Chr2_37_IS407/	10*Chr2_57a_IS407A_A		D-R	Bm_PRL20	10	622831-624066	Chr2	IS407A	CCCTT(	TGACCTGCCCCC~TAC	ATGAAGAAGCGCTTTACG(	GCCCGCAAGCCAAACGCG,	TGTTGGC(	AGGGACTTTGCTAGAA(	CGCCCGC(	ACCT	CGCC	N
8*Chr2_49_IS407A_A			N	Bm_200272128	8	630594-631829	Chr2	IS407A	CCGAC	TGACCTGCCCCC~TAC	ATGAAGAAGCGCTTTACG(	GCCCGCAAGCCAAACGCG,	TGTTGGC(	AGGGACTTTGCTAGAA(	AGCGACG(	AGCG	AGCG	Y
3*Chr2_34_IS407A	3*Chr2_54_IS407A_A		D-R	Bm_JHU	3	631977-633212	Chr2	IS407A	CGCGG	TGACCTGCCCCC~TAC	ATGAAGAAGCGCTTTACG(	GCCCGCAAGCCAAACGCG,	TGTTGGC(	AGGGACTTTGCTAGAA(	GGATAGA(	GAGG	GGAT	N
5*Chr2_83_IS407A_C			N	Bm_ATCC_1039	5	6354-7589	Chr2	IS407A	ACGGC	TGACCTGCCCCC~TAC	ATGAAGAAGCGCTTTACG(	GCCCGCAAGCCAAACGCG,	TGTTGGC(	AGGGACTTTGCTAGAA(	CGGCCAC(	CGGC	CGGC	Y
9*Chr2_57a_IS407A_A			D-R	Bm_SAVP1	9	639673-640908	Chr2	IS407A	CCCTT(	TGACCTGCCCCC~TAC	ATGAAGAAGCGCTTTACG(	GCCCGCAAGCCAAACGCG,	TGTTGGC(	AGGGACTTTGCTAGAA(	CAGAGAC.	ACCT	CAGA	N
4*Chr2_35_IS407A_A			N	Bm_gb8_horse_	4	655266-656501	Chr2	IS407A	CGATC(	TGACCTGCCCCC~TAC	ATGAAGAAGCGCTTTACG(	GCCCGCAAGCCAAACGCG,	TGTTGGC(	AGGGACTTTGCTAGAA(	CATCAGC/	CATC	CATC	Y
1*Chr2_35_IS407A_A			N	Bm_ATCC_2334	1	656707-657942	Chr2	IS407A	CGATC(	TGACCTGCCCCC~TAC	ATGAAGAAGCGCTTTACG(	GCCCGCAAGCCAAACGCG,	TGTTGGC(	AGGGACTTTGCTAGAA(	CATCAGC/	CATC	CATC	Y
8*Chr2_48_IS407A_A			N	Bm_200272128	8	658424-659659	Chr2	IS407A	CATTC(	TGACCTGCCCCC~TAC	ATGAAGAAGCGCTTTACG(	GCCCGCAAGCCAAACGCG,	TGTTGGC(	AGGGACTTTGCTAGAA(	GCCGCTC(	GCCG	GCCG	Y
2*Chr2_35_IS407A_A			N	Bm_fmh	2	668220-669455	Chr2	IS407A	CGATC(	TGACCTGCCCCC~TAC	ATGAAGAAGCGCTTTACG(	GCCCGCAAGCCAAACGCG,	TGTTGGC(	AGGGACTTTGCTAGAA(	CATCAGC/	CATC	CATC	Y
6*Chr2_38_IS407A	6*Chr2_56_IS407A_A		D-R	Bm_NCTC_102:	6	675587-676822	Chr2	IS407A	GTCCA(	TGACCTGCCCCC~TAC	ATGAAGAAGCGCTTTACG(	GCCCGCAAGCCAAACGCG,	TGTTGGC(	AGGGACTTTGCTAGAA(	CATCGGC1	GAAC	CATC	N
9*Chr2_23_IS407A_A			D-R	Bm_SAVP1	9	686954-688189	Chr2	IS407A	AATGG.	TGACCTGCCCCC~TAC	ATGAAGAAGCGCTTTACG(	GCCCGCAAGCCAAACGCG,	TGTTGGC(	AGGGACTTTGCTAGAA(	ATCGGCC(	CCAT	ATCG	N
5*Chr2_37_IS407A	5*Chr2_57a_IS407A_A		D-R	Bm_ATCC_1039	5	689319-690554	Chr2	IS407A	CCCTT(	TGACCTGCCCCC~TAC	ATGAAGAAGCGCTTTACG(	GCCCGCAAGCCAAACGCG,	TGTTGGC(	AGGGACTTTGCTAGAA(	CGCCCGC(	ACCT	CGCC	N
3*Chr2_35_IS407A_A			N	Bm_JHU	3	696599-697834	Chr2	IS407A	CGATC(	TGACCTGCCCCC~TAC	ATGAAGAAGCGCTTTACG(	GCCCGCAAGCCAAACGCG,	TGTTGGC(	AGGGACTTTGCTAGAA(	CATCAGC/	CATC	CATC	Y
7*Chr2_13b_IS407.	7*Chr2_57b_IS407A_A		R	Bm_NCTC_1024	7	714504-715739	Chr2	IS407A	CAAGC	TGACCTGCCCCC~TAC	ATGAAGAAGCGCTTTACG(	GCCCGCAAGCCAAACGCG,	TGTTGGC(	AGGGACTTTGCTAGAA(	ATCTTCCC	GGTT	ATCT	N
7*Chr2_6_IS407A_A			N	Bm_NCTC_1024	7	721251-722486	Chr2	IS407A	GCGCA	TGACCTGCCCCC~TAC	ATGAAGAAGCGCTTTACG(	GCCCGCAAGCCAAACGCG,	TGTTGGC(	AGGGACTTTGCTAGAA(	CGTGAAC(	CGTG	CGTG	Y
10*Chr2_31b_IS40	10*Chr2_56_IS407A_A		D-R	Bm_PRL20	10	726908-728143	Chr2	IS407A	GAACG	TGACCTGCCCCC~TAC	ATGAAGAAGCGCTTTACG(	GCCCGCAAGCCAAACGCG,	TGTTGGC(	AGGGACTTTGCTAGAA(	CATCGGC1	GATC	CATC	N
4*Chr2_37_IS407A	4*Chr2_57a_IS407A_A		D-R	Bm_gb8_horse_	4	732439-733674	Chr2	IS407A	CCCTT(	TGACCTGCCCCC~TAC	ATGAAGAAGCGCTTTACG(	GCCCGCAAGCCAAACGCG,	TGTTGGC(	AGGGACTTTGCTAGAA(	CGCCCGC(	ACCT	CGCC	N
1*Chr2_37_IS407A	1*Chr2_57a_IS407A_A		D-R	Bm_ATCC_2334	1	733880-735115	Chr2	IS407A	CCCTT(	TGACCTGCCCCC~TAC	ATGAAGAAGCGCTTTACG(	GCCCGCAAGCCAAACGCG,	TGTTGGC(	AGGGACTTTGCTAGAA(	CGCCCGC(	ACCT	CGCC	N
2*Chr2_37_IS407A	2*Chr2_57a_IS407A_A		D-R	Bm_fmh	2	746706-747941	Chr2	IS407A	CCCTT(	TGACCTGCCCCC~TAC	ATGAAGAAGCGCTTTACG(	GCCCGCAAGCCAAACGCG,	TGTTGGC(	AGGGACTTTGCTAGAA(	CGCCCGC(	ACCT	CGCC	N
10*Chr2_9a_IS407/	10*Chr2_24_IS40	10*Chr2_32a_	D-R	Bm_PRL20	10	749164-750399	Chr2	IS407A	CGGGA	TGACCTGCCCCC~TAC	ATGAAGAAGCGCTTTACG(	GCCCGCAAGCCAAACGCG,	TGTTGGC(	AGGGACTTTGCTAGAA(	CAAGCAG(	CCAC	CAAG	N
9*Chr2_19_IS407A_B			N	Bm_SAVP1	9	764597-765832	Chr2	IS407A	TTATG1	TGACCTGCCCCC~TAC	ATGAAGAAGCGCTTTACG(	GCCCGCAAGCCAAACGCG,	TGTTGGC(	AGGGACTTTGCTAGAA(	GGGTATTT	GGGT	GGGT	Y
8*Chr2_44a_IS407A_A			N	Bm_200272128	8	769677-770912	Chr2	IS407A	GACAG	TGACCTGCCCCC~TAC	ATGAAGAAGCGCTTTACG(	GCCCGCAAGCCAAACGCG,	TGTTGGC(	AGGGACTTTGCTAGAA(	AGGGGTA.	CGCC	AGGG	N
3*Chr2_37_IS407A	3*Chr2_57a_IS407A_A		D-R	Bm_JHU	3	773773-775008	Chr2	IS407A	CCCTT(	TGACCTGCCCCC~TAC	ATGAAGAAGCGCTTTACG(	GCCCGCAAGCCAAACGCG,	TGTTGGC(	AGGGACTTTGCTAGAA(	CGCCCGC(	ACCT	CGCC	N
10*Chr2_25_IS407A_C			N	Bm_PRL20	10	778318-779553	Chr2	IS407A	GGTTA.	TGACCTGCCCCC~TAC	ATGAAGAAGCGCTTTACG(	GCCCGCAAGCCAAACGCG,	TGTTGGC(	AGGGACTTTGCTAGAA(	ATCGACG(	ATCG	ATCG	Y
6*Chr2_37_IS407A	6*Chr2_57a_IS407A_A		D-R	Bm_NCTC_102:	6	780241-781476	Chr2	IS407A	CCCTT(	TGACCTGCCCCC~TAC	ATGAAGAAGCGCTTTACG(	GCCCGCAAGCCAAACGCG,	TGTTGGC(	AGGGACTTTGCTAGAA(	CGCCCGC(	ACCT	CGCC	N
10*Chr2_26_IS407/	10*Chr2_27_IS407A_A		D	Bm_PRL20	10	783043-784278	Chr2	IS407A	GGCGT	TGACCTGCCCCC~TAC	ATGAAGAAGCGCTTTACG(	GCCCGCAAGCCAAACGCG,	TGTTGGC(	AGGGACTTTGCTAGAA(	CTCGGCG(	GAAA	CTCG	N
5*Chr2_44b_IS407.	5*Chr2_56_IS407A_A		D-R	Bm_ATCC_1039	5	793863-795098	Chr2	IS407A	GACAG	TGACCTGCCCCC~TAC	ATGAAGAAGCGCTTTACG(	GCCCGCAAGCCAAACGCG,	TGTTGGC(	AGGGACTTTGCTAGAA(	CATCGGC1	CGCC	CATC	N
9*Chr2_15a_IS407/	9*Chr2_18_IS407A_A		D-R	Bm_SAVP1	9	799543-800778	Chr2	IS407A	AGACC	TGACCTGCCCCC~TAC	ATGAAGAAGCGCTTTACG(	GCCCGCAAGCCAAACGCG,	TGTTGGC(	AGGGACTTTGCTAGAA(	GGATCAG(	AGAT	GGAT	N
4*Chr2_2_IS407A_/	4*Chr2_73_IS407A_A		D-R	Bm_gb8_horse_	4	80307-81542	Chr2	IS407A	CGCTG(	TGACCTGCCCCC~TAC	ATGAAGAAGCGCTTTACG(	GCCCGCAAGCCAAACGCG,	TGTTGGC(	AGGGACTTTGCTAGAA(	CAACGCA1	GTGT	CAAC	N
1*Chr2_2_IS407A_/	1*Chr2_73_IS407A_A		D-R	Bm_ATCC_2334	1	80328-81563	Chr2	IS407A	CGCTG(	TGACCTGCCCCC~TAC	ATGAAGAAGCGCTTTACG(	GCCCGCAAGCCAAACGCG,	TGTTGGC(	AGGGACTTTGCTAGAA(	CAACGCA1	GTGT	CAAC	N
7*Chr2_2_IS407A_/	7*Chr2_73_IS407A_A		D-R	Bm_NCTC_1024	7	81697-82932	Chr2	IS407A	CGCTG(	TGACCTGCCCCC~TAC	ATGAAGAAGCGCTTTACG(	GCCCGCAAGCCAAACGCG,	TGTTGGC(	AGGGACTTTGCTAGAA(	CAACGCA1	GTGT	CAAC	N
5*Chr2_45_IS407A_B			N	Bm_ATCC_1039	5	828859-830094	Chr2	IS407A	GCCGC	TGACCTGCCCCC~TAC	ATGAAGAAGCGCTTTACG(	GCCCGCAAGCCAAACGCG,	TGTTGGC(	AGGGACTTTGCTAGAA(	GAAAGGC	GGGC	GAAA	N
4*Chr2_24_IS407A	4*Chr2_56_IS407A_A		D-R	Bm_gb8_horse_	4	836729-837964	Chr2	IS407A	CGGGA	TGACCTGCCCCC~TAC	ATGAAGAAGCGCTTTACG(	GCCCGCAAGCCAAACGCG,	TGTTGGC(	AGGGACTTTGCTAGAA(	CATCGGC1	CCAC	CATC	N
1*Chr2_24_IS407A	1*Chr2_56_IS407A_A		D-R	Bm_ATCC_2334	1	838658-839893	Chr2	IS407A	CGGGA	TGACCTGCCCCC~TAC	ATGAAGAAGCGCTTTACG(	GCCCGCAAGCCAAACGCG,	TGTTGGC(	AGGGACTTTGCTAGAA(	CATCGGC1	CCAC	CATC	N
7*Chr2_9a_IS407A_A			N	Bm_NCTC_1024	7	840090-841325	Chr2	IS407A	CATGC(	TGACCTGCCCCC~TAC	ATGAAGAAGCGCTTTACG(	GCCCGCAAGCCAAACGCG,	TGTTGGC(	AGGGACTTTGCTAGAA(	CAAGCAG(	CAAG	CAAG	Y
10*Chr2_9b_IS407.	10*Chr2_31a_IS407A_A		R	Bm_PRL20	10	845139-846374	Chr2	IS407A	CATGC(	TGACCTGCCCCC~TAC	ATGAAGAAGCGCTTTACG(	GCCCGCAAGCCAAACGCG,	TGTTGGC(	AGGGACTTTGCTAGAA(	GATCGAG(	CAAG	GATC	N
2*Chr2_24_IS407A	2*Chr2_56_IS407A_A		D-R	Bm_fmh	2	852556-853791	Chr2	IS407A	CGGGA	TGACCTGCCCCC~TAC	ATGAAGAAGCGCTTTACG(	GCCCGCAAGCCAAACGCG,	TGTTGGC(	AGGGACTTTGCTAGAA(	CATCGGC1	CCAC	CATC	N
6*Chr2_35_IS407A_A			N	Bm_NCTC_102:	6	859532-860767	Chr2	IS407A	CGATC(	TGACCTGCCCCC~TAC	ATGAAGAAGCGCTTTACG(	GCCCGCAAGCCAAACGCG,	TGTTGGC(	AGGGACTTTGCTAGAA(	CATCAGC/	CATC	CATC	Y
7*Chr2_10a_IS407/	7*Chr2_13a_IS407A_A		R	Bm_NCTC_1024	7	862482-863717	Chr2	IS407A	GAGCC	TGACCTGCCCCC~TAC	ATGAAGAAGCGCTTTACG(	GCCCGCAAGCCAAACGCG,	TGTTGGC(	AGGGACTTTGCTAGAA(	AAGCCGT1	CTCG	AAGC	N
4*Chr2_9b_IS407A	4*Chr2_26_IS407A_A		D-R	Bm_gb8_horse_	4	865037-866272	Chr2	IS407A	CATGC(	TGACCTGCCCCC~TAC	ATGAAGAAGCGCTTTACG(	GCCCGCAAGCCAAACGCG,	TGTTGGC(	AGGGACTTTGCTAGAA(	CTGGATA(	CAAG	CTGG	N
10*Chr2_10a_IS40	10*Chr2_13a_IS407A_A		D-R	Bm_PRL20	10	867383-868618	Chr2	IS407A	GAGCC	TGACCTGCCCCC~TAC	ATGAAGAAGCGCTTTACG(	GCCCGCAAGCCAAACGCG,	TGTTGGC(	AGGGACTTTGCTAGAA(	AAGCCGT1	CTCG	AAGC	N
1*Chr2_9b_IS407A	1*Chr2_26_IS407A_A		D-R	Bm_ATCC_2334	1	869730-870965	Chr2	IS407A	CATGC(	TGACCTGCCCCC~TAC	ATGAAGAAGCGCTTTACG(	GCCCGCAAGCCAAACGCG,	TGTTGGC(	AGGGACTTTGCTAGAA(	CTGGATA(	CAAG	CTGG	N
8*Chr2_42_IS407A_A			N	Bm_200272128	8	875084-876319	Chr2	IS407A	ATCGA(	TGACCTGCCCCC~TAC	ATGAAGAAGCGCTTTACG(	GCCCGCAAGCCAAACGCG,	TGTTGGC(	AGGGACTTTGCTAGAA(	CTGCGGC(	CTGC	CTGC	Y
7*Chr2_10b_IS407A_A			R	Bm_NCTC_1024	7	876441-877676	Chr2	IS407A	GCCTC(	TGACCTGCCCCC~TAC	ATGAAGAAGCGCTTTACG(	GCCCGCAAGCCAAACGCG,	TGTTGGC(	AGGGACTTTGCTAGAA(	CCGGGCG	GCGG	CCGG	N
10*Chr2_32b_IS407A_C			R	Bm_PRL20	10	878187-878956	Chr2	IS407A	GTTAA(	TGACCTGCCCCC~TAC	ATGAAGAAGCGCTTTACG(	GCCCGCAAGCCAAACGCG,	TGTTGGC(	#	#contig_br	AGTG	#con	N.D.
7*Chr2_38_IS407A_A			D-R	Bm_NCTC_1024	7	878410-879645	Chr2	IS407A	GTCCA(	TGACCTGCCCCC~TAC	ATGAAGAAGCGCTTTACG(	GCCCGCAAGCCAAACGCG,	TGTTGGC(	AGGGACTTTGCTAGAA(	GGAGTTC1	GAAC	GGAG	N
3*Chr2_24_IS407A	3*Chr2_56_IS407A_A		D-R	Bm_JHU	3	881257-882492	Chr2	IS407A	CGGGA	TGACCTGCCCCC~TAC	ATGAAGAAGCGCTTTACG(	GCCCGCAAGCCAAACGCG,	TGTTGGC(	AGGGACTTTGCTAGAA(	CATCGGC1	CCAC	CATC	N
2*Chr2_9b_IS407A	2*Chr2_26_IS407A_A		D-R	Bm_fmh	2	883719-884954	Chr2	IS407A	CATGC(	TGACCTGCCCCC~TAC	ATGAAGAAGCGCTTTACG(	GCCCGCAAGCCAAACGCG,	TGTTGGC(	AGGGACTTTGCTAGAA(	CTGGATA(	CAAG	CTGG	N
4*Chr2_10a_IS407A_A			N	Bm_gb8_horse_	4	887110-888345	Chr2	IS407A	GTTAA(	TGACCTGCCCCC~TAC	ATGAAGAAGCGCTTTACG(	GCCCGCAAGCCAAACGCG,	TGTTGGC(	AGGGACTTTGCTAGAA(	AAGCCGT1	AGTG	AAGC	N
1*Chr2_10a_IS407A_A			N	Bm_ATCC_2334	1	891974-893209	Chr2	IS407A	GTTAA(	TGACCTGCCCCC~TAC	ATGAAGAAGCGCTTTACG(	GCCCGCAAGCCAAACGCG,	TGTTGGC(	AGGGACTTTGCTAGAA(	AAGCCGT1	AGTG	AAGC	N
10*Chr2_33_IS407/	10*Chr2_53_IS407A_A		D-R	Bm_PRL20	10	894908-896143	Chr2	IS407A	GCGAC	TGACCTGCCCCC~TAC	ATGAAGAAGCGCTTTACG(	GCCCGCAAGCCAAACGCG,	TGTTGGC(	AGGGACTTTGCTAGAA(	GTCCAGC/	CCTG	GTCC	N
2*Chr2_2_IS407A_/	2*Chr2_73_IS407A_A		D-R	Bm_fmh	2	89553-90788	Chr2	IS407A	CGCTG(	TGACCTGCCCCC~TAC	ATGAAGAAGCGCTTTACG(	GCCCGCAAGCCAAACGCG,	TGTTGGC(	AGGGACTTTGCTAGAA(	CAACGCA1	GTGT	CAAC	N
4*Chr2_13a_IS407A_A			R	Bm_gb8_horse_	4	903023-904258	Chr2	IS407A	GAGCC	TGACCTGCCCCC~TAC	ATGAAGAAGCGCTTTACG(	GCCCGCAAGCCAAACGCG,	TGTTGGC(	AGGGACTTTGCTAGAA(	CCGGGCG	CTCG	CCGG	N
4*Chr2_53_IS407A_A			D-R	Bm_gb8_horse_	4	904992-906227	Chr2	IS407A	GCGAC	TGACCTGCCCCC~TAC	ATGAAGAAGCGCTTTACG(	GCCCGCAAGCCAAACGCG,	TGTTGGC(	AGGGACTTTGCTAGAA(	GGAGTTC1	CCTG	GGAG	N
2*Chr2_10a_IS407A_A			N	Bm_fmh	2	905698-906933	Chr2	IS407A	GTTAA(	TGACCTGCCCCC~TAC	ATGAAGAAGCGCTTTACG(	GCCCGCAAGCCAAACGCG,	TGTTGGC(	AGGGACTTTGCTAGAA(	AAGCCGT1	AGTG	AAGC	N
5*Chr2_44a_IS407A_A			R	Bm_ATCC_1039	5	906115-907350	Chr2	IS407A	CGATC.	TGACCTGCCCCC~TAC	ATGAAGAAGCGCTTTACG(	GCCCGCAAGCCAAACGCG,	TGTTGGC(	AGGGACTTTGCTAGAA(	AGGGGTA.	GCGG	AGGG	N
3*Chr2_2_IS407A_/	3*Chr2_73_IS407A_A		D-R	Bm_JHU	3	90720-91955	Chr2	IS407A	CGCTG(	TGACCTGCCCCC~TAC	ATGAAGAAGCGCTTTACG(	GCCCGCAAGCCAAACGCG,	TGTTGGC(	AGGGACTTTGCTAGAA(	CAACGCA1	GTGT	CAAC	N

1*Chr2_13a_IS407A_A	R	Bm_ATCC_2334	1	907887-909122	Chr2	IS407A	GAGCC	TGACCTGCCCCC~TAC	ATGAAGAAGCGCTTTACGC	GCCCGCAAGCCAAACGCG/	TGTTGGCC	AGGGACTTTGCTAGAA(	CCGGGCG	CTCG	CCGG	N
1*Chr2_53_IS407A_A	D-R	Bm_ATCC_2334	1	909856-911091	Chr2	IS407A	GCGAC	TGACCTGCCCCC~TAC	ATGAAGAAGCGCTTTACGC	GCCCGCAAGCCAAACGCG/	TGTTGGCC	AGGGACTTTGCTAGAA(	GGAGTTCT	CCTG	GGAG	N
10*Chr2_21_IS407/ 10*Chr2_52_IS407A_A	N	Bm_PRL20	10	920084-921319	Chr2	IS407A	GAATT	TGACCTGCCCCC~TAC	ATGAAGAAGCGCTTTACGC	GCCCGCAAGCCAAACGCG/	TGTTGGCC	AGGGACTTTGCTAGAA(	CCGTCAC(	GGAT	CCGT	N
2*Chr2_13a_IS407A_A	R	Bm_fmh	2	922047-923283	Chr2	IS407A	GAGCC	TGACCTGCCCCC~TAC	ATGAAGAAGCGCTTTACGC	GCCCGCAAGCCAAACGCG/	TGTTGGCC	AGGGACTTTGCTAGAA(	CCGGGCG	CTCG	CCGG	N
2*Chr2_53_IS407A_A	D-R	Bm_fmh	2	924017-925252	Chr2	IS407A	GCGAC	TGACCTGCCCCC~TAC	ATGAAGAAGCGCTTTACGC	GCCCGCAAGCCAAACGCG/	TGTTGGCC	AGGGACTTTGCTAGAA(	GGAGTTCT	CCTG	GGAG	N
6*Chr2_34_IS407A 6*Chr2_54_IS407A_A	D-R	Bm_NCTC_102	6	924390-925625	Chr2	IS407A	CGCGG	TGACCTGCCCCC~TAC	ATGAAGAAGCGCTTTACGC	GCCCGCAAGCCAAACGCG/	TGTTGGCC	AGGGACTTTGCTAGAA(	GGATAGA(	GAGG	GGAT	N
8*Chr2_13a_IS407A_A	R	Bm_200272128	8	92450-93685	Chr2	IS407A	GAGCC	TGACCTGCCCCC~TAC	ATGAAGAAGCGCTTTACGC	GCCCGCAAGCCAAACGCG/	TGTTGGCC	AGGGACTTTGCTAGAA(	AAGCCGT	CTCG	AAGC	N
4*Chr2_52_IS407A_A	N	Bm_gb8_horse_	4	930168-931403	Chr2	IS407A	GAATT	TGACCTGCCCCC~TAC	ATGAAGAAGCGCTTTACGC	GCCCGCAAGCCAAACGCG/	TGTTGGCC	AGGGACTTTGCTAGAA(	CCGTCAC(	GGAT	CCGT	N
1*Chr2_52_IS407A_A	N	Bm_ATCC_2334	1	935032-936267	Chr2	IS407A	GAATT	TGACCTGCCCCC~TAC	ATGAAGAAGCGCTTTACGC	GCCCGCAAGCCAAACGCG/	TGTTGGCC	AGGGACTTTGCTAGAA(	CCGTCAC(	GGAT	CCGT	N
3*Chr2_9b_IS407A 3*Chr2_26_IS407A_A	D-R	Bm_JHU	3	944285-945520	Chr2	IS407A	CATGC(	TGACCTGCCCCC~TAC	ATGAAGAAGCGCTTTACGC	GCCCGCAAGCCAAACGCG/	TGTTGGCC	AGGGACTTTGCTAGAA(	CTGGATA(	CAAG	CTGG	N
7*Chr2_40_IS407A_A	N	Bm_NCTC_1024	7	946055-947290	Chr2	IS407A	CGCGT	TGACCTGCCCCC~TAC	ATGAAGAAGCGCTTTACGC	GCCCGCAAGCCAAACGCG/	TGTTGGCC	AGGGACTTTGCTAGAA(	CGAACTG(	CAGG	CGAA	N
2*Chr2_52_IS407A_A	N	Bm_fmh	2	949193-950428	Chr2	IS407A	GAATT	TGACCTGCCCCC~TAC	ATGAAGAAGCGCTTTACGC	GCCCGCAAGCCAAACGCG/	TGTTGGCC	AGGGACTTTGCTAGAA(	CCGTCAC(	GGAT	CCGT	N
6*Chr2_17_IS407A 6*Chr2_55_IS407A_A	D-R	Bm_NCTC_102	6	950458-951693	Chr2	IS407A	AGCGT(	TGACCTGCCCCC~TAC	ATGAAGAAGCGCTTTACGC	GCCCGCAAGCCAAACGCG/	TGTTGGCC	AGGGACTTTGCTAGAA(	GTGCGCTT	GCCC	GTGC	N
6*Chr2_15b_IS407A_A	R	Bm_NCTC_102	6	966292-967527	Chr2	IS407A	CGCGC	TGACCTGCCCCC~TAC	ATGAAGAAGCGCTTTACGC	GCCCGCAAGCCAAACGCG/	TGTTGGCC	AGGGACTTTGCTAGAA(	ACAGTTTT	GCTT	ACAG	N
3*Chr2_10a_IS407A_A	N	Bm_JHU	3	967328-968563	Chr2	IS407A	GTTAA(	TGACCTGCCCCC~TAC	ATGAAGAAGCGCTTTACGC	GCCCGCAAGCCAAACGCG/	TGTTGGCC	AGGGACTTTGCTAGAA(	AAGCCGT	AGTG	AAGC	N
8*Chr2_40_IS407A_A	N	Bm_200272128	8	968920-970155	Chr2	IS407A	CGCGT	TGACCTGCCCCC~TAC	ATGAAGAAGCGCTTTACGC	GCCCGCAAGCCAAACGCG/	TGTTGGCC	AGGGACTTTGCTAGAA(	CGAACTG(	CAGG	CGAA	N
9*Chr2_3_IS407A_/ 9*Chr2_76_IS407A_A	D-R	Bm_SAVP1	9	977425-978660	Chr2	IS407A	AGTCG	TGACCTGCCCCC~TAC	ATGAAGAAGCGCTTTACGC	GCCCGCAAGCCAAACGCG/	TGTTGGCC	AGGGACTTTGCTAGAA(	TCTTCCTT(	GGGT	TCTT	N
3*Chr2_13a_IS407A_A	R	Bm_JHU	3	983241-984476	Chr2	IS407A	GAGCC	TGACCTGCCCCC~TAC	ATGAAGAAGCGCTTTACGC	GCCCGCAAGCCAAACGCG/	TGTTGGCC	AGGGACTTTGCTAGAA(	CCGGGCG	CTCG	CCGG	N
3*Chr2_53_IS407A_A	D-R	Bm_JHU	3	986093-987328	Chr2	IS407A	GCGAC	TGACCTGCCCCC~TAC	ATGAAGAAGCGCTTTACGC	GCCCGCAAGCCAAACGCG/	TGTTGGCC	AGGGACTTTGCTAGAA(	GGAGTTCT	CCTG	GGAG	N

IS *Bma* 1

							5'-ext	5'-IR	ORF	3'-IR	3'-ext	5'-DR	3'-DR	rget duplicates
								GGTTCATCGC	GCGATGA ACC					
7*Chr2_43_ISBma1_A	N	Bm_NCTC_1024	7	1053553-1054857	Chr2	ISBma1	GCGCT(	GGTTCATCGCAGAAAA	TTGCTCGATCGCAAGGCAC	ACC	CAAGGAA.	CAAGGAAA	CAAGGAA.	Y
9*Chr2_79_ISBma1_A	N	Bm_SAVP1	9	1107451-1108755	Chr2	ISBma1	GTTTGT	GGTTCATCGCAGAAAA	TTGCTCGATCGCAAGGCAC	ACC	CTTTTTTC(	CTTTTTTC	CTTTTTTC	Y
4*Chr2_29_ISBma1_B	N	Bm_gb8_horse_	4	1169546-1170852	Chr2	ISBma1	GCGCT	GGTTCATCGCAGAAAA	TTGCTCGATCGCAAGGCAC	ACC	AAATTTTT(	AAATTTTT	AAATTTTT	Y
1*Chr2_29_ISBma1_B	N	Bm_ATCC_2334	1	1175066-1176372	Chr2	ISBma1	GCGCT	GGTTCATCGCAGAAAA	TTGCTCGATCGCAAGGCAC	ACC	AAATTTTT(	AAATTTTT	AAATTTTT	Y
2*Chr2_29_ISBma1_B	N	Bm_fmh	2	1194495-1195801	Chr2	ISBma1	GCGCT	GGTTCATCGCAGAAAA	TTGCTCGATCGCAAGGCAC	ACC	AAATTTTT(	AAATTTTT	AAATTTTT	Y
10*Chr2_43_ISBma1_A	N	Bm_PRL20	10	1229130-1230350	Chr2	ISBma1	GCGCT(	GGTTCATCGCAGAAAA	TTGCTCGATCGCAAGGCAC	ACC	CAAGGAA.	CAAGGAAA	CAAGGAA.	Y
5*Chr2_69-1_ISBma1_A	N	Bm_ATCC_1039	5	125354-125421	Chr2	ISBma1	#IS407/	~~~~~~~~~~~~~~~	~~~~~~~~~~~~~~~~~~	ACC	TAAAAAA/	#IS407A	TAAAAAA/	N.D.
3*Chr2_29_ISBma1_B	N	Bm_JHU	3	1258947-1260253	Chr2	ISBma1	GCGCT	GGTTCATCGCAGAAAA	TTGCTCGATCGCAAGGCAC	ACC	AAATTTTT(	AAATTTTT	AAATTTTT	Y
5*Chr2_69_ISBma1_A	N	Bm_ATCC_1039	5	126655-127898	Chr2	ISBma1	CAGGC	GGTTCATCGCAGAAAA	TTGCTCGATCGCAAGGCAC	#	#IS407A	TAAAAAAA	#IS407A	N.D.
6*Chr2_4_ISBma1_B	N	Bm_NCTC_102	6	1284442-1285746	Chr2	ISBma1	TCCGT(	GGTTCATCGCAGAAAA	TTGCTCGATCGCAAGGCAC	ACC	TTTTTTAAT	TTTTTTAA	TTTTTTAA	Y
4*Chr2_43_ISBma1_A	N	Bm_gb8_horse_	4	1304618-1305922	Chr2	ISBma1	GCGCT(	GGTTCATCGCAGAAAA	TTGCTCGATCGCAAGGCAC	ACC	CAAGGAA.	CAAGGAAA	CAAGGAA.	Y
1*Chr2_43_ISBma1_A	N	Bm_ATCC_2334	1	1310749-1312053	Chr2	ISBma1	GCGCT(	GGTTCATCGCAGAAAA	TTGCTCGATCGCAAGGCAC	ACC	CAAGGAA.	CAAGGAAA	CAAGGAA.	Y
10*Chr2_69_ISBma1_A	N	Bm_PRL20	10	133392-134635	Chr2	ISBma1	CAGGC	GGTTCATCGCAGAAAA	TTGCTCGATCGCAAGGCAC	#	#IS407A	TAAAAAAA	#IS407A	N.D.
9*Chr2_69-1_ISBma1_A	N	Bm_SAVP1	9	1334706-1334773	Chr2	ISBma1	#IS407/	~~~~~~~~~~~~~~~	~~~~~~~~~~~~~~~~~~	ACC	TAAAAAA/	#IS407A	TAAAAAA/	Y
2*Chr2_43_ISBma1_A	N	Bm_fmh	2	1335542-1336846	Chr2	ISBma1	GCGCT(	GGTTCATCGCAGAAAA	TTGCTCGATCGCAAGGCAC	ACC	CAAGGAA.	CAAGGAAA	CAAGGAA.	Y
9*Chr2_69_ISBma1_A	N	Bm_SAVP1	9	1336007-1337250	Chr2	ISBma1	CAGGC	GGTTCATCGCAGAAAA	TTGCTCGATCGCAAGGCAC	#	#IS407A	TAAAAAAA	#IS407A	N.D.
6*Chr2_79_ISBma1_A	N	Bm_NCTC_102	6	1436997-1438301	Chr2	ISBma1	GTTTGT	GGTTCATCGCAGAAAA	TTGCTCGATCGCAAGGCAC	ACC	CTTTTTTC(	CTTTTTTC	CTTTTTTC	Y
6*Chr2_69_ISBma1_A	N	Bm_NCTC_102	6	1600893-1602136	Chr2	ISBma1	CAGGC	GGTTCATCGCAGAAAA	TTGCTCGATCGCAAGGCAC	#	#IS407A	TAAAAAAA	#IS407A	N.D.
8*Chr2_69-1_ISBma1_A	N	Bm_200272128	8	1609838-1609906	Chr2	ISBma1	#IS407/	~~~~~~~~~~~~~~~	~~~~~~~~~~~~~~~~~~	ACC	TAAAAAA/	#IS407A	TAAAAAA/	N.D.
4*Chr2_69-1_ISBma1_A	N	Bm_gb8_horse_	4	170220-170747	Chr2	ISBma1	#IS407/	~~~~~~~~~~~~~~~	~~~~~~~~~~~~~~~~~~	ACC	TAAAAAA/	#IS407A	TAAAAAA/	N.D.
1*Chr2_69-1_ISBma1_A	N	Bm_ATCC_2334	1	170527-170594	Chr2	ISBma1	#IS407/	~~~~~~~~~~~~~~~	~~~~~~~~~~~~~~~~~~	ACC	TAAAAAA/	#IS407A	TAAAAAA/	N.D.
4*Chr2_69_ISBma1_A	N	Bm_gb8_horse_	4	171521-172764	Chr2	ISBma1	CAGGC	GGTTCATCGCAGAAAA	TTGCTCGATCGCAAGGCAC	#	#IS407A	TAAAAAAA	#IS407A	N.D.
7*Chr2_69-1_ISBma1_A	N	Bm_NCTC_1024	7	171689-171756	Chr2	ISBma1	#IS407/	~~~~~~~~~~~~~~~	~~~~~~~~~~~~~~~~~~	ACC	TAAAAAA/	#IS407A	TAAAAAA/	N.D.
1*Chr2_69_ISBma1_A	N	Bm_ATCC_2334	1	171828-173071	Chr2	ISBma1	CAGGC	GGTTCATCGCAGAAAA	TTGCTCGATCGCAAGGCAC	#	#IS407A	TAAAAAAA	#IS407A	N.D.
7*Chr2_69_ISBma1_A	N	Bm_NCTC_1024	7	172990-174233	Chr2	ISBma1	CAGGC	GGTTCATCGCAGAAAA	TTGCTCGATCGCAAGGCAC	#	#IS407A	TAAAAAAA	#IS407A	N.D.
2*Chr2_69-1_ISBma1_A	N	Bm_fmh	2	180027-180094	Chr2	ISBma1	#IS407/	~~~~~~~~~~~~~~~	~~~~~~~~~~~~~~~~~~	ACC	TAAAAAA/	#IS407A	TAAAAAA/	N.D.
2*Chr2_69_ISBma1_A	N	Bm_fmh	2	181328-182571	Chr2	ISBma1	CAGGC	GGTTCATCGCAGAAAA	TTGCTCGATCGCAAGGCAC	#	#IS407A	TAAAAAAA	#IS407A	N.D.
8*Chr2_4_ISBma1_B	N	Bm_200272128	8	2007904-2009208	Chr2	ISBma1	TCCGT(	GGTTCATCGCAGAAAA	TTGCTCGATCGCAAGGCAC	ACC	TTTTTTAAT	TTTTTTAA	TTTTTTAA	Y
3*Chr2_69-1_ISBma1_A	N	Bm_JHU	3	207599-207666	Chr2	ISBma1	#IS407/	~~~~~~~~~~~~~~~	~~~~~~~~~~~~~~~~~~	ACC	TAAAAAA/	#IS407A	TAAAAAA/	N.D.
3*Chr2_69_ISBma1_A	N	Bm_JHU	3	208900-210143	Chr2	ISBma1	CAGGC	GGTTCATCGCAGAAAA	TTGCTCGATCGCAAGGCAC	#	#IS407A	TAAAAAAA	#IS407A	N.D.
9*Chr2_43_ISBma1_A	N	Bm_SAVP1	9	209882-211186	Chr2	ISBma1	GCGCT(	GGTTCATCGCAGAAAA	TTGCTCGATCGCAAGGCAC	ACC	CAAGGAA.	CAAGGAAA	CAAGGAA.	Y
10*Chr2_79_ISBma1_A	N	Bm_PRL20	10	2104725-2106029	Chr2	ISBma1	GTTTGT	GGTTCATCGCAGAAAA	TTGCTCGATCGCAAGGCAC	ACC	CTTTTTTC(	CTTTTTTC	CTTTTTTC	Y
7*Chr2_4_ISBma1_B	N	Bm_NCTC_1024	7	2142029-2143333	Chr2	ISBma1	TCCGT(	GGTTCATCGCAGAAAA	TTGCTCGATCGCAAGGCAC	ACC	TTTTTTAAT	TTTTTTAA	TTTTTTAA	Y
5*Chr2_79_ISBma1_A	N	Bm_ATCC_1039	5	2227856-2229160	Chr2	ISBma1	GTTTGT	GGTTCATCGCAGAAAA	TTGCTCGATCGCAAGGCAC	ACC	CTTTTTTC(	CTTTTTTC	CTTTTTTC	Y
4*Chr2_79_ISBma1_A	N	Bm_gb8_horse_	4	2257635-2258939	Chr2	ISBma1	GTTTGT	GGTTCATCGCAGAAAA	TTGCTCGATCGCAAGGCAC	ACC	CTTTTTTC(	CTTTTTTC	CTTTTTTC	Y

1*Chr2_79_ISBma1_A		N	Bm_ATCC_2334	1	2265680-2266984	Chr2	ISBma1	GTTTGT	GGTTCATCGCAGAAAA	TTGCTCGATCGCAAGGCAC	ACC	CTTTTTTC(	CTTTTTTC	CTTTTTTC	Y
3*Chr2_79_ISBma1_A		N	Bm_JHU	3	2280857-2282161	Chr2	ISBma1	GTTTGT	GGTTCATCGCAGAAAA	TTGCTCGATCGCAAGGCAC	ACC	CTTTTTTC(	CTTTTTTC	CTTTTTTC	Y
7*Chr2_79_ISBma1_A		N	Bm_NCTC_1024	7	2294384-2295688	Chr2	ISBma1	GTTTGT	GGTTCATCGCAGAAAA	TTGCTCGATCGCAAGGCAC	ACC	CTTTTTTC(	CTTTTTTC	CTTTTTTC	Y
2*Chr2_79_ISBma1_A		N	Bm_fmh	2	2306093-2307397	Chr2	ISBma1	GTTTGT	GGTTCATCGCAGAAAA	TTGCTCGATCGCAAGGCAC	ACC	CTTTTTTC(	CTTTTTTC	CTTTTTTC	Y
6*Chr2_43_ISBma1_A		N	Bm_NCTC_1022	6	500364-501668	Chr2	ISBma1	GCGCT(	GGTTCATCGCAGAAAA	TTGCTCGATCGCAAGGCAC	ACC	CAAGGAA	CAAGGAAA	CAAGGAA	Y
8*Chr2_43_ISBma1_A		N	Bm_200272128	8	861441-862745	Chr2	ISBma1	GCGCT(	GGTTCATCGCAGAAAA	TTGCTCGATCGCAAGGCAC	ACC	CAAGGAA	CAAGGAAA	CAAGGAA	Y
5*Chr2_43_ISBma1_A		N	Bm_ATCC_1039	5	991262-992566	Chr2	ISBma1	GCGCT(	GGTTCATCGCAGAAAA	TTGCTCGATCGCAAGGCAC	ACC	CAAGGAA	CAAGGAAA	CAAGGAA	Y

IS *Bma* 2

								5'-ext	5'-IR	ORF	3'-IR	3'-ext	5'-DR	3'-DR	rget duplicates
									CAGATTGCTGACAAACCC		GGGTTCGTCAGCAGTCTG				
8*Chr2_12_ISBma2_A		N	Bm_200272128	8	101363-102937	Chr2	ISBma2	GCCGC	CAGATTGCTGACAAAC	ATGCTGAAGACGCCCATG(	TCCCAGAAAGACAAAACC(	GCGCTCC/	GCGCTCCATTCGGAGC(	GCGCTCC/	Y
9*Chr2_80a_ISBma2_A		N	Bm_SAVP1	9	1108780-1110352	Chr2	ISBma2	GCCCT	CAGATTGCTGACAAAC	ATGCTGAAGACGCCCATG(	TCCCAGAAAGACAAAACC(	AGGGCGC	CGAAAAAAGGGCGCCA	AGGGCGC	N
10*Chr2_47_ISBma2_A		N	Bm_PRL20	10	1133963-1135535	Chr2	ISBma2	CGCGG	CAGATTGCTGACAAAC	ATGCTGAAGACGCCCATG(	TCCCAGAAAGACAAAACC(	AGCGCAC(	GCGGATGCAGCGCACG	AGCGCAC(	N
6*Chr2_5a_ISBma2	6*Chr2_14b_ISBma2_A	R	Bm_NCTC_1022	6	1142945-1144517	Chr2	ISBma2	TAAAA(	CAGATTGCTGACAAAC	ATGCTGAAGACGCCCATG(	TCCCAGAAAGACAAAACC(	GGCCTTC/	AAACGCCCCGCACGAA	GGCCTTC/	N
9*Chr2_47_ISBma2_A		D-R	Bm_SAVP1	9	116165-117737	Chr2	ISBma2	CGCGG	CAGATTGCTGACAAAC	ATGCTGAAGACGCCCATG(	TCCCAGAAAGACAAAACC(	AGCGCAC(	GCGGATGCAGCGCACG	AGCGCAC(	N
4*Chr2_30_ISBma2	4*Chr2_47_ISBma2_A	D-R	Bm_gb8_horse_	4	1166671-1168243	Chr2	ISBma2	CGCGG	CAGATTGCTGACAAAC	ATGCTGAAGACGCCCATG(	TCCCAGAAAGACAAAACC(	AACAGCC(	GCGGATGCAGCGCACG	AACAGCC(	N
1*Chr2_30_ISBma2	1*Chr2_47_ISBma2_A	D-R	Bm_ATCC_2334	1	1172191-1173763	Chr2	ISBma2	CGCGG	CAGATTGCTGACAAAC	ATGCTGAAGACGCCCATG(	TCCCAGAAAGACAAAACC(	AACAGCC(	GCGGATGCAGCGCACG	AACAGCC(	N
8*Chr2_5b_ISBma2	8*Chr2_14a_ISBma2_A	R	Bm_200272128	8	1189961-1191534	Chr2	ISBma2	TGTGC(	CAGATTGCTGACAAAC	ATGCTGAAGACGCCCATG(	TCCCAGAAAGACAAAACC(	ACGCCCC(	AGGCCTTCATCTGGCG/	ACGCCCC(	N
2*Chr2_30_ISBma2	2*Chr2_47_ISBma2_A	D-R	Bm_fmh	2	1191620-1193192	Chr2	ISBma2	CGCGG	CAGATTGCTGACAAAC	ATGCTGAAGACGCCCATG(	TCCCAGAAAGACAAAACC(	AACAGCC(	GCGGATGCAGCGCACG	AACAGCC(	N
5*Chr2_12_ISBma2_A		N	Bm_ATCC_1039	5	1209160-1210732	Chr2	ISBma2	GCCGC	CAGATTGCTGACAAAC	ATGCTGAAGACGCCCATG(	TCCCAGAAAGACAAAACC(	GCGCTCC/	GCGCTCCATTCGGAGC(	GCGCTCC/	Y
5*Chr2_11_ISBma2_B		N	Bm_ATCC_1039	5	1214269-1214325	Chr2	ISBma2	TCAGC	CAGATTGCTGACAAAC	ATGACCTGCCCCCTTCGAT.#		#IS407A	ACAAAGCCCCGTCAAT/	#IS407A	N.D.
5*Chr2_71a_ISBma	5*Chr2_80b_ISBma2_A	R	Bm_ATCC_1039	5	121539-123111	Chr2	ISBma2	CGCGC	CAGATTGCTGACAAAC	ATGCTGAAGACGCCCATG(	TCCCAGAAAGACAAAACC(	AGGGCGC	AGGGCCGCTCGTAGCG	AGGGCGC	N
5*Chr2_30_ISBma2_A		N	Bm_ATCC_1039	5	1246578-1248150	Chr2	ISBma2	ACAGG	CAGATTGCTGACAAAC	ATGCTGAAGACGCCCATG(	TCCCAGAAAGACAAAACC(	AACAGCC(	AAAACAGCCGGCCCGA	AACAGCC(	N
7*Chr2_47_ISBma2_A		N	Bm_NCTC_1024	7	1255366-1256940	Chr2	ISBma2	CGCGG	CAGATTGCTGACAAAC	ATGCTGAAGACGCCCATG(	TCCCAGAAAGACAAAACC(	AGCGCAC(	GCGGATGCAGCGCACG	AGCGCAC(	N
3*Chr2_30_ISBma2	3*Chr2_47_ISBma2_A	D-R	Bm_JHU	3	1256072-1257644	Chr2	ISBma2	CGCGG	CAGATTGCTGACAAAC	ATGCTGAAGACGCCCATG(	TCCCAGAAAGACAAAACC(	AACAGCC(	GCGGATGCAGCGCACG	AACAGCC(	N
6*Chr2_77_ISBma2	6*Chr2_78_ISBma2_A	D	Bm_NCTC_1022	6	1328449-1330024	Chr2	ISBma2	TGACC(	CAGATTGCTGACAAAC	ATGCTGAAGACGCCCATG(	TCCCAGAAAGACAAAACC(	AGGCGTT(	GCGGGAAGCGCCGCG(	AGGCGTT(	N
9*Chr2_71b_ISBma2_A		N	Bm_SAVP1	9	1330891-1332463	Chr2	ISBma2	CGCGC	CAGATTGCTGACAAAC	ATGCTGAAGACGCCCATG(	TCCCAGAAAGACAAAACC(	AGGGCCG	AGGGCCGCTCGTAGCG	AGGGCCG	Y
6*Chr2_80a_ISBma2_A		N	Bm_NCTC_1022	6	1438326-1439898	Chr2	ISBma2	GCCCT	CAGATTGCTGACAAAC	ATGCTGAAGACGCCCATG(	TCCCAGAAAGACAAAACC(	AGGGCGC	CGAAAAAAGGGCGCCA	AGGGCGC	N
9*Chr2_65_ISBma2_B		N	Bm_SAVP1	9	1478060-1479632	Chr2	ISBma2	CTCTA(	CAGATTGCTGACAAAC	ATGCTGAAGACGCCCATG(	TCCCAGAAAGACAAAACC(	AGACGGG	AGACGGGCTGCAGCCC	AGACGGG	Y
6*Chr2_82_ISBma2_B		N	Bm_NCTC_1022	6	1490409-1491981	Chr2	ISBma2	TCGCA(	CAGATTGCTGACAAAC	ATGCTGAAGACGCCCATG(	TCCCAGAAAGACAAAACC(	CTGGGCC(	GAAACGGGGCCCGCTG	CTGGGCC(	N
9*Chr2_63_ISBma2_B		N	Bm_SAVP1	9	1493768-1495340	Chr2	ISBma2	CCGCG	CAGATTGCTGACAAAC	ATGCTGAAGACGCCCATG(	TCCCAGAAAGACAAAACC(	AAGCCGG	AAAAAGCCGGCGTGCA	AAGCCGG	N
10*Chr2_5b_ISBma	10*Chr2_14a_ISBma2_A	R	Bm_PRL20	10	1506314-1507887	Chr2	ISBma2	TGTGC(	CAGATTGCTGACAAAC	ATGCTGAAGACGCCCATG(	TCCCAGAAAGACAAAACC(	ACGCCCC(	AGGCCTTCATCTGGCG/	ACGCCCC(	N
6*Chr2_12_ISBma2_A		N	Bm_NCTC_1022	6	15095-16668	Chr2	ISBma2	GCCGC	CAGATTGCTGACAAAC	ATGCTGAAGACGCCCATG(	TCCCAGAAAGACAAAACC(	GCGCTCC/	GCGCTCCATTCGGAGC(	GCGCTCC/	Y
8*Chr2_65_ISBma2_B		N	Bm_200272128	8	1592660-1594232	Chr2	ISBma2	CGCGC	CAGATTGCTGACAAAC	ATGCTGAAGACGCCCATG(	TCCCAGAAAGACAAAACC(	GACGGGC	GACGGGCTGCAGCCCG	GACGGGC	Y
5*Chr2_7_ISBma2_	5*Chr2_14a_ISBma2_A	D-R	Bm_ATCC_1039	5	1600353-1601926	Chr2	ISBma2	TGTGC(	CAGATTGCTGACAAAC	ATGCTGAAGACGCCCATG(	TCCCAGAAAGACAAAACC(	GGCCGAC(	AGGCCTTCATCTGGCG/	GGCCGAC(	N
8*Chr2_71b_ISBma2_A		N	Bm_200272128	8	1612147-1613720	Chr2	ISBma2	CGCGC	CAGATTGCTGACAAAC	ATGCTGAAGACGCCCATG(	TCCCAGAAAGACAAAACC(	AGGGCCG	AGGGCCGCTCGTAGCG	AGGGCCG	Y
4*Chr2_5b_ISBma2	4*Chr2_14a_ISBma2_A	R	Bm_gb8_horse_	4	1631568-1633144	Chr2	ISBma2	TGTGC(	CAGATTGCTGACAAAC	ATGCTGAAGACGCCCATG(	TCCCAGAAAGACAAAACC(	ACGCCCC(	AGGCCTTCATCTGGCG/	ACGCCCC(	N
3*Chr2_5b_ISBma2	3*Chr2_14a_ISBma2_A	R	Bm_JHU	3	1632858-1634434	Chr2	ISBma2	TGTGC(	CAGATTGCTGACAAAC	ATGCTGAAGACGCCCATG(	TCCCAGAAAGACAAAACC(	ACGCCCC(	AGGCCTTCATCTGGCG/	ACGCCCC(	N
1*Chr2_5b_ISBma2	1*Chr2_14a_ISBma2_A	R	Bm_ATCC_2334	1	1638049-1639625	Chr2	ISBma2	TGTGC(	CAGATTGCTGACAAAC	ATGCTGAAGACGCCCATG(	TCCCAGAAAGACAAAACC(	ACGCCCC(	AGGCCTTCATCTGGCG/	ACGCCCC(	N
7*Chr2_36_ISBma2_B		N	Bm_NCTC_1024	7	1655136-1656709	Chr2	ISBma2	ACGCC	CAGATTGCTGACAAAC	ATGCTGAAGACGCCCATG(	TCCCAGAAAGACAAAACC(	AAGGCGC	CAAGGCGCTCGGCAAC	AAGGCGC	N
4*Chr2_71b_ISBma2_A		N	Bm_gb8_horse_	4	166405-167977	Chr2	ISBma2	CGCGC	CAGATTGCTGACAAAC	ATGCTGAAGACGCCCATG(	TCCCAGAAAGACAAAACC(	AGGGCCG	AGGGCCGCTCGTAGCG	AGGGCCG	Y
1*Chr2_71b_ISBma2_A		N	Bm_ATCC_2334	1	166712-168284	Chr2	ISBma2	CGCGC	CAGATTGCTGACAAAC	ATGCTGAAGACGCCCATG(	TCCCAGAAAGACAAAACC(	AGGGCCG	AGGGCCGCTCGTAGCG	AGGGCCG	Y
2*Chr2_5b_ISBma2	2*Chr2_14a_ISBma2_A	R	Bm_fmh	2	1668531-1670107	Chr2	ISBma2	TGTGC(	CAGATTGCTGACAAAC	ATGCTGAAGACGCCCATG(	TCCCAGAAAGACAAAACC(	ACGCCCC(	AGGCCTTCATCTGGCG/	ACGCCCC(	N
7*Chr2_71b_ISBma2_A		N	Bm_NCTC_1024	7	167874-169446	Chr2	ISBma2	CGCGC	CAGATTGCTGACAAAC	ATGCTGAAGACGCCCATG(	TCCCAGAAAGACAAAACC(	AGGGCCG	AGGGCCGCTCGTAGCG	AGGGCCG	Y
9*Chr2_12_ISBma2_A		D-R	Bm_SAVP1	9	1707496-1709043	Chr2	ISBma2	GCCGC	CAGATTGCTGACAAAC	ATGCTGAAGACGCCCATG(	TCCCAGAAAGACAAAACC(	AAGCCCC(	GCGCTCCATTCGGAGC(	AAGCCCC(	N
6*Chr2_65_ISBma2_B		N	Bm_NCTC_1022	6	1744134-1745709	Chr2	ISBma2	CGCGC	CAGATTGCTGACAAAC	ATGCTGAAGACGCCCATG(	TCCCAGAAAGACAAAACC(	GACGGGC	GACGGGCTGCAGCCCG	GACGGGC	Y
2*Chr2_71b_ISBma2_A		N	Bm_fmh	2	176212-177784	Chr2	ISBma2	CGCGC	CAGATTGCTGACAAAC	ATGCTGAAGACGCCCATG(	TCCCAGAAAGACAAAACC(	AGGGCCG	AGGGCCGCTCGTAGCG	AGGGCCG	Y
8*Chr2_82_ISBma2_B		N	Bm_200272128	8	1784937-1785906	Chr2	ISBma2	#contig	~~~~~~~~~~~~~~	~~~~~~~~~~~~~~~~~~	TCCCAGAAAGACAAAACC(	CTGGGCC(	#contig_break	CTGGGCC(	N.D.
10*Chr2_5a_ISBma	10*Chr2_14b_ISBma2_A	R	Bm_PRL20	10	1809463-1811035	Chr2	ISBma2	TAAAA(	CAGATTGCTGACAAAC	ATGCTGAAGACGCCCATG(	TCCCAGAAAGACAAAACC(	GGCCTTC/	AAACGCCCCGCACGAA	GGCCTTC/	N
8*Chr2_5a_ISBma2	8*Chr2_14b_ISBma2_A	R	Bm_200272128	8	1866602-1868174	Chr2	ISBma2	TAAAA(	CAGATTGCTGACAAAC	ATGCTGAAGACGCCCATG(	TCCCAGAAAGACAAAACC(	GGCCTTC/	AAACGCCCCGCACGAA	GGCCTTC/	N
5*Chr2_5a_ISBma2	5*Chr2_14b_ISBma2_A	D-R	Bm_ATCC_1039	5	1933847-1935419	Chr2	ISBma2	TAAAA(	CAGATTGCTGACAAAC	ATGCTGAAGACGCCCATG(	TCCCAGAAAGACAAAACC(	GGCCTTC/	AAACGCCCCGCACGAA	GGCCTTC/	N
4*Chr2_5a_ISBma2	4*Chr2_14b_ISBma2_A	R	Bm_gb8_horse_	4	1964034-1965606	Chr2	ISBma2	TAAAA(	CAGATTGCTGACAAAC	ATGCTGAAGACGCCCATG(	TCCCAGAAAGACAAAACC(	GGCCTTC/	AAACGCCCCGCACGAA	GGCCTTC/	N
1*Chr2_5a_ISBma2	1*Chr2_14b_ISBma2_A	R	Bm_ATCC_2334	1	1971717-1973289	Chr2	ISBma2	TAAAA(	CAGATTGCTGACAAAC	ATGCTGAAGACGCCCATG(	TCCCAGAAAGACAAAACC(	GGCCTTC/	AAACGCCCCGCACGAA	GGCCTTC/	N
3*Chr2_5a_ISBma2	3*Chr2_14b_ISBma2_A	R	Bm_JHU	3	1979924-1981496	Chr2	ISBma2	TAAAA(	CAGATTGCTGACAAAC	ATGCTGAAGACGCCCATG(	TCCCAGAAAGACAAAACC(	GGCCTTC/	AAACGCCCCGCACGAA	GGCCTTC/	N
10*Chr2_77_ISBma	10*Chr2_78_ISBma2_A	D	Bm_PRL20	10	1995877-1997449	Chr2	ISBma2	TGACC(	CAGATTGCTGACAAAC	ATGCTGAAGACGCCCATG(	TCCCAGAAAGACAAAACC(	AGGCGTT(	GCGGGAAGCGCCGCG(	AGGCGTT(	N
7*Chr2_5a_ISBma2	7*Chr2_14b_ISBma2_A	R	Bm_NCTC_1024	7	2000703-2002275	Chr2	ISBma2	TAAAA(	CAGATTGCTGACAAAC	ATGCTGAAGACGCCCATG(	TCCCAGAAAGACAAAACC(	GGCCTTC/	AAACGCCCCGCACGAA	GGCCTTC/	N

2*Chr2_5a_ISBma2	2*Chr2_14b_ISBma2_A	R	Bm_fmh	2	2007940-2009512	Chr2	ISBma2	TAAAA	CAGATTGCTGACAAAC	ATGCTGAAGACGCCCATG	TCCCAGAAAGACAAAACC	GGCCTTC	AAACGCCCCGCACGAA	GGCCTTC	N
3*Chr2_71b_ISBma2_A		N	Bm_JHU	3	203784-205356	Chr2	ISBma2	CGCGC	CAGATTGCTGACAAAC	ATGCTGAAGACGCCCATG	TCCCAGAAAGACAAAACC	AGGGCCG	AGGGCCGCTCGTAGCG	AGGGCCG	Y
8*Chr2_77_ISBma2	8*Chr2_80a_ISBma2_A	D-R	Bm_200272128	8	2051909-2053481	Chr2	ISBma2	TGCCG	CAGATTGCTGACAAAC	ATGCTGAAGACGCCCATG	TCCCAGAAAGACAAAACC	AGGGCGC	AAAAAGCGGCGTTCGC	AGGGCGC	N
10*Chr2_80a_ISBma2_A		N	Bm_PRL20	10	2106054-2107626	Chr2	ISBma2	GCCCT	CAGATTGCTGACAAAC	ATGCTGAAGACGCCCATG	TCCCAGAAAGACAAAACC	AGGGCGC	CGAAAAAAGGGCGCC	AGGGCGC	N
5*Chr2_77_ISBma2	5*Chr2_78_ISBma2_A	D	Bm_ATCC_103	5	2119130-2120702	Chr2	ISBma2	TGACC	CAGATTGCTGACAAAC	ATGCTGAAGACGCCCATG	TCCCAGAAAGACAAAACC	AGGCGTT	GCGGGAAGCGCCGCG	AGGCGTT	N
6*Chr2_5b_ISBma2	6*Chr2_14a_ISBma2_A	R	Bm_NCTC_102	6	2146943-2148516	Chr2	ISBma2	TGTGC	CAGATTGCTGACAAAC	ATGCTGAAGACGCCCATG	TCCCAGAAAGACAAAACC	ACGCCCC	AGGCCTTCATCTGGCG	ACGCCCC	N
4*Chr2_77_ISBma2	4*Chr2_78_ISBma2_A	D	Bm_gb8_horse_	4	2149249-2150821	Chr2	ISBma2	TGACC	CAGATTGCTGACAAAC	ATGCTGAAGACGCCCATG	TCCCAGAAAGACAAAACC	AGGCGTT	GCGGGAAGCGCCGCG	AGGCGTT	N
1*Chr2_77_ISBma2	1*Chr2_78_ISBma2_A	D	Bm_ATCC_233	1	2157201-2158773	Chr2	ISBma2	TGACC	CAGATTGCTGACAAAC	ATGCTGAAGACGCCCATG	TCCCAGAAAGACAAAACC	AGGCGTT	GCGGGAAGCGCCGCG	AGGCGTT	N
3*Chr2_77_ISBma2	3*Chr2_78_ISBma2_A	D	Bm_JHU	3	2168372-2169944	Chr2	ISBma2	TGACC	CAGATTGCTGACAAAC	ATGCTGAAGACGCCCATG	TCCCAGAAAGACAAAACC	AGGCGTT	GCGGGAAGCGCCGCG	AGGCGTT	N
7*Chr2_77_ISBma2	7*Chr2_78_ISBma2_A	D	Bm_NCTC_102	7	2186019-2187594	Chr2	ISBma2	TGACC	CAGATTGCTGACAAAC	ATGCTGAAGACGCCCATG	TCCCAGAAAGACAAAACC	AGGCGTT	GCGGGAAGCGCCGCG	AGGCGTT	N
2*Chr2_77_ISBma2	2*Chr2_78_ISBma2_A	D	Bm_fmh	2	2194588-2196160	Chr2	ISBma2	TGACC	CAGATTGCTGACAAAC	ATGCTGAAGACGCCCATG	TCCCAGAAAGACAAAACC	AGGCGTT	GCGGGAAGCGCCGCG	AGGCGTT	N
5*Chr2_71b_ISBma	5*Chr2_80a_ISBma2_A	R	Bm_ATCC_103	5	2229185-2230757	Chr2	ISBma2	TTTTCT	CAGATTGCTGACAAAC	ATGCTGAAGACGCCCATG	TCCCAGAAAGACAAAACC	AGGGCCG	AGGGCGCCAAAGCGC	AGGGCCG	N
4*Chr2_80a_ISBma2_A		N	Bm_gb8_horse_	4	2258964-2259023	Chr2	ISBma2	GCCCT	CAGATTGCTGACAAAC	~TGACCTGCCCCCTTCGAT	#	#IS407A	AGGGCGCCAAAGCGC	#IS407A	N.D.
4*Chr2_80a-1_ISBma2_A		N	Bm_gb8_horse_	4	2260253-2261776	Chr2	ISBma2	#IS407	~~~~~~~~~~~~~~~	ATGCTGAAGACGCCCATG	TCCCAGAAAGACAAAACC	AGGGCGC	#IS407A	AGGGCGC	N.D.
1*Chr2_80a_ISBma2_A		N	Bm_ATCC_233	1	2267009-2267068	Chr2	ISBma2	GCCCT	CAGATTGCTGACAAAC	~TGACCTGCCCCCTTCGAT	#	#IS407A	AGGGCGCCAAAGCGC	#IS407A	N.D.
1*Chr2_80a-1_ISBma2_A		N	Bm_ATCC_233	1	2268298-2269821	Chr2	ISBma2	#IS407	~~~~~~~~~~~~~~~	ATGCTGAAGACGCCCATG	TCCCAGAAAGACAAAACC	AGGGCGC	#IS407A	AGGGCGC	N.D.
3*Chr2_80a_ISBma2_A		N	Bm_JHU	3	2282186-2282245	Chr2	ISBma2	GCCCT	CAGATTGCTGACAAAC	~TGACCTGCCCCCTTCGAT	#	#IS407A	AGGGCGCCAAAGCGC	#IS407A	N.D.
3*Chr2_80a-1_ISBma2_A		N	Bm_JHU	3	2283475-2284998	Chr2	ISBma2	#IS407	~~~~~~~~~~~~~~~	ATGCTGAAGACGCCCATG	TCCCAGAAAGACAAAACC	AGGGCGC	#IS407A	AGGGCGC	N.D.
7*Chr2_80a_ISBma2_A		N	Bm_NCTC_102	7	2295713-2297285	Chr2	ISBma2	GCCCT	CAGATTGCTGACAAAC	ATGCTGAAGACGCCCATG	TCCCAGAAAGACAAAACC	AGGGCGC	CGAAAAAAGGGCGCC	AGGGCGC	N
2*Chr2_80a_ISBma2_A		N	Bm_fmh	2	2307422-2307481	Chr2	ISBma2	GCCCT	CAGATTGCTGACAAAC	~TGACCTGCCCCCTTCGAT	#	#IS407A	AGGGCGCCAAAGCGC	#IS407A	N.D.
2*Chr2_80a-1_ISBma2_A		N	Bm_fmh	2	2308711-2310234	Chr2	ISBma2	#IS407	~~~~~~~~~~~~~~~	ATGCTGAAGACGCCCATG	TCCCAGAAAGACAAAACC	AGGGCGC	#IS407A	AGGGCGC	N.D.
4*Chr2_82_ISBma2_B		N	Bm_gb8_horse_	4	2310538-2312110	Chr2	ISBma2	CGCAC	CAGATTGCTGACAAAC	ATGCTGAAGACGCCCATG	TCCCAGAAAGACAAAACC	AAACGGG	AAACGGGGCCCGCTGG	AAACGGG	Y
1*Chr2_82_ISBma2_B		N	Bm_ATCC_233	1	2320471-2322043	Chr2	ISBma2	CGCAC	CAGATTGCTGACAAAC	ATGCTGAAGACGCCCATG	TCCCAGAAAGACAAAACC	AAACGGG	AAACGGGGCCCGCTGG	AAACGGG	Y
7*Chr2_82_ISBma2_B		N	Bm_NCTC_102	7	2347787-2349359	Chr2	ISBma2	TCGCA	CAGATTGCTGACAAAC	ATGCTGAAGACGCCCATG	TCCCAGAAAGACAAAACC	CTGGGCC	CTGGGCCCCGTT	CTGGGCC	Y
8*Chr2_36_ISBma2_B		N	Bm_200272128	8	260811-262383	Chr2	ISBma2	ACGCC	CAGATTGCTGACAAAC	ATGCTGAAGACGCCCATG	TCCCAGAAAGACAAAACC	AAGGCGC	CAAGGCGCTCGGCAAC	AAGGCGC	N
10*Chr2_65_ISBma2_B		N	Bm_PRL20	10	274212-275784	Chr2	ISBma2	CTCTA	CAGATTGCTGACAAAC	ATGCTGAAGACGCCCATG	TCCCAGAAAGACAAAACC	AGACGGG	AGACGGGCTGCAGCCC	AGACGGG	Y
5*Chr2_64_ISBma2_B		N	Bm_ATCC_103	5	274296-275868	Chr2	ISBma2	CCACG	CAGATTGCTGACAAAC	ATGCTGAAGACGCCCATG	TCCCAGAAAGACAAAACC	AGGCCGT	AGGCCGTTCGATTCGA	AGGCCGT	Y
10*Chr2_63_ISBma2_B		N	Bm_PRL20	10	289920-291492	Chr2	ISBma2	CCGCG	CAGATTGCTGACAAAC	ATGCTGAAGACGCCCATG	TCCCAGAAAGACAAAACC	AAGCCGG	AAAAAGCCGGCGTGCA	AAGCCGG	N
6*Chr2_47_ISBma2_A		N	Bm_NCTC_102	6	298358-299930	Chr2	ISBma2	CGCGG	CAGATTGCTGACAAAC	ATGCTGAAGACGCCCATG	TCCCAGAAAGACAAAACC	AGCGCAC	GCGGATGCAGCGCACG	AGCGCAC	N
7*Chr2_65_ISBma2_B		N	Bm_NCTC_102	7	316219-317791	Chr2	ISBma2	CGCGC	CAGATTGCTGACAAAC	ATGCTGAAGACGCCCATG	TCCCAGAAAGACAAAACC	GACGGGC	GACGGGCTGCAGCCCG	GACGGGC	Y
4*Chr2_64_ISBma2_B		N	Bm_gb8_horse_	4	318688-320260	Chr2	ISBma2	CCACG	CAGATTGCTGACAAAC	ATGCTGAAGACGCCCATG	TCCCAGAAAGACAAAACC	AGGCCGT	AGGCCGTTCGATTCGA	AGGCCGT	Y
1*Chr2_64_ISBma2_B		N	Bm_ATCC_233	1	319482-321054	Chr2	ISBma2	CCACG	CAGATTGCTGACAAAC	ATGCTGAAGACGCCCATG	TCCCAGAAAGACAAAACC	AGGCCGT	AGGCCGTTCGATTCGA	AGGCCGT	Y
2*Chr2_64_ISBma2_B		N	Bm_fmh	2	328979-330551	Chr2	ISBma2	CCACG	CAGATTGCTGACAAAC	ATGCTGAAGACGCCCATG	TCCCAGAAAGACAAAACC	AGGCCGT	AGGCCGTTCGATTCGA	AGGCCGT	Y
3*Chr2_64_ISBma2_B		N	Bm_JHU	3	357598-359170	Chr2	ISBma2	CCACG	CAGATTGCTGACAAAC	ATGCTGAAGACGCCCATG	TCCCAGAAAGACAAAACC	AGGCCGT	AGGCCGTTCGATTCGA	AGGCCGT	Y
3*Chr2_82_ISBma2_B		N	Bm_JHU	3	3773-5345	Chr2	ISBma2	CGCAC	CAGATTGCTGACAAAC	ATGCTGAAGACGCCCATG	TCCCAGAAAGACAAAACC	AAACGGG	AAACGGGGCCCGCTGG	AAACGGG	Y
2*Chr2_82_ISBma2_B		N	Bm_fmh	2	4374-5946	Chr2	ISBma2	CGCAC	CAGATTGCTGACAAAC	ATGCTGAAGACGCCCATG	TCCCAGAAAGACAAAACC	AAACGGG	AAACGGGGCCCGCTGG	AAACGGG	Y
8*Chr2_47_ISBma2_A		N	Bm_200272128	8	659779-661351	Chr2	ISBma2	CGCGG	CAGATTGCTGACAAAC	ATGCTGAAGACGCCCATG	TCCCAGAAAGACAAAACC	AGCGCAC	GCGGATGCAGCGCACG	AGCGCAC	N
7*Chr2_5b_ISBma2	7*Chr2_14a_ISBma2_A	R	Bm_NCTC_102	7	719172-720746	Chr2	ISBma2	TGTGC	CAGATTGCTGACAAAC	ATGCTGAAGACGCCCATG	TCCCAGAAAGACAAAACC	ACGCCCC	AGGCCTTCATCTGGCG	ACGCCCC	N
6*Chr2_36_ISBma2_B		N	Bm_NCTC_102	6	797475-799047	Chr2	ISBma2	ACGCC	CAGATTGCTGACAAAC	ATGCTGAAGACGCCCATG	TCCCAGAAAGACAAAACC	AAGGCGC	CAAGGCGCTCGGCAAC	AAGGCGC	N
9*Chr2_5a_ISBma2	9*Chr2_14b_ISBma2_A	D-R	Bm_SAVP1	9	813639-815211	Chr2	ISBma2	TAAAA	CAGATTGCTGACAAAC	ATGCTGAAGACGCCCATG	TCCCAGAAAGACAAAACC	GGCCTTC	AAACGCCCCGCACGAA	GGCCTTC	N
10*Chr2_30_ISBma2_A		N	Bm_PRL20	10	834777-836349	Chr2	ISBma2	ACAGG	CAGATTGCTGACAAAC	ATGCTGAAGACGCCCATG	TCCCAGAAAGACAAAACC	AACAGCC	AAAACAGCCGGCCCGA	AACAGCC	N
7*Chr2_12_ISBma2_A		N	Bm_NCTC_102	7	871394-872967	Chr2	ISBma2	GCCGC	CAGATTGCTGACAAAC	ATGCTGAAGACGCCCATG	TCCCAGAAAGACAAAACC	GCGCTCC	GCGCTCCATTCGGAGC	GCGCTCC	Y
10*Chr2_12_ISBma2_A		D-R	Bm_PRL20	10	876295-877868	Chr2	ISBma2	GCCGC	CAGATTGCTGACAAAC	ATGCTGAAGACGCCCATG	TCCCAGAAAGACAAAACC	AAGCCCC	GCGCTCCATTCGGAGC	AAGCCCC	N
4*Chr2_11_ISBma2_B		N	Bm_gb8_horse_	4	888664-890236	Chr2	ISBma2	TCAGC	CAGATTGCTGACAAAC	ATGCTGAAGACGCCCATG	TCCCAGAAAGACAAAACC	AAGCCCC	ACAAAGCCCCGTCAAT	AAGCCCC	N
1*Chr2_11_ISBma2_B		N	Bm_ATCC_233	1	893528-895100	Chr2	ISBma2	TCAGC	CAGATTGCTGACAAAC	ATGCTGAAGACGCCCATG	TCCCAGAAAGACAAAACC	AAGCCCC	ACAAAGCCCCGTCAAT	AAGCCCC	N
4*Chr2_12_ISBma2_A		N	Bm_gb8_horse_	4	893773-895346	Chr2	ISBma2	GCCGC	CAGATTGCTGACAAAC	ATGCTGAAGACGCCCATG	TCCCAGAAAGACAAAACC	GCGCTCC	GCGCTCCATTCGGAGC	GCGCTCC	Y
1*Chr2_12_ISBma2_A		N	Bm_ATCC_233	1	898637-900210	Chr2	ISBma2	GCCGC	CAGATTGCTGACAAAC	ATGCTGAAGACGCCCATG	TCCCAGAAAGACAAAACC	GCGCTCC	GCGCTCCATTCGGAGC	GCGCTCC	Y
5*Chr2_47_ISBma2_A		N	Bm_ATCC_103	5	904424-905996	Chr2	ISBma2	CGCGG	CAGATTGCTGACAAAC	ATGCTGAAGACGCCCATG	TCCCAGAAAGACAAAACC	AGCGCAC	GCGGATGCAGCGCACG	AGCGCAC	N
2*Chr2_11_ISBma2_B		N	Bm_fmh	2	907252-909260	Chr2	ISBma2	TCAGC	CAGATTGCTGACAAAC	ATGCTGAAGACGCCCATG	TCCCAGAAAGACAAAACC	AAGCCCC	ACAAAGCCCCGTCAAT	AAGCCCC	N
2*Chr2_12_ISBma2_A		N	Bm_fmh	2	912797-914370	Chr2	ISBma2	GCCGC	CAGATTGCTGACAAAC	ATGCTGAAGACGCCCATG	TCCCAGAAAGACAAAACC	GCGCTCC	GCGCTCCATTCGGAGC	GCGCTCC	Y
3*Chr2_11_ISBma2_B		N	Bm_JHU	3	968882-970454	Chr2	ISBma2	TCAGC	CAGATTGCTGACAAAC	ATGCTGAAGACGCCCATG	TCCCAGAAAGACAAAACC	AAGCCCC	ACAAAGCCCCGTCAAT	AAGCCCC	N
3*Chr2_12_ISBma2_A		N	Bm_JHU	3	973991-975564	Chr2	ISBma2	GCCGC	CAGATTGCTGACAAAC	ATGCTGAAGACGCCCATG	TCCCAGAAAGACAAAACC	GCGCTCC	GCGCTCCATTCGGAGC	GCGCTCC	Y
9*Chr2_77_ISBma2	9*Chr2_78_ISBma2_A	D	Bm_SAVP1	9	998682-1000254	Chr2	ISBma2	TGACC	CAGATTGCTGACAAAC	ATGCTGAAGACGCCCATG	TCCCAGAAAGACAAAACC	AGGCGTT	GCGGGAAGCGCCGCG	AGGCGTT	N
