# Supplementary material for: The Early Stage of Bacterial Genome-Reductive Evolution in the Host
Source: PLoS Pathog. 2010 May 27;6(5):e1000922. doi: 10.1371/journal.ppat.1000922 (PMC2877748; doi:10.1371/journal.ppat.1000922)
Supplement: Table S4 — Disrupted genes that resulted from SSR-mediated frameshift mutations in B. mallei and B. pseudomallei. (0.34 MB PDF) [file ppat.1000922.s006.pdf]

Table S4. Disrupted genes that resulted from SSR-mediated frameshift mutations in *B. mallei* and *B. pseudomallei.*

IS*407*A	disrupted by IS*407*A
genomic-del	removed by large genomic deletion
o	disrupted gene
**absent**	either deletion in the strain or insertion in K96243

		B. mallei										*B. pseudomallei*								
locus	protein	**ATCC23344**	**FMH**	**JHU**	**GB8 horse**	**ATCC 10399**	**10229**	**10247**	**2002721280**	**SAVP1**	**PRL-20**	**1106a**	**1106b**	**1710a**	**1710b**	**406e**	**668**	**Pasteur**	**S13**	**1655**
BPSL0006	hypothetical protein BPSL0006	o	o	o	o	o	o	o	o	o	o									
BPSL0035	hypothetical protein BPSL0035																	o		
BPSL0223	putative acyl-CoA dehydrogenase	o	o	o	o	o	o	o	o	o	o									
BPSL0258	hypothetical protein BPSL0258	o	o	o	o	o	o	o	o	o	o									
BPSL0269	flagellar basal body P-ring biosynthesis p	o	o	o	o	o	o	o	o	o	o									
BPSL0276	flagellar L-ring protein precursor	o	o	o	o															
BPSL0308	hypothetical protein BPSL0308				o															
BPSL0321	hypothetical protein BPSL0321								o											
BPSL0347	putative insertion element protein	genomic-del	genomic-del	genomic-del	genomic-del	genomic-del	genomic-del	genomic-del	genomic-del	genomic-del	genomic-del	o	o			o	**absent**		o	
BPSL0371	acetyl-CoA acetyltransferase									o	o									
BPSL0377	hypothetical protein BPSL0377	genomic-del	genomic-del	genomic-del	genomic-del	genomic-del	genomic-del	genomic-del	genomic-del	genomic-del	genomic-del			o	o				o	
BPSL0405	hypothetical protein BPSL0405													o						
BPSL0426	C4-dicarboxylate transport sensor protein	o	o	o	o	o	o	o	genomic-del	o	o									
BPSL0492	hypothetical protein BPSL0492											o	o	o	o	o		o	o	o
BPSL0578	dienelactone hydrolase family protein	genomic-del	genomic-del	genomic-del	genomic-del	genomic-del	genomic-del	genomic-del	genomic-del	genomic-del	genomic-del									o
BPSL0589	hypothetical protein BPSL0589	IS*407A*	IS*408A*	IS*409A*	IS*410A*	IS*411A*	IS*412A*	IS*413A*	IS*414A*	IS*415A*	IS*416A*			o	o	o		o		
BPSL0662	hypothetical protein BPSL0662	o	o	o	o	o	o	o	o	o	o									
BPSL0681	hypothetical protein BPSL0681	o	o	o	o	o	o	o	o	o	o									
BPSL0703	putative two-component sensor histidine kinase																		o	
BPSL0726	hypothetical protein BPSL0726	genomic-del	genomic-del	genomic-del	genomic-del	genomic-del	genomic-del	genomic-del	genomic-del	genomic-del	genomic-del			o	o			o		o
BPSL0735	hypothetical protein BPSL0735	genomic-del	genomic-del	genomic-del	genomic-del	genomic-del	genomic-del	genomic-del	genomic-del	genomic-del	genomic-del									o
BPSL0787	hypothetical protein BPSL0787	o	o	o	o															
BPSL0828	putative tagatose 6-phosphate kinase pro	o	o	o	o	o	o	o	o	o	o									
BPSL0860	putative hydrolase										o									
BPSL0862	putative methylated-DNA--protein-cysteine methyltransferase											o	o	o	o		o	o	o	
BPSL0878	tetraacyldisaccharide 4'-kinase	o																		
BPSL0882	putative chromate transporter protein		o				genomic-del													
BPSL0898a	ATP-dependent Clp protease adaptor protein ClpS								o											
BPSL0917	putative DNA repair protein													o		**absent**				
BPSL0922	putative ABC transporter ATP-binding protein								genomic-del		o					**absent**				
BPSL0954	hypothetical protein BPSL0954													o	o	**absent**		o		
BPSL0967	hypothetical protein BPSL0967											o				**absent**				
BPSL0970	putative isoleucine biosynthesis transcript	o	o	o	o	o	o	o	o	o	o									
BPSL0976	putative outer membrane receptor protein												o							
BPSL0977	putative transmembrane ABC transporter permease															o				
BPSL1007	putative fimbriae usher protein												o							
BPSL1014	hypothetical protein BPSL1014															o				
BPSL1038a	hypothetical protein BPSL1038a																o			
BPSL1041	hypothetical protein BPSL1041	genomic-del	genomic-del	genomic-del	genomic-del	genomic-del	genomic-del	genomic-del	genomic-del	genomic-del	genomic-del			o	o			o		
BPSL1114	putative ATP-dependent RNA helicase 2													o						
BPSL1117	DNA polymerase III subunit alpha														o					
BPSL1128	putative lipoprotein															o				
BPSL1133	hypothetical protein BPSL1133																o			o
BPSL1174	two-component system, sensor kinase pro	o	o	o	o	o	o	o	o	o	o									
BPSL1179	hypothetical protein BPSL1179													o						

BPSL1179	hypothetical protein BPSL1179	o	o	o	o	o	o	o	o	o	o									
BPSL1201	2-isopropylmalate synthase	o	o	o	o	o	o	o	o	o	o									
BPSL1202	putative transport-related membrane protein										o									
BPSL1236	putative phosphoglycerate mutase													o						
BPSL1239	putative aminotransferase																			o
BPSL1245	putative transcriptional regulator				genomic-del												o			
BPSL1246	hypothetical protein BPSL1246					o														
BPSL1270	putative exported alkaline phosphatase					o														
BPSL1274	hypothetical protein BPSL1274	o	o	o	o	o	o	o	o	o	o									
BPSL1286	hypothetical protein BPSL1286	o	o	o	o	o	o	o	o	o	o									
BPSL1290a	hypothetical protein BPSL1290a																o			
BPSL1317	putative lipoprotein	o	o	o	o	o				o	o									
BPSL1334	putative coenzyme-A synthase								o											
BPSL1347	hypothetical protein BPSL1347																			o
BPSL1368	hypothetical protein BPSL1368													o						
BPSL1393	putative exported avidin family protein	genomic-del	genomic-del	genomic-del	genomic-del	genomic-del	genomic-del	genomic-del	genomic-del	genomic-del	genomic-del					o			o	
BPSL1396	hypothetical protein BPSL1396							o												
BPSL1427	hypothetical protein BPSL1427	o	o	o	o	o	o	o	o	o	o			o	o					
BPSL1443	hypothetical protein BPSL1443											o	o	o	o		o	o	o	o
BPSL1446	hypothetical protein BPSL1446	o	o	o	o	o	o	o	o	o	o	o	o	o	o			o	o	o
BPSL1476	aspartate aminotransferase	o	o	o	o	o	o	o	o	o	o									
BPSL1498	DNA polymerase III subunits gamma and tau			o							o									
BPSL1500	recombination protein RecR										o									
BPSL1534	poly-beta-hydroxybutyrate polymerase										o									
BPSL1551	putative 2-nitropropane dioxygenase								o											
BPSL1591	hypothetical protein BPSL1591						genomic-del	genomic-del		genomic-del				o						
BPSL1593	putative metal ion transport protein						genomic-del	genomic-del		genomic-del									o	
BPSL1593	putative metal ion transport protein						genomic-del	genomic-del		genomic-del	o									
BPSL1597	hypothetical protein BPSL1597						genomic-del	genomic-del		genomic-del		o	o	o	o	o	incomp	o	o	o
BPSL1598	putative transport-related, membrane protein						genomic-del	genomic-del		genomic-del								o		
BPSL1599	putative membrane linked regulatory protein						genomic-del	genomic-del		genomic-del				o	o	o				
BPSL1607	nitrous-oxide reductase precurser			o			genomic-del	genomic-del		genomic-del										
BPSL1612	hypothetical protein BPSL1612	o	o	o	o	o	genomic-del	genomic-del	o	genomic-del	o	o	o	o	o				o	o
BPSL1618	hypothetical protein BPSL1618	o	o	o	o	o	genomic-del	genomic-del	o	genomic-del	o									
BPSL1621	hypothetical protein BPSL1621						genomic-del	genomic-del		genomic-del									absent	o
BPSL1624	hypothetical protein BPSL1624	o	o	o	o	o	genomic-del	genomic-del	o	genomic-del	o	o	o	o	o	o		o	o	
BPSL1668	putative adenylylsulfate kinase	genomic-del	genomic-del	genomic-del	genomic-del	genomic-del	genomic-del	genomic-del	genomic-del	genomic-del	genomic-del	o	o				absent		o	
BPSL1670	putative transposase	genomic-del	genomic-del	genomic-del	genomic-del	genomic-del	genomic-del	genomic-del	genomic-del	genomic-del	genomic-del						absent	o absent	o insertion	
BPSL1679	hypothetical protein BPSL1679	genomic-del	genomic-del	genomic-del	genomic-del	genomic-del	genomic-del	genomic-del	genomic-del	genomic-del	genomic-del						absent		o	
BPSL1705	hypothetical protein BPSL1705	genomic-del	genomic-del	genomic-del	genomic-del	genomic-del	genomic-del	genomic-del	genomic-del	genomic-del	genomic-del	absent	absent				absent	o	absent	absent
BPSL1707	putative exported oxidase	genomic-del	genomic-del	genomic-del	genomic-del	genomic-del	genomic-del	genomic-del	genomic-del	genomic-del	genomic-del	absent	absent				absent	o	absent	
BPSL1712	putative non-ribosomal antibiotic-related	genomic-del	genomic-del	genomic-del	genomic-del	genomic-del	genomic-del	genomic-del	genomic-del	genomic-del	genomic-del									o
BPSL1727	putative non-ribosomal peptide synthase	o	o	o	o	o	o	o	o	o	o									
BPSL1729	putative AraC-family transcriptional regulator										o									
BPSL1734	acyl-CoA synthase																		o	o
BPSL1734	acyl-CoA synthase																		o	
BPSL1739	putative ABC transport system, ATP-binding protein										o									
BPSL1767	putative magnesium chelatase protein																	o		
BPSL1770	high-affinity nickel transport protein						o	o	o											
BPSL1771	cobalamin biosynthesis protein CbiG							o												
BPSL1789	short chain dehydrogenase																			o
BPSL1812	hypothetical protein BPSL1812	genomic-del	genomic-del	genomic-del	genomic-del	o	o	o	o	o	genomic-del									
BPSL1815	putative fimbriae assembly-related protei	genomic-del	genomic-del	genomic-del	genomic-del						genomic-del			o						
BPSL1819	putative fimbriae-assembly related protei	genomic-del	genomic-del	genomic-del	genomic-del						genomic-del		o							
BPSL1829	putative methyl-accepting chemotaxis pr	genomic-del	genomic-del	genomic-del	genomic-del	genomic-del	genomic-del	genomic-del	genomic-del	genomic-del	genomic-del					o				
BPSL1833	putative ribose transport system, ATP-bin	genomic-del	genomic-del	genomic-del	genomic-del	genomic-del	genomic-del	genomic-del	genomic-del	genomic-del	genomic-del					o				
BPSL1838	sulfate transport system permease protein					o					genomic-del									
BPSL1857	putative aliphatic sulfonates transport permease protein																			o

BPSL1881	putative metabolite transport, membrane protein																			o
BPSL1884	hypothetical protein BPSL1884							o												
BPSL1888	hypothetical protein BPSL1888																		o	
BPSL1936	hypothetical protein BPSL1936	genomic-del	genomic-del	genomic-del	genomic-del	genomic-del	genomic-del	genomic-del	genomic-del	genomic-del	genomic-del									o
BPSL1963	hypothetical protein BPSL1963	o	o	o	o	o	o	o	o	o	o									
BPSL1988	putative oxidoreductase									o										
BPSL2006	subfamily S1C non-peptidase homologue	o	o	o	o	o	o	o	o	o	o									
BPSL2022	putative drug-resistance related outer-me	o	o	o	o	o	o	o	o	o	o									
BPSL2027	putative fimbriae-related protein	o	o	o	o	o		o	o		o									
BPSL2043	putative lipoprotein	genomic-del	genomic-del	genomic-del	genomic-del	genomic-del	genomic-del	genomic-del	genomic-del	genomic-del	genomic-del					o				
BPSL2047	hypothetical protein BPSL2047	genomic-del	genomic-del	genomic-del	genomic-del	genomic-del	genomic-del	genomic-del	genomic-del	genomic-del	genomic-del								o	absent
BPSL2048/	hypothetical protein BPSL2048A	genomic-del	genomic-del	genomic-del	genomic-del	genomic-del	genomic-del	genomic-del	genomic-del	genomic-del	genomic-del			o	o			o		
BPSL2053	hypothetical protein BPSL2053							o												
BPSL2054	hypothetical protein BPSL2054	o	o	o	o	o	o	o	o	o	o	o	o				absent		o	o
BPSL2064	hypothetical protein BPSL2064													o	o			o		
BPSL2064	hypothetical protein BPSL2064	o	o	o	o	o				o	o									
BPSL2161	PII uridylyl-transferase																	o		
BPSL2191	putative dehalogenase																o			
BPSL2198	putative exported phospholipase																	o		
BPSL2209	molybdenum cofactor biosynthesis protein A													o	o	o	o	o		o
BPSL2211	putative TetR-family regulatory protein								o											
BPSL2229	putative siderophore non-ribosomal pept	o	o	o	o	o	o	o	o	o	o									
BPSL2230	hypothetical protein BPSL2230									o	o									
BPSL2236	hypothetical protein BPSL2236																	o		
BPSL2236	hypothetical protein BPSL2236									o										
BPSL2254	putative FkbP-type peptidyl-prolyl cis-tra	o	o	o	o	o	o	o	o	o	o									
BPSL2285	chaperone protein HscA																	o		
BPSL2330	LysR family regulatory protein				o															
BPSL2351	hypothetical protein BPSL2351								o											
BPSL2358	putative pyruvate radical-activating enzyme								o											
BPSL2367	putative methyl-accepting chemotaxis protein								o											
BPSL2369	hypothetical protein BPSL2369	o	o	o	o	o	o	o	o	o	o									
BPSL2407	hypothetical protein BPSL2407					o														
BPSL2424	beta-hexosaminidase								o											
BPSL2455	putative molybdopterin biosynthesis protein																			o
BPSL2460	hypothetical protein BPSL2460																	o		
BPSL2498	putative acyl-CoA dehydrogenase	o	o	o	o	o	o	o	o	o	o									
BPSL2532	putative oxygenase												o							
BPSL2548	hypothetical protein BPSL2548																o			
BPSL2559	outer membrane porin protein					genomic-del	genomic-del			genomic-del										o
BPSL2561	guanosine-3',5'-bis(diphosphate) 3'-pyrophosphohydrolase													o						
BPSL2588	hypothetical protein BPSL2588																o			
BPSL2589	putative lipoprotein								o											
BPSL2606	hypothetical protein BPSL2606													o						
BPSL2606	hypothetical protein BPSL2606			o																
BPSL2606	hypothetical protein BPSL2606		o																	
BPSL2621	hypothetical protein BPSL2621											o	o							o
BPSL2641	Sir2 family protein																			o
BPSL2641	Sir2 family protein													o	o		o			
BPSL2641	Sir2 family protein																o			
BPSL2672	putative epimerase/dehydratase polysaccharide-related biosynthesis protein																			o
BPSL2683	dTDP-4-dehydrorhamnose reductase																o			
BPSL2707	hypothetical protein BPSL2707	o	o	o	o	o	o	o	o	o	o	o	o	o	o			o		
BPSL2718	hypothetical protein BPSL2718																o			
BPSL2722	membrane transport solute-binding protein																			o
BPSL2722	membrane transport solute-binding prote	o	o	o	o	o	o	o	o	o	o									
BPSL2768	hypothetical protein BPSL2768	genomic-del	genomic-del	genomic-del	genomic-del	genomic-del	genomic-del	genomic-del	genomic-del	genomic-del	genomic-del			o	o	o				o
BPSL2770	putative capsule expression protein	genomic-del	genomic-del	genomic-del	genomic-del	genomic-del	genomic-del	genomic-del	genomic-del	genomic-del	genomic-del							o		

BPSL2784	hypothetical protein BPSL2784	genomic-del	genomic-del	genomic-del	genomic-del	genomic-del	genomic-del	genomic-del	genomic-del	genomic-del	genomic-del						o			
BPSL2827	molecular chaperone DnaK								o											
BPSL2832	heat-inducible transcription repressor													o						
BPSL2862	hypothetical protein BPSL2862											o	o							
BPSL2863	putative ferritin DPS-family DNA binding protein					o														
BPSL2901	putative lipoprotein																			o
BPSL2905	hypothetical protein BPSL2905																			o
BPSL2933	putative regulatory protein								genomic-del							o				
BPSL2944	ureidoglycolate hydrolase	o	o	o	o	o	o	o	genomic-del	o	o									
BPSL2996	hypoxanthine-guanine phosphoribosyltransferase								genomic-del									o		
BPSL3103	hypothetical protein BPSL3103							o												
BPSL3104	hypothetical protein BPSL3104					o														
BPSL3118	putative restriction modification system	genomic-del	genomic-del	genomic-del	genomic-del	genomic-del	genomic-del	genomic-del	genomic-del	genomic-del	genomic-del	absent	absent	absent	absent	o		absent		absent
BPSL3119	putative stringent starvation protein B																o			
BPSL3156	hypothetical protein BPSL3156				o															
BPSL3205	50S ribosomal protein L29					o														
BPSL3225	50S ribosomal protein L11													o						
BPSL3229	hypothetical protein BPSL3229																o			
BPSL3237	hypothetical protein BPSL3237	o	o	o	o	o	o	o	o	o	o	o	o			o				
BPSL3237	hypothetical protein BPSL3237													o	o					
BPSL3239	4-hydroxyphenylpyruvate dioxygenase								o											
BPSL3293	flagellar biosynthesis protein	o	o	o	o	o	o	o	o	o	o									
BPSL3301	chemotaxis-specific methylesterase							o												
BPSL3336	putative lipoprotein	o	o	o	o	o	o	o	o	o	o									
BPSL3337	putative hydrolase												o							
BPSL3338	putative methyl-accepting chemotaxis protein						o	o												
BPSL3375	hypothetical protein BPSL3375																	o		
BPSL3378	putative heavy metal resistance membrane ATPase								o											
BPSL3410	putative branched-chain amino acid ABC transporter								o											
BPSL3413	hypothetical protein BPSL3413																			o
BPSS0003	hypothetical protein BPSS0003	o	o	o	o	o	o	o	o	o	o	o	o	o	o	o	o	o	o	o
BPSS0061	alpha-ketoglutarate permease								o											
BPSS0065	hypothetical protein BPSS0065								genomic-del								o			
BPSS0068	hypothetical protein BPSS0068	IS*407A*	IS*408A*	IS*409A*	IS*410A*	IS*411A*	IS*412A*	IS*413A*	IS*414A*	IS*415A*	IS*416A*	absent	absent	absent	absent	absent		absent	o	absent
BPSS0078	hypothetical protein BPSS0078	genomic-del	genomic-del	genomic-del	genomic-del	genomic-del	genomic-del	genomic-del	genomic-del	genomic-del	genomic-del									o
BPSS0078	hypothetical protein BPSS0078	genomic-del	genomic-del	genomic-del	genomic-del	genomic-del	genomic-del	genomic-del	genomic-del	genomic-del	genomic-del					o				
BPSS0128	pyruvate kinase	genomic-del	genomic-del	genomic-del	genomic-del	genomic-del	genomic-del	genomic-del	genomic-del	genomic-del	genomic-del							o		
BPSS0147	transporter protein																		o	
BPSS0147	transporter protein	o	o	o	o	o	o	o	o	o	o									
BPSS0167	hypothetical protein BPSS0167																			o
BPSS0167	hypothetical protein BPSS0167	o	o	o	o	o	o	o	o	o	o									
BPSS0174	chaperone	o	o	o	o	o	o	o	o	o	o									
BPSS0179	hypothetical protein BPSS0179	o																		
BPSS0190	O-acetylhomoserine								genomic-del								o			
BPSS0202	hypothetical protein BPSS0202											o	o	o	o		o	o	o	o
BPSS0208	aconitate hydratase										o									
BPSS0216	hypothetical protein BPSS0216	o	o	o	o	o	o	o	o	o	o	o	o							
BPSS0224	cation-transporting ATPase membrane protein							o												
BPSS0225	hypothetical protein BPSS0225										o									
BPSS0237	chromosome replication initiation inhibitor protein					o														
BPSS0238	penicillin-binding protein							o	o											
BPSS0252	porin membrane protein								genomic-del								o			
BPSS0262	aminoglycoside 6'-N-acetyltransferase								genomic-del			o	o	o	o			o		
BPSS0286	hypothetical protein BPSS0286									genomic-del				o	o			o		absent
BPSS0306	multifunctional polyketide-peptide syntase									genomic-del									o	absent
BPSS0311	multifunctional polyketide-peptide syntase							o		genomic-del										absent
BPSS0320	GntR-family transcriptional regulator								o	genomic-del										
BPSS0338	insertion element protein	IS*407A*	IS*408A*	IS*409A*	IS*410A*	IS*411A*	IS*412A*	IS*413A*	IS*414A*	IS*415A*	IS*416A*					o			o	o

BPSS0343	aminotransferase protein								genomic-del					o				absent		
BPSS0349	NADP-dependent glyceraldehyde-3-phosphate dehydrogenase								genomic-del							o				
BPSS0359	hypothetical protein BPSS0359								o											
BPSS0374	hypothetical protein BPSS0374	o	o	o	o	o	o	o	o	o	o									
BPSS0376	hypothetical protein BPSS0376	o	o	o	o	o	o	o	o	o	o									
BPSS0400	bacteriophage protein	genomic-del	genomic-del	genomic-del	genomic-del	genomic-del	genomic-del	genomic-del	genomic-del	genomic-del	genomic-del			o		absent	absent	absent	absent	absent
BPSS0403	hypothetical protein BPSS0403								o	genomic-del							absent			
BPSS0404	methylamine utilization protein	o	o	o	o	o	o	o	o	genomic-del	o						absent			
BPSS0406	hypothetical protein BPSS0406	genomic-del	genomic-del	genomic-del	genomic-del	genomic-del				genomic-del	genomic-del	o	o				absent		o	
BPSS0407	insertion element hypothetical protein	genomic-del	genomic-del	genomic-del	genomic-del	genomic-del				genomic-del	genomic-del						o			
BPSS0407	insertion element hypothetical protein	genomic-del	genomic-del	genomic-del	genomic-del	genomic-del				genomic-del	genomic-del						o			
BPSS0412	hypothetical protein BPSS0412	genomic-del	genomic-del	genomic-del	genomic-del	genomic-del	genomic-del	genomic-del	genomic-del	genomic-del	genomic-del	o	o					o		
BPSS0452	phosphoesterase	genomic-del	genomic-del	genomic-del	genomic-del	genomic-del	genomic-del	genomic-del	genomic-del	genomic-del	genomic-del	o	o							
BPSS0480	hypothetical protein BPSS0480	genomic-del	genomic-del	genomic-del	genomic-del	genomic-del	genomic-del	genomic-del	genomic-del	genomic-del	genomic-del			o	o	o		o		o
BPSS0508	thiamine pyrophosphate enzyme	o	o	o	o	o	o	o	o	o	o									
BPSS0510	hypothetical protein BPSS0510								o											
BPSS0512	hypothetical protein BPSS0512	o	o	o	o	o	o	o	o	o	o									
BPSS0514	acetyl-CoA hydrolase/transferase																o			
BPSS0522	ATPase										o									
BPSS0532	hypothetical protein BPSS0532									o										
BPSS0536	3-hydroxydecanoyl-ACP:CoA transacylase																		o	
BPSS0539	hypothetical protein BPSS0539															o				
BPSS0541	HlyD family secretion protein													o						
BPSS0581	salicylate biosynthesis isochorismate synt	genomic-del	genomic-del	genomic-del	genomic-del	genomic-del	genomic-del	genomic-del	genomic-del	genomic-del	genomic-del					o				
BPSS0655	hypothetical protein BPSS0655	genomic-del	genomic-del	genomic-del	genomic-del	genomic-del	genomic-del			genomic-del	genomic-del			o						
BPSS0664	response regulator protein	genomic-del	genomic-del	genomic-del	genomic-del	genomic-del	genomic-del	genomic-del	genomic-del	genomic-del	genomic-del						o			
BPSS0681	AraC family regulatory protein		o							genomic-del										
BPSS0686a	hypothetical protein BPSS0686a	o	o	o	o	o	o	o	o	genomic-del	o	o	o			o		o	o	o
BPSS0699	AraC family regulatory protein									genomic-del				o	o					
BPSS0703	transmembrane ABC transporter protein	o	o	o	o	o	o	o	o	genomic-del	o									
BPSS0733	methyl-accepting chemotaxis protein																o			
BPSS0733	methyl-accepting chemotaxis protein	o	o	o	o	o		o	o	o	o									
BPSS0735	glycosyl transferase					o														
BPSS0737	hypothetical protein BPSS0737	o	o	o	o	o	o	o	o	o	o									
BPSS0781	feruloyl-CoA synthase	o	o	o	o	o	o	o	o	genomic-del	genomic-del						absent			
BPSS0788	transport system permease protein								o	genomic-del	genomic-del									
BPSS0812	hypothetical protein BPSS0812	o	o	o	o	o	o	o	o	genomic-del	o			o	o	o	o	o	o	o
BPSS0816	penicillin-binding protein						o	o	o	genomic-del										
BPSS0820	tRNA synthetase	o	o	o	o	o	o	o	o	genomic-del	o									
BPSS0820	tRNA synthetase			o						genomic-del										
BPSS0837	hypothetical protein BPSS0837						o	o		genomic-del										
BPSS0847	hypothetical protein BPSS0847								o	genomic-del										
BPSS0857	hypothetical protein BPSS0857																		o	
BPSS0857	hypothetical protein BPSS0857	o	o	o	o	o	o	o	o	o	o									
BPSS0874	hypothetical protein BPSS0874	o	o	o	o	o	o	o	o	o	o									
BPSS0875	LysE type translocator																			o
BPSS0879	porin protein			o																
BPSS0885	N-acylhomoserine lactone synthase															o				
BPSS0899	hydrolase															o				
BPSS0902	hypothetical protein BPSS0902										o									
BPSS0919	hypothetical protein BPSS0919	o	o	o	o	o	o	o	o	o	o									
BPSS0933	methyl-accepting chemotaxis protein																	o		
BPSS0933	methyl-accepting chemotaxis protein	o	o	o	o	o	o	o	o	o	o									
BPSS0936	hypothetical protein BPSS0936			o																
BPSS0940	ornithine cyclodeaminase															o				
BPSS0946	beta-lactamase precursor		o																	
BPSS0993	catalase precursor			genomic-del			o	o	o											
BPSS0995	AraC family regulatory protein			genomic-del															o	

BPSS1006	polyketide synthase			genomic-del														o		
BPSS1006	polyketide synthase	o	o	genomic-del	o	o	o	o		o										
BPSS1007	polyketide synthase			genomic-del											o					
BPSS1007	polyketide synthase	o	o	genomic-del	o	o	o	o	o	o	o									
BPSS1007	polyketide synthase		o	genomic-del																
BPSS1008	polyketide synthase			genomic-del										o						
BPSS1008	polyketide synthase			genomic-del			o	o												
BPSS1020	AraC family transcriptional regulator			genomic-del												o				
BPSS1028	iron uptake regulator protein	o	o	genomic-del	o	o	o	o	o	o	o							absent		
BPSS1084	bacteriophage major tail sheath protein	genomic-del	genomic-del	genomic-del	genomic-del	genomic-del	genomic-del	genomic-del	genomic-del	genomic-del	genomic-del	absent	absent	absent	absent	absent	absent	absent	o	absent
BPSS1089	bacteriophage late control gene D protei	genomic-del	genomic-del	genomic-del	genomic-del	genomic-del	genomic-del	genomic-del	genomic-del	genomic-del	genomic-del	absent	absent	absent	absent	absent	absent	absent	o	absent
BPSS1094	hypothetical protein BPSS1094	genomic-del	genomic-del	genomic-del	genomic-del	genomic-del	genomic-del	genomic-del	genomic-del	genomic-del	genomic-del					o				
BPSS1103	periplasmic thiamine binding protein	genomic-del	genomic-del	genomic-del	genomic-del	genomic-del	genomic-del	genomic-del	genomic-del	genomic-del	genomic-del				o					absent
BPSS1115	hypothetical protein BPSS1115	o	o	o	o	o				o	o									absent
BPSS1116	hypothetical protein BPSS1116			o																absent
BPSS1120	outer membrane efflux protein																	o		absent
BPSS1174	non-ribosomal peptide/polyketide syntha	genomic-del	genomic-del	genomic-del	genomic-del	genomic-del	genomic-del	genomic-del	genomic-del	genomic-del	genomic-del					o				absent
BPSS1193	non-ribosomal peptide/polyketide syntha	genomic-del	genomic-del	genomic-del	genomic-del	genomic-del	genomic-del	genomic-del	genomic-del	genomic-del	genomic-del			o						absent
BPSS1194	peptide synthase/polyketide synthase	genomic-del	genomic-del	genomic-del	genomic-del	genomic-del	genomic-del	genomic-del	genomic-del	genomic-del	genomic-del	o	o	o	o	o	o	o	o	absent
BPSS1213	hypothetical protein BPSS1213	genomic-del	genomic-del	genomic-del	genomic-del	genomic-del	genomic-del	genomic-del	genomic-del	genomic-del	genomic-del							o		
BPSS1224	hypothetical protein BPSS1224		o																	
BPSS1240	penicillin-binding protein															o	o		o	
BPSS1243	nitrite reductase (NAD(P)H)															o				
BPSS1253	LysR family transcriptional regulator	o	o	o	o	o	o	o	o	o	o									
BPSS1255	LysR family transcriptional regulator	o	o	o	o	o	o	o	o	o	o									
BPSS1266	peptide/siderophore synthetase		o																	
BPSS1270	hypothetical protein BPSS1270									genomic-del	genomic-del			o	o		o			o
BPSS1282	lipoprotein	o	o	o	o					genomic-del	genomic-del									
BPSS1299	LysR family transcriptional regulator									o	o									
BPSS1318	D-lactate dehydrogenase																	o		
BPSS1327	MFS family sugar transporter protein	o	o	o	o	o	o	o	o	o	o									
BPSS1332	cytochrome-c methylamine utilization protein		o																	
BPSS1341	cysteine desulfurase													o						
BPSS1347	HlyD family efflux pump protein															o				
BPSS1347	HlyD family efflux pump protein													o						
BPSS1347	HlyD family efflux pump protein												o							
BPSS1348	outer membrane efflux protein					o														
BPSS1360	two-component sensor kinase protein													o	o		o	o	o	
BPSS1366	sarcosine oxidase gamma subunit	o	o	o	o	o	o	o	o	o	o									
BPSS1379	transmembrane transporter protein	genomic-del	genomic-del	genomic-del	genomic-del	genomic-del	genomic-del	genomic-del	genomic-del	genomic-del	genomic-del			o	o		o		o	
BPSS1381	transmembrane phospholipase protein	genomic-del	genomic-del	genomic-del	genomic-del	genomic-del	genomic-del	genomic-del	genomic-del	genomic-del	genomic-del			o						
BPSS1382	Endonuclease/Exonuclease/phosphatase f	genomic-del	genomic-del	genomic-del	genomic-del	genomic-del	genomic-del	genomic-del	genomic-del	genomic-del	genomic-del					o				
BPSS1421	hypothetical protein BPSS1421									genomic-del	genomic-del									o
BPSS1422	hypothetical protein BPSS1422	o	o	o	o	o	o	o	o	genomic-del	genomic-del								o	
BPSS1445	biotin carboxylase					o														
BPSS1458	MFS family transport protein						o	o	o											
BPSS1464	transmembrane NADPH flavoprotein	o	o	o	o	o	o	o	o	o	o									
BPSS1467	aminotransferase protein																	o		
BPSS1474	DeoR family transcriptional regulator																	o		
BPSS1494	two-component response regulator protein																			o
BPSS1495	two-component regulator sensor kinase										o									
BPSS1502	Clp-type ATPase chaperone protein										o									
BPSS1511	hypothetical protein BPSS1511										o									
BPSS1513	hypothetical protein BPSS1513																	o	o	
BPSS1541	type Ⅲ secretion system ATPase									genomic-del						o				
BPSS1545	Type Ⅲ secretion system protein									genomic-del									o	
BPSS1545	Type Ⅲ secretion system protein									genomic-del					o					
BPSS1579	hypothetical protein BPSS1579									genomic-del								o		

BPSS1580	cellulose biosynthesis protein									genomic-del			o							
BPSS1582a	hypothetical protein BPSS1582a	o	o	o	o	o	o	o	o	genomic-del	o					o				o
BPSS1600	type IV pilus biosynthesis protein					o				genomic-del										
BPSS1602	twitching motility protein									genomic-del				o						
BPSS1614	type III secretion protein	o	o	genomic-del	o	o	o	o	o	genomic-del	o									
BPSS1622	type III secretion protein								o	genomic-del										
BPSS1627	type III secretion system ATPase								o	genomic-del										
BPSS1634	probable non-ribosomal peptide syntheta	non ribosomal	non ribosomal	non ribosomal	non ribosomal	non ribosomal	non ribosomal	non ribosomal	non ribosomal	non ribosomal	non ribosomal					o	absent			absent
BPSS1653	hypothetical protein BPSS1653											o	o	o	o	o		o	o	
BPSS1659	hypothetical protein BPSS1659																o			o
BPSS1689	UDP-glucose 4-epimerase	o	o	o	o	o	o	o	o	o	o									
BPSS1702	hypothetical protein BPSS1702	o																		
BPSS1735	ABC transport system, membrane protein	o	o	o	o	o	o	o	o	genomic-del	o									
BPSS1741	Lipase precursor	o	o	o	o	o	o	o	o	genomic-del	o									
BPSS1758	30S ribosomal protein S21						o													
BPSS1792	LysR-family transcriptional regulator								genomic-del					o						
BPSS1844	hypothetical protein BPSS1844								genomic-del		o									
BPSS1869	dehalogenase	o	o	o	o	o	o	o	genomic-del	o	o	o	o				o			
BPSS1869	dehalogenase								genomic-del								o			
BPSS1879	hypothetical protein BPSS1879								genomic-del										o	
BPSS1882	acetyltransferase	o	o	o	o	o	o	o	genomic-del	o	o	o	o	o	o					o
BPSS1889	AraC-family transcriptional regulator								genomic-del								o			o
BPSS1936	outer membrane efflux protein						genomic-del		o		genomic-del									
BPSS1937	ABC transport system, exported protein	o	o	o	o	o	genomic-del	o	o	o	genomic-del									
BPSS1940	sensor kinase/response regulator fusion protein						genomic-del		o		genomic-del									
BPSS1964	hypothetical protein BPSS1964						genomic-del				genomic-del					o				
BPSS1973	sedolisin-B																	o		
BPSS1974	lipoprotein													o			absent			
BPSS2010	hypothetical protein BPSS2010	genomic-del	genomic-del	genomic-del	genomic-del	genomic-del	genomic-del	genomic-del	genomic-del	genomic-del	genomic-del	o	o							
BPSS2033	enoyl-CoA hydratase/isomerase	genomic-del	genomic-del	genomic-del	genomic-del	genomic-del	genomic-del	genomic-del	genomic-del	genomic-del	genomic-del							o		
BPSS2058	ATP-binding inner membrane transport p	genomic-del	genomic-del	genomic-del	genomic-del	genomic-del	genomic-del	genomic-del	genomic-del	genomic-del	genomic-del	absent	absent	absent	absent		absent	absent	absent	o
BPSS2086	hypothetical protein BPSS2086	genomic-del	genomic-del	genomic-del	genomic-del	genomic-del	genomic-del	genomic-del	genomic-del	genomic-del	genomic-del			o	o		absent	o		
BPSS2095	hypothetical protein BPSS2095											o	o			o			o	o
BPSS2096	hypothetical protein BPSS2096										o									
BPSS2111	hypothetical protein BPSS2111	o	o	o	o	o	o	o	o	o	o									
BPSS2113	hypothetical protein BPSS2113																o			
BPSS2115	LysR family transcriptional regulator																	o		
BPSS2122	hypothetical protein BPSS2122															o			o	
BPSS2129	hypothetical protein BPSS2129								o											
BPSS2130	acetyl-CoA synthetase												o							
BPSS2130	acetyl-CoA synthetase								o											
BPSS2130	acetyl-CoA synthetase				o															
BPSS2135	DedA family transmembrane protein								o											
BPSS2137	oligopeptide transport ATP-binding ABC transport protein								o											
BPSS2179	hypothetical protein BPSS2179	genomic-del	genomic-del	genomic-del	genomic-del	genomic-del	genomic-del	genomic-del	genomic-del	genomic-del	genomic-del									o
BPSS2183	hypothetical protein BPSS2183	genomic-del	genomic-del	genomic-del	genomic-del	genomic-del	genomic-del	genomic-del	genomic-del	genomic-del	genomic-del							o		
BPSS2225	hypothetical protein BPSS2225	genomic-del	genomic-del	genomic-del	genomic-del	genomic-del	genomic-del	genomic-del	genomic-del	genomic-del	genomic-del						o			
BPSS2233	DNA glycosylase	genomic-del	genomic-del	genomic-del	genomic-del	genomic-del	genomic-del	genomic-del	genomic-del	genomic-del	genomic-del									o
BPSS2240	hypothetical protein BPSS2240	o	o	o	o	o	o	o	genomic-del	o	o									
BPSS2243	lipoprotein	o	o	o	o	o	o	o	genomic-del	o	o									
BPSS2248	transferase								genomic-del									o		
BPSS2256	haloacid dehalogenase-like hydrolase	o	o	o	o	o	o	o	genomic-del	o	o									
BPSS2259	fusion protein, ATP-binding transmembra	o	o	o	o				genomic-del											
BPSS2260	hypothetical protein BPSS2260			o					genomic-del											
BPSS2286	hypothetical protein BPSS2286								genomic-del			o	o							o
BPSS2297	hypothetical protein BPSS2297	o	o	o	o				genomic-del								absent			
BPSS2301	hypothetical protein BPSS2301								genomic-del											o
BPSS2320	4'-phosphopantetheinyl transferase super	o	o	o	o	o	o	o	o	o	o									

BPSS2326	flavin-binding monooxygenase-like protein										o									
BPSS2327	cytochrome P450 family protein																	o		
BPSS2328	multi-domain beta keto-acyl synthase		o																	
