# Supplementary material for: The Early Stage of Bacterial Genome-Reductive Evolution in the Host
Source: PLoS Pathog. 2010 May 27;6(5):e1000922. doi: 10.1371/journal.ppat.1000922 (PMC2877748; doi:10.1371/journal.ppat.1000922)
Supplement: Table S5 — Potential regulatory genes that survived the genomic reduction in B. mallei. (0.18 MB PDF) [file ppat.1000922.s007.pdf]

Table S5. Potential regulatory genes that survived the genomic reduction in*B. mallei*

Bp-V	variations among *B. pseudomallei* genes
FS	frameshift mutation
IS*407*A	disrupted by IS*407*A
missense	missense mutation
nonsense	nonsense mutation
genomic-del	removed by large genomic deletion

locus	protein	ATCC23344	FMH	JHU	GB8 horse 4	ATCC 10399	10229	10247	2002721280	SAVP1	PRL-20
BPSS1027	RNA polymerase sigma factor	Bp-V	Bp-V	genomic-del	Bp-V	Bp-V	Bp-V	Bp-V	Bp-V	Bp-V	Bp-V
BPSL0283	LysR family regulatory protein	FS	FS	FS	FS	FS	FS	FS	FS	FS	FS
BPSL0970	putative isoleucine biosynthesis transcriptional activator	FS	FS	FS	FS	FS	FS	FS	FS	FS	FS
BPSS1253	LysR family transcriptional regulator	FS	FS	FS	FS	FS	FS	FS	FS	FS	FS
BPSS0856	hypothetical protein BPSS0856	IS*407*A	IS*407*A	IS*407*A	IS*407*A	IS*407*A	IS*407*A	IS*407*A	IS*407*A	IS*407*A	IS*407*A
BPSL0060	AraC family transcriptional regulator	genomic-del	genomic-del	genomic-del	genomic-del	genomic-del	Bp-V	Bp-V	Bp-V	Bp-V	genomic-del
BPSL1805	TetR family regulatory protein	genomic-del	genomic-del	genomic-del	genomic-del	Bp-V	Bp-V	Bp-V	Bp-V	Bp-V	Bp-V
BPSS1255	LysR family transcriptional regulator	missense	missense	missense	FS	FS	FS	FS	FS	FS	FS
BPSL2947	putative GntR-family regulatory protein	missense	missense	missense	missense	missense	missense	missense	genomic-del	missense	missense
BPSL2948	cyn operon transcriptional activator (LysR-family)	missense	missense	missense	missense	missense	missense	missense	genomic-del	missense	missense
BPSL2977	putative exoribonuclease II	missense	missense	missense	missense	missense	missense	missense	genomic-del	missense	missense
BPSS0030	chromosome replication initiation inhibitor protein	missense	missense	missense	missense	missense	missense	missense	FS	missense	missense
BPSS0145	ATP-independent RNA helicase	missense	missense	missense	missense		nonsense	nonsense	nonsense		
BPSS0237	chromosome replication initiation inhibitor protein	missense	missense	missense	missense	FS	missense	missense	missense	missense	missense
BPSS0282	GntR-family regulatory protein	missense	missense	missense	missense	missense	missense	missense	missense	genomic-del	missense
BPSS0284	IclR-family transcriptional regulator	missense	missense	missense	missense	missense	missense	missense	missense	genomic-del	missense
BPSS0365	LysR-family transcriptional regulator	missense	missense	missense	missense	missense	missense	missense	missense	genomic-del	missense
BPSS0502	GntR family regulatory protein	missense	missense	missense	missense	nonsense	missense	missense	missense	missense	missense
BPSS0648	transcriptional activator FtrA	missense	missense	missense	missense	missense	missense	missense	missense	genomic-del	missense
BPSS0651	hypothetical protein BPSS0651	missense	missense	missense	missense	missense	missense	missense	missense	genomic-del	missense
BPSS0688	response regulator protein	missense	missense	missense	missense	missense	missense	missense	missense	genomic-del	missense
BPSS0691	MarR family regulator protein	missense	missense	missense	missense	missense	missense	missense	missense	genomic-del	missense
BPSS0705	response regulator protein	missense	missense	missense	missense	missense	missense	missense	missense	genomic-del	missense
BPSS0755	LysR family regulatory protein	missense	missense	missense	missense	missense	missense	missense	missense	genomic-del	genomic-del
BPSS0765	DNA-binding protein	missense	missense	missense	missense	missense	missense	missense	missense	genomic-del	genomic-del
BPSS0770	GerE family regulatory protein	missense	missense	missense	missense	missense	missense	missense	missense	genomic-del	genomic-del
BPSS0797	IclR family regulatory protein	missense	missense	missense	missense	missense	missense	missense	missense	genomic-del	missense
BPSS1299	LysR family transcriptional regulator	missense	missense	missense	missense	missense	missense	missense	missense	FS	FS
BPSS1309	RpiR family regulatory protein	missense	missense	missense	missense	missense	missense	missense	FS	missense	missense
BPSS1424	AraC transcriptional regulatory protein	missense	missense	missense	missense	missense	missense	missense	missense	genomic-del	genomic-del
BPSS1477	GntR family transcriptional regulator protein	missense	missense	missense	missense	missense	missense	missense	missense	missense	missense
BPSS1483	TetR family transcriptional regulator	missense	missense	missense	missense	missense	missense	missense	FS	missense	missense
BPSS1553	hypothetical protein BPSS1553	missense	missense	missense	missense	missense	missense	missense	missense	genomic-del	missense
BPSS1556	MarR-family transcriptional regulator	missense	missense	missense	missense	missense	missense	missense	missense	genomic-del	missense
BPSS1583	transcription regulator	missense	missense	missense	missense	missense	missense	missense	missense	genomic-del	missense
BPSS1739	GntR-family regulatory protein	missense	missense	missense	missense	missense	missense	missense	missense	genomic-del	missense
BPSS1751	NAD-dependent deacetylase	missense	missense	missense	missense	missense	missense	missense	missense	genomic-del	missense
BPSS1781	MarR-family regulatory protein	missense	missense	missense	missense	missense	missense	missense	genomic-del	missense	missense
BPSS1841	RNA polymerase sigma factor	missense	missense	missense	missense	missense	missense	missense	genomic-del	missense	missense
BPSS1858	LysR-family transcriptional regulator	missense	missense	missense	missense	missense	missense	missense	genomic-del	missense	missense
BPSS1864	AraC-family regulatory protein	missense	missense	missense	missense	missense	missense	missense	genomic-del	missense	missense
BPSS1890	cat operon LysR-family transcriptional activator	missense	missense	missense	missense	missense	missense	missense	genomic-del	missense	missense
BPSS1902	LysR-family transcriptional regulator	missense	missense	missense	missense	missense	missense	missense	genomic-del	missense	missense
BPSL1315	putative LysR-family transcriptional regulator	nonsense	nonsense	nonsense	nonsense	missense	missense	missense	missense	missense	missense
BPSL3385	putative TetR-family transcriptional regulator	nonsense	nonsense	nonsense	nonsense	nonsense	nonsense	nonsense	nonsense	nonsense	nonsense

BPSS1900	LysR-family transcriptional regulator	nonsense	nonsense	nonsense	nonsense	nonsense	nonsense	nonsense	genomic-del	nonsense	nonsense
BPSS1967	TetR-family regulatory protein	nonsense	nonsense	nonsense	nonsense	nonsense	nonsense	nonsense	nonsense	nonsense	nonsense
BPSS1015	LysR family transcriptional regulator			genomic-del							
BPSS1022	RNA polymerase sigma factor			genomic-del							
BPSL0418	LysR family regulatory protein								genomic-del		
BPSL0891	GntR family transcriptional regulator						genomic-del				
BPSL1569	putative TetR-family regulatory protein						genomic-del	genomic-del		genomic-del	
BPSL1572	putative LysR-family transcriptional regulator						genomic-del	genomic-del		genomic-del	
BPSL1578	putative LacI-family transcriptional regulator						genomic-del	genomic-del		genomic-del	
BPSL1588	putative AsnC-family transcriptional regulator						genomic-del	genomic-del		genomic-del	
BPSL1633	putative two-component system, response regulator						genomic-del	genomic-del		genomic-del	
BPSL1835	transcriptional regulator Cbl										deletion, not by IS
BPSL2926	putative LysR-family transcriptional regulator								genomic-del		
BPSL2972	putative IclR-family regulatory protein								genomic-del		
BPSS0320	GntR-family transcriptional regulator								FS	genomic-del	
BPSS0630	response regulator protein									genomic-del	
BPSS0643	response regulator protein									genomic-del	missense
BPSS0644	DNA-binding protein						missense			genomic-del	
BPSS0675	GerR family regulatory protein									genomic-del	
BPSS0749	GerE familyregulatory protein									genomic-del	genomic-del
BPSS0772	MarR family regulatory protein									genomic-del	genomic-del
BPSS0785	IclR family regulatory protein									genomic-del	genomic-del
BPSS0800	TetR family regulatory protein									genomic-del	
BPSS0806	leucine-responsive regulatory protein									genomic-del	
BPSS0835	response regulator protein									genomic-del	
BPSS1275	RNA polymerase sigma factor									genomic-del	genomic-del
BPSS1278a	TetR family regulatory protein									genomic-del	genomic-del
BPSS1414	GntR family transcriptional regulator									genomic-del	genomic-del
BPSS1430	ArsR family regulatory protein									genomic-del	genomic-del
BPSS1522	two-component response regulator									genomic-del	
BPSS1559	LysR-family transcriptional regulator									genomic-del	
BPSS1564	AsnC-family transcriptional regulator									genomic-del	
BPSS1569	N-acylhomoserine lactone-dependent regulatory protein									genomic-del	
BPSS1586	LysR-family transcriptional regulator					missense				genomic-del	
BPSS1604	probable two-component system response regulator									genomic-del	
BPSS2276	LysR family regulatory protein								genomic-del		
BPSS2315	response regulator protein								genomic-del		
BPSS0718	TetR-family regulatory protein										
BPSS0741	LysR family regulatory protein										
BPSS0878	ArsR family regulatory protein										
BPSS0880	transcription elongation factor										
BPSS0900	IclR family regulatory protein										
BPSS0912	AsnC family regulatory protein										
BPSS0929	hypothetical protein BPSS0929										
BPSS0944	LysR family regulatory protein										
BPSS0956	hypothetical protein BPSS0956										
BPSS1040	transcriptional activator protein										
BPSS1229	transcriptional regulator										
BPSS0505	AraC family regulatory protein										
BPSS0546	AraC family regulatory protein										
BPSL1748	putative LysR-family transcriptional regulator										
BPSL1752	putative MarR-family regulatory protein										
BPSL1840	LexA repressor										
BPSL1850	hypothetical protein BPSL1850										
BPSL1870	putative GntR-family regulatory protein										
BPSL1874	putative ribonuclease										
BPSL1912	putative transcriptional regulatory protein										

BPSL1919	transcription elongation factor NusA						
BPSL1923	putative transcriptional regulator						
BPSL1938	putative transcriptional regulator						
BPSL1956	putative LuxR-family regulatory protein						
BPSL1985	putative two component system, response regulator						
BPSL1990	putative transcriptional regulatory protein						
BPSL2008	putative LysR-family transcriptional regulator						
BPSL2024	putative two component regulatory system, response regulator						
BPSL2032	putative LysR-family transcriptional regulator						
BPSL2065	putative two component system, response regulator						
BPSL2068	putative two component system, response regulator						
BPSL2094	oxidative stress related two component system, transcriptional regulator						
BPSL2121	putative GntR-family transcriptional regulator						
BPSL2187	putative ATP-dependent RNA helicase						
BPSL2190	putative LysR-family regulatory protein						
BPSL2290	hypothetical protein BPSL2290						
BPSL2314	putative response regulator protein						
BPSL2343	putative histidine utilization repressor		**missense**	**missense**	**missense**		
BPSL2417	LysR family regulatory protein	**missense**					
BPSL2436	RNA polymerase sigma-70 factor						
BPSL2471	ArsR family regulatory protein						
BPSL2546	transcriptional activator protein						
BPSL2560	transcription elongation factor GreB						
BPSL2562	DNA-directed RNA polymerase omega subunit						
BPSL2628	transcription antitermination protein NusB						
BPSL2662	urease accessory protein						
BPSL2693	hypothetical protein BPSL2693						
BPSL2700	hypothetical protein BPSL2700						
BPSL2733	putative LysR-family transcriptional regulator		**missense**				
BPSL2746	IclR-family transcriptional regulator						
BPSL2884	hypothetical protein BPSL2884						
BPSL2895	DNA-binding protein Fis						
BPSL3142	BolA-like protein						
BPSL3187	DNA-directed RNA polymerase alpha subunit						
BPSL3221	DNA-directed RNA polymerase beta subunit					**missense**	**missense**
BPSL3238	putativeAsnC-family transcriptional regulator						
BPSL3279	putative AsnC-family transcriptional regulator						
BPSL3291	flagellar biosynthesis sigma factor FliA						
BPSL3379	MerR family regulatory protein						
BPSL3380	putative PadR transcriptional regulator	**missense**					
BPSL3423	putative AsnC-family transcriptional regulator						
BPSL3426	putative two-component response regulator						
BPSS0004	DNA-binding protein						
BPSS0008	TetR-family regulatory protein	**missense**					
BPSS0012	LysR-family regulatory protein						
BPSS0022	PadR-like family regulatory protein						
BPSS0041	LysR-family transcriptional regulator						
BPSS1325	AsnC family leucine-responsive regulatory protein						
BPSS1328	GntR-family transcriptional regulator						
BPSS1339	ArsR family regulatory protein						
BPSS1343	DNA-binding protein						
BPSS1449	TetR family transcriptional regulator						
BPSS1461	two-component response regulator						
BPSS1469	LysR family transcriptional regulator						
BPSS1471	TetR family transcriptional regulator						
BPSS1643	LysR-family transcriptional regulator						
BPSS1721	GntR-family transcriptional regulator						

BPSS1755	RNA polymerase sigma factor										
BPSS1966	LysR-family transcriptional regulator										
BPSS2127	IclR transcriptional regulator protein										
BPSS2142	MerR family transcriptional regulator										
BPSS2158	IclR family transcriptional regulator										
BPSL0137	hypothetical protein BPSL0137	Bp-V	Bp-V	Bp-V	Bp-V	Bp-V	Bp-V	Bp-V	Bp-V	Bp-V	Bp-V
BPSL0141	putative phage DNA-binding protein	Bp-V	Bp-V	Bp-V	Bp-V	Bp-V	Bp-V	Bp-V	Bp-V	Bp-V	Bp-V
BPSL0174	putative phage DNA-binding protein	Bp-V	Bp-V	Bp-V	Bp-V	Bp-V	Bp-V	Bp-V	Bp-V	Bp-V	Bp-V
BPSL0932	LysR family transcriptional regulator	Bp-V	Bp-V	Bp-V	Bp-V	Bp-V	Bp-V	Bp-V	Bp-V	Bp-V	Bp-V
BPSL0933	LysR family transcriptional regulator	Bp-V	Bp-V	Bp-V	Bp-V	Bp-V	Bp-V	Bp-V	Bp-V	Bp-V	Bp-V
BPSL0938	putative DNA-binding protein	Bp-V	Bp-V	Bp-V	Bp-V	Bp-V	Bp-V	Bp-V	Bp-V	Bp-V	Bp-V
BPSL0953	hypothetical protein BPSL0953	Bp-V	Bp-V	Bp-V	Bp-V	Bp-V	Bp-V	Bp-V	Bp-V	Bp-V	Bp-V
BPSL1114	putative ATP-dependent RNA helicase 2	Bp-V	Bp-V	Bp-V	Bp-V	Bp-V	Bp-V	Bp-V	Bp-V	Bp-V	Bp-V
BPSL1245	putative transcriptional regulator	Bp-V	Bp-V	Bp-V	Bp-V	Bp-V	Bp-V	Bp-V	Bp-V	Bp-V	Bp-V
BPSL1269	putative IclR-family transcriptional regulatory protein	Bp-V	Bp-V	Bp-V	Bp-V	Bp-V	Bp-V	Bp-V	Bp-V	Bp-V	Bp-V
BPSL1599	putative membrane linked regulatory protein	Bp-V	Bp-V	Bp-V	Bp-V	Bp-V	Bp-V	Bp-V	Bp-V	Bp-V	Bp-V
BPSL1750	putative MarR-family transcriptional regulator	Bp-V	Bp-V	Bp-V	Bp-V	Bp-V	Bp-V	Bp-V	Bp-V	Bp-V	Bp-V
BPSL2084	putative bifunctional protein	Bp-V	Bp-V	Bp-V	Bp-V	Bp-V	Bp-V	Bp-V	Bp-V	Bp-V	Bp-V
BPSL2561	guanosine-3',5'-bis(diphosphate) 3'-pyrophosphohydrolase	Bp-V	Bp-V	Bp-V	Bp-V	Bp-V	Bp-V	Bp-V	Bp-V	Bp-V	Bp-V
BPSL2582	hypothetical protein BPSL2582	Bp-V	Bp-V	Bp-V	Bp-V	Bp-V	Bp-V	Bp-V	Bp-V	Bp-V	Bp-V
BPSL2641	Sir2 family protein	Bp-V	Bp-V	Bp-V	Bp-V	Bp-V	Bp-V	Bp-V	Bp-V	Bp-V	Bp-V
BPSL2707	hypothetical protein BPSL2707	Bp-V	Bp-V	Bp-V	Bp-V	Bp-V	Bp-V	Bp-V	Bp-V	Bp-V	Bp-V
BPSL2832	heat-inducible transcription repressor	Bp-V	Bp-V	Bp-V	Bp-V	Bp-V	Bp-V	Bp-V	Bp-V	Bp-V	Bp-V
BPSL2862	hypothetical protein BPSL2862	Bp-V	Bp-V	Bp-V	Bp-V	Bp-V	Bp-V	Bp-V	Bp-V	Bp-V	Bp-V
BPSL2933	putative regulatory protein	Bp-V	Bp-V	Bp-V	Bp-V	Bp-V	Bp-V	Bp-V	Bp-V	Bp-V	Bp-V
BPSS0270	LysR-family transcriptional regulator	Bp-V	Bp-V	Bp-V	Bp-V	Bp-V	Bp-V	Bp-V	Bp-V	Bp-V	Bp-V
BPSS0273	TetR-family regulatory protein	Bp-V	Bp-V	Bp-V	Bp-V	Bp-V	Bp-V	Bp-V	Bp-V	Bp-V	Bp-V
BPSS0274	IclR-family transcriptional regulator	Bp-V	Bp-V	Bp-V	Bp-V	Bp-V	Bp-V	Bp-V	Bp-V	Bp-V	Bp-V
BPSS0290	LysR-family transcriptional regulator	Bp-V	Bp-V	Bp-V	Bp-V	Bp-V	Bp-V	Bp-V	Bp-V	Bp-V	Bp-V
BPSS0296	GntR-family transcriptional regulator	Bp-V	Bp-V	Bp-V	Bp-V	Bp-V	Bp-V	Bp-V	Bp-V	Bp-V	Bp-V
BPSS0312	LuxR-family transcriptional regulator	Bp-V	Bp-V	Bp-V	Bp-V	Bp-V	Bp-V	Bp-V	Bp-V	Bp-V	Bp-V
BPSS0383	DNA-binding protein	Bp-V	Bp-V	Bp-V	Bp-V	Bp-V	Bp-V	Bp-V	Bp-V	Bp-V	Bp-V
BPSS1061	DNA binding protein	Bp-V	Bp-V	Bp-V	Bp-V	Bp-V	Bp-V	Bp-V	Bp-V	Bp-V	Bp-V
BPSS1117	hypothetical protein BPSS1117	Bp-V	Bp-V	Bp-V	Bp-V	Bp-V	Bp-V	Bp-V	Bp-V	Bp-V	Bp-V
BPSS1131	LuxR family transcriptional regulator	Bp-V	Bp-V	Bp-V	Bp-V	Bp-V	Bp-V	Bp-V	Bp-V	Bp-V	Bp-V
BPSS1134	PadR-like family regulatory protein	Bp-V	Bp-V	Bp-V	Bp-V	Bp-V	Bp-V	Bp-V	Bp-V	Bp-V	Bp-V
BPSS1161	response regulator protein	Bp-V	Bp-V	Bp-V	Bp-V	Bp-V	Bp-V	Bp-V	Bp-V	Bp-V	Bp-V
BPSS1176	N-acyl-homoserine lactone dependent regulatory protein	Bp-V	Bp-V	Bp-V	Bp-V	Bp-V	Bp-V	Bp-V	Bp-V	Bp-V	Bp-V
BPSS1200	GntR family regulatory protein	Bp-V	Bp-V	Bp-V	Bp-V	Bp-V	Bp-V	Bp-V	Bp-V	Bp-V	Bp-V
BPSS1214	transcriptional regulator	Bp-V	Bp-V	Bp-V	Bp-V	Bp-V	Bp-V	Bp-V	Bp-V	Bp-V	Bp-V
BPSS1467	aminotransferase protein	Bp-V	Bp-V	Bp-V	Bp-V	Bp-V	Bp-V	Bp-V	Bp-V	Bp-V	Bp-V
BPSS1474	DeoR family transcriptional regulator	Bp-V	Bp-V	Bp-V	Bp-V	Bp-V	Bp-V	Bp-V	Bp-V	Bp-V	Bp-V
BPSS1494	two-component response regulator protein	Bp-V	Bp-V	Bp-V	Bp-V	Bp-V	Bp-V	Bp-V	Bp-V	Bp-V	Bp-V
BPSS1792	LysR-family transcriptional regulator	Bp-V	Bp-V	Bp-V	Bp-V	Bp-V	Bp-V	Bp-V	Bp-V	Bp-V	Bp-V
BPSS2115	LysR family transcriptional regulator	Bp-V	Bp-V	Bp-V	Bp-V	Bp-V	Bp-V	Bp-V	Bp-V	Bp-V	Bp-V
BPSS2118	ArsR family transcriptional regulator	Bp-V	Bp-V	Bp-V	Bp-V	Bp-V	Bp-V	Bp-V	Bp-V	Bp-V	Bp-V
BPSS2132	sigma-54 dependent transcriptional regulator	Bp-V	Bp-V	Bp-V	Bp-V	Bp-V	Bp-V	Bp-V	Bp-V	Bp-V	Bp-V
BPSS2291	GerE family regulatory protein	Bp-V	Bp-V	Bp-V	Bp-V	Bp-V	Bp-V	Bp-V	Bp-V	Bp-V	Bp-V
BPSS0887	N-acylhomoserine lactone dependent regulatory protein	Bp-V	Bp-V	Bp-V	Bp-V	Bp-V	Bp-V	Bp-V	Bp-V	Bp-V	Bp-V
BPSL3083	putative TetR-family transcriptional regulator	missense	missense	missense	missense	missense	missense	missense	missense	missense	missense
BPSL3220	DNA-directed RNA polymerase beta' subunit	missense	missense	missense	missense	missense	missense	missense	missense	missense	missense
BPSL3226	transcription antitermination protein NusG	missense	missense	missense	missense	missense	missense	missense	missense	missense	missense
BPSL3236	putative TetR-family transcriptional regulator	missense	missense	missense	missense	missense	missense	missense	missense	missense	missense
BPSL3405	chromosome partitioning protein ParB	missense	missense	missense	missense	missense	missense	missense	missense	missense	missense
BPSL3421	putative LysR-family transcriptional regulator	missense	missense	missense	missense	missense	missense	missense	missense	missense	missense
BPSL3431	putative MarR-family transcriptional regulator	missense	missense	missense	missense	missense	missense	missense	missense	missense	missense
BPSS0057	RNA polymerase sigma-70 factor	missense	missense	missense	missense	missense	missense	missense	missense	missense	missense

BPSS0059	hypothetical protein BPSS0059	missense	missense	missense	missense	missense	missense	missense	missense	missense	missense
BPSS0149	LysR-family transcriptional regulator	missense	missense	missense	missense	missense	missense	missense	missense	missense	missense
BPSS0155	LysR-family transcriptional regulator	missense	missense	missense	missense	missense	missense	missense	missense	missense	missense
BPSS0166	LysR family regulatory protein	missense	missense	missense	missense	missense	missense	missense	missense	missense	missense
BPSS0187	LysR-family transcriptional regulator	missense	missense	missense	missense	missense	missense	missense	missense	missense	missense
BPSS0199	LysR-family transcriptional regulator	missense	missense	missense	missense	missense	missense	missense	missense	missense	missense
BPSS0205	sigma interaction-related Fis-family transcriptional regulator	missense	missense	missense	missense	missense	missense	missense	missense	missense	missense
BPSS0217	LysR-family transcriptional regulator	missense	missense	missense	missense	missense	missense	missense	missense	missense	missense
BPSS0246	LysR-family transcriptional regulator	missense	missense	missense	missense	missense	missense	missense	missense	missense	missense
BPSS0253	DNA-binding protein	missense	missense	missense	missense	missense	missense	missense	missense	missense	missense
BPSS0544	LacI family regulatory protein	missense	missense	missense	missense	missense	missense	missense	missense	missense	missense
BPSS0559	LysR family regulatory protein	missense	missense	missense	missense	missense	missense	missense	missense	missense	missense
BPSS0565	AraC family regulatory protein	missense	missense	missense	missense	missense	missense	missense	missense	missense	missense
BPSS0726	GntR family regulatory protein	missense	missense	missense	missense	missense	missense	missense	missense	missense	missense
BPSS0861	LysR family regulatory protein	missense	missense	missense	missense	missense	missense	missense	missense	missense	missense
BPSS0867	GerE family regulatory protein	missense	missense	missense	missense	missense	missense	missense	missense	missense	missense
BPSS0892	IclR family regulatory protein	missense	missense	missense	missense	missense	missense	missense	missense	missense	missense
BPSS0918	DNA-binding protein	missense	missense	missense	missense	missense	missense	missense	missense	missense	missense
BPSS0935	hypothetical protein BPSS0935	missense	missense	missense	missense	missense	missense	missense	missense	missense	missense
BPSS0948	LysR family regulatory protein	missense	missense	missense	missense	missense	missense	missense	missense	missense	missense
BPSS0970	GntR family regulatory protein	missense	missense	missense	missense	missense	missense	missense	missense	missense	missense
BPSS1013	hypothetical protein BPSS1013	missense	missense	missense	missense	missense	missense	missense	missense	missense	missense
BPSS1353	transcriptional regulator BetI	missense	missense	missense	missense	missense					
BPSS1640	LysR-family transcriptional regulator	missense	missense	missense	missense	missense	missense	missense	missense	missense	missense
BPSS1648	probable two-component response regulator	missense	missense	missense	missense	missense	missense	missense	missense	missense	missense
BPSS2102	protein kinase	missense	missense	missense	missense	missense	missense	missense	missense	missense	missense
BPSS2117	LysR family transcriptional regulator	missense	missense	missense	missense	missense	missense	missense	missense	missense	missense
BPSS2124	LysR family transcriptional regulator	missense	missense	missense	missense	missense	missense	missense	missense	missense	missense
BPSS2146	LysR family transcriptional regulator	missense	missense	missense	missense	missense	missense	missense	missense	missense	missense
BPSS2345	sensory kinase protein	missense	missense	missense	missense	missense	missense	missense	missense	missense	missense
BPSS2347	RNA polymerase sigma-70 factor	missense	missense	missense	missense	missense	missense	missense	missense	missense	missense
BPSL0021	MarR family protein	missense	missense	missense	missense	missense					
BPSL0056	PadR-like family transcriptional regulator	missense	missense	missense	missense	missense	missense	missense	missense	missense	missense
BPSL0103	RNA polymerase sigma factor	missense	missense	missense	missense	missense	missense	missense	missense	missense	missense
BPSL0107	LysR family transcription regulatory protein	missense	missense	missense	missense	missense	missense	missense	missense	missense	missense
BPSL0198	TetR family transcription regulatory protein	missense	missense	missense	missense	missense	missense	missense	missense	missense	missense
BPSL0397	Bordetella pertussis Bvg accessory factor family protein	missense	missense	missense	missense						
BPSL0495	GntR family regulatory protein	missense	missense	missense	missense	missense	missense	missense	missense	missense	missense
BPSL0504	RNA polymerase sigma factor	missense	missense	missense	missense	missense	missense	missense	missense	missense	missense
BPSL0597	putative protein kinase	missense	missense	missense	missense	missense	missense	missense	missense	missense	missense
BPSL0629	RpiR family regulatory protein	missense	missense	missense	missense						
BPSL0675	putative LysR family transcriptional regulator	missense	missense	missense	missense	missense	missense	missense	missense	missense	missense
BPSL0775	response regulator transcription regulatory protein	missense	missense	missense	missense	missense	missense	missense	missense	missense	missense
BPSL0812	TetR family regulatory protein	missense	missense	missense	missense	missense	missense	missense	missense	missense	missense
BPSL0819	AraC family transcriptional regulator	missense	missense	missense	missense	missense	missense	missense	missense	missense	missense
BPSL0838	putative transcriptional regulator	missense	missense	missense	missense						
BPSL0841	putative LysR family transcriptional regulator	missense	missense	missense	missense	missense	missense	missense	missense	missense	missense
BPSL1036	putative two-component system, response regulator	missense	missense	missense	missense	missense	missense	missense	missense	missense	missense
BPSL1072	putative LysR-family transcriptional regulatory protein	missense	missense	missense	missense	missense	missense	missense	missense	missense	missense
BPSL1074	putative LysR family transcriptional regulator	missense	missense	missense	missense	missense	missense	missense	missense	missense	missense
BPSL1083	putative aminotransferase	missense	missense	missense	missense	missense	missense	missense	missense	missense	missense
BPSL1086	putative GntR family regulatory protein	missense	missense	missense	missense	missense	missense	missense	missense	missense	missense
BPSL1188	putative TetR-family transcriptional regulator	missense	missense	missense	missense	missense				missense	missense
BPSL1195	RNA polymerase sigma-70 factor	missense	missense	missense	missense	missense	missense	missense	missense	missense	missense
BPSL1309	hypothetical protein BPSL1309	missense	missense	missense	missense	missense	missense	missense	missense	missense	missense
BPSL1310	putative GntR-family regulatory protein	missense	missense	missense	missense	missense				missense	missense
BPSL1311	putative AsnC-family regulatory protein	missense	missense	missense	missense	missense	missense	missense	missense	missense	missense

BPSL1321	putative GntR-family transcriptional regulator	missense	missense	missense	missense	missense	missense	missense	missense	missense	missense
BPSL1364	phosphate regulon two-component system response regulat	missense	missense	missense	missense	missense	missense	missense	missense	missense	missense
BPSL1398	putative GerE-family transcriptional regulator	missense	missense	missense	missense	missense	missense	missense	missense	missense	missense
BPSL1456	RNA polymerase sigma-70 factor	missense	missense	missense	missense	missense	missense	missense	missense	missense	missense
BPSL1457	hypothetical protein BPSL1457	missense	missense	missense	missense	missense	missense	missense	missense	missense	missense
BPSL1494	putative MerR-family transcriptional regulator	missense	missense	missense	missense	missense	missense	missense	missense	missense	missense
BPSL1495	transcription termination factor Rho	missense	missense	missense	missense	missense	missense	missense	missense	missense	missense
BPSL1527	putative transcription accessory protein	missense	missense	missense	missense	missense	missense	missense	missense	missense	missense
BPSL1787	RNA polymerase sigma-70 factor	missense	missense	missense	missense						
BPSL1882	putative TetR-family regulatory protein	missense	missense	missense	missense	missense	missense	missense	missense	missense	missense
BPSL1887	putative sigma-54 related transcriptional regulatory protein	missense	missense	missense	missense	missense	missense	missense	missense	missense	missense
BPSL1946	GTP pyrophosphokinase	missense	missense	missense	missense	missense	missense	missense	missense	missense	missense
BPSL1950	putative LysR-family transcriptional regulator	missense	missense	missense	missense	missense	missense	missense	missense	missense	missense
BPSL1971	putative LysR-family transcriptional regulator	missense	missense	missense	missense	missense	missense	missense	missense	missense	missense
BPSL2069	putative LysR-family transcriptional regulator	missense	missense	missense	missense	missense	missense	missense	missense	missense	missense
BPSL2083	putative GntR-family transcriptional regulator	missense	missense	missense	missense	missense	missense	missense	missense	missense	missense
BPSL2100	transcription-repair coupling factor	missense	missense	missense	missense	missense	missense	missense	missense	missense	missense
BPSL2105	hypothetical protein BPSL2105	missense	missense	missense	missense	missense	missense	missense	missense	missense	missense
BPSL2295	IclR regulatory protein	missense	missense	missense	missense	missense	missense	missense	missense	missense	missense
BPSL2330	LysR family regulatory protein	missense	missense	missense	missense	missense	missense	missense	missense	missense	missense
BPSL2347	LuxR family regulatory protein	missense	missense	missense	missense	missense	missense	missense	missense	missense	missense
BPSL2376	putative DNA-binding protein	missense	missense	missense	missense	missense	missense	missense	missense	missense	missense
BPSL2391	AraC family regulatory protein	missense	missense	missense	missense	missense	missense	missense	missense	missense	missense
BPSL2429	ribonuclease III	missense	missense	missense	missense	missense	missense	missense	missense	missense	missense
BPSL2496	leucine-responsive regulatory protein	missense	missense	missense	missense	missense	missense	missense	missense	missense	missense
BPSL2614	glucokinase	missense	missense	missense	missense	missense	missense	missense	missense	missense	missense
BPSL2709	putative DNA-binding protein	missense	missense	missense	missense	missense	missense	missense	missense	missense	missense
BPSL2720	LysR-family regultor	missense	missense	missense	missense	missense					
BPSL2858	putative GntR family transcriptional regulator	missense	missense	missense	missense	missense	missense	missense	missense	missense	missense
BPSL2866	oxidative stress regulatory protein	missense	missense	missense	missense	missense	missense	missense	missense	missense	missense
BPSL2867	putative ATP-dependent DNA helicase	missense	missense	missense	missense	missense	missense	missense	missense	missense	missense
BPSS1710	AsnC-family transcriptional regulator			missense							
BPSL0023	LysR family transcription regulatory protein					missense					
BPSL0033	MerR family transcription regulatory protein										
BPSL0040	response regulator protein										
BPSL0117	LysR family transcription regulatory protein										
BPSL0202	response regulator protein										
BPSL0239	response regulator protein										
BPSL0260	hypothetical protein BPSL0260										
BPSL0322	LacI family regulatory protein										
BPSL0378	MarR family regulatory protein										
BPSL0389	IclR family regulatory protein										
BPSL0411	LysR family regulatory protein										
BPSL0494	LysR family regulatory protein										
BPSL0533	DNA-directed RNA polymerase subunit N										
BPSL0626	MarR family regulatory protein										
BPSL0647	TetR family regulatory protein										
BPSL0691	putative DeoR family glycerol-3-phosphate regulon repressor										
BPSL0695	putative ATP-dependent RNA helicase 1										
BPSL0704	putative two-component response regulator protein										
BPSL0806	response regulator protein										
BPSL0835	LysR family transcriptional regulator										
BPSL0849	AsnC family transcriptional regulator										
BPSL0955	cys regulon transcriptional activator										
BPSL1105	putative TetR family regulatory protein										
BPSL1130	putative sigma factor										missense
BPSL1175	two-component system, regulatory protein KdpE										

BPSL1272	hypothetical protein BPSL1272
BPSL1275	putative AsnC-family transcriptional regulator
BPSL1304	putative LacI-family transcriptional regulator
BPSL1326	putative LysR-family transcriptional regulator
BPSL1352	transcription elongation factor GreA
BPSL1400	putative MarR-family transcriptional regulator
BPSL1483	putative DNA-binding protein
BPSL1487	putative MerR-family transcriptional regulator
BPSL1505	RNA polymerase sigma factor
BPSL1530	putative ATP-dependent DNA helicase-related protein
BPSS2350	partitioning protein ParB
