# Supplementary material for: The Early Stage of Bacterial Genome-Reductive Evolution in the Host
Source: PLoS Pathog. 2010 May 27;6(5):e1000922. doi: 10.1371/journal.ppat.1000922 (PMC2877748; doi:10.1371/journal.ppat.1000922)
Supplement: Table S6 — Comparison of the upstream regions of genes containing potential promoters. (2.92 MB PDF) [file ppat.1000922.s008.pdf]

Table S6. Comparison of the upstream regions of genes containing potential promoters.

locus	protein	start pos	end pos	strand	aa len	gi	geneID	name	COGs	Upstream sequence	romosor	qlen	qstart	qEnd	ngth mat	subject	sLen	sStart	sEnd	iden	score	evalue	strand
BPSL0002	hypothetical protein BPSL0002	1161	2375	-	404	53717641	3094484	-	COG0025P	CAACGAACGACGGGGCAACCCCTC	1	103	1	103	100	Bm_ATCC23344_chr1	3510148	3007138	3007240	100	515	1.20E-18	+
BPSL0003	hypothetical protein BPSL0003	3114	5168	-	684	53717642	3092684	-	COG0665E, COG4121S	GCGAATTTTGTCGAGTTAGATGCGT	1	103	1	103	100	Bm_ATCC23344_chr1	3510148	3004345	3004447	100	515	1.20E-18	+
BPSL0004	DNA-binding protein HU-alpha	5488	5766	+	92	53717643	3091972	hupA	NULL	GCGCCGTGCCGTTGCCACACACTA	1	103	103	1	100	Bm_ATCC23344_chr1	3510148	3004123	3004225	100	515	1.20E-18	-
BPSL0005	putative cobalamin synthesis protein/P47K	5869	7209	-	446	53717644	3092685	-	COG0523R	CGGCTTTCCCGCCGTCCGTTCGCG	1	103	1	103	100	Bm_ATCC23344_chr1	3510148	2878393	2878495	100	515	1.20E-18	+
BPSL0006	hypothetical protein BPSL0006	7528	8013	-	161	53717645	3092172	-	COG0741M	AGGTATGATACGGGCGGCCGGAG	1	103	1	103	100	Bm_ATCC23344_chr1	3510148	2877590	2877692	100	515	1.20E-18	+
BPSL0007	general secretory pathway protein D	8395	10668	+	757	53717646	3091427	gspD	COG1450NU	GATATTTCGGACATGAAAAACTGCT	1	103	103	1	100	Bm_ATCC23344_chr1	3510148	2877307	2877409	100	515	1.20E-18	-
BPSL0010	putative general secretory pathway protein	13452	13862	-	136	53717649	3092040	gspC	NULL	CGCGGCGCGAGCCGCGCGGCGTG	1	103	1	103	100	Bm_ATCC23344_chr1	3510148	2871742	2871844	100	515	1.20E-18	+
BPSL0011	general secretory pathway protein G	14010	14462	+	150	53717650	3092989	gspG	COG2165NU	CGGAGCAAGTCGCGCCTTACAATG	1	103	103	1	100	Bm_ATCC23344_chr1	3510148	2871692	2871794	100	515	1.20E-18	-
BPSL0012	general secretory pathway protein H	14617	15096	+	159	53717651	3092990	gspH	COG2165NU	ACCCGGCACGATTTTCGTTTGCGAT	1	103	103	1	100	Bm_ATCC23344_chr1	3510148	2871085	2871187	98	497	7.60E-18	-
BPSL0015	general secretory pathway protein K	16391	17461	+	356	53717654	3094007	gspK	COG3156U	CGCGCAGCCGGCGCCGGCGCGTTC	1	103	103	1	100	Bm_ATCC23344_chr1	3510148	2869311	2869413	98	497	7.60E-18	-
BPSL0019	outer membrane efflux lipoprotein	20480	22135	-	551	53717658	3092173	-	COG1538MU	CCCGCACCCGTCAGTTACCATTTCG	1	103	1	103	100	Bm_ATCC23344_chr1	3510148	2863368	2863470	100	515	1.20E-18	+
BPSL0020	hypothetical protein BPSL0020	22357	22629	-	90	53717659	3091507	-	NULL	AGTCAATGAGTTACGCGCTTAATGC	1	103	1	103	100	Bm_ATCC23344_chr1	3510148	2862865	2862967	100	515	1.20E-18	+
BPSL0021	MarR family protein	22828	23319	+	163	53717660	3091508	-	COG1846K	CTGAAATAATATTGACTATGCAATA	1	103	103	1	100	Bm_ATCC23344_chr1	3510148	2862764	2862866	100	515	1.20E-18	-
BPSL0022	putative transporter protein	23466	25028	+	520	53717661	3091830	-	NULL	TATTTATCTGTCTGGGCAGAAGCTG	1	103	103	1	100	Bm_ATCC23344_chr1	3510148	2862125	2862227	100	515	1.20E-18	-
BPSL0023	LysR family transcription regulatory protein	25088	26011	-	307	53717662	3091831	-	COG0583K	AGTATAGGATTCACGCGTCCATAA	1	103	1	103	100	Bm_ATCC23344_chr1	3510148	2859482	2859584	100	515	1.20E-18	+
BPSL0024	LrgA family protein	26223	26621	+	132	53717663	3093775	-	COG1380R	GTAAAGCAAGGCATTTCGGGAGCA	1	103	103	1	100	Bm_ATCC23344_chr1	3510148	2859368	2859470	99	506	3.00E-18	-
BPSL0025	hypothetical protein BPSL0025	26690	27412	+	240	53717664	3093776	-	NULL	CGTGGCACGGTTTCCCCTTTCCCCA	1	71	71	1	100	Bm_ATCC23344_chr1	3510148	2858901	2858971	100	355	2.00E-11	-
BPSL0029	flagellar protein	30426	30920	+	164	53717668	3093248	fliO	COG3190N	CCGGGCAGCCGAACGCGCGCCGC	1	103	103	1	100	Bm_ATCC23344_chr1	3510148	2855166	2855268	100	515	1.20E-18	-
BPSL0032	flagellar biosynthetic protein	32169	32951	+	260	53717671	3091494	fliR	COG1684NU	GCCCGCCGCGCCGGGCGTCGCATC	1	103	103	1	100	Bm_ATCC23344_chr1	3510148	2785436	2785538	99	506	3.00E-18	-
BPSL0033	MerR family transcription regulatory protein	33271	33699	-	142	53717672	3091890	-	COG0789K	CGTCGTTCTCCAGTGCGAATGCTGG	1	84	1	84	100	Bm_ATCC23344_chr1	3510148	2783827	2783910	100	420	2.30E-14	+
BPSL0034	hypothetical protein BPSL0034	33781	34656	+	291	53717673	3092235	-	COG0500QR	GCGCTTGACCTCAAGTGGACTTGA	1	84	84	1	100	Bm_ATCC23344_chr1	3510148	2783824	2783907	100	420	2.30E-14	-
BPSL0036	putative transport protein	35328	36341	+	337	53717675	3094577	-	COG0715P	AAGCCGCGCCCGCGAAAGGTTGCG	1	103	103	1	100	Bm_ATCC23344_chr1	3510148	2782277	2782379	100	515	1.20E-18	-
BPSL0037	putative ATP-binding ABC transporter protein	36425	37222	+	265	53717676	3094578	-	COG1116P	CAGCGCCGCGAGTTTTGAACCAGG	1	86	86	1	100	Bm_ATCC23344_chr1	3510148	2781180	2781265	100	430	8.00E-15	-
BPSL0039	sensor kinase protein	38176	39555	+	459	53717678	3094682	-	COG0642T	AGACGATGCATCAACGAAACGGCG	1	80	80	1	100	Bm_ATCC23344_chr1	3510148	2779429	2779508	100	400	1.80E-13	-
BPSL0041	outer membrane porin lipoprotein precursor	40566	41693	+	375	53717680	3093987	-	COG3203M	CGATGGCGTGGCCGTTCGACGCGC	1	103	103	1	100	Bm_ATCC23344_chr1	3510148	2777029	2777131	100	515	1.20E-18	-
BPSL0042	type III restriction-modification system methylase	42169	44181	+	670	53717681	3092538	-	COG2189L	GCGCGTGACGGATCGCTAGCGTTT	1	103	103	1	100	Bm_ATCC23344_chr1	3510148	2775426	2775528	100	515	1.20E-18	-
BPSL0044	putative outer membrane porin protein	47789	48889	+	366	53717683	3093858	-	COG3203M	GAAAGATGCGCGCCGCACGCTGCT	1	103	103	1	100	Bm_ATCC23344_chr1	3510148	2769806	2769908	100	515	1.20E-18	-
BPSL0045	AraC family regulatory protein	49289	50248	+	319	53717684	3093859	-	NULL	ACCGGATTCAGTTCGCGCCAAATAT	1	103	103	1	100	Bm_ATCC23344_chr1	3510148	2768306	2768408	99	506	3.00E-18	-
BPSL0046	ABC transport system ATP-binding protein	50371	51102	-	243	53717685	3092441	-	COG0410E	GCCGCCCGGCACGCGCGGCGATGC	1	103	1	103	100	Bm_ATCC23344_chr1	3510148	2766395	2766497	100	515	1.20E-18	+
BPSL0051	putative phenylacetaldehyde dehydrogenase	55599	57104	-	501	53717690	3094742	-	COG1012C	GCGCCTTCGTCCGTTCGTTCAACCA	1	58	1	58	100	Bm_ATCC23344_chr1	3510148	2760438	2760495	98	281	4.30E-08	+
BPSL0054	putative short chain dehydrogenase	58418	59215	-	265	53717693	3093902	-	COG1028IQR	GCGCCGCGCGCTCGCCGTCGCGCC	1	87	1	87	100	Bm_ATCC23344_chr1	3510148	2758298	2758384	100	435	4.80E-15	+
BPSL0056	PadR-like family transcriptional regulator	61697	62038	+	113	53717695	3093581	-	COG1695K	GTGTTGCTATAGTCCGCCCATGTTC	1	103	103	1	100	Bm_ATCC23344_chr1	3510148	2755918	2756020	99	506	3.00E-18	-
BPSL0073	DNA gyrase subunit B	81428	83896	-	822	53717712	3091483	-	COG0187L	GCCGGCCCCCGAAAGGGGCGAAA	1	103	1	103	100	Bm_ATCC23344_chr1	3510148	2981	3083	100	515	1.20E-18	+
BPSL0074	DNA polymerase III subunit beta	84085	85188	-	367	53717713	3092204	dnaN	COG0592L	GGCACAATACTGGTTTGACCGCCC	1	103	1	103	100	Bm_ATCC23344_chr1	3510148	1689	1791	100	515	1.20E-18	+
BPSL0075	chromosomal replication initiation protein	85351	86952	-	533	53717714	3093573	dnaA	COG0593L	GACCGAGGCACGTCCGCACGGCCG	1	103	1	103	100	Bm_ATCC23344_chr1	3510148	3510073	27	100	380	1.50E-12	+
BPSL0075a	50S ribosomal protein L34	87398	87532	+	44	53717715	3094113	-	NULL	ACCAATCCTGTTATTTTCGGCGATT	1	103	103	1	100	Bm_ATCC23344_chr1	3510148	3509725	3509827	100	515	1.20E-18	-
BPSL0076	ribonuclease P protein component	87606	88016	+	136	53717716	3092170	rnpA	COG0594J	TAAGGGCGGGCCGCGCGCCGTGTC	1	79	79	1	100	Bm_ATCC23344_chr1	3510148	3509517	3509595	100	395	3.10E-13	-
BPSL0077	hypothetical protein BPSL0077	88096	88365	+	89	53717717	3094114	-	NULL	CGCGCCGACGCCCGGCGCGGACG	1	82	82	1	100	Bm_ATCC23344_chr1	3510148	3509027	3509108	97	392	4.20E-13	-
BPSL0079	hypothetical protein BPSL0079	90207	90668	-	153	53717719	3093553	-	NULL	CTGATTGGTTGTCTTCCTGGCAGCG	1	103	1	103	100	Bm_ATCC23344_chr1	3510148	3506453	3506555	98	497	7.60E-18	+
BPSL0080	tRNA modification GTPase	90964	92367	+	467	53717720	3093677	trmE	COG0486R	TGGTCTGCGCAATACTATTGCGTGG	1	103	103	1	100	Bm_ATCC23344_chr1	3510148	3506159	3506261	99	506	3.00E-18	-
BPSL0098	hypothetical protein BPSL0098	108943	109497	+	184	53717738	3093577	-	NULL	GCCGGCGGCGGCAATTCCGCGCGA	1	103	1	103	100	Bm_ATCC23344_chr1	3510148	125192	125294	100	515	1.20E-18	+
BPSL0102	glutamate--cysteine ligase	112703	114304	+	533	53717742	3094155	-	COG2918H	TTCGGCGAGATGTTAAAGTGCCCG	1	103	1	103	100	Bm_ATCC23344_chr1	3510148	128967	129070	98	496	8.40E-18	+
BPSL0103	RNA polymerase sigma factor	114323	115771	-	482	53717743	3093184	-	COG0568K	TAAGGCTCATCGGCGTGGACGGGC	1	103	103	1	100	Bm_ATCC23344_chr1	3510148	132134	132236	98	497	7.60E-18	-
BPSL0104	hypothetical protein BPSL0104	116034	116582	-	182	53717744	3093185	-	NULL	CAGCCCCCGTAAGGCCCGCCGCAG	1	103	103	1	100	Bm_ATCC23344_chr1	3510148	132946	133048	99	506	3.00E-18	-
BPSL0105	hypothetical protein BPSL0105	116974	117090	+	38	53717745	3094117	-	NULL	GCACCGCCGGCGCGCGTGCCGTTC	1	103	1	103	100	Bm_ATCC23344_chr1	3510148	133240	133342	99	506	3.00E-18	+
BPSL0106	hypothetical protein BPSL0106	117179	117748	-	189	53717746	3094118	-	COG3678UNTP	ACGGTCAAGCGGCGCTTCTCCACG	1	103	103	1	100	Bm_ATCC23344_chr1	3510148	134108	134210	97	488	1.90E-17	-
BPSL0107	LysR family transcription regulatory protein	117851	118819	-	322	53717747	3092142	-	COG0583K	CGCGCGCTGCATCGGCGAACGTTC	1	103	103	1	100	Bm_ATCC23344_chr1	3510148	135179	135281	100	515	1.20E-18	-
BPSL0108	putative extracellular solute-binding lipoprotein	118962	120056	+	364	53717748	3092143	-	COG0687E	AATCGATTTTTTTCTCTACTTACGAT	1	103	1	103	100	Bm_ATCC23344_chr1	3510148	135224	135326	100	515	1.20E-18	+
BPSL0114	hypothetical protein BPSL0114	127678	128082	+	134	53717755	3091909	-	COG2050Q	TCCGAACGCGCACGATCCGTCGCA	1	103	1	103	100	Bm_ATCC23344_chr1	3510148	144182	144284	100	515	1.20E-18	+
BPSL0116	D-isomer specific 2-hydroxyacid dehydrogenase	129110	130126	-	338	53717757	3092423	-	COG0111HE	AGGATCATTACGGCCCTCAAACCG	1	103	103	1	100	Bm_ATCC23344_chr1	3510148	146728	146830	99	506	3.00E-18	-
BPSL0118	DNA topoisomerase III	131843	134452	-	869	53717759	3092351	-	COG0550L	CTTTTTTGCTCACGCCGGCCGCCAT	1	103	103	1	100	Bm_ATCC23344_chr1	3510148	151064	151166	100	515	1.20E-18	-
BPSL0119	putative thioredoxin	134663	135034	-	123	53717760	3093728	-	COG0526OC	GCGTCCCGCCGGGCGTTCGCCCGA	1	62	62	1	100	Bm_ATCC23344_chr1	3510148	151646	151707	98	301	5.40E-09	-
BPSL0120	SMF family protein	135094	136284	-	396	53717761	3093729	-	COG0758LU	CAGCCCGCAGGCCGCGCTTTTCGA	1	103	103	1	100	Bm_ATCC23344_chr1	3510148	152830	152932	100	515	1.20E-18	-
BPSL0121	peptide deformylase	136513	137016	+	167	53717762	3093438	def	NULL	GGCGCGCGTCGTCGAGTCCATCTT	1	103	1	103	100	Bm_ATCC23344_chr1	3510148	153027	153129	100	515	1.20E-18	+
BPSL0123	putative LysE type translocator protein	138152	138787	+	211	53717764	3093926	-	COG1280E	TCATCGGAATCGCGCGCGCCGCGC	1	103	1	103	100	Bm_ATCC23344_chr1	3510148	154666	154768	100	515	1.20E-18	+
BPSL0124	heat shock protein HtpX	138897	139754	+	285	53717765	3093927	-	COG0501O	TTTCTCGAAACGTTCTTATCTAACAG	1	103	1	103	100	Bm_ATCC23344_chr1	3510148	155411	155513	100	515	1.20E-18	+
BPSL0125	NOL1/NOP2/Sun family protein	140228	141637	+	469	53717766	3092372	sun	COG0144J	GCGCGCTACAATATCGCGCTGACC	1	103	1	103	100	Bm_ATCC23344_chr1	3510148	156646	156748	99	506	3.00E-18	+
BPSL0177	hypothetical protein BPSL0177	182072	182806	+	244	53717819	3093810	-	COG0603R	CCGTTTCGGGCCGCGCGCACCTGC	1	103	1	103	100	Bm_ATCC23344_chr1	3510148	162204	162306	100	515	1.20E-18	+
BPSL0178	hypothetical protein BPSL0178	182900	183532	+	210	53717820	3093742	-	COG0602O	CGCGCGGCTGGCACGGCGCACGC	1	96	1	96	100	Bm_ATCC23344_chr1	3510148	163039	163134	100	480	4.40E-17	+
BPSL0180	HpcH/HpaI aldolase family protein	184424	185209	+	261	53717822	3094620	-	COG3836G	TCGCGCGCGCCTGTTACCGCGCAG	1	103	1	103	100	Bm_ATCC23344_chr1	3510148	164558	164660	99	506	3.00E-18	+
BPSL0183	penicillin-binding protein	187471	189927	-	818	53717825	3091495	mrdA	COG0768M	GCCGCGCGGGCGGCCCGCCCGGC	1	103	103	1	100	Bm_ATCC23344_chr1	3510148	170196	170298	98	497	7.60E-18	-
BPSL0185	putative rod shape-determining protein	190620	191693	-	357	53717827	3094617	-	COG1792M	GCGCTCGCGCGGCGTGGCAGGCTC	1	103	103	1	100	Bm_ATCC23344_chr1	3510148	171962	172064	99	506	3.00E-18	-
BPSL0186	putative rod shape-determining protein	191813	192856	-	347	53717828	3091496	mreB	COG1077D	GAGCGGCCGCTCCCGCCCGATCCG	1	103	103	1	100	Bm_ATCC23344_chr1	3510148	173125	173228	99	505	3.30E-18	-
BPSL0187	aspartyl/glutamyl-tRNA amidotransferase subunit C	193227	193520	+	97	53717829	3092492	gatC	COG0721J	GCCCCCGATTTTTGAGGGTTCGATC	1	103	1	103	100	Bm_ATCC23344_chr1	3510148	173399	173501	100	515	1.20E-18	+
BPSL0188	glutamyl-tRNA amidotransferase subunit A	193633	195123	+	496	53717830	3094691	gatA	NULL	GCGCGCGGCCGCGCAAGCGGCTC	1	103	1	103	100	Bm_ATCC23344_chr1	3510148	173805	173907	100	515	1.20E-18	+
BPSL0192	NADP-dependent alcohol dehydrogenase	198655	199719	-	354	53717834	3094049	adhC	COG1064R	CAGGTATTCCGGGCATCCCGCCGC	1	103	103	1	100	Bm_ATCC23344_chr1	3510148	3349660	3349760	98	485	2.60E-17	-
BPSL0193	AraC family transcription regulatory protein	199931	200875	+	314	53717835	3093871	-	NULL	GGTTTGCCTATTTCTCTTGAACTTGC	1	103	23	103	78	Bm_ATCC23344_chr1	3510148	3351106	3351187	91	341	8.40E-11	+
BPSL0194	hypothetical protein BPSL0194	201249	202193	-	314	53717836	3093872	-	COG0501O	GCTGCGCGTCGCCCGCGCCGCGCT	1	Not found											
BPSL0197	homoserine O-acetyltransferase	204227	205372	-	381	53717839	3094355	-	COG2021E	GGGTTCACTACAAATACGGCTAAA	1	103	103	1	100	Bm_ATCC23344_chr1	3510148	3356606	3356709	99	505	3.30E-18	-
BPSL0200	acetylglutamate kinase	207177	208076	-	299	53717842	3092545	argB	COG0548E	GCCCGCCGCCCATCGTCCGAACGG	1	103	103	1	100	Bm_ATCC23344_chr1	3510148	3359314	3359416	100	515	1.20E-18	-

BPSL0201	sensor kinase protein	208670	209950	+	426	53717843	3092250	-	COG0642T	CGTGCCTCGCCGACGAGATCGCCC	1	103	1	103	100	Bm_ATCC23344_chr1	3510148	3359810	3359912	100	515	1.20E-18	+
BPSL0204	ATP-dependent protease peptidase subunit	212283	212819	-	178	53717846	3091995	hslV	COG5405O	AATGCGCGCCGAGCCGGCCGGCC	1	103	103	1	100	Bm_ATCC23344_chr1	3510148	3364066	3364168	99	506	3.00E-18	-
BPSL0205	dksA/traR C4-type zinc finger family protein	213074	213490	-	138	53717847	3093397	-	COG1734T	CCCGCCACAGCGAATGGCAAACAA	1	103	103	1	100	Bm_ATCC23344_chr1	3510148	3364737	3364839	99	506	3.00E-18	-
BPSL0206	hypothetical protein BPSL0206	214040	215152	-	370	53717848	3091841	-	COG0523R	GCGCCGGCCGTTCGGCCGACAGCG	1	103	103	1	100	Bm_ATCC23344_chr1	3510148	3366402	3366504	100	515	1.20E-18	-
BPSL0207	hypothetical protein BPSL0207	215294	216571	-	425	53717849	3091842	-	COG1092R	CCCGCGCCGCCGCGCGCGCCGCAT	1	91	91	1	100	Bm_ATCC23344_chr1	3510148	3367821	3367911	97	437	3.90E-15	-
BPSL0211	lipid A biosynthesis lauroyl acyltransferase	219301	220185	-	294	53717853	3093529	-	COG1560M	CTTTCTCGAATTGTCACAGAGCGTC	1	103	103	1	100	Bm_ATCC23344_chr1	3510148	3371442	3371544	100	515	1.20E-18	-
BPSL0212	S-adenosylmethionine synthetase	220451	221638	+	395	53717854	3091760	metK	COG0192H	AGCACCCACGGCGCAGGAAATCCG	1	103	1	103	100	Bm_ATCC23344_chr1	3510148	3371610	3371712	100	515	1.20E-18	+
BPSL0213	hypothetical protein BPSL0213	222402	223295	+	297	53717856	3094753	-	COG5285Q	GGCGCTTCGTGACGCCAGGAAGTC	1	103	1	103	100	Bm_ATCC23344_chr1	3510148	3373577	3373679	98	497	7.60E-18	+
BPSL0214	hypothetical protein BPSL0214	223564	223818	+	84	53717857	3094754	-	NULL	TGCGCTTCGGCGGCAGTCGCGACG	1	103	1	103	100	Bm_ATCC23344_chr1	3510148	3374746	3374848	98	497	7.60E-18	+
BPSL0215	aldo/keto reductase family protein	223950	224993	+	347	53717858	3093767	-	COG0667C	GGCGTTGCTGCACGATTCGCGACG	1	103	1	103	100	Bm_ATCC23344_chr1	3510148	3375132	3375234	100	515	1.20E-18	+
BPSL0216	hypothetical protein BPSL0216	225080	225265	-	61	53717859	3093768	-	NULL	GCGAAGCTTCGGATAACCAAAAAA	1	103	103	1	100	Bm_ATCC23344_chr1	3510148	3376501	3376603	99	506	3.00E-18	-
BPSL0217	hypothetical protein BPSL0217	225533	226396	-	287	53717860	3092144	-	NULL	TCTCCGTTCGCCGCTGATCGAACCG	1	103	103	1	100	Bm_ATCC23344_chr1	3510148	3377632	3377734	100	515	1.20E-18	-
BPSL0218	putative transporter protein	226751	228040	-	429	53717861	3092145	-	NULL	CATTCGGAAATGCGGCCAAAAGCC	1	103	103	1	100	Bm_ATCC23344_chr1	3510148	3379258	3379360	100	515	1.20E-18	-
BPSL0219	hypothetical protein BPSL0219	228394	228606	+	70	53717862	3092801	-	NULL	ATGAACAATGGAATGCGTGCGCTT	1	103	1	103	100	Bm_ATCC23344_chr1	3510148	3379514	3379616	100	515	1.20E-18	+
BPSL0224	putative GMC oxidoreductase	233741	235384	+	547	53717866	3092388	-	COG2303E	TTTTGCGTTCCTAAAATGCGCGAAC	1	103	1	103	100	Bm_ATCC23344_chr1	3510148	3384839	3384941	100	515	1.20E-18	+
BPSL0230	flagellar M-ring protein	240547	242343	-	598	53717872	3094419	fliF	COG1766NU	CGCCCGATCGGGCTTGACCGCGCT	1	103	103	1	100	Bm_ATCC23344_chr1	3510148	3393530	3393632	100	515	1.20E-18	-
BPSL0231	flagellar hook-basal body complex protein	242591	242932	+	113	53717873	3092006	fliE	COG1677NU	GGCTGCTATGCTTTTTCGCAATCCG	1	103	1	103	100	Bm_ATCC23344_chr1	3510148	3393680	3393783	99	505	3.30E-18	+
BPSL0232	flagellar protein	243065	243499	+	144	53717874	3094559	fliS	COG1516NUO	AAGCTTTCGGGCTTCCTTACCGATA	1	103	1	103	100	Bm_ATCC23344_chr1	3510148	3394155	3394257	100	515	1.20E-18	+
BPSL0234	hypothetical protein BPSL0234	243932	245443	+	503	53717876	3093455	-	NULL	CGCGCCGCCGCCTTCGGGCGCGCC	1	103	1	103	100	Bm_ATCC23344_chr1	3510148	3395022	3395124	100	515	1.20E-18	+
BPSL0236	hypothetical protein BPSL0236	245950	246696	+	248	53717878	3094778	-	COG3295S	CCCGCGTCCGGCGACATTGAGTCC	1	103	1	103	100	Bm_ATCC23344_chr1	3510148	3397018	3397120	100	515	1.20E-18	+
BPSL0237	hypothetical protein BPSL0237	246793	247818	-	341	53717879	3094779	-	COG1975O	TGCGCGGCCGGGCGCGCCTGCACG	1	103	103	1	100	Bm_ATCC23344_chr1	3510148	3398978	3399080	99	506	3.00E-18	-
BPSL0238	putative amino-acid transporter transmembrane pro	248017	249420	-	467	53717880	3092628	-	COG0531E	TCGCGCATCGTCCGGCGCGGGCAT	1	103	103	1	100	Bm_ATCC23344_chr1	3510148	3400580	3400682	100	515	1.20E-18	-
BPSL0241	ferredoxin--NADP reductase	251607	252377	-	256	53717882	3092154	fpr	COG1018C	GGCGCGTGTCCGACGCGCCTGCCG	1	103	103	1	100	Bm_ATCC23344_chr1	3510148	3404995	3405097	100	515	1.20E-18	-
BPSL0242	LysE type translocator	252590	253243	-	217	53717883	3093993	-	COG1280E	GCACGGCGGCGCGCGGCGCGCTA	1	68	68	1	100	Bm_ATCC23344_chr1	3510148	3405861	3405928	100	340	9.30E-11	-
BPSL0244	hypothetical protein BPSL0244	254128	254691	-	187	53717885	3093816	-	NULL	TTGAAAAATCCGCGCGTCGCGCAC	1	103	103	1	100	Bm_ATCC23344_chr1	3510148	3407309	3407411	100	515	1.20E-18	-
BPSL0245	putative ATPase	255025	256032	+	335	53717886	3092515	-	COG0466O	TCGCGTGCAGCATCGCGCGCATGT	1	103	1	103	100	Bm_ATCC23344_chr1	3510148	3407545	3407647	100	515	1.20E-18	+
BPSL0246	putative FAD dependent oxidoreductase	256104	257225	+	373	53717887	3092516	-	COG0579R	CGGGCGGCGCGCGCGTGCGCTTCG	1	74	1	74	100	Bm_ATCC23344_chr1	3510148	3408653	3408726	100	370	4.10E-12	+
BPSL0247	putative iron-sulfur protein	257360	257674	+	104	53717888	3093330	-	NULL	TCTTCGGAATTACTCGCGACACATC	1	103	1	103	100	Bm_ATCC23344_chr1	3510148	3409880	3409982	100	515	1.20E-18	+
BPSL0248	putative transporter protein	257907	259199	+	430	53717889	3093331	-	NULL	CGCCCGGCGCGGCGTTCGCTTTTT	1	103	1	103	100	Bm_ATCC23344_chr1	3510148	3410427	3410528	99	500	5.50E-18	+
BPSL0249	putative periplasmic dipeptide transport protein	259630	261258	+	542	53717890	3091878	dppA	COG0747E	ACGGATGGTTTTTCTCCAGCTTGTT	1	103	1	103	100	Bm_ATCC23344_chr1	3510148	3412125	3412227	100	515	1.20E-18	+
BPSL0250	putative dipeptide transport system permease protei	261370	262380	+	336	53717891	3091879	dppB	COG0601EP	CGCCGTTAGCCCGACTGCACGATA	1	103	1	103	100	Bm_ATCC23344_chr1	3510148	3413865	3413967	100	515	1.20E-18	+
BPSL0256	hypothetical protein BPSL0256	267975	268727	-	250	53717897	3092328	-	NULL	GAGCCGCGAAGGCCCATCGGCGC	1	103	103	1	100	Bm_ATCC23344_chr1	3510148	3421183	3421285	100	515	1.20E-18	-
BPSL0257	hypothetical protein BPSL0257	269350	270114	-	254	53717898	3092329	-	COG1540R	TTCCGGAAGATCGGACGCCGCCGC	1	61	61	1	100	Bm_ATCC23344_chr1	3510148	3422567	3422627	100	305	3.60E-09	-
BPSL0259	hypothetical protein BPSL0259	271249	271905	-	218	53717900	3093401	-	NULL	CGTCCGCGCGACGCCGCGGCAGAT	1	103	103	1	100	Bm_ATCC23344_chr1	3510148	3424356	3424458	99	506	3.00E-18	-
BPSL0260	hypothetical protein BPSL0260	272055	272567	+	170	53717901	3092063	-	COG5631K	AAATGTCGATAACTTCTCGCCAAAA	1	103	1	103	100	Bm_ATCC23344_chr1	3510148	3424408	3424510	100	515	1.20E-18	+
BPSL0263	hypothetical protein BPSL0263	275534	276493	+	319	53717904	3093790	-	COG0702MG	GCGTGCCGCGCACGCACTGTCCGG	1	103	1	103	100	Bm_ATCC23344_chr1	3510148	3427847	3427949	98	497	7.60E-18	+
BPSL0264	hypothetical protein BPSL0264	276548	277204	+	218	53717905	3091442	-	COG0625O	GCAGGCGGGCGACCGCGGGCGCC	1	57	1	57	100	Bm_ATCC23344_chr1	3510148	3428907	3428963	100	285	2.80E-08	+
BPSL0267	putative flagella synthesis protein	279820	280260	-	146	53717908	3094413	-	COG3418NUO	ACCGGCTTCGCGGCCGAAAGACGG	1	82	82	1	100	Bm_ATCC23344_chr1	3510148	3432635	3432716	100	410	6.40E-14	-
BPSL0268	putative negative regulator of flagellin synthesis	280340	280684	-	114	53717909	3094414	-	NULL	TGCAAACTTTTGTTGATTCAGGGGG	1	103	103	1	100	Bm_ATCC23344_chr1	3510148	3433059	3433161	100	515	1.20E-18	-
BPSL0269	flagellar basal body P-ring biosynthesis protein	280788	281711	-	307	53717910	3094150	-	COG1261NO	TCGTTCCGCCGGCTCCGCGTCGGC	1	103	103	1	100	Bm_ATCC23344_chr1	3510148	3434082	3434184	100	515	1.20E-18	-
BPSL0271	flagellar basal body rod protein	283152	283577	+	141	53717912	3094664	flgC	COG1558N	CGCGCCGCTTCGGCGCTTTTCCCGC	1	86	1	86	100	Bm_ATCC23344_chr1	3510148	3435430	3435515	100	430	8.00E-15	+
BPSL0279	hypothetical protein BPSL0279	290455	291213	+	252	53717920	3094383	-	NULL	TGCCGCTAAACGTGAGAGACCAGC	1	103	1	103	100	Bm_ATCC23344_chr1	3510148	3442696	3442798	99	506	3.00E-18	+
BPSL0280	flagellar hook-associated protein	291419	293422	+	667	53717921	3093210	-	COG1256N	TTTTGCGCGTGCGGTCCCTCAATTT	1	103	1	103	100	Bm_ATCC23344_chr1	3510148	3443660	3443762	100	515	1.20E-18	+
BPSL0282	putative permease	295038	296345	+	435	53717923	3093674	-	COG2233F	CCGCTTGTCAGCGCGATTCCCGCTT	1	103	1	103	100	Bm_ATCC23344_chr1	3510148	3447279	3447381	100	515	1.20E-18	+
BPSL0283	LysR family regulatory protein	296548	297429	-	293	53717924	3093675	-	COG0583K	GGCAGTCCTCGTGATTTCCACGAG	1	100	100	1	100	Bm_ATCC23344_chr1	3510148	3449755	3449854	100	500	5.50E-18	-
BPSL0284	putative chromate transport protein	297527	298126	+	199	53717925	3093131	-	COG2059P	GCTTGTGATTCTAGCTCACAAGGGC	1	100	1	100	100	Bm_ATCC23344_chr1	3510148	3449758	3449857	100	500	5.50E-18	+
BPSL0286	hypothetical protein BPSL0286	298984	299322	-	112	53717927	3094537	-	NULL	TGTTTGCAGTTGATCGAATACGTGA	1	103	103	1	100	Bm_ATCC23344_chr1	3510148	3451667	3451769	100	515	1.20E-18	-
BPSL0287	hypothetical protein BPSL0287	299436	299780	+	114	53717928	3094538	-	COG2824P	GCGGTGAATCGTGAGACGGAGCGC	1	103	1	103	100	Bm_ATCC23344_chr1	3510148	3451683	3451785	100	515	1.20E-18	+
BPSL0289	putative outer membrane porin protein	301079	302212	+	377	53717930	3094401	-	COG3203M	AGCTTTCATCGTCATATGATTCGTG	1	103	1	103	100	Bm_ATCC23344_chr1	3510148	3453299	3453401	100	515	1.20E-18	+
BPSL0291	hypothetical protein BPSL0291	303251	303814	-	187	53717932	3092426	-	NULL	GTTTGCAAGCCGCTGGTGGAGACG	1	103	103	1	100	Bm_ATCC23344_chr1	3510148	3456132	3456234	100	515	1.20E-18	-
BPSL0293	putative glucosyltransferase	305245	306513	-	422	53717934	3094737	-	COG1215M	CGTCGCACTCACGCGGAGCCATCA	1	103	102	1	100	Bm_ATCC23344_chr1	3510148	3460072	3460173	100	510	2.00E-18	-
BPSL0294	hypothetical protein BPSL0294	306895	308424	-	509	53717935	3094738	-	COG3659M	GGTGGTTCTCGTAAGCGCTCGCCG	1	103	103	1	100	Bm_ATCC23344_chr1	3510148	3461982	3462084	98	497	7.60E-18	-
BPSL0297	glutathione reductase	312715	314076	+	453	53717938	3092896	gor	COG1249C	GGCTGATGATTGCGCTGCATGACA	1	103	1	103	100	Bm_ATCC23344_chr1	3510148	3467766	3467868	100	515	1.20E-18	+
BPSL0298	argininosuccinate synthase	314669	316009	+	446	53717939	3091609	argG	COG0137E	CGGCCGGGGCCGCGCGCGAGACTT	1	103	1	103	100	Bm_ATCC23344_chr1	3510148	3469720	3469822	100	515	1.20E-18	+
BPSL0300	hypothetical protein BPSL0300	316896	317621	+	241	53717941	3091773	-	NULL	CGCGCCACGTGCGTCGCGGCGCGC	1	92	3	92	97.7528	Bm_ATCC23344_chr1	3510148	3471958	3472049	95	412	5.20E-14	+
BPSL0301	putative cheavy metal binding protein	317943	318191	+	82	53717942	3093380	-	NULL	CCACGCGCATGTCTTGGGCACGCTT	1	103	1	103	100	Bm_ATCC23344_chr1	3510148	3472994	3473096	99	506	3.00E-18	+
BPSL0302	putative cation transporting P-type ATPase	318749	321172	-	807	53717943	3094207	silP	COG2217P	CGCCGCGATGCCGCGGCGCGGCG	1	103	103	1	100	Bm_ATCC23344_chr1	3510148	3476334	3476436	100	515	1.20E-18	-
BPSL0307	outer membrane efflux protein	324453	325754	+	433	53717948	3093884	-	NULL	CCCCGCTTCCTTCCTACAGGTTCGC	1	101	1	101	100	Bm_ATCC23344_chr1	3510148	3479501	3479601	100	505	3.30E-18	+
BPSL0311	avidin family protein	331093	331539	+	148	53717952	3092301	-	NULL	GCGCGGGCGGCGGCGCGATTCGC	1	103	1	103	100	Bm_ATCC23344_chr1	3510148	3486124	3486226	100	515	1.20E-18	+
BPSL0312	D-fructose-6-phosphate amidotransferase	331614	333446	-	610	53717953	3091987	glmS1	COG0449M	ACCGTTTTTCGGGCGCGCAGCGGC	1	66	66	1	100	Bm_ATCC23344_chr1	3510148	3488575	3488640	100	330	2.60E-10	-
BPSL0313	bifunctional glmU protein	333510	334871	-	453	53717954	3093120	glmU	COG1207M	GGCCGCCGCCGCGCCCGGCCCGCC	1	103	103	1	100	Bm_ATCC23344_chr1	3510148	3490000	3490102	99	506	3.00E-18	-
BPSL0316	hypothetical protein BPSL0316	336953	337834	-	293	53717957	3092800	-	NULL	CCCGGCATTGTAAATGACCGTCCCA	1	70	70	1	100	Bm_ATCC23344_chr1	3510148	3492934	3493003	100	350	3.30E-11	-
BPSL0317	hypothetical protein BPSL0317	337902	339092	+	396	53717958	3092448	-	COG1565S	GACCCGCGCGGTGCGGGCCGCGG	1	70	1	70	100	Bm_ATCC23344_chr1	3510148	3492937	3493006	100	350	3.30E-11	+
BPSL0322	LacI family regulatory protein	342488	343507	-	339	53717963	3091777	-	COG1609K	ATAAATAAGCGCTTATAAGCAGCA	1	103	103	1	100	Bm_ATCC23344_chr1	3510148	3498601	3498703	97	488	1.90E-17	-
BPSL0323	putative methyl-accepting chemotaxis protein	343678	345237	-	519	53717964	3093691	-	COG0840NT	GAAAAATTCATTCAACCACGGTTCC	1	103	103	1	100	Bm_ATCC23344_chr1	3510148	3500331	3500433	100	515	1.20E-18	-
BPSL0324	putative sodium bile acid symporter family protein	345813	346853	-	346	53717965	3093692	-	COG0385R	GCCTATTCAATCGCCGGCCACGAC	1	103	103	1	100	Bm_ATCC23344_chr1	3510148	3501867	3501969	100	515	1.20E-18	-
BPSL0325	hypothetical protein BPSL0325	347284	347712	+	142	53717966	3093037	-	NULL	TGCGCGCGATTCAGGTGCGTGCGC	1	103	1	103	100	Bm_ATCC23344_chr1	3510148	3502198	3502300	99	506	3.00E-18	+
BPSL0359	hypothetical protein BPSL0359	387068	387448	+	126	53717999	3093115	-	NULL	GCGTTGCATTTTGCGAACGCGAGG	1	103	103	1	100	Bm_ATCC23344_chr1	3510148	122798	122900	99	506	3.00E-18	-
BPSL0360	putative alkaline phosphatase	387744	389183	+	479	53718000	3092645	-	NULL	GCCATTCCAAATGCTTTCAATTCAA	1	103	103	2	99	Bm_ATCC23344_chr1	3510148	122127	122223	95	435	4.80E-15	-
BPSL0363	CutC family protein	391828	392541	+	237	53718003	3092475	-	COG3142P	CCGCACGGGCCCCCTTCGGCCGTC	1	103	103	1	100	Bm_ATCC23344_chr1	3510148	118031	118132	99	500	5.50E-18	-
BPSL0364	biotin synthase	392668	393687	-	339	53718004	3094556	bioB	COG0502H	CGTTTTAGCCGCTCGTTTCAACCGC	1	66	1	66	100	Bm_ATCC23344_chr1	3510148	116111	116176	98	321	6.70E-10	+
BPSL0367	adenosylmethionine--8-amino-7-oxononanoate tran:	395651	396997	-	448	53718007	3092139	bioA	COG0161H	GCGTTTTTCTTCGCCATCCGATCGT	1	103	1	103	100	Bm_ATCC23344_chr1	3510148	112764	112866	100	515	1.20E-18	+
BPSL0368	subfamily S9C unassigned peptidase	397561	398844	-	427	53718008	3091532	-	COG1506E	CCGGGTAATTCCGGCGTTGCACAA	1	103	1	103	100	Bm_ATCC23344_chr1	3510148	110924	111026	100	515	1.20E-18	+

BPSL0372	putative carbonic anhydrase	401445	402215 -	256	53718012	3092418 -	NULL	GCCGCGCCCGCCCCGCGCTCGAAC	1	60	1	60	100 Bm_ATCC23344_chr1	3510148	107590	107649	98	291	1.50E-08 +
BPSL0373	bifunctional isocitrate dehydrogenase kinase/phosph	402273	404084 -	603	53718013	3092419 aceK	NULL	GGCCGCGCCCGCCGCCTGCGCCGA	1	103	1	103	100 Bm_ATCC23344_chr1	3510148	105678	105780	100	515	1.20E-18 +
BPSL0374	metallo-beta-lactamase superfamily protein	404374	405453 +	359	53718014	3093229 -	COG0491R	CGAAGGCGGCCCGGCGCGCCTTCG	1	103	103	1	100 Bm_ATCC23344_chr1	3510148	105486	105588	100	515	1.20E-18 -
BPSL0375	ABC transport system ATP-binding protein	405732	407378 -	548	53718015	3093230 -	COG0488R	CCTTCCCCCGAATGGTTTCGCCGCT	1	103	1	103	100 Bm_ATCC23344_chr1	3510148	102368	102470	100	515	1.20E-18 +
BPSL0376	extracellular solute-binding protein	407612	409198 -	528	53718016	3092891 -	COG0747E	TAACGGCGAGCGCCCATTGACGGT	1	103	1	103	100 Bm_ATCC23344_chr1	3510148	100548	100650	100	515	1.20E-18 +
BPSL0378	MarR family regulatory protein	410175	410642 -	155	53718018	3093026 -	COG1846K	CGCATCGATTCGGCCAATGGCGCG	1	103	1	103	100 Bm_ATCC23344_chr1	3510148	99570	99672	100	515	1.20E-18 +
BPSL0379	short chain dehydrogenase	410881	411594 +	237	53718019	3093027 -	COG1028IQR	CCGTGCGCCGCGGCGTTCGACGCG	1	103	103	1	100 Bm_ATCC23344_chr1	3510148	99428	99531	97	487	2.10E-17 -
BPSL0381	thiol:disulfide interchange protein	412535	413173 -	212	53718021	3093149 dsbA	NULL	GCTGCGCCGTCATCCGACTTCGTTA	1	54	1	54	100 Bm_ATCC23344_chr1	3510148	97079	97132	98	261	3.40E-07 +
BPSL0382	hypothetical protein BPSL0382	413225	414073 -	282	53718022	3092048 -	COG3087D	ATCCGAGACCCTCGGCCAAGCGCC	1	81	1	81	100 Bm_ATCC23344_chr1	3510148	96152	96232	100	405	1.10E-13 +
BPSL0383	arginyl-tRNA synthetase	414152	415936 -	594	53718023	3093875 argS	COG0018J	ATTAGCACCTGTTAAAATTCAGCGA	1	103	1	103	100 Bm_ATCC23344_chr1	3510148	94267	94369	100	515	1.20E-18 +
BPSL0384	hypothetical protein BPSL0384	416139	416462 +	107	53718024	3092817 -	NULL	GCCGCTCAGGTGCTAAGCGCCCCC	1	103	103	1	100 Bm_ATCC23344_chr1	3510148	94162	94264	99	506	3.00E-18 -
BPSL0385	putative 5-methyltetrahydrofolate--homocysteine me	416706	419408 -	900	53718025	3091951 metH1	COG1410E	CGCGCGCGGCCCTCCCCACTCCAA	1	63	1	63	100 Bm_ATCC23344_chr1	3510148	89243	89305	100	315	1.30E-09 +
BPSL0386	putative 5-methyltetrahydrofolate--homocysteine me	419469	420548 -	359	53718026	3093220 metH2	COG0646E	CCCGGCCCGCCGCGCGGGGGCTTT	1	103	1	103	100 Bm_ATCC23344_chr1	3510148	88064	88165	99	500	5.50E-18 +
BPSL0387	hypothetical protein BPSL0387	420737	420994 -	85	53718027	3092818 -	NULL	AAAAACGACCGGCGTGCAGCGCCA	1	103	1	103	100 Bm_ATCC23344_chr1	3510148	87618	87720	100	515	1.20E-18 +
BPSL0389	IclR family regulatory protein	422575	423438 -	287	53718029	3093933 -	COG1414K	AGCCGTTGTAGTGACGATGGAAGG	1	103	1	103	100 Bm_ATCC23344_chr1	3510148	85174	85276	99	506	3.00E-18 +
BPSL0390	fumarylacetoacetate (FAA) hydrolase family protein	423548	424540 +	330	53718030	3092780 -	COG0179Q	GGAAGAATGGGGAGGGGGCGGCT	1	103	103	1	100 Bm_ATCC23344_chr1	3510148	85162	85264	98	497	7.60E-18 -
BPSL0391	enoyl-CoA hydratase	424759	425586 +	275	53718031	3092781 -	COG1024I	GCGCCGGGTTTCGCCGGATGACCC	1	103	103	1	100 Bm_ATCC23344_chr1	3510148	83973	84075	98	497	7.60E-18 -
BPSL0392	hypothetical protein BPSL0392	425676	426467 +	263	53718032	3091454 -	NULL	GCGCCGCGCGCGCTATGATCGATC	1	92	92	1	100 Bm_ATCC23344_chr1	3510148	83056	83147	100	460	3.50E-16 -
BPSL0394	hypothetical protein BPSL0394	428396	429271 +	291	53718034	3093845 -	NULL	GCGTAAAATTATCGGCTTGGCTGCG	1	103	103	1	100 Bm_ATCC23344_chr1	3510148	80402	80504	99	506	3.00E-18 -
BPSL0395	putative cytidylyltransferase	429412	429897 +	161	53718035	3093846 -	COG2870M	CGGCGGGCTTTTCTTTTGCCGATTC	1	103	103	1	100 Bm_ATCC23344_chr1	3510148	79386	79488	99	506	3.00E-18 -
BPSL0396	hypothetical protein BPSL0396	429943	430365 -	140	53718036	3092275 -	NULL	CACGCGCAGGCGGCTCGCGCGCCC	1	103	1	103	100 Bm_ATCC23344_chr1	3510148	78323	78425	100	515	1.20E-18 +
BPSL0398	biotin--protein ligase	431251	432210 -	319	53718038	3093583 -	COG0340H	GACCGAGCCGCGCAAGGCCGCGA	1	103	1	103	100 Bm_ATCC23344_chr1	3510148	76478	76580	100	515	1.20E-18 +
BPSL0400	putative 2',3'-cyclic-nucleotide 2'-phosphodiesterase	433552	435612 -	686	53718040	3091766 cpdB	COG0737F	GTTTCCTTTCGATACTCTCACGCGC	1	103	1	103	100 Bm_ATCC23344_chr1	3510148	73071	73173	100	515	1.20E-18 +
BPSL0401	hypothetical protein BPSL0401	435878	437002 +	374	53718041	3094331 -	NULL	CGCGCGGCGCGCGCCGCGCCGGC	1	103	103	1	100 Bm_ATCC23344_chr1	3510148	72899	73005	96	475	7.40E-17 -
BPSL0406	putative ferredoxin	440800	441129 +	109	53718046	3092794 -	COG3411C	CACGACTAGCCTGCCGATGCCGGC	1	103	103	1	100 Bm_ATCC23344_chr1	3510148	67990	68091	99	500	5.50E-18 -
BPSL0408	penicillin-binding protein 6	442663	443874 +	403	53718048	3091693 -	COG1686M	GCGTCTGTCTTCCCTCGGCCTCACG	1	103	103	1	100 Bm_ATCC23344_chr1	3510148	66105	66207	100	515	1.20E-18 -
BPSL0409	putative D-amino acid aminotransferase	443964	444911 +	315	53718049	3091605 -	COG0115EH	GCTGTCCGCTCGCGTCGAGCGCGG	1	92	92	1	100 Bm_ATCC23344_chr1	3510148	64804	64895	100	460	3.50E-16 -
BPSL0411	LysR family regulatory protein	445227	446192 -	321	53718051	3092092 -	COG0583K	GACAGCCTCCGCGAAATCGCTTCA	1	103	1	103	100 Bm_ATCC23344_chr1	3510148	62478	62580	99	506	3.00E-18 +
BPSL0412	hypothetical protein BPSL0412	446293	446619 +	108	53718052	3092093 -	NULL	CGGTTAGTTTTTCTCACAAGCACGT	1	103	103	1	100 Bm_ATCC23344_chr1	3510148	62475	62577	99	506	3.00E-18 -
BPSL0416	family T3 non-peptidase homologue	449010	450893 -	627	53718055	3091829 -	NULL	GACACGGCGACAATACCCCGCCCG	1	103	1	103	100 Bm_ATCC23344_chr1	3510148	3344236	3344338	100	515	1.20E-18 +
BPSL0418	LysR family regulatory protein	452345	453247 -	300	53718057	3093256 -	COG0583K	CCACGATTCTCGATTGATCTAGATT	1	103	1	103	100 Bm_ATCC23344_chr1	3510148	3342040	3342142	99	506	3.00E-18 +
BPSL0419	putative trifunctional protein	453402	455528 +	708	53718058	3093098 -	COG1024I, COG1250I	AGAATGCTGGAATTGTCAATCAATC	1	103	103	1	100 Bm_ATCC23344_chr1	3510148	3341983	3342085	100	515	1.20E-18 -
BPSL0420	hypothetical protein BPSL0420	455583	456791 +	402	53718059	3093099 -	COG1960I	CGCCGCGCCGCGCCGCGCGCCGG	1	57	57	1	100 Bm_ATCC23344_chr1	3510148	3339802	3339858	100	285	2.80E-08 -
BPSL0425	C4-dicarboxylate transport protein	462003	463289 +	428	53718064	3094703 dctA	COG1301C	GGCACGGCGGCCGCGCGGGCGAA	1	103	103	1	100 Bm_ATCC23344_chr1	3510148	3333536	3333638	100	515	1.20E-18 -
BPSL0428	hypothetical protein BPSL0428	466845	467381 -	178	53718067	3093439 -	COG0526OC	GGTGCGCACGCCCGATTGACGGTA	1	103	1	103	100 Bm_ATCC23344_chr1	3510148	3328130	3328232	99	506	3.00E-18 +
BPSL0429	hypothetical protein BPSL0429	467674	468264 +	196	53718068	3093440 -	NULL	TATGCGCCTGCTTCGTTCCCGTTCG	1	103	103	1	100 Bm_ATCC23344_chr1	3510148	3327935	3328037	99	506	3.00E-18 -
BPSL0430	putative thioesterase	468950	469729 -	259	53718069	3092430 -	COG3208Q	GCCCTTCGGCGGGCATCGCCCCGC	1	103	1	103	100 Bm_ATCC23344_chr1	3510148	3325801	3325905	98	495	9.30E-18 +
BPSL0432	putative magnesium chelatase subunit	471119	472705 -	528	53718071	3092499 -	COG0606O	GCAGCTCGCGCCACGCCGGCCAAG	1	103	1	103	100 Bm_ATCC23344_chr1	3510148	3322839	3322941	100	515	1.20E-18 +
BPSL0433	hypothetical protein BPSL0433	472843	473094 -	83	53718072	3092500 -	NULL	AAAACGGGCCCGCGCGCGCACCAA	1	103	1	103	100 Bm_ATCC23344_chr1	3510148	3322450	3322552	100	515	1.20E-18 +
BPSL0434	nitrogen regulatory protein P-II 1	473463	473801 +	112	53718073	3093665 glnB1	COG0347E	TTTCGATACCGGCGTGAGCCGACA	1	103	103	1	100 Bm_ATCC23344_chr1	3510148	3322179	3322281	100	515	1.20E-18 -
BPSL0436	glutamate--cysteine ligase	475712	477040 +	442	53718075	3093803 gshA	NULL	CGGGCCGCGCGCGATAACCCTTCG	1	103	103	1	100 Bm_ATCC23344_chr1	3510148	3318483	3318585	99	506	3.00E-18 -
BPSL0438	sugar transport PTS system IIa component	478197	478670 +	157	53718077	3094449 -	COG2893G	CCGGACCGGCTCGACCGGCCTGCT	1	103	103	1	100 Bm_ATCC23344_chr1	3510148	3315998	3316100	100	515	1.20E-18 -
BPSL0439	phosphocarrier protein HPr	478747	479016 +	89	53718078	3094543 ptsH	COG1925G	GCCCGCGCACCGATTCGTATCATCC	1	79	79	1	100 Bm_ATCC23344_chr1	3510148	3315448	3315526	97	377	2.00E-12 -
BPSL0440	putative phosphoenolpyruvate-protein phosphotrans	479212	480984 +	590	53718079	3091621 -	COG1080G	CGCACGCAGTCATGCGCGTATGCG	1	103	103	1	100 Bm_ATCC23344_chr1	3510148	3314983	3315085	100	515	1.20E-18 -
BPSL0441	molybdopterin biosynthesis protein MoeB	481272	482030 -	252	53718080	3091622 -	COG0476H	GCGACGCCGTGCACGGGCCATCGC	1	103	1	103	100 Bm_ATCC23344_chr1	3510148	3312133	3312235	100	515	1.20E-18 +
BPSL0442	C-terminal processing protease-3	482135	483709 -	524	53718081	3092719 -	COG0793M	CGATCCACAAGGCGTCCGGACGAA	1	103	1	103	100 Bm_ATCC23344_chr1	3510148	3310454	3310556	100	515	1.20E-18 +
BPSL0443	phosphoglyceromutase	483930	484679 -	249	53718082	3093540 gpmA	COG0588G	GCAATCCCGCCATTATAAAATAACC	1	103	1	103	100 Bm_ATCC23344_chr1	3510148	3309484	3309586	100	515	1.20E-18 +
BPSL0444	hypothetical protein BPSL0444	484813	485220 +	135	53718083	3092720 -	COG0607P	GCACGGATGCGGACCCGGCTCCGG	1	103	103	1	100 Bm_ATCC23344_chr1	3510148	3309448	3309550	100	515	1.20E-18 -
BPSL0446	export protein SecB	485609	486088 +	159	53718085	3091642 secB	COG1952U	GCGCGGCCGCCGTCTTGCGGGCGG	1	103	103	1	100 Bm_ATCC23344_chr1	3510148	3308652	3308754	100	515	1.20E-18 -
BPSL0448	Appr-1-p processing enzyme family protein	487265	487798 +	177	53718087	3092510 -	NULL	TGCACGGCGGCGCGCCCGCGCTTG	1	103	103	1	100 Bm_ATCC23344_chr1	3510148	3306996	3307098	100	515	1.20E-18 -
BPSL0450	hypothetical protein BPSL0450	488744	489472 -	242	53718089	3093817 -	COG1040R	CCGAAGTATACTCGCTCGCTTCGCG	1	103	1	103	100 Bm_ATCC23344_chr1	3510148	3304691	3304793	99	506	3.00E-18 +
BPSL0451	hypothetical protein BPSL0451	489595	490560 +	321	53718090	3093818 -	COG0500QR	ACGGCCGGATTCGCCATCGCCGGG	1	103	103	1	100 Bm_ATCC23344_chr1	3510148	3304666	3304768	100	515	1.20E-18 -
BPSL0452	hypothetical protein BPSL0452	490758	491273 +	171	53718091	3092265 -	COG5488S	CGCCGGGCGATGGCCGTTTGCTTG	1	103	103	1	100 Bm_ATCC23344_chr1	3510148	3303503	3303605	100	515	1.20E-18 -
BPSL0455	hypothetical protein BPSL0455	494620	494760 +	46	53718094	3092603 -	NULL	TGATTTGACGGCGTTCGCCGCCCGT	1	71	71	1	100 Bm_ATCC23344_chr1	3510148	3299641	3299711	98	346	5.00E-11 -
BPSL0458	putative cytochrome c oxidase subunit III	495742	496599 +	285	53718097	3093868 -	COG1845C	CGTTCGCGAGCGCAAACAAAGCGA	1	103	103	1	100 Bm_ATCC23344_chr1	3510148	3298519	3298621	100	515	1.20E-18 -
BPSL0458a	hypothetical protein BPSL0458a	496759	496968 -	69	53718098	3092337 -	NULL	CGCGCCGCTCCCCGGGCGCGACGA	1	90	1	90	100 Bm_ATCC23344_chr1	3510148	3297208	3297297	100	450	1.00E-15 +
BPSL0459	hypothetical protein BPSL0459	497056	497769 +	237	53718099	3092338 -	COG3346S	GAAGCTAACCGGAAACCGGCAGTT	1	90	90	1	100 Bm_ATCC23344_chr1	3510148	3297205	3297294	100	450	1.00E-15 -
BPSL0460	hypothetical protein BPSL0460	497831	498475 +	214	53718100	3093165 -	NULL	CGGCGGCGCCGGCGAATCGCGCG	1	64	64	1	100 Bm_ATCC23344_chr1	3510148	3296430	3296493	100	320	7.50E-10 -
BPSL0461	putative cytochrome oxidase assembly protein	498579	499628 +	349	53718101	3093166 -	COG1612O	CCGGCCCGGGCCGGCGATAGACGC	1	103	103	1	100 Bm_ATCC23344_chr1	3510148	3295682	3295784	100	515	1.20E-18 -
BPSL0464	hypothetical protein BPSL0464	501260	501553 -	97	53718104	3094571 -	NULL	CCGGCTTACAACTTGAATGGAAGC	1	103	1	103	100 Bm_ATCC23344_chr1	3510148	3292610	3292712	98	497	7.60E-18 +
BPSL0465	putative methyl-accepting chemotaxis protein	501740	503380 +	546	53718105	3094572 -	COG0840NT	AAGTTGTAAGCCGGCGTGCCGTAA	1	103	103	1	100 Bm_ATCC23344_chr1	3510148	3292521	3292623	100	515	1.20E-18 -
BPSL0466	hypothetical protein BPSL0466	503530	504348 +	272	53718106	3091607 -	COG1464P	TTGTGACACCATTTGCGCTTTCGTC	1	103	103	1	100 Bm_ATCC23344_chr1	3510148	3290731	3290833	100	515	1.20E-18 -
BPSL0467	hypothetical protein BPSL0467	504599	505570 +	323	53718107	3091608 -	COG2984R	GACGCTTGCCGCCGCGCCCGTTTC	1	103	1	103	100 Bm_ATCC23344_chr1	3510148	3237496	3237598	100	515	1.20E-18 +
BPSL0468	putative branched-chain amino acid transport systen	505879	506775 +	298	53718108	3093750 -	COG4120R	GCCCGGCCGGGCCGCCGCGCGGC	1	103	1	103	100 Bm_ATCC23344_chr1	3510148	3238816	3238918	100	515	1.20E-18 +
BPSL0474	putative transporter protein	514322	515515 -	397	53718113	3093352 -	NULL	CCGCCATTGCGCATCCGGTATCAAT	1	103	103	1	100 Bm_ATCC23344_chr1	3510148	3248514	3248616	100	515	1.20E-18 -
BPSL0477	hypothetical protein BPSL0477	518191	518724 -	177	53718116	3093252 -	NULL	GCAGATCGGGACCGAGCAGGACTG	1	77	77	1	100 Bm_ATCC23344_chr1	3510148	3251723	3251799	100	385	8.70E-13 -
BPSL0488	hypothetical protein BPSL0488	530545	531486 -	313	53718127	3093167 -	NULL	CCGACCATGGAAGCCACTATGCAA	1	63	63	1	100 Bm_ATCC23344_chr1	3510148	3264512	3264574	100	315	1.30E-09 -
BPSL0491	putative acyl carrier protein	533030	533278 -	82	53718130	3092642 -	NULL	TCCGCCGTTCACCGTTGTTCGCCGT	1	58	58	1	100 Bm_ATCC23344_chr1	3510148	3266304	3266361	100	290	1.70E-08 -
BPSL0493	putative AMP-binding enzyme	534524	536290 -	588	53718132	3093092 -	COG0318IQ	ACTGTATGGAAATTGTCAGGCAAG	1	103	103	1	100 Bm_ATCC23344_chr1	3510148	3269307	3269409	99	506	3.00E-18 -
BPSL0494	LysR family regulatory protein	536724	537647 +	307	53718133	3094239 -	COG0583K	TCGGAAATGTCGATAACTCGCGAC	1	103	1	103	100 Bm_ATCC23344_chr1	3510148	3269643	3269745	100	515	1.20E-18 +
BPSL0495	GntR family regulatory protein	538360	539124 +	254	53718134	3094240 -	COG2188K	GGGCAACCGTTTCGGCGATTTCGA	1	103	1	103	100 Bm_ATCC23344_chr1	3510148	3271272	3271374	98	497	7.60E-18 +
BPSL0496	putative N-acetylglucosamine-6-phosphate deacetyla	539223	540326 +	367	53718135	3093684 -	COG1820G	CACGACAGACTGTCCGCGGCGGCC	1	101	1	101	100 Bm_ATCC23344_chr1	3510148	3272137	3272237	100	505	3.30E-18 +
BPSL0499	phosphotransferase system, IIbc component	544079	545851 +	590	53718138	3093341 -	COG1263G	TGCGTGTCCGTCGTCCGGCGAATC	1	103	2	103	99 Bm_ATCC23344_chr1	3510148	3276992	3277093	100	510	2.00E-18 +

BPSL0503	hypothetical protein BPSL0503	553330	553614	-	94	53718143	3092140	-	NULL	GCCCTCCTACAATTGGGAGGACCG	1	103	103	1	100	Bm_ATCC23344_chr1	3510148	3286529	3286631	100	515	1.20E-18	-
BPSL0504	RNA polymerase sigma factor	554070	555005	-	311	53718144	3094670	rpoH	COG0568K	TCCTTCTAAGAGTTAGCACTCCAGT	1	103	1	103	100	Bm_ATCC23344_chr1	3510148	3234700	3234802	100	515	1.20E-18	+
BPSL0505	hypothetical protein BPSL0505	555370	555939	+	189	53718145	3092141	-	NULL	TGCCGATCGGAAAAGACCTTTACCA	1	103	103	1	100	Bm_ATCC23344_chr1	3510148	3234433	3234535	100	515	1.20E-18	-
BPSL0507	2-isopropylmalate synthase	556694	558334	-	546	53718147	3092728	-	COG0119E	TGCGCGCCGTCGTGCCATCGGGCA	1	103	1	103	100	Bm_ATCC23344_chr1	3510148	3231371	3231473	100	515	1.20E-18	+
BPSL0508	acetyltransferase (GNAT) family protein	558769	559617	-	282	53718148	3092599	-	NULL	CACGCACAGACGACGCTCGCCGCC	1	103	1	103	100	Bm_ATCC23344_chr1	3510148	3230087	3230189	98	497	7.60E-18	+
BPSL0511	putative nitrite reductase small subunit	564060	564455	-	131	53718151	3094128	-	COG2146PR	TGCCGATGCGCGATGCGTGACGCG	1	78	1	78	100	Bm_ATCC23344_chr1	3510148	3225274	3225351	93	338	1.10E-10	+
BPSL0512	putative nitrite reductase	564531	566975	-	814	53718152	3092067	-	COG1251C	TTTTAAATAGCGGCCAAATAAAAAG	1	103	1	103	100	Bm_ATCC23344_chr1	3510148	3222736	3222838	100	515	1.20E-18	+
BPSL0513	putative maleylacetoacetate isomerase	567360	568004	+	214	53718153	3092068	-	COG0625O	GGCGCGGGCGGCTTTTCGCCCGTC	1	103	103	1	100	Bm_ATCC23344_chr1	3510148	3222449	3222551	99	506	3.00E-18	-
BPSL0514	putative cell division protein	568161	569480	-	439	53718154	3092707	-	COG0552U	GCAGCGCGCGTGCCGCCGCACGCC	1	103	1	103	100	Bm_ATCC23344_chr1	3510148	3220231	3220333	99	506	3.00E-18	+
BPSL0515	hypothetical protein BPSL0515	569732	570358	+	208	53718155	3092708	-	COG0742L	ACGGCGCGCCGCTTGTGGTAACGT	1	103	103	1	100	Bm_ATCC23344_chr1	3510148	3220077	3220179	100	515	1.20E-18	-
BPSL0516	phosphopantetheine adenylyltransferase	570469	570969	+	166	53718156	3092046	coaD	COG0669H	AAACGAGGCGCCGCGTGCAGTCGC	1	103	103	1	100	Bm_ATCC23344_chr1	3510148	3219340	3219442	100	515	1.20E-18	-
BPSL0517	ferredoxin	571032	571298	+	88	53718157	3094859	fdx	NULL	GCGCCCGCGCGCGGCGCGCGGCC	1	65	65	1	100	Bm_ATCC23344_chr1	3510148	3218777	3218841	100	325	4.40E-10	-
BPSL0518	histidinol-phosphate aminotransferase	571468	572538	-	356	53718158	3094062	-	COG0079E	GCCGCCGCGGGTGCCGCCCGCGCC	1	91	1	91	100	Bm_ATCC23344_chr1	3510148	3217176	3217266	100	455	6.00E-16	+
BPSL0519	peptidyl-tRNA hydrolase	572627	573232	-	201	53718159	3093142	pth	NULL	CGTTTCGCACGACGGTCGACGAGA	1	103	1	103	100	Bm_ATCC23344_chr1	3510148	3216470	3216572	100	515	1.20E-18	+
BPSL0520	50S ribosomal protein L25	573346	573972	-	208	53718160	3094063	-	COG1825J	CTTTTTGCACAATCGGCCGGGACCG	1	103	1	103	100	Bm_ATCC23344_chr1	3510148	3215730	3215832	100	515	1.20E-18	+
BPSL0525	hypothetical protein BPSL0525	576793	578613	-	606	53718164	3093198	-	NULL	GTTTTACGGGTCTCTATCTGGCTAAT	1	91	1	91	100	Bm_ATCC23344_chr1	3510148	3211077	3211167	100	455	6.00E-16	+
BPSL0529	hypothetical protein BPSL0529	581696	582589	-	297	53718168	3093646	-	COG1660R	CCCGCGTAACCCGCGCCGGCTTCG	1	88	1	88	100	Bm_ATCC23344_chr1	3510148	3207122	3207209	97	422	1.80E-14	+
BPSL0530	HPr kinase/phosphorylase	582675	583643	-	322	53718169	3093647	-	COG1493T	CGTCGCGGCCCGCGCCGCCGAGCC	1	103	1	103	100	Bm_ATCC23344_chr1	3510148	3206053	3206155	100	515	1.20E-18	+
BPSL0531	putative nitrogen regulatory IIA protein	583772	584227	-	151	53718170	3092867	-	COG1762GT	GCGCTTCACAGGTCTCCGTCGCCTA	1	103	1	103	100	Bm_ATCC23344_chr1	3510148	3205469	3205571	100	515	1.20E-18	+
BPSL0532	putatve PTS system, EIIa component	584514	584873	-	119	53718171	3092868	-	COG1544J	AAGCCGCCGACCGACACCTGCTCG	1	103	1	103	100	Bm_ATCC23344_chr1	3510148	3204823	3204925	100	515	1.20E-18	+
BPSL0533	DNA-directed RNA polymerase subunit N	585023	586531	-	502	53718172	3094832	rpoN	COG1508K	GGCTCGCGCCGCGCGTGCGCGTCC	1	103	1	103	100	Bm_ATCC23344_chr1	3510148	3203165	3203267	99	506	3.00E-18	+
BPSL0538	hypothetical protein BPSL0538	589316	590299	-	327	53718177	3092678	-	COG0517R, COG0794M	CGCCGCGCAACGGACGCCGGGGT	1	103	1	103	100	Bm_ATCC23344_chr1	3510148	3199397	3199499	100	515	1.20E-18	+
BPSL0539	sodium/hydrogen exchanger family protein	590427	592436	+	669	53718178	3092679	-	COG0475P, COG1226P	CGCCGCGCCGGGCCCCTTGCGCCC	1	103	103	1	100	Bm_ATCC23344_chr1	3510148	3199367	3199469	100	515	1.20E-18	-
BPSL0540	adenine phosphoribosyltransferase	592520	593083	+	187	53718179	3094203	-	COG0503F	CGCCGGGCGGCCGCCGCTTGTTCA	1	86	86	1	100	Bm_ATCC23344_chr1	3510148	3197274	3197359	100	430	8.00E-15	-
BPSL0541	LysE type translocator	593177	593791	+	204	53718180	3094204	-	COG1280E	GCGCGCCGGCCGCGCGCCGGCCG	1	96	96	1	100	Bm_ATCC23344_chr1	3510148	3196618	3196713	98	471	1.10E-16	-
BPSL0543	formyltetrahydrofolate deformylase	594713	595594	+	293	53718182	3094057	-	COG0788F	GCGCGGCGCATTGTTCGGAAGATT	1	54	54	1	100	Bm_ATCC23344_chr1	3510148	3195112	3195165	100	270	1.40E-07	-
BPSL0544	hypothetical protein BPSL0544	595893	596927	-	344	53718183	3093773	-	COG0628R	CGCTTGCGGCCACAAGCGCGCCGA	1	103	1	103	100	Bm_ATCC23344_chr1	3510148	3192803	3192905	99	506	3.00E-18	+
BPSL0545	excinuclease ABC subunit A	597179	600046	-	955	53718184	3091749	uvrA	COG0178L	CCGCTGCCGGAGGGCGCGGGAATT	1	103	1	103	100	Bm_ATCC23344_chr1	3510148	3189666	3189768	100	515	1.20E-18	+
BPSL0546	putative transporter protein	600260	601447	+	395	53718185	3093774	-	NULL	CCGGGCCGCACCAGGGCGGCGCG	1	103	103	1	100	Bm_ATCC23344_chr1	3510148	3189550	3189652	100	515	1.20E-18	-
BPSL0591	hypothetical protein BPSL0591	660133	663417	-	1094	53718234	3092207	-	NULL	TCGATGCCGTCATCGCTGTTCGCGC	1	103	103	1	100	Bm_ATCC23344_chr1	3510148	17167	17269	100	515	1.20E-18	-
BPSL0595	hypothetical protein BPSL0595	666528	666893	-	121	53718238	3093649	-	NULL	AATGGATTTTGACGATCATCTCGCC	1	103	103	1	100	Bm_ATCC23344_chr1	3510148	20639	20741	100	515	1.20E-18	-
BPSL0596	putative DNA-binding protein	667141	668148	+	335	53718239	3094462	-	COG1716T	ACAGAATCTGATATTCGACCGTCAA	1	103	1	103	100	Bm_ATCC23344_chr1	3510148	20789	20891	99	506	3.00E-18	+
BPSL0601	hypothetical protein BPSL0601	675423	676199	+	258	53718244	3092919	-	COG0235G	CGCGGGCCCGCCGCCGGCGGGCTT	1	72	1	72	100	Bm_ATCC23344_chr1	3510148	29093	29164	100	360	1.20E-11	+
BPSL0602	hypothetical protein BPSL0602	676682	679045	+	787	53718245	3092194	-	COG5001T	GTGCTCGAAATGTTCGCGGCTTGTC	1	103	1	103	100	Bm_ATCC23344_chr1	3510148	30321	30423	98	497	7.60E-18	+
BPSL0603	putative polysaccharide biosynthesis protein	679223	680491	-	422	53718246	3092195	-	COG2244R	CCGATCCGGCTCACCTCGCCGCGC	1	79	79	1	100	Bm_ATCC23344_chr1	3510148	34228	34306	100	395	3.10E-13	-
BPSL0606	hypothetical protein BPSL0606	683388	684038	-	216	53718249	3094502	-	COG2153R	TTGCGACCATAGGCCGCATCGGAA	1	103	103	1	100	Bm_ATCC23344_chr1	3510148	37775	37877	100	515	1.20E-18	-
BPSL0607	putative glycosyl transferase	684416	685657	+	413	53718250	3092454	-	COG0438M	TGCGATGACGGCGCGCAGCCGTGC	1	103	1	103	100	Bm_ATCC23344_chr1	3510148	38055	38157	100	515	1.20E-18	+
BPSL0609	fis family regulatory protein	687164	688552	+	462	53718252	3094535	-	COG2204T	TCCCATCTCGCGCTGCGTCGCCGGT	1	103	1	103	100	Bm_ATCC23344_chr1	3510148	40803	40905	99	506	3.00E-18	+
BPSL0615	acyl carrier protein	696372	696674	-	100	53718258	3094361	-	COG0236IQ	ACGGCGCGCGGCGCGCGGCACGC	1	103	103	2	99	Bm_ATCC23344_chr1	3510148	50411	50512	99	501	5.00E-18	-
BPSL0616	hypothetical protein BPSL0616	696875	697669	-	264	53718259	3094362	-	COG0664T	ATAAAAATCGTACCTGTCTAGCCAG	1	103	103	1	100	Bm_ATCC23344_chr1	3510148	51399	51501	100	515	1.20E-18	-
BPSL0617	hypothetical protein BPSL0617	698536	699402	+	288	53718260	3092616	-	COG0664T	TCACCCCCGCCAAGAGGGTGGTGC	1	103	1	103	100	Bm_ATCC23344_chr1	3510148	52168	52270	100	515	1.20E-18	+
BPSL0619	putative polysaccharide biosynthesis/export protein	700889	702178	+	429	53718262	3094301	-	NULL	GCCGGCGCGCGTTCGCCGCGAACG	1	74	1	74	100	Bm_ATCC23344_chr1	3510148	54550	54623	100	370	4.10E-12	+
BPSL0620	glycosyl transferase group 1 protein	702458	703603	+	381	53718263	3094302	-	COG0438M	CATGAATCTGTCTTCCCCGTTATCCA	1	103	1	103	100	Bm_ATCC23344_chr1	3510148	56090	56192	99	506	3.00E-18	+
BPSL0621	hypothetical protein BPSL0621	703632	704531	-	299	53718264	3091729	-	NULL	CCCGCTTTCTCACGTCGCCGCGTCC	1	103	103	1	100	Bm_ATCC23344_chr1	3510148	58261	58363	99	506	3.00E-18	-
BPSL0623	putative hydrolase	705775	706740	+	321	53718266	3092112	-	COG0657I	GCCGCGCAGCCGGATTTAGAGGAG	1	94	1	94	100	Bm_ATCC23344_chr1	3510148	181068	181161	100	470	1.30E-16	+
BPSL0624	putative protease	706710	707624	-	304	53718267	3092113	-	NULL	TTCGTGCAACTTTTGATGCGTCAAC	1	103	103	1	100	Bm_ATCC23344_chr1	3510148	183006	183108	100	515	1.20E-18	-
BPSL0625	putative transport protein	707731	709326	+	531	53718268	3094261	-	NULL	CCTTCGCGACGCGCGCGTCGCATG	1	103	1	103	100	Bm_ATCC23344_chr1	3510148	183015	183117	100	515	1.20E-18	+
BPSL0627	family C56 unassigned peptidase	709927	710508	-	193	53718270	3092409	-	COG0693R	GTCACGCCCGCTTCACGCCCCCGTC	1	103	103	1	100	Bm_ATCC23344_chr1	3510148	185890	185992	100	515	1.20E-18	-
BPSL0629	RpiR family regulatory protein	712076	712969	+	297	53718272	3093819	-	COG1737K	GTTGACTTCGCGATAGTACAGAAAC	1	103	1	103	100	Bm_ATCC23344_chr1	3510148	187345	187447	99	506	3.00E-18	+
BPSL0633	threonine dehydratase	716147	717670	-	507	53718276	3092379	ilvA	COG1171E	CGTCCGGACGGTCGGTAGCCATTC	1	103	103	1	100	Bm_ATCC23344_chr1	3510148	192989	193091	100	515	1.20E-18	-
BPSL0634	putative oxidoreductase	718126	722205	+	1359	53718277	3092290	-	COG0247C, COG0277C	GGCACGCGCGCTGCGCGCGATCGT	1	103	1	103	100	Bm_ATCC23344_chr1	3510148	193347	193449	100	515	1.20E-18	+
BPSL0638	hypothetical protein BPSL0638	723962	724933	+	323	53718281	3091452	-	COG4395S	GGGGTTCCATCCATACATTTTCCTTT	1	92	1	92	100	Bm_ATCC23344_chr1	3510148	199194	199285	100	460	3.50E-16	+
BPSL0639	hypothetical protein BPSL0639	725014	725688	+	224	53718282	3091453	-	NULL	CGGAAGTCGGGCCGCGCGTCGTGC	1	83	1	83	100	Bm_ATCC23344_chr1	3510148	200255	200337	100	415	3.80E-14	+
BPSL0642	hypothetical protein BPSL0642	728344	728685	+	113	53718285	3092398	-	COG2331S	CGGCGCGCCCGCGCGGACCGGGG	1	103	1	65	63.1068	Bm_ATCC23344_chr1	3510148	203556	203620	100	325	4.50E-08	+
												103	40	103	62.1359	Bm_ATCC23344_chr1	3510148	203608	203673	92	273	3.40E-06	+
BPSL0643	hypothetical protein BPSL0643	728756	729403	+	215	53718286	3092399	-	COG2928S	TCGCCGCGCCGCCCGTGTCGGCGG	1	73	1	73	100	Bm_ATCC23344_chr1	3510148	204013	204085	100	365	6.90E-12	+
BPSL0644	aspartyl-tRNA synthetase	729466	731262	+	598	53718287	3092849	aspS	COG0173J	TGCCCGGGAGAGAGTGCGGCTCTT	1	59	1	59	100	Bm_ATCC23344_chr1	3510148	204731	204789	100	295	1.00E-08	+
BPSL0645	dATP pyrophosphohydrolase	731470	731946	+	158	53718288	3094352	ntpA	COG0494LR	ATGCGCCCTTTTCGTTTTTTTGCGGT	1	103	1	103	100	Bm_ATCC23344_chr1	3510148	206698	206800	100	515	1.20E-18	+
BPSL0647	TetR family regulatory protein	733407	734006	+	199	53718290	3091725	-	COG1309K	ACCGATTTCTAATAATTTCCTTGTTT	1	103	1	103	100	Bm_ATCC23344_chr1	3510148	208635	208737	100	515	1.20E-18	+
BPSL0648	putative acyl-CoA dehydrogenase oxidoreductase pr	734064	735851	+	595	53718291	3091726	-	COG1960I	CGGCGGCACGACCGCCGCCGGGG	1	60	1	60	100	Bm_ATCC23344_chr1	3510148	209335	209394	100	300	6.00E-09	+
BPSL0649	putative 3-hydroxyacyl-CoA dehydrogenase oxidored	736009	738444	+	811	53718292	3094624	-	COG1024I, COG1250I	CCGACAGAGGGGGCGCTGCCTTCCC	1	103	1	103	100	Bm_ATCC23344_chr1	3510148	211237	211339	100	515	1.20E-18	+
BPSL0651	enoyl-CoA hydratase	739918	740682	+	254	53718294	3091688	-	COG1024I	TCGGAGCGCGCGACGGATGACGAC	1	97	1	97	100	Bm_ATCC23344_chr1	3510148	215152	215248	97	467	1.70E-16	+
BPSL0652	FdhD/NarQ family protein	740796	741620	-	274	53718295	3091689	-	NULL	GGCCCGTCTGGAGTATTTTAGAACC	1	103	103	1	100	Bm_ATCC23344_chr1	3510148	216946	217047	99	500	5.50E-18	-
BPSL0653	hypothetical protein BPSL0653	741755	742234	+	159	53718296	3092116	-	NULL	GCGGGGGCGCGACGCCGGGCGGC	1	103	4	103	97	Bm_ATCC23344_chr1	3510148	216985	217084	100	500	5.50E-18	+
BPSL0654	thioesterase superfamily protein	742279	742692	-	137	53718297	3092117	-	COG1607I	CACTTTCTCGTACAATTTCAGAACG	1	103	103	1	100	Bm_ATCC23344_chr1	3510148	218017	218119	100	515	1.20E-18	-
BPSL0655	ATP-binding transmembrane ABC transporter protei	742999	744870	+	623	53718298	3092132	-	COG5265O	GCGCTGCATGGCCGGATCGCGCCG	1	103	1	103	100	Bm_ATCC23344_chr1	3510148	218226	218328	99	506	3.00E-18	+
BPSL0657	hypothetical protein BPSL0657	746086	747120	-	344	53718300	3091651	-	COG3178R	ATGGCGGGCTGCGGGCCCGCGCCC	1	103	78	1	75.7282	Bm_ATCC23344_chr1	3510148	222483	222560	100	390	5.10E-13	-
												103	103	65	37.8641	Bm_ATCC23344_chr1	3510148	222445	222483	100	195	0.00063	-
BPSL0658	hypothetical protein BPSL0658	747333	749696	+	787	53718301	3091652	-	COG1452M	CCCGTTTCCCCCGGTATGCGCGAAC	1	103	1	103	100	Bm_ATCC23344_chr1	3510148	222573	222675	100	515	1.20E-18	+
BPSL0659	putative peptidyl-prolyl cis-trans isomerase	749757	751109	+	450	53718302	3094814	-	COG0760O	CCGCCGACGAGGCGTTCGCGGCGA	1	63	1	63	100	Bm_ATCC23344_chr1	3510148	225037	225099	100	315	1.30E-09	+
BPSL0662	hypothetical protein BPSL0662	753301	754239	-	312	53718305	3094815	-	NULL	TGCGAACCTTCTTCCTGCTCTTCGTC	1	103	103	1	100	Bm_ATCC23344_chr1	3510148	229545	229647	100	515	1.20E-18	-
BPSL0663	lactoylglutathione lyase	754617	755006	+	129	53718306	3092375	gloA	COG0346E	TATCGGCCCTGCTGCGCGGATGGG	1	103	1	103	100	Bm_ATCC23344_chr1	3510148	229825	229927	99	506	3.00E-18	+
BPSL0664	hypothetical protein BPSL0664	755219	756064	-	281	53718307	3093196	-	COG1451R	AGTCAAGCGAGTTTCACCCATTGCG	1	83	83	1	100	Bm_ATCC23344_chr1	3510148	231370	231452	100	415	3.80E-14	-

BPSL0668	glycyl-tRNA synthetase alpha subunit	759664	760671	-	335	53718311	3094758	glyQ	NULL	AGCACAGACCCAAGCCGCGCGCCG	1	103	103	1	100	Bm_ATCC23344_chr1	3510148	235977	236079	100	515	1.20E-18	-
BPSL0670	putative cation transporter efflux protein	762676	763563	-	295	53718313	3093851	-	COG4535P	TTCGGCCCGCGCGCCGCCCGCCGA	1	103	103	1	100	Bm_ATCC23344_chr1	3510148	238869	238970	99	500	5.50E-18	-
BPSL0672	hypothetical protein BPSL0672	764729	765544	-	271	53718315	3093081	-	COG0319R	CCGCGCGGCGGCCCCTCCTCGCCC	1	103	103	1	100	Bm_ATCC23344_chr1	3510148	240839	240940	99	500	5.50E-18	-
BPSL0674	putative tRNA thiotransferase protein MiaB	767263	768636	-	457	53718317	3093127	miaB	COG0621J	GTACGCGGCGCGCATCGCGCGGCT	1	103	103	1	100	Bm_ATCC23344_chr1	3510148	243972	244074	100	515	1.20E-18	-
BPSL0675	putative LysR family transcriptional regulator	769122	770054	-	310	53718318	3092721	-	COG0583K	CGCATCAAGAAATCTGACGAGCGC	1	103	103	1	100	Bm_ATCC23344_chr1	3510148	245390	245492	99	506	3.00E-18	-
BPSL0676	putative transmembrane transporter protein	770200	771372	+	390	53718319	3092722	-	COG2814G	TTATCAAAAAACTTCGGATTGGGGA	1	103	1	103	100	Bm_ATCC23344_chr1	3510148	245438	245540	99	506	3.00E-18	+
BPSL0677	putative DNA binding protein	771506	772174	+	222	53718320	3094245	-	NULL	TAACAGTGTGAAGATAACTTTGTTT	1	103	1	103	100	Bm_ATCC23344_chr1	3510148	246744	246846	100	515	1.20E-18	+
BPSL0678	putative 3,4-dihydroxy-2-butanone 4-phosphate syn	772598	773320	+	240	53718321	3092772	ribB	NULL	GGCTTCGTCCGCGCGGCGCGGAAG	1	103	1	103	100	Bm_ATCC23344_chr1	3510148	247831	247933	99	506	3.00E-18	+
BPSL0679	putative hydrolase protein	773388	774074	-	228	53718322	3094246	-	COG0637R	TCATGGCGGCGCGTGTCGCCGCGC	1	103	103	1	100	Bm_ATCC23344_chr1	3510148	249405	249507	100	515	1.20E-18	-
BPSL0680	hypothetical protein BPSL0680	774204	775058	-	284	53718323	3093650	-	NULL	GCCGCGCGGCGCGCCGGCGCCGA	1	91	91	1	100	Bm_ATCC23344_chr1	3510148	250389	250479	100	455	6.00E-16	-
BPSL0685	hypothetical protein BPSL0685	780710	781132	-	140	53718328	3093001	-	COG0251J	CAAGTCCGACATTCTGAAATCTTCA	1	103	103	1	100	Bm_ATCC23344_chr1	3510148	256458	256560	100	515	1.20E-18	-
BPSL0686	putative glycerol uptake facilitator protein	782049	782765	-	238	53718329	3093044	glpF	NULL	GGCATCCGTATCGATCACATTTCAA	1	103	103	1	100	Bm_ATCC23344_chr1	3510148	258091	258193	99	506	3.00E-18	-
BPSL0687	glycerol kinase	782878	784380	-	500	53718330	3092942	glpK	COG0554C	GCGCACGACGCCGGCACCGACGA	1	103	103	1	100	Bm_ATCC23344_chr1	3510148	259706	259808	100	515	1.20E-18	-
BPSL0688	putative glycerol-3-phosphate dehydrogenase	784486	786018	-	510	53718331	3093043	glpD	COG0578C	TCAAAAGCGAACTTTACTTTTTCGA	1	103	103	1	100	Bm_ATCC23344_chr1	3510148	261344	261446	100	515	1.20E-18	-
BPSL0689	hypothetical protein BPSL0689	786186	786557	+	123	53718332	3094507	-	NULL	GAACATAATCGAAAAAGTAAAGTT	1	103	1	103	100	Bm_ATCC23344_chr1	3510148	261414	261516	100	515	1.20E-18	+
BPSL0690	hypothetical protein BPSL0690	786624	786833	-	69	53718333	3094508	-	NULL	ACGTCAATAACGCCAAGGCCGGGA	1	103	103	1	100	Bm_ATCC23344_chr1	3510148	262159	262261	100	515	1.20E-18	-
BPSL0691	putative DeoR family glycerol-3-phosphate regulon r	787140	787976	-	278	53718334	3092943	glpR	COG1349KG	ATGAATGCGCGCAAGACATGAATC	1	103	103	1	100	Bm_ATCC23344_chr1	3510148	263304	263406	100	515	1.20E-18	-
BPSL0692	gamma-glutamyltransferase 2	788147	789820	+	557	53718335	3091752	-	NULL	AGACTCGATTCATGTCTTGCGCGCA	1	103	1	103	100	Bm_ATCC23344_chr1	3510148	263377	263479	100	515	1.20E-18	+
BPSL0693	hypothetical protein BPSL0693	790123	790956	+	277	53718336	3091753	-	NULL	GTGTCGAATAACCGATTTCAACGCG	1	103	1	103	100	Bm_ATCC23344_chr1	3510148	265353	265455	100	515	1.20E-18	+
BPSL0694	hypothetical protein BPSL0694	791026	791526	+	166	53718337	3092944	-	NULL	GCGTCGGGTGCGTGGCGCGCGGTA	1	72	1	72	100	Bm_ATCC23344_chr1	3510148	266287	266358	100	360	1.20E-11	+
BPSL0697	putative periplasmic cytochrome c protein	794567	795328	-	253	53718340	3092201	-	COG2863C	ATAGAATTCCGCGTCGCAGCAGCC	1	103	103	1	100	Bm_ATCC23344_chr1	3510148	270656	270758	97	488	1.90E-17	-
BPSL0698	putative transmembrane transporter protein	795753	796679	+	308	53718341	3092202	-	NULL	CTTTTTCCTATTTGCCTGAAAACAAC	1	103	1	103	100	Bm_ATCC23344_chr1	3510148	270980	271082	100	515	1.20E-18	+
BPSL0699	putative galactonate dehydratase protein	797270	798418	-	382	53718342	3093666	-	COG4948MR	TCACTATGTGCACAACGGCGCGAT	1	103	103	1	100	Bm_ATCC23344_chr1	3510148	273587	273689	100	515	1.20E-18	-
BPSL0700	hypothetical protein BPSL0700	798874	799221	+	115	53718343	3093667	-	COG3791S	TTGCGATTCGCCCGGCCGGGCGTTA	1	103	1	103	100	Bm_ATCC23344_chr1	3510148	273932	274034	100	515	1.20E-18	+
BPSL0702	calcineurin-like phosphoesterase	800104	801786	+	560	53718345	3094055	-	COG1409R	CGCGTCACGCATCCCGTCATAAGGT	1	103	1	103	100	Bm_ATCC23344_chr1	3510148	275193	275295	99	506	3.00E-18	+
BPSL0704	putative two-component response regulator protein	803697	805016	-	439	53718347	3093007	-	COG3437KT	GCCCATCGCGCCGGATGCGCCGCA	1	103	103	1	100	Bm_ATCC23344_chr1	3510148	280203	280305	100	515	1.20E-18	-
BPSL0705	hypothetical protein BPSL0705	805804	806385	+	193	53718348	3092324	-	NULL	CGCGGCGACGGCATCGAGTGCTTC	1	103	1	103	100	Bm_ATCC23344_chr1	3510148	280894	280996	100	515	1.20E-18	+
BPSL0773	putative transmembrane transport protein	898930	900600	-	556	53718412	3094588	-	NULL	GCGCTTTTTTGACGCGCCGAGTCAG	1	103	103	1	100	Bm_ATCC23344_chr1	3510148	287426	287528	100	515	1.20E-18	-
BPSL0775	response regulator transcription regulatory protein	902391	903134	-	247	53718414	3093154	-	COG0745TK	CAGTGGTTTATGCAAGCCCGCGTTG	1	103	103	1	100	Bm_ATCC23344_chr1	3510148	289921	290023	100	515	1.20E-18	-
BPSL0776	recombinase A	903338	904408	+	356	53718415	3094325	recA	COG0468L	TTTTATACAGTGTCTGCATTCGCGC	1	103	1	103	100	Bm_ATCC23344_chr1	3510148	290027	290129	100	515	1.20E-18	+
BPSL0777	RecA regulator RecX	904774	905391	+	205	53718416	3094326	recX	COG2137R	CGGTGGCGCGGAGGGCGTTCGCGC	1	103	1	103	100	Bm_ATCC23344_chr1	3510148	291463	291565	100	515	1.20E-18	+
BPSL0778	hypothetical protein BPSL0778	905683	906294	+	203	53718417	3093914	-	NULL	TTCCCCCAAATCCCTCGAAACCCCG	1	103	1	103	100	Bm_ATCC23344_chr1	3510148	292372	292474	100	515	1.20E-18	+
BPSL0779	succinyl-CoA synthetase subunit beta	906374	907540	+	388	53718418	3093010	sucC	COG0045C	GGGCGAACGCTTGAATCGTCGGAA	1	82	1	82	100	Bm_ATCC23344_chr1	3510148	293084	293165	100	410	6.40E-14	+
BPSL0780	succinyl-CoA synthetase alpha subunit	907685	908566	+	293	53718419	3092908	sucD	COG0074C	CGGGAAGACTCGGGGCAGCCCCGC	1	103	1	103	100	Bm_ATCC23344_chr1	3510148	294374	294476	100	515	1.20E-18	+
BPSL0781	hypothetical protein BPSL0781	908618	909430	+	270	53718420	3093915	-	COG0861P	GCATCGGTTTCATCGCCGCAAAAAA	1	54	1	54	100	Bm_ATCC23344_chr1	3510148	295356	295409	100	270	1.40E-07	+
BPSL0782	putative type 4 fimbrial pilin protein	909649	910167	+	172	53718421	3092148	-	COG4969NU	CGATGTTCTTTCCGTTTTACCGCCGC	1	103	1	103	100	Bm_ATCC23344_chr1	3510148	296338	296440	100	515	1.20E-18	+
BPSL0783	hypothetical protein BPSL0783	910375	912192	+	605	53718422	3092149	-	NULL	GCGCGGGCGGCGCGGCGGCCGCG	1	103	1	103	100	Bm_ATCC23344_chr1	3510148	297064	297166	100	515	1.20E-18	+
BPSL0784	hypothetical protein BPSL0784	912788	913261	+	157	53718423	3094398	-	NULL	CATCGCGCGCCGCAGCGCGCGTAT	1	103	1	103	100	Bm_ATCC23344_chr1	3510148	299420	299522	100	515	1.20E-18	+
BPSL0785	putative lipoprotein	913498	913863	+	121	53718424	3094399	-	NULL	AAACATCGGCGAACAAAACGGGGC	1	103	1	103	100	Bm_ATCC23344_chr1	3510148	300130	300232	100	515	1.20E-18	+
BPSL0787	hypothetical protein BPSL0787	914698	916422	+	574	53718426	3093163	-	COG4783R	GCCGGCCCGGCGCACGCGGCAGCC	1	103	1	103	100	Bm_ATCC23344_chr1	3510148	302659	302761	100	515	1.20E-18	+
BPSL0790	hypothetical protein BPSL0790	918418	918864	-	148	53718429	3092710	-	NULL	CCGCGCACCCGCACCCGATTCACC	1	103	103	1	100	Bm_ATCC23344_chr1	3510148	306915	307017	100	515	1.20E-18	-
BPSL0792	hypothetical protein BPSL0792	920164	920361	-	65	53718431	3092094	-	COG4391S	GCCCGAATGCGGCGCGGCGCCACC	1	68	68	1	100	Bm_ATCC23344_chr1	3510148	308412	308479	100	340	9.30E-11	-
BPSL0793	branched-chain amino acid aminotransferase	920427	921350	-	307	53718432	3093889	ilvE	COG0115EH	CACGTACAAAAACGCTAAAATGAG	1	103	103	1	100	Bm_ATCC23344_chr1	3510148	309401	309503	100	515	1.20E-18	-
BPSL0794	AzlC family protein	921572	922321	+	249	53718433	3092095	-	COG1296E	ACGGCGCTATAATCAACGCGCAAT	1	103	1	103	100	Bm_ATCC23344_chr1	3510148	309525	309627	100	515	1.20E-18	+
BPSL0796	phosphoglycerate kinase	922908	924101	+	397	53718435	3092746	pgk	NULL	CGCGGGCCTCGCCGCGCAAAGCCG	1	103	1	103	100	Bm_ATCC23344_chr1	3510148	310861	310963	100	515	1.20E-18	+
BPSL0797	pyruvate kinase	924436	925872	+	478	53718436	3093639	pykA	NULL	CGCACCGGCGCGCGTTCCGAAGCG	1	103	1	103	100	Bm_ATCC23344_chr1	3510148	312389	312491	100	515	1.20E-18	+
BPSL0798	fructose-bisphosphate aldolase	926153	927217	+	354	53718437	3092411	cbbA	COG0191G	GCTATTCGACGCTCGGCCGCGCCG	1	103	1	103	100	Bm_ATCC23344_chr1	3510148	314106	314208	100	515	1.20E-18	+
BPSL0799	phosphoribosylaminoimidazole-succinocarboxamide	927407	928297	+	296	53718438	3094817	hemH	NULL	CGCCCGGCCGGTTCGCCGAAGTAT	1	103	1	103	100	Bm_ATCC23344_chr1	3510148	315360	315462	100	515	1.20E-18	+
BPSL0801	phosphoribosylaminoimidazole carboxylase	928931	930127	+	398	53718440	3091911	purK	COG0026F	GGCCGCCGCGCGAGCCGCTTGCAA	1	82	1	82	100	Bm_ATCC23344_chr1	3510148	316905	316986	100	410	6.40E-14	+
BPSL0803	putative acylhydrolase	931480	932619	-	379	53718442	3094179	-	COG3240IR	ACGAGGCTTCACAACCATTGCGCC	1	103	103	1	100	Bm_ATCC23344_chr1	3510148	320608	320710	100	515	1.20E-18	-
BPSL0804	hypothetical protein BPSL0804	932724	933641	-	305	53718443	3094180	-	COG3000I	TGCGTACCCTTTTGTCTGCAATTGA	1	103	103	1	100	Bm_ATCC23344_chr1	3510148	321630	321732	100	515	1.20E-18	-
BPSL0805	family S13 unassigned peptidase	934041	935492	-	483	53718444	3093045	-	COG2027M	GCATTCGTCGATGAATCACCACGCC	1	103	103	1	100	Bm_ATCC23344_chr1	3510148	323469	323571	100	515	1.20E-18	-
BPSL0806	response regulator protein	935760	936422	+	220	53718445	3093046	-	COG0745TK	GGCGCGCGAGCAGCGCCGAAATC	1	103	1	103	100	Bm_ATCC23344_chr1	3510148	323639	323741	100	515	1.20E-18	+
BPSL0808	subfamily S1C unassigned peptidase	938273	939691	+	472	53718447	3092688	-	COG0265O	GCTTCAAGCCATTAGATGAGGAGA	1	103	1	103	100	Bm_ATCC23344_chr1	3510148	326152	326254	100	515	1.20E-18	+
BPSL0809	hypothetical protein BPSL0809	939851	940273	+	140	53718448	3093613	-	NULL	GCGGCGGCCTGCCGCGCTGCGCGG	1	103	1	103	100	Bm_ATCC23344_chr1	3510148	327730	327832	99	506	3.00E-18	+
BPSL0810	hypothetical protein BPSL0810	940401	940766	+	121	53718449	3093614	-	NULL	TTTGCGGCGGTTTGGCCGATAATGC	1	103	1	103	100	Bm_ATCC23344_chr1	3510148	328280	328382	100	515	1.20E-18	+
BPSL0812	TetR family regulatory protein	941844	942479	-	211	53718451	3094029	bpeR	COG1309K	TTACAACAGGCAGGCCAAGCAAGC	1	103	103	1	100	Bm_ATCC23344_chr1	3510148	330461	330563	99	506	3.00E-18	-
BPSL0814	putative RND family acriflavine resistance protein A p	942889	944151	+	420	53718452	3094743	bpeA	COG0845M	AGTCAAGCAATTCAGTTGTCCGATC	1	103	1	103	100	Bm_ATCC23344_chr1	3510148	330773	330875	100	515	1.20E-18	+
BPSL0819	AraC family transcriptional regulator	952965	953999	-	344	53718457	3094112	-	COG4977K	GTTGTCAGGCCCGGGCGGCCGCGC	1	103	103	1	100	Bm_ATCC23344_chr1	3510148	342460	342562	100	515	1.20E-18	-
BPSL0820	glutathione-dependent formaldehyde dehydrogenas	954204	955310	+	368	53718458	3091474	flhA	COG1062C	CGCCGATTCCCGCCAATTTCCGGCC	1	103	1	103	100	Bm_ATCC23344_chr1	3510148	342567	342669	99	506	3.00E-18	+
BPSL0824	putative periplasmic solute-binding protein	958008	958883	-	291	53718462	3094299	-	COG0803P	CACGCCGTCAACCCAATCGAGGAT	1	103	103	1	100	Bm_ATCC23344_chr1	3510148	347362	347464	96	472	1.00E-16	-
BPSL0825	putative Fur family transcriptional regulator	959141	959605	-	154	53718463	3094300	-	COG0735P	GCGGGTGAGGACGGAGGACAGGC	1	103	103	1	100	Bm_ATCC23344_chr1	3510148	348084	348186	99	506	3.00E-18	-
BPSL0826	sorbitol dehydrogenase	959899	960675	+	258	53718464	3093880	-	COG1028IQR	TGGCCGATCATTCGTTCGAAAATAG	1	103	1	103	100	Bm_ATCC23344_chr1	3510148	348280	348382	98	497	7.60E-18	+
BPSL0829	putative periplasmic ABC transporter substrate-bindi	963171	964499	+	442	53718467	3093412	-	COG1653G	AGCAGTCCGCCCGCCGGGCGGCGA	1	53	1	53	100	Bm_ATCC23344_chr1	3510148	351595	351647	100	265	2.30E-07	+
BPSL0835	LysR family transcriptional regulator	970247	971131	-	294	53718473	3094509	-	COG0583K	GCAGCCGGCGAATGAAGAAAAACT	1	103	103	1	100	Bm_ATCC23344_chr1	3510148	359515	359617	97	488	1.90E-17	-
BPSL0836	family S12 unassigned peptidase	971269	972465	+	398	53718474	3094510	-	COG1680V	TGCGGACTTCAATGTCCGCAGTGTC	1	103	1	103	100	Bm_ATCC23344_chr1	3510148	359555	359657	98	497	7.60E-18	+
BPSL0838	putative transcriptional regulator	973846	974793	-	315	53718476	3094835	-	COG2390K	GCGCGTTGCGCTACAGTGCGAATTT	1	89	89	1	100	Bm_ATCC23344_chr1	3510148	363166	363254	100	445	1.70E-15	-
BPSL0839	putative carbohydrate kinase	974880	976361	-	493	53718477	3092433	dalK	COG1070G	CGTCACGCCGCCGCGCGTTGCTCG	1	103	103	1	100	Bm_ATCC23344_chr1	3510148	364725	364827	100	515	1.20E-18	-
BPSL0840	putative D-arabinitol 4-dehydrogenase	976472	977914	-	480	53718478	3092432	dalD	NULL	CGCGCATCGCGCCGATTGGCGCGC	1	103	103	1	100	Bm_ATCC23344_chr1	3510148	366278	366380	100	515	1.20E-18	-
BPSL0841	putative LysR family transcriptional regulator	978086	979000	-	304	53718479	3094739	-	COG0583K	CCTCATCGTCATCATCGGTTTCCAC	1	103	103	1	100	Bm_ATCC23344_chr1	3510148	367334	367436	100	515	1.20E-18	-
BPSL0842	benzoylformate decarboxylase	979101	980720	+	539	53718480	3094740	-	COG0028EH	GCTGCCGTGTAATGGCTTCTTGCAA	1	103	1	103	100	Bm_ATCC23344_chr1	3510148	367349	367451	100	515	1.20E-18	+
BPSL0846	putative tryptophan 2,3-dioxygenase	985394	986314	-	306	53718485	3092668	-	COG3483E	GCCCGCGCGAGCGCCGCGCGAAG	1	101	101	1	100	Bm_ATCC23344_chr1	3510148	374668	374768	99	496	8.40E-18	-
BPSL0848	hypothetical protein BPSL0848	987705	988346	-	213	53718487	3094257	-	COG1878R	ATTATCTGCCATATTGGTGGATTGT	1	103	103	1	100	Bm_ATCC23344_chr1	3510148	376700	376802	100	515	1.20E-18	-

BPSL0849	AsnC family transcriptional regulator	988476	988979	+	167	53718488	3094258	-	COG1522K	CATGATAGGCAAAGCCGCGAAGCA	1	103	1	103	100	Bm_ATCC23344_chr1	3510148	376732	376834	100	515	1.20E-18	+
BPSL0850	hypothetical protein BPSL0850	988966	989481	-	171	53718489	3094784	-	COG1853R	TCCCGATGACCGCCCTGCTCCCTTC	1	103	103	1	100	Bm_ATCC23344_chr1	3510148	377835	377937	100	515	1.20E-18	-
BPSL0853	putative cyclopropane-fatty-acyl-phospholipid syntha	992374	993594	-	406	53718492	3092925	-	COG2230M	GCGTTTCGTTTGACTCGCGGGCGGC	1	103	103	1	100	Bm_ATCC23344_chr1	3510148	381939	382041	100	515	1.20E-18	-
BPSL0854	pyridoxamine 5'-phosphate oxidase	993726	994388	-	220	53718493	3094226	pdxH	COG0259H	GGCAACGGATAGCCCGCTATTTTGC	1	103	103	1	100	Bm_ATCC23344_chr1	3510148	382733	382835	100	515	1.20E-18	-
BPSL0855	ThiF family protein	994504	995370	+	288	53718494	3091819	-	COG1179H	CGAAGGTGGAAAAAACCTGCGTCG	1	103	1	103	100	Bm_ATCC23344_chr1	3510148	382751	382853	100	515	1.20E-18	+
BPSL0857	hypothetical protein BPSL0857	996545	997426	-	293	53718496	3093200	-	COG1741R	GCGCGCCGCCGCATCCGCCAGGAC	1	103	103	1	100	Bm_ATCC23344_chr1	3510148	385770	385872	99	506	3.00E-18	-
BPSL0864	putative AMP-binding enzyme	1004177	1005742	-	521	53718503	3093176	-	COG0318IQ	AGACTATTGTCCGAACGGCCGATTC	1	103	103	1	100	Bm_ATCC23344_chr1	3510148	394071	394173	100	515	1.20E-18	-
BPSL0865	hypothetical protein BPSL0865	1005872	1006363	+	163	53718504	3093177	-	COG2606S	GTGCCGACCGTGTGGCGACGATCG	1	103	1	103	100	Bm_ATCC23344_chr1	3510148	394103	394205	100	515	1.20E-18	+
BPSL0866	hypothetical protein BPSL0866	1006472	1007083	+	203	53718505	3094044	-	COG0344S	CCCGCGTGGCCCGCTGCGCCGCTTC	1	103	1	103	100	Bm_ATCC23344_chr1	3510148	394703	394805	100	515	1.20E-18	+
BPSL0867	hypothetical protein BPSL0867	1007313	1007798	-	161	53718506	3094045	-	NULL	TTGGGCAAATGTAGGGGCGCCGGT	1	103	103	1	100	Bm_ATCC23344_chr1	3510148	396127	396229	99	506	3.00E-18	-
BPSL0868	putative UDP-N-acetylenolpyruvoylglucosamine redu	1007935	1008984	+	349	53718507	3091554	murB	COG0812M	GCATGGGCACTCGCTATAATTGCGC	1	103	1	103	100	Bm_ATCC23344_chr1	3510148	396166	396268	99	506	3.00E-18	+
BPSL0869	ornithine carbamoyltransferase	1009176	1010105	-	309	53718508	3092982	argF	COG0078E	CAAAGCGTAAAAAATCCTTTATGCT	1	103	103	1	100	Bm_ATCC23344_chr1	3510148	398435	398537	100	515	1.20E-18	-
BPSL0870	hypothetical protein BPSL0870	1010519	1010854	+	111	53718509	3093781	-	NULL	TGCACGGTCGGGCGCGCTCGTCGC	1	103	1	103	100	Bm_ATCC23344_chr1	3510148	398751	398853	99	506	3.00E-18	+
BPSL0871	30S ribosomal protein S20	1011098	1011376	-	92	53718510	3094377	rpsT	COG0268J	TTTTGCGCTCCCGGGCAATCGCTCC	1	103	103	1	100	Bm_ATCC23344_chr1	3510148	399691	399793	100	515	1.20E-18	-
BPSL0872	MviN-like protein	1011781	1013331	+	516	53718511	3093782	-	COG0728R	GGTGAGCGGGGCGTCCCGAGCCGC	1	103	1	103	100	Bm_ATCC23344_chr1	3510148	399998	400100	100	515	1.20E-18	+
BPSL0874	putative short-chain dehydrogenase family protein	1014452	1015210	-	252	53718513	3091633	-	COG1028IQR	CGGGCCTCGGCGCCCGCTCGTACA	1	103	103	1	100	Bm_ATCC23344_chr1	3510148	403514	403616	99	506	3.00E-18	-
BPSL0875	adenylate kinase	1015408	1016070	-	220	53718514	3091823	adk	COG0563F	CAGTCAGCCCGGCCGATCGCGCTTC	1	103	1	103	100	Bm_ATCC23344_chr1	3510148	2361240	2361342	100	515	1.20E-18	+
BPSL0876	3-deoxy-manno-octulosonate cytidylyltransferase	1016279	1017115	-	278	53718515	3091448	kdsB	COG1212M	CGCGCCGTCTCGCACGGCGCCGGC	1	103	1	103	100	Bm_ATCC23344_chr1	3510148	2360195	2360297	100	515	1.20E-18	+
BPSL0878	tetraacyldisaccharide 4'-kinase	1017431	1018459	-	342	53718517	3092050	-	COG1663M	GCCGCCTTTCGAAGACCGCGCGGC	1	103	1	103	100	Bm_ATCC23344_chr1	3510148	2358850	2358952	100	515	1.20E-18	+
BPSL0879	exodeoxyribonuclease VII large subunit	1018910	1020292	+	460	53718518	3091647	-	COG1570L	GTTTCGCGCGACGTGTGGAAACCTC	1	103	103	1	100	Bm_ATCC23344_chr1	3510148	2358490	2358592	100	515	1.20E-18	-
BPSL0880	putative superoxide dismutase	1020489	1021067	+	192	53718519	3094563	sodB	COG0605P	AAGGCCCGCGAGTTTGCGGGGTTA	1	103	103	1	100	Bm_ATCC23344_chr1	3510148	2356911	2357013	100	515	1.20E-18	-
BPSL0882	putative chromate transporter protein	1023539	1024744	-	401	53718521	3092758	-	COG2059P	CCGCAGCCCCCATCCGATCGGCGA	1	103	1	103	100	Bm_ATCC23344_chr1	3510148	2352758	2352860	96	479	4.90E-17	+
BPSL0883	putative regulatory protein	1024978	1025733	-	251	53718522	3092759	-	COG0664T	CGTGTCTCCTCCCGATGCGAGTGAC	1	87	1	87	100	Bm_ATCC23344_chr1	3510148	2351785	2351871	100	435	4.80E-15	+
BPSL0884	hypothetical protein BPSL0884	1025818	1026345	+	175	53718523	3093944	-	COG4539S	CGGCCGTGGCGGTGTCGGGTAGCT	1	87	87	1	100	Bm_ATCC23344_chr1	3510148	2351782	2351868	100	435	4.80E-15	-
BPSL0886	hypothetical protein BPSL0886	1027910	1028515	+	201	53718525	3091854	-	NULL	AACGGCCCGCGAAGCATGAAATCC	1	103	103	1	100	Bm_ATCC23344_chr1	3510148	2349693	2349795	97	488	1.90E-17	-
BPSL0887	hypothetical protein BPSL0887	1028626	1029900	-	424	53718526	3091855	-	NULL	GCGCCGCGCGCGCAGCCGCGGCGT	1	103	1	103	100	Bm_ATCC23344_chr1	3510148	2347584	2347686	100	515	1.20E-18	+
BPSL0888	putative transporter protein	1030572	1032011	-	479	53718527	3093849	-	NULL	CCGCCGCGCAACGCGCCCGCATGC	1	103	1	103	100	Bm_ATCC23344_chr1	3510148	2345502	2345604	100	515	1.20E-18	+
BPSL0889	hypothetical protein BPSL0889	1032308	1032643	+	111	53718528	3093850	-	NULL	TACAGTGCGGGAGACGGCGCGCGA	1	103	103	1	100	Bm_ATCC23344_chr1	3510148	2345303	2345405	100	515	1.20E-18	-
BPSL0891	GntR family transcriptional regulator	1034684	1035430	-	248	53718530	3093798	-	COG2188K	AGCGATTCGGCGCGCGCCGGGCCG	1	103	1	103	100	Bm_ATCC23344_chr1	3510148	2342139	2342241	98	497	7.60E-18	+
BPSL0892	putative aldo/keto reductase family oxidoreductase	1035599	1036444	+	281	53718531	3094218	-	COG0656R	GCGCGAGGCGGCCCGGCGCGCGCC	1	103	103	1	100	Bm_ATCC23344_chr1	3510148	2342068	2342170	97	488	1.90E-17	-
BPSL0892a	putative lipoprotein	1036989	1038575	-	528	53718532	3094219	-	NULL	AGACGATCGGCAATTGGCAGGGCA	1	103	1	103	100	Bm_ATCC23344_chr1	3510148	2339042	2339144	100	515	1.20E-18	+
BPSL0893	elongation factor EF-2	1039748	1041862	-	704	53718533	3092467	fusA	COG0480J	CAAAAGCCACCAGCAAGCGCTATG	1	103	1	103	100	Bm_ATCC23344_chr1	3510148	2335755	2335857	100	515	1.20E-18	+
BPSL0894	hypothetical protein BPSL0894	1042163	1042672	-	169	53718534	3092782	-	COG1430S	TTTTTGCCGCCCTCGCCCGCCCGAG	1	103	1	103	100	Bm_ATCC23344_chr1	3510148	2334939	2335047	94	455	6.00E-16	+
BPSL0895	putative pseudouridine synthase	1043345	1043923	-	192	53718535	3092783	-	COG1187J	ACAAAAGACTCGCCGTTCCGTATTA	1	103	103	1	100	Bm_ATCC23344_chr1	3510148	513601	513703	100	515	1.20E-18	-
BPSL0896	isocitrate dehydrogenase	1044191	1045450	+	419	53718536	3092199	icd	COG0538C	ATAGCGGCTCGCCGTTGCGGACGCA	1	103	1	103	100	Bm_ATCC23344_chr1	3510148	513771	513873	100	515	1.20E-18	+
BPSL0897	putative multicopper oxidase	1045618	1047237	+	539	53718537	3093301	-	COG2132Q	TGGTGCCGCGCGTGCGCGCGTTGTC	1	103	1	103	100	Bm_ATCC23344_chr1	3510148	515198	515300	100	515	1.20E-18	+
BPSL0898	cold shock-like protein	1047367	1047570	-	67	53718538	3093302	-	NULL	CGAGGTGAGCTTGTGAGGGGGGAA	1	103	103	1	100	Bm_ATCC23344_chr1	3510148	2363885	2363987	100	515	1.20E-18	-
BPSL0898a	ATP-dependent Clp protease adaptor protein ClpS	1048103	1048417	+	104	53718539	3091700	clpS	NULL	AGAGGGACGCTGCCGCACGTGGGC	1	103	1	103	100	Bm_ATCC23344_chr1	3510148	2364320	2364422	100	515	1.20E-18	+
BPSL0969	dihydroxy-acid dehydratase	1126530	1128203	-	557	53718611	3093888	ilvD	NULL	TTCTCTAACAGAAACTGCATTCTTGC	1	103	103	1	100	Bm_ATCC23344_chr1	3510148	702418	702520	100	515	1.20E-18	-
BPSL0970	putative isoleucine biosynthesis transcriptional activa	1128381	1129376	+	331	53718612	3092725	ilvR	COG0583K	AGAATGCAGTTTCTGTTAGAGAATG	1	103	1	103	100	Bm_ATCC23344_chr1	3510148	702498	702600	98	497	7.60E-18	+
BPSL0971	prolipoprotein diacylglyceryl transferase	1129483	1130373	+	296	53718613	3094086	lgt	NULL	GCGCGGCGCGCGCGTCGAATCGCC	1	103	1	103	100	Bm_ATCC23344_chr1	3510148	703604	703706	99	506	3.00E-18	+
BPSL0972	hypothetical protein BPSL0972	1130741	1131478	-	245	53718614	3093686	-	COG3471S	TGCCGTGCCGCGTTCGCCGGGCGT	1	103	103	1	100	Bm_ATCC23344_chr1	3510148	705682	705784	100	515	1.20E-18	-
BPSL0973	hypothetical protein BPSL0973	1131677	1132138	-	153	53718615	3093687	-	COG2947S	GCCTAGCGGCTAAGGCGGGGGCCT	1	103	103	1	100	Bm_ATCC23344_chr1	3510148	706342	706444	100	515	1.20E-18	-
BPSL0975	hypothetical protein BPSL0975	1132789	1133139	-	116	53718617	3092529	-	NULL	TTCGCGGCGAGAAGCGGGGGTGTT	1	103	103	1	100	Bm_ATCC23344_chr1	3510148	707343	707445	100	515	1.20E-18	-
BPSL0976	putative outer membrane receptor protein	1133678	1135735	+	685	53718618	3093408	-	COG4206H	CCCGGATACCGGCCGAGGCGGGGA	1	103	1	103	100	Bm_ATCC23344_chr1	3510148	707785	707887	100	515	1.20E-18	+
BPSL0977	putative transmembrane ABC transporter permease	1135814	1136815	+	333	53718619	3093409	-	COG0609P	CGGCCGGCCGCGTCGCCGGCGCGC	1	81	1	81	100	Bm_ATCC23344_chr1	3510148	709943	710023	100	405	1.10E-13	+
BPSL0982	hypothetical protein BPSL0982	1140236	1141072	-	278	53718624	3092134	-	NULL	CGCGGCGGCCCGCGCACGCCGATG	1	103	103	1	100	Bm_ATCC23344_chr1	3510148	715277	715379	99	506	3.00E-18	-
BPSL0986	putative bifunctional cobalamin biosynthesis protein	1144159	1144716	-	185	53718628	3091756	-	COG2087H	TGGCGTTGGCGTTGGCAATCATCGT	1	103	103	1	100	Bm_ATCC23344_chr1	3510148	718909	719011	100	515	1.20E-18	-
BPSL0989	ParA family ATPase	1147399	1148022	+	207	53718631	3094388	-	COG1192D	GCAGCCGCATTTGCCGATATCATCG	1	103	1	103	100	Bm_ATCC23344_chr1	3510148	721494	721596	100	515	1.20E-18	+
BPSL0990	aspartate 1-decarboxylase precursor	1148137	1148523	-	128	53718632	3093259	panD	NULL	CGGCCGGCCACGCAGCCGCCGCCT	1	55	55	1	100	Bm_ATCC23344_chr1	3510148	722721	722775	100	275	8.00E-08	-
BPSL0991	pantoate--beta-alanine ligase	1148576	1149415	-	279	53718633	3093228	panC	NULL	ATCGCCGACGCTCGACGCCCTGCGC	1	103	103	1	100	Bm_ATCC23344_chr1	3510148	723613	723715	100	515	1.20E-18	-
BPSL0993	hypothetical protein BPSL0993	1151047	1151235	-	62	53718635	3094637	-	NULL	TCGACGCCGTTTGCCGCCGGCTGTT	1	103	103	1	100	Bm_ATCC23344_chr1	3510148	725437	725541	96	477	6.10E-17	-
BPSL0994	hypothetical protein BPSL0994	1151442	1153259	+	605	53718636	3094638	-	COG0729M	GAGCGTCGTGCGCGAATGTGGCCG	1	103	1	103	100	Bm_ATCC23344_chr1	3510148	725548	725650	99	506	3.00E-18	+
BPSL0996	hypothetical protein BPSL0996	1157739	1158449	+	236	53718638	3092185	-	NULL	CATGGCAGCGCGCGTGGGCCGGCC	1	103	1	103	100	Bm_ATCC23344_chr1	3510148	731845	731950	95	467	1.70E-16	+
BPSL0998	methionyl-tRNA synthetase	1159815	1161992	-	725	53718640	3091950	metG	COG0073R, COG0143J	GCGCGATTCCGCGCGGTCCGCCCG	1	103	103	1	100	Bm_ATCC23344_chr1	3510148	736192	736294	100	515	1.20E-18	-
BPSL0999	putative OmpA family transmembrane protein	1162474	1163121	-	215	53718641	3093683	-	COG2885M	GCCCGCGCGCCCGCACGATGGTCG	1	103	103	1	100	Bm_ATCC23344_chr1	3510148	737322	737425	98	496	8.40E-18	-
BPSL1000	putative ATP-binding protein	1163502	1164590	+	362	53718642	3093990	-	COG0489D	GGCGTGCAATAAATTCTTTCACTTTT	1	103	1	103	100	Bm_ATCC23344_chr1	3510148	737606	737708	100	515	1.20E-18	+
BPSL1001	putative membrane-attached superoxide dismutase	1164723	1165262	+	179	53718643	3093991	-	NULL	GCGGCGCCGCGAGTCACGGTATCA	1	103	1	103	100	Bm_ATCC23344_chr1	3510148	738827	738929	99	506	3.00E-18	+
BPSL1002	deoxycytidine triphosphate deaminase	1165366	1165935	+	189	53718644	3094702	dcd	COG0717F	GAGTCGGTGCGTTCACGATCGCGTA	1	103	1	103	100	Bm_ATCC23344_chr1	3510148	739470	739572	100	515	1.20E-18	+
BPSL1003	putative ornithine decarboxylase	1166038	1168317	+	759	53718645	3093996	-	COG1982E	TGCTTGCATATCGGTCTGACCTATC	1	103	1	103	100	Bm_ATCC23344_chr1	3510148	740142	740244	100	515	1.20E-18	+
BPSL1006	argininosuccinate lyase	1169883	1171292	-	469	53718648	3092554	argH	COG0165E	GCCGGGCGAGCGCCGGCATCGGCC	1	103	103	1	100	Bm_ATCC23344_chr1	3510148	745494	745596	100	515	1.20E-18	-
BPSL1007	putative fimbriae usher protein	1171461	1173761	-	766	53718649	3091591	-	COG3188NU	CCTATTCGCATCGCCGTCACTCCCG	1	103	103	1	100	Bm_ATCC23344_chr1	3510148	747963	748059	94	425	1.40E-14	-
BPSL1009	hypothetical protein BPSL1009	1175234	1175752	-	172	53718651	3091869	-	COG5430S	GTCTTCAATTTATCGCTCGCCCGTTC	1	103	103	1	100	Bm_ATCC23344_chr1	3510148	751143	751245	99	506	3.00E-18	-
BPSL1010	putative lipoprotein	1176147	1177136	+	329	53718652	3092911	-	COG5430S	GGTGTGGATCGAACCTCTTTTTTATC	1	103	1	103	100	Bm_ATCC23344_chr1	3510148	751440	751542	100	515	1.20E-18	+
BPSL1011	putative DNA-binding protein	1177309	1178124	-	271	53718653	3092912	-	NULL	AGGTACCGGTACAAGCCAATCAGT	1	103	103	1	100	Bm_ATCC23344_chr1	3510148	753515	753617	98	497	7.60E-18	-
BPSL1012	putative biotin biosyntehsis related protein	1178289	1179062	+	257	53718654	3091472	-	COG0500QR	GATACCAGGATAACTGATTGGCTTG	1	103	1	103	100	Bm_ATCC23344_chr1	3510148	753582	753684	100	515	1.20E-18	+
BPSL1013	phosphoenolpyruvate carboxylase	1179480	1182464	-	994	53718655	3091473	-	COG2352C	GTCGCCAGCCGTGCGGCATGCGCC	1	103	103	1	100	Bm_ATCC23344_chr1	3510148	759422	759524	97	488	1.90E-17	-
BPSL1014	hypothetical protein BPSL1014	1182577	1182756	+	59	53718656	3093060	-	NULL	TGGGAAAGCACGAGTGAACGGGAA	1	103	1	103	100	Bm_ATCC23344_chr1	3510148	759437	759539	97	488	1.90E-17	+
BPSL1019	3-ketoacyl-(acyl-carrier-protein) reductase	1188469	1189227	-	252	53718661	3092104	-	COG1028IQR	GCCGGGCGGTGACGCGCGCGCTGC	1	91	91	1	100	Bm_ATCC23344_chr1	3510148	766197	766287	97	437	3.90E-15	-
BPSL1020	putative aldehyde dehydrogenase	1189316	1190755	-	479	53718662	3092105	-	COG1012C	ATGGAAAGCGCATTATAGCCAATCC	1	103	103	1	100	Bm_ATCC23344_chr1	3510148	767725	767827	100	515	1.20E-18	-
BPSL1021	inorganic pyrophosphatase	1190916	1191443	+	175	53718663	3094000	ppa	COG0221C	GCAAGCGCCATCGGGCGATTGGCT	1	103	1	103	100	Bm_ATCC23344_chr1	3510148	767788	767890	100	515	1.20E-18	+
BPSL1022	hypothetical protein BPSL1022	1191502	1192266	-	254	53718664	3093404	-	COG2827L	GGCGCGGCGTCGAGCCGCTATCGC	1	103	103	1	100	Bm_ATCC23344_chr1	3510148	769368	769470	100	515	1.20E-18	-
BPSL1025	hypothetical protein BPSL1025	1193265	1194443	-	392	53718665	3094827	-	COG3146S	GGCGCGAACGGCTTTGGCAAACGC	1	93	93	1	100	Bm_ATCC23344_chr1	3510148	771546	771638	98	456	5.40E-16	-

BPSL1026	NAD(+) synthase	1194534	1196264 +	576	53718666	3092495 -	COG0171H, COG0388R	CGATTCGAATCCGTTTCGTTCGTAC	1	93	1	93	100	Bm_ATCC23344_chr1	3510148	771549	771641	98	456	5.40E-16 +
BPSL1027	nitrogen regulatory protein P-II 2	1196325	1196663 +	112	53718667	3093736 glnB2	COG0347E	CGCGGCGCGCGGCGTAGAATCGG	1	63	1	63	100	Bm_ATCC23344_chr1	3510148	773370	773432	100	315	1.30E-09 +
BPSL1029	putative exported outer membrane porin protein	1198437	1199537 -	366	53718669	3092496 -	COG3203M	CGCCGAACCGGCTGCACGCGGTCT	1	103	103	1	100	Bm_ATCC23344_chr1	3510148	775335	775437	100	515	1.20E-18 -
BPSL1032	histidine transport system permease protein	1201931	1202620 -	229	53718672	3094529 hisQ	COG4215E	GCCCGCGAAAGCGGAGCGGCGAT	1	103	103	1	100	Bm_ATCC23344_chr1	3510148	778419	778521	100	515	1.20E-18 -
BPSL1033	histidine-binding periplasmic protein precursor	1202831	1203607 -	258	53718673	3094022 hisJ	COG0834ET	GATCGACGAACGCGAATTTTGGCC	1	103	103	1	100	Bm_ATCC23344_chr1	3510148	779406	779508	100	515	1.20E-18 -
BPSL1035	hypothetical protein BPSL1035	1205046	1205744 -	232	53718675	3091915 -	NULL	GGCGTGAAGTTTACCGTGTGGGGT	1	91	91	1	100	Bm_ATCC23344_chr1	3510148	781532	781622	100	455	6.00E-16 -
BPSL1038	hypothetical protein BPSL1038	1207929	1208195 -	88	53718678	3093799 -	NULL	CGGCGAGCGCGGGCGAGGCCTTC	1	103	103	1	100	Bm_ATCC23344_chr1	3510148	783956	784058	100	515	1.20E-18 -
BPSL1038a	hypothetical protein BPSL1038a	1208322	1208597 -	91	53718679	3093800 -	NULL	CAGGCGCCCTCCCCGGCAAGGCCG	1	97	97	1	100	Bm_ATCC23344_chr1	3510148	784358	784454	100	485	2.60E-17 -
BPSL1039	putative ABC transport system, membrane protein	1208692	1210449 +	585	53718680	3091509 -	COG4986P	AACGAATCACCGCACGAGCGCGCC	1	97	1	97	100	Bm_ATCC23344_chr1	3510148	784361	784457	100	485	2.60E-17 +
BPSL1040	putative ABC transport system, ATP-binding protein	1210503	1211843 +	446	53718681	3091510 -	COG1116P, COG3842E	CACAGATTTGAGACCGATGTCAGC	1	56	1	56	100	Bm_ATCC23344_chr1	3510148	786213	786268	100	280	4.80E-08 +
BPSL1043	putative lipoprotein	1212731	1213282 -	183	53718684	3093837 -	NULL	TGTCAATTGTTTGCATTTCTCGTCGG	1	103	103	1	100	Bm_ATCC23344_chr1	3510148	3112296	3112397	97	482	3.60E-17 -
BPSL1044	alpha,alpha-trehalose-phosphate synthase	1213901	1215319 +	472	53718685	3092835 otsA	COG0380G	ACGTTCGAAATGTCGCGAGGTGGC	1	103	1	103	100	Bm_ATCC23344_chr1	3510148	3112816	3112920	97	486	2.40E-17 +
BPSL1046	hypothetical protein BPSL1046	1216087	1216698 -	203	53718687	3093344 -	NULL	CCGGCTGTCCAATACATCGTGCCC	1	103	103	1	100	Bm_ATCC23344_chr1	3510148	3115713	3115815	100	515	1.20E-18 -
BPSL1047	hypothetical protein BPSL1047	1217068	1217325 +	85	53718688	3093345 -	NULL	GTGCGGGAGGTGGCGATCGCTTCG	1	103	1	103	100	Bm_ATCC23344_chr1	3510148	3115974	3116076	98	497	7.60E-18 +
BPSL1048	putative sugar transport, integral membrane protein	1217526	1218953 -	475	53718689	3094165 -	NULL	TCCGCCCCGGCCGGGCGCTGCGCC	1	72	72	1	100	Bm_ATCC23344_chr1	3510148	3117890	3117961	100	360	1.20E-11 -
BPSL1049	hypothetical protein BPSL1049	1219023	1219535 -	170	53718690	3094166 -	COG1881R	GCGCCGGTGATACACTCGACCGGC	1	103	103	1	100	Bm_ATCC23344_chr1	3510148	3118472	3118574	100	515	1.20E-18 -
BPSL1050	hypothetical protein BPSL1050	1220110	1220490 -	126	53718691	3091599 -	NULL	AATAGATGCAGCAAGGGGGTAACG	1	103	103	1	100	Bm_ATCC23344_chr1	3510148	3119427	3119529	98	497	7.60E-18 -
BPSL1053	putative lipoprotein	1221524	1221904 -	126	53718692	3094633 -	NULL	CTTACGTGATTGTAATGTGCGGACA	1	103	103	1	100	Bm_ATCC23344_chr1	3510148	3120841	3120943	100	515	1.20E-18 -
BPSL1054	ecotin precursor	1222077	1222616 +	179	53718693	3094320 eco	NULL	TTGTGTCCGCACATTACAATCACGT	1	103	1	103	100	Bm_ATCC23344_chr1	3510148	3120916	3121018	100	515	1.20E-18 +
BPSL1055	murein-DD-endopeptidase	1222714	1223850 -	378	53718694	3094634 -	COG1686M	ACACCATCAATATCGCCGTCGCCG	1	103	103	1	100	Bm_ATCC23344_chr1	3510148	3122787	3122889	100	515	1.20E-18 -
BPSL1057	hypothetical protein BPSL1057	1224788	1225264 +	158	53718695	3093766 -	NULL	ATGCCGCAATCGCGCGACGGGCCG	1	103	1	103	100	Bm_ATCC23344_chr1	3510148	3123627	3123729	98	497	7.60E-18 +
BPSL1062	translation initiation factor IF-1	1228340	1228606 +	88	53718699	3092726 infA	COG0361J	TTCCAGATAACGCGGGCGCTCGCC	1	103	1	103	100	Bm_ATCC23344_chr1	3510148	2283970	2284072	100	515	1.20E-18 +
BPSL1065	hypothetical protein BPSL1065	1230018	1231604 -	528	53718702	3093116 -	NULL	CGGCCCGACGACGTTTGAAGGTAT	1	103	103	1	100	Bm_ATCC23344_chr1	3510148	2287332	2287434	99	506	3.00E-18 -
BPSL1067	hypothetical protein BPSL1067	1232690	1233145 +	151	53718704	3093117 -	NULL	GTGTGGCGCCCGCCGCCTGGGCGC	1	103	1	103	100	Bm_ATCC23344_chr1	3510148	2288236	2288338	97	488	1.90E-17 +
BPSL1068	putative ABC transport system, ATP-binding protein	1233313	1235259 +	648	53718705	3093263 -	COG0488R	CGGCGTTGCGCGTTTCGCGCCCGG	1	103	1	103	100	Bm_ATCC23344_chr1	3510148	2288859	2288961	100	515	1.20E-18 +
BPSL1069	DNA topoisomerase IV subunit B	1235465	1237447 +	660	53718706	3093443 parE	COG0187L	TTGTTTCGACTCGGATTTTTATCGAA	1	103	1	103	100	Bm_ATCC23344_chr1	3510148	2291011	2291113	100	515	1.20E-18 +
BPSL1070	DNA topoisomerase IV subunit A	1237506	1239824 +	772	53718707	3093260 parC	COG0188L	GCGCGGCCGGGCGCGCGCCCGGC	1	61	1	61	100	Bm_ATCC23344_chr1	3510148	2293094	2293154	100	305	3.60E-09 +
BPSL1071	hypothetical protein BPSL1071	1240517	1240975 +	152	53718708	3093264 -	COG5615S	AACCGCCGCGCGTCTCGGCTGTCG	1	103	1	103	100	Bm_ATCC23344_chr1	3510148	2296063	2296165	96	479	4.90E-17 +
BPSL1072	putative LysR-family transcriptional regulatory protei	1241121	1242005 -	294	53718709	3093453 -	COG0583K	CAATTAACGAGACGGAGCCATTTTC	1	96	96	1	100	Bm_ATCC23344_chr1	3510148	2297626	2297721	96	453	7.30E-16 -
BPSL1073	hypothetical protein BPSL1073	1242099	1242566 +	155	53718710	3093454 -	NULL	CGCGAATGCTCCTTTGATATATCGG	1	96	1	96	100	Bm_ATCC23344_chr1	3510148	2297629	2297724	96	453	7.30E-16 +
BPSL1074	putative LysR family transcriptional regulator	1242775	1243737 -	320	53718711	3091778 -	COG0583K	CGTTTTATCGCCGTCCATTAACTATT	1	103	103	1	100	Bm_ATCC23344_chr1	3510148	2299373	2299475	100	515	1.20E-18 -
BPSL1075	putative transport system, integral membrane protei	1243953	1245266 +	437	53718712	3091779 -	NULL	CCCGCGTGCGGCGCAGGCCGGGC	1	103	1	103	100	Bm_ATCC23344_chr1	3510148	2299491	2299593	99	506	3.00E-18 +
BPSL1079	putative metal transport integral membrane protein	1248218	1249411 -	397	53718715	3093557 -	COG2059P	CAACGAACGGTTTTCAGCGTGCAC	1	103	103	1	100	Bm_ATCC23344_chr1	3510148	2305016	2305118	100	515	1.20E-18 -
BPSL1080	hypothetical protein BPSL1080	1249558	1251918 -	786	53718716	3093558 -	COG5001T	CATTCAGTAATATCGGGCCTCGTCT	1	103	103	1	100	Bm_ATCC23344_chr1	3510148	2307523	2307625	99	506	3.00E-18 -
BPSL1082	hypothetical protein BPSL1082	1253042	1253509 -	155	53718718	3094041 -	COG0251J	CGCGCCTCGTTCGCTCACGCATTCA	1	58	58	4	94.5455	Bm_ATCC23344_chr1	3510148	2309123	2309177	96	257	5.20E-07 -
BPSL1085	hypothetical protein BPSL1085	1255816	1256499 -	227	53718721	3094532 -	NULL	CTGTGCCGGTCGCCGCGATTACAG	1	103	1	103	100	Bm_ATCC23344_chr1	3510148	2048506	2048608	100	515	1.20E-18 +
BPSL1086	putative GntR family regulatory protein	1256635	1258104 +	489	53718722	3094533 -	COG1167KE	AACGCGCGCGGCGCCCCCGCGCCC	1	103	103	1	100	Bm_ATCC23344_chr1	3510148	2048468	2048570	100	515	1.20E-18 -
BPSL1087	heat shock protein 90	1258240	1260138 +	632	53718723	3091971 htpG	COG0326O	AGCGTTGCGGCGGGCGGCACGCCC	1	103	103	1	100	Bm_ATCC23344_chr1	3510148	2046863	2046965	99	506	3.00E-18 -
BPSL1091	hypothetical protein BPSL1091	1261467	1262396 +	309	53718726	3093821 -	COG0421E	CCCGATGAAGCGGGCCAGCCGGC	1	103	103	1	100	Bm_ATCC23344_chr1	3510148	2043636	2043738	100	515	1.20E-18 -
BPSL1092	hypothetical protein BPSL1092	1262588	1262827 +	79	53718727	3093822 -	NULL	CGCCAGGGGAGTCGCAACAATATA	1	103	103	1	100	Bm_ATCC23344_chr1	3510148	2042515	2042617	100	515	1.20E-18 -
BPSL1094	hypothetical protein BPSL1094	1264502	1264936 -	144	53718729	3093488 -	COG2764S	CCGCTTCTCACGATCCCCGTCACTG	1	103	1	103	100	Bm_ATCC23344_chr1	3510148	2040071	2040173	100	515	1.20E-18 +
BPSL1095	transaldolase	1265301	1266254 -	317	53718730	3094101 talB	NULL	CCGGCGACGCGCGCCCAGCGCGTC	1	103	1	103	100	Bm_ATCC23344_chr1	3510148	2038752	2038854	100	515	1.20E-18 +
BPSL1097	hypothetical protein BPSL1097	1267966	1269141 -	391	53718732	3092613 -	COG3825S	CGCCCGACGCGGGCGCGCTGCGG	1	103	1	103	100	Bm_ATCC23344_chr1	3510148	2035874	2035976	99	506	3.00E-18 +
BPSL1098	hypothetical protein BPSL1098	1269354	1270196 -	280	53718733	3091862 -	COG0714R	ATGCGGCCCTGCCGAGCACGCCCC	1	103	1	103	100	Bm_ATCC23344_chr1	3510148	2034819	2034921	100	515	1.20E-18 +
BPSL1099	putative cytochrome C precursor-related protein	1270430	1270789 +	119	53718734	3091863 -	COG2863C	TGGCCGTTTTTTTGGCCTGCGTGGC	1	103	103	1	100	Bm_ATCC23344_chr1	3510148	2034683	2034785	100	515	1.20E-18 -
BPSL1101	hypothetical protein BPSL1101	1271697	1272248 -	183	53718736	3094149 -	NULL	CCGCCCCGACGCCGGGCGCGCCG	1	103	1	103	100	Bm_ATCC23344_chr1	3510148	2031461	2031563	100	515	1.20E-18 +
BPSL1102	hypothetical protein BPSL1102	1272686	1273756 +	356	53718737	3091536 -	NULL	GCGGGGCGCCGCGCGGGCTGCAA	1	103	103	1	100	Bm_ATCC23344_chr1	3510148	2031121	2031223	98	497	7.60E-18 -
BPSL1104	ferredoxin	1274538	1275410 -	290	53718739	3091537 -	COG2878C	TGCGAAAAATCGGCGATAATTGTTG	1	103	1	103	100	Bm_ATCC23344_chr1	3510148	2028299	2028401	100	515	1.20E-18 +
BPSL1105	putative TetR family regulatory protein	1275668	1276300 +	210	53718740	3093813 -	COG1309K	GGTGTCCCTTAACGATGGCGCGCC	1	103	103	1	100	Bm_ATCC23344_chr1	3510148	2028139	2028241	100	515	1.20E-18 -
BPSL1106	putative depolymerase/histone-like protein	1276435	1277901 -	488	53718741	3093814 -	COG4553I	ATTCGATTTCACCCCCCGAATCGGC	1	103	1	103	100	Bm_ATCC23344_chr1	3510148	2025800	2025902	100	515	1.20E-18 +
BPSL1107	hypothetical protein BPSL1107	1278121	1279953 -	610	53718742	3091503 -	COG3387G	CGCCGTCCGCCGAGTATACGGGCC	1	103	1	103	100	Bm_ATCC23344_chr1	3510148	2023748	2023850	100	515	1.20E-18 +
BPSL1108	hypothetical protein BPSL1108	1280130	1281365 +	411	53718743	3091504 -	COG2706G	GGCCCGTATACTCGGCGGACGGCG	1	103	103	1	100	Bm_ATCC23344_chr1	3510148	2023669	2023771	100	515	1.20E-18 -
BPSL1112	putative lipoprotein	1290012	1290233 +	73	53718747	3092291 -	NULL	AGTGCTGACGAGTGCTGACGTGTG	1	103	103	2	99	Bm_ATCC23344_chr1	3510148	2014131	2014232	98	492	1.30E-17 -
BPSL1113	hypothetical protein BPSL1113	1290292	1290678 +	128	53718748	3092292 -	COG3895R	TGGCGCGGCGCGCGGTTTGCGCGC	1	61	61	1	100	Bm_ATCC23344_chr1	3510148	2013851	2013911	100	305	3.60E-09 -
BPSL1114	putative ATP-dependent RNA helicase 2	1291364	1292812 +	482	53718749	3092874 rhlE2	COG0513LKJ	CGCGGCTTCATGTTCATTCCGAAGT	1	103	103	1	100	Bm_ATCC23344_chr1	3510148	2012839	2012941	100	515	1.20E-18 -
BPSL1116	hypothetical protein BPSL1116	1294247	1295146 -	299	53718751	3092537 -	COG1054R	CCCGTTTTCGTCCGCTTGCGCCCAG	1	103	1	103	100	Bm_ATCC23344_chr1	3510148	2008927	2009029	100	515	1.20E-18 +
BPSL1117	DNA polymerase III subunit alpha	1295759	1299283 +	1174	53718752	3093623 dnaE	COG0587L	CGGCCCGCCGCCCGCGCCGGTCTT	1	103	103	1	100	Bm_ATCC23344_chr1	3510148	2008403	2008505	98	497	7.60E-18 -
BPSL1119	putative LPS biosynthesis-related protein	1301167	1302249 +	360	53718754	3091748 -	COG0438M	GCGGCGCACGCCCGGCCGAGCGA	1	56	56	1	100	Bm_ATCC23344_chr1	3510148	2002995	2003050	100	280	4.80E-08 -
BPSL1120	putative transferase (LPS biosynthesis-related)	1302531	1303676 +	381	53718755	3092407 -	COG0859M	TATGAATCGTGGAAATTCTGCATGA	1	103	103	1	100	Bm_ATCC23344_chr1	3510148	2001631	2001733	100	515	1.20E-18 -
BPSL1123	putative LPS core biosynthesis-related protein	1306305	1307489 +	394	53718758	3094542 -	COG0859M	GCTCGCTCGCTTCGCACGGGCGCG	1	77	77	1	100	Bm_ATCC23344_chr1	3510148	1997857	1997933	98	376	2.20E-12 -
BPSL1124	putative LPS biosynthesis-related protein	1307754	1308518 -	254	53718760	3093371 -	COG0463M	GCCCGCTCGTACGATCGCGCCGGC	1	103	1	103	100	Bm_ATCC23344_chr1	3510148	1995544	1995646	99	506	3.00E-18 +
BPSL1125	hypothetical protein BPSL1125	1308976	1309356 +	126	53718761	3093885 -	COG5455S	ATCGACCCGCCATTCCCGGCGAAT	1	103	103	1	100	Bm_ATCC23344_chr1	3510148	1995184	1995286	99	506	3.00E-18 -
BPSL1126	hypothetical protein BPSL1126	1309637	1310230 -	197	53718762	3093886 -	NULL	CCCACGGTCGCCACCGCCGCTCGC	1	103	1	103	100	Bm_ATCC23344_chr1	3510148	1993832	1993934	100	515	1.20E-18 +
BPSL1128	putative lipoprotein	1311772	1312755 -	327	53718764	3093796 -	NULL	CTCACAGGTTAAACTCTGTCCTCCC	1	103	1	103	100	Bm_ATCC23344_chr1	3510148	1991346	1991448	100	515	1.20E-18 +
BPSL1129	hypothetical protein BPSL1129	1313442	1313894 -	150	53718765	3091501 -	COG2128S	ATCTCCCGCGCGCGCGGCGAGCTG	1	103	1	103	100	Bm_ATCC23344_chr1	3510148	1990216	1990316	98	485	2.60E-17 +
BPSL1130	putative sigma factor	1314397	1315290 -	297	53718766	3091502 -	COG1595K	CCCCGCCGCGCCCGCGGCTGTCAC	1	103	1	103	100	Bm_ATCC23344_chr1	3510148	1988827	1988929	100	515	1.20E-18 +
BPSL1132	hypothetical protein BPSL1132	1316037	1316330 -	97	53718767	3094623 -	NULL	CGCGCCGGGCATCGCCTAGAATTT	1	103	1	103	100	Bm_ATCC23344_chr1	3510148	1987787	1987889	100	515	1.20E-18 +
BPSL1134	hypothetical protein BPSL1134	1318558	1319238 +	226	53718769	3093357 -	NULL	TTCGACATAGAACCTACACGAAAG	1	103	103	1	100	Bm_ATCC23344_chr1	3510148	1985665	1985767	99	506	3.00E-18 -
BPSL1135	hypothetical protein BPSL1135	1319300	1320697 +	465	53718770	3092970 -	NULL	GCGAACCGGCGTGCGCGTTGCCGT	1	64	64	1	100	Bm_ATCC23344_chr1	3510148	1984923	1984986	100	320	7.50E-10 -
BPSL1136	hypothetical protein BPSL1136	1320725	1322113 -	462	53718771	3092971 -	COG2233F	CGTCGAACACGCGCGCGGCCAATC	1	103	1	103	100	Bm_ATCC23344_chr1	3510148	1982012	1982114	99	506	3.00E-18 +
BPSL1159	Maf-like protein	1339113	1339742 -	209	53718795	3093041 maf	COG0424D	CGGTGCGTCGCCCCGACGCGCCGC	1	55	1	55	100	Bm_ATCC23344_chr1	3510148	1979638	1979692	100	275	8.00E-08 +
BPSL1160	hypothetical protein BPSL1160	1339795	1340265 -	156	53718796	3093042 -	COG1576S	GCGCGCGCCGCCGTTTCGTCGCCT	1	59	1	59	100	Bm_ATCC23344_chr1	3510148	1979111	1979169	98	286	2.60E-08 +
BPSL1163	coproporphyrinogen III oxidase	1341540	1342463 -	307	53718799	3092344 hemF	NULL	CAAGCCGGCCGGCGCGCCCTCTCC	1	103	1	103	100	Bm_ATCC23344_chr1	3510148	1976869	1976971	100	515	1.20E-18 +

BPSL1164	phosphoribosylamine--glycine ligase	1342594	1343871	-	425	53718800	3094818	purD	COG0151F	GCCGCCCTTTCGCGCGAAGGACGG	1	103	1	103	100	Bm_ATCC23344_chr1	3510148	1975461	1975563	100	515	1.20E-18	+
BPSL1165	hypothetical protein BPSL1165	1343982	1344710	-	242	53718801	3093513	-	NULL	GGCCCAAGCGGCACTCGCCCGCTC	1	103	1	103	100	Bm_ATCC23344_chr1	3510148	1974622	1974724	100	515	1.20E-18	+
BPSL1166	uracil phosphoribosyltransferase	1345220	1345870	-	216	53718802	3091557	upp	COG0035F	CGGATCGCGCGCGCCCCATCTTCG(	1	103	1	103	100	Bm_ATCC23344_chr1	3510148	1973477	1973579	100	515	1.20E-18	+
BPSL1167	putative dehydrogenase	1346128	1346907	+	259	53718803	3093514	-	COG1028IQR	CAGCGCGCGGGGGCGGCGGCAAC(	1	103	103	1	100	Bm_ATCC23344_chr1	3510148	1973317	1973419	100	515	1.20E-18	-
BPSL1170	hypothetical protein BPSL1170	1349820	1349912	+	30	53718806	3093535	-	NULL	ATGCGGTTTACATAGGCGGTCTGG(	1	103	103	1	100	Bm_ATCC23344_chr1	3510148	1969646	1969748	100	515	1.20E-18	-
BPSL1172	potassium-transporting ATPase b chain	1351813	1353873	+	686	53718808	3092345	kdpB	COG2216P	GCGCGCATCACGACACGAGAGGAC	1	98	98	1	100	Bm_ATCC23344_chr1	3510148	1967653	1967750	100	490	1.60E-17	-
BPSL1174	two-component system, sensor kinase protein KdpD	1354752	1357733	+	993	53718810	3094181	kdpD	COG2205T	CTCGAACGCCGGACGGCGCGCCTG	1	103	103	1	100	Bm_ATCC23344_chr1	3510148	1964713	1964815	99	506	3.00E-18	-
BPSL1176	hypothetical protein BPSL1176	1358812	1359108	+	98	53718812	3093536	-	COG3042R	CTCCGGGGCGGCCCGAGCACGTTT	1	103	103	1	100	Bm_ATCC23344_chr1	3510148	1960661	1960763	99	506	3.00E-18	-
BPSL1177	putative chaperone	1359324	1359644	+	106	53718813	3092893	sugE	COG2076P	AGGCTGTAAGCCTGTTGTTATACTT	1	103	103	1	100	Bm_ATCC23344_chr1	3510148	1960149	1960251	100	515	1.20E-18	-
BPSL1178	hypothetical protein BPSL1178	1359836	1360453	+	205	53718814	3094526	-	NULL	CGCGCGTGAATCGGCGGGGCCGG(	1	103	103	1	100	Bm_ATCC23344_chr1	3510148	1959637	1959739	98	497	7.60E-18	-
BPSL1180	putative globin	1363156	1363602	+	148	53718816	3093896	-	NULL	AGCGCGCGCACGCGGTATCTTCGG(	1	66	66	1	100	Bm_ATCC23344_chr1	3510148	1956390	1956455	100	330	2.60E-10	-
BPSL1181	putative alanyl-tRNA synthetase	1363713	1364444	+	243	53718817	3093897	-	COG2872R	CGCTTCGGGGCGTGCCGTGCGCAA	1	103	103	1	100	Bm_ATCC23344_chr1	3510148	1955833	1955935	99	506	3.00E-18	-
BPSL1183	amidase	1365426	1366820	+	464	53718819	3094688	-	NULL	CCGCCGCCGTTTTTGCGTTGCTATC(	1	103	103	1	100	Bm_ATCC23344_chr1	3510148	1954120	1954222	100	515	1.20E-18	-
BPSL1185	hypothetical protein BPSL1185	1368695	1370602	-	635	53718821	3092923	-	COG1807M	GACTGCGGCGCAAGCCGCGCACCT	1	103	1	103	100	Bm_ATCC23344_chr1	3510148	1947512	1947614	100	515	1.20E-18	+
BPSL1186	glucose-1-dehydrogenase	1371117	1371932	-	271	53718822	3093594	-	COG1028IQR	CCCGGCGGGGCGGTTTTCGCGGCG	1	103	1	103	100	Bm_ATCC23344_chr1	3510148	1946182	1946284	98	497	7.60E-18	+
BPSL1188	putative TetR-family transcriptional regulator	1372891	1373610	-	239	53718824	3092213	-	COG1309K	ATCGGCCGACAGCCTTCAAAACAG(	1	103	1	103	100	Bm_ATCC23344_chr1	3510148	1944518	1944620	99	506	3.00E-18	+
BPSL1189	putative kinase	1373847	1374317	-	156	53718825	3092214	-	NULL	AACCAGCCATGCGCGCGAAAGCGC	1	86	1	86	100	Bm_ATCC23344_chr1	3510148	1943828	1943913	98	421	2.00E-14	+
BPSL1191	hypothetical protein BPSL1191	1375457	1376395	-	312	53718827	3093909	-	COG2908S	TTCCCCGTAATAAGAACGTCTCGTG	1	93	1	93	100	Bm_ATCC23344_chr1	3510148	1941743	1941835	100	465	2.10E-16	+
BPSL1195	RNA polymerase sigma-70 factor	1378207	1378767	-	186	53718831	3093318	-	COG1595K	ACAGGATGCCGCGCGCCGCGAAAA	1	103	1	103	100	Bm_ATCC23344_chr1	3510148	1939361	1939463	100	515	1.20E-18	+
BPSL1196	acetolactate synthase III large subunit	1379163	1380926	+	587	53718832	3093947	ilvI	COG0028EH	TGAAGCCCGCCTTTCAACTGGACG(	1	103	103	1	100	Bm_ATCC23344_chr1	3510148	1939063	1939165	100	515	1.20E-18	-
BPSL1197	acetolactate synthase III small subunit	1381038	1381529	+	163	53718833	3094328	ilvH	COG0440E	GCTCGAAGCCGCGGGCGGCCGCCT	1	103	103	1	100	Bm_ATCC23344_chr1	3510148	1937188	1937290	100	515	1.20E-18	-
BPSL1198	ketol-acid reductoisomerase	1381612	1382628	+	338	53718834	3092052	ilvC	COG0059EH	CGCCGCCGCGACCAACGAGCCGG(	1	85	85	1	100	Bm_ATCC23344_chr1	3510148	1936614	1936698	100	425	1.40E-14	-
BPSL1199	phosphatidylserine decarboxylase	1382750	1383400	+	216	53718835	3093663	-	COG0688I	CTCGAATCGAGGGTCGGCATCAAA	1	103	103	1	100	Bm_ATCC23344_chr1	3510148	1935476	1935578	100	515	1.20E-18	-
BPSL1201	2-isopropylmalate synthase	1384648	1386195	+	515	53718837	3091966	leuA	COG0119E	CCGTTTTCGCCCTCCGTCGAGCTTC(	1	103	103	1	100	Bm_ATCC23344_chr1	3510148	1933578	1933680	100	515	1.20E-18	-
BPSL1202	putative transport-related membrane protein	1386330	1387877	-	515	53718838	3093654	-	COG0659P	GCCGCGGCCGCCGCGACATCGCAC	1	60	1	60	100	Bm_ATCC23344_chr1	3510148	1929051	1929109	98	285	2.80E-08	+
BPSL1203	putative carbonic anhydrase	1387935	1388570	-	211	53718839	3093655	-	NULL	CTAATATCACTCACAACAACCTTTC/	1	103	1	103	100	Bm_ATCC23344_chr1	3510148	1928315	1928417	100	515	1.20E-18	+
BPSL1204	putative lipoprotein	1389014	1389514	-	166	53718840	3092299	-	NULL	CGGCATATGAATGCCGCGGCGCAA	1	103	1	103	100	Bm_ATCC23344_chr1	3510148	1927370	1927472	99	506	3.00E-18	+
BPSL1205	putative amino acid transport system, exported prot	1389893	1391170	+	425	53718841	3092300	-	COG0683E	GGCGCGAGGTGTCCGCCGGCGCGC	1	103	103	1	100	Bm_ATCC23344_chr1	3510148	1927093	1927195	100	515	1.20E-18	-
BPSL1206	30S ribosomal protein S15	1391276	1391545	+	89	53718842	3093559	rpsO	NULL	GGGTCGCCGGGCGGGCGACTTGT	1	103	103	1	100	Bm_ATCC23344_chr1	3510148	1925710	1925812	100	515	1.20E-18	-
BPSL1207	polyribonucleotide nucleotidyltransferase	1391849	1393990	+	713	53718843	3092633	pnp	COG1185J	GCATAAAAAACACACCTCTTCCCAT	1	103	103	1	100	Bm_ATCC23344_chr1	3510148	1925137	1925239	100	515	1.20E-18	-
BPSL1208	putative oxidoreductase	1394106	1395125	+	339	53718844	3091522	-	COG0604CR	CCGGCAGCGCGAATGCGCGCCGGC	1	103	103	1	100	Bm_ATCC23344_chr1	3510148	1922880	1922982	100	515	1.20E-18	-
BPSL1209	triosephosphate isomerase	1395230	1395985	+	251	53718845	3092857	tpiA	COG0149G	GGTCGTTGACCGGTTCTTCAGCGT(	1	103	103	1	100	Bm_ATCC23344_chr1	3510148	1921756	1921858	99	506	3.00E-18	-
BPSL1210	protein-export membrane protein	1396065	1396442	+	125	53718846	3093077	secG	COG1314U	GCGTTCGCGAGACGCGCGCGACGA	1	82	82	1	100	Bm_ATCC23344_chr1	3510148	1920921	1921002	100	410	6.40E-14	-
BPSL1211	NADH dehydrogenase alpha subunit	1396791	1397150	+	119	53718847	3094307	nuoA	COG0838C	CCTTGCGCTCAGCTTGGGTTCTCAG	1	103	103	1	100	Bm_ATCC23344_chr1	3510148	1920195	1920297	100	515	1.20E-18	-
BPSL1215	ATP synthase subunit E	1399748	1400233	+	161	53718851	3091523	-	COG1905C	CGCCGTCGCAACAAGCTGTCAACA(	1	103	103	1	100	Bm_ATCC23344_chr1	3510148	1917238	1917340	100	515	1.20E-18	-
BPSL1217	NADH dehydrogenase gamma subunit	1401623	1403953	+	776	53718853	3091552	-	COG1034C	CGGCGCAACCCGATTGCAGAATTC(	1	85	85	1	100	Bm_ATCC23344_chr1	3510148	1915363	1915447	100	425	1.40E-14	-
BPSL1220	NADH dehydrogenase subunit J	1405632	1406288	+	218	53718856	3091498	nuoJ	COG0839C	CGGCCCGGCGAGCGGGCCGGCAA(	1	103	103	1	100	Bm_ATCC23344_chr1	3510148	1911354	1911456	100	515	1.20E-18	-
BPSL1227	hypothetical protein BPSL1227	1412756	1413178	+	140	53718863	3092015	-	COG5319S	ACGGTCGGCGCGATCGGGGTCCTC	1	103	103	1	100	Bm_ATCC23344_chr1	3510148	1904230	1904332	100	515	1.20E-18	-
BPSL1228	putative acyl-CoA dehydrogenase family protein	1413239	1414435	+	398	53718864	3092016	-	COG1960I	TCGCCCGCTGATCGCTGCGCGGCC(	1	63	63	1	100	Bm_ATCC23344_chr1	3510148	1903747	1903809	100	315	1.30E-09	-
BPSL1233	putative lipoprotein	1416651	1417913	+	420	53718868	3091611	-	NULL	TGAGCGGGTTCGCGCGCGATGCGT.	1	98	98	1	100	Bm_ATCC23344_chr1	3510148	1900335	1900432	100	490	1.60E-17	-
BPSL1237	enoyl-CoA hydratase	1421317	1422180	-	287	53718872	3093130	-	COG1024I	AAAGCACGTTCATGCTAGTCTAAAG	1	103	1	103	100	Bm_ATCC23344_chr1	3510148	1894704	1894806	100	515	1.20E-18	+
BPSL1238	putative glutathione S-transferase related protein	1422330	1423004	+	224	53718873	3093261	-	COG0625O	GGACGTCGCAGCGCGCTTCGAAGT	1	103	103	1	100	Bm_ATCC23344_chr1	3510148	1894652	1894754	100	515	1.20E-18	-
BPSL1239	putative aminotransferase	1423224	1424378	-	384	53718874	3093262	-	COG0436E	GCGACGCACTCGCCCGAGCCGCCC	1	69	1	69	100	Bm_ATCC23344_chr1	3510148	1892529	1892597	98	336	1.40E-10	+
BPSL1240	hypothetical protein BPSL1240	1424445	1425113	-	222	53718875	3094810	-	COG1569R	AGAGGCTCGCCGGCACGCCGTGCC	1	60	1	60	100	Bm_ATCC23344_chr1	3510148	1891803	1891862	100	300	6.00E-09	+
BPSL1241	hypothetical protein BPSL1241	1425171	1425953	+	260	53718876	3094811	-	COG3022S	TCGTTACAATCGGTGGTTTCGTCT(	1	60	60	1	100	Bm_ATCC23344_chr1	3510148	1891800	1891859	100	300	6.00E-09	-
BPSL1242	family M14 unassigned peptidase	1426012	1427166	+	384	53718877	3091880	-	COG2866E	ATAGGCGGCTATCGGCGTATTCGG(	1	61	61	1	100	Bm_ATCC23344_chr1	3510148	1890959	1891019	100	305	3.60E-09	-
BPSL1243	hypothetical protein BPSL1243	1427377	1428036	-	219	53718878	3091881	-	NULL	GCGCCATCGTACGCGCGCGCAGGC	1	103	1	103	100	Bm_ATCC23344_chr1	3510148	1888837	1888939	100	515	1.20E-18	+
BPSL1244	hypothetical protein BPSL1244	1428157	1428831	-	224	53718879	3093058	-	COG3235S	CCGGTAAAACCCGCGTAGAGGGTG	1	69	1	69	100	Bm_ATCC23344_chr1	3510148	1888076	1888144	100	345	5.50E-11	+
BPSL1245	putative transcriptional regulator	1428898	1429806	-	302	53718880	3093059	-	COG2197TK	GCACCGCCGAAGGCCGCCTCGAGG	1	103	1	103	100	Bm_ATCC23344_chr1	3510148	1887067	1887169	99	506	3.00E-18	+
BPSL1270	putative exported alkaline phosphatase	1472111	1474072	-	653	53718905	3091945	-	COG3211R	TTTCATTTACAAAAACTAAACTTTCA	1	103	1	103	100	Bm_ATCC23344_chr1	3510148	1878539	1878641	100	515	1.20E-18	+
BPSL1271	hypothetical protein BPSL1271	1474323	1474844	+	173	53718906	3093525	-	NULL	GCCGGCCCGTTTTTTCCGGCGGAAT	1	103	103	1	100	Bm_ATCC23344_chr1	3510148	1878386	1878488	99	506	3.00E-18	-
BPSL1272	hypothetical protein BPSL1272	1474934	1475395	+	153	53718907	3093526	-	COG1959K	ATCCGCTTGCATTCCCGGCTAAATG	1	92	92	1	100	Bm_ATCC23344_chr1	3510148	1877775	1877866	100	460	3.50E-16	-
BPSL1273	hypothetical protein BPSL1273	1475662	1476288	+	208	53718908	3093841	-	COG2910R	GACTATGATGGGGATCGTGGATCC(	1	103	103	1	100	Bm_ATCC23344_chr1	3510148	1877047	1877149	99	506	3.00E-18	-
BPSL1274	hypothetical protein BPSL1274	1477083	1477739	-	218	53718909	3093842	-	COG5553R	ATGAGGGAACAATATGGAATCCGT	1	103	1	103	100	Bm_ATCC23344_chr1	3510148	1874874	1874976	100	515	1.20E-18	+
BPSL1275	putative AsnC-family transcriptional regulator	1477886	1478353	+	155	53718910	3094265	-	COG1522K	GTACGTATTTATACCGGTCGACTTG(	1	103	103	1	100	Bm_ATCC23344_chr1	3510148	1874825	1874927	100	515	1.20E-18	-
BPSL1278	putative ABC transport system, iron-binding exporte	1480968	1482023	-	351	53718913	3093931	-	COG1840P	TTACAATCCAAAGCTTTCTATTTGTA	1	103	1	103	100	Bm_ATCC23344_chr1	3510148	1870586	1870688	99	506	3.00E-18	+
BPSL1279	hypothetical protein BPSL1279	1482263	1482442	+	59	53718914	3093555	-	NULL	AAAAGACTGACGAATCCCGCCGCG	1	103	103	1	100	Bm_ATCC23344_chr1	3510148	1870444	1870546	100	515	1.20E-18	-
BPSL1280	putative amino acid transport system, membrane pr	1482584	1483948	-	454	53718915	3093556	-	COG1113E	TTGGTATGATCTATCCCGCGCCAAG	1	103	1	103	100	Bm_ATCC23344_chr1	3510148	1868661	1868763	100	515	1.20E-18	+
BPSL1281	hypothetical protein BPSL1281	1484133	1486409	-	758	53718916	3091577	-	COG1289S	CGCCGGCCCCGAAACACGGCTTTC(	1	103	1	103	100	Bm_ATCC23344_chr1	3510148	1866200	1866302	100	515	1.20E-18	+
BPSL1282	putative exodeoxyribonuclease V gamma chain	1487013	1490357	+	1114	53718917	3091578	-	COG1330L	CGAGCGCCCGGCCGATCAGCCAGC	1	103	103	1	100	Bm_ATCC23344_chr1	3510148	1865309	1865411	100	515	1.20E-18	-
BPSL1287	hypothetical protein BPSL1287	1499926	1500858	-	310	53718922	3094649	-	NULL	CCCGTATCTTGCTGTTTCATCCCGG(	1	103	1	103	100	Bm_ATCC23344_chr1	3510148	1851321	1851423	100	515	1.20E-18	+
BPSL1288	putative lipoprotein	1501151	1501375	-	74	53718923	3094650	-	NULL	CGGAAATGAGCCGTTACCGTTTCAC	1	103	1	103	100	Bm_ATCC23344_chr1	3510148	1850826	1850928	100	515	1.20E-18	+
BPSL1289	osmotically inducible lipoprotein B precursor	1501627	1501839	-	70	53718924	3093217	osmB	NULL	CAAGTCCTTACAGCGCAAAACGGA'	1	103	1	103	100	Bm_ATCC23344_chr1	3510148	1850362	1850464	99	506	3.00E-18	+
BPSL1290	thiamine biosynthesis protein ThiC	1502063	1503994	-	643	53718925	3092226	thiC	NULL	CGCGTCGCGAATCTCGTCTCCTGCT	1	103	1	103	100	Bm_ATCC23344_chr1	3510148	3183884	3183986	100	515	1.20E-18	+
BPSL1292	hypothetical protein BPSL1292	1505095	1505919	+	274	53718928	3091727	-	NULL	GGCGGCCTTCGTCTTGCGGCGCGG'	1	103	103	1	100	Bm_ATCC23344_chr1	3510148	3182866	3182968	100	515	1.20E-18	-
BPSL1295	hypothetical protein BPSL1295	1508343	1509278	+	311	53718931	3094525	-	NULL	GCCGCCGTTTCCCGTCATTCCGGTT	1	103	103	1	100	Bm_ATCC23344_chr1	3510148	3179654	3179756	99	506	3.00E-18	-
BPSL1299	putative phosphodiesterase	1512862	1513689	-	275	53718935	3093387	-	COG1409R	GGCGACACATGATGCGCGAAGGC(	1	103	1	103	100	Bm_ATCC23344_chr1	3510148	3174285	3174387	100	515	1.20E-18	+
BPSL1300	putative ABC transport system, ATP-binding protein	1513884	1514912	-	342	53718936	3091741	-	COG3842E	TTGCGCCGCCGTTGTTTTCGGCCTT(	1	103	1	103	100	Bm_ATCC23344_chr1	3510148	3173020	3173122	98	497	7.60E-18	+
BPSL1303	hypothetical protein BPSL1303	1518129	1519190	-	353	53718939	3091485	-	COG1840P	ACCGTGCTTTCGCTTTCCGTCGCTTT	1	59	1	59	100	Bm_ATCC23344_chr1	3510148	3168817	3168875	98	286	2.60E-08	+
BPSL1305	hypothetical protein BPSL1305	1522632	1523786	-	384	53718943	3094732	-	NULL	TCCCGGTCGCCGCCGATAACCCTG/	1	103	2	103	99	Bm_ATCC23344_chr1	3510148	3164202	3164303	99	501	5.00E-18	+
BPSL1306	hypothetical protein BPSL1306	1524128	1525492	-	454	53718944	3094471	-	NULL	TCGCCGCCCGGCGTCCCGCGCGGA	1	103	1	103	100	Bm_ATCC23344_chr1	3510148	3162471	3162573	100	515	1.20E-18	+
BPSL1307	hypothetical protein BPSL1307	1526205	1526807	+	200	53718945	3094472	-	NULL	TCATATGTCGCGAAGGCACCGCCA(	1	103	103	1	100	Bm_ATCC23344_chr1	3510148	3161882	3161984	100	515	1.20E-18	-
BPSL1309	hypothetical protein BPSL1309	1528053	1528691	-	212	53718947	3094141	-	COG2808K	ATTCAAACTGGTTCTACAGAATAAA	1	103	1	103	100	Bm_ATCC23344_chr1	3510148	3159301	3159403	100	515	1.20E-18	+

BPSL1310	putative GntR-family regulatory protein	1528813	1530354	+	513	53718948	3094229	-	COG1167KE	CGTTCGCGCGGCGCATCGGGATTG	1	103	103	1	100	Bm_ATCC23344_chr1	3510148	3159277	3159379	100	515	1.20E-18	-
BPSL1311	putative AsnC-family regulatory protein	1530602	1531096	-	164	53718949	3094230	-	COG1522K	AATGGAATTTAATTTCAAAAATATC	1	103	1	103	100	Bm_ATCC23344_chr1	3510148	3156938	3157040	99	506	3.00E-18	+
BPSL1312	glucosamine--fructose-6-phosphate aminotransferas	1531255	1533156	+	633	53718950	3091988	glmS2	COG0449M	GGAAATTTCAGAGAATCGATATTTT	1	103	103	1	100	Bm_ATCC23344_chr1	3510148	3156877	3156979	100	515	1.20E-18	-
BPSL1314	hypothetical protein BPSL1314	1534719	1535423	-	234	53718952	3093745	-	COG3644S	GCCGGGCGGCGACGTCGAACGAA	1	103	1	103	100	Bm_ATCC23344_chr1	3510148	3152592	3152694	99	506	3.00E-18	+
BPSL1315	putative LysR-family transcriptional regulator	1535587	1536486	-	299	53718953	3091704	-	COG0583K	GCCGCGATGACTCCGCTTGCGTCCC	1	103	1	103	100	Bm_ATCC23344_chr1	3510148	3151529	3151631	100	515	1.20E-18	+
BPSL1316	short chain dehydrogenase	1536737	1537507	+	256	53718954	3091705	-	COG1028IQR	TGTCAGAACAGGTGACTTTATAAGG	1	103	103	1	100	Bm_ATCC23344_chr1	3510148	3151376	3151478	100	515	1.20E-18	-
BPSL1319	putative nitrite/sulfite reductase	1539904	1541721	-	605	53718957	3094334	-	COG0155P	GCCGCGCGCCCTACGCTTGGTGAT	1	103	1	103	100	Bm_ATCC23344_chr1	3510148	3146257	3146359	100	515	1.20E-18	+
BPSL1321	putative GntR-family transcriptional regulator	1542534	1544009	-	491	53718959	3094070	-	COG1167KE	GGCGTCGCCTCCCGATCGCGCCGC	1	103	1	103	100	Bm_ATCC23344_chr1	3510148	3143993	3144095	98	497	7.60E-18	+
BPSL1323	putative heat shock protein	1545147	1545584	-	145	53718961	3094350	-	COG0071O	TGCGCGTTTCGACCGATTTGTCGCG	1	103	1	103	100	Bm_ATCC23344_chr1	3510148	3142418	3142520	100	515	1.20E-18	+
BPSL1323a	hypothetical protein BPSL1323a	1545977	1546300	+	107	53718962	3094351	-	NULL	ATCGGAGCCGTGTCGCCGAAGCCG	1	103	103	1	100	Bm_ATCC23344_chr1	3510148	3142126	3142225	96	461	3.20E-16	-
BPSL1324	phosphoenolpyruvate carboxykinase (GTP)	1547220	1549085	+	621	53718963	3093468	pckG	COG1274C	GCGTCGCCCCCAGCCATCGCGTAG	1	103	103	1	100	Bm_ATCC23344_chr1	3510148	3140876	3140978	100	515	1.20E-18	-
BPSL1325	putative 3-hydroxyacyl-CoA dehydrogenase	1549366	1550298	+	310	53718964	3094011	-	COG1250I	TGCGCGGGCGGCCGCGCGCGTGCC	1	103	103	2	99	Bm_ATCC23344_chr1	3510148	3138731	3138832	97	483	3.20E-17	-
BPSL1327	putative malonate transport-related system membra	1551987	1552382	+	131	53718966	3091661	-	NULL	ATTGCCGACGTACCGCCATCGCCG	1	103	103	1	100	Bm_ATCC23344_chr1	3510148	3136109	3136211	100	515	1.20E-18	-
BPSL1329	putative malonate decarboxylase alpha-subunit	1553456	1555102	+	548	53718968	3094323	-	COG4670I	GCGCCACTGAGTCGTATCGCGCAA	1	103	103	1	100	Bm_ATCC23344_chr1	3510148	3134613	3134715	100	515	1.20E-18	-
BPSL1337	hypothetical protein BPSL1337	1561322	1562236	+	304	53718976	3094591	-	COG4757R	GACCGAAGAGGCCGGCAAGTCTTG	1	103	103	1	100	Bm_ATCC23344_chr1	3510148	3126984	3127086	100	515	1.20E-18	-
BPSL1339	putative proline/betaine transporter	1563720	1565207	-	495	53718978	3094767	-	NULL	CGCGACCGACCCTCGTCCGATCGC	1	103	103	1	100	Bm_ATCC23344_chr1	3510148	791674	791776	100	515	1.20E-18	-
BPSL1340	hypothetical protein BPSL1340	1565742	1566074	+	110	53718979	3094768	-	NULL	GTCATGCGGCGGTTTGGCGGGCGT	1	103	1	103	100	Bm_ATCC23344_chr1	3510148	792111	792213	98	497	7.60E-18	+
BPSL1341	DNA polymerase III subunit epsilon	1566419	1567141	-	240	53718980	3092205	dnaQ	COG0847L	GCCCGCTCGCGCCCCCCTTTGCCCT	1	60	60	1	100	Bm_ATCC23344_chr1	3510148	793696	793755	100	300	6.00E-09	-
BPSL1345	putative exported transglycosylase protein	1569365	1571026	+	553	53718984	3091560	-	COG0741M	GCCCGTGCAATAGTCGGCCAAGCT	1	86	1	86	100	Bm_ATCC23344_chr1	3510148	795839	795924	98	421	2.00E-14	+
BPSL1346	hypothetical protein BPSL1346	1571107	1572399	+	430	53718985	3091561	-	NULL	GACGAAAGGGCGCGCATGCCGCGC	1	83	1	83	100	Bm_ATCC23344_chr1	3510148	797584	797666	100	415	3.80E-14	+
BPSL1349	carbamoyl-phosphate synthase small subunit	1575304	1576449	+	381	53718988	3092517	carA	COG0505EF	CGCGCGAAAGCGCCCACGCGCGCC	1	103	1	103	100	Bm_ATCC23344_chr1	3510148	801772	801874	100	515	1.20E-18	+
BPSL1351	carbamoyl-phosphate synthase large subunit	1577195	1580449	+	1084	53718990	3092518	carB	COG0458EF	GCGCCGCCCGCGGATCGCGCATCA	1	54	1	54	100	Bm_ATCC23344_chr1	3510148	803712	803765	98	261	3.40E-07	+
BPSL1352	transcription elongation factor GreA	1580568	1581044	+	158	53718991	3093169	greA	COG0782K	AAGCAACACGTGCCGCGATTAAGC	1	103	1	103	100	Bm_ATCC23344_chr1	3510148	807036	807138	100	515	1.20E-18	+
BPSL1354	hypothetical protein BPSL1354	1581618	1582172	-	184	53718993	3092734	-	NULL	CAAGCCGCGATTTTACGTGGTTTCG	1	103	103	1	100	Bm_ATCC23344_chr1	3510148	808738	808840	98	497	7.60E-18	-
BPSL1355	putative ribosomal RNA large subunit methyltransfer	1582366	1583028	+	220	53718994	3092735	-	COG0293J	CGGCTTGGGCCCTTCGGGGCCGAA	1	103	1	103	100	Bm_ATCC23344_chr1	3510148	808834	808936	100	515	1.20E-18	+
BPSL1356	FtsH endopeptidase	1583196	1585082	+	628	53718995	3093403	ftsH	COG0465O	AGCGAATCCTTATGGCGAGGGGCT	1	103	1	103	100	Bm_ATCC23344_chr1	3510148	809664	809766	100	515	1.20E-18	+
BPSL1357	dihydropteroate synthase	1585195	1586085	+	296	53718996	3092153	folP	COG0294H	TGGGTTCACGGGCTGGTGTGGCTG	1	103	1	103	100	Bm_ATCC23344_chr1	3510148	811663	811765	100	515	1.20E-18	+
BPSL1365	putative phosphate regulon two-component system	1593304	1594614	+	436	53719004	3093459	-	COG0642T	CGGCCGCGCGGCGCGCGGGGTTC	1	72	1	72	100	Bm_ATCC23344_chr1	3510148	819803	819874	100	360	1.20E-11	+
BPSL1366	polyphosphate kinase	1594813	1596873	-	686	53719005	3093143	ppk	NULL	TTTGCCGACAAACATCAAACTGTCA	1	103	103	1	100	Bm_ATCC23344_chr1	3510148	823441	823543	100	515	1.20E-18	-
BPSL1367	putative exopolyphosphatase	1597104	1598663	+	519	53719006	3093460	-	COG0248FP	AAGCATCCTCTAGATACAATGAAA	1	103	1	103	100	Bm_ATCC23344_chr1	3510148	823574	823676	99	506	3.00E-18	+
BPSL1368	hypothetical protein BPSL1368	1598976	1599869	-	297	53719007	3094861	-	NULL	GATTCACTCTGAGGCGGGCGGACG	1	68	68	1	100	Bm_ATCC23344_chr1	3510148	826437	826503	98	325	4.40E-10	-
BPSL1369	hypothetical protein BPSL1369	1599935	1601008	+	357	53719008	3094862	-	NULL	GGCGAACCGCCGAAACCATGCTGG	1	68	1	68	100	Bm_ATCC23344_chr1	3510148	826440	826506	98	325	4.40E-10	+
BPSL1373	hypothetical protein BPSL1373	1603310	1603720	-	136	53719012	3094436	-	NULL	TCGTCACATCTTTCGGGGGAAATGA	1	103	103	1	100	Bm_ATCC23344_chr1	3510148	830289	830392	98	496	8.40E-18	-
BPSL1374	putative hydrolase	1604281	1604742	+	153	53719013	3093970	-	COG2062T	CGCTTGCACGCGGACTGCGCAACA	1	103	1	103	100	Bm_ATCC23344_chr1	3510148	830753	830855	100	515	1.20E-18	+
BPSL1375	hypothetical protein BPSL1375	1604894	1605712	+	272	53719014	3093971	-	COG3176R	GACACGTCACCGAATGGACACCGT	1	103	1	103	100	Bm_ATCC23344_chr1	3510148	831366	831468	100	515	1.20E-18	+
BPSL1376	hypothetical protein BPSL1376	1606063	1606326	-	87	53719015	3094812	-	NULL	GGATTTCCTTGGAGTGATCGGTCGA	1	77	77	1	100	Bm_ATCC23344_chr1	3510148	832896	832972	100	385	8.70E-13	-
BPSL1377	putative lipoprotein	1606401	1606592	+	63	53719016	3094813	-	NULL	CGATTTCCAGTAGACTCGCCGGGC	1	77	1	77	100	Bm_ATCC23344_chr1	3510148	832899	832975	100	385	8.70E-13	+
BPSL1379	hypothetical protein BPSL1379	1608253	1608603	-	116	53719018	3093286	-	COG5626S	GCCTCATCGGCTTCATCCACGTGCC	1	103	103	1	100	Bm_ATCC23344_chr1	3510148	835166	835268	100	515	1.20E-18	-
BPSL1381	subfamily M23B unassigned peptidase	1610849	1611562	-	237	53719020	3093551	-	COG0739M	GCCTTCAGCGCCTCCGCGGCCCCG	1	103	103	1	100	Bm_ATCC23344_chr1	3510148	838092	838194	99	506	3.00E-18	-
BPSL1382	hypothetical protein BPSL1382	1611847	1612755	-	302	53719021	3094293	-	COG2017G	TCGCGCGGCAGTCACCTGCCGCAC	1	Not found											
BPSL1394	putative exported endonuclease	1620853	1621476	-	207	53719030	3093399	-	COG1502I	CGGCGATGCGCGTCGACAGGAACT	1	103	1	103	100	Bm_ATCC23344_chr1	3510148	1531185	1531287	100	515	1.20E-18	+
BPSL1397	putative porin signal peptide protein	1624054	1625121	-	355	53719033	3094565	-	COG3203M	CCCGCACCGTTCATCTGCATGGCTT	1	103	1	103	100	Bm_ATCC23344_chr1	3510148	1527540	1527642	100	515	1.20E-18	+
BPSL1398	putative GerE-family transcriptional regulator	1625452	1626600	+	382	53719034	3094566	-	COG2771K	GCGCGGGCGGTTTTCAAAAAAGAC	1	103	103	1	100	Bm_ATCC23344_chr1	3510148	1527307	1527409	100	515	1.20E-18	-
BPSL1399	2-dehydropantoate 2-reductase	1626699	1627685	+	328	53719035	3093239	-	COG1893H	CGCGCGGTGCGCGCGGCCGGATG	1	101	101	1	100	Bm_ATCC23344_chr1	3510148	1526060	1526160	100	505	3.30E-18	-
BPSL1400	putative MarR-family transcriptional regulator	1628075	1628524	-	149	53719036	3093240	-	COG1846K	GACCCGCCGCGCACGCGGCGAGC	1	103	1	103	100	Bm_ATCC23344_chr1	3510148	1524151	1524253	100	515	1.20E-18	+
BPSL1401	putative kinase	1628627	1629790	-	387	53719037	3092186	-	NULL	TCGCCCGCCCGCACGGCTCGGGCG	1	103	1	103	100	Bm_ATCC23344_chr1	3510148	1522885	1522987	99	506	3.00E-18	+
BPSL1402	trigger factor	1630243	1631592	+	449	53719038	3092748	tig	COG0544O	CGCGTGAGCGAGGCGGCGGCGCG	1	103	103	1	100	Bm_ATCC23344_chr1	3510148	1522530	1522632	100	515	1.20E-18	-
BPSL1403	ATP-dependent Clp protease proteolytic subunit	1631727	1632380	+	217	53719039	3094271	clpP	COG0740OU	ACGACGGCCCGCACGCCGTTTTTTC	1	103	103	1	100	Bm_ATCC23344_chr1	3510148	1521046	1521148	99	506	3.00E-18	-
BPSL1404	ATP-dependent protease ATP-binding subunit	1632520	1633812	+	430	53719040	3093205	clpX	COG1219O	CACACGCCCCGAGCATCTGCGGCA	1	103	103	1	100	Bm_ATCC23344_chr1	3510148	1520253	1520355	99	506	3.00E-18	-
BPSL1405	ATP-dependent protease	1634008	1636425	+	805	53719041	3092784	lon	COG0466O	GGTGACTTTTTTACTGAATATTTCCC	1	103	103	1	100	Bm_ATCC23344_chr1	3510148	1518765	1518867	100	515	1.20E-18	-
BPSL1410	putative peptidyl-prolyl cis-trans isomerase	1640747	1642681	+	644	53719046	3094072	-	NULL	CGTCCGCTCCGCCATCATCCCGAA	1	103	103	1	100	Bm_ATCC23344_chr1	3510148	1512102	1512204	100	515	1.20E-18	-
BPSL1413	glucose-6-phosphate isomerase	1644934	1646556	-	540	53719049	3093521	pgi	COG0166G	GTCCCGCCGCGCAGGCGGGCTCCT	1	103	1	103	100	Bm_ATCC23344_chr1	3510148	1506291	1506393	100	515	1.20E-18	+
BPSL1415	putative D-amino acid dehydrogenase small subunit	1648302	1649606	-	434	53719051	3093732	-	COG0665E	CTTCATTTGGCGCTATCATGCGCGC	1	103	1	103	100	Bm_ATCC23344_chr1	3510148	1503241	1503343	100	515	1.20E-18	+
BPSL1420	putative intracellular septation transmembrane prote	1655689	1656219	-	176	53719056	3094013	-	COG2917D	GCACGGGCCGGCCGAAGAGCCGG	1	95	1	95	100	Bm_ATCC23344_chr1	3510148	1496645	1496739	98	466	1.90E-16	+
BPSL1421	peptide methionine sulfoxide reductase	1656312	1656743	-	143	53719057	3091857	msrB	NULL	CGCCGCCGGCACGCCGTGCGTTGC	1	53	1	53	100	Bm_ATCC23344_chr1	3510148	1496163	1496215	100	265	2.30E-07	+
BPSL1422	hypothetical protein BPSL1422	1656794	1658371	-	525	53719058	3094014	-	NULL	TGAAGTATTTGTCCGATACGGTCTG	1	103	1	103	100	Bm_ATCC23344_chr1	3510148	1494485	1494587	100	515	1.20E-18	+
BPSL1423	acyl-CoA synthase	1658487	1660127	+	546	53719059	3094023	-	COG0318IQ	TGTAAGTCCGCGCCGCGCGGGGCG	1	103	103	1	100	Bm_ATCC23344_chr1	3510148	1494467	1494569	100	515	1.20E-18	-
BPSL1424	putative fatty acid degradation protein (possibly trifu	1660379	1662463	+	694	53719060	3094024	-	COG1024I, COG1250I	ACGGCGCATCGACCGCACGGTTTC	1	103	103	1	100	Bm_ATCC23344_chr1	3510148	1492581	1492683	99	506	3.00E-18	-
BPSL1426	acetyl-CoA acetyltransferase	1663202	1664380	+	392	53719062	3094082	-	COG0183I	CCGCCGTCGTTCATTTCATCGCATA	1	61	61	1	100	Bm_ATCC23344_chr1	3510148	1489758	1489818	98	296	9.10E-09	-
BPSL1427	hypothetical protein BPSL1427	1664742	1665281	-	179	53719063	3094105	-	NULL	CGACGGCGCGCATCGCCGCGCGAT	1	103	1	103	100	Bm_ATCC23344_chr1	3510148	1487570	1487672	100	515	1.20E-18	+
BPSL1428	hypothetical protein BPSL1428	1665958	1666272	+	104	53719064	3094106	-	NULL	CGCCGCGGCGAAGCACGCGAGAG	1	103	103	1	100	Bm_ATCC23344_chr1	3510148	1486994	1487096	100	515	1.20E-18	-
BPSL1430	putative oxidoreductase	1666843	1667841	+	332	53719066	3094678	-	COG2130R	CGCGAGCGGGCGGCCCCGCCCGTC	1	94	94	1	100	Bm_ATCC23344_chr1	3510148	1486109	1486202	100	470	1.30E-16	-
BPSL1431	putative esterase/lipase	1667911	1668906	+	331	53719067	3094698	-	COG0657I	CGCGCCGTGGCATCATACGGCGCT	1	72	72	1	100	Bm_ATCC23344_chr1	3510148	1485041	1485112	98	351	3.00E-11	-
BPSL1432	putative acetyltransferase	1669270	1669710	+	146	53719068	3094699	-	NULL	GACGCGAATGACGACGCGGGCGTG	1	103	103	1	100	Bm_ATCC23344_chr1	3510148	1483674	1483776	100	515	1.20E-18	-
BPSL1433	hypothetical protein BPSL1433	1669746	1670597	-	283	53719069	3092672	-	COG3332S	CGGCCGCGCGCCGCCGGAGCGCG	1	73	1	73	100	Bm_ATCC23344_chr1	3510148	1482279	1482351	100	365	6.90E-12	+
BPSL1434	hypothetical protein BPSL1434	1670668	1671621	-	317	53719070	3092673	-	COG0354R	TCATTCCATGAGCACACCGATCGCC	1	103	1	103	100	Bm_ATCC23344_chr1	3510148	1481225	1481327	100	515	1.20E-18	+
BPSL1435	putative lipoprotein	1671793	1672812	+	339	53719071	3094599	-	NULL	GGGAGGCGATCGGTGTGCTCATGG	1	103	103	1	100	Bm_ATCC23344_chr1	3510148	1481151	1481253	100	515	1.20E-18	-
BPSL1439	putative deoxyribonuclease	1675112	1675921	+	269	53719075	3092237	-	COG0084L	GCGCGGCCCGGCGCGGCGAGCGG	1	78	78	1	100	Bm_ATCC23344_chr1	3510148	1477832	1477909	98	381	1.30E-12	-
BPSL1441	hypothetical protein BPSL1441	1676737	1677087	+	116	53719077	3093249	-	NULL	GGGTTTGGGGCGCGGCGGCTTGCC	1	79	79	1	100	Bm_ATCC23344_chr1	3510148	1476228	1476306	100	395	3.10E-13	-
BPSL1442	hypothetical protein BPSL1442	1677154	1678341	+	395	53719078	3093250	-	COG0668M, COG3264M	TCCGGGCCGGCCGCCGCCCCGGCG	1	69	69	1	100	Bm_ATCC23344_chr1	3510148	1475811	1475879	100	345	5.50E-11	-
BPSL1444	putative sugar transferase	1680141	1681205	+	354	53719080	3094298	-	COG0438M	AATCGGCCCGCTGGCGTGCGCGCC	1	103	103	1	100	Bm_ATCC23344_chr1	3510148	1472813	1472915	99	506	3.00E-18	-
BPSL1445	putative lipoprotein	1681413	1682000	-	195	53719081	3092363	-	COG2930S	CTTACAGCAGTAAGGCAGGGTACT	1	103	1	103	100	Bm_ATCC23344_chr1	3510148	1470857	1470959	100	515	1.20E-18	+
BPSL1446	hypothetical protein BPSL1446	1682238	1682750	-	170	53719082	3092364	-	NULL	GCGCGCGCCGACTGCGGCGTTTGG	1	103	1	103	100	Bm_ATCC23344_chr1	3510148	1470109	1470211	100	515	1.20E-18	+

BPSL1447	hypothetical protein BPSL1447	1682926	1683216	-	96	53719083	3091723	-	NULL	CGTTTCAGCGCCGATTTGCCATAAT	1	103	1	103	100	Bm_ATCC23344_chr1	3510148	1469643	1469745	100	515	1.20E-18	+
BPSL1448	L-asparaginase II precursor	1683714	1684757	+	347	53719084	3092601	ansB	NULL	GTCGCGCAACCGGCGGGAGCGGC	1	103	103	1	100	Bm_ATCC23344_chr1	3510148	1469243	1469345	100	515	1.20E-18	-
BPSL1455	hypothetical protein BPSL1455	1691494	1692414	-	306	53719091	3094551	-	COG1409R	GTTTATTCCCGCGATCCACGCCCGG	1	103	1	103	100	Bm_ATCC23344_chr1	3510148	1464653	1464754	99	500	5.50E-18	+
BPSL1456	RNA polymerase sigma-70 factor	1692620	1693327	+	235	53719092	3094552	-	COG1595K	CGACGGGCGGTAAGGATGCCTGAG	1	103	103	1	100	Bm_ATCC23344_chr1	3510148	1464545	1464647	100	515	1.20E-18	-
BPSL1458	30S ribosomal protein S6	1694743	1695117	+	124	53719094	3093548	rpsF	COG0360J	TGAGCCTTGCATGCGACCCATATAC	1	103	103	1	100	Bm_ATCC23344_chr1	3510148	1462588	1462690	98	497	7.60E-18	-
BPSL1462	replicative DNA helicase	1696355	1697737	+	460	53719098	3093450	dnaB	COG0305L	AGGGCAGGGGCTCGGGCAACCGG	1	103	103	1	100	Bm_ATCC23344_chr1	3510148	1460976	1461078	100	515	1.20E-18	-
BPSL1463	hypothetical protein BPSL1463	1697876	1698502	+	208	53719099	3094583	-	COG1392P	CCGTCGCCGTCGTTGCGATCGGGC	1	103	103	1	100	Bm_ATCC23344_chr1	3510148	1459455	1459557	100	515	1.20E-18	-
BPSL1466	putative phosphorous metabolism-related protein	1701212	1702603	-	463	53719102	3094545	-	COG1875T	GCGCTCGTCGAAGACCGATGCGGC	1	103	1	103	100	Bm_ATCC23344_chr1	3510148	1454620	1454722	98	497	7.60E-18	+
BPSL1467	putative bacterioferritin comigratory protein (detoxif	1703337	1703798	-	153	53719103	3094546	-	COG1225O	ACGAAAGCCGGTGCCGGCTTTCGC	1	92	1	92	100	Bm_ATCC23344_chr1	3510148	1453436	1453527	100	460	3.50E-16	+
BPSL1472	putative LPS biosynthesis-related protein	1707809	1708960	-	383	53719106	3093574	-	COG0399M	CGGACCGACAAAAAGTGACGTACC	1	103	1	103	100	Bm_ATCC23344_chr1	3510148	1448262	1448364	99	506	3.00E-18	+
BPSL1474	hypothetical protein BPSL1474	1709479	1711185	-	568	53719108	3092098	-	COG1807M	CGCCCCGCTTCGCGCCGCGGCCCG	1	103	1	103	100	Bm_ATCC23344_chr1	3510148	1446037	1446139	100	515	1.20E-18	+
BPSL1475	hypothetical protein BPSL1475	1711354	1711728	-	124	53719109	3092099	-	COG3737S	GGCCGCACGCATCGCGGCAGGCG	1	103	1	103	100	Bm_ATCC23344_chr1	3510148	1445494	1445596	100	515	1.20E-18	+
BPSL1476	aspartate aminotransferase	1712067	1713305	+	412	53719110	3092368	-	COG0436E	AGCCGCATGAAGCCATTCCTAGAC	1	103	103	1	100	Bm_ATCC23344_chr1	3510148	1445253	1445355	100	515	1.20E-18	-
BPSL1482	hypothetical protein BPSL1482	1719333	1719875	-	180	53719116	3092369	-	NULL	AAGATGCAGACCGTCAGCACTTTTA	1	103	1	103	100	Bm_ATCC23344_chr1	3510148	1437371	1437473	99	506	3.00E-18	+
BPSL1483	putative DNA-binding protein	1720059	1720556	+	165	53719117	3093311	-	COG1959K	CTGACGGTCTGCATCTTATGACGCG	1	103	103	1	100	Bm_ATCC23344_chr1	3510148	1437285	1437387	100	515	1.20E-18	-
BPSL1484	ClpB heat-shock protein	1720727	1723357	+	876	53719118	3094211	clpB	COG0542O	CCGCGTCCCGCTCTCGTTCGCCGCC	1	103	103	1	100	Bm_ATCC23344_chr1	3510148	1436617	1436719	100	515	1.20E-18	-
BPSL1485	hypothetical protein BPSL1485	1723606	1724028	-	140	53719119	3093312	-	COG3753S	GTCGGGCGCCGCGCGACGCGCGG	1	81	1	81	100	Bm_ATCC23344_chr1	3510148	1433250	1433330	98	396	2.80E-13	+
BPSL1486	hypothetical protein BPSL1486	1724107	1724802	-	231	53719120	3093390	-	COG1555L	ATGGTCAAAACCCGCCCGCATCCG	1	103	1	103	100	Bm_ATCC23344_chr1	3510148	1432454	1432556	100	515	1.20E-18	+
BPSL1490	hypothetical protein BPSL1490	1727666	1729504	-	612	53719124	3093011	-	COG1807M	CGGGCCGGTTCCGCAAGAAAGGCA	1	103	1	103	100	Bm_ATCC23344_chr1	3510148	1427754	1427856	100	515	1.20E-18	+
BPSL1491	50S ribosomal protein L31	1729638	1729901	-	87	53719125	3093012	-	COG0254J	GCGCCAGTACGAATGCGCGGCC	1	103	1	103	100	Bm_ATCC23344_chr1	3510148	1427357	1427459	99	506	3.00E-18	+
BPSL1494	putative MerR-family transcriptional regulator	1732557	1733057	-	166	53719128	3094530	-	COG0789K	CTCGCCGGGCGGTTTTTTTATCGCC	1	103	1	103	100	Bm_ATCC23344_chr1	3510148	1424201	1424303	100	515	1.20E-18	+
BPSL1495	transcription termination factor Rho	1733194	1734456	-	420	53719129	3094080	rho	COG1158K	TGAGCGCTTCCGCTCGCCCTCCTCC	1	69	1	69	100	Bm_ATCC23344_chr1	3510148	1422836	1422904	100	345	5.50E-11	+
BPSL1497	thioredoxin 1	1734652	1734978	-	108	53719131	3091475	trxA	COG0526OC	CCCTTCTTTTCGCAAGAGCGGAGCT	1	103	1	103	100	Bm_ATCC23344_chr1	3510148	1422280	1422382	100	515	1.20E-18	+
BPSL1498	DNA polymerase III subunits gamma and tau	1735645	1738122	+	825	53719132	3094276	dnaX	COG2812L	TCCTTCTCTTTTGCTTCGCTTCTCCG	1	103	103	1	100	Bm_ATCC23344_chr1	3510148	1421714	1421816	99	506	3.00E-18	-
BPSL1499	hypothetical protein BPSL1499	1738219	1738545	+	108	53719133	3092295	-	COG0718S	CGCGAGCCGGGCGCCGCGCGCGT	1	99	99	1	100	Bm_ATCC23344_chr1	3510148	1419140	1419238	100	495	9.30E-18	-
BPSL1501	putative transferase	1739249	1740454	+	401	53719135	3092296	-	COG1804C	CCGGCCGCGCGCCGGCGCCGCGC	1	72	72	1	100	Bm_ATCC23344_chr1	3510148	1418110	1418181	100	360	1.20E-11	-
BPSL1502	acid phosphatase	1740559	1741320	+	253	53719136	3092285	surE	COG0496R	CGAGGGCCGCGCGGCATGCGTTTG	1	103	103	1	100	Bm_ATCC23344_chr1	3510148	1416800	1416902	98	497	7.60E-18	-
BPSL1507	hypothetical protein BPSL1507	1745236	1746096	-	286	53719141	3094153	-	COG3568R	CAGCTTTTCCTGACGAACGATTGTG	1	80	1	80	100	Bm_ATCC23344_chr1	3510148	1411188	1411267	100	400	1.80E-13	+
BPSL1508	23S rRNA (uracil-5-)-methyltransferase	1746174	1747571	+	465	53719142	3092076	rumA	COG2265J	CGTCGAATCTCCGTTGCTTGGCGGC	1	80	80	1	100	Bm_ATCC23344_chr1	3510148	1411185	1411264	100	400	1.80E-13	-
BPSL1509	hypothetical protein BPSL1509	1747645	1748358	-	237	53719143	3092077	-	COG0670R	GTTGAAACCTCCGCCGTCGAAACCT	1	103	1	103	100	Bm_ATCC23344_chr1	3510148	1408903	1409005	98	497	7.60E-18	+
BPSL1510	nucleoside diphosphate kinase	1748592	1749017	+	141	53719144	3091685	ndk	NULL	CCTTTCGATCGTGTAAGGGTTCAAT	1	103	103	1	100	Bm_ATCC23344_chr1	3510148	1408767	1408869	100	515	1.20E-18	-
BPSL1512	hypothetical protein BPSL1512	1750337	1751425	+	362	53719146	3093086	-	COG1426S	CGGGGGCGGCGCGCGGCGCCGCG	1	103	103	1	100	Bm_ATCC23344_chr1	3510148	1407022	1407124	100	515	1.20E-18	-
BPSL1513	4-hydroxy-3-methylbut-2-en-1-yl diphosphate synth	1751569	1752819	+	416	53719147	3094863	-	NULL	TGCGCCGCCGGGCAGGCGGCGCG	1	103	103	1	100	Bm_ATCC23344_chr1	3510148	1405790	1405892	100	515	1.20E-18	-
BPSL1515	hypothetical protein BPSL1515	1754231	1754860	+	209	53719149	3094864	-	COG2976S	GCGCCGCGCATTCGCTCGACAAAA	1	55	55	1	100	Bm_ATCC23344_chr1	3510148	1403128	1403182	98	266	2.10E-07	-
BPSL1517	GTP-binding protein EngA	1756326	1757663	+	445	53719151	3092256	-	COG1160R	GCGCCGACACGGCGCGAATTCGTG	1	103	103	1	100	Bm_ATCC23344_chr1	3510148	1401034	1401136	100	515	1.20E-18	-
BPSL1518	RNA-binding protein Hfq	1757798	1758037	+	79	53719152	3092453	hfq	COG1923R	GGGCGGGGTGGACAAAATCGGCTA	1	103	103	1	100	Bm_ATCC23344_chr1	3510148	1399562	1399664	100	515	1.20E-18	-
BPSL1519	putative GTP-binding protein	1758094	1759272	+	392	53719153	3092452	hflX	COG2262R	CCCATTGCCGCGGCTGGCTTGCGTC	1	59	59	1	100	Bm_ATCC23344_chr1	3510148	1399266	1399324	100	295	1.00E-08	-
BPSL1523	ATP phosphoribosyltransferase regulatory subunit	1761911	1763059	+	382	53719157	3092401	-	COG3705E	GCGCCCGTTCGGCGCCGGCCGGCG	1	103	103	1	100	Bm_ATCC23344_chr1	3510148	1395449	1395551	100	515	1.20E-18	-
BPSL1524	adenylosuccinate synthetase	1763184	1764530	+	448	53719158	3093829	purA	NULL	ATGCGCGTTCGGCGTGCAATGTCG	1	103	103	1	100	Bm_ATCC23344_chr1	3510148	1394176	1394278	100	515	1.20E-18	-
BPSL1526	kup system potassium uptake protein	1765422	1767314	+	630	53719160	3091956	kup	COG3158P	AGCAAGTGGTGTGCTGACAACACC	1	103	103	1	100	Bm_ATCC23344_chr1	3510148	1391939	1392041	100	515	1.20E-18	-
BPSL1527	putative transcription accessory protein	1767550	1769874	-	774	53719161	3091541	tex	COG2183K	TTATGGCAAAATGCCCCGCTCCATT	1	103	1	103	100	Bm_ATCC23344_chr1	3510148	1387390	1387492	100	515	1.20E-18	+
BPSL1528	hypothetical protein BPSL1528	1770014	1770763	+	249	53719162	3092312	-	NULL	CGGCGTCGCGCAGACGCCGGCACC	1	103	103	1	100	Bm_ATCC23344_chr1	3510148	1387150	1387252	99	506	3.00E-18	-
BPSL1529	hypothetical protein BPSL1529	1770872	1771087	+	71	53719163	3091985	-	NULL	CGGCTGGCAGCAGGGGTACACGCC	1	96	96	1	100	Bm_ATCC23344_chr1	3510148	1386505	1386600	100	480	4.40E-17	-
BPSL1530	putative ATP-dependent DNA helicase-related prote	1771348	1773408	+	686	53719164	3091986	-	COG1199KL	GAGCGTAAAGCGTATCGTCGAGCT	1	103	103	1	100	Bm_ATCC23344_chr1	3510148	1386014	1386116	99	506	3.00E-18	-
BPSL1531	putative lipoprotein	1773584	1774408	-	274	53719165	3091887	-	COG4105R	CGGTTTGACTTGCCCGCACGGGCA	1	103	1	103	100	Bm_ATCC23344_chr1	3510148	1382856	1382958	100	515	1.20E-18	+
BPSL1532	putative ribosomal large subunit pseudouridine synt	1774462	1775649	+	395	53719166	3091888	-	COG0564J	CGCTTCTGCGGGCGATTCGCTCGAC	1	103	103	1	100	Bm_ATCC23344_chr1	3510148	1382720	1382822	100	515	1.20E-18	-
BPSL1534	poly-beta-hydroxybutyrate polymerase	1777009	1778814	+	601	53719168	3093057	phbC	COG3243I	CACCGTTAAGGCAGGTATGACAGC	1	103	103	1	100	Bm_ATCC23344_chr1	3510148	1380353	1380455	99	506	3.00E-18	-
BPSL1535	acetyl-CoA acetyltransferase	1778911	1780092	+	393	53719169	3094224	phbA	COG0183I	CGAACGGCGCAAGGCGGAGCACG	1	99	99	1	100	Bm_ATCC23344_chr1	3510148	1378442	1378540	100	495	9.30E-18	-
BPSL1536	acetoacetyl-CoA reductase	1780218	1780958	+	246	53719170	3093056	phbB	COG1028IQR	GGCGCGCCGGCGGGCGCGGCCCG	1	103	103	1	100	Bm_ATCC23344_chr1	3510148	1377135	1377237	100	515	1.20E-18	-
BPSL1537	hypothetical protein BPSL1537	1781095	1781667	+	190	53719171	3091519	-	COG5394S	TGGGTCGGATGTTCCGGGAACGCC	1	103	103	1	100	Bm_ATCC23344_chr1	3510148	1376258	1376360	100	515	1.20E-18	-
BPSL1538	hypothetical protein BPSL1538	1782265	1783644	+	459	53719172	3093910	-	COG0621J	AGAAACGCCGCCGCGCGGAGGGT	1	103	103	1	100	Bm_ATCC23344_chr1	3510148	1375103	1375205	100	515	1.20E-18	-
BPSL1540	beta-ketothiolase	1784669	1785853	+	394	53719174	3093104	-	COG0183I	GCCCGCGCGCGCGGCGCGCGATCC	1	98	98	1	100	Bm_ATCC23344_chr1	3510148	1372699	1372796	100	490	1.60E-17	-
BPSL1542	cystathionine beta-lyase	1786752	1787936	+	394	53719176	3092176	-	COG0626E	CGGCCGCGCCGATTGTACCGGGCC	1	103	103	1	100	Bm_ATCC23344_chr1	3510148	1370616	1370718	99	506	3.00E-18	-
BPSL1543	putative phosphoserine phosphatase	1788200	1789045	-	281	53719177	3092177	-	COG0560E	CTGGCGCCCATCCGCTGGCGTCCG	1	103	1	103	100	Bm_ATCC23344_chr1	3510148	1368225	1368327	100	515	1.20E-18	+
BPSL1546	putative ABC transport system, ATP-binding protein	1792027	1792722	-	231	53719180	3093241	-	COG4619R	CGTGCCGCGCAGCGTGCGCGACGC	1	103	1	103	100	Bm_ATCC23344_chr1	3510148	1364733	1364835	100	515	1.20E-18	+
BPSL1548	putative ABC transport system, ATP-binding protein	1795789	1797381	+	530	53719182	3093243	-	COG0488R	CGTGCGTTTTCGGGGCCCGAAGCG	1	103	103	1	100	Bm_ATCC23344_chr1	3510148	1361747	1361849	100	515	1.20E-18	-
BPSL1549	hypothetical protein BPSL1549	1797528	1798163	-	211	53719183	3093244	-	NULL	GTTTCATCCGGCAGAGGTCGCCGG	1	103	1	103	100	Bm_ATCC23344_chr1	3510148	1359276	1359378	100	515	1.20E-18	+
BPSL1550	putative betaine aldehyde dehydrogenase	1798479	1799915	+	478	53719184	3094163	-	COG1012C	GGGATAACGTGCGCGCACGGCCCG	1	103	103	1	100	Bm_ATCC23344_chr1	3510148	1359058	1359160	100	515	1.20E-18	-
BPSL1551	putative 2-nitropropane dioxygenase	1800114	1801304	+	396	53719185	3094164	-	COG2070R	GCGTACATGCGCATGCGTGCGTTT	1	103	103	1	100	Bm_ATCC23344_chr1	3510148	1357423	1357525	100	515	1.20E-18	-
BPSL1552	putative outer membrane protein	1801377	1802021	+	214	53719186	3093192	-	COG3047M	TGCTTGATGTCGGTCAATATAAGTA	1	75	75	1	100	Bm_ATCC23344_chr1	3510148	1356160	1356234	97	357	1.60E-11	-
BPSL1553	hypothetical protein BPSL1553	1802147	1802350	-	67	53719187	3093193	-	COG3313R	CCTTGCGGGCCGCCGCGGCCGGCT	1	103	1	103	100	Bm_ATCC23344_chr1	3510148	1355089	1355191	100	515	1.20E-18	+
BPSL1554	putative metal-ion transport system, membrane prot	1802768	1804075	-	435	53719188	3092464	-	COG1914P	GCCCGTTCGTCGTTCCGGGTAGGAC	1	103	1	103	100	Bm_ATCC23344_chr1	3510148	1353371	1353473	100	515	1.20E-18	+
BPSL1555	putrescine-binding periplasmic protein precursor	1804461	1805555	+	364	53719189	3091817	potF	COG0687E	ATTAGTGGTAGCTTTCGGGGTTTTC	1	103	103	1	100	Bm_ATCC23344_chr1	3510148	1353082	1353184	100	515	1.20E-18	-
BPSL1556	putrescine transport ATP-binding protein	1805670	1806806	+	378	53719190	3091818	potG	COG3842E	CATCGCGGCAAAACCGAGAAGCCC	1	103	103	1	100	Bm_ATCC23344_chr1	3510148	1351873	1351975	99	506	3.00E-18	-
BPSL1568	putative lipoprotein	1822892	1824007	-	371	53719202	3094780	-	COG0845M	CCGATTGTTTTGGTTTTTTGCCGCGC	1	103	103	1	100	Bm_ATCC23344_chr1	3510148	1007546	1007648	100	515	1.20E-18	-
BPSL1569	putative TetR-family regulatory protein	1824198	1824836	+	212	53719203	3094781	-	COG1309K	AAACAATCGGCGCGCACGTTGATG	1	103	1	103	100	Bm_ATCC23344_chr1	3510148	1007639	1007741	100	515	1.20E-18	+
BPSL1571	hypothetical protein BPSL1571	1826975	1828045	-	356	53719205	3094590	-	NULL	TATCGGATTTCCGGATATTTTACGC	1	103	103	1	100	Bm_ATCC23344_chr1	3510148	1011587	1011689	100	515	1.20E-18	-
BPSL1575	putative sugar kinase	1830638	1831627	+	329	53719209	3093367	-	COG0524G	AACCCATCCACCGTCGCCCGGCTC	1	86	1	86	100	Bm_ATCC23344_chr1	3510148	1014090	1014175	98	421	2.00E-14	+
BPSL1576	putative transport related, membrane protein	1831712	1832989	+	425	53719210	3092376	-	NULL	ACCGCCTGTCCCGACGCAGGACGG	1	87	1	87	100	Bm_ATCC23344_chr1	3510148	1015163	1015249	100	435	4.80E-15	+
BPSL1578	putative LacI-family transcriptional regulator	1834579	1835622	+	347	53719212	3092377	-	COG1609K	AATCGGCCGACAGGAGACATCGTC	1	103	1	103	100	Bm_ATCC23344_chr1	3510148	1018004	1018106	100	515	1.20E-18	+
BPSL1579	hypothetical protein BPSL1579	1835680	1836468	+	262	53719213	3093531	-	COG4377S	GTTGAGTATCATTCGGGGCACGCAC	1	60	1	60	100	Bm_ATCC23344_chr1	3510148	1019148	1019207	100	300	6.00E-09	+
BPSL1581	hypothetical protein BPSL1581	1837334	1837768	-	144	53719215	3093560	-	NULL	TCACTTCATCTTTGTCGCAACGCCT	1	103	103	2	99	Bm_ATCC23344_chr1	3510148	1021168	1021269	98	492	1.30E-17	-
BPSL1582	putative depolymerase	1837965	1838984	+	339	53719216	3093561	-	COG3509Q	CGCATTCACGATCACGCTCGCCGCC	1	103	1	103	100	Bm_ATCC23344_chr1	3510148	1021402	1021504	100	515	1.20E-18	+

BPSL1583	hypothetical protein BPSL1583	1839642	1840157 -	171	53719217	3091719 -	NULL	CCGCCGTGCTACTAATAGCGCGGA	1	103	103	1	100	Bm_ATCC23344_chr1	3510148	1023337	1023439	100	515	1.20E-18 -
BPSL1588	putative AsnC-family transcriptional regulator	1842086	1842547 +	153	53719218	3093966 -	COG1522K	CGGCGAGCGCGCCCGCGCGCAAA	1	103	1	103	100	Bm_ATCC23344_chr1	3510148	1025163	1025265	98	497	7.60E-18 +
BPSL1589	hypothetical protein BPSL1589	1842602	1843684 +	360	53719219	3093967 -	NULL	CGCGCGCCGCGCGCGACGACGGA	1	57	1	57	100	Bm_ATCC23344_chr1	3510148	1025725	1025781	100	285	2.80E-08 +
BPSL1590	hypothetical protein BPSL1590	1843714	1844196 -	160	53719220	3093590 -	NULL	GCCCGTCTCGATGCAGCGCATCGC	1	103	103	1	100	Bm_ATCC23344_chr1	3510148	1027371	1027473	100	515	1.20E-18 -
BPSL1591	hypothetical protein BPSL1591	1844497	1845597 +	366	53719221	3093591 -	COG1748E	CCGCATCAATTTTCGTCTATAAAGT	1	103	1	103	100	Bm_ATCC23344_chr1	3510148	1027574	1027676	100	515	1.20E-18 +
BPSL1593	putative metal ion transport protein	1846747	1847757 +	336	53719223	3091673 -	COG0598P	GCGGCGGGCGACGAGCGCGTGCC	1	103	1	103	100	Bm_ATCC23344_chr1	3510148	1029827	1029929	99	506	3.00E-18 +
BPSL1594	hypothetical protein BPSL1594	1847765	1848118 -	117	53719224	3091674 -	NULL	CGCGGTTCGCGCCGACGATGCGCG	1	103	103	1	100	Bm_ATCC23344_chr1	3510148	1031296	1031398	99	506	3.00E-18 -
BPSL1596	hypothetical protein BPSL1596	1849709	1849972 -	87	53719226	3092014 -	NULL	GCCCGCCGTGCCGATATCGAACCC	1	95	95	1	100	Bm_ATCC23344_chr1	3510148	1033137	1033231	100	475	7.40E-17 -
BPSL1597	hypothetical protein BPSL1597	1850065	1850436 +	123	53719227	3094321 -	NULL	AGCGCGATTGCGCCCTGCGCGCCT	1	95	1	95	100	Bm_ATCC23344_chr1	3510148	1033140	1033234	100	475	7.40E-17 +
BPSL1598	putative transport-related, membrane protein	1850780	1853209 -	809	53719228	3094322 -	COG3264M	TGTTTCGGATTGTCGTCGGCGATCC	1	103	103	1	100	Bm_ATCC23344_chr1	3510148	1036371	1036473	100	515	1.20E-18 -
BPSL1607	nitrous-oxide reductase precurser	1860683	1862650 -	655	53719237	3093662 nosZ	COG4263C	ATGCGGCGCAAATCGTCACCGGCC	1	103	103	1	100	Bm_ATCC23344_chr1	3510148	1045790	1045892	100	515	1.20E-18 -
BPSL1608	putative thiamine biosynthesis lipoprotein	1863332	1864390 +	352	53719238	3094171 -	NULL	CACTCGGGCGCGCGGCCCGGAAAA	1	103	1	103	100	Bm_ATCC23344_chr1	3510148	1046343	1046445	100	515	1.20E-18 +
BPSL1611	putative methyl-accepting chemotaxis transmembrar	1866328	1867917 +	529	53719241	3094498 -	COG0840NT	CCGTGGCCCGCGCCGCGCCGTAAA	1	103	1	103	100	Bm_ATCC23344_chr1	3510148	1049339	1049441	99	506	3.00E-18 +
BPSL1612	hypothetical protein BPSL1612	1868038	1868523 +	161	53719242	3093237 -	NULL	GTTCGCCCGCGCGCACATAGGTCG	1	103	1	103	100	Bm_ATCC23344_chr1	3510148	1051049	1051151	98	497	7.60E-18 +
BPSL1613	putative glycerol utilisation-related protein	1868738	1870450 +	570	53719243	3093238 -	COG2376G	GCGGGCTCGCCCACGAACGGCCGC	1	103	3	103	98	Bm_ATCC23344_chr1	3510148	1051751	1051851	100	505	3.30E-18 +
BPSL1618	hypothetical protein BPSL1618	1875876	1876574 -	232	53719248	3092088 -	NULL	CCGGATATGCACCTAGACTCGGTCT	1	103	103	1	100	Bm_ATCC23344_chr1	3510148	1059685	1059787	100	515	1.20E-18 -
BPSL1621	hypothetical protein BPSL1621	1878828	1879178 +	116	53719249	3092749 -	NULL	CATTGAACGCACATCGAGCGCGTA	1	103	1	103	100	Bm_ATCC23344_chr1	3510148	1061853	1061954	97	482	3.60E-17 +
BPSL1623	subfamily S9C non-peptidase homologue	1880385	1881473 -	362	53719251	3092283 -	COG3509Q	GGCTGCGAACGTCCTCCGAAGCGC	1	103	103	1	100	Bm_ATCC23344_chr1	3510148	1064585	1064687	100	515	1.20E-18 -
BPSL1624	hypothetical protein BPSL1624	1881764	1881955 -	63	53719252	3092284 -	NULL	GGCGTGGGCTCCTGTGGGCGATTG	1	103	103	1	100	Bm_ATCC23344_chr1	3510148	1065068	1065170	98	497	7.60E-18 -
BPSL1625	hypothetical protein BPSL1625	1882057	1882524 +	155	53719253	3091575 -	NULL	GAGCTTCGTCGGGCGAGCCACGTT	1	103	1	103	100	Bm_ATCC23344_chr1	3510148	1065072	1065174	98	497	7.60E-18 +
BPSL1626	putative fimbrial subunit type 1 precursor	1883679	1884209 +	176	53719254	3091576 -	COG3539NU	ATCGCGGTTTTTGTCGAAGAATTTT	1	103	1	103	100	Bm_ATCC23344_chr1	3510148	1068007	1068109	100	515	1.20E-18 +
BPSL1627	putative fimbrial assembly chaperone precursor	1884308	1885078 +	256	53719255	3092023 -	COG3121NU	CGGGCGCCGAAGCGTTCCTGCATG	1	101	1	101	100	Bm_ATCC23344_chr1	3510148	1068637	1068737	100	505	3.30E-18 +
BPSL1630	hypothetical protein BPSL1630	1889376	1889627 +	83	53719258	3094244 -	NULL	TTGAGCCGCTTATGCGGCTTATGTC	1	103	1	102	100	Bm_ATCC23344_chr1	3510148	1073703	1073804	99	501	5.00E-18 +
BPSL1632	hypothetical protein BPSL1632	1894492	1894977 +	161	53719260	3094820 -	NULL	CGATTCCCGCTCGATTCGGCGAGC	1	103	1	103	100	Bm_ATCC23344_chr1	3510148	1078492	1078594	100	515	1.20E-18 +
BPSL1633	putative two-component system, response regulator	1895264	1895929 +	221	53719261	3092021 -	COG2197TK	ACGCAGGCAATCGTTATCGTTTTGC	1	103	1	103	100	Bm_ATCC23344_chr1	3510148	1079275	1079377	100	515	1.20E-18 +
BPSL1710	putative penicillin amidase	1994317	1996689 -	790	53719324	3091709 -	NULL	CCGCCGCCTGCCTCCGGAGCGCCG	1	103	103	1	100	Bm_ATCC23344_chr1	3510148	1084001	1084102	99	500	5.50E-18 -
BPSL1724	putative histidinol-phosphate aminotransferase	2030608	2031618 -	336	53719338	3092858 -	COG0079E	AGCCCGTGCGCACGATGCATCGGG	1	64	64	1	100	Bm_ATCC23344_chr1	3510148	1162379	1162442	100	320	7.50E-10 -
BPSL1726	hypothetical protein BPSL1726	2032551	2032775 -	74	53719340	3094659 -	NULL	ATCCGGCGCGTCTTTCCAGCCGATG	1	103	103	1	100	Bm_ATCC23344_chr1	3510148	1163536	1163638	99	506	3.00E-18 -
BPSL1728	putative exported porin	2036054	2037277 -	407	53719342	3092778 -	COG3203M	CTCCGTCCGCCAATCGGCGCCCGG	1	103	103	1	100	Bm_ATCC23344_chr1	3510148	1168083	1168185	100	515	1.20E-18 -
BPSL1729	putative AraC-family transcriptional regulator	2037625	2038425 +	266	53719343	3092779 -	NULL	CGCCGCGGGTCGCGCGCCGCAAAC	1	103	1	103	100	Bm_ATCC23344_chr1	3510148	1168333	1168435	100	515	1.20E-18 +
BPSL1730	putative transmembrane protein	2038590	2039528 +	312	53719344	3093918 -	NULL	ATGGTCCTCGCCATCGCCCGCTCCC	1	103	1	103	100	Bm_ATCC23344_chr1	3510148	1169298	1169400	100	515	1.20E-18 +
BPSL1733	hypothetical protein BPSL1733	2042724	2042912 -	62	53719347	3091918 -	NULL	TCATAGAGCGATTCAATTTGCGCCG	1	103	103	1	100	Bm_ATCC23344_chr1	3510148	1173718	1173821	97	487	2.10E-17 -
BPSL1735	putative transport system membrane protein	2045352	2046755 +	467	53719349	3093699 -	NULL	TCGTGAACGCCGCGCCGCGCTGCC	1	79	1	79	100	Bm_ATCC23344_chr1	3510148	1176088	1176166	100	395	3.10E-13 +
BPSL1736	putative methyltransferase	2047096	2047866 -	256	53719350	3093700 -	COG0500QR	CGCGCATGGAACGGATATTGCGCT	1	103	103	1	100	Bm_ATCC23344_chr1	3510148	1178820	1178922	100	515	1.20E-18 -
BPSL1737	putative ABC transport system, exported protein	2048228	2049166 +	312	53719351	3093342 -	COG1732M	GCGCGCGGCCCCGCTTCACGCCCG	1	103	1	103	100	Bm_ATCC23344_chr1	3510148	1179139	1179241	100	515	1.20E-18 +
BPSL1738	putative ABC transport system, membrane protein	2049223	2050383 +	386	53719352	3093343 -	COG1174E	CGCCGCGCACGGCGGGCGGCGCG	1	59	1	59	100	Bm_ATCC23344_chr1	3510148	1180178	1180236	100	295	1.00E-08 +
BPSL1741	hypothetical protein BPSL1741	2053356	2053688 +	110	53719355	3093374 -	NULL	ACGCGAGAACCGGGATTCAAAGCC	1	103	1	103	100	Bm_ATCC23344_chr1	3510148	1184267	1184369	100	515	1.20E-18 +
BPSL1742	arginine/ornithine antiporter	2053849	2055309 +	486	53719356	3092544 arcD	COG0531E	AGGCGGCAAACCGCCGCGCTGCCT	1	103	1	103	100	Bm_ATCC23344_chr1	3510148	1184760	1184862	98	497	7.60E-18 +
BPSL1744	ornithine carbamoyltransferase	2056648	2057658 +	336	53719358	3092910 arcB	COG0078E	GCGCGAACCCAAGCGTCGCGGCGC	1	67	1	67	100	Bm_ATCC23344_chr1	3510148	1187595	1187661	100	335	1.60E-10 +
BPSL1745	carbamate kinase	2057747	2058685 +	312	53719359	3091935 arcC	COG0549E	AGGCGCGCCGCGCGCGATCGAGA	1	91	1	91	100	Bm_ATCC23344_chr1	3510148	1188670	1188760	100	455	6.00E-16 +
BPSL1747	hypothetical protein BPSL1747	2059719	2060168 -	149	53719361	3094233 -	COG3193R	TAGAAAAACTTGATACGTTTGTGAG	1	100	100	1	100	Bm_ATCC23344_chr1	3510148	1191177	1191276	100	500	5.50E-18 -
BPSL1748	putative LysR-family transcriptional regulator	2060266	2061186 +	306	53719362	3094234 -	COG0583K	CGGCGGCTCCTTGTGCGGTTCGACT	1	100	1	100	100	Bm_ATCC23344_chr1	3510148	1191180	1191279	100	500	5.50E-18 +
BPSL1749	putative glutathione S-transferase	2061471	2062175 +	234	53719363	3092879 -	COG0625O	ACGCGCGGGATCGGCTACATTGTG	1	103	1	103	100	Bm_ATCC23344_chr1	3510148	1192382	1192484	98	497	7.60E-18 +
BPSL1750	putative MarR-family transcriptional regulator	2062731	2063270 +	179	53719364	3092880 -	COG1846K	TGCGAACGCGGTACGCGGGGACGC	1	103	1	103	100	Bm_ATCC23344_chr1	3510148	1193639	1193741	100	515	1.20E-18 +
BPSL1751	putative amino-acid transport-related exported prot	2063491	2064606 +	371	53719365	3093906 -	COG0683E	GTCGATCCATGCCGAAAGCGATTG	1	103	1	103	100	Bm_ATCC23344_chr1	3510148	1194398	1194500	100	515	1.20E-18 +
BPSL1752	putative MarR-family regulatory protein	2064914	2065408 -	164	53719366	3093907 -	COG1846K	GGACGGTTGCCGGACACGCGCAGT	1	103	103	1	100	Bm_ATCC23344_chr1	3510148	1196400	1196502	99	506	3.00E-18 -
BPSL1753	putative transport-related membrane protein	2065532	2066716 +	394	53719367	3092318 -	COG2807P	ATCTTGACGTAGAGACAAGTCGAAT	1	103	1	103	100	Bm_ATCC23344_chr1	3510148	1196426	1196528	99	506	3.00E-18 +
BPSL1754	putative lipoprotein	2066841	2068412 +	523	53719368	3092319 -	COG1649S	CGCAACGCCCGGCCGCGCGCCGCC	1	103	5	103	96	Bm_ATCC23344_chr1	3510148	1197725	1197826	91	411	5.80E-14 +
BPSL1755	precorrin-4 C11-methyltransferase	2068491	2069216 -	241	53719369	3094850 cobM	COG2875H	GCGGCTTGCGACGCAGGCGTGAGC	1	103	103	1	100	Bm_ATCC23344_chr1	3510148	1200197	1200299	100	515	1.20E-18 -
BPSL1758	precorrin-6Y C5,15-methyltransferase	2071377	2072603 -	408	53719372	3092208 cobL	COG2241H, COG2242H	ACGGACGCTGTATTATGCCTTTTTTC	1	103	103	1	100	Bm_ATCC23344_chr1	3510148	1203584	1203686	100	515	1.20E-18 -
BPSL1759	putative oxidoreductase	2073001	2074449 +	482	53719373	3094470 -	COG0155P	CCTTCATCGGGCGGGGTGTACCGA	1	103	1	103	100	Bm_ATCC23344_chr1	3510148	1203884	1203986	100	515	1.20E-18 +
BPSL1763	putative exported chitinase	2077979	2079352 +	457	53719377	3093420 -	COG3979R	GCGGTGGATTCCTCCATGATGTGCA	1	103	1	103	100	Bm_ATCC23344_chr1	3510148	1208855	1208957	100	515	1.20E-18 +
BPSL1764	hypothetical protein BPSL1764	2079715	2080041 +	108	53719378	3093421 -	NULL	TGCAGGCTCGCGCCAAGCGGCGGG	1	103	1	103	100	Bm_ATCC23344_chr1	3510148	1210591	1210693	100	515	1.20E-18 +
BPSL1766	hypothetical protein BPSL1766	2081458	2082072 -	204	53719380	3093425 -	COG1240H	TGAGCCATCGGGCGGATGCCGCCG	1	86	86	1	100	Bm_ATCC23344_chr1	3510148	1212987	1213072	100	430	8.00E-15 -
BPSL1768	cobaltochelatase	2084041	2087886 -	1281	53719382	3091604 -	NULL	CGCGGCGGCGGGCGACGCGCGGC	1	69	69	1	100	Bm_ATCC23344_chr1	3510148	1218576	1218644	100	345	5.50E-11 -
BPSL1769	putative cobalamin biosynthesis-related protein	2087953	2089026 -	357	53719383	3093490 cobW	COG0523R	CATGAGCTCATGCGCCGCGCGCGT	1	103	103	1	100	Bm_ATCC23344_chr1	3510148	1219716	1219818	100	515	1.20E-18 -
BPSL1771	cobalamin biosynthesis protein CbiG	2091314	2091766 +	150	53719385	3093779 -	NULL	GCCCGGCCGGCAAGCCGGGCGCC	1	103	1	103	100	Bm_ATCC23344_chr1	3510148	1221890	1221992	100	515	1.20E-18 +
BPSL1772	cob(I)yrinic acid a,c-diamide adenosyltransferase	2091825	2092427 +	200	53719386	3094851 cobO	COG2109H	TGACGCGCGCCGCGCGCGGTCTTC	1	64	1	64	100	Bm_ATCC23344_chr1	3510148	1222440	1222503	100	320	7.50E-10 +
BPSL1774	putative siderophore biosynthesis related protein	2094346	2095185 -	279	53719388	3093780 -	NULL	GCGCGTTTCATCACCCGATGGGCG	1	67	67	1	100	Bm_ATCC23344_chr1	3510148	1225868	1225934	100	335	1.60E-10 -
BPSL1776	putative L-ornithine 5-monooxygenase	2097511	2098917 -	468	53719390	3094194 -	COG3486Q	GGCCGCATCGGGCGCATCGATGAA	1	103	103	1	100	Bm_ATCC23344_chr1	3510148	1229597	1229699	100	515	1.20E-18 -
BPSL1779	putative siderophore biosynthesis related ABC transp	2114773	2116518 +	581	53719393	3091954 -	COG4615QP	GCCCATTCGTCTAGCAGACAGAGA	1	103	1	103	100	Bm_ATCC23344_chr1	3510148	1245357	1245457	98	485	2.60E-17 +
BPSL1785	hypothetical protein BPSL1785	2122834	2123850 -	338	53719399	3091936 -	NULL	TGCGCCGCGCGCGCCGCTCGTTTC	1	61	61	1	100	Bm_ATCC23344_chr1	3510148	1254502	1254562	100	305	3.60E-09 -
BPSL1786	hypothetical protein BPSL1786	2123909	2124151 -	80	53719400	3091937 -	NULL	ACGCGGCGCACGCGGTGCGCCGTC	1	58	58	1	100	Bm_ATCC23344_chr1	3510148	1254803	1254860	100	290	1.70E-08 -
BPSL1792	hypothetical protein BPSL1792	2130598	2132127 -	509	53719405	3093022 -	NULL	GCGGCCCGCGCGACGAGGCCGAC	1	64	64	1	100	Bm_ATCC23344_chr1	3510148	1262806	1262869	100	320	7.50E-10 -
BPSL1793	putative sugar-binding exported protein	2132189	2133133 -	314	53719406	3092676 -	COG1879G	GCCCGCGTTCCCGAGCAATCCAGC	1	103	103	1	100	Bm_ATCC23344_chr1	3510148	1263812	1263914	99	506	3.00E-18 -
BPSL1794	putative AraC-family transcriptional regulator	2133238	2134254 -	338	53719407	3092677 -	NULL	GTGGCCGGCCGGATGGCCGGCGAC	1	103	103	1	100	Bm_ATCC23344_chr1	3510148	1264933	1265035	99	506	3.00E-18 -
BPSL1796	hypothetical protein BPSL1796	2135807	2137408 +	533	53719409	3094822 -	COG0367E	ACGGCGCGCGTGGACGGACGCGG	1	79	1	79	100	Bm_ATCC23344_chr1	3510148	1266371	1266449	97	377	2.00E-12 +
BPSL1835	transcriptional regulator Cbl	2188911	2189837 -	308	53719448	3094539 cbl	COG0583K	CGGCGCTTCCGCGAGCGACTCGCG	1	72	72	1	100	Bm_ATCC23344_chr1	3510148	1270241	1270312	100	360	1.20E-11 -
BPSL1839	sulfate-binding protein precursor	2193033	2194016 -	327	53719452	3093697 sbp	COG1613P	AACGAACGAACCGCAACGAACGAC	1	103	103	1	100	Bm_ATCC23344_chr1	3510148	1274432	1274534	100	515	1.20E-18 -
BPSL1840	LexA repressor	2194373	2195020 +	215	53719453	3094085 lexA	COG1974KT	CGGCGGAATCGCCGCGATAGCGCC	1	103	1	103	100	Bm_ATCC23344_chr1	3510148	1274691	1274793	99	506	3.00E-18 +
BPSL1841	hypothetical protein BPSL1841	2195083	2195409 +	108	53719454	3093447 -	NULL	TCGCCTTCGGCCAGCTGCAGCCCG	1	65	1	65	100	Bm_ATCC23344_chr1	3510148	1275439	1275503	96	307	2.90E-09 +
BPSL1843	putative stress-related protein	2196592	2197083 -	163	53719456	3092258 -	COG0589T	TTCGCGGTGCAACGCGAATCAGCG	1	103	103	1	100	Bm_ATCC23344_chr1	3510148	1277502	1277604	100	515	1.20E-18 -
BPSL1844	putative ABC transport system, ATP-binding protein	2197307	2198227 +	306	53719457	3092478 -	COG1131V	GCAGCGGCATCAGCGCCGGGATGA	1	103	65	103	36	Bm_ATCC23344_chr1	3510148	1278932	1278970	100	195	0.0011 +

BPSL1846	hypothetical protein BPSL1846	2199174	2199671	-	165	53719459	3092209	-	NULL	GTGCGCCGGCGCACGATGCACGGC	1	103	103	1	100	Bm_ATCC23344_chr1	3510148	1281303	1281405	98	497	7.60E-18	-
BPSL1847	hypothetical protein BPSL1847	2199911	2200972	-	353	53719460	3092210	-	COG0701R	CGCCGCCCGCCGCGCGATGGTGCG	1	103	103	1	100	Bm_ATCC23344_chr1	3510148	1282604	1282706	99	506	3.00E-18	-
BPSL1848	putative oxidoreductase	2201328	2202380	-	350	53719461	3094315	-	COG0667C	CGAGCGCTGCGCCGGCAGCGGCTG	1	103	103	1	100	Bm_ATCC23344_chr1	3510148	1284012	1284114	99	506	3.00E-18	-
BPSL1849	hypothetical protein BPSL1849	2202667	2203113	-	148	53719462	3094316	-	COG2050Q	GTGTTGACAGCCGATGAACGTCTGC	1	103	103	1	100	Bm_ATCC23344_chr1	3510148	1284734	1284836	99	506	3.00E-18	-
BPSL1850	hypothetical protein BPSL1850	2203286	2203762	+	158	53719463	3094338	-	COG1733K	TTGGCAGACGTTCATCGGCTGTCAA	1	103	1	103	100	Bm_ATCC23344_chr1	3510148	1284809	1284911	100	515	1.20E-18	+
BPSL1853	hypothetical protein BPSL1853	2208821	2209075	-	84	53719466	3093393	-	NULL	CGGGGGCGGCGGACGCCGGGCGA	1	76	76	1	100	Bm_ATCC23344_chr1	3510148	1293257	1293332	98	371	3.70E-12	-
BPSL1854	hypothetical protein BPSL1854	2209149	2209394	-	81	53719467	3094673	-	NULL	GCGGGCGCCGCCGCGCCCCCCGTG	1	83	83	1	100	Bm_ATCC23344_chr1	3510148	1293576	1293659	98	405	1.10E-13	-
BPSL1855	molybdenum-pterin binding protein II	2209475	2209690	-	71	53719468	3092330	mopII	COG3585H	CGGGCCGCGCGGCGACGCGCCGCC	1	91	91	1	100	Bm_ATCC23344_chr1	3510148	1293873	1293963	100	455	6.00E-16	-
BPSL1859	hypothetical protein BPSL1859	2213101	2213565	+	154	53719472	3094674	-	NULL	GGTTTTCCGCTTTCGTGCATAGCGC	1	103	1	103	100	Bm_ATCC23344_chr1	3510148	1297200	1297302	99	506	3.00E-18	+
BPSL1862	hypothetical protein BPSL1862	2215537	2216034	-	165	53719475	3093981	-	NULL	TCGCCGGCCGAGCCGCCGAGGCTA	1	103	103	1	100	Bm_ATCC23344_chr1	3510148	1300231	1300333	100	515	1.20E-18	-
BPSL1863	hypothetical protein BPSL1863	2216754	2217299	+	181	53719476	3091582	-	COG1051F	CCCGGGCCGCGCCCGCCCGCATGG	1	103	1	103	100	Bm_ATCC23344_chr1	3510148	1300852	1300954	100	515	1.20E-18	+
BPSL1866	dihydroorotate dehydrogenase	2219044	2220072	+	342	53719479	3093839	pyrD	COG0167F	CCTGGGCCGCTTGCGCCGCCGCGC	1	103	1	103	100	Bm_ATCC23344_chr1	3510148	1303142	1303244	99	506	3.00E-18	+
BPSL1867	putative ABC transport system, cystine-binding prote	2220192	2220986	+	264	53719480	3091964	-	COG0834ET	AACAAACGGTATTTAGACATCGATT	1	103	1	103	100	Bm_ATCC23344_chr1	3510148	1304290	1304392	100	515	1.20E-18	+
BPSL1868	putative ABC transport system, membrane protein	2221116	2221775	+	219	53719481	3092263	-	COG0765E	CGACATCCGGCGGCGCGCGAGGGC	1	103	1	103	100	Bm_ATCC23344_chr1	3510148	1305214	1305316	99	506	3.00E-18	+
BPSL1869	putative oxidoreductase	2222175	2223158	-	327	53719482	3092264	-	COG0673R	GCGCCATGCGTGTCGCGCGCCGCG	1	102	102	1	100	Bm_ATCC23344_chr1	3510148	1308466	1308567	100	510	2.00E-18	-
BPSL1870	putative GntR-family regulatory protein	2223258	2223971	-	237	53719483	3092305	-	COG2186K	CATAAGAACAAAGGCTGATAGTCGT	1	103	103	1	100	Bm_ATCC23344_chr1	3510148	1309279	1309381	100	515	1.20E-18	-
BPSL1871	ribose-5-phosphate isomerase A	2224309	2225004	-	231	53719484	3094103	rpiA	COG0120G	GCGATGCGGCGGCGCGCCGCCGGC	1	103	103	1	100	Bm_ATCC23344_chr1	3510148	1310312	1310414	100	515	1.20E-18	-
BPSL1872	putative N-acetylmuramoyl-L-alanine amidase	2225273	2226172	-	299	53719485	3092306	-	COG3023V	CACGAAACGCGCAGGAATGCGAGC	1	103	103	1	100	Bm_ATCC23344_chr1	3510148	1311480	1311582	100	515	1.20E-18	-
BPSL1873	putative tRNA/rRNA methyltransferase	2226546	2227289	-	247	53719486	3091946	-	COG0566J	CGAAACGCCGCGCGCCCCGCCAGC	1	103	103	1	100	Bm_ATCC23344_chr1	3510148	1312597	1312699	100	515	1.20E-18	-
BPSL1874	putative ribonuclease	2227421	2229955	-	844	53719487	3091947	-	COG0557K	AGACGGCCAAACGAACCTGTCAAC	1	103	103	1	100	Bm_ATCC23344_chr1	3510148	1315263	1315365	100	515	1.20E-18	-
BPSL1875	putative methyl-accepting chemotaxis protein	2230595	2232307	-	570	53719488	3094733	-	COG0840NT	TTGCTACGGAATTGCCGCATTAGGA	1	103	103	1	100	Bm_ATCC23344_chr1	3510148	1319206	1319308	100	515	1.20E-18	-
BPSL1876	putative phospholipase	2232808	2234328	+	506	53719489	3094734	-	NULL	CGTATAACGAACACAGGCTCAATGC	1	103	1	103	100	Bm_ATCC23344_chr1	3510148	1319610	1319712	99	506	3.00E-18	+
BPSL1877	hypothetical protein BPSL1877	2234635	2236161	+	508	53719490	3094689	-	NULL	AACGCTCGCCCAGGGGCGCGCCGC	1	103	1	103	100	Bm_ATCC23344_chr1	3510148	1321417	1321519	100	515	1.20E-18	+
BPSL1881	putative metabolite transport, membrane protein	2240052	2241272	+	406	53719494	3094066	-	NULL	GGCGAGCGGCGTACACTCGCGTTA	1	103	1	103	100	Bm_ATCC23344_chr1	3510148	1326798	1326900	100	515	1.20E-18	+
BPSL1882	putative TetR-family regulatory protein	2241489	2242445	+	318	53719495	3094067	-	COG1309K	GCCACCCTGCGTCGCTTTTTATCTTA	1	103	1	103	100	Bm_ATCC23344_chr1	3510148	1328235	1328337	100	515	1.20E-18	+
BPSL1883	putative acyl-CoA synthetase	2242630	2244402	+	590	53719496	3094522	-	COG0318IQ	TTTCGTCGTTCACCCCCGCACGCGC	1	103	1	103	100	Bm_ATCC23344_chr1	3510148	1329376	1329478	100	515	1.20E-18	+
BPSL1884	hypothetical protein BPSL1884	2244462	2245421	+	319	53719497	3094523	-	NULL	TCCGGGCGAACCGCGCAAGCATGA	1	62	1	62	100	Bm_ATCC23344_chr1	3510148	1331249	1331310	100	310	2.10E-09	+
BPSL1885	hypothetical protein BPSL1885	2245609	2245881	-	90	53719498	3091754	-	NULL	GTCGGGTTACAGGTCTCGCACGGC	1	103	103	1	100	Bm_ATCC23344_chr1	3510148	1332725	1332827	99	506	3.00E-18	-
BPSL1886	hypothetical protein BPSL1886	2246987	2247718	-	243	53719500	3091423	-	NULL	AAGCAGGCGGCGAGCCGGCCGACC	1	75	75	1	100	Bm_ATCC23344_chr1	3510148	1334523	1334597	100	375	2.40E-12	-
BPSL1888	hypothetical protein BPSL1888	2249199	2250905	-	568	53719502	3093705	-	COG4655S	GCCGTTAGCCCGCCGCCGCGCCGA	1	103	103	1	100	Bm_ATCC23344_chr1	3510148	1337710	1337812	99	506	3.00E-18	-
BPSL1894	putative fimbriae assembly protein	2255773	2257011	-	412	53719508	3093182	-	COG4963U	GCGCGCGACGCCACGGCCGCGCGC	1	69	69	1	100	Bm_ATCC23344_chr1	3510148	1343712	1343780	100	345	5.50E-11	-
BPSL1895	putative exported fimbriae assembly protein	2257078	2258403	-	441	53719509	3093183	-	COG4964U	TCGACAAGCGGTTTCGAACAGTGAC	1	66	66	1	100	Bm_ATCC23344_chr1	3510148	1345104	1345169	100	330	2.60E-10	-
BPSL1896	putative membrane fimbriae assembly protein	2258467	2259366	-	299	53719510	3094657	-	NULL	AGTACCCGAGCGACTAGGCCGCAG	1	82	82	1	100	Bm_ATCC23344_chr1	3510148	1346067	1346146	97	380	1.50E-12	-
BPSL1898	family A24A unassigned peptidase	2259910	2260410	-	166	53719512	3093974	-	COG4960OU	CGTTCACATCCAATTAGCGCGTGAC	1	85	85	1	100	Bm_ATCC23344_chr1	3510148	1347109	1347193	100	425	1.40E-14	-
BPSL1899	putative fimbriae assembly related protein	2260493	2260663	-	56	53719513	3093975	-	NULL	ACGAAACCGCCCGAGCCGGCATTA	1	Not found											
BPSL1903	putative exported heme utilisation related protein	2264383	2266062	+	559	53719517	3094580	-	COG2831U	GTCGGATCGGCACAACGCAAGACC	1	103	1	103	100	Bm_ATCC23344_chr1	3510148	1089936	1090038	100	515	1.20E-18	+
BPSL1904	hypothetical protein BPSL1904	2266285	2266767	+	160	53719518	3093668	-	COG4731S	ACTTGTATCAAACGCCGCGCGCGG	1	103	1	103	100	Bm_ATCC23344_chr1	3510148	1091838	1091940	100	515	1.20E-18	+
BPSL1905	putative transposase	2267004	2267414	-	136	53719519	3093669	-	COG3293L	AATTCGGTATTAACTATTCTAAATC	1	103	103	1	100	Bm_ATCC23344_chr1	3510148	1093065	1093167	100	515	1.20E-18	-
BPSL1906	hypothetical protein BPSL1906	2267610	2268707	-	365	53719520	3093678	-	COG1485R	CAACGCAACACCGCGCGCCGGCCC	1	103	103	1	100	Bm_ATCC23344_chr1	3510148	1094357	1094459	100	515	1.20E-18	-
BPSL1907	dihydrolipoamide dehydrogenase	2268814	2270244	-	476	53719521	3094406	odhL	COG1249C	GCACGCTTTTCATACCGTATCGAAC	1	102	102	1	100	Bm_ATCC23344_chr1	3510148	1095894	1095995	100	510	2.00E-18	-
BPSL1909	2-oxoglutarate dehydrogenase E1 component	2271756	2274620	-	954	53719523	3094519	sucA	COG0567C	CAAGACTTGATTCGCGCAATCCGCT	1	103	103	1	100	Bm_ATCC23344_chr1	3510148	1100260	1100362	100	515	1.20E-18	-
BPSL1911	GTP-binding protein	2276550	2278376	-	608	53719525	3091717	typA	COG1217T	TGACGTGCTATAATTTCAGGCTTTCC	1	103	103	1	100	Bm_ATCC23344_chr1	3510148	1102425	1102527	100	515	1.20E-18	-
BPSL1912	putative transcriptional regulatory protein	2278669	2279166	+	165	53719526	3092472	-	COG1846K	TGCAGCCCTGCAACCAAATCGCCGT	1	103	1	103	100	Bm_ATCC23344_chr1	3510148	1102620	1102722	100	515	1.20E-18	+
BPSL1913	hypothetical protein BPSL1913	2279266	2280762	+	498	53719527	3092473	-	COG1538MU	ATATTGCCATAACAAGCAATTTAGA	1	102	1	102	100	Bm_ATCC23344_chr1	3510148	1103218	1103319	100	510	2.00E-18	+
BPSL1914	putative multidrug resistance protein A	2280830	2282065	+	411	53719528	3093432	emrA	COG1566V	ACCGAATCATTTCCGGCATGCCGGA	1	70	1	70	100	Bm_ATCC23344_chr1	3510148	1104814	1104883	100	350	3.30E-11	+
BPSL1918	translation initiation factor IF-2	2285354	2288281	-	975	53719532	3093280	infB	COG0532J	GAAATGACCATGGCGCACTGATTTC	1	94	94	1	100	Bm_ATCC23344_chr1	3510148	1112330	1112423	100	470	1.30E-16	-
BPSL1920	hypothetical protein BPSL1920	2289845	2290306	-	153	53719534	3093802	-	COG0779S	GAAACGGTTGGGCATCTCGGGTAG	1	103	103	1	100	Bm_ATCC23344_chr1	3510148	1114355	1114457	100	515	1.20E-18	-
BPSL1921	hypothetical protein BPSL1921	2290611	2292275	-	554	53719535	3092190	-	COG1187J	GACGGGCGGCCGAACCCCAGCCGT	1	66	66	1	100	Bm_ATCC23344_chr1	3510148	1116324	1116389	100	330	2.60E-10	-
BPSL1923	putative transcriptional regulator	2294125	2295027	-	300	53719537	3091898	-	COG0583K	TGCGTTTCAGCCGAGTGCTGCACCC	1	103	103	1	100	Bm_ATCC23344_chr1	3510148	1119037	1119139	100	515	1.20E-18	-
BPSL1924	hypothetical protein BPSL1924	2295449	2295847	+	132	53719538	3091899	-	COG3293L	GAGCCGACGCCTCACACCGGCAC	1	103	1	103	100	Bm_ATCC23344_chr1	3510148	1119361	1119463	100	515	1.20E-18	+
BPSL1925	hypothetical protein BPSL1925	2296086	2296310	+	74	53719539	3093711	-	NULL	CGCTCCCCCGCTTAACGTAGCGTTA	1	103	1	103	100	Bm_ATCC23344_chr1	3510148	1119998	1120100	100	515	1.20E-18	+
BPSL1926	hypothetical protein BPSL1926	2296471	2296788	-	105	53719540	3093712	-	NULL	TATAAGGACCCCTCTTGAGCTGCGT	1	103	103	1	100	Bm_ATCC23344_chr1	3510148	1120796	1120898	100	515	1.20E-18	-
BPSL1927	hypothetical protein BPSL1927	2296989	2297711	+	240	53719541	3091702	-	COG1434S	ATCCCTCCACTCCTTCCGACCCGCG	1	103	1	103	100	Bm_ATCC23344_chr1	3510148	1120899	1121001	100	515	1.20E-18	+
BPSL1929	hypothetical protein BPSL1929	2297950	2298228	+	92	53719542	3091703	-	NULL	TGCACTGATCCGTCACTTTTGATAA	1	103	1	103	100	Bm_ATCC23344_chr1	3510148	1121860	1121962	100	515	1.20E-18	+
BPSL1930	hypothetical protein BPSL1930	2298377	2298634	+	85	53719543	3094794	-	NULL	TCGGCTTTTTCGTTGGCGTGCCCGA	1	103	1	103	100	Bm_ATCC23344_chr1	3510148	1122287	1122389	100	515	1.20E-18	+
BPSL1931	hypothetical protein BPSL1931	2298684	2298974	-	96	53719544	3094795	-	COG1359S	AACCTATTTTTTTGAGGCGGCGGCT	1	103	103	1	100	Bm_ATCC23344_chr1	3510148	1122982	1123083	98	491	1.40E-17	-
BPSL1933	hypothetical protein BPSL1933	2300849	2302075	-	408	53719546	3091550	-	COG3317M	CCCGGGCGGCGAACAAGCGGGCG	1	103	103	1	100	Bm_ATCC23344_chr1	3510148	1126548	1126650	100	515	1.20E-18	-
BPSL1937	hypothetical protein BPSL1937	2306519	2308639	+	706	53719550	3091894	-	NULL	GATGTGGACACCGGTCCAGCGCGT	1	Not found											
BPSL1938	putative transcriptional regulator	2308761	2309165	-	134	53719551	3091895	-	COG0789K	GCCGGCGTTTCGCCTCCTGCGCGCC	1	61	61	1	100	Bm_ATCC23344_chr1	3510148	1133092	1133152	100	305	3.60E-09	-
BPSL1939	integration host factor alpha subunit	2309224	2309571	-	115	53719552	3092200	ihfA	NULL	TGCCGGTGTTTTTGTCCAAGTTTGAC	1	103	103	1	100	Bm_ATCC23344_chr1	3510148	1133498	1133600	100	515	1.20E-18	-
BPSL1940	phenylalanyl-tRNA synthetase beta subunit	2309701	2312133	-	810	53719553	3091565	pheT	COG0072J, COG0073R	CGGCGCGACCGTCCCGCTCCGCGC	1	90	90	1	100	Bm_ATCC23344_chr1	3510148	1136060	1136149	100	450	1.00E-15	-
BPSL1941	phenylalanyl-tRNA synthetase alpha subunit	2312221	2313234	-	337	53719554	3092764	pheS	COG0016J	GCGGCCGCTGCAGCGAAAACGGGC	1	103	103	1	100	Bm_ATCC23344_chr1	3510148	1137161	1137263	100	515	1.20E-18	-
BPSL1943	50S ribosomal protein L35	2313773	2313970	-	65	53719556	3092639	rpmI	COG0291J	CGTACGCGCCGTACACAAGTGGAC	1	103	103	1	100	Bm_ATCC23344_chr1	3510148	1137897	1137999	100	515	1.20E-18	-
BPSL1945	threonyl-tRNA synthetase	2314737	2316644	-	635	53719558	3092747	thrS	COG0441J	AGCGGGGCCGCATTTTTTTTGTTGCC	1	103	103	1	100	Bm_ATCC23344_chr1	3510148	1140571	1140672	99	500	5.50E-18	-
BPSL1948	putative hydrolase	2319812	2320717	-	301	53719561	3094676	-	COG0596R	GCGTGACGAAGCGGTTTACAATCGC	1	103	103	1	100	Bm_ATCC23344_chr1	3510148	1144644	1144746	100	515	1.20E-18	-
BPSL1950	putative LysR-family transcriptional regulator	2322043	2322948	-	301	53719562	3092824	-	COG0583K	ATTCATGTTAGTTTTCCGATTTCGAT	1	103	103	1	100	Bm_ATCC23344_chr1	3510148	1146880	1146982	99	506	3.00E-18	-
BPSL1951	hypothetical protein BPSL1951	2323136	2323693	+	185	53719563	3094629	-	COG5485R	AACTAACATGAATGCCGCCGCCGTA	1	103	1	103	100	Bm_ATCC23344_chr1	3510148	1146970	1147072	98	497	7.60E-18	+
BPSL1955	succinyl-CoA:3-ketoacid-coenzyme A transferase sub	2326613	2327317	-	234	53719567	3093698	scoA	COG1788I	GGCAGTATTCGCCGCGCGTGCTTAC	1	103	103	1	100	Bm_ATCC23344_chr1	3510148	1151243	1151345	100	515	1.20E-18	-
BPSL1956	putative LuxR-family regulatory protein	2327570	2328115	+	181	53719568	3094632	-	COG2771K	GTGAAAGGGTACGATGCGCGGCGC	1	103	1	103	100	Bm_ATCC23344_chr1	3510148	1151398	1151500	100	515	1.20E-18	+
BPSL1958	hypothetical protein BPSL1958	2331168	2332241	+	357	53719570	3094819	-	NULL	CCCACCAAACACGCAAAGGAGGCC	1	103	1	103	100	Bm_ATCC23344_chr1	3510148	1154975	1155077	100	515	1.20E-18	+
BPSL1960	short chain dehydrogenase	2333095	2333874	-	259	53719572	3094596	-	COG1028IQR	TCCTAGATTAGCACGATCGTTCTATT	1	103	103	1	100	Bm_ATCC23344_chr1	3510148	1157777	1157879	100	515	1.20E-18	-
BPSL1961	putative electron transport protein	2334055	2335728	+	557	53719573	3094771	-	COG0644C	ACGATCGTGCTAATCTAGGATTCCG	1	103	1	103	100	Bm_ATCC23344_chr1	3510148	1157860	1157962	100	515	1.20E-18	+
BPSL1962	chorismate synthase	2335915	2337024	-	369	53719574	3092463	aroC	NULL	GTCGCGCGGCGCCGCCCGATCGCG	1	103	1	103	100	Bm_ATCC23344_chr1	3510148	997575	997677	100	515	1.20E-18	+

BPSL1963	hypothetical protein BPSL1963	2337389	2339323 -	644	53719575	3094772 -	COG0204I	ACCCGCGCGCGGGCCGCCGCGCG	1	102	1	102	100	Bm_ATCC23344_chr1	3510148	995281	995382	100	510	2.00E-18 +
BPSL1964	hypothetical protein BPSL1964	2339423	2339887 -	154	53719576	3094295 -	COG0517R	CCGGGCAGTTGCCGCAGTCGAGCG	1	103	1	103	100	Bm_ATCC23344_chr1	3510148	994716	994818	100	515	1.20E-18 +
BPSL1965	hypothetical protein BPSL1965	2340104	2340424 +	106	53719577	3094296 -	COG4323S	CCACGGCGGCGGCTGACGCGGTTG	1	103	103	1	100	Bm_ATCC23344_chr1	3510148	994597	994699	100	515	1.20E-18 -
BPSL1966	putative tRNA processing exoribonuclease	2340641	2341945 -	434	53719578	3094823 -	COG1295S	AGCGGCTATTATAGGGGCTGAAGC	1	103	1	103	100	Bm_ATCC23344_chr1	3510148	992703	992805	100	515	1.20E-18 +
BPSL1967	putative Trp repressor binding protein	2342060	2342668 +	202	53719579	3094824 -	COG0655R	TTCTCCTATTGCTGCACGTGCCGCG	1	103	103	1	100	Bm_ATCC23344_chr1	3510148	992686	992788	100	515	1.20E-18 -
BPSL1971	putative LysR-family transcriptional regulator	2345922	2346863 -	313	53719583	3093268 -	COG0583K	CCGAATAAACCGAGAATGGTGCAA	1	103	1	103	100	Bm_ATCC23344_chr1	3510148	987778	987880	100	515	1.20E-18 +
BPSL1972	putative antibiotic resistance membrane protein	2347021	2348973 +	650	53719584	3092307 -	COG1538MU	CAATGTCCGTAAGAATGCGTTGCAC	1	103	103	1	100	Bm_ATCC23344_chr1	3510148	987718	987820	100	515	1.20E-18 -
BPSL1976	putative chemotaxis methyl-accepting membrane pr	2352657	2354333 -	558	53719588	3092697 -	COG0840NT	GCCTGCCGTAGAACCCGTCGAGAC	1	103	1	103	100	Bm_ATCC23344_chr1	3510148	980510	980612	99	506	3.00E-18 +
BPSL1977	putative fosmidomycin resistance protein	2354685	2355935 +	416	53719589	3093901 fsr	NULL	TGCCGATGAGACACATTACACTTCG	1	103	103	1	100	Bm_ATCC23344_chr1	3510148	980256	980358	100	515	1.20E-18 -
BPSL1979	putative helicase	2357081	2359444 -	787	53719591	3094437 -	COG0210L	ACCTTCGATTATAAATGGATGAGCA	1	103	1	103	100	Bm_ATCC23344_chr1	3510148	975422	975524	100	515	1.20E-18 +
BPSL1980	valyl-tRNA synthetase	2359584	2362451 +	955	53719592	3094083 valS	COG0525J	TTATAAATCGCGCGCGGCCCGCGC	1	103	103	1	100	Bm_ATCC23344_chr1	3510148	975380	975482	100	515	1.20E-18 -
BPSL1981	UTP--glucose-1-phosphate uridylyltransferase	2362538	2363419 +	293	53719593	3092713 galU	COG1210M	CAATCCGTAAGGAATCTTGCGTTCA	1	89	89	1	100	Bm_ATCC23344_chr1	3510148	972426	972514	100	445	1.70E-15 -
BPSL1982	putative lipoprotein	2363480	2364097 -	205	53719594	3094438 -	NULL	CGACACAGGCATTCGGCCGCCCGC	1	91	1	91	100	Bm_ATCC23344_chr1	3510148	970781	970871	100	455	6.00E-16 +
BPSL1985	putative two component system, response regulator	2367951	2368679 -	242	53719597	3094683 -	COG0745TK	CCGGACAAACCTTCATCATTCAGCC	1	103	1	103	100	Bm_ATCC23344_chr1	3510148	966187	966289	100	515	1.20E-18 +
BPSL1986	hypothetical protein BPSL1986	2368983	2369369 -	128	53719598	3094684 -	NULL	TCTCGTCTGCAGAATCGCGCCGCG	1	103	1	103	100	Bm_ATCC23344_chr1	3510148	965497	965599	100	515	1.20E-18 +
BPSL1990	putative transcriptional regulatory protein	2372102	2372983 -	293	53719602	3093645 -	COG1737K	GTGCCTATACTCGCTGCACGACAAC	1	103	1	103	100	Bm_ATCC23344_chr1	3510148	961883	961985	100	515	1.20E-18 +
BPSL1991	putative sugar ABC transport system, substrate-bind	2373228	2374277 +	349	53719603	3093898 -	COG1879G	AAATTTTCTTTGCAATCGGCGGCGA	1	103	103	1	100	Bm_ATCC23344_chr1	3510148	961736	961838	100	515	1.20E-18 -
BPSL1992	putative sugar ABC transport system, membrane pro	2374378	2375523 +	381	53719604	3093899 -	COG1172G	CGTCCATCGCAGCGATTCGCACGA	1	103	103	1	100	Bm_ATCC23344_chr1	3510148	960586	960688	99	506	3.00E-18 -
BPSL2002	putative iron-containing alcohol dehydrogenase	2386847	2387995 -	382	53719614	3091834 -	COG1454C	CCCGGACGGCGGCCGCTTGTCCAG	1	103	1	103	100	Bm_ATCC23344_chr1	3510148	946925	947027	99	506	3.00E-18 +
BPSL2003	hypothetical protein BPSL2003	2388211	2388546 +	111	53719615	3094867 -	NULL	GGGCGCGGCCGCGCGGGGCGTGC	1	103	103	1	100	Bm_ATCC23344_chr1	3510148	946807	946909	99	506	3.00E-18 -
BPSL2004	putative phosphatase	2388646	2389275 -	209	53719616	3094868 -	COG1051F	CAGCGCGGCGCGATCGCTCGACGG	1	103	1	103	100	Bm_ATCC23344_chr1	3510148	945645	945747	99	506	3.00E-18 +
BPSL2005	hypothetical protein BPSL2005	2389489	2390166 +	225	53719617	3091940 -	NULL	TCGGGCCTGCCGCGCCGATGCCCG	1	103	103	1	100	Bm_ATCC23344_chr1	3510148	945529	945631	100	515	1.20E-18 -
BPSL2006	subfamily S1C non-peptidase homologue	2390396	2391952 -	518	53719618	3091941 -	COG0265O	TTCGCCGGGCGCTATGCAATAATC	1	103	1	103	100	Bm_ATCC23344_chr1	3510148	942987	943089	100	515	1.20E-18 +
BPSL2008	putative LysR-family transcriptional regulator	2394125	2395090 +	321	53719620	3093973 -	COG0583K	GGCCGGTCAAGATCATGGTCGAAG	1	103	103	1	100	Bm_ATCC23344_chr1	3510148	940132	940234	100	515	1.20E-18 -
BPSL2009	alanyl-tRNA synthetase	2395633	2398257 -	874	53719621	3094503 alaS	COG0013J	TCTATCCCGTATAATCGATTGTTTCA	1	103	1	103	100	Bm_ATCC23344_chr1	3510148	935941	936043	100	515	1.20E-18 +
BPSL2010	putative lipid metabolism-related protein	2398792	2400012 +	406	53719622	3093635 -	COG1804C	ATGCGGCGGGAGACGATTCACCGA	1	103	103	1	100	Bm_ATCC23344_chr1	3510148	935371	935473	100	515	1.20E-18 -
BPSL2011	putative osmosis-related lipoprotein	2400341	2400547 -	68	53719623	3093636 -	NULL	GATTTCGCTTTTTTAAGATGCGCTCA	1	103	1	103	100	Bm_ATCC23344_chr1	3510148	933523	933625	99	506	3.00E-18 +
BPSL2012	glutaminyl-tRNA synthetase	2400830	2402539 +	569	53719624	3091627 glnS	COG0008J	GCAAGGGGCGCCGCTCCTCATGAC	1	103	103	1	100	Bm_ATCC23344_chr1	3510148	933340	933440	98	485	2.60E-17 -
BPSL2014	hypothetical protein BPSL2014	2403398	2403784 -	128	53719626	3094196 -	NULL	GGTTTCAAATACCGTTCGTTGCAGT	1	103	1	103	100	Bm_ATCC23344_chr1	3510148	930314	930416	100	515	1.20E-18 +
BPSL2016	hypothetical protein BPSL2016	2406423	2407133 +	236	53719628	3091656 -	NULL	CGCGCAGCATAGCACGGTACTTTTC	1	103	103	1	100	Bm_ATCC23344_chr1	3510148	927784	927886	98	497	7.60E-18 -
BPSL2017	Di-haem cytochrome c peroxidase	2407186	2408571 -	461	53719629	3094838 -	COG1858P	GCTTTCGTTCAGCTGTCGATCGGGC	1	103	1	103	100	Bm_ATCC23344_chr1	3510148	925521	925623	100	515	1.20E-18 +
BPSL2018	putative acid phosphatase	2408727	2410400 +	557	53719630	3094839 -	NULL	CCGCATGACAGTTCGATGACGCCC	1	103	103	1	100	Bm_ATCC23344_chr1	3510148	925463	925565	99	506	3.00E-18 -
BPSL2020	HlyD family secretion protein	2412415	2413602 +	395	53719632	3091471 -	COG1566V	GGCAAACGGGAAGCGAGCAGCGA	1	103	103	1	100	Bm_ATCC23344_chr1	3510148	921786	921888	99	506	3.00E-18 -
BPSL2025	putative two component regulatory system, sensor k	2418051	2420132 -	693	53719636	3093611 -	COG0642T	CCGTACCCATAAGCGGAACGCCGC	1	103	1	103	100	Bm_ATCC23344_chr1	3510148	914005	914107	99	506	3.00E-18 +
BPSL2027	putative fimbriae-related protein	2421524	2423872 -	782	53719638	3093013 -	COG3188NU	CACGACACAAGCGACAATGTCGCG	1	103	1	103	100	Bm_ATCC23344_chr1	3510148	910264	910366	100	515	1.20E-18 +
BPSL2029	hypothetical protein BPSL2029	2424837	2425361 -	174	53719640	3093364 -	COG5430S	GCGAGCGCGCCGTCCCGTTTGGCC	1	79	1	79	100	Bm_ATCC23344_chr1	3510148	908799	908877	100	395	3.10E-13 +
BPSL2030	hypothetical protein BPSL2030	2425438	2425998 -	186	53719641	3093365 -	COG5430S	TCCGGGCAGGTATGCAGCAGCAGT	1	55	1	55	100	Bm_ATCC23344_chr1	3510148	908186	908240	100	275	8.00E-08 +
BPSL2031	hypothetical protein BPSL2031	2426051	2426596 -	181	53719642	3092962 -	COG5430S	GGTACGGGCATCTGGCGCGCGTGG	1	103	1	103	100	Bm_ATCC23344_chr1	3510148	907540	907642	100	515	1.20E-18 +
BPSL2032	putative LysR-family transcriptional regulator	2427413	2428306 -	297	53719643	3092963 -	COG0583K	GGGGCGAGCGCGGAATGAAAGCG	1	103	1	103	100	Bm_ATCC23344_chr1	3510148	905830	905932	100	515	1.20E-18 +
BPSL2033	putative transport-related membrane protein	2428723	2430009 +	428	53719644	3092589 -	NULL	CTTTCGTGATGCATATGTCGTTTACC	1	103	103	1	100	Bm_ATCC23344_chr1	3510148	905511	905613	100	515	1.20E-18 -
BPSL2035	putative regulatory protein	2431505	2431786 +	93	53719646	3092674 -	COG2916R	CGGCGGTCGGCTTGCCGTCTTTCAT	1	103	103	1	100	Bm_ATCC23344_chr1	3510148	902720	902822	100	515	1.20E-18 -
BPSL2036	hypothetical protein BPSL2036	2432049	2432318 -	89	53719647	3092675 -	NULL	CGCGGCATACTTCCGCCATGTCTCA	1	103	1	103	100	Bm_ATCC23344_chr1	3510148	901819	901921	99	506	3.00E-18 +
BPSL2050	hypothetical protein BPSL2050	2451504	2454302 -	932	53719663	3093282 -	COG3501S, COG4253S	CGGGATGATCGCGCTGCTCAGTGG	1	103	1	103	100	Bm_ATCC23344_chr1	3510148	893867	893969	100	515	1.20E-18 +
BPSL2051	hypothetical protein BPSL2051	2454823	2455089 +	88	53719664	3093693 -	COG4104S	TGGCGATTAGCCGGTAAAATCGGC	1	103	103	1	100	Bm_ATCC23344_chr1	3510148	893444	893546	99	506	3.00E-18 -
BPSL2053	hypothetical protein BPSL2053	2456556	2458406 -	616	53719666	3092313 -	COG3519S	CGCGTCGGCTCCAGCCGTTCGCGC	1	103	1	103	100	Bm_ATCC23344_chr1	3510148	889763	889865	100	515	1.20E-18 +
BPSL2055	hypothetical protein BPSL2055	2459672	2460508 -	278	53719668	3092165 -	COG0657I	ATAGCGCGGTGCCGAACCGGCGCA	1	103	1	103	100	Bm_ATCC23344_chr1	3510148	887639	887741	100	515	1.20E-18 +
BPSL2056	putative oxidase	2460880	2463447 +	855	53719669	3092166 -	NULL	CGCGTGATTCAGAAAAGTTCAAAC	1	103	103	1	100	Bm_ATCC23344_chr1	3510148	887365	887467	100	515	1.20E-18 -
BPSL2062	OmpA family protein	2467187	2467897 -	236	53719675	3094075 -	COG2885M	CCGAGTTCACCGGCGATTCATCGAC	1	83	1	83	100	Bm_ATCC23344_chr1	3510148	880303	880385	100	415	3.80E-14 +
BPSL2063	hypothetical protein BPSL2063	2467978	2471250 -	1090	53719676	3093202 -	NULL	GAATGTTCAGATTCGGATTGCGCCT	1	73	1	73	100	Bm_ATCC23344_chr1	3510148	876945	877017	98	356	1.80E-11 +
BPSL2065	putative two component system, response regulator	2471650	2472360 -	236	53719678	3093071 -	COG0745TK	AGTCCTTCGGATTCCTGGCTGTTCA	1	103	1	103	100	Bm_ATCC23344_chr1	3510148	875843	875946	99	505	3.30E-18 +
BPSL2068	putative two component system, response regulator	2474112	2474894 -	260	53719681	3093710 -	COG0745TK	GAATATATTAAACAATAAACGGCC	1	103	1	103	100	Bm_ATCC23344_chr1	3510148	873302	873404	100	515	1.20E-18 +
BPSL2069	putative LysR-family transcriptional regulator	2475308	2476240 -	310	53719682	3093695 -	COG0583K	ATGGCCGGTTGGACGTTTGGCGGT	1	103	1	103	100	Bm_ATCC23344_chr1	3510148	871956	872058	100	515	1.20E-18 +
BPSL2070	hypothetical protein BPSL2070	2476552	2477943 -	463	53719683	3093696 -	NULL	TTACAACGACAGGAGACATAGCAT	1	103	1	103	100	Bm_ATCC23344_chr1	3510148	870253	870355	100	515	1.20E-18 +
BPSL2073	poly(3-hydroxybutyrate) depolymerase precursor	2480505	2481983 +	492	53719686	3093222 phaZ	COG3509Q, COG3979R	AATCGAAACGCGACGAACGACGCC	1	103	103	1	100	Bm_ATCC23344_chr1	3510148	867806	867908	99	506	3.00E-18 -
BPSL2074	putative alpha-amylase-related protein	2482438	2485884 +	1148	53719687	3094840 -	NULL	CTGCGCCGTCGATCCTCGCCTCGCC	1	103	103	1	100	Bm_ATCC23344_chr1	3510148	865873	865975	100	515	1.20E-18 -
BPSL2077	putative glycogen operon related protein	2491542	2493650 +	702	53719690	3094846 -	COG1523G	CGCCGCGCGGCGCCCGCCGCACGC	1	55	55	1	100	Bm_ATCC23344_chr1	3510148	856773	856827	100	275	8.00E-08 -
BPSL2081	hypothetical protein BPSL2081	2500774	2501262 -	162	53719694	3094877 -	COG0655R	GCCGAGGCCGATGAACGGACGTCG	1	103	1	103	100	Bm_ATCC23344_chr1	3510148	847087	847189	99	506	3.00E-18 +
BPSL2082	hypothetical protein BPSL2082	2501376	2501753 -	125	53719695	3094878 -	NULL	GGGCGCCGCCGGCGCCGTCGCACC	1	103	1	103	100	Bm_ATCC23344_chr1	3510148	846596	846698	99	506	3.00E-18 +
BPSL2083	putative GntR-family transcriptional regulator	2501976	2503436 +	486	53719696	3091569 -	COG1167KE	GCGCCGCGAAGGCGCGCGCCGCG	1	103	103	1	100	Bm_ATCC23344_chr1	3510148	846471	846573	100	515	1.20E-18 -
BPSL2084	putative bifunctional protein	2503503	2505482 -	659	53719697	3091570 -	COG0789K, COG3315Q	GGCCGATAGACGCATCGCGCAACC	1	103	1	103	100	Bm_ATCC23344_chr1	3510148	842867	842969	98	497	7.60E-18 +
BPSL2086	hypothetical protein BPSL2086	2506560	2507069 +	169	53719698	3093762 -	NULL	GATCGGCAGCCGCGGCGGCGGCC	1	103	103	1	100	Bm_ATCC23344_chr1	3510148	841884	841989	96	476	6.70E-17 -
BPSL2090	putative transferase	2509753	2510595 +	280	53719701	3093960 -	COG1562I	CTAGAATTCGCCGTTGTCGCCGCAA	1	103	1	103	100	Bm_ATCC23344_chr1	3510148	1535115	1535217	100	515	1.20E-18 +
BPSL2091	hypothetical protein BPSL2091	2510636	2511892 -	418	53719702	3093961 -	COG4102S	AGAACATGAACACGATGACGATGA	1	85	85	1	100	Bm_ATCC23344_chr1	3510148	1537334	1537418	97	407	8.80E-14 -
BPSL2092	hypothetical protein BPSL2092	2511975	2513390 -	471	53719703	3093083 -	COG5267S	CCTCGCGCATCGCGAGCCGGCGTG	1	103	103	1	100	Bm_ATCC23344_chr1	3510148	1538832	1538934	99	506	3.00E-18 -
BPSL2093	hypothetical protein BPSL2093	2513634	2513972 -	112	53719704	3093084 -	NULL	ACTCGAAGACGACATTCGGTGGAT	1	103	103	1	100	Bm_ATCC23344_chr1	3510148	1539414	1539516	100	515	1.20E-18 -
BPSL2094	oxidative stress related two component system, tran	2514194	2514919 +	241	53719705	3094372 ompR	COG0745TK	CTTTACTCCGCACAATAGCGCGGGT	1	103	1	103	100	Bm_ATCC23344_chr1	3510148	1539538	1539640	100	515	1.20E-18 +
BPSL2095	putative oxidative stress related two component syst	2514986	2516344 +	452	53719706	3093607 -	COG0642T	TATTCGTTTCGACGCAAAACACGTC	1	69	1	69	100	Bm_ATCC23344_chr1	3510148	1540364	1540432	100	345	5.50E-11 +
BPSL2096	putative hydroperoxide reductase	2516543	2517091 +	182	53719707	3093608 -	COG0450O	TAGCGTTATAGTCTTCCCCATGCTG	1	103	1	103	100	Bm_ATCC23344_chr1	3510148	1541887	1541989	99	506	3.00E-18 +
BPSL2097	hypothetical protein BPSL2097	2517169	2517696 +	175	53719708	3093476 -	NULL	GCGTCCGATGCACGAAAGCCCGCG	1	80	1	80	100	Bm_ATCC23344_chr1	3510148	1542536	1542615	100	400	1.80E-13 +
BPSL2102	putative threonine dehydratase	2525367	2526398 +	343	53719713	3094380 -	COG1171E	GCCCGCGCCGCACTCCGTTCCGGC	1	64	1	64	100	Bm_ATCC23344_chr1	3510148	1550758	1550821	96	302	4.90E-09 +
BPSL2103	hypothetical protein BPSL2103	2526906	2527313 +	135	53719714	3094381 -	NULL	GCGCGCTTGCCCGGCTAATCGAAT	1	103	1	103	100	Bm_ATCC23344_chr1	3510148	1552258	1552360	99	506	3.00E-18 +
BPSL2104	putative penicillin-binding protein	2527868	2530426 -	852	53719715	3093585 -	COG5009M	TGCGGACCGGATAGCCGTATGGAC	1	103	103	1	100	Bm_ATCC23344_chr1	3510148	1555876	1555978	100	515	1.20E-18 -
BPSL2105	hypothetical protein BPSL2105	2530906	2531586 +	226	53719716	3093586 -	COG1842KT	CGGTGGTGTAATATCCGCCGACGC	1	103	1	103	100	Bm_ATCC23344_chr1	3510148	1556257	1556359	100	515	1.20E-18 +

BPSL2107	large-conductance mechanosensitive channel	2533099	2533530 +	143	53719718	3092570 mscL	NULL	ATCGATAAAGGATCGGGTTCGTTAT	1	103	1	103	100 Bm_ATCC23344_chr1	3510148	1560045	1560147	99	506	3.00E-18 +
BPSL2108	hypothetical protein BPSL2108	2533842	2534339 +	165	53719719	3093806 -	NULL	CCGGTTCGGACGACGCACGGCAAA	1	103	1	103	100 Bm_ATCC23344_chr1	3510148	1560789	1560891	100	515	1.20E-18 +
BPSL2116	putative allantoicase	2542680	2543693 -	337	53719727	3094422 -	NULL	GCGCGCCAGACGCGCCGCCTCTGT	1	103	103	1	100 Bm_ATCC23344_chr1	3510148	1565380	1565482	99	506	3.00E-18 -
BPSL2118	hypothetical protein BPSL2118	2544443	2545396 -	317	53719729	3093925 -	NULL	CTACACTGGGCCATCCGTCGCTTCG	1	103	103	1	100 Bm_ATCC23344_chr1	3510148	1567083	1567185	97	488	1.90E-17 -
BPSL2120	putative transport-related membrane protein	2546527	2548059 -	510	53719731	3091738 -	COG1953FH	CGCCGAACGCGCTCACTGAACTGA	1	103	103	1	100 Bm_ATCC23344_chr1	3510148	1569710	1569812	99	506	3.00E-18 -
BPSL2121	putative GntR-family transcriptional regulator	2548172	2548876 -	234	53719732	3093416 -	COG1802K	CACCGAAATGAAGCGCGCCTGCGC	1	103	103	1	100 Bm_ATCC23344_chr1	3510148	1570527	1570629	100	515	1.20E-18 -
BPSL2122	muramoyl-tetrapeptide carboxypeptidase	2549191	2550132 -	313	53719733	3093417 -	COG1619V	ACCGCGCGGCGCGGGACGCCTCTA	1	58	58	1	100 Bm_ATCC23344_chr1	3510148	1573096	1573153	100	290	1.70E-08 -
BPSL2123	putative deaminase	2550188	2550712 -	174	53719734	3091900 -	COG0590FJ	GGCCCGCCGGCGCGTCCGCGTCCG	1	71	71	1	100 Bm_ATCC23344_chr1	3510148	1573676	1573746	100	355	2.00E-11 -
BPSL2124	hypothetical protein BPSL2124	2550781	2551182 -	133	53719735	3091901 -	NULL	TTCTCGCGTCCGGGCCCGGATATCG	1	103	103	1	100 Bm_ATCC23344_chr1	3510148	1574146	1574248	100	515	1.20E-18 -
BPSL2125	hypothetical protein BPSL2125	2551984	2552490 -	168	53719736	3091514 -	COG3812S	GCGCCATCGAGCGTGGCTATCCGA	1	103	103	1	100 Bm_ATCC23344_chr1	3510148	1575452	1575554	100	515	1.20E-18 -
BPSL2126	putative transport-related, membrane protein	2552858	2554078 +	406	53719737	3091515 -	NULL	CTTCGCATGGTTTCAATTTGCAACC	1	103	1	103	100 Bm_ATCC23344_chr1	3510148	1575722	1575824	100	515	1.20E-18 +
BPSL2127	bifunctional GMP synthase/glutamine amidotransfer	2555410	2557053 -	547	53719738	3093632 guaA	COG0518F, COG0519F	GCGCGGTTTTCCGGCCCGTCAGGC	1	103	103	1	100 Bm_ATCC23344_chr1	3510148	1580016	1580118	100	515	1.20E-18 -
BPSL2129	inositol-5-monophosphate dehydrogenase	2557817	2559277 -	486	53719740	3092041 guaB	COG0516F, COG0517R	ATCCGAACGATTCGGGGCCCAAAC	1	103	103	1	100 Bm_ATCC23344_chr1	3510148	1582240	1582342	100	515	1.20E-18 -
BPSL2130	hypothetical protein BPSL2130	2559694	2560458 -	254	53719741	3091513 -	COG4312S	AGCCCGCGACTAATCTACCACCAC	1	103	103	1	100 Bm_ATCC23344_chr1	3510148	1583422	1583524	100	515	1.20E-18 -
BPSL2134	hypothetical protein BPSL2134	2562571	2563539 +	322	53719743	3093758 -	NULL	CATCGACCAAGCGCAGGGCCGAAT	1	103	1	103	100 Bm_ATCC23344_chr1	3510148	1585730	1585832	100	515	1.20E-18 +
BPSL2136	hypothetical protein BPSL2136	2564020	2564457 -	145	53719745	3094452 -	COG2867I	GAAGGGAAATGGCCAACTCGTTTA	1	100	100	1	100 Bm_ATCC23344_chr1	3510148	1587707	1587806	100	500	5.50E-18 -
BPSL2137	SsrA-binding protein	2564555	2565001 +	148	53719746	3092303 smpB	COG0691O	AAATCGCGCGGCATCGCAAAGGTA	1	100	1	100	100 Bm_ATCC23344_chr1	3510148	1587710	1587809	100	500	5.50E-18 +
BPSL2138	hypothetical protein BPSL2138	2565179	2566126 -	315	53719747	3094453 -	COG0330O	GCGCGCGCATTTTCATACGCACTTT	1	82	82	1	100 Bm_ATCC23344_chr1	3510148	1589367	1589448	100	410	6.40E-14 -
BPSL2139	hypothetical protein BPSL2139	2566206	2566640 -	144	53719748	3094444 -	NULL	AATTGCCCGATTGCCGCCCGGATG	1	103	103	1	100 Bm_ATCC23344_chr1	3510148	1589881	1589983	98	497	7.60E-18 -
BPSL2140	phosphoenolpyruvate synthase	2566768	2569167 -	799	53719749	3092220 ppsA	COG0574G	TTATCCGACAGGTTGTGCAATGCGC	1	103	103	1	100 Bm_ATCC23344_chr1	3510148	1592408	1592510	99	506	3.00E-18 -
BPSL2142	family S53 unassigned peptidase	2571036	2572925 -	629	53719751	3092352 -	COG4934O	GAGAGCGGCGTCGTTCGCCCTCGG	1	103	103	1	100 Bm_ATCC23344_chr1	3510148	1596118	1596220	100	515	1.20E-18 -
BPSL2143	hypothetical protein BPSL2143	2573475	2574290 +	271	53719752	3092353 -	NULL	ACCGCTTCGCCCATCGCGCCGGGT	1	103	1	103	100 Bm_ATCC23344_chr1	3510148	1596555	1596657	100	515	1.20E-18 +
BPSL2147	UDP-N-acetylglucosamine acyltransferase	2577091	2577879 -	262	53719756	3093538 lpxA	COG1043M	GCGCGCGGCACGCAAGGCTACGC	1	58	58	1	100 Bm_ATCC23344_chr1	3510148	1601057	1601114	100	290	1.70E-08 -
BPSL2148	(3R)-hydroxymyristoyl ACP dehydratase	2577935	2578444 -	169	53719757	3094303 fabZ	COG0764I	CACGTCGGCCGCAACCGCGCGCCG	1	103	103	1	100 Bm_ATCC23344_chr1	3510148	1601622	1601724	99	506	3.00E-18 -
BPSL2151	putative outer membrane protein	2580221	2582530 -	769	53719760	3092935 -	COG4775M	CGCGTCGGGCGGCAGCCGTAACGG	1	67	67	1	100 Bm_ATCC23344_chr1	3510148	1605708	1605774	100	335	1.60E-10 -
BPSL2156	ribosome releasing factor	2586860	2587420 -	186	53719765	3093900 frr	NULL	ACTCTCGCCGATGCGGGAGGCCGC	1	93	93	1	100 Bm_ATCC23344_chr1	3510148	1610598	1610690	100	465	2.10E-16 -
BPSL2157	uridylate kinase	2587511	2588224 -	237	53719766	3093161 pyrH	COG0528F	GACGGGGCTCTCAGCCAGCCCGGC	1	103	103	1	100 Bm_ATCC23344_chr1	3510148	1611402	1611504	98	497	7.60E-18 -
BPSL2158	elongation factor Ts	2588403	2589284 -	293	53719767	3092745 tsf	NULL	CCGGCGCCGCGCAAAAAATGCGCG	1	103	103	1	100 Bm_ATCC23344_chr1	3510148	1612462	1612564	100	515	1.20E-18 -
BPSL2159	30S ribosomal protein S2	2589467	2590207 -	246	53719768	3093074 rpsB	NULL	ACCCAGGGTGTCGCGGCGCGGACG	1	103	103	1	100 Bm_ATCC23344_chr1	3510148	1613385	1613486	99	500	5.50E-18 -
BPSL2160	methionine aminopeptidase	2590690	2591505 +	271	53719769	3094064 map	COG0024J	GAGCCGGCCGCGATTCGCGCGTCG	1	103	1	103	100 Bm_ATCC23344_chr1	3510148	1613769	1613871	100	515	1.20E-18 +
BPSL2161	PII uridylyl-transferase	2591671	2594247 +	858	53719770	3093139 -	COG2844O	GCTCGCCCCGCCTGAGCGGCGCGA	1	103	1	103	100 Bm_ATCC23344_chr1	3510148	1614750	1614852	100	515	1.20E-18 +
BPSL2164	DNA ligase	2596711	2598786 -	691	53719773	3092689 ligA	COG0272L	CCGCGATTTCCACGCTCGAGCGGC	1	103	103	1	100 Bm_ATCC23344_chr1	3510148	1621930	1622032	99	506	3.00E-18 -
BPSL2165	hypothetical protein BPSL2165	2599226	2600512 -	428	53719774	3093959 -	NULL	CGTATGACGAATGGCACGCGGGCG	1	69	69	1	100 Bm_ATCC23344_chr1	3510148	1623639	1623707	100	345	5.50E-11 -
BPSL2166	putative chromosome partition protein	2600579	2604091 -	1170	53719775	3091528 -	COG1196D	CGAATCTTGGGCGTGCGGCGCGCG	1	103	103	1	100 Bm_ATCC23344_chr1	3510148	1627218	1627322	98	495	9.30E-18 -
BPSL2167	hypothetical protein BPSL2167	2604240	2605187 -	315	53719776	3091529 -	COG0697GER	GGGCGCGGCGACGGCGCTCGATTA	1	103	103	1	100 Bm_ATCC23344_chr1	3510148	1628316	1628418	100	515	1.20E-18 -
BPSL2168	hypothetical protein BPSL2168	2605452	2606684 +	410	53719777	3093039 -	COG0436E	GCCCGCGCAAGGCGGCGCCAGCC	1	103	1	103	100 Bm_ATCC23344_chr1	3510148	1628483	1628585	100	515	1.20E-18 +
BPSL2169	2,3,4,5-tetrahydropyridine-2,6-dicarboxylate N-succir	2606741	2607568 +	275	53719778	3091791 dapD	COG2171E	ACGCGCGCCGAAACGCCCCGATTC	1	59	1	59	100 Bm_ATCC23344_chr1	3510148	1629816	1629874	100	295	1.00E-08 +
BPSL2171	succinyl-diaminopimelate desuccinylase	2608004	2609155 +	383	53719780	3091792 dapE	COG0624E	GCGCCGCGCGCCGCCGCCCCCAAC	1	64	1	64	100 Bm_ATCC23344_chr1	3510148	1631068	1631131	100	320	7.50E-10 +
BPSL2175	glutathione peroxidase	2611872	2612351 -	159	53719784	3094481 gpo	COG0386O	TTCGGGGGATCGTCCGGATGGCGG	1	103	103	1	100 Bm_ATCC23344_chr1	3510148	1635429	1635531	100	515	1.20E-18 -
BPSL2176	putative ABC transport system, ATP-binding protein	2612497	2614437 +	646	53719785	3091506 -	COG0488R	GTCTAGGCGAATTCGGCGCGGCGC	1	103	1	103	100 Bm_ATCC23344_chr1	3510148	1635477	1635579	100	515	1.20E-18 +
BPSL2177	hypothetical protein BPSL2177	2614525	2614857 -	110	53719786	3091617 -	NULL	GCGCGGCGCGCGGCGGTTGCGCCC	1	100	100	1	100 Bm_ATCC23344_chr1	3510148	1637935	1638034	100	500	5.50E-18 -
BPSL2179	alanine racemase	2616342	2617412 -	356	53719788	3092804 alr	COG0787M	CGGACTAGCAAGATCCCCCGATCG	1	103	103	1	100 Bm_ATCC23344_chr1	3510148	1640490	1640592	99	506	3.00E-18 -
BPSL2180	hypothetical protein BPSL2180	2617624	2618931 +	435	53719789	3091618 -	NULL	TTTCGTGATATAAAGCCGTGCGCAC	1	103	1	103	100 Bm_ATCC23344_chr1	3510148	1640604	1640706	100	515	1.20E-18 +
BPSL2181	phosphomethylpyrimidine kinase	2619042	2619851 +	269	53719790	3091851 thiD	COG0351H	GCGCCGCCCATGCCGCGCCGCGTG	1	103	1	103	100 Bm_ATCC23344_chr1	3510148	1642022	1642124	100	515	1.20E-18 +
BPSL2186	putative acyl-CoA-binding protein	2624009	2624278 +	89	53719795	3093962 -	COG4281I	GCGAATCGGTTTGGAAGGAACGCT	1	103	1	103	100 Bm_ATCC23344_chr1	3510148	1646953	1647055	100	515	1.20E-18 +
BPSL2187	putative ATP-dependent RNA helicase	2624724	2626274 +	516	53719796	3093963 -	COG0513LKJ	CGAGGCCGGCGACGCCTGCGACGC	1	103	1	103	100 Bm_ATCC23344_chr1	3510148	1647857	1647959	100	515	1.20E-18 +
BPSL2188	isocitrate lyase	2626666	2627973 +	435	53719797	3092651 aceA	COG2224C	GACTTCACGAACGAAACCGCCGAA	1	103	1	103	100 Bm_ATCC23344_chr1	3510148	1651202	1651304	100	515	1.20E-18 +
BPSL2189	universal stress protein family protein	2628233	2628667 -	144	53719798	3092701 -	COG0589T	CAATCGAATCACTCGGCGTCCGCG	1	103	103	1	100 Bm_ATCC23344_chr1	3510148	1653301	1653403	100	515	1.20E-18 -
BPSL2190	putative LysR-family regulatory protein	2628947	2629849 -	300	53719799	3092702 -	COG0583K	AAAAGACAGTAATCATCACTTGATC	1	103	103	1	100 Bm_ATCC23344_chr1	3510148	1654483	1654585	100	515	1.20E-18 -
BPSL2191	putative dehalogenase	2630009	2630791 +	260	53719800	3093354 -	COG1011R	ATATCGAAAAGTAAAAGATCAAGT	1	103	1	103	100 Bm_ATCC23344_chr1	3510148	1654545	1654647	100	515	1.20E-18 +
BPSL2192	malate synthase	2630844	2632436 +	530	53719801	3092656 aceB	COG2225C	TTCATGCAATTGCACCACTCCGTAA	1	55	1	55	100 Bm_ATCC23344_chr1	3510148	1655428	1655482	100	275	8.00E-08 +
BPSL2194	ribonuclease activity regulator protein RraA	2633488	2633985 -	165	53719803	3093172 menG	NULL	TCCGGCTATTGTAGGGCGAGCCTTC	1	103	103	1	100 Bm_ATCC23344_chr1	3510148	1658898	1659000	100	515	1.20E-18 -
BPSL2195	putative AraC-family transcriptional regulator	2634115	2634978 +	287	53719804	3093293 -	NULL	TCGATCCGGCATTATCGGCGAAGA	1	103	1	103	100 Bm_ATCC23344_chr1	3510148	1658930	1659032	98	497	7.60E-18 +
BPSL2196	putative acetyltransferase	2635582	2636049 -	155	53719805	3093294 -	NULL	CGAACGGCGCCGCATGCCGCTGCC	1	103	103	1	100 Bm_ATCC23344_chr1	3510148	1660976	1661078	99	506	3.00E-18 -
BPSL2197	glutamyl-tRNA synthetase	2637135	2638544 -	469	53719806	3093847 gltX	COG0008J	CTTTCCGTTCCGGTCGCGTGCGCAC	1	103	103	1	100 Bm_ATCC23344_chr1	3510148	1663177	1663279	100	515	1.20E-18 -
BPSL2198	putative exported phospholipase	2639117	2640052 +	311	53719807	3093563 -	COG1752R	GCCGCGCGCGGCCGCCCGACGTCG	1	103	1	103	100 Bm_ATCC23344_chr1	3510148	1663646	1663748	100	515	1.20E-18 +
BPSL2199	family C40 unassigned peptidase	2640218	2640919 -	233	53719808	3093564 -	COG0791M	GAAAATATCAATTCCTTTTGACAAA	1	103	103	1	100 Bm_ATCC23344_chr1	3510148	1665549	1665651	100	515	1.20E-18 -
BPSL2202	putative ABC transport system, membrane protein	2643800	2644840 -	346	53719811	3092316 -	COG4174R	GCAGGCGCGGCGAAAGACGCGGC	1	103	103	1	100 Bm_ATCC23344_chr1	3510148	1669470	1669572	100	515	1.20E-18 -
BPSL2203	putative ABC transport system, substrate-binding pr	2645029	2646981 -	650	53719812	3092317 -	COG4166E	CCCGCGCCCGCACGGTCGGCGGG	1	103	103	1	100 Bm_ATCC23344_chr1	3510148	1671611	1671713	100	515	1.20E-18 -
BPSL2204	enoyl-(acyl carrier protein) reductase	2647102	2647893 +	263	53719813	3091461 fabI	COG0623I	GAGGATGGCGATGTGAGAGAATTC	1	103	1	103	100 Bm_ATCC23344_chr1	3510148	1671634	1671736	100	515	1.20E-18 +
BPSL2205	hypothetical protein BPSL2205	2648146	2648499 -	117	53719814	3094715 -	NULL	CGGCTGCGATCCCGCGCATCAGTT	1	103	103	1	100 Bm_ATCC23344_chr1	3510148	1673128	1673230	100	515	1.20E-18 -
BPSL2206	hypothetical protein BPSL2206	2648753	2648974 +	73	53719815	3094716 -	NULL	GCGTGCCCGCGCGCGGCGCGACG	1	103	1	103	100 Bm_ATCC23344_chr1	3510148	1673284	1673386	100	515	1.20E-18 +
BPSL2207	putative molybdopterin oxidoreductase	2649224	2651545 -	773	53719816	3092493 -	COG0243C	TGTTCGATTGACTCCGATCAATACA	1	103	103	1	100 Bm_ATCC23344_chr1	3510148	1676142	1676244	100	515	1.20E-18 -
BPSL2209	molybdenum cofactor biosynthesis protein A	2652831	2653820 -	329	53719818	3092494 -	COG2896H	GGCGCGTGATGCCGCCTCGTTCGG	1	86	86	1	100 Bm_ATCC23344_chr1	3510148	1678417	1678502	98	421	2.00E-14 -
BPSL2210	ferredoxin I	2653904	2654242 -	112	53719819	3092480 fdxA	COG1146C	CGGAACGATGCGCGGGCGCGGTG	1	103	103	1	100 Bm_ATCC23344_chr1	3510148	1678839	1678941	100	515	1.20E-18 -
BPSL2211	putative TetR-family regulatory protein	2654721	2655287 +	188	53719820	3094386 -	NULL	CATCGGCGAAGGCCCATTGAATAA	1	103	1	103	100 Bm_ATCC23344_chr1	3510148	1679222	1679324	100	515	1.20E-18 +
BPSL2213	hypothetical protein BPSL2213	2656179	2656403 -	74	53719822	3094387 -	NULL	AGGCTGACCGCAAAAGGCTAGGGC	1	103	103	1	100 Bm_ATCC23344_chr1	3510148	1681002	1681104	100	515	1.20E-18 -
BPSL2214	argininosuccinate lyase	2656629	2659325 -	898	53719823	3091920 -	COG0165E, COG0439I	CGCGCGGCGCGCGGCCGGCATTCG	1	82	82	2	98.7342 Bm_ATCC23344_chr1	3510148	1683976	1684056	98	396	2.80E-13 -
BPSL2217	sulfate adenylyltransferase subunit 2	2661696	2662661 -	321	53719826	3092792 -	COG0175EH	TCGCCGCGGGCCCCATCGGCGGGC	1	103	103	1	100 Bm_ATCC23344_chr1	3510148	1687312	1687414	100	515	1.20E-18 -
BPSL2218	hypothetical protein BPSL2218	2662817	2663635 -	272	53719827	3094433 -	COG0463M	CGCGCGGCCGGCACCCGGCGCGC	1	85	85	1	100 Bm_ATCC23344_chr1	3510148	1688286	1688370	98	416	3.40E-14 -
BPSL2220	hypothetical protein BPSL2220	2664853	2665980 -	375	53719829	3092251 -	COG1835I	GCGGCCGCCCGCCGGCCGCCGCG	1	54	54	1	100 Bm_ATCC23344_chr1	3510148	1690631	1690684	100	270	1.40E-07 -
BPSL2226	hypothetical protein BPSL2226	2670822	2671670 -	282	53719835	3094828 -	COG1092R	GGCGCGGGCGGCAGACACGCTCG	1	72	72	1	100 Bm_ATCC23344_chr1	3510148	1696285	1696356	100	360	1.20E-11 -
BPSL2230	hypothetical protein BPSL2230	2682440	2683840 -	466	53719839	3093108 -	COG4529S	CGGCGGCGACGCGGCGGCGCGCG	1	103	103	1	100 Bm_ATCC23344_chr1	3510148	1708451	1708553	98	497	7.60E-18 -

BPSL2231	hypothetical protein BPSL2231	2684134	2685435	-	433	53719840	3093109	-	NULL	TCGGCCTCGCGGCCGCGGACATGG	1	70	70	1	100	Bm_ATCC23344_chr1	3510148	1709969	1710038	100	350	3.30E-11	-
BPSL2234	hypothetical protein BPSL2234	2690294	2691574	+	426	53719843	3093295	-	COG0845M	CGCGCGCCCGCTGAGCCCCGGCGC	1	103	1	103	100	Bm_ATCC23344_chr1	3510148	1714721	1714823	100	515	1.20E-18	+
BPSL2236	hypothetical protein BPSL2236	2694795	2696015	+	406	53719845	3092819	-	NULL	CGGGCATGCGCGCCGCATCACGCG	1	80	1	80	100	Bm_ATCC23344_chr1	3510148	1719242	1719321	100	400	1.80E-13	+
BPSL2237	putative exported lipase	2696054	2697886	-	610	53719846	3092820	-	COG3240IR, COG4625S	GCCGCCGGCTTTCAACCGTGATTCC	1	103	103	1	100	Bm_ATCC23344_chr1	3510148	1722408	1722509	98	491	1.40E-17	-
BPSL2239	aspartate kinase	2700393	2701643	-	416	53719848	3094028	ask	COG0527E	GTTCTTCCCGTCGTCCGTCCACAGC	1	103	103	1	100	Bm_ATCC23344_chr1	3510148	1726175	1726277	100	515	1.20E-18	-
BPSL2241	acetyl-CoA carboxylase alpha subunit	2703497	2704468	-	323	53719850	3092968	accA	COG0825I	GAGCTATTGCTTTTCGCTAATCGAT	1	103	103	1	100	Bm_ATCC23344_chr1	3510148	1729000	1729102	100	515	1.20E-18	-
BPSL2243	cysteinyl-tRNA synthetase	2705558	2706955	-	465	53719852	3093095	cysS	COG0215J	GAGGAAGCGCACGGCACGCGGCC	1	103	103	1	100	Bm_ATCC23344_chr1	3510148	1731487	1731589	100	515	1.20E-18	-
BPSL2244	hypothetical protein BPSL2244	2707244	2707909	+	221	53719853	3093298	-	NULL	TTTTGTTAGAATGGCTCGGAGTATA	1	103	1	103	100	Bm_ATCC23344_chr1	3510148	1731678	1731780	100	515	1.20E-18	+
BPSL2245	peptidyl-prolyl cis-trans isomerase A precursor	2707966	2708541	+	191	53719854	3094185	ppiA	COG0652O	CGCAATGACGCGCGGCCGCCGCCG	1	59	1	59	100	Bm_ATCC23344_chr1	3510148	1732444	1732502	100	295	1.00E-08	+
BPSL2246	peptidyl-prolyl cis-trans isomerase B	2708622	2709113	+	163	53719855	3094186	ppiB	COG0652O	GCCGGCCCGAGCCTCTCGCGCGGC	1	83	1	83	100	Bm_ATCC23344_chr1	3510148	1733076	1733158	100	415	3.80E-14	+
BPSL2247	UDP-2,3-diacylglucosamine hydrolase	2709177	2709938	+	253	53719856	3094136	-	COG2908S	AGCCGGCGCGACGGGGGCAGCGG	1	66	1	66	100	Bm_ATCC23344_chr1	3510148	1733648	1733713	100	330	2.60E-10	+
BPSL2248	putative serine acetyltransferase	2710271	2711056	-	261	53719857	3094137	-	COG1045E	CGTCCGCATGTCATAAGCAAAGCT	1	103	103	1	100	Bm_ATCC23344_chr1	3510148	1735591	1735693	100	515	1.20E-18	-
BPSL2249	putative tRNA/rRNA methyltransferase protein	2711247	2712158	-	303	53719858	3093206	-	COG0565J	AAACGGTTGCGCGGGCCGTTCGAT	1	103	103	1	100	Bm_ATCC23344_chr1	3510148	1736693	1736795	99	506	3.00E-18	-
BPSL2250	inositol-1-monophosphatase	2712459	2713262	+	267	53719859	3092894	suhB	COG0483G	CGAAACGCCCGCCTGGCGCCCCTC	1	103	1	103	100	Bm_ATCC23344_chr1	3510148	1736896	1736998	100	515	1.20E-18	+
BPSL2251	hypothetical protein BPSL2251	2713542	2713814	-	90	53719860	3093207	-	NULL	CACTTCAGAATGTCCGAATATCGTT	1	103	103	1	100	Bm_ATCC23344_chr1	3510148	1738349	1738451	100	515	1.20E-18	-
BPSL2252	DNA mismatch repair protein	2715154	2717826	+	890	53719861	3094284	mutS	COG0249L	GCGACCGAATCGACACATTGGCCG	1	103	1	103	100	Bm_ATCC23344_chr1	3510148	1739593	1739695	100	515	1.20E-18	+
BPSL2254	putative FkbP-type peptidyl-prolyl cis-trans isomeras	2719091	2719636	-	181	53719863	3091962	-	COG1047O	CGGCGCGCGAGTGCGTTCGCGCAC	1	95	95	1	100	Bm_ATCC23344_chr1	3510148	1744175	1744269	100	475	7.40E-17	-
BPSL2255	hypothetical protein BPSL2255	2719729	2720892	+	387	53719864	3092468	-	COG2850S	CCAATTCTCCCAAGTACAACAGCCA	1	95	1	95	100	Bm_ATCC23344_chr1	3510148	1744178	1744272	100	475	7.40E-17	+
BPSL2257	putative lipoprotein	2722400	2723542	-	380	53719867	3092580	-	COG3317M	GGCCGCCCGGGCGGCCGCACCGC	1	103	103	1	100	Bm_ATCC23344_chr1	3510148	1748114	1748216	99	506	3.00E-18	-
BPSL2261	hypothetical protein BPSL2261	2726425	2727090	-	221	53719871	3093628	-	COG1994R	GCGCCGCCGCGCCGTCCCGGCGCA	1	101	101	1	100	Bm_ATCC23344_chr1	3510148	1751662	1751762	100	505	3.30E-18	-
BPSL2262	hypothetical protein BPSL2262	2727189	2727818	-	209	53719872	3093629	-	COG0009J	CGGCGTTTGCCGGCGGCACCTCCG	1	67	67	1	100	Bm_ATCC23344_chr1	3510148	1752390	1752456	100	335	1.60E-10	-
BPSL2263	hypothetical protein BPSL2263	2727883	2728722	-	279	53719873	3094473	-	COG0613R	GGAAGGGCGAACGCGCGGGCGCG	1	103	103	1	100	Bm_ATCC23344_chr1	3510148	1753294	1753396	100	515	1.20E-18	-
BPSL2264	putative hydrolase	2728968	2729858	-	296	53719874	3094474	-	COG0596R	CTAGAGAAGTGCCTCGACACGCGT	1	103	103	1	100	Bm_ATCC23344_chr1	3510148	1754409	1754512	99	505	3.30E-18	-
BPSL2265	hypothetical protein BPSL2265	2730013	2730864	-	283	53719875	3092946	-	NULL	CGGTGGCGTCGCGCTTGGATGCGG	1	103	103	1	100	Bm_ATCC23344_chr1	3510148	1755416	1755518	100	515	1.20E-18	-
BPSL2266	hypothetical protein BPSL2266	2731041	2731565	+	174	53719876	3092947	-	COG0663R	CCGCATCCAAGCGCGACGCCACCG	1	103	1	103	100	Bm_ATCC23344_chr1	3510148	1755495	1755597	100	515	1.20E-18	+
BPSL2268	putative lipoprotein	2732638	2732985	+	115	53719878	3094228	-	NULL	TTCGCCCGCCCGCCTGCGTGCGGG	1	86	1	86	100	Bm_ATCC23344_chr1	3510148	1757109	1757194	100	430	8.00E-15	+
BPSL2269	cell divison protein FtsB	2733211	2733642	-	143	53719879	3091612	ftsB	COG2919D	CACGCGCTGACGCGTTACTATCGG	1	94	94	1	100	Bm_ATCC23344_chr1	3510148	1758182	1758275	100	470	1.30E-16	-
BPSL2270	phosphopyruvate hydratase	2733734	2735017	-	427	53719880	3091594	eno	COG0148G	ACTCGGCGGCCGTAGCGCGCGTCA	1	103	103	1	100	Bm_ATCC23344_chr1	3510148	1759557	1759659	100	515	1.20E-18	-
BPSL2272	CTP synthetase	2735972	2737633	-	553	53719882	3091437	pyrG	COG0504F	CGAGCGCGGACGGCGAAGGGAAG	1	103	103	1	100	Bm_ATCC23344_chr1	3510148	1762173	1762275	99	506	3.00E-18	-
BPSL2277	putative lipoprotein releasing system transmembran	2742870	2744123	-	417	53719887	3093214	-	COG4591M	TTAAGCATTGTGTGGCACACTGCGC	1	103	103	1	100	Bm_ATCC23344_chr1	3510148	1768663	1768765	100	515	1.20E-18	-
BPSL2278	hypothetical protein BPSL2278	2744691	2745767	+	358	53719888	3092074	-	COG4255S	TACCCACGCACGCCGCACGCTCCA	1	103	1	103	100	Bm_ATCC23344_chr1	3510148	1769137	1769239	97	488	1.90E-17	+
BPSL2280	peptide chain release factor 2	2747838	2748942	+	367	53719890	3093204	supK	COG1186J	CCCCCCGCCACGCGCGGCCGCCGG	1	103	1	103	100	Bm_ATCC23344_chr1	3510148	1772286	1772388	100	515	1.20E-18	+
BPSL2281	lysyl-tRNA synthetase	2749035	2750561	+	508	53719891	3092883	lysS	COG1190J	GCGCCCCTTCGCGCGGGCGCGGCG	1	95	1	95	100	Bm_ATCC23344_chr1	3510148	1773491	1773587	97	455	6.00E-16	+
BPSL2282	hypothetical protein BPSL2282	2750912	2751637	+	241	53719892	3092259	-	NULL	CGCAGCGAATCGTTACAACTTGAA	1	103	1	103	100	Bm_ATCC23344_chr1	3510148	1775371	1775473	100	515	1.20E-18	+
BPSL2284	ferredoxin, 2Fe-2S	2752301	2752642	-	113	53719894	3094860	fdx	COG0633C	ATACGCACCGCGCCGCGCGGCCGA	1	95	95	1	100	Bm_ATCC23344_chr1	3510148	1777177	1777271	100	475	7.40E-17	-
BPSL2285	chaperone protein HscA	2752735	2754603	-	622	53719895	3092392	hscA	COG0443O	CCGCGCGCTTTACGCACGCGATGC	1	60	60	1	100	Bm_ATCC23344_chr1	3510148	1779138	1779197	100	300	6.00E-09	-
BPSL2286	co-chaperone HscB	2754661	2755188	-	175	53719896	3094409	hscB	COG1076O	CGGGGCGGGCAAGAGGCGGCGCG	1	103	103	1	100	Bm_ATCC23344_chr1	3510148	1779731	1779833	100	515	1.20E-18	-
BPSL2287	HesB family protein	2755301	2755624	-	107	53719897	3094410	-	COG0316S	GCGGCGTCTCGGGTTCGACGCACG	1	85	85	1	100	Bm_ATCC23344_chr1	3510148	1780167	1780251	98	416	3.40E-14	-
BPSL2288	hypothetical protein BPSL2288	2755707	2756114	-	135	53719898	3092118	-	COG0822C	GCGCGGCGACTTTTTCGCGGGCGC	1	68	68	1	100	Bm_ATCC23344_chr1	3510148	1780657	1780724	100	340	9.30E-11	-
BPSL2289	cysteine desulfurase	2756180	2757403	-	407	53719899	3094249	iscS	COG1104E	TGGCCGCGCACTGAACGCGGCCCG	1	71	71	1	100	Bm_ATCC23344_chr1	3510148	1781946	1782016	100	355	2.00E-11	-
BPSL2290	hypothetical protein BPSL2290	2757472	2758011	-	179	53719900	3092119	-	COG1959K	GCGCCGCATACAACCCCTTTTAGCG	1	103	103	1	100	Bm_ATCC23344_chr1	3510148	1782554	1782656	99	506	3.00E-18	-
BPSL2291	low molecular weight phosphotyrosine protein phos	2758113	2758592	-	159	53719901	3091669	-	COG0394T	CGCGGCACCCGGCGCAAGCGGGA	1	94	94	1	100	Bm_ATCC23344_chr1	3510148	1783135	1783228	100	470	1.30E-16	-
BPSL2292	hypothetical protein BPSL2292	2758684	2758944	-	86	53719902	3091670	-	NULL	CTCGGCCGCGACTGTTCATCGCGCC	1	103	103	1	100	Bm_ATCC23344_chr1	3510148	1783487	1783589	100	515	1.20E-18	-
BPSL2293	putative iron-sulfur binding protein	2759055	2760506	-	483	53719903	3093480	-	COG1139C	CGGCCCGCCGCTACGAGACACGGA	1	103	103	2	99	Bm_ATCC23344_chr1	3510148	1785049	1785157	93	440	2.80E-15	-
BPSL2294	hypothetical protein BPSL2294	2760648	2761370	-	240	53719904	3093481	-	COG0247C	GTCCTCGCTCGATGCCGCGAGCGG	1	103	103	1	100	Bm_ATCC23344_chr1	3510148	1785920	1786022	100	515	1.20E-18	-
BPSL2295	IclR regulatory protein	2761553	2762365	+	270	53719905	3092232	-	COG1414K	GCGGCATCGAGCGAGGACTTCCTC	1	103	1	103	100	Bm_ATCC23344_chr1	3510148	1786005	1786107	100	515	1.20E-18	+
BPSL2297	family S11 unassigned peptidase	2764077	2765156	-	359	53719907	3091882	-	COG1686M	TATTTTTTCAGCTTTTACTACACTTG	1	103	1	103	100	Bm_ATCC23344_chr1	3510148	1789799	1789901	100	515	1.20E-18	+
BPSL2298	phasin-like protein	2765868	2766437	+	189	53719908	3093221	phaP	NULL	GGAACCATTGTTGCGTTGCACAAA	1	103	103	1	100	Bm_ATCC23344_chr1	3510148	1789185	1789287	100	515	1.20E-18	-
BPSL2299	putative dihydrolipoamide dehydrogenase	2766719	2768488	-	589	53719909	3091883	-	COG1249C	GTACTGCCCAGGTTCGCGTTCTTTG	1	103	103	1	100	Bm_ATCC23344_chr1	3510148	1794585	1794687	100	515	1.20E-18	-
BPSL2301	pyruvate dehydrogenase E1 component	2770569	2773265	-	898	53719911	3093625	pdhA	COG2609C	CCCGCAACGCTCTTTACAATCCGCG	1	103	103	2	99	Bm_ATCC23344_chr1	3510148	1799311	1799412	99	501	5.00E-18	-
BPSL2302	sensor kinase protein	2773508	2776048	+	846	53719912	3093892	-	COG0642T, COG2202T	AATGTGGAATATTGCTGCGCCGCA	1	103	1	103	100	Bm_ATCC23344_chr1	3510148	1799456	1799558	99	506	3.00E-18	+
BPSL2304	FolD bifunctional protein	2777000	2777857	+	285	53719914	3094333	folD	COG0190H	TCGGCTGACGACAGGCGGGCGGTA	1	103	1	103	100	Bm_ATCC23344_chr1	3510148	1802948	1803050	100	515	1.20E-18	+
BPSL2305	oligopeptidase A	2778566	2780665	+	699	53719915	3093501	-	COG0339E	AGCGCACGATGCGCCCCAATAATG	1	103	1	103	100	Bm_ATCC23344_chr1	3510148	1804483	1804585	100	515	1.20E-18	+
BPSL2306	DNA polymerase IV	2780897	2782117	-	406	53719916	3093502	-	COG0389L	GCTTTCGCCGCAATCGCCTCAATCG	1	103	103	1	100	Bm_ATCC23344_chr1	3510148	1808112	1808214	96	479	4.90E-17	-
BPSL2307	putative nitrite extrusion protein	2782966	2784246	+	426	53719917	3092694	-	COG2223P	CGACCCAGCGACACGCGCGCGCCC	1	103	1	103	100	Bm_ATCC23344_chr1	3510148	1808737	1808839	99	506	3.00E-18	+
BPSL2309	respiratory nitrate reductase alpha chain	2785926	2789651	+	1241	53719919	3094439	narG	COG5013C	ATGGGCGTGCGAGCGCGCGCGCCG	1	103	1	103	100	Bm_ATCC23344_chr1	3510148	1811697	1811799	99	506	3.00E-18	+
BPSL2315	endonuclease/exonuclease/phosphatase family prote	2795573	2796349	+	258	53719925	3093999	-	COG0708L	CGCCGGCGGCCCGCCGCGGCGGC	1	103	1	103	100	Bm_ATCC23344_chr1	3510148	1821344	1821446	100	515	1.20E-18	+
BPSL2317	putative nitrogen regulation protein NR(II)	2798211	2799305	-	364	53719927	3091626	glnL	COG3852T	CCCTTTTTTGTTCCGCCCGCGACGG	1	103	103	1	100	Bm_ATCC23344_chr1	3510148	1826764	1826866	100	515	1.20E-18	-
BPSL2318	glutamine synthetase	2799520	2800935	-	471	53719928	3093121	glnA	NULL	GTTCGAATCGGTTGTACGCGGCAA	1	103	103	1	100	Bm_ATCC23344_chr1	3510148	1829988	1830090	100	515	1.20E-18	-
BPSL2319	hypothetical protein BPSL2319	2801316	2801804	+	162	53719929	3091745	-	NULL	GCTCCTCTCGCATTTTGCACCGCTT	1	103	1	103	100	Bm_ATCC23344_chr1	3510148	1830271	1830373	98	497	7.60E-18	+
BPSL2321	hypothetical protein BPSL2321	2802872	2803705	-	277	53719931	3094311	-	NULL	CTTTCGCCGCCTGGTGCGCTGCCGC	1	103	103	1	100	Bm_ATCC23344_chr1	3510148	1832758	1832860	98	497	7.60E-18	-
BPSL2323	hypothetical protein BPSL2323	2805244	2806236	-	330	53719933	3092797	-	COG3391S	CGCCGTTCAAGGTATGCGGGTTCA	1	103	103	1	100	Bm_ATCC23344_chr1	3510148	1835300	1835402	100	515	1.20E-18	-
BPSL2325	N-acetylglutamate synthase	2810489	2811865	+	458	53719935	3092976	-	COG0548E, COG1246E	GCAGCGGCGCCGGCGGGCTGCGG	1	103	1	103	100	Bm_ATCC23344_chr1	3510148	1839455	1839557	100	515	1.20E-18	+
BPSL2326	hypothetical protein BPSL2326	2811918	2812193	+	91	53719936	3092977	-	NULL	GCGGCGCCGCGCGCCCTTCCCCCC	1	55	1	55	100	Bm_ATCC23344_chr1	3510148	1840932	1840986	100	275	8.00E-08	+
BPSL2327	putative transporter protein	2812366	2813709	-	447	53719937	3092136	-	NULL	GTCTTCGGCCGAACGGTTCGCGCC	1	103	103	1	100	Bm_ATCC23344_chr1	3510148	1842773	1842875	100	515	1.20E-18	-
BPSL2328	CAIB/BAIF family protein	2813906	2815288	-	460	53719938	3092137	-	COG1804C	TGTCCGCCCTGGCGCGCTCGCCGG	1	103	103	2	99	Bm_ATCC23344_chr1	3510148	1844070	1844171	100	510	2.00E-18	-
BPSL2329	putative acyl-CoA dehydrogenase	2815456	2817249	-	597	53719939	3094830	-	COG1960I	CATTCTGGCGATCGATAAATAGCGT	1	103	103	1	100	Bm_ATCC23344_chr1	3510148	1846286	1846388	100	515	1.20E-18	-
BPSL2330	LysR family regulatory protein	2817358	2818260	+	300	53719940	3094831	-	COG0583K	TCCTGGTCCGACGAGCGTAGAACC	1	103	1	103	100	Bm_ATCC23344_chr1	3510148	1846297	1846399	100	515	1.20E-18	+
BPSL2338	HutG protein	2825728	2826531	-	267	53719948	3092885	-	COG3741E	CGAGCGTTTCGCGCGCACGATACC	1	103	1	103	100	Bm_ATCC23344_chr1	3510148	678505	678607	98	497	7.60E-18	+
BPSL2344	histidine ammonia-lyase	2832481	2834004	-	507	53719954	3091780	hutH	NULL	GGGAAACTCCTAGCCCCGATTTTTG	1	103	1	103	100	Bm_ATCC23344_chr1	3510148	671032	671134	99	506	3.00E-18	+
BPSL2345	hypothetical protein BPSL2345	2834673	2835464	-	263	53719955	3092556	-	COG2091H	CGCGAGACGCGCGGCGGCCGCCG	1	103	1	103	100	Bm_ATCC23344_chr1	3510148	669572	669674	99	506	3.00E-18	+
BPSL2346	hypothetical protein BPSL2346	2835569	2836579	-	336	53719956	3092557	-	COG4757R	CCCGCCGATTCGCAGCCGACCGAC	1	103	1	103	100	Bm_ATCC23344_chr1	3510148	668457	668559	100	515	1.20E-18	+
BPSL2347	LuxR family regulatory protein	2837796	2838521	-	241	53719957	3091912	-	COG2771K	ATCATTCGGGCCGGTTGATCTTGCC	1	103	1	103	100	Bm_ATCC23344_chr1	3510148	666515	666616	99	500	5.50E-18	+

BPSL2349	putative DNA photolyase	2840120	2841580	-	486	53719958	3093055	-	COG0415L	TCGTCCGCCGAGCGTATTCGATCGC	1	103	1	103	100	Bm_ATCC23344_chr1	3510148	663450	663552	99	506	3.00E-18	+
BPSL2350	putative alkane monooxygenase	2842000	2843166	+	388	53719959	3092483	-	NULL	TCAGCGCAACGGGCACGAACCCGT	1	103	103	1	100	Bm_ATCC23344_chr1	3510148	663129	663231	100	515	1.20E-18	-
BPSL2351	hypothetical protein BPSL2351	2843904	2846192	+	762	53719960	3092484	-	COG3256P	GCTAAAACATACATTCTTTGTACATC	1	103	103	1	100	Bm_ATCC23344_chr1	3510148	661241	661343	100	515	1.20E-18	-
BPSL2355	hypothetical protein BPSL2355	2847949	2849172	+	407	53719962	3091952	-	COG3213P	CGCGGACAACGCCGCGGCCGCGCA	1	103	103	1	100	Bm_ATCC23344_chr1	3510148	657205	657307	99	506	3.00E-18	-
BPSL2356	anaerobic ribonucleoside triphosphate reductase	2849770	2851533	+	587	53719963	3091953	-	COG1328F	CGCGTCAAACTTCGCCGGATCGCCC	1	103	103	1	100	Bm_ATCC23344_chr1	3510148	655384	655486	99	506	3.00E-18	-
BPSL2360	hypothetical protein BPSL2360	2853220	2853699	-	159	53719967	3091798	-	COG3154I	GGCCGCGGCGCGGCGTGCGCCGCC	1	58	1	58	100	Bm_ATCC23344_chr1	3510148	651397	651452	94	251	9.80E-07	+
BPSL2362	family U32 unassigned peptidase	2854683	2855717	-	344	53719969	3092168	-	NULL	CGGCGCCCGCGCCGGGCGCCCGCC	1	91	1	91	100	Bm_ATCC23344_chr1	3510148	649346	649436	100	455	6.00E-16	+
BPSL2363	putative 2-nitropropane dioxygenase	2855806	2857023	-	405	53719970	3092162	-	COG2070R	GCGATCCGGACCGAGACCTTGACG	1	103	1	103	100	Bm_ATCC23344_chr1	3510148	648028	648130	99	506	3.00E-18	+
BPSL2364	hypothetical protein BPSL2364	2857243	2858559	+	438	53719971	3092163	-	NULL	ATCAGAATACCGAAACATGGGTGCC	1	103	103	1	100	Bm_ATCC23344_chr1	3510148	647906	648008	99	506	3.00E-18	-
BPSL2366	coproporphyrinogen III oxidase	2859237	2860682	-	481	53719973	3092079	-	COG0635H	CGGCGTCGGAGGTTGATTTGCATCA	1	103	1	103	100	Bm_ATCC23344_chr1	3510148	644369	644471	100	515	1.20E-18	+
BPSL2367	putative methyl-accepting chemotaxis protein	2860837	2862759	-	640	53719974	3091619	-	COG0840NT	CGTGATCGGATGATTGATCTTGCTTA	1	103	1	103	100	Bm_ATCC23344_chr1	3510148	642292	642394	100	515	1.20E-18	+
BPSL2369	hypothetical protein BPSL2369	2864978	2866432	+	484	53719976	3091762	-	NULL	CCATATACAACAATTAAGGCCAACC	1	103	103	1	100	Bm_ATCC23344_chr1	3510148	640171	640273	100	515	1.20E-18	-
BPSL2375	hypothetical protein BPSL2375	2871037	2871336	-	99	53719982	3094281	-	COG4309S	CATGTTGCAAGCCGGACGAACGCCC	1	103	1	103	100	Bm_ATCC23344_chr1	3510148	633920	634022	99	506	3.00E-18	+
BPSL2376	putative DNA-binding protein	2871527	2872015	+	162	53719983	3094282	-	COG1959K	TTGCAACATGCAGCCGTTAACTATA	1	103	103	1	100	Bm_ATCC23344_chr1	3510148	633827	633929	98	497	7.60E-18	-
BPSL2378	ubiquinol oxidase polypeptide II precursor	2873037	2873927	+	296	53719985	3092531	cyoA	COG1622C	CTACATTCCGCCGCCAAAGCGACTC	1	103	103	1	100	Bm_ATCC23344_chr1	3510148	632297	632399	98	497	7.60E-18	-
BPSL2383	putative histidine-binding periplasmic protein precur	2878543	2879337	-	264	53719990	3091630	-	COG0834ET	ACGGCCGCCCCGCGCGGTTGTCCG	1	103	1	103	100	Bm_ATCC23344_chr1	3510148	625922	626024	99	506	3.00E-18	+
BPSL2390	bifunctional N-succinyldiaminopimelate-aminotransfe	2886121	2887353	-	410	53719996	3093428	argD	COG4992E	GAACCCCGTCCGATAAATCCCGTCC	1	103	1	103	100	Bm_ATCC23344_chr1	3510148	617910	618012	100	515	1.20E-18	+
BPSL2398	hypothetical protein BPSL2398	2894877	2896298	-	473	53720004	3092192	-	COG1032C	CTCATACACAGGATGCGCGGGCGG	1	103	103	1	100	Bm_ATCC23344_chr1	3510148	604129	604231	100	515	1.20E-18	-
BPSL2399	putative glycosyltransferase	2896410	2897591	-	393	53720005	3092193	-	COG1215M	AAGCAAGTCAGAAAGGAGTTGGGA	1	103	103	1	100	Bm_ATCC23344_chr1	3510148	605419	605521	100	515	1.20E-18	-
BPSL2400	luciferase-like monooxygenase	2898218	2899276	+	352	53720006	3094285	-	COG2141C	GTCGTCAGGGATATCCATCCGGCCC	1	103	1	103	100	Bm_ATCC23344_chr1	3510148	605948	606050	100	515	1.20E-18	+
BPSL2402	pyridoxamine kinase	2901316	2902179	+	287	53720008	3094845	pdxY	COG2240H	CCCTCCGATACCGTATTACCGGATG	1	103	1	103	100	Bm_ATCC23344_chr1	3510148	608993	609095	100	515	1.20E-18	+
BPSL2403	non-hemolytic phospholipase C precursor	2902870	2904972	+	700	53720009	3093316	plcN	NULL	GGCGCTGCCGCGCAAGACGGCAGC	1	103	1	103	100	Bm_ATCC23344_chr1	3510148	610526	610628	100	515	1.20E-18	+
BPSL2404	periplasmic ligand binding lipoprotein	2905792	2906940	+	382	53720010	3094724	-	COG0683E	AGCCGGCGCCGCTCCGGCGGCCCG	1	103	103	1	100	Bm_ATCC23344_chr1	3510148	598799	598901	100	515	1.20E-18	-
BPSL2405	putative FAD dependent oxidoreductase	2907039	2908211	+	390	53720011	3093338	-	COG0665E	CCGGCCGCCCATGTATGGAGCGGA	1	101	101	1	100	Bm_ATCC23344_chr1	3510148	597552	597652	99	496	8.40E-18	-
BPSL2406	hypothetical protein BPSL2406	2908367	2908828	-	153	53720012	3093339	-	COG3556S	CGCCGGCGGGCCGGCCCGCGCCGC	1	58	1	58	100	Bm_ATCC23344_chr1	3510148	595762	595819	100	290	1.70E-08	+
BPSL2407	hypothetical protein BPSL2407	2908884	2909855	-	323	53720013	3091904	-	NULL	GCCGATTTGCGGCGCTGGGGCATCA	1	103	1	103	100	Bm_ATCC23344_chr1	3510148	594729	594831	100	515	1.20E-18	+
BPSL2408	glycosyl transferase group 1 protein	2910122	2911186	-	354	53720014	3091905	-	COG0438M	AGCCGGCTCGCCCGACGTCCAACCC	1	103	1	103	100	Bm_ATCC23344_chr1	3510148	593398	593500	100	515	1.20E-18	+
BPSL2409	ABC transporter ATP binding protein	2911753	2913618	+	621	53720015	3092643	-	COG1132V	GCGCGACGCACCGGCTTGATTACAA	1	103	103	1	100	Bm_ATCC23344_chr1	3510148	592931	593033	100	515	1.20E-18	-
BPSL2411	putative trehalose-phosphatase	2915053	2915862	-	269	53720017	3093035	otsB	COG1877G	ACCGCGCACCGAACCGGGGCAAAA	1	103	1	103	100	Bm_ATCC23344_chr1	3510148	588724	588826	99	506	3.00E-18	+
BPSL2412	SCO1/SenC family protein	2916222	2916881	+	219	53720018	3092644	-	COG1999R	CGATGCGTCCTGGATCGATGCGGGC	1	103	103	1	100	Bm_ATCC23344_chr1	3510148	588462	588564	100	515	1.20E-18	-
BPSL2415	hypothetical protein BPSL2415	2918372	2918875	-	167	53720021	3093269	-	COG2153R	GGCGCAGGCGGGCGGCGTTCGCGT	1	103	1	103	100	Bm_ATCC23344_chr1	3510148	585712	585814	99	506	3.00E-18	+
BPSL2416	hypothetical protein BPSL2416	2919089	2919955	-	288	53720022	3093270	-	COG1741R	TGAGTGTGATTTTCGCGGTCTACTGA	1	103	1	103	100	Bm_ATCC23344_chr1	3510148	584612	584714	100	515	1.20E-18	+
BPSL2417	LysR family regulatory protein	2920082	2920990	+	302	53720023	3093640	-	COG0583K	AGCGAACGCTTCGATCGATGGATAC	1	103	103	1	100	Bm_ATCC23344_chr1	3510148	584583	584685	100	515	1.20E-18	-
BPSL2418	hypothetical protein BPSL2418	2921155	2921667	+	170	53720024	3093641	-	COG1956T	GCCCATGCGGCGACGCCGCGCGCG	1	103	103	1	100	Bm_ATCC23344_chr1	3510148	583508	583610	99	506	3.00E-18	-
BPSL2419	putative CDP-diacylglycerol--glycerol-3-phosphate 3	2923370	2923963	-	197	53720025	3092636	-	COG0558I	CGCGGCTCGCGCCGGGCGGCGCGC	1	103	1	103	100	Bm_ATCC23344_chr1	3510148	580594	580696	100	515	1.20E-18	+
BPSL2422	elongation factor P	2927947	2928504	+	185	53720028	3092950	-	COG0231J	TCGCGCCTGCGCTCGCCGGGCTCGC	1	103	103	1	100	Bm_ATCC23344_chr1	3510148	576687	576789	99	506	3.00E-18	-
BPSL2423	sigma-54 interacting response regulator protein	2928775	2930172	+	465	53720029	3092540	-	COG2204T	ACGCCAAGTGCGCGATGGGGCATA	1	103	103	1	100	Bm_ATCC23344_chr1	3510148	575859	575961	100	515	1.20E-18	-
BPSL2428	GTP-binding protein Era	2933396	2934295	-	299	53720034	3093107	era	COG1159R	CATCGTCCCAATCGACATCGCGGTC	1	72	1	72	100	Bm_ATCC23344_chr1	3510148	570272	570343	100	360	1.20E-11	+
BPSL2429	ribonuclease III	2934365	2935768	-	467	53720035	3092548	rnc	COG0571K	CGAACTACTTTAAGAACCGGCGGCA	1	103	1	103	100	Bm_ATCC23344_chr1	3510148	568768	568870	100	515	1.20E-18	+
BPSL2431	GTP-binding protein LepA	2936828	2938621	-	597	53720037	3091676	lepA	COG0481M	CCTGGCGGGCCTTTTCGGCTAAAAT	1	103	1	103	100	Bm_ATCC23344_chr1	3510148	565915	566017	100	515	1.20E-18	+
BPSL2433	subfamily S1C unassigned peptidase	2939113	2940570	-	485	53720039	3094614	-	COG0265O	CCCTTCGGTTTCCGACATGATGAATC	1	70	1	70	100	Bm_ATCC23344_chr1	3510148	563999	564068	100	350	3.30E-11	+
BPSL2435	putative sigma E factor negative regulatory protein	2941696	2942298	-	200	53720041	3091492	-	COG3073T	GCGCCTGCGGCGAATCGAAGCGGC	1	86	1	86	100	Bm_ATCC23344_chr1	3510148	562255	562340	100	430	8.00E-15	+
BPSL2436	RNA polymerase sigma-70 factor	2942382	2942981	-	199	53720042	3092349	rpoE	COG1595K	GCGCGGCAGGCCGCTTCCGGTCGA	1	103	1	103	100	Bm_ATCC23344_chr1	3510148	561555	561657	100	515	1.20E-18	+
BPSL2438	3-oxoacyl-(acyl carrier protein) synthase	2943572	2944810	-	412	53720044	3092217	fabF	COG0304IQ	GCGTTCTCGCCGGCCGAGCTAACAC	1	103	1	103	100	Bm_ATCC23344_chr1	3510148	559726	559828	100	515	1.20E-18	+
BPSL2439	acyl carrier protein	2944967	2945206	-	79	53720045	3092505	acpP	COG0236IQ	TCGGCGCATGTTTGGCCGCAAAGCC	1	103	1	103	100	Bm_ATCC23344_chr1	3510148	559330	559432	100	515	1.20E-18	+
BPSL2440	3-ketoacyl-(acyl-carrier-protein) reductase	2945350	2946099	-	249	53720046	3091516	fabG	COG1028IQR	CGCCGTCAGCGCGCGAGCTTGCCG	1	53	1	53	100	Bm_ATCC23344_chr1	3510148	558487	558539	100	265	2.30E-07	+
BPSL2441	acyl-carrier-protein S-malonyltransferase	2946150	2947082	-	310	53720047	3092873	fabD	COG0331I	CGGCCCTTCTTCGCATCCCATCGGC	1	73	1	73	100	Bm_ATCC23344_chr1	3510148	557484	557556	100	365	6.90E-12	+
BPSL2443	fatty acid/phospholipid synthesis protein	2948142	2949248	-	368	53720049	3094277	-	COG0416I	GGCAGCTCGCGCGACAACCGGTTC	1	103	1	103	100	Bm_ATCC23344_chr1	3510148	555289	555390	98	491	1.40E-17	+
BPSL2444	50S ribosomal protein L32	2949388	2949567	-	59	53720050	3092457	rpmF	COG0333J	GCAGTTGGGGCGCGGGAAGGCGTC	1	99	1	99	100	Bm_ATCC23344_chr1	3510148	554974	555072	100	495	9.30E-18	+
BPSL2445	hypothetical protein BPSL2445	2949664	2950296	-	210	53720051	3094278	-	COG1399R	TTTTTCATCGGCAGGGGTTTTGCGGC	1	103	2	103	99	Bm_ATCC23344_chr1	3510148	554242	554343	98	492	1.30E-17	+
BPSL2446	Maf-like protein	2950540	2951187	+	215	53720052	3092699	maf	COG0424D	GGCCTCGCCAGGCACGCGGCCGCC	1	103	103	1	100	Bm_ATCC23344_chr1	3510148	554095	554197	99	506	3.00E-18	-
BPSL2451	ribosomal large subunit pseudouridine synthase C	2954013	2955020	-	335	53720057	3094628	rluC	COG0564J	AAGTAAAATAACAGTTTGCGCTTGC	1	103	3	103	98	Bm_ATCC23344_chr1	3510148	549883	549983	99	496	8.40E-18	+
BPSL2452	putative ribonuclease E	2955743	2959009	+	1088	53720058	3091436	-	COG1530J	AGCGTGCTTGTGACAACACAACAAC	1	103	103	1	100	Bm_ATCC23344_chr1	3510148	549215	549317	100	515	1.20E-18	-
BPSL2459	2-hydroxyacid dehydrogenase	2964859	2965848	-	329	53720065	3091694	-	COG1052CHR	CGGCCGCACGGGCGATGCGCGGCC	1	68	1	68	100	Bm_ATCC23344_chr1	3510148	539021	539088	100	340	9.30E-11	+
BPSL2460	hypothetical protein BPSL2460	2965914	2967356	-	480	53720066	3091695	-	COG1055P	TGCGGCCGCCGCCGCGTCACGCGC	1	58	1	58	100	Bm_ATCC23344_chr1	3510148	537523	537580	100	290	1.70E-08	+
BPSL2466	hypothetical protein BPSL2466	2972515	2973039	-	174	53720072	3091990	-	NULL	AACTGCGCACGAAAGCGGCTGGTC	1	103	1	103	100	Bm_ATCC23344_chr1	3510148	531762	531864	100	515	1.20E-18	+
BPSL2467	hypothetical protein BPSL2467	2973359	2973580	+	73	53720073	3094001	-	COG2501S	GCGCGCGCCGCGCATCGCTTATGAT	1	103	103	1	100	Bm_ATCC23344_chr1	3510148	531540	531642	100	515	1.20E-18	-
BPSL2468	putative multidrug resistance protein	2973705	2975111	+	468	53720074	3091686	norM	COG0534V	ATCGGCGGGTCTCGTCGACAGCGC	1	103	103	1	100	Bm_ATCC23344_chr1	3510148	531194	531296	100	515	1.20E-18	-
BPSL2469	fumarate hydratase	2975700	2977094	+	464	53720075	3091813	fumC	COG0114C	TGCTTGCCCATCAGTGGATGCCGTC	1	103	103	1	100	Bm_ATCC23344_chr1	3510148	525409	525511	100	515	1.20E-18	-
BPSL2472	putative prolin-rich exported protein	2979995	2982568	+	857	53720079	3094643	-	NULL	CACGTCAAATCGCATAACAAAACCA	1	103	103	1	100	Bm_ATCC23344_chr1	3510148	521211	521313	100	515	1.20E-18	-
BPSL2473	putative thymidylate synthase	2982658	2983629	-	323	53720080	3094644	-	NULL	CGCGGGCCTTGTTAAAATGGCGGGC	1	59	59	1	100	Bm_ATCC23344_chr1	3510148	406271	406329	100	295	1.00E-08	-
BPSL2474	sigma-54 interacting regulatory protein	2983686	2985071	-	461	53720081	3093920	-	COG2204T	TGAAAAATGACGAGCCGAGTCCTTC	1	103	103	1	100	Bm_ATCC23344_chr1	3510148	407713	407815	100	515	1.20E-18	-
BPSL2474a	hypothetical protein BPSL2474A	2985594	2985962	+	122	53720082	3093921	-	NULL	GCGTCGATGACGGTGCGTTCGCGCC	1	103	1	103	100	Bm_ATCC23344_chr1	3510148	408413	408515	100	515	1.20E-18	+
BPSL2478	microcin-processing peptidase	2991780	2993150	-	456	53720087	3094761	-	COG0312R	GTCGCCGACCCGCGTCGCGCGCGC	1	103	103	1	100	Bm_ATCC23344_chr1	3510148	415743	415845	99	506	3.00E-18	-
BPSL2479	hypothetical protein BPSL2479	2993296	2993904	+	202	53720088	3094762	-	NULL	TGTGCGTATTCCGCGATCATAGCAA	1	103	1	103	100	Bm_ATCC23344_chr1	3510148	415791	415893	100	515	1.20E-18	+
BPSL2481	oligoribonuclease	2994786	2995391	-	201	53720090	3093419	orn	COG1949A	TCTATGCGAGAATTATATGTTCTTGC	1	103	103	1	100	Bm_ATCC23344_chr1	3510148	417951	418053	100	515	1.20E-18	-
BPSL2482	subfamily M48A unassigned peptidase	2995510	2996769	+	419	53720091	3092309	-	COG0501O	GGCGGACGGCGCGCGCCGCCGC	1	103	1	103	100	Bm_ATCC23344_chr1	3510148	417972	418074	100	515	1.20E-18	+
BPSL2484	hypothetical protein BPSL2484	2997779	2998096	+	105	53720093	3091634	-	NULL	CCGGGCAGCGCAAGCGGGCCGCAT	1	71	1	71	100	Bm_ATCC23344_chr1	3510148	420279	420349	100	355	2.00E-11	+
BPSL2489	50S ribosomal protein L19	3003117	3003506	-	129	53720098	3094791	rplS	NULL	GCATGCGTGCGCGGCGCCTGATGTC	1	103	103	1	100	Bm_ATCC23344_chr1	3510148	424763	424865	100	515	1.20E-18	-
BPSL2491	16S rRNA-processing protein	3004440	3005075	-	211	53720100	3094627	rimM	COG0806J	AGCATGGCGCGGCGTGCCGGAAGC	1	101	101	1	100	Bm_ATCC23344_chr1	3510148	426332	426432	100	505	3.30E-18	-
BPSL2492	30S ribosomal protein S16	3005174	3005428	-	84	53720101	3094357	rpsP	NULL	CCGGGTTTTTTGGCGGGTAAGTGTT	1	103	103	1	100	Bm_ATCC23344_chr1	3510148	426685	426787	100	515	1.20E-18	-
BPSL2493	hypothetical protein BPSL2493	3005644	3006762	+	372	53720102	3093825	-	COG2133G	GCTTACAATCGGCCTGTCGCGCGCC	1	103	1	103	100	Bm_ATCC23344_chr1	3510148	426803	426905	100	515	1.20E-18	+
BPSL2494	hypothetical protein BPSL2494	3006869	3007336	+	155	53720103	3093826	-	NULL	CGTGGCGTGACCGCGCCGCACCGC	1	103	1	103	100	Bm_ATCC23344_chr1	3510148	428028	428130	100	515	1.20E-18	+

BPSL2495	hypothetical protein BPSL2495	3007371	3008528 -	385	53720104	3092450 -	COG1533L	GCGGGGCCGAAGCGCTTGGCACGA	1	103	103	1	100 Bm_ATCC23344_chr1	3510148	429785	429887	100	515	1.20E-18 -
BPSL2496	leucine-responsive regulatory protein	3008637	3009125 -	162	53720105	3092406 lrp	COG1522K	TGCGCTCTGCGATCTCGATCCGACC	1	103	103	1	100 Bm_ATCC23344_chr1	3510148	430382	430484	100	515	1.20E-18 -
BPSL2497	D-amino acid dehydrogenase small subunit	3009273	3010559 +	428	53720106	3091489 dadA	COG0665E	CTCAACCATACCATAGCGCAAAAA	1	103	1	103	100 Bm_ATCC23344_chr1	3510148	430432	430534	100	515	1.20E-18 +
BPSL2498	putative acyl-CoA dehydrogenase	3010673	3012463 -	596	53720107	3092451 -	COG1960I	CGGCGCGCGTCGCGTTCAGCGCAC	1	103	103	1	100 Bm_ATCC23344_chr1	3510148	433719	433820	99	500	5.50E-18 -
BPSL2500	electron transfer flavoprotein beta-subunit	3013528	3014277 -	249	53720109	3091793 etfB	COG2086C	CTCGGTGCAGCCGCAAATTGAATG	1	103	103	1	100 Bm_ATCC23344_chr1	3510148	435532	435634	100	515	1.20E-18 -
BPSL2501	hypothetical protein BPSL2501	3014504	3015298 -	264	53720110	3094263 -	COG1464P	GAAGACCATCGAAGACGCACGAGC	1	76	76	1	100 Bm_ATCC23344_chr1	3510148	436553	436628	100	380	1.50E-12 -
BPSL2503	ABC transporter, ATP-binding component	3016015	3017049 -	344	53720112	3092979 -	COG1135P	ATGTGGCGAATGCGGTAAACTCGC	1	103	103	1	100 Bm_ATCC23344_chr1	3510148	438304	438406	100	515	1.20E-18 -
BPSL2504	putative hydrolase	3017358	3018200 +	280	53720113	3092980 -	COG2267I	TTCGTCACGACACGCCACGGAGAC	1	103	1	103	100 Bm_ATCC23344_chr1	3510148	438515	438617	100	515	1.20E-18 +
BPSL2505	putative histone deacetylase-family protein	3018543	3019466 -	307	53720114	3094456 -	NULL	GTGATGTGGTAAGTTAGCGGCGAA	1	103	103	1	100 Bm_ATCC23344_chr1	3510148	440721	440823	100	515	1.20E-18 -
BPSL2506	putative exported transglycosylase	3019594	3020946 +	450	53720115	3094457 -	COG2951M	GCGCCGCAGGTCTCCGTCAAGGAT	1	103	1	103	100 Bm_ATCC23344_chr1	3510148	440751	440853	100	515	1.20E-18 +
BPSL2507	cysteine synthase	3021173	3022075 -	300	53720116	3092757 cysM	COG0031E	CCGGGGCGCTCGGCGGCGGGCTTG	1	103	103	1	100 Bm_ATCC23344_chr1	3510148	443323	443426	99	505	3.30E-18 -
BPSL2508	hypothetical protein BPSL2508	3022411	3022728 -	105	53720118	3093097 -	COG1555L	GCGGCGCGCGCGCCGCCTTAGACT	1	74	74	1	100 Bm_ATCC23344_chr1	3510148	443964	444037	100	370	4.10E-12 -
BPSL2509	putative ADP-glycero-mannoheptose epimerase	3022800	3023792 -	330	53720119	3092933 gmhD	COG0451MG	ACGACAGCGGCGCGCGCCGCCCGC	1	61	61	1	100 Bm_ATCC23344_chr1	3510148	445028	445088	100	305	3.60E-09 -
BPSL2511	putative UDP-glucose dehydrogenase	3024806	3026206 -	466	53720121	3093002 udg	COG1004M	AATGAAGCGGTAATGAGGCAGGCC	1	103	103	1	100 Bm_ATCC23344_chr1	3510148	447442	447544	100	515	1.20E-18 -
BPSL2512	hypothetical protein BPSL2512	3026327	3027496 -	389	53720122	3093004 -	COG2956G	CGATGCCGACGCCGCGCACGCCGC	1	55	55	1	100 Bm_ATCC23344_chr1	3510148	448732	448786	100	275	8.00E-08 -
BPSL2515	30S ribosomal protein S1	3028413	3030125 -	570	53720125	3093073 rpsA	COG0539J	CGTGTGTTAAACCCTTAACCCCGTG	1	103	103	1	100 Bm_ATCC23344_chr1	3510148	452954	453056	100	515	1.20E-18 -
BPSL2519	phosphoserine aminotransferase	3034582	3035664 -	360	53720129	3094241 serC	COG1932HE	GATAATGGCCGTTTGCGCGACAGC	1	103	103	1	100 Bm_ATCC23344_chr1	3510148	458431	458533	100	515	1.20E-18 -
BPSL2520	hypothetical protein BPSL2520	3035838	3036434 -	198	53720130	3092000 -	NULL	GTCAGGTTGTGACCGTCGAGCCGG	1	101	101	1	100 Bm_ATCC23344_chr1	3510148	459201	459301	100	505	3.30E-18 -
BPSL2521	DNA gyrase subunit A	3036533	3039133 -	866	53720131	3092481 gyrA	COG0188L	TGGGGCGGGTTGGGGCGCATGTTA	1	103	103	1	100 Bm_ATCC23344_chr1	3510148	461900	462002	100	515	1.20E-18 -
BPSL2522	outer membrane protein a precursor	3039644	3040318 +	224	53720132	3093812 ompA	COG2885M	TTTGTGCCGCACCAATGTTATACTT	1	103	1	103	100 Bm_ATCC23344_chr1	3510148	462313	462415	100	515	1.20E-18 +
BPSL2523	3-demethylubiquinone-9 3-methyltransferase	3040487	3041185 +	232	53720133	3094669 ubiG	COG2227H	CTCACCCGCGGCCGCGTTCCTGGC	1	103	1	103	100 Bm_ATCC23344_chr1	3510148	463156	463258	100	515	1.20E-18 +
BPSL2525	hypothetical protein BPSL2525	3042586	3043272 +	228	53720135	3094167 -	COG3963I	GCGCGAGCGCAACATAGCGGGTAA	1	103	1	103	100 Bm_ATCC23344_chr1	3510148	467698	467800	100	515	1.20E-18 +
BPSL2526	hypothetical protein BPSL2526	3043453	3043716 -	87	53720136	3094168 -	NULL	GAAACGGGCGGGCGCACGGCGCT	1	103	103	1	100 Bm_ATCC23344_chr1	3510148	468926	469028	100	515	1.20E-18 -
BPSL2527	possible regulatory protein	3043818	3044924 -	368	53720137	3093218 -	COG1910P, COG2005R	ATAGAGCGCAAACGGACCTTGAGA	1	91	91	1	100 Bm_ATCC23344_chr1	3510148	470134	470224	100	455	6.00E-16 -
BPSL2528	NAD-dependent formate dehydrogenase gamma sul	3045013	3045528 +	171	53720138	3091500 fdsG	COG1905C	TTATGCAATTCCGTTTCCTATTGCGA	1	91	1	91	100 Bm_ATCC23344_chr1	3510148	470137	470227	100	455	6.00E-16 +
BPSL2532	putative oxygenase	3050669	3052336 -	555	53720142	3093219 -	COG0654HC	CCATACCTCGAACCGCGCCGGCCG	1	103	103	1	100 Bm_ATCC23344_chr1	3510148	477580	477682	100	515	1.20E-18 -
BPSL2533	hypothetical protein BPSL2533	3053003	3054313 +	436	53720143	3093173 -	COG0457R	CGCGCTCAACGCGCTCGGCCGGCA	1	103	1	103	100 Bm_ATCC23344_chr1	3510148	478149	478251	100	515	1.20E-18 +
BPSL2534	citrate-proton symporter	3054702	3055997 -	431	53720144	3092760 citA	NULL	TCGGTCTGCGCGGGCCCCGGCGCG	1	103	103	1	100 Bm_ATCC23344_chr1	3510148	481241	481343	99	506	3.00E-18 -
BPSL2536	amidase	3058425	3059801 +	458	53720146	3094608 -	NULL	CTACAATGCGCGAACCGGCCGCCG	1	103	1	103	100 Bm_ATCC23344_chr1	3510148	483569	483671	100	515	1.20E-18 +
BPSL2537	disulfide bond formation protein B	3059910	3060419 +	169	53720147	3093150 dsbB	COG1495O	CACCCGCGCGCCGGATGCCGCGCC	1	103	1	103	100 Bm_ATCC23344_chr1	3510148	485054	485156	100	515	1.20E-18 +
BPSL2538	hypothetical protein BPSL2538	3060961	3061983 +	340	53720148	3094609 -	COG1975O	GAGCCTCCGATGCCGCCGCCCCCG	1	103	1	103	100 Bm_ATCC23344_chr1	3510148	486072	486174	98	497	7.60E-18 +
BPSL2540	hypothetical protein BPSL2540	3063112	3064524 +	470	53720150	3093934 -	COG2252R	CGGCCGCGCAACGCGCGCGCCCCC	1	64	1	61	96 Bm_ATCC23344_chr1	3510148	488262	488321	96	281	4.30E-08 +
BPSL2542	hypothetical protein BPSL2542	3066348	3066920 +	190	53720152	3093935 -	COG2119S	CAGGGCCTGGCGAGACCGATGGTT	1	103	1	103	100 Bm_ATCC23344_chr1	3510148	491449	491551	100	515	1.20E-18 +
BPSL2543	putative lipoprotein	3067254	3067967 -	237	53720153	3094775 -	NULL	GTCTTCTCCATATCGGCGCGGCGTT	1	103	103	1	100 Bm_ATCC23344_chr1	3510148	493166	493268	100	515	1.20E-18 -
BPSL2544	alanyl aminopeptidase	3068188	3070890 +	900	53720154	3094776 -	COG0308E	TCGTCGCGCGCGCCTTGCGCAAAG	1	103	1	103	100 Bm_ATCC23344_chr1	3510148	493289	493391	100	515	1.20E-18 +
BPSL2545	5-methyltetrahydropteroyltriglutamate--homocystein	3071238	3073532 -	764	53720155	3092004 metE	COG0620E	GCGAATGATATTGAAAAAAATTCAT	1	103	103	1	100 Bm_ATCC23344_chr1	3510148	498731	498833	100	515	1.20E-18 -
BPSL2546	transcriptional activator protein	3073649	3074581 +	310	53720156	3091761 metR	COG0583K	TGACCGGCAGTATCGGATTTGCCCT	1	103	1	103	100 Bm_ATCC23344_chr1	3510148	498750	498852	100	515	1.20E-18 +
BPSL2547	fructose-1,6-bisphosphatase	3074711	3075727 +	338	53720157	3092110 fbp	COG0158G	TTTTCGCCGAAAAACCGTCGGCCGC	1	103	1	103	100 Bm_ATCC23344_chr1	3510148	499812	499914	100	515	1.20E-18 +
BPSL2548	hypothetical protein BPSL2548	3076607	3077083 +	158	53720159	3094092 -	NULL	AGCGCGCTACCGGCCGATCCATCG	1	103	1	103	100 Bm_ATCC23344_chr1	3510148	503021	503123	100	515	1.20E-18 +
BPSL2549	hypothetical protein BPSL2549	3077252	3077500 +	82	53720160	3094250 -	NULL	TCCTTCGGTATCCGGATCAATTGGT	1	103	1	103	100 Bm_ATCC23344_chr1	3510148	503666	503768	100	515	1.20E-18 +
BPSL2550	hypothetical protein BPSL2550	3078156	3078503 +	115	53720161	3094251 -	NULL	TACGCACATCGAAATCCGTTCGACA	1	103	1	103	100 Bm_ATCC23344_chr1	3510148	504572	504674	99	506	3.00E-18 +
BPSL2553	putative siderophore receptor protein	3080543	3082879 -	778	53720164	3092253 -	COG1629P	CGCGACGTCCGGCGTCGTGCCCGC	1	103	103	1	100 Bm_ATCC23344_chr1	3510148	509384	509486	100	515	1.20E-18 -
BPSL2554	hypothetical protein BPSL2554	3083026	3083502 -	158	53720165	3092254 -	NULL	AGTGATTATCGAACGCAAACTTTGC	1	103	103	1	100 Bm_ATCC23344_chr1	3510148	510007	510109	99	506	3.00E-18 -
BPSL2555	chorismate mutase	3084028	3084621 +	197	53720166	3092729 -	COG1605E	TGATCTTCCGCTTGCGCCGCGTCGC	1	103	1	103	100 Bm_ATCC23344_chr1	3510148	510435	510537	100	515	1.20E-18 +
BPSL2559	outer membrane porin protein	3087604	3088743 +	379	53720170	3092648 -	COG3203M	CGAGCCATGCCCCGGCTCCGTTTCT	1	103	1	103	100 Bm_ATCC23344_chr1	3510148	2181422	2181524	100	515	1.20E-18 +
BPSL2560	transcription elongation factor GreB	3088971	3089534 -	187	53720171	3094279 greB	COG0782K	ATAGCAAACAAATTTAAAAAGGGA	1	103	103	1	100 Bm_ATCC23344_chr1	3510148	2184763	2184865	100	515	1.20E-18 -
BPSL2561	guanosine-3',5'-bis(diphosphate) 3'-pyrophosphohyd	3090073	3092442 -	789	53720172	3093604 spoT	COG0317TK	CGCGCGCCCGGTTTCTTCAGCCATG	1	69	69	1	100 Bm_ATCC23344_chr1	3510148	2187670	2187738	100	345	5.50E-11 -
BPSL2562	DNA-directed RNA polymerase omega subunit	3092509	3092712 -	67	53720173	3092978 rpoZ	COG1758K	CGGCGGGCAACGAGACACATAAGC	1	63	63	1	100 Bm_ATCC23344_chr1	3510148	2187940	2188002	100	315	1.30E-09 -
BPSL2564	hypothetical protein BPSL2564	3093463	3094386 -	307	53720175	3092715 -	COG1561S	GCAAAAATGGGATAATGGATGTTTC	1	103	103	1	100 Bm_ATCC23344_chr1	3510148	2189614	2189716	99	506	3.00E-18 -
BPSL2565	ribonuclease PH	3094558	3095289 +	243	53720176	3094131 rph	COG0689J	GCGCGAAACATCCATTATCCCATTT	1	103	1	103	100 Bm_ATCC23344_chr1	3510148	2189688	2189790	100	515	1.20E-18 +
BPSL2588	hypothetical protein BPSL2588	3108136	3108714 -	192	53720198	3094562 -	NULL	ACGATCTCCCTTTTCCCGAAGAGGG	1	103	103	1	100 Bm_ATCC23344_chr1	3510148	2194170	2194272	98	497	7.60E-18 -
BPSL2589	putative lipoprotein	3108888	3109382 +	164	53720199	3091774 -	NULL	AACCCTCTTCGGGAAAAGGGAGAT	1	103	1	103	100 Bm_ATCC23344_chr1	3510148	2194246	2194348	98	497	7.60E-18 +
BPSL2590	probable membrane transport protein	3109801	3111114 +	437	53720200	3091775 -	NULL	CCCGACTCTCGATTCGAACAAAGG	1	103	1	103	100 Bm_ATCC23344_chr1	3510148	2195189	2195291	100	515	1.20E-18 +
BPSL2591	hypothetical protein BPSL2591	3111295	3111477 +	60	53720201	3091991 -	NULL	TTTCGGCACGCACCGCCCTGTCTAT	1	103	1	103	100 Bm_ATCC23344_chr1	3510148	2196683	2196785	98	497	7.60E-18 +
BPSL2592	lipopolysaccharide heptosyltransferase-1	3111512	3112534 -	340	53720202	3093376 waaC2	COG0859M	CGACGTTGTGACAAATGTAAATTCT	1	103	103	1	100 Bm_ATCC23344_chr1	3510148	2198020	2198122	100	515	1.20E-18 -
BPSL2593	putative chloride-channel protein	3112696	3114357 +	553	53720203	3091992 -	COG0038P	ACCACAAATTGCGAAGAATTTACAT	1	103	1	103	100 Bm_ATCC23344_chr1	3510148	2198084	2198186	100	515	1.20E-18 +
BPSL2594	hypothetical protein BPSL2594	3114474	3114893 -	139	53720204	3092560 -	NULL	CGCGGCCGCGCCGCCGCCGCGCGC	1	103	103	1	100 Bm_ATCC23344_chr1	3510148	2200373	2200475	99	506	3.00E-18 -
BPSL2596	septum site-determining protein	3115282	3116097 -	271	53720206	3093025 minD	COG2894D	CACGCGTGGCGCGACAAACGGGTC	1	61	61	1	100 Bm_ATCC23344_chr1	3510148	2201577	2201637	100	305	3.60E-09 -
BPSL2597	septum formation inhibitor	3116156	3116968 -	270	53720207	3093128 minC	NULL	CGGGCGGGCGGGGCACCGCGCGC	1	103	103	1	100 Bm_ATCC23344_chr1	3510148	2202448	2202550	100	515	1.20E-18 -
BPSL2599	hypothetical protein BPSL2599	3118611	3118883 +	90	53720209	3093414 -	NULL	CCCGCGCATTTCGATTGACCCAATC	1	103	1	103	100 Bm_ATCC23344_chr1	3510148	2203954	2204056	100	515	1.20E-18 +
BPSL2600	seryl-tRNA synthetase	3119313	3120614 -	433	53720210	3094242 serS	COG0172J	ACGGACGCCCGGTTTGCGGCATCG	1	103	103	1	100 Bm_ATCC23344_chr1	3510148	2206055	2206157	100	515	1.20E-18 -
BPSL2601	hypothetical protein BPSL2601	3120727	3121038 +	103	53720211	3093415 -	COG5460S	GAATCGTTGTGTAAGGGGAGAATT	1	103	1	103	100 Bm_ATCC23344_chr1	3510148	2206070	2206172	100	515	1.20E-18 +
BPSL2603	outer-membrane lipoprotein carrier protein precursc	3122575	3123291 -	238	53720213	3092565 lolA	COG2834M	ATCCGCGCCGGCGCGCCTGCCGAA	1	60	60	1	100 Bm_ATCC23344_chr1	3510148	2208743	2208802	100	300	6.00E-09 -
BPSL2604	putative cell division protein	3123349	3125817 -	822	53720214	3091542 -	COG1674D	GATACCGTCGCAGATGGGTGCAAA	1	103	103	1	100 Bm_ATCC23344_chr1	3510148	2211269	2211371	100	515	1.20E-18 -
BPSL2605	thioredoxin reductase	3125983	3126945 +	320	53720215	3092744 trxB	COG0492O	ATAATGCGGGCTTTGCACCCATCTG	1	103	1	103	100 Bm_ATCC23344_chr1	3510148	2211337	2211439	100	515	1.20E-18 +
BPSL2608	ABC transporter, ATP-binding component	3128727	3129845 -	372	53720218	3092683 -	COG3839G	CGTCGGCACGCACGACGCGAACAC	1	103	103	1	100 Bm_ATCC23344_chr1	3510148	2215299	2215401	100	515	1.20E-18 -
BPSL2610	ABC transporter, membrane permease	3130793	3131731 -	312	53720220	3092595 -	COG1175G	CATCCGCAGCAAACGGGCCGCGAG	1	103	103	1	100 Bm_ATCC23344_chr1	3510148	2217185	2217287	99	506	3.00E-18 -
BPSL2611	maltose-binding protein	3131888	3133135 -	415	53720221	3093125 malE	COG1653G	CGCGGCTCGATTTCCCGTGTCGTTC	1	103	103	1	100 Bm_ATCC23344_chr1	3510148	2218589	2218691	100	515	1.20E-18 -
BPSL2612	glucose-6-phosphate 1-dehydrogenase	3133594	3135063 +	489	53720222	3094730 zwf	NULL	ACCCCTTGCGGCGGATTGTAGTTAA	1	103	1	103	100 Bm_ATCC23344_chr1	3510148	2218950	2219052	100	515	1.20E-18 +
BPSL2613	6-phosphogluconolactonase	3135151	3135831 +	226	53720223	3094109 pgl	COG0363G	TGCAATGCGGCGCGCGGCTGCCGT	1	90	1	90	100 Bm_ATCC23344_chr1	3510148	2220520	2220609	100	450	1.00E-15 +
BPSL2615	ABC transporter, substrate binding component	3138237	3139037 +	266	53720225	3093954 -	COG0834ET	CCGCGCGCCTGCCGATCATGCGGC	1	103	1	103	100 Bm_ATCC23344_chr1	3510148	2223561	2223663	99	506	3.00E-18 +
BPSL2616	putative ABC-family amino acid transporter, permeas	3139123	3139782 +	219	53720226	3093955 -	COG0765E	CCGGGCGAGCCCCGCCGCCCGCTC	1	88	1	88	100 Bm_ATCC23344_chr1	3510148	2224456	2224543	98	431	7.20E-15 +
BPSL2618	D-cysteine desulfhydrase	3140625	3141644 +	339	53720228	3094122 -	COG2515E	GCCGCGCCGCGCGCGCCGTTCGTC	1	73	1	73	100 Bm_ATCC23344_chr1	3510148	2225973	2226045	95	338	1.10E-10 +

BPSL2620	MFS-family transport protein	3142218	3143453 -	411	53720230	3093939 -	NULL	CTCGCGGCGGGCCGACCGGCCGC	1	89	89	1	100 Bm_ATCC23344_chr1	3510148	2228836	2228924	100	445	1.70E-15 -
BPSL2621	hypothetical protein BPSL2621	3143540	3144160 +	206	53720231	3094773 -	NULL	TGGAATTCCACGGAAGCGGCGGCC	1	89	1	89	100 Bm_ATCC23344_chr1	3510148	2228839	2228927	100	445	1.70E-15 +
BPSL2623	glutamate-1-semialdehyde 2,1-aminomutase	3145160	3146443 +	427	53720233	3091548 hemL	COG0001H	ACCCGTCGCGCGCGTCTCCGGTAC	1	103	1	103	100 Bm_ATCC23344_chr1	3510148	2230437	2230539	99	506	3.00E-18 +
BPSL2626	bifunctional 3,4-dihydroxy-2-butanone 4-phosphate	3148701	3149837 +	378	53720236	3092773 ribB	COG0807H	TCGGCCGTCCGGCCAACGCGGCGG	1	103	1	103	100 Bm_ATCC23344_chr1	3510148	2233983	2234085	99	506	3.00E-18 +
BPSL2627	riboflavin synthase subunit beta	3149917	3150438 +	173	53720237	3091867 ribH	NULL	CGGGTTTCGCGTCATCGCCACACTG	1	82	1	82	100 Bm_ATCC23344_chr1	3510148	2235220	2235301	98	401	1.60E-13 +
BPSL2630	putative lipoprotein	3152745	3153812 -	355	53720240	3094018 -	COG0741M	TGTCTCGCTTCGAGCACCGAGCGA	1	103	103	1	100 Bm_ATCC23344_chr1	3510148	2239234	2239336	100	515	1.20E-18 -
BPSL2631	3-octaprenyl-4-hydroxybenzoate carboxy-lyase	3154437	3155996 +	519	53720241	3094696 ubiD	NULL	CGCCTGGCCGTGCGGCCGGGTAAA	1	103	1	103	100 Bm_ATCC23344_chr1	3510148	2239761	2239863	100	515	1.20E-18 +
BPSL2633	serine/threonine dehydratase	3157166	3158149 -	327	53720243	3094461 -	COG1171E	TGTCCGTGCCGCGCCGTTTTCTTTT	1	103	103	1	100 Bm_ATCC23344_chr1	3510148	2243570	2243672	99	506	3.00E-18 -
BPSL2634	hypothetical protein BPSL2634	3158311	3158604 -	97	53720244	3094201 -	NULL	TGGCTCGCGATTCCGGATTTCGCTA	1	103	103	1	100 Bm_ATCC23344_chr1	3510148	2244025	2244127	100	515	1.20E-18 -
BPSL2635	hypothetical protein BPSL2635	3159029	3160165 +	378	53720245	3094202 -	COG5495S	GCTCCGCCCCCATCATCGACGCGA	1	103	1	103	100 Bm_ATCC23344_chr1	3510148	2244352	2244455	99	505	3.30E-18 +
BPSL2636	hypothetical protein BPSL2636	3160248	3160811 +	187	53720246	3094511 -	NULL	TAAGGTGACGCCCGATGCACGCCG	1	85	1	85	100 Bm_ATCC23344_chr1	3510148	2245590	2245674	100	425	1.40E-14 +
BPSL2638	CrcB-family inner membrane protein	3161215	3161601 -	128	53720248	3094255 -	COG0239D	TGTCCGACGCCGCGCAGCCATGCG	1	103	103	1	100 Bm_ATCC23344_chr1	3510148	2247023	2247125	100	515	1.20E-18 -
BPSL2639	family S53 non-peptidase homologue	3162211	3163431 +	406	53720249	3094256 -	COG4934O	TCGGGGAATTCCCTCGGCTTGCCG	1	103	1	103	100 Bm_ATCC23344_chr1	3510148	2247535	2247637	100	515	1.20E-18 +
BPSL2640	hypothetical protein BPSL2640	3163699	3164427 -	242	53720250	3093518 -	COG0500QR	CGCCCCCGCCCGCGCCCGCATCCA	1	103	103	1	100 Bm_ATCC23344_chr1	3510148	2249849	2249951	98	497	7.60E-18 -
BPSL2641	Sir2 family protein	3164671	3165921 -	416	53720251	3093519 -	COG0846K	GAGCCGGCCTCACGACACGGCGCC	1	103	103	1	100 Bm_ATCC23344_chr1	3510148	2251331	2251433	100	515	1.20E-18 -
BPSL2642	tRNA (guanine-N(7)-)-methyltransferase	3166316	3167113 +	265	53720252	3093291 -	COG0220R	TCGTATTTCATACGCAGTAAGGGCG	1	103	1	103	100 Bm_ATCC23344_chr1	3510148	2251628	2251730	100	515	1.20E-18 +
BPSL2643	undecaprenyl pyrophosphate phosphatase	3167269	3168099 -	276	53720253	3093292 uppP	NULL	CATCGCGCCGCGCGCGGCCGGAG	1	79	79	1	100 Bm_ATCC23344_chr1	3510148	2253510	2253588	100	395	3.10E-13 -
BPSL2645	hypothetical protein BPSL2645	3168754	3169065 -	103	53720255	3094466 -	COG0727R	GATGGCACAATACGGATTCCGTGC	1	103	103	1	100 Bm_ATCC23344_chr1	3510148	2254476	2254578	100	515	1.20E-18 -
BPSL2646	coproporphyrinogen III oxidase	3169253	3170647 +	464	53720256	3091549 hemN	COG0635H	GTATTGTGCCATCCGCCGCTGCGA	1	103	1	103	100 Bm_ATCC23344_chr1	3510148	2254566	2254668	100	515	1.20E-18 +
BPSL2648	probable protein-L-isoaspartate O-methyltransferase	3171093	3171746 +	217	53720258	3093624 pcm	COG2518O	CGGATCGCAGGCGTTCTGAAGGCA	1	103	1	103	100 Bm_ATCC23344_chr1	3510148	2256406	2256508	100	515	1.20E-18 +
BPSL2650	beta alanine-pyruvate transaminase	3172177	3173505 -	442	53720260	3094317 -	COG0161H	AGGCGAATTCCCATCGATGCGGCG	1	103	103	1	100 Bm_ATCC23344_chr1	3510148	2258907	2259010	97	487	2.10E-17 -
BPSL2651	hypothetical protein BPSL2651	3173859	3175166 +	435	53720261	3094318 -	COG0683E	GCACGCGCGCCGGCCCGCATCTGG	1	103	1	103	100 Bm_ATCC23344_chr1	3510148	2259164	2259266	99	506	3.00E-18 +
BPSL2652	putative branched-chain amino acid transport systen	3175261	3176883 +	540	53720262	3092033 -	COG0559E	CGCGCGTGCGGGCGCCGCGCCAG	1	97	1	97	100 Bm_ATCC23344_chr1	3510148	2260572	2260668	100	485	2.60E-17 +
BPSL2656	UreD-family accessory protein	3179763	3180761 +	332	53720266	3092926 UreD	NULL	TCGGCGCGCAGGGCAGGCGACGC	1	103	1	102	100 Bm_ATCC23344_chr1	3510148	2265010	2265111	96	474	8.30E-17 +
BPSL2657	urease gamma subunit	3180983	3181285 +	100	53720267	3093579 ureA	NULL	GCGGGCGTCGCCGCACGCGCACGT	1	103	1	103	100 Bm_ATCC23344_chr1	3510148	2266250	2266352	100	515	1.20E-18 +
BPSL2658	urease beta subunit	3181375	3181680 +	101	53720268	3093580 ureB	NULL	AGGCGCGTCATGATTCACCGCATG	1	92	1	92	100 Bm_ATCC23344_chr1	3510148	2266653	2266744	100	460	3.50E-16 +
BPSL2659	urease alpha subunit	3181745	3183451 +	568	53720269	3092913 ureC	COG0804E	GCGCCGGCGCTTCGCCGCGCTGCG	1	67	1	67	100 Bm_ATCC23344_chr1	3510148	2267048	2267114	100	335	1.60E-10 +
BPSL2665	lipopolysaccharide heptosyltransferase-1	3187994	3188989 -	331	53720275	3093034 waaC1	COG0859M	GCATGCGTGCGTTCGCCGCTTTTTG	1	103	103	1	100 Bm_ATCC23344_chr1	3510148	2274280	2274382	100	515	1.20E-18 -
BPSL2666	phosphoglucomutase	3189170	3190564 -	464	53720276	3094110 pgm	COG1109G	GCGTTGCGTTGCCCCCTCGCCGGT	1	103	103	1	100 Bm_ATCC23344_chr1	3510148	2275855	2275958	98	496	8.40E-18 -
BPSL2667	putative lipopolysaccharide biosynthesis protein	3190822	3192081 +	419	53720277	3094876 -	COG2244R	CGATCGCGCCCATTTTAATGCCTGA	1	103	1	103	100 Bm_ATCC23344_chr1	3510148	2276016	2276118	99	506	3.00E-18 +
BPSL2675	putative glycosyl transferase	3200555	3201385 -	276	53720285	3092845 wbiF	COG1216R	TGCATTGGATGGCTGGCTGCCAGT	1	77	77	1	100 Bm_ATCC23344_chr1	3510148	2076487	2076563	98	376	2.20E-12 -
BPSL2676	putative glycosyl transferase	3201460	3203301 -	613	53720286	3092805 wbiE	COG0463M, COG1216R	TTCGTCGATGCCGGCGGATGCGTG	1	54	54	1	100 Bm_ATCC23344_chr1	3510148	2078403	2078456	100	270	1.40E-07 -
BPSL2686	dTDP-glucose 4,6-dehydratase	3212944	3214005 -	353	53720296	3091967 rmlB	COG1088M	GCGGTGTCATTCGAGCGACCAACG	1	103	103	1	100 Bm_ATCC23344_chr1	3510148	2089107	2089209	99	506	3.00E-18 -
BPSL2687	diadenosinetetraphosphatase	3214383	3215231 +	282	53720297	3092602 apaH	NULL	GAGACCGATGCCAGGCCGGATTTT	1	103	1	103	100 Bm_ATCC23344_chr1	3510148	2089387	2089489	100	515	1.20E-18 +
BPSL2689	dihydroorotase	3216245	3217522 -	425	53720299	3092915 pyrX	COG0044F	CGGGGCGCGGGCGTCGCGGGCAA	1	94	94	1	100 Bm_ATCC23344_chr1	3510148	2092624	2092717	98	461	3.20E-16 -
BPSL2690	aspartate carbamoyltransferase catalytic subunit	3217614	3218645 -	343	53720300	3093493 pyrB	COG0540F	ACGCGCGCGACGCATTCCGGCCGC	1	83	83	1	100 Bm_ATCC23344_chr1	3510148	2093747	2093829	100	415	3.80E-14 -
BPSL2693	hypothetical protein BPSL2693	3219668	3220246 -	192	53720303	3093866 -	COG1678K	GCGTGATAGAGTGAGCGTATGTCG	1	103	103	1	100 Bm_ATCC23344_chr1	3510148	2095348	2095450	100	515	1.20E-18 -
BPSL2694	hypothetical protein BPSL2694	3220452	3221783 -	443	53720304	3093855 -	NULL	CCGTCTCGCGTCGGCGGTTCGGCA	1	101	101	1	100 Bm_ATCC23344_chr1	3510148	2096885	2096985	100	505	3.30E-18 -
BPSL2695	rubredoxin	3221882	3222052 -	56	53720305	3092277 rubA	COG1773C	GCGAGCAAACGCGACTAGAGGCC	1	103	103	1	100 Bm_ATCC23344_chr1	3510148	2097154	2097256	100	515	1.20E-18 -
BPSL2696	putative kinase	3222272	3223120 +	282	53720306	3093856 -	COG0351H	GCTTCGGCCCGCCCGAATCCGCCG	1	103	1	103	100 Bm_ATCC23344_chr1	3510148	2097276	2097378	100	515	1.20E-18 +
BPSL2698	co-chaperonin GroES	3225167	3225460 -	97	53720308	3094607 groES	COG0234O	GCACTCGCTGCACGAGAGTGCTAA	1	103	103	1	100 Bm_ATCC23344_chr1	3510148	2100646	2100748	100	515	1.20E-18 -
BPSL2700	hypothetical protein BPSL2700	3229267	3229701 -	144	53720310	3091877 -	COG1733K	CTCGGCCGATGGCGGATGTGCGAA	1	103	103	1	100 Bm_ATCC23344_chr1	3510148	2103927	2104029	99	506	3.00E-18 -
BPSL2701	probable alcohol dehydrogenase	3229950	3230957 +	335	53720311	3094446 -	COG0604CR	CGGCACACGATACGCGCGCCGATC	1	103	1	103	100 Bm_ATCC23344_chr1	3510148	2104078	2104180	100	515	1.20E-18 +
BPSL2703	hypothetical protein BPSL2703	3232494	3233009 -	171	53720313	3094189 -	NULL	CGCCGGCGCCCGGGCTTCCTGTGA	1	103	103	1	100 Bm_ATCC23344_chr1	3510148	2107181	2107283	100	515	1.20E-18 -
BPSL2705	putative lipoprotein	3234027	3235280 -	417	53720315	3093984 -	NULL	CGCATGACTTCGCGGCGGCGCCGG	1	62	62	1	100 Bm_ATCC23344_chr1	3510148	2109452	2109513	100	310	2.10E-09 -
BPSL2706	putative lipoprotein	3235340	3236938 -	532	53720316	3093985 -	NULL	CGAATTACCGCGTCGCCACCGGCG	1	103	103	1	100 Bm_ATCC23344_chr1	3510148	2111110	2111212	99	506	3.00E-18 -
BPSL2707	hypothetical protein BPSL2707	3237675	3238121 -	148	53720317	3091690 -	COG1733K	CGGCGGGCGCCGATCGGCGCCGC	1	103	103	1	100 Bm_ATCC23344_chr1	3510148	2112294	2112396	99	506	3.00E-18 -
BPSL2708	putative exported Metallo-beta-lactamase-family prc	3238401	3239633 +	410	53720318	3091691 -	COG0491R	TGATACGCCCGGAAACCGTTTCGA	1	103	1	103	100 Bm_ATCC23344_chr1	3510148	2112477	2112579	100	515	1.20E-18 +
BPSL2711	LysE-family membrane transport protein	3241273	3241896 +	207	53720321	3093194 -	COG1280E	CCGCCGGCGTCGACGCATCACCGC	1	103	1	103	100 Bm_ATCC23344_chr1	3510148	2115352	2115454	100	515	1.20E-18 +
BPSL2713	putative membrane transport protein	3243615	3245057 +	480	53720323	3094407 -	NULL	CCGCGGCCCGCACGCGAGCGAAC	1	103	1	103	100 Bm_ATCC23344_chr1	3510148	2117715	2117817	99	506	3.00E-18 +
BPSL2714	hypothetical protein BPSL2714	3245554	3246342 +	262	53720324	3094408 -	NULL	GCTTTTTACGCTTTCCCGCATCGAC	1	103	1	103	100 Bm_ATCC23344_chr1	3510148	2119654	2119756	100	515	1.20E-18 +
BPSL2715	putative lipoprotein	3246675	3248522 -	615	53720325	3092215 -	NULL	GGAGTGCGCGTCGCATAACGGTCG	1	103	103	1	100 Bm_ATCC23344_chr1	3510148	2122720	2122822	100	515	1.20E-18 -
BPSL2716	probable exported hydrolase	3249128	3251227 -	699	53720326	3092216 -	COG1073R	CAGCGACGCCGCCCGCCGCGCGC	1	103	103	1	100 Bm_ATCC23344_chr1	3510148	2125425	2125527	100	515	1.20E-18 -
BPSL2717	putative lipoprotein	3251347	3251715 -	122	53720327	3093869 -	NULL	CGGCACGGCGCGGCGCGACGACG	1	103	103	6	95 Bm_ATCC23344_chr1	3510148	2125913	2126010	100	490	1.60E-17 -
BPSL2719	putative isomerase	3253725	3254363 +	212	53720329	3094046 -	COG3917Q	GCCGAAAATCCGTCGCGGCGGCTG	1	100	1	100	100 Bm_ATCC23344_chr1	3510148	2127821	2127920	100	500	5.50E-18 +
BPSL2720	LysR-family regultor	3254437	3255384 +	315	53720330	3094047 -	COG0583K	TGCCGCCCGCCGCGCCGCATCGCG	1	76	1	76	100 Bm_ATCC23344_chr1	3510148	2128557	2128632	100	380	1.50E-12 +
BPSL2724	putative TonB-dependent outer-membrane receptor	3258434	3260704 -	756	53720334	3094843 -	COG1629P	GAGCGAAGCGGGCGGGAAACGTT	1	103	103	1	100 Bm_ATCC23344_chr1	3510148	2134900	2135002	100	515	1.20E-18 -
BPSL2726	putative solute-binding periplasmic protein	3261786	3262568 -	260	53720336	3092262 -	COG0725P	AGTGAACCGATCAGTACAAACCTG	1	103	103	1	100 Bm_ATCC23344_chr1	3510148	2136762	2136864	99	506	3.00E-18 -
BPSL2728	xanthine dehydrogenase	3265344	3266861 -	505	53720338	3093191 xdhA	COG4630F	AACATGACGACGACGGCCGCGGCG	1	103	103	1	100 Bm_ATCC23344_chr1	3510148	2141054	2141156	98	497	7.60E-18 -
BPSL2729	hypothetical protein BPSL2729	3267141	3268364 +	407	53720339	3092273 -	NULL	TTGCGTGGTGTTTAACTAAGCGGTA	1	103	1	103	100 Bm_ATCC23344_chr1	3510148	2141236	2141338	100	515	1.20E-18 +
BPSL2732	putative aminotransferase	3270894	3272108 -	404	53720342	3091650 -	COG0075E	CCCGACGCGTATGTCGATCAAAAC	1	103	103	1	100 Bm_ATCC23344_chr1	3510148	2146228	2146330	100	515	1.20E-18 -
BPSL2735	putative oxidoreductase	3275577	3276236 +	219	53720345	3092161 -	COG0778C	CCGCGCCGATACGAATCGGCAGCA	1	103	1	103	100 Bm_ATCC23344_chr1	3510148	2149487	2149589	100	515	1.20E-18 +
BPSL2736	MFS-family membrane transport protein	3276352	3277521 +	389	53720346	3092770 -	COG2814G	AAGGGTTTTCCCTGAGATAGAATCG	1	103	1	103	100 Bm_ATCC23344_chr1	3510148	2150262	2150364	99	506	3.00E-18 +
BPSL2737	MFS-family membrane transport protein	3277789	3278985 +	398	53720347	3092771 -	NULL	GCAGCGCGGCGCGTATCGCTCGCC	1	103	1	103	100 Bm_ATCC23344_chr1	3510148	2151699	2151801	98	497	7.60E-18 +
BPSL2743	putative oxidoreductase	3285381	3286337 -	318	53720353	3093209 -	COG0491R	ACTATTCGTAAATCGTGACGTTGAC	1	103	103	1	100 Bm_ATCC23344_chr1	3510148	2160324	2160426	100	515	1.20E-18 -
BPSL2744	hypothetical protein BPSL2744	3286594	3287400 +	268	53720354	3093793 -	NULL	ATATCGATATGAGCAACTCACGCA	1	103	1	103	100 Bm_ATCC23344_chr1	3510148	2160483	2160585	100	515	1.20E-18 +
BPSL2745	hypothetical protein BPSL2745	3287868	3288323 -	151	53720355	3093794 -	COG3791S	TTTCGACGCCCCGCATGCAGGCGC	1	103	103	1	100 Bm_ATCC23344_chr1	3510148	2162202	2162304	99	506	3.00E-18 -
BPSL2748	putative oxidoreductase	3291026	3291664 -	212	53720358	3091891 -	COG0450O	TAATCGTTCGCGCCGGCCGCAGGA	1	103	103	1	100 Bm_ATCC23344_chr1	3510148	2165599	2165701	100	515	1.20E-18 -
BPSL2749	hypothetical protein BPSL2749	3291992	3292294 +	100	53720359	3091892 -	COG4575S	TCTTGCGCCACCTGCGCGCCGTGC	1	103	1	103	100 Bm_ATCC23344_chr1	3510148	2165829	2165931	100	515	1.20E-18 +
BPSL2750	hypothetical protein BPSL2750	3292418	3292807 +	129	53720360	3094765 -	COG5393S	AGCCCCCGGCTCGCCGCCGCGTGA	1	103	1	103	100 Bm_ATCC23344_chr1	3510148	2166255	2166357	100	515	1.20E-18 +
BPSL2756	hypothetical protein BPSL2756	3295677	3296237 -	186	53720366	3094396 -	NULL	TGTCGTGTGCCCGGTCGCGGCGCG	1	103	103	1	100 Bm_ATCC23344_chr1	3510148	2170172	2170274	100	515	1.20E-18 -
BPSL2758	serine hydroxymethyltransferase	3296909	3298156 -	415	53720368	3094757 glyA	COG0112E	GTTCCTCGGAATCCCTCCGCCACGT	1	103	103	1	100 Bm_ATCC23344_chr1	3510148	2172091	2172193	100	515	1.20E-18 -
BPSL2759	putative short-chain dehydrogenase	3298608	3299354 +	248	53720369	3091464 -	COG4221R	ACATGCCCGCCAGCGCGCGCCGCC	1	103	1	103	100 Bm_ATCC23344_chr1	3510148	2172445	2172547	100	515	1.20E-18 +

BPSL2760	putative thioesterase	3299451	3299945 +	164	53720370	3091465 -	COG0824R	GCGCGCCGCCGGGGCGCGCACGC	1	99	1	99	100 Bm_ATCC23344_chr1	3510148	2173292	2173390	100	495	9.30E-18 +
BPSL2761	putative TolQ transport transmembrane protein	3300011	3300799 +	262	53720371	3093135 tolQ	COG0811U	ACAAAGCCGATGAACTCGACCCCA	1	68	1	68	100 Bm_ATCC23344_chr1	3510148	2173883	2173950	100	340	9.30E-11 +
BPSL2764	translocation protein TolB precursor	3302378	3303679 +	433	53720374	3093134 tolB	COG0823U	GCGCATTTGCGCTTACACTCCCGCC	1	99	1	99	100 Bm_ATCC23344_chr1	3510148	2176219	2176317	100	495	9.30E-18 +
BPSL2787	putative acyl-CoA transferase	3327179	3328498 -	439	53720396	3091459 wcbT	COG0156H	AACCGCGCTTTTTCAAAATGCACGG	1	67	67	1	100 Bm_ATCC23344_chr1	3510148	2371964	2372030	100	335	1.60E-10 -
BPSL2792	putative capsule polysaccharide biosynthesis/export	3339439	3340641 -	400	53720401	3092365 wcbO	NULL	TGAAACGGCGTGGGTGACGGGCTG	1	103	103	1	100 Bm_ATCC23344_chr1	3510148	2384107	2384209	100	515	1.20E-18 -
BPSL2806	putative capsule polysaccharide export ABC transpor	3353701	3354849 -	382	53720415	3094162 wcbD	COG3524M	CATTTCCGTCGACGAATTGCTTCCC	1	103	103	1	100 Bm_ATCC23344_chr1	3510148	2398315	2398417	100	515	1.20E-18 -
BPSL2807	putative capsular polysaccharide biosynthesis/export	3355330	3356493 -	387	53720416	3093048 wcbC	NULL	CACCGCGTTAGGCTTGCTTTGGTAA	1	103	103	1	100 Bm_ATCC23344_chr1	3510148	2399959	2400061	100	515	1.20E-18 -
BPSL2808	putative capsular polysaccharide glycosyltransferase	3356674	3357825 +	383	53720417	3093047 wcbB	COG0438M	AAAGCAAGCCTAACGCGGTGTGAA	1	103	1	103	100 Bm_ATCC23344_chr1	3510148	2400042	2400144	100	515	1.20E-18 +
BPSL2810	putative GDP-mannose pyrophosphorylase	3360240	3361667 +	475	53720419	3093126 manC	COG0662G, COG0836M	CCCCTTGGCCAGCGGTGCGACCGC	1	103	1	103	100 Bm_ATCC23344_chr1	3510148	2403607	2403709	99	506	3.00E-18 +
BPSL2811	hypothetical protein BPSL2811	3361929	3362774 +	281	53720420	3093359 -	COG0121R	ATGGCTCGCGCGGCAAAAATCGGC	1	103	1	103	100 Bm_ATCC23344_chr1	3510148	2405296	2405398	97	488	1.90E-17 +
BPSL2812	hypothetical protein BPSL2812	3362840	3363712 +	290	53720421	3093715 -	COG0668M	CGCGCGGCGCGCATGGCGAAGGTT	1	68	1	68	100 Bm_ATCC23344_chr1	3510148	2406242	2406309	100	340	9.30E-11 +
BPSL2813	putative DedA family transmembrane protein	3364033	3364776 -	247	53720422	3093716 -	COG0586S	GCGTGGCGCGGCGCGTGCCGCGTT	1	103	103	1	100 Bm_ATCC23344_chr1	3510148	2408241	2408343	100	515	1.20E-18 -
BPSL2818	phosphoribosylaminoimidazole synthetase	3369943	3370998 -	351	53720427	3092877 purM	COG0150F	CGGGGCAAGTTGCTTTCCCTGAAGT	1	103	103	1	100 Bm_ATCC23344_chr1	3510148	2413150	2413252	100	515	1.20E-18 -
BPSL2819	hypothetical protein BPSL2819	3371297	3372022 +	241	53720428	3093778 -	COG0593L	CGCCCGCCCGCGTTGCGCCCGGCC	1	103	1	103	100 Bm_ATCC23344_chr1	3510148	2413351	2413453	100	515	1.20E-18 +
BPSL2824	3-methyl-2-oxobutanoate hydroxymethyltransferase	3375583	3376398 +	271	53720433	3093227 panB	COG0413H	CGAACGCGCGCGGCGTCGCCGATC	1	67	1	67	100 Bm_ATCC23344_chr1	3510148	2417673	2417739	100	335	1.60E-10 +
BPSL2826	putative DnaJ chaperone protein	3378622	3379752 -	376	53720435	3093637 dnaJ	COG0484O	CTCGTGCGGGCCGGCGCACGCGGC	1	103	103	1	100 Bm_ATCC23344_chr1	3510148	2421896	2421998	100	515	1.20E-18 -
BPSL2827	molecular chaperone DnaK	3380019	3381971 -	650	53720436	3093638 dnaK	COG0443O	GGCCGCACTTATTTCGAGTGCAGA	1	103	103	1	100 Bm_ATCC23344_chr1	3510148	2424115	2424217	100	515	1.20E-18 -
BPSL2829	putative heat shock protein	3382564	3383121 -	185	53720437	3093283 -	COG0576O	CCATTTGCACCGATATTGTGCGGCG	1	103	103	1	100 Bm_ATCC23344_chr1	3510148	2425265	2425367	100	515	1.20E-18 -
BPSL2831	ferrochelatase	3384031	3385134 -	367	53720439	3093284 -	NULL	TTCCGCTCCGGCTACGTCATCGCCA	1	103	103	1	100 Bm_ATCC23344_chr1	3510148	2427278	2427380	100	515	1.20E-18 -
BPSL2832	heat-inducible transcription repressor	3385307	3386329 -	340	53720440	3092424 -	COG1420K	AACCGCCCGCCGGCGGCCGGCCG	1	103	103	1	100 Bm_ATCC23344_chr1	3510148	2428473	2428575	100	515	1.20E-18 -
BPSL2833	inorganic polyphosphate/ATP-NAD kinase	3386463	3387365 +	300	53720441	3092425 ppnK	COG0061G	GCGAGCGCCGCTTGCGCGCCTCGC	1	103	1	103	100 Bm_ATCC23344_chr1	3510148	2428509	2428611	100	515	1.20E-18 +
BPSL2836	hypothetical protein BPSL2836	3392387	3396580 +	1397	53720444	3094554 -	COG3164S	CGGCCCGCACGGACGGCGTCGCG	1	103	1	103	100 Bm_ATCC23344_chr1	3510148	2434433	2434535	100	515	1.20E-18 +
BPSL2837	putative carbon-nitrogen hydrolase protein	3396632	3397459 +	275	53720445	3091839 -	COG0388R	AGCGGCCGCCGGGCCCGCACCCTT	1	54	1	54	100 Bm_ATCC23344_chr1	3510148	2438727	2438780	100	270	1.40E-07 +
BPSL2838	putative DNA gyrase control protein	3397539	3399035 +	498	53720446	3092359 tldD	COG0312R	GCGGCGATCCGCCCGACCGGAAA	1	82	1	82	100 Bm_ATCC23344_chr1	3510148	2439606	2439687	100	410	6.40E-14 +
BPSL2839	3-deoxy-7-phosphoheptulonate synthase	3399445	3400518 +	357	53720447	3092395 aroG	COG0722E	CGGGCCGATCCACCGCTTTACCGA	1	103	1	103	100 Bm_ATCC23344_chr1	3510148	2441491	2441593	99	506	3.00E-18 +
BPSL2840	flavohemoprotein	3400797	3402005 -	402	53720448	3092234 hmpA	COG1017C, COG1018C	GCAATCGCGGGCCCGTTATGGCGA	1	103	1	103	100 Bm_ATCC23344_chr1	3510148	2523652	2523754	100	515	1.20E-18 +
BPSL2841	hypothetical protein BPSL2841	3402117	3402689 -	190	53720449	3091840 -	COG2096S	ACGCTCGAATGGATGCGCGCCGCC	1	103	1	103	100 Bm_ATCC23344_chr1	3510148	2522968	2523070	100	515	1.20E-18 +
BPSL2842	putative FAD-binding oxidase	3402853	3404262 +	469	53720450	3094160 -	COG0277C	CAGCCGCGTTCGGGGCGGCGCGCA	1	103	103	1	100 Bm_ATCC23344_chr1	3510148	2522902	2523004	100	515	1.20E-18 -
BPSL2843	putative glycolate oxidase subunit GlcD	3404327	3405820 +	497	53720451	3093324 glcD	COG0277C	AAACGACGGCGCCCGCGACGAGC	1	67	67	1	100 Bm_ATCC23344_chr1	3510148	2521428	2521494	100	335	1.60E-10 -
BPSL2844	putative glycolate oxidase subunit GlcE	3405922	3407010 +	362	53720452	3092816 glcE	COG0277C	GCCGCGCGCGGGCCCCGAGCGGA	1	103	103	1	100 Bm_ATCC23344_chr1	3510148	2519833	2519935	99	506	3.00E-18 -
BPSL2846	hypothetical protein BPSL2846	3408489	3409187 +	232	53720454	3094161 -	COG0325R	CGGTTGTCCGTATAATTCCCGCATC	1	103	103	1	100 Bm_ATCC23344_chr1	3510148	2517293	2517395	100	515	1.20E-18 -
BPSL2850	putative phosphonates transport ATP-binding protei	3412347	3413318 -	323	53720458	3091665 phnC	COG3638P	CTCGCCCGTTCGCGGCGCGGCCTT	1	103	1	103	100 Bm_ATCC23344_chr1	3510148	2512351	2512453	99	506	3.00E-18 +
BPSL2857	putative phosphonate metabolism PhnG protein	3419033	3419581 -	182	53720465	3093458 phnG	COG3624P	AATTGCCGTATGCGTCGACGGATC	1	103	1	103	100 Bm_ATCC23344_chr1	3510148	2506085	2506187	98	497	7.60E-18 +
BPSL2858	putative GntR family transcriptional regulator	3419714	3420532 +	272	53720466	3093457 phnF	COG2188K	ACCACCCAGTTGTCTAAACGTCTAG	1	103	103	1	100 Bm_ATCC23344_chr1	3510148	2506050	2506152	98	497	7.60E-18 -
BPSL2861	4-hydroxybenzoate octaprenyltransferase	3422301	3423251 -	316	53720469	3094205 ubiA	COG0382H	CGCGGCGGCGCCGCACGCGCCTC	1	103	1	103	100 Bm_ATCC23344_chr1	3510148	2502446	2502548	99	506	3.00E-18 +
BPSL2863	putative ferritin DPS-family DNA binding protein	3424448	3424936 -	162	53720471	3093452 -	COG0783P	GCTGCCCATGCGCATGTCGCCTGC	1	103	1	103	100 Bm_ATCC23344_chr1	3510148	2500798	2500900	100	515	1.20E-18 +
BPSL2864	hypothetical protein BPSL2864	3425136	3425309 -	57	53720472	3092019 -	NULL	TCGCGCCGCCGCGCCGCCGGAGC	1	101	1	101	100 Bm_ATCC23344_chr1	3510148	2500428	2500527	99	490	1.60E-17 +
BPSL2865	catalase-peroxidase protein	3425408	3427654 -	748	53720473	3092072 katG	COG0376P	AGCGCCGCCGCGTGCCGGGCCGCT	1	103	1	103	100 Bm_ATCC23344_chr1	3510148	2498081	2498183	99	506	3.00E-18 +
BPSL2866	oxidative stress regulatory protein	3428080	3429039 -	319	53720474	3093036 oxyR	COG0583K	GCGCGCTTCGGACAGGCTTGTTTTC	1	103	1	103	100 Bm_ATCC23344_chr1	3510148	2496696	2496798	100	515	1.20E-18 +
BPSL2868	S-adenosylmethionine:tRNA ribosyltransferase-isome	3432038	3433120 +	360	53720476	3092055 queA	NULL	GAGCGACGCCGGCCAAGCCGCGC	1	103	103	1	100 Bm_ATCC23344_chr1	3510148	2493795	2493897	100	515	1.20E-18 -
BPSL2869	queuine tRNA-ribosyltransferase	3433421	3434614 +	397	53720477	3092225 tgt	COG0343J	CGCGAGCCGCGCTTCACTTTTTTCC	1	103	103	1	100 Bm_ATCC23344_chr1	3510148	2492452	2492554	100	515	1.20E-18 -
BPSL2870	preprotein translocase subunit YajC	3435044	3435370 +	108	53720478	3092020 yajC	COG1862U	AACAGCTTGAAACCAAAGCGCATT	1	103	103	1	100 Bm_ATCC23344_chr1	3510148	2490874	2490976	100	515	1.20E-18 -
BPSL2871	protein export protein SecD	3435478	3437502 +	674	53720479	3091980 secD	COG0342U	GGCGCGTCCGGCCGCCCGACGCCC	1	103	103	1	100 Bm_ATCC23344_chr1	3510148	2490440	2490542	100	515	1.20E-18 -
BPSL2873	hypothetical protein BPSL2873	3438714	3439337 -	207	53720481	3092293 -	NULL	GCGTTTTCGCGAGGCCGCCGCGTG	1	103	1	103	100 Bm_ATCC23344_chr1	3510148	2486490	2486592	99	506	3.00E-18 +
BPSL2874	hypothetical protein BPSL2874	3439438	3440016 -	192	53720482	3092294 -	NULL	CGCGCGCGGCGGTTCGCACCACCC	1	69	1	69	100 Bm_ATCC23344_chr1	3510148	2485845	2485913	100	345	5.50E-11 +
BPSL2876	putative cytochrome b-561 membrane protein	3440655	3441218 -	187	53720484	3094292 -	COG3038C	CTGCCATTCGTCGGCGGTTGCGGT	1	103	1	103	100 Bm_ATCC23344_chr1	3510148	2484609	2484711	100	515	1.20E-18 +
BPSL2877	putative paraquat-inducible protein	3441483	3442139 +	218	53720485	3092120 -	COG2995S	CGCCGACGCTTGACACCTCGAAAA	1	103	103	1	100 Bm_ATCC23344_chr1	3510148	2484442	2484544	99	506	3.00E-18 -
BPSL2882	putative RNA-methylase protein	3448434	3449726 +	430	53720490	3092339 -	COG0116L	CAAGACGTGGCGGCACGGCGGCCT	1	103	103	1	100 Bm_ATCC23344_chr1	3510148	2477649	2477751	100	515	1.20E-18 -
BPSL2883	hypothetical protein BPSL2883	3451155	3451565 +	136	53720491	3092340 -	COG3324R	CGTCATGCTGCTGACAATACGTTGT	1	103	103	1	100 Bm_ATCC23344_chr1	3510148	2475034	2475136	99	506	3.00E-18 -
BPSL2887	NAD(P) transhydrogenase subunit alpha	3455033	3456172 -	379	53720495	3093541 pntAA	COG3288C	TCGGTTAAAGTGGCGCCTGAAACA	1	103	1	103	100 Bm_ATCC23344_chr1	3510148	2470044	2470146	100	515	1.20E-18 +
BPSL2888	putative hydrolase	3456416	3456925 +	169	53720496	3092417 -	COG0494LR	GCGGGTGCGCGCCGGCCACACGG	1	103	103	1	100 Bm_ATCC23344_chr1	3510148	2469898	2470000	100	515	1.20E-18 -
BPSL2890	putative glutamate synthase	3458195	3459835 +	546	53720498	3092460 -	COG0069E	CGGCACGCGACGCACGCCGCGCC	1	Not found									
BPSL2892	aminopeptidase P	3461269	3462678 +	469	53720500	3093835 -	COG0006E	AATCGCGGCGCGCGCCGCATCGCA	1	Not found									
BPSL2894	putative nitrogen regulation-related protein	3464124	3465188 +	354	53720502	3094392 -	COG0042J	GACAGTTGCCTAATTCGGCGGCAC	1	103	103	1	100 Bm_ATCC23344_chr1	3510148	2460788	2460890	100	515	1.20E-18 -
BPSL2897	Holliday junction resolvase	3467395	3467937 +	180	53720505	3092930 ruvC	COG0817L	GCCGCCGCGCCCGATGCGCCCGGC	1	103	103	1	100 Bm_ATCC23344_chr1	3510148	2457517	2457619	99	506	3.00E-18 -
BPSL2898	Holliday junction DNA helicase motor protein	3468011	3468592 +	193	53720506	3092278 ruvA	COG0632L	CGCGCGACGCACGCCGACGCGCG	1	76	76	1	100 Bm_ATCC23344_chr1	3510148	2456901	2456976	100	380	1.50E-12 -
BPSL2899	Holliday junction DNA helicase RuvB	3468774	3469844 +	356	53720507	3093138 ruvB	COG2255L	ACGGCGCGTCGTCGAAAACAGCTT	1	103	103	1	100 Bm_ATCC23344_chr1	3510148	2456152	2456254	100	515	1.20E-18 -
BPSL2901	putative lipoprotein	3470958	3472100 +	380	53720509	3093110 -	COG4318S	TCACATCGATTCGTGATGCTGACGG	1	103	103	1	100 Bm_ATCC23344_chr1	3510148	2453968	2454070	99	506	3.00E-18 -
BPSL2903	D-tyrosyl-tRNA deacylase	3472953	3473432 -	159	53720511	3094756 dtd	NULL	GCGCGCGCATCGCCGCTTGGCCGG	1	73	1	73	100 Bm_ATCC23344_chr1	3510148	2451426	2451498	100	365	6.90E-12 +
BPSL2904	tyrosyl-tRNA synthetase	3473503	3474744 -	413	53720512	3091718 tyrZ	COG0162J	TTATCCGCGCAAATCCGCGCGAAC	1	103	1	103	100 Bm_ATCC23344_chr1	3510148	2450084	2450186	100	515	1.20E-18 +
BPSL2905	hypothetical protein BPSL2905	3474875	3476086 +	403	53720513	3092869 -	COG2377O	AGCGATTTTCCCATAAGTTGCACGC	1	103	103	1	100 Bm_ATCC23344_chr1	3510148	2450051	2450153	100	515	1.20E-18 -
BPSL2907	putative glutathione S-transferase like protein	3476900	3477604 -	234	53720515	3091916 -	COG0625O	GCAATTGAGATTTATCGACGCATAT	1	95	1	95	100 Bm_ATCC23344_chr1	3510148	2447232	2447326	100	475	7.40E-17 +
BPSL2908	putative AraC_family transcriptional regulator	3477697	3478599 +	300	53720516	3091917 -	NULL	CGATTGGGCCCTCCTCGTCATGCTG	1	95	95	1	100 Bm_ATCC23344_chr1	3510148	2447229	2447323	100	475	7.40E-17 -
BPSL2909	putative HesB-like protein	3478769	3479137 -	122	53720517	3093015 -	COG0316S	ATGCGCGAAAAACGGCGGCGCGC	1	103	1	103	100 Bm_ATCC23344_chr1	3510148	2445693	2445795	100	515	1.20E-18 +
BPSL2913	hypothetical protein BPSL2913	3481935	3482777 -	280	53720521	3094601 -	NULL	GGGCGATCCGCGCGGCGCTTGCGG	1	103	103	1	100 Bm_ATCC23344_chr1	3510148	2528925	2529027	100	515	1.20E-18 -
BPSL2914	dihydroorotase	3483172	3484230 -	352	53720522	3093494 pyrC	COG0418F	GCGAGCGGTTCCGTGTTGCGGTTTG	1	103	103	1	100 Bm_ATCC23344_chr1	3510148	2530333	2530435	100	515	1.20E-18 -
BPSL2915	family C44 non-peptidase homologue	3484440	3485354 +	304	53720523	3094602 -	COG0121R	TCGCCTTTTGCCGCTTAGGGCTCGG	1	103	1	103	100 Bm_ATCC23344_chr1	3510148	2530445	2530547	100	515	1.20E-18 +
BPSL2917	putative heat shock Hsp20-related protein	3486496	3486915 -	139	53720525	3094752 -	COG0071O	CGGCGGCGCGCGGTGCCATATGCA	1	68	68	1	100 Bm_ATCC23344_chr1	3510148	2533047	2533114	100	340	9.30E-11 -
BPSL2918	putative heat shock Hsp20-related protein	3486981	3487421 -	146	53720526	3092837 -	COG0071O	GTTTCGATTTGTGTGTCGGCGGGCT	1	103	103	1	100 Bm_ATCC23344_chr1	3510148	2533553	2533655	100	515	1.20E-18 -
BPSL2919	10 kDa chaperonin	3487884	3488201 +	105	53720527	3094581 groES2	COG0234O	CGGCTCCGCTCGCATCCCGCCGCT	1	103	1	103	100 Bm_ATCC23344_chr1	3510148	2533918	2534020	100	515	1.20E-18 +
BPSL2923	glutamate/aspartate transport system permease prot	3490259	3490999 -	246	53720531	3093707 gltJ	COG0765E	GCGGACGGCCGTTCGAGCTAACAA	1	85	85	1	100 Bm_ATCC23344_chr1	3510148	2537131	2537215	100	425	1.40E-14 -
BPSL2924	glutamate/aspartate periplasmic binding protein pre	3491082	3491984 -	300	53720532	3092902 gltI	COG0834ET	GCATGTCGAAATGCGTATGCCGCC	1	103	103	1	100 Bm_ATCC23344_chr1	3510148	2539431	2539533	100	515	1.20E-18 -
BPSL2925	putative glutamate dehydrogenase	3492208	3493512 -	434	53720533	3094709 -	COG0334E	CCGATCATGCACGAAACGCATAAT	1	103	103	1	100 Bm_ATCC23344_chr1	3510148	2540959	2541061	100	515	1.20E-18 -

BPSL2926	putative LysR-family transcriptional regulator	3493893	3494843 -	316	53720534	3094710 -	COG0583K	TTCGCGCGCGCCGCACGTCGATGC	1	103	103	1	100	Bm_ATCC23344_chr1	3510148	2542290	2542392	98	497	7.60E-18 -
BPSL2927	putative nitrate transporter	3495261	3496718 +	485	53720535	3092269 -	COG2223P	GCCCGGCGGGCATTCACGCCGTTC	1	103	1	103	100	Bm_ATCC23344_chr1	3510148	2542610	2542712	100	515	1.20E-18 +
BPSL2930	putative gluconate permease	3498867	3500228 -	453	53720538	3092428 -	COG2610GE	AGCGTCCCGACGGGCGGCGAACC	1	103	103	1	100	Bm_ATCC23344_chr1	3510148	2547645	2547747	100	515	1.20E-18 -
BPSL2932	phosphogluconate dehydratase	3501096	3502949 -	617	53720540	3093431 edd	NULL	GTAGTTTTTCTACTAAACTCGCGCC	1	103	103	1	100	Bm_ATCC23344_chr1	3510148	2550366	2550468	100	515	1.20E-18 -
BPSL2933	putative regulatory protein	3503247	3504245 +	332	53720541	3092429 -	COG1737K	GATCGATATCGGGAAATTCGCTAG	1	103	1	103	100	Bm_ATCC23344_chr1	3510148	2550566	2550668	99	506	3.00E-18 +
BPSL2938	leucyl-tRNA synthetase	3508297	3510891 -	864	53720546	3094550 leuS	COG0495J	CCCCGGCCGCCGGGGAAGCGCGA	1	103	103	1	100	Bm_ATCC23344_chr1	3510148	2558279	2558381	99	506	3.00E-18 -
BPSL2942	putative outer membrane protein	3513232	3514056 -	274	53720550	3093811 omlA	COG2913J	AGAGTACAATGCAGGGCTTCAATA	1	103	103	1	100	Bm_ATCC23344_chr1	3510148	2561468	2561570	100	515	1.20E-18 -
BPSL2943	ferric uptake regulator	3514171	3514599 +	142	53720551	3091814 fur	COG0735P	TGGCCCTTGTCCAAGGCCGCGCGA	1	103	1	103	100	Bm_ATCC23344_chr1	3510148	2561485	2561587	100	515	1.20E-18 +
BPSL2945	putative allantoicase	3515274	3516284 -	336	53720553	3094036 -	NULL	AGCTTCGCGCCGGCGCTTCGTTTTT	1	103	103	1	100	Bm_ATCC23344_chr1	3510148	2563692	2563794	100	515	1.20E-18 -
BPSL2946	C4-dicarboxylate transport protein	3516422	3517744 -	440	53720554	3094344 dctA	COG1301C	CTTGACTCGACTATATCGGCTTCCT	1	103	103	1	100	Bm_ATCC23344_chr1	3510148	2565152	2565254	98	497	7.60E-18 -
BPSL2947	putative GntR-family regulatory protein	3517990	3518682 +	230	53720555	3094037 -	COG2186K	CGGGCCCGGCGCACGCCGGATCAT	1	103	1	103	100	Bm_ATCC23344_chr1	3510148	2565300	2565402	98	497	7.60E-18 +
BPSL2948	cyn operon transcriptional activator (LysR-family)	3519305	3520240 -	311	53720556	3092007 cynR	COG0583K	TCGATGGCGAGTGCCGATGACCGA	1	103	103	1	100	Bm_ATCC23344_chr1	3510148	2567659	2567761	99	506	3.00E-18 -
BPSL2949	carbonic anhydrase	3520355	3521014 +	219	53720557	3092530 cynT	NULL	GCGTTGCCTATGCGAACAATAATA	1	103	1	103	100	Bm_ATCC23344_chr1	3510148	2567676	2567778	99	506	3.00E-18 +
BPSL2950	cyanate hydratase	3521101	3521571 +	156	53720558	3092008 cynS	COG1513P	CGCATGACACCACGCGCGCCGCCC	1	89	1	89	100	Bm_ATCC23344_chr1	3510148	2568436	2568524	98	436	4.30E-15 +
BPSL2951	hypothetical protein BPSL2951	3521744	3522943 +	399	53720559	3092833 -	NULL	TTTCGGCATTCCGGCGCTTCCGGTA	1	103	1	103	100	Bm_ATCC23344_chr1	3510148	2569072	2569175	96	471	1.10E-16 +
BPSL2952	glyceraldehyde 3-phosphate dehydrogenase 1	3523351	3524361 -	336	53720560	3092714 gapA	COG0057G	CCCATTCGACGCCCGGGCGGCCGT	1	93	93	1	100	Bm_ATCC23344_chr1	3510148	2571789	2571881	100	465	2.10E-16 -
BPSL2953	transketolase	3524452	3526479 -	675	53720561	3094803 tktA	COG0021G	CGCCTCCCGCCGGCGCCGCACCGC	1	103	103	1	100	Bm_ATCC23344_chr1	3510148	2573907	2574009	100	515	1.20E-18 -
BPSL2954	spermidine synthase	3526742	3527599 +	285	53720562	3092834 speE	COG0421E	GGCCTTGTCGGAATCCGCCCGCGC	1	103	1	103	100	Bm_ATCC23344_chr1	3510148	2574072	2574174	99	506	3.00E-18 +
BPSL2957	hypothetical protein BPSL2957	3529221	3529784 -	187	53720565	3094052 -	NULL	CAACCTGTTCCAGCGCGTCATGCA	1	103	103	1	100	Bm_ATCC23344_chr1	3510148	2577047	2577149	100	515	1.20E-18 -
BPSL2958	putative exported ribonuclease	3529801	3530232 -	143	53720566	3094053 -	COG4290F	TTTCATTTTCCTAACGAGCATTGATT	1	103	103	1	100	Bm_ATCC23344_chr1	3510148	2577660	2577762	100	515	1.20E-18 -
BPSL2959	phosphate acetyltransferase	3530561	3532870 -	769	53720567	3093937 maeB	COG0280C, COG0281C	GGTCGCGGCTGTCAAACCCTAATC	1	103	103	1	100	Bm_ATCC23344_chr1	3510148	2580297	2580399	100	515	1.20E-18 -
BPSL2960	thiamine monophosphate kinase	3533072	3534073 +	333	53720568	3093144 -	COG0611H	AAACGTCACGAATCGGCCGCGCGC	1	103	1	103	100	Bm_ATCC23344_chr1	3510148	2580401	2580503	100	515	1.20E-18 +
BPSL2961	putative phosphatidylglycerophosphatase	3534207	3534680 +	157	53720569	3093145 -	COG1267I	CGCCAGCCAGCCCGGCGCCACGCC	1	103	1	103	100	Bm_ATCC23344_chr1	3510148	2581536	2581638	100	515	1.20E-18 +
BPSL2963	orotidine 5'-phosphate decarboxylase	3535507	3536334 -	275	53720571	3093383 pyrF	COG0284F	CGGCGCGGCCGGCGCCGGCCGTG	1	99	99	1	100	Bm_ATCC23344_chr1	3510148	2583761	2583859	97	477	6.10E-17 -
BPSL2964	putative epimerase	3536431	3537414 -	327	53720572	3092985 -	COG2017G	GGACCGAATCATGACGGCGACGCA	1	103	103	1	100	Bm_ATCC23344_chr1	3510148	2584841	2584943	99	506	3.00E-18 -
BPSL2966	L-arabinose transport system, permease protein	3538396	3539400 -	334	53720574	3094069 araH	COG1172G	GCGCGCCGCCCGGCGCGATGACGT	1	103	103	1	100	Bm_ATCC23344_chr1	3510148	2586827	2586929	99	506	3.00E-18 -
BPSL2967	L-arabinose transport system, ATP-binding protein	3539515	3541047 -	510	53720575	3094068 araG	COG1129G	GCGAAGTCCGCCGCGCGTCGCGCG	1	70	70	1	100	Bm_ATCC23344_chr1	3510148	2588474	2588543	100	350	3.30E-11 -
BPSL2968	putative L-arabinose transport system, exported prot	3541115	3542110 -	331	53720576	3092606 -	COG1879G	GTGCACGCGCGAAGGCGGCGCGC	1	103	103	1	100	Bm_ATCC23344_chr1	3510148	2589537	2589639	100	515	1.20E-18 -
BPSL2970	keto-hydroxyglutarate-aldolase/keto-deoxy-phospho	3543016	3543645 -	209	53720578	3093435 -	COG0800G	GCCCGCGTCTCTACACGAAGTGGG	1	100	100	1	100	Bm_ATCC23344_chr1	3510148	2591072	2591171	100	500	5.50E-18 -
BPSL2972	putative IclR-family regulatory protein	3544753	3545670 -	305	53720580	3093994 -	COG1414K	CGCCCCCCTACAATCCGAAGCACA	1	103	103	1	100	Bm_ATCC23344_chr1	3510148	2593097	2593199	99	506	3.00E-18 -
BPSL2973	hypothetical protein BPSL2973	3545935	3546366 -	143	53720581	3093995 -	NULL	CGCCGAACGATGCCGCGGCGCCCG	1	63	63	1	100	Bm_ATCC23344_chr1	3510148	2593793	2593855	100	315	1.30E-09 -
BPSL2974	putative lipoprotein	3546427	3548061 -	544	53720582	3092542 -	NULL	GCGTATATTGGCCGACGCCTTGGCT	1	103	103	1	100	Bm_ATCC23344_chr1	3510148	2595488	2595590	100	515	1.20E-18 -
BPSL2977	putative exoribonuclease II	3550187	3552265 -	692	53720585	3093843 -	COG0557K	GTGCGCGGCACGTCTGCACCCTAT	1	103	103	1	100	Bm_ATCC23344_chr1	3510148	2599692	2599794	100	515	1.20E-18 -
BPSL2979	UDP-N-acetylmuramate:L-alanyl-gamma-D-glutamyl-	3552955	3554439 -	494	53720587	3091976 mpl	COG0773M	CGGTGCGGAGCGACCCGGCGGTTG	1	103	103	1	100	Bm_ATCC23344_chr1	3510148	2601866	2601968	100	515	1.20E-18 -
BPSL2980	hypothetical protein BPSL2980	3554561	3555217 +	218	53720588	3093461 -	NULL	GCCGCGCAAAGAGGGCTATTGTAA	1	103	1	103	100	Bm_ATCC23344_chr1	3510148	2601890	2601992	100	515	1.20E-18 +
BPSL2981	putative thioredoxin-related protein	3555288	3555770 +	160	53720589	3093462 -	COG0526OC	CAAAGGCATTTTCGCCGGCATCGTG	1	73	1	73	100	Bm_ATCC23344_chr1	3510148	2602647	2602719	98	356	1.80E-11 +
BPSL2982	3-dehydroquinate dehydratase	3555966	3556418 +	150	53720590	3094027 aroQ1	COG0757E	GCCGCGCGCTCGCCGCCCCCGTCG	1	103	1	103	100	Bm_ATCC23344_chr1	3510148	2603295	2603397	100	515	1.20E-18 +
BPSL2983	acetyl-CoA carboxylase	3556486	3556959 +	157	53720591	3092969 accB	COG0511I	TTTTCAAGAAACTCGATTCAGCGCC	1	70	1	70	100	Bm_ATCC23344_chr1	3510148	2603848	2603917	100	350	3.30E-11 +
BPSL2984	acetyl-CoA carboxylase	3557044	3558411 +	455	53720592	3093093 accC	COG0439I	GACCCGCCGCGCGGCGCGCCGCC	1	87	1	87	100	Bm_ATCC23344_chr1	3510148	2604389	2604475	100	435	4.80E-15 +
BPSL2987	thiol peroxidase	3560841	3561344 +	167	53720595	3092953 tpx	COG2077O	CGCGCCGCGACGCGGCGCGCGGC	1	70	1	70	100	Bm_ATCC23344_chr1	3510148	2608221	2608290	100	350	3.30E-11 +
BPSL2988	putative sugar kinase	3561445	3562383 +	312	53720596	3093473 -	COG0524G	GCCGCACCGAACGGCCGCCGCGC	1	103	1	103	100	Bm_ATCC23344_chr1	3510148	2608792	2608894	99	506	3.00E-18 +
BPSL2991	ribonucleotide-diphosphate reductase beta subunit	3564144	3565196 -	350	53720599	3093394 nrdB	COG0208F	TGCGATGCGCGACCCCGCAGGAAT	1	103	103	1	100	Bm_ATCC23344_chr1	3510148	2612509	2612611	100	515	1.20E-18 -
BPSL2992	ribonucleotide-diphosphate reductase alpha subunit	3565723	3568710 -	995	53720600	3091740 -	COG0209F	CGCCGCTTCTTCCACGTCTTACCGC	1	103	103	1	100	Bm_ATCC23344_chr1	3510148	2616023	2616125	99	506	3.00E-18 -
BPSL2994	hypothetical protein BPSL2994	3570102	3571001 -	299	53720603	3091559 -	COG4137R	GGGGAGCGATGGACAGTGAAGCC	1	90	90	1	100	Bm_ATCC23344_chr1	3510148	2618314	2618403	100	450	1.00E-15 -
BPSL2995	signal recognition particle protein	3571089	3572456 +	455	53720604	3092670 ffh	COG0541U	GTTCGAAGTTTACACTAGGCCCCTT	1	90	1	90	100	Bm_ATCC23344_chr1	3510148	2618317	2618406	100	450	1.00E-15 +
BPSL2996	hypoxanthine-guanine phosphoribosyltransferase	3572554	3573105 +	183	53720605	3094711 -	COG0634F	TTCGCCGCGGCGCCGCGCACGGGT	1	100	1	100	100	Bm_ATCC23344_chr1	3510148	2619772	2619871	100	500	5.50E-18 +
BPSL2998	prolyl-tRNA synthetase	3573930	3575666 -	578	53720607	3091660 proS	COG0442J	GGTATGTCATCTGCGCCGCATACTG	1	103	103	1	100	Bm_ATCC23344_chr1	3510148	2622979	2623081	100	515	1.20E-18 -
BPSL2999	dinucleoside polyphosphate hydrolase	3575884	3576534 +	216	53720608	3092881 -	COG0494LR	TGTAGTTGACACGCTCCGCCCGGA	1	103	1	103	100	Bm_ATCC23344_chr1	3510148	2623099	2623201	100	515	1.20E-18 +
BPSL3000	putative lipoprotein	3576590	3577177 +	195	53720609	3092882 -	NULL	CGCGCGGGCTGGTTCGACGGGAAT	1	58	1	58	100	Bm_ATCC23344_chr1	3510148	2623850	2623907	100	290	1.70E-08 +
BPSL3002	gamma-glutamyl kinase	3578983	3580101 -	372	53720611	3091659 proB	COG0263E	GCGTCTCGTCCATCGCCCATGAAG	1	87	87	1	100	Bm_ATCC23344_chr1	3510148	2625817	2625903	100	435	4.80E-15 -
BPSL3003	putative conserved GTP-binding protein	3580186	3581304 -	372	53720612	3092862 -	COG0536R	AAGCATCCGGTGCGCGCGTCGGCC	1	103	103	1	100	Bm_ATCC23344_chr1	3510148	2627020	2627122	100	515	1.20E-18 -
BPSL3005	50S ribosomal protein L21	3581781	3582092 -	103	53720614	3093772 rplU	NULL	GCAGACGCGGATGAGCTTTGACTG	1	103	103	1	100	Bm_ATCC23344_chr1	3510148	2627808	2627910	100	515	1.20E-18 -
BPSL3006	octaprenyl-diphosphate synthase	3582308	3583306 +	332	53720615	3092512 ispB	COG0142H	CGCGGACATCGGCCGAACGGCCTA	1	103	1	103	100	Bm_ATCC23344_chr1	3510148	2627926	2628028	100	515	1.20E-18 +
BPSL3007	hypothetical protein BPSL3007	3583587	3584882 +	431	53720616	3092385 -	COG4536P	CGGCACCGGGCGGTACGGCCGATG	1	103	1	103	100	Bm_ATCC23344_chr1	3510148	2633829	2633931	100	515	1.20E-18 +
BPSL3008	putative type IV pilus assembly protein	3584982	3586241 +	419	53720617	3092386 -	COG2804NU	CGGCCGGCGCGGTTGGCCGTTCGG	1	102	1	102	100	Bm_ATCC23344_chr1	3510148	2635225	2635326	99	501	5.00E-18 +
BPSL3012	hypothetical protein BPSL3012	3589178	3589933 +	251	53720621	3092347 -	NULL	CATCGTCAGCGCCGAAAAATGGCG	1	103	1	103	100	Bm_ATCC23344_chr1	3510148	2639420	2639522	100	515	1.20E-18 +
BPSL3016	translocase	3593040	3595835 -	931	53720626	3091641 secA	COG0653U	CGACGTTTGAATTCACTTCTACGCT	1	103	103	1	100	Bm_ATCC23344_chr1	3510148	2646175	2646277	100	515	1.20E-18 -
BPSL3018	UDP-3-O-	3597311	3598228 -	305	53720628	3093106 envA	NULL	GAAACGTCGATCATCACGACGCGA	1	103	103	1	100	Bm_ATCC23344_chr1	3510148	2648680	2648782	100	515	1.20E-18 -
BPSL3019	putative redoxin	3598470	3598976 -	168	53720629	3091581 -	COG0678O	CGCGACGCCGGGCCGGGCGAGTC	1	103	103	1	100	Bm_ATCC23344_chr1	3510148	2649428	2649530	99	506	3.00E-18 -
BPSL3020	cell division protein FtsZ	3599162	3600358 -	398	53720630	3092827 ftsZ	COG0206D	AAATTCGCGCCGGTGCCGGCGGCC	1	103	103	1	100	Bm_ATCC23344_chr1	3510148	2650810	2650912	100	515	1.20E-18 -
BPSL3025	N-acetylglucosaminyl transferase	3604833	3605936 -	367	53720635	3094394 murG	COG0707M	TTGCGGCGAAGGCGCATTGCGTGC	1	103	103	1	100	Bm_ATCC23344_chr1	3510148	2656388	2656490	99	506	3.00E-18 -
BPSL3034	hypothetical protein BPSL3034	3616147	3616575 -	142	53720644	3091938 mraZ	COG2001S	AGTGTGAGAAAGTGTATTTTTGTGT	1	103	103	1	100	Bm_ATCC23344_chr1	3510148	2667027	2667129	99	506	3.00E-18 -
BPSL3035	hypothetical protein BPSL3035	3616964	3617590 -	208	53720645	3094287 -	COG2941H	CGGGTTAACCGCTATTTTCGAGCGC	1	103	103	1	100	Bm_ATCC23344_chr1	3510148	2668041	2668143	99	506	3.00E-18 -
BPSL3036	outer membrane porin	3617871	3619031 +	386	53720646	3094288 -	COG3203M	CTACATTACGAGCGGCTTCATTCGA	1	103	1	103	100	Bm_ATCC23344_chr1	3510148	2668224	2668326	100	515	1.20E-18 +
BPSL3040	enoyl-CoA hydratase	3629441	3630235 -	264	53720649	3092157 paaF	COG1024I	GGTGAACGTTGAGGGAAGCGGTG	1	103	1	103	100	Bm_ATCC23344_chr2	2325379	553502	553604	98	497	5.00E-18 +
BPSL3041	putative phenylacetic acid degradation oxidoreducta	3630348	3632054 +	568	53720650	3092152 paaZ	COG1012C	TGCCGCACACGCACGGATGATTTG	1	103	103	1	100	Bm_ATCC23344_chr2	2325379	553487	553589	98	497	5.00E-18 -
BPSL3042	acetyl-CoA acetyltransferase	3632128	3633345 +	405	53720651	3093770 paaJ	COG0183I	CCCGTTCGGCCGAGCTGCCGATCG	1	76	76	1	100	Bm_ATCC23344_chr2	2325379	551707	551782	100	380	9.60E-13 -
BPSL3044	phenylacetic acid degradation protein PaaI	3634318	3634710 +	130	53720653	3093769 paaI	COG2050Q	GATGGCTGCACGCTCTCCCGAACG	1	88	88	1	100	Bm_ATCC23344_chr2	2325379	549554	549641	98	431	4.80E-15 -
BPSL3046	putative outer membrane-bound lytic murein transg	3636374	3637492 -	372	53720655	3092381 -	COG2821M	GAGCAGGGCGAGCGCCAGCAACG	1	103	1	103	100	Bm_ATCC23344_chr2	2325379	546284	546386	100	515	7.70E-19 +
BPSL3047	hypothetical protein BPSL3047	3637709	3638083 -	124	53720656	3091750 -	NULL	GGGGCCTCATCAAGCAGAATGCCG	1	103	1	103	100	Bm_ATCC23344_chr2	2325379	545685	545787	100	515	7.70E-19 +
BPSL3048	ribulose-phosphate 3-epimerase	3638220	3638951 +	243	53720657	3091751 -	COG0036G	CGAGGCGCCCGCCCCGCGCGGCC	1	103	103	1	100	Bm_ATCC23344_chr2	2325379	545646	545748	100	515	7.70E-19 -
BPSL3050	anthranilate synthase component I	3640048	3641541 +	497	53720659	3092035 trpE	COG0147EH	TCGTCCGATCCACCCCGCGAGGGG	1	103	103	1	100	Bm_ATCC23344_chr2	2325379	543818	543920	100	515	7.70E-19 -
BPSL3053	indole-3-glycerol phosphate synthase	3643257	3644042 +	261	53720662	3092690 trpC	COG0134E	TCCGGCGCCCCGCGCGCGCCTCAA	1	65	65	1	100	Bm_ATCC23344_chr2	2325379	540609	540673	100	325	2.90E-10 -

BPSL3055	uracil-DNA glycosylase	3644787	3645524	+	245	53720664	3091556	ung	COG0692L	CCACCTCTTTGCGCATGACCAAGCC	1	95	82	1	89.1304	Bm_ATCC23344_chr2	2325379	539092	539173	97	392	2.80E-13	-
BPSL3056	acyl carrier protein phosphodiesterase	3645743	3646339	-	198	53720665	3094516	acpD	COG1182I	CAATCCGTCACCCGGCCACCGCTTT	1	103	1	103	100	Bm_ATCC23344_chr2	2325379	537437	537539	99	506	2.00E-18	+
BPSL3057	family M61 unassigned peptidase	3646478	3648316	-	612	53720666	3094618	-	COG3975R	CGCGGCGTTTCCTGCATCGCGAAG	1	102	1	102	100	Bm_ATCC23344_chr2	2325379	535470	535571	99	501	3.30E-18	+
BPSL3058	thiol:disulfide interchange protein DsbC	3648416	3649153	-	245	53720667	3094725	dsbC	COG1651O	CGCGCGGCGCGAACCTTCGCGCGG	1	95	1	95	100	Bm_ATCC23344_chr2	2325379	534640	534734	100	475	4.90E-17	+
BPSL3059	hypothetical protein BPSL3059	3649246	3650427	-	393	53720668	3094619	-	COG0654HC	CGCGGCCGCCGCGAAACCGGCACC	1	103	1	103	100	Bm_ATCC23344_chr2	2325379	533358	533460	100	515	7.70E-19	+
BPSL3060	putative lipoprotein	3650742	3650870	+	42	53720669	3094267	-	NULL	GCCGCGCATTACGCGCCACACGTT	1	103	103	1	100	Bm_ATCC23344_chr2	2325379	533141	533243	99	506	2.00E-18	-
BPSL3061	hypothetical protein BPSL3061	3651327	3651692	+	121	53720670	3094268	-	NULL	CTTATCTGATTCCTCCGATCGCGTG	1	103	103	1	100	Bm_ATCC23344_chr2	2325379	532556	532658	99	506	2.00E-18	-
BPSL3062	putative GTP-binding protein	3652194	3653288	+	364	53720671	3094493	-	COG0012J	CCGCCCCGGATTTCGTCCAGCGAC	1	103	103	1	100	Bm_ATCC23344_chr2	2325379	531721	531823	100	515	7.70E-19	-
BPSL3064	hypothetical protein BPSL3064	3654507	3655250	+	247	53720673	3091973	-	NULL	CCGACAATCCATGCCCGCCTATCGA	1	103	103	1	100	Bm_ATCC23344_chr2	2325379	529413	529515	99	506	2.00E-18	-
BPSL3065	putative amino acid permease	3655322	3656725	-	467	53720674	3091974	-	COG1113E	CGGCGCGCATTGCGCCGCGCACGG	1	103	1	103	100	Bm_ATCC23344_chr2	2325379	527097	527199	99	506	2.00E-18	+
BPSL3066	putative hydrolase	3657986	3658966	-	326	53720675	3091837	-	COG0584C	GCCCGACGGCATCTTAACCGCGGC	1	103	1	103	100	Bm_ATCC23344_chr2	2325379	524862	524964	100	515	7.70E-19	+
BPSL3067	putative ABC transporter ATP-binding subunit	3659704	3661371	+	555	53720676	3091838	-	COG0488R	GCACGCGCCGCGGCCGCTTCCATG	1	103	103	1	100	Bm_ATCC23344_chr2	2325379	524222	524324	99	506	2.00E-18	-
BPSL3069	putative hydrolase	3662327	3662854	-	175	53720678	3094708	-	COG1335Q	CGATGTCGATGTCGGTGCAGGTGT	1	103	1	103	100	Bm_ATCC23344_chr2	2325379	520974	521076	98	497	5.00E-18	+
BPSL3071	hypothetical protein BPSL3071	3664866	3665120	-	84	53720680	3091684	-	NULL	CCGCCAGGCGGCATCGCGCGGCGT	1	103	1	103	100	Bm_ATCC23344_chr2	2325379	518726	518828	99	506	2.00E-18	+
BPSL3072	glutamyl-tRNA reductase	3665689	3667014	+	441	53720681	3094369	hemA	COG0373H	ACGGCCGAGCGCCGGCCGGAAGA	1	103	103	1	100	Bm_ATCC23344_chr2	2325379	518251	518353	98	497	5.00E-18	-
BPSL3073	peptide chain release factor 1	3667081	3668163	+	360	53720682	3092221	prfA	COG0216J	CGGCGCCCGCCGGCCCGCCTCTCC	1	69	69	1	100	Bm_ATCC23344_chr2	2325379	516859	516927	100	345	3.70E-11	-
BPSL3075	hypothetical protein BPSL3075	3669144	3669452	+	102	53720684	3093730	-	COG0278O	CACGCCGCCCGGCGCCCGCCGGCC	1	82	82	1	100	Bm_ATCC23344_chr2	2325379	514796	514877	100	410	4.30E-14	-
BPSL3078	putative amino-acid permease	3671584	3673185	-	533	53720687	3091697	-	COG0531E	CCCGATCGGGCAACCCGTCCTCCA	1	103	1	103	100	Bm_ATCC23344_chr2	2325379	510733	510835	100	515	7.70E-19	+
BPSL3079	cold shock-like protein CspD	3673619	3673822	+	67	53720688	3094215	cspD	NULL	TTCGCCCATCAACGGTACTCCGGTT	1	103	103	1	100	Bm_ATCC23344_chr1	3510148	2881740	2881842	100	515	1.20E-18	-
BPSL3081	putative oxidoreductase	3675933	3676550	-	205	53720690	3094253	-	COG0778C	TACGCCGATTTGCATTCGCGCGCAC	1	103	103	1	100	Bm_ATCC23344_chr1	3510148	2892841	2892943	100	515	1.20E-18	-
BPSL3082	hypothetical protein BPSL3082	3677024	3678307	-	427	53720691	3092025	-	NULL	GCAACAACTTGTGACCGATCGGTA	1	103	103	1	100	Bm_ATCC23344_chr1	3510148	2894609	2894711	100	515	1.20E-18	-
BPSL3083	putative TetR-family transcriptional regulator	3678466	3679110	+	214	53720692	3092026	-	COG1309K	ACATGCGCCGATCTTTGTTACCGAT	1	103	1	103	100	Bm_ATCC23344_chr1	3510148	2894670	2894772	99	506	3.00E-18	+
BPSL3084	hypothetical protein BPSL3084	3679455	3680078	+	207	53720693	3092839	-	NULL	CACGCGTCGTATCACGTCGCATCCT	1	103	1	103	100	Bm_ATCC23344_chr1	3510148	2895652	2895754	99	506	3.00E-18	+
BPSL3085	hypothetical protein BPSL3085	3680163	3681059	-	298	53720694	3092840	-	NULL	GGCGCGGATGGTTTTCGGTAAACT	1	103	103	1	100	Bm_ATCC23344_chr1	3510148	2897354	2897456	100	515	1.20E-18	-
BPSL3086	hypothetical protein BPSL3086	3681187	3682377	-	396	53720695	3094309	-	COG2890J	TTGCCCGCCGCCGGTGCGTCGACC	1	103	103	1	100	Bm_ATCC23344_chr1	3510148	2898672	2898774	97	488	1.90E-17	-
BPSL3087	putative acyltransferase	3682680	3683882	-	400	53720696	3094310	-	COG1835I	TGCCGCCTGACTGATCGATCCTGCC	1	103	103	1	100	Bm_ATCC23344_chr1	3510148	2900177	2900279	99	506	3.00E-18	-
BPSL3088	hypothetical protein BPSL3088	3684236	3684682	-	148	53720697	3094612	-	NULL	TTAAAATGGCGGTTCCTGCGCCCCG	1	103	103	1	100	Bm_ATCC23344_chr1	3510148	2900942	2901045	99	505	3.30E-18	-
BPSL3089	family M1 unassigned peptidase	3685343	3687508	+	721	53720698	3094613	-	COG0308E	CCGGGATAATTCATTTCGCTGCCGA	1	103	1	103	100	Bm_ATCC23344_chr1	3510148	2901506	2901608	99	506	3.00E-18	+
BPSL3090	hypothetical protein BPSL3090	3688531	3688800	+	89	53720699	3094158	-	NULL	GCGGTGCGCAGCGCACCGCCTCGC	1	103	1	103	100	Bm_ATCC23344_chr1	3510148	2904694	2904796	100	515	1.20E-18	+
BPSL3091	hypothetical protein BPSL3091	3688903	3689133	+	76	53720700	3094159	-	NULL	GGCATGCCGATGATCGATTGAGCG	1	103	1	103	100	Bm_ATCC23344_chr1	3510148	2905066	2905168	100	515	1.20E-18	+
BPSL3092	putative bacteriocin secretion protein	3689749	3691017	+	422	53720701	3094505	-	NULL	GCCGCGGCGCACGCCGCCGCCCGC	1	103	1	103	100	Bm_ATCC23344_chr1	3510148	2905912	2906014	99	506	3.00E-18	+
BPSL3096	putative bacteriophage-related peptidase	3696333	3696911	+	192	53720705	3092435	-	NULL	GGCAAGAATGTCGCGCTCCACTTTA	1	103	1	103	100	Bm_ATCC23344_chr1	3510148	2912467	2912569	100	515	1.20E-18	+
BPSL3100	hypothetical protein BPSL3100	3703205	3704326	-	373	53720709	3093360	-	COG3515S	GGCCGGCGCGCGGCGCACGTCACC	1	92	92	1	100	Bm_ATCC23344_chr1	3510148	2920551	2920642	100	460	3.50E-16	-
BPSL3103	hypothetical protein BPSL3103	3708183	3710009	-	608	53720712	3092705	-	COG3519S	GCCGTATCGCGCGGGCTGCCGGGC	1	94	94	1	100	Bm_ATCC23344_chr1	3510148	2926258	2926351	100	470	1.30E-16	-
BPSL3120	putative stringent starvation protein A	3725547	3726158	-	203	53720727	3092038	-	COG0625O	TCACGCCGAGACTGGCGTGGGGCC	1	95	95	1	100	Bm_ATCC23344_chr1	3510148	2792052	2792146	100	475	7.40E-17	-
BPSL3123	ubiquinol-cytochrome c reductase iron-sulfur subuni	3728415	3729038	-	207	53720730	3094363	petA	COG0723C	AGGTATTTAACGTCCGGGTCTTGTA	1	103	103	1	100	Bm_ATCC23344_chr1	3510148	2794932	2795034	100	515	1.20E-18	-
BPSL3128	twin argininte translocase protein A	3732947	3733180	-	77	53720735	3094102	tatA	COG1826U	GATGGCGGGCGGCCCGCCATCGGA	1	91	91	1	100	Bm_ATCC23344_chr1	3510148	2799065	2799155	100	455	6.00E-16	-
BPSL3130	hypothetical protein BPSL3130	3733663	3734052	-	129	53720737	3091601	-	COG3671S	GCCAATGCGGCCACGCGCCGCGTA	1	55	55	1	100	Bm_ATCC23344_chr1	3510148	2799937	2799991	100	275	8.00E-08	-
BPSL3133	imidazole glycerol phosphate synthase subunit HisF	3734883	3735671	-	262	53720740	3091767	hisF	COG0107E	CGCGCCGTCTCGGCACGACGCCTT	1	70	70	1	100	Bm_ATCC23344_chr1	3510148	2801556	2801625	100	350	3.30E-11	-
BPSL3134	1-(5-phosphoribosyl)-5-	3735739	3736494	-	251	53720741	3093946	hisA	COG0106E	CAGAAAGAGTTGTACTAAACTAGC	1	103	103	1	100	Bm_ATCC23344_chr1	3510148	2802379	2802481	100	515	1.20E-18	-
BPSL3136	hypothetical protein BPSL3136	3737300	3737920	-	206	53720743	3091602	-	COG2095U	TTTCCGGCCGCAGCGAACACGACC	1	58	58	1	100	Bm_ATCC23344_chr1	3510148	2803805	2803862	100	290	1.70E-08	-
BPSL3137	imidazoleglycerol-phosphate dehydratase	3737976	3738563	-	195	53720744	3092887	hisB	NULL	CGGGCCACCCCGGGGCGCCGCCTT	1	66	66	1	100	Bm_ATCC23344_chr1	3510148	2804448	2804513	100	330	2.60E-10	-
BPSL3138	histidinol-phosphate aminotransferase	3738627	3739697	-	356	53720745	3092888	hisC	COG0079E	ACCCGAGACCGCCCGGCGAGGCG	1	103	103	1	100	Bm_ATCC23344_chr1	3510148	2805582	2805684	99	506	3.00E-18	-
BPSL3142	BolA-like protein	3743248	3743487	-	79	53720749	3091486	-	COG5007K	TATGAAGCGACGGCCGCCCGCCGC	1	88	88	1	100	Bm_ATCC23344_chr1	3510148	2809371	2809458	100	440	2.80E-15	-
BPSL3144	putative ABC transporter ATP-binding subunit	3744325	3745266	-	313	53720751	3091438	-	COG1131V	GCCTGGCCGGTTTGCCCTATAATCA	1	103	103	1	100	Bm_ATCC23344_chr1	3510148	2811150	2811252	100	515	1.20E-18	-
BPSL3146	hypothetical protein BPSL3146	3745813	3746445	-	210	53720753	3094199	-	COG2854Q	CGGTTACAGCCGGAAACGATTCTG	1	103	103	1	100	Bm_ATCC23344_chr1	3510148	2812329	2812431	100	515	1.20E-18	-
BPSL3150	putative ABC transporter ATP-binding subunit	3748904	3749722	-	272	53720757	3094146	-	COG1127Q	TTTGACAATTTCTGACTGGAGCGCC	1	102	102	1	100	Bm_ATCC23344_chr1	3510148	2815606	2815707	99	501	5.00E-18	-
BPSL3152	thiazole synthase	3750913	3751728	-	271	53720759	3092619	thiG	COG2022H	CGCGCCGCCGCGCGCGCCTCGCCC	1	55	55	1	100	Bm_ATCC23344_chr1	3510148	2817621	2817675	98	266	2.10E-07	-
BPSL3154	putative thiamine biosynthesis oxidoreductase ThiO	3752027	3753154	-	375	53720761	3092334	thiO	COG0665E	GTTCTTTTGCGTCGCTGCGAGCCGT	1	103	103	1	100	Bm_ATCC23344_chr1	3510148	2819047	2819149	99	506	3.00E-18	-
BPSL3155	putative ABC transporter	3753709	3755529	+	606	53720762	3094147	-	COG4178R	GGAATCGGCGCGCGGGCGGCATCC	1	103	1	103	100	Bm_ATCC23344_chr1	3510148	2819504	2819606	100	515	1.20E-18	+
BPSL3157	putative sodium:amino acid symporter	3756315	3757826	-	503	53720764	3092183	-	COG1115E	CATTTTGTAAGAGTCGGTGCAACCT	1	103	103	1	100	Bm_ATCC23344_chr1	3510148	2823750	2823852	97	488	1.90E-17	-
BPSL3159	glutamate synthase large subunit	3759667	3764370	-	1567	53720766	3092803	glt1	COG0067E, COG0069E, CC	CCTGCCCGGCCGGCCACGTCGCGA	1	103	103	1	100	Bm_ATCC23344_chr1	3510148	2830295	2830396	99	500	5.50E-18	-
BPSL3160	hypothetical protein BPSL3160	3764662	3765375	-	237	53720767	3093030	-	COG1943L	CGCACGACCGTGCGTATTCCCGCAC	1	103	103	1	100	Bm_ATCC23344_chr1	3510148	2831299	2831401	100	515	1.20E-18	-
BPSL3161	putative outer membrane protein	3765568	3766299	+	243	53720768	3093031	-	COG3047M	GTCGTGCGAATTCTAGGGAAAATG	1	103	1	103	100	Bm_ATCC23344_chr1	3510148	2831394	2831496	100	515	1.20E-18	+
BPSL3165	putative ABC transporter permease	3769201	3770085	-	294	53720772	3093368	-	COG1175G	CGCGGCCTTTTCGCGCATGCGGTTG	1	103	103	1	100	Bm_ATCC23344_chr1	3510148	2836009	2836111	100	515	1.20E-18	-
BPSL3166	putative ABC transporter extracellular solute-binding	3770216	3771541	-	441	53720773	3093369	-	COG1653G	ACTGTCCGGCTCGCCGCATCCGGC	1	87	87	1	100	Bm_ATCC23344_chr1	3510148	2837465	2837551	100	435	4.80E-15	-
BPSL3169	shikimate kinase	3773983	3774537	-	184	53720776	3092396	aroK	COG0703E	CGCCCGTGGGAGCCAGGCTCGACA	1	83	83	1	100	Bm_ATCC23344_chr1	3510148	2840461	2840543	100	415	3.80E-14	-
BPSL3178	hypothetical protein BPSL3178	3784587	3785582	-	331	53720786	3094826	-	COG2041R	CGCTACGCTGCCGAAACGGCCGGC	1	103	103	46	55	Bm_ATCC23344_chr1	3510148	2853100	2853158	96	271	7.10E-07	-
BPSL3180	putative cytochrome C biogenesis protein	3786955	3789054	-	699	53720788	3091445	-	COG1333O	TTTGCTGTGAGTTGGAGTTTGAATG	1	103	103	1	100	Bm_ATCC23344_chr1	3510148	2709977	2710079	100	515	1.20E-18	-
BPSL3181	cytochrome C	3789263	3789910	-	215	53720789	3094749	-	COG2863C	GCAGGCGGTGGAAGCTTCGGAGCG	1	103	103	1	100	Bm_ATCC23344_chr1	3510148	2710833	2710935	100	515	1.20E-18	-
BPSL3182	GTP-binding protein	3790127	3790786	+	219	53720790	3093266	engB	COG0218R	TGTACAATACCCCGTTGAAAGCCTT	1	103	1	103	100	Bm_ATCC23344_chr1	3510148	2710952	2711054	100	515	1.20E-18	+
BPSL3183	delta-aminolevulinic acid dehydratase	3791004	3792068	+	354	53720791	3092987	hemB	NULL	CGGGTTAAACAGCCTAATCGAAAA	1	103	1	103	100	Bm_ATCC23344_chr1	3510148	2711829	2711931	99	506	3.00E-18	+
BPSL3185	putative periplasmic cytochrome biogenesis protein	3794160	3794510	-	116	53720793	3094750	-	COG1324P	ATGCTTGGCCCTTTTTGTTTTTCGTT	1	103	103	1	100	Bm_ATCC23344_chr1	3510148	2715433	2715535	100	515	1.20E-18	-
BPSL3186	50S ribosomal protein L17	3794645	3795040	-	131	53720794	3092315	rplQ	COG0203J	TTTAAAATCCGCATCTTGCTTTTTTC	1	103	103	1	100	Bm_ATCC23344_chr1	3510148	2715963	2716065	99	506	3.00E-18	-
BPSL3187	DNA-directed RNA polymerase alpha subunit	3795181	3796158	-	325	53720795	3094313	rpoA	COG0202K	TATTGCGTCCCGCAAGGCAGGACC	1	103	103	1	100	Bm_ATCC23344_chr1	3510148	2717081	2717183	100	515	1.20E-18	-
BPSL3188	30S ribosomal protein S4	3796286	3796909	-	207	53720796	3091836	rpsD	COG0522J	TTTTGTTGCGCATTTGATTGATTAAG	1	103	103	1	100	Bm_ATCC23344_chr1	3510148	2717832	2717934	99	506	3.00E-18	-
BPSL3203	50S ribosomal protein L14	3803365	3803733	-	122	53720811	3093603	rplN	NULL	TCGCTGCTGTTCCAACCCAAGCAGC	1	103	103	1	100	Bm_ATCC23344_chr1	3510148	2724656	2724758	100	515	1.20E-18	-
BPSL3213	50S ribosomal protein L3	3808136	3808795	-	219	53720821	3091930	rplC	NULL	TTCGCGTATTCGCGTAAAAAAGCCG	1	103	103	1	100	Bm_ATCC23344_chr1	3510148	2729718	2729819	99	500	5.50E-18	-
BPSL3216	elongation factor EF-2	3810679	3812781	-	700	53720824	3092466	fusA	COG0480J	GAAAAATTCCGGGCGGGTGCGCTT	1	103	103	1	100	Bm_ATCC23344_chr1	3510148	2733703	2733805	100	515	1.20E-18	-
BPSL3217	30S ribosomal protein S7	3812903	3813373	-	156	53720825	3093549	rpsG	COG0049J	AGTGGTCACCCGGCCAAGCTGGTT	1	103	103	1	100	Bm_ATCC23344_chr1	3510148	2734295	2734397	100	515	1.20E-18	-
BPSL3218	30S ribosomal protein S12	3813544	3813924	-	126	53720826	3091469	rpsL	NULL	AAGCTCAGGCGTCGCGCACGCGTT	1	103	103	1	100	Bm_ATCC23344_chr1	3510148	2734846	2734948	100	515	1.20E-18	-
BPSL3221	DNA-directed RNA polymerase beta subunit	3820448	3824554	-	1368	53720829	3092566	-	COG0085K	CGTGCCGTCGGACGGCCATCGGGT	1	103	103	1	100	Bm_ATCC23344_chr1	3510148	2746789	2746891	100	515	1.20E-18	-
BPSL3222	50S ribosomal protein L7/L12	3824918	3825250	-	110	53720830	3093065	rplL	COG0222J	CGCACATCAGCGTCACTGATCGCTC	1	103	103	1	100	Bm_ATCC23344_chr1	3510148	2747485	2747587	100	515	1.20E-18	-

BPSL3223	50S ribosomal protein L10	3825356	3825853	-	165	53720831	3093181	rplJ	COG0244J	TCGCTGGTCTCCAGCGGAAATACT	1	103	103	1	100	Bm_ATCC23344_chr1	3510148	2748088	2748190	99	506	3.00E-18	-
BPSL3225	50S ribosomal protein L11	3826850	3827281	-	143	53720833	3092790	rplK	NULL	ACGGCCCGCGTTAATGGCCGTTGA	1	103	103	1	100	Bm_ATCC23344_chr1	3510148	2749516	2749618	100	515	1.20E-18	-
BPSL3227	translocase	3827986	3828366	-	126	53720835	3092983	secE	COG0690U	GCAGAGCGTCGGTCTCCAAAACCG	1	103	103	1	100	Bm_ATCC23344_chr1	3510148	2750601	2750703	99	506	3.00E-18	-
BPSL3228	elongation factor Tu	3828544	3829734	-	396	53720836	3092631	tuf	COG0050J	GGGAGAGGTCGGCAGTTCGATCCT	1	103	103	1	100	Bm_ATCC23344_chr1	3510148	2751969	2752071	100	515	1.20E-18	-
BPSL3229	hypothetical protein BPSL3229	3836722	3837999	-	425	53720837	3092567	-	COG2755E	AGCAATGCCCCGCCCGCCACCGGC	1	103	103	1	100	Bm_ATCC23344_chr1	3510148	2679638	2679740	100	515	1.20E-18	-
BPSL3230	phenylacetic acid degradation protein PaaA	3838612	3839610	+	332	53720838	3091478	paaA	COG3396S	CTCGCACCGCGAATCTTGCCCATCC	1	103	1	103	100	Bm_ATCC23344_chr1	3510148	2680727	2680829	97	488	1.90E-17	+
BPSL3231	phenylacetic acid degradation protein PaaB	3839673	3839957	+	94	53720839	3091479	paaB	NULL	CCGGCATGACGCGCGGCAAGCGC	1	65	1	65	100	Bm_ATCC23344_chr1	3510148	2681826	2681890	100	325	4.40E-10	+
BPSL3237	hypothetical protein BPSL3237	3844299	3844985	+	228	53720845	3094132	-	NULL	GCTTACGCTCACCAATCGGTGACTT	1	103	1	103	100	Bm_ATCC23344_chr1	3510148	2686414	2686516	98	497	7.60E-18	+
BPSL3238	putativeAsnC-family transcriptional regulator	3845104	3845619	-	171	53720846	3094133	-	COG1522K	AAGTGTAGCGGGGAGGGCCGAGT	1	103	103	1	100	Bm_ATCC23344_chr1	3510148	2687831	2687933	99	506	3.00E-18	-
BPSL3239	4-hydroxyphenylpyruvate dioxygenase	3845771	3846868	+	365	53720847	3091429	-	COG3185ER	TTGCGCGACCAAGTTCGCAAGTTTC	1	103	1	103	100	Bm_ATCC23344_chr1	3510148	2687885	2687987	100	515	1.20E-18	+
BPSL3240	putative outer membrane protein	3847104	3847301	-	65	53720848	3091430	-	NULL	CGTTTACCGTTAGTCTCTAGGGCCC	1	103	103	1	100	Bm_ATCC23344_chr1	3510148	2689513	2689615	99	506	3.00E-18	-
BPSL3241	indolepyruvate ferredoxin oxidoreductase	3847621	3851202	+	1193	53720849	3091567	-	COG1014C, COG4231C	ATCGAACGAAACCCGCCAATATTC	1	103	1	103	100	Bm_ATCC23344_chr1	3510148	2689735	2689837	100	515	1.20E-18	+
BPSL3242	phosphate acetyltransferase	3851722	3853992	-	756	53720850	3091568	-	COG0280C, COG0281C	CGGACGCCGCAGTCGACCATCCCT	1	103	103	1	100	Bm_ATCC23344_chr1	3510148	2696169	2696271	100	515	1.20E-18	-
BPSL3243	orotate phosphoribosyltransferase	3854521	3855207	+	228	53720851	3093840	pyrE	COG0461F	ACGCCCTATCGGCCCGCCACCCGG	1	103	1	103	100	Bm_ATCC23344_chr1	3510148	2696600	2696702	100	515	1.20E-18	+
BPSL3244	putative lipoprotein	3855508	3856104	+	198	53720852	3091870	-	COG1881R	CCCCCAATGCTAGACTTGCCCTCAT	1	103	1	103	100	Bm_ATCC23344_chr1	3510148	2697588	2697690	100	515	1.20E-18	+
BPSL3245	putative flavoprotein (regulator)	3856491	3857066	+	191	53720853	3091871	-	COG0655R	TCCGCCAGCCGTTCGCGCACCCAG	1	103	1	103	100	Bm_ATCC23344_chr1	3510148	2698567	2698669	100	515	1.20E-18	+
BPSL3246	N-acetyl-gamma-glutamyl-phosphate reductase	3857219	3858163	+	314	53720854	3092981	argC	COG0002E	TTTTTTCGGCCGAACCGGTTACGGC	1	103	1	103	100	Bm_ATCC23344_chr1	3510148	2699295	2699397	100	515	1.20E-18	+
BPSL3247	putative lipoprotein	3858608	3860011	+	467	53720855	3091902	-	NULL	AATTGTTTAAATCGACTCGGTTAGA	1	103	1	103	100	Bm_ATCC23344_chr1	3510148	2700684	2700786	100	515	1.20E-18	+
BPSL3248	putative lipoprotein	3860137	3861387	+	416	53720856	3091903	-	NULL	ACACGCGTCGCGCCGGGCCGCGCC	1	103	1	103	100	Bm_ATCC23344_chr1	3510148	2702213	2702316	99	505	3.30E-18	+
BPSL3256	putative amino acid permease	3871231	3872637	+	468	53720866	3093735	-	COG1113E	CTCCCGCTCGCCGTCATCGAGACG	1	103	1	103	100	Bm_ATCC23344_chr1	3510148	2883528	2883630	99	506	3.00E-18	+
BPSL3273	putative phosphosugar isomerase	3889496	3890074	-	192	53720883	3092692	-	COG0279G	TGCGGCGACGCTCGGGCGGCGGG	1	103	103	1	100	Bm_ATCC23344_chr1	3510148	2887105	2887207	100	515	1.20E-18	-
BPSL3276	putative RlpA-like lipoprotein	3891660	3892295	-	211	53720886	3092131	-	COG0797M	CGCGCCAATGCAAGCCGCCCGCGC	1	103	1	103	100	Bm_ATCC23344_chr1	3510148	3001421	3001523	100	515	1.20E-18	+
BPSL3277	metallo-beta-lactamase superfamily protein	3892896	3893540	+	214	53720887	3093505	-	COG0491R	TGATTCTCTCTATGATCGAAACAAT	1	103	103	1	100	Bm_ATCC23344_chr1	3510148	3000918	3001020	100	515	1.20E-18	-
BPSL3279	putative AsnC-family transcriptional regulator	3894251	3894742	+	163	53720889	3093067	-	COG1522K	CGAATATCGCTCGTTTCAACGGCTC	1	60	60	1	100	Bm_ATCC23344_chr1	3510148	2999563	2999622	98	291	1.50E-08	-
BPSL3280	putative cation efflux protein	3894921	3896111	-	396	53720890	3093068	-	COG0053P	TCATAAGCGATTGATTTTGTTTGTTT	1	103	1	103	100	Bm_ATCC23344_chr1	3510148	2997605	2997707	99	506	3.00E-18	+
BPSL3281	putative HNS-like transcriptional regulator	3896351	3896644	-	97	53720891	3092211	-	COG2916R	TTTTTTGTGCTAACCTTTGACCCAAT	1	103	1	103	100	Bm_ATCC23344_chr1	3510148	2997072	2997174	100	515	1.20E-18	+
BPSL3282	hypothetical protein BPSL3282	3897029	3897835	-	268	53720892	3092212	-	COG0748P	AGCGCGGTGCACTTCGCTTGCGCT	1	103	1	103	100	Bm_ATCC23344_chr1	3510148	2995881	2995983	100	515	1.20E-18	+
BPSL3283	hypothetical protein BPSL3283	3898112	3899230	+	372	53720893	3094655	-	NULL	CCAGCTTCCCGTGTCCGACCAGCC	1	103	103	1	100	Bm_ATCC23344_chr1	3510148	2995702	2995804	100	515	1.20E-18	-
BPSL3284	putative secreted substrate binding protein	3899419	3900561	-	380	53720894	3094656	-	COG0683E	AATGCAACCGGCGAATGTCCAGTG	1	103	1	103	100	Bm_ATCC23344_chr1	3510148	2993153	2993255	100	515	1.20E-18	+
BPSL3286	putative dienelactone hydrolase	3902445	3903137	+	230	53720896	3092010	-	COG0412Q	TTCGCGGCGCTGCAACGACGCGGG	1	103	1	103	100	Bm_ATCC23344_chr1	3510148	2929369	2929471	100	515	1.20E-18	+
BPSL3287	putative amidase	3903233	3904723	+	496	53720897	3093499	-	NULL	GCCGCCGGCGATCGCCGCGCTCGC	1	98	1	98	100	Bm_ATCC23344_chr1	3510148	2930162	2930260	98	480	4.40E-17	+
BPSL3289	hypothetical protein BPSL3289	3905755	3906108	-	117	53720899	3093500	-	COG1950S	GCGCGCAGTCGGCACGGCAACGCC	1	103	103	1	100	Bm_ATCC23344_chr1	3510148	2933131	2933233	99	506	3.00E-18	-
BPSL3290	S-adenosyl-L-homocysteine hydrolase	3906272	3907693	-	473	53720900	3092196	ahcY	COG0499H	CATCGTCGCATAGCGGCGATCGTTT	1	103	103	1	100	Bm_ATCC23344_chr1	3510148	2934716	2934818	99	506	3.00E-18	-
BPSL3295	flagellar biosynthetic protein FlhB	3913455	3914672	-	405	53720905	3091457	flhB	COG1377NU	CGAGAAATACCCCGGTTTCCCCGCT	1	103	103	1	100	Bm_ATCC23344_chr1	3510148	2941687	2941789	100	515	1.20E-18	-
BPSL3296	hypothetical protein BPSL3296	3915368	3915868	-	166	53720906	3094060	-	COG3865S	TTGGTTTGGCATTCGACTATCGAAG	1	103	103	1	100	Bm_ATCC23344_chr1	3510148	2942883	2942985	100	515	1.20E-18	-
BPSL3298	hypothetical protein BPSL3298	3917382	3917885	-	167	53720908	3093670	-	NULL	TGAAAGCTTTCTTTTCGCTTTCGATT	1	103	103	1	100	Bm_ATCC23344_chr1	3510148	2944846	2944948	100	515	1.20E-18	-
BPSL3300	chemotaxis protein CheY	3918813	3919241	-	142	53720910	3092753	cheY	NULL	GTCGCCTGTTTGGCTCGGCCAAAC	1	103	103	1	100	Bm_ATCC23344_chr1	3510148	2946202	2946304	100	515	1.20E-18	-
BPSL3309	flagellar motor protein	3928654	3929514	-	286	53720919	3092331	motA	COG1291N	ACCGGCGAGCCCGCGCGAGGCGTT	1	103	103	1	100	Bm_ATCC23344_chr1	3510148	2956533	2956635	100	515	1.20E-18	-
BPSL3310	flagellar regulon master regulator subunit FlhC	3929708	3930259	-	183	53720920	3092108	flhC	NULL	CCGGAAGGCCGCGCGCCGCGCCC	1	96	96	1	100	Bm_ATCC23344_chr1	3510148	2957278	2957373	98	471	1.10E-16	-
BPSL3311	transcriptional activator FlhD	3930353	3930673	-	106	53720921	3092109	flhD	NULL	TATAAACAAGCCGATTGCCCCTTTT	1	103	103	1	100	Bm_ATCC23344_chr1	3510148	2957692	2957794	100	515	1.20E-18	-
BPSL3312	putative glycosyltransferase	3931267	3932331	+	354	53720922	3092717	-	COG0438M	ACCGCCCCGTCTGCCTGCCGGCAG	1	103	1	103	100	Bm_ATCC23344_chr1	3510148	2958188	2958290	99	506	3.00E-18	+
BPSL3313	HNS-like transcriptional regulator	3932435	3932731	-	98	53720923	3092718	-	COG2916R	CGAAATAATTTTGGTGGATTTCGTA	1	103	103	1	100	Bm_ATCC23344_chr1	3510148	2959750	2959852	100	515	1.20E-18	-
BPSL3314	aquaporin Z	3933037	3933741	+	234	53720924	3093879	aqpZ	NULL	AACCCGTAGGATTTCCTATAATCCA	1	103	1	103	100	Bm_ATCC23344_chr1	3510148	2959958	2960060	100	515	1.20E-18	+
BPSL3315	putative Cof-family hydrolase	3933930	3934772	-	280	53720925	3093598	-	COG0561R	ACCCGATCGGAGCGGCGCGGCCCG	1	99	99	1	100	Bm_ATCC23344_chr1	3510148	2961791	2961891	95	448	1.20E-15	-
BPSL3316	hypothetical protein BPSL3316	3934869	3935759	-	296	53720926	3093599	-	COG2971G	CGCGCAAGCCTGGCCATTCGGCTG	1	103	103	1	100	Bm_ATCC23344_chr1	3510148	2962780	2962882	99	506	3.00E-18	-
BPSL3317	DNA-3-methyladenine glycosylase I	3935910	3936518	+	202	53720927	3092286	tag	NULL	TAACCGCAACGGCTCGCCTCGCCC	1	103	1	103	100	Bm_ATCC23344_chr1	3510148	2962833	2962935	98	497	7.60E-18	+
BPSL3318	30S ribosomal protein S21	3936905	3937117	-	70	53720928	3094653	rpsU	COG0828J	GCTACCCGCTCGTTTCGCCTGTTCG	1	103	103	1	100	Bm_ATCC23344_chr1	3510148	2964138	2964240	100	515	1.20E-18	-
BPSL3319	flagellin	3937675	3938841	+	388	53720929	3094015	fliC	COG1344N	ACGCCGAAATCCTGCACAAGGCGG	1	103	1	103	100	Bm_ATCC23344_chr1	3510148	2964598	2964700	100	515	1.20E-18	+
BPSL3320	flagellar hook-associated protein	3938991	3940511	+	506	53720930	3094016	fliD	COG1345N	CGGAGCAACACCCTCGTGGCCGCC	1	103	1	103	100	Bm_ATCC23344_chr1	3510148	2965914	2966016	100	515	1.20E-18	+
BPSL3322	putative TPR domain protein	3941062	3943392	+	776	53720932	3091873	-	COG0457R, COG3914O	TTCGCCAAATTGGCGGCTCTTTCCT	1	103	1	103	100	Bm_ATCC23344_chr1	3510148	2967985	2968087	100	515	1.20E-18	+
BPSL3331	putative outer membrane protein	3950469	3950867	-	132	53720941	3091768	-	NULL	CACGGCGCGACGAGGATATCGCGC	1	103	103	1	100	Bm_ATCC23344_chr1	3510148	2977891	2977993	100	515	1.20E-18	-
BPSL3332	putative amino acid efflux protein	3951436	3952059	+	207	53720942	3091769	-	COG1280E	GTCGACCATCCCGAGCCCGCTCGC	1	103	1	103	100	Bm_ATCC23344_chr1	3510148	2978360	2978462	99	506	3.00E-18	+
BPSL3335	putative lipoprotein	3955243	3955671	-	142	53720945	3093618	-	COG4259S	GCGCGCCGCGCGGCGCGGACGAA	1	62	62	1	100	Bm_ATCC23344_chr1	3510148	2982686	2982747	100	310	2.10E-09	-
BPSL3337	putative hydrolase	3957454	3958275	+	273	53720947	3091785	-	COG0596R	CAATCACGACGATTGCCGCCGATT	1	103	1	103	100	Bm_ATCC23344_chr1	3510148	2984360	2984462	98	497	7.60E-18	+
BPSL3338	putative methyl-accepting chemotaxis protein	3958328	3960145	-	605	53720948	3094879	-	COG0840NT	CATCGCGTAAACCCTAGAGCGAAA	1	103	103	1	100	Bm_ATCC23344_chr1	3510148	2987149	2987251	100	515	1.20E-18	-
BPSL3340	putative chitin-binding protein	3962264	3962965	-	233	53720950	3093787	-	COG3397S	TCTTTAAGGTGAAATATAAAACCAG	1	103	103	1	100	Bm_ATCC23344_chr1	3510148	2989963	2990065	98	497	7.60E-18	-
BPSL3354	putative cytochrome	3976225	3977124	+	299	53720963	3094567	-	COG3245C	CGCCGCCATGTAAACGTTCCGTCTA	1	103	103	1	100	Bm_ATCC23344_chr1	3510148	3109149	3109251	100	515	1.20E-18	-
BPSL3355	ATP-dependent DNA helicase Rep	3977437	3979524	-	695	53720964	3093146	rep	COG0210L	GTCCCGCGAGCCGTTCGCCGCCGC	1	103	1	103	100	Bm_ATCC23344_chr1	3510148	3105729	3105831	100	515	1.20E-18	+
BPSL3356	hypothetical protein BPSL3356	3979774	3980622	+	282	53720965	3094568	-	NULL	GAACGCCGCGTCGGCCGTTCGGGC	1	103	103	1	100	Bm_ATCC23344_chr1	3510148	3105577	3105679	99	506	3.00E-18	-
BPSL3358	putative oxidoreductase	3981885	3982988	+	367	53720967	3093673	-	COG0673R	CCACCTGACGTTTCGCTTTCGCACG	1	103	103	1	100	Bm_ATCC23344_chr1	3510148	3103472	3103574	100	515	1.20E-18	-
BPSL3359	putative lipoprotein	3983160	3983519	+	119	53720968	3092991	-	NULL	ACAAACGGTTACAAACAATCCGGA	1	103	103	1	100	Bm_ATCC23344_chr1	3510148	3102197	3102299	100	515	1.20E-18	-
BPSL3360	aminomethyltransferase	3984376	3985494	+	372	53720969	3092626	gcvT	COG0404E	TGCACGCATCGCAGGCGGCTTCAT	1	103	103	1	100	Bm_ATCC23344_chr1	3510148	3101031	3101133	100	515	1.20E-18	-
BPSL3361	glycine cleavage system H protein	3985588	3985968	+	126	53720970	3092123	gcvH	COG0509E	CGGGCCGGGCGGCGCGCCGCCCG	1	96	96	1	100	Bm_ATCC23344_chr1	3510148	3099819	3099914	100	480	4.40E-17	-
BPSL3362	glycine dehydrogenase	3986567	3989494	+	975	53720971	3092605	gcvP	COG0403E, COG1003E	TGGGACCGGAGTAAGCGCTAAAGC	1	103	103	1	100	Bm_ATCC23344_chr1	3510148	3099134	3099236	91	434	5.30E-15	-
BPSL3363	hypothetical protein BPSL3363	3989646	3990764	+	372	53720972	3092992	-	NULL	CCGTCCGCAATACGCCGTTGCGGA	1	103	103	1	100	Bm_ATCC23344_chr1	3510148	3096055	3096157	100	515	1.20E-18	-
BPSL3365	hypothetical protein BPSL3365	3992221	3993402	-	393	53720974	3092863	-	NULL	GACAGAAAGTGACTGAGAGAGGC	1	103	1	103	100	Bm_ATCC23344_chr1	3510148	3092197	3092303	94	457	4.80E-16	+
BPSL3366	acetolactate synthase II large subunit	3993626	3995329	+	567	53720975	3094327	ilvG	COG0028EH	CCGCAGCCTCGATATTAAGAAACA	1	103	103	1	100	Bm_ATCC23344_chr1	3510148	3092071	3092173	99	506	3.00E-18	-
BPSL3368	putative AraC-family transcriptional regulator	3996544	3997623	-	359	53720977	3094237	-	NULL	GCGCCATTGCTGAACACTCGCCGC	1	103	1	103	100	Bm_ATCC23344_chr1	3510148	3087976	3088078	99	506	3.00E-18	+
BPSL3369	acetaldehyde dehydrogenase	3997868	3999388	+	506	53720978	3092657	acoD	COG1012C	GATGCACCGCGGCGCGGCGTGCTG	1	103	103	1	100	Bm_ATCC23344_chr1	3510148	3087829	3087931	100	515	1.20E-18	-
BPSL3370	hypothetical protein BPSL3370	3999653	3999898	-	81	53720979	3094238	-	NULL	CGGGCGCCCGCCGGGCGGCGGCC	1	68	1	68	100	Bm_ATCC23344_chr1	3510148	3085734	3085801	100	340	9.30E-11	+
BPSL3372	ethanolamine ammonia-lyase heavy chain	4000764	4002161	-	465	53720981	3091794	eutB	NULL	CAGGCGTGCGCCGCGCGCCGGAG	1	59	1	59	100	Bm_ATCC23344_chr1	3510148	3081889	3081947	98	286	2.60E-08	+
BPSL3373	putative ethanolamine permease	4002218	4003627	-	469	53720982	3094269	-	COG0531E	ATTTTCGATAACACGTCGATTAATG	1	103	1	103	100	Bm_ATCC23344_chr1	3510148	3080379	3080481	100	515	1.20E-18	+
BPSL3374	hypothetical protein BPSL3374	4004245	4005423	+	392	53720983	3094270	-	COG3594G	GCGAACCCCTACATTGTCCATGTGC	1	103	103	1	100	Bm_ATCC23344_chr1	3510148	3079860	3079962	100	515	1.20E-18	-

BPSL3376	hypothetical protein BPSL3376	4005796	4007307 -	503	53720985	3092848 -	NULL	AGATTAAAGTACGAGTACAGTGAA	1	103	1	103	100 Bm_ATCC23344_chr1	3510148	3076700	3076802	100	515	1.20E-18 +
BPSL3378	putative heavy metal resistance membrane ATPase	4008608	4011118 -	836	53720986	3094359 -	COG2217P	TCGAATCTCCTTACAAAAGAGTTG	1	85	1	85	100 Bm_ATCC23344_chr1	3510148	3072926	3073010	100	425	1.40E-14 +
BPSL3379	MerR family regulatory protein	4011201	4011632 +	143	53720987	3094360 -	COG0789K	GGCAACCTCCTCAGTAACTGACGG	1	85	85	1	100 Bm_ATCC23344_chr1	3510148	3072923	3073007	100	425	1.40E-14 -
BPSL3380	putative PadR transcriptional regulator	4011732	4012373 +	213	53720988	3091546 -	COG1695K	CGCGCAAGCCGCGTCACGCCCGGC	1	102	102	1	100 Bm_ATCC23344_chr1	3510148	3072392	3072493	99	501	5.00E-18 -
BPSL3383	amino acid-binding periplasmic transport protein	4013381	4014175 -	264	53720989	3091948 -	COG0834ET	CGCGCGCGGTGCCCGCCGCTGCGG	1	56	1	56	100 Bm_ATCC23344_chr1	3510148	3069898	3069953	100	280	4.80E-08 +
BPSL3387	putative fatty-acid CoA ligase	4018770	4020317 +	515	53720993	3093894 -	COG0318IQ	GGCGGCTTGCCCGCGCCCGGCCGC	1	103	103	1	100 Bm_ATCC23344_chr1	3510148	3065458	3065560	100	515	1.20E-18 -
BPSL3388	putative periplasmic amino acid binding transport p	4020565	4021704 -	379	53720994	3093895 -	COG0683E	GCAATCCTTGCCCTTCGATTCAAGA	1	87	1	87	100 Bm_ATCC23344_chr1	3510148	3062450	3062536	100	435	4.80E-15 +
BPSL3389	bifunctional proline dehydrogenase/delta-1-pyrroline	4021789	4025718 -	1309	53720995	3092967 putA	COG4230C	CCGGGTGTGAAATCTGGTTGCACCT	1	103	1	103	100 Bm_ATCC23344_chr1	3510148	3058420	3058522	100	515	1.20E-18 +
BPSL3390	primosome assembly protein PriA	4026183	4028450 -	755	53720997	3092247 priA	COG1198L	ATCCAAAACTTAGCGCCGAAAGTG	1	103	1	103	100 Bm_ATCC23344_chr1	3510148	3055688	3055790	100	515	1.20E-18 +
BPSL3391	uroporphyrinogen decarboxylase	4028891	4029985 -	364	53720998	3092343 hemE	COG0407H	TCATAACGGACGGCCGGCGAGCGT	1	103	1	103	100 Bm_ATCC23344_chr1	3510148	3054153	3054255	100	515	1.20E-18 +
BPSL3392	cyclohexadienyl dehydratase	4030125	4030910 +	261	53720999	3092973 pheC	COG0834ET	CGGACGCGGCGCGCCGCCCGGCG	1	103	103	1	100 Bm_ATCC23344_chr1	3510148	3054111	3054213	100	515	1.20E-18 -
BPSL3393	hypothetical protein BPSL3393	4031110	4031424 -	104	53721000	3093512 -	COG1832R	CCGAGAAAGATTTTGAAACGCGAT	1	103	1	103	100 Bm_ATCC23344_chr1	3510148	3052714	3052816	99	506	3.00E-18 +
BPSL3394	acyl-CoA synthase	4032011	4033741 -	576	53721001	3094034 -	COG0318IQ	TTGATGTCAGAGTGCCGTAAGCGG	1	103	1	103	100 Bm_ATCC23344_chr1	3510148	3050397	3050499	99	506	3.00E-18 +
BPSL3395	ATP synthase subunit epsilon	4033974	4034399 -	141	53721002	3093523 atpC	NULL	CGCGGGGCCAGAGTAAAGCGCTGA	1	103	1	103	100 Bm_ATCC23344_chr1	3510148	3049739	3049841	99	506	3.00E-18 +
BPSL3396	ATP synthase subunit B	4034643	4036037 -	464	53721003	3093524 atpD	COG0055C	CGCGGCACGCGCGACGCCGGCTGC	1	82	1	82	100 Bm_ATCC23344_chr1	3510148	3047936	3048017	100	410	6.40E-14 +
BPSL3400	ATP synthase subunit B	4039194	4039634 -	146	53721007	3092776 atpF	COG0711C	CGCTGATCGAATGCAACTCGATGA	1	103	1	103	100 Bm_ATCC23344_chr1	3510148	3044318	3044420	100	515	1.20E-18 +
BPSL3401	ATP synthase subunit C	4039792	4040067 -	91	53721008	3094555 atpE	COG0636C	AGCGCTGCGGGCAAAAGATAGTTT	1	73	1	73	100 Bm_ATCC23344_chr1	3510148	3043915	3043987	100	365	6.90E-12 +
BPSL3402	ATP synthase subunit A	4040138	4041007 -	289	53721009	3092611 atpB	NULL	AAAACAGCGGCGCGCGGGTTCGAC	1	97	1	97	100 Bm_ATCC23344_chr1	3510148	3042951	3043047	100	485	2.60E-17 +
BPSL3403	putative ATP synthase protein I AtpI	4041102	4041632 -	176	53721010	3094035 -	COG3312C	GCAACTATCGCTTACAATCGCCCGC	1	103	1	103	100 Bm_ATCC23344_chr1	3510148	3042320	3042422	100	515	1.20E-18 +
BPSL3408	glucose-inhibited division protein A	4045485	4047458 -	657	53721015	3092627 gidA	COG0445D	TCGGCCGTTCGAACAAGATTCGGG	1	103	1	103	100 Bm_ATCC23344_chr1	3510148	3036494	3036596	100	515	1.20E-18 +
BPSL3411	putative branched-chain amino acid ABC transporter	4050167	4051222 -	351	53721018	3094686 -	COG0559E	GTATGCCGCGCCGGGCGCGTTTCG	1	103	1	103	100 Bm_ATCC23344_chr1	3510148	3032730	3032832	100	515	1.20E-18 +
BPSL3412	putative branched-chain amino acid ABC transporter	4051535	4052692 -	385	53721019	3093378 -	COG0683E	CGTCGAACGTGAAACCGGCGCGGC	1	103	1	103	100 Bm_ATCC23344_chr1	3510148	3031260	3031362	100	515	1.20E-18 +
BPSL3413	hypothetical protein BPSL3413	4053013	4054248 -	411	53721020	3093379 -	NULL	GGCGGTGTACATATCGCTTTCGCTA	1	103	1	103	100 Bm_ATCC23344_chr1	3510148	3029704	3029806	100	515	1.20E-18 +
BPSL3415	putative branched-chain amino acid ABC transporter	4055632	4056516 -	294	53721022	3093113 -	COG0559E	GCGAATGCACGCGCGACGGCGGA	1	103	1	103	100 Bm_ATCC23344_chr1	3510148	3027436	3027538	100	515	1.20E-18 +
BPSL3416	putative branched-chain amino acid ABC transporter	4056656	4057852 -	398	53721023	3093118 -	COG0683E	CGCCGCTTTGCCGTTCCCCCGATGT	1	70	1	70	100 Bm_ATCC23344_chr1	3510148	3026133	3026202	100	350	3.30E-11 +
BPSL3418	putative branched-chain amino acid ABC transporter	4058672	4059451 -	259	53721025	3094089 -	COG0411E	AACGATACAAAGCTCGGGGGCAGC	1	103	1	103	100 Bm_ATCC23344_chr1	3510148	3024501	3024603	100	515	1.20E-18 +
BPSL3419	putative GMC oxidoreductase	4059951	4061636 -	561	53721026	3094090 -	COG2303E	GCGCGCCGGCGATCTGCGATCGGC	1	103	1	103	100 Bm_ATCC23344_chr1	3510148	3022316	3022418	100	515	1.20E-18 +
BPSL3420	methylmalonic acid semialdehyde dehydrogenase	4061811	4063337 -	508	53721027	3091856 msdA	COG1012C	CTCATTGGATCGCGCATGGGGGC	1	103	1	103	100 Bm_ATCC23344_chr1	3510148	3020615	3020717	100	515	1.20E-18 +
BPSL3421	putative LysR-family transcriptional regulator	4063478	4064377 +	299	53721028	3094692 -	COG0583K	CGCGTATCGCGCGCTTCGGGAATG	1	103	103	1	100 Bm_ATCC23344_chr1	3510148	3020572	3020674	100	515	1.20E-18 -
BPSL3422	putative adenylate cyclase	4064769	4065299 +	176	53721029	3094693 -	COG1437F	CGCGGCTTTCCGCGTCGCGTCAAA	1	103	103	1	100 Bm_ATCC23344_chr1	3510148	3019282	3019384	99	506	3.00E-18 -
BPSL3423	putative AsnC-family transcriptional regulator	4065400	4065951 -	183	53721030	3092624 -	COG1522K	GGGGTAGCCCTGCTTCGCATCAGT	1	86	1	86	100 Bm_ATCC23344_chr1	3510148	3018019	3018104	100	430	8.00E-15 +
BPSL3424	phenylalanine-4-hydroxylase	4066035	4066928 +	297	53721031	3091566 phhA	COG3186E	TCGCCCATTTTGCGCCCAAACCGCG	1	86	86	1	100 Bm_ATCC23344_chr1	3510148	3018016	3018101	100	430	8.00E-15 -
BPSL3425	pterin-4-alpha-carbinolamine dehydratase	4067012	4067317 +	101	53721032	3092625 -	NULL	CGAACAATTATGAAAGAATGGCCG	1	86	86	1	100 Bm_ATCC23344_chr1	3510148	3017039	3017124	100	430	8.00E-15 -
BPSL3426	putative two-component response regulator	4067752	4068444 +	230	53721033	3093752 -	COG0745TK	CCGTCCTGCTTGCCGCCGGGCTTCC	1	103	103	1	100 Bm_ATCC23344_chr1	3510148	3016299	3016401	100	515	1.20E-18 -
BPSL3427	putative two-component sensor kinase	4068551	4070035 +	494	53721034	3093753 -	COG0642T	GACGGTCTGACGCCTTGCGTGCGG	1	103	103	1	100 Bm_ATCC23344_chr1	3510148	3015500	3015602	99	506	3.00E-18 -
BPSL3428	hypothetical protein BPSL3428	4070477	4070989 -	170	53721035	3091595 -	COG2940R	ATGATGGCCGCCTGCGGCGCGACG	1	103	1	103	100 Bm_ATCC23344_chr1	3510148	3012961	3013063	100	515	1.20E-18 +
BPSL3431	putative MarR-family transcriptional regulator	4073388	4074029 -	213	53721038	3091796 -	COG1846K	GACGCGCTCGTCCGGGTGCGCCCT	1	103	1	103	100 Bm_ATCC23344_chr1	3510148	3010026	3010128	100	515	1.20E-18 +
BPSS0001	hypothetical protein BPSS0001	1	1188 +	395	53721040	3095690 -	NULL	CAACAGACAAACCAAAAGCGTATC	2	103	1	100	100 Bm_ATCC23344_chr2	2325379	1063	1162	97	473	6.10E-17 +
BPSS0002	integrase	1282	2325 -	347	53721041	3095691 -	NULL	GGCGGTTCGCCCGCCGCCGGGTTC	2	103	103	1	100 Bm_ATCC23344_chr2	2325379	3461	3563	100	515	7.70E-19 -
BPSS0004	DNA-binding protein	2946	3554 -	202	53721043	3094949 -	COG1396K	GCTTGCTACTGTTATAGTCGGCCGT	2	103	103	1	100 Bm_ATCC23344_chr2	2325379	4692	4794	100	515	7.70E-19 -
BPSS0007	hypothetical protein BPSS0007	6300	6953 -	217	53721046	3094950 -	NULL	GCGTTTACGGTAGAACGATCATTCT	2	92	92	1	100 Bm_ATCC23344_chr2	2325379	8091	8182	100	460	2.30E-16 -
BPSS0008	TetR-family regulatory protein	7043	7660 +	205	53721047	3097084 -	COG1309K	CCGTCCTCCCCTTTTGCCGAATGTG	2	92	1	92	100 Bm_ATCC23344_chr2	2325379	8094	8185	100	460	2.30E-16 +
BPSS0009	hypothetical protein BPSS0009	7761	8240 +	159	53721048	3095092 -	NULL	AGCCCCGCCTTTTCATTCGACCGAA	2	103	1	103	100 Bm_ATCC23344_chr2	2325379	8801	8903	100	515	7.70E-19 +
BPSS0010	hypothetical protein BPSS0010	8455	9471 -	338	53721049	3095093 -	COG3608R	CGCGGTGGAGGCATGTTCGGCCCG	2	103	103	1	100 Bm_ATCC23344_chr2	2325379	10616	10718	99	506	2.00E-18 -
BPSS0011	tartrate dehydrogenase	9774	10859 -	361	53721050	3096144 -	COG0473CE	TCGGCGATCGCGCAAGCTCCGCGC	2	103	103	1	100 Bm_ATCC23344_chr2	2325379	12004	12106	100	515	7.70E-19 -
BPSS0012	LysR-family regulatory protein	11028	11945 +	305	53721051	3096145 -	COG0583K	AAGCGATCGCGCGGAGCTTGCGCG	2	103	1	103	100 Bm_ATCC23344_chr2	2325379	12075	12177	100	515	7.70E-19 +
BPSS0013	hypothetical protein BPSS0013	12536	13213 +	225	53721052	3095048 -	COG2020O	AATTTACCGCTCGTCACGCGCGTCG	2	103	1	103	100 Bm_ATCC23344_chr2	2325379	13478	13580	100	515	7.70E-19 +
BPSS0014	glutathione S-transferase like protein	13448	14071 +	207	53721053	3096935 -	COG0625O	GGGCAAGCGCATCGGTCGATAGA	2	103	1	103	100 Bm_ATCC23344_chr2	2325379	14370	14472	100	515	7.70E-19 +
BPSS0015	hypothetical protein BPSS0015	14506	14970 -	154	53721054	3096936 -	NULL	GTCTCGCCACATGCGGCCGGCGCG	2	82	82	1	100 Bm_ATCC23344_chr2	2325379	15990	16071	98	401	1.10E-13 -
BPSS0016	phospholipase	15050	16222 -	390	53721055	3096001 -	COG1752R	AGCCGCGCGCCCGCACCGAGAAGC	2	65	65	1	100 Bm_ATCC23344_chr2	2325379	17242	17306	100	325	2.90E-10 -
BPSS0018	acetoacetate decarboxylase	17117	17857 -	246	53721057	3096265 adc	NULL	ATTCCGCAATAATCTTTTCCAGTTGC	2	103	103	1	100 Bm_ATCC23344_chr2	2325379	18877	18979	100	515	7.70E-19 -
BPSS0019	transport-related membrane protein	18316	18912 +	198	53721058	3097142 -	NULL	CTCGCGGCGCTTCGGGGTTAAGCCT	2	103	1	103	100 Bm_ATCC23344_chr2	2325379	19238	19340	100	515	7.70E-19 +
BPSS0020	hypothetical protein BPSS0020	18935	19228 -	97	53721059	3097005 -	NULL	CTATGTCGACGGCACATGGCGACG	2	103	103	1	100 Bm_ATCC23344_chr2	2325379	20248	20350	100	515	7.70E-19 -
BPSS0022	PadR-like family regulatory protein	20804	21508 -	234	53721061	3095972 -	COG1695K	GCCATCGCACTCCGCCCCCATCGTT	2	103	103	1	100 Bm_ATCC23344_chr2	2325379	22531	22632	99	500	3.70E-18 -
BPSS0023	cytochrome monooxygenase related protein	21911	23317 +	468	53721062	3096410 -	COG2124Q	GGCGGCTTCGGAAGACGTGATACT	2	103	1	103	100 Bm_ATCC23344_chr2	2325379	22841	22943	100	515	7.70E-19 +
BPSS0024	hypothetical protein BPSS0024	23467	23667 +	66	53721063	3097099 -	NULL	CGACGGGATTTTTTCGGTCATCGAT	2	103	1	103	100 Bm_ATCC23344_chr2	2325379	24397	24499	99	506	2.00E-18 +
BPSS0025	hypothetical protein BPSS0025	23841	24692 +	283	53721064	3097100 -	NULL	AGCCGCGCCGCCGAACGCGAGGCC	2	103	1	103	100 Bm_ATCC23344_chr2	2325379	24771	24873	100	515	7.70E-19 +
BPSS0029	transport-related membrane protein	28637	29257 -	206	53721068	3097043 -	COG1279R	CCTTAATCGGCATTAGATCAATTAG	2	103	103	1	100 Bm_ATCC23344_chr2	2325379	30285	30387	99	506	2.00E-18 -
BPSS0030	chromosome replication initiation inhibitor protein	29365	30258 +	297	53721069	3097061 -	COG0583K	GCGGCTTCCGGCGGTGGACTAACG	2	103	1	103	100 Bm_ATCC23344_chr2	2325379	30295	30397	99	506	2.00E-18 +
BPSS0031	anaerobic growth regulatory protein	30314	31069 -	251	53721070	3097062 -	COG0664T	ACAAGGAAATCGCACGGGGAGTCG	2	103	103	1	100 Bm_ATCC23344_chr2	2325379	32088	32190	100	515	7.70E-19 -
BPSS0032	universal stress-related protein	31219	31686 -	155	53721071	3097166 -	COG0589T	CGCTGCACCTTCGTCAGTCGTCGTT	2	103	103	1	100 Bm_ATCC23344_chr2	2325379	32705	32807	100	515	7.70E-19 -
BPSS0033	hypothetical protein BPSS0033	32074	32340 +	88	53721072	3097030 -	NULL	ACACTTCGGGCGAGAGCGTTCCCT	2	103	1	103	100 Bm_ATCC23344_chr2	2325379	32995	33097	100	515	7.70E-19 +
BPSS0034	fatty aldehyde dehydrogenase	33115	34695 +	526	53721073	3097031 -	COG1012C	ACTCGTTCAATTCGACCCATTCGAC	2	103	1	103	100 Bm_ATCC23344_chr2	2325379	34027	34129	100	515	7.70E-19 +
BPSS0036	hypothetical protein BPSS0036	36388	36804 -	138	53721075	3095159 -	NULL	GGTAAGCGCGACGCGCGGTGCGGC	2	103	103	1	100 Bm_ATCC23344_chr2	2325379	37846	37948	100	515	7.70E-19 -
BPSS0037	hypothetical protein BPSS0037	37059	37307 +	82	53721076	3095160 -	NULL	ATAGCGGTGCGCGACATGCGGCGC	2	103	1	103	100 Bm_ATCC23344_chr2	2325379	38003	38105	100	515	7.70E-19 +
BPSS0040	GTP cyclohydrolase I	39410	39964 -	184	53721079	3095266 folE	COG0302H	GAACAAAGCGAGCAAGACGGCGA	2	103	103	1	100 Bm_ATCC23344_chr2	2325379	40943	41045	100	515	7.70E-19 -
BPSS0041	LysR-family transcriptional regulator	40276	41202 -	308	53721080	3096400 -	COG0583K	AACATTGATTCGAATAGTGAATCGA	2	103	103	1	100 Bm_ATCC23344_chr2	2325379	42181	42283	100	515	7.70E-19 -
BPSS0042	4-hydroxybenzoate 3-monooxygenase	41316	42539 +	407	53721081	3095552 pobA	COG0654HC	GATATCCGTATACAACTTATCTCCT	2	103	1	103	100 Bm_ATCC23344_chr2	2325379	42197	42299	100	515	7.70E-19 +
BPSS0043	3-oxoadipate CoA-transferase subunit A	42747	43460 +	237	53721082	3095334 pcaI	COG1788I	TCGATATTCGAACCAATCGGATCGT	2	103	1	103	100 Bm_ATCC23344_chr2	2325379	43628	43730	98	497	5.00E-18 +
BPSS0045	3-carboxy-cis,cis-muconate cycloisomerase	44232	45629 +	465	53721084	3095228 pcaB	COG0015F	GACGCCGACGCGCGGCGGCGCGC	2	99	1	99	100 Bm_ATCC23344_chr2	2325379	45117	45215	98	486	1.60E-17 +
BPSS0048	4-hydroxybenzoate transporter	47008	48363 +	451	53721087	3095372 pcaK	NULL	CGGCGCGCCGGACGGCCGCCGCCC	2	103	1	103	100 Bm_ATCC23344_chr2	2325379	47889	47991	100	515	7.70E-19 +
BPSS0049	hypothetical protein BPSS0049	48431	48679 -	82	53721088	3095340 -	NULL	AATGCCCAAGCCCGTTGTGATCGA	2	103	103	1	100 Bm_ATCC23344_chr2	2325379	49658	49760	100	515	7.70E-19 -
BPSS0050	hypothetical protein BPSS0050	48780	49397 -	205	53721089	3095341 -	COG1280E	GGCAACCATCCTCAAAACTGTATCG	2	103	103	1	100 Bm_ATCC23344_chr2	2325379	50376	50478	100	515	7.70E-19 -

BPSS0052	hypothetical protein BPSS0052	50835	51263	-	142	53721091	3097155 -	COG4275S	GGCGGTGCCGGAGCGTCTCGGCCG	2	103	103	1	100	Bm_ATCC23344_chr2	2325379	52242	52344	100	515	7.70E-19	-
BPSS0054	chromate resistance exported protein	52852	53847	-	331	53721093	3096597 chrB	COG4275S	CGCCACGACGAACAGCGGACGTTC	2	103	102	1	100	Bm_ATCC23344_chr2	2325379	54827	54928	95	465	1.40E-16	-
BPSS0056	hypothetical protein BPSS0056	55366	55668	+	100	53721095	3096093 -	COG3794C	CGCCGTGCGCCCGTCGGCGCGATG	2	55	1	55	100	Bm_ATCC23344_chr2	2325379	56352	56406	100	275	5.30E-08	+
BPSS0058	excinuclease ABC subunit A	56852	62767	+	1971	53721097	3096641 uvrA	COG0178L	CACCGGTGCCCGCATCGCGGCGCG	2	103	1	103	100	Bm_ATCC23344_chr2	2325379	57787	57889	99	506	2.00E-18	+
BPSS0059	hypothetical protein BPSS0059	63800	64060	-	86	53721098	3095446 -	COG3609K	CATATCGCCGCGCACAGCCCTCCA	2	103	103	1	100	Bm_ATCC23344_chr2	2325379	65091	65193	100	515	7.70E-19	-
BPSS0061	alpha-ketoglutarate permease	66124	67476	+	450	53721101	3097120 kgtP	NULL	AGAGGCTTATGAAGGGCTCGACAG	2	103	1	103	100	Bm_ATCC23344_chr2	2325379	67040	67142	100	515	7.70E-19	+
BPSS0065	hypothetical protein BPSS0065	73093	75045	+	650	53721105	3096942 -	NULL	CACCGGCGGTAACGACGAAGATTT	2	103	1	103	100	Bm_ATCC23344_chr2	2325379	73936	74038	100	515	7.70E-19	+
BPSS0066	AraC family regulatory protein	75296	75802	+	168	53721106	3096943 -	NULL	GCGTCGGCCTTCGGCCGGTTCCGG	2	103	1	103	100	Bm_ATCC23344_chr2	2325379	76139	76241	100	515	7.70E-19	+
BPSS0067	phospholipase C precursor	76174	78369	+	731	53721107	3096819 -	NULL	GCTCGATGTGCAACGCCGCCGCAG	2	103	1	103	100	Bm_ATCC23344_chr2	2325379	77017	77119	99	506	2.00E-18	+
BPSS0145	ATP-independent RNA helicase	190115	191512	-	465	53721181	3096710 dbpA	COG0513LKJ	TGCGGCGGCGCCGTCGCGCGAGCG	2	103	3	103	98	Bm_ATCC23344_chr2	2325379	2133396	2133497	96	468	1.00E-16	+
BPSS0148	subfamily M20D non-peptidase homologue	193695	194885	-	396	53721184	3096644 -	COG1473R	TGAGCACCGACGCAATTTATCAGCT	2	103	1	103	100	Bm_ATCC23344_chr2	2325379	2129994	2130096	100	515	7.70E-19	+
BPSS0151	desaturase	197293	198459	+	388	53721187	3096555 -	COG3239I	CGCCCCGAACGCCGCGTCGCCCGG	2	103	103	1	100	Bm_ATCC23344_chr2	2325379	2127664	2127766	100	515	7.70E-19	-
BPSS0152	hypothetical protein BPSS0152	198951	199391	+	146	53721188	3095697 -	NULL	TCTCTTACCAGCGCCGTGCCCAAAG	2	103	103	1	100	Bm_ATCC23344_chr2	2325379	2125996	2126098	100	515	7.70E-19	-
BPSS0153	glutamate/aspartate periplasmic binding protein pre	199973	200872	-	299	53721189	3096477 gltI	COG0834ET	CCCGCCCCGGCGCCGCGCGCATGC	2	103	1	103	100	Bm_ATCC23344_chr2	2325379	2123953	2124055	100	515	7.70E-19	+
BPSS0154	D-amino acid dehydrogenase small subunit	201036	202283	-	415	53721190	3096536 dadA	COG0665E	CACTTGCCGCGGTCGTTTCGGTCGG	2	103	1	103	100	Bm_ATCC23344_chr2	2325379	2122542	2122644	100	515	7.70E-19	+
BPSS0155	LysR-family transcriptional regulator	202436	203368	-	310	53721191	3095698 -	COG0583K	CGCCGCCTCCCAGCCGCCTTCCGG	2	103	1	103	99	Bm_ATCC23344_chr2	2325379	2121457	2121559	99	506	2.00E-18	+
BPSS0157	hypothetical protein BPSS0157	205043	205462	+	139	53721193	3096326 -	NULL	ATGATCGCGTGCTAGCCTTGATTTC	2	103	103	1	100	Bm_ATCC23344_chr2	2325379	2119880	2119982	100	515	7.70E-19	-
BPSS0158	hypothetical protein BPSS0158	205904	207820	+	638	53721194	3096327 -	NULL	GACCAATCAACGAGGGCGGCGACG	2	103	103	1	100	Bm_ATCC23344_chr2	2325379	2119019	2119121	99	506	2.00E-18	-
BPSS0159	hypothetical protein BPSS0159	208096	210993	+	965	53721195	3095945 -	COG3537G	CGGTGTCCTAGTCTTCCAGCGCAGT	2	103	103	1	100	Bm_ATCC23344_chr2	2325379	2116828	2116930	100	515	7.70E-19	-
BPSS0160	siderophore biosynthesis-related protein	211595	212653	+	352	53721196	3096465 -	NULL	TCTCCGAACGGTTATTTCAATTAATT	2	103	103	1	100	Bm_ATCC23344_chr2	2325379	2113329	2113431	100	515	7.70E-19	-
BPSS0165	dioxygenase	218043	218969	+	308	53721201	3096416 -	COG3384S	GCCCCCACCGCTCGCCCGCTCATTC	2	57	57	1	100	Bm_ATCC23344_chr2	2325379	2106896	2106952	98	276	4.80E-08	-
BPSS0166	LysR family regulatory protein	219213	220175	-	320	53721202	3096735 -	COG0583K	TATCCAGCACCGGGCTCATGTCGCC	2	103	1	103	100	Bm_ATCC23344_chr2	2325379	2104667	2104769	99	506	2.00E-18	+
BPSS0168	hypothetical protein BPSS0168	225502	226767	-	421	53721204	3096620 -	COG1360N, COG3455S	CGGCGAGTGCGCGCAGCGGCCGG	2	91	1	91	100	Bm_ATCC23344_chr2	2325379	2098090	2098180	100	455	3.90E-16	+
BPSS0170	lipoprotein	228224	228736	-	170	53721206	3096592 -	COG3521S	GCGGCGCCCGCGCGGGGCCCGCC	2	100	1	100	100	Bm_ATCC23344_chr2	2325379	2096112	2096211	100	500	3.70E-18	+
BPSS0171	hypothetical protein BPSS0171	228834	229319	-	161	53721207	3096593 -	NULL	CGTCCCCGGTCGGCCGGCTTCGCG	2	103	1	103	100	Bm_ATCC23344_chr2	2325379	2095526	2095628	99	506	2.00E-18	+
BPSS0175	hypothetical protein BPSS0175	235059	235778	+	239	53721211	3096077 -	NULL	GAGATAAGGCGGAGTGCGCCGGAT	2	103	103	1	100	Bm_ATCC23344_chr2	2325379	2089900	2090002	100	515	7.70E-19	-
BPSS0181	hypothetical protein BPSS0181	241527	243854	+	775	53721217	3096254 -	COG3501S	GCGCGGCCGGCGCGCGAGATGCG	2	74	74	1	100	Bm_ATCC23344_chr2	2325379	2083475	2083548	100	370	2.70E-12	-
BPSS0184	hypothetical protein BPSS0184	247624	248283	+	219	53721220	3095906 -	NULL	TGCGTTGTCGGCGCGTTGCCGATGT	2	103	103	1	100	Bm_ATCC23344_chr2	2325379	2077378	2077480	97	488	1.30E-17	-
BPSS0186	hypothetical protein BPSS0186	249406	251322	-	638	53721222	3095747 -	NULL	CAATCACGCCGCTGTTTGAGCCAAT	2	103	1	103	100	Bm_ATCC23344_chr2	2325379	2073564	2073666	100	515	7.70E-19	+
BPSS0187	LysR-family transcriptional regulator	251662	252555	-	297	53721223	3096255 -	COG0583K	ATCGGGAGAATCGATGGATTGACT	2	98	1	98	100	Bm_ATCC23344_chr2	2325379	2072336	2072433	100	490	1.00E-17	+
BPSS0188	dehydrogenase	252651	253427	+	258	53721224	3095845 -	COG2249R	TGCAATACCTGGAATATTGGAAATC	2	98	98	1	100	Bm_ATCC23344_chr2	2325379	2072333	2072430	100	490	1.00E-17	-
BPSS0190	O-acetylhomoserine	254427	255722	-	431	53721225	3096143 -	COG2873E	TCACCAAACAAGCTAAAAGCGCGC	2	103	1	103	100	Bm_ATCC23344_chr2	2325379	2069164	2069266	100	515	7.70E-19	+
BPSS0193	hypothetical protein BPSS0193	258412	259767	-	451	53721228	3096290 -	COG1457F	GACGTTGTGACGCGCCCCGCGTCG	2	103	1	103	100	Bm_ATCC23344_chr2	2325379	2065260	2065362	99	506	2.00E-18	+
BPSS0198	hypothetical protein BPSS0198	263527	264327	-	266	53721233	3096371 -	COG1540R	ATAGAACTATTTTGGCGACCGCGCC	2	103	1	103	100	Bm_ATCC23344_chr2	2325379	2060700	2060802	98	497	5.00E-18	+
BPSS0199	LysR-family transcriptional regulator	264595	265509	+	304	53721234	3096372 -	COG0583K	GACCGGGCGCGAGCGGCGGGCTG	2	103	103	1	100	Bm_ATCC23344_chr2	2325379	2060530	2060632	98	497	5.00E-18	-
BPSS0201	prolyl iminopeptidase	269128	270066	+	312	53721236	3095394 pip	COG0596R	CATCGCATGGGGCGCACGCATGCG	2	103	103	1	100	Bm_ATCC23344_chr2	2325379	2055910	2056012	100	515	7.70E-19	-
BPSS0202	hypothetical protein BPSS0202	270241	271134	+	297	53721237	3096621 -	COG0500QR	CGTCGTTGCCGGTGCGCGAAGCGG	2	103	103	1	100	Bm_ATCC23344_chr2	2325379	2054811	2054913	100	515	7.70E-19	-
BPSS0204	methyl-accepting chemotaxis protein I	274149	275777	-	542	53721239	3095098 tsr	COG0840NT	CCGCGGCCATTCGTCTGTTTTAAGG	2	103	1	103	100	Bm_ATCC23344_chr2	2325379	2049107	2049209	100	515	7.70E-19	+
BPSS0205	sigma interaction-related Fis-family transcriptional re	276127	278211	-	694	53721240	3095843 prpR	COG3829KT	ATATTGAAATTAAAATTCCATAATTG	2	103	1	103	100	Bm_ATCC23344_chr2	2325379	2046673	2046775	98	497	5.00E-18	+
BPSS0206	probable methylisocitrate lyase	278428	279321	+	297	53721241	3095582 prpB	COG2513G	GTGCGCCGAATGCGGAAAGCGCGC	2	103	103	1	100	Bm_ATCC23344_chr2	2325379	2046554	2046656	99	506	2.00E-18	-
BPSS0208	aconitate hydratase	280616	283210	+	864	53721243	3095728 acnA	COG1048C	TCGGCGGCACCCGGCGGCCGGGCC	2	76	76	1	100	Bm_ATCC23344_chr2	2325379	2044366	2044441	100	380	9.60E-13	-
BPSS0209	hypothetical protein BPSS0209	283368	284558	+	396	53721244	3096880 -	NULL	CGTTCGCCGCCGAGGCGAACGGGC	2	103	103	1	100	Bm_ATCC23344_chr2	2325379	2041613	2041716	99	505	2.20E-18	-
BPSS0212	hypothetical protein BPSS0212	286414	287052	+	212	53721247	3096247 -	NULL	CCGCGAATCCGCCGCTTCGGCGAG	2	95	95	1	100	Bm_ATCC23344_chr2	2325379	2037969	2038063	98	466	1.30E-16	-
BPSS0213	hypothetical protein BPSS0213	287105	287695	+	196	53721248	3096906 -	NULL	GCGGCGCGGCCTTGCATCGCCATC	2	55	55	1	100	Bm_ATCC23344_chr2	2325379	2037278	2037332	98	266	1.40E-07	-
BPSS0214	heme/metallo cofactor biosynthesis related protein	287774	289249	+	491	53721249	3096907 -	COG0535R	GCCCGCGCGCGCCGTTTGCCGCAC	2	81	81	1	100	Bm_ATCC23344_chr2	2325379	2036609	2036689	98	396	1.80E-13	-
BPSS0215	methyl-accepting chemotaxis protein	289471	291084	+	537	53721250	3096604 tar	COG0840NT	GGCCGCAGAGCCCCTACGCCGCGT	2	103	103	1	100	Bm_ATCC23344_chr2	2325379	2034962	2035064	100	515	7.70E-19	-
BPSS0216	hypothetical protein BPSS0216	291447	293084	-	545	53721251	3096037 -	NULL	ATTGCTTTTTCATGCAATAAAGATG	2	103	1	103	100	Bm_ATCC23344_chr2	2325379	2031426	2031528	100	515	7.70E-19	+
BPSS0217	LysR-family transcriptional regulator	293213	294070	+	285	53721252	3096243 -	COG0583K	CGGCATGTTAGGCTGAGCGGCTGT	2	103	103	1	100	Bm_ATCC23344_chr2	2325379	2031395	2031497	100	515	7.70E-19	-
BPSS0218	isomerase	294153	295256	-	367	53721253	3096244 -	COG2942G	CCATGTTCGACGGCGGCAGCCCGC	2	103	1	103	100	Bm_ATCC23344_chr2	2325379	2029244	2029347	97	487	1.40E-17	+
BPSS0219	MgtC family membrane protein	295367	296062	+	231	53721254	3096098 -	COG1285S	GGCGCGGTTGACGGGGGAAGAAC	2	103	103	1	100	Bm_ATCC23344_chr2	2325379	2029231	2029334	97	487	1.40E-17	-
BPSS0223	hypothetical protein BPSS0223	299703	300371	-	222	53721258	3096389 -	NULL	GCGGGCGACAAATTGCGACACGGC	2	103	1	103	100	Bm_ATCC23344_chr2	2325379	2024395	2024497	100	515	7.70E-19	+
BPSS0226	lipoprotein	305951	307372	+	473	53721261	3096089 -	COG5361S	ACAGCCGGCCGGCACGCACGCTAC	2	103	103	1	100	Bm_ATCC23344_chr2	2325379	2018657	2018759	100	515	7.70E-19	-
BPSS0229	hypothetical protein BPSS0229	312200	313861	-	553	53721264	3096456 -	COG3119P	GACAGACAAACGAAAGCGGCGCGT	2	103	1	103	100	Bm_ATCC23344_chr2	2325379	2010684	2010786	100	515	7.70E-19	+
BPSS0230	Glyoxalase/Bleomycin resistance protein/Dioxygenas	314442	314834	-	130	53721265	3096457 -	COG0346E	CACGGCGACGTTGCGCAGGGGCGC	2	103	1	103	100	Bm_ATCC23344_chr2	2325379	2009711	2009813	100	515	7.70E-19	+
BPSS0231	hypothetical protein BPSS0231	314959	315243	-	94	53721266	3095687 -	NULL	GCAGGGTGCGACCGGGCGCGCCGC	2	103	1	103	100	Bm_ATCC23344_chr2	2325379	2009302	2009404	100	515	7.70E-19	+
BPSS0232	squalene/phytoene synthase	315412	316482	+	356	53721267	3096335 -	COG1562I	CGCGCCGTGCGGCGCGCCCGGTCG	2	103	103	1	100	Bm_ATCC23344_chr2	2325379	2009231	2009333	100	515	7.70E-19	-
BPSS0235	cytochrome oxidase subunit I	319052	320464	-	470	53721269	3095529 -	COG1271C	GCGATACTCGGCTCCGACTCTACGC	2	103	1	103	100	Bm_ATCC23344_chr2	2325379	2004078	2004180	100	515	7.70E-19	+
BPSS0236	L-allo-threonine aldolase	321065	322078	+	337	53721270	3097028 ltaE	COG2008E	GACGCCGAATTGCGTACACTCGAA	2	103	103	1	100	Bm_ATCC23344_chr2	2325379	2003580	2003682	100	515	7.70E-19	-
BPSS0237	chromosome replication initiation inhibitor protein	322047	323024	-	325	53721271	3095530 -	COG0583K	GTCCGGGGAGGATCCCGATACCGA	2	103	1	103	100	Bm_ATCC23344_chr2	2325379	2001523	2001625	100	515	7.70E-19	+
BPSS0238	penicillin-binding protein	323180	325342	+	720	53721272	3095959 -	COG0744M	CAACTTGAGCTAAATATTGTATCGG	2	103	103	1	100	Bm_ATCC23344_chr2	2325379	2001465	2001567	100	515	7.70E-19	-
BPSS0243	hemin ABC transport system-related protein	329697	330848	-	383	53721277	3095000 -	COG3720P	TGCGGCCCGCACGCTTTTTTCCCAA	2	59	1	59	100	Bm_ATCC23344_chr2	2325379	1993890	1993948	100	295	6.70E-09	+
BPSS0244	exported heme receptor protein	330905	333184	-	759	53721278	3096221 -	COG1629P	CGCCGTCCAGACGCGCGGGCGCCA	2	103	1	103	100	Bm_ATCC23344_chr2	2325379	1991486	1991588	100	515	7.70E-19	+
BPSS0245	hypothetical protein BPSS0245	333659	333886	-	75	53721279	3096316 -	NULL	CCGCGCGCCGCTCGGCTTTTGCGCC	2	103	1	103	100	Bm_ATCC23344_chr2	2325379	1990783	1990885	98	497	5.00E-18	+
BPSS0246	LysR-family transcriptional regulator	334048	334944	-	298	53721280	3096317 -	COG0583K	ATCGCGACGACGGCCGCCAGTGTG	2	103	1	103	100	Bm_ATCC23344_chr2	2325379	1989725	1989827	99	506	2.00E-18	+
BPSS0247	ABC transport system, membrane protein	335111	335959	+	282	53721281	3096126 -	COG1177E	GTCGCGCGCCCACACTGGCGGCCG	2	103	103	1	100	Bm_ATCC23344_chr2	2325379	1989656	1989758	100	515	7.70E-19	-
BPSS0252	porin membrane protein	339553	340752	+	399	53721286	3095081 -	COG3203M	CCGCATCGGGGGCGGGGGAATGA	2	69	69	1	100	Bm_ATCC23344_chr2	2325379	1985214	1985282	98	336	9.40E-11	-
BPSS0254	ribose 5-phosphate isomerase	341809	342303	-	164	53721288	3096194 -	COG0698G	ATGAGCAAAATTTCGCCCTTTGCCA	2	103	1	103	100	Bm_ATCC23344_chr2	2325379	1982370	1982472	99	506	2.00E-18	+
BPSS0255	ribose ABC transport system, ATP-binding protein	342542	344059	+	505	53721289	3096195 -	COG1129G	AAAGTAGGGTGGTGTGGTGCATGT	2	103	103	1	100	Bm_ATCC23344_chr2	2325379	1982229	1982331	99	506	2.00E-18	-
BPSS0259	dehydrogenase	346953	348089	+	378	53721293	3095744 -	COG1063ER	GGCGGCGGCCGCGCAATCATCGCC	2	68	68	1	100	Bm_ATCC23344_chr2	2325379	1977900	1977967	100	340	6.20E-11	-
BPSS0263	hypothetical protein BPSS0263	350258	350713	-	151	53721297	3096310 -	NULL	ATTGCGTTGGAATCCCTTTGCCCGC	2	103	1	103	100	Bm_ATCC23344_chr2	2325379	1974042	1974144	100	515	7.70E-19	+
BPSS0264	hypothetical protein BPSS0264	351133	351393	-	86	53721298	3096122 -	NULL	TCACACCCCCCCTGTTGGCCGCCTG	2	103	1	103	100	Bm_ATCC23344_chr2	2325379	1973361	1973463	99	506	2.00E-18	+
BPSS0266	hypothetical protein BPSS0266	353062	353862	+	266	53721300	3095082 -	COG1464P	GGAATATATGTATGAAAATTGCTTG	2	103	103	1	100	Bm_ATCC23344_chr2	2325379	1635286	1635388	100	515	7.70E-19	-
BPSS0267	hypothetical protein BPSS0267	354335	355450	-	371	53721301	3096044 -	COG3608R	TCGAACGGCACAGGTTTGGTGCGT	2	103	1	103	100	Bm_ATCC23344_chr2	2325379	1632800	1632902	100	515	7.70E-19	+

BPSS0268	O-antigen acetylase	355722	357836 +	704	53721302	3096045 -	COG1835I	TTTTTAATCGATACACTGTTCGCCGC	2	103	103	1	100	Bm_ATCC23344_chr2	2325379	1632626	1632728	100	515	7.70E-19 -
BPSS0285	hypothetical protein BPSS0285	381038	381946 -	302	53721320	3096046 -	COG3741E	AAGCACTTGACCGGCCAATTTGGG	2	103	1	103	100	Bm_ATCC23344_chr2	2325379	1606053	1606155	100	515	7.70E-19 +
BPSS0318	hypothetical protein BPSS0318	445815	446642 -	275	53721353	3096051 -	NULL	TCGGCGCGAAAACCGCCGGCCGTC	2	103	1	103	100	Bm_ATCC23344_chr2	2325379	1541743	1541845	100	515	7.70E-19 +
BPSS0324	glycolate permease	452083	453801 +	572	53721359	3095424 glcA	COG1620C	CGCCGCACCGACAACAAAGCGCCG	2	103	103	1	100	Bm_ATCC23344_chr2	2325379	1536463	1536565	100	515	7.70E-19 -
BPSS0325	hypothetical protein BPSS0325	454103	454540 +	145	53721360	3096205 -	COG2259S	CTTTTGCTTGGAACGACGGACGAG	2	103	103	1	100	Bm_ATCC23344_chr2	2325379	1534416	1534518	100	515	7.70E-19 -
BPSS0327	sugar transport-related, membrane protein	456588	457880 -	430	53721362	3096012 -	NULL	CCACCCCGCGCGTTGCACCGTCTG	2	103	1	103	100	Bm_ATCC23344_chr2	2325379	1530590	1530692	100	515	7.70E-19 +
BPSS0329	fatty aldehyde dehydrogenase	459223	460815 -	530	53721364	3095117 -	COG1012C	CGCGCGGCGCGGGCGCATCCGCTT	2	78	1	78	100	Bm_ATCC23344_chr2	2325379	1527654	1527731	100	390	3.40E-13 +
BPSS0331	dihydrodipicolinate synthase	462961	463881 -	306	53721366	3096903 -	COG0329EM	CGGTTGGAAAAGGCTCAAGCCTCG	2	103	1	103	100	Bm_ATCC23344_chr2	2325379	1524613	1524715	100	515	7.70E-19 +
BPSS0332	hypothetical protein BPSS0332	464132	465064 +	310	53721367	3097013 -	COG3938E	GGGCTGTGCCGATTTCGTCGCGCG	2	103	103	1	100	Bm_ATCC23344_chr2	2325379	1524460	1524562	100	515	7.70E-19 -
BPSS0336	AraC-family transcriptional regulator	467905	468621 +	238	53721371	3095560 -	NULL	CACGGCCGCCGGCCCGACGCCGGC	2	103	103	1	100	Bm_ATCC23344_chr2	2325379	1520678	1520780	99	506	2.00E-18 -
BPSS0337	hypothetical protein BPSS0337	468818	469087 -	89	53721372	3095561 -	NULL	ATGCCGTAAACCTTTCGCCGGGCC	2	103	1	103	100	Bm_ATCC23344_chr2	2325379	1519397	1519499	100	515	7.70E-19 +
BPSS0339	amino acid dioxygenase	470668	472722 -	684	53721374	3096498 -	COG1082G, COG3185ER	CTCCGACCTTCGACGGCACGCGGG	2	103	103	1	100	Bm_ATCC23344_chr2	2325379	873971	874073	100	515	7.70E-19 -
BPSS0340	3-dehydroquinate dehydratase	472982	473437 +	151	53721375	3097223 aroQ2	COG0757E	AAGACATTGCGCGAGCGCAAGCGC	2	103	1	103	100	Bm_ATCC23344_chr2	2325379	874133	874235	100	515	7.70E-19 +
BPSS0345	ABC transport system, ATP-binding protein	478571	479668 +	365	53721380	3096022 -	COG3842E	GCGGCCGCCGCGGCCGCGCCAAG	2	59	1	59	100	Bm_ATCC23344_chr2	2325379	879617	879675	100	295	6.70E-09 +
BPSS0352	hypothetical protein BPSS0352	492322	493167 -	281	53721387	3096091 -	COG2961R	GCCGCGTCGGTCGGCCATGTCCAC	2	103	103	1	100	Bm_ATCC23344_chr2	2325379	896389	896491	100	515	7.70E-19 -
BPSS0353	potassium channel subunit	493316	494287 -	323	53721388	3095964 -	COG0667C	TATCGCCGCGCGGCCGACACCGCT	2	103	103	1	100	Bm_ATCC23344_chr2	2325379	897509	897611	100	515	7.70E-19 -
BPSS0354	3-hydroxybutyrate dehydrogenase	494475	495263 +	262	53721389	3095965 -	COG1028IQR	CCGCGCGGCGATAGCCGGCCATTC	2	103	1	103	100	Bm_ATCC23344_chr2	2325379	897599	897701	100	515	7.70E-19 +
BPSS0355	aspartate aminotransferase	495489	496688 -	399	53721390	3095099 tyrB	COG1448E	TCTCGCCGGAGTTGCCGCGGCTCG	2	103	103	1	100	Bm_ATCC23344_chr2	2325379	901496	901598	100	515	7.70E-19 -
BPSS0356	excinuclease ABC subunit B	496976	499066 +	696	53721391	3096546 uvrB	COG0556L	TTCGGTCAGCCAATCCCGAGCGCG	2	103	1	103	100	Bm_ATCC23344_chr2	2325379	901686	901788	100	515	7.70E-19 +
BPSS0357	hypothetical protein BPSS0357	499290	499841 +	183	53721392	3096107 -	COG3470P	ATAGTTCGCATTAACGTTCTTCATCC	2	103	1	103	100	Bm_ATCC23344_chr2	2325379	904000	904102	100	515	7.70E-19 +
BPSS0359	hypothetical protein BPSS0359	500290	501132 +	280	53721394	3095950 -	COG0672P	CGGTTTTCGCGTTTGACGCGCGGCA	2	62	1	62	100	Bm_ATCC23344_chr2	2325379	905041	905102	100	310	1.40E-09 +
BPSS0361	hypothetical protein BPSS0361	502589	502999 +	136	53721396	3096720 -	COG3193R	CGCGCTTCGGGCGGTATGATGCAT	2	62	1	62	100	Bm_ATCC23344_chr2	2325379	907340	907401	100	310	1.40E-09 +
BPSS0362	hypothetical protein BPSS0362	503302	503565 -	87	53721397	3096721 -	COG4256P	CTATCGACATTCGGTTTTCGATCGA	2	103	1	103	100	Bm_ATCC23344_chr2	2325379	1642554	1642656	99	506	2.00E-18 +
BPSS0363	hypothetical protein BPSS0363	503709	504194 -	161	53721398	3095788 -	NULL	GCCGAACGGCGAAGGGGGAAGCT	2	103	1	103	100	Bm_ATCC23344_chr2	2325379	1641925	1642027	100	515	7.70E-19 +
BPSS0364	chromosome condensation protein	504313	505185 -	290	53721399	3095894 -	COG1741R	GATCCATTGCGAATCGAGCGGATTT	2	103	1	103	100	Bm_ATCC23344_chr2	2325379	1640934	1641036	100	515	7.70E-19 +
BPSS0365	LysR-family transcriptional regulator	505308	506279 +	323	53721400	3095895 -	COG0583K	AAATAGCGTTATTTTTGCAACGCAA	2	103	103	1	100	Bm_ATCC23344_chr2	2325379	1640909	1641011	100	515	7.70E-19 -
BPSS0368	TonB-like transport protein	507869	508564 -	231	53721403	3096504 -	COG0810M	CAGCACTTCGCGGCGCGCCTCGCG	2	103	1	103	100	Bm_ATCC23344_chr2	2325379	1969628	1969730	100	515	7.70E-19 +
BPSS0369	bacterioferritin ferredoxin protein	508840	509079 -	79	53721404	3095842 -	COG2906P	AAGCATCTGGTTTTGTTACAAAAAA	2	103	1	103	100	Bm_ATCC23344_chr2	2325379	1969113	1969215	100	515	7.70E-19 +
BPSS0371	bacterioferritin	510130	510606 -	158	53721406	3097235 bfr	COG2193P	CGTTCCATCTTTCGGATATCGAACG	2	103	1	103	100	Bm_ATCC23344_chr2	2325379	1967586	1967688	100	515	7.70E-19 +
BPSS0373	hypothetical protein BPSS0373	512336	512944 -	202	53721408	3094938 -	COG2862S	CGCGATGCCGCGCCGCTGCAAGCC	2	103	1	103	100	Bm_ATCC23344_chr2	2325379	1965248	1965350	99	506	2.00E-18 +
BPSS0374	hypothetical protein BPSS0374	513177	514076 -	299	53721409	3095533 -	COG0697GER	GAACTTACCCGTCACTTACGCGACA	2	103	1	103	100	Bm_ATCC23344_chr2	2325379	1964120	1964222	99	506	2.00E-18 +
BPSS0375	acetyl-coenzyme A synthetase	514366	516348 +	660	53721410	3095797 acoE	COG0365I	GCGCGCAGGGTGGGAGACAGGCG	2	103	103	1	100	Bm_ATCC23344_chr2	2325379	1963928	1964030	100	515	7.70E-19 -
BPSS0492	alkyl hydroperoxide reductase protein	669449	670012 -	187	53721528	3096133 ahpC	COG0450O	AATACCGATTCCGATCATTGAAACA	2	103	1	103	100	Bm_ATCC23344_chr2	2325379	1955780	1955882	99	506	2.00E-18 +
BPSS0493	chitin-binding protein	670272	671369 -	365	53721529	3096851 -	COG3397S	CGCATCACATTTCGATATCCAAAAC	2	103	1	103	100	Bm_ATCC23344_chr2	2325379	1954423	1954525	99	506	2.00E-18 +
BPSS0494	hypothetical protein BPSS0494	671929	672432 -	167	53721530	3096852 -	NULL	CGGCCGCGCCGCCCGACACCCACG	2	77	1	77	100	Bm_ATCC23344_chr2	2325379	1953388	1953464	100	385	5.70E-13 +
BPSS0495	nitroreductase family protein	672507	673130 -	207	53721531	3095734 -	COG0778C	GTATCGAATCAGCGACATTGATAAA	2	103	1	103	100	Bm_ATCC23344_chr2	2325379	1952664	1952766	100	515	7.70E-19 +
BPSS0496	hypothetical protein BPSS0496	673843	675279 +	478	53721532	3096563 -	COG2308S	CCAAAGTGCCAAATCAGACGGAAG	2	103	103	1	100	Bm_ATCC23344_chr2	2325379	1952049	1952151	100	515	7.70E-19 -
BPSS0497	hypothetical protein BPSS0497	675374	676315 +	313	53721533	3096564 -	COG2307S	GCGCACGCGCACGGCGGCGCTCGC	2	97	97	1	100	Bm_ATCC23344_chr2	2325379	1950518	1950614	98	476	4.40E-17 -
BPSS0499	transglutaminase-like superfamily protein	677417	680839 +	1140	53721535	3096215 -	COG1305E, COG4196S	TGCGGCGCGCGATGCCGCCGCGCG	2	103	103	1	100	Bm_ATCC23344_chr2	2325379	1948475	1948577	100	515	7.70E-19 -
BPSS0500	hypothetical protein BPSS0500	681027	683675 +	882	53721536	3096216 -	COG2307S, COG2308S	ACGTCGCGCTTGCGGCGTGCGGCA	2	103	103	1	100	Bm_ATCC23344_chr2	2325379	1944865	1944967	100	515	7.70E-19 -
BPSS0502	GntR family regulatory protein	684891	686393 -	500	53721538	3095927 -	COG1167KE	GCGAGGTTCTCGCGGCCTGGATCG	2	103	1	103	100	Bm_ATCC23344_chr2	2325379	1939416	1939518	98	497	5.00E-18 +
BPSS0503	transporter protein	686521	687708 +	395	53721539	3096742 -	NULL	GGTTCTTCGGCTTATACCACATTGC	2	103	103	1	100	Bm_ATCC23344_chr2	2325379	1939386	1939488	99	506	2.00E-18 -
BPSS0504	hypothetical protein BPSS0504	688046	689119 -	357	53721540	3095305 -	COG1289S	GCGATGGACAGCGCATCATGCTTA	2	103	1	103	100	Bm_ATCC23344_chr2	2325379	1936714	1936816	100	515	7.70E-19 +
BPSS0505	AraC family regulatory protein	689358	690323 -	321	53721541	3095306 -	COG4977K	CGAGGCGACGGTGCGCATCGCCCC	2	103	1	103	100	Bm_ATCC23344_chr2	2325379	1935510	1935612	98	497	5.00E-18 +
BPSS0506	DJ-1/PfpI family protein	690465	691163 +	232	53721542	3096430 -	NULL	CGCCAATCGGTCGCGCGCTACGCT	2	103	103	1	100	Bm_ATCC23344_chr2	2325379	1935466	1935568	96	479	3.30E-17 -
BPSS0513	CDP-alcohol phosphatidyltransferase	697828	698517 -	229	53721549	3096807 -	COG0558I	CAGCCGACGCCAAACCTTACACTTT	2	103	2	103	99	Bm_ATCC23344_chr2	2325379	1927328	1927429	100	510	1.30E-18 +
BPSS0514	acetyl-CoA hydrolase/transferase	698792	700396 +	534	53721550	3096808 -	COG0427C	ATATCCAGCAAATCCGGCAAGTTAC	2	103	103	1	100	Bm_ATCC23344_chr2	2325379	1927150	1927252	100	515	7.70E-19 -
BPSS0515	hypothetical protein BPSS0515	702116	703054 +	312	53721551	3095506 -	COG3515S	CTAATACATTCGAAGAAATAAGTTT	2	103	103	1	100	Bm_ATCC23344_chr2	2325379	1923837	1923939	100	515	7.70E-19 -
BPSS0518	hypothetical protein BPSS0518	705288	705815 +	175	53721554	3096785 -	NULL	GTGTTCGAGGCGCTGCGGGATGGA	2	103	1	103	100	Bm_ATCC23344_chr2	2325379	436115	436217	99	506	2.00E-18 +
BPSS0524	hypothetical protein BPSS0524	714509	716797 +	762	53721560	3095481 -	COG3501S	ACAGTAGCGTCGCCGCGCCTGAAC	2	103	1	103	100	Bm_ATCC23344_chr2	2325379	445291	445393	98	497	5.00E-18 +
BPSS0531	hypothetical protein BPSS0531	723278	724936 +	552	53721567	3095834 -	COG1360N, COG3455S	CCCACGACATGAGACGCAATCTGC	2	85	1	85	100	Bm_ATCC23344_chr2	2325379	454078	454162	100	425	8.90E-15 +
BPSS0533	hypothetical protein BPSS0533	728495	728854 +	119	53721569	3096749 -	NULL	TGCGTCGAGCATCGGTATTCATGCG	2	61	1	61	100	Bm_ATCC23344_chr2	2325379	459319	459379	100	305	2.40E-09 +
BPSS0535	hypothetical protein BPSS0535	729527	729898 +	123	53721571	3095636 -	NULL	GCATAAAACTATTGTATAAACACCG	2	103	1	103	100	Bm_ATCC23344_chr2	2325379	460309	460411	100	515	7.70E-19 +
BPSS0536	3-hydroxydecanoyl-ACP:CoA transacylase	730449	731348 +	299	53721572	3096849 -	COG0596R	CAGGGCGGGCAGATCGCGCGGGA	2	103	1	103	100	Bm_ATCC23344_chr2	2325379	461231	461333	100	515	7.70E-19 +
BPSS0537	UDP-glucoronosyl and UDP-glucosyl transferase	731570	732889 +	439	53721573	3096379 -	COG1819GC	CCGGCGGGGCGCATCCCGGCCGGC	2	103	1	103	100	Bm_ATCC23344_chr2	2325379	462352	462454	100	515	7.70E-19 +
BPSS0539	hypothetical protein BPSS0539	734725	735720 +	331	53721575	3095614 -	COG1216R	GACGAAGCCGAACGGAGAAGCAT	2	103	1	103	100	Bm_ATCC23344_chr2	2325379	465519	465621	100	515	7.70E-19 +
BPSS0541	HlyD family secretion protein	738188	739354 +	388	53721577	3096737 -	COG1566V	GCACGCCACCATCTTGAACGACAC	2	103	1	103	100	Bm_ATCC23344_chr2	2325379	468982	469084	100	515	7.70E-19 +
BPSS0542	glycosyl hydrolase	739867	741531 -	554	53721578	3095630 -	NULL	GCCTTCCTGCGCGCGGACGGCCGG	2	103	103	1	100	Bm_ATCC23344_chr2	2325379	472512	472614	100	515	7.70E-19 -
BPSS0543	levansucrase precursor	741664	743229 -	521	53721579	3095602 -	NULL	ACTTTTGACGCCCAAACCGGTTTGA	2	103	103	1	100	Bm_ATCC23344_chr2	2325379	474210	474312	100	515	7.70E-19 -
BPSS0544	LacI family regulatory protein	743418	744434 +	338	53721580	3095603 -	COG1609K	GGCGTCAAAAGTAATTTATTTGACG	2	103	1	103	100	Bm_ATCC23344_chr2	2325379	474301	474403	100	515	7.70E-19 +
BPSS0545	glutathione-independent formaldehyde dehydrogen	744930	746129 +	399	53721581	3095054 fdhA	COG1063ER	CGGACTACCCTCGAAAGGCTCGCT	2	103	1	103	100	Bm_ATCC23344_chr2	2325379	475757	475859	100	515	7.70E-19 +
BPSS0546	AraC family regulatory protein	746307	747332 -	341	53721582	3094917 -	COG4977K	TCTAGGGTTTAGCCGTATGGCACGC	2	103	103	1	100	Bm_ATCC23344_chr2	2325379	478257	478359	100	515	7.70E-19 -
BPSS0547	serine hydroxymethyltransferase	747751	749040 +	429	53721583	3096429 glyA	COG0112E	TCCGAACGACAAGAATAGAGCCGT	2	103	1	103	100	Bm_ATCC23344_chr2	2325379	478578	478680	100	515	7.70E-19 +
BPSS0548	family M19 non-peptidase homologue	749113	750084 +	323	53721584	3095707 -	COG2355E	CACGCCGGAGCGGAACGAACGCC	2	75	1	75	100	Bm_ATCC23344_chr2	2325379	479968	480042	100	375	1.60E-12 +
BPSS0549	hypothetical protein BPSS0549	750235	750768 +	177	53721585	3095708 -	COG1719R	ATCCGATACAGGACACGCGCGTGC	2	103	1	103	100	Bm_ATCC23344_chr2	2325379	481062	481164	100	515	7.70E-19 +
BPSS0550	NADH:flavin oxidoreductase / NADH oxidase family	750829	752892 +	687	53721586	3095620 -	COG0446R, COG1902C	TCCGCCGACGCCGAACCCCACTCG	2	63	1	63	100	Bm_ATCC23344_chr2	2325379	481696	481758	100	315	8.30E-10 +
BPSS0556	hypothetical protein BPSS0556	759348	760211 +	287	53721592	3095782 -	COG2113E	CGCTGCGCAAGAACGTGTCGTAAA	2	103	1	103	100	Bm_ATCC23344_chr2	2325379	490175	490277	100	515	7.70E-19 +
BPSS0557	hypothetical protein BPSS0557	760392	762047 +	551	53721593	3095539 -	COG0531E	TTGCGGGTTTTTCCGCGGGAATTTC	2	103	1	103	100	Bm_ATCC23344_chr2	2325379	491219	491321	100	515	7.70E-19 +
BPSS0558	formyltetrahydrofolate deformylase	762134	763009 -	291	53721594	3095540 -	COG0788F	GGTTCTATCCGCATCCGATCTGGCT	2	103	103	1	100	Bm_ATCC23344_chr2	2325379	493934	494036	99	506	2.00E-18 -
BPSS0559	LysR family regulatory protein	763153	764052 -	299	53721595	3096278 -	COG0583K	CGGCGGCGCGACAGGCTGCCGCCC	2	103	103	1	100	Bm_ATCC23344_chr2	2325379	494977	495079	99	506	2.00E-18 -
BPSS0560	choline-sulfatase	764188	765741 +	517	53721596	3096948 -	COG3119P	TCACGGCCGCATTACGGCCGGCAA	2	103	1	103	100	Bm_ATCC23344_chr2	2325379	495015	495117	99	506	2.00E-18 +
BPSS0562	porin protein	767003	768145 +	380	53721598	3096945 -	COG3203M	GGTCGTCGGAGTGGTGCGTCGTTTC	2	103	1	103	100	Bm_ATCC23344_chr2	2325379	497686	497788	99	506	2.00E-18 +
BPSS0564	thermolysin metallopeptidase	769544	771247 -	567	53721600	3096280 -	COG3227E	CGGTTGCATCGGCCAGTCAAGGCC	2	103	103	1	100	Bm_ATCC23344_chr2	2325379	502028	502130	100	515	7.70E-19 -

BPSS0565	AraC family regulatory protein	771719	772663 -	314	53721601	3095853 -	COG4977K	GTCTTGGCGCGGCTTCGGCCGCGA	2	103	103	1	100	Bm_ATCC23344_chr2	2325379	503444	503546	100	515	7.70E-19 -
BPSS0566	lipoprotein	772931	773881 +	316	53721602	3095242 -	COG2113E	CGCCTCGCCGCGAATCTGGCCCGT	2	103	1	103	100	Bm_ATCC23344_chr2	2325379	503614	503716	100	515	7.70E-19 +
BPSS0567	hypothetical protein BPSS0567	773964	774893 +	309	53721603	3095243 -	COG3246S	ACGGTGCGGTTCGCCGAGCGCGCG	2	85	1	85	100	Bm_ATCC23344_chr2	2325379	504665	504749	98	416	2.30E-14 +
BPSS0568	3-hydroxybutyryl-CoA dehydrogenase	775008	775973 +	321	53721604	3095253 -	COG1250I	GCATCGACGGCCGCGCAACGTGGC	2	103	1	103	100	Bm_ATCC23344_chr2	2325379	505691	505793	100	515	7.70E-19 +
BPSS0571	hypothetical protein BPSS0571	777815	778123 -	102	53721607	3095861 -	NULL	AACCGAGCGTTTACCCGTAGGACC	2	103	103	1	100	Bm_ATCC23344_chr2	2325379	508904	509006	100	515	7.70E-19 -
BPSS0630	response regulator protein	858794	859504 +	236	53721666	3094956 -	COG0745TK	AATGTTTCAGGATCTTACAAAGTTG	2	103	1	103	100	Bm_ATCC23344_chr2	2325379	841882	841984	100	515	7.70E-19 +
BPSS0631	sensor protein	859892	861007 +	371	53721667	3095623 -	COG0642T	GCCGGCGCTGATGCCGATGCCCGC	2	103	1	103	100	Bm_ATCC23344_chr2	2325379	842980	843082	99	506	2.00E-18 +
BPSS0633	rod shape-determining protein	864490	865554 +	354	53721669	3095136 mreB	COG1077D	AGCGACTGAGTGGATTGTGCTCGT	2	103	1	103	100	Bm_ATCC23344_chr2	2325379	847530	847632	100	515	7.70E-19 +
BPSS0643	response regulator protein	876575	877237 -	220	53721677	3095245 -	COG0745TK	CCGACGCCGCGCCGCTTTAAGCCG	2	103	103	1	100	Bm_ATCC23344_chr2	2325379	860251	860353	99	506	2.00E-18 -
BPSS0644	DNA-binding protein	877398	878258 +	286	53721678	3095224 -	COG1737K	GGTGGATGCTTAGCGGCGGCTTAA	2	103	1	103	100	Bm_ATCC23344_chr2	2325379	860314	860416	99	506	2.00E-18 +
BPSS0645	hypothetical protein BPSS0645	878644	878913 +	89	53721679	3095225 -	COG5475S	CGGTTATTCAGACGATTGCTAATCT	2	103	1	103	100	Bm_ATCC23344_chr2	2325379	861560	861662	100	515	7.70E-19 +
BPSS0647	hypothetical protein BPSS0647	879561	880028 -	155	53721681	3095859 -	COG0607P	ATGTTACCGGAAGTTGTCGATCGCG	2	103	103	1	100	Bm_ATCC23344_chr2	2325379	863039	863141	99	506	2.00E-18 -
BPSS0648	transcriptional activator FtrA	880135	881091 +	318	53721682	3095860 ftrA	COG4977K	TCTCCTCGGGACGGTGACGGTGGC	2	103	1	103	100	Bm_ATCC23344_chr2	2325379	863048	863150	98	497	5.00E-18 +
BPSS0650	peptidase	882183	883634 +	483	53721684	3095610 -	COG0624E	TCGTCCGCATCGGCGCTATCATCGC	2	66	1	66	100	Bm_ATCC23344_chr2	2325379	865157	865222	100	330	1.70E-10 +
BPSS0651	hypothetical protein BPSS0651	883744	884112 +	122	53721685	3095611 -	COG3070K	CGGCGGGCATGTCCGTTGCGATGA	2	103	1	103	100	Bm_ATCC23344_chr2	2325379	866681	866783	96	479	3.30E-17 +
BPSS0652	hypothetical protein BPSS0652	884644	885222 +	192	53721686	3095595 -	COG4094S	ACGGAGGATGCGAACCCTGGCGGC	2	103	1	103	100	Bm_ATCC23344_chr2	2325379	867576	867678	99	506	2.00E-18 +
BPSS0669	hypothetical protein BPSS0669	905454	906683 -	409	53721698	3095202 -	NULL	TGTCAGACGCCCGCGCGGCAATGC	2	103	1	103	100	Bm_ATCC23344_chr2	2325379	1222461	1222563	100	515	7.70E-19 +
BPSS0670	transporter protein	907100	908497 -	465	53721699	3095203 -	NULL	CGGCGCGCGAACGGCAACGCGTGA	2	103	1	103	100	Bm_ATCC23344_chr2	2325379	1220647	1220749	100	515	7.70E-19 +
BPSS0672	hypothetical protein BPSS0672	911273	912085 +	270	53721701	3096127 -	NULL	ATTCGCTTGCCGGCGCCGCGGTCGA	2	103	103	1	100	Bm_ATCC23344_chr2	2325379	1217972	1218074	100	515	7.70E-19 -
BPSS0673	cytochrome c oxidase subunit Ⅲ protein	912150	912743 +	197	53721702	3096128 -	COG1845C	ACGGGCGCGCGACGCGCGGCGGC	2	67	67	1	100	Bm_ATCC23344_chr2	2325379	1217095	1217161	100	335	1.00E-10 -
BPSS0675	GerR family regulatory protein	913160	914050 -	296	53721704	3095090 -	COG2197TK, COG2771K	GCCGCACACGCGTTCGTCACGAGC	2	103	1	103	100	Bm_ATCC23344_chr2	2325379	1215098	1215200	100	515	7.70E-19 +
BPSS0676	hypothetical protein BPSS0676	914574	915398 +	274	53721705	3095091 -	NULL	CCGCGCACGGCAGCGAGACGAAA	2	103	103	1	100	Bm_ATCC23344_chr2	2325379	1214672	1214774	100	515	7.70E-19 -
BPSS0677	asparagine synthetase	915616	917586 +	656	53721706	3095912 -	COG0367E	GATTAATTAACGAATATTTTCTTCGT	2	103	103	1	100	Bm_ATCC23344_chr2	2325379	1213630	1213732	98	497	5.00E-18 -
BPSS0680	oxidoreductase alpha subunit	920987	921448 -	153	53721708	3095200 -	COG2080C	ATATTTTTGCAGGATTGTGTAAACG	2	103	1	103	100	Bm_ATCC23344_chr2	2325379	1207726	1207828	100	515	7.70E-19 +
BPSS0681	AraC family regulatory protein	921649	922632 +	327	53721709	3095215 -	NULL	TGAACAATCCTGCAAATCCCGGGG	2	103	103	1	100	Bm_ATCC23344_chr2	2325379	1207623	1207725	99	506	2.00E-18 -
BPSS0682	hypothetical protein BPSS0682	922851	923249 +	132	53721710	3095216 -	NULL	GCATCGGCGCACGCGCGCATCGGC	2	103	103	1	100	Bm_ATCC23344_chr2	2325379	1206419	1206521	100	515	7.70E-19 -
BPSS0683	hypothetical protein BPSS0683	923681	924037 -	118	53721711	3095466 -	NULL	ATGCGACGGACGTCAATCTTTGCG	2	103	1	103	100	Bm_ATCC23344_chr2	2325379	1205135	1205237	100	515	7.70E-19 +
BPSS0684	hypothetical protein BPSS0684	924442	925680 +	412	53721712	3095807 -	COG1960I	CGTATTTGCAGCGCTCCGGAATAA	2	103	103	1	100	Bm_ATCC23344_chr2	2325379	1204828	1204930	98	497	5.00E-18 -
BPSS0688	response regulator protein	929989	930711 -	240	53721717	3095461 -	COG0745TK	TGTTGCGCCGCCCCCGGCGCGCG	2	103	1	103	100	Bm_ATCC23344_chr2	2325379	1198455	1198557	100	515	7.70E-19 +
BPSS0689	hypothetical protein BPSS0689	930919	931698 +	259	53721718	3095483 -	NULL	ACAATCGGACACAAATCCGCACAT	2	103	103	1	100	Bm_ATCC23344_chr2	2325379	1198345	1198447	99	506	2.00E-18 -
BPSS0692	fumarylacetoacetate (FAA) hydrolase family protein	933556	934245 +	229	53721721	3095248 -	COG0179Q	ATCGGGGTTATCAGGGTTATTGCG	2	103	103	1	100	Bm_ATCC23344_chr2	2325379	1195708	1195810	99	506	2.00E-18 -
BPSS0695	3,4-dihydroxyphenylacetate 2,3-dioxygenase	936566	937414 +	282	53721724	3094970 hpcB	COG3384S	CGCCCGCCGACAACGAACGATACG	2	74	74	1	100	Bm_ATCC23344_chr2	2325379	1192701	1192772	95	331	1.60E-10 -
BPSS0696	5-carboxymethyl-2-hydroxymuconate delta-isomeras	937472	937864 +	130	53721725	3094886 hpcD	COG3232E	GCGGCGCGGCGGTTCGCTTCGAGC	2	60	60	1	100	Bm_ATCC23344_chr2	2325379	1191795	1191854	100	300	4.00E-09 -
BPSS0699	AraC family regulatory protein	939500	940342 -	280	53721728	3095824 -	NULL	CAGCGCCCCGCGCGCCGTATCCAC	2	103	1	103	100	Bm_ATCC23344_chr2	2325379	1188885	1188987	100	515	7.70E-19 +
BPSS0700	zinc-binding dehydrogenase	940619	941584 +	321	53721729	3095287 -	COG0604CR	CTTGCCGTGCGCATGGGCGCGCGC	2	103	103	1	100	Bm_ATCC23344_chr2	2325379	1188706	1188808	100	515	7.70E-19 -
BPSS0701	outer membrane porin protein	941677	942768 -	363	53721730	3095288 -	COG3203M	GCCCGCGCCGGCCCGCACCGGCG	2	103	1	103	100	Bm_ATCC23344_chr2	2325379	1186459	1186561	99	506	2.00E-18 +
BPSS0703	transmembrane ABC transporter protein	944313	946082 -	589	53721732	3095475 -	COG1178P	GCCCGCGCGCGTGACGGTTCGCGC	2	102	1	102	100	Bm_ATCC23344_chr2	2325379	1183154	1183256	97	482	2.40E-17 +
BPSS0704	periplasmic solute-binding protein	946182	947270 -	362	53721733	3095476 -	COG1840P	AAAAGAAAGCGATCGTGACGAAAA	2	103	1	103	100	Bm_ATCC23344_chr2	2325379	1181965	1182067	100	515	7.70E-19 +
BPSS0705	response regulator protein	947399	948070 +	223	53721734	3095674 -	COG0745TK	TCGAGTGACGCGGATGGTAAGCGG	2	103	103	1	100	Bm_ATCC23344_chr2	2325379	1181934	1182036	100	515	7.70E-19 -
BPSS0708	porin protein	953131	954333 +	400	53721737	3095521 -	COG3203M	CGTTTTGCAACACGGGCGAATCGG	2	103	103	72	29	Bm_ATCC23344_chr2	2325379	1175035	1175066	100	160	0.019 -
BPSS0717	hypothetical protein BPSS0717	964746	966038 -	430	53721746	3095281 -	COG3447T	TCATTCGCGCATCGCGCATCCGCA	2	103	1	103	100	Bm_ATCC23344_chr2	2325379	1516241	1516343	99	506	2.00E-18 +
BPSS0718	TetR-family regulatory protein	967024	967668 +	214	53721747	3096272 -	COG1309K	TTTGCGTTGGCGCTCGCGTGCGCC	2	103	103	1	100	Bm_ATCC23344_chr2	2325379	1515353	1515455	100	515	7.70E-19 -
BPSS0719	family C56 unassigned peptidase	968213	968893 +	226	53721748	3095395 -	COG0693R	GCGCGCGCGAGCGGGACGCCGGC	2	103	103	2	99	Bm_ATCC23344_chr2	2325379	1514164	1514265	99	501	3.30E-18 -
BPSS0721	enoyl-(acyl carrier protein) reductase	971275	972036 +	253	53721750	3096283 -	COG0623I	CATACGGCGCGTGAATGCGCCGTA	2	103	103	1	100	Bm_ATCC23344_chr2	2325379	1511126	1511228	100	515	7.70E-19 -
BPSS0723	hypothetical protein BPSS0723	972923	973993 +	356	53721751	3095454 -	COG0628R	CGAATGTCCTCGAGCGTCCTTGGG	2	103	103	1	100	Bm_ATCC23344_chr2	2325379	1509484	1509586	99	506	2.00E-18 -
BPSS0724	hypothetical protein BPSS0724	974124	974519 +	131	53721752	3096482 -	NULL	TTTCTGACTGTTTTTAGAAAATTCAA	2	103	103	1	100	Bm_ATCC23344_chr2	2325379	1508288	1508385	95	440	1.90E-15 -
BPSS0725	short-chain dehydrogenase	974797	975591 -	264	53721753	3095421 -	COG1028IQR	CTCGCCCGACTGCCGACTTCGCGG	2	103	1	103	100	Bm_ATCC23344_chr2	2325379	1506723	1506825	100	515	7.70E-19 +
BPSS0726	GntR family regulatory protein	975934	977442 -	502	53721754	3095422 -	COG1167KE	CGGGGCTCCTGCATCGGCATGTTC	2	103	1	103	100	Bm_ATCC23344_chr2	2325379	1504872	1504974	100	515	7.70E-19 +
BPSS0727	hypothetical protein BPSS0727	977546	978265 +	239	53721755	3096524 -	COG3467R	GGCTCCAAAATTCAGTAATCGCATG	2	103	103	1	100	Bm_ATCC23344_chr2	2325379	1504866	1504968	100	515	7.70E-19 -
BPSS0731	acyl-CoA synthase	980533	982194 +	553	53721757	3095220 -	COG0318IQ	CGCTTGCATGACAGACGGCTCACA	2	103	103	1	100	Bm_ATCC23344_chr2	2325379	1501852	1501954	100	515	7.70E-19 -
BPSS0734	outer membrane efflux protein	985782	987641 -	619	53721760	3097257 oprM	COG1538MU	ACGCCGCGAGCGCCGCTTGCCCAC	2	60	1	60	100	Bm_ATCC23344_chr2	2325379	1495510	1495569	100	300	4.00E-09 +
BPSS0738	DNA-binding protein	993595	993924 +	109	53721764	3097226 -	NULL	TCGGGGACGAGGACCGCGCCGGC	2	103	103	3	98	Bm_ATCC23344_chr2	2325379	1489620	1489720	100	505	2.20E-18 -
BPSS0739	hypothetical protein BPSS0739	994808	995302 -	164	53721766	3097073 -	NULL	GTTCGGCGAACCTTGCGCGCGCCG	2	103	1	103	100	Bm_ATCC23344_chr2	2325379	1487814	1487916	98	497	5.00E-18 +
BPSS0740	hypothetical protein BPSS0740	995453	996238 -	261	53721767	3095143 -	NULL	GCACGCTCGCGGATCTCCACGCGA	2	103	1	103	100	Bm_ATCC23344_chr2	2325379	1486877	1486980	96	478	3.60E-17 +
BPSS0741	LysR family regulatory protein	997157	998107 -	316	53721768	3095124 -	COG0583K	GGTCGGCGCGGCGATGCGCGATTG	2	103	1	103	100	Bm_ATCC23344_chr2	2325379	1484974	1485076	99	506	2.00E-18 +
BPSS0742	beta alanine-pyruvate transaminase	998211	999566 +	451	53721769	3095125 -	COG0161H	TGCTCAAGTAAACATTGATATTGCG	2	103	103	1	100	Bm_ATCC23344_chr2	2325379	1484968	1485070	99	506	2.00E-18 -
BPSS0743	methylmalonate-semialdehyde dehydrogenase	999777	1001294 +	505	53721770	3096016 -	COG1012C	TCGGTCCGCTTCGATCCGCGCGGT	2	103	103	1	100	Bm_ATCC23344_chr2	2325379	1483402	1483504	96	479	3.30E-17 -
BPSS0746	lipase	1003007	1004239 -	410	53721772	3097116 -	NULL	GATGTGGGACGACACGGTCGAAAT	2	103	103	1	100	Bm_ATCC23344_chr2	2325379	594433	594535	100	515	7.70E-19 -
BPSS0747	outer membrane porin protein	1004758	1005855 +	365	53721773	3095213 -	COG3203M	GTTTCGAATAATAAATAAGACGGT	2	103	1	103	100	Bm_ATCC23344_chr2	2325379	594854	594956	100	515	7.70E-19 +
BPSS0748	hypothetical protein BPSS0748	1006475	1006846 -	123	53721774	3095214 -	COG4315S	CCTATGGTTTGAAAGGGTGCGCGA	2	103	103	1	100	Bm_ATCC23344_chr2	2325379	597039	597141	100	515	7.70E-19 -
BPSS0749	GerE familyregulatory protein	1007013	1007813 -	266	53721775	3096315 -	COG2771K	TTTTTGAACGCCCATACTCGGGCAA	2	103	103	1	100	Bm_ATCC23344_chr2	2325379	598006	598108	100	515	7.70E-19 -
BPSS0750	amino acid permease	1007935	1009344 +	469	53721776	3095118 -	COG0531E	CCCCAAGGGGGAATGGCGGCGCG	2	103	1	103	100	Bm_ATCC23344_chr2	2325379	598030	598132	100	515	7.70E-19 +
BPSS0752	lipoprotein	1010507	1012153 -	548	53721778	3095201 -	NULL	CAAAAACCTTTGCGAGACTGCGCA	2	103	103	1	100	Bm_ATCC23344_chr2	2325379	602346	602448	100	515	7.70E-19 -
BPSS0754	hypothetical protein BPSS0754	1012673	1014904 -	743	53721780	3095294 -	COG1289S	GGCTCCTACAATCGGCGCCTCTCC	2	103	103	1	100	Bm_ATCC23344_chr2	2325379	605103	605205	99	506	2.00E-18 -
BPSS0755	LysR family regulatory protein	1015115	1016125 +	336	53721781	3096111 -	COG0583K	GCATTAACGCGGGTGCGGGGAAAC	2	103	1	103	100	Bm_ATCC23344_chr2	2325379	605216	605318	99	506	2.00E-18 +
BPSS0756	hypothetical protein BPSS0756	1016647	1016949 -	100	53721782	3097006 -	NULL	CGGCCGGTTTTTCCGCGTGCTTCGG	2	103	103	1	100	Bm_ATCC23344_chr2	2325379	607129	607231	99	506	2.00E-18 -
BPSS0757	porin protein	1017268	1018422 +	384	53721783	3097007 -	COG3203M	AAGCGCTGCACGCCGGGCCGCAGA	2	103	1	103	100	Bm_ATCC23344_chr2	2325379	607350	607452	100	515	7.70E-19 +
BPSS0758	hypothetical protein BPSS0758	1018536	1019000 +	154	53721784	3097066 -	COG2764S	GCATCGCGCGCTGCCGGGGACAGA	2	103	1	103	100	Bm_ATCC23344_chr2	2325379	608618	608720	100	515	7.70E-19 +
BPSS0759	cytosine permease	1019550	1020812 +	420	53721785	3095400 -	COG1457F	CCCGAGTTCGATTTCGGTATTAATT	2	103	1	103	100	Bm_ATCC23344_chr2	2325379	609632	609734	100	515	7.70E-19 +
BPSS0762	transporter protein	1023400	1024737 -	445	53721788	3095350 -	NULL	AGCGCCGCCGGCGCGCGACCCGCC	2	103	103	1	100	Bm_ATCC23344_chr2	2325379	614889	614991	100	515	7.70E-19 -
BPSS0764	short chain dehydrogenase	1025907	1026704 -	265	53721790	3096626 -	COG1028IQR	CATGCGGCGCCGCCCGCCTCGCGC	2	103	103	1	100	Bm_ATCC23344_chr2	2325379	616856	616958	99	506	2.00E-18 -
BPSS0765	DNA-binding protein	1026819	1027535 -	238	53721791	3095161 -	COG1802K	CCTGATCGTCGCGCGCTTGACTGCG	2	103	103	1	100	Bm_ATCC23344_chr2	2325379	617687	617782	93	410	4.30E-14 -
BPSS0766	voltage gated chloride channel	1027889	1029235 +	448	53721792	3095328 -	COG0038P	AAAATCCATTTGTTTCATGATTTCCC	2	103	1	103	100	Bm_ATCC23344_chr2	2325379	617936	618038	99	506	2.00E-18 +

BPSS0767	hypothetical protein BPSS0767	1029559	1029882 -	107	53721793	3095329 -	NULL	TTGTTCGCCCGACGACGCGATGCCT	2	103	103	1	100	Bm_ATCC23344_chr2	2325379	620027	620129	100	515	7.70E-19 -
BPSS0769	hypothetical protein BPSS0769	1030401	1031381 -	326	53721795	3095308 -	COG0657I	GCCGCGCGACGCGACGTCCGGCGC	2	103	103	1	100	Bm_ATCC23344_chr2	2325379	625166	625268	100	515	7.70E-19 -
BPSS0770	GerE family regulatory protein	1031917	1032555 -	212	53721797	3095096 -	COG2197TK	GGATCGCCGCATGCGCTCGCGCGC	2	103	103	1	100	Bm_ATCC23344_chr2	2325379	626340	626442	99	506	2.00E-18 -
BPSS0772	MarR family regulatory protein	1034568	1035050 +	160	53721799	3094955 -	COG1846K	CGAATCACCGCGCATCATGCCGTG	2	103	1	103	100	Bm_ATCC23344_chr2	2325379	628240	628342	100	515	7.70E-19 +
BPSS0776	hypothetical protein BPSS0776	1036929	1037864 +	311	53721800	3096873 -	COG3386G	CGGAGCAGCCGGGCGAGCGCATG	2	103	1	103	100	Bm_ATCC23344_chr2	2325379	630563	630665	100	515	7.70E-19 +
BPSS0778	hypothetical protein BPSS0778	1038984	1039424 -	146	53721802	3097229 -	COG5649S	ACGCCTGAGCGTGCCCGTGCATGC	2	103	103	1	100	Bm_ATCC23344_chr2	2325379	633226	633328	100	515	7.70E-19 -
BPSS0785	IclR family regulatory protein	1050707	1051531 -	274	53721809	3096707 -	COG1414K	ACTTTTTGAAGTCAAGGAGACCGTG	2	103	103	1	100	Bm_ATCC23344_chr2	2325379	645321	645423	100	515	7.70E-19 -
BPSS0786	ABC transporter, periplasmic binding protein	1051720	1052733 +	337	53721810	3095419 -	COG1879G	ACTTCAAAAAGTCCGTATATAAATA	2	103	1	103	100	Bm_ATCC23344_chr2	2325379	645412	645514	100	515	7.70E-19 +
BPSS0792	short chain dehydrogenase	1058226	1058969 +	247	53721816	3095165 -	COG1028IQR	CCGCGGCGAACGGCGAACTGGCG	2	103	1	103	100	Bm_ATCC23344_chr2	2325379	651918	652020	100	515	7.70E-19 +
BPSS0793	fumarylacetoacetate (FAA) hydrolase family protein	1059045	1059887 +	280	53721817	3095375 -	COG0179Q	CGCGCGCCGCGCGGCGGCCGCGA	2	78	1	78	100	Bm_ATCC23344_chr2	2325379	652762	652839	100	390	3.40E-13 +
BPSS0794	short chain dehydrogenase	1060055	1060831 +	258	53721818	3095376 -	COG1028IQR	GCATGGACGCCCGGGCCGCGGTCG	2	103	1	103	100	Bm_ATCC23344_chr2	2325379	653731	653833	100	515	7.70E-19 +
BPSS0797	IclR family regulatory protein	1069699	1070439 -	246	53721822	3095069 -	COG1414K	ATCCGGCGGCAGACAAACACGAAT	2	103	103	1	100	Bm_ATCC23344_chr2	2325379	665116	665218	100	515	7.70E-19 -
BPSS0800	TetR family regulatory protein	1073131	1073745 -	204	53721825	3095067 -	COG1309K	CGATTGAAACAGAACAATGTTCTGT	2	103	103	1	100	Bm_ATCC23344_chr2	2325379	668422	668524	98	497	5.00E-18 -
BPSS0801	hypothetical protein BPSS0801	1073860	1074297 +	145	53721826	3095741 -	COG2050Q	GAAAACAAGGACGCGCAATGGTAC	2	103	1	103	100	Bm_ATCC23344_chr2	2325379	668439	668541	98	497	5.00E-18 +
BPSS0802	outative extracellular ligand binding protein	1074446	1075588 +	380	53721827	3095742 -	COG0683E	GCAGCCTGCGAACGAACCGGTGCG	2	103	1	103	100	Bm_ATCC23344_chr2	2325379	669025	669127	98	497	5.00E-18 +
BPSS0803	hemolysin	1076062	1076697 +	211	53721828	3094994 -	COG1272R	AATCAATGGCCCGCGGACGTGGCC	2	103	1	103	100	Bm_ATCC23344_chr2	2325379	670629	670731	98	497	5.00E-18 +
BPSS0804	hypothetical protein BPSS0804	1076755	1077132 -	125	53721829	3095330 -	NULL	GATCCCGGCCACTCAATCGCACGC	2	103	103	1	100	Bm_ATCC23344_chr2	2325379	671797	671899	100	515	7.70E-19 -
BPSS0805	hypothetical protein BPSS0805	1077778	1079844 -	688	53721830	3095331 -	COG3300T, COG5001T	CGGCGCGAATCGCCCCTCGCCGAT	2	103	103	1	100	Bm_ATCC23344_chr2	2325379	674499	674601	100	515	7.70E-19 -
BPSS0806	leucine-responsive regulatory protein	1080171	1080638 -	155	53721831	3095387 -	COG1522K	GTTTATCGCCGGCGGGATGAAATG	2	103	103	1	100	Bm_ATCC23344_chr2	2325379	675293	675395	99	506	2.00E-18 -
BPSS0807	aromatic amino acid transport protein	1080971	1082356 +	461	53721832	3096175 aroP	COG1113E	CGCCGCGACATCCGCGCGGGCGGC	2	103	1	103	100	Bm_ATCC23344_chr2	2325379	675528	675630	100	515	7.70E-19 +
BPSS0810	hypothetical protein BPSS0810	1085445	1086068 +	207	53721835	3095858 -	COG1670J	GCGCGCTCGCCGGCTCGCGCGGGA	2	103	1	103	100	Bm_ATCC23344_chr2	2325379	680012	680114	100	515	7.70E-19 +
BPSS0818	hypothetical protein BPSS0818	1102008	1102562 +	184	53721844	3096109 -	NULL	ATCAGCTGGCCGCAATCGCCGAAT	2	103	1	103	100	Bm_ATCC23344_chr2	2325379	696550	696652	100	515	7.70E-19 +
BPSS0827	subfamily M9A unassigned peptidase	1113438	1115375 +	645	53721853	3096790 -	NULL	TGAAATGAACTCGCATATTATCGCT	2	103	1	103	100	Bm_ATCC23344_chr2	2325379	708008	708110	99	506	2.00E-18 +
BPSS0828	hypothetical protein BPSS0828	1115663	1116001 -	112	53721854	3096527 -	NULL	ACACATCGCAACATCCGATACGTTG	2	103	103	1	100	Bm_ATCC23344_chr2	2325379	710670	710772	100	515	7.70E-19 -
BPSS0829	hypothetical protein BPSS0829	1116654	1117820 -	388	53721855	3095140 -	COG0654HC	CCGGCGCTTTCATAGACCTGCATTG	2	103	103	1	100	Bm_ATCC23344_chr2	2325379	712482	712584	99	506	2.00E-18 -
BPSS0830	DNA-binding protein	1118418	1119161 -	247	53721856	3095141 -	NULL	CATCATCGCCGCGCCGCGCCCGCG	2	103	103	1	100	Bm_ATCC23344_chr2	2325379	713823	713925	100	515	7.70E-19 -
BPSS0831	hypothetical protein BPSS0831	1119533	1119721 +	62	53721857	3095804 -	NULL	AAAGGCTGGACGGCAAGCACGAG	2	103	1	103	100	Bm_ATCC23344_chr2	2325379	714097	714199	98	497	5.00E-18 +
BPSS0832	hypothetical protein BPSS0832	1119990	1120478 -	162	53721858	3096691 -	NULL	TCAACCGGGCGCGCGCGGCGCGC	2	103	103	1	100	Bm_ATCC23344_chr2	2325379	715129	715231	99	506	2.00E-18 -
BPSS0833	hypothetical protein BPSS0833	1120668	1120958 +	96	53721859	3096692 -	NULL	CGCCCGGTTGACGTGGGTCAAGCG	2	103	1	103	100	Bm_ATCC23344_chr2	2325379	715221	715323	100	515	7.70E-19 +
BPSS0835	response regulator protein	1121342	1121989 -	215	53721861	3094906 -	COG2197TK	GCGGCTCGCAGCCGCGCAACGGCC	2	103	103	1	100	Bm_ATCC23344_chr2	2325379	716633	716735	99	506	2.00E-18 -
BPSS0836	universal stress protein	1122557	1123015 +	152	53721862	3094907 -	COG0589T	ACGCTTGATCCTCGTCAACGGTGGG	2	103	1	103	100	Bm_ATCC23344_chr2	2325379	717097	717199	100	515	7.70E-19 +
BPSS0839	hypothetical protein BPSS0839	1124884	1125723 -	279	53721865	3095018 -	COG0589T	GCGCTTTGATCTGAGTCAAGCGCG	2	103	103	1	100	Bm_ATCC23344_chr2	2325379	720371	720473	100	515	7.70E-19 -
BPSS0840	zinc-binding dehydrogenase	1125973	1127013 +	346	53721866	3095097 -	COG1063ER	CGCGCGCCGCGCCGTTTGACGCGC	2	103	1	103	100	Bm_ATCC23344_chr2	2325379	720523	720625	99	506	2.00E-18 +
BPSS0841	hypothetical protein BPSS0841	1127458	1128504 +	348	53721867	3095145 -	COG0586S	GATGCCGATCCACGCCGCGCACGC	2	103	1	103	100	Bm_ATCC23344_chr2	2325379	722044	722146	100	515	7.70E-19 +
BPSS0842	transporter protein	1128693	1129901 +	402	53721868	3095146 -	NULL	ATTGCTTCGAGACACTGCTTCGAGA	2	103	1	103	100	Bm_ATCC23344_chr2	2325379	723279	723381	99	506	2.00E-18 +
BPSS0845	proline-specific permease	1133021	1134406 +	461	53721871	3095836 proY	COG1113E	GCGATGAGCATGCAACATTGAGCG	2	103	1	103	100	Bm_ATCC23344_chr2	2325379	727463	727565	100	515	7.70E-19 +
BPSS0845a	DNA-binding protein	1134960	1135202 -	80	53721872	3095137 msr0960	NULL	ACCATGTTGTTATGCTGAATAGTGC	2	103	103	1	100	Bm_ATCC23344_chr2	2325379	729742	729844	100	515	7.70E-19 -
BPSS0846	hypothetical protein BPSS0846	1135347	1135643 +	98	53721873	3096188 -	COG2916R	ATTGGGCTATGATAGCCGAAAACTC	2	103	1	103	100	Bm_ATCC23344_chr2	2325379	729789	729891	100	515	7.70E-19 +
BPSS0847	hypothetical protein BPSS0847	1136037	1137353 -	438	53721874	3095447 -	COG1960I	CGTTCGCGCCGCATAAGCATCGCA	2	103	103	1	100	Bm_ATCC23344_chr2	2325379	731893	731995	100	515	7.70E-19 -
BPSS0848	hypothetical protein BPSS0848	1137775	1138272 -	165	53721875	3096230 -	NULL	ACGCGCGCGGCGCGCCTGCATGCG	2	103	103	1	100	Bm_ATCC23344_chr2	2325379	732812	732914	100	515	7.70E-19 -
BPSS0860	flagellar hook-associated protein	1151257	1152780 +	507	53721885	3096005 -	COG1345N	CTTACAACAATCTTCACAAACCGAT	2	103	103	1	100	Bm_ATCC23344_chr2	2325379	1476657	1476759	100	515	7.70E-19 -
BPSS0861	LysR family regulatory protein	1153177	1154076 -	299	53721886	3097176 -	COG0583K	GAAAAATGAAAGTGAAACGAGTCT	2	92	1	92	100	Bm_ATCC23344_chr2	2325379	1473751	1473842	98	451	6.00E-16 +
BPSS0862	hypothetical protein BPSS0862	1154166	1154864 +	232	53721887	3097177 -	COG3531O	ATTCGCTCAATCCATGCAACAAATT	2	92	92	1	100	Bm_ATCC23344_chr2	2325379	1473748	1473839	98	451	6.00E-16 -
BPSS0864	aldo/keto reductase family protein	1155494	1156441 +	315	53721888	3096517 -	COG0667C	AATCGGGTTTAATCGGTTTGAAGCG	2	103	103	1	100	Bm_ATCC23344_chr2	2325379	1472420	1472522	100	515	7.70E-19 -
BPSS0865	hypothetical protein BPSS0865	1156869	1157735 +	288	53721889	3096518 -	NULL	CGTTCAACCTTTCGGTTGATACCGC	2	103	103	1	100	Bm_ATCC23344_chr2	2325379	1470991	1471093	100	515	7.70E-19 -
BPSS0866	short chain dehydrogenase	1157810	1158565 +	251	53721890	3096883 -	COG1028IQR	ACGGCGTCGATTCGAGGCGCGCGG	2	77	77	1	100	Bm_ATCC23344_chr2	2325379	1470050	1470126	100	385	5.70E-13 -
BPSS0867	GerE family regulatory protein	1159092	1159799 +	235	53721891	3096754 -	COG2771K	GAAGAACGTCCGCACGATTTGCCG	2	103	103	1	100	Bm_ATCC23344_chr2	2325379	1468852	1468954	98	497	5.00E-18 -
BPSS0868	phenylacetaldehyde dehydrogenase	1160056	1161558 +	500	53721892	3095384 feaB	COG1012C	GCTAACCGGCCGCGAAACCGCGTC	2	103	103	1	100	Bm_ATCC23344_chr2	2325379	1467888	1467990	100	515	7.70E-19 -
BPSS0870	amino acid permease	1163154	1164644 +	496	53721894	3095068 -	COG0531E	GGCGAAGGCGCGGCTCCGGCCGTT	2	103	103	1	100	Bm_ATCC23344_chr2	2325379	1464790	1464892	100	515	7.70E-19 -
BPSS0873	porin protein	1166507	1167709 +	400	53721897	3096602 -	COG3203M	GGCCCAAATTTTCGCCGGATAGAG	2	103	103	1	100	Bm_ATCC23344_chr2	2325379	1461437	1461539	100	515	7.70E-19 -
BPSS0875	LysE type translocator	1168399	1169010 -	203	53721899	3096877 -	COG1280E	TCACTGGTGAATGGCGCATGGTCG	2	103	1	103	100	Bm_ATCC23344_chr2	2325379	1458846	1458948	100	515	7.70E-19 +
BPSS0876	GNAT family acetyltransferase	1169182	1169661 +	159	53721900	3095180 -	NULL	CCGATCGACCATGCGCCATTCACC	2	103	103	1	100	Bm_ATCC23344_chr2	2325379	1458772	1458874	97	488	1.30E-17 -
BPSS0878	ArsR family regulatory protein	1170913	1171218 -	101	53721902	3097093 -	COG0640K	GGATTCCATCGCCCCTAGTCAATAG	2	103	1	103	100	Bm_ATCC23344_chr2	2325379	1456638	1456740	100	515	7.70E-19 +
BPSS0879	porin protein	1172547	1173671 +	374	53721903	3096508 -	COG3203M	CAGGTAGTTTCGCAAGCCGATATGC	2	103	103	1	100	Bm_ATCC23344_chr2	2325379	1455406	1455508	100	515	7.70E-19 -
BPSS0880	transcription elongation factor	1173890	1174300 -	136	53721904	3096884 -	COG0782K	CCGCGGCCCGGCGCTTGATCTGAC	2	103	1	103	100	Bm_ATCC23344_chr2	2325379	1453550	1453652	97	488	1.30E-17 +
BPSS0881	hypothetical protein BPSS0881	1174807	1175148 +	113	53721905	3096885 -	NULL	GTAGGGGTTTCCCCTTGTTGTTCCG	2	103	103	1	100	Bm_ATCC23344_chr2	2325379	1453140	1453242	100	515	7.70E-19 -
BPSS0882	hypothetical protein BPSS0882	1175353	1175817 -	154	53721906	3095478 -	COG0517R	GACGCACCCGCCCGCGGACGCCG	2	103	1	103	100	Bm_ATCC23344_chr2	2325379	1452032	1452134	99	506	2.00E-18 +
BPSS0883	GTP cyclohydrolase II	1175994	1176647 +	217	53721907	3095954 ribA	COG0807H	TCGGCGTCCGCGGGCGGGTGCGTC	2	103	103	1	100	Bm_ATCC23344_chr2	2325379	1451953	1452055	99	506	2.00E-18 -
BPSS0884	hypothetical protein BPSS0884	1176610	1177458 -	282	53721908	3095910 -	COG1257I	GCGCGCCCGGTCGCCCCGCTTCGG	2	64	1	64	100	Bm_ATCC23344_chr2	2325379	1450430	1450493	100	320	4.90E-10 +
BPSS0885	N-acylhomoserine lactone synthase	1177520	1178131 -	203	53721909	3097087 pmlI	COG3916TQ	CACCAGCCCCGCTTTCCCGACCCTG	2	103	1	103	100	Bm_ATCC23344_chr2	2325379	1449718	1449820	100	515	7.70E-19 +
BPSS0886	hypothetical protein BPSS0886	1178356	1178799 -	147	53721910	3095911 -	NULL	TCGTTCTCCAGCAGCTTCACGCTGT	2	76	1	76	100	Bm_ATCC23344_chr2	2325379	1449077	1449152	100	380	9.60E-13 +
BPSS0887	N-acylhomoserine lactone dependent regulatory pro	1178873	1179592 +	239	53721911	3095551 pmlR	COG2771K	GCCGCGGAATGTATCAGCCCGCGC	2	76	76	1	100	Bm_ATCC23344_chr2	2325379	1449074	1449149	100	380	9.60E-13 -
BPSS0888	MgtC family protein	1179628	1180344 -	238	53721912	3095386 -	COG1285S	TCGCGCGCTAAAGTGGCGCGCGAG	2	103	1	103	100	Bm_ATCC23344_chr2	2325379	1447505	1447607	96	479	3.30E-17 +
BPSS0889	calcineurin-like phosphoesterase	1181017	1182222 -	401	53721913	3096981 -	COG1408R	GCGCGCGACGGCGGCGCGTTCGCC	2	65	1	65	100	Bm_ATCC23344_chr2	2325379	1445645	1445708	98	310	1.40E-09 +
BPSS0890	hypothetical protein BPSS0890	1182285	1182869 -	194	53721914	3096982 -	COG0778C	CGCGGAATGGCGCGAACCGCAACC	2	103	1	103	100	Bm_ATCC23344_chr2	2325379	1444960	1445062	99	506	2.00E-18 +
BPSS0891	2OG-Fe(II) oxygenase superfamily protein	1183002	1183628 +	208	53721915	3096643 -	COG3145L	GGCGTTCGCGCGGCTAGAATTTGA	2	103	103	1	100	Bm_ATCC23344_chr2	2325379	1444925	1445027	99	506	2.00E-18 -
BPSS0892	IclR family regulatory protein	1183715	1184665 +	316	53721916	3096969 -	COG1414K	CGCGGGCCCGCCCGCGCGCCGAA	2	89	89	1	100	Bm_ATCC23344_chr2	2325379	1444212	1444300	100	445	1.10E-15 -
BPSS0893	porin protein	1184982	1186070 -	362	53721917	3096970 -	COG3203M	ACAAAAGAAAGCCAAAAACTCAGC	2	103	1	103	100	Bm_ATCC23344_chr2	2325379	1441768	1441870	99	506	2.00E-18 +
BPSS0894	Rieske	1186505	1186822 +	105	53721918	3094948 -	COG2146PR	TTATTTTGATCTTCGGATGACGCTCC	2	103	103	1	100	Bm_ATCC23344_chr2	2325379	1441431	1441533	99	506	2.00E-18 -
BPSS0908	surface-exposed protein	1200501	1202816 +	771	53721932	3096654 -	COG5295UW	CAAGTGTTCGACGACGCAAGTCGT	2	103	103	1	100	Bm_ATCC23344_chr2	2325379	1427429	1427531	99	506	2.00E-18 -
BPSS0909	OmpA family protein	1202875	1203705 +	276	53721933	3095347 -	COG2885M, COG2913J	CGCGAGTCGCAACGGCGGCCGGC	2	61	61	1	100	Bm_ATCC23344_chr2	2325379	1425055	1425115	100	305	2.40E-09 -
BPSS0910	hypothetical protein BPSS0910	1203866	1204300 -	144	53721934	3095039 -	NULL	GGCGTTTACGACATTTTTACGCCGC	2	103	1	103	100	Bm_ATCC23344_chr2	2325379	1423532	1423634	100	515	7.70E-19 +
BPSS0911	amidase	1204825	1206318 +	497	53721935	3095040 -	NULL	TTATATAATCGTCGGCATCCCGCCG	2	103	103	1	100	Bm_ATCC23344_chr2	2325379	1423096	1423198	100	515	7.70E-19 -

BPSS0912	AsnC family regulatory protein	1206483	1206965	-	160	53721936	3094914	-	COG1522K	AGAAAGCTAGCAGGAAATATGCTTT	2	103	1	103	100	Bm_ATCC23344_chr2	2325379	1420858	1420960	98	497	5.00E-18	+
BPSS0913	methionine gamma-lyase	1207153	1208454	+	433	53721937	3096921	-	COG0626E	CTGCTAGCTTTCTTACACTGAGTCG/	2	103	103	1	100	Bm_ATCC23344_chr2	2325379	1420768	1420870	99	506	2.00E-18	-
BPSS0914	hypothetical protein BPSS0914	1208622	1208846	-	74	53721938	3096922	-	NULL	TCATTCGGCGAATCGAACATCGCA/	2	103	1	103	100	Bm_ATCC23344_chr2	2325379	1418977	1419079	100	515	7.70E-19	+
BPSS0915	hypothetical protein BPSS0915	1209283	1209984	+	233	53721939	3096282	-	NULL	GCGGCGAAGTCTGTCAATACGTTTC	2	103	103	1	100	Bm_ATCC23344_chr2	2325379	1418638	1418740	99	506	2.00E-18	-
BPSS0916	N-hydroxyarylamine O-acetyltransferase	1210101	1210973	+	290	53721940	3095584	-	COG2162Q	GCGCGCCGATATGGCCGCCGGCGC	2	103	103	1	100	Bm_ATCC23344_chr2	2325379	1417820	1417922	100	515	7.70E-19	-
BPSS0917	hypothetical protein BPSS0917	1211259	1211816	+	185	53721941	3095585	-	COG3554S	TACCGTTCAAGATCCGCGTGACGG(	2	103	103	1	100	Bm_ATCC23344_chr2	2325379	1416652	1416754	97	488	1.30E-17	-
BPSS0918	DNA-binding protein	1211936	1212397	-	153	53721942	3095408	-	COG1959K	AGTTACGAAAGAACGCGACGTTTG1	2	103	1	103	100	Bm_ATCC23344_chr2	2325379	1415416	1415518	100	515	7.70E-19	+
BPSS0919	hypothetical protein BPSS0919	1212527	1213438	+	303	53721943	3096911	-	COG0492O	ATCTACCACTGCGTCGCACACGTAA	2	103	103	1	100	Bm_ATCC23344_chr2	2325379	1415384	1415486	99	506	2.00E-18	-
BPSS0927	hypothetical protein BPSS0927	1223235	1223885	-	216	53721951	3095052	-	NULL	GCCTTCTTGCCGATAATGAACGGCC	2	103	1	103	100	Bm_ATCC23344_chr2	2325379	1411132	1411234	100	515	7.70E-19	+
BPSS0928	aliphatic sulfonates binding protein precursor	1224086	1225069	-	327	53721952	3095053	-	COG0715P	GGCAATGCTTGGTTCGCGCGGGCG(	2	103	1	103	100	Bm_ATCC23344_chr2	2325379	1409962	1410064	100	515	7.70E-19	+
BPSS0929	hypothetical protein BPSS0929	1225194	1226000	-	268	53721953	3095715	-	COG0789K	CACGCGCGGTCGCGCTAAAATCGC(	2	81	1	81	100	Bm_ATCC23344_chr2	2325379	1409053	1409133	100	405	7.20E-14	+
BPSS0930	phosphinothricin N-acetyltransferase	1226079	1226645	-	188	53721954	3097010	-	COG1247M	CGCCCGCCGCGCCCACCGTTCGAC(	2	103	1	103	100	Bm_ATCC23344_chr2	2325379	1408386	1408488	97	488	1.30E-17	+
BPSS0932	membrane transport protein	1228206	1229720	-	504	53721956	3095364	-	NULL	GGCCGGTAACCAATGGAAATTCTTC	2	103	1	103	100	Bm_ATCC23344_chr2	2325379	1405319	1405421	100	515	7.70E-19	+
BPSS0933	methyl-accepting chemotaxis protein	1229959	1231515	+	518	53721957	3097215	-	COG0840NT	GCCGCATCGCTTGAGGCGCCTCAA(	2	103	103	1	100	Bm_ATCC23344_chr2	2325379	1405178	1405280	100	515	7.70E-19	-
BPSS0934	hypothetical protein BPSS0934	1231748	1233412	-	554	53721958	3097216	-	COG0210L	CGACGCTGGTCGCCCCGCAAGCCG	2	103	1	103	100	Bm_ATCC23344_chr2	2325379	1401628	1401730	99	506	2.00E-18	+
BPSS0935	hypothetical protein BPSS0935	1234055	1234963	-	302	53721959	3096206	-	COG0745TK	ACACTAGTGCGCAGGACGAACTTT(	2	103	1	103	100	Bm_ATCC23344_chr2	2325379	1400077	1400179	100	515	7.70E-19	+
BPSS0936	hypothetical protein BPSS0936	1235142	1236470	+	442	53721960	3094952	-	COG0665E	AGTTCGTCCTGCGCACTAGTGTGCG	2	103	103	1	100	Bm_ATCC23344_chr2	2325379	1399996	1400098	100	515	7.70E-19	-
BPSS0937	hypothetical protein BPSS0937	1236747	1237856	+	369	53721961	3094953	-	COG1226P	GAGCCCGGCGCTCCCACCGCACCC	2	103	103	1	100	Bm_ATCC23344_chr2	2325379	1398400	1398502	99	506	2.00E-18	-
BPSS0939	carboxymuconolactone decarboxylase	1239185	1239583	+	132	53721963	3097273	-	COG0599S	GACCTCGAGCACGAAGCGCCGGCC	2	103	103	1	100	Bm_ATCC23344_chr2	2325379	1395964	1396066	98	497	5.00E-18	-
BPSS0942	hypothetical protein BPSS0942	1242298	1243464	-	388	53721966	3096029	-	COG4645S	TAGCAATGCCGCACTTCGTCATGCG	2	103	1	103	100	Bm_ATCC23344_chr2	2325379	1391869	1391971	100	515	7.70E-19	+
BPSS0943	porin protein	1243885	1245039	+	384	53721967	3096030	-	COG3203M	GGATGCGCTGGACATGCCCCGCGA	2	103	103	1	100	Bm_ATCC23344_chr2	2325379	1391546	1391648	100	515	7.70E-19	-
BPSS0944	LysR family regulatory protein	1245541	1246452	-	303	53721968	3096786	-	COG0583K	ATGCGGAACCGCGAAGCGGACCT	2	103	1	103	100	Bm_ATCC23344_chr2	2325379	1388881	1388983	99	506	2.00E-18	+
BPSS0945	subfamily M23B unassigned peptidase	1247073	1248020	+	315	53721969	3094901	-	COG0739M	TGCCCGCTGCGATTTTGTCATCATT(	2	103	103	1	100	Bm_ATCC23344_chr2	2325379	1388361	1388463	99	506	2.00E-18	-
BPSS0948	LysR family regulatory protein	1249773	1250645	+	290	53721971	3094902	-	COG0583K	CAACGTCCGCATTTCGCTTATCGTG/	2	103	103	1	100	Bm_ATCC23344_chr2	2325379	1385621	1385723	100	515	7.70E-19	-
BPSS0949	hypothetical protein BPSS0949	1250756	1251091	-	111	53721972	3096121	-	NULL	AAGTTTTCGCATGCCCTGCCGTTTA(	2	103	1	103	100	Bm_ATCC23344_chr2	2325379	1384206	1384308	100	515	7.70E-19	+
BPSS0956	hypothetical protein BPSS0956	1258000	1258890	-	296	53721979	3095513	-	COG1737K	GCTCATAACGATAGATTTTTGGGTA	2	103	1	103	100	Bm_ATCC23344_chr2	2325379	1376413	1376515	100	515	7.70E-19	+
BPSS0957	hypothetical protein BPSS0957	1259881	1261092	-	403	53721980	3095514	-	NULL	TTATCTCGAAATCCACGATTCCACA(	2	103	1	103	100	Bm_ATCC23344_chr2	2325379	1374211	1374313	99	506	2.00E-18	+
BPSS0958	hypothetical protein BPSS0958	1261382	1263367	+	661	53721981	3095509	-	COG3501S	TGAAAAGGCGCTATTTAAATCTTGT	2	103	103	1	100	Bm_ATCC23344_chr2	2325379	1374019	1374121	100	515	7.70E-19	-
BPSS0959	hypothetical protein BPSS0959	1263459	1263899	+	146	53721982	3096496	-	COG5435S	CCGGCCGGGCGGCGGGCCGCGGA	2	94	94	1	100	Bm_ATCC23344_chr2	2325379	1371942	1372035	100	470	8.30E-17	-
BPSS0960	Rhs-related membrane protein	1263951	1268576	+	1541	53721983	3096497	-	COG3209M	TGCCGGGAGGCGACGCCGTTTTTC(	2	54	54	1	100	Bm_ATCC23344_chr2	2325379	1371456	1371509	100	270	9.00E-08	-
BPSS0962	extracellular serine protease precursor	1270105	1273500	+	1131	53721985	3095427	-	COG4625S	AAAGAGAGTGAGGCGGCTCAATTG	2	103	103	1	100	Bm_ATCC23344_chr2	2325379	1365302	1365404	100	515	7.70E-19	-
BPSS0964	periplasmic solute-binding protein	1274424	1276016	-	530	53721987	3095768	-	COG0747E	TGACGCGGCGCAGCGGGGTCTCGC	2	103	1	103	100	Bm_ATCC23344_chr2	2325379	1359295	1359397	99	506	2.00E-18	+
BPSS0965	oxalate decarboxylase	1276302	1277564	+	420	53721988	3095557	-	COG2140GR	TTTGACGGTTTGTATTTAACTTGCGT	2	103	103	1	100	Bm_ATCC23344_chr2	2325379	1359107	1359209	98	497	5.00E-18	-
BPSS0967	hypothetical protein BPSS0967	1279448	1279900	-	150	53721990	3096307	-	COG4682S	CATCCAATTTCCAGGCCGCCGCAGT	2	103	1	103	100	Bm_ATCC23344_chr2	2325379	1355447	1355549	100	515	7.70E-19	+
BPSS0969	molybdopterin oxidoreductase	1281070	1283394	-	774	53721992	3096966	-	COG0243C	TCTTTTTCTTTCCGGCAAGGTGTGCT	2	103	1	103	100	Bm_ATCC23344_chr2	2325379	1351953	1352055	100	515	7.70E-19	+
BPSS0970	GntR family regulatory protein	1283573	1284991	+	472	53721993	3096058	-	COG1167KE	CACCTTGCCGGAAAGAAAAAGAGC	2	103	103	1	100	Bm_ATCC23344_chr2	2325379	1351872	1351974	100	515	7.70E-19	-
BPSS0971	DNA-binding protein	1285062	1285421	+	119	53721994	3095231	-	NULL	GGGCGGGGGGATGAAGACGAACG(	2	73	73	1	100	Bm_ATCC23344_chr2	2325379	1350383	1350455	98	356	1.20E-11	-
BPSS0972	hypothetical protein BPSS0972	1285528	1286271	-	247	53721995	3095232	-	NULL	CGCGAAGCGTTTCGCTCCCGCATCC	2	78	1	78	100	Bm_ATCC23344_chr2	2325379	1349049	1349126	100	390	3.40E-13	+
BPSS0973	Mg(2+) transport ATPase, P-type 2	1286347	1289133	-	928	53721996	3095370	mgtB	COG0474P	GATTCAGCCGAACGCGCGCGACGC	2	103	1	103	100	Bm_ATCC23344_chr2	2325379	1346162	1346264	100	515	7.70E-19	+
BPSS0975	hypothetical protein BPSS0975	1293047	1293562	+	171	53721998	3096900	-	NULL	TTGTTCAAATCACGGCGAACGCGTA	2	103	103	1	100	Bm_ATCC23344_chr2	2325379	1342360	1342462	100	515	7.70E-19	-
BPSS0979	porin protein	1297191	1298306	+	371	53722002	3095833	-	COG3203M	GCGGCGCGCCGGACGTGTTTTGAA(	2	55	55	1	100	Bm_ATCC23344_chr2	2325379	1338216	1338270	100	275	5.30E-08	-
BPSS0980	hypothetical protein BPSS0980	1298505	1299308	-	267	53722003	3095066	-	NULL	GCGGAACACGGCACGCACGCGCG(	2	103	1	103	100	Bm_ATCC23344_chr2	2325379	1336001	1336103	100	515	7.70E-19	+
BPSS0981	histidine transport system permease protein	1299775	1300464	+	229	53722004	3094969	hisQ	COG4215E	GTCGAACCGGTTCGCAAGCTTTCAC	2	103	103	1	100	Bm_ATCC23344_chr2	2325379	1335632	1335734	100	515	7.70E-19	-
BPSS0983	histidine transport ATP-binding protein	1301243	1302010	+	255	53722006	3095049	hisP	COG4598E	CCGGCGCGCGCGACGCGCGCGGG(	2	71	71	1	100	Bm_ATCC23344_chr2	2325379	1334164	1334234	100	355	1.30E-11	-
BPSS0985	hypothetical protein BPSS0985	1303632	1304126	+	164	53722008	3096585	-	COG3791S	GGTGGCGATAAGGCCGGGCGCTTC	2	103	103	1	100	Bm_ATCC23344_chr2	2325379	1331791	1331893	98	497	5.00E-18	-
BPSS0986	hypothetical protein BPSS0986	1304382	1305131	+	249	53722009	3096469	-	NULL	GGTGAAACGGTTGAGGCGATTGAC(	2	103	103	1	100	Bm_ATCC23344_chr2	2325379	1331043	1331145	100	515	7.70E-19	-
BPSS0987	hypothetical protein BPSS0987	1305249	1305593	-	114	53722010	3095847	-	NULL	TGTTTCCGAATTGCCGGAACATCGC	2	103	1	103	100	Bm_ATCC23344_chr2	2325379	1329734	1329836	99	506	2.00E-18	+
BPSS0993	catalase precursor	1310767	1312284	+	505	53722014	3095083	katB	COG0753P	GCGCGATAGAAATATAGAAATTCAT	2	103	103	1	100	Bm_ATCC23344_chr2	2325379	1323450	1323552	100	515	7.70E-19	-
BPSS0994	hypothetical protein BPSS0994	1312677	1314146	-	489	53722015	3095065	-	COG0500QR	CGGCTGCCGGCACGGCGCGCCGCC	2	103	1	103	100	Bm_ATCC23344_chr2	2325379	1319901	1320003	99	506	2.00E-18	+
BPSS0995	AraC family regulatory protein	1314324	1315883	-	519	53722016	3096304	-	NULL	CGCCGCCCGTCATTCGCCGGCTCAT	2	103	1	103	100	Bm_ATCC23344_chr2	2325379	1318164	1318266	99	506	2.00E-18	+
BPSS0996	Rieske iron-sulphur domain protein	1316050	1317054	-	334	53722017	3096639	-	COG4638PR	GCCGGGCCGCGTCGCGGCGGAAC(	2	103	1	103	100	Bm_ATCC23344_chr2	2325379	1316993	1317095	100	515	7.70E-19	+
BPSS0999	acyl carrier protein	1320172	1320411	-	79	53722020	3095102	-	NULL	GTCATCCAACCCGATCGCCGCCCG1	2	56	1	56	100	Bm_ATCC23344_chr2	2325379	1313749	1313804	100	280	3.20E-08	+
BPSS1004	acyl transferase	1324682	1325866	-	394	53722025	3095813	-	COG0331I	CGTCGACCGACGCGGGCAATCGAC	2	103	1	103	100	Bm_ATCC23344_chr2	2325379	1306952	1307054	100	515	7.70E-19	+
BPSS1013	hypothetical protein BPSS1013	1385187	1385600	-	137	53722034	3095210	-	COG1846K	GAGGCGGGACTCGACACCATGAAT	2	103	1	103	100	Bm_ATCC23344_chr2	2325379	1248144	1248246	95	470	8.30E-17	+
BPSS1015	LysR family transcriptional regulator	1387287	1388258	+	323	53722036	3095451	-	COG0583K	GGCCCGCGCATCCGGTCGATCAGT(	2	103	103	1	100	Bm_ATCC23344_chr2	2325379	1246555	1246657	100	515	7.70E-19	-
BPSS1018	hypothetical protein BPSS1018	1391733	1392641	-	302	53722039	3096729	-	NULL	CGCCCGGGCCCCGCACGGGCGGC/	2	103	1	103	100	Bm_ATCC23344_chr2	2325379	1241139	1241241	100	515	7.70E-19	+
BPSS1020	AraC family transcriptional regulator	1393691	1394737	-	348	53722041	3095996	-	COG4753T	CCTCCCCTGCCCGCGCGTTTTTAGC(	2	103	1	103	100	Bm_ATCC23344_chr2	2325379	1239049	1239151	99	506	2.00E-18	+
BPSS1024	hypothetical protein BPSS1024	1397146	1397958	+	270	53722045	3096718	-	NULL	TGCGGGCATCGCGCAGACGGCGTG	2	103	103	1	100	Bm_ATCC23344_chr2	2325379	1236694	1236796	100	515	7.70E-19	-
BPSS1027	RNA polymerase sigma factor	1398855	1399364	+	169	53722048	3095415	-	COG1595K	CGTGCGGACGCATTCACCGTCCGG1	2	103	103	1	100	Bm_ATCC23344_chr2	2325379	1234985	1235087	98	497	5.00E-18	-
BPSS1038	hypothetical protein BPSS1038	1414651	1414923	+	90	53722059	3096532	-	NULL	TGACGGATTTGTCATCGCACGCTCA	2	103	1	103	100	Bm_ATCC23344_chr2	2325379	1063231	1063333	100	515	7.70E-19	+
BPSS1043	cobalt-zinc-cadmium resistance protein	1421864	1423222	-	452	53722064	3096566	czcC	COG1538MU	GTCATGTTCGCCTCATGTTTGCATT(	2	103	103	1	100	Bm_ATCC23344_chr2	2325379	1071918	1072020	100	515	7.70E-19	-
BPSS1045	hypothetical protein BPSS1045	1424960	1425448	-	162	53722066	3095670	-	COG1839S	AACGAACCGGAACAAAACGCGCC(	2	103	103	1	100	Bm_ATCC23344_chr2	2325379	1074144	1074241	95	440	1.90E-15	-
BPSS1046	hypothetical protein BPSS1046	1425780	1426265	+	161	53722067	3095671	-	COG2606S	GGAGATGCCGCATTGTTCGGACGA(	2	103	1	103	100	Bm_ATCC23344_chr2	2325379	1074373	1074475	100	515	7.70E-19	+
BPSS1219	penicillin-binding protein	1647742	1649526	+	594	53722242	3097077	-	COG0768M	CCGACACCGCGCGCTTCTTGAACAT	2	103	1	103	100	Bm_ATCC23344_chr2	2325379	1098043	1098145	96	479	3.30E-17	+
BPSS1220	L-arabinose-binding periplasmic protein precursor	1649914	1650915	+	333	53722243	3095667	araF	COG1879G	CCGCCGACGCGCCTCCTACAATCA(	2	103	1	103	100	Bm_ATCC23344_chr2	2325379	1100259	1100361	99	506	2.00E-18	+
BPSS1221	short chain dehydrogenase	1650974	1651747	+	257	53722244	3097078	-	COG1028IQR	AGCGCGCCGACGCCGCACGGCTGC	2	61	1	61	100	Bm_ATCC23344_chr2	2325379	1101361	1101421	100	305	2.40E-09	+
BPSS1222	efflux pump/antiporter	1651782	1653533	-	583	53722245	3097201	-	COG1226P, COG4651P	CCGCAATCGTCGACGACAAATTTTG	2	103	103	1	100	Bm_ATCC23344_chr2	2325379	1103958	1104060	100	515	7.70E-19	-
BPSS1223	hypothetical protein BPSS1223	1654763	1655665	+	300	53722246	3095929	-	COG3687R	ACGACGTCGCGCCGCGCACGCAAT	2	103	1	103	100	Bm_ATCC23344_chr2	2325379	1105097	1105199	100	515	7.70E-19	+
BPSS1224	hypothetical protein BPSS1224	1655766	1657739	+	657	53722247	3095930	-	COG4257V	GCCCGCCCCCGCCTGAAGCGAGCC	2	103	1	103	100	Bm_ATCC23344_chr2	2325379	1106100	1106202	100	515	7.70E-19	+
BPSS1226	hypothetical protein BPSS1226	1658338	1658616	+	92	53722249	3097014	-	NULL	GGGAAGGTCATCGTGTTTCATGGC(	2	103	1	103	100	Bm_ATCC23344_chr2	2325379	1108680	1108782	98	497	5.00E-18	+
BPSS1227	hypothetical protein BPSS1227	1658768	1659241	-	157	53722250	3097015	-	COG3865S	TTTTCCGCGCGACGTGTCGATTCCG	2	103	103	1	100	Bm_ATCC23344_chr2	2325379	1109687	1109789	99	506	2.00E-18	-
BPSS1229	transcriptional regulator	1660932	1661714	+	260	53722252	3096395	-	COG1349KG	CGCGCGGCGTTGCGATAACGGCGC	2	103	1	103	100	Bm_ATCC23344_chr2	2325379	1111280	1111382	100	515	7.70E-19	+
BPSS1232	transporter periplasmic binding protein	1664562	1665842	+	426	53722255	3096060	-	COG1653G	TTCTTTTGACGTAGTGGAGCGCCGT	2	103	1	103	100	Bm_ATCC23344_chr2	2325379	1114888	1114990	100	515	7.70E-19	+

BPSS1239	family S11 unassigned peptidase	1673068	1674069 +	333	53722262	3097132 -	COG1686M	GCCGGCGATTTGCGCTTCGCCGTTC	2	103	1	103	100 Bm_ATCC23344_chr2	2325379	1123337	1123439	100	515	7.70E-19 +
BPSS1240	penicillin-binding protein	1674424	1676136 +	570	53722263	3095351 ftsI	COG0768M	TGCCCGCCGAATGCGCGTGCCCCG	2	103	1	103	100 Bm_ATCC23344_chr2	2325379	1124660	1124762	99	506	2.00E-18 +
BPSS1241	bifunctional reductase	1676705	1680961 -	1418	53722264	3095935 -	COG0243C, COG0369P	CGCGCCGCATCGGCCGATAGCGAC	2	61	61	1	100 Bm_ATCC23344_chr2	2325379	1131329	1131389	100	305	2.40E-09 -
BPSS1244	nitrate transporter	1683992	1685317 -	441	53722267	3096246 -	COG2223P	TCCGCCCGGCAACGGGCTTCCTGG	2	103	103	1	100 Bm_ATCC23344_chr2	2325379	1135700	1135802	100	515	7.70E-19 -
BPSS1246	response regulator protein	1687254	1687850 +	198	53722269	3096027 -	COG3707T	CGGCGCGAGCCGGCGCGGCGCAC	2	103	1	103	100 Bm_ATCC23344_chr2	2325379	1137523	1137625	100	515	7.70E-19 +
BPSS1249	lipoprotein	1691427	1693619 +	730	53722272	3095693 -	NULL	CGATTCAAGCGTCATTGCGCAGTGC	2	103	1	103	100 Bm_ATCC23344_chr2	2325379	1141665	1141767	100	515	7.70E-19 +
BPSS1252	inner membrane transport protein	1696611	1697945 -	444	53722275	3095027 -	NULL	ATTCTTAAGATAATAACTTAGAATTT	2	103	103	1	100 Bm_ATCC23344_chr2	2325379	1148290	1148392	100	515	7.70E-19 -
BPSS1253	LysR family transcriptional regulator	1698134	1699072 +	312	53722276	3095028 -	COG0583K	TATCTTAAGAATAAATTTCATTAATC	2	103	1	103	100 Bm_ATCC23344_chr2	2325379	1148381	1148483	99	506	2.00E-18 +
BPSS1255	LysR family transcriptional regulator	1699715	1700722 -	335	53722277	3095633 -	COG0583K	TGGATGGGACGAATGATCGGCCAT	2	103	103	1	100 Bm_ATCC23344_chr2	2325379	1151028	1151130	100	515	7.70E-19 -
BPSS1256	short-chain type dehydrogenase/reductase	1700846	1701589 +	247	53722278	3095634 -	COG1028IQR	TAAAGCAAATGGAATGATGATATCC	2	103	1	103	100 Bm_ATCC23344_chr2	2325379	1151054	1151156	100	515	7.70E-19 +
BPSS1258	oxidoreductase/dehydrogenase	1704390	1705427 -	345	53722280	3095455 -	COG2130R	GCGCGCCGCGCTTCGCCCCGTTCGC	2	67	67	1	100 Bm_ATCC23344_chr2	2325379	1155692	1155758	100	335	1.00E-10 -
BPSS1260	hypothetical protein BPSS1260	1708563	1710377 +	604	53722282	3095492 -	NULL	CGTCGCCGCCTCGCTCTCCGCCCTG	2	103	1	103	100 Bm_ATCC23344_chr2	2325379	1158641	1158743	100	515	7.70E-19 +
BPSS1261	hypothetical protein BPSS1261	1710619	1710951 -	110	53722283	3097112 -	COG4390S	GGCGTGGCGGCGGCGCGGCCGAGC	2	103	103	1	100 Bm_ATCC23344_chr2	2325379	1161127	1161229	98	497	5.00E-18 -
BPSS1262	hypothetical protein BPSS1262	1711200	1711982 +	260	53722284	3097113 -	NULL	CGCGCCGAAGCCGCCCGTCGCCCG	2	103	1	103	100 Bm_ATCC23344_chr2	2325379	1161293	1161395	99	506	2.00E-18 +
BPSS1277	cobalt-nickel-resistance system transmembrane prot	1743703	1744773 +	356	53722299	3096702 cnrT	NULL	GGCGAATCGCGGCCCGCCGCCGCA	2	103	103	1	100 Bm_ATCC23344_chr2	2325379	1040195	1040297	100	515	7.70E-19 -
BPSS1285	family C39 unassigned peptidase	1755323	1757467 -	714	53722308	3095622 -	COG2274V	TCGTCTCTCGCCGTGTCCCGCCCCG	2	103	1	103	100 Bm_ATCC23344_chr2	2325379	1027561	1027663	100	515	7.70E-19 +
BPSS1286	hypothetical protein BPSS1286	1757583	1757801 -	72	53722309	3095038 -	NULL	CTTTGGTTGGCAATCGGAGGAAATC	2	103	1	103	100 Bm_ATCC23344_chr2	2325379	1027227	1027329	100	515	7.70E-19 +
BPSS1286a	hypothetical protein BPSS1286a	1758015	1758188 -	57	53722310	3095982 -	NULL	AATCGCAGCGATAAAAAATAAACGT	2	103	1	103	100 Bm_ATCC23344_chr2	2325379	1026840	1026942	100	515	7.70E-19 +
BPSS1287	outer membrane efflux protein	1758557	1759897 +	446	53722311	3095983 -	COG1538MU	GGAGACGGAGATGTTTGAAACAGA	2	103	103	1	100 Bm_ATCC23344_chr2	2325379	1026569	1026671	100	515	7.70E-19 -
BPSS1288	gamma-glutamyltransferase 1	1760116	1761837 -	573	53722312	3095085 -	NULL	ACGAAACCCCATTTTTGTATACGCG	2	103	1	103	100 Bm_ATCC23344_chr2	2325379	1023213	1023315	98	497	5.00E-18 +
BPSS1289	hypothetical protein BPSS1289	1762739	1763257 +	172	53722313	3095284 -	NULL	GCTCCGAGAATTTCCCTCGCCGCCG	2	103	103	1	100 Bm_ATCC23344_chr2	2325379	1022443	1022545	100	515	7.70E-19 -
BPSS1290	hypothetical protein BPSS1290	1763600	1764118 +	172	53722314	3095285 -	NULL	CGCGCGGTGCGCGGCGCGGTGCTC	2	103	103	1	100 Bm_ATCC23344_chr2	2325379	1021595	1021697	100	515	7.70E-19 -
BPSS1292	amino acid permease	1770212	1771507 +	431	53722316	3095434 -	COG0531E	CCGCCGCTCGCCGCCCGCTACCGG	2	103	103	1	100 Bm_ATCC23344_chr2	2325379	1013887	1013989	100	515	7.70E-19 -
BPSS1295	3-deoxy-7-phosphoheptulonate synthase	1773286	1774404 +	372	53722319	3096312 aroG	COG0722E	CGCGTTTGCGGCAGCGCAGCGCGA	2	103	103	1	100 Bm_ATCC23344_chr2	2325379	1010739	1010841	100	515	7.70E-19 -
BPSS1297	regulatory protein	1776176	1777645 -	489	53722321	3094958 -	NULL	TCGGCGCGCGCCGTTTCGATGCGC	2	103	4	103	97 Bm_ATCC23344_chr2	2325379	1006180	1006279	100	500	3.70E-18 +
BPSS1298	hypothetical protein BPSS1298	1778359	1779030 +	223	53722322	3095336 -	NULL	GCGCTCGAGGCGCCTCGTCGCCGG	2	103	103	1	100 Bm_ATCC23344_chr2	2325379	1005605	1005707	99	506	2.00E-18 -
BPSS1299	LysR family transcriptional regulator	1779761	1780765 -	334	53722323	3094910 -	COG0583K	GTCTCCAGATTGATGATTGTCAAGG	2	103	1	103	100 Bm_ATCC23344_chr2	2325379	1003089	1003191	99	506	2.00E-18 +
BPSS1300	protocatechuate 3,4-dioxygenase	1780873	1781577 +	234	53722324	3095333 pcaH	COG3485Q	AGTTATACGATTCGCGATAAAACAT	2	103	103	1	100 Bm_ATCC23344_chr2	2325379	1003079	1003181	100	515	7.70E-19 -
BPSS1305	ABC transport permease	1785706	1786626 -	306	53722329	3097026 -	COG0601EP	ATCGTCGCGATTTCGCCCGCCCGGC	2	103	1	103	100 Bm_ATCC23344_chr2	2325379	997417	997519	99	506	2.00E-18 +
BPSS1309	RpiR family regulatory protein	1791684	1792646 -	320	53722333	3095872 -	COG1737K	GGCGGGGTCTGCGGCGATCGGACC	2	103	1	103	100 Bm_ATCC23344_chr2	2325379	991429	991532	99	505	2.20E-18 +
BPSS1310	BCCT (betaine/carnitine/choline) family transporter	1792979	1795009 +	676	53722334	3097064 -	COG1292M	GCGCTTCGCGCGCGCCGCCGCATC	2	103	103	1	100 Bm_ATCC23344_chr2	2325379	991194	991296	100	515	7.70E-19 -
BPSS1311	esterase	1795244	1796458 -	404	53722335	3095112 estA	COG1680V	TCGCGCGACGCGCGCGCAGCTTGC	2	103	1	103	100 Bm_ATCC23344_chr2	2325379	987456	987558	100	515	7.70E-19 +
BPSS1312	hypothetical protein BPSS1312	1797052	1798512 +	486	53722337	3095031 -	NULL	GCTGCGGCGCGGGCGCCCGTAGGC	2	103	103	1	100 Bm_ATCC23344_chr2	2325379	986960	987062	98	497	5.00E-18 -
BPSS1313	hypothetical protein BPSS1313	1799023	1799664 +	213	53722338	3096066 -	NULL	ATCCGGCATGATAGGTGCCTGAAAC	2	103	103	1	100 Bm_ATCC23344_chr2	2325379	985012	985114	97	488	1.30E-17 -
BPSS1315	isoleucyl-tRNA synthetase	1800598	1803501 -	967	53722340	3094966 ileS2	COG0060J	TCCGGCCGGGCCGCGGGCGGCCCC	2	86	1	86	100 Bm_ATCC23344_chr2	2325379	980465	980550	100	430	5.30E-15 +
BPSS1318	D-lactate dehydrogenase	1805789	1807282 -	497	53722343	3095158 -	COG0277C	CGACCGGCGCGAACGCGGCCCGGA	2	85	1	85	100 Bm_ATCC23344_chr2	2325379	976604	976688	96	398	1.50E-13 +
BPSS1319	hypothetical protein BPSS1319	1807422	1807718 -	98	53722344	3095299 -	NULL	CGATGCCGGGCACGGCGCCGCGCC	2	63	1	63	100 Bm_ATCC23344_chr2	2325379	976253	976315	100	315	8.30E-10 +
BPSS1321	hypothetical protein BPSS1321	1808641	1809741 -	366	53722346	3095932 -	NULL	GGGCCCGCGGGATCGAGCCGGTCC	2	103	1	103	100 Bm_ATCC23344_chr2	2325379	974189	974291	98	497	5.00E-18 +
BPSS1322	AraC family transcriptional regulatory protein	1810477	1811349 +	290	53722347	3094941 -	NULL	GACATCGAAACACCCGCCGGCGTG	2	103	103	1	100 Bm_ATCC23344_chr2	2325379	973551	973653	99	506	2.00E-18 -
BPSS1323	hypothetical protein BPSS1323	1811570	1812529 -	319	53722348	3096632 -	NULL	CCCGCGCGATTCGCGATTCGCGACA	2	103	1	103	100 Bm_ATCC23344_chr2	2325379	971428	971530	98	497	5.00E-18 +
BPSS1324	D-cysteine desulfhydrase	1812770	1813786 -	338	53722349	3096633 -	COG2515E	ATCGTTTTTAACATGAGGCATCGCT	2	103	1	103	100 Bm_ATCC23344_chr2	2325379	970171	970273	100	515	7.70E-19 +
BPSS1325	AsnC family leucine-responsive regulatory protein	1813979	1814488 +	169	53722350	3097167 lrp	COG1522K	AAAACGATCGGGAATAACAGGTT	2	103	103	1	100 Bm_ATCC23344_chr2	2325379	970076	970178	100	515	7.70E-19 -
BPSS1326	hypothetical protein BPSS1326	1814887	1816338 +	483	53722351	3096550 -	COG1432S	GGCGCGCGCCCGCACGGATTTCCG	2	103	103	4	97 Bm_ATCC23344_chr2	2325379	969137	969236	99	491	9.30E-18 -
BPSS1327	MFS family sugar transporter protein	1816540	1817895 -	451	53722352	3096778 -	NULL	CCAAATTCATCTATATGTTTGCATGA	2	103	1	103	100 Bm_ATCC23344_chr2	2325379	966024	966126	99	506	2.00E-18 +
BPSS1328	GntR-family transcriptional regulator	1818003	1818728 +	241	53722353	3096779 -	COG2188K	TTACCCGTGCAGCCTGCCTGAATTG	2	103	103	1	100 Bm_ATCC23344_chr2	2325379	966014	966116	99	506	2.00E-18 -
BPSS1329	MFS family transport protein	1818788	1820212 +	474	53722354	3096551 -	NULL	CGGCCGGTTCGCGCGATAGCGGAG	2	62	62	1	100 Bm_ATCC23344_chr2	2325379	965229	965290	96	292	9.10E-09 -
BPSS1330	hypothetical protein BPSS1330	1820264	1821151 +	295	53722355	3097088 -	COG3618R	AGTTCGCGCCGGATTCAGGTCGGC	2	54	54	1	100 Bm_ATCC23344_chr2	2325379	963753	963806	100	270	9.00E-08 -
BPSS1331	phosphatase protein	1821570	1823045 +	491	53722356	3097089 -	NULL	CACGCCCCCCGAGCGCGCCGCCCG	2	103	103	1	100 Bm_ATCC23344_chr2	2325379	962517	962618	97	482	2.40E-17 -
BPSS1333	hypothetical protein BPSS1333	1824843	1825703 +	286	53722358	3097199 -	COG3315Q	GCATGCCGAAGCTCAGGGCGACAT	2	103	103	1	100 Bm_ATCC23344_chr2	2325379	959172	959274	99	506	2.00E-18 -
BPSS1334	hypothetical protein BPSS1334	1826391	1826663 +	90	53722359	3097200 -	NULL	CTAATCGCCGCCCCTCCATCCCACC	2	103	103	1	100 Bm_ATCC23344_chr2	2325379	957635	957737	97	488	1.30E-17 -
BPSS1335	hypothetical protein BPSS1335	1827019	1827231 +	70	53722360	3096197 -	NULL	GCGCGCGTCGCGCCTTTTCCAGCCC	2	103	103	1	100 Bm_ATCC23344_chr2	2325379	956996	957097	98	491	9.30E-18 -
BPSS1339	ArsR family regulatory protein	1829021	1829368 -	115	53722364	3095311 -	COG0640K	GCGACCCTTGCATTTCCGTCATTCAT	2	103	1	103	100 Bm_ATCC23344_chr2	2325379	954583	954685	100	515	7.70E-19 +
BPSS1340	transmembrane cytochrome b561	1829882	1830415 +	177	53722365	3095312 -	COG3038C	CCATAAGAATCGCCCGCGATAATCC	2	103	103	1	100 Bm_ATCC23344_chr2	2325379	954140	954242	99	506	2.00E-18 -
BPSS1344	serine acetyltransferase	1834030	1835019 -	329	53722369	3097027 -	COG1045E	GCGCCGGGATCGCCGGCGCGCGGC	2	103	1	103	100 Bm_ATCC23344_chr2	2325379	948913	949015	99	506	2.00E-18 +
BPSS1345	hypothetical protein BPSS1345	1835134	1835601 -	155	53722370	3095318 -	COG0607P	CGGCTCTTTCATAGTGAACTCCCGA	2	103	1	103	100 Bm_ATCC23344_chr2	2325379	948331	948433	99	506	2.00E-18 +
BPSS1346	subfamily S9A unassigned peptidase	1835968	1838085 +	705	53722371	3095319 -	COG1505E	CGCCACGCGCGCGCCGCGAATGCC	2	103	103	2	99 Bm_ATCC23344_chr2	2325379	948080	948181	98	492	8.40E-18 -
BPSS1347	HlyD family efflux pump protein	1838603	1839856 -	417	53722372	3097138 -	COG1566V	CGCCGCCGAGCATCGAAGCGCCCC	2	103	6	103	95 Bm_ATCC23344_chr2	2325379	943957	944054	97	472	6.70E-17 +
BPSS1349	dTDP-rhamnosyl transferase	1842237	1843232 -	331	53722374	3096814 -	COG1216R	GACGAAGCCGAACGGAGAAGCATC	2	103	1	103	100 Bm_ATCC23344_chr2	2325379	940576	940678	100	515	7.70E-19 +
BPSS1352	fatty acid biosynthetic protein	1846596	1847495 -	299	53722377	3097052 -	COG0596R	CAGGGCGGGCAGATCGCGCGGGAC	2	103	1	103	100 Bm_ATCC23344_chr2	2325379	936288	936390	100	515	7.70E-19 +
BPSS1353	transcriptional regulator BetI	1848477	1849064 +	195	53722378	3096015 betI	COG1309K	CCCGATGCGATCGCCTCCTACTTTT	2	103	103	1	100 Bm_ATCC23344_chr2	2325379	934740	934842	100	515	7.70E-19 -
BPSS1356	hypothetical protein BPSS1356	1853417	1856794 +	1125	53722381	3096459 -	COG3246S	AAACACCAGATGCCCGGGGCCGGC	2	103	103	1	100 Bm_ATCC23344_chr2	2325379	929844	929946	100	515	7.70E-19 -
BPSS1357	MFS sugar transporter	1857019	1858455 +	478	53722382	3095250 -	NULL	ATGCCGCTTTCCTTCTATGGCGGCG	2	103	103	1	100 Bm_ATCC23344_chr2	2325379	926242	926344	100	515	7.70E-19 -
BPSS1360	two-component sensor kinase protein	1860467	1862680 -	737	53722385	3094977 -	COG0642T, COG4251T, CC	CCGCCCCGCACGACGGCGCGCTTC	2	103	1	103	100 Bm_ATCC23344_chr2	2325379	920483	920585	100	515	7.70E-19 +
BPSS1362	2-hydroxyacid dehydrogenase	1863764	1864696 -	310	53722387	3097270 -	COG1052CHR	CTTCGGGGCACGAGCGACATTCGC	2	103	1	103	100 Bm_ATCC23344_chr2	2325379	918430	918532	100	515	7.70E-19 +
BPSS1363	hypothetical protein BPSS1363	1865456	1865911 +	151	53722388	3096007 -	NULL	ATGACGGTACTGGCGCAGGCTTTGC	2	103	103	1	100 Bm_ATCC23344_chr2	2325379	917819	917921	99	506	2.00E-18 -
BPSS1364	cold shock-like protein	1866688	1866891 -	67	53722389	3096537 cspD	NULL	TTCCCGCCGCTCGCGCGGAATTCGT	2	103	1	103	100 Bm_ATCC23344_chr2	2325379	916308	916411	99	505	2.20E-18 +
BPSS1364a	30S ribosomal protein S21	1867543	1867755 +	70	53722390	3095643 rpsU	COG0828J	GCACCGTCGTTGCCTCTTCATGGCC	2	103	103	1	100 Bm_ATCC23344_chr2	2325379	915754	915856	100	515	7.70E-19 -
BPSS1413	hydrolase protein	1925626	1926240 -	204	53722440	3094909 -	COG1011R	GAGCATTCGGGATAACCCTTGGGG	2	103	1	103	100 Bm_ATCC23344_chr2	2325379	589001	589103	100	515	7.70E-19 +
BPSS1414	GntR family transcriptional regulator	1926431	1927135 +	234	53722441	3096292 -	COG2186K	CCGGAGCCGTTTCGCCAAAATCCGC	2	103	103	1	100 Bm_ATCC23344_chr2	2325379	588947	589049	99	506	2.00E-18 -
BPSS1415	dehydrogenase/oxidoreductase protein	1927214	1928104 +	296	53722442	3096293 -	COG2084I	GCCGCGATCCGCGAACGGCCGCGC	2	81	81	1	100 Bm_ATCC23344_chr2	2325379	588164	588244	100	405	7.20E-14 -
BPSS1418	transmembrane permease transporter	1930260	1931582 +	440	53722445	3096559 -	NULL	CGCAGTCCTGCCCGCCGTGTGCGC	2	91	91	1	100 Bm_ATCC23344_chr2	2325379	585157	585247	100	455	3.90E-16 -
BPSS1419	hydroxypyruvate isomerase	1931664	1932440 +	258	53722446	3094965 hyi	COG3622G	ATAATCGACCGTCGGCGGCGCCGC	2	84	84	1	100 Bm_ATCC23344_chr2	2325379	583738	583821	100	420	1.50E-14 -
BPSS1421	hypothetical protein BPSS1421	1934223	1935014 +	263	53722448	3095591 -	NULL	GCACGGCTGCCCCGCCCGTTCCGA	2	103	103	1	100 Bm_ATCC23344_chr2	2325379	581074	581176	99	506	2.00E-18 -

BPSS1422	hypothetical protein BPSS1422	1935909	1936808 -	299	53722449	3095592 -	NULL	CCGCATGGACGGCGCCCGCCGTCC	2	103	1	103	100	Bm_ATCC23344_chr2	2325379	578356	578458	99	506	2.00E-18 +
BPSS1423	glycine betaine/L-proline-binding protein ABC transp	1937183	1938133 -	316	53722450	3096295 -	COG2113E	ACGCATTGAAACAAGCGCGTGGCC	2	103	1	103	100	Bm_ATCC23344_chr2	2325379	577072	577174	100	515	7.70E-19 +
BPSS1424	AraC transcriptional regulatory protein	1938234	1939232 -	332	53722451	3096864 -	COG4977K	CGCCGGTTTTTTCGCCTTCCTCGTTT	2	103	1	103	100	Bm_ATCC23344_chr2	2325379	575973	576075	100	515	7.70E-19 +
BPSS1426	glycine betaine/L-proline transport ATP-binding prot	1940250	1941419 -	389	53722453	3095234 -	COG4175E	AAGCGTTTGTCGCAATTCGTCGGCT	2	103	1	103	100	Bm_ATCC23344_chr2	2325379	573786	573888	100	515	7.70E-19 +
BPSS1428	hypothetical protein BPSS1428	1943447	1943662 +	71	53722455	3095879 -	NULL	CGTTCGTCCGCGCGGGATGATTTGC	2	103	103	1	100	Bm_ATCC23344_chr2	2325379	571839	571941	100	515	7.70E-19 -
BPSS1429	hypothetical protein BPSS1429	1943761	1944660 +	299	53722456	3096411 -	NULL	GCCGGCCGGCGGCATGTGCGCCGA	2	101	101	1	100	Bm_ATCC23344_chr2	2325379	571525	571625	100	505	2.20E-18 -
BPSS1433	sodium bile acid symporter family protein	1946525	1947595 +	356	53722460	3096313 -	COG0798P	CCGTTCGCATCGCGGCCGCCGAAC	2	65	65	1	100	Bm_ATCC23344_chr2	2325379	568751	568815	100	325	2.90E-10 -
BPSS1444	hypothetical protein BPSS1444	1967536	1968717 -	393	53722467	3095412 -	NULL	CGGCGCGCGCGGCGTACGCGGCGT	2	103	1	103	100	Bm_ATCC23344_chr2	2325379	833041	833143	100	515	7.70E-19 +
BPSS1445	biotin carboxylase	1968819	1970813 -	664	53722468	3095238 -	COG4770I	GACCTTCGCGGCGGCGCGCGGCGC	2	103	1	103	100	Bm_ATCC23344_chr2	2325379	830945	831047	100	515	7.70E-19 +
BPSS1448	acyl-coA dehydrogenase	1973410	1974591 -	393	53722471	3095407 -	COG1960I	GAAAATTTCCGCAGCATATGGCAAT	2	103	1	103	100	Bm_ATCC23344_chr2	2325379	827148	827250	98	497	5.00E-18 +
BPSS1449	TetR family transcriptional regulator	1974799	1975563 +	254	53722472	3097213 -	COG1309K	AACCGCCCGACGCCGCGGGACAAC	2	103	103	1	100	Bm_ATCC23344_chr2	2325379	826966	827068	100	515	7.70E-19 -
BPSS1452	copper nitrite reductase protein	1978963	1980426 +	487	53722473	3095134 -	COG2132Q	GCACGAGCCGCATCCGCCGCCTGC	2	103	103	1	100	Bm_ATCC23344_chr2	2325379	822859	822961	99	506	2.00E-18 -
BPSS1454	hypothetical protein BPSS1454	1984227	1985591 +	454	53722475	3095104 -	NULL	TCTGCGCGAGCTGACGCGGCAACA	2	103	103	1	100	Bm_ATCC23344_chr2	2325379	817460	817562	100	515	7.70E-19 -
BPSS1456	hypothetical protein BPSS1456	1986772	1987392 +	206	53722477	3095005 -	NULL	CAAATCTCCACACGAATCCGATCGA	2	103	103	1	100	Bm_ATCC23344_chr2	2325379	815077	815179	99	506	2.00E-18 -
BPSS1457	hypothetical protein BPSS1457	1987533	1988546 +	337	53722478	3095006 -	NULL	AGGCGTCTTCGCCGTTCATGCGCGC	2	103	103	1	100	Bm_ATCC23344_chr2	2325379	814316	814418	100	515	7.70E-19 -
BPSS1458	MFS family transport protein	1989047	1990465 -	472	53722479	3097090 -	NULL	TGCCCGAACCCCGGCGGCCACGCG	2	103	1	103	100	Bm_ATCC23344_chr2	2325379	811207	811309	99	506	2.00E-18 +
BPSS1459	acetyltransferase protein	1990568	1991350 -	260	53722480	3097091 -	COG1670J	TCGCCGCCACCCGTTGGCGACCGC	2	103	1	103	100	Bm_ATCC23344_chr2	2325379	810289	810391	100	515	7.70E-19 +
BPSS1461	two-component response regulator	1993328	1993987 -	219	53722482	3095218 -	COG0745TK	CGGTACCTGAAGCGAAGCTTGCGA	2	103	1	103	100	Bm_ATCC23344_chr2	2325379	807652	807754	99	506	2.00E-18 +
BPSS1462	hypothetical protein BPSS1462	1994219	1994575 +	118	53722483	3095219 -	COG5591S	CGAGCGCATTGTAGAACCCAACGA	2	103	103	1	100	Bm_ATCC23344_chr2	2325379	807551	807653	99	506	2.00E-18 -
BPSS1466	aldehyde dehydrogenase family protein	1999417	2000928 -	503	53722487	3096623 -	COG1012C	GCCGGTGCGCTTGCATGCGGCGCG	2	103	1	103	100	Bm_ATCC23344_chr2	2325379	800637	800739	100	515	7.70E-19 +
BPSS1468	hypothetical protein BPSS1468	2002377	2003396 -	339	53722489	3096260 -	NULL	GTGAATTTCGCGCACCGAGAATCC	2	103	1	103	100	Bm_ATCC23344_chr2	2325379	798289	798391	99	506	2.00E-18 +
BPSS1469	LysR family transcriptional regulator	2003571	2004479 +	302	53722490	3096072 -	COG0583K	ATGGATTCTCGGTGCGCGAAATTCA	2	103	103	1	100	Bm_ATCC23344_chr2	2325379	798212	798314	100	515	7.70E-19 -
BPSS1471	TetR family transcriptional regulator	2006630	2007328 +	232	53722493	3095626 -	COG1309K	AGCCCGGAGTCGGGCAAAAGCCGC	2	55	55	1	100	Bm_ATCC23344_chr2	2325379	795174	795228	100	275	5.30E-08 -
BPSS1473	hypothetical protein BPSS1473	2009252	2011057 -	601	53722495	3096613 -	COG1502I	ACTTCATCGGAGCGTCACACGCAC	2	103	1	103	100	Bm_ATCC23344_chr2	2325379	792118	792220	100	515	7.70E-19 +
BPSS1474	DeoR family transcriptional regulator	2011759	2012517 -	252	53722496	3096929 -	COG1349KG	GCGGGCGATCGGCGGCCGGCGCG	2	103	1	103	100	Bm_ATCC23344_chr2	2325379	790633	790735	100	515	7.70E-19 +
BPSS1476	mannitol dehydrogenase protein	2014766	2016235 -	489	53722498	3096662 -	NULL	AGAGGATGTAGCGTTTACACCGAT	2	103	1	103	100	Bm_ATCC23344_chr2	2325379	786877	786979	98	497	5.00E-18 +
BPSS1480	hypothetical protein BPSS1480	2020393	2020578 +	61	53722502	3095632 -	NULL	AACCTGCAAGCAATTCAGTATTTCT	2	103	103	1	100	Bm_ATCC23344_chr2	2325379	782812	782914	99	506	2.00E-18 -
BPSS1481	hypothetical protein BPSS1481	2020691	2021569 +	292	53722503	3095004 -	NULL	GCAGGCCGCGCGCAGTGCAGGTTT	2	103	103	1	100	Bm_ATCC23344_chr2	2325379	782514	782616	100	515	7.70E-19 -
BPSS1482	NAD(+) synthetase	2021925	2022779 +	284	53722504	3097154 nadE	COG0171H	CGACACCCGACGACCGGATCGACA	2	103	103	1	100	Bm_ATCC23344_chr2	2325379	781191	781293	100	515	7.70E-19 -
BPSS1483	TetR family transcriptional regulator	2023034	2024068 -	344	53722505	3095007 -	COG1309K	CGCCAATCGTTCGTTTGTCGCACGA	2	103	1	103	100	Bm_ATCC23344_chr2	2325379	778959	779061	100	515	7.70E-19 +
BPSS1484	hypothetical protein BPSS1484	2024180	2025397 +	405	53722506	3095008 -	NULL	GGACGACCGCTGAGACGGCCGTCT	2	103	103	1	100	Bm_ATCC23344_chr2	2325379	778945	779047	100	515	7.70E-19 -
BPSS1487	anaerobically induced outer membrane protein	2027135	2028697 +	520	53722509	3096319 -	COG2010C, COG2132Q	CGGCCCGCGCCGCCCCTTAAAGAT	2	103	103	1	100	Bm_ATCC23344_chr2	2325379	775989	776091	97	488	1.30E-17 -
BPSS1490	N-acetylmuramoyl-L-alanine amidase	2031208	2032233 +	341	53722512	3095176 -	COG3023V	ACTGCCGATGCCGTTCCATCGGCGC	2	103	103	1	100	Bm_ATCC23344_chr2	2325379	771916	772018	100	515	7.70E-19 -
BPSS1491	hypothetical protein BPSS1491	2032502	2033761 +	419	53722513	3096138 -	NULL	AAGCGCGCACGAGCCGCCGAAGGT	2	103	103	1	100	Bm_ATCC23344_chr2	2325379	770603	770705	99	506	2.00E-18 -
BPSS1495	two-component regulator sensor kinase	2038711	2040555 -	614	53722517	3096752 -	COG0642T	GCCGCCTTCGTTGCGGATGAAAAG	2	103	1	103	100	Bm_ATCC23344_chr2	2325379	762832	762934	100	515	7.70E-19 +
BPSS1496	hypothetical protein BPSS1496	2040815	2041309 +	164	53722518	3096753 -	NULL	CTTGCCGCGCCGATGCGGCTTTTGA	2	103	103	1	100	Bm_ATCC23344_chr2	2325379	762670	762772	100	515	7.70E-19 -
BPSS1498	hypothetical protein BPSS1498	2043052	2043561 +	169	53722520	3095204 -	NULL	CCGGCATCGCACGCGCGACGCCGC	2	103	103	1	100	Bm_ATCC23344_chr2	2325379	760433	760535	99	506	2.00E-18 -
BPSS1508	hypothetical protein BPSS1508	2057929	2058606 +	225	53722530	3096370 -	COG3521S	CCCGCCGGCCGCGACGCGCGCGCC	2	103	103	1	100	Bm_ATCC23344_chr2	2325379	745502	745604	100	515	7.70E-19 -
BPSS1512	hypothetical protein BPSS1512	2065185	2066609 +	474	53722534	3096758 -	NULL	TGCCGCTTCGCCCTCCGCGCCATCC	2	103	103	1	100	Bm_ATCC23344_chr2	2325379	737587	737689	100	515	7.70E-19 -
BPSS1513	hypothetical protein BPSS1513	2066773	2067066 +	97	53722535	3096713 -	NULL	CAGCGCTCGTGCGCGGCTTCGCGT	2	103	103	1	100	Bm_ATCC23344_chr2	2325379	736569	736671	98	497	5.00E-18 -
BPSS1521	hypothetical protein BPSS1521	2073665	2074108 -	147	53722542	3095465 -	NULL	CGTACGCCGCGCGAGGCCCCGGGC	2	103	103	1	100	Bm_ATCC23344_chr2	2325379	1648417	1648519	100	515	7.70E-19 -
BPSS1522	two-component response regulator	2074346	2075017 -	223	53722543	3096722 -	COG2197TK	AGGGCATGCGCTGGCGCGGTGGGC	2	103	103	1	100	Bm_ATCC23344_chr2	2325379	1649344	1649446	99	506	2.00E-18 -
BPSS1523	chaperone	2075253	2075708 -	151	53722544	3096082 bicP	NULL	GCCGGGCGCGCGCAGCCGTGCGTC	2	103	103	1	100	Bm_ATCC23344_chr2	2325379	1650043	1650145	98	497	5.00E-18 -
BPSS1524	intercellular spread protein	2075849	2077387 -	512	53722545	3096275 bopA	NULL	GCGCGCCCGGCGGGCGACGGCCTC	2	103	103	1	100	Bm_ATCC23344_chr2	2325379	1651733	1651835	100	515	7.70E-19 -
BPSS1529	membrane antigen	2082841	2083773 -	310	53722550	3096115 bipD	NULL	AGGCGGGCGCCGCGCATCGCGCGC	2	64	64	1	100	Bm_ATCC23344_chr2	2325379	1658160	1658223	100	320	4.90E-10 -
BPSS1530	HNS-like regulatory protein	2083835	2084125 -	96	53722551	3096413 bprA	COG2916R	GGCGGGCGTCGCCCGCCGCGCGCC	2	103	103	1	100	Bm_ATCC23344_chr2	2325379	1658524	1658626	100	515	7.70E-19 -
BPSS1533	surface presentation of antigens protein	2087441	2087956 -	171	53722554	3096042 bicA	NULL	ATTTTCCCCGGCGCGCGGCGCGTA	2	103	103	1	100	Bm_ATCC23344_chr2	2325379	1662343	1662445	100	515	7.70E-19 -
BPSS1546	AraC-family regulator of type III secretion system	2100565	2101206 -	213	53722567	3096570 bsaN	NULL	TAAGTTCGATTGCGTGGTGGTTTCA	2	103	103	1	100	Bm_ATCC23344_chr2	2325379	1675607	1675709	100	515	7.70E-19 -
BPSS1547	type III secretion system protein	2101717	2103003 +	428	53722568	3096220 -	NULL	ATTATCCGGTTCACGATCGACGGCA	2	103	1	103	100	Bm_ATCC23344_chr2	2325379	1676020	1676122	100	515	7.70E-19 +
BPSS1549	Type III secretion system protein	2103329	2103631 +	100	53722570	3095550 -	NULL	CCCTCTCTACCGCTCCGGGACGACC	2	62	1	62	100	Bm_ATCC23344_chr2	2325379	1677673	1677729	91	235	3.40E-06 +
BPSS1553	hypothetical protein BPSS1553	2106912	2107883 +	323	53722574	3095781 -	COG3710K	GCGCGGCAGCGCGGCTTCCACACG	2	103	1	103	100	Bm_ATCC23344_chr2	2325379	1681225	1681327	100	515	7.70E-19 +
BPSS1555	family M4 unassigned peptidase	2109379	2111076 +	565	53722576	3095651 -	COG3227E	CTAGCATGCCTCTGCAGTTCGAAGC	2	103	1	103	100	Bm_ATCC23344_chr2	2325379	1683658	1683760	100	515	7.70E-19 +
BPSS1556	MarR-family transcriptional regulator	2112198	2112686 -	162	53722577	3095003 -	COG1846K	CGACGCGCCCCGCCGCGCCGAACC	2	103	103	1	100	Bm_ATCC23344_chr2	2325379	1687047	1687149	97	488	1.30E-17 -
BPSS1557	glyoxalase	2113012	2113470 +	152	53722578	3096288 -	COG0346E	GTTAACGTACTATACTCGACCCGAG	2	103	1	103	100	Bm_ATCC23344_chr2	2325379	1687275	1687378	96	478	3.60E-17 +
BPSS1558	alpha-ketoglutarate permease	2114037	2115356 +	439	53722579	3094946 kgtP	NULL	CTGCGCCCCCTCTTACCCTTGCTGA	2	103	1	103	100	Bm_ATCC23344_chr2	2325379	1688298	1688400	98	497	5.00E-18 +
BPSS1559	LysR-family transcriptional regulator	2115742	2116629 -	295	53722580	3096289 -	COG0583K	TGTTGGGCTTTCCTGACGCGACGCA	2	103	103	1	100	Bm_ATCC23344_chr2	2325379	1690963	1691065	100	515	7.70E-19 -
BPSS1560	L(+)-mandelate dehydrogenase	2116730	2117953 +	407	53722581	3095127 mdlB	COG1304C	GATTATTCGCTTTTATCGAACGATTT	2	103	1	103	100	Bm_ATCC23344_chr2	2325379	1690966	1691068	100	515	7.70E-19 +
BPSS1561	carboxypeptidase	2118077	2119741 -	554	53722582	3096104 -	NULL	CGCAGCGGTAGCGGCGCGCGCGAC	2	103	103	1	100	Bm_ATCC23344_chr2	2325379	1694075	1694177	99	506	2.00E-18 -
BPSS1562	kumamolisin	2120444	2122033 -	529	53722583	3097003 -	COG4934O	CGGCGTGTGTGCGCCGTCCGACAT	2	103	103	1	100	Bm_ATCC23344_chr2	2325379	1696406	1696508	100	515	7.70E-19 -
BPSS1563	LysE-family translocator	2122464	2123090 -	208	53722584	3097004 -	COG1280E	TAGTGTATTCCAATCAGTTTGGTGC	2	103	103	1	100	Bm_ATCC23344_chr2	2325379	1697451	1697553	99	506	2.00E-18 -
BPSS1564	AsnC-family transcriptional regulator	2123224	2123688 +	154	53722585	3095576 -	COG1522K	ACCCGCTCGACGAGCGCTTTTTGGC	2	103	2	103	99	Bm_ATCC23344_chr2	2325379	1697488	1697589	100	510	1.30E-18 +
BPSS1565	hypothetical protein BPSS1565	2124036	2124440 +	134	53722586	3094891 -	COG3832S	GCGCTCCGCGGGCAGCGCGTCGCG	2	103	1	103	100	Bm_ATCC23344_chr2	2325379	1698275	1698377	99	506	2.00E-18 +
BPSS1566	probable phosphate transporter	2124793	2126379 +	528	53722587	3094892 -	COG0306P	CCGAGTCGCACACCCGAATCGCCC	2	103	1	103	100	Bm_ATCC23344_chr2	2325379	1699071	1699175	98	495	6.20E-18 +
BPSS1567	hypothetical protein BPSS1567	2126529	2127311 +	260	53722588	3095655 -	COG3826S	ATGCGCGGCCGAATTGCAGCGCAA	2	103	1	103	100	Bm_ATCC23344_chr2	2325379	1700809	1700911	100	515	7.70E-19 +
BPSS1568	probable AMP-binding rotein	2127907	2129514 +	535	53722589	3095342 -	COG0318IQ	AGCCAGGATCGGCCGCGTTCGCGC	2	103	1	103	100	Bm_ATCC23344_chr2	2325379	1702171	1702273	100	515	7.70E-19 +
BPSS1569	N-acylhomoserine lactone-dependent regulatory prc	2130282	2130974 +	230	53722590	3095343 -	COG2771K	AACGGGGGCATGCAAATTGAGCAG	2	103	1	103	100	Bm_ATCC23344_chr2	2325379	1704546	1704648	100	515	7.70E-19 +
BPSS1570	N-acylhomoserine lactone synthase	2131287	2131895 +	202	53722591	3096892 -	COG3916TQ	CGAGACCGCGCACGCTGCTTCTCG	2	103	1	103	100	Bm_ATCC23344_chr2	2325379	1705563	1705666	99	505	2.20E-18 +
BPSS1571	probable NADH oxidoreductase	2132198	2133562 +	454	53722592	3095256 -	COG1902C	CCCGGCACGCCCATCGCCGTGCGG	2	103	1	103	100	Bm_ATCC23344_chr2	2325379	1706466	1706568	100	515	7.70E-19 +
BPSS1578	hypothetical protein BPSS1578	2142234	2143097 -	287	53722599	3096014 -	NULL	GCTTCGATCGCAGCGACCCGGTTCC	2	103	103	1	100	Bm_ATCC23344_chr2	2325379	1716362	1716464	100	515	7.70E-19 -
BPSS1582	cyclic di-GMP binding protein	2151584	2153980 -	798	53722604	3096080 bcsB	NULL	GCCGGCGCTTCAGCCGCCGCGCGC	2	103	103	1	100	Bm_ATCC23344_chr2	2325379	1727266	1727368	100	515	7.70E-19 -
BPSS1582a	hypothetical protein BPSS1582a	2154301	2154891 +	196	53722605	3096075 -	NULL	CGCGTGCGTGACCGCGCGCCGTCA	2	103	1	103	100	Bm_ATCC23344_chr2	2325379	1727732	1727834	100	515	7.70E-19 +
BPSS1585	hypothetical protein BPSS1585	2156968	2157420 -	150	53722608	3096076 -	NULL	GCCAGGTCAAGCCGGGCGGCTCCG	2	103	103	1	100	Bm_ATCC23344_chr2	2325379	1730937	1731039	100	515	7.70E-19 -
BPSS1586	LysR-family transcriptional regulator	2157837	2158751 -	304	53722609	3094920 -	COG0583K	CGCCAGAATGGCGACTTGAATCCC	2	103	103	1	100	Bm_ATCC23344_chr2	2325379	1732261	1732363	100	515	7.70E-19 -

BPSS1587	agmatinase	2158918	2159871 +	317	53722610	3096603 speB	COG0010E	TTCGAACTTCGGGATTCAAGTCGCC	2	103	1	103	100 Bm_ATCC23344_chr2	2325379	1732330	1732432	99	506	2.00E-18 +
BPSS1588	hypothetical protein BPSS1588	2160486	2161886 +	466	53722611	3097129 -	NULL	CGTTCGCCCGCCCATCGATCCGAA	2	103	1	103	100 Bm_ATCC23344_chr2	2325379	1733894	1733996	99	506	2.00E-18 +
BPSS1591	hypothetical protein BPSS1591	2164673	2165557 +	294	53722614	3095725 -	NULL	CCCCGCCTCGACGATGCGGCGGCG	2	103	1	103	100 Bm_ATCC23344_chr2	2325379	1738067	1738169	99	506	2.00E-18 +
BPSS1592	type III secretion system protein	2165625	2167430 -	601	53722615	3095726 -	COG1450NU	CGTGGCCGCCGCCGCGCGGCGCTG	2	103	103	1	100 Bm_ATCC23344_chr2	2325379	1740916	1741018	100	515	7.70E-19 -
BPSS1601	type IV pilus biosynthesis protein	2176601	2177170 -	189	53722624	3096323 -	NULL	CGAAGGCAGTCCGACGCGTCGGAC	2	103	103	1	100 Bm_ATCC23344_chr2	2325379	1750657	1750759	100	515	7.70E-19 -
BPSS1602	twitching motility protein	2177487	2178593 +	368	53722625	3095406 pilT	COG2805NU, COG5008NU	CAAAAGCTAGTTGGCATGCAAAATT	2	103	1	103	100 Bm_ATCC23344_chr2	2325379	1750876	1750978	100	515	7.70E-19 +
BPSS1605	probable two-component system sensor kinase	2181394	2182479 -	361	53722628	3095321 -	COG0642T	CTGGACAAGCGCTTTCGAATCGGG	2	103	103	1	100 Bm_ATCC23344_chr2	2325379	1755973	1756075	100	515	7.70E-19 -
BPSS1606	hypothetical protein BPSS1606	2182607	2183251 -	214	53722629	3096533 -	NULL	CGCGGCGCCGGCGCCGGCGCATG	2	103	103	1	100 Bm_ATCC23344_chr2	2325379	1756745	1756848	96	478	3.60E-17 -
BPSS1607	hypothetical protein BPSS1607	2183466	2183954 +	162	53722630	3096161 -	NULL	TTCGCCGCGCACGCCTTCGCTTCGC	2	103	1	103	100 Bm_ATCC23344_chr2	2325379	1756856	1756958	98	497	5.00E-18 +
BPSS1608	hypothetical protein BPSS1608	2184499	2185161 -	220	53722631	3096162 -	COG1670J	GCGAAGCGATGCAGCCGCGCACG	2	103	103	1	100 Bm_ATCC23344_chr2	2325379	1758628	1758730	100	515	7.70E-19 -
BPSS1609	hypothetical protein BPSS1609	2185370	2186017 +	215	53722632	3096937 -	NULL	CGGCCGGATCGCCCTCGCCGGCGT	2	103	1	103	100 Bm_ATCC23344_chr2	2325379	1758739	1758841	100	515	7.70E-19 +
BPSS1610	AraC-family transcriptional regulator	2186573	2188018 -	481	53722633	3095884 -	NULL	CATCCATGCGCTGCGCCCCGCGAC	2	103	103	1	100 Bm_ATCC23344_chr2	2325379	1761463	1761565	100	515	7.70E-19 -
BPSS1611	hypothetical protein BPSS1611	2188230	2188685 -	151	53722634	3095885 -	NULL	GCCGGCTGCTGCTCGGCATCGTGTT	2	95	95	1	100 Bm_ATCC23344_chr2	2325379	1762130	1762224	100	475	4.90E-17 -
BPSS1614	type III secretion protein	2189407	2190390 -	327	53722637	3096788 -	NULL	GGCGCGATCGACGCGGCGCATCGC	2	103	103	1	100 Bm_ATCC23344_chr2	2325379	1763832	1763934	99	506	2.00E-18 -
BPSS1621	type III secretion protein	2196386	2197468 -	360	53722644	3096071 -	COG1377NU	CGCTTCGCATCCGGCGGCGCGGCT	2	103	103	1	100 Bm_ATCC23344_chr2	2325379	1770920	1771022	99	506	2.00E-18 -
BPSS1622	type III secretion protein	2197704	2198285 +	193	53722645	3095189 -	NULL	ATCGCCGCGCCCCCGCGCCCGGCG	2	103	1	103	100 Bm_ATCC23344_chr2	2325379	1771058	1771160	100	515	7.70E-19 +
BPSS1630	hypothetical protein BPSS1630	2204186	2205952 +	588	53722653	3096210 -	NULL	CCGCGAGCCGTTCGGTCGCCGGCC	2	103	1	103	100 Bm_ATCC23344_chr2	2325379	1777505	1777607	100	515	7.70E-19 +
BPSS1631	hypothetical protein BPSS1631	2206192	2207145 -	317	53722654	3095627 -	NULL	ACGAACCGAACCGCGCCGGCGGC	2	58	58	1	100 Bm_ATCC23344_chr2	2325379	1780579	1780636	98	281	2.90E-08 -
BPSS1635	probable class III aminotransferase	2248989	2251772 -	927	53722658	3097151 -	COG0160E	TGTCTCGCCGTCTCGCCTTTGCCGA	2	103	103	1	100 Bm_ATCC23344_chr2	2325379	1799523	1799625	99	506	2.00E-18 -
BPSS1639	citrate lyase beta chain	2257163	2258044 -	293	53722662	3096759 -	COG2301G	CCGATTCGCCGAAGGCTGCCCCCG	2	103	103	1	100 Bm_ATCC23344_chr2	2325379	1805699	1805801	100	515	7.70E-19 -
BPSS1640	LysR-family transcriptional regulator	2258149	2259060 -	303	53722663	3096760 -	COG0583K	TCGTTCGGCACGTTGTGAAAACCCC	2	103	103	1	100 Bm_ATCC23344_chr2	2325379	1806715	1806817	100	515	7.70E-19 -
BPSS1643	LysR-family transcriptional regulator	2259622	2260542 -	306	53722664	3095831 -	COG0583K	TCGGGCGACGTAGTGTAAAGTCCG	2	103	103	1	100 Bm_ATCC23344_chr2	2325379	1808188	1808290	99	506	2.00E-18 -
BPSS1644	hypothetical protein BPSS1644	2260651	2262048 +	465	53722665	3097123 -	NULL	CGATTGTCAAAATTTTTCGATCAGT	2	103	1	103	100 Bm_ATCC23344_chr2	2325379	1808208	1808310	99	506	2.00E-18 +
BPSS1647	hypothetical protein BPSS1647	2263768	2264946 -	392	53722668	3096241 -	COG3287S	TACATCACGGCCGGCGACGGCGAA	2	75	75	1	100 Bm_ATCC23344_chr2	2325379	1812601	1812675	100	375	1.60E-12 -
BPSS1648	probable two-component response regulator	2265019	2266446 -	475	53722669	3096242 -	COG3437KT	ATTTCTCATGTTTTTCGCGTTTCCCA	2	103	103	1	100 Bm_ATCC23344_chr2	2325379	1814101	1814202	99	500	3.70E-18 -
BPSS1649	sugar-binding protein	2266833	2269253 +	806	53722670	3094967 -	NULL	GCCGGCCGTCGTCCGGTTCGTCATT	2	103	1	103	100 Bm_ATCC23344_chr2	2325379	1814389	1814491	99	506	2.00E-18 +
BPSS1653	hypothetical protein BPSS1653	2272044	2272613 -	189	53722674	3095458 -	NULL	CTGTAGCGTGAGGCTCGTCGCCCC	2	70	70	1	100 Bm_ATCC23344_chr2	2325379	1820285	1820354	97	332	1.40E-10 -
BPSS1654	cytochrome P450	2272681	2275035 -	784	53722675	3094930 -	COG1018C, COG2124Q	GGCGATGCAGGTTAACGCGATCAT	2	103	103	1	100 Bm_ATCC23344_chr2	2325379	1822707	1822809	100	515	7.70E-19 -
BPSS1656	hypothetical protein BPSS1656	2275525	2276781 +	418	53722677	3095556 -	NULL	CGCTCGCCGCATGGCCGGCCGATT	2	74	1	74	100 Bm_ATCC23344_chr2	2325379	1823129	1823202	100	370	2.70E-12 +
BPSS1657	beta-glucosidase	2277526	2279721 +	731	53722678	3097236 bglB	NULL	ATCCGTCACACGCTTGACGGCGCC	2	103	1	103	100 Bm_ATCC23344_chr2	2325379	1825095	1825197	100	515	7.70E-19 +
BPSS1658	hypothetical protein BPSS1658	2280113	2280697 +	194	53722679	3096980 -	NULL	CTCGAGCATGGCCCGCCTGCGGCG	2	103	1	103	100 Bm_ATCC23344_chr2	2325379	1827775	1827877	99	506	2.00E-18 +
BPSS1659	hypothetical protein BPSS1659	2280838	2281125 +	95	53722680	3096460 -	NULL	ATCCGGCGGATGCGCCGCATGCAG	2	103	1	103	100 Bm_ATCC23344_chr2	2325379	1828407	1828509	100	515	7.70E-19 +
BPSS1667	formate dehydrogenase-O, major subunit	2288604	2291675 -	1022	53722688	3094982 fdoG	COG0243C	CGCGCGCCCGGAGCGGCCGCGCC	2	103	103	1	100 Bm_ATCC23344_chr2	2325379	1839342	1839444	100	515	7.70E-19 -
BPSS1669	hypothetical protein BPSS1669	2292615	2293382 -	255	53722689	3095163 -	COG1434S	TTTTCCTCACGACACGCGCAGCCA	2	103	103	1	100 Bm_ATCC23344_chr2	2325379	1841050	1841152	100	515	7.70E-19 -
BPSS1671	ABC transport system, ATP-binding protein	2295168	2297078 -	636	53722691	3096511 -	COG1126E	CGGCGCTGCGCCGCATGCATGCCG	2	103	103	1	100 Bm_ATCC23344_chr2	2325379	1844666	1844768	99	506	2.00E-18 -
BPSS1672	monooxygenase	2297194	2298516 -	440	53722692	3095043 -	COG2141C	GCGCCCGAGGCGGCGCGCGCATC	2	58	58	1	100 Bm_ATCC23344_chr2	2325379	1846104	1846161	100	290	1.10E-08 -
BPSS1673	hypothetical protein BPSS1673	2298572	2299573 -	333	53722693	3095044 -	COG2141C	CGCGCGCAGGCCGGGCGGCCGGG	2	103	103	1	100 Bm_ATCC23344_chr2	2325379	1847161	1847263	100	515	7.70E-19 -
BPSS1674	hypothetical protein BPSS1674	2299893	2300846 -	317	53722694	3096057 -	COG0834ET	CGGGCCGCGCGTCTCGCGCCCGGC	2	88	88	1	100 Bm_ATCC23344_chr2	2325379	1848417	1848504	98	431	4.80E-15 -
BPSS1676	hypothetical protein BPSS1676	2302418	2303440 -	340	53722696	3097038 -	COG3608R	GCGCCGCCGGCGCTCCGCTCCGAG	2	103	103	1	100 Bm_ATCC23344_chr2	2325379	1851110	1851212	98	497	5.00E-18 -
BPSS1678	ribonuclease	2304652	2305575 +	307	53722698	3096670 -	COG1295S	GCCCTGCTCCGTGGAATTGAATAA	2	103	1	103	100 Bm_ATCC23344_chr2	2325379	1852186	1852288	100	515	7.70E-19 +
BPSS1679	porin related exported protein	2306033	2307187 +	384	53722699	3096671 -	COG3203M	TTTGAGACACTTTATTTTTTTGGATT	2	103	1	103	100 Bm_ATCC23344_chr2	2325379	1853567	1853669	100	515	7.70E-19 +
BPSS1680	histone-like protein	2309027	2309449 -	140	53722700	3096774 -	NULL	CGGCGCTTGTAGAATCCGGCGTTG	2	103	103	1	100 Bm_ATCC23344_chr2	2325379	1857019	1857121	100	515	7.70E-19 -
BPSS1681	hypothetical protein BPSS1681	2309745	2310872 +	375	53722701	3096587 -	COG1835I	GCAGCGGTTTTTGCGTCGCGTATCG	2	103	1	103	100 Bm_ATCC23344_chr2	2325379	1857217	1857319	100	515	7.70E-19 +
BPSS1682	UTP-glucose-1-phosphate uridylyltransferase	2311424	2312311 -	295	53722702	3095010 gtaB	COG1210M	CCGAATGCCGATGCGCGCCGTGCG	2	103	103	1	100 Bm_ATCC23344_chr2	2325379	1859860	1859962	100	515	7.70E-19 -
BPSS1683	lipopolysaccharide biosynthesis-related membrane p	2312964	2314121 +	385	53722703	3096588 -	COG1835I	TTTTTTATTCTCCCGCCCGTATTTCTT	2	103	1	103	100 Bm_ATCC23344_chr2	2325379	1860415	1860517	100	515	7.70E-19 +
BPSS1684	lipopolysaccharide biosynthesys-related glycosyltran	2314235	2316697 -	820	53722704	3096455 -	COG0438M	CGCGACGTTTCAGGACGTCGAGCG	2	103	103	1	100 Bm_ATCC23344_chr2	2325379	1864109	1864211	99	506	2.00E-18 -
BPSS1687	hypothetical protein BPSS1687	2320944	2322002 +	352	53722707	3096186 -	COG1835I	TTTCATTTATTTCAGCGGCCGCACG	2	103	1	103	100 Bm_ATCC23344_chr2	2325379	1868242	1868344	100	515	7.70E-19 +
BPSS1691	O-succinylhomoserine sulfhydrylase	2324782	2325999 -	405	53722711	3095378 metZ	COG0626E	GTCGATAGCTGTTCGTACCGCACAC	2	103	103	1	100 Bm_ATCC23344_chr2	2325379	1873309	1873411	100	515	7.70E-19 -
BPSS1692	amidophosphoribosyltransferase	2326228	2327763 -	511	53722712	3095844 purF	COG0034F	TCCGCGCGAGCGCACCGACCGAAC	2	55	55	1	100 Bm_ATCC23344_chr2	2325379	1875073	1875127	100	275	5.30E-08 -
BPSS1694	hypothetical protein BPSS1694	2328317	2329057 -	246	53722714	3095486 -	NULL	CGGGTAGCTTCGGGTTCAGCCATA	2	103	103	1	100 Bm_ATCC23344_chr2	2325379	1876367	1876469	99	506	2.00E-18 -
BPSS1695	folylpolyglutamate synthase/dihydrofolate synthase	2329162	2330475 -	437	53722715	3095339 folC	COG0285H	ATGATCGCACGTCCGCGCGTCGCT	2	103	103	1	100 Bm_ATCC23344_chr2	2325379	1877785	1877887	100	515	7.70E-19 -
BPSS1696	acetyl-CoA carboxylase beta subunit	2330587	2331459 -	290	53722716	3095672 accD	COG0777I	GCCGCGCGACACGTCAACACAAGC	2	82	82	1	100 Bm_ATCC23344_chr2	2325379	1878769	1878850	100	410	4.30E-14 -
BPSS1699	tryptophan synthase subunit beta	2333256	2334449 -	397	53722719	3096325 trpB	COG0133E	TGCCAGTCCGCGCGGCGGGCGCCG	2	74	74	1	100 Bm_ATCC23344_chr2	2325379	1881759	1881832	100	370	2.70E-12 -
BPSS1704	aspartate-semialdehyde dehydrogenase	2338910	2340031 -	373	53722724	3097224 asd	COG0136E	GATCACGAAAATCTGATCGTCGTCC	2	103	103	1	100 Bm_ATCC23344_chr2	2325379	1887287	1887389	100	515	7.70E-19 -
BPSS1706	isopropylmalate isomerase small subunit	2341492	2342142 -	216	53722726	3095029 leuD	COG0066E	TGACGGCGCGCGCGAGCGCGCCG	2	83	83	1	100 Bm_ATCC23344_chr2	2325379	1889395	1889477	100	415	2.50E-14 -
BPSS1707	isopropylmalate isomerase large subunit	2342366	2343775 -	469	53722728	3095020 leuC	NULL	TATGGGTTTTTTCGTTTCCCTGGTAC	2	103	103	1	100 Bm_ATCC23344_chr2	2325379	1891028	1891130	100	515	7.70E-19 -
BPSS1709	hypothetical protein BPSS1709	2345455	2346402 -	315	53722730	3096708 -	NULL	CATCGATCGGTCCGCTGGCGACGG	2	103	103	1	100 Bm_ATCC23344_chr2	2325379	1893385	1893487	98	497	5.00E-18 -
BPSS1710	AsnC-family transcriptional regulator	2347122	2347649 -	175	53722731	3096709 -	COG1522K	ATCCATCGAGCCGCAGAATGTGCA	2	103	103	1	100 Bm_ATCC23344_chr2	2325379	1894902	1895004	99	506	2.00E-18 -
BPSS1711	pyruvate dehydrogenase E1 component	2347810	2350533 +	907	53722732	3095727 aceE	COG2609C	TTCCAATCAAAAGAGATGCACATTC	2	103	1	103	100 Bm_ATCC23344_chr2	2325379	1894965	1895067	99	506	2.00E-18 +
BPSS1712	hypothetical protein BPSS1712	2350787	2352031 -	414	53722733	3096502 -	NULL	GGCGCGATCGTATCGCTTCGAACG	2	103	103	1	100 Bm_ATCC23344_chr2	2325379	1899282	1899384	100	515	7.70E-19 -
BPSS1714	AraC-family transcriptional regulator	2353354	2354328 +	324	53722735	3096669 -	NULL	TTGCGAACCTCCTGTATCGTCGAAA	2	78	1	78	100 Bm_ATCC23344_chr2	2325379	1901771	1901848	100	390	3.40E-13 +
BPSS1715	citrate synthase	2354425	2355726 -	433	53722736	3096977 gltA	COG0372C	GAGCGTTGTGCCCGTTATCGAATCC	2	55	55	1	100 Bm_ATCC23344_chr2	2325379	1904216	1904270	100	275	5.30E-08 -
BPSS1720	succinate dehydrogenase cytochrome b-556 subunit	2358929	2359345 -	138	53722741	3096507 -	COG2009C	ACCGTGAATTGCAACGCTCCAGAG	2	103	103	1	100 Bm_ATCC23344_chr2	2325379	1907835	1907937	100	515	7.70E-19 -
BPSS1721	GntR-family transcriptional regulator	2359545	2360345 -	266	53722742	3097204 -	COG2188K	GACGGGTAAAGTCAAACGGTATCT	2	103	103	1	100 Bm_ATCC23344_chr2	2325379	1908835	1908937	100	515	7.70E-19 -
BPSS1722	malate dehydrogenase	2360644	2361627 +	327	53722743	3095126 mdh	COG0039C	GCCGCGAGTCCTCCGCCCTGCTTTA	2	103	1	103	100 Bm_ATCC23344_chr2	2325379	1909036	1909138	100	515	7.70E-19 +
BPSS1723	lyase	2361825	2362844 +	339	53722744	3096700 -	COG2301G	CCCCGGCGCCCGCGCAGAGCCCGC	2	103	1	103	100 Bm_ATCC23344_chr2	2325379	1910241	1910343	99	506	2.00E-18 +
BPSS1724	hypothetical protein BPSS1724	2363001	2363510 +	169	53722745	3096701 -	NULL	GCCGCTGCGCGCTTCCGGGGCGCG	2	103	1	103	100 Bm_ATCC23344_chr2	2325379	1911393	1911495	100	515	7.70E-19 +
BPSS1725	2-methylcitrate dehydratase	2363587	2365038 +	483	53722746	3095704 prpD	NULL	GGCCGCCCGACAGCCACCTTTGCG	2	79	1	79	100 Bm_ATCC23344_chr2	2325379	1912003	1912081	98	386	5.20E-13 +
BPSS1728	secretion/activator protein	2370877	2372568 -	563	53722749	3095194 -	COG2831U	ATCGGCGGCCCGCCAATCGGATGA	2	103	103	1	100 Bm_ATCC23344_chr2	2325379	1921049	1921151	98	497	5.00E-18 -
BPSS1736	ABC transport system, membrane protein	2383826	2385094 -	422	53722757	3095980 -	COG1176E	CGGCCGCGTGAGCCGGTGAAGCA	2	94	1	94	100 Bm_ATCC23344_chr2	2325379	430115	430208	98	461	2.10E-16 +
BPSS1737	ABC transport system, exported protein	2385186	2386232 -	348	53722758	3095981 -	COG0687E	ACGCGAAGCCGCGCGTGGCGCGC	2	77	1	77	100 Bm_ATCC23344_chr2	2325379	428994	429070	100	385	5.70E-13 +
BPSS1738	ABC transport system ATP-binding protein	2386307	2387407 -	366	53722759	3095673 -	COG3842E	TACCGGAAATATTGAAATGGTTCCC	2	103	1	103	100 Bm_ATCC23344_chr2	2325379	427793	427895	100	515	7.70E-19 +
BPSS1739	GntR-family regulatory protein	2387516	2389045 +	509	53722760	3097070 -	COG1167KE	CCCCTTTTCGTCTTGGCTCGATATTG	2	103	103	1	100 Bm_ATCC23344_chr2	2325379	427782	427884	100	515	7.70E-19 -

BPSS1741	Lipase precursor	2390190	2391284 -	364	53722762	3095030 lipA1	COG1075R	GGGTCGGCGTGCATGCCGCGTGTG	2	103	1	103	100	Bm_ATCC23344_chr2	2325379	423922	424024	99	506	2.00E-18 +
BPSS1742	outer membrane copper receptor	2391608	2393857 -	749	53722763	3097071 -	COG1629P	CCACGCGCGCCGCCGGCGGCGCG	2	103	1	103	100	Bm_ATCC23344_chr2	2325379	421505	421607	100	515	7.70E-19 +
BPSS1743	hypothetical protein BPSS1743	2393945	2394340 -	131	53722764	3097168 -	NULL	GCGCGCACCGCCGGCCCCGCGACC	2	103	1	103	100	Bm_ATCC23344_chr2	2325379	420878	420980	100	515	7.70E-19 +
BPSS1744	cytochrome b561	2394501	2395037 -	178	53722765	3097263 -	COG3038C	GCCACGGGGCCCGCCCATCGCGCG	2	103	1	103	100	Bm_ATCC23344_chr2	2325379	420181	420283	100	515	7.70E-19 +
BPSS1745	hypothetical protein BPSS1745	2395306	2395941 +	211	53722766	3097264 -	NULL	TCGGCTCGGCGATGCTTGAACTATT	2	103	103	1	100	Bm_ATCC23344_chr2	2325379	420010	420112	100	515	7.70E-19 -
BPSS1746	lipoprotein	2396072	2396836 -	254	53722767	3097222 -	COG0739M	TGACGCAGCCGAATCGGTGCGCCG	2	103	1	103	100	Bm_ATCC23344_chr2	2325379	418382	418484	100	515	7.70E-19 +
BPSS1747	hypothetical protein BPSS1747	2397116	2397433 -	105	53722768	3095783 -	COG0662G	GCCGATCCCCGGCAACTTACGTTC	2	103	1	103	100	Bm_ATCC23344_chr2	2325379	417786	417888	100	515	7.70E-19 +
BPSS1748	hypothetical protein BPSS1748	2397872	2398204 -	110	53722769	3095784 -	NULL	TTTTCGTTGATACATGGTTTTCCCTA	2	103	1	103	100	Bm_ATCC23344_chr2	2325379	417009	417111	99	506	2.00E-18 +
BPSS1749	6-phosphogluconate dehydrogenase	2398403	2399818 -	471	53722770	3097024 gnd	COG0362G	CGTCTTTCATTCGCGGCGCGCTCGC	2	103	1	103	100	Bm_ATCC23344_chr2	2325379	415401	415503	100	515	7.70E-19 +
BPSS1752	transport-related, membrane protein	2401848	2403272 -	474	53722773	3096049 -	COG1457F	GGATGTCGGTACAAACCCGCATCC	2	103	1	103	100	Bm_ATCC23344_chr2	2325379	411946	412048	100	515	7.70E-19 +
BPSS1753	hypothetical protein BPSS1753	2403909	2404769 +	286	53722774	3096059 -	NULL	ACATTGGGGACAGAGTTATTTCTCG	2	103	103	1	100	Bm_ATCC23344_chr2	2325379	411407	411509	100	515	7.70E-19 -
BPSS1755	RNA polymerase sigma factor	2406884	2408926 -	680	53722776	3096687 -	COG0568K	GGCTGCCGCGCCGGACGAAACGG	2	103	1	103	100	Bm_ATCC23344_chr2	2325379	327025	327127	100	515	7.70E-19 +
BPSS1756	DNA primase	2409370	2411244 -	624	53722777	3096768 dnaG	COG0358L	CTTCCCGCCGGCACGGCGCGCGAG	2	96	1	96	100	Bm_ATCC23344_chr2	2325379	324713	324809	98	470	8.30E-17 +
BPSS1757	hypothetical protein BPSS1757	2411338	2411784 -	148	53722778	3096576 -	COG1610S	GCCGAATTCGAGCAAACCCGCTTG	2	103	1	103	100	Bm_ATCC23344_chr2	2325379	324166	324268	100	515	7.70E-19 +
BPSS1758	30S ribosomal protein S21	2411951	2412163 -	70	53722779	3095644 rpsU	COG0828J	TGCGTTTGTGCAAAGCGTCTGCTAT	2	82	1	82	100	Bm_ATCC23344_chr2	2325379	323808	323889	100	410	4.30E-14 +
BPSS1759	hypothetical protein BPSS1759	2412243	2413466 -	407	53722780	3095896 -	COG2081R	ACGGCCGGAAAATCAACGTAACAG	2	103	1	103	100	Bm_ATCC23344_chr2	2325379	322484	322586	99	506	2.00E-18 +
BPSS1760	O-sialoglycoprotein endopeptidase	2413606	2414646 +	346	53722781	3095273 gcp	COG0533O	GCGCCGGCTGGCGAAGCGGCCGC	2	103	103	1	100	Bm_ATCC23344_chr2	2325379	322442	322544	100	515	7.70E-19 -
BPSS1761	hypothetical protein BPSS1761	2415068	2415877 -	269	53722782	3095897 -	COG1469S	TCCCCCGAAAGCATGGGCCTGCGG	2	103	1	103	100	Bm_ATCC23344_chr2	2325379	318508	318610	100	515	7.70E-19 +
BPSS1762	1-deoxy-D-xylulose-5-phosphate synthase	2416028	2417932 -	634	53722783	3096803 dxs	COG1154HI	CGCGAAAGCCCGCCGCCGCGCCCA	2	86	1	86	100	Bm_ATCC23344_chr2	2325379	316470	316555	100	430	5.30E-15 +
BPSS1764	exodeoxyribonuclease VII small subunit	2418897	2419190 -	97	53722785	3096525 xseB	COG1722L	GGCGCGCGGCGGCCAAGCCCCGC	2	103	1	103	100	Bm_ATCC23344_chr2	2325379	315195	315297	100	515	7.70E-19 +
BPSS1765	osmotic adaptation-related protein	2419460	2420566 +	368	53722786	3096723 -	COG4638PR	TACCCCACCCCAAGAATGTCGATC	2	103	103	1	100	Bm_ATCC23344_chr2	2325379	315029	315131	100	515	7.70E-19 -
BPSS1767	carboxymethylenebutenolidase	2421642	2422517 -	291	53722788	3096363 -	COG0412Q	CGCGCGGCGCGACTGCATCGCGCC	2	103	4	103	97	Bm_ATCC23344_chr2	2325379	311871	311970	100	500	3.70E-18 +
BPSS1768	hypothetical protein BPSS1768	2423237	2425030 +	597	53722789	3096026 -	COG4655S	CGGATGCGCCGCGGACGACCACAC	2	103	103	1	100	Bm_ATCC23344_chr2	2325379	311252	311355	99	505	2.20E-18 -
BPSS1769	NADH dehydrogenase	2425270	2426652 -	460	53722790	3097258 ndh	COG1252C	CGCCCAAAGTGTCCGAACTAGTTA	2	103	2	103	99	Bm_ATCC23344_chr2	2325379	307611	307712	100	510	1.30E-18 +
BPSS1772	hypothetical protein BPSS1772	2430911	2431195 +	94	53722793	3096491 -	NULL	CTTCCTGCAGCGTACGCGTGGAAA	2	103	103	1	100	Bm_ATCC23344_chr2	2325379	303440	303542	99	506	2.00E-18 -
BPSS1777	AMP nucleosidase	2436429	2437955 -	508	53722796	3095963 amn	COG0775F	CCGTTCGACGATGCGCGAGCGGCC	2	103	1	103	100	Bm_ATCC23344_chr2	2325379	296286	296388	100	515	7.70E-19 +
BPSS1778	hypothetical protein BPSS1778	2438111	2438509 +	132	53722797	3096631 -	NULL	CGATGCCCATCGCGGCGGCAAGGC	2	103	103	1	100	Bm_ATCC23344_chr2	2325379	296228	296330	100	515	7.70E-19 -
BPSS1779	homoserine kinase	2438603	2439598 +	331	53722798	3096847 thrB	COG2334R	CGCACCGCCGGCGCCGCGCGGCG	2	96	96	1	100	Bm_ATCC23344_chr2	2325379	295736	295831	100	480	2.90E-17 -
BPSS1781	MarR-family regulatory protein	2440904	2441356 +	150	53722800	3096256 -	COG1846K	GCCCTTTTCCGGCCGACGCTCGTG	2	103	103	1	100	Bm_ATCC23344_chr2	2325379	293435	293537	100	515	7.70E-19 -
BPSS1782	organic hydroperoxide resistance protein	2441494	2441913 +	139	53722801	3097161 ohr	COG1764O	AATTAATTTGCACGCTAATGATTAG	2	103	103	1	100	Bm_ATCC23344_chr2	2325379	292845	292947	98	497	5.00E-18 -
BPSS1783	hypothetical protein BPSS1783	2442026	2442184 -	52	53722802	3096257 -	NULL	CGACGCACGCTCGGGAACGGCTG	2	99	1	99	100	Bm_ATCC23344_chr2	2325379	292061	292159	100	495	6.20E-18 +
BPSS1784	depolymerase	2442281	2443327 -	348	53722803	3096172 -	COG3509Q	GACCAAAAGTCTGACGAAATTCTG	2	103	1	103	100	Bm_ATCC23344_chr2	2325379	290914	291016	100	515	7.70E-19 +
BPSS1785	hypothetical protein BPSS1785	2443689	2444378 +	229	53722804	3096771 -	NULL	TGAGCGCTCGCGCTTGTCTTCGTTG	2	103	103	1	100	Bm_ATCC23344_chr2	2325379	290650	290752	99	506	2.00E-18 -
BPSS1786	molybdenum transport-related, exported protein	2444947	2445747 +	266	53722805	3096772 -	COG0725P	CTGCGGCGTCATGCAGCACCCGCA	2	103	103	1	100	Bm_ATCC23344_chr2	2325379	289521	289623	100	515	7.70E-19 -
BPSS1789	molybdenum transport protein	2447680	2448468 -	262	53722808	3096486 modE	COG2005R	CTATAATGGCCTCCCGTCCTGGTTC	2	97	1	97	100	Bm_ATCC23344_chr2	2325379	285907	286003	100	485	1.70E-17 +
BPSS1790	hypothetical protein BPSS1790	2448563	2450620 -	685	53722809	3096899 -	COG5607S	AACTCGGCGCATTCGGGCTGGTCG	2	65	1	65	100	Bm_ATCC23344_chr2	2325379	283787	283851	100	325	2.90E-10 +
BPSS1791	hypothetical protein BPSS1791	2450683	2451066 -	127	53722810	3095357 -	NULL	TTCAAATGGAAATGGATGAAATATT	2	100	1	100	100	Bm_ATCC23344_chr2	2325379	283306	283405	98	482	2.40E-17 +
BPSS1792	LysR-family transcriptional regulator	2451164	2452039 +	291	53722811	3096139 -	COG0583K	CGGGTTTCTCCTTGACGGTTGGGAC	2	100	100	1	100	Bm_ATCC23344_chr2	2325379	283303	283402	98	482	2.40E-17 -
BPSS1793	hypothetical protein BPSS1793	2452106	2453026 +	306	53722812	3096140 -	NULL	ACTGTCAGGTTTTCGTTGGCCGACC	2	69	69	1	100	Bm_ATCC23344_chr2	2325379	282361	282429	98	336	9.40E-11 -
BPSS1795	hypothetical protein BPSS1795	2453984	2455324 +	446	53722814	3097205 -	COG1253R	TCGGCCGCGCGCGTCGCGACGCTC	2	88	88	1	100	Bm_ATCC23344_chr2	2325379	280483	280570	100	440	1.90E-15 -
BPSS1797	trehalose trehalohydrolase	2456109	2457752 -	547	53722816	3096087 -	COG0296G	GGCGCATACTGGGTGCCCCTTCTTC	2	103	1	103	100	Bm_ATCC23344_chr2	2325379	276617	276719	100	515	7.70E-19 +
BPSS1799	hypothetical protein BPSS1799	2459079	2459534 +	151	53722818	3095968 -	COG2606S	AGGGTTCACCAAAGATGCGGGAGC	2	103	103	1	100	Bm_ATCC23344_chr2	2325379	275430	275532	100	515	7.70E-19 -
BPSS1800	dehydrogenase	2459599	2460837 -	412	53722819	3095089 -	COG1304C	ATCGCGGTGCGCCGGTGTGAGCGC	2	103	1	103	100	Bm_ATCC23344_chr2	2325379	273574	273676	99	506	2.00E-18 +
BPSS1801	hypothetical transposon protein	2461063	2461500 +	145	53722820	3095615 -	COG3415L	TCCAGCAGGATTTCCACCTCGACTG	2	103	103	1	100	Bm_ATCC23344_chr2	2325379	273446	273548	99	506	2.00E-18 -
BPSS1802	hypothetical protein BPSS1802	2461742	2462368 -	208	53722821	3095616 -	COG1280E	GACAGCCTCCCTGACGCTTCCATAA	2	65	1	65	100	Bm_ATCC23344_chr2	2325379	272081	272145	100	325	2.90E-10 +
BPSS1803	AraC-family transcriptional regulator	2462431	2463285 +	284	53722822	3095107 -	NULL	CGAAGCTCCAGCGCGCCATCTTTTT	2	65	65	1	100	Bm_ATCC23344_chr2	2325379	272078	272142	100	325	2.90E-10 -
BPSS1836	hypothetical protein BPSS1836	2497575	2498579 -	334	53722855	3095240 -	COG3293L	ACCGAATAGCGTCCCTGTGCAACC	2	103	1	103	100	Bm_ATCC23344_chr2	2325379	264210	264312	100	515	7.70E-19 +
BPSS1839	oxidative stress related rubrerythrin protein	2501208	2501630 -	140	53722858	3096815 -	COG1592C	GCACGGTGCCCCGGCGGCGGCAG	2	103	1	103	100	Bm_ATCC23344_chr2	2325379	261155	261257	100	515	7.70E-19 +
BPSS1840	N-acetylmuramoyl-L-alanine amidase	2501901	2502935 -	344	53722859	3096352 -	COG3023V	GCGGACTCCCACGCTCGCGCGTCG	2	103	1	103	100	Bm_ATCC23344_chr2	2325379	259844	259946	100	515	7.70E-19 +
BPSS1843	hypothetical protein BPSS1843	2505333	2505746 -	137	53722862	3095289 -	NULL	CCTTAATTTTTCGCGTCGCGTGTCG	2	103	1	103	100	Bm_ATCC23344_chr2	2325379	257059	257162	99	505	2.20E-18 +
BPSS1844	hypothetical protein BPSS1844	2505912	2506265 -	117	53722863	3095290 -	NULL	GCAGCCCGCTCAAAATAGAGCCGC	2	103	1	103	100	Bm_ATCC23344_chr2	2325379	256540	256642	99	506	2.00E-18 +
BPSS1845	endonuclease/exonuclease/phosphatase family prote	2506642	2508516 +	624	53722864	3095094 -	COG2374R	CCAAAACCTGGCCAGATTACATATT	2	103	103	1	100	Bm_ATCC23344_chr2	2325379	256277	256379	100	515	7.70E-19 -
BPSS1846	hypothetical protein BPSS1846	2509305	2509685 +	126	53722865	3095095 -	COG4575S	GCGGCTTGCCGCCGCGGCCGCGCG	2	103	103	1	100	Bm_ATCC23344_chr2	2325379	253625	253727	100	515	7.70E-19 -
BPSS1848	hypothetical protein BPSS1848	2510510	2511604 +	364	53722867	3094990 -	COG0628R	CAGCCCGAACGACATGCGCTCGGG	2	103	103	1	100	Bm_ATCC23344_chr2	2325379	252420	252522	100	515	7.70E-19 -
BPSS1849	hypothetical protein BPSS1849	2511820	2512296 -	158	53722868	3094991 -	NULL	GGGGAGGGGCCCGCCGCCCGGCT	2	103	1	103	100	Bm_ATCC23344_chr2	2325379	250538	250638	98	485	1.70E-17 +
BPSS1850	hypothetical protein BPSS1850	2512421	2514517 -	698	53722869	3095485 -	COG1629P	TTCCTGTGCGCTGATATTGATACGT	2	103	1	103	100	Bm_ATCC23344_chr2	2325379	248317	248419	99	506	2.00E-18 +
BPSS1851	transport-related membrane protein	2514984	2516189 +	401	53722870	3096896 -	NULL	CGCGTCGCCCGTCGCCGCAAACGC	2	103	103	1	100	Bm_ATCC23344_chr2	2325379	247948	248050	98	497	5.00E-18 -
BPSS1852	oxidoreductase	2516831	2517847 -	338	53722871	3096897 -	COG2130R	CGCGCCTCGCGCCTCGCGCGAATG	2	103	1	103	100	Bm_ATCC23344_chr2	2325379	244981	245083	100	515	7.70E-19 +
BPSS1853	2,5-dichloro-2,5-cyclohexadiene-1,4-diol dehydroger	2518209	2519006 -	265	53722872	3094931 -	COG1028IQR	CCGCATCGCCGCCGCCCGCCGCTC	2	103	2	103	99	Bm_ATCC23344_chr2	2325379	243795	243896	100	510	1.30E-18 +
BPSS1857	acyl-CoA dehydrogenase	2521809	2523041 -	410	53722876	3096468 -	COG1960I	CATCAATGGCATCTATGCCATCGCA	2	103	1	103	100	Bm_ATCC23344_chr2	2325379	239777	239879	100	515	7.70E-19 +
BPSS1859	hypothetical protein BPSS1859	2524416	2524817 +	133	53722878	3095282 -	COG1765O	GCGCCGCTGCAGGCGCGCGCGAA	2	94	94	1	100	Bm_ATCC23344_chr2	2325379	238459	238552	100	470	8.30E-17 -
BPSS1862	ABC transport system, ATP-binding protein	2528589	2530598 +	669	53722881	3095871 -	COG1132V	CTTCGCAGCGCGCGCTGACGCGCG	2	103	103	1	100	Bm_ATCC23344_chr2	2325379	234266	234368	100	515	7.70E-19 -
BPSS1863	DJ-1/PfpI family protein	2530933	2531631 -	232	53722882	3095108 -	COG0693R	ACGGTAATTGCGTACAGTCGGTTTG	2	81	1	81	100	Bm_ATCC23344_chr2	2325379	231114	231194	98	396	1.80E-13 +
BPSS1864	AraC-family regulatory protein	2531740	2532780 +	346	53722883	3096381 -	COG4977K	GTCAACATCATGAGCGTCGCGCCG	2	81	81	1	100	Bm_ATCC23344_chr2	2325379	231111	231191	98	396	1.80E-13 -
BPSS1865	tryptophanyl-tRNA synthetase	2533352	2534392 +	346	53722884	3097179 trpS	COG0180J	ACATCCCGATCACGACCATGCCGC	2	103	103	1	100	Bm_ATCC23344_chr2	2325379	229617	229719	98	497	5.00E-18 -
BPSS1867	hypothetical protein BPSS1867	2536426	2537370 +	314	53722887	3095023 -	COG1266R	TACCATTGCGCCCCGTACGCGCCA	2	103	103	1	100	Bm_ATCC23344_chr2	2325379	226587	226689	99	506	2.00E-18 -
BPSS1868	voltage gated chloride channel membrane protein	2537573	2538856 -	427	53722888	3095024 -	COG0038P	GATGATGCCTACGCGTTCCTGGGTG	2	103	1	103	100	Bm_ATCC23344_chr2	2325379	224059	224161	100	515	7.70E-19 +
BPSS1875	chemotaxis-related protein	2547224	2548900 -	558	53722895	3097202 -	COG0840NT	TGCCCGGGGCGCGCATCGCGCCGG	2	103	1	103	100	Bm_ATCC23344_chr2	2325379	213975	214077	98	497	5.00E-18 +
BPSS1876	sensor kinase/response regulator fusion protein	2549071	2550168 -	365	53722896	3097203 -	COG0642T, COG3706T	GGGCTCGCGCTTACGCGATCGCAT	2	103	1	103	100	Bm_ATCC23344_chr2	2325379	212707	212809	100	515	7.70E-19 +
BPSS1877	hypothetical protein BPSS1877	2550984	2551319 +	111	53722898	3097145 -	COG2030I	TATCGGCAGTCGCGGCCGTCGATTT	2	103	103	1	100	Bm_ATCC23344_chr2	2325379	211989	212091	99	506	2.00E-18 -
BPSS1879	hypothetical protein BPSS1879	2553124	2554764 -	546	53722900	3097059 -	COG0028EH	GTCGAAGGAAAAAGGCCCGTGCGC	2	103	1	103	100	Bm_ATCC23344_chr2	2325379	208110	208213	99	505	2.20E-18 +
BPSS1880	Na+/H+ antiporter membrane protein	2555071	2556651 -	526	53722901	3097060 -	COG0025P	ATCGCCGCGCGCGAGCATCAGGCC	2	103	1	103	100	Bm_ATCC23344_chr2	2325379	206184	206286	99	506	2.00E-18 +
BPSS1883	hypothetical protein BPSS1883	2558842	2560494 +	550	53722904	3096184 -	COG1502I	GCGTCGGCGGCGCCACTTAGAATG	2	103	103	1	100	Bm_ATCC23344_chr2	2325379	204093	204195	100	515	7.70E-19 -

BPSS1884	hypothetical protein BPSS1884	2560561	2560977 -	138	53722905	3097008 -	COG0537FGR	GCGCGGCTTCGTGCCGCCGTATCGCT	2	103	1	103	100	Bm_ATCC23344_chr2	2325379	201857	201959	99	506	2.00E-18 +
BPSS1888	aromatic oxygenase	2563271	2564527 -	418	53722909	3094897 -	COG4638PR	GCGGATTGTCCAGCGCCTCAACCG	2	103	1	103	100	Bm_ATCC23344_chr2	2325379	198308	198410	100	515	7.70E-19 +
BPSS1889	AraC-family transcriptional regulator	2564727	2565767 +	346	53722910	3097234 -	NULL	CCTGACGAGACCGATTTTTGAAACG	2	103	103	1	100	Bm_ATCC23344_chr2	2325379	198206	198308	100	515	7.70E-19 -
BPSS1890	cat operon LysR-family transcriptional activator	2565817	2566704 -	295	53722911	3096978 -	COG0583K	CGGAACCCTCCTGTCGCACACATG	2	92	1	92	100	Bm_ATCC23344_chr2	2325379	196142	196233	100	460	2.30E-16 +
BPSS1891	muconate cycloisomerase I	2566794	2567927 +	377	53722912	3096549 catB	COG4948MR	GCATACCTCGAAAGTATGGAAGTA	2	92	92	1	100	Bm_ATCC23344_chr2	2325379	196139	196230	100	460	2.30E-16 -
BPSS1897	ubiquinol oxidase polypeptide II precursor	2572268	2573155 -	295	53722918	3096608 cyoA	COG1622C	GGATTTTCATCTCGTTCGCCGGCCG	2	103	1	103	100	Bm_ATCC23344_chr2	2325379	189686	189788	100	515	7.70E-19 +
BPSS1898	2,4-dienoyl-CoA reductase (NADPH)	2573880	2575913 -	677	53722919	3094934 fadH	COG0446R, COG1902C	AATGACAAGCATTATGGCTATTTGC	2	103	1	103	100	Bm_ATCC23344_chr2	2325379	186928	187030	100	515	7.70E-19 +
BPSS1899	AraC-family regulatory protein	2576063	2577115 +	350	53722920	3096979 -	NULL	CGCGCGCACCGCTTGTCCGTGAGG	2	103	103	1	100	Bm_ATCC23344_chr2	2325379	186876	186978	100	515	7.70E-19 -
BPSS1900	LysR-family transcriptional regulator	2577069	2578016 -	315	53722921	3095653 -	COG0583K	CGCCTGCTCCCGAACGTGATCGAC	2	97	1	97	100	Bm_ATCC23344_chr2	2325379	184831	184927	98	476	4.40E-17 +
BPSS1901	hypothetical protein BPSS1901	2578111	2579112 +	333	53722922	3095654 -	COG0451MG	ACGAAAATGTATAGCCACCAAACG	2	97	97	1	100	Bm_ATCC23344_chr2	2325379	184828	184924	98	476	4.40E-17 -
BPSS1902	LysR-family transcriptional regulator	2579291	2580205 -	304	53722923	3096458 -	COG0583K	TAGTGCTCGACGACAGCGCGGTCA	2	103	1	103	100	Bm_ATCC23344_chr2	2325379	182640	182742	100	515	7.70E-19 +
BPSS1903	benzoate 1,2-dioxygenase alpha subunit	2580429	2581796 +	455	53722924	3096099 benA	COG4638PR	ACCCGGGGTTTCCCATGCGACGCG	2	103	103	1	100	Bm_ATCC23344_chr2	2325379	182514	182616	100	515	7.70E-19 -
BPSS1907	hypothetical protein BPSS1907	2584182	2585090 -	302	53722928	3095515 -	COG0657I	CCATGCGGCGTTTGAATCGTTCGAA	2	103	1	103	100	Bm_ATCC23344_chr2	2325379	177785	177887	99	506	2.00E-18 +
BPSS1908	MarR-family regulatory protein	2585463	2585948 +	161	53722929	3095516 -	NULL	CGCCGCGTCGCCCTCCCCCGCATC	2	103	103	1	100	Bm_ATCC23344_chr2	2325379	177480	177582	100	515	7.70E-19 -
BPSS1913	lysine-specific permease	2591285	2592847 -	520	53722934	3097029 lysP	COG0833E	CACCGGGCGGCGCGCCGCGGATCG	2	103	1	103	100	Bm_ATCC23344_chr2	2325379	168779	168881	100	515	7.70E-19 +
BPSS1914	hypothetical protein BPSS1914	2593487	2594377 -	296	53722935	3096552 -	COG3687R	GATGTGAGATAGCGACTGCGCCCC	2	103	1	103	100	Bm_ATCC23344_chr2	2325379	165654	165756	100	515	7.70E-19 +
BPSS1915	metallo-beta-lactamase family protein	2595091	2596470 +	459	53722936	3096553 -	COG1236J	TGCCGGCGCTTCGCCGCGCGGATT	2	103	103	1	100	Bm_ATCC23344_chr2	2325379	165038	165140	100	515	7.70E-19 -
BPSS1916	acetoacetyl-CoA reductase	2597063	2597809 -	248	53722937	3095326 phbB	COG1028IQR	CAACACATTTTTCAACCCATGCCCG	2	103	1	103	100	Bm_ATCC23344_chr2	2325379	162221	162323	99	506	2.00E-18 +
BPSS1917	crp-family transcriptional regulator	2598474	2599256 +	260	53722938	3097133 -	COG0664T	GCTTCGTCGACGCCGTCGCGCAAA	2	103	103	1	100	Bm_ATCC23344_chr2	2325379	161654	161756	100	515	7.70E-19 -
BPSS1920	hypothetical protein BPSS1920	2601413	2601841 +	142	53722941	3097074 -	NULL	CAGGCGCGCGCAGCGGGCGACAC	2	103	103	1	100	Bm_ATCC23344_chr2	2325379	158694	158796	99	506	2.00E-18 -
BPSS1921	hypothetical protein BPSS1921	2602227	2602550 -	107	53722942	3097232 -	NULL	ATATCAAGCGGCGTCCGTCGAAGA	2	103	1	103	100	Bm_ATCC23344_chr2	2325379	157459	157561	100	515	7.70E-19 +
BPSS1922	hypothetical protein BPSS1922	2602830	2603777 +	315	53722943	3097233 -	COG1611R	GTCGACTCGATCCTCCGTACCGGA	2	103	98	1	98	Bm_ATCC23344_chr2	2325379	157213	157310	96	463	1.70E-16 -
BPSS1923	hypothetical protein BPSS1923	2604114	2604503 +	129	53722944	3096349 -	NULL	GCGGGTTCCCCCGTAAGGGGCTTT	2	103	103	1	100	Bm_ATCC23344_chr2	2325379	155991	156093	100	515	7.70E-19 -
BPSS1924	hypothetical protein BPSS1924	2605105	2605755 -	216	53722945	3096350 -	COG2823R	GCCGGTCCGCACTTGACGTGCATC	2	103	1	103	100	Bm_ATCC23344_chr2	2325379	154252	154354	100	515	7.70E-19 +
BPSS1925	hypothetical protein BPSS1925	2606114	2607292 -	392	53722946	3095579 -	NULL	CGCGCACCCGCGCCGACGCACCCC	2	103	1	103	100	Bm_ATCC23344_chr2	2325379	152759	152861	99	506	2.00E-18 +
BPSS1926	denylylsulfate kinase	2607590	2608234 -	214	53722947	3095059 -	COG0529P	CCGCGCCGCCCGTCCATCGCAAGG	2	103	1	103	100	Bm_ATCC23344_chr2	2325379	151817	151919	100	515	7.70E-19 +
BPSS1927	methyl-accepting chemotaxis protein	2608475	2610166 -	563	53722948	3095060 -	COG0840NT	TATCCAGGAACGGCACGACGGCCC	2	103	1	103	100	Bm_ATCC23344_chr2	2325379	149876	149978	99	506	2.00E-18 +
BPSS1928	hypothetical protein BPSS1928	2611158	2611997 +	279	53722949	3097076 -	NULL	GGCGCATGCTAGTGCATACATGCTT	2	103	103	1	100	Bm_ATCC23344_chr2	2325379	149083	149186	99	505	2.20E-18 -
BPSS1934	universal stress protein	2616694	2617164 -	156	53722955	3096674 -	COG0589T	CGGCAAGCGAATCCGGTGCGCGAA	2	103	1	103	100	Bm_ATCC23344_chr2	2325379	142893	142995	100	515	7.70E-19 +
BPSS1935	TetR-family transcriptional regulator	2617380	2618246 -	288	53722956	3096675 -	NULL	CATGCCCGATGCGGGTGCGCCCGC	2	103	1	103	100	Bm_ATCC23344_chr2	2325379	141811	141913	100	515	7.70E-19 +
BPSS1936	outer membrane efflux protein	2618540	2620066 +	508	53722957	3096705 -	COG1538MU	CGCGCGGCGCGCGGCGCTTCTCCG	2	103	103	24	77	Bm_ATCC23344_chr2	2325379	141642	141721	98	391	2.00E-12 -
BPSS1940	sensor kinase/response regulator fusion protein	2625634	2627469 +	611	53722961	3095123 -	COG0642T	CGCCTCGTCGCATCGCGCGCCGAT	2	103	103	1	100	Bm_ATCC23344_chr2	2325379	134561	134663	100	515	7.70E-19 -
BPSS1941	methyl-accepting chemotaxis transducer transmemb	2627825	2629390 -	521	53722962	3097050 -	COG0840NT	TCGGGTTTAACAGTACGCATCCGCG	2	103	1	103	100	Bm_ATCC23344_chr2	2325379	130719	130821	97	488	1.30E-17 +
BPSS1942	hypothetical protein BPSS1942	2629931	2630440 +	169	53722963	3097136 -	NULL	CGAACGGCGCGCCCGTCCCGCCCT	2	103	103	1	100	Bm_ATCC23344_chr2	2325379	130276	130378	99	506	2.00E-18 -
BPSS1944	alcohol dehydrogenase	2631078	2632103 -	341	53722965	3096266 adhA	COG1064R	CGCAATCGCGCCTCGCGCCGGGCG	2	103	1	103	100	Bm_ATCC23344_chr2	2325379	128006	128108	99	506	2.00E-18 +
BPSS1953	ATP synthase subunit B	2638096	2639631 -	511	53722974	3096070 atpD	COG0055C	CAGCCAGACGGGAGCGACGGTGG	2	103	1	103	100	Bm_ATCC23344_chr2	2325379	120487	120589	99	506	2.00E-18 +
BPSS1954	polymerase	2640073	2641866 +	597	53722975	3095531 -	COG3243I	AGCGGCGACGCCCGCGTCGCGGCT	2	103	103	1	100	Bm_ATCC23344_chr2	2325379	120143	120245	100	515	7.70E-19 -
BPSS1957	6-phosphofructokinase	2645187	2646128 -	313	53722978	3095075 -	COG1105G	CGGCGCGTGCCCGCCGCGCAGCAC	2	103	1	103	100	Bm_ATCC23344_chr2	2325379	114089	114191	100	515	7.70E-19 +
BPSS1958	hypothetical protein BPSS1958	2646621	2647268 -	215	53722979	3096375 -	COG2823R	GGTCCGCGCCGCGTAATGCGCGAT	2	103	1	103	100	Bm_ATCC23344_chr2	2325379	112851	112953	98	497	5.00E-18 +
BPSS1960	thymidine phosphorylase	2648584	2649906 -	440	53722981	3097221 deoA	COG0213F	CGGTTTCGGAAACGGCTGGGCCGC	2	103	1	103	100	Bm_ATCC23344_chr2	2325379	110333	110435	100	515	7.70E-19 +
BPSS1961	hypothetical protein BPSS1961	2650594	2650773 +	59	53722982	3096376 -	NULL	CCGGCCCCCGGAAATTCGGCATAT	2	103	103	1	100	Bm_ATCC23344_chr2	2325379	109752	109854	99	506	2.00E-18 -
BPSS1963	nucleoside transport related membrane protein	2651869	2653143 -	424	53722984	3095752 -	NULL	GATGAATAAACGGCGCGCGCGCGC	2	103	1	103	100	Bm_ATCC23344_chr2	2325379	107105	107207	100	515	7.70E-19 +
BPSS1964	hypothetical protein BPSS1964	2653661	2654914 -	417	53722985	3095753 -	NULL	GATGTCGCGTGATGCGGCCCGACA	2	103	1	103	100	Bm_ATCC23344_chr2	2325379	105330	105432	100	515	7.70E-19 +
BPSS1965	glutathione S-transferase protein	2655043	2655687 -	214	53722986	3095850 -	COG0625O	GCCGCCGCAGCGCCGCGTCGTGAC	2	103	1	103	100	Bm_ATCC23344_chr2	2325379	104557	104659	100	515	7.70E-19 +
BPSS1966	LysR-family transcriptional regulator	2655961	2656866 +	301	53722987	3095916 -	COG0583K	GGCGGTCCCGCCGCCGACACATGC	2	103	103	51	50	Bm_ATCC23344_chr2	2325379	103141	103193	96	247	2.10E-06 -
BPSS1967	TetR-family regulatory protein	2657077	2657730 -	217	53722988	3095917 -	COG1309K	GACGGCCCTCCCTAAGGGTGTCTC	2	103	1	103	100	Bm_ATCC23344_chr2	2325379	101274	101376	100	515	7.70E-19 +
BPSS1968	dehydrogenase	2657833	2658741 +	302	53722989	3096358 -	COG1028IQR	CGCTATTTGACATCAGTCAATGTCA	2	103	103	1	100	Bm_ATCC23344_chr2	2325379	101269	101371	100	515	7.70E-19 -
BPSS1971	two component system fusin protein	2662056	2663579 +	507	53722992	3097107 -	NULL	CGAATGCTCAAGAAAATGCGGCGT	2	103	103	1	100	Bm_ATCC23344_chr2	2325379	97106	97208	100	515	7.70E-19 -
BPSS1972	hypothetical protein BPSS1972	2663739	2664188 +	149	53722993	3097108 -	NULL	CCATGCCGGCAACCGCACCCGAAA	2	103	103	1	100	Bm_ATCC23344_chr2	2325379	95360	95462	100	515	7.70E-19 -
BPSS1973	sedolisin-B	2665124	2667058 +	644	53722994	3095105 -	COG4934O	CCGCCTCGCCGCATCGCCATGCAT	2	103	103	1	100	Bm_ATCC23344_chr2	2325379	93975	94077	100	515	7.70E-19 -
BPSS1975	hypothetical protein BPSS1975	2669668	2670951 +	427	53722996	3097219 -	COG2020O	CGCGCAGGCAAGCCGAACGGCGC	2	93	93	1	100	Bm_ATCC23344_chr2	2325379	89311	89403	100	465	1.40E-16 -
BPSS1976	hypothetical protein BPSS1976	2671756	2673126 +	456	53722997	3095868 -	NULL	GCCGGCCGCGCGAACGCGTCCACG	2	103	103	1	100	Bm_ATCC23344_chr2	2325379	87211	87313	100	515	7.70E-19 -
BPSS1978	EPS transport-related membrane protein kinase	2674611	2676974 +	787	53722999	3095490 -	COG0489D, COG3206M	CACGAAGCCGGATTCGCTCGACGC	2	103	103	1	100	Bm_ATCC23344_chr2	2325379	84356	84458	100	515	7.70E-19 -
BPSS2098	hypothetical protein BPSS2098	2839991	2840482 -	163	53723112	3095171 -	NULL	GCGGCAAACGCTCGGGATTGGACT	2	62	1	62	100	Bm_ATCC23344_chr2	2325379	397685	397746	98	301	3.60E-09 +
BPSS2100	hypothetical protein BPSS2100	2842043	2842621 -	192	53723114	3096765 -	NULL	TGCGGGGGCGCCGCTGCGGGCGG	2	65	1	65	100	Bm_ATCC23344_chr2	2325379	395543	395607	98	316	7.50E-10 +
BPSS2101	hypothetical protein BPSS2101	2842684	2843763 -	359	53723115	3095432 -	COG3515S	AGCCGACGCGCCGGCGCGCGCCGG	2	55	1	55	100	Bm_ATCC23344_chr2	2325379	394411	394465	100	275	5.30E-08 +
BPSS2107	hypothetical protein BPSS2107	2853944	2854462 -	172	53723121	3095961 -	COG3521S	AATAGCCCCAATAGATCGTCACCTT	2	103	1	103	100	Bm_ATCC23344_chr2	2325379	383708	383810	99	506	2.00E-18 +
BPSS2108	lipoprotein	2855102	2855503 +	133	53723122	3097247 -	NULL	ACGGCATGGCGCATCGCCCGTCCA	2	103	103	1	100	Bm_ATCC23344_chr2	2325379	383115	383217	100	515	7.70E-19 -
BPSS2109	FHA-family protein	2855558	2856994 +	478	53723123	3097248 -	COG3456T	ACGACTCGGATCGGTTTCCATGCGA	2	57	57	1	100	Bm_ATCC23344_chr2	2325379	382710	382766	100	285	1.90E-08 -
BPSS2110	hypothetical protein BPSS2110	2857037	2858941 -	634	53723124	3095064 -	NULL	GCGGCCGACACTGGCTCGACCGAC	2	103	1	103	100	Bm_ATCC23344_chr2	2325379	379235	379337	100	515	7.70E-19 +
BPSS2114	oxidoreductase/short-chain dehydrogenase protein	2861989	2862693 -	234	53723127	3095077 -	COG1028IQR	TCGACAGCGGGGGTTGCCGCGCCT	2	103	1	103	100	Bm_ATCC23344_chr2	2325379	375404	375506	100	515	7.70E-19 +
BPSS2115	LysR family transcriptional regulator	2862875	2863789 +	304	53723128	3094962 -	COG0583K	GTGGTAATTTAGGCGCGGCAACCC	2	103	103	1	100	Bm_ATCC23344_chr2	2325379	375335	375437	100	515	7.70E-19 -
BPSS2116	D-serine dehydratase	2864045	2865382 -	445	53723129	3096802 dsdA	COG3048E	TAGCGTGGCTTATGCATGGGCAAG	2	99	1	99	100	Bm_ATCC23344_chr2	2325379	372826	372924	96	468	1.00E-16 +
BPSS2117	LysR family transcriptional regulator	2865479	2866420 +	313	53723130	3097152 -	COG0583K	GATCTGAAATAAAATGTGATGGAC	2	99	99	1	100	Bm_ATCC23344_chr2	2325379	372823	372921	96	468	1.00E-16 -
BPSS2122	hypothetical protein BPSS2122	2871821	2872219 -	132	53723136	3097191 -	NULL	TCGTGCGCGCTGAGATTGACGGAG	2	103	1	103	100	Bm_ATCC23344_chr2	2325379	365998	366100	100	515	7.70E-19 +
BPSS2123	transporter protein	2872672	2874006 +	444	53723137	3097053 -	NULL	CATCCCGAAGCCCCGGCATCACGC	2	103	103	1	100	Bm_ATCC23344_chr2	2325379	365592	365694	100	515	7.70E-19 -
BPSS2125	L-lactate dehydrogenase	2875583	2876725 +	380	53723139	3095025 lldA	COG1304C	AGATATTTCCGGGCGGGCAGCCGG	2	67	67	1	100	Bm_ATCC23344_chr2	2325379	362555	362621	100	335	1.00E-10 -
BPSS2126	FAA-hydrolase family protein	2876902	2877750 -	282	53723140	3095071 -	COG0179Q	CGATGGTACAAATGCCGCAACGGG	2	103	1	103	100	Bm_ATCC23344_chr2	2325379	360283	360385	100	515	7.70E-19 +
BPSS2127	IclR transcriptional regulator protein	2878261	2879085 +	274	53723141	3094923 -	COG1414K	GACCTATGAATCAACCATCCATCGA	2	103	103	1	100	Bm_ATCC23344_chr2	2325379	359870	359972	98	497	5.00E-18 -
BPSS2129	hypothetical protein BPSS2129	2880980	2881531 +	183	53723143	3094992 -	NULL	CGCGGGTCTCGCGCACGAATCGGC	2	103	103	1	100	Bm_ATCC23344_chr2	2325379	357166	357268	100	515	7.70E-19 -
BPSS2130	acetyl-CoA synthetase	2882057	2884420 -	787	53723144	3094993 -	COG1042C	GATGATGCGGGCGCTTGCGTGGTT	2	103	1	103	100	Bm_ATCC23344_chr2	2325379	353601	353703	98	497	5.00E-18 +
BPSS2132	sigma-54 dependent transcriptional regulator	2887571	2889091 -	506	53723148	3095355 -	COG2204T, COG3829KT	GCGATGCTCGGCGATTCATGGCCG	2	103	1	103	100	Bm_ATCC23344_chr2	2325379	348986	349089	99	505	2.20E-18 +
BPSS2133	hypothetical protein BPSS2133	2889396	2890799 +	467	53723149	3095356 -	COG2610GE	AGCGTCGCGCCGTCCGCCGGGCGC	2	103	103	1	100	Bm_ATCC23344_chr2	2325379	348741	348842	99	500	3.70E-18 -

BPSS2134	hypothetical protein BPSS2134	2891157	2891402	+	81	53723150	3095193	-	NULL	CCGGTTCGGTTAAACTGCGTTCAAG	2	103	103	1	100	Bm_ATCC23344_chr2	2325379	347019	347121	99	506	2.00E-18	-
BPSS2135	DedA family transmembrane protein	2891557	2892549	+	330	53723151	3095429	-	COG0586S	TCGCTCGCGGCATGAACTCCTTGCC	2	103	103	1	100	Bm_ATCC23344_chr2	2325379	346619	346721	99	506	2.00E-18	-
BPSS2136	family S43 non-peptidase homologue	2892881	2894323	-	480	53723152	3095430	-	NULL	GTCGCTACGGCGGCGCGTCGGGGT	2	103	1	103	100	Bm_ATCC23344_chr2	2325379	343755	343857	100	515	7.70E-19	+
BPSS2141	periplasmic oligopeptide-binding protein precursor	2898490	2900154	-	554	53723157	3095325	oppA	COG4166E	TGTCGAAAGGAACGTAAGGATGAC	2	103	1	103	100	Bm_ATCC23344_chr2	2325379	337924	338026	100	515	7.70E-19	+
BPSS2142	MerR family transcriptional regulator	2900290	2900871	-	193	53723158	3095015	-	COG1396K	GAGGCGGCGGCGCAGCTCGGGAC	2	103	1	103	100	Bm_ATCC23344_chr2	2325379	337207	337309	100	515	7.70E-19	+
BPSS2143	POT-family proton dependent transporter	2901507	2903009	+	500	53723159	3095016	-	COG3104E	CGCCCGGCCGCGCCGGCCGCCGC	2	103	103	1	100	Bm_ATCC23344_chr2	2325379	336669	336771	99	506	2.00E-18	-
BPSS2144	hypothetical protein BPSS2144	2903449	2903652	+	67	53723160	3095149	-	NULL	ACATGTGACTTGCGCGCCACATTCT	2	103	103	1	100	Bm_ATCC23344_chr2	2325379	334728	334830	100	515	7.70E-19	-
BPSS2146	LysR family transcriptional regulator	2905720	2906661	-	313	53723162	3095444	-	COG0583K	ATCCTTTCCGGCGCGTGACGGAAA	2	103	1	103	100	Bm_ATCC23344_chr2	2325379	331385	331487	100	515	7.70E-19	+
BPSS2157	Acyl-CoA transferase/carnitine dehydratase protein	2918645	2919922	-	425	53723173	3095891	-	COG1804C	CGTGAGGACGGCGGGCTCCGGCAT	2	76	76	1	100	Bm_ATCC23344_chr2	2325379	2139233	2139308	100	380	9.60E-13	-
BPSS2158	IclR family transcriptional regulator	2919996	2920745	-	249	53723174	3095638	-	COG1414K	TCGTATAATGAAAGTAACTTTCAAA	2	103	103	1	100	Bm_ATCC23344_chr2	2325379	2140056	2140158	100	515	7.70E-19	-
BPSS2159	citrate lyase beta chain	2920989	2921894	+	301	53723175	3095537	-	COG2301G	GTTGTTTACCCGACACTCCTATTTCT	2	103	1	103	100	Bm_ATCC23344_chr2	2325379	2140202	2140304	100	515	7.70E-19	+
BPSS2160	outer membrane porin protein	2922210	2923289	+	359	53723176	3095538	-	COG3203M	CGGCAACGGCTGTAACGGCTGTAA	2	103	1	103	100	Bm_ATCC23344_chr2	2325379	2141423	2141525	96	479	3.30E-17	+
BPSS2162	hypothetical protein BPSS2162	2924882	2926009	-	375	53723178	3095941	-	COG4645S	GGCAATCGCATCCCGCCGCAGCCG	2	103	103	1	100	Bm_ATCC23344_chr2	2325379	2145325	2145427	100	515	7.70E-19	-
BPSS2164	acylphosphatase protein	2927298	2927594	-	98	53723180	3095820	-	COG1254C	TGCTCGCCCGGGATGCGCGGCGCG	2	103	103	1	100	Bm_ATCC23344_chr2	2325379	2146911	2147013	100	515	7.70E-19	-
BPSS2165	hypothetical protein BPSS2165	2927755	2928762	+	335	53723181	3095821	-	COG0451MG	TGCTCGATGCAGCCCGCGCGCCGC	2	103	1	103	100	Bm_ATCC23344_chr2	2325379	2146974	2147076	100	515	7.70E-19	+
BPSS2168	4-hydroxy-3-methylbut-2-enyl diphosphate reductas	2932395	2933336	+	313	53723184	3095062	ispH	NULL	TGCCGGGCCCGTTCCAGCATTCATA	2	103	1	103	100	Bm_ATCC23344_chr2	2325379	2151494	2151596	100	515	7.70E-19	+
BPSS2170	hypothetical protein BPSS2170	2934853	2935206	+	117	53723186	3095750	-	COG3805Q	CGGCGCCGAGACGATGCGCCGCCC	2	103	1	103	100	Bm_ATCC23344_chr2	2325379	2153953	2154055	100	515	7.70E-19	+
BPSS2171	glutamate-1-semialdehyde 2,1-aminomutase	2935765	2937123	+	452	53723187	3095751	-	COG0001H	CGCTCGCGATCGCTCACGGCCGGA	2	103	1	103	100	Bm_ATCC23344_chr2	2325379	2154855	2154957	98	497	5.00E-18	+
BPSS2240	hypothetical protein BPSS2240	3013274	3013585	-	103	53723258	3096681	-	NULL	CGGCTCGCGCCGCGTGGCGCGCCG	2	103	103	1	100	Bm_ATCC23344_chr2	2325379	2162403	2162505	100	515	7.70E-19	-
BPSS2243	lipoprotein	3014799	3015290	-	163	53723261	3096573	-	COG2823R	GGCCGGCGGACGGTGGCACGGCG	2	103	103	1	100	Bm_ATCC23344_chr2	2325379	2164103	2164205	100	515	7.70E-19	-
BPSS2244	transferase	3015521	3016909	+	462	53723262	3096716	-	COG0438M	CCGCGCGCGGCGCGCGCGGCGCC	2	103	1	103	100	Bm_ATCC23344_chr2	2325379	2164236	2164338	100	515	7.70E-19	+
BPSS2246	sensor kinase/response regulator fusion protein	3018081	3020396	-	771	53723264	3095951	-	COG0642T	GGATGCGCGGCTTGCCCGTGCGGA	2	103	103	1	100	Bm_ATCC23344_chr2	2325379	2169212	2169314	100	515	7.70E-19	-
BPSS2247	hypothetical protein BPSS2247	3020531	3021223	+	230	53723265	3096427	-	NULL	GCGCGCGCGCAGCCGGACGCGCC	2	103	1	103	100	Bm_ATCC23344_chr2	2325379	2169246	2169348	100	515	7.70E-19	+
BPSS2249	sigma-54 interacting response regulator	3022173	3023543	-	456	53723267	3095235	-	COG2204T	CGCACATCGGCCGGCATCGTTCGG	2	103	103	1	100	Bm_ATCC23344_chr2	2325379	2172356	2172458	100	515	7.70E-19	-
BPSS2250	sigma-54 interacting transcriptional regulator	3023932	3024900	+	322	53723268	3095236	-	COG2204T	AACGCCGTTCGTTCGCACGCTTGTT	2	103	1	103	100	Bm_ATCC23344_chr2	2325379	2172647	2172749	99	506	2.00E-18	+
BPSS2257	short chain dehydrogenase	3032424	3033179	-	251	53723275	3097173	-	COG1028IQR	GCTCGCGCCGCGCCGGGCACGCA	2	103	103	1	100	Bm_ATCC23344_chr2	2325379	2181998	2182100	100	515	7.70E-19	-
BPSS2261	phosphatidylserine decarboxylase	3038859	3040160	-	433	53723279	3096568	-	COG0688I	GCCGACCGGAGCCGGCGCTTCGCT	2	103	103	1	100	Bm_ATCC23344_chr2	2325379	2188791	2188893	99	506	2.00E-18	-
BPSS2262	succinate dehydrogenase catalytic subunit	3040322	3041029	-	235	53723280	3095755	sdhB	COG0479C	TCCGCGGCGCGGCCGCTCGCGCGC	2	85	85	1	100	Bm_ATCC23344_chr2	2325379	2189660	2189744	98	416	2.30E-14	-
BPSS2263	hypothetical protein BPSS2263	3041112	3041357	-	81	53723281	3096569	-	NULL	CAGGCATGCCCGCTGCGCGCAGCG	2	103	103	1	100	Bm_ATCC23344_chr2	2325379	2189988	2190090	99	506	2.00E-18	-
BPSS2264	dehydrogenase	3041653	3042540	-	295	53723282	3096985	-	COG2084I	GTCGCCAGCCCGGGCGCCCGGCCG	2	103	103	1	100	Bm_ATCC23344_chr2	2325379	2191195	2191297	99	506	2.00E-18	-
BPSS2265	monooxygenase protein	3042681	3044030	-	449	53723283	3096986	-	COG2141C	CCGCGGCGTGGCGCGCGGCCTTCC	2	103	103	1	100	Bm_ATCC23344_chr2	2325379	2192685	2192787	100	515	7.70E-19	-
BPSS2266	4'-phosphopantetheinyl transferase superfamily prot	3044311	3045126	-	271	53723284	3095361	-	COG2091H	GCGGTGCCGACATCGCGAATGCGA	2	103	103	1	100	Bm_ATCC23344_chr2	2325379	2193781	2193883	99	506	2.00E-18	-
BPSS2267	hypothetical protein BPSS2267	3045344	3046597	-	417	53723285	3096387	-	NULL	GCGCACCGTGCGATCGAGGATGCG	2	103	103	1	100	Bm_ATCC23344_chr2	2325379	2195252	2195354	100	515	7.70E-19	-
BPSS2269	transcriptional regulator	3048340	3049497	-	385	53723287	3095606	-	NULL	CGCAATTTGACCTGCCGCCGTGAG	2	103	103	1	100	Bm_ATCC23344_chr2	2325379	2198137	2198239	99	506	2.00E-18	-
BPSS2273	2-oxoisovalerate dehydrogenase alpha subunit	3054057	3055289	-	410	53723291	3096116	bkdA1	COG1071C	TCGGGGCGACTCCGAGTCCGAAGA	2	103	103	1	100	Bm_ATCC23344_chr2	2325379	2203929	2204031	100	515	7.70E-19	-
BPSS2274	hypothetical protein BPSS2274	3056541	3056981	+	146	53723292	3095607	-	NULL	GCTATCGTGACGCACCGCGGCATC	2	103	1	103	100	Bm_ATCC23344_chr2	2325379	2205084	2205186	100	515	7.70E-19	+
BPSS2275	hypothetical protein BPSS2275	3057306	3058421	-	371	53723293	3095185	-	NULL	TCACGATTGTCCATTGGAGAAATCA	2	103	103	1	100	Bm_ATCC23344_chr2	2325379	2207072	2207174	100	515	7.70E-19	-
BPSS2276	LysR family regulatory protein	3058719	3059654	+	311	53723294	3095186	-	COG0583K	CGCGCCCACCGTGCGCGCGCAAGC	2	103	1	103	100	Bm_ATCC23344_chr2	2325379	2207272	2207374	100	515	7.70E-19	+
BPSS2277	hypothetical protein BPSS2277	3059829	3060260	+	143	53723295	3095876	-	NULL	GATCGCGGCCGCCGCGATCGGCGC	2	103	1	102	100	Bm_ATCC23344_chr2	2325379	2208382	2208484	97	482	2.40E-17	+
BPSS2278	hypothetical protein BPSS2278	3060560	3062218	+	552	53723296	3095902	-	COG2303E	AGGCCGTGCGCCGCATCGCGCAAC	2	103	1	103	100	Bm_ATCC23344_chr2	2325379	2209111	2209213	99	506	2.00E-18	+
BPSS2279	fusion protein, pyridine nucleotide-disulphide oxidor	3062508	3064781	-	757	53723297	3095903	-	COG0446R, COG1752R	GCAGTCGCCGCACGCGCCGCGTCT	2	103	103	1	100	Bm_ATCC23344_chr2	2325379	2213460	2213562	100	515	7.70E-19	-
BPSS2283	hypothetical protein BPSS2283	3070172	3071881	+	569	53723301	3095816	-	COG0645R, COG2187S	GACGACGGGCGCCGTCGCGGCGC	2	103	1	103	100	Bm_ATCC23344_chr2	2325379	2218789	2218891	100	515	7.70E-19	+
BPSS2284	hypothetical protein BPSS2284	3072406	3073437	+	343	53723302	3096601	-	COG0778C	TGAAATCGATGCCGCGCAGCCCGA	2	103	1	103	100	Bm_ATCC23344_chr2	2325379	2221017	2221119	99	506	2.00E-18	+
BPSS2286	hypothetical protein BPSS2286	3075738	3076256	-	172	53723304	3095830	-	COG0517R	GCAGCGCGGGAAGCGGCTTCGGCC	2	103	103	1	100	Bm_ATCC23344_chr2	2325379	2224873	2224975	100	515	7.70E-19	-
BPSS2287	hypothetical protein BPSS2287	3076641	3077294	+	217	53723305	3097094	-	NULL	GATCCGGATCAGAGCGGCGCGCCG	2	103	1	103	100	Bm_ATCC23344_chr2	2325379	2225160	2225262	100	515	7.70E-19	+
BPSS2288	HSP20/alpha crystallin family protein	3077355	3077789	-	144	53723306	3097095	-	COG0071O	CCGGTCAGCATCATCGATCAACGG	2	103	103	1	100	Bm_ATCC23344_chr2	2325379	2226413	2226515	100	515	7.70E-19	-
BPSS2299	molybdopterin oxidoreductase	3092021	3094897	+	958	53723317	3095001	-	COG0243C	CGACCAACGTCGCGCTCGCTTCGA	2	103	1	103	100	Bm_ATCC23344_chr2	2325379	2239891	2239993	100	515	7.70E-19	+
BPSS2301	hypothetical protein BPSS2301	3095766	3096716	+	316	53723319	3097188	-	COG3302R	GGGGCGCCGGCGCCCCCGACAAC	2	60	1	60	100	Bm_ATCC23344_chr2	2325379	2243679	2243738	100	300	4.00E-09	+
BPSS2302	hypothetical protein BPSS2302	3097926	3098816	+	296	53723320	3097189	-	NULL	CGCGAAGCGCACGTCGCGCCGAGC	2	103	1	103	100	Bm_ATCC23344_chr2	2325379	2245717	2245819	99	506	2.00E-18	+
BPSS2304	penicillin-binding protein	3100875	3103394	-	839	53723322	3096190	-	COG5009M	CACGCGCCGAATCCGCCCTCCCGC	2	103	103	1	100	Bm_ATCC23344_chr2	2325379	2251271	2251373	99	506	2.00E-18	-
BPSS2305	lipoprotein	3104258	3105826	-	522	53723323	3096196	-	NULL	AGAACGGAGCGCGCGGCCGGCGC	2	103	103	1	100	Bm_ATCC23344_chr2	2325379	2253537	2253639	100	515	7.70E-19	-
BPSS2306	porin protein	3106526	3107698	-	390	53723324	3095436	-	COG3203M	TAATCATATCCATCGCAGAATTAAT	2	103	103	1	100	Bm_ATCC23344_chr2	2325379	2255404	2255506	99	506	2.00E-18	-
BPSS2314	sensor kinase protein	3115766	3117166	+	466	53723331	3095719	-	COG4564T	GCGGCGCGCGGCCGACCCGACGG	2	103	1	103	100	Bm_ATCC23344_chr2	2325379	2263391	2263493	99	506	2.00E-18	+
BPSS2316	hypothetical protein BPSS2316	3118004	3118600	-	198	53723333	3095037	-	COG2854Q	CGTCACGGCGCCGCGCGGGCGCG	2	103	103	1	100	Bm_ATCC23344_chr2	2325379	2270467	2270569	100	515	7.70E-19	-
BPSS2318	hypothetical protein BPSS2318	3119667	3122108	-	813	53723335	3096973	-	NULL	CGGGCCGCCGCCGGTTCGTTTCCG	2	103	103	1	100	Bm_ATCC23344_chr2	2325379	2273975	2274077	100	515	7.70E-19	-
BPSS2319	lipase precursor	3122425	3123495	-	356	53723336	3095019	lipA2	COG1075R	CGCGCGCGGCGAAGGGTGCACCG	2	103	103	1	100	Bm_ATCC23344_chr2	2325379	2275362	2275464	100	515	7.70E-19	-
BPSS2320	4'-phosphopantetheinyl transferase superfamily prot	3123622	3124386	-	254	53723337	3096967	-	COG2091H	TGCCCGCGCCGCGCGGCCCGCACG	2	65	65	1	100	Bm_ATCC23344_chr2	2325379	2276254	2276318	100	325	2.90E-10	-
BPSS2323	hypothetical protein BPSS2323	3126182	3126964	-	260	53723340	3096923	-	NULL	ATCGCGGAAGTCTTCAGTTTGAACA	2	59	59	1	100	Bm_ATCC23344_chr2	2325379	2278832	2278890	100	295	6.70E-09	-
BPSS2330	hypothetical protein BPSS2330	3143858	3146491	+	877	53723347	3094894	-	COG1033R	CCGGCTGGATTCACGCGCATCGGA	2	103	1	103	100	Bm_ATCC23344_chr2	2325379	2295904	2296006	99	506	2.00E-18	+
BPSS2336	glutamine ABC transporter permease component	3151446	3152102	-	218	53723353	3095377	glnP	COG0765E	GCGACGCATCGGGGTGGCGCGGC	2	67	67	1	100	Bm_ATCC23344_chr2	2325379	2304258	2304324	100	335	1.00E-10	-
BPSS2337	glutamine ABC transporter periplasmic-binding prote	3152167	3152919	-	250	53723354	3095223	glnH	COG0834ET	CAAATTACAATCGCCGCATCGCGTG	2	103	103	1	100	Bm_ATCC23344_chr2	2325379	2305075	2305177	98	497	5.00E-18	-
BPSS2339	squalene--hopene cyclase	3154080	3156053	-	657	53723356	3095684	shc	COG1657I	CGGGCGCTTGGACGGCCGCAGGCC	2	103	103	1	100	Bm_ATCC23344_chr2	2325379	2308210	2308312	99	506	2.00E-18	-
BPSS2340	squalene/phytoene synthase	3156381	3157229	-	282	53723357	3094989	-	COG1562I	TGTCTGCGCCGATCGTTCGCGCATC	2	103	103	1	100	Bm_ATCC23344_chr2	2325379	2309386	2309488	100	515	7.70E-19	-
BPSS2341	esterase	3157746	3158651	+	301	53723358	3095087	estC	COG0596R	GCACGATCGAACGCCACGGCACCG	2	103	1	103	100	Bm_ATCC23344_chr2	2325379	2309833	2309935	100	515	7.70E-19	+
BPSS2344	response regulator protein	3160246	3160614	-	122	53723361	3094944	-	NULL	GCGGTCCGTCCCTCGTATCGTCAGT	2	103	103	1	100	Bm_ATCC23344_chr2	2325379	2312799	2312901	100	515	7.70E-19	-
BPSS2345	sensory kinase protein	3161120	3163402	+	760	53723362	3094945	-	COG0642T, COG0745TK	GGAAAACCGGCCCCGCGCAAGCTC	2	103	1	103	100	Bm_ATCC23344_chr2	2325379	2313207	2313309	100	515	7.70E-19	+
BPSS2348	arsenate reductase	3165814	3166170	-	118	53723366	3095275	-	COG1393P	GACGGAGACGGAGACGGAGACGG	2	103	103	1	100	Bm_ATCC23344_chr2	2325379	2318243	2318346	96	478	3.60E-17	-
BPSS2349	partition protein ParA	3166744	3167406	+	220	53723367	3095276	-	COG1192D	ACCATCGGCCAGATGGCCAACACG	2	103	1	103	100	Bm_ATCC23344_chr2	2325379	2318574	2318676	100	515	7.70E-19	+
BPSS2351	replication protein	3168589	3170031	-	480	53723369	3095381	-	NULL	GCCTGCAAAGGTGAGTCGACGATG	2	103	103	1	100	Bm_ATCC23344_chr2	2325379	2323566	2323668	100	515	7.7E-19	-
