# Supplementary material for: The Early Stage of Bacterial Genome-Reductive Evolution in the Host
Source: PLoS Pathog. 2010 May 27;6(5):e1000922. doi: 10.1371/journal.ppat.1000922 (PMC2877748; doi:10.1371/journal.ppat.1000922)
Supplement: Table S7 — Putative regulatory genes deleted from B. mallei as IS-bounded genomic fragments. (0.04 MB PDF) [file ppat.1000922.s009.pdf]

Table S7. Putative regulatory genes deleted from *B. mallei* as IS-bounded genomic fragments

Locus tag	Common name	5'-end	3'-end	COG
Chromosome 1				
BPSL0069	putative transmembrane regulator	78278	77520	COG5662K
BPSL0070	putative RNA polymerase sigma factor	78790	78275	COG1595K
BPSL0327	LysR family regulatory protein	349356	350318	COG0583K
BPSL0330	AsnC family regulatory protein	353512	353024	COG1522K
BPSL0340	AsnC family regulatory protein	362642	362184	COG1522K
BPSL0350	Fis family regulatory protein	373423	374079	COG3829KT
BPSL0549A	putative DNA-binding protein	607069	606593	COG1396K
BPSL0562	putative DNA-binding protein	615702	616079	COG1396K
BPSL0571	hypothetical protein BPSL0571	626686	628548	COG0515RTKL
BPSL0709	putative LysR family transcriptional regulator	811759	812583	COG0583K
BPSL0731	LysR family transcriptional regulator	844113	845339	COG0583K
BPSL0734	putative two-component transcriptional response regulator	848805	847843	COG0745TK
BPSL0756	putative DNA-binding protein	873708	873400	COG1396K
BPSL0763	putative helicase SNF2 family protein	878619	881801	COG0553KL
BPSL0765	putative helicase family protein	886079	889639	COG1200LK, COG1205R
BPSL0939	putative DeoR family regulatory protein	1091418	1092440	COG2378K
BPSL1453	putative LysR-family transcriptional regulator	1690008	1690919	COG0583K
BPSL1562	putative transcriptional regulatory protein	1812459	1813862	COG3829KT
BPSL1564	putative transcriptional regulatory protein	1814749	1814973	COG3655K
BPSL1565	putative MerR-family transcriptional regulator	1817363	1816314	COG0789K
BPSL1642	putative GntR-family regulatory protein	1904965	1905735	COG1802K
BPSL1653	putative GntR-family regulatory protein	1915444	1916208	COG1802K
BPSL1669	putative two component system, response regulator	1948084	1948797	COG0745TK
BPSL1680	putative LysR-family transcriptional regulatory protein	1961861	1960971	COG0583K
BPSL1686	RNA polymerase sigma-70 factor	1966393	1966929	COG1595K
BPSL1695	putative GntR-family transcriptional regulator	1973254	1972586	COG1802K
BPSL1831	putative ribose operon repressor	2184766	2183735	COG1609K
BPSL2111	putative LysR-family transcriptonal regulator	2536505	2537434	COG0583K
BPSL2782	putative AraC family transcriptional regulator	3321881	3320835	COG4977K

BPSL3115	putative transcriptional regulator	3719241	3720311	COG1396K, COG2856E
BPSL3250	putative LysR-family transcriptional regulator	3863362	3862424	COG0583K
Chromosome 2				
BPSS0119	two-component system response regulator	152618	151956	COG2197TK
BPSS0124	response regulator	159632	160324	COG2197TK
BPSS0129	transcriptional regulator	165876	167096	COG3835KT
BPSS0134	LysR-family transcriptional regulator	174673	173732	COG0583K
BPSS0143	ROK family transcriptional regulator	185761	184511	COG1940KG
BPSS0431	LysR-family transcriptional regulator	594292	593393	COG0583K
BPSS0438	LysR-family transcriptional regulator	601354	602265	COG0583K
BPSS0449	GntR-family regulatory protein	615297	613963	COG1167KE
BPSS0472	MerR-family transcriptional regulator	642281	642832	COG1396K
BPSS0596	sigma-54 activated regulatory protein	819431	817491	COG3284QK
BPSS0606	LysR family regulatory protein	827920	828819	COG0583K
BPSS0664	response regulator protein	898946	899656	COG0745TK
BPSS1371	AraC family transcriptional regulator	1876037	1877203	COG4977K
BPSS1816	regulatory protein	2475821	2475528	COG0640K
BPSS1994	metal-related two-component system, response regulator	2698676	2699368	COG0745TK
BPSS1995	metal-related two-component system, histidine kinase	2699365	2700819	COG0642T
BPSS2017	LysR-family transcriptional regulator	2728954	2728076	COG0583K
BPSS2028	TetR-family transcriptional regulator	2741325	2740651	COG1309K
BPSS2073	GntR family regulator protein	2809190	2808477	COG2186K
BPSS2087	LacI family regulatory protein	2826375	2825317	COG1609K
BPSS2177	LysR-family transcriptional regulator	2944785	2945741	COG0583K
BPSS2199	AsnC-family regulatory protein	2967135	2966659	COG1522K
BPSS2204	GntR-family regulatory protein	2972798	2972031	COG2186K
BPSS2207	LysR-family transcriptional regulator	2976263	2977177	COG0583K
BPSS2218	DNA-directed RNA polymerase subunit N	2992777	2991302	COG1508K
